# Supplementary material for: Burkholderia PglL enzymes are Serine preferring oligosaccharyltransferases which target conserved proteins across the Burkholderia genus
Source: Commun Biol. 2021 Sep 7;4:1045. doi: 10.1038/s42003-021-02588-y (PMC8423747; doi:10.1038/s42003-021-02588-y)
Supplement: Supplementary file 2 — Supplementary Information [file 42003_2021_2588_MOESM2_ESM.pdf]

Burkholderia PglL enzymes are Serine preferring oligosaccharyltransferases which target conserved proteins across the _Burkholderia_ genus

Andrew J. Hayes[1], Jessica M. Lewis[1], Mark R. Davies[1] and Nichollas E. Scott[1#]

[1]Department of Microbiology and Immunology, University of Melbourne at the Peter Doherty Institute for Infection and Immunity, Melbourne 3000, Australia

[#]To whom correspondence and requests for materials should be addressed N.E.S (Nichollas.scott@unimelb.edu.au).

Key words: Glycosylation, *Burkholderia cenocepacia*, *Burkholderia*, Post-translational modifications, Proteomics, Glycosyltransferases, PglL

Table of Contents

Title	Page
**Supplementary Table 1: Strain list**	**3**
**Supplementary Table 2: Plasmid list**	**4**
**Supplementary Table 3: Primer list**	**5**
**Supplementary Figure 1. Glycoproteins identified across *B. cenocepacia* datasets**	**6**
**Supplementary Figure 2. Byonic assigned glycopeptide scores**	**7**
**Supplementary Figure 3. Comparison of glycopeptide identification performance with different proteases.**	**8**
**Supplementary Figure 4. *O*-linked glycosylation occurs on Serine residues across the *B. cenocepacia* glycoproteome**	**9**
**Supplementary Figure 5. Misassignment of the Threonine glycosylation event within BCAM0996**	**10**
**Supplementary Figure 6. O-Pair analysis of glycoproteomic datasets reveal a strong preference for glycosylation at Serine residues.**	**11**
**Supplementary Figure 7. Confirmation of S^{36} as the site of glycosylation within DsbA1$_{Nm}$-his$_{6}$.**	**12**
**Supplementary Figure 8. MS analysis of DsbA1$_{Nm}$-his$_{6}$ variants within K56-2 WT reveals low levels of glycosylation within ^{23}VQTSVPADSAPAATAAAAPAGLVEGQNYTVLANPIPQQQAGK^{64}**	**13**
**Supplementary Figure 9. Conservation of *B. cenocepacia* glycosylation site within BCAL0039 to BCAL0426.**	**14**
**Supplementary Figure 10. Conservation of *B. cenocepacia* glycosylation site within BCAL0525 to BCAL1389.**	**15**
**Supplementary Figure 11. Conservation of *B. cenocepacia* glycosylation site within BCAL1453 to BCAL2466.**	**16**
**Supplementary Figure 12. Conservation of *B. cenocepacia* glycosylation site within BCAL2466 to BCAL3469.**	**17**
**Supplementary Figure 13. Conservation of *B. cenocepacia* glycosylation site within BCAM0505 to BCAM2055.**	**18**
**Supplementary Figure 14. Conservation of *B. cenocepacia* glycosylation site within BCAM2063 to BCAS0773.**	**19**
**Supplementary Figure 15. Comparison of PglL protein sequences across the Burkholderia genus.**	**20**
**Supplementary Figure 16. Glycopeptide analysis of DsbA1$_{Nm}$-his$_{6}$ variants within *B. humptydooensis* MSMB43 and *B. ubonensis* MSMB22 supports ^{23}VQTSVPADSAPAA TAAAAPAGLVEGQNYTVLANPIPQQQAGK^{64} is poorly glycosylated.**	**21**
**Supplementary Figure 17. Conservation of glycosylation sites across *Burkholderia* species.**	**22**
**Supplementary Figure 18. Glycoproteins observed with the *B. cenocepacia* H111 and K56-2 proteomes.**	**23**
**Supplementary Figure 19. Uncropped Western blotting images.**	**24**

Supplementary Tables

Supplementary Table 1: Strain list

Strain name	Description	Source/ Reference
***E. coli* strain**		
E. coli pir2	F⁻ Δ*lac169 rpoS*(*Am*) *robA1 creC510 hsdR514 endA recA1 uidA*(Δ*MluI*)::*pir-116*	Thermo Scientific
***B. cenocepacia* strains**		
B. cenocepacia K56-2	Clinical isolate of the ET12 lineage (Darling P 1998, Mahenthiralingam E *et al* 2005)	Canadian *B. cepacia* research and referral repository [1]
B. cenocepacia K56-2 Δ*pglL*	Δ*pglL* (*BCAL0960*) derivative of K56-2 created using pYM8	[2]
B. cenocepacia K56-2 Δ*pglL amrAB::S7-pglL-his*$_{10}$	*amrAB::S7-pglL-his*$_{10}$ chromosomal complement derivative of Δ*pglL* (*BCAL0960*) overexpressing *pglL* from the *S7* promoter	[2]
B. cenocepacia H111	Clinically non-ET12 lineage isolate	[3]
B. cenocepacia H111 Δ*pglL canidatate 1*	Δ*pglL* (I35_RS13570) derivative of H111 created using pYM8 candidate	This study
B. cenocepacia H111 Δ*pglL canidatate 2*	Δ*pglL* (I35_RS13570) derivative of H111 created using pYM8 candidate	This study

Supplementary Table 2: Plasmid list

Plasmid	Description	Source/ Reference
pRK2013	ori_{colE1}, RK2 derivative, Kan^R mob^+ tra^+	[4]
pDAI-SceI-SacB	ori_{pBBR1}, Tet^R, P_{dhfr}, mob^+, expressing ISce-I and the negative selection marker SacB	[5,6]
pYM8	pGPI-SceI with fragments flanking *pglL* (BCAL0960)	[7]
pSCrhaB2	ori_{pBBR1}, *rhaR, rhaS,* P_{rhaB} Tp^R mob^+ (Addgene: #113634)	[8]
pSCrhaB2-BCAL2466-his_6	Tp^R pSCrhaB2 Rhamnose inducible plasmid containing C-terminal his_6-tagged BCAL2466 (Ecotin)	This study
pSCrhaB2-BCAL2345-his_6	Tp^R pSCrhaB2 Rhamnose inducible plasmid containing C-terminal his_6-tagged BCAL2345 (SecG)	This study
pKM4 ($DsbA1_{Nm}$-his_6)	Tp^R pMLBad-based plasmid containing C-terminal his_6-tagged DsbA1 from *N. meningitidis* MC58	[9]
$pKM4^{S31A}$ ($DsbA1_{Nm}$-his_6 A^{31})	Tp^R pMLBad-based plasmid containing C-terminal his_6-tagged DsbA1-his_6 from *N. meningitidis* MC58 site directed mutant of S^{31} to A^{31}	This study
$pKM4^{S31T}$ ($DsbA1_{Nm}$-his_6 T^{31})	Tp^R pMLBad-based plasmid containing C-terminal his_6-tagged DsbA1-his_6 from *N. meningitidis* MC58 site directed mutant of S^{31} to T^{31}	This study
$pKM4^{S36A}$ ($DsbA1_{Nm}$-his_6 A^{36})	Tp^R pMLBad-based plasmid containing C-terminal his_6-tagged DsbA1-his_6 from *N. meningitidis* MC58 site directed mutant of S^{36} to A^{36}	This study
$pKM4^{S36T}$ ($DsbA1_{Nm}$-his_6 T^{36})	Tp^R pMLBad-based plasmid containing C-terminal his_6-tagged DsbA1-his_6 from *N. meningitidis* MC58 site directed mutant of S^{36} to T^{36}	This study

Supplementary Table 3: Primer list

Primers name	Description	Sequence
NS323	*BCAL2466*-his_6 forward	GAAATTCAGCAGGATCACATATGGATGAAATTCGCGATCCGGGCC
NS324	*BCAL2466*-his_6 reverse	CATGCCTGCAGGTCGACTCTAGATCAGTGGTGGTGGTGGTGGTGGTGGTGGTGCTGCTTCACAGCTTGCTGCACG
NS329	*BCAL2345*-his_6 forward	GAAATTCAGCAGGATCACATATGGATGCTGTTATTCAAGACGCTG
NS330	*BCAL2345*-his_6 reverse	CATGCCTGCAGGTCGACTCTAGATCAGTGGTGGTGGTGGTGGTGGTGGTGGTGTTTCGGGACATCCTGGCCCGGC
NS416	*dsbA1*$_{Nm}$-his_6 site directed mutagenesis S36 to T36 forward	GCGCGCCTGCCGCTACCGCAGCCGCCGCCCCGGCAG
NS417	*dsbA1*$_{Nm}$-his_6 site directed mutagenesis S36 to T36 reverse	CTGCCGGGGCGGCGGCTGCGGTAGCGGCAGGCGCGC
NS418	*dsbA1*$_{Nm}$-his_6 site directed mutagenesis S36 to A36 forward	GCGCGCCTGCCGCTGCGGCAGCCGCCGCCCCGGCAG
NS419	*dsbA1*$_{Nm}$-his_6 site directed mutagenesis S36 to A36 Reverse	CTGCCGGGGCGGCGGCTGCCGCAGCGGCAGGCGCGC
NS426	*dsbA1*$_{Nm}$-his_6 site directed mutagenesis S31 to T31 forward	CCAGCGTCCCCGCCGACACCGCGCCTGCCGCT
NS427	*dsbA1*$_{Nm}$-his_6 site directed mutagenesis S31 to T31 reverse	AGCGGCAGGCGCGGTGTCGGCGGGGACGCTGG
NS428	*dsbA1*$_{Nm}$-his_6 site directed mutagenesis S31 to A31 forward	CCAGCGTCCCCGCCGACGCGGCGCCTGCCGCT
NS429	*dsbA1*$_{Nm}$-his_6 site directed mutagenesis S31 to A31 reverse	AGCGGCAGGCGCCGCGTCGGCGGGGACGCTGG

Supplementary Figures

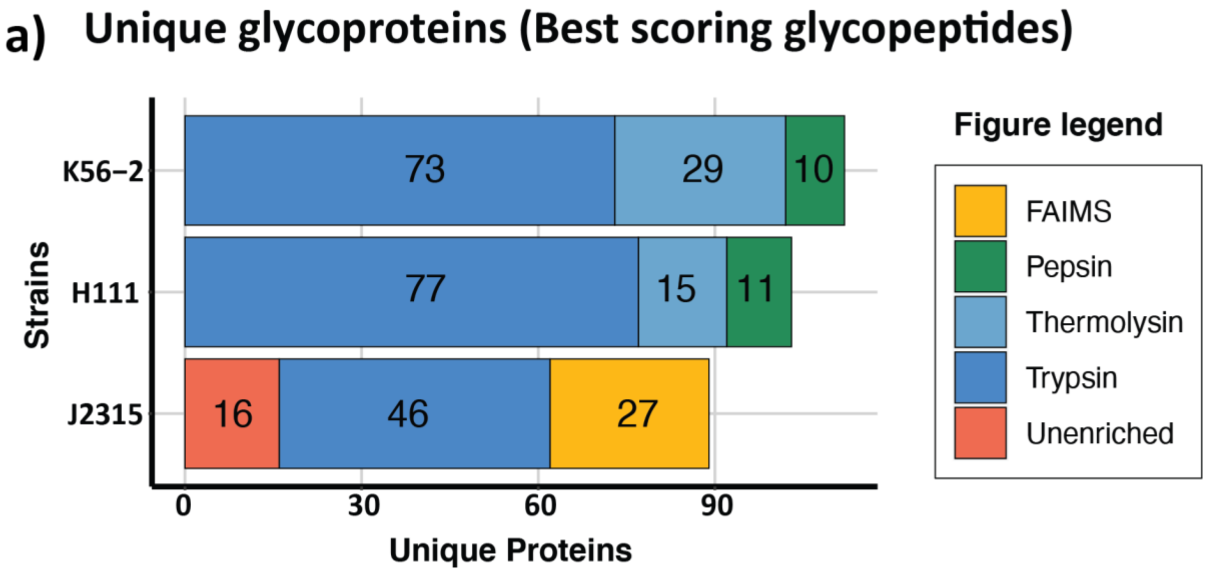

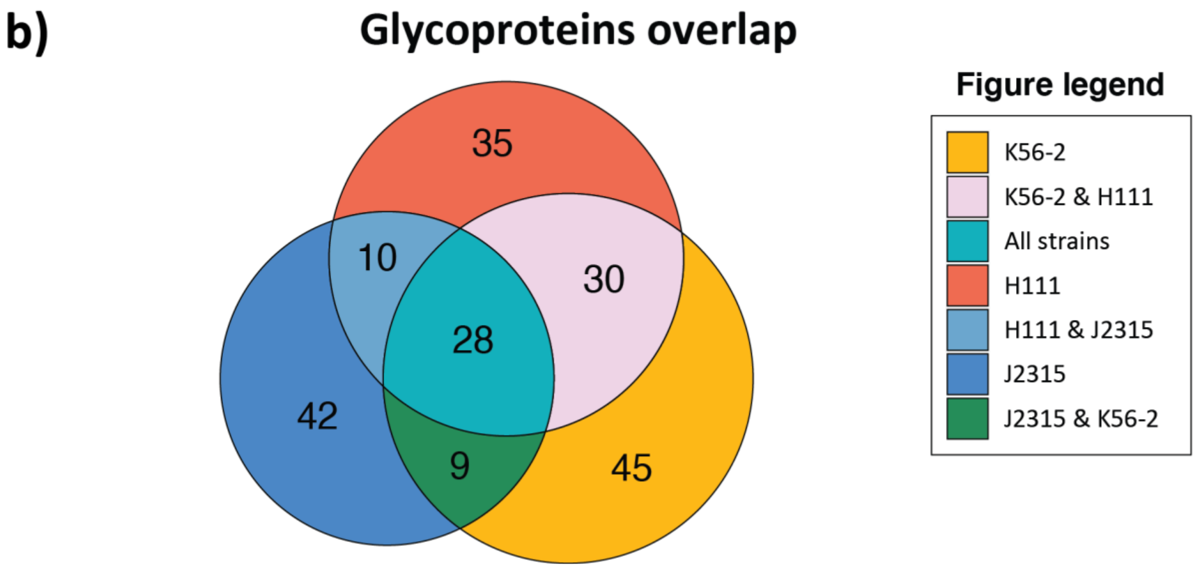

Supplementary Figure 1. Glycoproteins identified across *B. cenocepacia* datasets. a) Analysis of the K56-2 & H111 glycoproteome observed using three different proteases compared to previously published J2315 datasets reveals an overall increase in the number of glycoproteins identified within *B. cenocepacia* K56-2 & H111 compared to *B. cenocepacia* J2315. **b)** Venn diagram of glycoproteins observed across strains revealing the majority of glycoproteins were identified within a single strain supporting either a high degree of glycosylation heterogeneity between strains or the presence of erroneous glycoprotein assignments within datasets.

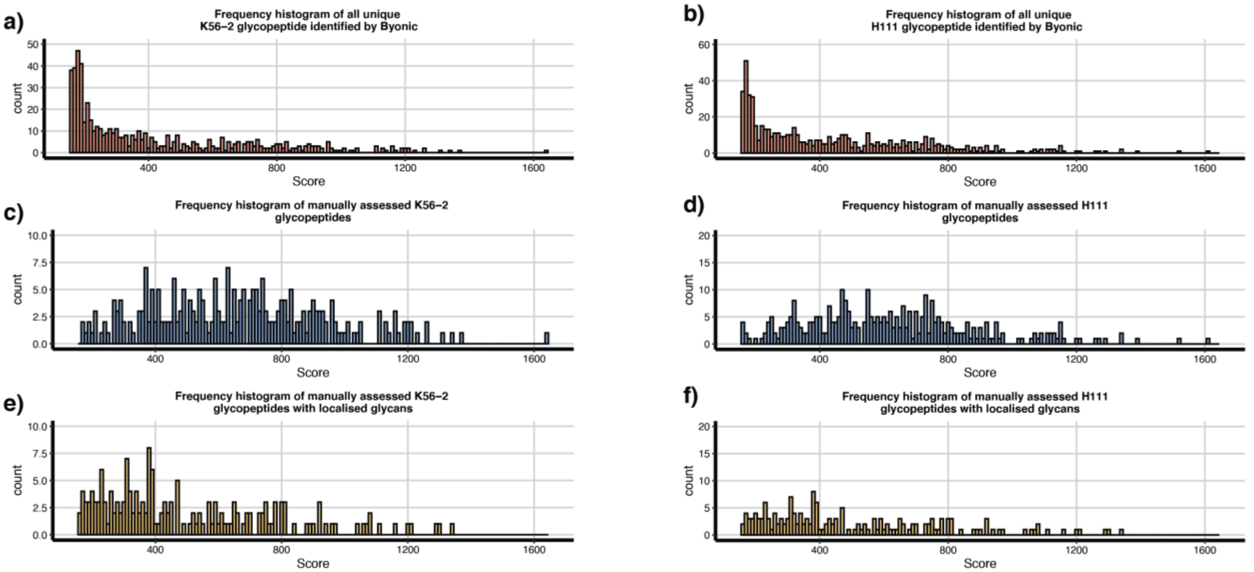

Supplementary Figure 2. Byonic assigned glycopeptide scores. a and b) Score distributions of glycopeptides with a Byonic score >300 reveal a strong bias for low scoring glycopeptides suggesting a high rate of erroneous glycopeptide assignments. **c and d)** The manual curation of datasets resulted in the removal of predominately low scoring glycopeptides revealing a gaussian distribution of assigned glycopeptides**. e and f)** The score distribution of unique glycopeptides for which partial or complete site localisation could be achieved reveals score alone does not correlate with if localisation information is observed within a peptide spectral match.

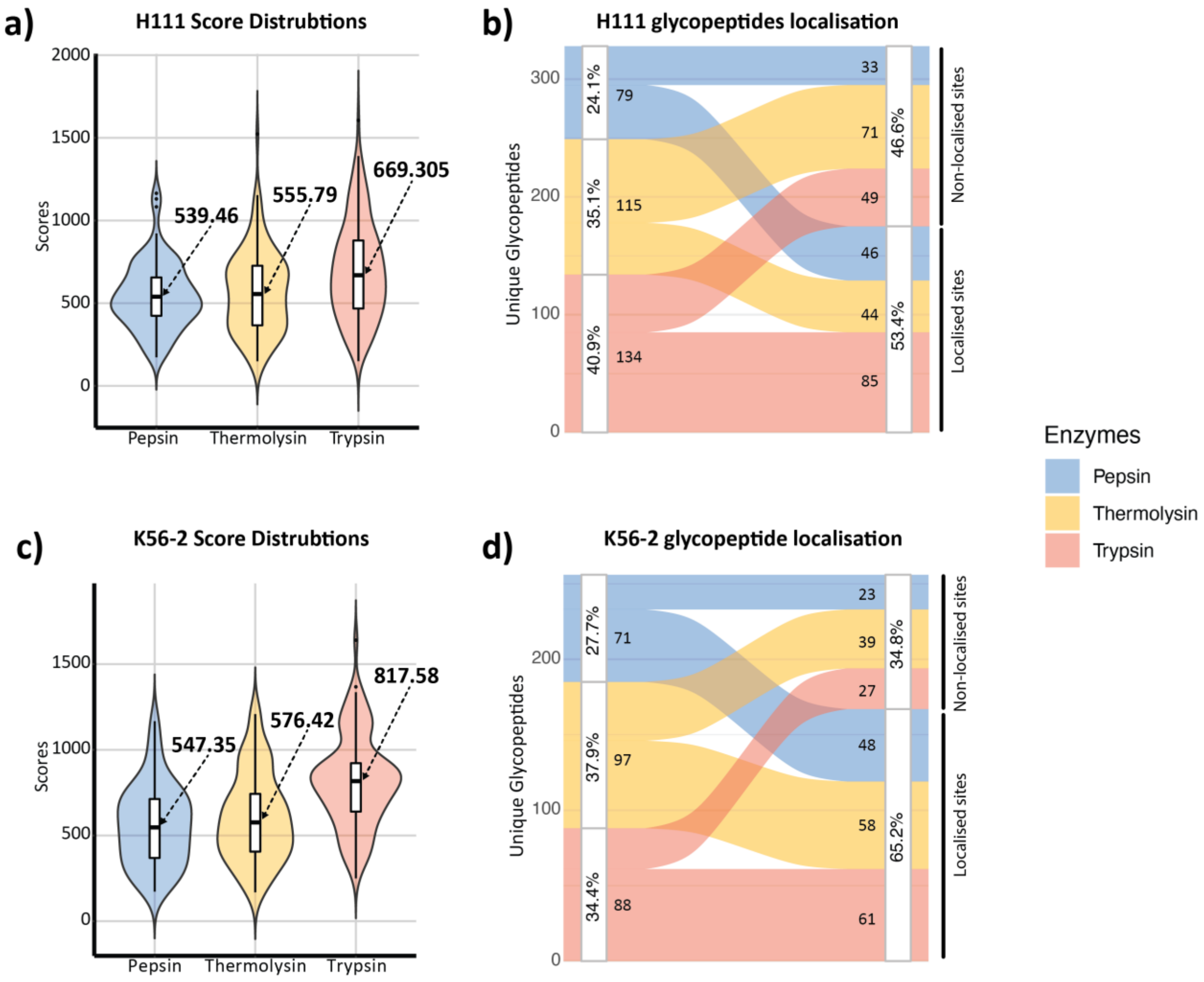

Supplementary Figure 3. Comparison of glycopeptide identification performance with different proteases. a and c) Score distributions of proteases revealing Pepsin and Thermolysin glycopeptides are typically scored lower than Tryptic glycopeptides. The medium scores are provided for each enzyme. **b and d)** Alluvial plots of curated glycopeptides demonstrating that each protease generates a similar number of unique glycopeptides and >50% allow the localisation of glycosylation events.

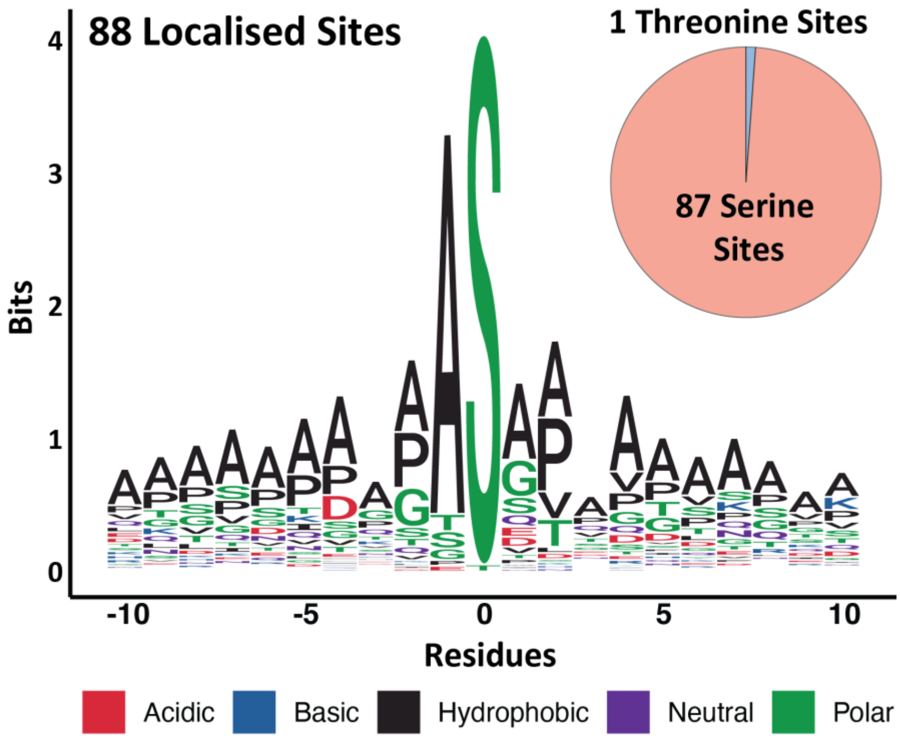

Supplementary Figure 4. *O*-linked glycosylation occurs on Serine residues across the *B. cenocepacia* glycoproteome. Sequence analysis of the initially localised glycosylation sites across *B. cenocepacia* strains revealed the majority of assigned sites were Serine with a single site assigned to Threonine.

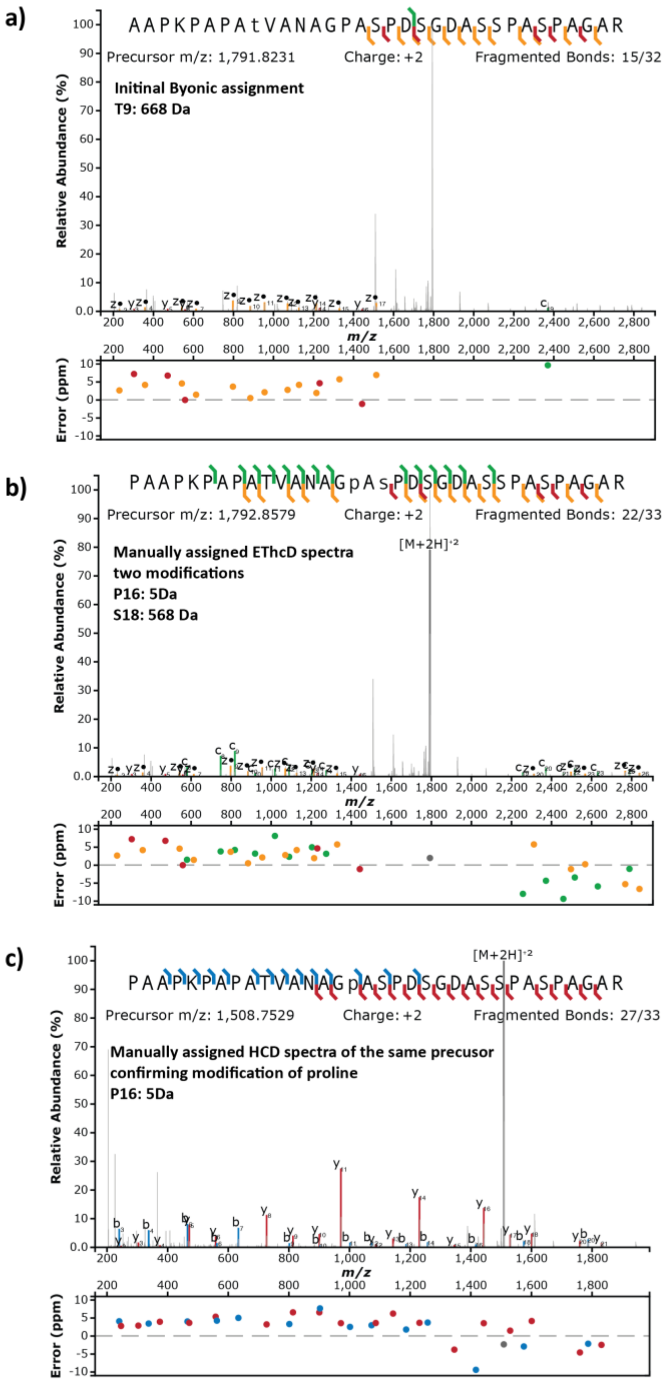

Supplementary Figure 5. Misassignment of the Threonine glycosylation event within BCAM0996. **a)** The glycopeptide ^{151}AAPKPAPATVANAGPASPDSGDASSPASPAGAR^{183} is assigned by Byonic yet within the EThcD spectra no sequence coverage is obtained for the majority of the N-termini. **b)** Manual analysis of the EThcD spectra supports the amino acid sequence to be ^{150}PAAPKPAPATVANAGPASPDSGDASSPASPAGAR^{183} with S^{178} modified with the +568Da *Burkholderia* glycan and P^{176} modified by +5Da. **c)** HCD fragmentation data of the identical precursor supports the modification of proline with an unexpected +5Da modification. Modified amino acids within peptides denoted by underlining.

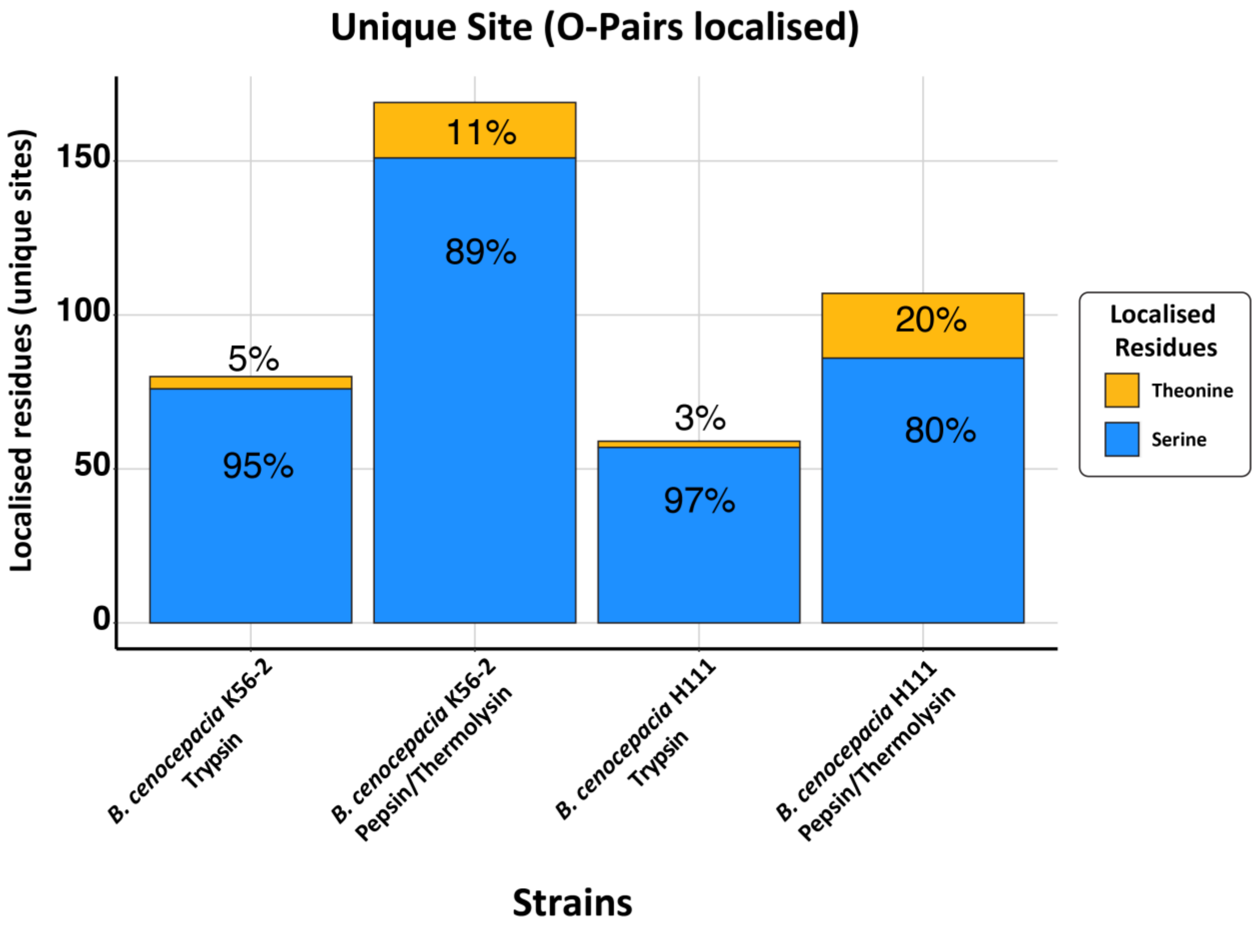

Supplementary Figure 6. O-Pair analysis of glycoproteomic datasets reveal a strong preference for glycosylation at Serine residues. Re-analysis of *B. cenocepacia* glycopeptide datasets revealed the majority of assigned glycosylation sites are localised to Serine residues.

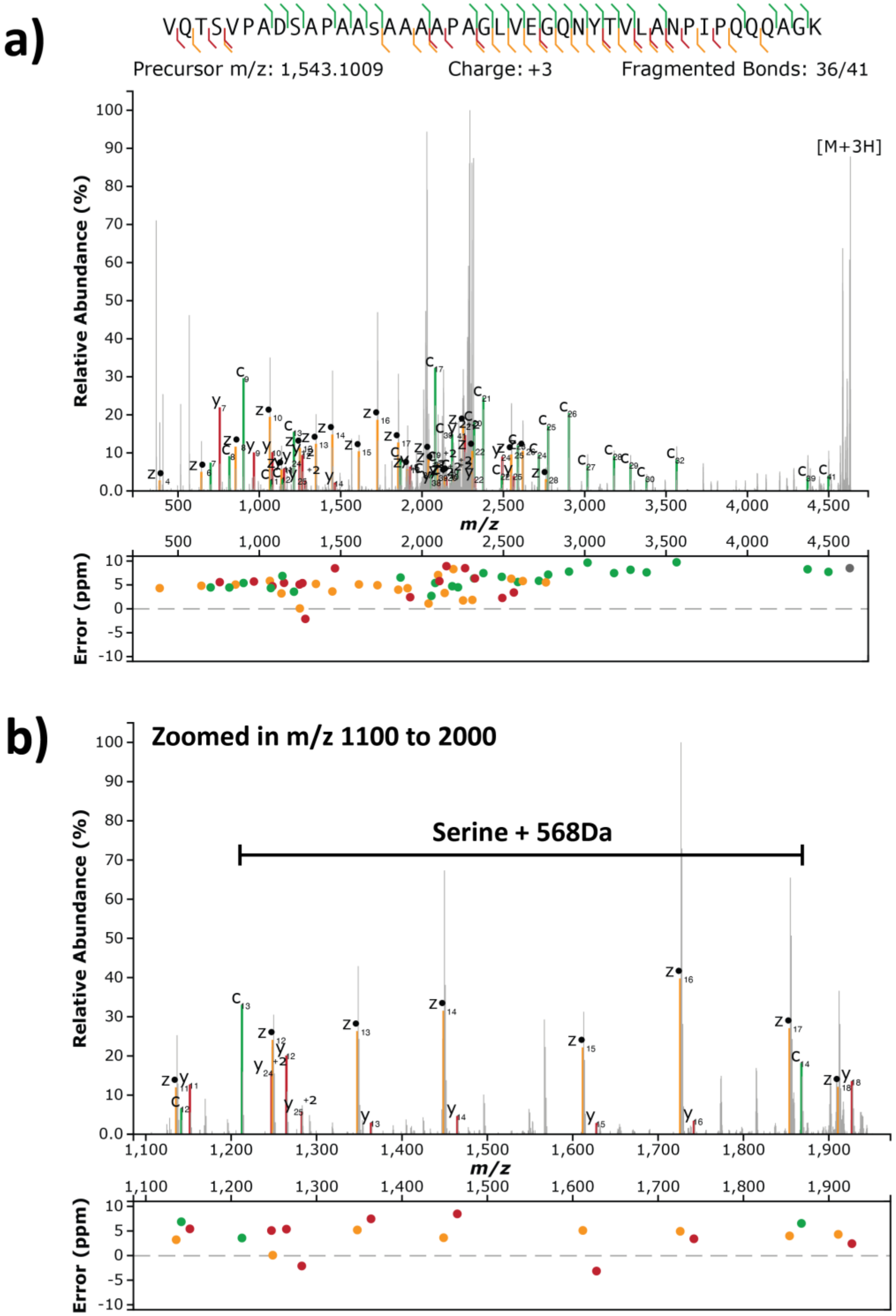

Supplementary Figure 7. Confirmation of S^{36} as the site of glycosylation within DsbA1$_{Nm}$-his$_6$. a and b) EThcD fragmentation of the glycopeptide ^{23}VQTSVPADSAPAA<u>S</u>AAAAPA GLVEGQNYTVLANPIPQQQAGK^{64} confirms glycosylation occurs at S^{36}. Glycosylation site within peptides denoted by underlining.

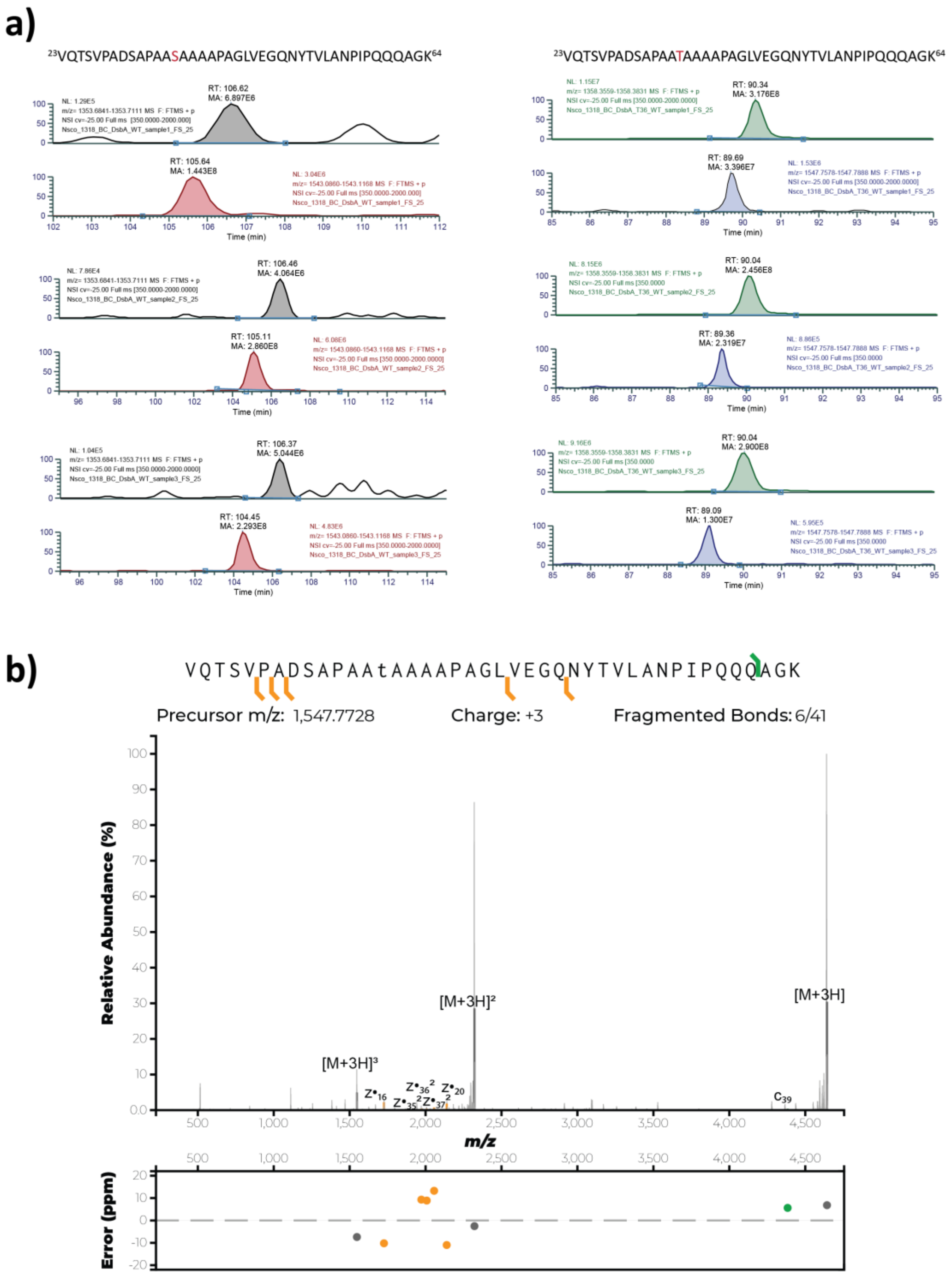

Supplementary Figure 8. MS analysis of DsbA1$_{Nm}$-his$_6$ variants within K56-2 WT reveals low levels of glycosylation within ^{23}VQTSVPADSAPAA<u>T</u>AAAAPAGLVEGQNYTVLANPIPQQQAGK^{64}. a) Extracted ion chromatograms used to construct figure 2C. **b)** PRM analysis of K56-2 WT expressing DsbA1$_{Nm}$-his$_6$ T^{36}, EThcD data supports the identity of this peptide yet failed to provide site localisation. Glycosylation site within peptides denoted by underlining.

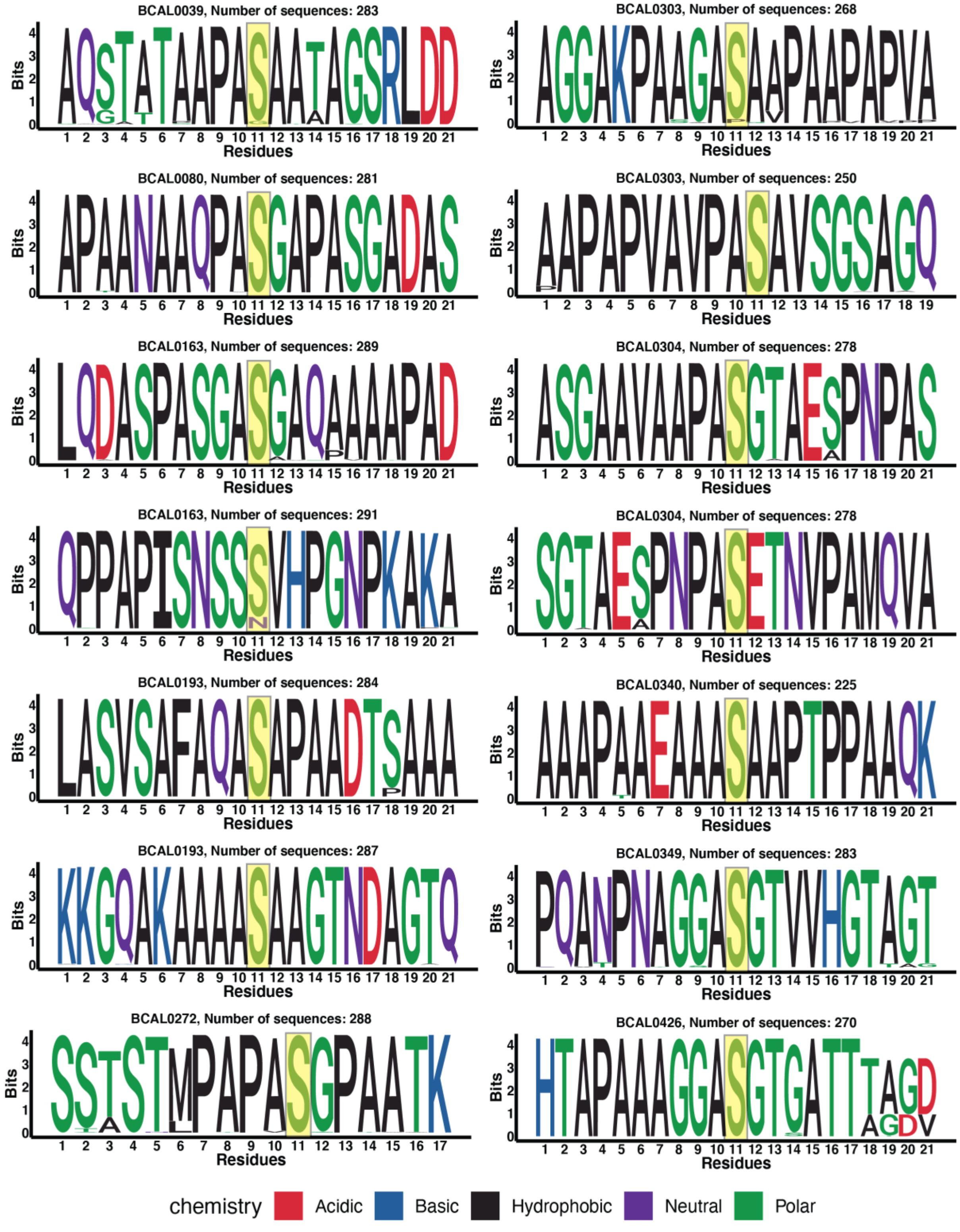

Supplementary Figure 9. Conservation of *B. cenocepacia* glycosylation site within BCAL0039 to BCAL0426. Glycosylation sites are highlighted in yellow and conserved across the majority of *B. cenocepacia* strains.

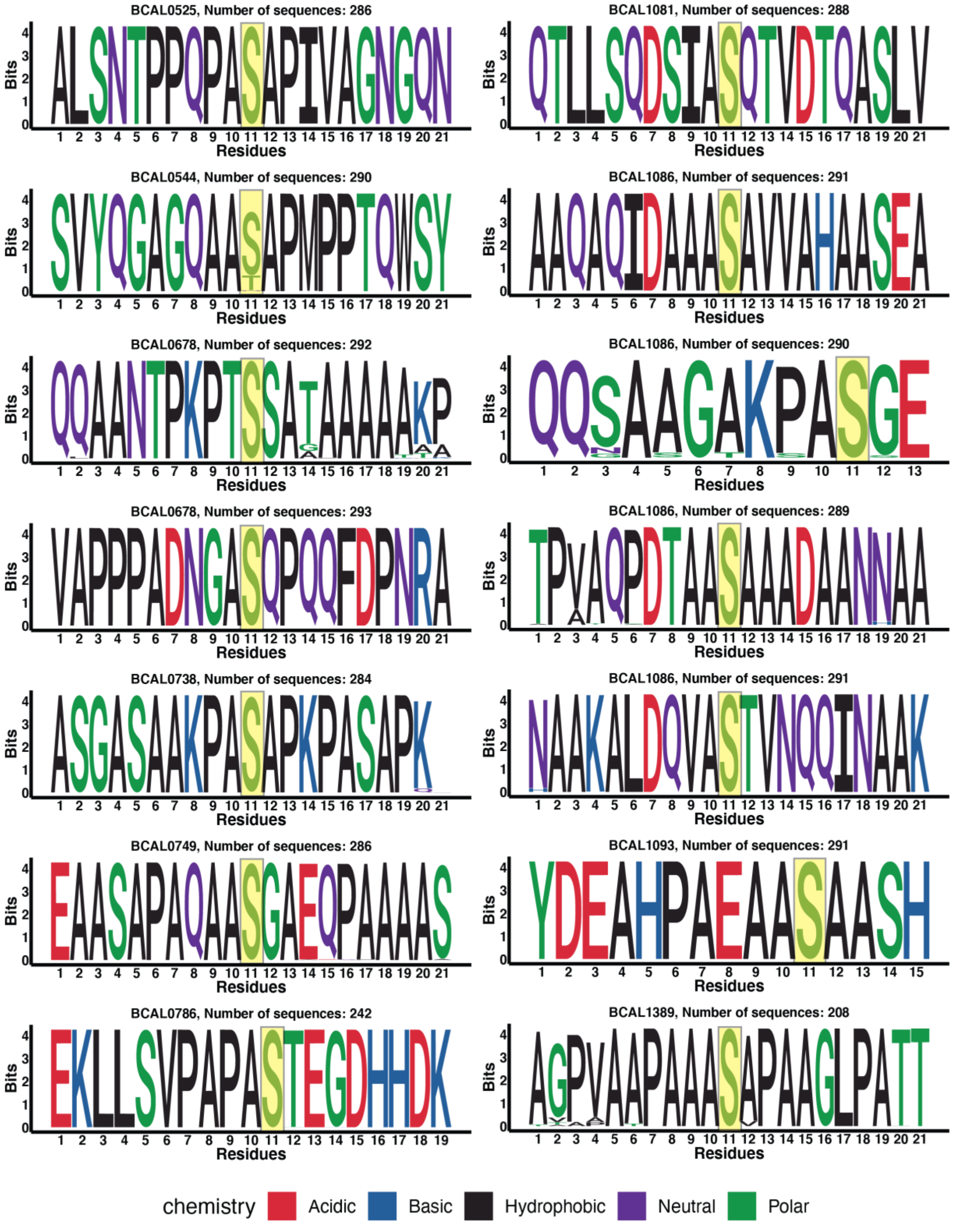

Supplementary Figure 10. Conservation of *B. cenocepacia* glycosylation site within BCAL0525 to BCAL1389. Glycosylation sites are highlighted in yellow and conserved across the majority of *B. cenocepacia* strains.

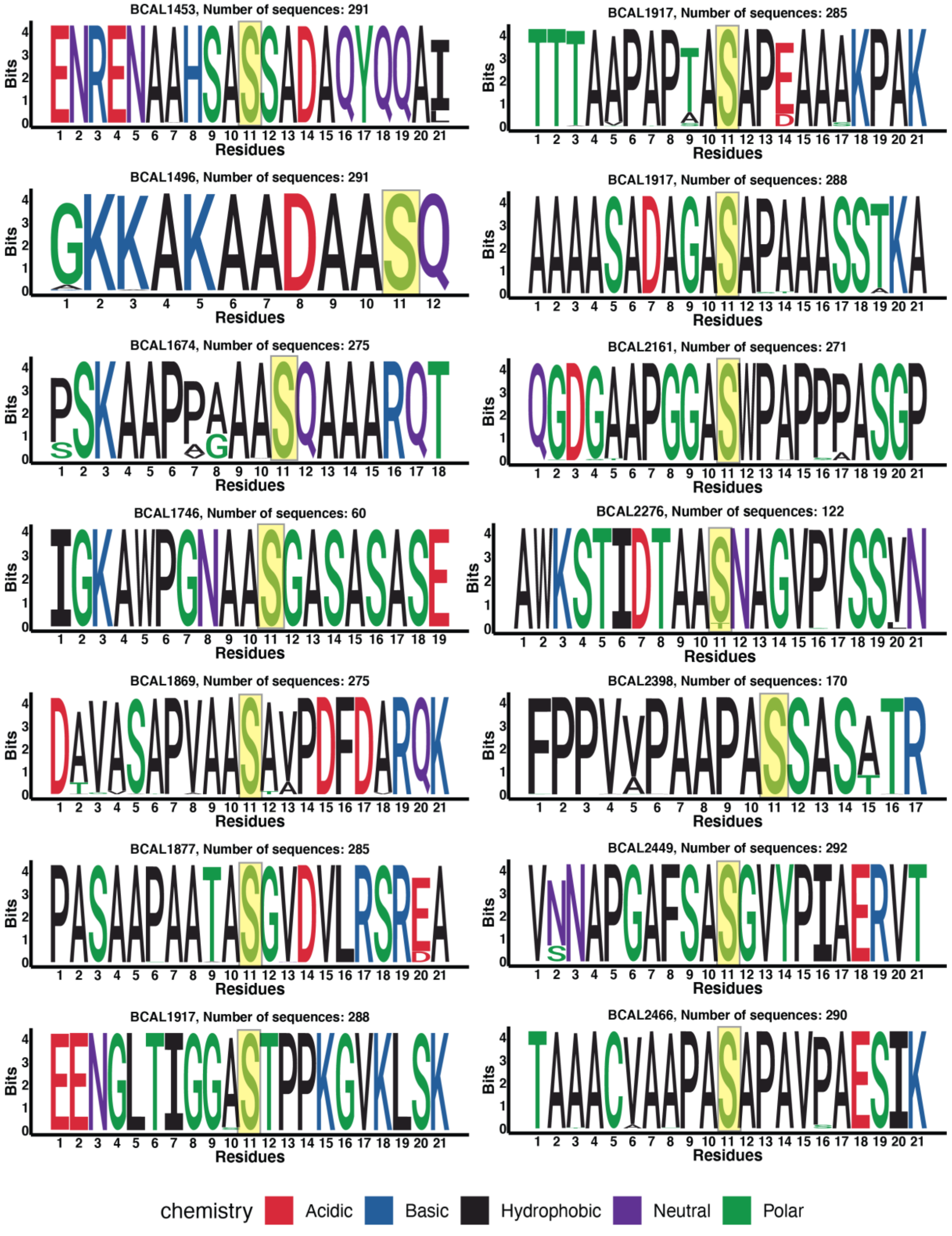

Supplementary Figure 11. Conservation of *B. cenocepacia* glycosylation site within BCAL1453 to BCAL2466. Glycosylation sites are highlighted in yellow and conserved across the majority of *B. cenocepacia* strains.

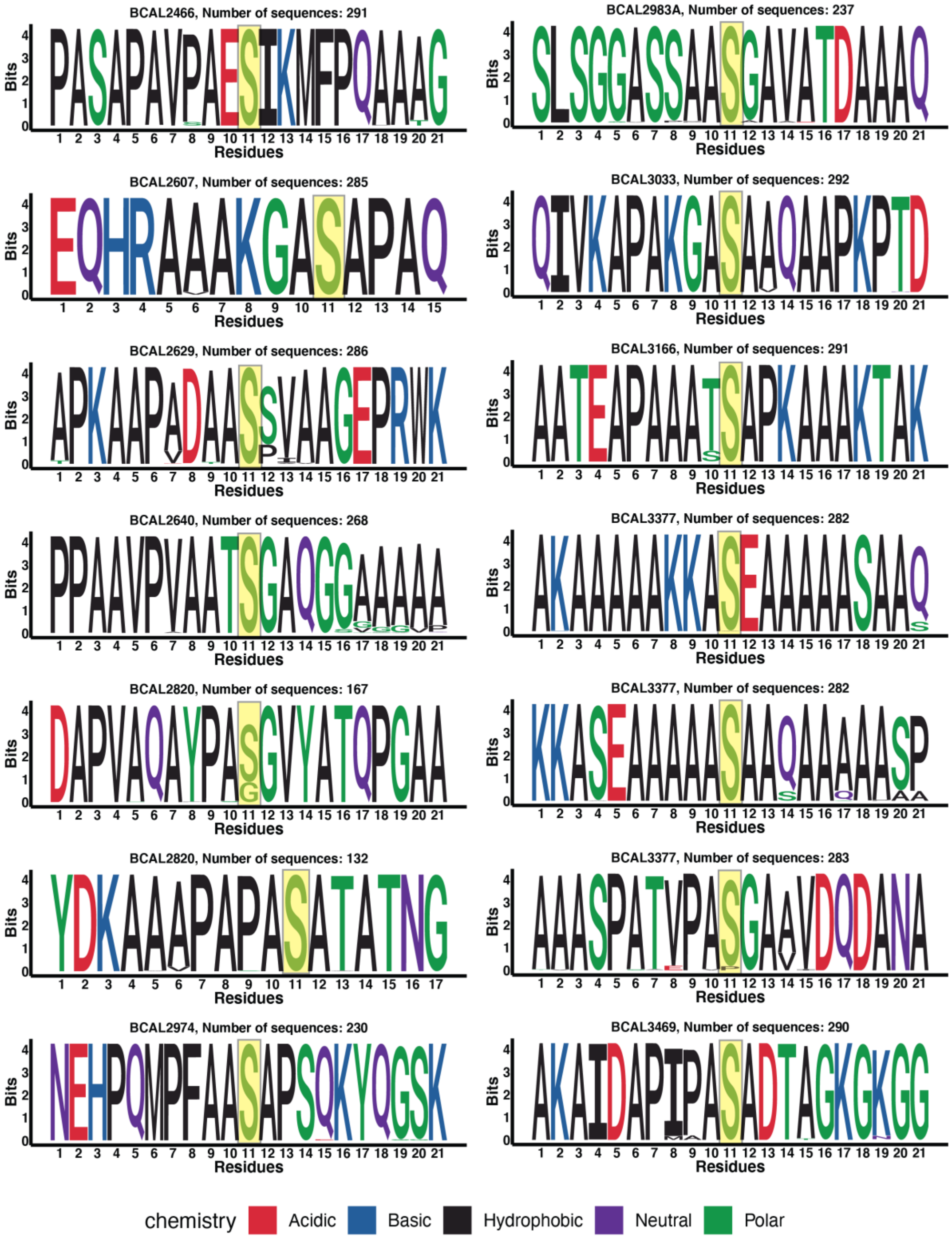

Supplementary Figure 12. Conservation of *B. cenocepacia* glycosylation site within BCAL2466 to BCAL3469. Glycosylation sites are highlighted in yellow and conserved across the majority of *B. cenocepacia* strains.

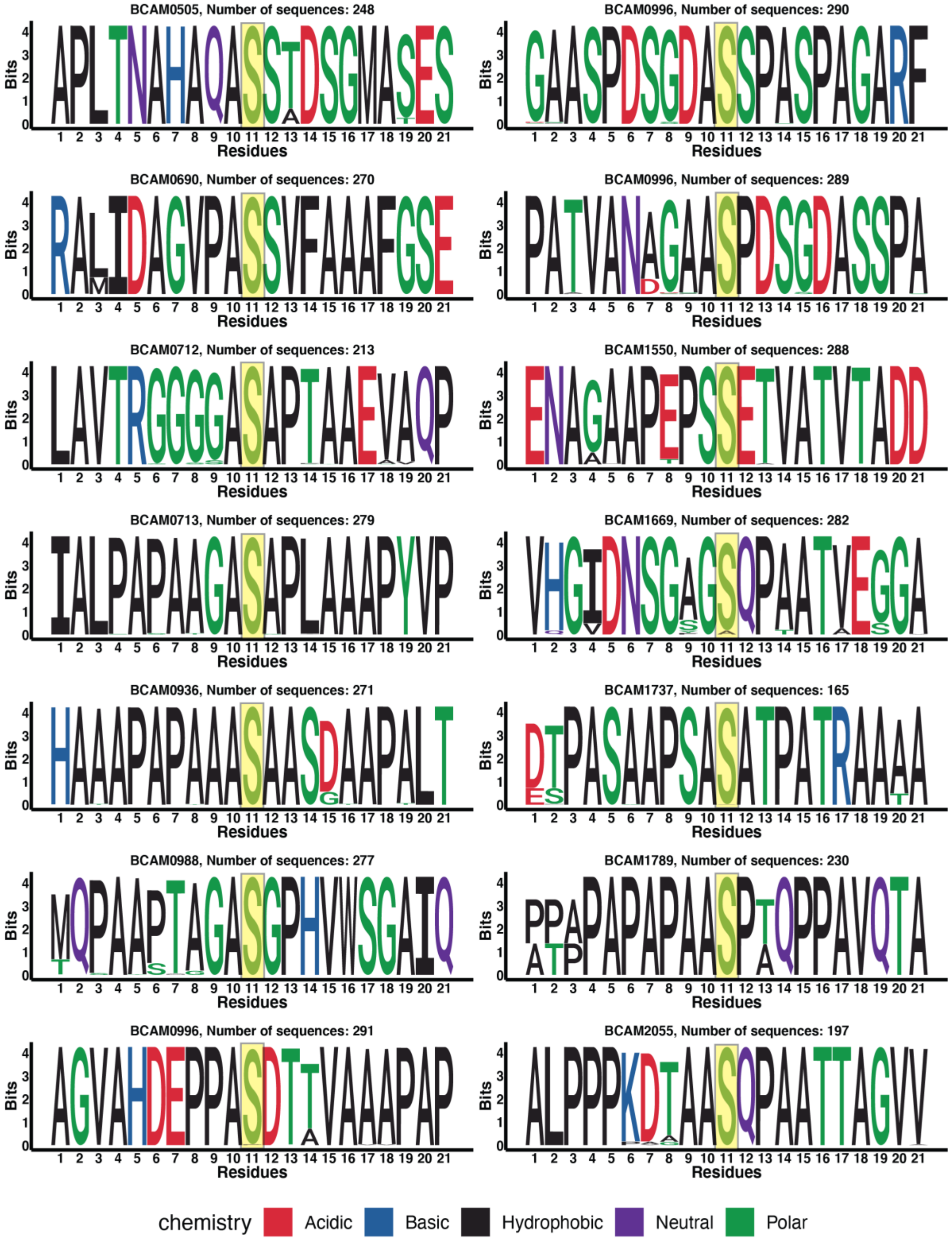

Supplementary Figure 13. Conservation of *B. cenocepacia* glycosylation site within BCAM0505 to BCAM2055. Glycosylation sites are highlighted in yellow and conserved across the majority of *B. cenocepacia* strains.

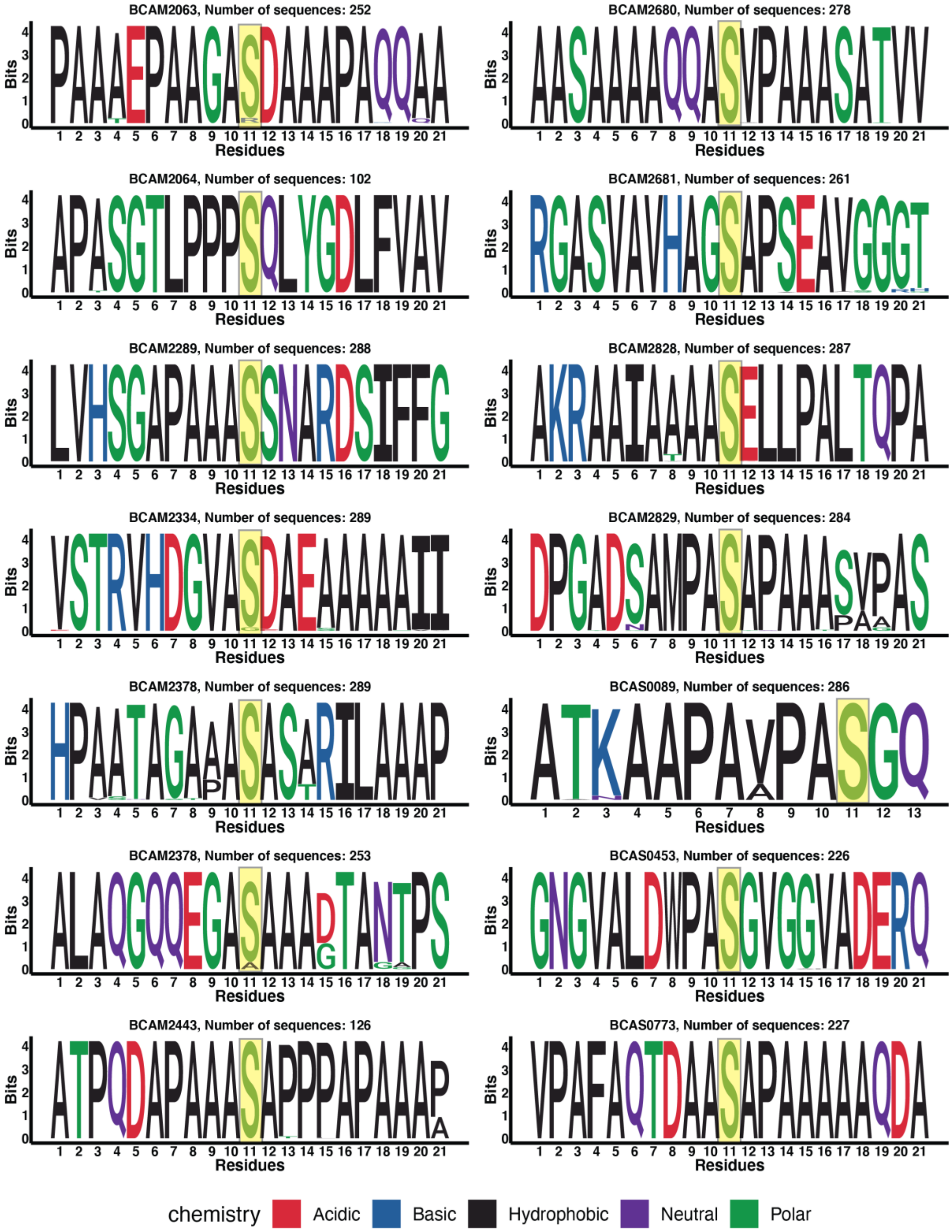

Supplementary Figure 14. Conservation of *B. cenocepacia* glycosylation site within BCAM2063 to BCAS0773. Glycosylation sites are highlighted in yellow and conserved across the majority of *B. cenocepacia* strains.

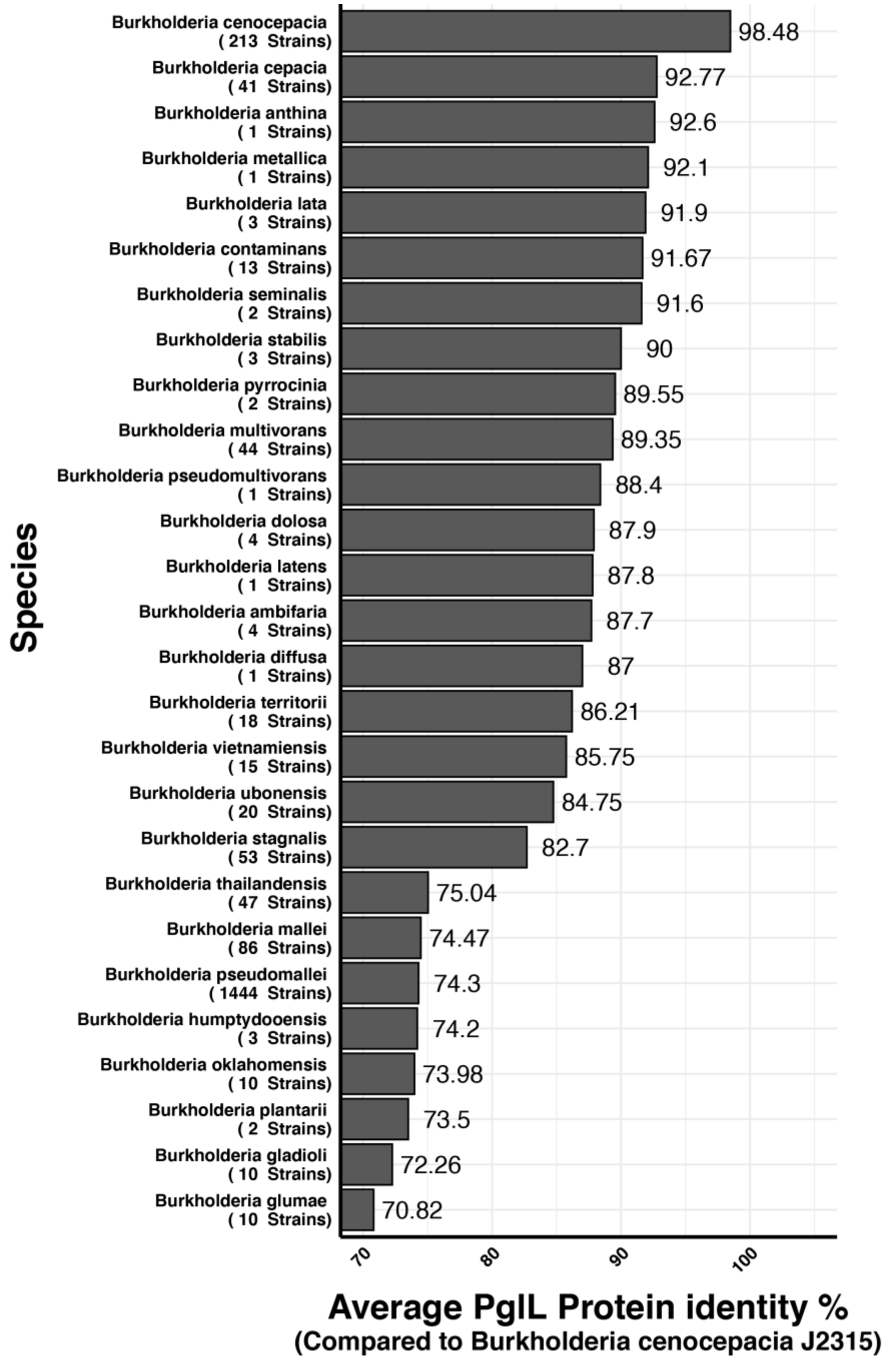

Supplementary Figure 15. Comparison of PglL protein sequences across the Burkholderia genus. Diamond BlastX analysis of the *pglL* genes within the Burkholderia.com database against the *B. cenocepacia* J2315 *pglL* sequence (BCAL0960) demonstrates that PglL homologs have high protein identity across *Burkholderia* species.

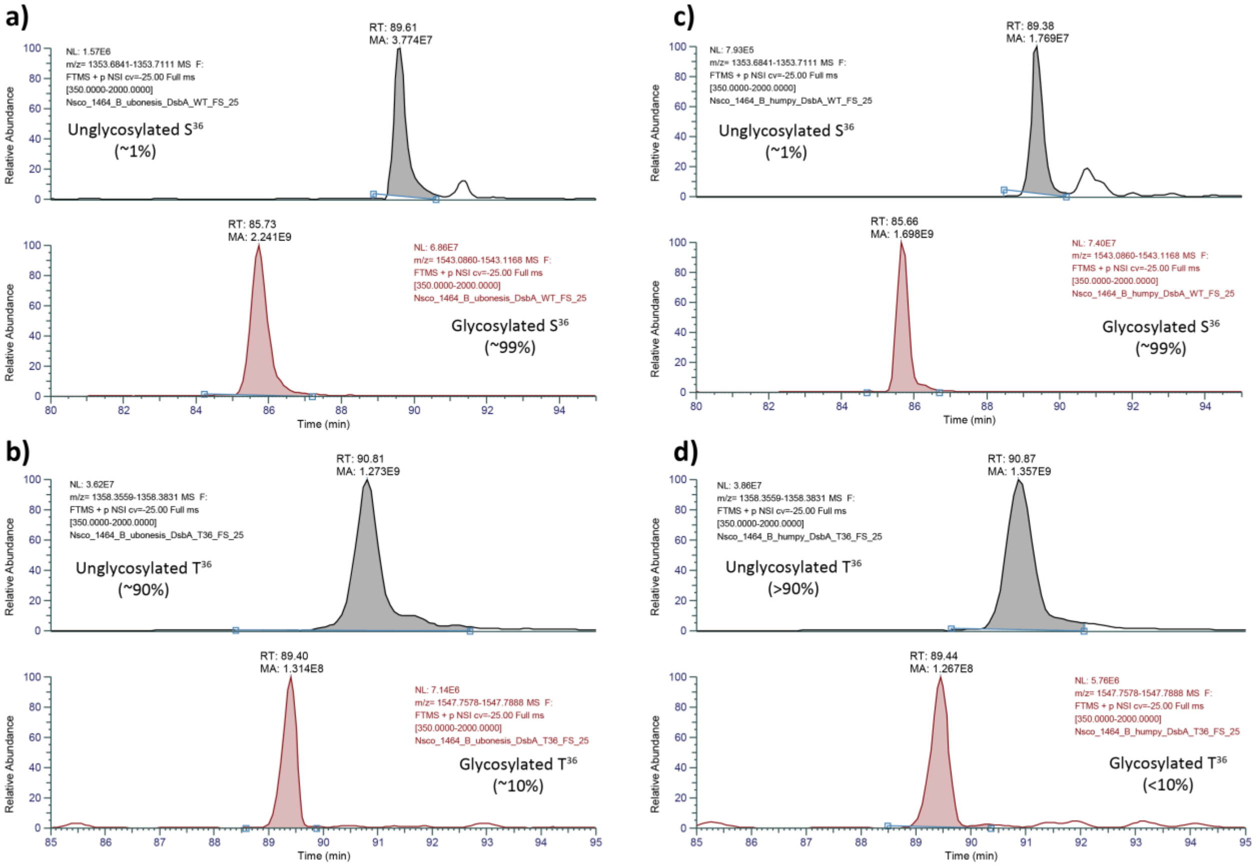

Supplementary Figure 16. Glycopeptide analysis of DsbA1$_{Nm}$-his$_6$ variants within *B. humptydooensis* MSMB43 and *B. ubonensis* MSMB22 supports ^{23}VQTSVPADSAPAATAAAAP AGLVEGQNYTVLANPIPQQQAGK^{64} is poorly glycosylated. a) Extracted ion chromatograms of DsbA1$_{Nm}$-his$_6$ WT in *B. ubonensis* MSMB22. **b)** Extracted ion chromatograms of DsbA1$_{Nm}$-his$_6$ T^{36} in *B. ubonensis* MSMB22. **c)** Extracted ion chromatograms of DsbA1$_{Nm}$-his$_6$ WT in *B. humptydooensis* MSMB43. **d)** Extracted ion chromatograms of DsbA1$_{Nm}$-his$_6$ T^{36} in *B. humptydooensis* MSMB43.

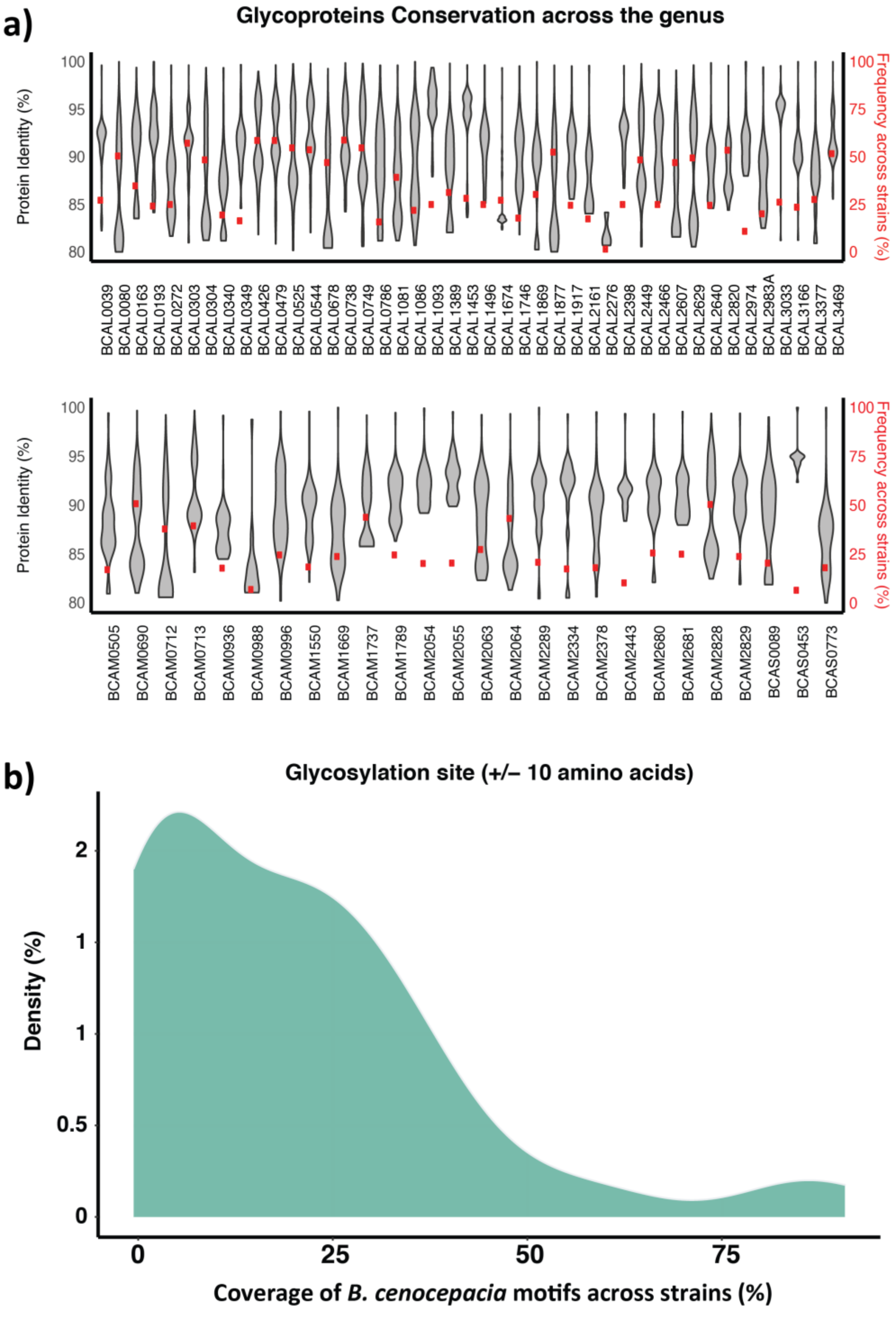

Supplementary Figure 17. Conservation of glycosylation sites across *Burkholderia* species. a). Homologues of *B. cenocepacia* glycoproteins are observed across the *Burkholderia* genus. **b)** At the glycosylation site level, the majority of sites appear to diverge from the confirmed sequences in *B. cenocepacia* yet still highlight some sites are highly conserved. Raw data provided within Supplementary Data 12.

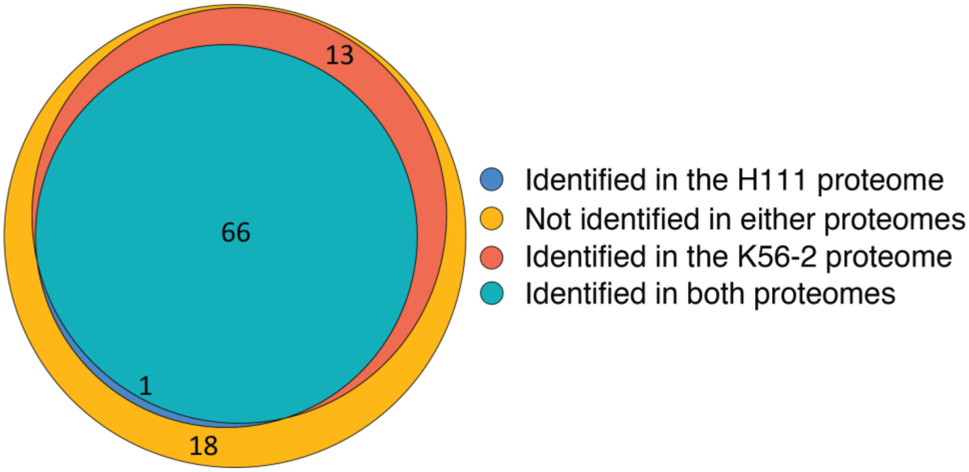

Supplementary Figure 18. Glycoproteins observed with the *B. cenocepacia* H111 and K56-2 proteomes. Venn diagram of the overlaps in the observed glycoproteomes and proteomes of H111 and K56-2.

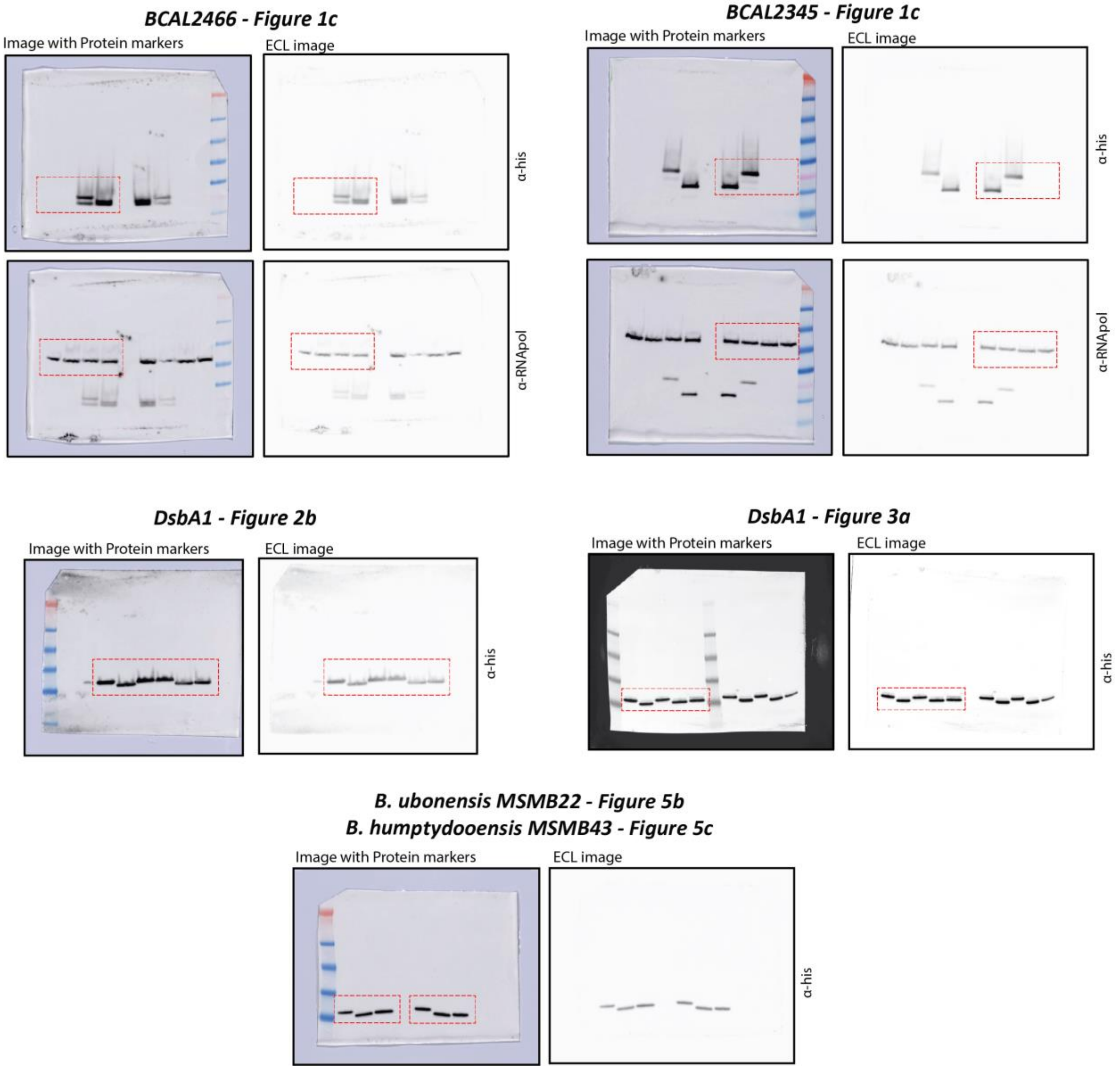

Supplementary Figure 19. Uncropped Western blotting images. The uncropped ECL and membrane images for all western shown with the manuscript are provided.

References

1 Darling, P., Chan, M., Cox, A. D. & Sokol, P. A. Siderophore production by cystic fibrosis isolates of Burkholderia cepacia. *Infect Immun* **66**, 874-877 doi: 10.1128/IAI.66.2.874-877.1998. (1998).

2 Oppy, C. C. *et al.* Loss of O-linked protein glycosylation in Burkholderia cenocepacia impairs biofilm formation, siderophore activity and alters transcriptional regulators. *mSphere* **4**, e00660-19. doi: 10.1128/mSphere.00660-19. (2019).

3 Romling, U. *et al.* Epidemiology of chronic Pseudomonas aeruginosa infections in cystic fibrosis. *J Infect Dis* **170**, 1616-1621, doi:10.1093/infdis/170.6.1616 (1994).

4 Figurski, D. H. & Helinski, D. R. Replication of an origin-containing derivative of plasmid RK2 dependent on a plasmid function provided in trans. *Proc Natl Acad Sci U S A* **76**, 1648-1652, doi:10.1073/pnas.76.4.1648 (1979).

5 Hamad, M. A., Di Lorenzo, F., Molinaro, A. & Valvano, M. A. Aminoarabinose is essential for lipopolysaccharide export and intrinsic antimicrobial peptide resistance in Burkholderia cenocepacia. *Mol Microbiol* **85**, 962-974, doi:10.1111/j.1365-2958.2012.08154.x (2012).

6 Flannagan, R. S., Linn, T. & Valvano, M. A. A system for the construction of targeted unmarked gene deletions in the genus Burkholderia. *Environ Microbiol* **10**, 1652-1660, doi:10.1111/j.1462-2920.2008.01576.x (2008).

7 Fathy Mohamed, Y. *et al.* A general protein O-glycosylation machinery conserved in Burkholderia species improves bacterial fitness and elicits glycan immunogenicity in humans. *J Biol Chem*, **294**, 13248-13268. doi: 10.1074/jbc.RA119.009671. (2019).

8 Cardona, S. T. & Valvano, M. A. An expression vector containing a rhamnose-inducible promoter provides tightly regulated gene expression in Burkholderia cenocepacia. *Plasmid* **54**, 219-228, doi:10.1016/j.plasmid.2005.03.004 (2005).

9 Gebhart, C. *et al.* Characterization of exogenous bacterial oligosaccharyltransferases in Escherichia coli reveals the potential for O-linked protein glycosylation in Vibrio cholerae and Burkholderia thailandensis. *Glycobiology* **22**, 962-974, doi:10.1093/glycob/cws059 (2012).
