# Supplementary material for: Burkholderia PglL enzymes are Serine preferring oligosaccharyltransferases which target conserved proteins across the Burkholderia genus
Source: Commun Biol. 2021 Sep 7;4:1045. doi: 10.1038/s42003-021-02588-y (PMC8423747; doi:10.1038/s42003-021-02588-y)
Supplement: Supplementary file 3 — Description of additional supplementary items [file 42003_2021_2588_MOESM3_ESM.pdf]

Description of Additional Supplementary Files

File name: Supplementary Data 1

Description: Combined Byonic search results of *B. cenocepacia* H111 glycopeptide enrichments. The combined Byonic search results from all proteases across each of the three biological replicates of *B. cenocepacia* H111 are provided. The complete list of all glycopeptides identified, the best unique glycopeptides filtered for glycopeptides with a Byonic score over 300 and the curated list of glycopeptides associated with Supplementary Data 3A are provided.

File name: Supplementary Data 2

Description: Combined Byonic search results of *B. cenocepacia* K56-2 glycopeptide enrichments. The combined Byonic search results from all proteases across each of the three biological replicates of *B. cenocepacia* K56-2 are provided. The complete list of all glycopeptides identified, the best unique glycopeptides filtered for glycopeptides with a Byonic score over 300 and the curated list of glycopeptides associated with Supplementary Data 3B are provided.

File name: Supplementary Data 3

3A) Description: Manually curated *B. cenocepacia* H111 glycopeptides (Best scoring unique glycopeptides). For each of the best scoring unique glycopeptide, the Byonic assigned spectra is provided in addition to the assigned J2315 gene number, protein name, peptide assignment, glycan assignment, if the spectra enables localisation of the glycan within the sequence, assignment associated metrics (m/z; mass error, score, delta score, delta mod score, scan number within the LC-MS run and scan time), enzymatic digest for which the best scoring glycopeptide was observed in, replicate for which the best scoring glycopeptide was observed in and page within the pdf the spectra can be found.

3B) Description: Manually curated *B. cenocepacia* K56-2 glycopeptides (Best scoring unique glycopeptides). For each of the best scoring unique glycopeptide, the Byonic assigned spectra is provided in addition to the assigned J2315 gene number, protein name, peptide assignment, glycan assignment, if the spectra enables localisation of the glycan within the sequence, assignment associated metrics (m/z; mass error, score, delta score, delta mod score, scan number within the LC-MS run and scan time), enzymatic digest for which the best scoring glycopeptide was observed in, replicate for which the best scoring glycopeptide was observed in and page within the pdf the spectra can be found.

3C) Description: Manually curated *B. cenocepacia* H111 glycopeptides (Best localised unique glycopeptides). For each of the best localised unique glycopeptide, the Byonic assigned spectra

is provided in addition to the assigned J2315 gene number, protein name, peptide assignment, glycan assignment, if the spectra enables localisation of the glycan within the sequence, assignment associated metrics (m/z; mass error, score, delta score, delta mod score, scan number within the LC-MS run and scan time), enzymatic digest for which the best scoring glycopeptide was observed in, replicate for which the best scoring glycopeptide was observed in and page within the pdf the spectra can be found.

3D) Description: Manually curated *B. cenocepacia* K56-2 glycopeptides (Best localised unique glycopeptides). For each of the best localised unique glycopeptide, the Byonic assigned spectra is provided in addition to the assigned J2315 gene number, protein name, peptide assignment, glycan assignment, if the spectra enables localisation of the glycan within the sequence, assignment associated metrics (m/z; mass error, score, delta score, delta mod score, scan number within the LC-MS run and scan time), enzymatic digest for which the best scoring glycopeptide was observed in, replicate for which the best scoring glycopeptide was observed in and page within the pdf the spectra can be found.

File name: Supplementary Data 4

Description: LFQ proteome analysis of *B. cenocepacia* strains H111 WT vs H111 Δ*pglL* candidate 1 vs H111 Δ*pglL* candidate 8. The Perseus processed MaxQuant search results for the protein analysis of four biological replicates of strains H111 WT, H111 Δ*pglL* candidate 1 and H111 Δ*pglL* candidate 8 are provided. For each identified protein, the log2 LFQ protein values, the T-test information including the $-\log_{10}(p\text{-value})$, difference in the mean between the groups and if the resulting *p*-values is below the multiple hypothesis corrected *p*-value are provided. Categorical information used for enrichment analysis including GO terms and if proteins were considered to be altered within K56-2 Δ*pglL* within Oppy *et al.* 2019 [5] are provided. For each protein the protein score, number of MS/MS for the corresponding protein, expected molecular weight, number of peptides identified, peptide sequence coverage and corresponding J2315 gene number for the protein are provided.

File name: Supplementary Data 5

Description: Enrichment analysis of global proteome changes in response to loss of glycosylation in *B. cenocepacia* H111. Using Perseus, proteins determined to undergo statistically significant changes were assessed for co-occurrence of statistically significant changes across strains as well as enrichment for proteins considered to be altered within K56-2 Δ*pglL* within Oppy *et al.* 2019.

File name: Supplementary Data 6

Description: Curated *B. cenocepacia* glycosylation sites. The identified glycosylation sites from *B. cenocepacia* K56-2 and H111 are provided. For each site the J2315 Gene name, position of the site within the J2315 version of the protein, residue the glycan was localised to, amino acid sequence +/- 10 amino acid either side of the localised site and which strain these sites were observed in are provided.

File name: Supplementary Data 7

Description: O-Pair analysis of *B. cenocepacia* glycosylation sites. O-pair site localisation information is provided with only class "level 1" glycopeptides with a Q-value less then 0.01 and a site-specific probability of >0.75 considered as localised and used for data visualisation.

File name: Supplementary Data 8

Description: Proteome analysis of the 25-35kDa of *B. cenocepacia* lysates expressing DsbA1$_{Nm}$-his$_6$ variants. MaxQuant peptide search results of induced K56-2 expressing DsbA1$_{Nm}$-his$_6$ WT or DsbA1$_{Nm}$-his$_6$ T^{36}. For each peptide the mass, Protein Group, Modification status, identification type (by MS/MS or matching), Retention time, Charge, PEP, MS/MS scan number for the best scoring peptide spectral match, Raw file of the best scoring peptide spectral match, Score, Delta Score, Intensity and number of MS/MS are provided.

File name: Supplementary Data 9

Description: Glycoprotein and glycosylation site conservation across Burkholderia genomes. Summary of sequence identity percentages of glycoproteins and glycosylation motifs identified across *B. cenocepacia* strains and representative *Burkholderia* species.

File name: Supplementary Data 10

Description: O-Pair analysis of multiple *Burkholderia* species. O-pair site localisation information is provided with only class "level 1" glycopeptides with a Q-value less than 0.01 and a site-specific probability of >0.75 considered as localised and used for data visualisation.

File name: Supplementary Data 11

Description: Proteome analysis of the 25-35kDa of *B. ubonensis* MSMB22 and *B. humptydooensis* MSMB43 lysates expressing DsbA1$_{Nm}$-his$_6$ variants. MaxQuant peptide search results of induced K56-2 expressing DsbA1$_{Nm}$-his$_6$ WT or DsbA1$_{Nm}$-his$_6$ T^{36}. For each peptide the mass, Protein Group, Modification status, identification type (by MS/MS or matching),

Retention time, Charge, PEP, MS/MS scan number for the best scoring peptide spectral match, Raw file of the best scoring peptide spectral match, Score, Delta Score, Intensity and number of MS/MS are provided.

File name: Supplementary Data 12

Description: Glycoprotein homologues identified across *Burkholderia* species. Summary of glycoprotein homologues identified, and enrichment analysis of glycoproteins identified across *Burkholderia* species.
