# Supplementary material for: Burkholderia PglL enzymes are Serine preferring oligosaccharyltransferases which target conserved proteins across the Burkholderia genus
Source: Commun Biol. 2021 Sep 7;4:1045. doi: 10.1038/s42003-021-02588-y (PMC8423747; doi:10.1038/s42003-021-02588-y)
Supplement: Supplementary file 6 — Supplementary Data 3 [file 42003_2021_2588_MOESM6_ESM.zip › Supplementary_Data_3D_K56_2_Best_Localised_Unique_Glycopeptides.pdf]

Burkholderia cenocepacia K56-2 Best Localised unique glycopeptides

J2315 Gene name	Protein Name	Peptide < ProteinMetrics Confidential >	Glycans NHFAGNa	Localised site (Yes / No / Partial) ?	Observed m/z	z	Observed (M+H)	Calc. mass (M+H)	Mass error (ppm)	Cleavage	Score	Delta	Delta Mod	Comment	Scan Time	Enzyme	Replicate	Site	Page
BCAL0039	>gi|443605781|gb|ELT73605.1| ABC transporter, substrate-binding protein, family 3 [Burkholderia cenocepacia K56-2Valvano]	T.AAPAS[+568.212]AATAGSRLDDVLARGA.L	HexNAc(2)Hex(1)	Yes	837.0821	3	2509.2316	2509.226	2.1	Non	226.4	110.9	67.13	Nsco_20191108_BC_ZIC_HILIC_K56_2_thermolysin_B1.32913.32913.3	66.64	Thermolysin	B1	S33	12
BCAL0080	>gi|443593207|gb|ELT61961.1| cytochrome C domain protein, partial [Burkholderia cenocepacia K56-2Valvano]	N.AAQPAS[+568.212]GAPASGADASNAQA.-	HexNAc(2)Hex(1)	Partial	1141.0048	2	2281.0023	2280.995	3.2	Non	319.9	187.1	187.05	Nsco_20191108_BC_ZIC_HILIC_K56_2_thermolysin_B1.12889.12889.2	31.01	Thermolysin	B1	NA	13
BCAL0080	>gi|443593207|gb|ELT61961.1| cytochrome C domain protein, partial [Burkholderia cenocepacia K56-2Valvano]	Y.MANNDGANFPEPAAPAANAAQPAS[+568.212]GAPASGADASNAQ.A	HexNAc(2)Hex(1)	Yes	1321.9149	3	3963.7302	3962.711	4.1	Non	498.8	403.1	58.83	Nsco_20191108_BC_ZIC_HILIC_K56_2_thermolysin_B2_.45836.45836.3	83.76	Thermolysin	B2	S176	14
BCAL0080	>gi|443593207|gb|ELT61961.1| cytochrome C domain protein, partial [Burkholderia cenocepacia K56-2Valvano]	Y.MANNDGANFPEPAAPAANAAQPAS[+568.212]GAPASGADASNAQA.-	HexNAc(2)Hex(1)	Yes	1345.9263	3	4035.7644	4033.748	2.5	Non	237.4	215.4	42.78	Nsco_20191108_BC_ZIC_HILIC_K56_2_thermolysin_B1.42150.42150.3	86.91	Thermolysin	B1	S176	15
BCAL0163	>gi|443595598|gb|ELT64173.1| BON domain protein [Burkholderia cenocepacia K56-2Valvano]	V.KVFQYVKPQDAQALQDASPASGAS[+568.212]GAQAAAAPADNATVGAVPDASVQSTPLQPPAPISNSSSVHPGNPK.A	HexNAc(2)Hex(1)	Yes	1460.9239	5	7300.5903	7300.568	3	NRagged	1310	1268	36.68	Nsco_20191108_BC_ZIC_HILIC_K56_2_typsin_B2.46071.46071.5	93.66	Trypsin	B2	S224	16
BCAL0163	>gi|443595598|gb|ELT64173.1| BON domain protein [Burkholderia cenocepacia K56-2Valvano]	A.LQDASPASGAS[+568.212]GAQAAAAPADN.A	HexNAc(2)Hex(1)	Partial	1255.0619	2	2509.1166	2509.106	4.2	Non	346.5	235.7	8.44	Nsco_20191108_BC_ZIC_HILIC_K56_2_pepsin_B2.22492.22492.2	49.04	Pepsin	B2	Na	17
BCAL0163	>gi|443595598|gb|ELT64173.1| BON domain protein [Burkholderia cenocepacia K56-2Valvano]	K.PQDAQALQDASPASGAS[+568.212]GAQAAAAPADNATVGAVPDASVQSTPLQPPAPISNSSSVHPGNPK.A	HexNAc(2)Hex(1)	Yes	1602.7728	4	6408.0695	6408.051	2.9	Specific	830.5	830.5	81.53	Nsco_20191108_BC_ZIC_HILIC_K56_2_typsin_B2.48571.48571.4	97.84	Trypsin	B2	S224	18
BCAL0163	>gi|443595598|gb|ELT64173.1| BON domain protein [Burkholderia cenocepacia K56-2Valvano]	K.VFQYVKPQDAQALQDASPASGAS[+568.212]GAQAAAAPADNATVGAVPDASVQSTPLQPPAPISNSSSVHPGNPK.A	HexNAc(2)Hex(1)	Yes	1435.303	5	7172.486	7172.473	1.8	Specific	1194	1194	111.85	Nsco_20191108_BC_ZIC_HILIC_K56_2_typsin_B1.51502.51502.5	101.1	Trypsin	B1	S224	19
BCAL0163	>gi|443595598|gb|ELT64173.1| BON domain protein [Burkholderia cenocepacia K56-2Valvano]	Y.VKPQDAQALQDASPASGAS[+568.212]GAQAAAAPADNATVGAVPDASVQSTPLQPPAPISNSSSVHPGNPK.A	HexNAc(2)Hex(1)	Yes	1659.8165	4	6636.2443	6635.214	4	NRagged	963.7	963.7	93.72	Nsco_20191108_BC_ZIC_HILIC_K56_2_typsin_B3.45475.45475.4	91.03	Trypsin	B3	S224	20
BCAL0163	>gi|443595598|gb|ELT64173.1| BON domain protein [Burkholderia cenocepacia K56-2Valvano]	S.VQSTPLQPPAPISNSSS[+568.212]VHPGNPKAKAQ.-	HexNAc(2)Hex(1)	Partial	852.1829	4	3405.7097	3405.702	2.3	Non	314.8	314.8	170.78	Nsco_20191108_BC_ZIC_HILIC_K56_2_pepsin_B1.20403.20403.4	44.79	Pepsin	B1	S262	21
BCAL0193	>gi|443590944|gb|ELT59881.1| hypothetical protein BURCENK562V_2433 [Burkholderia cenocepacia K56-2Valvano]	S.AAAPAPAKKDHSKPKHQLKHHGSKKGQAKAAAAS[+568.212][+100.064]AAGTNDAGTQN.-	HexNAc(2)Hex(1) 100.064	Partial	1025.3224	5	5122.5828	5121.606	-5.3	Non	459.5	459.5	51.91	Nsco_20191108_BC_ZIC_HILIC_K56_2_pepsin_B1.3536.3536.5	16.51	Pepsin	B1	Na	22

BCAL0193	>gi|443590944|gb|ELT59881.1| hypothetical protein BURCENK562V_2433 [Burkholderia cenocepacia K56-2Valvano]	A.AAPAPAKKDHSKPKHQLKHHGSKKGQAKAAAAS[+568.212]AAGT[+568.212]ND.A	HexNAc(2)Hex(1),HexNAc(2)Hex(1)	Partial	1010.7127	5	5049.5344	5047.509	3.7	Non	199.3	199.3	143.14	Nsco_20191108_BC_ZIC_HILIC_K56_2_pepsin_B3_reinject.3350.3350.5	16.23	Pepsin	B3	Na	23
BCAL0193	>gi|443590944|gb|ELT59881.1| hypothetical protein BURCENK562V_2433 [Burkholderia cenocepacia K56-2Valvano]	Q.AS[+568.212]APAADTSAAAPAPAKK.D	HexNAc(2)Hex(1)	Yes	1082.5332	2	2164.0591	2164.05	4.1	NRagged	360	184.2	184.16	Nsco_20191108_BC_ZIC_HILIC_K56_2_typsin_B1.6823.6823.2	22.75	Trypsin	B1	S25	24
BCAL0193	>gi|443590944|gb|ELT59881.1| hypothetical protein BURCENK562V_2433 [Burkholderia cenocepacia K56-2Valvano]	A.DTSAAAPAPAKKDHSKPKHQLKHHGSKKGQAKAAAASAAGTNDAGT[+568.212]QN.-	HexNAc(2)Hex(1)	Partial	1066.1395	5	5326.6685	5324.649	2.4	Non	785	785	785.04	Nsco_20191108_BC_ZIC_HILIC_K56_2_pepsin_B3_reinject.3443.3443.5	16.38	Pepsin	B3	NA	25
BCAL0193	>gi|443590944|gb|ELT59881.1| hypothetical protein BURCENK562V_2433 [Burkholderia cenocepacia K56-2Valvano]	T.SAAAPAPAKKDHSKPKHQLKHHGSKKGQAKAAAAS[+568.212]AAGT[+568.212]ND.A	HexNAc(2)Hex(1),HexNAc(2)Hex(1)	Partial	1042.5261	5	5208.6015	5205.578	2.5	Non	254.9	254.9	166.1	Nsco_20191108_BC_ZIC_HILIC_K56_2_pepsin_B3_reinject.3735.3735.5	16.81	Pepsin	B3	NA	26
BCAL0193	>gi|443590944|gb|ELT59881.1| hypothetical protein BURCENK562V_2433 [Burkholderia cenocepacia K56-2Valvano]	T.SAAAPAPAKKDHSKPKHQLKHHGSKKGQAKAAAASAAGTNDAGT[+568.212][+100.064]QN.-	HexNAc(2)Hex(1)	Partial	1042.9348	5	5210.645	5208.638	0	Non	238.5	238.5	238.53	Nsco_20191108_BC_ZIC_HILIC_K56_2_pepsin_B1.3351.3351.5	16.26	Pepsin	B1	NA	27
BCAL0303	>gi|443596149|gb|ELT64670.1| hypothetical protein BURCENK562V_3632 [Burkholderia cenocepacia K56-2Valvano]	S.KAADAGGAKPAAGAS[+568.212]AAPAAPAPVAV.P	HexNAc(2)Hex(1)	Yes	909.4652	3	2726.381	2726.373	2.9	Non	745.7	742.6	742.58	Nsco_20191108_BC_ZIC_HILIC_K56_2_pepsin_B1.22517.22517.3	48.78	Pepsin	B1	S164	28
BCAL0303	>gi|443596149|gb|ELT64670.1| hypothetical protein BURCENK562V_3632 [Burkholderia cenocepacia K56-2Valvano]	L.YSKAADAGGAKPAAGAS[+568.212]AAPAAPAPVAV.P	HexNAc(2)Hex(1)	Yes	992.8305	3	2976.4769	2976.468	2.8	CRagged	663.7	565.7	328.45	Nsco_20191108_BC_ZIC_HILIC_K56_2_pepsin_B3_reinject.24722.24722.3	51.66	Pepsin	B3	S164	29
BCAL0303	>gi|443596149|gb|ELT64670.1| hypothetical protein BURCENK562V_3632 [Burkholderia cenocepacia K56-2Valvano]	L.YSKAADAGGAKPAAGAS[+568.212]AAPAAPAPVAVPASAVSG.S	HexNAc(2)Hex(1)	Partial	1182.9243	3	3546.7582	3545.749	1.6	CRagged	275.2	256.1	19.2	Nsco_20191108_BC_ZIC_HILIC_K56_2_pepsin_B3_reinject.31278.31278.3	64.13	Pepsin	B3	NA	30
BCAL0426	>gi|443602087|gb|ELT70191.1| membrane protein insertase, YidC/Oxa1 family [Burkholderia cenocepacia K56-2Valvano]	R.DHGRPSMFFPSATHTAPAAAGGAS[+568.212]GTGATTAGDVPAAAAGAAPSTTAPAAQAQLVK.F	HexNAc(2)Hex(1)	Yes	1422.4382	4	5686.7311	5683.709	2.1	Specific	1216	733.3	78.31	Nsco_20191108_BC_ZIC_HILIC_K56_2_typsin_B2.40769.40769.4	84.14	Trypsin	B2	S49	31
BCAL0426	>gi|443602087|gb|ELT70191.1| membrane protein insertase, YidC/Oxa1 family [Burkholderia cenocepacia K56-2Valvano]	F.FPSATHTAPAAAGGAS[+568.212][+100.064]GTGATTAGDVPAAAAGAAPSTTAPAAQ.A	HexNAc(2)Hex(1)100.064	Partial	1080.2585	4	4318.0124	4317.028	-4.4	CRagged	416	416	20.65	Nsco_20191108_BC_ZIC_HILIC_K56_2_pepsin_B2.36655.36655.4	78.73	Pepsin	B2	NA	32
BCAL0426	>gi|443602087|gb|ELT70191.1| membrane protein insertase, YidC/Oxa1 family [Burkholderia cenocepacia K56-2Valvano]	T.HTAPAAAGGAS[+568.212]GTGATTAGDVPAAAAGAAPSTTAPAAQ.A	HexNAc(2)Hex(1)	Yes	1238.5833	3	3713.7354	3713.726	2.5	Non	692.7	661.1	37.63	Nsco_20191108_BC_ZIC_HILIC_K56_2_pepsin_B3_reinject.30627.30627.3	62.86	Pepsin	B3	S49	33
BCAL0525	>gi|443597648|gb|ELT66063.1| flagellar M-ring protein FliF, partial [Burkholderia cenocepacia K56-2Valvano]	L.SNTPPQPAS[+568.212]APIVAGNGQNAPQTTPVSD.R	HexNAc(2)Hex(1)	Yes	1095.5191	3	3284.5427	3284.529	4.2	CRagged	535.3	449.8	35.51	Nsco_20191108_BC_ZIC_HILIC_K56_2_pepsin_B3_reinject.37980.37980.3	78.38	Pepsin	B3	S358	34
BCAL0525	>gi|443597648|gb|ELT66063.1| flagellar M-ring protein FliF, partial [Burkholderia cenocepacia K56-2Valvano]	R.SQQTSSATELAQGGAS[+568.212]GVPGALSNTPPQPASAPIVAGNGQNAPQTT[+568.212]PVSDRK.D	HexNAc(2)Hex(1),HexNAc(2)Hex(1)	Partial	1534.984	4	6136.9142	6134.902	0.9	Specific	328.6	328.6	81.93	Nsco_20191108_BC_ZIC_HILIC_K56_2_typsin_B2.40291.40291.4	83.32	Trypsin	B2	NA	35
BCAL0544	>gi|443601095|gb|ELT69255.1| ABC transporter, substrate-binding protein, family 5 [Burkholderia cenocepacia K56-2Valvano]	K.KAILESVYQGAGQAAS[+568.212]APMPPTQWSYDK.N	HexNAc(2)Hex(1)	Localised	1188.2346	3	3562.6891	3562.678	3.1	Non	438.6	425.7	120.52	Nsco_20191108_BC_ZIC_HILIC_K56_2_thermolysin_B3.48291.48291.3	96.95	Thermolysin	B3	S343	36
BCAL0678	>gi|443605648|gb|ELT73487.1| sporulation and cell division repeat protein [Burkholderia cenocepacia K56-2Valvano]	Q.AAAQKQQQQQQAANT[+568.212]PKPTSSAT.A	HexNAc(2)Hex(1)	Partial	984.4776	3	2951.4183	2951.408	3.6	Non	418.1	342.9	11.7	Nsco_20191108_BC_ZIC_HILIC_K56_2_thermolysin_B1.4855.4855.3	18.56	Thermolysin	B1	NA	37

BCAL0678	>gi|443605648|gb|ELT73487.1| sporulation and cell division repeat protein [Burkholderia cenocepacia K56-2Valvano]	Q.AAAQKQQQQQQAANT[+568.212]PKPTSSATA.A	HexNAc(2)Hex(1)	Partial	1008.1566	3	3022.4553	3022.445	3.5	Non	460.6	420	4.15	Nsco_20191108_BC_ZIC_HILIC_K56_2_thermolysin_B2_.5227.5229.3	20.13	Thermolysin	B2	NA	38
BCAL0678	>gi|443605648|gb|ELT73487.1| sporulation and cell division repeat protein [Burkholderia cenocepacia K56-2Valvano]	S.KVAPPPADNGAS[+568.212]QPQQFDPNRA.L	HexNAc(2)Hex(1)	Yes	958.4554	3	2873.3517	2873.344	2.8	Non	630.6	630.6	630.58	Nsco_20191108_BC_ZIC_HILIC_K56_2_pepsin_B3_reinject.18300.18300.3	40.82	Pepsin	B3	S39	39
BCAL0678	>gi|443605648|gb|ELT73487.1| sporulation and cell division repeat protein [Burkholderia cenocepacia K56-2Valvano]	S.KVAPPPADNGAS[+568.212]QPQQFDPNRALQG.K	HexNAc(2)Hex(1)	Yes	1057.8449	3	3171.5201	3171.508	3.9	Non	621.4	611.9	611.92	Nsco_20191108_BC_ZIC_HILIC_K56_2_pepsin_B1.27694.27694.3	59.06	Pepsin	B1	S39	40
BCAL0678	>gi|443605648|gb|ELT73487.1| sporulation and cell division repeat protein [Burkholderia cenocepacia K56-2Valvano]	S.KVAPPPADNGAS[+568.212]QPQQFDPNRALQGKTPGQPVPQA.A	HexNAc(2)Hex(1)	Yes	1044.5223	4	4175.0672	4175.053	3.5	Non	743.2	743.2	488.41	Nsco_20191108_BC_ZIC_HILIC_K56_2_pepsin_B3_reinject.31852.31852.4	65.21	Pepsin	B3	S39	41
BCAL0678	>gi|443605648|gb|ELT73487.1| sporulation and cell division repeat protein [Burkholderia cenocepacia K56-2Valvano]	S.KVAPPPADNGAS[+568.212]QPQQFDPNRALQGKTPGQPVPQAAQPAPPNTA.P	HexNAc(2)Hex(1)	Yes	1256.3777	4	5022.4889	5022.472	3.5	Non	665.4	665.4	320.9	Nsco_20191108_BC_ZIC_HILIC_K56_2_pepsin_B1.33145.33145.4	71.36	Pepsin	B1	S39	42
BCAL0678	>gi|443605648|gb|ELT73487.1| sporulation and cell division repeat protein [Burkholderia cenocepacia K56-2Valvano]	S.KVAPPPADNGAS[+568.212]QPQQFDPNRALQGKTPGQPVPQAAQPAPPNTAPGQ.A	HexNAc(2)Hex(1)	Yes	1326.9113	4	5304.6232	5304.604	3.6	Non	836.8	836.8	393.47	Nsco_20191108_BC_ZIC_HILIC_K56_2_pepsin_B3_reinject.35229.35229.4	72.36	Pepsin	B3	S39	43
BCAL0678	>gi|443605648|gb|ELT73487.1| sporulation and cell division repeat protein [Burkholderia cenocepacia K56-2Valvano]	S.KVAPPPADNGAS[+568.212]QPQQFDPNRALQGKTPGQPVPQAAQPAPPNTAPGQA.A	HexNAc(2)Hex(1)	Yes	1075.9371	5	5375.6566	5375.642	2.8	Non	942	942	522.63	Nsco_20191108_BC_ZIC_HILIC_K56_2_pepsin_B2.34589.34589.5	73.89	Pepsin	B2	S39	44
BCAL0678	>gi|443605648|gb|ELT73487.1| sporulation and cell division repeat protein [Burkholderia cenocepacia K56-2Valvano]	S.KVAPPPADNGAS[+568.212]QPQQFDPNRALQGKTPGQPVPQAAQPAPPNTAPGQAA.N	HexNAc(2)Hex(1)	Yes	1362.4287	4	5446.693	5446.679	2.7	Non	807.9	807.9	435.58	Nsco_20191108_BC_ZIC_HILIC_K56_2_pepsin_B1.33981.33981.4	73.55	Pepsin	B1	S39	45
BCAL0678	>gi|443605648|gb|ELT73487.1| sporulation and cell division repeat protein [Burkholderia cenocepacia K56-2Valvano]	S.KVAPPPADNGAS[+568.212]QPQQFDPNRALQGKTPGQPVPQAAQPAPPNTAPGQAAN.Q	HexNAc(2)Hex(1)	Yes	1113.3529	5	5562.7354	5560.722	1.3	Non	1015	1001	483.05	Nsco_20191108_BC_ZIC_HILIC_K56_2_pepsin_B1.33588.33588.5	72.44	Pepsin	B1	S39	46
BCAL0678	>gi|443605648|gb|ELT73487.1| sporulation and cell division repeat protein [Burkholderia cenocepacia K56-2Valvano]	S.KVAPPPADNGAS[+568.212]QPQQFDPNRALQGKTPGQPVPQAAQPAPPNTAPGQAANQ.T	HexNAc(2)Hex(1)	Yes	1422.9518	4	5688.7853	5688.78	0.9	Non	680.9	672.1	302.36	Nsco_20191108_BC_ZIC_HILIC_K56_2_pepsin_B3_reinject.35184.35184.4	72.26	Pepsin	B3	S39	47
BCAL0678	>gi|443605648|gb|ELT73487.1| sporulation and cell division repeat protein [Burkholderia cenocepacia K56-2Valvano]	K.PT[+568.212]SSATAAAAAKPPTANDANTGYFLQVGAYK.T	HexNAc(2)Hex(1)	Partial	1208.9186	3	3624.7412	3622.728	1.7	Specific	447.8	406.4	1.8	Nsco_20191108_BC_ZIC_HILIC_K56_2_typsin_B1.39530.39530.3	81.3	Trypsin	B1	NA	48
BCAL0678	>gi|443605648|gb|ELT73487.1| sporulation and cell division repeat protein [Burkholderia cenocepacia K56-2Valvano]	Q.QAANT[+568.212][+100.064]PKPTSS[+568.212][+100.064]ATAAAAAKPPTANDANTGYFLQVGAYK.T	HexNAc(2)Hex(1) 100.064,HexNAc(2)Hex(1)	Partial	1276.3612	4	5102.423	5101.439	-3.8	NRagged	178.7	178.7	178.68	Nsco_20191108_BC_ZIC_HILIC_K56_2_typsin_B1.36658.36658.4	76.27	Trypsin	B1	NA	49
BCAL0678	>gi|443605648|gb|ELT73487.1| sporulation and cell division repeat protein [Burkholderia cenocepacia K56-2Valvano]	Q.QAANT[+568.212][+100.064]PKPTSSATAAAAAKPPT[+568.212]ANDANTGYFLQVGAYKTEGDAEQQR.A	HexNAc(2)Hex(1) 100.064,HexNAc(2)Hex(1) 100.064	Partial	1204.7736	5	6019.8387	6015.812	2.3	NRagged	275.4	234.3	39.05	Nsco_20191108_BC_ZIC_HILIC_K56_2_typsin_B1.34882.34882.5	73.1	Trypsin	B1	NA	50
BCAL0678	>gi|443605648|gb|ELT73487.1| sporulation and cell division repeat protein [Burkholderia cenocepacia K56-2Valvano]	A.QKQQQQQQAANT[+568.212]PKPTSSATAAA.A	HexNAc(2)Hex(1)	Partial	1476.2091	2	2951.4109	2951.408	1.1	Non	209.4	187.4	13.8	Nsco_20191108_BC_ZIC_HILIC_K56_2_pepsin_B1.7370.7370.2	22.89	Pepsin	B1	NA	51

BCAL0678	>gi|443605648|gb|ELT73487.1| sporulation and cell division repeat protein [Burkholderia cenocepacia K56-2Valvano]	Q.QQAANT[+568.212]PKPTS[+568.212]SATAAAAAKPPTANDANTGYFLQVGAYK.T	HexNAc(2)Hex(1),HexNAc(2)Hex(1)	Partial	1258.1008	4	5029.3815	5029.37	2.4	NRagged	262.2	262.2	6.08	Nsco_20191108_BC_ZIC_HILIC_K56_2_typsin_B2.35560.35560.4	74.65	Trypsin	B2	NA	52
BCAL0678	>gi|443605648|gb|ELT73487.1| sporulation and cell division repeat protein [Burkholderia cenocepacia K56-2Valvano]	Q.QQAANTPKPTS[+568.212]SATAAAAAKPPT[+568.212]ANDANTGYFLQVGAYKTEGDAEQQR.A	HexNAc(2)Hex(1),HexNAc(2)Hex(1)	Partial	1209.5704	5	6043.8231	6043.806	2.8	NRagged	538.3	520.2	22.54	Nsco_20191108_BC_ZIC_HILIC_K56_2_typsin_B1.33924.33924.5	71.43	Trypsin	B1	NA	53
BCAL0678	>gi|443605648|gb|ELT73487.1| sporulation and cell division repeat protein [Burkholderia cenocepacia K56-2Valvano]	K.QQQQQQAANTPKPTS[+568.212]SATAAAAAKPPTANDANTGYFLQV.G	HexNAc(2)Hex(1)	Yes	1519.0698	3	4555.1947	4554.175	3.5	Non	206.8	132	1.06	Nsco_20191108_BC_ZIC_HILIC_K56_2_thermolysin_B3.38670.38670.3	79.71	Thermolysin	B3	S180	54
BCAL0678	>gi|443605648|gb|ELT73487.1| sporulation and cell division repeat protein [Burkholderia cenocepacia K56-2Valvano]	K.QQQQQQAANTPKPTS[+568.212]SATAAAAAKPPTANDANTGYFLQVGAYK.T	HexNAc(2)Hex(1)	Yes	1244.1025	4	4973.3883	4973.392	-0.8	Specific	1231	607.5	26.24	Nsco_20191108_BC_ZIC_HILIC_K56_2_typsin_B3.35416.35416.4	73.18	Trypsin	B3	S180	55
BCAL0678	>gi|443605648|gb|ELT73487.1| sporulation and cell division repeat protein [Burkholderia cenocepacia K56-2Valvano]	K.QQQQQQAANTPKPTSSATAAAAAKPPT[+568.212]ANDANTGYFLQVGAYKTEGDAQQR.A	HexNAc(2)Hex(1)	Partial	1198.5741	5	5988.8414	5987.829	1.5	Specific	798	508.6	34.71	Nsco_20191108_BC_ZIC_HILIC_K56_2_typsin_B1.34007.34007.5	71.56	Trypsin	B1	NA	56
BCAL0678	>gi|443605648|gb|ELT73487.1| sporulation and cell division repeat protein [Burkholderia cenocepacia K56-2Valvano]	K.VAPPPADNGAS[+568.212]QPQQFDPNR.A	HexNAc(2)Hex(1)	Yes	1337.6141	2	2674.2208	2674.212	3.5	Specific	607.5	540	540.04	Nsco_20191108_BC_ZIC_HILIC_K56_2_typsin_B2.21592.21592.2	48.6	Trypsin	B2	S39	57
BCAL0678	>gi|443605648|gb|ELT73487.1| sporulation and cell division repeat protein [Burkholderia cenocepacia K56-2Valvano]	K.VAPPPADNGAS[+568.212]QPQQFDPNRALQGK.T	HexNAc(2)Hex(1)	Yes	1057.8439	3	3171.5171	3171.508	3	Specific	699.6	699.6	699.57	Nsco_20191108_BC_ZIC_HILIC_K56_2_typsin_B3.27804.27804.3	60.07	Trypsin	B3	S39	58
BCAL0678	>gi|443605648|gb|ELT73487.1| sporulation and cell division repeat protein [Burkholderia cenocepacia K56-2Valvano]	F.VSKVAPPPADNGAS[+568.212]QPQQFDPNRA.L	HexNAc(2)Hex(1)	Yes	1020.4881	3	3059.4496	3059.444	1.8	CRagged	233.1	216.8	126.3	Nsco_20191108_BC_ZIC_HILIC_K56_2_pepsin_B1.20586.20586.3	45.12	Pepsin	B1	S39	59
BCAL0738	>gi|443604531|gb|ELT72458.1| peptidase, S41 family [Burkholderia cenocepacia K56-2Valvano]	K.ASGASAAKPAS[+568.212]APKPASAPK.-	HexNAc(2)Hex(1)	Yes	778.3989	3	2333.1821	2333.172	4.4	Specific	265.2	187.9	46.49	Nsco_20191108_BC_ZIC_HILIC_K56_2_typsin_B3.4910.4910.4	19.47	Trypsin	B3	S508	60
BCAL0749	>gi|443604502|gb|ELT72429.1| cytochrome c oxidase, subunit II [Burkholderia cenocepacia K56-2Valvano]	R.NGKLPEDTAGAAT[+568.212]AAAPAEAASAPAQAAS[+568.212]GAEQPAAAASAALSTIYFETGK.S	HexNAc(2)Hex(1),HexNAc(2)Hex(1)	Partial (1 of 2)	1463.693	4	5851.7502	5851.73	3.4	Specific	1107	1018	111.99	Nsco_20191108_BC_ZIC_HILIC_K56_2_typsin_B3.61271.61271.4	117.8	Trypsin	B3	S410	61
BCAL0749	>gi|443604502|gb|ELT72429.1| cytochrome c oxidase, subunit II [Burkholderia cenocepacia K56-2Valvano]	L.PEDTAGAAT[+568.212][+100.064]AAAPAEAASAPAQAAS[+568.212][+100.064]GAEQPAAAASAALSTIYFETGK.S	HexNAc(2)Hex(1) 100.064,HexNAc(2)Hex(1) 100.064	Partial (1 of 2)	1410.6604	4	5639.6198	5639.615	0.9	CRagged	273.1	268.3	3.02	Nsco_20191108_BC_ZIC_HILIC_K56_2_thermolysin_B3.60729.60729.4	120.7	Thermolysin	B3	S410	62
BCAL0749	>gi|443604502|gb|ELT72429.1| cytochrome c oxidase, subunit II [Burkholderia cenocepacia K56-2Valvano]	K.QVADARNGKLPEDTAGAATAAAPAEAAS[+568.212][+100.064]APAQAASGAEQPAAAASAALSTIYFETGK.S	HexNAc(2)Hex(1)	Partial	1506.9807	4	6024.901	6023.912	-2.4	Specific	362.8	362.8	47.23	Nsco_20191108_BC_ZIC_HILIC_K56_2_typsin_B2.61326.61326.4	122.3	Trypsin	B2	NA	63
BCAL0786	>gi|443598725|gb|ELT67055.1| hypothetical protein BURCENK562V_1882 [Burkholderia cenocepacia K56-2Valvano]	K.LLSVPAPAS[+568.212]TEGDHHDK.-	HexNAc(2)Hex(1)	Yes	586.2788	4	2342.0932	2342.088	2.2	Specific	499.2	439.7	35.82	Nsco_20191108_BC_ZIC_HILIC_K56_2_typsin_B3.14440.14440.4	35.98	Trypsin	B3	S80	64
BCAL1086	>gi|443597491|gb|ELT65913.1| putative lipoprotein [Burkholderia cenocepacia K56-2Valvano]	K.ALDQVAS[+568.212]TVNQQINAAK.A	HexNAc(2)Hex(1)	Partial	1170.0798	2	2339.1522	2339.146	2.7	Specific	339.4	163	163.02	Nsco_20191108_BC_ZIC_HILIC_K56_2_typsin_B1.35454.35454.2	74	Trypsin	B1	NA	65
BCAL1086	>gi|443597491|gb|ELT65913.1| putative lipoprotein [Burkholderia cenocepacia K56-2Valvano]	Q.IDAAAS[+568.212]AVVAH.A	HexNAc(2)Hex(1)	Yes	796.8835	2	1592.7597	1592.754	3.7	Non	394.4	147.3	147.31	Nsco_20191108_BC_ZIC_HILIC_K56_2_thermolysin_B2_.18308.18308.2	39.2	Thermolysin	B2	S104	66

BCAL1086	>gi|443597491|gb|ELT65913.1| putative lipoprotein [Burkholderia cenocepacia K56-2Valvano]	K.KLQQWSQQSAAGAKPAS[+568.212]GE.-	HexNAc(2)Hex(1)	Yes	1270.6071	2	2540.2069	2540.2	2.8	CRagged	331.7	204.8	204.84	Nsco_20191108_BC_ZIC_HILIC_K56_2_typsin_B2.11121.11121.2	29.91	Trypsin	B2	S140	67
BCAL1086	>gi|443597491|gb|ELT65913.1| putative lipoprotein [Burkholderia cenocepacia K56-2Valvano]	E.KSGTPVAQPDTAAS[+568.212]AAAD.A	HexNAc(2)Hex(1)	Yes	1113.5132	2	2226.0191	2226.014	2.1	Non	253.6	143	143	Nsco_20191108_BC_ZIC_HILIC_K56_2_pepsin_B1.13983.13983.2	33.95	Pepsin	B1	S45	68
BCAL1086	>gi|443597491|gb|ELT65913.1| putative lipoprotein [Burkholderia cenocepacia K56-2Valvano]	E.KSGTPVAQPDTAAS[+568.212]AAADAANNAAKAL.D	HexNAc(2)Hex(1)	Yes	1017.4953	3	3050.4713	3050.465	2.1	NRagged	380.7	265.4	30.76	Nsco_20191108_BC_ZIC_HILIC_K56_2_pepsin_B1.37649.37649.4	82.74	Pepsin	B1	S45	69
BCAL1086	>gi|443597491|gb|ELT65913.1| putative lipoprotein [Burkholderia cenocepacia K56-2Valvano]	K.LQQWSQQSAAGAKPAS[+568.212]GE.-	HexNAc(2)Hex(1)	Yes	1206.559	2	2412.1107	2412.105	2.4	CRagged	283.9	121.9	67.95	Nsco_20191108_BC_ZIC_HILIC_K56_2_typsin_B1.15007.15007.2	36.76	Trypsin	B1	S140	70
BCAL1093	>gi|443599421|gb|ELT67702.1| hypothetical protein BURCENK562V_2687 [Burkholderia cenocepacia K56-2Valvano]	K.HAYDEAHPAEAAS[+568.212]AASH.-	HexNAc(2)Hex(1)	Yes	768.3271	3	2302.9666	2302.958	3.6	CRagged	304.4	251.1	92.55	Nsco_20191108_BC_ZIC_HILIC_K56_2_typsin_B3.7340.7340.3	24.24	Trypsin	B3	S49	71
BCAL1453	>gi|443604751|gb|ELT72660.1| HlyD family secretion protein [Burkholderia cenocepacia K56-2Valvano]	R.ENAAHSAS[+568.212]SADAQYQQAIAALDAAK.L	HexNAc(2)Hex(1)	Yes	1024.4741	3	3071.4078	3070.397	2.4	Specific	263.2	209.4	14.32	Nsco_20191108_BC_ZIC_HILIC_K56_2_typsin_B2.39533.39533.3	82	Trypsin	B2	S130	72
BCAL1453	>gi|443604751|gb|ELT72660.1| HlyD family secretion protein [Burkholderia cenocepacia K56-2Valvano]	K.ENRENAAHSAS[+568.212]SADAQYQQAIAALDAAK.L	HexNAc(2)Hex(1)	Yes	868.1537	4	3469.5931	3469.584	2.7	Specific	1112	987.1	56.26	Nsco_20191108_BC_ZIC_HILIC_K56_2_typsin_B3.34661.34661.4	71.93	Trypsin	B3	S130	73
BCAL1496	>gi|443597624|gb|ELT66041.1| hypothetical protein BURCENK562V_3510 [Burkholderia cenocepacia K56-2Valvano]	K.AAAKKAGKKAKAADAAS[+568.212]Q.-	HexNAc(2)Hex(1)	Yes	564.3015	4	2254.1841	2254.177	3	Non	480.3	448.6	448.6	Nsco_20191108_BC_ZIC_HILIC_K56_2_thermolysin_B2_.2250.2250.4	13.92	Thermolysin	B2	S90	74
BCAL1496	>gi|443597624|gb|ELT66041.1| hypothetical protein BURCENK562V_3510 [Burkholderia cenocepacia K56-2Valvano]	A.AAKKAGKKAKAADAAS[+568.212]Q.-	HexNAc(2)Hex(1)	Yes	546.5424	4	2183.1477	2183.14	3.5	Non	455.2	455.2	455.21	Nsco_20191108_BC_ZIC_HILIC_K56_2_thermolysin_B2_.2204.2204.4	13.71	Thermolysin	B2	S90	75
BCAL1496	>gi|443597624|gb|ELT66041.1| hypothetical protein BURCENK562V_3510 [Burkholderia cenocepacia K56-2Valvano]	K.AGKKAKAADAAS[+568.212]Q.-	HexNAc(2)Hex(1)	Yes	595.632	3	1784.8815	1784.876	3.1	Non	193.6	183.9	183.89	Nsco_20191108_BC_ZIC_HILIC_K56_2_thermolysin_B1.2214.2214.3	13.4	Thermolysin	B1	S90	76
BCAL1496	>gi|443597624|gb|ELT66041.1| hypothetical protein BURCENK562V_3510 [Burkholderia cenocepacia K56-2Valvano]	K.AKAADAAS[+568.212]Q.-	HexNAc(2)Hex(1)	Yes	700.8192	2	1400.631	1400.628	2.5	CRagged	403.4	208.7	208.72	Nsco_20191108_BC_ZIC_HILIC_K56_2_typsin_B3.2167.2167.2	14.41	Trypsin	B3	S90	77
BCAL1496	>gi|443597624|gb|ELT66041.1| hypothetical protein BURCENK562V_3510 [Burkholderia cenocepacia K56-2Valvano]	H.KAAAKKAGKKAKAADAAS[+568.212]Q.-	HexNAc(2)Hex(1)	Yes	596.3253	4	2382.2795	2382.272	3.1	Non	709.7	648.2	648.15	Nsco_20191108_BC_ZIC_HILIC_K56_2_pepsin_B3_reinject.2004.2004.4	13.57	Pepsin	B3	S90	78
BCAL1496	>gi|443597624|gb|ELT66041.1| hypothetical protein BURCENK562V_3510 [Burkholderia cenocepacia K56-2Valvano]	K.KAGKKAKAADAAS[+568.212]Q.-	HexNAc(2)Hex(1)	Yes	638.3299	3	1912.9752	1912.971	2.2	Non	346.5	250.2	250.15	Nsco_20191108_BC_ZIC_HILIC_K56_2_pepsin_B3_reinject.1926.1926.3	13.28	Pepsin	B3	S90	79
BCAL1496	>gi|443597624|gb|ELT66041.1| hypothetical protein BURCENK562V_3510 [Burkholderia cenocepacia K56-2Valvano]	A.KKAGKKAKAADAAS[+568.212]Q.-	HexNAc(2)Hex(1)	Yes	511.0234	4	2041.0719	2041.066	2.9	Non	349.8	268.4	268.41	Nsco_20191108_BC_ZIC_HILIC_K56_2_pepsin_B2.1914.1914.4	13.27	Pepsin	B2	S90	80
BCAL1496	>gi|443597624|gb|ELT66041.1| hypothetical protein BURCENK562V_3510 [Burkholderia cenocepacia K56-2Valvano]	G.KKAKAADAAS[+568.212]Q.-	HexNAc(2)Hex(1)	Yes	552.9457	3	1656.8225	1656.817	3.1	Non	367.4	216.1	216.13	Nsco_20191108_BC_ZIC_HILIC_K56_2_pepsin_B1.1926.1926.3	13.11	Pepsin	B1	S90	81
BCAL1674	>gi|443605593|gb|ELT73435.1| periplasmic multidrug efflux lipoprotein [Burkholderia cenocepacia K56-2Valvano]	P.VEKAPSSKAAPPAAAS[+568.212]QA.A	HexNAc(2)Hex(1)	Yes	1125.0594	2	2249.1115	2249.103	3.7	Non	388	231.4	187.03	Nsco_20191108_BC_ZIC_HILIC_K56_2_thermolysin_B1.7802.7802.2	23.32	Thermolysin	B1	S404	82
BCAL1746	>gi|443592790|gb|ELT61568.1| putative lipoprotein [Burkholderia cenocepacia K56-2Valvano]	L.IDHIGKAWPGNAAS[+568.212]GASASASE.-	HexNAc(2)Hex(1)	Yes	889.4119	3	2666.221	2665.211	2.5	CRagged	334.5	226.7	49.17	Nsco_20191108_BC_ZIC_HILIC_K56_2_pepsin_B1.22279.22279.3	48.33	Pepsin	B1	S140	83

BCAL1798	>gi|443598613|gb|ELT66949.1| hypothetical protein BURCENK562V_2315 [Burkholderia cenocepacia K56-2Valvano]	T.AATPAAASAPS[+568.212]AEQRAARHEARIEQ.R	HexNAc(2)Hex(1)	Partial	1043.5109	3	3128.5181	3128.509	2.9	Non	503.5	426	7.2	Nsco_20191108_BC_ZIC_HILIC_K56_2_pepsin_B3_reinject.12565.12565.3	31.5	Pepsin	B3	NA	84
BCAL1869	>gi|443596292|gb|ELT64810.1| hypothetical protein BURCENK562V_2581 [Burkholderia cenocepacia K56-2Valvano]	P.VAAS[+568.212]AVPDFDARQK.V	HexNAc(2)Hex(1)	Yes	681.6658	3	2042.9828	2042.976	3.1	Non	394.4	175.1	175.06	Nsco_20191108_BC_ZIC_HILIC_K56_2_thermolysin_B1.18482.18482.3	39.94	Thermolysin	B1	S29	85
BCAL1877	>gi|443594658|gb|ELT63293.1| HflK protein [Burkholderia cenocepacia K56-2Valvano]	A.ATAS[+568.212]GVDVLRSRE.A	HexNAc(2)Hex(1)	Yes	964.9725	2	1928.9376	1928.93	4.2	Non	171.9	59.51	59.51	Nsco_20191108_BC_ZIC_HILIC_K56_2_thermolysin_B1.14431.14431.2	33.54	Thermolysin	B1	S427	86
BCAL1877	>gi|443594658|gb|ELT63293.1| HflK protein [Burkholderia cenocepacia K56-2Valvano]	L.VEQGRQNAAAST[+568.212]GASAADAAS[+568.212]APAATVPSAA.A	HexNAc(2)Hex(1),HexNAc(2)Hex(1)	Partial	1302.5969	3	3905.776	3905.763	3.3	CRagged	179.8	79.69	16.04	Nsco_20191108_BC_ZIC_HILIC_K56_2_pepsin_B2.26026.26026.3	55.76	Pepsin	B2	NA	87
BCAL1917	>gi|443605668|gb|ELT73505.1| hypothetical protein BURCENK562V_0636 [Burkholderia cenocepacia K56-2Valvano]	K.AAAAAASADAGAS[+568.212]APAAASST[+568.212]K.A	HexNAc(2)Hex(1),HexNAc(2)Hex(1)	Partial (1 of 2)	975.7731	3	2925.3047	2925.295	3.2	Specific	399.5	259.7	93.53	Nsco_20191108_BC_ZIC_HILIC_K56_2_typsin_B2.9570.9570.3	27.51	Trypsin	B2	S184	88
BCAL1917	>gi|443605668|gb|ELT73505.1| hypothetical protein BURCENK562V_0636 [Burkholderia cenocepacia K56-2Valvano]	T.AAPAPTAS[+568.212]APEAAAKPAKTKR.A	HexNAc(2)Hex(1)	Yes	644.0907	4	2573.341	2573.331	4.1	Non	928.6	779.6	128.8	Nsco_20191108_BC_ZIC_HILIC_K56_2_thermolysin_B1.5828.5828.4	20.43	Thermolysin	B1	S151	89
BCAL1917	>gi|443605668|gb|ELT73505.1| hypothetical protein BURCENK562V_0636 [Burkholderia cenocepacia K56-2Valvano]	T.AAPAPTAS[+568.212]APEAAAKPAKTKRA.S	HexNAc(2)Hex(1)	Yes	882.1293	3	2644.3733	2644.368	2.2	Non	263.7	185	50.94	Nsco_20191108_BC_ZIC_HILIC_K56_2_thermolysin_B2_.5922.5922.3	21.24	Thermolysin	B2	S151	90
BCAL1917	>gi|443605668|gb|ELT73505.1| hypothetical protein BURCENK562V_0636 [Burkholderia cenocepacia K56-2Valvano]	T.AAPAPTAS[+568.212]APEAAAKPAKTKRASKKEKA.A	HexNAc(2)Hex(1)	Yes	663.9601	5	3315.7712	3315.764	2.1	Non	1029	993.3	140.99	Nsco_20191108_BC_ZIC_HILIC_K56_2_thermolysin_B2_.3999.3999.5	18.07	Thermolysin	B2	S151	91
BCAL1917	>gi|443605668|gb|ELT73505.1| hypothetical protein BURCENK562V_0636 [Burkholderia cenocepacia K56-2Valvano]	T.AAPAPTAS[+568.212]APEAAAKPAKTKRASKKEKAA.A	HexNAc(2)Hex(1)	Yes	678.167	5	3386.8058	3386.801	1.3	Non	940.1	902.6	127.47	Nsco_20191108_BC_ZIC_HILIC_K56_2_thermolysin_B2_.4052.4052.4	18.15	Thermolysin	B2	S151	92
BCAL1917	>gi|443605668|gb|ELT73505.1| hypothetical protein BURCENK562V_0636 [Burkholderia cenocepacia K56-2Valvano]	P.AATTSATTSTTTSAGTASTSTATAGTTTAAPAPTAS[+568.212]APEAAAKPAKTKR.A	HexNAc(2)Hex(1)	Partial	1027.9116	5	5135.529	5135.509	4	Non	652.6	652.6	1.99	Nsco_20191108_BC_ZIC_HILIC_K56_2_thermolysin_B2_.17866.17866.5	38.5	Thermolysin	B2	NA	93
BCAL1917	>gi|443605668|gb|ELT73505.1| hypothetical protein BURCENK562V_0636 [Burkholderia cenocepacia K56-2Valvano]	T.AGTTTAAPAPTAS[+568.212]APEAAAKPAKTKR.A	HexNAc(2)Hex(1)	Yes	751.8917	4	3004.5449	3004.532	4.3	Non	907.5	830.6	99.82	Nsco_20191108_BC_ZIC_HILIC_K56_2_thermolysin_B2_.8205.8205.4	24.64	Thermolysin	B2	S151	94
BCAL1917	>gi|443605668|gb|ELT73505.1| hypothetical protein BURCENK562V_0636 [Burkholderia cenocepacia K56-2Valvano]	T.ATAGTTTAAPAPTAS[+568.212]APEAAAKPAKTKR.A	HexNAc(2)Hex(1)	Yes	1059.5459	3	3176.6232	3176.617	2	Non	242.4	240.3	40.27	Nsco_20191108_BC_ZIC_HILIC_K56_2_thermolysin_B2_.8996.8996.3	25.75	Thermolysin	B2	S151	95
BCAL1917	>gi|443605668|gb|ELT73505.1| hypothetical protein BURCENK562V_0636 [Burkholderia cenocepacia K56-2Valvano]	A.ATTSATTSTTTSAGTASTSTTAGTTTAAPAPTASAPEAAAKPAKT[+568.212]KR.A	HexNAc(2)Hex(1)	Partial	1013.9047	5	5065.4942	5064.471	3.8	Non	375.1	375.1	38.72	Nsco_20191108_BC_ZIC_HILIC_K56_2_thermolysin_B2_.17346.17346.5	37.74	Thermolysin	B2	NA	96
BCAL1917	>gi|443605668|gb|ELT73505.1| hypothetical protein BURCENK562V_0636 [Burkholderia cenocepacia K56-2Valvano]	S.ATTSTTTSAGTASTSTTATATTAAPAPTAS[+568.212]APEAAAK.P	HexNAc(2)Hex(1)	Yes	1341.9662	3	4023.884	4022.878	0.6	Non	603.2	547.8	37.41	Nsco_20191108_BC_ZIC_HILIC_K56_2_thermolysin_B3.24381.24381.3	53.09	Thermolysin	B3	S151	97
BCAL1917	>gi|443605668|gb|ELT73505.1| hypothetical protein BURCENK562V_0636 [Burkholderia cenocepacia K56-2Valvano]	S.ATTSTTTSAGTASTSTTATATTAAPAPTAS[+568.212]APEAAAKPAK.T	HexNAc(2)Hex(1)	Yes	1440.3646	3	4319.0791	4319.063	3.7	NRagged	349.6	270.5	51.3	Nsco_20191108_BC_ZIC_HILIC_K56_2_typsin_B3.20286.20286.3	46.2	Trypsin	B3	S151	98
BCAL1917	>gi|443605668|gb|ELT73505.1| hypothetical protein BURCENK562V_0636 [Burkholderia cenocepacia K56-2Valvano]	A.GTASTSTTATAGTTTAAPAPTAS[+568.212]APEAAAKPAK.T	HexNAc(2)Hex(1)	Yes	1166.5707	3	3497.6977	3497.687	3.2	Non	314.1	293.6	41.62	Nsco_20191108_BC_ZIC_HILIC_K56_2_thermolysin_B3.17558.17558.3	40.46	Thermolysin	B3	S151	99
BCAL1917	>gi|443605668|gb|ELT73505.1| hypothetical protein BURCENK562V_0636 [Burkholderia cenocepacia K56-2Valvano]	K.LSKPAATTSATTSTTTSAGTASTSTTATAGTTTAAPAPTAS[+568.212]APEAAAK.P	HexNAc(2)Hex(1)	Yes	1220.595	4	4879.3582	4879.344	3	Non	743.2	678.9	64.85	Nsco_20191108_BC_ZIC_HILIC_K56_2_thermolysin_B3.24903.24903.4	54.02	Thermolysin	B3	S151	100

BCAL1917	>gi|443605668|gb|ELT73505.1| hypothetical protein BURCENK562V_0636 [Burkholderia cenocepacia K56-2Valvano]	K.LSKPAATTSATTSTTTSAGTA STSTTATAGTTTAAPAPTAS[+ 568.212]APEAAAKPA.K	HexNAc(2)Hex(1)	Yes	1262.6184	4	5047.4518	5047.434	3.6	Non	1202	1202	99.55	Nsco_20191108_BC_ZIC_HILIC_K56_2_ther molysin_B3.26332.26332.4	56.76	Thermolysin	B3	S151	101
BCAL1917	>gi|443605668|gb|ELT73505.1| hypothetical protein BURCENK562V_0636 [Burkholderia cenocepacia K56-2Valvano]	K.LSKPAATTSATTSTTTSAGTA STSTTATAGTTTAAPAPTAS[+ 568.212]APEAAAKPAK.T	HexNAc(2)Hex(1)	Yes	1294.6409	4	5175.5417	5175.529	2.5	Non	1556	1556	95.66	Nsco_20191108_BC_ZIC_HILIC_K56_2_ther molysin_B3.21919.21919.4	48.63	Thermolysin	B3	S151	102
BCAL1917	>gi|443605668|gb|ELT73505.1| hypothetical protein BURCENK562V_0636 [Burkholderia cenocepacia K56-2Valvano]	K.LSKPAATTSATTSTTTSAGTA STSTTATAGTTTAAPAPTASAP EAAAKPAKT[+568.212].K	HexNAc(2)Hex(1)	Partial	1056.3196	5	5277.5688	5276.576	-2.1	Non	1203	1203	118.24	Nsco_20191108_BC_ZIC_HILIC_K56_2_ther molysin_B3.23031.23031.5	50.72	Thermolysin	B3	NA	103
BCAL1917	>gi|443605668|gb|ELT73505.1| hypothetical protein BURCENK562V_0636 [Burkholderia cenocepacia K56-2Valvano]	K.PAATTSATTSTTTSAGTASTS TATAGTTTAAPAPTAS[+568. 212]APEAAAKPAK.T	HexNAc(2)Hex(1)	Yes	1616.7836	3	4848.3362	4847.318	3.2	Specific	284.7	231.1	16.42	Nsco_20191108_BC_ZIC_HILIC_K56_2_typs in_B1.23256.23256.3	52.28	Trypsin	B1	S151	104
BCAL1917	>gi|443605668|gb|ELT73505.1| hypothetical protein BURCENK562V_0636 [Burkholderia cenocepacia K56-2Valvano]	T.SAGTASTSTTATAGTTTAAPA PTAS[+568.212]APEAAAK PAK.T	HexNAc(2)Hex(1)	Yes	1219.5968	3	3656.7759	3655.756	4.6	NRagged	186.1	186.1	37.59	Nsco_20191108_BC_ZIC_HILIC_K56_2_typs in_B1.17687.17687.3	41.65	Trypsin	B1	S151	105
BCAL1917	>gi|443605668|gb|ELT73505.1| hypothetical protein BURCENK562V_0636 [Burkholderia cenocepacia K56-2Valvano]	L.SKPAATTSATTSTTTSAGTAS STTATAGTTTAAPAPTAS[+56 8.212]APEAAAKPAK.T	HexNAc(2)Hex(1)	Yes	1266.371	4	5062.4621	5062.445	3.5	NRagged	1230	1230	53.78	Nsco_20191108_BC_ZIC_HILIC_K56_2_typs in_B2.19359.19359.4	44.35	Trypsin	B2	S151	106
BCAL1917	>gi|443605668|gb|ELT73505.1| hypothetical protein BURCENK562V_0636 [Burkholderia cenocepacia K56-2Valvano]	T.STTATAGTTTAAPAPTAS[+5 68.212]APEAAAKPAK.T	HexNAc(2)Hex(1)	Yes	1027.8418	3	3081.5108	3080.501	2.3	NRagged	173.3	149.7	89.39	Nsco_20191108_BC_ZIC_HILIC_K56_2_typs in_B3.14962.14962.3	36.8	Trypsin	B3	S151	107
BCAL1917	>gi|443605668|gb|ELT73505.1| hypothetical protein BURCENK562V_0636 [Burkholderia cenocepacia K56-2Valvano]	T.STTTSAGTASTSTTATAGTTT APAPTAS[+568.212]APEA AAKPAK.T	HexNAc(2)Hex(1)	Yes	1349.6518	3	4046.9408	4045.931	1.7	NRagged	342.1	258.8	47.02	Nsco_20191108_BC_ZIC_HILIC_K56_2_typs in_B2.19517.19517.3	44.64	Trypsin	B2	S151	108
BCAL1917	>gi|443605668|gb|ELT73505.1| hypothetical protein BURCENK562V_0636 [Burkholderia cenocepacia K56-2Valvano]	K.SVGHLEENGLTIGGAS[+56 8.212]TPPK.G	HexNAc(2)Hex(1)	Yes	844.7462	3	2532.224	2532.22	1.6	Specific	257.6	195.8	11.82	Nsco_20191108_BC_ZIC_HILIC_K56_2_typs in_B2.24677.24677.3	54.28	Trypsin	B2	S104	109
BCAL1917	>gi|443605668|gb|ELT73505.1| hypothetical protein BURCENK562V_0636 [Burkholderia cenocepacia K56-2Valvano]	A.TAGTTTAAPAPT[+568.21 2]ASAPEAAAKPAK.T	HexNAc(2)Hex(1)	Partial	907.4528	3	2720.3437	2720.336	2.8	NRagged	164	111.5	49.17	Nsco_20191108_BC_ZIC_HILIC_K56_2_typs in_B2.12753.12753.3	32.39	Trypsin	B2	NA	110
BCAL1917	>gi|443605668|gb|ELT73505.1| hypothetical protein BURCENK562V_0636 [Burkholderia cenocepacia K56-2Valvano]	A.TTSTTTSAGTASTSTTATAGT TAAPAPTAS[+568.212]AP EAAAKPAK.T	HexNAc(2)Hex(1)	Yes	1416.6862	3	4248.0439	4248.026	4.2	NRagged	273.6	207.2	53	Nsco_20191108_BC_ZIC_HILIC_K56_2_typs in_B2.20180.20180.3	45.99	Trypsin	B2	S151	111
BCAL2161	>gi|443595154|gb|ELT63756.1| hypothetical protein BURCENK562V_1535 [Burkholderia cenocepacia K56-2Valvano]	S.SVAPPLQGDGAAPGGAS[+ 568.212]WPAPPPASGPA PGLPASSVQGT[+568.212] P.-	HexNAc(2)Hex(1) ,HexNAc(2)Hex(1)	Yes	1570.0799	3	4708.2251	4708.205	4.3	Non	281.7	234.9	31.41	Nsco_20191108_BC_ZIC_HILIC_K56_2_pep sin_B3_reinject.52747.52747.3	119.4	Pepsin	B3	S391	112
BCAL2161	>gi|443595154|gb|ELT63756.1| hypothetical protein BURCENK562V_1535 [Burkholderia cenocepacia K56-2Valvano]	S.VAPPLQGDGAAPGGAS[+ 568.212]WPAPPPASGPA PGLPASSVQGT[+568.212] P.-	HexNAc(2)Hex(1)	Partial (1 of 2)	1541.0661	3	4621.1837	4621.173	2.4	Non	295.7	239.6	17.02	Nsco_20191108_BC_ZIC_HILIC_K56_2_pep sin_B3_reinject.52161.52161.3	117.7	Pepsin	B3	S391	113
BCAL2276	>gi|443595650|gb|ELT64220.1| hypothetical protein BURCENK562V_2512 [Burkholderia cenocepacia K56-2Valvano]	K.STIDTAAS[+568.212]NA GVPVSSVNYIVHDAGK.G	HexNAc(2)Hex(1)	Yes	1047.8408	3	3141.508	3141.496	3.9	Non	346.6	279.4	18.21	Nsco_20191108_BC_ZIC_HILIC_K56_2_ther molysin_B3.44492.44492.3	90.18	Thermolysin	B3	S353	114
BCAL2398	>gi|443599855|gb|ELT68098.1| PRC-barrel domain protein [Burkholderia cenocepacia K56-2Valvano]	K.ASPPYAADKPIVAVFPPV VPAAPAS[+568.212]SASA TR.-	HexNAc(2)Hex(1)	Yes	1191.9507	3	3573.8377	3573.821	4.6	Non	288.5	248.9	19.9	Nsco_20191108_BC_ZIC_HILIC_K56_2_ther molysin_B3.52470.52470.3	104.7	Thermolysin	B3	S315	115

BCAL2449	>gi|443591735|gb|ELT60603.1| diguanylate cyclase (GGDEF) domain protein [Burkholderia cenocepacia K56-2Valvano]	R.RTGSVNNAPGAFSAS[+568.212]GVYPIAER.V	HexNAc(2)Hex(1)	Yes	964.1344	3	2890.3886	2889.375	3.6	Specific	405.8	312.8	33.91	Nsco_20191108_BC_ZIC_HILIC_K56_2_typsin_B3.31102.31102.3	65.91	Trypsin	B3	S254	116
BCAL2466	>gi|443591728|gb|ELT60596.1| ecotin [Burkholderia cenocepacia K56-2Valvano]	A.APAS[+568.212]APAVPAESIKMFPQAAAGQQR.V	HexNAc(2)Hex(1)	Yes	1021.842	3	3063.5114	3062.499	3.1	NRagged	168.4	159.9	159.94	Nsco_20191108_BC_ZIC_HILIC_K56_2_typsin_B1.36665.36665.3	76.28	Trypsin	B1	S13	117
BCAL2629	>gi|443603168|gb|ELT71192.1| bifunctional uroporphyrinogen-III synthetase/uroporphyrin-III C-methyltransferase, partial [Burkholderia cenocepacia K56-2Valvano]	K.AAPADAAS[+568.212]SVAAGEPR.W	HexNAc(2)Hex(1)	Yes	1004.9679	2	2008.9285	2008.919	4.6	Non	194.2	80.45	27.14	Nsco_20191108_BC_ZIC_HILIC_K56_2_thermolysin_B3.16955.16955.2	39.37	Thermolysin	B3	S546	118
BCAL2629	>gi|443603168|gb|ELT71192.1| bifunctional uroporphyrinogen-III synthetase/uroporphyrin-III C-methyltransferase, partial [Burkholderia cenocepacia K56-2Valvano]	K.AAPADAAS[+568.212]SVAAGEPRWK.V	HexNAc(2)Hex(1)	Yes	775.0395	3	2323.1038	2323.094	4.4	Non	255	123.2	34.56	Nsco_20191108_BC_ZIC_HILIC_K56_2_thermolysin_B2_.25473.25473.3	50.65	Thermolysin	B2	S546	119
BCAL2629	>gi|443603168|gb|ELT71192.1| bifunctional uroporphyrinogen-III synthetase/uroporphyrin-III C-methyltransferase, partial [Burkholderia cenocepacia K56-2Valvano]	H.AAPKAAPADAAS[+568.212]SVAAGEPRWK.V	HexNAc(2)Hex(1)	Yes	897.4465	3	2690.325	2690.316	3.5	Non	242.7	185.5	25.72	Nsco_20191108_BC_ZIC_HILIC_K56_2_thermolysin_B1.20913.20913.3	44.23	Thermolysin	B1	S546	120
BCAL2629	>gi|443603168|gb|ELT71192.1| bifunctional uroporphyrinogen-III synthetase/uroporphyrin-III C-methyltransferase, partial [Burkholderia cenocepacia K56-2Valvano]	E.AIVPHAAPKAAPADAAS[+568.212]SVAAGEPRW.K	HexNAc(2)Hex(1)	Partial	770.6379	4	3079.5298	3079.522	2.6	Non	198.5	198.5	17.54	Nsco_20191108_BC_ZIC_HILIC_K56_2_pepsin_B3_reinject.31319.31319.4	64.21	Pepsin	B3	NA	121
BCAL2820	>gi|443594108|gb|ELT62784.1| outer membrane multidrug efflux protein OprB [Burkholderia cenocepacia K56-2Valvano]	K.RPDAPVAQAYPAS[+568.212]GVYATQPGAAGAR.S	HexNAc(2)Hex(1)	Yes	1037.84	3	3111.5053	3110.491	3.4	Non	590.7	510.1	170.93	Nsco_20191108_BC_ZIC_HILIC_K56_2_thermolysin_B3.26573.26573.3	57.17	Thermolysin	B3	S39	122
BCAL2820	>gi|443594108|gb|ELT62784.1| outer membrane multidrug efflux protein OprB [Burkholderia cenocepacia K56-2Valvano]	D.YDKAAAPAPAS[+568.212]ATATNG.-	HexNAc(2)Hex(1)	Yes	1072.9926	2	2144.9779	2144.972	2.9	Non	223.1	100.2	97.92	Nsco_20191108_BC_ZIC_HILIC_K56_2_pepsin_B2.12672.12672.2	32.21	Pepsin	B2	S501	123
BCAL2974	>gi|443594432|gb|ELT63086.1| hypothetical protein BURCENK562V_0304 [Burkholderia cenocepacia K56-2Valvano]	P.FAAS[+568.212]APSQKYQGSKK.S	HexNAc(2)Hex(1)	Yes	1083.5312	2	2166.0551	2166.045	4.7	Non	204.3	71.42	14.81	Nsco_20191108_BC_ZIC_HILIC_K56_2_thermolysin_B1.5607.5607.2	20.06	Thermolysin	B1	S91	124
BCAL2974	>gi|443594432|gb|ELT63086.1| hypothetical protein BURCENK562V_0304 [Burkholderia cenocepacia K56-2Valvano]	P.FAAS[+568.212]APSQKYQGSKKSA.L	HexNAc(2)Hex(1)	Yes	581.786	4	2324.1221	2324.114	3.5	Non	691.1	577.5	116.79	Nsco_20191108_BC_ZIC_HILIC_K56_2_thermolysin_B2_.5731.5731.4	20.96	Thermolysin	B2	S91	125
BCAL2974	>gi|443594432|gb|ELT63086.1| hypothetical protein BURCENK562V_0304 [Burkholderia cenocepacia K56-2Valvano]	R.LNEHPQMPFAAS[+568.212]APSQK.Y	HexNAc(2)Hex(1)	Yes	1211.0643	2	2421.1212	2421.113	3.6	Specific	515.3	320	93.9	Nsco_20191108_BC_ZIC_HILIC_K56_2_typsin_B3.22620.22620.2	50.4	Trypsin	B3	S91	126
BCAL2974	>gi|443594432|gb|ELT63086.1| hypothetical protein BURCENK562V_0304 [Burkholderia cenocepacia K56-2Valvano]	L.NEHPQMPFAAS[+568.212]APSQKYQG.S	HexNAc(2)Hex(1)	Yes	886.0642	3	2656.178	2656.172	2.3	CRagged	257.8	178.7	21.09	Nsco_20191108_BC_ZIC_HILIC_K56_2_pepsin_B2.23934.23934.3	51.68	Pepsin	B2	S91	127
BCAL2974	>gi|443594432|gb|ELT63086.1| hypothetical protein BURCENK562V_0304 [Burkholderia cenocepacia K56-2Valvano]	L.NEHPQMPFAAS[+568.212]APSQKYQGSKKSAL.R	HexNAc(2)Hex(1)	Yes	818.3949	4	3270.5576	3270.547	3.2	Specific	1143	1119	147.53	Nsco_20191108_BC_ZIC_HILIC_K56_2_pepsin_B3_reinject.18154.18154.4	40.6	Pepsin	B3	S91	128
BCAL2974	>gi|443594432|gb|ELT63086.1| hypothetical protein BURCENK562V_0304 [Burkholderia cenocepacia K56-2Valvano]	M.PFAAS[+568.212]APSQKYQGSKKSAL.R	HexNAc(2)Hex(1)	Yes	634.5717	4	2535.2651	2534.251	4.3	NRagged	432.1	412.2	96.69	Nsco_20191108_BC_ZIC_HILIC_K56_2_pepsin_B1.12465.12465.4	31.43	Pepsin	B1	S91	129
BCAL2974	>gi|443594432|gb|ELT63086.1| hypothetical protein BURCENK562V_0304 [Burkholderia cenocepacia K56-2Valvano]	F.RLNEHPQMPFAAS[+568.212]APSQ.K	HexNAc(2)Hex(1)	Yes	817.0473	3	2449.1274	2449.119	3.5	CRagged	263.8	162	63.15	Nsco_20191108_BC_ZIC_HILIC_K56_2_pepsin_B1.25394.25394.3	54.25	Pepsin	B1	S91	130

BCAL2974	>gi|443594432|gb|ELT63086.1| hypothetical protein BURCENK562V_0304 [Burkholderia cenocepacia K56-2Valvano]	F.RLNEHPQMPFAAS[+568.212]APSQKYQGSKKSAL.R	HexNAc(2)Hex(1)	Yes	708.7548	5	3539.7451	3539.732	3.6	Specific	1159	1138	125.6	Nsco_20191108_BC_ZIC_HILIC_K56_2_pepsin_B3_reinject.18210.18210.5	40.69	Pepsin	B3	S91	131
BCAL2983A	>gi|443599451|gb|ELT67730.1| PF11180 family protein [Burkholderia cenocepacia K56-2Valvano]	S.ADASAPVAGT[+568.212]RPAVTSLSGGASSAASGAVATDAAAQGNVAELTQMLHDGR.I	HexNAc(2)Hex(1)	Partial	1301.8793	4	5204.4953	5204.477	3.5	NRagged	310	303.4	139.62	Nsco_20191108_BC_ZIC_HILIC_K56_2_typsin_B3.55925.55925.4	108.8	Trypsin	B3	NA	132
BCAL3017	>gi|443591015|gb|ELT59947.1| hypothetical protein BURCENK562V_1182 [Burkholderia cenocepacia K56-2Valvano]	A.DTASGS[+568.212]DAQASC[+57.021]AIAYVTGVGGSPR.G	HexNAc(2)Hex(1)	Partial	1483.6553	2	2966.3033	2966.306	-0.8	NRagged	294.6	196.1	51.03	Nsco_20191108_BC_ZIC_HILIC_K56_2_typsin_B2.48932.48932.2	98.49	Trypsin	B2	NA	133
BCAL3033	>gi|443591005|gb|ELT59937.1| outer-membrane lipoprotein carrier protein [Burkholderia cenocepacia K56-2Valvano]	K.GAS[+568.212]AAQAAPKPTDNSSGTFVFAR.P	HexNAc(2)Hex(1)	Yes	1410.1691	2	2819.3309	2819.322	3.2	Specific	458.8	247.8	247.82	Nsco_20191108_BC_ZIC_HILIC_K56_2_typsin_B1.25150.25150.2	55.93	Trypsin	B1	S68	134
BCAL3033	>gi|443591005|gb|ELT59937.1| outer-membrane lipoprotein carrier protein [Burkholderia cenocepacia K56-2Valvano]	K.GAS[+568.212]AAQAAPKPTDNSSGTFVFARPGK.F	HexNAc(2)Hex(1)	Yes	776.1305	4	3101.5	3101.491	2.9	Non	655.3	655.3	655.26	Nsco_20191108_BC_ZIC_HILIC_K56_2_thermolysin_B3.22747.22747.4	50.25	Thermolysin	B3	S68	135
BCAL3033	>gi|443591005|gb|ELT59937.1| outer-membrane lipoprotein carrier protein [Burkholderia cenocepacia K56-2Valvano]	Q.IVKAPAKGAS[+568.212]AAQAAPKPTDN.S	HexNAc(2)Hex(1)	Yes	859.1134	3	2575.3257	2574.315	3.1	Non	184.6	144.6	144.61	Nsco_20191108_BC_ZIC_HILIC_K56_2_pepsin_B2.8772.8772.3	25.73	Pepsin	B2	S68	136
BCAL3033	>gi|443591005|gb|ELT59937.1| outer-membrane lipoprotein carrier protein [Burkholderia cenocepacia K56-2Valvano]	Q.IVKAPAKGAS[+568.212]AAQAAPKPTDNSSGT.F	HexNAc(2)Hex(1)	Yes	727.3692	4	2906.4548	2906.448	2.5	Non	907.1	606.5	449.61	Nsco_20191108_BC_ZIC_HILIC_K56_2_thermolysin_B2_.9118.9118.4	25.97	Thermolysin	B2	S68	137
BCAL3033	>gi|443591005|gb|ELT59937.1| outer-membrane lipoprotein carrier protein [Burkholderia cenocepacia K56-2Valvano]	Q.IVKAPAKGAS[+568.212]AAQAAPKPTDNSSGTF.V	HexNAc(2)Hex(1)	Yes	764.1369	4	3053.5259	3053.516	3.2	NRagged	1186	1133	528.59	Nsco_20191108_BC_ZIC_HILIC_K56_2_pepsin_B2.15586.15586.4	36.98	Pepsin	B2	S68	138
BCAL3033	>gi|443591005|gb|ELT59937.1| outer-membrane lipoprotein carrier protein [Burkholderia cenocepacia K56-2Valvano]	Q.IVKAPAKGAS[+568.212]AAQAAPKPTDNSSGTFV.F	HexNAc(2)Hex(1)	Yes	788.9039	4	3152.5937	3152.585	2.9	Non	855.2	855.2	397.28	Nsco_20191108_BC_ZIC_HILIC_K56_2_thermolysin_B2_.19314.19314.4	40.68	Thermolysin	B2	S68	139
BCAL3166	>gi|443602480|gb|ELT70558.1| BON domain protein [Burkholderia cenocepacia K56-2Valvano]	A.ATS[+568.212]APKAAAKTAKKANRKLGY.A	HexNAc(2)Hex(1)	Yes	905.826	3	2715.4635	2714.457	1.1	Non	186.7	115.5	15.17	Nsco_20191108_BC_ZIC_HILIC_K56_2_pepsin_B2.4419.4419.3	18.47	Pepsin	B2	S40	140
BCAL3166	>gi|443602480|gb|ELT70558.1| BON domain protein [Burkholderia cenocepacia K56-2Valvano]	A.PAAATS[+568.212]APKAAAKTAKKANRKLGY.A	HexNAc(2)Hex(1)	Yes	739.1546	4	2953.5967	2953.584	4.3	Non	392.3	392.3	68.75	Nsco_20191108_BC_ZIC_HILIC_K56_2_pepsin_B2.6995.6995.4	23.02	Pepsin	B2	S40	141
BCAL3166	>gi|443602480|gb|ELT70558.1| BON domain protein [Burkholderia cenocepacia K56-2Valvano]	A.TEAPAAATS[+568.212]APKAAAKTAKKANRKLGY.A	HexNAc(2)Hex(1)	Yes	1085.9101	3	3255.7156	3254.711	0.3	Non	285.4	157.3	27.28	Nsco_20191108_BC_ZIC_HILIC_K56_2_pepsin_B3_reinject.11243.11243.3	29.3	Pepsin	B3	S40	142
BCAL3377	>gi|443595379|gb|ELT63971.1| SmpA/OmlA family protein, partial [Burkholderia cenocepacia K56-2Valvano]	A.AAKKASEAAAAAS[+568.212]AAQA.A	HexNAc(2)Hex(1)	Yes	1028.5033	2	2055.9993	2055.993	3.2	Non	181.1	71.41	64.93	Nsco_20191108_BC_ZIC_HILIC_K56_2_thermolysin_B2_.6537.6537.2	22.1	Thermolysin	B2	S152	143
BCAL3377	>gi|443595379|gb|ELT63971.1| SmpA/OmlA family protein, partial [Burkholderia cenocepacia K56-2Valvano]	D.IDGDRGGKKAKAAAAAKKAS[+568.212]EAAA.A	HexNAc(2)Hex(1)	Yes	706.8718	4	2824.4656	2824.453	4.3	Non	240.3	240.3	240.3	Nsco_20191108_BC_ZIC_HILIC_K56_2_pepsin_B2.3463.3463.3	17.05	Pepsin	B2	S145	144
BCAL3469	>gi|443597454|gb|ELT65879.1| cell division protein FtsL [Burkholderia cenocepacia K56-2Valvano]	A.IDAPIPAS[+568.212]ADTAGKGKGGAR.-	HexNAc(2)Hex(1)	Yes	606.0576	4	2421.2087	2421.199	4	Non	780.2	649.5	197.28	Nsco_20191108_BC_ZIC_HILIC_K56_2_thermolysin_B1.12654.12654.4	30.68	Thermolysin	B1	S101	145
BCAM0505	>gi|443591075|gb|ELT60005.1| BON domain protein [Burkholderia cenocepacia K56-2Valvano]	A.HAQAS[+568.212]STDSGMASESNQPVTDTWITTK.V	HexNAc(2)Hex(1)	Yes	1140.1741	3	3418.5077	3418.496	3.4	Non	505.8	485.6	37.17	Nsco_20191108_BC_ZIC_HILIC_K56_2_thermolysin_B3.30160.30160.3	63.86	Thermolysin	B3	S32	146
BCAM0505	>gi|443591075|gb|ELT60005.1| BON domain protein [Burkholderia cenocepacia K56-2Valvano]	A.QAS[+568.212]STDSGMASESNQPVTDTW.I	HexNAc(2)Hex(1)	Yes	1384.0723	2	2767.1373	2767.126	4.2	Non	269.6	185.6	21.49	Nsco_20191108_BC_ZIC_HILIC_K56_2_thermolysin_B1.53831.53831.2	119.6	Thermolysin	B1	S32	147

<table>
<tr><td>BCAM0505</td><td>>gi|443591075|gb|ELT60005.1| BON domain protein [Burkholderia cenocepacia K56-2Valvano]</td><td>A.QAS[+568.212]STDSGMASESNQPVTDTWITTK.V</td><td>HexNAc(2)Hex(1)</td><td>Yes</td><td>1606.2004</td><td>2</td><td>3211.3934</td><td>3210.4</td><td>-3.1</td><td>Non</td><td>327.9</td><td>214</td><td>41.1</td><td>Nsco_20191108_BC_ZIC_HILIC_K56_2_thermolysin_B3.39356.39356.2</td><td>80.95</td><td>Thermolysin</td><td>B3</td><td>S32</td><td>148</td></tr>
<tr><td>BCAM0690</td><td>>gi|443592239|gb|ELT61058.1| OmpA family protein [Burkholderia cenocepacia K56-2Valvano]</td><td>R.ALIDAGVPAS[+568.212]SVFAAAFGSEQPVSSNADDE[illegible]RAK.N</td><td>HexNAc(2)Hex(1)</td><td>Partial</td><td>1301.9476</td><td>3</td><td>3903.8281</td><td>3902.83</td><td>-1.4</td><td>Specific</td><td>159.3</td><td>114.1</td><td>15.7</td><td>Nsco_20191108_BC_ZIC_HILIC_K56_2_typsin_B2.58333.58333.3</td><td>115.8</td><td>Trypsin</td><td>B2</td><td>NA</td><td>149</td></tr>
<tr><td>BCAM0713</td><td>>gi|443605703|gb|ELT73537.1| cation efflux system protein CzcA [Burkholderia cenocepacia K56-2Valvano]</td><td>K.RLPIALPAPAAGAS[+568.212]APLAAAPYVPLAELATIDVAPGPNQISREDGK.R</td><td>HexNAc(2)Hex(1)</td><td>Yes</td><td>1275.4307</td><td>4</td><td>5098.7008</td><td>5098.683</td><td>3.4</td><td>Specific</td><td>1132</td><td>1132</td><td>379.2</td><td>Nsco_20191108_BC_ZIC_HILIC_K56_2_typsin_B2.70574.70574.4</td><td>143.2</td><td>Trypsin</td><td>B2</td><td>S802</td><td>150</td></tr>
<tr><td>BCAM0996</td><td>>gi|443592743|gb|ELT61524.1| sporulation and cell division repeat protein [Burkholderia cenocepacia K56-2Valvano]</td><td>R.DDDVSDVQAGVAHDEPPAS[+568.212]DTTVAAAPAPAPK.D</td><td>HexNAc(2)Hex(1)</td><td>Yes</td><td>1228.2304</td><td>3</td><td>3682.6767</td><td>3682.661</td><td>4.1</td><td>Non</td><td>472.5</td><td>446.1</td><td>66.25</td><td>Nsco_20191108_BC_ZIC_HILIC_K56_2_thermolysin_B3.35728.35728.3</td><td>74.11</td><td>Thermolysin</td><td>B3</td><td>S104</td><td>151</td></tr>
<tr><td>BCAM0996</td><td>>gi|443592743|gb|ELT61524.1| sporulation and cell division repeat protein [Burkholderia cenocepacia K56-2Valvano]</td><td>K.PAAPAAKPAAPKPAPATVANAGAASPDSGDAS[+568.212]SPASPAGAR.F</td><td>HexNAc(2)Hex(1)</td><td>Yes</td><td>1040.7653</td><td>4</td><td>4160.0394</td><td>4160.027</td><td>3.1</td><td>Specific</td><td>1003</td><td>1003</td><td>77.16</td><td>Nsco_20191108_BC_ZIC_HILIC_K56_2_typsin_B2.19309.19309.4</td><td>44.26</td><td>Trypsin</td><td>B2</td><td>S174</td><td>152</td></tr>
<tr><td>BCAM0996</td><td>>gi|443592743|gb|ELT61524.1| sporulation and cell division repeat protein [Burkholderia cenocepacia K56-2Valvano]</td><td>K.PAAPKPAPATVANAGAASPDSGDAS[+568.212]SPASPAGAR.F</td><td>HexNAc(2)Hex(1)</td><td>Yes</td><td>1185.2319</td><td>3</td><td>3553.6811</td><td>3553.678</td><td>1</td><td>Specific</td><td>456</td><td>436.8</td><td>46.57</td><td>Nsco_20191108_BC_ZIC_HILIC_K56_2_typsin_B3.20127.20127.3</td><td>45.91</td><td>Trypsin</td><td>B3</td><td>S174</td><td>153</td></tr>
<tr><td>BCAM0996</td><td>>gi|443592743|gb|ELT61524.1| sporulation and cell division repeat protein [Burkholderia cenocepacia K56-2Valvano]</td><td>P.VTDDIAIDIPNRPAHQAVAPRDDDVSDVQAGVAHDEPPAS[+568.212]DTTVAAAPAPAPK.D</td><td>HexNAc(2)Hex(1)</td><td>Yes</td><td>1187.7791</td><td>5</td><td>5934.8662</td><td>5932.848</td><td>1.9</td><td>NRagged</td><td>727.3</td><td>559.4</td><td>65.07</td><td>Nsco_20191108_BC_ZIC_HILIC_K56_2_typsin_B3.38823.38823.5</td><td>79.6</td><td>Trypsin</td><td>B3</td><td>S104</td><td>154</td></tr>
<tr><td>BCAM1550</td><td>>gi|443602195|gb|ELT70283.1| peptidoglycan-associated lipoprotein [Burkholderia cenocepacia K56-2Valvano]</td><td>K.TPENAGAAPEPSS[+568.212]ETVATVTADDLNNPNSPLAK.R</td><td>HexNAc(2)Hex(1)</td><td>Yes</td><td>1282.936</td><td>3</td><td>3846.7934</td><td>3846.778</td><td>4.1</td><td>Specific</td><td>824.7</td><td>381.5</td><td>28.07</td><td>Nsco_20191108_BC_ZIC_HILIC_K56_2_typsin_B2.45430.45430.3</td><td>92.51</td><td>Trypsin</td><td>B2</td><td>S37</td><td>155</td></tr>
<tr><td>BCAM1550</td><td>>gi|443602195|gb|ELT70283.1| peptidoglycan-associated lipoprotein [Burkholderia cenocepacia K56-2Valvano]</td><td>K.TPENAGAAPEPSS[+568.212]ETVATVTADDLNNPNSPLAKR.S</td><td>HexNAc(2)Hex(1)</td><td>Yes</td><td>1334.9677</td><td>3</td><td>4002.8884</td><td>4002.879</td><td>2.5</td><td>Specific</td><td>339.5</td><td>201.2</td><td>18.88</td><td>Nsco_20191108_BC_ZIC_HILIC_K56_2_typsin_B1.40337.40337.3</td><td>82.58</td><td>Trypsin</td><td>B1</td><td>S37</td><td>156</td></tr>
<tr><td>BCAM1669</td><td>>gi|443598083|gb|ELT66474.1| hypothetical protein BURCENK562V_A2079 [Burkholderia cenocepacia K56-2Valvano]</td><td>R.VHGIDNSGAGS[+568.212]QPAATVEGGAPVV.R</td><td>HexNAc(2)Hex(1)</td><td>Yes</td><td>1380.1554</td><td>2</td><td>2759.3035</td><td>2758.29</td><td>3.6</td><td>Non</td><td>426.9</td><td>259.6</td><td>9.99</td><td>Nsco_20191108_BC_ZIC_HILIC_K56_2_thermolysin_B3.34068.34068.2</td><td>70.94</td><td>Thermolysin</td><td>B3</td><td>S73</td><td>157</td></tr>
<tr><td>BCAM1669</td><td>>gi|443598083|gb|ELT66474.1| hypothetical protein BURCENK562V_A2079 [Burkholderia cenocepacia K56-2Valvano]</td><td>R.VHGIDNSGAGS[+568.212]QPAATVEGGAPVVR.A</td><td>HexNAc(2)Hex(1)</td><td>Yes</td><td>972.1378</td><td>3</td><td>2914.3988</td><td>2914.391</td><td>2.6</td><td>Non</td><td>621.3</td><td>532.4</td><td>132.54</td><td>Nsco_20191108_BC_ZIC_HILIC_K56_2_thermolysin_B3.24364.24364.3</td><td>53.07</td><td>Thermolysin</td><td>B3</td><td>S73</td><td>158</td></tr>
<tr><td>BCAM1789</td><td>>gi|443592202|gb|ELT61024.1| putative esterase [Burkholderia cenocepacia K56-2Valvano]</td><td>A.TPPAPAPAAS[+568.212]PAQPPAVQTATTPSTAQEPSVNPGSSVVLR.T</td><td>HexNAc(2)Hex(1)</td><td>Yes</td><td>1473.0701</td><td>3</td><td>4417.1957</td><td>4416.194</td><td>-0.4</td><td>Non</td><td>570.4</td><td>217.4</td><td>66.06</td><td>Nsco_20191108_BC_ZIC_HILIC_K56_2_thermolysin_B3.44672.44672.3</td><td>90.46</td><td>Thermolysin</td><td>B3</td><td>S32</td><td>159</td></tr>
<tr><td>BCAM2055</td><td>>gi|443591388|gb|ELT60286.1| type III secretion outer membrane pore, YscC/HrcC-like family protein [Burkholderia cenocepacia K56-2Valvano]</td><td>K.DTAAS[+568.212]QPAATTAGVVTHVDEHH.-</td><td>HexNAc(2)Hex(1)</td><td>Yes</td><td>696.5697</td><td>4</td><td>2783.2568</td><td>2783.249</td><td>2.8</td><td>Non</td><td>373.3</td><td>327.1</td><td>26.78</td><td>Nsco_20191108_BC_ZIC_HILIC_K56_2_thermolysin_B3.20172.20172.4</td><td>45.22</td><td>Thermolysin</td><td>B3</td><td>S664</td><td>160</td></tr>
<tr><td>BCAM2063</td><td>>gi|443595086|gb|ELT63692.1| carbohydrate-selective porin, OprB family [Burkholderia cenocepacia K56-2Valvano]</td><td>A.SSPAAAEPAAGAS[+568.212]DAAAPAQQAADAAAPAPTGFWER.S</td><td>HexNAc(2)Hex(1)</td><td>Yes</td><td>1306.934</td><td>3</td><td>3918.7875</td><td>3918.779</td><td>2.2</td><td>NRagged</td><td>384.3</td><td>376.9</td><td>134.79</td><td>Nsco_20191108_BC_ZIC_HILIC_K56_2_typsin_B3.48808.48808.3</td><td>96.67</td><td>Trypsin</td><td>B3</td><td>S46</td><td>161</td></tr>
<tr><td>BCAM2064</td><td>>gi|443595102|gb|ELT63708.1| alpha,alpha-trehalase [Burkholderia cenocepacia K56-2Valvano]</td><td>A.DNANQAAAQAVGQSAIPATTAAAAAPASGTLPPPPSQLYGDLFVAVQT[+568.212]AQLYPDQK.T</td><td>HexNAc(2)Hex(1)</td><td>Partial</td><td>1526.2583</td><td>4</td><td>6102.0114</td><td>6101.999</td><td>2</td><td>NRagged</td><td>595.4</td><td>553.5</td><td>29.48</td><td>Nsco_20191108_BC_ZIC_HILIC_K56_2_typsin_B2.77596.77596.4</td><td>160.7</td><td>Trypsin</td><td>B2</td><td>NA</td><td>162</td></tr>
<tr><td>BCAM2289</td><td>>gi|443599704|gb|ELT67963.1| hypothetical protein BURCENK562V_A2967 [Burkholderia cenocepacia K56-2Valvano]</td><td>R.VHGADTSGYGAQPAPLVHSGAPAAAS[+568.212]SNAR.D</td><td>HexNAc(2)Hex(1)</td><td>Yes</td><td>1129.2016</td><td>3</td><td>3385.5901</td><td>3385.578</td><td>3.6</td><td>Specific</td><td>471.9</td><td>429.5</td><td>40.53</td><td>Nsco_20191108_BC_ZIC_HILIC_K56_2_typsin_B1.16993.16993.3</td><td>40.39</td><td>Trypsin</td><td>B1</td><td>S63</td><td>163</td></tr>
</table>

BCAM2334	>gi|443606050|gb|ELT73855.1| auxiliary transport protein, membrane fusion protein (MFP) domain protein, partial [Burkholderia cenocepacia K56-2Valvano]	R.VHDGVAS[+568.212]DAEAAAAAIIRENQGG.-	HexNAc(2)Hex(1)	Yes	930.7708	3	2790.298	2790.291	2.4	Non	592.5	592.5	592.51	Nsco_20191108_BC_ZIC_HILIC_K56_2_thermolysin_B3.49647.49647.3	99.38	Thermolysin	B3	S380	164
BCAM2378	>gi|443594879|gb|ELT63498.1| X-Pro dipeptidyl-peptidase (S15 family) [Burkholderia cenocepacia K56-2Valvano]	A.LAQGQQEGAS[+568.212]AAAGTANTPS.L	HexNAc(2)Hex(1)	Yes	1199.5439	2	2398.0806	2398.074	2.8	Non	437.4	304.9	139.31	Nsco_20191108_BC_ZIC_HILIC_K56_2_thermolysin_B1.16412.16412.2	36.59	Thermolysin	B1	S48	165
BCAM2378	>gi|443594879|gb|ELT63498.1| X-Pro dipeptidyl-peptidase (S15 family) [Burkholderia cenocepacia K56-2Valvano]	R.TPVIVLASPYYAGLADSPNHDVDVELDGTPHPAATAGAAAS[+568.212]ASAR.I	HexNAc(2)Hex(1)	Yes	1246.8543	4	4984.3952	4984.386	1.9	Specific	1038	1038	76.39	Nsco_20191108_BC_ZIC_HILIC_K56_2_typsin_B1.56596.56596.4	109.8	Trypsin	B1	S174	166
BCAM2680	>gi|443603690|gb|ELT71680.1| hypothetical protein BURCENK562V_A2523, partial [Burkholderia cenocepacia K56-2Valvano]	A.AAQQAS[+568.212]VPAAASAT[+568.212]VVVK.A	HexNAc(2)Hex(1),HexNAc(2)Hex(1)	Partial (1 of 2)	1403.1835	2	2805.3598	2805.351	3.1	Non	351.2	187.3	187.32	Nsco_20191108_BC_ZIC_HILIC_K56_2_thermolysin_B1.24775.24775.2	51.53	Thermolysin	B1	S858	167
BCAM2680	>gi|443603690|gb|ELT71680.1| hypothetical protein BURCENK562V_A2523, partial [Burkholderia cenocepacia K56-2Valvano]	A.AAQQASVPAAAS[+568.212]AT[+568.212]VVVKAAPQPQNP.V	HexNAc(2)Hex(1),HexNAc(2)Hex(1)	Partial (1 of 2)	1203.9231	3	3609.7547	3608.744	2.1	Non	194.9	194.9	74.58	Nsco_20191108_BC_ZIC_HILIC_K56_2_thermolysin_B2_.36530.36530.3	68.44	Thermolysin	B2	S858	168
BCAM2829	>gi|443591146|gb|ELT60069.1| hypothetical protein BURCENK562V_A2847 [Burkholderia cenocepacia K56-2Valvano]	D.SAMPAS[+568.212]APAAASAPASSGAT[+568.212]TAPAANGAPIA.P	HexNAc(2)Hex(1),HexNAc(2)Hex(1)	Partial (1 of 2)	1248.9023	3	3744.6923	3744.69	0.5	Non	369.3	262.4	6.65	Nsco_20191108_BC_ZIC_HILIC_K56_2_pepsin_B3_reinject.37501.37501.3	77.29	Pepsin	B3	S296	169
BCAS0089	>gi|443598997|gb|ELT67309.1| hypothetical protein BURCENK562V_B0207 [Burkholderia cenocepacia K56-2Valvano]	N.AVQQAPATKAAPAVPAS[+568.212]GQ.-	HexNAc(2)Hex(1)	Yes	1166.0856	2	2331.164	2331.156	3.3	Non	298.1	168.3	78.72	Nsco_20191108_BC_ZIC_HILIC_K56_2_thermolysin_B1.15802.15802.2	35.63	Thermolysin	B1	S99	170
BCAS0089	>gi|443598997|gb|ELT67309.1| hypothetical protein BURCENK562V_B0207 [Burkholderia cenocepacia K56-2Valvano]	A.VQQAPATKAAPAVPAS[+568.212]GQ.-	HexNAc(2)Hex(1)	Yes	1130.5662	2	2260.1251	2260.119	2.7	Non	503.1	244.5	150.76	Nsco_20191108_BC_ZIC_HILIC_K56_2_thermolysin_B1.14539.14539.2	33.71	Thermolysin	B1	S99	171
BURCENK562V_3104	>gi|443598112|gb|ELT66499.1| hypothetical protein BURCENK562V_3104 [Burkholderia cenocepacia K56-2Valvano]	G.AAADGAKDTTSS[+568.212]AVHSTKKHTKHAAKSAKSHAGSA.K	HexNAc(2)Hex(1)	Partial	797.6005	5	3983.9733	3983.954	4.8	Non	613.4	594.4	35.12	Nsco_20191108_BC_ZIC_HILIC_K56_2_pepsin_B3_reinject.3843.3843.5	16.95	Pepsin	B3	NA	172
BURCENK562V_3104	>gi|443598112|gb|ELT66499.1| hypothetical protein BURCENK562V_3104 [Burkholderia cenocepacia K56-2Valvano]	N.ASGNATGAATDAVGAAADGAKDTTSS[+568.212]AVHS.T	HexNAc(2)Hex(1)	Yes	1067.4822	3	3200.4321	3200.42	3.9	Non	302.2	245.4	8.44	Nsco_20191108_BC_ZIC_HILIC_K56_2_thermolysin_B1.29677.29677.3	60.55	Thermolysin	B1	S83	173
BURCENK562V_3104	>gi|443598112|gb|ELT66499.1| hypothetical protein BURCENK562V_3104 [Burkholderia cenocepacia K56-2Valvano]	N.ASGNATGAATDAVGAAADGAKDTTSS[+568.212]AVHSTKKH.T	HexNAc(2)Hex(1)	Yes	739.751	5	3694.7259	3694.716	2.6	Non	493.6	493.6	20.41	Nsco_20191108_BC_ZIC_HILIC_K56_2_thermolysin_B1.19879.19879.5	42.31	Thermolysin	B1	S83	174
BURCENK562V_3104	>gi|443598112|gb|ELT66499.1| hypothetical protein BURCENK562V_3104 [Burkholderia cenocepacia K56-2Valvano]	G.VGAGAQGGAGANASGNATGAATDAVGAAADGAKDTTSS[+568.212]AVHSTKK.H	HexNAc(2)Hex(1)	Yes	894.4257	5	4468.0996	4468.083	3.7	Non	1193	979.4	27.83	Nsco_20191108_BC_ZIC_HILIC_K56_2_thermolysin_B1.26977.26977.5	55.63	Thermolysin	B1	S83	175
BURCENK562V_3104	>gi|443598112|gb|ELT66499.1| hypothetical protein BURCENK562V_3104 [Burkholderia cenocepacia K56-2Valvano]	G.VGAGAQGGAGANASGNATGAATDAVGAAADGAKDTTSS[+568.212]AVHSTKKH.T	HexNAc(2)Hex(1)	Partial	768.5336	6	4606.165	4605.142	4.3	Non	957.8	881.9	18.94	Nsco_20191108_BC_ZIC_HILIC_K56_2_thermolysin_B1.24815.24815.6	51.6	Thermolysin	B1	NA	176
BURCENK562V_3104	>gi|443598112|gb|ELT66499.1| hypothetical protein BURCENK562V_3104 [Burkholderia cenocepacia K56-2Valvano]	G.VQAQTPAAGVGAGAQGGAGANASGNATGAATDAVGAAADGAKDTTSS[+568.212]AVHSTKKH.T	HexNAc(2)Hex(1)	Partial	1086.9199	5	5430.5705	5428.561	0.6	Non	335.9	335.6	6.22	Nsco_20191108_BC_ZIC_HILIC_K56_2_thermolysin_B2_.31006.31006.5	59.67	Thermolysin	B2	NA	177
BCAS0773	>gi|443593562|gb|ELT62293.1| hypothetical protein BURCENK562V_B0326 [Burkholderia cenocepacia K56-2Valvano]	A.QTDAAS[+568.212]APAAAAAQDAK.A	HexNAc(2)Hex(1)	Yes	1063.4875	2	2125.9678	2125.962	2.8	Non	295.6	96.23	71.49	Nsco_20191108_BC_ZIC_HILIC_K56_2_thermolysin_B3.10971.10971.2	29.38	Thermolysin	B3	S33	178

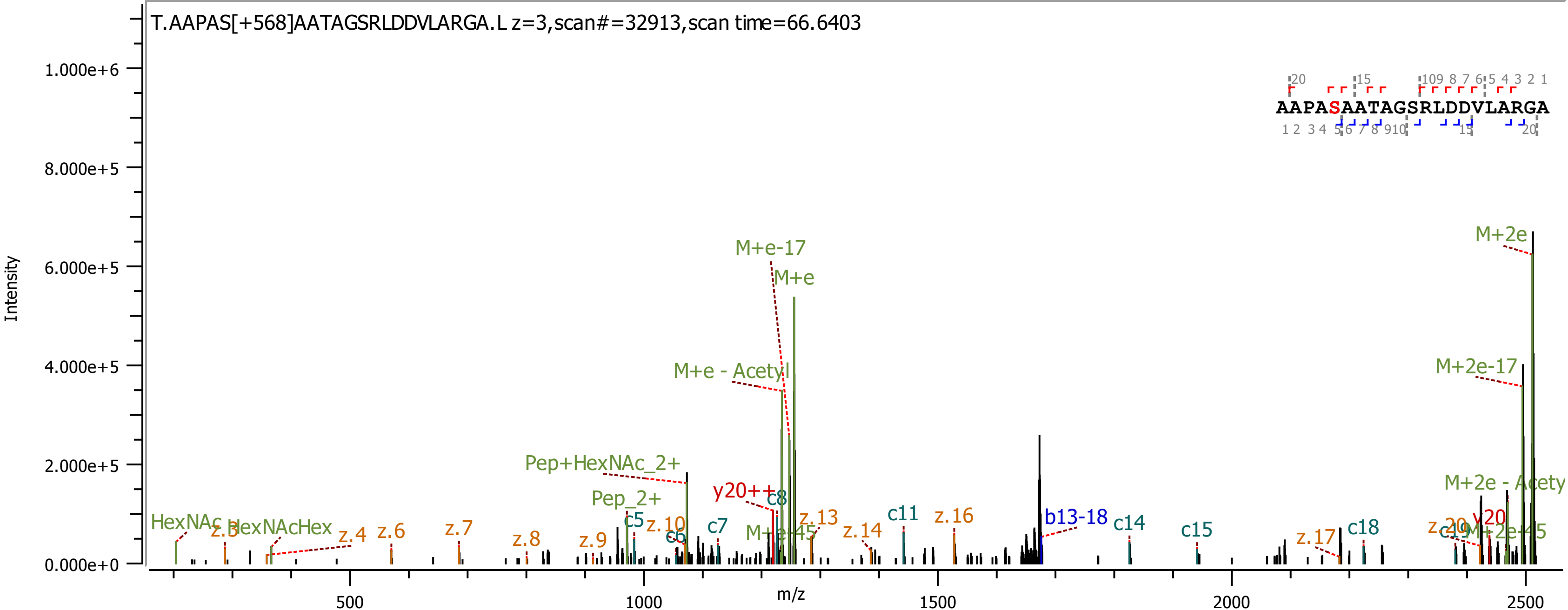
T.AAPAS[+568]AATAGSRLDDVLARGA.L z=3,scan#=32913,scan time=66.6403
AAPASAATAGSRLDDVLARGA
Intensity
m/z
1.000e+6
8.000e+5
6.000e+5
4.000e+5
2.000e+5
0.000e+0
500
1000
1500
2000
2500
M+2e
M+e-17
M+e
M+e - Acetyl
M+2e-17
Pep+HexNAc_2+
Pep_2+
y20++
c8
c5
z.10
c6
c7
M+e-45
z.13
c11
z.16
b13-18
c14
c15
c18
z.17
y20
M+2e - Acety
M+2e-45
HexNAc
z.3
HexNAcHex
z.4
z.6
z.7
z.8
z.9
z.14

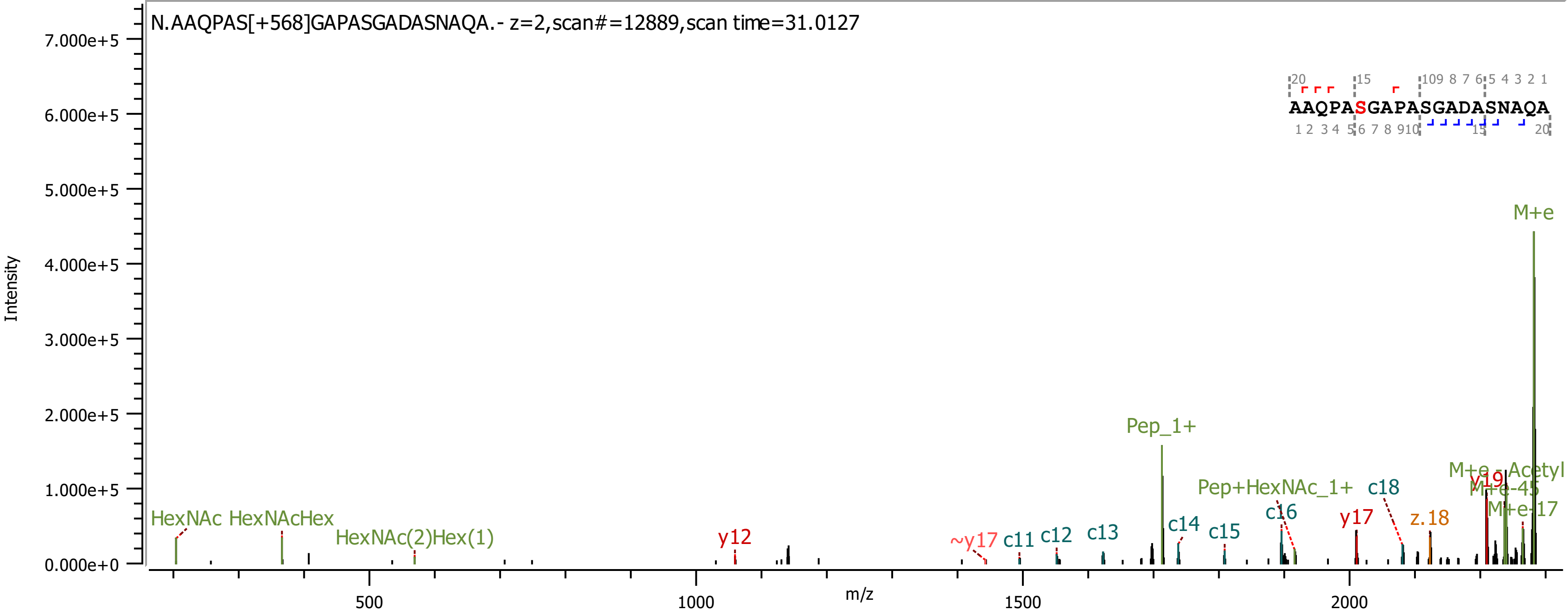

N.AAQPAS[+568]GAPASGADASNAQA.- z=2,scan#=12889,scan time=31.0127
AAQPASGAPASGADASNAQA
Intensity
m/z
HexNAc HexNAcHex
HexNAc(2)Hex(1)
y12
~y17
c11
c12
c13
Pep_1+
c14
c15
c16
Pep+HexNAc_1+
c18
y17
z.18
y19
M+e - Acetyl
M+e-45
M+e-17
M+e

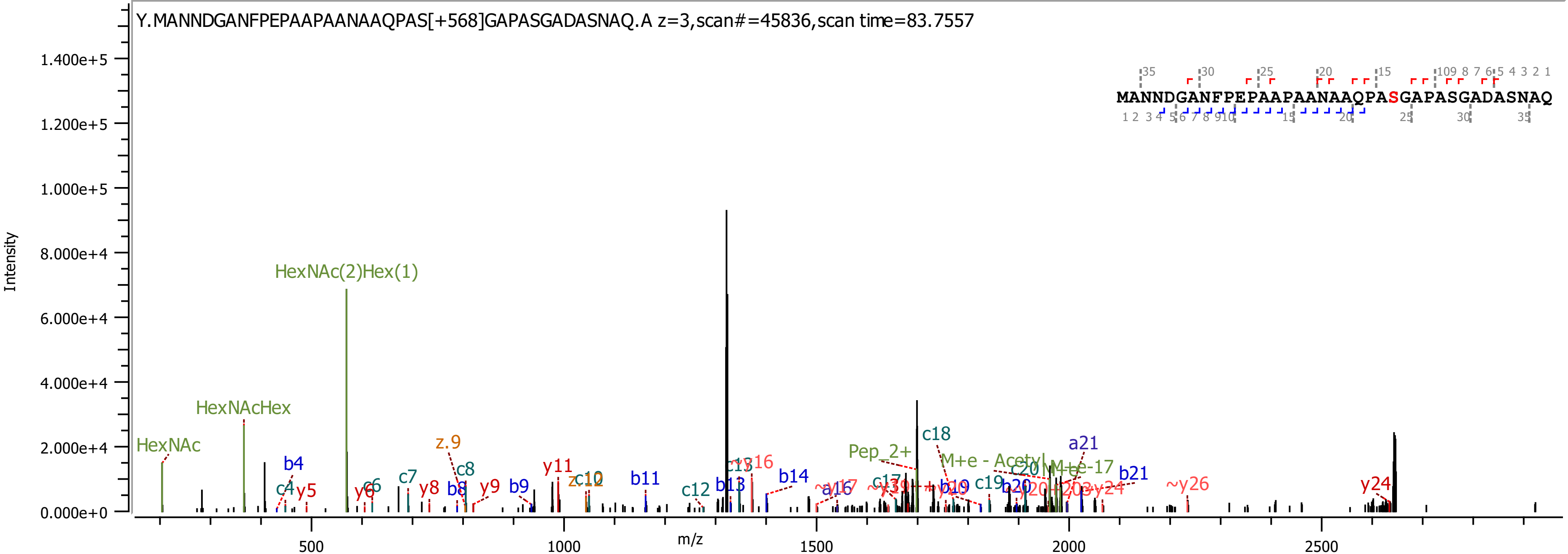

Y.MANNDGANFPEPAAPAANAAQPAS[+568]GAPASGADASNAQ.A z=3,scan#=45836,scan time=83.7557
MANNDGANFPEPAAPAANAAQPASGAPASGADASNAQ
Intensity
m/z
0.000e+0
2.000e+4
4.000e+4
6.000e+4
8.000e+4
1.000e+5
1.200e+5
1.400e+5
500
1000
1500
2000
2500
HexNAc
HexNAcHex
HexNAc(2)Hex(1)
b4
c4
y5
y6
c6
c7
y8
b8
c8
z.9
y9
b9
y11
c12
b11
c12
b13
c13
b14
c18
Pep_2+
M+e - Acetyl
M+e-17
a21
b21
c19
b20
~y26
y24

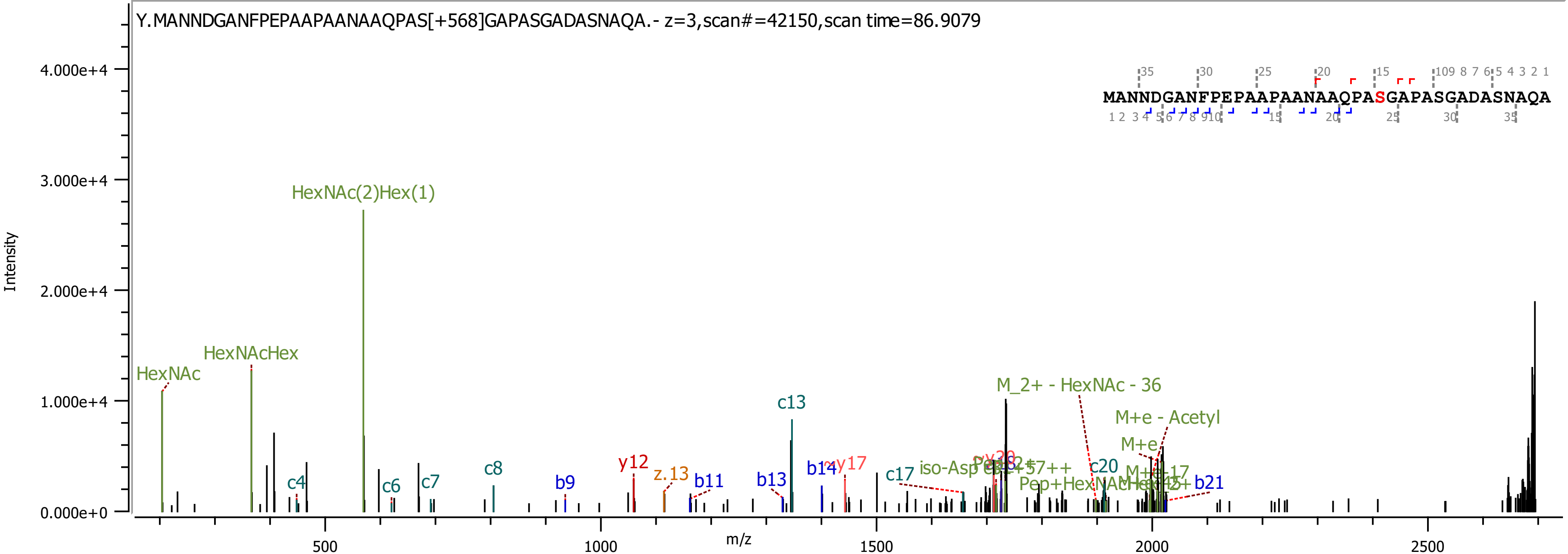

Y.MANNDGANFPEPAAPAANAAQPAS[+568]GAPASGADASNAQA.- z=3,scan#=42150,scan time=86.9079
MANNDGANFPEPAAPAANAAQPASGAPASGADASNAQA
Intensity
m/z
HexNAc
HexNAcHex
HexNAc(2)Hex(1)
c4
c6
c7
c8
b9
y12
z.13
b11
b13
c13
b14
y17
c17
c20
b21
M_2+ - HexNAc - 36
M+e - Acetyl
M+e

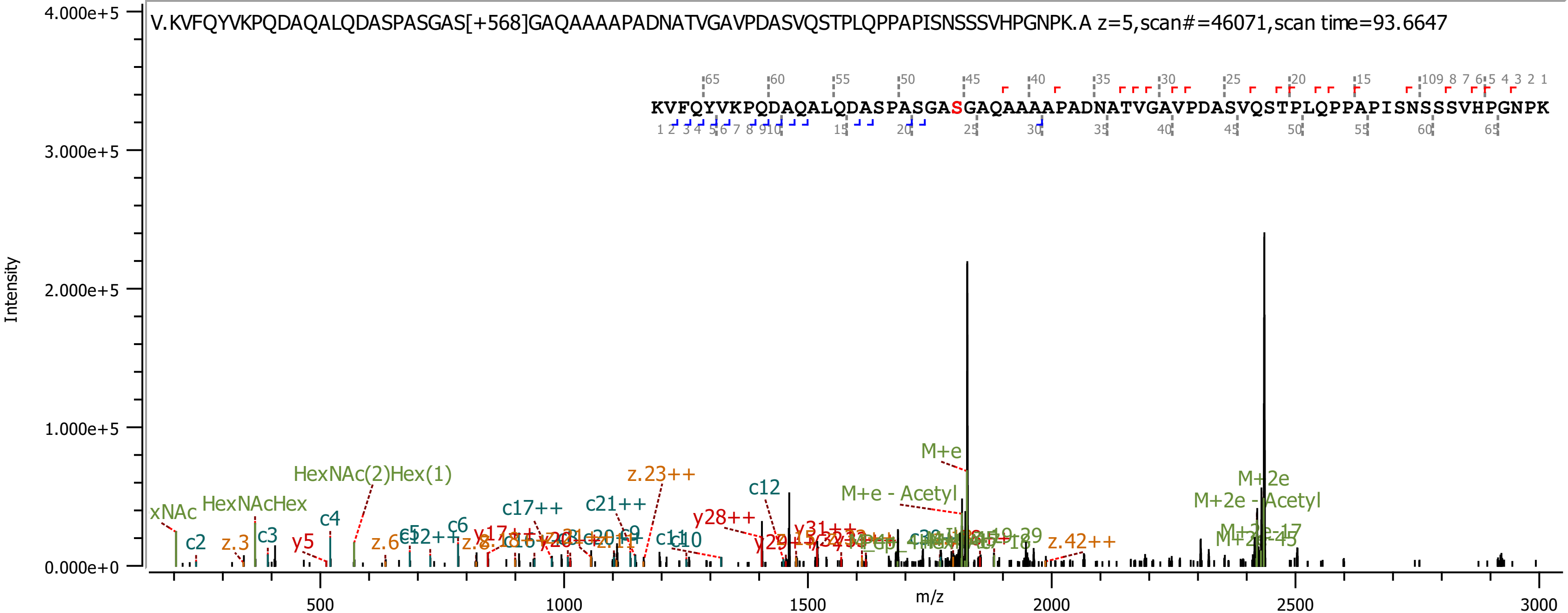
V.KVFQYVKPQDAQALQDASPASGAS[+568]GAQAAAAPADNATVGAVPDASVQSTPLQPPAPISNSSSVHPGNPK.A z=5,scan#=46071,scan time=93.6647
KVFQYVKPQDAQALQDASPASGASGAQAAAAPADNATVGAVPDASVQSTPLQPPAPISNSSSVHPGNPK
Intensity
m/z
M+e
M+e - Acetyl
M+2e
M+2e - Acetyl
HexNAc(2)Hex(1)
HexNAcHex
z.23++
z.42++
c12
y28++
c17++
c21++
c2
c3
c4
c5
c6
c9
c10
y5
z.3
z.6
0.000e+0
1.000e+5
2.000e+5
3.000e+5
4.000e+5
500
1000
1500
2000
2500
3000

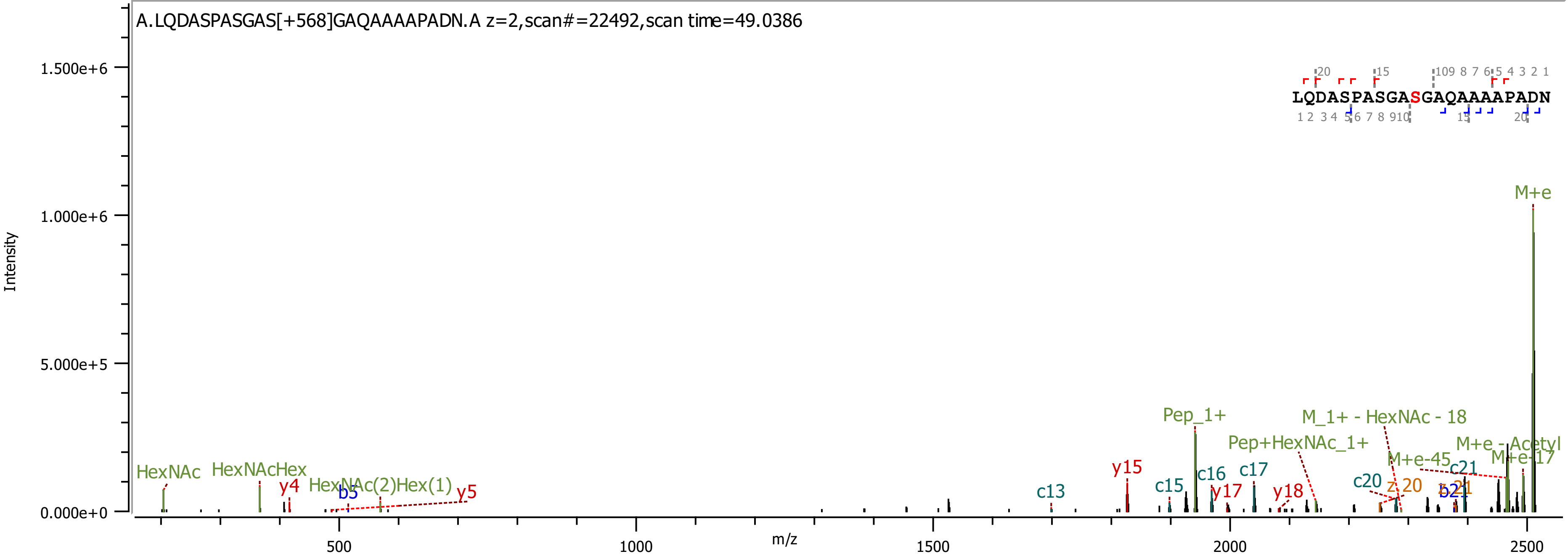
A.LQDASPASGAS[+568]GAQAAAAPADN.A z=2,scan#=22492,scan time=49.0386
LQDASPASGASGAQAAAAPADN
Intensity
1.500e+6
1.000e+6
5.000e+5
0.000e+0
m/z
500
1000
1500
2000
2500
M+e
Pep_1+
M_1+ - HexNAc - 18
Pep+HexNAc_1+
M+e - Acetyl
M+e-45
M+e-17
c21
c20
z 20
b21
z 21
c17
c16
y17
y18
c15
y15
c13
HexNAc
HexNAcHex
y4
HexNAc(2)Hex(1)
b5
y5

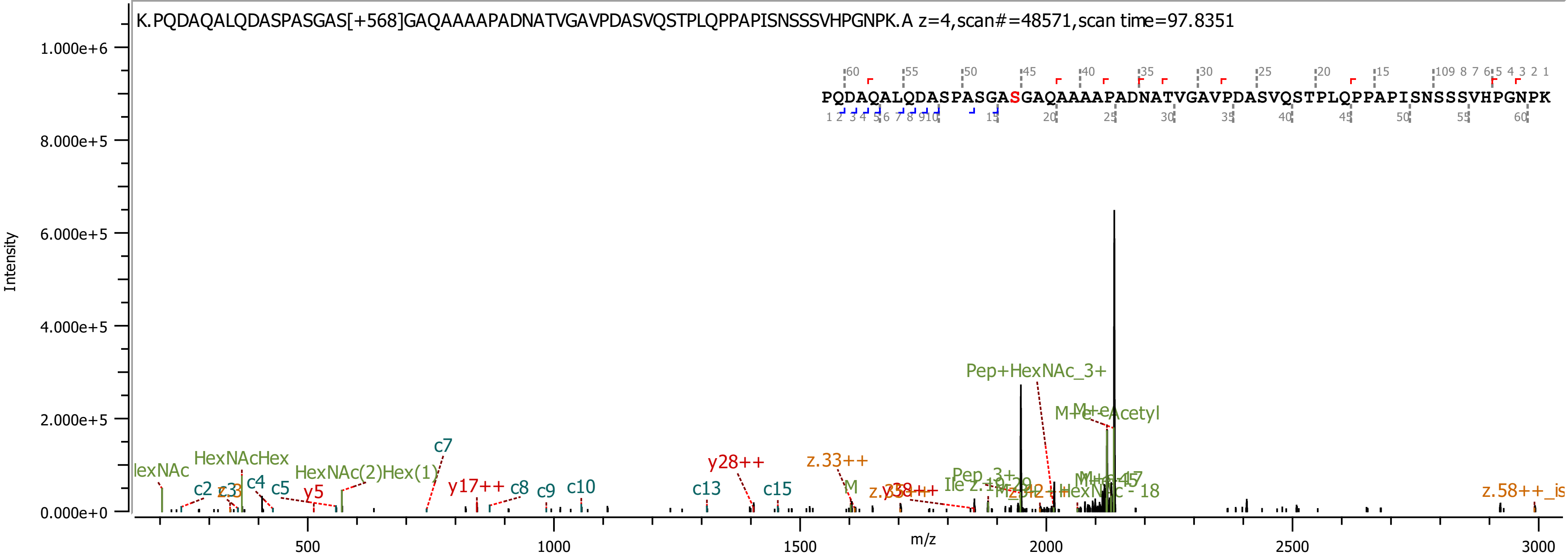
K.PQDAQALQDASPASGAS[+568]GAQAAAAPADNATVGAVPDASVQSTPLQPPAPISNSSSVHPGNPK.A z=4,scan#=48571,scan time=97.8351
PQDAQALQDASPASGASGAQAAAAPADNATVGAVPDASVQSTPLQPPAPISNSSSVHPGNPK
Intensity
m/z
1.000e+6
8.000e+5
6.000e+5
4.000e+5
2.000e+5
0.000e+0
500
1000
1500
2000
2500
3000
HexNAcHex
HexNAc(2)Hex(1)
c2
c4
c5
y5
c7
y17++
c8
c9
c10
y28++
c13
c15
z.33++
Pep+HexNAc_3+
M+e-Acetyl
z.58++_is

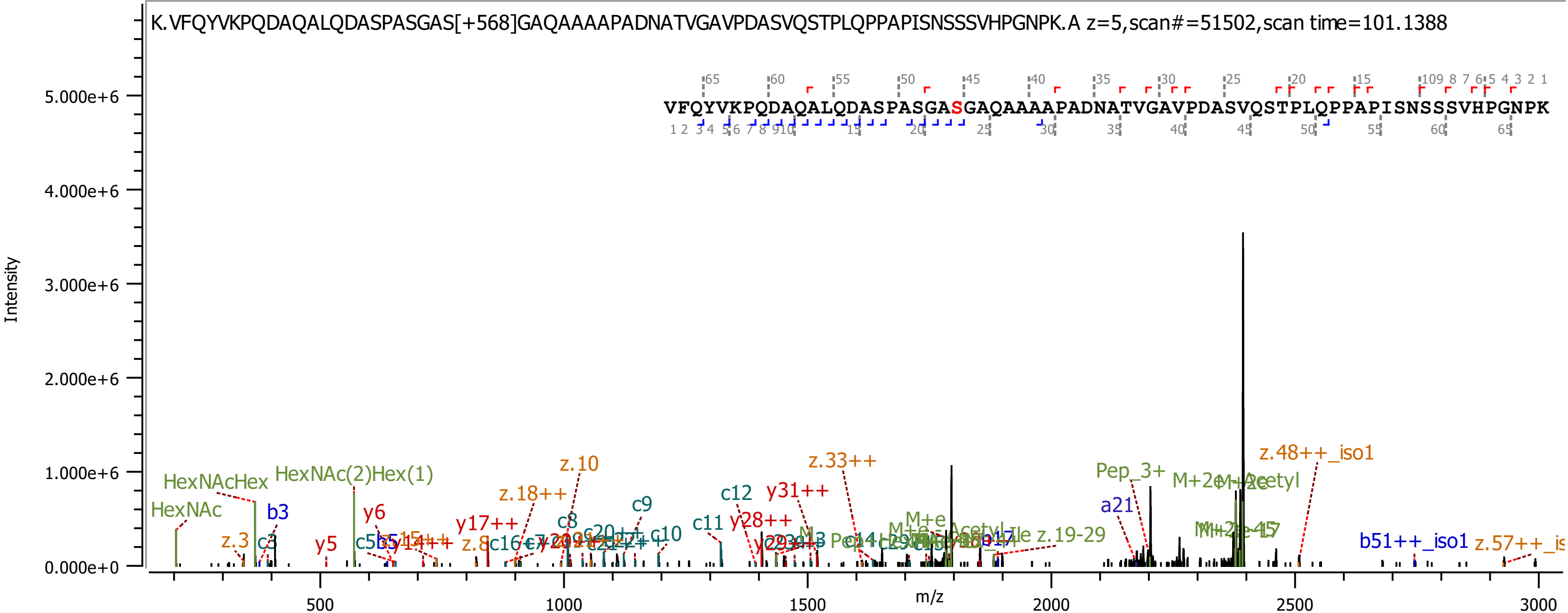
K.VFQYVKPQDAQALQDASPASGAS[+568]GAQAAAAPADNATVGAVPDASVQSTPLQPPAPISNSSSVHPGNPK.A z=5,scan#=51502,scan time=101.1388
VFQYVKPQDAQALQDASPASGASGAQAAAAPADNATVGAVPDASVQSTPLQPPAPISNSSSVHPGNPK
Intensity
m/z
5.000e+6
4.000e+6
3.000e+6
2.000e+6
1.000e+6
0.000e+0
500
1000
1500
2000
2500
3000
HexNAc
HexNAcHex
HexNAc(2)Hex(1)
b3
c3
z.3
y5
y6
z.18++
z.10
y17++
c8
c9
c10
c11
c12
y31++
y28++
z.33++
z.19-29
Pep_3+
a21
z.48++_iso1
b51++_iso1

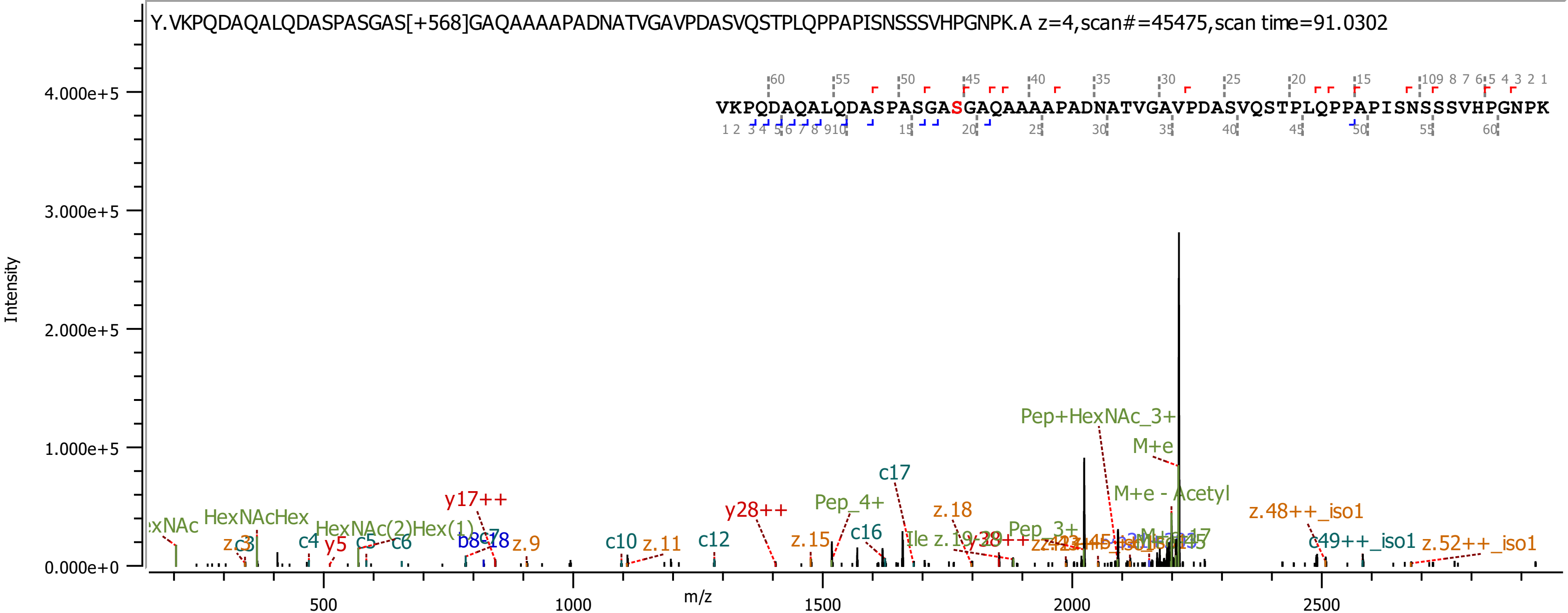
Y.VKPQDAQALQDASPASGAS[+568]GAQAAAAPADNATVGAVPDASVQSTPLQPPAPISNSSSVHPGNPK.A z=4,scan#=45475,scan time=91.0302
VKPQDAQALQDASPASGASGAQAAAAPADNATVGAVPDASVQSTPLQPPAPISNSSSVHPGNPK
Intensity
4.000e+5
3.000e+5
2.000e+5
1.000e+5
0.000e+0
m/z
500
1000
1500
2000
2500
HexNAcHex
HexNAc(2)Hex(1)
y17++
y28++
Pep_4+
c17
z.18
Pep+HexNAc_3+
M+e
M+e - Acetyl
z.48++_iso1
c49++_iso1
z.52++_iso1
c4
y5
c5
c6
z.9
c10
z.11
c12
z.15
c16
Pep_3+

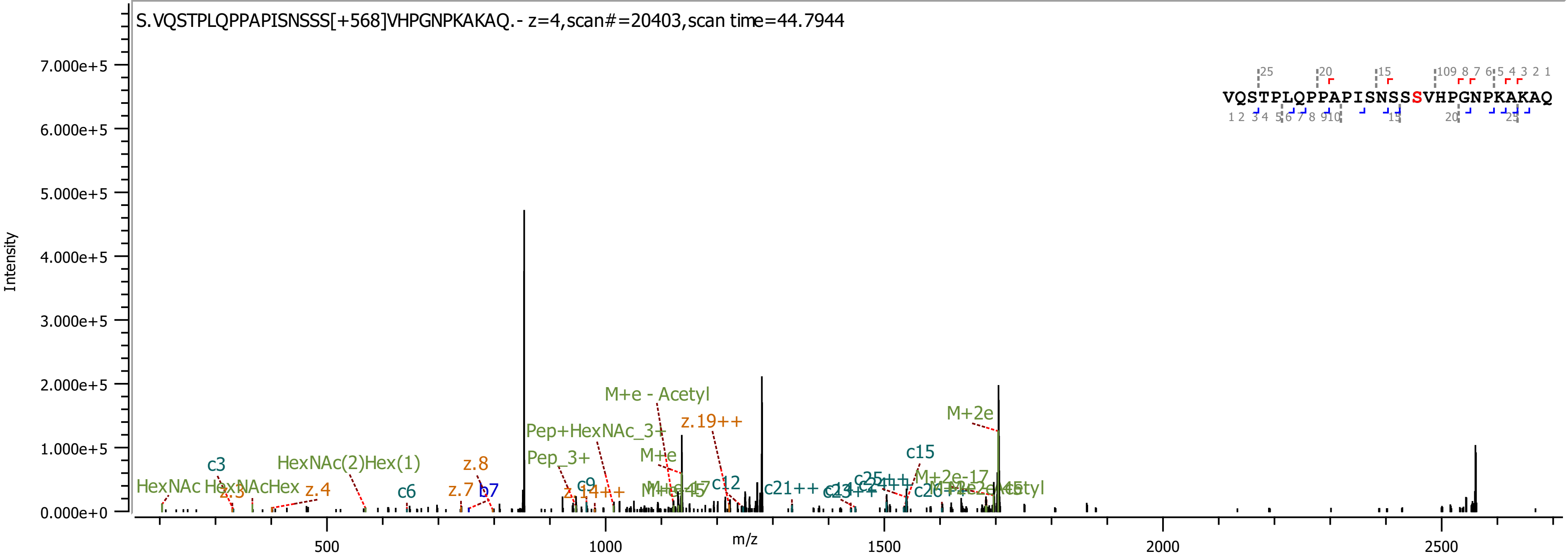
S.VQSTPLQPPAPISNSSS[+568]VHPGNPKAKAQ.- z=4,scan#=20403,scan time=44.7944
VQSTPLQPPAPISNSSSVHPGNPKAKAQ
Intensity
m/z
M+e - Acetyl
z.19++
Pep+HexNAc_3+
M+2e
Pep_3+
M+e
c15
c3
HexNAc(2)Hex(1)
z.8
HexNAc HexNAcHex
z.4
c6
z.7
b7
c9
z.14++
c12
c21++
M+2e-17

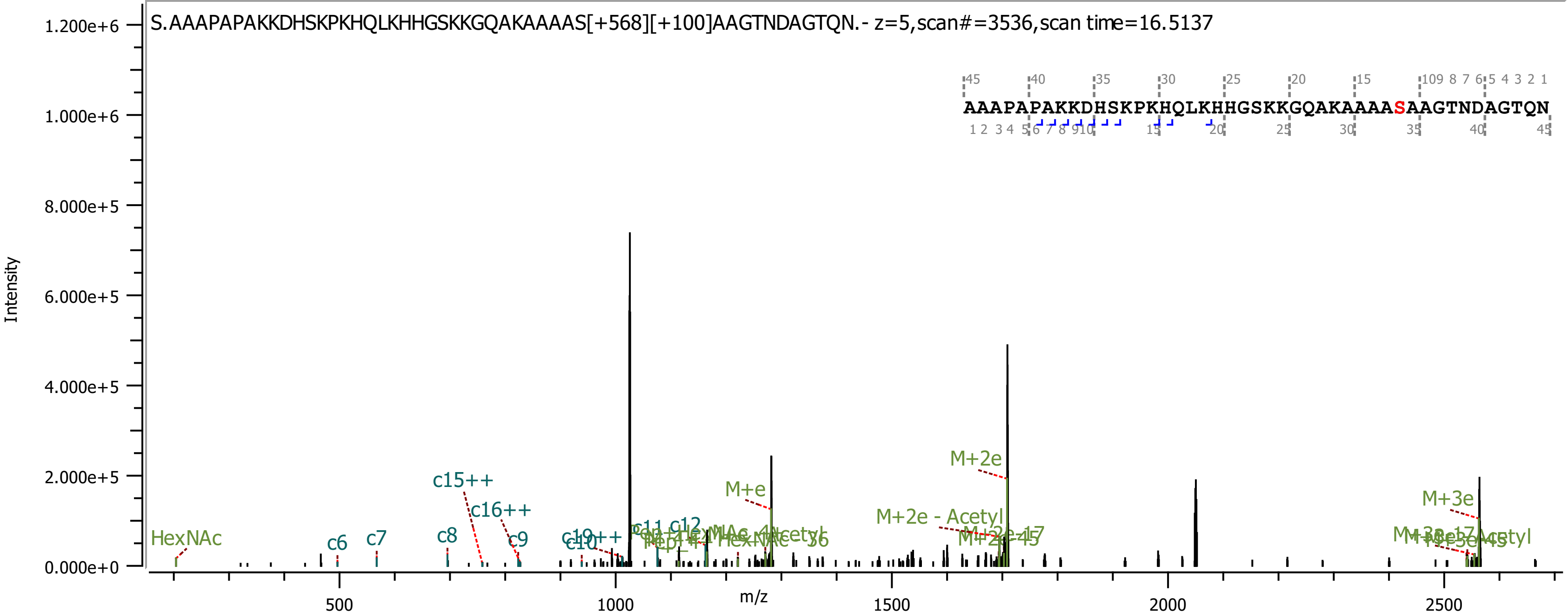
S.AAAPAPAKKDHSKPKHQLKHHGSKKGQAKAAAAS[+568][+100]AAGTNDAGTQN.- z=5,scan#=3536,scan time=16.5137
AAAPAPAKKDHSKPKHQLKHHGSKKGQAKAAAASAAGTNDAGTQN
Intensity
m/z
HexNAc
c6
c7
c8
c15++
c16++
c9
c10
c11
c12
M+e
M+2e - Acetyl
M+2e
M+3e

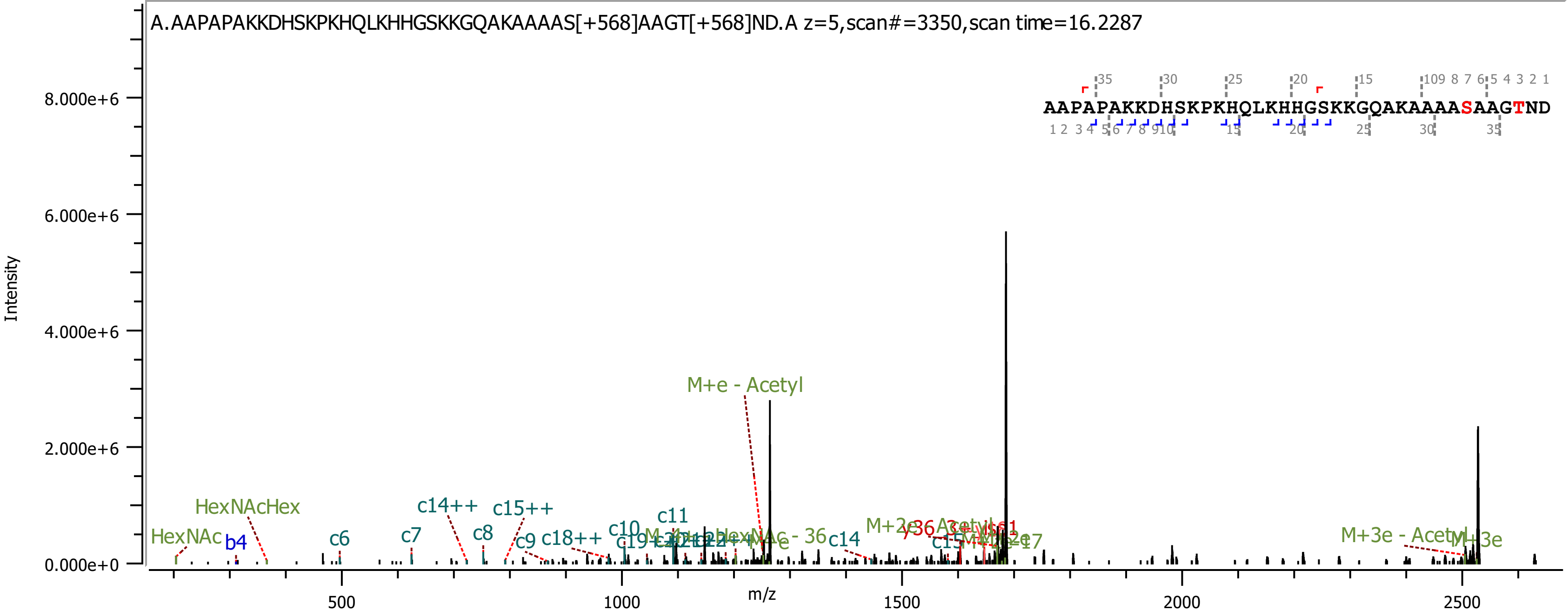
A.AAPAPAKKDHSKPKHQLKHHGSKKGQAKAAAAS[+568]AAGT[+568]ND.A z=5,scan#=3350,scan time=16.2287
AAPAPAKKDHSKPKHQLKHHGSKKGQAKAAAASAAGTND
Intensity
m/z
M+e - Acetyl
HexNAcHex
HexNAc
b4
c6
c7
c8
c9
c10
c11
c14
c14++
c15++
c18++
M+3e - Acetyl
M+3e

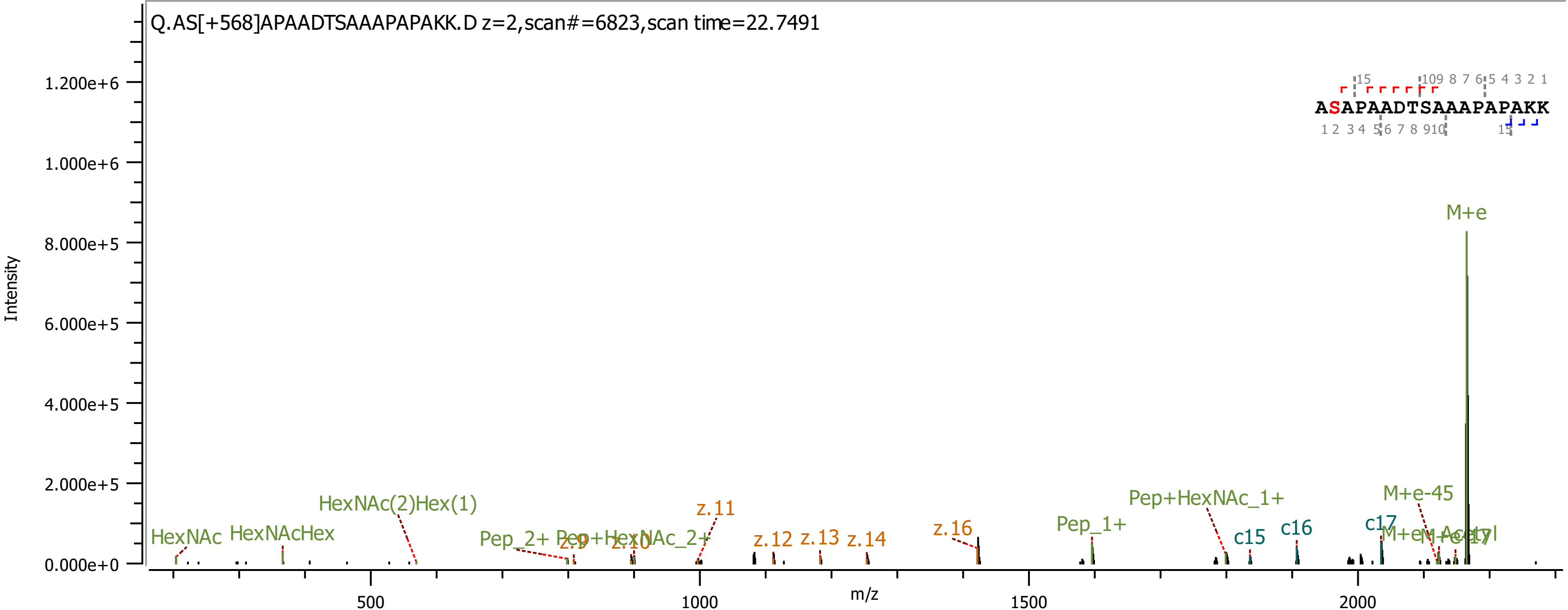

Q.AS[+568]APAADTSAAAPAPAKK.D z=2,scan#=6823,scan time=22.7491
ASAPAADTSAAAPAPAKK
Intensity
m/z
1.200e+6
1.000e+6
8.000e+5
6.000e+5
4.000e+5
2.000e+5
0.000e+0
500
1000
1500
2000
HexNAc
HexNAcHex
HexNAc(2)Hex(1)
Pep_2+
Pep+HexNAc_2+
z.9
z.10
z.11
z.12
z.13
z.14
z.16
Pep_1+
Pep+HexNAc_1+
c15
c16
c17
M+e-45
M+e

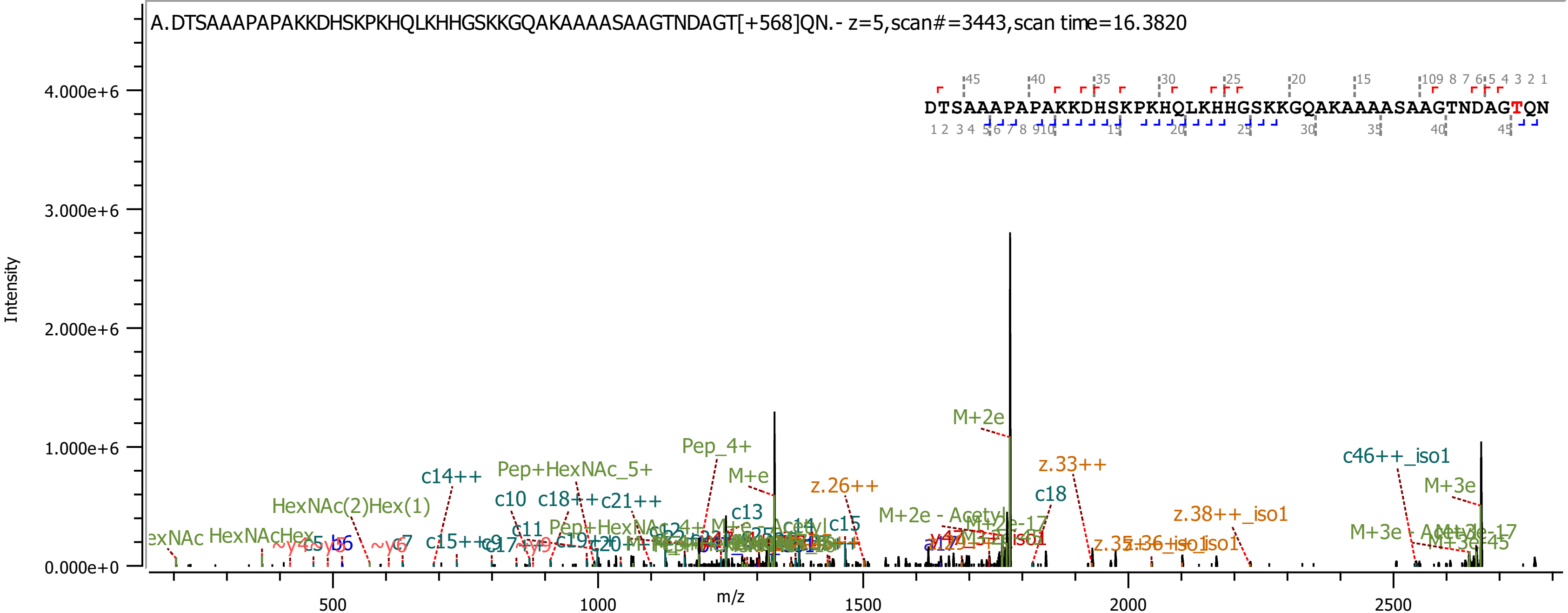
A.DTSAAAPAPAKKDHSKPKHQLKHHGSKKGQAKAAAASAAGTNDAGT[+568]QN.- z=5,scan#=3443,scan time=16.3820
DTSAAAPAPAKKDHSKPKHQLKHHGSKKGQAKAAAASAAGTNDAGTQN
Intensity
4.000e+6
3.000e+6
2.000e+6
1.000e+6
0.000e+0
m/z
500
1000
1500
2000
2500
M+2e
Pep_4+
M+e
z.26++
z.33++
c46++_iso1
M+3e
c18
c14++
Pep+HexNAc_5+
HexNAc(2)Hex(1)
c10
c18++
c21++
c13
c15
M+2e - Acetyl
z.38++_iso1
M+3e - Acetyl
c11
HexNAcHex
c7
c15++
c9
c17++
c19++
c20++

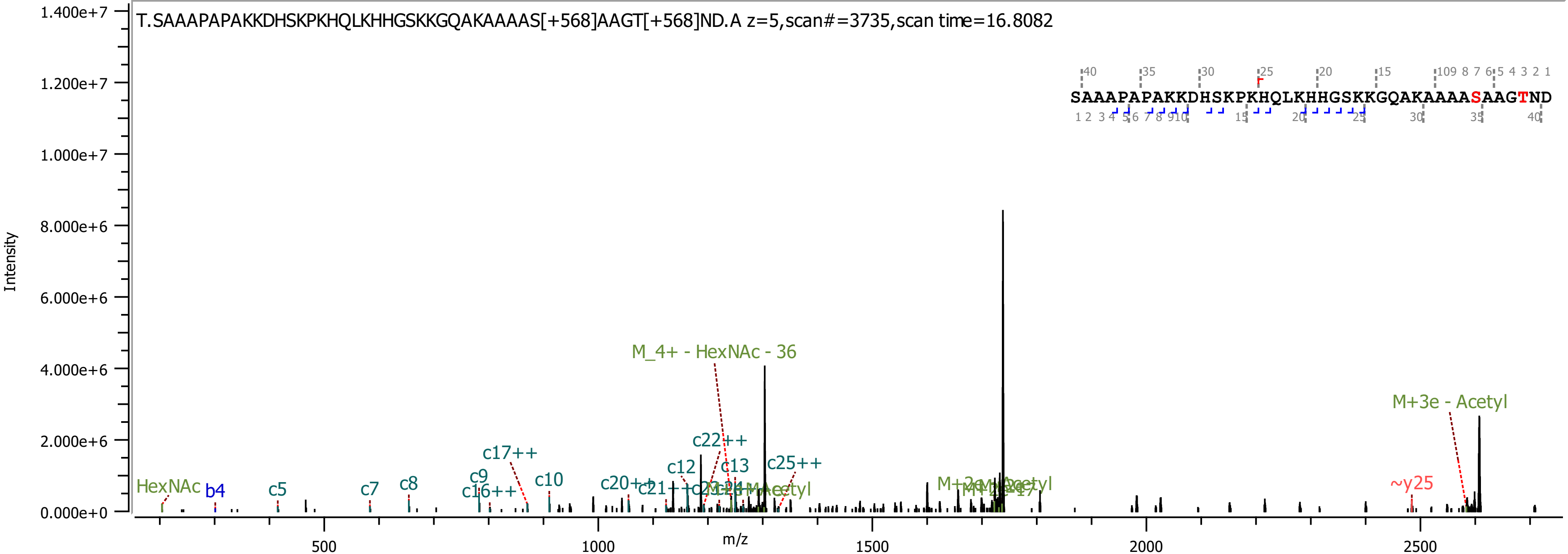
T.SAAAPAPAKKDHSKPKHQLKHHGSKKGQAKAAAAS[+568]AAGT[+568]ND.A z=5,scan#=3735,scan time=16.8082
SAAAPAPAKKDHSKPKHQLKHHGSKKGQAKAAAASAAGTND
Intensity
m/z
M_4+ - HexNAc - 36
M+3e - Acetyl
HexNAc
b4
c5
c7
c8
c9
c16++
c17++
c10
c12
c13
c22++
c25++
~y25

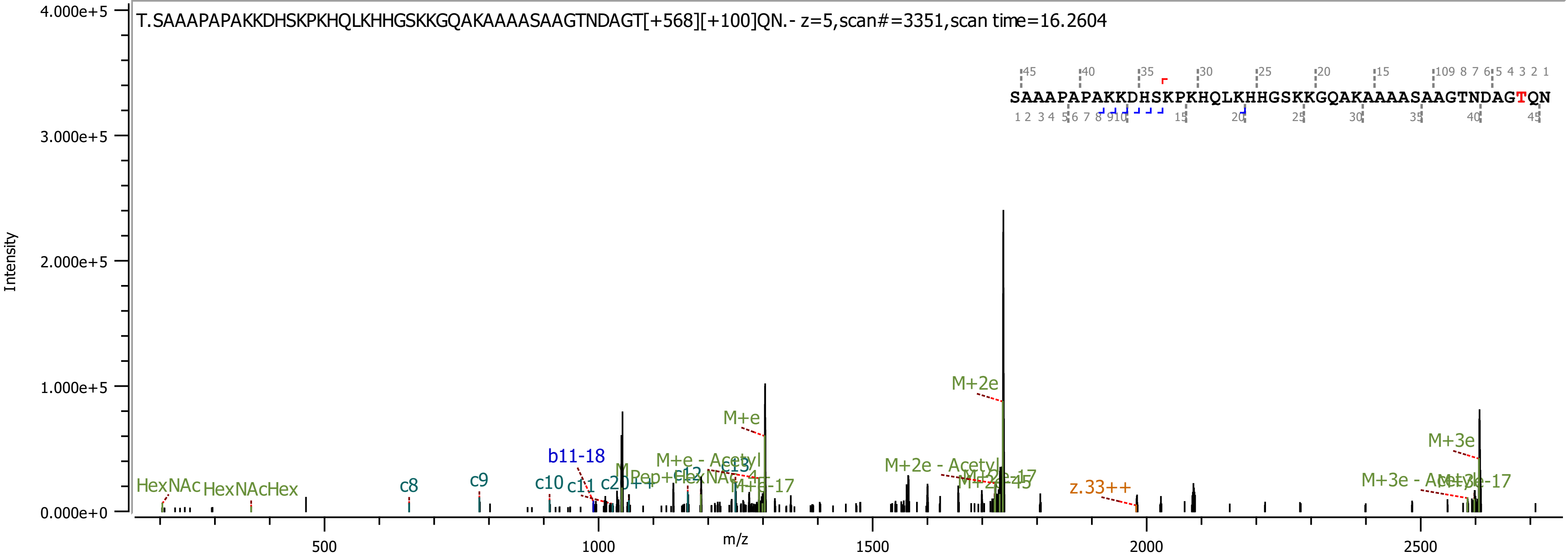
T.SAAAPAPAKKDHSKPKHQLKHHGSKKGQAKAAAASAAGTNDAGT[+568][+100]QN.- z=5,scan#=3351,scan time=16.2604
SAAAPAPAKKDHSKPKHQLKHHGSKKGQAKAAAASAAGTNDAGTQN
Intensity
4.000e+5
3.000e+5
2.000e+5
1.000e+5
0.000e+0
m/z
500
1000
1500
2000
2500
HexNAc
HexNAcHex
c8
c9
c10
b11-18
M+e
M+2e
M+3e
M+e - Acetyl
M+2e - Acetyl
M+e-17
z.33++

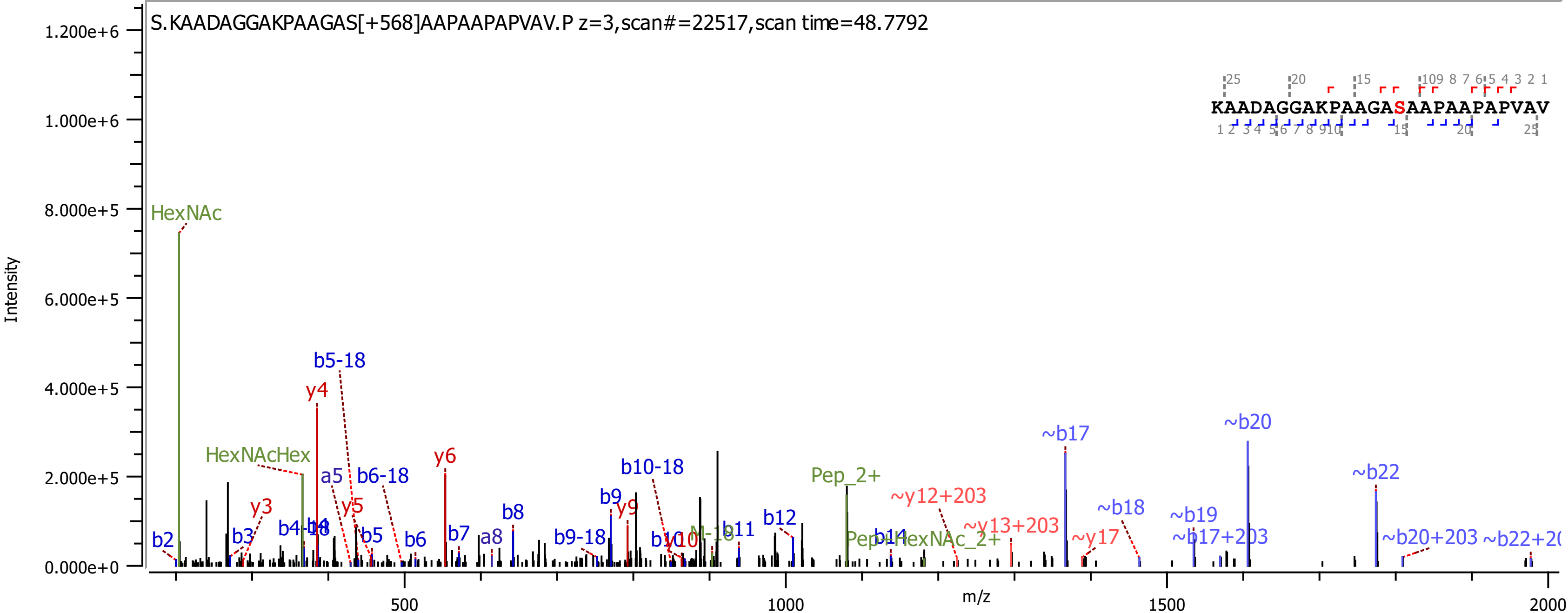
S.KAADAGGAKPAAGAS[+568]AAPAAPAPVAV.P z=3,scan#=22517,scan time=48.7792
KAADAGGAKPAAGASAAPAAPAPVAV
HexNAc
HexNAcHex
b5-18
y4
y6
a5
b6-18
b10-18
Pep_2+
~y12+203
~y13+203
~b17
~b20
~b22
~b18
~b19
~b17+203
~b20+203
~y17
b2
b3
y3
y5
b5
b6
b7
a8
b8
b9
y9
b9-18
b11
b12
M-18
Pep+HexNAc_2+
Intensity
m/z
1.200e+6
1.000e+6
8.000e+5
6.000e+5
4.000e+5
2.000e+5
0.000e+0
500
1000
1500
2000

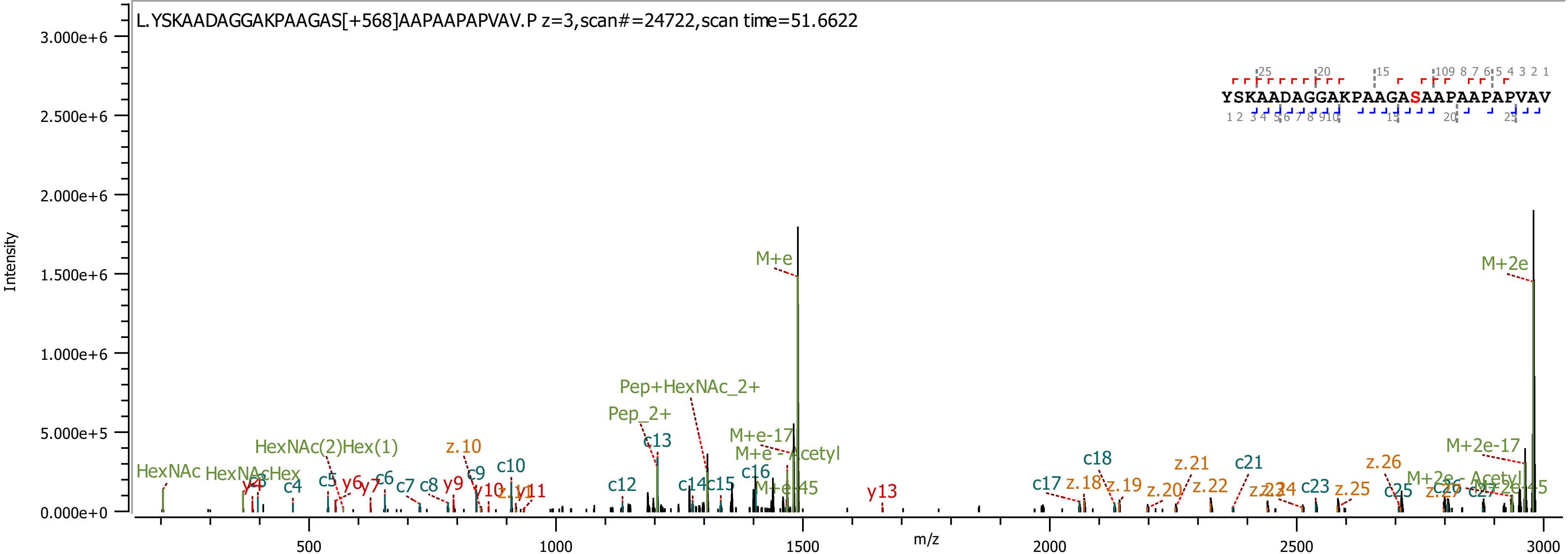
L.YSKAADAGGAKPAAGAS[+568]AAPAAPAPVAV.P z=3,scan#=24722,scan time=51.6622
YSKAADAGGAKPAAGASAAPAAPAPVAV
Intensity
m/z
M+e
M+2e
Pep+HexNAc_2+
Pep_2+
HexNAc(2)Hex(1)
HexNAc
HexNAcHex
M+e-17
M+e - Acetyl
M+e-45
M+2e-17
M+2e - Acetyl
M+2e-45
c4
c5
c6
c7
c8
c9
c10
c12
c13
c14
c15
c16
c17
c18
c21
c23
c25
c26
c27
y4
y6
y7
y9
y10
y11
y13
z.10
z.18
z.19
z.20
z.21
z.22
z.24
z.25
z.26
z.27

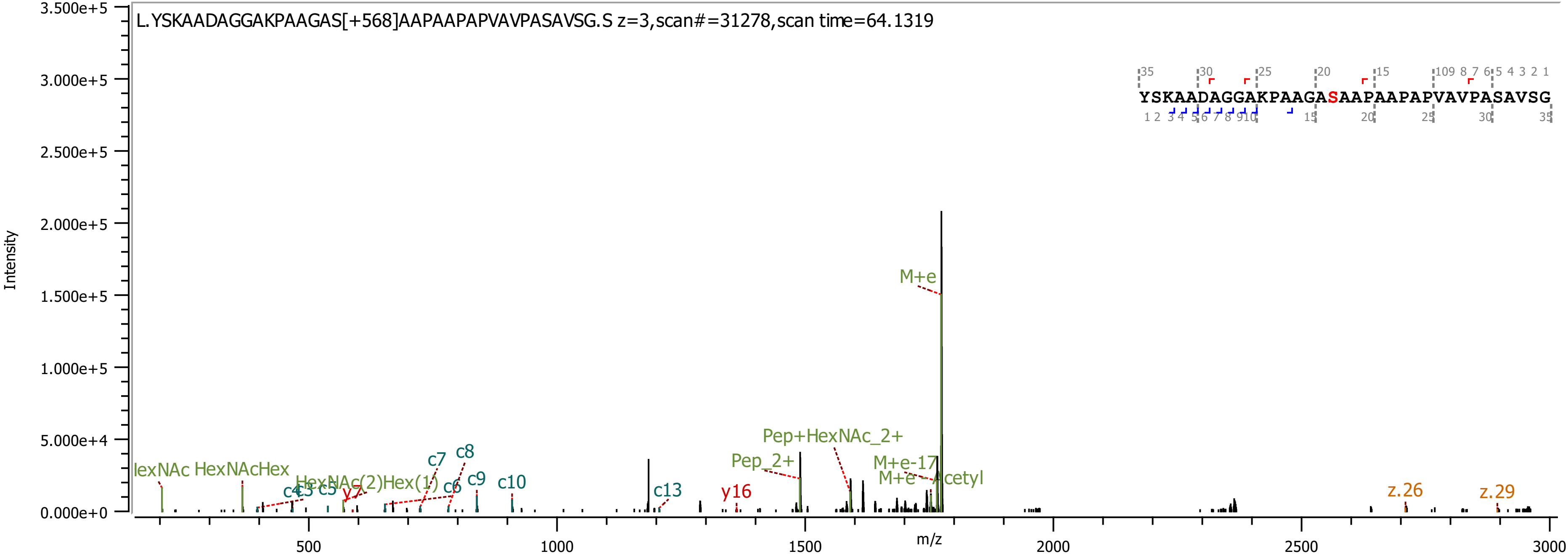

L.YSKAADAGGAKPAAGAS[+568]AAPAAPAPVAVPASAVSG.S z=3,scan#=31278,scan time=64.1319
YSKAADAGGAKPAAGASAAPAAPAPVAVPASAVSG
Intensity
m/z
M+e
Pep+HexNAc_2+
Pep_2+
M+e-17
M+e - Acetyl
HexNAcHex
HexNAc(2)Hex(1)
c4
c5
c6
c7
c8
c9
c10
c13
y7
y16
z.26
z.29

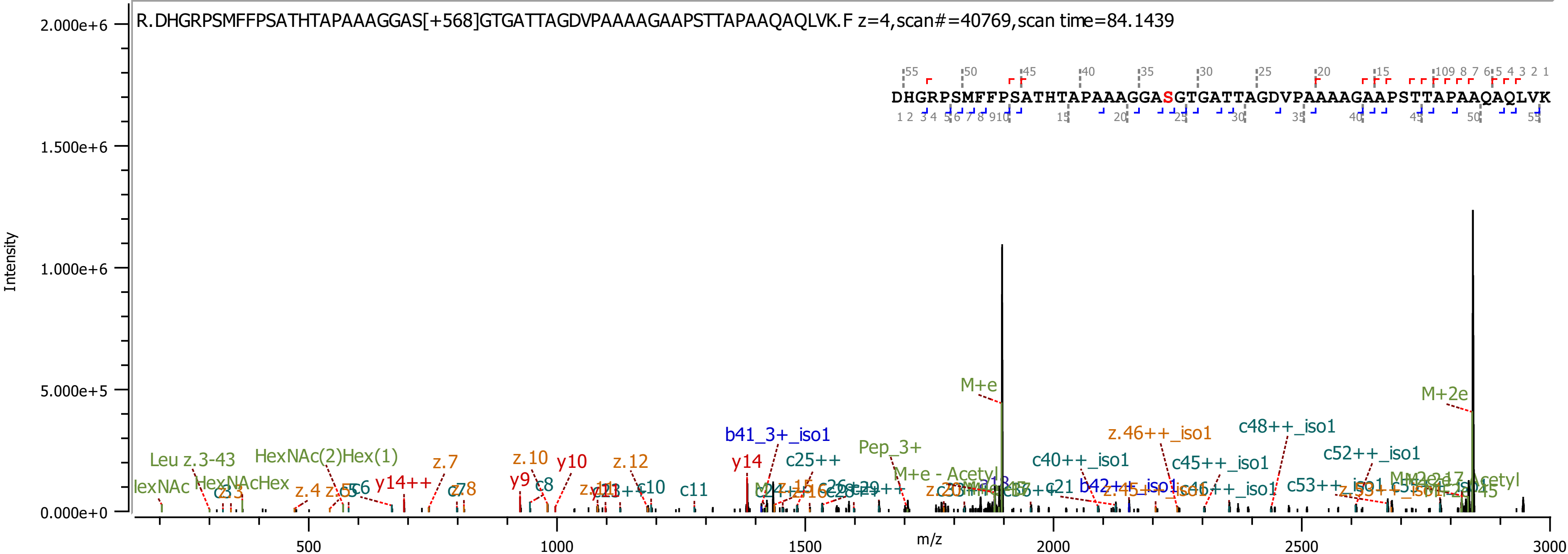
R.DHGRPSMFFPSATHTAPAAAGGAS[+568]GTGATTAGDVPAAAAGAAPSTTAPAAQAQLVK.F z=4,scan#=40769,scan time=84.1439
DHGRPSMFFPSATHTAPAAAGGASGTGATTAGDVPAAAAGAAPSTTAPAAQAQLVK
Intensity
m/z
M+e
M+2e
b41_3+_iso1
c25++
Pep_3+
z.46++_iso1
c48++_iso1
c40++_iso1
c45++_iso1
c52++_iso1
HexNAc(2)Hex(1)
Leu z.3-43
HexNAcHex
y14++
y14
y10
y9
c8
z.10
z.12
z.7
c11
c10
c21
M+e - Acetyl
c53++_iso1

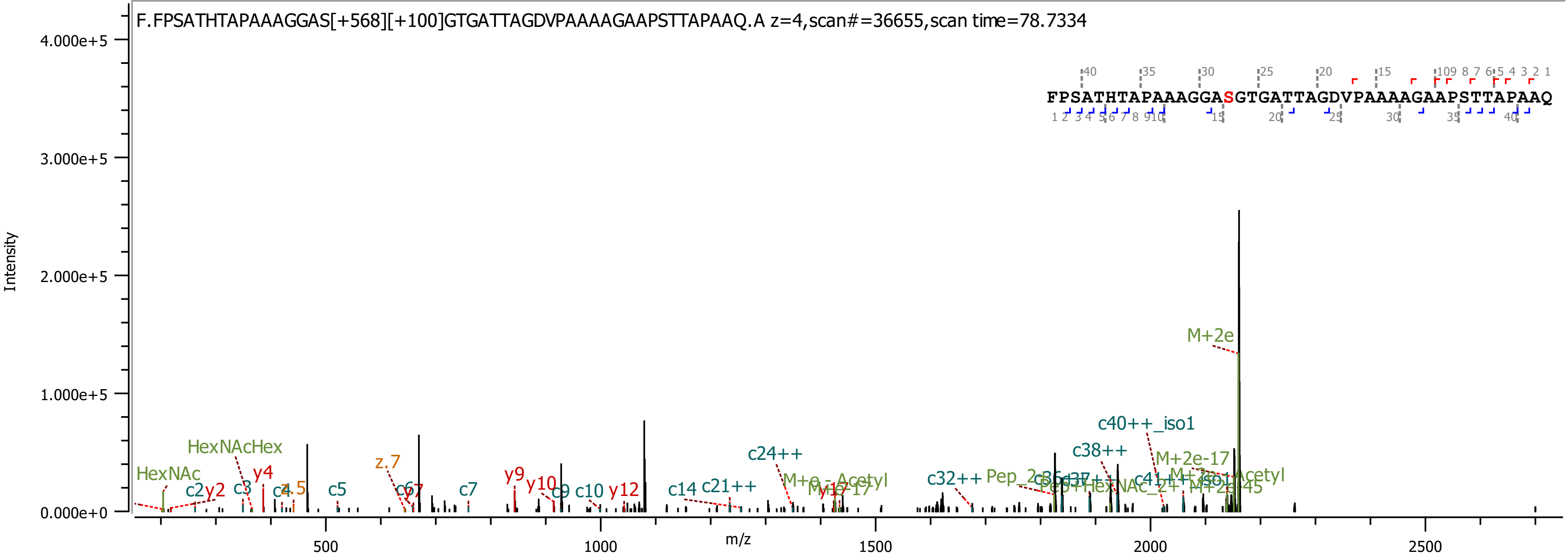

F.FPSATHTAPAAAGGAS[+568][+100]GTGATTAGDVPAAAAGAAPSTTAPAAQ.A z=4,scan#=36655,scan time=78.7334
FPSATHTAPAAAGGASGTGATTAGDVPAAAAGAAPSTTAPAAQ
Intensity
4.000e+5
3.000e+5
2.000e+5
1.000e+5
0.000e+0
m/z
500
1000
1500
2000
2500
M+2e
c40++_iso1
c38++
M+2e-17
c24++
HexNAcHex
HexNAc
y4
z.7
y9
y10
c2
y2
c3
c4
z.5
c5
c6
y7
c7
c9
c10
y12
c14
c21++
M+e - Acetyl
y12
M+2e-17
c32++
c41++_iso1
M+2e-45

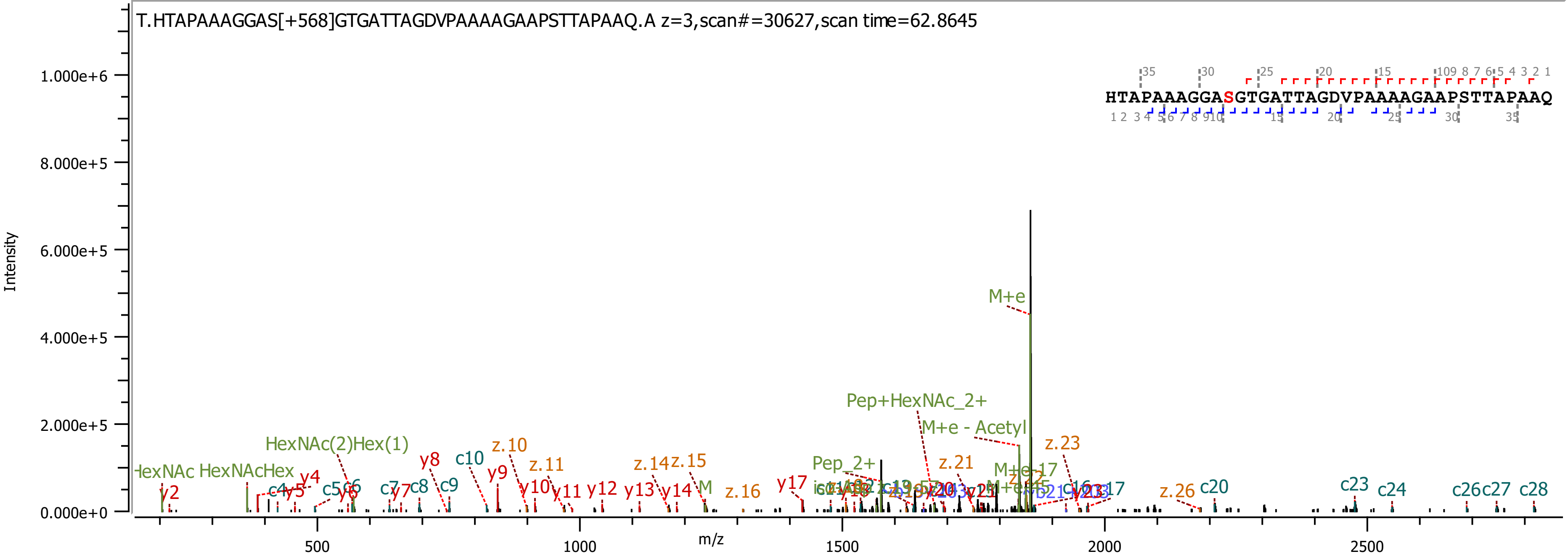
T.HTAPAAAGGAS[+568]GTGATTAGDVPAAAAGAAPSTTAPAAQ.A z=3,scan#=30627,scan time=62.8645
HTAPAAAGGASGTGATTAGDVPAAAAGAAPSTTAPAAQ
Intensity
m/z
1.000e+6
8.000e+5
6.000e+5
4.000e+5
2.000e+5
0.000e+0
500
1000
1500
2000
2500
M+e
Pep+HexNAc_2+
M+e - Acetyl
Pep_2+
HexNAc(2)Hex(1)
HexNAcHex
z.10
z.11
z.14
z.15
z.16
z.21
z.23
z.26
y2
y4
y5
y6
y7
y8
y9
y10
y11
y12
y13
y14
y17
c4
c5
c7
c8
c9
c10
c20
c23
c24
c26
c27
c28

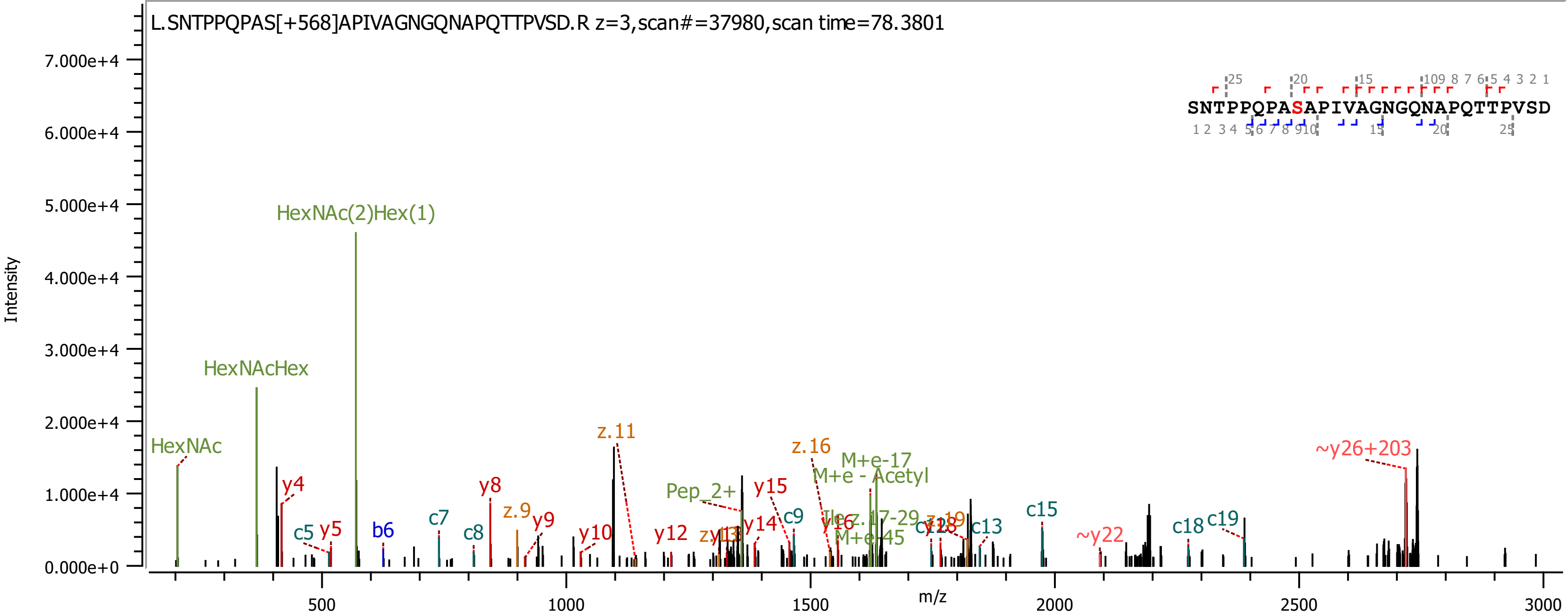

L.SNTPPQPAS[+568]APIVAGNGQNAPQTTPVSD.R z=3,scan#=37980,scan time=78.3801
SNTPPQPASAPIVAGNGQNAPQTTPVSD
Intensity
m/z
HexNAc(2)Hex(1)
HexNAcHex
HexNAc
z.11
z.16
M+e-17
M+e - Acetyl
~y26+203
y4
y5
c5
b6
c7
c8
y8
z.9
y9
y10
y12
Pep_2+
y15
y14
c9
y16
c13
c15
~y22
c18
c19
M+e-45

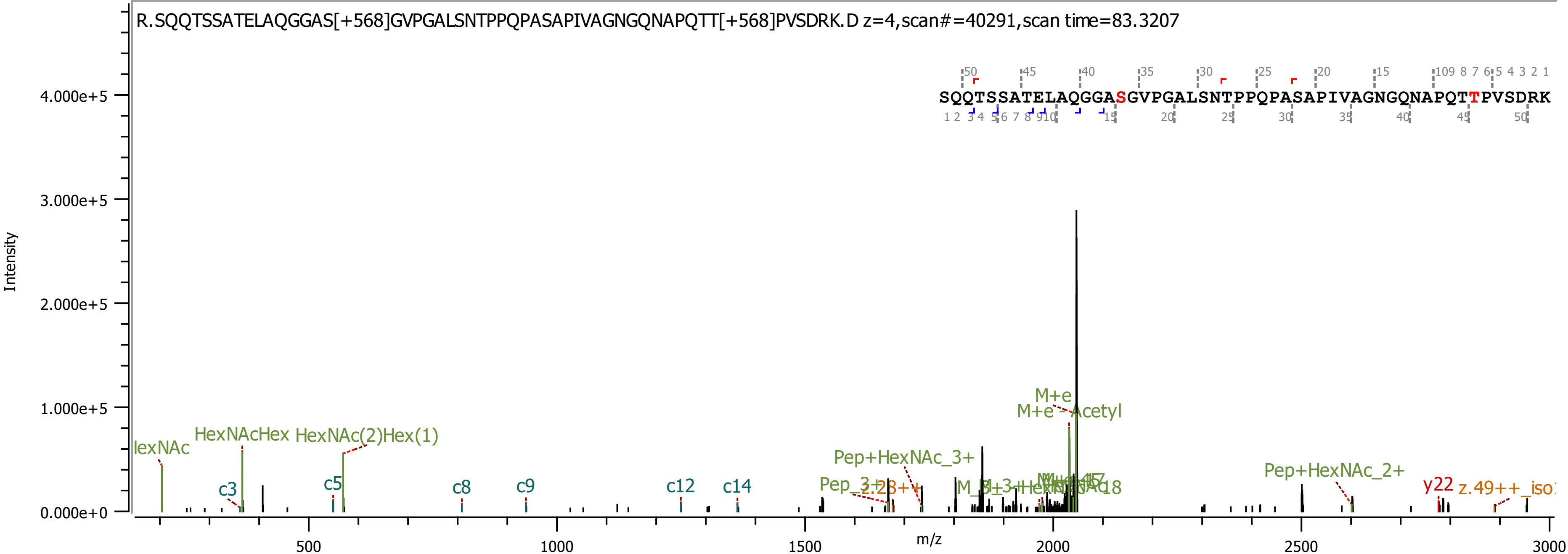

R.SQQTSSATELAQGGAS[+568]GVPGALSNTPPQPASAPIVAGNGQNAPQTT[+568]PVSDRK.D z=4,scan#=40291,scan time=83.3207
SQQTSSATELAQGGASGVPGALSNTPPQPASAPIVAGNGQNAPQTTPVSDRK
Intensity
m/z
M+e
M+e - Acetyl
HexNAcHex
HexNAc(2)Hex(1)
Pep+HexNAc_3+
Pep+HexNAc_2+
c3
c5
c8
c9
c12
c14
y22

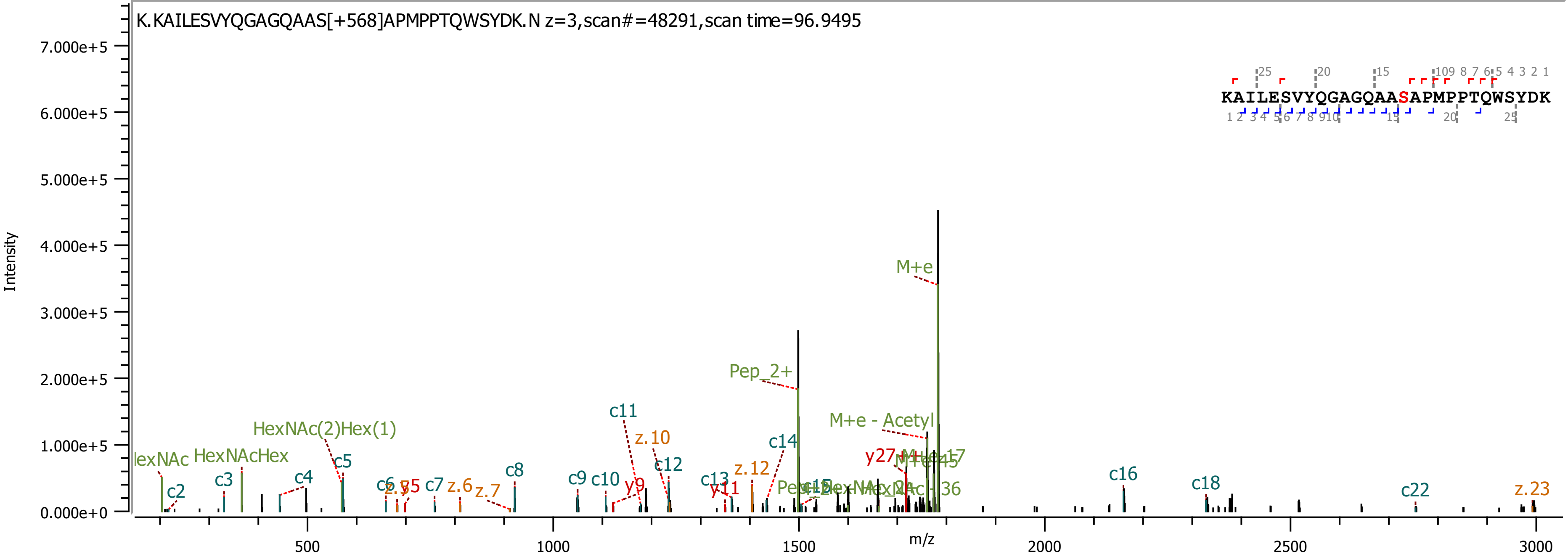
K.KAILESVYQGAGQAAS[+568]APMPPTQWSYDK.N z=3,scan#=48291,scan time=96.9495
KAILESVYQGAGQAASAPMPPTQWSYDK
Intensity
m/z
M+e
Pep_2+
M+e - Acetyl
HexNAc(2)Hex(1)
HexNAcHex
c2
c3
c4
c5
c7
c8
c9
c10
c11
c12
c13
c14
c16
c18
c22
z.6
z.7
z.10
z.12
z.23
y9
y11
y27

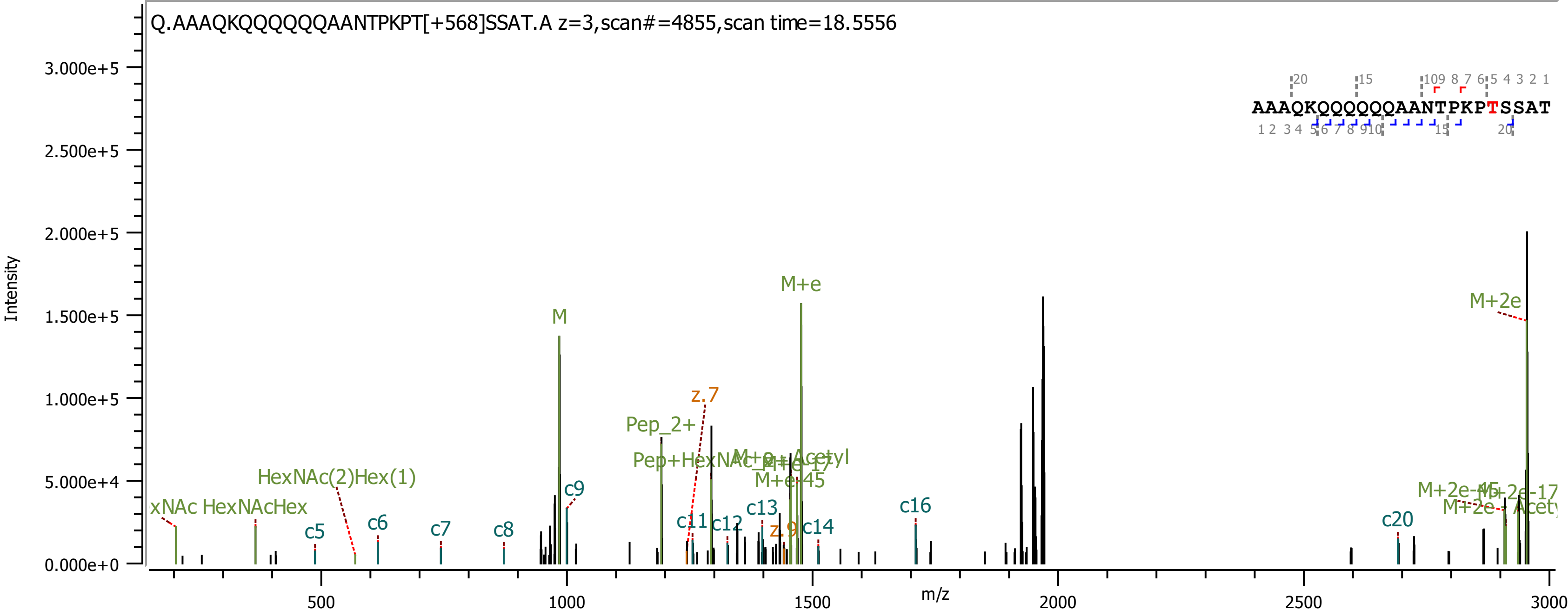

Q.AAAQKQQQQQQAANTPKPT[+568]SSAT.A z=3,scan#=4855,scan time=18.5556
AAAQKQQQQQQAANTPKPTSSAT
Intensity
m/z
M+e
M
M+2e
z.7
Pep_2+
Pep+HexNAc
M+e-45
HexNAc(2)Hex(1)
HexNAcHex
c5
c6
c7
c8
c9
c11
c12
c13
c14
c16
c20
M+2e-45
M+2e-17

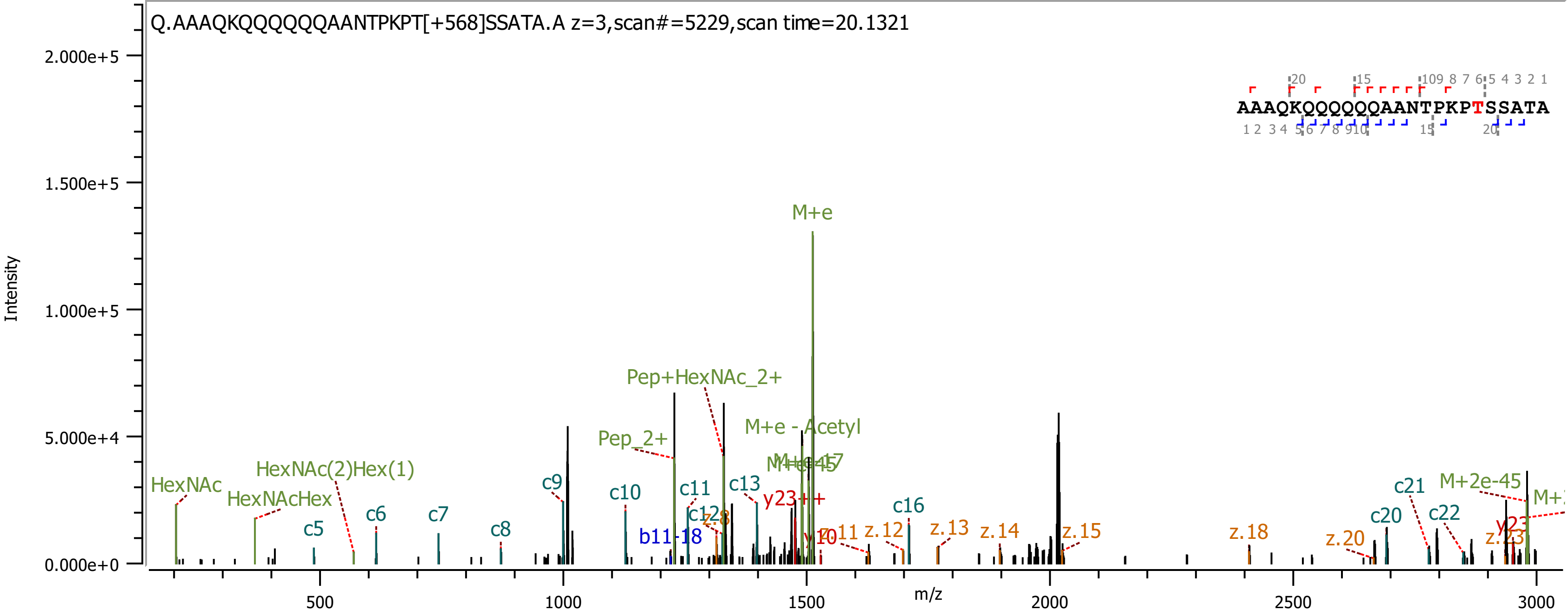

Q.AAAQKQQQQQQAANTPKPT[+568]SSATA.A z=3,scan#=5229,scan time=20.1321
AAAQKQQQQQQAANTPKPTSSATA
Intensity
m/z
2.000e+5
1.500e+5
1.000e+5
5.000e+4
0.000e+0
500
1000
1500
2000
2500
3000
M+e
Pep+HexNAc_2+
M+e - Acetyl
Pep_2+
HexNAc
HexNAcHex
HexNAc(2)Hex(1)
c5
c6
c7
c8
c9
c10
c11
c13
c16
c20
c21
c22
b11-18
y23++
z.11
z.12
z.13
z.14
z.15
z.18
z.20
M+2e-45

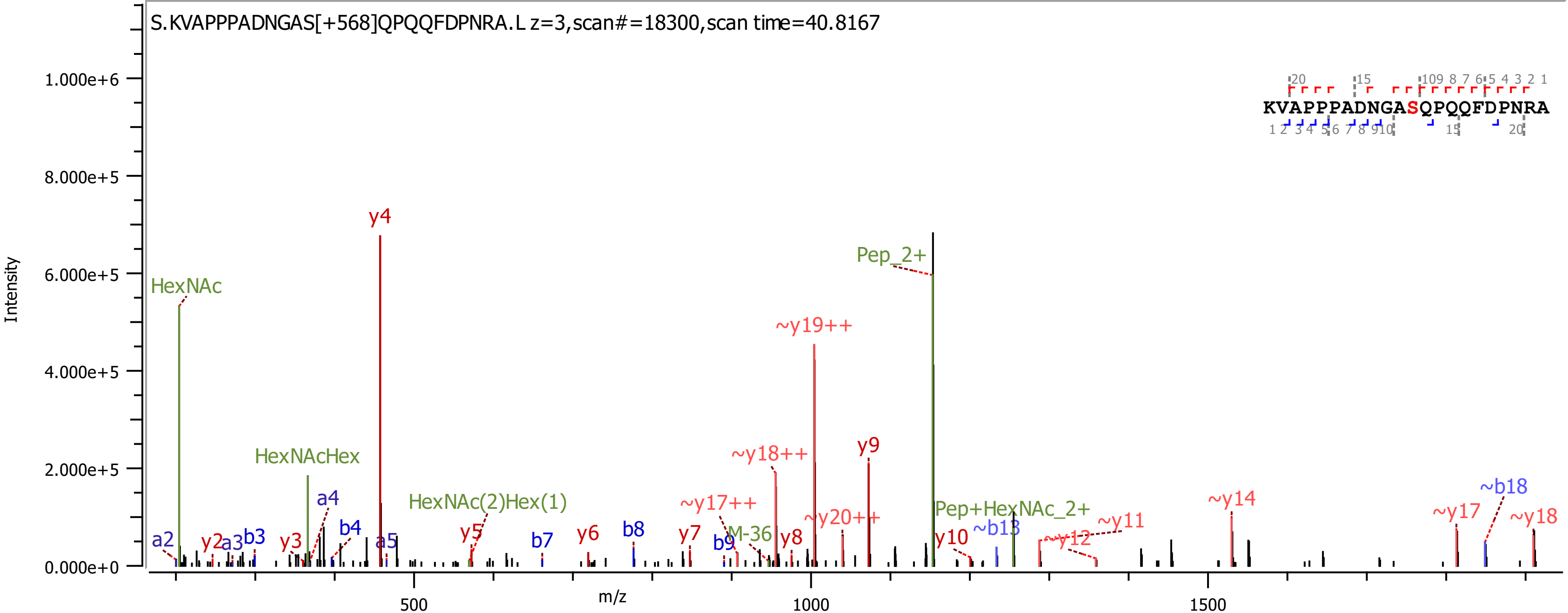

S.KVAPPPADNGAS[+568]QPQQFDPNRA.L z=3,scan#=18300,scan time=40.8167
KVAPPPADNGASQPQQFDPNRA
Intensity
m/z
1.000e+6
8.000e+5
6.000e+5
4.000e+5
2.000e+5
0.000e+0
500
1000
1500
HexNAc
HexNAcHex
HexNAc(2)Hex(1)
Pep_2+
Pep+HexNAc_2+
M-36
y4
~y19++
~y18++
y9
~y17++
~y20++
~y14
~y17
~b18
~y18
~y11
~y12
~b13
y10
y8
y7
y6
y5
y3
y2
a2
a3
a4
a5
b3
b4
b7
b8
b9

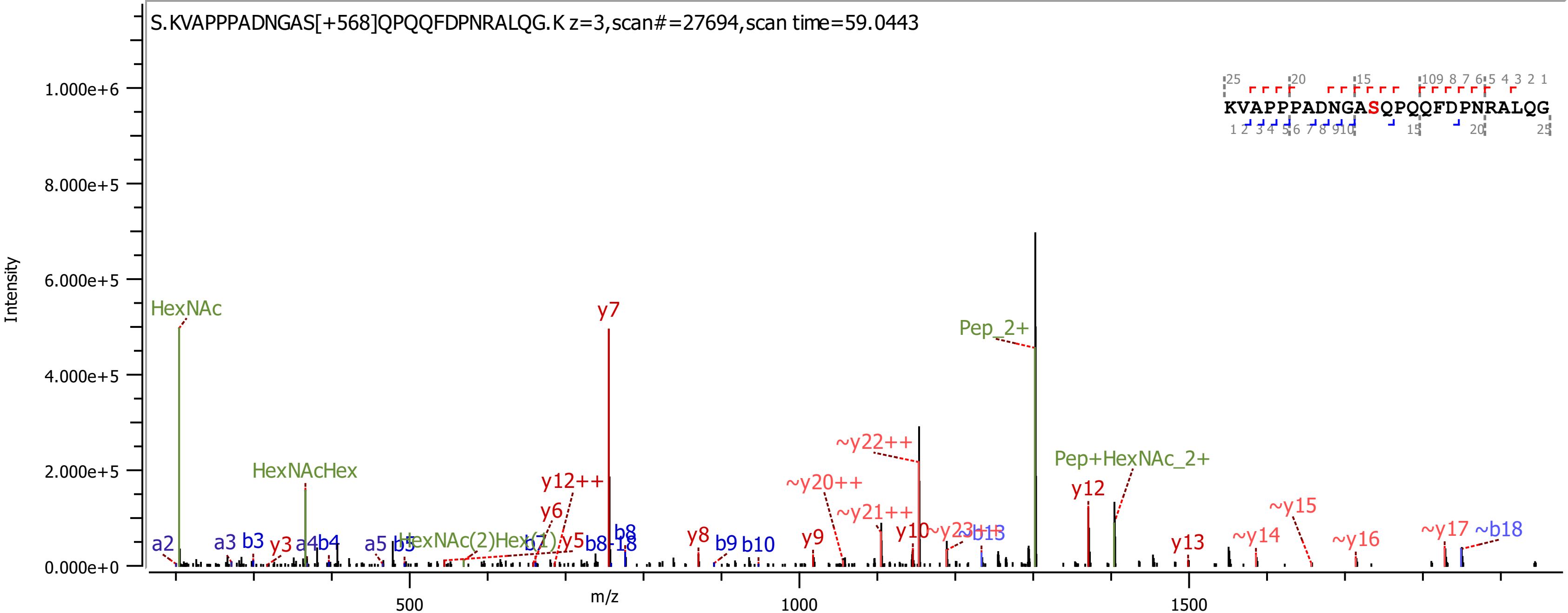
S.KVAPPPADNGAS[+568]QPQQFDPNRALQG.K z=3,scan#=27694,scan time=59.0443
KVAPPPADNGASQPQQFDPNRALQG
Intensity
m/z
HexNAc
HexNAcHex
Pep_2+
Pep+HexNAc_2+
y7
y12++
y6
y5
y3
y8
y9
y10
y12
y13
~y22++
~y20++
~y21++
~y23++
~y14
~y15
~y16
~y17
~b18
a2
a3
b3
a4
b4
a5
b5
b7
b8
b9
b10
HexNAc(2)Hex(1)
1.000e+6
8.000e+5
6.000e+5
4.000e+5
2.000e+5
0.000e+0
500
1000
1500

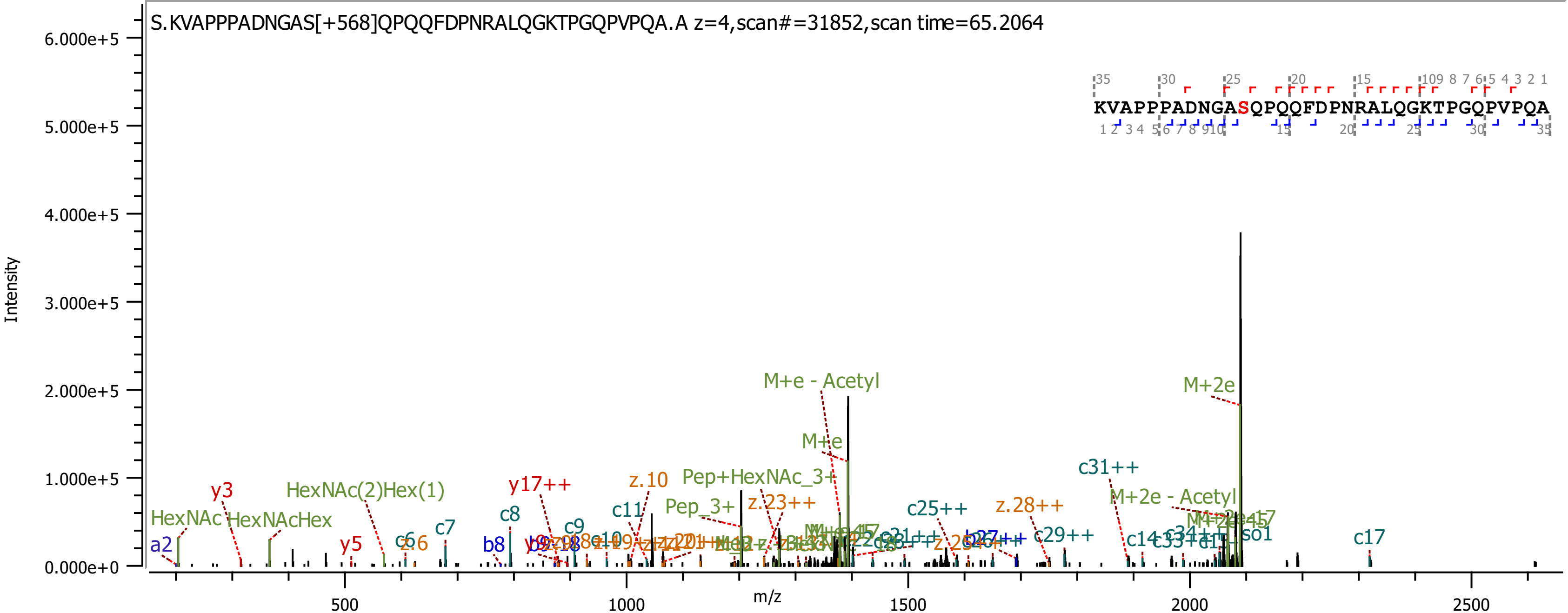
S.KVAPPPADNGAS[+568]QPQQFDPNRALQGKTPGQPVPQA.A z=4,scan#=31852,scan time=65.2064
KVAPPPADNGASQPQQFDPNRALQGKTPGQPVPQA
Intensity
6.000e+5
5.000e+5
4.000e+5
3.000e+5
2.000e+5
1.000e+5
0.000e+0
m/z
500
1000
1500
2000
2500
M+e - Acetyl
M+2e
M+e
Pep+HexNAc_3+
c31++
M+2e - Acetyl
y17++
z.10
HexNAc(2)Hex(1)
y3
Pep_3+
z.23++
c25++
z.28++
HexNAc
HexNAcHex
c11
c8
c9
c7
c29++
c17
a2
y5
c6
z.6
b8
c10
c14

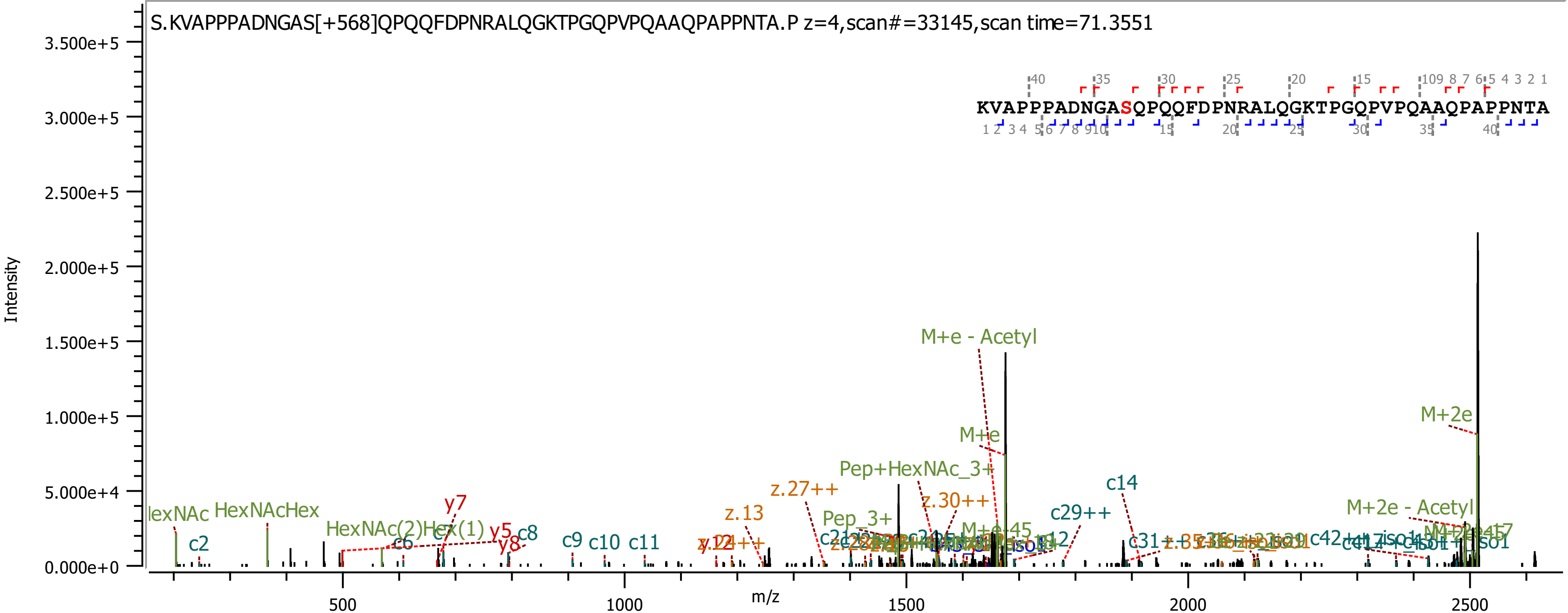
S.KVAPPPADNGAS[+568]QPQQFDPNRALQGKTPGQPVPQAAQPAPPNTA.P z=4,scan#=33145,scan time=71.3551
KVAPPPADNGASQPQQFDPNRALQGKTPGQPVPQAAQPAPPNTA
Intensity
m/z
M+e - Acetyl
M+2e
M+e
Pep+HexNAc_3+
M+2e - Acetyl
c14
c29++
z.30++
z.27++
z.13
Pep_3+
HexNAcHex
HexNAc(2)Hex(1)
y7
y5
y8
c8
c9
c10
c11
c2
c6
c12
3.500e+5
3.000e+5
2.500e+5
2.000e+5
1.500e+5
1.000e+5
5.000e+4
0.000e+0
500
1000
1500
2000
2500

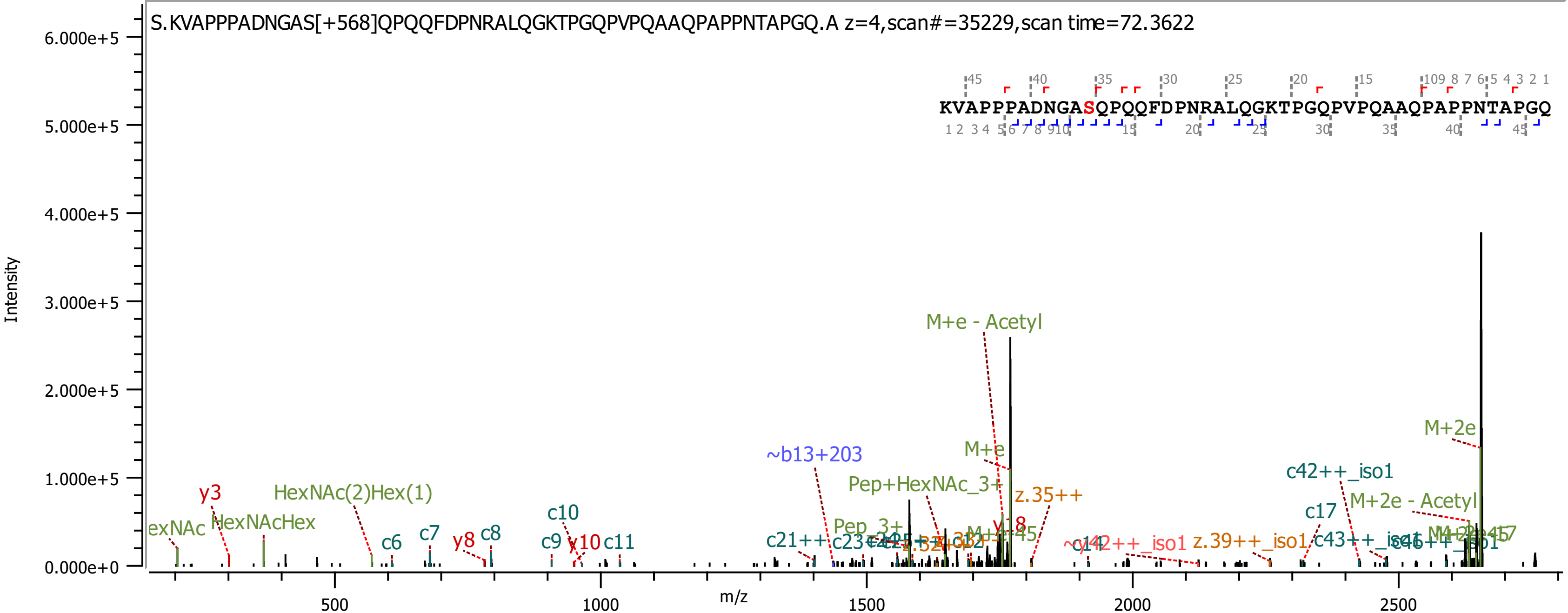

S.KVAPPPADNGAS[+568]QPQQFDPNRALQGKTPGQPVPQAAQPAPPNTAPGQ.A z=4,scan#=35229,scan time=72.3622
KVAPPPADNGASQPQQFDPNRALQGKTPGQPVPQAAQPAPPNTAPGQ
Intensity
6.000e+5
5.000e+5
4.000e+5
3.000e+5
2.000e+5
1.000e+5
0.000e+0
m/z
500
1000
1500
2000
2500
M+e - Acetyl
M+e
M+2e
M+2e - Acetyl
~b13+203
Pep+HexNAc_3+
Pep_3+
z.35++
c42++_iso1
c17
c43++_iso1
z.39++_iso1
y18
HexNAc(2)Hex(1)
HexNAcHex
y3
y8
y10
c6
c7
c8
c9
c10
c11
c21++

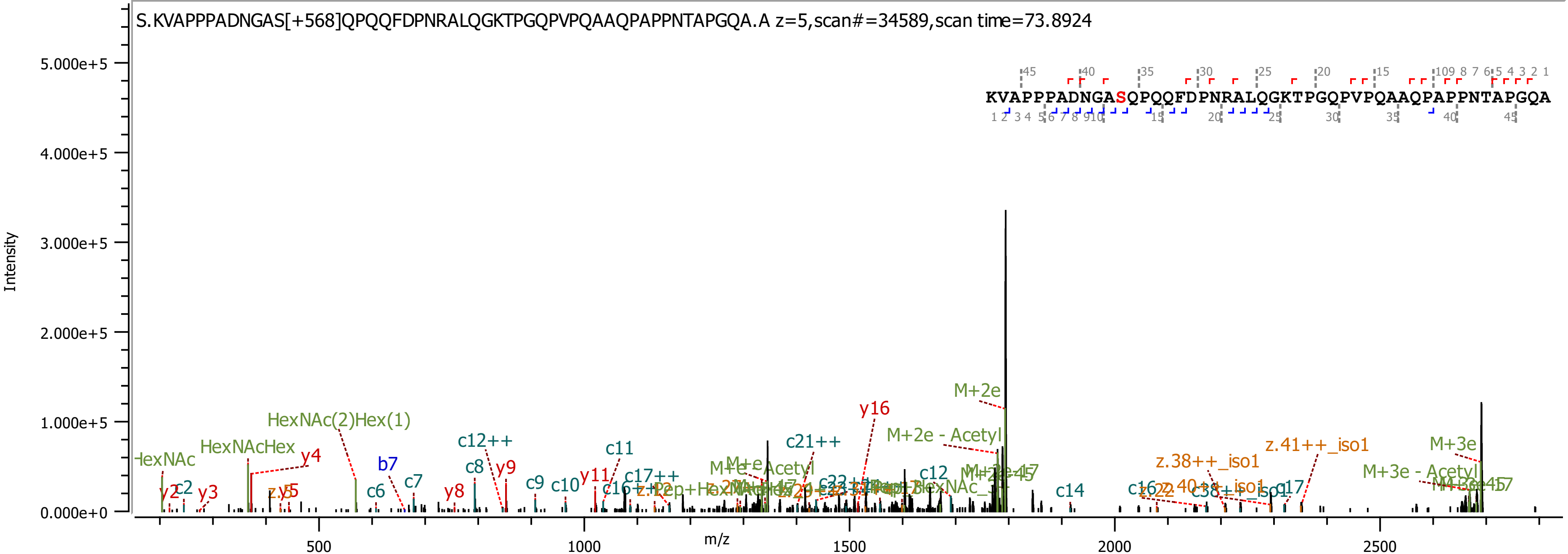
S.KVAPPPADNGAS[+568]QPQQFDPNRALQGKTPGQPVPQAAQPAPPNTAPGQA.A z=5,scan#=34589,scan time=73.8924
KVAPPPADNGASQPQQFDPNRALQGKTPGQPVPQAAQPAPPNTAPGQA
Intensity
5.000e+5
4.000e+5
3.000e+5
2.000e+5
1.000e+5
0.000e+0
m/z
500
1000
1500
2000
2500
HexNAcHex
HexNAc(2)Hex(1)
y2
c2
y3
y4
b7
c6
c7
y8
c8
c12++
y9
c9
c10
y11
c11
c17++
y16
c21++
M+2e
M+2e - Acetyl
c12
c14
z.38++_iso1
z.41++_iso1
M+3e
M+3e - Acetyl

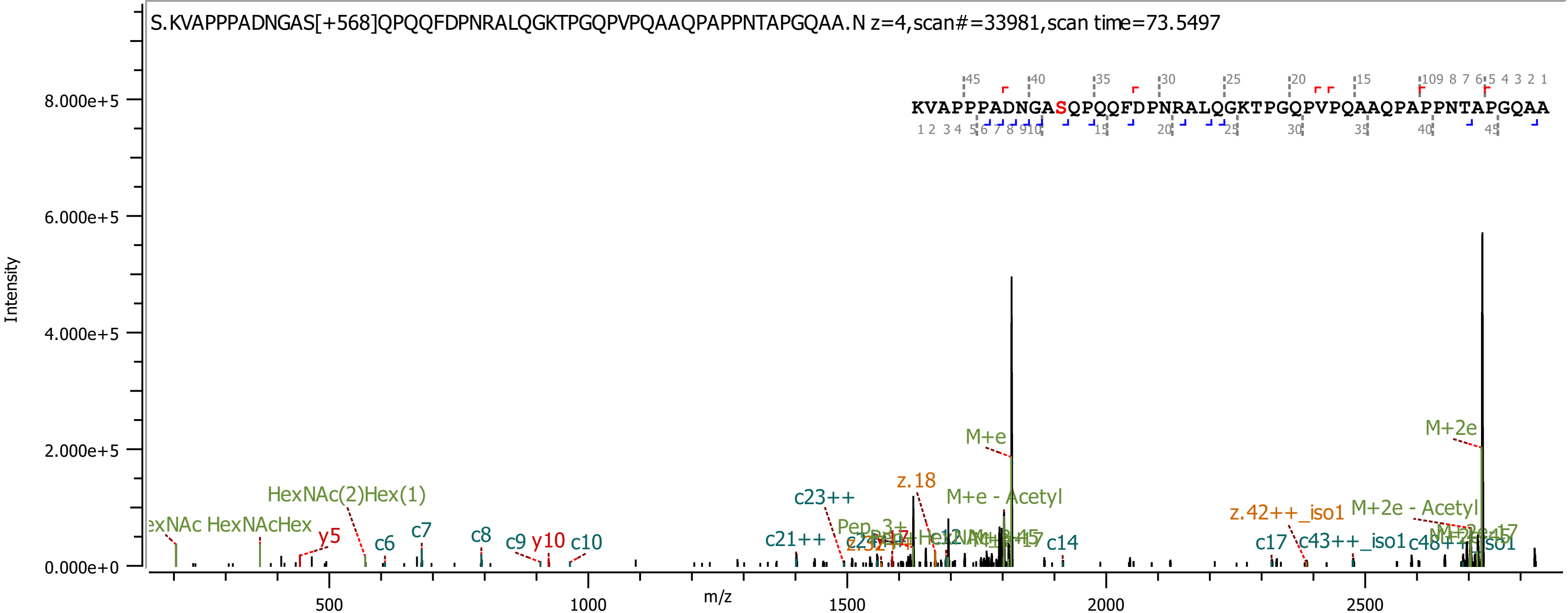
S.KVAPPPADNGAS[+568]QPQQFDPNRALQGKTPGQPVPQAAQPAPPNTAPGQAA.N z=4,scan#=33981,scan time=73.5497
KVAPPPADNGASQPQQFDPNRALQGKTPGQPVPQAAQPAPPNTAPGQAA
Intensity
8.000e+5
6.000e+5
4.000e+5
2.000e+5
0.000e+0
m/z
500
1000
1500
2000
2500
HexNAc(2)Hex(1)
HexNAcHex
y5
c6
c7
c8
c9
y10
c10
c21++
c23++
z.18
M+e
M+e - Acetyl
c14
c17
z.42++_iso1
c43++_iso1
M+2e - Acetyl
M+2e

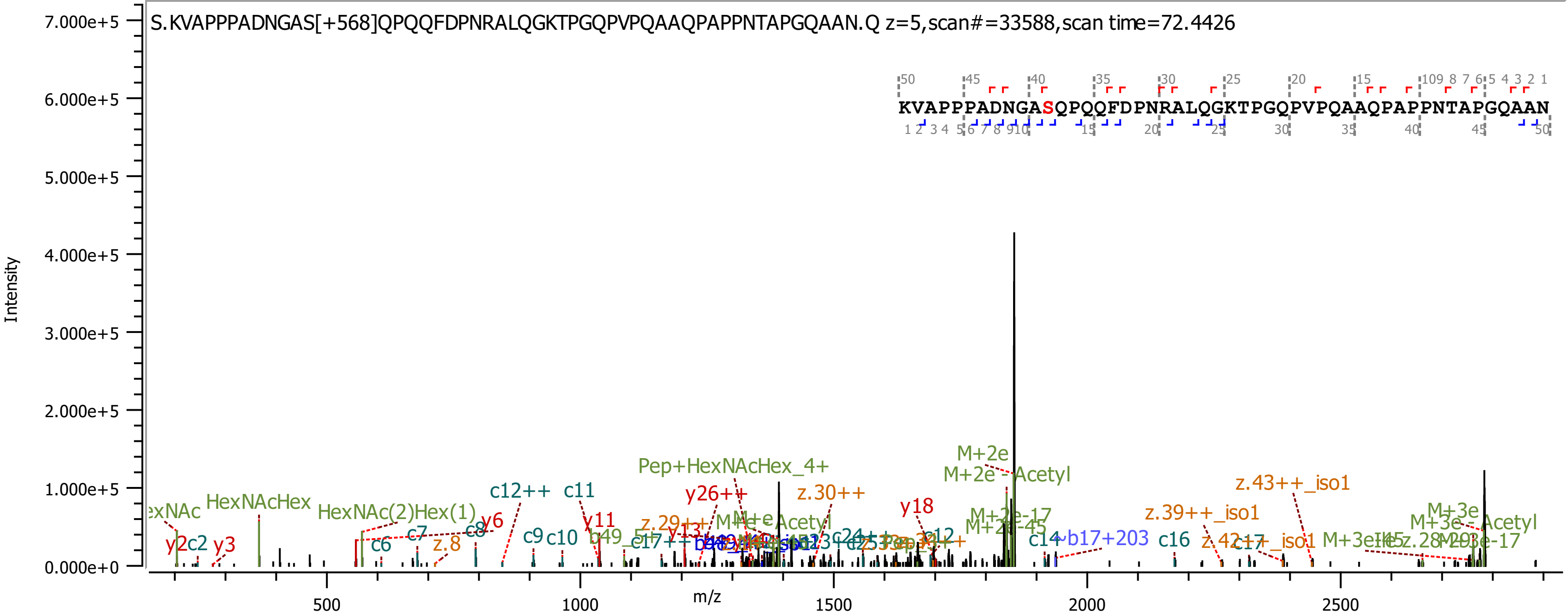
S.KVAPPPADNGAS[+568]QPQQFDPNRALQGKTPGQPVPQAAQPAPPNTAPGQAAN.Q z=5,scan#=33588,scan time=72.4426
Intensity
m/z
KVAPPPADNGASQPQQFDPNRALQGKTPGQPVPQAAQPAPPNTAPGQAAN
HexNAcHex
HexNAc(2)Hex(1)
Pep+HexNAcHex_4+
M+2e
M+2e - Acetyl
z.43++_iso1
z.39++_iso1
z.30++
y26++
y18
y11
y6
y3
c12++
c11
c10
c9
c8
c7
c6
c2
z.8
c14
b17+203
c16
M+3e
M+3e - Acetyl

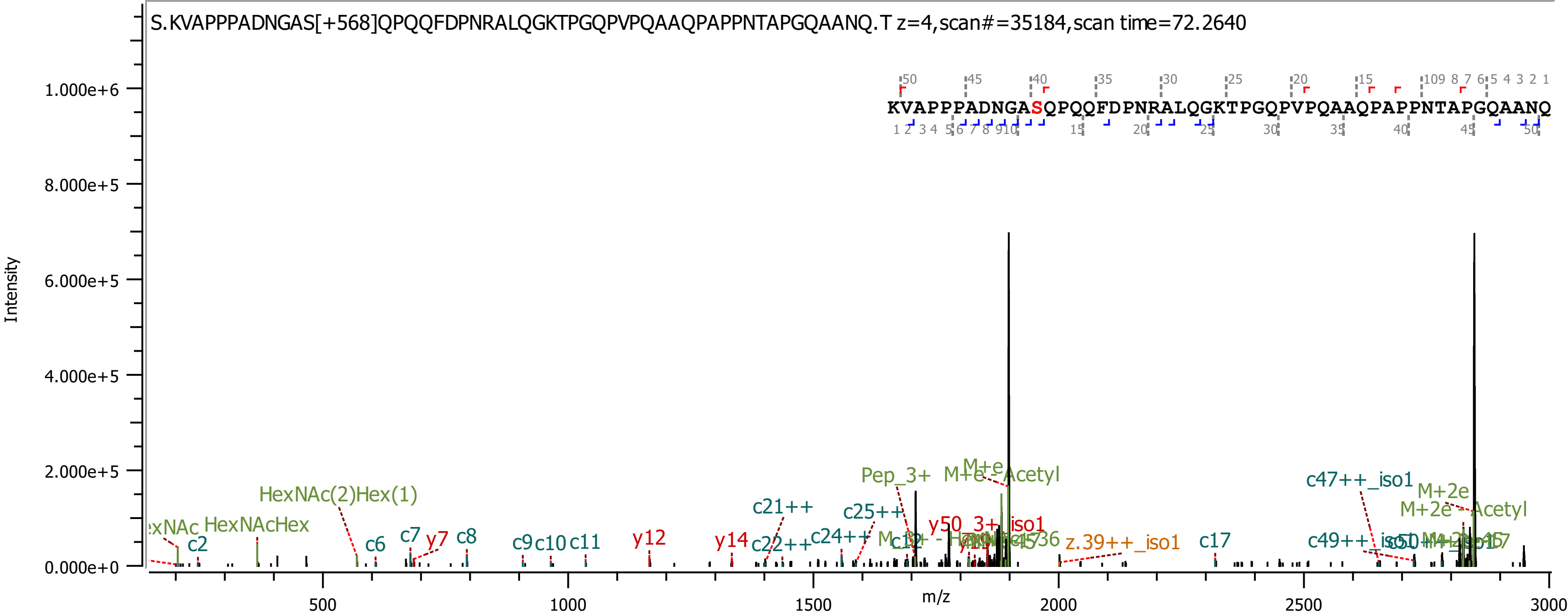

S.KVAPPPADNGAS[+568]QPQQFDPNRALQGKTPGQPVPQAAQPAPPNTAPGQAANQ.T z=4,scan#=35184,scan time=72.2640
KVAPPPADNGASQPQQFDPNRALQGKTPGQPVPQAAQPAPPNTAPGQAANQ
Intensity
m/z
HexNAc(2)Hex(1)
HexNAcHex
c2
c6
c7
y7
c8
c9
c10
c11
y12
y14
c21++
c22++
c24++
c25++
Pep_3+
M+e
M+e - Acetyl
y50_3+_iso1
z.39++_iso1
c17
c47++_iso1
c49++
M+2e
M+2e - Acetyl

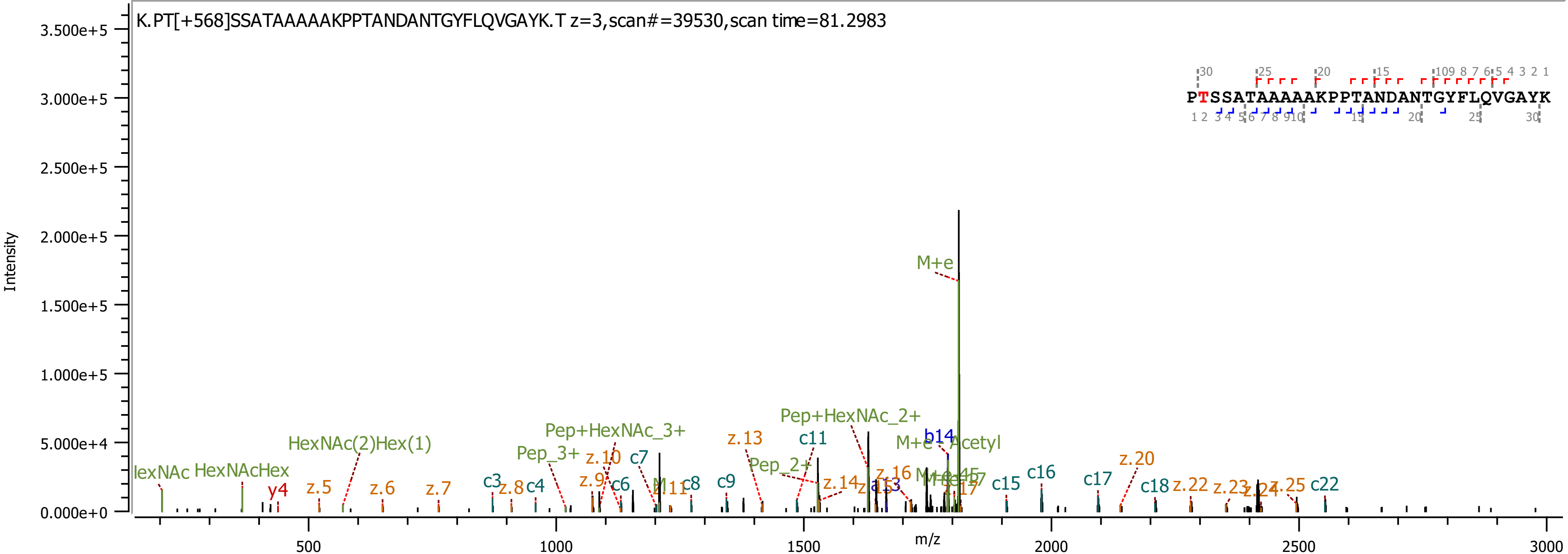

K.PT[+568]SSATAAAAAKPPTANDANTGYFLQVGAYK.T z=3,scan#=39530,scan time=81.2983
PTSSATAAAAAKPPTANDANTGYFLQVGAYK
Intensity
m/z
M+e
Pep+HexNAc_2+
Pep+HexNAc_3+
HexNAc(2)Hex(1)
HexNAcHex
Pep_3+
Pep_2+
M+e - Acetyl
b14
y4
c3
c4
c6
c7
c8
c9
c11
c15
c16
c17
c18
c22
z.5
z.6
z.7
z.8
z.9
z.10
z.13
z.14
z.16
z.17
z.20
z.22
z.23
z.24
z.25

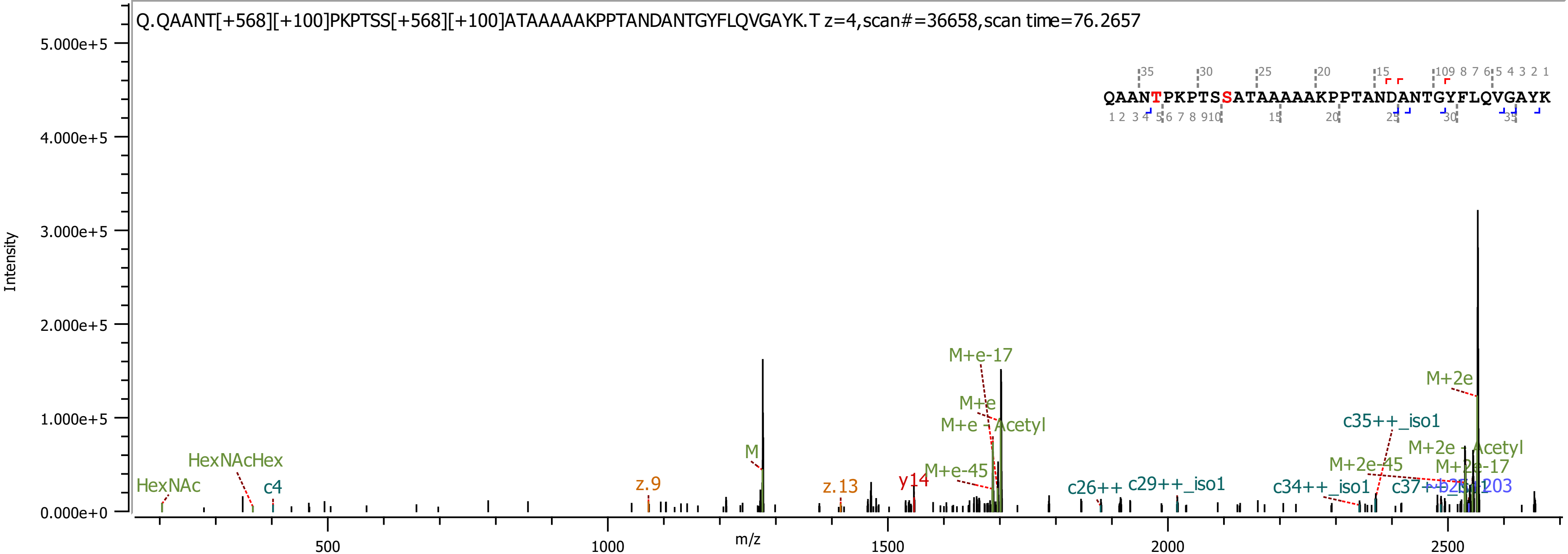

Q.QAANT[+568][+100]PKPTSS[+568][+100]ATAAAAAKPPTANDANTGYFLQVGAYK.T z=4,scan#=36658,scan time=76.2657
QAANTPKPTSSATAAAAAKPPTANDANTGYFLQVGAYK
Intensity
m/z
5.000e+5
4.000e+5
3.000e+5
2.000e+5
1.000e+5
0.000e+0
500
1000
1500
2000
2500
HexNAc
HexNAcHex
c4
z.9
M
z.13
y14
M+e-45
M+e-17
M+e
M+e - Acetyl
c26++
c29++_iso1
c34++_iso1
c35++_iso1
M+2e-45
M+2e
M+2e - Acetyl
M+2e-17
c37++

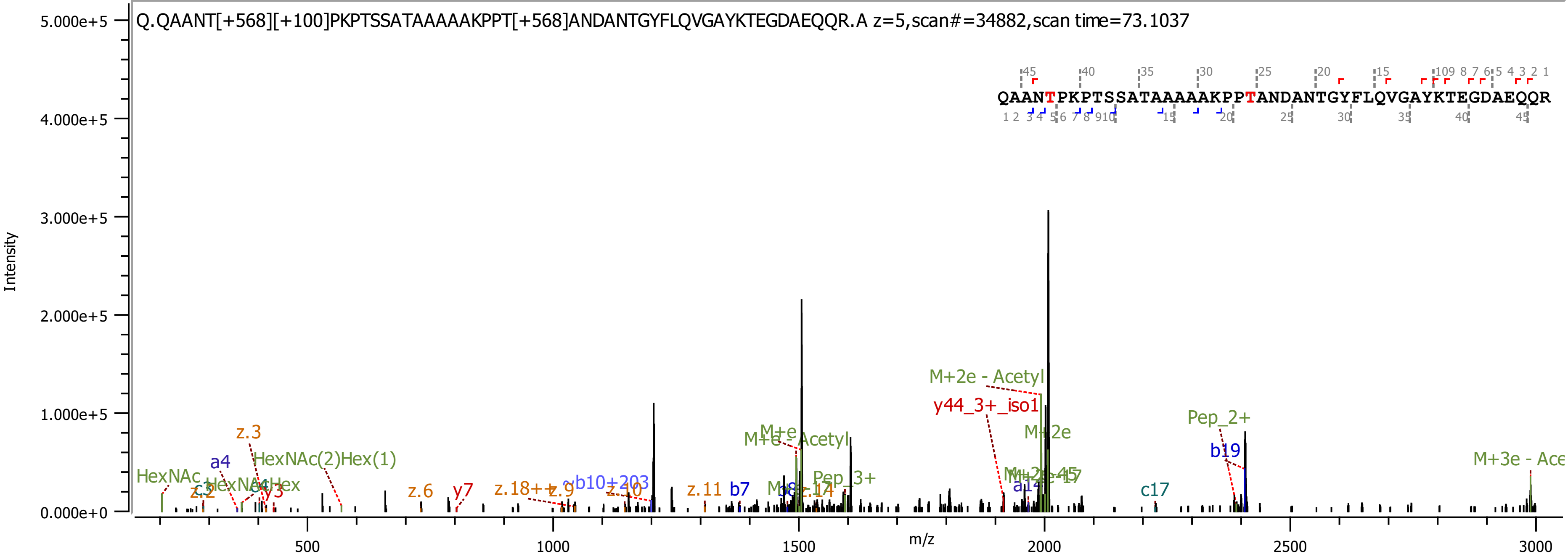

Q.QAANT[+568][+100]PKPTSSATAAAAAKPPT[+568]ANDANTGYFLQVGAYKTEGDAEQQR.A z=5,scan#=34882,scan time=73.1037
QAANTPKPTSSATAAAAAKPPTANDANTGYFLQVGAYKTEGDAEQQR
Intensity
m/z
5.000e+5
4.000e+5
3.000e+5
2.000e+5
1.000e+5
0.000e+0
500
1000
1500
2000
2500
3000
M+2e - Acetyl
y44_3+_iso1
M+2e
Pep_2+
b19
M+3e - Ace
c17
M+e - Acetyl
M+e
Pep_3+
z.3
a4
HexNAc(2)Hex(1)
HexNAc
z.6
y7
z.11
b7
c3
y3

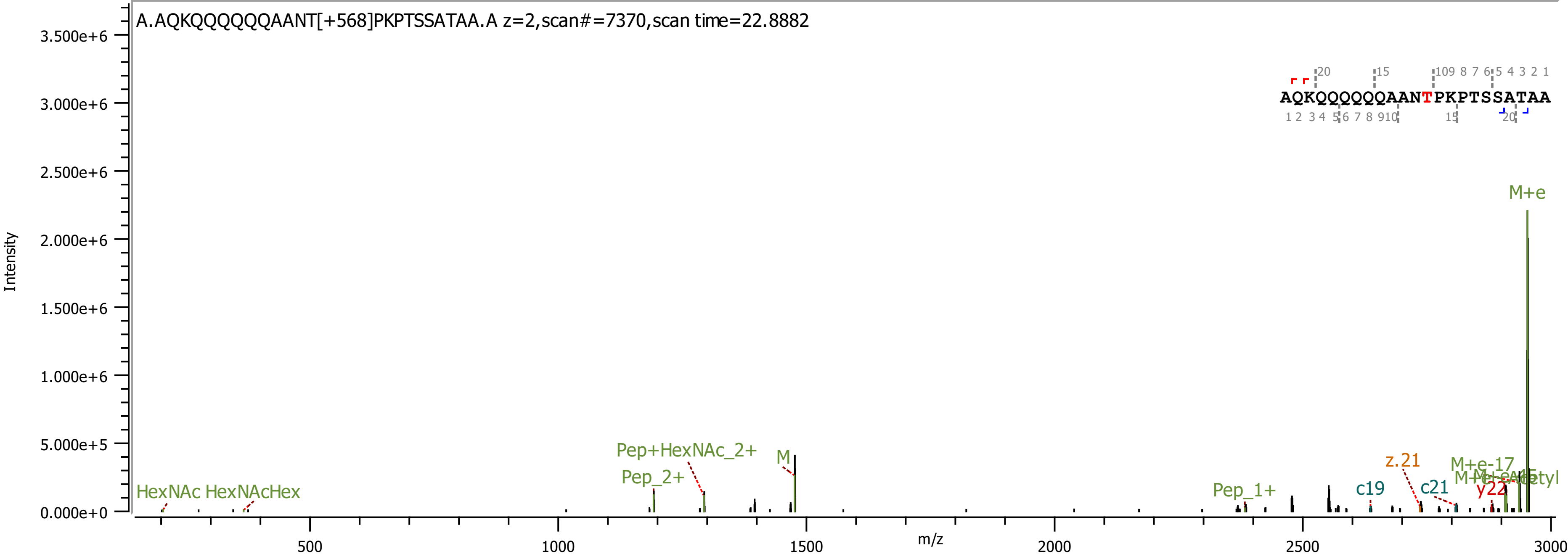
A.AQKQQQQQQAANT[+568]PKPTSSATAA.A z=2,scan#=7370,scan time=22.8882
AQKQQQQQQAANTPKPTSSATAA
Intensity
m/z
M+e
M+e-17
Pep+HexNAc_2+
Pep_2+
M
HexNAc
HexNAcHex
Pep_1+
c19
c21
z.21
y22
3.500e+6
3.000e+6
2.500e+6
2.000e+6
1.500e+6
1.000e+6
5.000e+5
0.000e+0
500
1000
1500
2000
2500
3000

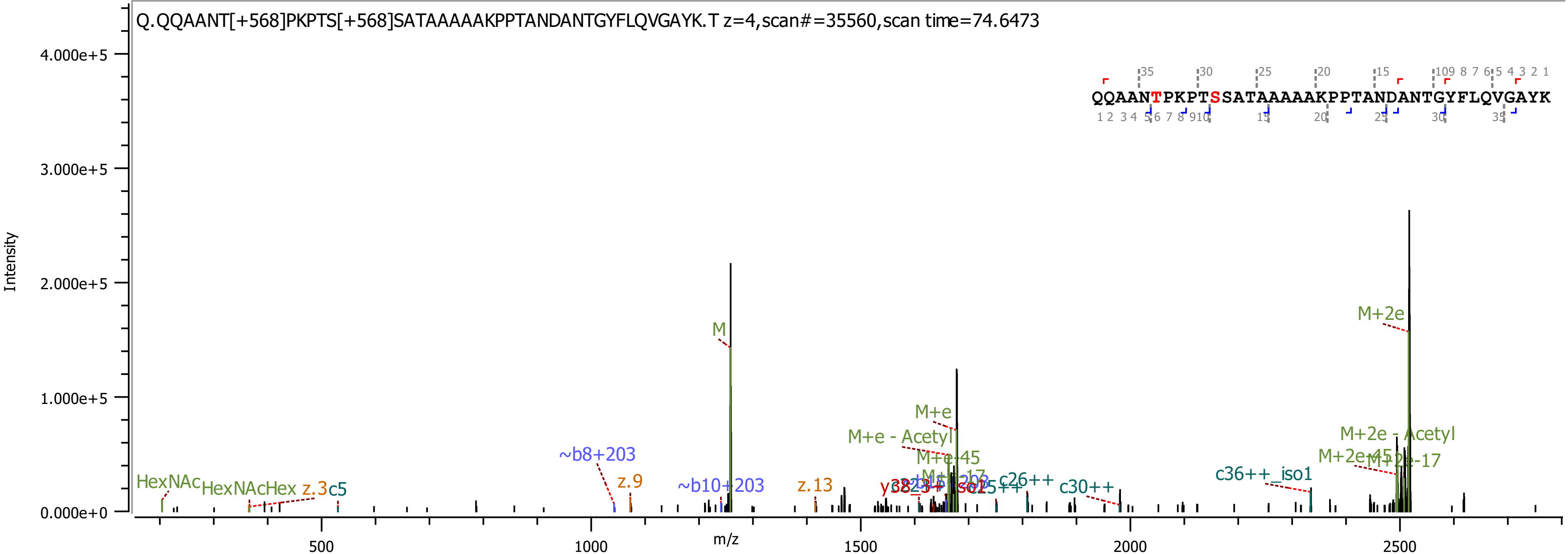
Q.QQAANT[+568]PKPTS[+568]SATAAAAAKPPTANDANTGYFLQVGAYK.T z=4,scan#=35560,scan time=74.6473
QQAANTPKPTSSATAAAAAKPPTANDANTGYFLQVGAYK
Intensity
m/z
4.000e+5
3.000e+5
2.000e+5
1.000e+5
0.000e+0
500
1000
1500
2000
2500
M
M+2e
M+e
M+e - Acetyl
M+e-45
M+2e - Acetyl
M+2e-45
M+2e-17
HexNAc
HexNAcHex
z.3
c5
~b8+203
z.9
~b10+203
z.13
c26++
c30++
c36++_iso1

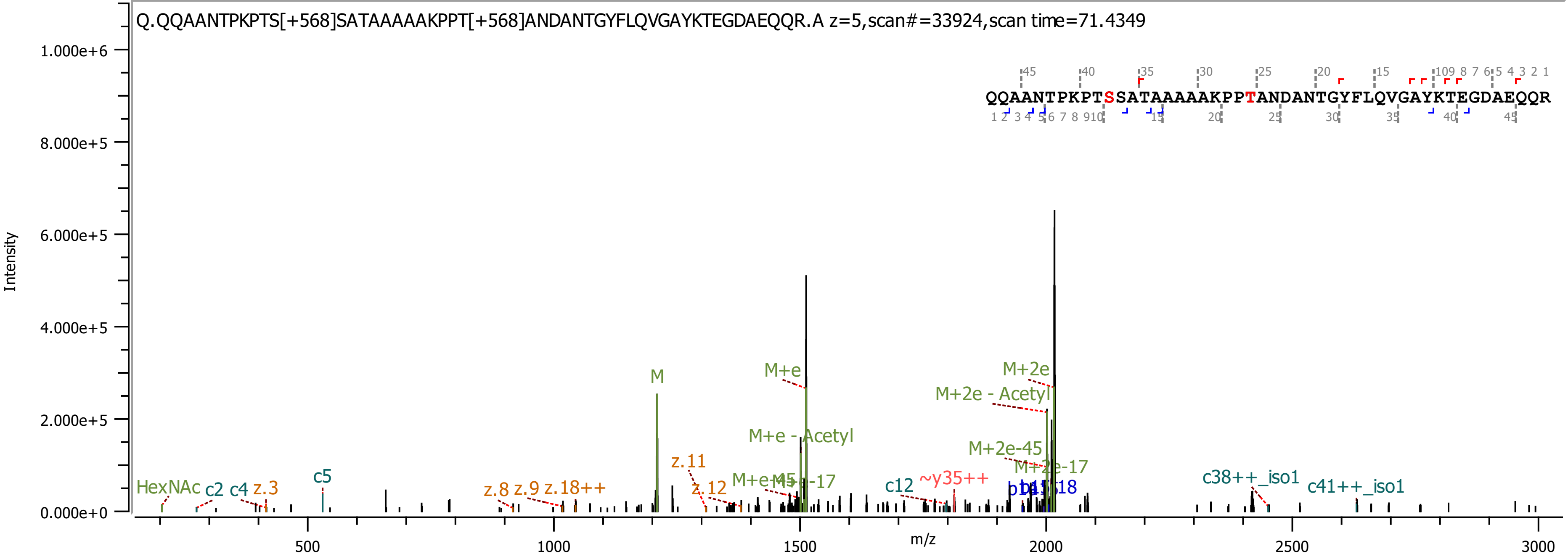
Q.QQAANTPKPTS[+568]SATAAAAAKPPT[+568]ANDANTGYFLQVGAYKTEGDAEQQR.A z=5,scan#=33924,scan time=71.4349
QQAANTPKPTSSATAAAAAKPPTANDANTGYFLQVGAYKTEGDAEQQR
Intensity
m/z
M
M+e
M+2e
M+2e - Acetyl
M+e - Acetyl
M+2e-45
M+2e-17
M+e-45
HexNAc
c2
c4
z.3
c5
z.8
z.9
z.18++
z.11
z.12
c12
~y35++
c38++_iso1
c41++_iso1

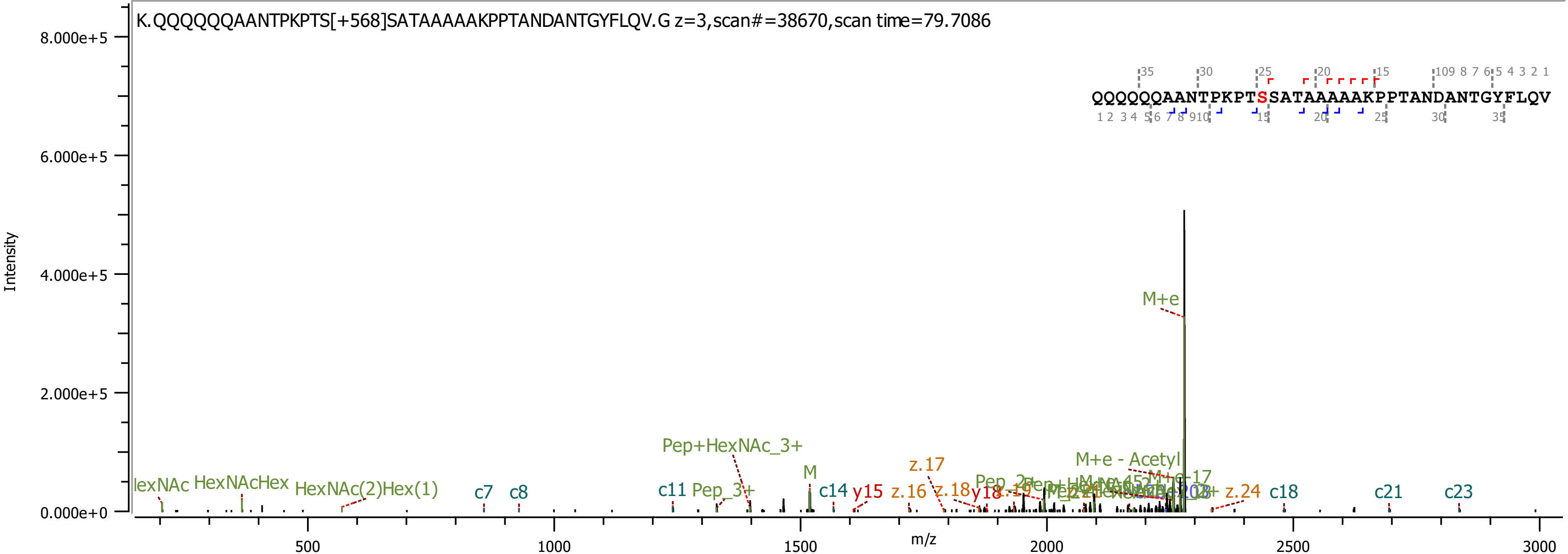

K.QQQQQQAANTPKPTS[+568]SATAAAAAKPPTANDANTGYFLQV.G z=3,scan#=38670,scan time=79.7086
QQQQQQAANTPKPTSSATAAAAAKPPTANDANTGYFLQV
Intensity
m/z
M+e
Pep+HexNAc_3+
M
M+e - Acetyl
z.17
HexNAcHex
HexNAc(2)Hex(1)
c7
c8
c11
Pep_3+
c14
y15
z.16
z.18
z.24
c18
c21
c23

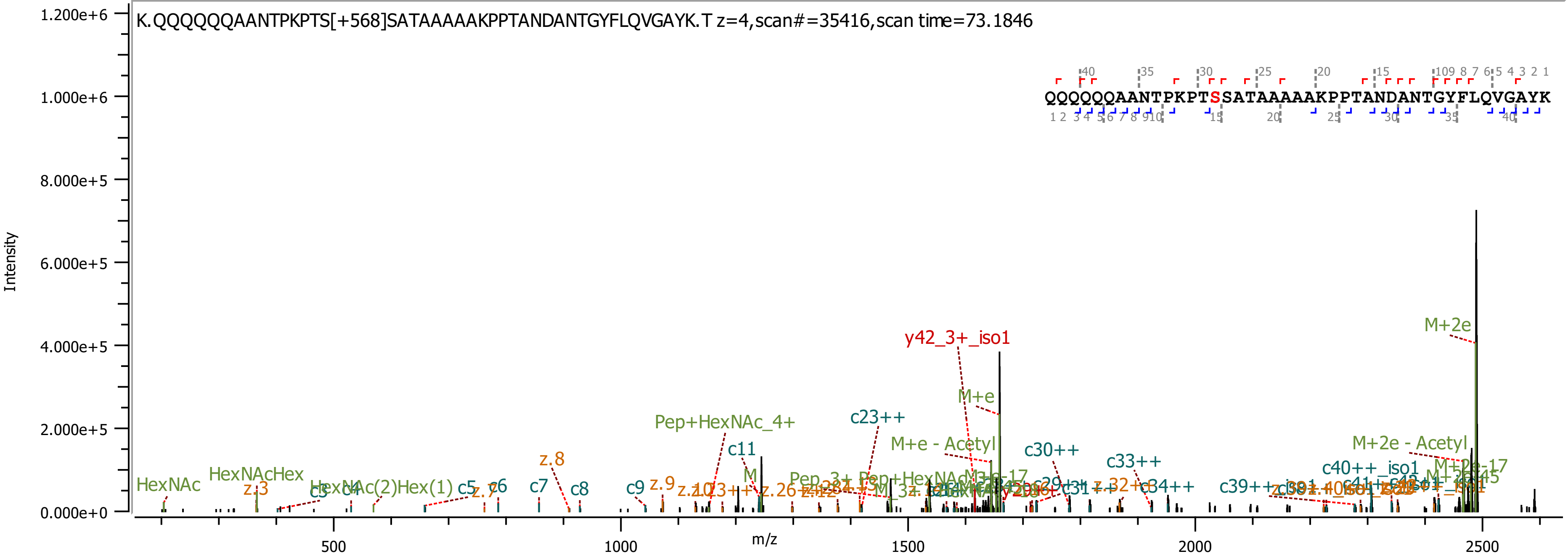
K.QQQQQQAANTPKPTS[+568]SATAAAAAKPPTANDANTGYFLQVGAYK.T z=4,scan#=35416,scan time=73.1846
QQQQQQAANTPKPTSSATAAAAAKPPTANDANTGYFLQVGAYK
Intensity
m/z
1.200e+6
1.000e+6
8.000e+5
6.000e+5
4.000e+5
2.000e+5
0.000e+0
500
1000
1500
2000
2500
M+2e
y42_3+_iso1
M+e
Pep+HexNAc_4+
c23++
M+e - Acetyl
c30++
c33++
M+2e - Acetyl
c40++_iso1
c11
z.8
HexNAc
HexNAcHex
z.3
c3
c4
HexNAc(2)Hex(1)
c5
z.7
c6
c7
c8
c9
z.9
c34++
c39++

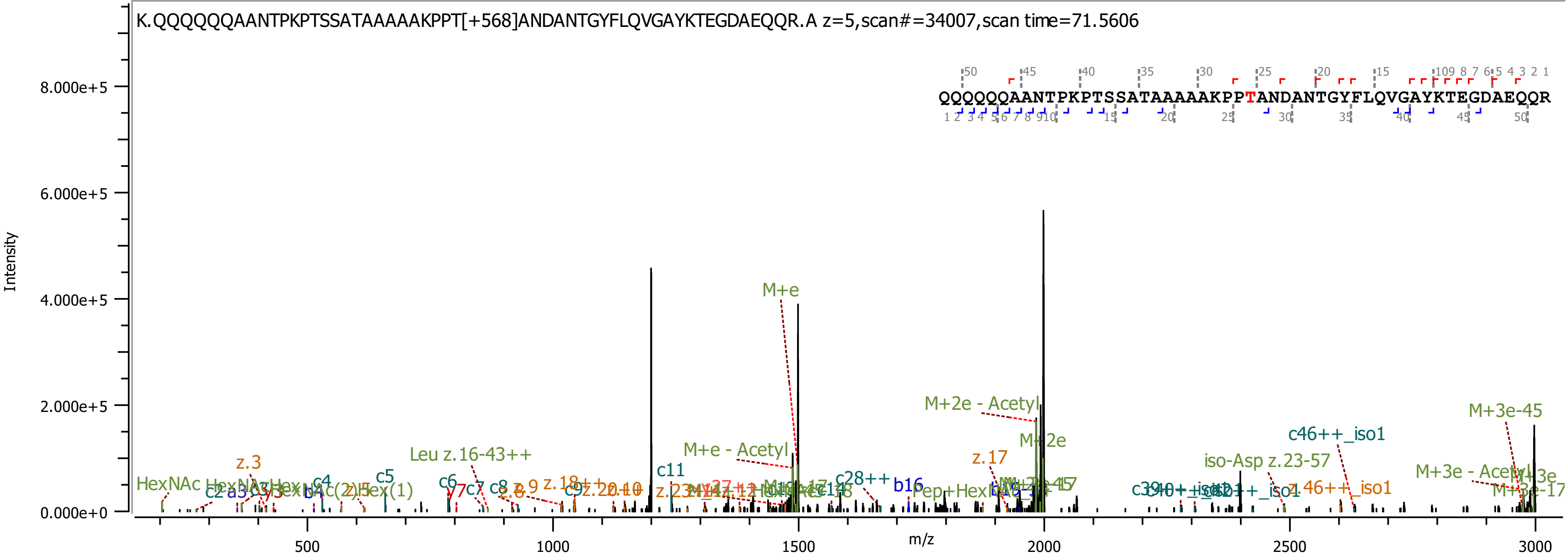

K.QQQQQQAANTPKPTSSATAAAAAKPPT[+568]ANDANTGYFLQVGAYKTEGDAEQQR.A z=5,scan#=34007,scan time=71.5606
QQQQQQAANTPKPTSSATAAAAAKPPTANDANTGYFLQVGAYKTEGDAEQQR
Intensity
m/z
8.000e+5
6.000e+5
4.000e+5
2.000e+5
0.000e+0
500
1000
1500
2000
2500
3000
M+e
M+e - Acetyl
M+2e - Acetyl
M+2e
M+3e-45
M+3e - Acetyl
c46++_iso1
iso-Asp z.23-57
z.46++_iso1
z.17
Leu z.16-43++
z.3
HexNAc
c4
c5
c6
y7
c7
c8
c11
c28++
b16

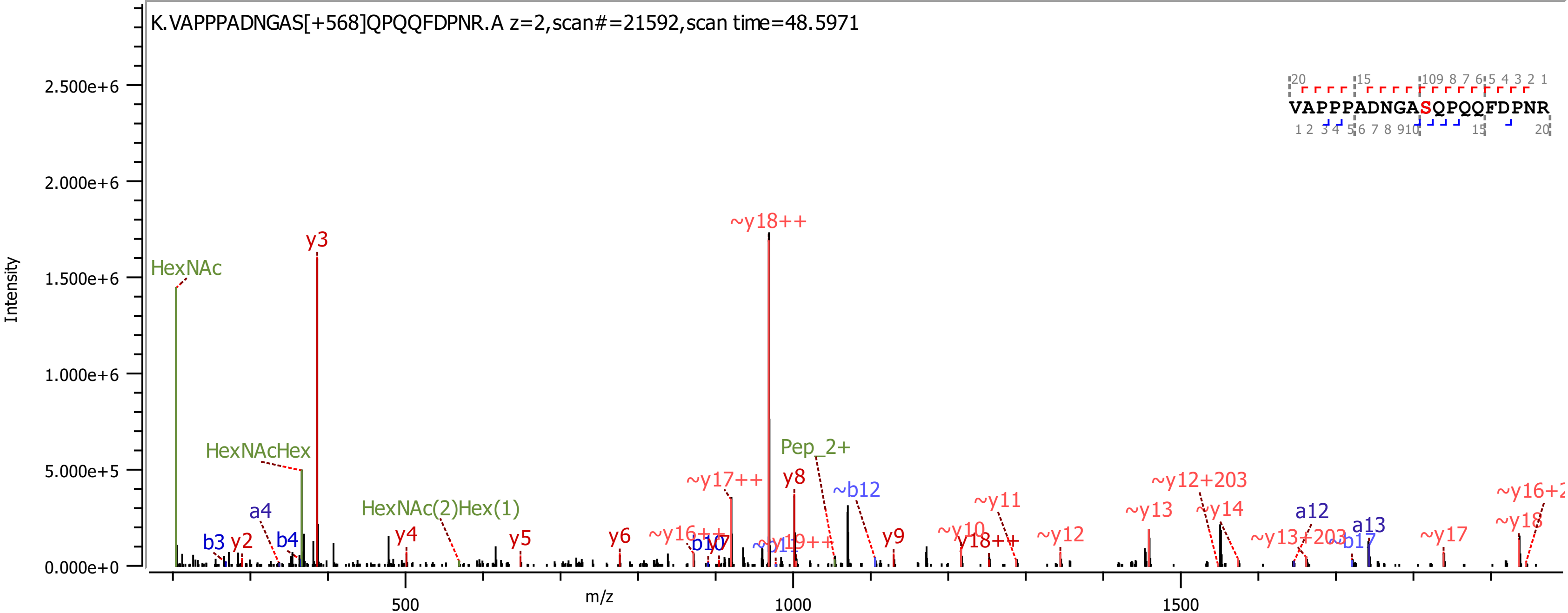
K.VAPPPADNGAS[+568]QPQQFDPNR.A z=2,scan#=21592,scan time=48.5971
VAPPPADNGASQPQQFDPNR
Intensity
m/z
2.500e+6
2.000e+6
1.500e+6
1.000e+6
5.000e+5
0.000e+0
500
1000
1500
HexNAc
HexNAcHex
HexNAc(2)Hex(1)
Pep_2+
y3
~y18++
y2
y4
y5
y6
y8
y9
b3
b4
a4
~y17++
~b12
~y11
~y13
~y14
~y12+203
~y12
a12
a13
~y17
~y18
~y13+203
~b17
y18++

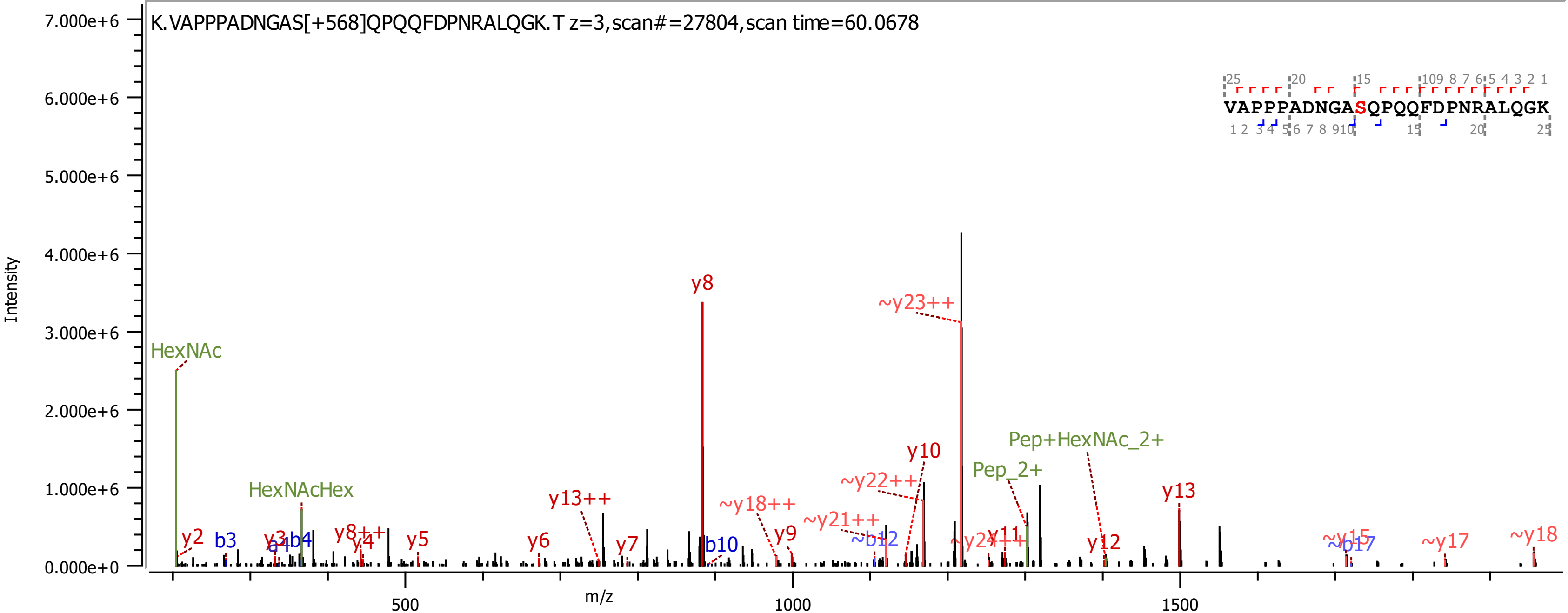
K.VAPPPADNGAS[+568]QPQQFDPNRALQGK.T z=3,scan#=27804,scan time=60.0678
VAPPPADNGASQPQQFDPNRALQGK
Intensity
m/z
HexNAc
HexNAcHex
y8
~y23++
y10
~y22++
Pep+HexNAc_2+
Pep_2+
y13
y13++
~y18++
~y21++
y2
b3
y3
a4
b4
y8++
y4
y5
y6
y7
b10
y9
~b12
~y24++
y11
y12
~y15
~b17
~y17
~y18

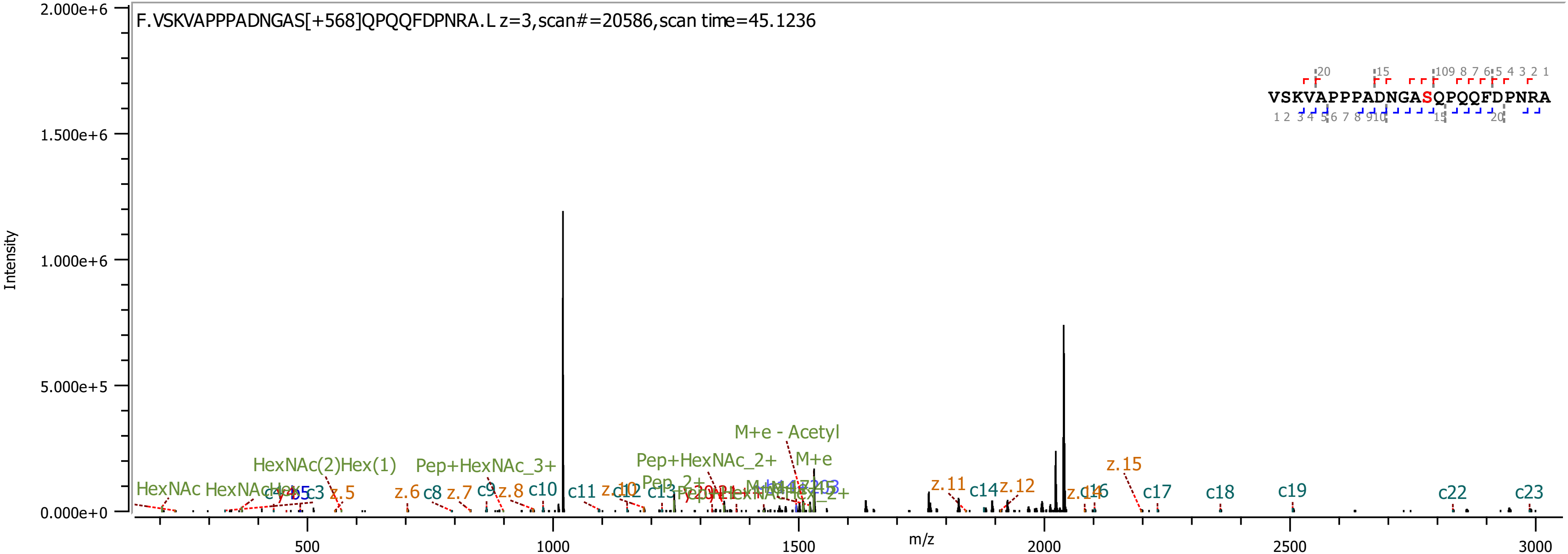
F.VSKVAPPPADNGAS[+568]QPQQFDPNRA.L z=3,scan#=20586,scan time=45.1236
VSKVAPPPADNGASQPQQFDPNRA
Intensity
2.000e+6
1.500e+6
1.000e+6
5.000e+5
0.000e+0
m/z
500
1000
1500
2000
2500
3000
HexNAc
HexNAc(2)Hex(1)
Pep+HexNAc_3+
Pep+HexNAc_2+
M+e - Acetyl
M+e
z.5
z.6
c8
z.7
c9
z.8
c10
c11
z.11
c14
z.12
z.15
c17
c18
c19
c22
c23

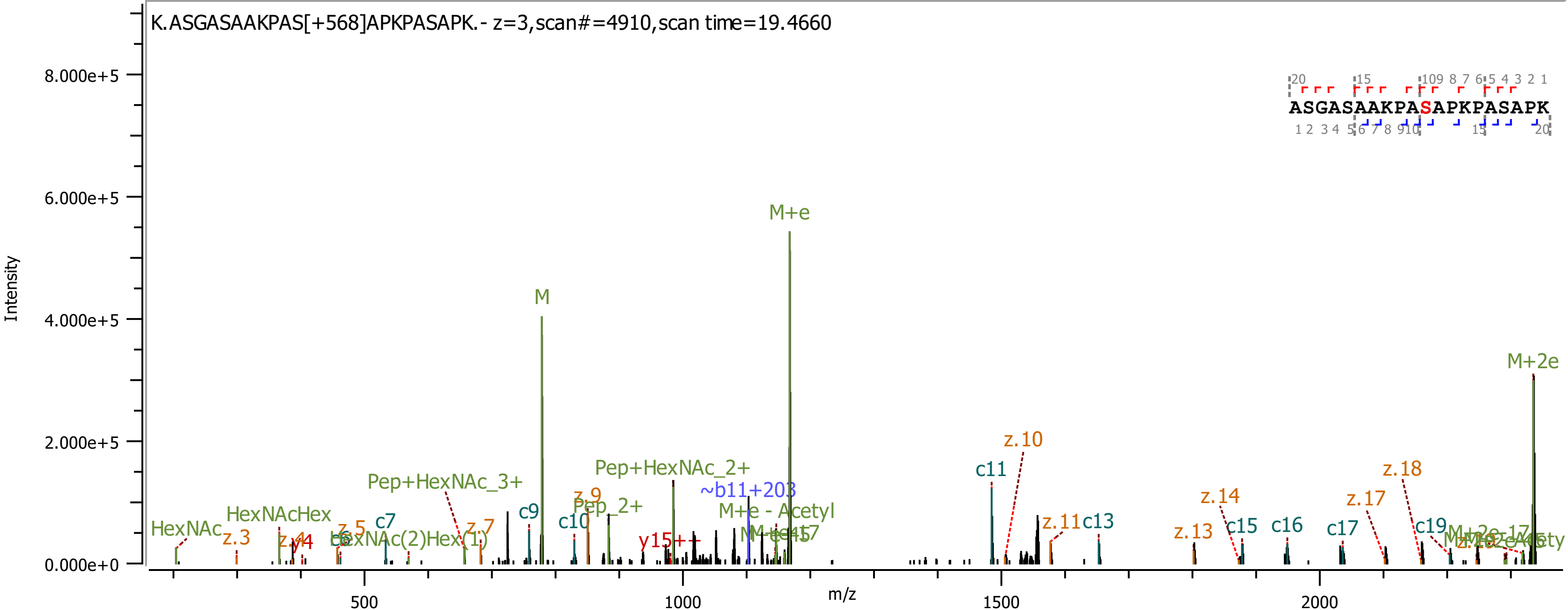
K.ASGASAAKPAS[+568]APKPASAPK.- z=3,scan#=4910,scan time=19.4660
Intensity
m/z
ASGASAAKPASAPKPASAPK
M+e
M
M+2e
z.10
c11
z.18
z.14
z.17
Pep+HexNAc_2+
Pep+HexNAc_3+
~b11+203
M+e - Acetyl
HexNAcHex
HexNAc
c9
c10
c7
z.9
Pep_2+
z.7
z.5
z.3
y15++
z.11
c13
z.13
c15
c16
c17
c19

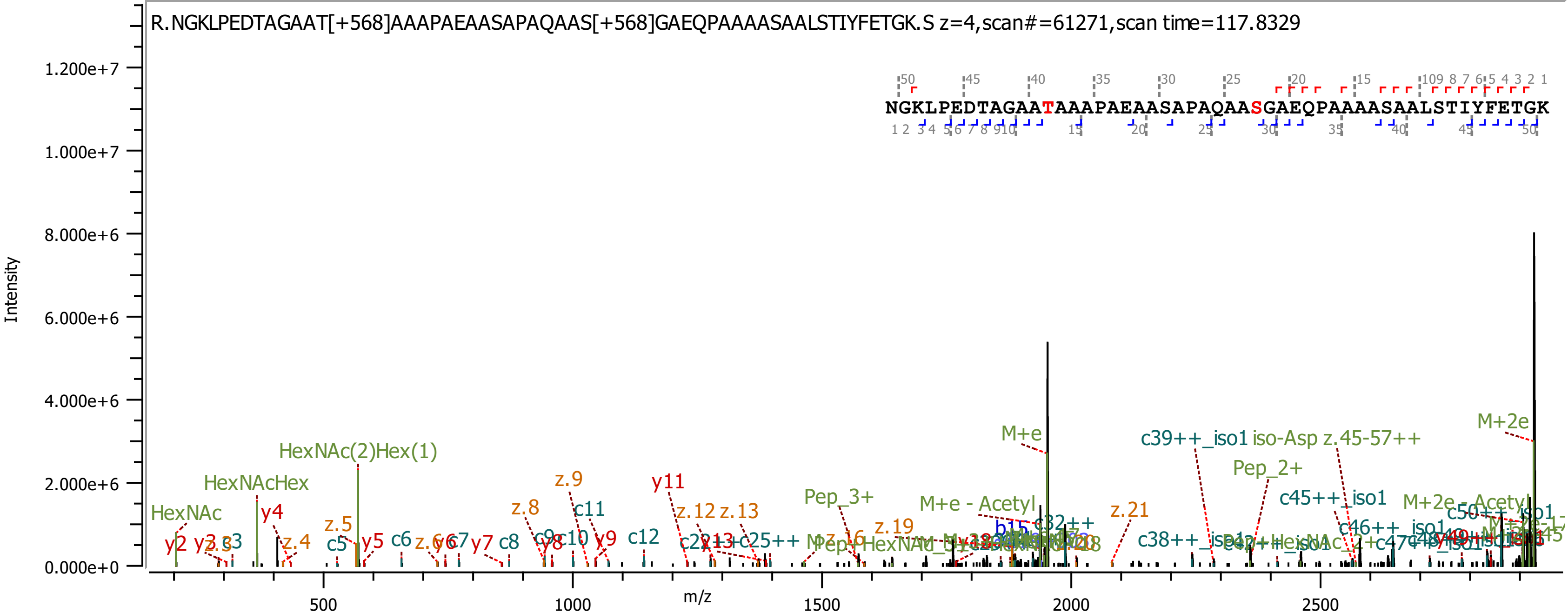
R.NGKLPEDTAGAAT[+568]AAAPAEAASAPAQAAS[+568]GAEQPAAAASAALSTIYFETGK.S z=4,scan#=61271,scan time=117.8329
NGKLPEDTAGAATAAAPAEAASAPAQAASGAEQPAAAASAALSTIYFETGK
Intensity
m/z
1.200e+7
1.000e+7
8.000e+6
6.000e+6
4.000e+6
2.000e+6
0.000e+0
500
1000
1500
2000
2500
HexNAc(2)Hex(1)
HexNAcHex
HexNAc
M+e
M+2e
M+e - Acetyl
M+2e - Acetyl
Pep_3+
Pep_2+
c39++_iso1
iso-Asp z.45-57++
c45++_iso1
c46++_iso1
z.21
y11
z.9
z.8
c11
z.12
z.13
z.19
c12
y9
c10
y8
c9
c8
y7
c7
y6
z.6
c6
y5
z.5
c5
z.4
y4
c3
z.3
y3
y2

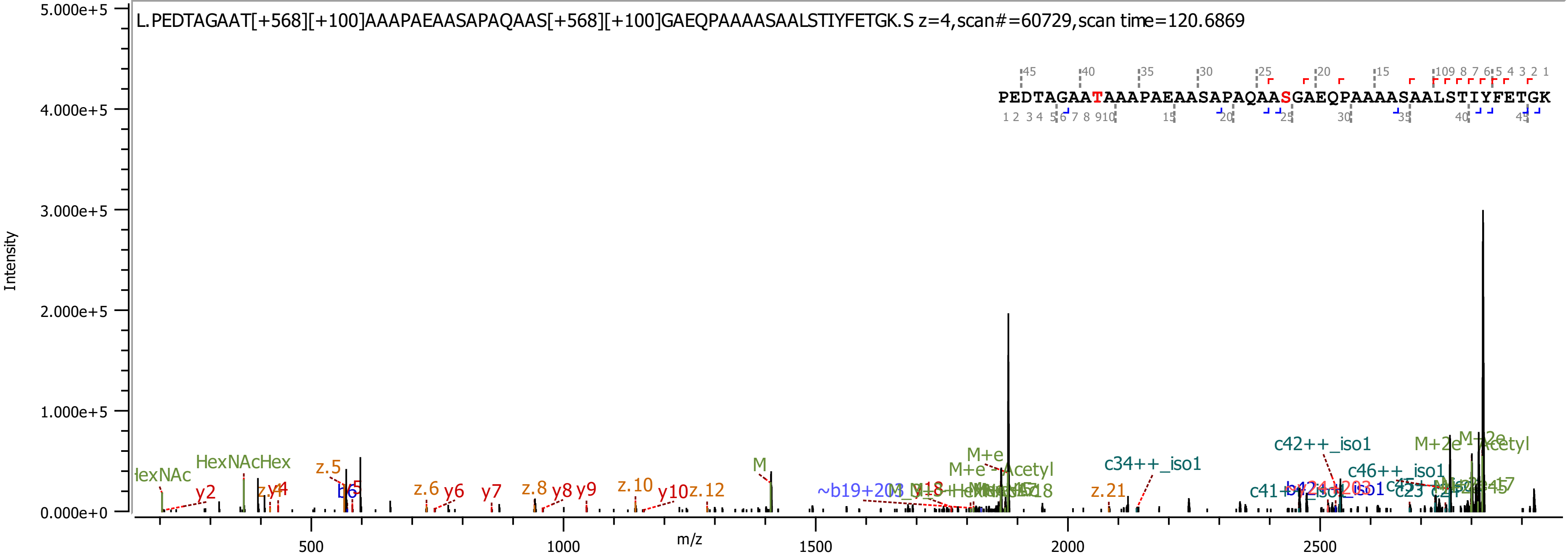
L.PEDTAGAAT[+568][+100]AAAPAEAASAPAQAAS[+568][+100]GAEQPAAAASAALSTIYFETGK.S z=4,scan#=60729,scan time=120.6869
PEDTAGAATAAAPAEAASAPAQAASGAEQPAAAASAALSTIYFETGK
Intensity
m/z
HexNAcHex
M+e
M+e-Acetyl
c42++_iso1
c34++_iso1
c46++_iso1
M+2e
M+2e-Acetyl
~b19+203
z.21
z.12
y10
z.10
y9
y8
z.8
y7
y6
z.6
b6
z.5
y4
y2

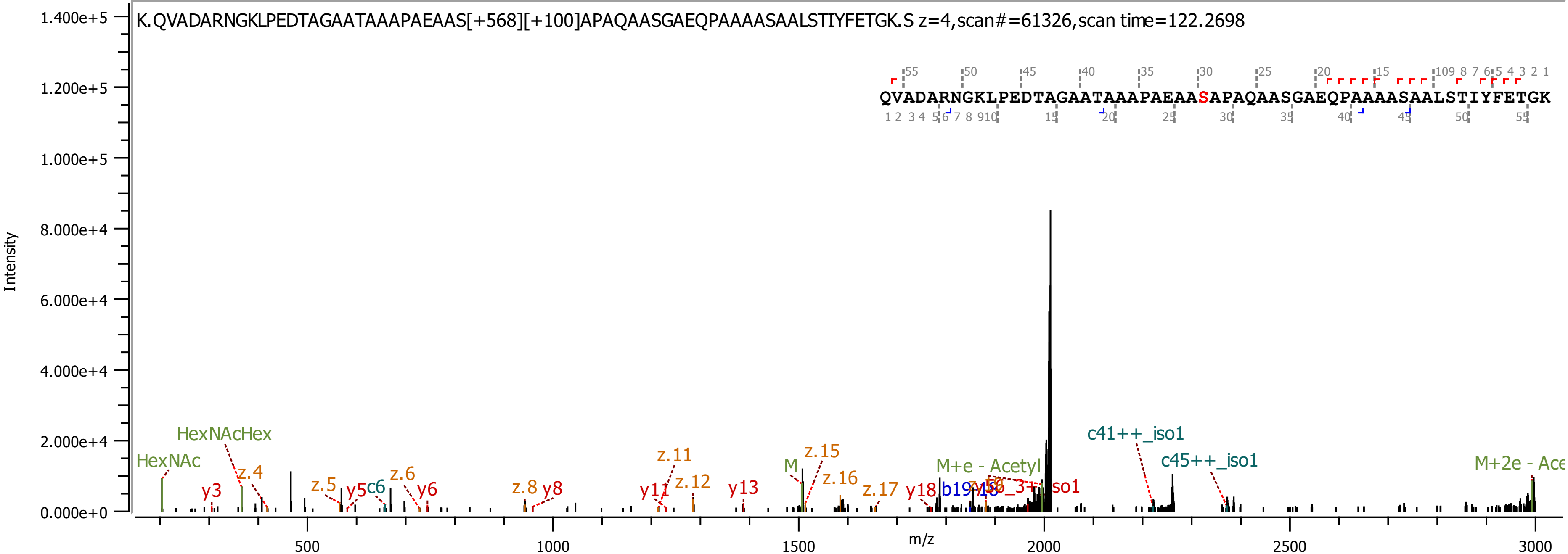

K.QVADARNGKLPEDTAGAATAAAPAEAAS[+568][+100]APAQAASGAEQPAAAASAALSTIYFETGK.S z=4,scan#=61326,scan time=122.2698
QVADARNGKLPEDTAGAATAAAPAEAASAPAQAASGAEQPAAAASAALSTIYFETGK
Intensity
m/z
1.400e+5
1.200e+5
1.000e+5
8.000e+4
6.000e+4
4.000e+4
2.000e+4
0.000e+0
500
1000
1500
2000
2500
3000
HexNAc
HexNAcHex
y3
z.4
z.5
y5
c6
z.6
y6
z.8
y8
y11
z.11
z.12
y13
M
z.15
z.16
z.17
y18
b19
M+e - Acetyl
c41++_iso1
c45++_iso1
M+2e - Ace

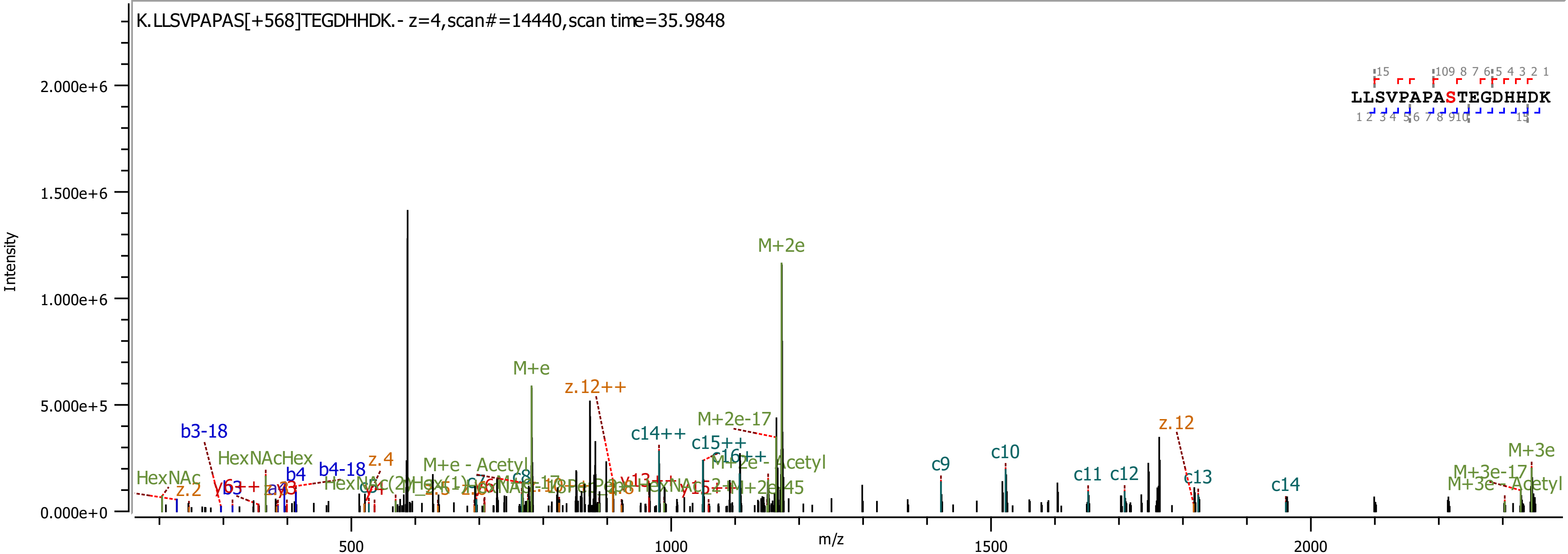
K.LLSVPAPAS[+568]TEGDHHDK.- z=4,scan#=14440,scan time=35.9848
Intensity
m/z
LLSVPAPASTEGDHHDK
M+2e
M+e
z.12++
M+2e-17
c14++
c15++
c16++
M+2e - Acetyl
z.12
c9
c10
c11
c12
c13
c14
M+3e
M+3e-17
M+3e - Acetyl
b3-18
HexNAcHex
HexNAc
z.2
b3
b4
b4-18
z.4
M+e - Acetyl
c8
0.000e+0
5.000e+5
1.000e+6
1.500e+6
2.000e+6
500
1000
1500
2000

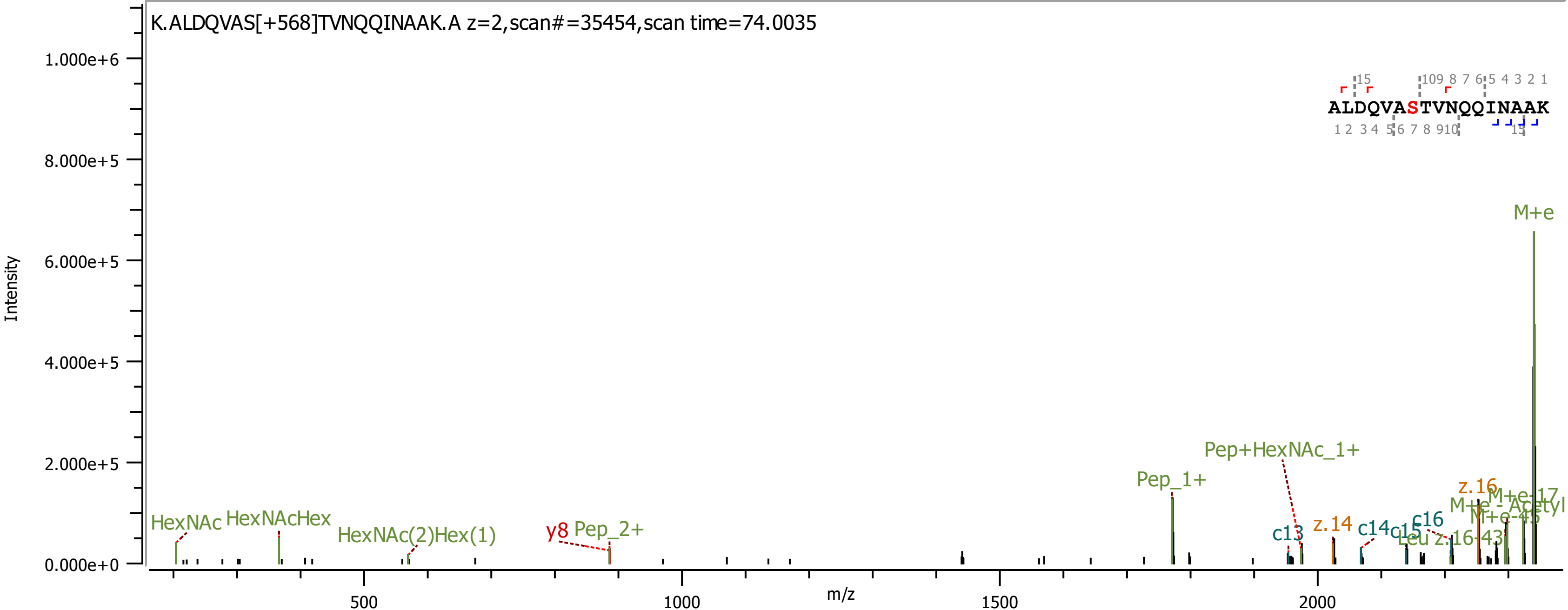
K.ALDQVAS[+568]TVNQQINAAK.A z=2,scan#=35454,scan time=74.0035
ALDQVASTVNQQINAAK
Intensity
m/z
M+e
Pep+HexNAc_1+
Pep_1+
HexNAc
HexNAcHex
HexNAc(2)Hex(1)
y8
Pep_2+
c13
z.14
c14
c15
c16
z.16
Leu
M+e-17
M+e - Acetyl
M+e-45
z.16-43

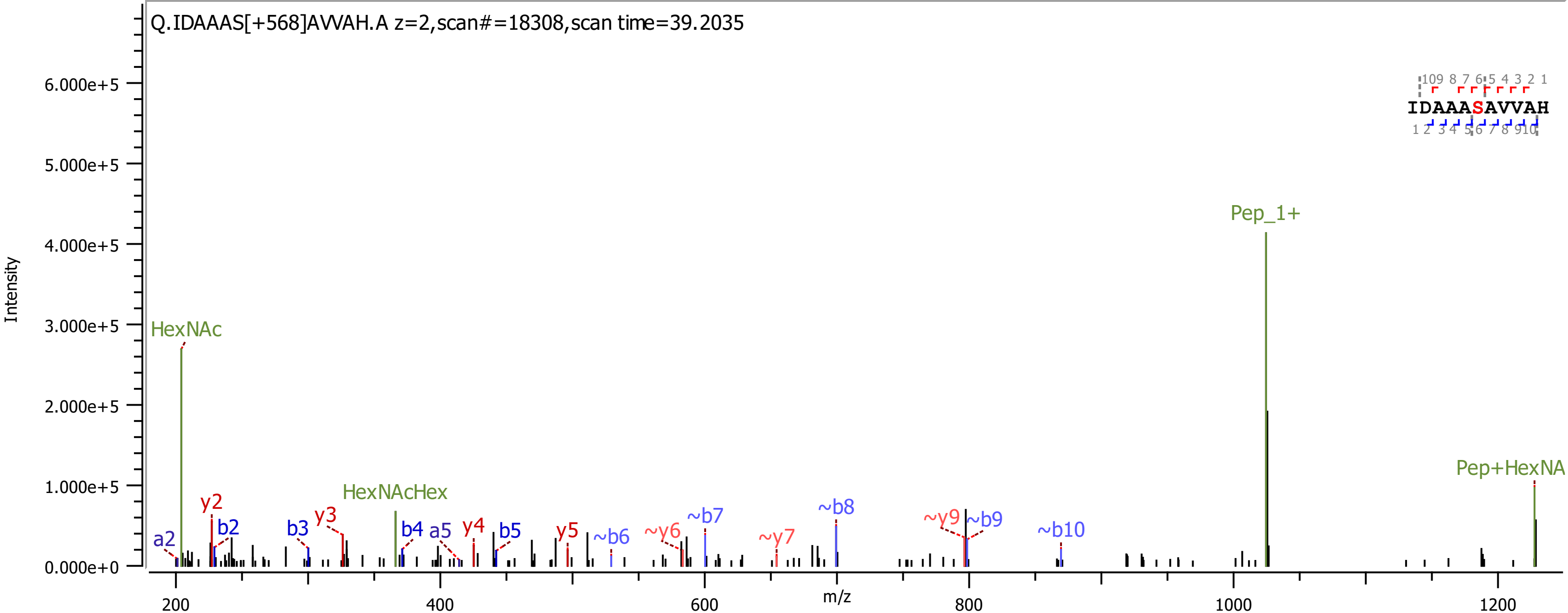

Q.IDAAAS[+568]AVVAH.A z=2,scan#=18308,scan time=39.2035
Intensity
m/z
HexNAc
HexNAcHex
Pep_1+
Pep+HexNA
a2
y2
b2
b3
y3
b4
a5
y4
b5
y5
~b6
~y6
~b7
~y7
~b8
~y9
~b9
~b10
IDAAASAVVAH

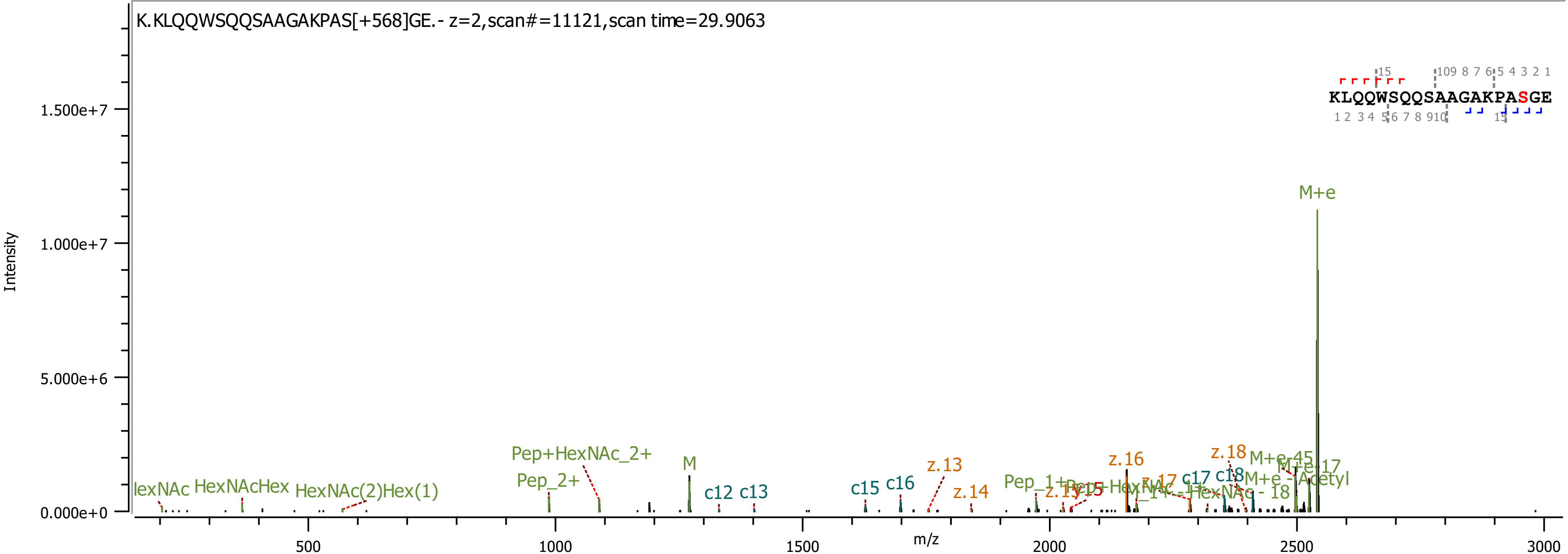

K.KLQQWSQQSAAGAKPAS[+568]GE.- z=2,scan#=11121,scan time=29.9063
KLQQWSQQSAAGAKPASGE
Intensity
m/z
M+e
z.16
z.18
M+e-45
M+e-17
M+e - Acetyl
c17 c18
c15 c16
c12 c13
z.13
z.14
M
Pep+HexNAc_2+
Pep_2+
HexNAcHex
HexNAc(2)Hex(1)
Pep_1+

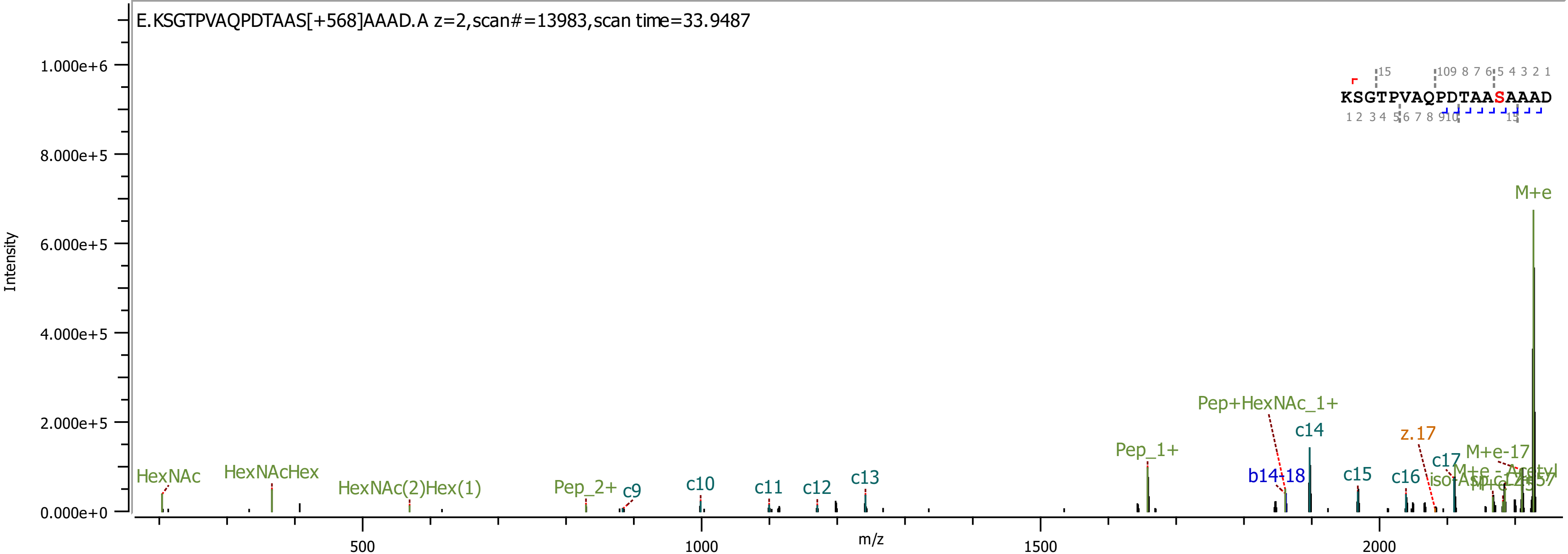

E.KSGTPVAQPDTAAS[+568]AAAD.A z=2,scan#=13983,scan time=33.9487
KSGTPVAQPDTAASAAAD
Intensity
m/z
HexNAc
HexNAcHex
HexNAc(2)Hex(1)
Pep_2+
c9
c10
c11
c12
c13
Pep_1+
Pep+HexNAc_1+
b14-18
c14
c15
c16
z.17
c17
M+e-17
M+e

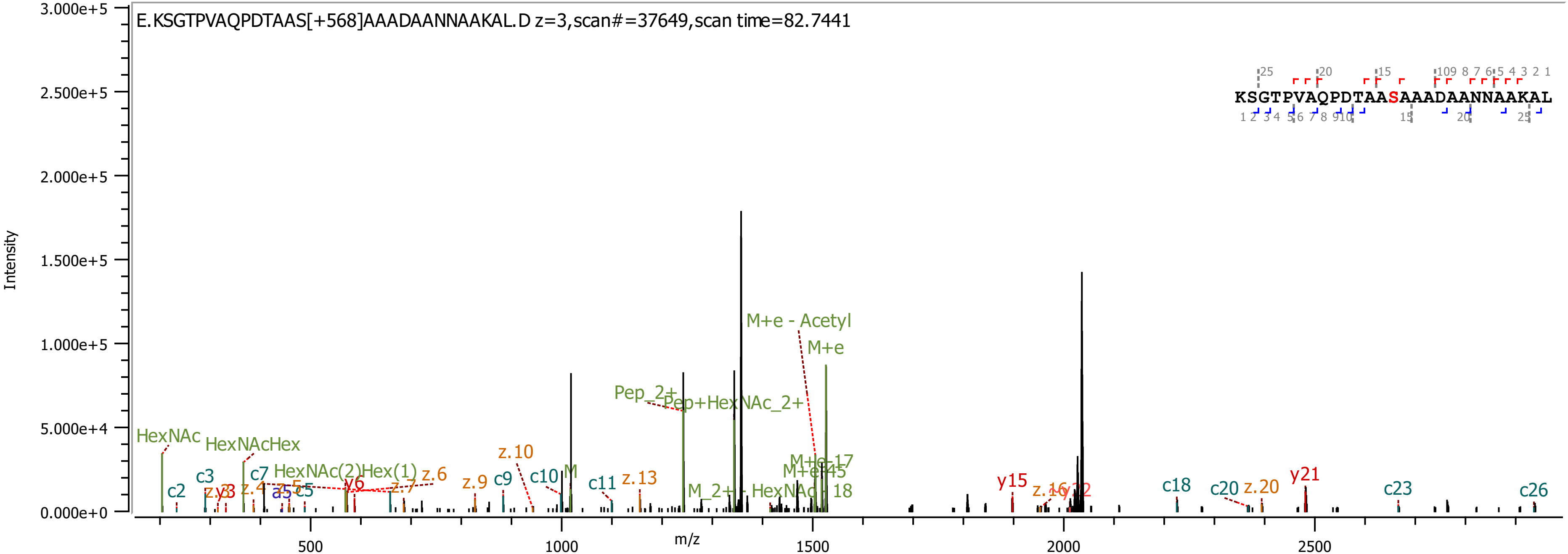
E.KSGTPVAQPDTAAS[+568]AAADAANNAAKAL.D z=3,scan#=37649,scan time=82.7441
KSGTPVAQPDTAASAAADAANNAAKAL
Intensity
m/z
3.000e+5
2.500e+5
2.000e+5
1.500e+5
1.000e+5
5.000e+4
0.000e+0
500
1000
1500
2000
2500
HexNAc
HexNAcHex
HexNAc(2)Hex(1)
Pep_2+
Pep+HexNAc_2+
M+e - Acetyl
M+e
M_2+
c2
c3
c5
c7
c9
c10
c11
c18
c20
c23
c26
a5
y6
y15
y21
z.4
z.5
z.6
z.7
z.9
z.10
z.13
z.20

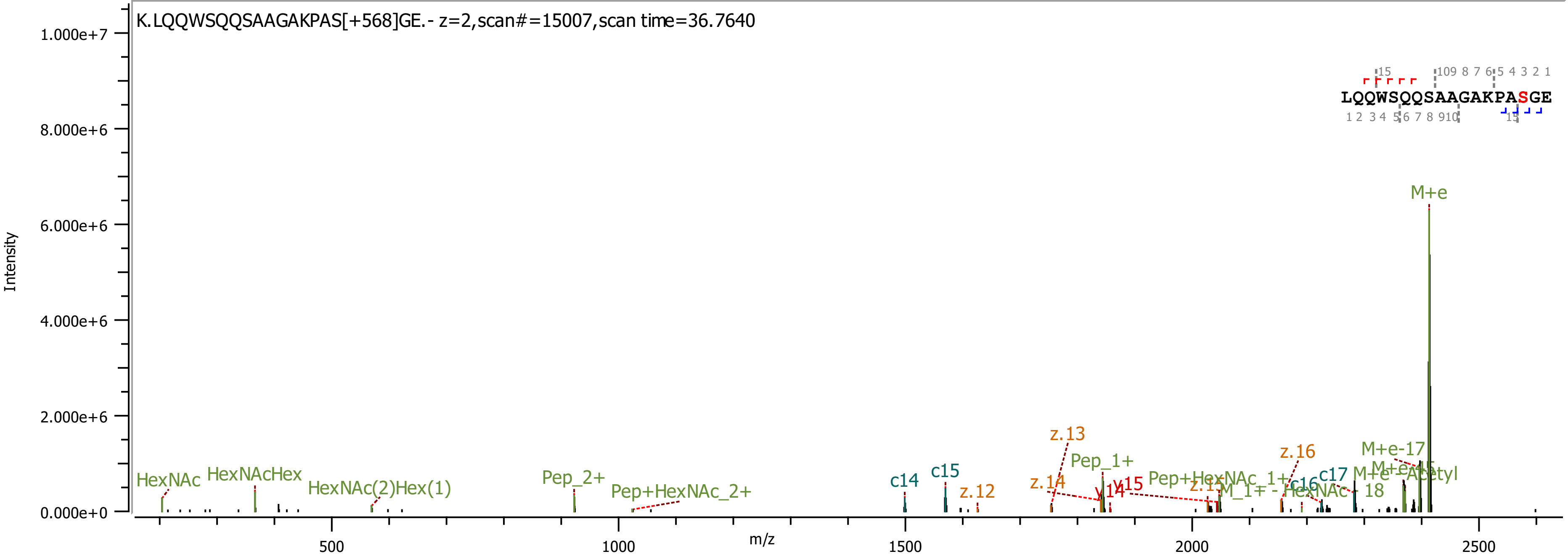
K.LQQWSQQSAAGAKPAS[+568]GE.- z=2,scan#=15007,scan time=36.7640
LQQWSQQSAAGAKPASGE
Intensity
m/z
1.000e+7
8.000e+6
6.000e+6
4.000e+6
2.000e+6
0.000e+0
500
1000
1500
2000
2500
HexNAc
HexNAcHex
HexNAc(2)Hex(1)
Pep_2+
Pep+HexNAc_2+
c14
c15
z.12
z.13
z.14
Pep_1+
y14
y15
Pep+HexNAc_1+
z.16
c16
c17
M+e-17
M+e
M+e-Acetyl

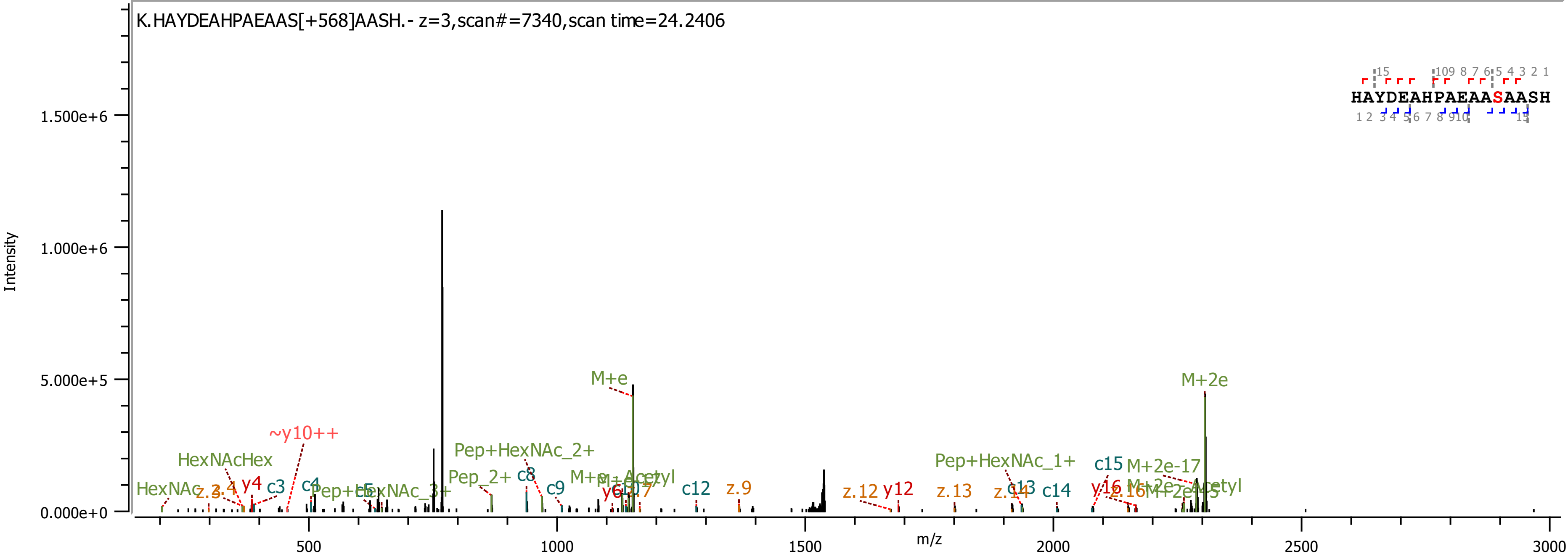
K.HAYDEAHPAEAAS[+568]AASH.- z=3,scan#=7340,scan time=24.2406
HAYDEAHPAEAASAASH
Intensity
m/z
1.500e+6
1.000e+6
5.000e+5
0.000e+0
500
1000
1500
2000
2500
3000
M+e
M+2e
~y10++
HexNAcHex
HexNAc
Pep+HexNAc_2+
Pep_2+
Pep+HexNAc_1+
M+2e-17
c15
c3
c4
c8
c9
c12
c13
c14
y4
y12
y16
z.9
z.12
z.13
z.14

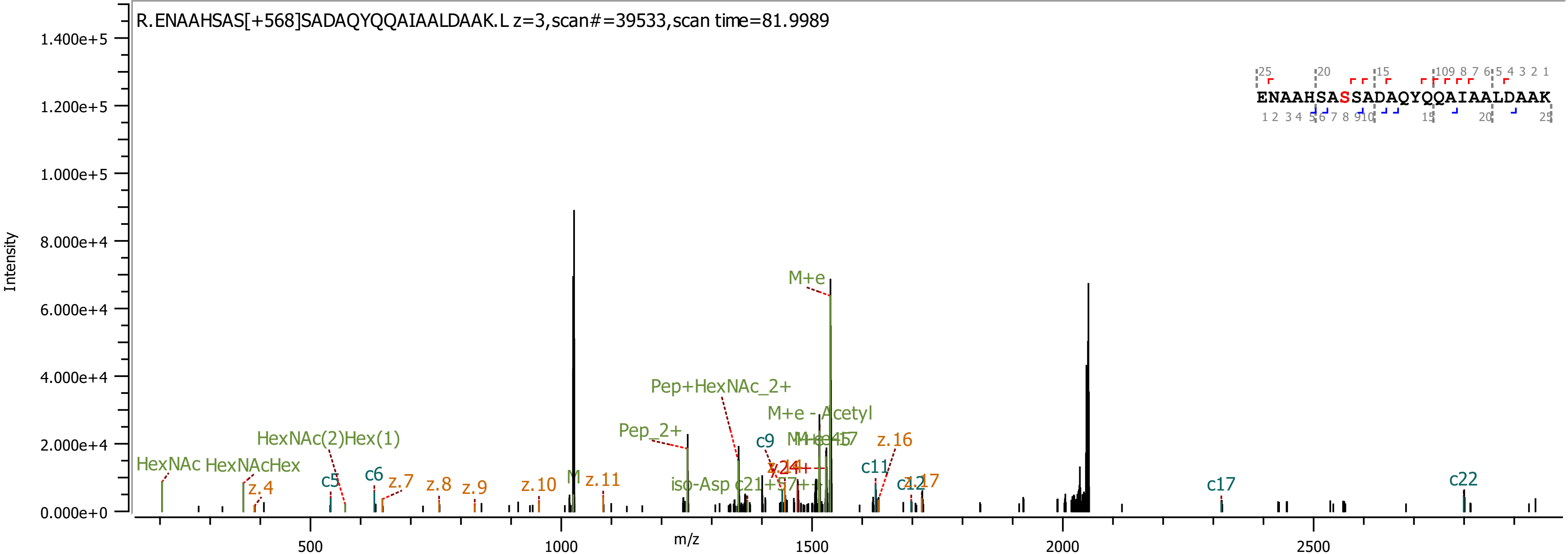
R.ENAAHSAS[+568]SADAQYQQAIAALDAAK.L z=3,scan#=39533,scan time=81.9989
ENAAHSASSADAQYQQAIAALDAAK
Intensity
m/z
1.400e+5
1.200e+5
1.000e+5
8.000e+4
6.000e+4
4.000e+4
2.000e+4
0.000e+0
500
1000
1500
2000
2500
M+e
Pep+HexNAc_2+
M+e - Acetyl
Pep_2+
HexNAc(2)Hex(1)
HexNAc
HexNAcHex
c5
c6
c9
c11
c17
c22
z.4
z.7
z.8
z.9
z.10
z.11
z.16
M
iso-Asp

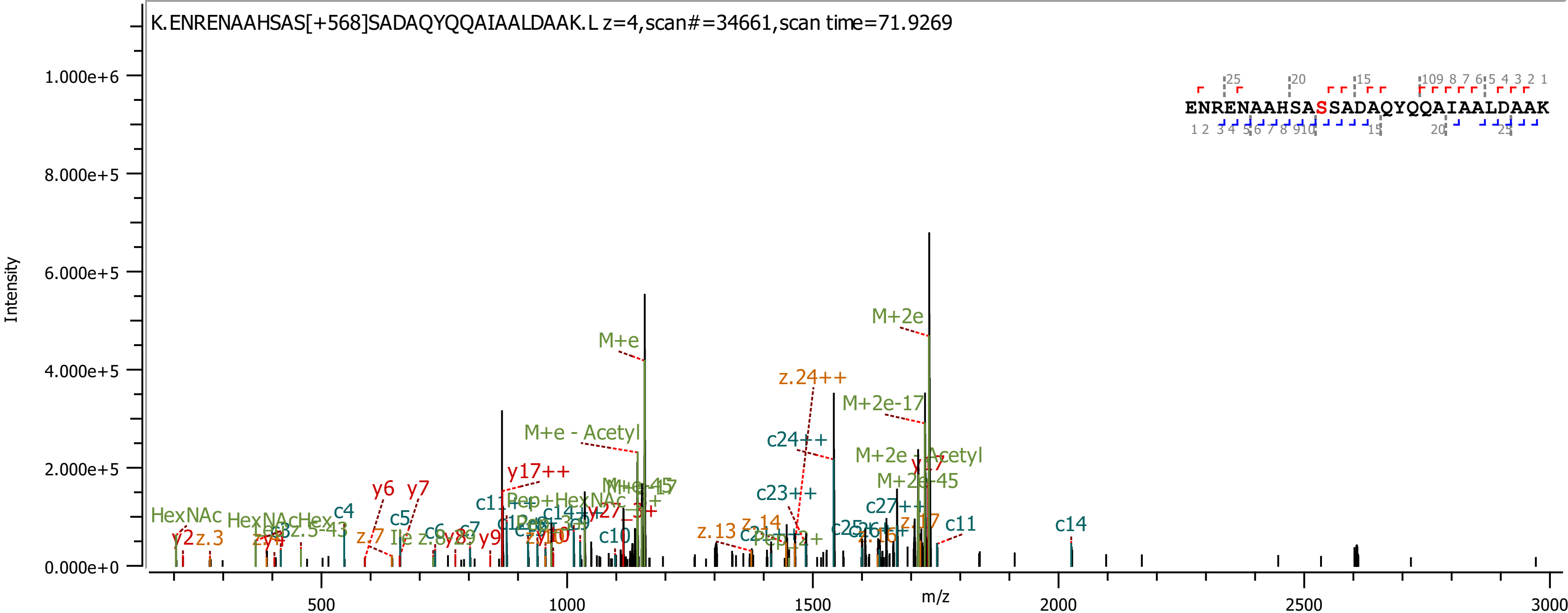
K.ENRENAAHSAS[+568]SADAQYQQAIAALDAAK.L z=4,scan#=34661,scan time=71.9269
ENRENAAHSASSADAQYQQAIAALDAAK
Intensity
m/z
1.000e+6
8.000e+5
6.000e+5
4.000e+5
2.000e+5
0.000e+0
500
1000
1500
2000
2500
3000
M+2e
M+e
z.24++
M+2e-17
M+e - Acetyl
c24++
M+2e - Acetyl
M+2e-45
y17++
c23++
c27++
y6
y7
c4
c5
HexNAc
HexNAcHex
y2
z.3
z.7
c6
c7
y9
c10
c11
c14
z.13
z.14
z.17

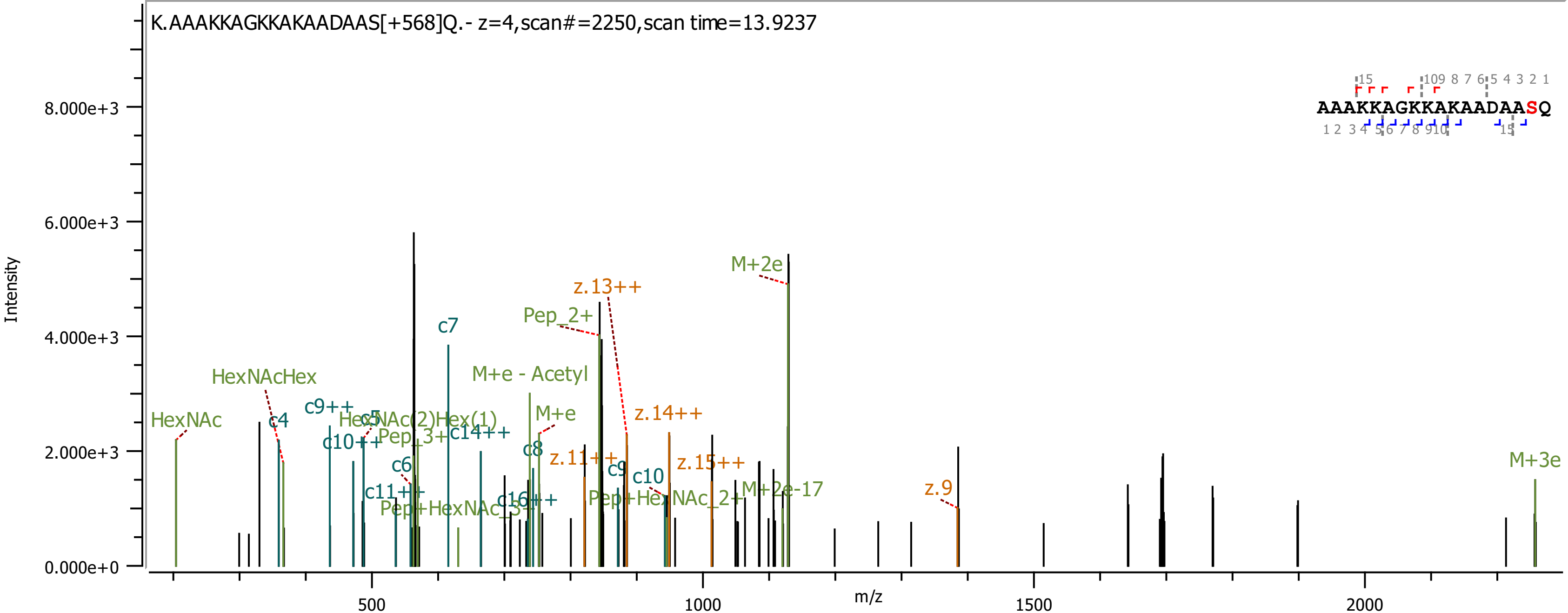
K.AAAKKAGKKAKAADAAS[+568]Q.- z=4,scan#=2250,scan time=13.9237
Intensity
m/z
0.000e+0
2.000e+3
4.000e+3
6.000e+3
8.000e+3
500
1000
1500
2000
AAAKKAGKKAKAADAASQ
HexNAc
HexNAcHex
c4
c9++
c5
HexNAc(2)Hex(1)
c10++
Pep_3+
c14++
c6
c11++
Pep+HexNAc_3+
c7
c16++
M+e - Acetyl
M+e
c8
Pep_2+
z.13++
z.11++
c9
Pep+HexNAc_2+
c10
z.14++
z.15++
M+2e-17
M+2e
z.9
M+3e

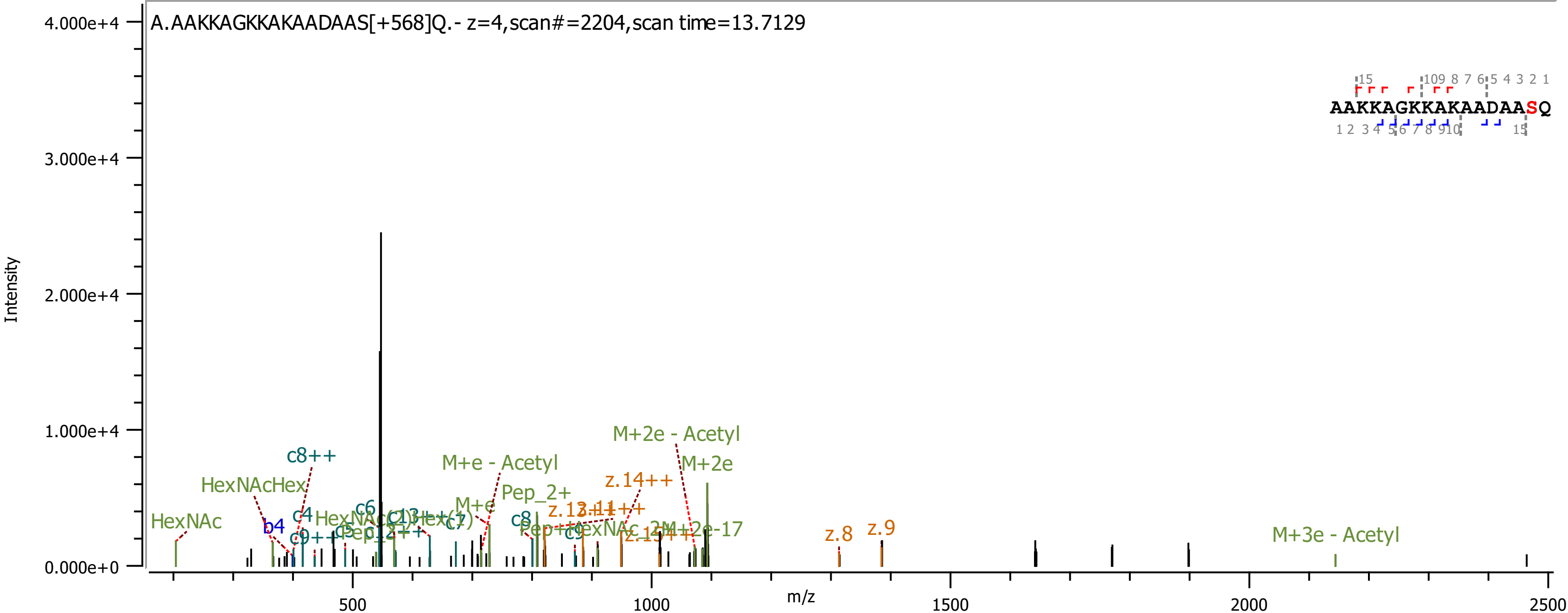
A.AAKKAGKKAKAADAAS[+568]Q.- z=4,scan#=2204,scan time=13.7129
Intensity
m/z
4.000e+4
3.000e+4
2.000e+4
1.000e+4
0.000e+0
500
1000
1500
2000
2500
AAKKAGKKAKAADAASQ
HexNAc
HexNAcHex
b4
c4
c8++
c9++
c5
c6
HexNAc(1)Hex(1)
Pep_2++
c7
M+e - Acetyl
M+e
Pep_2+
c8
c9
Pep+HexNAc_2+
z.14++
z.12++
z.11++
z.13++
M+2e - Acetyl
M+2e
M+2e-17
z.8
z.9
M+3e - Acetyl

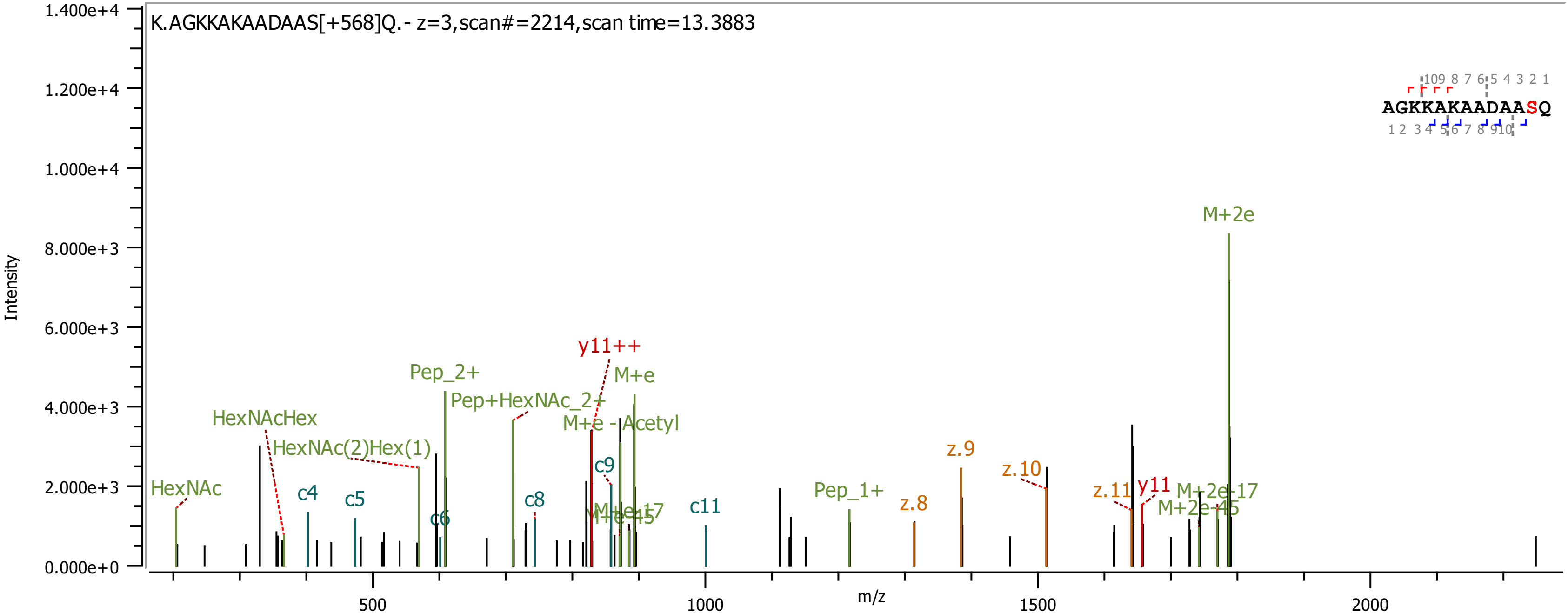

K.AGKKAKAADAAS[+568]Q.- z=3,scan#=2214,scan time=13.3883
Intensity
m/z
AGKKAKAADAASQ
HexNAc
HexNAcHex
HexNAc(2)Hex(1)
c4
c5
c6
Pep_2+
Pep+HexNAc_2+
c8
y11++
M+e
M+e - Acetyl
c9
c11
Pep_1+
z.8
z.9
z.10
z.11
y11
M+2e-17
M+2e-45
M+2e

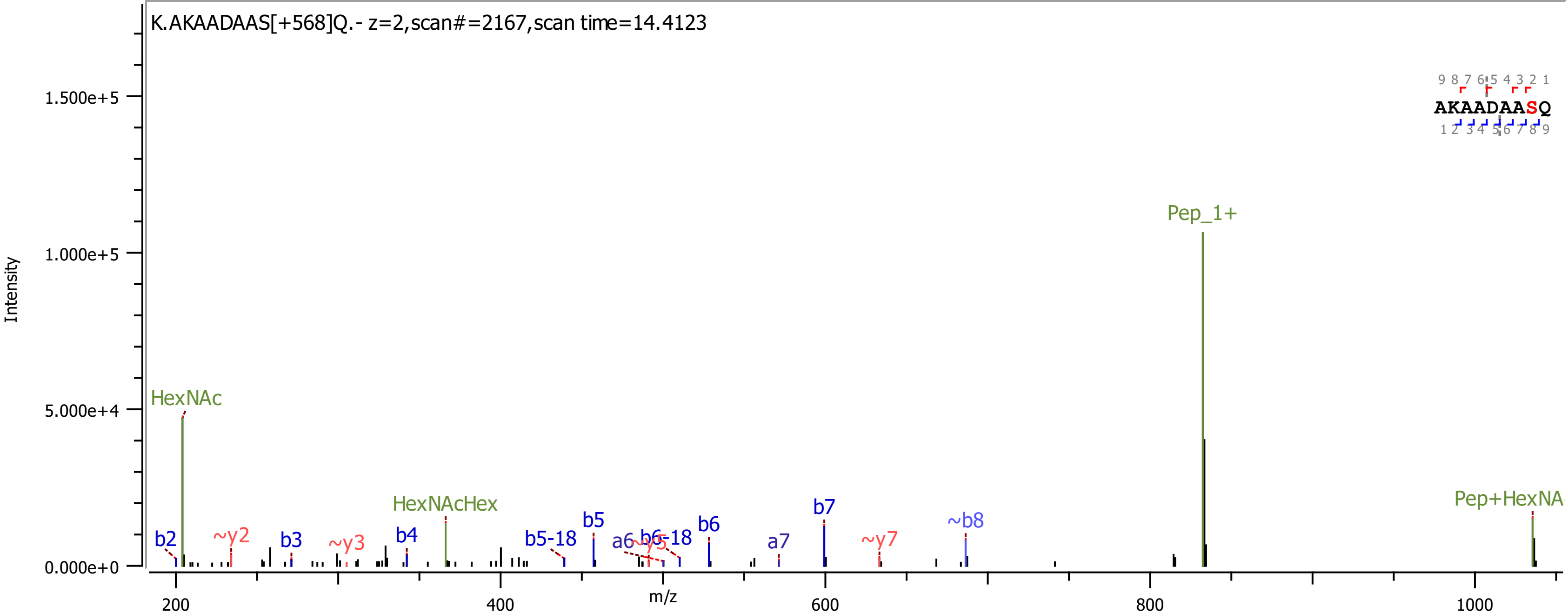
K.AKAADAAS[+568]Q.- z=2,scan#=2167,scan time=14.4123
Intensity
m/z
1.500e+5
1.000e+5
5.000e+4
0.000e+0
200
400
600
800
1000
HexNAc
HexNAcHex
Pep_1+
b2
~y2
b3
~y3
b4
b5-18
b5
a6
~y5
b6-18
b6
a7
b7
~y7
~b8
9 8 7 6 5 4 3 2 1
AKAADAASQ
1 2 3 4 5 6 7 8 9

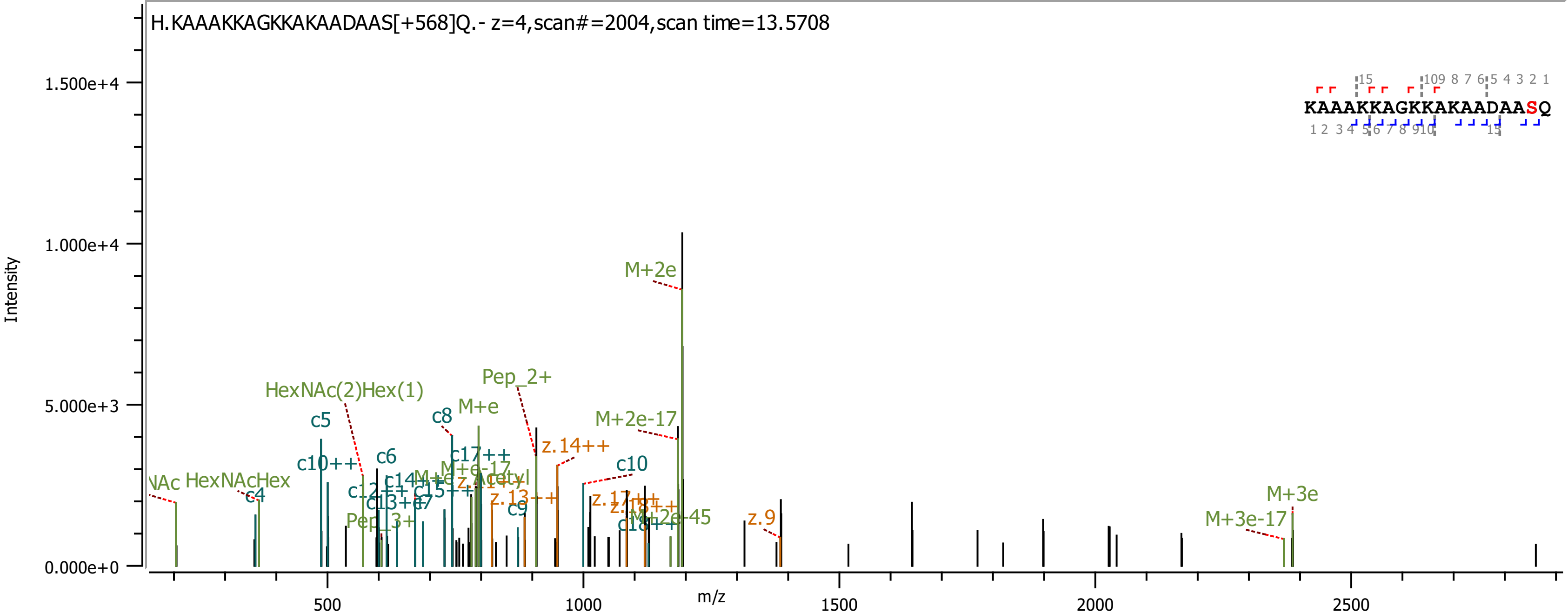
H.KAAAKKAGKKAKAADAAS[+568]Q.- z=4,scan#=2004,scan time=13.5708
KAAAKKAGKKAKAADAASQ
Intensity
m/z
M+2e
M+2e-17
Pep_2+
HexNAc(2)Hex(1)
c5
c8
M+e
c6
c10++
c10
z.14++
z.9
M+3e
M+3e-17
HexNAcHex
c4
c9
z.13++
Pep_3+

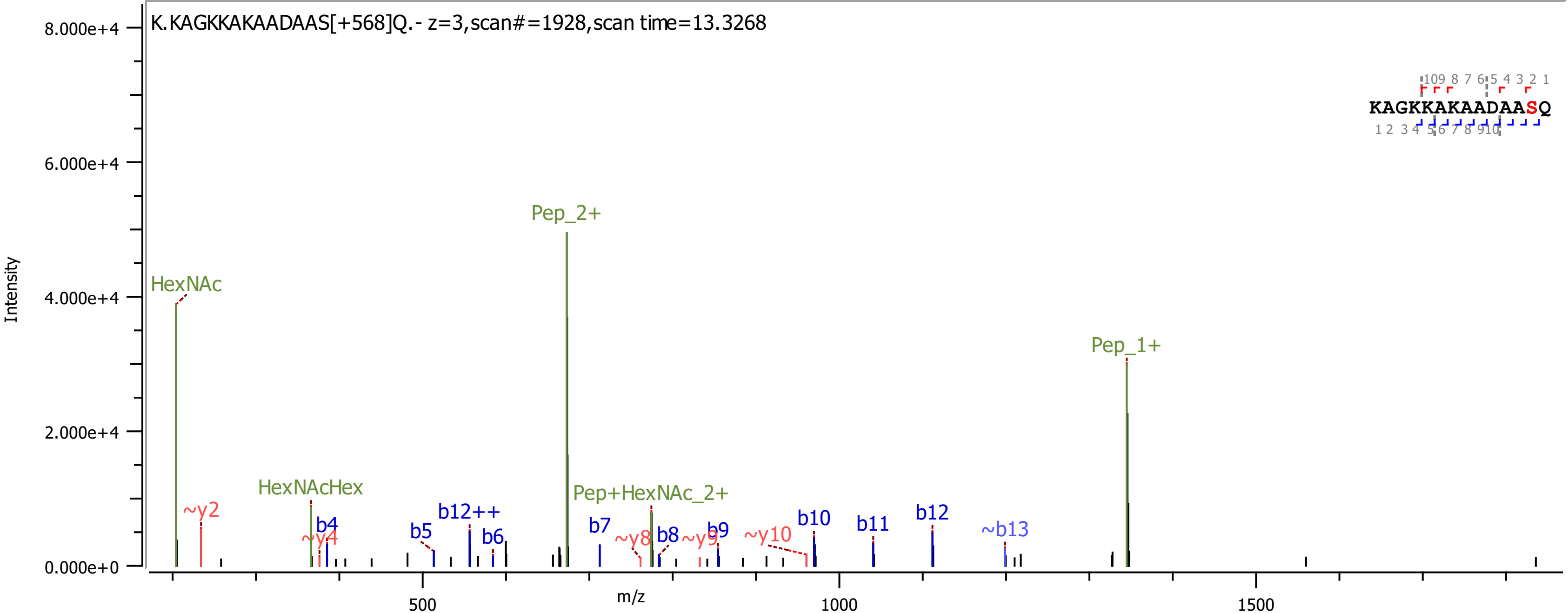
K.KAGKKAKAADAAS[+568]Q.- z=3,scan#=1928,scan time=13.3268
KAGKKAKAADAASQ
Intensity
m/z
8.000e+4
6.000e+4
4.000e+4
2.000e+4
0.000e+0
500
1000
1500
HexNAc
~y2
HexNAcHex
~y4
b4
b5
b12++
b6
Pep_2+
Pep+HexNAc_2+
b7
~y8
b8
~y9
b9
~y10
b10
b11
b12
~b13
Pep_1+

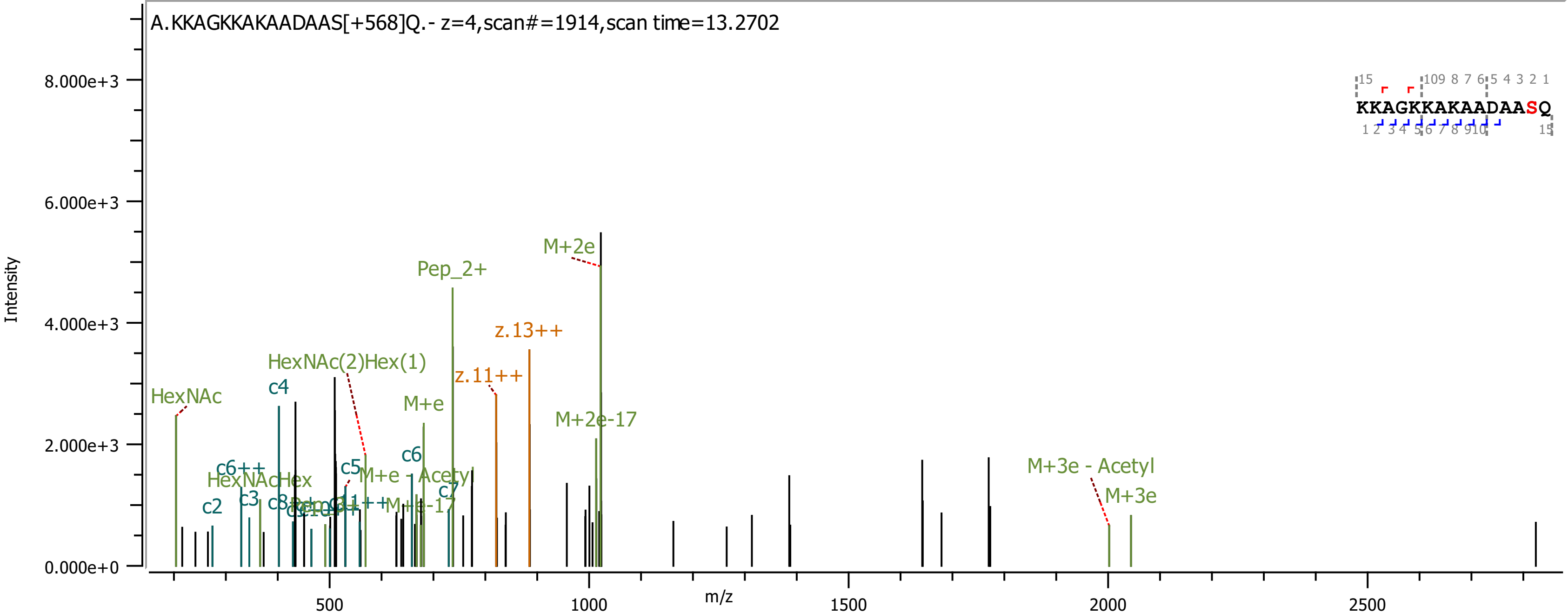
A.KKAGKKAKAADAAS[+568]Q.- z=4,scan#=1914,scan time=13.2702
KKAGKKAKAADAASQ
Intensity
m/z
HexNAc
HexNAc(2)Hex(1)
Pep_2+
M+2e
z.13++
z.11++
M+e
M+2e-17
M+3e - Acetyl
M+3e
M+e - Acetyl
HexNAcHex
c2
c3
c4
c5
c6
c6++
c7
8.000e+3
6.000e+3
4.000e+3
2.000e+3
0.000e+0
500
1000
1500
2000
2500

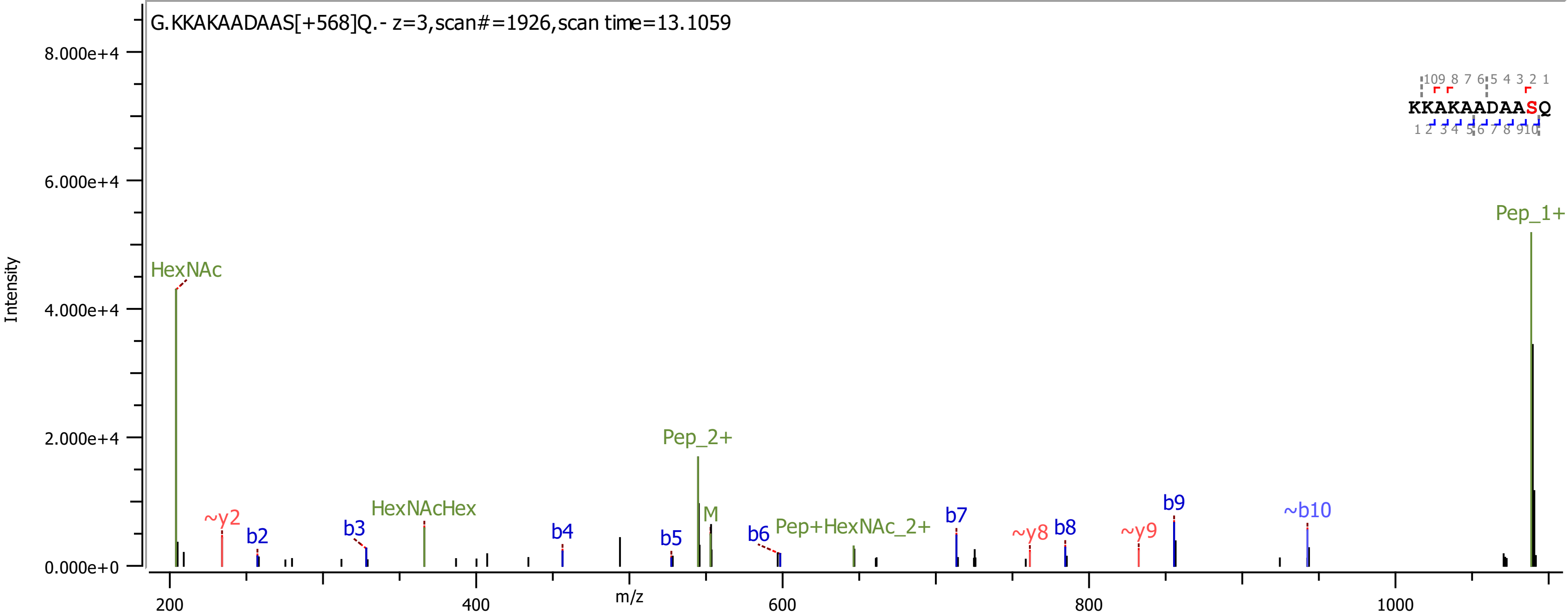
G.KKAKAADAAS[+568]Q.- z=3,scan#=1926,scan time=13.1059
Intensity
m/z
8.000e+4
6.000e+4
4.000e+4
2.000e+4
0.000e+0
200
400
600
800
1000
HexNAc
~y2
b2
b3
HexNAcHex
b4
b5
Pep_2+
M
b6
Pep+HexNAc_2+
b7
~y8
b8
~y9
b9
~b10
Pep_1+
KKAKAADAASQ

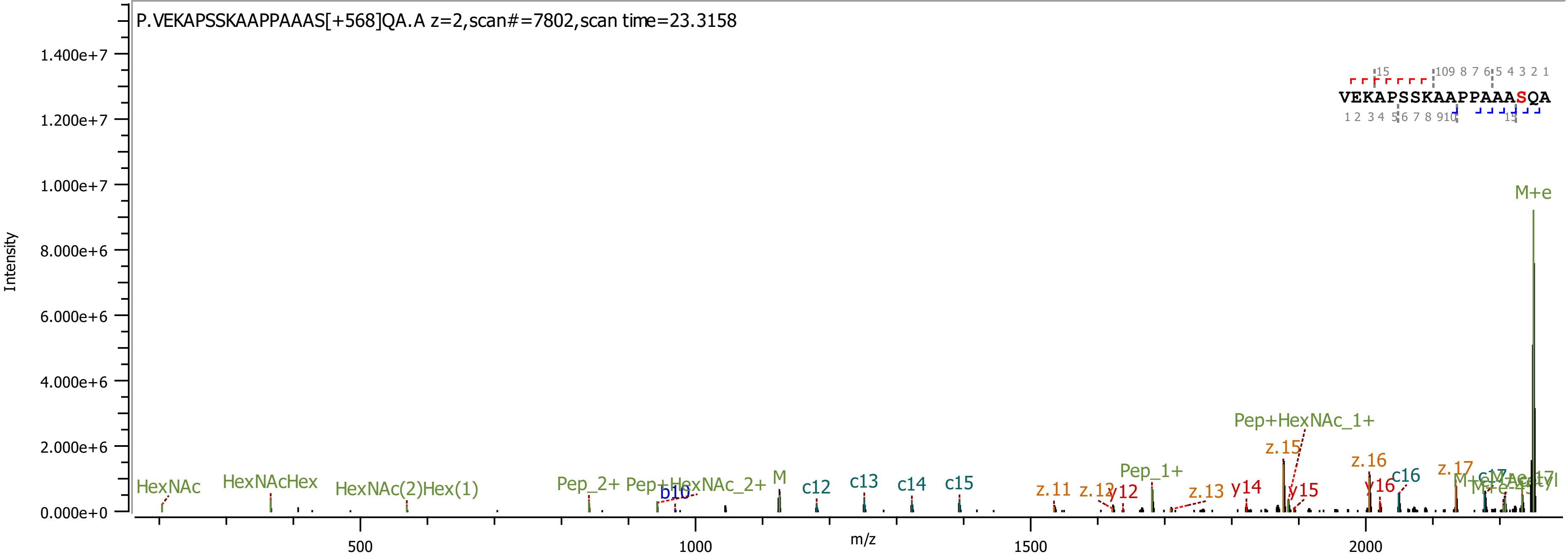
P.VEKAPSSKAAPPAAAS[+568]QA.A z=2,scan#=7802,scan time=23.3158
VEKAPSSKAAPPAAASQA
Intensity
m/z
1.400e+7
1.200e+7
1.000e+7
8.000e+6
6.000e+6
4.000e+6
2.000e+6
0.000e+0
500
1000
1500
2000
HexNAc
HexNAcHex
HexNAc(2)Hex(1)
Pep_2+
Pep+HexNAc_2+
b10
M
c12
c13
c14
c15
z.11
z.12
y12
Pep_1+
z.13
y14
Pep+HexNAc_1+
z.15
y15
z.16
y16
c16
z.17
M+e

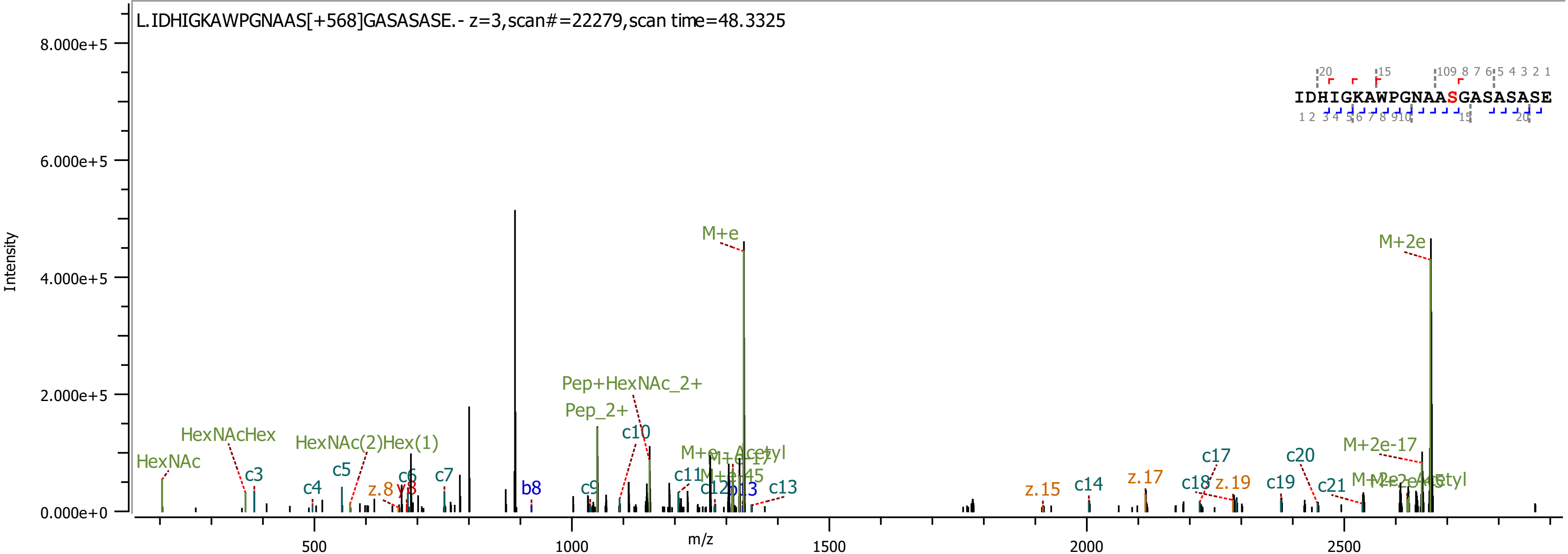

L.IDHIGKAWPGNAAS[+568]GASASASE.- z=3,scan#=22279,scan time=48.3325
Intensity
m/z
8.000e+5
6.000e+5
4.000e+5
2.000e+5
0.000e+0
500
1000
1500
2000
2500
IDHIGKAWPGNAASGASASASE
M+e
M+2e
Pep+HexNAc_2+
Pep_2+
HexNAcHex
HexNAc(2)Hex(1)
HexNAc
c3
c4
c5
c6
c7
c9
c10
c11
c12
c13
c14
c17
c18
c19
c20
c21
z.8
y8
b8
z.15
z.17
z.19
M+2e-17

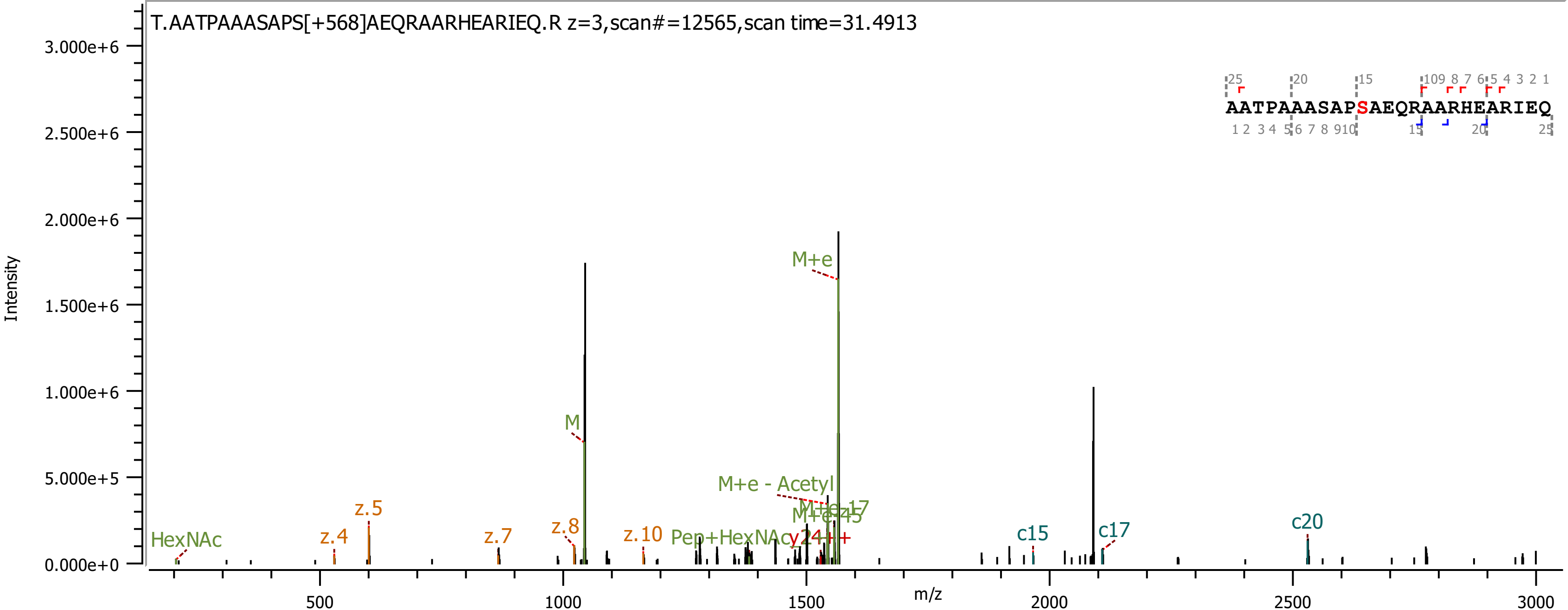
T.AATPAAASAPS[+568]AEQRAARHEARIEQ.R z=3,scan#=12565,scan time=31.4913
AATPAAASAPSAEQRAARHEARIEQ
Intensity
m/z
M+e
M
M+e - Acetyl
M+e-17
M+e-45
HexNAc
z.4
z.5
z.7
z.8
z.10
Pep+HexNAc
y24+++
c15
c17
c20

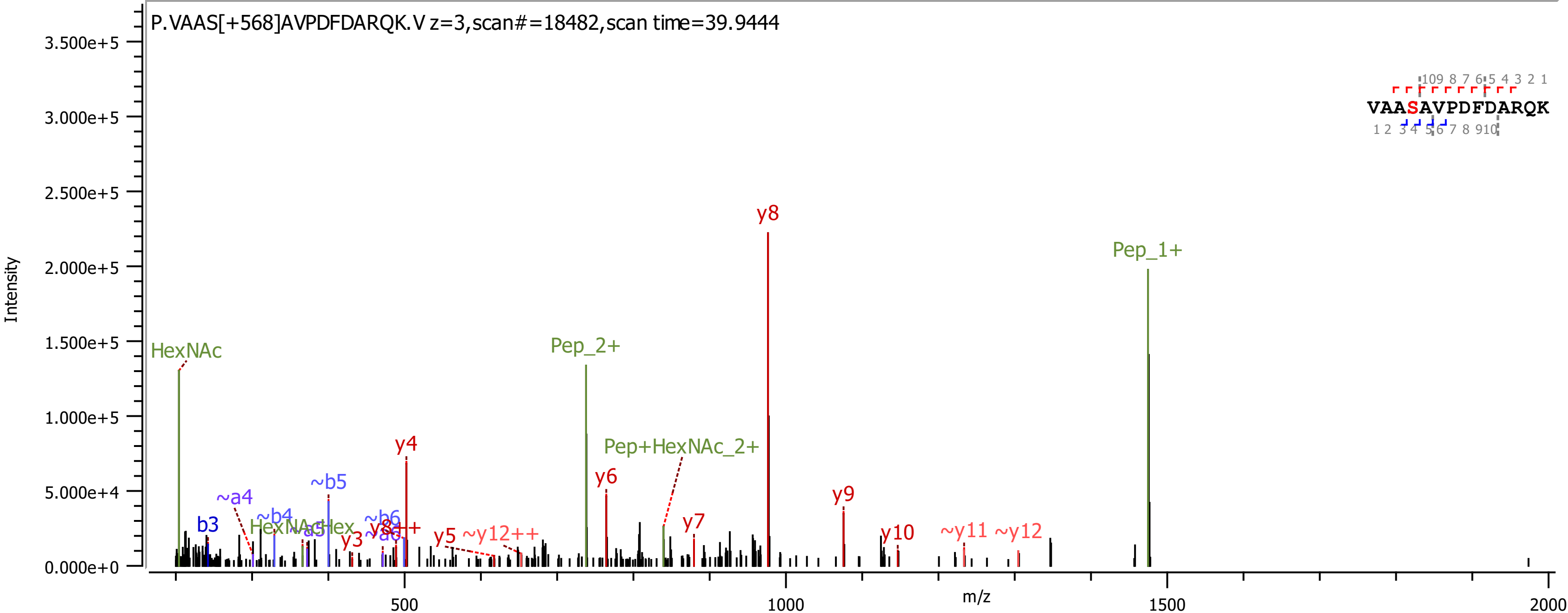

P.VAAS[+568]AVPDFDARQK.V z=3,scan#=18482,scan time=39.9444
Intensity
m/z
VAASAVPDFDARQK
HexNAc
Pep_2+
Pep_1+
Pep+HexNAc_2+
HexNAc-Hex
y8
y4
y6
y7
y9
y10
y3
y5
y8++
~y12++
~y11
~y12
b3
~a4
~b4
~a5
~b5
~b6
~a6

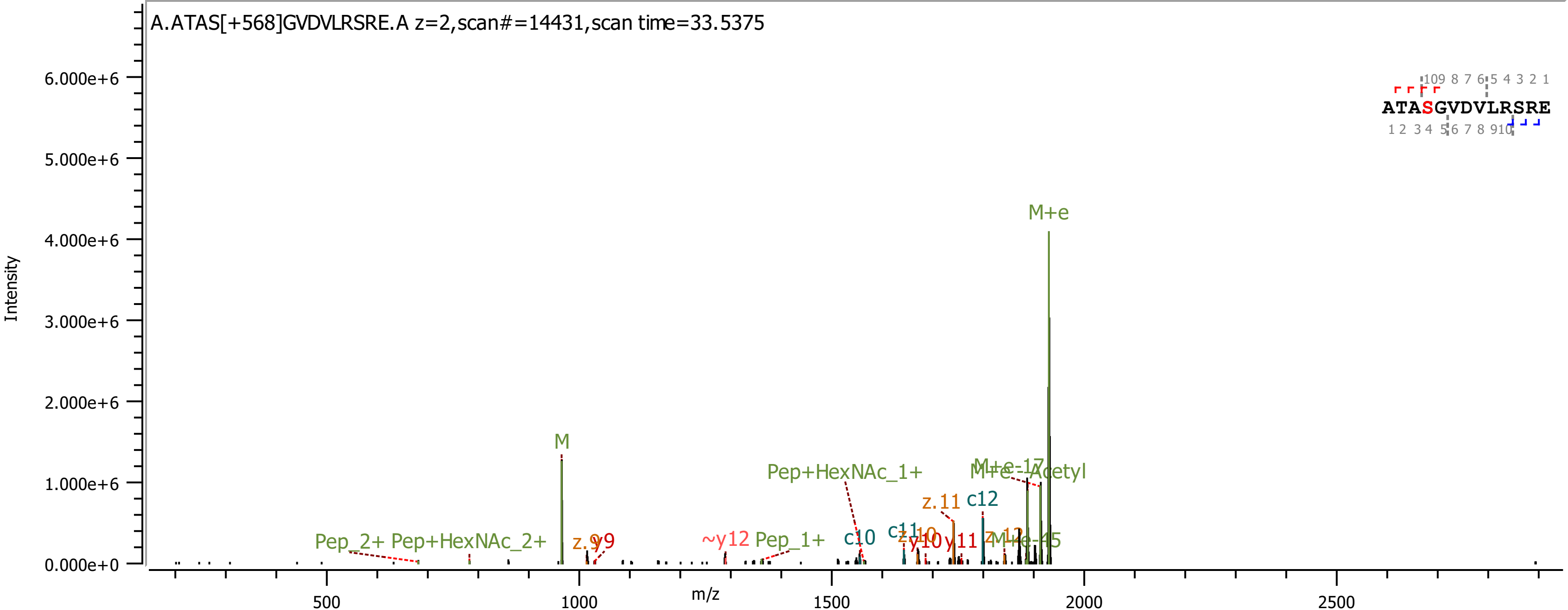

A.ATAS[+568]GVDVLRSRE.A z=2,scan#=14431,scan time=33.5375
ATASGVDVLRSRE
Intensity
m/z
M+e
M
Pep+HexNAc_1+
M+e-17
M+e-Acetyl
z.11
c12
Pep_2+
Pep+HexNAc_2+
z.9
y9
~y12
Pep_1+
c10
c11
z.10
y10
y11
z.12
M+e-45
6.000e+6
5.000e+6
4.000e+6
3.000e+6
2.000e+6
1.000e+6
0.000e+0
500
1000
1500
2000
2500

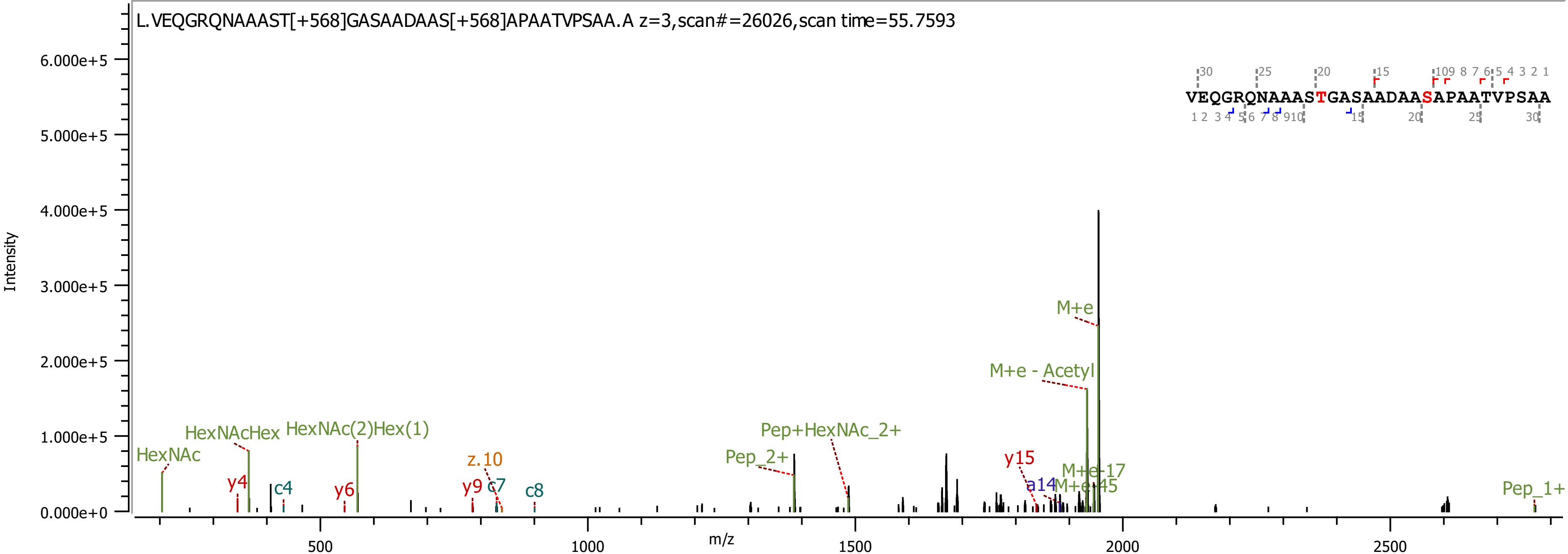

L.VEQGRQNAAAST[+568]GASAADAAS[+568]APAATVPSAA.A z=3,scan#=26026,scan time=55.7593
VEQGRQNAAASTGASAADAASAPAATVPSAA
Intensity
m/z
6.000e+5
5.000e+5
4.000e+5
3.000e+5
2.000e+5
1.000e+5
0.000e+0
500
1000
1500
2000
2500
M+e
M+e - Acetyl
HexNAc
HexNAcHex
HexNAc(2)Hex(1)
z.10
Pep+HexNAc_2+
Pep_2+
y15
M+e-17
a14
M+e-45
y4
c4
y6
y9
c7
c8
Pep_1+

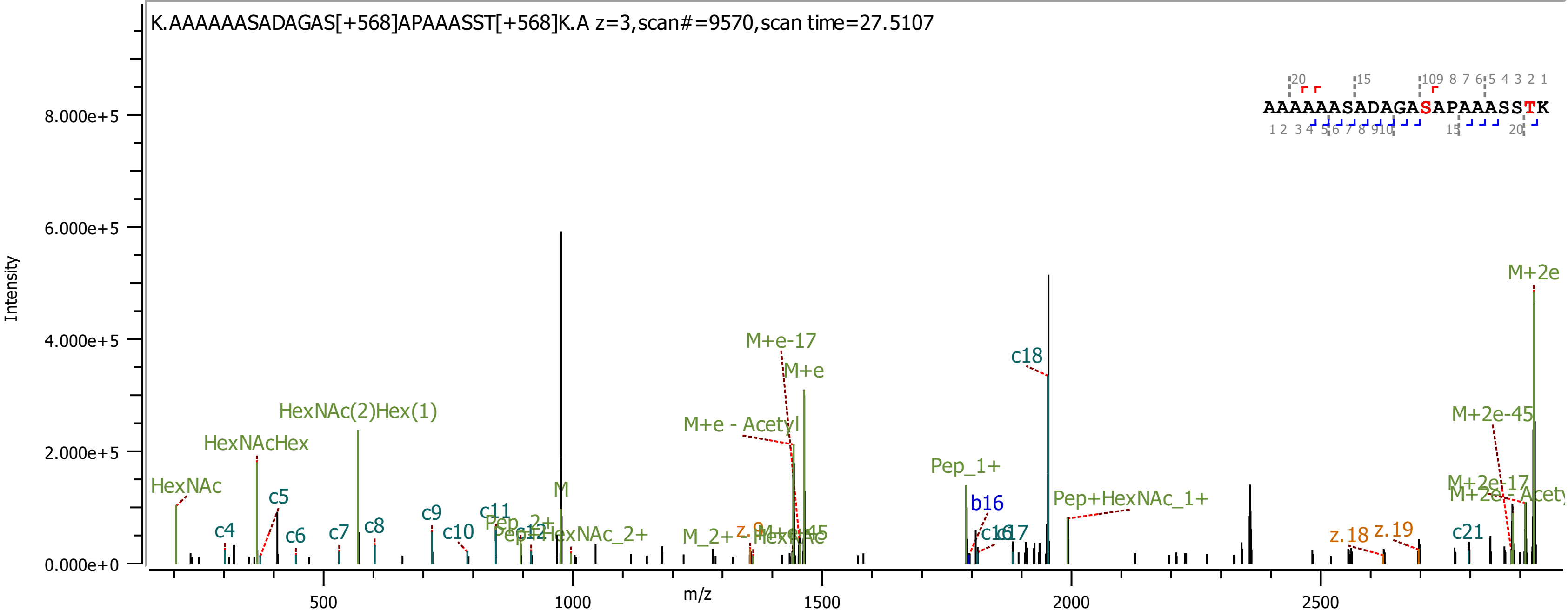
K.AAAAAASADAGAS[+568]APAAASST[+568]K.A z=3,scan#=9570,scan time=27.5107
AAAAAASADAGASAPAAASSTK
Intensity
m/z
8.000e+5
6.000e+5
4.000e+5
2.000e+5
0.000e+0
500
1000
1500
2000
2500
HexNAc
HexNAcHex
HexNAc(2)Hex(1)
c4
c5
c6
c7
c8
c9
c10
c11
c18
c21
M
M+e-17
M+e
M+e - Acetyl
Pep_1+
b16
Pep+HexNAc_1+
z.18
z.19
M+2e
M+2e-45
M+2e-17

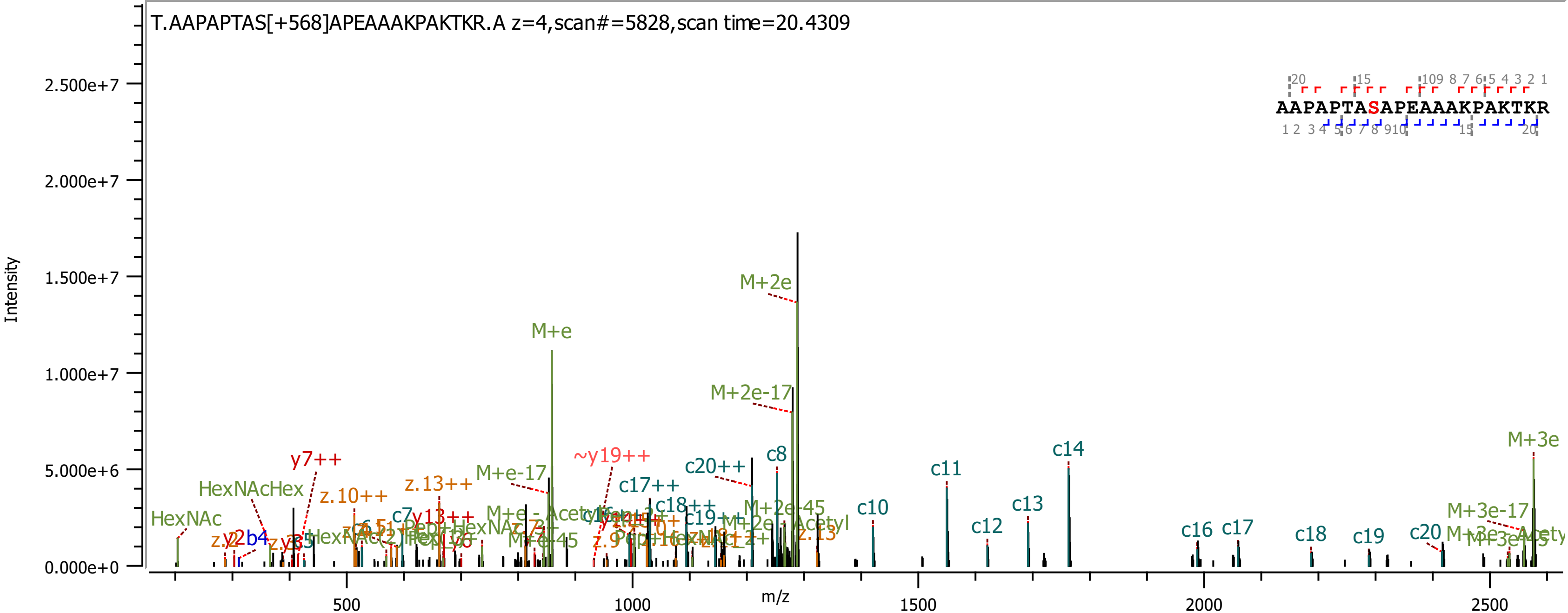
T.AAPAPTAS[+568]APEAAAKPAKTKR.A z=4,scan#=5828,scan time=20.4309
AAPAPTASAPEAAAKPAKTKR
Intensity
m/z
2.500e+7
2.000e+7
1.500e+7
1.000e+7
5.000e+6
0.000e+0
500
1000
1500
2000
2500
M+2e
M+e
M+2e-17
M+e-17
M+3e
M+3e-17
HexNAc
HexNAcHex
y7++
~y19++
z.10++
z.13++
c20++
c17++
c18++
c19++
M+2e-45
c8
c10
c11
c12
c13
c14
c16
c17
c18
c19
c20
b4

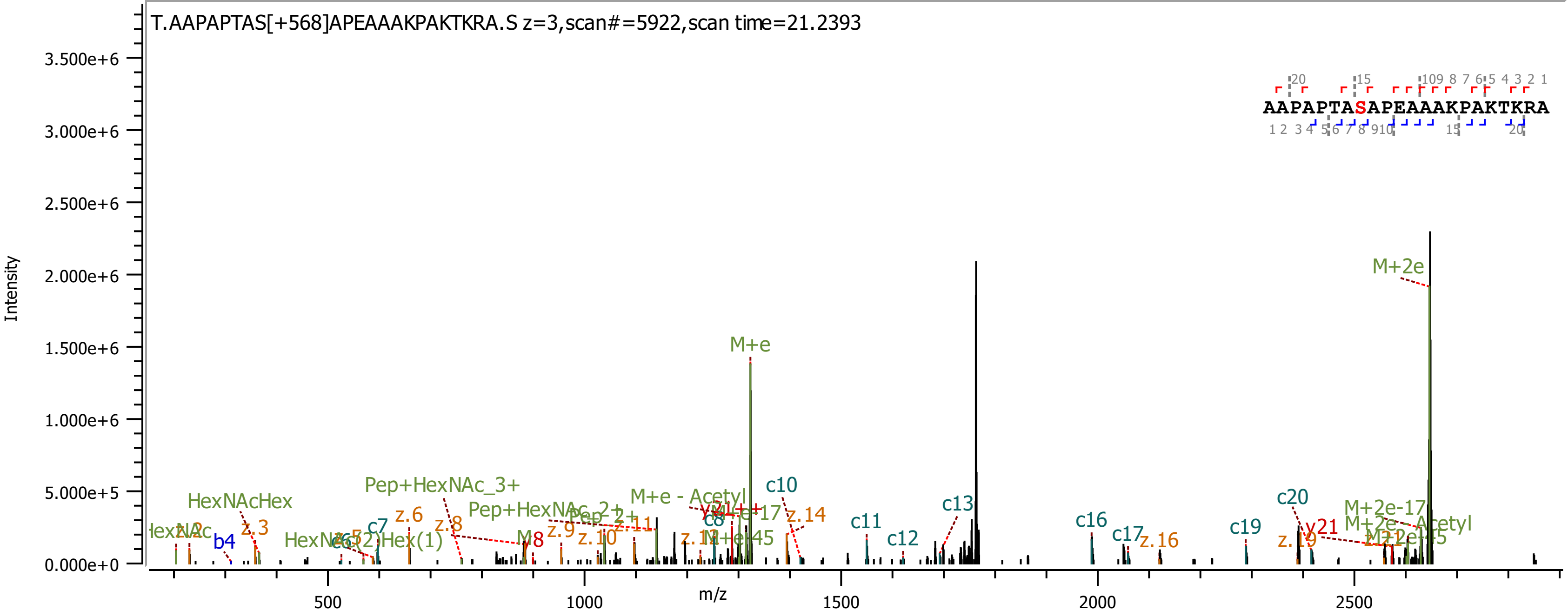

T.AAPAPTAS[+568]APEAAAKPAKTKRA.S z=3,scan#=5922,scan time=21.2393
AAPAPTASAPEAAAKPAKTKRA
Intensity
m/z
M+2e
M+e
c10
c11
c12
c13
c16
c17
c19
c20
z.16
y21
HexNAcHex
Pep+HexNAc_3+
Pep+HexNAc_2+
M+e - Acetyl
b4
c7
c8
z.3
z.6
z.8
z.9
z.10
z.11
z.14
M+2e-17
M+2e - Acetyl

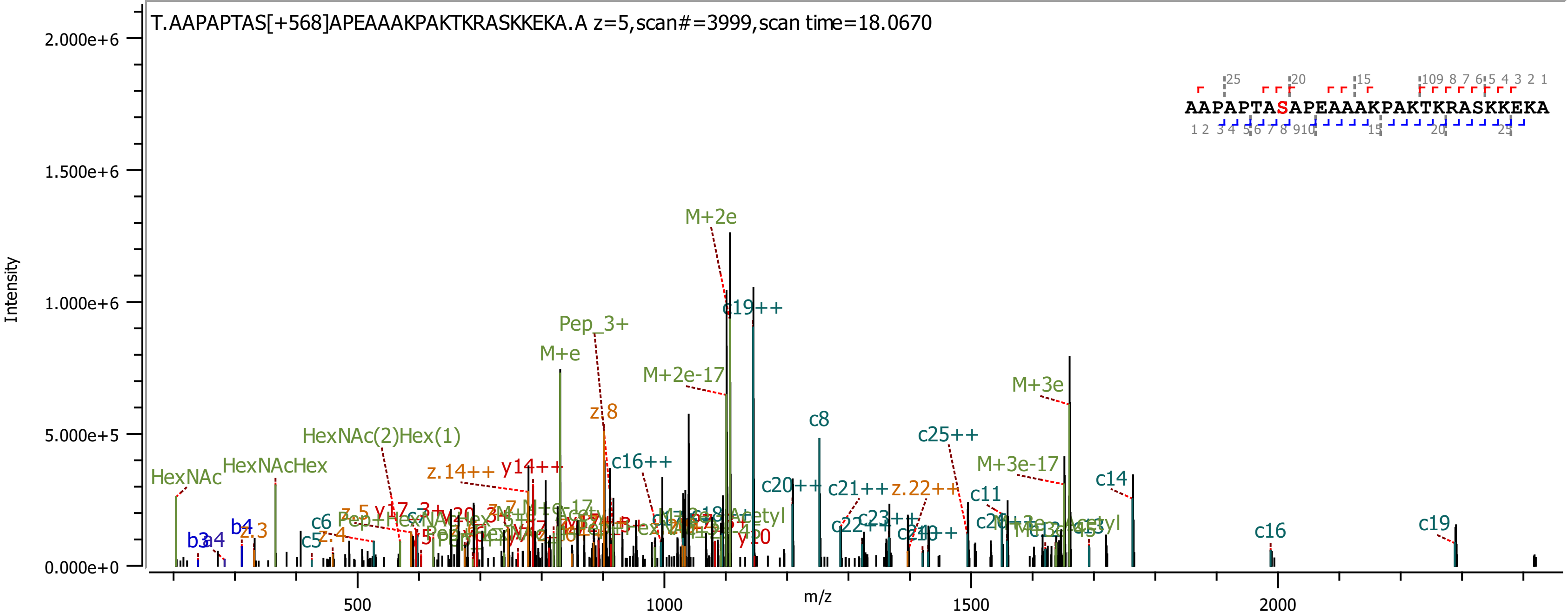

T.AAPAPTAS[+568]APEAAAKPAKTKRASKKEKA.A z=5,scan#=3999,scan time=18.0670
AAPAPTASAPEAAAKPAKTKRASKKEKA
Intensity
m/z
2.000e+6
1.500e+6
1.000e+6
5.000e+5
0.000e+0
500
1000
1500
2000
M+2e
c19++
Pep_3+
M+e
M+2e-17
z.8
c8
c25++
M+3e
M+3e-17
HexNAc(2)Hex(1)
HexNAcHex
HexNAc
z.14++
y14++
c16++
c20++
c21++
z.22++
c11
c14
c16
c19
b3
a4
b4
z.3
c5
z.4
c6
y20

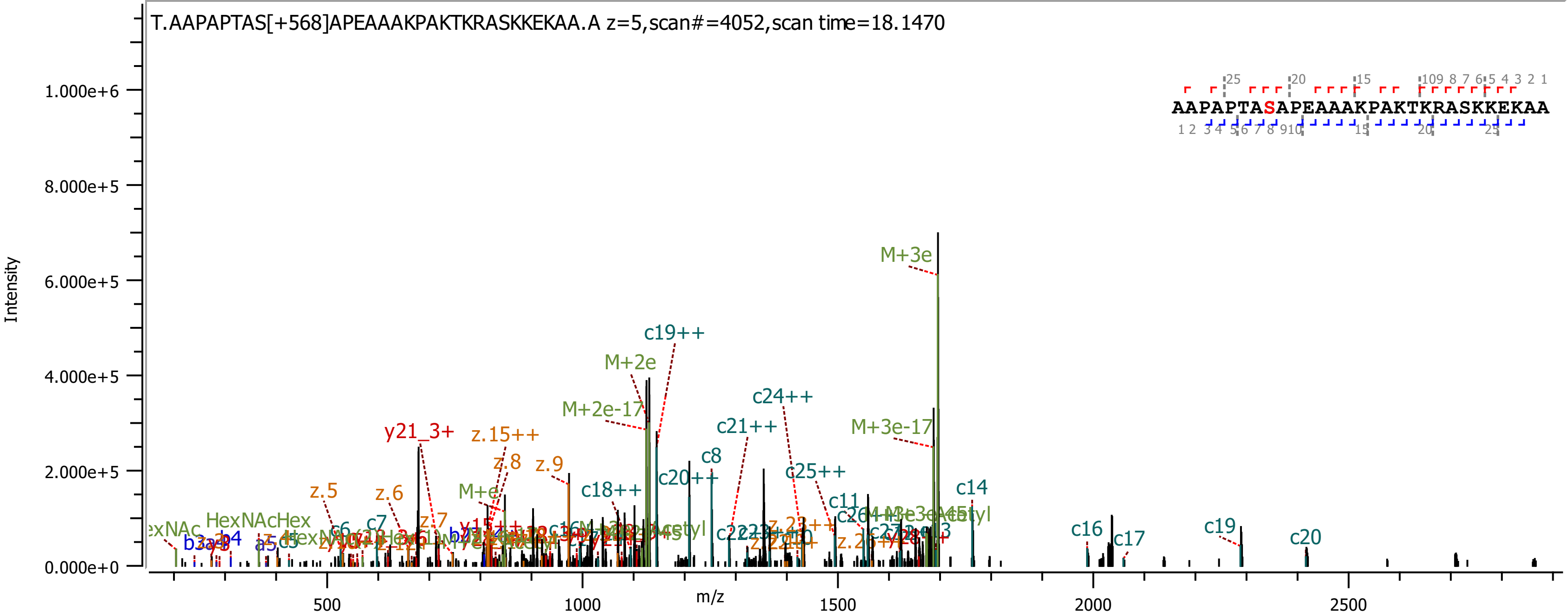
T.AAPAPTAS[+568]APEAAAKPAKTKRASKKEKAA.A z=5,scan#=4052,scan time=18.1470
AAPAPTASAPEAAAKPAKTKRASKKEKAA
Intensity
m/z
1.000e+6
8.000e+5
6.000e+5
4.000e+5
2.000e+5
0.000e+0
500
1000
1500
2000
2500
M+3e
c19++
M+2e
M+2e-17
c24++
M+3e-17
c21++
y21_3+
z.15++
z.8
z.9
c8
c20++
c25++
c14
z.5
z.6
M+e
c18++
c11
c16
c17
c19
c20
HexNAcHex
c6
c7
z.7
b4
a5
c5

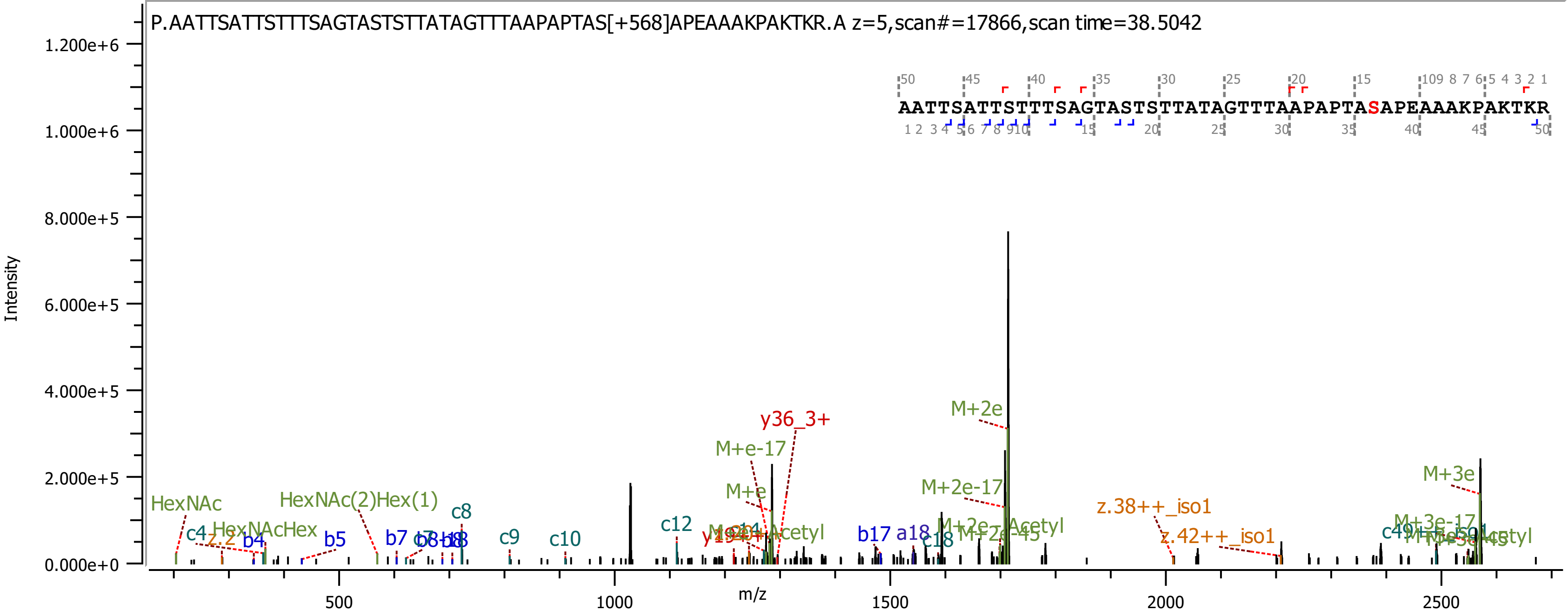
P.AATTSATTSTTTSAGTASTSTTATAGTTTAAPAPTAS[+568]APEAAAKPAKTKR.A z=5,scan#=17866,scan time=38.5042
AATTSATTSTTTSAGTASTSTTATAGTTTAAPAPTASAPEAAAKPAKTKR
Intensity
m/z
1.200e+6
1.000e+6
8.000e+5
6.000e+5
4.000e+5
2.000e+5
0.000e+0
500
1000
1500
2000
2500
HexNAc
HexNAc(2)Hex(1)
HexNAcHex
c4
b4
b5
b7
c8
c9
c10
c12
y36_3+
M+e-17
M+e
M+2e
M+2e-17
b17
a18
c18
z.38++_iso1
z.42++_iso1
M+3e
M+3e-17

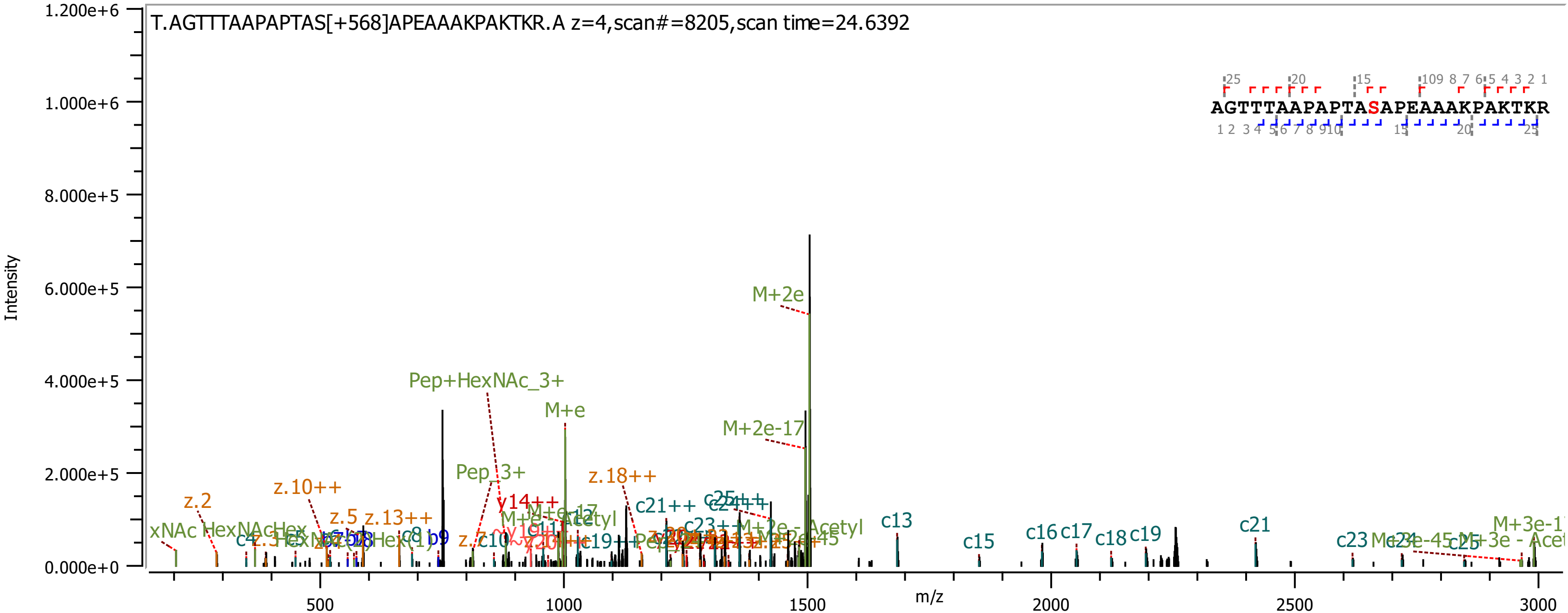
T.AGTTTAAPAPTAS[+568]APEAAAKPAKTKR.A z=4,scan#=8205,scan time=24.6392
AGTTTAAPAPTASAPEAAAKPAKTKR
Intensity
m/z
M+2e
Pep+HexNAc_3+
M+e
M+2e-17
Pep_3+
z.18++
z.10++
z.2
z.5
z.13++
y14++
c21++
c13
c15
c16
c17
c18
c19
c21
c23
c25
c4
c8
b9
c10
M+3e-1

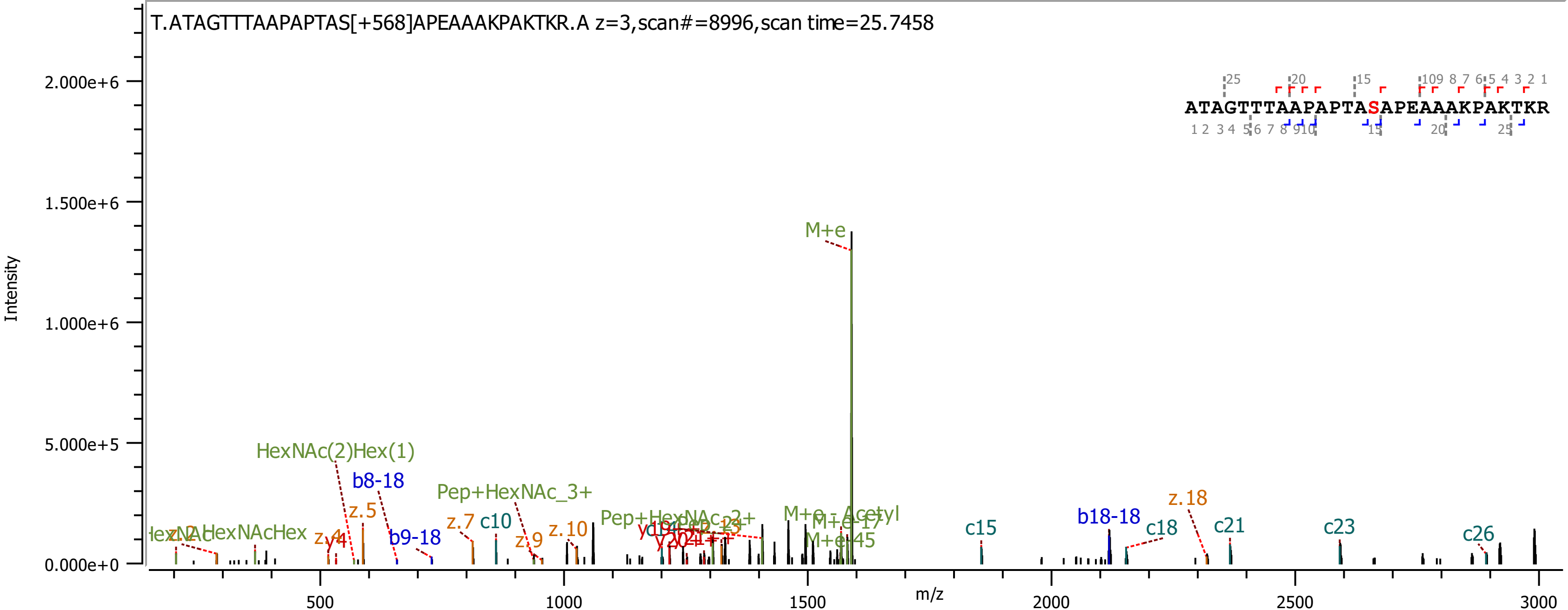
T.ATAGTTTAAPAPTAS[+568]APEAAAKPAKTKR.A z=3,scan#=8996,scan time=25.7458
ATAGTTTAAPAPTASAPEAAAKPAKTKR
Intensity
m/z
2.000e+6
1.500e+6
1.000e+6
5.000e+5
0.000e+0
500
1000
1500
2000
2500
3000
M+e
HexNAc(2)Hex(1)
b8-18
Pep+HexNAc_3+
z.5
z.7
c10
b9-18
z.9
z.10
Pep+HexNAc_2+
M+e-Acetyl
M+e-17
M+e-45
c15
b18-18
c18
z.18
c21
c23
c26
HexNAcHex
z.4

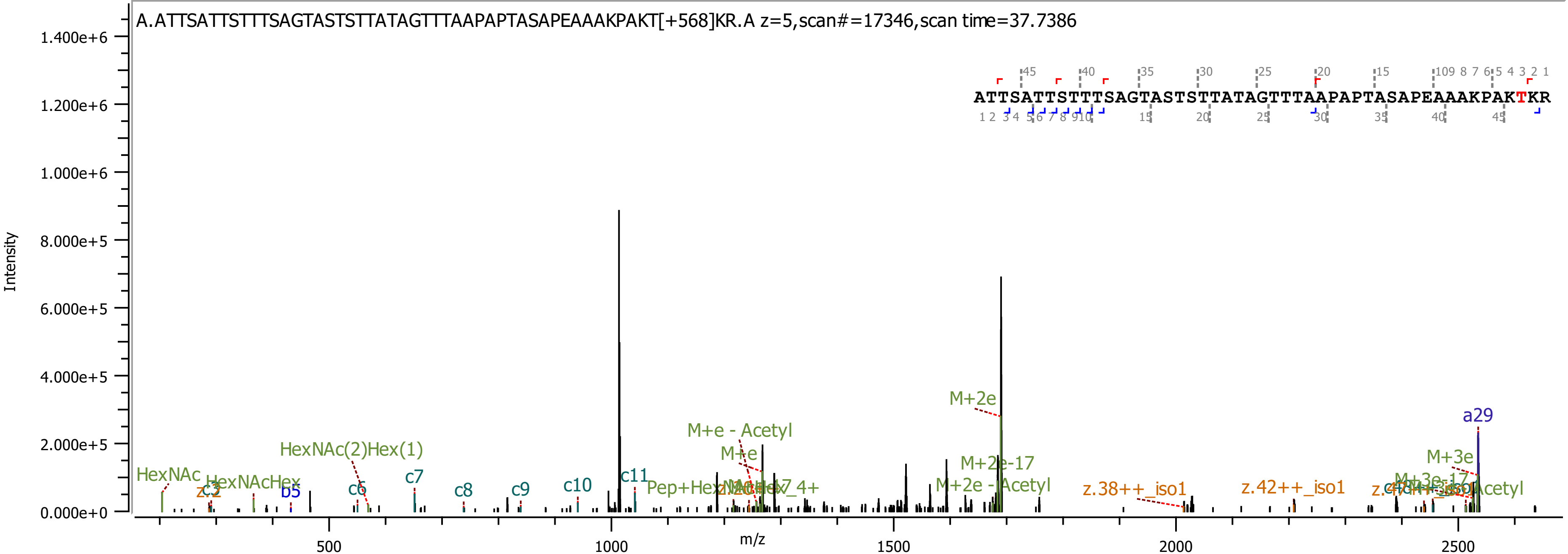

A.ATTSATTSTTTSAGTASTSTTATAGTTTAAPAPTASAPEAAAKPAKT[+568]KR.A z=5,scan#=17346,scan time=37.7386
ATTSATTSTTTSAGTASTSTTATAGTTTAAPAPTASAPEAAAKPAKTKR
Intensity
m/z
M+2e
a29
M+e - Acetyl
M+e
HexNAc(2)Hex(1)
M+2e-17
M+3e
M+2e - Acetyl
HexNAc
HexNAcHex
c7
c8
c9
c10
c11
c6
b5
z.38++_iso1
z.42++_iso1

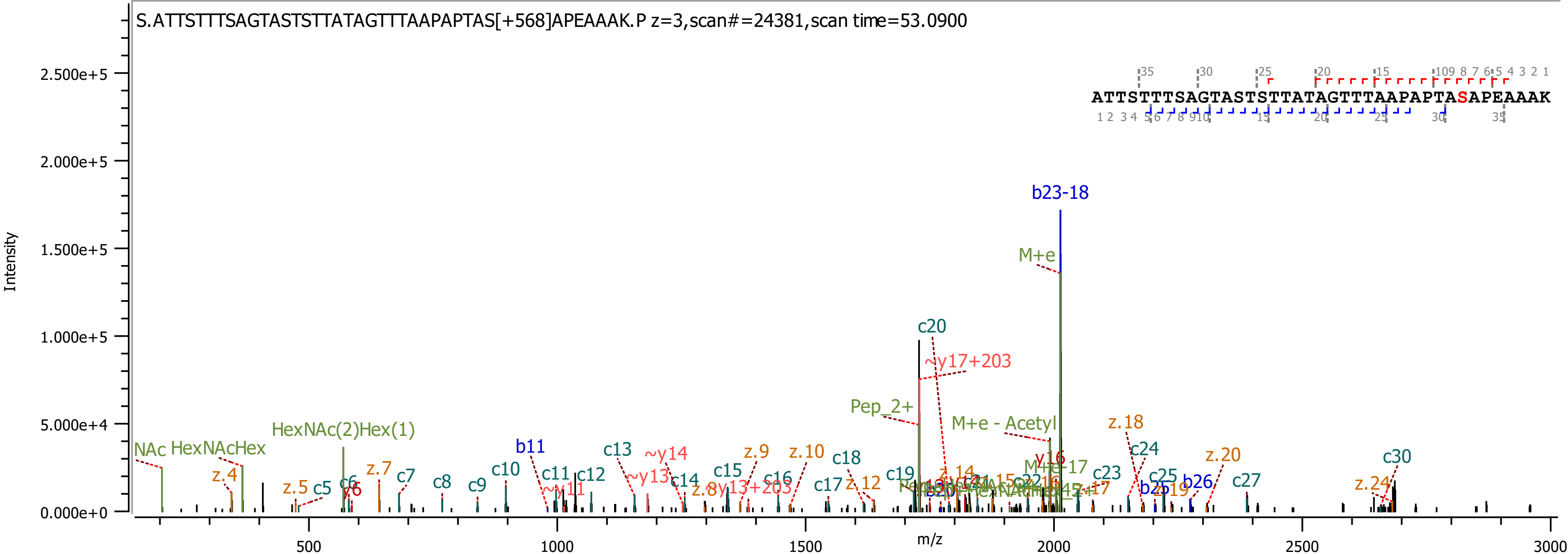
S.ATTSTTTSAGTASTSTTATAGTTTAAPAPTAS[+568]APEAAAK.P z=3,scan#=24381,scan time=53.0900
ATTSTTTSAGTASTSTTATAGTTTAAPAPTASAPEAAAK
Intensity
m/z
b23-18
M+e
c20
~y17+203
Pep_2+
M+e - Acetyl
z.18
HexNAc(2)Hex(1)
NAc
HexNAcHex
z.4
z.5
c5
c6
y6
z.7
c7
c8
c9
c10
b11
c11
~y11
c12
c13
~y14
~y13
c14
c15
z.8
~y13+203
z.9
c16
z.10
c17
c18
z.12
c19
y16
M+e-17
c23
c24
c25
b25
z.19
b26
z.20
c27
c30
z.24
0.000e+0
5.000e+4
1.000e+5
1.500e+5
2.000e+5
2.500e+5
500
1000
1500
2000
2500
3000

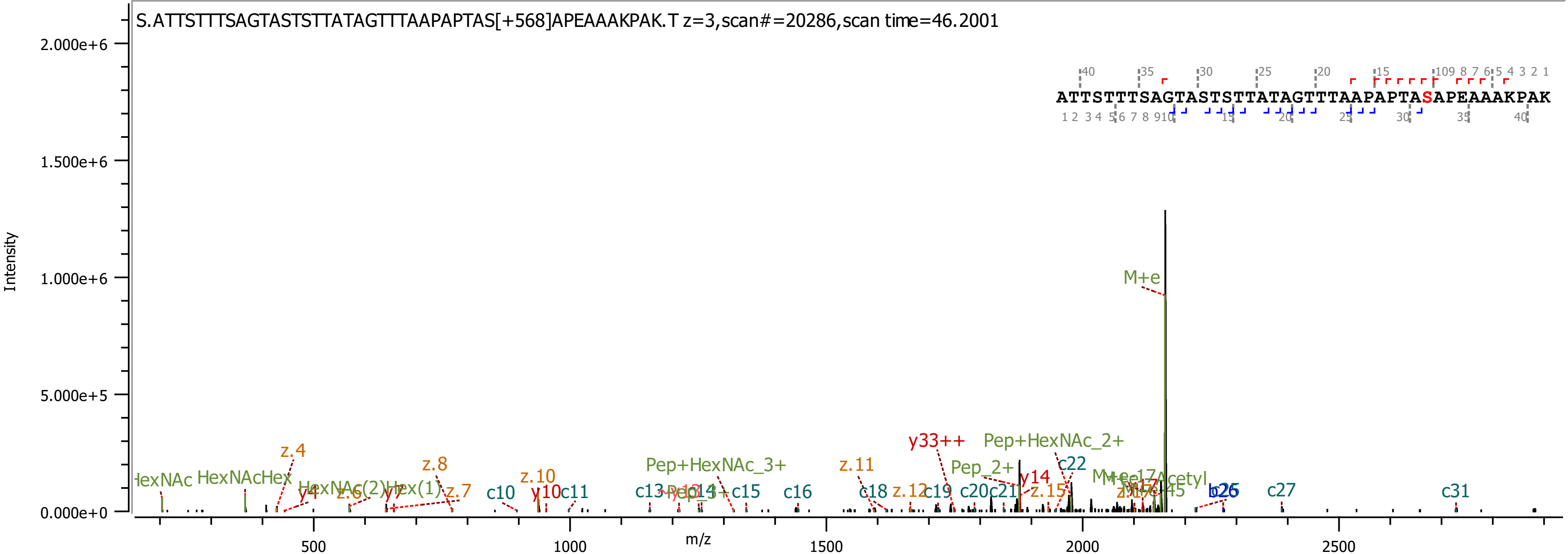
S.ATTSTTTSAGTASTSTTATAGTTTAAPAPTAS[+568]APEAAAKPAK.T z=3,scan#=20286,scan time=46.2001
ATTSTTTSAGTASTSTTATAGTTTAAPAPTASAPEAAAKPAK
Intensity
m/z
2.000e+6
1.500e+6
1.000e+6
5.000e+5
0.000e+0
500
1000
1500
2000
2500
M+e
HexNAcHex
z.4
z.8
z.10
c10
y10
c11
Pep+HexNAc_3+
c15
c16
z.11
c18
z.12
c19
y33++
Pep+HexNAc_2+
Pep_2+
y14
c22
z.15
c27
c31

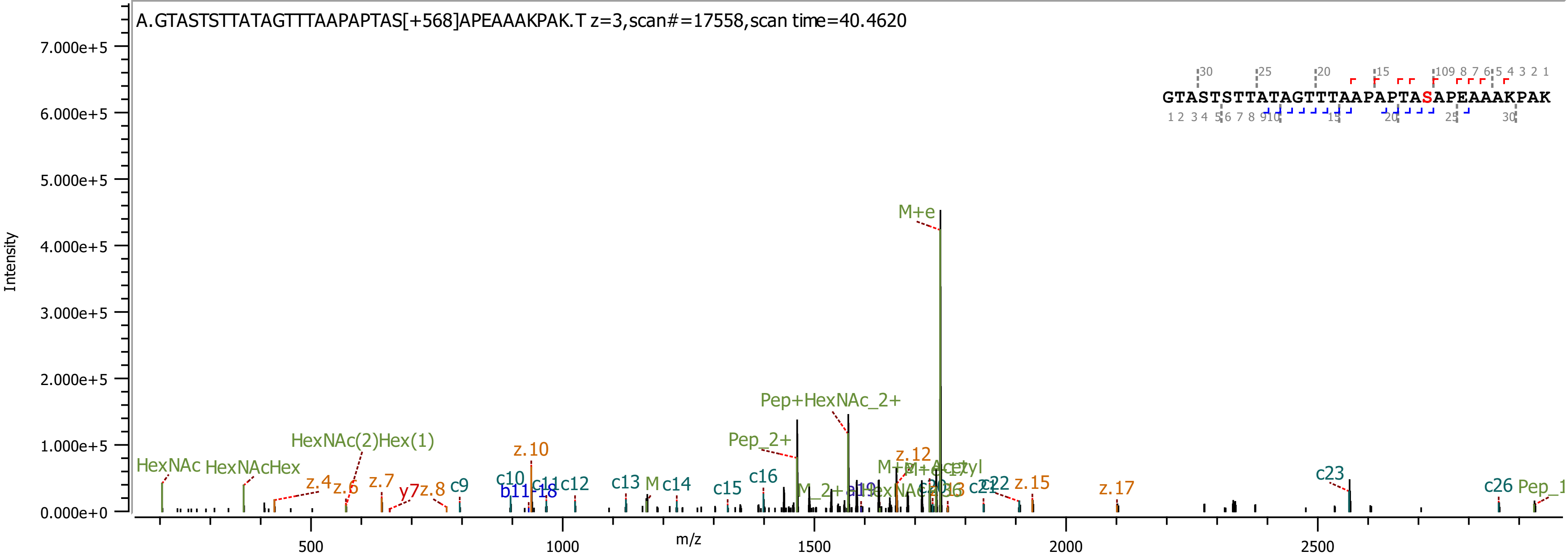
A.GTASTSTTATAGTTTAAPAPTAS[+568]APEAAAKPAK.T z=3,scan#=17558,scan time=40.4620
GTASTSTTATAGTTTAAPAPTASAPEAAAKPAK
Intensity
m/z
M+e
Pep+HexNAc_2+
Pep_2+
HexNAc(2)Hex(1)
HexNAc
HexNAcHex
z.10
z.12
z.15
z.17
c9
c10
c12
c13
c14
c15
c16
c23
c26
y7
z.4
z.6
z.7
z.8

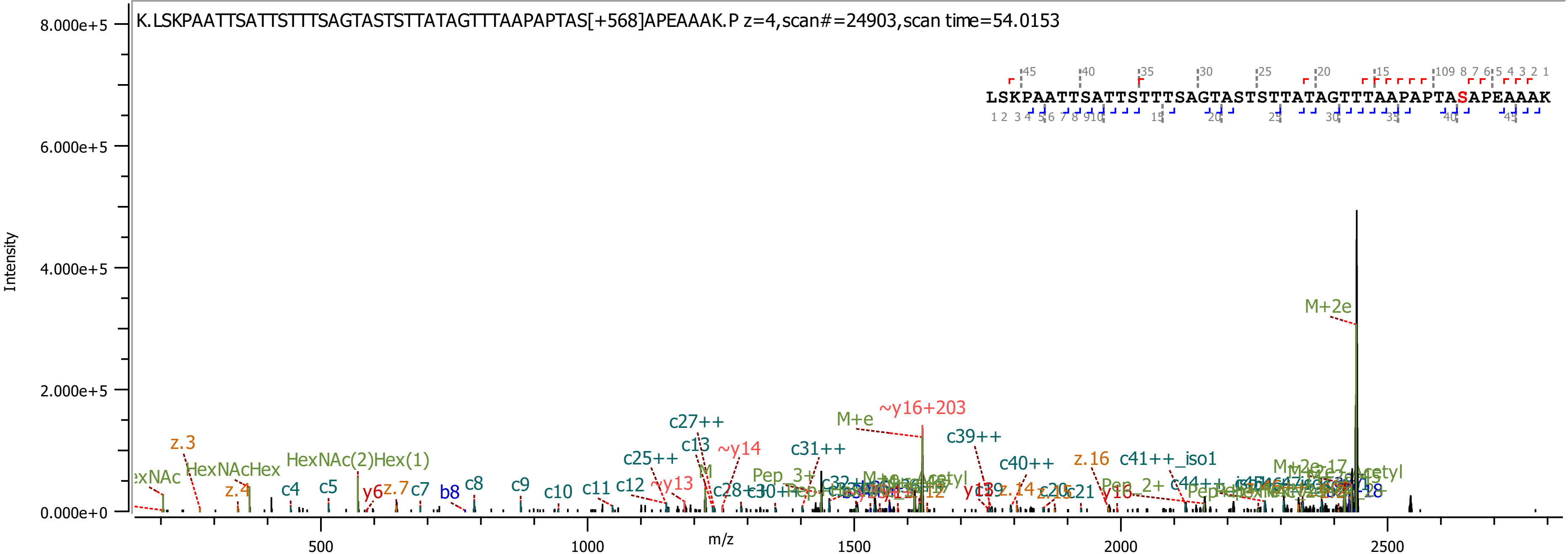

K.LSKPAATTSATTSTTTSAGTASTSTTATAGTTTAAPAPTAS[+568]APEAAAK.P z=4,scan#=24903,scan time=54.0153
LSKPAATTSATTSTTTSAGTASTSTTATAGTTTAAPAPTASAPEAAAK
Intensity
m/z
8.000e+5
6.000e+5
4.000e+5
2.000e+5
0.000e+0
500
1000
1500
2000
2500
M+2e
~y16+203
M+e
c39++
c27++
c13
~y14
c31++
c25++
z.3
HexNAc(2)Hex(1)
HexNAcHex
c40++
z.16
c41++_iso1
M+2e-17
z.4
c4
c5
y6
z.7
c7
b8
c8
c9
c10
c11
c12
~y13
c28++
Pep_3+
Pep_2+
c44++
y16
c21

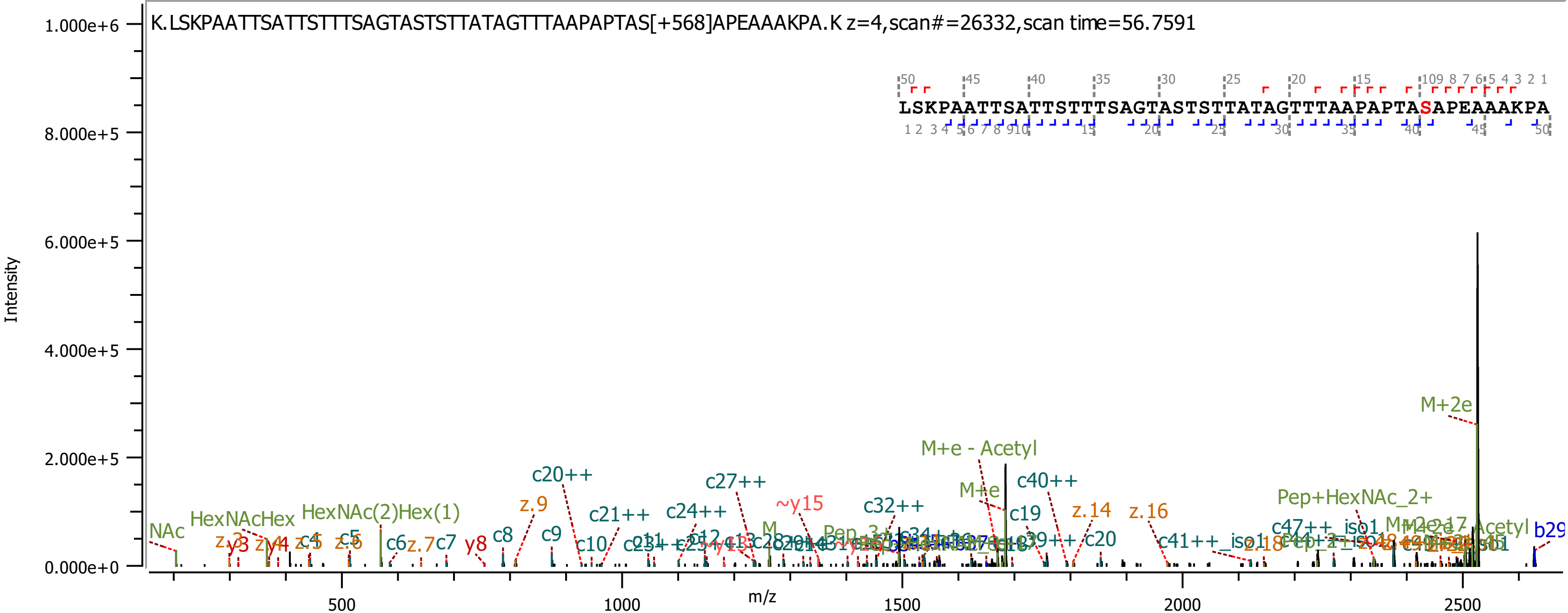
K.LSKPAATTSATTSTTTSAGTASTSTTATAGTTTAAPAPTAS[+568]APEAAAKPA.K z=4,scan#=26332,scan time=56.7591
LSKPAATTSATTSTTTSAGTASTSTTATAGTTTAAPAPTASAPEAAAKPA
Intensity
m/z
1.000e+6
8.000e+5
6.000e+5
4.000e+5
2.000e+5
0.000e+0
500
1000
1500
2000
2500
M+2e
M+e - Acetyl
M+e
c20++
c27++
c21++
c24++
c32++
c40++
~y15
z.9
z.14
z.16
c19
c20
c8
c9
c10
c7
c6
y8
z.7
HexNAcHex
HexNAc(2)Hex(1)
NAc
Pep+HexNAc_2+
c47++_iso1
c41++_iso1
z.18
b29

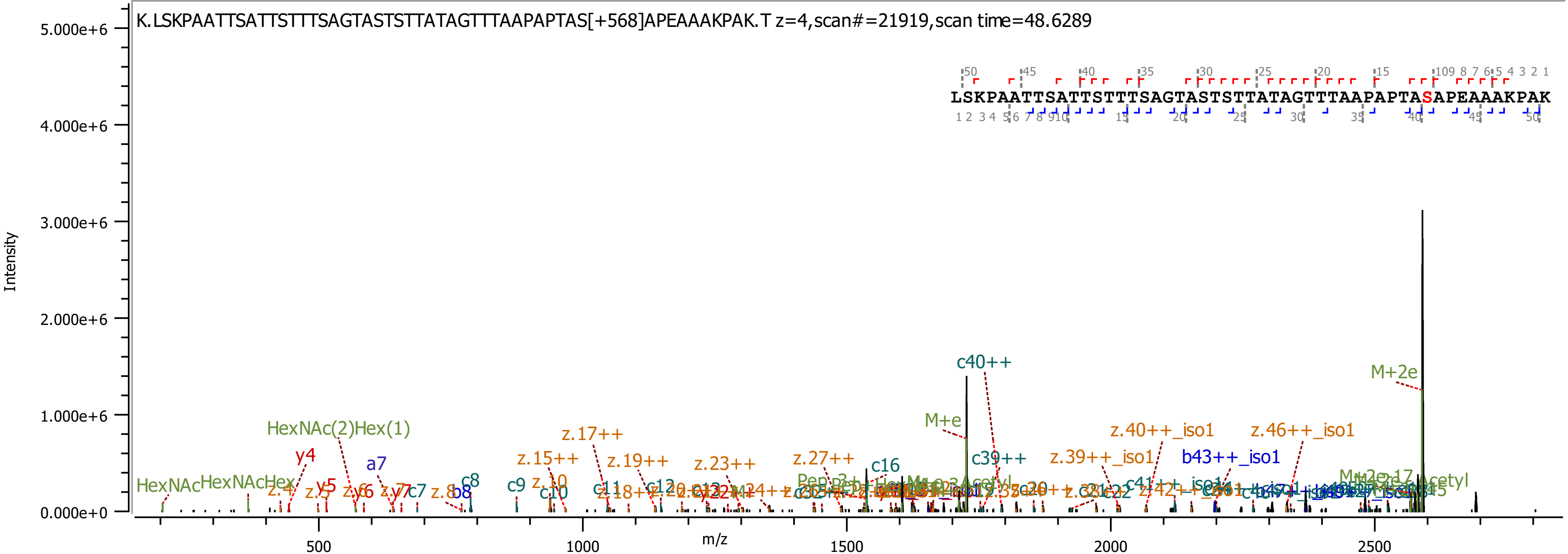
K.LSKPAATTSATTSTTTSAGTASTSTTATAGTTTAAPAPTAS[+568]APEAAAKPAK.T z=4,scan#=21919,scan time=48.6289
LSKPAATTSATTSTTTSAGTASTSTTATAGTTTAAPAPTASAPEAAAKPAK
Intensity
m/z
5.000e+6
4.000e+6
3.000e+6
2.000e+6
1.000e+6
0.000e+0
500
1000
1500
2000
2500
c40++
M+2e
M+e
z.40++_iso1
z.46++_iso1
HexNAc(2)Hex(1)
z.17++
c39++
z.39++_iso1
b43++_iso1
z.15++
z.19++
z.23++
z.27++
c16
y4
a7
HexNAc
HexNAcHex
y5
c7
c8
b8
c9
c10
c11
c12

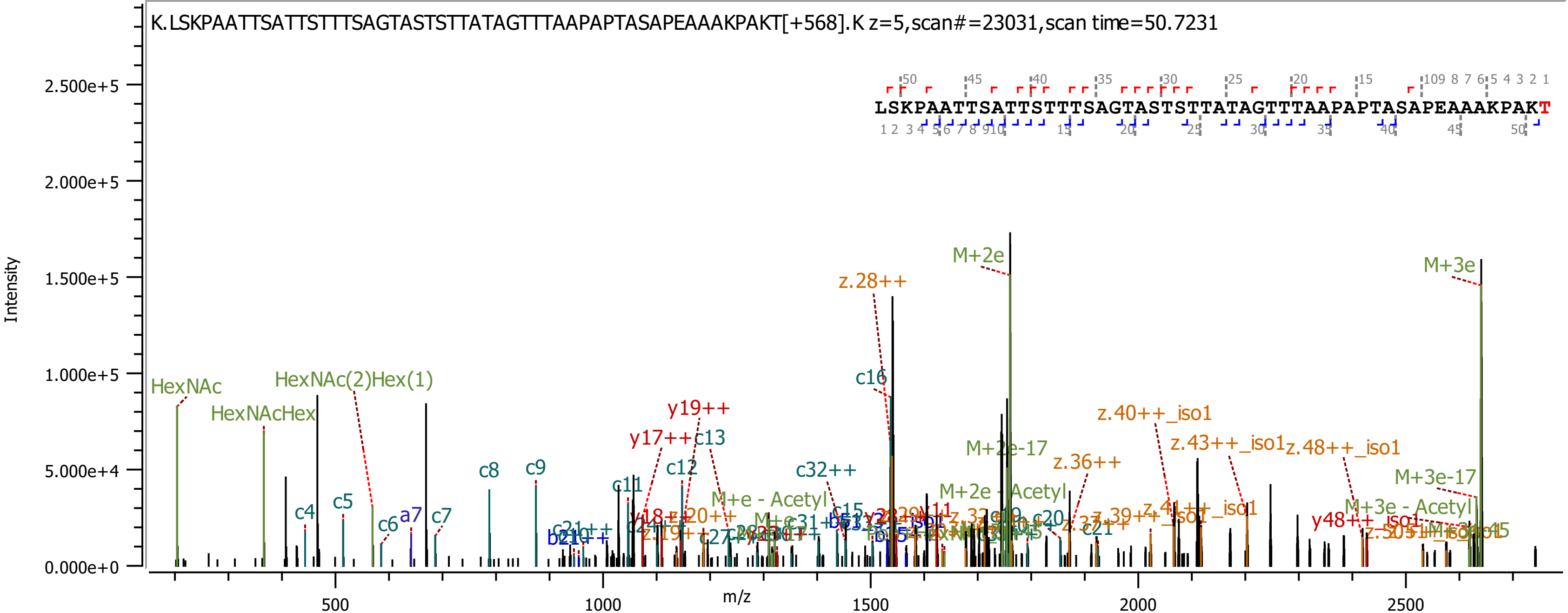
K.LSKPAATTSATTSTTTSAGTASTSTTATAGTTTAAPAPTASAPEAAAKPAKT[+568].K z=5,scan#=23031,scan time=50.7231
LSKPAATTSATTSTTTSAGTASTSTTATAGTTTAAPAPTASAPEAAAKPAKT
Intensity
m/z
HexNAc
HexNAcHex
HexNAc(2)Hex(1)
M+2e
M+3e
z.28++
c16
y19++
y17++
c13
c12
c11
c8
c9
c5
c4
c6
a7
c7
c32++
M+2e-17
z.36++
z.40++_iso1
z.43++_iso1
z.48++_iso1
M+3e-17
M+e - Acetyl
M+2e - Acetyl
M+3e - Acetyl
y48++
z.37++
c21

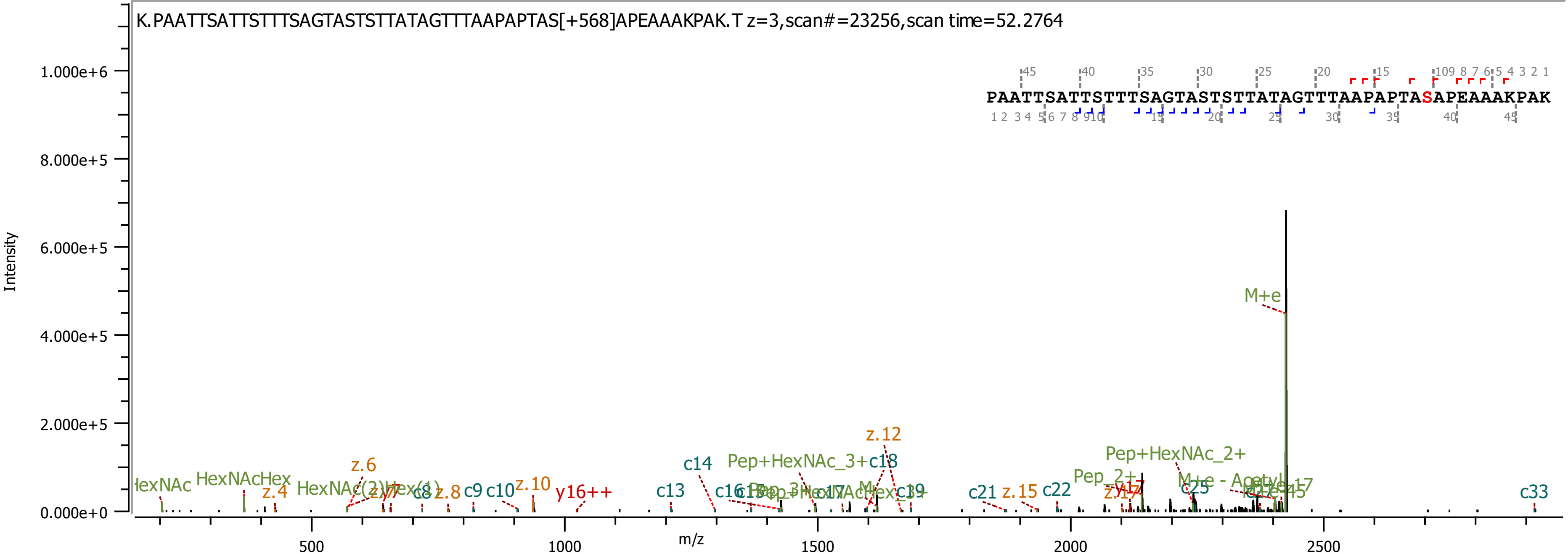
K.PAATTSATTSTTTSAGTASTSTTATAGTTTAAPAPTAS[+568]APEAAAKPAK.T z=3,scan#=23256,scan time=52.2764
PAATTSATTSTTTSAGTASTSTTATAGTTTAAPAPTASAPEAAAKPAK
Intensity
m/z
1.000e+6
8.000e+5
6.000e+5
4.000e+5
2.000e+5
0.000e+0
500
1000
1500
2000
2500
M+e
z.12
Pep+HexNAc_2+
z.6
c14
Pep+HexNAc_3+c18
Pep_2+
HexNAcHex
z.4
z.10
y16++
c13
c9
c10
z.8
z.15
c21
c22
c25
c33

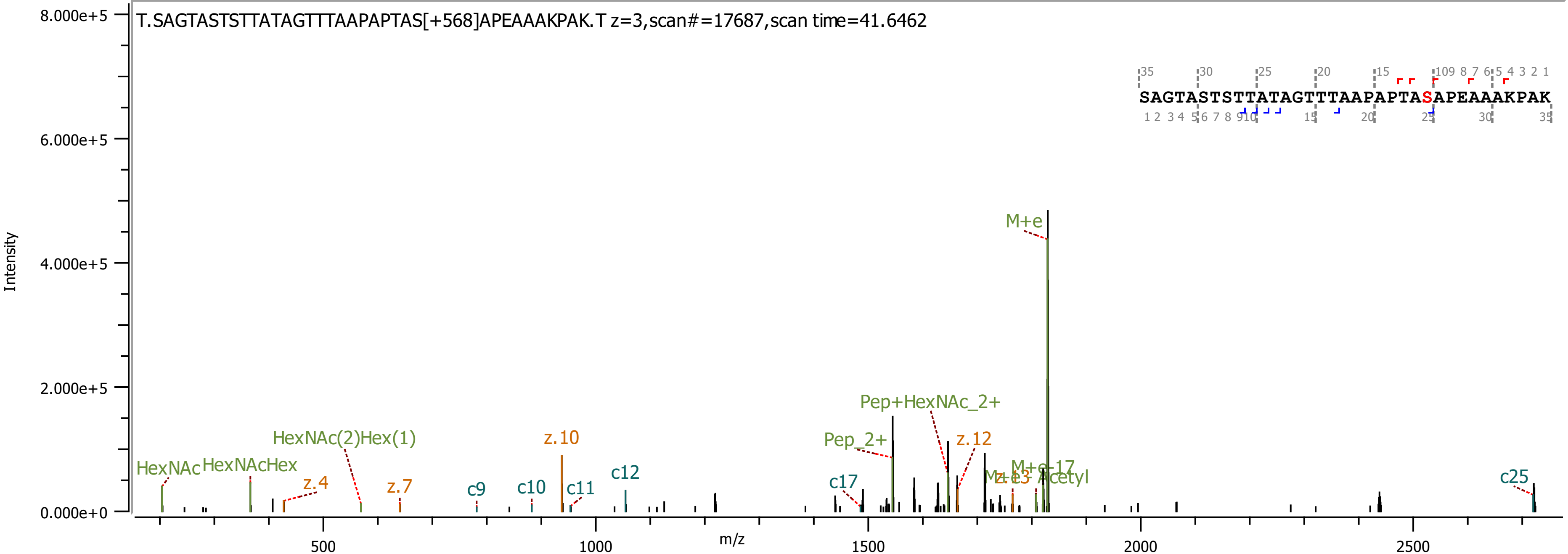

T.SAGTASTSTTATAGTTTAAPAPTAS[+568]APEAAAKPAK.T z=3,scan#=17687,scan time=41.6462
SAGTASTSTTATAGTTTAAPAPTASAPEAAAKPAK
Intensity
m/z
8.000e+5
6.000e+5
4.000e+5
2.000e+5
0.000e+0
500
1000
1500
2000
2500
M+e
Pep+HexNAc_2+
Pep_2+
HexNAc(2)Hex(1)
HexNAc
HexNAcHex
z.4
z.7
z.10
z.12
c9
c10
c11
c12
c17
c25
M+e-17
Acetyl

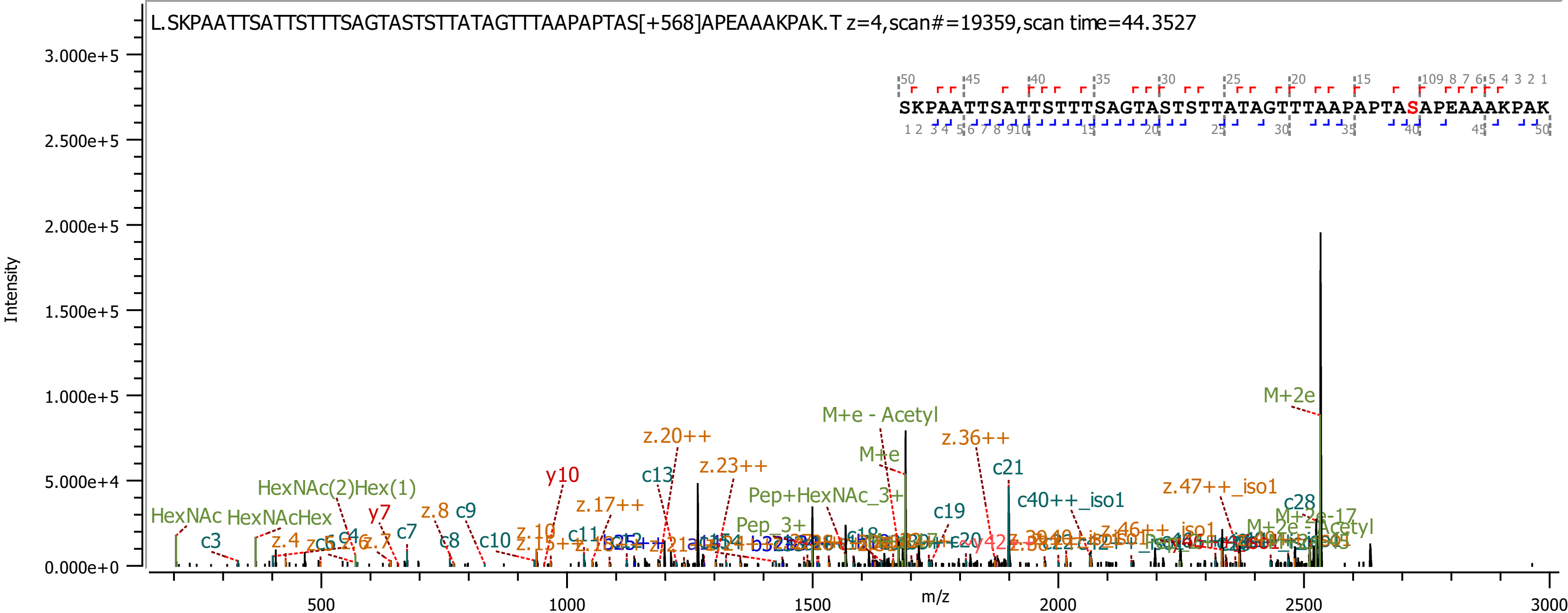

L.SKPAATTSATTSTTTSAGTASTSTTATAGTTTAAPAPTAS[+568]APEAAAKPAK.T z=4,scan#=19359,scan time=44.3527
SKPAATTSATTSTTTSAGTASTSTTATAGTTTAAPAPTASAPEAAAKPAK
Intensity
m/z
3.000e+5
2.500e+5
2.000e+5
1.500e+5
1.000e+5
5.000e+4
0.000e+0
500
1000
1500
2000
2500
3000
M+2e
M+e - Acetyl
M+e
z.36++
z.20++
z.23++
c21
c13
y10
c40++_iso1
z.47++_iso1
HexNAc(2)Hex(1)
Pep+HexNAc_3+
c19
c28
z.17++
HexNAc
HexNAcHex
y7
z.8
c9
c7
c3
z.4
c8
c10
z.10
c11
Pep_3+
M+2e-17
M+2e - Acetyl

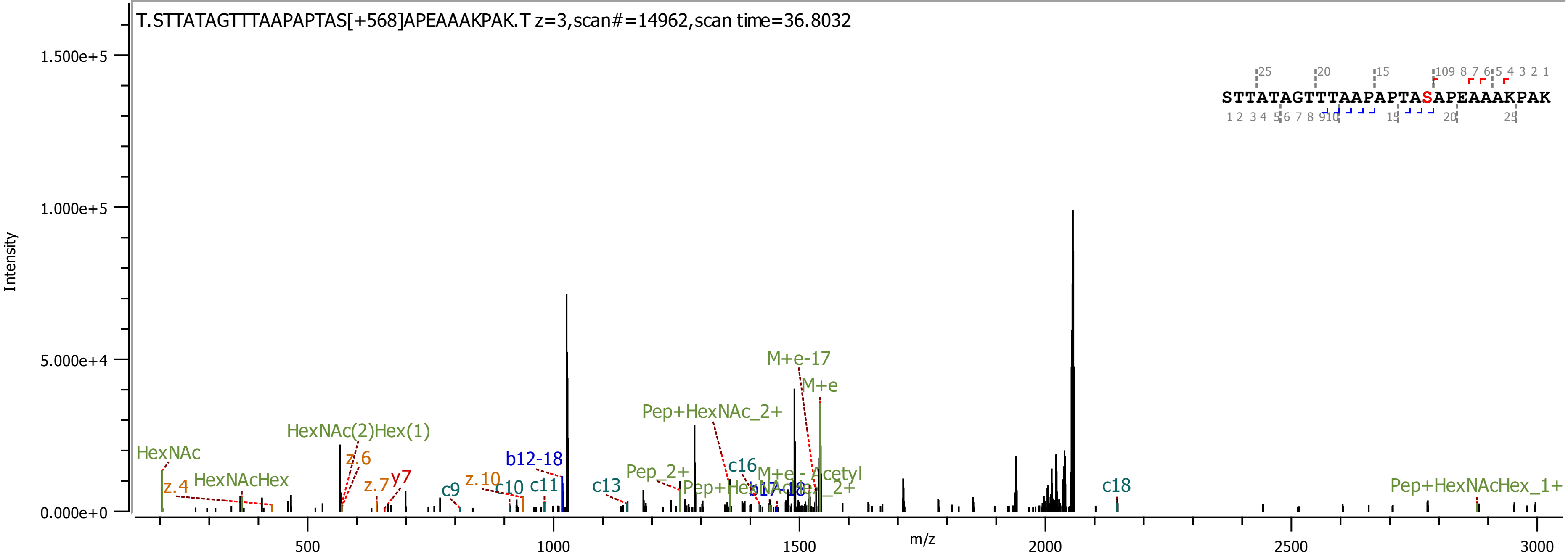

T.STTATAGTTTAAPAPTAS[+568]APEAAAKPAK.T z=3,scan#=14962,scan time=36.8032
STTATAGTTTAAPAPTASAPEAAAKPAK
Intensity
m/z
1.500e+5
1.000e+5
5.000e+4
0.000e+0
500
1000
1500
2000
2500
3000
HexNAc
z.4
HexNAcHex
HexNAc(2)Hex(1)
z.6
z.7
y7
c9
z.10
c10
c11
b12-18
c13
Pep_2+
Pep+HexNAc_2+
c16
M+e-17
M+e
M+e - Acetyl
c18
Pep+HexNAcHex_1+

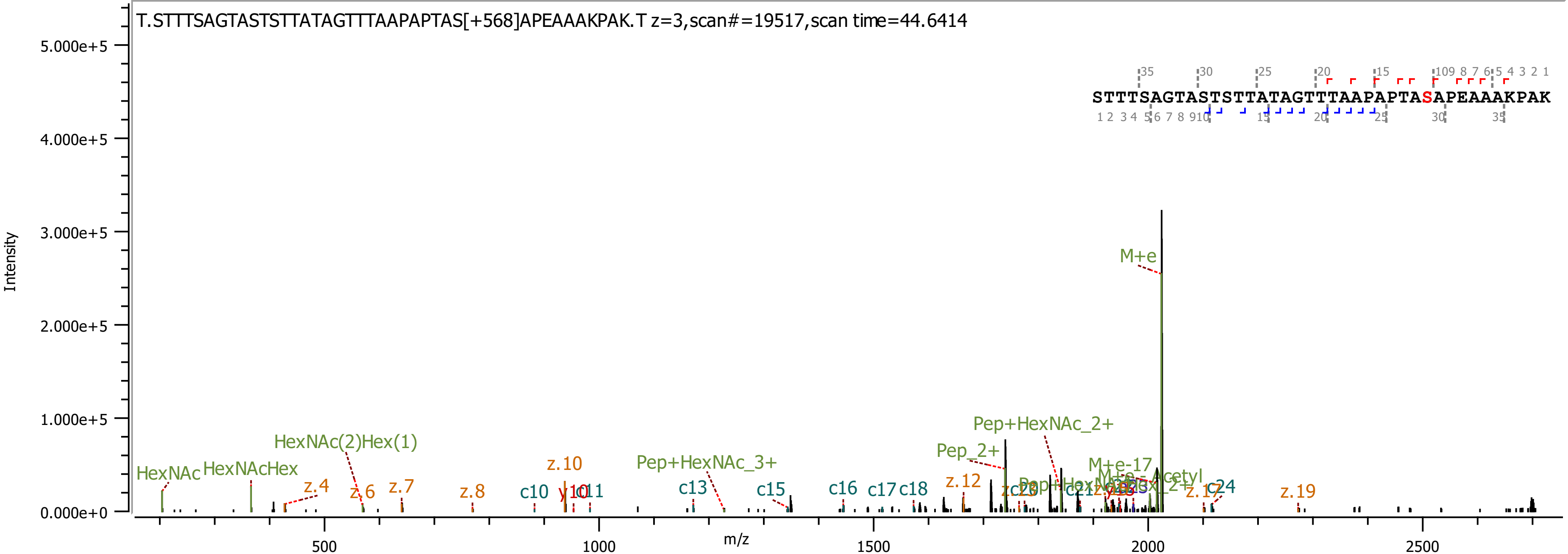

T.STTTSAGTASTSTTATAGTTTAAPAPTAS[+568]APEAAAKPAK.T z=3,scan#=19517,scan time=44.6414
STTTSAGTASTSTTATAGTTTAAPAPTASAPEAAAKPAK
Intensity
m/z
M+e
Pep+HexNAc_2+
Pep_2+
M+e-17
HexNAc(2)Hex(1)
HexNAc
HexNAcHex
Pep+HexNAc_3+
z.4
z.6
z.7
z.8
z.10
c10
y10
c11
c13
c15
c16
c17
c18
z.12
z.17
c24
z.19

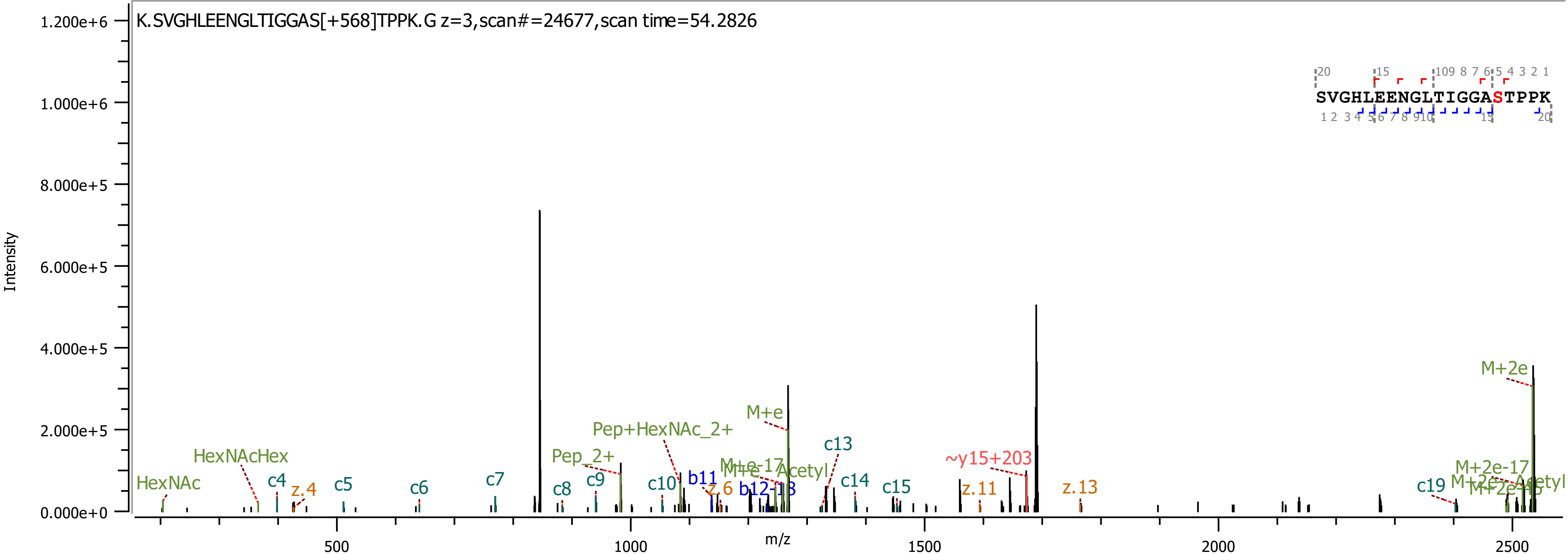
K.SVGHLEENGLTIGGAS[+568]TPPK.G z=3,scan#=24677,scan time=54.2826
SVGHLEENGLTIGGASTPPK
Intensity
m/z
HexNAc
HexNAcHex
c4
z.4
c5
c6
c7
c8
c9
Pep_2+
Pep+HexNAc_2+
c10
b11
z.6
b12-18
M+e-17
M+e-Acetyl
M+e
c13
c14
c15
~y15+203
z.11
z.13
c19
M+2e-17
M+2e-Acetyl
M+2e-45
M+2e

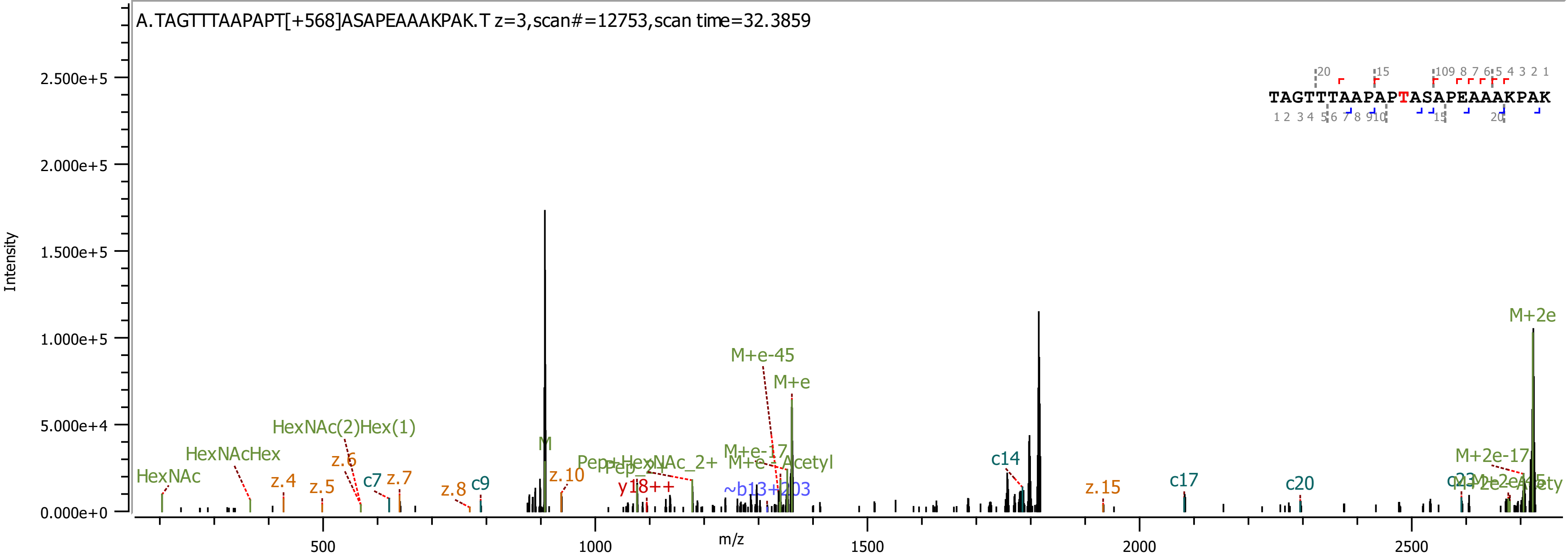

A.TAGTTTAAPAPT[+568]ASAPEAAAKPAK.T z=3,scan#=12753,scan time=32.3859
TAGTTTAAPAPTASAPEAAAKPAK
Intensity
m/z
M+2e
M+e-45
M+e
HexNAc(2)Hex(1)
HexNAcHex
HexNAc
M
c14
c17
c20
c9
c7
z.4
z.5
z.6
z.7
z.8
z.10
z.15
y18++
M+e-17
M+2e-17

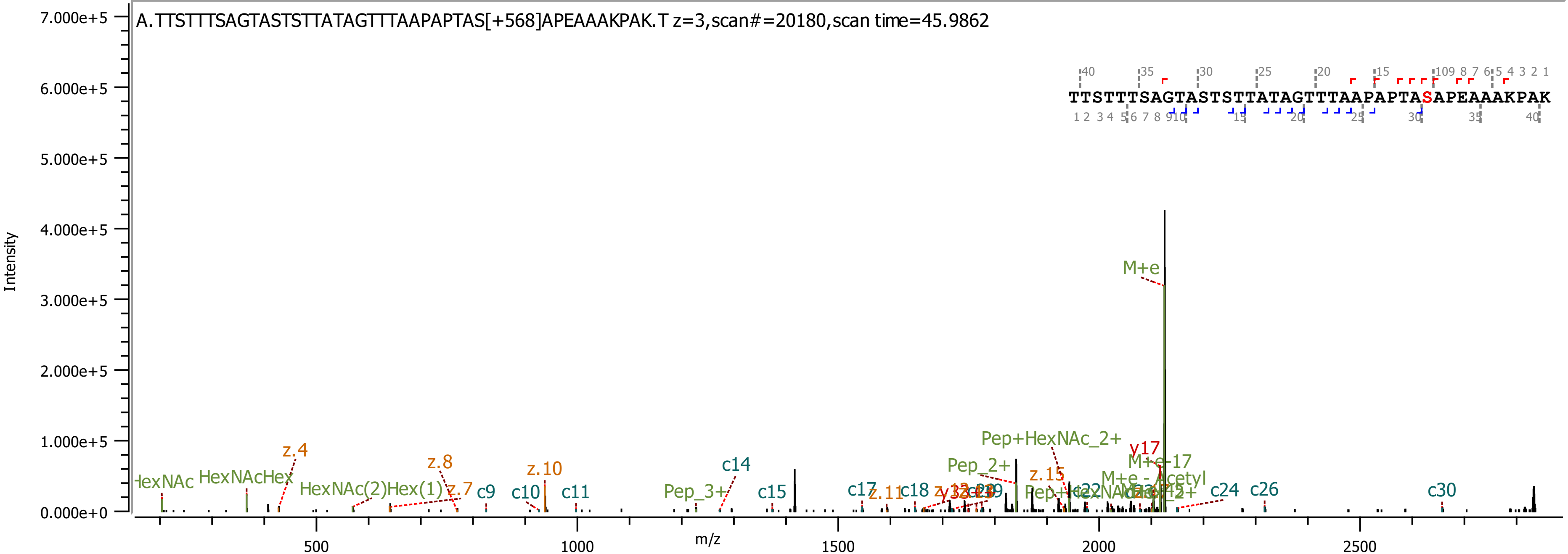

A.TTSTTTSAGTASTSTTATAGTTTAAPAPTAS[+568]APEAAAKPAK.T z=3,scan#=20180,scan time=45.9862
TTSTTTSAGTASTSTTATAGTTTAAPAPTASAPEAAAKPAK
Intensity
m/z
M+e
Pep+HexNAc_2+
y17
M+e-17
Pep_2+
M+e - Acetyl
z.15
HexNAc
HexNAcHex
HexNAc(2)Hex(1)
z.4
z.8
z.7
z.10
c9
c10
c11
Pep_3+
c14
c15
c17
z.11
c18
c24
c26
c30

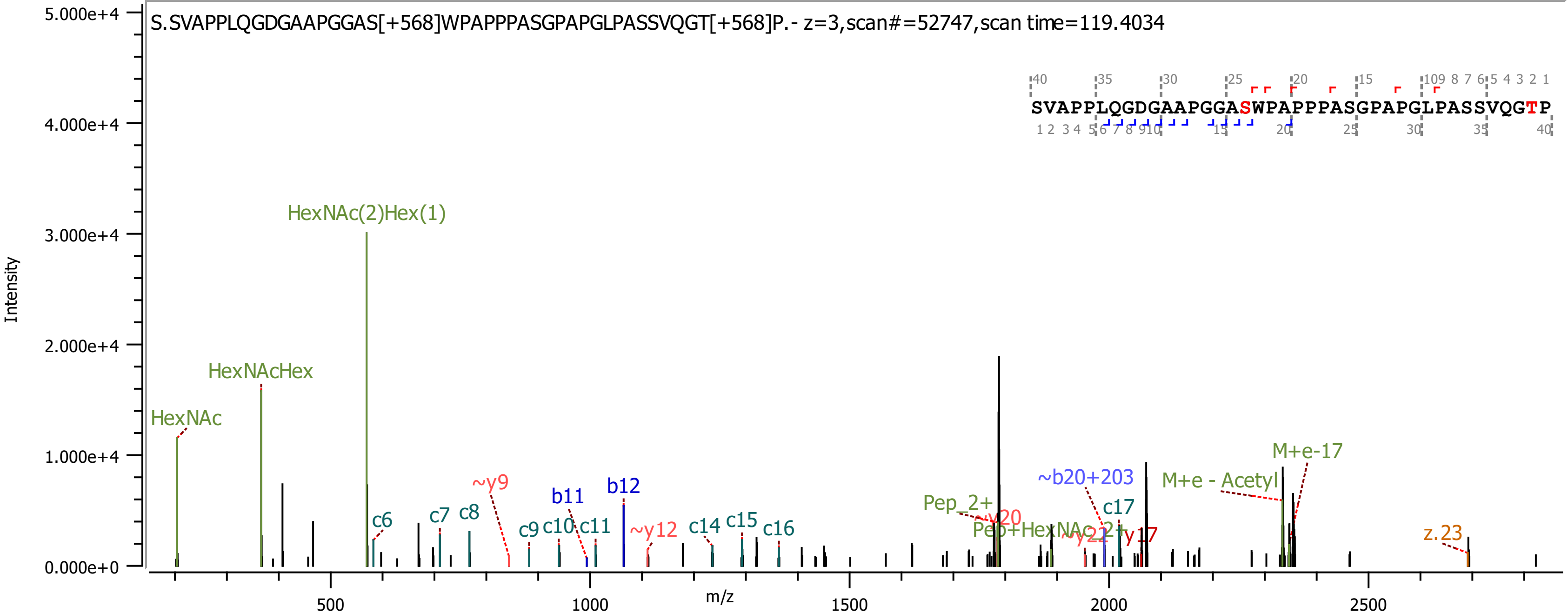
S.SVAPPLQGDGAAPGGAS[+568]WPAPPPASGPAPGLPASSVQGT[+568]P.- z=3,scan#=52747,scan time=119.4034
SVAPPLQGDGAAPGGASWPAPPPASGPAPGLPASSVQGTP
Intensity
m/z
5.000e+4
4.000e+4
3.000e+4
2.000e+4
1.000e+4
0.000e+0
500
1000
1500
2000
2500
HexNAc
HexNAcHex
HexNAc(2)Hex(1)
c6
c7
c8
~y9
c9
c10
c11
b11
b12
~y12
c14
c15
c16
Pep_2+
~y20
Pep+HexNAc_2+
~y22
y17
~b20+203
c17
M+e - Acetyl
M+e-17
z.23

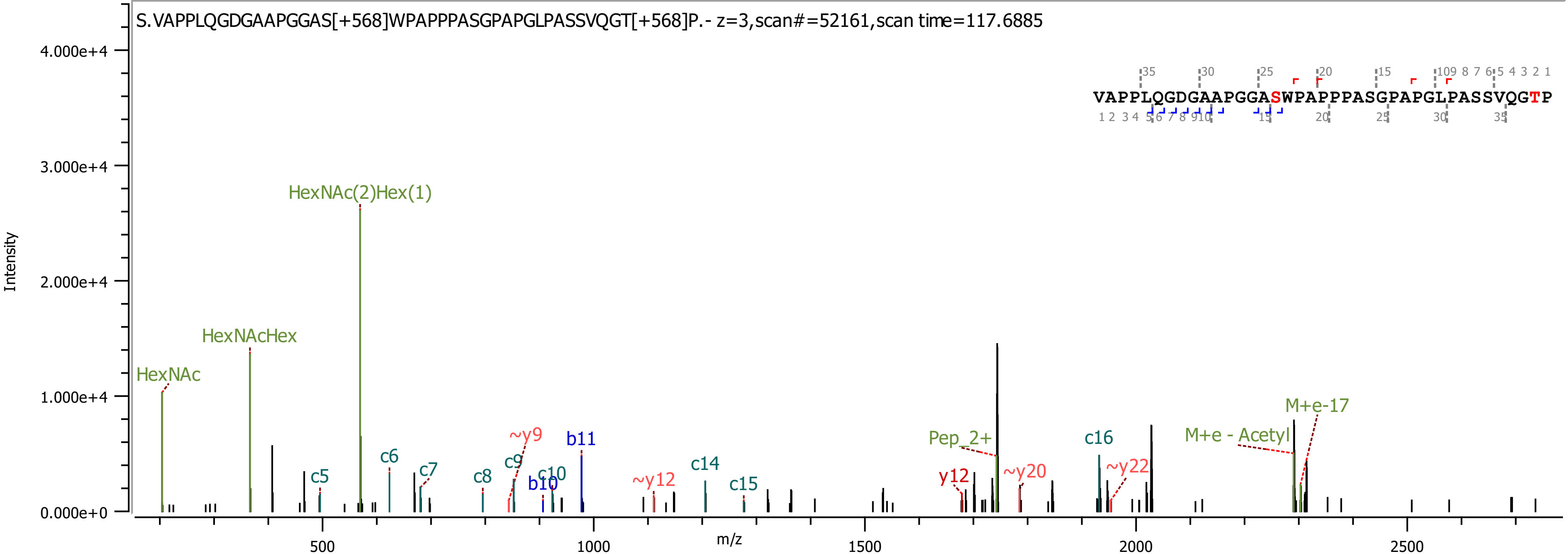

S.VAPPLQGDGAAPGGAS[+568]WPAPPPASGPAPGLPASSVQGT[+568]P.- z=3,scan#=52161,scan time=117.6885
VAPPLQGDGAAPGGASWPAPPPASGPAPGLPASSVQGTP
Intensity
m/z
4.000e+4
3.000e+4
2.000e+4
1.000e+4
0.000e+0
500
1000
1500
2000
2500
HexNAc(2)Hex(1)
HexNAcHex
HexNAc
c5
c6
c7
c8
c9
~y9
b10
c10
b11
~y12
c14
c15
Pep_2+
y12
~y20
c16
~y22
M+e - Acetyl
M+e-17

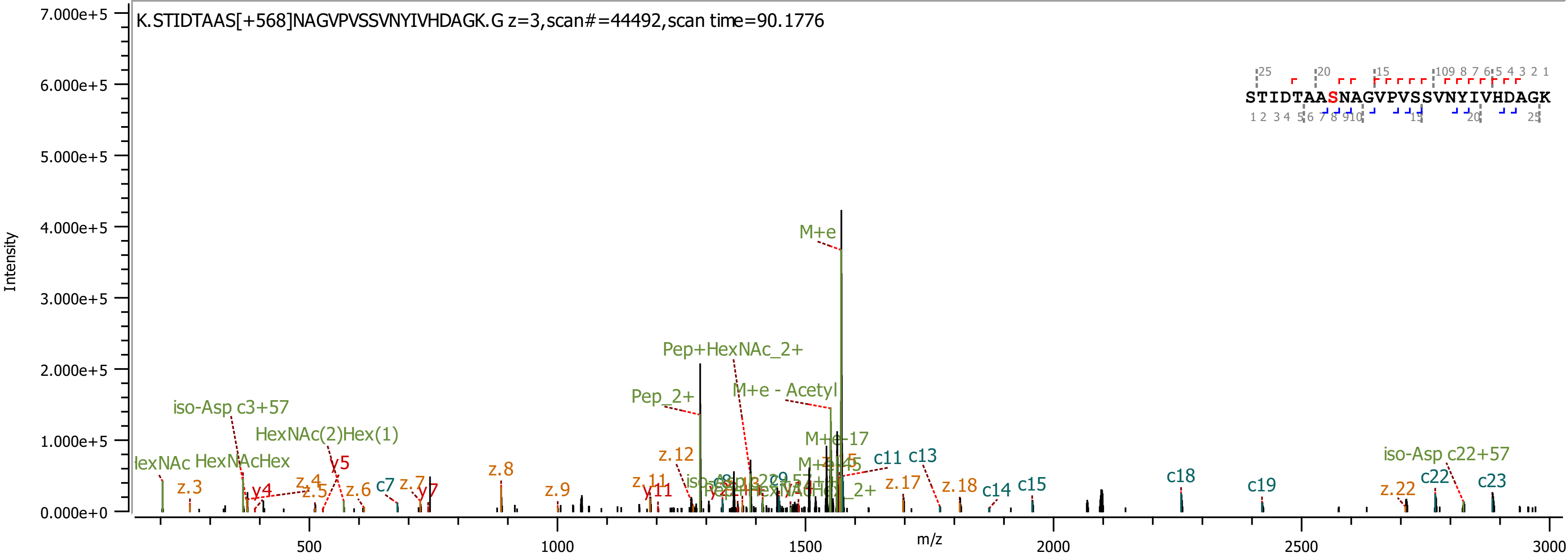

K.STIDTAAS[+568]NAGVPVSSVNYIVHDAGK.G z=3,scan#=44492,scan time=90.1776
STIDTAASNAGVPVSSVNYIVHDAGK
Intensity
m/z
7.000e+5
6.000e+5
5.000e+5
4.000e+5
3.000e+5
2.000e+5
1.000e+5
0.000e+0
500
1000
1500
2000
2500
3000
M+e
Pep+HexNAc_2+
M+e - Acetyl
Pep_2+
iso-Asp c3+57
HexNAc(2)Hex(1)
HexNAcHex
M+e-17
z.12
z.8
z.3
y4
z.4
y5
z.5
z.6
c7
z.7
y7
z.9
z.11
y11
c11
c13
z.17
z.18
c14
c15
c18
c19
iso-Asp c22+57
z.22
c22
c23

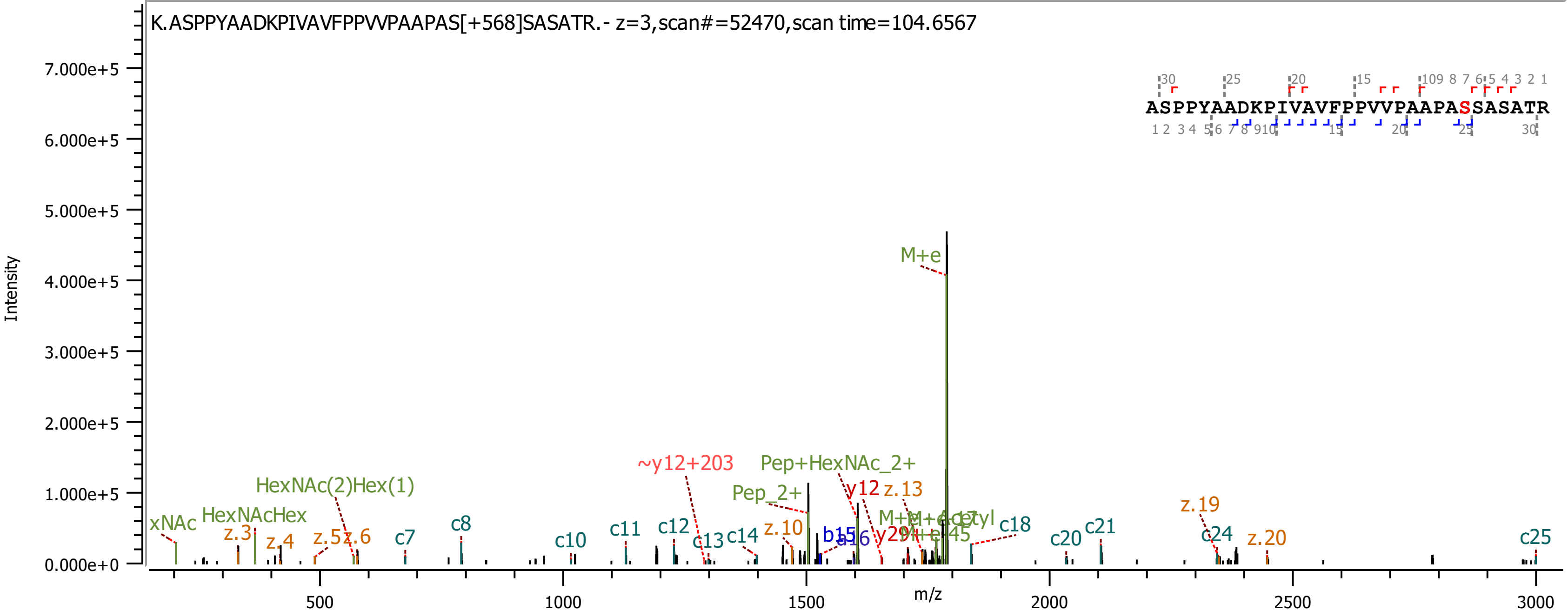

K.ASPPYAADKPIVAVFPPVVPAAPAS[+568]SASATR.- z=3,scan#=52470,scan time=104.6567
ASPPYAADKPIVAVFPPVVPAAPASSASATR
Intensity
m/z
M+e
~y12+203
Pep+HexNAc_2+
Pep_2+
HexNAc(2)Hex(1)
HexNAcHex
xNAc
y12
z.13
z.19
z.20
c7
c8
c10
c11
c12
c13
c14
c18
c20
c21
c24
c25
z.3
z.4
z.5
z.6
z.10
b15
a16
y20

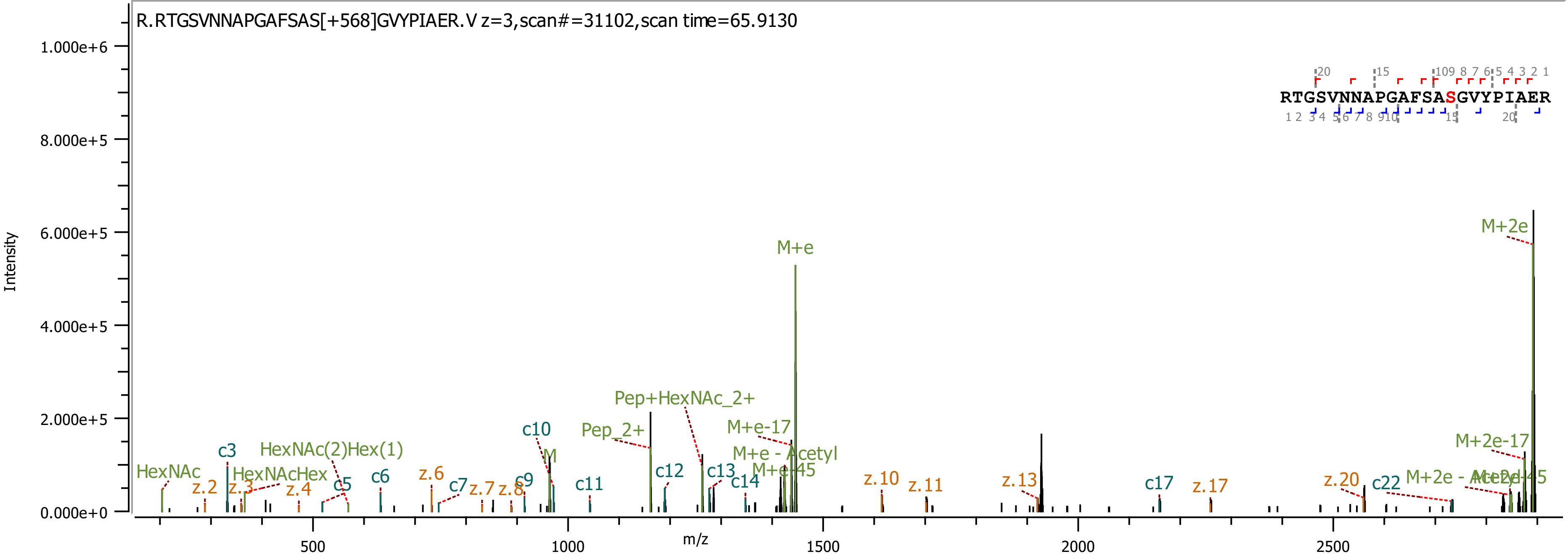

R.RTGSVNNAPGAFSAS[+568]GVYPIAER.V z=3,scan#=31102,scan time=65.9130
RTGSVNNAPGAFSASGVYPIAER
Intensity
m/z
M+2e
M+e
Pep+HexNAc_2+
Pep_2+
M+e-17
M+e - Acetyl
M+e-45
M+2e-17
HexNAc
HexNAcHex
HexNAc(2)Hex(1)

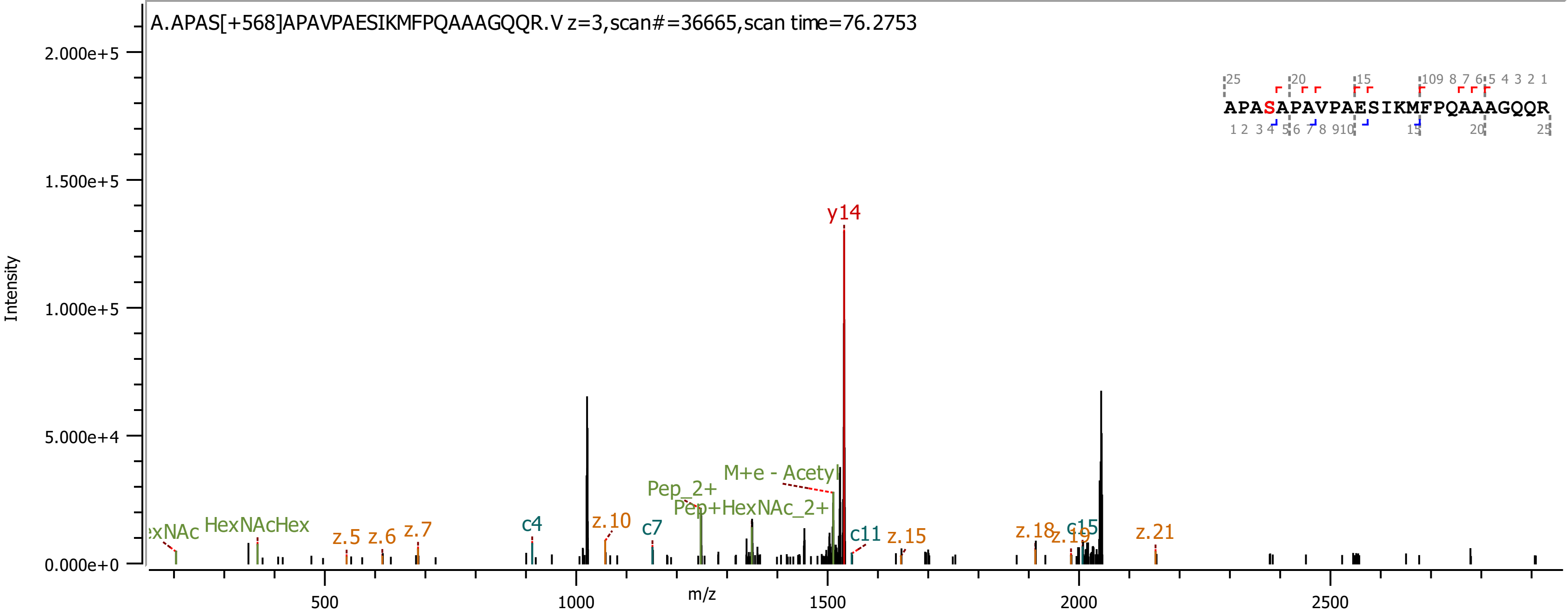

A.APAS[+568]APAVPAESIKMFPQAAAGQQR.V z=3,scan#=36665,scan time=76.2753
APASAPAVPAESIKMFPQAAAGQQR
Intensity
m/z
y14
M+e - Acetyl
Pep_2+
Pep+HexNAc_2+
HexNAcHex
z.5
z.6
z.7
c4
z.10
c7
c11
z.15
z.18
z.19
c15
z.21

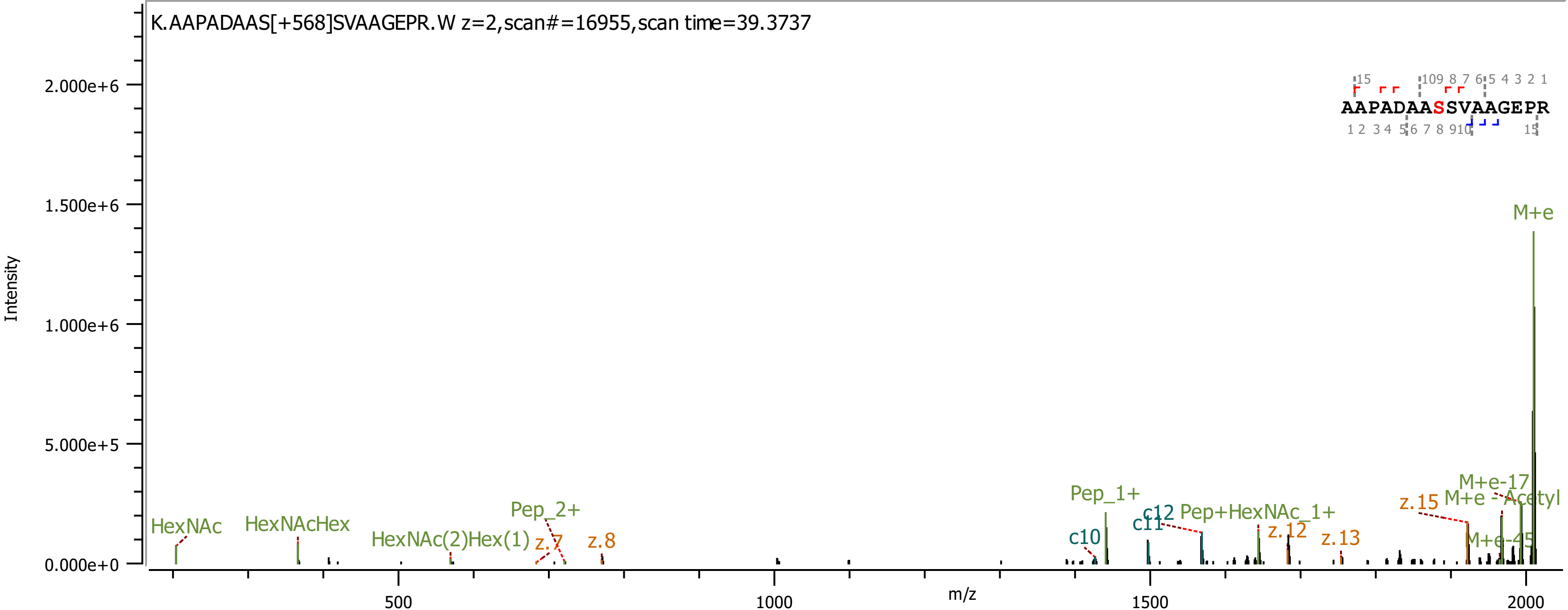
K.AAPADAAS[+568]SVAAGEPR.W z=2,scan#=16955,scan time=39.3737
Intensity
m/z
AAPADAASSVAAGEPR
M+e
M+e-17
M+e - Acetyl
M+e-45
z.15
z.13
z.12
Pep+HexNAc_1+
c12
c11
c10
Pep_1+
Pep_2+
HexNAc
HexNAcHex
HexNAc(2)Hex(1)
z.7
z.8

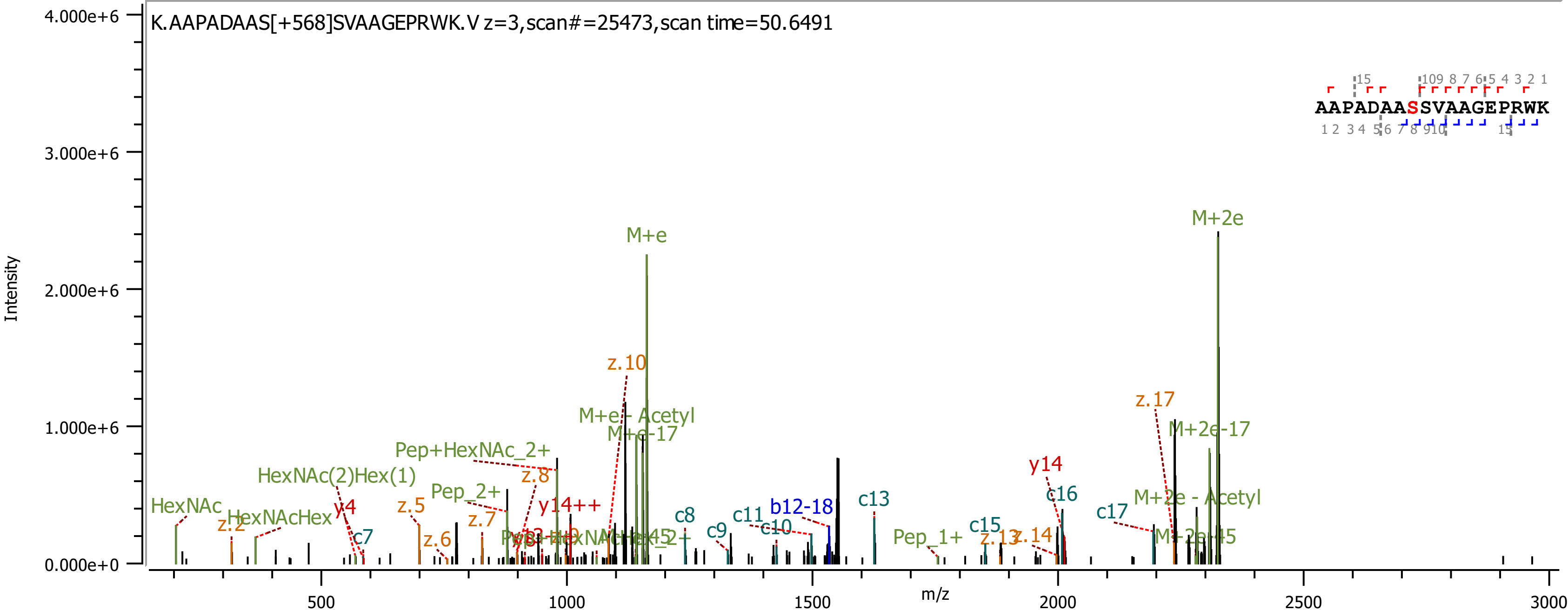
K.AAPADAAS[+568]SVAAGEPRWK.V z=3,scan#=25473,scan time=50.6491
AAPADAASSVAAGEPRWK
Intensity
m/z
4.000e+6
3.000e+6
2.000e+6
1.000e+6
0.000e+0
500
1000
1500
2000
2500
3000
M+2e
M+e
z.10
z.17
M+e - Acetyl
M+e-17
M+2e-17
Pep+HexNAc_2+
y14
HexNAc(2)Hex(1)
z.8
Pep_2+
c16
M+2e - Acetyl
c13
c17
HexNAc
z.5
y4
y14++
b12-18
c8
c11
HexNAcHex
z.2
z.7
c10
c9
c15
z.14
z.13
M+2e-45
Pep_1+
c7
z.6

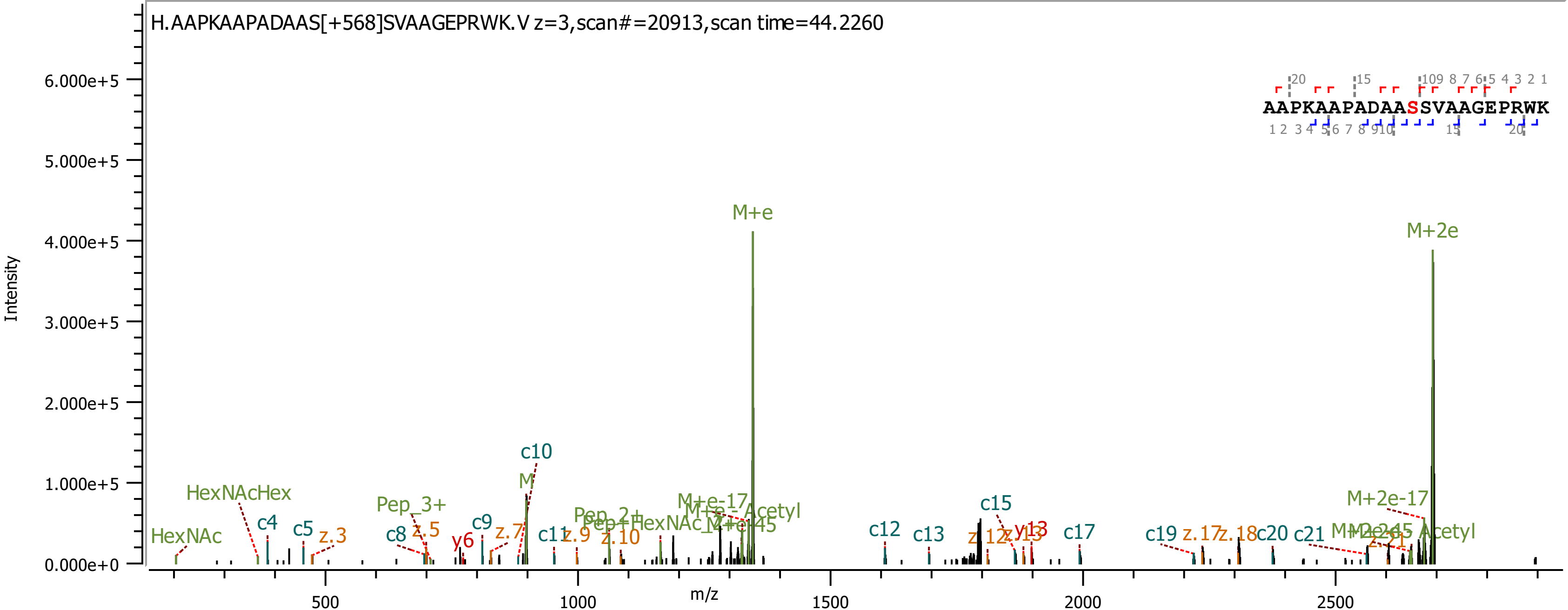

H.AAPKAAPADAAS[+568]SVAAGEPRWK.V z=3,scan#=20913,scan time=44.2260
AAPKAAPADAASSVAAGEPRWK
Intensity
m/z
M+e
M+2e
c10
M
HexNAcHex
HexNAc
c4
c5
z.3
Pep_3+
c8
z.5
y6
c9
z.7
c11
z.9
z.10
Pep_2+
Pep+HexNAc_2+
M+e-17
M+e-Acetyl
M+e-45
c12
c13
c15
z.12
z.13
y13
c17
c19
z.17
z.18
c20
c21
M+2e-17
M+2e-45
z.21
Acetyl

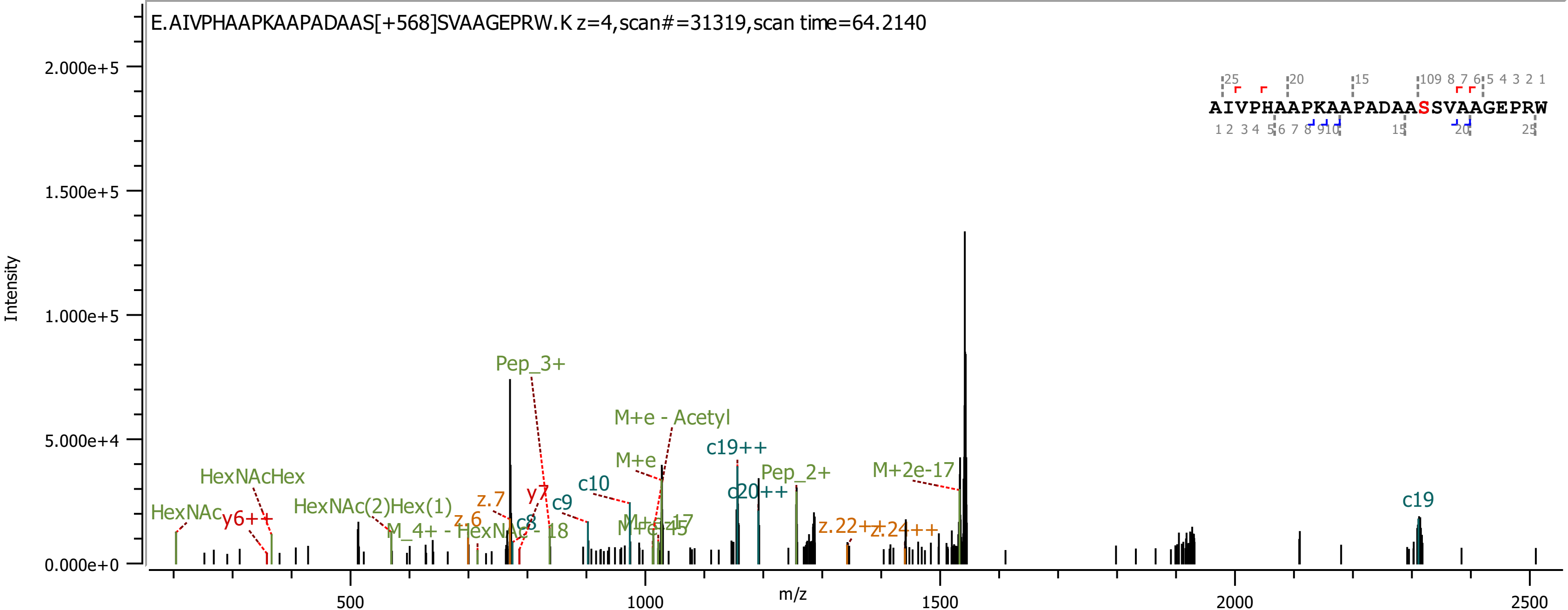

E.AIVPHAAPKAAPADAAS[+568]SVAAGEPRW.K z=4,scan#=31319,scan time=64.2140
Intensity
m/z
AIVPHAAPKAAPADAASSVAAGEPRW
Pep_3+
M+e - Acetyl
M+e
c19++
Pep_2+
M+2e-17
c10
c20++
c19
HexNAcHex
HexNAc
y6++
HexNAc(2)Hex(1)
z.7
z.6
y7
c9
c8
M_4+ - HexNAc - 18
z.22++
z.24++

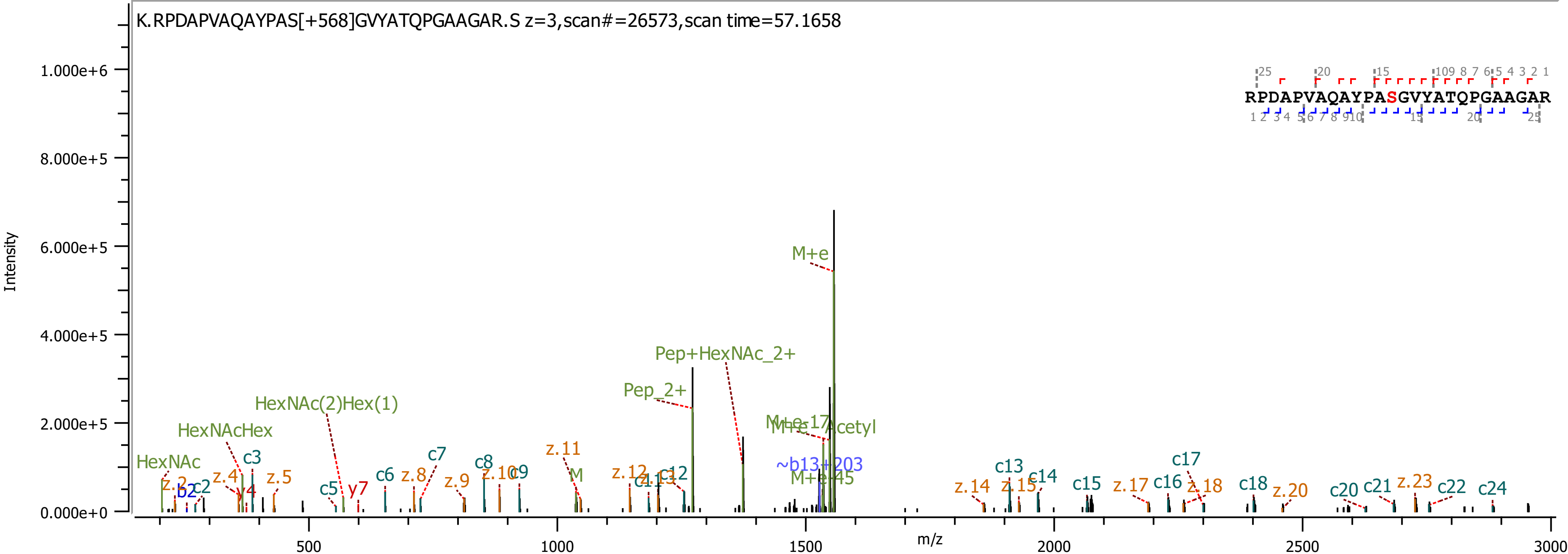
K.RPDAPVAQAYPAS[+568]GVYATQPGAAGAR.S z=3,scan#=26573,scan time=57.1658
Intensity
m/z
RPDAPVAQAYPASGVYATQPGAAGAR
M+e
Pep+HexNAc_2+
Pep_2+
HexNAc(2)Hex(1)
HexNAcHex
HexNAc
~b13+203
M+e-45
c13
c14
c15
c16
c17
c18
c20
c21
c22
c24
z.14
z.15
z.17
z.18
z.20
z.23
z.11
z.12
c12
c7
c8
c9
c6
c5
c3
c2
b2
y4
y7
z.2
z.4
z.5
z.8
z.9
z.10

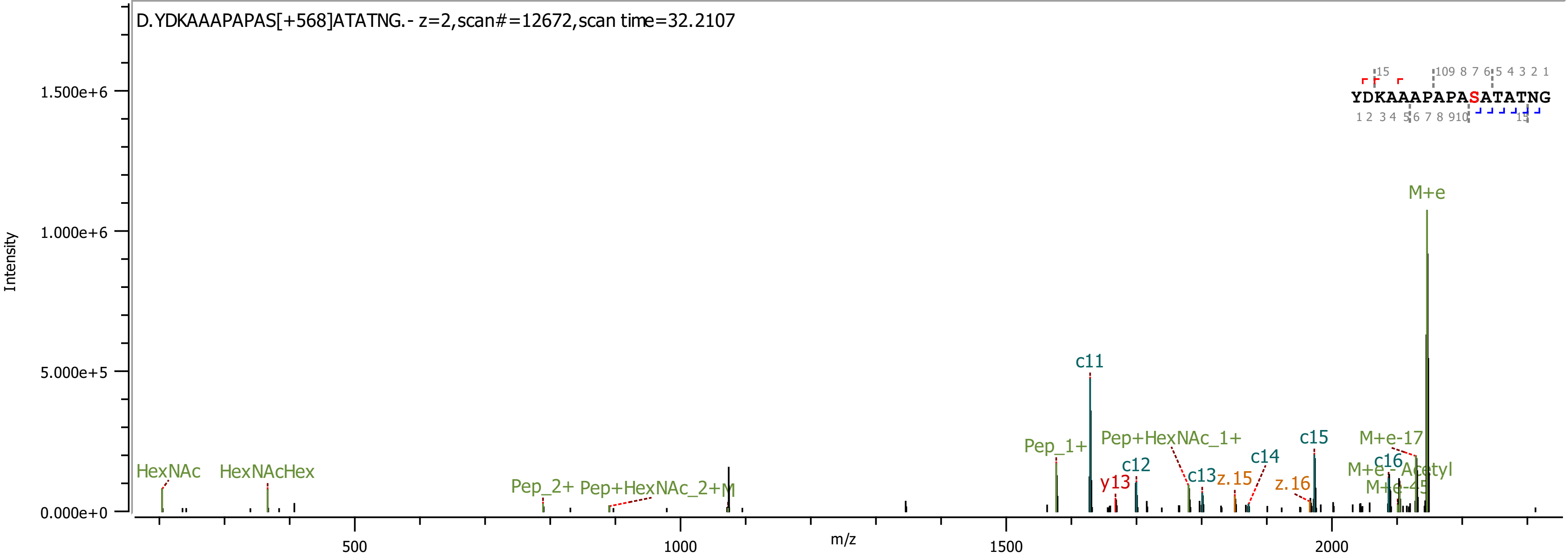
D.YDKAAAPAPAS[+568]ATATNG.- z=2,scan#=12672,scan time=32.2107
YDKAAAPAPASATATNG
Intensity
m/z
M+e
c11
Pep_1+
Pep+HexNAc_1+
c12
c13
c14
c15
c16
y13
z.15
z.16
M+e-17
M+e-Acetyl
M+e-45
HexNAc
HexNAcHex
Pep_2+
Pep+HexNAc_2+M

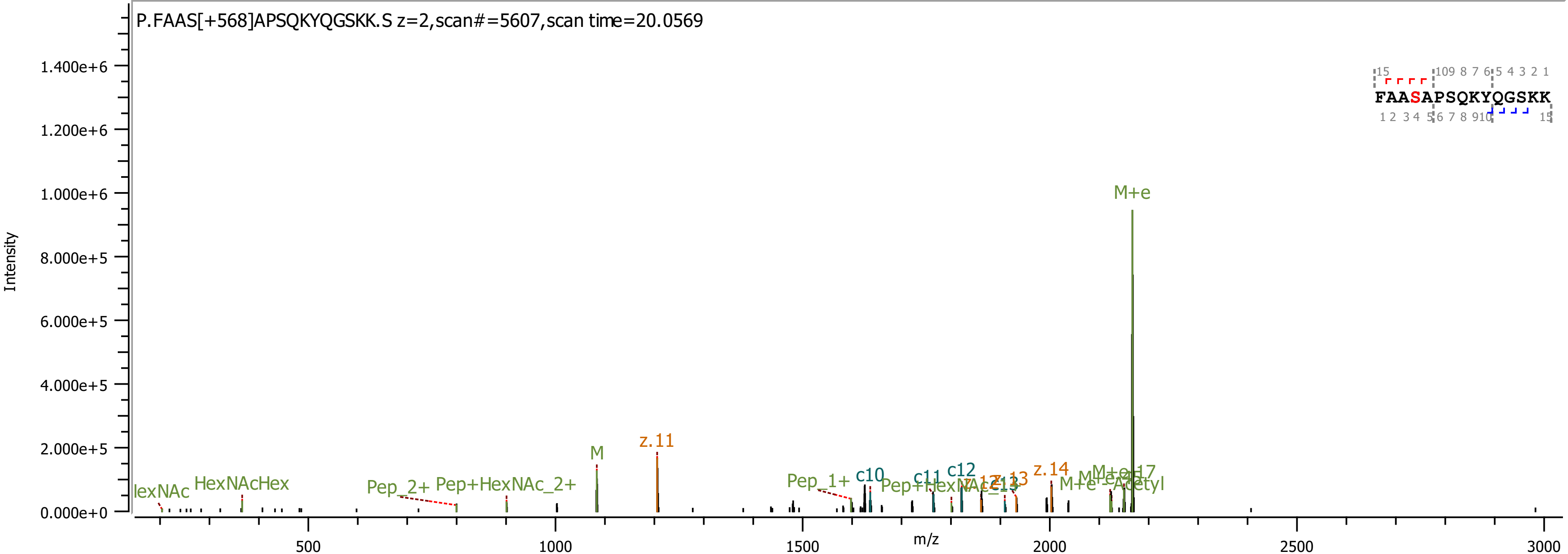

P.FAAS[+568]APSQKYQGSKK.S z=2,scan#=5607,scan time=20.0569
Intensity
m/z
M+e
M
z.11
HexNAcHex
Pep_2+
Pep+HexNAc_2+
Pep_1+
c10
c11
c12
z.14
M+e-Acetyl
FAASAPSQKYQGSKK

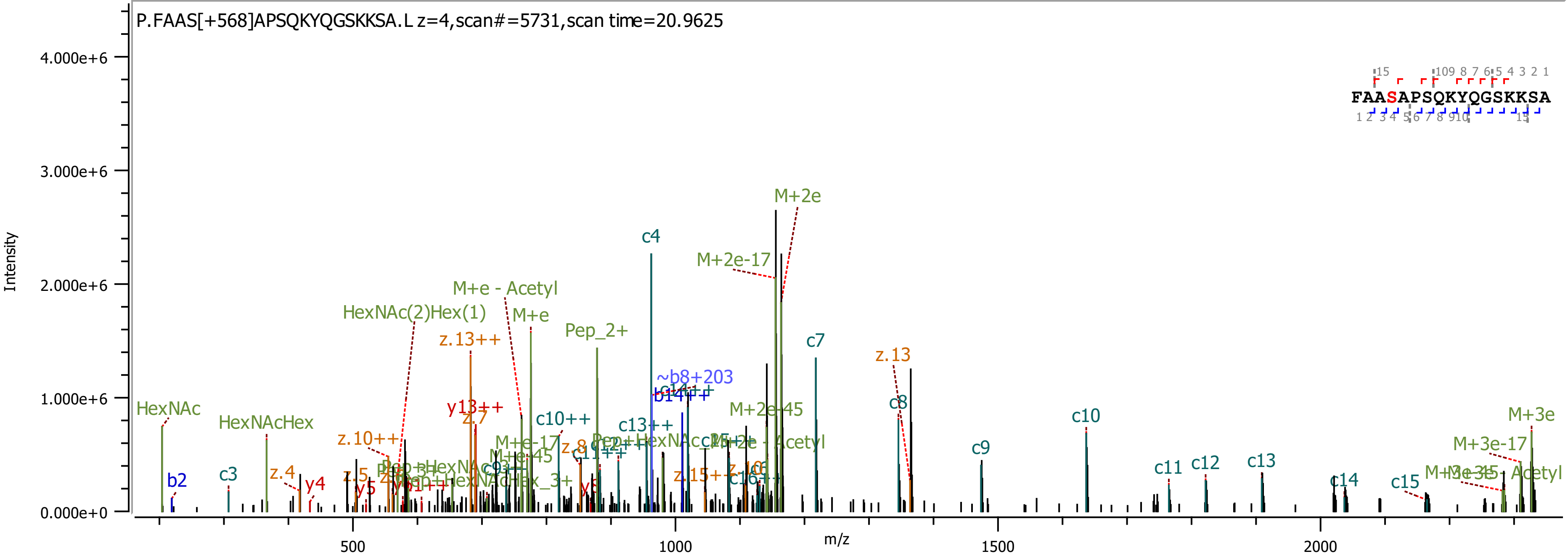
P.FAAS[+568]APSQKYQGSKKSA.L z=4,scan#=5731,scan time=20.9625
Intensity
4.000e+6
3.000e+6
2.000e+6
1.000e+6
0.000e+0
500
1000
1500
2000
m/z
FAASAPSQKYQGSKKSA
M+2e
M+2e-17
c4
M+e - Acetyl
HexNAc(2)Hex(1)
M+e
Pep_2+
z.13++
c7
z.13
~b8+203
y13++
c8
c10
HexNAc
HexNAcHex
z.10++
c10++
c13++
M+2e-45
M+3e
M+3e-17
c9
c11
c12
c13
c14
c15
b2
c3
z.4
y4
z.5
y5
z.7
z.8
M+e-17
M+e-45
b14++
y6
z.15++
c16++
M+3e-45 - Acetyl

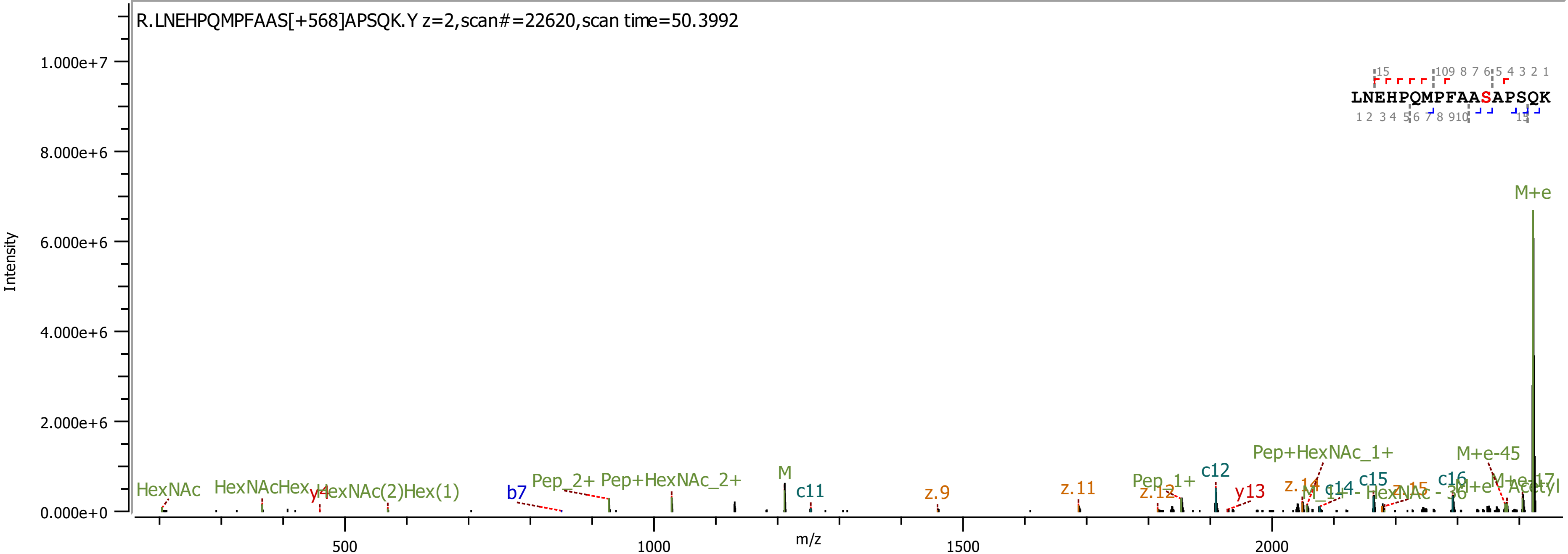
R.LNEHPQMPFAAS[+568]APSQK.Y z=2,scan#=22620,scan time=50.3992
LNEHPQMPFAASAPSQK
Intensity
m/z
1.000e+7
8.000e+6
6.000e+6
4.000e+6
2.000e+6
0.000e+0
500
1000
1500
2000
M+e
HexNAc
HexNAcHex
y4
HexNAc(2)Hex(1)
b7
Pep_2+
Pep+HexNAc_2+
M
c11
z.9
z.11
Pep_1+
z.12
c12
y13
Pep+HexNAc_1+
z.14
c14
c15
c16
M+e-45
M+e-17

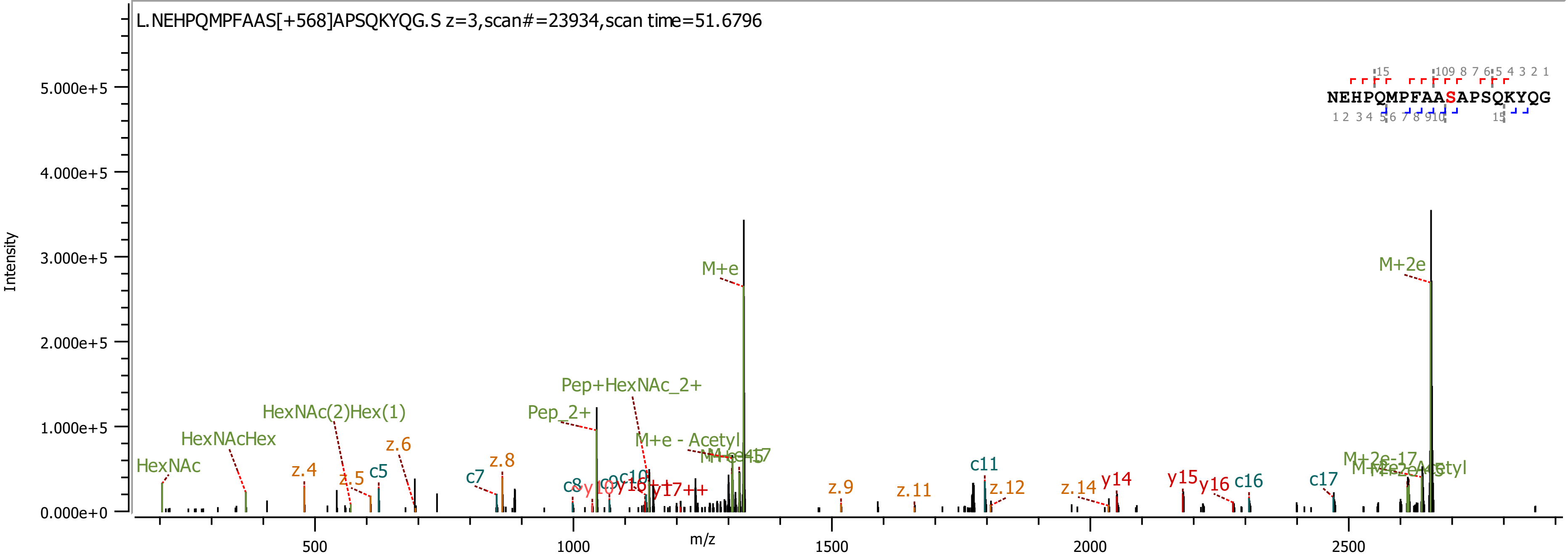

L.NEHPQMPFAAS[+568]APSQKYQG.S z=3,scan#=23934,scan time=51.6796
NEHPQMPFAASAPSQKYQG
Intensity
m/z
0.000e+0
1.000e+5
2.000e+5
3.000e+5
4.000e+5
5.000e+5
500
1000
1500
2000
2500
M+e
M+2e
HexNAc
HexNAcHex
HexNAc(2)Hex(1)
Pep_2+
Pep+HexNAc_2+
M+e - Acetyl
z.4
z.5
z.6
z.8
z.9
z.11
z.12
z.14
c5
c7
c8
c11
c16
c17
y14
y15
y16
y17++
M+2e-17

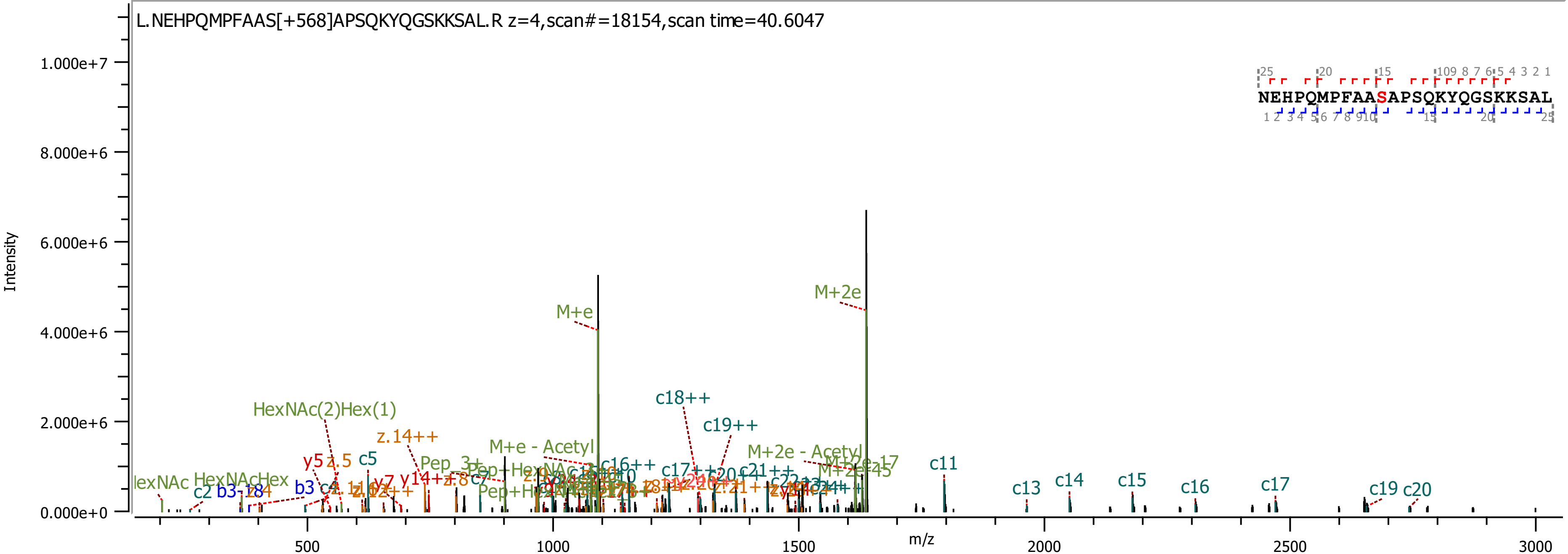
L.NEHPQMPFAAS[+568]APSQKYQGSKKSAL.R z=4,scan#=18154,scan time=40.6047
NEHPQMPFAASAPSQKYQGSKKSAL
Intensity
m/z
M+e
M+2e
c18++
c19++
HexNAc(2)Hex(1)
z.14++
M+e - Acetyl
M+2e - Acetyl
c11
c13
c14
c15
c16
c17
c19
c20
c5
y5
z.5
c2
b3
b3
y7
c7
c16++
c17++
Pep_3+
HexNAcHex

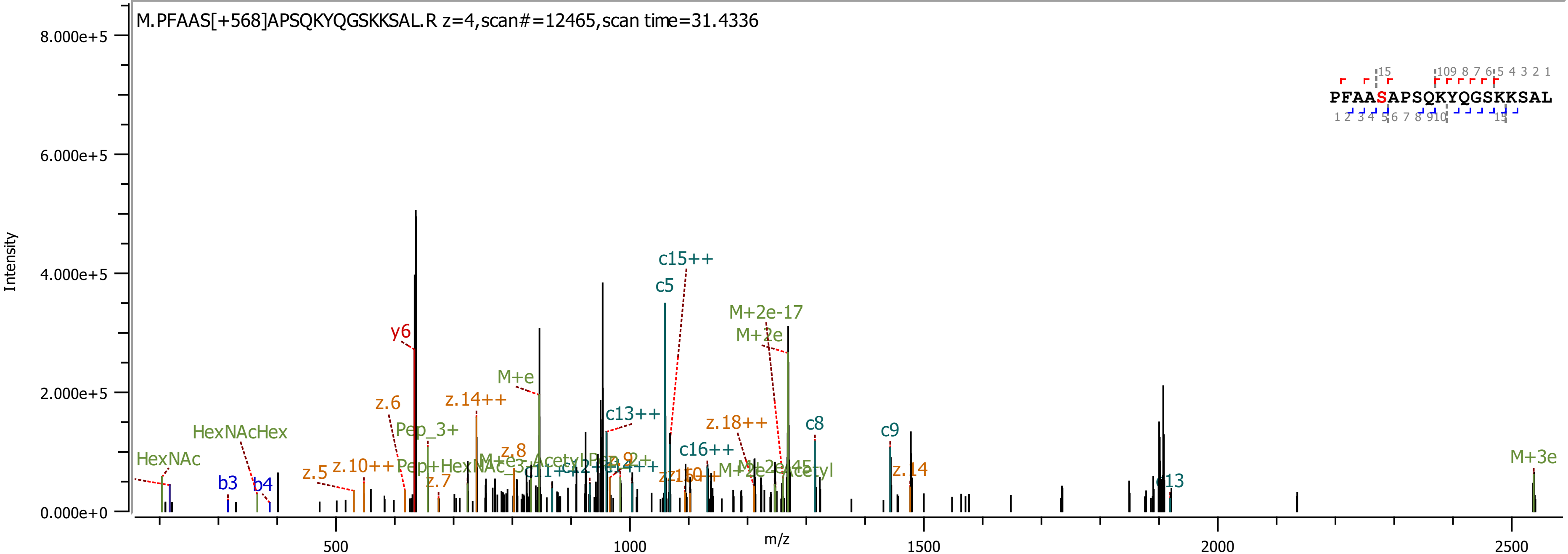
M.PFAAS[+568]APSQKYQGSKKSAL.R z=4,scan#=12465,scan time=31.4336
Intensity
m/z
PFAASAPSQKYQGSKKSAL
y6
c15++
c5
M+2e-17
M+2e
M+e
z.6
z.14++
c13++
z.18++
c8
c9
HexNAcHex
Pep_3+
c16++
HexNAc
z.5
z.10++
z.8
z.14
M+3e
b3
b4
z.7
c13

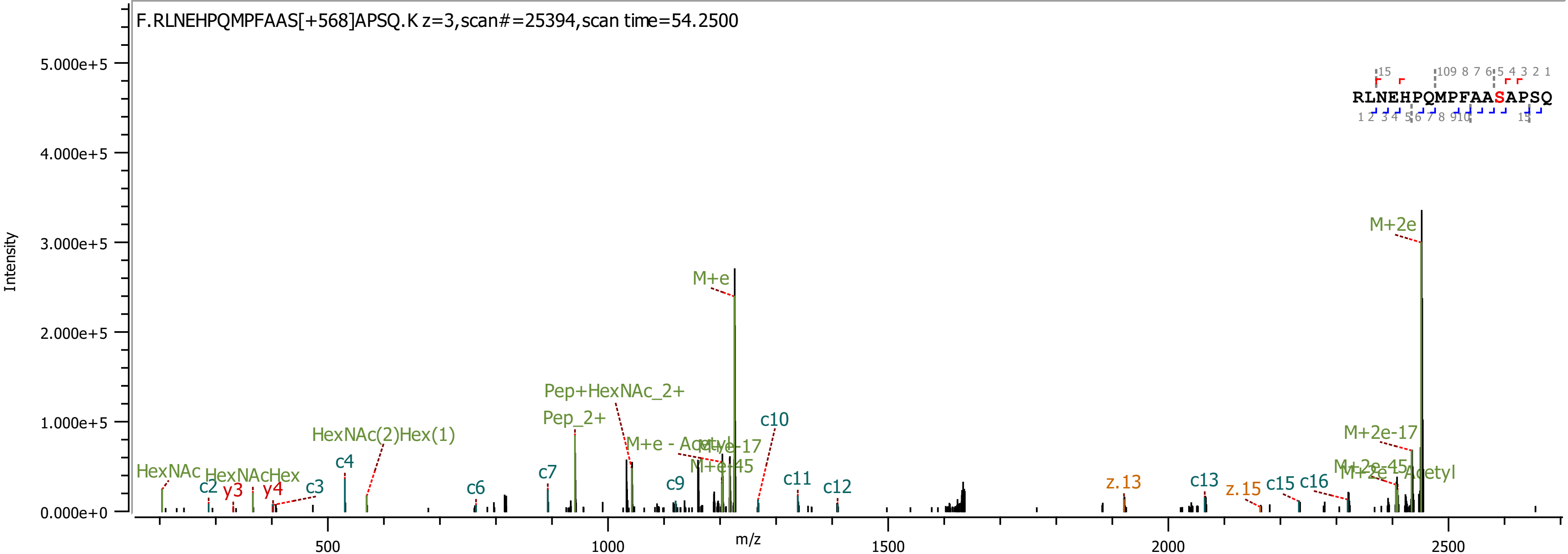

F.RLNEHPQMPFAAS[+568]APSQ.K z=3,scan#=25394,scan time=54.2500
Intensity
m/z
M+2e
M+e
Pep+HexNAc_2+
Pep_2+
HexNAc(2)Hex(1)
HexNAc
HexNAcHex
c2
y3
y4
c3
c4
c6
c7
c9
c10
c11
c12
c13
c15
c16
z.13
z.15
M+2e-17
M+e-17
M+e-45
M+e - Acetyl
RLNEHPQMPFAASAPSQ

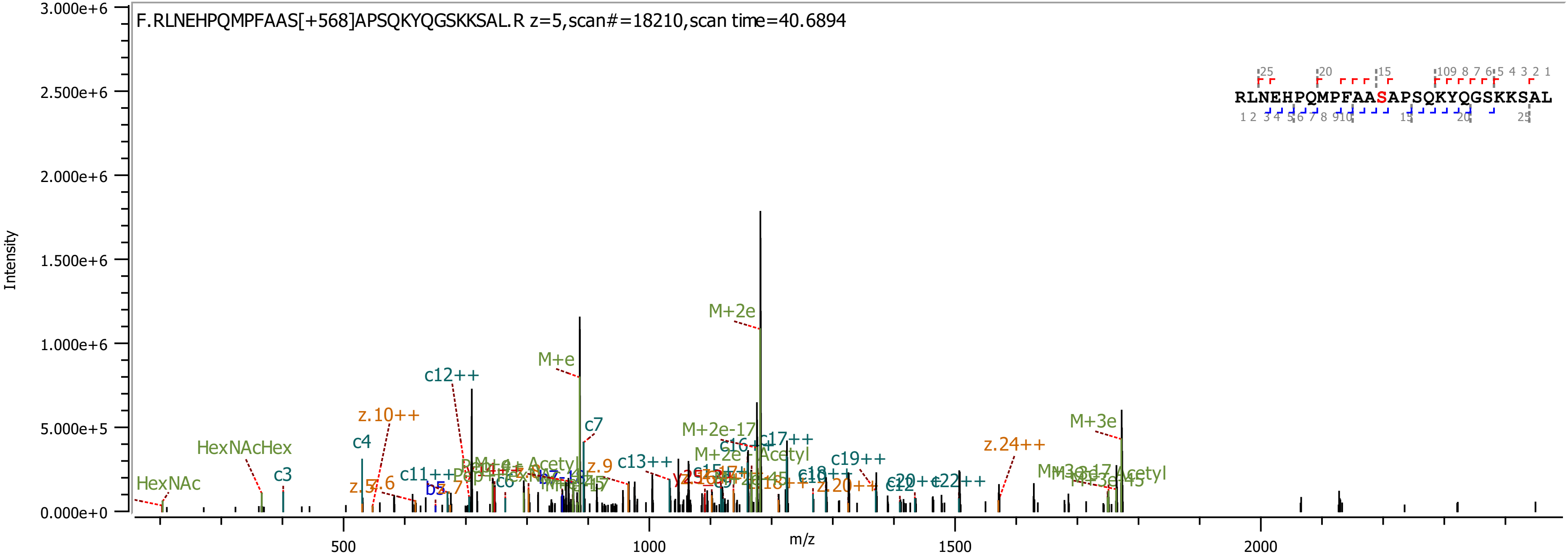

F.RLNEHPQMPFAAS[+568]APSQKYQGSKKSAL.R z=5,scan#=18210,scan time=40.6894
RLNEHPQMPFAASAPSQKYQGSKKSAL
Intensity
m/z
3.000e+6
2.500e+6
2.000e+6
1.500e+6
1.000e+6
5.000e+5
0.000e+0
500
1000
1500
2000
HexNAc
HexNAcHex
c3
c4
z.10++
c12++
M+e
c7
M+2e
M+2e-17
c17++
M+2e-Acetyl
c13++
c19++
z.24++
M+3e
z.9
c11++
c6
c22++
z.20++

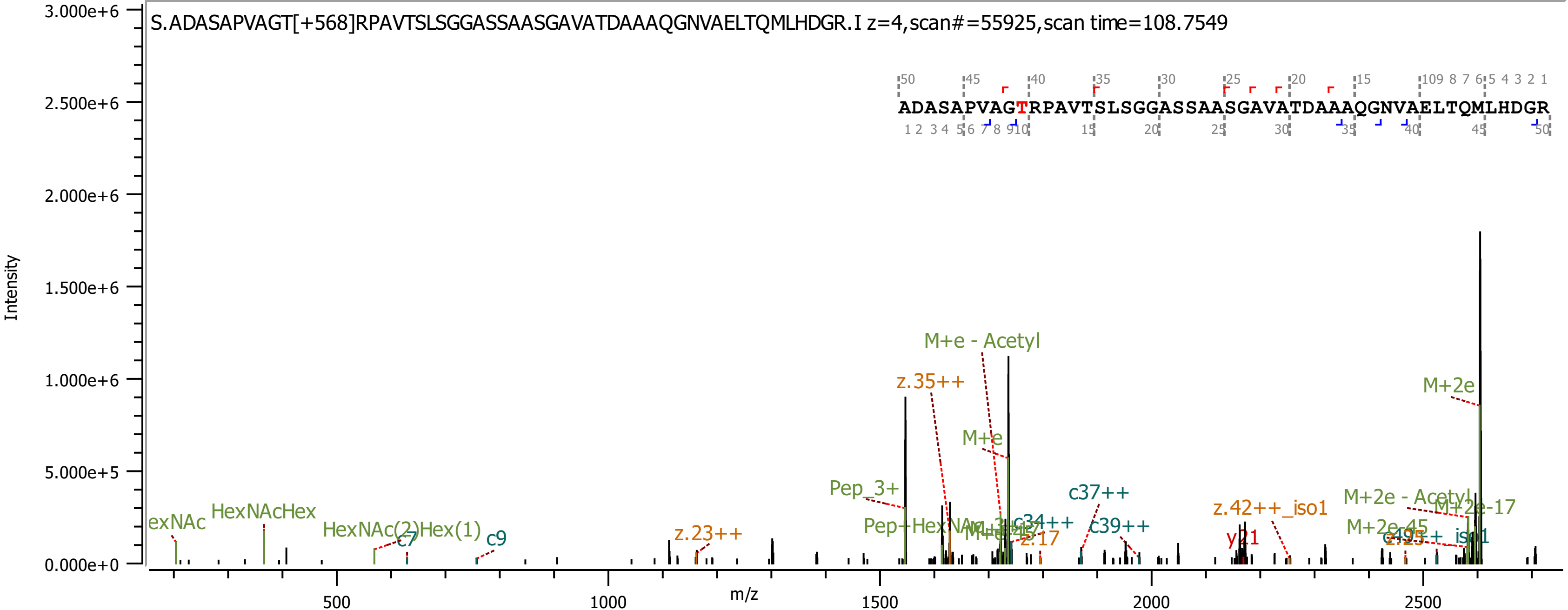
S.ADASAPVAGT[+568]RPAVTSLSGGASSAASGAVATDAAAQGNVAELTQMLHDGR.I z=4,scan#=55925,scan time=108.7549
ADASAPVAGTRPAVTSLSGGASSAASGAVATDAAAQGNVAELTQMLHDGR
Intensity
m/z
3.000e+6
2.500e+6
2.000e+6
1.500e+6
1.000e+6
5.000e+5
0.000e+0
500
1000
1500
2000
2500
M+e - Acetyl
z.35++
M+2e
M+e
c37++
Pep_3+
M+2e - Acetyl
z.42++_iso1
M+2e-17
HexNAcHex
exNAc
HexNAc(2)Hex(1)
c9
c7
z.23++
c39++
c34++
z.17
y21
M+2e-45
iso1

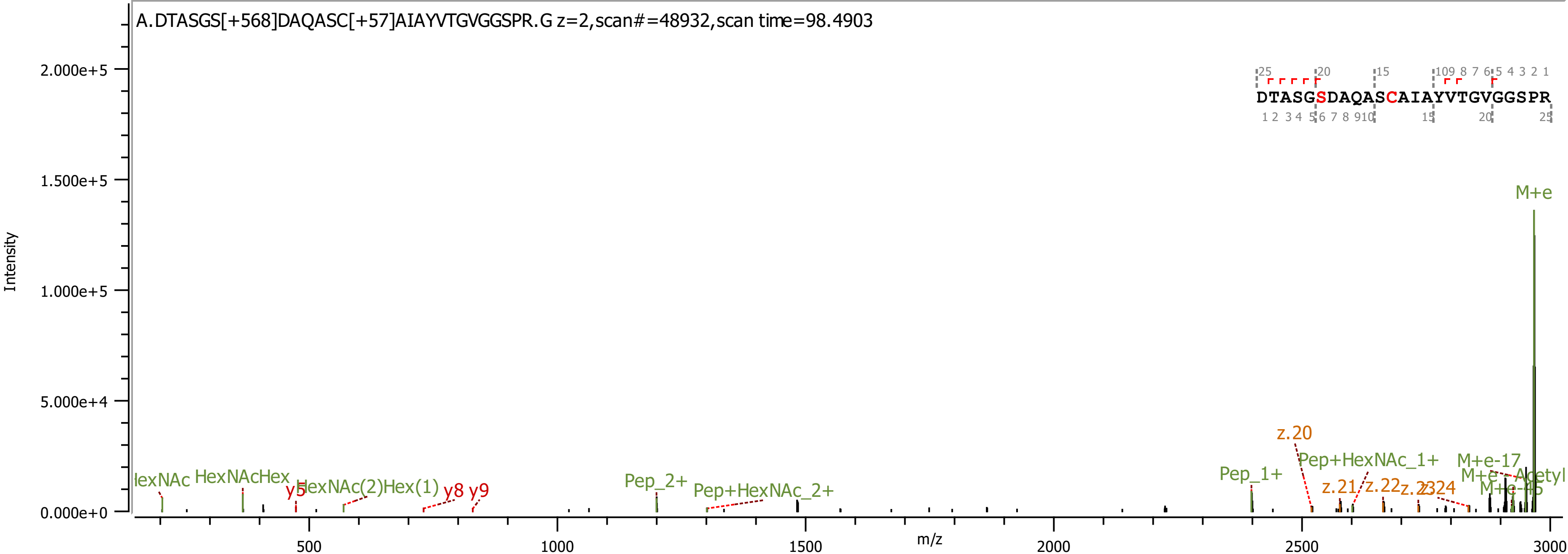

A.DTASGS[+568]DAQASC[+57]AIAYVTGVGGSPR.G z=2,scan#=48932,scan time=98.4903
DTASGSDAQASCAIAYVTGVGGSPR
Intensity
m/z
2.000e+5
1.500e+5
1.000e+5
5.000e+4
0.000e+0
500
1000
1500
2000
2500
3000
M+e
z.20
Pep+HexNAc_1+
M+e-17
Pep_1+
HexNAc
HexNAcHex
y5
HexNAc(2)Hex(1)
y8
y9
Pep_2+
Pep+HexNAc_2+
z.21
z.22

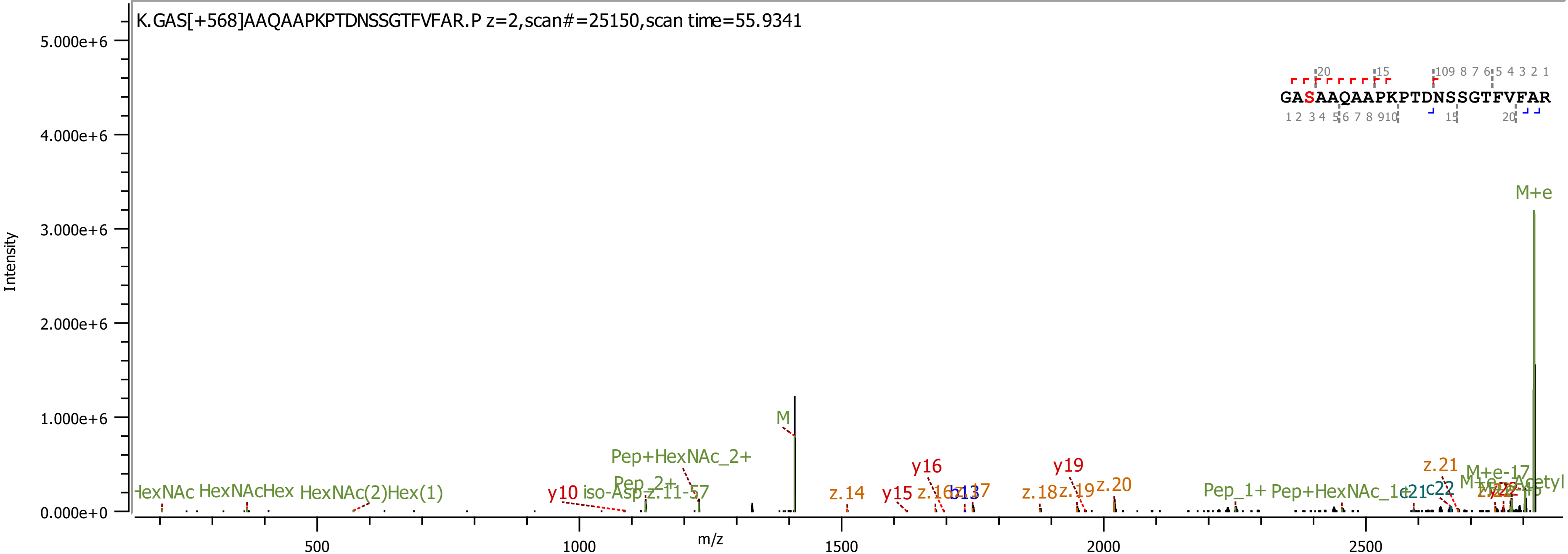
K.GAS[+568]AAQAAPKPTDNSSGTFVFAR.P z=2,scan#=25150,scan time=55.9341
GASAAQAAPKPTDNSSGTFVFAR
Intensity
m/z
M+e
M
Pep+HexNAc_2+
Pep_2+
HexNAcHex
HexNAc(2)Hex(1)
y10
y15
y16
y19
z.14
z.18
z.19
z.20
z.21
Pep_1+
c21
c22
M+e-17
5.000e+6
4.000e+6
3.000e+6
2.000e+6
1.000e+6
0.000e+0
500
1000
1500
2000
2500

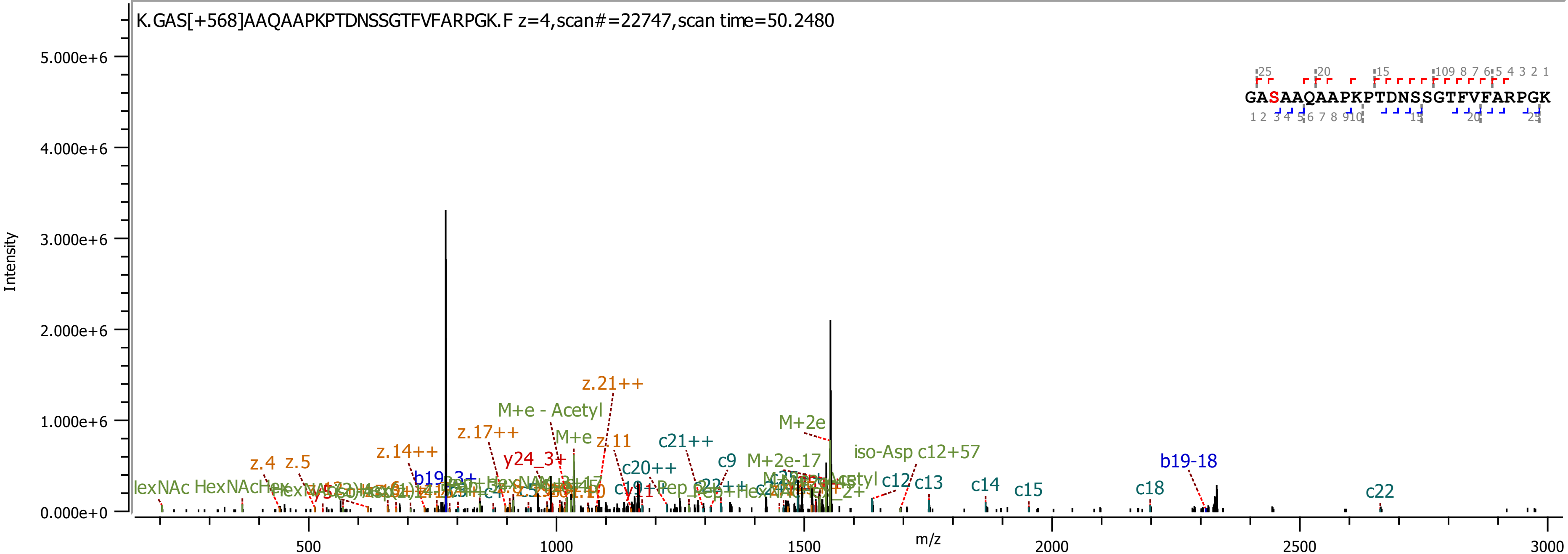

K.GAS[+568]AAQAAPKPTDNSSGTFVFARPGK.F z=4,scan#=22747,scan time=50.2480
GASAAQAAPKPTDNSSGTFVFARPGK
Intensity
m/z
5.000e+6
4.000e+6
3.000e+6
2.000e+6
1.000e+6
0.000e+0
500
1000
1500
2000
2500
3000
z.21++
M+e - Acetyl
z.17++
M+e
z.11
c21++
M+2e
z.14++
y24_3+
c9
M+2e-17
iso-Asp c12+57
b19-18
z.4
z.5
c20++
c12
c13
c14
c15
c18
c22

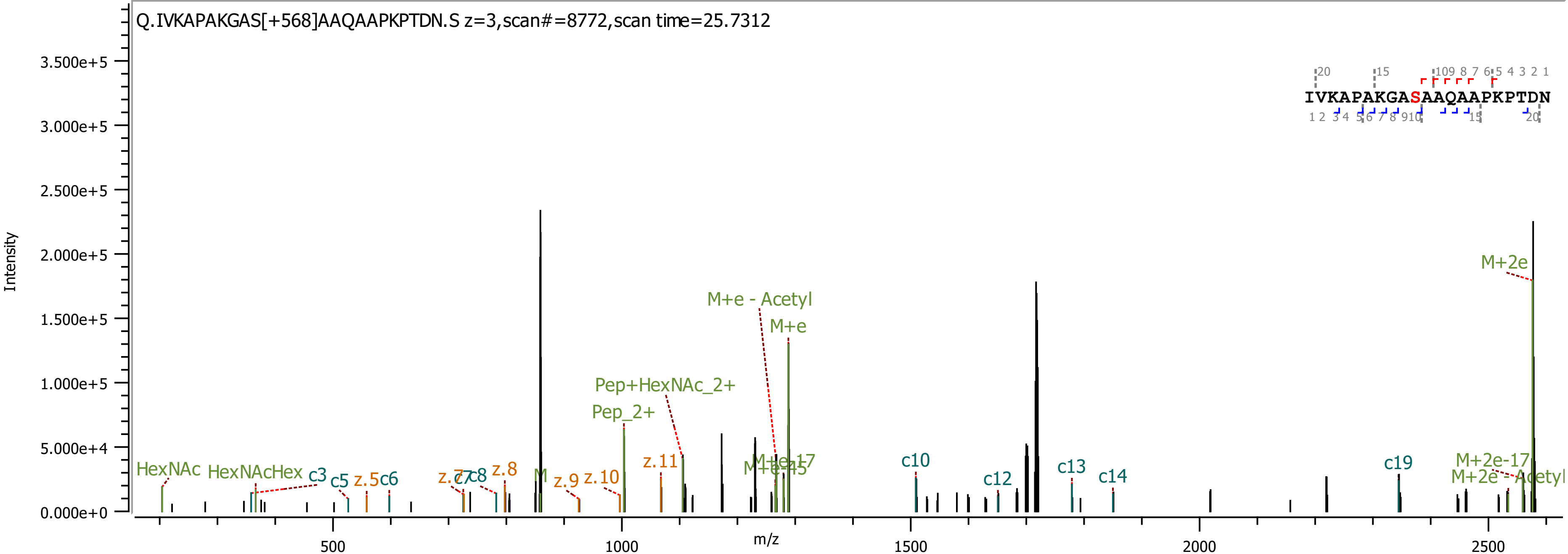

Q.IVKAPAKGAS[+568]AAQAAPKPTDN.S z=3,scan#=8772,scan time=25.7312
IVKAPAKGASAAQAAPKPTDN
Intensity
m/z
3.500e+5
3.000e+5
2.500e+5
2.000e+5
1.500e+5
1.000e+5
5.000e+4
0.000e+0
500
1000
1500
2000
2500
HexNAc
HexNAcHex
c3
c5
z.5
c6
z.7
c7
c8
z.8
M
z.9
z.10
Pep_2+
Pep+HexNAc_2+
z.11
M+e - Acetyl
M+e
M+e-17
M+e-45
c10
c12
c13
c14
c19
M+2e
M+2e-17
M+2e - Acetyl

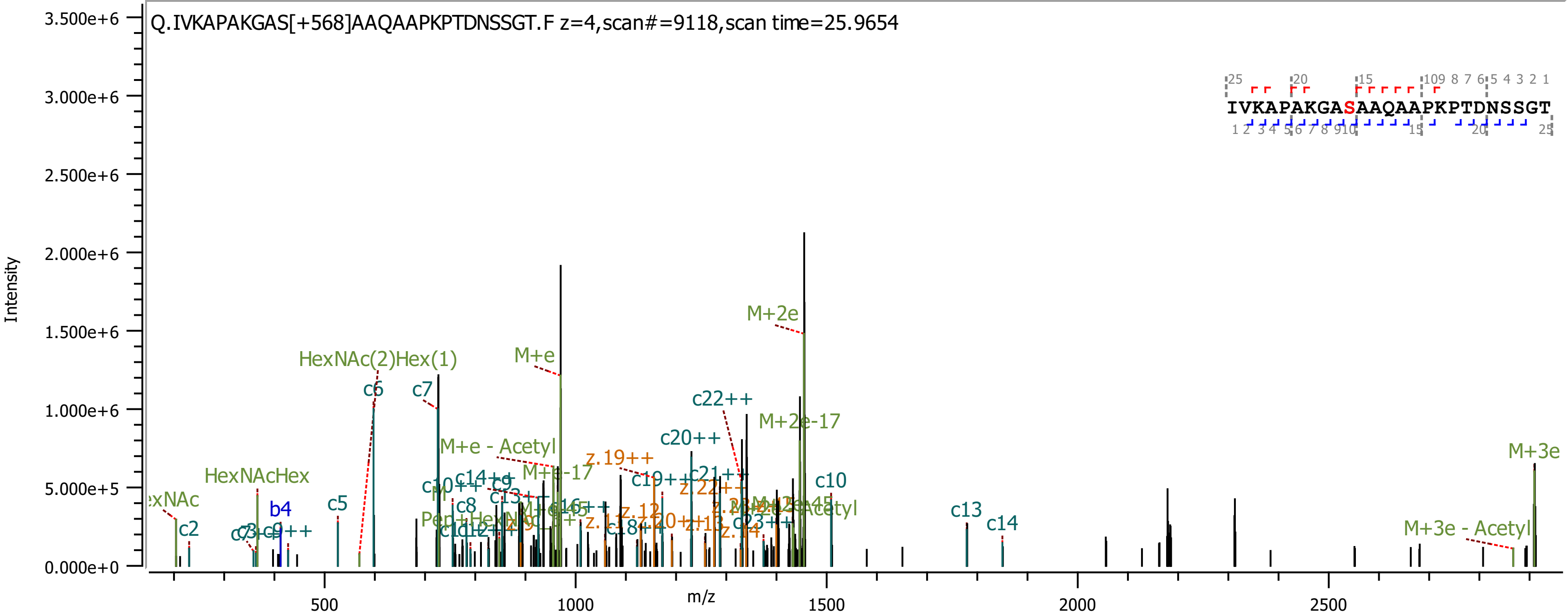

Q.IVKAPAKGAS[+568]AAQAAPKPTDNSSGT.F z=4,scan#=9118,scan time=25.9654
IVKAPAKGASAAQAAPKPTDNSSGT
Intensity
m/z
3.500e+6
3.000e+6
2.500e+6
2.000e+6
1.500e+6
1.000e+6
5.000e+5
0.000e+0
500
1000
1500
2000
2500
HexNAc(2)Hex(1)
HexNAcHex
b4
c2
c5
c6
c7
c8
c9
c10
c13
c14
c20++
c22++
M+e
M+2e
M+2e-17
M+3e
M+3e - Acetyl
M+e - Acetyl
M+e-17
z.19++

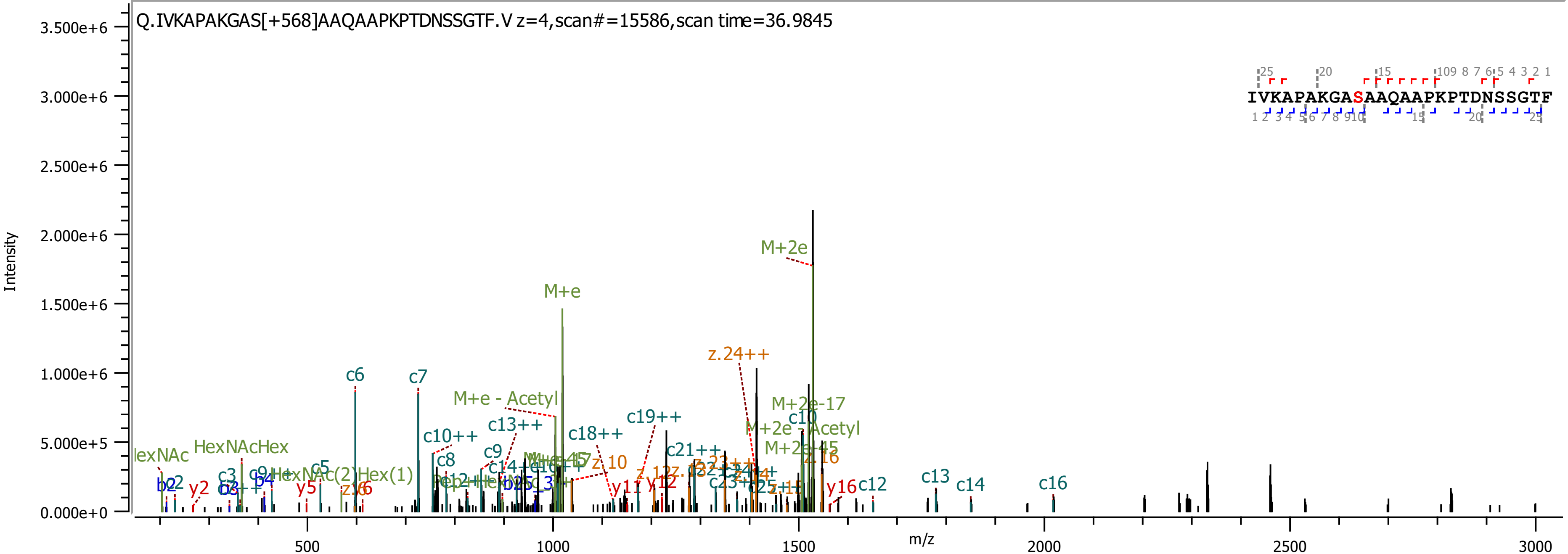
Q.IVKAPAKGAS[+568]AAQAAPKPTDNSSGTF.V z=4,scan#=15586,scan time=36.9845
IVKAPAKGASAAQAAPKPTDNSSGTF
Intensity
m/z
M+2e
M+e
z.24++
c6
c7
M+e - Acetyl
c19++
c18++
c13++
c10++
M+2e-17
M+2e - Acetyl
M+2e-45
HexNAcHex
c8
c9
c5
y5
y2
y11
y12
y16
c12
c13
c14
c16
z.10
z.16
HexNAc(2)Hex(1)
c21++
c23++
c25
0.000e+0
5.000e+5
1.000e+6
1.500e+6
2.000e+6
2.500e+6
3.000e+6
3.500e+6
500
1000
1500
2000
2500
3000

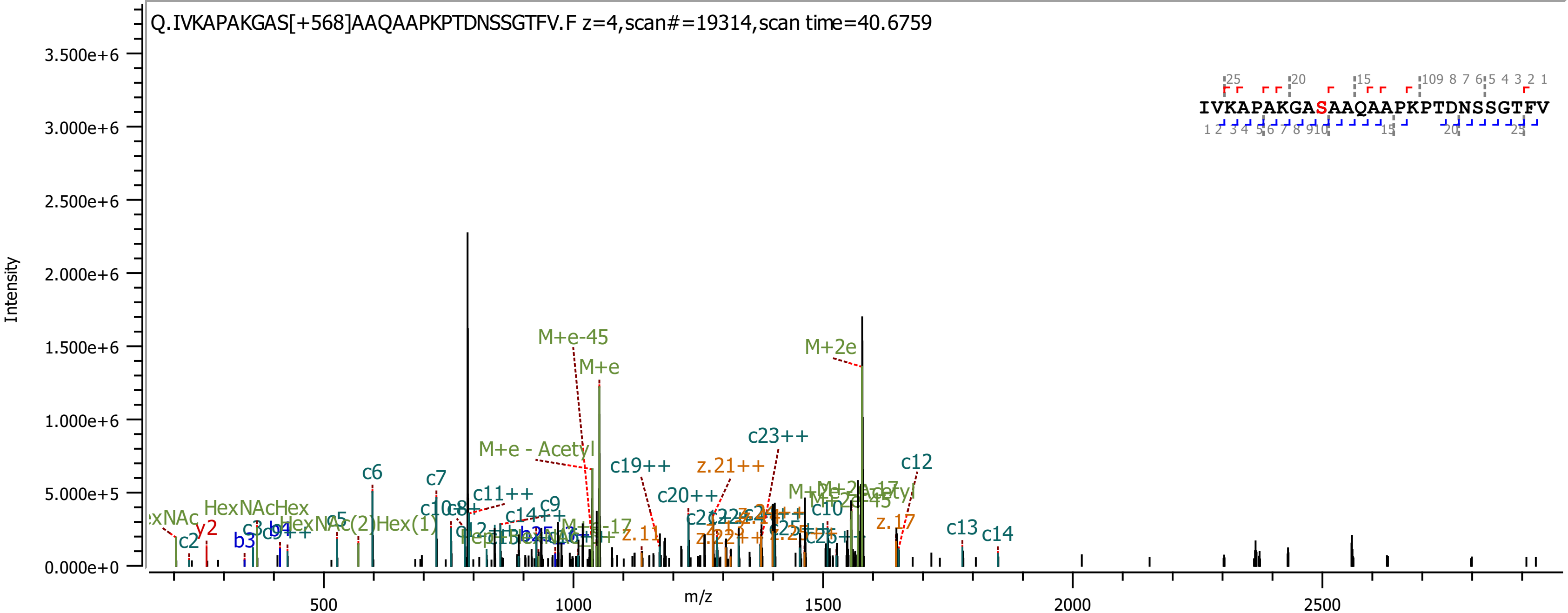

Q.IVKAPAKGAS[+568]AAQAAPKPTDNSSGTFV.F z=4,scan#=19314,scan time=40.6759
IVKAPAKGASAAQAAPKPTDNSSGTFV
Intensity
m/z
M+e-45
M+e
M+2e
M+e - Acetyl
c23++
c19++
z.21++
c12
c6
c7
c11++
c20++
c9
c13
c14
z.17
HexNAcHex
HexNAc(2)Hex(1)
y2
c2
b3
c5
z.11

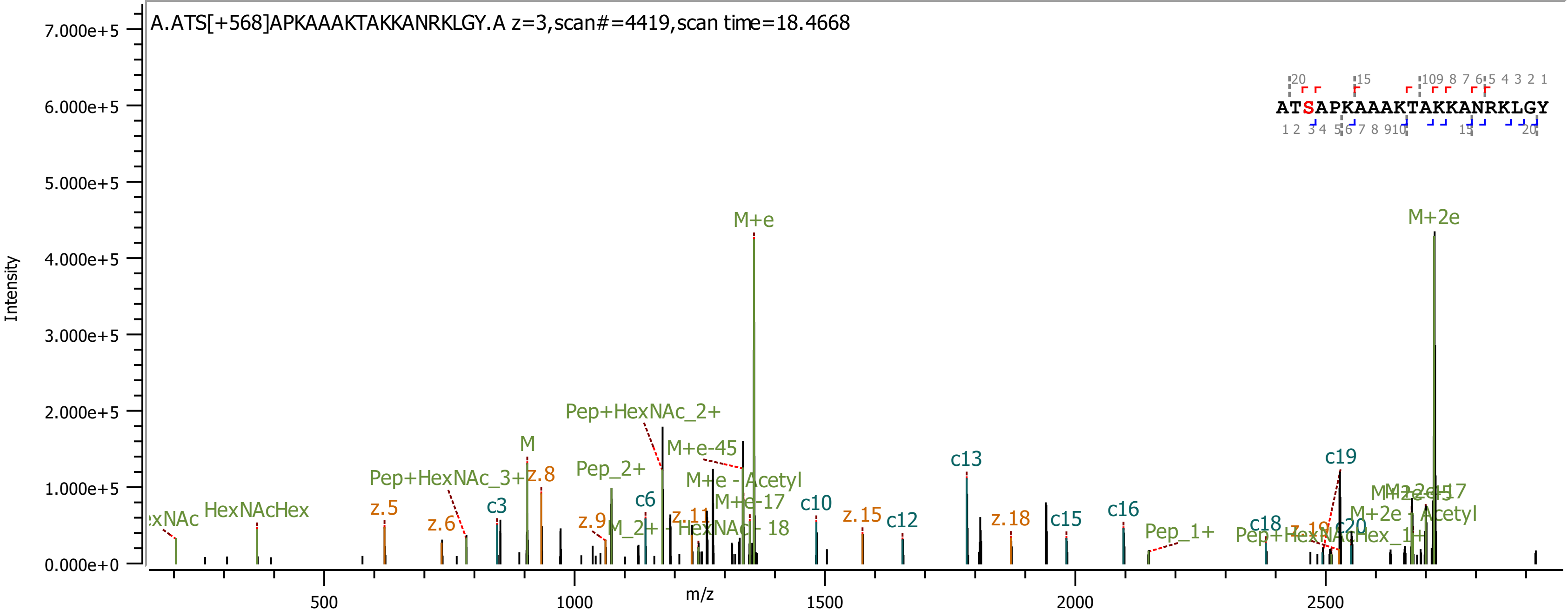

A.ATS[+568]APKAAAKTAKKANRKLGY.A z=3,scan#=4419,scan time=18.4668
ATSAPKAAAKTAKKANRKLGY
Intensity
m/z
M+e
M+2e
Pep+HexNAc_2+
M+e-45
M
Pep+HexNAc_3+
z.8
Pep_2+
M+e - Acetyl
M+e-17
c13
c19
c3
c6
c10
c12
c15
c16
c18
c20
z.5
z.6
z.9
z.11
z.15
z.18
z.19
HexNAcHex
Pep_1+
M+2e - Acetyl

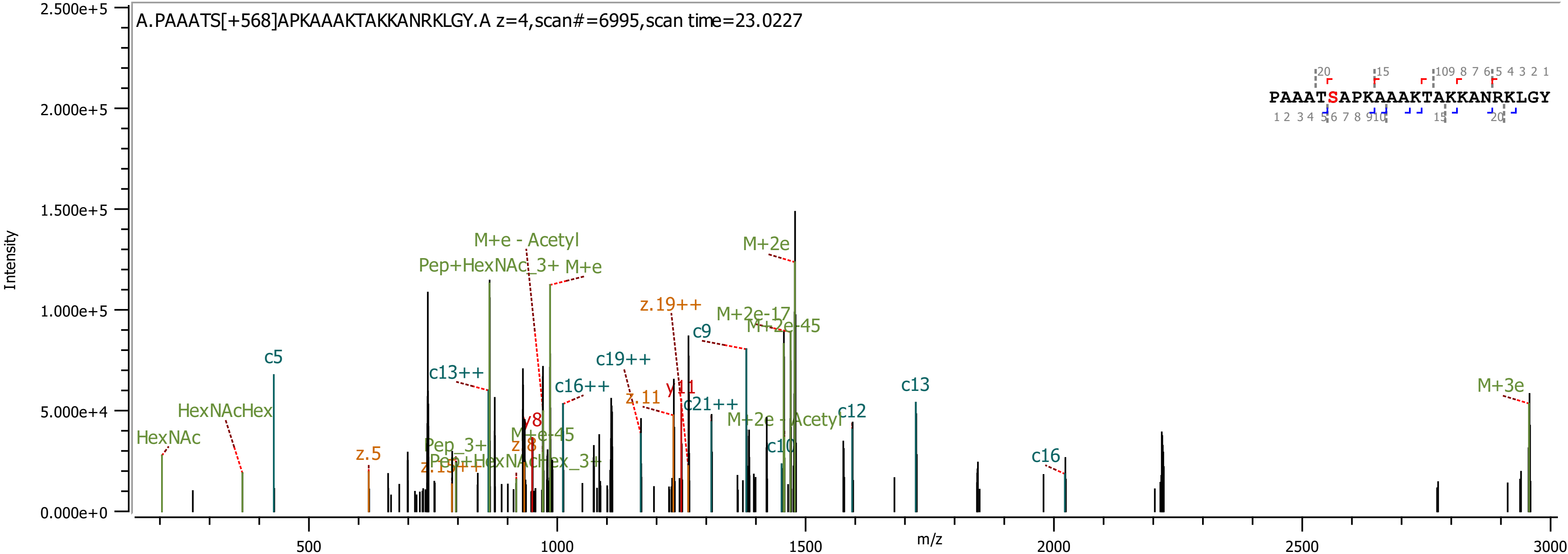
A.PAAATS[+568]APKAAAKTAKKANRKLGY.A z=4,scan#=6995,scan time=23.0227
PAAATSAPKAAAKTAKKANRKLGY
Intensity
m/z
HexNAc
HexNAcHex
c5
z.5
z.13++
Pep_3+
Pep+HexNAcHex_3+
c13++
Pep+HexNAc_3+
M+e - Acetyl
M+e
y8
z.8
M+e-45
c16++
c19++
z.11
y11
z.19++
c21++
c9
M+2e-17
M+2e-45
M+2e
M+2e - Acetyl
c10
c12
c13
c16
M+3e

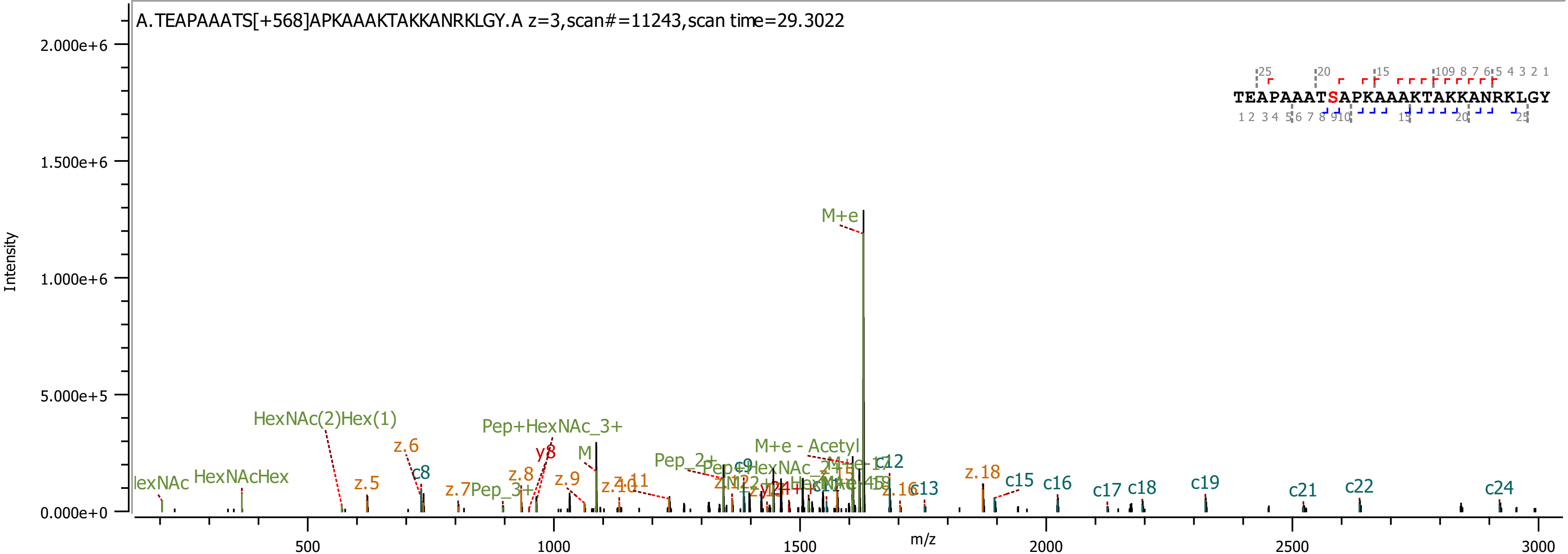
A.TEAPAAATS[+568]APKAAAKTAKKANRKLGY.A z=3,scan#=11243,scan time=29.3022
TEAPAAATSAPKAAAKTAKKANRKLGY
Intensity
m/z
2.000e+6
1.500e+6
1.000e+6
5.000e+5
0.000e+0
500
1000
1500
2000
2500
3000
M+e
HexNAc(2)Hex(1)
Pep+HexNAc_3+
M+e - Acetyl
HexNAcHex
z.5
z.6
c8
y8
M
z.7
z.8
z.9
z.10
z.11
Pep_2+
c9
c12
c13
z.16
z.18
c15
c16
c17
c18
c19
c21
c22
c24

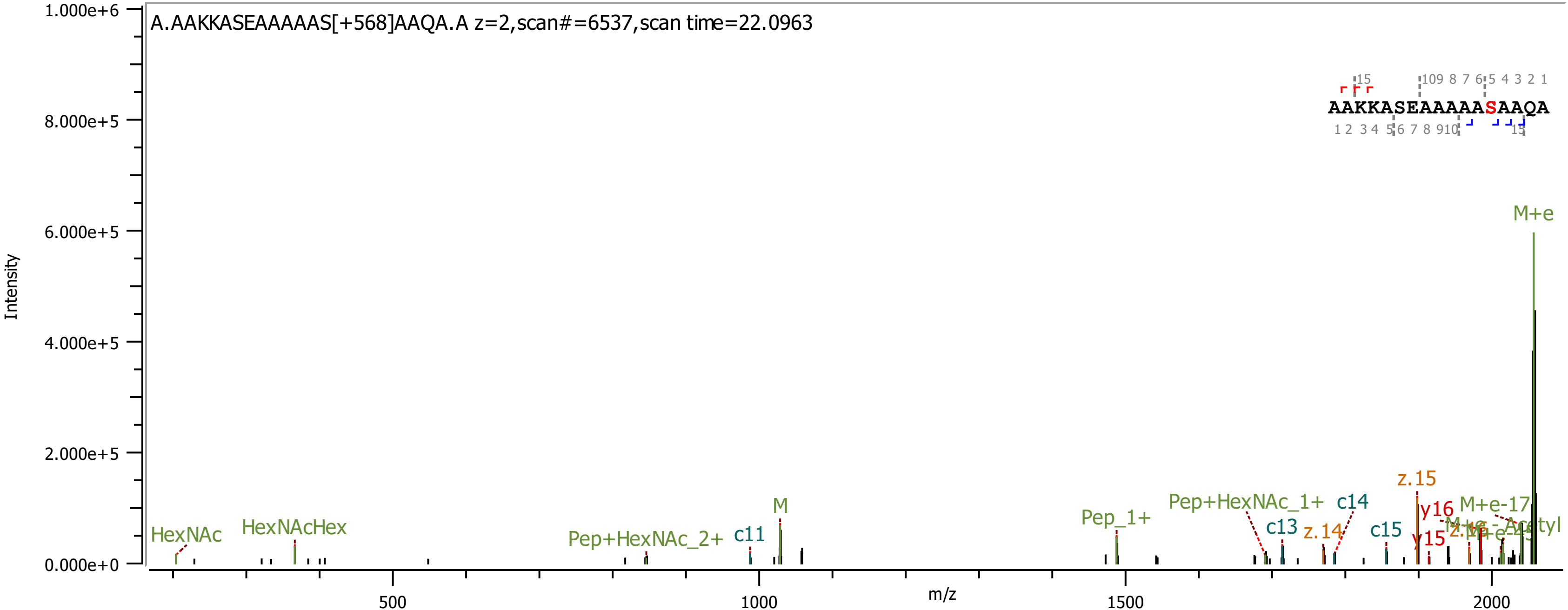
A.AAKKASEAAAAAS[+568]AAQA.A z=2,scan#=6537,scan time=22.0963
AAKKASEAAAAASAAQA
Intensity
m/z
1.000e+6
8.000e+5
6.000e+5
4.000e+5
2.000e+5
0.000e+0
500
1000
1500
2000
M+e
z.15
y16
M+e-17
c14
Pep+HexNAc_1+
c13
z.14
c15
y15
Pep_1+
M
c11
Pep+HexNAc_2+
HexNAcHex
HexNAc

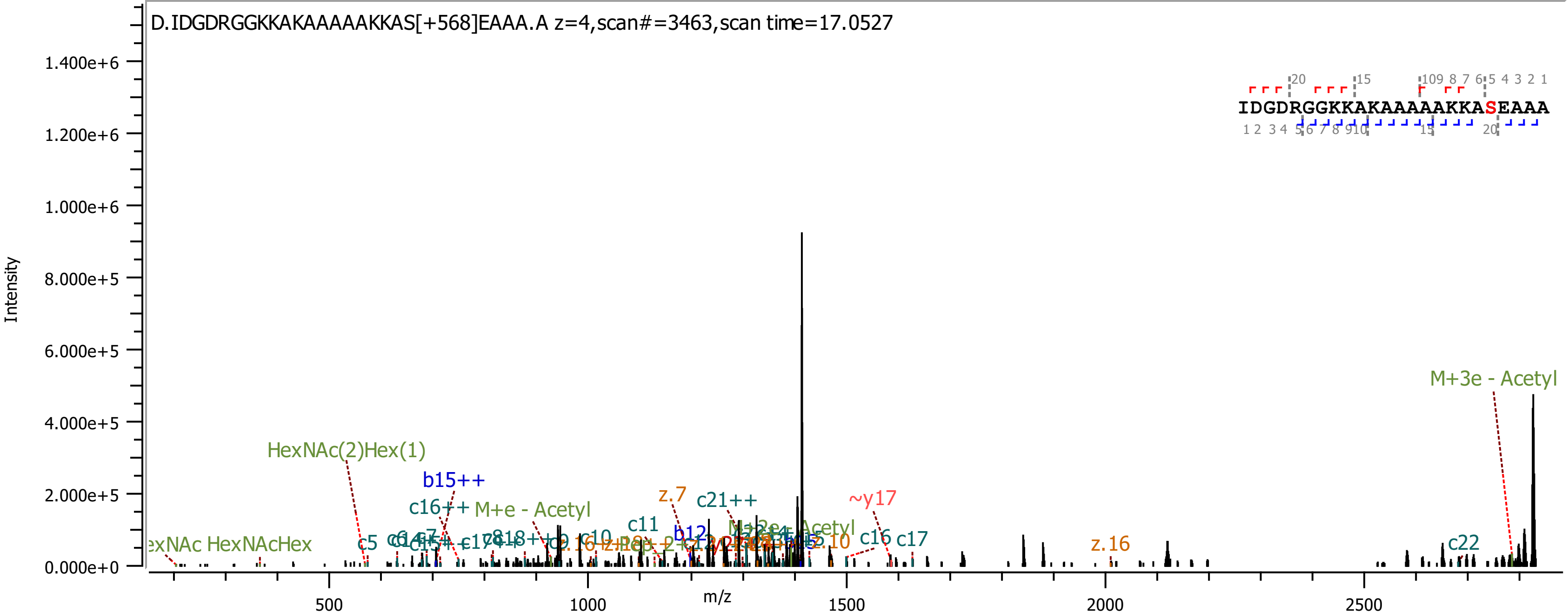
D.IDGDRGGKKAKAAAAAKKAS[+568]EAAA.A z=4,scan#=3463,scan time=17.0527
IDGDRGGKKAKAAAAAKKASEAAA
Intensity
1.400e+6
1.200e+6
1.000e+6
8.000e+5
6.000e+5
4.000e+5
2.000e+5
0.000e+0
m/z
500
1000
1500
2000
2500
M+3e - Acetyl
HexNAc(2)Hex(1)
HexNAcHex
b15++
c16++
M+e - Acetyl
z.7
c21++
~y17
c11
b12
c5
c16
c17
z.16
c22
c10

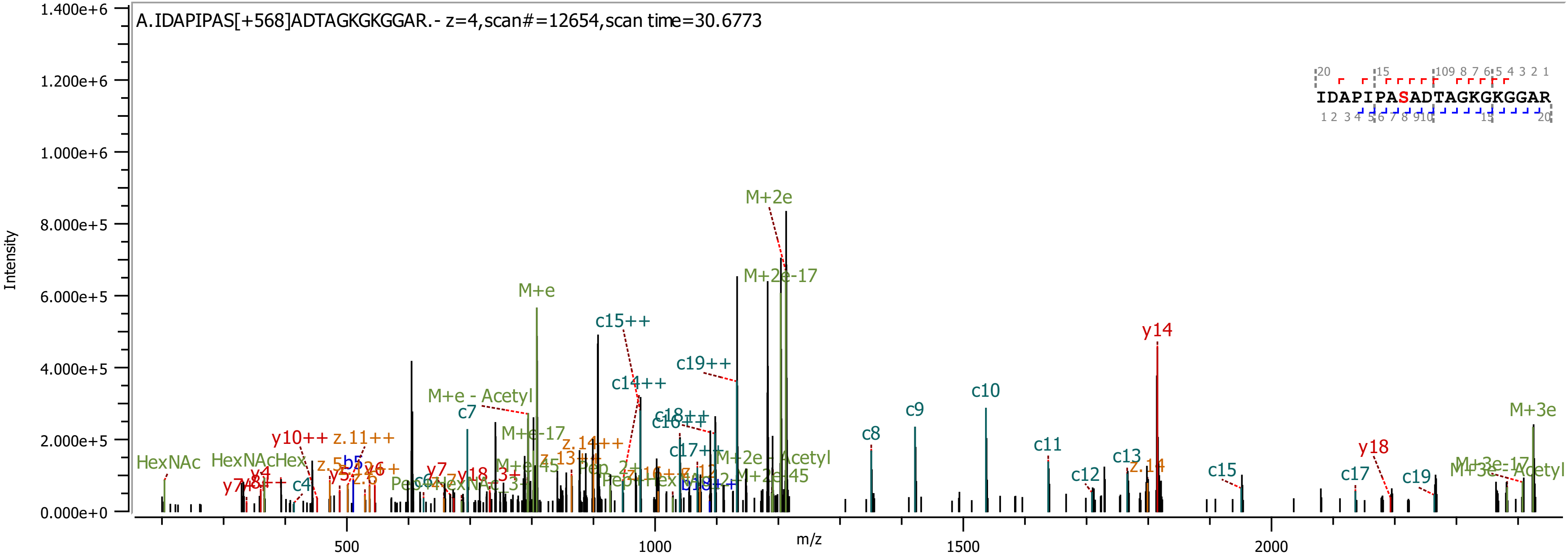

A.IDAPIPAS[+568]ADTAGKGKGGAR.- z=4,scan#=12654,scan time=30.6773
IDAPIPASADTAGKGKGGAR
Intensity
m/z
1.400e+6
1.200e+6
1.000e+6
8.000e+5
6.000e+5
4.000e+5
2.000e+5
0.000e+0
500
1000
1500
2000
M+2e
M+2e-17
M+e
c15++
c19++
c14++
M+e - Acetyl
c7
c18++
c16++
M+e-17
z.14++
c17++
M+2e - Acetyl
M+2e-45
y10++
z.11++
HexNAc
HexNAcHex
y4
c4
b5
y7
y18
c8
c9
c10
c11
c12
c13
y14
c15
c17
y18
c19
M+3e
M+3e-17
M+3e - Acetyl

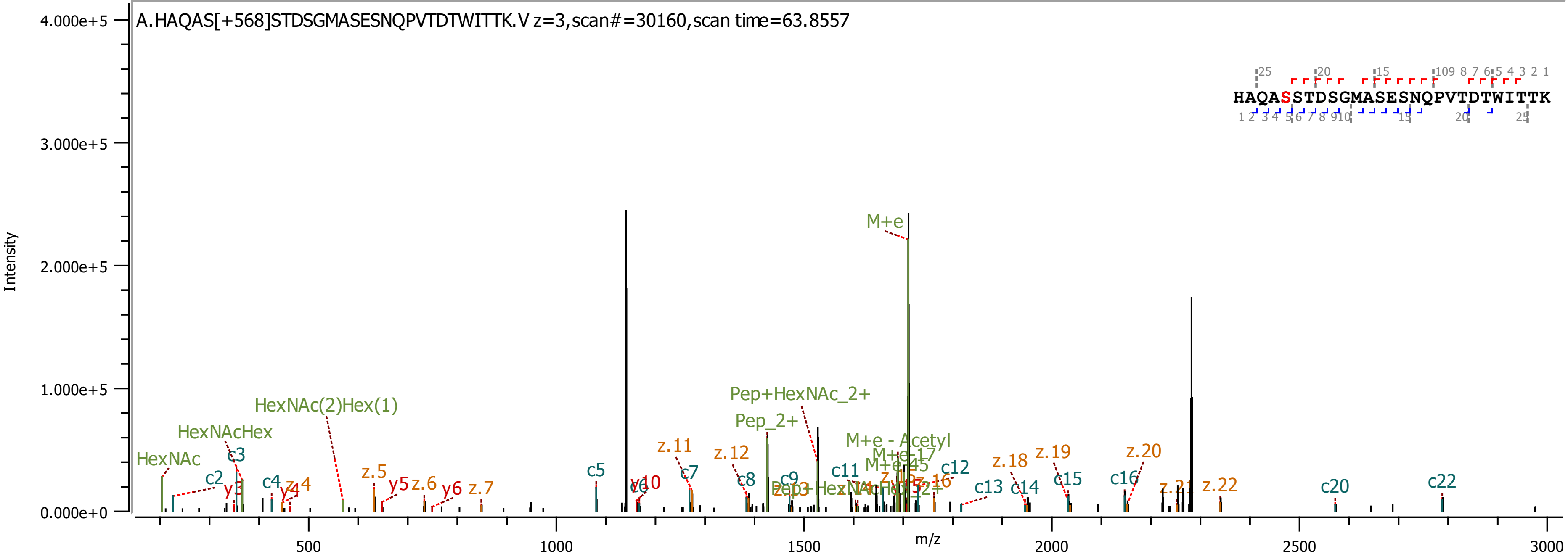
A.HAQAS[+568]STDSGMASESNQPVTDTWITTK.V z=3,scan#=30160,scan time=63.8557
HAQASSTDSGMASESNQPVTDTWITTK
Intensity
m/z
4.000e+5
3.000e+5
2.000e+5
1.000e+5
0.000e+0
500
1000
1500
2000
2500
3000
M+e
HexNAc(2)Hex(1)
Pep+HexNAc_2+
Pep_2+
HexNAcHex
HexNAc
M+e - Acetyl
M+e-17
M+e-45
c2
c3
c4
c5
c7
c8
c9
c11
c12
c13
c14
c15
c16
c20
c22
y3
y4
y5
y6
y10
z.4
z.5
z.6
z.7
z.11
z.12
z.16
z.18
z.19
z.20
z.21
z.22

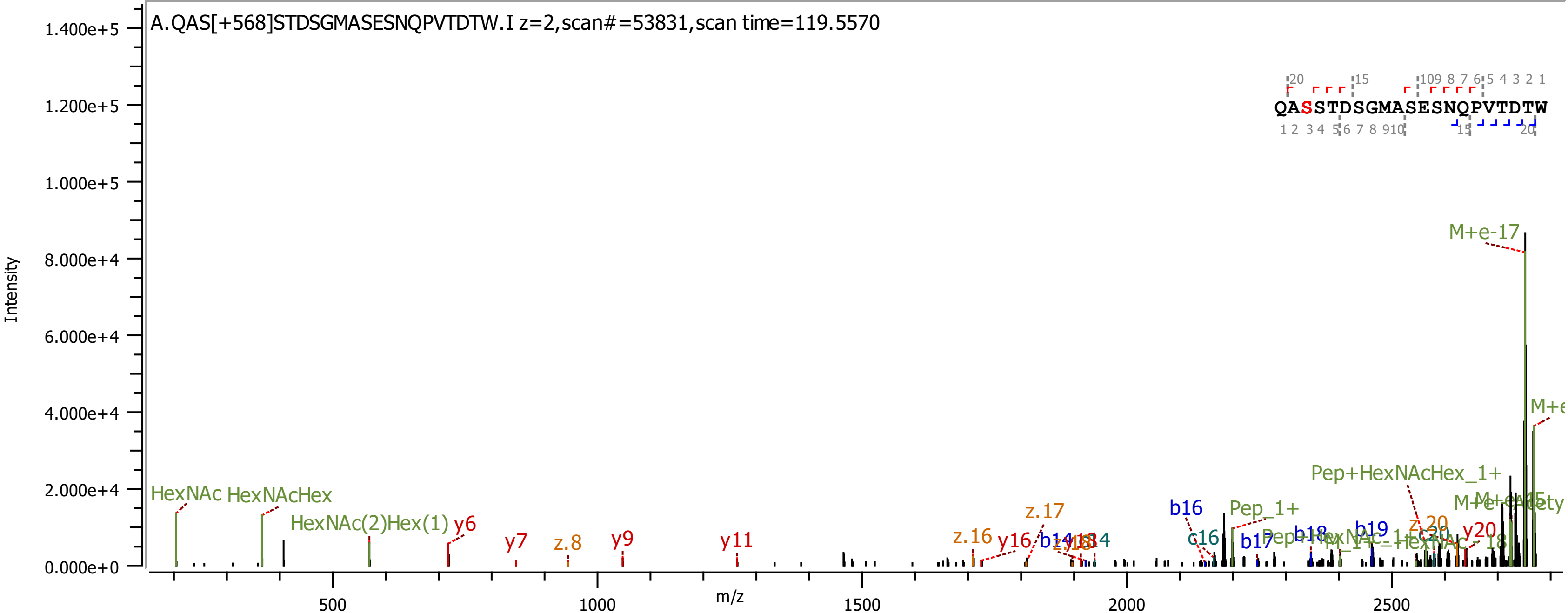
A.QAS[+568]STDSGMASESNQPVTDTW.I z=2,scan#=53831,scan time=119.5570
QASSTDSGMASESNQPVTDTW
Intensity
m/z
HexNAc
HexNAcHex
HexNAc(2)Hex(1)
y6
y7
z.8
y9
y11
z.16
y16
z.17
b16
c16
Pep_1+
b17
b18
b19
Pep+HexNAcHex_1+
z.20
y20
M+e-17

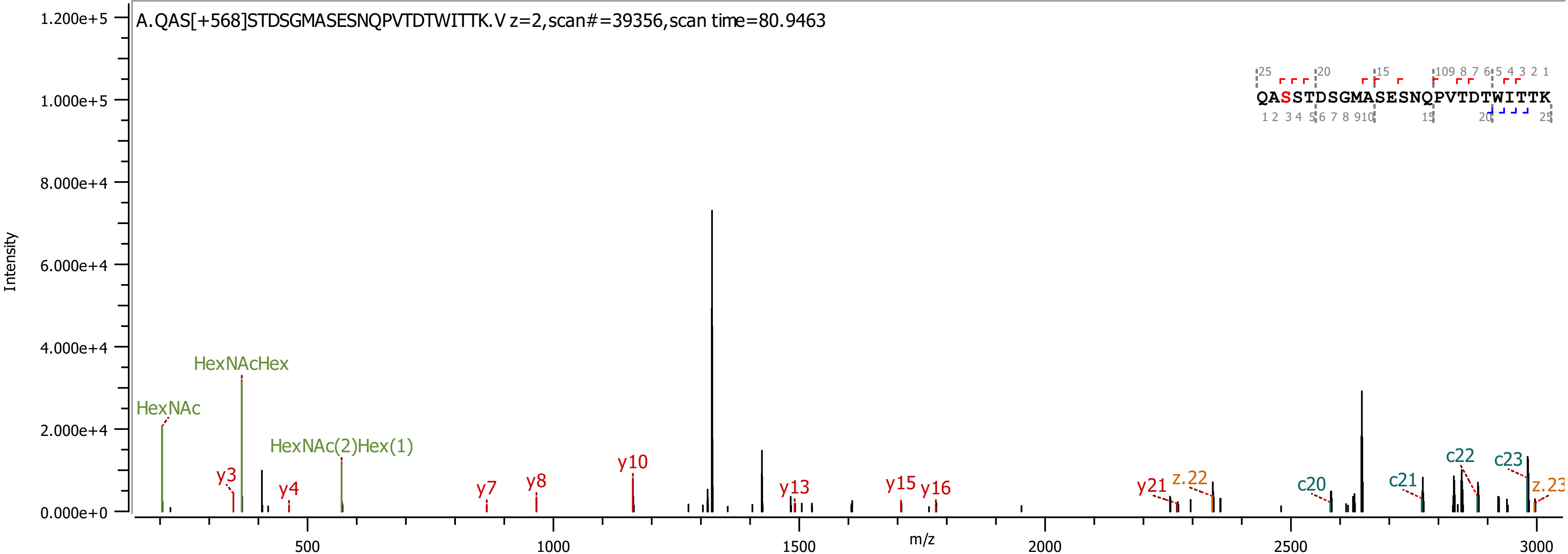
A.QAS[+568]STDSGMASESNQPVTDTWITTK.V z=2,scan#=39356,scan time=80.9463
QASSTDSGMASESNQPVTDTWITTK
Intensity
m/z
HexNAc
HexNAcHex
HexNAc(2)Hex(1)
y3
y4
y7
y8
y10
y13
y15
y16
y21
z.22
c20
c21
c22
c23
z.23

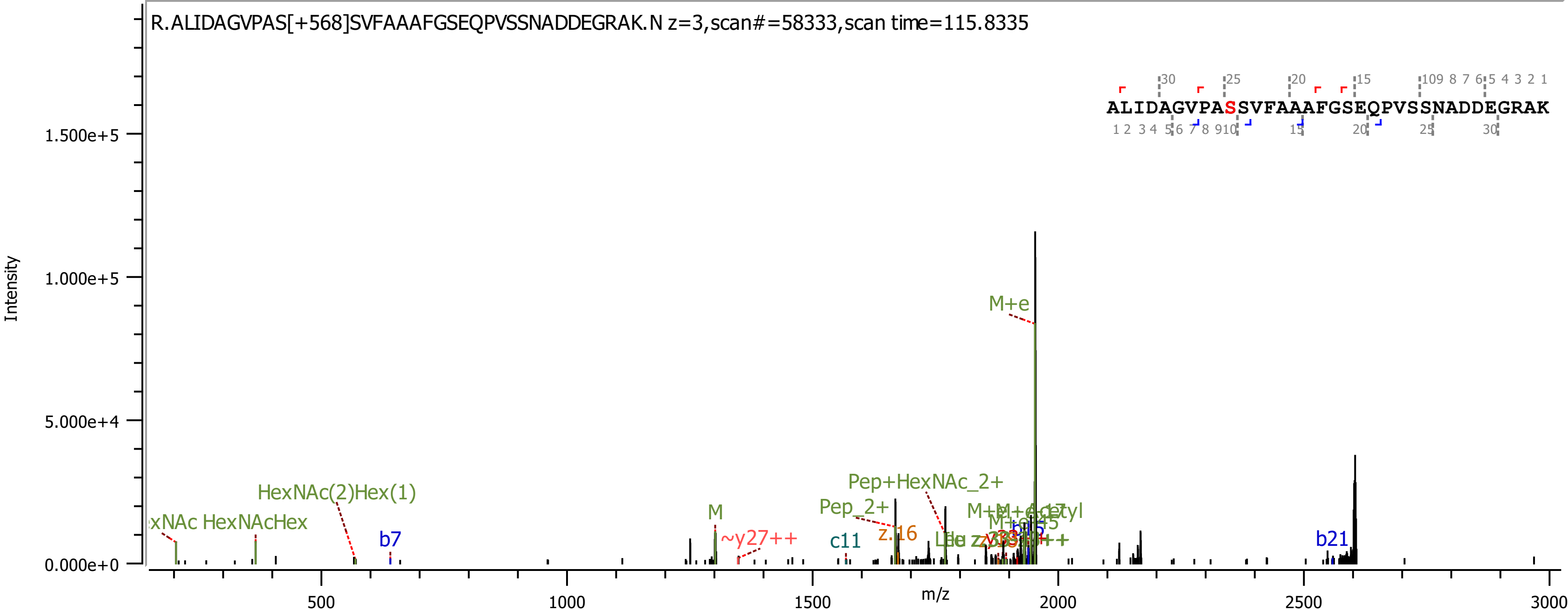
R.ALIDAGVPAS[+568]SVFAAAFGSEQPVSSNADDEGRAK.N z=3,scan#=58333,scan time=115.8335
ALIDAGVPASSVFAAAFGSEQPVSSNADDEGRAK
Intensity
m/z
M+e
HexNAc(2)Hex(1)
HexNAcHex
b7
M
~y27++
c11
Pep_2+
Pep+HexNAc_2+
z.16
b21

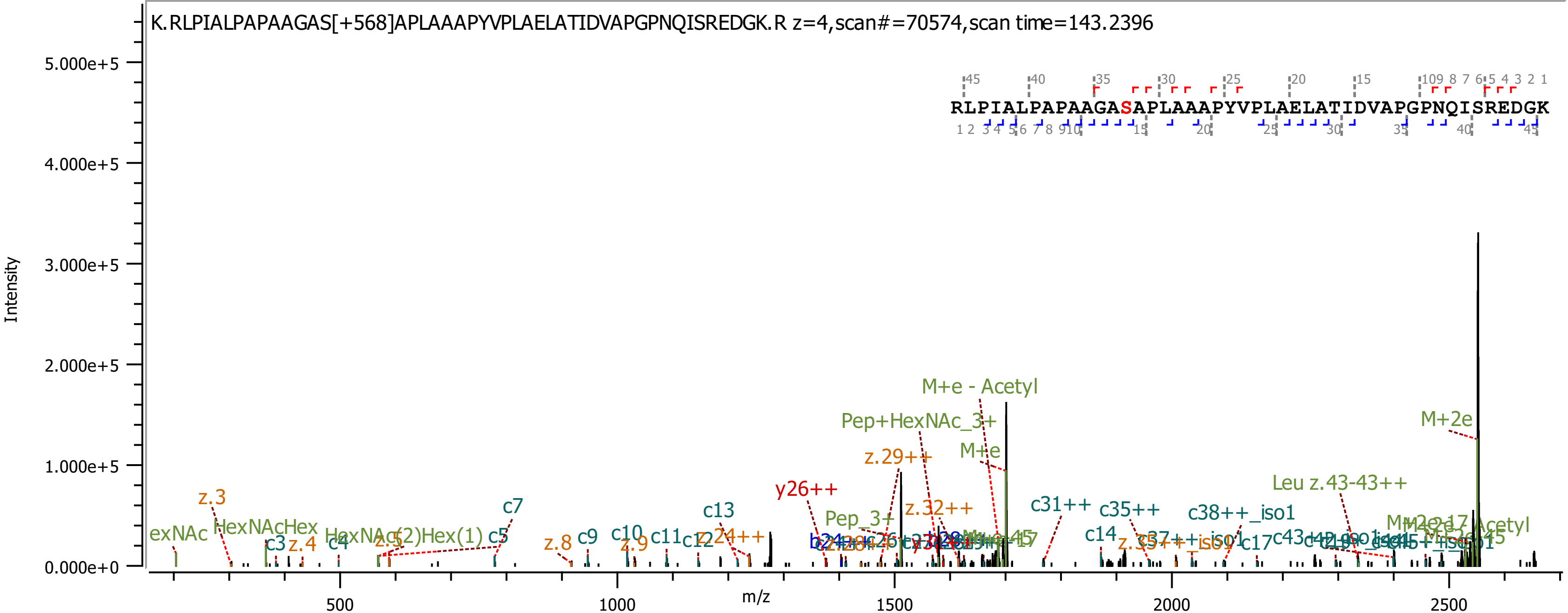

K.RLPIALPAPAAGAS[+568]APLAAAPYVPLAELATIDVAPGPNQISREDGK.R z=4,scan#=70574,scan time=143.2396
RLPIALPAPAAGASAPLAAAPYVPLAELATIDVAPGPNQISREDGK
Intensity
5.000e+5
4.000e+5
3.000e+5
2.000e+5
1.000e+5
0.000e+0
m/z
500
1000
1500
2000
2500
M+e - Acetyl
Pep+HexNAc_3+
M+e
z.29++
M+2e
Leu z.43-43++
y26++
z.32++
z.3
c7
c13
c31++
c35++
c38++_iso1
Pep_3+
HexNAcHex
exNAc
c3
z.4
c4
HexNAc(2)Hex(1)
c5
z.8
c9
c10
z.9
c11
c12
z.24++
c14
c17

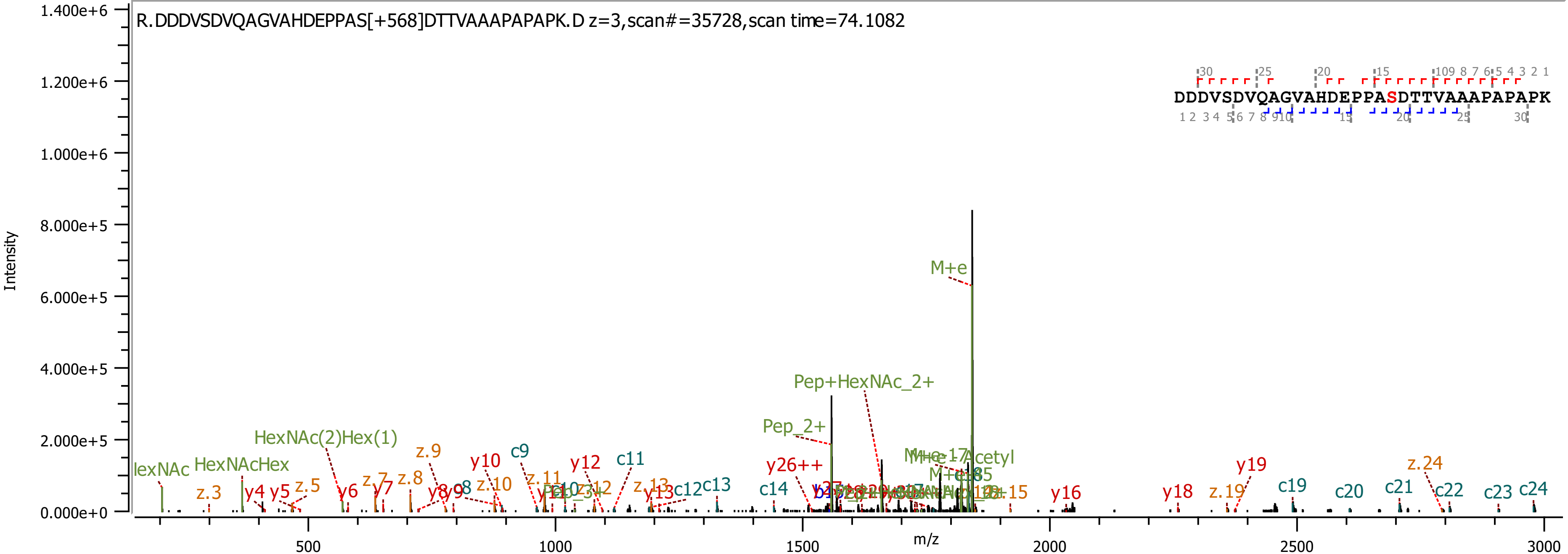
R.DDDVSDVQAGVAHDEPPAS[+568]DTTVAAAPAPAPK.D z=3,scan#=35728,scan time=74.1082
DDDVSDVQAGVAHDEPPASDTTVAAAPAPAPK
Intensity
m/z
M+e
Pep+HexNAc_2+
Pep_2+
HexNAc(2)Hex(1)
HexNAcHex
y26++
y19
z.24
c19
c20
c21
c22
c23
c24
y18
z.19
y16
c14
c13
c12
c11
c9
y12
y10
z.9
z.8
z.7
z.5
z.3
y4
y5
y6
y7
z.10
z.11
z.13
y13
c8
y8
y9
y11
c10
z.12
z.15
M+e-17
M+e-Acetyl

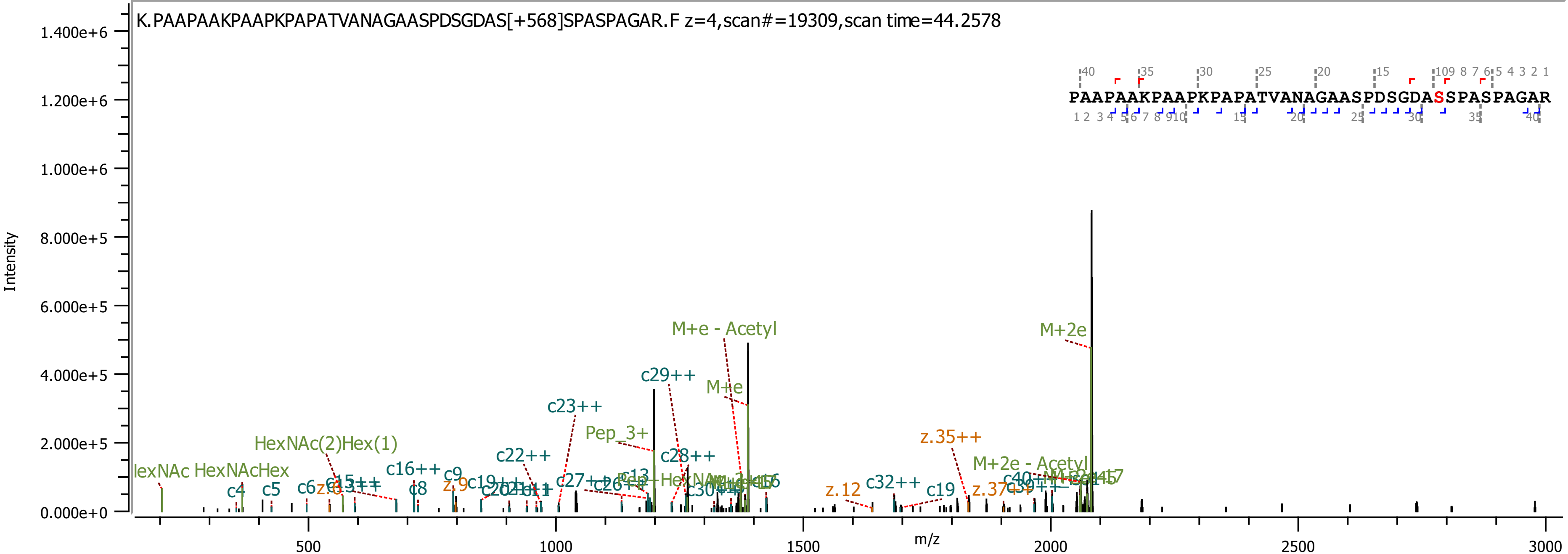
K.PAAPAAKPAAPKPAPATVANAGAASPDSGDAS[+568]SPASPAGAR.F z=4,scan#=19309,scan time=44.2578
PAAPAAKPAAPKPAPATVANAGAASPDSGDASSPASPAGAR
Intensity
1.400e+6
1.200e+6
1.000e+6
8.000e+5
6.000e+5
4.000e+5
2.000e+5
0.000e+0
m/z
500
1000
1500
2000
2500
3000
M+2e
M+e - Acetyl
c29++
M+e
c23++
z.35++
Pep_3+
HexNAc(2)Hex(1)
c22++
c28++
M+2e - Acetyl
HexNAc
c16++
c4
c5
c6
c9
c8
z.9
c19++
c27++
c26++
c13
c32++
c19
z.12
z.37++
c40++
c30++

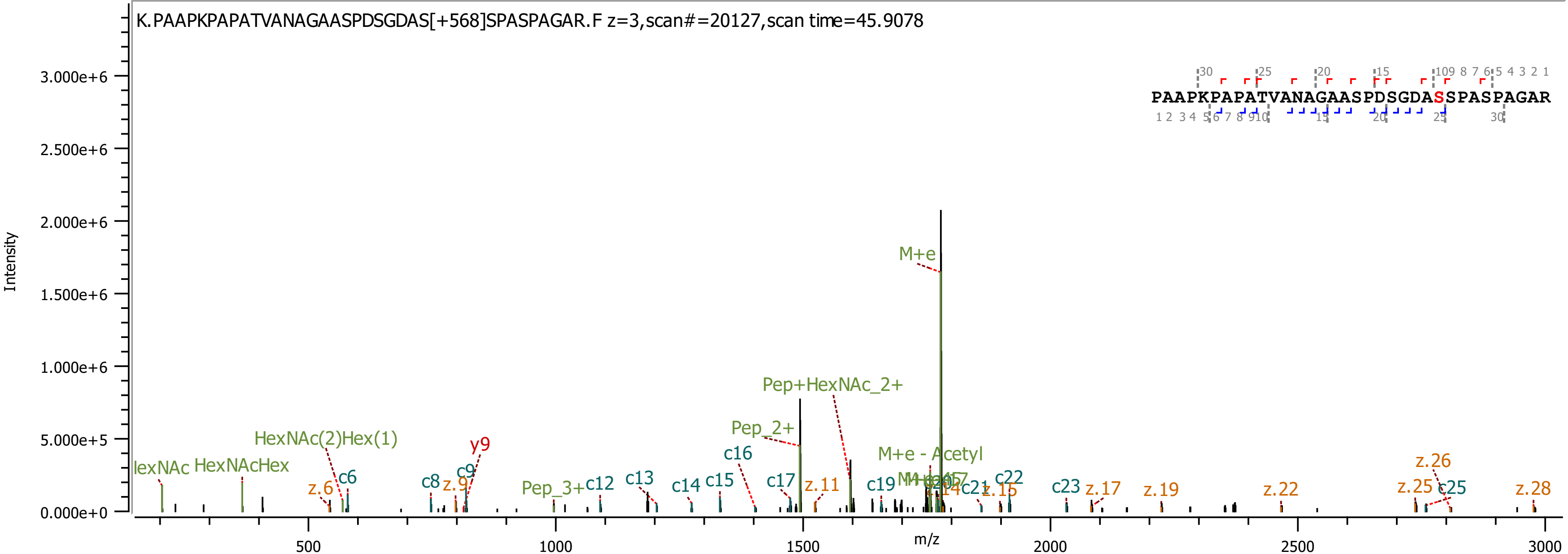

K.PAAPKPAPATVANAGAASPDSGDAS[+568]SPASPAGAR.F z=3,scan#=20127,scan time=45.9078
PAAPKPAPATVANAGAASPDSGDASSPASPAGAR
Intensity
m/z
M+e
Pep+HexNAc_2+
Pep_2+
M+e - Acetyl
HexNAc(2)Hex(1)
HexNAcHex
y9
c6
c8
c9
z.6
z.9
Pep_3+
c12
c13
c14
c15
c16
c17
z.11
c19
c22
c23
z.15
z.17
z.19
z.22
z.25
z.26
c25
z.28

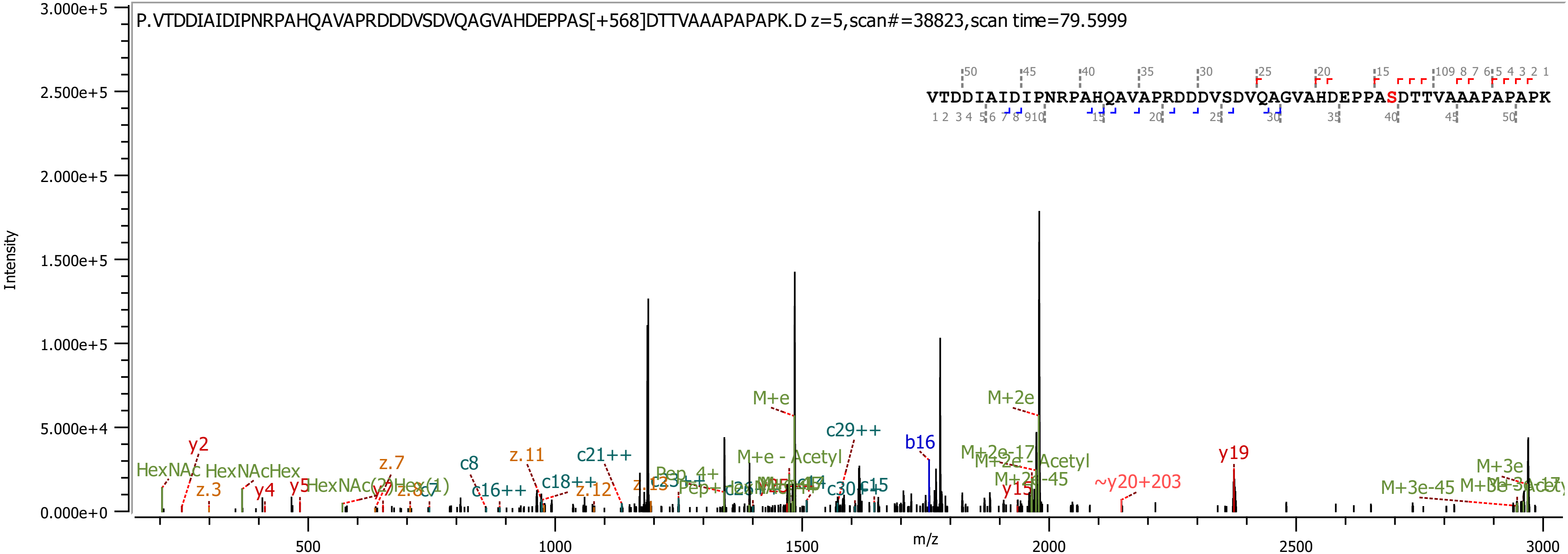

P.VTDDIAIDIPNRPAHQAVAPRDDDVSDVQAGVAHDEPPAS[+568]DTTVAAAPAPAPK.D z=5,scan#=38823,scan time=79.5999
VTDDIAIDIPNRPAHQAVAPRDDDVSDVQAGVAHDEPPASDTTVAAAPAPAPK
Intensity
m/z
3.000e+5
2.500e+5
2.000e+5
1.500e+5
1.000e+5
5.000e+4
0.000e+0
500
1000
1500
2000
2500
3000
HexNAc
y2
z.3
HexNAcHex
y4
y5
z.7
c8
z.11
c16++
c18++
z.12
c21++
M+e
M+e - Acetyl
c29++
b16
M+2e
M+2e-45
y15
~y20+203
y19
M+3e
M+3e-45

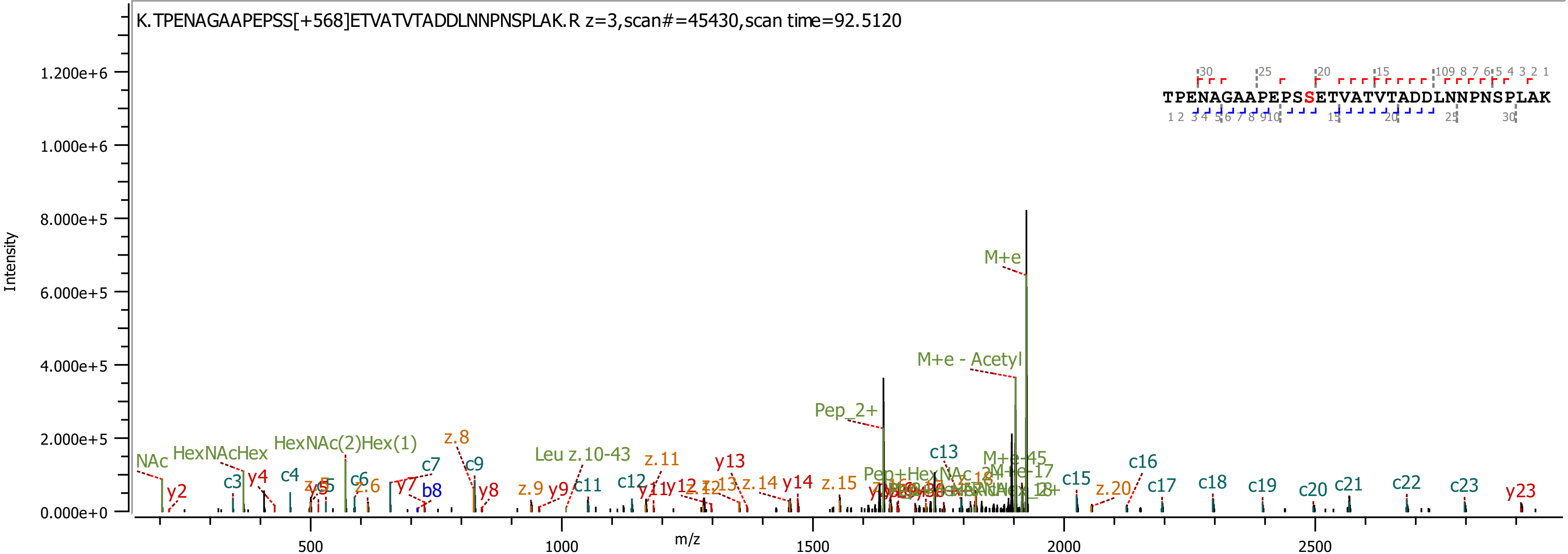
K.TPENAGAAPEPSS[+568]ETVATVTADDLNNPNSPLAK.R z=3,scan#=45430,scan time=92.5120
TPENAGAAPEPSSETVATVTADDLNNPNSPLAK
Intensity
m/z
M+e
M+e - Acetyl
Pep_2+
NAc
HexNAcHex
HexNAc(2)Hex(1)
Leu z.10-43
c3
c4
c6
c7
c9
c11
c12
c13
c15
c16
c17
c18
c19
c20
c21
c22
c23
y2
y4
y7
y8
y9
y11
y12
y13
y14
y23
b8
z.5
z.6
z.8
z.9
z.11
z.12
z.13
z.14
z.15
z.20
M+e-45
M+e-17
0.000e+0
2.000e+5
4.000e+5
6.000e+5
8.000e+5
1.000e+6
1.200e+6
500
1000
1500
2000
2500

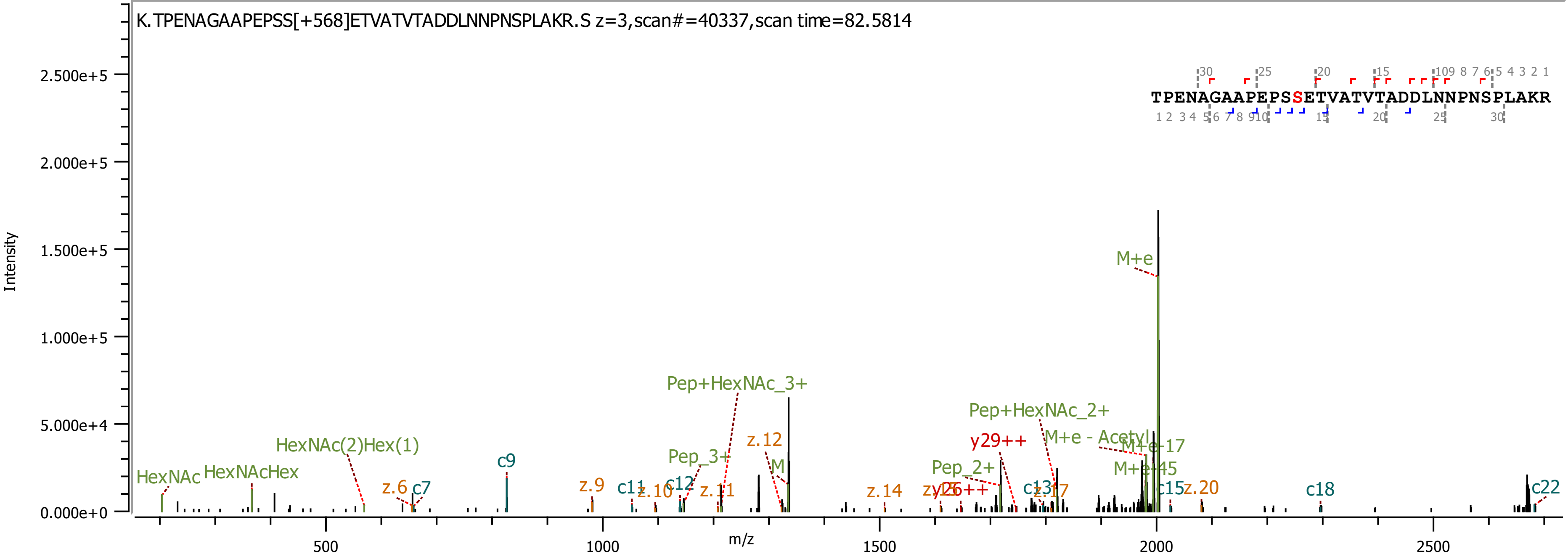

K.TPENAGAAPEPSS[+568]ETVATVTADDLNNPNSPLAKR.S z=3,scan#=40337,scan time=82.5814
TPENAGAAPEPSSETVATVTADDLNNPNSPLAKR
Intensity
m/z
M+e
Pep+HexNAc_3+
Pep+HexNAc_2+
HexNAc(2)Hex(1)
HexNAc
HexNAcHex
c9
z.6
c7
z.9
c11
z.10
c12
Pep_3+
z.11
z.12
M
z.14
y29++
Pep_2+
M+e - Acetyl
M+e-17
M+e+45
c13
z.17
c15
z.20
c18
c22

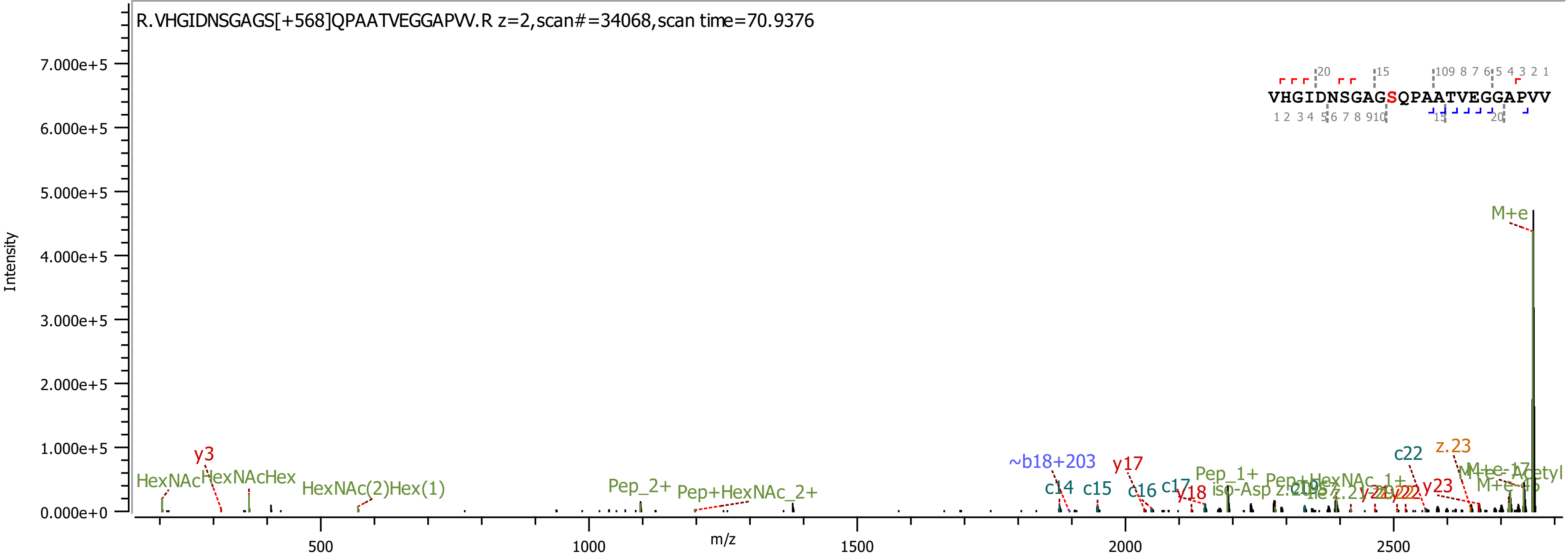

R.VHGIDNSGAGS[+568]QPAATVEGGAPVV.R z=2,scan#=34068,scan time=70.9376
VHGIDNSGAGSQPAATVEGGAPVV
Intensity
7.000e+5
6.000e+5
5.000e+5
4.000e+5
3.000e+5
2.000e+5
1.000e+5
0.000e+0
m/z
500
1000
1500
2000
2500
M+e
HexNAc
y3
HexNAcHex
HexNAc(2)Hex(1)
Pep_2+
Pep+HexNAc_2+
~b18+203
c14
c15
y17
c16
c17
y18
Pep_1+
iso-Asp
Pep+HexNAc_1+
c22
z.23
y23

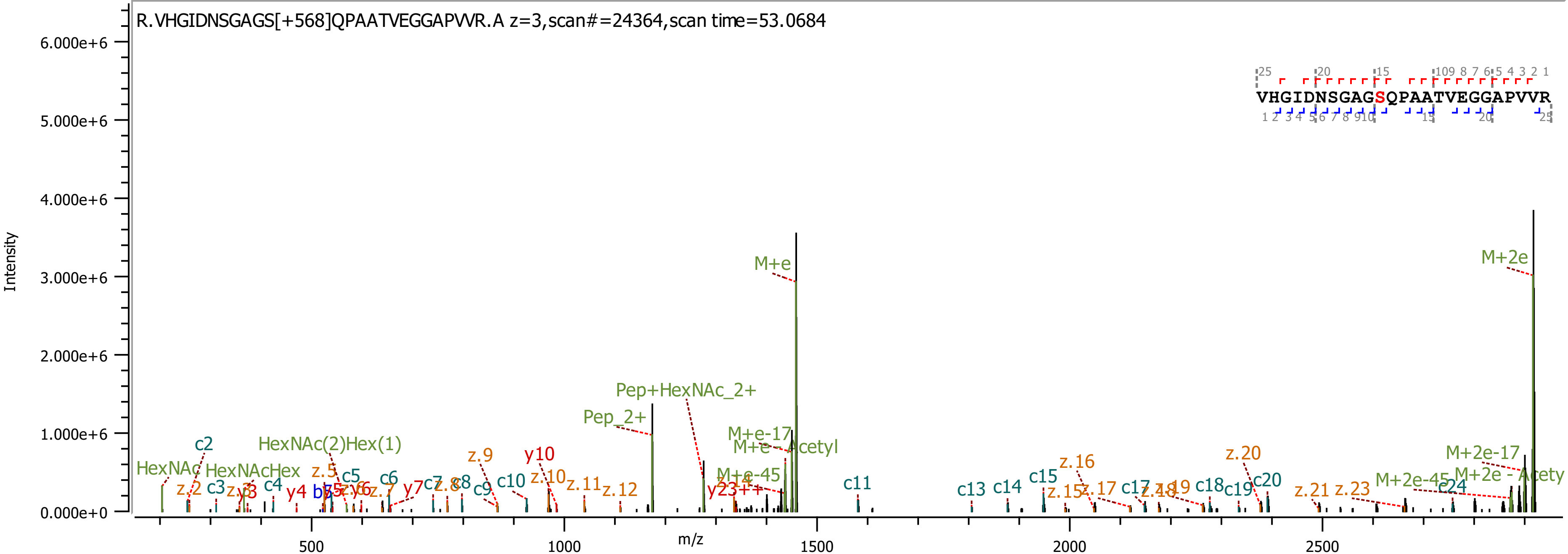
R.VHGIDNSGAGS[+568]QPAATVEGGAPVVR.A z=3,scan#=24364,scan time=53.0684
VHGIDNSGAGSQPAATVEGGAPVVR
Intensity
m/z
6.000e+6
5.000e+6
4.000e+6
3.000e+6
2.000e+6
1.000e+6
0.000e+0
500
1000
1500
2000
2500
M+e
M+2e
Pep+HexNAc_2+
Pep_2+
M+e-17
M+e - Acetyl
M+e-45
M+2e-17
M+2e - Acety
M+2e-45
HexNAc
HexNAcHex
HexNAc(2)Hex(1)
y10
y23++
c11
c13
c14
c15
c20
c24
z.16
z.20
z.21
z.23

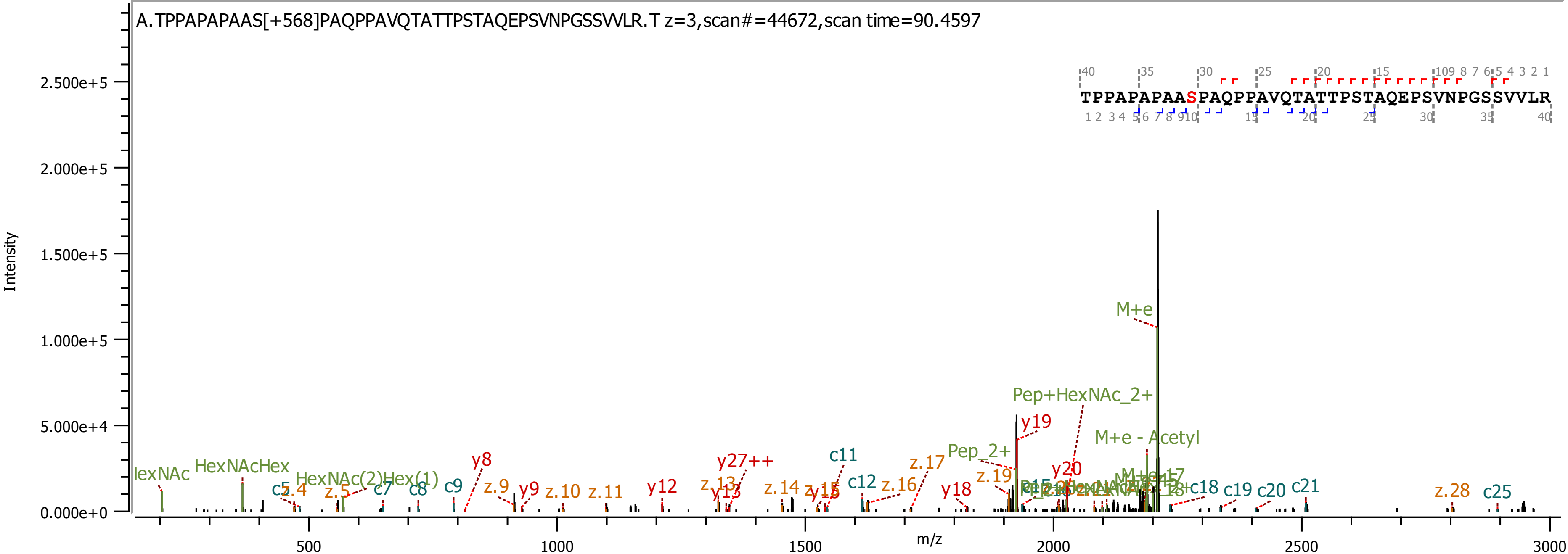
A.TPPAPAPAAS[+568]PAQPPAVQTATTPSTAQEPSVNPGSSVVLR.T z=3,scan#=44672,scan time=90.4597
TPPAPAPAASPAQPPAVQTATTPSTAQEPSVNPGSSVVLR
Intensity
m/z
2.500e+5
2.000e+5
1.500e+5
1.000e+5
5.000e+4
0.000e+0
500
1000
1500
2000
2500
3000
M+e
Pep+HexNAc_2+
M+e - Acetyl
Pep_2+
y19
y20
y8
y9
y12
y27++
y18
c11
c12
c7
c8
c9
c18
c19
c20
c21
c25
z.9
z.10
z.11
z.14
z.16
z.17
z.19
z.28
HexNAcHex
HexNAc(2)Hex(1)

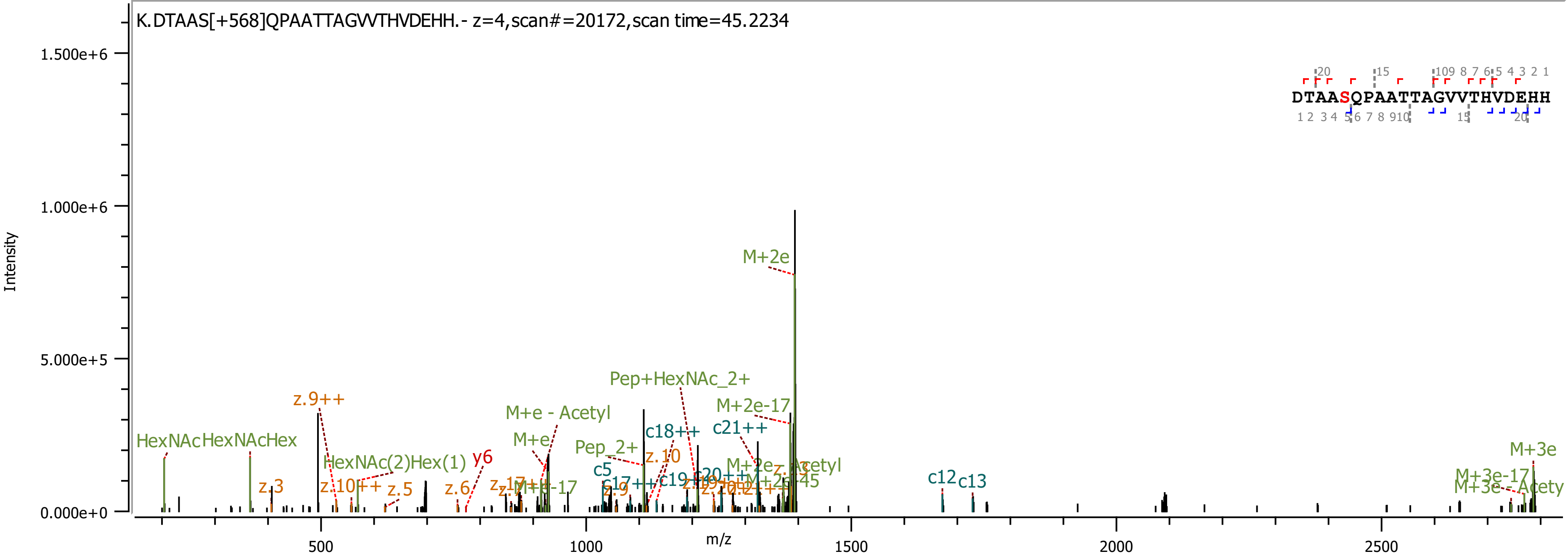

K.DTAAS[+568]QPAATTAGVVTHVDEHH.- z=4,scan#=20172,scan time=45.2234
DTAASQPAATTAGVVTHVDEHH
Intensity
m/z
1.500e+6
1.000e+6
5.000e+5
0.000e+0
500
1000
1500
2000
2500
M+2e
Pep+HexNAc_2+
z.9++
M+e - Acetyl
M+2e-17
HexNAc
HexNAcHex
M+e
c18++
c21++
Pep_2+
z.10
y6
HexNAc(2)Hex(1)
c5
z.3
z.10++
z.5
z.6
c12
c13
M+3e
M+3e-17
M+3e - Acety

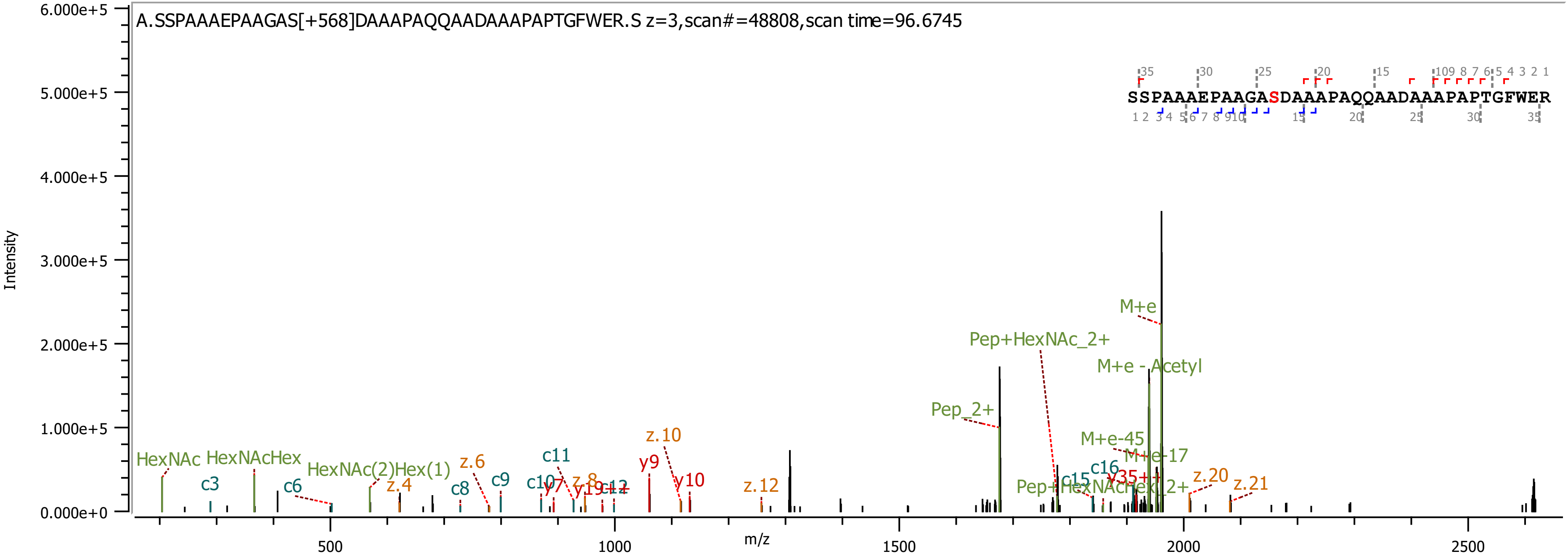
A.SSPAAAEPAAGAS[+568]DAAAPAQQAADAAAPAPTGFWER.S z=3,scan#=48808,scan time=96.6745
SSPAAAEPAAGASDAAAPAQQAADAAAPAPTGFWER
Intensity
m/z
M+e
Pep+HexNAc_2+
M+e - Acetyl
Pep_2+
M+e-45
M+e-17
HexNAc
HexNAcHex
HexNAc(2)Hex(1)
c3
c6
z.4
z.6
c8
c9
c10
c11
y7
z.8
z.10
y9
y10
z.12
c15
c16
y35++
Pep+HexNAcHex_2+
z.20
z.21

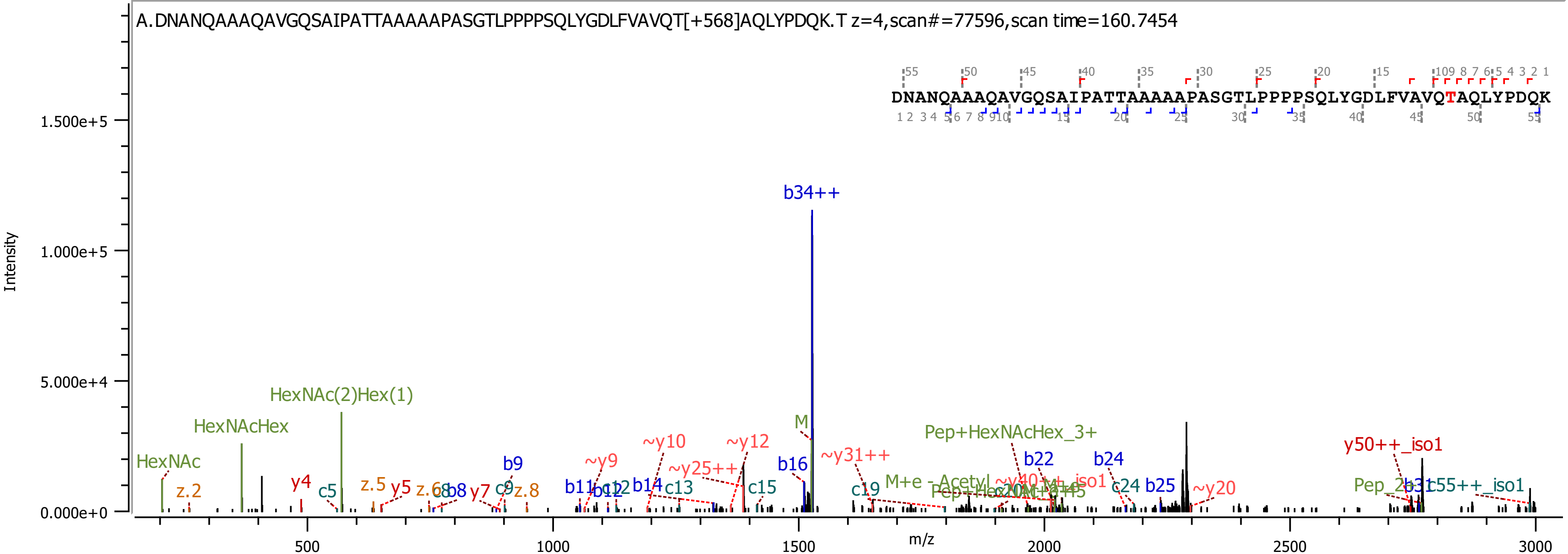

A.DNANQAAAQAVGQSAIPATTAAAAAPASGTLPPPPSQLYGDLFVAVQT[+568]AQLYPDQK.T z=4,scan#=77596,scan time=160.7454
DNANQAAAQAVGQSAIPATTAAAAAPASGTLPPPPSQLYGDLFVAVQTAQLYPDQK
Intensity
m/z
b34++
HexNAc(2)Hex(1)
HexNAcHex
HexNAc
Pep+HexNAcHex_3+
y50++_iso1
b22
b24
b25
b16
b9
~y10
~y12
~y9
~y31++
~y25++
~y20
y4
z.2
z.5
y5
b8
y7
z.8
c5
c9
b11
b12
b14
c13
c15
c19
c24

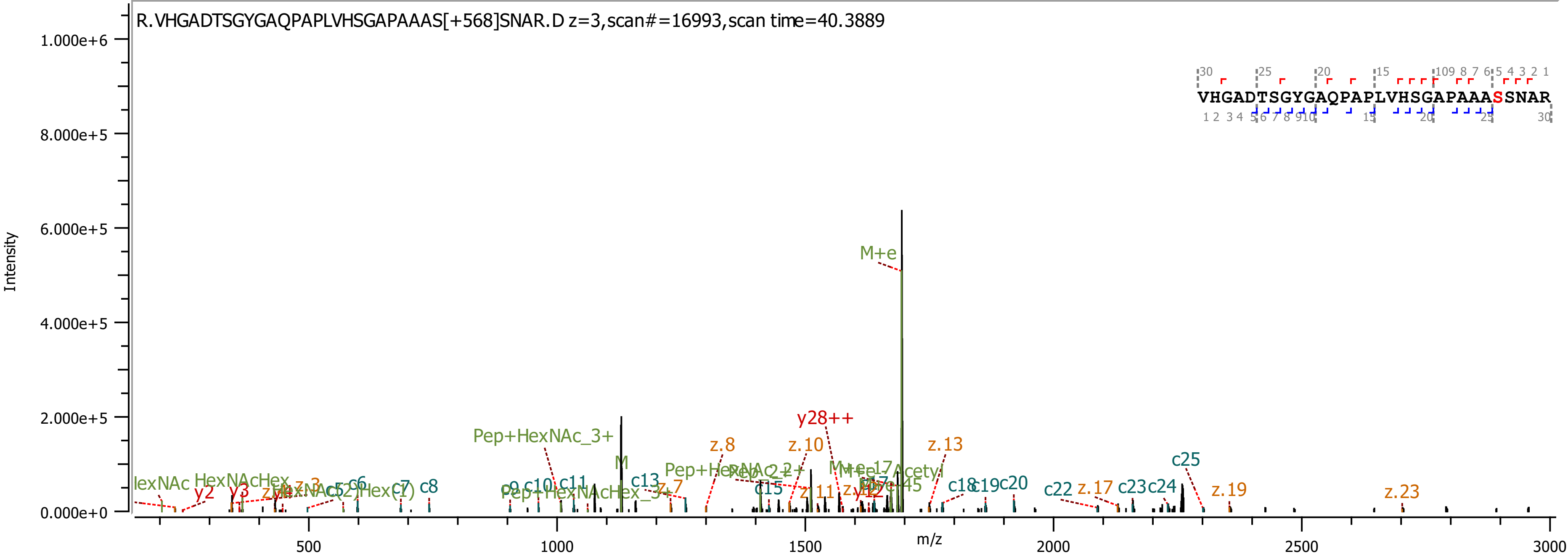
R.VHGADTSGYGAQPAPLVHSGAPAAAS[+568]SNAR.D z=3,scan#=16993,scan time=40.3889
Intensity
m/z
VHGADTSGYGAQPAPLVHSGAPAAASSNAR
M+e
y28++
z.8
z.10
z.13
Pep+HexNAc_3+
c25
c6
c7
c8
c10
c11
c13
c15
c18
c19
c20
c22
z.17
c23
c24
z.19
z.23
y2
y3
z.7
z.11
M

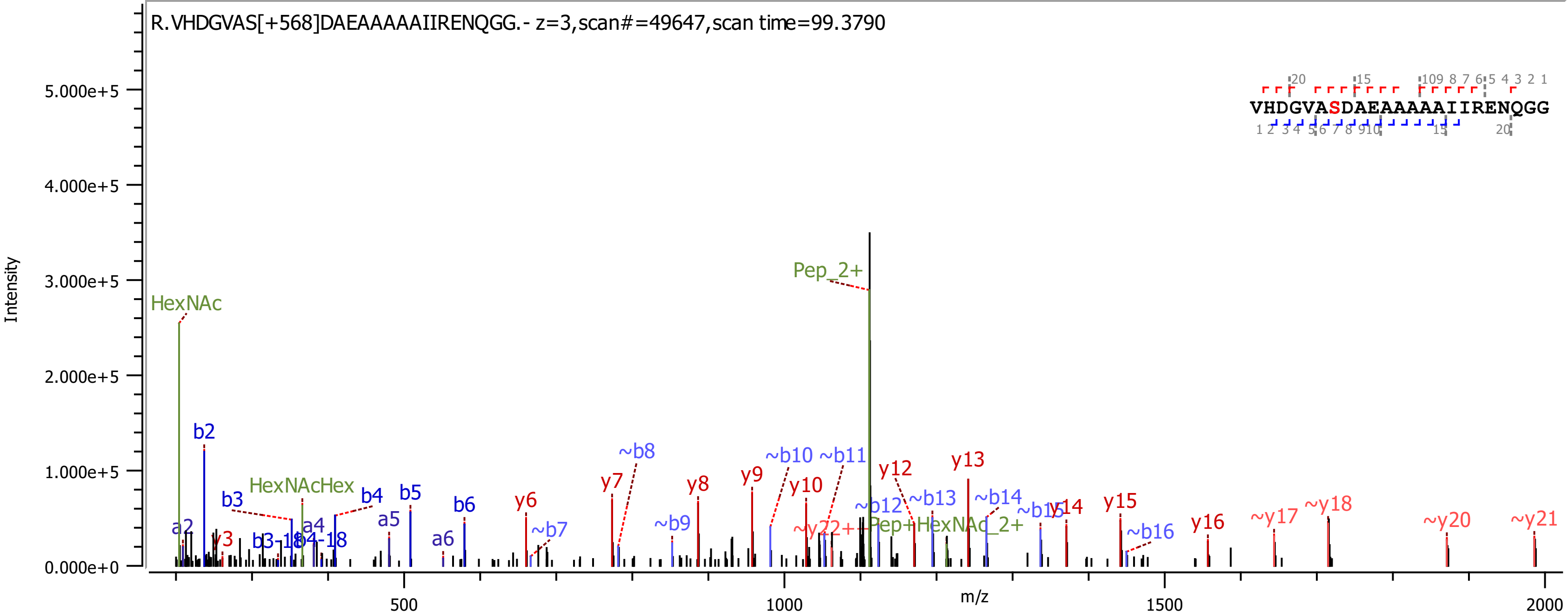
R.VHDGVAS[+568]DAEAAAAAIIRENQGG.- z=3,scan#=49647,scan time=99.3790
VHDGVASDAEAAAAAIIRENQGG
Intensity
5.000e+5
4.000e+5
3.000e+5
2.000e+5
1.000e+5
0.000e+0
m/z
500
1000
1500
2000
HexNAc
HexNAcHex
Pep_2+
Pep+HexNAc_2+
a2
a4
a5
a6
b2
b3
b4
b5
b6
b3-18
b4-18
~b7
~b8
~b9
~b10
~b11
~b12
~b13
~b14
~b15
~b16
y3
y6
y7
y8
y9
y10
y12
y13
y14
y15
y16
~y17
~y18
~y20
~y21
~y22++

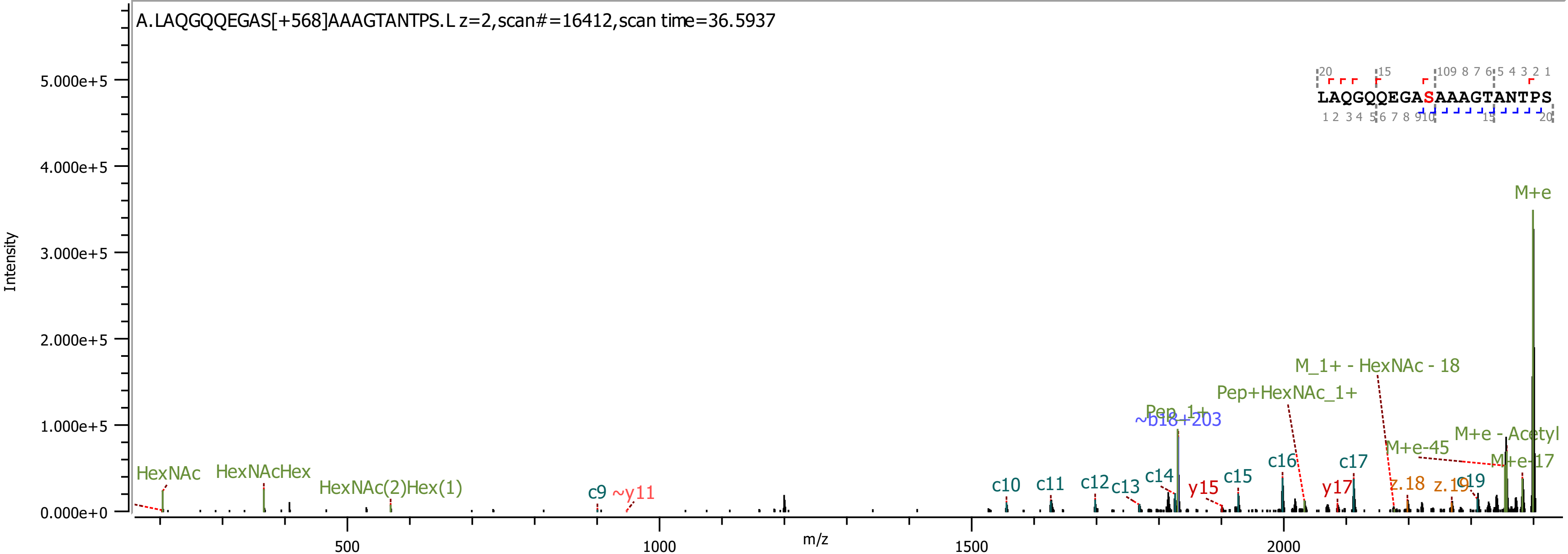

A.LAQGQQEGAS[+568]AAAGTANTPS.L z=2,scan#=16412,scan time=36.5937
LAQGQQEGASAAAGTANTPS
Intensity
m/z
M+e
M_1+ - HexNAc - 18
Pep+HexNAc_1+
Pep_1+
~b18+203
M+e - Acetyl
M+e-45
M+e-17
HexNAc
HexNAcHex
HexNAc(2)Hex(1)
c9
~y11
c10
c11
c12
c13
c14
y15
c15
c16
y17
c17
z.18
z.19
c19

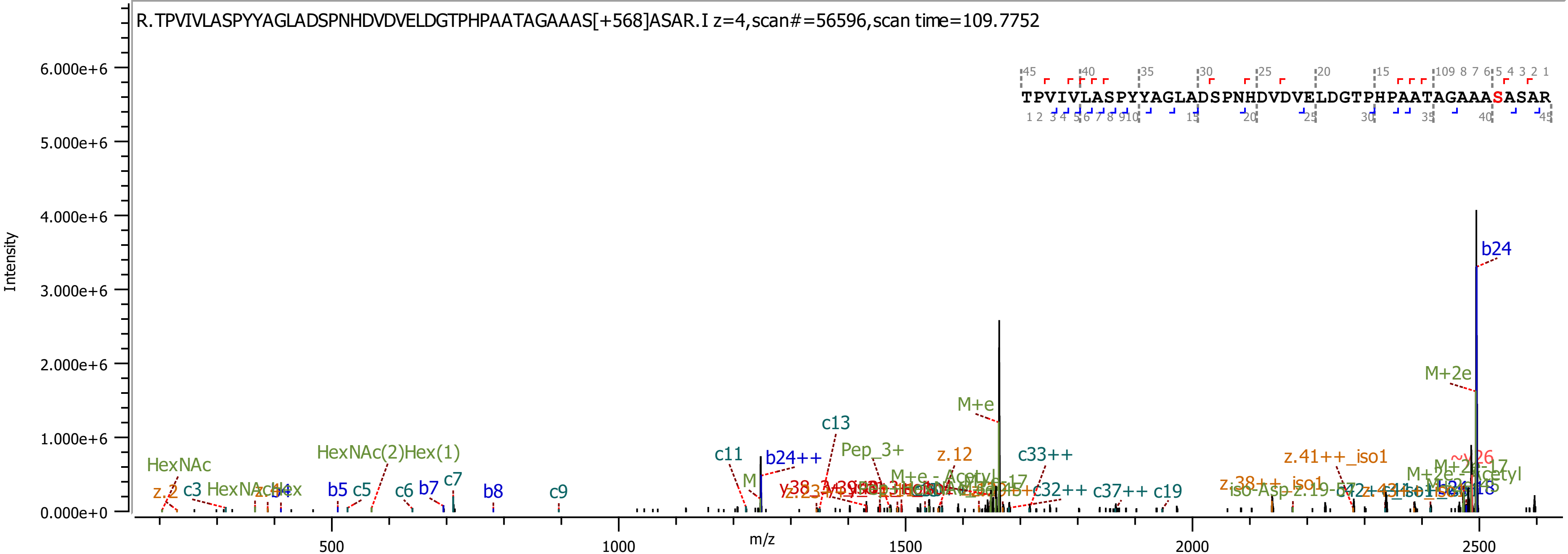

R.TPVIVLASPYYAGLADSPNHDVDVELDGTPHPAATAGAAAS[+568]ASAR.I z=4,scan#=56596,scan time=109.7752
TPVIVLASPYYAGLADSPNHDVDVELDGTPHPAATAGAAASASAR
Intensity
m/z
6.000e+6
5.000e+6
4.000e+6
3.000e+6
2.000e+6
1.000e+6
0.000e+0
500
1000
1500
2000
2500
b24
M+2e
M+e
c13
c11
b24++
Pep_3+
z.12
c33++
z.41++_iso1
HexNAc(2)Hex(1)
HexNAc
z.2
c3
b4
b5
c5
c6
b7
c7
b8
c9
c32++
c37++
c19

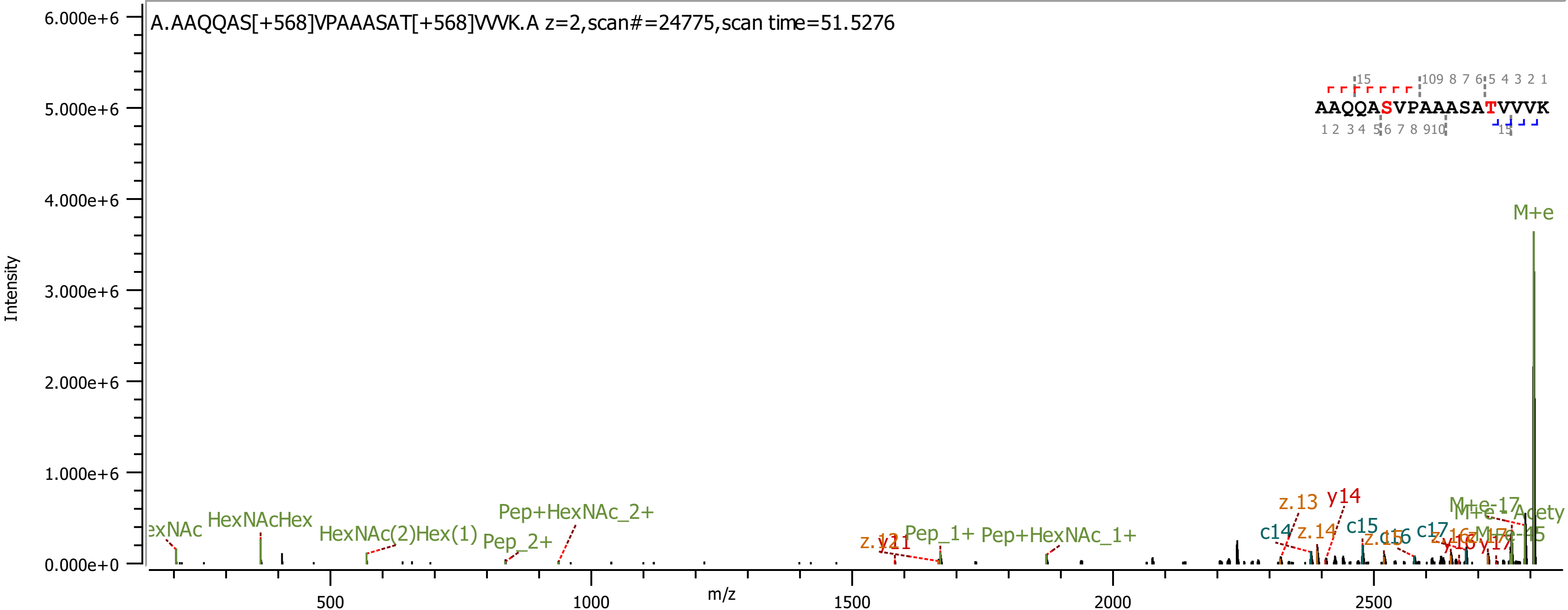
A.AAQQAS[+568]VPAAASAT[+568]VVVK.A z=2,scan#=24775,scan time=51.5276
AAQQASVPAAASATVVVK
Intensity
m/z
M+e
HexNAcHex
HexNAc(2)Hex(1)
Pep_2+
Pep+HexNAc_2+
Pep_1+
Pep+HexNAc_1+
z.13
y14
z.14
c14
c15
c17
M+e-17

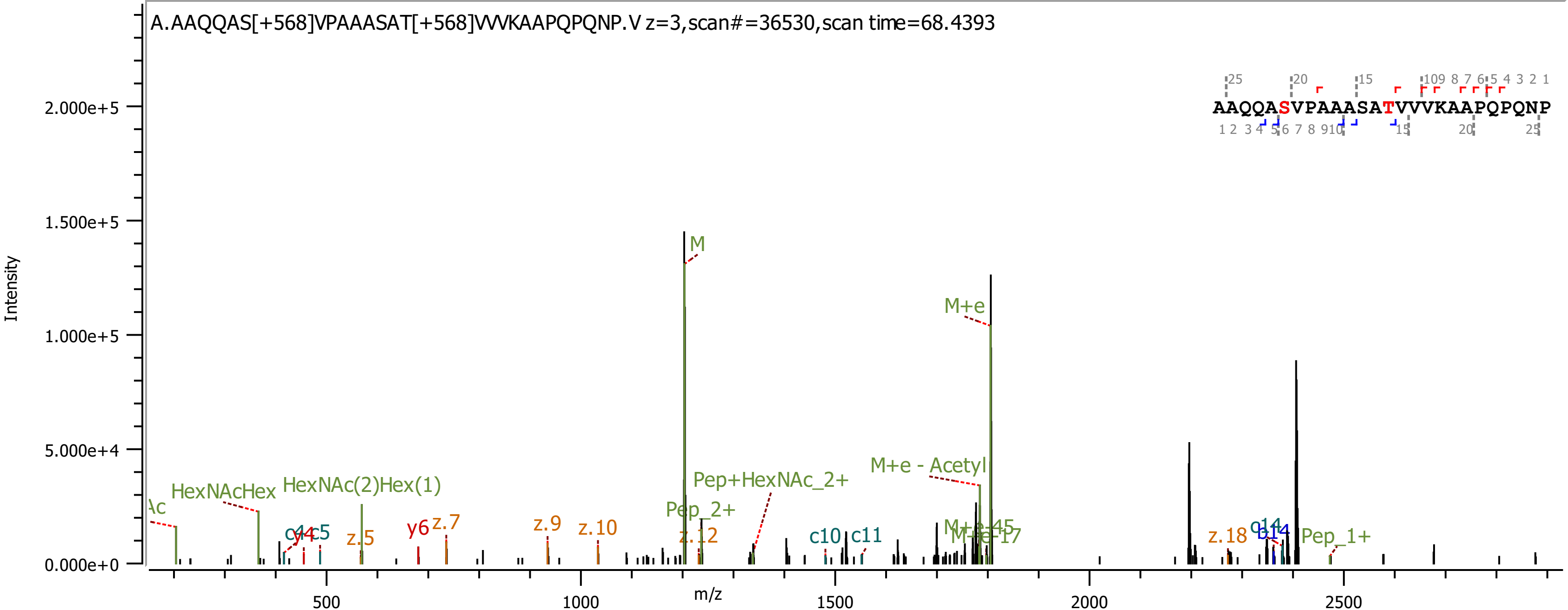
A.AAQQAS[+568]VPAAASAT[+568]VVVKAAPQPQNP.V z=3,scan#=36530,scan time=68.4393
AAQQASVPAAASATVVVKAAPQPQNP
Intensity
m/z
M
M+e
HexNAcHex
HexNAc(2)Hex(1)
Pep+HexNAc_2+
M+e - Acetyl
Pep_2+
z.12
z.5
z.7
z.9
z.10
y6
c10
c11
z.18
Pep_1+
M+e-17
2.000e+5
1.500e+5
1.000e+5
5.000e+4
0.000e+0
500
1000
1500
2000
2500

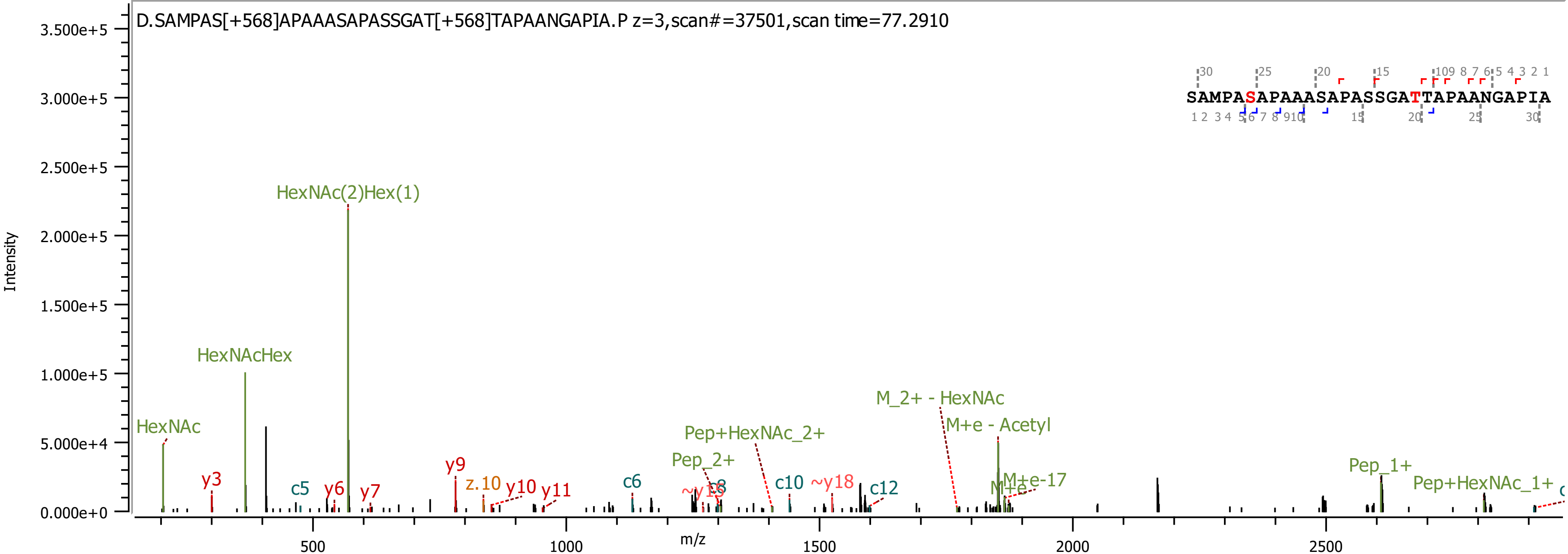
D.SAMPAS[+568]APAAASAPASSGAT[+568]TAPAANGAPIA.P z=3,scan#=37501,scan time=77.2910
SAMPASAPAAASAPASSGATTAPAANGAPIA
Intensity
m/z
3.500e+5
3.000e+5
2.500e+5
2.000e+5
1.500e+5
1.000e+5
5.000e+4
0.000e+0
500
1000
1500
2000
2500
HexNAc(2)Hex(1)
HexNAcHex
HexNAc
y3
c5
y6
y7
y9
z.10
y10
y11
c6
Pep_2+
Pep+HexNAc_2+
c10
~y18
c12
M_2+ - HexNAc
M+e - Acetyl
M+e-17
Pep_1+
Pep+HexNAc_1+

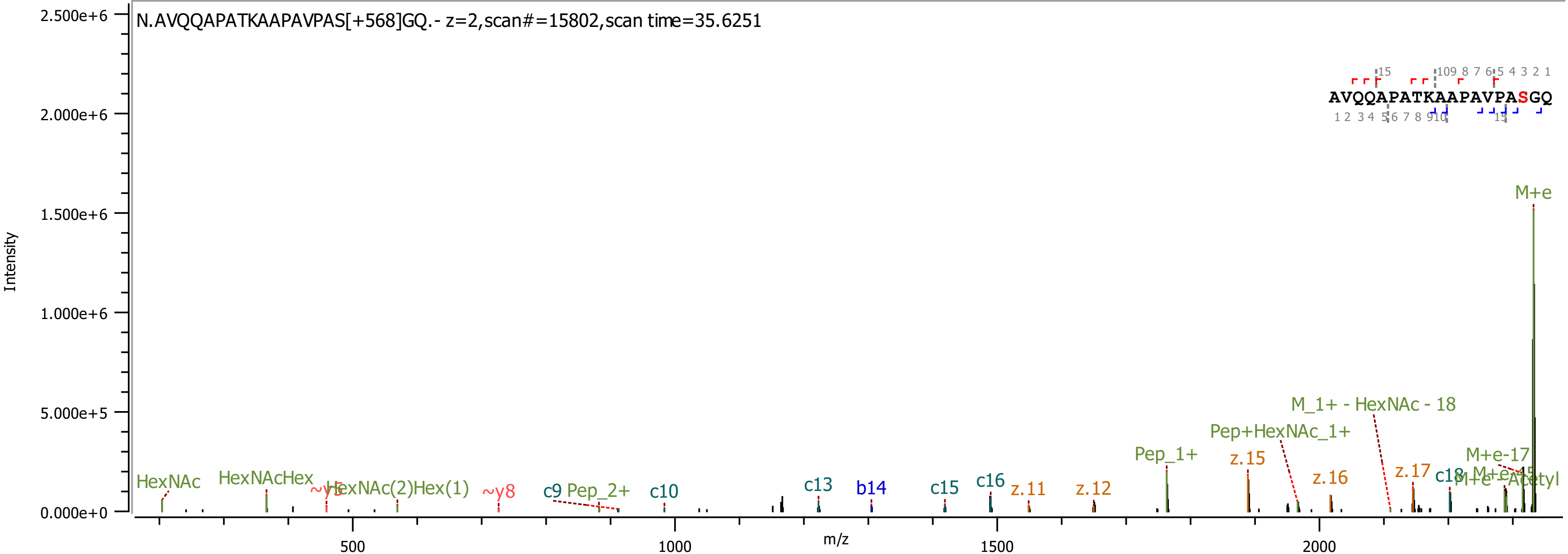

N.AVQQAPATKAAPAVPAS[+568]GQ.- z=2,scan#=15802,scan time=35.6251
AVQQAPATKAAPAVPASGQ
Intensity
m/z
M+e
M_1+ - HexNAc - 18
Pep+HexNAc_1+
Pep_1+
z.15
M+e-17
z.17
z.16
c18
M+e-45
M+e - Acetyl
HexNAc
HexNAcHex
~y5
HexNAc(2)Hex(1)
~y8
c9
Pep_2+
c10
c13
b14
c15
c16
z.11
z.12

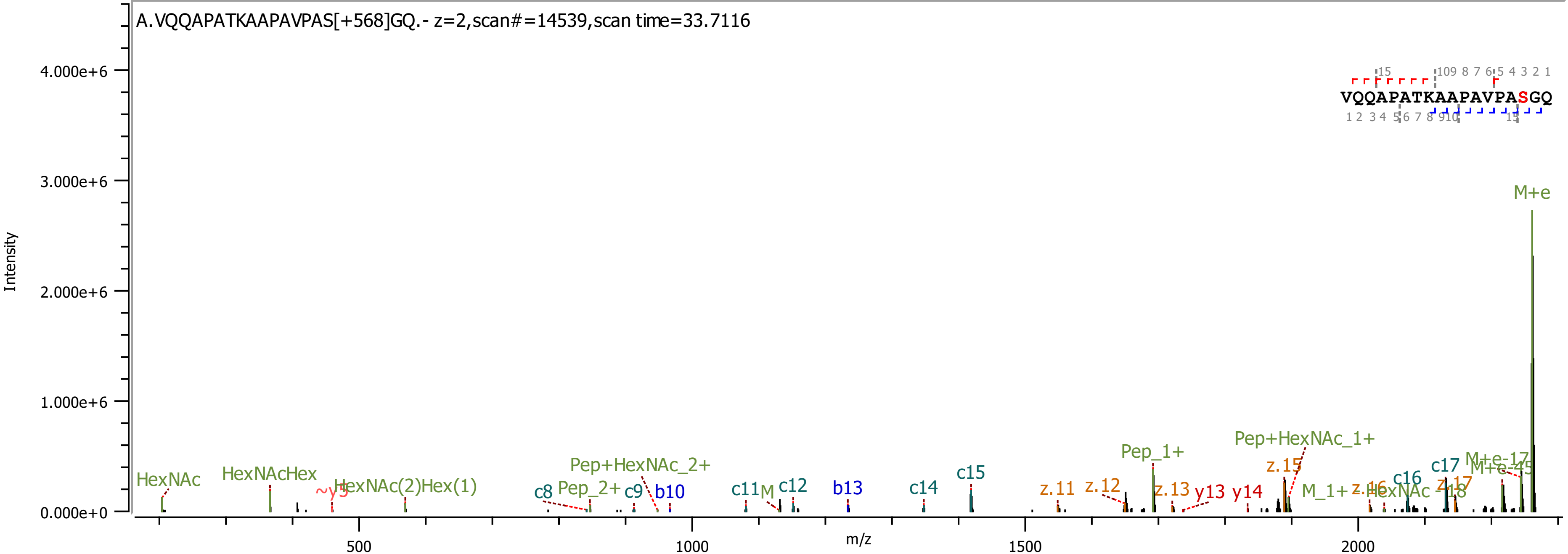
A.VQQAPATKAAPAVPAS[+568]GQ.- z=2,scan#=14539,scan time=33.7116
VQQAPATKAAPAVPASGQ
Intensity
m/z
M+e
Pep_1+
Pep+HexNAc_1+
Pep+HexNAc_2+
Pep_2+
HexNAc
HexNAcHex
HexNAc(2)Hex(1)
c8
c9
b10
c11
c12
b13
c14
c15
c16
c17
z.11
z.12
z.13
z.15
z.17
y13
y14
M_1+
M+e-17

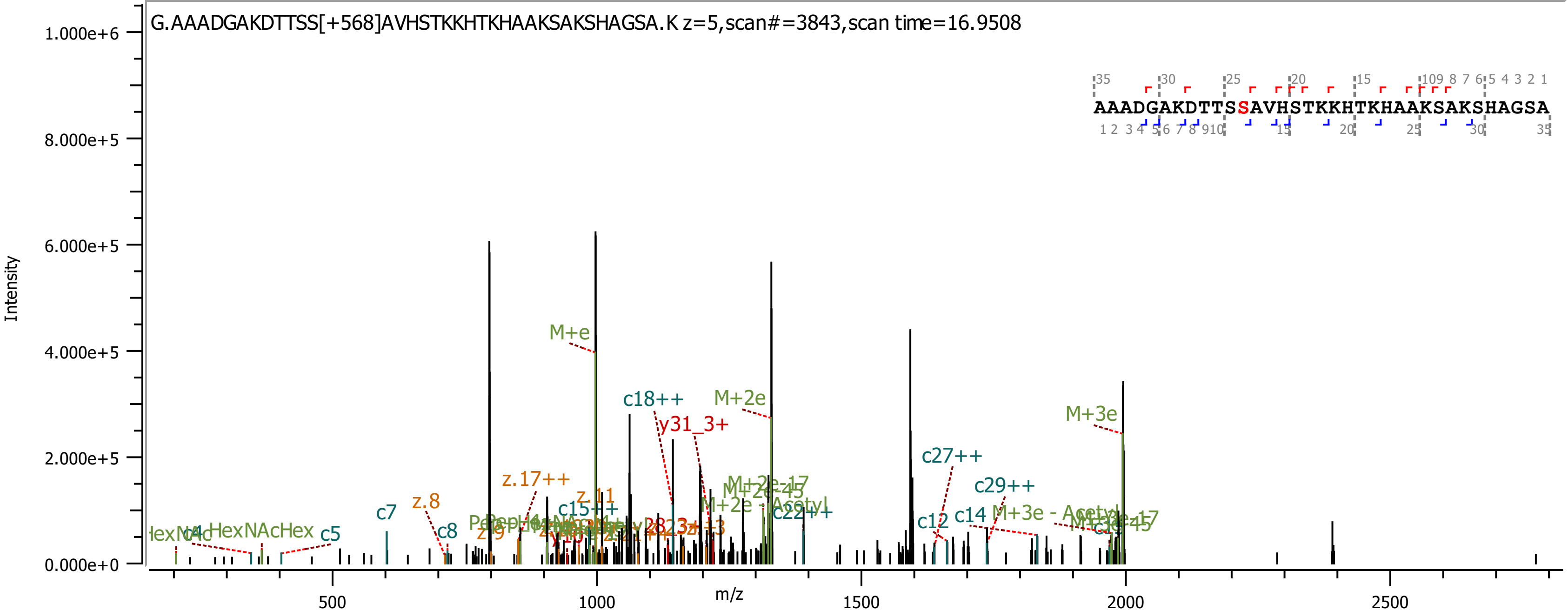
G.AAADGAKDTTSS[+568]AVHSTKKHTKHAAKSAKSHAGSA.K z=5,scan#=3843,scan time=16.9508
AAADGAKDTTSSAVHSTKKHTKHAAKSAKSHAGSA
Intensity
m/z
M+e
c18++
M+2e
y31_3+
M+3e
c27++
c29++
z.17++
z.8
c7
c8
c5
HexNAcHex
c15++
c22++
c12
c14
M+3e - Acetyl
M+2e - Acetyl
M+2e-17
M+2e-45

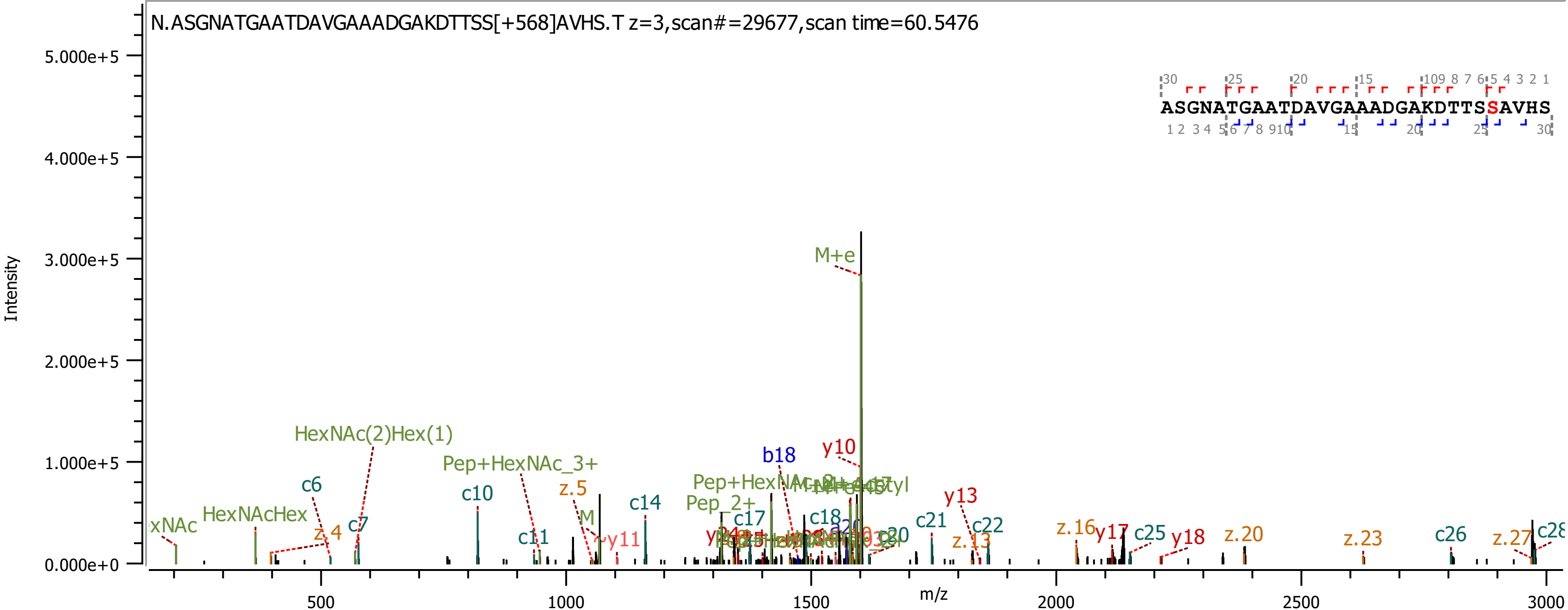
N.ASGNATGAATDAVGAAADGAKDTTSS[+568]AVHS.T z=3,scan#=29677,scan time=60.5476
ASGNATGAATDAVGAAADGAKDTTSSAVHS
Intensity
m/z
5.000e+5
4.000e+5
3.000e+5
2.000e+5
1.000e+5
0.000e+0
500
1000
1500
2000
2500
3000
M+e
HexNAc(2)Hex(1)
Pep+HexNAc_3+
HexNAcHex
b18
y10
c6
c7
z.4
c10
c11
z.5
c14
c17
c18
c21
c22
y13
z.13
z.16
y17
c25
y18
z.20
z.23
c26
z.27
Pep_2+
y11

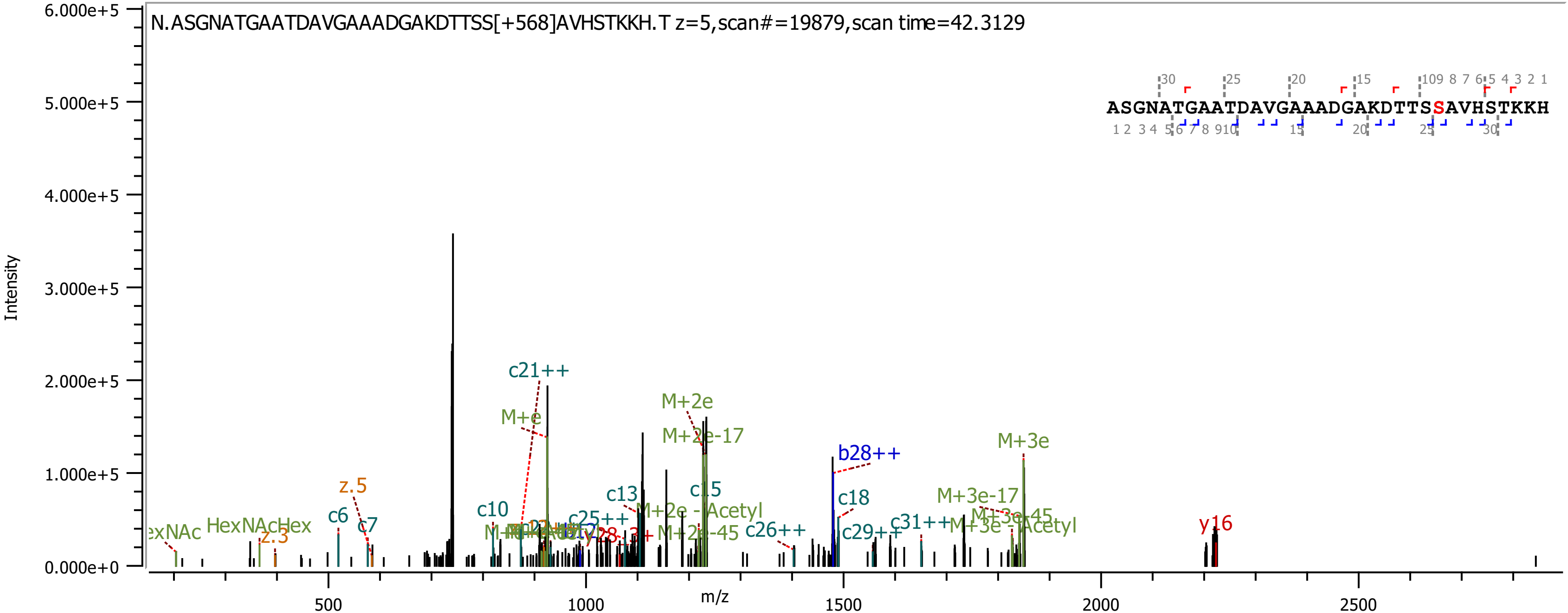

N.ASGNATGAATDAVGAAADGAKDTTSS[+568]AVHSTKKH.T z=5,scan#=19879,scan time=42.3129
ASGNATGAATDAVGAAADGAKDTTSSAVHSTKKH
Intensity
m/z
6.000e+5
5.000e+5
4.000e+5
3.000e+5
2.000e+5
1.000e+5
0.000e+0
500
1000
1500
2000
2500
c21++
M+e
M+2e
M+2e-17
b28++
M+3e
z.5
c6
c7
z.3
HexNAcHex
c10
c13
c15
M+2e - Acetyl
M+2e-45
c18
c26++
c29++
c31++
M+3e-17
M+3e-45
M+3e - Acetyl
y16
c25++

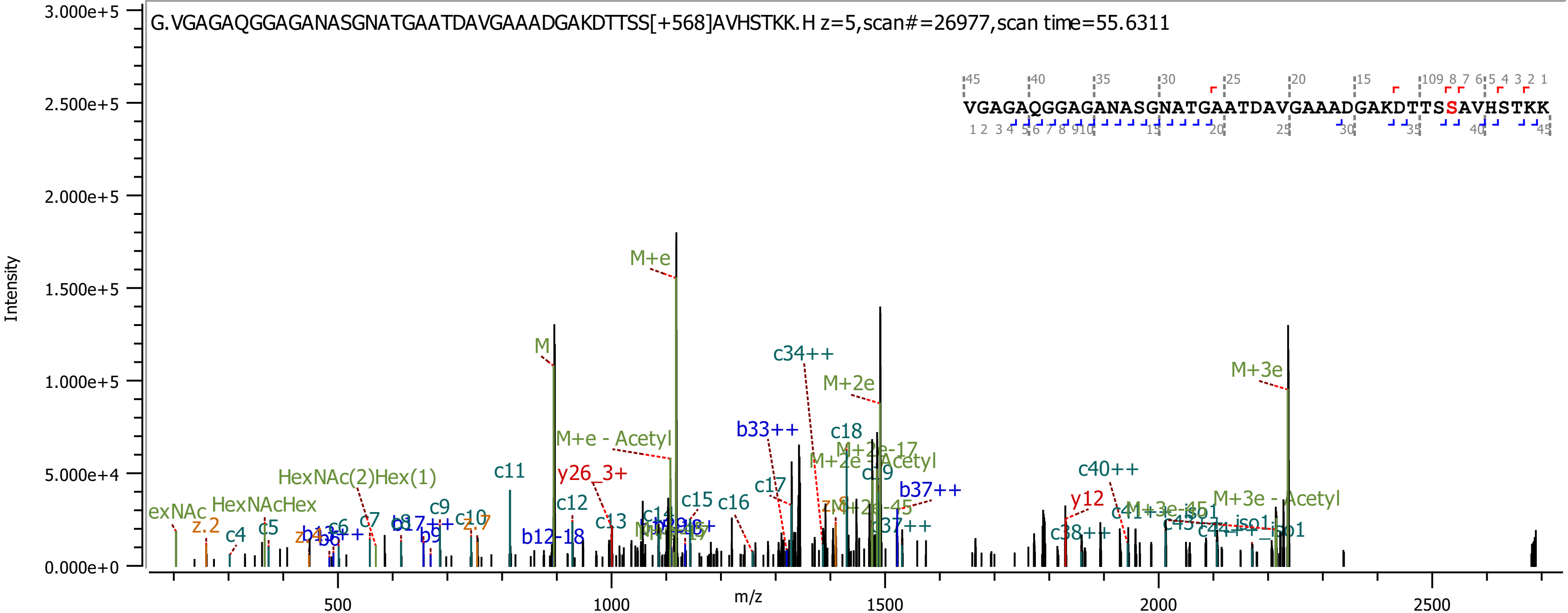
G.VGAGAQGGAGANASGNATGAATDAVGAAADGAKDTTSS[+568]AVHSTKK.H z=5,scan#=26977,scan time=55.6311
VGAGAQGGAGANASGNATGAATDAVGAAADGAKDTTSSAVHSTKK
Intensity
m/z
3.000e+5
2.500e+5
2.000e+5
1.500e+5
1.000e+5
5.000e+4
0.000e+0
500
1000
1500
2000
2500
M+e
M
M+e - Acetyl
c34++
M+2e
b33++
c18
M+3e
M+3e - Acetyl
c40++
y12
c11
y26_3+
c12
c13
c15
c16
c17
b37++
HexNAc(2)Hex(1)
HexNAcHex
z.2
c4
c5
c7
c9
b12-18
c38++

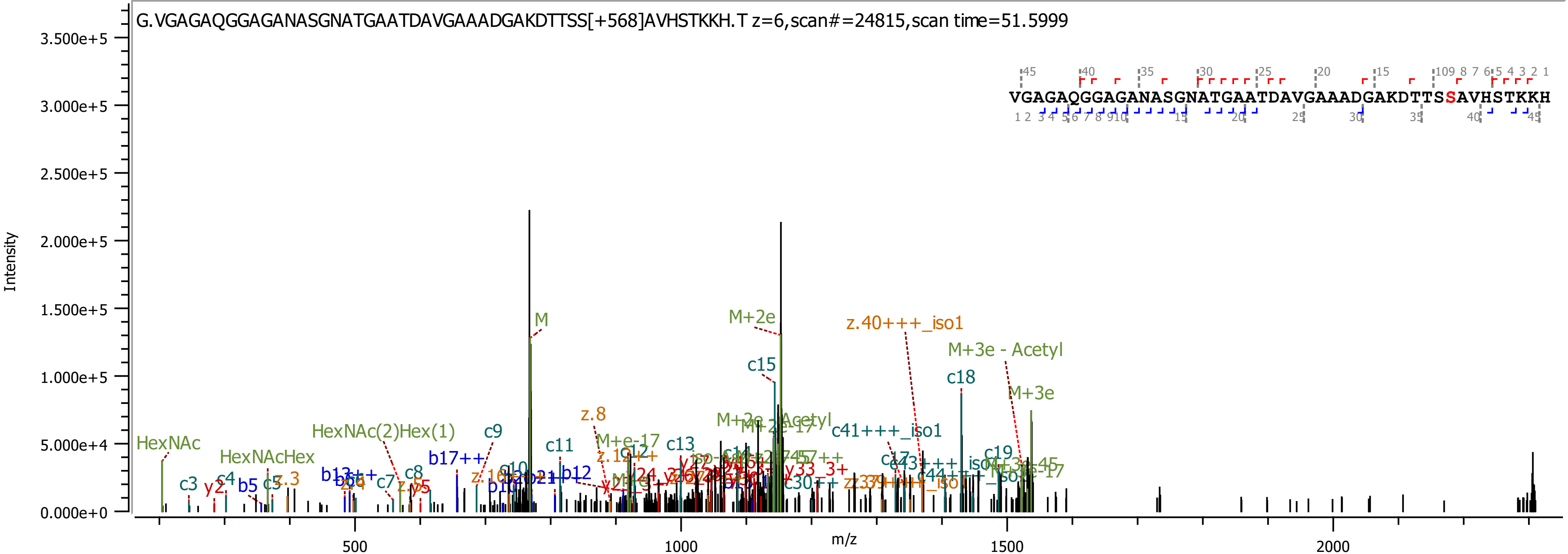
G.VGAGAQGGAGANASGNATGAATDAVGAAADGAKDTTSS[+568]AVHSTKKH.T z=6,scan#=24815,scan time=51.5999
VGAGAQGGAGANASGNATGAATDAVGAAADGAKDTTSSAVHSTKKH
Intensity
m/z
3.500e+5
3.000e+5
2.500e+5
2.000e+5
1.500e+5
1.000e+5
5.000e+4
0.000e+0
500
1000
1500
2000
M
M+2e
z.40+++_iso1
M+3e - Acetyl
c15
c18
M+3e
z.8
HexNAc
HexNAc(2)Hex(1)
c9
c11
c13
b17++
c41+++_iso1
HexNAcHex
c3
y2
c4
b5
z.3
c7
c8
b12
c19
y33_3+

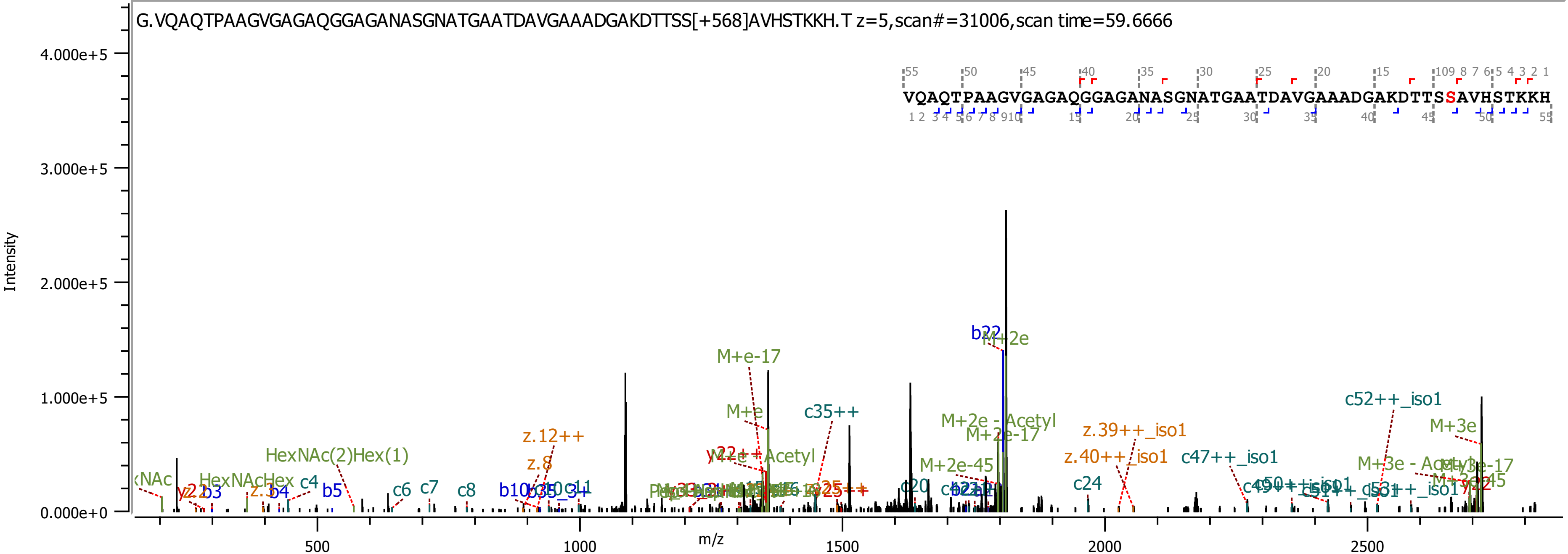
G.VQAQTPAAGVGAGAQGGAGANASGNATGAATDAVGAAADGAKDTTSS[+568]AVHSTKKH.T z=5,scan#=31006,scan time=59.6666
VQAQTPAAGVGAGAQGGAGANASGNATGAATDAVGAAADGAKDTTSSAVHSTKKH
Intensity
m/z
4.000e+5
3.000e+5
2.000e+5
1.000e+5
0.000e+0
500
1000
1500
2000
2500
b22
M+2e
M+e-17
M+e
c35++
M+2e - Acetyl
M+2e-17
M+2e-45
z.39++_iso1
z.40++_iso1
c47++_iso1
c52++_iso1
M+3e
M+3e - Acetyl
c24
c20
z.12++
z.8
HexNAc(2)Hex(1)
HexNAcHex
c4
b5
c6
c7
c8
b3
b4
y2
z.3

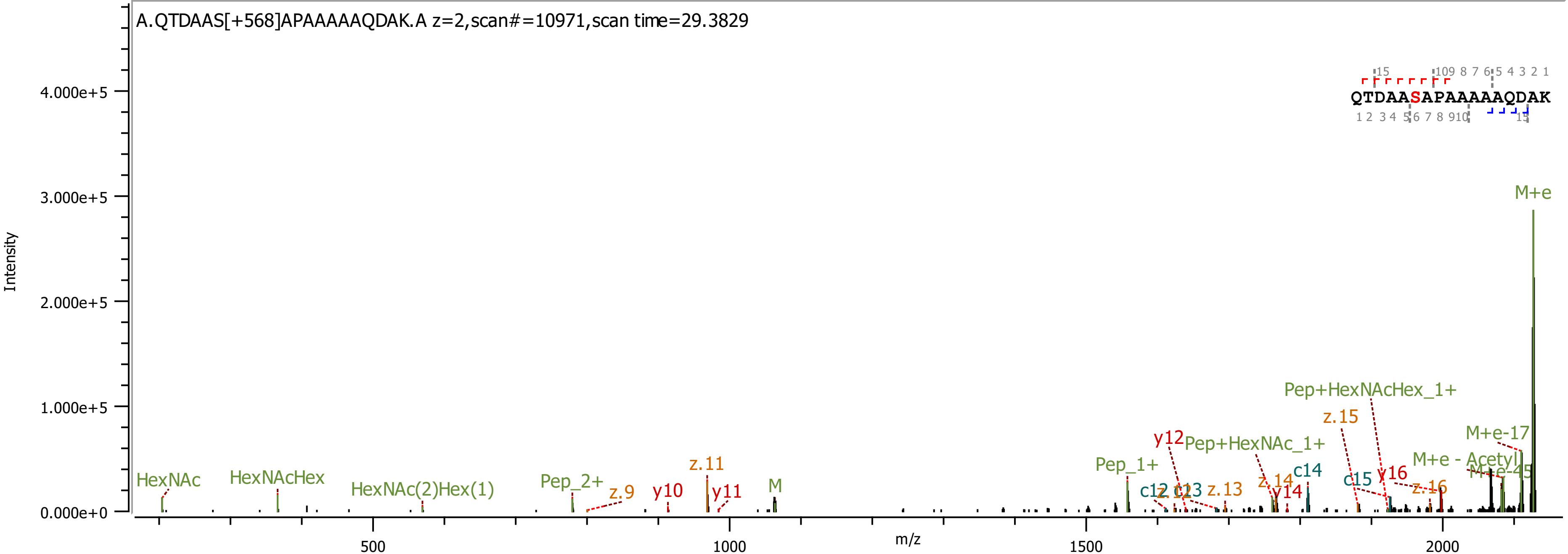
A.QTDAAS[+568]APAAAAAQDAK.A z=2,scan#=10971,scan time=29.3829
QTDAASAPAAAAAQDAK
Intensity
m/z
4.000e+5
3.000e+5
2.000e+5
1.000e+5
0.000e+0
500
1000
1500
2000
M+e
HexNAc
HexNAcHex
HexNAc(2)Hex(1)
Pep_2+
z.9
y10
z.11
y11
M
Pep_1+
y12
Pep+HexNAc_1+
Pep+HexNAcHex_1+
z.15
c12
z.12
c13
z.13
z.14
y14
c14
c15
y16
z.16
M+e - Acetyl
M+e-45
M+e-17
