# Supplementary material for: Burkholderia PglL enzymes are Serine preferring oligosaccharyltransferases which target conserved proteins across the Burkholderia genus
Source: Commun Biol. 2021 Sep 7;4:1045. doi: 10.1038/s42003-021-02588-y (PMC8423747; doi:10.1038/s42003-021-02588-y)
Supplement: Supplementary file 6 — Supplementary Data 3 [file 42003_2021_2588_MOESM6_ESM.zip › Supplementary_Data_3A_H111_Best_Scoring_Unique_Glycopeptides.pdf]

Burkholderia cenocepacia H111 Best Scoring Unique glycopeptides

<table>
<tr><th>J2315 numbers</th><th>Protein Name</th><th>Peptide < ProteinMetrics Confidential ></th><th>Glycans NHFAGNa</th><th>Localised site (Yes/No)</th><th>Observed m/z</th><th>z</th><th>Observed (M+H)</th><th>Calc. mass (M+H)</th><th>Mass error (ppm)</th><th>Cleavage</th><th>Score</th><th>Delta</th><th>Delta Mod</th><th>Comment</th><th>Scan Time</th><th>Enzyme</th><th>Replicate</th><th>Page</th></tr>
<tr><td>BCAL0080</td><td>>tr|A0A1V2WZJ2|A0A1V2WZJ2_9BURK Class I cytochrome c OS=Burkholderia cenocepacia OX=95486 GN=A8E72_32710 PE=4 SV=1</td><td>Y.MANNDGANFPEPAAPAANAAQPAS[+568.212]GAPASGADASNAQA.A</td><td>HexNAc(2)Hex(1)</td><td>Yes</td><td>1345.2598</td><td>3</td><td>4033.7649</td><td>4033.7476</td><td>4.3</td><td>Non</td><td>661.09</td><td>592.27</td><td>106.51</td><td>Nsco_20191108_BC_ZIC_HILIC_H111_thermolysin_B3.42890.42890.3</td><td>84.7311</td><td>Thermolysin</td><td>B3</td><td>28</td></tr>
<tr><td>BCAL0080</td><td>>tr|A0A1V2WZJ2|A0A1V2WZJ2_9BURK Class I cytochrome c OS=Burkholderia cenocepacia OX=95486 GN=A8E72_32710 PE=4 SV=1</td><td>Y.MANNDGANFPEPAAPAANAAQPAS[+568.212]GAPASGADASNAQAA.A</td><td>HexNAc(2)Hex(1)</td><td>Yes</td><td>1369.2733</td><td>3</td><td>4105.8054</td><td>4104.7847</td><td>4.2</td><td>Non</td><td>466.66</td><td>430.12</td><td>35.3</td><td>Nsco_20191108_BC_ZIC_HILIC_H111_thermolysin_B3.43275.43275.3</td><td>85.4568</td><td>Thermolysin</td><td>B3</td><td>29</td></tr>
<tr><td>BCAL0080</td><td>>tr|A0A1V2WZJ2|A0A1V2WZJ2_9BURK Class I cytochrome c OS=Burkholderia cenocepacia OX=95486 GN=A8E72_32710 PE=4 SV=1</td><td>Y.MANNDGANFPEPAAPAANAAQPAS[+568.212][+100.064]GAPAS[+568.212]GADASNAQAAAA.M</td><td>HexNAc(2)Hex(1) 100.064,HexNAc(2)Hex(1)</td><td>No</td><td>1639.0439</td><td>3</td><td>4915.1171</td><td>4915.1345</td><td>-3.5</td><td>Non</td><td>268.13</td><td>247.1</td><td>2.69</td><td>Nsco_20191108_BC_ZIC_HILIC_H111_thermolysin_B3.44017.44017.3</td><td>86.817</td><td>Thermolysin</td><td>B3</td><td>30</td></tr>
<tr><td>BCAL0080</td><td>>tr|A0A1V2WZJ2|A0A1V2WZJ2_9BURK Class I cytochrome c OS=Burkholderia cenocepacia OX=95486 GN=A8E72_32710 PE=4 SV=1</td><td>R.AIVYMANNDGANFPEPAAPAANAAQPAS[+568.212]GAPAS[+568.212][+100.064]GADASNAQAAAAMAAIAAIPK.A</td><td>HexNAc(2)Hex(1), HexNAc(2)Hex(1) 100.064</td><td>No</td><td>1557.7234</td><td>4</td><td>6227.8717</td><td>6227.8922</td><td>-3.3</td><td>Specific</td><td>806.09</td><td>806.09</td><td>0</td><td>Nsco_20191108_BC_ZIC_HILIC_H111_typsin_B2.71129.71129.4</td><td>140.2105</td><td>Trypsin</td><td>B2</td><td>31</td></tr>
<tr><td>BCAL0080</td><td>>tr|A0A1V2WZJ2|A0A1V2WZJ2_9BURK Class I cytochrome c OS=Burkholderia cenocepacia OX=95486 GN=A8E72_32710 PE=4 SV=1</td><td>A.IVYMANNDGANFPEPAAPAANAAQPAS[+568.212][+100.064]GAPAS[+568.212]GADASNAQAAAAMAAIAAIPK.A</td><td>HexNAc(2)Hex(1) 100.064,HexNAc(2)Hex(1)</td><td>No</td><td>1540.4741</td><td>4</td><td>6158.8747</td><td>6156.8551</td><td>2.1</td><td>NRagged</td><td>464.47</td><td>464.47</td><td>0</td><td>Nsco_20191108_BC_ZIC_HILIC_H111_typsin_B3.66894.66894.4</td><td>139.2643</td><td>Trypsin</td><td>B3</td><td>32</td></tr>
<tr><td>BCAL0163</td><td>>tr|A0A144S4M8|A0A144S4M8_9BURK BON domain protein OS=Burkholderia cenocepacia OX=95486 GN=A8E72_32405 PE=4 SV=1</td><td>A.LQDASPASGAS[+568.212]GAQAAAAPADN.A</td><td>HexNAc(2)Hex(1)</td><td>No</td><td>1255.0607</td><td>2</td><td>2509.1141</td><td>2509.106</td><td>3.2</td><td>Non</td><td>554.16</td><td>266.54</td><td>0</td><td>Nsco_20191108_BC_ZIC_HILIC_H111_pepsin_B1.22640.22640.2</td><td>48.7976</td><td>Pepsin</td><td>B1</td><td>33</td></tr>
<tr><td>BCAL0163</td><td>>tr|A0A144S4M8|A0A144S4M8_9BURK BON domain protein OS=Burkholderia cenocepacia OX=95486 GN=A8E72_32405 PE=4 SV=1</td><td>S.VQSTPLQPPAPISNSS[+568.212]SVHPGNPKAKAQ.-</td><td>HexNAc(2)Hex(1)</td><td>Yes</td><td>852.1834</td><td>4</td><td>3405.7117</td><td>3405.702</td><td>2.8</td><td>Non</td><td>783.02</td><td>702.53</td><td>22.83</td><td>Nsco_20191108_BC_ZIC_HILIC_H111_pepsin_B2.21640.21640.4</td><td>45.2027</td><td>Pepsin</td><td>B2</td><td>34</td></tr>
<tr><td>BCAL0163</td><td>>tr|A0A144S4M8|A0A144S4M8_9BURK BON domain protein OS=Burkholderia cenocepacia OX=95486 GN=A8E72_32405 PE=4 SV=1</td><td>A.SVQSTPLQPPAPISNS[+568.212]SSVHPGNPKAKAQ.-</td><td>HexNAc(2)Hex(1)</td><td>No</td><td>873.9426</td><td>4</td><td>3492.7486</td><td>3492.734</td><td>4.2</td><td>Non</td><td>404.08</td><td>404.08</td><td>0</td><td>Nsco_20191108_BC_ZIC_HILIC_H111_pepsin_B3.22640.22640.4</td><td>46.758</td><td>Pepsin</td><td>B3</td><td>35</td></tr>
<tr><td>BCAL0163</td><td>>tr|A0A144S4M8|A0A144S4M8_9BURK BON domain protein OS=Burkholderia cenocepacia OX=95486 GN=A8E72_32405 PE=4 SV=1</td><td>A.LQDASPASGASGAQAAAAPADNATVGAVPDASVQSTPLQPPAPIS[+568.212]NSSSVHPGNPKAKAQ.-</td><td>HexNAc(2)Hex(1)</td><td>No</td><td>1549.7634</td><td>4</td><td>6196.0319</td><td>6196.0076</td><td>3.9</td><td>Non</td><td>830.97</td><td>830.97</td><td>0</td><td>Nsco_20191108_BC_ZIC_HILIC_H111_thermolysin_B1.44267.44267.4</td><td>83.5595</td><td>Thermolysin</td><td>B1</td><td>36</td></tr>
<tr><td>BCAL0163</td><td>>tr|A0A144S4M8|A0A144S4M8_9BURK BON domain protein OS=Burkholderia cenocepacia OX=95486 GN=A8E72_32405 PE=4 SV=1</td><td>Y.VKPQDAQALQDASPASGASGAQAAAAPADNAT[+568.212]VGAVPDASVQSTPLQPPAP.I</td><td>HexNAc(2)Hex(1)</td><td>No</td><td>1778.5364</td><td>3</td><td>5333.5946</td><td>5330.567</td><td>3.3</td><td>Non</td><td>148.88</td><td>148.88</td><td>0</td><td>Nsco_20191108_BC_ZIC_HILIC_H111_thermolysin_B1.57277.57277.3</td><td>107.1961</td><td>Thermolysin</td><td>B1</td><td>37</td></tr>
</table>

BCAL0163	>tr|A0A144S4M8|A0A144S4M8_9BURK BON domain protein OS=Burkholderia cenocepacia OX=95486 GN=A8E72_32405 PE=4 SV=1	Y.VKPQDAQALQDASPAS[+568.212]GASGAQA.A	HexNAc(2)Hex(1)	No	1368.6446	2	2736.2819	2736.2694	4.6	Non	539.84	480.2	4.11	Nsco_20191108_BC_ZIC_HILIC_H111_thermolysin_B2.25007.25007.2	52.3677	Thermolysin	B2	38
BCAL0163	>tr|A0A144S4M8|A0A144S4M8_9BURK BON domain protein OS=Burkholderia cenocepacia OX=95486 GN=A8E72_32405 PE=4 SV=1	A.LQDASPASGAS[+568.212]GAQAAAAPADNAT.V	HexNAc(2)Hex(1)	No	1341.606	2	2682.2047	2681.1908	3.9	Non	545.39	207.92	2.4	Nsco_20191108_BC_ZIC_HILIC_H111_thermolysin_B2.26277.26277.2	54.5842	Thermolysin	B2	39
BCAL0163	>tr|A0A144S4M8|A0A144S4M8_9BURK BON domain protein OS=Burkholderia cenocepacia OX=95486 GN=A8E72_32405 PE=4 SV=1	A.LQDASPASGAS[+568.212]GAQAAAAPADNATVGAVPDASVQSTP.L	HexNAc(2)Hex(1)	Yes	1297.2759	3	3889.8133	3889.7945	4.8	Non	260.83	253.64	45.47	Nsco_20191108_BC_ZIC_HILIC_H111_thermolysin_B2.46669.46669.3	92.8997	Thermolysin	B2	40
BCAL0163	>tr|A0A144S4M8|A0A144S4M8_9BURK BON domain protein OS=Burkholderia cenocepacia OX=95486 GN=A8E72_32405 PE=4 SV=1	Y.VKPQDAQALQDAS[+568.212][+100.064]PASGASGAQAAAAPADNA.T	HexNAc(2)Hex(1) 100.064	No	1759.8258	2	3518.6443	3517.6416	-0.2	Non	276.03	115.52	2.12	Nsco_20191108_BC_ZIC_HILIC_H111_thermolysin_B3.32267.32267.2	64.9265	Thermolysin	B3	41
BCAL0163	>tr|A0A144S4M8|A0A144S4M8_9BURK BON domain protein OS=Burkholderia cenocepacia OX=95486 GN=A8E72_32405 PE=4 SV=1	Y.VKPQDAQALQDASPAS[+568.212]GASGAQ.A	HexNAc(2)Hex(1)	No	1333.1246	2	2665.242	2665.2323	3.6	Non	494.43	461.19	4.68	Nsco_20191108_BC_ZIC_HILIC_H111_thermolysin_B3.23059.23059.2	49.2567	Thermolysin	B3	42
BCAL0163	>tr|A0A144S4M8|A0A144S4M8_9BURK BON domain protein OS=Burkholderia cenocepacia OX=95486 GN=A8E72_32405 PE=4 SV=1	Y.VKPQDAQALQDASPAS[+568.212]GASGAQAAAAPADNAT.V	HexNAc(2)Hex(1)	No	1759.825	2	3518.6428	3518.6253	5	Non	579.7	300.08	3.43	Nsco_20191108_BC_ZIC_HILIC_H111_thermolysin_B3.32243.32243.2	64.8849	Thermolysin	B3	43
BCAL0163	>tr|A0A144S4M8|A0A144S4M8_9BURK BON domain protein OS=Burkholderia cenocepacia OX=95486 GN=A8E72_32405 PE=4 SV=1	S.GASGAQAAAAPADNATVGAVPDASVQST[+568.212][+100.064]PLQPPAPIS[+568.212][+100.064]NSSSVHPGNPKAKAQ.-	HexNAc(2)Hex(1) 100.064,HexNAc(2)Hex(1) 100.064	No	1549.5074	4	6195.008	6194.9866	3.5	Non	457.38	457.38	0	Nsco_20191108_BC_ZIC_HILIC_H111_thermolysin_B3.42433.42433.4	83.785	Thermolysin	B3	44
BCAL0163	>tr|A0A144S4M8|A0A144S4M8_9BURK BON domain protein OS=Burkholderia cenocepacia OX=95486 GN=A8E72_32405 PE=4 SV=1	Y.VKPQDAQALQDASPASGAS[+568.212]GAQAAAAPADNATVGAVPDASVQSTPLQPPAPISNSSSVHPGNPK.A	HexNAc(2)Hex(1)	Yes	1659.8156	4	6636.2404	6635.2143	3.4	NRagged	901.28	550.13	85.05	Nsco_20191108_BC_ZIC_HILIC_H111_typsin_B2.47144.47144.4	91.2822	Trypsin	B2	45
BCAL0163	>tr|A0A144S4M8|A0A144S4M8_9BURK BON domain protein OS=Burkholderia cenocepacia OX=95486 GN=A8E72_32405 PE=4 SV=1	K.PQDAQALQDASPASGASGAQAAAAPADNATVGAVPDASVQST[+568.212]PLQPPAPISNSSSVHPGNPK.A	HexNAc(2)Hex(1)	No	1603.0234	4	6409.0719	6408.051	2.7	Specific	1091.36	1068.39	0	Nsco_20191108_BC_ZIC_HILIC_H111_typsin_B2.50885.50885.4	97.7338	Trypsin	B2	46
BCAL0163	>tr|A0A144S4M8|A0A144S4M8_9BURK BON domain protein OS=Burkholderia cenocepacia OX=95486 GN=A8E72_32405 PE=4 SV=1	V.KPQDAQALQDASPASGAS[+568.212]GAQAAAAPADNAT[+568.212][+100.064]VGAVPDASVQST[+568.212]PLQPPAPISNSSSVHPGNPK.A	HexNAc(2)Hex(1), HexNAc(2)Hex(1) 100.064,HexNAc(2)Hex(1)	No	1297.1181	6	7777.6722	7772.6331	2.9	NRagged	287.53	287.53	0	Nsco_20191108_BC_ZIC_HILIC_H111_typsin_B2.51562.51562.6	98.9045	Trypsin	B2	47
BCAL0163	>tr|A0A144S4M8|A0A144S4M8_9BURK BON domain protein OS=Burkholderia cenocepacia OX=95486 GN=A8E72_32405 PE=4 SV=1	V.FQYVKPQDAQALQDAS[+568.212]PASGASGAQAAAAPADNATVGAVPDASVQSTPLQPPAPISNSSSVHPGNPK.A	HexNAc(2)Hex(1)	No	1769.8647	4	7076.4372	7073.4047	3.2	NRagged	590.81	590.81	1.2	Nsco_20191108_BC_ZIC_HILIC_H111_typsin_B2.51800.51800.4	99.3283	Trypsin	B2	48
BCAL0163	>tr|A0A144S4M8|A0A144S4M8_9BURK BON domain protein OS=Burkholderia cenocepacia OX=95486 GN=A8E72_32405 PE=4 SV=1	K.VFQYVKPQDAQALQDASPASGASGAQAAAAPADNATVGAVPDASVQST[+568.212]PLQPPAPISNSSSVHPGNPK.A	HexNAc(2)Hex(1)	No	1793.8806	4	7172.5005	7172.4731	3.8	Specific	1198.94	1198.94	0	Nsco_20191108_BC_ZIC_HILIC_H111_typsin_B2.54057.54057.4	103.675	Trypsin	B2	49

BCAL0163	>tr|A0A144S4M8|A0A144S4M8_9BURK BON domain protein OS=Burkholderia cenocepacia OX=95486 GN=A8E72_32405 PE=4 SV=1	V.KVFQYVKPQDAQALQDASPASGAS[+568.212]GAQAAAAPADNATVGAVPDASVQSTPLQPPAPISNSSSVHPGNPK.A	HexNAc(2)Hex(1)	Yes	1460.9267	5	7300.6043	7300.568	5	NRagged	1341.58	1341.58	22.45	Nsco_20191108_BC_ZIC_HILIC_H111_typsin_B3.45311.45311.5	93.2228	Trypsin	B3	50
BCAL0193	>tr|A0A071MDE2|A0A071MDE2_9BURK Uncharacterized protein OS=Burkholderia cenocepacia OX=95486 GN=A8E72_00390 PE=4 SV=1	Q.LKHHGSKKGQAKAAAAS[+568.212]AAGTNDAGTQN.-	HexNAc(2)Hex(1)	Yes	652.5254	5	3258.5977	3258.5833	4.4	Non	882.97	882.97	102.81	Nsco_20191108_BC_ZIC_HILIC_H111_thermolysin_B1.2860.2860.5	16.0864	Thermolysin	B1	51
BCAL0193	>tr|A0A071MDE2|A0A071MDE2_9BURK Uncharacterized protein OS=Burkholderia cenocepacia OX=95486 GN=A8E72_00390 PE=4 SV=1	P.AADTSAAAPAPAKKDHS[+568.212][+100.064]KPKHQ.L	HexNAc(2)Hex(1) 100.064	No	966.1519	3	2896.4413	2895.4334	1.6	Non	360.56	293.32	9.6	Nsco_20191108_BC_ZIC_HILIC_H111_thermolysin_B3.5940.5940.3	21.4368	Thermolysin	B3	52
BCAL0193	>tr|A0A071MDE2|A0A071MDE2_9BURK Uncharacterized protein OS=Burkholderia cenocepacia OX=95486 GN=A8E72_00390 PE=4 SV=1	Q.ASAPAADT[+568.212]SAAAPAPAKK.D	HexNAc(2)Hex(1)	No	1082.5309	2	2164.0546	2164.0503	2	NRagged	769.06	615.58	0	Nsco_20191108_BC_ZIC_HILIC_H111_typsin_B1.6903.6903.2	22.6365	Trypsin	B1	53
BCAL0272	>tr|A0A142PJB0|A0A142PJB0_9BURK Lipoprotein OS=Burkholderia cenocepacia OX=95486 GN=A8E72_20920 PE=4 SV=1	K.PIQPQNTPPSDVKPTDENASSDESPDTSGSPLTLSPELSSTSTMPPAS[+568.212]GPAATK.-	HexNAc(2)Hex(1)	Yes	1515.9615	4	6060.8244	6058.798	3.2	Specific	944.57	888.07	96.92	Nsco_20191108_BC_ZIC_HILIC_H111_typsin_B2.51007.51007.4	97.9364	Trypsin	B2	54
BCAL0303	>tr|A0A144S0T9|A0A144S0T9_9BURK Outer membrane lipid asymmetry maintenance protein MlaD OS=Burkholderia cenocepacia OX=95486 GN=yrbD PE=4 SV=1	F.LYS[+568.212]KAADAGGAKPAAGASAAPAAPAPVAV.P	HexNAc(2)Hex(1)	No	1030.5266	3	3089.5654	3089.5525	4.2	CRagged	222.88	189.14	2.91	Nsco_20191108_BC_ZIC_HILIC_H111_pepsin_B1.28060.28060.3	59.3181	Pepsin	B1	55
BCAL0303	>tr|A0A144S0T9|A0A144S0T9_9BURK Outer membrane lipid asymmetry maintenance protein MlaD OS=Burkholderia cenocepacia OX=95486 GN=yrbD PE=4 SV=1	L.YSKAADAGGAKPAAGAS[+568.212]AAPAAPAPVAVPASAVSGSAGQ.-	HexNAc(2)Hex(1)	No	1296.9756	3	3888.9124	3888.8985	3.6	CRagged	785.85	782.53	7.13	Nsco_20191108_BC_ZIC_HILIC_H111_pepsin_B1.31112.31112.3	65.6963	Pepsin	B1	56
BCAL0303	>tr|A0A144S0T9|A0A144S0T9_9BURK Outer membrane lipid asymmetry maintenance protein MlaD OS=Burkholderia cenocepacia OX=95486 GN=yrbD PE=4 SV=1	S.KAADAGGAKPAAGAS[+568.212]AAPAAPAPVAV.P	HexNAc(2)Hex(1)	Yes	909.4655	3	2726.3819	2726.3731	3.2	Non	741.46	731.06	731.06	Nsco_20191108_BC_ZIC_HILIC_H111_pepsin_B2.23564.23564.3	48.9597	Pepsin	B2	57
BCAL0303	>tr|A0A144S0T9|A0A144S0T9_9BURK Outer membrane lipid asymmetry maintenance protein MlaD OS=Burkholderia cenocepacia OX=95486 GN=yrbD PE=4 SV=1	Y.SKAADAGGAKPAAGAS[+568.212]AAPAAPAPVAV.P	HexNAc(2)Hex(1)	No	938.4753	3	2813.4114	2813.4051	2.2	Non	621.17	600	47.88	Nsco_20191108_BC_ZIC_HILIC_H111_pepsin_B2.23611.23611.3	49.0427	Pepsin	B2	58
BCAL0303	>tr|A0A144S0T9|A0A144S0T9_9BURK Outer membrane lipid asymmetry maintenance protein MlaD OS=Burkholderia cenocepacia OX=95486 GN=yrbD PE=4 SV=1	L.YSKAADAGGAKPAAGAS[+568.212]AAPAAPAPVAV.P	HexNAc(2)Hex(1)	Yes	744.8754	4	2976.48	2976.4684	3.9	CRagged	843.54	806.59	509.24	Nsco_20191108_BC_ZIC_HILIC_H111_pepsin_B3.25168.25168.4	51.9446	Pepsin	B3	59
BCAL0303	>tr|A0A144S0T9|A0A144S0T9_9BURK Outer membrane lipid asymmetry maintenance protein MlaD OS=Burkholderia cenocepacia OX=95486 GN=yrbD PE=4 SV=1	F.LYSKAADAGGAKPAAGAS[+568.212]AAPAAPAPVAVPASAVSGSAGQ.-	HexNAc(2)Hex(1)	No	1334.671	3	4001.9983	4001.9826	3.9	CRagged	752	752	18	Nsco_20191108_BC_ZIC_HILIC_H111_thermolysin_B1.36046.36046.3	69.1968	Thermolysin	B1	60

BCAL0303	>tr|A0A144S0T9|A0A144S0T9_9BURK Outer membrane lipid asymmetry maintenance protein MlaD OS=Burkholderia cenocepacia OX=95486 GN=yrbD PE=4 SV=1	K.AADAGGAKPAAGASAAPAAPAPVAVPAS[+568.212]AVSGSAGQ.-	HexNAc(2)Hex(1)	No	1755.8662	2	3510.7251	3510.7082	4.8	CRagged	800.05	686.18	1.17	Nsco_20191108_BC_ZIC_HILIC_H111_typsin_B1.36885.36885.2	74.4191	Trypsin	B1	61
BCAL0303	>tr|A0A144S0T9|A0A144S0T9_9BURK Outer membrane lipid asymmetry maintenance protein MlaD OS=Burkholderia cenocepacia OX=95486 GN=yrbD PE=4 SV=1	A.ADAGGAKPAAGAS[+568.212][+100.064]AAPAAPAPVAVPASAVSGSAGQ.-	HexNAc(2)Hex(1) 100.064	No	1181.246	3	3541.7234	3539.7351	-5.2	Non	279.51	279.51	13.16	Nsco_20191108_BC_ZIC_HILIC_H111_typsin_B2.39186.39186.3	77.2324	Trypsin	B2	62
BCAL0303	>tr|A0A144S0T9|A0A144S0T9_9BURK Outer membrane lipid asymmetry maintenance protein MlaD OS=Burkholderia cenocepacia OX=95486 GN=yrbD PE=4 SV=1	K.PAAGASAAPAAPAPVAVPAS[+568.212][+100.064]AVS[+568.212]GSAGQ.-	HexNAc(2)Hex(1) 100.064,HexNAc(2)Hex(1)	Yes	1180.2254	3	3538.6616	3537.6705	-3.4	CRagged	247.45	206.29	1.76	Nsco_20191108_BC_ZIC_HILIC_H111_typsin_B2.42457.42457.3	82.9779	Trypsin	B2	63
BCAL0304	>tr|A0A2N9CYS8|A0A2N9CYS8_9BURK Surface lipoprotein OS=Burkholderia cenocepacia OX=95486 GN=F01_50090 PE=4 SV=1	A.PASGTAESPNPAS[+568.212]ET[+568.212]NV.P	HexNAc(2)Hex(1), HexNAc(2)Hex(1)	No	1383.091	2	2765.1747	2765.163	4.2	Non	683.52	496.16	9.94	Nsco_20191108_BC_ZIC_HILIC_H111_pepsin_B1.21155.21155.2	45.8986	Pepsin	B1	64
BCAL0304	>tr|A0A2N9CYS8|A0A2N9CYS8_9BURK Surface lipoprotein OS=Burkholderia cenocepacia OX=95486 GN=F01_50090 PE=4 SV=1	A.VAAPASGTAESPNPAS[+568.212]ET[+568.212]NV.P	HexNAc(2)Hex(1), HexNAc(2)Hex(1)	No	1002.7762	3	3006.3141	3006.3056	2.8	Non	549.86	421.72	8.83	Nsco_20191108_BC_ZIC_HILIC_H111_pepsin_B1.26846.26846.3	56.911	Pepsin	B1	65
BCAL0304	>tr|A0A2N9CYS8|A0A2N9CYS8_9BURK Surface lipoprotein OS=Burkholderia cenocepacia OX=95486 GN=F01_50090 PE=4 SV=1	A.PASGTAESPNPAS[+568.212][+100.064]ET[+568.212]NVPA.M	HexNAc(2)Hex(1) 100.064,HexNAc(2)Hex(1)	No	1518.1598	2	3035.3124	3033.3169	-3.7	Non	314.5	153.64	8.22	Nsco_20191108_BC_ZIC_HILIC_H111_pepsin_B2.29530.29530.2	60.8298	Pepsin	B2	66
BCAL0304	>tr|A0A2N9CYS8|A0A2N9CYS8_9BURK Surface lipoprotein OS=Burkholderia cenocepacia OX=95486 GN=F01_50090 PE=4 SV=1	A.VAAPASGTAESPNPAS[+568.212]ET[+568.212]NVPAM.Q	HexNAc(2)Hex(1), HexNAc(2)Hex(1)	No	1102.4886	3	3305.4511	3305.436	4.6	Non	480.43	256.57	2.4	Nsco_20191108_BC_ZIC_HILIC_H111_pepsin_B3.35544.35544.3	76.6557	Pepsin	B3	67
BCAL0304	>tr|A0A2N9CYS8|A0A2N9CYS8_9BURK Surface lipoprotein OS=Burkholderia cenocepacia OX=95486 GN=F01_50090 PE=4 SV=1	A.PASGTAESPNPAS[+568.212]ET[+568.212]NVPAMQ.V	HexNAc(2)Hex(1), HexNAc(2)Hex(1)	No	1064.792	3	3192.3615	3192.3519	3	Non	334.39	334.39	1.2	Nsco_20191108_BC_ZIC_HILIC_H111_pepsin_B3.31204.31204.3	65.5756	Pepsin	B3	68
BCAL0304	>tr|A0A2N9CYS8|A0A2N9CYS8_9BURK Surface lipoprotein OS=Burkholderia cenocepacia OX=95486 GN=F01_50090 PE=4 SV=1	A.VAAPASGTAESPNPAS[+568.212]ET[+568.212]NVPA.M	HexNAc(2)Hex(1), HexNAc(2)Hex(1)	No	1587.7082	2	3174.4091	3174.3955	4.3	Non	472.31	305.68	6.36	Nsco_20191108_BC_ZIC_HILIC_H111_thermolysin_B2.30194.30194.2	61.2273	Thermolysin	B2	69
BCAL0304	>tr|A0A2N9CYS8|A0A2N9CYS8_9BURK Surface lipoprotein OS=Burkholderia cenocepacia OX=95486 GN=F01_50090 PE=4 SV=1	A.VAAPASGTAES[+568.212][+100.064]PNPAS[+568.212]ETNVP.A	HexNAc(2)Hex(1) 100.064,HexNAc(2)Hex(1)	No	1602.2084	2	3203.4095	3203.4224	-4	Non	349.49	208.59	3.63	Nsco_20191108_BC_ZIC_HILIC_H111_thermolysin_B2.32625.32625.2	65.2288	Thermolysin	B2	70
BCAL0304	>tr|A0A2N9CYS8|A0A2N9CYS8_9BURK Surface lipoprotein OS=Burkholderia cenocepacia OX=95486 GN=F01_50090 PE=4 SV=1	A.APASGTAESPNPAS[+568.212][+100.064]ET[+568.212][+100.064]NVPAMQ.V	HexNAc(2)Hex(1) 100.064,HexNAc(2)Hex(1) 100.064	No	1732.26	2	3463.5127	3463.517	-1.2	Non	338.9	87.16	2.4	Nsco_20191108_BC_ZIC_HILIC_H111_thermolysin_B2.38282.38282.2	75.6943	Thermolysin	B2	71
BCAL0304	>tr|A0A2N9CYS8|A0A2N9CYS8_9BURK Surface lipoprotein OS=Burkholderia cenocepacia OX=95486 GN=F01_50090 PE=4 SV=1	A.VAAPASGT[+568.212]AES[+568.212]PNPASETNVPAMQ.V	HexNAc(2)Hex(1), HexNAc(2)Hex(1)	No	1717.2554	2	3433.5036	3433.4946	2.6	Non	482.57	394.87	2.47	Nsco_20191108_BC_ZIC_HILIC_H111_thermolysin_B2.36740.36740.2	72.7165	Thermolysin	B2	72

BCAL0304	>tr|A0A2N9CYS8|A0A2N9CYS8_9BURK Surface lipoprotein OS=Burkholderia cenocepacia OX=95486 GN=F01_50090 PE=4 SV=1	L.PEDGAAPAAAGAAGT[+568.212][+100.064]AGAAAVGGAAPAGAAVAAPASGT[+568.212]AES[+568.212]PNPASETNVPAMQVAPPSPGGFRFPSIR.L	HexNAc(2)Hex(1)100.064,HexNAc(2)Hex(1),HexNAc(2)Hex(1)	No	1589.3426	5	7942.6838	7938.7048	-4.3	NRagged	316.83	316.83	0	Nsco_20191108_BC_ZIC_HILIC_H111_typsin_B2.65020.65020.5	126.0041	Trypsin	B2	73
BCAL0332	>tr|A0A2N9CYY5|A0A2N9CYY5_9BURK ClpXP protease specificity-enhancing factor OS=Burkholderia cenocepacia OX=95486 GN=sspB PE=4 SV=1	E.DSGAFDDEQADDAQRDES[+568.212]VSPLAPVADSGANEEPSEGADEPPKTDGDGSK.G	HexNAc(2)Hex(1)	No	1412.1012	4	5645.383	5643.3656	1.9	NRagged	572.55	572.55	0	Nsco_20191108_BC_ZIC_HILIC_H111_typsin_B2.43714.43714.4	85.2165	Trypsin	B2	74
BCAL0332	>tr|A0A2N9CYY5|A0A2N9CYY5_9BURK ClpXP protease specificity-enhancing factor OS=Burkholderia cenocepacia OX=95486 GN=sspB PE=4 SV=1	A.DDAQRDESVSPLAPVADS[+568.212]GANEEPSEGADEPPKTDGDGSK.G	HexNAc(2)Hex(1)	No	1153.5083	4	4611.0115	4607.9875	3	NRagged	634.53	634.53	1.2	Nsco_20191108_BC_ZIC_HILIC_H111_typsin_B3.35283.35283.4	73.8531	Trypsin	B3	75
BCAL0340	>tr|A0A1V2W5P4|A0A1V2W5P4_9BURK Uncharacterized protein OS=Burkholderia cenocepacia OX=95486 GN=A8E72_11900 PE=4 SV=1	K.AAAAPAAEAAASAAPT[+568.212]PPAAQK.G	HexNAc(2)Hex(1)	No	1236.6064	2	2472.2055	2472.1988	2.7	Specific	817.86	747.77	19.34	Nsco_20191108_BC_ZIC_HILIC_H111_typsin_B1.21382.21382.2	47.1614	Trypsin	B1	76
BCAL0349 (typ	>tr|A0A2N9D2R1|A0A2N9D2R1_9BURK Outer membrane protein OS=Burkholderia cenocepacia OX=95486 GN=F01_70026 PE=3 SV=1	T.TALPQANPNAGGAS[+568.212]GTVVHGTAGTLTPPPANAAPGQ.V	HexNAc(2)Hex(1)	Yes	1277.9608	3	3831.8677	3831.8519	4.1	Non	548.84	334.26	39.38	Nsco_20191108_BC_ZIC_HILIC_H111_pepsin_B1.36350.36350.3	77.6999	Pepsin	B1	77
BCAL0349 (typ	>tr|A0A2N9D2R1|A0A2N9D2R1_9BURK Outer membrane protein OS=Burkholderia cenocepacia OX=95486 GN=F01_70026 PE=3 SV=1	L.PQANPNAGGASGTVVHGT[+568.212]AGTLTPPPANAAPGQ.V	HexNAc(2)Hex(1)	No	1182.9037	3	3546.6967	3546.6831	3.8	CRagged	556.85	505.61	3.6	Nsco_20191108_BC_ZIC_HILIC_H111_pepsin_B2.30910.30910.3	63.7214	Pepsin	B2	78
BCAL0349 (typ	>tr|A0A2N9D2R1|A0A2N9D2R1_9BURK Outer membrane protein OS=Burkholderia cenocepacia OX=95486 GN=F01_70026 PE=3 SV=1	A.TTALPQANPNAGGASGT[+568.212]VVHGTAGTLTPPPANAAPGQ.V	HexNAc(2)Hex(1)	No	1311.6434	3	3932.9155	3932.8996	4.1	Non	327.12	308.22	1.2	Nsco_20191108_BC_ZIC_HILIC_H111_pepsin_B2.37363.37363.3	78.598	Pepsin	B2	79
BCAL0349 (typ	>tr|A0A2N9D2R1|A0A2N9D2R1_9BURK Outer membrane protein OS=Burkholderia cenocepacia OX=95486 GN=F01_70026 PE=3 SV=1	T.ALPQANPNAGGAS[+568.212]GTVVHGTAGTLTPPPANAAPGQVVVGGKVPDEATKA.A	HexNAc(2)Hex(1)	Yes	1271.3992	4	5082.5749	5081.5549	3.3	Non	1019.7	1019.7	77.15	Nsco_20191108_BC_ZIC_HILIC_H111_thermolysin_B1.41139.41139.4	78.3066	Thermolysin	B1	80
BCAL0349 (typ	>tr|A0A2N9D2R1|A0A2N9D2R1_9BURK Outer membrane protein OS=Burkholderia cenocepacia OX=95486 GN=F01_70026 PE=3 SV=1	T.ALPQANPNAGGAS[+568.212][+100.064]GTVVHGTAGTLTPPPANAAPGQVVVGGKVPDEATKAAVLQK.L	HexNAc(2)Hex(1) 100.064	Yes	1145.1952	5	5721.9468	5720.9621	-3.2	Non	803.05	467.38	82.36	Nsco_20191108_BC_ZIC_HILIC_H111_thermolysin_B1.44302.44302.5	83.627	Thermolysin	B1	81
BCAL0349 (typ	>tr|A0A2N9D2R1|A0A2N9D2R1_9BURK Outer membrane protein OS=Burkholderia cenocepacia OX=95486 GN=F01_70026 PE=3 SV=1	T.VATTALPQANPNAGGAS[+568.212]GTVVHGTAGTLTPPPANAAPGQVVVGGKVPDEATKAAVLQK.L	HexNAc(2)Hex(1)	No	1199.4313	5	5993.1273	5993.099	4.7	Non	896.04	896.04	29.65	Nsco_20191108_BC_ZIC_HILIC_H111_thermolysin_B1.45401.45401.5	85.4774	Thermolysin	B1	82
BCAL0349 (typ	>tr|A0A2N9D2R1|A0A2N9D2R1_9BURK Outer membrane protein OS=Burkholderia cenocepacia OX=95486 GN=F01_70026 PE=3 SV=1	T.VATTALPQANPNAGGAS[+568.212][+100.064]GTVVHGTAGTLTPPPANAAPGQVVVGGKVPDEATKAA.V	HexNAc(2)Hex(1) 100.064	No	1406.964	4	5624.8341	5624.8569	-4.1	Non	832.16	832.16	76.86	Nsco_20191108_BC_ZIC_HILIC_H111_thermolysin_B1.45794.45794.4	86.1307	Thermolysin	B1	83
BCAL0349 (typ	>tr|A0A2N9D2R1|A0A2N9D2R1_9BURK Outer membrane protein OS=Burkholderia cenocepacia OX=95486 GN=F01_70026 PE=3 SV=1	A.TVTPVGNGTVATTALPQANPNAGGASGTVVHGT[+568.212]AGTLTPPPANAAPGQVVVGGKVPDEATKA.A	HexNAc(2)Hex(1)	No	1571.046	4	6281.1623	6280.1743	-2.5	Non	717.57	717.57	0	Nsco_20191108_BC_ZIC_HILIC_H111_thermolysin_B1.52394.52394.4	97.6529	Thermolysin	B1	84

BCAL0349 (typ	>tr|A0A2N9D2R1|A0A2N9D2R1_9BURK Outer membrane protein OS=Burkholderia cenocepacia OX=95486 GN=F01_70026 PE=3 SV=1	T.VTPVGNGTVATTALPQANPNAGGASGTVVHGTAGTLT[+568.212][+100.064]PPPANAAPGQVVVGGKVPDEATKAAVLQK.L	HexNAc(2)Hex(1) 100.064	No	1365.1086	5	6821.5141	6818.5338	-4.4	Non	905.81	176.65	0	Nsco_20191108_BC_ZIC_HILIC_H111_thermolysin_B1.50091.50091.5	93.6016	Thermolysin	B1	85
BCAL0349 (typ	>tr|A0A2N9D2R1|A0A2N9D2R1_9BURK Outer membrane protein OS=Burkholderia cenocepacia OX=95486 GN=F01_70026 PE=3 SV=1	T.VTPVGNGTVATTALPQANPNAGGAS[+568.212]GTVVHGTAGTLTPPPANAAPGQVVVGGKVPDEATKA.A	HexNAc(2)Hex(1)	No	1545.5436	4	6179.1525	6179.1266	4.2	Non	947.6	947.6	0	Nsco_20191108_BC_ZIC_HILIC_H111_thermolysin_B1.48566.48566.4	91.0348	Thermolysin	B1	86
BCAL0349 (typ	>tr|A0A2N9D2R1|A0A2N9D2R1_9BURK Outer membrane protein OS=Burkholderia cenocepacia OX=95486 GN=F01_70026 PE=3 SV=1	T.TALPQANPNAGGAS[+568.212]GTVVHGTAGTLTPPPANAAPGQVVVGGKVPDEATKA.A	HexNAc(2)Hex(1)	Yes	1297.1617	4	5185.6251	5182.6026	2.4	Non	851.32	245.93	84.48	Nsco_20191108_BC_ZIC_HILIC_H111_thermolysin_B1.42189.42189.4	80.0561	Thermolysin	B1	87
BCAL0349 (typ	>tr|A0A2N9D2R1|A0A2N9D2R1_9BURK Outer membrane protein OS=Burkholderia cenocepacia OX=95486 GN=F01_70026 PE=3 SV=1	T.VATTALPQANPNAGGAS[+568.212]GTVVHGTAGTLTPPPANAAPGQVVVGGKVPDEATKA.A	HexNAc(2)Hex(1)	Yes	1364.1989	4	5453.7736	5453.7558	3.3	Non	1069.56	1032.4	116.78	Nsco_20191108_BC_ZIC_HILIC_H111_thermolysin_B1.43972.43972.4	83.1087	Thermolysin	B1	88
BCAL0349 (typ	>tr|A0A2N9D2R1|A0A2N9D2R1_9BURK Outer membrane protein OS=Burkholderia cenocepacia OX=95486 GN=F01_70026 PE=3 SV=1	T.VATTALPQANPNAGGAS[+568.212]GTVVHGTAGTLTPPPAN.A	HexNAc(2)Hex(1)	Yes	1227.2776	3	3679.8184	3678.7981	4.6	Non	367.42	345.16	47.16	Nsco_20191108_BC_ZIC_HILIC_H111_thermolysin_B2.39792.39792.3	78.7213	Thermolysin	B2	89
BCAL0349 (typ	>tr|A0A2N9D2R1|A0A2N9D2R1_9BURK Outer membrane protein OS=Burkholderia cenocepacia OX=95486 GN=F01_70026 PE=3 SV=1	A.LPQANPNAGGASGTVVHGT[+568.212][+100.064]AGTLTPPPANAAPGQVVVGGKVPDEATKA.A	HexNAc(2)Hex(1) 100.064	No	1278.6484	4	5111.5719	5110.5818	-2.6	Non	335.82	323.31	0	Nsco_20191108_BC_ZIC_HILIC_H111_thermolysin_B2.40376.40376.4	79.8677	Thermolysin	B2	90
BCAL0349 (typ	>tr|A0A2N9D2R1|A0A2N9D2R1_9BURK Outer membrane protein OS=Burkholderia cenocepacia OX=95486 GN=F01_70026 PE=3 SV=1	T.VTPVGNGTVATTALPQANPNAGGAS[+568.212]GTVVHGTAGTLTPPPAN.A	HexNAc(2)Hex(1)	No	1469.0619	3	4405.1711	4404.1689	-0.3	Non	299.6	138.55	2.4	Nsco_20191108_BC_ZIC_HILIC_H111_thermolysin_B2.46162.46162.3	91.7994	Thermolysin	B2	91
BCAL0349 (typ	>tr|A0A2N9D2R1|A0A2N9D2R1_9BURK Outer membrane protein OS=Burkholderia cenocepacia OX=95486 GN=F01_70026 PE=3 SV=1	T.ALPQANPNAGGASGTVVHGTAGT[+568.212]LTPPPANAAPGQVVVGGK.V	HexNAc(2)Hex(1)	No	1424.0583	3	4270.1603	4270.1474	3	NRagged	598.25	598.25	0	Nsco_20191108_BC_ZIC_HILIC_H111_typsin_B1.38428.38428.3	77.2536	Trypsin	B1	92
BCAL0349 (typ	>tr|A0A2N9D2R1|A0A2N9D2R1_9BURK Outer membrane protein OS=Burkholderia cenocepacia OX=95486 GN=F01_70026 PE=3 SV=1	V.GNGTVATTALPQANPNAGGAS[+568.212]GTVVHGTAGTLTPPPANAAPGQVVVGGKVPDEATK.A	HexNAc(2)Hex(1)	No	1428.7255	4	5711.88	5711.8523	4.9	NRagged	1116.29	1116.29	0	Nsco_20191108_BC_ZIC_HILIC_H111_typsin_B1.42452.42452.4	83.9668	Trypsin	B1	93
BCAL0349 (typ	>tr|A0A2N9D2R1|A0A2N9D2R1_9BURK Outer membrane protein OS=Burkholderia cenocepacia OX=95486 GN=F01_70026 PE=3 SV=1	T.ALPQANPNAGGAS[+568.212]GTVVHGTAGTLTPPPANAAPGQVVVGGKVPDEATK.A	HexNAc(2)Hex(1)	Yes	1253.3899	4	5010.5378	5010.5178	4	NRagged	974.71	974.71	93.18	Nsco_20191108_BC_ZIC_HILIC_H111_typsin_B2.39145.39145.4	77.1581	Trypsin	B2	94
BCAL0349 (typ	>tr|A0A2N9D2R1|A0A2N9D2R1_9BURK Outer membrane protein OS=Burkholderia cenocepacia OX=95486 GN=F01_70026 PE=3 SV=1	A.QNTGATVTPVGNGTVATTAL QANPNAGGASGTVVHGTAGTL [+568.212]PPPANAAPGQVVVGGKVPDEATK.A	HexNAc(2)Hex(1)	No	1670.8411	4	6680.3428	6680.345	-0.3	NRagged	615.65	198.73	0	Nsco_20191108_BC_ZIC_HILIC_H111_typsin_B2.55798.55798.4	106.9982	Trypsin	B2	95
BCAL0349 (typ	>tr|A0A2N9D2R1|A0A2N9D2R1_9BURK Outer membrane protein OS=Burkholderia cenocepacia OX=95486 GN=F01_70026 PE=3 SV=1	V.TPVGNGTVATTALPQANPNAGGAS[+568.212][+100.064]GTVVHGTAGTLT[+568.212][+100.064]PPPANAAPGQVVVGGKVPDEATK.A	HexNAc(2)Hex(1) 100.064,HexNAc(2)Hex(1) 100.064	No	1695.8493	4	6780.3755	6777.3607	0.7	NRagged	458.14	63.6	0	Nsco_20191108_BC_ZIC_HILIC_H111_typsin_B2.56710.56710.4	108.7073	Trypsin	B2	96

<table>
<tr><td>BCAL0358</td><td>>tr|A0A142PJ25|A0A142PJ25_9BURK Aminopeptidase N OS=Burkholderia cenocepacia OX=95486 GN=pepN_1 PE=4 SV=1</td><td>L.SSAGAPHTGSSPT[+568.212]VAAPPSAS.N</td><td>HexNAc(2)Hex(1)</td><td>No</td><td>1203.049</td><td>2</td><td>2405.0906</td><td>2405.0838</td><td>2.8</td><td>CRagged</td><td>388.69</td><td>354.52</td><td>0</td><td>Nsco_20191108_BC_ZIC_HILIC_H111_pepsin_B2.16836.16836.2</td><td>37.0932</td><td>Pepsin</td><td>B2</td><td>97</td></tr>
<tr><td>BCAL0358</td><td>>tr|A0A142PJ25|A0A142PJ25_9BURK Aminopeptidase N OS=Burkholderia cenocepacia OX=95486 GN=pepN_1 PE=4 SV=1</td><td>L.SSAGAPHTGS[+568.212]SPTVAAPPSASN.V</td><td>HexNAc(2)Hex(1)</td><td>No</td><td>1260.0722</td><td>2</td><td>2519.1371</td><td>2519.1267</td><td>4.1</td><td>CRagged</td><td>635.65</td><td>631.94</td><td>0</td><td>Nsco_20191108_BC_ZIC_HILIC_H111_pepsin_B2.16231.16231.2</td><td>36.2053</td><td>Pepsin</td><td>B2</td><td>98</td></tr>
<tr><td>BCAL0426</td><td>>tr|A0A1V6KWZ1|A0A1V6KWZ1_9BURK Membrane protein insertase YidC OS=Burkholderia cenocepacia OX=95486 GN=yidC PE=3 SV=1</td><td>T.HTAPAAAGGASGTGATTT[+568.212]AGDVPAAAAGAAPSTTAPAAQ.A</td><td>HexNAc(2)Hex(1)</td><td>No</td><td>1272.2641</td><td>3</td><td>3814.7777</td><td>3814.7737</td><td>1.1</td><td>Non</td><td>172.9</td><td>172.9</td><td>1.2</td><td>Nsco_20191108_BC_ZIC_HILIC_H111_pepsin_B2.31114.31114.3</td><td>64.1861</td><td>Pepsin</td><td>B2</td><td>99</td></tr>
<tr><td>BCAL0426</td><td>>tr|A0A1V6KWZ1|A0A1V6KWZ1_9BURK Membrane protein insertase YidC OS=Burkholderia cenocepacia OX=95486 GN=yidC PE=3 SV=1</td><td>S.ATHTAPAAAGGASGT[+568.212]GATTTAGDVPAA.A</td><td>HexNAc(2)Hex(1)</td><td>No</td><td>1411.1507</td><td>2</td><td>2821.2941</td><td>2821.2858</td><td>2.9</td><td>Non</td><td>732.51</td><td>500.51</td><td>0</td><td>Nsco_20191108_BC_ZIC_HILIC_H111_thermolysin_B2.19464.19464.2</td><td>42.59</td><td>Thermolysin</td><td>B2</td><td>100</td></tr>
<tr><td>BCAL0426</td><td>>tr|A0A1V6KWZ1|A0A1V6KWZ1_9BURK Membrane protein insertase YidC OS=Burkholderia cenocepacia OX=95486 GN=yidC PE=3 SV=1</td><td>S.ATHTAPAAAGGASGTGATTT[568.212]AGDVPA.A</td><td>HexNAc(2)Hex(1)</td><td>No</td><td>1375.6329</td><td>2</td><td>2750.2584</td><td>2750.2486</td><td>3.6</td><td>Non</td><td>685.64</td><td>508.9</td><td>1.67</td><td>Nsco_20191108_BC_ZIC_HILIC_H111_thermolysin_B3.17670.17670.2</td><td>39.8571</td><td>Thermolysin</td><td>B3</td><td>101</td></tr>
<tr><td>BCAL0426</td><td>>tr|A0A1V6KWZ1|A0A1V6KWZ1_9BURK Membrane protein insertase YidC OS=Burkholderia cenocepacia OX=95486 GN=yidC PE=3 SV=1</td><td>S.ATHTAPAAAGGASGTGATTT[568.212]AGDVPAAAAGAAPSTTAPAAQAQ.L</td><td>HexNAc(2)Hex(1)</td><td>No</td><td>1396.6617</td><td>3</td><td>4187.9707</td><td>4185.9542</td><td>2.3</td><td>Non</td><td>631.07</td><td>574.7</td><td>1.2</td><td>Nsco_20191108_BC_ZIC_HILIC_H111_thermolysin_B3.32236.32236.3</td><td>64.8714</td><td>Thermolysin</td><td>B3</td><td>102</td></tr>
<tr><td>BCAL0426</td><td>>tr|A0A1V6KWZ1|A0A1V6KWZ1_9BURK Membrane protein insertase YidC OS=Burkholderia cenocepacia OX=95486 GN=yidC PE=3 SV=1</td><td>R.DHGRPSMFFPSATHTAPAAAGGAS[+568.212]GTGATTTAGDVPAAAAGAAPSTTAPAAQAQLVK.F</td><td>HexNAc(2)Hex(1)</td><td>Yes</td><td>1157.7617</td><td>5</td><td>5784.7795</td><td>5784.757</td><td>3.9</td><td>Specific</td><td>1146.79</td><td>1145.41</td><td>48.09</td><td>Nsco_20191108_BC_ZIC_HILIC_H111_typsin_B2.43258.43258.5</td><td>84.3869</td><td>Trypsin</td><td>B2</td><td>103</td></tr>
<tr><td>BCAL0479</td><td>>tr|A0A2N9CSF4|A0A2N9CSF4_9BURK Peptidoglycan D,D-transpeptidase MrdA OS=Burkholderia cenocepacia OX=95486 GN=mrdA PE=3 SV=1</td><td>A.VAAAASATEPVSAPVVGDT[+568.212]S[+568.212]KPAT.I</td><td>HexNAc(2)Hex(1), HexNAc(2)Hex(1)</td><td>No</td><td>1667.287</td><td>2</td><td>3333.5667</td><td>3333.5578</td><td>2.7</td><td>Non</td><td>660.44</td><td>357.82</td><td>9.42</td><td>Nsco_20191108_BC_ZIC_HILIC_H111_thermolysin_B1.30870.30870.2</td><td>60.3488</td><td>Thermolysin</td><td>B1</td><td>104</td></tr>
<tr><td>BCAL0479</td><td>>tr|A0A2N9CSF4|A0A2N9CSF4_9BURK Peptidoglycan D,D-transpeptidase MrdA OS=Burkholderia cenocepacia OX=95486 GN=mrdA PE=3 SV=1</td><td>R.RPAS[+568.212]DAQPVVATPR.D</td><td>HexNAc(2)Hex(1)</td><td>Yes</td><td>1017.0083</td><td>2</td><td>2033.0094</td><td>2033.0033</td><td>3</td><td>Specific</td><td>632.91</td><td>349.99</td><td>39.47</td><td>Nsco_20191108_BC_ZIC_HILIC_H111_typsin_B1.9404.9404.2</td><td>27.4075</td><td>Trypsin</td><td>B1</td><td>105</td></tr>
<tr><td>BCAL0481</td><td>>tr|A0A2N9CS00|A0A2N9CS00_9BURK Rod shape-determining protein MreC OS=Burkholderia cenocepacia OX=95486 GN=DFS07_10564 PE=4 SV=1</td><td>K.PAAAAPPAGANPAPAPAAPAKPAAAPAKPAAGQS[+568.212][+100.064]GAQR.-</td><td>HexNAc(2)Hex(1) 100.064</td><td>No</td><td>1326.3508</td><td>3</td><td>3977.0379</td><td>3977.0366</td><td>0.3</td><td>Specific</td><td>474.1</td><td>474.1</td><td>474.1</td><td>Nsco_20191108_BC_ZIC_HILIC_H111_typsin_B2.16250.16250.3</td><td>36.756</td><td>Trypsin</td><td>B2</td><td>106</td></tr>
<tr><td>BCAL0525</td><td>>tr|A0A119PFA4|A0A119PFA4_9BURK Flagellar M-ring protein OS=Burkholderia cenocepacia OX=95486 GN=fliF PE=3 SV=1</td><td>L.SNTPPQPAS[+568.212]APIVAGNGQNAPQTTPVSD.R</td><td>HexNAc(2)Hex(1)</td><td>Yes</td><td>1095.5194</td><td>3</td><td>3284.5438</td><td>3284.5288</td><td>4.5</td><td>CRagged</td><td>539.46</td><td>429.01</td><td>14.55</td><td>Nsco_20191108_BC_ZIC_HILIC_H111_pepsin_B1.36617.36617.3</td><td>78.3735</td><td>Pepsin</td><td>B1</td><td>107</td></tr>
<tr><td>BCAL0525</td><td>>tr|A0A119PFA4|A0A119PFA4_9BURK Flagellar M-ring protein OS=Burkholderia cenocepacia OX=95486 GN=fliF PE=3 SV=1</td><td>A.LSNTPPQPASAPIVAGNGQNAPQTT[+568.212]PVSDRKDQTTNYE.L</td><td>HexNAc(2)Hex(1)</td><td>No</td><td>1512.0564</td><td>3</td><td>4534.1546</td><td>4533.1387</td><td>2.8</td><td>Non</td><td>753.41</td><td>704.3</td><td>0</td><td>Nsco_20191108_BC_ZIC_HILIC_H111_thermolysin_B2.33278.33278.3</td><td>66.396</td><td>Thermolysin</td><td>B2</td><td>108</td></tr>
<tr><td>BCAL0525</td><td>>tr|A0A119PFA4|A0A119PFA4_9BURK Flagellar M-ring protein OS=Burkholderia cenocepacia OX=95486 GN=fliF PE=3 SV=1</td><td>R.SQQTSSATELAQGGASGVPGALS[+568.212]NTPPQPAS[+568.212][+100.064]APIVAGNGQNAPQTTPVSDRK.D</td><td>HexNAc(2)Hex(1), HexNAc(2)Hex(1) 100.064</td><td>No</td><td>1559.7397</td><td>4</td><td>6235.9372</td><td>6234.9659</td><td>-5.1</td><td>Specific</td><td>695.91</td><td>695.91</td><td>0</td><td>Nsco_20191108_BC_ZIC_HILIC_H111_typsin_B3.41487.41487.4</td><td>86.0175</td><td>Trypsin</td><td>B3</td><td>109</td></tr>
</table>

BCAL0544	>tr|A0A2N9CS86|A0A2N9CS86_9BURK Dipeptide transport system substrate-binding protein OS=Burkholderia cenocepacia OX=95486 GN=dppA PE=4 SV=1	S.VYQGAGQAASAPMPPT[+568.212]QWSYDKN.L	HexNAc(2)Hex(1)	No	1518.1818	2	3035.3563	3035.3463	3.3	Non	479.1	418.9	6	Nsco_20191108_BC_ZIC_HILIC_H111_thermolysin_B2.39343.39343.2	77.7918	Thermolysin	B2	110
BCAL0544	>tr|A0A2N9CS86|A0A2N9CS86_9BURK Dipeptide transport system substrate-binding protein OS=Burkholderia cenocepacia OX=95486 GN=dppA PE=4 SV=1	K.AILESVYQGAGQAAS[+568.212]APMPPTQWSYDK.N	HexNAc(2)Hex(1)	No	1718.3034	2	3435.5995	3434.5832	3.8	Specific	782.03	749.56	8.4	Nsco_20191108_BC_ZIC_HILIC_H111_typsin_B1.57958.57958.2	109.4793	Trypsin	B1	111
BCAL0544	>tr|A0A2N9CS86|A0A2N9CS86_9BURK Dipeptide transport system substrate-binding protein OS=Burkholderia cenocepacia OX=95486 GN=dppA PE=4 SV=1	K.KAILESVYQGAGQAAS[+568.212]APMPPTQWSYDK.N	HexNAc(2)Hex(1)	No	1188.2358	3	3562.6928	3562.6782	4.1	Specific	754.77	754.77	9.6	Nsco_20191108_BC_ZIC_HILIC_H111_typsin_B2.50150.50150.3	96.4702	Trypsin	B2	112
BCAL0678	>tr|A0A142PL18|A0A142PL18_9BURK Cell division protein OS=Burkholderia cenocepacia OX=95486 GN=A8E72_03155 PE=4 SV=1	S.KVAPPPADNGAS[+568.212]QPQQFDPNRALQ.G	HexNAc(2)Hex(1)	Yes	1038.8367	3	3114.4955	3114.4862	3	Non	609.94	549.65	549.65	Nsco_20191108_BC_ZIC_HILIC_H111_pepsin_B1.27083.27083.3	57.3842	Pepsin	B1	113
BCAL0678	>tr|A0A142PL18|A0A142PL18_9BURK Cell division protein OS=Burkholderia cenocepacia OX=95486 GN=A8E72_03155 PE=4 SV=1	S.KVAPPPADNGAS[+568.212]QPQQFDPNRAL.Q	HexNAc(2)Hex(1)	Yes	996.151	3	2986.4384	2986.4276	3.6	NRagged	652.56	631.5	631.5	Nsco_20191108_BC_ZIC_HILIC_H111_pepsin_B1.27669.27669.3	58.5354	Pepsin	B1	114
BCAL0678	>tr|A0A142PL18|A0A142PL18_9BURK Cell division protein OS=Burkholderia cenocepacia OX=95486 GN=A8E72_03155 PE=4 SV=1	F.VSKVAPPPADNGASQPQQFDPNRALQGKT[+568.212]PGQPVPQAAQPAPPNTAPGQA.A	HexNAc(2)Hex(1)	No	1391.4462	4	5562.7629	5561.7419	3.2	CRagged	263.81	247.51	3.6	Nsco_20191108_BC_ZIC_HILIC_H111_pepsin_B1.35328.35328.4	75.1743	Pepsin	B1	115
BCAL0678	>tr|A0A142PL18|A0A142PL18_9BURK Cell division protein OS=Burkholderia cenocepacia OX=95486 GN=A8E72_03155 PE=4 SV=1	S.KVAPPPADNGAS[+568.212]QPQQFDPNRALQGKTPGQPVPQAAQPAPPNTAPGQA.A	HexNAc(2)Hex(1)	Yes	1344.6699	4	5375.6579	5375.6415	3.1	Non	753.23	753.23	297.94	Nsco_20191108_BC_ZIC_HILIC_H111_pepsin_B1.34700.34700.4	73.7034	Pepsin	B1	116
BCAL0678	>tr|A0A142PL18|A0A142PL18_9BURK Cell division protein OS=Burkholderia cenocepacia OX=95486 GN=A8E72_03155 PE=4 SV=1	S.KVAPPPADNGASQPQQFDPNRALQGKT[+568.212][+100.064]PGQPVPQAAQPAPPNTAPGQAANQ.T	HexNAc(2)Hex(1) 100.064	No	1447.9617	4	5788.8249	5788.8441	-3.3	Non	616.03	481.95	9.3	Nsco_20191108_BC_ZIC_HILIC_H111_pepsin_B1.35407.35407.4	75.3781	Pepsin	B1	117
BCAL0678	>tr|A0A142PL18|A0A142PL18_9BURK Cell division protein OS=Burkholderia cenocepacia OX=95486 GN=A8E72_03155 PE=4 SV=1	S.KVAPPPADNGASQPQQFDPNRALQGKT[+568.212]PGQPVPQAAQPAPPNTAP.G	HexNAc(2)Hex(1)	No	1281.3821	4	5122.5065	5119.5243	-5.4	Non	253.73	253.73	10.07	Nsco_20191108_BC_ZIC_HILIC_H111_pepsin_B2.35513.35513.4	74.1341	Pepsin	B2	118
BCAL0678	>tr|A0A142PL18|A0A142PL18_9BURK Cell division protein OS=Burkholderia cenocepacia OX=95486 GN=A8E72_03155 PE=4 SV=1	S.KVAPPPADNGAS[+568.212]QPQQFDPNRA.L	HexNAc(2)Hex(1)	Yes	958.4571	3	2873.3566	2873.3436	4.6	Non	589.42	589.42	589.42	Nsco_20191108_BC_ZIC_HILIC_H111_pepsin_B2.18898.18898.3	40.4584	Pepsin	B2	119
BCAL0678	>tr|A0A142PL18|A0A142PL18_9BURK Cell division protein OS=Burkholderia cenocepacia OX=95486 GN=A8E72_03155 PE=4 SV=1	S.KVAPPPADNGAS[+568.212]QPQQFDPNRALQG.K	HexNAc(2)Hex(1)	Yes	1057.8449	3	3171.5201	3171.5077	3.9	Non	509.23	509.23	509.23	Nsco_20191108_BC_ZIC_HILIC_H111_pepsin_B2.28821.28821.3	59.3228	Pepsin	B2	120
BCAL0678	>tr|A0A142PL18|A0A142PL18_9BURK Cell division protein OS=Burkholderia cenocepacia OX=95486 GN=A8E72_03155 PE=4 SV=1	S.KVAPPPADNGAS[+568.212]QPQQFDPNRALQGKTPGQPVPQA.A	HexNAc(2)Hex(1)	Yes	1044.523	4	4175.0701	4175.0527	4.2	Non	776.89	776.89	543.75	Nsco_20191108_BC_ZIC_HILIC_H111_pepsin_B2.31728.31728.4	65.5889	Pepsin	B2	121

BCAL0678	>tr|A0A142PL18|A0A142PL18_9BURK Cell division protein OS=Burkholderia cenocepacia OX=95486 GN=A8E72_03155 PE=4 SV=1	S.KVAPPPADNGAS[+568.212][+100.064]QPQQFDPNRALQGKTPGQPVPQAAQ.P	HexNAc(2)Hex(1) 100.064	No	1119.5551	4	4475.1986	4474.2124	-3.9	Non	366.59	282.54	5.72	Nsco_20191108_BC_ZIC_HILIC_H111_pepsin_B2.32825.32825.2	67.8781	Pepsin	B2	122
BCAL0678	>tr|A0A142PL18|A0A142PL18_9BURK Cell division protein OS=Burkholderia cenocepacia OX=95486 GN=A8E72_03155 PE=4 SV=1	K.VAPPPADNGAS[+568.212][+100.064]QPQQFDPNRALQGKTPGQPVPQAAQPAPPNTAPGQA.A	HexNAc(2)Hex(1) 100.064	Yes	1337.6516	4	5347.5846	5347.6105	-4.8	Non	560.62	560.62	232.52	Nsco_20191108_BC_ZIC_HILIC_H111_pepsin_B2.39476.39476.4	83.9714	Pepsin	B2	123
BCAL0678	>tr|A0A142PL18|A0A142PL18_9BURK Cell division protein OS=Burkholderia cenocepacia OX=95486 GN=A8E72_03155 PE=4 SV=1	S.KVAPPPADNGAS[+568.212]QPQQFDPNRALQGKTPGQPVPQAAQPAPPNTAPGQAAN.Q	HexNAc(2)Hex(1)	Yes	1390.9406	4	5560.7404	5560.7215	3.4	Non	753.49	753.49	363.84	Nsco_20191108_BC_ZIC_HILIC_H111_pepsin_B2.34965.34965.4	72.7835	Pepsin	B2	124
BCAL0678	>tr|A0A142PL18|A0A142PL18_9BURK Cell division protein OS=Burkholderia cenocepacia OX=95486 GN=A8E72_03155 PE=4 SV=1	S.KVAPPPADNGAS[+568.212]QPQQFDPNRALQGKTPGQPVPQAAQPAPPNTAPGQ.A	HexNAc(2)Hex(1)	Yes	1326.9128	4	5304.6296	5304.6044	4.7	Non	726.93	726.93	266.35	Nsco_20191108_BC_ZIC_HILIC_H111_pepsin_B3.34094.34094.4	72.7935	Pepsin	B3	125
BCAL0678	>tr|A0A142PL18|A0A142PL18_9BURK Cell division protein OS=Burkholderia cenocepacia OX=95486 GN=A8E72_03155 PE=4 SV=1	S.KVAPPPADNGAS[+568.212]QPQQFDPNRALQGKTPGQPVPQAAQPAPPNTA.P	HexNAc(2)Hex(1)	Yes	1256.3777	4	5022.4889	5022.4716	3.5	Non	602.85	602.85	265.96	Nsco_20191108_BC_ZIC_HILIC_H111_pepsin_B3.33636.33636.4	71.5919	Pepsin	B3	126
BCAL0678	>tr|A0A142PL18|A0A142PL18_9BURK Cell division protein OS=Burkholderia cenocepacia OX=95486 GN=A8E72_03155 PE=4 SV=1	S.KVAPPPADNGAS[+568.212]QPQQFDPNRALQGKTPGQPVPQAAQPAPPNTAPGQAA.N	HexNAc(2)Hex(1)	Yes	1362.4308	4	5446.7013	5446.6786	4.2	Non	780.89	780.89	370.32	Nsco_20191108_BC_ZIC_HILIC_H111_pepsin_B3.34360.34360.4	73.5177	Pepsin	B3	127
BCAL0678	>tr|A0A142PL18|A0A142PL18_9BURK Cell division protein OS=Burkholderia cenocepacia OX=95486 GN=A8E72_03155 PE=4 SV=1	F.VSKVAPPPADNGAS[+568.212][+100.064]QPQQFDPNRALQGKTPGQPVPQAAQPAPPNTAPGQAA.N	HexNAc(2)Hex(1) 100.064	No	1434.4614	4	5734.8239	5732.843	-4.5	CRagged	477.51	477.51	229.78	Nsco_20191108_BC_ZIC_HILIC_H111_pepsin_B3.35710.35710.4	77.0909	Pepsin	B3	128
BCAL0678	>tr|A0A142PL18|A0A142PL18_9BURK Cell division protein OS=Burkholderia cenocepacia OX=95486 GN=A8E72_03155 PE=4 SV=1	A.QKQQQQQQAANTPKPTSSAT[+568.212]AAA.A	HexNAc(2)Hex(1)	No	1476.7124	2	2952.4176	2951.4076	2.3	Non	498.87	408.91	2.47	Nsco_20191108_BC_ZIC_HILIC_H111_pepsin_B3.7831.7831.2	22.0979	Pepsin	B3	129
BCAL0678	>tr|A0A142PL18|A0A142PL18_9BURK Cell division protein OS=Burkholderia cenocepacia OX=95486 GN=A8E72_03155 PE=4 SV=1	A.AAQKQQQQQQAANT[+568.212]PKPTSSATAAA.A	HexNAc(2)Hex(1)	No	1032.1702	3	3094.496	3093.4818	3.5	Non	378.39	378.39	14.7	Nsco_20191108_BC_ZIC_HILIC_H111_pepsin_B3.8210.8210.3	22.633	Pepsin	B3	130
BCAL0678	>tr|A0A142PL18|A0A142PL18_9BURK Cell division protein OS=Burkholderia cenocepacia OX=95486 GN=A8E72_03155 PE=4 SV=1	R.FAAQKQAQQAAAQKQQQQQQAANTPKPT[+568.212][+100.064]SSA.T	HexNAc(2)Hex(1) 100.064	No	1318.3182	3	3952.9402	3950.933	0.1	Non	170.22	145.54	0.88	Nsco_20191108_BC_ZIC_HILIC_H111_thermolysin_B1.12441.12441.3	30.8095	Thermolysin	B1	131
BCAL0678	>tr|A0A142PL18|A0A142PL18_9BURK Cell division protein OS=Burkholderia cenocepacia OX=95486 GN=A8E72_03155 PE=4 SV=1	K.VAPPPADNGAS[+568.212]QPQQFDPNRA.L	HexNAc(2)Hex(1)	No	1373.1332	2	2745.2591	2745.2486	3.8	Non	317.04	254.66	254.66	Nsco_20191108_BC_ZIC_HILIC_H111_thermolysin_B1.25881.25881.2	51.6772	Thermolysin	B1	132
BCAL0678	>tr|A0A142PL18|A0A142PL18_9BURK Cell division protein OS=Burkholderia cenocepacia OX=95486 GN=A8E72_03155 PE=4 SV=1	Q.AAAQKQQQQQQAANT[+568.212]PKPTSSATAA.A	HexNAc(2)Hex(1)	No	1031.8346	3	3093.4893	3093.4818	2.4	Non	385.59	283.64	3.62	Nsco_20191108_BC_ZIC_HILIC_H111_thermolysin_B1.6531.6531.3	21.9265	Thermolysin	B1	133
BCAL0678	>tr|A0A142PL18|A0A142PL18_9BURK Cell division protein OS=Burkholderia cenocepacia OX=95486 GN=A8E72_03155 PE=4 SV=1	Q.AAAQKQQQQQQAANT[+568.212]PKPTSSATAAA.A	HexNAc(2)Hex(1)	No	1055.5145	3	3164.529	3164.519	3.2	Non	458.21	366.96	1.56	Nsco_20191108_BC_ZIC_HILIC_H111_thermolysin_B2.7398.7398.3	23.3115	Thermolysin	B2	134

BCAL0678	>tr|A0A142PL18|A0A142PL18_9BURK Cell division protein OS=Burkholderia cenocepacia OX=95486 GN=A8E72_03155 PE=4 SV=1	K.QQQQQQAANTPKPTS[+568.212]SATAAAAAKPPTANDANTGYFLQVGAYK.T	HexNAc(2)Hex(1)	No	1244.1078	4	4973.4093	4973.3923	3.4	Specific	1270	1207.06	17.78	Nsco_20191108_BC_ZIC_HILIC_H111_typsin_B1.35724.35724.4	72.5132	Trypsin	B1	135
BCAL0678	>tr|A0A142PL18|A0A142PL18_9BURK Cell division protein OS=Burkholderia cenocepacia OX=95486 GN=A8E72_03155 PE=4 SV=1	Q.QAANTPKPTS[+568.212]SATAAAAAKPPT[+568.212][+100.064]ANDANTGYFLQVGAYKTEGDAEQQR.A	HexNAc(2)Hex(1), HexNAc(2)Hex(1) 100.064	No	1204.7749	5	6019.8454	6015.8116	3.4	NRagged	738.44	738.44	2.58	Nsco_20191108_BC_ZIC_HILIC_H111_typsin_B1.34157.34157.5	69.8041	Trypsin	B1	136
BCAL0678	>tr|A0A142PL18|A0A142PL18_9BURK Cell division protein OS=Burkholderia cenocepacia OX=95486 GN=A8E72_03155 PE=4 SV=1	K.QQQQQQAANTPKPTSS[+568.212][+100.064]ATAAAAAKPPTANDANTGYFLQVGAYKTEGDAEQQR.A	HexNAc(2)Hex(1) 100.064	No	1218.3798	5	6087.8697	6087.893	-3.8	Specific	1260.89	1260.89	0	Nsco_20191108_BC_ZIC_HILIC_H111_typsin_B2.35579.35579.5	70.8298	Trypsin	B2	137
BCAL0678	>tr|A0A142PL18|A0A142PL18_9BURK Cell division protein OS=Burkholderia cenocepacia OX=95486 GN=A8E72_03155 PE=4 SV=1	S.KVAPPPADNGAS[+568.212]QPQQFDPNR.A	HexNAc(2)Hex(1)	Yes	934.7765	3	2802.3148	2802.3064	3	NRagged	428.78	428.78	428.78	Nsco_20191108_BC_ZIC_HILIC_H111_typsin_B2.16578.16578.3	37.2989	Trypsin	B2	138
BCAL0678	>tr|A0A142PL18|A0A142PL18_9BURK Cell division protein OS=Burkholderia cenocepacia OX=95486 GN=A8E72_03155 PE=4 SV=1	K.VAPPPADNGAS[+568.212]QPQQFDPNRALQGK.T	HexNAc(2)Hex(1)	Yes	1057.8446	3	3171.5193	3171.5077	3.7	Specific	671.21	671.21	671.21	Nsco_20191108_BC_ZIC_HILIC_H111_typsin_B2.29596.29596.3	60.2503	Trypsin	B2	139
BCAL0678	>tr|A0A142PL18|A0A142PL18_9BURK Cell division protein OS=Burkholderia cenocepacia OX=95486 GN=A8E72_03155 PE=4 SV=1	Q.QQAANTPKPTSSATAAAAAKPPT[+568.212]ANDANT[+568.212][+100.064]GYFLQVGAYKTEGDAEQQR.A	HexNAc(2)Hex(1), HexNAc(2)Hex(1) 100.064	No	1229.9755	5	6145.8482	6143.8702	-4.7	NRagged	523.48	523.48	0	Nsco_20191108_BC_ZIC_HILIC_H111_typsin_B2.37796.37796.5	74.6971	Trypsin	B2	140
BCAL0678	>tr|A0A142PL18|A0A142PL18_9BURK Cell division protein OS=Burkholderia cenocepacia OX=95486 GN=A8E72_03155 PE=4 SV=1	Q.KQQQQQQAANTPKPTS[+568.212]SATAAAAAKPPTANDANTGYFLQVGAYKTEGDAEQQR.	HexNAc(2)Hex(1)	Yes	1020.6626	6	6118.9391	6115.9239	0.8	NRagged	1389.56	1103.5	15.81	Nsco_20191108_BC_ZIC_HILIC_H111_typsin_B3.29736.29736.6	63.1584	Trypsin	B3	141
BCAL0678	>tr|A0A142PL18|A0A142PL18_9BURK Cell division protein OS=Burkholderia cenocepacia OX=95486 GN=A8E72_03155 PE=4 SV=1	K.VAPPPADNGAS[+568.212]QPQQFDPNR.A	HexNAc(2)Hex(1)	Yes	1337.6127	2	2674.2182	2674.2115	2.5	Specific	557.94	510.14	510.14	Nsco_20191108_BC_ZIC_HILIC_H111_typsin_B3.22139.22139.2	48.3419	Trypsin	B3	142
BCAL0678	>tr|A0A142PL18|A0A142PL18_9BURK Cell division protein OS=Burkholderia cenocepacia OX=95486 GN=A8E72_03155 PE=4 SV=1	Q.KQQQQQQAANTPKPTS[+568.212]SATAAAAAKPPTANDANTGYFLQVGAYK.T	HexNAc(2)Hex(1)	Yes	1021.5052	5	5103.4971	5101.4873	0.6	NRagged	1343.42	1328.26	42.28	Nsco_20191108_BC_ZIC_HILIC_H111_typsin_B3.30947.30947.5	65.4326	Trypsin	B3	143
BCAL0738	>tr|A0A142PKY4|A0A142PKY4_9BURK C-terminal processing protease-3 OS=Burkholderia cenocepacia OX=95486 GN=A8E72_10915 PE=3 SV=1	A.T[+568.212]ASKAS[+568.212][+100.064]GASAAKPAS[+568.212]APKPASAPK.-	HexNAc(2)Hex(1), HexNAc(2)Hex(1) 100.064,HexNAc(2)Hex(1)	No	1319.6238	3	3956.8568	3956.8708	-3.5	Non	156.06	156.06	1.2	Nsco_20191108_BC_ZIC_HILIC_H111_thermolysin_B1.4774.4774.3	19.118	Thermolysin	B1	144
BCAL0749	>tr|A0A2N9D2V0|A0A2N9D2V0_9BURK Cytochrome c oxidase subunit 2 OS=Burkholderia cenocepacia OX=95486 GN=ctaC_1 PE=3 SV=1	Q.VADARNGKLPEDTAGAAT[+568.212][+100.064]AAAPAEAAS[+568.212][+100.064]A.P	HexNAc(2)Hex(1) 100.064,HexNAc(2)Hex(1) 100.064	No	1301.9514	3	3903.8395	3903.8207	4.8	Non	163.27	152.83	5.6	Nsco_20191108_BC_ZIC_HILIC_H111_thermolysin_B2.33883.33883.3	67.38	Thermolysin	B2	145
BCAL0749	>tr|A0A2N9D2V0|A0A2N9D2V0_9BURK Cytochrome c oxidase subunit 2 OS=Burkholderia cenocepacia OX=95486 GN=ctaC_1 PE=3 SV=1	G.AATAAAPAEAASAPAQAASGAEQPAAAASAALS[+568.212][+100.064]T[+568.212][+100.064]IYFETGK.S	HexNAc(2)Hex(1) 100.064,HexNAc(2)Hex(1) 100.064	No	1691.1403	3	5071.4065	5069.3863	2.7	NRagged	567.51	545.2	1.2	Nsco_20191108_BC_ZIC_HILIC_H111_typsin_B1.66797.66797.3	123.8368	Trypsin	B1	146
BCAL0749	>tr|A0A2N9D2V0|A0A2N9D2V0_9BURK Cytochrome c oxidase subunit 2 OS=Burkholderia cenocepacia OX=95486 GN=ctaC_1 PE=3 SV=1	A.GAATAAAPAEAASAPAQAAS[+568.212]GAEQPAAAAS[+568.212][+100.064]AALSTIYFETGK.S	HexNAc(2)Hex(1), HexNAc(2)Hex(1) 100.064	No	1676.4614	3	5027.3696	5026.3437	4.5	NRagged	433.97	433.97	0	Nsco_20191108_BC_ZIC_HILIC_H111_typsin_B1.67138.67138.3	124.4222	Trypsin	B1	147

BCAL0749	>tr|A0A2N9D2V0|A0A2N9D2V0_9BURK Cytochrome c oxidase subunit 2 OS=Burkholderia cenocepacia OX=95486 GN=ctaC_1 PE=3 SV=1	E.DTAGAATAAAPAEAASAPAQAAS[+568.212][+100.064]GAEQPAAAAS[+568.212]AALSTIYFETGK.S	HexNAc(2)Hex(1) 100.064,HexNAc(2)Hex(1)	No	1771.8249	3	5313.4602	5313.4555	0.9	NRagged	396.18	396.18	0	Nsco_20191108_BC_ZIC_HILIC_H111_typsin_B1.72265.72265.3	133.0596	Trypsin	B1	148
BCAL0749	>tr|A0A2N9D2V0|A0A2N9D2V0_9BURK Cytochrome c oxidase subunit 2 OS=Burkholderia cenocepacia OX=95486 GN=ctaC_1 PE=3 SV=1	G.KLPEDTAGAAT[+568.212][+100.064]AAAPAEAASAPAQAAS[+568.212]GAEQPAAAASAALSTIYFETGK.S	HexNAc(2)Hex(1) 100.064,HexNAc(2)Hex(1)	No	1447.1931	4	5785.7506	5780.7299	0.7	NRagged	243.78	243.78	0	Nsco_20191108_BC_ZIC_HILIC_H111_typsin_B2.63829.63829.4	123.3676	Trypsin	B2	149
BCAL0749	>tr|A0A2N9D2V0|A0A2N9D2V0_9BURK Cytochrome c oxidase subunit 2 OS=Burkholderia cenocepacia OX=95486 GN=ctaC_1 PE=3 SV=1	L.PEDTAGAATAAAPAEAASAPAQAASGAEQPAAAASAALS[+568.212][+100.064]T[+568.212][+100.064]IYFETGK.S	HexNAc(2)Hex(1) 100.064,HexNAc(2)Hex(1) 100.064	No	1410.6657	4	5639.6408	5639.6148	4.6	NRagged	493.11	493.11	1.2	Nsco_20191108_BC_ZIC_HILIC_H111_typsin_B2.62442.62442.4	120.3245	Trypsin	B2	150
BCAL0749	>tr|A0A2N9D2V0|A0A2N9D2V0_9BURK Cytochrome c oxidase subunit 2 OS=Burkholderia cenocepacia OX=95486 GN=ctaC_1 PE=3 SV=1	Q.PAAAASAALS[+568.212]T[+568.212]IYFETGK.S	HexNAc(2)Hex(1), HexNAc(2)Hex(1)	No	1454.1756	2	2907.3439	2905.3347	0.8	NRagged	388.67	378.93	15.6	Nsco_20191108_BC_ZIC_HILIC_H111_typsin_B3.59776.59776.4	122.6454	Trypsin	B3	151
BCAL0786	>tr|A0A1V2XQK6|A0A1V2XQK6_9BURK Membrane protein OS=Burkholderia cenocepacia OX=95486 GN=A8E72_26595 PE=4 SV=1	K.LLSVPAPAST[+568.212]EGDHHDK.-	HexNAc(2)Hex(1)	No	781.3706	3	2342.0973	2342.0882	3.9	Specific	542.13	542.13	0	Nsco_20191108_BC_ZIC_HILIC_H111_typsin_B2.15440.15440.3	35.4194	Trypsin	B2	152
BCAL1081	>tr|A0A1V2XQC3|A0A1V2XQC3_9BURK Multidrug efflux system membrane fusion protein OS=Burkholderia cenocepacia OX=95486 GN=mdtA PE=3 SV=1	K.RYQTLLSQDSIAS[+568.212]QTVDTQASLVK.Q	HexNAc(2)Hex(1)	No	1074.5406	3	3221.6074	3220.5955	2.7	Specific	440.52	357.48	1.2	Nsco_20191108_BC_ZIC_HILIC_H111_typsin_B3.39618.39618.3	82.4359	Trypsin	B3	153
BCAL1086	>tr|A0A107L3U1|A0A107L3U1_9BURK Putative lipoprotein OS=Burkholderia cenocepacia OX=95486 GN=A8E72_07105 PE=4 SV=1	S.AAQAQIDAAAS[+568.212]AVVAHAAS[+568.212][+100.064]EAGAK.I	HexNAc(2)Hex(1), HexNAc(2)Hex(1) 100.064	Yes	1139.5326	3	3416.5832	3415.5973	-5.1	Non	482.86	468.7	0	Nsco_20191108_BC_ZIC_HILIC_H111_thermolysin_B1.44403.44403.3	83.8155	Thermolysin	B1	154
BCAL1086	>tr|A0A107L3U1|A0A107L3U1_9BURK Putative lipoprotein OS=Burkholderia cenocepacia OX=95486 GN=A8E72_07105 PE=4 SV=1	K.LQQWSQQSAAGAKPAS[+568.212]GE.-	HexNAc(2)Hex(1)	No	1206.5597	2	2412.1122	2412.1049	3	CRagged	771.99	632.32	2.87	Nsco_20191108_BC_ZIC_HILIC_H111_typsin_B1.15216.15216.2	36.5522	Trypsin	B1	155
BCAL1086	>tr|A0A107L3U1|A0A107L3U1_9BURK Putative lipoprotein OS=Burkholderia cenocepacia OX=95486 GN=A8E72_07105 PE=4 SV=1	K.KLQQWSQQS[+568.212]AAGAKPASGE.-	HexNAc(2)Hex(1)	No	1270.6077	2	2540.2081	2540.1998	3.3	CRagged	820.17	701.91	2.83	Nsco_20191108_BC_ZIC_HILIC_H111_typsin_B1.11185.11185.2	30.1493	Trypsin	B1	156
BCAL1086	>tr|A0A107L3U1|A0A107L3U1_9BURK Putative lipoprotein OS=Burkholderia cenocepacia OX=95486 GN=A8E72_07105 PE=4 SV=1	K.ALDQVAST[+568.212]VNQQINAAK.A	HexNAc(2)Hex(1)	No	1170.0812	2	2339.1552	2339.146	3.9	Specific	757.76	635.78	3.22	Nsco_20191108_BC_ZIC_HILIC_H111_typsin_B3.35315.35315.2	73.9304	Trypsin	B3	157
BCAL1093	>tr|A0A2N9CGE7|A0A2N9CGE7_9BURK Signal peptide transmembrane protein OS=Burkholderia cenocepacia OX=95486 GN=DFS07_119133 PE=4 SV=1	K.HAYDEAHPAEAAS[+568.212]AASH.-	HexNAc(2)Hex(1)	No	768.3269	3	2302.9661	2302.9582	3.4	CRagged	733.24	729.7	1.54	Nsco_20191108_BC_ZIC_HILIC_H111_typsin_B1.7502.7502.3	24.0483	Trypsin	B1	158
BCAL1389	>tr|A0A2N9CG52|A0A2N9CG52_9BURK Cyclic di-GMP-binding protein OS=Burkholderia cenocepacia OX=95486 GN=bcsB PE=3 SV=1	P.AVPAAAS[+568.212]T[+568.212][+100.064]AAAGHAAAADVAPAAGPVAAPAAASAPAAGLPATTVHVPFASLGAFDPLR.L	HexNAc(2)Hex(1), HexNAc(2)Hex(1) 100.064	No	1598.0516	4	6389.1847	6389.1838	0.1	NRagged	954.87	371.84	0	Nsco_20191108_BC_ZIC_HILIC_H111_typsin_B2.65506.65506.4	127.0975	Trypsin	B2	159

BCAL1389	>tr|A0A2N9CG52|A0A2N9CG52_9BURK Cyclic di-GMP-binding protein OS=Burkholderia cenocepacia OX=95486 GN=bcsB PE=3 SV=1	A.APMPAVPAAAST[+568.212]AAAGHAAAADVAPAAGPVAAPAAAS[+568.212]APAAGLPATTVHVPFASLGAFDPLR.L	HexNAc(2)Hex(1), HexNAc(2)Hex(1)	Yes (1 of 2)	1672.0869	4	6685.3257	6685.3029	3.4	NRagged	1064.73	1061.81	159.46	Nsco_20191108_BC_ZIC_HILIC_H111_typsin_B2.67298.67298.4	131.0906	Trypsin	B2	160
BCAL1496	>tr|A0A0M1IFG9|A0A0M1IFG9_9BURK Signal peptide protein OS=Burkholderia cenocepacia OX=95486 GN=A8F55_16750 PE=4 SV=1	K.KAGKKAKAADAAS[+568.212]Q.-	HexNAc(2)Hex(1)	Yes	638.3309	3	1912.9781	1912.971	3.8	Non	431.43	251.88	251.88	Nsco_20191108_BC_ZIC_HILIC_H111_pepsin_B1.2102.2102.3	13.2946	Pepsin	B1	161
BCAL1496	>tr|A0A0M1IFG9|A0A0M1IFG9_9BURK Signal peptide protein OS=Burkholderia cenocepacia OX=95486 GN=A8F55_16750 PE=4 SV=1	H.KAAAKKAGKKAKAADAAS[+568.212]Q.-	HexNAc(2)Hex(1)	Yes	596.3257	4	2382.281	2382.2722	3.7	Non	638.78	549.42	549.42	Nsco_20191108_BC_ZIC_HILIC_H111_pepsin_B2.2867.2867.4	14.0627	Pepsin	B2	162
BCAL1496	>tr|A0A0M1IFG9|A0A0M1IFG9_9BURK Signal peptide protein OS=Burkholderia cenocepacia OX=95486 GN=A8F55_16750 PE=4 SV=1	G.KKAKAADAAS[+568.212]Q.-	HexNAc(2)Hex(1)	Yes	552.9459	3	1656.823	1656.8174	3.4	Non	326.6	179.24	179.24	Nsco_20191108_BC_ZIC_HILIC_H111_pepsin_B2.2605.2605.3	13.2624	Pepsin	B2	163
BCAL1496	>tr|A0A0M1IFG9|A0A0M1IFG9_9BURK Signal peptide protein OS=Burkholderia cenocepacia OX=95486 GN=A8F55_16750 PE=4 SV=1	A.KKAGKKAKAADAAS[+568.212]Q.-	HexNAc(2)Hex(1)	Yes	511.0242	4	2041.0748	2041.0659	4.4	Non	432.15	280.15	280.15	Nsco_20191108_BC_ZIC_HILIC_H111_pepsin_B2.2745.2745.4	13.726	Pepsin	B2	164
BCAL1496	>tr|A0A0M1IFG9|A0A0M1IFG9_9BURK Signal peptide protein OS=Burkholderia cenocepacia OX=95486 GN=A8F55_16750 PE=4 SV=1	A.AAKKAGKKAKAADAAS[+568.212]Q.-	HexNAc(2)Hex(1)	Yes	546.5425	4	2183.1482	2183.1401	3.7	Non	645.31	568.86	568.86	Nsco_20191108_BC_ZIC_HILIC_H111_thermolysin_B1.2030.2030.4	13.2481	Thermolysin	B1	165
BCAL1496	>tr|A0A0M1IFG9|A0A0M1IFG9_9BURK Signal peptide protein OS=Burkholderia cenocepacia OX=95486 GN=A8F55_16750 PE=4 SV=1	K.AAAKKAGKKAKAADAAS[+568.212]Q.-	HexNAc(2)Hex(1)	Yes	564.302	4	2254.186	2254.1773	3.9	Non	657.05	583.97	583.97	Nsco_20191108_BC_ZIC_HILIC_H111_thermolysin_B3.2327.2327.4	14.0219	Thermolysin	B3	166
BCAL1496	>tr|A0A0M1IFG9|A0A0M1IFG9_9BURK Signal peptide protein OS=Burkholderia cenocepacia OX=95486 GN=A8F55_16750 PE=4 SV=1	K.AKAADAAS[+568.212]Q.-	HexNAc(2)Hex(1)	Yes	700.819	2	1400.6308	1400.6275	2.4	CRagged	314.51	138.87	138.87	Nsco_20191108_BC_ZIC_HILIC_H111_typsin_B1.2165.2165.2	14.2832	Trypsin	B1	167
BCAL1537	>tr|A0A1V6KNH3|A0A1V6KNH3_9BURK Lipoprotein OS=Burkholderia cenocepacia OX=95486 GN=A8E72_13475 PE=4 SV=1	S.AVQQASAPVAAIPVIDSQAQT[+568.212]SVQPQAGETTGPSTVDDLQRQ.I	HexNAc(2)Hex(1)	No	1610.1277	3	4828.3685	4828.3494	3.9	Non	364.19	364.19	0	Nsco_20191108_BC_ZIC_HILIC_H111_thermolysin_B1.57459.57459.3	107.5788	Thermolysin	B1	168
BCAL1649	>tr|A0A1V6KP99|A0A1V6KP99_9BURK Membrane protein OS=Burkholderia cenocepacia OX=95486 GN=A8E72_01255 PE=4 SV=1	V.ASAASSPAPAPAAPASPS[+568.212][+100.064]EAT[+568.212][+100.064]QAPHQQQTSAGYRN.I	HexNAc(2)Hex(1) 100.064,HexNAc(2)Hex(1) 100.064	No	1571.7136	3	4713.1263	4712.146	-4.9	Non	230.26	29.55	2.4	Nsco_20191108_BC_ZIC_HILIC_H111_thermolysin_B1.23209.23209.3	47.0602	Thermolysin	B1	169
BCAL1674	>tr|A0A2N9CH15|A0A2N9CH15_9BURK Multidrug efflux system OS=Burkholderia cenocepacia OX=95486 GN=acrA PE=3 SV=1	Q.FAPDTAVKPVEKAPPS[+568.212]KAAPPAAASQA.A	HexNAc(2)Hex(1)	No	1062.5435	3	3185.6159	3185.61	1.9	Non	748.99	695.75	16.78	Nsco_20191108_BC_ZIC_HILIC_H111_thermolysin_B3.20755.20755.3	45.2678	Thermolysin	B3	170
BCAL1746	>tr|A0A1V2Y428|A0A1V2Y428_9BURK Uncharacterized protein OS=Burkholderia cenocepacia OX=95486 GN=A8F55_03700 PE=4 SV=1	L.IDHIGKAWPGNAAS[+568.212]GASASE.-	HexNAc(2)Hex(1)	No	836.3886	3	2507.1514	2507.142	3.7	CRagged	734.05	680.07	6	Nsco_20191108_BC_ZIC_HILIC_H111_pepsin_B2.22428.22428.3	46.795	Pepsin	B2	171

BCAL1877	>tr|A0A2N9CHN6|A0A2N9CHN6_9BURK Protein HflK OS=Burkholderia cenocepacia OX=95486 GN=F01_210017 PE=3 SV=1	A.AAST[+568.212]GAS[+568.212]AADAASAPAATVPSAA.A	HexNAc(2)Hex(1), HexNAc(2)Hex(1)	No	1008.4515	3	3023.34	3023.3322	2.6	Non	342.23	273.47	0	Nsco_20191108_BC_ZIC_HILIC_H111_pepsin_B2.29475.29475.3	60.7221	Pepsin	B2	172
BCAL1877	>tr|A0A2N9CHN6|A0A2N9CHN6_9BURK Protein HflK OS=Burkholderia cenocepacia OX=95486 GN=F01_210017 PE=3 SV=1	P.LDKLVEQGRQNAAAS[+568.212]T[+568.212]GASAADAASAPAATVPSA.A	HexNAc(2)Hex(1), HexNAc(2)Hex(1)	No	1435.6845	3	4305.039	4304.0159	4.6	Non	247.74	203.21	5.6	Nsco_20191108_BC_ZIC_HILIC_H111_thermolysin_B1.33074.33074.3	64.0234	Thermolysin	B1	173
BCAL1877	>tr|A0A2N9CHN6|A0A2N9CHN6_9BURK Protein HflK OS=Burkholderia cenocepacia OX=95486 GN=F01_210017 PE=3 SV=1	A.AASTGASAADAASAPAAT[+568.212]VPS[+568.212].A	HexNAc(2)Hex(1), HexNAc(2)Hex(1)	No	1441.1368	2	2881.2662	2881.2579	2.9	Non	621.81	258.83	0.76	Nsco_20191108_BC_ZIC_HILIC_H111_thermolysin_B2.26699.26699.2	55.2555	Thermolysin	B2	174
BCAL1877	>tr|A0A2N9CHN6|A0A2N9CHN6_9BURK Protein HflK OS=Burkholderia cenocepacia OX=95486 GN=F01_210017 PE=3 SV=1	P.LDKLVEQGRQNAAAST[+568.212]GAS[+568.212]AADAASAPAATVPS.A	HexNAc(2)Hex(1), HexNAc(2)Hex(1)	No	1411.6694	3	4232.9936	4232.9787	3.5	Non	306.31	283.72	6.13	Nsco_20191108_BC_ZIC_HILIC_H111_thermolysin_B2.30688.30688.3	62.0296	Thermolysin	B2	175
BCAL1885	>tr|A0A1V2XJM4|A0A1V2XJM4_9BURK Helix-turn-helix domain-containing protein OS=Burkholderia cenocepacia OX=95486 GN=A8F55_38750 PE=4 SV=1	A.AAST[+568.212]AAPAQPAS[+568.212]VVVA.A	HexNAc(2)Hex(1), HexNAc(2)Hex(1)	No	1274.099	2	2547.1908	2547.1819	3.5	Non	500.63	283.69	94.58	Nsco_20191108_BC_ZIC_HILIC_H111_pepsin_B1.29311.29311.2	61.8555	Pepsin	B1	176
BCAL1885	>tr|A0A1V2XJM4|A0A1V2XJM4_9BURK Helix-turn-helix domain-containing protein OS=Burkholderia cenocepacia OX=95486 GN=A8F55_38750 PE=4 SV=1	T.AAAST[+568.212]AAPAQPAS[+568.212]VVVAAGQS.M	HexNAc(2)Hex(1), HexNAc(2)Hex(1)	No	1482.1934	2	2963.3794	2961.3682	1.5	Non	320.1	240.96	20.27	Nsco_20191108_BC_ZIC_HILIC_H111_thermolysin_B3.33206.33206.2	66.5721	Thermolysin	B3	177
BCAL1893	>tr|A0A1V2VRW6|A0A1V2VRW6_9BURK Family M23 peptidase OS=Burkholderia cenocepacia OX=95486 GN=nlpD_1 PE=4 SV=1	R.VAPPGGAAVAGAPAAAPIVGGAVAT[+568.212]APLSSGPAAPAAGTSSALAATPPAAATGSSDTAAAPSGPVTFAWPAR.G	HexNAc(2)Hex(1)	No	1692.3637	4	6766.4331	6766.401	4.7	Specific	960.85	960.85	7.56	Nsco_20191108_BC_ZIC_HILIC_H111_typsin_B1.77573.77573.4	142.8025	Trypsin	B1	178
BCAL1917	>tr|A0A2N9CHU5|A0A2N9CHU5_9BURK Uncharacterized protein OS=Burkholderia cenocepacia OX=95486 GN=F01_210059 PE=4 SV=1	T.ATAGTT[+568.212]TAAPAPTASAPEA.A	HexNAc(2)Hex(1)	No	1113.0168	2	2225.0263	2225.0191	3.2	Non	479.56	271.22	0	Nsco_20191108_BC_ZIC_HILIC_H111_pepsin_B2.24784.24784.2	51.3388	Pepsin	B2	179
BCAL1917	>tr|A0A2N9CHU5|A0A2N9CHU5_9BURK Uncharacterized protein OS=Burkholderia cenocepacia OX=95486 GN=F01_210059 PE=4 SV=1	T.TATAGTTTAAPAPTAS[+568.212]APEAAAKPAKTKR.A	HexNAc(2)Hex(1)	No	1093.223	3	3277.6545	3277.6646	-3.1	Non	380.66	380.66	1.2	Nsco_20191108_BC_ZIC_HILIC_H111_thermolysin_B1.12081.12081.3	30.2274	Thermolysin	B1	180
BCAL1917	>tr|A0A2N9CHU5|A0A2N9CHU5_9BURK Uncharacterized protein OS=Burkholderia cenocepacia OX=95486 GN=F01_210059 PE=4 SV=1	K.LSKPAATTSATTSTTTSAGTASTTTATAGTTTAAPAPTAS[+568.212]APEAAAKPAKTKR.A	HexNAc(2)Hex(1)	No	1112.9653	5	5560.7976	5560.7723	4.5	Non	1523.41	1442.53	37.43	Nsco_20191108_BC_ZIC_HILIC_H111_thermolysin_B1.18180.18180.5	39.1099	Thermolysin	B1	181
BCAL1917	>tr|A0A2N9CHU5|A0A2N9CHU5_9BURK Uncharacterized protein OS=Burkholderia cenocepacia OX=95486 GN=F01_210059 PE=4 SV=1	K.LSKPAATTSATTSTTTSAGTASTTTATAGTTTAAPAPTAS[+568.212][+100.064]APEAAAKPA.K	HexNAc(2)Hex(1) 100.064	No	1717.4998	3	5150.4847	5147.4976	-4.5	Non	390.69	378.24	5.94	Nsco_20191108_BC_ZIC_HILIC_H111_thermolysin_B1.30680.30680.3	60.0593	Thermolysin	B1	182
BCAL1917	>tr|A0A2N9CHU5|A0A2N9CHU5_9BURK Uncharacterized protein OS=Burkholderia cenocepacia OX=95486 GN=F01_210059 PE=4 SV=1	T.AAPAPTAS[+568.212]APEAAAKPAKTKRASKKEK.A	HexNAc(2)Hex(1)	Yes	649.7541	5	3244.7412	3244.7271	4.4	Non	796.81	784.32	121.44	Nsco_20191108_BC_ZIC_HILIC_H111_thermolysin_B1.3500.3500.5	17.3463	Thermolysin	B1	183

BCAL1917	>tr|A0A2N9CHU5|A0A2N9CHU5_9BURK Uncharacterized protein OS=Burkholderia cenocepacia OX=95486 GN=F01_210059 PE=4 SV=1	G.TTTAAPAPT[+568.212]AS[+568.212][+100.064]APEAAAKPAKTKRASKKEKAA.A	HexNAc(2)Hex(1), HexNAc(2)Hex(1) 100.064	No	872.8513	5	4360.2276	4358.2199	0.2	Non	386.81	386.81	0	Nsco_20191108_BC_ZIC_HILIC_H111_thermolysin_B1.4588.4588.4	18.8915	Thermolysin	B1	184
BCAL1917	>tr|A0A2N9CHU5|A0A2N9CHU5_9BURK Uncharacterized protein OS=Burkholderia cenocepacia OX=95486 GN=F01_210059 PE=4 SV=1	T.TAAPAPTASAPEAAAKPAKT[+568.212]KRASKKEKA.A	HexNAc(2)Hex(1)	No	1139.9388	3	3417.802	3416.8119	-3.9	Non	161.58	161.58	9.6	Nsco_20191108_BC_ZIC_HILIC_H111_thermolysin_B1.5091.5091.3	19.6085	Thermolysin	B1	185
BCAL1917	>tr|A0A2N9CHU5|A0A2N9CHU5_9BURK Uncharacterized protein OS=Burkholderia cenocepacia OX=95486 GN=F01_210059 PE=4 SV=1	T.AAPAPTAS[+568.212]APEAAAKPAKTKRASKKEKA.A	HexNAc(2)Hex(1)	Yes	663.961	5	3315.7758	3315.7642	3.5	Non	1153.47	1053.48	140.09	Nsco_20191108_BC_ZIC_HILIC_H111_thermolysin_B1.3693.3693.5	17.6616	Thermolysin	B1	186
BCAL1917	>tr|A0A2N9CHU5|A0A2N9CHU5_9BURK Uncharacterized protein OS=Burkholderia cenocepacia OX=95486 GN=F01_210059 PE=4 SV=1	T.AAPAPTAS[+568.212]APEAAAKPAKTKRASKKEKAAAA.A	HexNAc(2)Hex(1)	Yes	882.9785	4	3528.8922	3528.8755	4.7	Non	761.25	761.25	127.74	Nsco_20191108_BC_ZIC_HILIC_H111_thermolysin_B1.4372.4372.4	18.6162	Thermolysin	B1	187
BCAL1917	>tr|A0A2N9CHU5|A0A2N9CHU5_9BURK Uncharacterized protein OS=Burkholderia cenocepacia OX=95486 GN=F01_210059 PE=4 SV=1	T.AGTTTAAPAPTAS[+568.212]APEAAAKPAKTKR.A	HexNAc(2)Hex(1)	No	1002.5198	3	3005.545	3004.5321	3.2	Non	486.62	400.84	2.4	Nsco_20191108_BC_ZIC_HILIC_H111_thermolysin_B1.8726.8726.3	24.9449	Thermolysin	B1	188
BCAL1917	>tr|A0A2N9CHU5|A0A2N9CHU5_9BURK Uncharacterized protein OS=Burkholderia cenocepacia OX=95486 GN=F01_210059 PE=4 SV=1	A.ATTSATTSTTTSAGTASTSTTATGTTT[+568.212][+100.064]AAPAPTASAPEAAAKPAKTKR.A	HexNAc(2)Hex(1) 100.064	No	1033.7095	5	5164.5183	5164.5354	-3.3	Non	298.51	298.51	0	Nsco_20191108_BC_ZIC_HILIC_H111_thermolysin_B2.17991.17991.5	40.013	Thermolysin	B2	189
BCAL1917	>tr|A0A2N9CHU5|A0A2N9CHU5_9BURK Uncharacterized protein OS=Burkholderia cenocepacia OX=95486 GN=F01_210059 PE=4 SV=1	T.AAPAPTAS[+568.212]APEAAAKPAKTKRAS.K	HexNAc(2)Hex(1)	Yes	683.6077	4	2731.4092	2731.3996	3.5	Non	695.41	663.67	96.64	Nsco_20191108_BC_ZIC_HILIC_H111_thermolysin_B2.5786.5786.4	20.9413	Thermolysin	B2	190
BCAL1917	>tr|A0A2N9CHU5|A0A2N9CHU5_9BURK Uncharacterized protein OS=Burkholderia cenocepacia OX=95486 GN=F01_210059 PE=4 SV=1	T.ATAGTTTAAPAPTAS[+568.212]APEAAAKPAKTKR.A	HexNAc(2)Hex(1)	No	1059.8811	3	3177.6288	3176.6169	2.7	Non	724.14	537.25	2.4	Nsco_20191108_BC_ZIC_HILIC_H111_thermolysin_B2.9225.9225.3	25.9324	Thermolysin	B2	191
BCAL1917	>tr|A0A2N9CHU5|A0A2N9CHU5_9BURK Uncharacterized protein OS=Burkholderia cenocepacia OX=95486 GN=F01_210059 PE=4 SV=1	T.AAPAPTAS[+568.212]APEAAAKPAKTKRASKKEKAAA.A	HexNAc(2)Hex(1)	Yes	692.3754	5	3457.8481	3457.8384	2.8	Non	1074.62	1074.62	140.46	Nsco_20191108_BC_ZIC_HILIC_H111_thermolysin_B3.4136.4136.5	18.4944	Thermolysin	B3	192
BCAL1917	>tr|A0A2N9CHU5|A0A2N9CHU5_9BURK Uncharacterized protein OS=Burkholderia cenocepacia OX=95486 GN=F01_210059 PE=4 SV=1	T.AAPAPTAS[+568.212]APEAAAKPAKTKRASKKEKAA.A	HexNAc(2)Hex(1)	Yes	678.1687	5	3386.8143	3386.8013	3.8	Non	1151.4	1103.63	130.25	Nsco_20191108_BC_ZIC_HILIC_H111_thermolysin_B3.3893.3893.5	18.0785	Thermolysin	B3	193
BCAL1917	>tr|A0A2N9CHU5|A0A2N9CHU5_9BURK Uncharacterized protein OS=Burkholderia cenocepacia OX=95486 GN=F01_210059 PE=4 SV=1	T.AAPAPTAS[+568.212]APEAAAKPAKTKR.A	HexNAc(2)Hex(1)	Yes	644.0909	4	2573.3418	2573.3305	4.4	Non	917.07	748.87	124.03	Nsco_20191108_BC_ZIC_HILIC_H111_thermolysin_B3.5571.5571.4	20.9475	Thermolysin	B3	194
BCAL1917	>tr|A0A2N9CHU5|A0A2N9CHU5_9BURK Uncharacterized protein OS=Burkholderia cenocepacia OX=95486 GN=F01_210059 PE=4 SV=1	P.AATTSATTSTTTSAGTASTS[+58.212]TTATAGTTTAAPAPTASAPEAAAKPAKTKR.A	HexNAc(2)Hex(1)	No	1285.1395	4	5137.5363	5135.5085	4.1	Non	698.47	689.16	0	Nsco_20191108_BC_ZIC_HILIC_H111_thermolysin_B3.17015.17015.4	38.7465	Thermolysin	B3	195
BCAL1917	>tr|A0A2N9CHU5|A0A2N9CHU5_9BURK Uncharacterized protein OS=Burkholderia cenocepacia OX=95486 GN=F01_210059 PE=4 SV=1	T.TAAPAPTASAPEAAAKPAKT[+568.212]KRASKKEKAAA.A	HexNAc(2)Hex(1)	No	712.5804	5	3558.8729	3558.8861	-3.7	Non	715.88	715.88	9.6	Nsco_20191108_BC_ZIC_HILIC_H111_thermolysin_B3.5230.5230.5	20.3847	Thermolysin	B3	196

BCAL1917	>tr|A0A2N9CHU5|A0A2N9CHU5_9BURK Uncharacterized protein OS=Burkholderia cenocepacia OX=95486 GN=F01_210059 PE=4 SV=1	T.TAAPAPTAS[+568.212]APEAAAKPAKTKR.A	HexNAc(2)Hex(1)	No	1337.6868	2	2674.3663	2674.3781	-4.4	Non	490.81	380.09	2.4	Nsco_20191108_BC_ZIC_HILIC_H111_thermolysin_B3.7211.7211.2	23.2425	Thermolysin	B3	197
BCAL1917	>tr|A0A2N9CHU5|A0A2N9CHU5_9BURK Uncharacterized protein OS=Burkholderia cenocepacia OX=95486 GN=F01_210059 PE=4 SV=1	A.AAAAAAAASADAGASAPAAASS[+568.212]T[+568.212]K.A	HexNAc(2)Hex(1), HexNAc(2)Hex(1)	No	1498.6727	2	2996.338	2996.3325	1.8	NRagged	855.07	790.62	1.2	Nsco_20191108_BC_ZIC_HILIC_H111_typsin_B1.11090.11090.2	30.0018	Trypsin	B1	198
BCAL1917	>tr|A0A2N9CHU5|A0A2N9CHU5_9BURK Uncharacterized protein OS=Burkholderia cenocepacia OX=95486 GN=F01_210059 PE=4 SV=1	T.STTATAGTTTAAPAPT[+568.212]ASAPEAAAKPAK.T	HexNAc(2)Hex(1)	No	1027.5093	3	3080.5134	3080.5005	4.2	NRagged	679.67	588.94	2.4	Nsco_20191108_BC_ZIC_HILIC_H111_typsin_B1.15161.15161.3	36.4723	Trypsin	B1	199
BCAL1917	>tr|A0A2N9CHU5|A0A2N9CHU5_9BURK Uncharacterized protein OS=Burkholderia cenocepacia OX=95486 GN=F01_210059 PE=4 SV=1	A.TAGTTTAAPAPTAS[+568.212]APEAAAKPAK.T	HexNAc(2)Hex(1)	No	907.453	3	2720.3444	2720.336	3.1	NRagged	708.75	145.82	7.02	Nsco_20191108_BC_ZIC_HILIC_H111_typsin_B1.12720.12720.3	32.3796	Trypsin	B1	200
BCAL1917	>tr|A0A2N9CHU5|A0A2N9CHU5_9BURK Uncharacterized protein OS=Burkholderia cenocepacia OX=95486 GN=F01_210059 PE=4 SV=1	T.SAGTASTSTTATAGTTTAAPAPAS[+568.212]APEAAAKPAK.T	HexNAc(2)Hex(1)	No	1219.262	3	3655.7715	3655.7556	4.3	NRagged	934.29	918.95	4.53	Nsco_20191108_BC_ZIC_HILIC_H111_typsin_B2.19051.19051.3	41.4334	Trypsin	B2	201
BCAL1917	>tr|A0A2N9CHU5|A0A2N9CHU5_9BURK Uncharacterized protein OS=Burkholderia cenocepacia OX=95486 GN=F01_210059 PE=4 SV=1	S.KPAATTSATTSTTTSAGTASTSTATAGTTTAAPAPTAS[+568.212]APEAAAKPAK.T	HexNAc(2)Hex(1)	No	1244.8641	4	4976.4347	4975.4125	3.8	NRagged	454.51	454.51	25.96	Nsco_20191108_BC_ZIC_HILIC_H111_typsin_B2.20241.20241.4	43.5398	Trypsin	B2	202
BCAL1917	>tr|A0A2N9CHU5|A0A2N9CHU5_9BURK Uncharacterized protein OS=Burkholderia cenocepacia OX=95486 GN=F01_210059 PE=4 SV=1	T.TSATTSTTTSAGTASTS[+568.212][+100.064]TTATAGTTTAAPAPT[+568.212][+100.064]ASAPEAAAKPAK.T	HexNAc(2)Hex(1) 100.064,HexNAc(2)Hex(1) 100.064	No	1319.631	4	5275.5021	5275.4824	3.7	NRagged	366.32	366.32	2.4	Nsco_20191108_BC_ZIC_HILIC_H111_typsin_B2.43076.43076.4	84.0457	Trypsin	B2	203
BCAL1917	>tr|A0A2N9CHU5|A0A2N9CHU5_9BURK Uncharacterized protein OS=Burkholderia cenocepacia OX=95486 GN=F01_210059 PE=4 SV=1	A.GTASTSTTATAGTTTAAPAPT[568.212]ASAPEAAAKPAK.T	HexNAc(2)Hex(1)	No	1166.5699	3	3497.6951	3497.6865	2.5	NRagged	863.61	803.48	3.18	Nsco_20191108_BC_ZIC_HILIC_H111_typsin_B2.18137.18137.3	39.9377	Trypsin	B2	204
BCAL1917	>tr|A0A2N9CHU5|A0A2N9CHU5_9BURK Uncharacterized protein OS=Burkholderia cenocepacia OX=95486 GN=F01_210059 PE=4 SV=1	K.LSKPAATTSATTSTTTSAGTASTTTATAGTTTAAPAPTAS[+568.212]APEAAAK.P	HexNAc(2)Hex(1)	No	1220.8451	4	4880.3587	4879.3437	2.4	Specific	915.76	906.55	60.93	Nsco_20191108_BC_ZIC_HILIC_H111_typsin_B3.24861.24861.4	53.4965	Trypsin	B3	205
BCAL1917	>tr|A0A2N9CHU5|A0A2N9CHU5_9BURK Uncharacterized protein OS=Burkholderia cenocepacia OX=95486 GN=F01_210059 PE=4 SV=1	K.PAATTSATTSTTTSAGTASTSTTAGTTTAAPAPT[+568.212]ASAPEAAAKPAK.T	HexNAc(2)Hex(1)	No	1213.089	4	4849.3341	4847.3175	2	Specific	1213.9	1213.9	4.8	Nsco_20191108_BC_ZIC_HILIC_H111_typsin_B3.24719.24719.4	53.2254	Trypsin	B3	206
BCAL1917	>tr|A0A2N9CHU5|A0A2N9CHU5_9BURK Uncharacterized protein OS=Burkholderia cenocepacia OX=95486 GN=F01_210059 PE=4 SV=1	A.AAAAAASADAGASAPAAAS[+568.212]S[+568.212][+100.064]T[+568.212]K.A	HexNAc(2)Hex(1), HexNAc(2)Hex(1) 100.064,HexNAc(2)Hex(1)	No	1797.2834	2	3593.5596	3593.571	-3.2	NRagged	582.16	537.26	0	Nsco_20191108_BC_ZIC_HILIC_H111_typsin_B3.12030.12030.2	30.1645	Trypsin	B3	207
BCAL1917	>tr|A0A2N9CHU5|A0A2N9CHU5_9BURK Uncharacterized protein OS=Burkholderia cenocepacia OX=95486 GN=F01_210059 PE=4 SV=1	A.STSTTATAGTTTAAPAPT[+568.212]ASAPEAAAKPAK.T	HexNAc(2)Hex(1)	No	1090.2005	3	3268.5869	3268.5802	2	NRagged	567.87	567.87	2.77	Nsco_20191108_BC_ZIC_HILIC_H111_typsin_B3.16581.16581.3	37.8916	Trypsin	B3	208
BCAL1917	>tr|A0A2N9CHU5|A0A2N9CHU5_9BURK Uncharacterized protein OS=Burkholderia cenocepacia OX=95486 GN=F01_210059 PE=4 SV=1	T.STTTSAGTASTSTTATAGTTTAAPT[+568.212]ASAPEAAAKPAK.T	HexNAc(2)Hex(1)	No	1349.3188	3	4045.9418	4045.9307	2.7	NRagged	720.97	718.16	4.8	Nsco_20191108_BC_ZIC_HILIC_H111_typsin_B3.20204.20204.3	44.5606	Trypsin	B3	209

BCAL1917	>tr|A0A2N9CHU5|A0A2N9CHU5_9BURK Uncharacterized protein OS=Burkholderia cenocepacia OX=95486 GN=F01_210059 PE=4 SV=1	A.TTSTTTSAGTASTSTTATAGTTAPAPT[+568.212]ASAPEAAAKPAK.T	HexNAc(2)Hex(1)	No	1417.6876	3	4251.0483	4248.026	2.9	NRagged	842.67	186.29	0.29	Nsco_20191108_BC_ZIC_HILIC_H111_typsin_B3.20809.20809.3	45.7375	Trypsin	B3	210
BCAL1917	>tr|A0A2N9CHU5|A0A2N9CHU5_9BURK Uncharacterized protein OS=Burkholderia cenocepacia OX=95486 GN=F01_210059 PE=4 SV=1	S.ATTSTTTSAGTASTSTTATAGTTAAPAPT[+568.212]ASAPEAAAKPAK.T	HexNAc(2)Hex(1)	No	1440.3658	3	4319.0828	4319.0631	4.5	NRagged	892.62	884.51	4.8	Nsco_20191108_BC_ZIC_HILIC_H111_typsin_B3.21061.21061.3	46.2839	Trypsin	B3	211
BCAL1917	>tr|A0A2N9CHU5|A0A2N9CHU5_9BURK Uncharacterized protein OS=Burkholderia cenocepacia OX=95486 GN=F01_210059 PE=4 SV=1	T.TSAGTASTSTTATAGTTTAAPATAS[+568.212]APEAAAKPAK.T	HexNAc(2)Hex(1)	No	1252.9331	3	3756.7848	3756.8033	-4.9	NRagged	608.21	601.73	3.99	Nsco_20191108_BC_ZIC_HILIC_H111_typsin_B3.21338.21338.3	46.7666	Trypsin	B3	212
BCAL1917	>tr|A0A2N9CHU5|A0A2N9CHU5_9BURK Uncharacterized protein OS=Burkholderia cenocepacia OX=95486 GN=F01_210059 PE=4 SV=1	K.SVGHLEENGLT[+568.212]IGGASTPPK.G	HexNAc(2)Hex(1)	No	844.7475	3	2532.2279	2532.2199	3.2	Specific	575.83	517.48	4.8	Nsco_20191108_BC_ZIC_HILIC_H111_typsin_B3.25309.25309.3	54.4126	Trypsin	B3	213
BCAL1917	>tr|A0A2N9CHU5|A0A2N9CHU5_9BURK Uncharacterized protein OS=Burkholderia cenocepacia OX=95486 GN=F01_210059 PE=4 SV=1	P.AATTSATTSTTTSAGTASTSTTAAGTTTAAPAPT[+568.212][+100.064]ASAPEAAAKPAK.T	HexNAc(2)Hex(1) 100.064	No	1617.4548	3	4850.3499	4850.3288	4.4	NRagged	594.2	582.41	4.8	Nsco_20191108_BC_ZIC_HILIC_H111_typsin_B3.25793.25793.3	55.4594	Trypsin	B3	214
BCAL1917	>tr|A0A2N9CHU5|A0A2N9CHU5_9BURK Uncharacterized protein OS=Burkholderia cenocepacia OX=95486 GN=F01_210059 PE=4 SV=1	K.PAATTSATTSTTTSAGTASTSTTTAGTTTAAPAPT[+568.212]ASAPEAAAK.P	HexNAc(2)Hex(1)	No	1518.0553	3	4552.1513	4551.1327	3.4	Specific	679.17	679.17	4.8	Nsco_20191108_BC_ZIC_HILIC_H111_typsin_B3.27783.27783.3	59.2997	Trypsin	B3	215
BCAL1917	>tr|A0A2N9CHU5|A0A2N9CHU5_9BURK Uncharacterized protein OS=Burkholderia cenocepacia OX=95486 GN=F01_210059 PE=4 SV=1	K.LSKPAATTSATTSTTTSAGTASTTTATAGTTTAAPAPTAS[+568.212]APEAAAKPAK.T	HexNAc(2)Hex(1)	yes	1294.6423	4	5175.5475	5175.5286	3.7	Specific	1605.91	1543.27	67.29	Nsco_20191108_BC_ZIC_HILIC_H111_typsin_B3.22646.22646.4	49.3785	Trypsin	B3	216
BCAL2097	>tr|A0A2N9CIF7|A0A2N9CIF7_9BURK Cell division protein ZipA OS=Burkholderia cenocepacia OX=95486 GN=F01_210252 PE=3 SV=1	T.PADLQAEATGVDTS[+568.212]AESSEPAAGEEAVPAATHEAAAEPAEPT[+568.212][+100.064]EPVMPAATTVSSAPPAIVDR.R	HexNAc(2)Hex(1), HexNAc(2)Hex(1) 100.064	No	1455.4792	5	7273.3667	7272.3334	4.1	NRagged	234.05	234.05	0	Nsco_20191108_BC_ZIC_HILIC_H111_typsin_B2.65630.65630.3	127.3905	Trypsin	B2	217
BCAL2097	>tr|A0A2N9CIF7|A0A2N9CIF7_9BURK Cell division protein ZipA OS=Burkholderia cenocepacia OX=95486 GN=F01_210252 PE=3 SV=1	G.GAAPADTPADLQAEATGVDTSAESS[+568.212][+100.064]EPAAGEEAVPAATHEAAAEPAEPTEPVMPAATTVSSAPPAIVDR.R	HexNAc(2)Hex(1) 100.064	No	1458.4841	5	7288.3912	7287.382	0.8	NRagged	243.47	243.47	0	Nsco_20191108_BC_ZIC_HILIC_H111_typsin_B2.65723.65723.5	127.6274	Trypsin	B2	218
BCAL2107	>tr|A0A2N9CIG6|A0A2N9CIG6_9BURK Fused putative transporter subunits of ABC superfamily: ATP-binding components OS=Burkholderia cenocepacia OX=95486 GN=yheS PE=4 SV=1	A.DS[+568.212]AAAK.A	HexNAc(2)Hex(1)	Yes	565.7523	2	1130.4974	1130.4947	2.4	NRagged	241.82	23.12	23.12	Nsco_20191108_BC_ZIC_HILIC_H111_typsin_B1.1961.1961.2	13.168	Trypsin	B1	219
BCAL2161	>tr|A0A2N9CIP1|A0A2N9CIP1_9BURK Putative exported protein OS=Burkholderia cenocepacia OX=95486 GN=F01_230051 PE=4 SV=1	S.VAPPLQGDGAAPGGAS[+568.212]WPAPPPASGPAPGLPASSVQGT[+568.212]P.-	HexNAc(2)Hex(1), HexNAc(2)Hex(1)	Yes (1 of 2)	1541.0685	3	4621.1911	4621.1727	4	Non	465.76	361.02	34.77	Nsco_20191108_BC_ZIC_HILIC_H111_thermolysin_B1.62166.62166.3	117.7316	Thermolysin	B1	220
BCAL2191	>tr|A0A119PBH5|A0A119PBH5_9BURK Glycine zipper 2TM protein OS=Burkholderia cenocepacia OX=95486 GN=A8E72_00900 PE=4 SV=1	K.ATSSNEQNGQTQAALIAS[+568.212]QPAVDT[+568.212][+100.064]AAAASAALAAQAQR.Q	HexNAc(2)Hex(1), HexNAc(2)Hex(1) 100.064	No	1631.4314	3	4892.2796	4890.2985	-5.2	Specific	294.5	294.5	0	Nsco_20191108_BC_ZIC_HILIC_H111_typsin_B3.50609.50609.3	103.5296	Trypsin	B3	221

BCAL2276	>tr|A0A2N9CJ70|A0A2N9CJ70_9BURK Putative membrane protein OS=Burkholderia cenocepacia OX=95486 GN=DFS07_11429 PE=4 SV=1	K.STIDTAASNAGVPVS[+568.212]SVNYIVHDAGK.G	HexNAc(2)Hex(1)	No	1047.841	3	3141.5084	3141.4958	4	Specific	598.71	598.71	1.2	Nsco_20191108_BC_ZIC_HILIC_H111_typsin_B2.46258.46258.3	89.8439	Trypsin	B2	222
BCAL2398	>tr|A0A2N9CKD9|A0A2N9CKD9_9BURK Periplasmic protein TonB, links inner and outer membranes OS=Burkholderia cenocepacia OX=95486 GN=F01_260105 PE=4 SV=1	K.ASPPYAADKPIVAVFPPVVPAAPAS[+568.212]SASATR.-	HexNAc(2)Hex(1)	No	1191.9495	3	3573.834	3573.8211	3.6	Specific	861.49	861.49	2.4	Nsco_20191108_BC_ZIC_HILIC_H111_typsin_B2.54229.54229.3	104.0211	Trypsin	B2	223
BCAL2449	>tr|A0A144SYR9|A0A144SYR9_9BURK Diguanylate cyclase (GGDEF) domain protein OS=Burkholderia cenocepacia OX=95486 GN=gmr_4 PE=4 SV=1	R.RTGSVNNAPGAFS[+568.212]ASGVYPIAER.V	HexNAc(2)Hex(1)	No	963.7993	3	2889.3835	2889.3749	3	Specific	465.63	396.16	2.4	Nsco_20191108_BC_ZIC_HILIC_H111_typsin_B1.31667.31667.3	65.5806	Trypsin	B1	224
BCAL2463	>tr|A0A1V2XLQ9|A0A1V2XLQ9_9BURK Putative exported protein OS=Burkholderia cenocepacia OX=95486 GN=A8F55_33885 PE=4 SV=1	V.SPGIANVT[+568.212]AS[+568.212]AAPRPEST.A	HexNAc(2)Hex(1), HexNAc(2)Hex(1)	No	1431.6581	2	2862.309	2862.2997	3.2	Non	312.22	144.26	0	Nsco_20191108_BC_ZIC_HILIC_H111_thermolysin_B3.16208.16208.2	37.5219	Thermolysin	B3	225
BCAL2466	>tr|A0A144SXW2|A0A144SXW2_9BURK Ecotin OS=Burkholderia cenocepacia OX=95486 GN=eco PE=4 SV=1	A.APASAPAVPAES[+568.212]IKMFPQ.A	HexNAc(2)Hex(1)	No	1190.5817	2	2380.1561	2380.1476	3.6	Non	620.21	426.33	65.03	Nsco_20191108_BC_ZIC_HILIC_H111_thermolysin_B1.47927.47927.2	89.9114	Thermolysin	B1	226
BCAL2466	>tr|A0A144SXW2|A0A144SXW2_9BURK Ecotin OS=Burkholderia cenocepacia OX=95486 GN=eco PE=4 SV=1	A.APASAPAVPAES[+568.212]IKMFPQA.A	HexNAc(2)Hex(1)	No	1226.5984	2	2452.1896	2451.1847	0.6	Non	552.79	324.07	39.88	Nsco_20191108_BC_ZIC_HILIC_H111_thermolysin_B1.49468.49468.2	92.5878	Thermolysin	B1	227
BCAL2466	>tr|A0A144SXW2|A0A144SXW2_9BURK Ecotin OS=Burkholderia cenocepacia OX=95486 GN=eco PE=4 SV=1	A.APASAPAVPAES[+568.212]IKMFPQAAAGQQR.V	HexNAc(2)Hex(1)	No	1021.8416	3	3063.5102	3062.4987	2.7	NRagged	702.19	648.84	88.78	Nsco_20191108_BC_ZIC_HILIC_H111_typsin_B2.38634.38634.3	76.2715	Trypsin	B2	228
BCAL2607	>tr|A0A1V2WZX0|A0A1V2WZX0_9BURK DNA repair ATPase OS=Burkholderia cenocepacia OX=95486 GN=F01_260195 PE=4 SV=1	M.KERWEQHRAAAKGAS[+568.212]APAQ.-	HexNAc(2)Hex(1)	Yes	665.8308	4	2660.3013	2660.2911	3.8	Non	659.69	477.63	477.63	Nsco_20191108_BC_ZIC_HILIC_H111_pepsin_B2.4597.4597.4	17.4269	Pepsin	B2	229
BCAL2640	>tr|A0A144SWF6|A0A144SWF6_9BURK Putative exported protein	A.LRGAADRYAPPPAAVPVAAT[+568.212]SGAQGGA.A	HexNAc(2)Hex(1)	No	1021.1807	3	3061.5276	3060.512	4	Non	155.97	107.63	2.4	Nsco_20191108_BC_ZIC_HILIC_H111_thermolysin_B2.29749.29749.3	60.5034	Thermolysin	B2	230
BCAL2640	>tr|A0A144SWF6|A0A144SWF6_9BURK Putative exported protein OS=Burkholderia cenocepacia OX=95486 GN=A8E72_18590 PE=4 SV=1	A.LRGAADRYAPPPAAVPVAAT[+568.212]SGAQGGAAA.A	HexNAc(2)Hex(1)	No	1068.2055	3	3202.6019	3202.5862	4.9	Non	472.56	280.84	2.4	Nsco_20191108_BC_ZIC_HILIC_H111_thermolysin_B2.30511.30511.3	61.7489	Thermolysin	B2	231
BCAL2640	>tr|A0A144SWF6|A0A144SWF6_9BURK Putative exported protein OS=Burkholderia cenocepacia OX=95486 GN=A8E72_18590 PE=4 SV=1	A.LRGAADRYAPPPAAVPVAAT[+568.212]SGAQGGAA.A	HexNAc(2)Hex(1)	No	1044.5257	3	3131.5624	3131.5491	4.2	Non	406.84	276.6	2.4	Nsco_20191108_BC_ZIC_HILIC_H111_thermolysin_B3.29780.29780.3	60.9241	Thermolysin	B3	232
BCAL2640	>tr|A0A144SWF6|A0A144SWF6_9BURK Putative exported protein OS=Burkholderia cenocepacia OX=95486 GN=A8E72_18590 PE=4 SV=1	R.GAADRYAPPPAAVPVAAT[+568.212]SGAQGGAAAAAAPAGTKPANAPR.E	HexNAc(2)Hex(1)	No	1052.53	4	4207.0981	4207.0902	1.9	Specific	1111.07	1111.07	0.8	Nsco_20191108_BC_ZIC_HILIC_H111_typsin_B1.29076.29076.3	60.9225	Trypsin	B1	233
BCAL2640	>tr|A0A144SWF6|A0A144SWF6_9BURK Putative exported protein OS=Burkholderia cenocepacia OX=95486 GN=A8E72_18590 PE=4 SV=1	R.YAPPPAAVPVAATSGAQGGAAAAAAPAGT[+568.212]KPANAPREPAVRR.V	HexNAc(2)Hex(1)	No	1112.5768	4	4447.2853	4445.2695	2	Specific	661.91	661.91	3.6	Nsco_20191108_BC_ZIC_HILIC_H111_typsin_B1.25357.25357.4	54.6382	Trypsin	B1	234
BCAL2640	>tr|A0A144SWF6|A0A144SWF6_9BURK Putative exported protein OS=Burkholderia cenocepacia OX=95486 GN=A8E72_18590 PE=4 SV=1	R.GAADRYAPPPAAVPVAAT[+568.212]SGAQGGAAAAAAPAGTKPANAPREPAVR.R	HexNAc(2)Hex(1)	No	1191.105	4	4761.3981	4759.3922	-0.2	Specific	1118.94	1118.94	0.8	Nsco_20191108_BC_ZIC_HILIC_H111_typsin_B1.28719.28719.4	60.3221	Trypsin	B1	235

BCAL2640	>tr|A0A144SWF6|A0A144SWF6_9BURK Putative exported protein OS=Burkholderia cenocepacia OX=95486 GN=A8E72_18590 PE=4 SV=1	R.YAPPPAAVPVAATSGAQGGAAAAAAPAGT[+568.212]KPANAPREPAVR.R	HexNAc(2)Hex(1)	No	1073.0513	4	4289.1832	4289.1684	3.5	Specific	1032.58	1032.58	1.2	Nsco_20191108_BC_ZIC_HILIC_H111_typsin_B2.30521.30521.4	62.0121	Trypsin	B2	236
BCAL2640	>tr|A0A144SWF6|A0A144SWF6_9BURK Putative exported protein OS=Burkholderia cenocepacia OX=95486 GN=A8E72_18590 PE=4 SV=1	R.GAADRYAPPPAAVPVAAT[+568.212]SGAQGGAAAAAAPAGTK.P	HexNAc(2)Hex(1)	No	1200.9278	3	3600.7688	3600.7664	0.7	Specific	491.08	469.86	2	Nsco_20191108_BC_ZIC_HILIC_H111_typsin_B2.32989.32989.5	66.322	Trypsin	B2	237
BCAL2640	>tr|A0A144SWF6|A0A144SWF6_9BURK Putative exported protein OS=Burkholderia cenocepacia OX=95486 GN=A8E72_18590 PE=4 SV=1	R.YAPPPAAVPVAATSGAQGGAAAAAAPAGT[+568.212]KPANAPR.E	HexNAc(2)Hex(1)	No	1246.2967	3	3736.8755	3736.8664	2.4	Specific	879.44	857.22	2.21	Nsco_20191108_BC_ZIC_HILIC_H111_typsin_B2.31307.31307.3	63.3668	Trypsin	B2	238
BCAL2820	>tr|A0A1V2WC66|A0A1V2WC66_9BURK Multidrug transporter OS=Burkholderia cenocepacia OX=95486 GN=oprM PE=3 SV=1	D.YDKAAAPAPASAT[+568.212]ATNG.-	HexNAc(2)Hex(1)	No	1072.9942	2	2144.9811	2144.9717	4.4	Non	551.21	411.92	1.2	Nsco_20191108_BC_ZIC_HILIC_H111_pepsin_B1.12913.12913.2	31.746	Pepsin	B1	239
BCAL2820	>tr|A0A1V2WC66|A0A1V2WC66_9BURK Multidrug transporter OS=Burkholderia cenocepacia OX=95486 GN=oprM PE=3 SV=1	A.LGGGWIQRAGETPRAPDAPVDYDKAAAPAPAS[+568.212]ATATNG.-	HexNAc(2)Hex(1)	Yes	1073.021	4	4289.0623	4289.048	3.3	Non	572.87	533.13	60.25	Nsco_20191108_BC_ZIC_HILIC_H111_thermolysin_B1.40111.40111.4	76.5565	Thermolysin	B1	240
BCAL2820	>tr|A0A1V2WC66|A0A1V2WC66_9BURK Multidrug transporter OS=Burkholderia cenocepacia OX=95486 GN=oprM PE=3 SV=1	R.AGETPRAPDAPVDYDKAAAPAPAS[+568.212]ATATNG.-	HexNAc(2)Hex(1)	No	1141.2008	3	3421.5879	3421.5765	3.3	Non	729.11	628.95	4.8	Nsco_20191108_BC_ZIC_HILIC_H111_thermolysin_B2.29377.29377.3	59.8556	Thermolysin	B2	241
BCAL2820	>tr|A0A1V2WC66|A0A1V2WC66_9BURK Multidrug transporter OS=Burkholderia cenocepacia OX=95486 GN=oprM PE=3 SV=1	P.VDYDKAAAPAPAS[+568.212]ATATNG.-	HexNAc(2)Hex(1)	No	1180.5431	2	2360.0789	2359.0671	3.6	Non	597.71	502.43	4	Nsco_20191108_BC_ZIC_HILIC_H111_thermolysin_B2.20925.20925.2	45.2202	Thermolysin	B2	242
BCAL2820	>tr|A0A1V2WC66|A0A1V2WC66_9BURK Multidrug transporter OS=Burkholderia cenocepacia OX=95486 GN=oprM PE=3 SV=1	W.IQRAGETPRAPDAPVDYDKAAAPAPAS[+568.212]ATATNG.-	HexNAc(2)Hex(1)	No	1273.6166	3	3818.8353	3818.8203	3.9	Non	676.21	638.85	4.8	Nsco_20191108_BC_ZIC_HILIC_H111_thermolysin_B2.25629.25629.3	53.4603	Thermolysin	B2	243
BCAL2820	>tr|A0A1V2WC66|A0A1V2WC66_9BURK Multidrug transporter OS=Burkholderia cenocepacia OX=95486 GN=oprM PE=3 SV=1	K.AAAPAPASAT[+568.212]ATNG.-	HexNAc(2)Hex(1)	No	869.9	2	1738.7927	1738.7865	3.5	CRagged	423.92	286.45	0	Nsco_20191108_BC_ZIC_HILIC_H111_typsin_B1.11032.11032.2	29.9075	Trypsin	B1	244
BCAL2820	>tr|A0A1V2WC66|A0A1V2WC66_9BURK Multidrug transporter OS=Burkholderia cenocepacia OX=95486 GN=oprM PE=3 SV=1	K.RPDAPVAQAYPAS[+568.212]GVYATQPGAAGAR.S	HexNAc(2)Hex(1)	No	1037.5055	3	3110.502	3110.4913	3.5	Specific	699.97	637.21	12.09	Nsco_20191108_BC_ZIC_HILIC_H111_typsin_B1.26451.26451.3	56.5269	Trypsin	B1	245
BCAL2974	>tr|A0A144U3Q1|A0A144U3Q1_9BURK Uncharacterized protein OS=Burkholderia cenocepacia OX=95486 GN=A8E72_12460 PE=4 SV=1	M.PFAASAPS[+568.212]QKYQGSKKSAL.R	HexNAc(2)Hex(1)	No	845.4247	3	2534.2597	2534.2508	3.5	NRagged	641.1	567.25	1.2	Nsco_20191108_BC_ZIC_HILIC_H111_pepsin_B1.12679.12679.3	31.416	Pepsin	B1	246
BCAL2974	>tr|A0A144U3Q1|A0A144U3Q1_9BURK Uncharacterized protein OS=Burkholderia cenocepacia OX=95486 GN=A8E72_12460 PE=4 SV=1	F.RLNEHPQMPFAAS[+568.212]APSQ.K	HexNAc(2)Hex(1)	No	817.0482	3	2449.1302	2449.1188	4.7	CRagged	435.18	421.83	3.39	Nsco_20191108_BC_ZIC_HILIC_H111_pepsin_B1.26120.26120.3	55.4949	Pepsin	B1	247
BCAL2974	>tr|A0A144U3Q1|A0A144U3Q1_9BURK Uncharacterized protein OS=Burkholderia cenocepacia OX=95486 GN=A8E72_12460 PE=4 SV=1	L.NEHPQMPFAASAPS[+568.212]QKYQG.S	HexNAc(2)Hex(1)	No	886.0625	3	2656.1728	2656.1719	0.4	CRagged	463.46	463.46	9.76	Nsco_20191108_BC_ZIC_HILIC_H111_pepsin_B2.24698.24698.3	51.1852	Pepsin	B2	248

BCAL2974	>tr|A0A144U3Q1|A0A144U3Q1_9BUR K Uncharacterized protein OS=Burkholderia cenocepacia OX=95486 GN=A8E72_12460 PE=4 SV=1	F.RLNEHPQMPFAAS[+568.212]APSQKYQGSKKSAL.R	HexNAc(2)Hex(1)	Yes	708.7547	5	3539.7445	3539.7323	3.5	Specific	1130.77	1103.56	104.29	Nsco_20191108_BC_ZIC_HILIC_H111_pepsin_B3.19692.19692.5	40.9669	Pepsin	B3	249
BCAL2974	>tr|A0A144U3Q1|A0A144U3Q1_9BUR K Uncharacterized protein OS=Burkholderia cenocepacia OX=95486 GN=A8E72_12460 PE=4 SV=1	L.NEHPQMPFAAS[+568.212]APSQKYQGSKKSAL.R	HexNAc(2)Hex(1)	Yes	818.3957	4	3270.5608	3270.5471	4.2	Specific	1164.9	1137.25	171.47	Nsco_20191108_BC_ZIC_HILIC_H111_pepsin_B3.19176.19176.4	40.0011	Pepsin	B3	250
BCAL2974	>tr|A0A144U3Q1|A0A144U3Q1_9BUR K Uncharacterized protein OS=Burkholderia cenocepacia OX=95486 GN=A8E72_12460 PE=4 SV=1	P.FAASAPSQKYQGS[+568.212]KKSA.L	HexNAc(2)Hex(1)	No	1162.5658	2	2324.1244	2324.114	4.5	Non	611.86	452.14	0.09	Nsco_20191108_BC_ZIC_HILIC_H111_thermolysin_B2.5849.5849.2	21.0158	Thermolysin	B2	251
BCAL2974	>tr|A0A144U3Q1|A0A144U3Q1_9BUR K Uncharacterized protein OS=Burkholderia cenocepacia OX=95486 GN=A8E72_12460 PE=4 SV=1	R.LNEHPQMPFAASAPS[+568.212]QK.Y	HexNAc(2)Hex(1)	No	1211.0638	2	2421.1203	2421.1126	3.2	Specific	870.93	774.64	4.79	Nsco_20191108_BC_ZIC_HILIC_H111_typsin_B1.22975.22975.2	50.0902	Trypsin	B1	252
BCAL2983A	>tr|A0A2N9CL86|A0A2N9CL86_9BURK Putative lipoprotein OS=Burkholderia cenocepacia OX=95486 GN=F01_310136 PE=4 SV=1	S.ADASAPVAGTRPAVTSLSGGASSAASGAVAT[+568.212]DAAAQGNVAELTQMLHDGR.I	HexNAc(2)Hex(1)	No	1301.8806	4	5204.5006	5204.4772	4.5	NRagged	604.47	604.47	0	Nsco_20191108_BC_ZIC_HILIC_H111_typsin_B3.53186.53186.5	108.5861	Trypsin	B3	253
BCAL3033	>tr|A0A2N9CM16|A0A2N9CM16_9BU RK Outer-membrane lipoprotein carrier protein OS=Burkholderia cenocepacia OX=95486 GN=lolA PE=3 SV=1	Q.IVKAPAKGAS[+568.212]AAQAAPKPTDN.S	HexNAc(2)Hex(1)	Yes	644.5867	4	2575.3249	2574.3145	2.8	Non	729.64	648.17	408.82	Nsco_20191108_BC_ZIC_HILIC_H111_pepsin_B3.10005.10005.4	25.4729	Pepsin	B3	254
BCAL3033	>tr|A0A2N9CM16|A0A2N9CM16_9BU RK Outer-membrane lipoprotein carrier protein OS=Burkholderia cenocepacia OX=95486 GN=lolA PE=3 SV=1	Q.IVKAPAKGAS[+568.212]AAQAAPKPTDNSSGTF.V	HexNAc(2)Hex(1)	Yes	764.1379	4	3053.5298	3053.5161	4.5	NRagged	1082.94	1032.62	506.95	Nsco_20191108_BC_ZIC_HILIC_H111_pepsin_B3.16451.16451.4	35.4703	Pepsin	B3	255
BCAL3033	>tr|A0A2N9CM16|A0A2N9CM16_9BU RK Outer-membrane lipoprotein carrier protein OS=Burkholderia cenocepacia OX=95486 GN=lolA PE=3 SV=1	Q.IVKAPAKGAS[+568.212]AAQAAPKPTDNSSGTFV.F	HexNAc(2)Hex(1)	Yes	788.9051	4	3152.5986	3152.5845	4.5	Non	805.9	805.9	378.72	Nsco_20191108_BC_ZIC_HILIC_H111_pepsin_B3.19759.19759.4	41.0925	Pepsin	B3	256
BCAL3033	>tr|A0A2N9CM16|A0A2N9CM16_9BU RK Outer-membrane lipoprotein carrier protein OS=Burkholderia cenocepacia OX=95486 GN=lolA PE=3 SV=1	Q.IVKAPAKGAS[+568.212]AAQAAPKPTDNSSGT.F	HexNAc(2)Hex(1)	Yes	727.371	4	2906.4621	2906.4477	5	Non	727.77	543.13	403.44	Nsco_20191108_BC_ZIC_HILIC_H111_thermolysin_B1.9738.9738.4	26.6693	Thermolysin	B1	257
BCAL3033	>tr|A0A2N9CM16|A0A2N9CM16_9BU RK Outer-membrane lipoprotein carrier protein OS=Burkholderia cenocepacia OX=95486 GN=lolA PE=3 SV=1	Q.IVKAPAKGAS[+568.212]AAQAAPKPTDNSSGTFVF.A	HexNAc(2)Hex(1)	Yes	825.6715	4	3299.6643	3299.6529	3.4	NRagged	451.66	424.44	313.42	Nsco_20191108_BC_ZIC_HILIC_H111_thermolysin_B3.26971.26971.4	55.9849	Thermolysin	B3	258
BCAL3033	>tr|A0A2N9CM16|A0A2N9CM16_9BU RK Outer-membrane lipoprotein carrier protein OS=Burkholderia cenocepacia OX=95486 GN=lolA PE=3 SV=1	K.GAS[+568.212]AAQAAPKPTDNSSGTFVFARPGK.F	HexNAc(2)Hex(1)	Yes	776.1301	4	3101.4988	3101.4909	2.5	Specific	1141.01	1022.65	329.49	Nsco_20191108_BC_ZIC_HILIC_H111_typsin_B1.21425.21425.4	47.2372	Trypsin	B1	259
BCAL3033	>tr|A0A2N9CM16|A0A2N9CM16_9BU RK Outer-membrane lipoprotein carrier protein OS=Burkholderia cenocepacia OX=95486 GN=lolA PE=3 SV=1	K.GASAAQAAPKPT[+568.212]DNSSGTFVFAR.P	HexNAc(2)Hex(1)	No	940.448	3	2819.3293	2819.3218	2.7	Specific	945.35	771.26	9.1	Nsco_20191108_BC_ZIC_HILIC_H111_typsin_B3.26104.26104.3	56.044	Trypsin	B3	260
BCAL3166	>tr|A0A1V2WZ65|A0A1V2WZ65_9BUR K BON domain-containing protein OS=Burkholderia cenocepacia OX=95486 GN=A8E72_33585 PE=4 SV=1	A.TEAPAAATSAPKAAAKT[+568.212]AKKANRKLGY.A	HexNAc(2)Hex(1)	No	1085.9139	3	3255.7273	3254.7114	3.8	Non	772.23	364.05	9.6	Nsco_20191108_BC_ZIC_HILIC_H111_pepsin_B3.12083.12083.3	28.6806	Pepsin	B3	261

BCAL3377	>tr|A0A142PIH6|A0A142PIH6_9BURK Outer membrane protein assembly factor BamE OS=Burkholderia cenocepacia OX=95486 GN=bamE PE=3 SV=1	D.IDGDRGGKKAKAAAAAKKAS[+568.212]EAAA.A	HexNAc(2)Hex(1)	Yes	565.6979	5	2824.4605	2824.4534	2.5	Non	649.03	597.74	597.74	Nsco_20191108_BC_ZIC_HILIC_H111_pepsin_B2.4588.4588.5	17.4183	Pepsin	B2	262
BCAL3377	>tr|A0A142PIH6|A0A142PIH6_9BURK Outer membrane protein assembly factor BamE OS=Burkholderia cenocepacia OX=95486 GN=bamE PE=3 SV=1	A.AAAASPATVPAS[+568.212]GAAVDQDANAQAARAANRATNQ.V	HexNAc(2)Hex(1)	No	1249.5988	3	3746.7819	3746.77	3.2	Non	766.21	698.32	9.6	Nsco_20191108_BC_ZIC_HILIC_H111_thermolysin_B1.28242.28242.3	55.8564	Thermolysin	B1	263
BCAL3377	>tr|A0A142PIH6|A0A142PIH6_9BURK Outer membrane protein assembly factor BamE OS=Burkholderia cenocepacia OX=95486 GN=bamE PE=3 SV=1	A.ASPATVPAS[+568.212]GAAVDQDANAQAARAANRATNQ.V	HexNAc(2)Hex(1)	No	1178.8954	3	3534.6716	3533.6586	2.7	Non	624.68	580.63	3.86	Nsco_20191108_BC_ZIC_HILIC_H111_thermolysin_B1.26493.26493.3	52.7385	Thermolysin	B1	264
BCAL3377	>tr|A0A142PIH6|A0A142PIH6_9BURK Outer membrane protein assembly factor BamE OS=Burkholderia cenocepacia OX=95486 GN=bamE PE=3 SV=1	A.AAAAKKASEAAAAASAAQAAAAAS[+568.212]PAT[+568.212]VPASGAAVDQDANAQ.A	HexNAc(2)Hex(1), HexNAc(2)Hex(1)	No	1601.7587	3	4803.2614	4801.2393	3.2	Non	375.64	169.53	4.8	Nsco_20191108_BC_ZIC_HILIC_H111_thermolysin_B1.38320.38320.3	73.5381	Thermolysin	B1	265
BCAL3377	>tr|A0A142PIH6|A0A142PIH6_9BURK Outer membrane protein assembly factor BamE OS=Burkholderia cenocepacia OX=95486 GN=bamE PE=3 SV=1	A.AASPATVPAS[+568.212]GAAVDQDANAQAARAANRATNQ.V	HexNAc(2)Hex(1)	No	1202.2415	3	3604.71	3604.6957	3.9	Non	754.14	628.56	9.6	Nsco_20191108_BC_ZIC_HILIC_H111_thermolysin_B1.27168.27168.3	53.8751	Thermolysin	B1	266
BCAL3377	>tr|A0A142PIH6|A0A142PIH6_9BURK Outer membrane protein assembly factor BamE OS=Burkholderia cenocepacia OX=95486 GN=bamE PE=3 SV=1	A.AAASPATVPAS[+568.212]GAAVDQDANAQAARAANR.A	HexNAc(2)Hex(1)	No	1088.1919	3	3262.5612	3261.5466	3.5	Non	670.85	352.84	9.6	Nsco_20191108_BC_ZIC_HILIC_H111_thermolysin_B2.22544.22544.3	48.0203	Thermolysin	B2	267
BCAL3377	>tr|A0A142PIH6|A0A142PIH6_9BURK Outer membrane protein assembly factor BamE OS=Burkholderia cenocepacia OX=95486 GN=bamE PE=3 SV=1	A.AAASPATVPAS[+568.212]GAAVDQDANAQ.A	HexNAc(2)Hex(1)	No	1276.0848	2	2551.1622	2551.153	3.6	Non	310.38	216.69	2.4	Nsco_20191108_BC_ZIC_HILIC_H111_thermolysin_B2.27766.27766.2	57.1181	Thermolysin	B2	268
BCAL3377	>tr|A0A142PIH6|A0A142PIH6_9BURK Outer membrane protein assembly factor BamE OS=Burkholderia cenocepacia OX=95486 GN=bamE PE=3 SV=1	Q.AAAAASPATVPAS[+568.212]GAAVDQDANAQAARAANRATNQ.V	HexNAc(2)Hex(1)	No	1273.9446	3	3819.8192	3817.8071	1.4	Non	725.88	651.03	2.45	Nsco_20191108_BC_ZIC_HILIC_H111_thermolysin_B3.27596.27596.3	57.259	Thermolysin	B3	269
BCAL3377	>tr|A0A142PIH6|A0A142PIH6_9BURK Outer membrane protein assembly factor BamE OS=Burkholderia cenocepacia OX=95486 GN=bamE PE=3 SV=1	A.AAAASPATVPAS[+568.212]GAAVDQDANAQ.A	HexNAc(2)Hex(1)	No	1311.6047	2	2622.2022	2622.1901	4.6	Non	398.76	278.3	12.86	Nsco_20191108_BC_ZIC_HILIC_H111_thermolysin_B3.28288.28288.2	58.4224	Thermolysin	B3	270
BCAL3377	>tr|A0A142PIH6|A0A142PIH6_9BURK Outer membrane protein assembly factor BamE OS=Burkholderia cenocepacia OX=95486 GN=bamE PE=3 SV=1	A.AAASPATVPAS[+568.212]GAAVDQDANAQAARAANRATNQ.V	HexNAc(2)Hex(1)	No	1225.9203	3	3675.7465	3675.7329	3.7	Non	710.7	638.62	5.75	Nsco_20191108_BC_ZIC_HILIC_H111_thermolysin_B3.26468.26468.3	55.226	Thermolysin	B3	271

BCAL3377	>tr|A0A142PIH6|A0A142PIH6_9BURK Outer membrane protein assembly factor BamE OS=Burkholderia cenocepacia OX=95486 GN=bamE PE=3 SV=1	K.KASEAAAAAS[+568.212]AAQAAAAASPATVPASGAAVDQDANAQAAR.A	HexNAc(2)Hex(1)	Yes	1030.498	4	4118.97	4118.9596	2.5	Specific	1133.19	1029.66	156.35	Nsco_20191108_BC_ZIC_HILIC_H111_typsin_B2.39556.39556.4	77.8852	Trypsin	B2	272
BCAL3377	>tr|A0A142PIH6|A0A142PIH6_9BURK Outer membrane protein assembly factor BamE OS=Burkholderia cenocepacia OX=95486 GN=bamE PE=3 SV=1	A.AAAAKKASEAAAAASAAQAAAAAS[+568.212][+100.064]PAT[+568.212][+100.064]VPASGAAVDQDANAQAAR.A	HexNAc(2)Hex(1) 100.064,HexNAc(2)Hex(1) 100.064	No	1326.395	4	5302.5583	5299.5426	1.1	NRagged	149.57	149.57	2.4	Nsco_20191108_BC_ZIC_HILIC_H111_typsin_B2.34416.34416.4	68.7503	Trypsin	B2	273
BCAL3377	>tr|A0A142PIH6|A0A142PIH6_9BURK Outer membrane protein assembly factor BamE OS=Burkholderia cenocepacia OX=95486 GN=bamE PE=3 SV=1	K.ASEAAAAASAAQAAAAASPAT[+568.212]VPAS[+568.212]GAAVDQDANAQAAR.A	HexNAc(2)Hex(1), HexNAc(2)Hex(1)	No	1520.3664	3	4559.0846	4559.0762	1.8	Specific	1089.29	1044.6	0	Nsco_20191108_BC_ZIC_HILIC_H111_typsin_B2.43338.43338.3	84.5161	Trypsin	B2	274
BCAL3377	>tr|A0A142PIH6|A0A142PIH6_9BURK Outer membrane protein assembly factor BamE OS=Burkholderia cenocepacia OX=95486 GN=bamE PE=3 SV=1	A.SEAAAAASAAQAAAAASPAT[+568.212][+100.064]VPAS[+568.212]GAAVDQDANAQAAR.A	HexNAc(2)Hex(1) 100.064,HexNAc(2)Hex(1)	No	1530.0365	3	4588.0949	4588.1031	-1.8	NRagged	801.32	801.32	0	Nsco_20191108_BC_ZIC_HILIC_H111_typsin_B2.44897.44897.3	87.3539	Trypsin	B2	275
BCAL3377	>tr|A0A142PIH6|A0A142PIH6_9BURK Outer membrane protein assembly factor BamE OS=Burkholderia cenocepacia OX=95486 GN=bamE PE=3 SV=1	A.KKASEAAAAAS[+568.212]AAQAAAAASPAT[+568.212]VPASGAAVDQDANAQAAR.A	HexNAc(2)Hex(1), HexNAc(2)Hex(1)	No	1204.5761	4	4815.2825	4815.2662	3.4	NRagged	879.73	879.73	6.08	Nsco_20191108_BC_ZIC_HILIC_H111_typsin_B3.30338.30338.4	64.2638	Trypsin	B3	276
BCAL3377	>tr|A0A142PIH6|A0A142PIH6_9BURK Outer membrane protein assembly factor BamE OS=Burkholderia cenocepacia OX=95486 GN=bamE PE=3 SV=1	A.SAAQAAAAAS[+568.212]PAT[+568.212]VPAS[+568.212][+100.064]GAAVDQDANAQAAR.A	HexNAc(2)Hex(1), HexNAc(2)Hex(1), HexNAc(2)Hex(1) 100.064	No	1529.6973	3	4587.0774	4585.0545	3.5	NRagged	374.52	374.52	0	Nsco_20191108_BC_ZIC_HILIC_H111_typsin_B3.42023.42023.3	87.0473	Trypsin	B3	277
BCAL3469	>tr|A0A142PJ18|A0A142PJ18_9BURK Cell division protein FtsL OS=Burkholderia cenocepacia OX=95486 GN=ftsL PE=3 SV=1	A.IDAPIPAS[+568.212]ADTAGKGKGGAR.-	HexNAc(2)Hex(1)	No	606.057	4	2421.206	2421.1991	2.9	Non	579.19	487.06	2.4	Nsco_20191108_BC_ZIC_HILIC_H111_thermolysin_B2.12797.12797.4	31.8597	Thermolysin	B2	278
BCAM0157	>tr|A0A2N9CQK4|A0A2N9CQK4_9BURK Putative exported protein OS=Burkholderia cenocepacia OX=95486 GN=F01_420142 PE=4 SV=1	R.AVDC[+57.021]GQLDAATSGPDDNFRPPAS[+568.212]GTVIGTGR.A	HexNAc(2)Hex(1)	No	1224.2327	3	3670.6834	3670.6661	4.7	Specific	746.57	746.57	4.8	Nsco_20191108_BC_ZIC_HILIC_H111_typsin_B1.39845.39845.3	79.8131	Trypsin	B1	279
BCAM0264	>tr|A0A2N9CNS4|A0A2N9CNS4_9BURK Receptor family ligand-binding protein OS=Burkholderia cenocepacia OX=95486 GN=F01_390005 PE=4 SV=1	I.ATAGAS[+568.212]T[+568.212][+100.064]GAIGVSSYF.M	HexNAc(2)Hex(1), HexNAc(2)Hex(1) 100.064	No	1348.6044	2	2696.2015	2696.1935	3	NRagged	244.27	96.45	0	Nsco_20191108_BC_ZIC_HILIC_H111_pepsin_B2.17819.17819.2	38.7448	Pepsin	B2	280
BCAM0505	>tr|A0A1V2WB29|A0A1V2WB29_9BURK BON domain protein OS=Burkholderia cenocepacia OX=95486 GN=osmY_2 PE=4 SV=1	D.SGMASES[+568.212]NQPVT[+568.212]DTWITTKVKGE.L	HexNAc(2)Hex(1), HexNAc(2)Hex(1)	No	1202.5412	3	3605.609	3602.6048	-1.6	Non	253.84	253.84	4.8	Nsco_20191108_BC_ZIC_HILIC_H111_thermolysin_B1.49324.49324.3	92.3335	Thermolysin	B1	281
BCAM0505	>tr|A0A1V2WB29|A0A1V2WB29_9BURK BON domain protein OS=Burkholderia cenocepacia OX=95486 GN=osmY_2 PE=4 SV=1	A.QAS[+568.212]STDSGMASESNQPVTDTWITTKVKGELATTDGVKSTDISVKT.V	HexNAc(2)Hex(1)	No	1311.1262	4	5241.4831	5240.4897	-1.9	Non	319.81	319.81	1.4	Nsco_20191108_BC_ZIC_HILIC_H111_thermolysin_B1.55291.55291.4	103.1577	Thermolysin	B1	282

BCAM0505	>tr|A0A1V2WB29|A0A1V2WB29_9BURK BON domain protein OS=Burkholderia cenocepacia OX=95486 GN=osmY_2 PE=4 SV=1	D.SGMASESNQPVTDTWIT[+568.212]TKVKGELATTDGVKS[+568.212]TDISVKT.V	HexNAc(2)Hex(1), HexNAc(2)Hex(1)	No	1306.3752	4	5222.4792	5219.4669	0.4	Non	555.79	555.79	0	Nsco_20191108_BC_ZIC_HILIC_H111_thermolysin_B3.52234.52234.4	103.1762	Thermolysin	B3	283
BCAM0505	>tr|A0A1V2WB29|A0A1V2WB29_9BURK BON domain protein OS=Burkholderia cenocepacia OX=95486 GN=osmY_2 PE=4 SV=1	A.QASSTDSGMAS[+568.212]ESNQPVTDTWITTK.V	HexNAc(2)Hex(1)	No	1607.2037	2	3213.4002	3210.4002	-3.1	NRagged	537.66	537.66	2.59	Nsco_20191108_BC_ZIC_HILIC_H111_typsin_B1.40204.40204.2	80.4063	Trypsin	B1	284
BCAM0505	>tr|A0A1V2WB29|A0A1V2WB29_9BURK BON domain protein OS=Burkholderia cenocepacia OX=95486 GN=osmY_2 PE=4 SV=1	Q.ASSTDSGMASES[+568.212]NQPVTDTWITTK.V	HexNAc(2)Hex(1)	No	1541.6788	2	3082.3502	3082.3416	2.8	NRagged	715.39	715.39	1.66	Nsco_20191108_BC_ZIC_HILIC_H111_typsin_B2.39570.39570.2	77.9153	Trypsin	B2	285
BCAM0505	>tr|A0A1V2WB29|A0A1V2WB29_9BURK BON domain protein OS=Burkholderia cenocepacia OX=95486 GN=osmY_2 PE=4 SV=1	A.HAQASSTDSGMASES[+568.212]NQPVTDTWITTK.V	HexNAc(2)Hex(1)	No	1140.1741	3	3418.5077	3418.4962	3.4	NRagged	759.04	759.04	2.4	Nsco_20191108_BC_ZIC_HILIC_H111_typsin_B3.29909.29909.3	63.4467	Trypsin	B3	286
BCAM0690	>tr|A0A1V2Y2A4|A0A1V2Y2A4_9BURK Flagellar motor protein OS=Burkholderia cenocepacia OX=95486 GN=A8F55_06330 PE=4 SV=1	R.ALIDAGVPASSVFAAAFGS[+568.212]EQPVSSNADDEGRAK.N	HexNAc(2)Hex(1)	No	1301.6159	3	3902.8332	3902.8302	0.8	Specific	635.27	600.91	3.7	Nsco_20191108_BC_ZIC_HILIC_H111_typsin_B1.61806.61806.3	115.5056	Trypsin	B1	287
BCAM0712	>tr|A0A2N9CPU2|A0A2N9CPU2_9BURK Cobalt-zinc-cadmium resistance protein CzcB OS=Burkholderia cenocepacia OX=95486 GN=czcB PE=3 SV=1	G.GGGASAPT[+568.212]AAEVAQPAAGAGAR.G	HexNAc(2)Hex(1)	No	1204.0726	2	2407.138	2406.1267	3.3	NRagged	657.6	562.44	48.38	Nsco_20191108_BC_ZIC_HILIC_H111_typsin_B2.19920.19920.2	42.9078	Trypsin	B2	288
BCAM0936	>tr|A0A1V2XJK6|A0A1V2XJK6_9BURK Mechanosensitive ion channel protein OS=Burkholderia cenocepacia OX=95486 GN=A8F55_37840 PE=4 SV=1	A.AAPAPAAASAAS[+568.212]DAAPALT[+568.212]PQQAR.Q	HexNAc(2)Hex(1), HexNAc(2)Hex(1)	No	1104.5209	3	3311.5482	3311.5384	2.9	NRagged	767.39	758.8	31.72	Nsco_20191108_BC_ZIC_HILIC_H111_typsin_B3.27082.27082.3	57.9011	Trypsin	B3	289
BCAM0988	>tr|A0A2N9CRH5|A0A2N9CRH5_9BURK Putative exported protein OS=Burkholderia cenocepacia OX=95486 GN=F01_420505 PE=4 SV=1	Q.PAAPTAGAS[+568.212]GPHVWSGA.I	HexNAc(2)Hex(1)	No	1051.4849	2	2101.9625	2101.956	3.1	Non	557.2	394.13	5.76	Nsco_20191108_BC_ZIC_HILIC_H111_pepsin_B2.25604.25604.2	52.8615	Pepsin	B2	290
BCAM0996	>tr|A0A2N9CRL1|A0A2N9CRL1_9BURK Sporulation related OS=Burkholderia cenocepacia OX=95486 GN=F01_420513 PE=4 SV=1	Q.AVAPRDDDVS[+568.212]DVQAGVAHDEPPASDTT.V	HexNAc(2)Hex(1)	No	1101.8256	3	3303.4622	3303.4507	3.5	Non	511.96	384.22	7.61	Nsco_20191108_BC_ZIC_HILIC_H111_thermolysin_B2.28438.28438.3	58.3006	Thermolysin	B2	291
BCAM0996	>tr|A0A2N9CRL1|A0A2N9CRL1_9BURK Sporulation related OS=Burkholderia cenocepacia OX=95486 GN=F01_420513 PE=4 SV=1	A.VAPRDDDVSDVQAGVAHDEPPAS[+568.212]DTT.V	HexNAc(2)Hex(1)	No	1078.1468	3	3232.4257	3232.4135	3.8	Non	609.61	485.16	1.1	Nsco_20191108_BC_ZIC_HILIC_H111_thermolysin_B2.27181.27181.3	56.0176	Thermolysin	B2	292
BCAM0996	>tr|A0A2N9CRL1|A0A2N9CRL1_9BURK Sporulation related OS=Burkholderia cenocepacia OX=95486 GN=F01_420513 PE=4 SV=1	K.DAAKPAAKPDTTTTASVTPPKPAPKPAAPAAKPAAPKPAPATVANAGPAS[+568.212]PDSGDASSPASPAGAR.F	HexNAc(2)Hex(1)	No	1106.2348	6	6632.3723	6628.3541	0.7	Specific	431.46	429.91	31.05	Nsco_20191108_BC_ZIC_HILIC_H111_typsin_B1.20631.20631.6	45.7781	Trypsin	B1	293
BCAM0996	>tr|A0A2N9CRL1|A0A2N9CRL1_9BURK Sporulation related OS=Burkholderia cenocepacia OX=95486 GN=F01_420513 PE=4 SV=1	P.APATVANAGPASPDS[+568.212][+100.064]GDASSPASPAGAR.F	HexNAc(2)Hex(1) 100.064	No	1560.214	2	3119.4208	3118.4298	-4	NRagged	426.55	417.46	2.4	Nsco_20191108_BC_ZIC_HILIC_H111_typsin_B1.22179.22179.2	48.6827	Trypsin	B1	294

BCAM0996	>tr|A0A2N9CRL1|A0A2N9CRL1_9BURK Sporulation related OS=Burkholderia cenocepacia OX=95486 GN=F01_420513 PE=4 SV=1	K.PAAPAAKPAAPKPAPAT[+568.212]VANAGPASPDSGDASSPASPAGAR.F	HexNAc(2)Hex(1)	No	1048.2685	4	4190.0521	4186.0422	-0.9	Specific	550.99	288.16	8	Nsco_20191108_BC_ZIC_HILIC_H111_typsin_B2.20495.20495.4	44.0185	Trypsin	B2	295
BCAM0996	>tr|A0A2N9CRL1|A0A2N9CRL1_9BURK Sporulation related OS=Burkholderia cenocepacia OX=95486 GN=F01_420513 PE=4 SV=1	R.DDDVSDVQAGVAHDEPPAS[+568.212]DTTVAAAPAPAPK.D	HexNAc(2)Hex(1)	No	1228.2275	3	3682.6679	3682.6614	1.8	Specific	1148.33	1148.33	4.8	Nsco_20191108_BC_ZIC_HILIC_H111_typsin_B2.37298.37298.3	73.8101	Trypsin	B2	296
BCAM0996	>tr|A0A2N9CRL1|A0A2N9CRL1_9BURK Sporulation related OS=Burkholderia cenocepacia OX=95486 GN=F01_420513 PE=4 SV=1	P.AAPKPAPAT[+568.212][+100.064]VANAGPASPDSGDASSPASPAGAR.F	HexNAc(2)Hex(1) 100.064	No	1195.2413	3	3583.7093	3582.7045	0.4	NRagged	293.98	293.98	2.1	Nsco_20191108_BC_ZIC_HILIC_H111_typsin_B2.21349.21349.3	45.5733	Trypsin	B2	297
BCAM0996	>tr|A0A2N9CRL1|A0A2N9CRL1_9BURK Sporulation related OS=Burkholderia cenocepacia OX=95486 GN=F01_420513 PE=4 SV=1	P.AAPAAKPAAPKPAPAT[+568.212][+100.064]VANAGPASPDSGDASSPASPAGAR.F	HexNAc(2)Hex(1) 100.064	No	1048.7671	4	4192.0467	4189.0535	-4	NRagged	299.3	299.3	7.6	Nsco_20191108_BC_ZIC_HILIC_H111_typsin_B3.19856.19856.2	43.877	Trypsin	B3	298
BCAM1550	>tr|A0A142PC85|A0A142PC85_9BURK Peptidoglycan-associated protein OS=Burkholderia cenocepacia OX=95486 GN=pal PE=3 SV=1	A.AKTPENAGAAPEPS[+568.212]SETVA.T	HexNAc(2)Hex(1)	No	1198.0492	2	2395.0911	2395.0882	1.2	Non	443.15	275.96	1.2	Nsco_20191108_BC_ZIC_HILIC_H111_pepsin_B1.16299.16299.2	37.1808	Pepsin	B1	299
BCAM1550	>tr|A0A142PC85|A0A142PC85_9BURK Peptidoglycan-associated protein OS=Burkholderia cenocepacia OX=95486 GN=pal PE=3 SV=1	H.NAAKTPENAGAAPEPS[+568.212]SETVA.T	HexNAc(2)Hex(1)	No	1290.5912	2	2580.1752	2580.1683	2.7	Non	468	427.26	1.2	Nsco_20191108_BC_ZIC_HILIC_H111_pepsin_B1.17269.17269.2	38.6954	Pepsin	B1	300
BCAM1550	>tr|A0A142PC85|A0A142PC85_9BURK Peptidoglycan-associated protein OS=Burkholderia cenocepacia OX=95486 GN=pal PE=3 SV=1	T.PENAGAAPEPSSETVAT[+568.212][+100.064]VTADDLNNPNSPLAK.R	HexNAc(2)Hex(1) 100.064	No	1282.9384	3	3846.8007	3845.7938	0.9	NRagged	581.29	581.29	2.4	Nsco_20191108_BC_ZIC_HILIC_H111_typsin_B1.47137.47137.3	92.0418	Trypsin	B1	301
BCAM1550	>tr|A0A142PC85|A0A142PC85_9BURK Peptidoglycan-associated protein OS=Burkholderia cenocepacia OX=95486 GN=pal PE=3 SV=1	K.TPENAGAAPEPSSETVATVT[+568.212]ADDLNNPNSPLAKR.S	HexNAc(2)Hex(1)	No	1335.6364	3	4004.8945	4002.8786	2.3	Specific	858	198.34	0	Nsco_20191108_BC_ZIC_HILIC_H111_typsin_B2.42072.42072.3	82.3448	Trypsin	B2	302
BCAM1550	>tr|A0A142PC85|A0A142PC85_9BURK Peptidoglycan-associated protein OS=Burkholderia cenocepacia OX=95486 GN=pal PE=3 SV=1	K.TPENAGAAPEPSSETVAT[+568.212]VTADDLNNPNSPLAK.R	HexNAc(2)Hex(1)	No	1282.9346	3	3846.7893	3846.7775	3.1	Specific	950.71	313.89	2.4	Nsco_20191108_BC_ZIC_HILIC_H111_typsin_B2.47780.47780.3	92.3752	Trypsin	B2	303
BCAM1550	>tr|A0A142PC85|A0A142PC85_9BURK Peptidoglycan-associated protein OS=Burkholderia cenocepacia OX=95486 GN=pal PE=3 SV=1	T.PENAGAAPEPSSETVAT[+568.212][+100.064]VTADDLNNPNSPLAKR.S	HexNAc(2)Hex(1) 100.064	No	1334.9743	3	4002.9083	4001.8949	2.5	NRagged	412.57	412.57	0	Nsco_20191108_BC_ZIC_HILIC_H111_typsin_B3.39599.39599.3	82.3936	Trypsin	B3	304
BCAM1669	>tr|A0A1V2X7H1|A0A1V2X7H1_9BURK Purine nucleoside phosphorylase OS=Burkholderia cenocepacia OX=95486 GN=A8E72_24650 PE=4 SV=1	A.AQARVHGIDNSGAGSQPAAT[+568.212]VEGGAPVVRAQNPRDSVY.F	HexNAc(2)Hex(1)	No	875.0314	5	4371.1279	4371.1084	4.5	Non	554.26	554.26	2.4	Nsco_20191108_BC_ZIC_HILIC_H111_pepsin_B1.26985.26985.5	57.1927	Pepsin	B1	305
BCAM1669	>tr|A0A1V2X7H1|A0A1V2X7H1_9BURK Purine nucleoside phosphorylase OS=Burkholderia cenocepacia OX=95486 GN=A8E72_24650 PE=4 SV=1	A.AQARVHGIDNS[+568.212]GAGSQPAA.T	HexNAc(2)Hex(1)	No	1188.5564	2	2376.1055	2375.0957	2.7	Non	375.97	339.11	5.6	Nsco_20191108_BC_ZIC_HILIC_H111_pepsin_B1.9852.9852.2	26.9269	Pepsin	B1	306
BCAM1669	>tr|A0A1V2X7H1|A0A1V2X7H1_9BURK Purine nucleoside phosphorylase OS=Burkholderia cenocepacia OX=95486 GN=A8E72_24650 PE=4 SV=1	Q.ARVHGIDNS[+568.212]GAGSQPAAT.V	HexNAc(2)Hex(1)	No	1139.0313	2	2277.0553	2277.0477	3.3	Non	473.51	147.67	0.63	Nsco_20191108_BC_ZIC_HILIC_H111_pepsin_B2.9736.9736.2	25.8538	Pepsin	B2	307

BCAM1669	>tr|A0A1V2X7H1|A0A1V2X7H1_9BURK Purine nucleoside phosphorylase OS=Burkholderia cenocepacia OX=95486 GN=A8E72_24650 PE=4 SV=1	A.AQARVHGIDNSGAGS[+568.212]QPAAT.V	HexNAc(2)Hex(1)	No	1238.5804	2	2476.1535	2476.1434	4.1	Non	500.02	157.54	1.63	Nsco_20191108_BC_ZIC_HILIC_H111_pepsin_B2.10761.10761.2	27.4903	Pepsin	B2	308
BCAM1669	>tr|A0A1V2X7H1|A0A1V2X7H1_9BURK Purine nucleoside phosphorylase OS=Burkholderia cenocepacia OX=95486 GN=A8E72_24650 PE=4 SV=1	A.RVHGIDNSGAGS[+568.212]QPAAT.V	HexNAc(2)Hex(1)	No	1103.5121	2	2206.0169	2206.0106	2.9	Non	465.26	365.35	3.12	Nsco_20191108_BC_ZIC_HILIC_H111_pepsin_B2.8843.8843.2	24.4525	Pepsin	B2	309
BCAM1669	>tr|A0A1V2X7H1|A0A1V2X7H1_9BURK Purine nucleoside phosphorylase OS=Burkholderia cenocepacia OX=95486 GN=A8E72_24650 PE=4 SV=1	A.RVHGIDNSGAGS[+568.212]QPAA.T	HexNAc(2)Hex(1)	No	1052.9897	2	2104.9721	2104.9629	4.3	Non	416.48	285.03	5.5	Nsco_20191108_BC_ZIC_HILIC_H111_pepsin_B3.8746.8746.2	23.5508	Pepsin	B3	310
BCAM1669	>tr|A0A1V2X7H1|A0A1V2X7H1_9BURK Purine nucleoside phosphorylase OS=Burkholderia cenocepacia OX=95486 GN=A8E72_24650 PE=4 SV=1	R.VHGIDNSGAGSQPAAT[+568.212]VEGGAPVVRAQNPRDSVYF.G	HexNAc(2)Hex(1)	No	1024.2471	4	4093.9665	4091.9429	4.1	NRagged	785.56	696.72	9.6	Nsco_20191108_BC_ZIC_HILIC_H111_thermolysin_B1.39135.39135.4	74.9032	Thermolysin	B1	311
BCAM1669	>tr|A0A1V2X7H1|A0A1V2X7H1_9BURK Purine nucleoside phosphorylase OS=Burkholderia cenocepacia OX=95486 GN=A8E72_24650 PE=4 SV=1	L.VQLENAGYKPSQSSPYYPADICAAQARVHGIDNSGAGS[+568.212]QPAAT[+568.212]VEGGAPVVRAQNPRDSVYF.G	HexNAc(2)Hex(1), HexNAc(2)Hex(1)	No	1266.6069	6	7594.6048	7593.5852	2.1	Specific	347.94	142.68	0	Nsco_20191108_BC_ZIC_HILIC_H111_thermolysin_B1.43288.43288.3	81.9145	Thermolysin	B1	312
BCAM1669	>tr|A0A1V2X7H1|A0A1V2X7H1_9BURK Purine nucleoside phosphorylase OS=Burkholderia cenocepacia OX=95486 GN=A8E72_24650 PE=4 SV=1	R.VHGIDNSGAGSQPAAT[+568.212]VEGGAPVVRAQNPRDSVY.F	HexNAc(2)Hex(1)	No	1316.637	3	3947.8963	3944.8744	3	Non	922.88	906.89	9.6	Nsco_20191108_BC_ZIC_HILIC_H111_thermolysin_B2.30783.30783.3	62.1659	Thermolysin	B2	313
BCAM1669	>tr|A0A1V2X7H1|A0A1V2X7H1_9BURK Purine nucleoside phosphorylase OS=Burkholderia cenocepacia OX=95486 GN=A8E72_24650 PE=4 SV=1	G.IDNSGAGSQPAAT[+568.212]VEGGAPVVRAQNPRDSVYF.G	HexNAc(2)Hex(1)	No	1266.9399	3	3798.8051	3798.7941	2.9	NRagged	755.68	683.26	12	Nsco_20191108_BC_ZIC_HILIC_H111_thermolysin_B2.41526.41526.3	82.134	Thermolysin	B2	314
BCAM1669	>tr|A0A1V2X7H1|A0A1V2X7H1_9BURK Purine nucleoside phosphorylase OS=Burkholderia cenocepacia OX=95486 GN=A8E72_24650 PE=4 SV=1	R.VHGIDNSGAGS[+568.212]QPAATVEGGAP.V	HexNAc(2)Hex(1)	No	1280.5865	2	2560.1657	2560.1533	4.8	Non	589.46	497.26	1.2	Nsco_20191108_BC_ZIC_HILIC_H111_thermolysin_B3.24499.24499.2	51.8666	Thermolysin	B3	315
BCAM1669	>tr|A0A1V2X7H1|A0A1V2X7H1_9BURK Purine nucleoside phosphorylase OS=Burkholderia cenocepacia OX=95486 GN=A8E72_24650 PE=4 SV=1	G.IDNSGAGSQPAAT[+568.212]VEGGAPVVRAQNPRD.S	HexNAc(2)Hex(1)	No	1101.8645	3	3303.579	3302.5619	4.2	Non	510.46	430.49	12	Nsco_20191108_BC_ZIC_HILIC_H111_thermolysin_B3.24567.24567.3	51.9771	Thermolysin	B3	316
BCAM1669	>tr|A0A1V2X7H1|A0A1V2X7H1_9BURK Purine nucleoside phosphorylase OS=Burkholderia cenocepacia OX=95486 GN=A8E72_24650 PE=4 SV=1	G.IDNSGAGSQPAAT[+568.212]VEGGAPVVRAQNPRDS.V	HexNAc(2)Hex(1)	No	1130.5406	3	3389.6071	3389.5939	3.9	Non	698.9	666.63	12	Nsco_20191108_BC_ZIC_HILIC_H111_thermolysin_B3.24570.24570.3	51.9841	Thermolysin	B3	317
BCAM1669	>tr|A0A1V2X7H1|A0A1V2X7H1_9BURK Purine nucleoside phosphorylase OS=Burkholderia cenocepacia OX=95486 GN=A8E72_24650 PE=4 SV=1	H.GIDNSGAGSQPAAT[+568.212]VEGGAPVVRAQNPRDS.V	HexNAc(2)Hex(1)	No	1149.5388	3	3446.6019	3446.6154	-3.9	Non	284.76	129.46	1.44	Nsco_20191108_BC_ZIC_HILIC_H111_thermolysin_B3.27776.27776.3	57.5507	Thermolysin	B3	318
BCAM1669	>tr|A0A1V2X7H1|A0A1V2X7H1_9BURK Purine nucleoside phosphorylase OS=Burkholderia cenocepacia OX=95486 GN=A8E72_24650 PE=4 SV=1	G.IDNSGAGSQPAAT[+568.212]VEGGAPVVRAQNPRDSVY.F	HexNAc(2)Hex(1)	No	1217.9178	3	3651.7388	3651.7256	3.6	Non	809.25	796.29	12	Nsco_20191108_BC_ZIC_HILIC_H111_thermolysin_B3.33078.33078.3	66.3496	Thermolysin	B3	319
BCAM1669	>tr|A0A1V2X7H1|A0A1V2X7H1_9BURK Purine nucleoside phosphorylase OS=Burkholderia cenocepacia OX=95486 GN=A8E72_24650 PE=4 SV=1	R.VHGIDNSGAGSQPAAT[+568.212]VEGGAPVVR.A	HexNAc(2)Hex(1)	No	1457.7007	2	2914.3942	2914.3912	1	Specific	974.46	925.6	0.05	Nsco_20191108_BC_ZIC_HILIC_H111_typsin_B1.25669.25669.2	55.2181	Trypsin	B1	320

BCAM1737	>tr|A0A2N9CVE8|A0A2N9CVE8_9BURK Putative exported glycoprotein OS=Burkholderia cenocepacia OX=95486 GN=F01_460192 PE=4 SV=1	W.SQAGSAAPADTPASAAPSAS[+568.212]AT[+568.212]PATRA.A	HexNAc(2)Hex(1), HexNAc(2)Hex(1)	No	1174.2	3	3520.5853	3519.5716	3	Non	468.72	468.72	3.01	Nsco_20191108_BC_ZIC_HILIC_H111_pepsin_B1.19245.19245.3	42.0253	Pepsin	B1	321
BCAM1737	>tr|A0A2N9CVE8|A0A2N9CVE8_9BURK Putative exported glycoprotein OS=Burkholderia cenocepacia OX=95486 GN=F01_460192 PE=4 SV=1	E.RAISWSQAGSAAPADT[+568.212]PASAAPSASATPATRA.A	HexNAc(2)Hex(1)	No	1189.2407	3	3565.7074	3564.6936	2.9	Non	331.43	311.67	2.4	Nsco_20191108_BC_ZIC_HILIC_H111_pepsin_B1.28994.28994.3	61.2309	Pepsin	B1	322
BCAM1737	>tr|A0A2N9CVE8|A0A2N9CVE8_9BURK Putative exported glycoprotein OS=Burkholderia cenocepacia OX=95486 GN=F01_460192 PE=4 SV=1	W.SQAGSAAPADTPASAAPSAS[+568.212]AT[+568.212]PATRAAA.A	HexNAc(2)Hex(1), HexNAc(2)Hex(1)	No	1221.5586	3	3662.6612	3661.6458	3.3	Non	478.39	444.26	3.6	Nsco_20191108_BC_ZIC_HILIC_H111_pepsin_B2.21264.21264.3	44.5412	Pepsin	B2	323
BCAM1737	>tr|A0A2N9CVE8|A0A2N9CVE8_9BURK Putative exported glycoprotein OS=Burkholderia cenocepacia OX=95486 GN=F01_460192 PE=4 SV=1	P.ADTPASAAPSAS[+568.212][+100.064]AT[+568.212]PAT[+568.212][+100.064]RA.A	HexNAc(2)Hex(1) 100.064,HexNAc(2)Hex(1),HexNAc(2)Hex(1) 100.064	No	1207.2055	3	3619.602	3618.6029	-1.2	Non	196.8	196.8	3.44	Nsco_20191108_BC_ZIC_HILIC_H111_pepsin_B3.23406.23406.3	48.2958	Pepsin	B3	324
BCAM1737	>tr|A0A2N9CVE8|A0A2N9CVE8_9BURK Putative exported glycoprotein OS=Burkholderia cenocepacia OX=95486 GN=F01_460192 PE=4 SV=1	S.WSQAGSAAPADTPASAAPSAS[+568.212]AT[+568.212]PATRA.A	HexNAc(2)Hex(1), HexNAc(2)Hex(1)	No	1236.2268	3	3706.6659	3705.6509	3.1	Non	180.07	180.07	2.4	Nsco_20191108_BC_ZIC_HILIC_H111_pepsin_B3.25874.25874.3	53.4267	Pepsin	B3	325
BCAM1737	>tr|A0A2N9CVE8|A0A2N9CVE8_9BURK Putative exported glycoprotein OS=Burkholderia cenocepacia OX=95486 GN=F01_460192 PE=4 SV=1	A.ISWSQAGSAAPADT[+568.212]PASAAPS[+568.212]ASATPATR.A	HexNAc(2)Hex(1), HexNAc(2)Hex(1)	No	1279.2545	3	3835.749	3834.7299	4.1	Non	514.82	230.7	2.27	Nsco_20191108_BC_ZIC_HILIC_H111_thermolysin_B2.34280.34280.3	68.0681	Thermolysin	B2	326
BCAM1737	>tr|A0A2N9CVE8|A0A2N9CVE8_9BURK Putative exported glycoprotein OS=Burkholderia cenocepacia OX=95486 GN=F01_460192 PE=4 SV=1	S.WSQAGSAAPADTPASAAPSAS[+568.212][+100.064]AT[+568.212][+100.064]PATRAA.A	HexNAc(2)Hex(1) 100.064,HexNAc(2)Hex(1) 100.064	No	1326.2803	3	3976.8264	3976.816	2.6	Non	399.36	399.36	2.4	Nsco_20191108_BC_ZIC_HILIC_H111_thermolysin_B2.35161.35161.3	69.6204	Thermolysin	B2	327
BCAM1737	>tr|A0A2N9CVE8|A0A2N9CVE8_9BURK Putative exported glycoprotein OS=Burkholderia cenocepacia OX=95486 GN=F01_460192 PE=4 SV=1	A.GSAAPADTPASAAPS[+568.212][+100.064]AS[+568.212]AT[+568.212][+100.064]PATRAA.A	HexNAc(2)Hex(1) 100.064,HexNAc(2)Hex(1),HexNAc(2)Hex(1) 100.064	No	1359.616	3	4076.8335	4072.8205	-0.1	Non	324.49	324.49	0	Nsco_20191108_BC_ZIC_HILIC_H111_thermolysin_B3.37010.37010.3	73.8046	Thermolysin	B3	328
BCAM1737	>tr|A0A2N9CVE8|A0A2N9CVE8_9BURK Putative exported glycoprotein OS=Burkholderia cenocepacia OX=95486 GN=F01_460192 PE=4 SV=1	S.WSQAGSAAPADTPASAAPSAS[+568.212][+100.064]AT[+568.212][+100.064]PATR.A	HexNAc(2)Hex(1) 100.064,HexNAc(2)Hex(1) 100.064	No	1279.2556	3	3835.7523	3834.7418	1.9	Non	319.11	319.11	2.4	Nsco_20191108_BC_ZIC_HILIC_H111_thermolysin_B3.34011.34011.3	67.9246	Thermolysin	B3	329
BCAM1737	>tr|A0A2N9CVE8|A0A2N9CVE8_9BURK Putative exported glycoprotein OS=Burkholderia cenocepacia OX=95486 GN=F01_460192 PE=4 SV=1	A.ISWSQAGSAAPADTPAS[+568.212]AAPSASAT[+568.212]PATRAA.A	HexNAc(2)Hex(1), HexNAc(2)Hex(1)	No	1326.6125	3	3977.8231	3976.8041	3.9	Non	684.89	302.89	5.88	Nsco_20191108_BC_ZIC_HILIC_H111_thermolysin_B3.34894.34894.3	69.4909	Thermolysin	B3	330
BCAM1737	>tr|A0A2N9CVE8|A0A2N9CVE8_9BURK Putative exported glycoprotein OS=Burkholderia cenocepacia OX=95486 GN=F01_460192 PE=4 SV=1	A.GSAAPADTPASAAPSAS[+568.212][+100.064]AT[+568.212]PAT[+568.212][+100.064]R.A	HexNAc(2)Hex(1) 100.064,HexNAc(2)Hex(1),HexNAc(2)Hex(1) 100.064	No	1311.9205	3	3933.7468	3930.7463	-2.4	NRagged	321.68	321.68	1.76	Nsco_20191108_BC_ZIC_HILIC_H111_typsin_B1.34574.34574.3	70.5281	Trypsin	B1	331
BCAM1737	>tr|A0A2N9CVE8|A0A2N9CVE8_9BURK Putative exported glycoprotein OS=Burkholderia cenocepacia OX=95486 GN=F01_460192 PE=4 SV=1	Q.AGSAAPADTPASAAPS[+568.212][+100.064]AS[+568.212]AT[+568.212][+100.064]PATR.A	HexNAc(2)Hex(1) 100.064,HexNAc(2)Hex(1),HexNAc(2)Hex(1) 100.064	No	1335.935	3	4005.7904	4001.7834	-1.6	NRagged	475.79	475.79	0	Nsco_20191108_BC_ZIC_HILIC_H111_typsin_B1.36212.36212.3	73.2799	Trypsin	B1	332

BCAM1737	>tr|A0A2N9CVE8|A0A2N9CVE8_9BURK Putative exported glycoprotein OS=Burkholderia cenocepacia OX=95486 GN=F01_460192 PE=4 SV=1	R.AISWSQAGSAAPADTPASAAPSAS[+568.212]ATPATR.A	HexNAc(2)Hex(1)	No	1669.2892	2	3337.5712	3337.5554	4.7	Specific	851.85	798.79	4.8	Nsco_20191108_BC_ZIC_HILIC_H111_typsin_B2.37171.37171.2	73.5891	Trypsin	B2	333
BCAM1737	>tr|A0A2N9CVE8|A0A2N9CVE8_9BURK Putative exported glycoprotein OS=Burkholderia cenocepacia OX=95486 GN=F01_460192 PE=4 SV=1	S.QAGSAAPADTPASAAPSAS[+568.212]AT[+568.212]PAT[+568.212][+100.064]R.A	HexNAc(2)Hex(1), HexNAc(2)Hex(1), HexNAc(2)Hex(1) 100.064	No	1345.2692	3	4033.7929	4029.778	0.4	NRagged	254.86	254.86	1.55	Nsco_20191108_BC_ZIC_HILIC_H111_typsin_B3.35430.35430.3	74.1491	Trypsin	B3	334
BCAM2054 (ty	>tr|A0A1V2XAF9|A0A1V2XAF9_9BURK Peptide-binding protein OS=Burkholderia cenocepacia OX=95486 GN=bcscD PE=4 SV=1	R.APEQAPAPPVAPLAS[+568.212]GAAAGVAQPVPGPTLLPREPAAGVSTK.E	HexNAc(2)Hex(1)	No	1493.1235	3	4477.3559	4477.3348	4.7	Specific	1112.68	1112.68	3.6	Nsco_20191108_BC_ZIC_HILIC_H111_typsin_B1.55514.55514.3	105.5981	Trypsin	B1	335
BCAM2055	>tr|A0A1V2VXC7|A0A1V2VXC7_9BURK EscC/YscC/HrcC family type III secretion system outer membrane ring protein OS=Burkholderia cenocepacia OX=95486 GN=A8E72_28425 PE=3 SV=1	D.VTVPLTPKPLPGTKFGPPALPPPKDTAAS[+568.212]QPAA.T	HexNAc(2)Hex(1)	No	958.2687	4	3830.053	3830.0361	4.4	Non	296.76	296.76	44.15	Nsco_20191108_BC_ZIC_HILIC_H111_pepsin_B1.40237.40237.4	88.1894	Pepsin	B1	336
BCAM2055	>tr|A0A1V2VXC7|A0A1V2VXC7_9BURK EscC/YscC/HrcC family type III secretion system outer membrane ring protein OS=Burkholderia cenocepacia OX=95486 GN=A8E72_28425 PE=3 SV=1	D.VTVPLTPKPLPGTKFGPPALPPPKDT[+568.212]AASQPAATTAG.V	HexNAc(2)Hex(1)	No	1040.8079	4	4160.2098	4160.1901	4.7	Non	439.15	398.15	6.47	Nsco_20191108_BC_ZIC_HILIC_H111_pepsin_B2.40975.40975.4	87.9263	Pepsin	B2	337
BCAM2055	>tr|A0A1V2VXC7|A0A1V2VXC7_9BURK EscC/YscC/HrcC family type III secretion system outer membrane ring protein OS=Burkholderia cenocepacia OX=95486 GN=A8E72_28425 PE=3 SV=1	K.DTAASQPAATT[+568.212]AGVVTHVDEHH.-	HexNAc(2)Hex(1)	No	928.7593	3	2784.2635	2783.249	4	CRagged	324.81	324.81	1.2	Nsco_20191108_BC_ZIC_HILIC_H111_typsin_B2.20983.20983.3	44.8364	Trypsin	B2	338
BCAM2063	>tr|A0A2N9CW94|A0A2N9CW94_9BURK Porin OS=Burkholderia cenocepacia OX=95486 GN=F01_460501 PE=3 SV=1	A.SSPAAAEPAAGASDAAAPAQQAADAAAPAPT[+568.212]GFWERSN.L	HexNAc(2)Hex(1)	No	1374.963	3	4122.8745	4119.8538	2.6	Non	635.81	612.9	4.83	Nsco_20191108_BC_ZIC_HILIC_H111_thermolysin_B3.49038.49038.3	96.7602	Thermolysin	B3	339
BCAM2064	>tr|A0A2N9CWA7|A0A2N9CWA7_9BURK Periplasmic trehalase OS=Burkholderia cenocepacia OX=95486 GN=treA PE=3 SV=1	A.DNANQAAAQAAGQSAIPATTAAAAAPASGT[+568.212]LPPPSQLYGDLFVAVQTAQLYPDQK.T	HexNAc(2)Hex(1)	No	1494.9893	4	5976.9352	5976.9149	3.4	NRagged	729.06	729.06	0	Nsco_20191108_BC_ZIC_HILIC_H111_typsin_B2.79285.79285.4	160.566	Trypsin	B2	340
BCAM2289	>tr|A0A142PAR8|A0A142PAR8_9BURK Purine nucleoside phosphorylase OS=Burkholderia cenocepacia OX=95486 GN=A8E72_27535 PE=4 SV=1	D.TSGYGAQPAPLVHSGAPAAAS[+568.212]SNARD.S	HexNAc(2)Hex(1)	No	1007.8045	3	3021.3989	3021.392	2.3	Non	921.88	333.53	2.4	Nsco_20191108_BC_ZIC_HILIC_H111_pepsin_B1.19535.19535.3	42.5368	Pepsin	B1	341
BCAM2289	>tr|A0A142PAR8|A0A142PAR8_9BURK Purine nucleoside phosphorylase OS=Burkholderia cenocepacia OX=95486 GN=A8E72_27535 PE=4 SV=1	R.VHGADTSGYGAQPAPLVHSGAPAAAS[+568.212]SNAR.D	HexNAc(2)Hex(1)	Yes	847.1534	4	3385.5918	3385.5779	4.1	Specific	1294.2	1230.17	71.23	Nsco_20191108_BC_ZIC_HILIC_H111_typsin_B3.17844.17844.4	40.1436	Trypsin	B3	342
BCAM2301	>tr|A0A1V2VWJ7|A0A1V2VWJ7_9BURK Amine dehydrogenase OS=Burkholderia cenocepacia OX=95486 GN=aauA PE=4 SV=1	A.DAASGPSGASGAAAAGAS[+568.212][+100.064]DDPMSC[+57.021]DYWK.Y	HexNAc(2)Hex(1) 100.064	No	1671.6795	2	3342.3517	3341.3584	-3	NRagged	447.12	447.12	6	Nsco_20191108_BC_ZIC_HILIC_H111_typsin_B2.40371.40371.2	79.3924	Trypsin	B2	343

BCAM2307	>tr|A0A2N9CXU5|A0A2N9CXU5_9BURK Bacillolysin OS=Burkholderia cenocepacia OX=95486 GN=F01_480197 PE=4 SV=1	F.EAVNVYYHIDTFLRYVNQT[+568.212][+100.064]LGIKALPYQYTGGVQYDPHGES[+568.212][+100.064]GDDNSSYSSSSGR.L	HexNAc(2)Hex(1) 100.064,HexNAc(2)Hex(1) 100.064	No	1472.6734	5	7359.3378	7355.3629	-5.2	NRagged	516.72	516.72	0	Nsco_20191108_BC_ZIC_HILIC_H111_typsin_B3.33778.33778.5	70.9602	Trypsin	B3	344
BCAM2334	>tr|A0A2N9CXX4|A0A2N9CXX4_9BURK Multidrug efflux system OS=Burkholderia cenocepacia OX=95486 GN=emrA PE=4 SV=1	R.VHDGVAS[+568.212]DAEAAAAAIIRENQGG.-	HexNAc(2)Hex(1)	Yes	930.7722	3	2790.302	2790.2912	3.9	CRagged	667.4	634.86	634.86	Nsco_20191108_BC_ZIC_HILIC_H111_typsin_B2.51636.51636.3	99.0316	Trypsin	B2	345
BCAM2443	>tr|A0A2N9CXV2|A0A2N9CXV2_9BURK Cytochrome c family protein OS=Burkholderia cenocepacia OX=95486 GN=F01_480355 PE=4 SV=1	G.ATPQDAPAAAS[+568.212]APPPAPAAAAAPAAKPFTPPPES.A	HexNAc(2)Hex(1)	No	1224.9368	3	3672.7959	3672.7803	4.3	Non	446.78	188.36	2.38	Nsco_20191108_BC_ZIC_HILIC_H111_pepsin_B2.38609.38609.3	81.695	Pepsin	B2	346
BCAM2443	>tr|A0A2N9CXV2|A0A2N9CXV2_9BURK Cytochrome c family protein OS=Burkholderia cenocepacia OX=95486 GN=F01_480355 PE=4 SV=1	A.TPQDAPAAASAPPPAPAAAAAPAAKPFT[+568.212]PPPESAIPADDFGK.T	HexNAc(2)Hex(1)	No	1506.4057	3	4517.2025	4516.193	1.4	NRagged	425.1	46.73	8.4	Nsco_20191108_BC_ZIC_HILIC_H111_typsin_B2.49021.49021.3	94.5171	Trypsin	B2	347
BCAM2443	>tr|A0A2N9CXV2|A0A2N9CXV2_9BURK Cytochrome c family protein OS=Burkholderia cenocepacia OX=95486 GN=F01_480355 PE=4 SV=1	T.PQDAPAAASAPPPAPAAAAAPAAKPFT[+568.212][+100.064]PPPESAIPADDFGK.T	HexNAc(2)Hex(1) 100.064	No	1506.0768	3	4516.216	4515.2093	0.7	NRagged	521.44	501.79	7.2	Nsco_20191108_BC_ZIC_HILIC_H111_typsin_B2.49077.49077.3	94.6144	Trypsin	B2	348
BCAM2681	>tr|A0A1V6L0G1|A0A1V6L0G1_9BURK Putative lipoprotein OS=Burkholderia cenocepacia OX=95486 GN=F01_490209 PE=4 SV=1	R.GASVAVHAGSAPSEAVGGGT[+568.212]PAEQVAALDPK.A	HexNAc(2)Hex(1)	No	1124.2145	3	3370.6289	3369.618	2.2	Specific	900.38	819.7	4.27	Nsco_20191108_BC_ZIC_HILIC_H111_typsin_B1.35986.35986.3	72.9199	Trypsin	B1	349
BCAM2828	>tr|A0A2N9CZJ3|A0A2N9CZJ3_9BURK Hopanoid biosynthesis associated RND transporter like protein HpnN OS=Burkholderia cenocepacia OX=95486 GN=hpnN PE=4 SV=1	K.RAAIAAAAS[+568.212]ELLPALTQPAAPPATDAQR.V	HexNAc(2)Hex(1)	No	1104.2425	3	3310.7128	3310.7013	3.5	Specific	662.67	648.76	36.7	Nsco_20191108_BC_ZIC_HILIC_H111_typsin_B2.51893.51893.3	99.4892	Trypsin	B2	350
BCAS0453	>tr|A0A142PSQ3|A0A142PSQ3_9BURK Uncharacterized protein OS=Burkholderia cenocepacia OX=95486 GN=A8E72_07995 PE=4 SV=1	R.DSLGNGVALDWPAS[+568.212]GVGGVADERQK.L	HexNAc(2)Hex(1)	No	1023.1548	3	3067.4498	3066.4386	2.6	Specific	547.29	535.04	61.99	Nsco_20191108_BC_ZIC_HILIC_H111_typsin_B1.47980.47980.3	93.4226	Trypsin	B1	351
BCAS0453	>tr|A0A142PSQ3|A0A142PSQ3_9BURK Uncharacterized protein OS=Burkholderia cenocepacia OX=95486 GN=A8E72_07995 PE=4 SV=1	R.DSLGNGVALDWPAS[+568.212]GVGGVADER.Q	HexNAc(2)Hex(1)	No	1405.6471	2	2810.287	2810.285	0.7	Specific	399.7	376.4	53.27	Nsco_20191108_BC_ZIC_HILIC_H111_typsin_B1.59228.59228.2	111.4013	Trypsin	B1	352
BCAS0773	>tr|A0A2N9CZW2|A0A2N9CZW2_9BURK Uncharacterized protein OS=Burkholderia cenocepacia OX=95486 GN=DFS07_107251 PE=4 SV=1	A.QTDAAS[+568.212]APAAAAAQDAK.A	HexNAc(2)Hex(1)	No	1063.4866	2	2125.9659	2125.9619	1.9	NRagged	491.58	432.38	29.17	Nsco_20191108_BC_ZIC_HILIC_H111_typsin_B1.11041.11041.2	29.9258	Trypsin	B1	353
I35_6143	>tr|A0A2N9CX34|A0A2N9CX34_9BURK Uncharacterized protein OS=Burkholderia cenocepacia OX=95486 GN=F01_480128 PE=4 SV=1	A.PAERSPFDTPDTRPLRVTS[+568.212]DAL.V	HexNAc(2)Hex(1)	No	753.1206	4	3009.4607	3009.4535	2.4	NRagged	684.21	615.43	14.88	Nsco_20191108_BC_ZIC_HILIC_H111_pepsin_B1.34820.34820.4	73.9991	Pepsin	B1	354
I35_6143	>tr|A0A2N9CX34|A0A2N9CX34_9BURK Uncharacterized protein OS=Burkholderia cenocepacia OX=95486 GN=F01_480128 PE=4 SV=1	F.AAPAERSPFDTPDTRPLRVT[+568.212]SDAL.V	HexNAc(2)Hex(1)	No	788.6396	4	3151.5366	3151.5277	2.8	Specific	550.31	537.55	3.39	Nsco_20191108_BC_ZIC_HILIC_H111_pepsin_B1.35214.35214.4	74.8912	Pepsin	B1	355

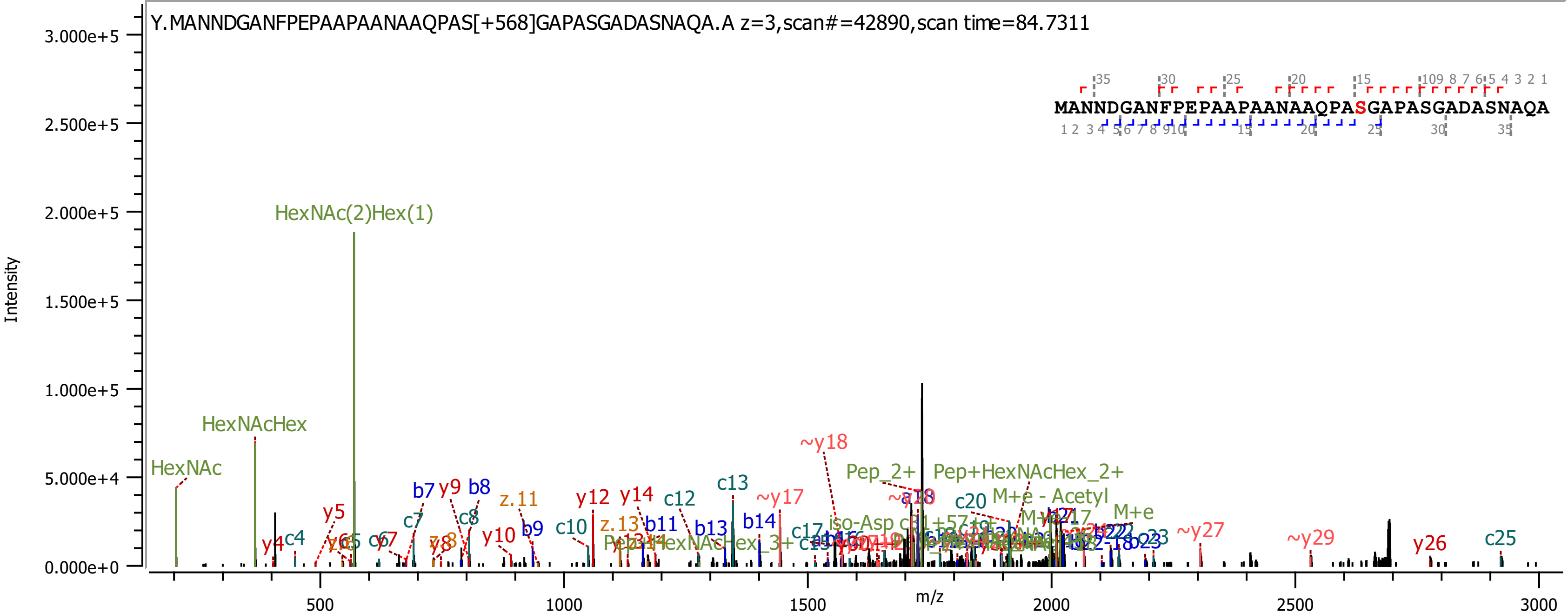
Y.MANNDGANFPEPAAPAANAAQPAS[+568]GAPASGADASNAQA.A z=3,scan#=42890,scan time=84.7311
MANNDGANFPEPAAPAANAAQPASGAPASGADASNAQA
Intensity
m/z
HexNAc(2)Hex(1)
HexNAcHex
HexNAc
~y18
Pep_2+
Pep+HexNAcHex_2+
M+e - Acetyl
M+e
~y17
~y27
~y29
y26
c25
c20
c13
c12
b14
b13
b11
y14
y12
c10
b9
y10
z.11
b8
y9
b7
c8
c7
y5
y4
c4
z.13

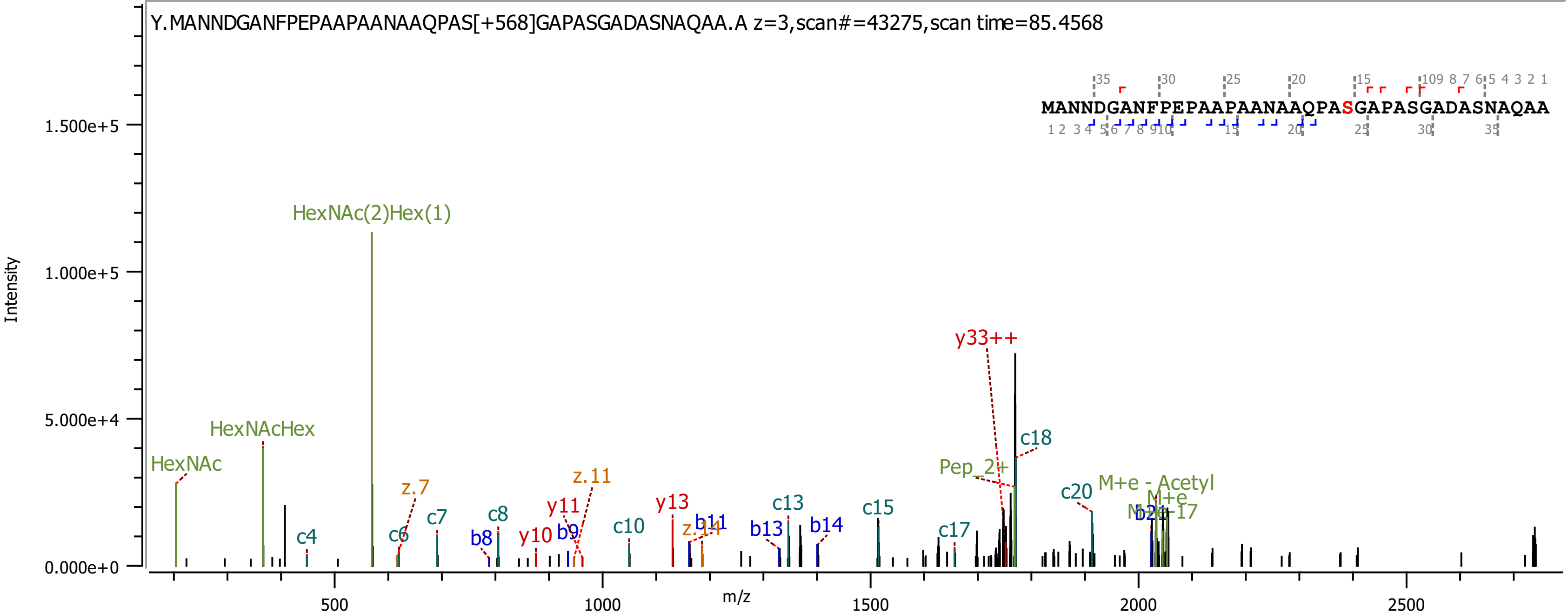

Y.MANNDGANFPEPAAPAANAAQPAS[+568]GAPASGADASNAQAA.A z=3,scan#=43275,scan time=85.4568
MANNDGANFPEPAAPAANAAQPASGAPASGADASNAQAA
Intensity
m/z
1.500e+5
1.000e+5
5.000e+4
0.000e+0
500
1000
1500
2000
2500
HexNAc(2)Hex(1)
HexNAcHex
HexNAc
y33++
c18
Pep_2+
M+e - Acetyl
c20
c4
c6
z.7
c7
b8
c8
y10
b9
y11
z.11
c10
y13
z.14
b11
b13
c13
b14
c15
c17

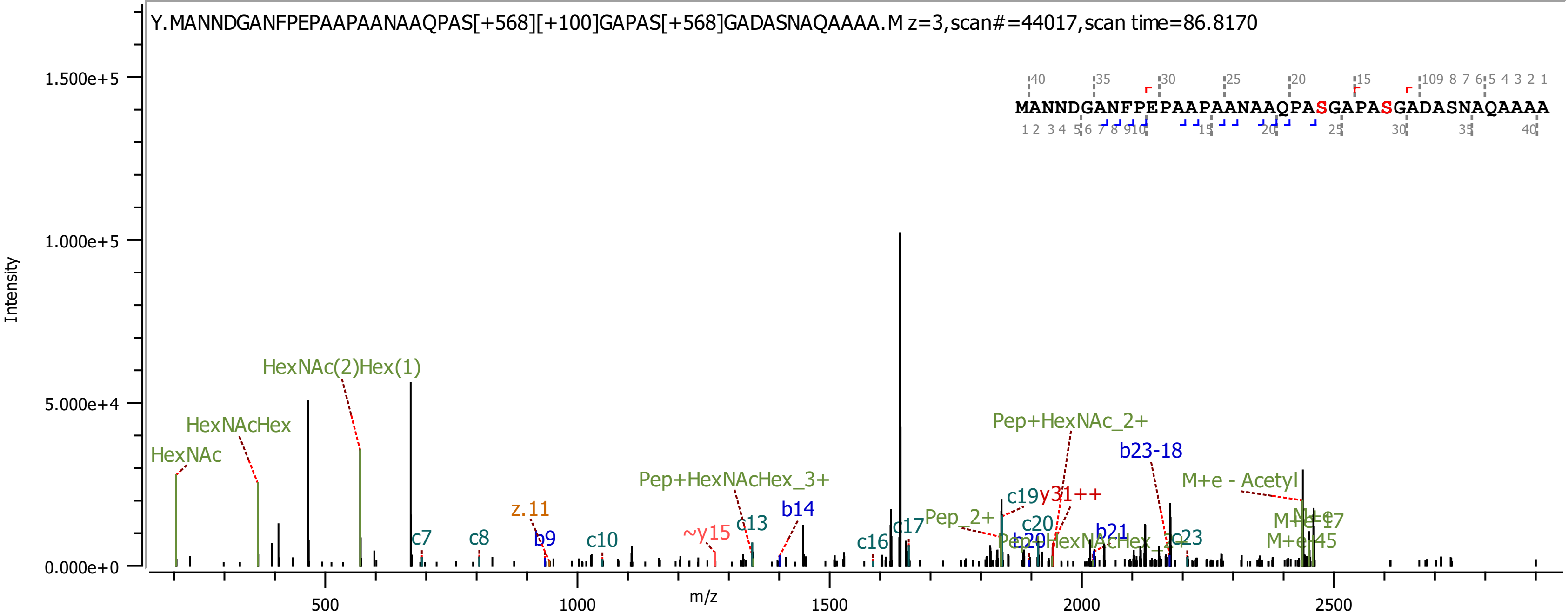

Y.MANNDGANFPEPAAPAANAAQPAS[+568][+100]GAPAS[+568]GADASNAQAAAA.M z=3,scan#=44017,scan time=86.8170
MANNDGANFPEPAAPAANAAQPASGAPASGADASNAQAAAA
Intensity
m/z
1.500e+5
1.000e+5
5.000e+4
0.000e+0
500
1000
1500
2000
2500
HexNAc
HexNAcHex
HexNAc(2)Hex(1)
c7
c8
z.11
b9
c10
~y15
Pep+HexNAcHex_3+
c13
b14
c16
c17
Pep_2+
c19
y31++
Pep+HexNAc_2+
c20
b20
Pep+HexNAcHex_2+
b21
b23-18
c23
M+e - Acetyl
M+e-17
M+e-45

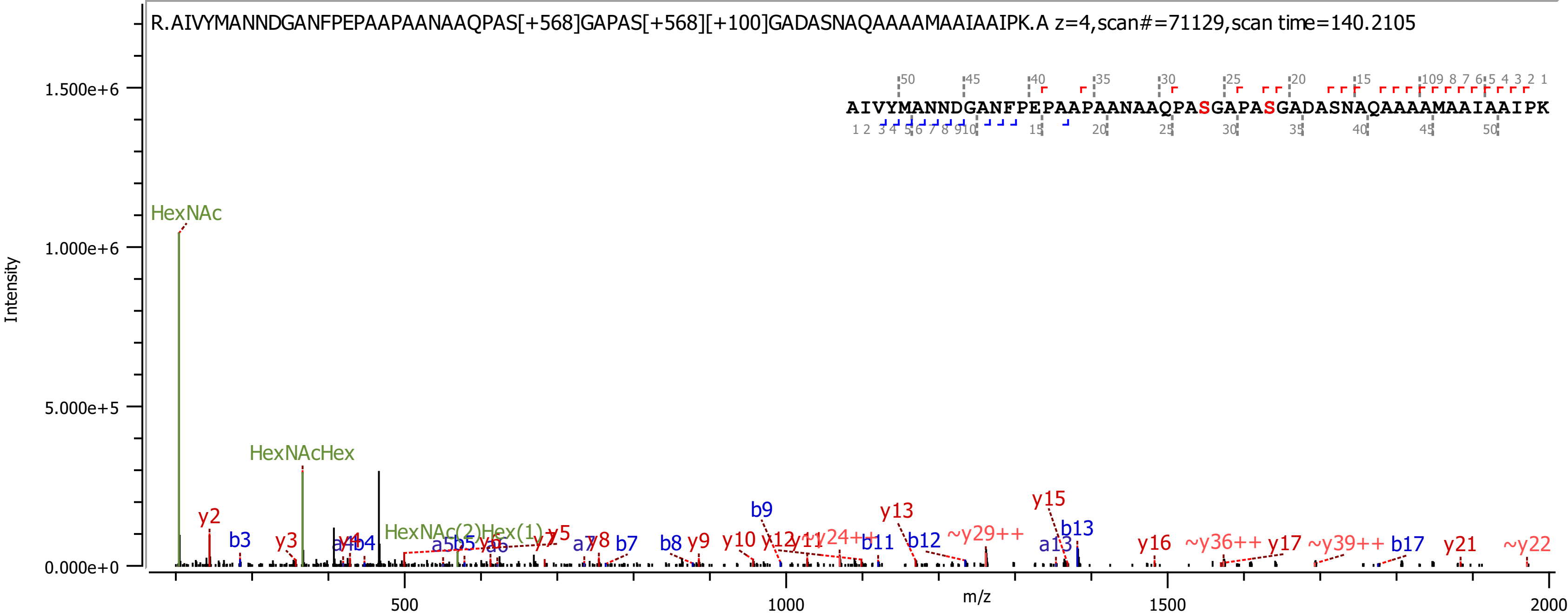
R.AIVYMANNDGANFPEPAAPAANAAQPAS[+568]GAPAS[+568][+100]GADASNAQAAAAMAAIAAIPK.A z=4,scan#=71129,scan time=140.2105
AIVYMANNDGANFPEPAAPAANAAQPASGAPASGADASNAQAAAAMAAIAAIPK
HexNAc
HexNAcHex
HexNAc(2)Hex(1)
Intensity
m/z
1.500e+6
1.000e+6
5.000e+5
0.000e+0
500
1000
1500
2000

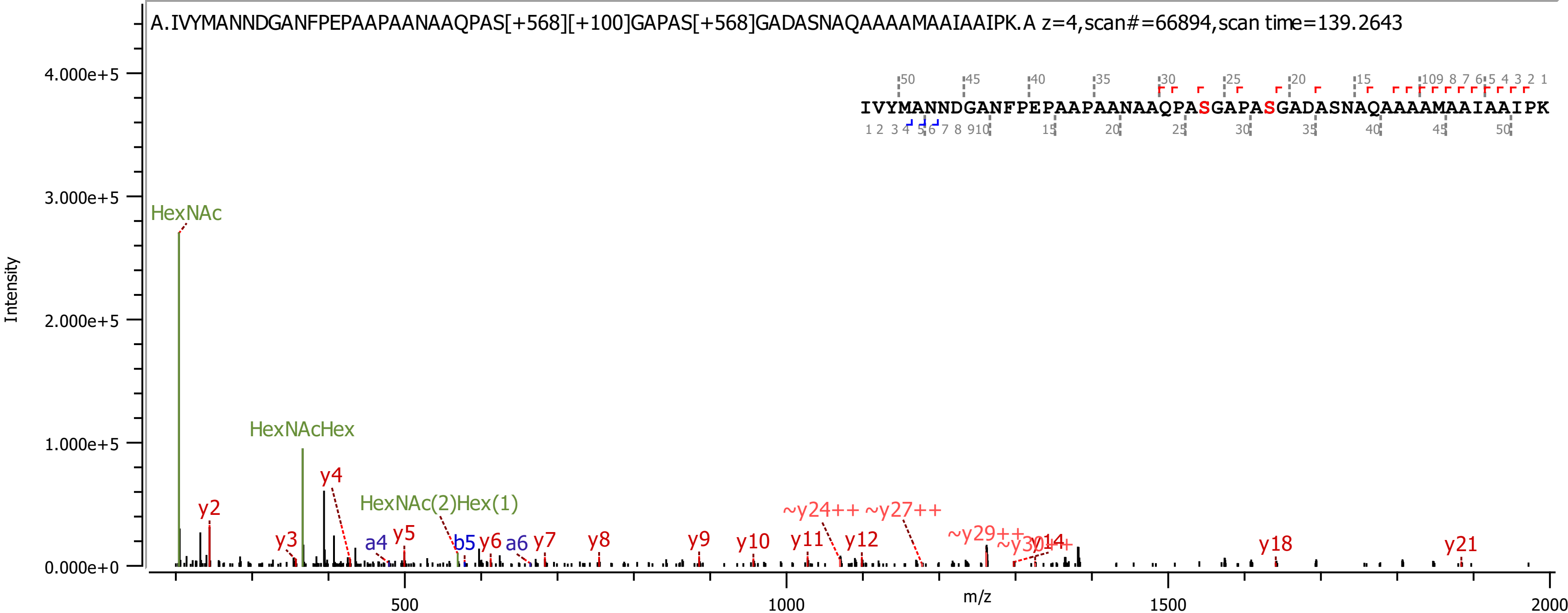
A.IVYMANNDGANFPEPAAPAANAAQPAS[+568][+100]GAPAS[+568]GADASNAQAAAAMAAIAAIPK.A z=4,scan#=66894,scan time=139.2643
IVYMANNDGANFPEPAAPAANAAQPASGAPASGADASNAQAAAAMAAIAAIPK
Intensity
4.000e+5
3.000e+5
2.000e+5
1.000e+5
0.000e+0
m/z
500
1000
1500
2000
HexNAc
HexNAcHex
HexNAc(2)Hex(1)
y2
y3
y4
a4
y5
b5
y6
a6
y7
y8
y9
y10
y11
y12
~y24++
~y27++
~y29++
~y30++
y14
y18
y21

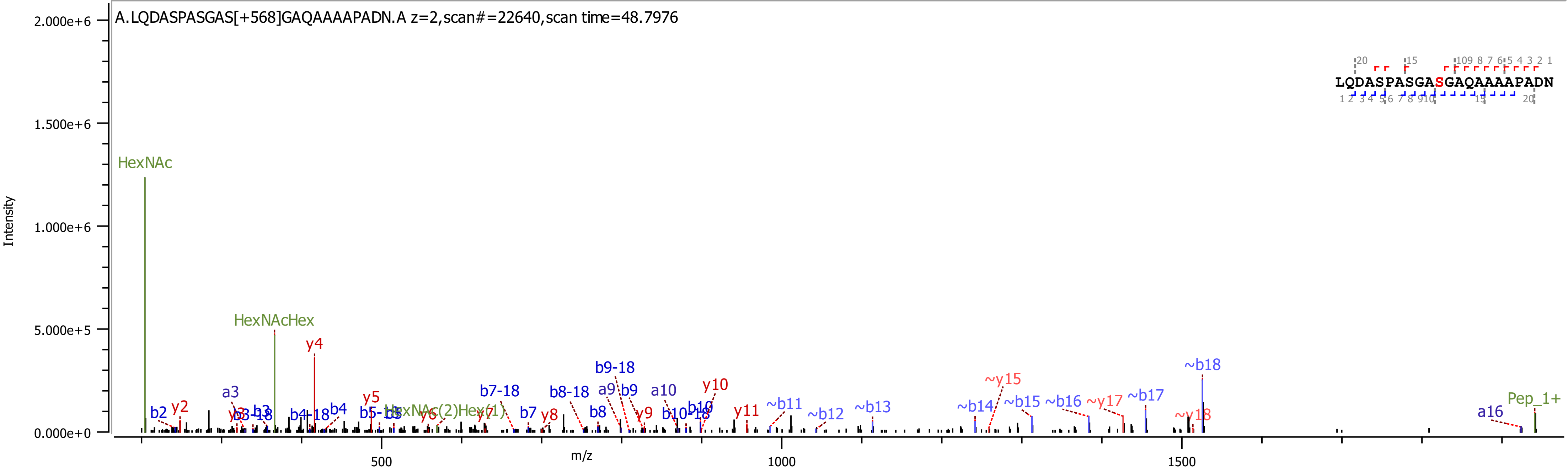

A.LQDASPASGAS[+568]GAQAAAAPADN.A z=2,scan#=22640,scan time=48.7976
LQDASPASGASGAQAAAAPADN
Intensity
m/z
HexNAc
HexNAcHex
HexNAc(2)Hex(1)
Pep_1+
y2
y3
y4
y5
y6
y7
y8
y9
y10
y11
~y15
~y17
~y18
b2
a3
b3
b3-18
b4
b4-18
b5
b5-18
b7
b7-18
b8
b8-18
a9
b9
b9-18
a10
b10
b10-18
~b11
~b12
~b13
~b14
~b15
~b16
~b17
~b18
a16

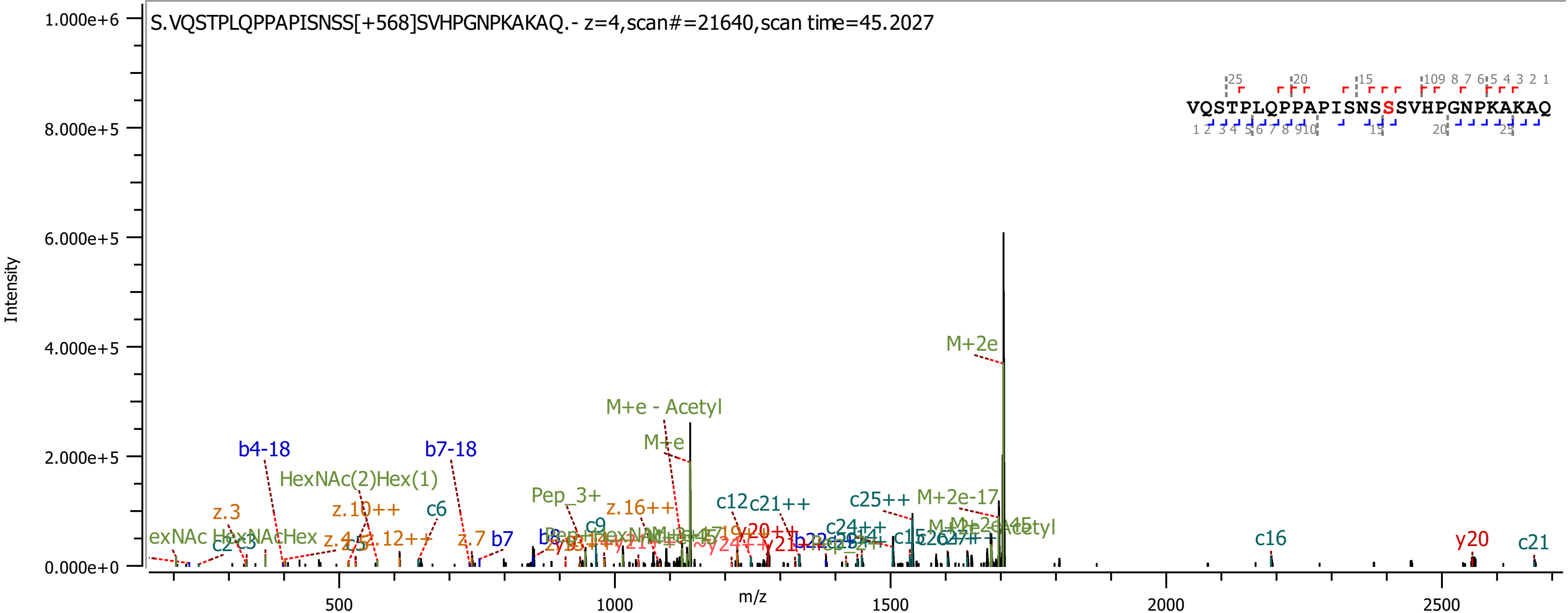

S.VQSTPLQPPAPISNSS[+568]SVHPGNPKAKAQ.- z=4,scan#=21640,scan time=45.2027
VQSTPLQPPAPISNSSSVHPGNPKAKAQ
Intensity
m/z
1.000e+6
8.000e+5
6.000e+5
4.000e+5
2.000e+5
0.000e+0
500
1000
1500
2000
2500
M+2e
M+e - Acetyl
M+e
b4-18
b7-18
HexNAc(2)Hex(1)
Pep_3+
z.3
z.10++
c6
z.16++
c12
c21++
c25++
M+2e-17
z.12++
z.7
b7
c16
y20
c21

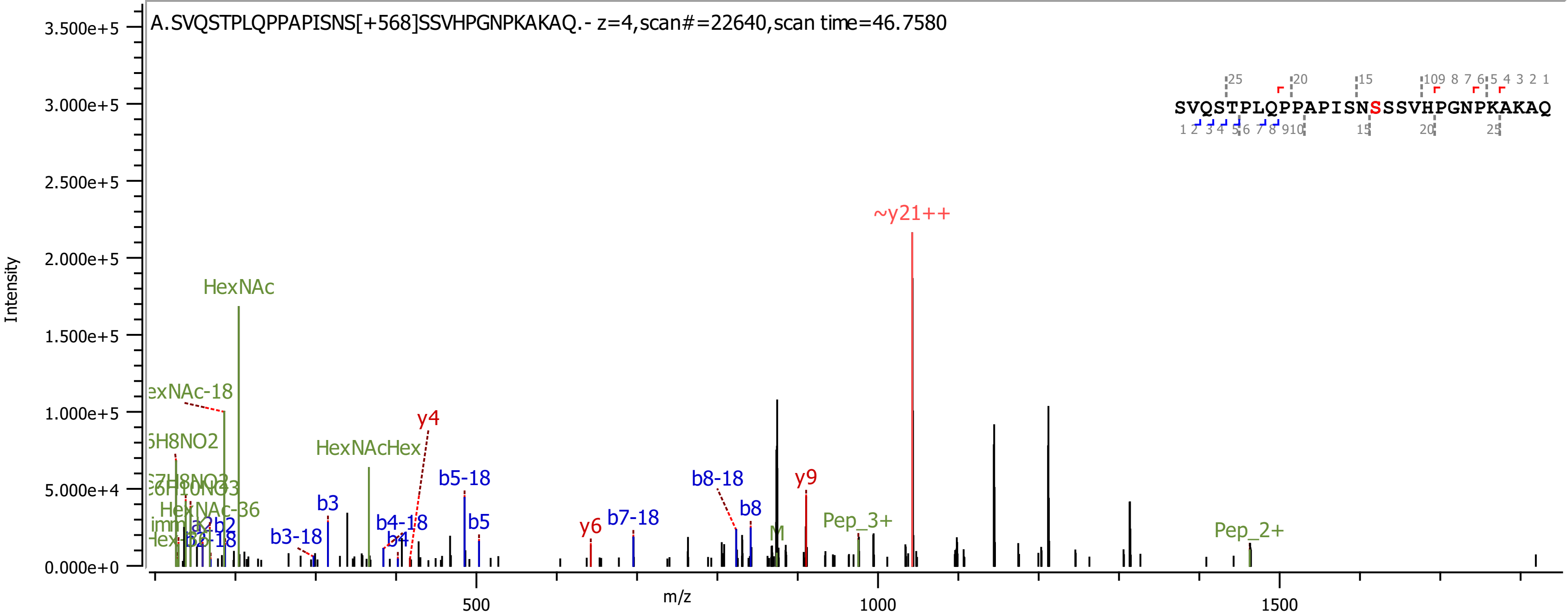
A.SVQSTPLQPPAPISNS[+568]SSVHPGNPKAKAQ.- z=4,scan#=22640,scan time=46.7580
SVQSTPLQPPAPISNSSSVHPGNPKAKAQ
Intensity
m/z
3.500e+5
3.000e+5
2.500e+5
2.000e+5
1.500e+5
1.000e+5
5.000e+4
0.000e+0
500
1000
1500
~y21++
HexNAc
HexNAcHex
y4
b5-18
b3
b4-18
b4
b5
b3-18
y6
b7-18
b8-18
b8
y9
Pep_3+
Pep_2+
M
HexNAc-36

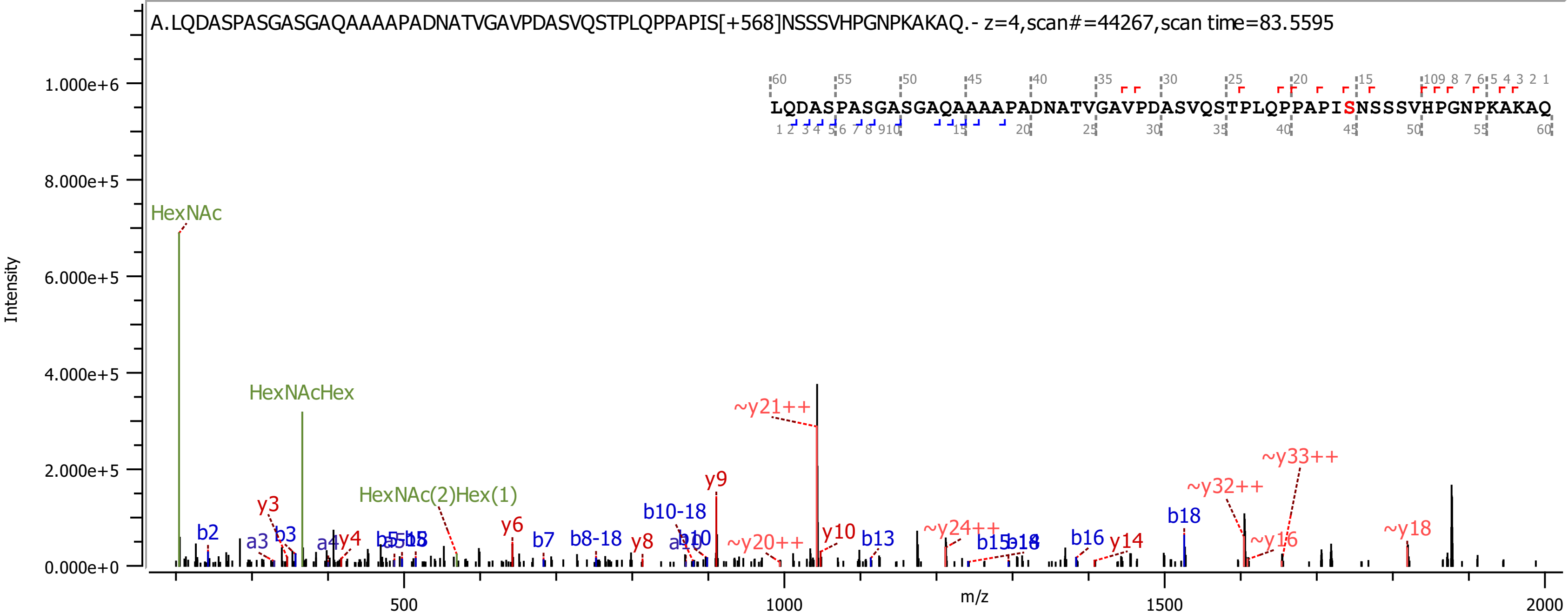
A.LQDASPASGASGAQAAAAPADNATVGAVPDASVQSTPLQPPAPIS[+568]NSSSVHPGNPKAKAQ.- z=4,scan#=44267,scan time=83.5595
LQDASPASGASGAQAAAAPADNATVGAVPDASVQSTPLQPPAPISNSSSVHPGNPKAKAQ
Intensity
m/z
0.000e+0
2.000e+5
4.000e+5
6.000e+5
8.000e+5
1.000e+6
500
1000
1500
2000
HexNAc
HexNAcHex
HexNAc(2)Hex(1)
b2
a3
b3
a4
y3
y4
y6
b7
b8-18
y8
b10-18
a10
b10
y9
~y20++
~y21++
y10
b13
~y24++
b15
b16
y14
b18
~y32++
~y33++
~y16
~y18

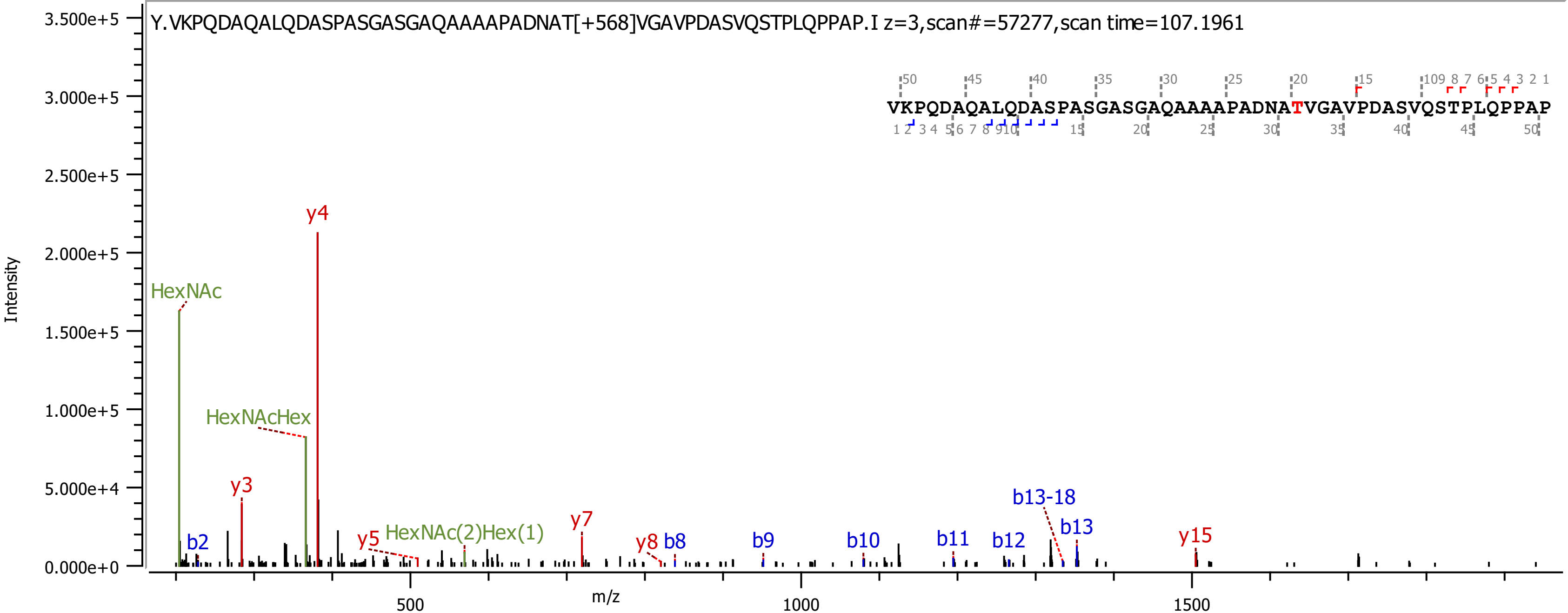

Y.VKPQDAQALQDASPASGASGAQAAAAPADNAT[+568]VGAVPDASVQSTPLQPPAP.I z=3,scan#=57277,scan time=107.1961
VKPQDAQALQDASPASGASGAQAAAAPADNATVGAVPDASVQSTPLQPPAP
Intensity
m/z
HexNAc
HexNAcHex
HexNAc(2)Hex(1)
y3
y4
y5
y7
y8
y15
b2
b8
b9
b10
b11
b12
b13-18
b13

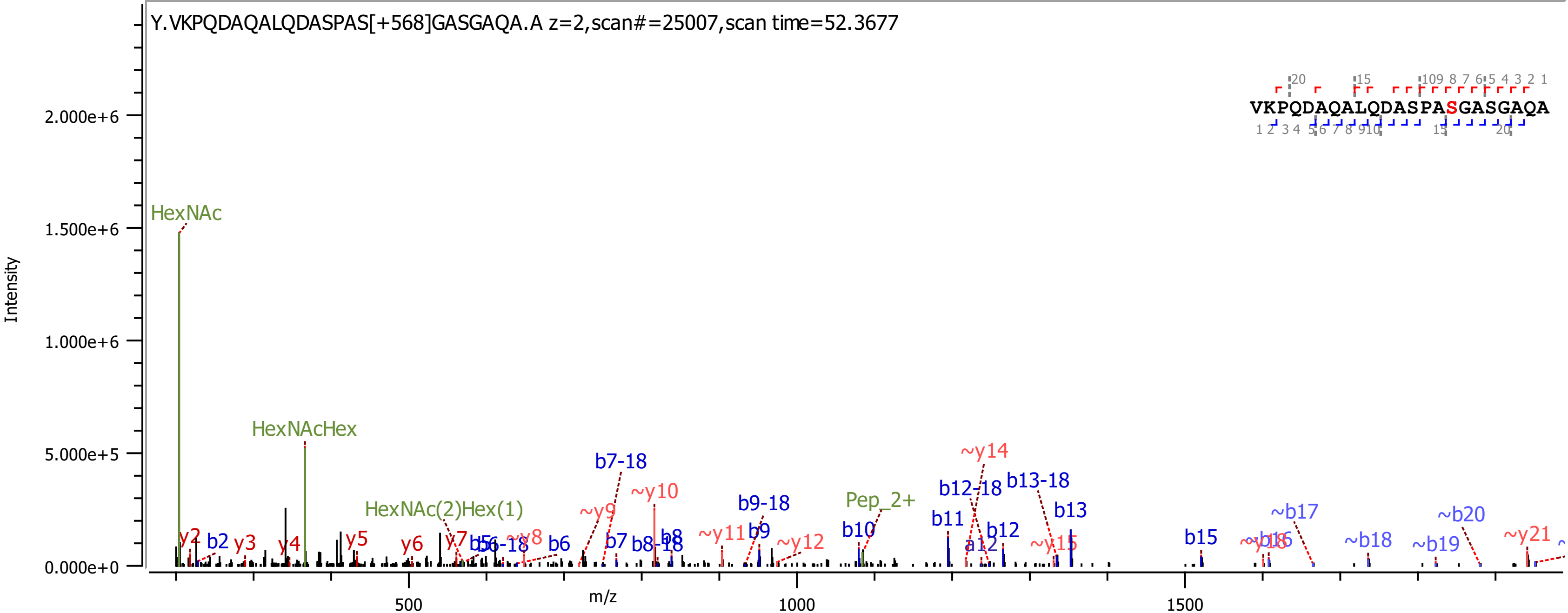

Y.VKPQDAQALQDASPAS[+568]GASGAQA.A z=2,scan#=25007,scan time=52.3677
VKPQDAQALQDASPASGASGAQA
Intensity
m/z
HexNAc
HexNAcHex
HexNAc(2)Hex(1)
Pep_2+

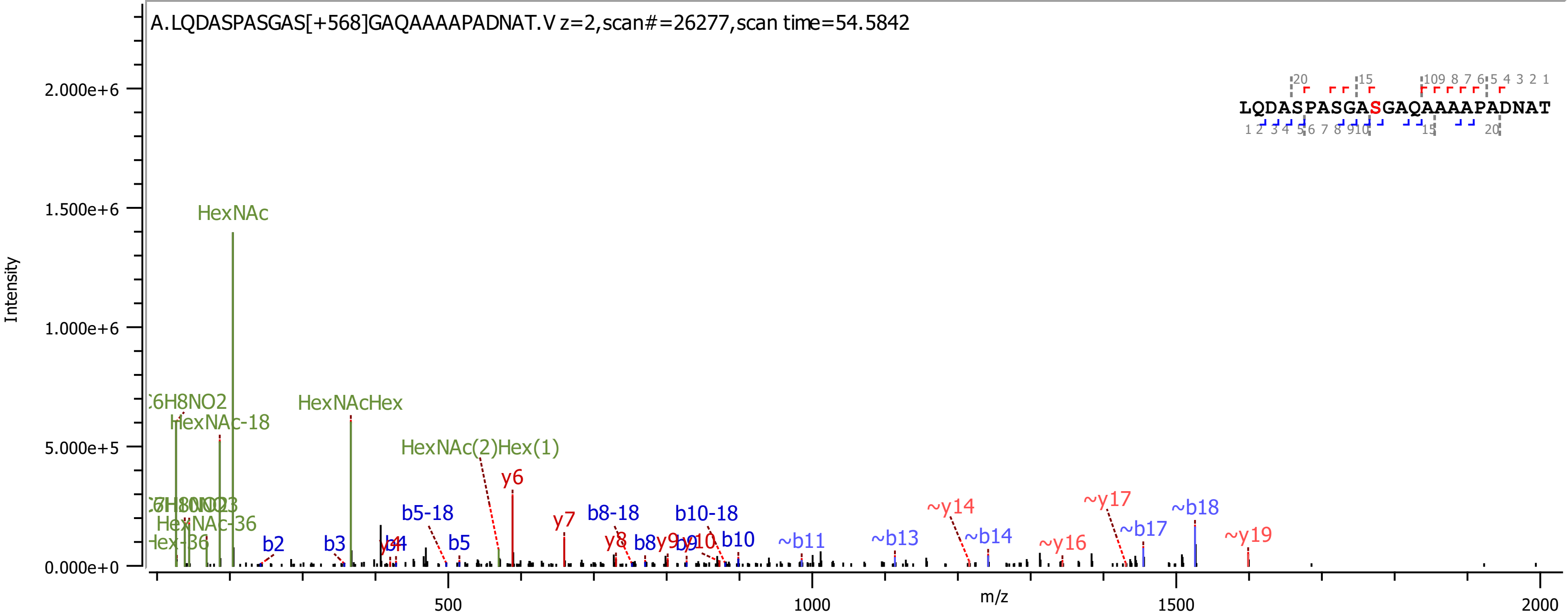
A.LQDASPASGAS[+568]GAQAAAAPADNAT.V z=2,scan#=26277,scan time=54.5842
LQDASPASGASGAQAAAAPADNAT
Intensity
m/z
2.000e+6
1.500e+6
1.000e+6
5.000e+5
0.000e+0
500
1000
1500
2000
HexNAc
HexNAc-18
HexNAc-36
HexNAcHex
HexNAc(2)Hex(1)
y6
y7
y8
b2
b3
b5
b5-18
b8-18
b8
b10-18
b10
~b11
~b13
~b14
~y14
~y16
~y17
~b17
~b18
~y19

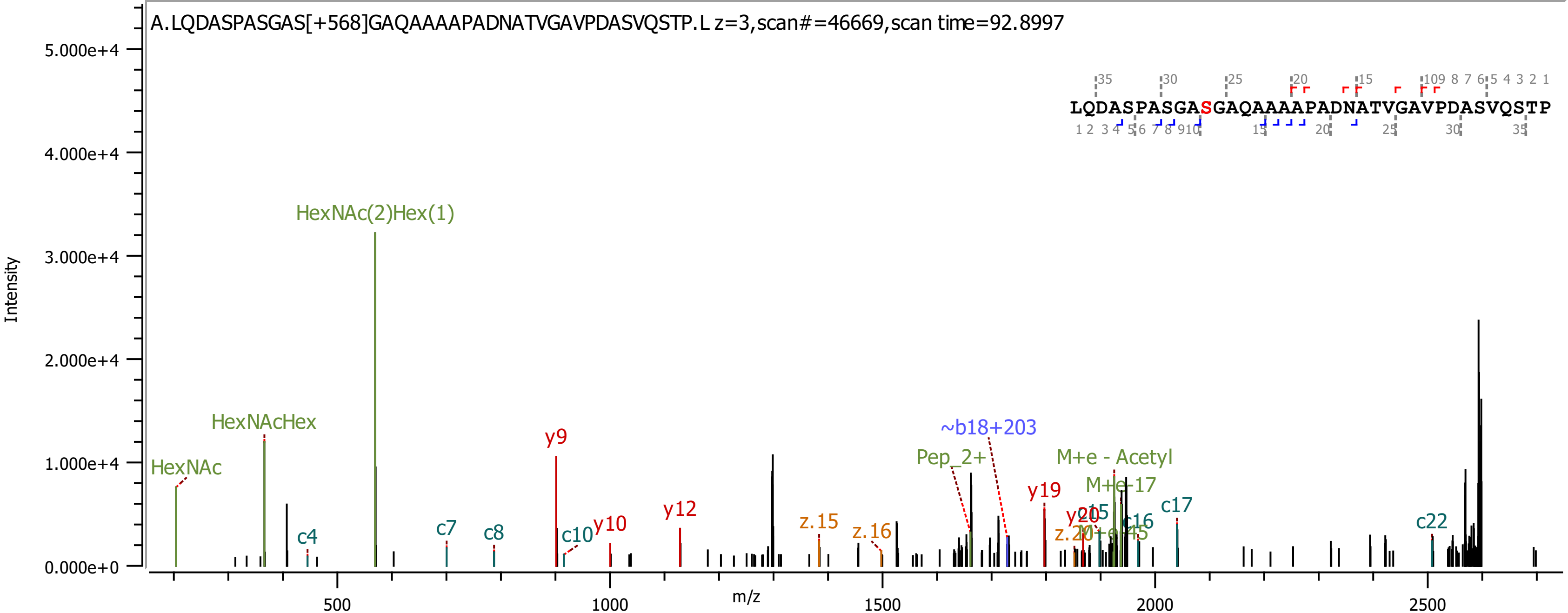
A.LQDASPASGAS[+568]GAQAAAAPADNATVGAVPDASVQSTP.L z=3,scan#=46669,scan time=92.8997
LQDASPASGASGAQAAAAPADNATVGAVPDASVQSTP
Intensity
m/z
5.000e+4
4.000e+4
3.000e+4
2.000e+4
1.000e+4
0.000e+0
500
1000
1500
2000
2500
HexNAc(2)Hex(1)
HexNAcHex
HexNAc
c4
c7
c8
y9
c10
y10
y12
z.15
z.16
~b18+203
Pep_2+
M+e - Acetyl
M+e-17
y19
y20
c15
c16
c17
c22

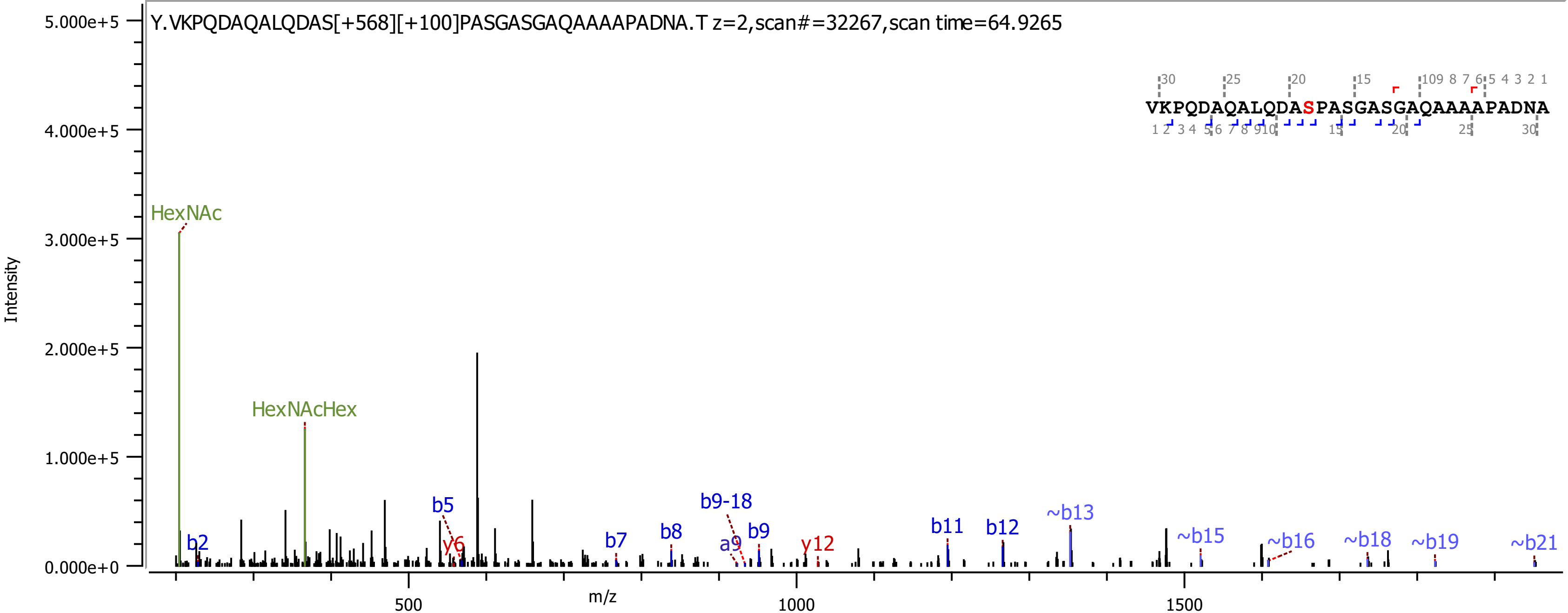

Y.VKPQDAQALQDAS[+568][+100]PASGASGAQAAAAPADNA.T z=2,scan#=32267,scan time=64.9265
VKPQDAQALQDASPASGASGAQAAAAPADNA
Intensity
m/z
HexNAc
HexNAcHex
b2
b5
y6
b7
b8
b9-18
a9
b9
y12
b11
b12
~b13
~b15
~b16
~b18
~b19
~b21

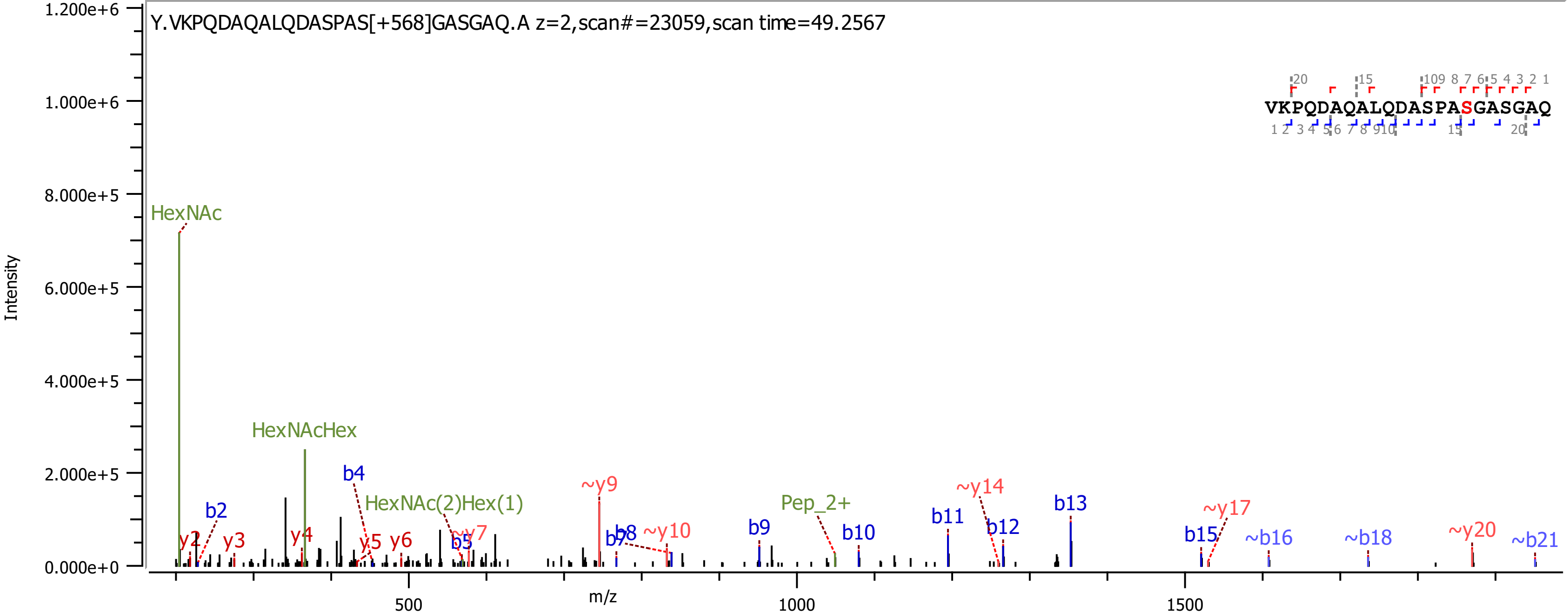

Y.VKPQDAQALQDASPAS[+568]GASGAQ.A z=2,scan#=23059,scan time=49.2567
VKPQDAQALQDASPASGASGAQ
Intensity
m/z
1.200e+6
1.000e+6
8.000e+5
6.000e+5
4.000e+5
2.000e+5
0.000e+0
500
1000
1500
HexNAc
HexNAcHex
HexNAc(2)Hex(1)
Pep_2+
b2
b4
b5
b7
b8
b9
b10
b11
b12
b13
b15
~b16
~b18
~b21
y2
y3
y4
y5
y6
~y7
~y9
~y10
~y14
~y17
~y20

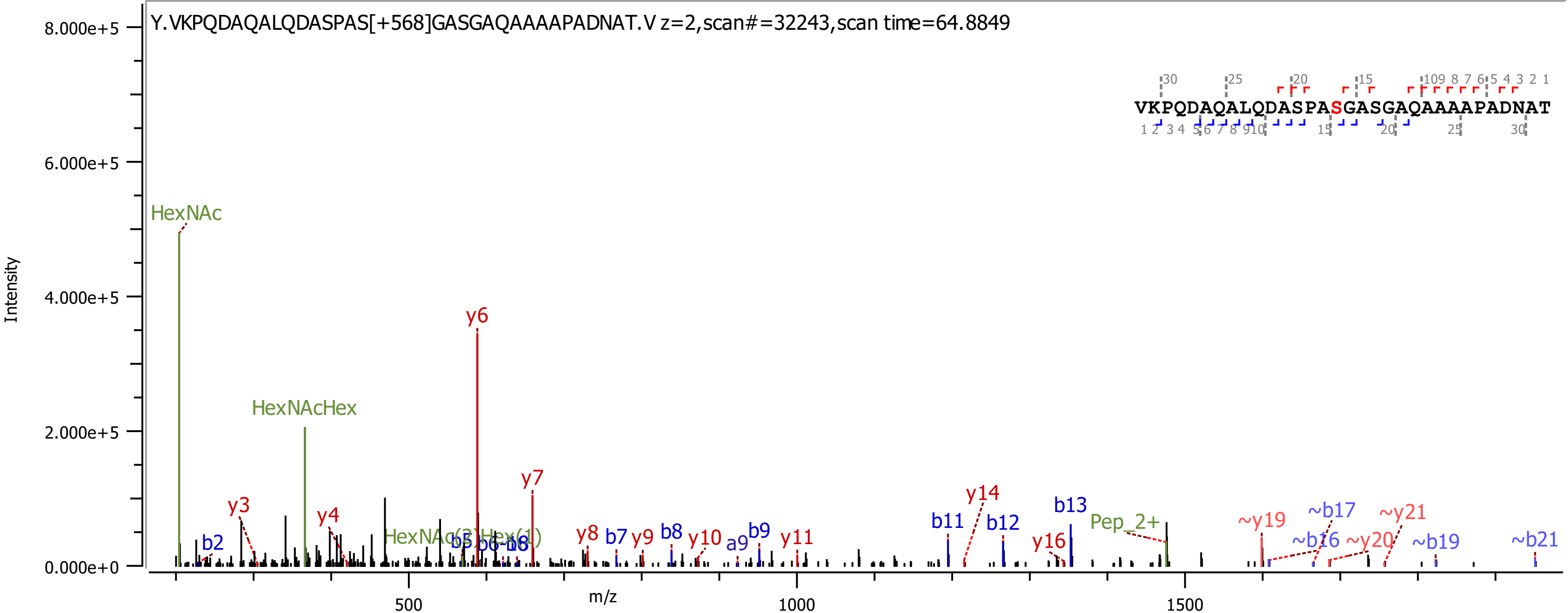

Y.VKPQDAQALQDASPAS[+568]GASGAQAAAAPADNAT.V z=2,scan#=32243,scan time=64.8849
VKPQDAQALQDASPASGASGAQAAAAPADNAT
Intensity
m/z
HexNAc
HexNAcHex
HexNAc(3)Hex(1)
Pep_2+
y3
y4
y6
y7
y8
y9
y10
y11
y14
y16
~y19
~y20
~y21
b2
b5
b6
b8
b7
b9
a9
b11
b12
b13
~b16
~b17
~b19
~b21

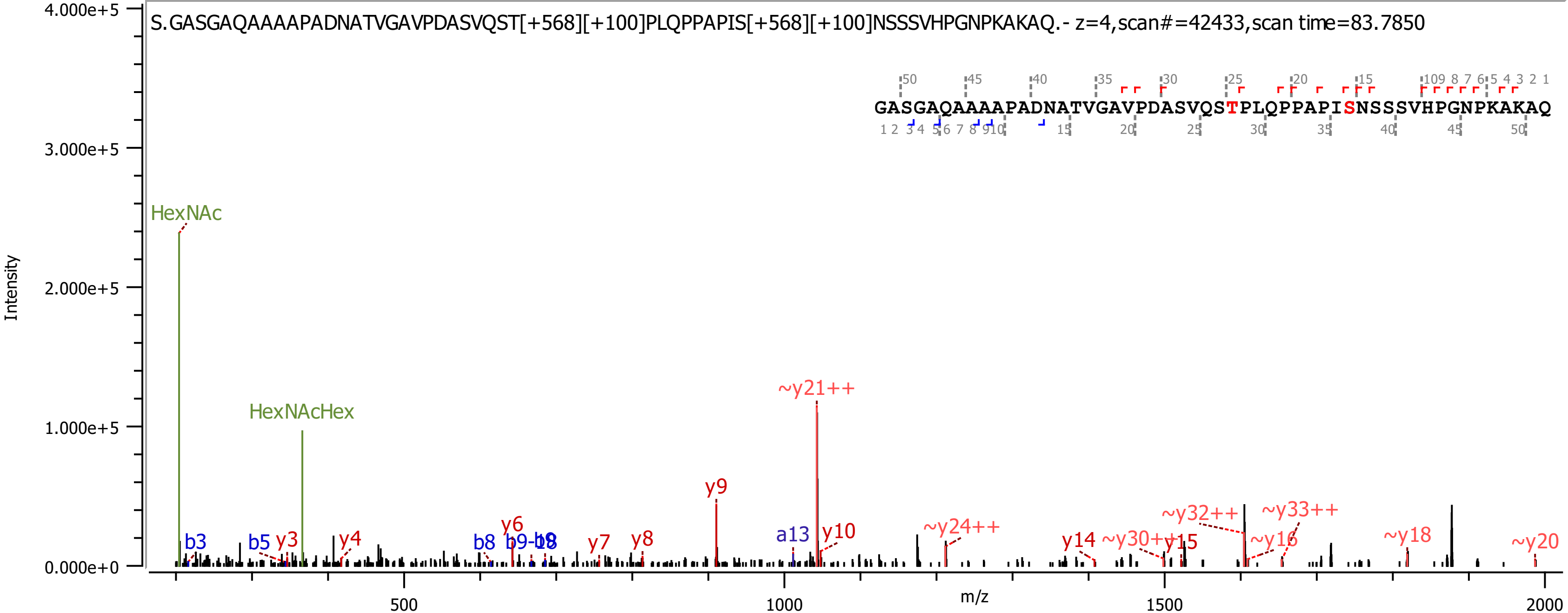
S.GASGAQAAAAPADNATVGAVPDASVQST[+568][+100]PLQPPAPIS[+568][+100]NSSSVHPGNPKAKAQ.- z=4,scan#=42433,scan time=83.7850
GASGAQAAAAPADNATVGAVPDASVQSTPLQPPAPISNSSSVHPGNPKAKAQ
Intensity
m/z
HexNAc
HexNAcHex
~y21++
y9
y10
a13
~y24++
~y32++
~y33++
~y30++
y14
y15
~y16
~y18
~y20
b3
b5
y3
y4
b8
y6
y7
y8
0.000e+0
1.000e+5
2.000e+5
3.000e+5
4.000e+5
500
1000
1500
2000

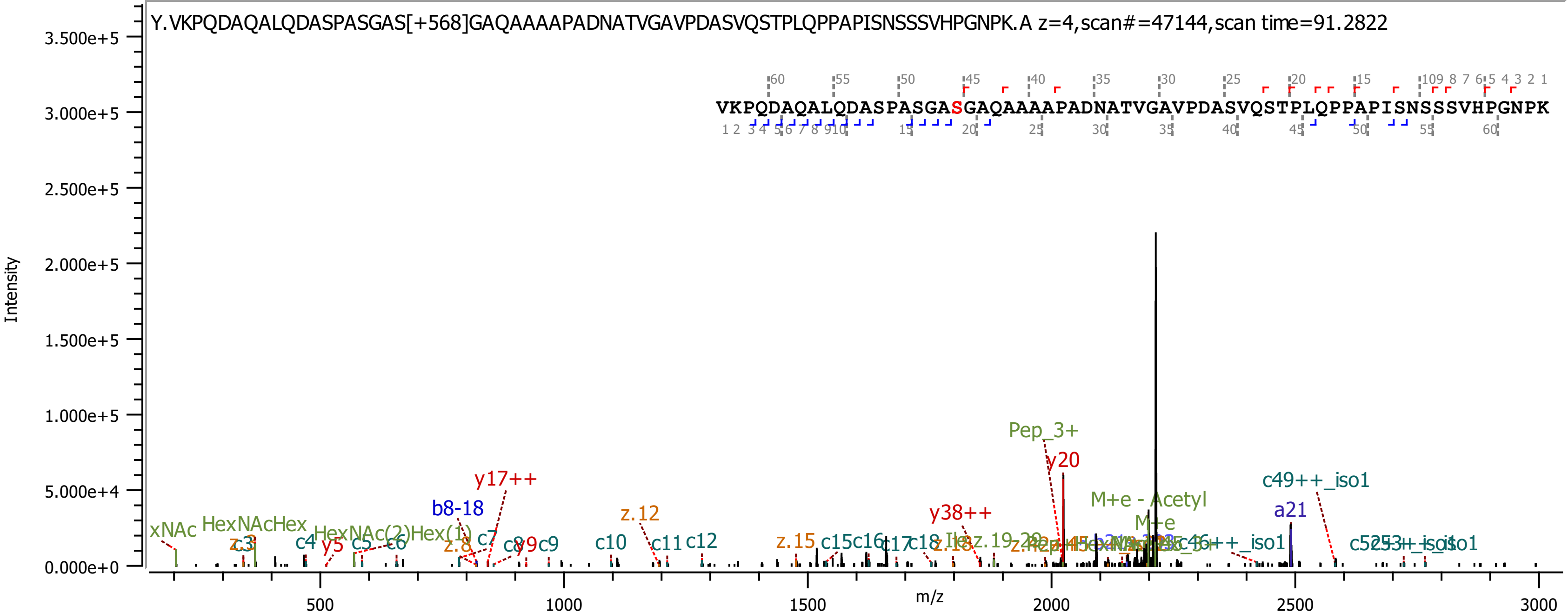

Y.VKPQDAQALQDASPASGAS[+568]GAQAAAAPADNATVGAVPDASVQSTPLQPPAPISNSSSVHPGNPK.A z=4,scan#=47144,scan time=91.2822
VKPQDAQALQDASPASGASGAQAAAAPADNATVGAVPDASVQSTPLQPPAPISNSSSVHPGNPK
Intensity
m/z
3.500e+5
3.000e+5
2.500e+5
2.000e+5
1.500e+5
1.000e+5
5.000e+4
0.000e+0
500
1000
1500
2000
2500
3000
Pep_3+
y20
c49++_iso1
M+e - Acetyl
M+e
a21
y38++
y17++
b8-18
z.12
HexNAcHex
HexNAc(2)Hex(1)
xNAc
c4
y5
c5
c6
c7
y9
c9
c10
c11
c12
z.15
c15
c16
c17
c18
z.18
z.8

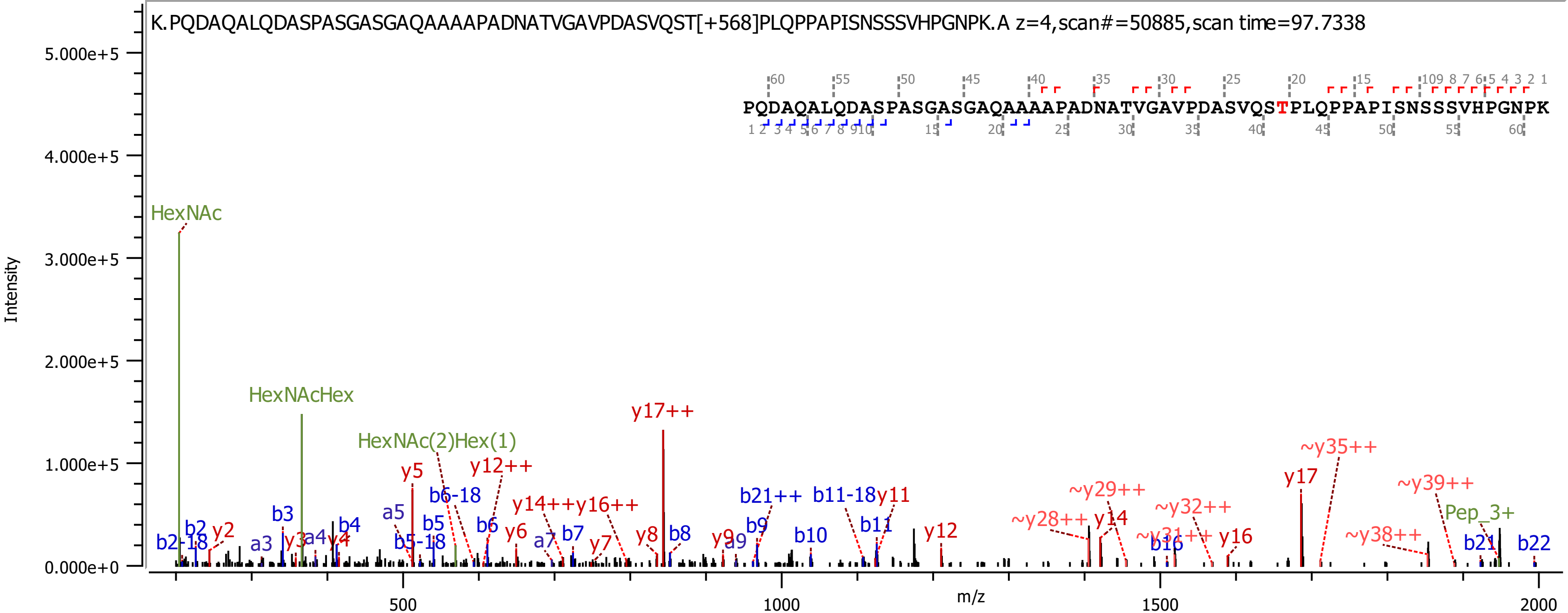
K.PQDAQALQDASPASGASGAQAAAAPADNATVGAVPDASVQST[+568]PLQPPAPISNSSSVHPGNPK.A z=4,scan#=50885,scan time=97.7338
PQDAQALQDASPASGASGAQAAAAPADNATVGAVPDASVQSTPLQPPAPISNSSSVHPGNPK
Intensity
m/z
HexNAc
HexNAcHex
HexNAc(2)Hex(1)
y17++
y5
y12++
b6-18
b21++
b11-18
y11
~y35++
y17
~y39++
~y29++
~y32++
Pep_3+
~y28++
y14
y16
~y31++
b16
~y38++
b21
b22
y12
b10
b11
b9
a9
y9
b8
y8
y7
b7
a7
y6
b6
y14++
y16++
b5
b5-18
a5
b4
y4
a4
y3
a3
b3
b2
b2-18
y2

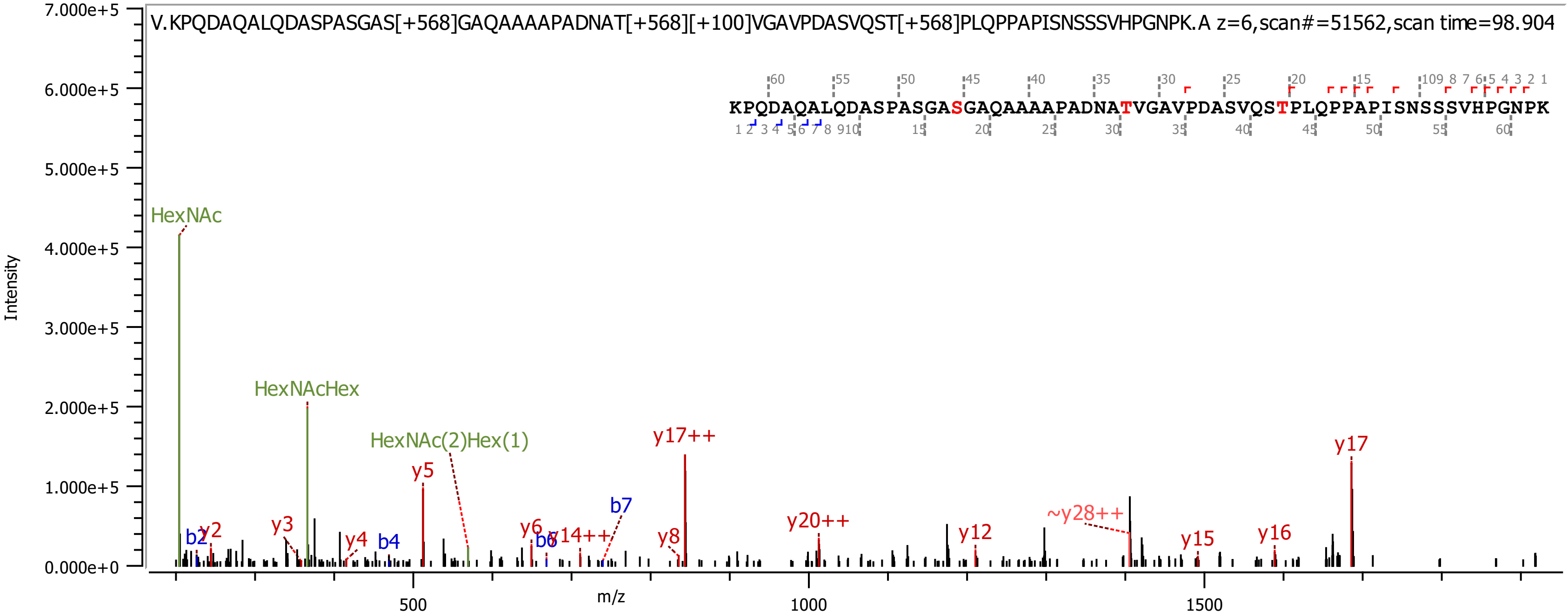

V.KPQDAQALQDASPASGAS[+568]GAQAAAAPADNAT[+568][+100]VGAVPDASVQST[+568]PLQPPAPISNSSSVHPGNPK.A z=6,scan#=51562,scan time=98.904
KPQDAQALQDASPASGASGAQAAAAPADNATVGAVPDASVQSTPLQPPAPISNSSSVHPGNPK
Intensity
m/z
HexNAc
HexNAcHex
HexNAc(2)Hex(1)
y17++
y17
y5
y20++
~y28++
y12
y15
y16
y8
y6
b6
y14++
b7
y2
b2
y3
y4
b4

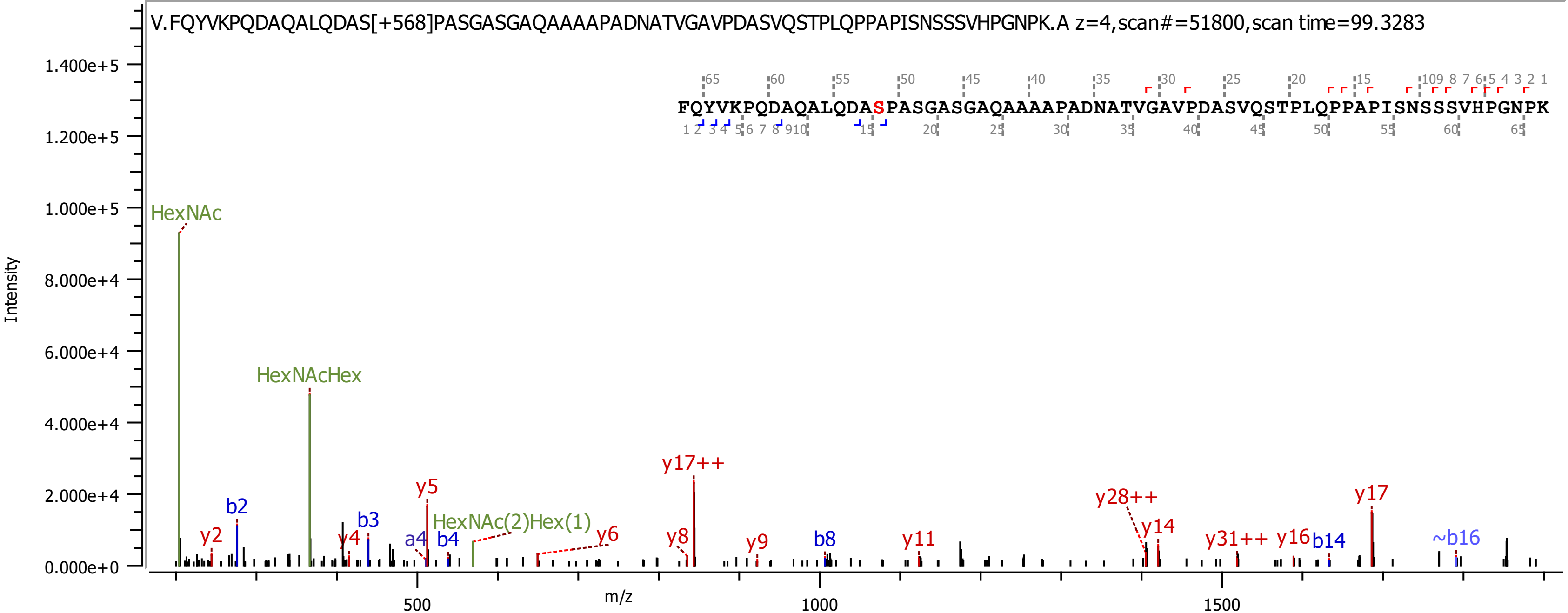
V.FQYVKPQDAQALQDAS[+568]PASGASGAQAAAAPADNATVGAVPDASVQSTPLQPPAPISNSSSVHPGNPK.A z=4,scan#=51800,scan time=99.3283
FQYVKPQDAQALQDASPASGASGAQAAAAPADNATVGAVPDASVQSTPLQPPAPISNSSSVHPGNPK
Intensity
m/z
HexNAc
HexNAcHex
HexNAc(2)Hex(1)
y2
b2
y4
b3
a4
b4
y5
y6
y8
y17++
y9
b8
y11
y28++
y14
y31++
y16
b14
y17
~b16

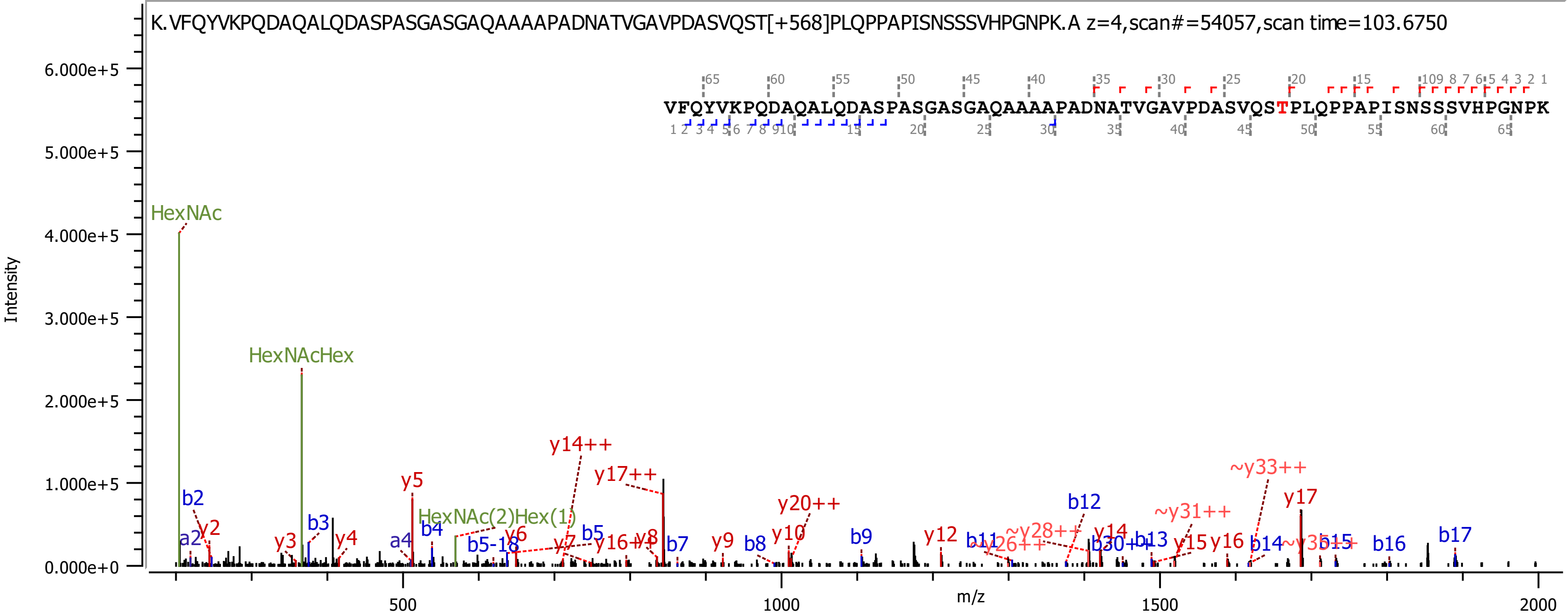

K.VFQYVKPQDAQALQDASPASGASGAQAAAAPADNATVGAVPDASVQST[+568]PLQPPAPISNSSSVHPGNPK.A z=4,scan#=54057,scan time=103.6750
VFQYVKPQDAQALQDASPASGASGAQAAAAPADNATVGAVPDASVQSTPLQPPAPISNSSSVHPGNPK
Intensity
m/z
HexNAc
HexNAcHex
HexNAc(2)Hex(1)
b2
y2
a2
y3
b3
y4
a4
y5
b4
b5-18
y6
y14++
y7
b5
y17++
y16++
y8
b7
y9
b8
y10
y20++
b9
y12
b11
~y26++
~y28++
b12
y14
b30++
b13
~y31++
y15
y16
b14
~y33++
y17
~y35++
b15
b16
b17

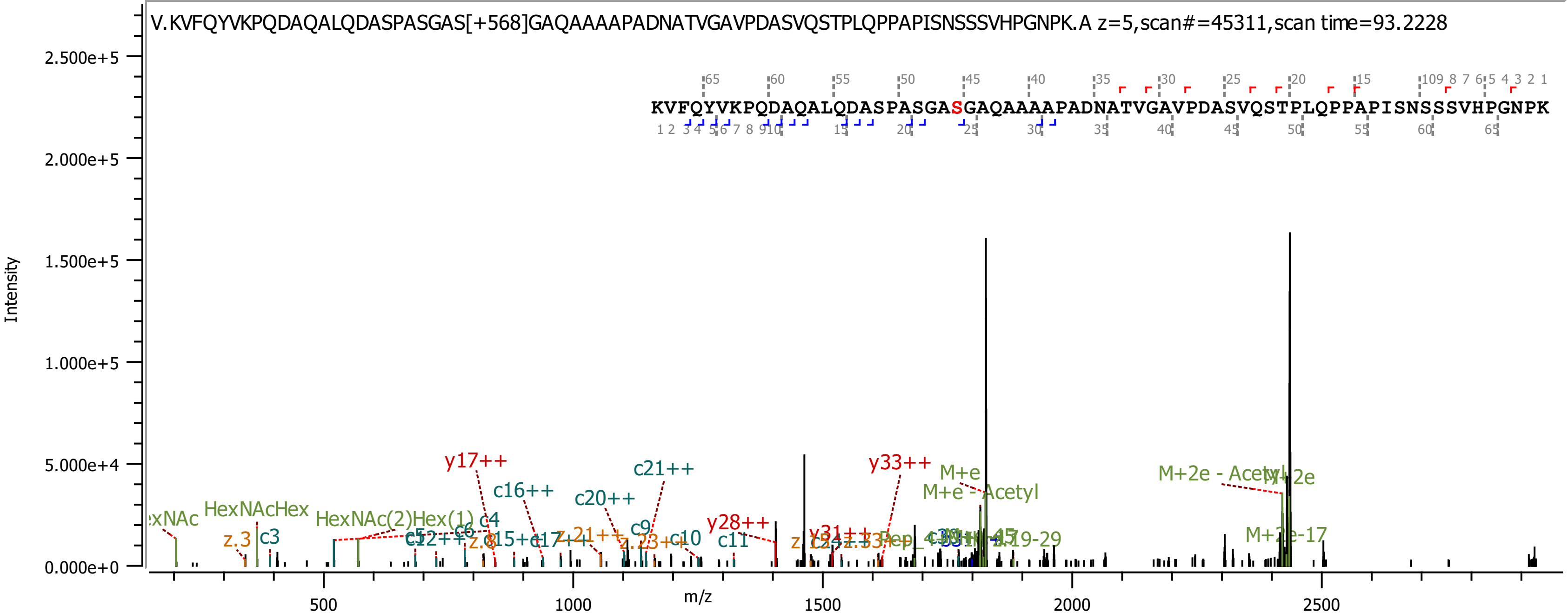
V.KVFQYVKPQDAQALQDASPASGAS[+568]GAQAAAAPADNATVGAVPDASVQSTPLQPPAPISNSSSVHPGNPK.A z=5,scan#=45311,scan time=93.2228
KVFQYVKPQDAQALQDASPASGASGAQAAAAPADNATVGAVPDASVQSTPLQPPAPISNSSSVHPGNPK
Intensity
m/z
2.500e+5
2.000e+5
1.500e+5
1.000e+5
5.000e+4
0.000e+0
500
1000
1500
2000
2500
y17++
c16++
c20++
c21++
y28++
y33++
M+e
M+e - Acetyl
M+2e - Acetyl
M+2e
M+2e-17
HexNAcHex
HexNAc(2)Hex(1)
c3
c4
c9
c10
c11
z.3
z.8
z.21++
z.23++
y31++

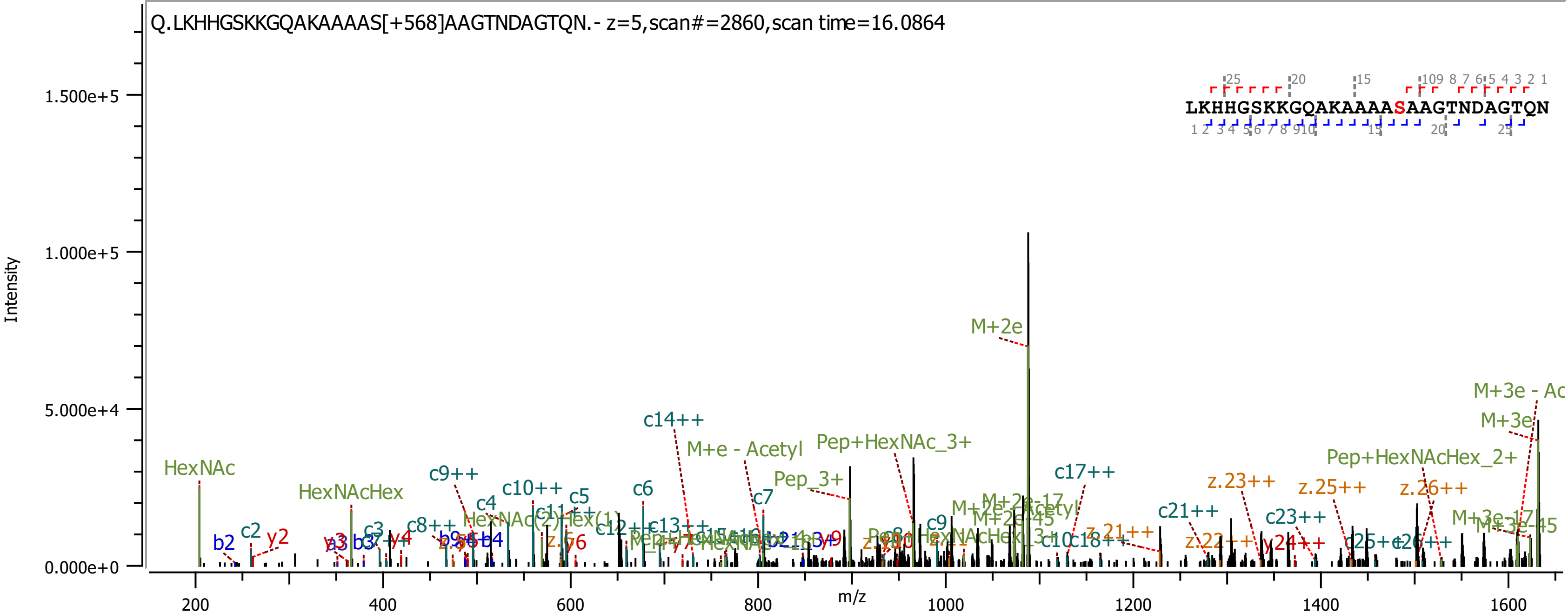

Q.LKHHGSKKGQAKAAAAS[+568]AAGTNDAGTQN.- z=5,scan#=2860,scan time=16.0864
LKHHGSKKGQAKAAAASAAGTNDAGTQN
Intensity
m/z
1.500e+5
1.000e+5
5.000e+4
0.000e+0
200
400
600
800
1000
1200
1400
1600
HexNAc
HexNAcHex
b2
c2
y2
c9++
c10++
c4
c5
c6
c14++
M+e - Acetyl
c7
Pep_3+
Pep+HexNAc_3+
M+2e
c9
c17++
c21++
c23++
z.23++
z.25++
z.26++
Pep+HexNAcHex_2+
M+3e - Ac
M+3e

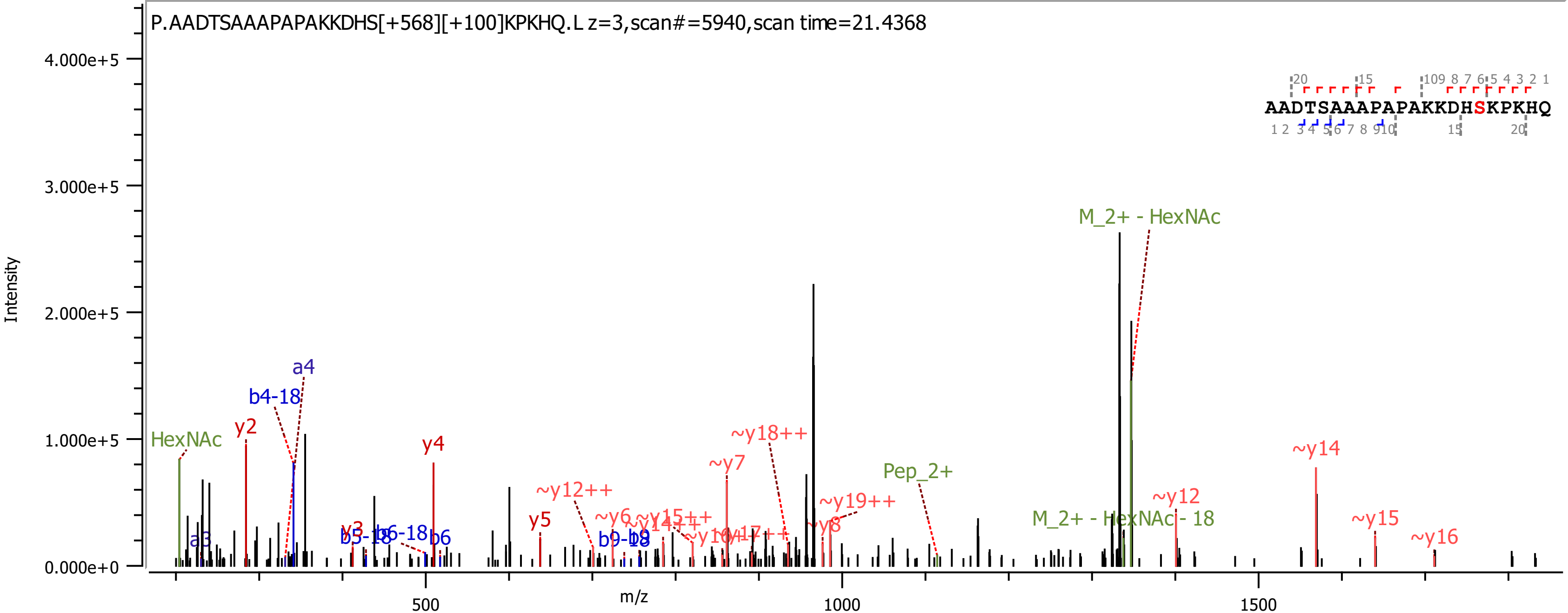
P.AADTSAAAPAPAKKDHS[+568][+100]KPKHQ.L z=3,scan#=5940,scan time=21.4368
AADTSAAAPAPAKKDHSKPKHQ
Intensity
m/z
0.000e+0
1.000e+5
2.000e+5
3.000e+5
4.000e+5
500
1000
1500
HexNAc
y2
b4-18
a4
a3
y3
b5-18
b6-18
b6
y4
y5
~y12++
~y6
~y15++
~y14++
b9-18
b9
~y16
~y17++
~y7
~y18++
~y8
~y19++
Pep_2+
M_2+ - HexNAc
M_2+ - HexNAc - 18
~y12
~y14
~y15
~y16

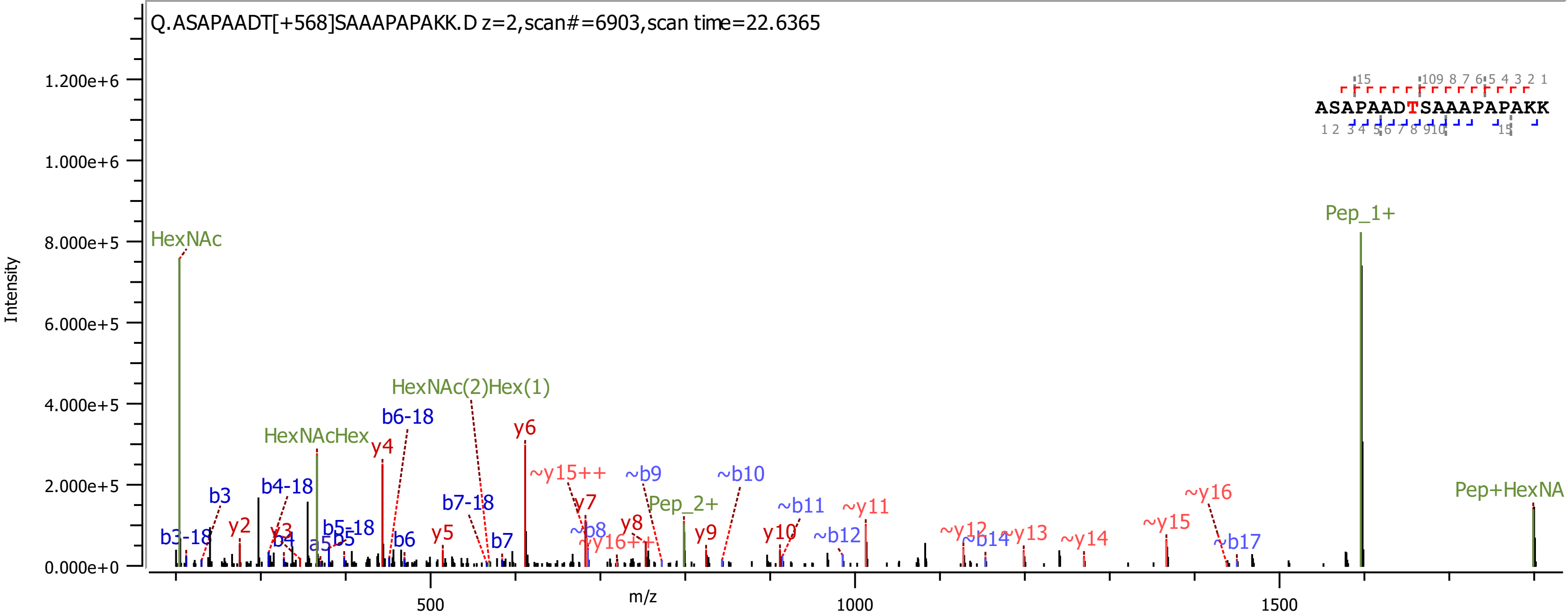
Q.ASAPAADT[+568]SAAAPAPAKK.D z=2,scan#=6903,scan time=22.6365
ASAPAADTSAAAPAPAKK
Intensity
m/z
HexNAc
HexNAcHex
HexNAc(2)Hex(1)
Pep_1+
Pep_2+
Pep+HexNA
b3-18
b3
y2
b4-18
y3
b4
a5
b5
b5-18
y4
b6-18
b6
y5
b7-18
b7
y6
~y15++
~b8
y7
~y16++
y8
~b9
y9
~b10
y10
~b11
~b12
~y11
~y12
~b14
~y13
~y14
~y15
~y16
~b17

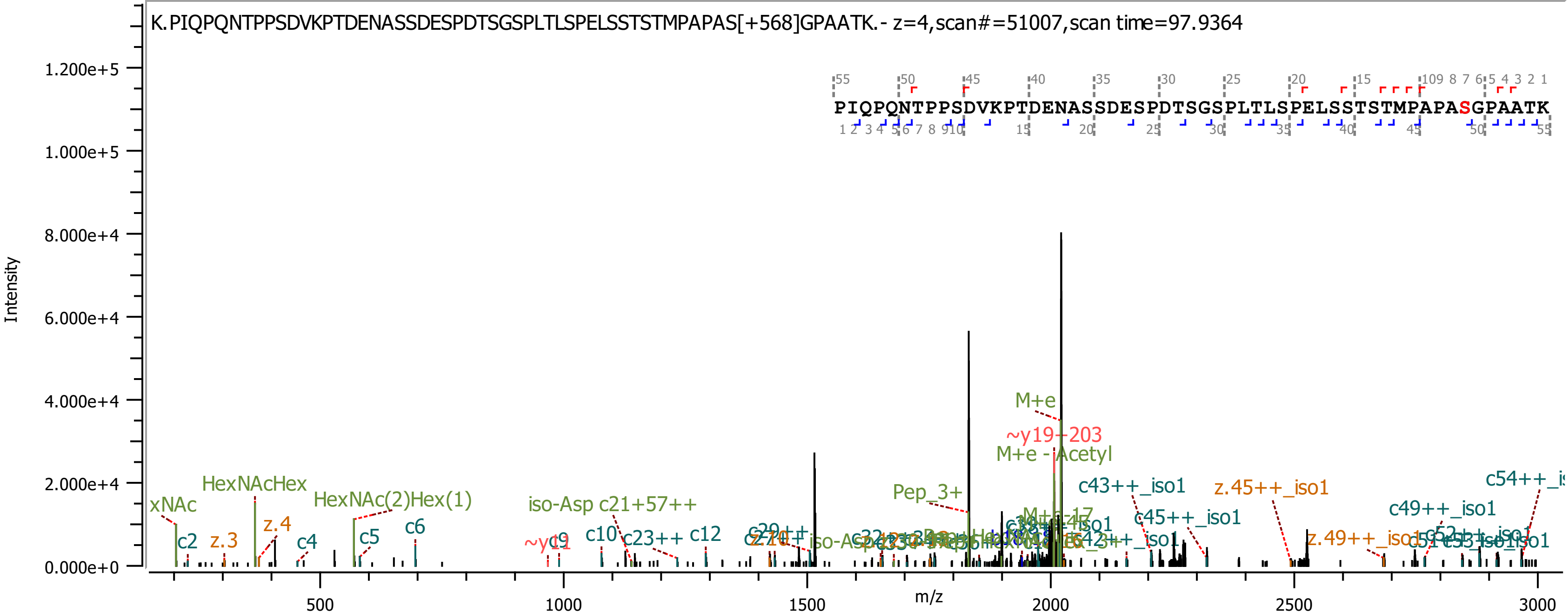

K.PIQPQNTPPSDVKPTDENASSDESPDTSGSPLTLSPELSSTSTMPAPAS[+568]GPAATK.- z=4,scan#=51007,scan time=97.9364
PIQPQNTPPSDVKPTDENASSDESPDTSGSPLTLSPELSSTSTMPAPASGPAATK
Intensity
m/z
1.200e+5
1.000e+5
8.000e+4
6.000e+4
4.000e+4
2.000e+4
0.000e+0
500
1000
1500
2000
2500
3000
HexNAcHex
HexNAc(2)Hex(1)
iso-Asp c21+57++
M+e
~y19-203
M+e - Acetyl
Pep_3+
c43++_iso1
z.45++_iso1
c45++_iso1
c49++_iso1
c2
z.3
z.4
c4
c5
c6
c10
c23++
c12
c29++
z.49++_iso1

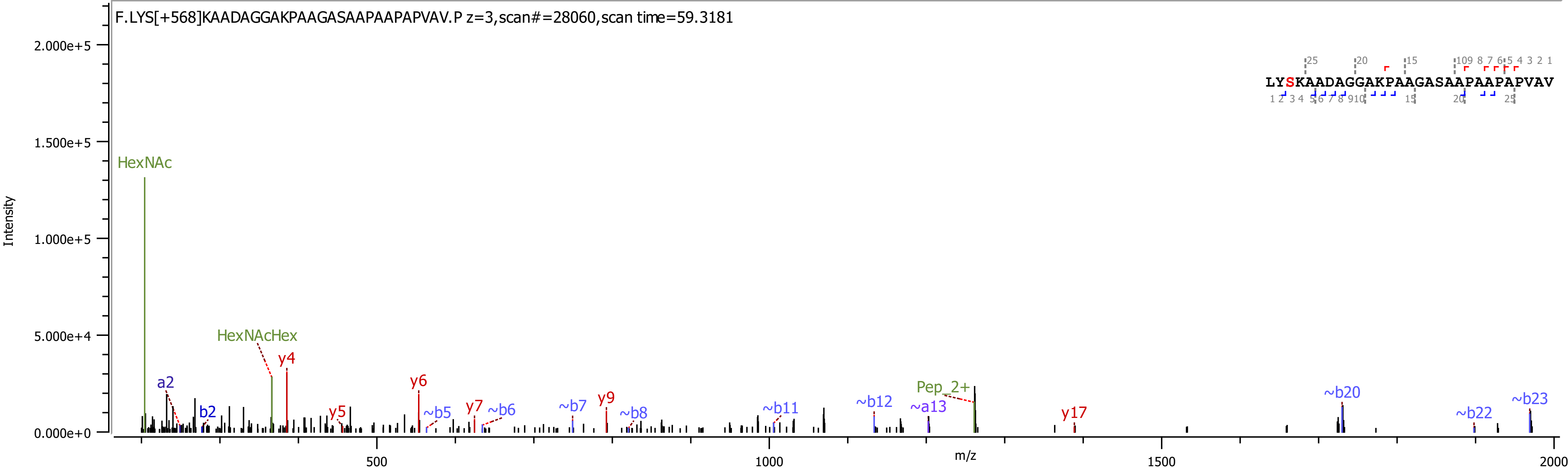

F.LYS[+568]KAADAGGAKPAAGASAAPAAPAPVAV.P z=3,scan#=28060,scan time=59.3181
LYSKAADAGGAKPAAGASAAPAAPAPVAV
Intensity
m/z
HexNAc
HexNAcHex
a2
b2
y4
y5
y6
~b5
y7
~b6
~b7
y9
~b8
~b11
~b12
~a13
Pep_2+
y17
~b20
~b22
~b23

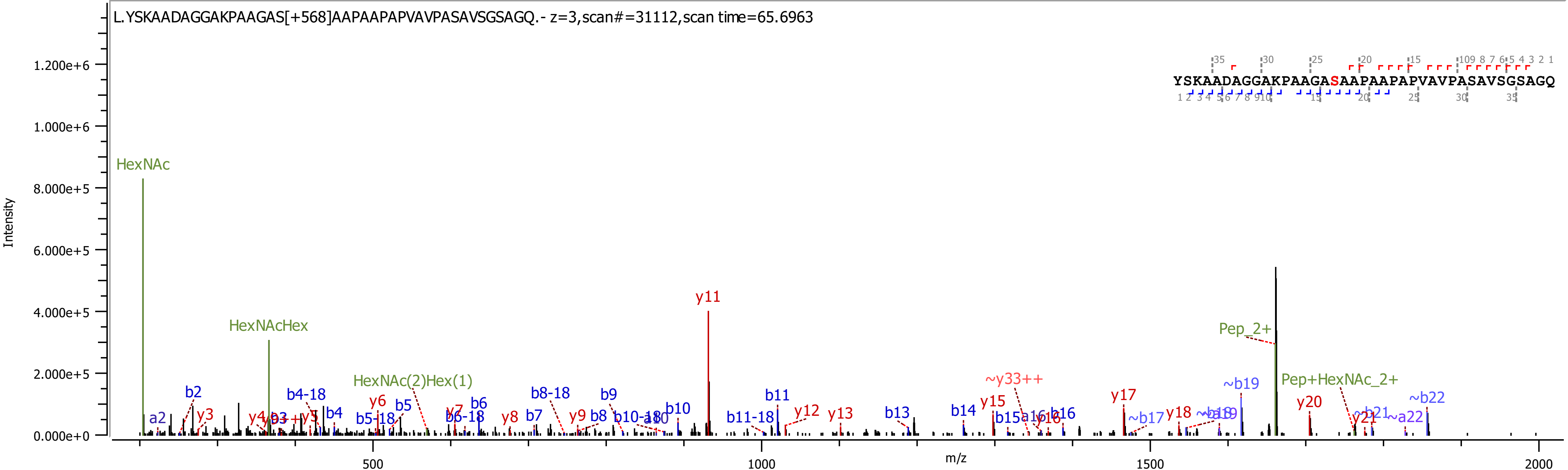

L.YSKAADAGGAKPAAGAS[+568]AAPAAPAPVAVPASAVSGSAGQ.- z=3,scan#=31112,scan time=65.6963
YSKAADAGGAKPAAGASAAPAAPAPVAVPASAVSGSAGQ
Intensity
m/z
HexNAc
HexNAcHex
HexNAc(2)Hex(1)
y11
Pep_2+
Pep+HexNAc_2+
~y33++
~b19
~b22

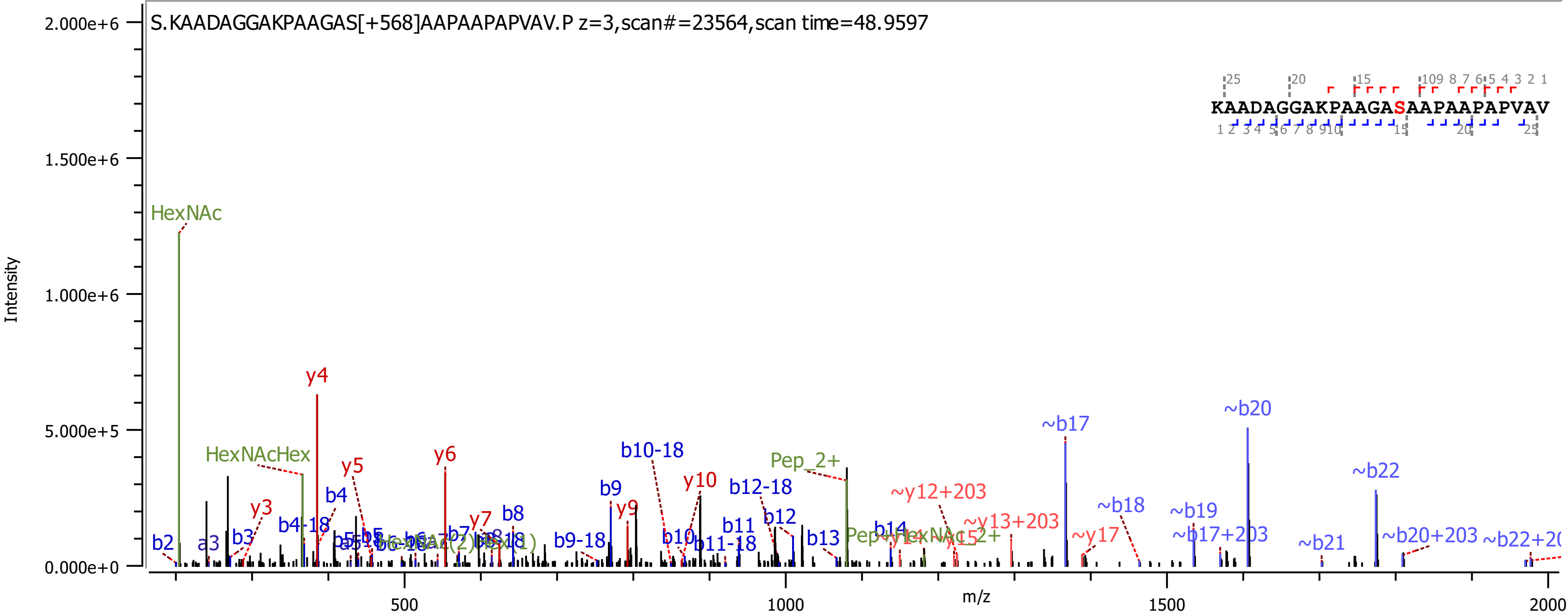
S.KAADAGGAKPAAGAS[+568]AAPAAPAPVAV.P z=3,scan#=23564,scan time=48.9597
KAADAGGAKPAAGASAAPAAPAPVAV
Intensity
m/z
2.000e+6
1.500e+6
1.000e+6
5.000e+5
0.000e+0
500
1000
1500
2000
HexNAc
HexNAcHex
y4
y6
y5
b4
y3
b4-18
b3
a3
b2
b10-18
b9
y9
y10
b12-18
b11
b12
b10
b11-18
b9-18
b8
y7
b13
b14
Pep_2+
~y12+203
~y13+203
~y17
~b17
~b18
~b19
~b17+203
~b20
~b21
~b22
~b20+203

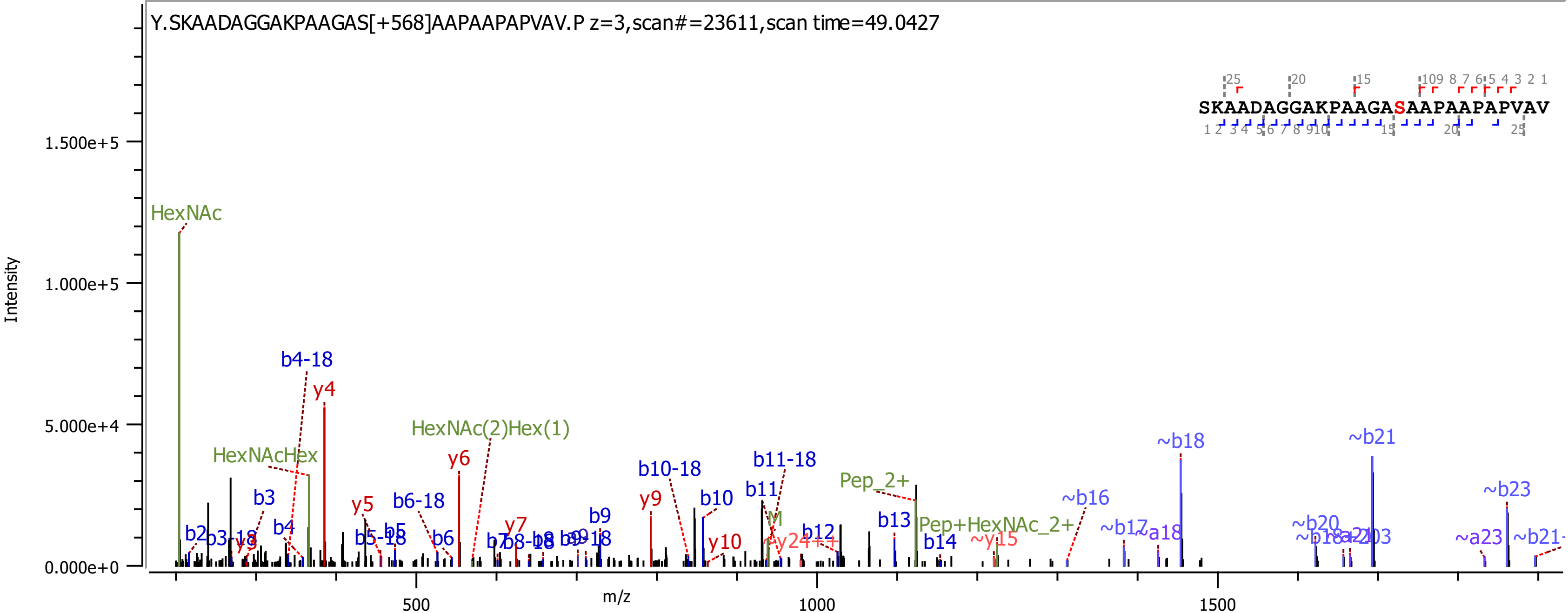
Y.SKAADAGGAKPAAGAS[+568]AAPAAPAPVAV.P z=3,scan#=23611,scan time=49.0427
SKAADAGGAKPAAGASAAPAAPAPVAV
Intensity
m/z
0.000e+0
5.000e+4
1.000e+5
1.500e+5
500
1000
1500
HexNAc
HexNAcHex
HexNAc(2)Hex(1)
Pep_2+
Pep+HexNAc_2+
b4-18
y4
y6
b10-18
b11-18
b10
b11
b9
y9
y5
b6-18
b3
b4
b2
b5
b6
y7
y10
b12
b13
b14
~y15
~b16
~b17
~a18
~b18
~b20
~b21
~b23
~a23
M

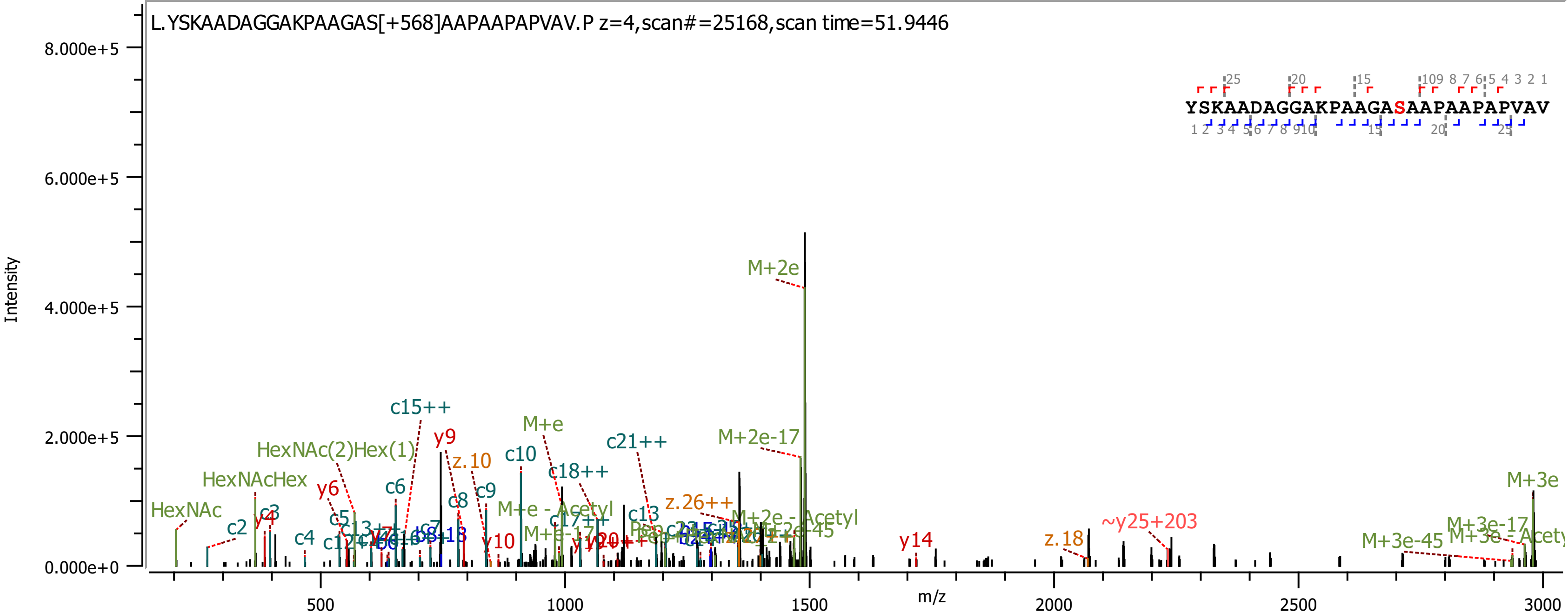

L.YSKAADAGGAKPAAGAS[+568]AAPAAPAPVAV.P z=4,scan#=25168,scan time=51.9446
Intensity
m/z
YSKAADAGGAKPAAGASAAPAAPAPVAV
M+2e
M+2e-17
M+e
c15++
y9
z.10
c10
c21++
c18++
HexNAc(2)Hex(1)
HexNAcHex
HexNAc
y6
c6
c8
c9
M+e - Acetyl
c13
z.26++
M+2e - Acetyl
c17++
~y25+203
y14
z.18
M+3e
M+3e-17
M+3e-45
c2
y4
c3
c4
c5
c7
y10

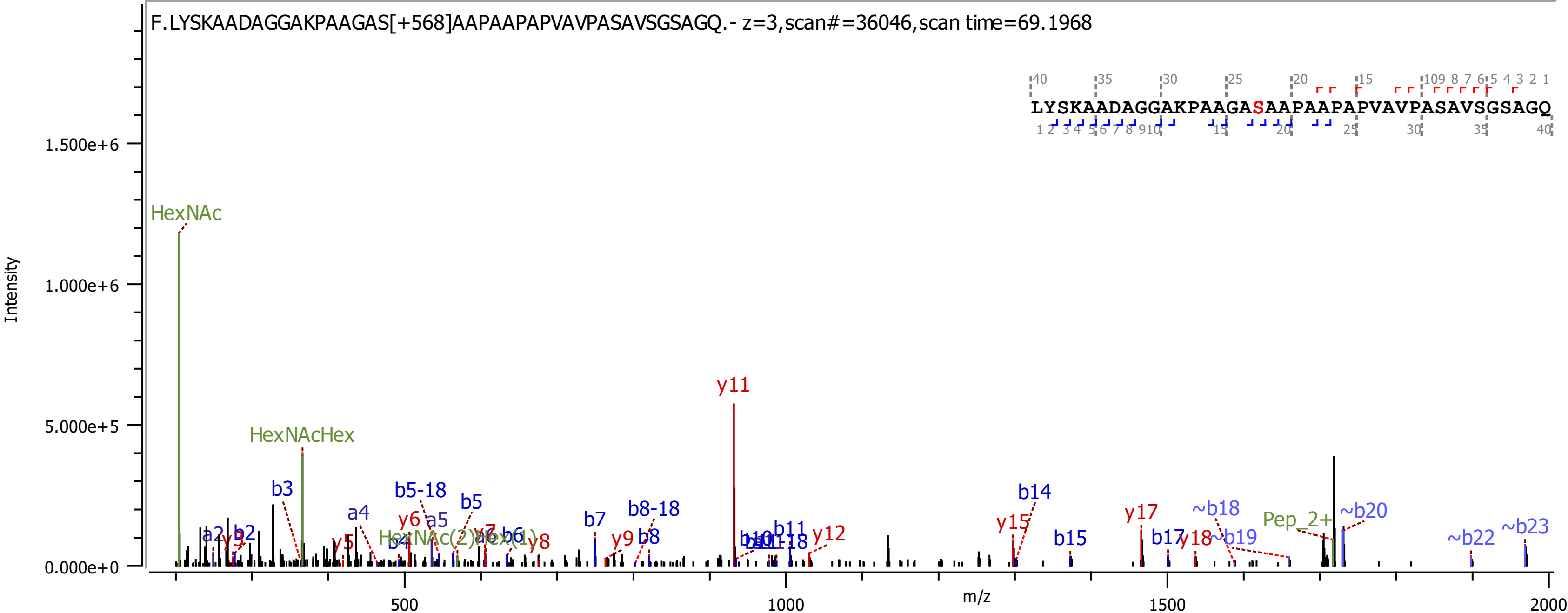
F.LYSKAADAGGAKPAAGAS[+568]AAPAAPAPVAVPASAVSGSAGQ.- z=3,scan#=36046,scan time=69.1968
LYSKAADAGGAKPAAGASAAPAAPAPVAVPASAVSGSAGQ
HexNAc
HexNAcHex
HexNAc(2)Hex(1)
Pep_2+
b3
b5-18
b5
b7
b8-18
b8
b11
b14
b15
b17
~b18
~b19
~b20
~b22
~b23
y11
y12
y15
y17
y18
y6
y9
y8
a4
a5
Intensity
m/z
0.000e+0
5.000e+5
1.000e+6
1.500e+6
500
1000
1500
2000

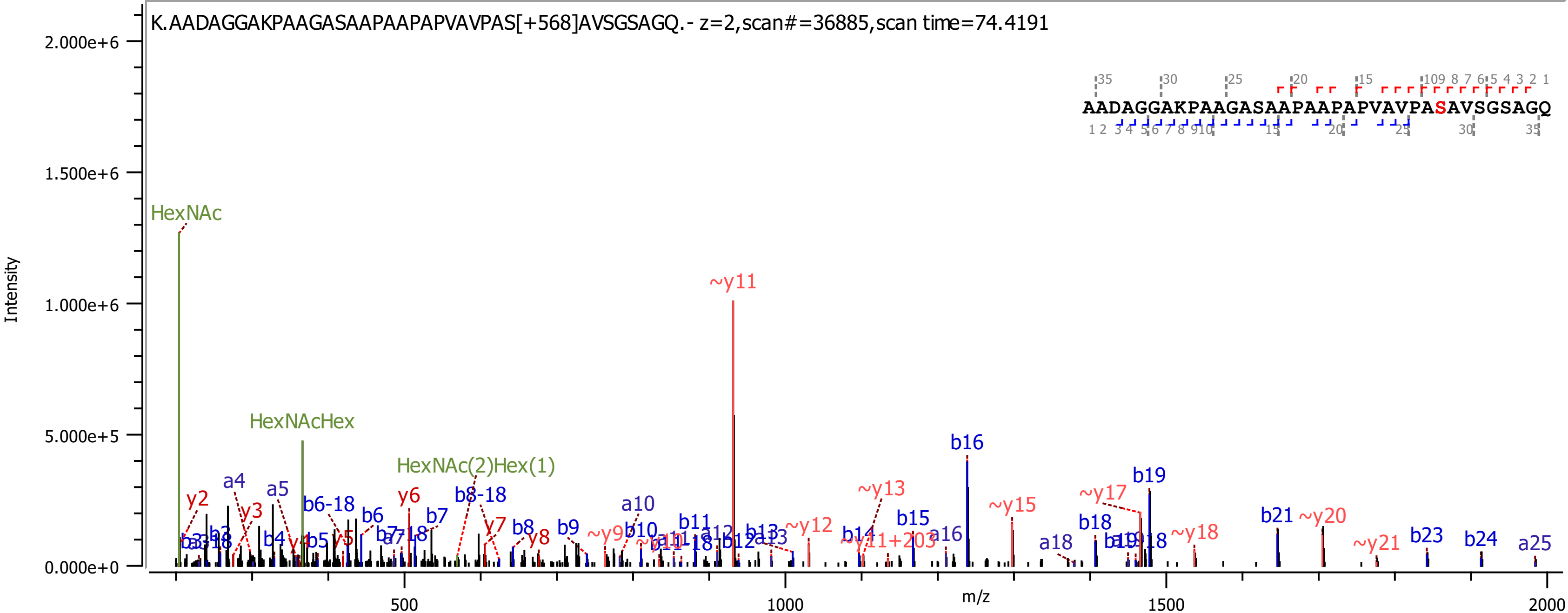
K.AADAGGAKPAAGASAAPAAPAPVAVPAS[+568]AVSGSAGQ.- z=2,scan#=36885,scan time=74.4191
AADAGGAKPAAGASAAPAAPAPVAVPASAVSGSAGQ
Intensity
m/z
2.000e+6
1.500e+6
1.000e+6
5.000e+5
0.000e+0
500
1000
1500
2000
HexNAc
HexNAcHex
HexNAc(2)Hex(1)
~y11
b16
b19
~y13
~y15
~y17
b18
b21
~y20
~y18
~y21
b23
b24
a25
b15
a16
b14
~y11+203
~y12
b13
a13
b12
a12
b11
a10
b10
~y9
b9
b8
y8
y7
b8-18
b7
b6
y6
b6-18
a5
a4
y3
y2
b4
y4
b5
y5
a18

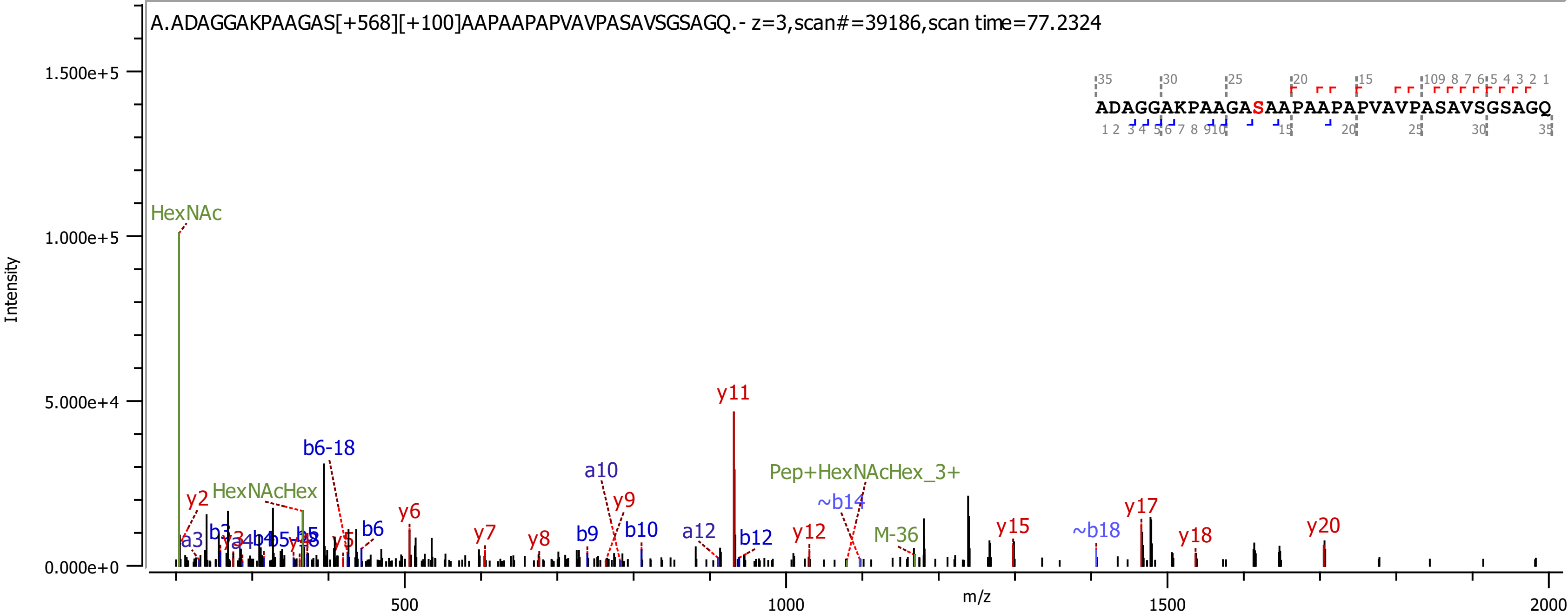

A.ADAGGAKPAAGAS[+568][+100]AAPAAPAPVAVPASAVSGSAGQ.- z=3,scan#=39186,scan time=77.2324
ADAGGAKPAAGASAAPAAPAPVAVPASAVSGSAGQ
Intensity
m/z
HexNAc
HexNAcHex
b6-18
y11
a10
y9
Pep+HexNAcHex_3+
~b14
y2
y6
b6
y7
y8
b9
b10
a12
b12
y12
M-36
y15
~b18
y17
y18
y20
a3
b3

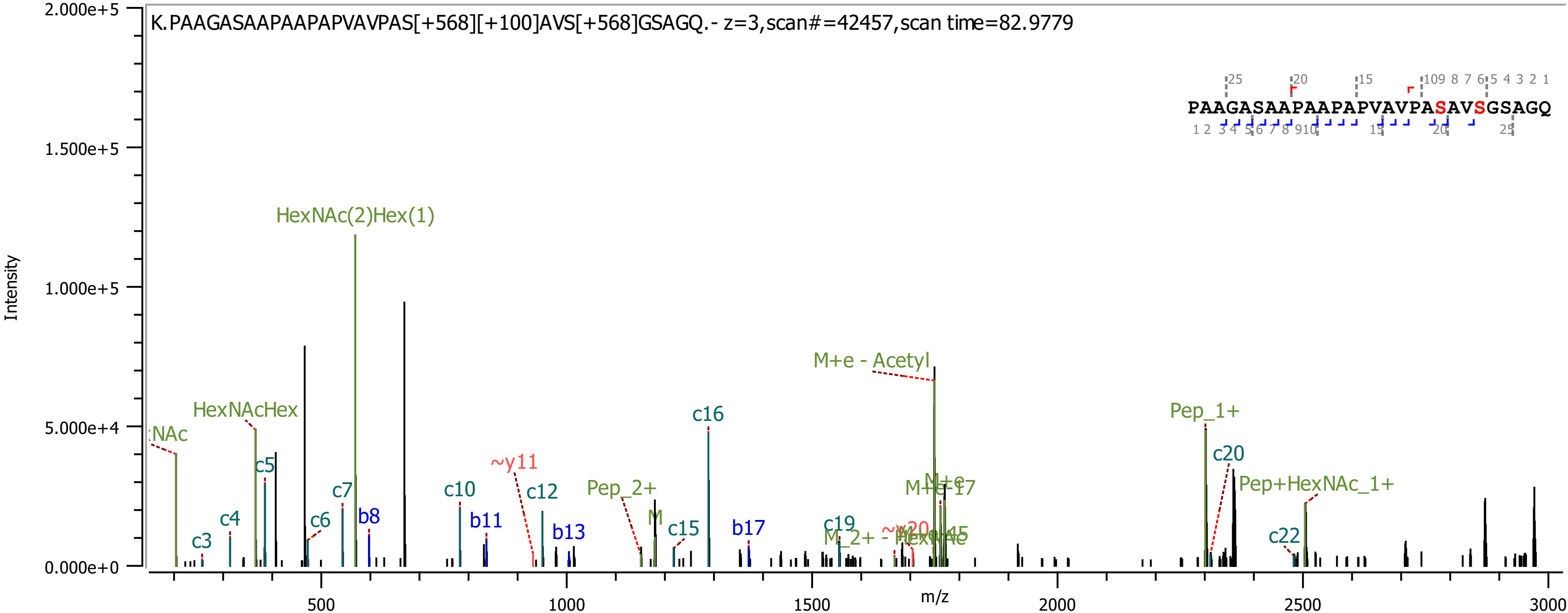
K.PAAGASAAPAAPAPVAVPAS[+568][+100]AVS[+568]GSAGQ.- z=3,scan#=42457,scan time=82.9779
PAAGASAAPAAPAPVAVPASAVSGSAGQ
Intensity
m/z
HexNAc(2)Hex(1)
HexNAcHex
M+e - Acetyl
Pep_1+
c20
Pep+HexNAc_1+
c16
c5
c7
c10
c12
~y11
Pep_2+
b8
b11
b13
b17
c15
c19
c22
c3
c4
c6
M+e-17

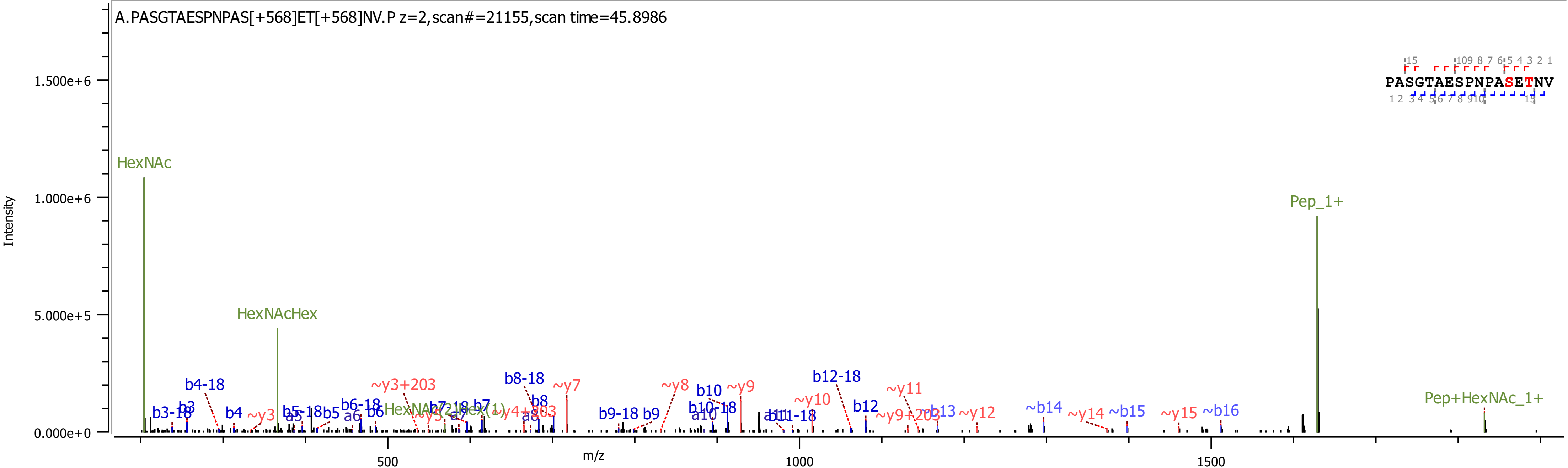

A.PASGTAESPNPAS[+568]ET[+568]NV.P z=2,scan#=21155,scan time=45.8986
PASGTAESPNPASETNV
Intensity
1.500e+6
1.000e+6
5.000e+5
0.000e+0
m/z
500
1000
1500
HexNAc
HexNAcHex
Pep_1+
Pep+HexNAc_1+
b3-18
b3
b4-18
b4
~y3
a5
b5-18
b5
a6
b6-18
b6
~y3+203
b7-18
b7
a7
~y4+203
b8-18
b8
a8
~y7
b9-18
b9
~y8
b10
b10-18
a10
~y9
~y10
a11
b11-18
b12-18
b12
~y11
~y9+203
~b13
~y12
~b14
~y14
~b15
~y15
~b16

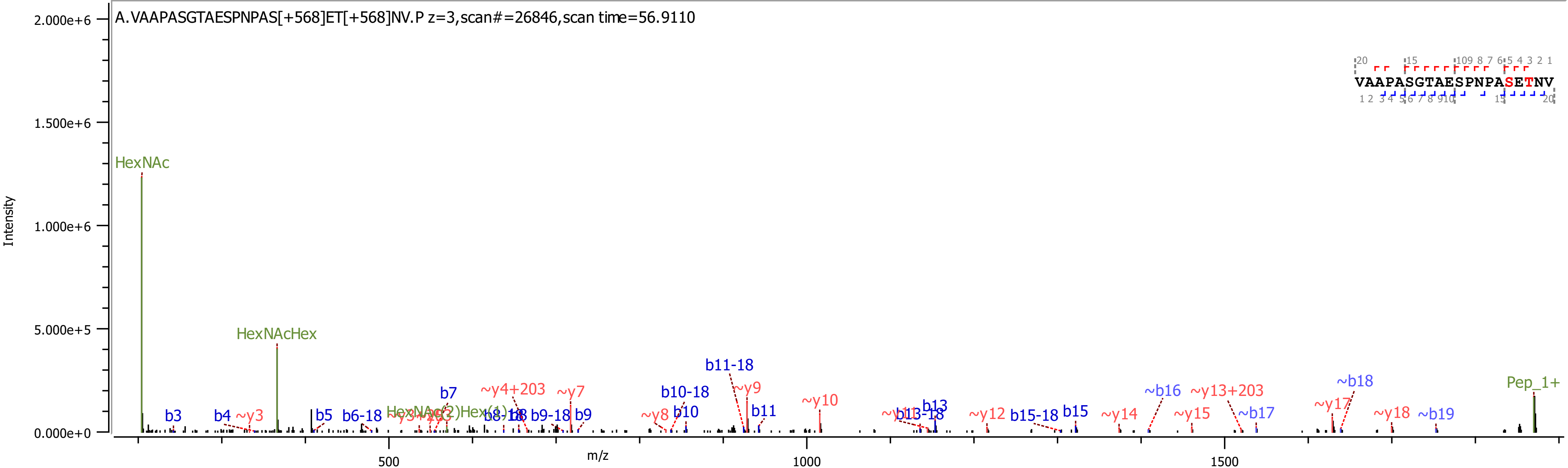
A.VAAPASGTAESPNPAS[+568]ET[+568]NV.P z=3,scan#=26846,scan time=56.9110
Intensity
m/z
HexNAc
HexNAcHex
Pep_1+
VAAPASGTAESPNPASETNV
b3
b4
~y3
b5
b6-18
b7
~y4+203
~y7
b8-18
b9-18
b9
~y8
b10
b10-18
b11-18
~y9
b11
~y10
b13
b13-18
~y12
b15-18
b15
~y14
~b16
~y13+203
~y15
~b17
~y17
~b18
~y18
~b19

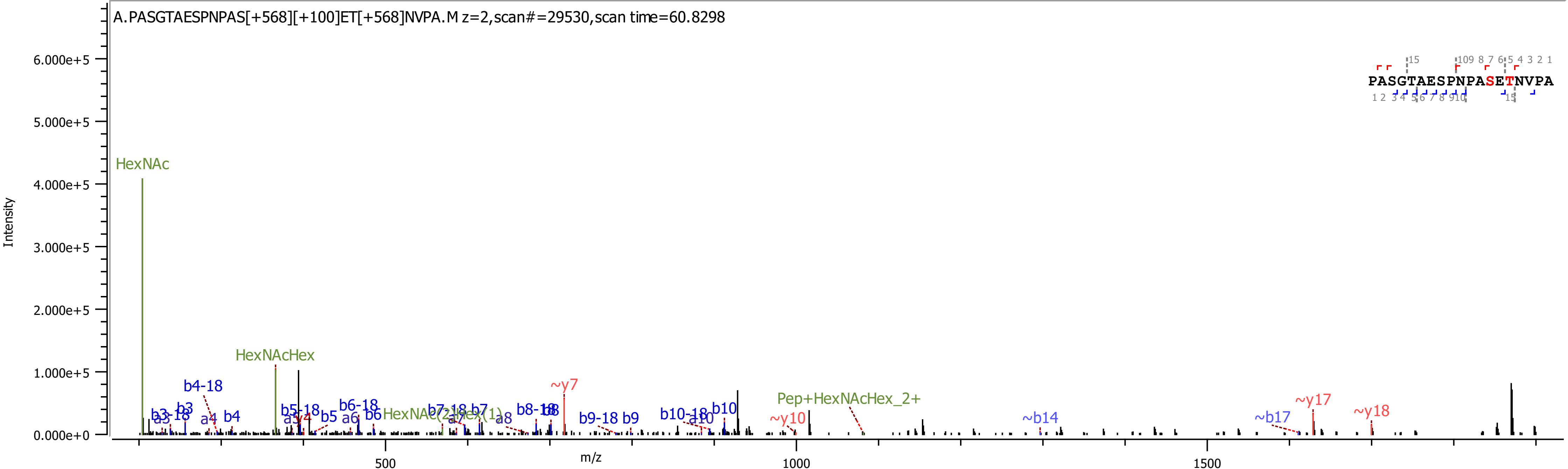

A.PASGTAESPNPAS[+568][+100]ET[+568]NVPA.M z=2,scan#=29530,scan time=60.8298
PASGTAESPNPASETNVPA
Intensity
m/z
HexNAc
HexNAcHex
b4-18
b3-18
a3-18
b3
a4
b4
b5-18
a5
y4
b5
b6-18
a6
b6
HexNAc(2)Hex(1)
b7-18
a7
b7
a8
b8-18
b8
~y7
b9-18
b9
b10-18
a10
b10
~y10
Pep+HexNAcHex_2+
~b14
~b17
~y17
~y18

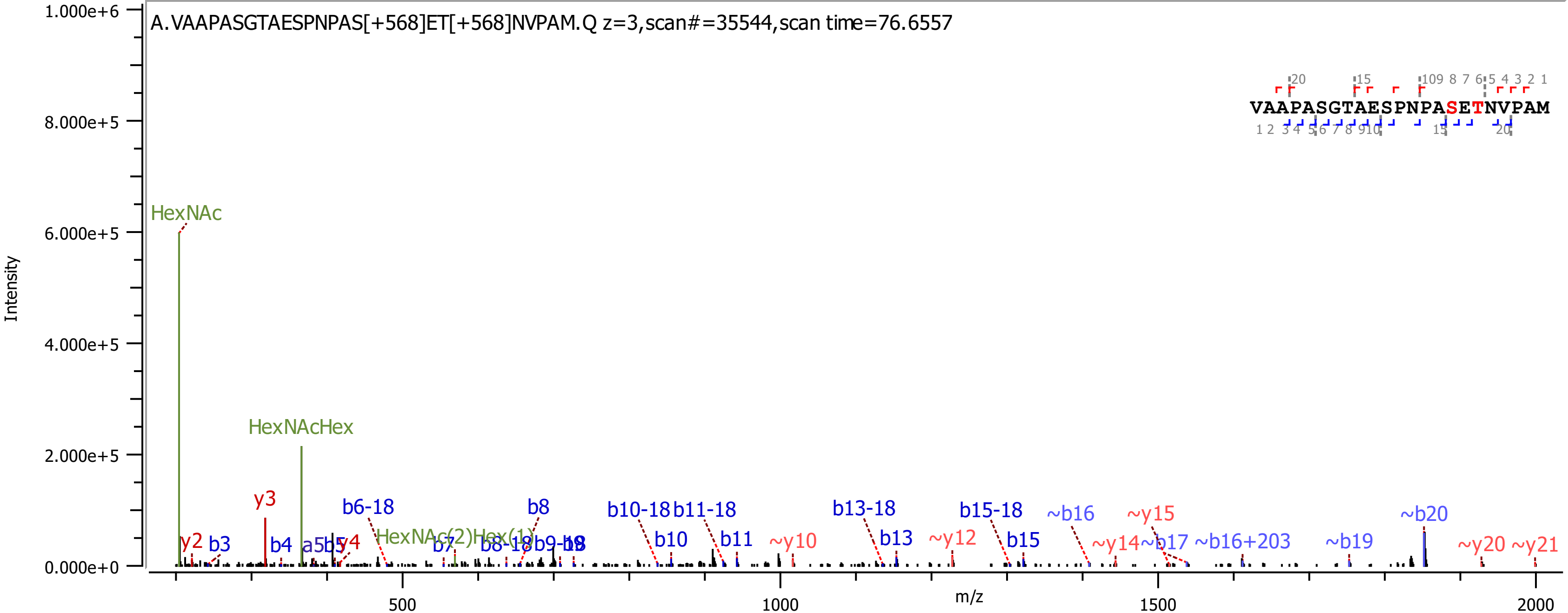

A.VAAPASGTAESPNPAS[+568]ET[+568]NVPAM.Q z=3,scan#=35544,scan time=76.6557
VAAPASGTAESPNPASETNVPAM
Intensity
m/z
1.000e+6
8.000e+5
6.000e+5
4.000e+5
2.000e+5
0.000e+0
500
1000
1500
2000
HexNAc
HexNAcHex
HexNAc(2)Hex(1)
y2
b3
y3
b4
a5
b5
y4
b6-18
b7
b8
b8-18
b9-18
b10-18
b10
b11-18
b11
~y10
b13-18
b13
~y12
b15-18
b15
~b16
~y14
~y15
~b17
~b16+203
~b19
~b20
~y20
~y21

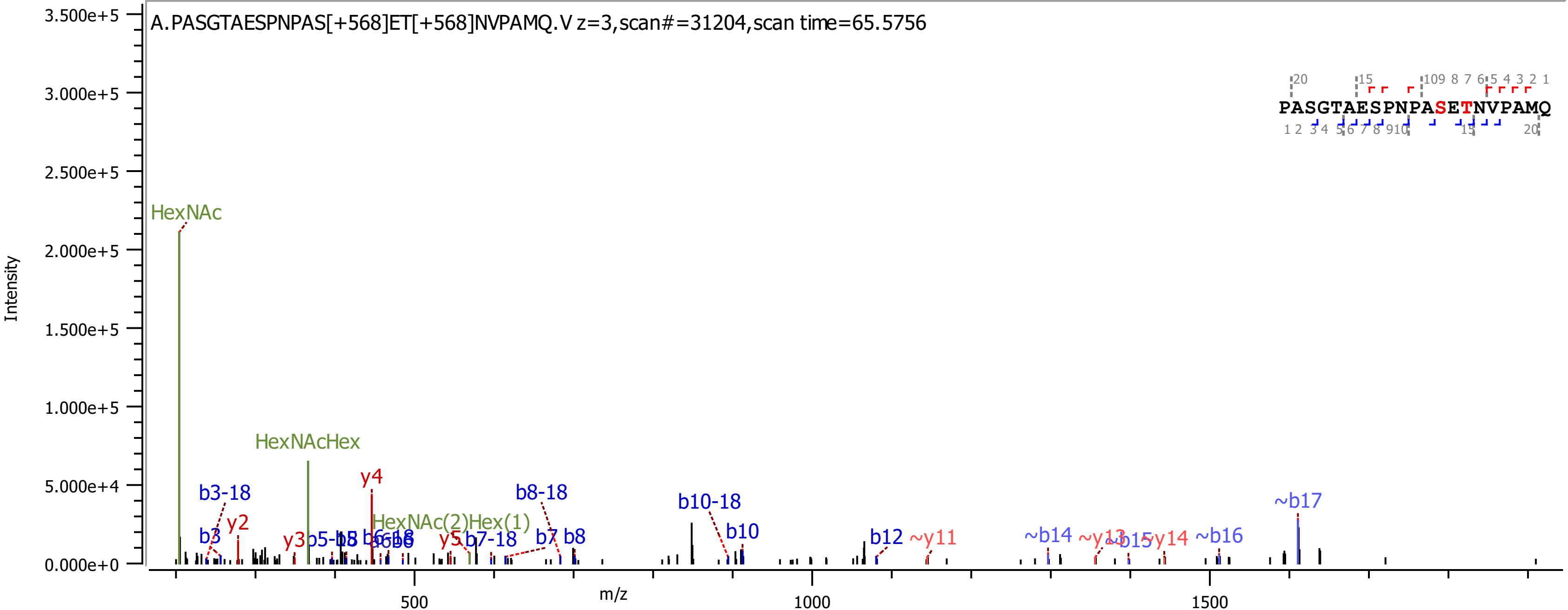

A.PASGTAESPNPAS[+568]ET[+568]NVPAMQ.V z=3,scan#=31204,scan time=65.5756
PASGTAESPNPASETNVPAMQ
Intensity
m/z
3.500e+5
3.000e+5
2.500e+5
2.000e+5
1.500e+5
1.000e+5
5.000e+4
0.000e+0
500
1000
1500
HexNAc
HexNAcHex
HexNAc(2)Hex(1)
b3-18
b3
y2
y3
y4
y5
b7-18
b8-18
b7
b8
b10-18
b10
b12
~y11
~b14
~y13
~b15
~y14
~b16
~b17

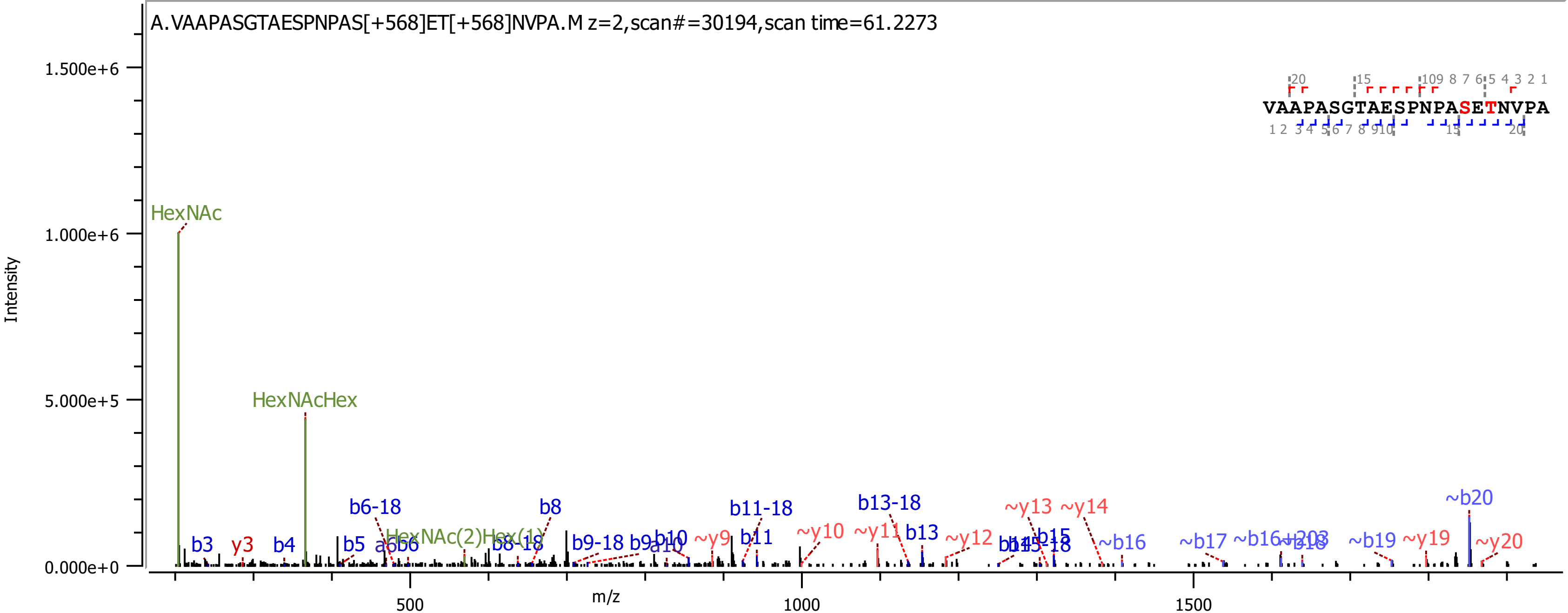

A.VAAPASGTAESPNPAS[+568]ET[+568]NVPA.M z=2,scan#=30194,scan time=61.2273
Intensity
m/z
1.500e+6
1.000e+6
5.000e+5
0.000e+0
500
1000
1500
VAAPASGTAESPNPASETNVPA
HexNAc
HexNAcHex
HexNAc(2)Hex(1)
b3
y3
b4
b5
b6-18
b8
b8-18
b9-18
b10
b11-18
b11
~y9
~y10
~y11
b13-18
b13
~y12
~y13
~y14
b15
~b16
~b17
~b19
~y19
~b20
~y20

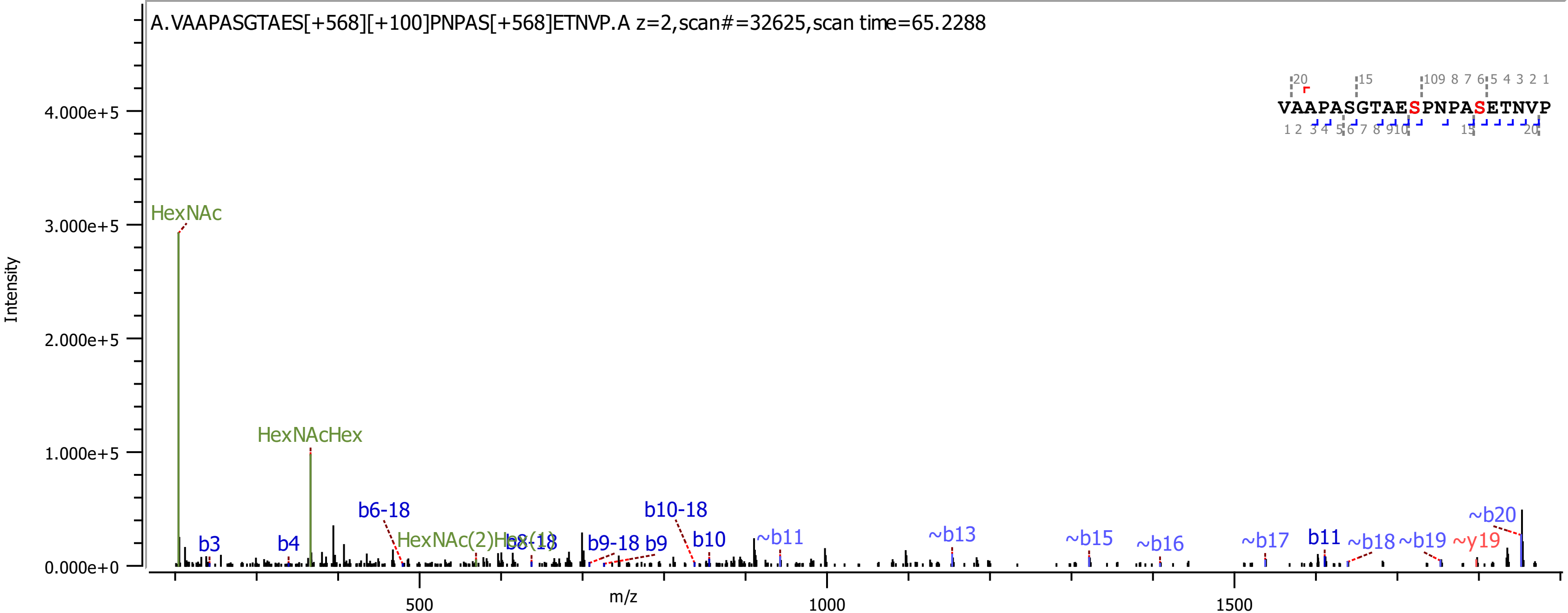
A.VAAPASGTAES[+568][+100]PNPAS[+568]ETNVP.A z=2,scan#=32625,scan time=65.2288
VAAPASGTAESPNPASETNVP
Intensity
m/z
HexNAc
HexNAcHex
HexNAc(2)Hex(1)
b3
b4
b6-18
b8-18
b9-18
b9
b10-18
b10
~b11
b11
~b13
~b15
~b16
~b17
~b18
~b19
~y19
~b20
0.000e+0
1.000e+5
2.000e+5
3.000e+5
4.000e+5
500
1000
1500

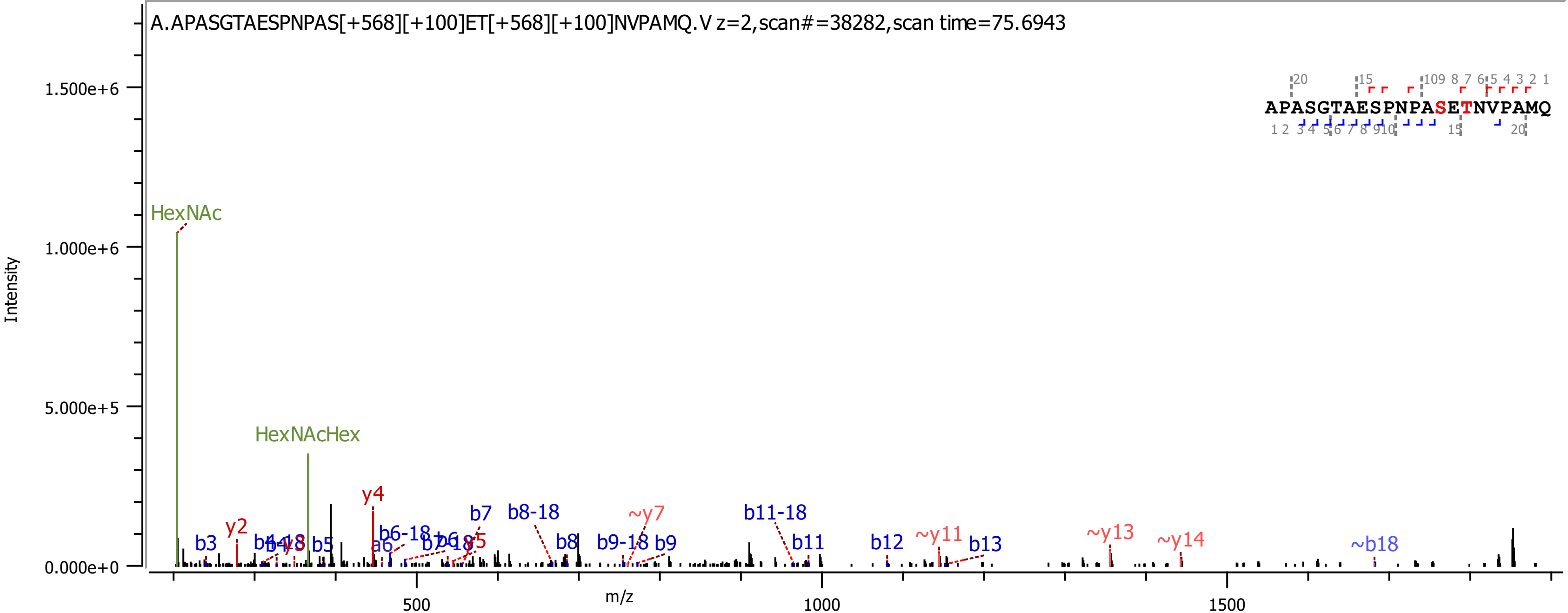

A.APASGTAESPNPAS[+568][+100]ET[+568][+100]NVPAMQ.V z=2,scan#=38282,scan time=75.6943
APASGTAESPNPASETNVPAMQ
Intensity
m/z
HexNAc
HexNAcHex
y2
y4
b3
b5
b7
b8-18
b8
b9-18
b9
~y7
b11-18
b11
b12
~y11
b13
~y13
~y14
~b18
b6-18
a6

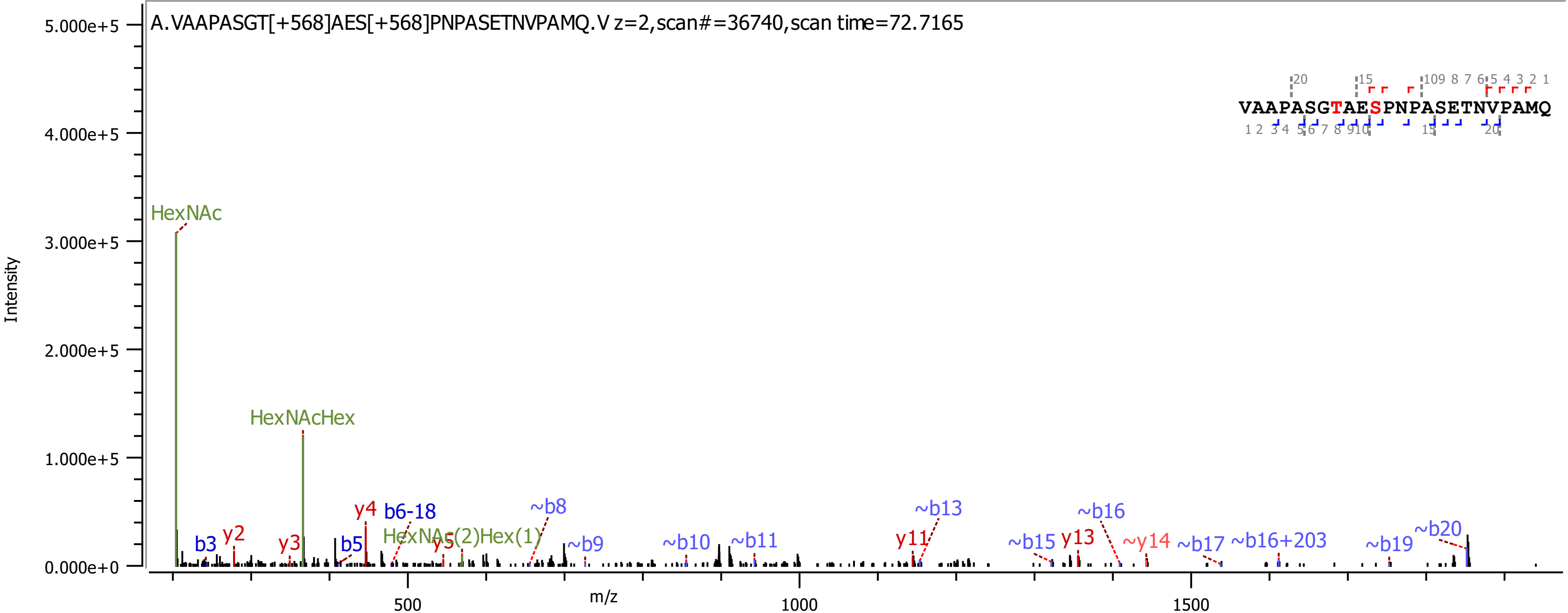
A.VAAPASGT[+568]AES[+568]PNPASETNVPAMQ.V z=2,scan#=36740,scan time=72.7165
VAAPASGTAESPNPASETNVPAMQ
Intensity
m/z
HexNAc
HexNAcHex
HexNAc(2)Hex(1)
b3
y2
y3
y4
b5
b6-18
y5
~b8
~b9
~b10
~b11
y11
~b13
~b15
y13
~b16
~y14
~b17
~b16+203
~b19
~b20

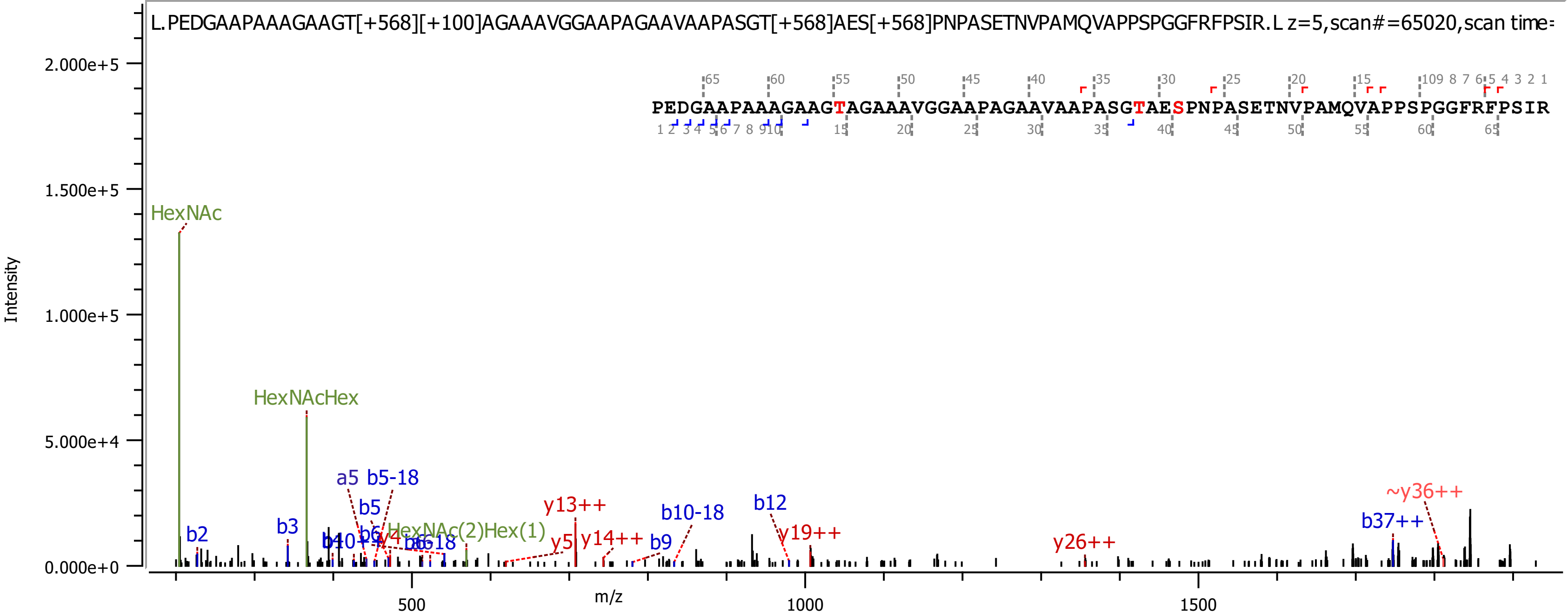
L.PEDGAAPAAAGAAGT[+568][+100]AGAAAVGGAAPAGAAVAAPASGT[+568]AES[+568]PNPASETNVPAMQVAPPSPGGFRFPSIR.L z=5,scan#=65020,scan time=
PEDGAAPAAAGAAGTAGAAAVGGAAPAGAAVAAPASGTAESPNPASETNVPAMQVAPPSPGGFRFPSIR
Intensity
m/z
2.000e+5
1.500e+5
1.000e+5
5.000e+4
0.000e+0
500
1000
1500
HexNAc
HexNAcHex
HexNAc(2)Hex(1)
a5
b5-18
b5
b2
b3
b9
b10-18
b12
b37++
y13++
y5
y14++
y19++
y26++
~y36++

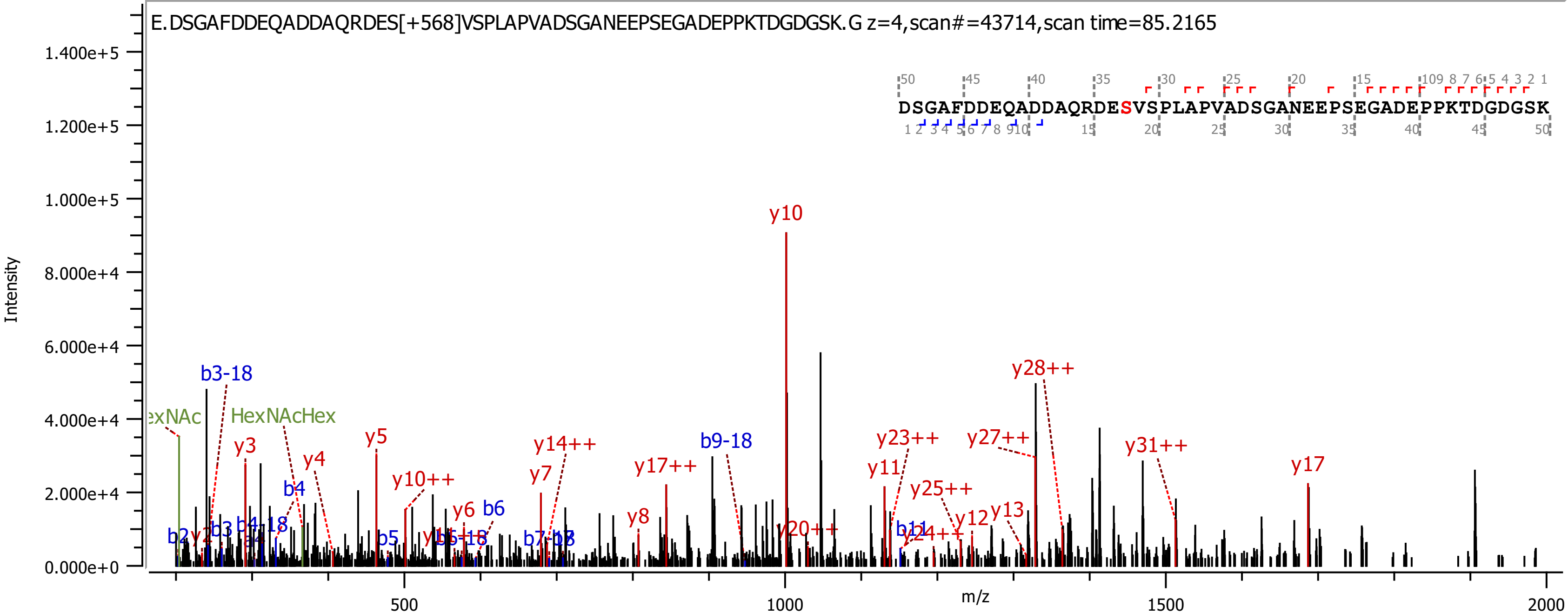
E.DSGAFDDEQADDAQRDES[+568]VSPLAPVADSGANEEPSEGADEPPKTDGDGSK.G z=4,scan#=43714,scan time=85.2165
DSGAFDDEQADDAQRDESVSPLAPVADSGANEEPSEGADEPPKTDGDGSK
Intensity
m/z
1.400e+5
1.200e+5
1.000e+5
8.000e+4
6.000e+4
4.000e+4
2.000e+4
0.000e+0
500
1000
1500
2000
y10
b3-18
HexNAcHex
y28++
y3
y5
y4
y14++
y27++
y23++
y31++
b9-18
y17++
y10++
y7
y11
y17
b4
y25++
y6
b6
y8
y13
y12
b2
b3
b5
y20++

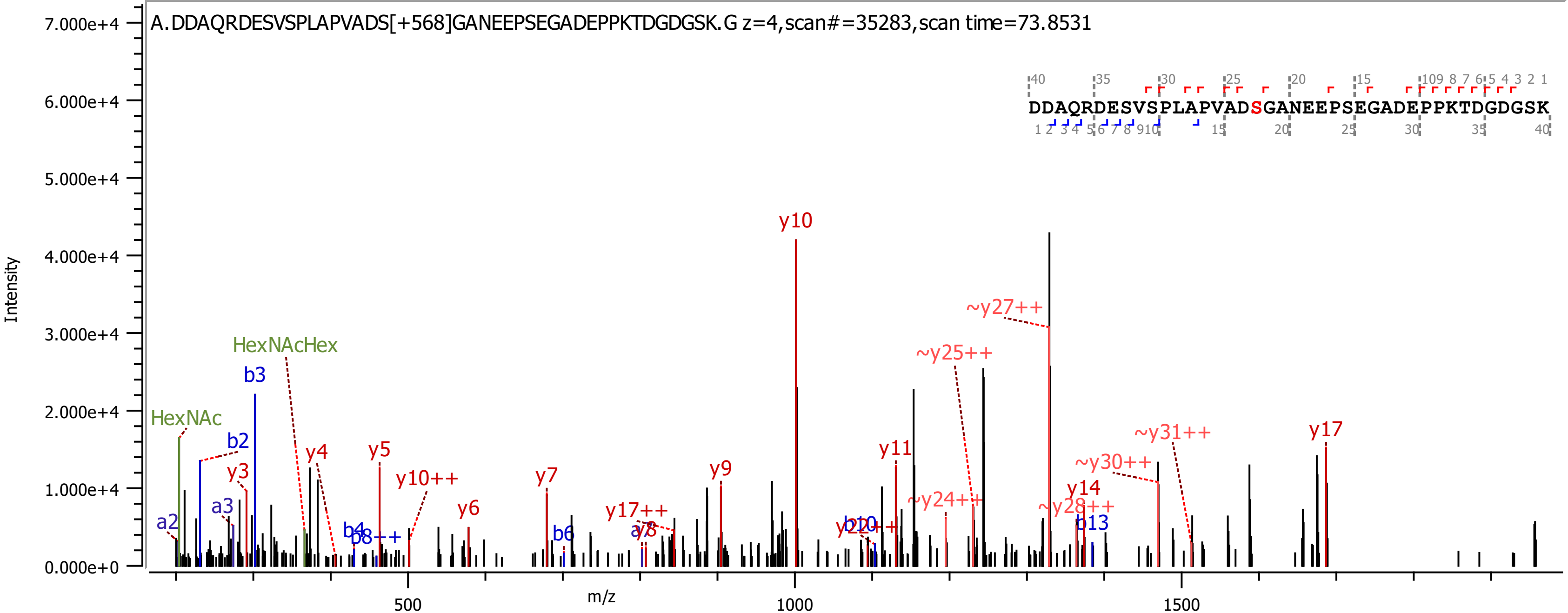

A.DDAQRDESVSPLAPVADS[+568]GANEEPSEGADEPPKTDGDGSK.G z=4,scan#=35283,scan time=73.8531
DDAQRDESVSPLAPVADSGANEEPSEGADEPPKTDGDGSK
Intensity
m/z
7.000e+4
6.000e+4
5.000e+4
4.000e+4
3.000e+4
2.000e+4
1.000e+4
0.000e+0
500
1000
1500
HexNAc
HexNAcHex
a2
a3
b2
b3
b4
b6
b8++
b10
b13
y3
y4
y5
y6
y7
y9
y10
y10++
y11
y14
y17
y17++
y22++
a8
~y24++
~y25++
~y27++
~y28++
~y30++
~y31++

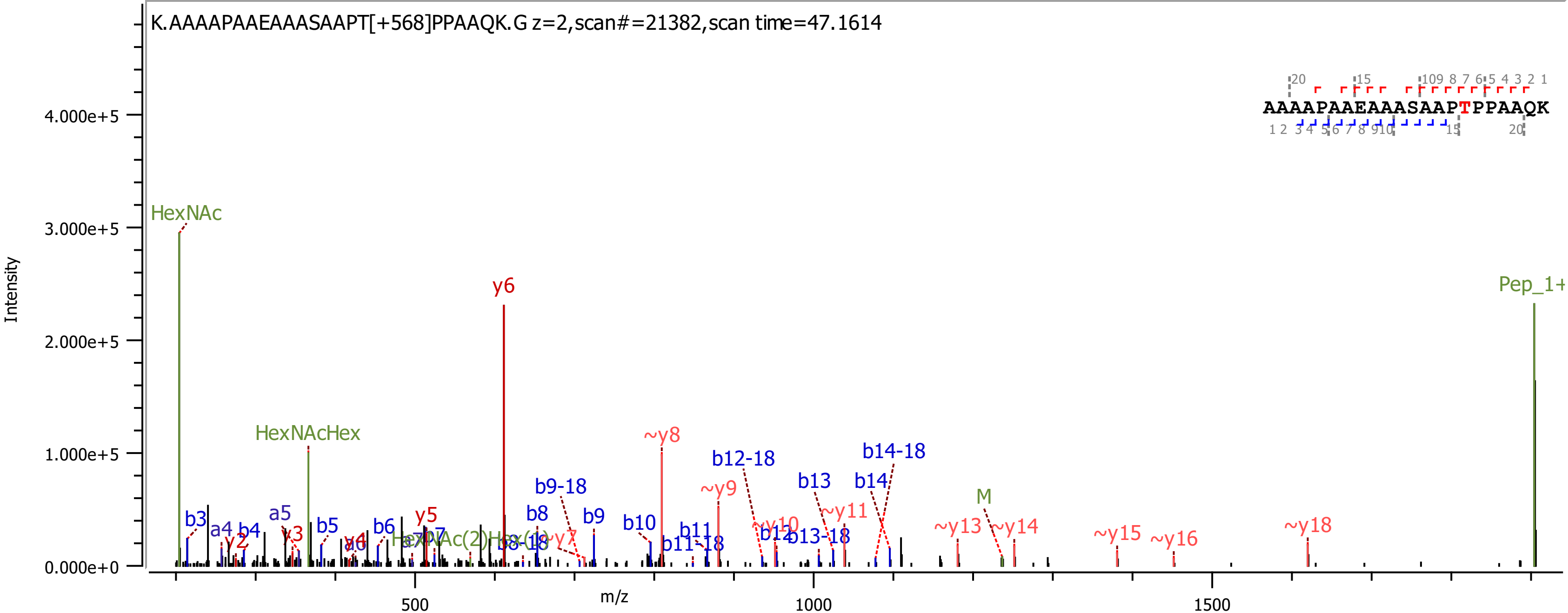
K.AAAAPAAEAAASAAPT[+568]PPAAQK.G z=2,scan#=21382,scan time=47.1614
AAAAPAAEAAASAAPTPPAAQK
Intensity
m/z
4.000e+5
3.000e+5
2.000e+5
1.000e+5
0.000e+0
500
1000
1500
HexNAc
HexNAcHex
y6
Pep_1+
~y8
b12-18
b14-18
~y9
b9-18
b13
b14
M
~y11
b3
a4
b4
a5
b5
b6
y5
b8
b9
b10
b11
b11-18
~y10
b12
b13-18
~y13
~y14
~y15
~y16
~y18
y2
y3
y4
a6
HexNAc(2)Hex(1)
b8-18
~y7

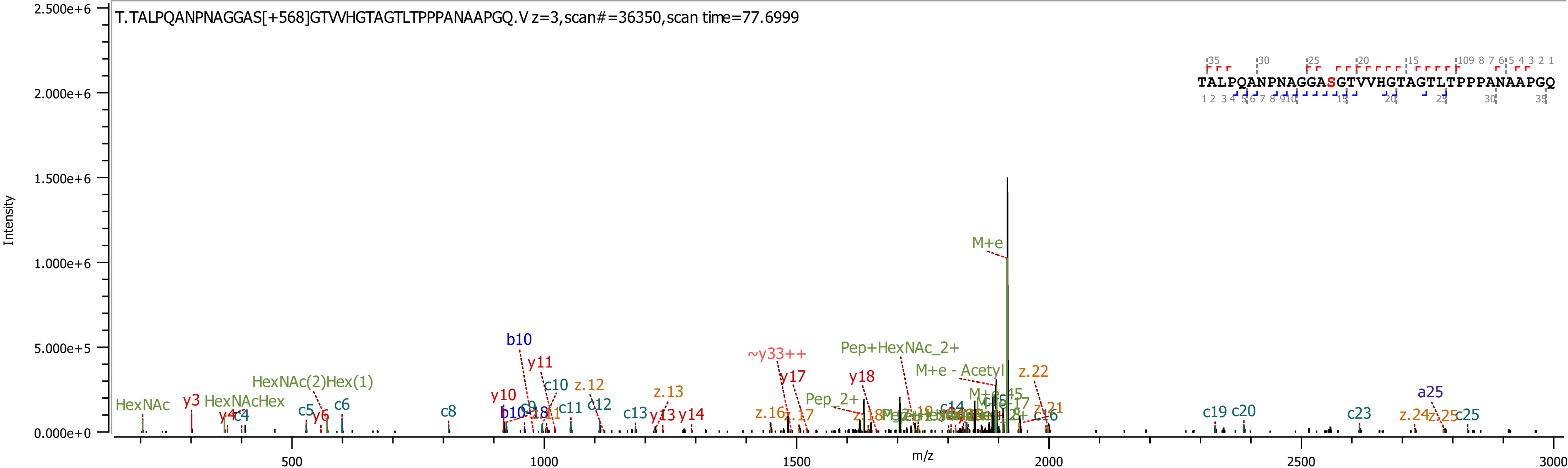
T.TALPQANPNAGGAS[+568]GTVVHGTAGTLTPPPANAAPGQ.V z=3,scan#=36350,scan time=77.6999
TALPQANPNAGGASGTVVHGTAGTLTPPPANAAPGQ
Intensity
m/z
2.500e+6
2.000e+6
1.500e+6
1.000e+6
5.000e+5
0.000e+0
500
1000
1500
2000
2500
3000
M+e
Pep+HexNAc_2+
M+e - Acetyl
~y33++
b10
y11
y10
y17
y18
z.22
z.12
z.13
c10
c11
c12
c13
y13
y14
a25
HexNAc
y3
HexNAcHex
HexNAc(2)Hex(1)
c5
c6
c8
c19
c20
c23
z.24
z.25
c25
Pep_2+
z.16
z.17
z.21

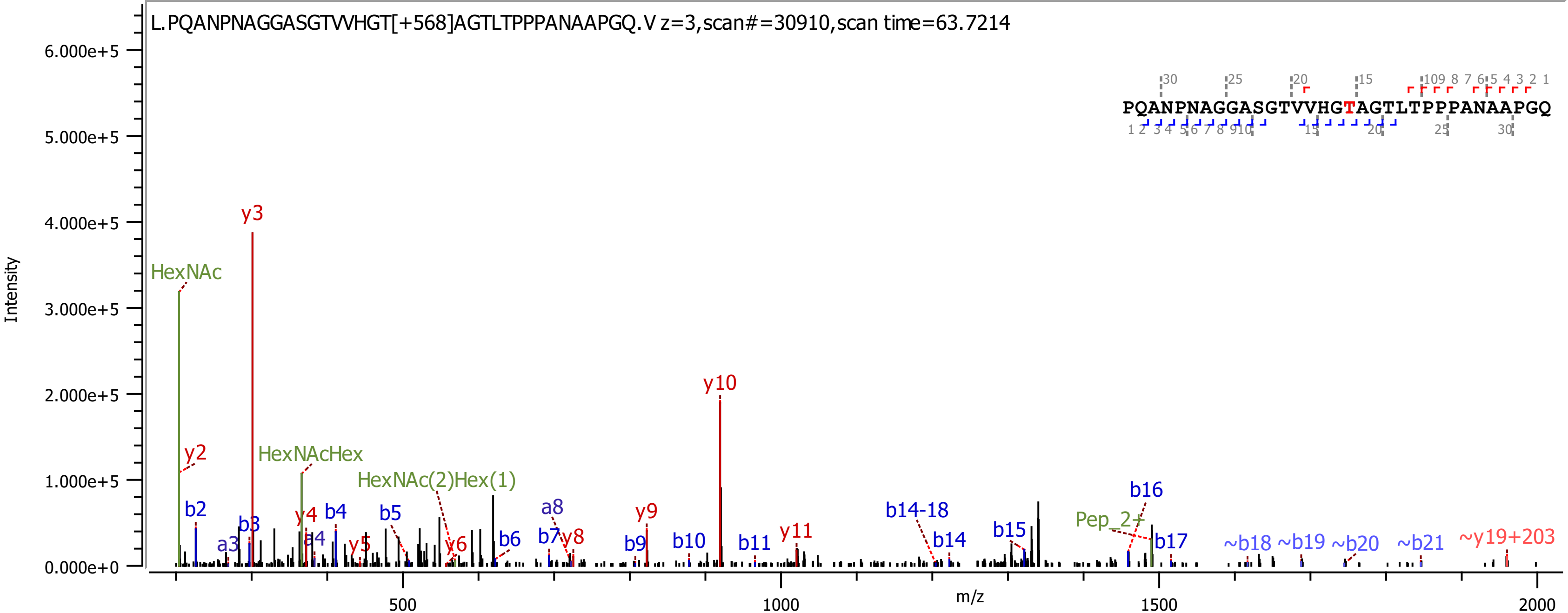
L.PQANPNAGGASGTVVHGT[+568]AGTLTPPPANAAPGQ.V z=3,scan#=30910,scan time=63.7214
PQANPNAGGASGTVVHGTAGTLTPPPANAAPGQ
Intensity
m/z
HexNAc
y3
y2
HexNAcHex
HexNAc(2)Hex(1)
b2
b3
a3
y4
b4
a4
y5
b5
y6
b6
a8
b7
y8
y9
b9
b10
y10
b11
y11
b14-18
b14
b15
Pep_2+
b16
b17
~b18
~b19
~b20
~b21
~y19+203

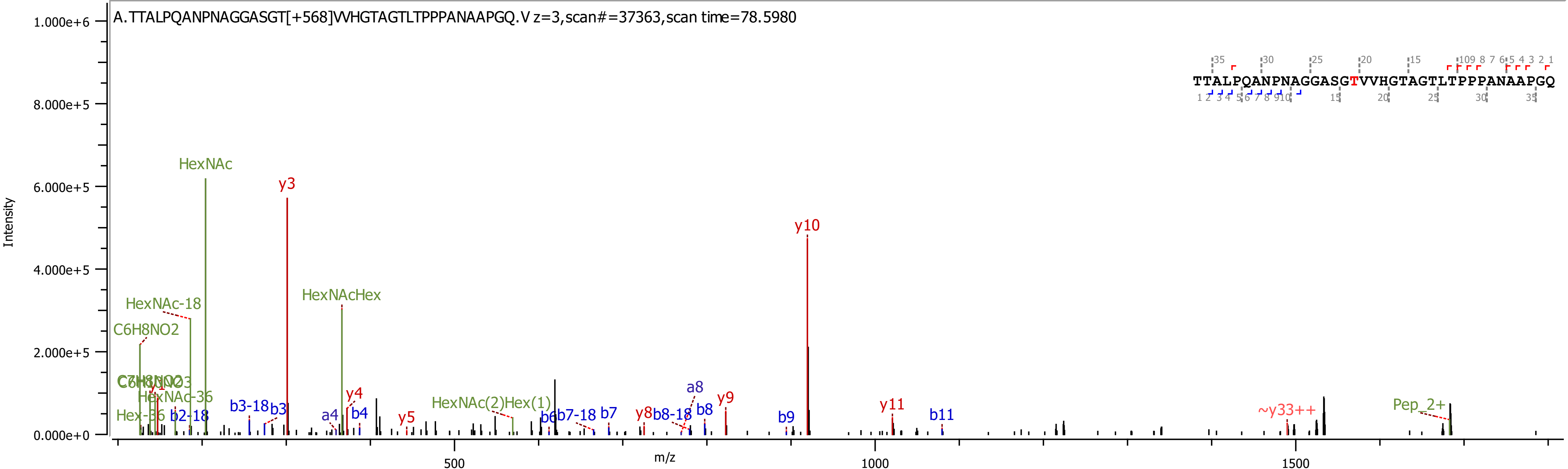

A.TTALPQANPNAGGASGT[+568]VVHGTAGTLTPPPANAAPGQ.V z=3,scan#=37363,scan time=78.5980
TTALPQANPNAGGASGTVVHGTAGTLTPPPANAAPGQ
Intensity
m/z
1.000e+6
8.000e+5
6.000e+5
4.000e+5
2.000e+5
0.000e+0
500
1000
1500
HexNAc
y3
y10
HexNAc-18
HexNAcHex
C6H8NO2
HexNAc-36
Hex-36
b2-18
b3-18
b3
a4
y4
b4
y5
HexNAc(2)Hex(1)
b6
b7-18
b7
y8
b8-18
a8
b8
y9
b9
y11
b11
~y33++
Pep_2+

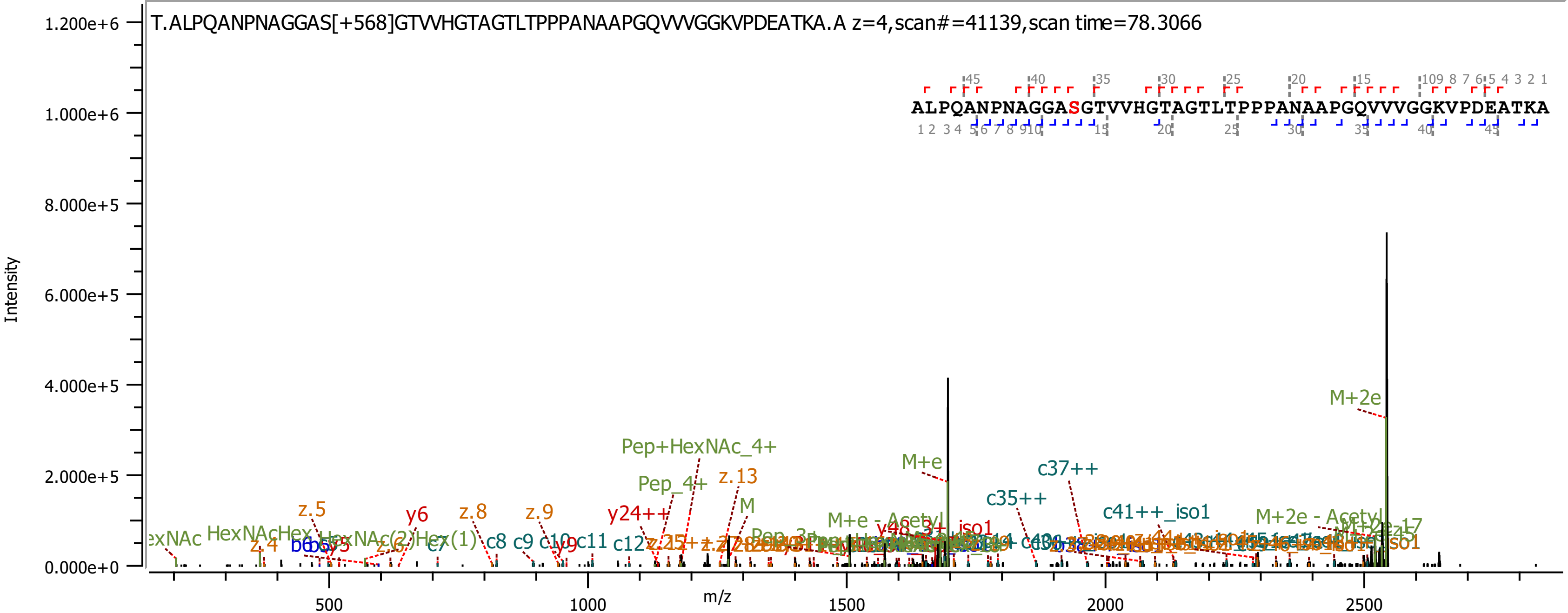
T.ALPQANPNAGGAS[+568]GTVVHGTAGTLTPPPANAAPGQVVVGGKVPDEATKA.A z=4,scan#=41139,scan time=78.3066
ALPQANPNAGGASGTVVHGTAGTLTPPPANAAPGQVVVGGKVPDEATKA
Intensity
m/z
1.200e+6
1.000e+6
8.000e+5
6.000e+5
4.000e+5
2.000e+5
0.000e+0
500
1000
1500
2000
2500
M+2e
Pep+HexNAc_4+
M+e
c37++
Pep_4+
z.13
c35++
c41++_iso1
M+2e - Acetyl
M+e - Acetyl
z.5
y6
z.8
z.9
y24++
M
HexNAcHex
HexNAc(2)Hex(1)
z.4
c7
c8
c9
c11
c12
y9
iso1

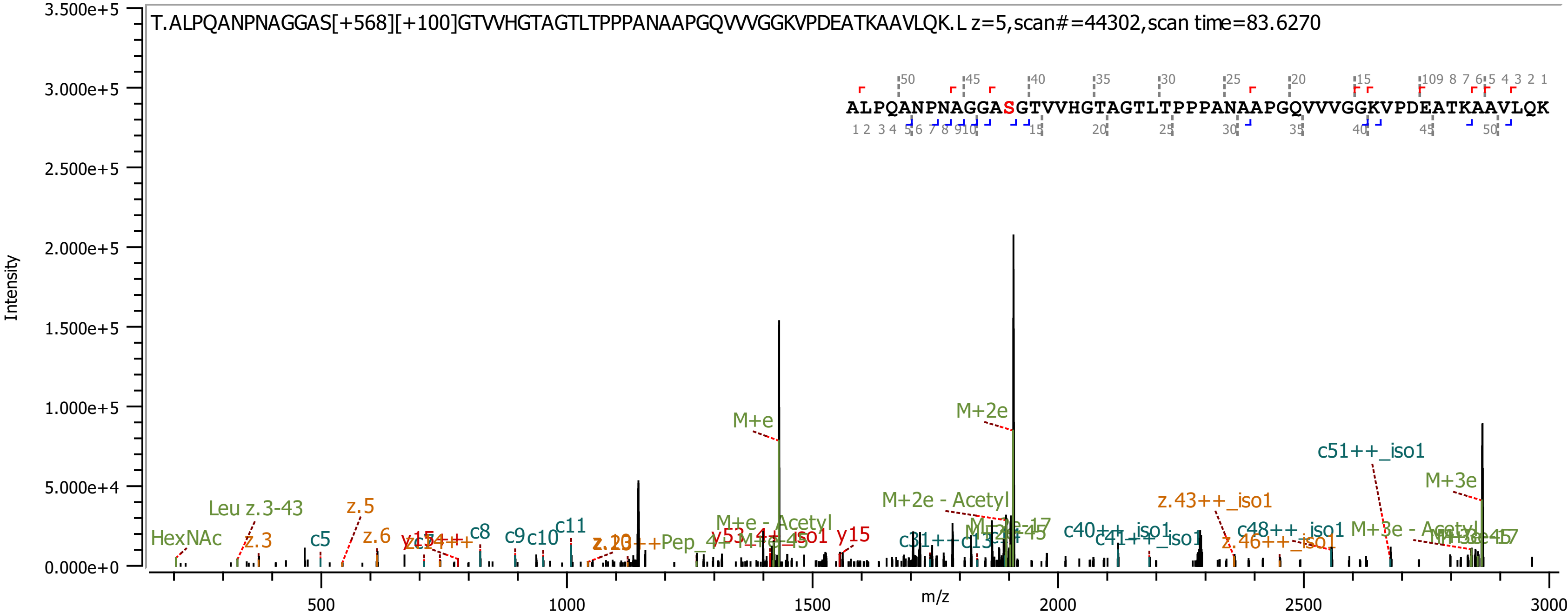
T.ALPQANPNAGGAS[+568][+100]GTVVHGTAGTLTPPPANAAPGQVVVGGKVPDEATKAAVLQK.L z=5,scan#=44302,scan time=83.6270
ALPQANPNAGGASGTVVHGTAGTLTPPPANAAPGQVVVGGKVPDEATKAAVLQK
Intensity
m/z
3.500e+5
3.000e+5
2.500e+5
2.000e+5
1.500e+5
1.000e+5
5.000e+4
0.000e+0
500
1000
1500
2000
2500
3000
M+e
M+2e
c51++_iso1
M+3e
M+2e - Acetyl
z.43++_iso1
M+e - Acetyl
Leu z.3-43
z.5
HexNAc
z.3
c5
z.6
c8
c9
c10
c11
y15
c40++_iso1
c41++_iso1
c48++_iso1
z.46++_iso1
M+3e - Acetyl

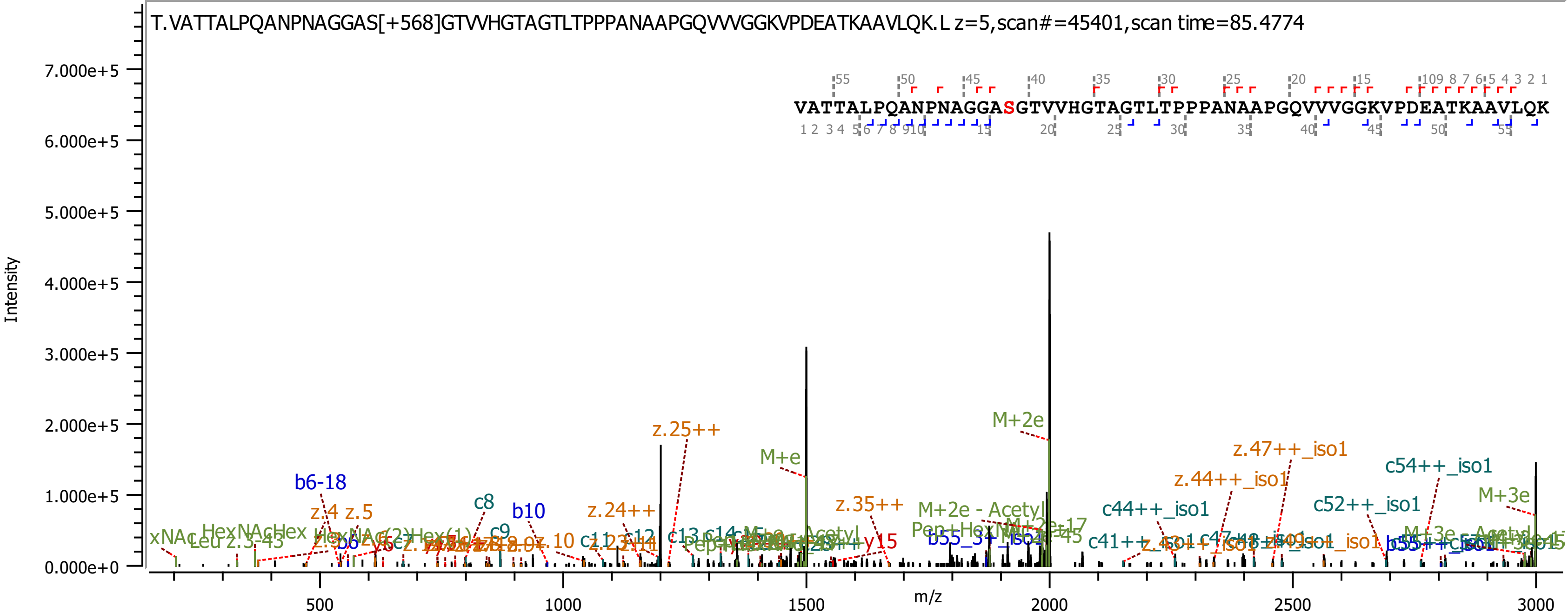
T.VATTALPQANPNAGGAS[+568]GTVVHGTAGTLTPPPANAAPGQVVVGGKVPDEATKAAVLQK.L z=5,scan#=45401,scan time=85.4774
VATTALPQANPNAGGASGTVVHGTAGTLTPPPANAAPGQVVVGGKVPDEATKAAVLQK
Intensity
m/z
z.25++
M+2e
M+e
b6-18
z.4 z.5
c8
b10
z.24++
z.35++
M+2e - Acetyl
c44++_iso1
z.47++_iso1
z.44++_iso1
c54++_iso1
c52++_iso1
M+3e
y15
c9

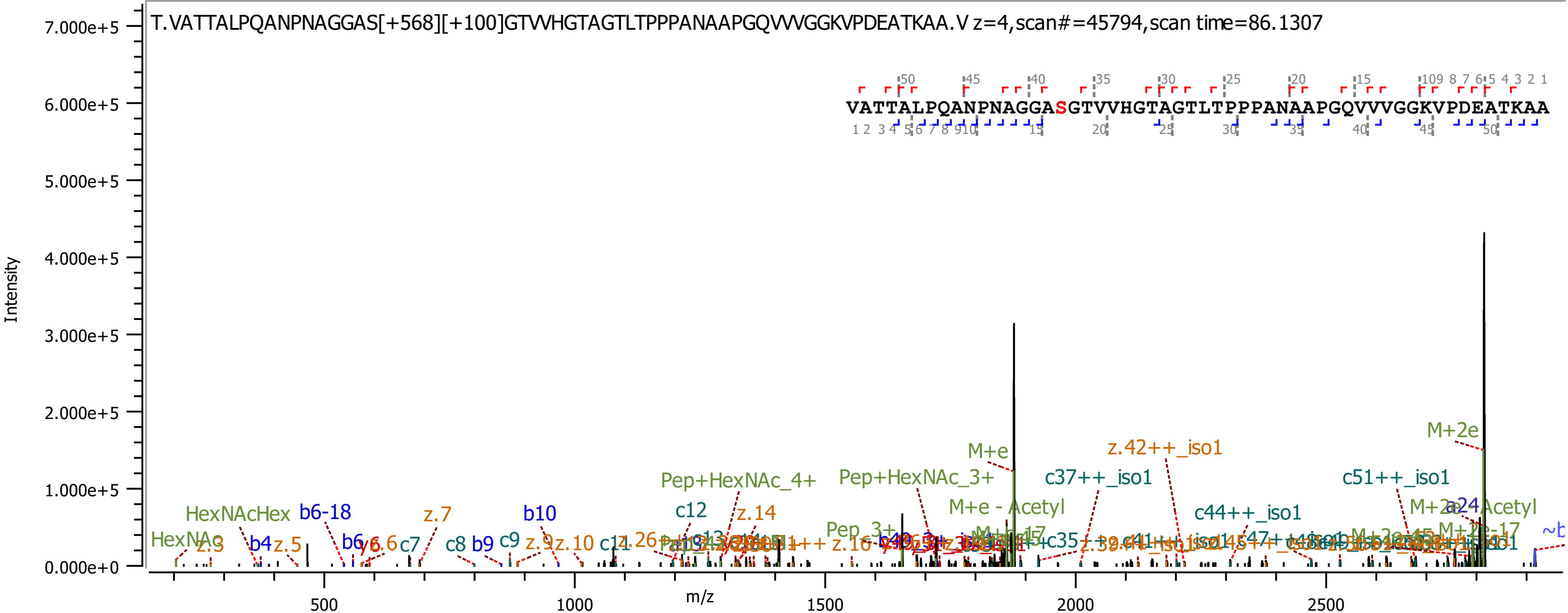

T.VATTALPQANPNAGGAS[+568][+100]GTVVHGTAGTLTPPPANAAPGQVVVGGKVPDEATKAA.V z=4,scan#=45794,scan time=86.1307
VATTALPQANPNAGGASGTVVHGTAGTLTPPPANAAPGQVVVGGKVPDEATKAA
Intensity
m/z
7.000e+5
6.000e+5
5.000e+5
4.000e+5
3.000e+5
2.000e+5
1.000e+5
0.000e+0
500
1000
1500
2000
2500
M+2e
M+e
z.42++_iso1
Pep+HexNAc_4+
Pep+HexNAc_3+
c37++_iso1
c51++_iso1
M+e - Acetyl
c44++_iso1
c12
z.14
HexNAcHex
b6-18
z.7
b10
HexNAc
z.3
b4
z.5
c7
c8
b9
c9
z.9
z.10

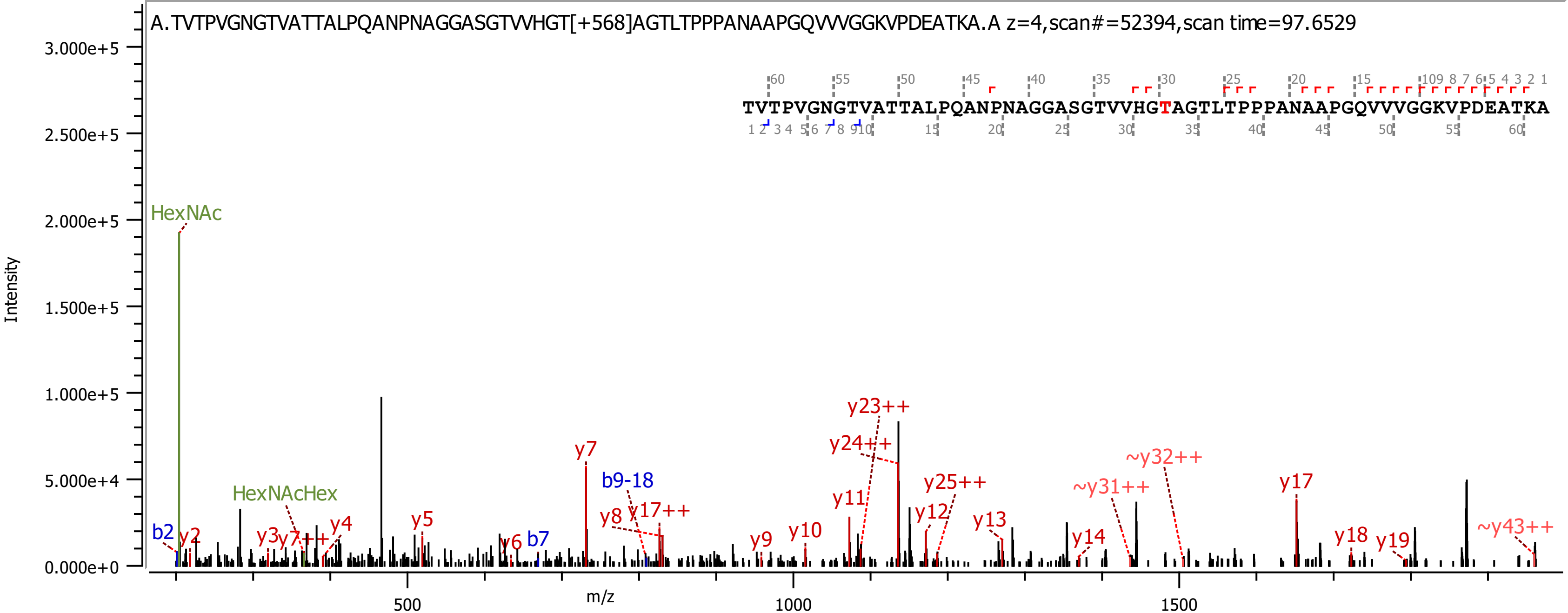

A.TVTPVGNGTVATTALPQANPNAGGASGTVVHGT[+568]AGTLTPPPANAAPGQVVVGGKVPDEATKA.A z=4,scan#=52394,scan time=97.6529
TVTPVGNGTVATTALPQANPNAGGASGTVVHGTAGTLTPPPANAAPGQVVVGGKVPDEATKA
Intensity
m/z
3.000e+5
2.500e+5
2.000e+5
1.500e+5
1.000e+5
5.000e+4
0.000e+0
500
1000
1500
HexNAc
HexNAcHex
b2
y2
y3
y7++
y4
y5
y6
b7
y7
b9-18
y8
y17++
y9
y10
y11
y23++
y24++
y12
y25++
y13
y14
~y31++
~y32++
y17
y18
y19
~y43++

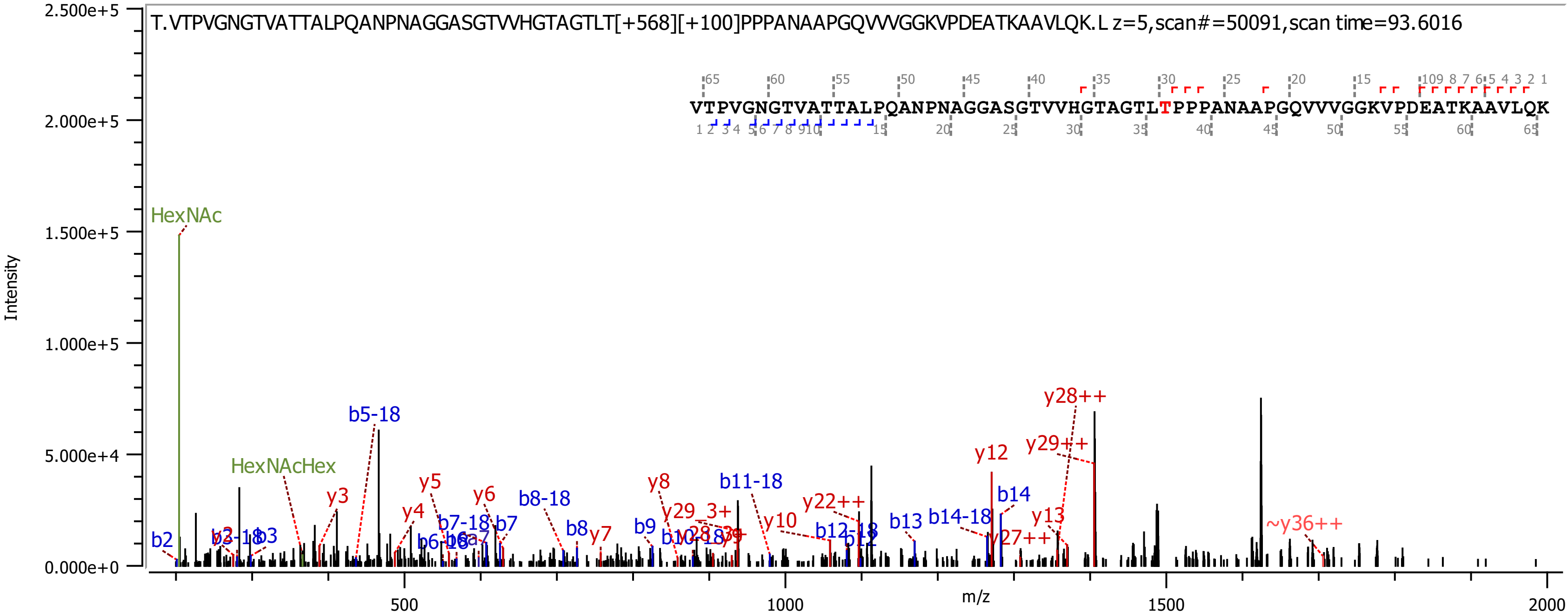
T.VTPVGNGTVATTALPQANPNAGGASGTVVHGTAGTLT[+568][+100]PPPANAAPGQVVVGGKVPDEATKAAVLQK.L z=5,scan#=50091,scan time=93.6016
VTPVGNGTVATTALPQANPNAGGASGTVVHGTAGTLTPPPANAAPGQVVVGGKVPDEATKAAVLQK
Intensity
m/z
HexNAc
HexNAcHex
b5-18
y28++
y29++
y12
b14
y13
~y36++
y3
y5
y6
y4
b8-18
b7-18
b7
b8
y7
y8
b9
y29_3+
b11-18
y22++
y10
b12-18
b13
b14-18
y27++
b2
b3
2.500e+5
2.000e+5
1.500e+5
1.000e+5
5.000e+4
0.000e+0
500
1000
1500
2000

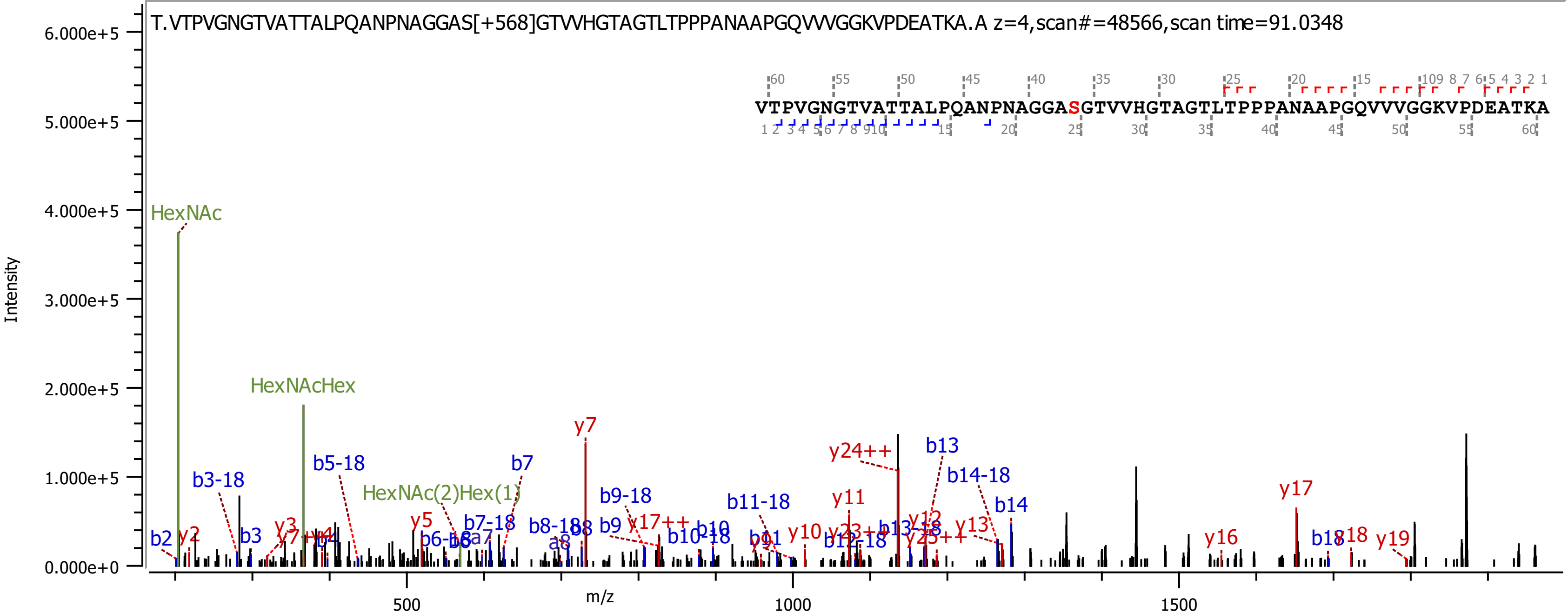
T.VTPVGNGTVATTALPQANPNAGGAS[+568]GTVVHGTAGTLTPPPANAAPGQVVVGGKVPDEATKA.A z=4,scan#=48566,scan time=91.0348
VTPVGNGTVATTALPQANPNAGGASGTVVHGTAGTLTPPPANAAPGQVVVGGKVPDEATKA
Intensity
m/z
HexNAc
HexNAcHex
HexNAc(2)Hex(1)
y7
y24++
b13
b14-18
b14
y17
b5-18
b7
b3-18
b9-18
b11-18
y11
y12
y13
y16
y18
y19
b18
y10
y9
b11
b12-18
b13-18
y23++
y25++
b10
b10-18
b9
y17++
b8-18
b8
a8
b7-18
a7
b6-18
b6
y5
y3
y7++
b4
y4
b3
b2
y2

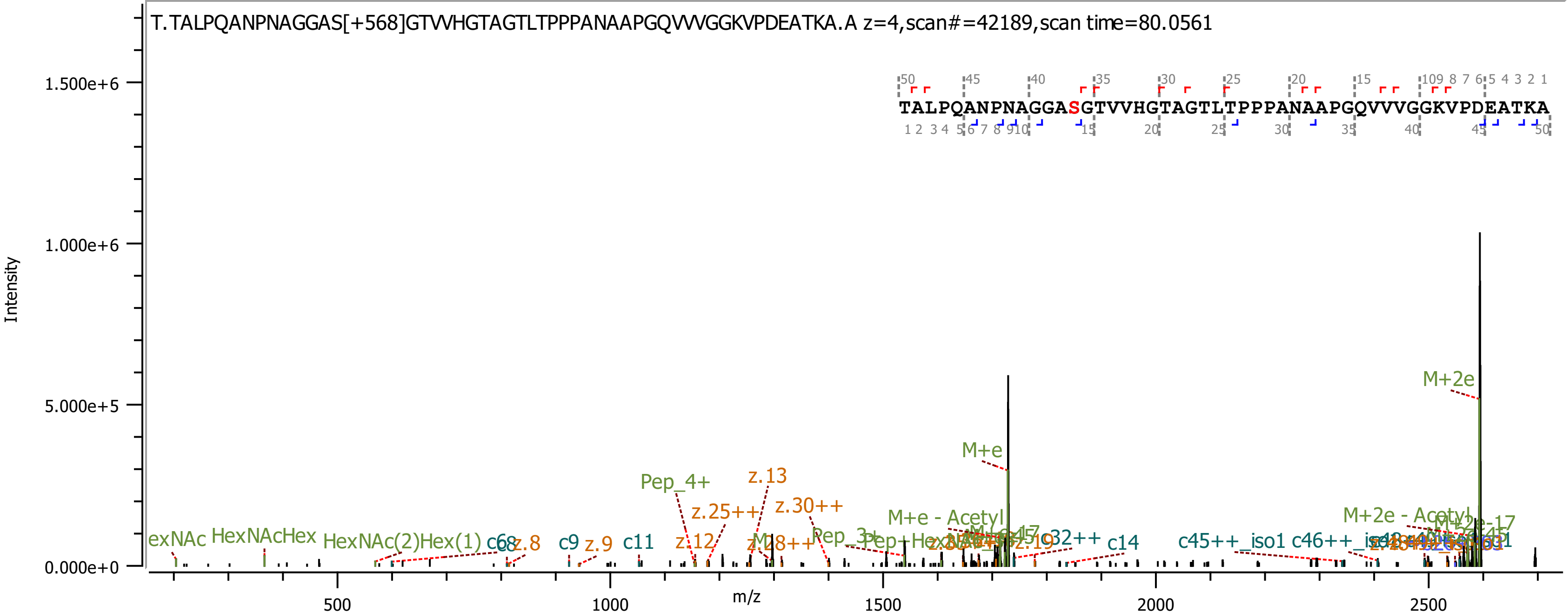
T.TALPQANPNAGGAS[+568]GTVVHGTAGTLTPPPANAAPGQVVVGGKVPDEATKA.A z=4,scan#=42189,scan time=80.0561
TALPQANPNAGGASGTVVHGTAGTLTPPPANAAPGQVVVGGKVPDEATKA
Intensity
1.500e+6
1.000e+6
5.000e+5
0.000e+0
m/z
500
1000
1500
2000
2500
M+2e
M+e
Pep_4+
z.13
z.25++
z.30++
z.12
c11
z.9
c9
z.8
c14
HexNAcHex
HexNAc(2)Hex(1)
M+e - Acetyl
M+2e - Acetyl
c45++_iso1
c32++
Pep_3+

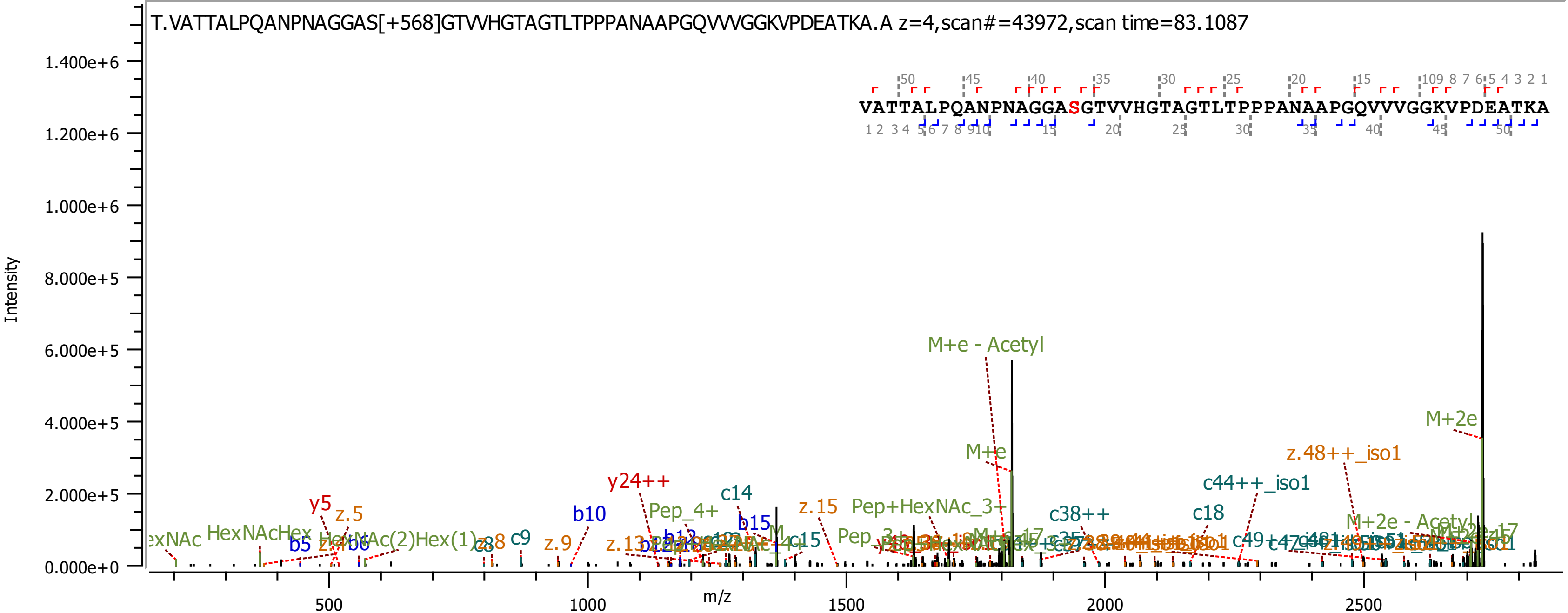
T.VATTALPQANPNAGGAS[+568]GTVVHGTAGTLTPPPANAAPGQVVVGGKVPDEATKA.A z=4,scan#=43972,scan time=83.1087
VATTALPQANPNAGGASGTVVHGTAGTLTPPPANAAPGQVVVGGKVPDEATKA
Intensity
m/z
1.400e+6
1.200e+6
1.000e+6
8.000e+5
6.000e+5
4.000e+5
2.000e+5
0.000e+0
500
1000
1500
2000
2500
M+e - Acetyl
M+2e
M+e
z.48++_iso1
c44++_iso1
y24++
c14
y5
z.5
b10
Pep_4+
z.15
Pep+HexNAc_3+
c38++
c18
b15
M+2e - Acetyl
HexNAcHex
HexNAc(2)Hex(1)
b5
b6
c9
z.9
z.13
c15
c8
z.8
Pep_3+

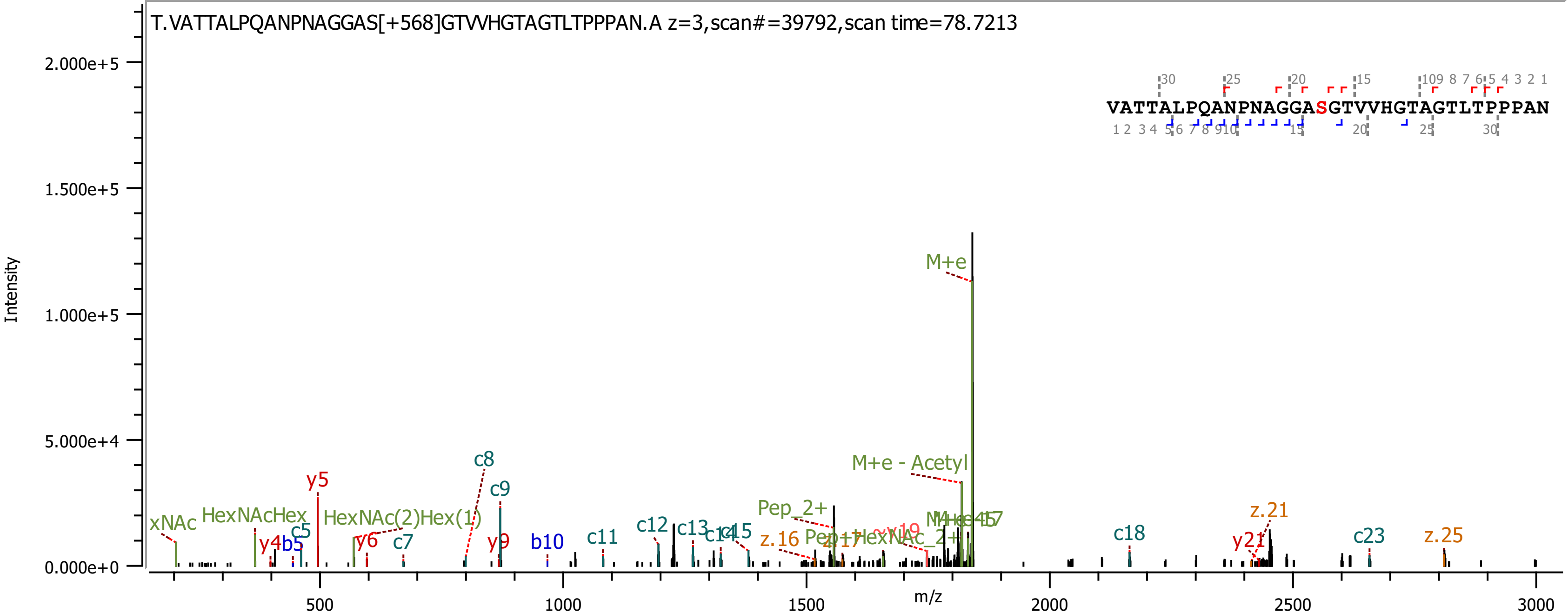

T.VATTALPQANPNAGGAS[+568]GTVVHGTAGTLTPPPAN.A z=3,scan#=39792,scan time=78.7213
VATTALPQANPNAGGASGTVVHGTAGTLTPPPAN
Intensity
2.000e+5
1.500e+5
1.000e+5
5.000e+4
0.000e+0
m/z
500
1000
1500
2000
2500
3000
M+e
M+e - Acetyl
Pep_2+
y5
c8
c9
xNAc
HexNAcHex
HexNAc(2)Hex(1)
y4
b5
c5
y6
c7
y9
b10
c11
c12
c13
c15
z.16
y19
c18
z.21
y21
c23
z.25

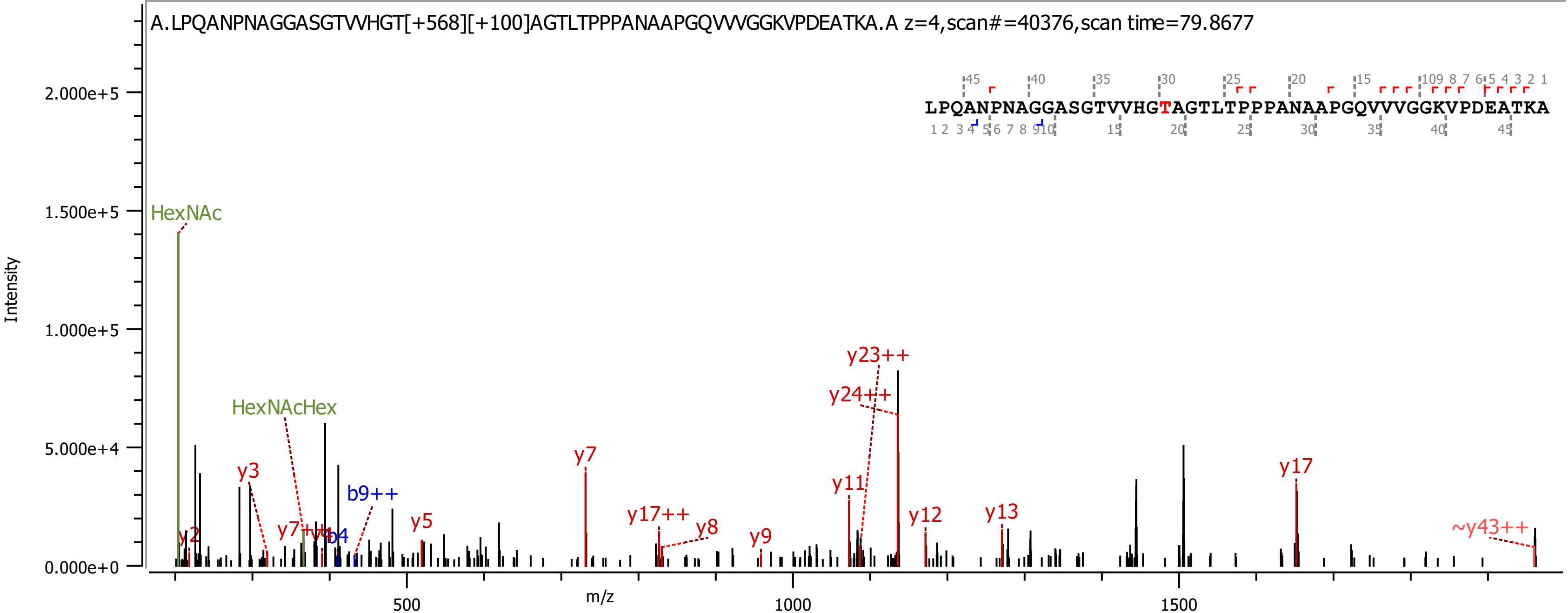
A.LPQANPNAGGASGTVVHGT[+568][+100]AGTLTPPPANAAPGQVVVGGKVPDEATKA.A z=4,scan#=40376,scan time=79.8677
LPQANPNAGGASGTVVHGTAGTLTPPPANAAPGQVVVGGKVPDEATKA
Intensity
m/z
2.000e+5
1.500e+5
1.000e+5
5.000e+4
0.000e+0
500
1000
1500
HexNAc
HexNAcHex
y2
y3
y7++
y4
b4
b9++
y5
y7
y17++
y8
y9
y11
y23++
y24++
y12
y13
y17
~y43++

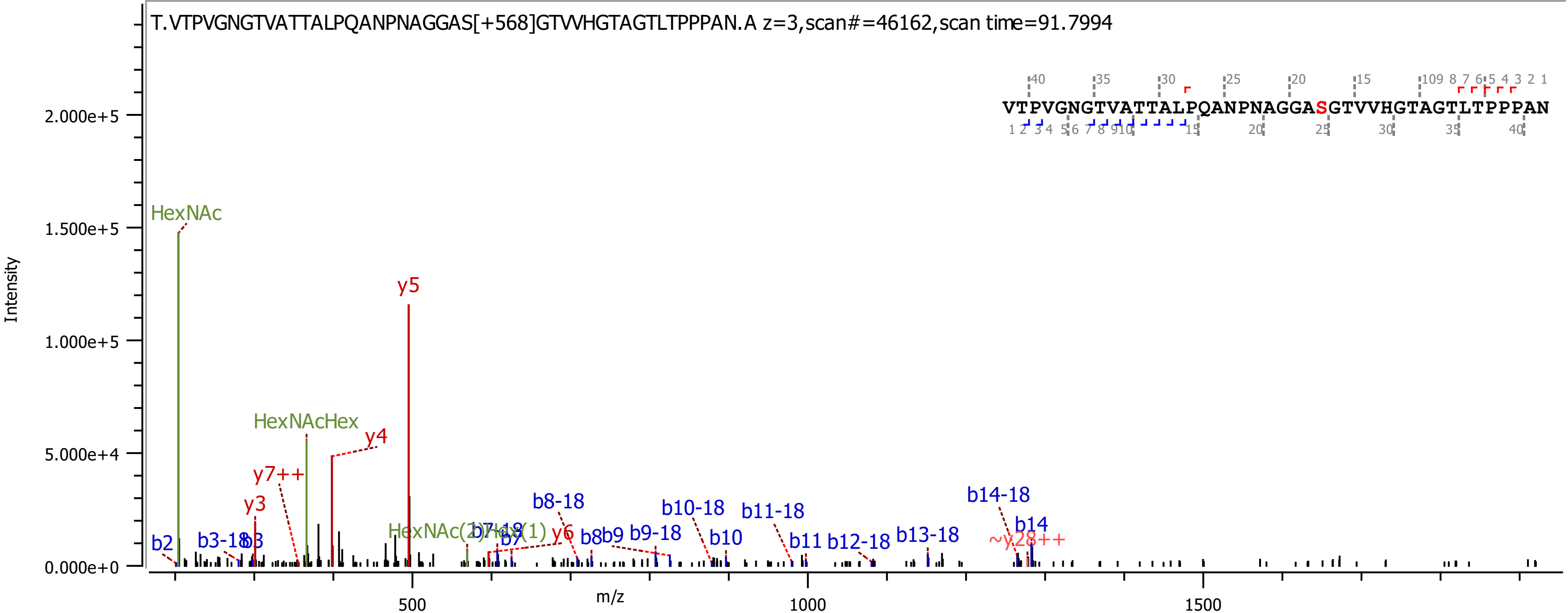
T.VTPVGNGTVATTALPQANPNAGGAS[+568]GTVVHGTAGTLTPPPAN.A z=3,scan#=46162,scan time=91.7994
VTPVGNGTVATTALPQANPNAGGASGTVVHGTAGTLTPPPAN
Intensity
m/z
HexNAc
HexNAcHex
y5
y4
y7++
y3
y6
b2
b3-18
b3
b7
b8-18
b8
b9
b9-18
b10-18
b10
b11-18
b11
b12-18
b13-18
b14-18
b14
~y28++
2.000e+5
1.500e+5
1.000e+5
5.000e+4
0.000e+0
500
1000
1500

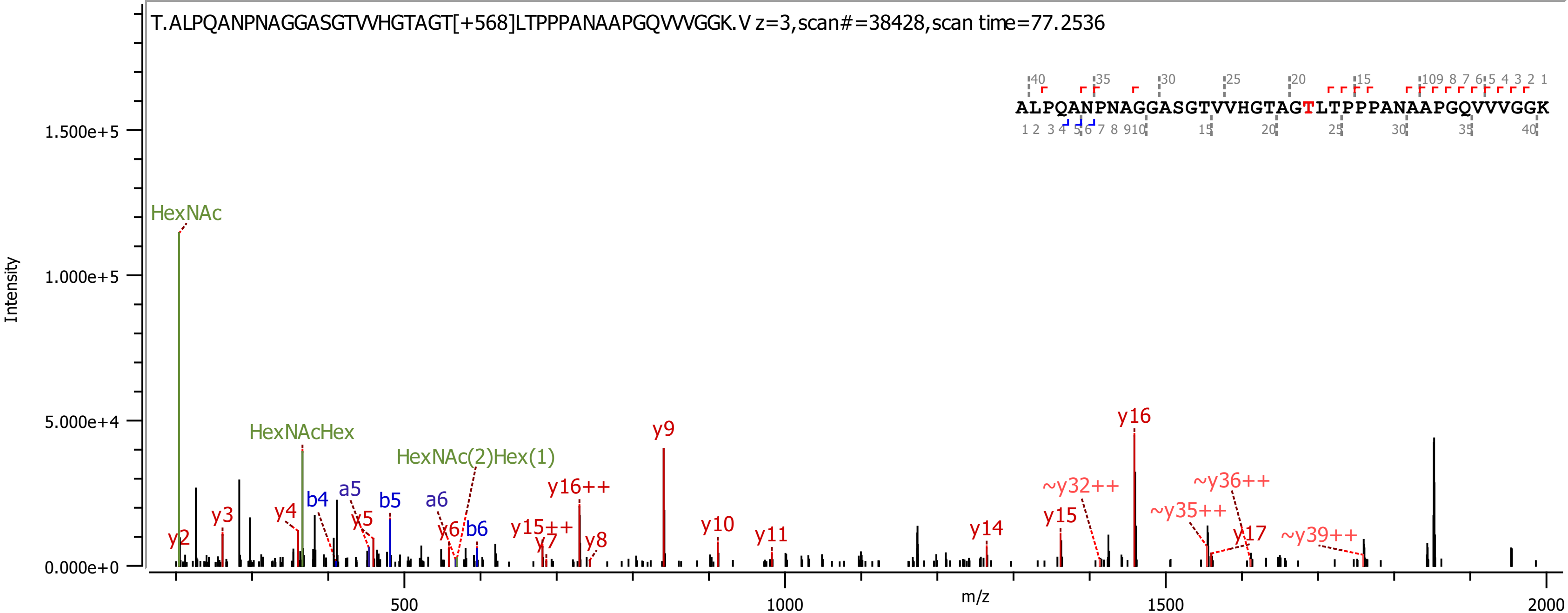
T.ALPQANPNAGGASGTVVHGTAGT[+568]LTPPPANAAPGQVVVGGK.V z=3,scan#=38428,scan time=77.2536
ALPQANPNAGGASGTVVHGTAGTLTPPPANAAPGQVVVGGK
Intensity
m/z
HexNAc
HexNAcHex
HexNAc(2)Hex(1)
y2
y3
y4
b4
a5
y5
b5
a6
y6
b6
y15++
y7
y16++
y8
y9
y10
y11
y14
y15
~y32++
y16
~y35++
~y36++
y17
~y39++
1.500e+5
1.000e+5
5.000e+4
0.000e+0
500
1000
1500
2000

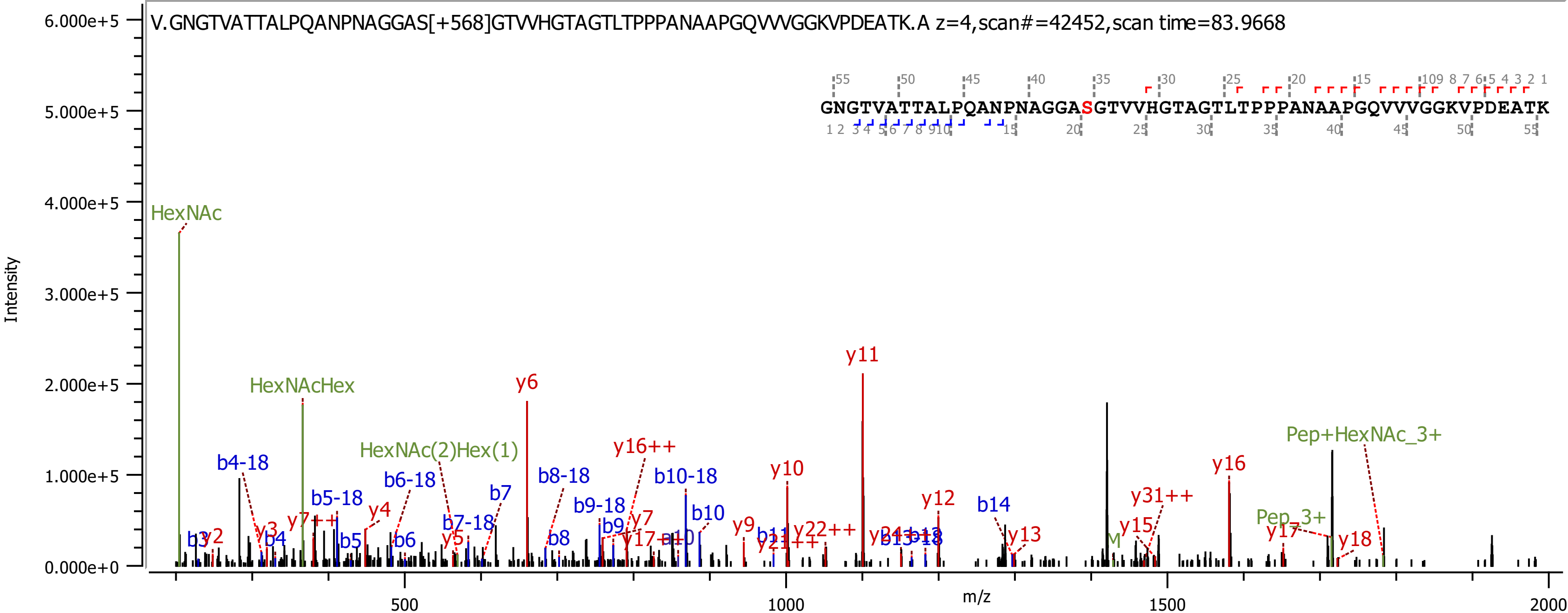
V.GNGTVATTALPQANPNAGGAS[+568]GTVVHGTAGTLTPPPANAAPGQVVVGGKVPDEATK.A z=4,scan#=42452,scan time=83.9668
GNGTVATTALPQANPNAGGASGTVVHGTAGTLTPPPANAAPGQVVVGGKVPDEATK
Intensity
m/z
HexNAc
HexNAcHex
HexNAc(2)Hex(1)
Pep+HexNAc_3+
Pep_3+
y6
y11
y10
y12
y16
y16++
y31++
b4-18
b5-18
b6-18
b7-18
b8-18
b9-18
b10-18
b10
b14
y4
y7
y9
y13
y15
y17
y18

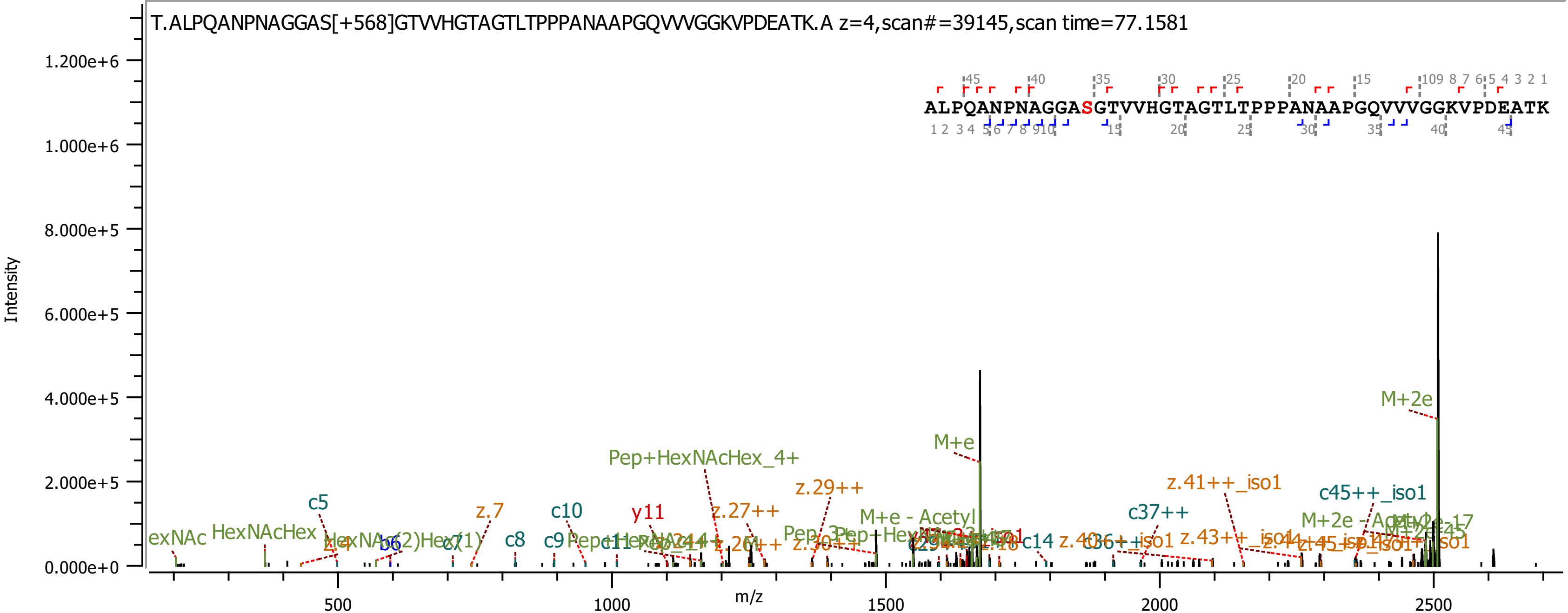

T.ALPQANPNAGGAS[+568]GTVVHGTAGTLTPPPANAAPGQVVVGGKVPDEATK.A z=4,scan#=39145,scan time=77.1581
ALPQANPNAGGASGTVVHGTAGTLTPPPANAAPGQVVVGGKVPDEATK
Intensity
m/z
1.200e+6
1.000e+6
8.000e+5
6.000e+5
4.000e+5
2.000e+5
0.000e+0
500
1000
1500
2000
2500
M+2e
M+e
Pep+HexNAcHex_4+
z.29++
z.41++_iso1
c45++_iso1
c37++
z.27++
y11
c10
z.7
c5
HexNAcHex
M+e - Acetyl
M+2e - Acetyl
c8
c14

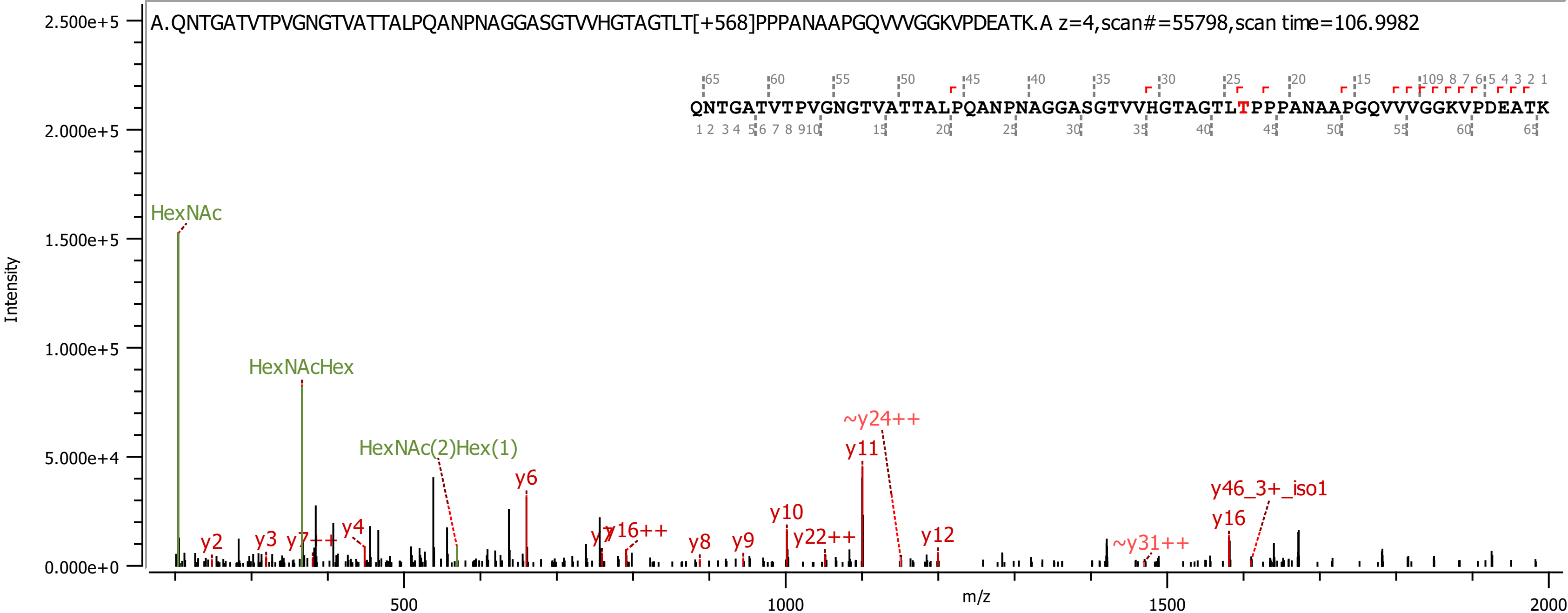

A.QNTGATVTPVGNGTVATTALPQANPNAGGASGTVVHGTAGTLT[+568]PPPANAAPGQVVVGGKVPDEATK.A z=4,scan#=55798,scan time=106.9982
QNTGATVTPVGNGTVATTALPQANPNAGGASGTVVHGTAGTLTPPPANAAPGQVVVGGKVPDEATK
HexNAc
HexNAcHex
HexNAc(2)Hex(1)
y2
y3
y7++
y4
y6
y7
y16++
y8
y9
y10
y22++
~y24++
y11
y12
~y31++
y16
y46_3+_iso1
Intensity
m/z
2.500e+5
2.000e+5
1.500e+5
1.000e+5
5.000e+4
0.000e+0
500
1000
1500
2000

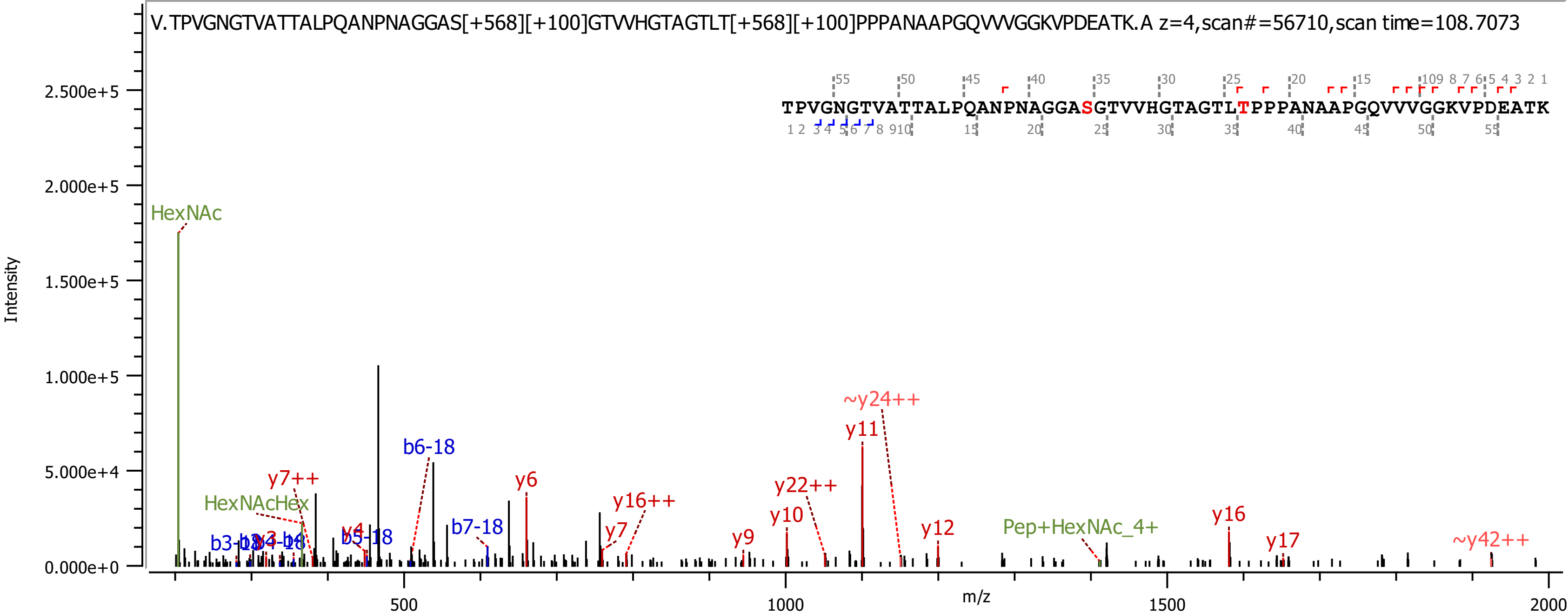

V.TPVGNGTVATTALPQANPNAGGAS[+568][+100]GTVVHGTAGTLT[+568][+100]PPPANAAPGQVVVGGKVPDEATK.A z=4,scan#=56710,scan time=108.7073
TPVGNGTVATTALPQANPNAGGASGTVVHGTAGTLTPPPANAAPGQVVVGGKVPDEATK
Intensity
m/z
HexNAc
HexNAcHex
y7++
b6-18
y6
y16++
y7
b7-18
b5-18
y4
y9
y10
y22++
y11
~y24++
y12
Pep+HexNAc_4+
y16
y17
~y42++

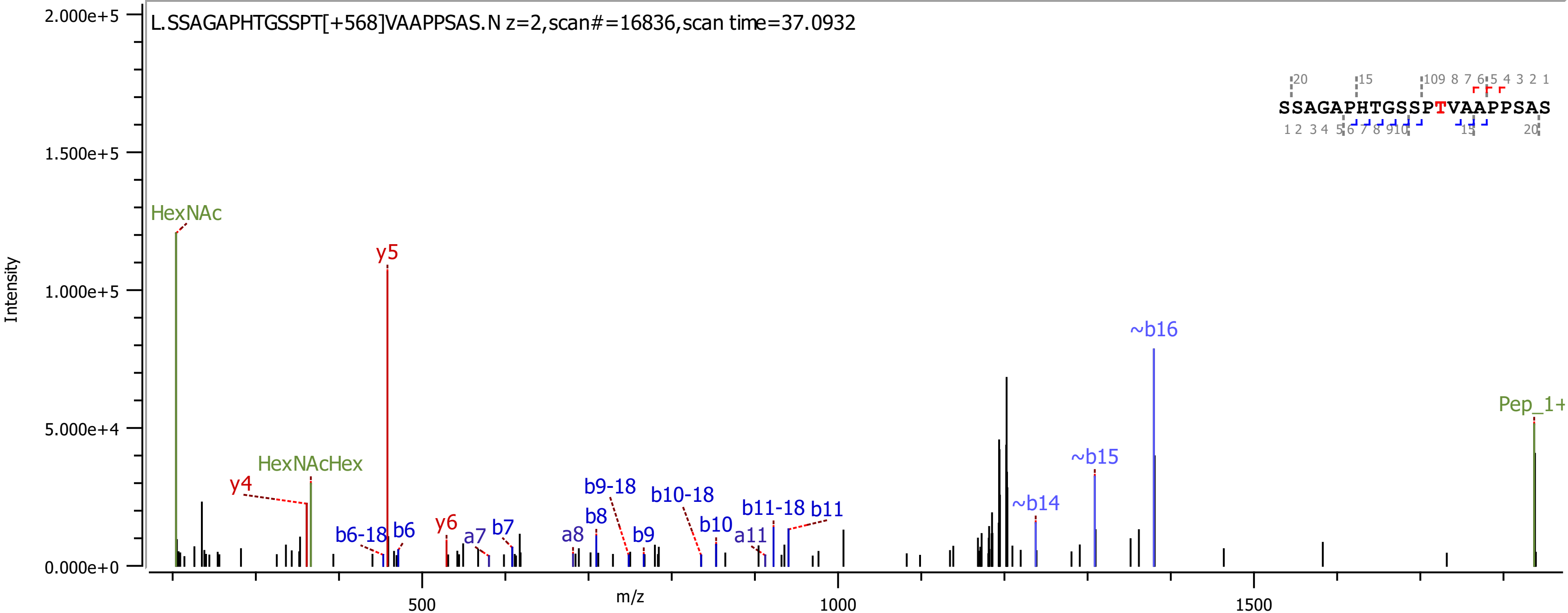
L.SSAGAPHTGSSPT[+568]VAAPPSAS.N z=2,scan#=16836,scan time=37.0932
SSAGAPHTGSSPTVAAPPSAS
Intensity
m/z
HexNAc
y5
~b16
~b15
~b14
Pep_1+
HexNAcHex
y4
b6-18
b6
y6
a7
b7
a8
b9-18
b8
b9
b10-18
b10
a11
b11-18
b11
2.000e+5
1.500e+5
1.000e+5
5.000e+4
0.000e+0
500
1000
1500

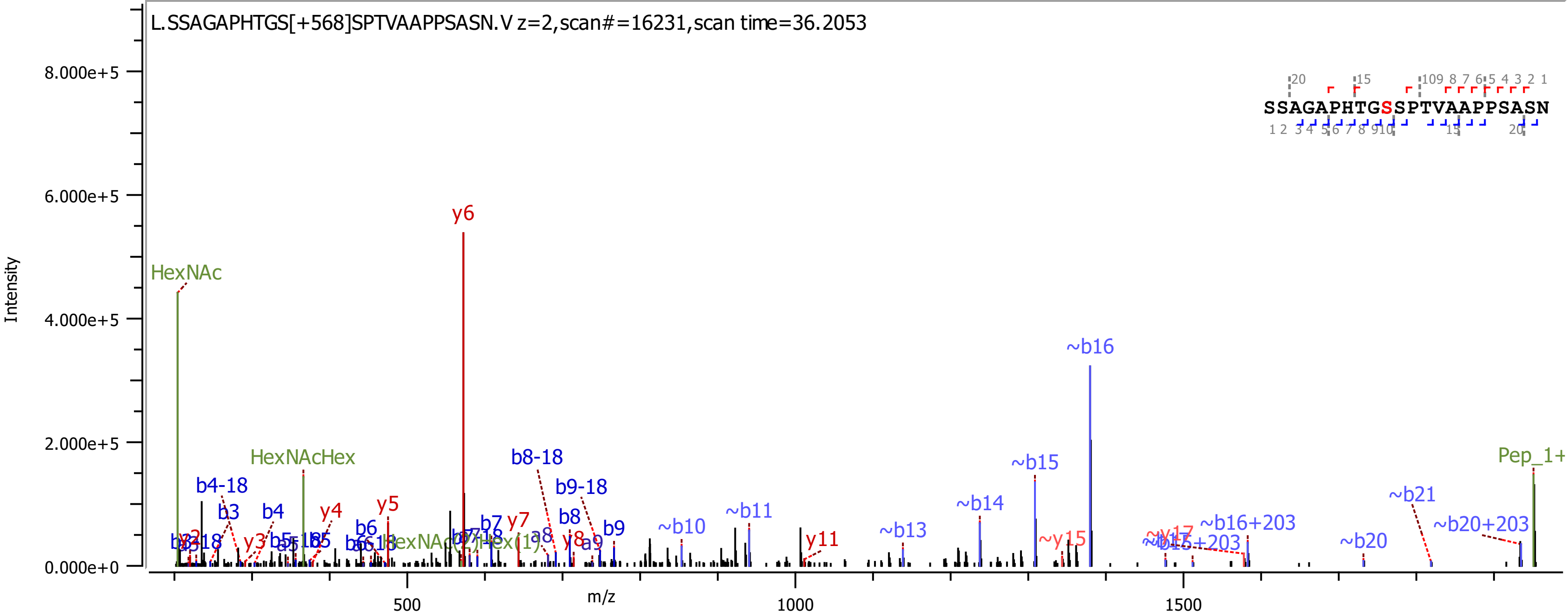
L.SSAGAPHTGS[+568]SPTVAAPPSASN.V z=2,scan#=16231,scan time=36.2053
SSAGAPHTGSSPTVAAPPSASN
Intensity
m/z
8.000e+5
6.000e+5
4.000e+5
2.000e+5
0.000e+0
500
1000
1500
HexNAc
HexNAcHex
y6
~b16
~b15
~b14
~b13
~b11
~b10
~b21
~b20
~b20+203
~b16+203
~y15
y11
b8-18
b9-18
b4-18
b3
b4
y4
y5
b6
b7
y7
b8
b9
y3
Pep_1+

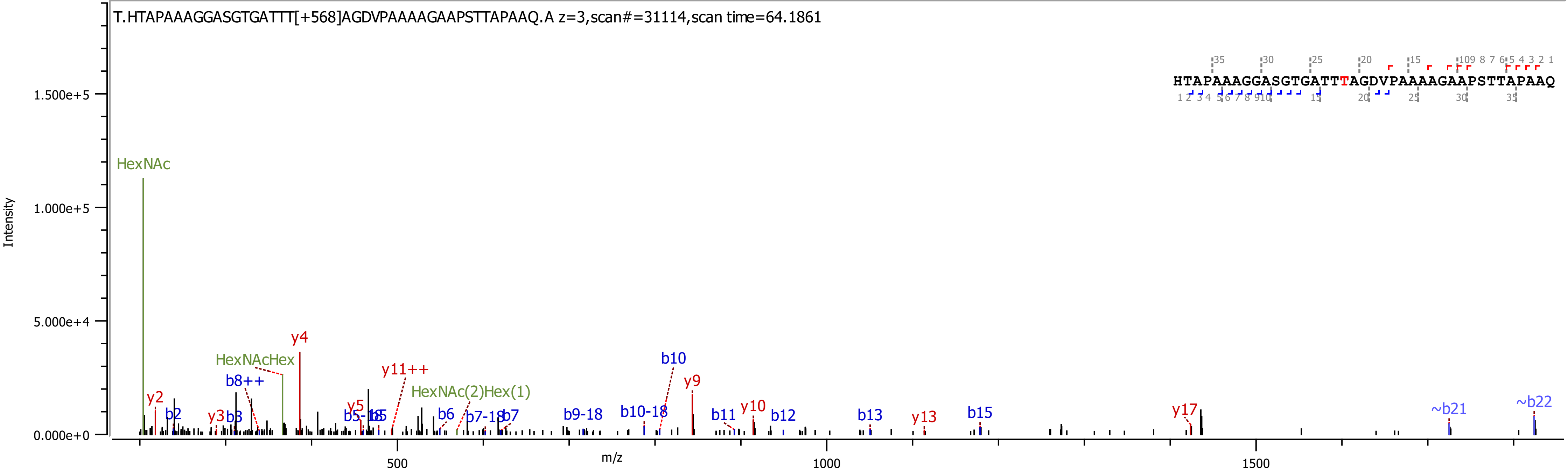
T.HTAPAAAGGASGTGATTT[+568]AGDVPAAAAGAAPSTTAPAAQ.A z=3,scan#=31114,scan time=64.1861
HTAPAAAGGASGTGATTTAGDVPAAAAGAAPSTTAPAAQ
Intensity
m/z
HexNAc
HexNAcHex
HexNAc(2)Hex(1)
y2
y3
y4
y5
y9
y10
y11++
y13
y17
b2
b3
b5-18
b5
b6
b7-18
b7
b8++
b9-18
b10-18
b10
b11
b12
b13
b15
~b21
~b22

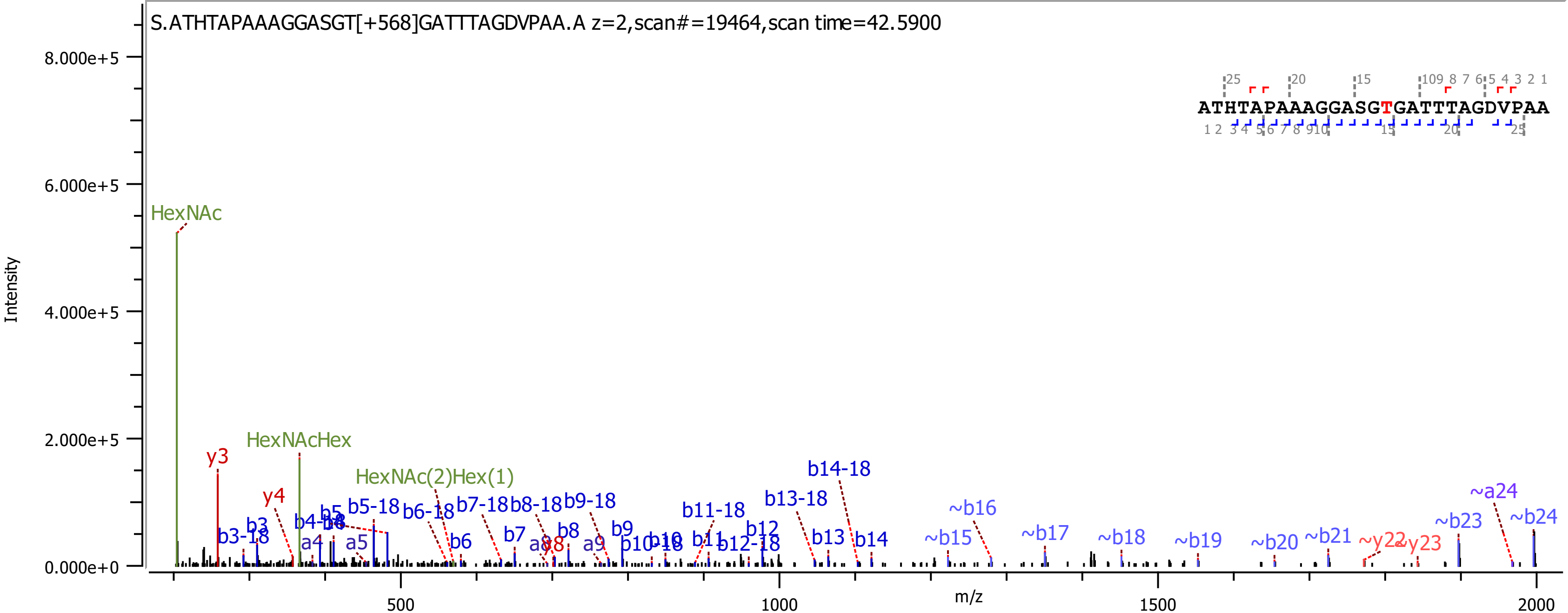

S.ATHTAPAAAGGASGT[+568]GATTTAGDVPAA.A z=2,scan#=19464,scan time=42.5900
ATHTAPAAAGGASGTGATTTAGDVPAA
Intensity
m/z
0.000e+0
2.000e+5
4.000e+5
6.000e+5
8.000e+5
500
1000
1500
2000
HexNAc
HexNAcHex
HexNAc(2)Hex(1)
y3
y4
b3-18
b3
b4-18
b5
b5-18
a4
a5
b6-18
b7-18
b8-18
b9-18
b6
b7
b8
b9
a8
y8
a9
b10-18
b10
b11-18
b11
b12-18
b12
b13-18
b13
b14-18
b14
~b15
~b16
~b17
~b18
~b19
~b20
~b21
~y22
~y23
~b23
~a24
~b24

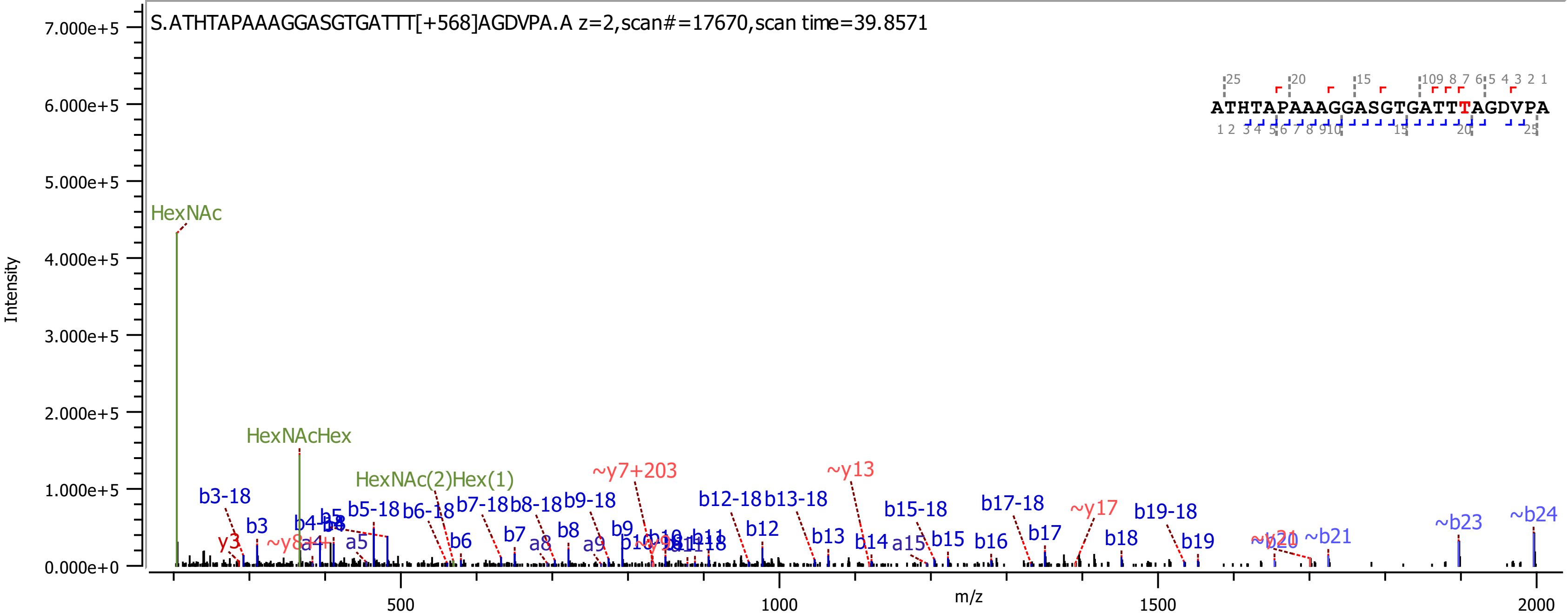
S.ATHTAPAAAGGASGTGATTT[+568]AGDVPA.A z=2,scan#=17670,scan time=39.8571
ATHTAPAAAGGASGTGATTTAGDVPA
Intensity
m/z
7.000e+5
6.000e+5
5.000e+5
4.000e+5
3.000e+5
2.000e+5
1.000e+5
0.000e+0
500
1000
1500
2000
HexNAc
HexNAcHex
HexNAc(2)Hex(1)
~y7+203
~y13
~y17
~b23
~b24
~b21
b3-18
b3
y3
b5-18
b6-18
b7-18
b8-18
b9-18
b12-18
b13-18
b15-18
b17-18
b19-18
a5
b6
b7
a8
b8
a9
b9
b12
b13
b14
a15
b15
b16
b17
b18
b19

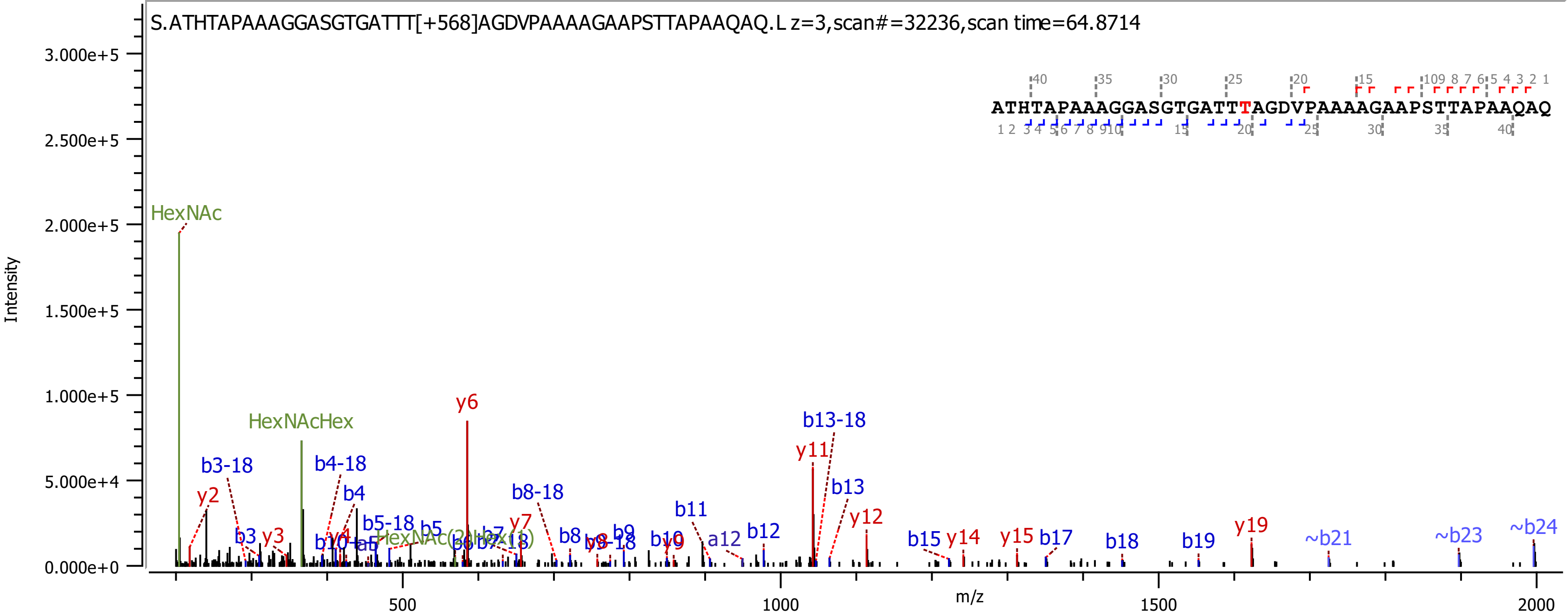

S.ATHTAPAAAGGASGTGATTT[+568]AGDVPAAAAGAAPSTTAPAAQAQ.L z=3,scan#=32236,scan time=64.8714
ATHTAPAAAGGASGTGATTTAGDVPAAAAGAAPSTTAPAAQAQ
Intensity
m/z
HexNAc
HexNAcHex
y6
b13-18
y11
b3-18
b4-18
y2
b4
b8-18
b13
b11
y12
b5-18
b5
y7
y3
b3
y19
b12
~b24
y15
y14
b17
~b23
b15
~b21
b18
b19
a12
b8
b9
b10
y9

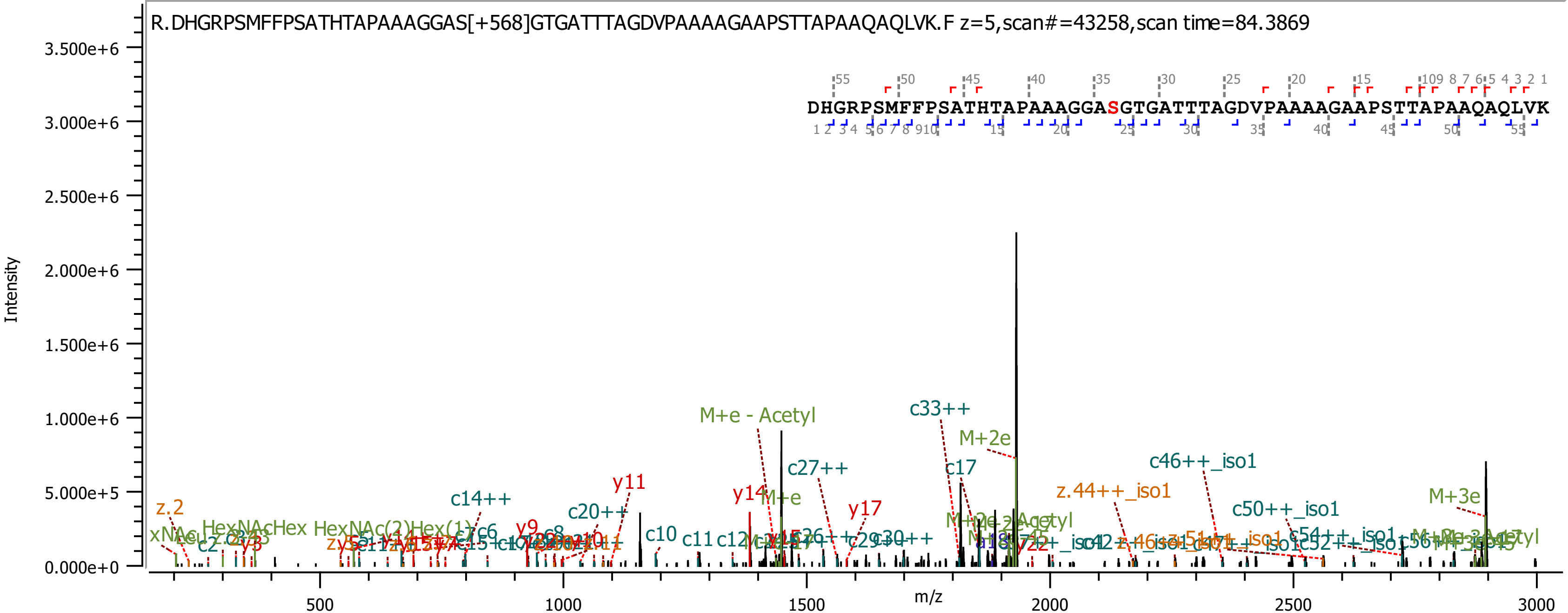

R.DHGRPSMFFPSATHTAPAAAGGAS[+568]GTGATTTAGDVPAAAAGAAPSTTAPAAQAQLVK.F z=5,scan#=43258,scan time=84.3869
DHGRPSMFFPSATHTAPAAAGGASGTGATTTAGDVPAAAAGAAPSTTAPAAQAQLVK
Intensity
m/z
M+e - Acetyl
c33++
M+2e
c27++
c17
c46++_iso1
y11
y14
M+e
y17
z.44++_iso1
c50++_iso1
M+3e
c14++
c20++
z.2
c10
y9
c8
c11
c12

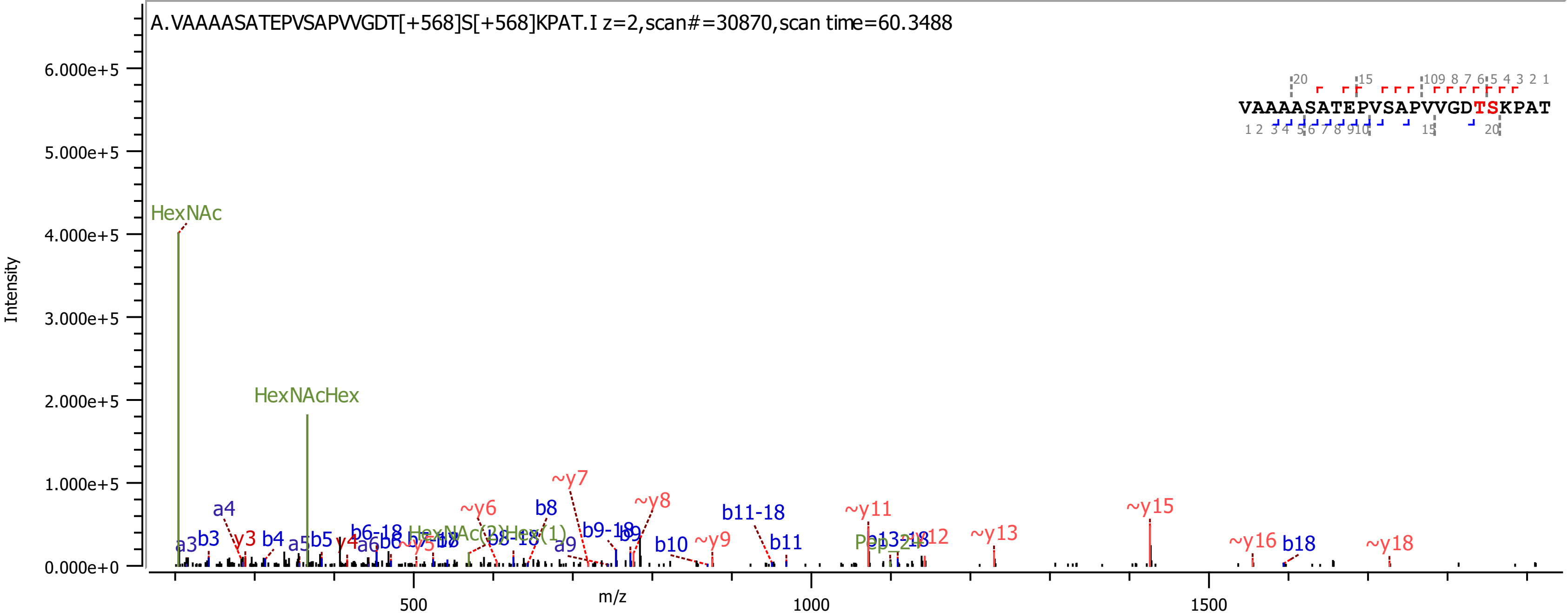
A.VAAAASATEPVSAPVVGDT[+568]S[+568]KPAT.I z=2,scan#=30870,scan time=60.3488
VAAAASATEPVSAPVVGDTSKPAT
Intensity
m/z
HexNAc
HexNAcHex
~y7
~y6
~y8
~y11
~y15
~y9
~y12
~y13
~y16
~y18
b8
b9-18
b10
b11-18
b11
b18
a4
b3
y3
b4
b5
y4
a3
a5
a9
Pep-2-

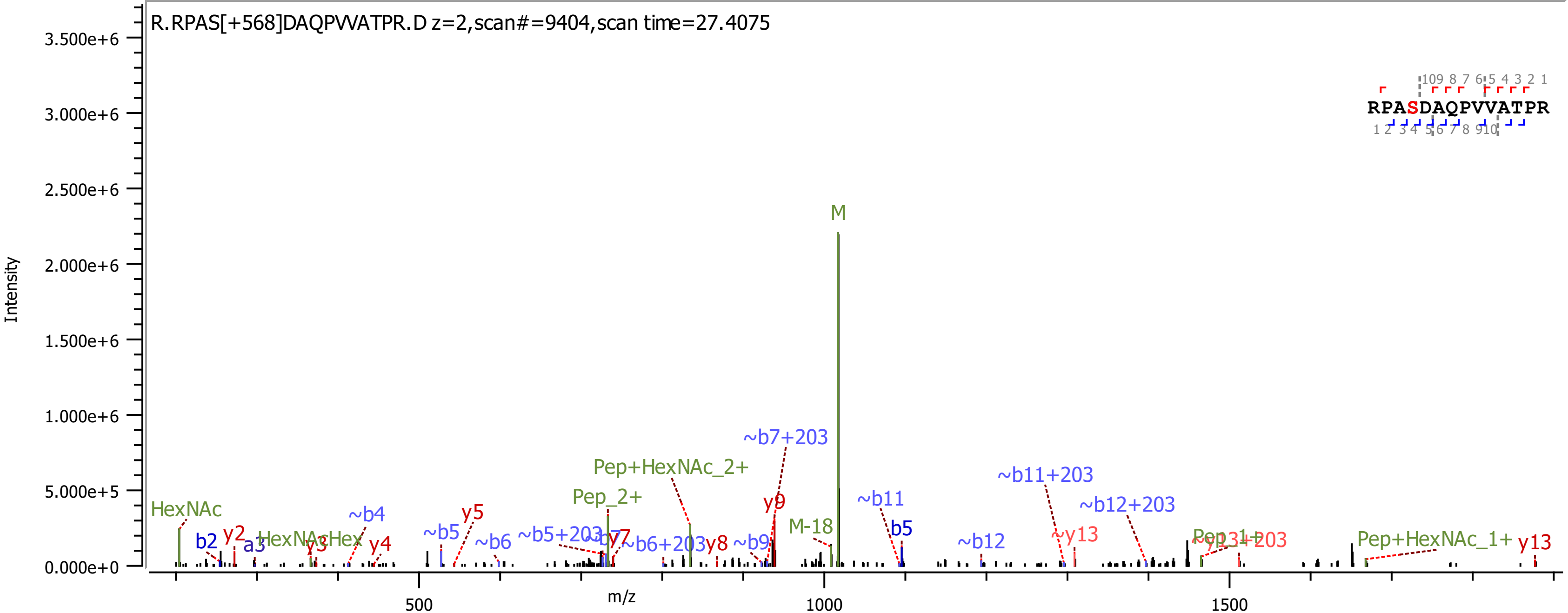

R.RPAS[+568]DAQPVVATPR.D z=2,scan#=9404,scan time=27.4075
RPASDAQPVVATPR
Intensity
m/z
M
~b7+203
Pep+HexNAc_2+
~b11+203
~b12+203
~b11
y9
Pep_2+
HexNAc
~b4
y5
M-18
b5
~b5
~b6
~b5+203
y7
~b6+203
y8
~b9
~b12
~y13
y13+203
Pep+HexNAc_1+
y13
b2
y2
a3
HexNAcHex
y3
y4

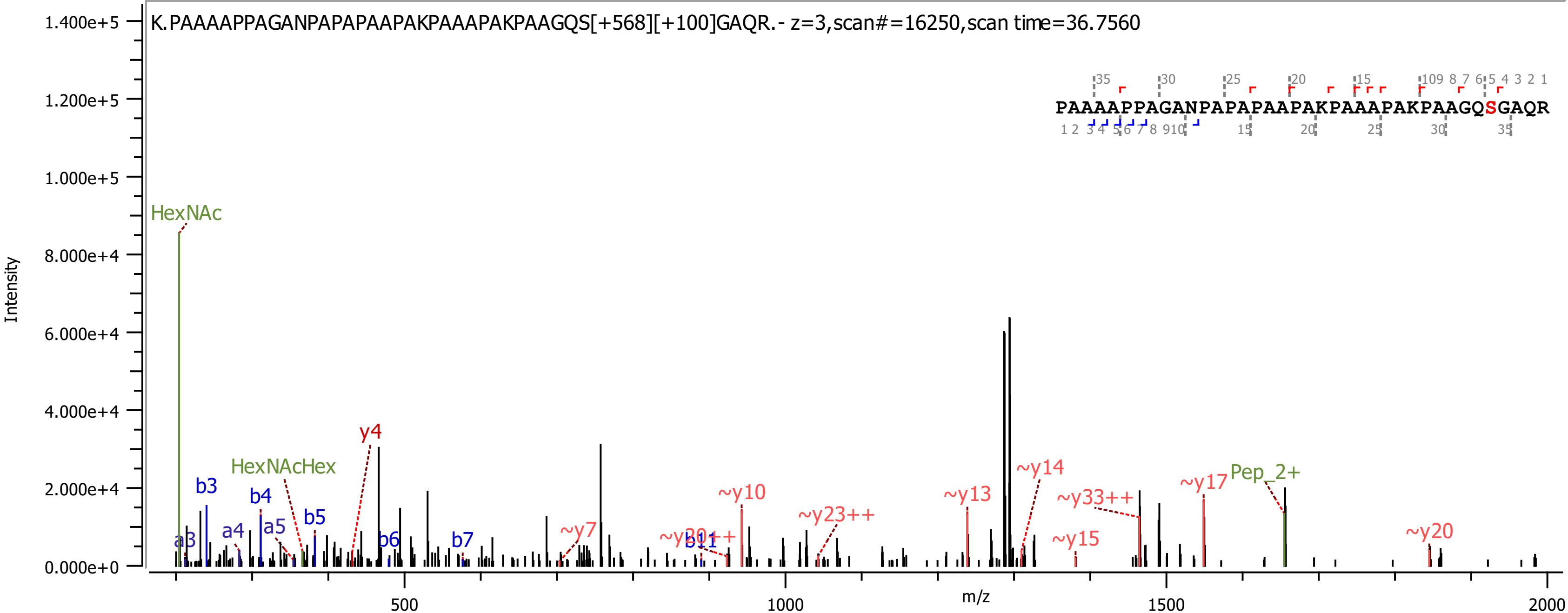
K.PAAAAPPAGANPAPAPAAPAKPAAAPAKPAAGQS[+568][+100]GAQR.- z=3,scan#=16250,scan time=36.7560
PAAAAPPAGANPAPAPAAPAKPAAAPAKPAAGQSGAQR
Intensity
m/z
HexNAc
HexNAcHex
Pep_2+
b3
b4
b5
b6
b7
b11
a3
a4
a5
y4
~y7
~y10
~y13
~y14
~y15
~y17
~y20
~y23++
~y33++
1.400e+5
1.200e+5
1.000e+5
8.000e+4
6.000e+4
4.000e+4
2.000e+4
0.000e+0
500
1000
1500
2000

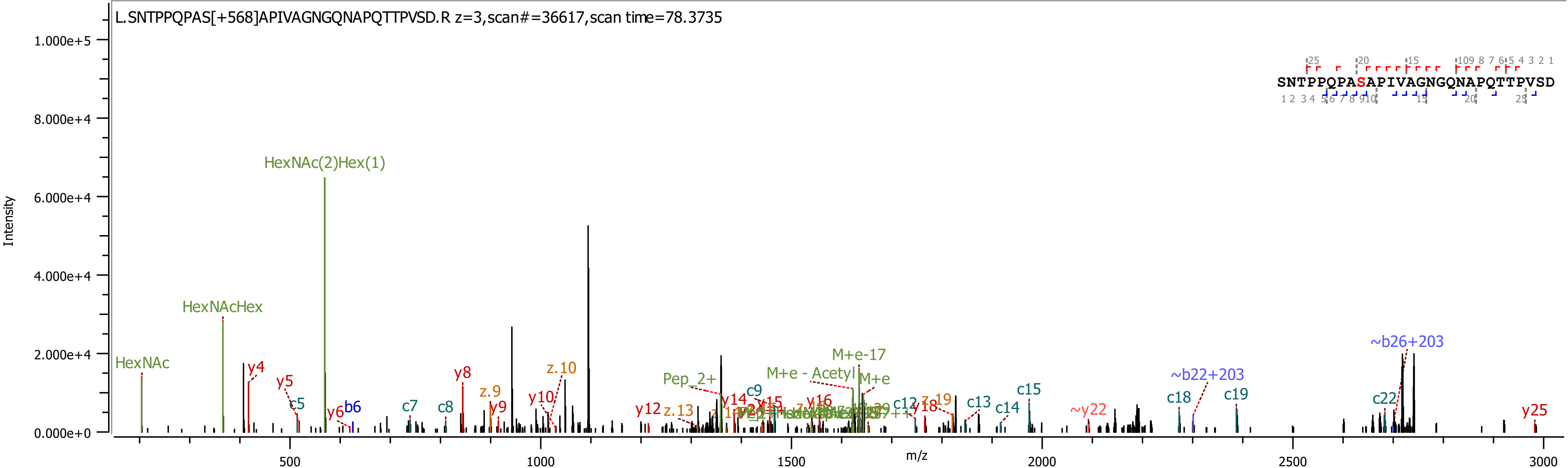

L.SNTPPQPAS[+568]APIVAGNGQNAPQTTPVSD.R z=3,scan#=36617,scan time=78.3735
SNTPPQPASAPIVAGNGQNAPQTTPVSD
Intensity
m/z
HexNAc(2)Hex(1)
HexNAcHex
HexNAc
y4
y5
c5
y6
b6
c7
c8
y8
z.9
y9
z.10
y10
y12
z.13
Pep_2+
y14
c9
y15
y16
M+e - Acetyl
M+e-17
M+e
c12
y18
z.19
c13
c14
c15
~y22
~b22+203
c18
c19
c22
~b26+203
y25

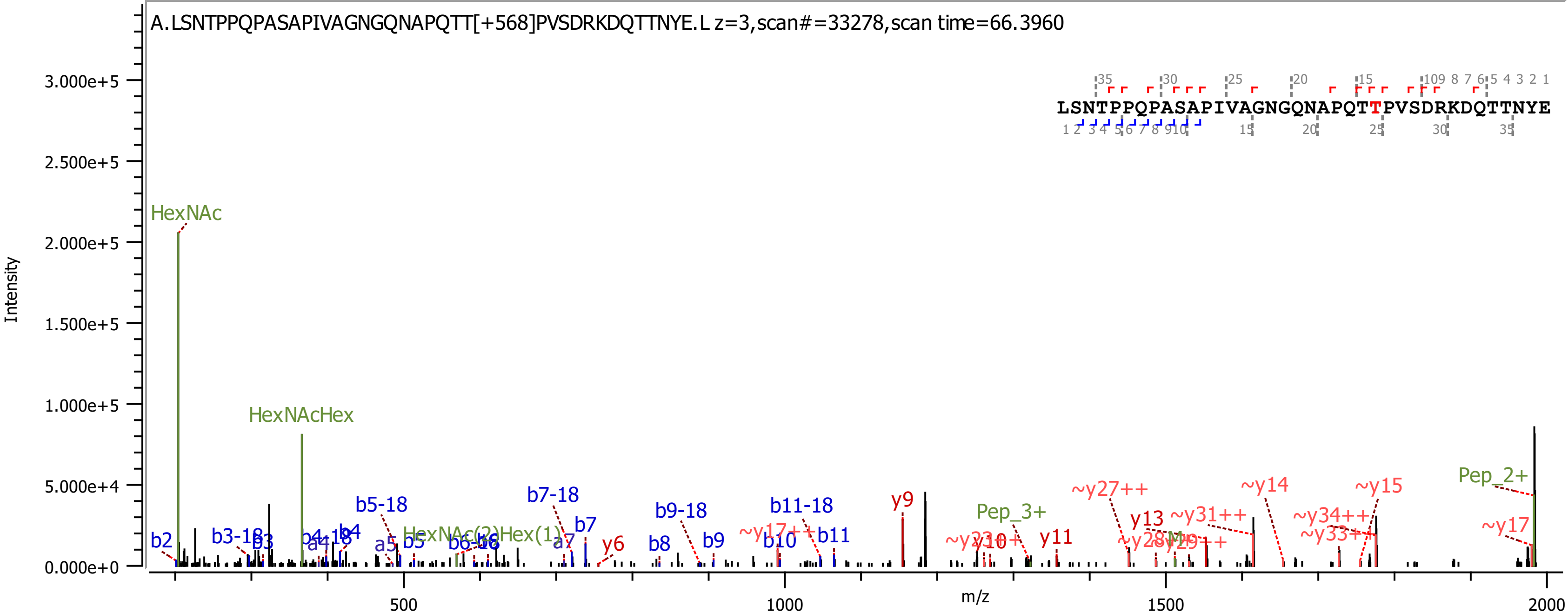

A.LSNTPPQPASAPIVAGNGQNAPQTT[+568]PVSDRKDQTTNYE.L z=3,scan#=33278,scan time=66.3960
LSNTPPQPASAPIVAGNGQNAPQTTPVSDRKDQTTNYE
Intensity
m/z
3.000e+5
2.500e+5
2.000e+5
1.500e+5
1.000e+5
5.000e+4
0.000e+0
500
1000
1500
2000
HexNAc
HexNAcHex
Pep_2+
Pep_3+
y9
y13
y11
y6
~y14
~y15
~y17
~y27++
~y31++
~y34++
~y33++
b5-18
b7-18
b9-18
b11-18
b2
b7
b8
b9
b10
b11
a5
a7

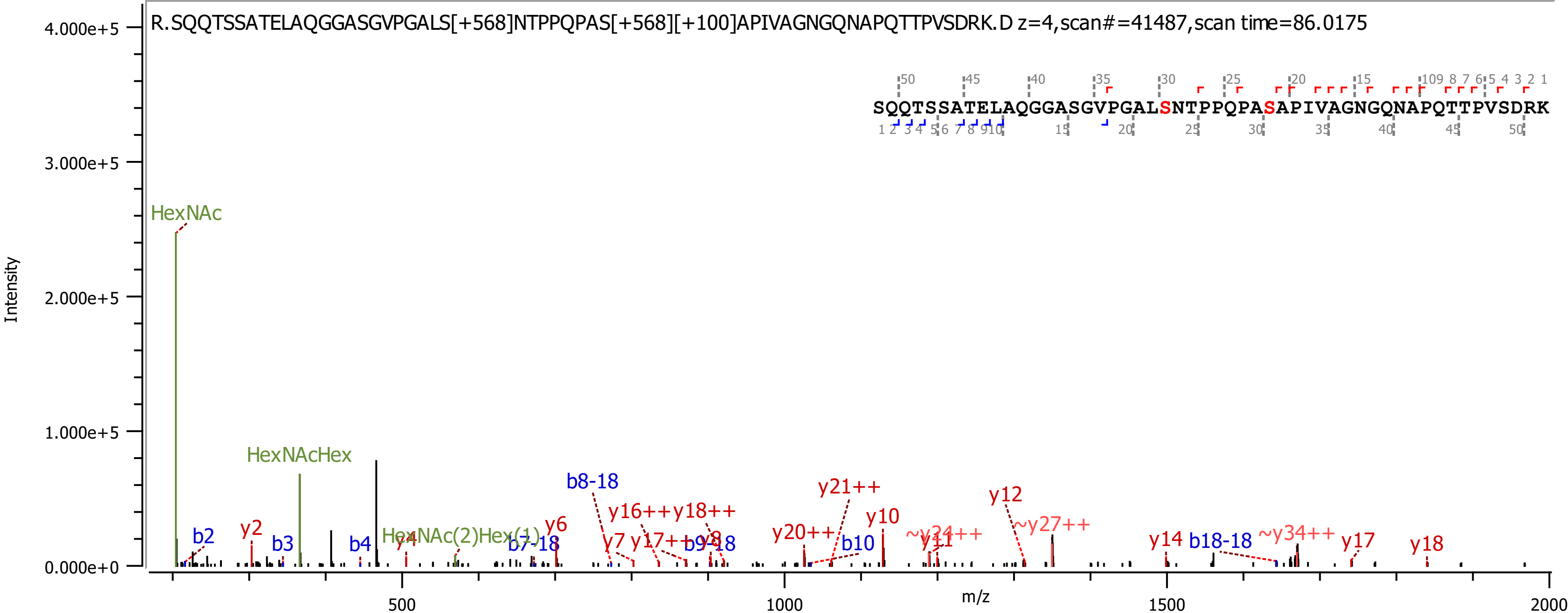
R.SQQTSSATELAQGGASGVPGALS[+568]NTPPQPAS[+568][+100]APIVAGNGQNAPQTTPVSDRK.D z=4,scan#=41487,scan time=86.0175
SQQTSSATELAQGGASGVPGALSNTPPQPASAPIVAGNGQNAPQTTPVSDRK
Intensity
m/z
4.000e+5
3.000e+5
2.000e+5
1.000e+5
0.000e+0
500
1000
1500
2000
HexNAc
HexNAcHex
HexNAc(2)Hex(1)
b2
b3
b4
b7-18
b8-18
b9-18
b10
b18-18
y2
y4
y6
y7
y8
y10
y11
y12
y14
y17
y18
y16++
y17++
y18++
y20++
y21++
~y24++
~y27++
~y34++

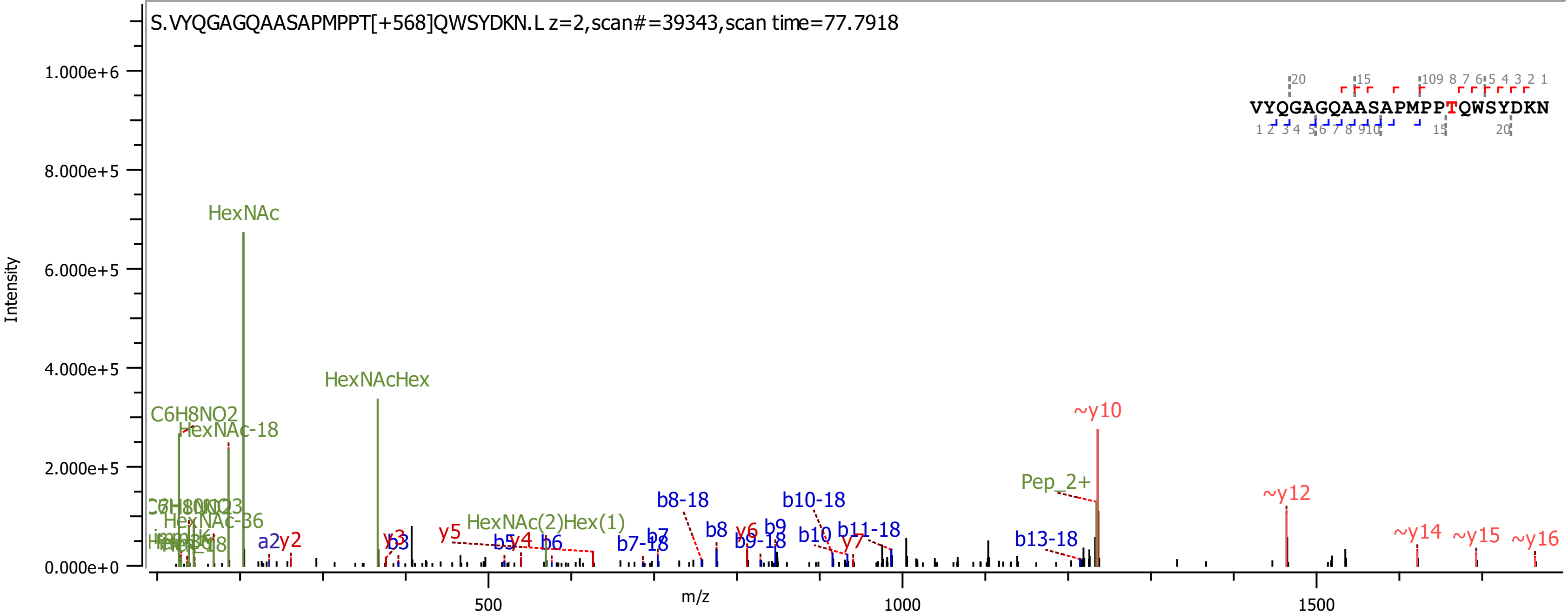

S.VYQGAGQAASAPMPPT[+568]QWSYDKN.L z=2,scan#=39343,scan time=77.7918
VYQGAGQAASAPMPPTQWSYDKN
Intensity
1.000e+6
8.000e+5
6.000e+5
4.000e+5
2.000e+5
0.000e+0
m/z
500
1000
1500
HexNAc
HexNAcHex
C6H8NO2
HexNAc-18
HexNAc-36
HexNAc(2)Hex(1)
Pep_2+
a2
y2
y3
b3
y5
b5
y4
b6
b7
b7-18
b8-18
b8
y6
b9
b9-18
b10-18
b10
b11-18
y7
b13-18
~y10
~y12
~y14
~y15
~y16

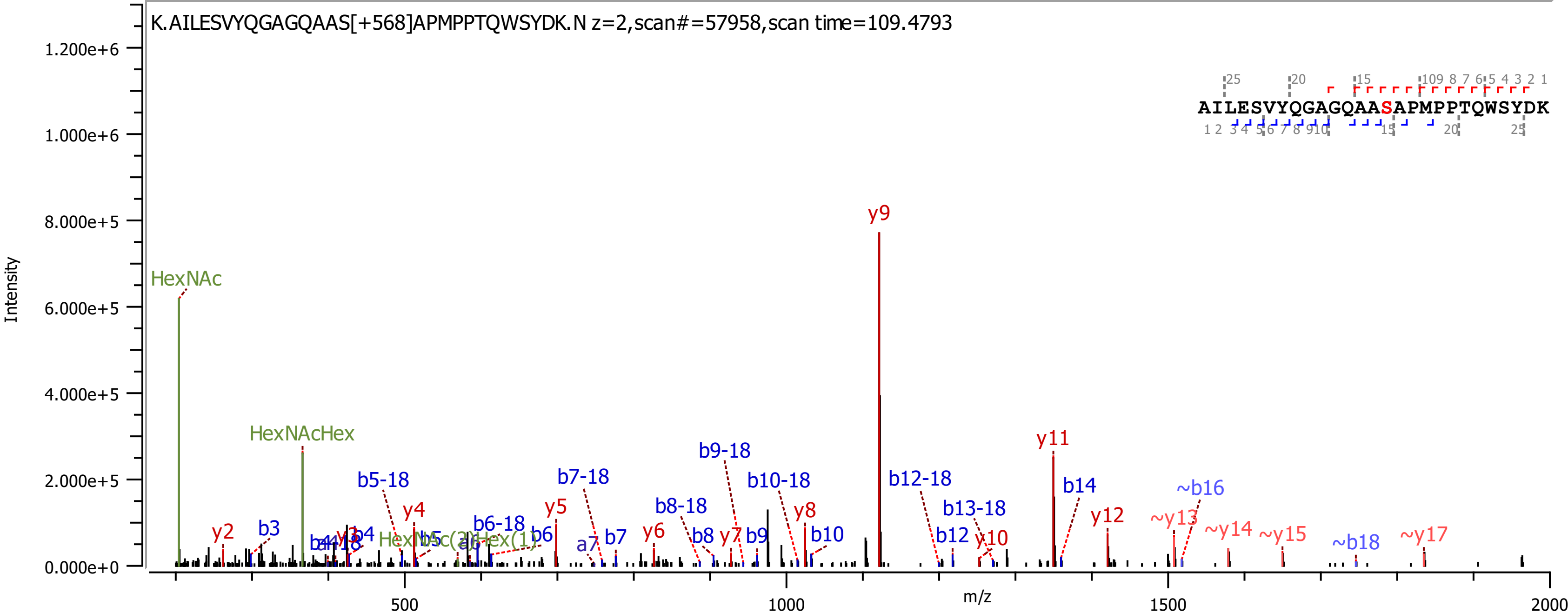

K.AILESVYQGAGQAAS[+568]APMPPTQWSYDK.N z=2,scan#=57958,scan time=109.4793
AILESVYQGAGQAASAPMPPTQWSYDK
Intensity
m/z
HexNAc
HexNAcHex
y9
y11
b14
~b16
y12
~y13
~y14
~y15
~b18
~y17
b12-18
b13-18
b12
y10
b9-18
b10-18
y8
b10
b8-18
b8
y7
b9
y6
b7-18
y5
b6-18
b6
a7
b7
b5-18
y4
y2
b3
b4

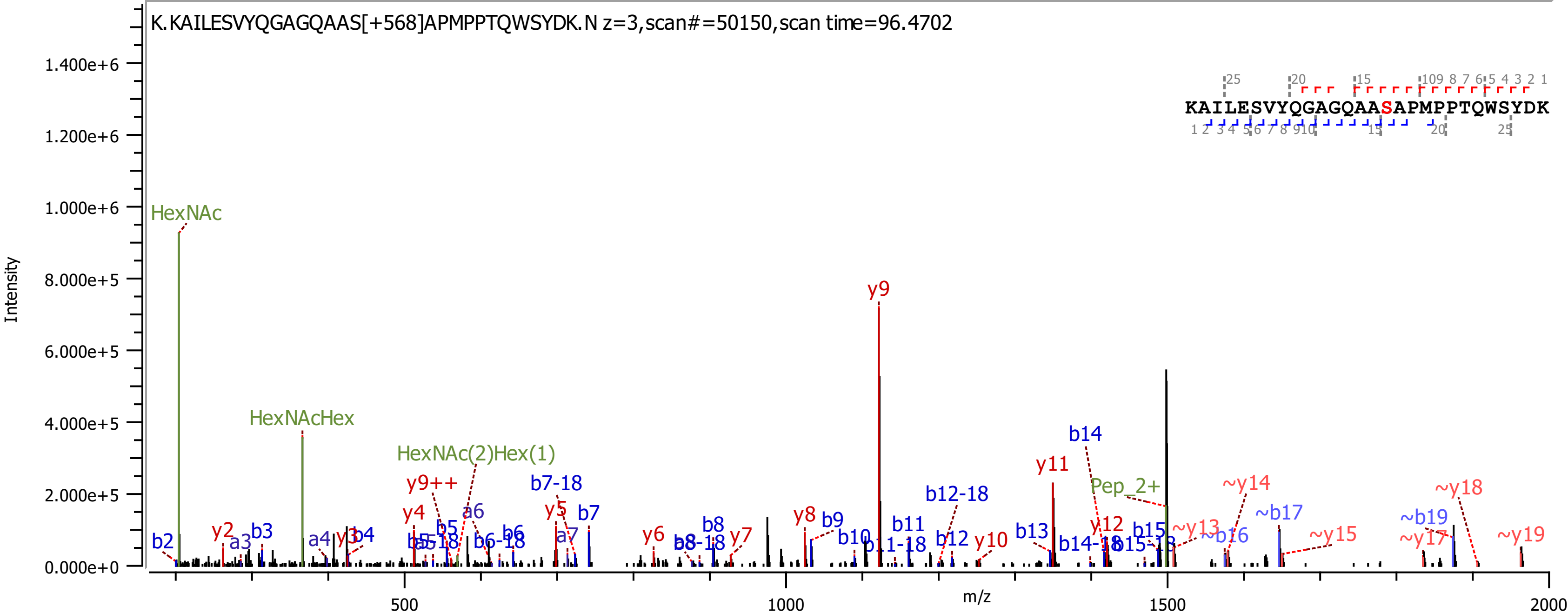
K.KAILESVYQGAGQAAS[+568]APMPPTQWSYDK.N z=3,scan#=50150,scan time=96.4702
KAILESVYQGAGQAASAPMPPTQWSYDK
Intensity
m/z
HexNAc
HexNAcHex
HexNAc(2)Hex(1)
y9
y9++
y11
b14
Pep_2+
~y14
~y18
~b17
~b19
b7-18
b12-18
y2
y4
y5
y6
y7
y8
y10
y12
b2
b3
b4
b5
b6
b7
b8
b9
b10
b11
b13
b15
a3
a4
a6
a7
~y13
~b16
~y15
~y17
~y19
0.000e+0
2.000e+5
4.000e+5
6.000e+5
8.000e+5
1.000e+6
1.200e+6
1.400e+6
500
1000
1500
2000

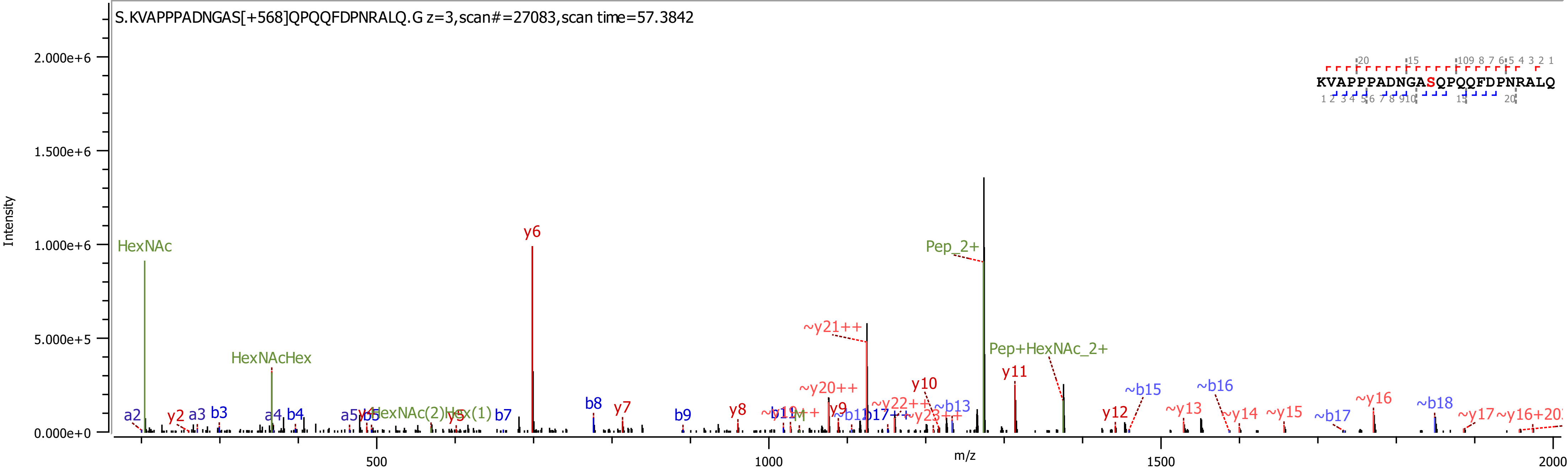

S.KVAPPPADNGAS[+568]QPQQFDPNRALQ.G z=3,scan#=27083,scan time=57.3842
Intensity
m/z
2.000e+6
1.500e+6
1.000e+6
5.000e+5
0.000e+0
500
1000
1500
2000
KVAPPPADNGASQPQQFDPNRALQ
HexNAc
HexNAcHex
y6
Pep_2+
~y21++
Pep+HexNAc_2+
y11
~y20++
y10
~b15
~b16
~y16
~b18
b8
y7
y8
y9
~y22++
~b13
y12
~y13
~y14
~y15
~b17
~y17
a2
y2
a3
b3
a4
b4
a5
b5
b7
b9
HexNAc(2)Hex(1)

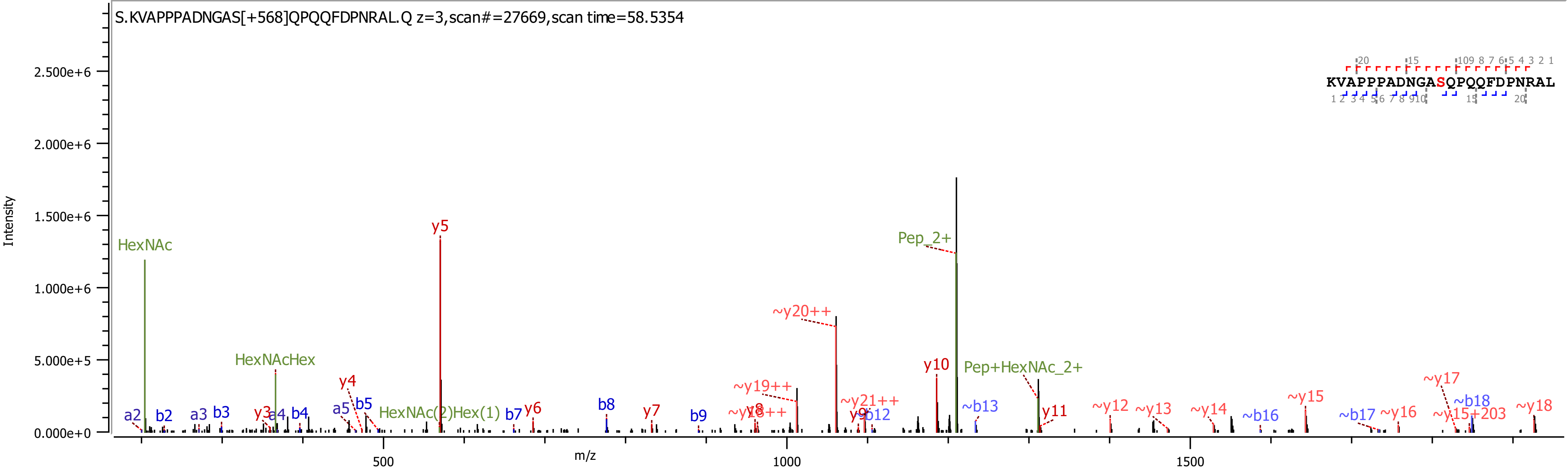

S.KVAPPPADNGAS[+568]QPQQFDPNRAL.Q z=3,scan#=27669,scan time=58.5354
Intensity
m/z
KVAPPPADNGASQPQQFDPNRAL
HexNAc
HexNAcHex
HexNAc(2)Hex(1)
Pep_2+
Pep+HexNAc_2+
y5
~y20++
y10

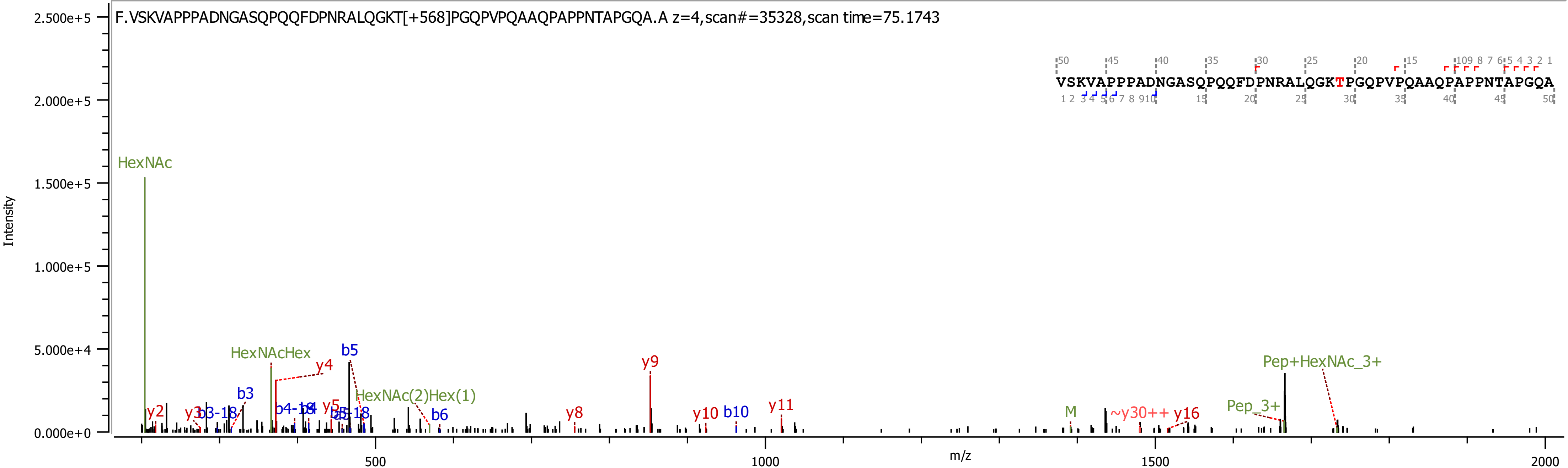

F.VSKVAPPPADNGASQPQQFDPNRALQGKT[+568]PGQPVPQAAQPAPPNTAPGQA.A z=4,scan#=35328,scan time=75.1743
VSKVAPPPADNGASQPQQFDPNRALQGKTPGQPVPQAAQPAPPNTAPGQA
Intensity
m/z
HexNAc
HexNAcHex
HexNAc(2)Hex(1)
Pep+HexNAc_3+
Pep_3+
M
~y30++
y2
y3
y4
y5
y8
y9
y10
y11
y16
b3
b3-18
b4-18
b5
b5-18
b6
b10

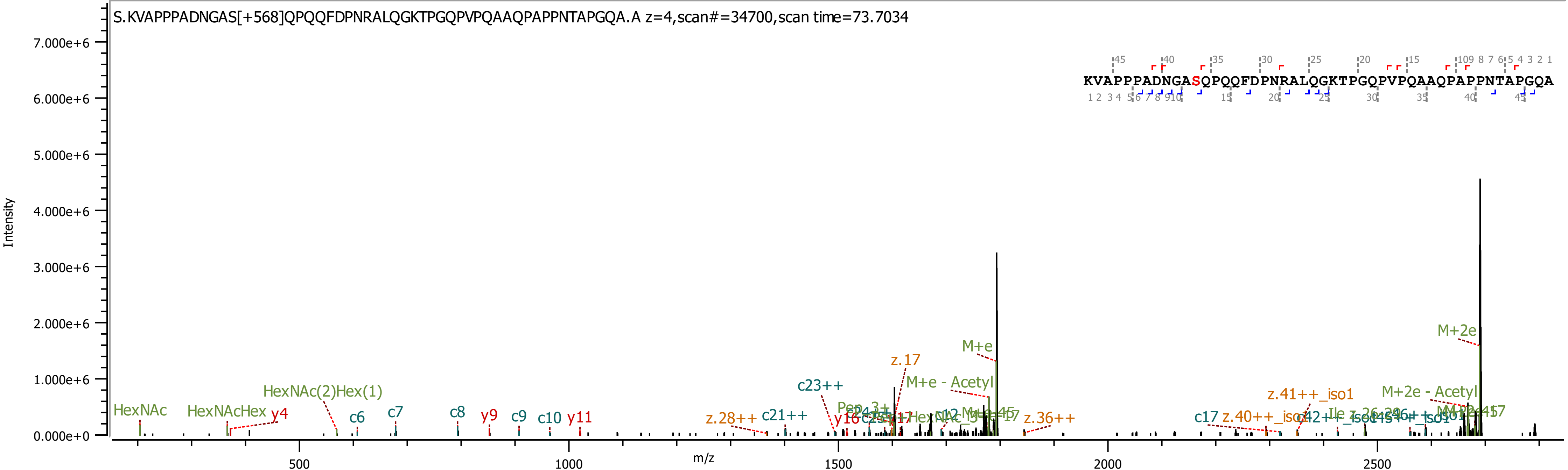

S.KVAPPPADNGAS[+568]QPQQFDPNRALQGKTPGQPVPQAAQPAPPNTAPGQA.A z=4,scan#=34700,scan time=73.7034
KVAPPPADNGASQPQQFDPNRALQGKTPGQPVPQAAQPAPPNTAPGQA
Intensity
m/z
7.000e+6
6.000e+6
5.000e+6
4.000e+6
3.000e+6
2.000e+6
1.000e+6
0.000e+0
500
1000
1500
2000
2500
HexNAc
HexNAcHex
y4
HexNAc(2)Hex(1)
c6
c7
c8
y9
c9
c10
y11
z.28++
c21++
c23++
y16
z.17
M+e - Acetyl
M+e
z.36++
c17
z.40++_iso1
z.41++_iso1
M+2e - Acetyl
M+2e

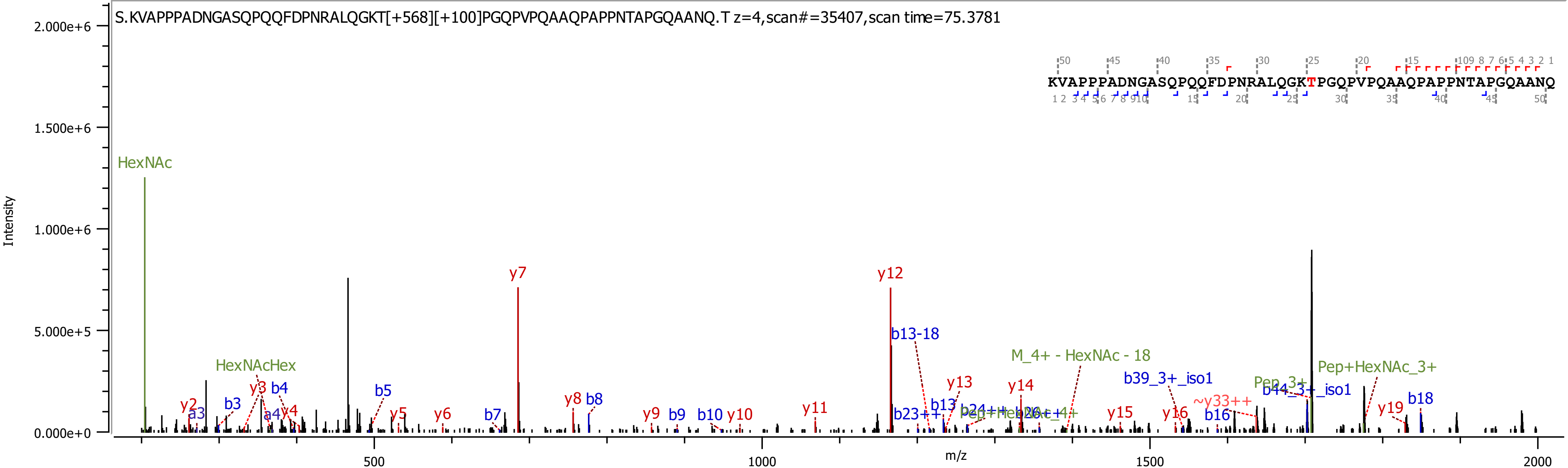
S.KVAPPPADNGASQPQQFDPNRALQGKT[+568][+100]PGQPVPQAAQPAPPNTAPGQAANQ.T z=4,scan#=35407,scan time=75.3781
KVAPPPADNGASQPQQFDPNRALQGKTPGQPVPQAAQPAPPNTAPGQAANQ
Intensity
m/z
HexNAc
HexNAcHex
y7
y12
b13-18
M_4+ - HexNAc - 18
Pep+HexNAc_3+
b39_3+_iso1
b44_3+_iso1
~y33++
y2
a3
b3
y3
b4
a4
y4
b5
y5
y6
b7
y8
b8
y9
b9
b10
y10
y11
b23++
b13
y13
y14
y15
y16
b16
y19
b18

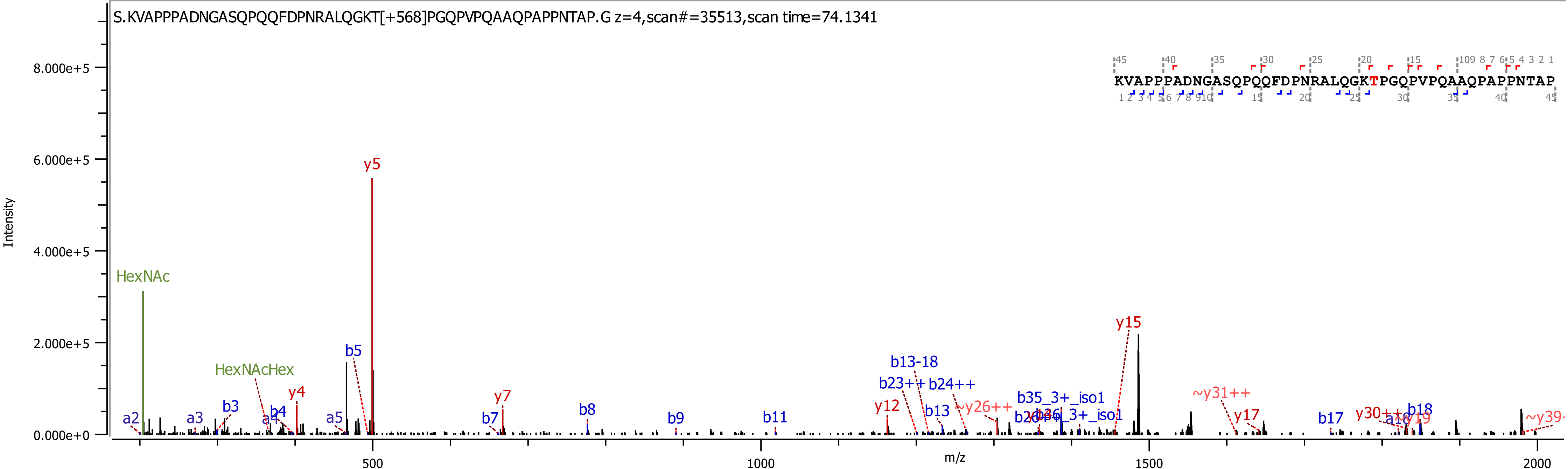

S.KVAPPPADNGASQPQQFDPNRALQGKT[+568]PGQPVPQAAQPAPPNTAP.G z=4,scan#=35513,scan time=74.1341
KVAPPPADNGASQPQQFDPNRALQGKTPGQPVPQAAQPAPPNTAP
Intensity
8.000e+5
6.000e+5
4.000e+5
2.000e+5
0.000e+0
m/z
500
1000
1500
2000
HexNAc
HexNAcHex
y5
y4
y7
y12
y15
y17
~y26++
~y31++
b3
b4
b5
b7
b8
b9
b11
b13
b13-18
b23++
b24++
b17
b18
a2
a3
a4
a5

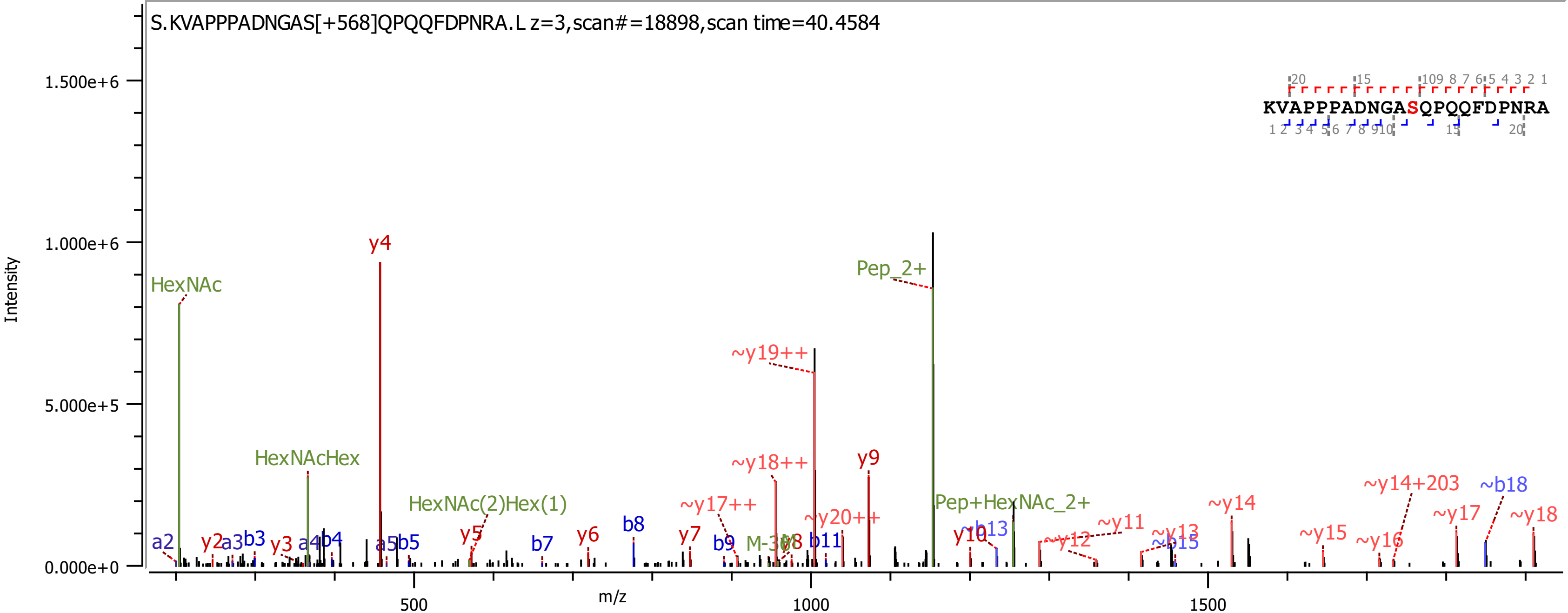
S.KVAPPPADNGAS[+568]QPQQFDPNRA.L z=3,scan#=18898,scan time=40.4584
KVAPPPADNGASQPQQFDPNRA
Intensity
m/z
HexNAc
HexNAcHex
HexNAc(2)Hex(1)
y4
Pep_2+
~y19++
~y18++
y9
~y17++
~y20++
Pep+HexNAc_2+
~y14+203
~b18
~y14
~y17
~y18
~y11
~y13
~b13
~b15
~y15
~y16
~y12
y10
a2
y2
a3
b3
y3
a4
b4
a5
b5
y5
b7
y6
b8
y7
b9
y8
b11

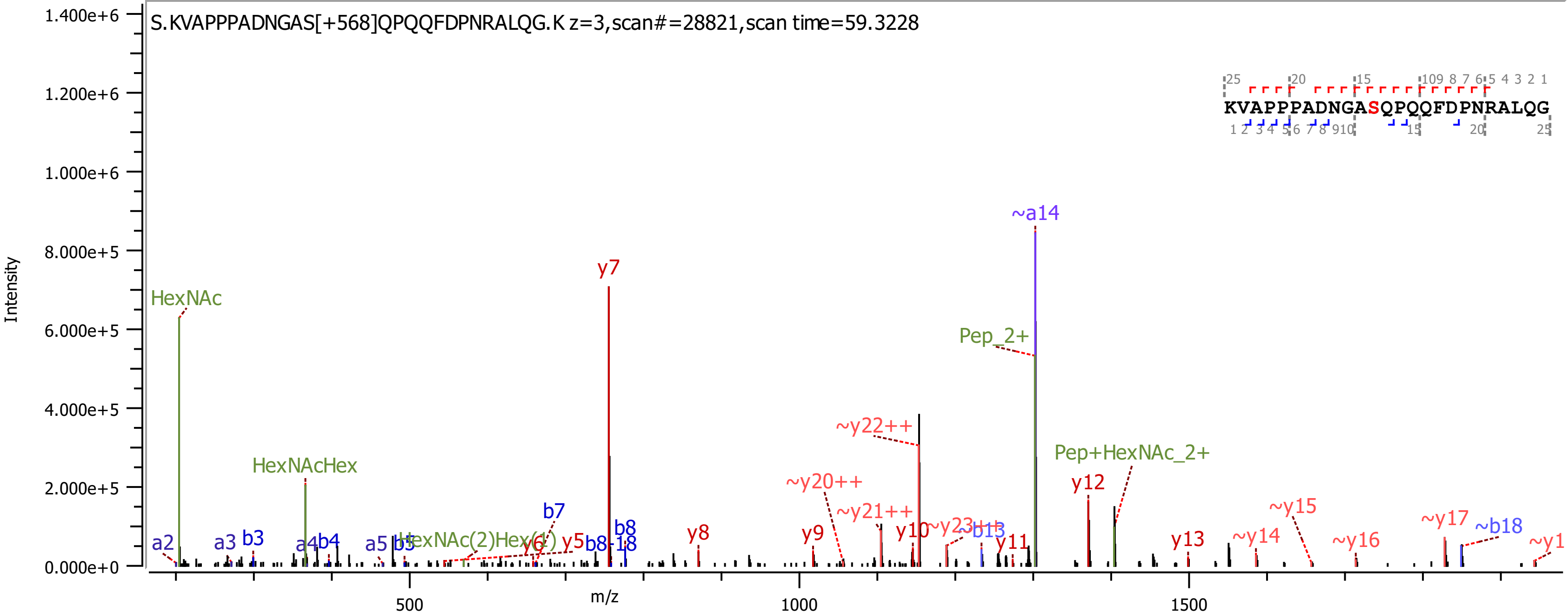
S.KVAPPPADNGAS[+568]QPQQFDPNRALQG.K z=3,scan#=28821,scan time=59.3228
KVAPPPADNGASQPQQFDPNRALQG
Intensity
m/z
1.400e+6
1.200e+6
1.000e+6
8.000e+5
6.000e+5
4.000e+5
2.000e+5
0.000e+0
500
1000
1500
HexNAc
HexNAcHex
HexNAc(2)Hex(1)
Pep_2+
Pep+HexNAc_2+
~a14
y7
y5
y6
y8
y9
y10
y11
y12
y13
~y14
~y15
~y16
~y17
~y20++
~y21++
~y22++
~y23++
a2
a3
a4
a5
b3
b4
b5
b7
b8
b8-18
~b13
~b18

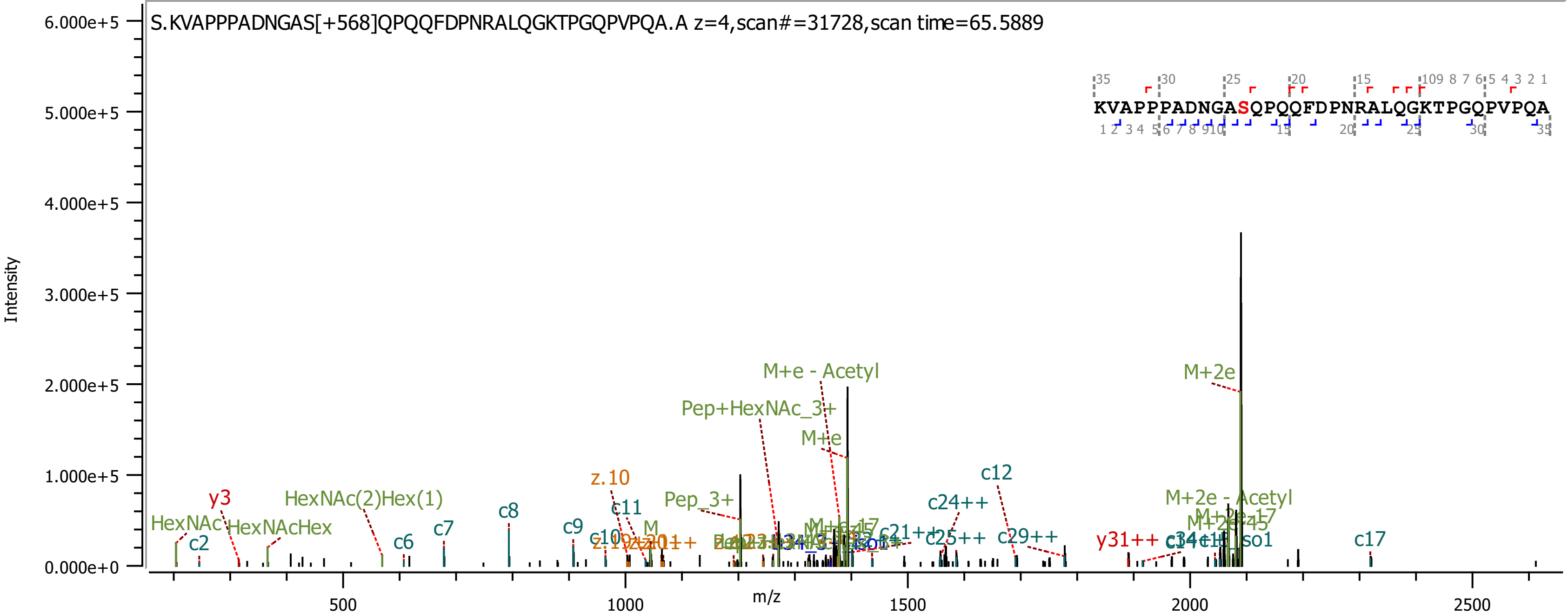

S.KVAPPPADNGAS[+568]QPQQFDPNRALQGKTPGQPVPQA.A z=4,scan#=31728,scan time=65.5889
KVAPPPADNGASQPQQFDPNRALQGKTPGQPVPQA
Intensity
m/z
6.000e+5
5.000e+5
4.000e+5
3.000e+5
2.000e+5
1.000e+5
0.000e+0
500
1000
1500
2000
2500
M+e - Acetyl
Pep+HexNAc_3+
M+e
M+2e
c12
z.10
y3
HexNAc(2)Hex(1)
c8
c11
Pep_3+
c24++
M+2e - Acetyl
HexNAc
HexNAcHex
c2
c6
c7
c9
c10
c21++
c25++
c29++
y31++
c17

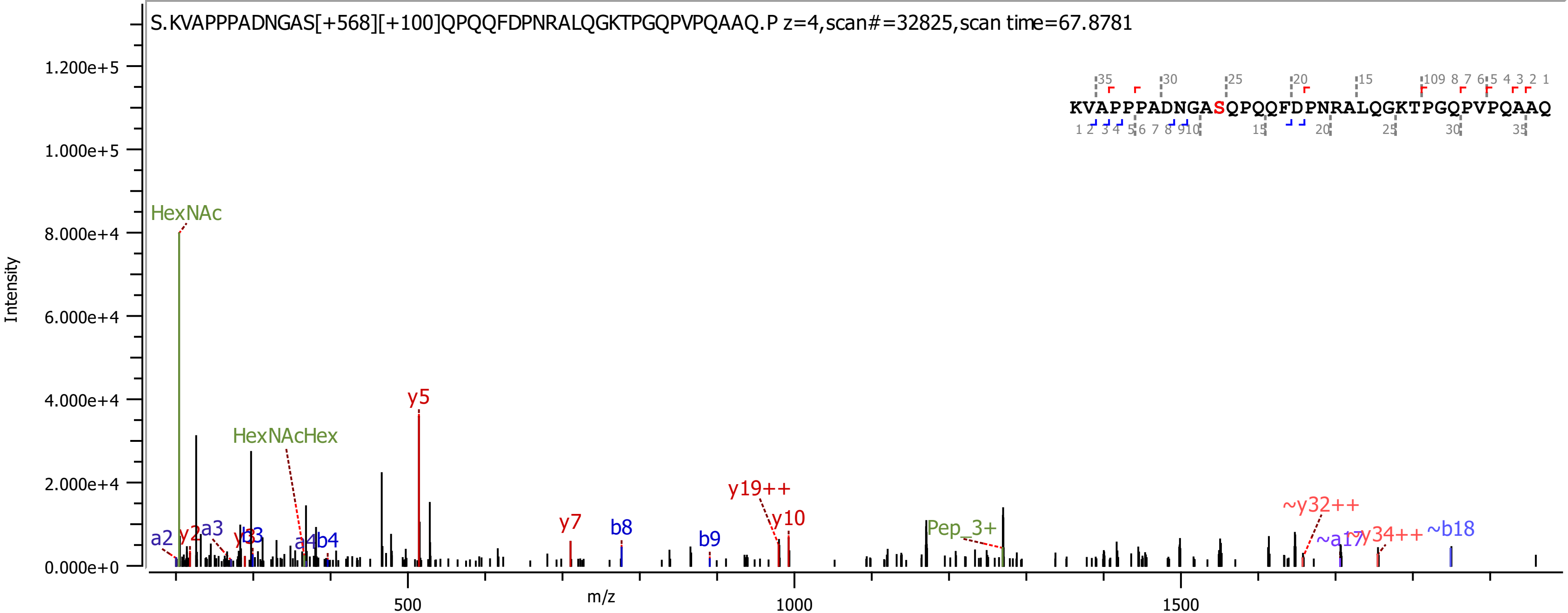
S.KVAPPPADNGAS[+568][+100]QPQQFDPNRALQGKTPGQPVPQAAQ.P z=4,scan#=32825,scan time=67.8781
KVAPPPADNGASQPQQFDPNRALQGKTPGQPVPQAAQ
Intensity
m/z
HexNAc
HexNAcHex
y5
y7
b8
b9
y19++
y10
Pep_3+
~y32++
~a17
~y34++
~b18
a2
y2
a3
y3
b3
a4
b4
0.000e+0
2.000e+4
4.000e+4
6.000e+4
8.000e+4
1.000e+5
1.200e+5
500
1000
1500

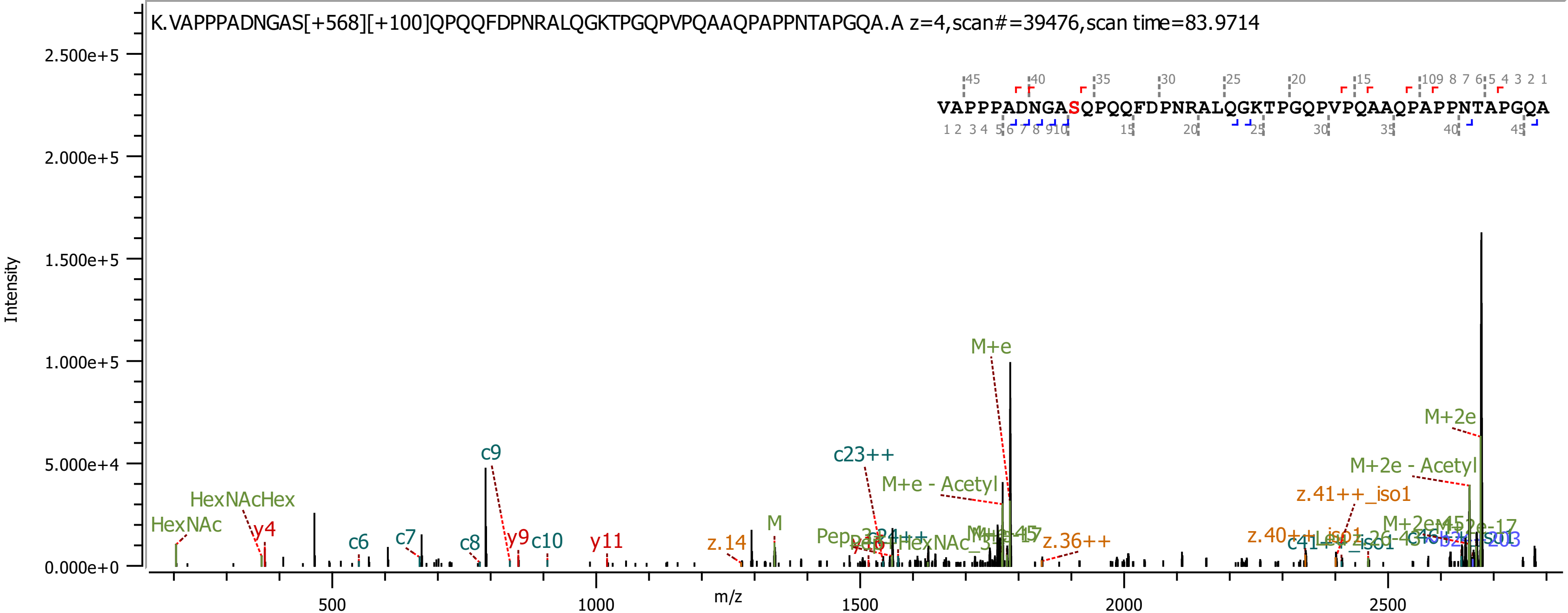
K.VAPPPADNGAS[+568][+100]QPQQFDPNRALQGKTPGQPVPQAAQPAPPNTAPGQA.A z=4,scan#=39476,scan time=83.9714
VAPPPADNGASQPQQFDPNRALQGKTPGQPVPQAAQPAPPNTAPGQA
Intensity
m/z
2.500e+5
2.000e+5
1.500e+5
1.000e+5
5.000e+4
0.000e+0
500
1000
1500
2000
2500
HexNAc
HexNAcHex
y4
c6
c7
c8
c9
y9
c10
y11
z.14
M
c23++
M+e - Acetyl
M+e
z.36++
M+2e
M+2e - Acetyl
z.41++_iso1
z.40++_iso1

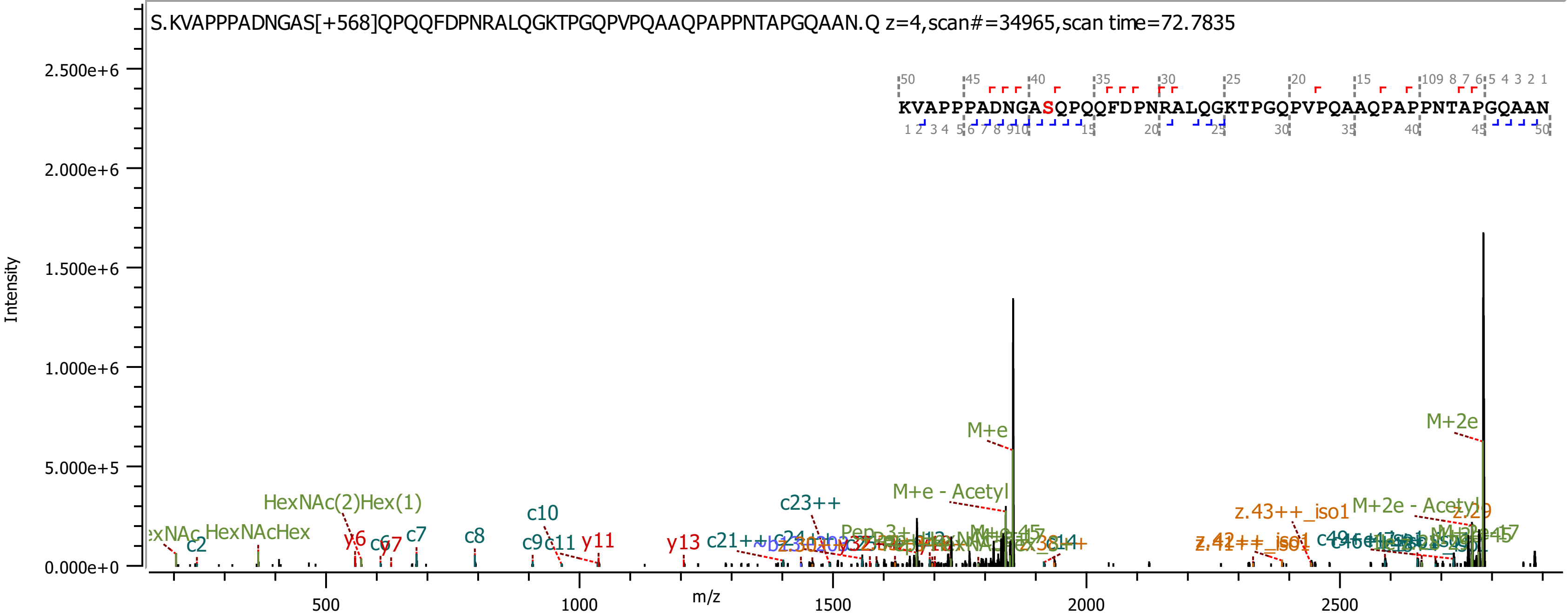

S.KVAPPPADNGAS[+568]QPQQFDPNRALQGKTPGQPVPQAAQPAPPNTAPGQAAN.Q z=4,scan#=34965,scan time=72.7835
KVAPPPADNGASQPQQFDPNRALQGKTPGQPVPQAAQPAPPNTAPGQAAN
Intensity
m/z
2.500e+6
2.000e+6
1.500e+6
1.000e+6
5.000e+5
0.000e+0
500
1000
1500
2000
2500
M+e
M+2e
M+e - Acetyl
M+2e - Acetyl
HexNAc(2)Hex(1)
HexNAcHex
c2
c7
c8
c10
c11
c23++
y6
y11
y13
z.43++_iso1

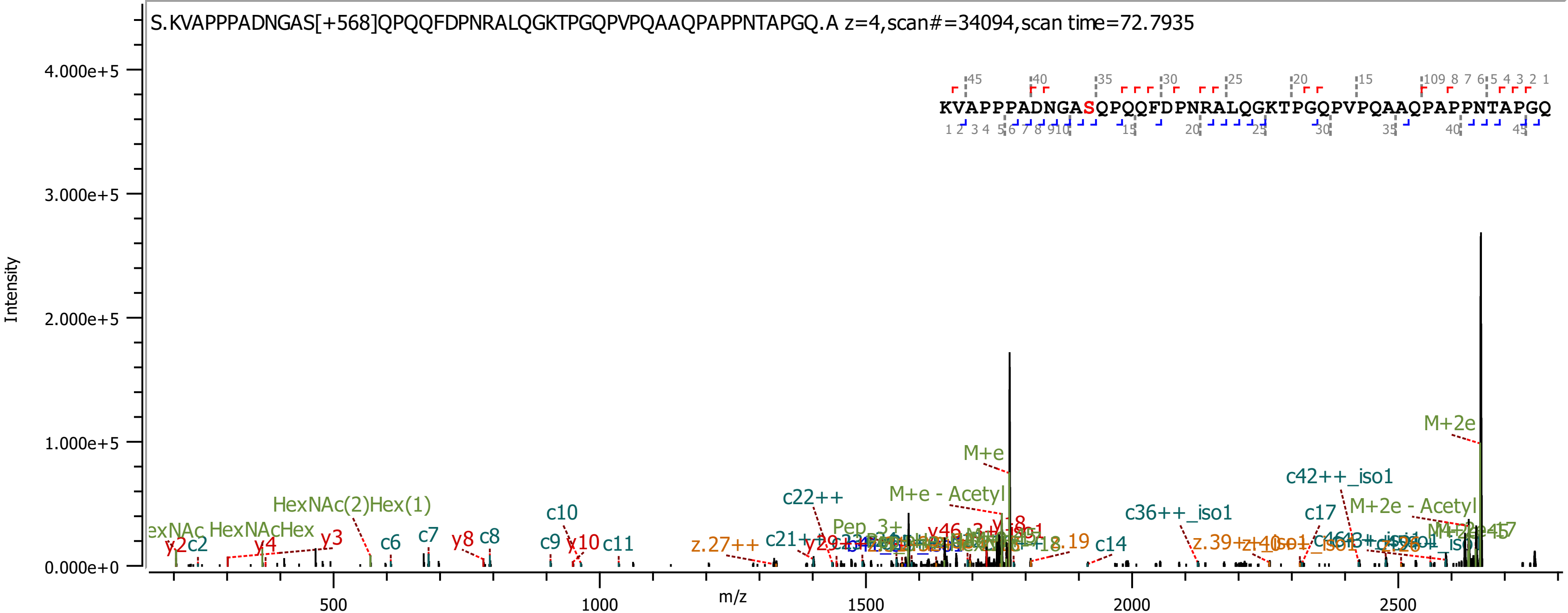
S.KVAPPPADNGAS[+568]QPQQFDPNRALQGKTPGQPVPQAAQPAPPNTAPGQ.A z=4,scan#=34094,scan time=72.7935
Intensity
4.000e+5
3.000e+5
2.000e+5
1.000e+5
0.000e+0
m/z
500
1000
1500
2000
2500
KVAPPPADNGASQPQQFDPNRALQGKTPGQPVPQAAQPAPPNTAPGQ
M+2e
M+e
M+e - Acetyl
M+2e - Acetyl
c42++_iso1
c36++_iso1
c22++
c17
c14
c11
c10
c9
c8
c7
c6
c2
y2
y3
y4
y8
y10
z.27++
HexNAc(2)Hex(1)
HexNAcHex
Pep_3+

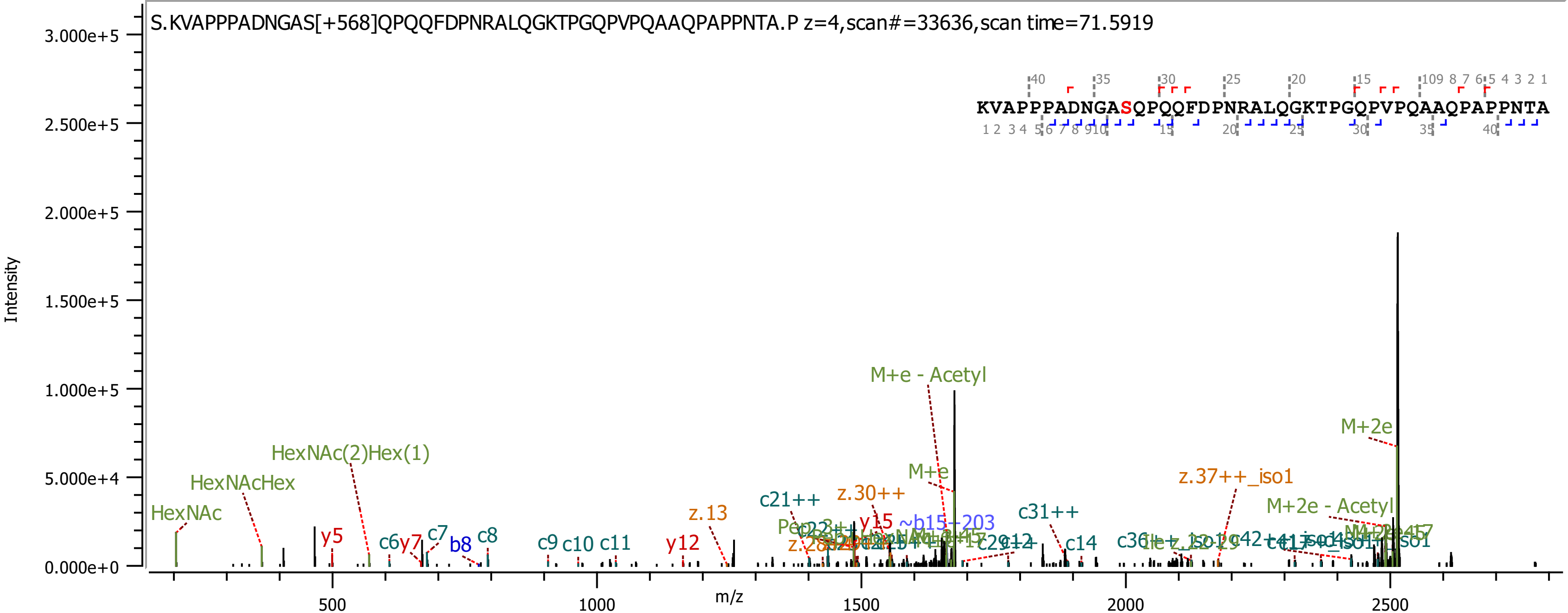

S.KVAPPPADNGAS[+568]QPQQFDPNRALQGKTPGQPVPQAAQPAPPNTA.P z=4,scan#=33636,scan time=71.5919
KVAPPPADNGASQPQQFDPNRALQGKTPGQPVPQAAQPAPPNTA
Intensity
m/z
3.000e+5
2.500e+5
2.000e+5
1.500e+5
1.000e+5
5.000e+4
0.000e+0
500
1000
1500
2000
2500
HexNAc
HexNAcHex
HexNAc(2)Hex(1)
y5
c6
y7
c7
b8
c8
c9
c10
c11
y12
z.13
c21++
z.30++
y15
~b15--203
M+e - Acetyl
M+e
c31++
c14
z.37++_iso1
M+2e - Acetyl
M+2e

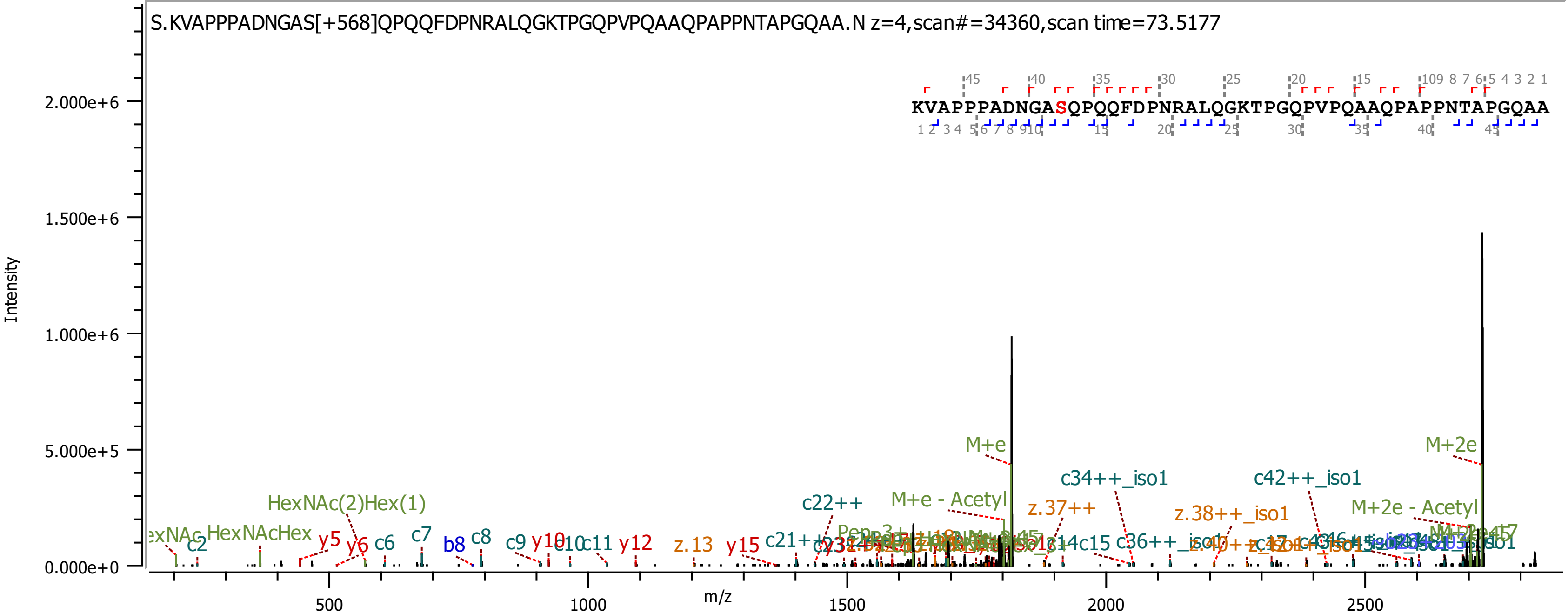
S.KVAPPPADNGAS[+568]QPQQFDPNRALQGKTPGQPVPQAAQPAPPNTAPGQAA.N z=4,scan#=34360,scan time=73.5177
KVAPPPADNGASQPQQFDPNRALQGKTPGQPVPQAAQPAPPNTAPGQAA
Intensity
2.000e+6
1.500e+6
1.000e+6
5.000e+5
0.000e+0
m/z
500
1000
1500
2000
2500
M+e
M+2e
M+e - Acetyl
M+2e - Acetyl
c34++_iso1
c42++_iso1
z.37++
z.38++_iso1
c22++
HexNAc(2)Hex(1)
HexNAcHex
y5
y6
c2
c6
c7
b8
c8
c9
y10
c10
c11
y12
z.13
y15
c14
c15
c36++_iso1

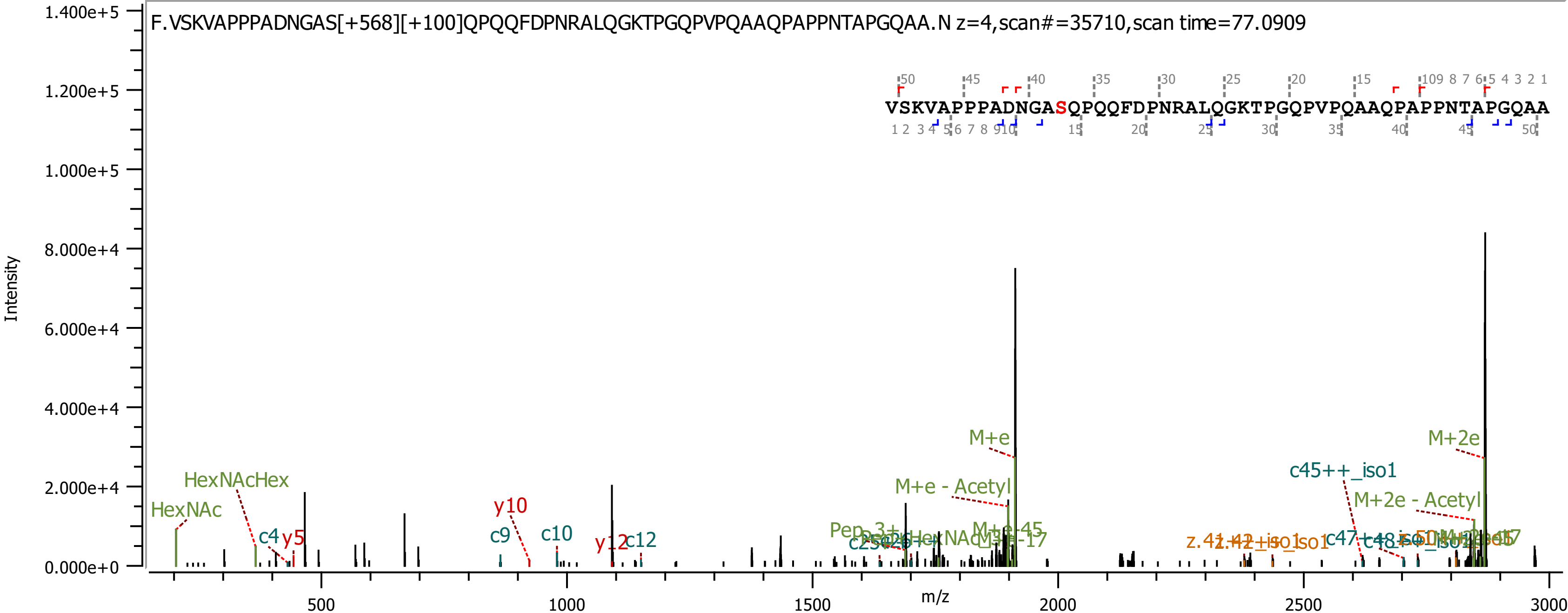
F.VSKVAPPPADNGAS[+568][+100]QPQQFDPNRALQGKTPGQPVPQAAQPAPPNTAPGQAA.N z=4,scan#=35710,scan time=77.0909
VSKVAPPPADNGASQPQQFDPNRALQGKTPGQPVPQAAQPAPPNTAPGQAA
Intensity
m/z
HexNAc
HexNAcHex
c4
y5
y10
c9
c10
y12
c12
M+e
M+e - Acetyl
M+2e
c45++_iso1
M+2e - Acetyl
Pep 3+
M+e-45
M+e-17
c47++

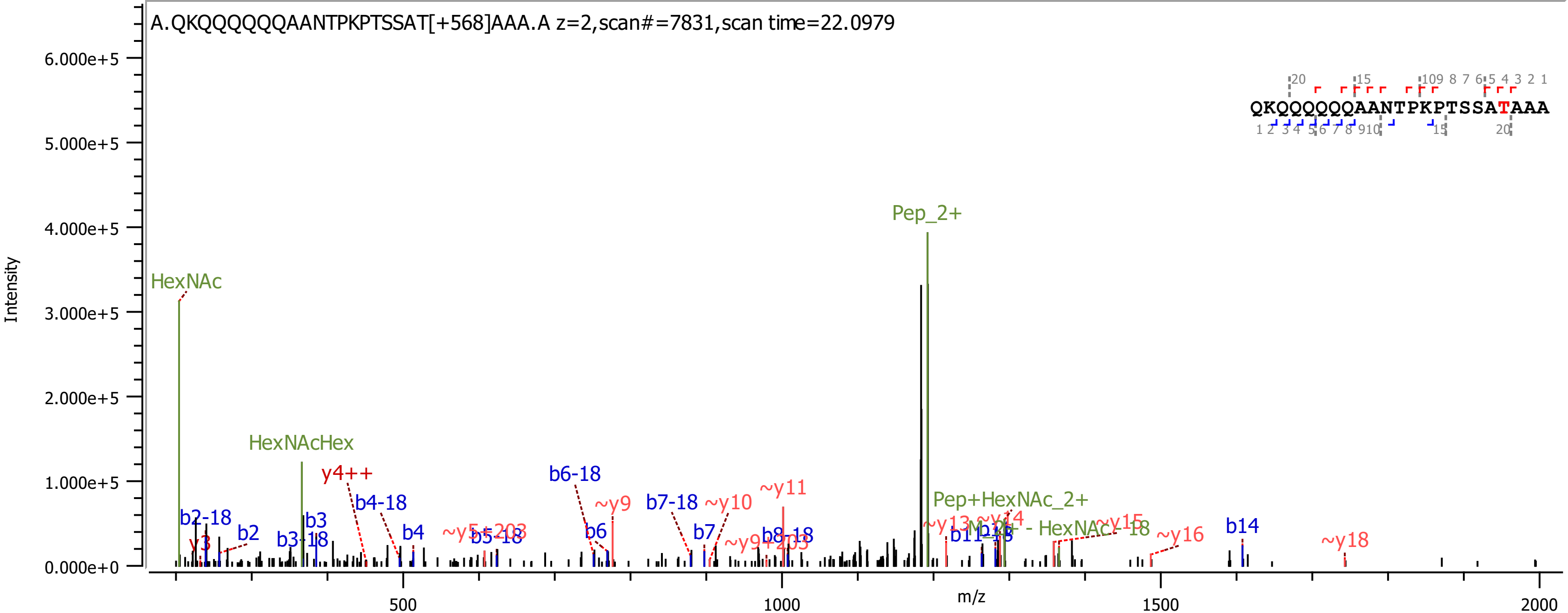
A.QKQQQQQQAANTPKPTSSAT[+568]AAA.A z=2,scan#=7831,scan time=22.0979
QKQQQQQQAANTPKPTSSATAAA
Intensity
m/z
6.000e+5
5.000e+5
4.000e+5
3.000e+5
2.000e+5
1.000e+5
0.000e+0
500
1000
1500
2000
HexNAc
HexNAcHex
Pep_2+
Pep+HexNAc_2+
y4++
b2-18
b2
y3
b3-18
b3
b4-18
b4
~y5+203
b5-18
b6-18
b6
~y9
b7-18
b7
~y10
~y9+203
~y11
b8-18
~y13
~y14
b11-18
b12
M-2H - HexNAc-18
~y15
~y16
b14
~y18

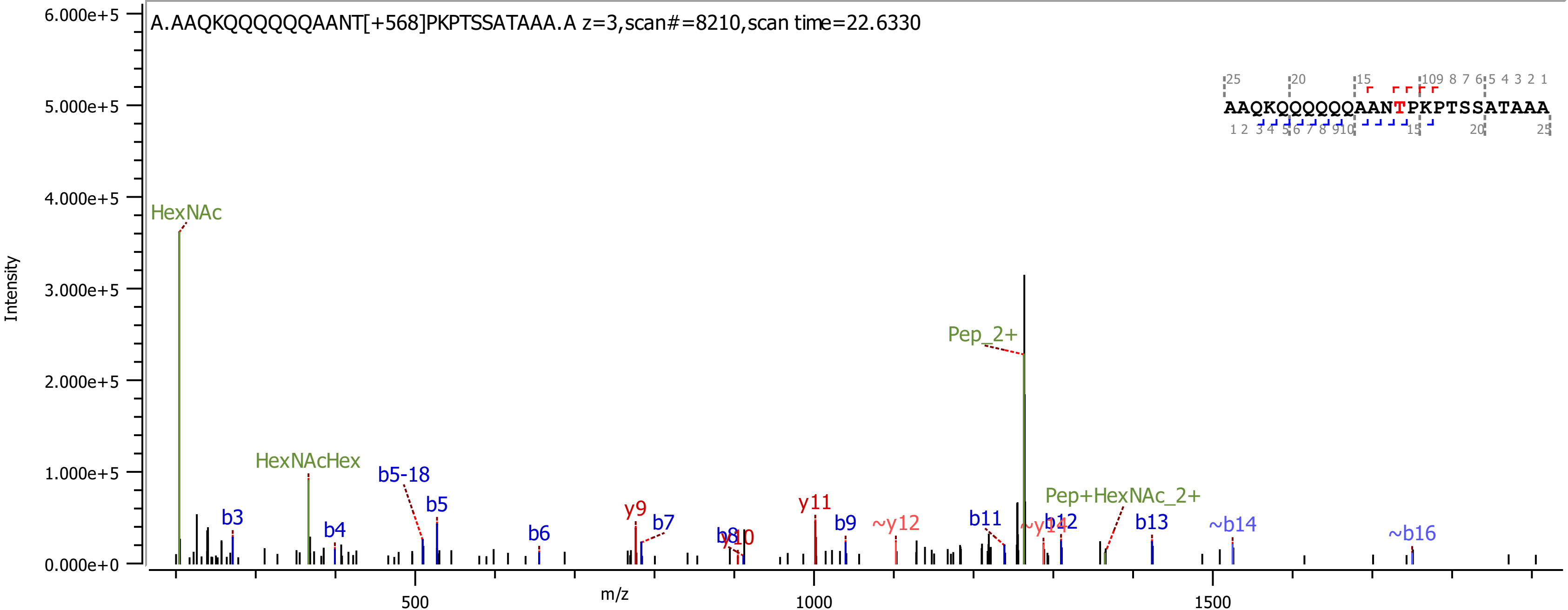
A.AAQKQQQQQQAANT[+568]PKPTSSATAAA.A z=3,scan#=8210,scan time=22.6330
AAQKQQQQQQAANTPKPTSSATAAA
Intensity
6.000e+5
5.000e+5
4.000e+5
3.000e+5
2.000e+5
1.000e+5
0.000e+0
m/z
500
1000
1500
HexNAc
HexNAcHex
b3
b4
b5-18
b5
b6
y9
b7
b8
y10
y11
b9
~y12
b11
~y14
b12
Pep_2+
Pep+HexNAc_2+
b13
~b14
~b16

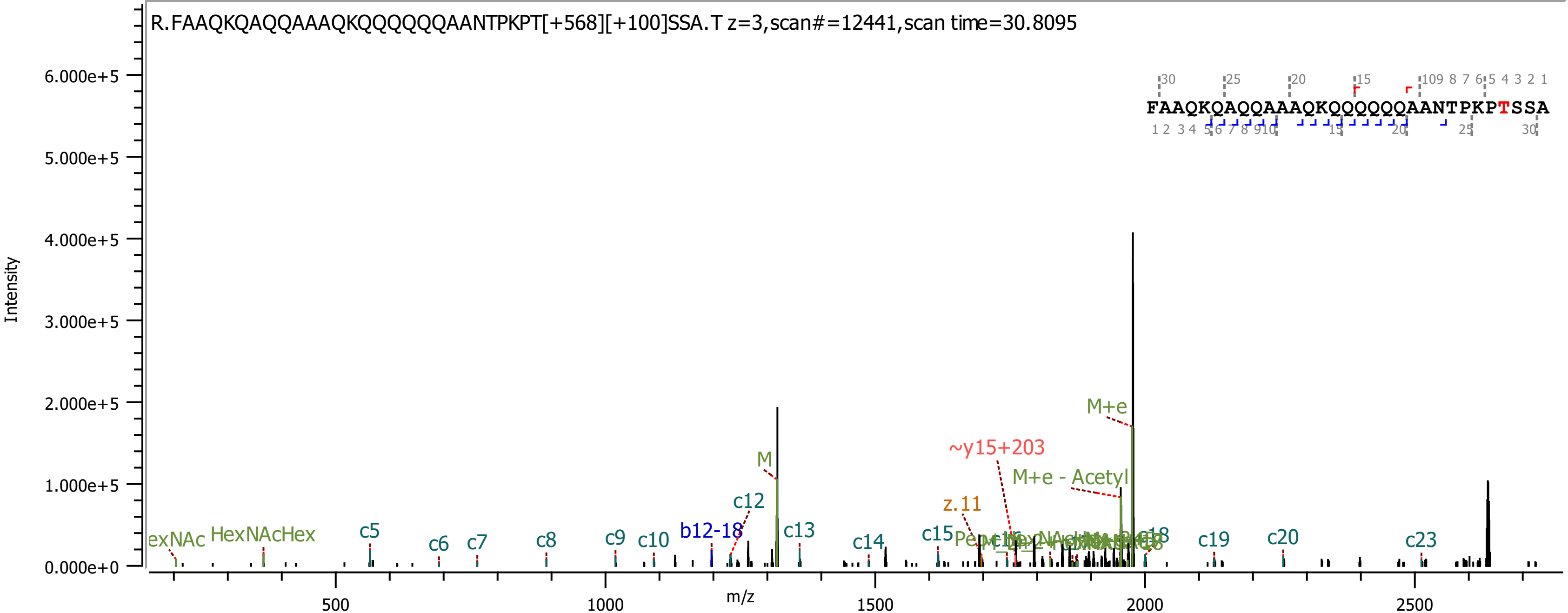
R.FAAQKQAQQAAAQKQQQQQQAANTPKPT[+568][+100]SSA.T z=3,scan#=12441,scan time=30.8095
FAAQKQAQQAAAQKQQQQQQAANTPKPTSSA
Intensity
6.000e+5
5.000e+5
4.000e+5
3.000e+5
2.000e+5
1.000e+5
0.000e+0
m/z
500
1000
1500
2000
2500
M+e
M
~y15+203
M+e - Acetyl
z.11
c12
b12-18
c5
c6
c7
c8
c9
c10
c13
c14
c15
c19
c20
c23
HexNAcHex

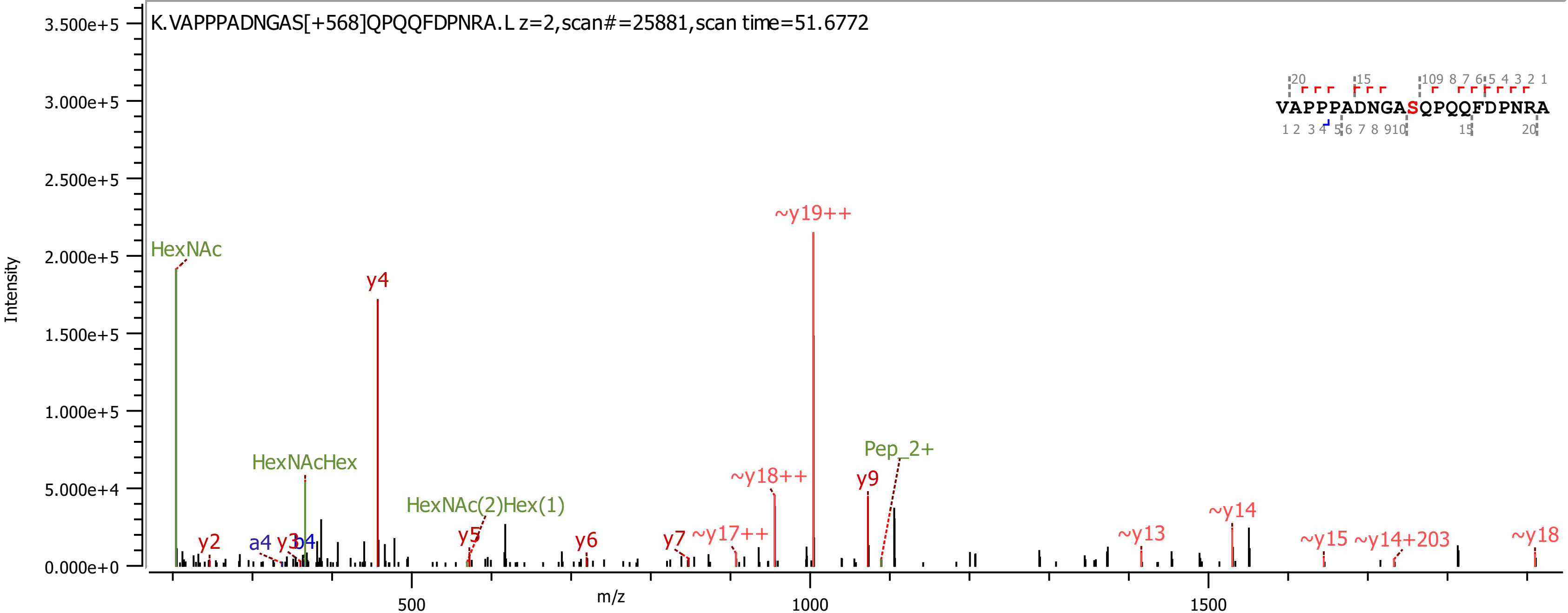
K.VAPPPADNGAS[+568]QPQQFDPNRA.L z=2,scan#=25881,scan time=51.6772
VAPPPADNGASQPQQFDPNRA
Intensity
m/z
HexNAc
HexNAcHex
HexNAc(2)Hex(1)
Pep_2+
~y19++
~y18++
~y17++
y2
a4
y3
b4
y4
y5
y6
y7
y9
~y13
~y14
~y15
~y14+203
~y18
3.500e+5
3.000e+5
2.500e+5
2.000e+5
1.500e+5
1.000e+5
5.000e+4
0.000e+0
500
1000
1500

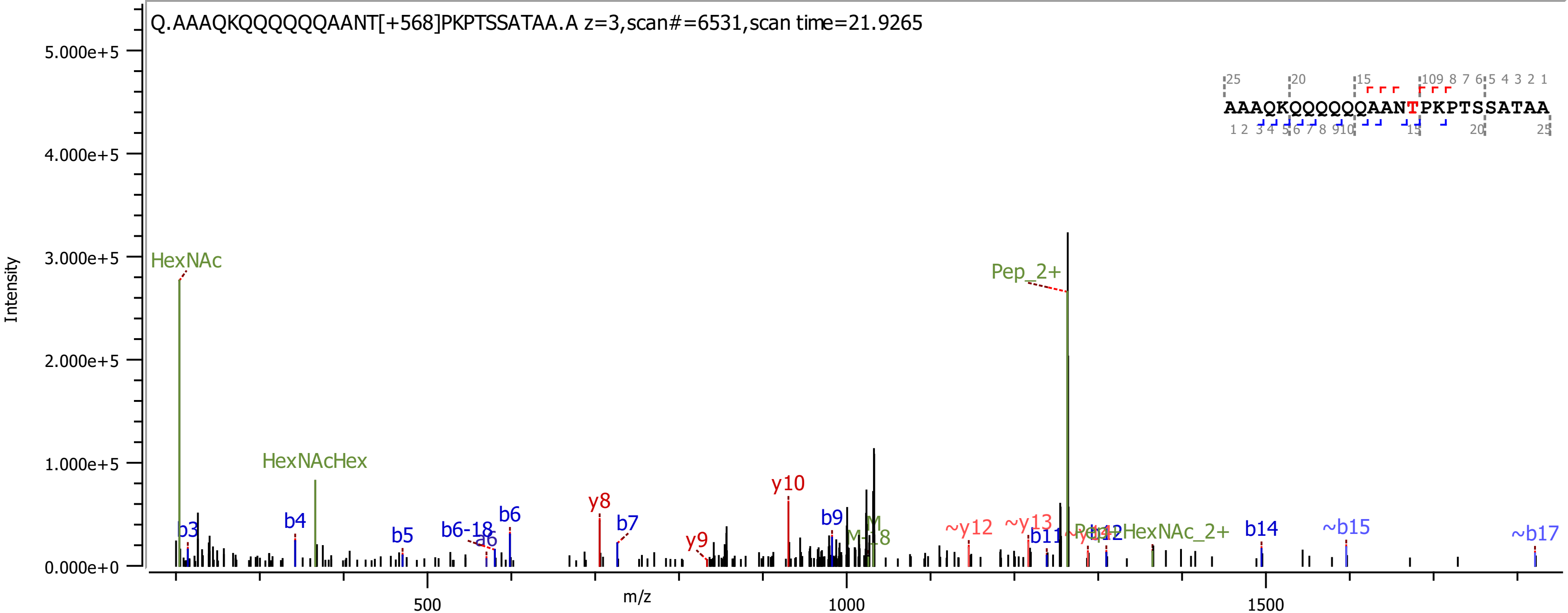
Q.AAAQKQQQQQQAANT[+568]PKPTSSATAA.A z=3,scan#=6531,scan time=21.9265
AAAQKQQQQQQAANTPKPTSSATAA
Intensity
m/z
5.000e+5
4.000e+5
3.000e+5
2.000e+5
1.000e+5
0.000e+0
500
1000
1500
HexNAc
HexNAcHex
Pep_2+
b3
b4
b5
b6-18
a6
b6
y8
b7
y9
y10
b9
M-18
~y12
~y13
b11
b14
~b15
~b17

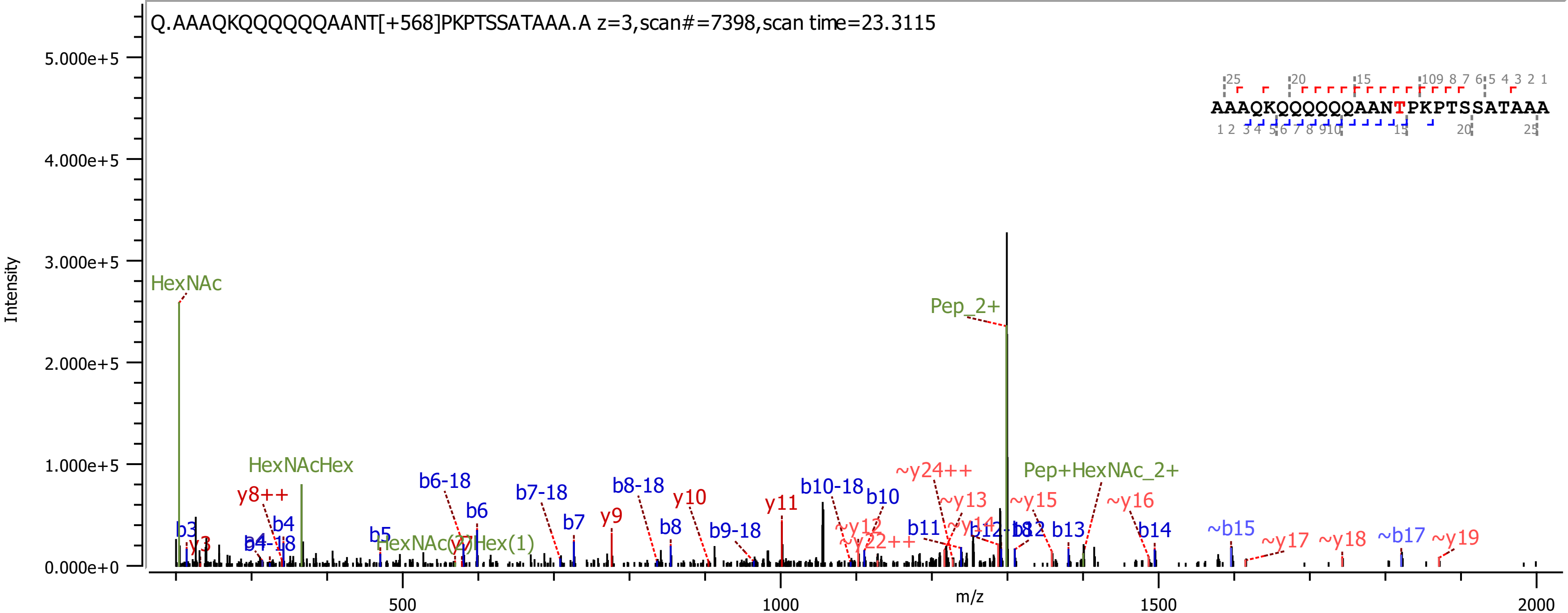

Q.AAAQKQQQQQQAANT[+568]PKPTSSATAAA.A z=3,scan#=7398,scan time=23.3115
AAAQKQQQQQQAANTPKPTSSATAAA
Intensity
m/z
5.000e+5
4.000e+5
3.000e+5
2.000e+5
1.000e+5
0.000e+0
500
1000
1500
2000
HexNAc
HexNAcHex
HexNAc(2)Hex(1)
Pep_2+
Pep+HexNAc_2+
b3
y3
b4
b4-18
y8++
b5
b6-18
b6
b7-18
b7
y9
b8-18
b8
y10
b9-18
y11
b10-18
b10
~y12
~y22++
~y24++
~y13
b11
~y14
b12-18
b12
~y15
b13
~y16
b14
~b15
~y17
~y18
~b17
~y19

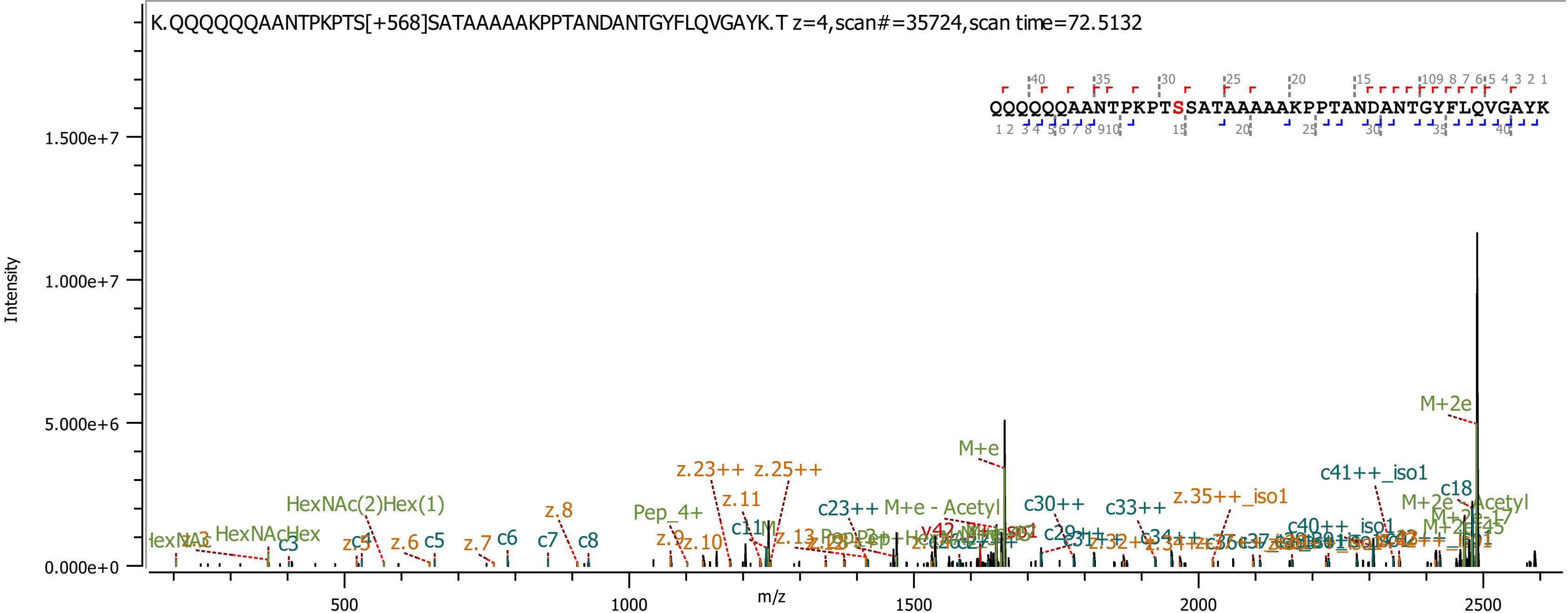
K.QQQQQQAANTPKPTS[+568]SATAAAAAKPPTANDANTGYFLQVGAYK.T z=4,scan#=35724,scan time=72.5132
QQQQQQAANTPKPTSSATAAAAAKPPTANDANTGYFLQVGAYK
Intensity
m/z
0.000e+0
5.000e+6
1.000e+7
1.500e+7
500
1000
1500
2000
2500
M+2e
M+e
c41++_iso1
c18
M+2e - Acetyl
z.35++_iso1
c33++
c30++
c23++
M+e - Acetyl
z.23++
z.25++
z.11
HexNAc(2)Hex(1)
z.8
Pep_4+
HexNAcHex
c3
c4
z.5
z.6
c5
z.7
c6
c7
c8
z.9
z.10
c40++_iso1

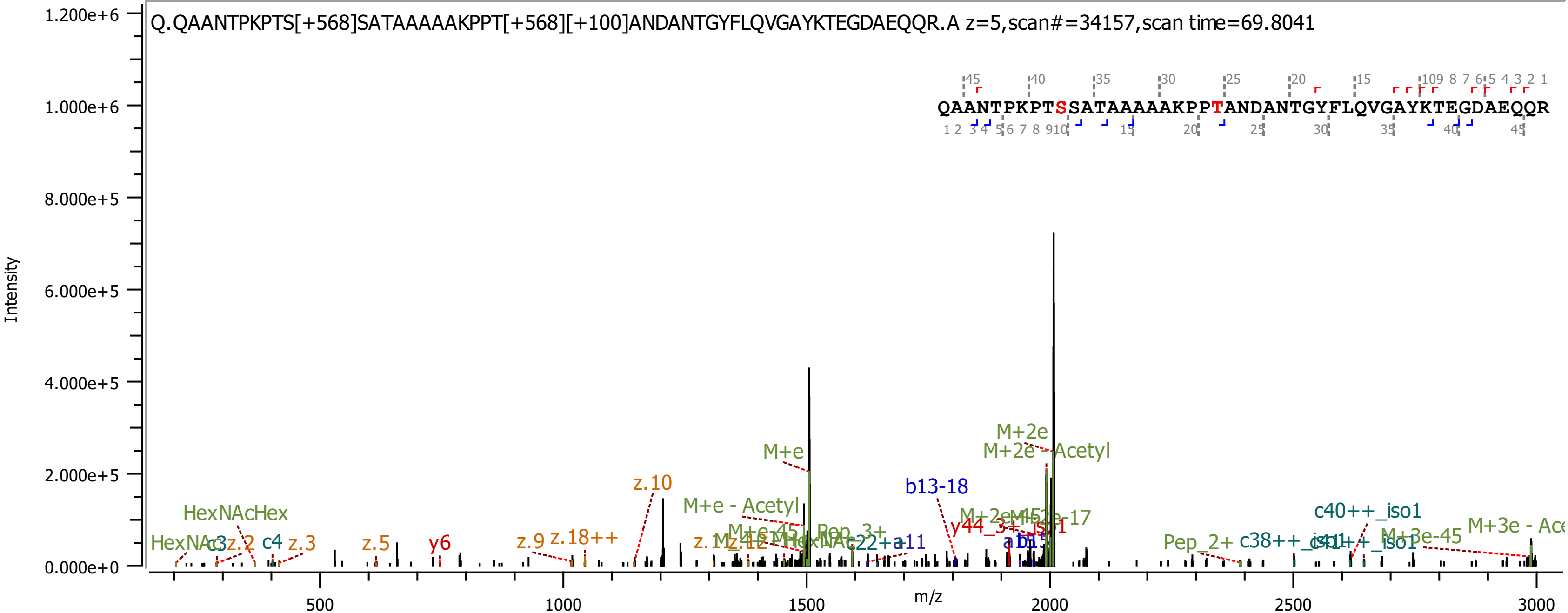
Q.QAANTPKPTS[+568]SATAAAAAKPPT[+568][+100]ANDANTGYFLQVGAYKTEGDAEQQR.A z=5,scan#=34157,scan time=69.8041
QAANTPKPTSSATAAAAAKPPTANDANTGYFLQVGAYKTEGDAEQQR
Intensity
m/z
M+2e
M+2e - Acetyl
M+e
b13-18
z.10
M+e - Acetyl
HexNAcHex
HexNAc
c3
z.2
c4
z.3
z.5
y6
z.9
z.18++
Pep_3+
c22+
a11
y44_3+
Pep_2+
c40++_iso1
c38++_iso1
M+3e-45
M+3e - Ace

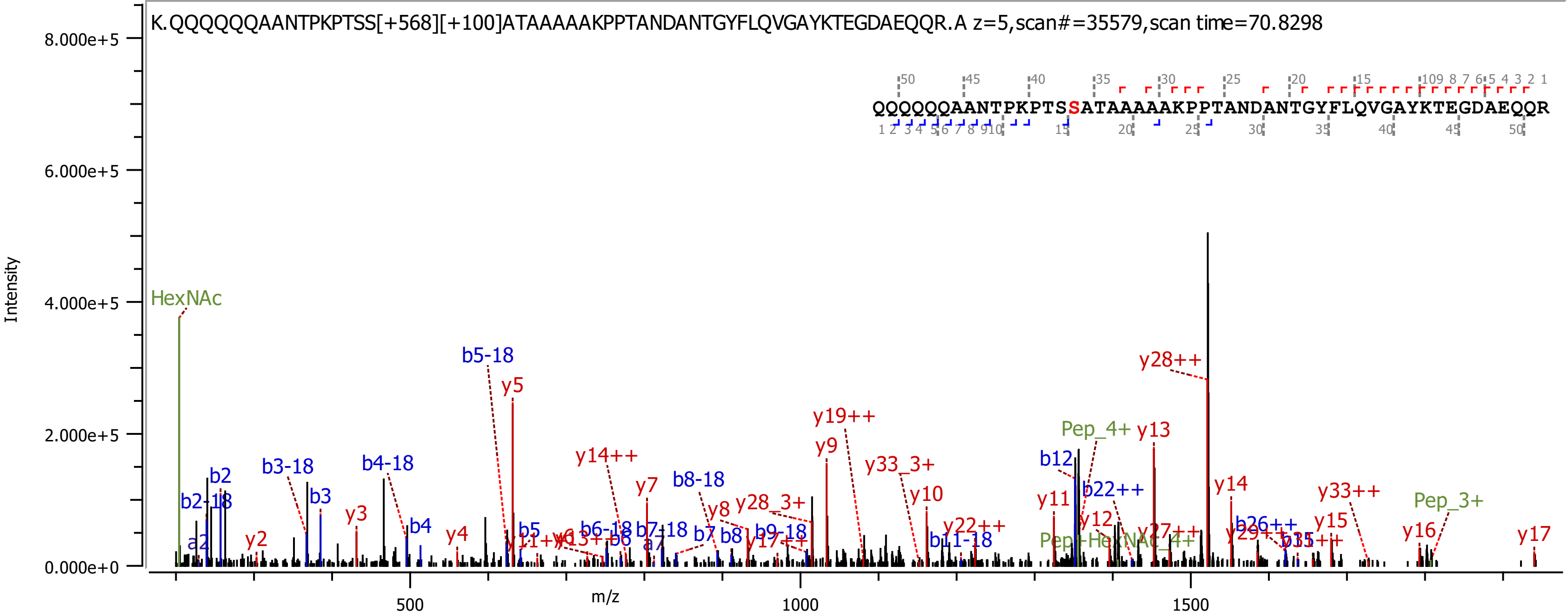
K.QQQQQQAANTPKPTSS[+568][+100]ATAAAAAKPPTANDANTGYFLQVGAYKTEGDAEQQR.A z=5,scan#=35579,scan time=70.8298
QQQQQQAANTPKPTSSATAAAAAKPPTANDANTGYFLQVGAYKTEGDAEQQR
Intensity
m/z
8.000e+5
6.000e+5
4.000e+5
2.000e+5
0.000e+0
500
1000
1500
HexNAc
b5-18
y5
y28++
y19++
y9
Pep_4+
y13
y14++
b3-18
b4-18
b2
b2-18
b3
y3
b4
y4
y7
b8-18
y8
y28_3+
y33_3+
y10
b12
b22++
y11
y12
y14
y33++
Pep_3+
y16
y17
y15
b26++
y2
b5
b6-18
b7-18
b7
b8
b9-18
b11-18
y22++
Pep-HexNAc_4+
y27++

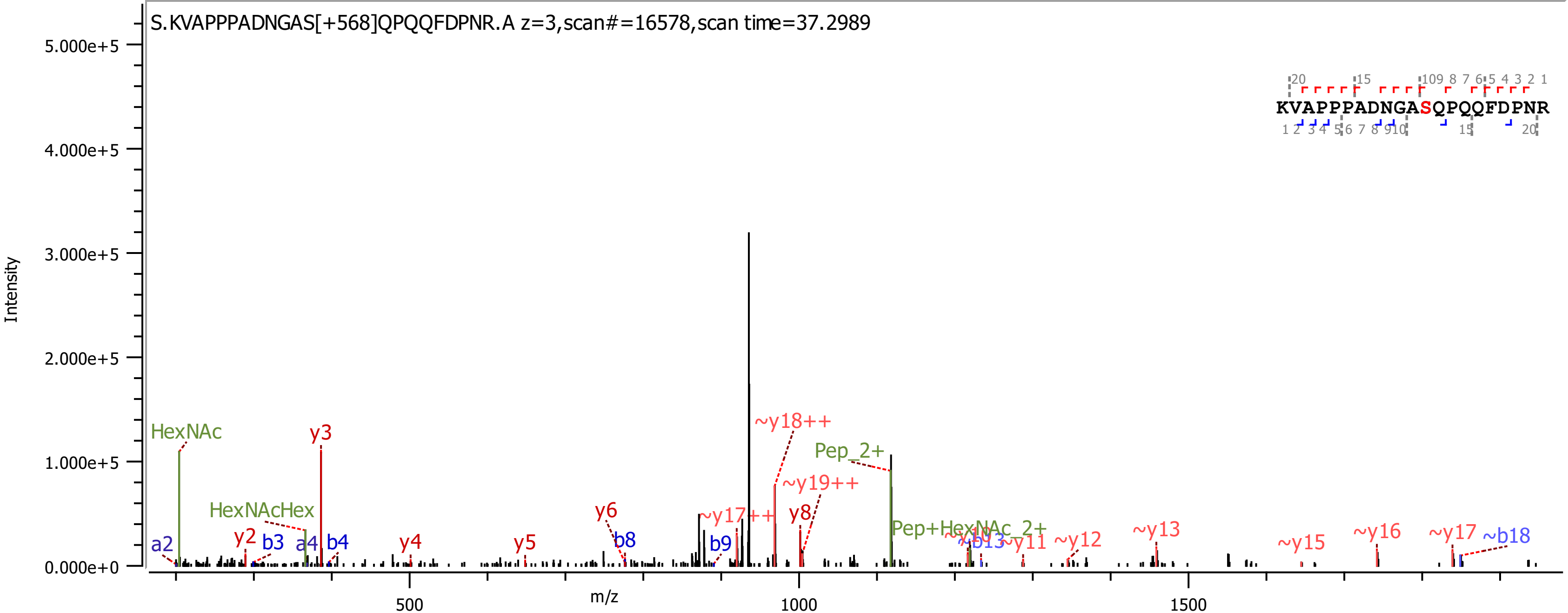
S.KVAPPPADNGAS[+568]QPQQFDPNR.A z=3,scan#=16578,scan time=37.2989
KVAPPPADNGASQPQQFDPNR
Intensity
m/z
5.000e+5
4.000e+5
3.000e+5
2.000e+5
1.000e+5
0.000e+0
500
1000
1500
HexNAc
HexNAcHex
a2
y2
b3
a4
b4
y3
y4
y5
y6
b8
b9
~y17++
~y18++
~y19++
y8
Pep_2+
Pep+HexNAc_2+
~b13
~y11
~y12
~y13
~y15
~y16
~y17
~b18

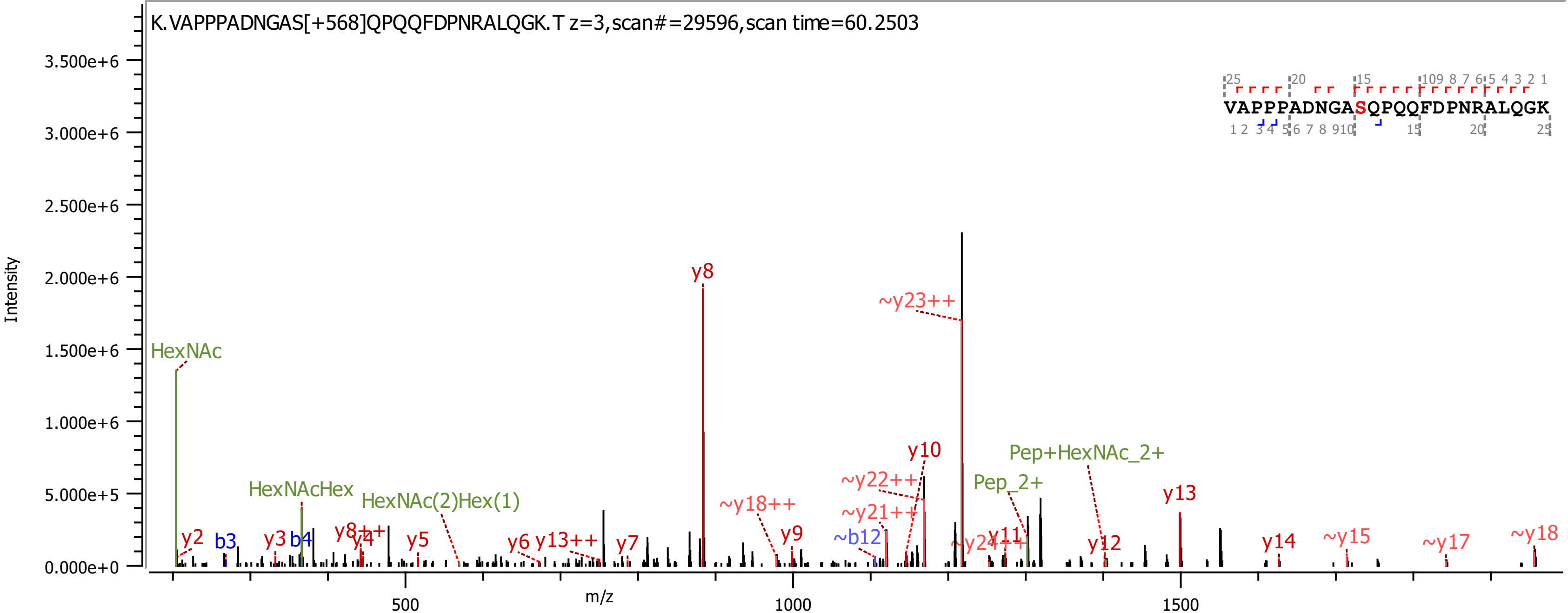

K.VAPPPADNGAS[+568]QPQQFDPNRALQGK.T z=3,scan#=29596,scan time=60.2503
VAPPPADNGASQPQQFDPNRALQGK
Intensity
m/z
HexNAc
HexNAcHex
HexNAc(2)Hex(1)
Pep+HexNAc_2+
Pep_2+
y8
~y23++
y10
~y22++
~y21++
~y18++
y9
~b12
y2
b3
y3
b4
y8++
y4
y5
y6
y13++
y7
~y24++
y11
y12
y13
y14
~y15
~y17
~y18

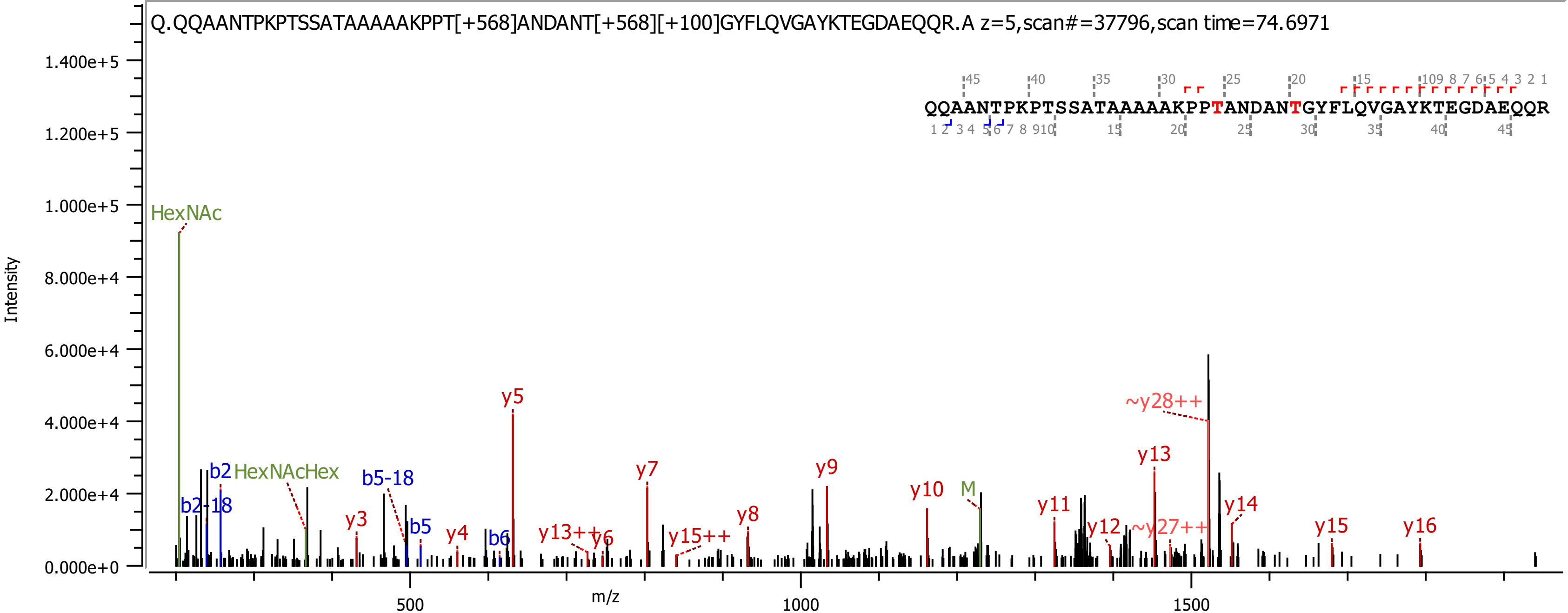

Q.QQAANTPKPTSSATAAAAAKPPT[+568]ANDANT[+568][+100]GYFLQVGAYKTEGDAEQQR.A z=5,scan#=37796,scan time=74.6971
QQAANTPKPTSSATAAAAAKPPTANDANTGYFLQVGAYKTEGDAEQQR
Intensity
m/z
HexNAc
HexNAcHex
b2
b2-18
b5-18
b5
b6
y3
y4
y5
y13++
y6
y7
y15++
y8
y9
y10
M
y11
y12
~y27++
y13
~y28++
y14
y15
y16

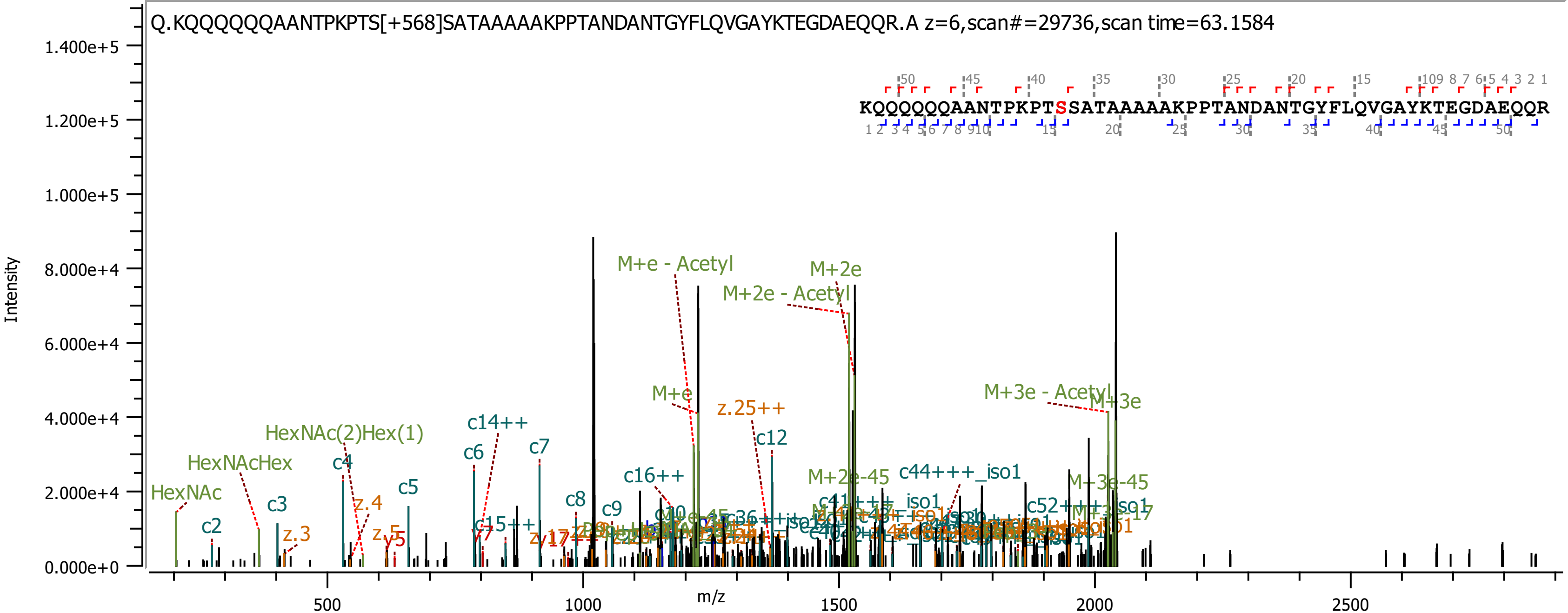

Q.KQQQQQQAANTPKPTS[+568]SATAAAAAKPPTANDANTGYFLQVGAYKTEGDAEQQR.A z=6,scan#=29736,scan time=63.1584
KQQQQQQAANTPKPTSSATAAAAAKPPTANDANTGYFLQVGAYKTEGDAEQQR
Intensity
m/z
M+e - Acetyl
M+2e
M+2e - Acetyl
M+e
z.25++
c12
M+3e - Acetyl
M+3e
HexNAc(2)Hex(1)
HexNAcHex
HexNAc
c14++
c6
c7
c4
c5
c3
c2
z.4
z.3
z.5
y5
y7
c8
c9
c16++
c15++
M+2e-45
c44+++_iso1
M+3e-45
c52+++_iso1

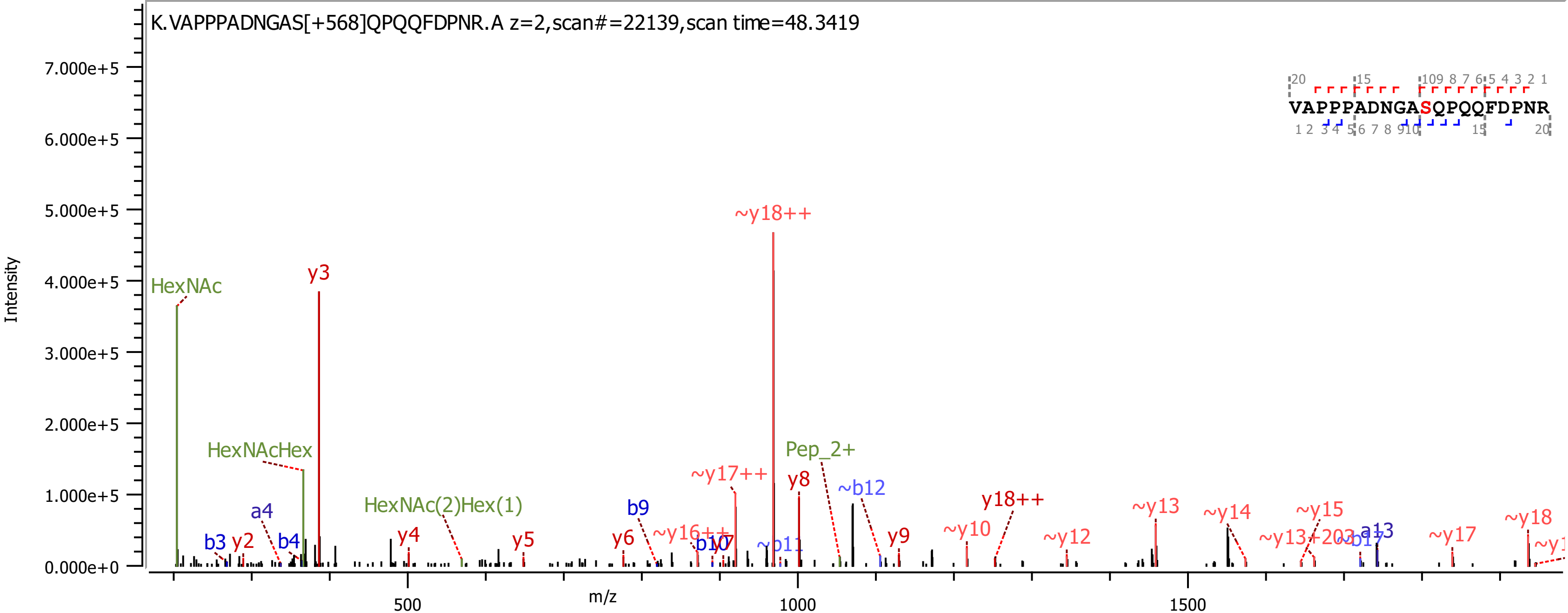
K.VAPPPADNGAS[+568]QPQQFDPNR.A z=2,scan#=22139,scan time=48.3419
VAPPPADNGASQPQQFDPNR
Intensity
m/z
7.000e+5
6.000e+5
5.000e+5
4.000e+5
3.000e+5
2.000e+5
1.000e+5
0.000e+0
500
1000
1500
HexNAc
y3
HexNAcHex
HexNAc(2)Hex(1)
~y18++
Pep_2+
~y17++
y8
~b12
y18++
b3
y2
a4
b4
y4
y5
y6
b9
~y16++
b10
y7
~b11
y9
~y10
~y12
~y13
~y14
~y15
~y13+203
a13
~b17
~y17
~y18

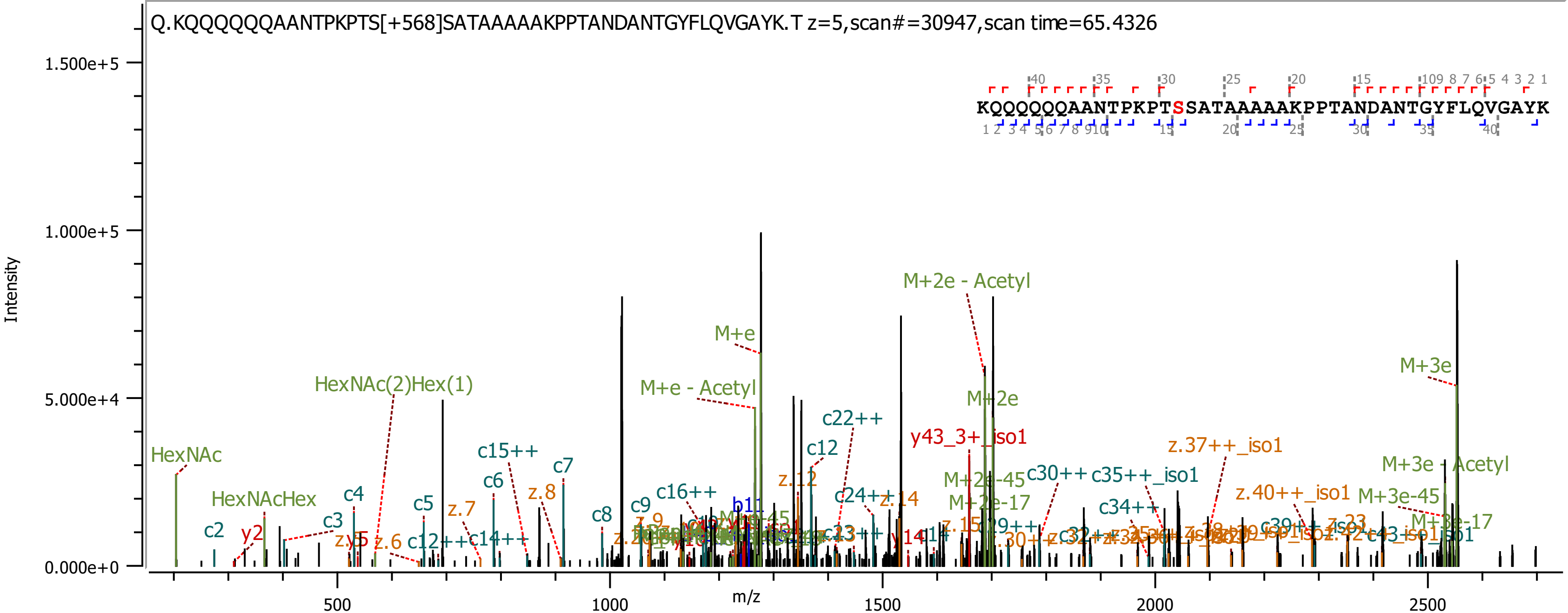

Q.KQQQQQQAANTPKPTS[+568]SATAAAAAKPPTANDANTGYFLQVGAYK.T z=5,scan#=30947,scan time=65.4326
KQQQQQQAANTPKPTSSATAAAAAKPPTANDANTGYFLQVGAYK
Intensity
m/z
1.500e+5
1.000e+5
5.000e+4
0.000e+0
500
1000
1500
2000
2500
HexNAc
HexNAcHex
HexNAc(2)Hex(1)
M+e
M+e - Acetyl
M+2e - Acetyl
M+2e
M+3e
M+3e - Acetyl
M+3e-45
M+3e-17
M+2e-45
M+2e-17
y43_3+_iso1
z.37++_iso1
z.40++_iso1
c22++
c12
c15++
c6
c7
c4
c5
c3
c2
y2
z.7
z.8
z.6
c8
c9
c16++
c12++
c14++
c24++
z.14
z.12
c30++
c35++_iso1
c34++
y14

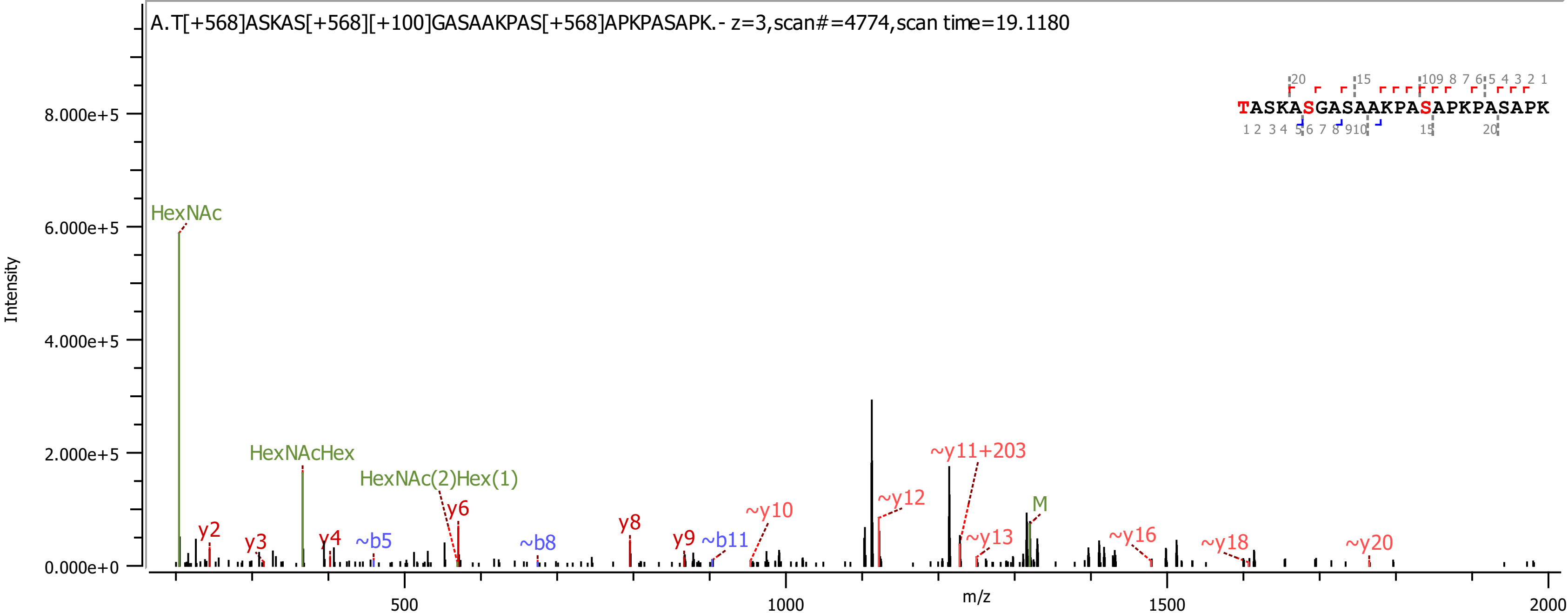
A.T[+568]ASKAS[+568][+100]GASAAKPAS[+568]APKPASAPK.- z=3,scan#=4774,scan time=19.1180
TASKASGASAAKPASAPKPASAPK
Intensity
m/z
HexNAc
HexNAcHex
HexNAc(2)Hex(1)
y2
y3
y4
~b5
y6
~b8
y8
y9
~b11
~y10
~y12
~y11+203
~y13
M
~y16
~y18
~y20

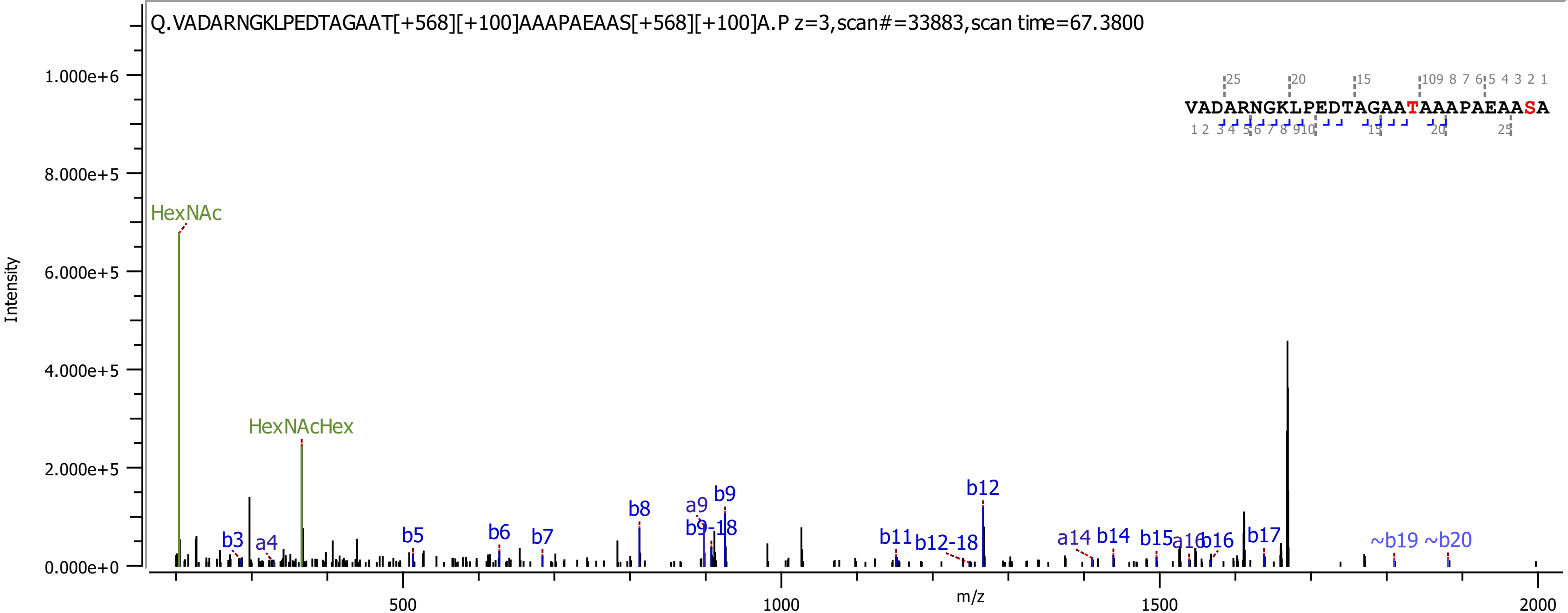

Q.VADARNGKLPEDTAGAAT[+568][+100]AAAPAEAAS[+568][+100]A.P z=3,scan#=33883,scan time=67.3800
VADARNGKLPEDTAGAATAAAPAEAASA
Intensity
m/z
HexNAc
HexNAcHex
b3
a4
b5
b6
b7
b8
a9
b9-18
b9
b11
b12-18
b12
a14
b14
b15
a16
b16
b17
~b19 ~b20

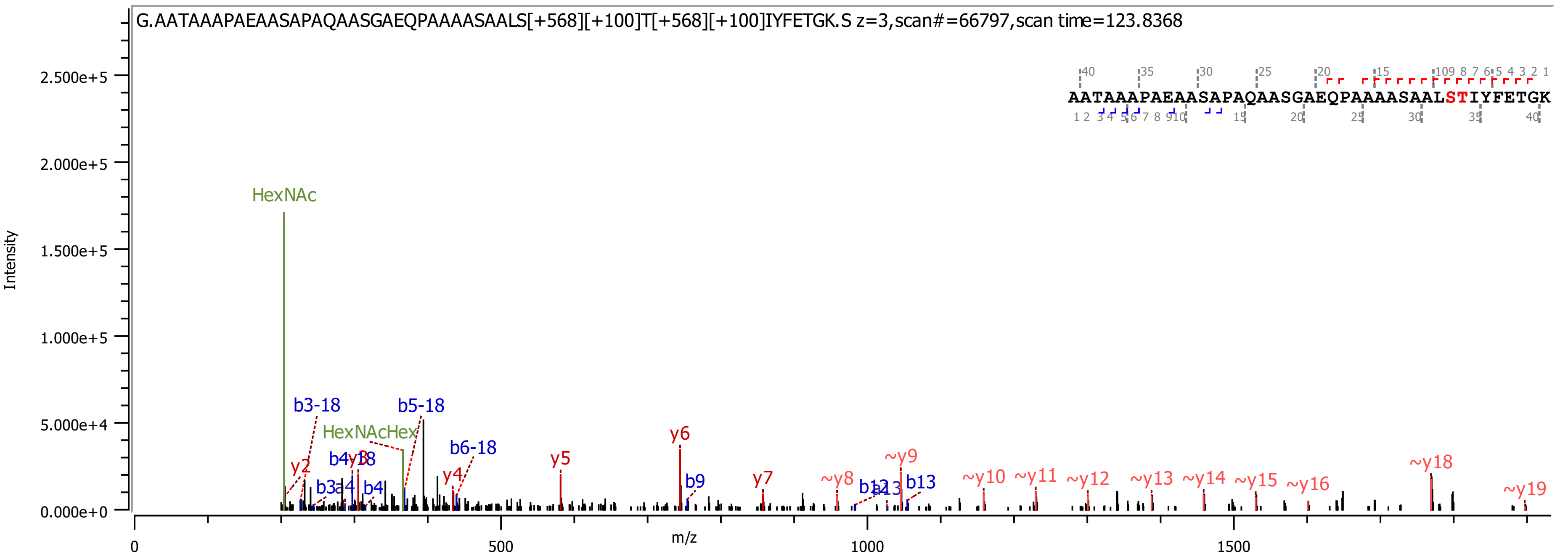
G.AATAAAPAEAASAPAQAASGAEQPAAAASAALS[+568][+100]T[+568][+100]IYFETGK.S z=3,scan#=66797,scan time=123.8368
AATAAAPAEAASAPAQAASGAEQPAAAASAALSTIYFETGK
Intensity
m/z
HexNAc
HexNAcHex
b3-18
b5-18
b6-18
b4-18
y2
y3
b3
a4
b4
y4
y5
y6
b9
y7
~y8
b12
b13
~y9
~y10
~y11
~y12
~y13
~y14
~y15
~y16
~y18
~y19

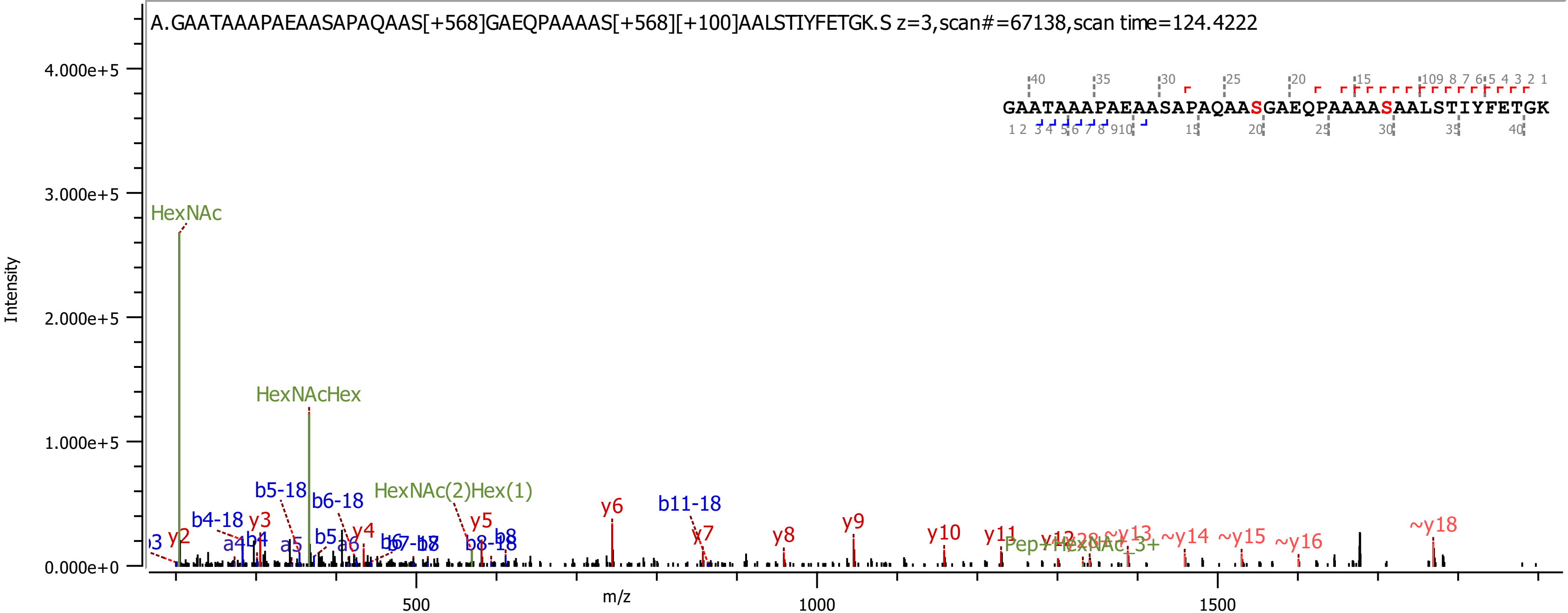
A.GAATAAAPAEAASAPAQAAS[+568]GAEQPAAAAS[+568][+100]AALSTIYFETGK.S z=3,scan#=67138,scan time=124.4222
GAATAAAPAEAASAPAQAASGAEQPAAAASAALSTIYFETGK
Intensity
m/z
HexNAc
HexNAcHex
HexNAc(2)Hex(1)
b4-18
b5-18
b6-18
b11-18
y2
y3
y4
y5
y6
y7
y8
y9
y10
y11
~y13
~y14
~y15
~y16
~y18
b4
b5
b8
b8-18
a5
a6
Pep+HexNAc_3+
0.000e+0
1.000e+5
2.000e+5
3.000e+5
4.000e+5
500
1000
1500

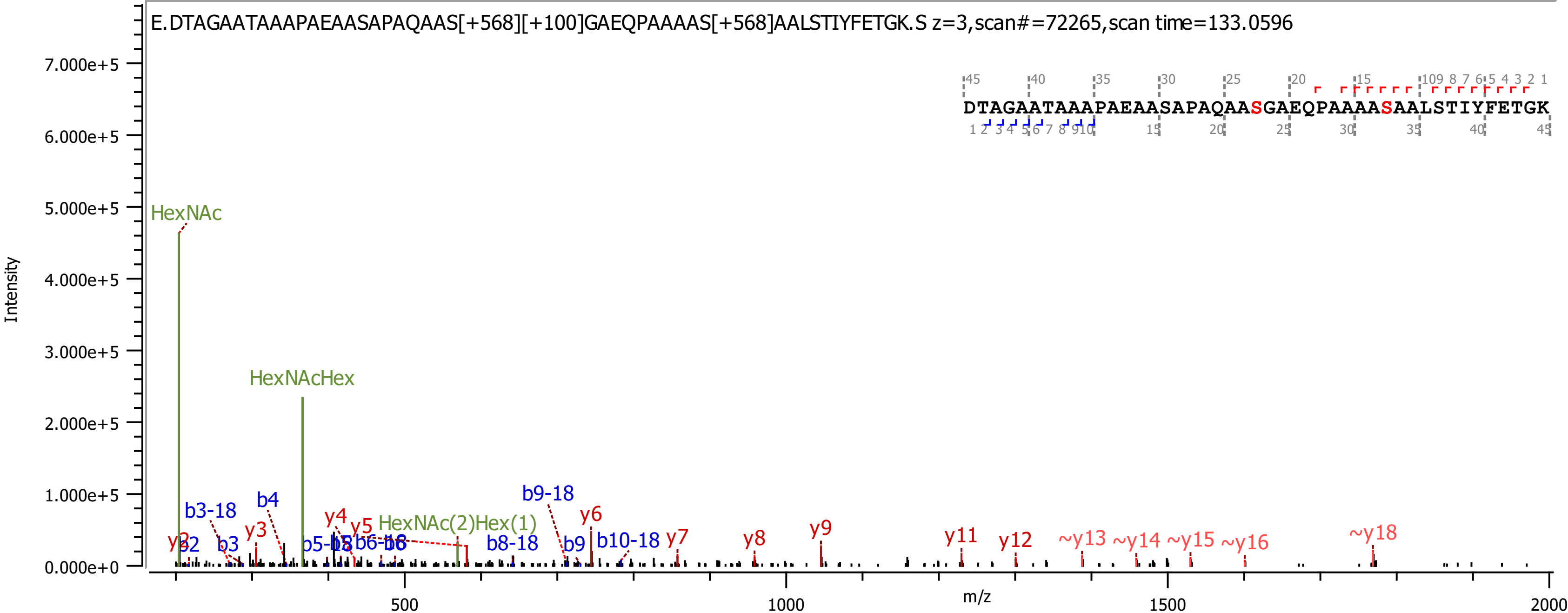
E.DTAGAATAAAPAEAASAPAQAAS[+568][+100]GAEQPAAAAS[+568]AALSTIYFETGK.S z=3,scan#=72265,scan time=133.0596
DTAGAATAAAPAEAASAPAQAASGAEQPAAAASAALSTIYFETGK
Intensity
m/z
HexNAc
HexNAcHex
HexNAc(2)Hex(1)
b3-18
b4
b3
b9-18
b8-18
b9
b10-18
y2
y3
y4
y5
y6
y7
y8
y9
y11
y12
~y13
~y14
~y15
~y16
~y18

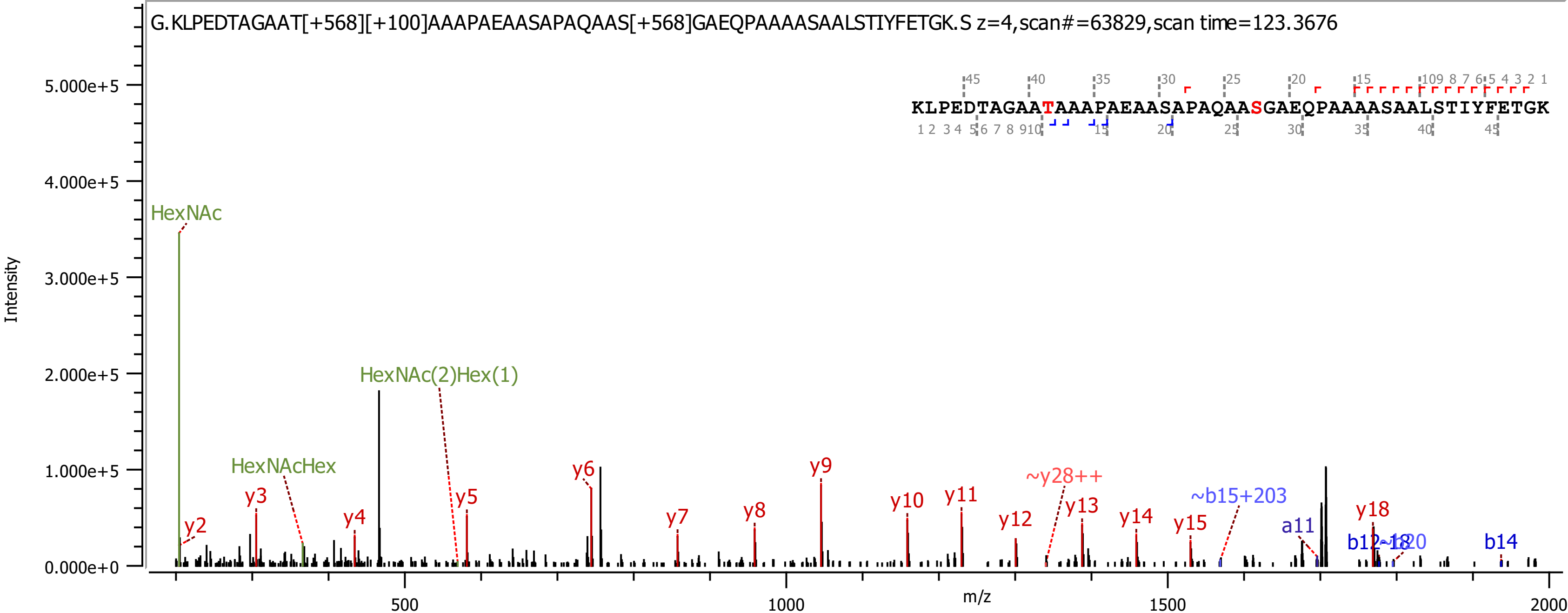

G.KLPEDTAGAAT[+568][+100]AAAPAEAASAPAQAAS[+568]GAEQPAAAASAALSTIYFETGK.S z=4,scan#=63829,scan time=123.3676
KLPEDTAGAATAAAPAEAASAPAQAASGAEQPAAAASAALSTIYFETGK
Intensity
5.000e+5
4.000e+5
3.000e+5
2.000e+5
1.000e+5
0.000e+0
HexNAc
HexNAc(2)Hex(1)
HexNAcHex
y2
y3
y4
y5
y6
y7
y8
y9
y10
y11
~y28++
y12
y13
y14
y15
~b15+203
a11
y18
b12
~120
b14
500
1000
1500
2000
m/z

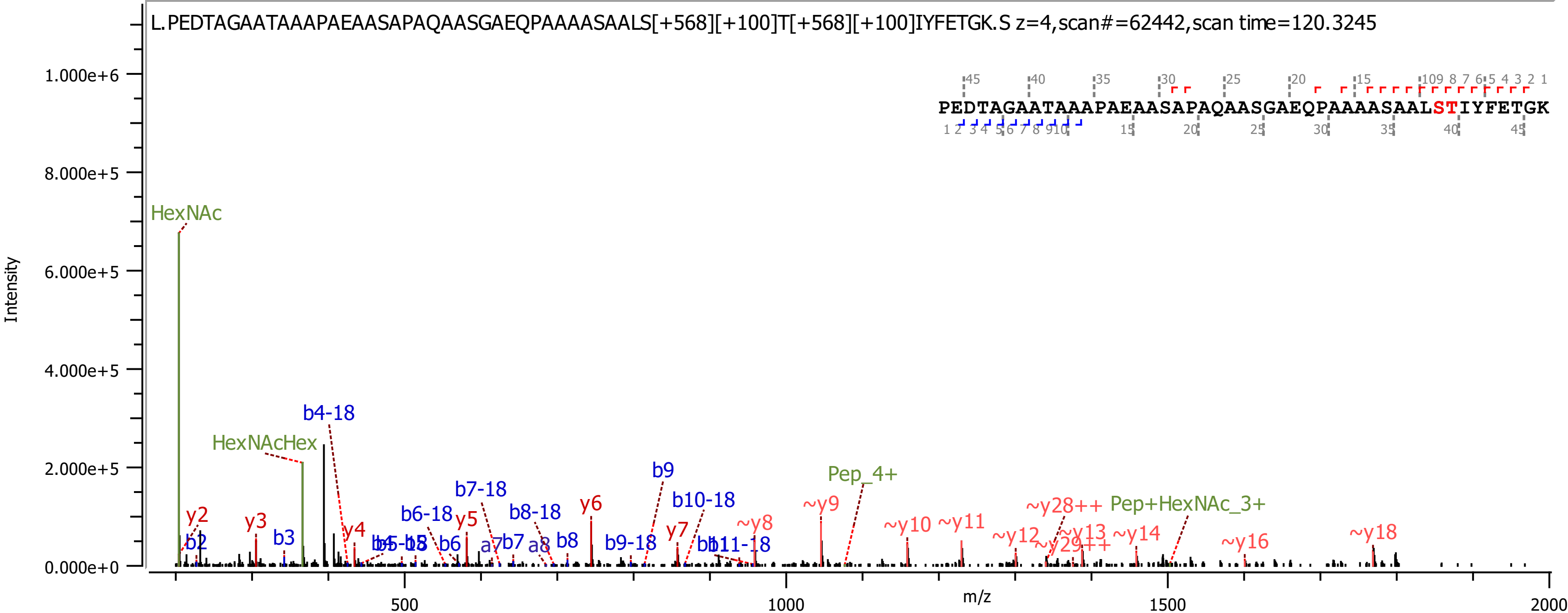

L.PEDTAGAATAAAPAEAASAPAQAASGAEQPAAAASAALS[+568][+100]T[+568][+100]IYFETGK.S z=4,scan#=62442,scan time=120.3245
PEDTAGAATAAAPAEAASAPAQAASGAEQPAAAASAALSTIYFETGK
Intensity
m/z
1.000e+6
8.000e+5
6.000e+5
4.000e+5
2.000e+5
0.000e+0
500
1000
1500
2000
HexNAc
HexNAcHex
b4-18
b9
Pep_4+
b7-18
b10-18
~y9
~y28++
Pep+HexNAc_3+
b6-18
b8-18
y6
y2
y3
y4
y5
y7
~y8
~y10
~y11
~y12
~y13
~y14
~y16
~y18
~y29++
b2
b3
b6
a7
b7
a8
b8
b9-18

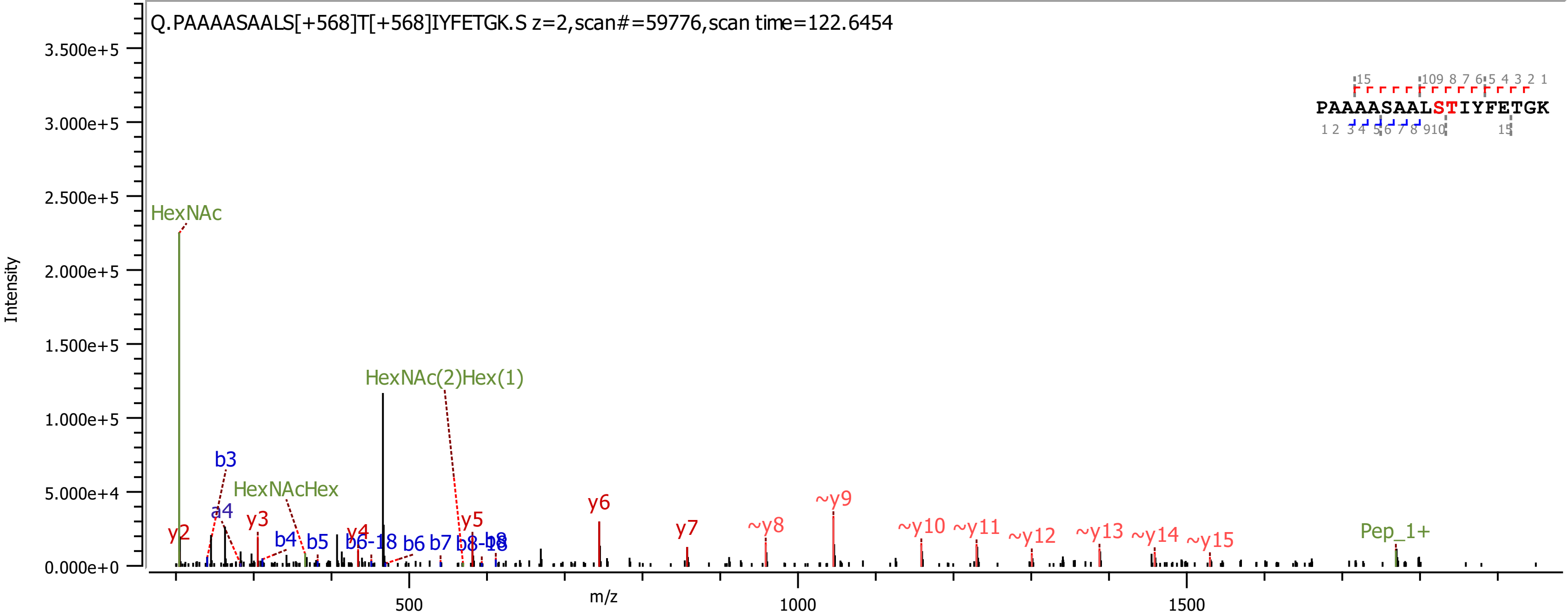
Q.PAAAASAALS[+568]T[+568]IYFETGK.S z=2,scan#=59776,scan time=122.6454
PAAAASAALSTIYFETGK
Intensity
m/z
3.500e+5
3.000e+5
2.500e+5
2.000e+5
1.500e+5
1.000e+5
5.000e+4
0.000e+0
500
1000
1500
HexNAc
HexNAc(2)Hex(1)
HexNAcHex
Pep_1+
y2
y3
y4
y5
y6
y7
~y8
~y9
~y10
~y11
~y12
~y13
~y14
~y15
b3
a4
b4
b5
b6-18
b6
b7
b8-18
b8

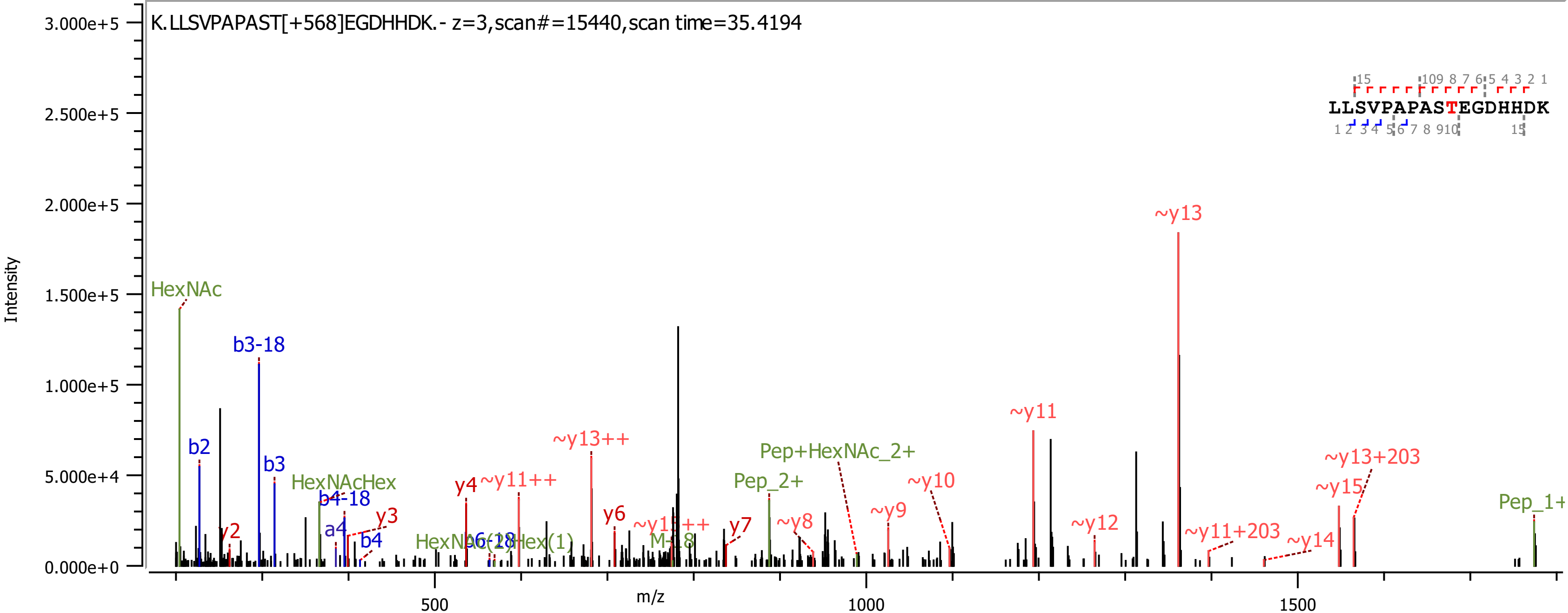

K.LLSVPAPAST[+568]EGDHHDK.- z=3,scan#=15440,scan time=35.4194
Intensity
m/z
3.000e+5
2.500e+5
2.000e+5
1.500e+5
1.000e+5
5.000e+4
0.000e+0
500
1000
1500
LLSVPAPASTEGDHHDK
HexNAc
b3-18
b2
b3
HexNAcHex
b4-18
y2
a4
y3
b4
y4
~y11++
~y13++
y6
~y13
~y11
Pep+HexNAc_2+
Pep_2+
~y10
~y9
~y8
y7
~y12
~y11+203
~y14
~y13+203
~y15
Pep_1+

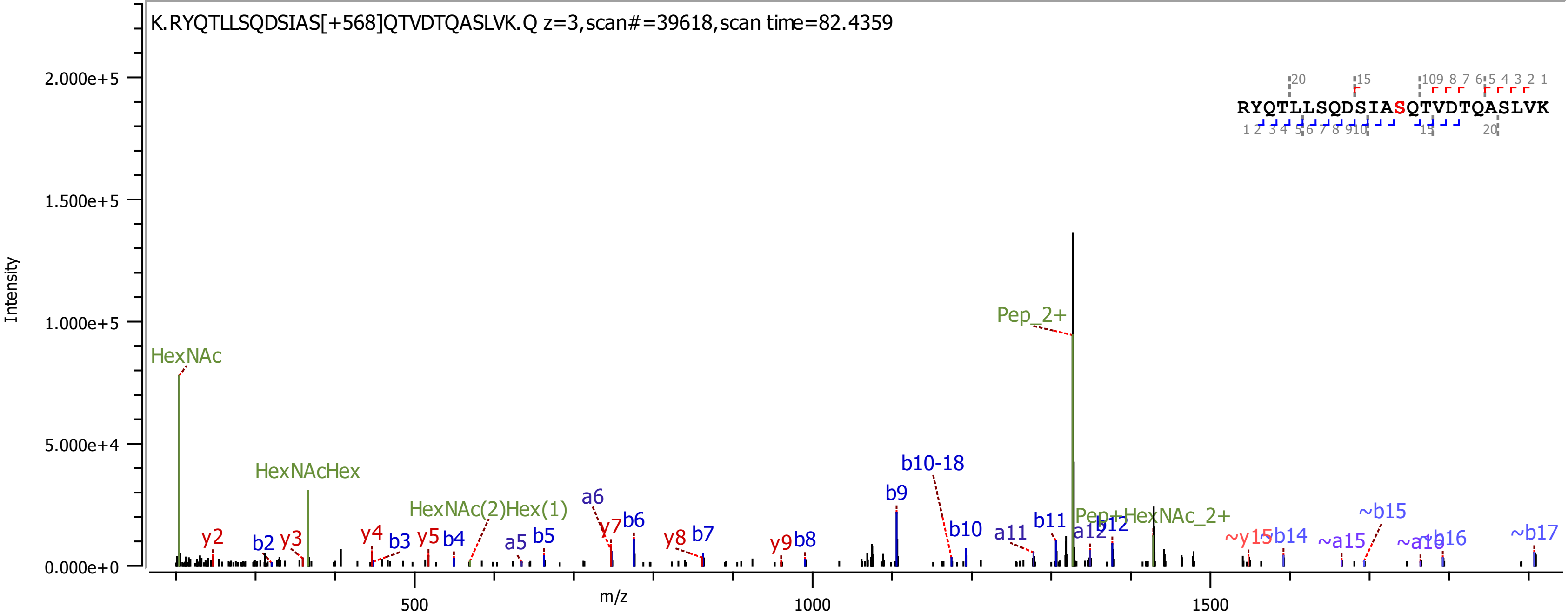

K.RYQTLLSQDSIAS[+568]QTVDTQASLVK.Q z=3,scan#=39618,scan time=82.4359
RYQTLLSQDSIASQTVDTQASLVK
Intensity
2.000e+5
1.500e+5
1.000e+5
5.000e+4
0.000e+0
m/z
500
1000
1500
HexNAc
HexNAcHex
HexNAc(2)Hex(1)
Pep_2+
Pep+HexNAc_2+
y2
y3
y4
y5
y7
y8
y9
b2
b3
b4
b5
b6
b7
b8
b9
b10-18
b10
b11
b12
a5
a6
a11
a12
~y15
~b14
~a15
~b15
~a16
~b16
~b17

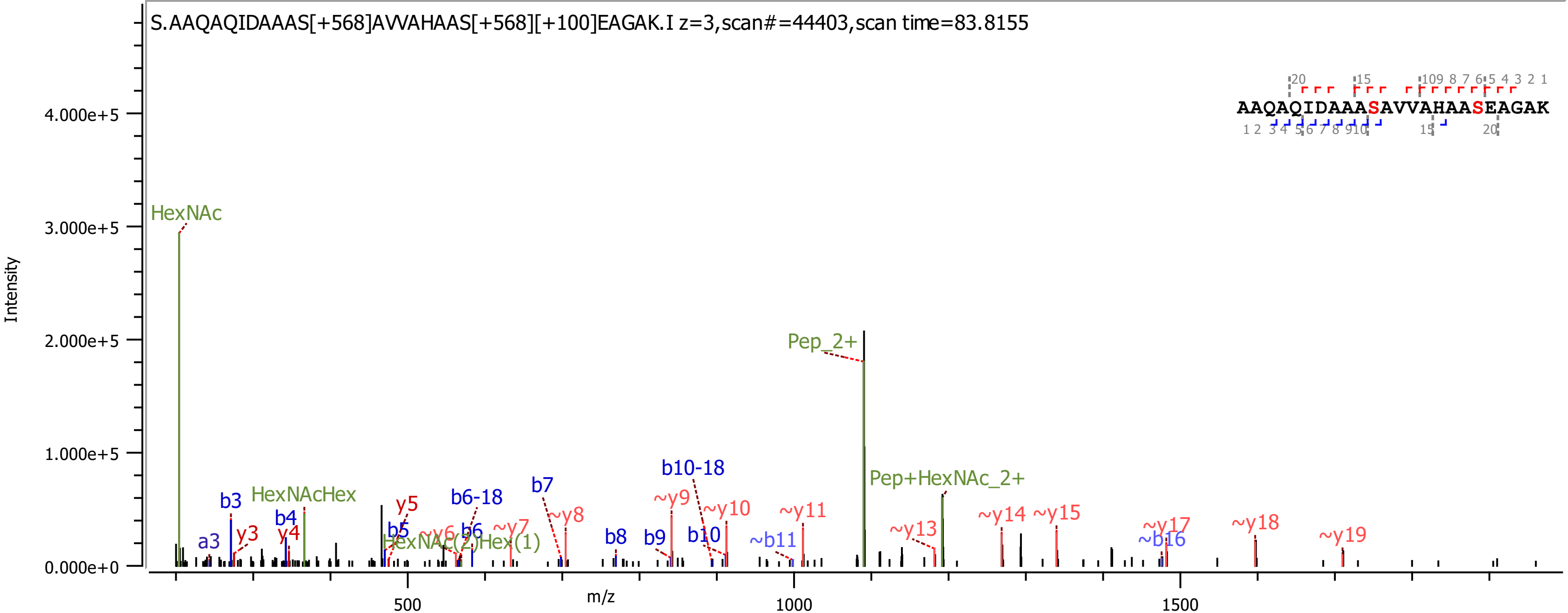
S.AAQAQIDAAAS[+568]AVVAHAAS[+568][+100]EAGAK.I z=3,scan#=44403,scan time=83.8155
AAQAQIDAAASAVVAHAASEAGAK
Intensity
m/z
4.000e+5
3.000e+5
2.000e+5
1.000e+5
0.000e+0
500
1000
1500
HexNAc
HexNAcHex
HexNAc(2)Hex(1)
Pep_2+
Pep+HexNAc_2+
a3
b3
b4
b5
b6
b6-18
b7
b8
b9
b10
b10-18
~b11
~b16
y3
y4
y5
~y6
~y7
~y8
~y9
~y10
~y11
~y13
~y14
~y15
~y17
~y18
~y19

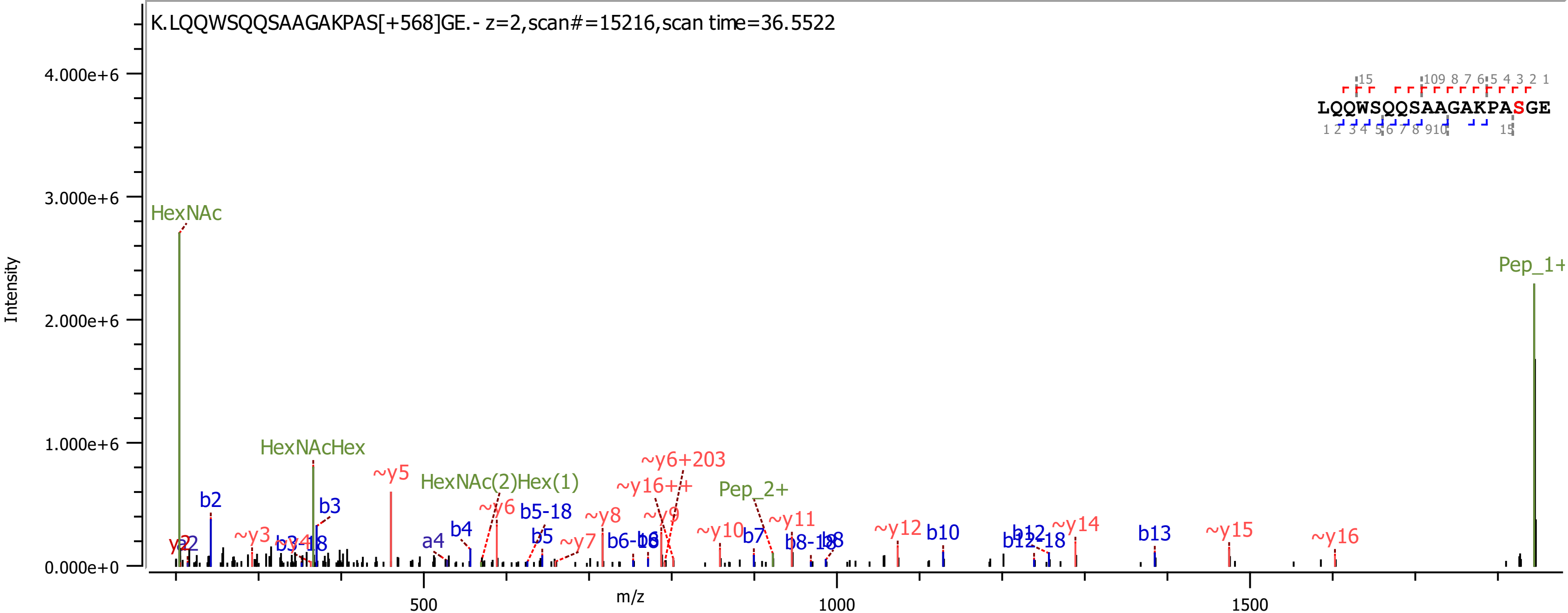

K.LQQWSQQSAAGAKPAS[+568]GE.- z=2,scan#=15216,scan time=36.5522
Intensity
m/z
4.000e+6
3.000e+6
2.000e+6
1.000e+6
0.000e+0
500
1000
1500
LQQWSQQSAAGAKPASGE
HexNAc
HexNAcHex
HexNAc(2)Hex(1)
Pep_2+
Pep_1+
b2
b3
b3-18
a4
b4
b5-18
b5
b6-18
b7
b8-18
b10
b12-18
b13
~y3
~y4
~y5
~y6
~y7
~y8
~y9
~y6+203
~y16++
~y10
~y11
~y12
~y14
~y15
~y16

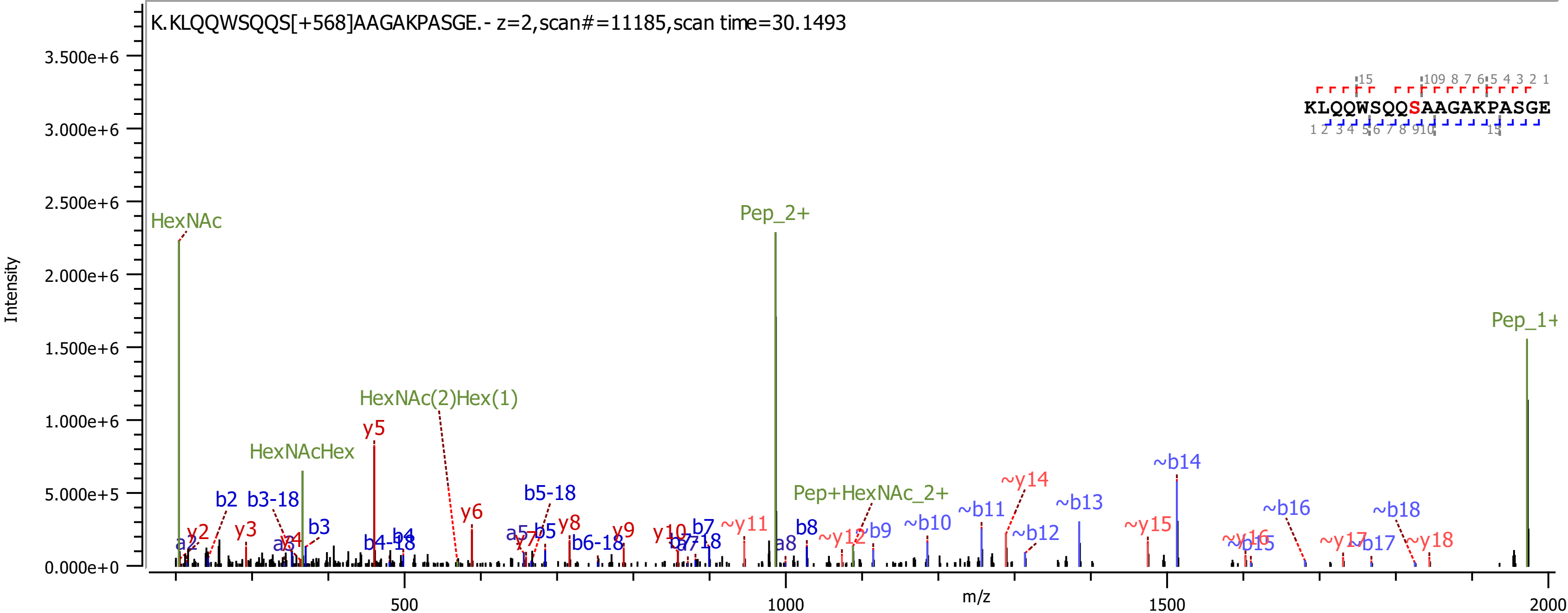

K.KLQQWSQQS[+568]AAGAKPASGE.- z=2,scan#=11185,scan time=30.1493
KLQQWSQQSAAGAKPASGE
Intensity
m/z
HexNAc
Pep_2+
Pep_1+
HexNAc(2)Hex(1)
HexNAcHex
Pep+HexNAc_2+
y5
~b14
~y14
~b13
~b11
~b16
~b18
b5-18
b2
b3-18
b3
y6
~y11
~y15
~b10
b8
b7
y9
y8
y10
~y12
~b9
~b12
~y16
~b15
~y17
~b17
~y18
b4-18
b4
b6-18
b5
a5
y7
y2
y3
a2
a3
y4
a8

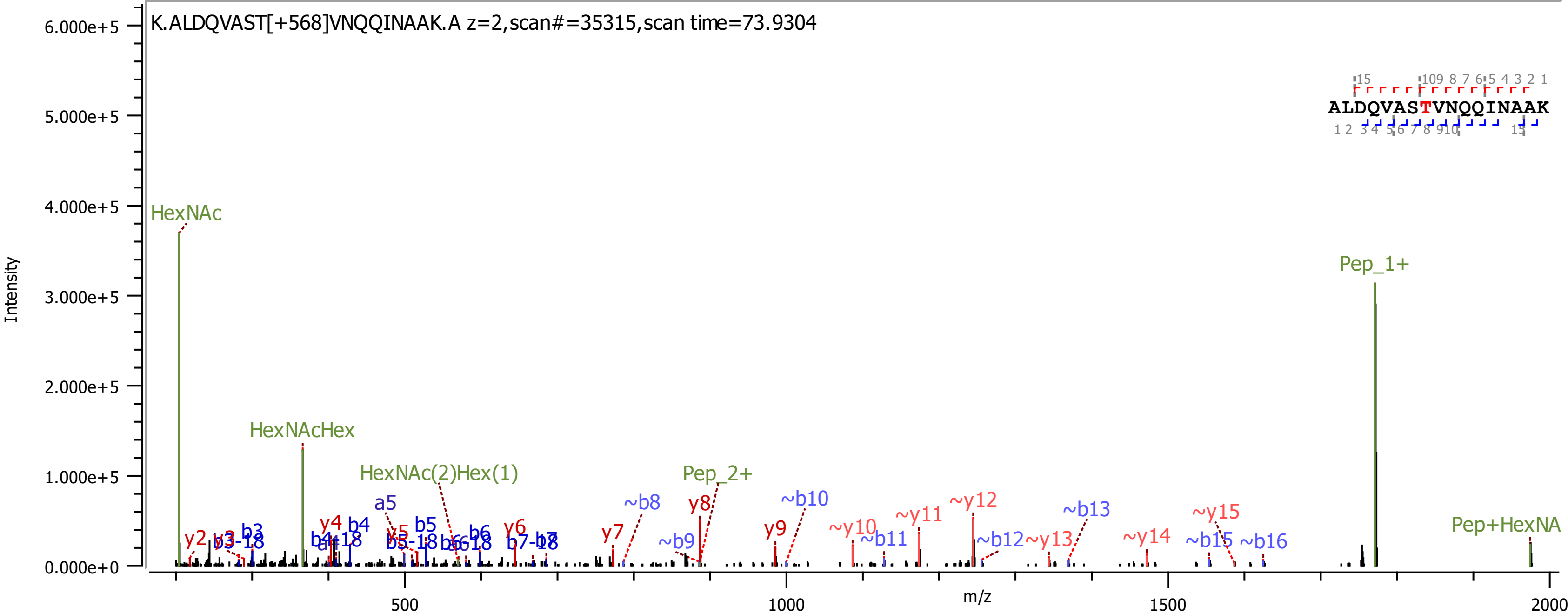

K.ALDQVAST[+568]VNQQINAAK.A z=2,scan#=35315,scan time=73.9304
ALDQVASTVNQQINAAK
Intensity
m/z
HexNAc
HexNAcHex
HexNAc(2)Hex(1)
Pep_2+
Pep_1+
Pep+HexNA
y2
y3
b3
b3-18
y4
b4
b4-18
a4
a5
y5
b5
b5-18
b6
b6-18
y6
b7
b7-18
y7
~b8
y8
~b9
y9
~b10
~y10
~b11
~y11
~y12
~b12
~y13
~b13
~y14
~y15
~b15
~b16

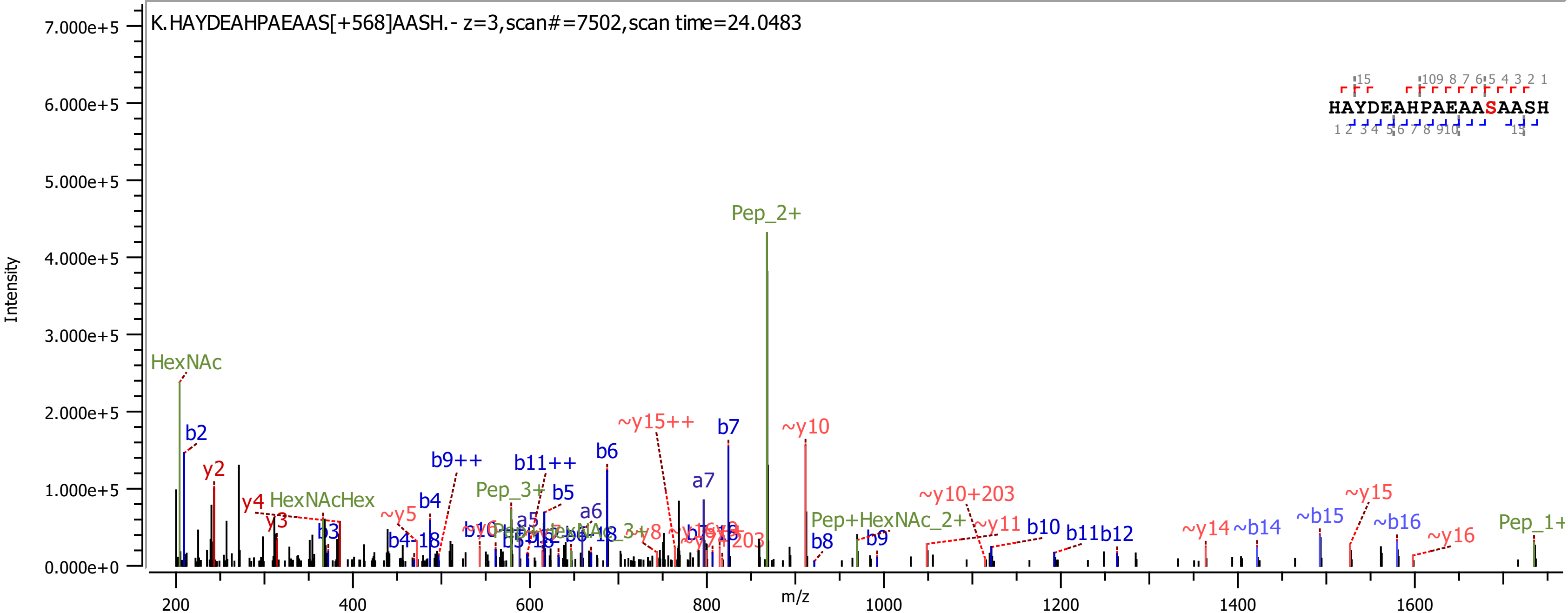

K.HAYDEAHPAEAAS[+568]AASH.- z=3,scan#=7502,scan time=24.0483
HAYDEAHPAEAASAASH
Intensity
m/z
7.000e+5
6.000e+5
5.000e+5
4.000e+5
3.000e+5
2.000e+5
1.000e+5
0.000e+0
200
400
600
800
1000
1200
1400
1600
Pep_2+
HexNAc
b2
y2
y4
HexNAcHex
y3
b3
~y5
b4
b4-18
b9++
b11++
Pep_3+
b5
a5
b6
a6
~y15++
a7
b7
~y10
Pep+HexNAc_2+
b8
b9
~y10+203
~y11
b10
b11
b12
~y14
~b14
~b15
~y15
~b16
~y16
Pep_1+

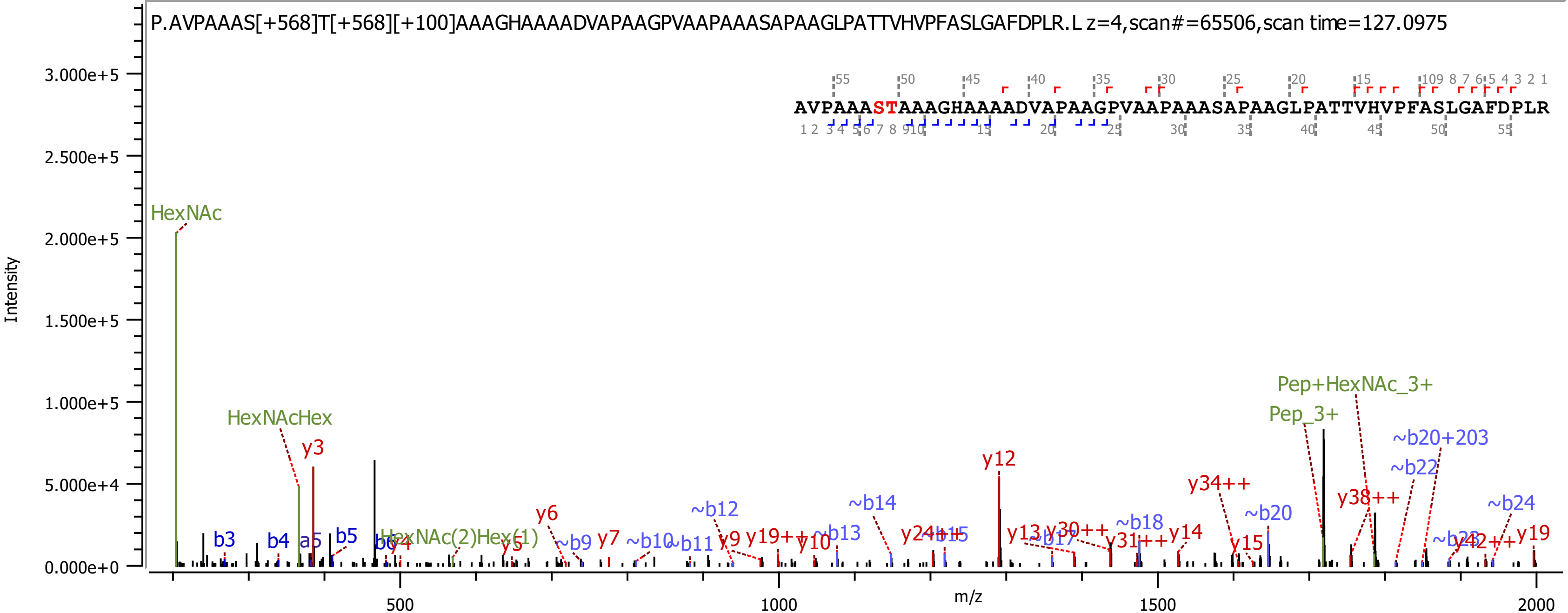

P.AVPAAAS[+568]T[+568][+100]AAAGHAAAADVAPAAGPVAAPAAASAPAAGLPATTVHVPFASLGAFDPLR.L z=4,scan#=65506,scan time=127.0975
AVPAAASTAAAGHAAAADVAPAAGPVAAPAAASAPAAGLPATTVHVPFASLGAFDPLR
Intensity
m/z
HexNAc
HexNAcHex
HexNAc(2)Hex(1)
Pep+HexNAc_3+
Pep_3+
y3
y12
y34++
y38++
~b20+203
~b22
~b24
~b20
~b18
~b14
~b12

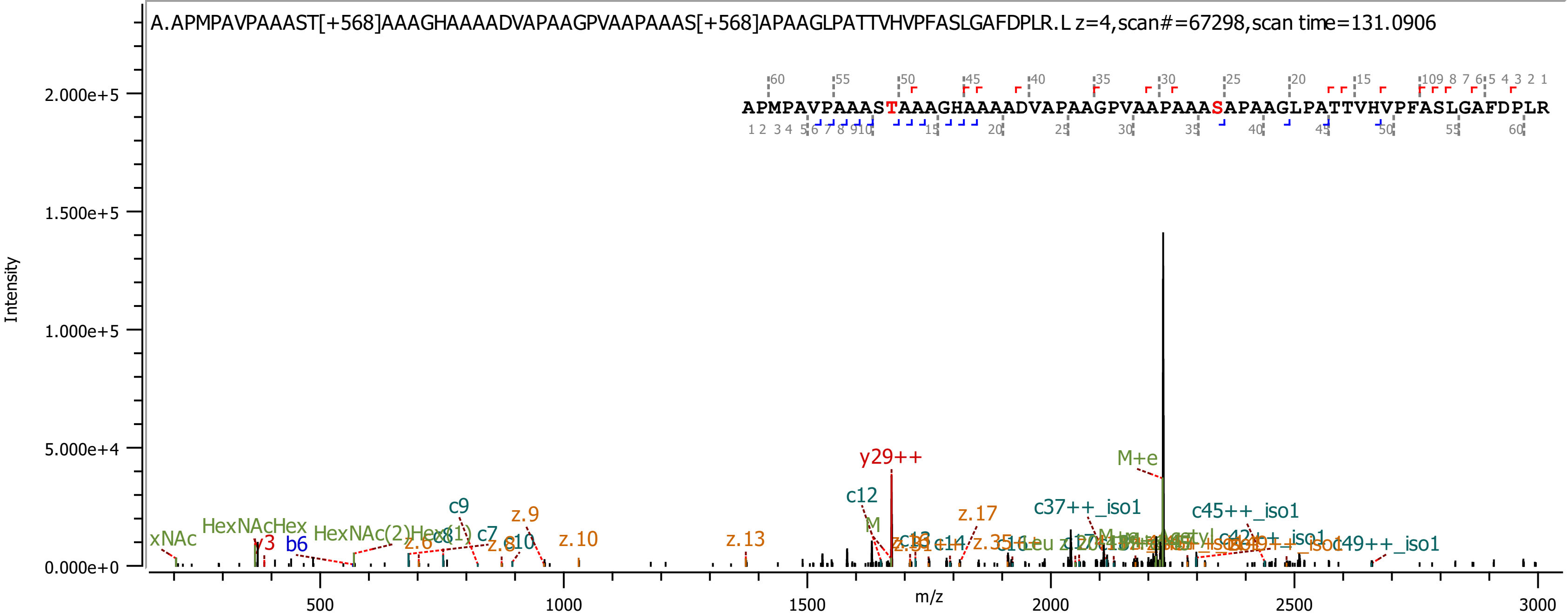
A.APMPAVPAAAST[+568]AAAGHAAAADVAPAAGPVAAPAAAS[+568]APAAGLPATTVHVPFASLGAFDPLR.L z=4,scan#=67298,scan time=131.0906
APMPAVPAAASTAAAGHAAAADVAPAAGPVAAPAAASAPAAGLPATTVHVPFASLGAFDPLR
Intensity
m/z
y29++
M+e
c12
c9
z.9
z.17
c37++_iso1
c45++_iso1
c49++_iso1
xNAc
HexNAcHex
y3
b6
z.10
z.13

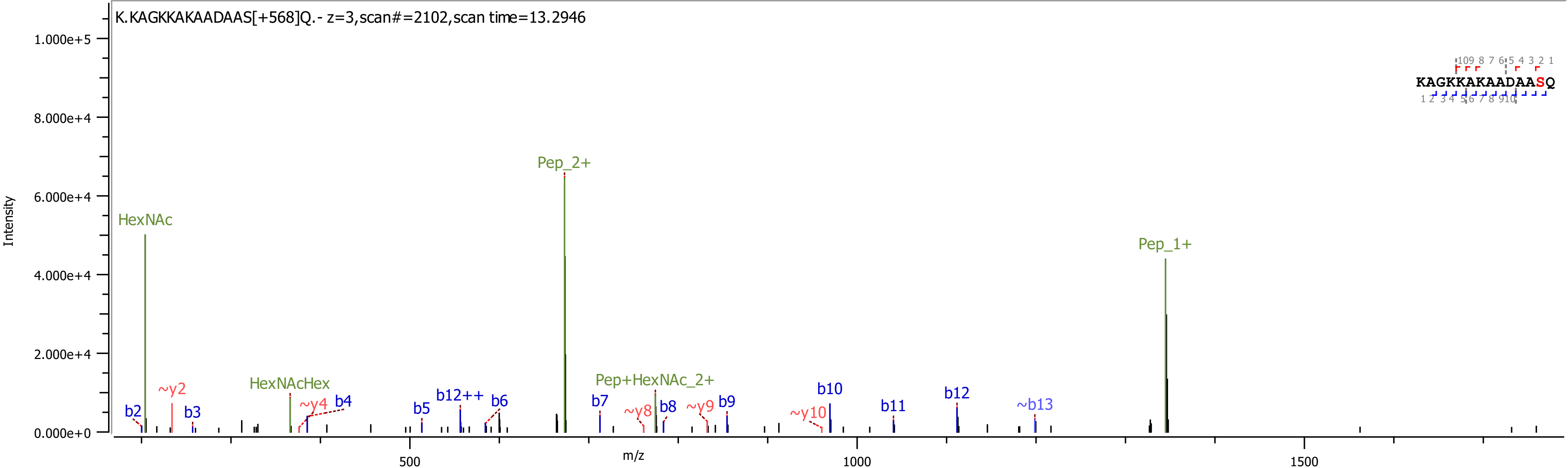

K.KAGKKAKAADAAS[+568]Q.- z=3,scan#=2102,scan time=13.2946
Intensity
m/z
1.000e+5
8.000e+4
6.000e+4
4.000e+4
2.000e+4
0.000e+0
500
1000
1500
KAGKKAKAADAASQ
HexNAc
~y2
b2
b3
HexNAcHex
~y4
b4
b5
b12++
b6
Pep_2+
Pep+HexNAc_2+
b7
~y8
b8
~y9
b9
~y10
b10
b11
b12
~b13
Pep_1+

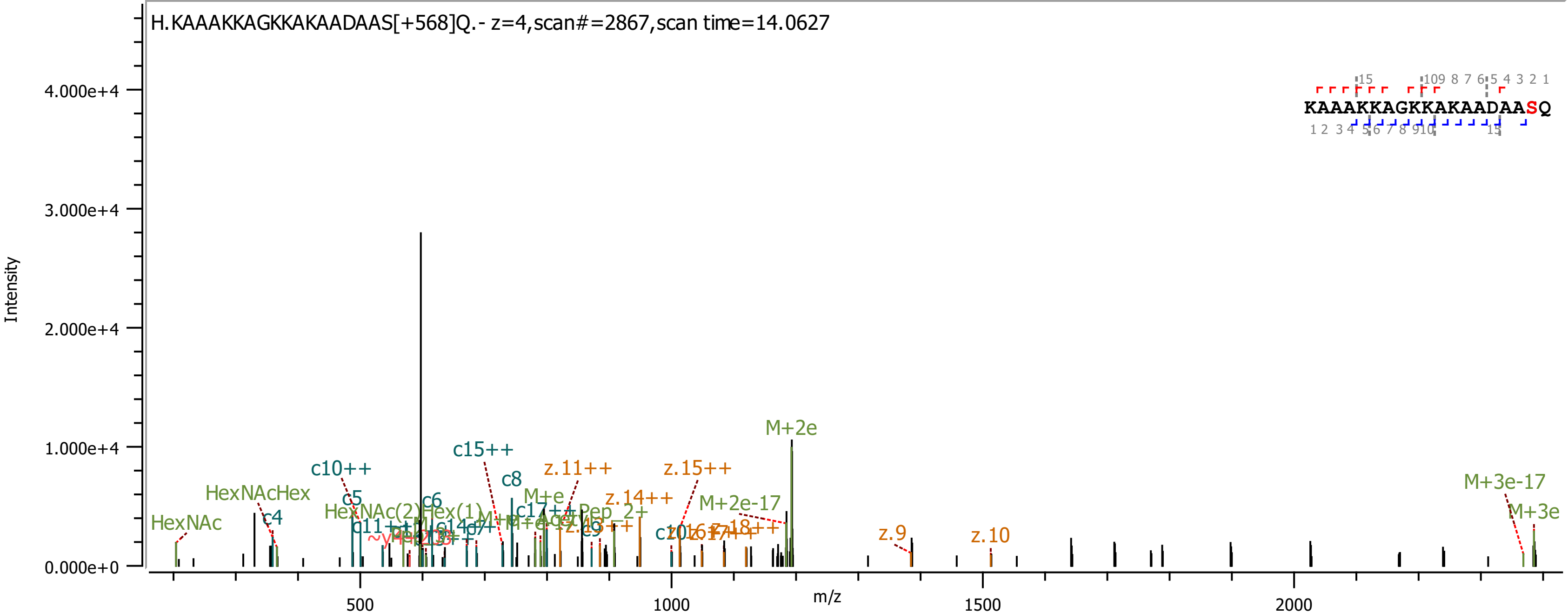

H.KAAAKKAGKKAKAADAAS[+568]Q.- z=4,scan#=2867,scan time=14.0627
KAAAKKAGKKAKAADAASQ
Intensity
m/z
4.000e+4
3.000e+4
2.000e+4
1.000e+4
0.000e+0
500
1000
1500
2000
HexNAc
HexNAcHex
c4
c5
c10++
c6
c8
c15++
M+e
z.11++
z.14++
z.15++
M+2e-17
M+2e
z.9
z.10
M+3e-17
M+3e

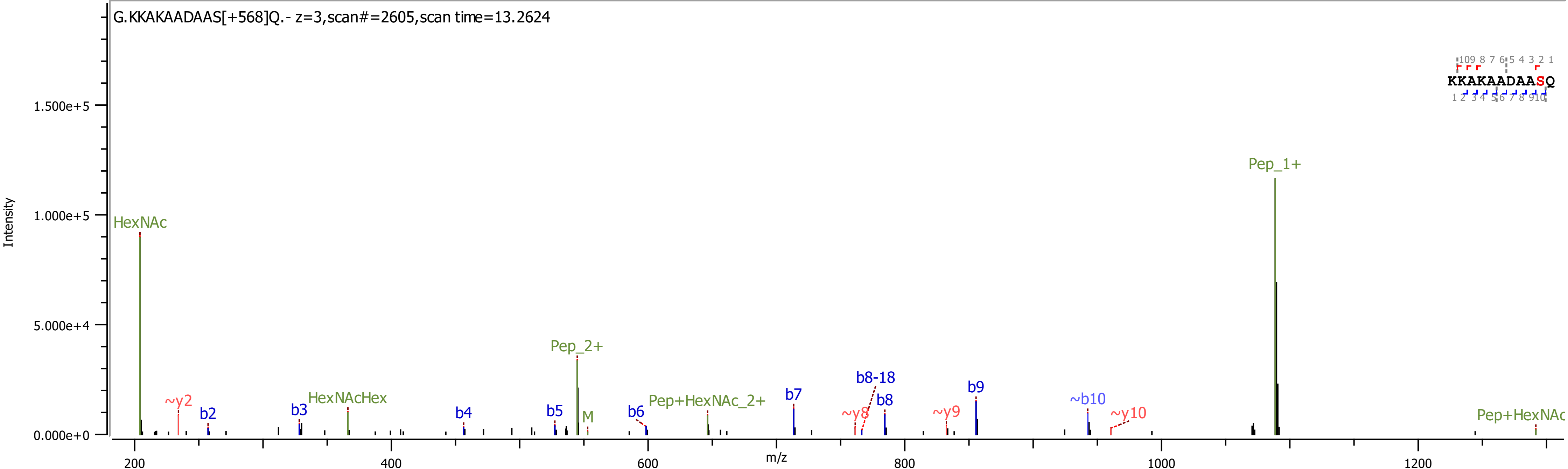

G.KKAKAADAAS[+568]Q.- z=3,scan#=2605,scan time=13.2624
KKAKAADAASQ
Intensity
1.500e+5
1.000e+5
5.000e+4
0.000e+0
HexNAc
~y2
b2
b3
HexNAcHex
b4
b5
Pep_2+
M
b6
Pep+HexNAc_2+
b7
~y8
b8-18
b8
~y9
b9
~b10
~y10
Pep_1+
Pep+HexNAc
200
400
600
800
1000
1200
m/z

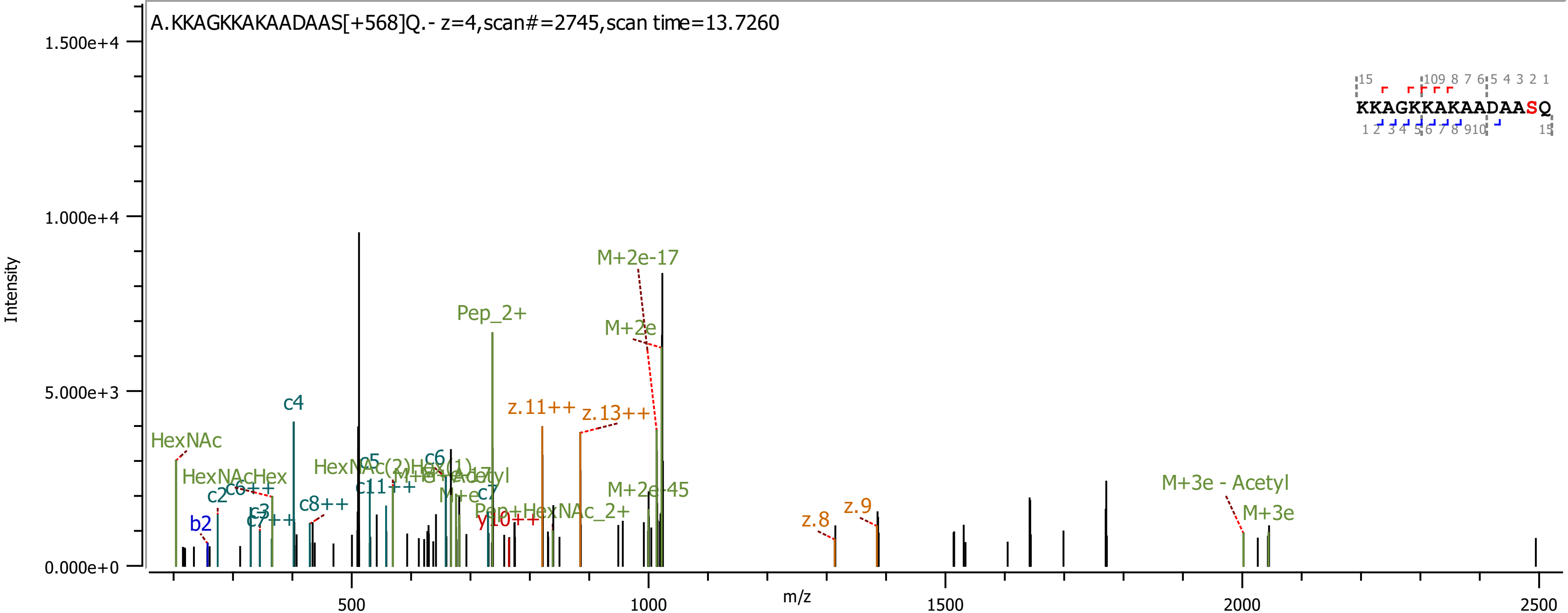

A.KKAGKKAKAADAAS[+568]Q.- z=4,scan#=2745,scan time=13.7260
KKAGKKAKAADAASQ
Intensity
1.500e+4
1.000e+4
5.000e+3
0.000e+0
m/z
500
1000
1500
2000
2500
M+2e-17
Pep_2+
M+2e
z.11++
z.13++
c4
HexNAc
HexNAcHex
c2
c6++
b2
c3
c7++
c8++
c5
HexNAc(2)Hex(1)
c6
c11++
M+e-Acetyl
M+e
c7
y10++
Pep+HexNAc_2+
M+2e-45
z.8
z.9
M+3e - Acetyl
M+3e

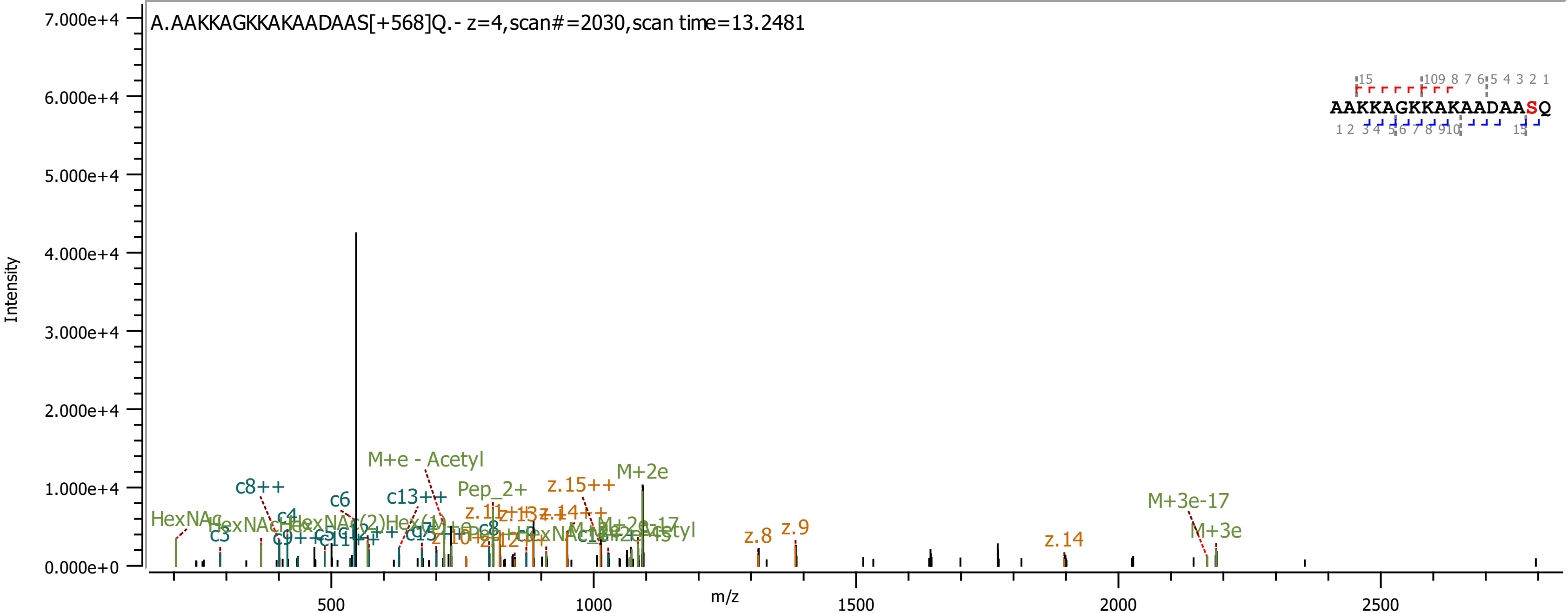

A.AAKKAGKKAKAADAAS[+568]Q.- z=4,scan#=2030,scan time=13.2481
AAKKAGKKAKAADAASQ
Intensity
m/z
7.000e+4
6.000e+4
5.000e+4
4.000e+4
3.000e+4
2.000e+4
1.000e+4
0.000e+0
500
1000
1500
2000
2500
HexNAc
c3
c8++
c4
c6
M+e - Acetyl
c13++
Pep_2+
z.15++
M+2e
z.8
z.9
z.14
M+3e-17
M+3e

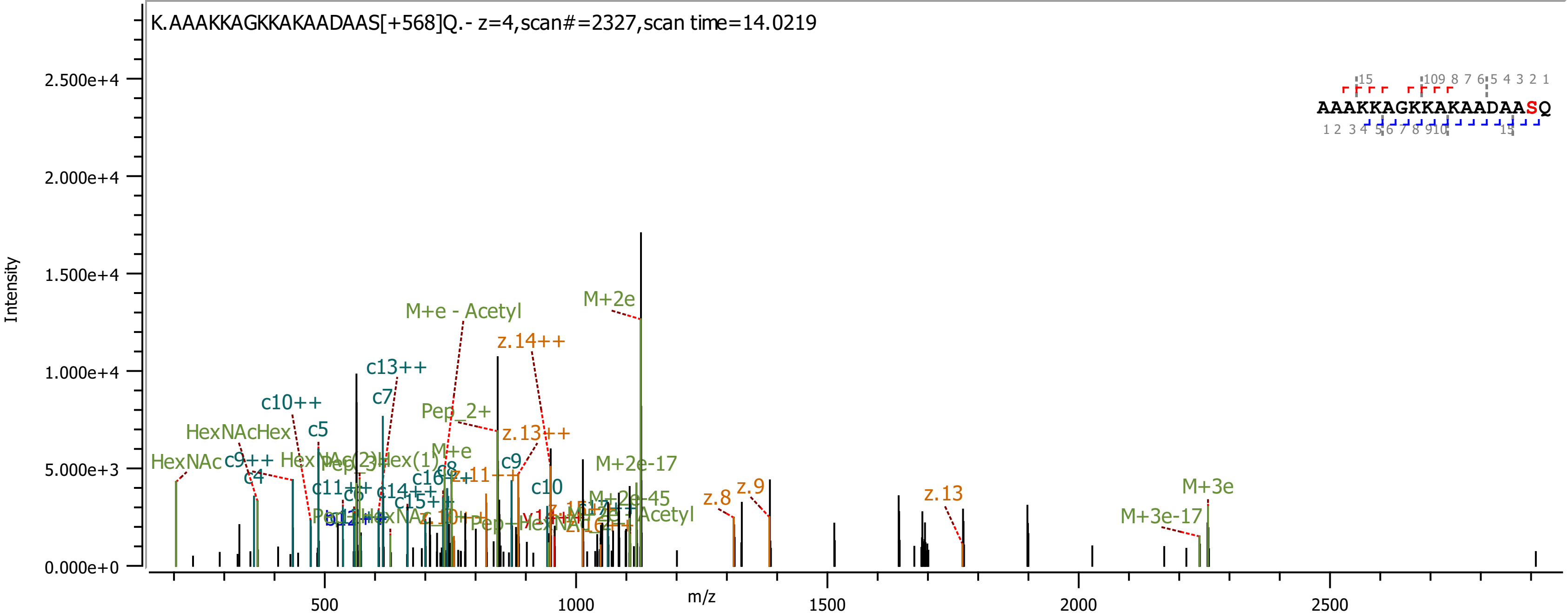

K.AAAKKAGKKAKAADAAS[+568]Q.- z=4,scan#=2327,scan time=14.0219
AAAKKAGKKAKAADAASQ
Intensity
m/z
0.000e+0
5.000e+3
1.000e+4
1.500e+4
2.000e+4
2.500e+4
500
1000
1500
2000
2500
M+2e
M+e - Acetyl
z.14++
c13++
c10++
c7
Pep_2+
HexNAcHex
c5
z.13++
HexNAc
c9++
M+e
c9
M+2e-17
c4
c16++
z.11++
c10
c11++
c14++
c15++
M+2e-45
z.8
z.9
z.13
M+3e
M+3e-17

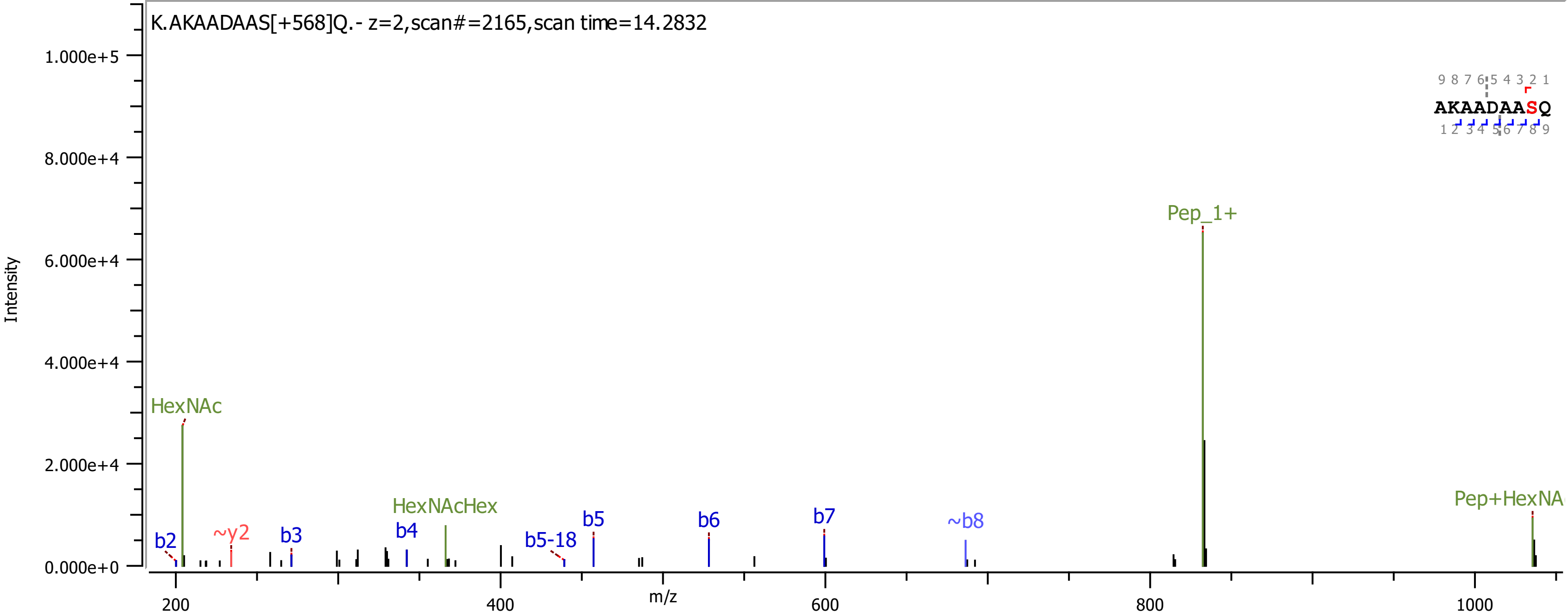
K.AKAADAAS[+568]Q.- z=2,scan#=2165,scan time=14.2832
Intensity
m/z
1.000e+5
8.000e+4
6.000e+4
4.000e+4
2.000e+4
0.000e+0
200
400
600
800
1000
HexNAc
b2
~y2
b3
b4
HexNAcHex
b5-18
b5
b6
b7
~b8
Pep_1+
Pep+HexNA
9 8 7 6 5 4 3 2 1
AKAADAASQ
1 2 3 4 5 6 7 8 9

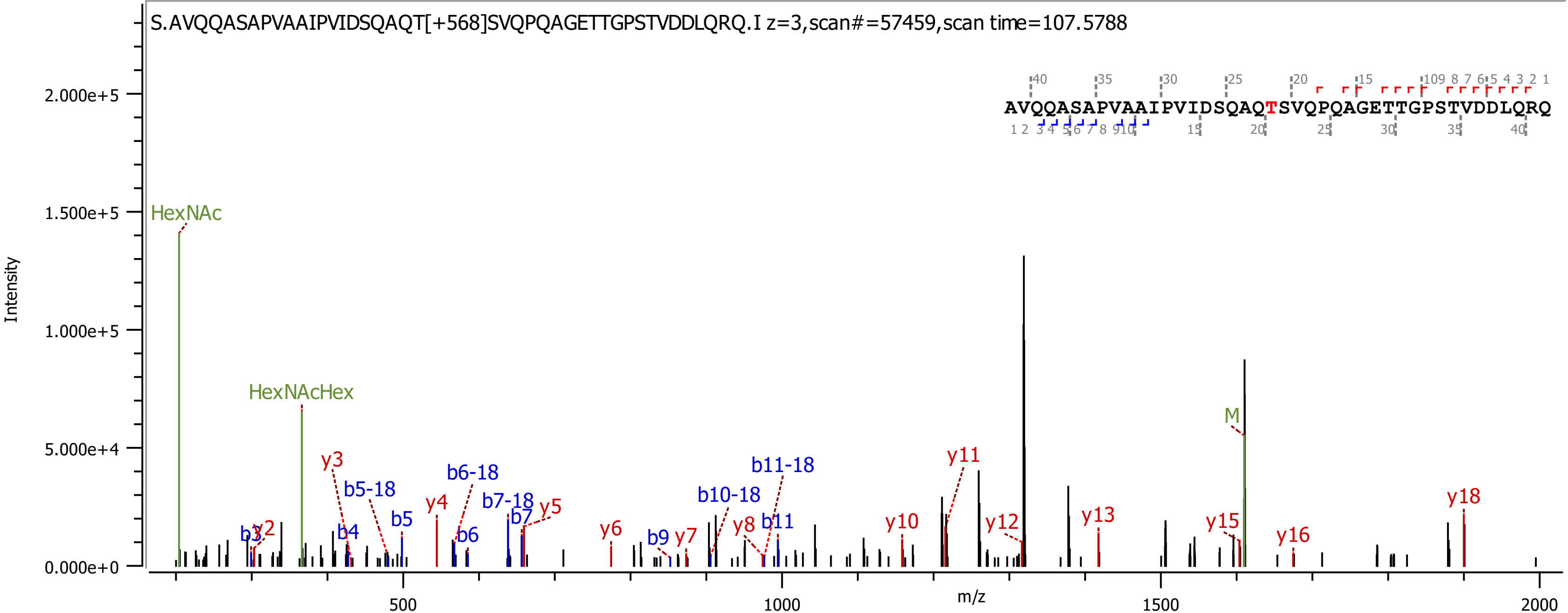
S.AVQQASAPVAAIPVIDSQAQT[+568]SVQPQAGETTGPSTVDDLQRQ.I z=3,scan#=57459,scan time=107.5788
AVQQASAPVAAIPVIDSQAQTSVQPQAGETTGPSTVDDLQRQ
Intensity
m/z
HexNAc
HexNAcHex
M
b3 b4 b5 b5-18 b6 b6-18 b7 b7-18 b9 b10-18 b11 b11-18
y2 y3 y4 y5 y6 y7 y8 y10 y11 y12 y13 y15 y16 y18
0.000e+0
5.000e+4
1.000e+5
1.500e+5
2.000e+5
500
1000
1500
2000

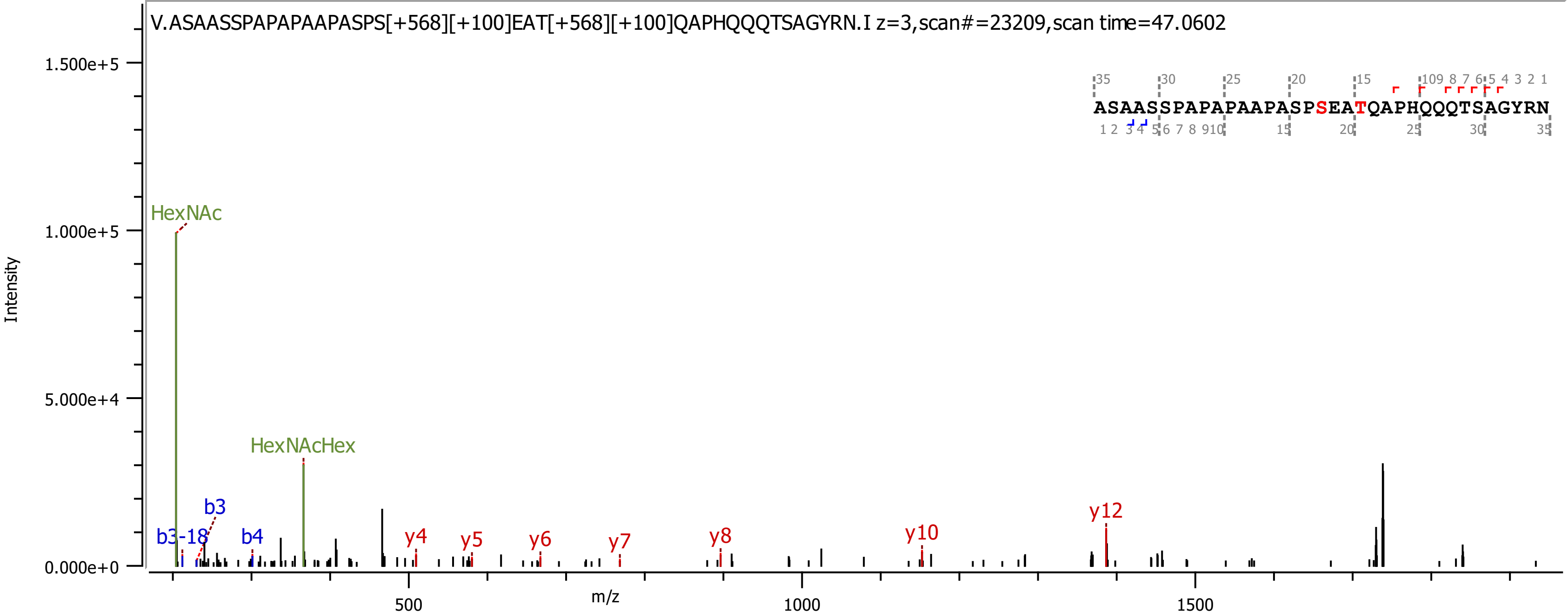

V.ASAASSPAPAPAAPASPS[+568][+100]EAT[+568][+100]QAPHQQQTSAGYRN.I z=3,scan#=23209,scan time=47.0602
Intensity
m/z
1.500e+5
1.000e+5
5.000e+4
0.000e+0
500
1000
1500
ASAASSPAPAPAAPASPSEATQAPHQQQTSAGYRN
HexNAc
HexNAcHex
b3-18
b3
b4
y4
y5
y6
y7
y8
y10
y12

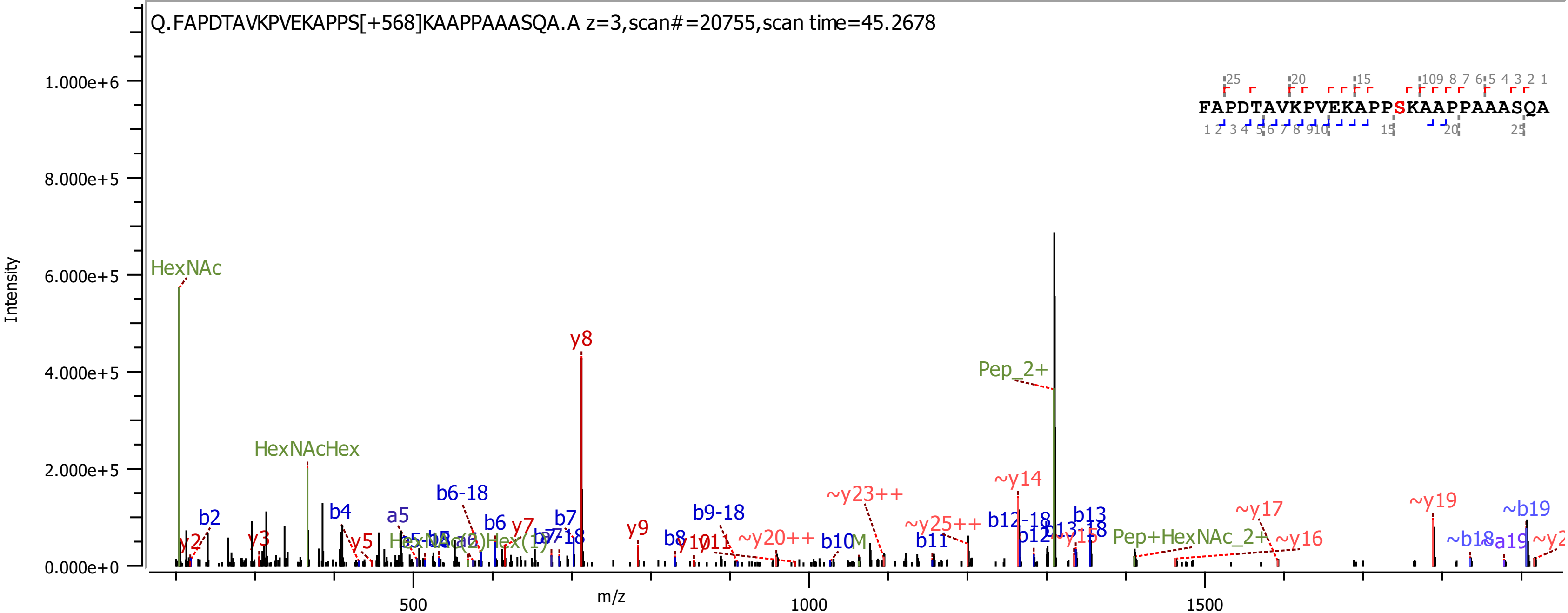

Q.FAPDTAVKPVEKAPPS[+568]KAAPPAAASQA.A z=3,scan#=20755,scan time=45.2678
FAPDTAVKPVEKAPPSKAAPPAAASQA
Intensity
m/z
1.000e+6
8.000e+5
6.000e+5
4.000e+5
2.000e+5
0.000e+0
500
1000
1500
HexNAc
HexNAcHex
y8
Pep_2+
~y14
~y23++
~y25++
~y20++
~y19
~b19
~y17
~y16
~b18
~a19
Pep+HexNAc_2+
b2
b4
a5
b6-18
b6
y7
b7
y9
b8
b9-18
b10
b11
b12-18
b12
b13
b13-18
~y15
y2
y3
y5

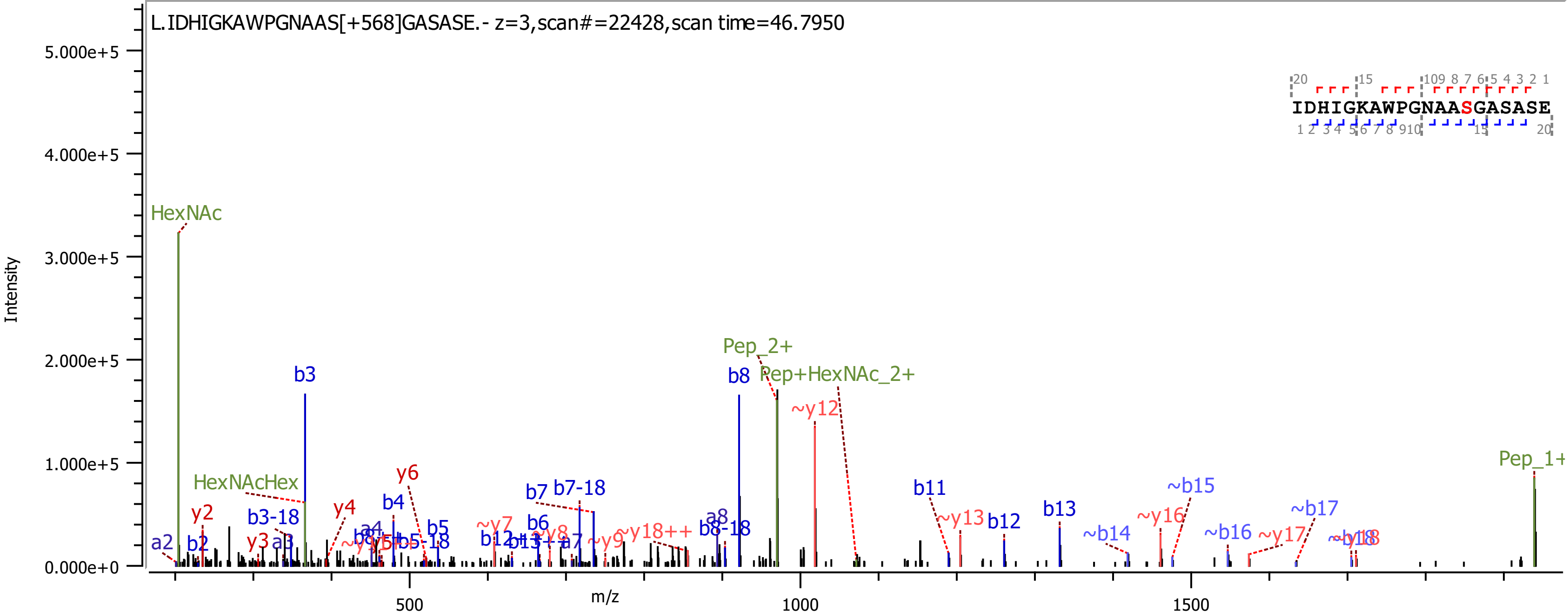
L.IDHIGKAWPGNAAS[+568]GASASE.- z=3,scan#=22428,scan time=46.7950
Intensity
m/z
IDHIGKAWPGNAASGASASE
HexNAc
HexNAcHex
Pep_2+
Pep+HexNAc_2+
Pep_1+
b3
b8
~y12
b11
b13
~b15
~y16
~b17
~y13
b12
~b14
~b16
~y17
y6
b4
y4
y2
a2
b2
y3
a3
b3-18
b7
b7-18
b6
b5
b5-18
a4
~y7
~y8
a7
~y9
~y18++
a8
b8-18
b12++
b13++

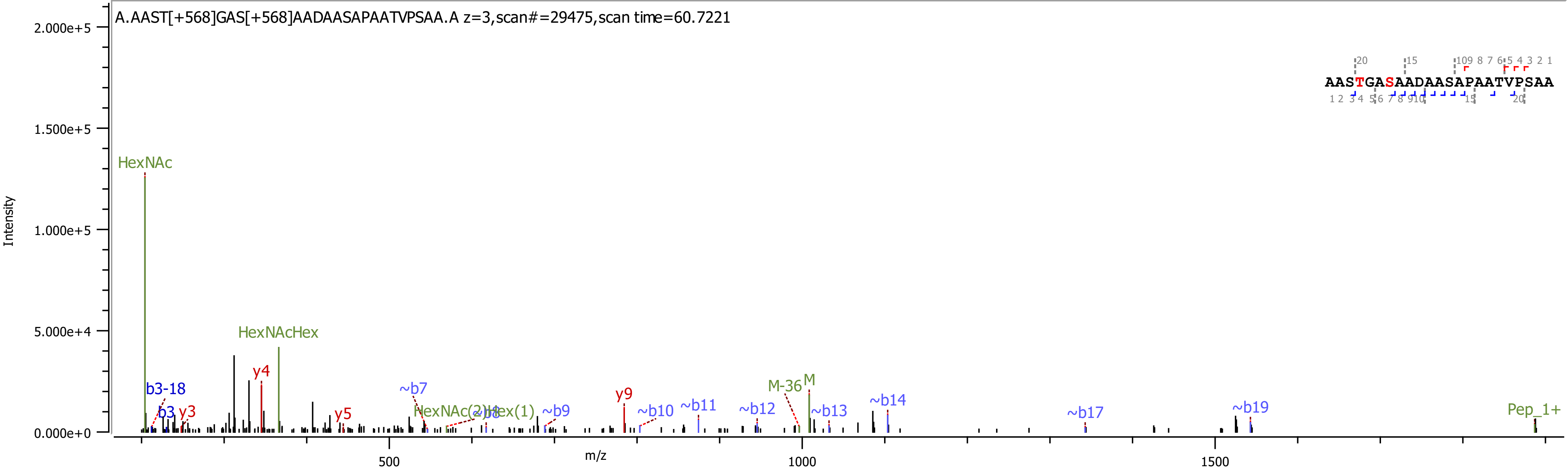

A.AAST[+568]GAS[+568]AADAASAPAATVPSAA.A z=3,scan#=29475,scan time=60.7221
AASTGASAADAASAPAATVPSAA
Intensity
m/z
HexNAc
HexNAcHex
b3-18
b3
y3
y4
y5
~b7
HexNAc(2)Hex(1)
~b8
~b9
y9
~b10
~b11
~b12
M-36
M
~b13
~b14
~b17
~b19
Pep_1+

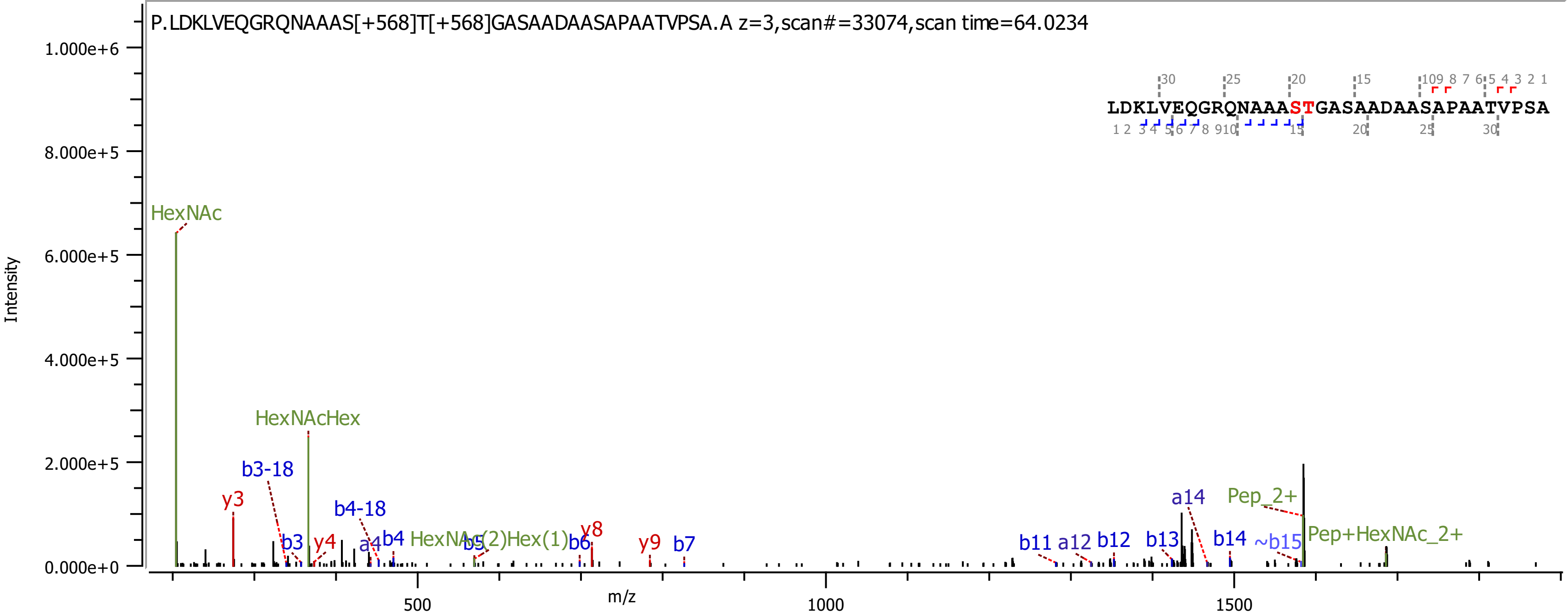
P.LDKLVEQGRQNAAAS[+568]T[+568]GASAADAASAPAATVPSA.A z=3,scan#=33074,scan time=64.0234
LDKLVEQGRQNAAASTGASAADAASAPAATVPSA
Intensity
m/z
HexNAc
HexNAcHex
b3-18
y3
b4-18
b3
y4
a4
b4
HexNAc(2)Hex(1)
b5
b6
y8
y9
b7
b11
a12
b12
b13
a14
b14
~b15
Pep_2+
Pep+HexNAc_2+
1.000e+6
8.000e+5
6.000e+5
4.000e+5
2.000e+5
0.000e+0
500
1000
1500

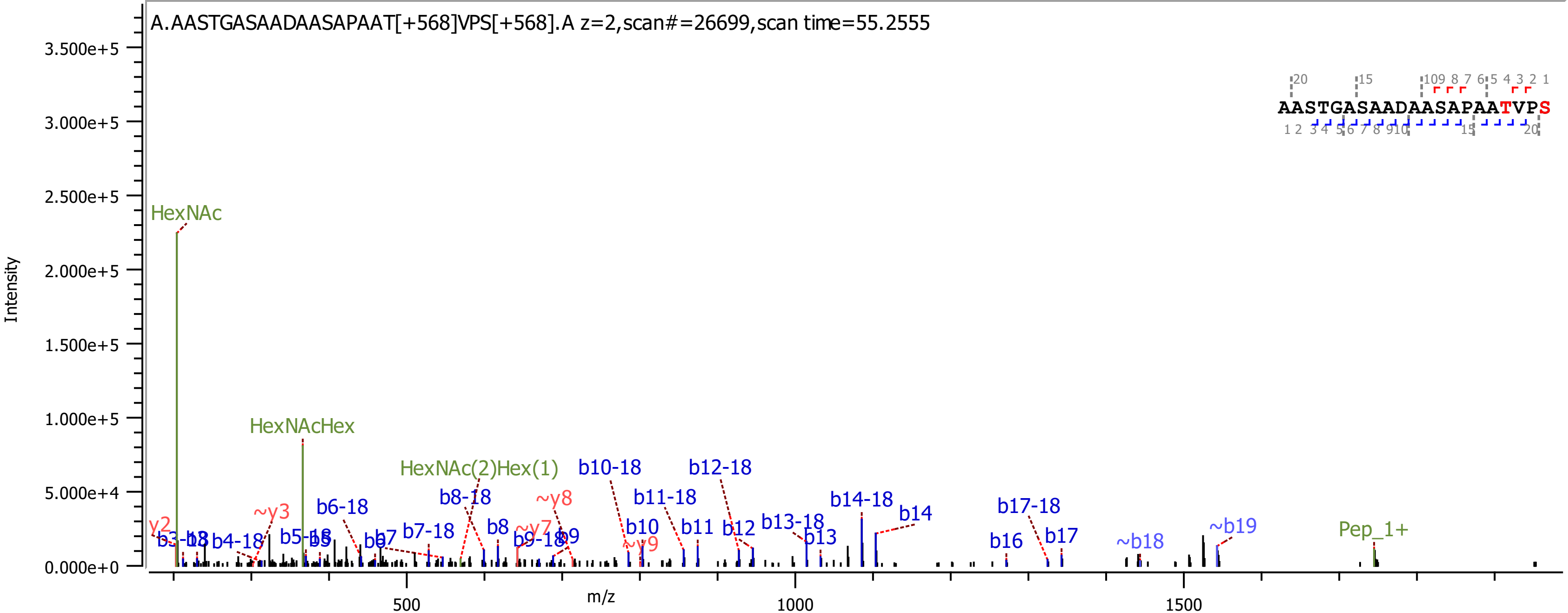
A.AASTGASAADAASAPAAT[+568]VPS[+568].A z=2,scan#=26699,scan time=55.2555
AASTGASAADAASAPAATVPS
Intensity
m/z
HexNAc
HexNAcHex
HexNAc(2)Hex(1)
Pep_1+
b10-18
b12-18
b11-18
b14-18
b14
b13-18
b13
b17-18
b17
b16
~b18
~b19
b8-18
b8
b6-18
b7-18
b7
b9-18
b9
b10
b11
b12
~y8
~y7
~y9
~y3
y2
b4-18
b5-18

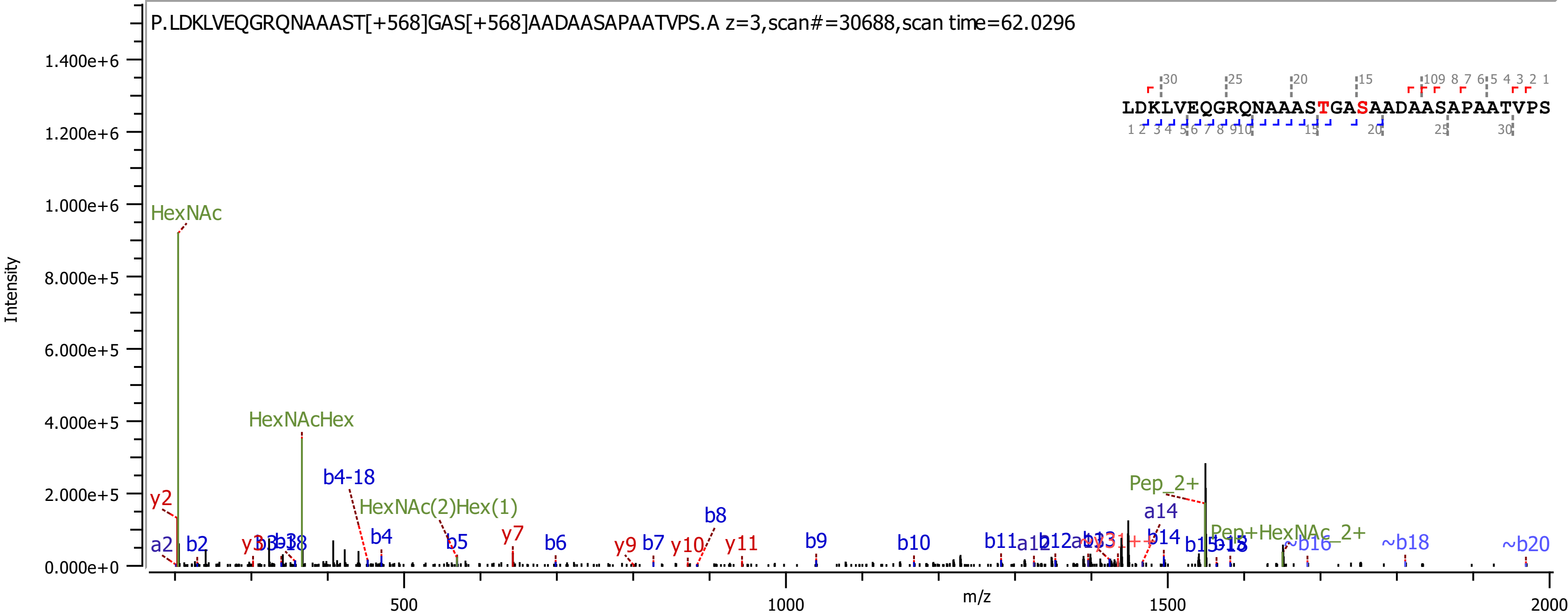
P.LDKLVEQGRQNAAAST[+568]GAS[+568]AADAASAPAATVPS.A z=3,scan#=30688,scan time=62.0296
LDKLVEQGRQNAAASTGASAADAASAPAATVPS
Intensity
m/z
HexNAc
HexNAcHex
HexNAc(2)Hex(1)
b4-18
Pep_2+
Pep+HexNAc_2+
y2
a2
b2
y3
b3
b3-18
b4
b5
y7
b6
y9
b7
y10
b8
y11
b9
b10
b11
a12
b12
b13
a14
b14
b15
~b16
~b18
~b20
0.000e+0
2.000e+5
4.000e+5
6.000e+5
8.000e+5
1.000e+6
1.200e+6
1.400e+6
500
1000
1500
2000

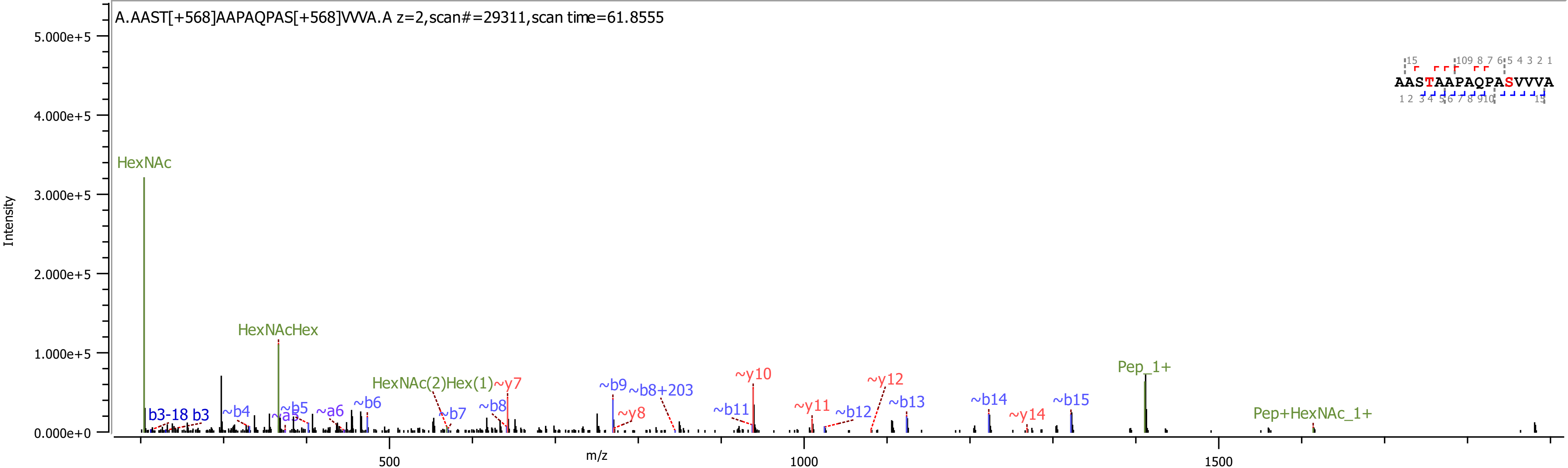

A.AAST[+568]AAPAQPAS[+568]VVVA.A z=2,scan#=29311,scan time=61.8555
Intensity
m/z
5.000e+5
4.000e+5
3.000e+5
2.000e+5
1.000e+5
0.000e+0
500
1000
1500
AASTAAPAQPASVVVA
HexNAc
HexNAcHex
HexNAc(2)Hex(1)
b3-18 b3
~b4
~a5
~b5
~a6
~b6
~b7
~y7
~b8
~b9
~b8+203
~y8
~b11
~y10
~y11
~b12
~y12
~b13
~b14
~y14
~b15
Pep_1+
Pep+HexNAc_1+

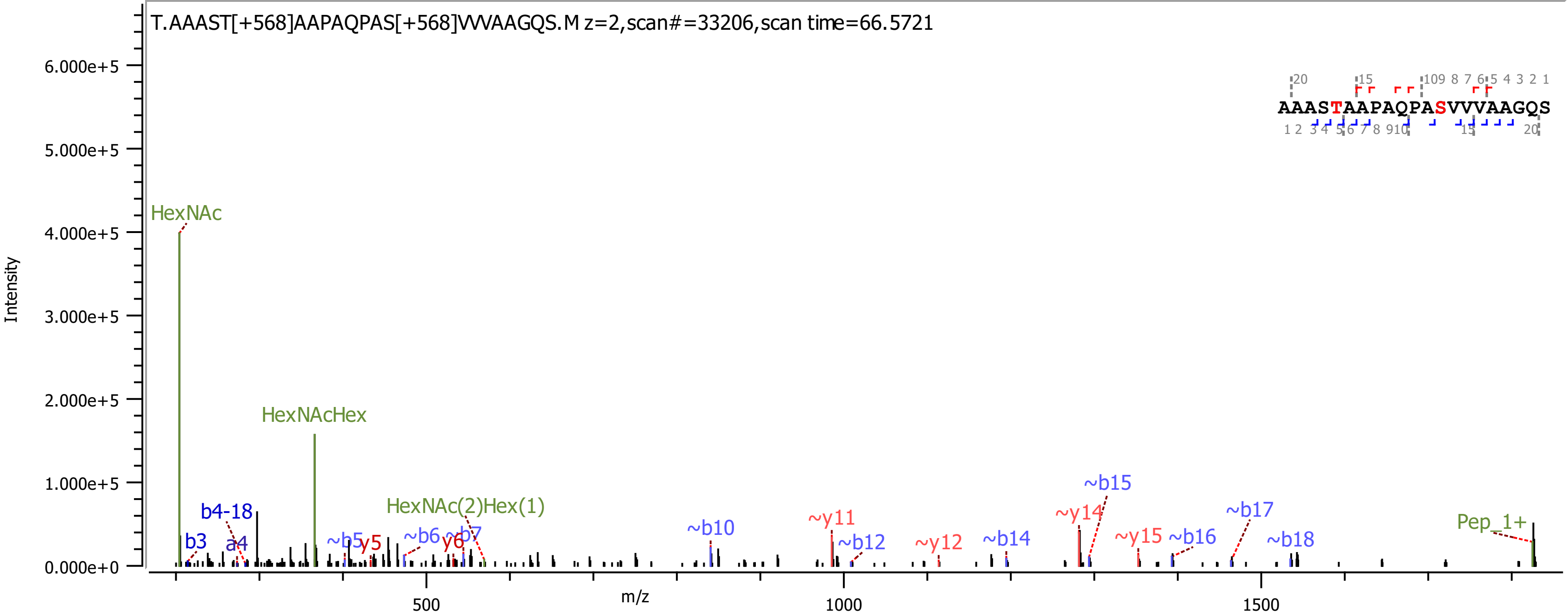
T.AAAST[+568]AAPAQPAS[+568]VVVAAGQS.M z=2,scan#=33206,scan time=66.5721
AAASTAAPAQPASVVVAAGQS
Intensity
6.000e+5
5.000e+5
4.000e+5
3.000e+5
2.000e+5
1.000e+5
0.000e+0
HexNAc
HexNAcHex
HexNAc(2)Hex(1)
b4-18
b3
a4
~b5
y5
~b6
y6
~b7
~b10
~y11
~b12
~y12
~b14
~y14
~b15
~y15
~b16
~b17
~b18
Pep_1+
500
1000
1500
m/z

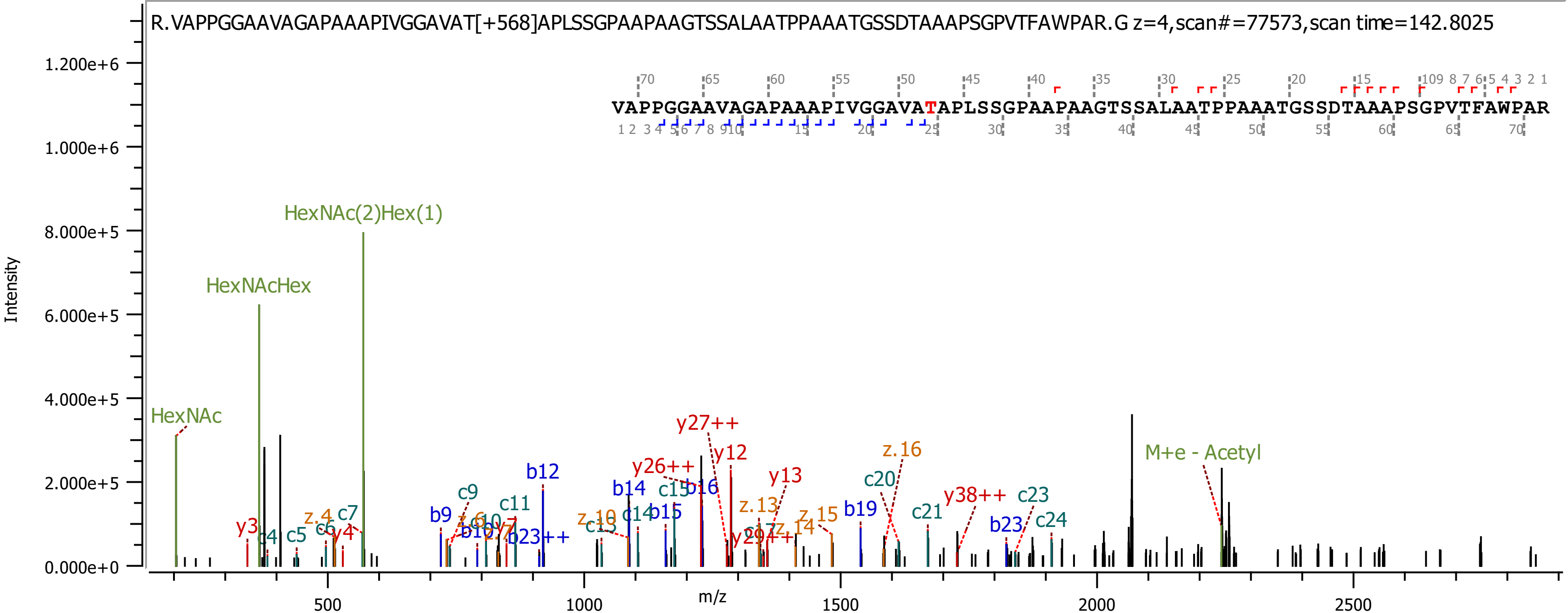

R.VAPPGGAAVAGAPAAAPIVGGAVAT[+568]APLSSGPAAPAAGTSSALAATPPAAATGSSDTAAAPSGPVTFAWPAR.G z=4,scan#=77573,scan time=142.8025
VAPPGGAAVAGAPAAAPIVGGAVATAPLSSGPAAPAAGTSSALAATPPAAATGSSDTAAAPSGPVTFAWPAR
Intensity
m/z
HexNAc(2)Hex(1)
HexNAcHex
HexNAc
M+e - Acetyl
y27++
y26++
y12
y13
y38++
y3
y4
b9
b12
b14
b15
b16
b19
b23
b23++
c4
c5
c7
c9
c11
c13
c14
c15
c20
c21
c23
c24
z.4
z.10
z.13
z.14
z.15
z.16

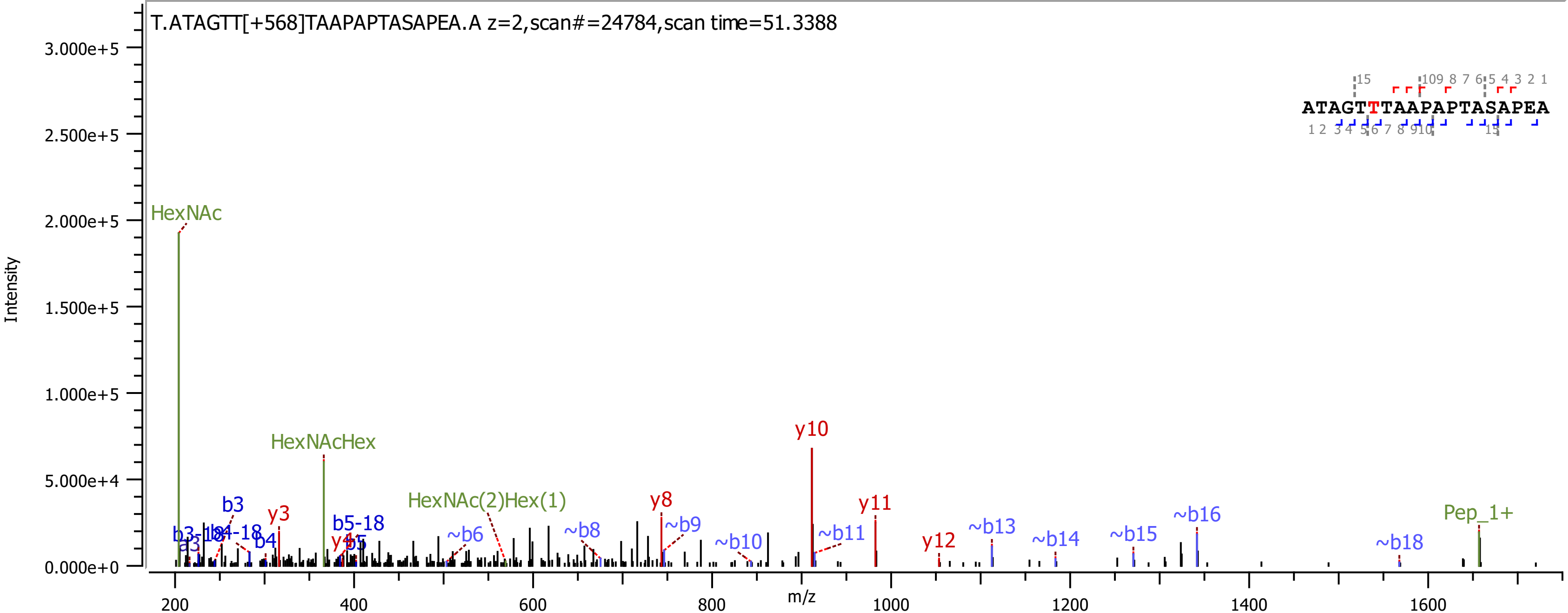
T.ATAGTT[+568]TAAPAPTASAPEA.A z=2,scan#=24784,scan time=51.3388
ATAGTTTAAPAPTASAPEA
Intensity
m/z
HexNAc
HexNAcHex
HexNAc(2)Hex(1)
Pep_1+
y3
y8
y10
y11
y12
b3
b4
b5-18
~b6
~b8
~b9
~b10
~b11
~b13
~b14
~b15
~b16
~b18

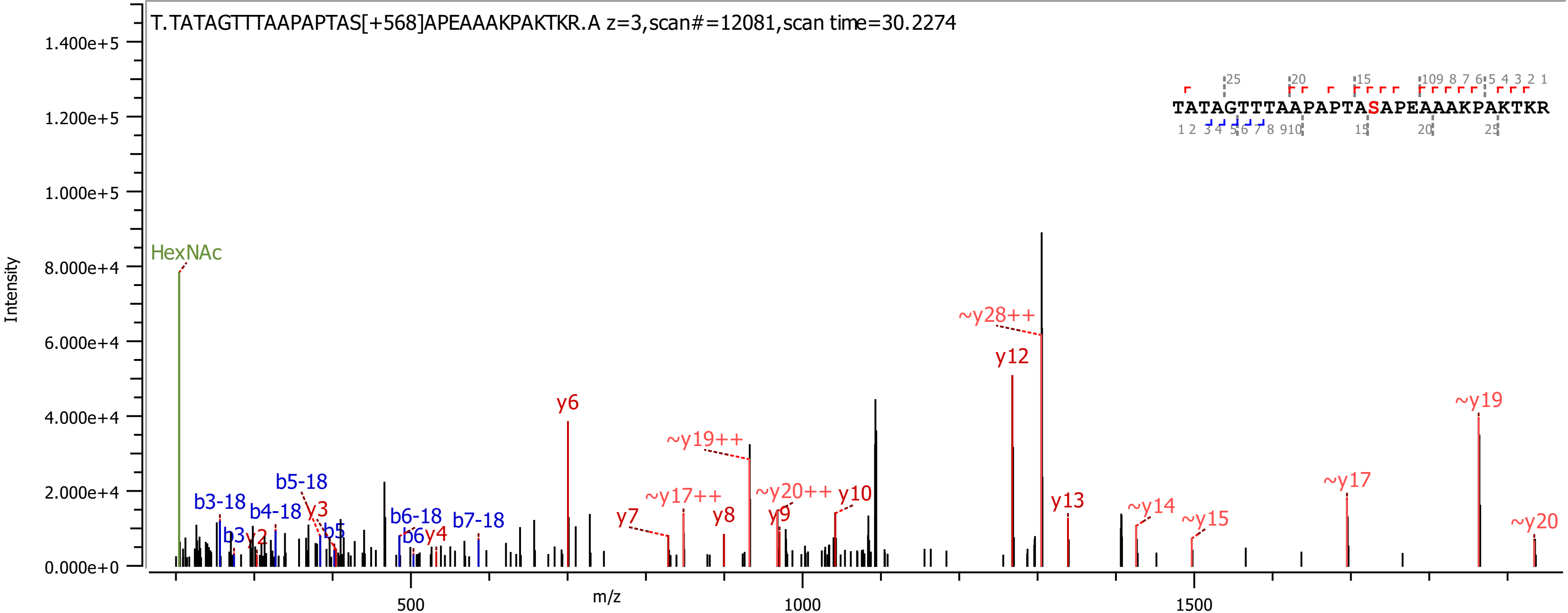

T.TATAGTTTAAPAPTAS[+568]APEAAAKPAKTKR.A z=3,scan#=12081,scan time=30.2274
TATAGTTTAAPAPTASAPEAAAKPAKTKR
Intensity
m/z
1.400e+5
1.200e+5
1.000e+5
8.000e+4
6.000e+4
4.000e+4
2.000e+4
0.000e+0
500
1000
1500
HexNAc
b3-18
b3
y2
b4-18
b5-18
y3
b5
b6-18
b6
y4
b7-18
y6
y7
~y17++
~y19++
y8
y9
~y20++
y10
~y28++
y12
y13
~y14
~y15
~y17
~y19
~y20

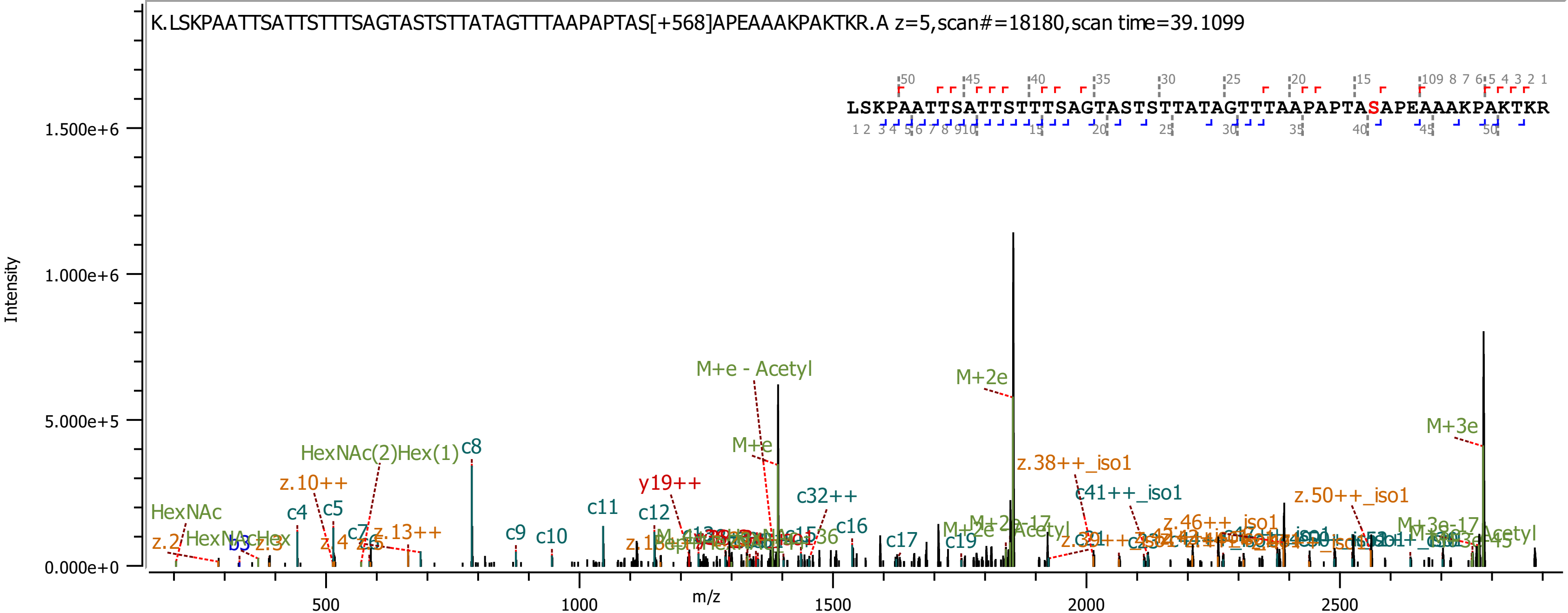
K.LSKPAATTSATTSTTTSAGTASTSTTATAGTTTAAPAPTAS[+568]APEAAAKPAKTKR.A z=5,scan#=18180,scan time=39.1099
LSKPAATTSATTSTTTSAGTASTSTTATAGTTTAAPAPTASAPEAAAKPAKTKR
Intensity
m/z
1.500e+6
1.000e+6
5.000e+5
0.000e+0
500
1000
1500
2000
2500
M+e - Acetyl
M+2e
M+3e
M+e
HexNAc(2)Hex(1)
c8
z.10++
y19++
c32++
z.38++_iso1
c41++_iso1
z.50++_iso1
HexNAc
c4
c5
c11
c12
c9
c10
c16
c17
c19
z.13++
z.46++_iso1
M+3e-17
M+2e-17

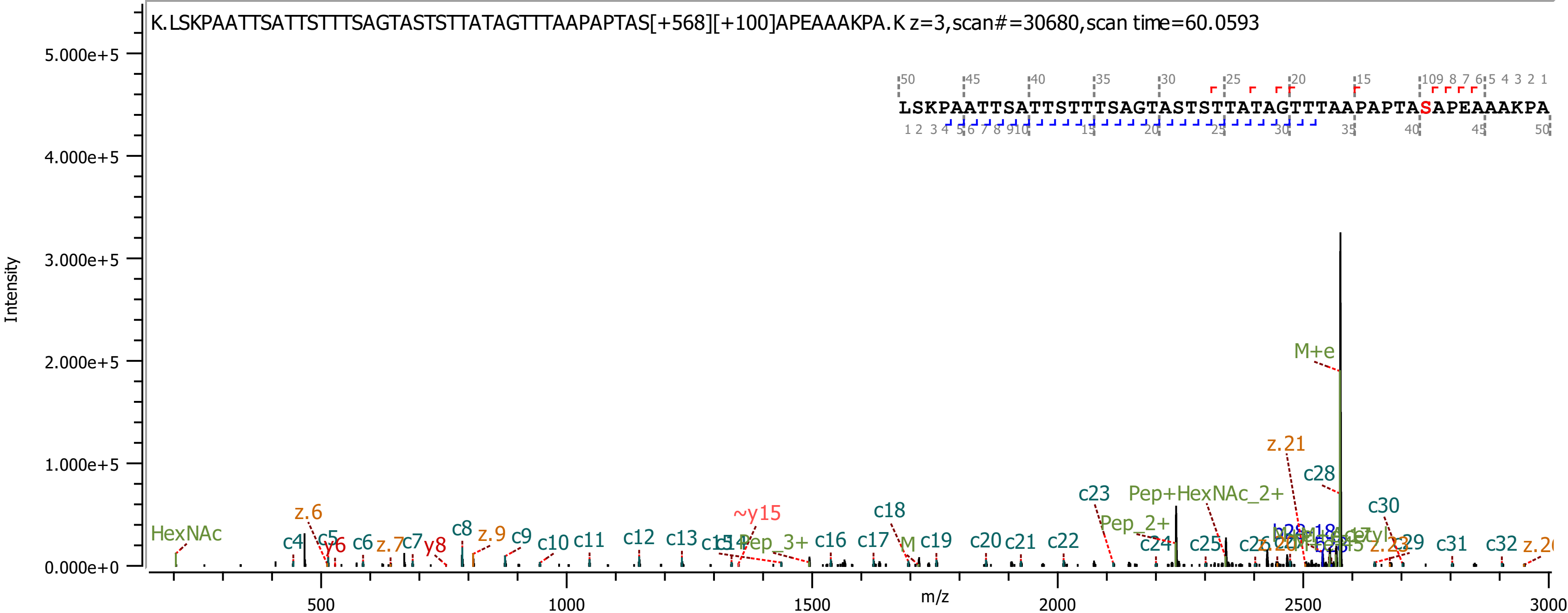

K.LSKPAATTSATTSTTTSAGTASTSTTATAGTTTAAPAPTAS[+568][+100]APEAAAKPA.K z=3,scan#=30680,scan time=60.0593
LSKPAATTSATTSTTTSAGTASTSTTATAGTTTAAPAPTASAPEAAAKPA
Intensity
m/z
5.000e+5
4.000e+5
3.000e+5
2.000e+5
1.000e+5
0.000e+0
500
1000
1500
2000
2500
3000
M+e
z.21
c28
c30
c23
Pep+HexNAc_2+
Pep_2+
c24
c25
~y15
c18
z.6
HexNAc
c4
c5
y6
c6
z.7
c7
y8
c8
z.9
c9
c10
c11
c12
c13
Pep_3+
c16
c17
M
c19
c20
c21
c22
c31
c32

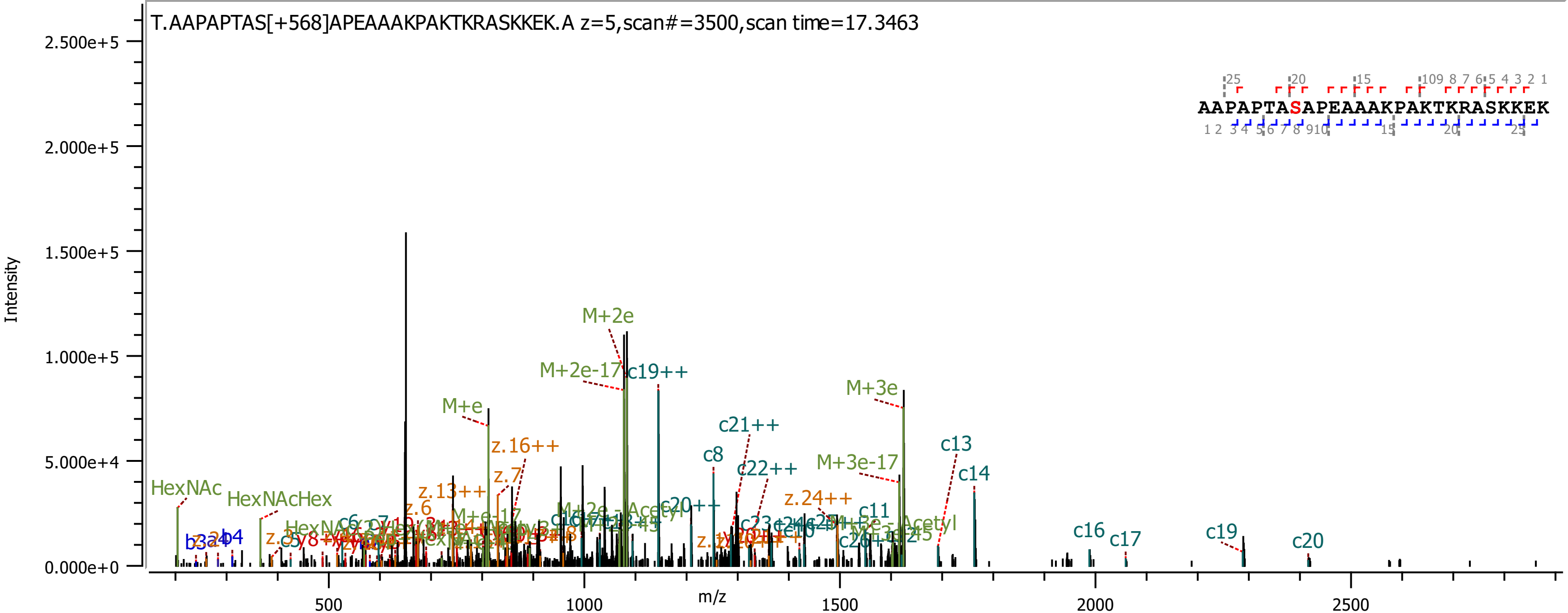
T.AAPAPTAS[+568]APEAAAKPAKTKRASKKEK.A z=5,scan#=3500,scan time=17.3463
AAPAPTASAPEAAAKPAKTKRASKKEK
Intensity
m/z
2.500e+5
2.000e+5
1.500e+5
1.000e+5
5.000e+4
0.000e+0
500
1000
1500
2000
2500
HexNAc
HexNAcHex
M+2e
M+2e-17
c19++
M+e
z.16++
z.7
z.13++
z.6
M+3e
c21++
c8
c22++
M+3e-17
c13
c14
z.24++
c11
c20++
c16
c17
c19
c20
b3
b4

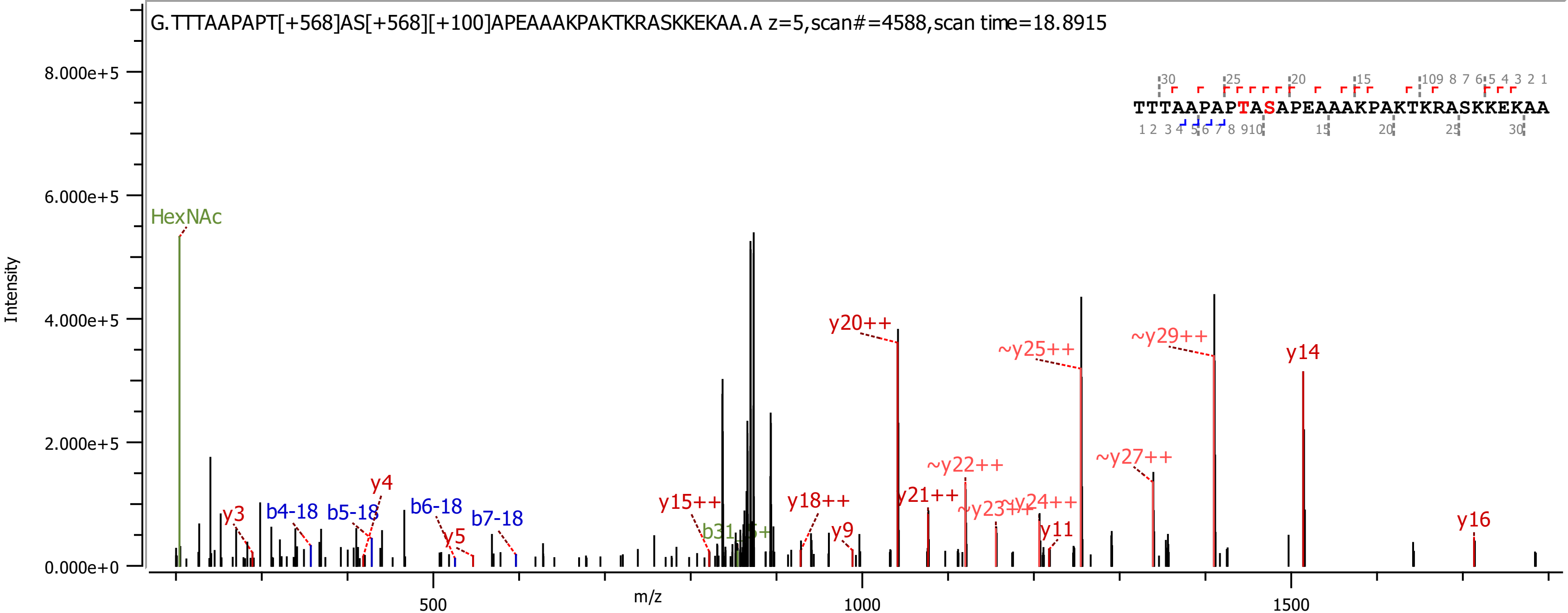

G.TTTAAPAPT[+568]AS[+568][+100]APEAAAKPAKTKRASKKEKAA.A z=5,scan#=4588,scan time=18.8915
Intensity
m/z
HexNAc
y3
b4-18
b5-18
y4
b6-18
y5
b7-18
y15++
y18++
y9
y20++
y21++
~y22++
~y23++
~y24++
y11
~y25++
~y27++
~y29++
y14
y16
TTTAAPAPTASAPEAAAKPAKTKRASKKEKAA

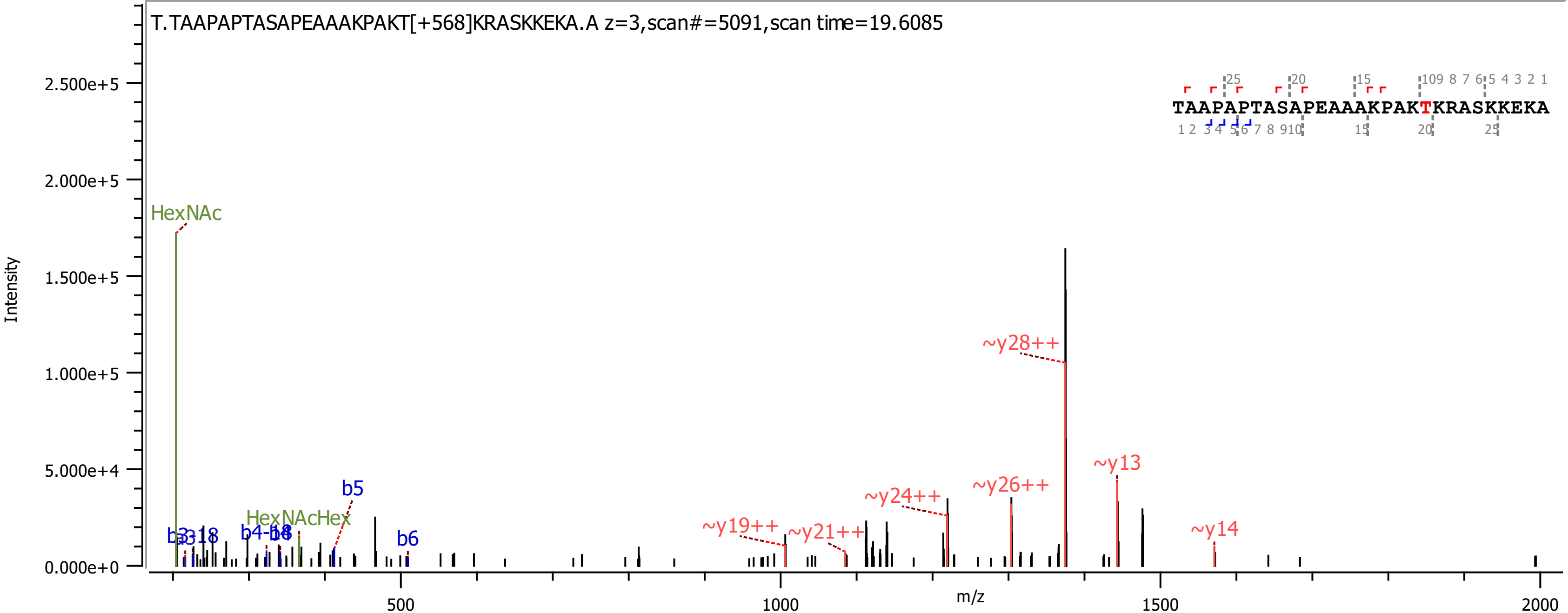
T.TAAPAPTASAPEAAAKPAKT[+568]KRASKKEKA.A z=3,scan#=5091,scan time=19.6085
TAAPAPTASAPEAAAKPAKTKRASKKEKA
Intensity
m/z
HexNAc
HexNAcHex
b3
b3-18
b4-18
b4
b5
b6
~y19++
~y21++
~y24++
~y26++
~y28++
~y13
~y14
0.000e+0
5.000e+4
1.000e+5
1.500e+5
2.000e+5
2.500e+5
500
1000
1500
2000

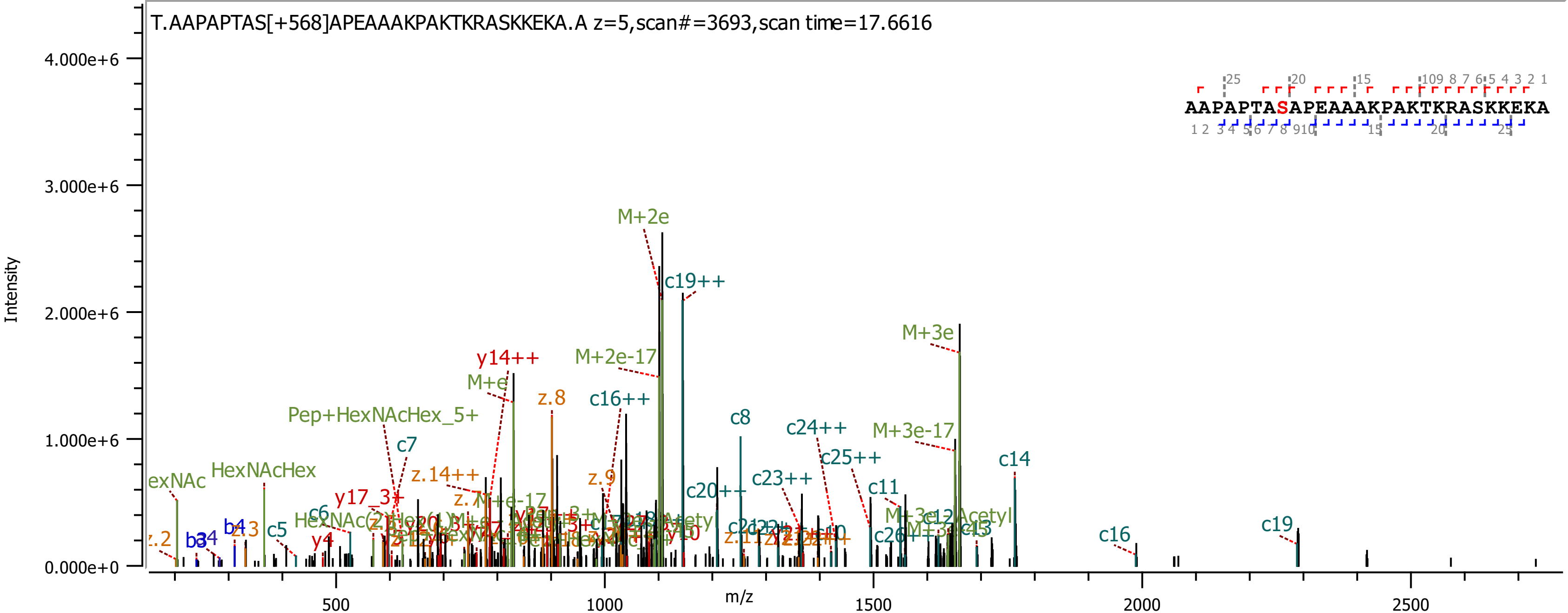
T.AAPAPTAS[+568]APEAAAKPAKTKRASKKEKA.A z=5,scan#=3693,scan time=17.6616
AAPAPTASAPEAAAKPAKTKRASKKEKA
Intensity
m/z
4.000e+6
3.000e+6
2.000e+6
1.000e+6
0.000e+0
500
1000
1500
2000
2500
M+2e
c19++
M+3e
M+2e-17
y14++
M+e
z.8
c16++
Pep+HexNAcHex_5+
c7
c8
c24++
M+3e-17
c25++
c14
HexNAcHex
z.14++
z.9
c20++
c23++
c11
y17_3+
c16
c19
c5
b4
y4

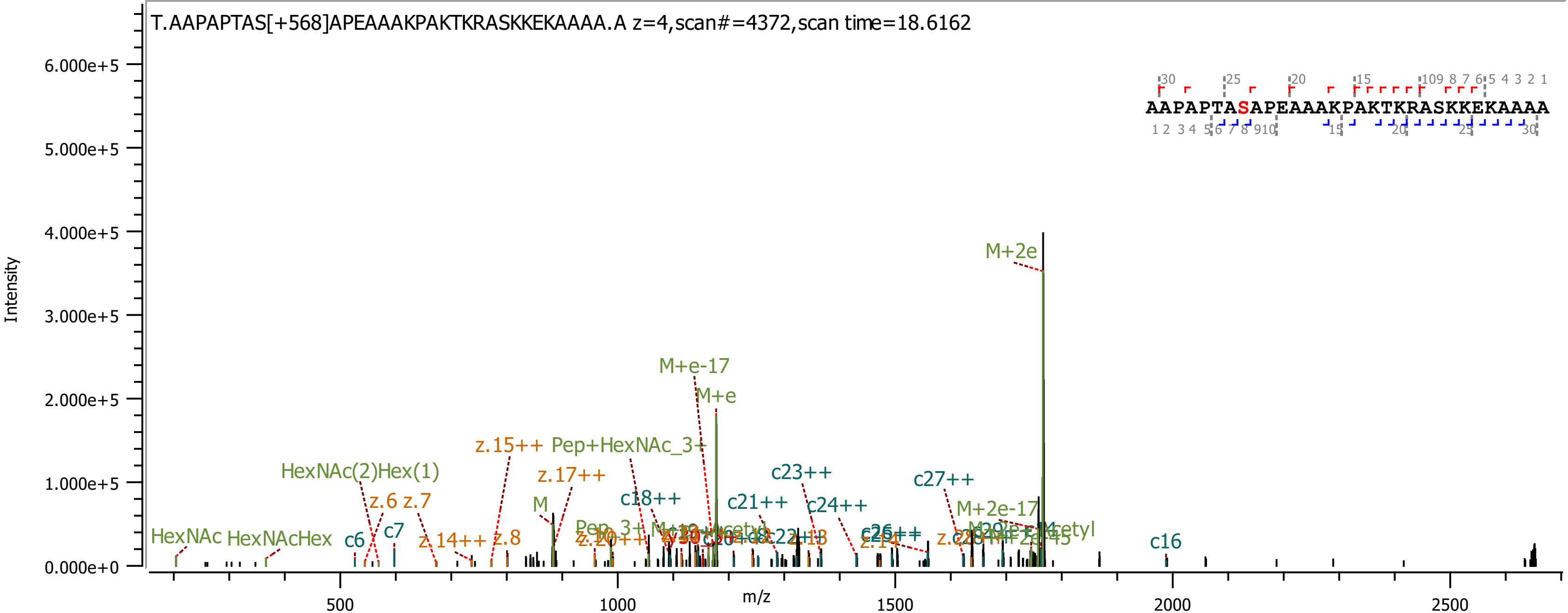

T.AAPAPTAS[+568]APEAAAKPAKTKRASKKEKAAAA.A z=4,scan#=4372,scan time=18.6162
AAPAPTASAPEAAAKPAKTKRASKKEKAAAA
Intensity
m/z
M+2e
M+e-17
M+e
z.15++
Pep+HexNAc_3+
z.17++
HexNAc(2)Hex(1)
z.6
z.7
c18++
c21++
c23++
c24++
c27++
M+2e-17
M
c7
c6
z.14++
z.8
HexNAc
HexNAcHex
c16

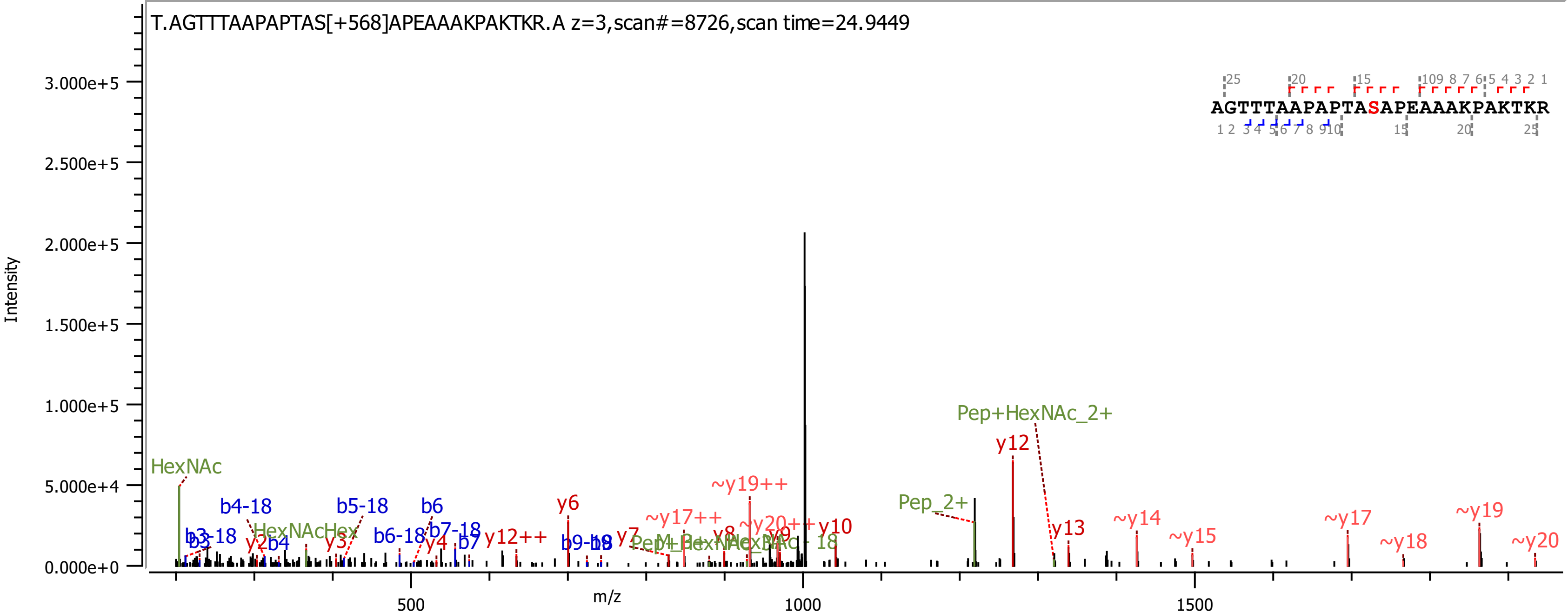

T.AGTTTAAPAPTAS[+568]APEAAAKPAKTKR.A z=3,scan#=8726,scan time=24.9449
AGTTTAAPAPTASAPEAAAKPAKTKR
Intensity
m/z
0.000e+0
5.000e+4
1.000e+5
1.500e+5
2.000e+5
2.500e+5
3.000e+5
500
1000
1500
HexNAc
b4-18
b5-18
b6
y6
~y19++
~y17++
~y20++
y10
Pep_2+
Pep+HexNAc_2+
y12
y13
~y14
~y15
~y17
~y18
~y19
~y20
b3-18
b3
y2
b4
HexNAcHex
y3
b6-18
b7-18
y4
b7
y12++
b9-18
b8
y7
y8
y9

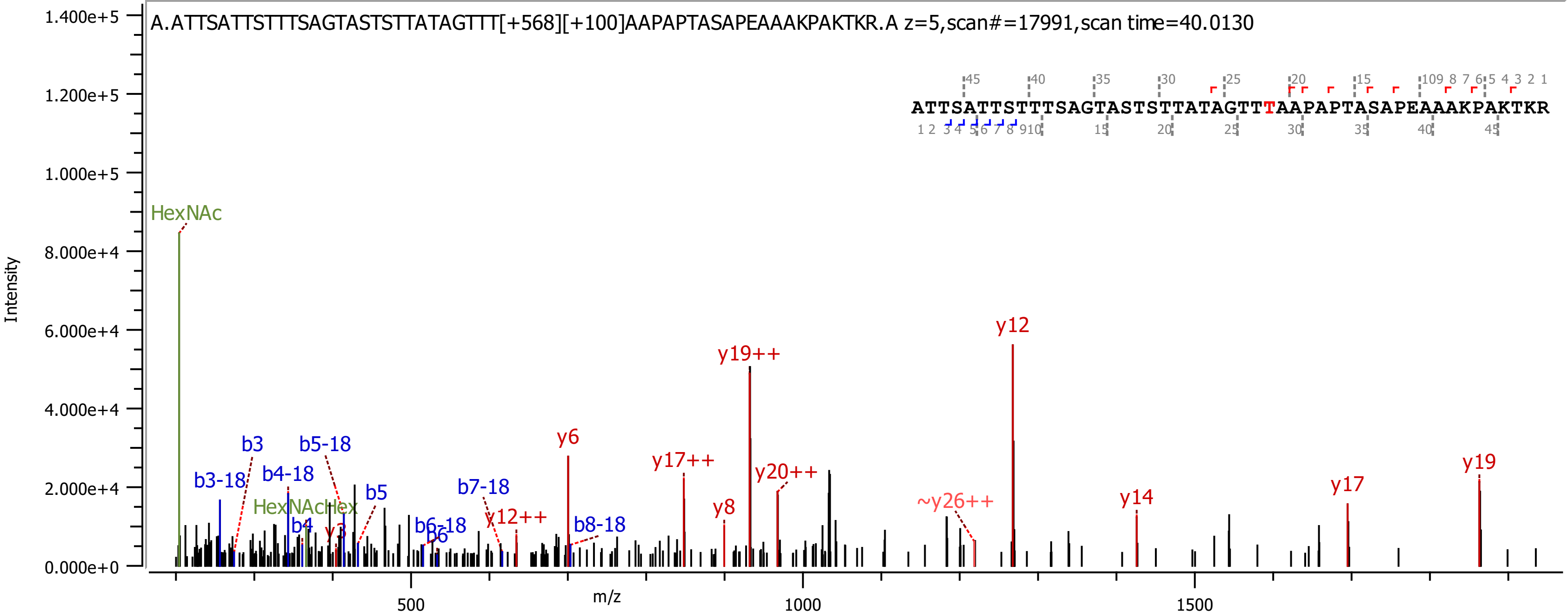

A.ATTSATTSTTTSAGTASTSTTATAGTTT[+568][+100]AAPAPTASAPEAAAKPAKTKR.A z=5,scan#=17991,scan time=40.0130
ATTSATTSTTTSAGTASTSTTATAGTTTAAPAPTASAPEAAAKPAKTKR
Intensity
m/z
HexNAc
HexNAcHex
b3-18
b3
b4-18
b4
b5-18
b5
b6-18
b6
b7-18
b8-18
y3
y6
y8
y12++
y17++
y19++
y20++
~y26++
y12
y14
y17
y19

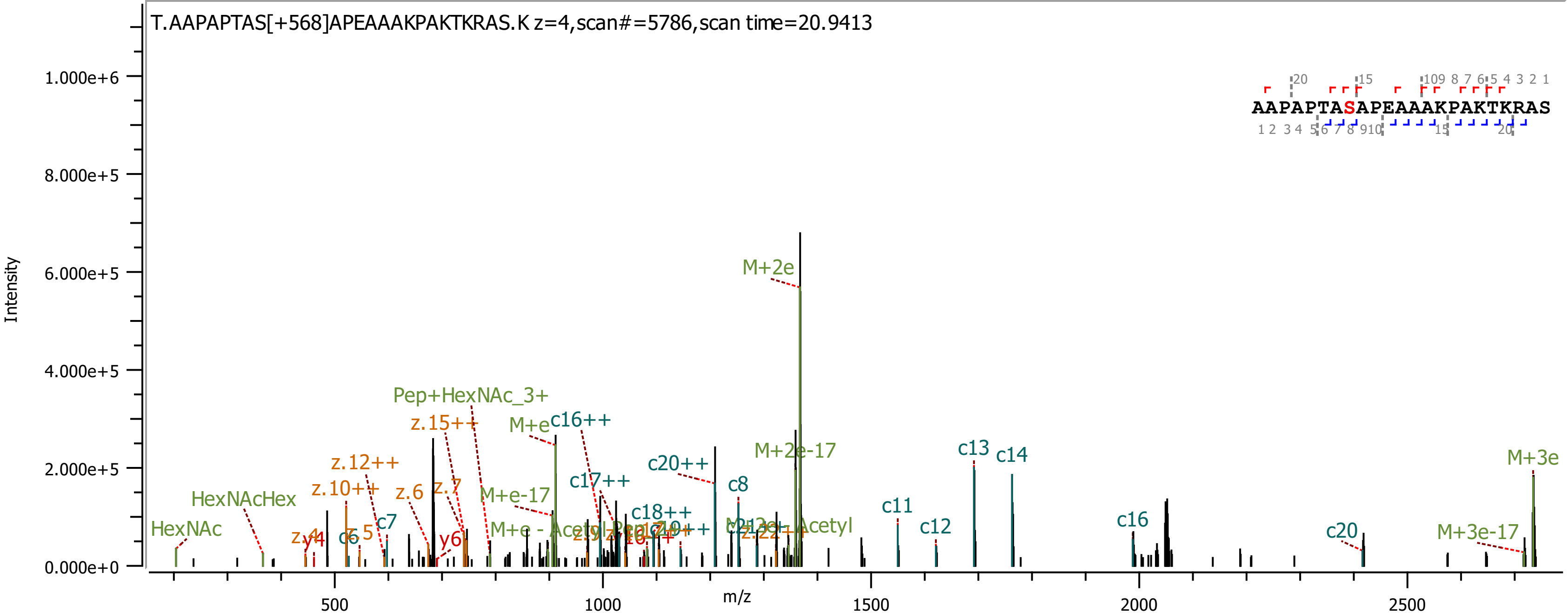

T.AAPAPTAS[+568]APEAAAKPAKTKRAS.K z=4,scan#=5786,scan time=20.9413
AAPAPTASAPEAAAKPAKTKRAS
Intensity
m/z
M+2e
M+3e
c13
c14
c16++
c20++
c8
c11
c12
c16
c20
M+2e-17
M+3e-17
Pep+HexNAc_3+
z.15++
z.12++
z.10++
z.6
z.7
M+e
M+e-17
HexNAcHex
HexNAc
c17++
c18++
c7
c6
y6
z.5

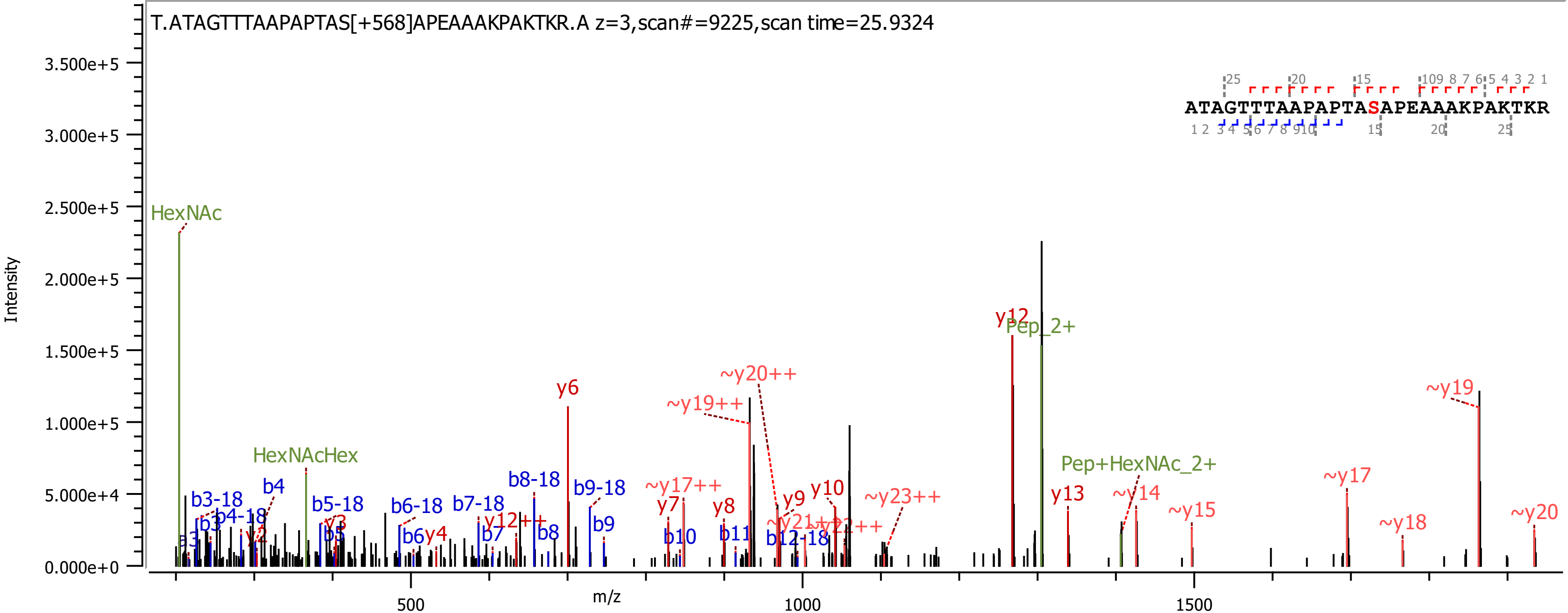

T.ATAGTTTAAPAPTAS[+568]APEAAAKPAKTKR.A z=3,scan#=9225,scan time=25.9324
ATAGTTTAAPAPTASAPEAAAKPAKTKR
Intensity
m/z
HexNAc
HexNAcHex
Pep_2+
Pep+HexNAc_2+
y12
y6
~y20++
~y19++
~y19
~y17
~y20
~y18
~y15
~y14
y13
y10
y9
y8
y7
~y17++
~y23++
b8-18
b9-18
b9
b8
b7-18
b7
b6-18
b6
y4
b5-18
b5
y3
b4
b3-18
b4-18
b3
y2
b10
b11
b12-18
y12++

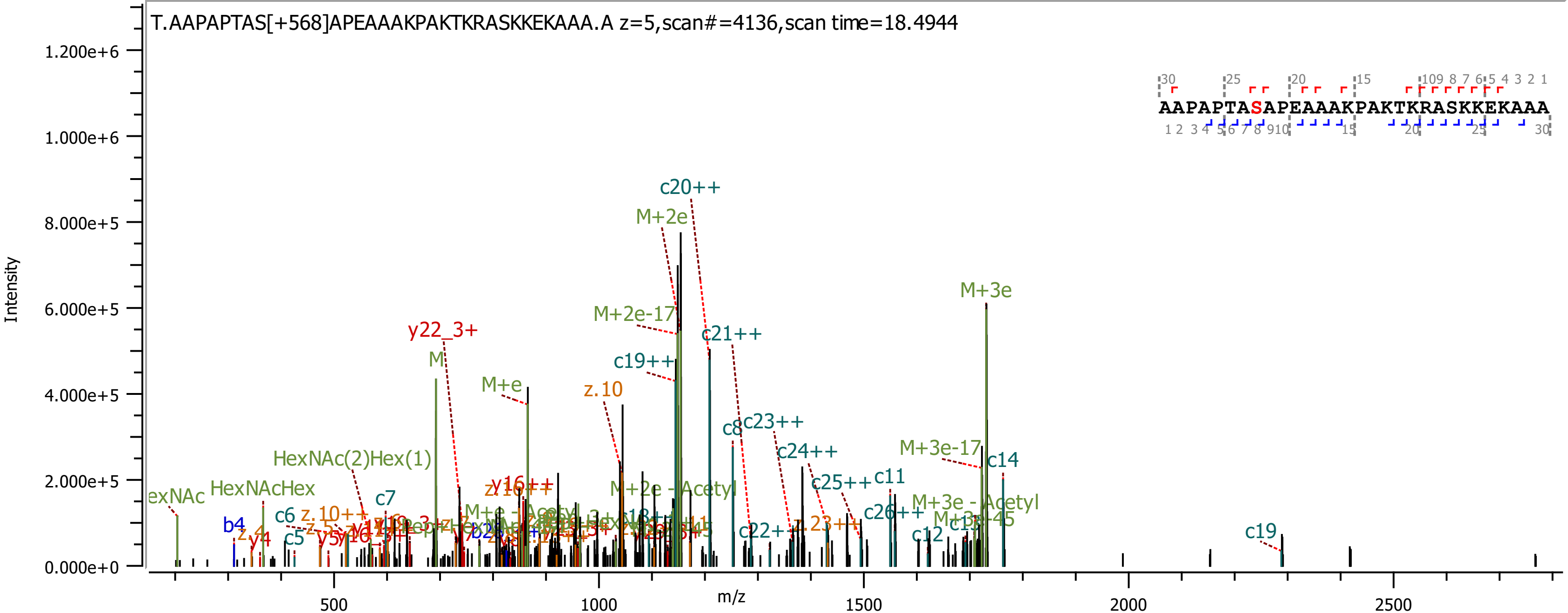
T.AAPAPTAS[+568]APEAAAKPAKTKRASKKEKAAA.A z=5,scan#=4136,scan time=18.4944
AAPAPTASAPEAAAKPAKTKRASKKEKAAA
Intensity
m/z
1.200e+6
1.000e+6
8.000e+5
6.000e+5
4.000e+5
2.000e+5
0.000e+0
500
1000
1500
2000
2500
c20++
M+2e
M+3e
y22_3+
M
M+2e-17
c21++
c19++
M+e
z.10
c8
c23++
c24++
M+3e-17
c14
HexNAc(2)Hex(1)
c11
c25++
HexNAcHex
M+2e - Acetyl
c7
c26++
M+3e - Acetyl
c6
b4
c5
c22++
z.23++
c12
c19

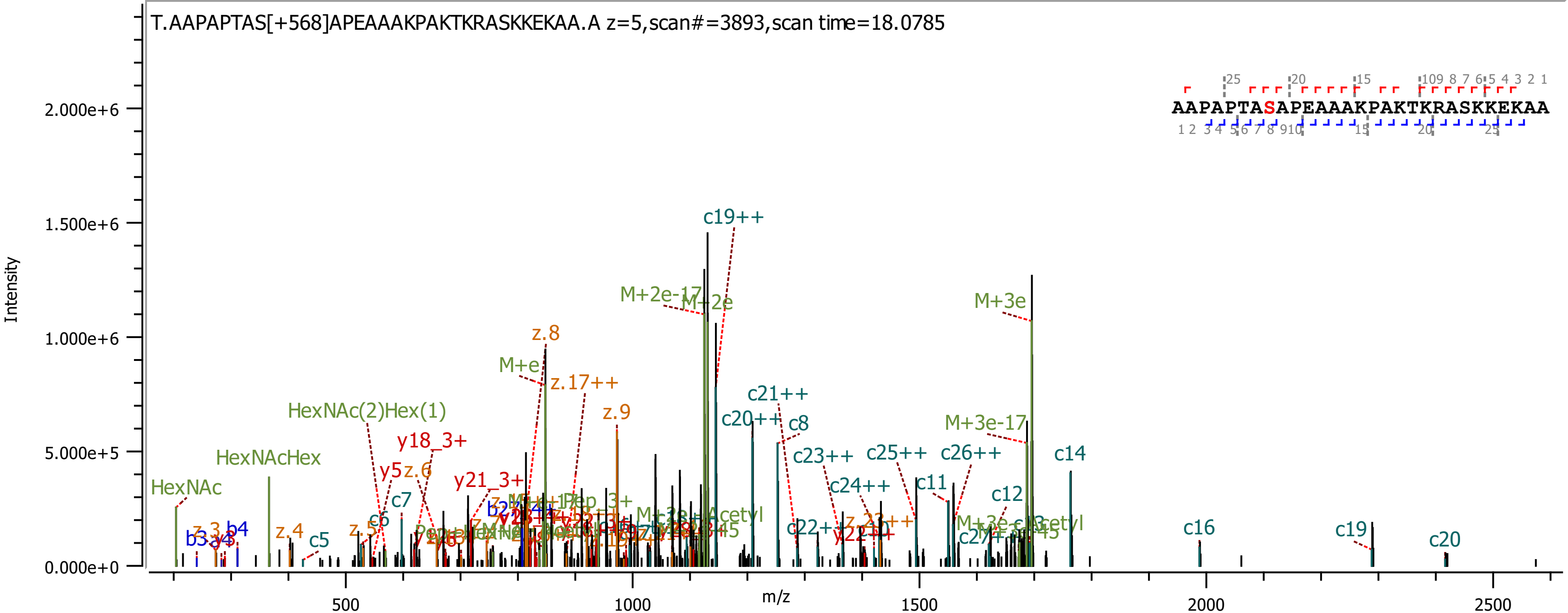

T.AAPAPTAS[+568]APEAAAKPAKTKRASKKEKAA.A z=5,scan#=3893,scan time=18.0785
AAPAPTASAPEAAAKPAKTKRASKKEKAA
Intensity
m/z
0.000e+0
5.000e+5
1.000e+6
1.500e+6
2.000e+6
500
1000
1500
2000
2500
c19++
M+2e-17
M+2e
M+3e
z.8
M+e
z.17++
HexNAc(2)Hex(1)
z.9
c21++
c20++
c8
y18_3+
M+3e-17
c14
HexNAcHex
y5
z.6
c23++
c25++
c26++
y21_3+
c24++
c11
HexNAc
c7
c12
c6
b4
z.4
z.3
c5
z.5
c22++
y22++
c16
c19
c20

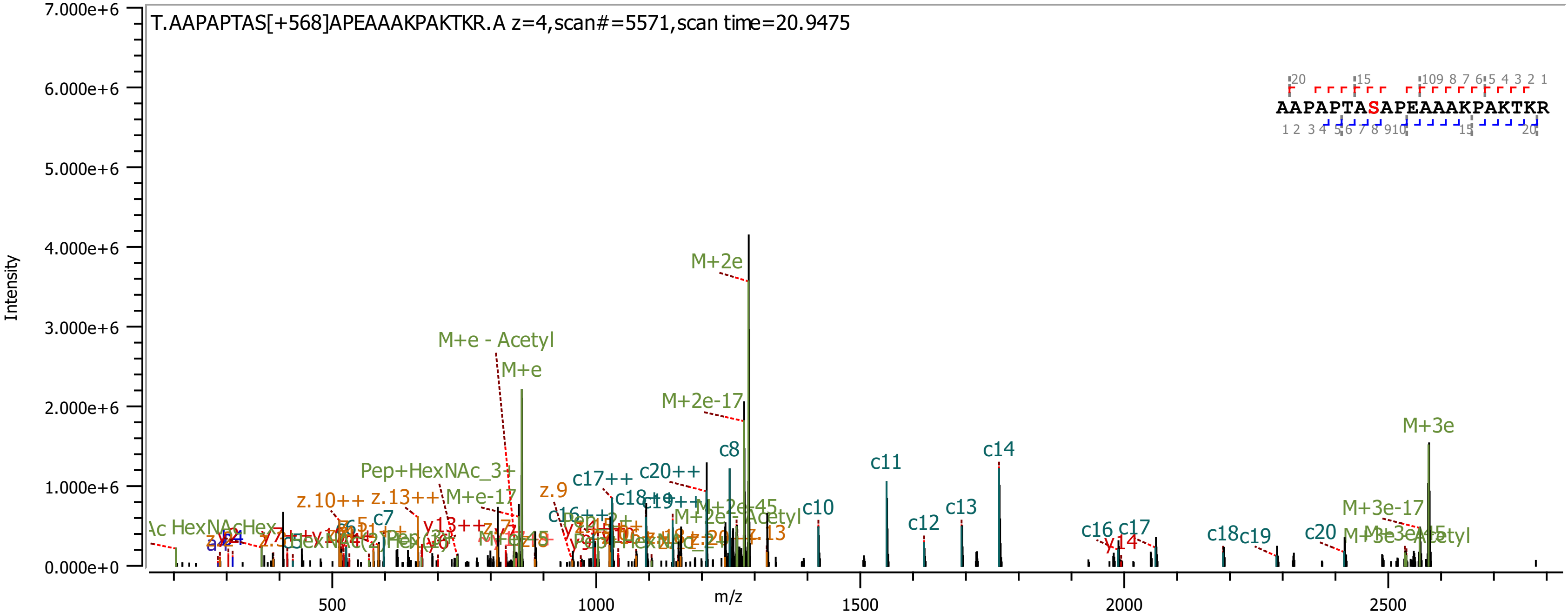
T.AAPAPTAS[+568]APEAAAKPAKTKR.A z=4,scan#=5571,scan time=20.9475
AAPAPTASAPEAAAKPAKTKR
Intensity
m/z
M+2e
M+e - Acetyl
M+e
M+2e-17
M+3e
M+3e-17
Pep+HexNAc_3+
c8
c10
c11
c12
c13
c14
c16
c17
c18
c19
c20
c7
c17++
c20++
z.9
z.10++
z.13++
M+e-17
y14
7.000e+6
6.000e+6
5.000e+6
4.000e+6
3.000e+6
2.000e+6
1.000e+6
0.000e+0
500
1000
1500
2000
2500

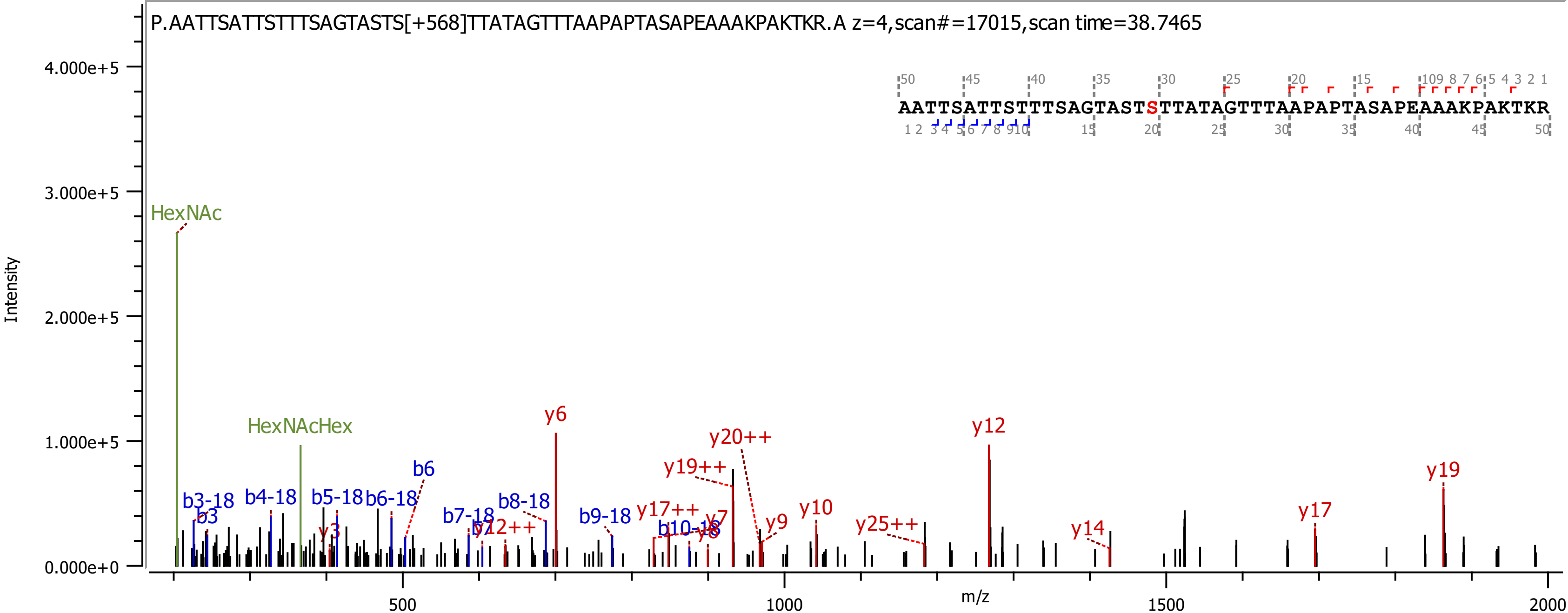

P.AATTSATTSTTTSAGTASTS[+568]TTATAGTTTAAPAPTASAPEAAAKPAKTKR.A z=4,scan#=17015,scan time=38.7465
AATTSATTSTTTSAGTASTSTTATAGTTTAAPAPTASAPEAAAKPAKTKR
Intensity
m/z
4.000e+5
3.000e+5
2.000e+5
1.000e+5
0.000e+0
500
1000
1500
2000
HexNAc
HexNAcHex
b3-18
b3
b4-18
b5-18
b6-18
b6
y3
b7-18
y12++
b8-18
y6
b9-18
y17++
b10-18
y7
y8
y19++
y20++
y9
y10
y25++
y12
y14
y17
y19

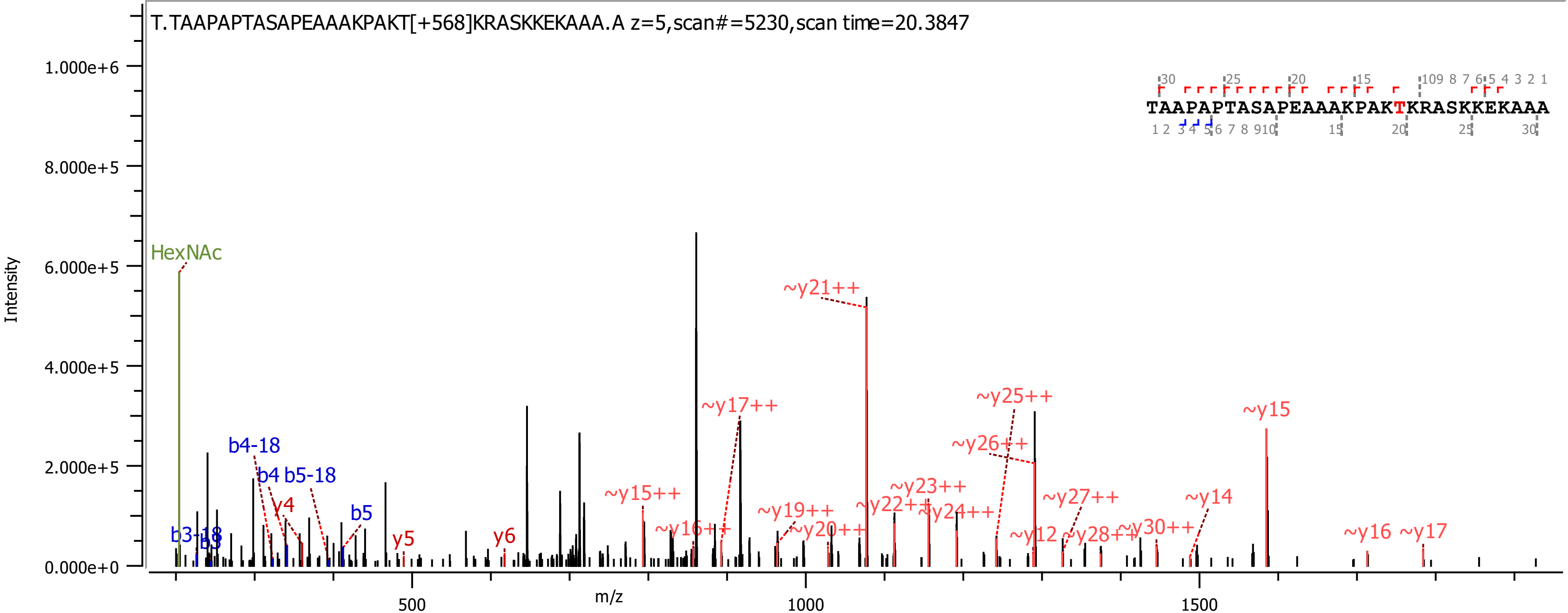

T.TAAPAPTASAPEAAAKPAKT[+568]KRASKKEKAAA.A z=5,scan#=5230,scan time=20.3847
Intensity
1.000e+6
8.000e+5
6.000e+5
4.000e+5
2.000e+5
0.000e+0
m/z
500
1000
1500
TAAPAPTASAPEAAAKPAKTKRASKKEKAAA
HexNAc
b3-18
b3
b4-18
b4
b5-18
b5
y4
y5
y6
~y15++
~y16++
~y17++
~y19++
~y20++
~y21++
~y22++
~y23++
~y24++
~y25++
~y26++
~y27++
~y28++
~y30++
~y12
~y14
~y15
~y16
~y17

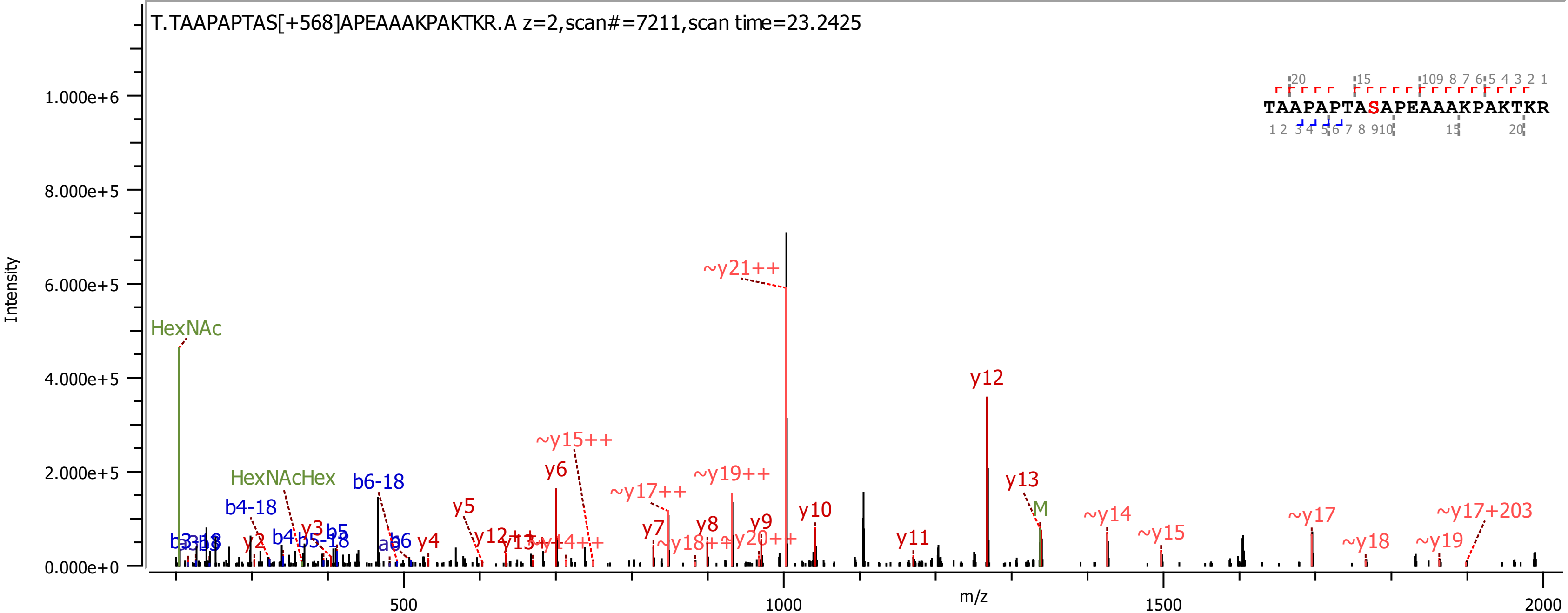
T.TAAPAPTAS[+568]APEAAAKPAKTKR.A z=2,scan#=7211,scan time=23.2425
TAAPAPTASAPEAAAKPAKTKR
Intensity
m/z
1.000e+6
8.000e+5
6.000e+5
4.000e+5
2.000e+5
0.000e+0
500
1000
1500
2000
HexNAc
HexNAcHex
~y21++
y12
~y15++
y6
b6-18
b4-18
~y17++
~y19++
y13
M
y5
y10
y9
y8
y7
y11
~y14
~y15
~y17
~y17+203
~y18
~y19
y4
y2
b4
b5-18
b6
y12++
~y18++
~y20++

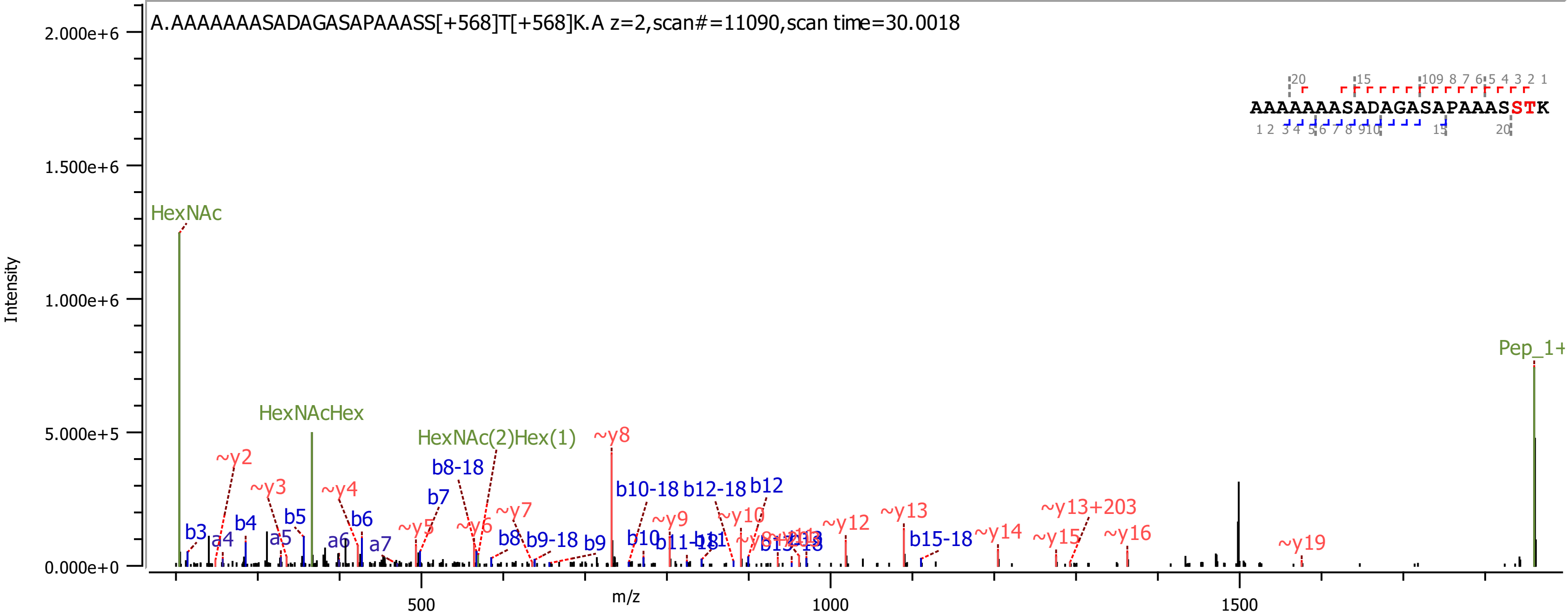
A.AAAAAAASADAGASAPAAASS[+568]T[+568]K.A z=2,scan#=11090,scan time=30.0018
AAAAAAASADAGASAPAAASSTK
Intensity
m/z
HexNAc
HexNAcHex
HexNAc(2)Hex(1)
Pep_1+
~y2
~y3
~y4
~y5
~y6
~y7
~y8
~y9
~y10
~y12
~y13
~y13+203
~y14
~y15
~y16
~y19
b3
b4
b5
b6
b7
b8
b8-18
b9
b9-18
b10
b10-18
b11-18
b12
b12-18
b15-18
a4
a5
a6
a7
0.000e+0
5.000e+5
1.000e+6
1.500e+6
2.000e+6
500
1000
1500

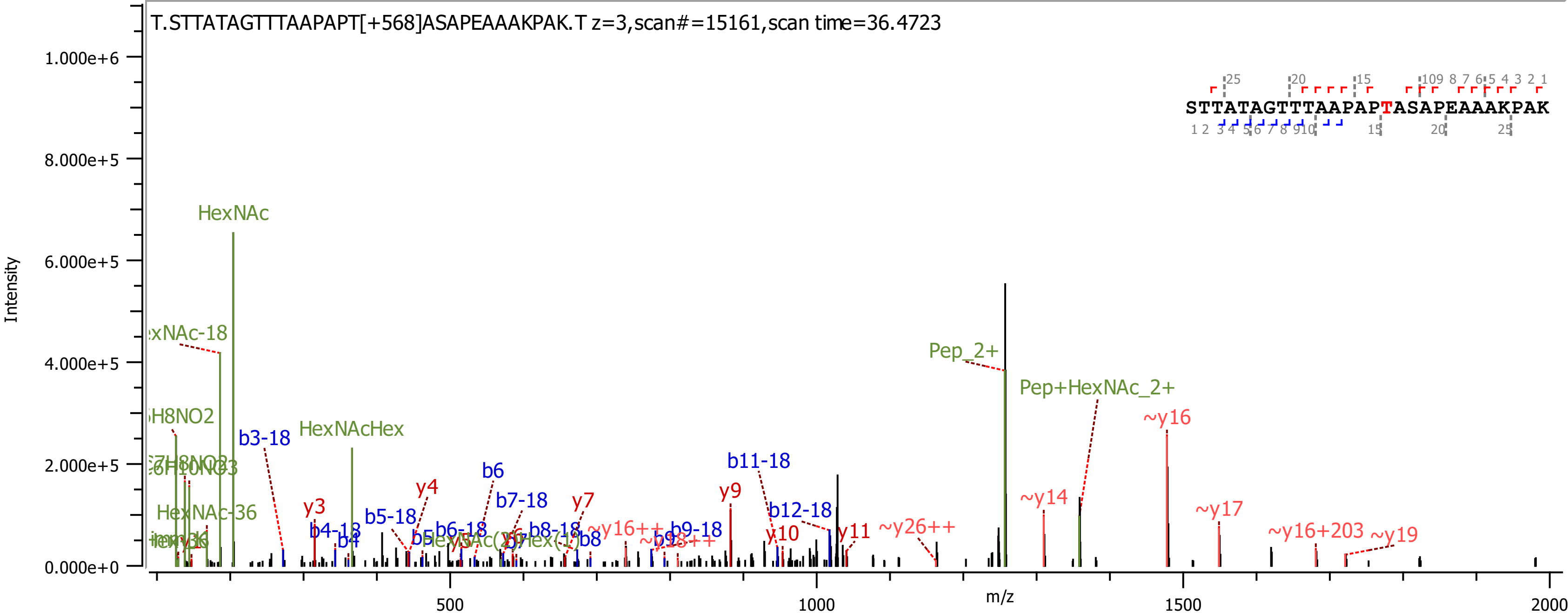

T.STTATAGTTTAAPAPT[+568]ASAPEAAAKPAK.T z=3,scan#=15161,scan time=36.4723
STTATAGTTTAAPAPTASAPEAAAKPAK
Intensity
m/z
1.000e+6
8.000e+5
6.000e+5
4.000e+5
2.000e+5
0.000e+0
500
1000
1500
2000
HexNAc
HexNAcHex
HexNAc-36
Pep_2+
Pep+HexNAc_2+
b3-18
b4-18
b4
b5-18
b6
b6-18
b7-18
b8-18
b8
b9-18
b11-18
b12-18
y3
y4
y7
y9
y10
y11
~y14
~y16
~y17
~y16+203
~y19
~y26++
~y16++

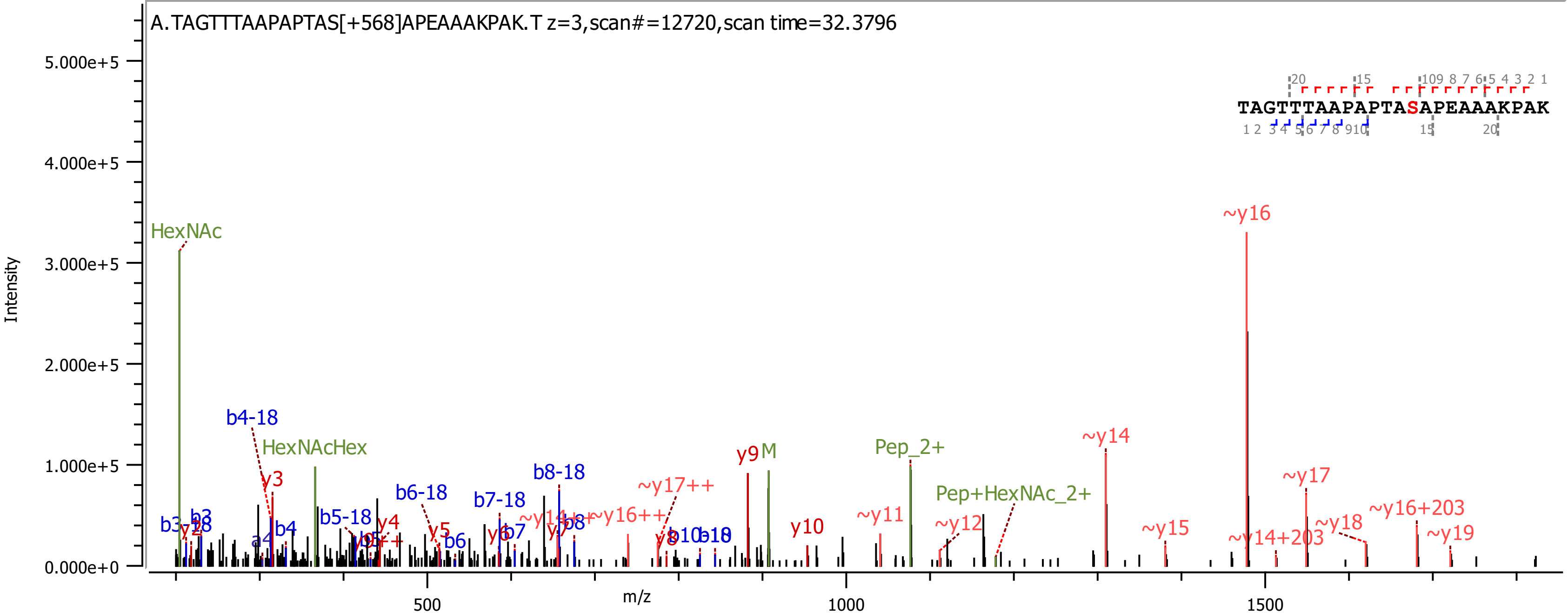

A.TAGTTTAAPAPTAS[+568]APEAAAKPAK.T z=3,scan#=12720,scan time=32.3796
TAGTTTAAPAPTASAPEAAAKPAK
Intensity
m/z
5.000e+5
4.000e+5
3.000e+5
2.000e+5
1.000e+5
0.000e+0
500
1000
1500
HexNAc
HexNAcHex
b4-18
y3
b5-18
b6-18
b7-18
b8-18
y4
y5
b6
y6
b7
y7
b8
~y17++
~y16++
y8
b10
y9
M
y10
~y11
Pep_2+
~y12
Pep+HexNAc_2+
~y14
~y15
~y16
~y14+203
~y17
~y18
~y16+203
~y19

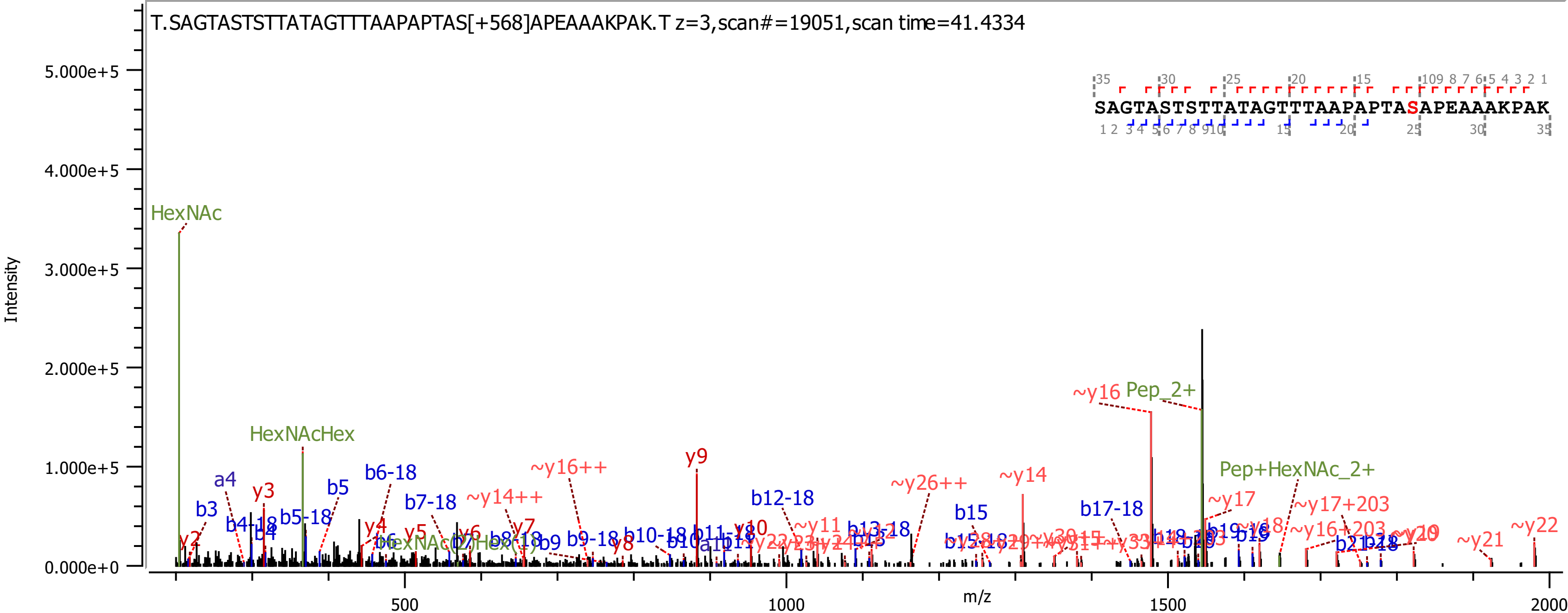
T.SAGTASTSTTATAGTTTAAPAPTAS[+568]APEAAAKPAK.T z=3,scan#=19051,scan time=41.4334
SAGTASTSTTATAGTTTAAPAPTASAPEAAAKPAK
Intensity
m/z
5.000e+5
4.000e+5
3.000e+5
2.000e+5
1.000e+5
0.000e+0
500
1000
1500
2000
HexNAc
HexNAcHex
Pep_2+
Pep+HexNAc_2+
~y16
~y14
~y16++
~y14++
~y26++
~y17
~y17+203
~y16+203
~y21
~y22
y9
y3
y4
y5
y6
y7
y8
y10
a4
b3
b4-18
b4
b5
b5-18
b6-18
b7-18
b12-18
b13-18
b15
b17-18
b9-18
b10-18
b11-18
b9
b19

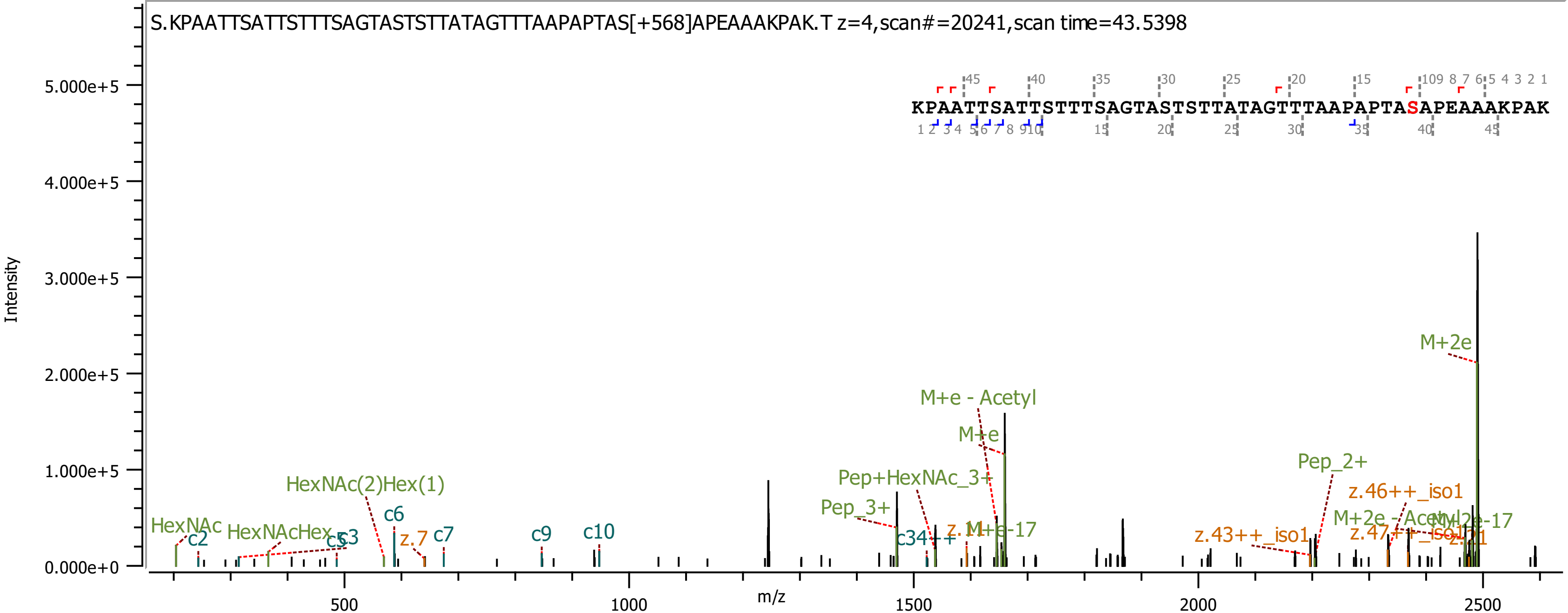

S.KPAATTSATTSTTTSAGTASTSTTATAGTTTAAPAPTAS[+568]APEAAAKPAK.T z=4,scan#=20241,scan time=43.5398
KPAATTSATTSTTTSAGTASTSTTATAGTTTAAPAPTASAPEAAAKPAK
Intensity
m/z
5.000e+5
4.000e+5
3.000e+5
2.000e+5
1.000e+5
0.000e+0
500
1000
1500
2000
2500
M+2e
M+e - Acetyl
M+e
Pep+HexNAc_3+
Pep_3+
Pep_2+
z.46++_iso1
z.43++_iso1
z.47++_iso1
HexNAc(2)Hex(1)
HexNAc
HexNAcHex
c2
c3
c5
c6
c7
c9
c10
z.7
c34++

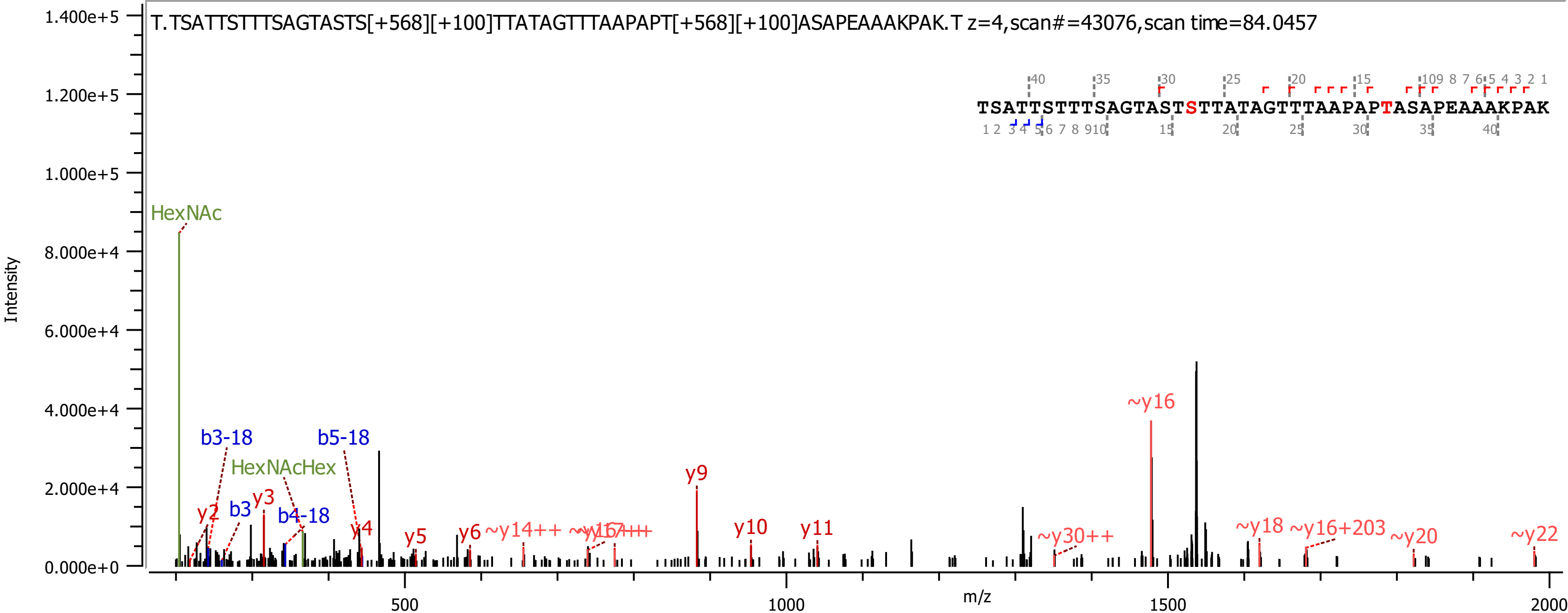
T.TSATTSTTTSAGTASTS[+568][+100]TTATAGTTTAAPAPT[+568][+100]ASAPEAAAKPAK.T z=4,scan#=43076,scan time=84.0457
TSATTSTTTSAGTASTSTTATAGTTTAAPAPTASAPEAAAKPAK
Intensity
m/z
HexNAc
HexNAcHex
b3-18
b5-18
b3
b4-18
y2
y3
y4
y5
y6
~y14++
y9
y10
y11
~y30++
~y16
~y18
~y16+203
~y20
~y22

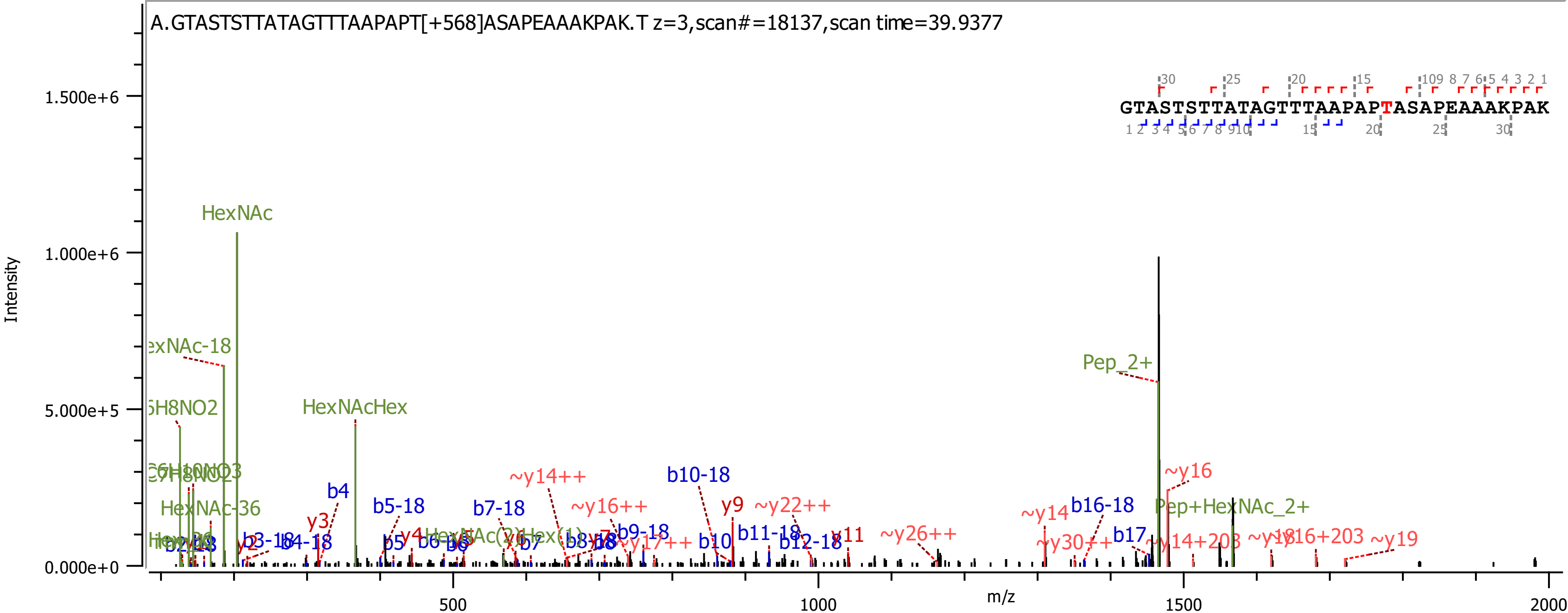
A.GTASTSTTATAGTTTAAPAPT[+568]ASAPEAAAKPAK.T z=3,scan#=18137,scan time=39.9377
GTASTSTTATAGTTTAAPAPTASAPEAAAKPAK
Intensity
m/z
1.500e+6
1.000e+6
5.000e+5
0.000e+0
500
1000
1500
2000
HexNAc
HexNAcHex
HexNAc-36
Pep_2+
Pep+HexNAc_2+
~y16
~y14++
~y16++
~y22++
~y26++
~y14
~y30++
~y19
b10-18
b16-18
b17
b4
b5-18
b7-18
b9-18
b11-18
b12-18
b10
y9
y11
y3
y2

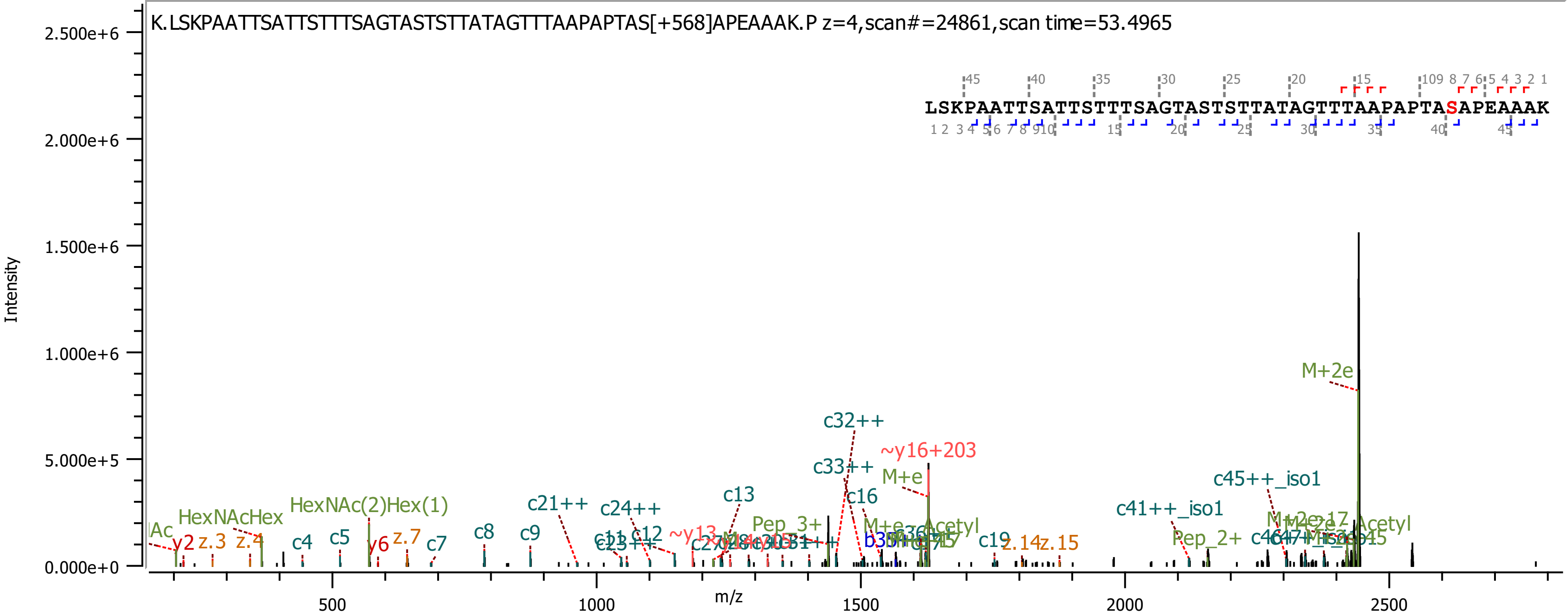
K.LSKPAATTSATTSTTTSAGTASTSTTATAGTTTAAPAPTAS[+568]APEAAAK.P z=4,scan#=24861,scan time=53.4965
LSKPAATTSATTSTTTSAGTASTSTTATAGTTTAAPAPTASAPEAAAK
Intensity
m/z
M+2e
c32++
~y16+203
c33++
M+e
c45++_iso1
c41++_iso1
c13
c16
HexNAc(2)Hex(1)
HexNAcHex
c21++
c24++
Pep_3+
Pep_2+
c4
c5
c7
c8
c9
c12
c19
z.14
z.15
z.3
z.4
z.7
y2
y6
~y13

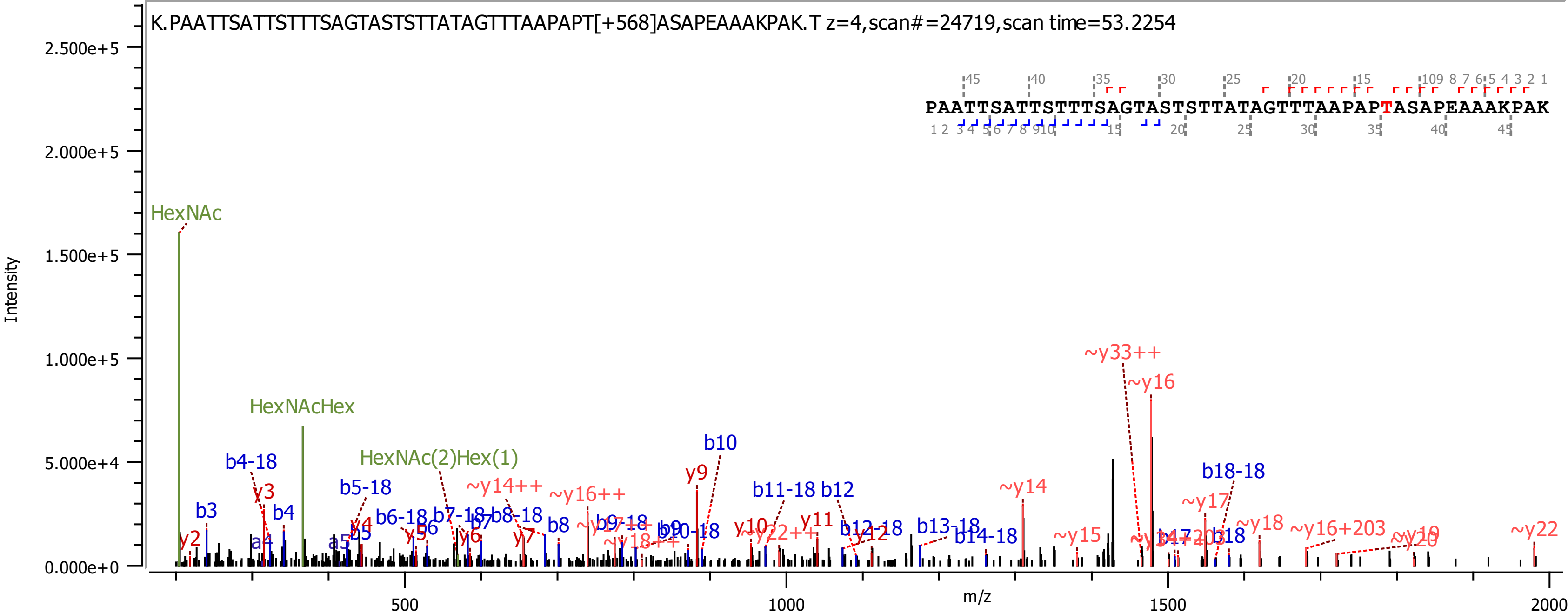
K.PAATTSATTSTTTSAGTASTSTTATAGTTTAAPAPT[+568]ASAPEAAAKPAK.T z=4,scan#=24719,scan time=53.2254
PAATTSATTSTTTSAGTASTSTTATAGTTTAAPAPTASAPEAAAKPAK
Intensity
m/z
2.500e+5
2.000e+5
1.500e+5
1.000e+5
5.000e+4
0.000e+0
500
1000
1500
2000
HexNAc
HexNAcHex
HexNAc(2)Hex(1)
y2
b3
b4-18
y3
a4
b4
a5
b5
y4
b5-18
b6-18
y5
b6
b7-18
y6
b7
b8-18
~y14++
y7
b8
~y16++
b9-18
b9
~y18++
b10-18
y9
b10
y10
~y22++
b11-18
b12
y11
b12-18
y12
b13-18
b14-18
~y14
~y15
~y33++
~y16
~y17
b18-18
b18
~y18
~y16+203
~y20
~y22

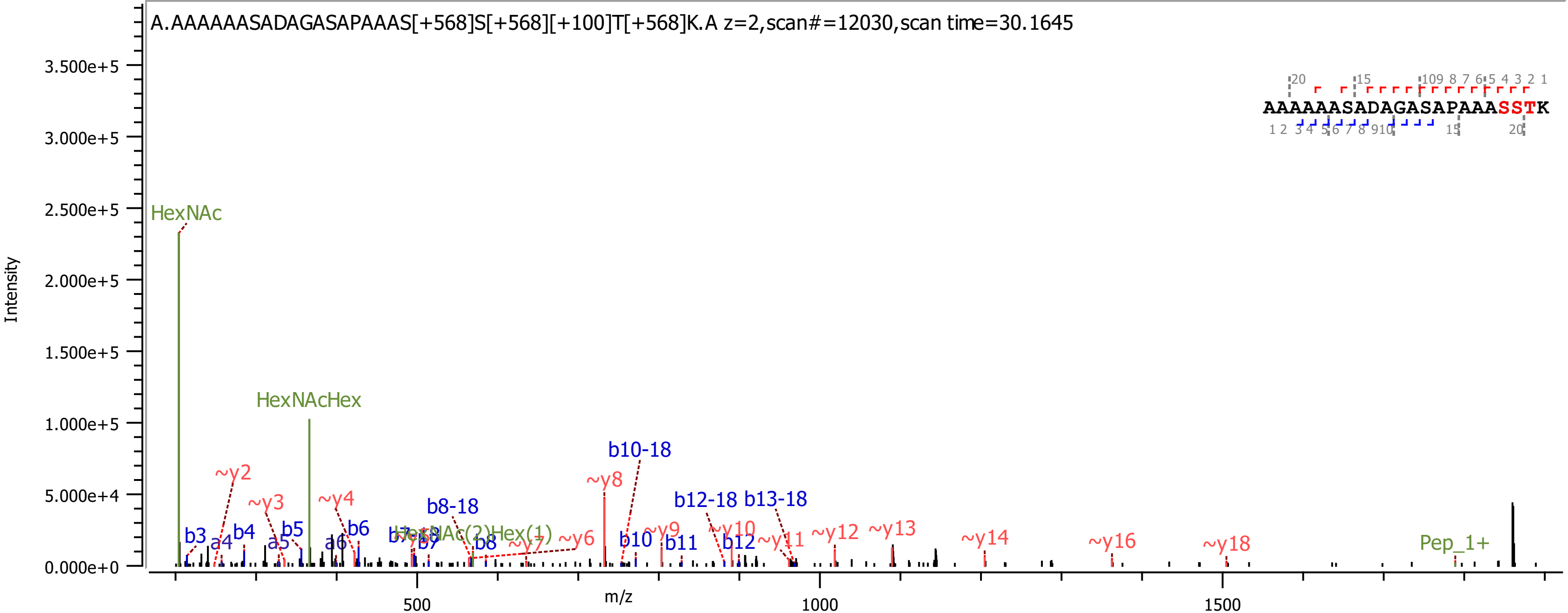

A.AAAAAASADAGASAPAAAS[+568]S[+568][+100]T[+568]K.A z=2,scan#=12030,scan time=30.1645
AAAAAASADAGASAPAAASSTK
Intensity
m/z
HexNAc
HexNAcHex
HexNAc(2)Hex(1)
Pep_1+
b3
a4
b4
a5
b5
a6
b6
b7
b8
b8-18
b10
b10-18
b11
b12
b12-18
b13-18
~y2
~y3
~y4
~y6
~y7
~y8
~y9
~y10
~y11
~y12
~y13
~y14
~y16
~y18

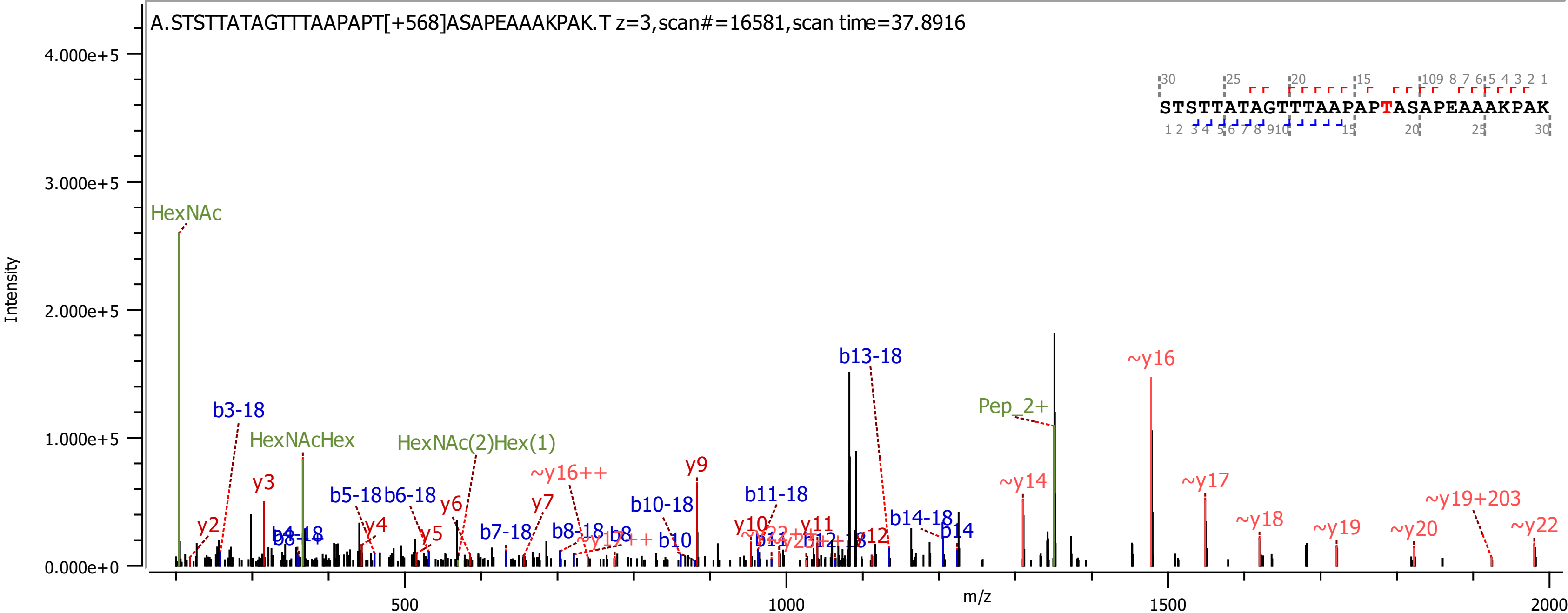

A.STSTTATAGTTTAAPAPT[+568]ASAPEAAAKPAK.T z=3,scan#=16581,scan time=37.8916
Intensity
4.000e+5
3.000e+5
2.000e+5
1.000e+5
0.000e+0
m/z
500
1000
1500
2000
STSTTATAGTTTAAPAPTASAPEAAAKPAK
HexNAc
HexNAcHex
HexNAc(2)Hex(1)
Pep_2+
b3-18
b5-18
b6-18
b7-18
b8-18
b8
b10
b10-18
b11-18
b13-18
b14-18
b14
y2
y3
y4
y5
y6
y7
y9
y10
y11
y12
~y14
~y16
~y16++
~y17
~y18
~y19
~y19+203
~y20
~y22

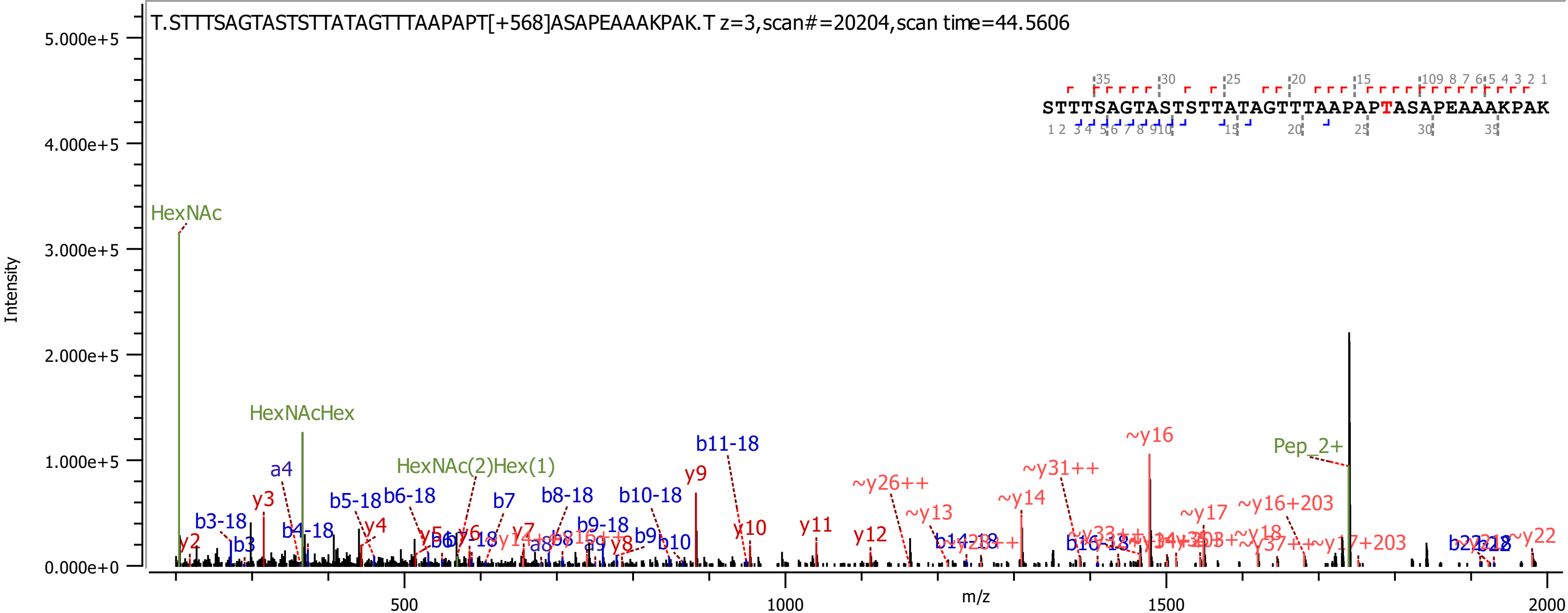
T.STTTSAGTASTSTTATAGTTTAAPAPT[+568]ASAPEAAAKPAK.T z=3,scan#=20204,scan time=44.5606
STTTSAGTASTSTTATAGTTTAAPAPTASAPEAAAKPAK
Intensity
m/z
HexNAc
HexNAcHex
HexNAc(2)Hex(1)
Pep_2+
~y16
~y31++
~y26++
~y14
~y13
~y17
~y16+203
b11-18
b10-18
b8-18
b9-18
b5-18
b6-18
b3-18
b4-18
a4
y3
y4
y9
y10
y11
y12
b7
b9
b10
y2
b3
y8
a8
a9

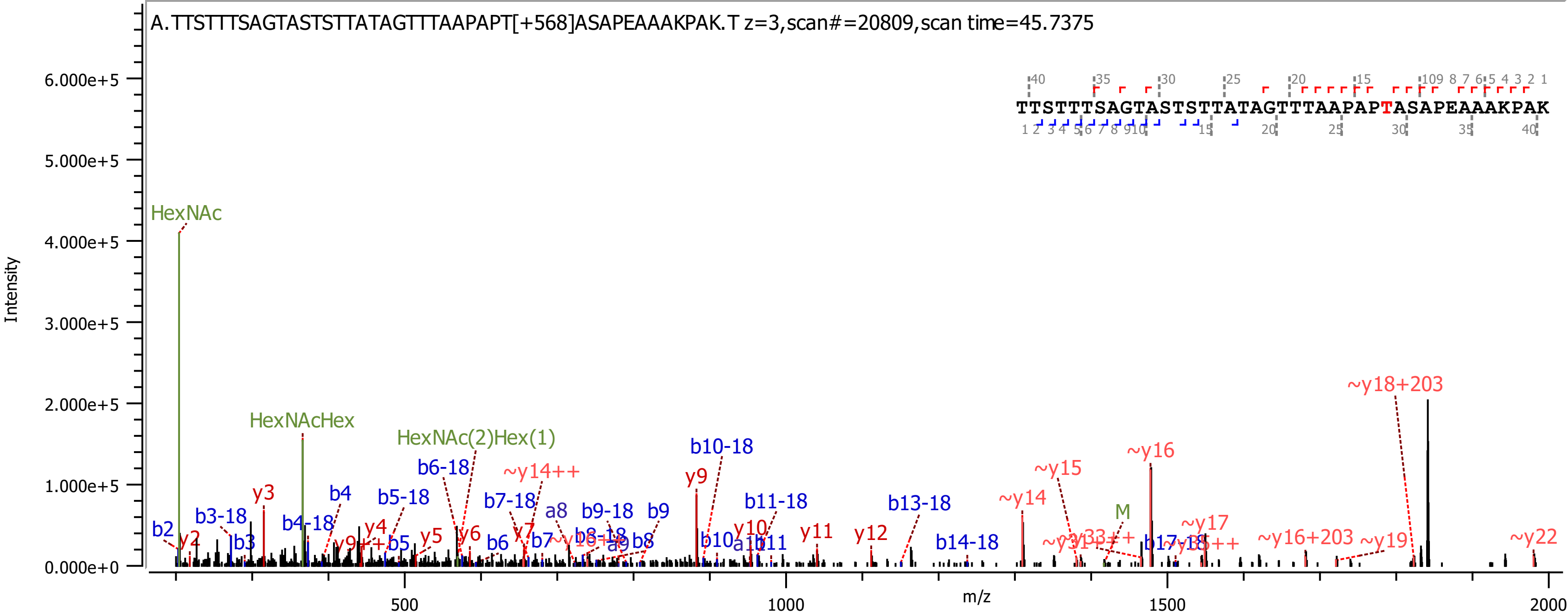
A.TTSTTTSAGTASTSTTATAGTTTAAPAPT[+568]ASAPEAAAKPAK.T z=3,scan#=20809,scan time=45.7375
TTSTTTSAGTASTSTTATAGTTTAAPAPTASAPEAAAKPAK
Intensity
m/z
HexNAc
HexNAcHex
HexNAc(2)Hex(1)
~y18+203
~y16
~y15
~y14
~y14++
~y17
~y16+203
~y19
~y22
b10-18
b11-18
b13-18
b14-18
b6-18
b5-18
b7-18
b9-18
b8-18
b4-18
b3-18
y9
y3
y4
y5
y6
y7
y10
y11
y12
b2
b3
b4
b5
b6
b7
b8
b9
b10
b11
a8
a9
M
6.000e+5
5.000e+5
4.000e+5
3.000e+5
2.000e+5
1.000e+5
0.000e+0
500
1000
1500
2000

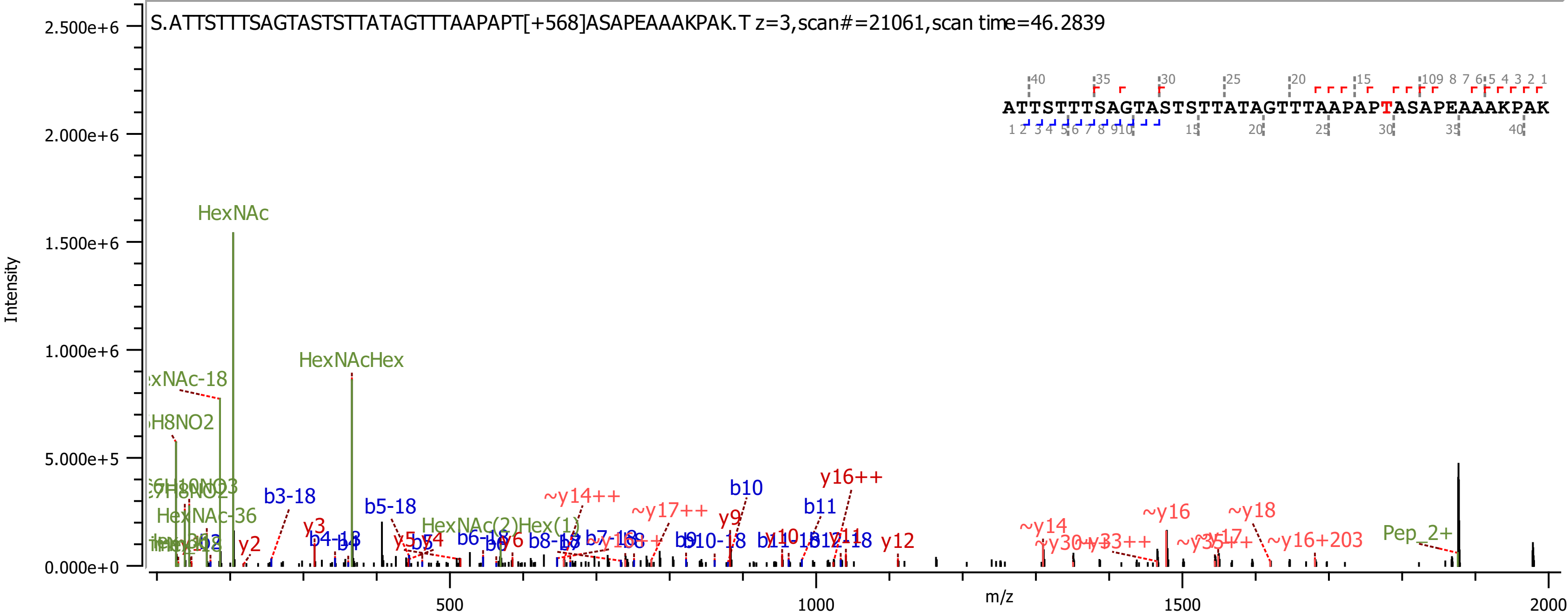
S.ATTSTTTSAGTASTSTTATAGTTTAAPAPT[+568]ASAPEAAAKPAK.T z=3,scan#=21061,scan time=46.2839
ATTSTTTSAGTASTSTTATAGTTTAAPAPTASAPEAAAKPAK
Intensity
m/z
HexNAc
HexNAcHex
HexNAc-36
HexNAc(2)Hex(1)
Pep_2+
b3-18
b5-18
b10
b11
y16++
~y14++
~y17++
y9
y3
y2
y12
~y14
~y16
~y18
~y16+203
0.000e+0
5.000e+5
1.000e+6
1.500e+6
2.000e+6
2.500e+6
500
1000
1500
2000

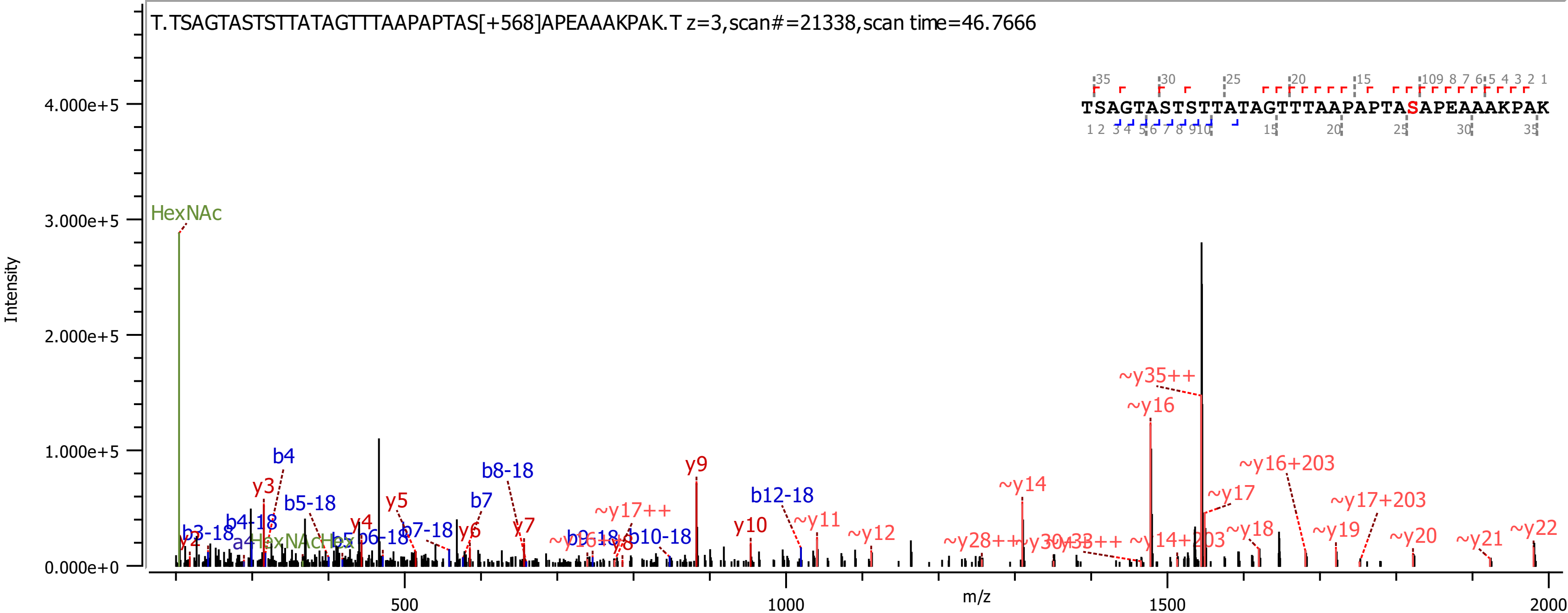
T.TSAGTASTSTTATAGTTTAAPAPTAS[+568]APEAAAKPAK.T z=3,scan#=21338,scan time=46.7666
TSAGTASTSTTATAGTTTAAPAPTASAPEAAAKPAK
Intensity
m/z
HexNAc
y9
b4
b8-18
b12-18
~y35++
~y16
~y16+203
~y17
~y17+203
~y14
~y17++
y10
~y11
~y12
~y18
~y19
~y20
~y21
~y22
y3
y5
b7
y7
b5-18
b4-18
b3-18
y4
b10-18
~y28++
~y14+203
4.000e+5
3.000e+5
2.000e+5
1.000e+5
0.000e+0
500
1000
1500
2000

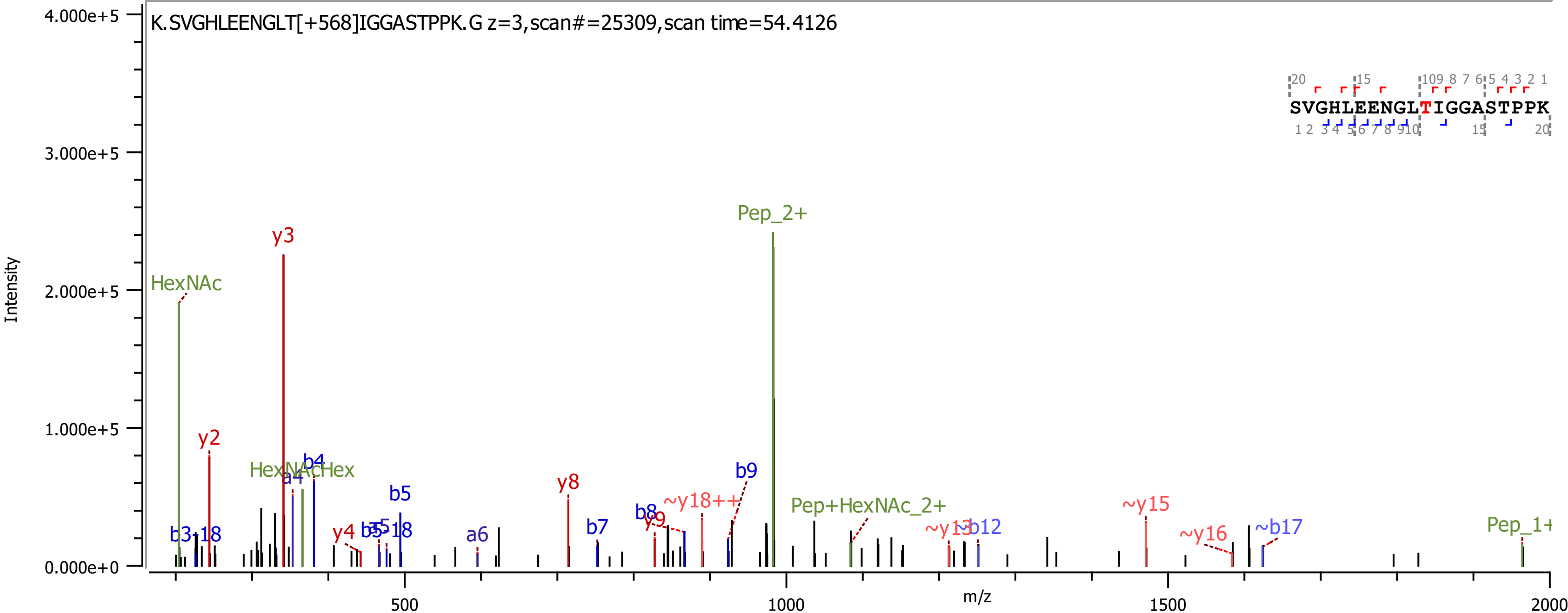

K.SVGHLEENGLT[+568]IGGASTPPK.G z=3,scan#=25309,scan time=54.4126
Intensity
m/z
4.000e+5
3.000e+5
2.000e+5
1.000e+5
0.000e+0
500
1000
1500
2000
HexNAc
y3
y2
HexNAcHex
a4
b4
b3-18
y4
b5-18
a5
b5
a6
y8
b7
b8
y9
~y18++
b9
Pep_2+
Pep+HexNAc_2+
~y13
~b12
~y15
~y16
~b17
Pep_1+
SVGHLEENGLTIGGASTPPK

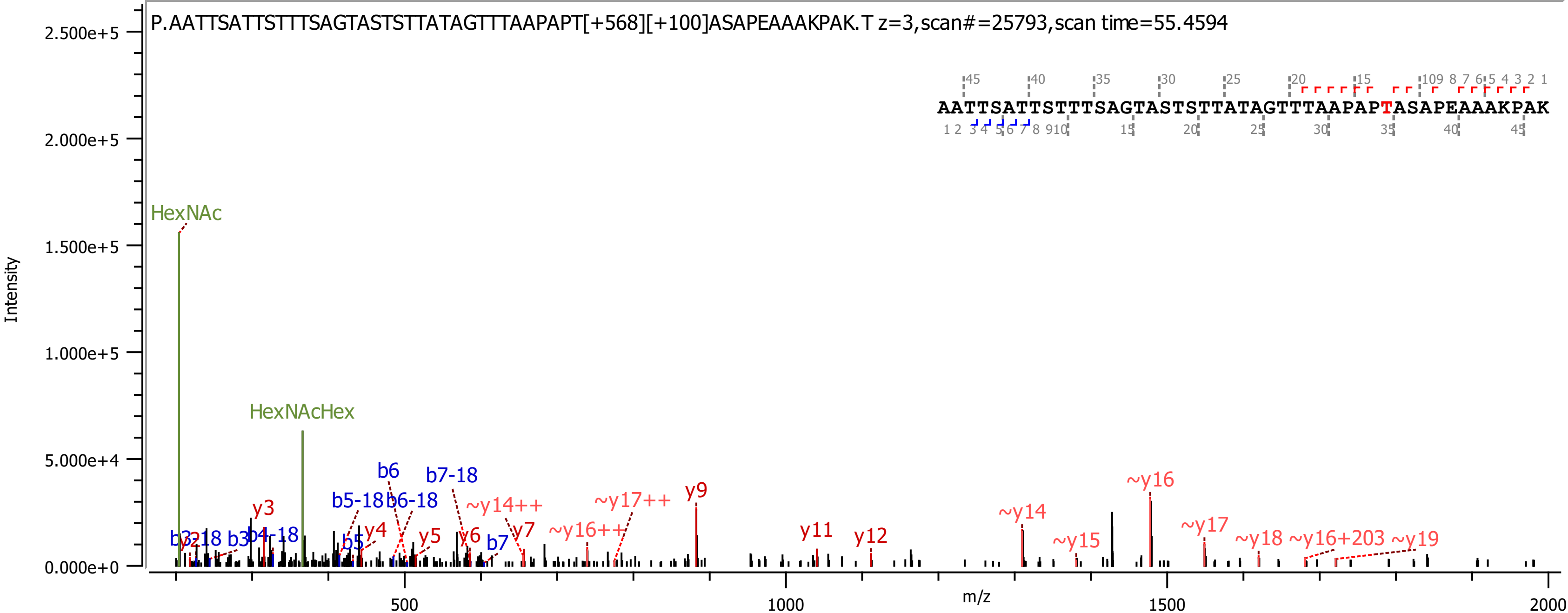

P.AATTSATTSTTTSAGTASTSTTATAGTTTAAPAPT[+568][+100]ASAPEAAAKPAK.T z=3,scan#=25793,scan time=55.4594
AATTSATTSTTTSAGTASTSTTATAGTTTAAPAPTASAPEAAAKPAK
Intensity
m/z
HexNAc
HexNAcHex
b6
b7-18
b5-18
b6-18
~y14++
~y17++
y9
~y16
~y14
~y17
y3
y4
y5
y6
y7
~y16++
y11
y12
~y15
~y18
~y16+203
~y19
b7
b5
b3
b4-18

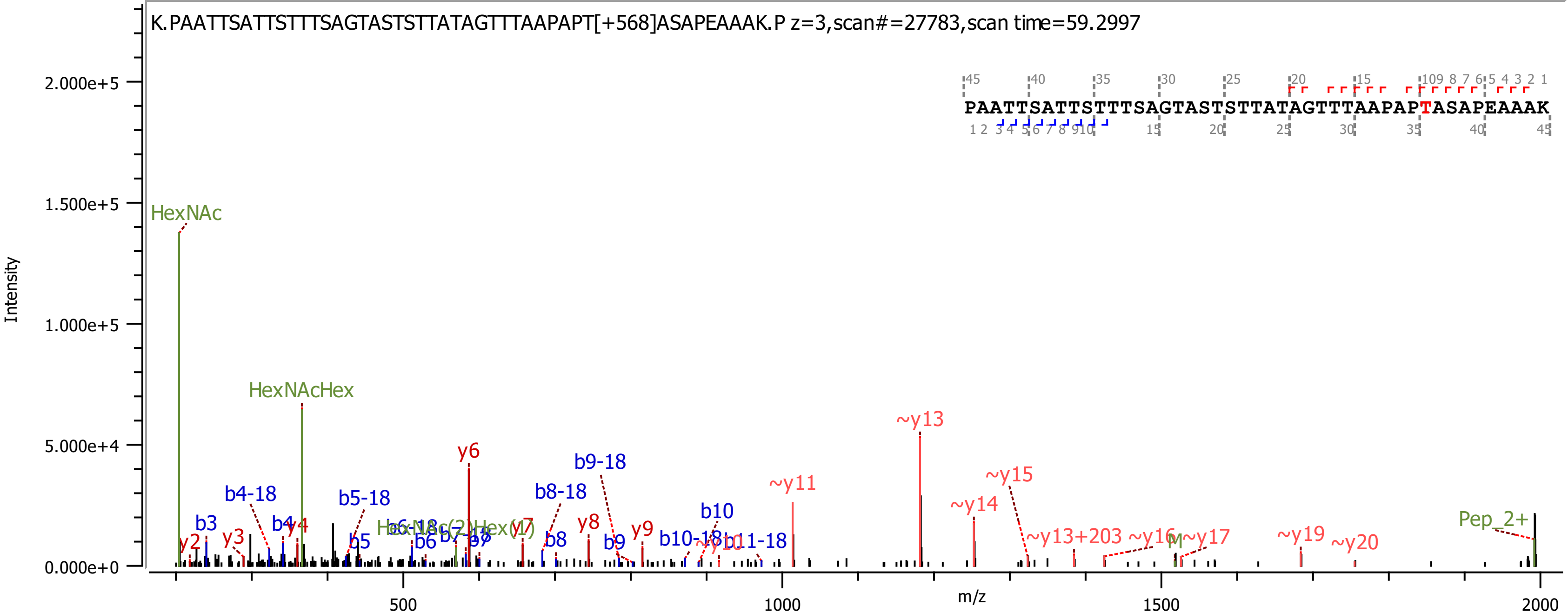
K.PAATTSATTSTTTSAGTASTSTTATAGTTTAAPAPT[+568]ASAPEAAAK.P z=3,scan#=27783,scan time=59.2997
PAATTSATTSTTTSAGTASTSTTATAGTTTAAPAPTASAPEAAAK
Intensity
m/z
HexNAc
HexNAcHex
y6
b9-18
~y13
~y11
~y15
b4-18
b8-18
b5-18
~y14
b10
b3
b4
y4
y2
y3
b5
b6
y8
y9
b8
b9
b10-18
b11-18
~y13+203
~y16
~y17
~y19
~y20
Pep_2+
M
2.000e+5
1.500e+5
1.000e+5
5.000e+4
0.000e+0
500
1000
1500
2000

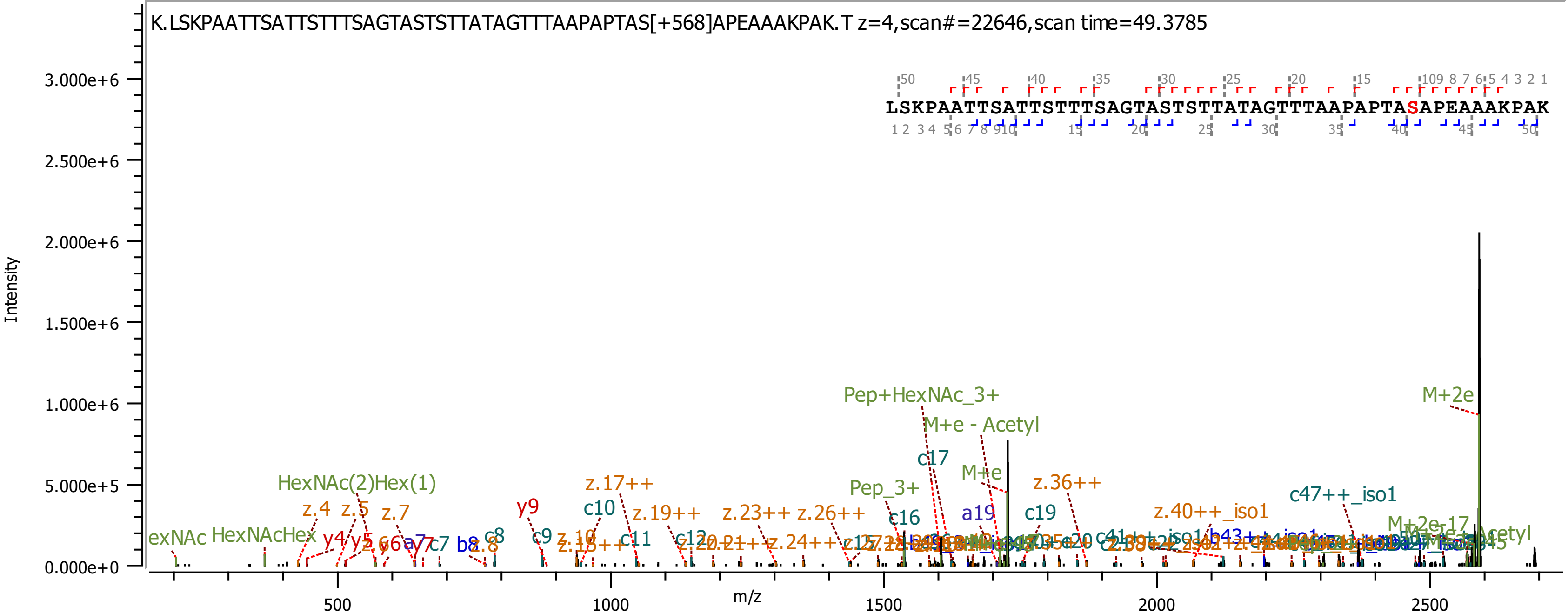
K.LSKPAATTSATTSTTTSAGTASTSTTATAGTTTAAPAPTAS[+568]APEAAAKPAK.T z=4,scan#=22646,scan time=49.3785
LSKPAATTSATTSTTTSAGTASTSTTATAGTTTAAPAPTASAPEAAAKPAK
Intensity
m/z
Pep+HexNAc_3+
M+e - Acetyl
M+2e
M+e
Pep_3+
HexNAc(2)Hex(1)
z.17++
z.36++
c47++_iso1
z.40++_iso1
z.19++
z.23++
z.26++
c17
c16
a19
c19
c10
y9
z.4
z.5
z.7
c8
c9
c11
y4
y5
y6
a7
c7
b8
z.8
HexNAcHex

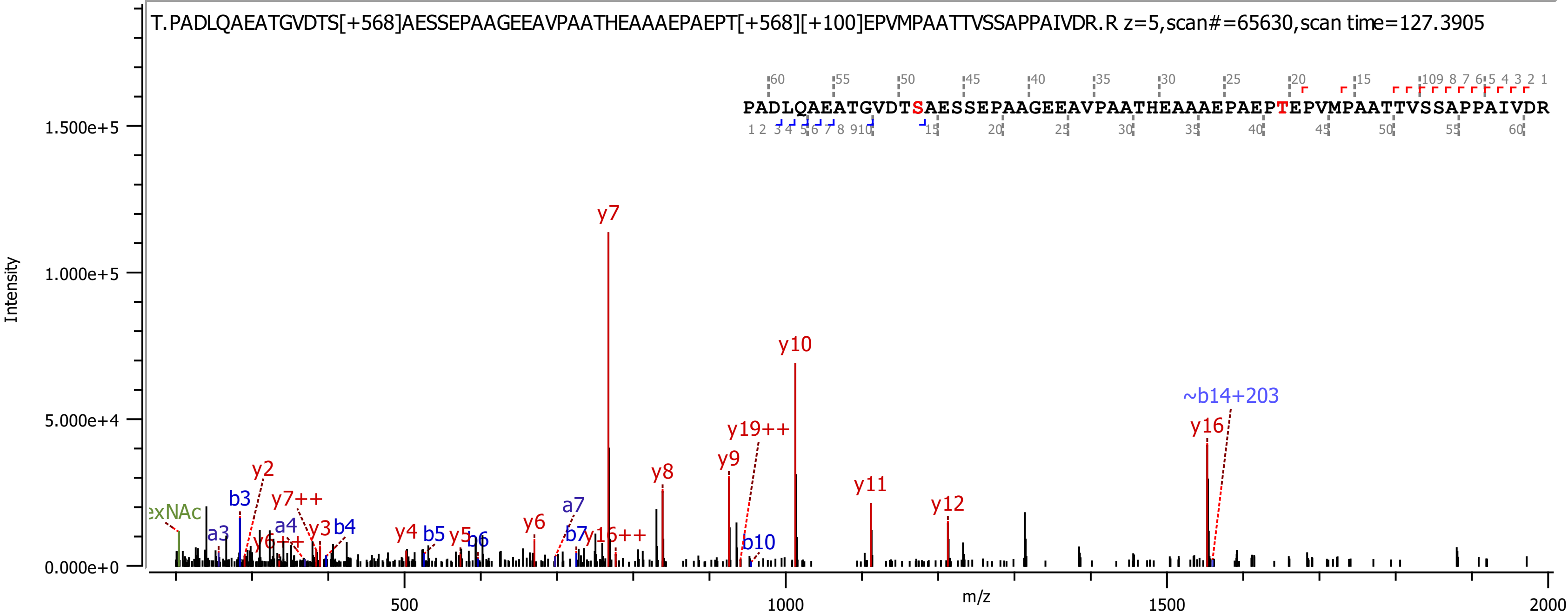

T.PADLQAEATGVDTS[+568]AESSEPAAGEEAVPAATHEAAAEPAEPT[+568][+100]EPVMPAATTVSSAPPAIVDR.R z=5,scan#=65630,scan time=127.3905
PADLQAEATGVDTSAESSEPAAGEEAVPAATHEAAAEPAEPTEPVMPAATTVSSAPPAIVDR
Intensity
m/z
y7
y10
~b14+203
y16
y19++
y9
y8
y11
y12
y2
b3
y7++
exNAc
a7
a4
y3
b4
a3
y6++
y4
b5
y5
b6
y6
b7
y16++
b10
1.500e+5
1.000e+5
5.000e+4
0.000e+0
500
1000
1500
2000

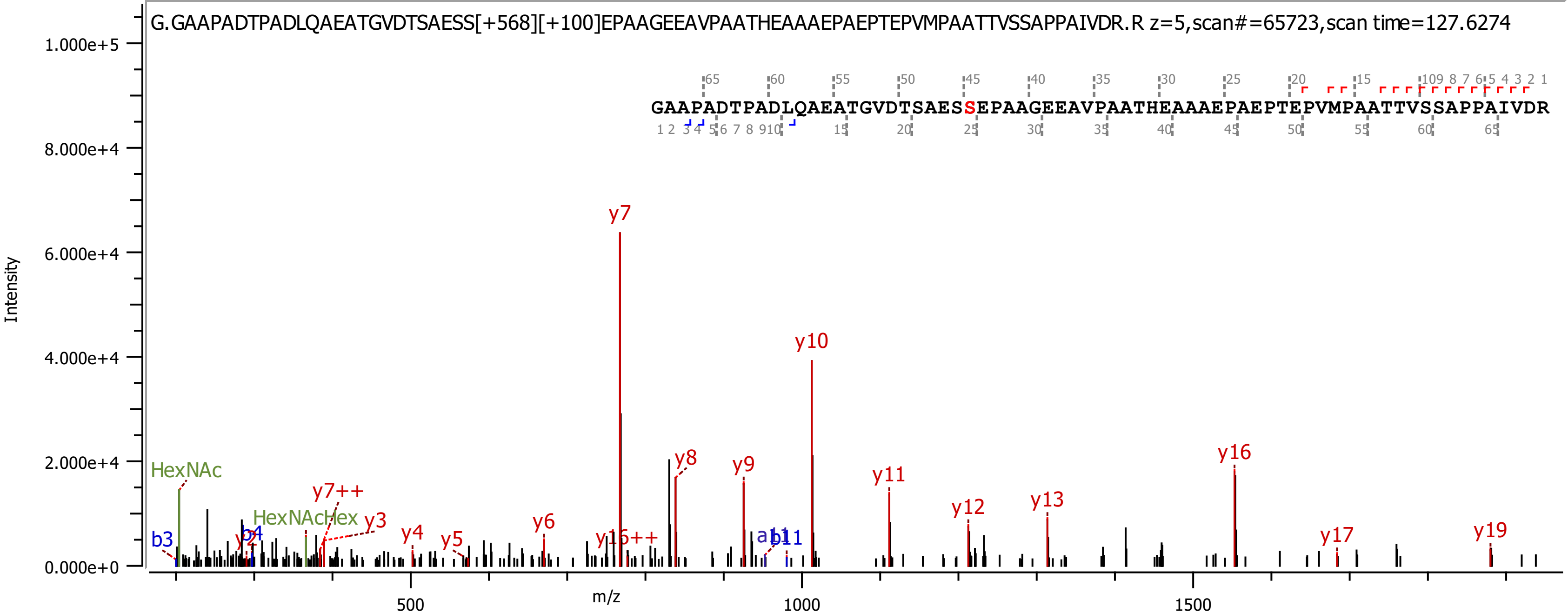
G.GAAPADTPADLQAEATGVDTSAESS[+568][+100]EPAAGEEAVPAATHEAAAEPAEPTEPVMPAATTVSSAPPAIVDR.R z=5,scan#=65723,scan time=127.6274
GAAPADTPADLQAEATGVDTSAESSEPAAGEEAVPAATHEAAAEPAEPTEPVMPAATTVSSAPPAIVDR
Intensity
m/z
1.000e+5
8.000e+4
6.000e+4
4.000e+4
2.000e+4
0.000e+0
500
1000
1500
HexNAc
HexNAcHex
b3
b4
y2
y7++
y3
y4
y5
y6
y7
y16++
y8
y9
a11
b11
y10
y11
y12
y13
y16
y17
y19

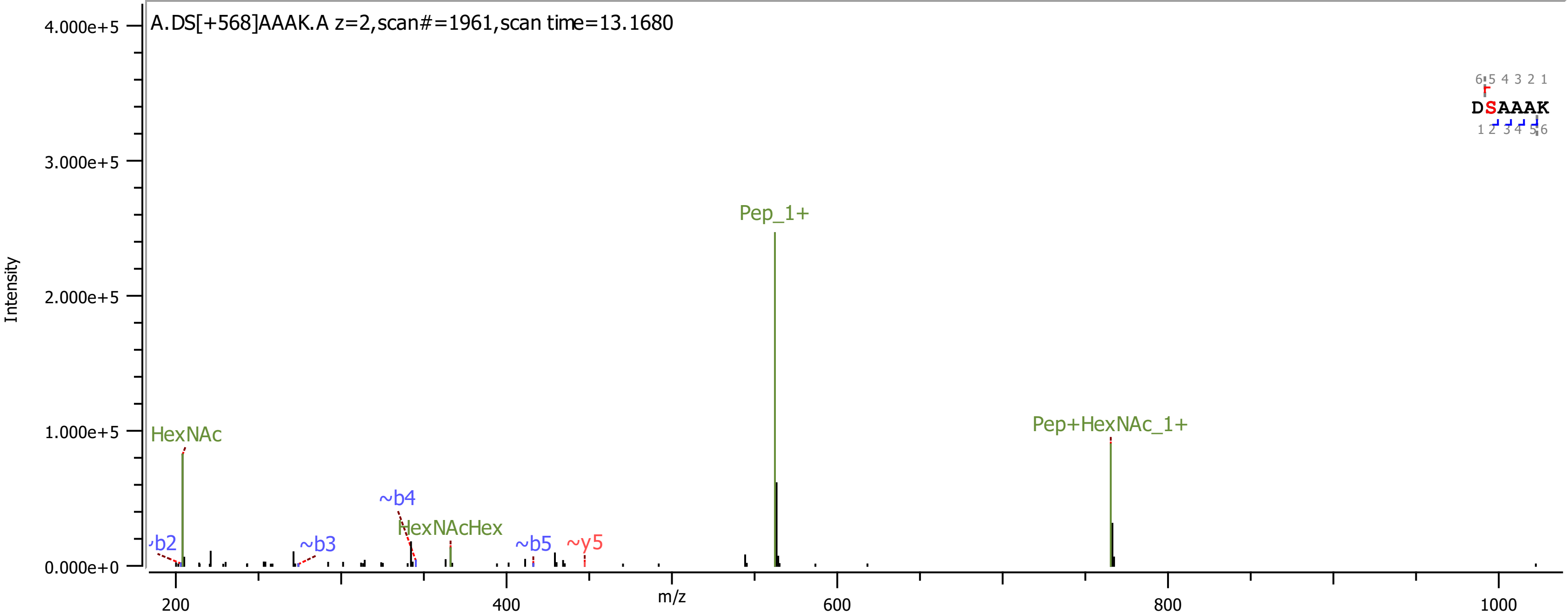
A.DS[+568]AAAK.A z=2,scan#=1961,scan time=13.1680
Intensity
4.000e+5
3.000e+5
2.000e+5
1.000e+5
0.000e+0
m/z
200
400
600
800
1000
HexNAc
~b2
~b3
~b4
HexNAcHex
~b5
~y5
Pep_1+
Pep+HexNAc_1+
DSAAAK
6 5 4 3 2 1
1 2 3 4 5 6

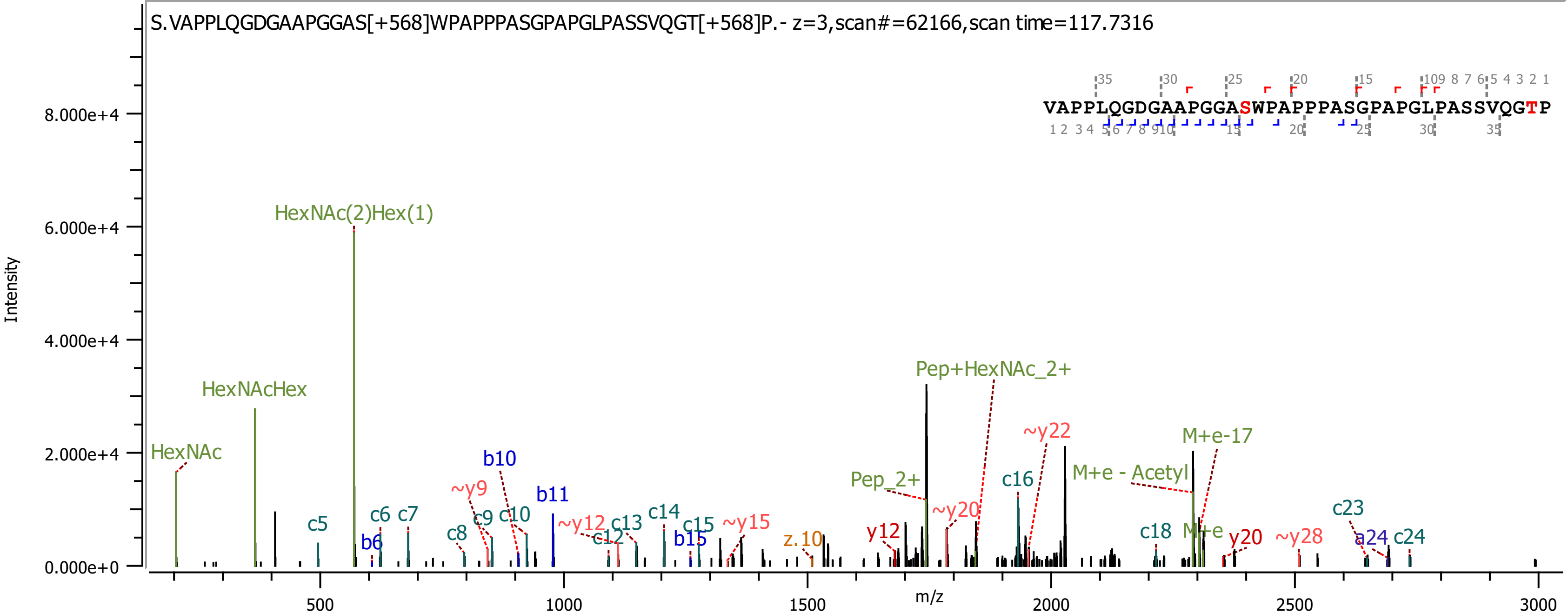

S.VAPPLQGDGAAPGGAS[+568]WPAPPPASGPAPGLPASSVQGT[+568]P.- z=3,scan#=62166,scan time=117.7316
VAPPLQGDGAAPGGASWPAPPPASGPAPGLPASSVQGTP
Intensity
m/z
HexNAc
HexNAcHex
HexNAc(2)Hex(1)
Pep+HexNAc_2+
Pep_2+
M+e-17
M+e - Acetyl
M+e
b6
b10
b11
b15
a24
c5
c6
c7
c8
c9
c10
c12
c13
c14
c15
c16
c18
c23
c24
~y9
~y12
~y15
~y20
~y22
~y28
y12
y20
z.10

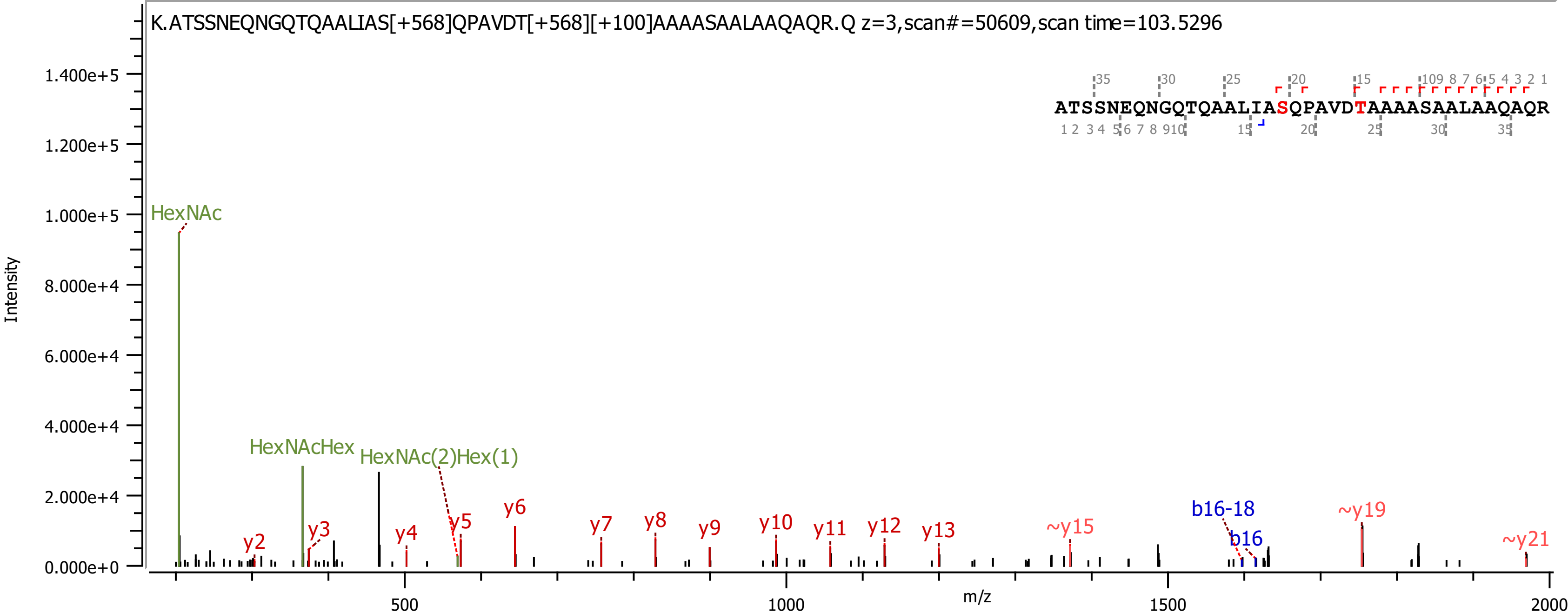
K.ATSSNEQNGQTQAALIAS[+568]QPAVDT[+568][+100]AAAASAALAAQAQR.Q z=3,scan#=50609,scan time=103.5296
ATSSNEQNGQTQAALIASQPAVDTAAAASAALAAQAQR
HexNAc
HexNAcHex
HexNAc(2)Hex(1)
y2
y3
y4
y5
y6
y7
y8
y9
y10
y11
y12
y13
~y15
b16-18
b16
~y19
~y21
Intensity
m/z

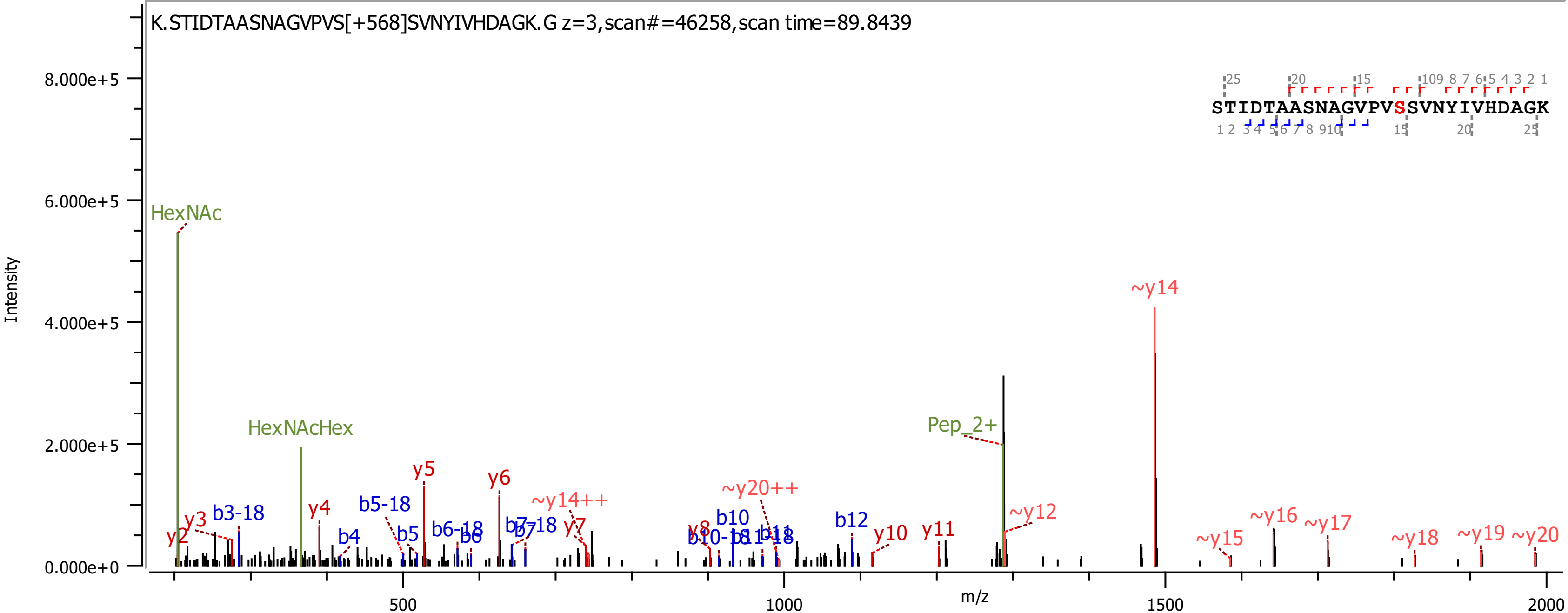

K.STIDTAASNAGVPVS[+568]SVNYIVHDAGK.G z=3,scan#=46258,scan time=89.8439
STIDTAASNAGVPVSSVNYIVHDAGK
Intensity
m/z
0.000e+0
2.000e+5
4.000e+5
6.000e+5
8.000e+5
500
1000
1500
2000
HexNAc
HexNAcHex
Pep_2+
~y14
~y14++
~y20++
~y12
~y15
~y16
~y17
~y18
~y19
~y20
y2
y3
y4
y5
y6
y7
y8
y10
y11
b3-18
b4
b5-18
b5
b6-18
b6
b7-18
b7
b10
b10-18
b11-18
b11
b12

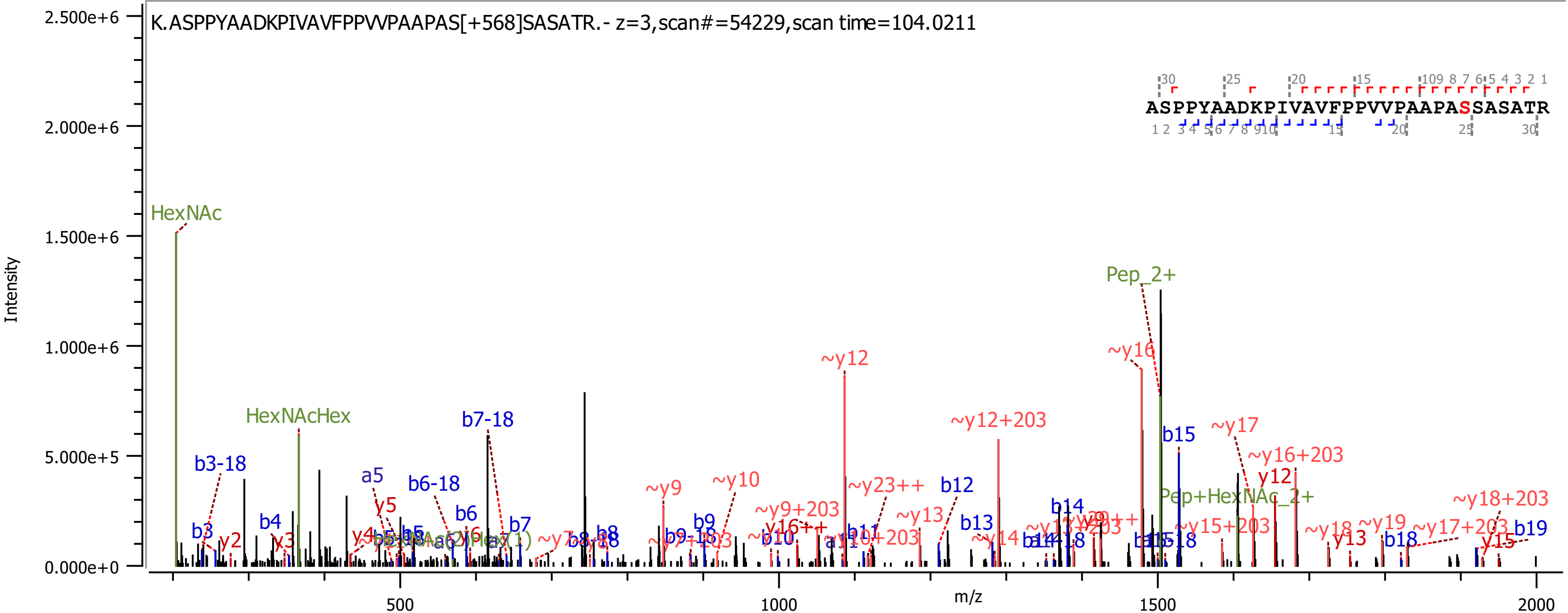
K.ASPPYAADKPIVAVFPPVVPAAPAS[+568]SASATR.- z=3,scan#=54229,scan time=104.0211
ASPPYAADKPIVAVFPPVVPAAPASSASATR
Intensity
m/z
2.500e+6
2.000e+6
1.500e+6
1.000e+6
5.000e+5
0.000e+0
500
1000
1500
2000
HexNAc
HexNAcHex
Pep_2+
Pep+HexNAc_2+
b3-18
b3
b4
a5
b5
b6-18
b6
b7-18
b7
b8
b9
b10
b11
b12
b13
b14
b15
b18
b19
y2
y3
y4
y5
y6
y8
y12
y13
y15
~y9
~y10
~y12
~y12+203
~y13
~y14
~y16
~y16+203
~y17
~y18
~y19
~y9+203
~y23++
~y15+203
~y17+203
~y18+203

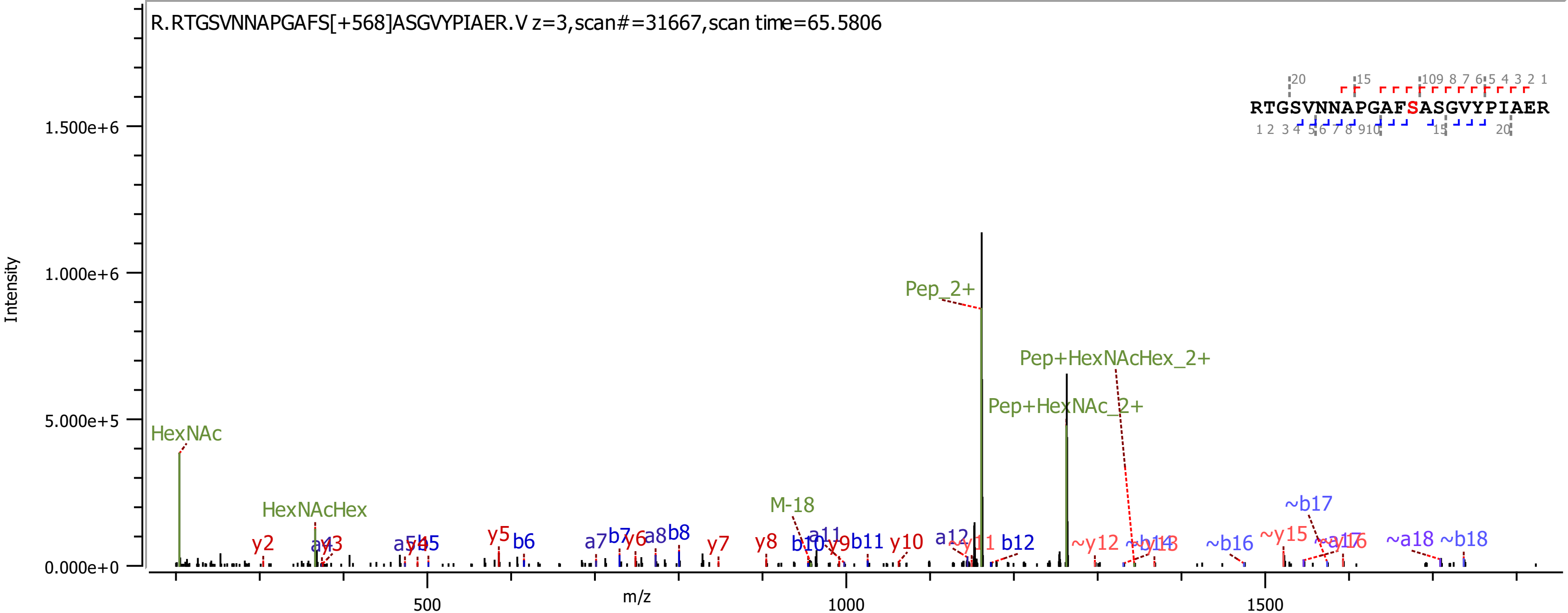

R.RTGSVNNAPGAFS[+568]ASGVYPIAER.V z=3,scan#=31667,scan time=65.5806
RTGSVNNAPGAFSASGVYPIAER
Intensity
m/z
Pep_2+
Pep+HexNAcHex_2+
Pep+HexNAc_2+
HexNAc
HexNAcHex
M-18
~b17
~y15
~a18 ~b18
~b16
~y12
b12
y10
y8
y7
b8
a8
y6
b7
a7
b6
y5
y3
y2
b11
a11
b10
y9
a12

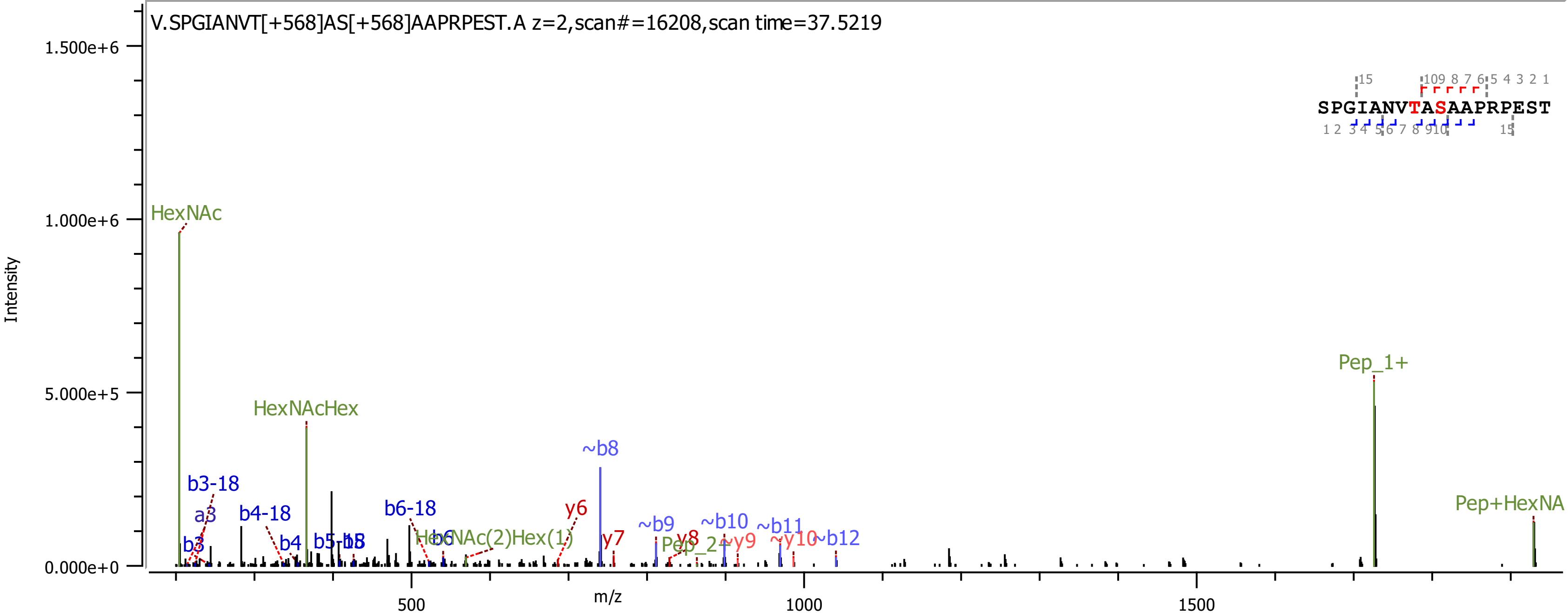

V.SPGIANVT[+568]AS[+568]AAPRPEST.A z=2,scan#=16208,scan time=37.5219
SPGIANVTASAAPRPEST
Intensity
m/z
HexNAc
HexNAcHex
~b8
b3-18
a3
b3
b4-18
b4
b5-18
b6-18
b6
HexNAc(2)Hex(1)
y6
y7
~b9
y8
Pep_2+
~b10
~y9
~b11
~y10
~b12
Pep_1+
Pep+HexNA

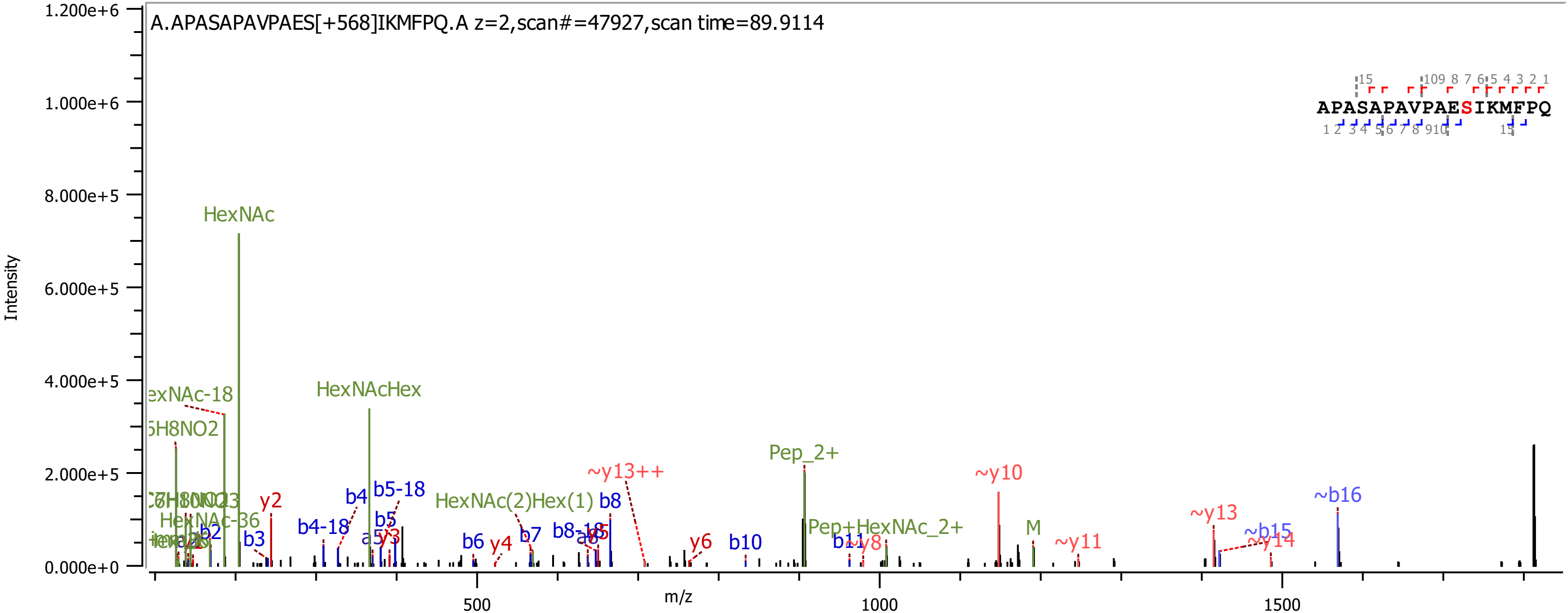
A.APASAPAVPAES[+568]IKMFPQ.A z=2,scan#=47927,scan time=89.9114
Intensity
m/z
APASAPAVPAESIKMFPQ
HexNAc
HexNAc-18
HexNAcHex
HexNAc-36
HexNAc(2)Hex(1)
Pep_2+
Pep+HexNAc_2+
M
y2
b2
b3
b4
b4-18
b5
b5-18
a5
y3
b6
y4
b7
b8
b8-18
a8
y5
~y13++
y6
b10
b11
~y8
~y10
~y11
~y13
~b15
~y14
~b16
0.000e+0
2.000e+5
4.000e+5
6.000e+5
8.000e+5
1.000e+6
1.200e+6
500
1000
1500

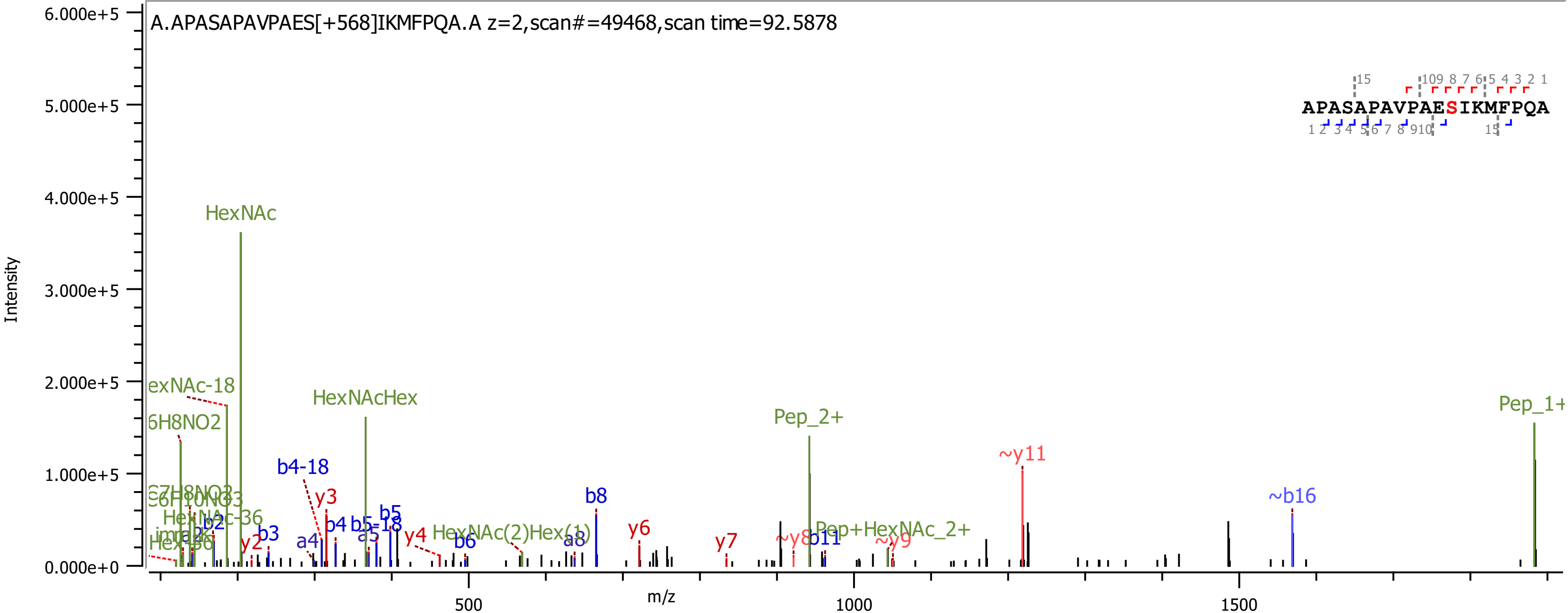
A.APASAPAVPAES[+568]IKMFPQA.A z=2,scan#=49468,scan time=92.5878
Intensity
m/z
6.000e+5
5.000e+5
4.000e+5
3.000e+5
2.000e+5
1.000e+5
0.000e+0
500
1000
1500
APASAPAVPAESIKMFPQA
HexNAc
HexNAcHex
Pep_2+
Pep_1+
~y11
~b16
b8
b4-18
y3
y6
y7
b5
b4
b5-18
a5
a4
b3
y2
y4
b6
a8
b11
~y9
Pep+HexNAc_2+
HexNAc(2)Hex(1)

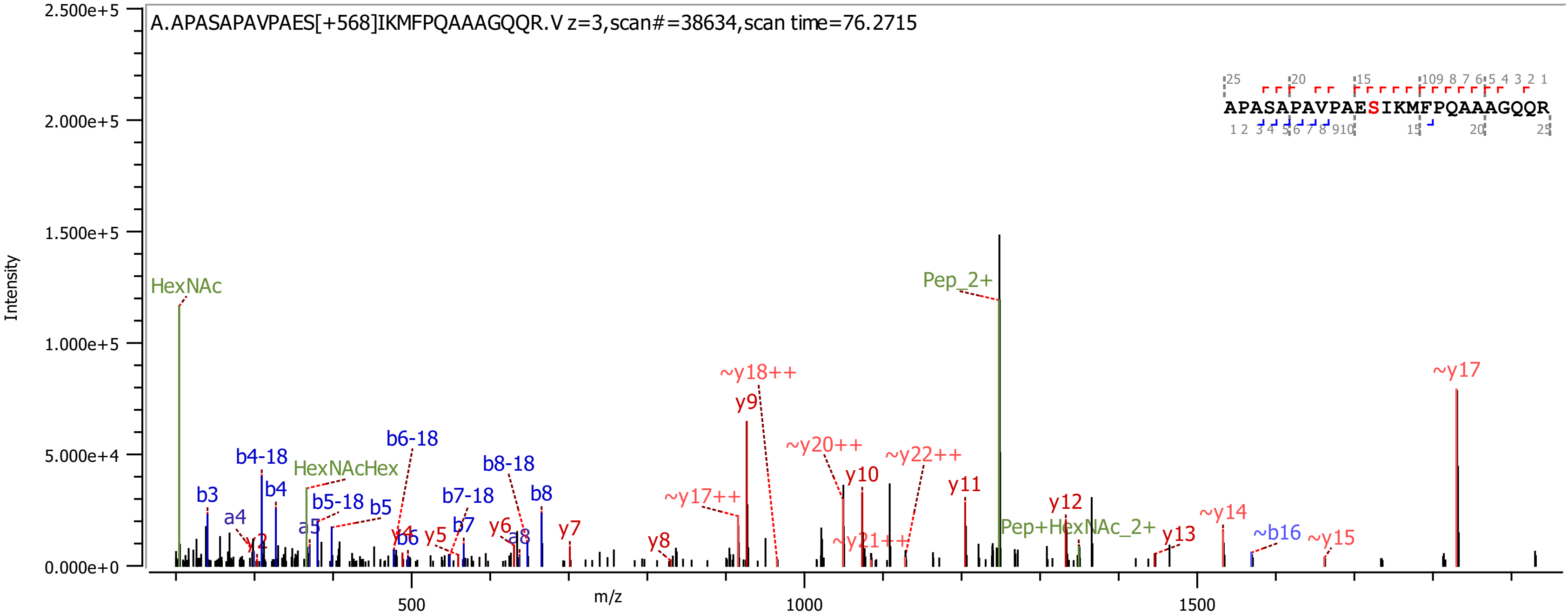

A.APASAPAVPAES[+568]IKMFPQAAAGQQR.V z=3,scan#=38634,scan time=76.2715
APASAPAVPAESIKMFPQAAAGQQR
Intensity
m/z
2.500e+5
2.000e+5
1.500e+5
1.000e+5
5.000e+4
0.000e+0
500
1000
1500
HexNAc
Pep_2+
b3
a4
y2
b4-18
b4
HexNAcHex
a5
b5-18
b5
b6-18
y4
b6
y5
b7-18
b7
y6
a8
b8-18
b8
y7
y8
~y17++
~y18++
y9
~y20++
y10
~y21++
~y22++
y11
Pep+HexNAc_2+
y12
y13
~y14
~b16
~y15
~y17

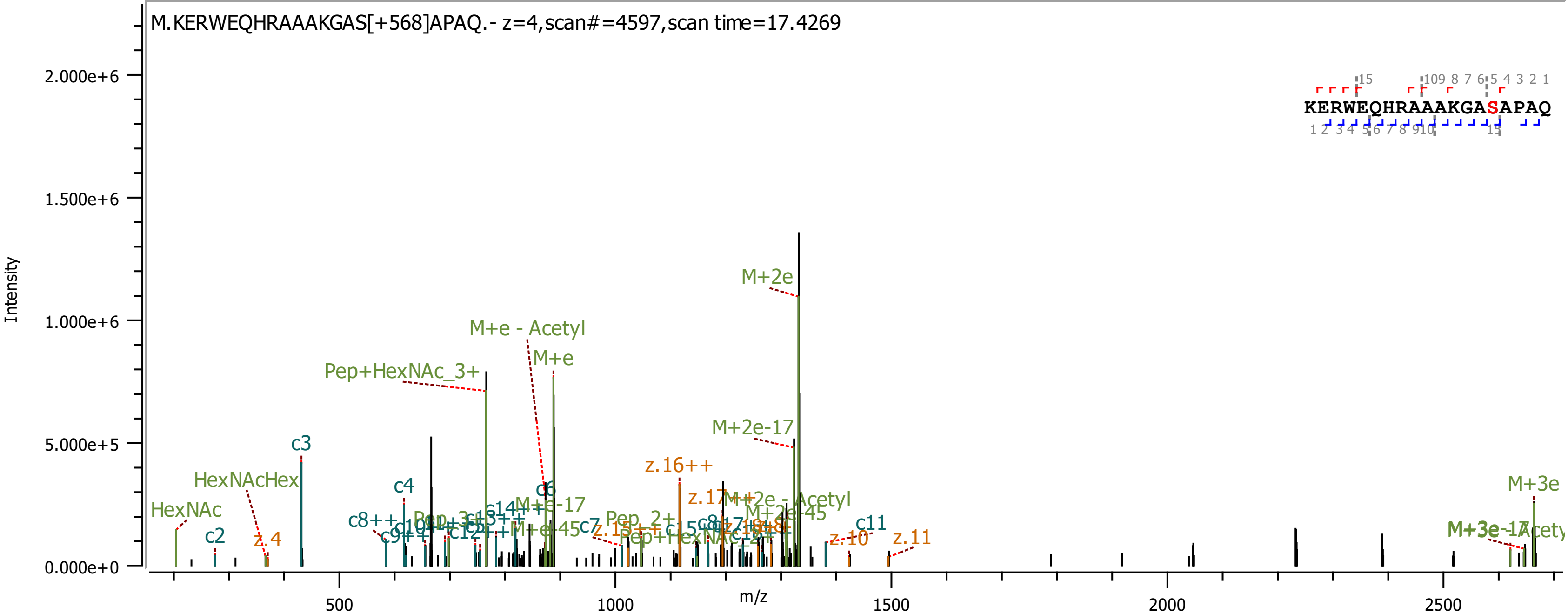

M.KERWEQHRAAAKGAS[+568]APAQ.- z=4,scan#=4597,scan time=17.4269
KERWEQHRAAAKGASAPAQ
Intensity
m/z
2.000e+6
1.500e+6
1.000e+6
5.000e+5
0.000e+0
500
1000
1500
2000
2500
M+2e
M+e - Acetyl
M+e
Pep+HexNAc_3+
M+2e-17
c3
HexNAcHex
HexNAc
c2
z.4
c4
c6
z.16++
M+e-17
c8++
c7
z.17
M+2e - Acetyl
M+2e-45
c11
z.10
z.11
M+3e

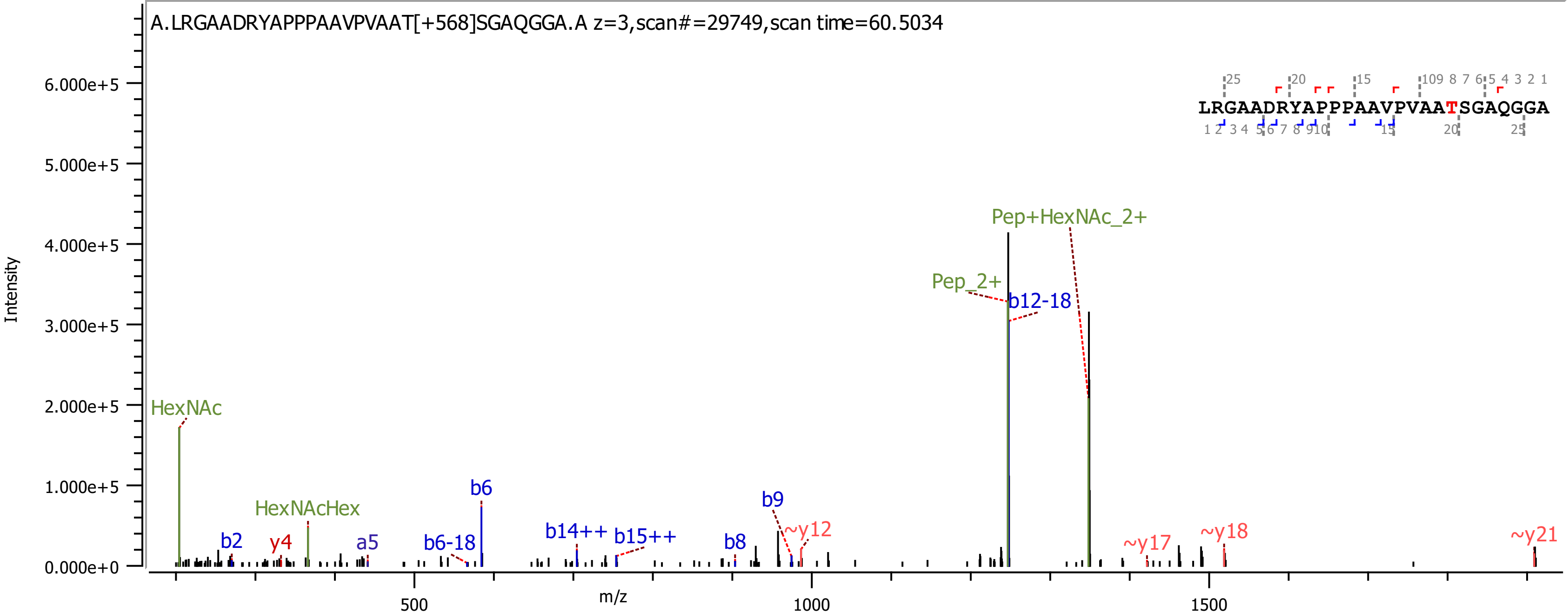
A.LRGAADRYAPPPAAVPVAAT[+568]SGAQGGA.A z=3,scan#=29749,scan time=60.5034
LRGAADRYAPPPAAVPVAATSGAQGGA
Intensity
6.000e+5
5.000e+5
4.000e+5
3.000e+5
2.000e+5
1.000e+5
0.000e+0
m/z
500
1000
1500
HexNAc
b2
y4
HexNAcHex
a5
b6-18
b6
b14++
b15++
b8
b9
~y12
Pep_2+
b12-18
Pep+HexNAc_2+
~y17
~y18
~y21

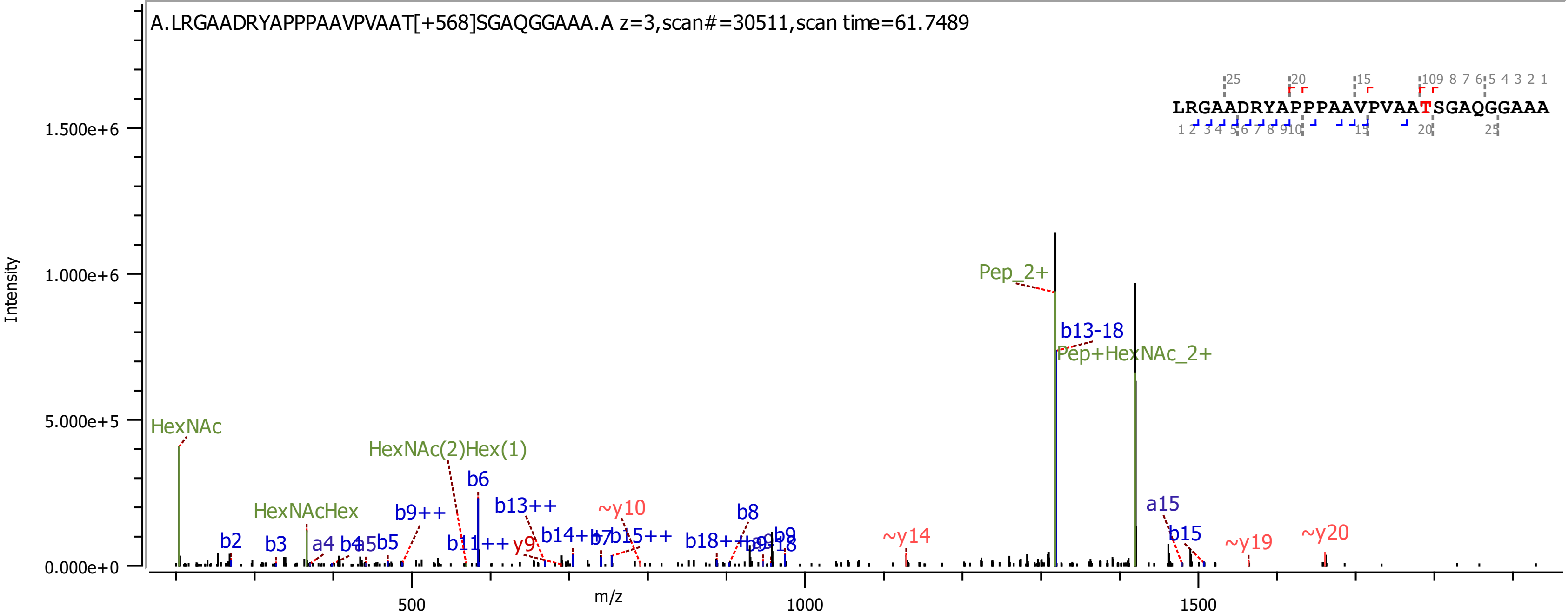
A.LRGAADRYAPPPAAVPVAAT[+568]SGAQGGAAA.A z=3,scan#=30511,scan time=61.7489
LRGAADRYAPPPAAVPVAATSGAQGGAAA
Intensity
m/z
0.000e+0
5.000e+5
1.000e+6
1.500e+6
500
1000
1500
HexNAc
HexNAcHex
HexNAc(2)Hex(1)
Pep_2+
b13-18
Pep+HexNAc_2+
b2
b3
a4
b6
b9++
b13++
~y10
b8
b14++
b15++
b18++
b11++
y9
~y14
a15
b15
~y19
~y20

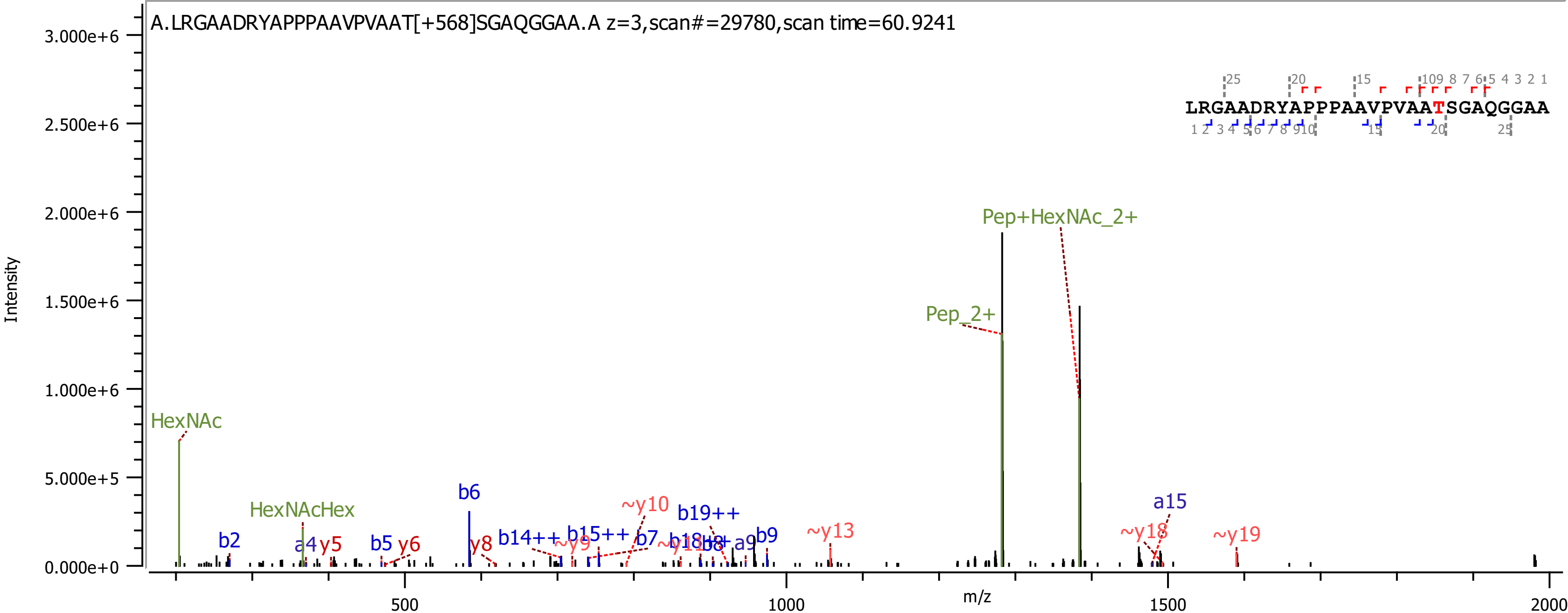

A.LRGAADRYAPPPAAVPVAAT[+568]SGAQGGAA.A z=3,scan#=29780,scan time=60.9241
LRGAADRYAPPPAAVPVAATSGAQGGAA
Intensity
m/z
3.000e+6
2.500e+6
2.000e+6
1.500e+6
1.000e+6
5.000e+5
0.000e+0
500
1000
1500
2000
Pep+HexNAc_2+
Pep_2+
HexNAc
HexNAcHex
b2
a4
y5
b5
y6
b6
y8
b14++
~y9
b15++
~y10
b7
b19++
b9
~y13
a15
~y18
~y19

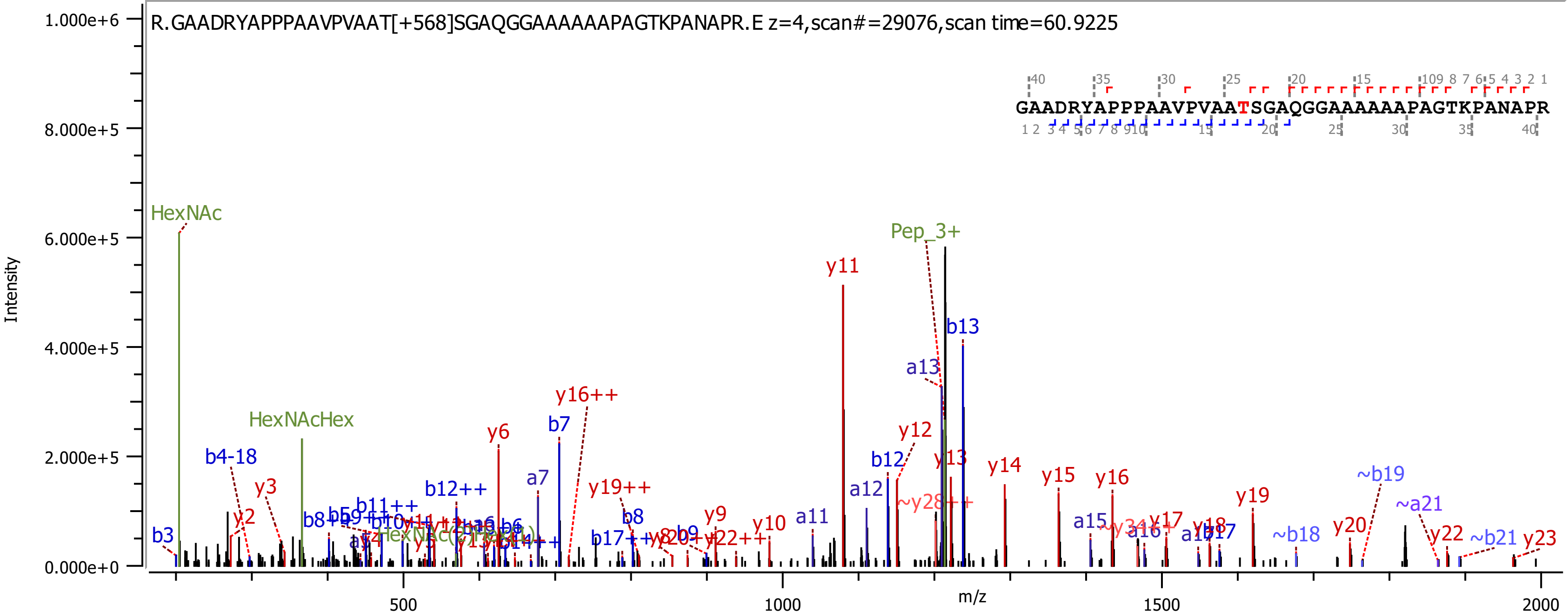
R.GAADRYAPPPAAVPVAAT[+568]SGAQGGAAAAAAPAGTKPANAPR.E z=4,scan#=29076,scan time=60.9225
GAADRYAPPPAAVPVAATSGAQGGAAAAAAPAGTKPANAPR
Intensity
m/z
1.000e+6
8.000e+5
6.000e+5
4.000e+5
2.000e+5
0.000e+0
500
1000
1500
2000
HexNAc
HexNAcHex
Pep_3+
y11
b13
a13
y16++
b7
y6
y12
b12
y13
y14
y15
y16
~b19
~a21
y19
y20
y22
y23
~b21
~b18
b4-18
y3
y2
b3
a7
y19++
b8
b12++
b11++
a12
a11
y10
y9
b9
~y28++
a15
y17
y18
b17
a16
a17
b17++

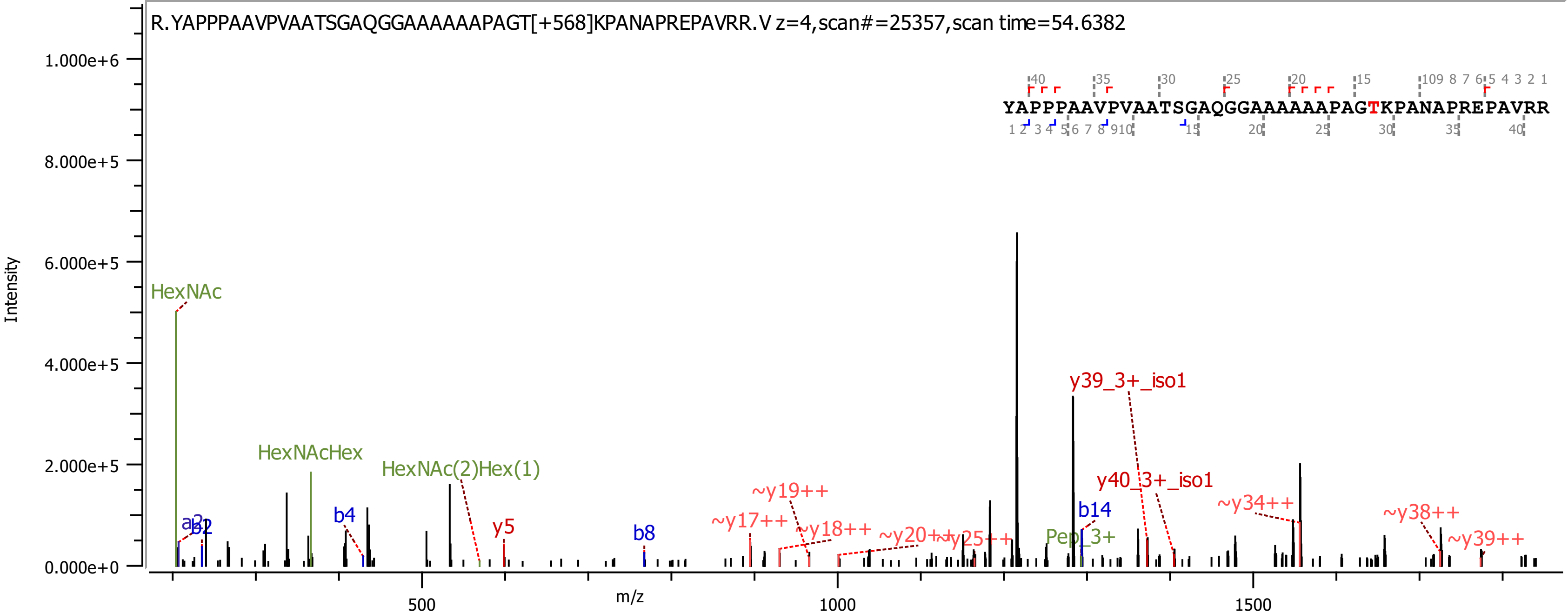

R.YAPPPAAVPVAATSGAQGGAAAAAAPAGT[+568]KPANAPREPAVRR.V z=4,scan#=25357,scan time=54.6382
YAPPPAAVPVAATSGAQGGAAAAAAPAGTKPANAPREPAVRR
Intensity
m/z
HexNAc
HexNAcHex
HexNAc(2)Hex(1)
a2
b2
b4
y5
b8
~y17++
~y19++
~y18++
~y20++
~y25++
Pep_3+
b14
y39_3+_iso1
y40_3+_iso1
~y34++
~y38++
~y39++

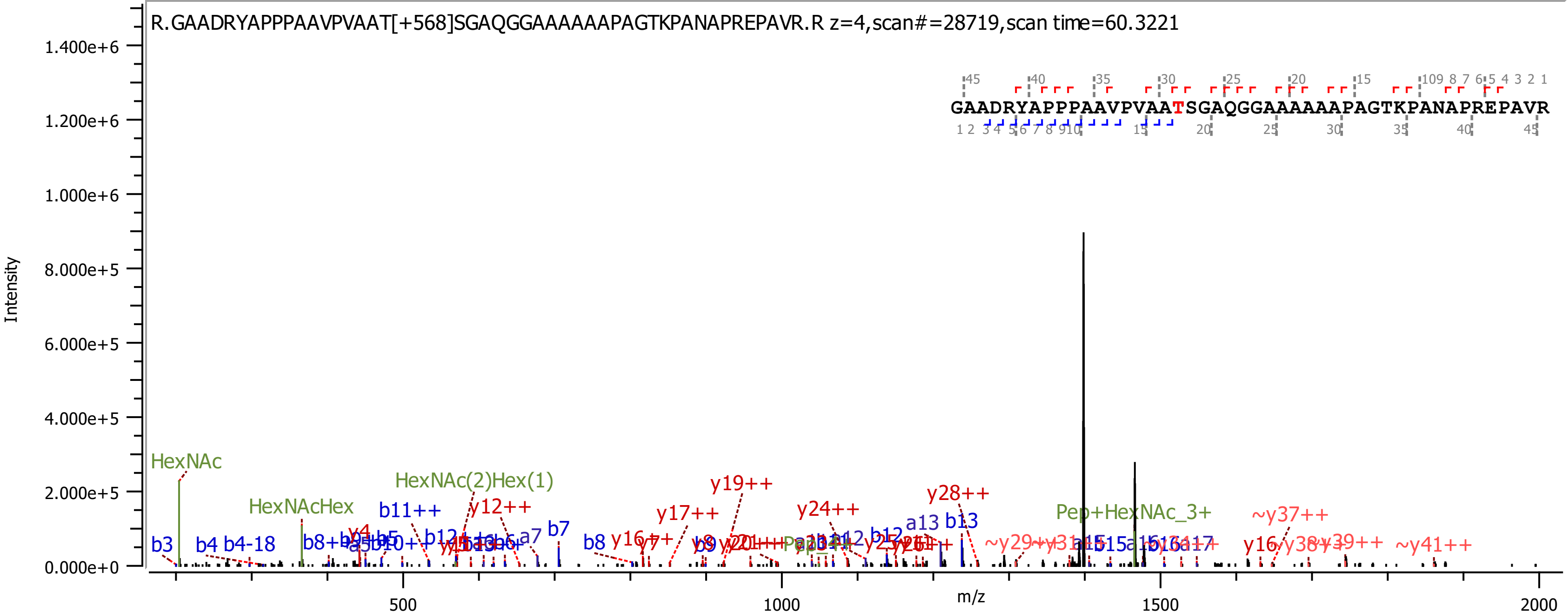
R.GAADRYAPPPAAVPVAAT[+568]SGAQGGAAAAAAPAGTKPANAPREPAVR.R z=4,scan#=28719,scan time=60.3221
GAADRYAPPPAAVPVAATSGAQGGAAAAAAPAGTKPANAPREPAVR
Intensity
m/z
0.000e+0
2.000e+5
4.000e+5
6.000e+5
8.000e+5
1.000e+6
1.200e+6
1.400e+6
500
1000
1500
2000
HexNAc
HexNAcHex
HexNAc(2)Hex(1)
Pep+HexNAc_3+
b3
b4
b4-18
b7
b8
b11++
b13
a13
a7
y4
y7
y12++
y16++
y17++
y19++
y24++
y28++
y16
~y29++
~y37++
~y41++

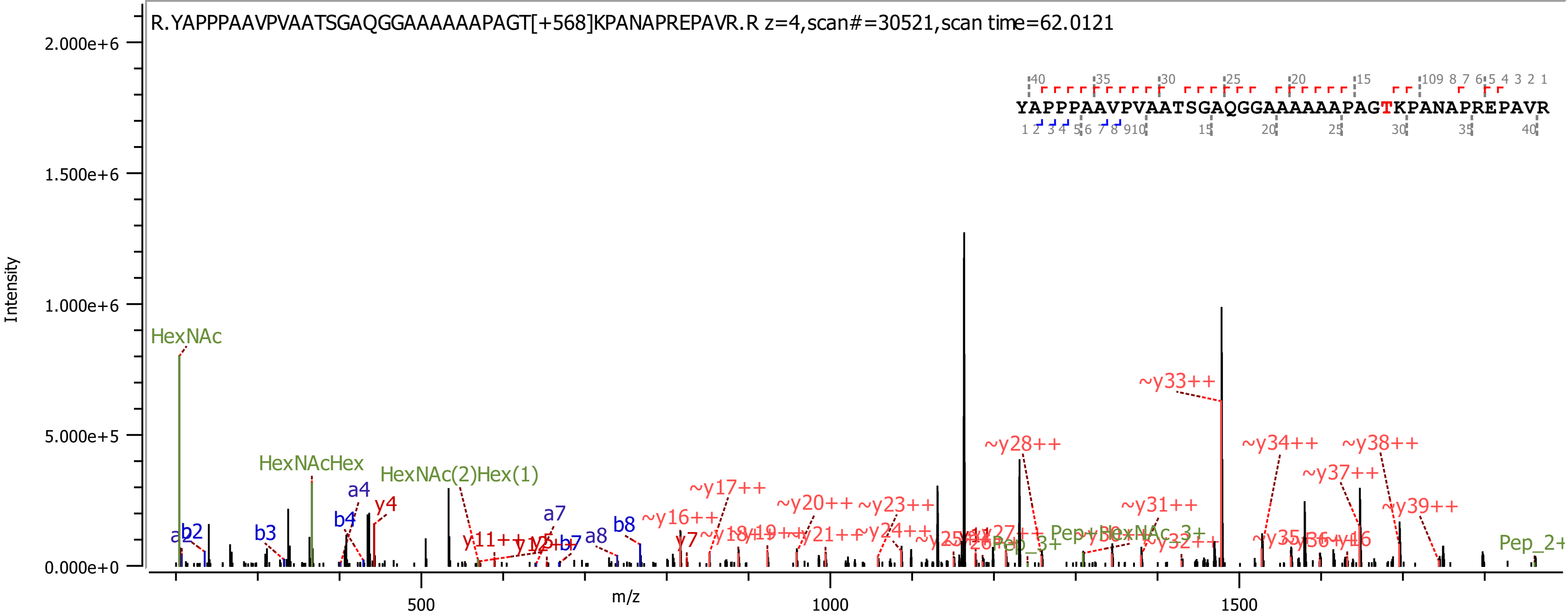
R.YAPPPAAVPVAATSGAQGGAAAAAAPAGT[+568]KPANAPREPAVR.R z=4,scan#=30521,scan time=62.0121
YAPPPAAVPVAATSGAQGGAAAAAAPAGTKPANAPREPAVR
Intensity
m/z
HexNAc
HexNAcHex
HexNAc(2)Hex(1)
~y33++
~y28++
~y34++
~y38++
~y37++
~y39++
~y31++
~y17++
~y20++
~y23++
~y16++
Pep_2+

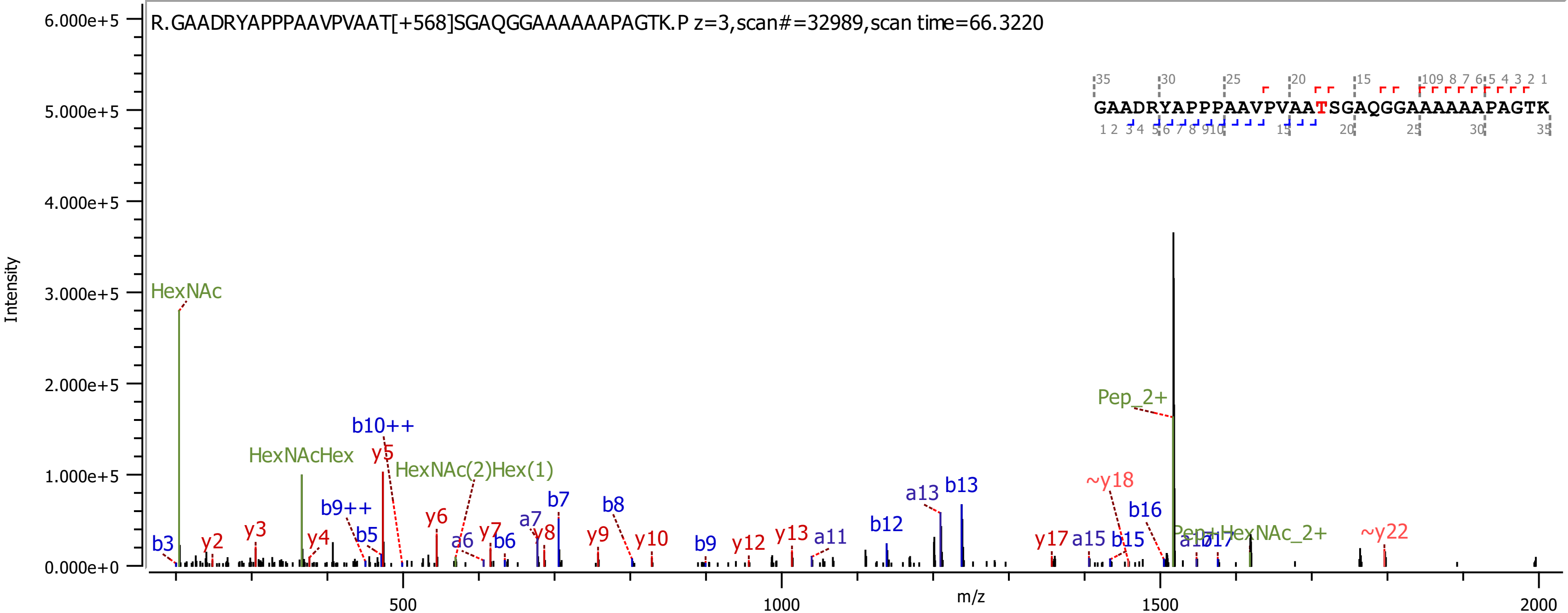

R.GAADRYAPPPAAVPVAAT[+568]SGAQGGAAAAAAPAGTK.P z=3,scan#=32989,scan time=66.3220
GAADRYAPPPAAVPVAATSGAQGGAAAAAAPAGTK
Intensity
m/z
HexNAc
HexNAcHex
HexNAc(2)Hex(1)
Pep_2+
Pep+HexNAc_2+
b3
y2
y3
y4
b9++
b5
b10++
y5
y6
a6
y7
b6
a7
y8
b7
b8
y9
y10
b9
y12
y13
a11
b12
a13
b13
y17
a15
b15
~y18
b16
~y22

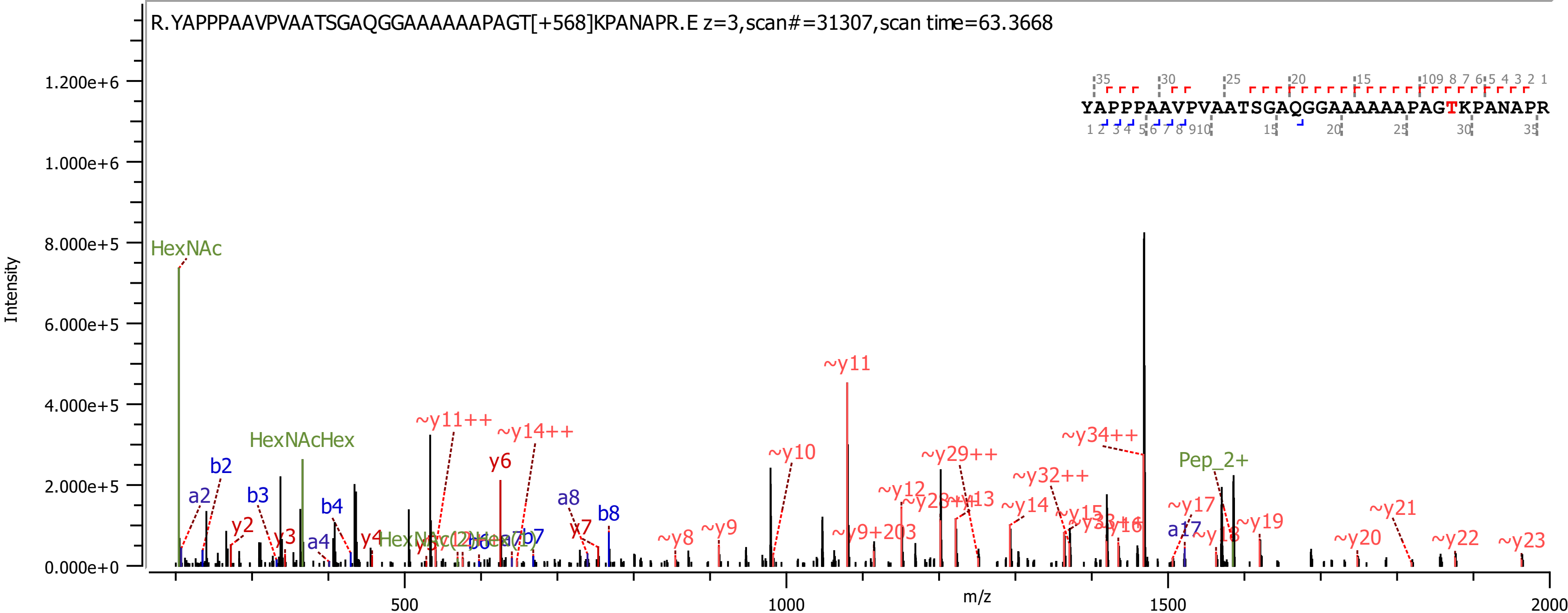
R.YAPPPAAVPVAATSGAQGGAAAAAAPAGT[+568]KPANAPR.E z=3,scan#=31307,scan time=63.3668
YAPPPAAVPVAATSGAQGGAAAAAAPAGTKPANAPR
Intensity
m/z
HexNAc
HexNAcHex
Pep_2+
~y11
~y11++
~y14++
~y34++
~y10
~y29++
~y32++
~y12
~y14
~y17
~y19
~y21
~y20
~y22
~y23
~y8
~y9
~y9+203
y6
y2
y3
y4
y7
a2
b2
b3
b4
a4
a8
b8
a17

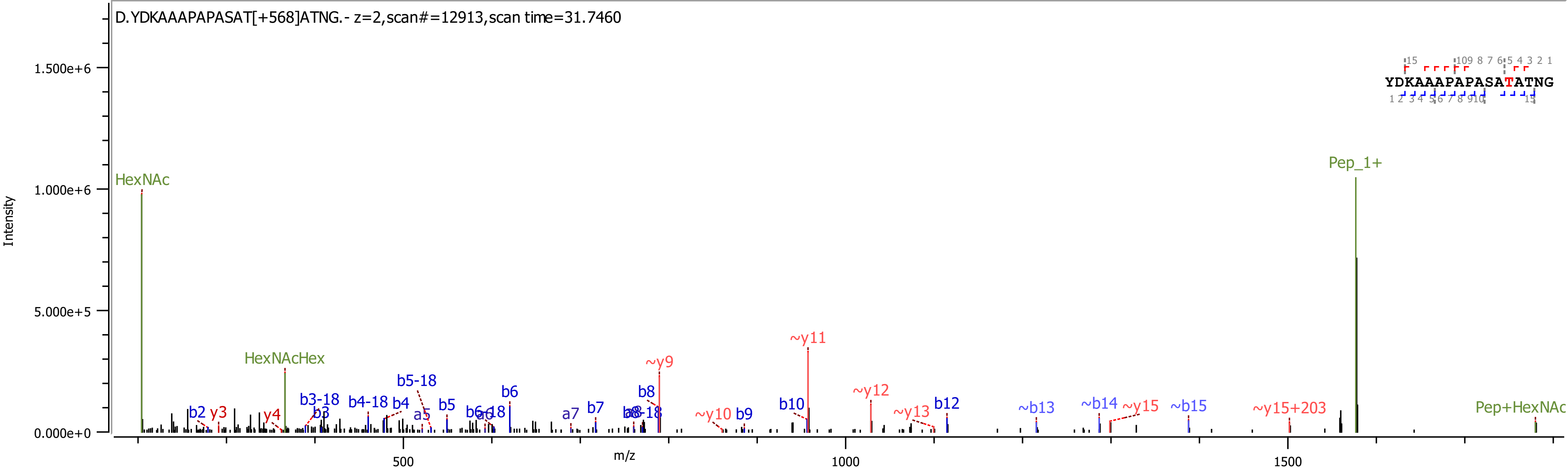
D.YDKAAAPAPASAT[+568]ATNG.- z=2,scan#=12913,scan time=31.7460
Intensity
m/z
1.500e+6
1.000e+6
5.000e+5
0.000e+0
500
1000
1500
HexNAc
HexNAcHex
Pep_1+
Pep+HexNAc
YDKAAAPAPASATATNG
b2
y3
y4
b3-18
b3
b4-18
b4
b5-18
a5
b5
b6
b6-18
a6
a7
b7
b8
b8-18
~y9
~y10
b9
b10
~y11
~y12
~y13
b12
~b13
~b14
~y15
~b15
~y15+203

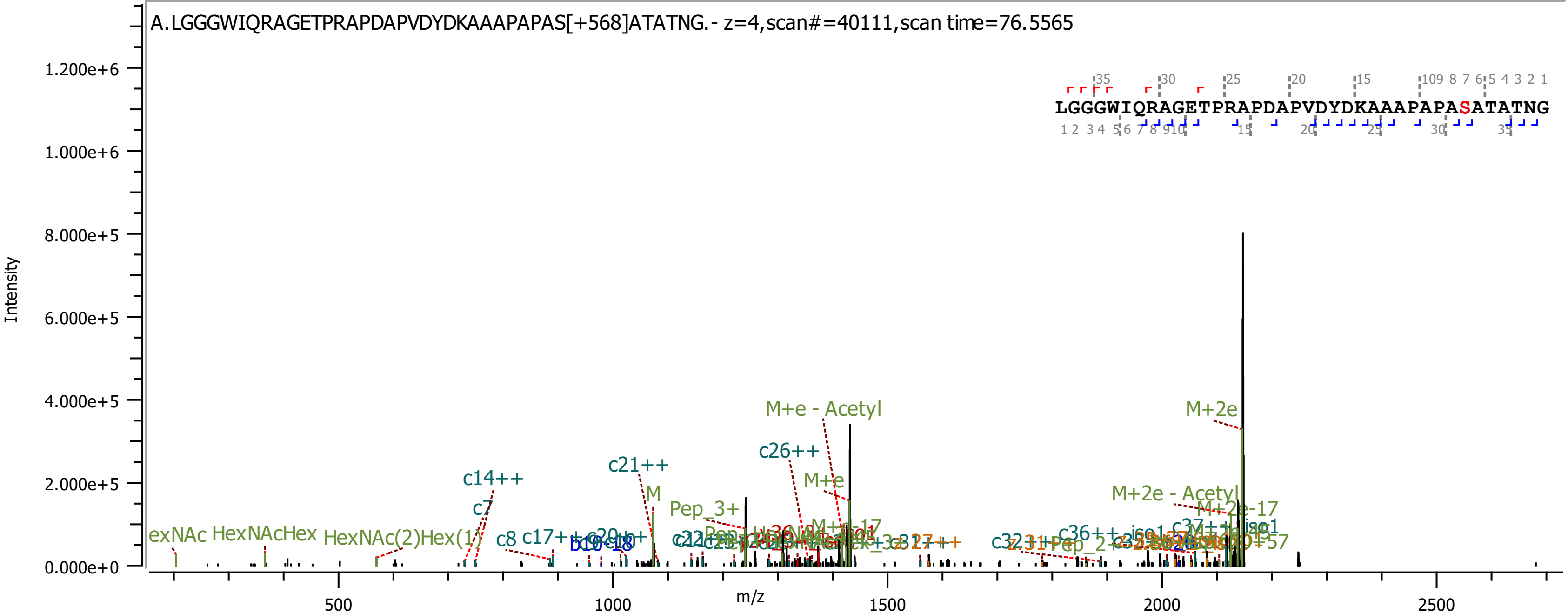

A.LGGGWIQRAGETPRAPDAPVDYDKAAAPAPAS[+568]ATATNG.- z=4,scan#=40111,scan time=76.5565
Intensity
m/z
0.000e+0
2.000e+5
4.000e+5
6.000e+5
8.000e+5
1.000e+6
1.200e+6
500
1000
1500
2000
2500
LGGGWIQRAGETPRAPDAPVDYDKAAAPAPASATATNG
M+e - Acetyl
M+2e
c26++
c21++
c14++
M+e
M
c7
Pep_3+
M+2e - Acetyl
M+2e-17
c8
c17++
HexNAcHex
HexNAc(2)Hex(1)
exNAc

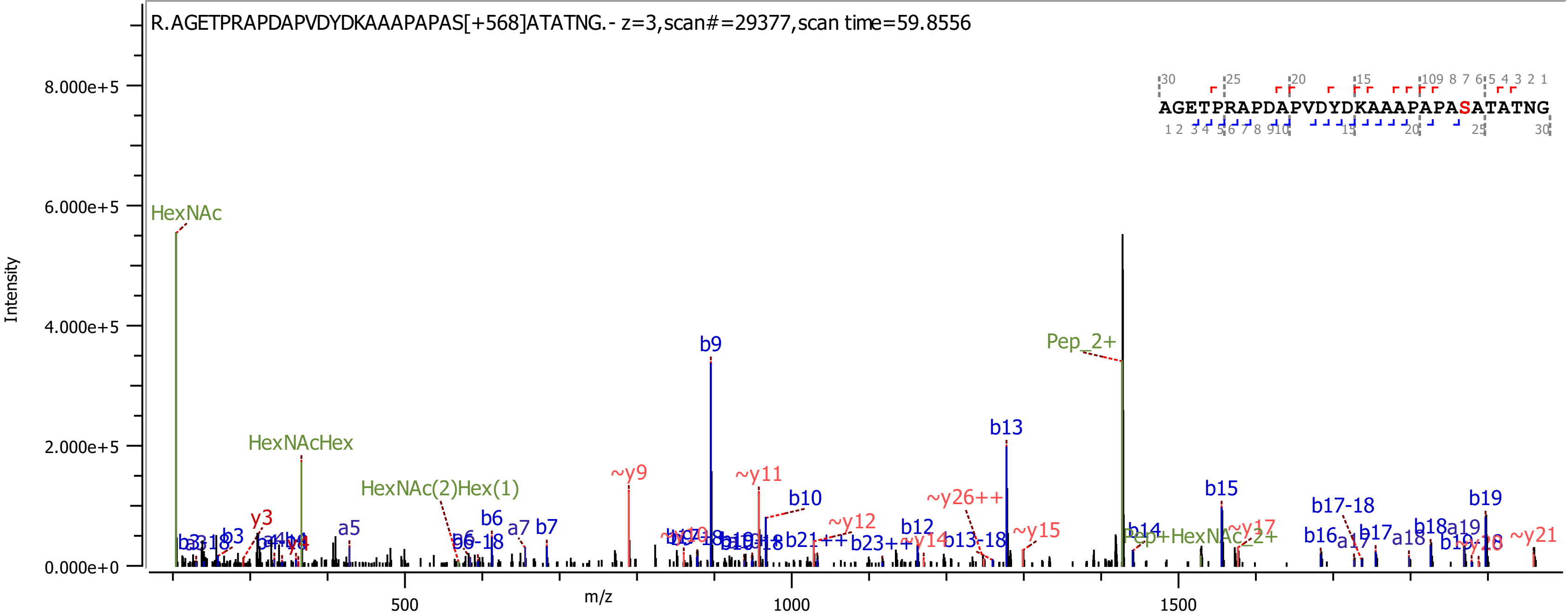
R.AGETPRAPDAPVDYDKAAAPAPAS[+568]ATATNG.- z=3,scan#=29377,scan time=59.8556
AGETPRAPDAPVDYDKAAAPAPASATATNG
Intensity
m/z
8.000e+5
6.000e+5
4.000e+5
2.000e+5
0.000e+0
500
1000
1500
HexNAc
HexNAcHex
HexNAc(2)Hex(1)
Pep_2+
Pep+HexNAc_2+
b9
b10
b13
b15
b19
~y9
~y11
~y12
~y15
~y17
~y21
~y26++
b12
b14
b16
b17-18
b18
a19
a5
a7
b6
b7
y3

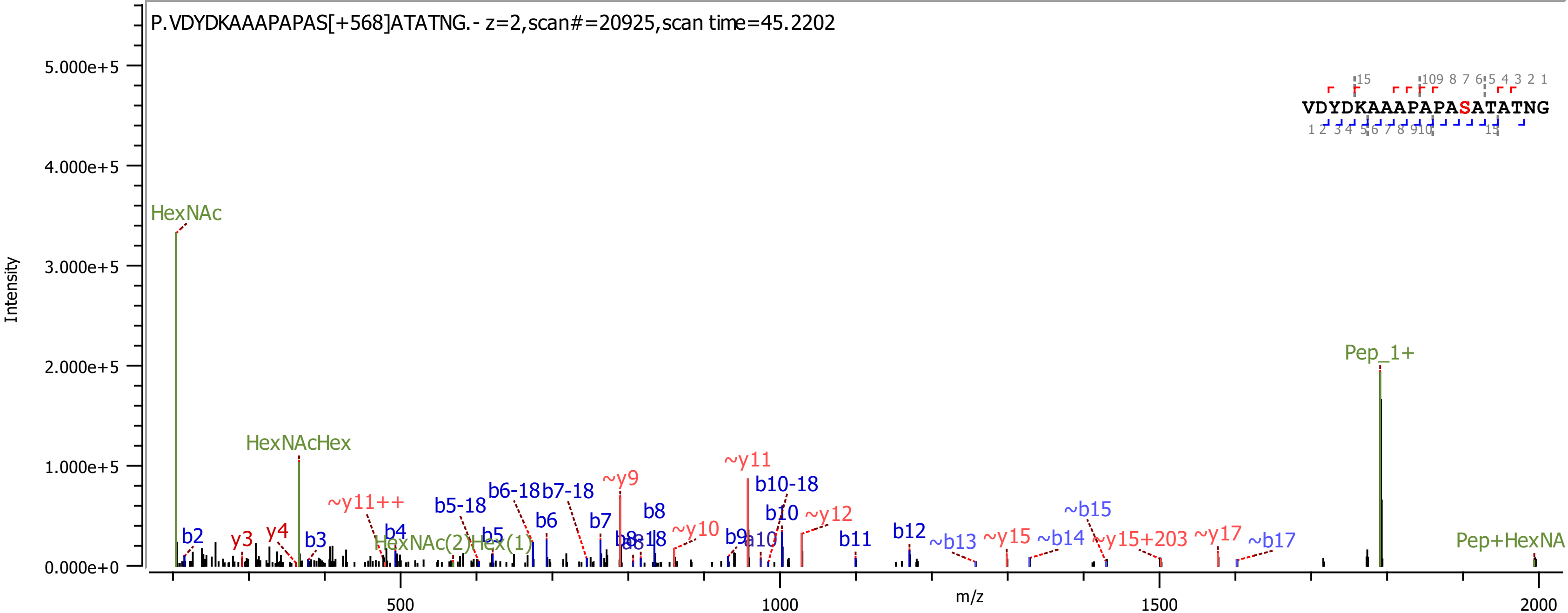
P.VDYDKAAAPAPAS[+568]ATATNG.- z=2,scan#=20925,scan time=45.2202
VDYDKAAAPAPASATATNG
Intensity
5.000e+5
4.000e+5
3.000e+5
2.000e+5
1.000e+5
0.000e+0
HexNAc
HexNAcHex
HexNAc(2)Hex(1)
Pep_1+
Pep+HexNA
b2
y3
y4
b3
~y11++
b4
b5-18
b5
b6-18
b7-18
b6
b7
~y9
b8
b8-18
~y10
b9
a10
~y11
b10-18
b10
~y12
b11
b12
~b13
~y15
~b14
~b15
~y15+203
~y17
~b17
500
1000
1500
2000
m/z

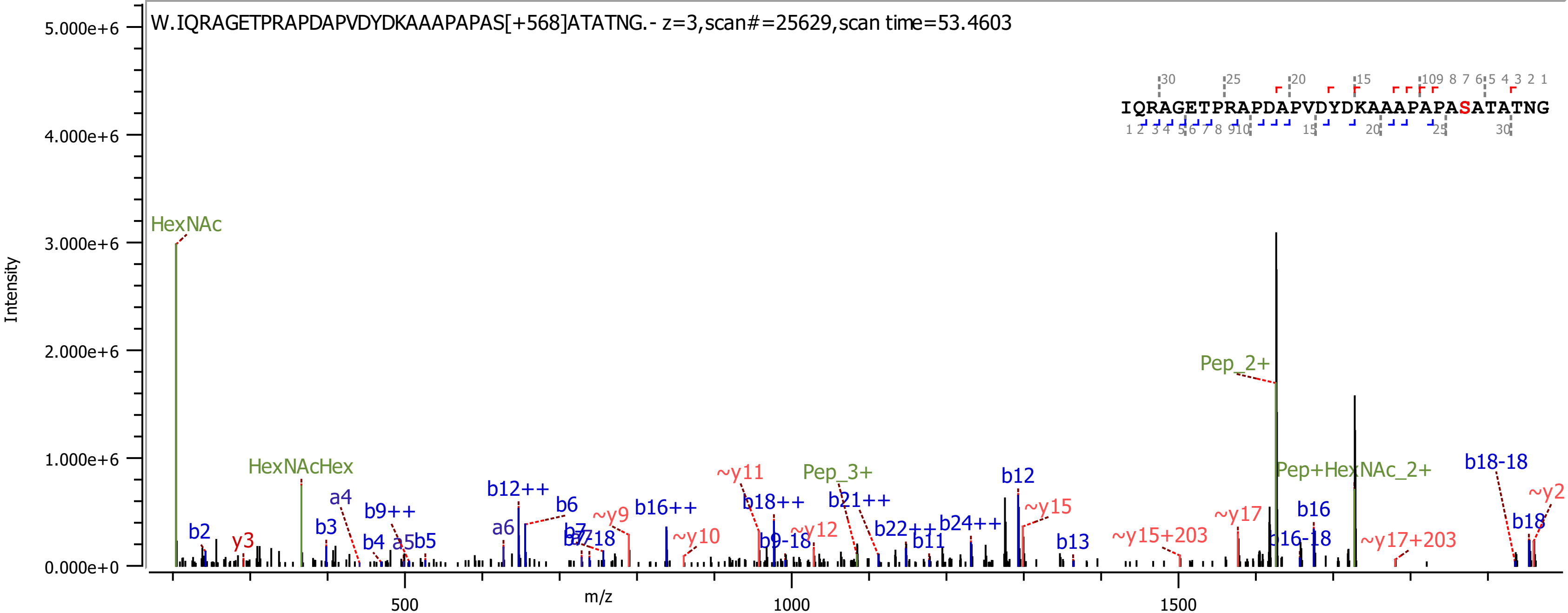
W.IQRAGETPRAPDAPVDYDKAAAPAPAS[+568]ATATNG.- z=3,scan#=25629,scan time=53.4603
IQRAGETPRAPDAPVDYDKAAAPAPASATATNG
Intensity
m/z
5.000e+6
4.000e+6
3.000e+6
2.000e+6
1.000e+6
0.000e+0
500
1000
1500
HexNAc
HexNAcHex
Pep_2+
Pep+HexNAc_2+
Pep_3+
b2
y3
b3
a4
b9++
b4
a5
b5
a6
b12++
b6
b7
~y9
b16++
~y10
~y11
b18++
b9-18
~y12
b21++
b22++
b11
b24++
b12
~y15
b13
~y15+203
~y17
b16
b16-18
~y17+203
b18-18
b18
~y2

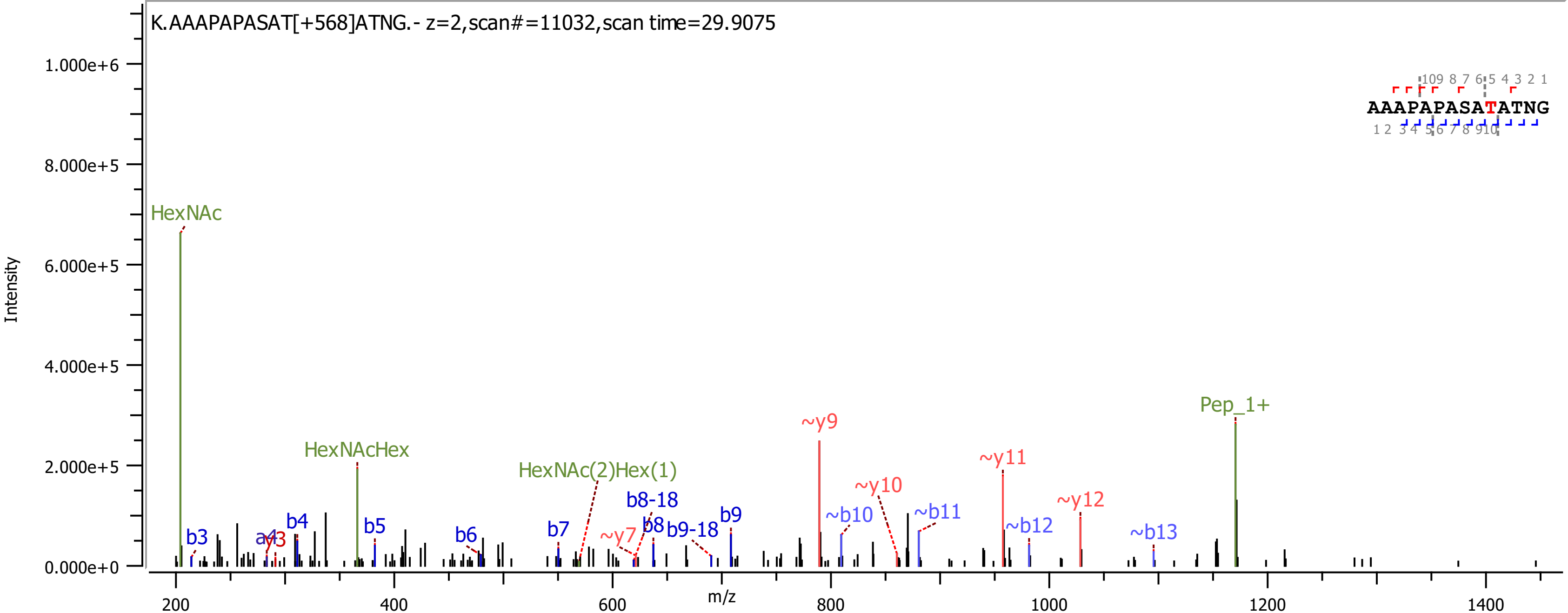

K.AAAPAPASAT[+568]ATNG.- z=2,scan#=11032,scan time=29.9075
Intensity
m/z
HexNAc
HexNAcHex
HexNAc(2)Hex(1)
Pep_1+
b3
a4
y3
b4
b5
b6
b7
~y7
b8-18
b8
b9-18
b9
~y9
~b10
~y10
~b11
~y11
~b12
~y12
~b13
AAAPAPASATATNG

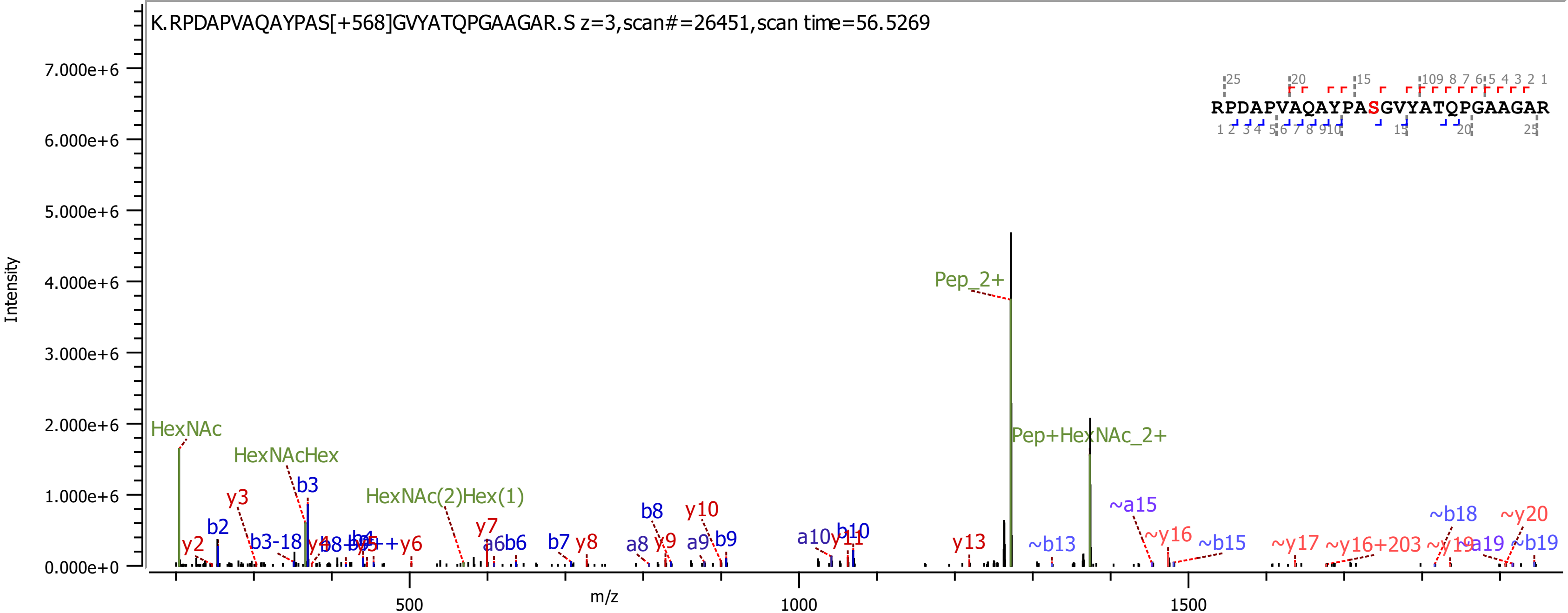
K.RPDAPVAQAYPAS[+568]GVYATQPGAAGAR.S z=3,scan#=26451,scan time=56.5269
RPDAPVAQAYPASGVYATQPGAAGAR
Intensity
m/z
Pep_2+
Pep+HexNAc_2+
HexNAc
HexNAcHex
HexNAc(2)Hex(1)
b2
b3
b3-18
b7
b8
b9
b10
y2
y3
y6
y7
y8
y10
y13
a6b6
a8
a9
a10
~a15
~b13
~b15
~b18
~b19
~y16
~y17
~y16+203
~y20

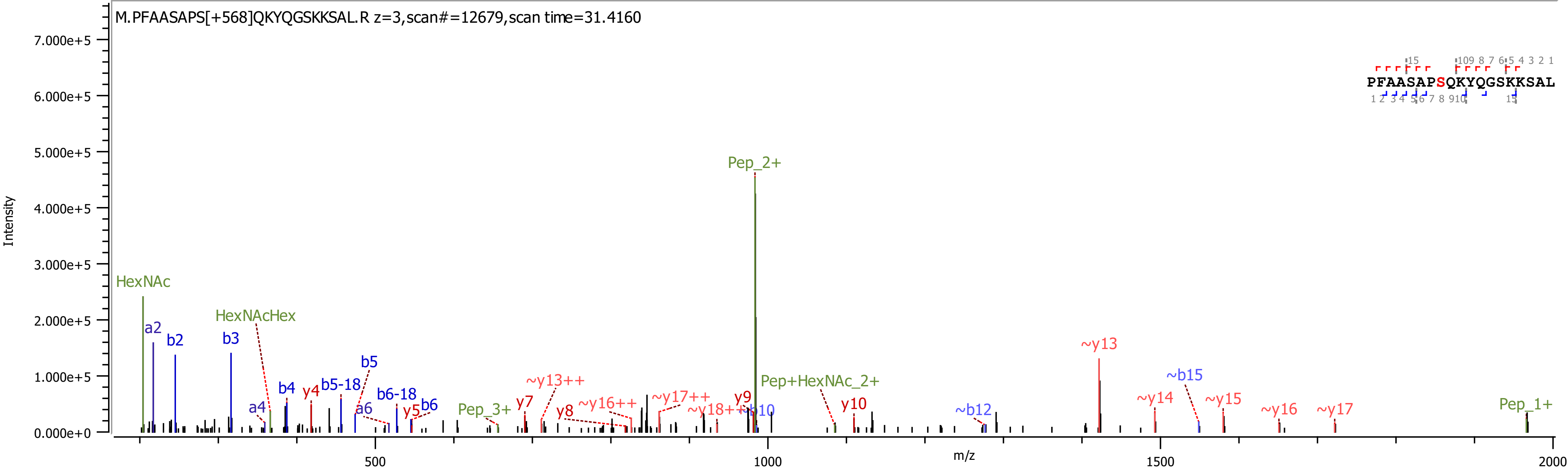
M.PFAASAPS[+568]QKYQGSKKSAL.R z=3,scan#=12679,scan time=31.4160
PFAASAPSQKYQGSKKSAL
Intensity
m/z
HexNAc
a2
b2
b3
HexNAcHex
a4
b4
y4
b5-18
b5
a6
b6-18
y5
b6
Pep_3+
y7
~y13++
y8
~y16++
~y17++
~y18++
y9
~b10
Pep_2+
Pep+HexNAc_2+
y10
~b12
~y13
~y14
~b15
~y15
~y16
~y17
Pep_1+

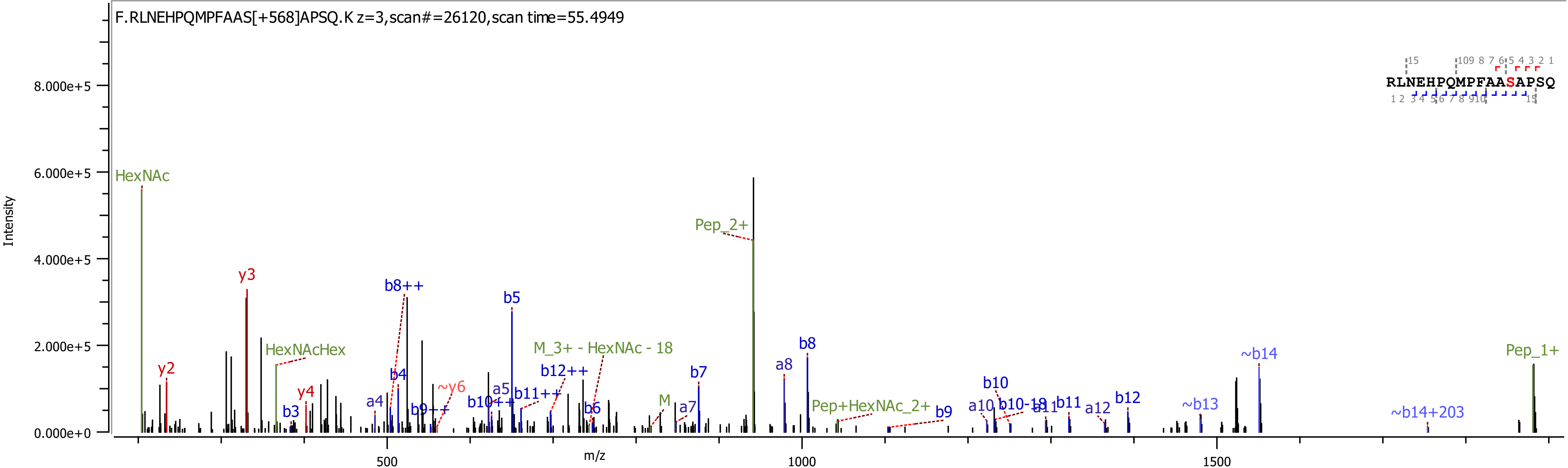
F.RLNEHPQMPFAAS[+568]APSQ.K z=3,scan#=26120,scan time=55.4949
Intensity
m/z
HexNAc
y2
y3
HexNAcHex
y4
b3
a4
b4
b8++
b9++
~y6
b10++
a5
b5
b11++
b12++
b6
M_3+ - HexNAc - 18
M
a7
b7
Pep_2+
a8
b8
Pep+HexNAc_2+
b9
a10
b10
b10-18
a11
b11
a12
b12
~b13
~b14
~b14+203
Pep_1+
RLNEHPQMPFAASAPSQ

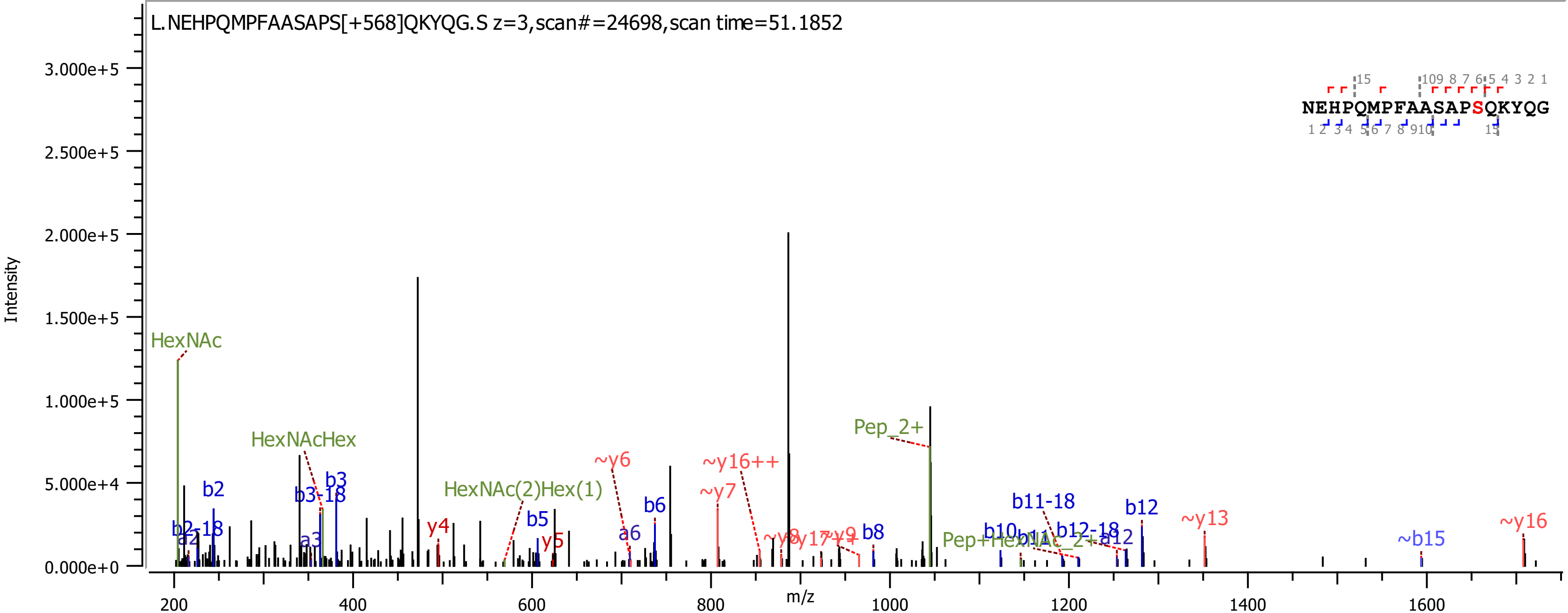
L.NEHPQMPFAASAPS[+568]QKYQG.S z=3,scan#=24698,scan time=51.1852
NEHPQMPFAASAPSQKYQG
Intensity
m/z
HexNAc
HexNAcHex
HexNAc(2)Hex(1)
Pep_2+
b2
b3
b3-18
b2-18
a2
a3
y4
b5
y5
a6
b6
~y6
~y7
~y16++
b8
b10
b11-18
b12
b12-18
a12
~y13
~b15
~y16

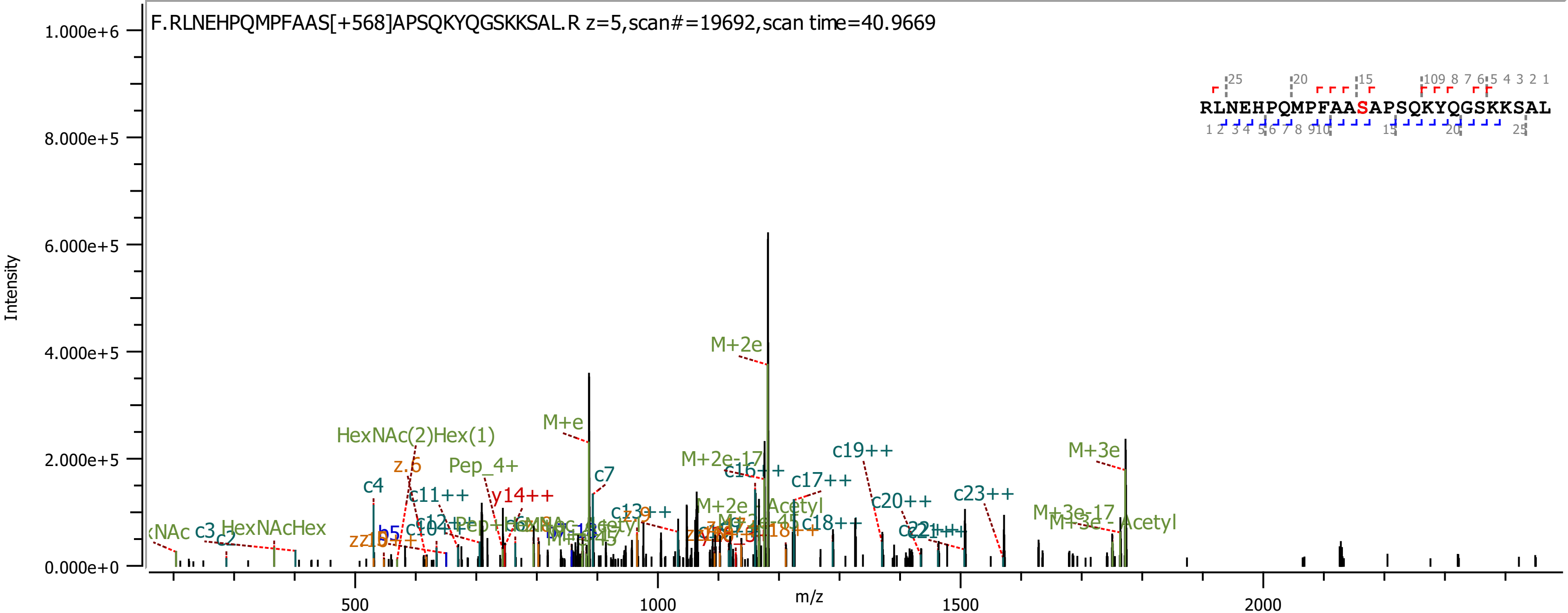

F.RLNEHPQMPFAAS[+568]APSQKYQGSKKSAL.R z=5,scan#=19692,scan time=40.9669
RLNEHPQMPFAASAPSQKYQGSKKSAL
Intensity
m/z
1.000e+6
8.000e+5
6.000e+5
4.000e+5
2.000e+5
0.000e+0
500
1000
1500
2000
M+2e
M+e
HexNAc(2)Hex(1)
M+3e
c19++
c17++
c23++
c20++
c4
c7
c11++
y14++
Pep_4+
M+2e-17
c16++
c18++
HexNAcHex
c3
c2
M+3e-17
M+3e - Acetyl
M+2e - Acetyl

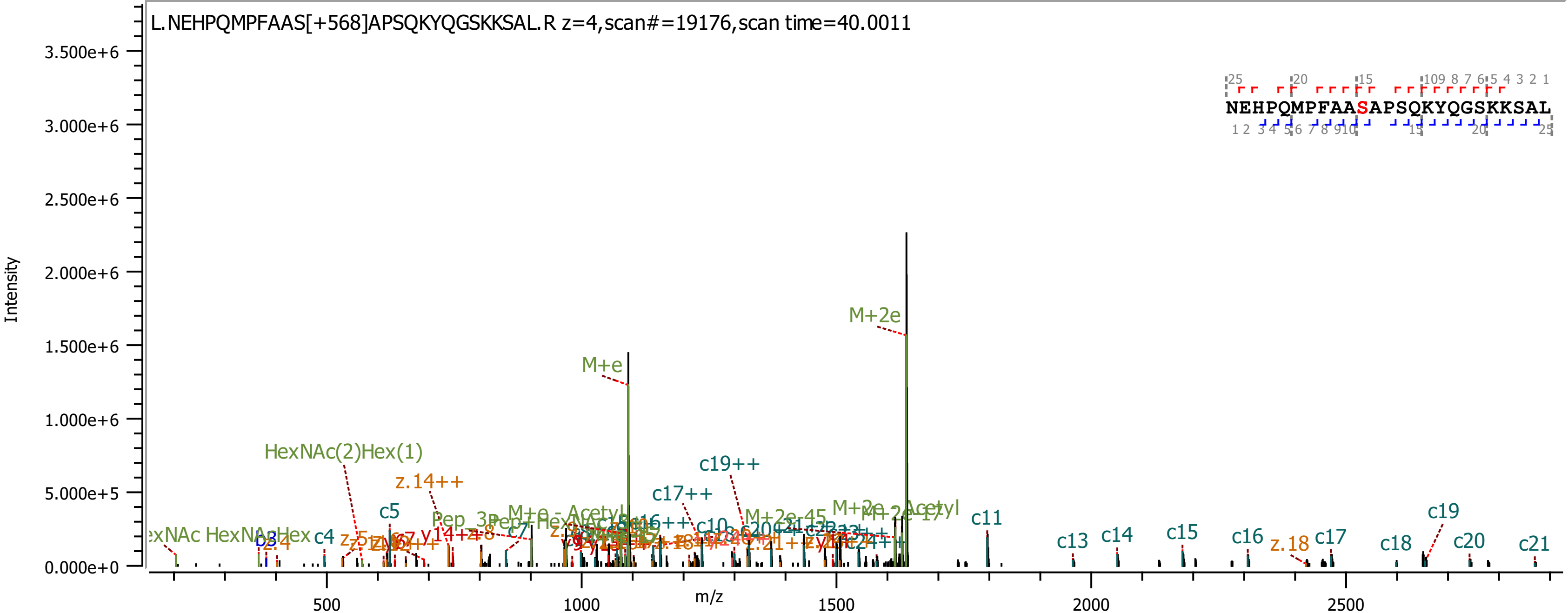
L.NEHPQMPFAAS[+568]APSQKYQGSKKSAL.R z=4,scan#=19176,scan time=40.0011
NEHPQMPFAASAPSQKYQGSKKSAL
Intensity
m/z
3.500e+6
3.000e+6
2.500e+6
2.000e+6
1.500e+6
1.000e+6
5.000e+5
0.000e+0
500
1000
1500
2000
2500
M+2e
M+e
HexNAc(2)Hex(1)
z.14++
c19++
c17++
c5
c4
c11
c13
c14
c15
c16
z.18
c17
c18
c19
c20
c21

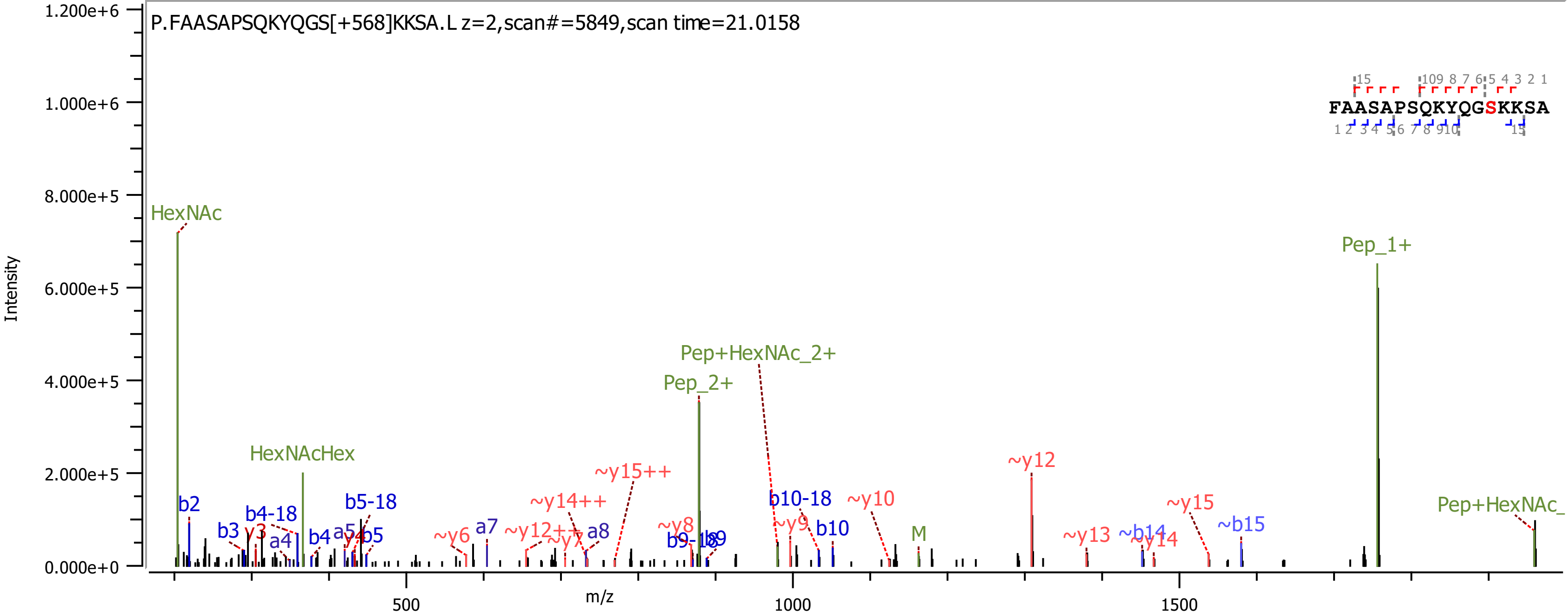
P.FAASAPSQKYQGS[+568]KKSA.L z=2,scan#=5849,scan time=21.0158
FAASAPSQKYQGSKKSA
Intensity
m/z
1.200e+6
1.000e+6
8.000e+5
6.000e+5
4.000e+5
2.000e+5
0.000e+0
500
1000
1500
HexNAc
HexNAcHex
Pep_1+
Pep+HexNAc_2+
Pep_2+
Pep+HexNAc_
b2
b3
y3
b4-18
a4
b4
a5
y4
b5
b5-18
~y6
a7
~y12++
~y7
a8
~y14++
~y15++
~y8
b9-18
b9
b10-18
~y9
b10
~y10
M
~y12
~y13
~b14
~y14
~y15
~b15

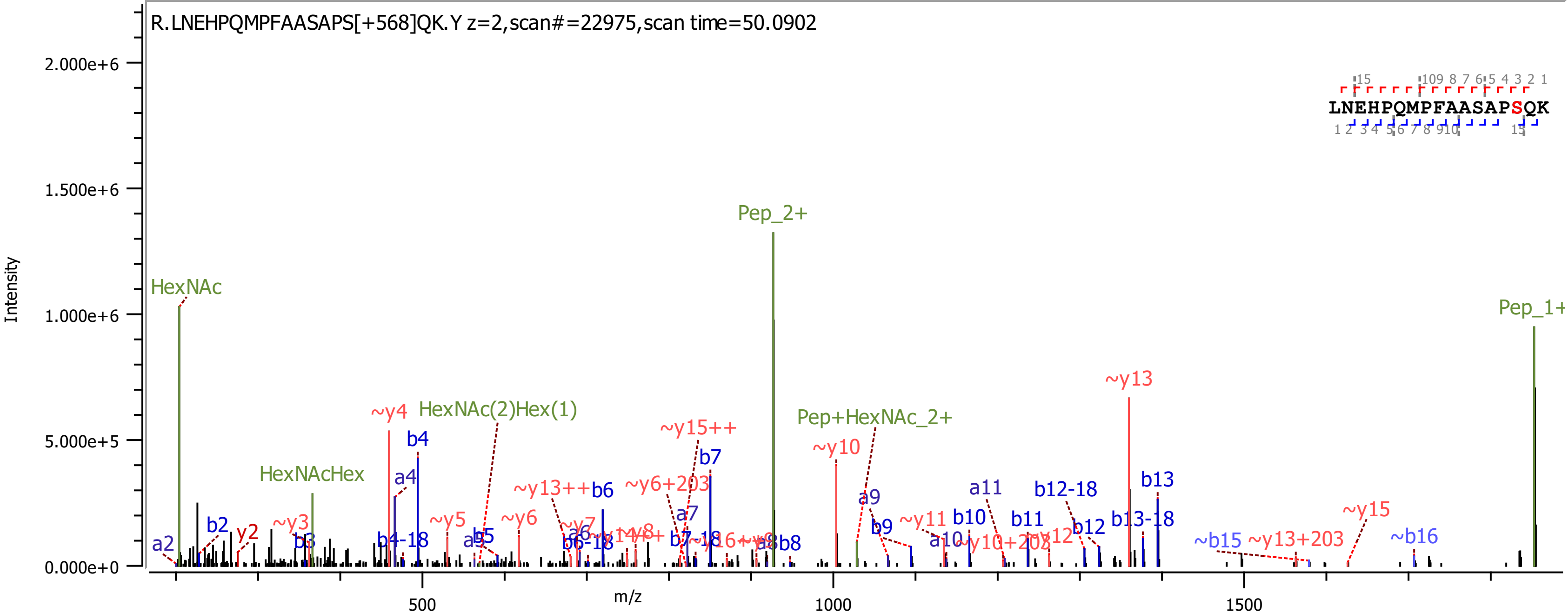

R.LNEHPQMPFAASAPS[+568]QK.Y z=2,scan#=22975,scan time=50.0902
Intensity
m/z
LNEHPQMPFAASAPSQK
HexNAc
HexNAcHex
HexNAc(2)Hex(1)
Pep_2+
Pep+HexNAc_2+
Pep_1+
~y4
~y13
~y10
~y15++
~y13++
~y6+203
~y15
~y13+203
~b15
~b16
~y10+203
b4
b7
b13
b13-18
b12-18
a11
a9

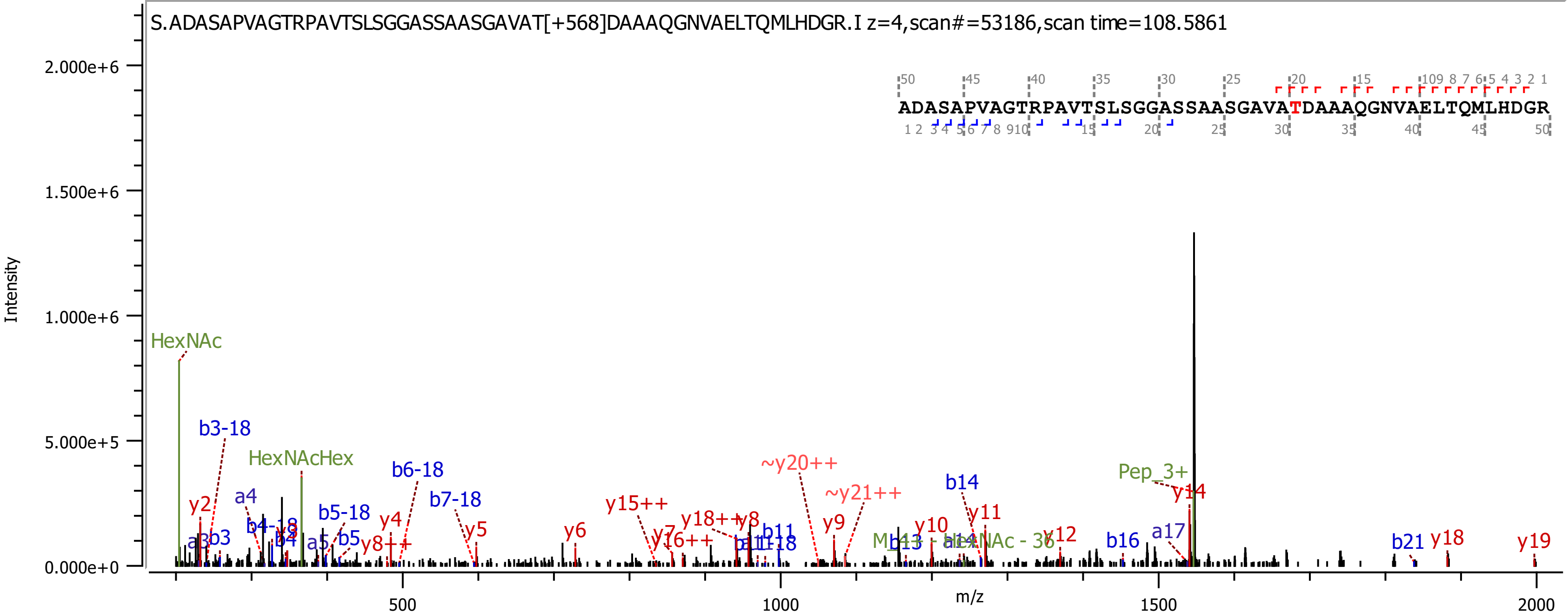
S.ADASAPVAGTRPAVTSLSGGASSAASGAVAT[+568]DAAAQGNVAELTQMLHDGR.I z=4,scan#=53186,scan time=108.5861
ADASAPVAGTRPAVTSLSGGASSAASGAVATDAAAQGNVAELTQMLHDGR
Intensity
m/z
HexNAc
HexNAcHex
b3-18
b6-18
b7-18
b5-18
y2
a4
b3
a3
a5
b5
y4
y8++
y5
y6
y15++
y18++
y8
y9
~y20++
~y21++
y10
b14
y11
y12
b16
a17
Pep_3+
y14
b21
y18
y19
0.000e+0
5.000e+5
1.000e+6
1.500e+6
2.000e+6
500
1000
1500
2000

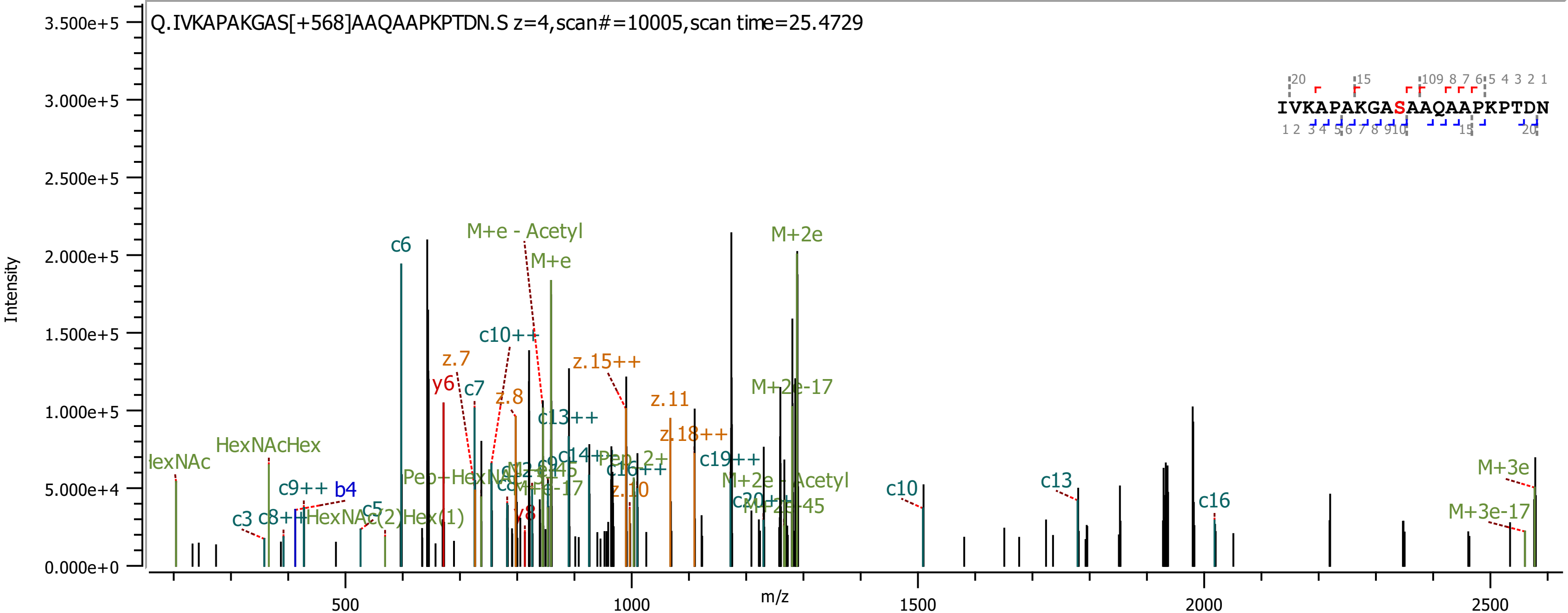
Q.IVKAPAKGAS[+568]AAQAAPKPTDN.S z=4,scan#=10005,scan time=25.4729
Intensity
m/z
IVKAPAKGASAAQAAPKPTDN
c6
M+e - Acetyl
M+e
M+2e
c10++
z.7
y6
c7
z.8
z.15++
c13++
z.11
M+2e-17
z.18++
c14++
Pep 2+
c16++
c19++
M+2e - Acetyl
HexNAcHex
c9++
b4
c3
c8++
HexNAc(2)Hex(1)
c5
Pep−HexNAc
z.10
y8
M+2e-45
c10
c13
c16
M+3e
M+3e-17

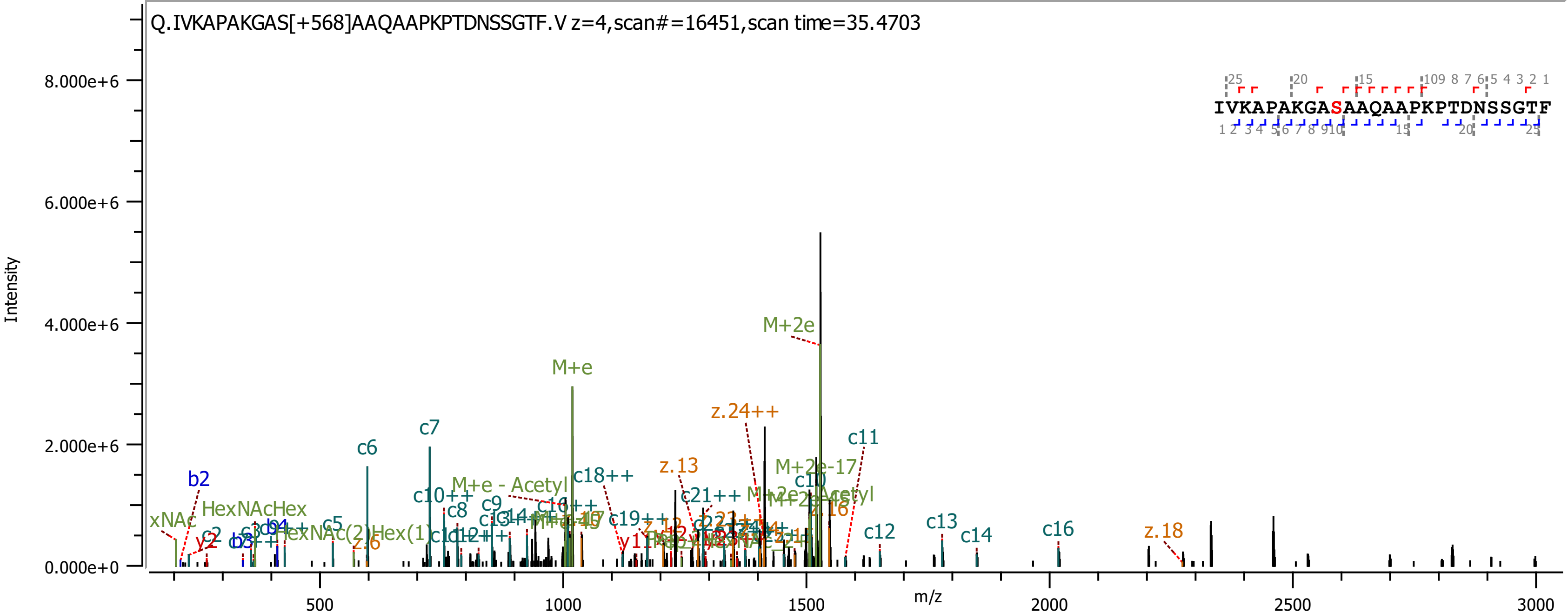
Q.IVKAPAKGAS[+568]AAQAAPKPTDNSSGTF.V z=4,scan#=16451,scan time=35.4703
IVKAPAKGASAAQAAPKPTDNSSGTF
Intensity
m/z
8.000e+6
6.000e+6
4.000e+6
2.000e+6
0.000e+0
500
1000
1500
2000
2500
3000
M+2e
M+e
z.24++
c7
c6
c11
b2
M+2e-17
c18++
z.13
M+e - Acetyl
HexNAcHex
c10++
c8
c9
c21++
c12
c13
c14
c16
z.18
c5
z.6
z.16

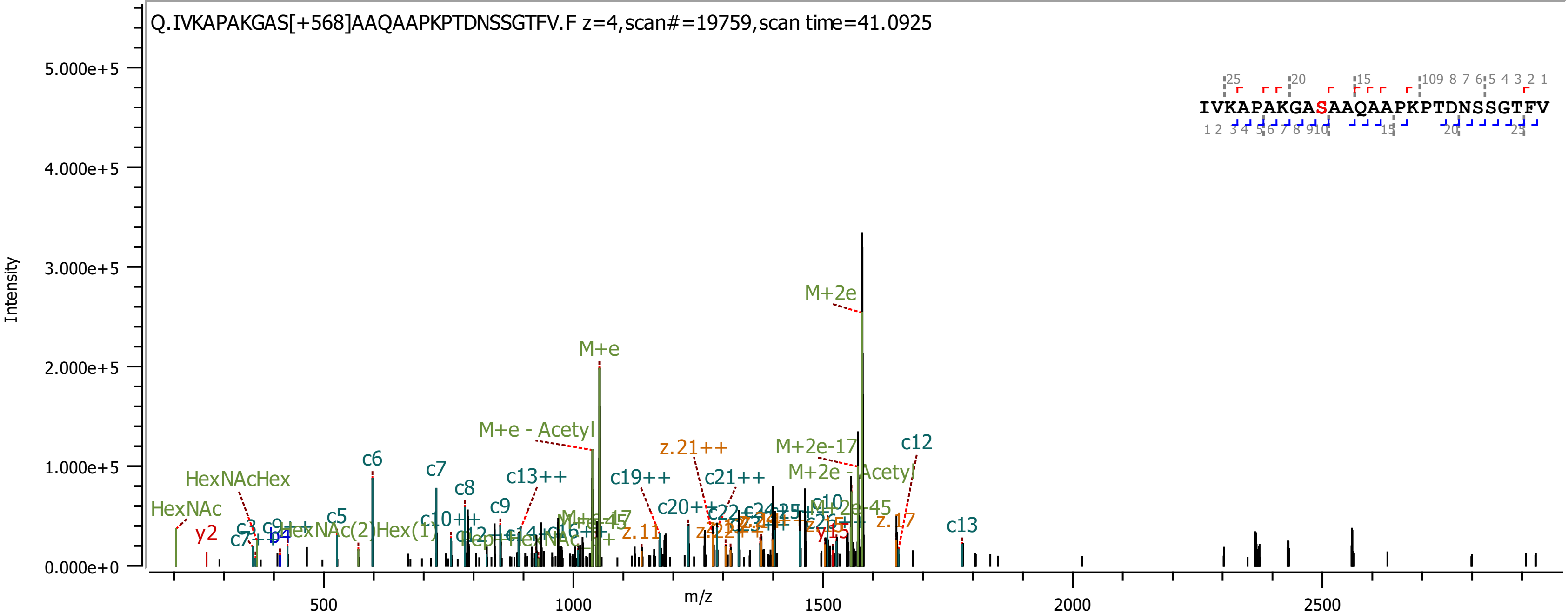
Q.IVKAPAKGAS[+568]AAQAAPKPTDNSSGTFV.F z=4,scan#=19759,scan time=41.0925
IVKAPAKGASAAQAAPKPTDNSSGTFV
Intensity
m/z
5.000e+5
4.000e+5
3.000e+5
2.000e+5
1.000e+5
0.000e+0
500
1000
1500
2000
2500
M+2e
M+e
M+e - Acetyl
z.21++
M+2e-17
c12
M+2e - Acetyl
c6
c7
HexNAcHex
c8
c13++
c19++
c21++
c9
c20++
HexNAc
c5
c10++
M+2e-45
z.17
c13
y2
y15
z.11

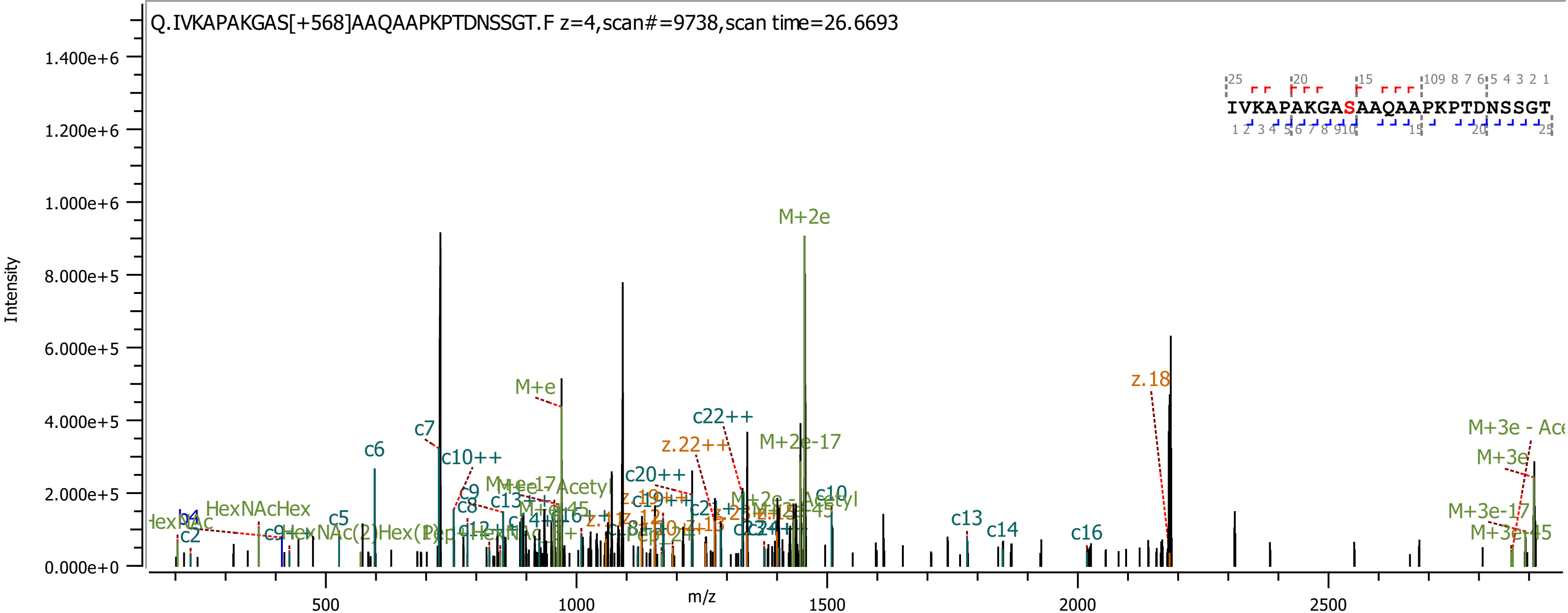

Q.IVKAPAKGAS[+568]AAQAAPKPTDNSSGT.F z=4,scan#=9738,scan time=26.6693
Intensity
1.400e+6
1.200e+6
1.000e+6
8.000e+5
6.000e+5
4.000e+5
2.000e+5
0.000e+0
m/z
500
1000
1500
2000
2500
IVKAPAKGASAAQAAPKPTDNSSGT
M+2e
M+e
z.18
c22++
z.22++
M+2e-17
c7
c6
c10++
c20++
c5
c2
HexNAcHex
c13
c14
c16
M+3e
M+3e-17
M+3e-45

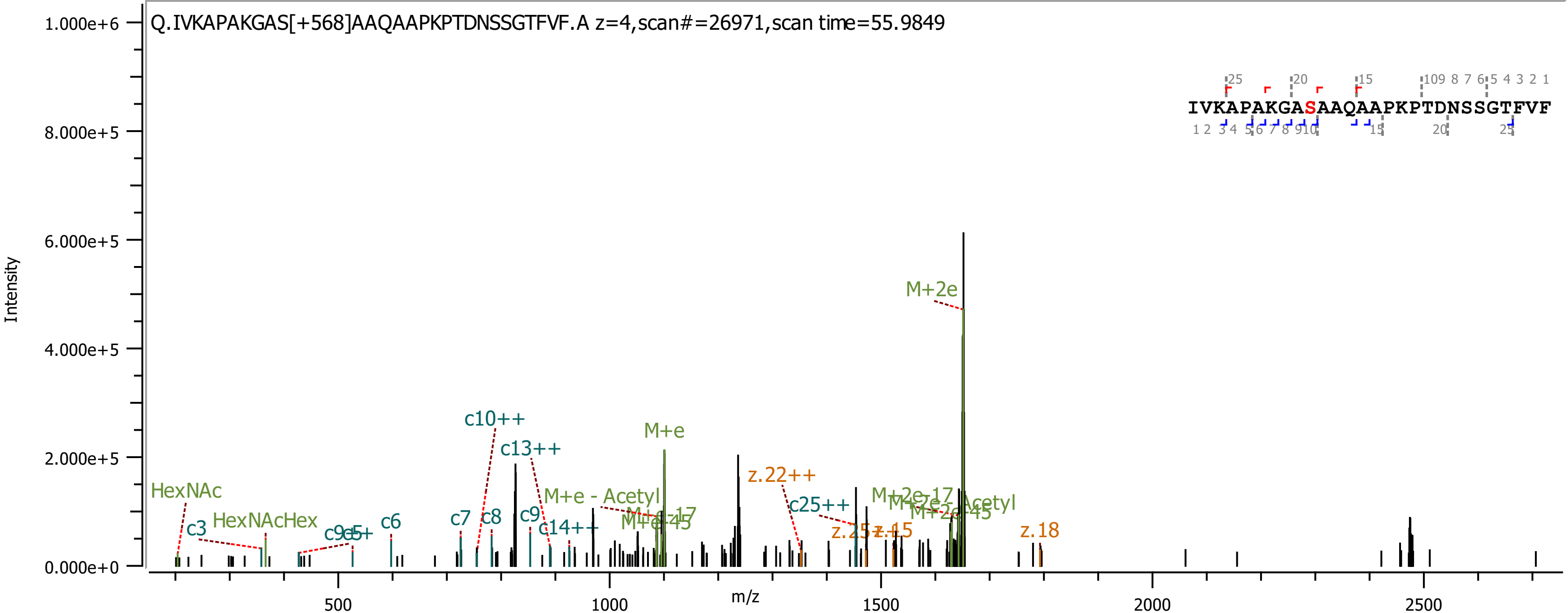

Q.IVKAPAKGAS[+568]AAQAAPKPTDNSSGTFVF.A z=4,scan#=26971,scan time=55.9849
IVKAPAKGASAAQAAPKPTDNSSGTFVF
Intensity
m/z
1.000e+6
8.000e+5
6.000e+5
4.000e+5
2.000e+5
0.000e+0
500
1000
1500
2000
2500
M+2e
c10++
c13++
M+e
z.22++
HexNAc
M+e - Acetyl
c25++
c3
HexNAcHex
c6
c7
c8
c9
c14++
z.18

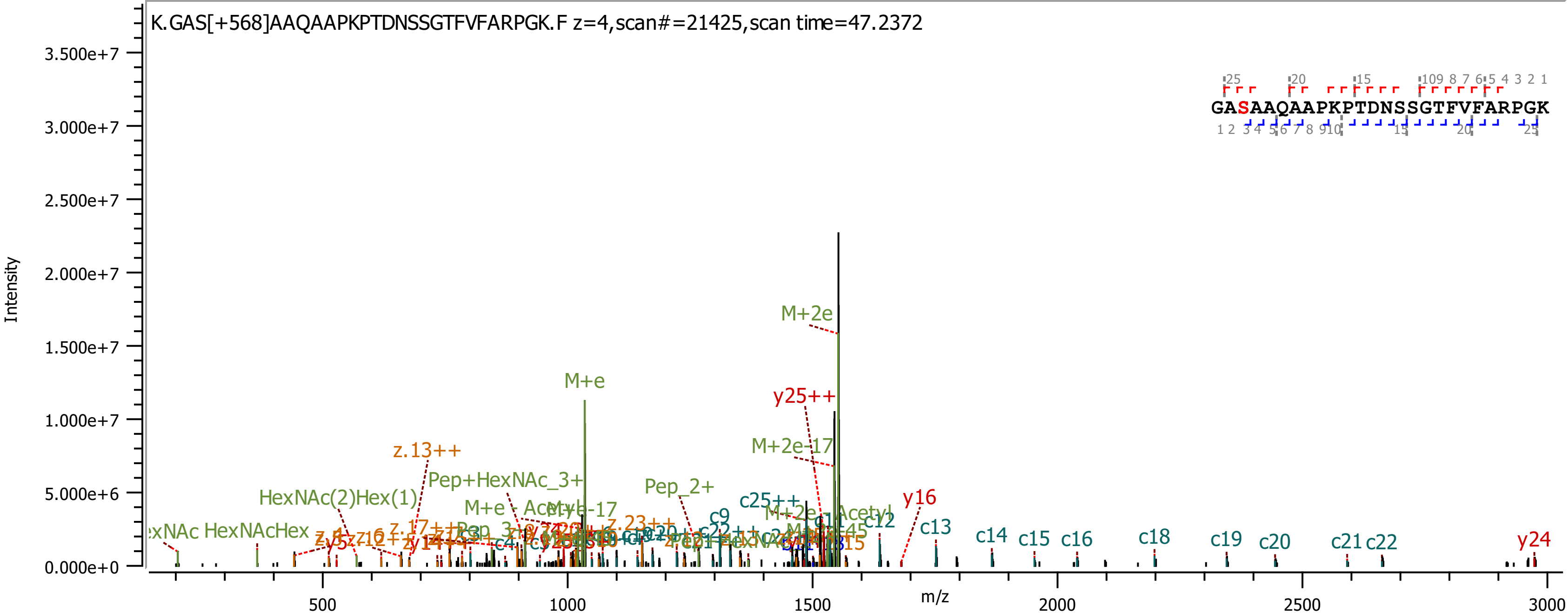
K.GAS[+568]AAQAAPKPTDNSSGTFVFARPGK.F z=4,scan#=21425,scan time=47.2372
GASAAQAAPKPTDNSSGTFVFARPGK
Intensity
m/z
3.500e+7
3.000e+7
2.500e+7
2.000e+7
1.500e+7
1.000e+7
5.000e+6
0.000e+0
500
1000
1500
2000
2500
3000
M+2e
M+e
y25++
M+2e-17
z.13++
Pep+HexNAc_3+
Pep_2+
HexNAc(2)Hex(1)
HexNAcHex
c25++
c9
c12
y16
c13
c14
c15
c16
c18
c19
c20
c21
c22
y24

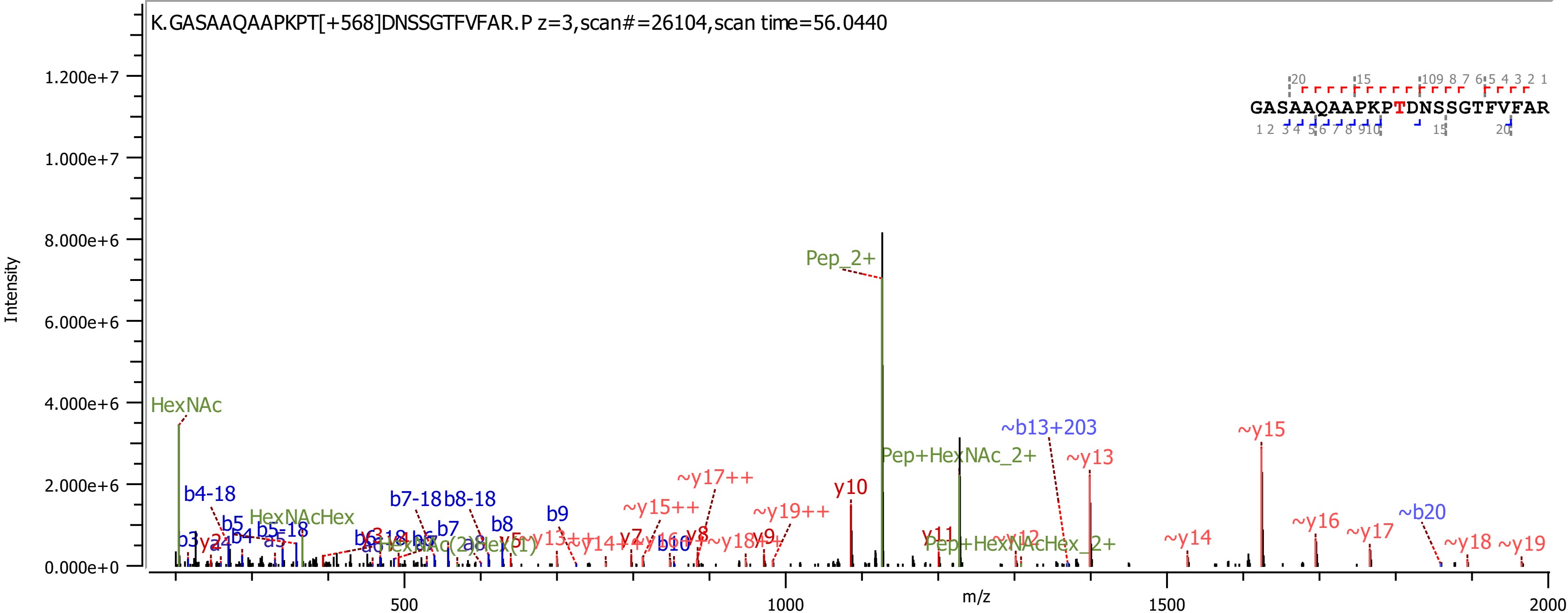
K.GASAAQAAPKPT[+568]DNSSGTFVFAR.P z=3,scan#=26104,scan time=56.0440
GASAAQAAPKPTDNSSGTFVFAR
Intensity
m/z
HexNAc
HexNAcHex
Pep_2+
Pep+HexNAc_2+
Pep+HexNAcHex_2+
~b13+203
~b20
~y13
~y15
~y16
~y17
~y18
~y19
~y14
~y12
y10
y11
~y17++
~y15++
~y19++
b4-18
b5
b5-18
b7-18
b8-18
b9
b8
b7
b10
y7
y8
y9

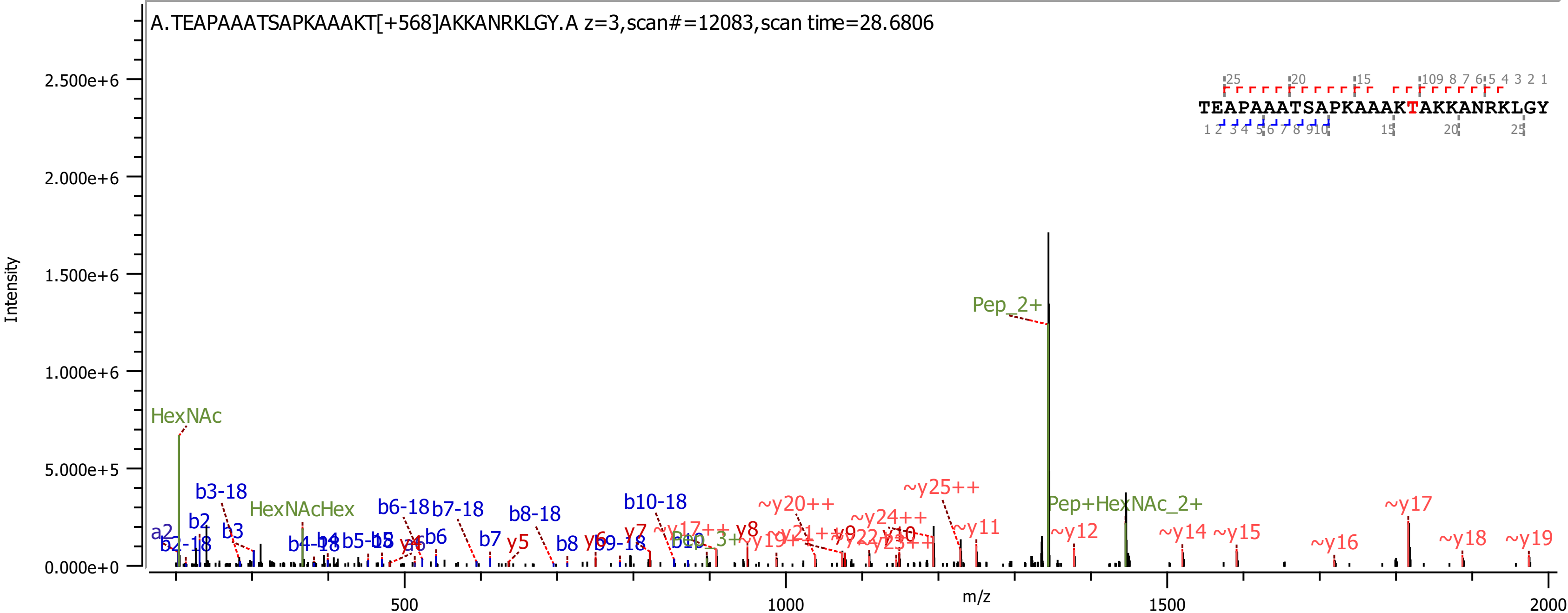
A.TEAPAAATSAPKAAAKT[+568]AKKANRKLGY.A z=3,scan#=12083,scan time=28.6806
TEAPAAATSAPKAAAKTAKKANRKLGY
Intensity
m/z
Pep_2+
HexNAc
HexNAcHex
Pep+HexNAc_2+
Pep_3+
~y25++
~y20++
~y24++
~y17++
~y11
~y12
~y14
~y15
~y16
~y17
~y18
~y19
b3-18
b2
b3
a2
b2-18
b6-18
b7-18
b8-18
b10-18
b6
b7
b8
y5
y7
y8
y10
b9-18

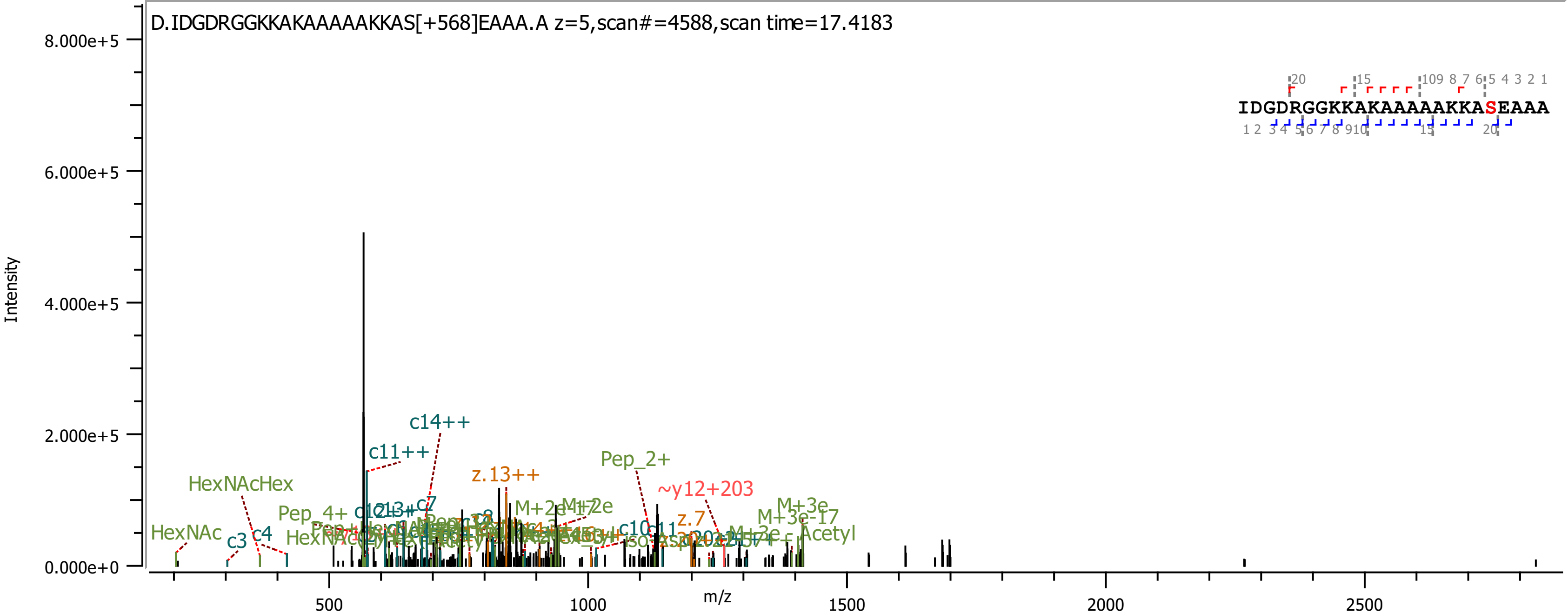

D.IDGDRGGKKAKAAAAAKKAS[+568]EAAA.A z=5,scan#=4588,scan time=17.4183
IDGDRGGKKAKAAAAAKKASEAAA
Intensity
m/z
8.000e+5
6.000e+5
4.000e+5
2.000e+5
0.000e+0
500
1000
1500
2000
2500
c14++
c11++
z.13++
Pep_2+
~y12+203
HexNAcHex
HexNAc
c3
c4
Pep_4+
c7
M+2e-17
M+2e
c10
c11
z.7
M+3e
M+3e-17
M+3e - Acetyl

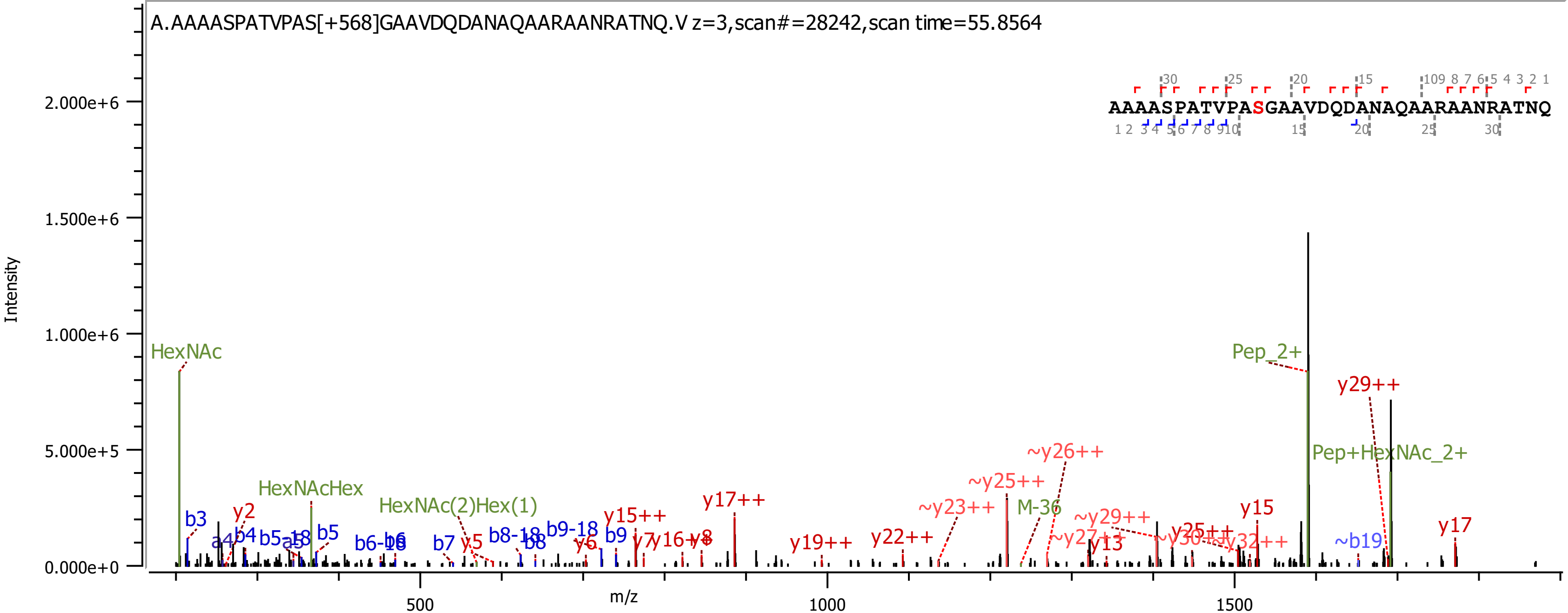
A.AAAASPATVPAS[+568]GAAVDQDANAQAARAANRATNQ.V z=3,scan#=28242,scan time=55.8564
AAAASPATVPASGAAVDQDANAQAARAANRATNQ
Intensity
m/z
HexNAc
HexNAcHex
HexNAc(2)Hex(1)
Pep_2+
Pep+HexNAc_2+
M-36
y29++
~y26++
~y25++
~y23++
~y29++
y17++
y15++
y19++
y22++
y15
y17
y13
~b19
b3
y2
b4
b5
b5-18
b6-18
b6
b7
y5
b8-18
b8
b9-18
b9
y6
y7
y16++
y8
0.000e+0
5.000e+5
1.000e+6
1.500e+6
2.000e+6
500
1000
1500

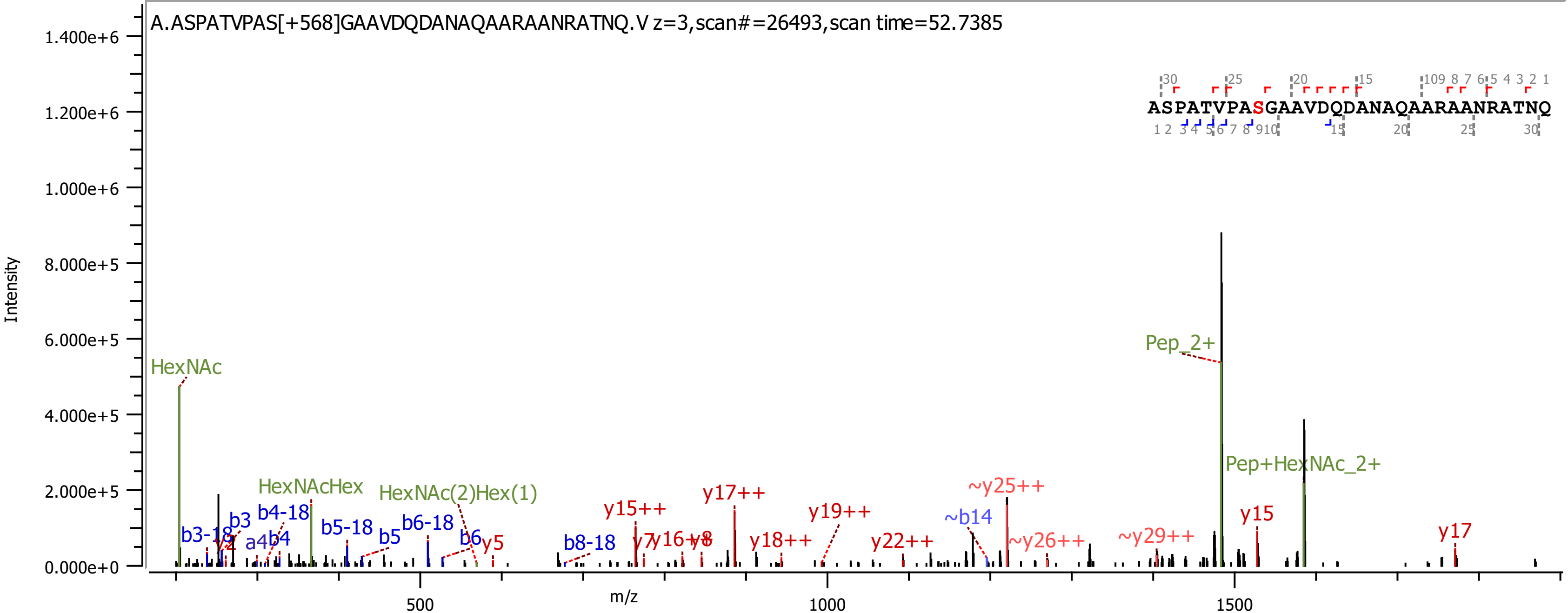
A.ASPATVPAS[+568]GAAVDQDANAQAARAANRATNQ.V z=3,scan#=26493,scan time=52.7385
ASPATVPASGAAVDQDANAQAARAANRATNQ
Intensity
m/z
HexNAc
HexNAcHex
HexNAc(2)Hex(1)
Pep_2+
Pep+HexNAc_2+
b3-18
b3
b4-18
a4
b4
b5-18
b5
b6-18
b6
y5
b8-18
y15++
y7
y16++
y8
y17++
y18++
y19++
y22++
~b14
~y25++
~y26++
~y29++
y15
y17

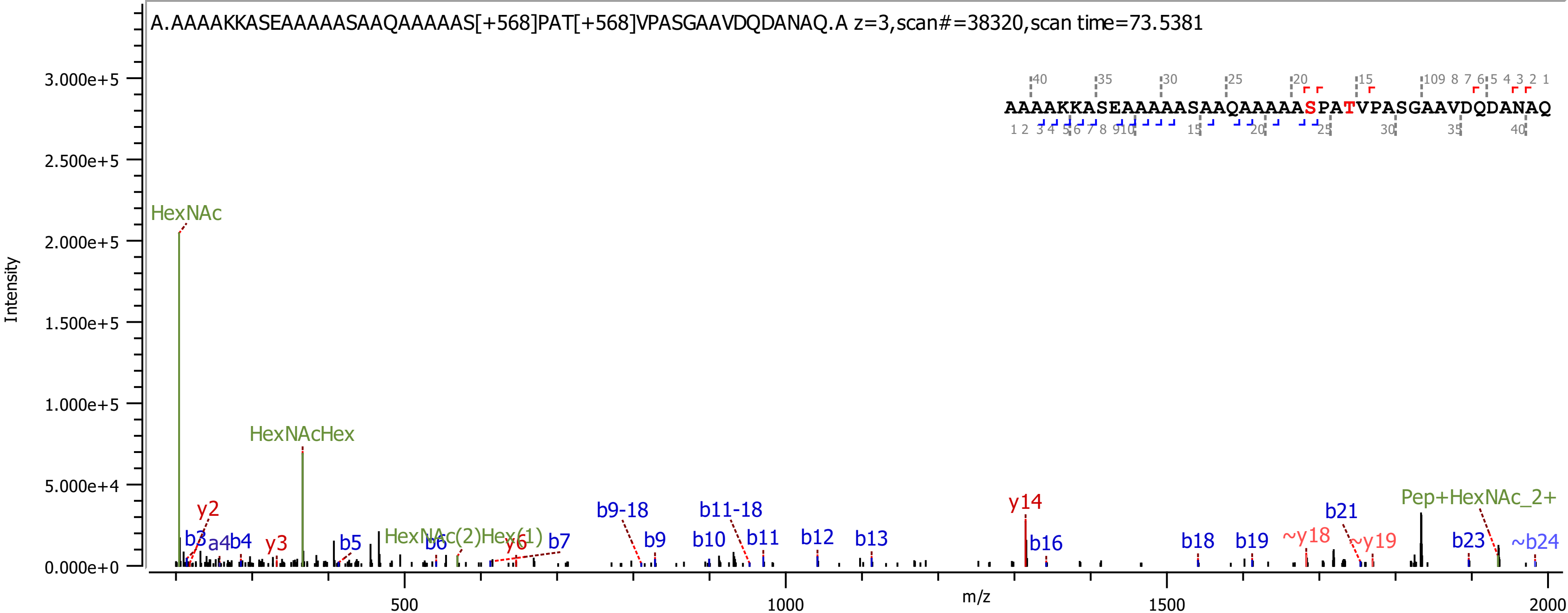
A.AAAAKKASEAAAAASAAQAAAAAS[+568]PAT[+568]VPASGAAVDQDANAQ.A z=3,scan#=38320,scan time=73.5381
AAAAKKASEAAAAASAAQAAAAASPATVPASGAAVDQDANAQ
Intensity
m/z
HexNAc
HexNAcHex
HexNAc(2)Hex(1)
Pep+HexNAc_2+
y2
y3
y6
y14
~y18
~y19
b3
a4
b4
b5
b6
b7
b9-18
b9
b10
b11-18
b11
b12
b13
b16
b18
b19
b21
b23
~b24

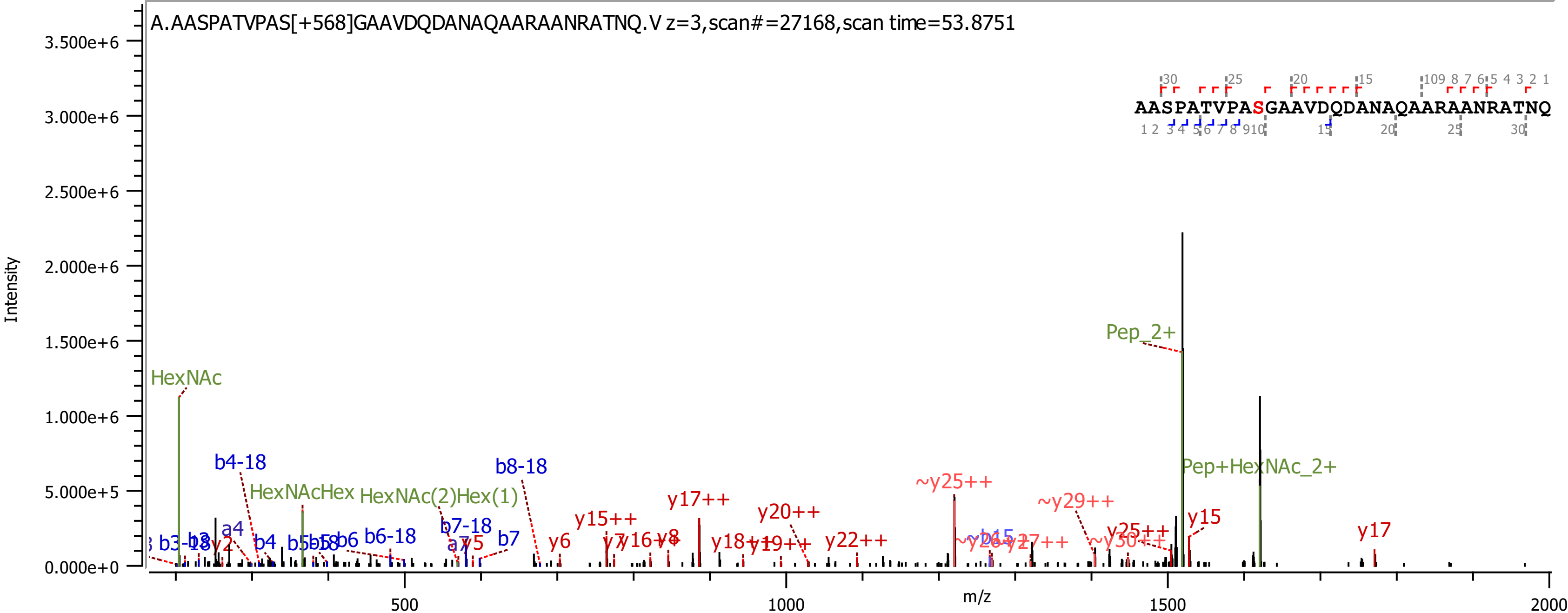
A.AASPATVPAS[+568]GAAVDQDANAQAARAANRATNQ.V z=3,scan#=27168,scan time=53.8751
AASPATVPASGAAVDQDANAQAARAANRATNQ
Intensity
m/z
HexNAc
HexNAcHex
HexNAc(2)Hex(1)
Pep_2+
Pep+HexNAc_2+
b4-18
b8-18
b6-18
b7-18
b6
b7
b4
a4
y2
y5
y6
y15++
y17++
y20++
y22++
y18++
y19++
~y25++
~y29++
y25++
~y30++
y15
y17

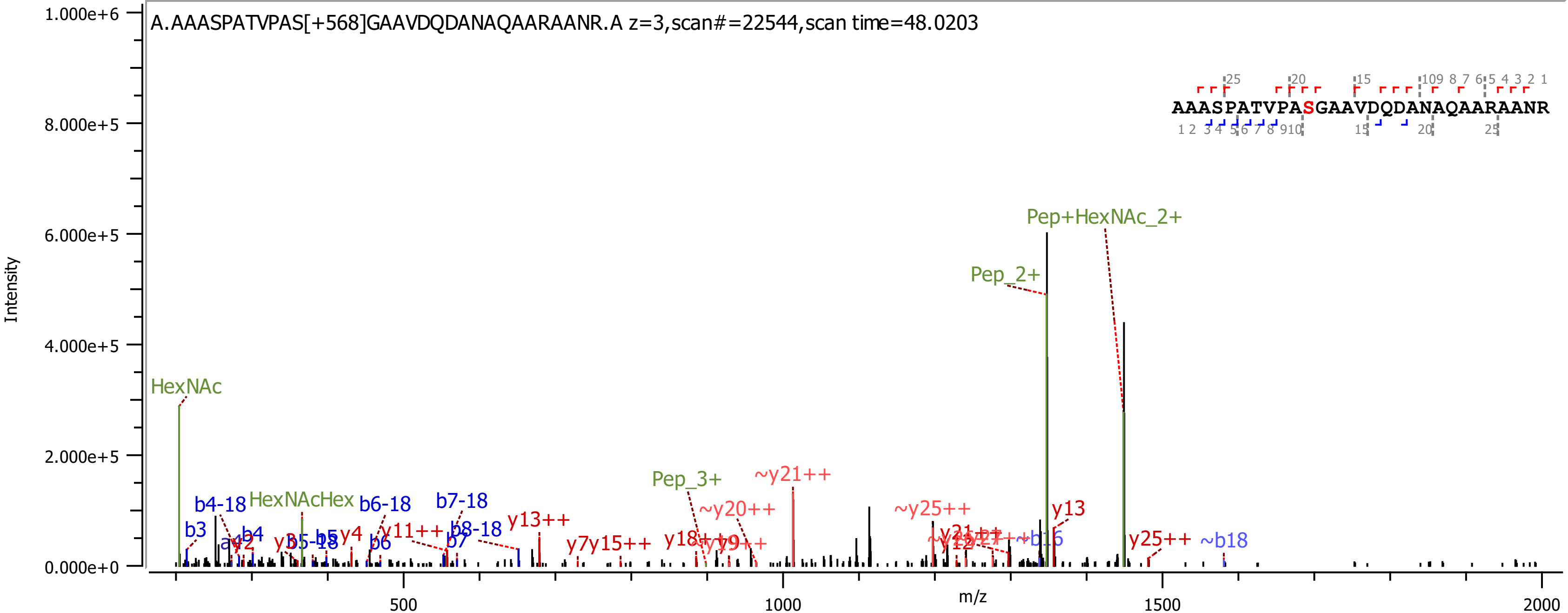
A.AAASPATVPAS[+568]GAAVDQDANAQAARAANR.A z=3,scan#=22544,scan time=48.0203
AAASPATVPASGAAVDQDANAQAARAANR
Intensity
m/z
HexNAc
HexNAcHex
Pep_2+
Pep+HexNAc_2+
Pep_3+
~y21++
~y20++
~y25++
y13
y25++
~b18
y13++
y11++
b7-18
b8-18
b6-18
b4-18
b3
b4
b6
b7
y4
y7
y15++
y18++
y9++

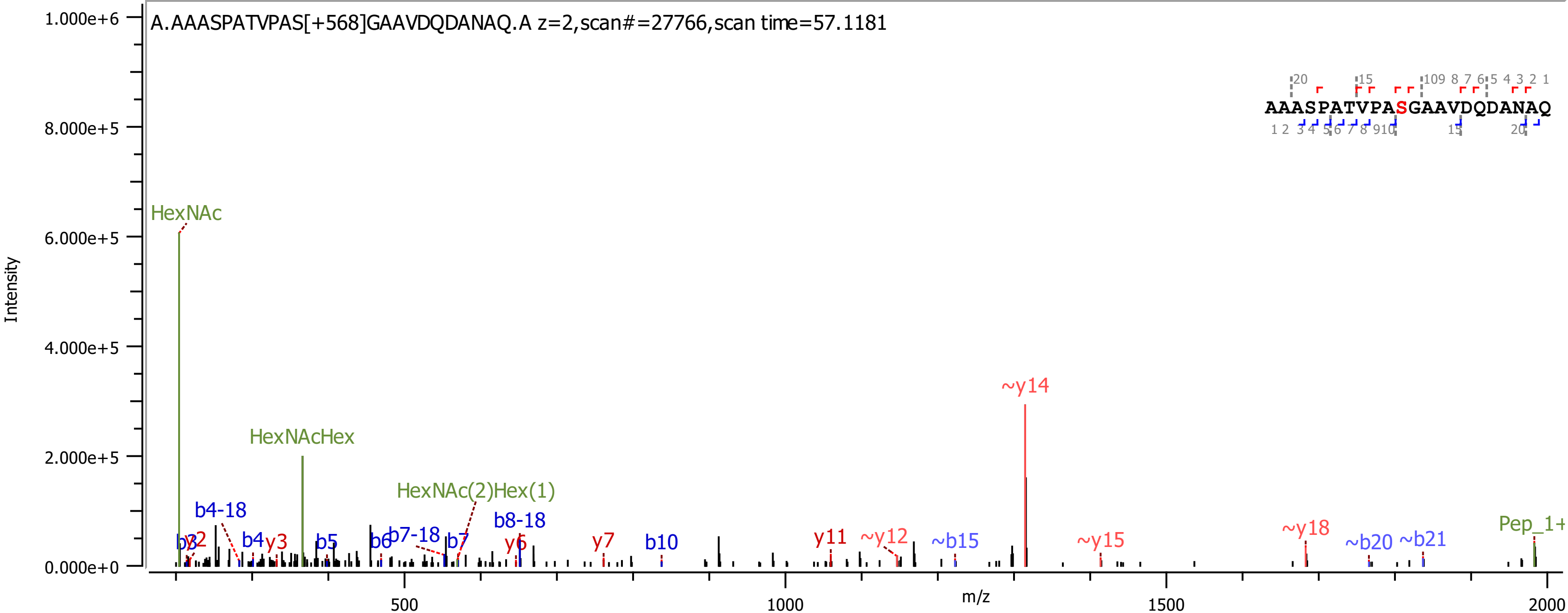
A.AAASPATVPAS[+568]GAAVDQDANAQ.A z=2,scan#=27766,scan time=57.1181
AAASPATVPASGAAVDQDANAQ
Intensity
m/z
HexNAc
HexNAcHex
HexNAc(2)Hex(1)
b4-18
b3
y2
b4
y3
b5
b6
b7-18
b7
b8-18
y6
y7
b10
y11
~y12
~b15
~y14
~y15
~y18
~b20
~b21
Pep_1+

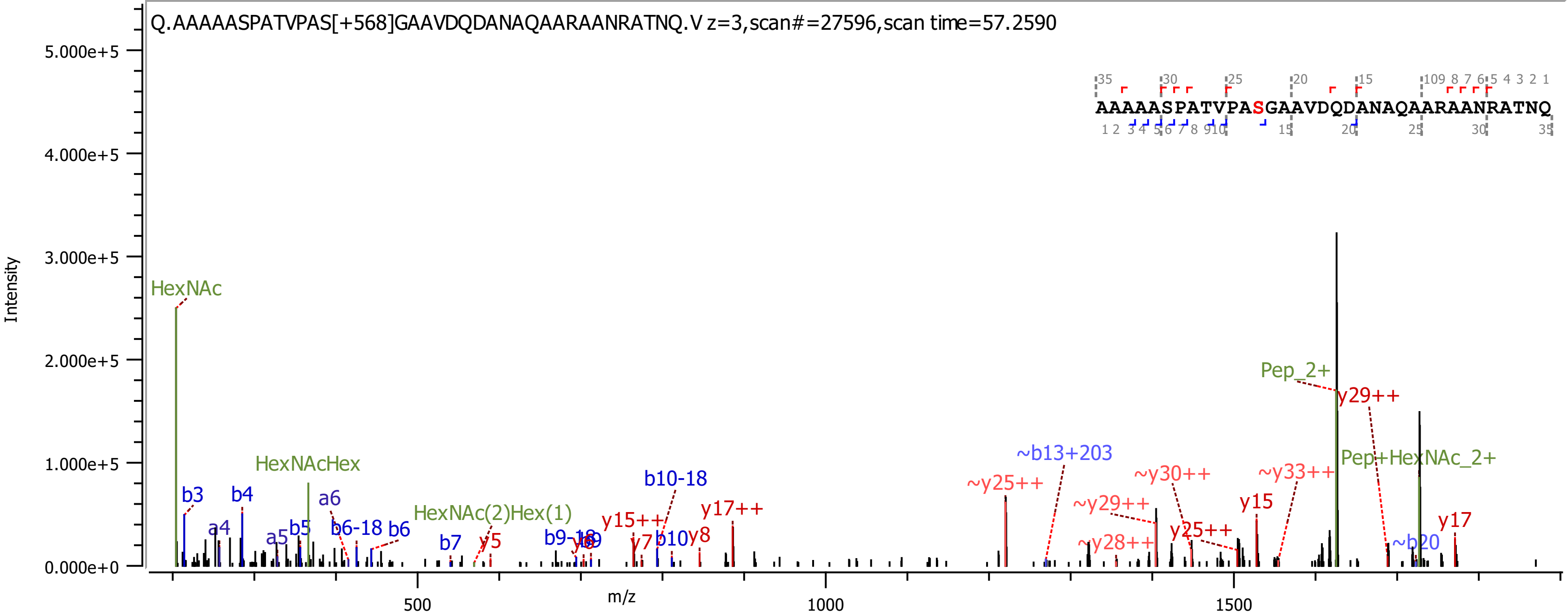

Q.AAAAASPATVPAS[+568]GAAVDQDANAQAARAANRATNQ.V z=3,scan#=27596,scan time=57.2590
AAAAASPATVPASGAAVDQDANAQAARAANRATNQ
Intensity
m/z
HexNAc
HexNAcHex
HexNAc(2)Hex(1)
Pep_2+
Pep+HexNAc_2+
b3
b4
a4
a5
b5
a6
b6-18
b6
b7
y5
b9-18
b10-18
y15++
y7
b10
y8
y17++
~y25++
~b13+203
~y29++
~y28++
~y30++
y25++
y15
~y33++
y29++
~b20
y17

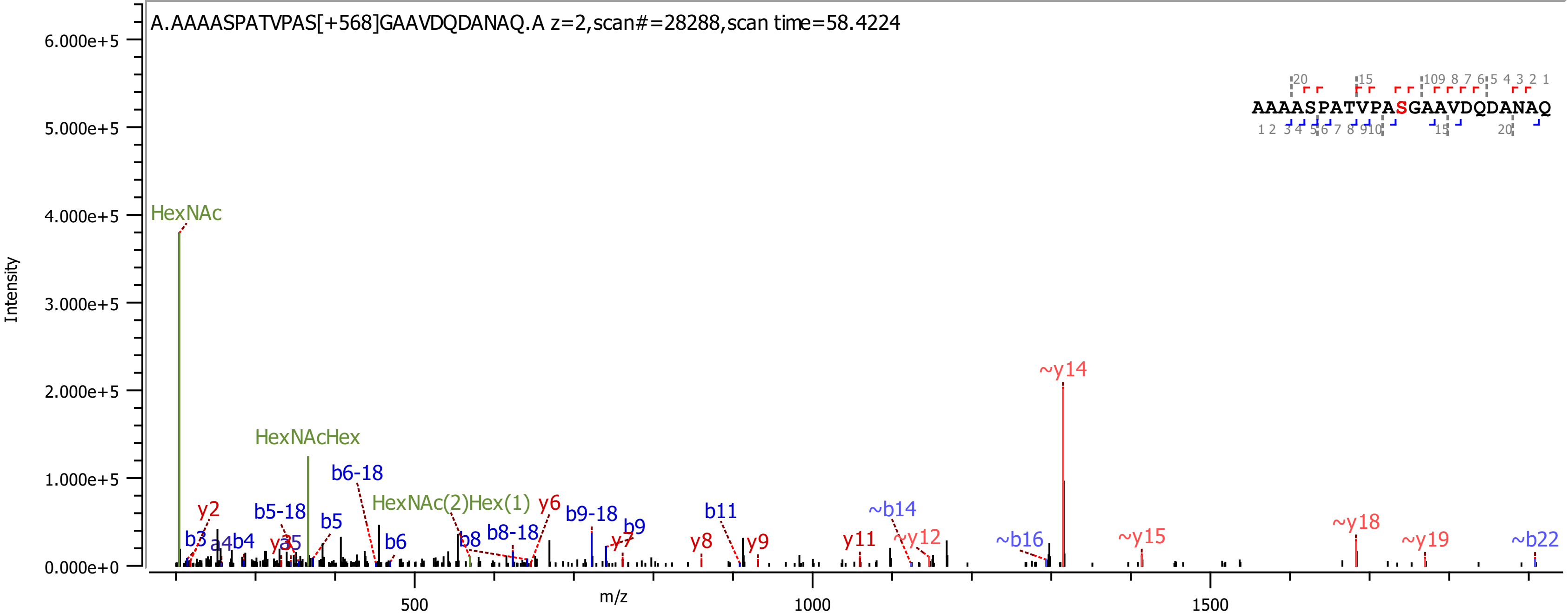

A.AAAASPATVPAS[+568]GAAVDQDANAQ.A z=2,scan#=28288,scan time=58.4224
AAAASPATVPASGAAVDQDANAQ
Intensity
m/z
HexNAc
HexNAcHex
HexNAc(2)Hex(1)
b3
a4
b4
y2
y3
b5-18
b5
b6-18
b6
y6
b8
b8-18
b9-18
b9
y7
b11
y8
y9
y11
~b14
~y12
~y14
~b16
~y15
~y18
~y19
~b22
0.000e+0
1.000e+5
2.000e+5
3.000e+5
4.000e+5
5.000e+5
6.000e+5
500
1000
1500

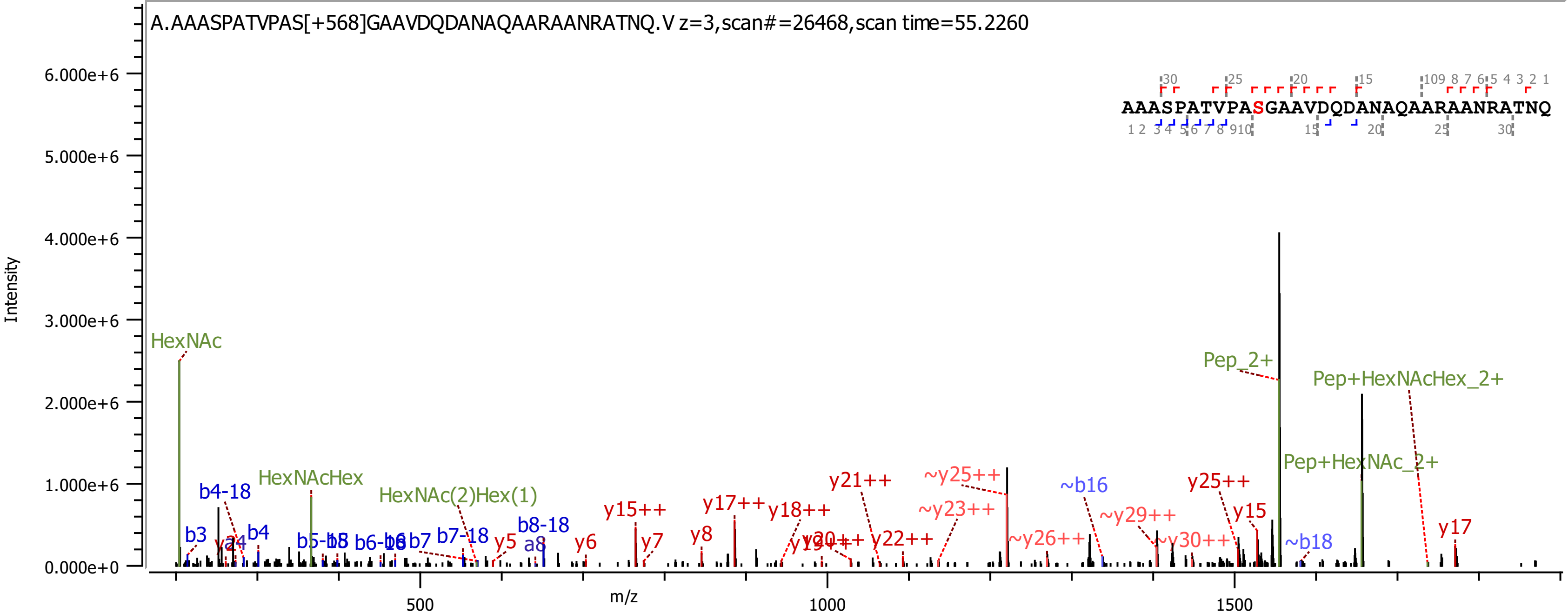
A.AAASPATVPAS[+568]GAAVDQDANAQAARAANRATNQ.V z=3,scan#=26468,scan time=55.2260
AAASPATVPASGAAVDQDANAQAARAANRATNQ
Intensity
m/z
HexNAc
HexNAcHex
HexNAc(2)Hex(1)
Pep_2+
Pep+HexNAcHex_2+
Pep+HexNAc_2+
b3
b4-18
b4
b7
b7-18
b8-18
a8
y5
y6
y7
y8
y15++
y17++
y18++
y21++
y22++
~y23++
~y25++
~y26++
~b16
~y29++
~y30++
y25++
y15
~b18
y17

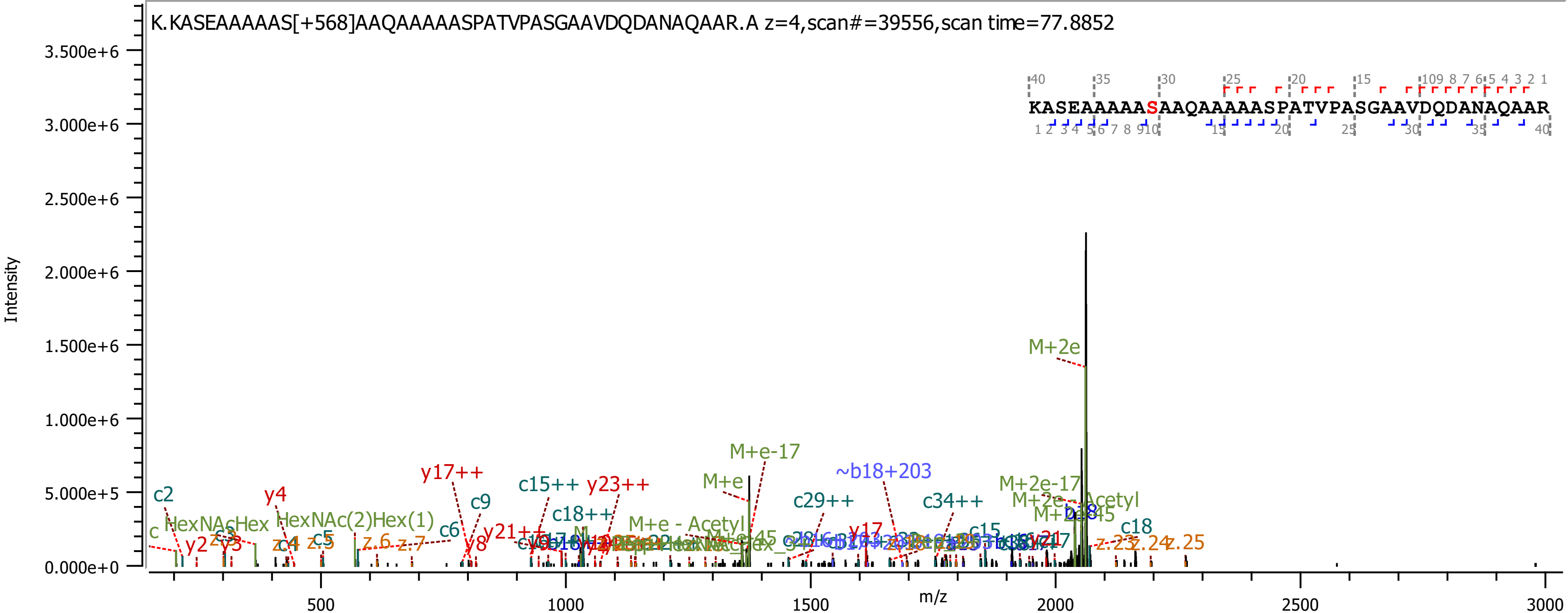
K.KASEAAAAAS[+568]AAQAAAAASPATVPASGAAVDQDANAQAAR.A z=4,scan#=39556,scan time=77.8852
KASEAAAAASAAQAAAAASPATVPASGAAVDQDANAQAAR
Intensity
m/z
3.500e+6
3.000e+6
2.500e+6
2.000e+6
1.500e+6
1.000e+6
5.000e+5
0.000e+0
500
1000
1500
2000
2500
3000
M+2e
M+e-17
M+e
~b18+203
M+2e-17
M+2e - Acetyl
M+2e-45
c2
y4
y17++
c9
c15++
y23++
c18++
c29++
c34++
HexNAcHex
HexNAc(2)Hex(1)
c6
y21++
M+e - Acetyl
y17
c15
c18
y2
y3
z.4
z.5
z.6
z.7
y8
z.23
z.24
z.25

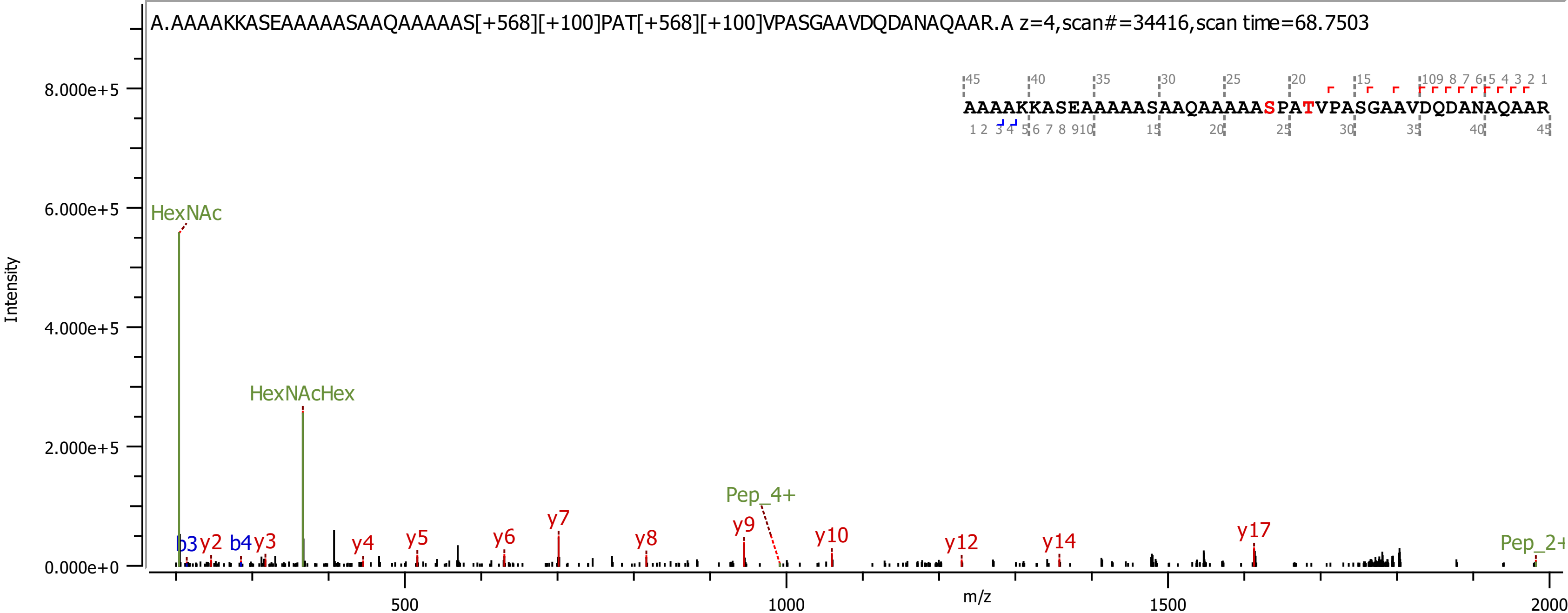
A.AAAAKKASEAAAAASAAQAAAAAS[+568][+100]PAT[+568][+100]VPASGAAVDQDANAQAAR.A z=4,scan#=34416,scan time=68.7503
AAAAKKASEAAAAASAAQAAAAASPATVPASGAAVDQDANAQAAR
Intensity
m/z
HexNAc
HexNAcHex
Pep_4+
Pep_2+
b3
b4
y2
y3
y4
y5
y6
y7
y8
y9
y10
y12
y14
y17
0.000e+0
2.000e+5
4.000e+5
6.000e+5
8.000e+5
500
1000
1500
2000

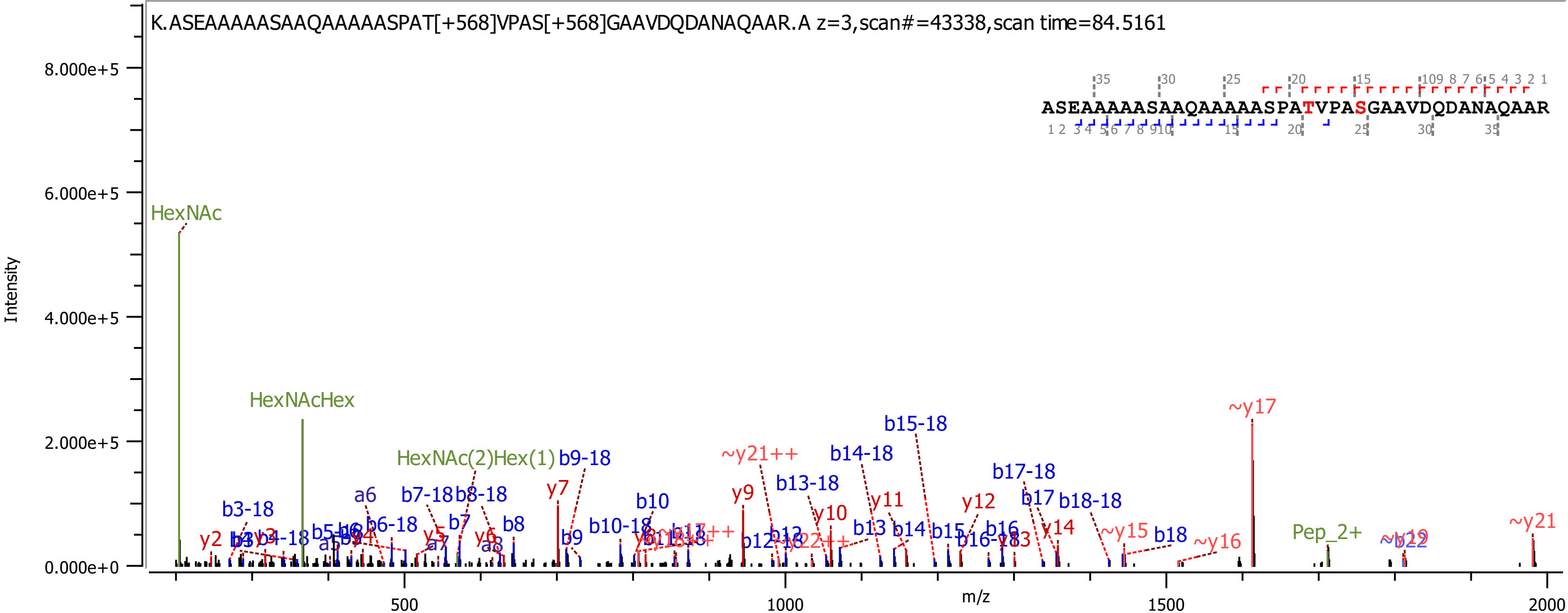
K.ASEAAAAASAAQAAAAASPAT[+568]VPAS[+568]GAAVDQDANAQAAR.A z=3,scan#=43338,scan time=84.5161
ASEAAAAASAAQAAAAASPATVPASGAAVDQDANAQAAR
Intensity
m/z
8.000e+5
6.000e+5
4.000e+5
2.000e+5
0.000e+0
500
1000
1500
2000
HexNAc
HexNAcHex
HexNAc(2)Hex(1)
b9-18
~y17
b15-18
~y21++
b14-18
b17-18
b13-18
b3-18
a6
b7-18
b8-18
y7
y9
b10
y10
y11
y12
b17
b18-18
b6-18
b7
b8
b10-18
b13
b14
b15
b16
y14
~y15
b18
~y16
Pep_2+
~y21
y2
b9
b12
y13

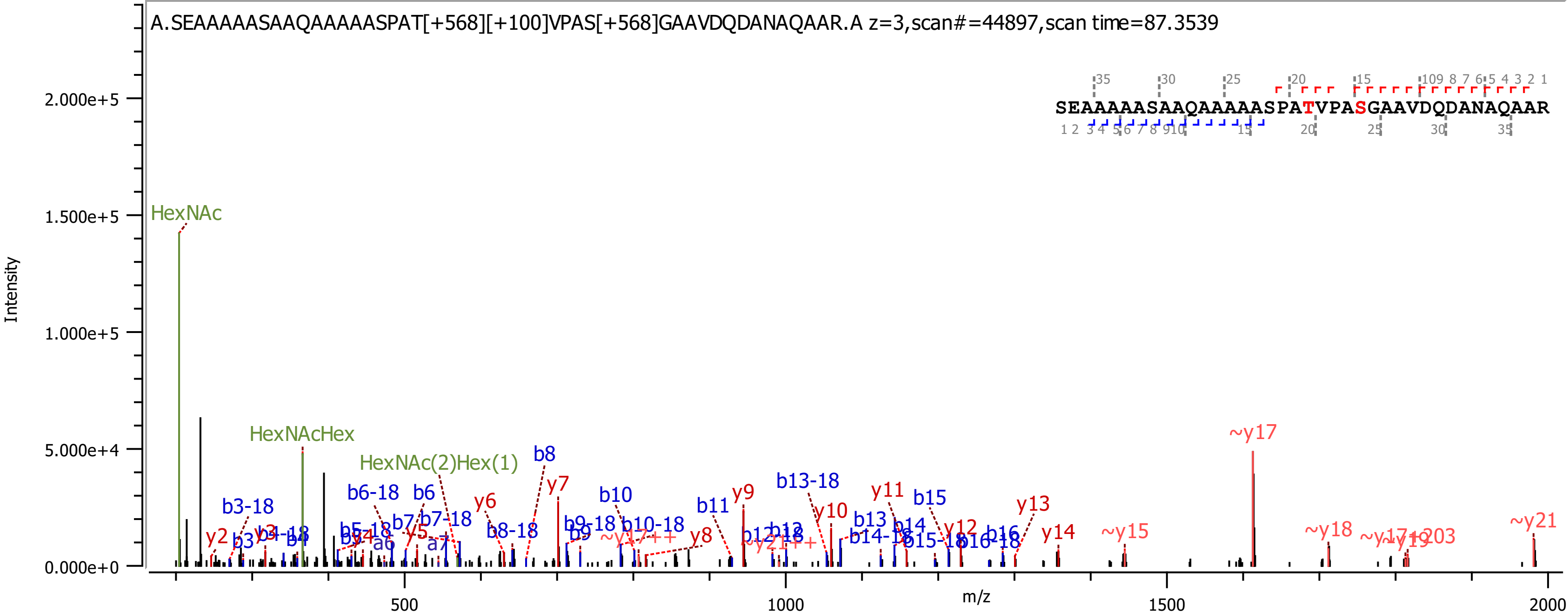
A.SEAAAAASAAQAAAAASPAT[+568][+100]VPAS[+568]GAAVDQDANAQAAR.A z=3,scan#=44897,scan time=87.3539
SEAAAAASAAQAAAAASPATVPASGAAVDQDANAQAAR
HexNAc
HexNAcHex
HexNAc(2)Hex(1)
b3-18
b6-18
b6
b8
y7
y6
b10
b11
y9
b13-18
y10
y11
b15
b13
b14
y12
y13
y14
~y15
~y17
~y18
~y21
b8-18
b9-18
b10-18
y8
b14-18
b15-18
b16-18
b16
Intensity
m/z
2.000e+5
1.500e+5
1.000e+5
5.000e+4
0.000e+0
500
1000
1500
2000

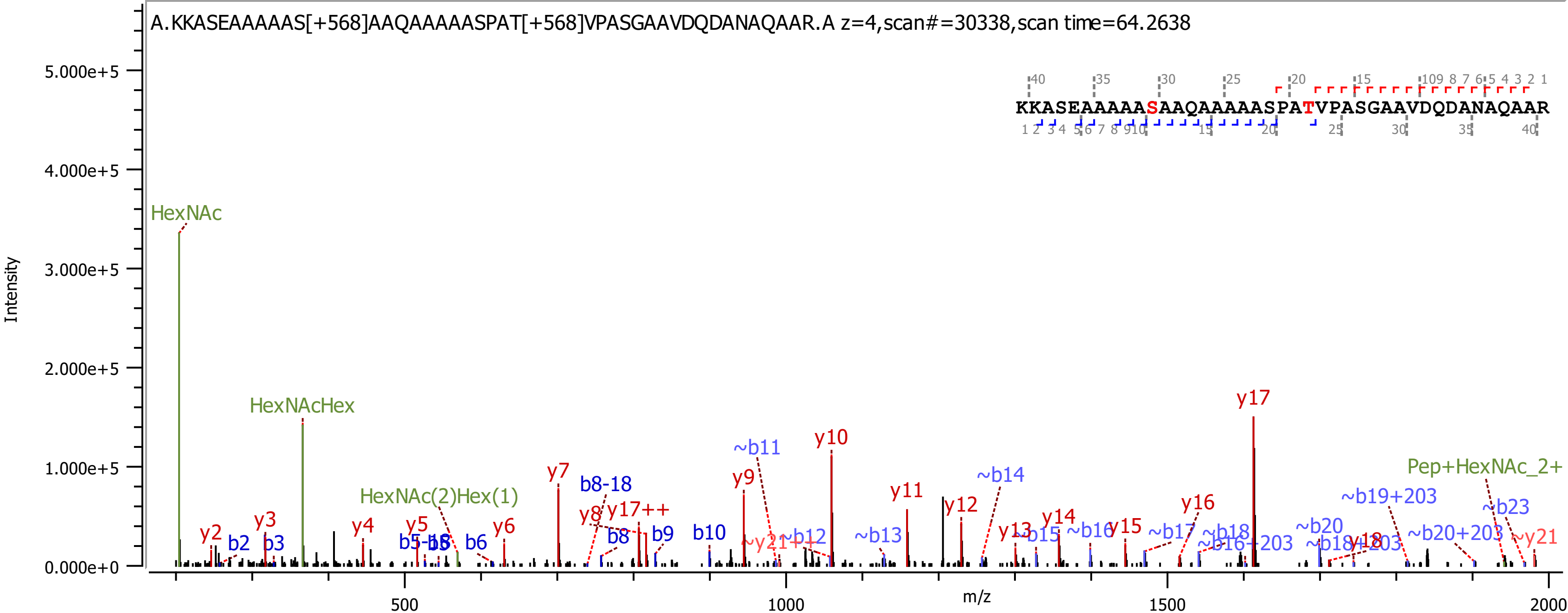
A.KKASEAAAAAS[+568]AAQAAAAASPAT[+568]VPASGAAVDQDANAQAAR.A z=4,scan#=30338,scan time=64.2638
KKASEAAAAASAAQAAAAASPATVPASGAAVDQDANAQAAR
Intensity
m/z
HexNAc
HexNAcHex
HexNAc(2)Hex(1)
Pep+HexNAc_2+
y2
y3
y4
y5
y6
y7
y8
y9
y10
y11
y12
y13
y14
y15
y16
y17
y17++
b2
b3
b6
b8
b8-18
b9
b10
~b11
~b12
~b13
~b14
~b15
~b16
~b17
~b18
~b20
~b23
~b19+203
~b20+203
~b16+203
~b18+203
~y21
~y21++
y18

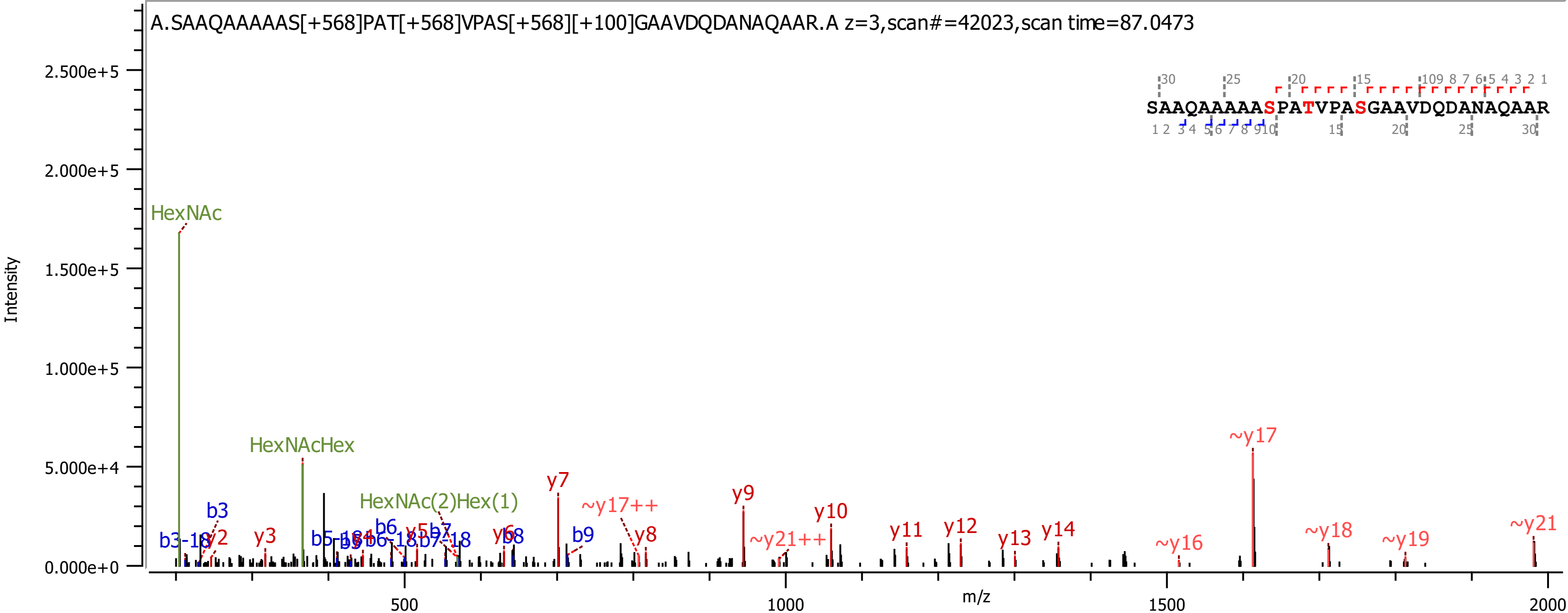
A.SAAQAAAAAS[+568]PAT[+568]VPAS[+568][+100]GAAVDQDANAQAAR.A z=3,scan#=42023,scan time=87.0473
SAAQAAAAASPATVPASGAAVDQDANAQAAR
Intensity
m/z
HexNAc
HexNAcHex
HexNAc(2)Hex(1)
b3
b3-18
y2
y3
b6
b9
b8
y6
y7
y8
y9
y10
y11
y12
y13
y14
~y16
~y17
~y17++
~y18
~y19
~y21
~y21++

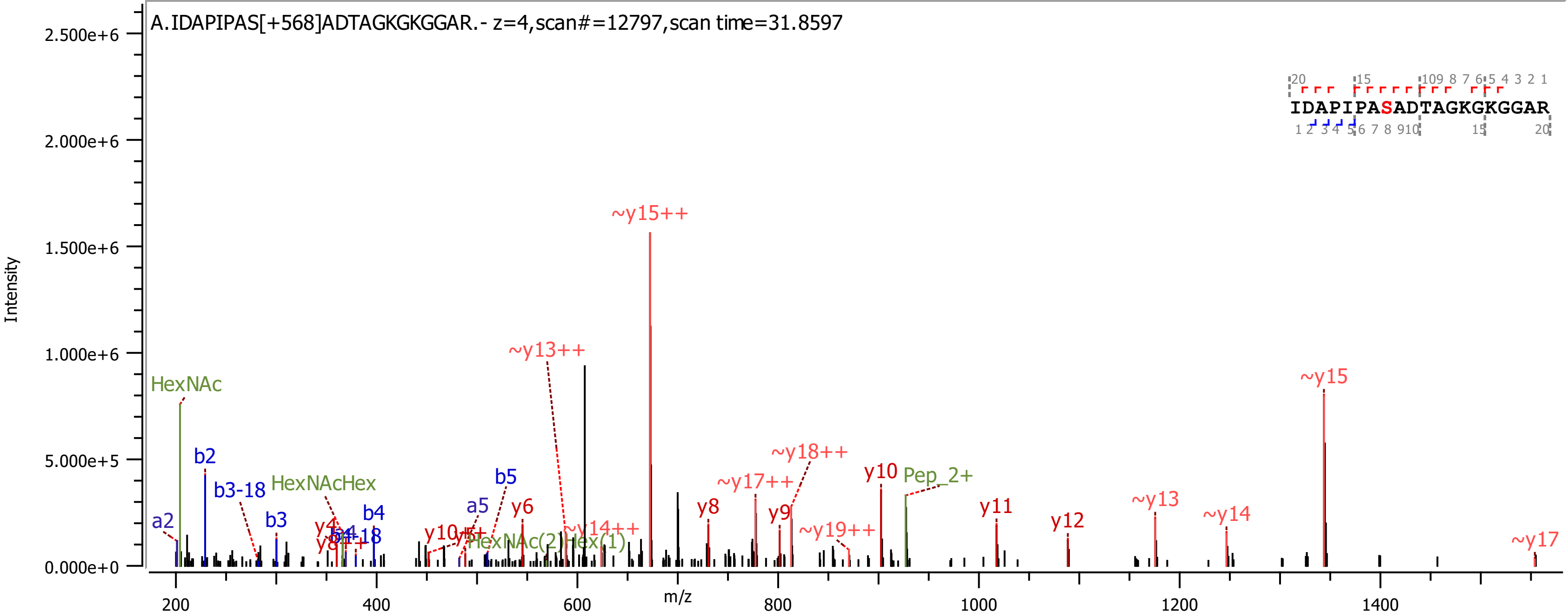
A.IDAPIPAS[+568]ADTAGKGKGGAR.- z=4,scan#=12797,scan time=31.8597
Intensity
m/z
IDAPIPASADTAGKGKGGAR
~y15++
~y13++
~y15
HexNAc
b2
b3-18
HexNAcHex
b3
a2
b4
b5
a5
y6
y4
y8++
y10++
y5++
HexNAc(2)Hex(1)
~y14++
y8
~y17++
~y18++
y9
~y19++
y10
Pep_2+
y11
y12
~y13
~y14
~y17
0.000e+0
5.000e+5
1.000e+6
1.500e+6
2.000e+6
2.500e+6
200
400
600
800
1000
1200
1400

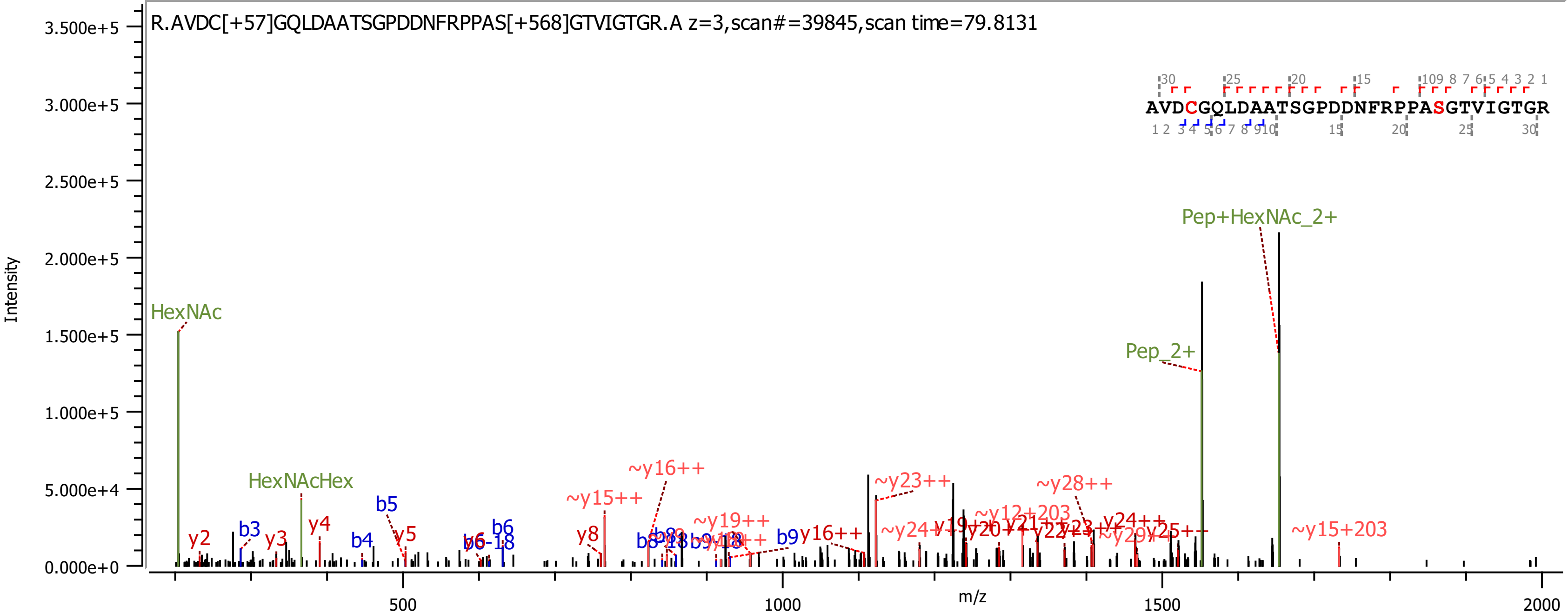
R.AVDC[+57]GQLDAATSGPDDNFRPPAS[+568]GTVIGTGR.A z=3,scan#=39845,scan time=79.8131
AVDCGQLDAATSGPDDNFRPPASGTVIGTGR
Intensity
m/z
3.500e+5
3.000e+5
2.500e+5
2.000e+5
1.500e+5
1.000e+5
5.000e+4
0.000e+0
500
1000
1500
2000
HexNAc
HexNAcHex
Pep+HexNAc_2+
Pep_2+
~y16++
~y15++
~y23++
~y28++
~y12+203
~y15+203
~y19++
y2
b3
y3
y4
b4
b5
y5
b6
y8
b9
y16++

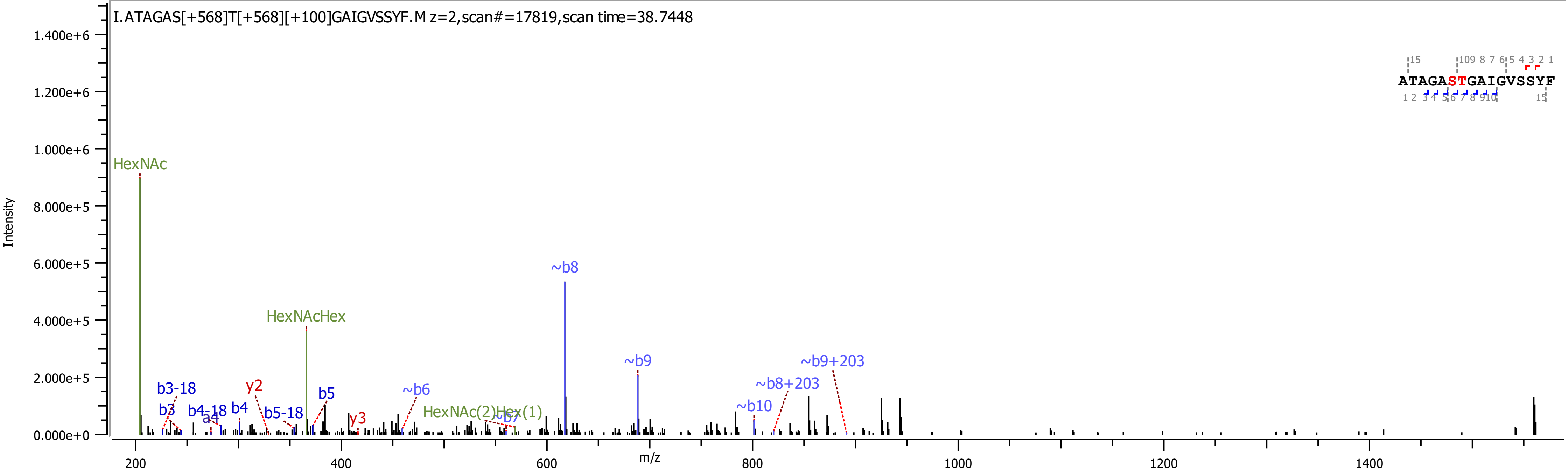

I.ATAGAS[+568]T[+568][+100]GAIGVSSYF.M z=2,scan#=17819,scan time=38.7448
Intensity
1.400e+6
1.200e+6
1.000e+6
8.000e+5
6.000e+5
4.000e+5
2.000e+5
0.000e+0
m/z
200
400
600
800
1000
1200
1400
HexNAc
HexNAcHex
HexNAc(2)Hex(1)
b3-18
b3
b4-18
a4
b4
y2
b5-18
b5
y3
~b6
~b7
~b8
~b9
~b10
~b8+203
~b9+203
ATAGASTGAIGVSSYF

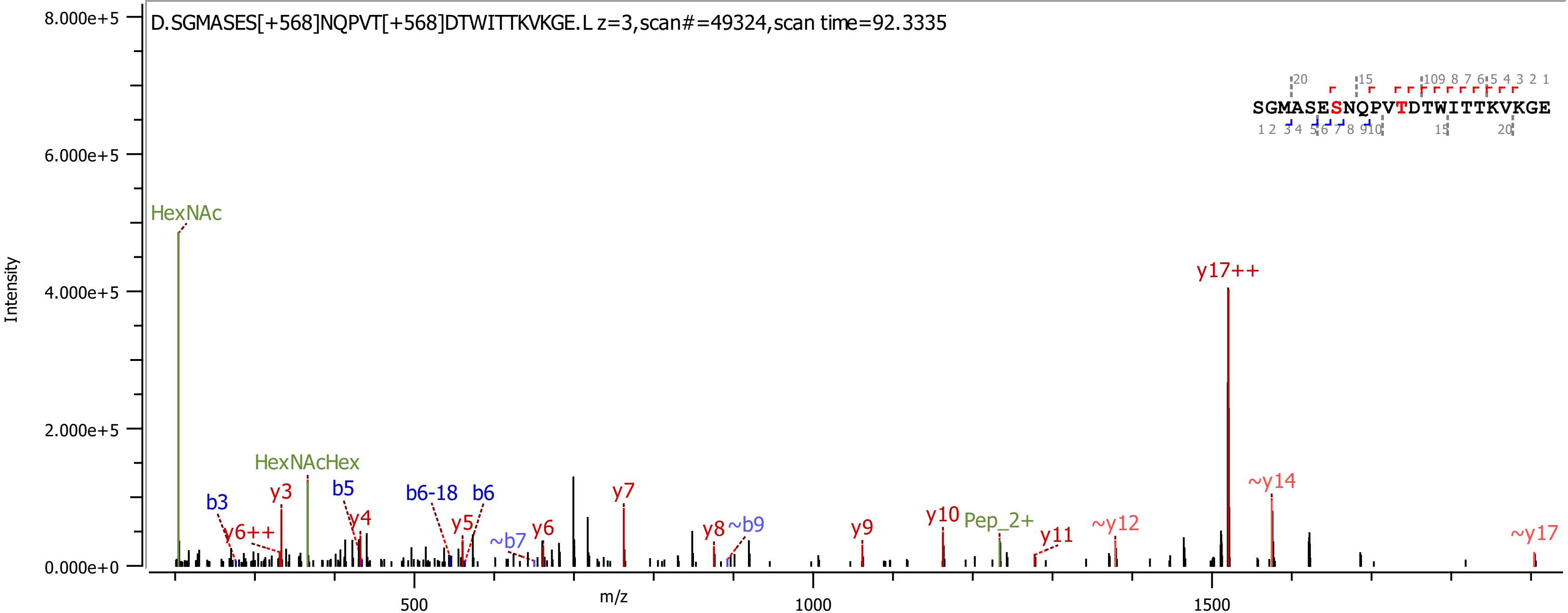

D.SGMASES[+568]NQPVT[+568]DTWITTKVKGE.L z=3,scan#=49324,scan time=92.3335
SGMASESNQPVTDTWITTKVKGE
Intensity
m/z
HexNAc
HexNAcHex
b3
y6++
y3
b5
y4
b6-18
b6
y5
~b7
y6
y7
y8
~b9
y9
y10
Pep_2+
y11
~y12
y17++
~y14
~y17

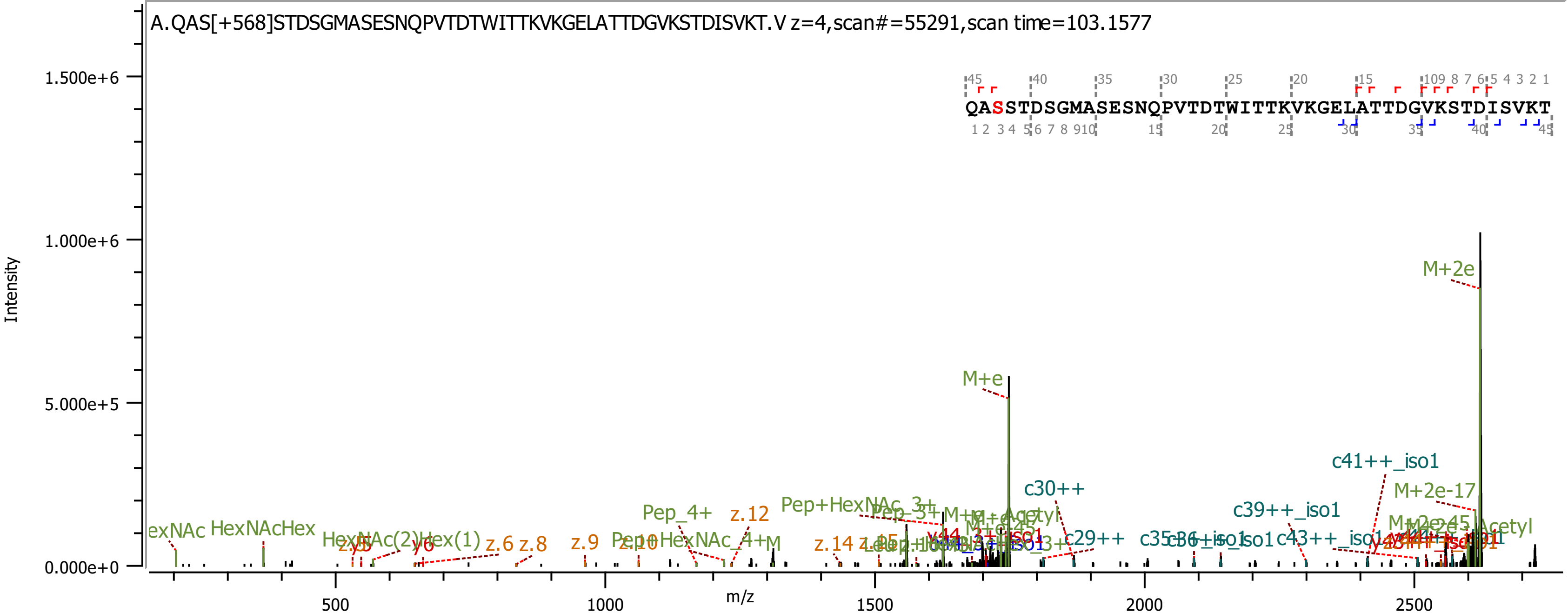
A.QAS[+568]STDSGMASESNQPVTDTWITTKVKGELATTDGVKSTDISVKT.V z=4,scan#=55291,scan time=103.1577
QASSTDSGMASESNQPVTDTWITTKVKGELATTDGVKSTDISVKT
Intensity
m/z
M+2e
M+e
c41++_iso1
M+2e-17
c30++
c39++_iso1
Pep+HexNAc_3+
Pep_4+
z.12
HexNAcHex
HexNAc(2)Hex(1)
z.6
z.8
z.9
z.14
c29++
y6

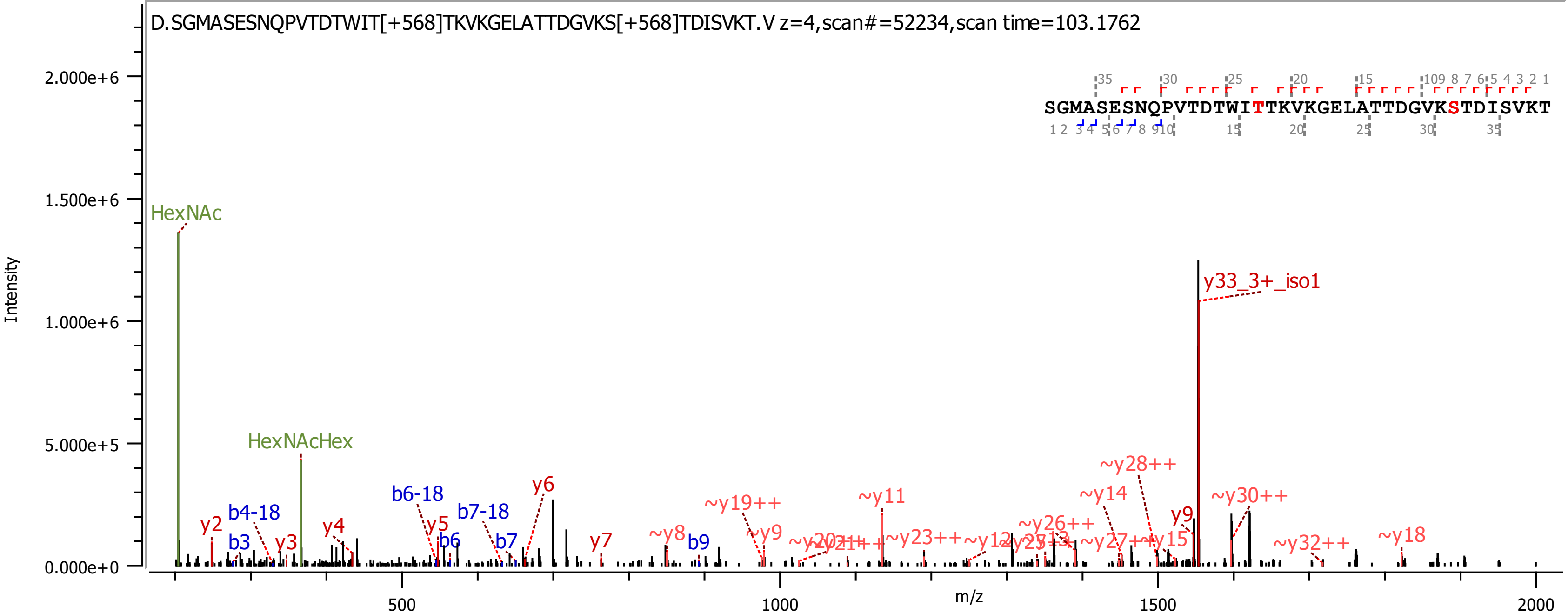
D.SGMASESNQPVTDTWIT[+568]TKVKGELATTDGVKS[+568]TDISVKT.V z=4,scan#=52234,scan time=103.1762
SGMASESNQPVTDTWITTKVKGELATTDGVKSTDISVKT
Intensity
m/z
HexNAc
HexNAcHex
y33_3+_iso1
y2
b4-18
b3
y3
y4
b6-18
y5
b6
b7-18
b7
y6
y7
~y8
b9
~y19++
~y9
~y11
~y23++
~y14
~y28++
~y26++
~y15
y9
~y30++
~y32++
~y18

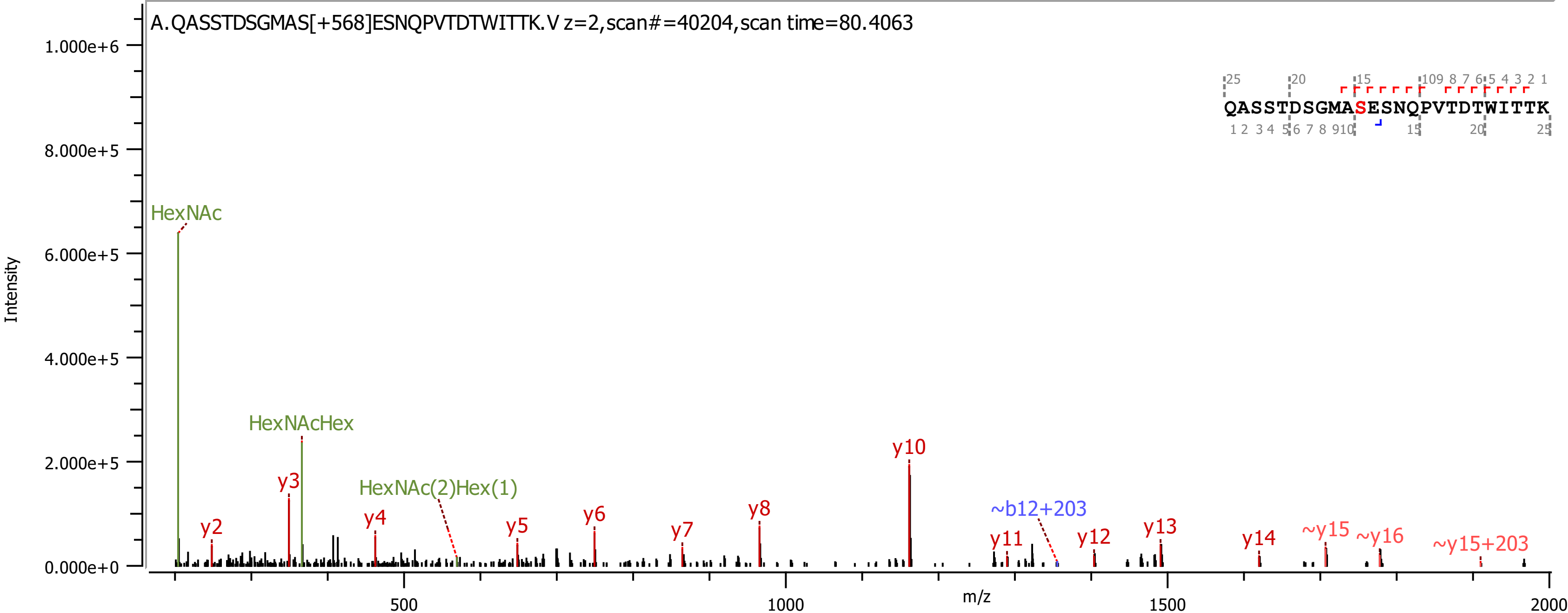
A.QASSTDSGMAS[+568]ESNQPVTDTWITTK.V z=2,scan#=40204,scan time=80.4063
QASSTDSGMASESNQPVTDTWITTK
HexNAc
HexNAcHex
HexNAc(2)Hex(1)
y2
y3
y4
y5
y6
y7
y8
y10
~b12+203
y11
y12
y13
y14
~y15
~y16
~y15+203
Intensity
m/z

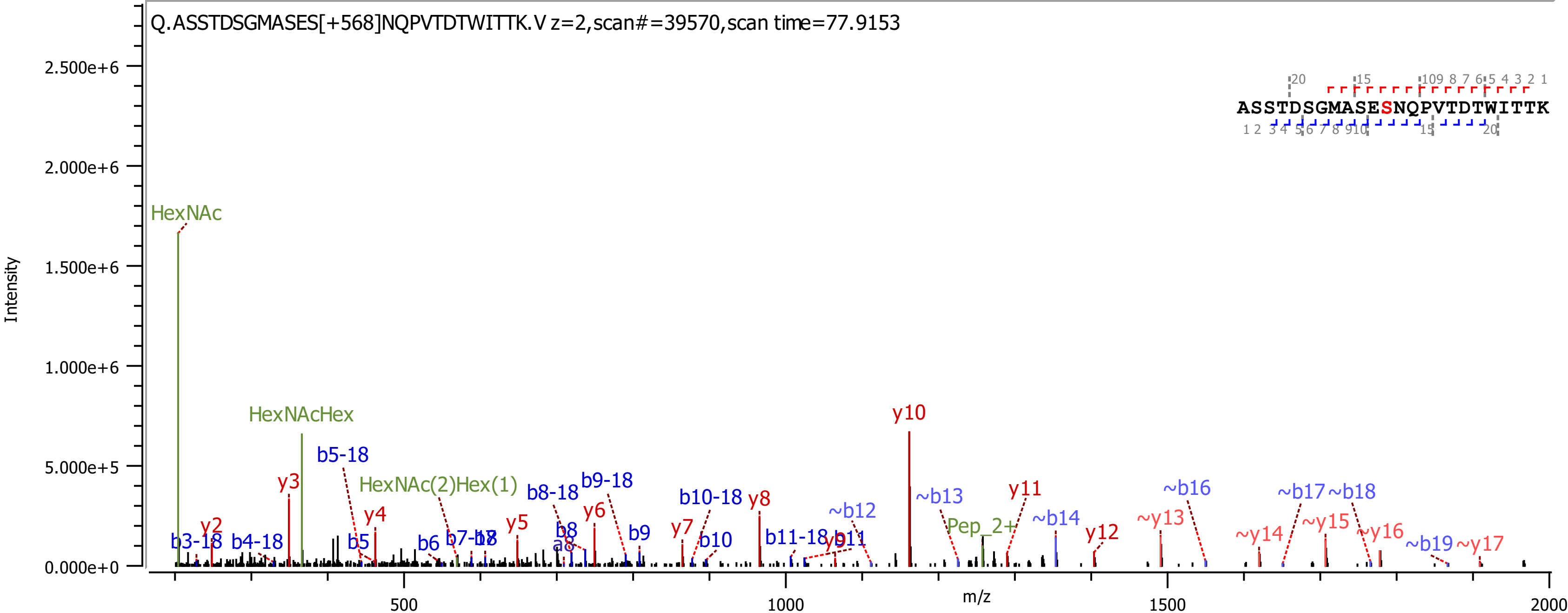
Q.ASSTDSGMASES[+568]NQPVTDTWITTK.V z=2,scan#=39570,scan time=77.9153
ASSTDSGMASESNQPVTDTWITTK
Intensity
m/z
HexNAc
HexNAcHex
HexNAc(2)Hex(1)
Pep_2+
y10
y3
y2
y4
y5
y6
y7
y8
y11
y12
~y13
~y14
~y15
~y16
~y17
b3-18
b4-18
b5-18
b5
b6
b8-18
b9-18
b8
a8
b9
b10-18
b10
b11-18
b11
~b12
~b13
~b14
~b16
~b17
~b18
~b19

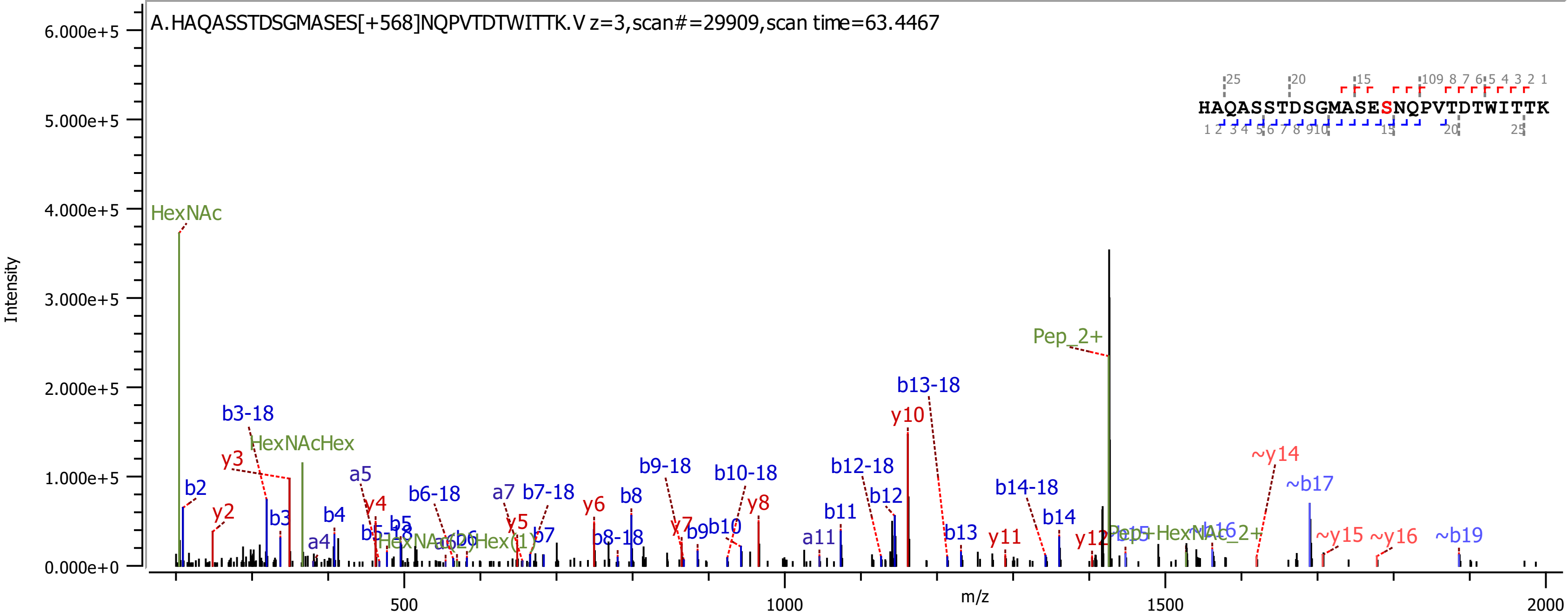

A.HAQASSTDSGMASES[+568]NQPVTDTWITTK.V z=3,scan#=29909,scan time=63.4467
HAQASSTDSGMASESNQPVTDTWITTK
Intensity
m/z
6.000e+5
5.000e+5
4.000e+5
3.000e+5
2.000e+5
1.000e+5
0.000e+0
500
1000
1500
2000
HexNAc
HexNAcHex
Pep_2+
b2
b3
b3-18
y2
y3
a4
b4
a5
y4
b5
b5-18
HexNAc(2)Hex(1)
a6
b6
b6-18
a7
b7-18
y5
b7
y6
b8
b8-18
b9-18
y7
b9
b10
b10-18
y8
a11
b11
b12-18
b12
y10
b13-18
b13
y11
b14-18
b14
y12
b15
Pep+HexNAc_2+
~b16
~y14
~b17
~y15
~y16
~b19

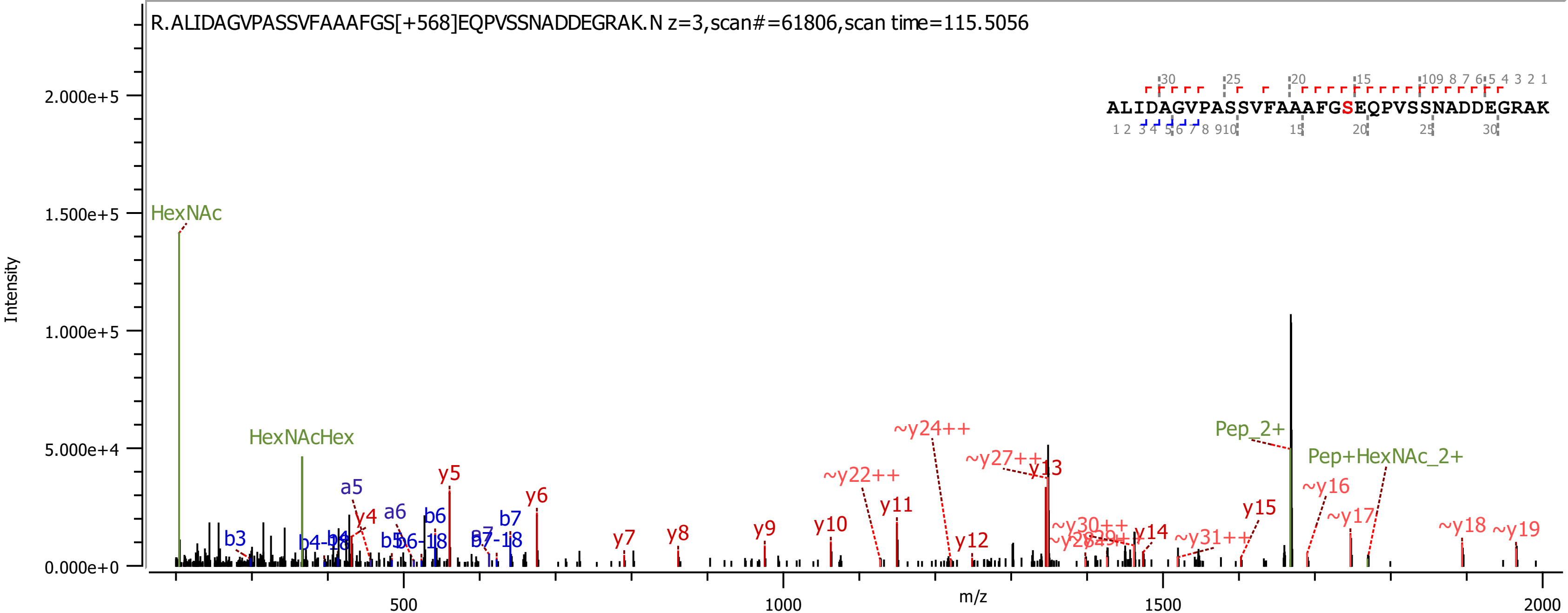

R.ALIDAGVPASSVFAAAFGS[+568]EQPVSSNADDEGRAK.N z=3,scan#=61806,scan time=115.5056
ALIDAGVPASSVFAAAFGSEQPVSSNADDEGRAK
Intensity
2.000e+5
1.500e+5
1.000e+5
5.000e+4
0.000e+0
m/z
500
1000
1500
2000
HexNAc
HexNAcHex
Pep_2+
Pep+HexNAc_2+
b3
a5
y4
a6
b6
b7
y5
y6
y7
y8
y9
y10
y11
y12
y13
y14
y15
~y16
~y17
~y18
~y19
~y22++
~y24++
~y27++
~y30++
~y31++

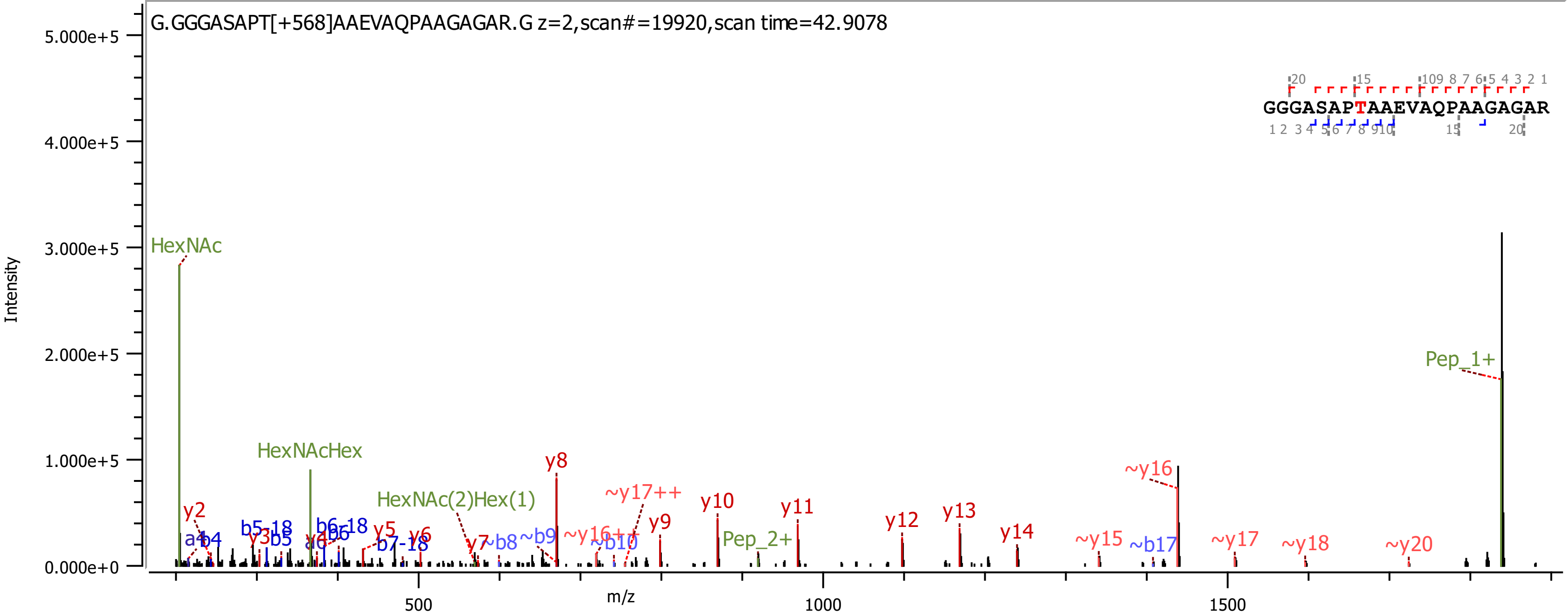

G.GGGASAPT[+568]AAEVAQPAAGAGAR.G z=2,scan#=19920,scan time=42.9078
GGGASAPTAAEVAQPAAGAGAR
Intensity
m/z
5.000e+5
4.000e+5
3.000e+5
2.000e+5
1.000e+5
0.000e+0
500
1000
1500
HexNAc
HexNAcHex
HexNAc(2)Hex(1)
Pep_2+
Pep_1+
y2
y3
y5
y6
y7
y8
y9
y10
y11
y12
y13
y14
~y15
~y16
~y17
~y18
~y20
~y16++
~y17++
b4
b5
b6
b5-18
b6-18
b7-18
~b8
~b9
~b10
~b17

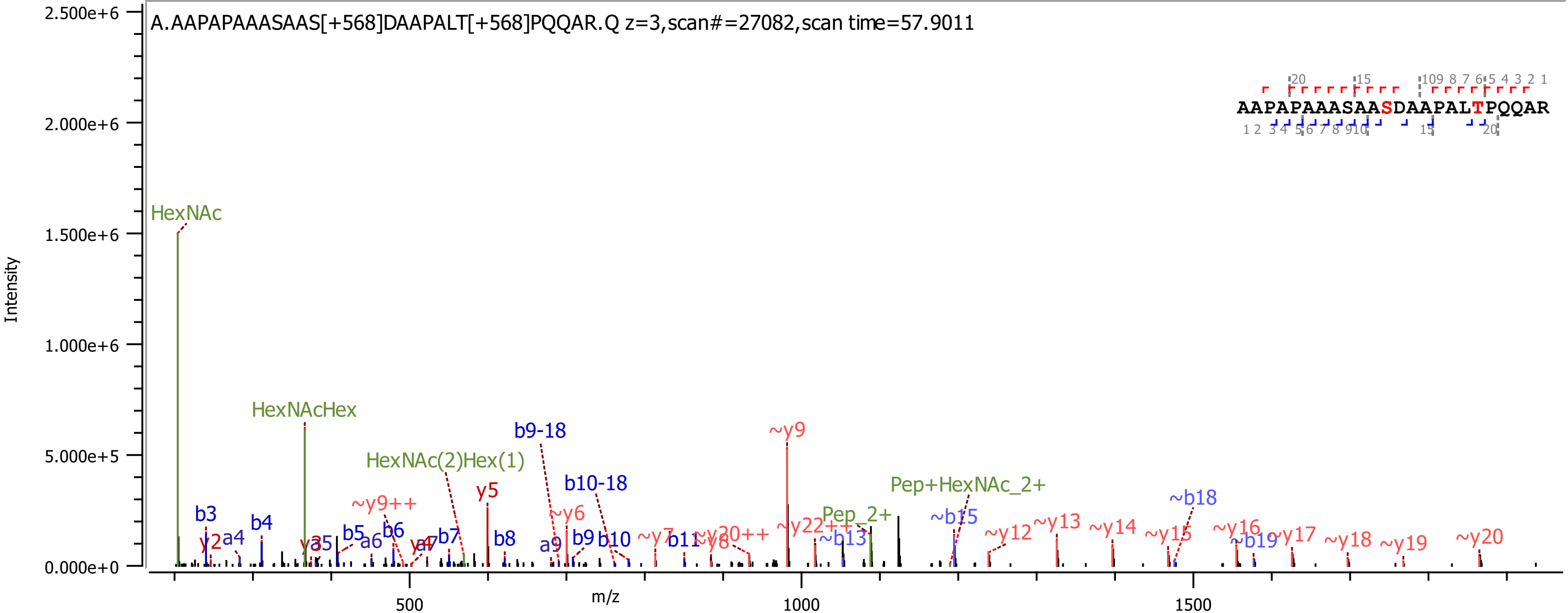
A.AAPAPAAASAAS[+568]DAAPALT[+568]PQQAR.Q z=3,scan#=27082,scan time=57.9011
AAPAPAAASAASDAAPALTPQQAR
Intensity
m/z
2.500e+6
2.000e+6
1.500e+6
1.000e+6
5.000e+5
0.000e+0
500
1000
1500
HexNAc
HexNAcHex
HexNAc(2)Hex(1)
b9-18
b10-18
~y9
~y9++
y5
~y6
Pep+HexNAc_2+
Pep_2+
~b18
~b15
b3
b4
y2
a4
a5
b5
a6
b6
b7
b8
a9
b9
b10
~y7
b11
~y8
~y20++
~y22++
~b13
~y12
~y13
~y14
~y15
~y16
~b19
~y17
~y18
~y19
~y20

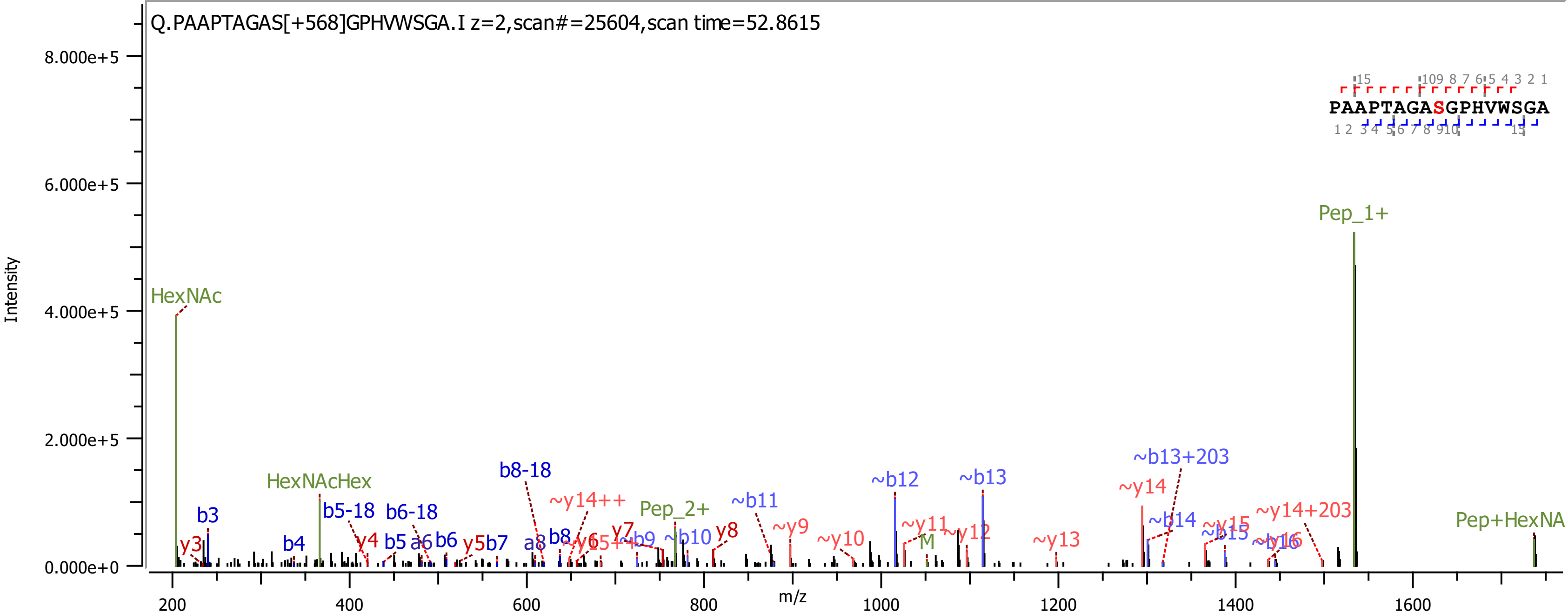

Q.PAAPTAGAS[+568]GPHVWSGA.I z=2,scan#=25604,scan time=52.8615
PAAPTAGASGPHVWSGA
Intensity
m/z
HexNAc
HexNAcHex
Pep_2+
Pep_1+
Pep+HexNA
b3
b4
b5-18
b6-18
b5
a6
b6
y5b7
b8-18
a8
b8
y3
y4
~y14++
y7
y8
~b9
~b10
~b11
~y9
~y10
~b12
~y11
~y12
~b13
~y13
~y14
~b13+203
~b14
~y15
~b15
~y16
~y14+203
M
8.000e+5
6.000e+5
4.000e+5
2.000e+5
0.000e+0
200
400
600
800
1000
1200
1400
1600

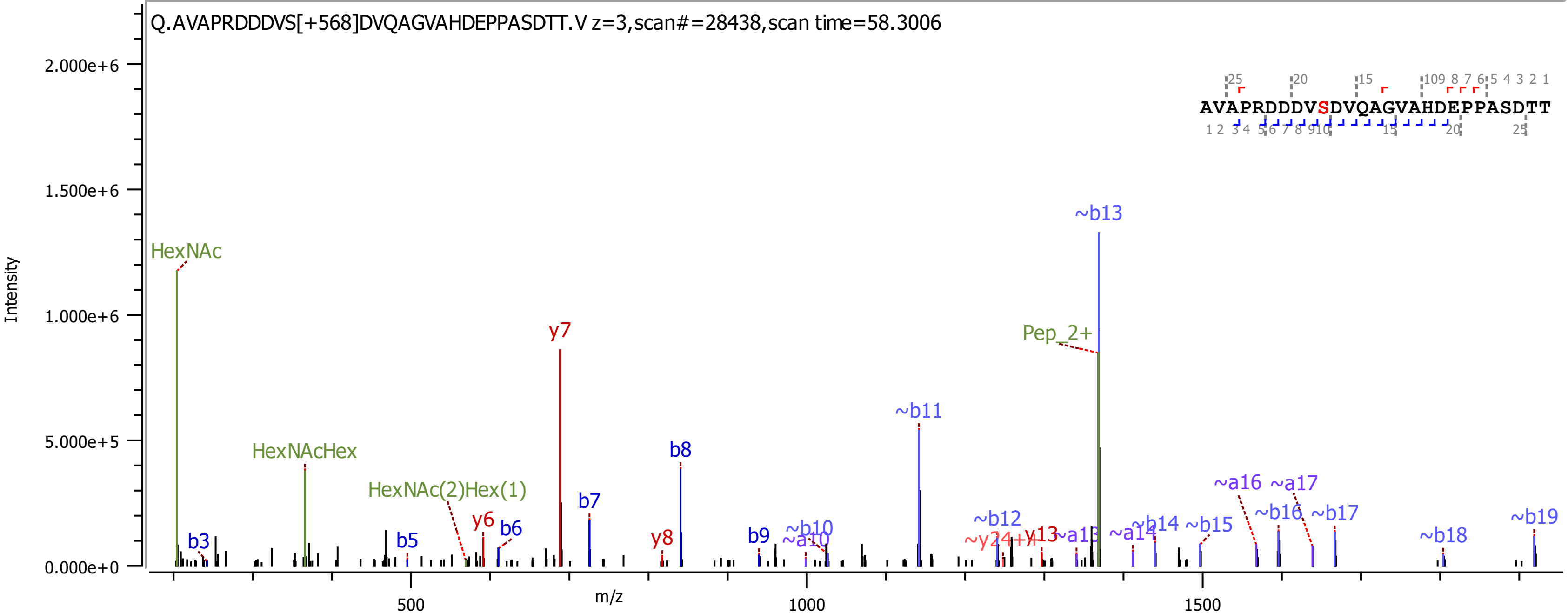

Q.AVAPRDDDVS[+568]DVQAGVAHDEPPASDTT.V z=3,scan#=28438,scan time=58.3006
AVAPRDDDVSDVQAGVAHDEPPASDTT
Intensity
m/z
2.000e+6
1.500e+6
1.000e+6
5.000e+5
0.000e+0
500
1000
1500
HexNAc
HexNAcHex
HexNAc(2)Hex(1)
Pep_2+
y6
y7
y8
y13
b3
b5
b6
b7
b8
b9
~b10
~a10
~b11
~b12
~b13
~a13
~a14
~b14
~b15
~a16
~b16
~a17
~b17
~b18
~b19

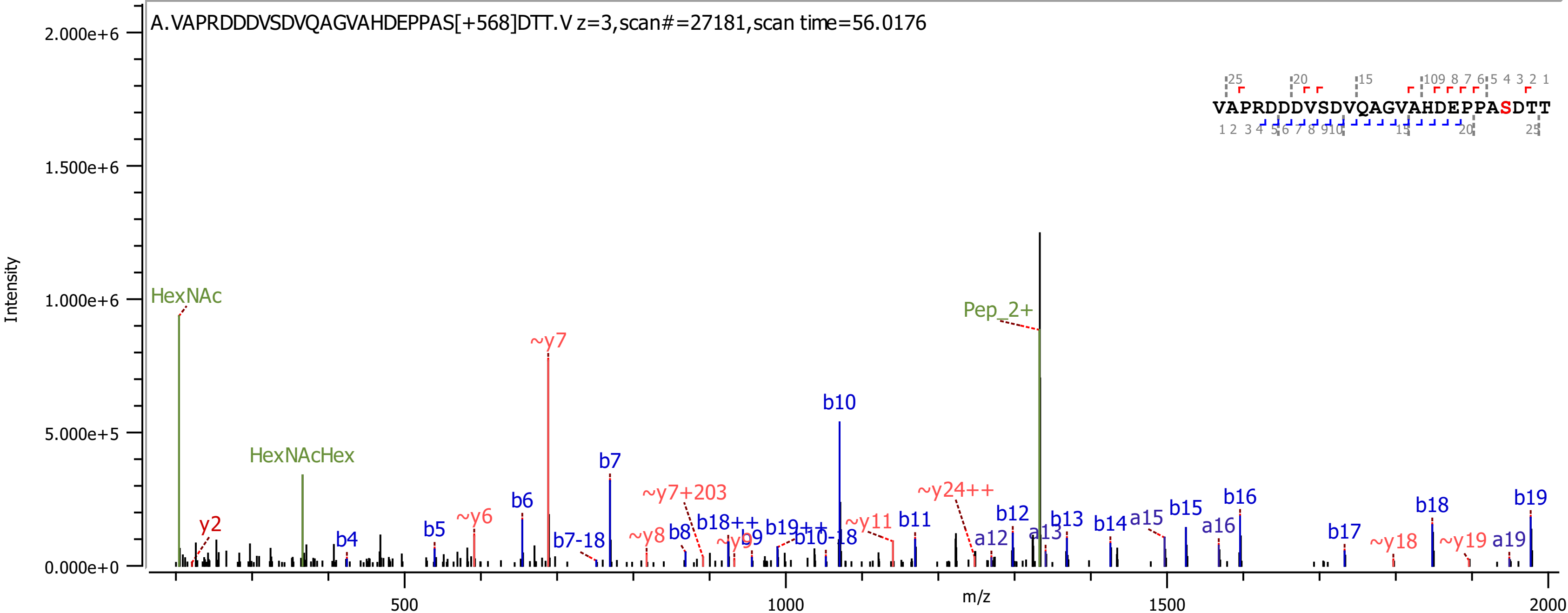
A.VAPRDDDVSDVQAGVAHDEPPAS[+568]DTT.V z=3,scan#=27181,scan time=56.0176
VAPRDDDVSDVQAGVAHDEPPASDTT
Intensity
m/z
HexNAc
HexNAcHex
Pep_2+
y2
b4
b5
~y6
b6
~y7
b7-18
b7
~y8
~y7+203
b8
b18++
~y9
b9
b19++
b10-18
b10
~y11
b11
~y24++
a12
b12
a13
b13
b14
a15
b15
a16
b16
b17
~y18
b18
~y19
a19
b19

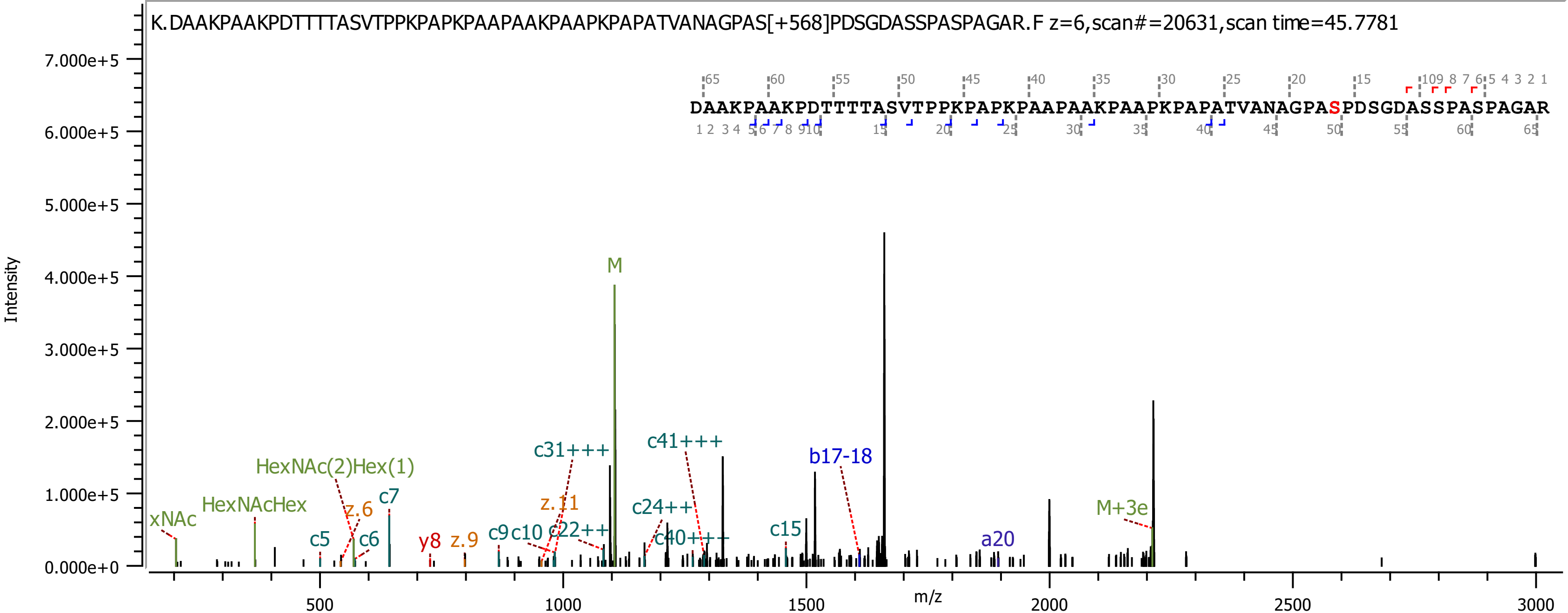
K.DAAKPAAKPDTTTTASVTPPKPAPKPAAPAAKPAAPKPAPATVANAGPAS[+568]PDSGDASSPASPAGAR.F z=6,scan#=20631,scan time=45.7781
DAAKPAAKPDTTTTASVTPPKPAPKPAAPAAKPAAPKPAPATVANAGPASPDSGDASSPASPAGAR
Intensity
m/z
M
M+3e
xNAc
HexNAcHex
HexNAc(2)Hex(1)
c5
c6
c7
z.6
y8
z.9
c9
c10
z.11
c22++
c31+++
c24++
c40+++
c41+++
c15
b17-18
a20

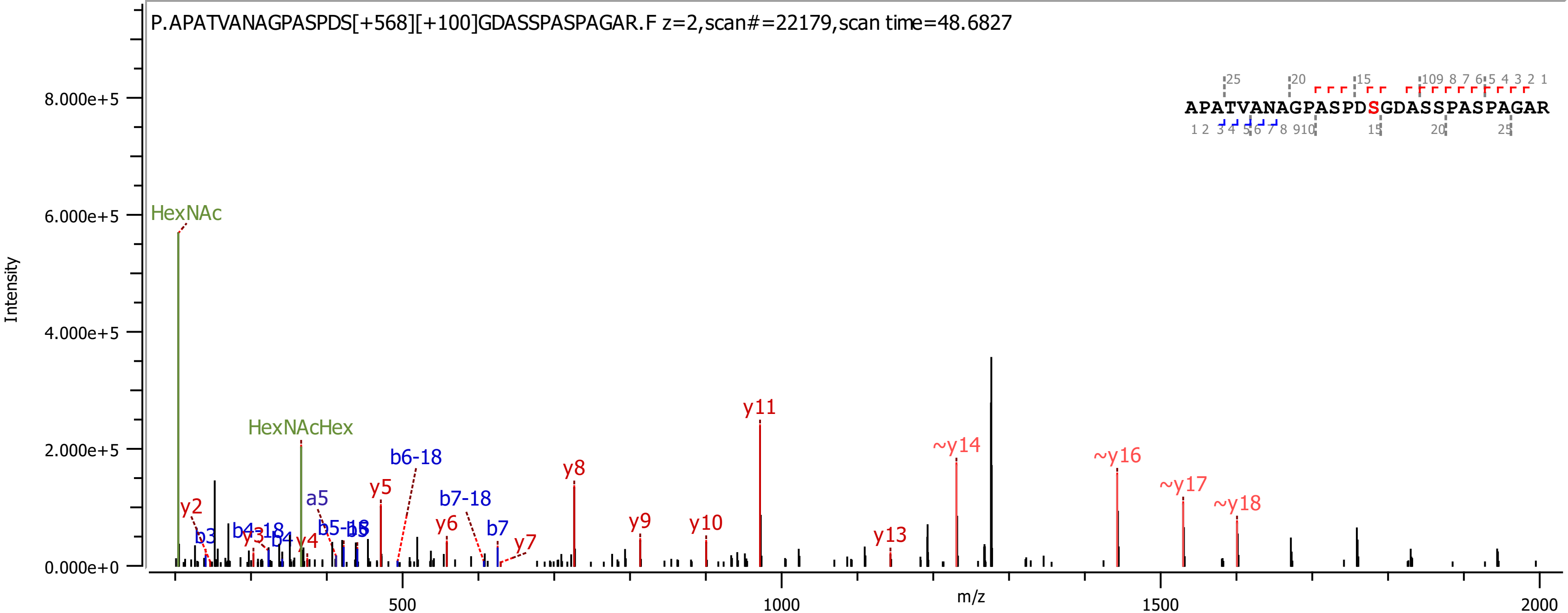
P.APATVANAGPASPDS[+568][+100]GDASSPASPAGAR.F z=2,scan#=22179,scan time=48.6827
APATVANAGPASPDSGDASSPASPAGAR
Intensity
m/z
8.000e+5
6.000e+5
4.000e+5
2.000e+5
0.000e+0
500
1000
1500
2000
HexNAc
HexNAcHex
y2
b3
b4-18
y3
b4
y4
a5
b5-18
b5
y5
b6-18
y6
b7-18
b7
y7
y8
y9
y10
y11
y13
~y14
~y16
~y17
~y18

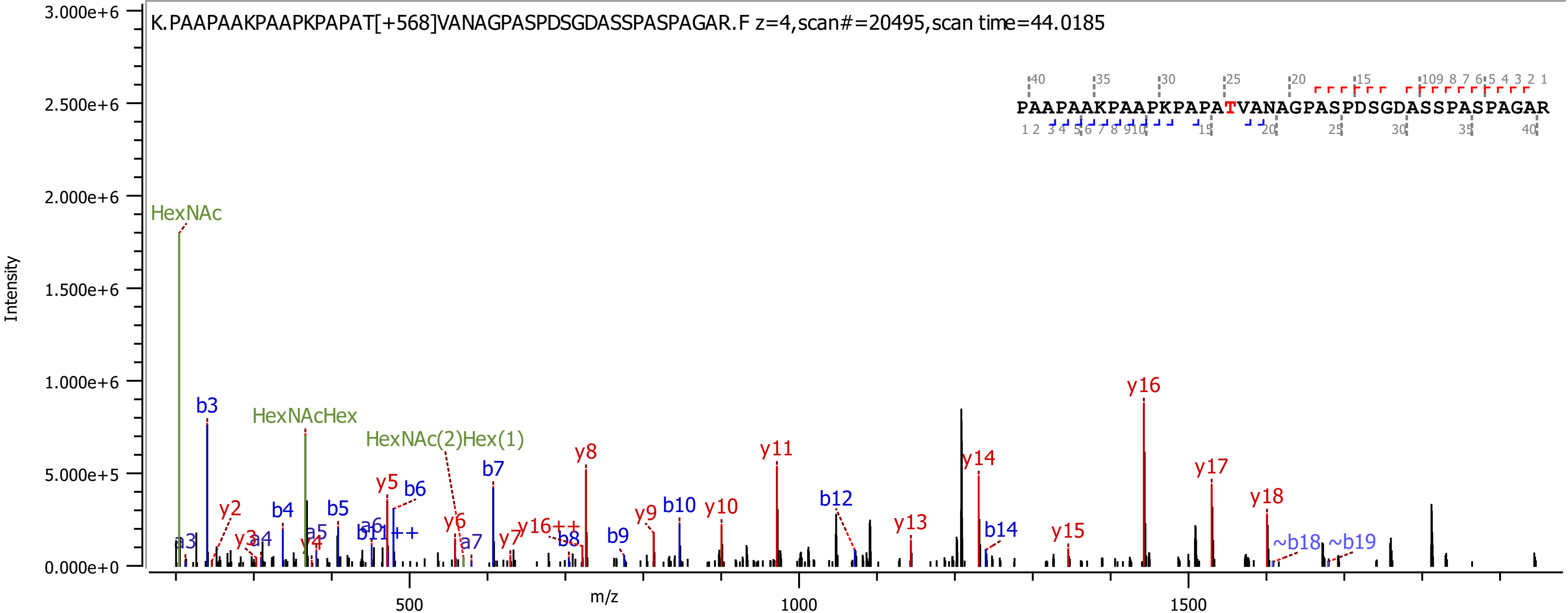

K.PAAPAAKPAAPKPAPAT[+568]VANAGPASPDSGDASSPASPAGAR.F z=4,scan#=20495,scan time=44.0185
Intensity
m/z
3.000e+6
2.500e+6
2.000e+6
1.500e+6
1.000e+6
5.000e+5
0.000e+0
500
1000
1500
PAAPAAKPAAPKPAPATVANAGPASPDSGDASSPASPAGAR
HexNAc
HexNAcHex
HexNAc(2)Hex(1)
b3
b4
b5
b6
b7
b8
b9
b10
b12
b14
b11++
~b18
~b19
a3
a4
a5
a6
a7
y2
y3
y4
y5
y6
y7
y8
y9
y10
y11
y13
y14
y15
y16
y16++
y17
y18

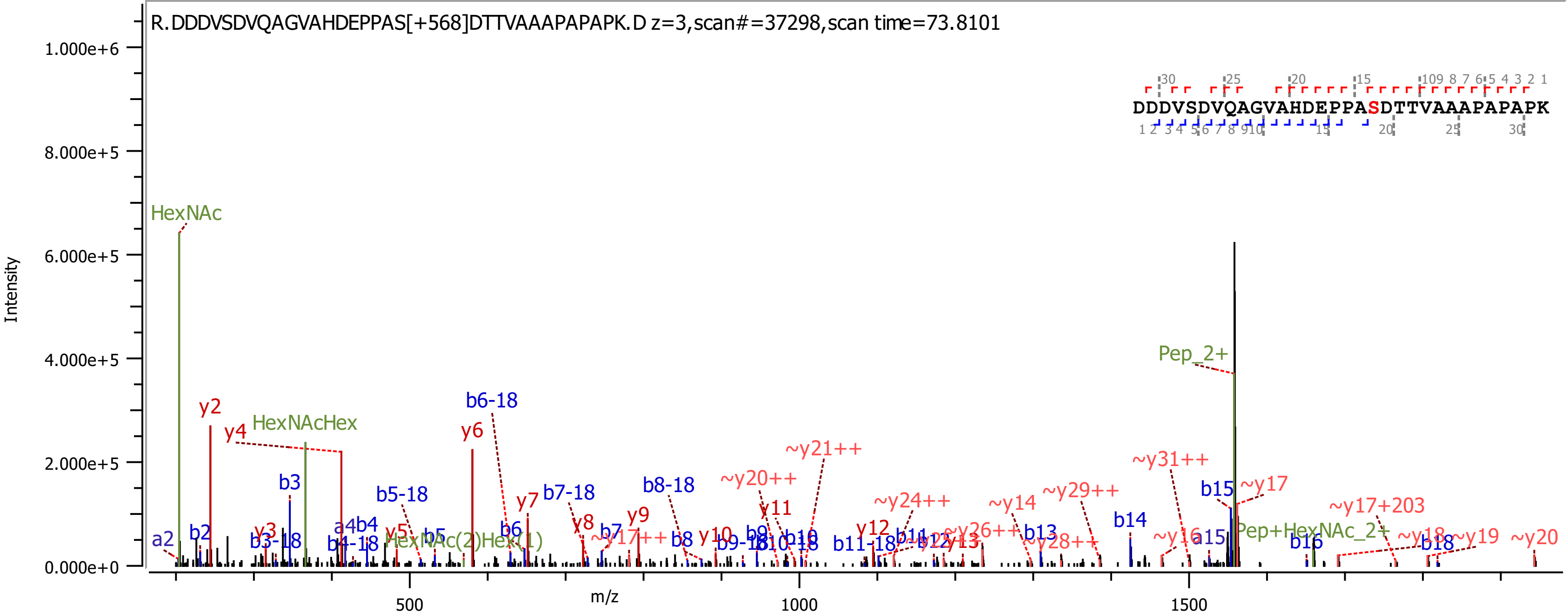
R.DDDVSDVQAGVAHDEPPAS[+568]DTTVAAAPAPAPK.D z=3,scan#=37298,scan time=73.8101
DDDVSDVQAGVAHDEPPASDTTVAAAPAPAPK
Intensity
m/z
HexNAc
HexNAcHex
Pep_2+
Pep+HexNAc_2+
HexNAc(2)Hex(1)
y2
y4
y6
y7
b6-18
b3
b5-18
b7-18
b8-18
~y20++
~y21++
~y31++
b15
~y17
~y17+203
b14
~y29++
~y24++
~y14
y11
y12
b13
~y26++
~y28++
~y16
a15
b16
~y18
b18
~y19
~y20
a2
b2
y3
b3-18
a4
b4
b4-18
y5
b5
b6
y8
b7
y9
~y17++
b8
y10
b9
b9-18
b10
b10-18
b11-18
b11
~y22
y13

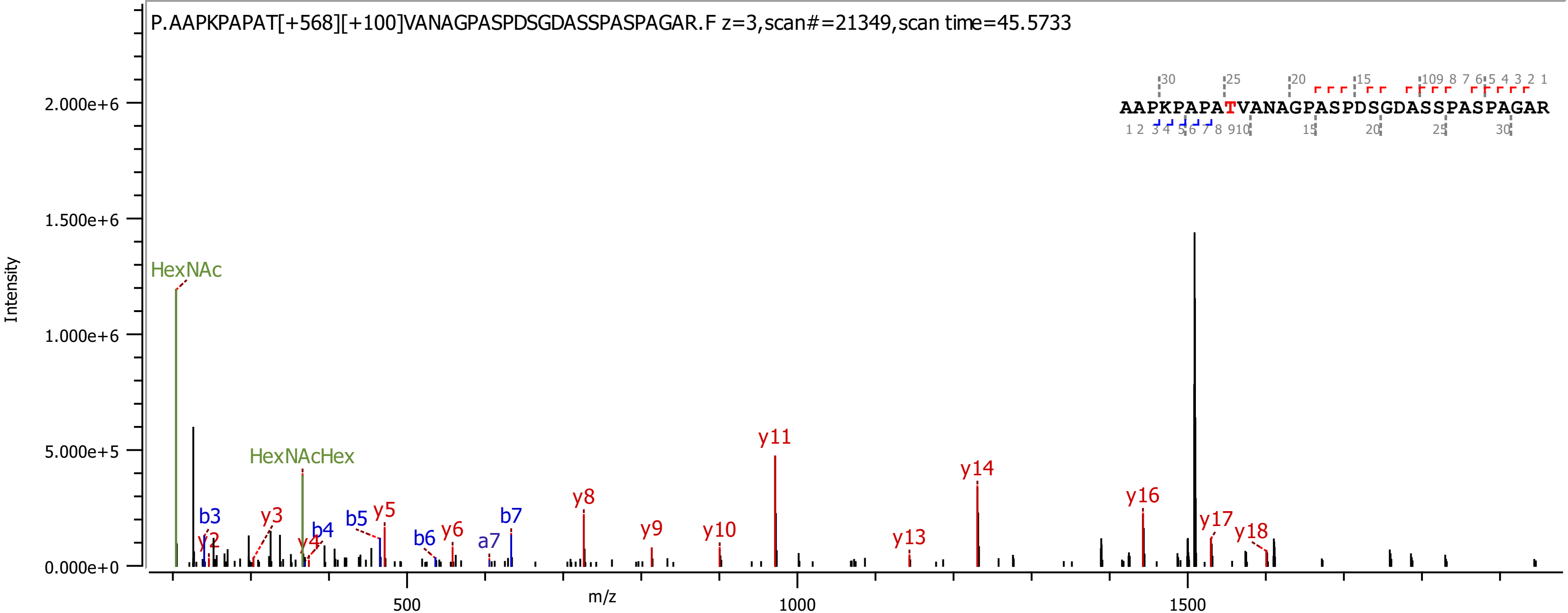

P.AAPKPAPAT[+568][+100]VANAGPASPDSGDASSPASPAGAR.F z=3,scan#=21349,scan time=45.5733
AAPKPAPATVANAGPASPDSGDASSPASPAGAR
Intensity
m/z
HexNAc
HexNAcHex
b3
y2
y3
y4
b4
b5
y5
b6
y6
a7
b7
y8
y9
y10
y11
y13
y14
y16
y17
y18

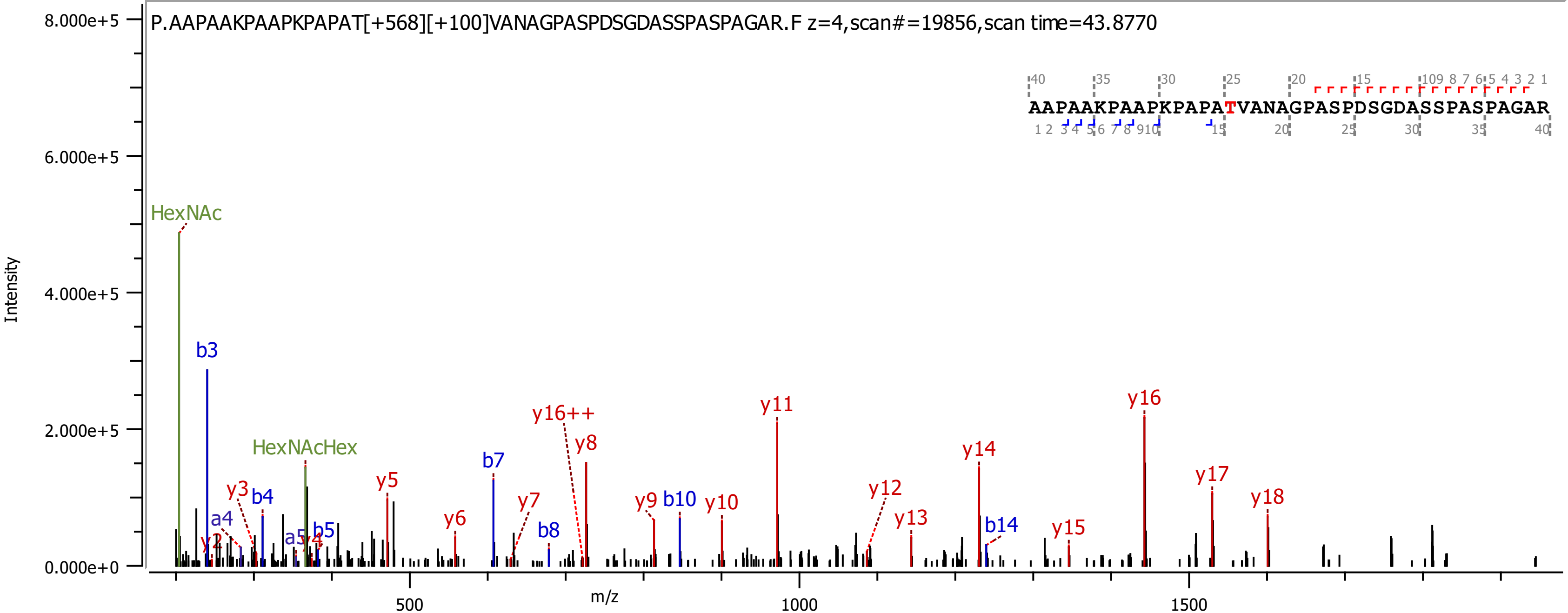
P.AAPAAKPAAPKPAPAT[+568][+100]VANAGPASPDSGDASSPASPAGAR.F z=4,scan#=19856,scan time=43.8770
AAPAAKPAAPKPAPATVANAGPASPDSGDASSPASPAGAR
Intensity
m/z
8.000e+5
6.000e+5
4.000e+5
2.000e+5
0.000e+0
500
1000
1500
HexNAc
HexNAcHex
b3
b4
b5
b7
b8
b10
b14
a4
a5
y2
y3
y4
y5
y6
y7
y8
y9
y10
y11
y12
y13
y14
y15
y16
y16++
y17
y18

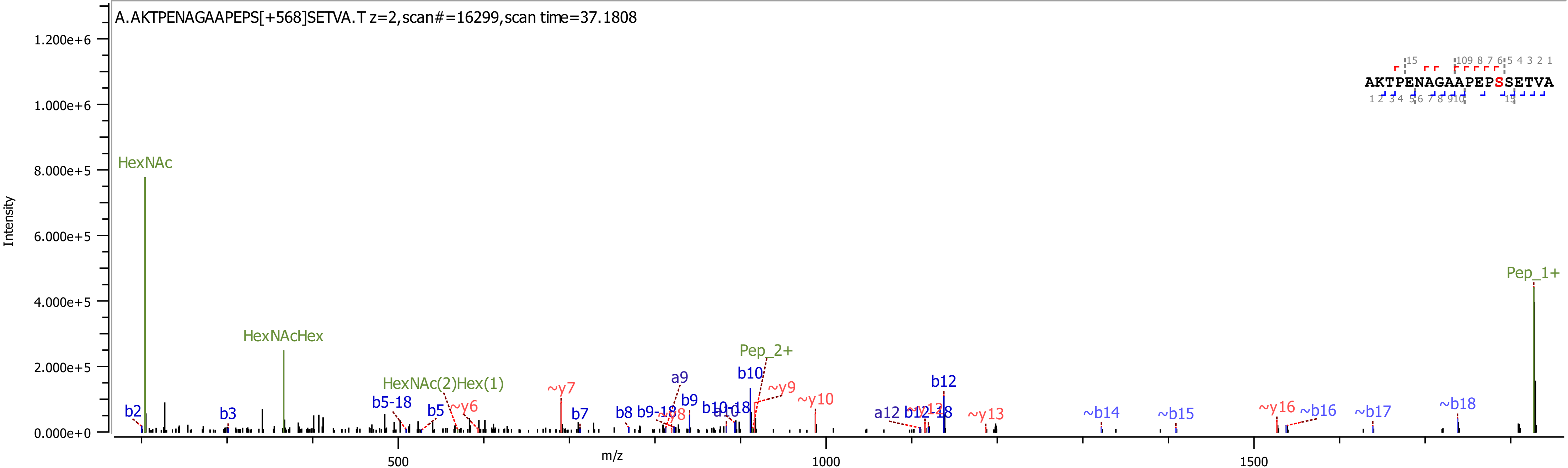

A.AKTPENAGAAPEPS[+568]SETVA.T z=2,scan#=16299,scan time=37.1808
AKTPENAGAAPEPSSETVA
Intensity
m/z
HexNAc
HexNAcHex
HexNAc(2)Hex(1)
Pep_2+
Pep_1+
b2
b3
b5-18
b5
~y6
~y7
b7
b8
b9-18
a9
b9
b10
b10-18
a10
~y9
~y10
a12
b12-18
b12
~y13
~b14
~b15
~y16
~b16
~b17
~b18

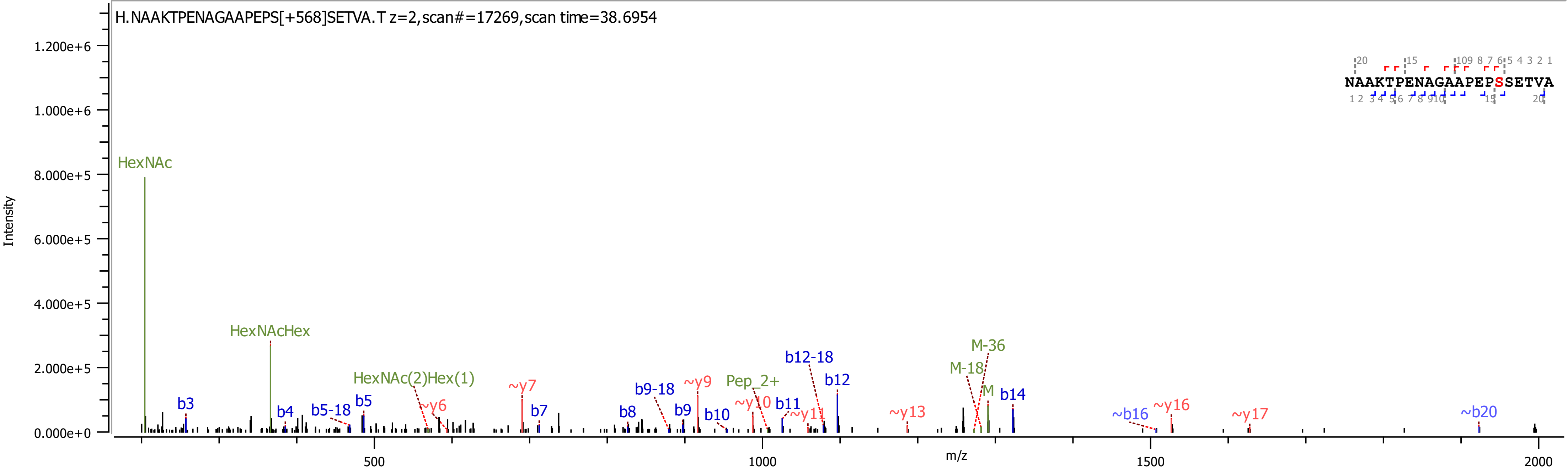

H.NAAKTPENAGAAPEPS[+568]SETVA.T z=2,scan#=17269,scan time=38.6954
Intensity
m/z
NAAKTPENAGAAPEPSSETVA
HexNAc
HexNAcHex
HexNAc(2)Hex(1)
b3
b4
b5-18
b5
~y6
~y7
b7
b8
b9-18
~y9
b9
b10
Pep_2+
~y10
b11
~y11
b12-18
b12
~y13
M-18
M-36
M
b14
~b16
~y16
~y17
~b20

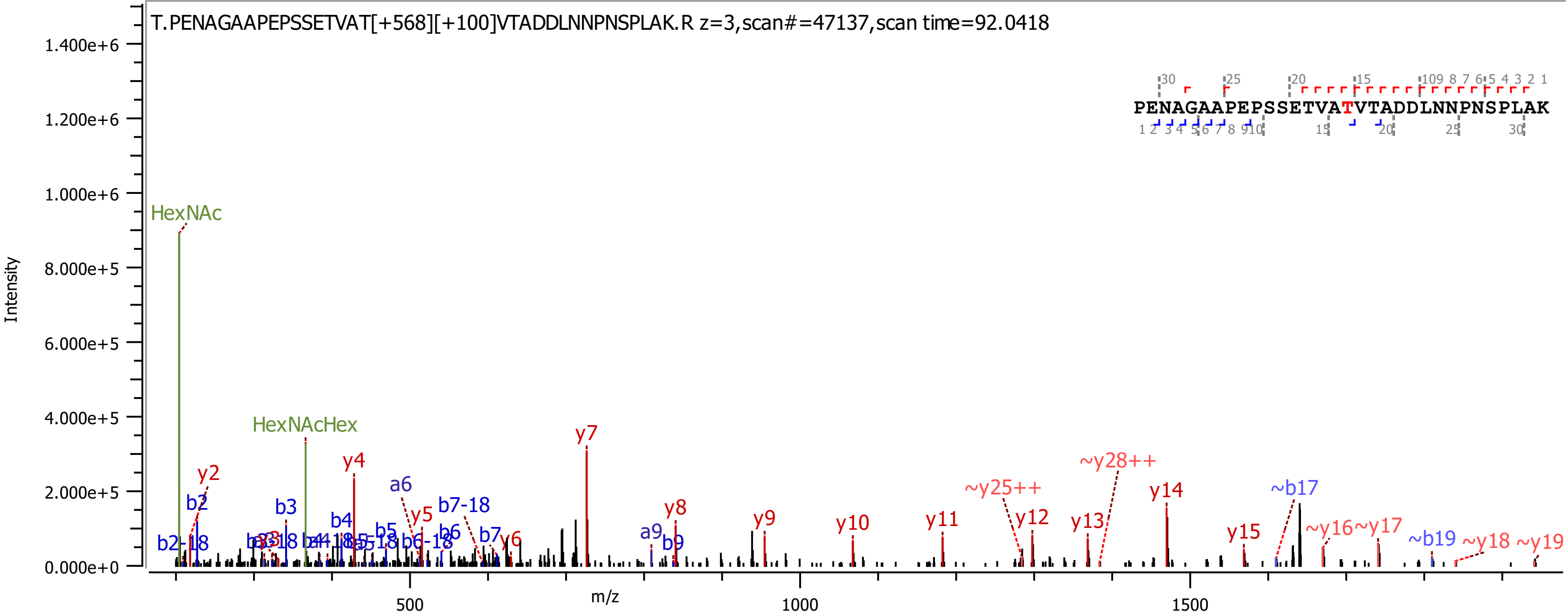

T.PENAGAAPEPSSETVAT[+568][+100]VTADDLNNPNSPLAK.R z=3,scan#=47137,scan time=92.0418
PENAGAAPEPSSETVATVTADDLNNPNSPLAK
Intensity
m/z
HexNAc
HexNAcHex
y2
b2
b2-18
b3
y4
a6
y5
b7-18
b4
b5
b6
b7
y6
y7
a9
b9
y8
y9
y10
y11
~y25++
y12
~y28++
y13
y14
y15
~b17
~y16
~y17
~b19
~y18
~y19

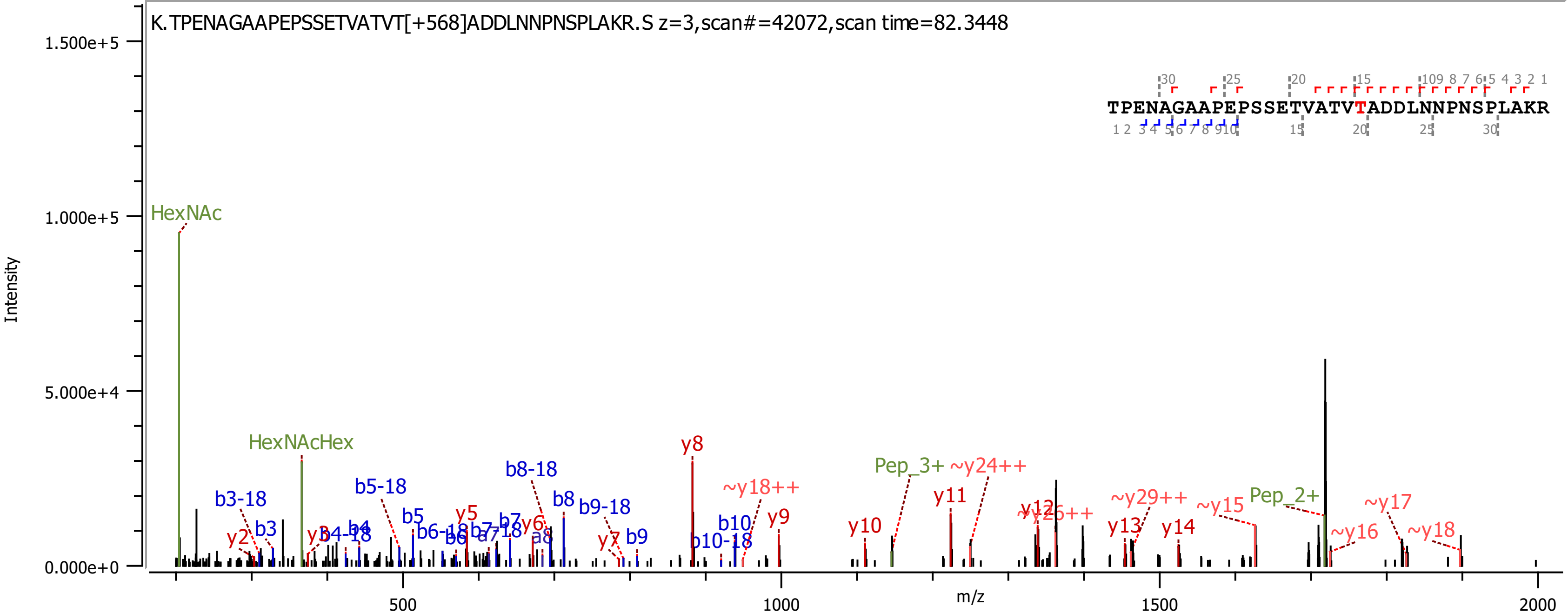

K.TPENAGAAPEPSSETVATVT[+568]ADDLNNPNSPLAKR.S z=3,scan#=42072,scan time=82.3448
TPENAGAAPEPSSETVATVTADDLNNPNSPLAKR
Intensity
m/z
1.500e+5
1.000e+5
5.000e+4
0.000e+0
500
1000
1500
2000
HexNAc
HexNAcHex
y8
Pep_3+
~y24++
Pep_2+
~y18++
y9
y10
y11
y12
y13
y14
~y15
~y16
~y17
~y18
~y29++
b3-18
b3
b4-18
b4
b5-18
b5
b6-18
b6
b7-18
b7
b8-18
b8
b9-18
b9
b10
b10-18
a7
a8
y2
y3
y5
y6
y7

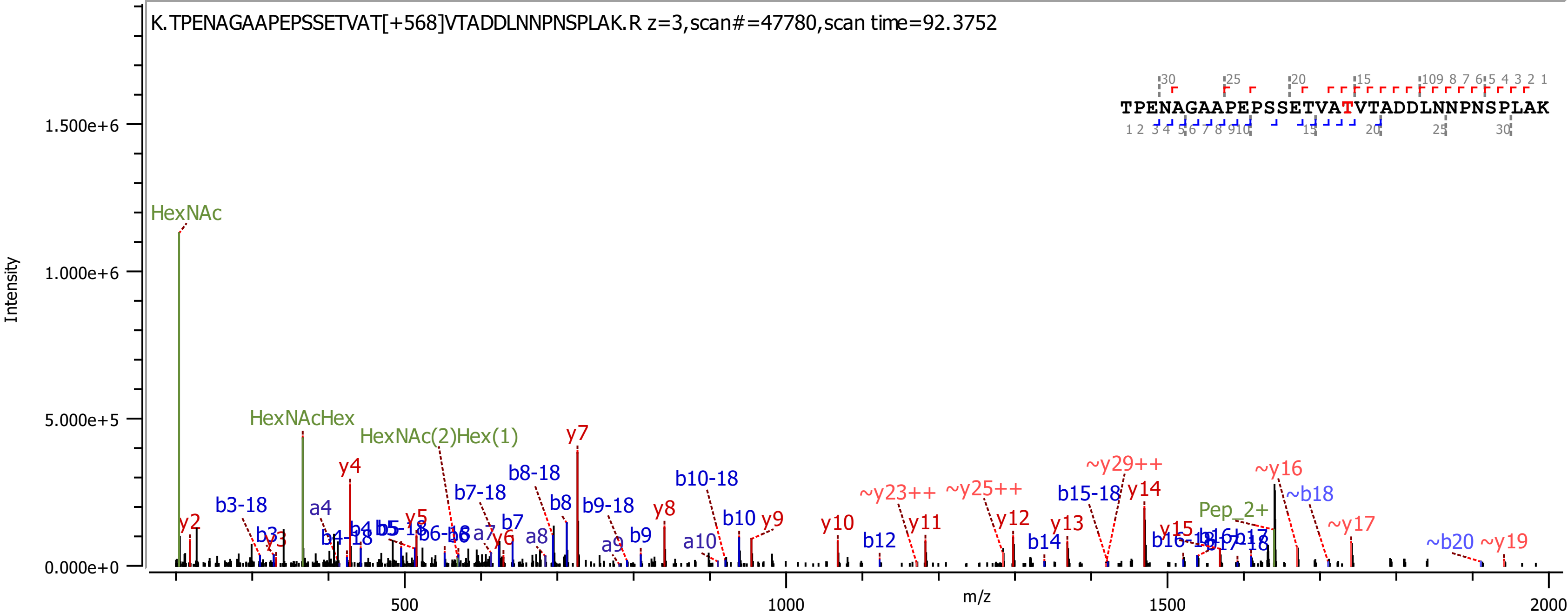

K.TPENAGAAPEPSSETVAT[+568]VTADDLNNPNSPLAK.R z=3,scan#=47780,scan time=92.3752
TPENAGAAPEPSSETVATVTADDLNNPNSPLAK
Intensity
m/z
HexNAc
HexNAcHex
HexNAc(2)Hex(1)
Pep_2+
y2
y3
y4
y5
y6
y7
y8
y9
y10
y11
y12
y13
y14
y15
b3-18
b3
a4
b4-18
b4
b5
b5-18
b6-18
b6
a7
b7-18
b7
a8
b8-18
b8
b9-18
a9
b9
a10
b10-18
b10
b12
b14
b15-18
b16-18
b16
b17-18
b17
~y23++
~y25++
~y29++
~y16
~b18
~y17
~b20
~y19

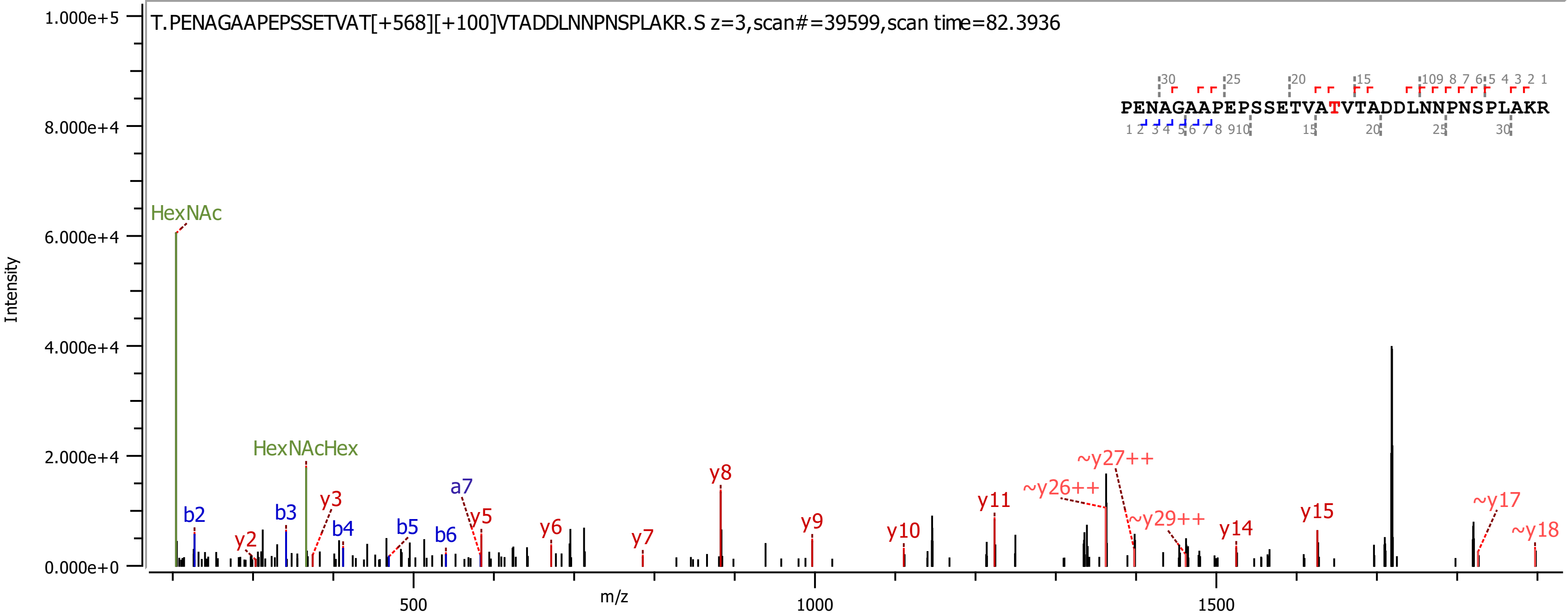
T.PENAGAAPEPSSETVAT[+568][+100]VTADDLNNPNSPLAKR.S z=3,scan#=39599,scan time=82.3936
Intensity
m/z
HexNAc
HexNAcHex
b2
b3
b4
b5
b6
a7
y2
y3
y5
y6
y7
y8
y9
y10
y11
~y26++
~y27++
~y29++
y14
y15
~y17
~y18
PENAGAAPEPSSETVATVTADDLNNPNSPLAKR

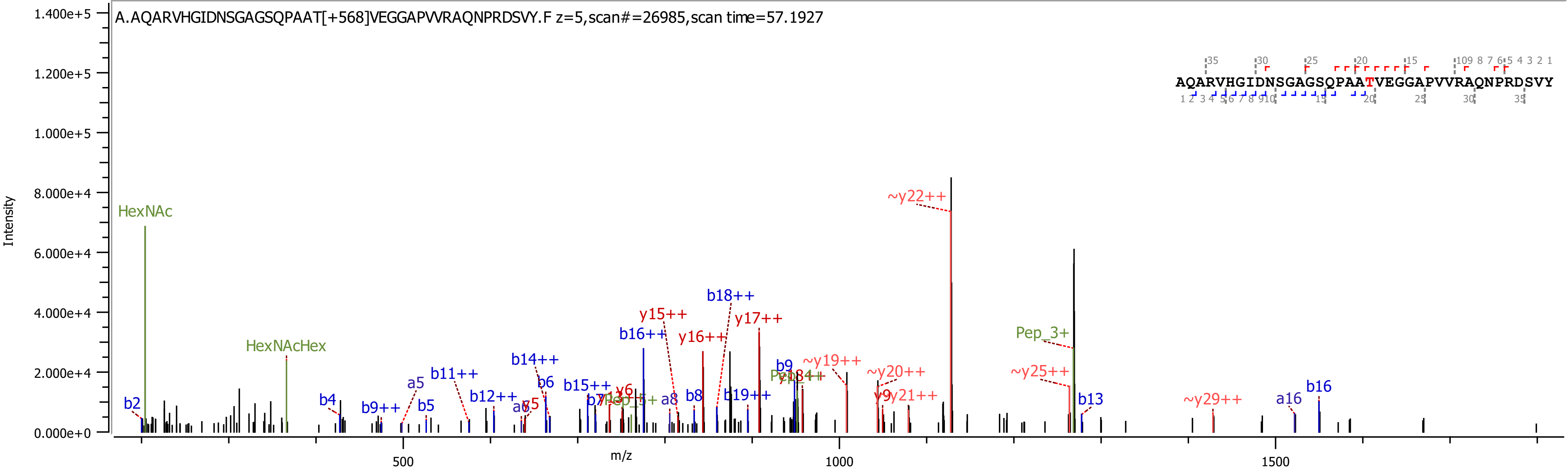

A.AQARVHGIDNSGAGSQPAAT[+568]VEGGAPVVRAQNPRDSVY.F z=5,scan#=26985,scan time=57.1927
AQARVHGIDNSGAGSQPAATVEGGAPVVRAQNPRDSVY
Intensity
m/z
1.400e+5
1.200e+5
1.000e+5
8.000e+4
6.000e+4
4.000e+4
2.000e+4
0.000e+0
500
1000
1500
HexNAc
HexNAcHex
~y22++
Pep_3+
b18++
y15++
y17++
b16++
y16++
b14++
b11++
~y19++
~y20++
~y25++
b16
a16
~y29++
b13
b2
b4
b9++
b5
a5
b12++
b6
b15++
b8
a8
b19++
b9
y9
y21++

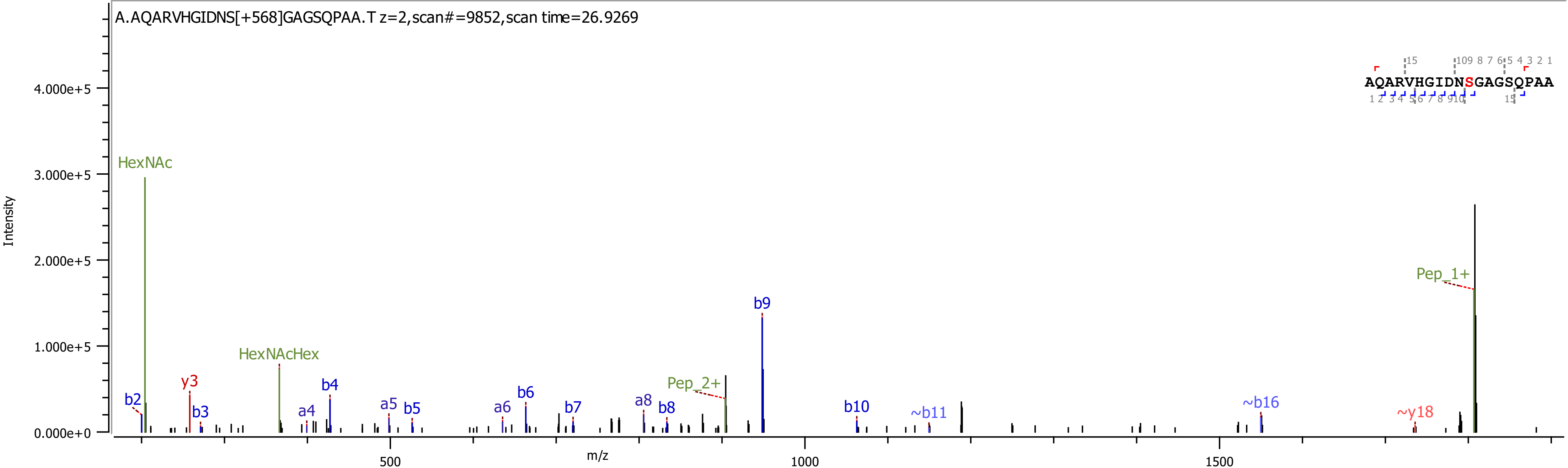

A.AQARVHGIDNS[+568]GAGSQPAA.T z=2,scan#=9852,scan time=26.9269
AQARVHGIDNSGAGSQPAA
Intensity
m/z
4.000e+5
3.000e+5
2.000e+5
1.000e+5
0.000e+0
500
1000
1500
HexNAc
HexNAcHex
Pep_2+
Pep_1+
b2
y3
b3
a4
b4
a5
b5
a6
b6
b7
a8
b8
b9
b10
~b11
~b16
~y18

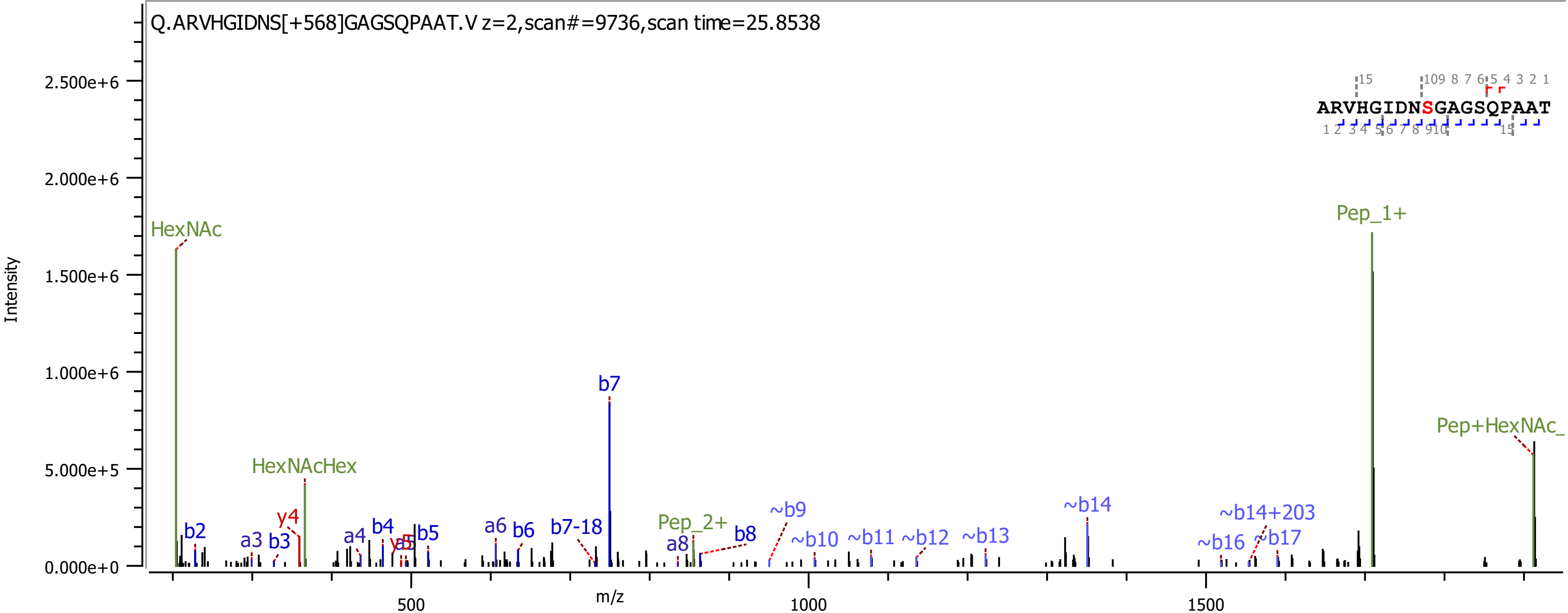
Q.ARVHGIDNS[+568]GAGSQPAAT.V z=2,scan#=9736,scan time=25.8538
ARVHGIDNSGAGSQPAAT
Intensity
m/z
HexNAc
HexNAcHex
Pep_1+
Pep+HexNAc_
Pep_2+
b2
a3
b3
y4
a4
b4
y5
b5
a6
b6
b7-18
b7
a8
b8
~b9
~b10
~b11
~b12
~b13
~b14
~b16
~b14+203
~b17

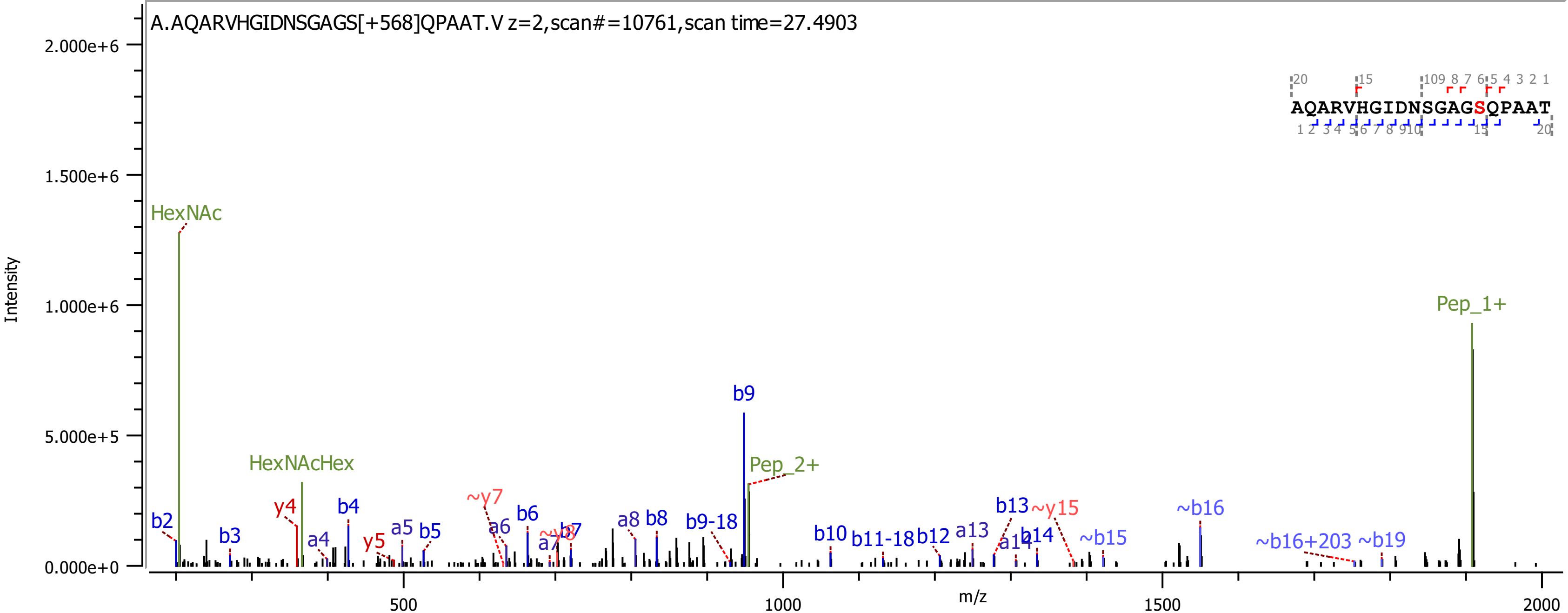

A.AQARVHGIDNSGAGS[+568]QPAAT.V z=2,scan#=10761,scan time=27.4903
AQARVHGIDNSGAGSQPAAT
Intensity
m/z
HexNAc
HexNAcHex
Pep_2+
Pep_1+
b2
b3
y4
a4
b4
y5
a5
b5
~y7
a6
b6
~y8
a7
b7
a8
b8
b9
b9-18
b10
b11-18
b12
a13
b13
~y15
a14
b14
~b15
~b16
~b16+203
~b19

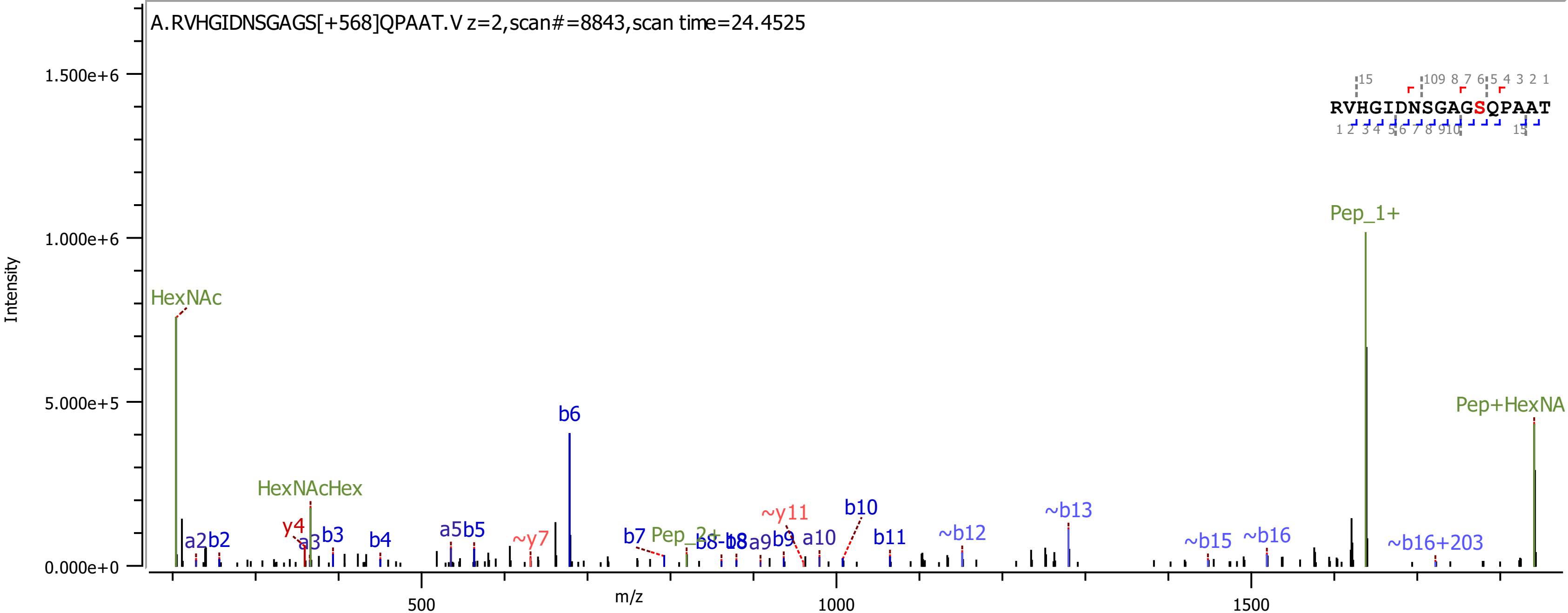
A.RVHGIDNSGAGS[+568]QPAAT.V z=2,scan#=8843,scan time=24.4525
RVHGIDNSGAGSQPAAT
Intensity
m/z
1.500e+6
1.000e+6
5.000e+5
0.000e+0
500
1000
1500
HexNAc
HexNAcHex
Pep_1+
Pep+HexNA
b6
a2b2
y4
a3
b3
b4
a5b5
~y7
b7
Pep_2+
b8-18
a9b9
~y11
a10
b10
b11
~b12
~b13
~b15
~b16
~b16+203

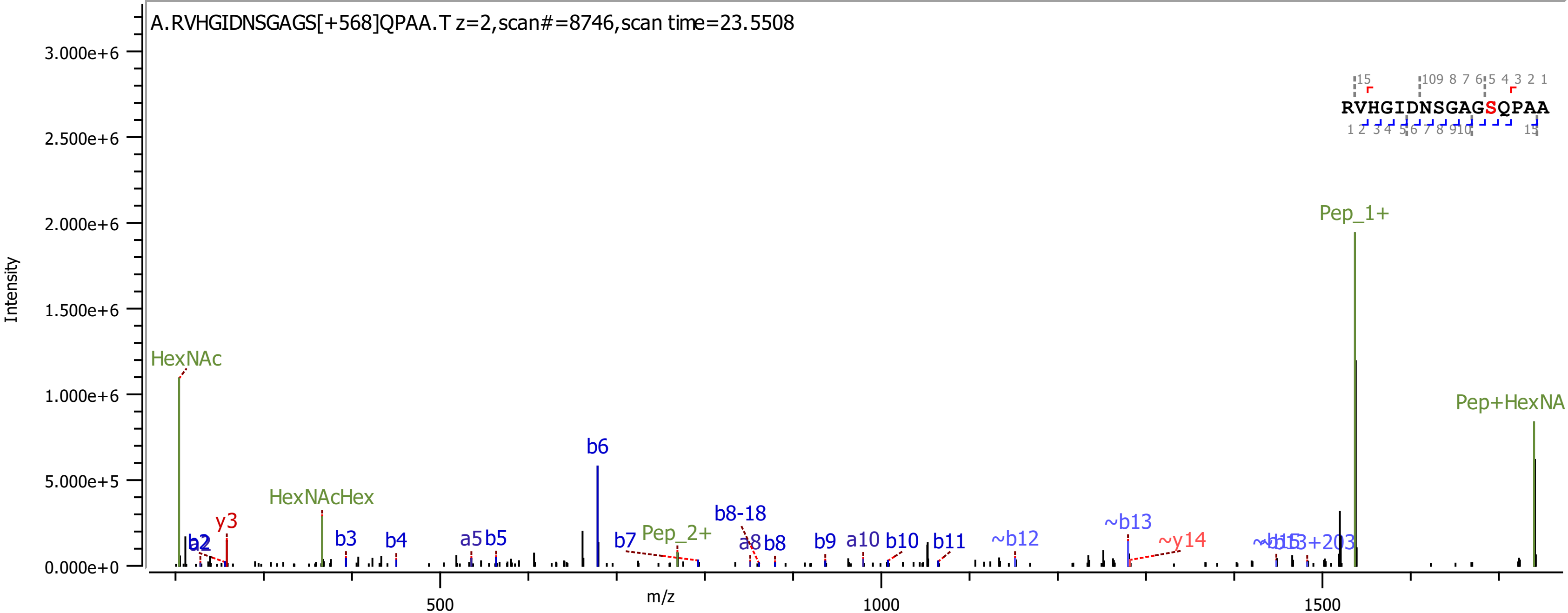
A.RVHGIDNSGAGS[+568]QPAA.T z=2,scan#=8746,scan time=23.5508
Intensity
m/z
3.000e+6
2.500e+6
2.000e+6
1.500e+6
1.000e+6
5.000e+5
0.000e+0
500
1000
1500
RVHGIDNSGAGSQPAA
HexNAc
HexNAcHex
b2
y3
b3
b4
a5
b5
b6
b7
Pep_2+
b8-18
a8
b8
b9
a10
b10
b11
~b12
~b13
~y14
Pep_1+
Pep+HexNA

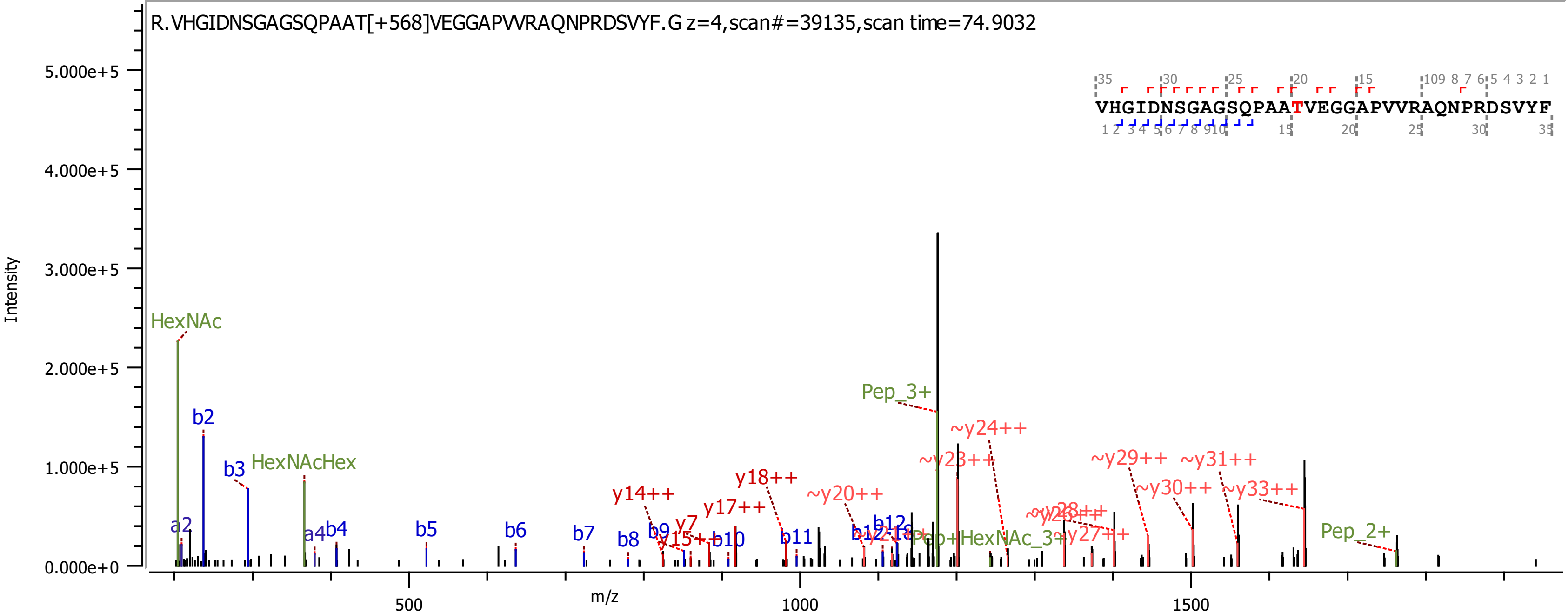
R.VHGIDNSGAGSQPAAT[+568]VEGGAPVVRAQNPRDSVYF.G z=4,scan#=39135,scan time=74.9032
VHGIDNSGAGSQPAATVEGGAPVVRAQNPRDSVYF
Intensity
m/z
HexNAc
b2
b3
HexNAcHex
a2
a4
b4
b5
b6
b7
b8
b9
y7
y14++
y15++
b10
y17++
y18++
b11
~y20++
b12
Pep_3+
~y23++
~y24++
Pep+HexNAc_3+
~y27++
~y29++
~y30++
~y31++
~y33++
Pep_2+

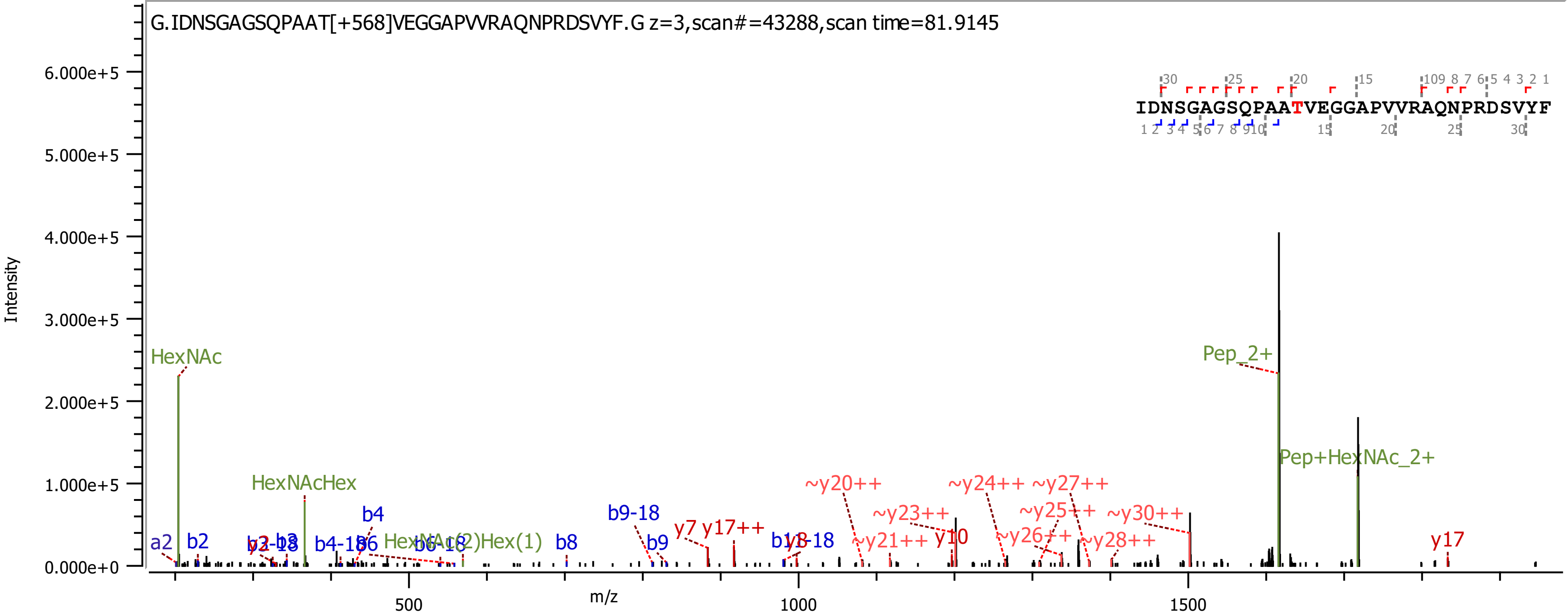

G.IDNSGAGSQPAAT[+568]VEGGAPVVRAQNPRDSVYF.G z=3,scan#=43288,scan time=81.9145
IDNSGAGSQPAATVEGGAPVVRAQNPRDSVYF
Intensity
m/z
6.000e+5
5.000e+5
4.000e+5
3.000e+5
2.000e+5
1.000e+5
0.000e+0
500
1000
1500
HexNAc
HexNAcHex
Pep_2+
Pep+HexNAc_2+
a2
b2
b4
b8
b9
b9-18
y7
y17++
y10
y17
~y20++
~y21++
~y23++
~y24++
~y25++
~y26++
~y27++
~y28++
~y30++

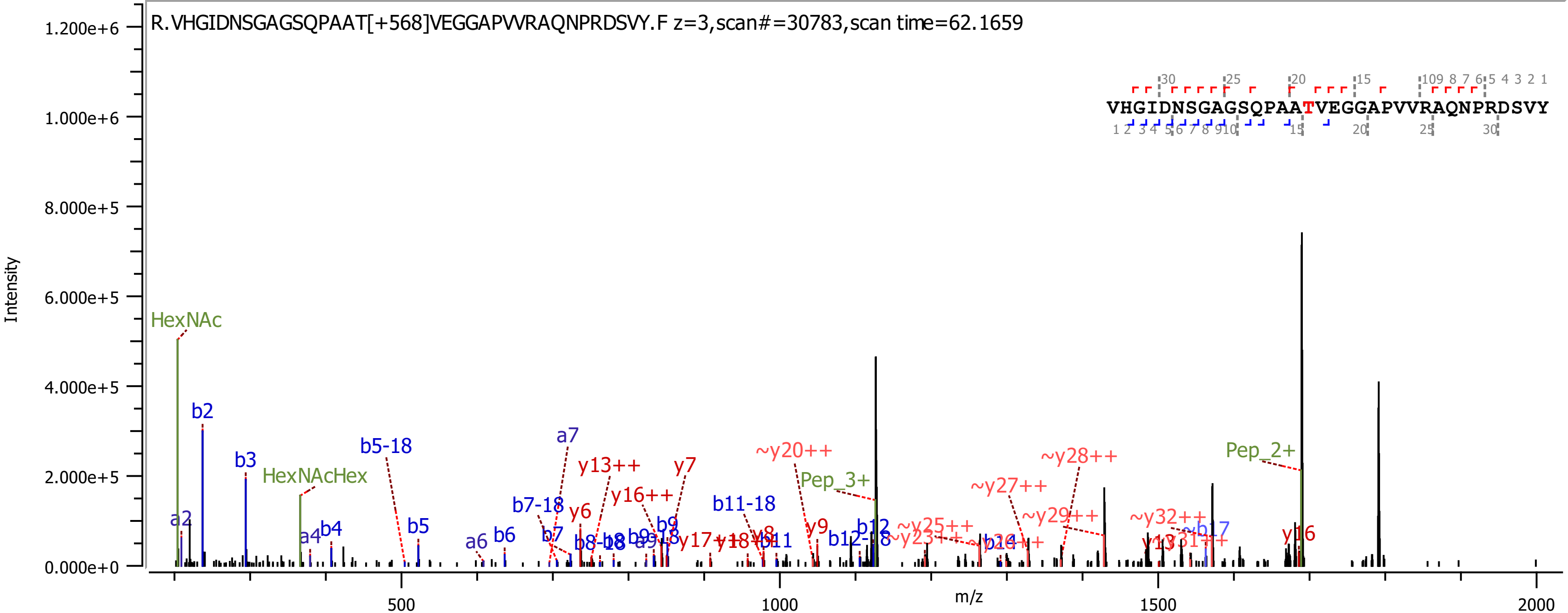

R.VHGIDNSGAGSQPAAT[+568]VEGGAPVVRAQNPRDSVY.F z=3,scan#=30783,scan time=62.1659
VHGIDNSGAGSQPAATVEGGAPVVRAQNPRDSVY
Intensity
m/z
1.200e+6
1.000e+6
8.000e+5
6.000e+5
4.000e+5
2.000e+5
0.000e+0
500
1000
1500
2000
HexNAc
HexNAcHex
Pep_3+
Pep_2+
b2
b3
a2
a4
b4
b5-18
b5
a6
b6
a7
b7-18
b7
y6
y13++
y16++
y7
b9
b11-18
~y20++
y9
b12
~y28++
~y27++
~y29++
~y32++
~b17
y16

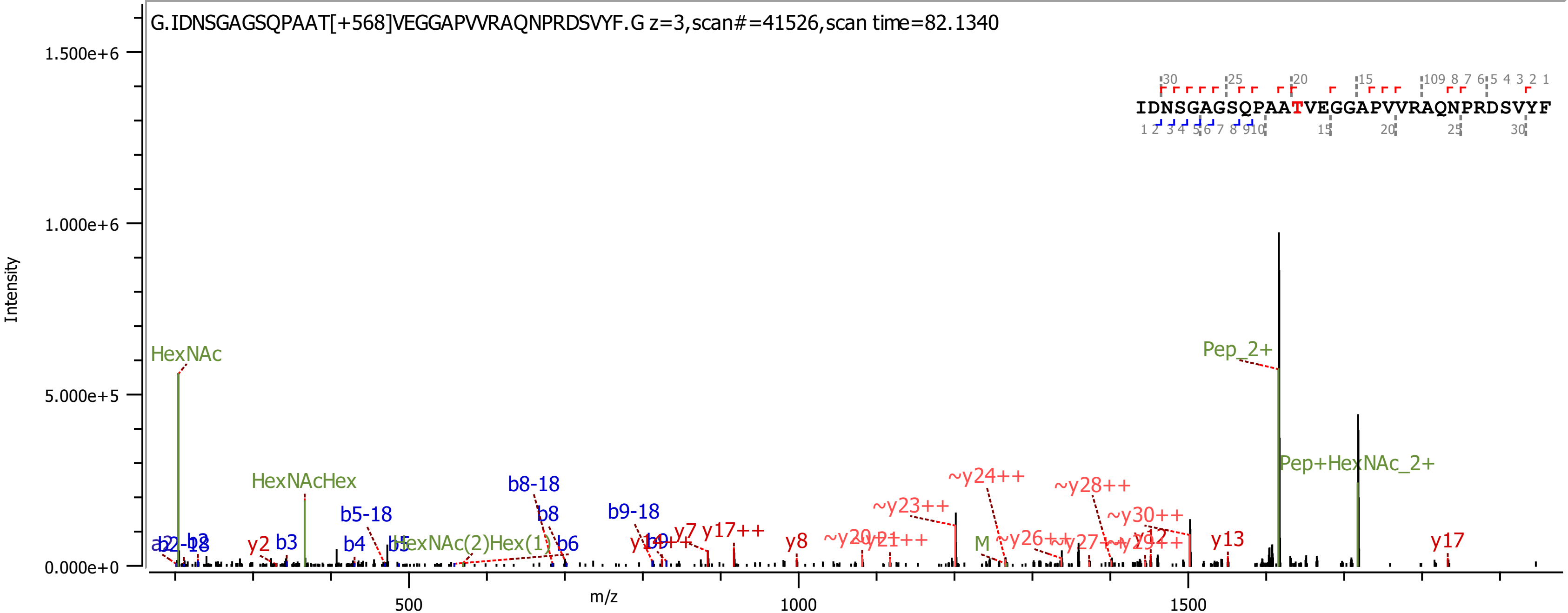
G.IDNSGAGSQPAAT[+568]VEGGAPVVRAQNPRDSVYF.G z=3,scan#=41526,scan time=82.1340
IDNSGAGSQPAATVEGGAPVVRAQNPRDSVYF
Intensity
m/z
1.500e+6
1.000e+6
5.000e+5
0.000e+0
500
1000
1500
HexNAc
HexNAcHex
Pep_2+
Pep+HexNAc_2+
b8-18
b5-18
b9-18
b8
b3
b4
b6
y2
y7
y17++
y8
y13
y17
~y24++
~y23++
~y28++
~y30++
M

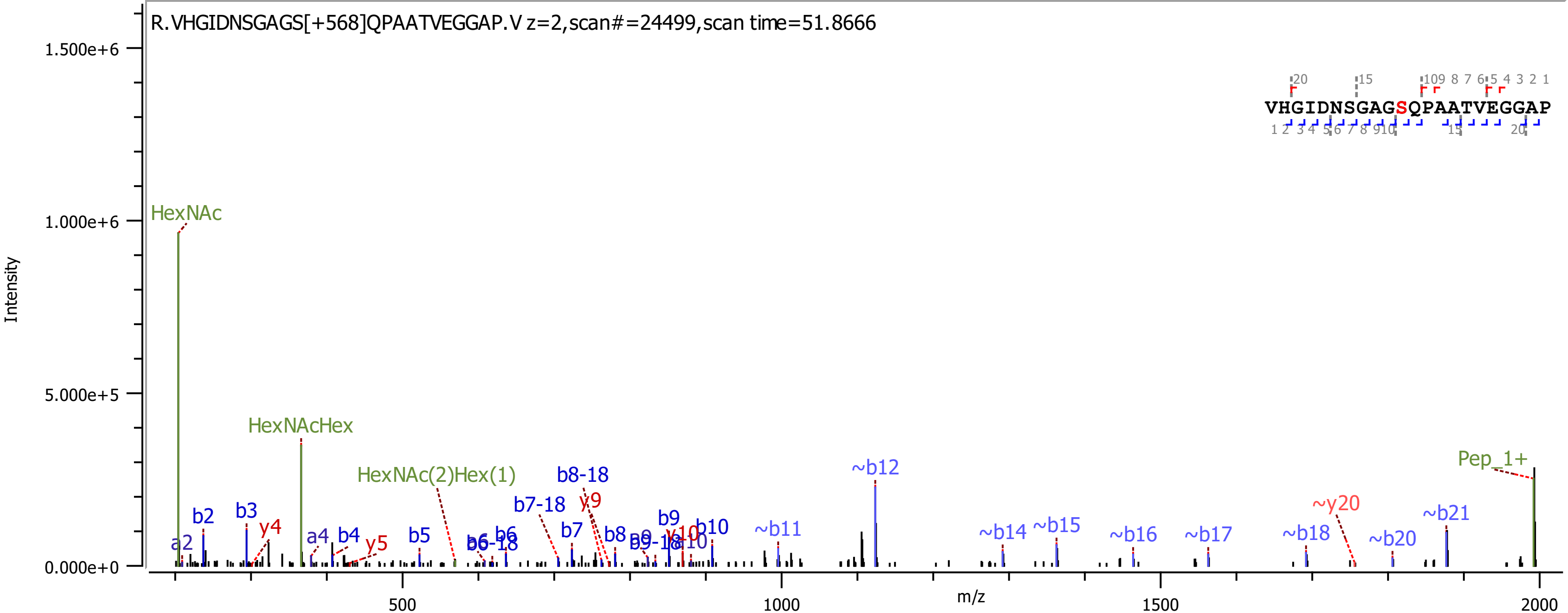
R.VHGIDNSGAGS[+568]QPAATVEGGAP.V z=2,scan#=24499,scan time=51.8666
VHGIDNSGAGSQPAATVEGGAP
Intensity
m/z
1.500e+6
1.000e+6
5.000e+5
0.000e+0
500
1000
1500
2000
HexNAc
HexNAcHex
HexNAc(2)Hex(1)
Pep_1+
a2
b2
b3
y4
a4
b4
y5
b5
b6-18
b6
b7-18
b7
b8-18
y9
b8
b9
b9-18
y10
b10
~b11
~b12
~b14
~b15
~b16
~b17
~b18
~y20
~b20
~b21

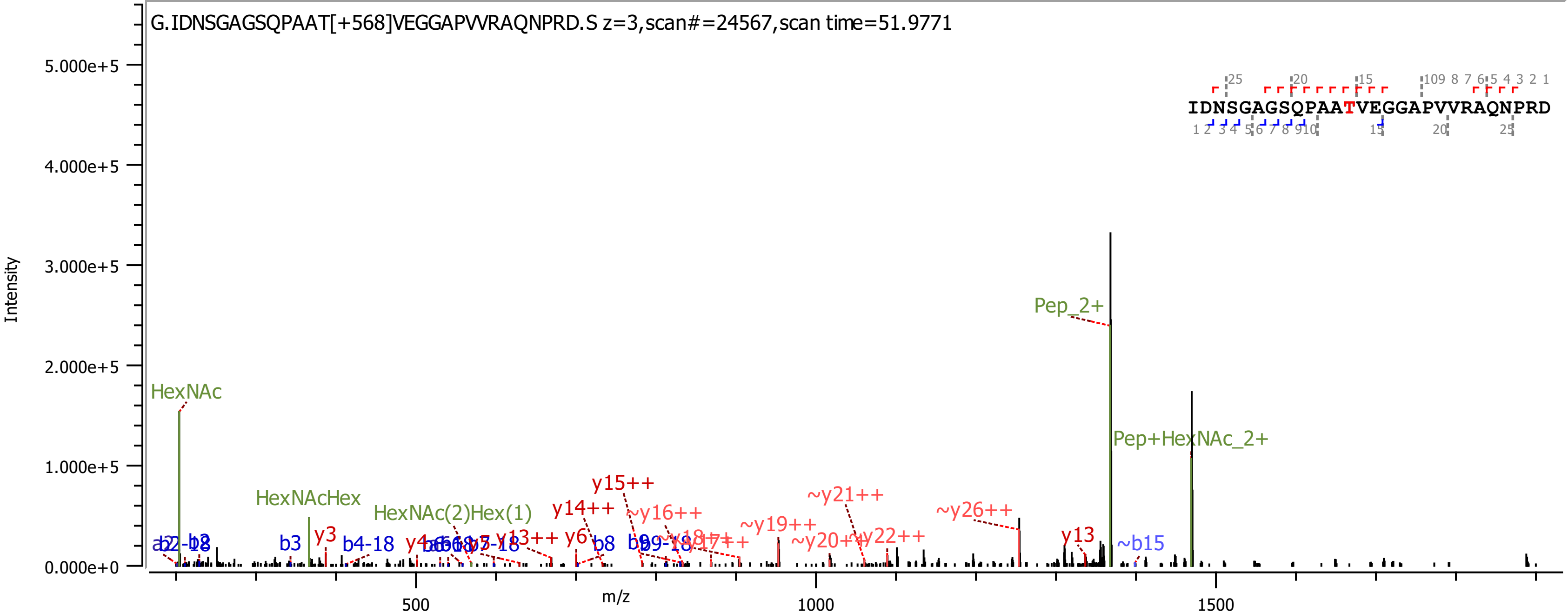
G.IDNSGAGSQPAAT[+568]VEGGAPVVRAQNPRD.S z=3,scan#=24567,scan time=51.9771
IDNSGAGSQPAATVEGGAPVVRAQNPRD
Intensity
m/z
0.000e+0
1.000e+5
2.000e+5
3.000e+5
4.000e+5
5.000e+5
500
1000
1500
HexNAc
HexNAcHex
HexNAc(2)Hex(1)
Pep_2+
Pep+HexNAc_2+
b3
y3
b4-18
y6
b8
y13
y13++
y14++
y15++
~y16++
~y19++
~y20++
~y21++
~y22++
~y26++
~b15

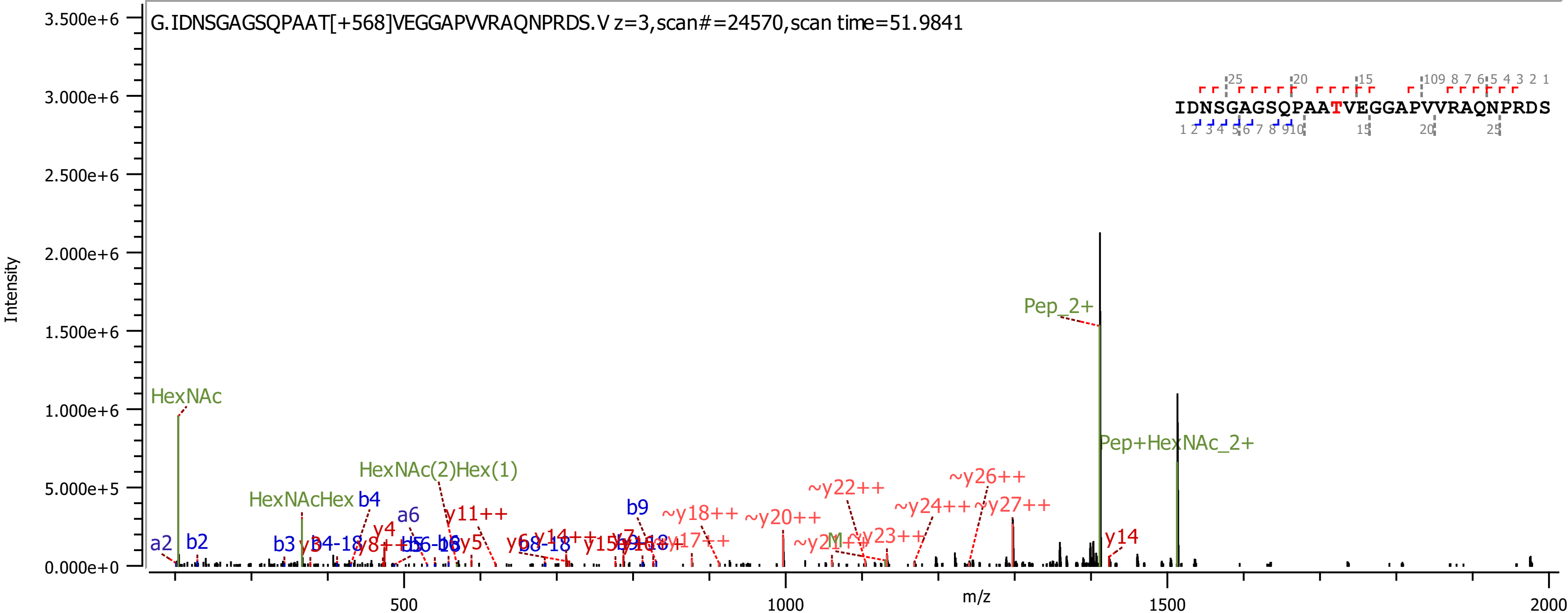
G.IDNSGAGSQPAAT[+568]VEGGAPVVRAQNPRDS.V z=3,scan#=24570,scan time=51.9841
IDNSGAGSQPAATVEGGAPVVRAQNPRDS
Intensity
m/z
HexNAc
HexNAcHex
HexNAc(2)Hex(1)
Pep_2+
Pep+HexNAc_2+
a2
b2
b3
b4
a6
b9
y4
y11++
y5
y14
~y18++
~y20++
~y22++
~y24++
~y26++
~y27++
~y23++

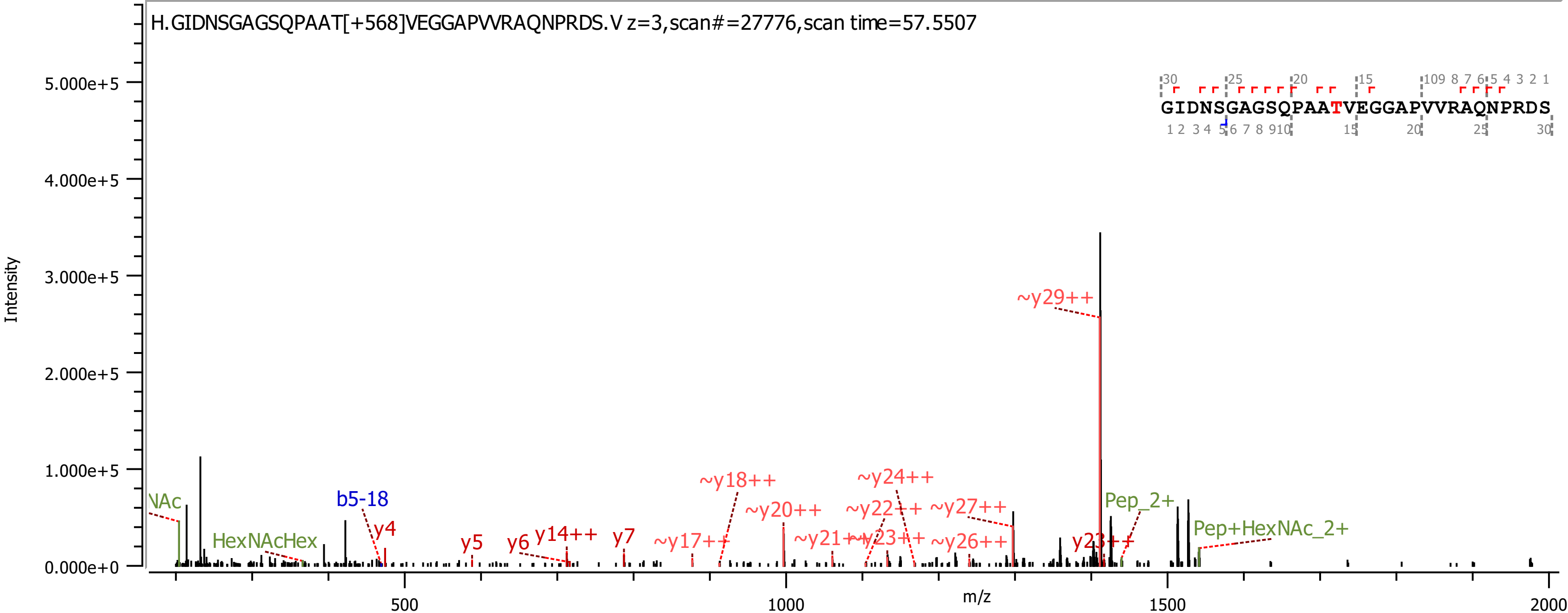
H.GIDNSGAGSQPAAT[+568]VEGGAPVVRAQNPRDS.V z=3,scan#=27776,scan time=57.5507
GIDNSGAGSQPAATVEGGAPVVRAQNPRDS
Intensity
m/z
5.000e+5
4.000e+5
3.000e+5
2.000e+5
1.000e+5
0.000e+0
500
1000
1500
2000
~y29++
NAc
HexNAcHex
b5-18
y4
y5
y6
y14++
y7
~y17++
~y18++
~y20++
~y21++
~y22++
~y23++
~y24++
~y26++
~y27++
y23++
Pep_2+
Pep+HexNAc_2+

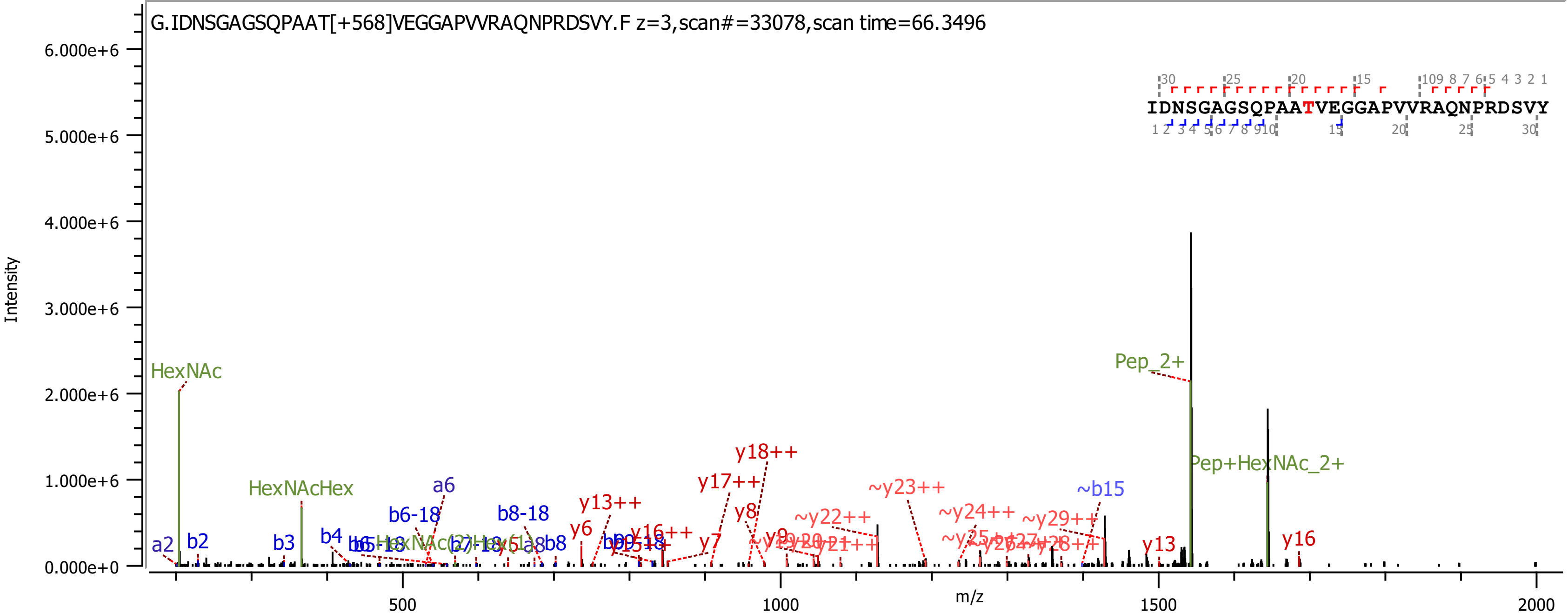
G.IDNSGAGSQPAAT[+568]VEGGAPVVRAQNPRDSVY.F z=3,scan#=33078,scan time=66.3496
IDNSGAGSQPAATVEGGAPVVRAQNPRDSVY
Intensity
m/z
HexNAc
HexNAcHex
Pep_2+
Pep+HexNAc_2+
~b15
y18++
y17++
y13++
~y23++
~y22++
~y24++
~y29++
y16
y13
y8
y7
y6
a6
b6-18
b8-18
a2
b2
b3
b4
b8

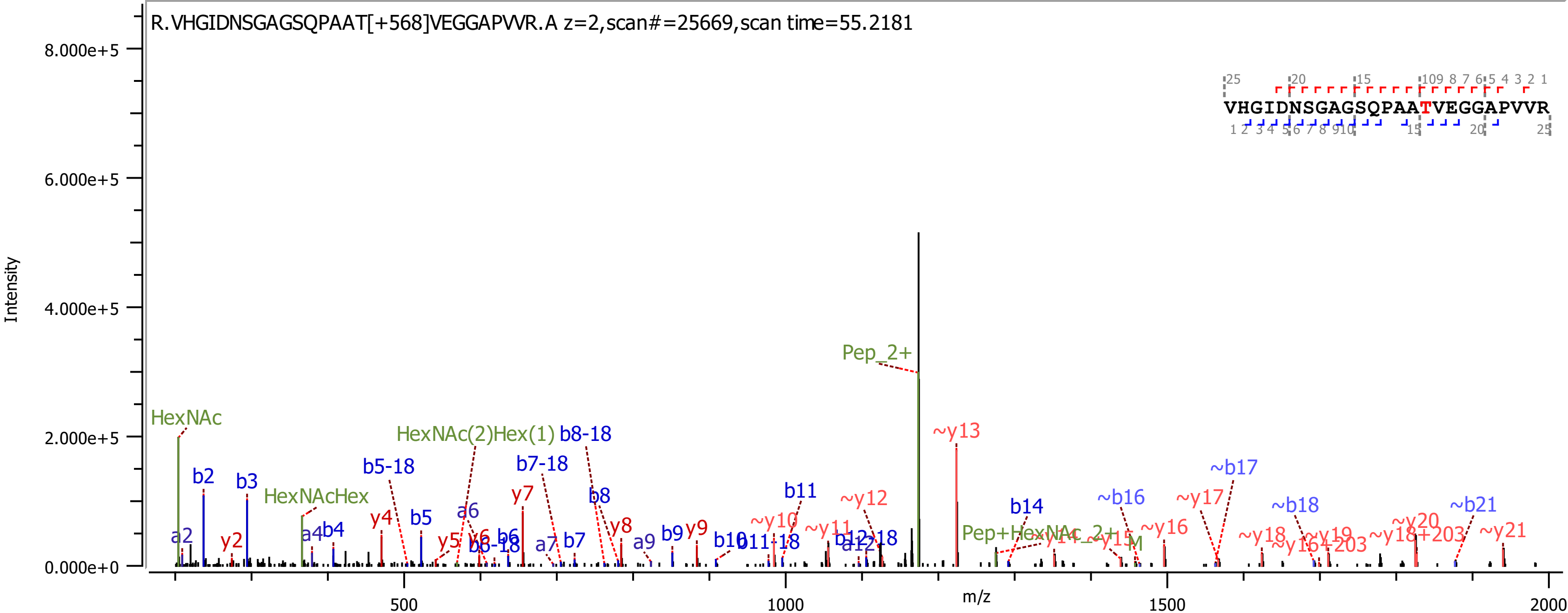

R.VHGIDNSGAGSQPAAT[+568]VEGGAPVVR.A z=2,scan#=25669,scan time=55.2181
Intensity
m/z
8.000e+5
6.000e+5
4.000e+5
2.000e+5
0.000e+0
500
1000
1500
2000
VHGIDNSGAGSQPAATVEGGAPVVR
Pep_2+
HexNAc
HexNAcHex
HexNAc(2)Hex(1)
Pep+HexNAc_2+
~y13
~b17
~b16
~y17
~y16
~b18
~b21
~y20
~y21
~y18
~y19
~y18+203
~y16+203
~y14
~y15
~y12
~y11
~y10
b11
b14
b12-18
a12
b11-18
b10
b9
y9
a9
y8
b8
b8-18
b7-18
b7
a7
b6
b6-18
y6
a6
y5
b5
b5-18
y4
y7
b4
a4
b3
b2
y2
a2

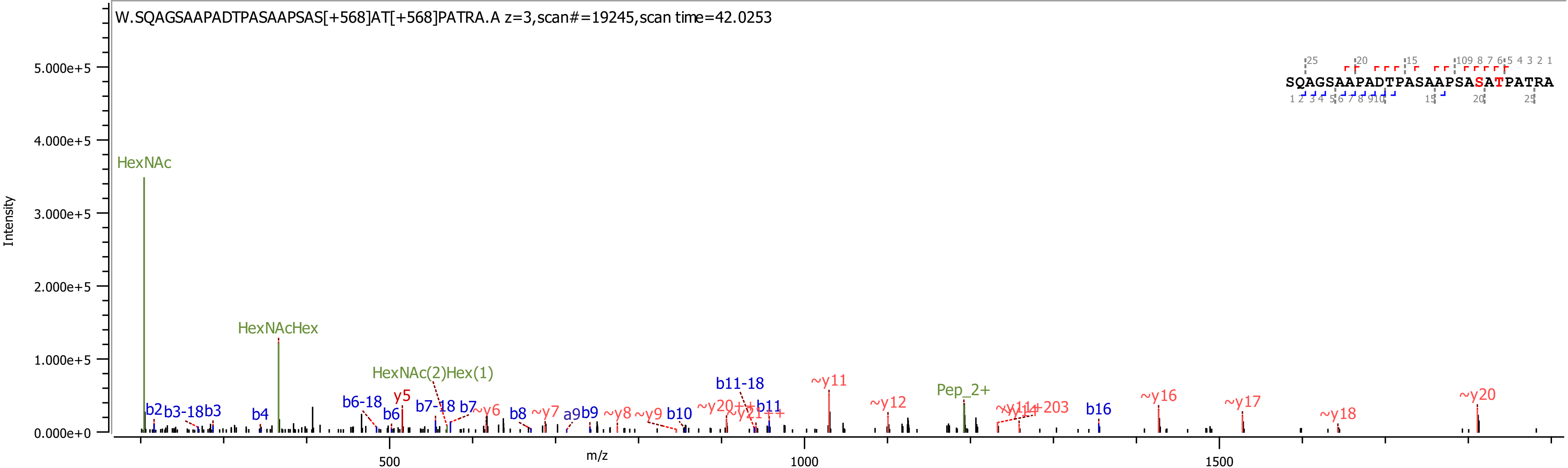

W.SQAGSAAPADTPASAAPSAS[+568]AT[+568]PATRA.A z=3,scan#=19245,scan time=42.0253
SQAGSAAPADTPASAAPSASATPATRA
Intensity
m/z
HexNAc
HexNAcHex
HexNAc(2)Hex(1)
Pep_2+
~y11
~y12
~y16
~y17
~y18
~y20
b11-18
b16

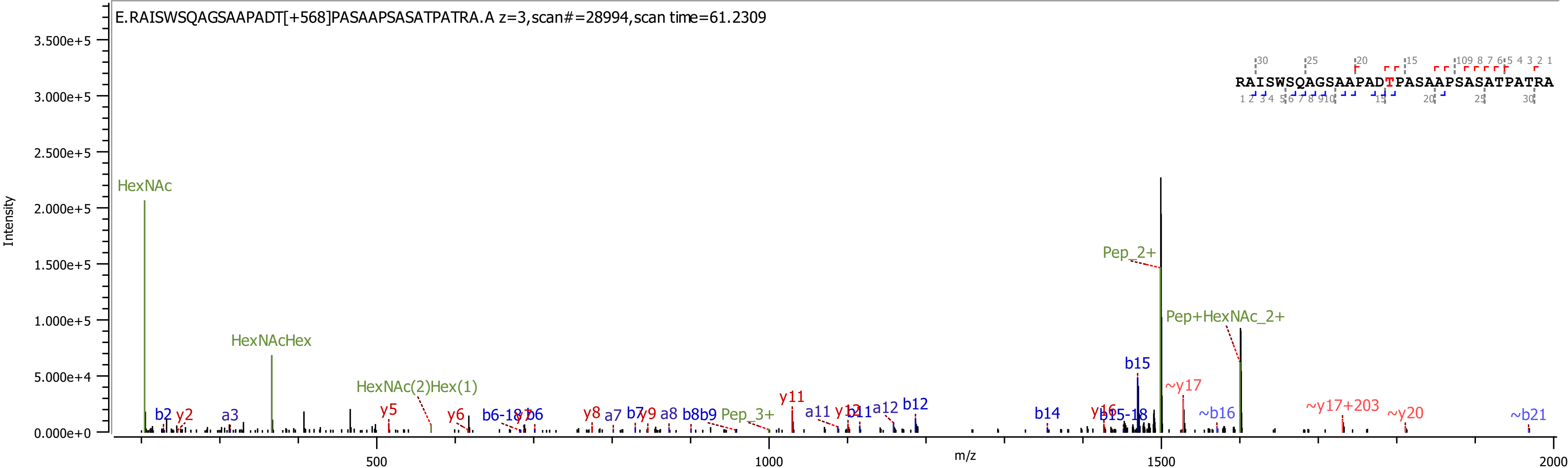

E.RAISWSQAGSAAPADT[+568]PASAAPSASATPATRA.A z=3,scan#=28994,scan time=61.2309
RAISWSQAGSAAPADTPASAAPSASATPATRA
Intensity
m/z
HexNAc
HexNAcHex
HexNAc(2)Hex(1)
Pep_2+
Pep+HexNAc_2+
Pep_3+
b15
~y17
~y17+203
~y20
~b16
~b21
b14
y16
b15-18
b12
a12
b11
y12
a11
y11
b8b9
a8
b7y9
a7
y8
b6-18
y7b6
y6
y5
a3
b2
y2

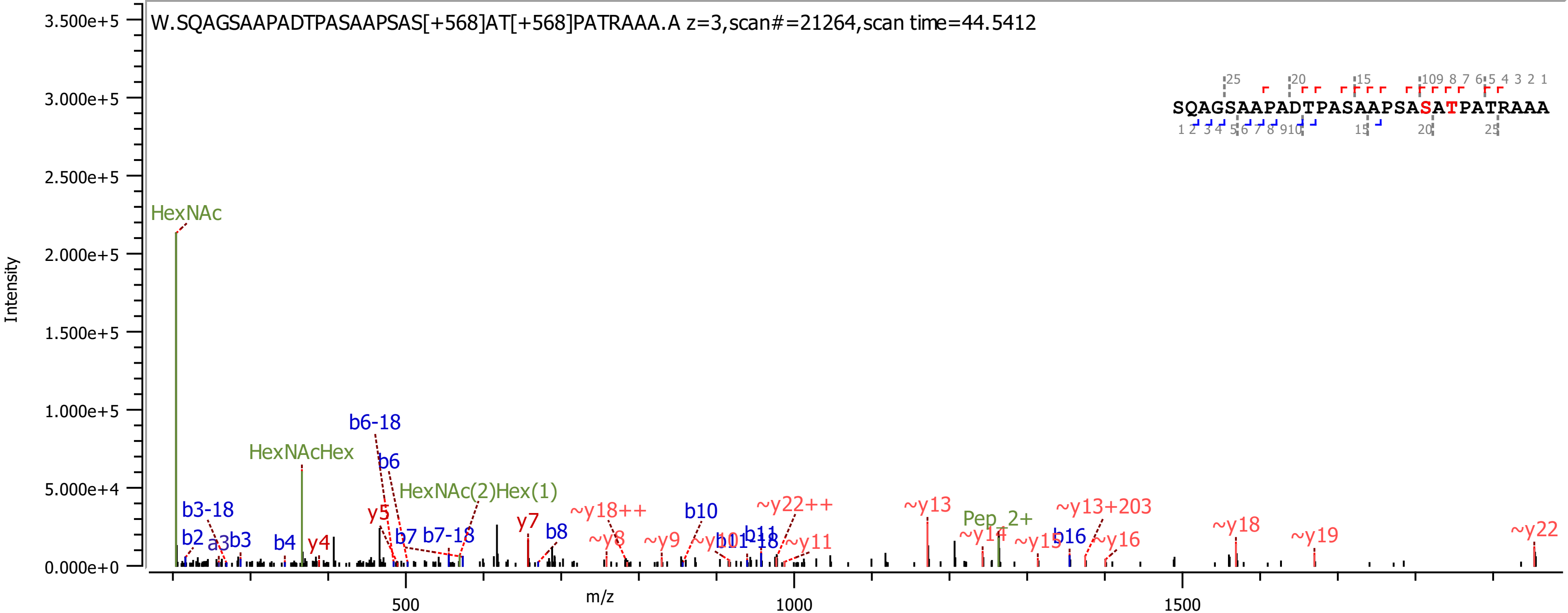

W.SQAGSAAPADTPASAAPSAS[+568]AT[+568]PATRAAA.A z=3,scan#=21264,scan time=44.5412
SQAGSAAPADTPASAAPSASATPATRAAA
Intensity
m/z
HexNAc
HexNAcHex
HexNAc(2)Hex(1)
b6-18
b6
b3-18
b2
a3
b3
b4
y4
y5
b7
b7-18
y7
b8
~y18++
~y8
~y9
b10
~y10
b11-18
b11
~y22++
~y11
~y13
Pep_2+
~y14
~y15
b16
~y13+203
~y16
~y18
~y19
~y22

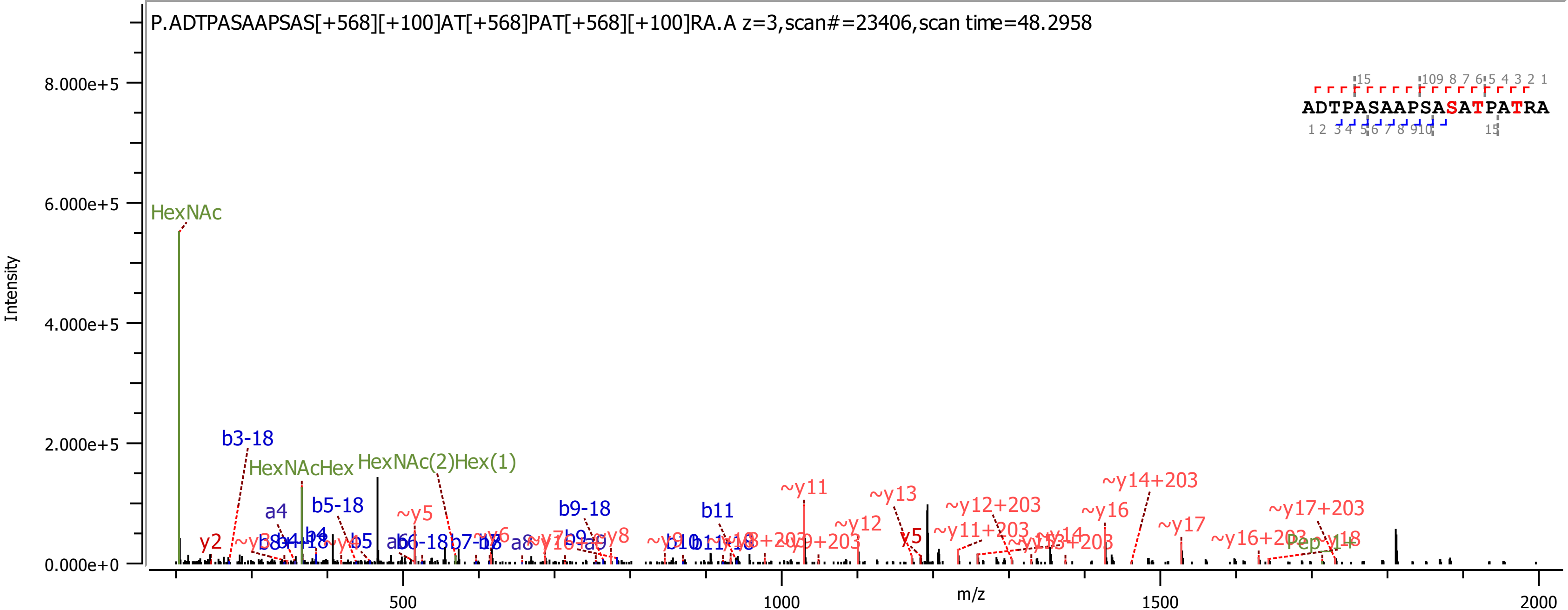
P.ADTPASAAPSAS[+568][+100]AT[+568]PAT[+568][+100]RA.A z=3,scan#=23406,scan time=48.2958
ADTPASAAPSASATPATRA
Intensity
m/z
HexNAc
HexNAcHex
HexNAc(2)Hex(1)
b3-18
a4
b5-18
~y5
b9-18
b11
~y11
~y13
~y12+203
~y14+203
~y16
~y12
~y17
~y11+203
~y17+203
~y16+203
y2
y5
Pep-18
0.000e+0
2.000e+5
4.000e+5
6.000e+5
8.000e+5
500
1000
1500
2000

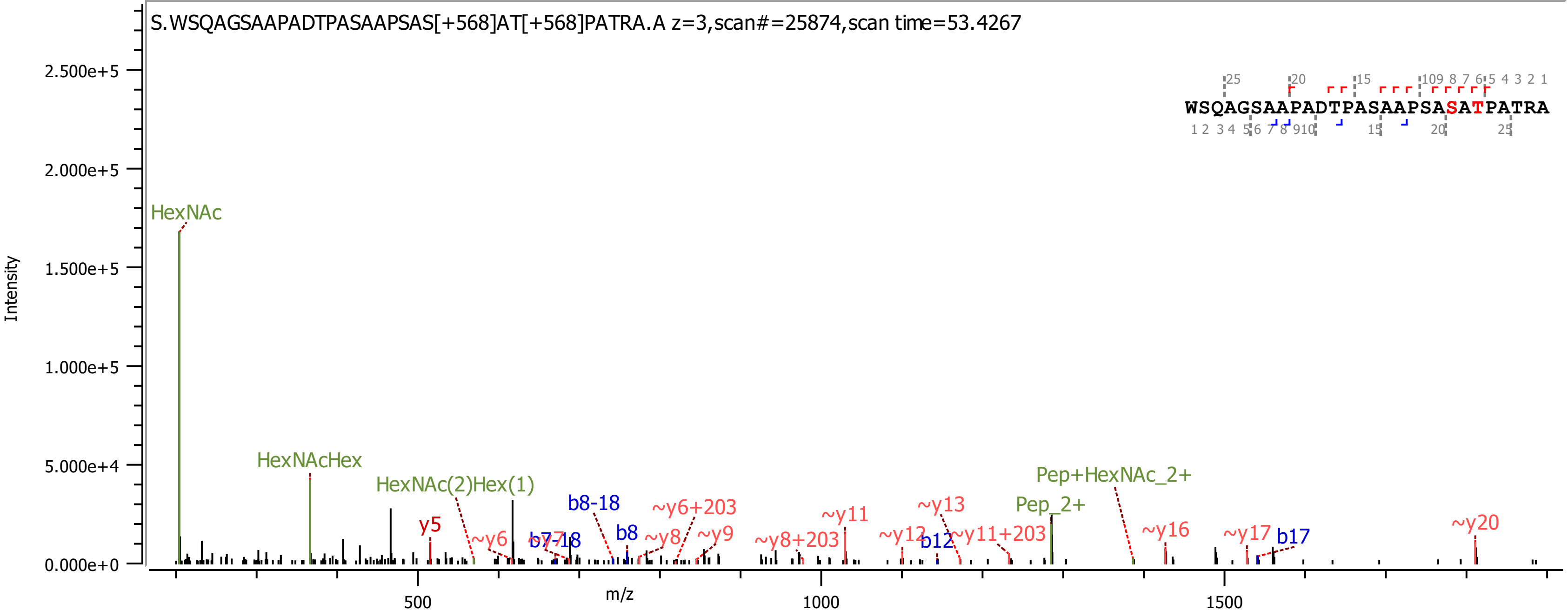

S.WSQAGSAAPADTPASAAPSAS[+568]AT[+568]PATRA.A z=3,scan#=25874,scan time=53.4267
WSQAGSAAPADTPASAAPSASATPATRA
Intensity
m/z
HexNAc
HexNAcHex
HexNAc(2)Hex(1)
y5
~y6
b7-18
b8-18
b8
~y6+203
~y8
~y9
~y8+203
~y11
~y12
b12
~y13
~y11+203
Pep_2+
Pep+HexNAc_2+
~y16
~y17
b17
~y20

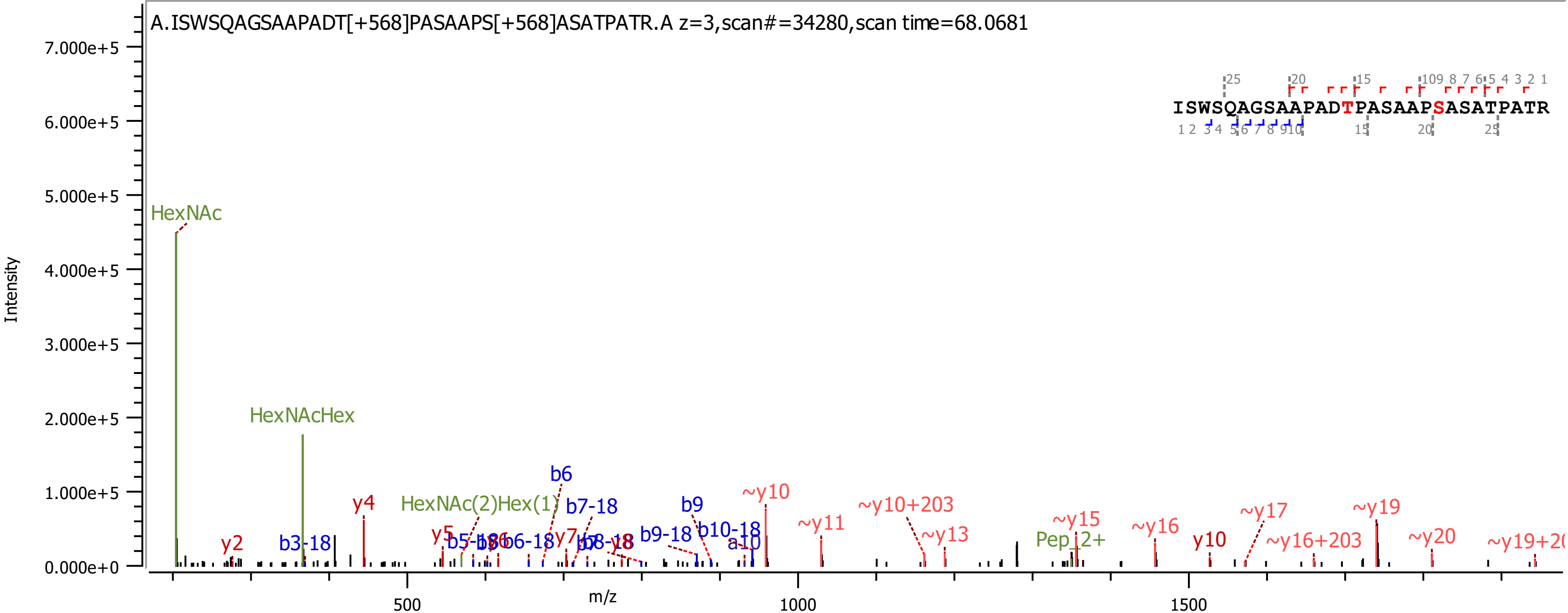
A.ISWSQAGSAAPADT[+568]PASAAPS[+568]ASATPATR.A z=3,scan#=34280,scan time=68.0681
ISWSQAGSAAPADTPASAAPSASATPATR
Intensity
m/z
HexNAc
HexNAcHex
HexNAc(2)Hex(1)
Pep_2+
y2
y4
y5
y7
y8
y10
b3-18
b5-18
b6
b6-18
b7-18
b8-18
b9
b9-18
b10-18
a10
~y10
~y11
~y10+203
~y13
~y15
~y16
~y17
~y16+203
~y19
~y20
0.000e+0
1.000e+5
2.000e+5
3.000e+5
4.000e+5
5.000e+5
6.000e+5
7.000e+5
500
1000
1500

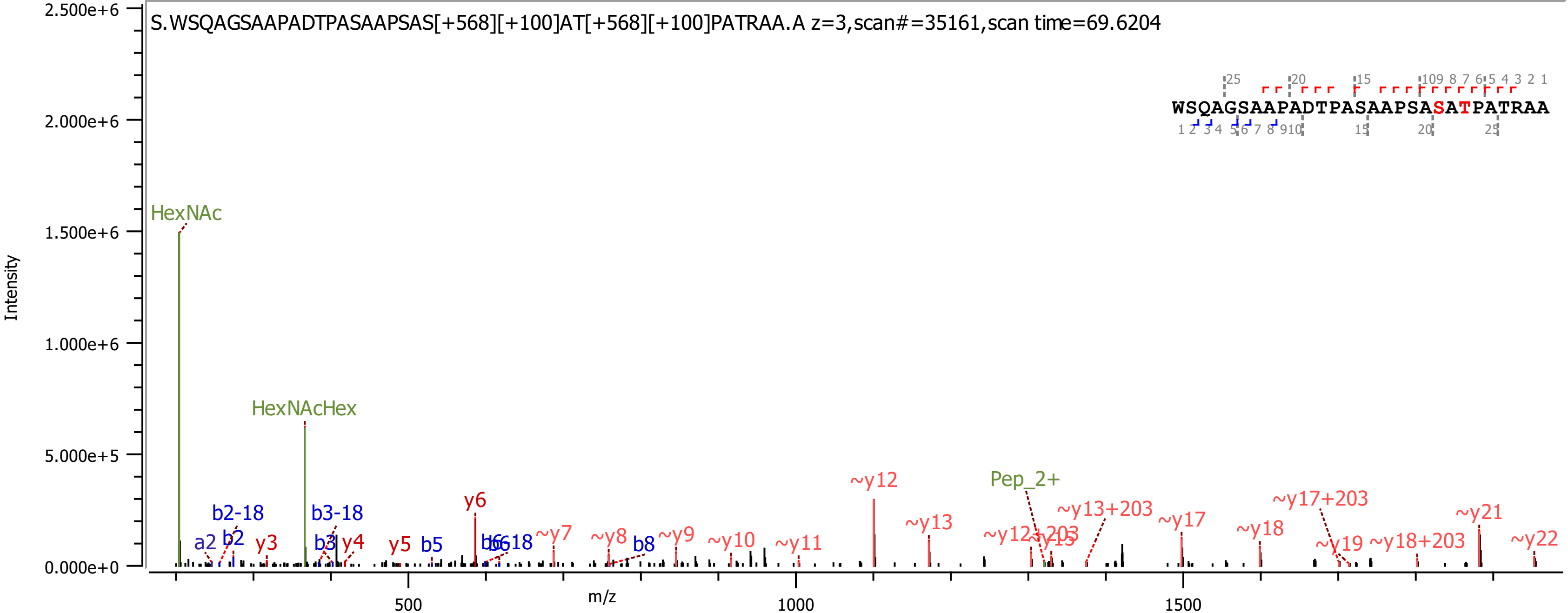
S.WSQAGSAAPADTPASAAPSAS[+568][+100]AT[+568][+100]PATRAA.A z=3,scan#=35161,scan time=69.6204
WSQAGSAAPADTPASAAPSASATPATRAA
Intensity
m/z
HexNAc
HexNAcHex
Pep_2+
a2
b2
b2-18
y3
b3-18
b3
y4
y5
b5
y6
b6
b6-18
~y7
~y8
b8
~y9
~y10
~y11
~y12
~y13
~y12+203
~y15
~y13+203
~y17
~y18
~y17+203
~y19
~y18+203
~y21
~y22
0.000e+0
5.000e+5
1.000e+6
1.500e+6
2.000e+6
2.500e+6
500
1000
1500

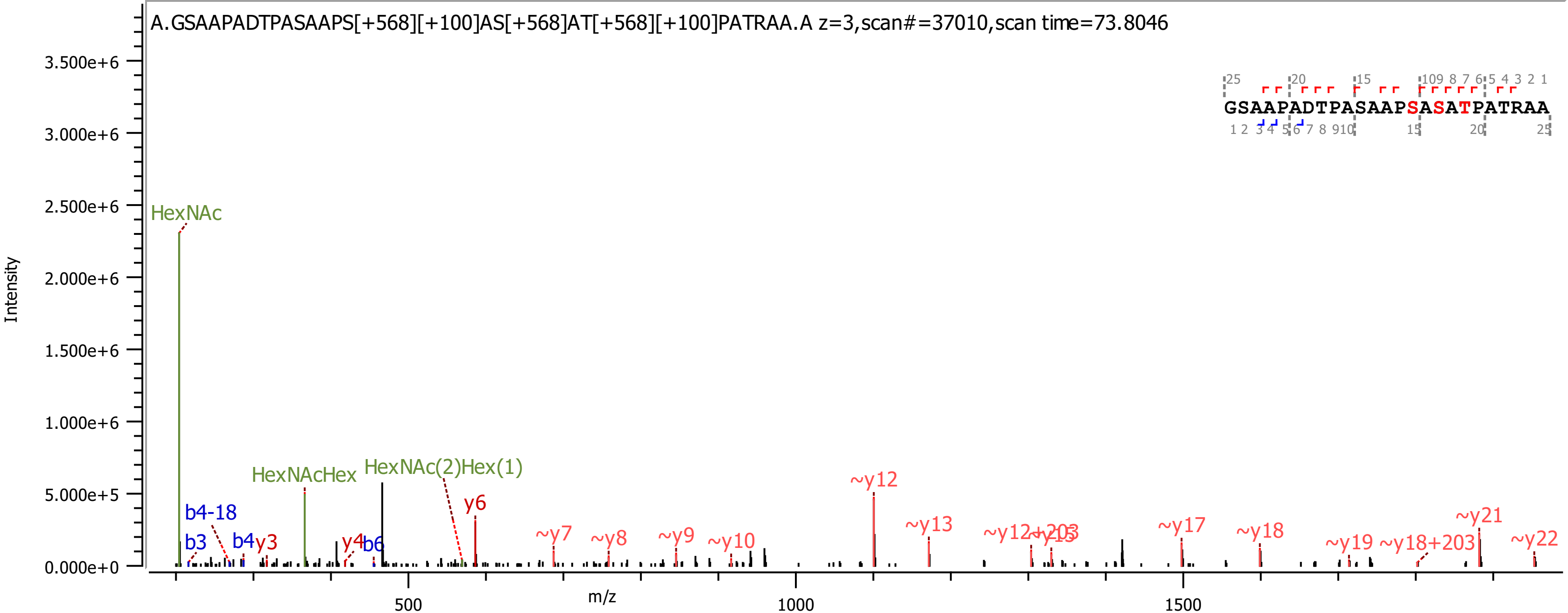
A.GSAAPADTPASAAPS[+568][+100]AS[+568]AT[+568][+100]PATRAA.A z=3,scan#=37010,scan time=73.8046
Intensity
m/z
HexNAc
HexNAcHex
HexNAc(2)Hex(1)
b4-18
b3
b4
y3
y4
b6
y6
~y7
~y8
~y9
~y10
~y12
~y13
~y12+203
~y15
~y17
~y18
~y19
~y18+203
~y21
~y22
GSAAPADTPASAAPSASATPATRAA

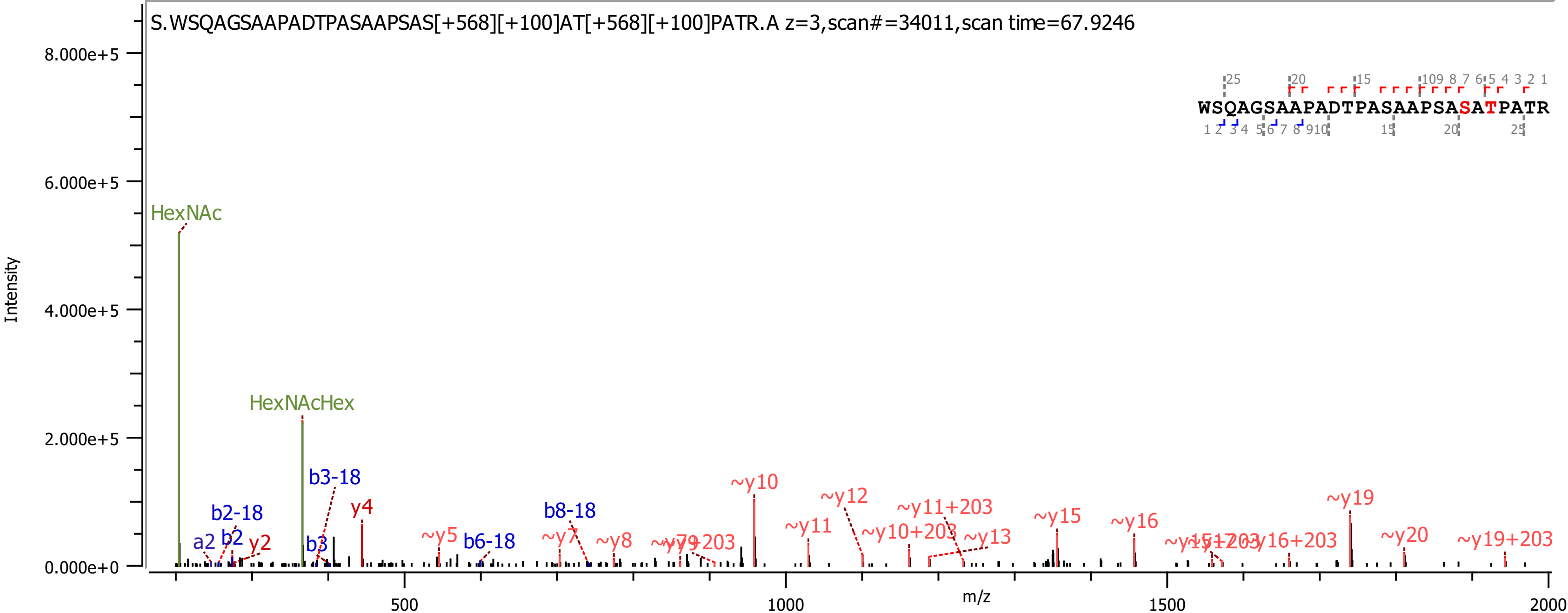
S.WSQAGSAAPADTPASAAPSAS[+568][+100]AT[+568][+100]PATR.A z=3,scan#=34011,scan time=67.9246
WSQAGSAAPADTPASAAPSASATPATR
Intensity
m/z
HexNAc
HexNAcHex
b3-18
b2-18
a2
b2
y2
b3
y4
~y5
b6-18
b8-18
~y7
~y8
~y10
~y11
~y12
~y11+203
~y10+203
~y13
~y15
~y16
~y16+203
~y19
~y20
~y19+203
0.000e+0
2.000e+5
4.000e+5
6.000e+5
8.000e+5
500
1000
1500
2000

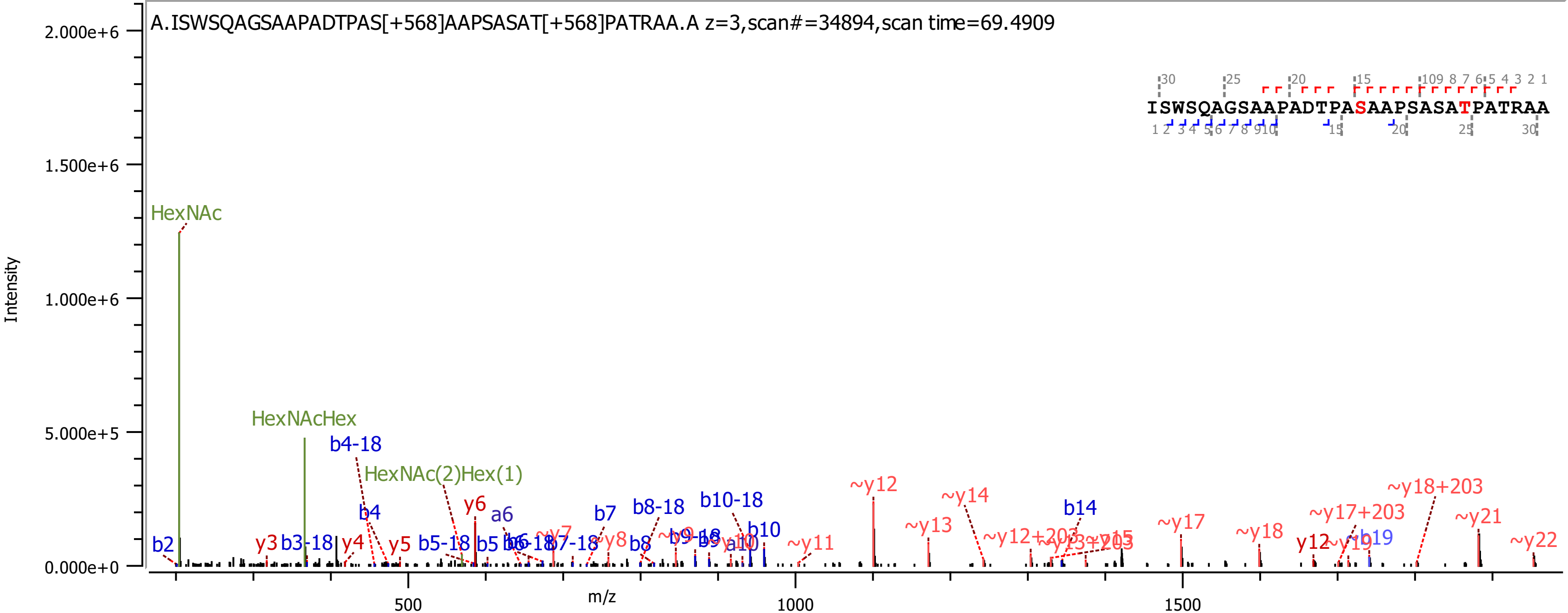

A.ISWSQAGSAAPADTPAS[+568]AAPSASAT[+568]PATRAA.A z=3,scan#=34894,scan time=69.4909
ISWSQAGSAAPADTPASAAPSASATPATRAA
Intensity
m/z
2.000e+6
1.500e+6
1.000e+6
5.000e+5
0.000e+0
500
1000
1500
HexNAc
HexNAcHex
HexNAc(2)Hex(1)
b4-18
b2
y3
b3-18
y4
b4
y5
b5-18
b5
y6
a6
b6-18
b7-18
~y7
b7
~y8
b8
b8-18
~y9
b10-18
b10
a10
~y10
~y11
~y12
~y13
~y14
~y12+203
~y15
b14
~y17
~y18
y12
~y17+203
~y19
b19
~y18+203
~y21
~y22

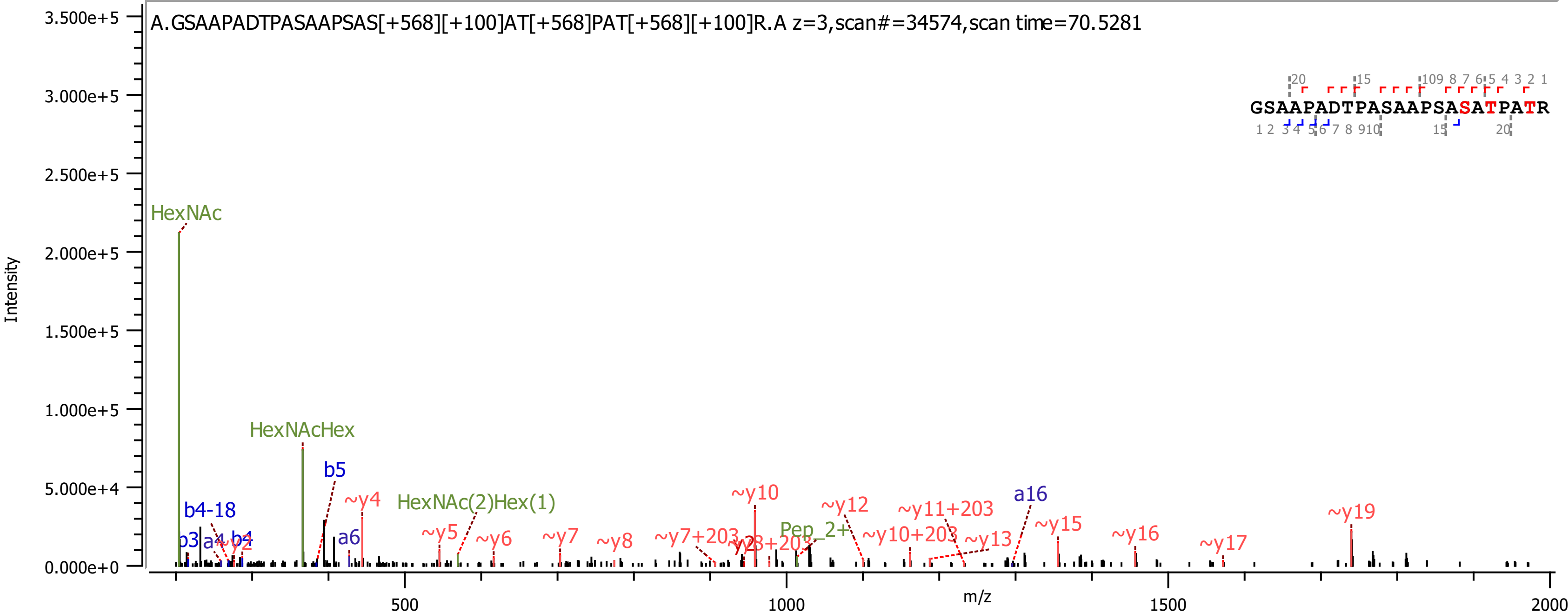

A.GSAAPADTPASAAPSAS[+568][+100]AT[+568]PAT[+568][+100]R.A z=3,scan#=34574,scan time=70.5281
GSAAPADTPASAAPSASATPATR
Intensity
m/z
HexNAc
HexNAcHex
HexNAc(2)Hex(1)
Pep_2+
b5
b4-18
a6
a16
~y4
~y5
~y6
~y7
~y8
~y7+203
~y10
~y12
~y10+203
~y11+203
~y13
~y15
~y16
~y17
~y19

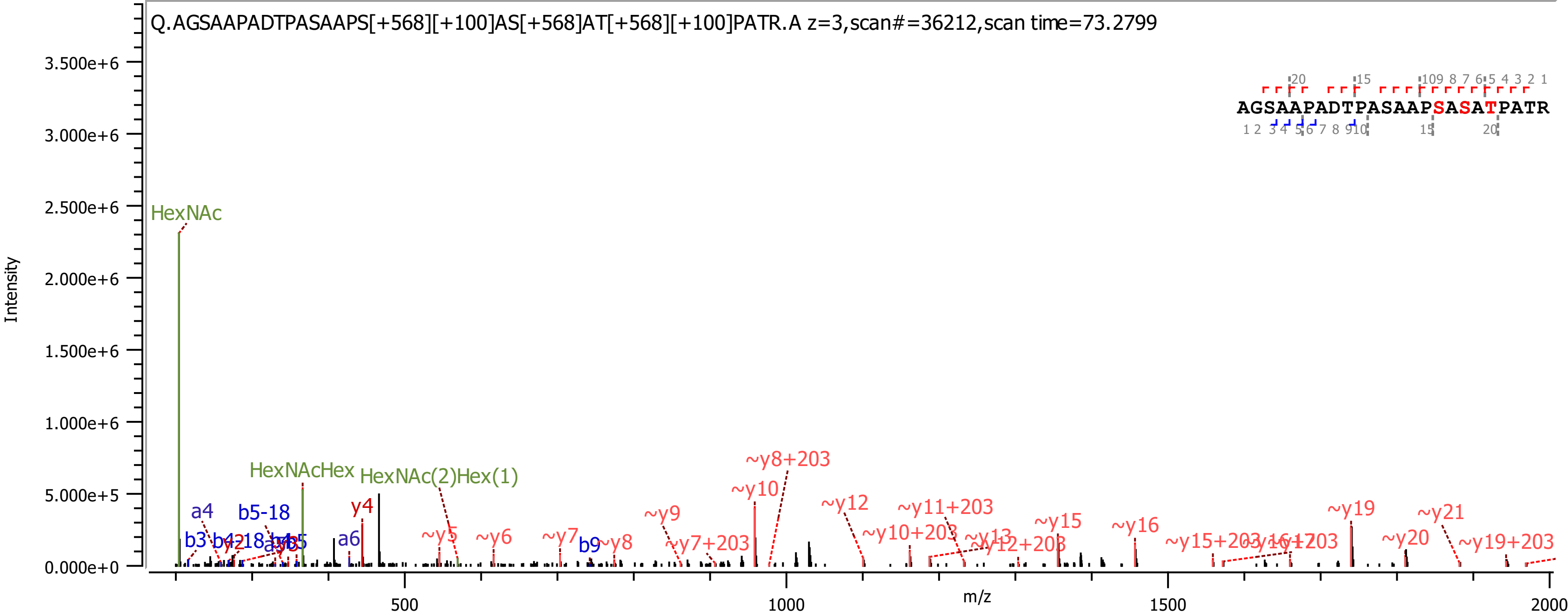

Q.AGSAAPADTPASAAPS[+568][+100]AS[+568]AT[+568][+100]PATR.A z=3,scan#=36212,scan time=73.2799
AGSAAPADTPASAAPSASATPATR
Intensity
m/z
HexNAc
HexNAcHex
HexNAc(2)Hex(1)
a4
b3
b5-18
a6
y4
b9
~y5
~y6
~y7
~y8
~y9
~y10
~y8+203
~y7+203
~y12
~y11+203
~y10+203
~y13
~y12+203
~y15
~y16
~y15+203
~y19
~y20
~y21
~y19+203
3.500e+6
3.000e+6
2.500e+6
2.000e+6
1.500e+6
1.000e+6
5.000e+5
0.000e+0
500
1000
1500
2000

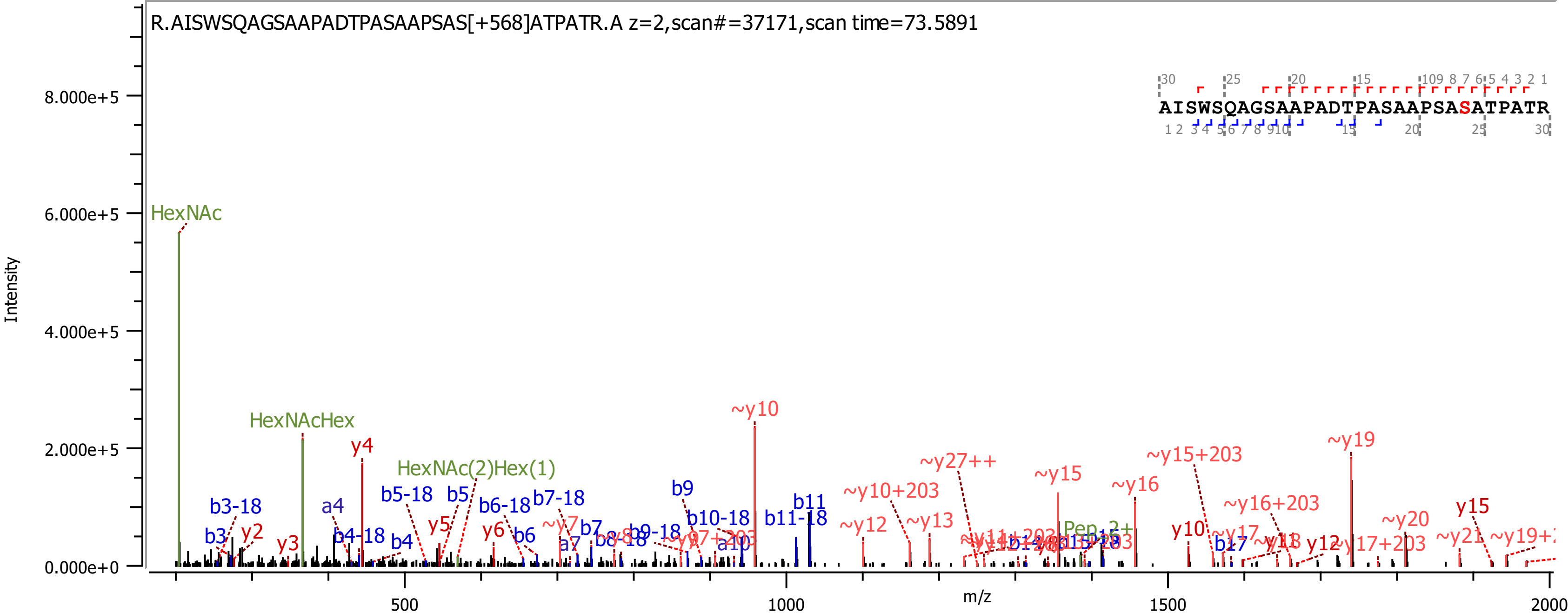

R.AISWSQAGSAAPADTPASAAPSAS[+568]ATPATR.A z=2,scan#=37171,scan time=73.5891
AISWSQAGSAAPADTPASAAPSASATPATR
Intensity
8.000e+5
6.000e+5
4.000e+5
2.000e+5
0.000e+0
m/z
500
1000
1500
2000
HexNAc
HexNAcHex
HexNAc(2)Hex(1)
y4
~y10
~y19
~y27++
~y15
~y15+203
~y16
~y10+203
~y16+203
y15
~y20
~y12
~y13
y10
~y17
~y21
b3-18
b3
y2
y3
a4
b4-18
b4
b5-18
b5
y5
b6-18
b6
y6
b7-18
b7
a7
b8-18
b9
b9-18
b10-18
a10
b11
b11-18
b17
y12
Pep 2+

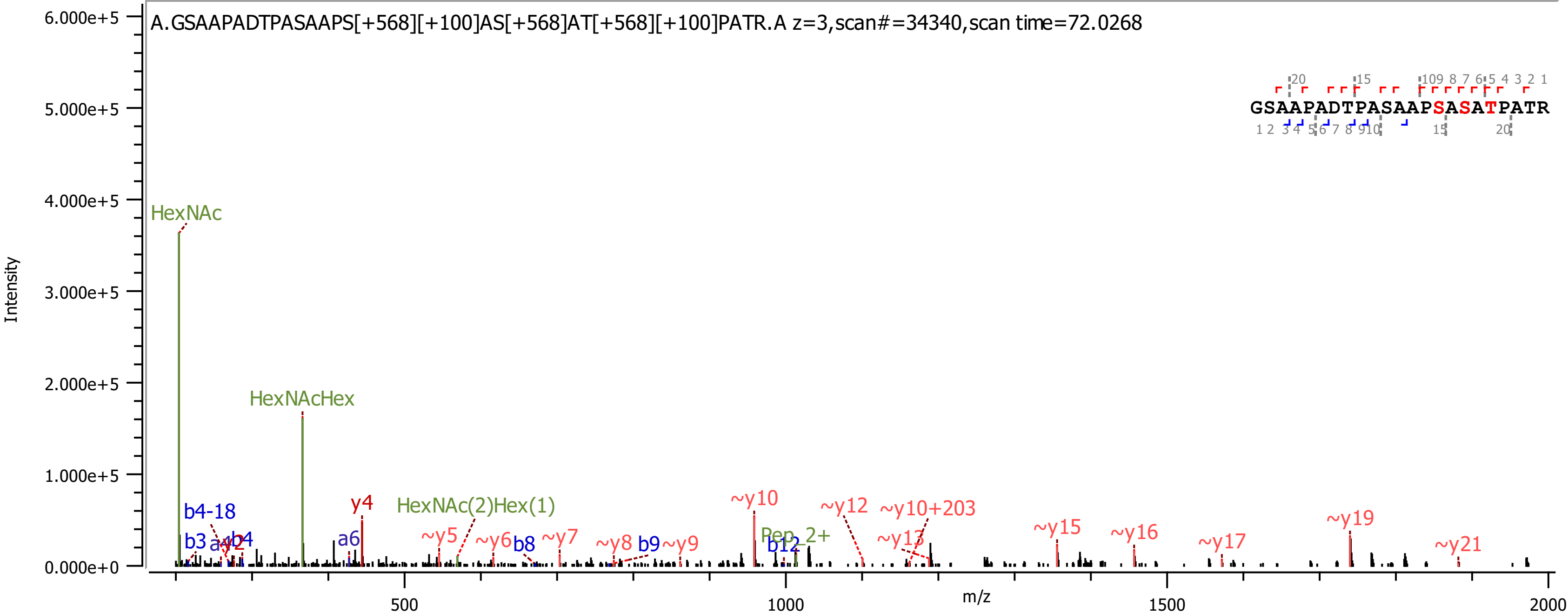

A.GSAAPADTPASAAPS[+568][+100]AS[+568]AT[+568][+100]PATR.A z=3,scan#=34340,scan time=72.0268
GSAAPADTPASAAPSASATPATR
Intensity
m/z
HexNAc
HexNAcHex
HexNAc(2)Hex(1)
Pep_2+
b4-18
b3
b4
a6
b8
b9
b12
y2
y4
~y5
~y6
~y7
~y8
~y9
~y10
~y12
~y10+203
~y13
~y15
~y16
~y17
~y19
~y21

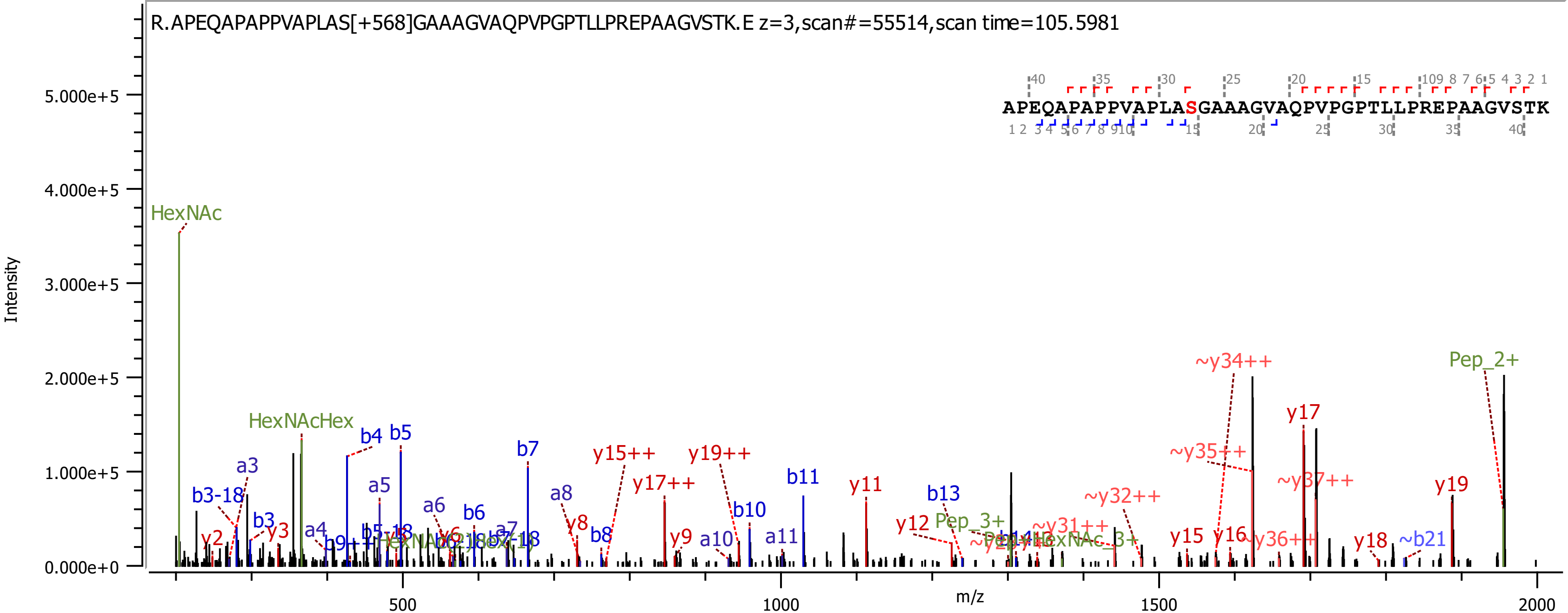
R.APEQAPAPPVAPLAS[+568]GAAAGVAQPVPGPTLLPREPAAGVSTK.E z=3,scan#=55514,scan time=105.5981
APEQAPAPPVAPLASGAAAGVAQPVPGPTLLPREPAAGVSTK
Intensity
m/z
5.000e+5
4.000e+5
3.000e+5
2.000e+5
1.000e+5
0.000e+0
500
1000
1500
2000
HexNAc
HexNAcHex
b4
b5
b7
a3
a5
b3-18
b3
y2
y3
a4
a6
b6
a8
y8
b8
y15++
y19++
y17++
y9
a10
b10
b11
a11
y11
b13
y12
Pep_3+
~y31++
~y32++
~y34++
~y35++
y15
y16
~y36++
y17
~y37++
y18
~b21
y19
Pep_2+

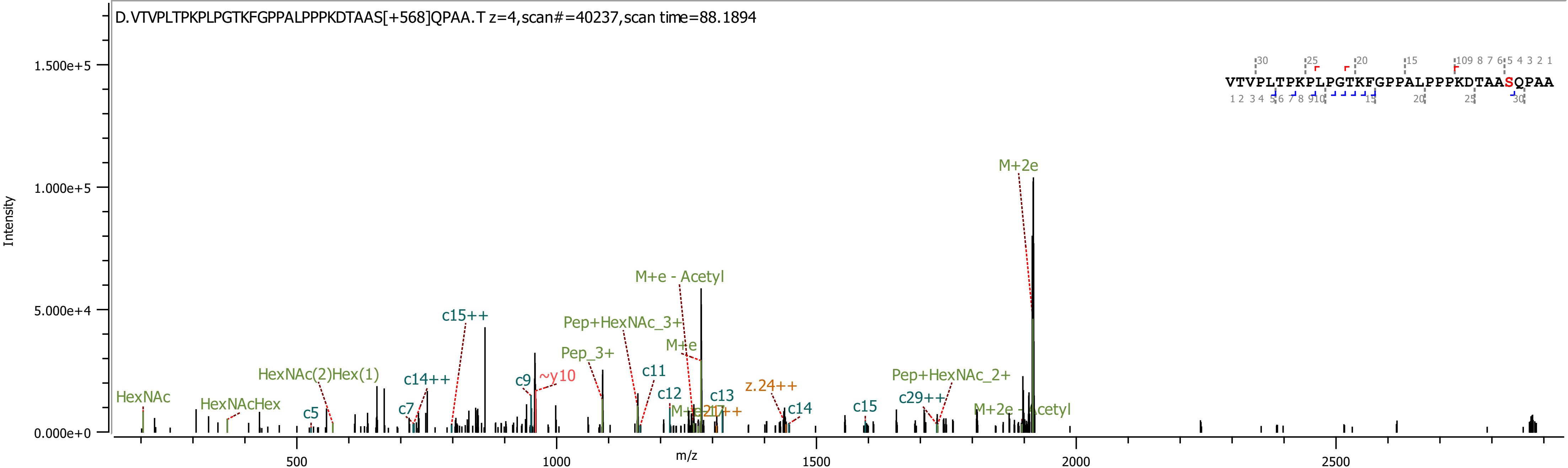

D.VTVPLTPKPLPGTKFGPPALPPPKDTAAS[+568]QPAA.T z=4,scan#=40237,scan time=88.1894
VTVPLTPKPLPGTKFGPPALPPPKDTAASQPAA
Intensity
m/z
M+2e
M+e - Acetyl
Pep+HexNAc_3+
M+e
Pep_3+
c15++
c14++
c11
c12
c13
z.24++
c14
c15
c29++
Pep+HexNAc_2+
M+2e - Acetyl
HexNAc(2)Hex(1)
HexNAc
HexNAcHex
c5
c7
c9
~y10

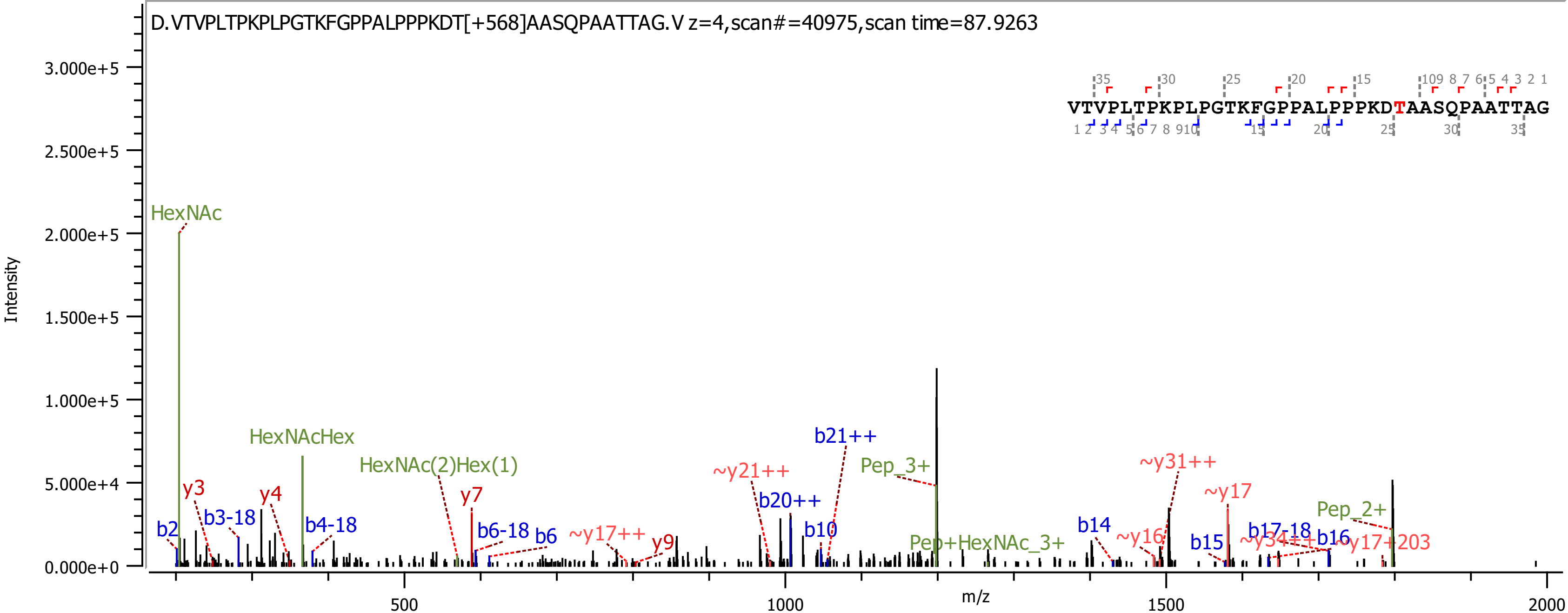
D.VTVPLTPKPLPGTKFGPPALPPPKDT[+568]AASQPAATTAG.V z=4,scan#=40975,scan time=87.9263
VTVPLTPKPLPGTKFGPPALPPPKDTAASQPAATTAG
Intensity
3.000e+5
2.500e+5
2.000e+5
1.500e+5
1.000e+5
5.000e+4
0.000e+0
m/z
500
1000
1500
2000
HexNAc
HexNAcHex
HexNAc(2)Hex(1)
y3
y4
y7
y9
b2
b3-18
b4-18
b6-18
b6
~y17++
~y21++
b20++
b21++
b10
Pep_3+
Pep+HexNAc_3+
b14
~y16
~y31++
~y17
b15
~y34++
b17-18
b16
~y17+203
Pep_2+

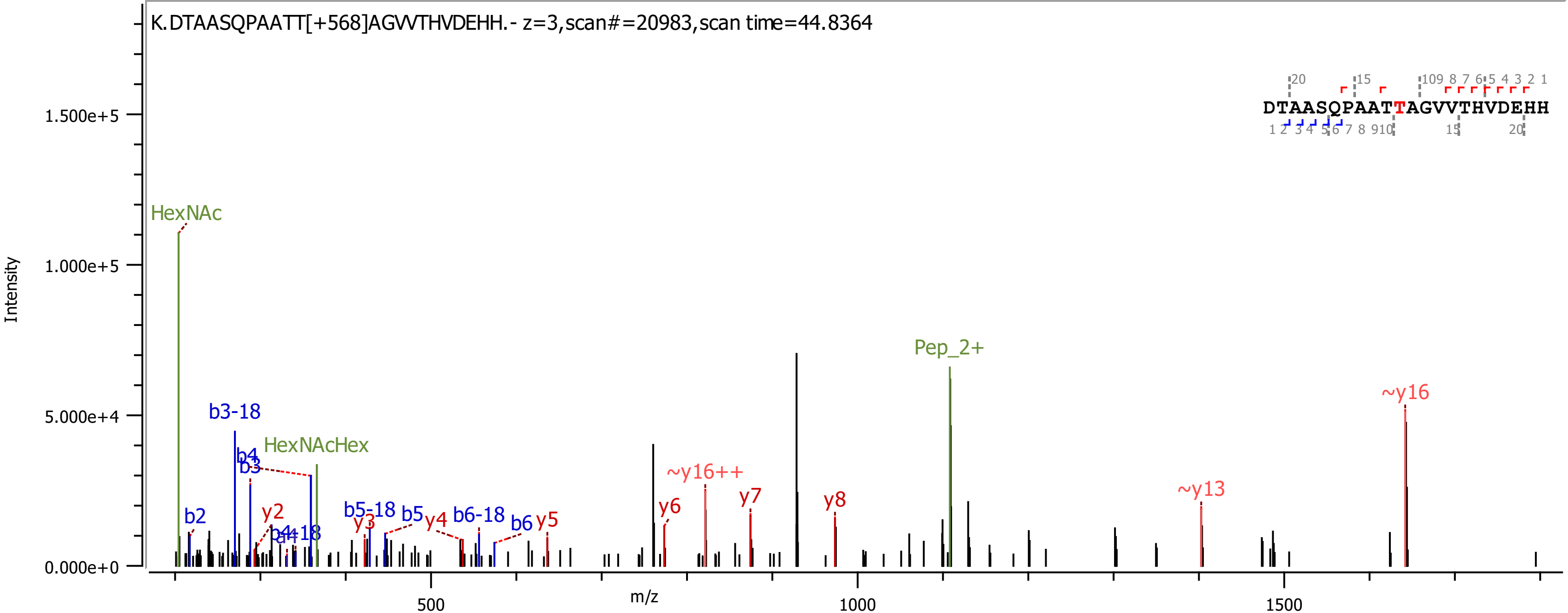
K.DTAASQPAATT[+568]AGVVTHVDEHH.- z=3,scan#=20983,scan time=44.8364
DTAASQPAATTAGVVTHVDEHH
Intensity
m/z
1.500e+5
1.000e+5
5.000e+4
0.000e+0
500
1000
1500
HexNAc
HexNAcHex
Pep_2+
b2
b3-18
b4
b3
y2
b4-18
b5-18
y3
b5
y4
b6-18
b6
y5
~y16++
y6
y7
y8
~y13
~y16

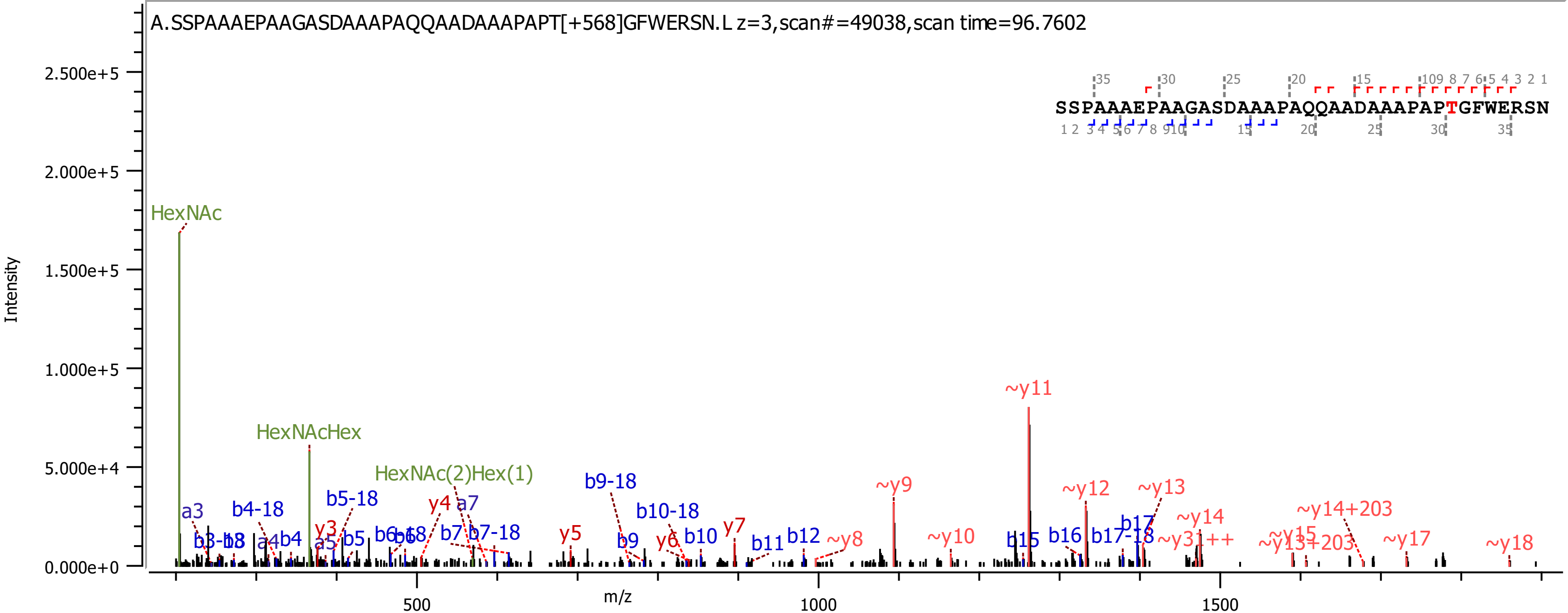
A.SSPAAAEPAAGASDAAAPAQQAADAAAPAPT[+568]GFWERSN.L z=3,scan#=49038,scan time=96.7602
SSPAAAEPAAGASDAAAPAQQAADAAAPAPTGFWERSN
Intensity
m/z
2.500e+5
2.000e+5
1.500e+5
1.000e+5
5.000e+4
0.000e+0
500
1000
1500
HexNAc
HexNAcHex
HexNAc(2)Hex(1)
a3
b3-18
a4
b4
b4-18
b5-18
y3
a5
b5
b6
b6-18
y4
a7
b7
b7-18
y5
b9-18
b9
b10-18
y6
b10
y7
b11
b12
~y8
~y9
~y10
~y11
b15
b16
~y12
b17-18
b17
~y13
~y14
~y31++
~y14+203
~y15
~y13+203
~y17
~y18

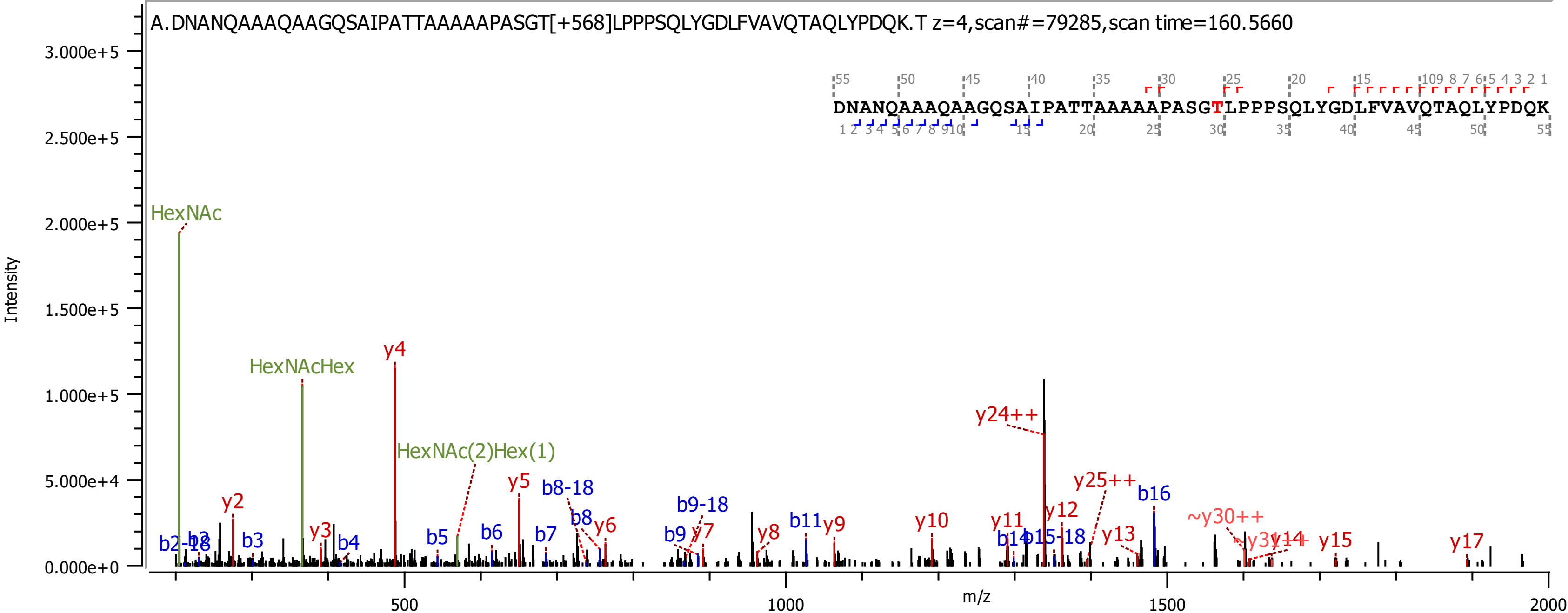
A.DNANQAAAQAAGQSAIPATTAAAAAPASGT[+568]LPPPSQLYGDLFVAVQTAQLYPDQK.T z=4,scan#=79285,scan time=160.5660
DNANQAAAQAAGQSAIPATTAAAAAPASGTLPPPSQLYGDLFVAVQTAQLYPDQK
Intensity
m/z
HexNAc
HexNAcHex
HexNAc(2)Hex(1)
y2
y3
y4
y5
y6
y7
y8
y9
y10
y11
y12
y13
y14
y15
y17
y24++
y25++
~y30++
b2-18
b3
b4
b5
b6
b7
b8
b8-18
b9
b9-18
b11
b14
b15-18
b16
3.000e+5
2.500e+5
2.000e+5
1.500e+5
1.000e+5
5.000e+4
0.000e+0
500
1000
1500
2000

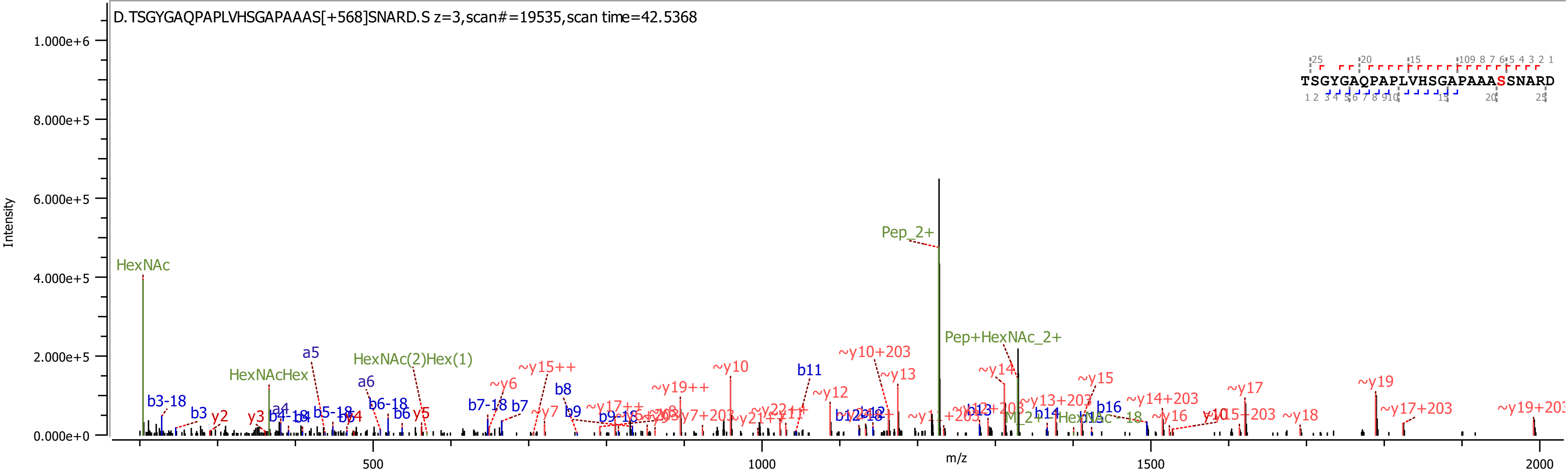

D.TSGYGAQPAPLVHSGAPAAAS[+568]SNARD.S z=3,scan#=19535,scan time=42.5368
Intensity
m/z
TSGYGAQPAPLVHSGAPAAASSNARD
HexNAc
HexNAcHex
HexNAc(2)Hex(1)
Pep_2+
Pep+HexNAc_2+
b3-18
b3
y2
y3
a4
a5
a6
b6-18
b6
y5
b7-18
b7
~y6
~y15++
b8
~y7
b9
~y17++
~y19++
~y10
b11
~y10+203
~y13
~y12
~y14
~y15
~y13+203
b16
~y14+203
~y16
~y17
~y18
~y19
~y17+203
~y19+203

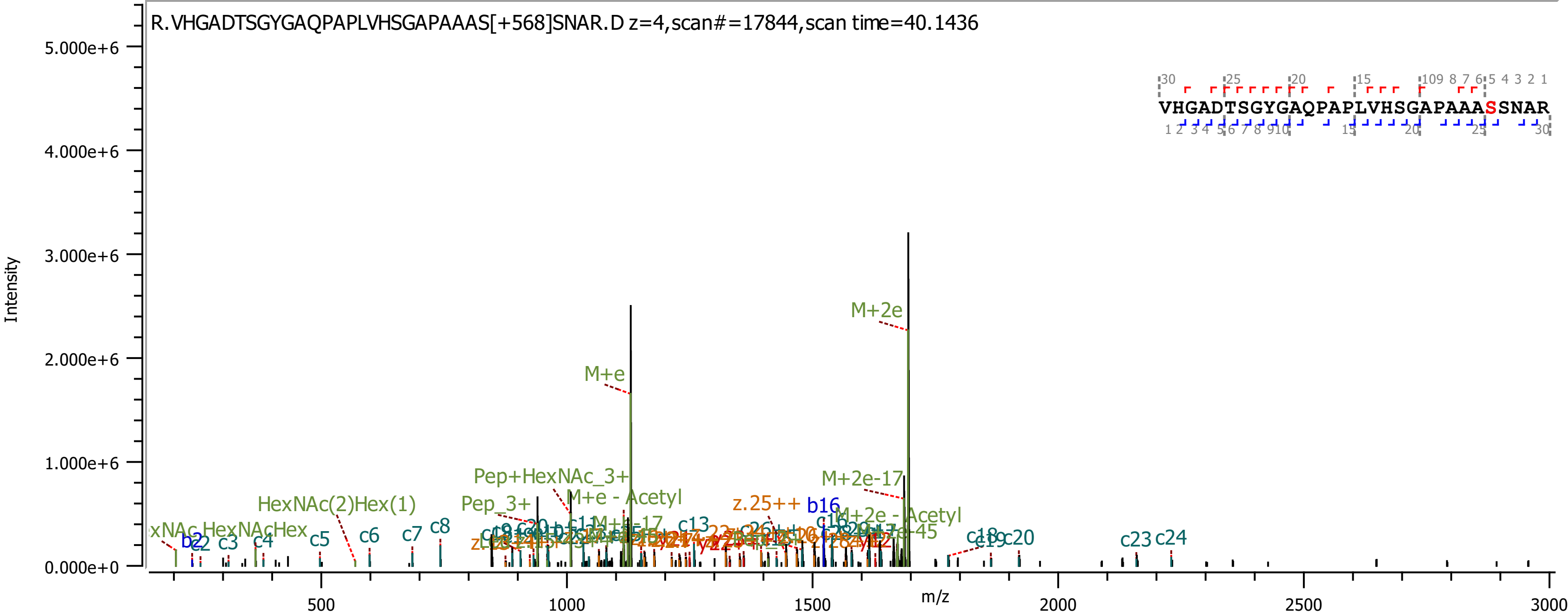
R.VHGADTSGYGAQPAPLVHSGAPAAAS[+568]SNAR.D z=4,scan#=17844,scan time=40.1436
VHGADTSGYGAQPAPLVHSGAPAAASSNAR
Intensity
5.000e+6
4.000e+6
3.000e+6
2.000e+6
1.000e+6
0.000e+0
m/z
500
1000
1500
2000
2500
3000
M+2e
M+e
Pep+HexNAc_3+
M+e - Acetyl
Pep_3+
HexNAc(2)Hex(1)
M+2e-17
z.25++
b16
M+2e - Acetyl
b2
c2
c3
c4
c5
c6
c7
c8
c13
c18
c19
c20
c23
c24

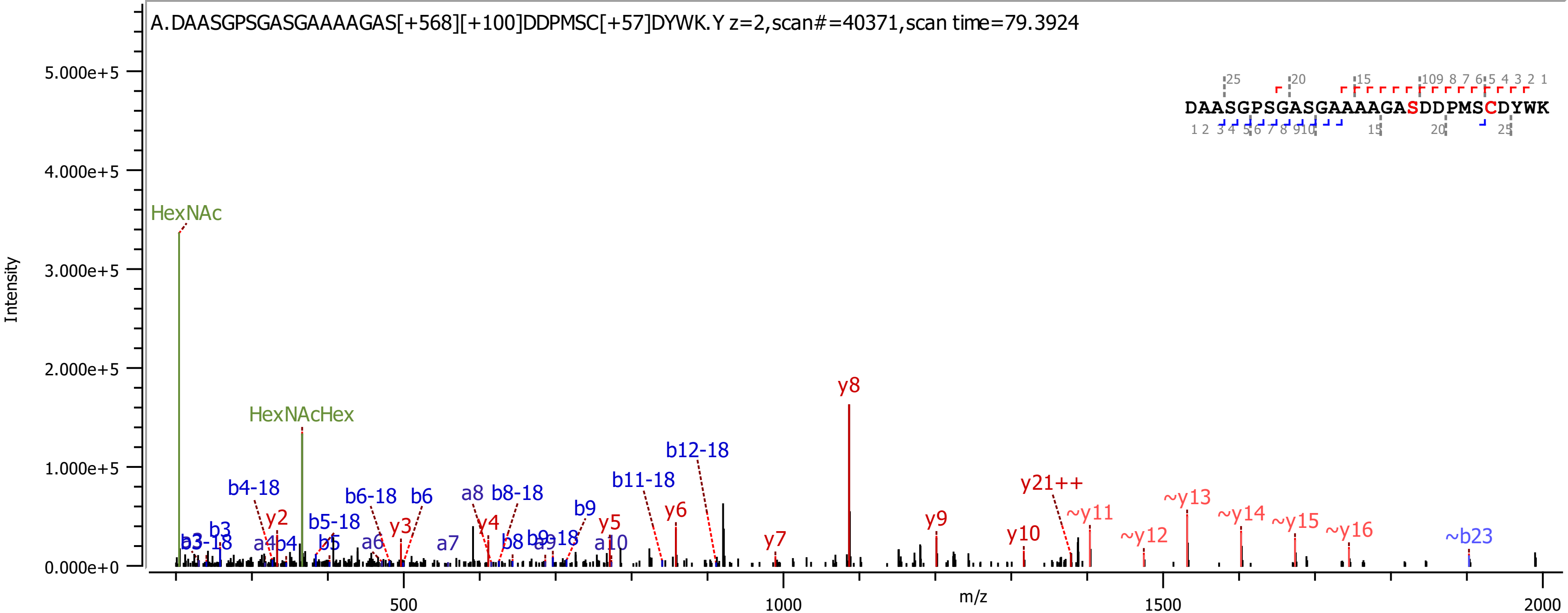

A.DAASGPSGASGAAAAGAS[+568][+100]DDPMSC[+57]DYWK.Y z=2,scan#=40371,scan time=79.3924
DAASGPSGASGAAAAGASDDPMSCDYWK
Intensity
m/z
HexNAc
HexNAcHex
y8
b12-18
b11-18
y21++
~y11
~y13
~y14
~y15
~y16
~y12
~b23
y9
y10
y7
y6
y5
b9
a10
a8
b8-18
b6-18
b6
b4-18
y2
y3
y4
b5-18
b3
b3-18
a4
b4
b5
a6
a7
b8
a9
b9-18
5.000e+5
4.000e+5
3.000e+5
2.000e+5
1.000e+5
0.000e+0
500
1000
1500
2000

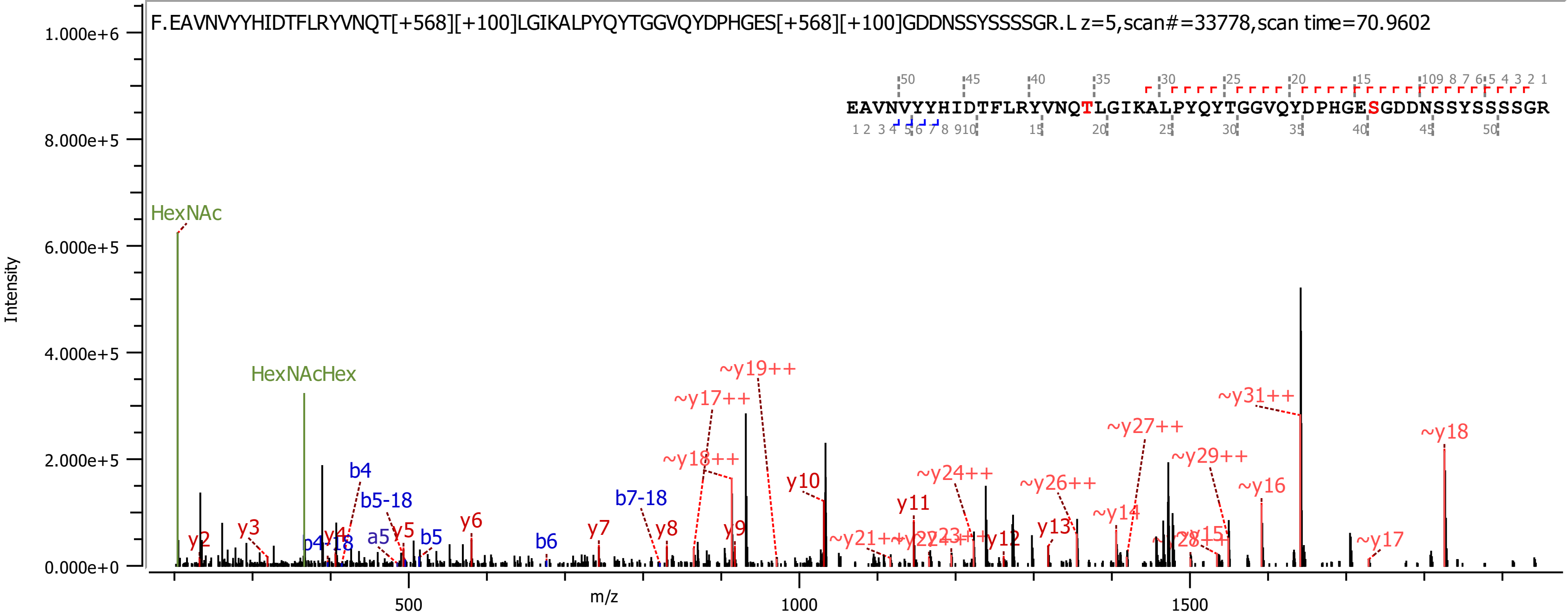

F.EAVNVYYHIDTFLRYVNQT[+568][+100]LGIKALPYQYTGGVQYDPHGES[+568][+100]GDDNSSYSSSSGR.L z=5,scan#=33778,scan time=70.9602
EAVNVYYHIDTFLRYVNQTLGIKALPYQYTGGVQYDPHGESGDDNSSYSSSSGR
Intensity
m/z
HexNAc
HexNAcHex
b4
b5-18
b5
b6
b7-18
a5
y2
y3
y5
y6
y7
y8
y9
y10
y11
y12
y13
~y14
~y16
~y17
~y18
~y17++
~y18++
~y19++
~y24++
~y26++
~y27++
~y29++
~y31++

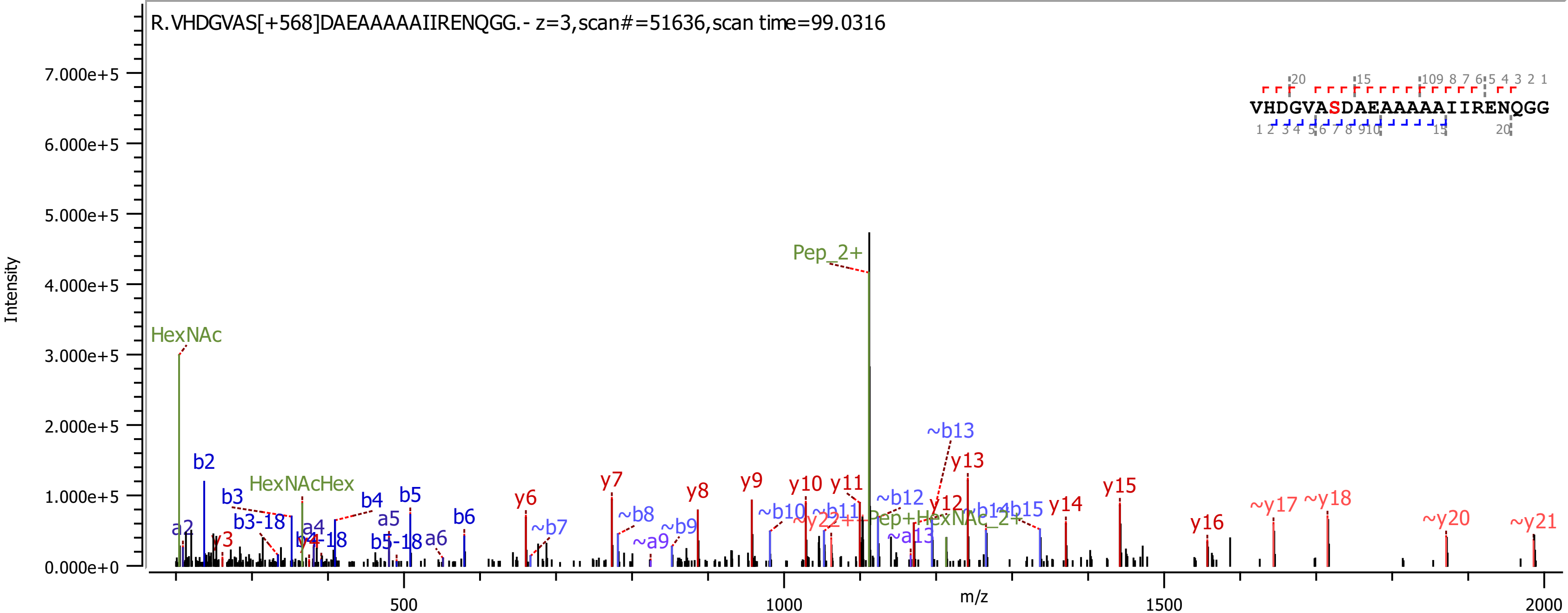

R.VHDGVAS[+568]DAEAAAAAIIRENQGG.- z=3,scan#=51636,scan time=99.0316
VHDGVASDAEAAAAAIIRENQGG
Intensity
m/z
HexNAc
HexNAcHex
Pep_2+
Pep+HexNAc_2+

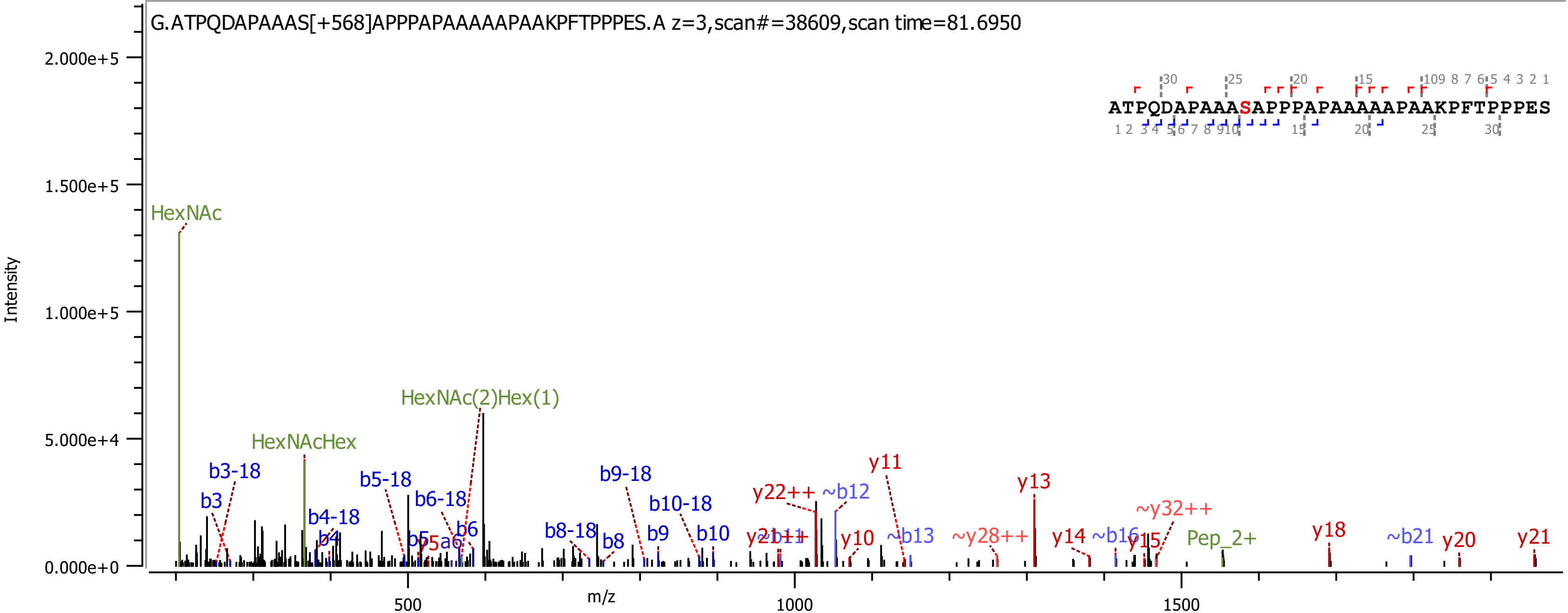

G.ATPQDAPAAAS[+568]APPPAPAAAAAPAAKPFTPPPES.A z=3,scan#=38609,scan time=81.6950
ATPQDAPAAASAPPPAPAAAAAPAAKPFTPPPES
Intensity
2.000e+5
1.500e+5
1.000e+5
5.000e+4
0.000e+0
m/z
500
1000
1500
HexNAc
HexNAcHex
HexNAc(2)Hex(1)
Pep_2+
b3
b3-18
b4
b4-18
b5
b5-18
b6
b6-18
b8
b8-18
b9
b9-18
b10
b10-18
~b12
~b13
~b16
~b21
y10
y11
y13
y14
y15
y18
y20
y21
y22++
~y28++
~y32++

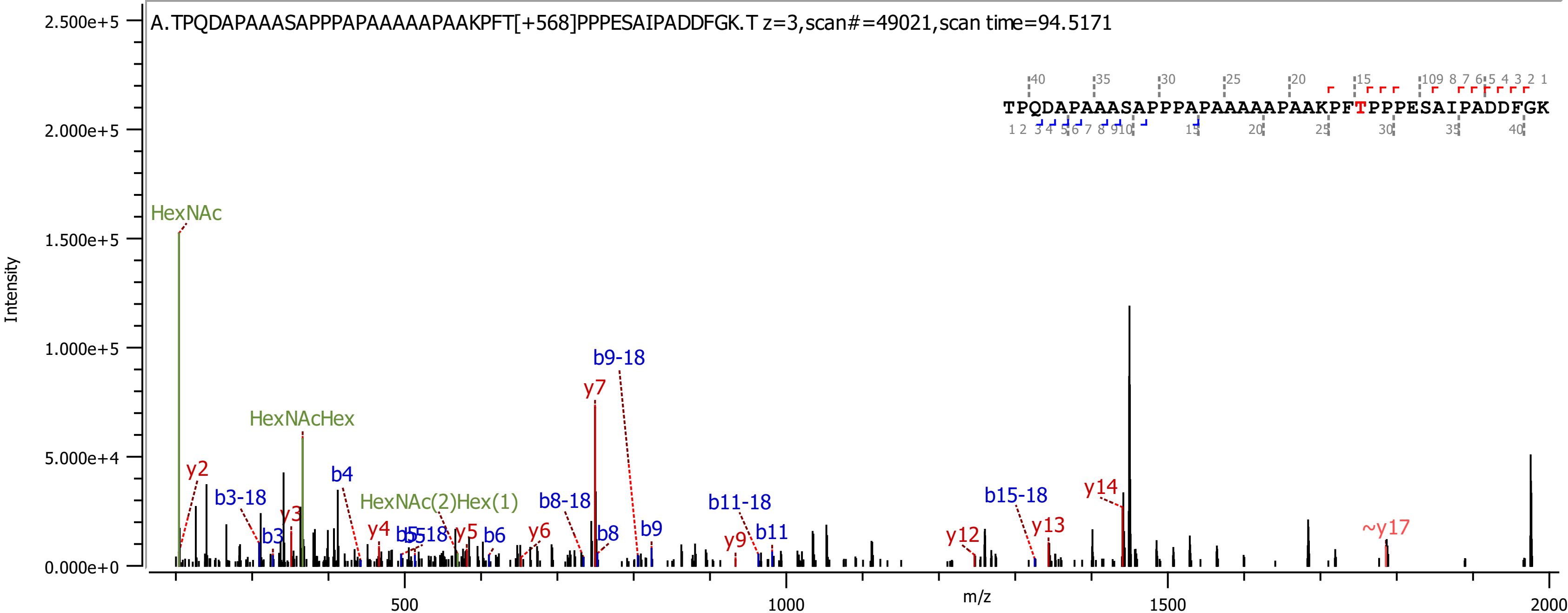

A.TPQDAPAAASAPPPAPAAAAAPAAKPFT[+568]PPPESAIPADDFGK.T z=3,scan#=49021,scan time=94.5171
TPQDAPAAASAPPPAPAAAAAPAAKPFTPPPESAIPADDFGK
Intensity
m/z
HexNAc
HexNAcHex
HexNAc(2)Hex(1)
y2
y3
y4
y5
y6
y7
y9
y12
y13
y14
~y17
b3-18
b3
b4
b5-18
b5
b6
b8-18
b8
b9-18
b9
b11-18
b11
b15-18
2.500e+5
2.000e+5
1.500e+5
1.000e+5
5.000e+4
0.000e+0
500
1000
1500
2000

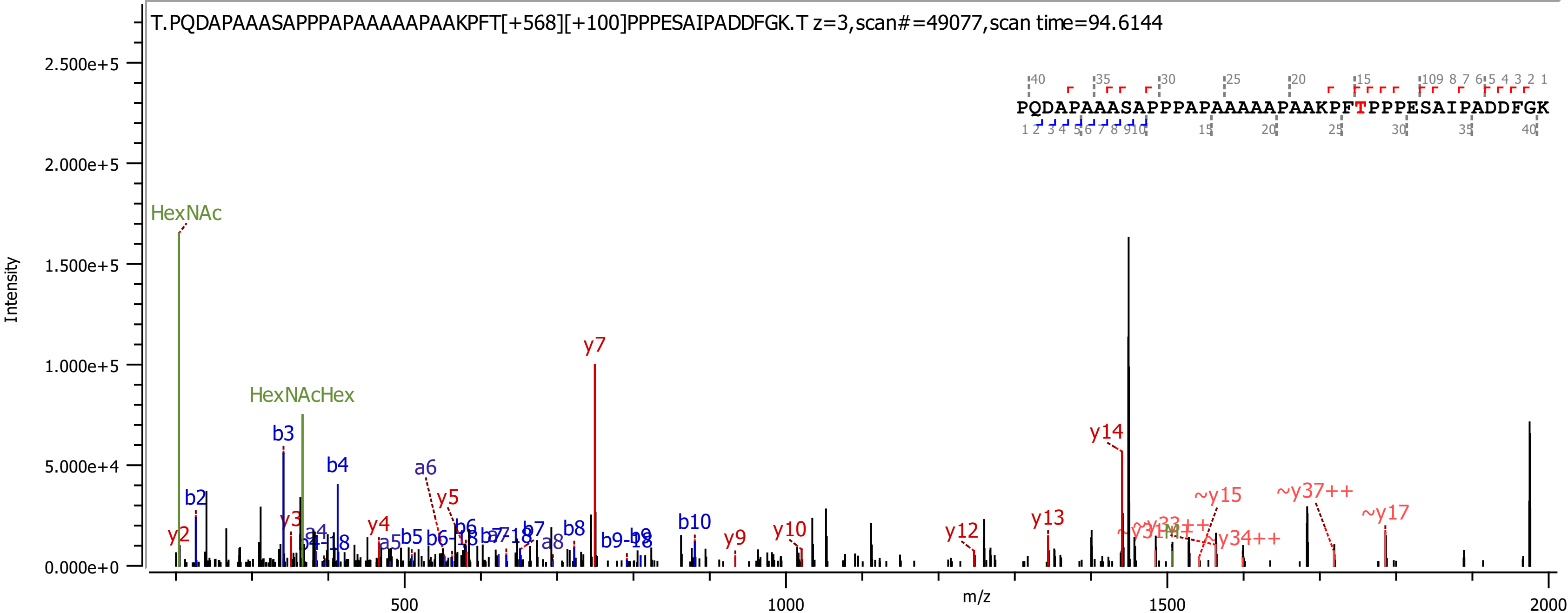

T.PQDAPAAASAPPPAPAAAAAPAAKPFT[+568][+100]PPPESAIPADDFGK.T z=3,scan#=49077,scan time=94.6144
PQDAPAAASAPPPAPAAAAAPAAKPFTPPPESAIPADDFGK
Intensity
m/z
HexNAc
HexNAcHex
y7
y14
b3
b4
a6
y5
b2
y3
y2
y4
a5
b5
b6
b7
a8
b8
b10
y9
y10
y12
y13
~y15
~y37++
~y17
~y34++

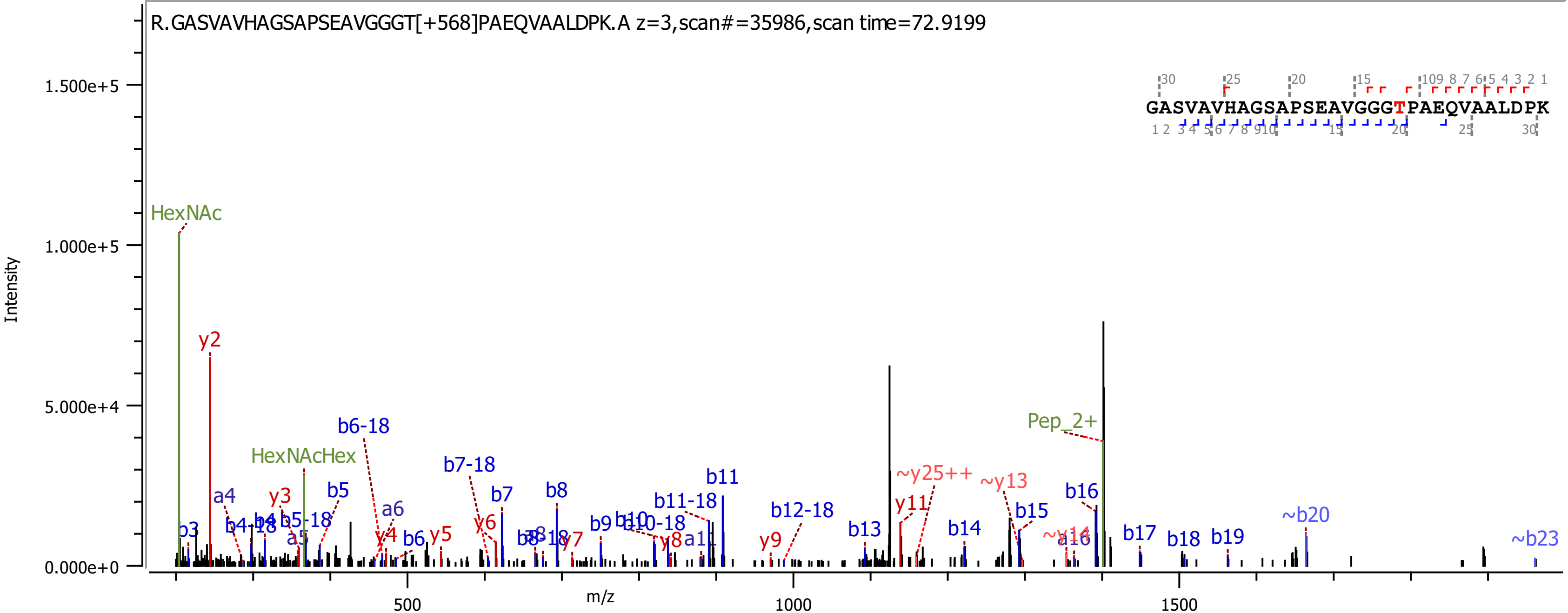

R.GASVAVHAGSAPSEAVGGGT[+568]PAEQVAALDPK.A z=3,scan#=35986,scan time=72.9199
Intensity
1.500e+5
1.000e+5
5.000e+4
0.000e+0
m/z
500
1000
1500
GASVAVHAGSAPSEAVGGGTPAEQVAALDPK
HexNAc
y2
HexNAcHex
b6-18
b7-18
b11
~y25++
~y13
Pep_2+
b16
b15
b14
b13
b12-18
b11-18
b10-18
b9
b8
b7
y6
y5
b6
a6
y4
b5
b5-18
a5
y3
a4
b4-18
b3
y7
y8
y9
y11
~y14
a16
b17
b18
b19
~b20
~b23

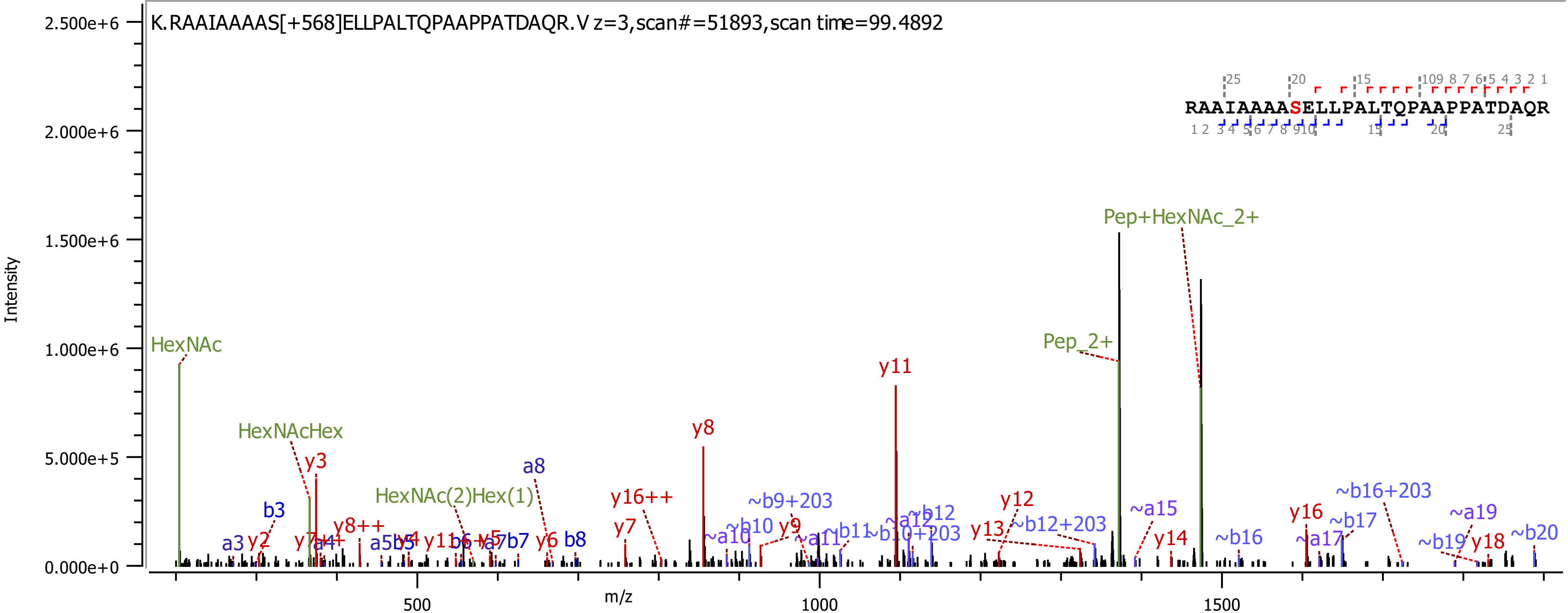
K.RAAIAAAAS[+568]ELLPALTQPAAPPATDAQR.V z=3,scan#=51893,scan time=99.4892
RAAIAAAASELLPALTQPAAPPATDAQR
Intensity
m/z
2.500e+6
2.000e+6
1.500e+6
1.000e+6
5.000e+5
0.000e+0
500
1000
1500
HexNAc
HexNAcHex
HexNAc(2)Hex(1)
Pep_2+
Pep+HexNAc_2+
y3
y8
y11
y16++
y7
y12
y13
y14
y16
y18
b3
b8
a8
~b9+203
~b16+203
~b12+203
~a15
~a19
~b16
~b17
~b20

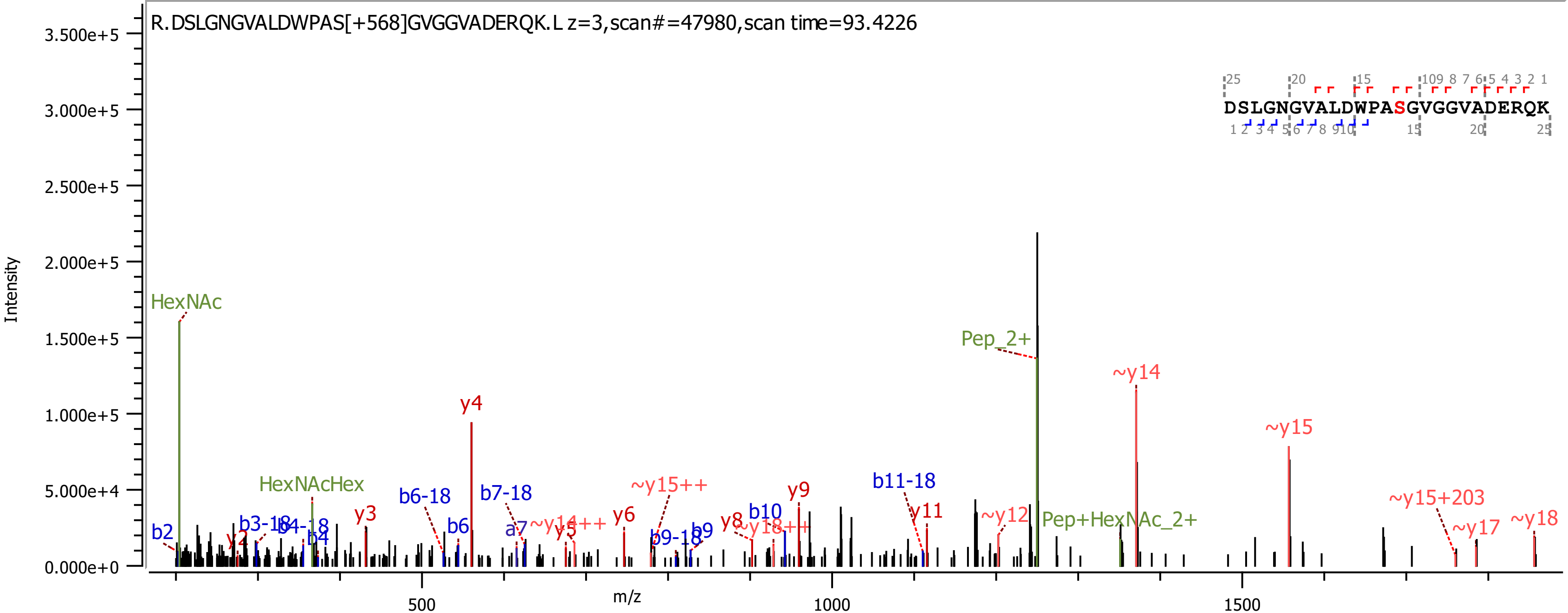
R.DSLGNGVALDWPAS[+568]GVGGVADERQK.L z=3,scan#=47980,scan time=93.4226
DSLGNGVALDWPASGVGGVADERQK
Intensity
m/z
HexNAc
HexNAcHex
Pep_2+
Pep+HexNAc_2+
y4
~y14
~y15
~y15++
~y15+203
~y17
~y18
~y12
y3
y6
y9
y11
y8
b2
b6
b10
b6-18
b7-18
b11-18
b9-18
a7

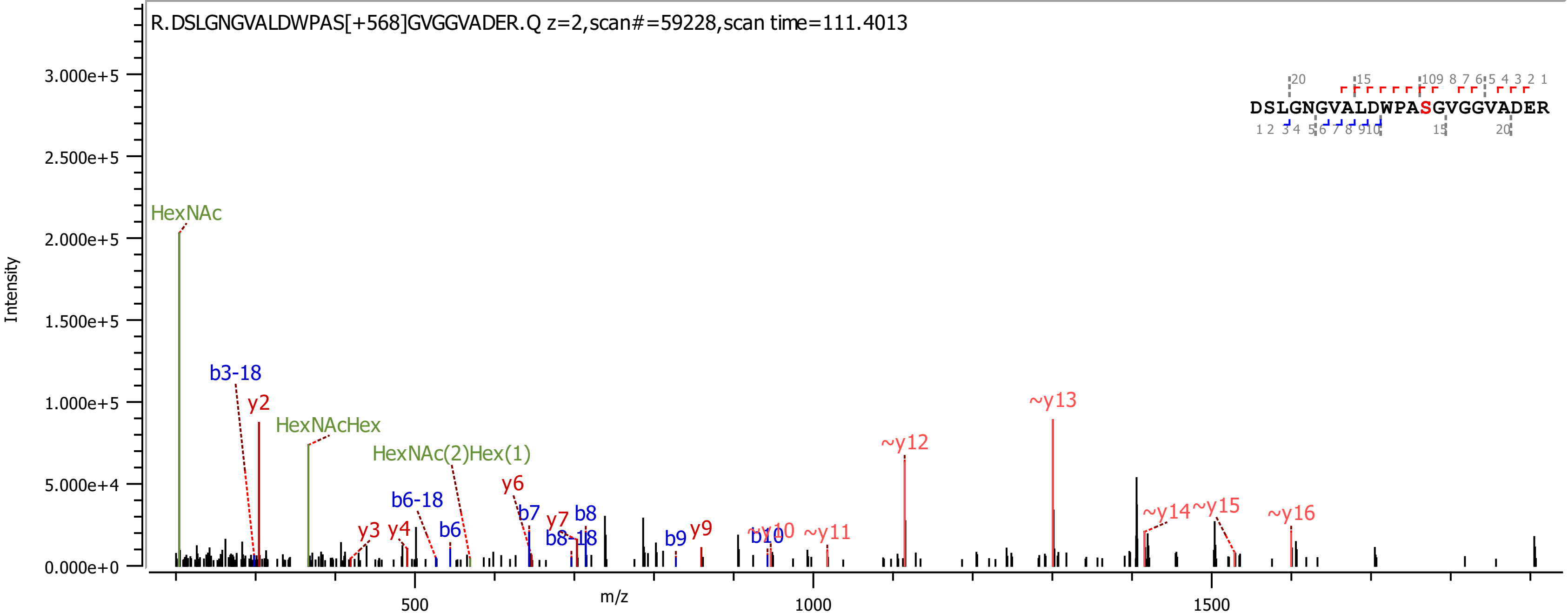
R.DSLGNGVALDWPAS[+568]GVGGVADER.Q z=2,scan#=59228,scan time=111.4013
DSLGNGVALDWPASGVGGVADER
Intensity
m/z
HexNAc
b3-18
y2
HexNAcHex
HexNAc(2)Hex(1)
b6-18
y3
y4
b6
y6
b7
y7
b8
b8-18
b9
y9
~y10
b10
~y11
~y12
~y13
~y14
~y15
~y16
3.000e+5
2.500e+5
2.000e+5
1.500e+5
1.000e+5
5.000e+4
0.000e+0
500
1000
1500

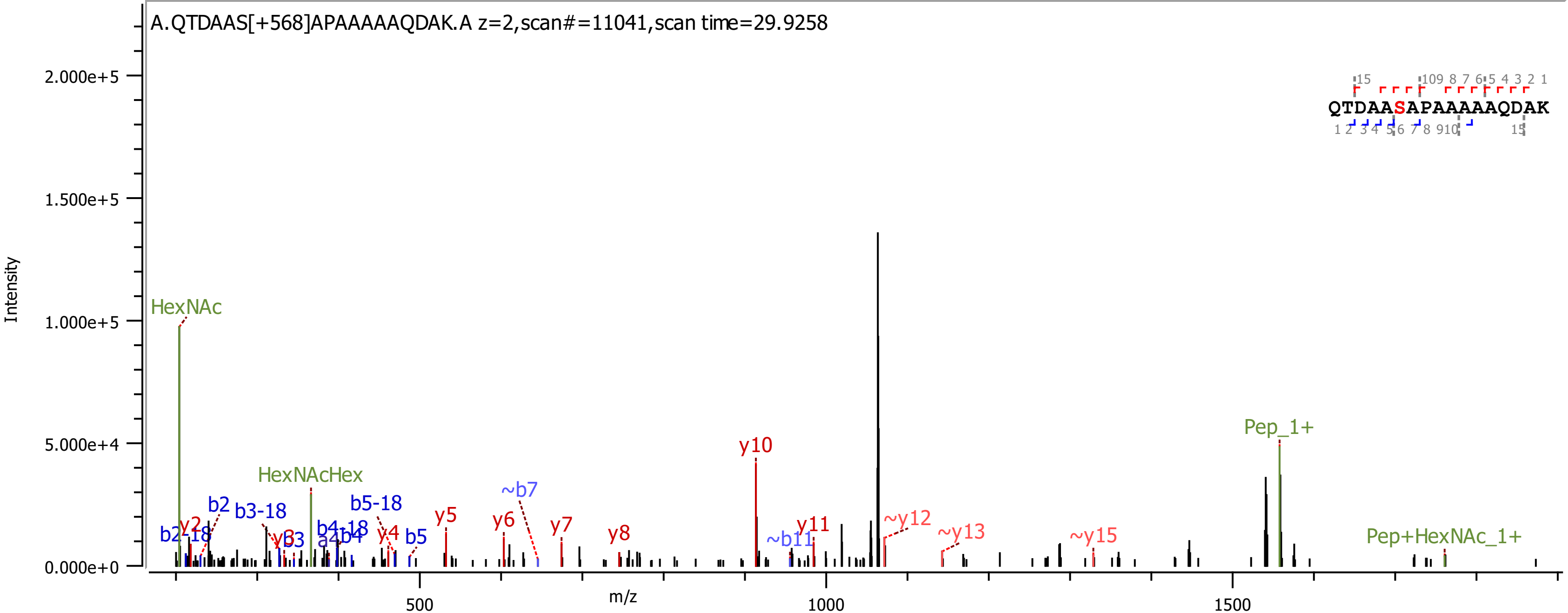
A.QTDAAS[+568]APAAAAAQDAK.A z=2,scan#=11041,scan time=29.9258
QTDAASAPAAAAAQDAK
Intensity
m/z
HexNAc
HexNAcHex
b2-18
y2
b2
b3-18
y3
b3
a4
b4-18
b4
b5-18
y4
b5
y5
y6
~b7
y7
y8
y10
~b11
y11
~y12
~y13
~y15
Pep_1+
Pep+HexNAc_1+

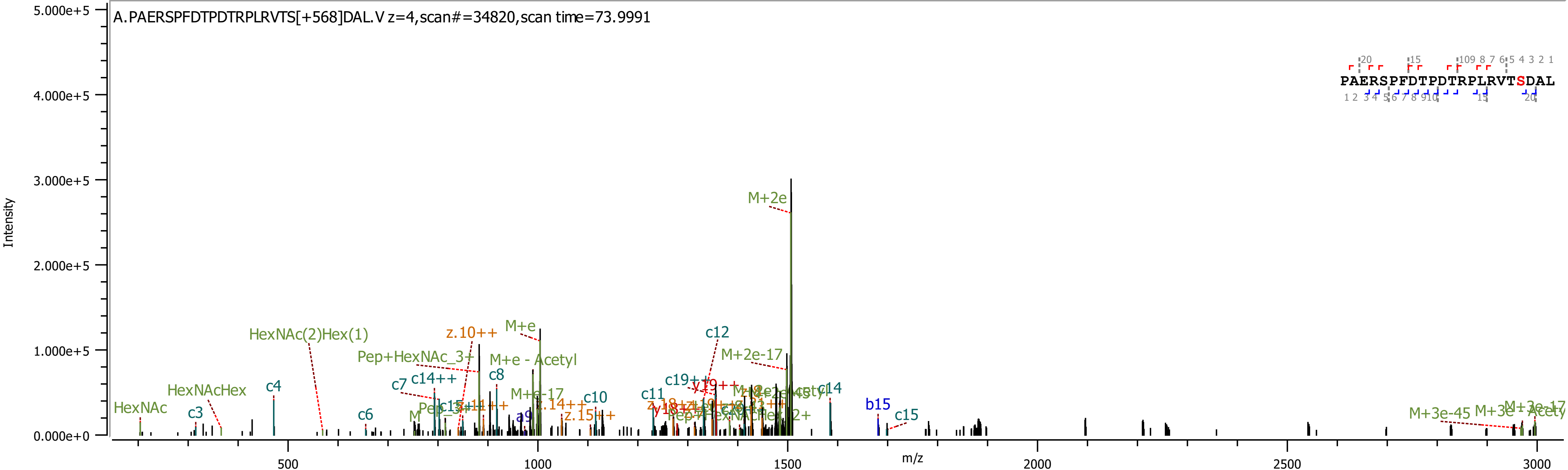
A.PAERSPFDTPDTRPLRVTS[+568]DAL.V z=4,scan#=34820,scan time=73.9991
PAERSPFDTPDTRPLRVTSDAL
Intensity
m/z
5.000e+5
4.000e+5
3.000e+5
2.000e+5
1.000e+5
0.000e+0
500
1000
1500
2000
2500
3000
M+2e
M+e
z.10++
HexNAc(2)Hex(1)
Pep+HexNAc_3+
M+e - Acetyl
c12
M+2e-17
c8
c7
c14++
c4
HexNAcHex
HexNAc
c3
c6
c10
c11
c14
b15
c15
a9
M+3e-45

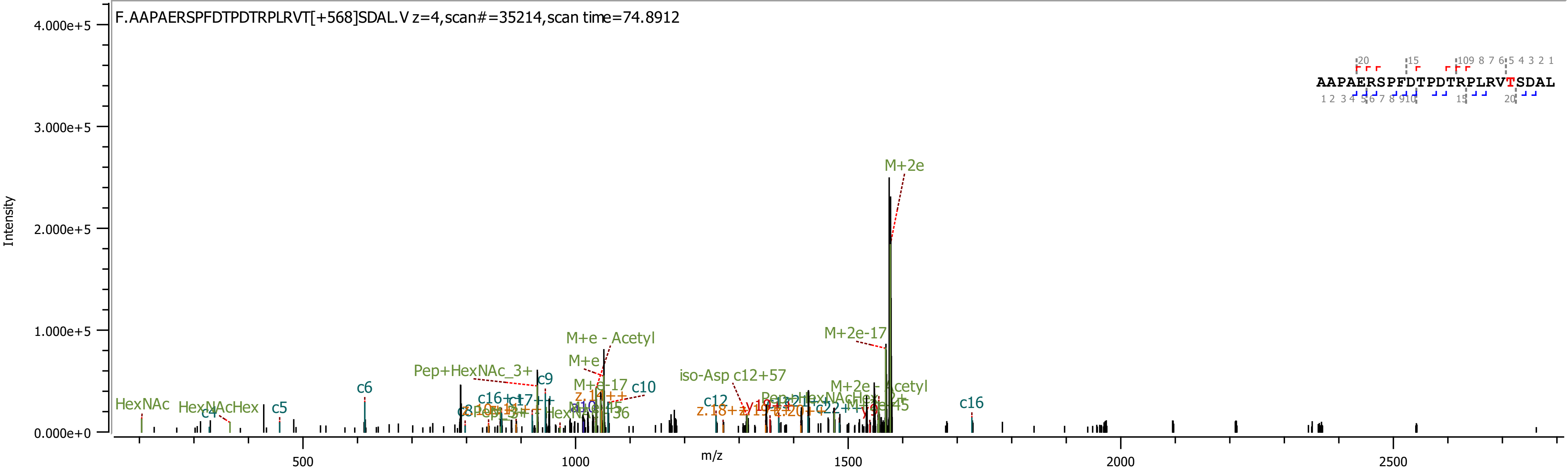

F.AAPAERSPFDTPDTRPLRVT[+568]SDAL.V z=4,scan#=35214,scan time=74.8912
Intensity
m/z
4.000e+5
3.000e+5
2.000e+5
1.000e+5
0.000e+0
500
1000
1500
2000
2500
AAPAERSPFDTPDTRPLRVTSDAL
M+2e
M+2e-17
M+e - Acetyl
M+e
Pep+HexNAc_3+
c9
c10
iso-Asp c12+57
c6
c5
c4
HexNAc
HexNAcHex
c12
c16
