# Supplementary material for: Burkholderia PglL enzymes are Serine preferring oligosaccharyltransferases which target conserved proteins across the Burkholderia genus
Source: Commun Biol. 2021 Sep 7;4:1045. doi: 10.1038/s42003-021-02588-y (PMC8423747; doi:10.1038/s42003-021-02588-y)
Supplement: Supplementary file 6 — Supplementary Data 3 [file 42003_2021_2588_MOESM6_ESM.zip › Supplementary_Data_3B_K56_2_Best_Scoring_Unique_glycopeptides.pdf]

Burkholderia cenocepacia K56-2 Best Scoring Unique glycopeptides

J2315 Gene name	Protein Name	Peptide < ProteinMetrics Confidential >	Glycans NHFAGNa	Localised site (Yes/No)	Observed m/z	z	Observed (M+H)	Calc. mass (M+H)	Mass error (ppm)	Cleavage	Score	Delta	Delta Mod	Comment	Scan Time	Enzyme	Replicate	Page
BCAL0039	>gi|443605781|gb|ELT73605.1| ABC transporter, substrate-binding protein, family 3 [Burkholderia cenocepacia K56-2Valvano]	T.AAPASAATAGS[+568.212]RLDDVLARGA.L	HexNAc(2)Hex(1)	No	837.0821	3	2509.2316	2509.2264	2.1	Non	412.99	336.37	1.2	Nsco_20191108_BC_ZIC_HILIC_K56_2_thermolysin_B1.32915.32915.3	66.6456	Thermolysin	B1	20
BCAL0080	>gi|443593207|gb|ELT61961.1| cytochrome C domain protein, partial [Burkholderia cenocepacia K56-2Valvano]	N.AAQPASGAPASGADAS[+568.212]NAQA.-	HexNAc(2)Hex(1)	No	1141.0048	2	2281.0023	2280.995	3.2	Non	421.08	283.51	0.87	Nsco_20191108_BC_ZIC_HILIC_K56_2_thermolysin_B1.12891.12891.2	31.0195	Thermolysin	B1	21
BCAL0080	>gi|443593207|gb|ELT61961.1| cytochrome C domain protein, partial [Burkholderia cenocepacia K56-2Valvano]	Y.MANNDGANFPEPAAPAANAAQPAS[+568.212]GAPASGADASNAQ.A	HexNAc(2)Hex(1)	No	1321.5789	3	3962.7222	3962.7105	3	Non	547.71	520.06	44.52	Nsco_20191108_BC_ZIC_HILIC_K56_2_thermolysin_B1.40978.40978.3	83.9361	Thermolysin	B1	22
BCAL0080	>gi|443593207|gb|ELT61961.1| cytochrome C domain protein, partial [Burkholderia cenocepacia K56-2Valvano]	Y.MANNDGANFPEPAAPAANAAQPAS[+568.212]GAPASGADASNAQA.-	HexNAc(2)Hex(1)	Yes (1 of 2)	1345.2585	3	4033.7609	4033.7476	3.3	Non	273.98	227.31	41.79	Nsco_20191108_BC_ZIC_HILIC_K56_2_thermolysin_B2_.46558.46558.3	85.0541	Thermolysin	B2	23
BCAL0163	>gi|443595598|gb|ELT64173.1| BON domain protein [Burkholderia cenocepacia K56-2Valvano]	V.KPQDAQALQDASPASGASGAQAAAAPADNATVGAVPDASVQSTPLQPPAPISNS[+568.212]S[+568.212][+100.064]SVHPGNPK.A	HexNAc(2)Hex(1), HexNAc(2)Hex(1) 100.064	No	1202.579	6	7210.4378	7204.4215	-0.5	Non	176.52	176.52	0	Nsco_20191108_BC_ZIC_HILIC_K56_2_thermolysin_B3.50702.50702.6	101.3486	Thermolysin	B3	24
BCAL0163	>gi|443595598|gb|ELT64173.1| BON domain protein [Burkholderia cenocepacia K56-2Valvano]	V.KVFQYVKPQDAQALQDASPASGAS[+568.212]GAQAAAAPADNATVGAVPDASVQSTPLQPPAPISNSSSVHPGNPK.A	HexNAc(2)Hex(1)	No	1460.9239	5	7300.5903	7300.568	3	NRagged	1310.09	1268.2	36.68	Nsco_20191108_BC_ZIC_HILIC_K56_2_typsin_B2.46071.46071.5	93.6647	Trypsin	B2	25
BCAL0163	>gi|443595598|gb|ELT64173.1| BON domain protein [Burkholderia cenocepacia K56-2Valvano]	A.LQDASPAS[+568.212]GASGAQAAAAPADN.A	HexNAc(2)Hex(1)	No	1255.0619	2	2509.1166	2509.106	4.2	Non	395.8	267.22	1.16	Nsco_20191108_BC_ZIC_HILIC_K56_2_pepsin_B2.22491.22491.2	49.0361	Pepsin	B2	26
BCAL0163	>gi|443595598|gb|ELT64173.1| BON domain protein [Burkholderia cenocepacia K56-2Valvano]	K.PQDAQALQDASPASGASGAQAAAAPADNATVGAVPDASVQST[+568.212]PLQPPAPISNSSSVHPGNPK.A	HexNAc(2)Hex(1)	No	1602.7728	4	6408.0695	6408.051	2.9	Specific	1162.92	1162.9	0	Nsco_20191108_BC_ZIC_HILIC_K56_2_typsin_B2.48573.48573.4	97.8402	Trypsin	B2	27
BCAL0163	>gi|443595598|gb|ELT64173.1| BON domain protein [Burkholderia cenocepacia K56-2Valvano]	K.VFQYVKPQDAQALQDASPASGAS[+568.212]GAQAAAAPADNATVGAVPDASVQSTPLQPPAPISNSSSVHPGNPK.A	HexNAc(2)Hex(1)	Yes	1435.303	5	7172.486	7172.4731	1.8	Specific	1194.18	1194.2	111.85	Nsco_20191108_BC_ZIC_HILIC_K56_2_typsin_B1.51502.51502.5	101.1388	Trypsin	B1	28
BCAL0163	>gi|443595598|gb|ELT64173.1| BON domain protein [Burkholderia cenocepacia K56-2Valvano]	Y.VKPQDAQALQDASPASGAS[+568.212]GAQAAAAPADNATVGAVPDASVQSTPLQPPAPISNSSSVHPGNPK.A	HexNAc(2)Hex(1)	Yes	1659.8165	4	6636.2443	6635.2143	4	NRagged	963.66	963.66	93.72	Nsco_20191108_BC_ZIC_HILIC_K56_2_typsin_B3.45475.45475.4	91.0302	Trypsin	B3	29
BCAL0163	>gi|443595598|gb|ELT64173.1| BON domain protein [Burkholderia cenocepacia K56-2Valvano]	S.VQSTPLQPPAPISNSS[+568.212]SVHPGNPKAKAQ.-	HexNAc(2)Hex(1)	Yes	852.1833	4	3405.7114	3405.702	2.8	Non	717.1	717.1	0	Nsco_20191108_BC_ZIC_HILIC_K56_2_pepsin_B1.20579.20579.4	45.1081	Pepsin	B1	30
BCAL0193	>gi|443590944|gb|ELT59881.1| hypothetical protein BURCENK562V_2433 [Burkholderia cenocepacia K56-2Valvano]	S.AAAPAPAKKDHSKPKHQLKHHGSKKGQAKAAAAS[+568.212][+100.064]AAGTNDAGTQN.-	HexNAc(2)Hex(1) 100.064	No	1025.3224	5	5122.5828	5121.6064	-5.3	Non	459.47	459.47	51.91	Nsco_20191108_BC_ZIC_HILIC_K56_2_pepsin_B1.3536.3536.5	16.5137	Pepsin	B1	31

BCAL0193	>gi|443590944|gb|ELT59881.1| hypothetical protein BURCENK562V_2433 [Burkholderia cenocepacia K56-2Valvano]	P.AADTSAAAPAPAKKDHS[+568.212][+100.064]KPKHQ.L	HexNAc(2)Hex(1) 100.064	No	965.8129	3	2895.4241	2895.4334	-3.2	Non	491.4	491.4	7.58	Nsco_20191108_BC_ZIC_HILIC_K56_2_thermolysin_B2_.5992.5992.3	21.3403	Thermolysin	B2	32
BCAL0193	>gi|443590944|gb|ELT59881.1| hypothetical protein BURCENK562V_2433 [Burkholderia cenocepacia K56-2Valvano]	A.AAPAPAKKDHSKPKHQLKHHGSKKGQAKAAAAS[+568.212]AAGT[+568.212]ND.A	HexNAc(2)Hex(1), HexNAc(2)Hex(1)	No	842.5956	6	5050.537	5047.5091	3.5	Non	299.37	299.37	137.37	Nsco_20191108_BC_ZIC_HILIC_K56_2_pepsin_B3_reinject.3333.3333.6	16.2103	Pepsin	B3	33
BCAL0193	>gi|443590944|gb|ELT59881.1| hypothetical protein BURCENK562V_2433 [Burkholderia cenocepacia K56-2Valvano]	Q.ASAPAADT[+568.212]SAAAPAPAKK.D	HexNAc(2)Hex(1)	No	1082.5332	2	2164.0591	2164.0503	4.1	NRagged	781.84	637.11	0	Nsco_20191108_BC_ZIC_HILIC_K56_2_typsin_B1.6825.6825.2	22.7563	Trypsin	B1	34
BCAL0193	>gi|443590944|gb|ELT59881.1| hypothetical protein BURCENK562V_2433 [Burkholderia cenocepacia K56-2Valvano]	A.DTSAAAPAPAKKDHSKPKHQLKHHGSKKGQAKAAAASAAGTNDAGT[+568.212]QN.-	HexNAc(2)Hex(1)	No	1066.1395	5	5326.6685	5324.649	2.4	Non	785.04	785.04	785.04	Nsco_20191108_BC_ZIC_HILIC_K56_2_pepsin_B3_reinject.3443.3443.5	16.382	Pepsin	B3	35
BCAL0193	>gi|443590944|gb|ELT59881.1| hypothetical protein BURCENK562V_2433 [Burkholderia cenocepacia K56-2Valvano]	A.PAADTSAAAPAPAKKDHS[+568.212]KPKHQ.L	HexNAc(2)Hex(1)	Yes (1 of 2)	724.3645	4	2894.4362	2892.4221	2.5	Non	283.84	283.84	8.4	Nsco_20191108_BC_ZIC_HILIC_K56_2_thermolysin_B2_.6117.6117.4	21.5289	Thermolysin	B2	36
BCAL0193	>gi|443590944|gb|ELT59881.1| hypothetical protein BURCENK562V_2433 [Burkholderia cenocepacia K56-2Valvano]	T.SAAAPAPAKKDHSKPKHQLKHHGSKKGQAKAAAAS[+568.212]AAGT[+568.212]ND.A	HexNAc(2)Hex(1), HexNAc(2)Hex(1)	No	868.9396	6	5208.601	5205.5782	2.4	Non	303.13	303.13	145.33	Nsco_20191108_BC_ZIC_HILIC_K56_2_pepsin_B3_reinject.3757.3757.6	16.838	Pepsin	B3	37
BCAL0193	>gi|443590944|gb|ELT59881.1| hypothetical protein BURCENK562V_2433 [Burkholderia cenocepacia K56-2Valvano]	T.SAAAPAPAKKDHSKPKHQLKHHGSKKGQAKAAAAS[+568.212]AAGTNDAGTQN.-	HexNAc(2)Hex(1)	No	639.4553	8	5108.5912	5108.5744	3.3	Non	467.89	467.89	16.28	Nsco_20191108_BC_ZIC_HILIC_K56_2_pepsin_B3_reinject.3233.3233.8	16.0795	Pepsin	B3	38
BCAL0303	>gi|443596149|gb|ELT64670.1| hypothetical protein BURCENK562V_3632 [Burkholderia cenocepacia K56-2Valvano]	K.AADAGGAKPAAGAS[+568.212]AAPAAPAPVAVPASAVSGSAGQ.-	HexNAc(2)Hex(1)	No	1755.8598	2	3510.7124	3510.7082	1.2	CRagged	830.5	764.54	9.2	Nsco_20191108_BC_ZIC_HILIC_K56_2_typsin_B1.35749.35749.2	74.5524	Trypsin	B1	39
BCAL0303	>gi|443596149|gb|ELT64670.1| hypothetical protein BURCENK562V_3632 [Burkholderia cenocepacia K56-2Valvano]	A.ADAGGAKPAAGASAAPAAPAPVAVPAS[+568.212]AVS[+568.212][+100.064]GSAGQ.-	HexNAc(2)Hex(1), HexNAc(2)Hex(1) 100.064	No	1369.9816	3	4107.9303	4107.9467	-4	Non	410.05	410.05	0	Nsco_20191108_BC_ZIC_HILIC_K56_2_typsin_B2.33198.33198.3	70.2605	Trypsin	B2	40
BCAL0303	>gi|443596149|gb|ELT64670.1| hypothetical protein BURCENK562V_3632 [Burkholderia cenocepacia K56-2Valvano]	S.KAADAGGAKPAAGAS[+568.212]AAPAAPAPVAV.P	HexNAc(2)Hex(1)	Yes	909.4652	3	2726.381	2726.3731	2.9	Non	745.72	742.58	742.58	Nsco_20191108_BC_ZIC_HILIC_K56_2_pepsin_B1.22517.22517.3	48.7792	Pepsin	B1	41
BCAL0303	>gi|443596149|gb|ELT64670.1| hypothetical protein BURCENK562V_3632 [Burkholderia cenocepacia K56-2Valvano]	S.KAADAGGAKPAAGAS[+568.212]AAPAAPAPVAVPASAVSGSAGQ.-	HexNAc(2)Hex(1)	No	1213.9411	3	3639.8087	3638.8032	0.6	Non	673.04	647.56	0.38	Nsco_20191108_BC_ZIC_HILIC_K56_2_pepsin_B1.29892.29892.3	63.7666	Pepsin	B1	42
BCAL0303	>gi|443596149|gb|ELT64670.1| hypothetical protein BURCENK562V_3632 [Burkholderia cenocepacia K56-2Valvano]	F.LYSKAADAGGAKPAAGASAAPAAPAPVAVPAS[+568.212]AVSGSAGQ.-	HexNAc(2)Hex(1)	No	1334.6696	3	4001.9941	4001.9826	2.9	CRagged	289.83	289.83	7.2	Nsco_20191108_BC_ZIC_HILIC_K56_2_pepsin_B2.33169.33169.3	70.6148	Pepsin	B2	43
BCAL0303	>gi|443596149|gb|ELT64670.1| hypothetical protein BURCENK562V_3632 [Burkholderia cenocepacia K56-2Valvano]	L.YSKAADAGGAKPAAGAS[+568.212]AAPAAPAPVAV.P	HexNAc(2)Hex(1)	No	992.8305	3	2976.4769	2976.4684	2.8	CRagged	794.53	737.06	49.7	Nsco_20191108_BC_ZIC_HILIC_K56_2_pepsin_B3_reinject.24724.24724.3	51.6664	Pepsin	B3	44

BCAL0303	>gi|443596149|gb|ELT64670.1| hypothetical protein BURCENK562V_3632 [Burkholderia cenocepacia K56-2Valvano]	L.YSKAADAGGAKPAAGAS[+568.212]AAPAAPAPVAVPASAVSG.S	HexNAc(2)Hex(1)	No	1182.9243	3	3546.7582	3545.7493	1.6	CRagged	783.83	744.61	16.57	Nsco_20191108_BC_ZIC_HILIC_K56_2_pepsin_B3_reinject.31280.31280.3	64.1422	Pepsin	B3	45
BCAL0303	>gi|443596149|gb|ELT64670.1| hypothetical protein BURCENK562V_3632 [Burkholderia cenocepacia K56-2Valvano]	L.YSKAADAGGAKPAAGASAAPAAPAPVAVPAS[+568.212]AVSGSAGQ.-	HexNAc(2)Hex(1)	No	1296.9753	3	3888.9113	3888.8985	3.3	CRagged	1045.52	937.06	1.2	Nsco_20191108_BC_ZIC_HILIC_K56_2_pepsin_B3_reinject.31719.31719.3	64.9573	Pepsin	B3	46
BCAL0332	>gi|443603144|gb|ELT71169.1| stringent starvation protein B [Burkholderia cenocepacia K56-2Valvano]	A.FQVDAVAGEGEDSGAFDDDAAQADDAQRDESVSPLAPVADS[+568.212][+100.064]GANEEPSEGADEPPKTDGDGSK.G	HexNAc(2)Hex(1) 100.064	No	1744.5016	4	6974.9844	6973.9813	0	NRagged	822.93	822.93	0	Nsco_20191108_BC_ZIC_HILIC_K56_2_typsin_B2.56297.56297.4	111.8126	Trypsin	B2	47
BCAL0332	>gi|443603144|gb|ELT71169.1| stringent starvation protein B [Burkholderia cenocepacia K56-2Valvano]	D.SGAFDDDAAQADDAQRDES[+568.212]VSPLAPVADSGANEEPS[+568.212][+100.064]EGADEPPKT[+568.212][+100.064]DGDGSK.G	HexNAc(2)Hex(1), HexNAc(2)Hex(1) 100.064,HexNAc(2)Hex(1) 100.064	No	1748.9992	4	6992.9751	6992.9484	3.8	Non	593.37	593.37	0	Nsco_20191108_BC_ZIC_HILIC_K56_2_thermolysin_B3.58745.58745.4	116.544	Thermolysin	B3	48
BCAL0348	>gi|443597060|gb|ELT65512.1| type VI secretion-associated protein, ImpA family [Burkholderia cenocepacia K56-2Valvano]	Q.AVQQAQPERIEPVFGQPIQT[+568.212]EET[+568.212][+100.064]HVQQQTASRPPVTQTIAGIQNR.A	HexNAc(2)Hex(1), HexNAc(2)Hex(1) 100.064	No	1250.6227	5	6249.0843	6243.0702	-1	Non	729.84	729.84	0	Nsco_20191108_BC_ZIC_HILIC_K56_2_thermolysin_B3.39945.39945.5	82.0146	Thermolysin	B3	49
BCAL0348	>gi|443597060|gb|ELT65512.1| type VI secretion-associated protein, ImpA family [Burkholderia cenocepacia K56-2Valvano]	T.GSAPHTQAVQQAQPERIEPVFGQPIQTEET[+568.212]HVQQQTASRPPVTQTIAGIQNR.A	HexNAc(2)Hex(1)	No	1251.6292	5	6254.1166	6253.1032	1.6	Non	469.57	469.57	0	Nsco_20191108_BC_ZIC_HILIC_K56_2_thermolysin_B3.44121.44121.5	89.5488	Thermolysin	B3	50
BCAL0358	>gi|443598967|gb|ELT67284.1| peptidase family M1 [Burkholderia cenocepacia K56-2Valvano]	S.SAGAPHTGSSPT[+568.212]VAAPPSAS[+568.212]NVDK.S	HexNAc(2)Hex(1), HexNAc(2)Hex(1)	No	1115.1758	3	3343.5128	3342.4966	3.8	Non	708.56	554.71	3.78	Nsco_20191108_BC_ZIC_HILIC_K56_2_thermolysin_B3.13859.13859.3	34.0672	Thermolysin	B3	51
BCAL0426	>gi|443602087|gb|ELT70191.1| membrane protein insertase, YidC/Oxa1 family [Burkholderia cenocepacia K56-2Valvano]	S.ATHTAPAAAGGASGT[+568.212]GATTAGDVPAA.A	HexNAc(2)Hex(1)	No	1360.6226	2	2720.2378	2720.2381	-0.1	Non	616.62	434.57	0	Nsco_20191108_BC_ZIC_HILIC_K56_2_thermolysin_B1.19339.19339.2	41.3403	Thermolysin	B1	52
BCAL0426	>gi|443602087|gb|ELT70191.1| membrane protein insertase, YidC/Oxa1 family [Burkholderia cenocepacia K56-2Valvano]	R.DHGRPSMFFPSATHTAPAAAGGAS[+568.212]GTGATTAGDVPAAAAGAAPSTTAPAAQAQLVK.F	HexNAc(2)Hex(1)	Yes	1137.5507	5	5683.7242	5683.7093	2.6	Specific	1336.17	1307.6	53.87	Nsco_20191108_BC_ZIC_HILIC_K56_2_typsin_B1.41182.41182.5	84.0593	Trypsin	B1	53
BCAL0426	>gi|443602087|gb|ELT70191.1| membrane protein insertase, YidC/Oxa1 family [Burkholderia cenocepacia K56-2Valvano]	F.FPSATHTAPAAAGGASGTGATT[+568.212][+100.064]AGDVPAAAAGAAPSTTAPAAQ.A	HexNAc(2)Hex(1) 100.064	No	1080.2585	4	4318.0124	4317.028	-4.4	CRagged	596.73	516.78	0	Nsco_20191108_BC_ZIC_HILIC_K56_2_pepsin_B2.36657.36657.4	78.7405	Pepsin	B2	54
BCAL0426	>gi|443602087|gb|ELT70191.1| membrane protein insertase, YidC/Oxa1 family [Burkholderia cenocepacia K56-2Valvano]	T.HTAPAAAGGASGTGATT[+568.212]AGDVPAAAAGAAPSTTAPAAQ.A	HexNAc(2)Hex(1)	No	1238.5833	3	3713.7354	3713.726	2.5	Non	804.69	742.1	1.2	Nsco_20191108_BC_ZIC_HILIC_K56_2_pepsin_B3_reinject.30629.30629.3	62.8704	Pepsin	B3	55
BCAL0479	>gi|443597239|gb|ELT65680.1| penicillin-binding protein 2, partial [Burkholderia cenocepacia K56-2Valvano]	A.AASAT[+568.212]EPVS[+568.212]AP.V	HexNAc(2)Hex(1), HexNAc(2)Hex(1)	No	1068.9668	2	2136.9264	2136.9177	4.1	Non	375.8	192.35	62.46	Nsco_20191108_BC_ZIC_HILIC_K56_2_thermolysin_B1.18700.18700.2	40.3138	Thermolysin	B1	56
BCAL0520	>gi|443592535|gb|ELT61330.1| hypothetical protein BURCENK562V_3076, partial [Burkholderia cenocepacia K56-2Valvano]	A.ARGSGAS[+568.212]AAGADAAAAT[+568.212]SAVP.F	HexNAc(2)Hex(1), HexNAc(2)Hex(1)	No	1433.6428	2	2866.2784	2866.2695	3.1	Non	357.63	283.69	2.4	Nsco_20191108_BC_ZIC_HILIC_K56_2_thermolysin_B1.20407.20407.2	43.3508	Thermolysin	B1	57

BCAL0520	>gi|443592535|gb|ELT61330.1| hypothetical protein BURCENK562V_3076, partial [Burkholderia cenocepacia K56-2Valvano]	R.GSGASAAGADAAAAT[+568.212]S[+568.212]AVPFAQTLK.Q	HexNAc(2)Hex(1), HexNAc(2)Hex(1)	No	1664.2688	2	3327.5304	3327.5221	2.5	Specific	968.77	869.46	14.4	Nsco_20191108_BC_ZIC_HILIC_K56_2_typsin_B2.39446.39446.2	81.8591	Trypsin	B2	58
BCAL0525	>gi|443597648|gb|ELT66063.1| flagellar M-ring protein FliF, partial [Burkholderia cenocepacia K56-2Valvano]	L.SNTPPQPAS[+568.212]APIVAGNGQNAPQTTPVSD.R	HexNAc(2)Hex(1)	Yes	1095.5191	3	3284.5427	3284.5288	4.2	CRagged	535.25	449.78	35.51	Nsco_20191108_BC_ZIC_HILIC_K56_2_pepsin_B3_reinject.37980.37980.3	78.3801	Pepsin	B3	59
BCAL0525	>gi|443597648|gb|ELT66063.1| flagellar M-ring protein FliF, partial [Burkholderia cenocepacia K56-2Valvano]	R.SQQTSSATELAQGGASGVPGALSNT[+568.212]PPQPAS[+568.212]APIVAGNGQNAPQTTPVSDRK.D	HexNAc(2)Hex(1), HexNAc(2)Hex(1)	No	1534.984	4	6136.9142	6134.9019	0.9	Specific	711.45	711.45	0	Nsco_20191108_BC_ZIC_HILIC_K56_2_typsin_B2.40293.40293.4	83.3257	Trypsin	B2	60
BCAL0544	>gi|443601095|gb|ELT69255.1| ABC transporter, substrate-binding protein, family 5 [Burkholderia cenocepacia K56-2Valvano]	K.KAILESVYQGAGQAAS[+568.212]APMPPTQWSYDK.N	HexNAc(2)Hex(1)	No	1188.2355	3	3562.692	3562.6782	3.9	Specific	898.13	898.13	6.78	Nsco_20191108_BC_ZIC_HILIC_K56_2_typsin_B2.47878.47878.3	96.7111	Trypsin	B2	61
BCAL0678	>gi|443605648|gb|ELT73487.1| sporulation and cell division repeat protein [Burkholderia cenocepacia K56-2Valvano]	Q.AAAQKQQQQQQAANT[+568.212]PKPTSSAT.A	HexNAc(2)Hex(1)	No	984.477	3	2951.4163	2951.4076	3	Non	463.13	421.62	5.62	Nsco_20191108_BC_ZIC_HILIC_K56_2_thermolysin_B1.4818.4818.3	18.5162	Thermolysin	B1	62
BCAL0678	>gi|443605648|gb|ELT73487.1| sporulation and cell division repeat protein [Burkholderia cenocepacia K56-2Valvano]	Q.AAAQKQQQQQQAANT[+568.212]PKPTSSATA.A	HexNAc(2)Hex(1)	No	1008.1566	3	3022.4553	3022.4447	3.5	Non	603.81	562.68	1.48	Nsco_20191108_BC_ZIC_HILIC_K56_2_thermolysin_B2_.5231.5231.3	20.1389	Thermolysin	B2	63
BCAL0678	>gi|443605648|gb|ELT73487.1| sporulation and cell division repeat protein [Burkholderia cenocepacia K56-2Valvano]	F.AAQKQAQQAAAQKQQQQQQAANT[568.212]PKPTSSATAAA.A	HexNAc(2)Hex(1)	No	1339.9922	3	4017.962	4017.9596	0.6	CRagged	527.89	389.69	9.6	Nsco_20191108_BC_ZIC_HILIC_K56_2_pepsin_B3_reinject.9448.9448.3	26.3294	Pepsin	B3	64
BCAL0678	>gi|443605648|gb|ELT73487.1| sporulation and cell division repeat protein [Burkholderia cenocepacia K56-2Valvano]	A.AAQKQQQQQQAANT[+568.212]PKPTSSATAAA.A	HexNAc(2)Hex(1)	No	1032.1697	3	3094.4945	3093.4818	3	Non	516.81	460.86	14.7	Nsco_20191108_BC_ZIC_HILIC_K56_2_pepsin_B2.6966.6966.3	22.9774	Pepsin	B2	65
BCAL0678	>gi|443605648|gb|ELT73487.1| sporulation and cell division repeat protein [Burkholderia cenocepacia K56-2Valvano]	Q.KQQQQQQAANTPKPTS[+568.212]SATAAAAAKPPTANDANTGYFLQVGAY.K	HexNAc(2)Hex(1)	No	1244.6036	4	4975.3927	4973.3923	-1.3	Non	586.51	547.75	15.42	Nsco_20191108_BC_ZIC_HILIC_K56_2_thermolysin_B3.37078.37078.4	76.7191	Thermolysin	B3	66
BCAL0678	>gi|443605648|gb|ELT73487.1| sporulation and cell division repeat protein [Burkholderia cenocepacia K56-2Valvano]	Q.KQQQQQQAANTPKPTS[+568.212]SATAAAAAKPPTANDANTGYFLQVGAYK.T	HexNAc(2)Hex(1)	No	1021.106	5	5101.501	5101.4873	2.7	NRagged	625.67	625.67	4.2	Nsco_20191108_BC_ZIC_HILIC_K56_2_typsin_B1.30503.30503.5	65.5598	Trypsin	B1	67
BCAL0678	>gi|443605648|gb|ELT73487.1| sporulation and cell division repeat protein [Burkholderia cenocepacia K56-2Valvano]	Q.KQQQQQQAANTPKPTS[+568.212]SATAAAAAKPPTANDANTGYFLQVGAYKTEGDAEQQR.A	HexNAc(2)Hex(1)	No	1020.3292	6	6116.939	6115.9239	1.9	NRagged	740.63	729.18	5	Nsco_20191108_BC_ZIC_HILIC_K56_2_typsin_B2.29453.29453.6	63.378	Trypsin	B2	68
BCAL0678	>gi|443605648|gb|ELT73487.1| sporulation and cell division repeat protein [Burkholderia cenocepacia K56-2Valvano]	S.KVAPPPADNGAS[+568.212]QPQQFDPNRA.L	HexNAc(2)Hex(1)	Yes	958.4554	3	2873.3517	2873.3436	2.8	Non	630.58	630.58	630.58	Nsco_20191108_BC_ZIC_HILIC_K56_2_pepsin_B3_reinject.18300.18300.3	40.8167	Pepsin	B3	69
BCAL0678	>gi|443605648|gb|ELT73487.1| sporulation and cell division repeat protein [Burkholderia cenocepacia K56-2Valvano]	S.KVAPPPADNGAS[+568.212]QPQQFDPNRAL.Q	HexNAc(2)Hex(1)	Yes	996.151	3	2986.4384	2986.4276	3.6	NRagged	681.76	616.02	616.02	Nsco_20191108_BC_ZIC_HILIC_K56_2_pepsin_B1.27173.27173.3	57.9729	Pepsin	B1	70
BCAL0678	>gi|443605648|gb|ELT73487.1| sporulation and cell division repeat protein [Burkholderia cenocepacia K56-2Valvano]	S.KVAPPPADNGAS[+568.212]QPQQFDPNRALQ.G	HexNAc(2)Hex(1)	No	1038.8365	3	3114.4948	3114.4862	2.8	Non	684.78	621.45	621.45	Nsco_20191108_BC_ZIC_HILIC_K56_2_pepsin_B1.26704.26704.3	56.8912	Pepsin	B1	71
BCAL0678	>gi|443605648|gb|ELT73487.1| sporulation and cell division repeat protein [Burkholderia cenocepacia K56-2Valvano]	S.KVAPPPADNGAS[+568.212]QPQQFDPNRALQG.K	HexNAc(2)Hex(1)	Yes	1057.8449	3	3171.5201	3171.5077	3.9	Non	621.38	611.92	611.92	Nsco_20191108_BC_ZIC_HILIC_K56_2_pepsin_B1.27694.27694.3	59.0443	Pepsin	B1	72
BCAL0678	>gi|443605648|gb|ELT73487.1| sporulation and cell division repeat protein [Burkholderia cenocepacia K56-2Valvano]	S.KVAPPPADNGAS[+568.212]QPQQFDPNRALQGKTPGQPVPQA.A	HexNAc(2)Hex(1)	Yes	1044.5223	4	4175.0672	4175.0527	3.5	Non	743.18	743.18	488.41	Nsco_20191108_BC_ZIC_HILIC_K56_2_pepsin_B3_reinject.31852.31852.4	65.2064	Pepsin	B3	73

BCAL0678	>gi|443605648|gb|ELT73487.1| sporulation and cell division repeat protein [Burkholderia cenocepacia K56-2Valvano]	S.KVAPPPADNGAS[+568.212]QPQQFDPNRALQGKTPGQPVPQAAQPAPPNTA.P	HexNAc(2)Hex(1)	Yes	1256.3777	4	5022.4889	5022.4716	3.5	Non	665.41	665.41	320.9	Nsco_20191108_BC_ZIC_HILIC_K56_2_pepsin_B1.33145.33145.4	71.3551	Pepsin	B1	74
BCAL0678	>gi|443605648|gb|ELT73487.1| sporulation and cell division repeat protein [Burkholderia cenocepacia K56-2Valvano]	S.KVAPPPADNGAS[+568.212]QPQQFDPNRALQGKTPGQPVPQAAQPAPPNTAPGQ.A	HexNAc(2)Hex(1)	Yes	1326.9113	4	5304.6232	5304.6044	3.6	Non	836.81	836.81	393.47	Nsco_20191108_BC_ZIC_HILIC_K56_2_pepsin_B3_reinject.35229.35229.4	72.3622	Pepsin	B3	75
BCAL0678	>gi|443605648|gb|ELT73487.1| sporulation and cell division repeat protein [Burkholderia cenocepacia K56-2Valvano]	S.KVAPPPADNGAS[+568.212]QPQQFDPNRALQGKTPGQPVPQAAQPAPPNTAPGQA.A	HexNAc(2)Hex(1)	Yes	1075.9376	5	5375.659	5375.6415	3.3	Non	957.7	957.7	499.25	Nsco_20191108_BC_ZIC_HILIC_K56_2_pepsin_B2.34545.34545.5	73.7842	Pepsin	B2	76
BCAL0678	>gi|443605648|gb|ELT73487.1| sporulation and cell division repeat protein [Burkholderia cenocepacia K56-2Valvano]	S.KVAPPPADNGAS[+568.212]QPQQFDPNRALQGKTPGQPVPQAAQPAPPNTAPGQAA.N	HexNAc(2)Hex(1)	Yes	1362.4287	4	5446.693	5446.6786	2.7	Non	807.9	807.9	435.58	Nsco_20191108_BC_ZIC_HILIC_K56_2_pepsin_B1.33981.33981.4	73.5497	Pepsin	B1	77
BCAL0678	>gi|443605648|gb|ELT73487.1| sporulation and cell division repeat protein [Burkholderia cenocepacia K56-2Valvano]	S.KVAPPPADNGAS[+568.212]QPQQFDPNRALQGKTPGQPVPQAAQPAPPNTAPGQAAN.Q	HexNAc(2)Hex(1)	Yes	1113.3529	5	5562.7354	5560.7215	1.3	Non	1014.59	1000.8	483.05	Nsco_20191108_BC_ZIC_HILIC_K56_2_pepsin_B1.33588.33588.5	72.4426	Pepsin	B1	78
BCAL0678	>gi|443605648|gb|ELT73487.1| sporulation and cell division repeat protein [Burkholderia cenocepacia K56-2Valvano]	S.KVAPPPADNGAS[+568.212]QPQQFDPNRALQGKTPGQPVPQAAQPAPPNTAPGQAANQ.T	HexNAc(2)Hex(1)	Yes	1422.9518	4	5688.7853	5688.7801	0.9	Non	680.89	672.05	302.36	Nsco_20191108_BC_ZIC_HILIC_K56_2_pepsin_B3_reinject.35184.35184.4	72.264	Pepsin	B3	79
BCAL0678	>gi|443605648|gb|ELT73487.1| sporulation and cell division repeat protein [Burkholderia cenocepacia K56-2Valvano]	S.KVAPPPADNGASQPQQFDPNRALQGKT[+568.212][+100.064]PGQPVPQAAQPAPPNTAPGQAANQTQGGL.L	HexNAc(2)Hex(1) 100.064	No	1562.0194	4	6245.0558	6245.0773	-3.4	NRagged	730.82	730.82	3.6	Nsco_20191108_BC_ZIC_HILIC_K56_2_pepsin_B3_reinject.39257.39257.4	81.4775	Pepsin	B3	80
BCAL0678	>gi|443605648|gb|ELT73487.1| sporulation and cell division repeat protein [Burkholderia cenocepacia K56-2Valvano]	T.PKPT[+568.212]S[+568.212]S[+568.212][+100.064]ATAAAAAKPPTANDANTGYFLQVGAYKTEGDAEQQR.A	HexNAc(2)Hex(1), HexNAc(2)Hex(1), HexNAc(2)Hex(1) 100.064	No	1525.4624	4	6098.8278	6098.7998	4.6	Non	496.51	496.51	0	Nsco_20191108_BC_ZIC_HILIC_K56_2_thermolysin_B3.40382.40382.4	82.8135	Thermolysin	B3	81
BCAL0678	>gi|443605648|gb|ELT73487.1| sporulation and cell division repeat protein [Burkholderia cenocepacia K56-2Valvano]	K.PTSSATAAAAAKPPT[+568.212]ANDANTGYFLQVGAYK.T	HexNAc(2)Hex(1)	No	1208.9186	3	3624.7412	3622.7283	1.7	Specific	915.91	852.98	1.2	Nsco_20191108_BC_ZIC_HILIC_K56_2_trypsin_B1.39532.39532.3	81.3052	Trypsin	B1	82
BCAL0678	>gi|443605648|gb|ELT73487.1| sporulation and cell division repeat protein [Burkholderia cenocepacia K56-2Valvano]	Q.QAANTPKPTSSATAAAAAKPPT[+568.212][+100.064]ANDANT[+568.212]GYFLQVGAYK.T	HexNAc(2)Hex(1) 100.064,HexNAc(2)Hex(1)	No	1251.8569	4	5004.4059	5001.375	4.2	NRagged	594.1	594.1	0	Nsco_20191108_BC_ZIC_HILIC_K56_2_trypsin_B2.34826.34826.4	73.3709	Trypsin	B2	83
BCAL0678	>gi|443605648|gb|ELT73487.1| sporulation and cell division repeat protein [Burkholderia cenocepacia K56-2Valvano]	Q.QAANTPKPTSS[+568.212][+100.064]AT[+568.212][+100.064]AAAAAKPPTANDANTGYFLQVGAYKTEGDAEQQR.A	HexNAc(2)Hex(1) 100.064,HexNAc(2)Hex(1) 100.064	No	1223.9764	5	6115.8531	6115.8756	-3.7	NRagged	820.45	820.45	0	Nsco_20191108_BC_ZIC_HILIC_K56_2_trypsin_B2.34844.34844.5	73.4024	Trypsin	B2	84
BCAL0678	>gi|443605648|gb|ELT73487.1| sporulation and cell division repeat protein [Burkholderia cenocepacia K56-2Valvano]	A.QKQQQQQQAANTPKPT[+568.212]SSATAAA.A	HexNAc(2)Hex(1)	No	1476.2091	2	2951.4109	2951.4076	1.1	Non	588.4	506.86	0.59	Nsco_20191108_BC_ZIC_HILIC_K56_2_pepsin_B1.7372.7372.2	22.8943	Pepsin	B1	85
BCAL0678	>gi|443605648|gb|ELT73487.1| sporulation and cell division repeat protein [Burkholderia cenocepacia K56-2Valvano]	Q.QQAANT[+568.212]PKPTS[+568.212]SATAAAAAKPPTANDANTGYFLQVGAYK.T	HexNAc(2)Hex(1), HexNAc(2)Hex(1)	No	1258.1035	4	5029.3922	5029.3696	4.5	NRagged	490.84	465.88	4.32	Nsco_20191108_BC_ZIC_HILIC_K56_2_trypsin_B2.36809.36809.4	77.0609	Trypsin	B2	86
BCAL0678	>gi|443605648|gb|ELT73487.1| sporulation and cell division repeat protein [Burkholderia cenocepacia K56-2Valvano]	Q.QQAANTPKPTSS[+568.212]ATAAAAAKPPT[+568.212]ANDANTGYFLQVGAYKTEGDAEQQR.A	HexNAc(2)Hex(1), HexNAc(2)Hex(1)	No	1209.9703	5	6045.8226	6043.8062	1.6	NRagged	854.96	854.96	0	Nsco_20191108_BC_ZIC_HILIC_K56_2_trypsin_B2.33550.33550.5	70.9351	Trypsin	B2	87
BCAL0678	>gi|443605648|gb|ELT73487.1| sporulation and cell division repeat protein [Burkholderia cenocepacia K56-2Valvano]	K.QQQQQQAANTPKPTS[+568.212][+100.064]SAT[+568.212][+100.064]AAAAAKPPTANDANTGYFL.Q	HexNAc(2)Hex(1) 100.064,HexNAc(2)Hex(1) 100.064	No	1275.8588	4	5100.4132	5095.388	1.7	NRagged	189.07	189.07	0	Nsco_20191108_BC_ZIC_HILIC_K56_2_thermolysin_B3.37083.37083.4	76.7329	Thermolysin	B3	88
BCAL0678	>gi|443605648|gb|ELT73487.1| sporulation and cell division repeat protein [Burkholderia cenocepacia K56-2Valvano]	K.QQQQQQAANTPKPTSS[+568.212]ATAAAAAKPPTANDANTGYFLQV.G	HexNAc(2)Hex(1)	No	1519.0698	3	4555.1947	4554.1754	3.5	Non	650.23	592.01	0	Nsco_20191108_BC_ZIC_HILIC_K56_2_thermolysin_B3.38672.38672.3	79.7172	Thermolysin	B3	89

BCAL0678	>gi|443605648|gb|ELT73487.1| sporulation and cell division repeat protein [Burkholderia cenocepacia K56-2Valvano]	K.QQQQQQAANTPKPTS[+568.212]SATAAAAAKPPTANDANTGYFLQVGAYK.T	HexNAc(2)Hex(1)	Yes	1244.1068	4	4973.4054	4973.3923	2.6	Specific	1261.39	1149.3	13.15	Nsco_20191108_BC_ZIC_HILIC_K56_2_typsin_B3.34982.34982.4	72.4325	Trypsin	B3	90
BCAL0678	>gi|443605648|gb|ELT73487.1| sporulation and cell division repeat protein [Burkholderia cenocepacia K56-2Valvano]	K.QQQQQQAANTPKPTS[+568.212]SATAAAAAKPPTANDANTGYFLQVGAYKT[+568.212][+100.064]EGDA.E	HexNAc(2)Hex(1), HexNAc(2)Hex(1) 100.064	No	1223.7767	5	6114.8546	6114.8437	1.8	Non	321.1	321.1	4.94	Nsco_20191108_BC_ZIC_HILIC_K56_2_thermolysin_B3.36187.36187.5	74.9504	Thermolysin	B3	91
BCAL0678	>gi|443605648|gb|ELT73487.1| sporulation and cell division repeat protein [Burkholderia cenocepacia K56-2Valvano]	K.QQQQQQAANTPKPTSSATAAAAAKPPTANDANT[+568.212]GYFLQVGAYKTEGDAEQQR.A	HexNAc(2)Hex(1)	No	1199.1738	5	5991.84	5987.829	-0.4	Specific	1367.58	458.19	0	Nsco_20191108_BC_ZIC_HILIC_K56_2_typsin_B2.33883.33883.5	71.5446	Trypsin	B2	92
BCAL0678	>gi|443605648|gb|ELT73487.1| sporulation and cell division repeat protein [Burkholderia cenocepacia K56-2Valvano]	K.VAPPPADNGAS[+568.212]QPQQFDPNR.A	HexNAc(2)Hex(1)	No	1337.6141	2	2674.2208	2674.2115	3.5	Specific	607.46	540.04	540.04	Nsco_20191108_BC_ZIC_HILIC_K56_2_typsin_B2.21592.21592.2	48.5971	Trypsin	B2	93
BCAL0678	>gi|443605648|gb|ELT73487.1| sporulation and cell division repeat protein [Burkholderia cenocepacia K56-2Valvano]	K.VAPPPADNGAS[+568.212]QPQQFDPNRA.L	HexNAc(2)Hex(1)	Yes	1373.1335	2	2745.2597	2745.2486	4	Non	430.75	319.55	319.55	Nsco_20191108_BC_ZIC_HILIC_K56_2_thermolysin_B1.25115.25115.2	52.1185	Thermolysin	B1	94
BCAL0678	>gi|443605648|gb|ELT73487.1| sporulation and cell division repeat protein [Burkholderia cenocepacia K56-2Valvano]	K.VAPPPADNGAS[+568.212]QPQQFDPNRALQGK.T	HexNAc(2)Hex(1)	No	1057.8439	3	3171.5171	3171.5077	3	Specific	699.57	699.57	699.57	Nsco_20191108_BC_ZIC_HILIC_K56_2_typsin_B3.27804.27804.3	60.0678	Trypsin	B3	95
BCAL0678	>gi|443605648|gb|ELT73487.1| sporulation and cell division repeat protein [Burkholderia cenocepacia K56-2Valvano]	K.VAPPPADNGASQPQQFDPNRALQGKT[+568.212][+100.064]PGQPVPQAAQPAPPNTAPGQA.A	HexNAc(2)Hex(1) 100.064	No	1337.9092	4	5348.6149	5347.6105	0.2	Non	207.24	207.24	2.56	Nsco_20191108_BC_ZIC_HILIC_K56_2_pepsin_B3_reinject.40181.40181.4	83.8184	Pepsin	B3	96
BCAL0678	>gi|443605648|gb|ELT73487.1| sporulation and cell division repeat protein [Burkholderia cenocepacia K56-2Valvano]	F.VSKVAPPPADNGAS[+568.212]QPQQFDPNRA.L	HexNAc(2)Hex(1)	No	1020.8231	3	3060.4548	3059.444	2.4	CRagged	557.21	180.84	80.42	Nsco_20191108_BC_ZIC_HILIC_K56_2_pepsin_B2.20692.20692.3	45.6549	Pepsin	B2	97
BCAL0678	>gi|443605648|gb|ELT73487.1| sporulation and cell division repeat protein [Burkholderia cenocepacia K56-2Valvano]	F.VSKVAPPPADNGAS[+568.212]QPQQFDPNRALQGKTPGQPVPQAAQPAPPNTAPGQA.A	HexNAc(2)Hex(1)	No	1391.1888	4	5561.7336	5561.7419	-1.5	CRagged	554.99	554.99	202.81	Nsco_20191108_BC_ZIC_HILIC_K56_2_pepsin_B2.35137.35137.4	75.2139	Pepsin	B2	98
BCAL0678	>gi|443605648|gb|ELT73487.1| sporulation and cell division repeat protein [Burkholderia cenocepacia K56-2Valvano]	F.VSKVAPPPADNGASQPQQFDPNRALQGKT[+568.212]PGQPVPQAAQPAPPNTAPGQAAN.Q	HexNAc(2)Hex(1)	No	1437.7139	4	5747.8337	5746.822	1.5	CRagged	357.31	287.71	3.6	Nsco_20191108_BC_ZIC_HILIC_K56_2_pepsin_B3_reinject.35940.35940.4	73.9241	Pepsin	B3	99
BCAL0738	>gi|443604531|gb|ELT72458.1| peptidase, S41 family [Burkholderia cenocepacia K56-2Valvano]	K.ASGASAAKPASAPKPAS[+568.212]APK.-	HexNAc(2)Hex(1)	No	778.3971	3	2333.1768	2333.1718	2.1	Specific	812.76	587.88	4.9	Nsco_20191108_BC_ZIC_HILIC_K56_2_typsin_B3.4955.4955.3	19.5407	Trypsin	B3	100
BCAL0749	>gi|443604502|gb|ELT72429.1| cytochrome c oxidase, subunit II [Burkholderia cenocepacia K56-2Valvano]	G.AATAAAPAEAASAPAQAASGAEQPAAAASAALS[+568.212][+100.064]T[+568.212][+100.064]IYFETGK.S	HexNAc(2)Hex(1) 100.064,HexNAc(2)Hex(1) 100.064	No	1691.4734	3	5072.4055	5069.3863	1.8	Non	287.24	225.55	1.2	Nsco_20191108_BC_ZIC_HILIC_K56_2_thermolysin_B3.63162.63162.3	125.8637	Thermolysin	B3	101
BCAL0749	>gi|443604502|gb|ELT72429.1| cytochrome c oxidase, subunit II [Burkholderia cenocepacia K56-2Valvano]	T.AGAATAAAPAEAASAPAQAAS[+568.212][+100.064]GAEQPAAAAS[+568.212]AALST[+568.212]IYFETGK.S	HexNAc(2)Hex(1) 100.064,HexNAc(2)Hex(1),HexNAc(2)Hex(1)	No	1418.1528	4	5669.5895	5665.5924	-2.9	Non	168.77	168.77	0	Nsco_20191108_BC_ZIC_HILIC_K56_2_thermolysin_B3.61428.61428.4	122.1804	Thermolysin	B3	102
BCAL0749	>gi|443604502|gb|ELT72429.1| cytochrome c oxidase, subunit II [Burkholderia cenocepacia K56-2Valvano]	R.NGKLPEDTAGAAT[+568.212]AAAPAEAASAPAQAAS[+568.212]GAEQPAAAASAALSTIYFETGK.S	HexNAc(2)Hex(1), HexNAc(2)Hex(1)	Yes (1 of 2)	1463.693	4	5851.7502	5851.7303	3.4	Specific	1107.43	1017.6	111.99	Nsco_20191108_BC_ZIC_HILIC_K56_2_typsin_B3.61271.61271.4	117.8329	Trypsin	B3	103
BCAL0749	>gi|443604502|gb|ELT72429.1| cytochrome c oxidase, subunit II [Burkholderia cenocepacia K56-2Valvano]	L.PEDTAGAATAAAPAEAASAPAQAASGAEQPAAAASAALS[+568.212][+100.064]T[+568.212][+100.064]IYFETGK.S	HexNAc(2)Hex(1) 100.064,HexNAc(2)Hex(1) 100.064	No	1410.6604	4	5639.6198	5639.6148	0.9	CRagged	510.79	70.27	1.2	Nsco_20191108_BC_ZIC_HILIC_K56_2_thermolysin_B3.60731.60731.4	120.695	Thermolysin	B3	104
BCAL0749	>gi|443604502|gb|ELT72429.1| cytochrome c oxidase, subunit II [Burkholderia cenocepacia K56-2Valvano]	K.QVADARNGKLPEDT[+568.212]AGAATAAAPAEAASAPAQAASGAEQPAAAASAALSTIYFETGK.S	HexNAc(2)Hex(1)	No	1481.9689	4	5924.8537	5923.848	0.4	Specific	880.62	880.62	1.6	Nsco_20191108_BC_ZIC_HILIC_K56_2_typsin_B3.62336.62336.4	119.6719	Trypsin	B3	105

BCAL0749	>gi|443604502|gb|ELT72429.1| cytochrome c oxidase, subunit II [Burkholderia cenocepacia K56-2Valvano]	Q.VADARNGKLPEDTAGAAT[+568.212]AAAPAEAAS[+568.212]APAQAAS GAEQPA.A	HexNAc(2)Hex(1), HexNAc(2)Hex(1)	Yes (1 of 2)	1595.0779	3	4783.2191	4782.1971	3.9	Non	226.43	204.84	0.8	Nsco_20191108_BC_ZIC_HILIC_K56_2_thermolysin_B2_.33508.33508.3	63.5736	Thermolysin	B2	106
BCAL0786	>gi|443598725|gb|ELT67055.1| hypothetical protein BURCENK562V_1882 [Burkholderia cenocepacia K56-2Valvano]	K.LLSVPAPAS[+568.212]TEGDHHDK.-	HexNAc(2)Hex(1)	No	1171.5508	2	2342.0944	2342.0882	2.6	Specific	726.94	522.22	0	Nsco_20191108_BC_ZIC_HILIC_K56_2_typsin_B3.14451.14451.4	36.0021	Trypsin	B3	107
BCAL1086	>gi|443597491|gb|ELT65913.1| putative lipoprotein [Burkholderia cenocepacia K56-2Valvano]	K.ALDQVAS[+568.212]TVNQQINAAK.A	HexNAc(2)Hex(1)	No	1170.0798	2	2339.1522	2339.146	2.7	Specific	684.32	642.21	0.8	Nsco_20191108_BC_ZIC_HILIC_K56_2_typsin_B1.35456.35456.2	74.0097	Trypsin	B1	108
BCAL1086	>gi|443597491|gb|ELT65913.1| putative lipoprotein [Burkholderia cenocepacia K56-2Valvano]	Q.IDAAAS[+568.212]AVVAH.A	HexNAc(2)Hex(1)	Yes	796.8835	2	1592.7597	1592.7538	3.7	Non	394.42	147.31	147.31	Nsco_20191108_BC_ZIC_HILIC_K56_2_thermolysin_B2_.18308.18308.2	39.2035	Thermolysin	B2	109
BCAL1086	>gi|443597491|gb|ELT65913.1| putative lipoprotein [Burkholderia cenocepacia K56-2Valvano]	K.KLQQWSQQSAAGAKPAS[+568.212]GE.-	HexNAc(2)Hex(1)	No	1270.6071	2	2540.2069	2540.1998	2.8	CRagged	814.72	675.6	2.34	Nsco_20191108_BC_ZIC_HILIC_K56_2_typsin_B2.11123.11123.2	29.9123	Trypsin	B2	110
BCAL1086	>gi|443597491|gb|ELT65913.1| putative lipoprotein [Burkholderia cenocepacia K56-2Valvano]	E.KSGTPVAQPDTAAS[+568.212]AAAD.A	HexNAc(2)Hex(1)	No	1113.5132	2	2226.0191	2226.0143	2.1	Non	524.82	394.52	0	Nsco_20191108_BC_ZIC_HILIC_K56_2_pepsin_B1.13985.13985.2	33.9556	Pepsin	B1	111
BCAL1086	>gi|443597491|gb|ELT65913.1| putative lipoprotein [Burkholderia cenocepacia K56-2Valvano]	E.KSGT[+568.212]PVAQPDTAASAAADAANN.A	HexNAc(2)Hex(1)	No	1298.5951	2	2596.183	2596.1744	3.3	Non	583.11	522.96	1.89	Nsco_20191108_BC_ZIC_HILIC_K56_2_pepsin_B2.20376.20376.2	44.9604	Pepsin	B2	112
BCAL1086	>gi|443597491|gb|ELT65913.1| putative lipoprotein [Burkholderia cenocepacia K56-2Valvano]	E.KSGTPVAQPDTAAS[+568.212]AAADAANNAAKAL.D	HexNAc(2)Hex(1)	No	1017.4953	3	3050.4713	3050.4648	2.1	NRagged	456.51	268.96	3.6	Nsco_20191108_BC_ZIC_HILIC_K56_2_pepsin_B1.37651.37651.4	82.7525	Pepsin	B1	113
BCAL1086	>gi|443597491|gb|ELT65913.1| putative lipoprotein [Burkholderia cenocepacia K56-2Valvano]	K.LQQWSQQSAAGAKPAS[+568.212]GE.-	HexNAc(2)Hex(1)	No	1206.5588	2	2412.1102	2412.1049	2.2	CRagged	750.26	616.49	1.06	Nsco_20191108_BC_ZIC_HILIC_K56_2_typsin_B2.15236.15236.2	36.5826	Trypsin	B2	114
BCAL1086	>gi|443597491|gb|ELT65913.1| putative lipoprotein [Burkholderia cenocepacia K56-2Valvano]	K.SGTPVAQPDTAAS[+568.212]AAADAANNAAK.A	HexNAc(2)Hex(1)	No	1369.6319	2	2738.2565	2738.2486	2.9	Specific	650.93	618.24	9.86	Nsco_20191108_BC_ZIC_HILIC_K56_2_typsin_B3.28764.28764.2	61.6522	Trypsin	B3	115
BCAL1093	>gi|443599421|gb|ELT67702.1| hypothetical protein BURCENK562V_2687 [Burkholderia cenocepacia K56-2Valvano]	K.HAYDEAHPAEAAS[+568.212]AASH.-	HexNAc(2)Hex(1)	No	768.3271	3	2302.9666	2302.9582	3.6	CRagged	642.3	642.3	6	Nsco_20191108_BC_ZIC_HILIC_K56_2_typsin_B3.7342.7342.3	24.2457	Trypsin	B3	116
BCAL1357	>gi|443602613|gb|ELT70680.1| PF11745 family protein [Burkholderia cenocepacia K56-2Valvano]	A.TDMSPAAS[+568.212]T[+568.212]AAGASSGAGAAAASGTMGLK.L	HexNAc(2)Hex(1), HexNAc(2)Hex(1)	No	1202.196	3	3604.5736	3604.5623	3.1	NRagged	493.66	231.83	7.8	Nsco_20191108_BC_ZIC_HILIC_K56_2_typsin_B3.30818.30818.3	65.4053	Trypsin	B3	117
BCAL1453	>gi|443604751|gb|ELT72660.1| HlyD family secretion protein [Burkholderia cenocepacia K56-2Valvano]	R.ENAAHSASS[+568.212]ADAQYQQAIAALDAAK.L	HexNAc(2)Hex(1)	No	1024.4731	3	3071.4046	3070.3971	1.3	Specific	915.41	915.41	2.4	Nsco_20191108_BC_ZIC_HILIC_K56_2_typsin_B1.39870.39870.3	81.8189	Trypsin	B1	118
BCAL1453	>gi|443604751|gb|ELT72660.1| HlyD family secretion protein [Burkholderia cenocepacia K56-2Valvano]	K.ENRENAAHSAS[+568.212]SADAQYQQAIAALDAAK.L	HexNAc(2)Hex(1)	Yes	868.1537	4	3469.5931	3469.5837	2.7	Specific	1111.53	987.06	56.26	Nsco_20191108_BC_ZIC_HILIC_K56_2_typsin_B3.34661.34661.4	71.9269	Trypsin	B3	119
BCAL1496	>gi|443597624|gb|ELT66041.1| hypothetical protein BURCENK562V_3510 [Burkholderia cenocepacia K56-2Valvano]	K.AAAKKAGKKAKAADAAS[+568.212]Q.-	HexNAc(2)Hex(1)	Yes	564.3015	4	2254.1841	2254.1773	3	Non	480.3	448.6	448.6	Nsco_20191108_BC_ZIC_HILIC_K56_2_thermolysin_B2_.2250.2250.4	13.9237	Thermolysin	B2	120
BCAL1496	>gi|443597624|gb|ELT66041.1| hypothetical protein BURCENK562V_3510 [Burkholderia cenocepacia K56-2Valvano]	A.AAKKAGKKAKAADAAS[+568.212]Q.-	HexNAc(2)Hex(1)	Yes	546.5424	4	2183.1477	2183.1401	3.5	Non	455.21	455.21	455.21	Nsco_20191108_BC_ZIC_HILIC_K56_2_thermolysin_B2_.2204.2204.4	13.7129	Thermolysin	B2	121

BCAL1496	>gi|443597624|gb|ELT66041.1| hypothetical protein BURCENK562V_3510 [Burkholderia cenocepacia K56-2Valvano]	K.AGKKAKAADAAS[+568.212]Q.-	HexNAc(2)Hex(1)	Yes	595.632	3	1784.8815	1784.876	3.1	Non	195.28	64.78	64.78	Nsco_20191108_BC_ZIC_HILIC_K56_2_thermolysin_B1.2214.2214.3	13.3883	Thermolysin	B1	122
BCAL1496	>gi|443597624|gb|ELT66041.1| hypothetical protein BURCENK562V_3510 [Burkholderia cenocepacia K56-2Valvano]	K.AKAADAAS[+568.212]Q.-	HexNAc(2)Hex(1)	Yes	700.8192	2	1400.631	1400.6275	2.5	CRagged	403.43	208.72	208.72	Nsco_20191108_BC_ZIC_HILIC_K56_2_trypsin_B3.2167.2167.2	14.4123	Trypsin	B3	123
BCAL1496	>gi|443597624|gb|ELT66041.1| hypothetical protein BURCENK562V_3510 [Burkholderia cenocepacia K56-2Valvano]	H.KAAAKKAGKKAKAADAAS[+568.212]Q.-	HexNAc(2)Hex(1)	Yes	596.3253	4	2382.2795	2382.2722	3.1	Non	709.68	648.15	648.15	Nsco_20191108_BC_ZIC_HILIC_K56_2_pepsin_B3_reinject.2004.2004.4	13.5708	Pepsin	B3	124
BCAL1496	>gi|443597624|gb|ELT66041.1| hypothetical protein BURCENK562V_3510 [Burkholderia cenocepacia K56-2Valvano]	K.KAGKKAKAADAAS[+568.212]Q.-	HexNAc(2)Hex(1)	Yes	638.3307	3	1912.9774	1912.971	3.4	Non	349.29	249.11	249.11	Nsco_20191108_BC_ZIC_HILIC_K56_2_pepsin_B2.1928.1928.3	13.3268	Pepsin	B2	125
BCAL1496	>gi|443597624|gb|ELT66041.1| hypothetical protein BURCENK562V_3510 [Burkholderia cenocepacia K56-2Valvano]	A.KKAGKKAKAADAAS[+568.212]Q.-	HexNAc(2)Hex(1)	Yes	511.0234	4	2041.0719	2041.0659	2.9	Non	349.79	268.41	268.41	Nsco_20191108_BC_ZIC_HILIC_K56_2_pepsin_B2.1914.1914.4	13.2702	Pepsin	B2	126
BCAL1496	>gi|443597624|gb|ELT66041.1| hypothetical protein BURCENK562V_3510 [Burkholderia cenocepacia K56-2Valvano]	G.KKAKAADAAS[+568.212]Q.-	HexNAc(2)Hex(1)	Yes	552.9457	3	1656.8225	1656.8174	3.1	Non	367.41	216.13	216.13	Nsco_20191108_BC_ZIC_HILIC_K56_2_pepsin_B1.1926.1926.3	13.1059	Pepsin	B1	127
BCAL1503	>gi|443597627|gb|ELT66044.1| segregation and condensation protein B [Burkholderia cenocepacia K56-2Valvano]	P.AGESAEASGDAAADVASTQEMPSRDT [+568.212][+100.064]LDADRGQTE QTEQVGAEAVPADVQPEPQPADWSEED RKPAAE.A	HexNAc(2)Hex(1) 100.064	No	1548.6949	5	7739.4452	7733.4351	-1.3	Non	437.87	437.87	0	Nsco_20191108_BC_ZIC_HILIC_K56_2_thermolysin_B1.39793.39793.5	81.2183	Thermolysin	B1	128
BCAL1503	>gi|443597627|gb|ELT66044.1| segregation and condensation protein B [Burkholderia cenocepacia K56-2Valvano]	P.AGESAEASGDAAADVASTQEMPSRDT [+568.212][+100.064]LDADRGQTE QTEQVGAEAVPADVQPEPQPADWSEED RKPAAEA.A	HexNAc(2)Hex(1) 100.064	No	1562.7001	5	7809.4715	7804.4722	-2.2	Non	274.45	274.45	0	Nsco_20191108_BC_ZIC_HILIC_K56_2_thermolysin_B1.40060.40060.5	81.8258	Thermolysin	B1	129
BCAL1503	>gi|443597627|gb|ELT66044.1| segregation and condensation protein B [Burkholderia cenocepacia K56-2Valvano]	T.EQVGAEAVPADVQPEPQPADWS[+568.212][+100.064]EEDRKPAAEAA AGDGAEAAGDMPGEADQADASR.S	HexNAc(2)Hex(1) 100.064	No	1551.9427	4	6204.7492	6198.724	0.8	NRagged	510.38	510.38	22.15	Nsco_20191108_BC_ZIC_HILIC_K56_2_trypsin_B2.45231.45231.4	92.148	Trypsin	B2	130
BCAL1503	>gi|443597627|gb|ELT66044.1| segregation and condensation protein B [Burkholderia cenocepacia K56-2Valvano]	T.LDADRGQTEQTEQVGAEAVPADVQPE PQPADWSEEDRKPAAEAAAGDGAEAA GDM[+15.995]PGEADQADAS[+568.212][+100.064]RSRPADDET[+568.212][+100.064]L.D	HexNAc(2)Hex(1) 100.064,HexNAc(2)Hex(1) 100.064	No	1515.848	6	9090.0514	9081.9984	2.9	NRagged	383.98	337.77	7.84	Nsco_20191108_BC_ZIC_HILIC_K56_2_thermolysin_B2_.53771.53771.6	99.391	Thermolysin	B2	131
BCAL1503	>gi|443597627|gb|ELT66044.1| segregation and condensation protein B [Burkholderia cenocepacia K56-2Valvano]	D.VASTQEMPSRDTLDADRGQTEQTEQV GAEAVPADVQPEPQPADWSEEDRKPA AEAAAGDGAEAAGDMPGEADQADASR S[+568.212][+100.064]RP.A	HexNAc(2)Hex(1) 100.064	No	1295.1584	7	9060.0649	9056.05	0.2	Non	374.51	226.21	17.98	Nsco_20191108_BC_ZIC_HILIC_K56_2_thermolysin_B2_.52190.52190.7	96.1447	Thermolysin	B2	132
BCAL1649	>gi|443605315|gb|ELT73173.1| PF11159 family protein [Burkholderia cenocepacia K56-2Valvano]	A.LMSGMPPRGNPGERPPEWS[+568.212]NQPPAGNAPGTTPAAPPANPAP ASAG.V	HexNAc(2)Hex(1)	No	1647.7775	3	4941.3178	4938.2945	2.7	Non	386.6	386.6	3.6	Nsco_20191108_BC_ZIC_HILIC_K56_2_thermolysin_B2_.42757.42757.3	78.5914	Thermolysin	B2	133
BCAL1674	>gi|443605593|gb|ELT73435.1| periplasmic multidrug efflux lipoprotein [Burkholderia cenocepacia K56-2Valvano]	T.AVKPVEKAPSSKAAPPAAAS[+568.212]QAAARQT.-	HexNAc(2)Hex(1)	No	1057.8885	3	3171.6508	3171.6379	4.1	Non	172.85	172.85	16.72	Nsco_20191108_BC_ZIC_HILIC_K56_2_pepsin_B3_reinject.10109.10109.3	27.5306	Pepsin	B3	134

BCAL1674	>gi|443605593|gb|ELT73435.1| periplasmic multidrug efflux lipoprotein [Burkholderia cenocepacia K56-2Valvano]	E.KAPSSKAAPPAAAS[+568.212]QAAARQT.-	HexNAc(2)Hex(1)	No	850.0991	3	2548.2826	2548.2737	3.5	Non	370.47	370.47	10.8	Nsco_20191108_BC_ZIC_HILIC_K56_2_pepsin_B1.6180.6180.3	20.969	Pepsin	B1	135
BCAL1674	>gi|443605593|gb|ELT73435.1| periplasmic multidrug efflux lipoprotein [Burkholderia cenocepacia K56-2Valvano]	D.TAVKPVEKAPSSKAAPPAAAS[+568.212]QAAARQT.-	HexNAc(2)Hex(1)	No	1091.5619	3	3272.6711	3272.6856	-4.4	Non	433.93	417.23	16.8	Nsco_20191108_BC_ZIC_HILIC_K56_2_pepsin_B2.11787.11787.3	30.799	Pepsin	B2	136
BCAL1674	>gi|443605593|gb|ELT73435.1| periplasmic multidrug efflux lipoprotein [Burkholderia cenocepacia K56-2Valvano]	P.VEKAPSSKAAPPAAAS[+568.212]QA.A	HexNAc(2)Hex(1)	No	1125.0602	2	2249.1132	2249.1031	4.5	Non	632.4	364.09	7.47	Nsco_20191108_BC_ZIC_HILIC_K56_2_thermolysin_B2_.7412.7412.2	23.3896	Thermolysin	B2	137
BCAL1746	>gi|443592790|gb|ELT61568.1| putative lipoprotein [Burkholderia cenocepacia K56-2Valvano]	L.IDHIGKAWPGNAASGAS[+568.212]ASASE.-	HexNAc(2)Hex(1)	No	889.0777	3	2665.2186	2665.2111	2.8	CRagged	722.83	722.83	0.53	Nsco_20191108_BC_ZIC_HILIC_K56_2_pepsin_B2.22484.22484.3	49.0176	Pepsin	B2	138
BCAL1798	>gi|443598613|gb|ELT66949.1| hypothetical protein BURCENK562V_2315 [Burkholderia cenocepacia K56-2Valvano]	T.AATPAAASAPS[+568.212]AEQRAARHEARIEQ.R	HexNAc(2)Hex(1)	No	1043.5105	3	3128.517	3128.5091	2.5	Non	610.85	521.74	6.51	Nsco_20191108_BC_ZIC_HILIC_K56_2_pepsin_B2.12045.12045.3	31.1659	Pepsin	B2	139
BCAL1798	>gi|443598613|gb|ELT66949.1| hypothetical protein BURCENK562V_2315 [Burkholderia cenocepacia K56-2Valvano]	S.AQAPATTAATPAAAS[+568.212]APSAEQR.A	HexNAc(2)Hex(1)	No	1304.1209	2	2607.2345	2607.2268	3	Non	684.62	339.9	3.6	Nsco_20191108_BC_ZIC_HILIC_K56_2_thermolysin_B1.17795.17795.2	38.7969	Thermolysin	B1	140
BCAL1798	>gi|443598613|gb|ELT66949.1| hypothetical protein BURCENK562V_2315 [Burkholderia cenocepacia K56-2Valvano]	S.AQAPATTAATPAAAS[+568.212]APSAEQRA.A	HexNAc(2)Hex(1)	No	1339.6401	2	2678.2728	2678.2639	3.3	Non	562.38	328.57	3.6	Nsco_20191108_BC_ZIC_HILIC_K56_2_thermolysin_B1.19018.19018.2	40.7989	Thermolysin	B1	141
BCAL1798	>gi|443598613|gb|ELT66949.1| hypothetical protein BURCENK562V_2315 [Burkholderia cenocepacia K56-2Valvano]	A.SAQAPATTAATPAAAS[+568.212]APSAEQR.A	HexNAc(2)Hex(1)	No	1348.1365	2	2695.2657	2694.2588	1.3	NRagged	892.33	839.31	3.6	Nsco_20191108_BC_ZIC_HILIC_K56_2_typsin_B3.16665.16665.2	39.6691	Trypsin	B3	142
BCAL1869	>gi|443596292|gb|ELT64810.1| hypothetical protein BURCENK562V_2581 [Burkholderia cenocepacia K56-2Valvano]	A.DASAPDAVAS[+568.212][+100.064]APVAAS[+568.212]AVPDFDARQKV.L	HexNAc(2)Hex(1) 100.064,HexNAc(2)Hex(1)	Yes (1 of 2)	1288.9348	3	3864.7899	3862.7979	-3.8	Non	293.29	293.29	2.4	Nsco_20191108_BC_ZIC_HILIC_K56_2_thermolysin_B2_.45532.45532.3	83.2144	Thermolysin	B2	143
BCAL1869	>gi|443596292|gb|ELT64810.1| hypothetical protein BURCENK562V_2581 [Burkholderia cenocepacia K56-2Valvano]	P.VAAS[+568.212]AVPDFDARQK.V	HexNAc(2)Hex(1)	Yes	681.6658	3	2042.9828	2042.9764	3.1	Non	394.43	175.06	175.06	Nsco_20191108_BC_ZIC_HILIC_K56_2_thermolysin_B1.18482.18482.3	39.9444	Thermolysin	B1	144
BCAL1877	>gi|443594658|gb|ELT63293.1| HflK protein [Burkholderia cenocepacia K56-2Valvano]	A.AAST[+568.212]GAS[+568.212]AADAASAPAATVPS.A	HexNAc(2)Hex(1), HexNAc(2)Hex(1)	No	1441.1372	2	2881.2672	2881.2579	3.2	Non	655.09	344.29	12.39	Nsco_20191108_BC_ZIC_HILIC_K56_2_thermolysin_B1.26761.26761.2	55.2382	Thermolysin	B1	145
BCAL1877	>gi|443594658|gb|ELT63293.1| HflK protein [Burkholderia cenocepacia K56-2Valvano]	A.AASTGASAADAASAPAAT[+568.212]VPS[+568.212][+100.064]A.A	HexNAc(2)Hex(1), HexNAc(2)Hex(1) 100.064	No	1526.6785	2	3052.3497	3052.359	-3.1	Non	372.17	372.17	0	Nsco_20191108_BC_ZIC_HILIC_K56_2_pepsin_B2.29721.29721.2	63.226	Pepsin	B2	146
BCAL1877	>gi|443594658|gb|ELT63293.1| HflK protein [Burkholderia cenocepacia K56-2Valvano]	A.AAST[+568.212]GAS[+568.212]AADAASAPAATVPSAA.A	HexNAc(2)Hex(1), HexNAc(2)Hex(1)	No	1512.1733	2	3023.3392	3023.3322	2.3	Non	829.49	314.77	16.52	Nsco_20191108_BC_ZIC_HILIC_K56_2_pepsin_B1.28514.28514.2	60.7583	Pepsin	B1	147
BCAL1877	>gi|443594658|gb|ELT63293.1| HflK protein [Burkholderia cenocepacia K56-2Valvano]	A.ATAS[+568.212]GVDVLRSRE.A	HexNAc(2)Hex(1)	No	964.9725	2	1928.9376	1928.9295	4.2	Non	483.57	209.95	0	Nsco_20191108_BC_ZIC_HILIC_K56_2_thermolysin_B1.14431.14431.2	33.5436	Thermolysin	B1	148
BCAL1877	>gi|443594658|gb|ELT63293.1| HflK protein [Burkholderia cenocepacia K56-2Valvano]	L.VEQGRQNAAAST[+568.212]GAS[+568.212]AADAASAPAATVPSAA.A	HexNAc(2)Hex(1), HexNAc(2)Hex(1)	No	1302.5969	3	3905.776	3905.7629	3.3	CRagged	458.8	458.8	1.53	Nsco_20191108_BC_ZIC_HILIC_K56_2_pepsin_B2.26028.26028.3	55.7646	Pepsin	B2	149

BCAL1917	>gi|443605668|gb|ELT73505.1| hypothetical protein BURCENK562V_0636 [Burkholderia cenocepacia K56-2Valvano]	K.AAAAAASADAGASAPAAASS[+568.212]T[+568.212]K.A	HexNAc(2)Hex(1), HexNAc(2)Hex(1)	No	1463.1529	2	2925.2985	2925.2954	1.1	Specific	871.39	776.45	1.2	Nsco_20191108_BC_ZIC_HILIC_K56_2_typsin_B1.9657.9657.2	28.075	Trypsin	B1	150
BCAL1917	>gi|443605668|gb|ELT73505.1| hypothetical protein BURCENK562V_0636 [Burkholderia cenocepacia K56-2Valvano]	T.AAPAPTAS[+568.212]APEAAAKPAKTKR.A	HexNAc(2)Hex(1)	Yes	644.0907	4	2573.341	2573.3305	4.1	Non	928.61	779.55	128.8	Nsco_20191108_BC_ZIC_HILIC_K56_2_thermolysin_B1.5828.5828.4	20.4309	Thermolysin	B1	151
BCAL1917	>gi|443605668|gb|ELT73505.1| hypothetical protein BURCENK562V_0636 [Burkholderia cenocepacia K56-2Valvano]	T.AAPAPTAS[+568.212]APEAAAKPAKTKRA.S	HexNAc(2)Hex(1)	No	1323.1912	2	2645.3751	2644.3676	1.6	Non	766.18	588.28	2.4	Nsco_20191108_BC_ZIC_HILIC_K56_2_thermolysin_B1.6283.6283.2	21.1178	Thermolysin	B1	152
BCAL1917	>gi|443605668|gb|ELT73505.1| hypothetical protein BURCENK562V_0636 [Burkholderia cenocepacia K56-2Valvano]	T.AAPAPTASAPEAAAKPAKT[+568.212]KRASKKEK.A	HexNAc(2)Hex(1)	No	649.7533	5	3244.7373	3244.7271	3.1	Non	526.01	499.99	9.6	Nsco_20191108_BC_ZIC_HILIC_K56_2_thermolysin_B2_.3798.3798.4	17.7541	Thermolysin	B2	153
BCAL1917	>gi|443605668|gb|ELT73505.1| hypothetical protein BURCENK562V_0636 [Burkholderia cenocepacia K56-2Valvano]	T.AAPAPTAS[+568.212]APEAAAKPAKTKRASKKEKA.A	HexNAc(2)Hex(1)	Yes	663.9601	5	3315.7712	3315.7642	2.1	Non	1029.46	993.3	140.99	Nsco_20191108_BC_ZIC_HILIC_K56_2_thermolysin_B2_.3999.3999.5	18.067	Thermolysin	B2	154
BCAL1917	>gi|443605668|gb|ELT73505.1| hypothetical protein BURCENK562V_0636 [Burkholderia cenocepacia K56-2Valvano]	T.AAPAPTAS[+568.212]APEAAAKPAKTKRASKKEKAA.A	HexNAc(2)Hex(1)	Yes	678.167	5	3386.8058	3386.8013	1.3	Non	940.1	902.62	127.47	Nsco_20191108_BC_ZIC_HILIC_K56_2_thermolysin_B2_.4052.4052.4	18.147	Thermolysin	B2	155
BCAL1917	>gi|443605668|gb|ELT73505.1| hypothetical protein BURCENK562V_0636 [Burkholderia cenocepacia K56-2Valvano]	A.AASADAGASAPAAASS[+568.212]T[+568.212]KA.T	HexNAc(2)Hex(1), HexNAc(2)Hex(1)	No	1356.5999	2	2712.1924	2712.184	3.1	Non	765.09	360.69	1.39	Nsco_20191108_BC_ZIC_HILIC_K56_2_thermolysin_B1.8292.8292.2	24.0281	Thermolysin	B1	156
BCAL1917	>gi|443605668|gb|ELT73505.1| hypothetical protein BURCENK562V_0636 [Burkholderia cenocepacia K56-2Valvano]	P.AATTSATTSTTTSAGTASTSTTATAGT[+68.212]TTAAPAPTASAPEAAAKPAKTKR.A	HexNAc(2)Hex(1)	No	1027.9116	5	5135.529	5135.5085	4	Non	904.63	904.63	0	Nsco_20191108_BC_ZIC_HILIC_K56_2_thermolysin_B2_.17868.17868.5	38.5085	Thermolysin	B2	157
BCAL1917	>gi|443605668|gb|ELT73505.1| hypothetical protein BURCENK562V_0636 [Burkholderia cenocepacia K56-2Valvano]	T.AGTTTAAPAPTAS[+568.212]APEAAAKPAKTKR.A	HexNAc(2)Hex(1)	Yes	751.8917	4	3004.5449	3004.5321	4.3	Non	907.53	830.57	99.82	Nsco_20191108_BC_ZIC_HILIC_K56_2_thermolysin_B2_.8205.8205.4	24.6392	Thermolysin	B2	158
BCAL1917	>gi|443605668|gb|ELT73505.1| hypothetical protein BURCENK562V_0636 [Burkholderia cenocepacia K56-2Valvano]	A.ASADAGASAPAAASS[+568.212]T[+568.212]KA.T	HexNAc(2)Hex(1), HexNAc(2)Hex(1)	No	1321.0827	2	2641.1581	2641.1469	4.2	Non	735.79	376.97	2.4	Nsco_20191108_BC_ZIC_HILIC_K56_2_thermolysin_B1.7704.7704.2	23.1603	Thermolysin	B1	159
BCAL1917	>gi|443605668|gb|ELT73505.1| hypothetical protein BURCENK562V_0636 [Burkholderia cenocepacia K56-2Valvano]	A.ASADAGASAPAAAS[+568.212]S[+568.212][+100.064]T[+568.212]KATKGSKKKSKKDKAASAA.A	HexNAc(2)Hex(1), HexNAc(2)Hex(1) 100.064,HexNAc(2)Hex(1)	No	718.6356	7	5024.4053	5024.4279	-4.5	Non	374.67	374.67	0	Nsco_20191108_BC_ZIC_HILIC_K56_2_thermolysin_B1.2759.2759.7	14.9823	Thermolysin	B1	160
BCAL1917	>gi|443605668|gb|ELT73505.1| hypothetical protein BURCENK562V_0636 [Burkholderia cenocepacia K56-2Valvano]	T.ATAGTTTAAPAPTAS[+568.212]APEAAAKPAKTKR.A	HexNAc(2)Hex(1)	No	1059.5459	3	3176.6232	3176.6169	2	Non	967.99	614.32	2.4	Nsco_20191108_BC_ZIC_HILIC_K56_2_thermolysin_B2_.8998.8998.3	25.7506	Thermolysin	B2	161
BCAL1917	>gi|443605668|gb|ELT73505.1| hypothetical protein BURCENK562V_0636 [Burkholderia cenocepacia K56-2Valvano]	A.ATTSATTSTTTSAGTASTSTTAT[+568.12]AGTTTAAPAPTASAPEAAAKPAKTKR.A	HexNAc(2)Hex(1)	No	1013.9047	5	5065.4942	5064.4714	3.8	Non	666.61	666.61	0	Nsco_20191108_BC_ZIC_HILIC_K56_2_thermolysin_B2_.17348.17348.5	37.743	Thermolysin	B2	162

BCAL1917	>gi|443605668|gb|ELT73505.1| hypothetical protein BURCENK562V_0636 [Burkholderia cenocepacia K56-2Valvano]	S.ATTSTTTSAGTASTSTTATAGTTTAAPA[+568.212]ASAPEAAAK.P	HexNAc(2)Hex(1)	No	1342.3036	3	4024.8962	4022.8783	2.8	Non	702.39	604.38	4.8	Nsco_20191108_BC_ZIC_HILIC_K56_2_thermolysin_B3.24355.24355.3	53.0549	Thermolysin	B3	163
BCAL1917	>gi|443605668|gb|ELT73505.1| hypothetical protein BURCENK562V_0636 [Burkholderia cenocepacia K56-2Valvano]	S.ATTSTTTSAGTASTSTTATAGTTTAAPA[+568.212]ASAPEAAAKPAK.T	HexNAc(2)Hex(1)	No	1440.3653	3	4319.0813	4319.0631	4.2	NRagged	885.76	816.33	4.8	Nsco_20191108_BC_ZIC_HILIC_K56_2_typsin_B1.20175.20175.3	46.4565	Trypsin	B1	164
BCAL1917	>gi|443605668|gb|ELT73505.1| hypothetical protein BURCENK562V_0636 [Burkholderia cenocepacia K56-2Valvano]	A.GTASTSTTATAGTTTAAPAPT[+568.212]ASAPEAAAKPAK.T	HexNAc(2)Hex(1)	No	1166.5695	3	3497.694	3497.6865	2.2	NRagged	873.9	854.34	4.8	Nsco_20191108_BC_ZIC_HILIC_K56_2_typsin_B2.17188.17188.3	40.2245	Trypsin	B2	165
BCAL1917	>gi|443605668|gb|ELT73505.1| hypothetical protein BURCENK562V_0636 [Burkholderia cenocepacia K56-2Valvano]	K.LSKPAATTSATTSTTTSAGTASTSTTATATTTAAPAPTAS[+568.212]APEAAAK.P	HexNAc(2)Hex(1)	No	1220.5935	4	4879.3524	4879.3437	1.8	Non	1001.69	1001.7	63.47	Nsco_20191108_BC_ZIC_HILIC_K56_2_thermolysin_B3.24738.24738.4	53.7309	Thermolysin	B3	166
BCAL1917	>gi|443605668|gb|ELT73505.1| hypothetical protein BURCENK562V_0636 [Burkholderia cenocepacia K56-2Valvano]	K.LSKPAATTSATTSTTTSAGTASTSTTATATTTAAPAPTAS[+568.212]APEAAAKP.A	HexNAc(2)Hex(1)	No	1245.3497	4	4978.3771	4976.3965	-5.2	Non	269.42	269.42	30.56	Nsco_20191108_BC_ZIC_HILIC_K56_2_thermolysin_B3.26131.26131.4	56.4092	Thermolysin	B3	167
BCAL1917	>gi|443605668|gb|ELT73505.1| hypothetical protein BURCENK562V_0636 [Burkholderia cenocepacia K56-2Valvano]	K.LSKPAATTSATTSTTTSAGTASTSTTATATTTAAPAPTAS[+568.212]APEAAAKPA.K	HexNAc(2)Hex(1)	Yes	1262.6184	4	5047.4518	5047.4336	3.6	Non	1201.96	1202	99.55	Nsco_20191108_BC_ZIC_HILIC_K56_2_thermolysin_B3.26332.26332.4	56.7591	Thermolysin	B3	168
BCAL1917	>gi|443605668|gb|ELT73505.1| hypothetical protein BURCENK562V_0636 [Burkholderia cenocepacia K56-2Valvano]	K.LSKPAATTSATTSTTTSAGTASTSTTATATTTAAPAPTAS[+568.212]APEAAAKPAK.T	HexNAc(2)Hex(1)	Yes	1036.1147	5	5176.5446	5175.5286	2.5	Specific	1639.98	1640	78.23	Nsco_20191108_BC_ZIC_HILIC_K56_2_typsin_B2.21791.21791.5	48.9606	Trypsin	B2	169
BCAL1917	>gi|443605668|gb|ELT73505.1| hypothetical protein BURCENK562V_0636 [Burkholderia cenocepacia K56-2Valvano]	K.LSKPAATTSATTSTTTSAGTASTSTTATATTTAAPAPTASAPEAAAKPAKT[+568.212].K	HexNAc(2)Hex(1)	No	1056.3196	5	5277.5688	5276.5763	-2.1	Non	1202.73	1202.7	118.24	Nsco_20191108_BC_ZIC_HILIC_K56_2_thermolysin_B3.23031.23031.5	50.7231	Thermolysin	B3	170
BCAL1917	>gi|443605668|gb|ELT73505.1| hypothetical protein BURCENK562V_0636 [Burkholderia cenocepacia K56-2Valvano]	K.PAATTSATTSTTTSAGTASTSTTATAGTAAPAPT[+568.212]ASAPEAAAKPAK.T	HexNAc(2)Hex(1)	No	1616.4483	3	4847.3303	4847.3175	2.6	Specific	1160.31	1160.3	4.8	Nsco_20191108_BC_ZIC_HILIC_K56_2_typsin_B2.25468.25468.3	55.8308	Trypsin	B2	171
BCAL1917	>gi|443605668|gb|ELT73505.1| hypothetical protein BURCENK562V_0636 [Burkholderia cenocepacia K56-2Valvano]	K.PAATTSATTSTTTSAGTASTSTTATAGTAAPAPTASAPEAAAKPAKT[+568.212].K	HexNAc(2)Hex(1)	No	1237.8422	4	4948.3468	4948.3652	-3.7	Non	667.31	667.31	75.16	Nsco_20191108_BC_ZIC_HILIC_K56_2_thermolysin_B3.26231.26231.4	56.5788	Thermolysin	B3	172
BCAL1917	>gi|443605668|gb|ELT73505.1| hypothetical protein BURCENK562V_0636 [Burkholderia cenocepacia K56-2Valvano]	A.SADAGASAPAAASST[+568.212][+100.064]KAT[+568.212]KGS[+568.212][+100.064]KKKSKKDKAASAAAASGA.-	HexNAc(2)Hex(1) 100.064,HexNAc(2)Hex(1),HexNAc(2)Hex(1) 100.064	No	903.1068	6	5413.6044	5410.6196	-4.7	Non	193.86	193.86	0	Nsco_20191108_BC_ZIC_HILIC_K56_2_pepsin_B1.2469.2469.6	14.4722	Pepsin	B1	173
BCAL1917	>gi|443605668|gb|ELT73505.1| hypothetical protein BURCENK562V_0636 [Burkholderia cenocepacia K56-2Valvano]	T.SAGTASTSTTATAGTTTAAPAPTAS[+58.212]APEAAAKPAK.T	HexNAc(2)Hex(1)	No	1219.9271	3	3657.7668	3655.7556	1.2	NRagged	758.67	715.82	4.22	Nsco_20191108_BC_ZIC_HILIC_K56_2_typsin_B1.17651.17651.3	41.5742	Trypsin	B1	174

BCAL1917	>gi|443605668|gb|ELT73505.1| hypothetical protein BURCENK562V_0636 [Burkholderia cenocepacia K56-2Valvano]	L.SKPAATTSATTSTTTSAGTASTSTTATAC TTAAPAPTAS[+568.212]APEAAAKP AK.T	HexNAc(2)Hex(1)	Yes	1266.371	4	5062.4621	5062.4445	3.5	NRagged	1229.78	1229.8	53.78	Nsco_20191108_BC_ZIC_HILIC_K56_2_typsi n_B2.19359.19359.4	44.3527	Trypsin	B2	175
BCAL1917	>gi|443605668|gb|ELT73505.1| hypothetical protein BURCENK562V_0636 [Burkholderia cenocepacia K56-2Valvano]	T.STTATAGTTTAAPAPT[+568.212]AS APEAAAK.P	HexNAc(2)Hex(1)	No	1392.665	2	2784.3228	2784.3157	2.6	NRagged	603.53	510.41	2.4	Nsco_20191108_BC_ZIC_HILIC_K56_2_typsi n_B3.18227.18227.2	42.3733	Trypsin	B3	176
BCAL1917	>gi|443605668|gb|ELT73505.1| hypothetical protein BURCENK562V_0636 [Burkholderia cenocepacia K56-2Valvano]	T.STTATAGTTTAAPAPT[+568.212]AS APEAAAKPAK.T	HexNAc(2)Hex(1)	No	1027.5076	3	3080.5082	3080.5005	2.5	NRagged	740.42	737.18	2.4	Nsco_20191108_BC_ZIC_HILIC_K56_2_typsi n_B1.14937.14937.3	36.647	Trypsin	B1	177
BCAL1917	>gi|443605668|gb|ELT73505.1| hypothetical protein BURCENK562V_0636 [Burkholderia cenocepacia K56-2Valvano]	T.STTTSAGTASTSTTATAGTTTAAPAPTA +568.212]APEAAAKPAK.T	HexNAc(2)Hex(1)	No	1349.652	3	4046.9414	4045.9307	1.8	NRagged	830.7	823.79	2.99	Nsco_20191108_BC_ZIC_HILIC_K56_2_typsi n_B2.19554.19554.3	44.715	Trypsin	B2	178
BCAL1917	>gi|443605668|gb|ELT73505.1| hypothetical protein BURCENK562V_0636 [Burkholderia cenocepacia K56-2Valvano]	K.SVGHLEENGLT[+568.212]IGGASTP PK.G	HexNAc(2)Hex(1)	No	844.7462	3	2532.224	2532.2199	1.6	Specific	550.22	550.22	4.8	Nsco_20191108_BC_ZIC_HILIC_K56_2_typsi n_B2.24679.24679.3	54.2878	Trypsin	B2	179
BCAL1917	>gi|443605668|gb|ELT73505.1| hypothetical protein BURCENK562V_0636 [Burkholderia cenocepacia K56-2Valvano]	A.TAGTTTAAPAPTAS[+568.212]APE AAAKPAK.T	HexNAc(2)Hex(1)	No	907.4528	3	2720.3437	2720.336	2.8	NRagged	413.69	118.08	0	Nsco_20191108_BC_ZIC_HILIC_K56_2_typsi n_B2.12755.12755.3	32.393	Trypsin	B2	180
BCAL1917	>gi|443605668|gb|ELT73505.1| hypothetical protein BURCENK562V_0636 [Burkholderia cenocepacia K56-2Valvano]	A.TAGTTTAAPAPTAS[+568.212]APE AAAKPAKTKR.A	HexNAc(2)Hex(1)	No	1036.1936	3	3106.5663	3105.5798	-5.4	Non	436.37	436.37	2.4	Nsco_20191108_BC_ZIC_HILIC_K56_2_ther molysin_B2_.10450.10450.3	28.0854	Thermolysin	B2	181
BCAL1917	>gi|443605668|gb|ELT73505.1| hypothetical protein BURCENK562V_0636 [Burkholderia cenocepacia K56-2Valvano]	T.TSTTTSAGTASTSTTATAGTTTAAPAPT [+568.212][+100.064]APEAAAKP AK.T	HexNAc(2)Hex(1) 100.064	No	1416.3502	3	4247.0359	4247.0424	-1.5	NRagged	628.08	628.08	4.43	Nsco_20191108_BC_ZIC_HILIC_K56_2_typsi n_B2.20176.20176.3	45.9778	Trypsin	B2	182
BCAL1917	>gi|443605668|gb|ELT73505.1| hypothetical protein BURCENK562V_0636 [Burkholderia cenocepacia K56-2Valvano]	A.TTSTTTSAGTASTSTTATAGTTTAAPAP AS[+568.212]APEAAAKPAK.T	HexNAc(2)Hex(1)	Yes	1417.3538	3	4250.0467	4248.026	3.3	NRagged	828.7	167.61	2.23	Nsco_20191108_BC_ZIC_HILIC_K56_2_typsi n_B1.19806.19806.3	45.8123	Trypsin	B1	183
BCAL2009	>gi|443598395|gb|ELT66759.1| PF08811 family protein [Burkholderia cenocepacia K56-2Valvano]	R.AHAMPVTDAAS[+568.212]AAGLR DTPAKPAR.G	HexNAc(2)Hex(1)	No	736.6168	4	2943.4452	2943.4364	3	Specific	321.81	274.8	1.6	Nsco_20191108_BC_ZIC_HILIC_K56_2_typsi n_B1.16553.16553.4	39.5274	Trypsin	B1	184
BCAL2161	>gi|443595154|gb|ELT63756.1| hypothetical protein BURCENK562V_1535 [Burkholderia cenocepacia K56-2Valvano]	S.SVAPPLQGDGAAPGGAS[+568.212]WPAPPPASGPAPGLPASSVQGT[+56 8.212]P.-	HexNAc(2)Hex(1), HexNAc(2)Hex(1)	Yes	1570.0799	3	4708.2251	4708.2047	4.3	Non	281.68	234.86	31.41	Nsco_20191108_BC_ZIC_HILIC_K56_2_pepsi n_B3_reinject.52747.52747.3	119.4034	Pepsin	B3	185
BCAL2161	>gi|443595154|gb|ELT63756.1| hypothetical protein BURCENK562V_1535 [Burkholderia cenocepacia K56-2Valvano]	S.VAPPLQGDGAAPGGASWPAPPPAS[+568.212]GPAPGLPASSVQGTP.-	HexNAc(2)Hex(1)	No	1351.6608	3	4052.9679	4052.9611	1.7	Non	405.9	362.09	14.46	Nsco_20191108_BC_ZIC_HILIC_K56_2_ther molysin_B1.55174.55174.3	123.2653	Thermolysin	B1	186
BCAL2276	>gi|443595650|gb|ELT64220.1| hypothetical protein BURCENK562V_2512 [Burkholderia cenocepacia K56-2Valvano]	K.STIDTAASNAGVPVS[+568.212]SVN YIVHDAGK.G	HexNAc(2)Hex(1)	No	1047.8413	3	3141.5095	3141.4958	4.4	Non	723.57	685.92	1.2	Nsco_20191108_BC_ZIC_HILIC_K56_2_ther molysin_B3.44475.44475.3	90.1581	Thermolysin	B3	187

BCAL2345	>gi|443599338|gb|ELT67627.1| preprotein translocase, SecG subunit [Burkholderia cenocepacia K56-2Valvano]	A.VT[+568.212]APASAPAAS[+568.212]APA.V	HexNAc(2)Hex(1), HexNAc(2)Hex(1)	No	1159.5265	2	2318.0458	2318.0392	2.8	Non	493.43	149.42	59.06	Nsco_20191108_BC_ZIC_HILIC_K56_2_thermolysin_B1.20443.20443.2	43.4074	Thermolysin	B1	188
BCAL2398	>gi|443599855|gb|ELT68098.1| PRC-barrel domain protein [Burkholderia cenocepacia K56-2Valvano]	K.ASPPYAADKPIVAVFPPVVPAAPAS[+568.212]SASATR.-	HexNAc(2)Hex(1)	No	1191.9501	3	3573.8358	3573.8211	4.1	Non	708.62	708.62	2.4	Nsco_20191108_BC_ZIC_HILIC_K56_2_thermolysin_B3.52294.52294.3	104.3649	Thermolysin	B3	189
BCAL2449	>gi|443591735|gb|ELT60603.1| diguanylate cyclase (GGDEF) domain protein [Burkholderia cenocepacia K56-2Valvano]	R.RTGSVNNAPGAFSAS[+568.212]GVYPIAER.V	HexNAc(2)Hex(1)	Yes	963.7997	3	2889.3846	2889.3749	3.4	Specific	447.58	341.35	22.33	Nsco_20191108_BC_ZIC_HILIC_K56_2_typsin_B3.31131.31131.3	65.9643	Trypsin	B3	190
BCAL2466	>gi|443591728|gb|ELT60596.1| ecotin [Burkholderia cenocepacia K56-2Valvano]	A.APASAPAVPAES[+568.212]IKMFPQAAAGQQR.V	HexNAc(2)Hex(1)	No	1021.8398	3	3063.5048	3062.4987	0.9	NRagged	751.55	672.06	81.86	Nsco_20191108_BC_ZIC_HILIC_K56_2_typsin_B2.36444.36444.3	76.3838	Trypsin	B2	191
BCAL2629	>gi|443603168|gb|ELT71192.1| bifunctional uroporphyrinogen-III synthetase/uroporphyrin-III C-methyltransferase, partial [Burkholderia cenocepacia K56-2Valvano]	K.AAPADAASS[+568.212]VAAGEPR.W	HexNAc(2)Hex(1)	Yes	1004.9679	2	2008.9285	2008.9193	4.6	Non	702.03	575.5	5.99	Nsco_20191108_BC_ZIC_HILIC_K56_2_thermolysin_B3.16957.16957.2	39.3801	Thermolysin	B3	192
BCAL2629	>gi|443603168|gb|ELT71192.1| bifunctional uroporphyrinogen-III synthetase/uroporphyrin-III C-methyltransferase, partial [Burkholderia cenocepacia K56-2Valvano]	K.AAPADAAS[+568.212]SVAAGEPRWK.V	HexNAc(2)Hex(1)	Yes	775.0395	3	2323.1038	2323.0936	4.4	Non	570.64	464.89	0.8	Nsco_20191108_BC_ZIC_HILIC_K56_2_thermolysin_B1.24528.24528.3	51.0012	Thermolysin	B1	193
BCAL2629	>gi|443603168|gb|ELT71192.1| bifunctional uroporphyrinogen-III synthetase/uroporphyrin-III C-methyltransferase, partial [Burkholderia cenocepacia K56-2Valvano]	H.AAPKAAPADAAS[+568.212]SVAAGEPRWK.V	HexNAc(2)Hex(1)	No	897.4465	3	2690.325	2690.3155	3.5	Non	540.27	387.31	0.8	Nsco_20191108_BC_ZIC_HILIC_K56_2_thermolysin_B1.20915.20915.3	44.2319	Thermolysin	B1	194
BCAL2629	>gi|443603168|gb|ELT71192.1| bifunctional uroporphyrinogen-III synthetase/uroporphyrin-III C-methyltransferase, partial [Burkholderia cenocepacia K56-2Valvano]	E.AIVPHAAPKAAPADAAS[+568.212]SVAAGEPRW.K	HexNAc(2)Hex(1)	No	770.6379	4	3079.5298	3079.5219	2.6	Non	291.36	291.36	2	Nsco_20191108_BC_ZIC_HILIC_K56_2_pepsin_B3_reinject.31321.31321.4	64.2187	Pepsin	B3	195
BCAL2820	>gi|443594108|gb|ELT62784.1| outer membrane multidrug efflux protein OprB [Burkholderia cenocepacia K56-2Valvano]	K.RPDAPVAQAYPAS[+568.212]GVYATQPGAAGAR.S	HexNAc(2)Hex(1)	No	1037.838	3	3111.4995	3110.4913	1.6	Specific	649.41	645.11	1.41	Nsco_20191108_BC_ZIC_HILIC_K56_2_typsin_B1.25608.25608.3	56.8	Trypsin	B1	196
BCAL2820	>gi|443594108|gb|ELT62784.1| outer membrane multidrug efflux protein OprB [Burkholderia cenocepacia K56-2Valvano]	D.YDKAAAPAPAS[+568.212]ATATNG.-	HexNAc(2)Hex(1)	No	1072.9926	2	2144.9779	2144.9717	2.9	Non	455.71	364.43	1.6	Nsco_20191108_BC_ZIC_HILIC_K56_2_pepsin_B2.12674.12674.2	32.2169	Pepsin	B2	197
BCAL2974	>gi|443594432|gb|ELT63086.1| hypothetical protein BURCENK562V_0304 [Burkholderia cenocepacia K56-2Valvano]	P.FAASAPS[+568.212]QKYQGSKK.S	HexNAc(2)Hex(1)	No	1083.5312	2	2166.0551	2166.0449	4.7	Non	594.76	434.49	0.25	Nsco_20191108_BC_ZIC_HILIC_K56_2_thermolysin_B1.5609.5609.2	20.0631	Thermolysin	B1	198
BCAL2974	>gi|443594432|gb|ELT63086.1| hypothetical protein BURCENK562V_0304 [Burkholderia cenocepacia K56-2Valvano]	P.FAASAPSQKYQGS[+568.212]KKSA.L	HexNAc(2)Hex(1)	No	1162.5644	2	2324.1215	2324.114	3.2	Non	743.28	499.05	1.89	Nsco_20191108_BC_ZIC_HILIC_K56_2_thermolysin_B1.6072.6072.2	20.8198	Thermolysin	B1	199
BCAL2974	>gi|443594432|gb|ELT63086.1| hypothetical protein BURCENK562V_0304 [Burkholderia cenocepacia K56-2Valvano]	R.LNEHPQMPFAASAPS[+568.212]QK.Y	HexNAc(2)Hex(1)	No	807.711	3	2421.1185	2421.1126	2.4	Specific	861.79	837.7	11.02	Nsco_20191108_BC_ZIC_HILIC_K56_2_typsin_B2.22692.22692.3	50.5873	Trypsin	B2	200

BCAL2974	>gi|443594432|gb|ELT63086.1| hypothetical protein BURCENK562V_0304 [Burkholderia cenocepacia K56-2Valvano]	L.NEHPQMPFAASAPS[+568.212]QKYQG.S	HexNAc(2)Hex(1)	No	886.0642	3	2656.178	2656.1719	2.3	CRagged	660.53	660.53	8.32	Nsco_20191108_BC_ZIC_HILIC_K56_2_pepsin_B2.23936.23936.3	51.6854	Pepsin	B2	201
BCAL2974	>gi|443594432|gb|ELT63086.1| hypothetical protein BURCENK562V_0304 [Burkholderia cenocepacia K56-2Valvano]	L.NEHPQMPFAAS[+568.212]APSQKYQGSKKSAL.R	HexNAc(2)Hex(1)	Yes	818.3941	4	3270.5544	3270.5471	2.2	Specific	1159.51	1149.4	137.04	Nsco_20191108_BC_ZIC_HILIC_K56_2_pepsin_B1.17741.17741.4	39.9659	Pepsin	B1	202
BCAL2974	>gi|443594432|gb|ELT63086.1| hypothetical protein BURCENK562V_0304 [Burkholderia cenocepacia K56-2Valvano]	M.PFAASAPS[+568.212]QKYQGSKKSAL.R	HexNAc(2)Hex(1)	No	845.4256	3	2534.2623	2534.2508	4.5	NRagged	705.99	676.5	1.2	Nsco_20191108_BC_ZIC_HILIC_K56_2_pepsin_B2.12496.12496.3	31.9139	Pepsin	B2	203
BCAL2974	>gi|443594432|gb|ELT63086.1| hypothetical protein BURCENK562V_0304 [Burkholderia cenocepacia K56-2Valvano]	F.RLNEHPQMPFAAS[+568.212]APSQ.K	HexNAc(2)Hex(1)	Yes	817.0473	3	2449.1274	2449.1188	3.5	CRagged	547.35	498.47	9.62	Nsco_20191108_BC_ZIC_HILIC_K56_2_pepsin_B1.25396.25396.3	54.2559	Pepsin	B1	204
BCAL2974	>gi|443594432|gb|ELT63086.1| hypothetical protein BURCENK562V_0304 [Burkholderia cenocepacia K56-2Valvano]	F.RLNEHPQMPFAAS[+568.212]APSQKYQGSKKSAL.R	HexNAc(2)Hex(1)	Yes	708.7539	5	3539.7405	3539.7323	2.3	Specific	1167.14	1140.8	122.97	Nsco_20191108_BC_ZIC_HILIC_K56_2_pepsin_B2.18532.18532.5	41.5782	Pepsin	B2	205
BCAL2983A	>gi|443599451|gb|ELT67730.1| PF11180 family protein [Burkholderia cenocepacia K56-2Valvano]	S.ADASAPVAGTRPAVTSLSGGASSAASGAVAT[+568.212]DAAAQGNVAELTQMLHDGR.I	HexNAc(2)Hex(1)	No	1301.8783	4	5204.4914	5204.4772	2.7	NRagged	898.91	855.7	0	Nsco_20191108_BC_ZIC_HILIC_K56_2_typsin_B3.55819.55819.4	108.6091	Trypsin	B3	206
BCAL3017	>gi|443591015|gb|ELT59947.1| hypothetical protein BURCENK562V_1182 [Burkholderia cenocepacia K56-2Valvano]	A.DTASGSDAQAS[+568.212]C[+57.021]AIAYVTGVGGSPR.G	HexNAc(2)Hex(1)	No	1483.6628	2	2966.3184	2966.3055	4.3	NRagged	792.59	792.59	3.6	Nsco_20191108_BC_ZIC_HILIC_K56_2_typsin_B1.50784.50784.2	100.0398	Trypsin	B1	207
BCAL3033	>gi|443591005|gb|ELT59937.1| outer-membrane lipoprotein carrier protein [Burkholderia cenocepacia K56-2Valvano]	K.GASAAQAAPKPT[+568.212]DNSSGTFVFAR.P	HexNAc(2)Hex(1)	No	940.4492	3	2819.3332	2819.3218	4.1	Specific	934.51	784.38	8.88	Nsco_20191108_BC_ZIC_HILIC_K56_2_typsin_B2.25616.25616.3	56.1071	Trypsin	B2	208
BCAL3033	>gi|443591005|gb|ELT59937.1| outer-membrane lipoprotein carrier protein [Burkholderia cenocepacia K56-2Valvano]	K.GAS[+568.212]AAQAAPKPTDNSSGTFVFARPGK.F	HexNAc(2)Hex(1)	Yes	776.1301	4	3101.4988	3101.4909	2.5	Non	1048.85	995.34	369.44	Nsco_20191108_BC_ZIC_HILIC_K56_2_thermolysin_B3.21596.21596.4	48.0474	Thermolysin	B3	209
BCAL3033	>gi|443591005|gb|ELT59937.1| outer-membrane lipoprotein carrier protein [Burkholderia cenocepacia K56-2Valvano]	Q.IVKAPAKGAS[+568.212]AAQAAPKPTDN.S	HexNAc(2)Hex(1)	No	859.1134	3	2575.3257	2574.3145	3.1	Non	407.82	407.82	23.8	Nsco_20191108_BC_ZIC_HILIC_K56_2_pepsin_B2.8774.8774.3	25.7362	Pepsin	B2	210
BCAL3033	>gi|443591005|gb|ELT59937.1| outer-membrane lipoprotein carrier protein [Burkholderia cenocepacia K56-2Valvano]	Q.IVKAPAKGAS[+568.212]AAQAAPKPTDNSSGT.F	HexNAc(2)Hex(1)	Yes	727.3692	4	2906.4548	2906.4477	2.5	Non	907.13	606.48	449.61	Nsco_20191108_BC_ZIC_HILIC_K56_2_thermolysin_B2_.9118.9118.4	25.9654	Thermolysin	B2	211
BCAL3033	>gi|443591005|gb|ELT59937.1| outer-membrane lipoprotein carrier protein [Burkholderia cenocepacia K56-2Valvano]	Q.IVKAPAKGAS[+568.212]AAQAAPKPTDNSSGTF.V	HexNAc(2)Hex(1)	Yes	764.1371	4	3053.5266	3053.5161	3.4	NRagged	1208.22	1174.3	519.79	Nsco_20191108_BC_ZIC_HILIC_K56_2_thermolysin_B1.15310.15310.4	34.8469	Thermolysin	B1	212
BCAL3033	>gi|443591005|gb|ELT59937.1| outer-membrane lipoprotein carrier protein [Burkholderia cenocepacia K56-2Valvano]	Q.IVKAPAKGAS[+568.212]AAQAAPKPTDNSSGTFV.F	HexNAc(2)Hex(1)	Yes	788.9039	4	3152.5937	3152.5845	2.9	Non	855.15	855.15	397.28	Nsco_20191108_BC_ZIC_HILIC_K56_2_thermolysin_B2_.19314.19314.4	40.6759	Thermolysin	B2	213
BCAL3166	>gi|443602480|gb|ELT70558.1| BON domain protein [Burkholderia cenocepacia K56-2Valvano]	A.ATSAPKAAAKT[+568.212]AKKANRKLGY.A	HexNAc(2)Hex(1)	No	905.826	3	2715.4635	2714.4571	1.1	Non	536.47	356.4	13.2	Nsco_20191108_BC_ZIC_HILIC_K56_2_pepsin_B2.4421.4421.3	18.4718	Pepsin	B2	214
BCAL3166	>gi|443602480|gb|ELT70558.1| BON domain protein [Burkholderia cenocepacia K56-2Valvano]	A.PAAATSAPKAAAKT[+568.212]AKKANRKLGY.A	HexNAc(2)Hex(1)	No	739.1546	4	2953.5967	2953.5841	4.3	Non	424.76	424.76	13.2	Nsco_20191108_BC_ZIC_HILIC_K56_2_pepsin_B2.6997.6997.4	23.0272	Pepsin	B2	215

BCAL3166	>gi|443602480|gb|ELT70558.1| BON domain protein [Burkholderia cenocepacia K56-2Valvano]	A.TEAPAAATSAPKAAAKT[+568.212]AKKANRKLGY.A	HexNAc(2)Hex(1)	No	1085.9101	3	3255.7156	3254.7114	0.3	Non	939.76	370.14	9.06	Nsco_20191108_BC_ZIC_HILIC_K56_2_pepsin_B3_reinject.11245.11245.3	29.3064	Pepsin	B3	216
BCAL3377	>gi|443595379|gb|ELT63971.1| SmpA/OmlA family protein, partial [Burkholderia cenocepacia K56-2Valvano]	A.AAKKAS[+568.212]EAAAAASAAQA.A	HexNAc(2)Hex(1)	No	1028.5033	2	2055.9993	2055.9928	3.2	Non	364.7	73.61	0.09	Nsco_20191108_BC_ZIC_HILIC_K56_2_thermolysin_B2_.6539.6539.2	22.1025	Thermolysin	B2	217
BCAL3377	>gi|443595379|gb|ELT63971.1| SmpA/OmlA family protein, partial [Burkholderia cenocepacia K56-2Valvano]	D.IDGDRGGKKAKAAAAAKKAS[+568.212]EAAA.A	HexNAc(2)Hex(1)	Yes	706.8718	4	2824.4656	2824.4534	4.3	Non	240.3	240.3	240.3	Nsco_20191108_BC_ZIC_HILIC_K56_2_pepsin_B2.3463.3463.3	17.0527	Pepsin	B2	218
BCAL3469	>gi|443597454|gb|ELT65879.1| cell division protein FtsL [Burkholderia cenocepacia K56-2Valvano]	A.IDAPIPAS[+568.212]ADTAGKGKGGAR.-	HexNAc(2)Hex(1)	No	807.7407	3	2421.2075	2421.1991	3.5	Non	796.3	567.41	2.4	Nsco_20191108_BC_ZIC_HILIC_K56_2_thermolysin_B1.12694.12694.3	30.7374	Thermolysin	B1	219
BCAM0157	>gi|443592579|gb|ELT61370.1| hypothetical protein BURCENK562V_A2231 [Burkholderia cenocepacia K56-2Valvano]	R.AVDC[+57.021]GQLDAATSGPDDNFRPPAS[+568.212]GTVIGTGR.A	HexNAc(2)Hex(1)	No	1224.2299	3	3670.6752	3670.6661	2.5	Specific	715.98	715.98	4.8	Nsco_20191108_BC_ZIC_HILIC_K56_2_typsin_B1.38785.38785.3	80.0577	Trypsin	B1	220
BCAM0175a	>gi|443598439|gb|ELT66798.1| putative lipoprotein [Burkholderia cenocepacia K56-2Valvano]	A.TPSSTPANASAPADT[+568.212]AAQAAPPR.V	HexNAc(2)Hex(1)	No	1359.6371	2	2718.2669	2718.2588	3	NRagged	795.66	290.44	0	Nsco_20191108_BC_ZIC_HILIC_K56_2_typsin_B3.18096.18096.2	42.1515	Trypsin	B3	221
BCAM0345	>gi|443597793|gb|ELT66203.1| hypothetical protein BURCENK562V_A1193 [Burkholderia cenocepacia K56-2Valvano]	R.SAPAGMQDGASS[+568.212]APQPALDR.L	HexNAc(2)Hex(1)	No	1248.0628	2	2495.1183	2495.109	3.7	Specific	804.86	763.61	6.85	Nsco_20191108_BC_ZIC_HILIC_K56_2_typsin_B3.21707.21707.2	48.7322	Trypsin	B3	222
BCAM0505	>gi|443591075|gb|ELT60005.1| BON domain protein [Burkholderia cenocepacia K56-2Valvano]	Q.ASSTDSGMASES[+568.212]NQPVTDTWITTK.V	HexNAc(2)Hex(1)	No	1541.681	2	3082.3546	3082.3416	4.2	NRagged	653.93	653.93	1.2	Nsco_20191108_BC_ZIC_HILIC_K56_2_typsin_B1.37726.37726.2	78.0391	Trypsin	B1	223
BCAM0505	>gi|443591075|gb|ELT60005.1| BON domain protein [Burkholderia cenocepacia K56-2Valvano]	A.HAQASSTDSGMASES[+568.212]NQPVTDTWITTK.V	HexNAc(2)Hex(1)	No	1140.1741	3	3418.5077	3418.4962	3.4	Non	850.7	850.7	2.4	Nsco_20191108_BC_ZIC_HILIC_K56_2_thermolysin_B3.30162.30162.3	63.8622	Thermolysin	B3	224
BCAM0505	>gi|443591075|gb|ELT60005.1| BON domain protein [Burkholderia cenocepacia K56-2Valvano]	A.QAS[+568.212]STDSGMASESNQPVTDTW.I	HexNAc(2)Hex(1)	Yes	1384.0723	2	2767.1373	2767.1258	4.2	Non	269.64	185.63	21.49	Nsco_20191108_BC_ZIC_HILIC_K56_2_thermolysin_B1.53831.53831.2	119.557	Thermolysin	B1	225
BCAM0505	>gi|443591075|gb|ELT60005.1| BON domain protein [Burkholderia cenocepacia K56-2Valvano]	A.QASSTDSGMASES[+568.212]NQPVTDTWITTK.V	HexNAc(2)Hex(1)	No	1606.7017	2	3212.396	3210.4002	-3.4	NRagged	518.98	518.98	3.6	Nsco_20191108_BC_ZIC_HILIC_K56_2_typsin_B3.39328.39328.2	80.5455	Trypsin	B3	226
BCAM0505	>gi|443591075|gb|ELT60005.1| BON domain protein [Burkholderia cenocepacia K56-2Valvano]	D.SGMASES[+568.212]NQPVT[+568.212]DTWITTK.V	HexNAc(2)Hex(1), HexNAc(2)Hex(1)	No	1064.7974	3	3192.3776	3189.3774	-3.1	NRagged	347.67	347.67	4.8	Nsco_20191108_BC_ZIC_HILIC_K56_2_typsin_B3.50916.50916.3	100.1249	Trypsin	B3	227
BCAM0505	>gi|443591075|gb|ELT60005.1| BON domain protein [Burkholderia cenocepacia K56-2Valvano]	A.SSTDSGMASES[+568.212]NQPVTDTWITTK.V	HexNAc(2)Hex(1)	No	1506.6624	2	3012.3174	3011.3045	3.2	Non	542.49	525.03	1.2	Nsco_20191108_BC_ZIC_HILIC_K56_2_thermolysin_B3.38287.38287.2	78.9073	Thermolysin	B3	228
BCAM0690	>gi|443592239|gb|ELT61058.1| OmpA family protein [Burkholderia cenocepacia K56-2Valvano]	R.ALIDAGVPASSVFAAAFGS[+568.212]EQPVSSNADDEGRAK.N	HexNAc(2)Hex(1)	No	1301.9484	3	3903.8307	3902.8302	-0.7	Specific	506.19	469.64	6	Nsco_20191108_BC_ZIC_HILIC_K56_2_typsin_B1.60279.60279.3	115.7062	Trypsin	B1	229
BCAM0713	>gi|443605703|gb|ELT73537.1| cation efflux system protein CzcA [Burkholderia cenocepacia K56-2Valvano]	K.RLPIALPAPAAGAS[+568.212]APLAAAPYVPLAELATIDVAPGPNQISREDGK.R	HexNAc(2)Hex(1)	Yes	1275.4307	4	5098.7008	5098.6834	3.4	Specific	1131.96	1132	379.2	Nsco_20191108_BC_ZIC_HILIC_K56_2_typsin_B2.70574.70574.4	143.2396	Trypsin	B2	230
BCAM0996	>gi|443592743|gb|ELT61524.1| sporulation and cell division repeat protein [Burkholderia cenocepacia K56-2Valvano]	R.DDDVSDVQAGVAHDEPPAS[+568.212]DTTVAAAPAPAPK.D	HexNAc(2)Hex(1)	No	1228.2274	3	3682.6675	3682.6614	1.7	Specific	1134.46	1134.5	4.8	Nsco_20191108_BC_ZIC_HILIC_K56_2_typsin_B1.35376.35376.3	73.8871	Trypsin	B1	231

BCAM0996	>gi|443592743|gb|ELT61524.1| sporulation and cell division repeat protein [Burkholderia cenocepacia K56-2Valvano]	K.PAAPAAKPAAPKPAPATVANAGAASPDS[+568.212]GDASSPASPAGAR.F	HexNAc(2)Hex(1)	No	1040.7653	4	4160.0394	4160.0266	3.1	Specific	1138.91	1138.9	0.4	Nsco_20191108_BC_ZIC_HILIC_K56_2_typsin_B2.19311.19311.4	44.2627	Trypsin	B2	232
BCAM0996	>gi|443592743|gb|ELT61524.1| sporulation and cell division repeat protein [Burkholderia cenocepacia K56-2Valvano]	K.PAAPKPAPATVANAGAASPDSGDAS[+568.212]SPASPAGAR.F	HexNAc(2)Hex(1)	No	1185.2342	3	3553.688	3553.6776	2.9	Specific	906.46	906.46	2.4	Nsco_20191108_BC_ZIC_HILIC_K56_2_typsin_B1.19901.19901.3	45.9826	Trypsin	B1	233
BCAM0996	>gi|443592743|gb|ELT61524.1| sporulation and cell division repeat protein [Burkholderia cenocepacia K56-2Valvano]	P.VTDDIAIDIPNRPAHQAVAPRDDDVSDVQAGVAHDEPPASDT[+568.212]TVAAAPAPAPK.D	HexNAc(2)Hex(1)	No	1187.7791	5	5934.8662	5932.8483	1.9	NRagged	903.05	347.07	2.4	Nsco_20191108_BC_ZIC_HILIC_K56_2_typsin_B3.38825.38825.5	79.6066	Trypsin	B3	234
BCAM1398	>gi|443598009|gb|ELT66406.1| gram-negative porin [Burkholderia cenocepacia K56-2Valvano]	L.GGGAWGDNSTVTSGGQPAVS[+568.212][+100.064]AINNGYQTAQSQQR.I	HexNAc(2)Hex(1) 100.064	No	1346.2901	3	4036.8557	4032.8293	3.2	CRagged	373.27	373.27	6	Nsco_20191108_BC_ZIC_HILIC_K56_2_thermolysin_B3.42045.42045.3	85.7221	Thermolysin	B3	235
BCAM1550	>gi|443602195|gb|ELT70283.1| peptidoglycan-associated lipoprotein [Burkholderia cenocepacia K56-2Valvano]	T.PENAGAAPEPSSETVAT[+568.212][+100.064]VTADDLNNPNSPLAK.R	HexNAc(2)Hex(1) 100.064	No	1282.6044	3	3845.7987	3845.7938	1.3	NRagged	631.68	631.68	2.4	Nsco_20191108_BC_ZIC_HILIC_K56_2_typsin_B1.45909.45909.3	92.3259	Trypsin	B1	236
BCAM1550	>gi|443602195|gb|ELT70283.1| peptidoglycan-associated lipoprotein [Burkholderia cenocepacia K56-2Valvano]	T.PENAGAAPEPSSET[+568.212][+100.064]VATVTADDLNNPNSPLAKR.S	HexNAc(2)Hex(1) 100.064	No	1334.9717	3	4002.9007	4001.8949	0.6	NRagged	737.92	705.13	0.82	Nsco_20191108_BC_ZIC_HILIC_K56_2_typsin_B1.40248.40248.3	82.4319	Trypsin	B1	237
BCAM1550	>gi|443602195|gb|ELT70283.1| peptidoglycan-associated lipoprotein [Burkholderia cenocepacia K56-2Valvano]	K.TPENAGAAPEPSSETVAT[+568.212]VTADDLNNPNSPLAK.R	HexNAc(2)Hex(1)	No	1282.9327	3	3846.7835	3846.7775	1.6	Specific	1037.88	260.78	2.4	Nsco_20191108_BC_ZIC_HILIC_K56_2_typsin_B3.46141.46141.3	92.2169	Trypsin	B3	238
BCAM1550	>gi|443602195|gb|ELT70283.1| peptidoglycan-associated lipoprotein [Burkholderia cenocepacia K56-2Valvano]	K.TPENAGAAPEPSSETVATVT[+568.212]ADDLNNPNSPLAKR.S	HexNAc(2)Hex(1)	No	1334.9677	3	4002.8884	4002.8786	2.5	Specific	1109.66	253.74	0	Nsco_20191108_BC_ZIC_HILIC_K56_2_typsin_B1.40339.40339.3	82.5901	Trypsin	B1	239
BCAM1669	>gi|443598083|gb|ELT66474.1| hypothetical protein BURCENK562V_A2079 [Burkholderia cenocepacia K56-2Valvano]	R.VHGIDNSGAGS[+568.212]QPAATVEGGAPVV.R	HexNAc(2)Hex(1)	No	1380.1554	2	2759.3035	2758.2901	3.6	Non	698.88	650.18	2.4	Nsco_20191108_BC_ZIC_HILIC_K56_2_thermolysin_B3.34070.34070.2	70.9495	Thermolysin	B3	240
BCAM1669	>gi|443598083|gb|ELT66474.1| hypothetical protein BURCENK562V_A2079 [Burkholderia cenocepacia K56-2Valvano]	R.VHGIDNSGAGS[+568.212]QPAATVEGGAPVVR.A	HexNAc(2)Hex(1)	No	972.1373	3	2914.3973	2914.3912	2.1	Specific	894.84	894.84	1.2	Nsco_20191108_BC_ZIC_HILIC_K56_2_typsin_B3.23965.23965.3	52.8858	Trypsin	B3	241
BCAM1789	>gi|443592202|gb|ELT61024.1| putative esterase [Burkholderia cenocepacia K56-2Valvano]	A.TPPAPAPAASPAQPPAVQTATTPS[+568.212]TAQEPSVNPGSSVVLR.T	HexNAc(2)Hex(1)	No	1473.0701	3	4417.1957	4416.194	-0.4	Non	975.65	248.91	0	Nsco_20191108_BC_ZIC_HILIC_K56_2_thermolysin_B3.44674.44674.3	90.4706	Thermolysin	B3	242
BCAM2055	>gi|443591388|gb|ELT60286.1| type III secretion outer membrane pore, YscC/HrcC-like family protein [Burkholderia cenocepacia K56-2Valvano]	K.DTAASQPAATT[+568.212]AGVVTHVDEHH.-	HexNAc(2)Hex(1)	No	696.5697	4	2783.2568	2783.249	2.8	Non	629.35	629.35	1.2	Nsco_20191108_BC_ZIC_HILIC_K56_2_thermolysin_B3.20174.20174.4	45.2282	Thermolysin	B3	243
BCAM2063	>gi|443595086|gb|ELT63692.1| carbohydrate-selective porin, OprB family [Burkholderia cenocepacia K56-2Valvano]	A.SSPAAAEPAAGAS[+568.212]DAAAPAQQAADAAAPAPTGFWER.S	HexNAc(2)Hex(1)	No	1306.9322	3	3918.782	3918.7788	0.8	NRagged	755.5	755.5	8.4	Nsco_20191108_BC_ZIC_HILIC_K56_2_typsin_B2.47912.47912.3	96.7611	Trypsin	B2	244
BCAM2064	>gi|443595102|gb|ELT63708.1| alpha,alpha-trehalase [Burkholderia cenocepacia K56-2Valvano]	Q.AAAQAVGQSAIPATTAAAAAPAS[+568.212]GT[+568.212]LPPPPSQLYGDLFVAVQTAQLYPDQK.T	HexNAc(2)Hex(1), HexNAc(2)Hex(1)	No	1533.5116	4	6131.0246	6128.0021	2	NRagged	367.41	367.41	0	Nsco_20191108_BC_ZIC_HILIC_K56_2_typsin_B2.77678.77678.4	160.9177	Trypsin	B2	245
BCAM2064	>gi|443595102|gb|ELT63708.1| alpha,alpha-trehalase [Burkholderia cenocepacia K56-2Valvano]	A.DNANQAAAQAVGQSAIPATTAAAAAPAS[+568.212]GTLPPPPSQLYGDLFVAVQTAQLYPDQK.T	HexNAc(2)Hex(1)	No	1526.2625	4	6102.028	6101.999	4.8	NRagged	932.43	887.63	5.77	Nsco_20191108_BC_ZIC_HILIC_K56_2_typsin_B3.85867.85867.4	160.5315	Trypsin	B3	246
BCAM2289	>gi|443599704|gb|ELT67963.1| hypothetical protein BURCENK562V_A2967 [Burkholderia cenocepacia K56-2Valvano]	R.VHGADTSGYGAQPAPLVHSGAPAAAS[+568.212]SNAR.D	HexNAc(2)Hex(1)	Yes	1129.1995	3	3385.5839	3385.5779	1.8	Specific	1258.46	1100.4	2.4	Nsco_20191108_BC_ZIC_HILIC_K56_2_typsin_B3.17140.17140.3	40.4898	Trypsin	B3	247

BCAM2334	>gi|443606050|gb|ELT73855.1| auxiliary transport protein, membrane fusion protein (MFP) domain protein, partial [Burkholderia cenocepacia K56-2Valvano]	R.VHDGVAS[+568.212]DAEAAAAAII RENQGG.-	HexNAc(2)Hex(1)	Yes	930.7708	3	2790.298	2790.2912	2.4	Non	592.51	592.51	592.51	Nsco_20191108_BC_ZIC_HILIC_K56_2_ther molysin_B3.49647.49647.3	99.379	Thermolysin	B3	248
BCAM2378	>gi|443594879|gb|ELT63498.1| X-Pro dipeptidyl-peptidase (S15 family) [Burkholderia cenocepacia K56-2Valvano]	L.AQGQQEGASAAAGT[+568.212]ANT PSL.A	HexNAc(2)Hex(1)	No	1199.5435	2	2398.0797	2398.074	2.4	Specific	333.01	260.81	2.4	Nsco_20191108_BC_ZIC_HILIC_K56_2_pepsi n_B2.27030.27030.2	57.6522	Pepsin	B2	249
BCAM2378	>gi|443594879|gb|ELT63498.1| X-Pro dipeptidyl-peptidase (S15 family) [Burkholderia cenocepacia K56-2Valvano]	A.LAQGQQEGASAAAGT[+568.212]A NTPS.L	HexNAc(2)Hex(1)	No	1199.5439	2	2398.0806	2398.074	2.8	Non	452.69	317.95	3.6	Nsco_20191108_BC_ZIC_HILIC_K56_2_ther molysin_B1.16414.16414.2	36.6046	Thermolysin	B1	250
BCAM2378	>gi|443594879|gb|ELT63498.1| X-Pro dipeptidyl-peptidase (S15 family) [Burkholderia cenocepacia K56-2Valvano]	T.PVIVLASPYYAGLADSPNHDVDVELDG TPHPAATAGAAAS[+568.212][+100. 064]ASAR.I	HexNAc(2)Hex(1) 100.064	No	1246.6063	4	4983.4035	4983.4021	0.3	NRagged	511.21	511.21	4.8	Nsco_20191108_BC_ZIC_HILIC_K56_2_typsi n_B1.56834.56834.4	110.1277	Trypsin	B1	251
BCAM2378	>gi|443594879|gb|ELT63498.1| X-Pro dipeptidyl-peptidase (S15 family) [Burkholderia cenocepacia K56-2Valvano]	R.TPVIVLASPYYAGLADSPNHDVDVELD GTPHPAATAGAAAS[+568.212]ASAR .I	HexNAc(2)Hex(1)	No	1246.8569	4	4984.4059	4984.3858	4	Specific	1206.74	471.98	2.36	Nsco_20191108_BC_ZIC_HILIC_K56_2_typsi n_B3.56510.56510.4	109.7632	Trypsin	B3	252
BCAM2443	>gi|443594187|gb|ELT62858.1| cytochrome C [Burkholderia cenocepacia K56-2Valvano]	G.ATPQDAPAAASAPPPAPAAAPAAK PFT[+568.212]PPPESAIPADDFGK.T	HexNAc(2)Hex(1)	No	1482.3965	3	4445.1751	4445.1559	4.3	NRagged	918.54	918.54	8.4	Nsco_20191108_BC_ZIC_HILIC_K56_2_typsi n_B1.46671.46671.3	93.5713	Trypsin	B1	253
BCAM2680	>gi|443603690|gb|ELT71680.1| hypothetical protein BURCENK562V_A2523, partial [Burkholderia cenocepacia K56-2Valvano]	A.AAQQASVPAAAS[+568.212]AT[+5 68.212]VVVK.A	HexNAc(2)Hex(1), HexNAc(2)Hex(1)	No	1403.1841	2	2805.3609	2805.351	3.5	Non	722.03	483.86	93.02	Nsco_20191108_BC_ZIC_HILIC_K56_2_ther molysin_B2_.25792.25792.2	51.1424	Thermolysin	B2	254
BCAM2680	>gi|443603690|gb|ELT71680.1| hypothetical protein BURCENK562V_A2523, partial [Burkholderia cenocepacia K56-2Valvano]	A.AAQQASVPAAAS[+568.212]AT[+5 68.212]VVVKAAPQPQNP.V	HexNAc(2)Hex(1), HexNAc(2)Hex(1)	No	1203.9231	3	3609.7547	3608.7436	2.1	Non	385.63	385.63	72.79	Nsco_20191108_BC_ZIC_HILIC_K56_2_ther molysin_B2_.36532.36532.3	68.4457	Thermolysin	B2	255
BCAM2680	>gi|443603690|gb|ELT71680.1| hypothetical protein BURCENK562V_A2523, partial [Burkholderia cenocepacia K56-2Valvano]	Q.AASAAAAQQAS[+568.212]VPAAA S[+568.212][+100.064]AT[+568.2 12]V.V	HexNAc(2)Hex(1), HexNAc(2)Hex(1) 100.064,HexNAc(2)Hex(1)	No	1173.8671	3	3519.5868	3518.5753	2.3	Non	242.35	44.1	0	Nsco_20191108_BC_ZIC_HILIC_K56_2_pepsi n_B3_reinject.32149.32149.3	65.8129	Pepsin	B3	256
BCAM2829	>gi|443591146|gb|ELT60069.1| hypothetical protein BURCENK562V_A2847 [Burkholderia cenocepacia K56-2Valvano]	D.SAMPASAPAAAS[+568.212][+10 0.064]APASSGATTA.P	HexNAc(2)Hex(1) 100.064	No	1258.0699	2	2515.1325	2514.1403	-4.4	Non	211.02	170.46	2.4	Nsco_20191108_BC_ZIC_HILIC_K56_2_pepsi n_B3_reinject.38438.38438.3	79.4583	Pepsin	B3	257
BCAM2829	>gi|443591146|gb|ELT60069.1| hypothetical protein BURCENK562V_A2847 [Burkholderia cenocepacia K56-2Valvano]	D.SAMPASAPAAASAPASS[+568.212]GAT[+568.212]TAPAANGAPIA.P	HexNAc(2)Hex(1), HexNAc(2)Hex(1)	No	1248.9059	3	3744.7033	3744.6903	3.5	Non	482.2	259.04	1.2	Nsco_20191108_BC_ZIC_HILIC_K56_2_pepsi n_B2.36147.36147.3	77.5477	Pepsin	B2	258
BCAM2829	>gi|443591146|gb|ELT60069.1| hypothetical protein BURCENK562V_A2847 [Burkholderia cenocepacia K56-2Valvano]	D.S[+568.212]AMPASAPAAASAPAS SGAT[+568.212]TAPAANGAPIAPAM .P	HexNAc(2)Hex(1), HexNAc(2)Hex(1)	No	1348.6181	3	4043.8397	4043.8207	4.7	Non	283.55	225.63	1.18	Nsco_20191108_BC_ZIC_HILIC_K56_2_pepsi n_B3_reinject.43487.43487.3	92.7742	Pepsin	B3	259
BCAS0089	>gi|443598997|gb|ELT67309.1| hypothetical protein BURCENK562V_B0207 [Burkholderia cenocepacia K56-2Valvano]	N.AVQQAPATKAAPAVPAS[+568.21 2]GQ.-	HexNAc(2)Hex(1)	No	1166.0856	2	2331.164	2331.1562	3.3	Non	576.42	463	48.07	Nsco_20191108_BC_ZIC_HILIC_K56_2_ther molysin_B1.15804.15804.2	35.6316	Thermolysin	B1	260
BCAS0089	>gi|443598997|gb|ELT67309.1| hypothetical protein BURCENK562V_B0207 [Burkholderia cenocepacia K56-2Valvano]	A.VQQAPATKAAPAVPAS[+568.212] GQ.-	HexNAc(2)Hex(1)	No	1130.5662	2	2260.1251	2260.1191	2.7	Non	626.38	388.77	65.96	Nsco_20191108_BC_ZIC_HILIC_K56_2_ther molysin_B1.14541.14541.2	33.7178	Thermolysin	B1	261

BCAS0321	>gi|443594043|gb|ELT62726.1| autotransporter-associated beta strand repeat (3 repeats) [Burkholderia cenocepacia K56-2Valvano]	W.NIT[+568.212]GASS[+568.212]AADASLA.G	HexNAc(2)Hex(1), HexNAc(2)Hex(1)	No	1193.0223	2	2385.0372	2385.0298	3.1	Non	210.12	1.04	1.04	Nsco_20191108_BC_ZIC_HILIC_K56_2_pepsin_B1.25683.25683.2	54.848	Pepsin	B1	262
BURCENK562V_3104	>gi|443598112|gb|ELT66499.1| hypothetical protein BURCENK562V_3104 [Burkholderia cenocepacia K56-2Valvano]	G.AAADGAKDTTSSAVHST[+568.212]KKHTKHAAKSAKSHAGSA.K	HexNAc(2)Hex(1)	No	797.6005	5	3983.9733	3983.9541	4.8	Non	652.05	652.05	1.2	Nsco_20191108_BC_ZIC_HILIC_K56_2_pepsin_B3_reinject.3845.3845.5	16.9543	Pepsin	B3	263
BURCENK562V_3104	>gi|443598112|gb|ELT66499.1| hypothetical protein BURCENK562V_3104 [Burkholderia cenocepacia K56-2Valvano]	S.AAFAQTGTT[+568.212]AQGAAGAGVQAQTPAAGVGAGAQGGAGANAS[+568.212]GNATGAATDAVGAAADGAK.D	HexNAc(2)Hex(1), HexNAc(2)Hex(1)	No	1513.7054	4	6051.8	6048.7672	3.8	NRagged	392.33	276.15	5.06	Nsco_20191108_BC_ZIC_HILIC_K56_2_typsin_B2.61923.61923.3	123.5184	Trypsin	B2	264
BURCENK562V_3104	>gi|443598112|gb|ELT66499.1| hypothetical protein BURCENK562V_3104 [Burkholderia cenocepacia K56-2Valvano]	K.AKADEATEGGAS[+568.212]VGAQGGASAQGATQ.-	HexNAc(2)Hex(1)	No	1429.6388	2	2858.2704	2858.2658	1.6	CRagged	834.52	757.98	2.47	Nsco_20191108_BC_ZIC_HILIC_K56_2_typsin_B1.11369.11369.2	30.6907	Trypsin	B1	265
BURCENK562V_3104	>gi|443598112|gb|ELT66499.1| hypothetical protein BURCENK562V_3104 [Burkholderia cenocepacia K56-2Valvano]	N.ASGNATGAATDAVGAAADGAKDT[+568.212]TSSAVHS.T	HexNAc(2)Hex(1)	No	1067.4822	3	3200.4321	3200.4197	3.9	Non	959.33	875.29	2.4	Nsco_20191108_BC_ZIC_HILIC_K56_2_thermolysin_B1.29679.29679.3	60.5536	Thermolysin	B1	266
BURCENK562V_3104	>gi|443598112|gb|ELT66499.1| hypothetical protein BURCENK562V_3104 [Burkholderia cenocepacia K56-2Valvano]	N.ASGNATGAATDAVGAAADGAKDTTSS[+568.212]AVHSTKKH.T	HexNAc(2)Hex(1)	Yes	739.751	5	3694.7259	3694.7162	2.6	Non	493.63	493.63	20.41	Nsco_20191108_BC_ZIC_HILIC_K56_2_thermolysin_B1.19879.19879.5	42.3129	Thermolysin	B1	267
BURCENK562V_3104	>gi|443598112|gb|ELT66499.1| hypothetical protein BURCENK562V_3104 [Burkholderia cenocepacia K56-2Valvano]	K.DT[+568.212]TSSAVHSTKK.H	HexNAc(2)Hex(1)	No	610.6228	3	1829.8539	1829.8498	2.2	Specific	250.3	191.7	0.8	Nsco_20191108_BC_ZIC_HILIC_K56_2_typsin_B1.2097.2097.3	14.3265	Trypsin	B1	268
BURCENK562V_3104	>gi|443598112|gb|ELT66499.1| hypothetical protein BURCENK562V_3104 [Burkholderia cenocepacia K56-2Valvano]	Q.GAAGAGVQAQTPAAGVGAGAQGGAGANAS[+568.212][+100.064]GNATGAATDAVGAAADGAK.D	HexNAc(2)Hex(1) 100.064	No	1512.0524	3	4534.1427	4533.1211	4	NRagged	867	854.17	4.8	Nsco_20191108_BC_ZIC_HILIC_K56_2_typsin_B2.63789.63789.3	127.5147	Trypsin	B2	269
BURCENK562V_3104	>gi|443598112|gb|ELT66499.1| hypothetical protein BURCENK562V_3104 [Burkholderia cenocepacia K56-2Valvano]	G.VGAGAQGGAGANASGNATGAAT[+568.212]DAVGAAADGAKDTTSSAVHS.T	HexNAc(2)Hex(1)	No	1371.2926	3	4111.8633	4110.8454	3.5	Non	959.2	917.8	3.6	Nsco_20191108_BC_ZIC_HILIC_K56_2_thermolysin_B1.33767.33767.3	68.324	Thermolysin	B1	270
BURCENK562V_3104	>gi|443598112|gb|ELT66499.1| hypothetical protein BURCENK562V_3104 [Burkholderia cenocepacia K56-2Valvano]	G.VGAGAQGGAGANASGNAT[+568.212]GAATDAVGAAADGAKDTTSSAVHST.K	HexNAc(2)Hex(1)	No	1405.3107	3	4213.9175	4211.8931	4.2	Non	539.37	424.76	0.81	Nsco_20191108_BC_ZIC_HILIC_K56_2_thermolysin_B2_.37230.37230.3	69.5539	Thermolysin	B2	271
BURCENK562V_3104	>gi|443598112|gb|ELT66499.1| hypothetical protein BURCENK562V_3104 [Burkholderia cenocepacia K56-2Valvano]	G.VGAGAQGGAGANASGNATGAATDAVGAAADGAKDTTSS[+568.212]AVHSTKK.H	HexNAc(2)Hex(1)	Yes	894.4257	5	4468.0996	4468.083	3.7	Non	1192.63	979.42	27.83	Nsco_20191108_BC_ZIC_HILIC_K56_2_thermolysin_B1.26977.26977.5	55.6311	Thermolysin	B1	272
BURCENK562V_3104	>gi|443598112|gb|ELT66499.1| hypothetical protein BURCENK562V_3104 [Burkholderia cenocepacia K56-2Valvano]	G.VGAGAQGGAGANASGNATGAATDAVGAAADGAKDTTSS[+568.212]AVHSTKKH.T	HexNAc(2)Hex(1)	No	922.0375	5	4606.1584	4605.1419	2.9	Non	992.62	905.74	10.81	Nsco_20191108_BC_ZIC_HILIC_K56_2_thermolysin_B2_.25510.25510.5	50.6999	Thermolysin	B2	273
BURCENK562V_3104	>gi|443598112|gb|ELT66499.1| hypothetical protein BURCENK562V_3104 [Burkholderia cenocepacia K56-2Valvano]	G.VQAQTPAAGVGAGAQGGAGANASGNATGAATDAVGAAADGAKDTTS[+568.212]SAVHSTKKH.T	HexNAc(2)Hex(1)	No	1086.9199	5	5430.5705	5428.5607	0.6	Non	1011.88	195.27	0.88	Nsco_20191108_BC_ZIC_HILIC_K56_2_thermolysin_B2_.31008.31008.5	59.674	Thermolysin	B2	274

BCAS0773	>gi|443593562|gb|ELT62293.1| hypothetical protein BURCENK562V_B0326 [Burkholderia cenocepacia K56-2Valvano]	A.QTDAAS[+568.212]APAAAAAQDAK.A	HexNAc(2)Hex(1)	No	1063.4875	2	2125.9678	2125.9619	2.8	Non	808.63	642.97	33.43	Nsco_20191108_BC_ZIC_HILIC_K56_2_thermolysin_B3.10973.10973.2	29.3941	Thermolysin	B3	275

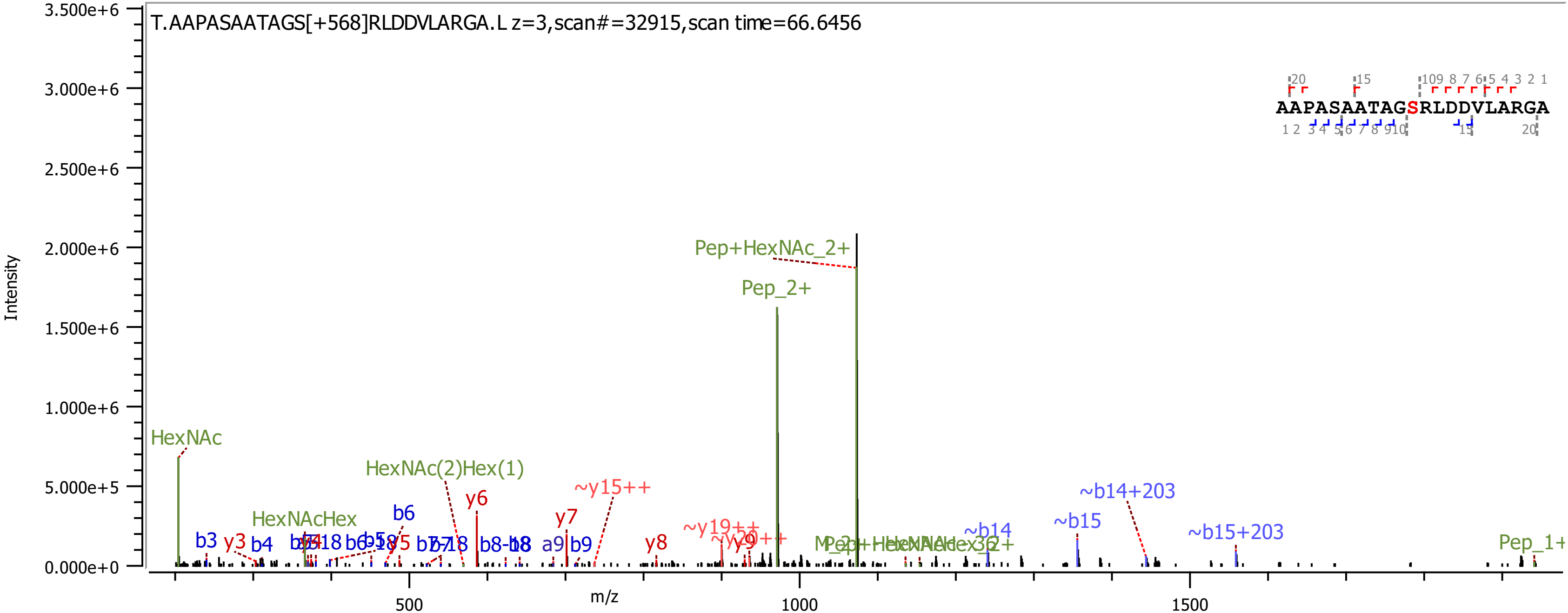
T.AAPASAATAGS[+568]RLDDVLARGA.L z=3,scan#=32915,scan time=66.6456
Intensity
m/z
AAPASAATAGSRLDDVLARGA
Pep+HexNAc_2+
Pep_2+
HexNAc
HexNAc(2)Hex(1)
HexNAcHex
~y15++
y6
y7
y8
b6
b3
y3
b4
b9
a9
~b14+203
~b15
~b15+203
~b14
Pep_1+

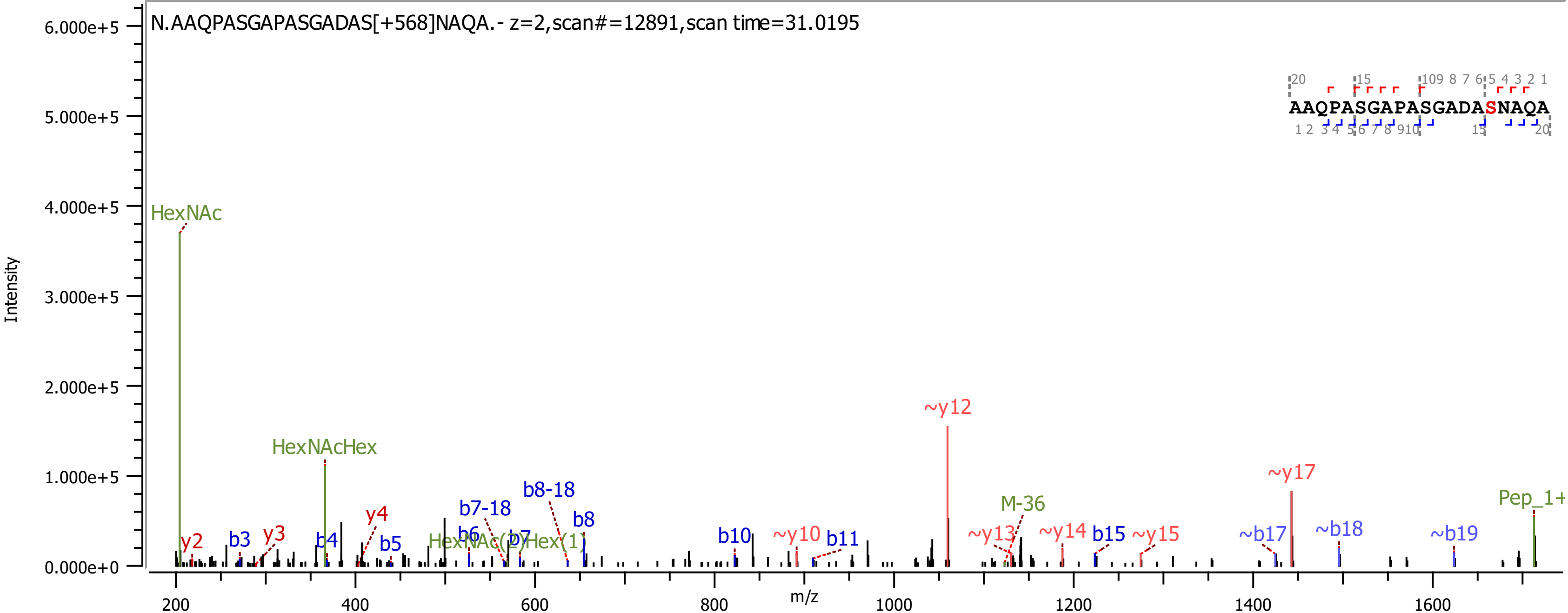

N.AAQPASGAPASGADAS[+568]NAQA.- z=2,scan#=12891,scan time=31.0195
AAQPASGAPASGADASNAQA
Intensity
m/z
HexNAc
HexNAcHex
HexNAc(2)Hex(1)
~y12
~y17
M-36
Pep_1+
y2
b3
y3
b4
y4
b5
b6
b7
b7-18
b8-18
b8
b10
~y10
b11
~y13
~y14
b15
~y15
~b17
~b18
~b19

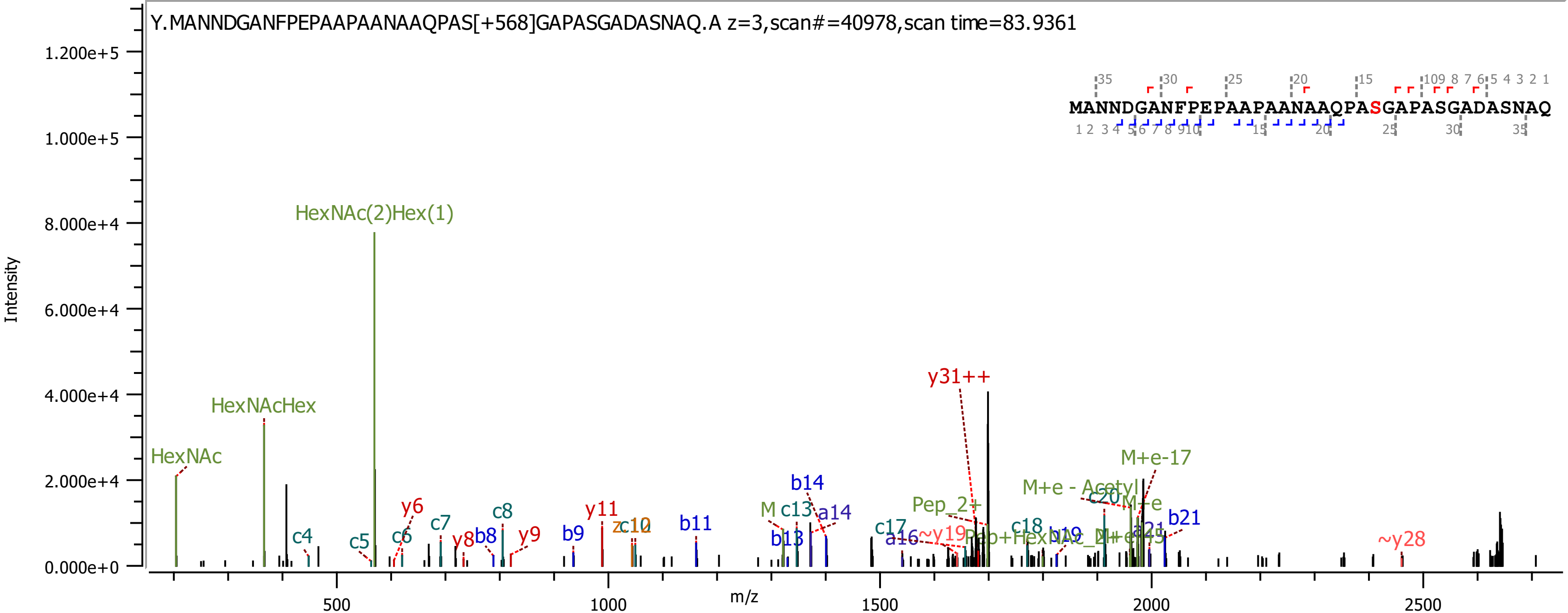

Y.MANNDGANFPEPAAPAANAAQPAS[+568]GAPASGADASNAQ.A z=3,scan#=40978,scan time=83.9361
MANNDGANFPEPAAPAANAAQPASGAPASGADASNAQ
Intensity
m/z
HexNAc(2)Hex(1)
HexNAcHex
HexNAc
y31++
M+e-17
M+e - Acetyl
Pep_2+
b14
a14
c13
b21
~y28

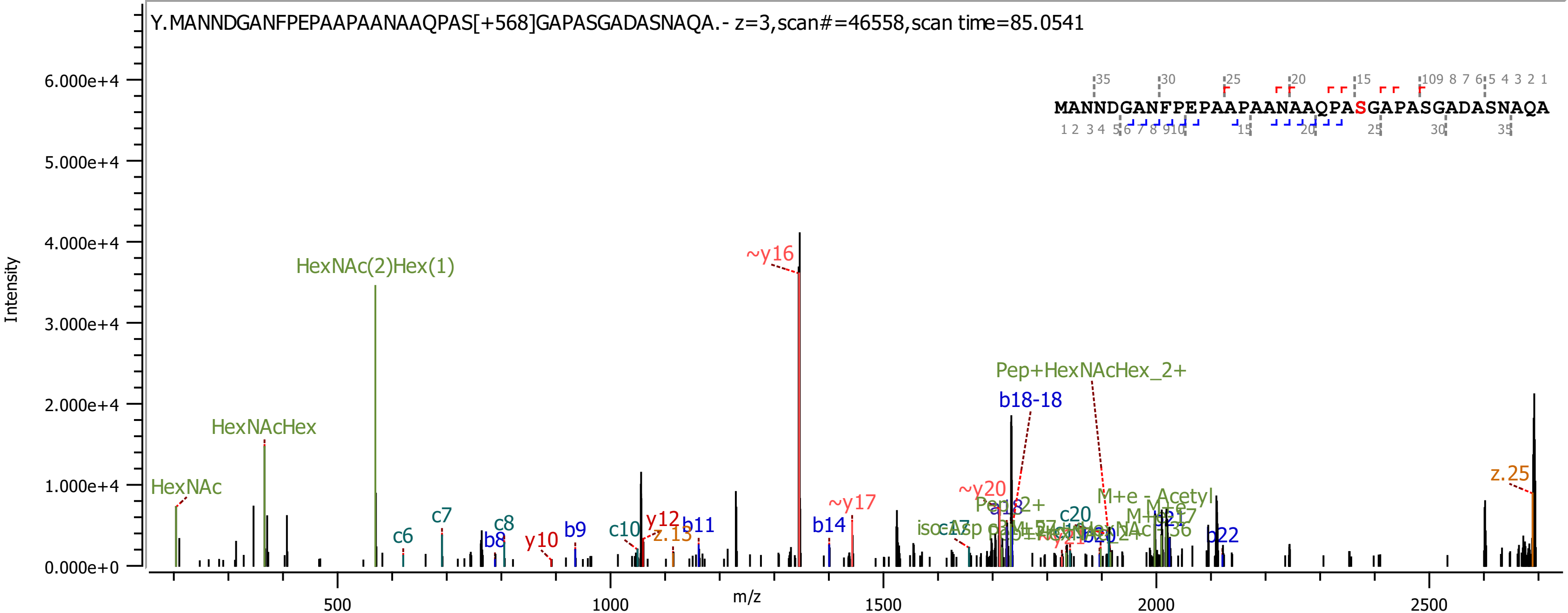

Y.MANNDGANFPEPAAPAANAAQPAS[+568]GAPASGADASNAQA.- z=3,scan#=46558,scan time=85.0541
MANNDGANFPEPAAPAANAAQPASGAPASGADASNAQA
Intensity
m/z
HexNAc(2)Hex(1)
HexNAcHex
HexNAc
~y16
Pep+HexNAcHex_2+
b18-18
z.25
~y20
~y17
b14
b11
y12
z.13
c10
b9
y10
c8
b8
c7
c6
b22
c20
M+e -Acetyl

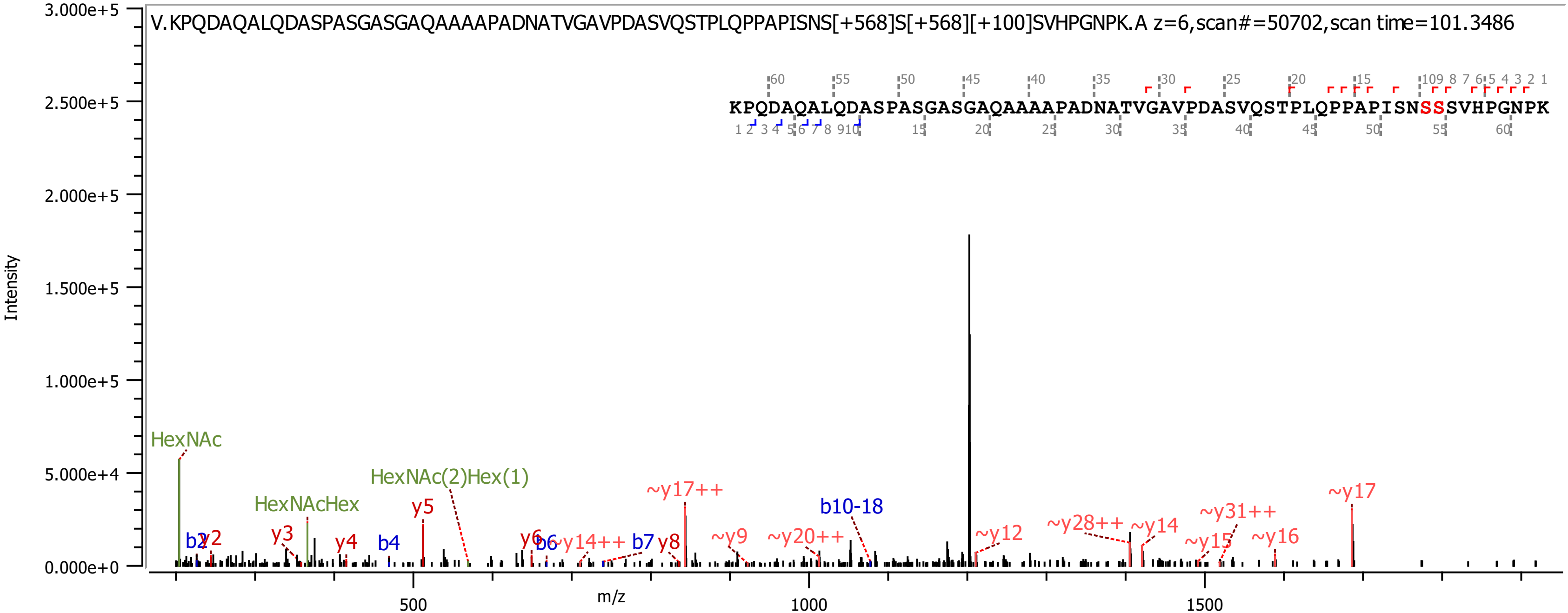
V.KPQDAQALQDASPASGASGAQAAAAPADNATVGAVPDASVQSTPLQPPAPISNS[+568]S[+568][+100]SVHPGNPK.A z=6,scan#=50702,scan time=101.3486
KPQDAQALQDASPASGASGAQAAAAPADNATVGAVPDASVQSTPLQPPAPISNSSSVHPGNPK
Intensity
m/z
HexNAc
HexNAcHex
HexNAc(2)Hex(1)
b2
y2
y3
y4
b4
y5
y6
b6
~y14++
b7
y8
~y17++
~y9
~y20++
b10-18
~y12
~y28++
~y14
~y15
~y31++
~y16
~y17

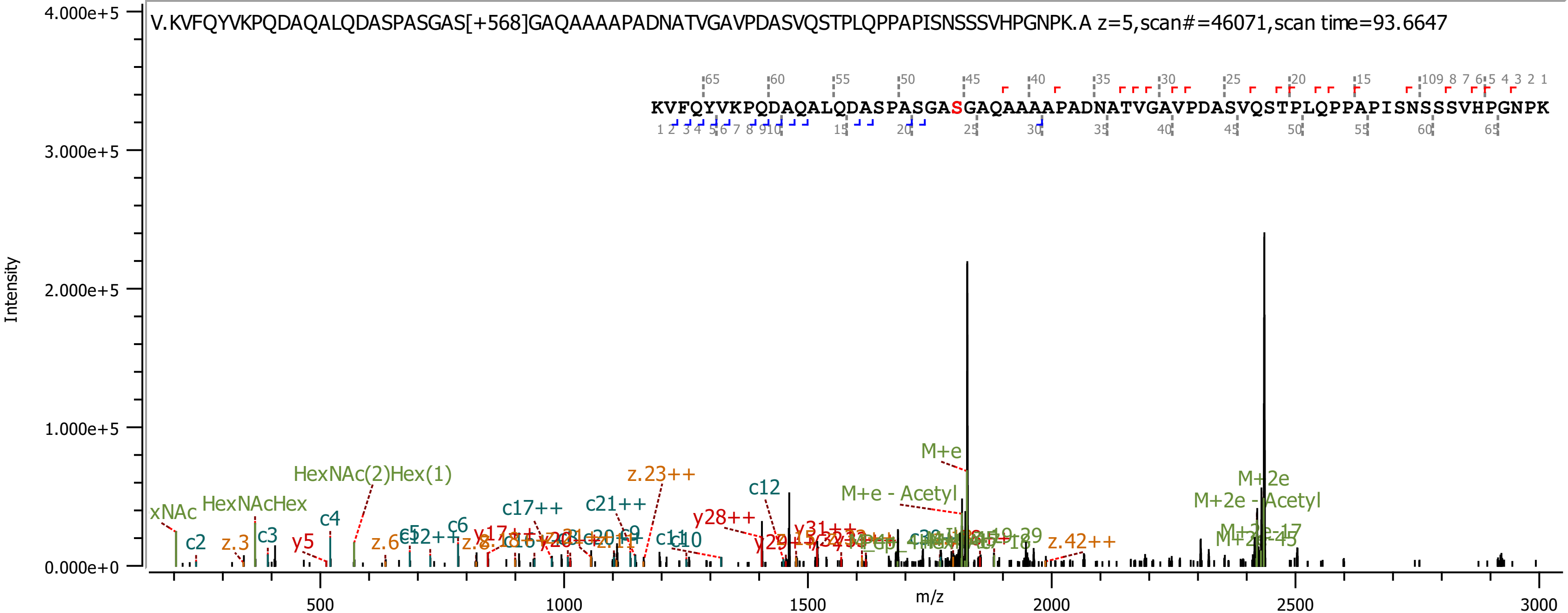
V.KVFQYVKPQDAQALQDASPASGAS[+568]GAQAAAAPADNATVGAVPDASVQSTPLQPPAPISNSSSVHPGNPK.A z=5,scan#=46071,scan time=93.6647
KVFQYVKPQDAQALQDASPASGASGAQAAAAPADNATVGAVPDASVQSTPLQPPAPISNSSSVHPGNPK
Intensity
m/z
4.000e+5
3.000e+5
2.000e+5
1.000e+5
0.000e+0
500
1000
1500
2000
2500
3000
M+e
M+e - Acetyl
M+2e
M+2e - Acetyl
HexNAc(2)Hex(1)
HexNAcHex
z.23++
c12
c17++
c21++
y28++
c2
z.3
c3
y5
c4
z.6
c6
c10
z.42++

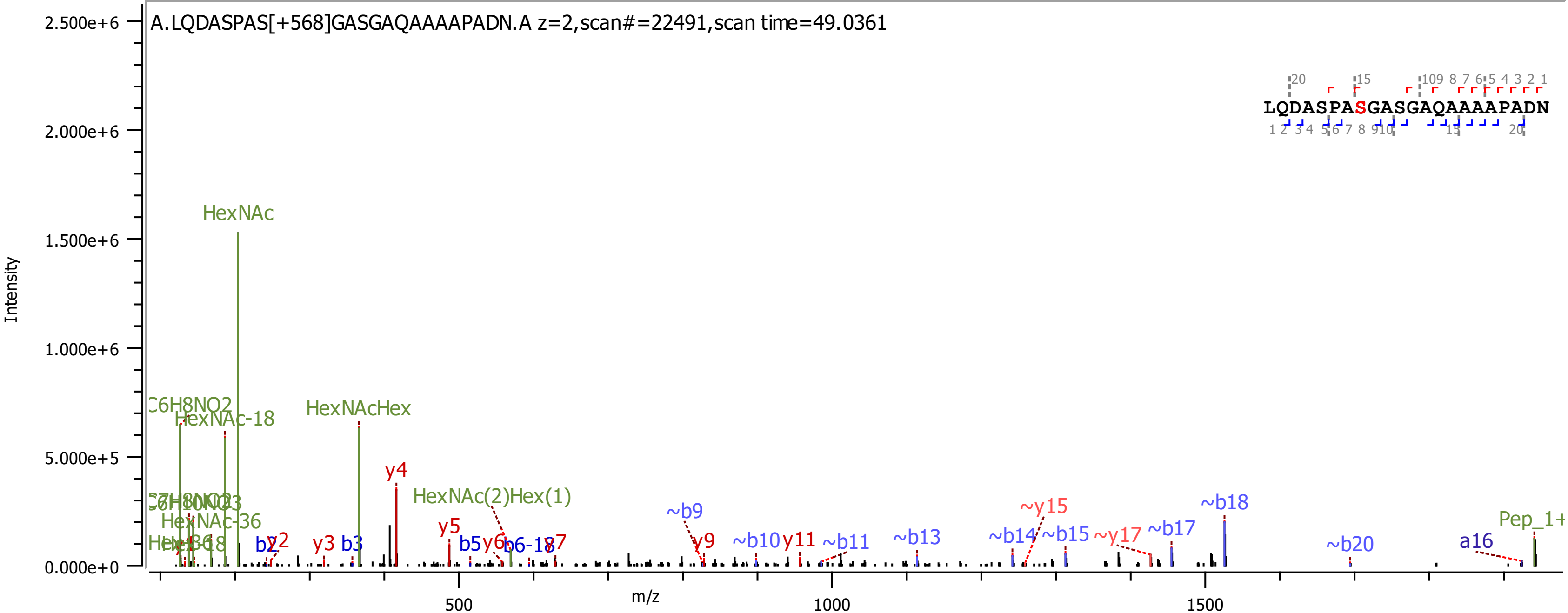

A.LQDASPAS[+568]GASGAQAAAAPADN.A z=2,scan#=22491,scan time=49.0361
LQDASPASGASGAQAAAAPADN
Intensity
m/z
HexNAc
HexNAcHex
HexNAc-18
C6H8NO2
HexNAc-36
HexNAc(2)Hex(1)
y4
y5
y3
b3
b5
y9
y11
~b9
~b10
~b11
~b13
~b14
~b15
~y15
~y17
~b17
~b18
~b20
a16
Pep_1+

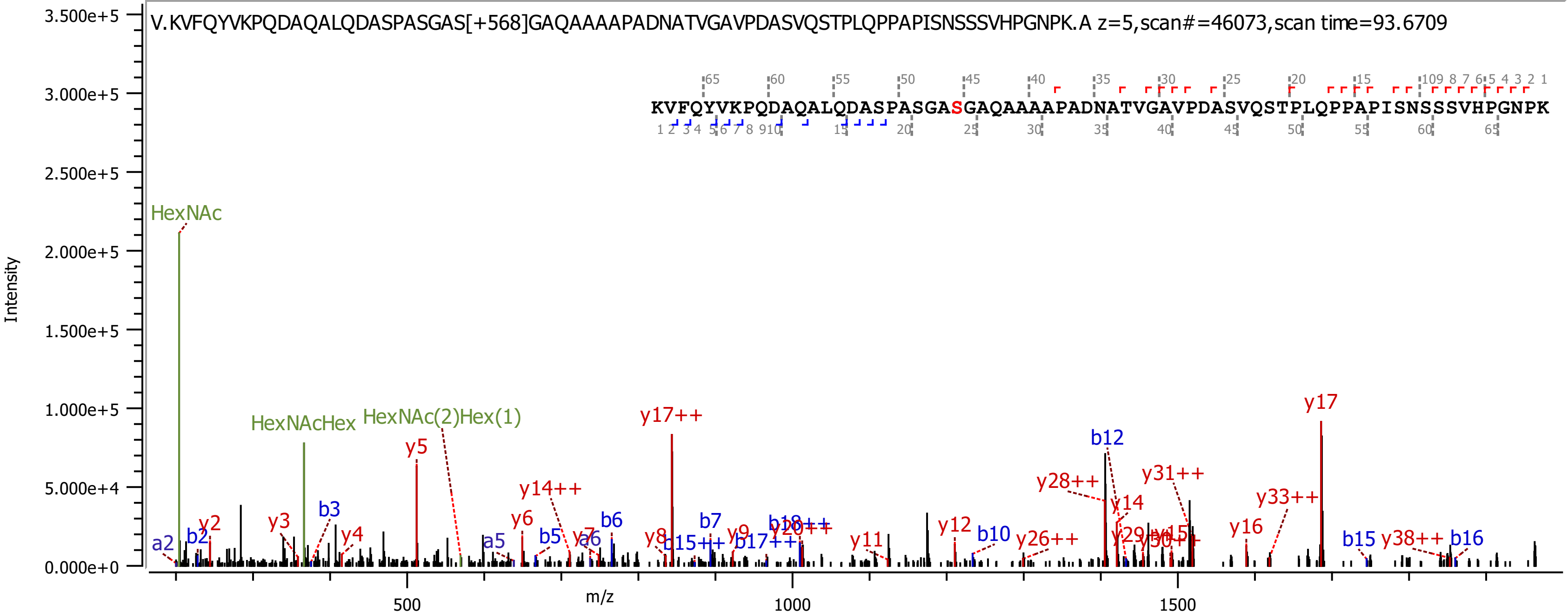

V.KVFQYVKPQDAQALQDASPASGAS[+568]GAQAAAAPADNATVGAVPDASVQSTPLQPPAPISNSSSVHPGNPK.A z=5,scan#=46073,scan time=93.6709
KVFQYVKPQDAQALQDASPASGASGAQAAAAPADNATVGAVPDASVQSTPLQPPAPISNSSSVHPGNPK
HexNAc
HexNAcHex
HexNAc(2)Hex(1)
y17++
y17
b12
y5
y28++
y31++
y14++
y33++
y14
y2
y3
b3
y6
b6
b7
y16
y12
a2
b2
y4
a5
b5
y7
a6
y8
b15++
y9
b18++
y20++
b17++
y11
b10
y26++
y29++
y15
y30++
b15
y38++
b16
Intensity
m/z
3.500e+5
3.000e+5
2.500e+5
2.000e+5
1.500e+5
1.000e+5
5.000e+4
0.000e+0
500
1000
1500

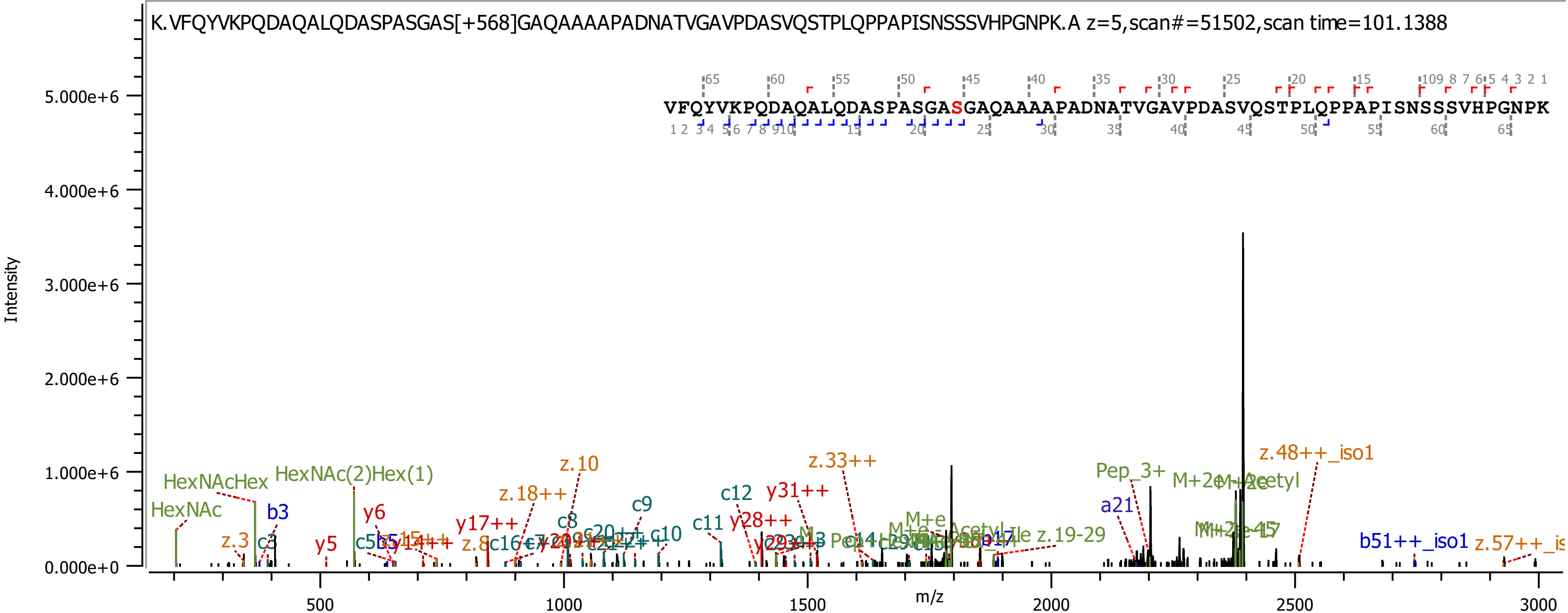
K.VFQYVKPQDAQALQDASPASGAS[+568]GAQAAAAPADNATVGAVPDASVQSTPLQPPAPISNSSSVHPGNPK.A z=5,scan#=51502,scan time=101.1388
VFQYVKPQDAQALQDASPASGASGAQAAAAPADNATVGAVPDASVQSTPLQPPAPISNSSSVHPGNPK
Intensity
5.000e+6
4.000e+6
3.000e+6
2.000e+6
1.000e+6
0.000e+0
m/z
500
1000
1500
2000
2500
3000
HexNAcHex
HexNAc(2)Hex(1)
HexNAc
b3
z.3
c3
y5
y6
z.10
z.18++
y17++
c8
c9
c12
y31++
c11
y28++
c10
z.33++
Pep_3+
a21
M+2e-Acetyl
z.48++_iso1
z.19-29
b51++_iso1

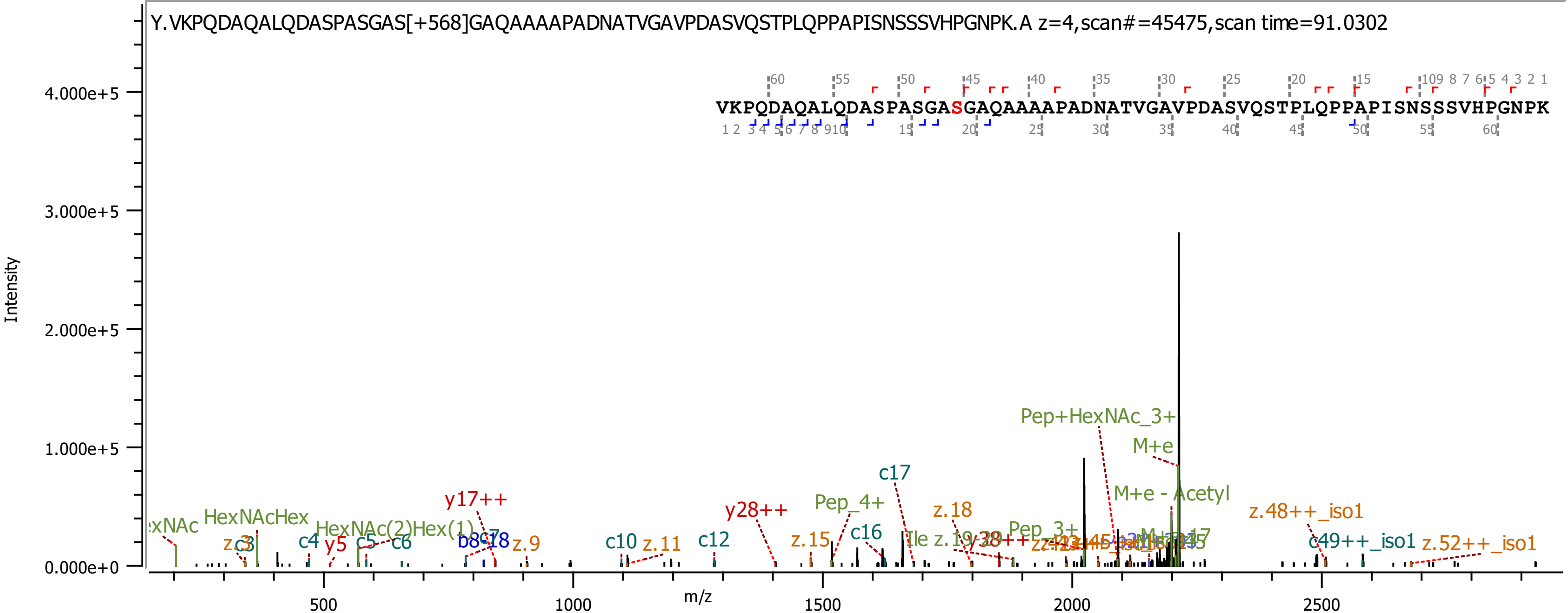
Y.VKPQDAQALQDASPASGAS[+568]GAQAAAAPADNATVGAVPDASVQSTPLQPPAPISNSSSVHPGNPK.A z=4,scan#=45475,scan time=91.0302
VKPQDAQALQDASPASGASGAQAAAAPADNATVGAVPDASVQSTPLQPPAPISNSSSVHPGNPK
Intensity
4.000e+5
3.000e+5
2.000e+5
1.000e+5
0.000e+0
m/z
500
1000
1500
2000
2500
Pep+HexNAc_3+
M+e
M+e - Acetyl
c17
Pep_4+
y17++
y28++
z.18
z.48++_iso1
c16
c49++_iso1
z.52++_iso1
HexNAcHex
HexNAc(2)Hex(1)
c4
y5
c5
c6
c10
z.11
c12
z.15
z.9
Pep_3+

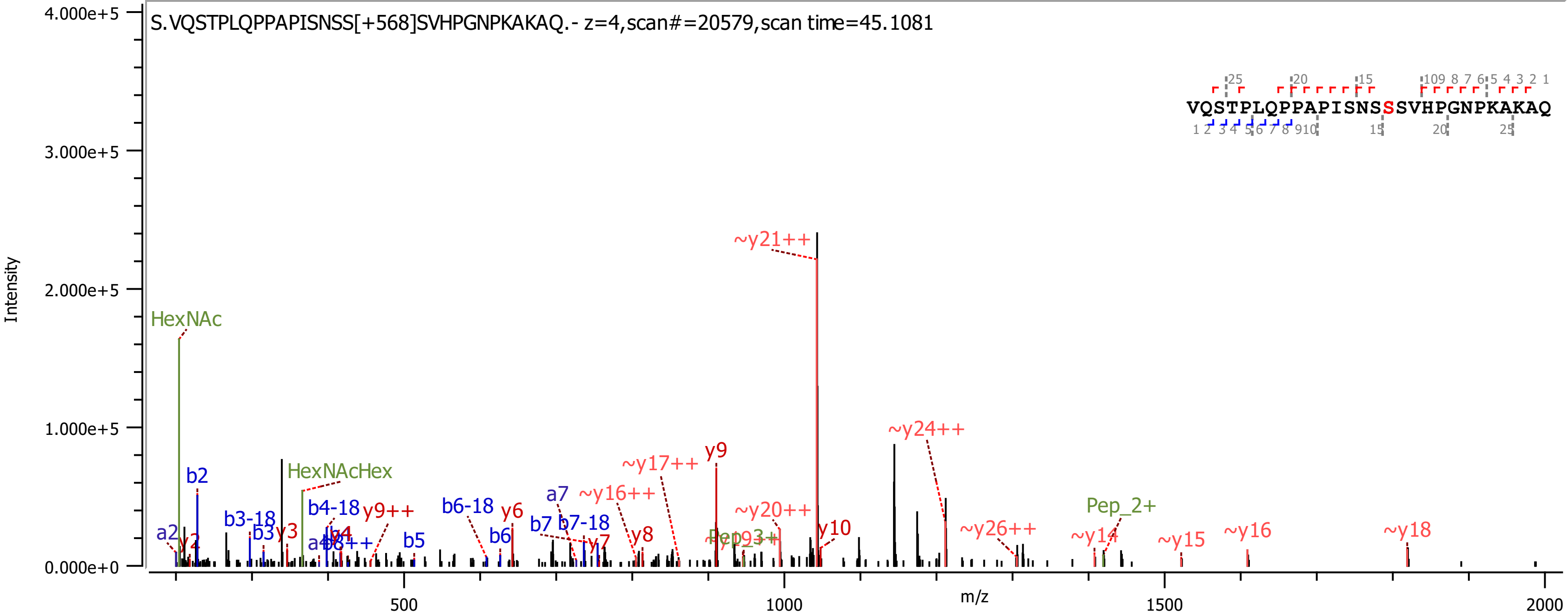

S.VQSTPLQPPAPISNSS[+568]SVHPGNPKAKAQ.- z=4,scan#=20579,scan time=45.1081
VQSTPLQPPAPISNSSSVHPGNPKAKAQ
Intensity
m/z
4.000e+5
3.000e+5
2.000e+5
1.000e+5
0.000e+0
500
1000
1500
2000
HexNAc
HexNAcHex
~y21++
~y24++
y9
~y17++
b2
a7
~y16++
b3-18
b4-18
y9++
b6-18
y6
~y20++
Pep_2+
y10
~y26++
a2
y2
b3
y3
a4
y4
b8++
b5
b6
b7
b7-18
y7
y8
Pep_3+
~y14
~y15
~y16
~y18

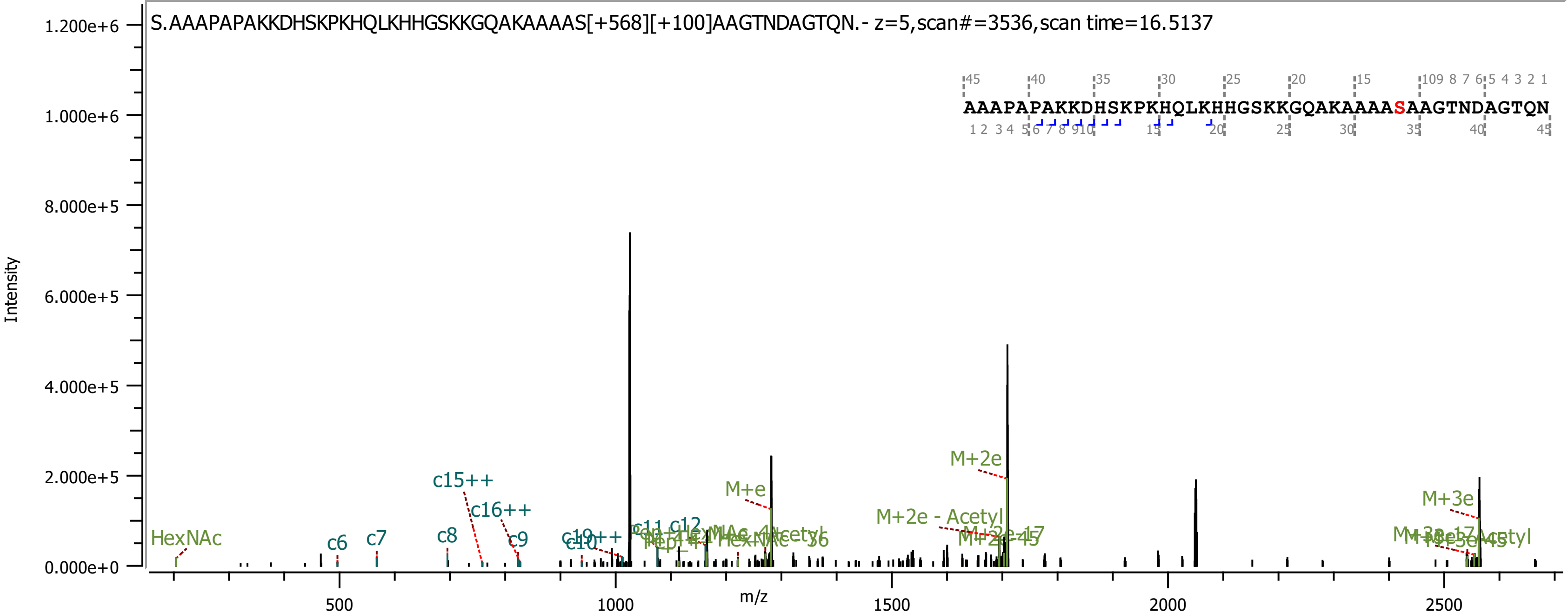
S.AAAPAPAKKDHSKPKHQLKHHGSKKGQAKAAAAS[+568][+100]AAGTNDAGTQN.- z=5,scan#=3536,scan time=16.5137
AAAPAPAKKDHSKPKHQLKHHGSKKGQAKAAAASAAGTNDAGTQN
Intensity
m/z
HexNAc
c6
c7
c8
c15++
c16++
c9
c19++
c10
c11
c12
M+e
M+2e
M+2e - Acetyl
M+3e
0.000e+0
2.000e+5
4.000e+5
6.000e+5
8.000e+5
1.000e+6
1.200e+6
500
1000
1500
2000
2500

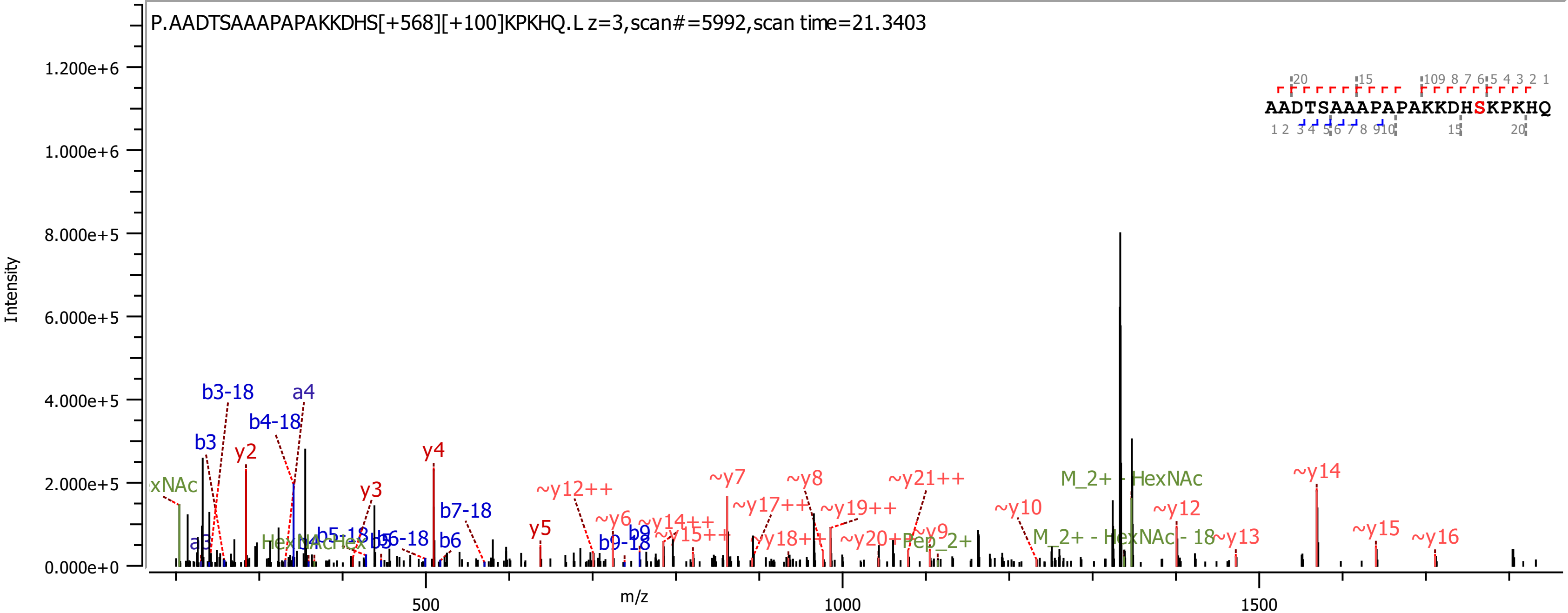

P.AADTSAAAPAPAKKDHS[+568][+100]KPKHQ.L z=3,scan#=5992,scan time=21.3403
AADTSAAAPAPAKKDHSKPKHQ
Intensity
m/z

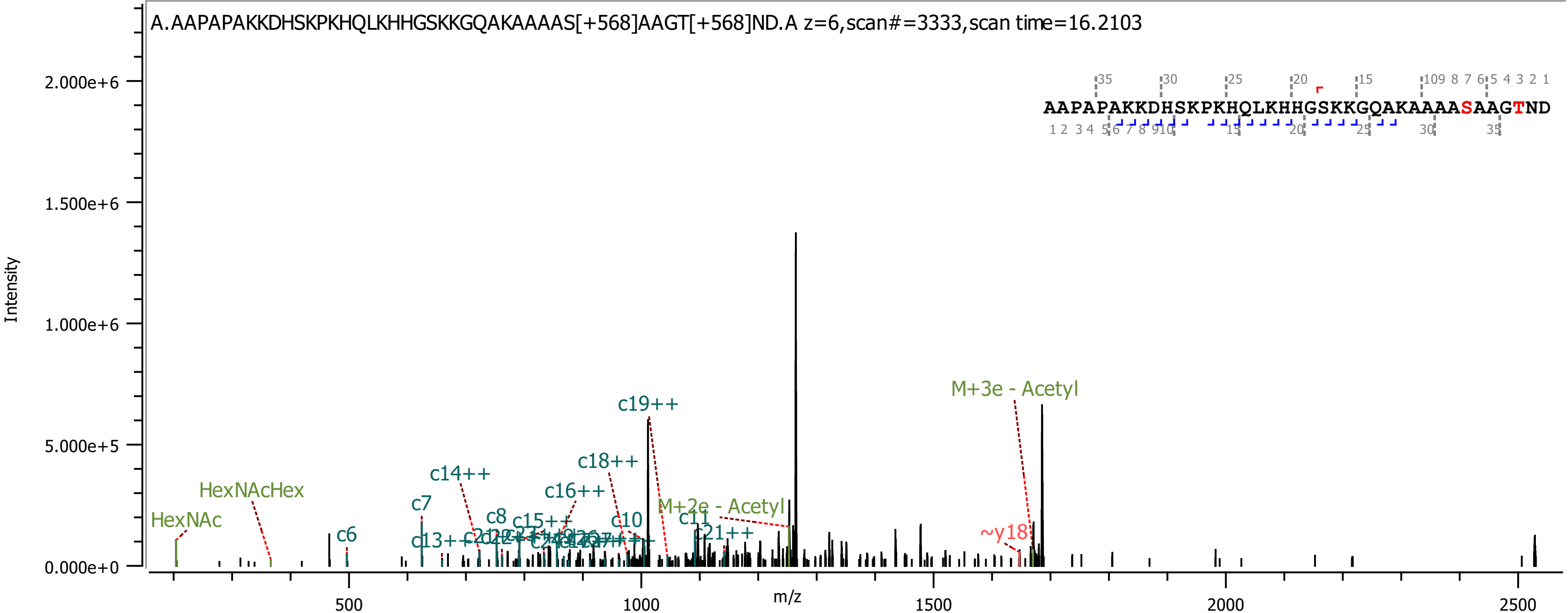
A.AAPAPAKKDHSKPKHQLKHHGSKKGQAKAAAAS[+568]AAGT[+568]ND.A z=6,scan#=3333,scan time=16.2103
AAPAPAKKDHSKPKHQLKHHGSKKGQAKAAAASAAGTND
Intensity
2.000e+6
1.500e+6
1.000e+6
5.000e+5
0.000e+0
m/z
500
1000
1500
2000
2500
c19++
c18++
c14++
c16++
c7
c8
c6
c10
c11
c21++
c15++
c13++
HexNAc
HexNAcHex
M+2e - Acetyl
M+3e - Acetyl
~y18

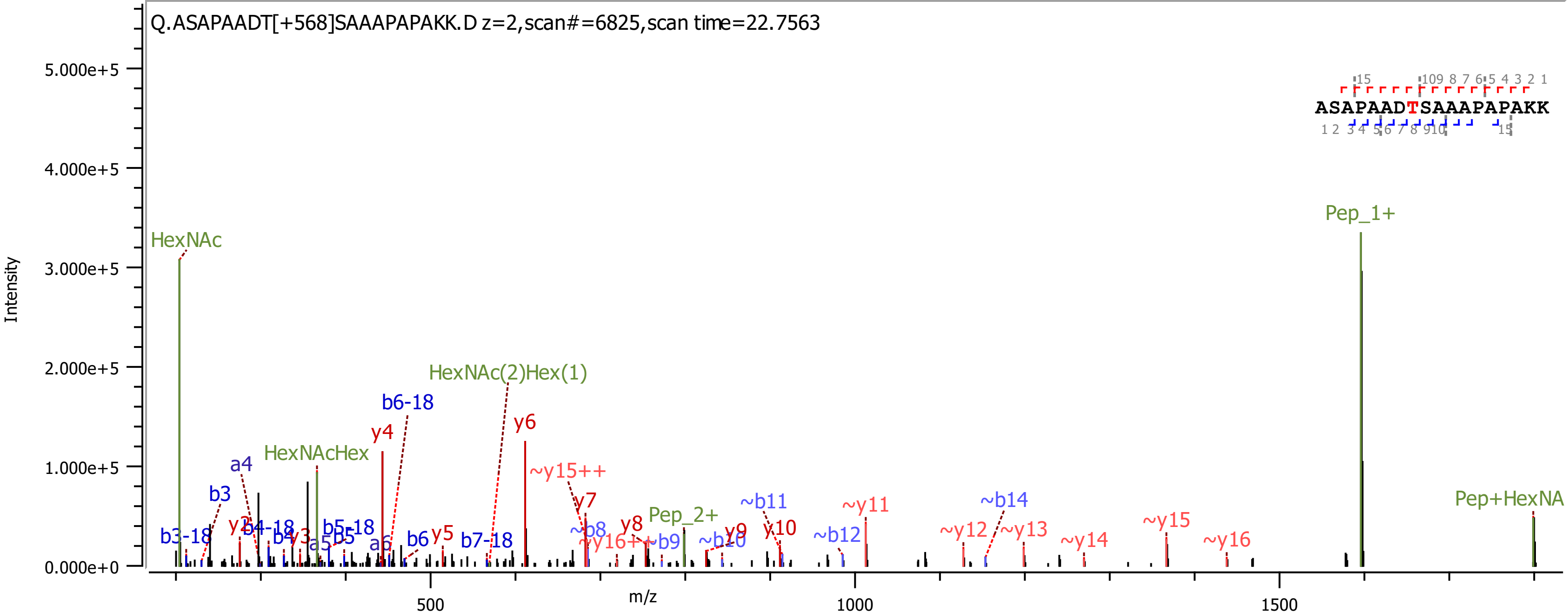
Q.ASAPAADT[+568]SAAAPAPAKK.D z=2,scan#=6825,scan time=22.7563
ASAPAADTSAAAPAPAKK
Intensity
m/z
HexNAc
Pep_1+
HexNAc(2)Hex(1)
HexNAcHex
Pep_2+
Pep+HexNA
b3-18
b3
a4
y2
b4-18
b4
y3
a5
b5-18
b5
a6
y4
b6-18
b6
y5
b7-18
y6
~y15++
y7
~b8
~y16++
y8
~b9
~b10
y9
y10
~b11
~b12
~y11
~y12
~b14
~y13
~y14
~y15
~y16
0.000e+0
1.000e+5
2.000e+5
3.000e+5
4.000e+5
5.000e+5
500
1000
1500

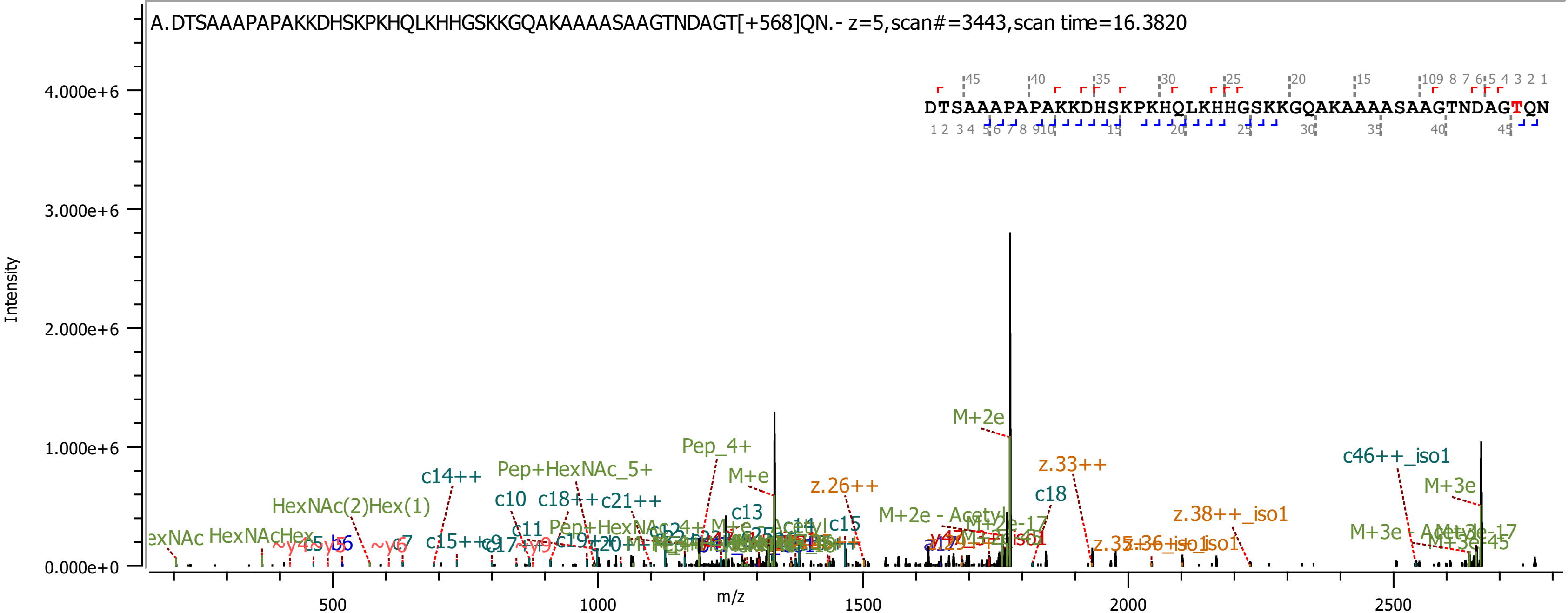
A.DTSAAAPAPAKKDHSKPKHQLKHHGSKKGQAKAAAASAAGTNDAGT[+568]QN.- z=5,scan#=3443,scan time=16.3820
DTSAAAPAPAKKDHSKPKHQLKHHGSKKGQAKAAAASAAGTNDAGTQN
Intensity
m/z
4.000e+6
3.000e+6
2.000e+6
1.000e+6
0.000e+0
500
1000
1500
2000
2500
M+2e
Pep_4+
M+e
c14++
Pep+HexNAc_5+
c10
c18++
c21++
HexNAc(2)Hex(1)
z.26++
z.33++
c18
c46++_iso1
M+3e
z.38++_iso1
c13
c15
M+2e - Acetyl
M+3e - Acetyl
HexNAcHex
c11
c7
~y6
b6
c15++
c17
c19

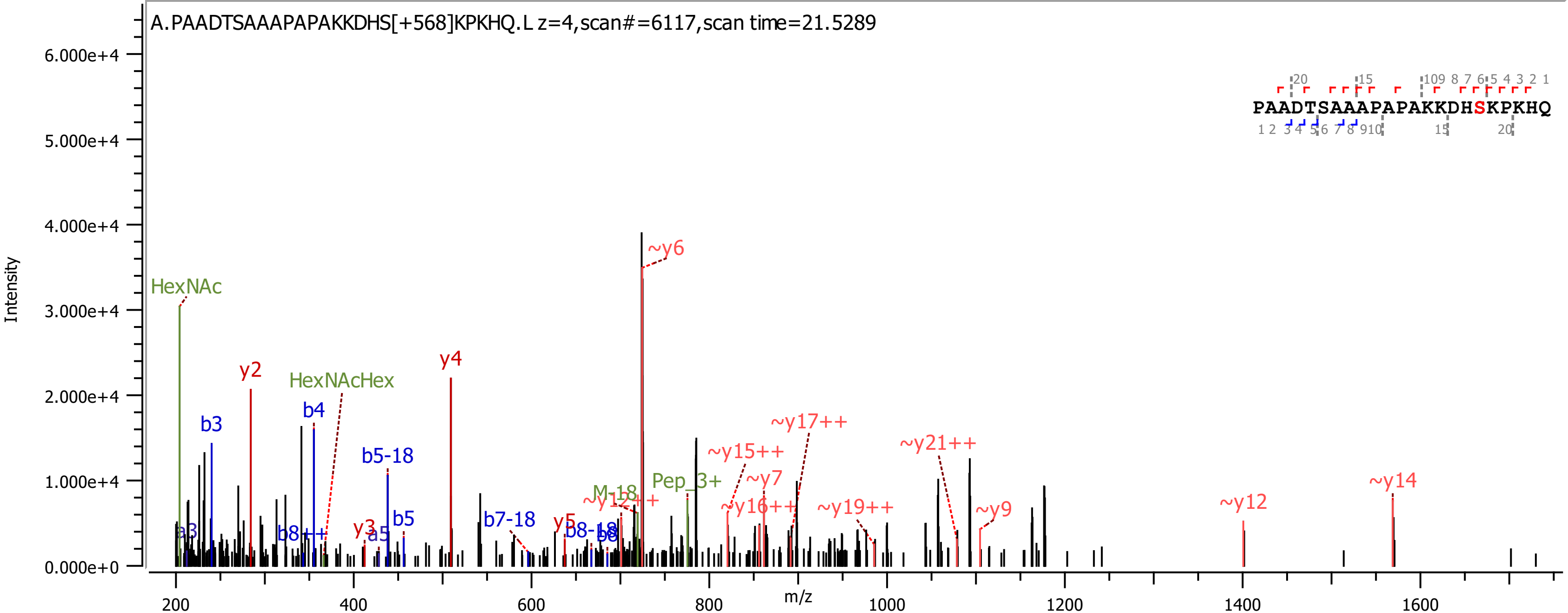

A.PAADTSAAAPAPAKKDHS[+568]KPKHQ.L z=4,scan#=6117,scan time=21.5289
PAADTSAAAPAPAKKDHSKPKHQ
Intensity
m/z
HexNAc
HexNAcHex
y2
y3
y4
y5
b3
b4
b5
b5-18
b7-18
b8-18
b8
b8++
a3
a5
~y6
~y7
~y9
~y12
~y12++
~y14
~y15++
~y16++
~y17++
~y19++
~y21++
M-18
Pep_3+

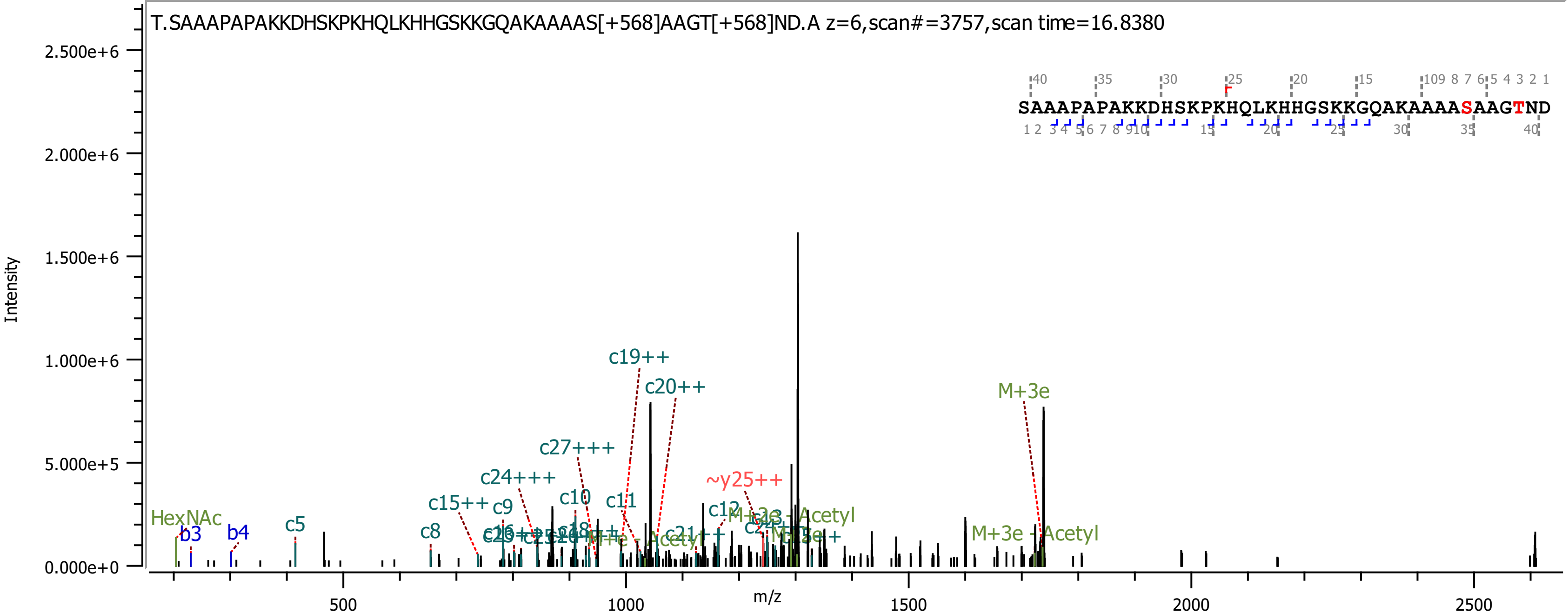

T.SAAAPAPAKKDHSKPKHQLKHHGSKKGQAKAAAAS[+568]AAGT[+568]ND.A z=6,scan#=3757,scan time=16.8380
SAAAPAPAKKDHSKPKHQLKHHGSKKGQAKAAAASAAGTND
Intensity
m/z
2.500e+6
2.000e+6
1.500e+6
1.000e+6
5.000e+5
0.000e+0
500
1000
1500
2000
2500
HexNAc
b3
b4
c5
c8
c9
c10
c11
c12
c15++
c19++
c20++
c24+++
c27+++
~y25++
M+3e
M+3e - Acetyl

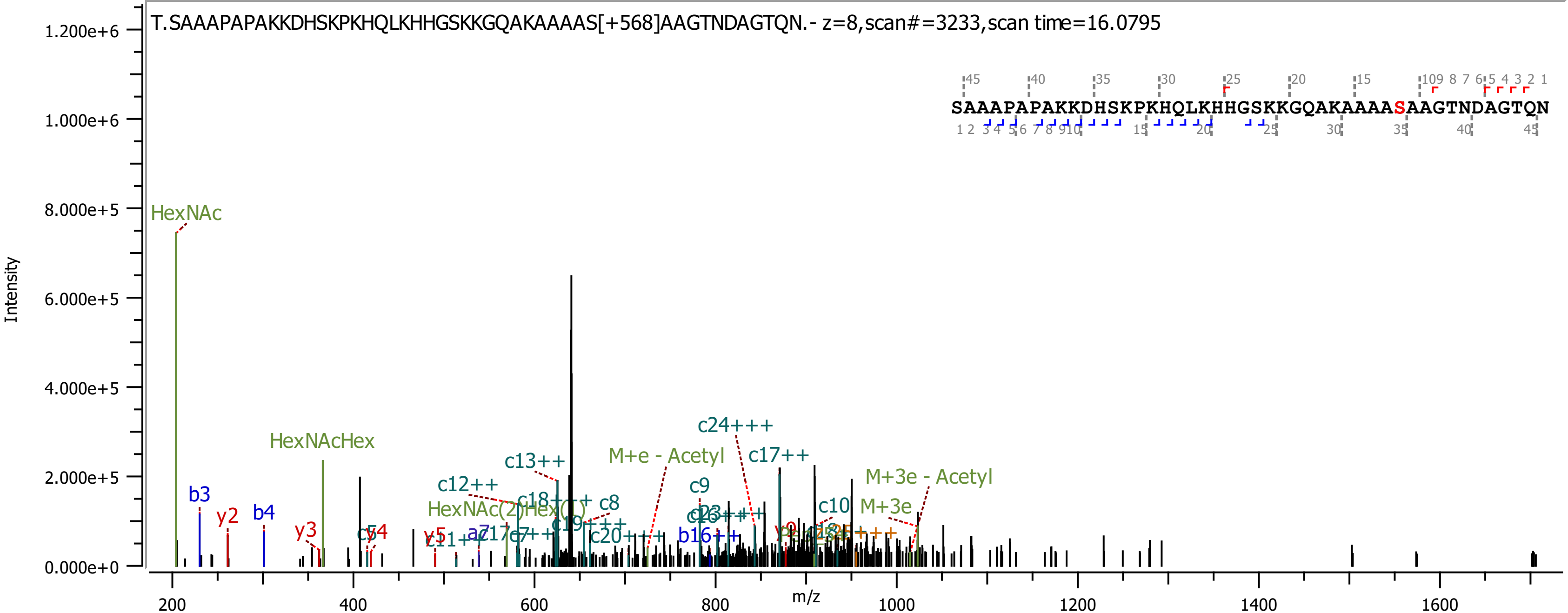
T.SAAAPAPAKKDHSKPKHQLKHHGSKKGQAKAAAAS[+568]AAGTNDAGTQN.- z=8,scan#=3233,scan time=16.0795
SAAAPAPAKKDHSKPKHQLKHHGSKKGQAKAAAASAAGTNDAGTQN
Intensity
m/z
1.200e+6
1.000e+6
8.000e+5
6.000e+5
4.000e+5
2.000e+5
0.000e+0
200
400
600
800
1000
1200
1400
1600
HexNAc
HexNAcHex
b3
y2
b4
y3
y5
c12++
c13++
HexNAc(2)Hex(1)
c8
c9
c10
c24+++
c17++
M+e - Acetyl
M+3e - Acetyl
M+3e
c20+++
b16++

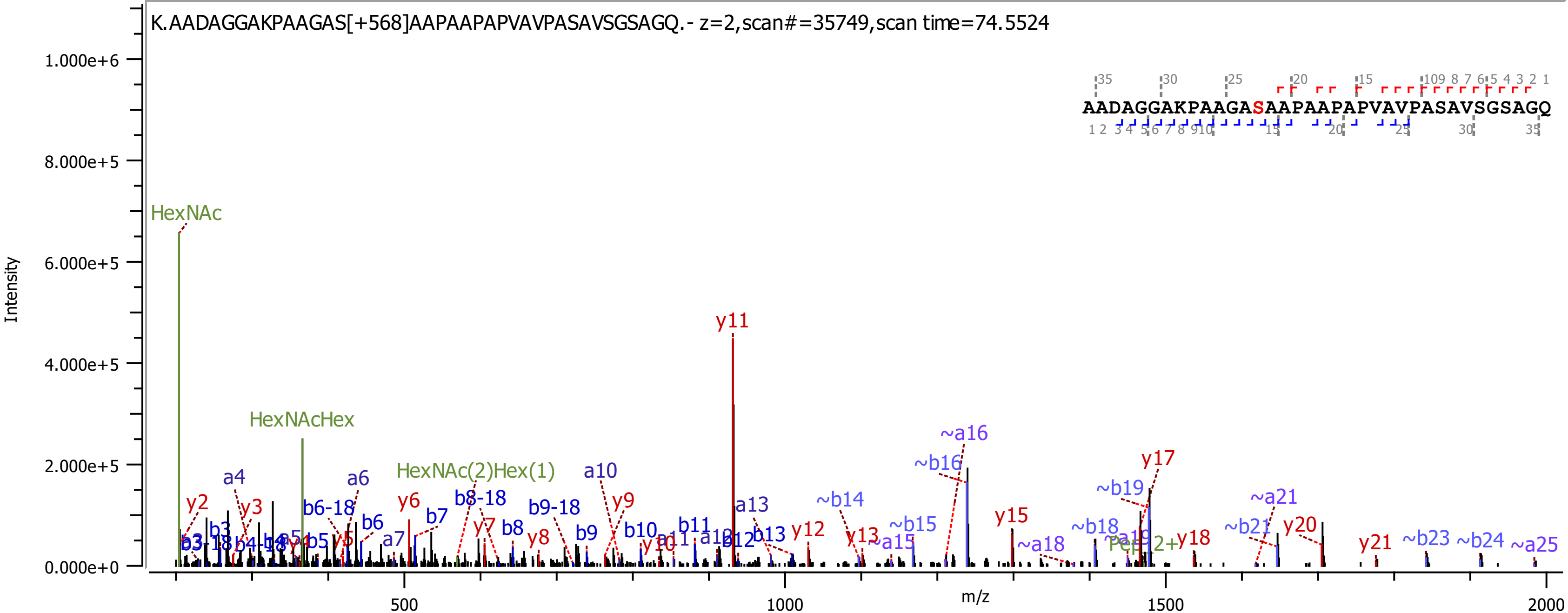

K.AADAGGAKPAAGAS[+568]AAPAAPAPVAVPASAVSGSAGQ.- z=2,scan#=35749,scan time=74.5524
AADAGGAKPAAGASAAPAAPAPVAVPASAVSGSAGQ
Intensity
1.000e+6
8.000e+5
6.000e+5
4.000e+5
2.000e+5
0.000e+0
m/z
500
1000
1500
2000
HexNAc
HexNAcHex
HexNAc(2)Hex(1)
y11
~a16
~b16
y17
~b19
~a21
y20
y2
y3
a4
a6
b6-18
b6
y6
b7
b8-18
y7
b8
b9-18
a10
y9
b9
b10
b11
a13
b13
y12
~b14
y13
~b15
~a15
y15
~a18
~b18
y18
~b21
y21
~b23
~b24
~a25

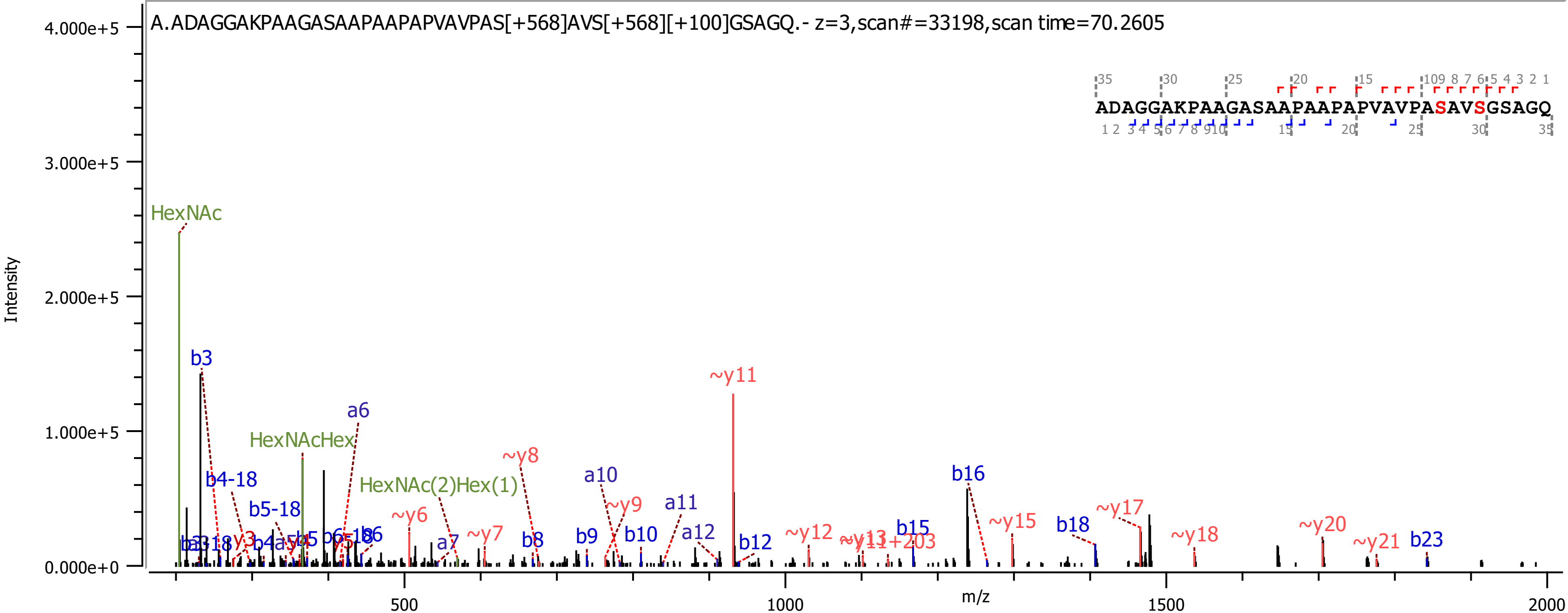

A.ADAGGAKPAAGASAAPAAPAPVAVPAS[+568]AVS[+568][+100]GSAGQ.- z=3,scan#=33198,scan time=70.2605
ADAGGAKPAAGASAAPAAPAPVAVPASAVSGSAGQ
Intensity
m/z
HexNAc
HexNAcHex
HexNAc(2)Hex(1)
b3
a6
~y11
b4-18
b5-18
~y8
a10
~y9
a11
a12
b10
b9
b8
~y7
a7
~y6
b12
~y12
~y13
~y11+203
b15
b16
~y15
b18
~y17
~y18
~y20
~y21
b23
4.000e+5
3.000e+5
2.000e+5
1.000e+5
0.000e+0
500
1000
1500
2000

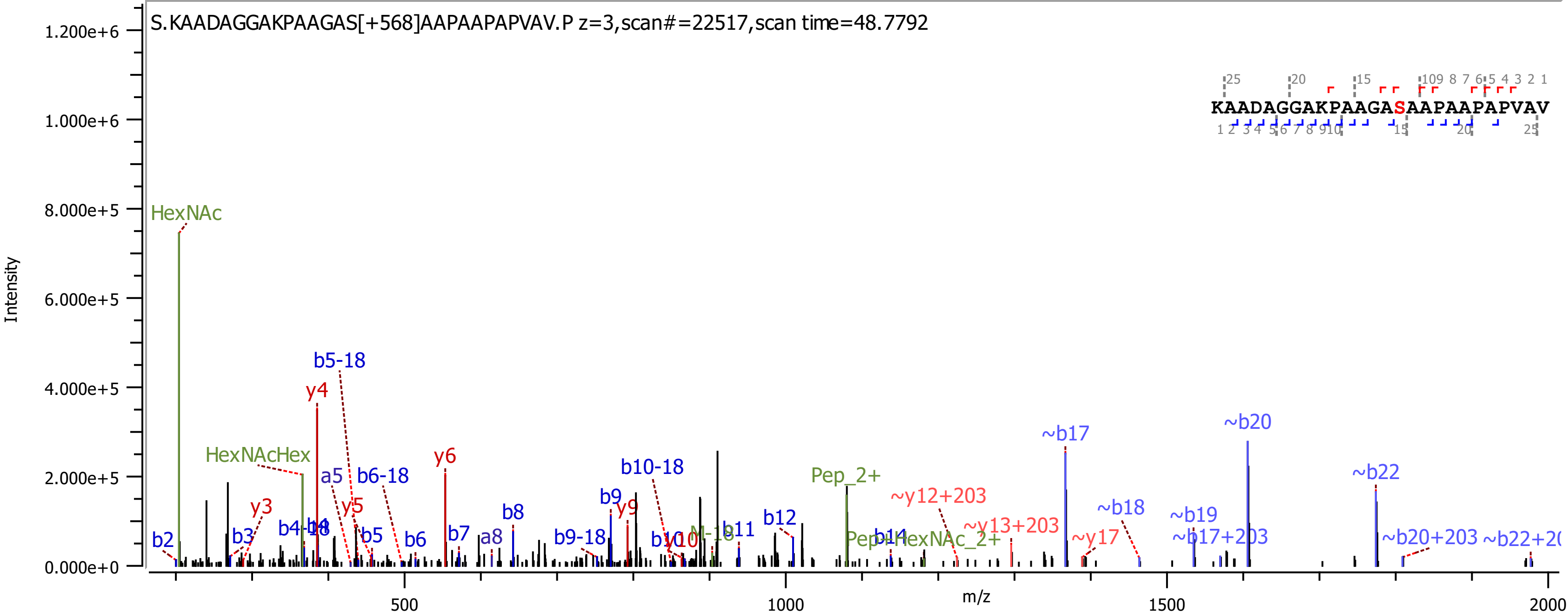
S.KAADAGGAKPAAGAS[+568]AAPAAPAPVAV.P z=3,scan#=22517,scan time=48.7792
KAADAGGAKPAAGASAAPAAPAPVAV
Intensity
m/z
HexNAc
HexNAcHex
b5-18
y4
y6
a5
b6-18
y3
y5
b2
b3
b4-18
b4
b5
b6
b7
a8
b8
b9
b9-18
y9
b10-18
b10
y10
M-18
b11
b12
Pep_2+
Pep+HexNAc_2+
b14
~y12+203
~y13+203
~b17
~y17
~b18
~b19
~b17+203
~b20
~b22
~b20+203

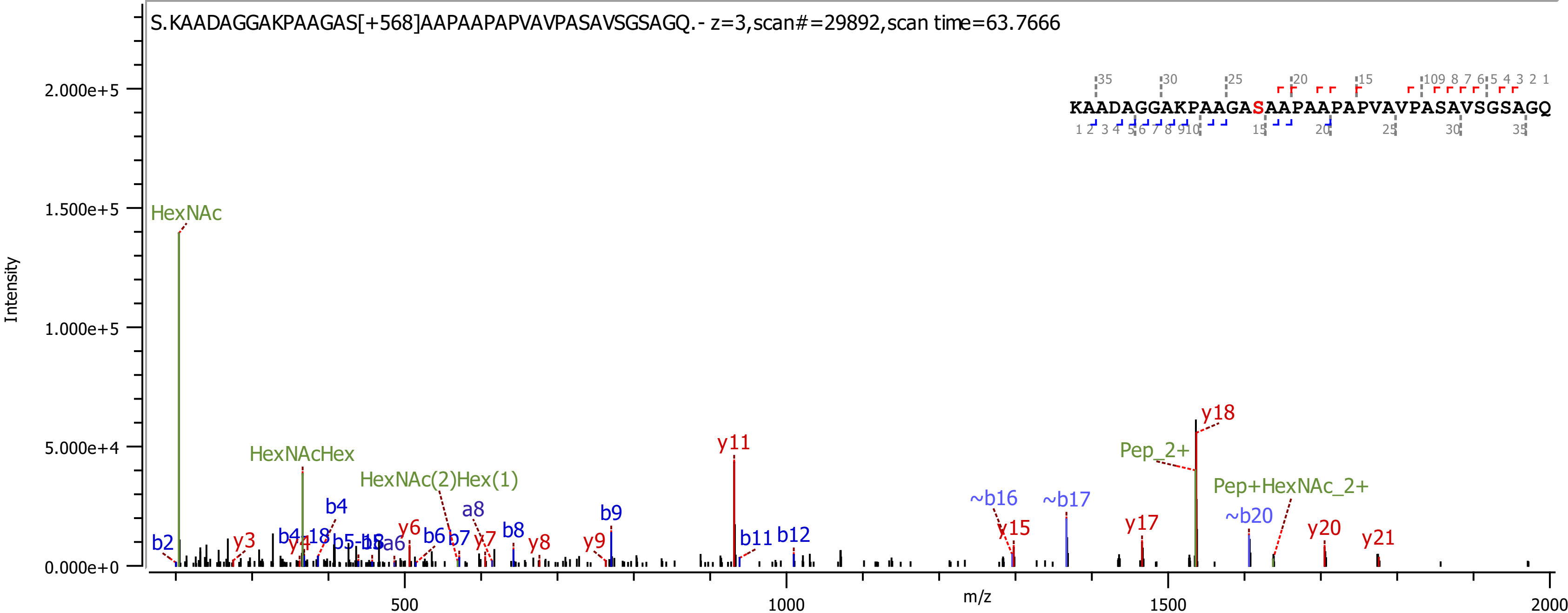

S.KAADAGGAKPAAGAS[+568]AAPAAPAPVAVPASAVSGSAGQ.- z=3,scan#=29892,scan time=63.7666
KAADAGGAKPAAGASAAPAAPAPVAVPASAVSGSAGQ
Intensity
2.000e+5
1.500e+5
1.000e+5
5.000e+4
0.000e+0
m/z
500
1000
1500
2000
HexNAc
HexNAcHex
HexNAc(2)Hex(1)
Pep_2+
Pep+HexNAc_2+
b2
y3
b4
b4-18
y4
b5-18
a6
y6
b6
b7
y7
a8
b8
y8
b9
y9
y11
b11
b12
~b16
y15
~b17
y17
y18
~b20
y20
y21

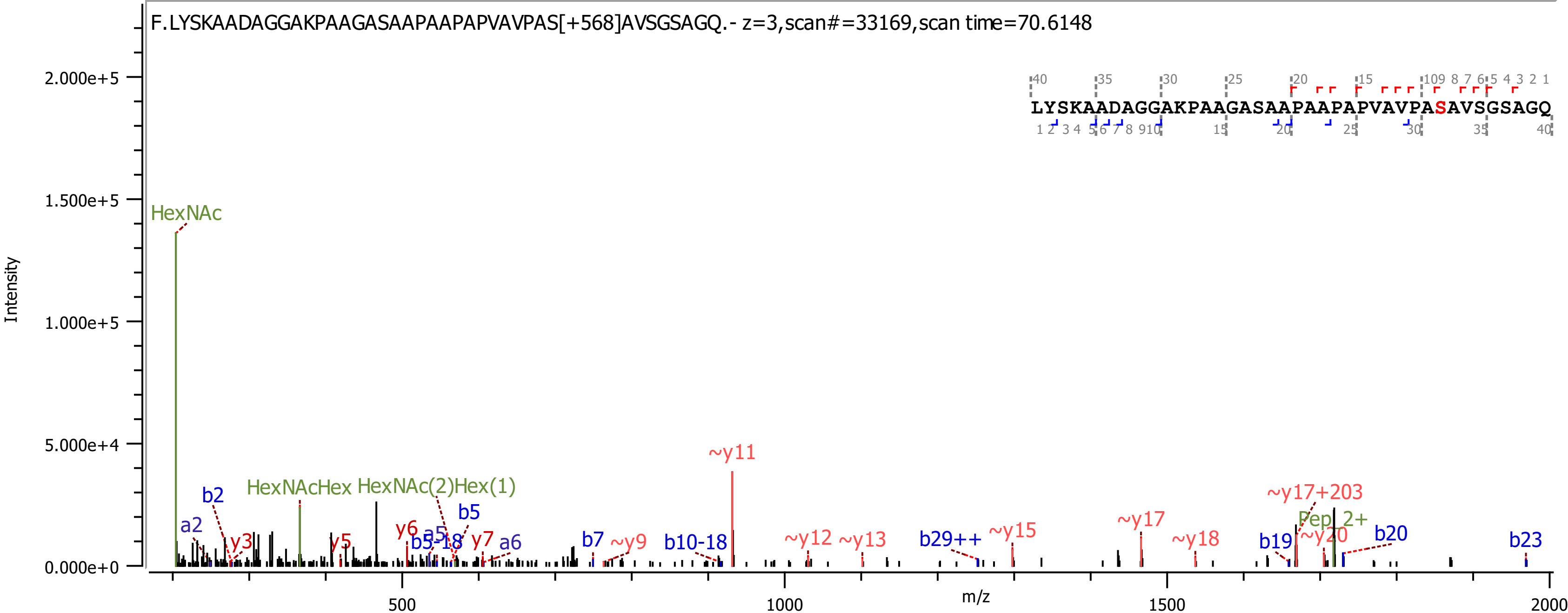

F.LYSKAADAGGAKPAAGASAAPAAPAPVAVPAS[+568]AVSGSAGQ.- z=3,scan#=33169,scan time=70.6148
LYSKAADAGGAKPAAGASAAPAAPAPVAVPASAVSGSAGQ
Intensity
m/z
HexNAc
HexNAcHex
HexNAc(2)Hex(1)
a2
b2
y3
y5
y6
a5
b5-18
b5
y7
a6
b7
~y9
b10-18
~y11
~y12
~y13
b29++
~y15
~y17
~y18
b19
~y17+203
Pep 2+
~y20
b20
b23
0.000e+0
5.000e+4
1.000e+5
1.500e+5
2.000e+5
500
1000
1500
2000

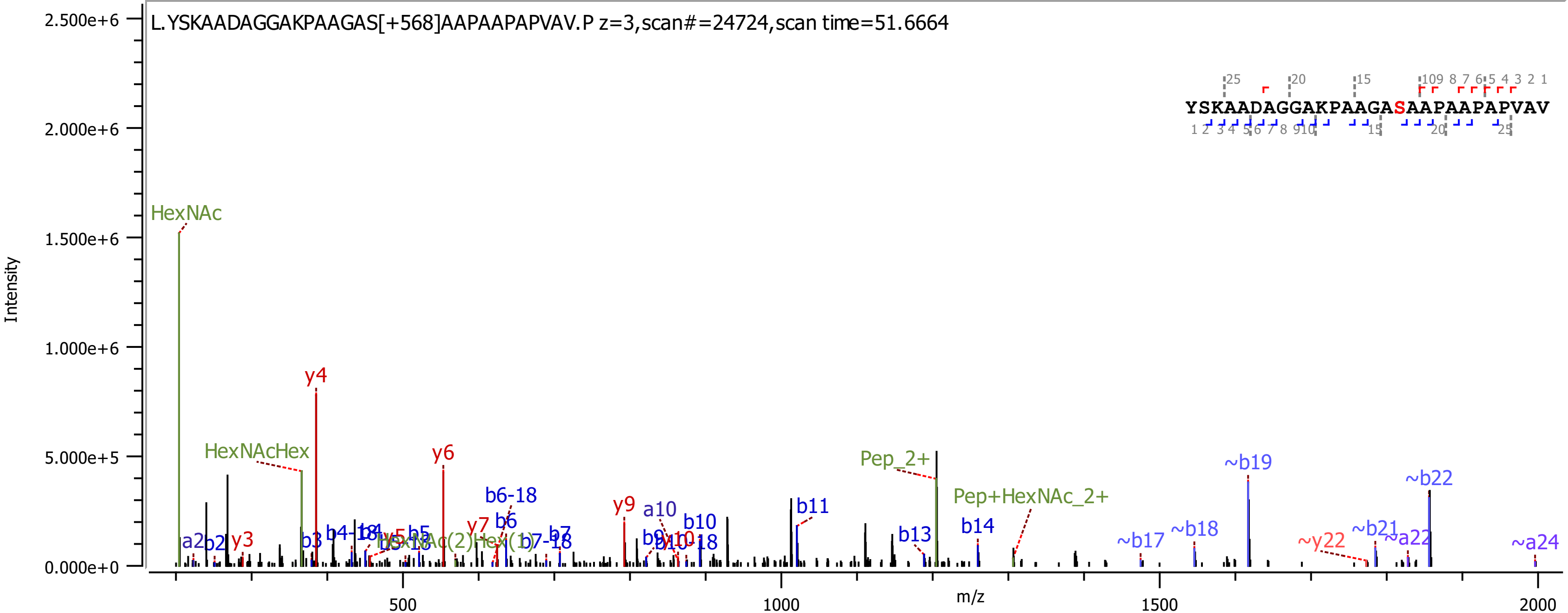

L.YSKAADAGGAKPAAGAS[+568]AAPAAPAPVAV.P z=3,scan#=24724,scan time=51.6664
YSKAADAGGAKPAAGASAAPAAPAPVAV
Intensity
m/z
HexNAc
HexNAcHex
y4
y6
y9
a10
b10
b11
Pep_2+
Pep+HexNAc_2+
b13
b14
~b17
~b18
~b19
~y22
~b21
~a22
~b22
~a24
b6-18
b6
y7
b7-18
b7
a2
b2
y3
b3
b5
b4-18
b10-18
b9

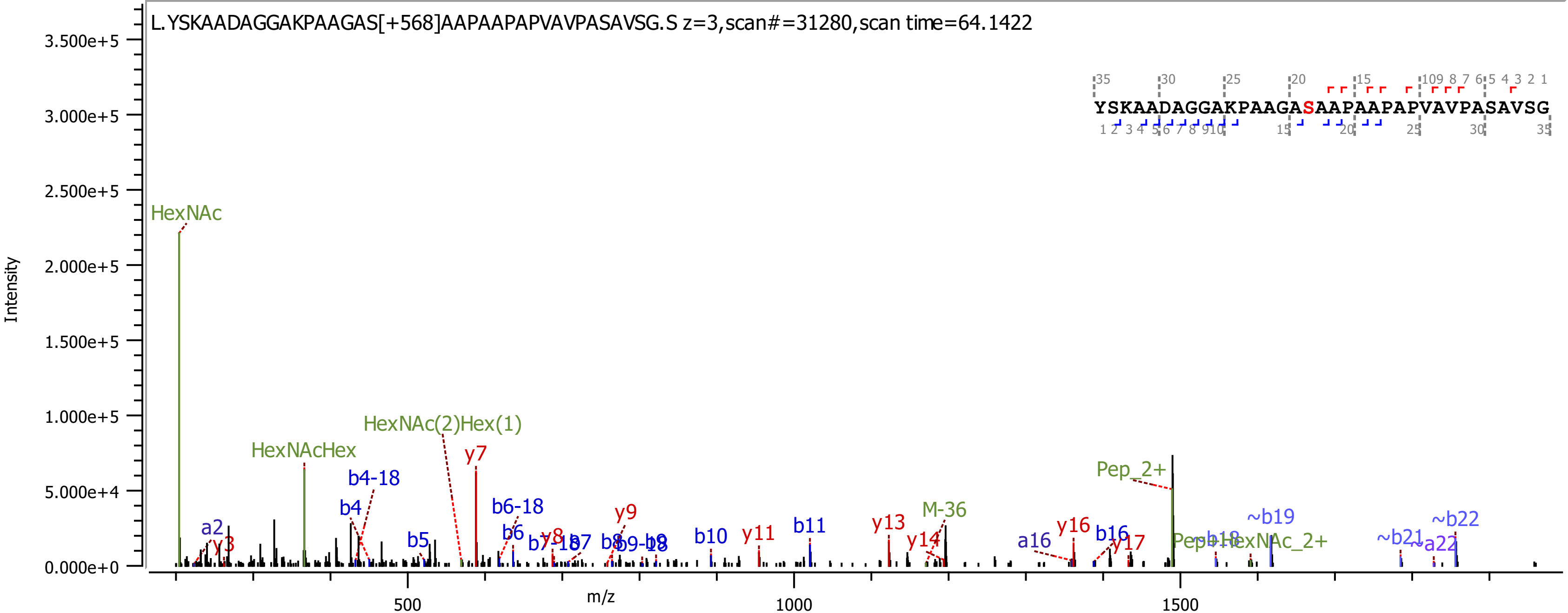

L.YSKAADAGGAKPAAGAS[+568]AAPAAPAPVAVPASAVSG.S z=3,scan#=31280,scan time=64.1422
YSKAADAGGAKPAAGASAAPAAPAPVAVPASAVSG
Intensity
m/z
HexNAc
HexNAcHex
HexNAc(2)Hex(1)
y7
b4-18
b4
b6-18
b6
a2
y3
b5
y8
y9
b10
y11
b11
y13
M-36
y14
a16
y16
b16
y17
Pep_2+
~b19
~b21
~b22

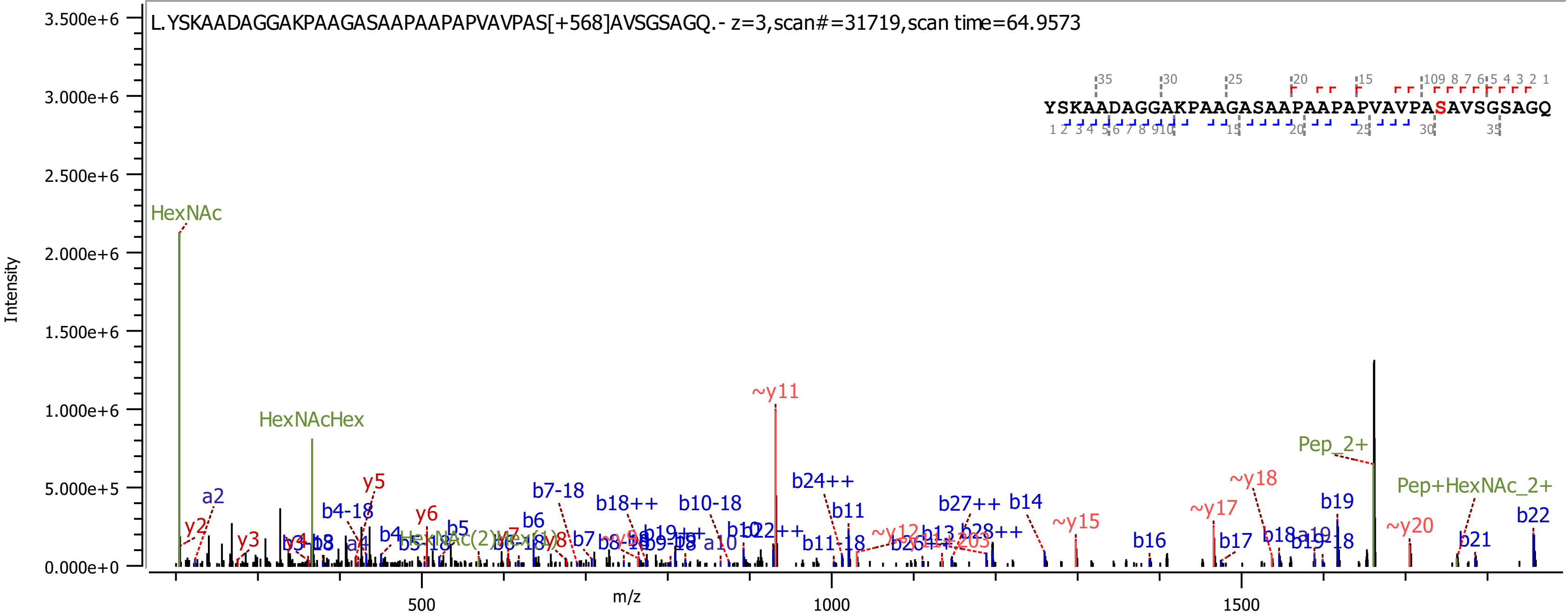

L.YSKAADAGGAKPAAGASAAPAAPAPVAVPAS[+568]AVSGSAGQ.- z=3,scan#=31719,scan time=64.9573
YSKAADAGGAKPAAGASAAPAAPAPVAVPASAVSGSAGQ
Intensity
m/z
HexNAc
HexNAcHex
Pep_2+
Pep+HexNAc_2+
~y11
~y18
~y17
~y15
~y20
b24++
b11
b27++
b14
b19
b22
b16
b17
b21
b7-18
b18++
b10-18
b4-18
y5
y6
a2
y2
y3
b5
b6
b7
a10
b11-18

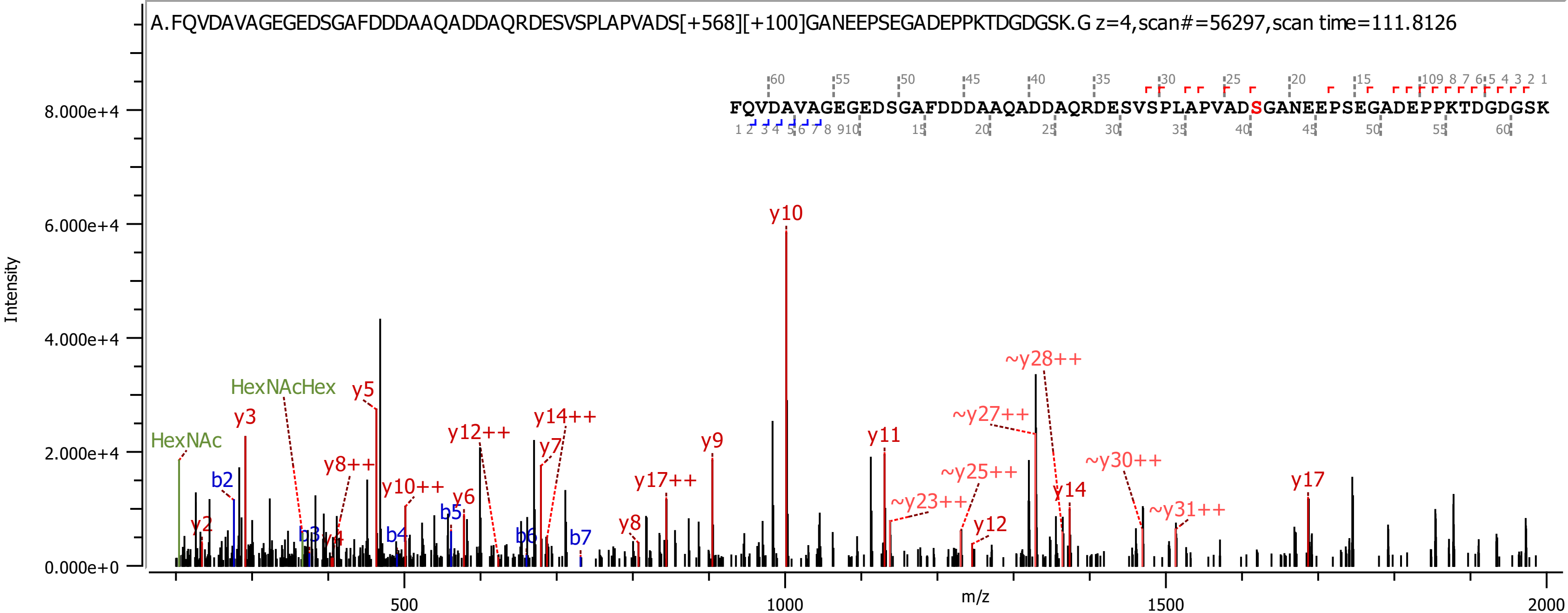

A.FQVDAVAGEGEDSGAFDDDAAQADDAQRDESVSPLAPVADS[+568][+100]GANEEPSEGADEPPKTDGDGSK.G z=4,scan#=56297,scan time=111.8126
FQVDAVAGEGEDSGAFDDDAAQADDAQRDESVSPLAPVADSGANEEPSEGADEPPKTDGDGSK
Intensity
m/z
8.000e+4
6.000e+4
4.000e+4
2.000e+4
0.000e+0
500
1000
1500
2000
HexNAc
HexNAcHex
y2
y3
y4
y5
y6
y7
y8
y9
y10
y11
y12
y14
y17
y8++
y10++
y12++
y14++
y17++
~y23++
~y25++
~y27++
~y28++
~y30++
~y31++
b2
b3
b4
b5
b6
b7

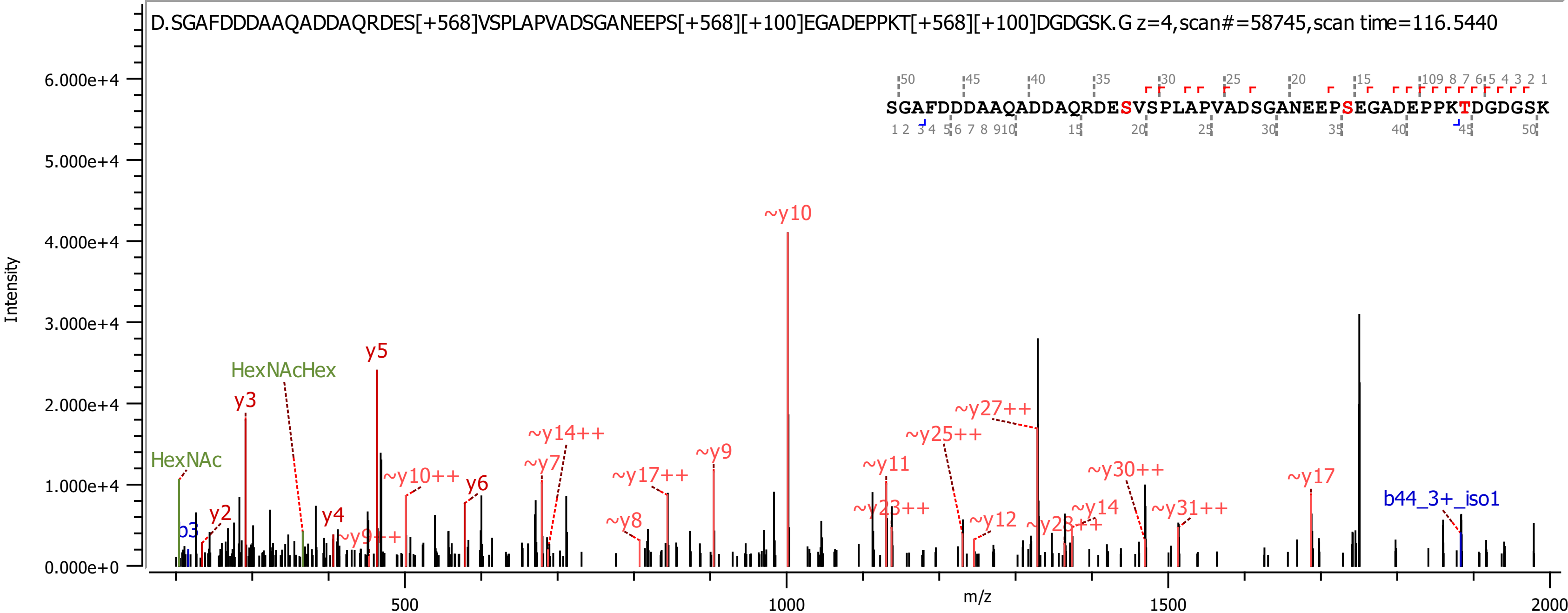
D.SGAFDDDAAQADDAQRDES[+568]VSPLAPVADSGANEEPS[+568][+100]EGADEPPKT[+568][+100]DGDGSK.G z=4,scan#=58745,scan time=116.5440
SGAFDDDAAQADDAQRDESVSPLAPVADSGANEEPSEGADEPPKTDGDGSK
Intensity
m/z
HexNAc
HexNAcHex
b3
y2
y3
y4
y5
y6
~y9++
~y10++
~y7
~y14++
~y8
~y17++
~y9
~y10
~y11
~y23++
~y25++
~y12
~y27++
~y28++
~y14
~y30++
~y31++
~y17
b44_3+_iso1

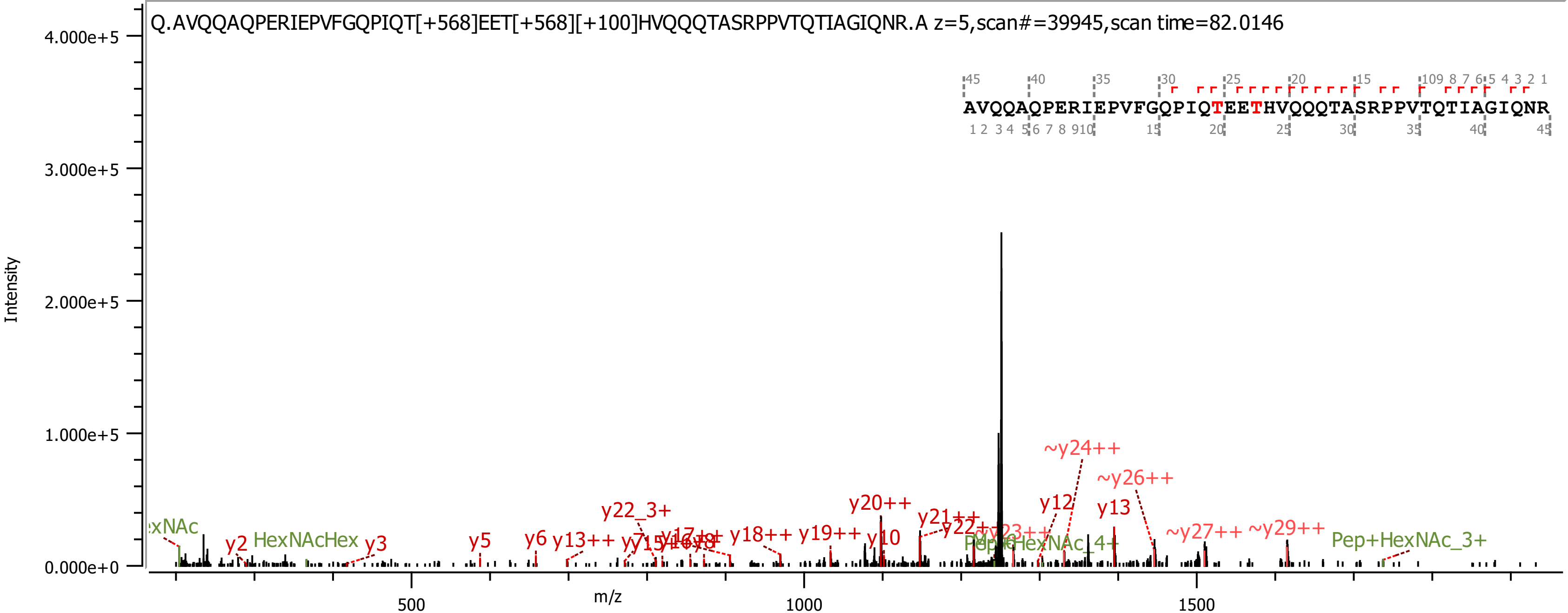

Q.AVQQAQPERIEPVFGQPIQT[+568]EET[+568][+100]HVQQQTASRPPVTQTIAGIQNR.A z=5,scan#=39945,scan time=82.0146
AVQQAQPERIEPVFGQPIQTEETHVQQQTASRPPVTQTIAGIQNR
Intensity
m/z
4.000e+5
3.000e+5
2.000e+5
1.000e+5
0.000e+0
500
1000
1500
y2
HexNAcHex
y3
y5
y6
y13++
y22_3+
y18++
y19++
y20++
y10
y21++
y12
y13
~y24++
~y26++
~y27++
~y29++
Pep+HexNAc_4+
Pep+HexNAc_3+

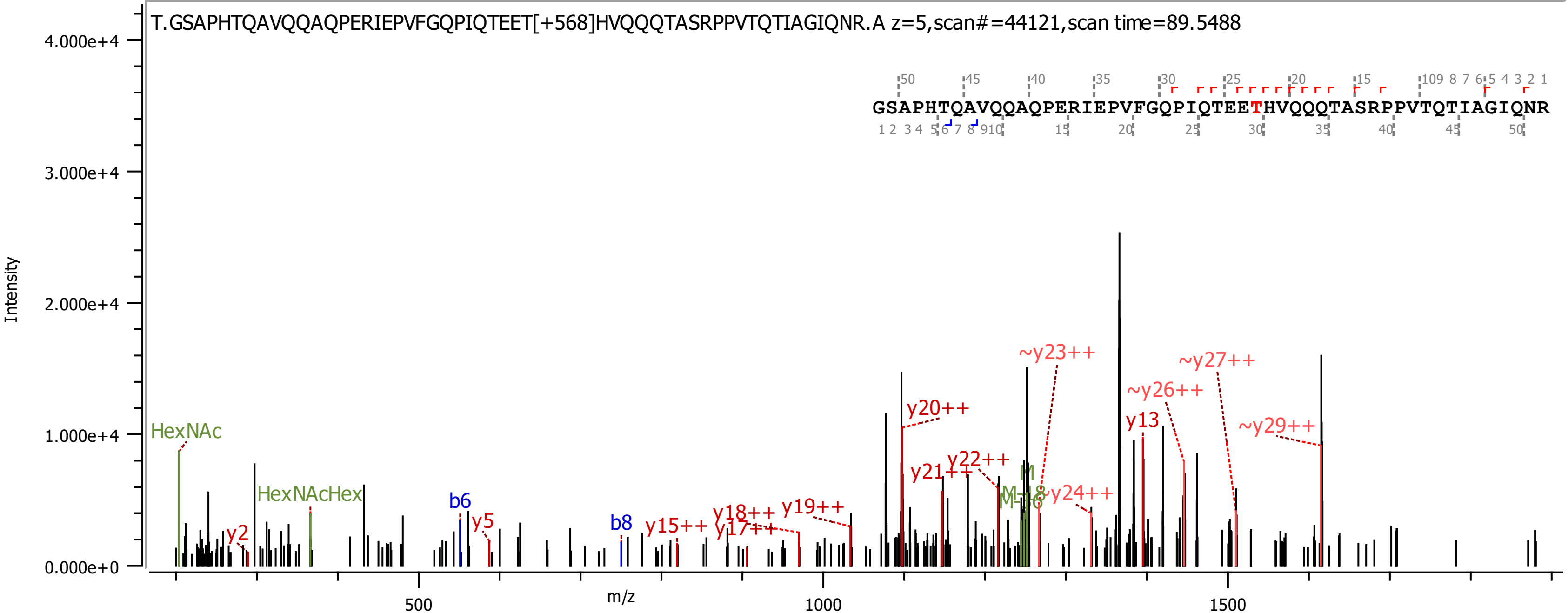

T.GSAPHTQAVQQAQPERIEPVFGQPIQTEET[+568]HVQQQTASRPPVTQTIAGIQNR.A z=5,scan#=44121,scan time=89.5488
GSAPHTQAVQQAQPERIEPVFGQPIQTEETHVQQQTASRPPVTQTIAGIQNR
Intensity
4.000e+4
3.000e+4
2.000e+4
1.000e+4
0.000e+0
m/z
500
1000
1500
HexNAc
HexNAcHex
y2
b6
y5
b8
y15++
y18++
y17++
y19++
y20++
y21++
y22++
~y23++
~y24++
y13
~y26++
~y27++
~y29++

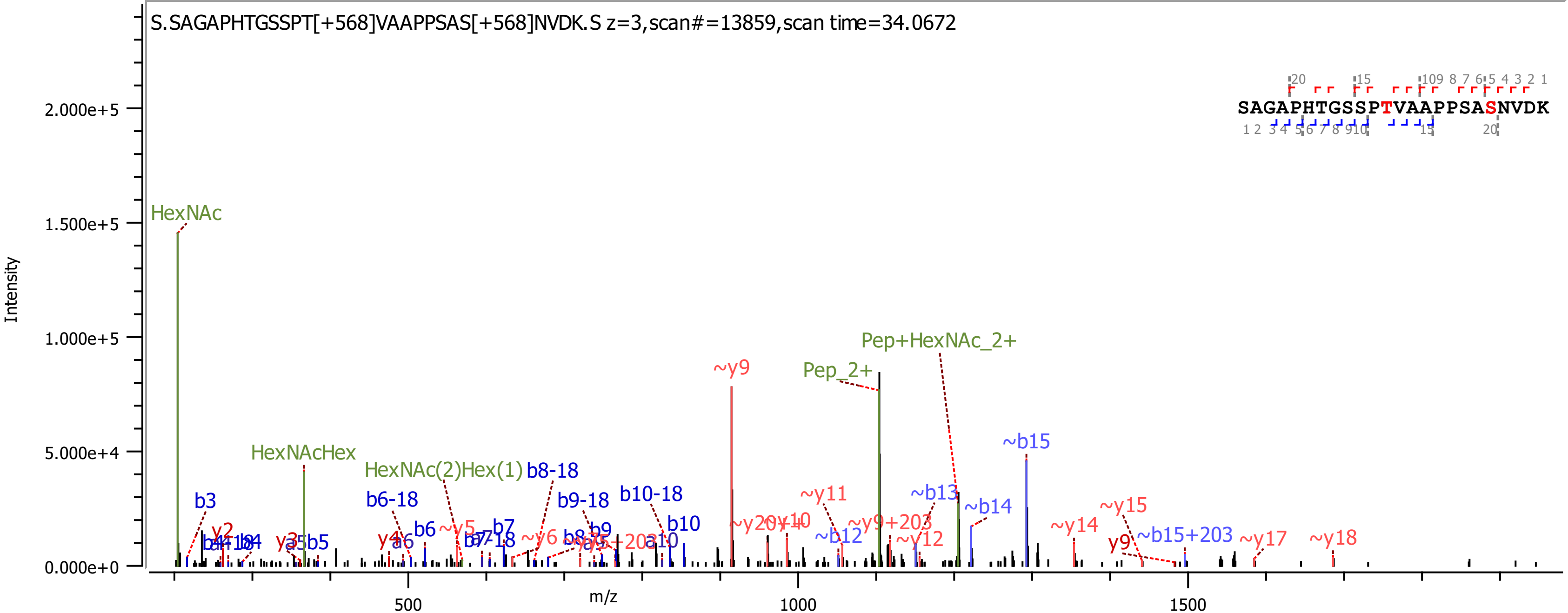

S.SAGAPHTGSSPT[+568]VAAPPSAS[+568]NVDK.S z=3,scan#=13859,scan time=34.0672
SAGAPHTGSSPTVAAPPSASNVDK
Intensity
m/z
0.000e+0
5.000e+4
1.000e+5
1.500e+5
2.000e+5
500
1000
1500
HexNAc
HexNAcHex
HexNAc(2)Hex(1)
Pep_2+
Pep+HexNAc_2+
b3
b5
b6
b6-18
b7
b7-18
b8-18
b9
b9-18
b10
b10-18
y2
y3
y4
~y5
~y6
~y9
~y10
~y11
~y12
~y9+203
~y14
~y15
~y17
~y18
y9
~b12
~b13
~b14
~b15
~b15+203

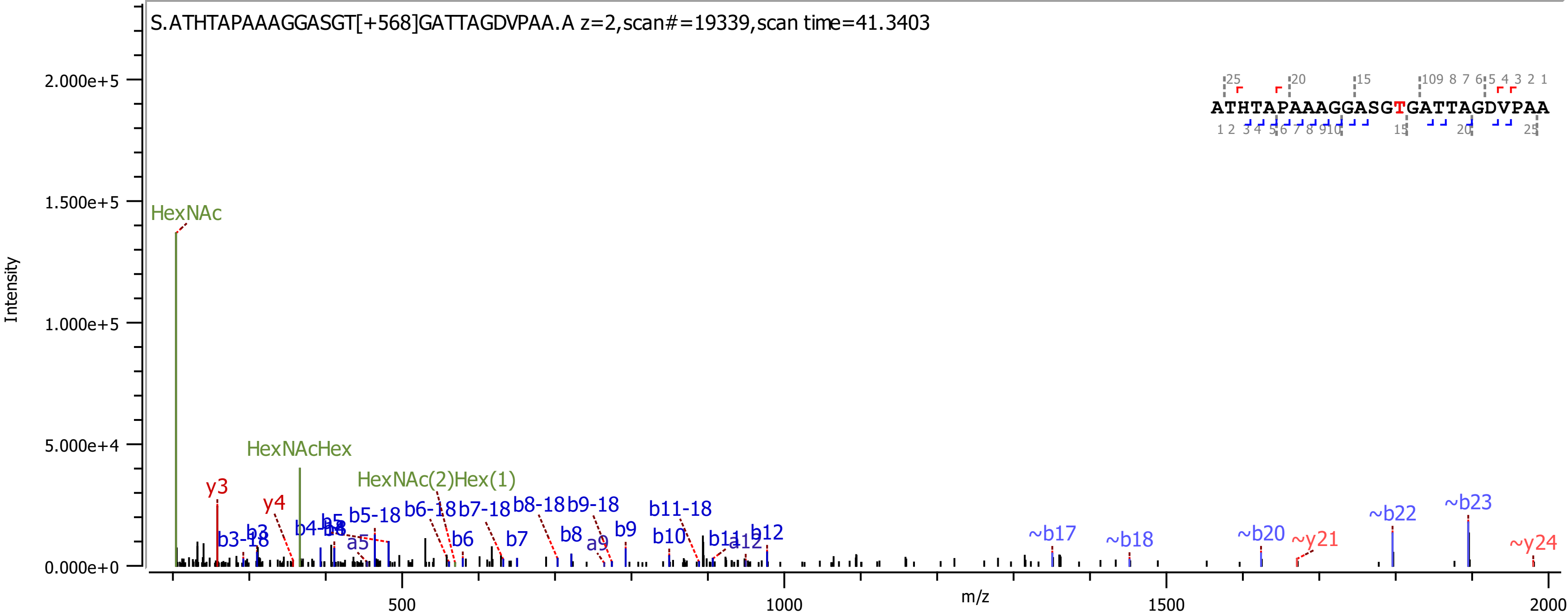

S.ATHTAPAAAGGASGT[+568]GATTAGDVPAA.A z=2,scan#=19339,scan time=41.3403
ATHTAPAAAGGASGTGATTAGDVPAA
Intensity
m/z
0.000e+0
5.000e+4
1.000e+5
1.500e+5
2.000e+5
500
1000
1500
2000
HexNAc
HexNAcHex
HexNAc(2)Hex(1)
y3
y4
b3-18
b4-18
b5-18
b6-18
b7-18
b8-18
b9-18
b11-18
a5
a9
a12
b6
b7
b8
b9
b10
b11
b12
~b17
~b18
~b20
~y21
~b22
~b23
~y24

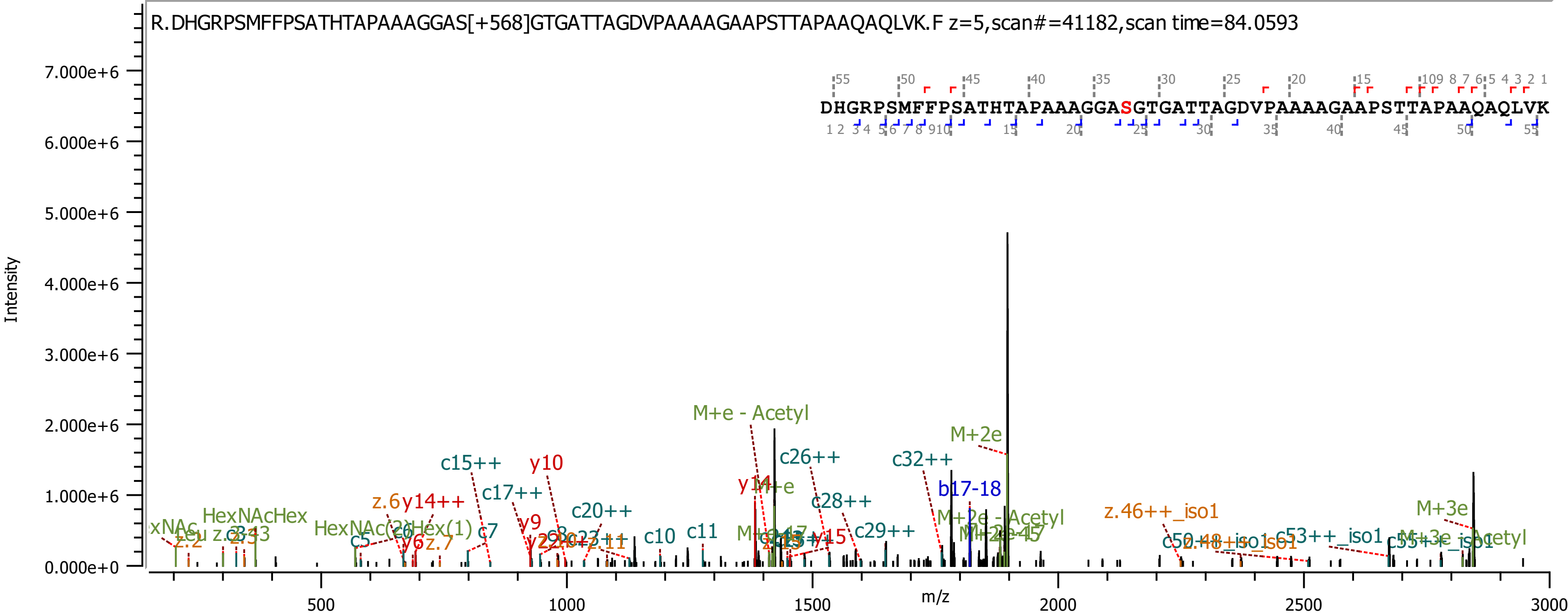
R.DHGRPSMFFPSATHTAPAAAGGAS[+568]GTGATTAGDVPAAAAGAAPSTTAPAAQAQLVK.F z=5,scan#=41182,scan time=84.0593
DHGRPSMFFPSATHTAPAAAGGASGTGATTAGDVPAAAAGAAPSTTAPAAQAQLVK
Intensity
7.000e+6
6.000e+6
5.000e+6
4.000e+6
3.000e+6
2.000e+6
1.000e+6
0.000e+0
m/z
500
1000
1500
2000
2500
3000
M+e - Acetyl
M+2e
c26++
c32++
c15++
y10
b17-18
c17++
y14
c28++
z.6
y14++
c20++
z.46++_iso1
M+3e
M+2e - Acetyl
HexNAcHex
HexNAc(2)Hex(1)
c29++
c7
y9
c10
c11
c5
y6
z.7
y15
z.2
c53++_iso1
M+3e - Acetyl

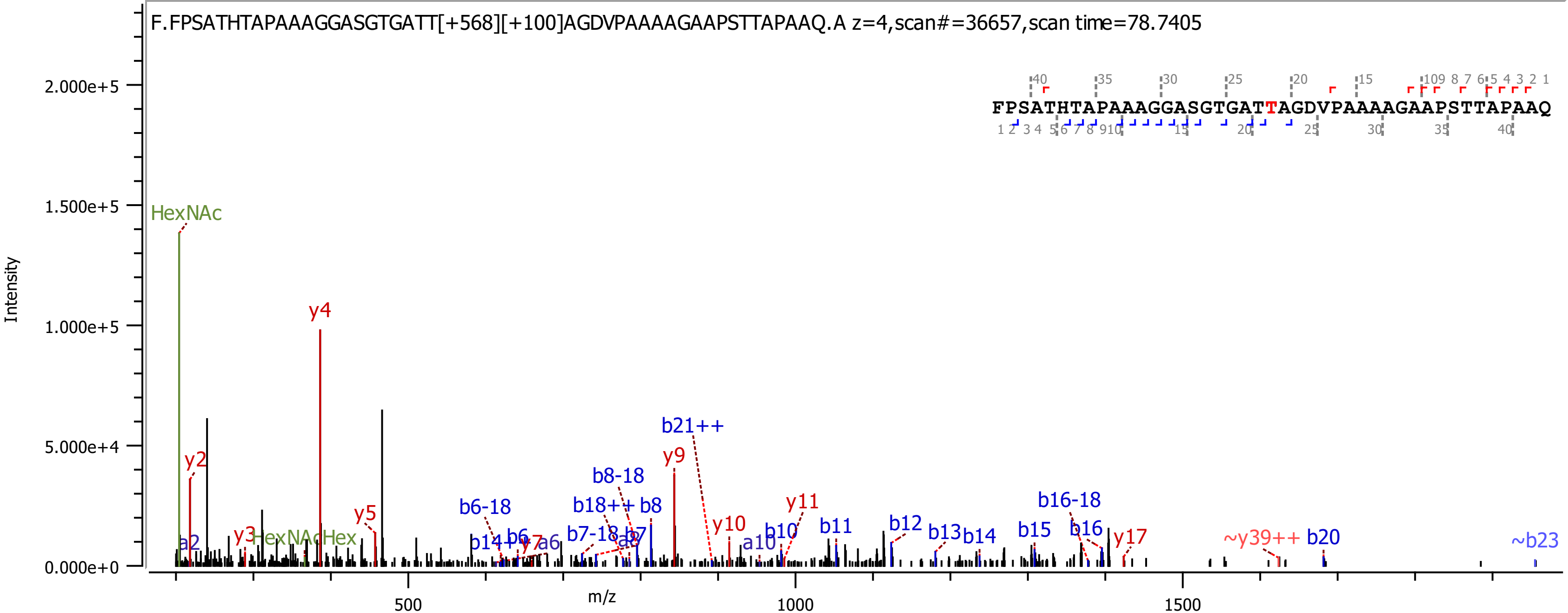

F.FPSATHTAPAAAGGASGTGATT[+568][+100]AGDVPAAAAGAAPSTTAPAAQ.A z=4,scan#=36657,scan time=78.7405
FPSATHTAPAAAGGASGTGATTAGDVPAAAAGAAPSTTAPAAQ
Intensity
m/z
2.000e+5
1.500e+5
1.000e+5
5.000e+4
0.000e+0
500
1000
1500
HexNAc
y4
y2
a2
y3
HexNAcHex
y5
b6-18
b14++
b6
y7
a6
b7-18
b8-18
b18++
b8
a8
b7
b21++
y9
y10
a10
b10
y11
b11
b12
b13
b14
b15
b16-18
b16
y17
~y39++
b20
~b23

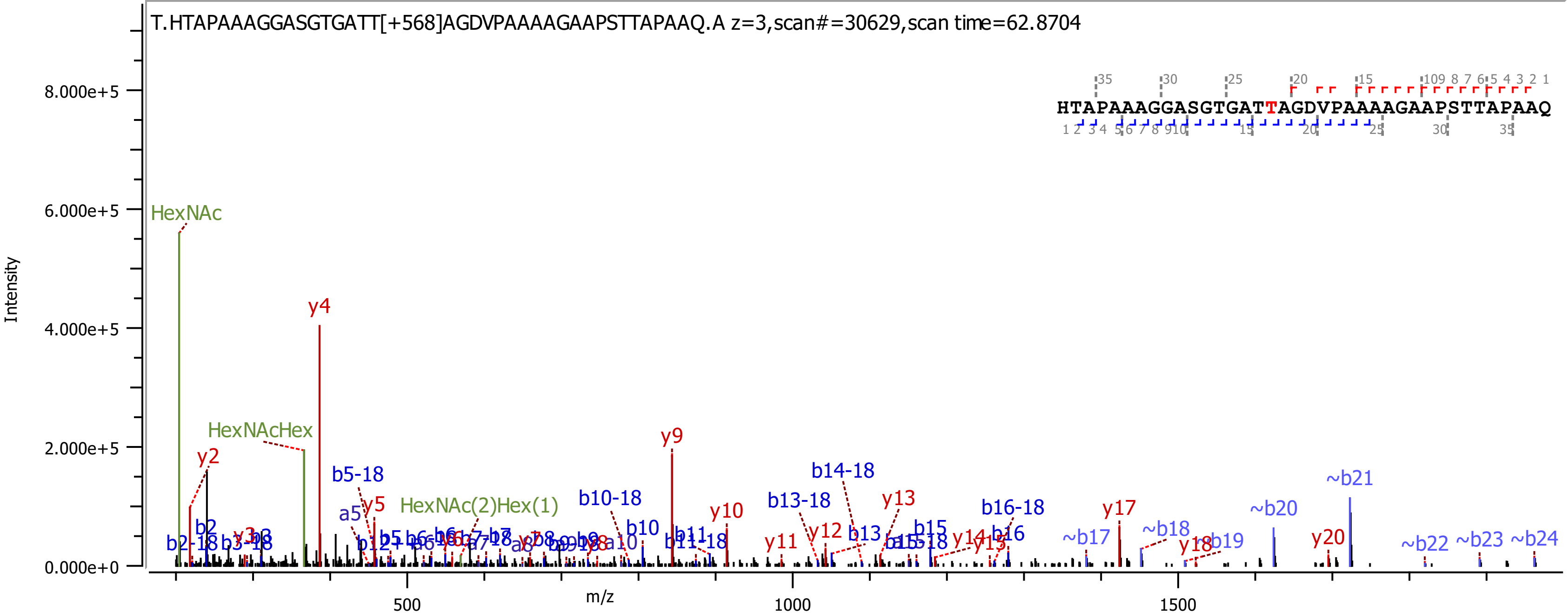
T.HTAPAAAGGASGTGATT[+568]AGDVPAAAAGAAPSTTAPAAQ.A z=3,scan#=30629,scan time=62.8704
HTAPAAAGGASGTGATTAGDVPAAAAGAAPSTTAPAAQ
Intensity
m/z
HexNAc
HexNAcHex
HexNAc(2)Hex(1)
y2
y4
y5
y9
y10
y11
y12
y13
y17
y20
b5-18
b10-18
b13-18
b14-18
b16-18
~b17
~b18
~b19
~b20
~b21
~b22
~b23
~b24

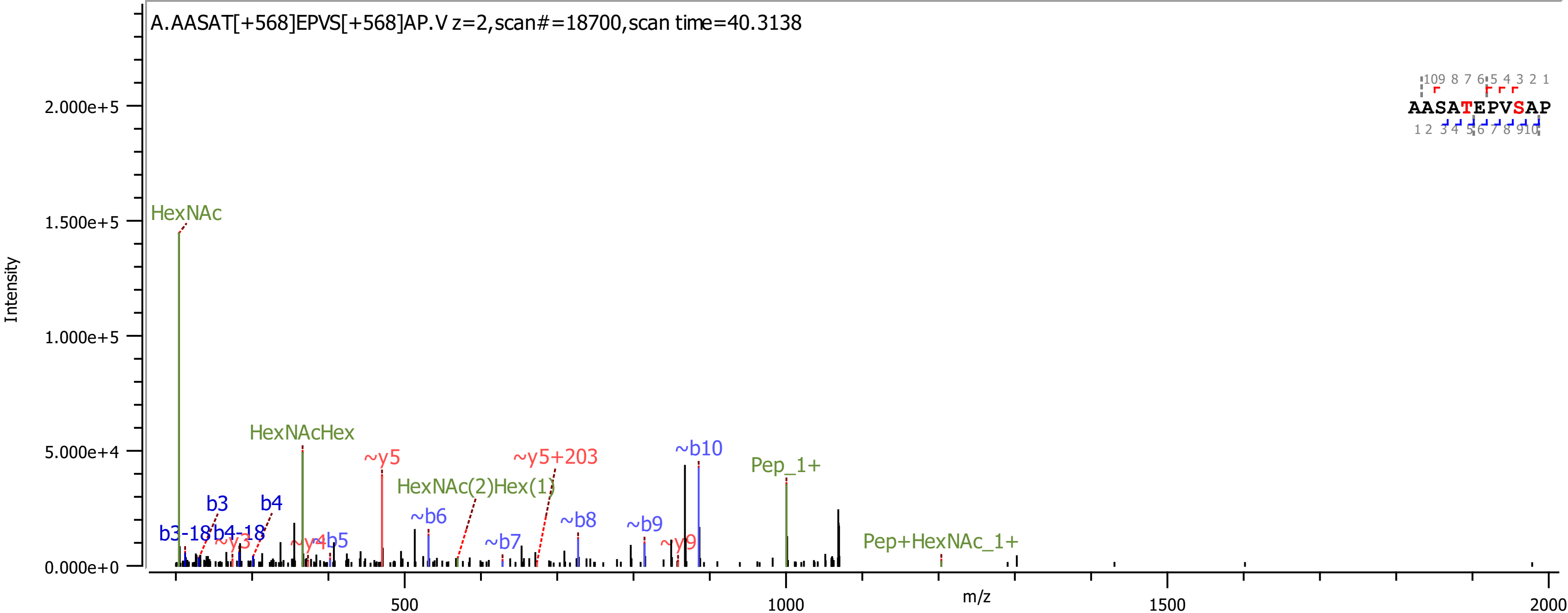

A.AASAT[+568]EPVS[+568]AP.V z=2,scan#=18700,scan time=40.3138
Intensity
m/z
HexNAc
HexNAcHex
~y5
~y5+203
~b10
Pep_1+
HexNAc(2)Hex(1)
b3
b4
~b6
~b8
~b9
b3-18
b4-18
~y3
~y4
~b5
~b7
~y9
Pep+HexNAc_1+
AASATEPVSAP
0.000e+0
5.000e+4
1.000e+5
1.500e+5
2.000e+5
500
1000
1500
2000

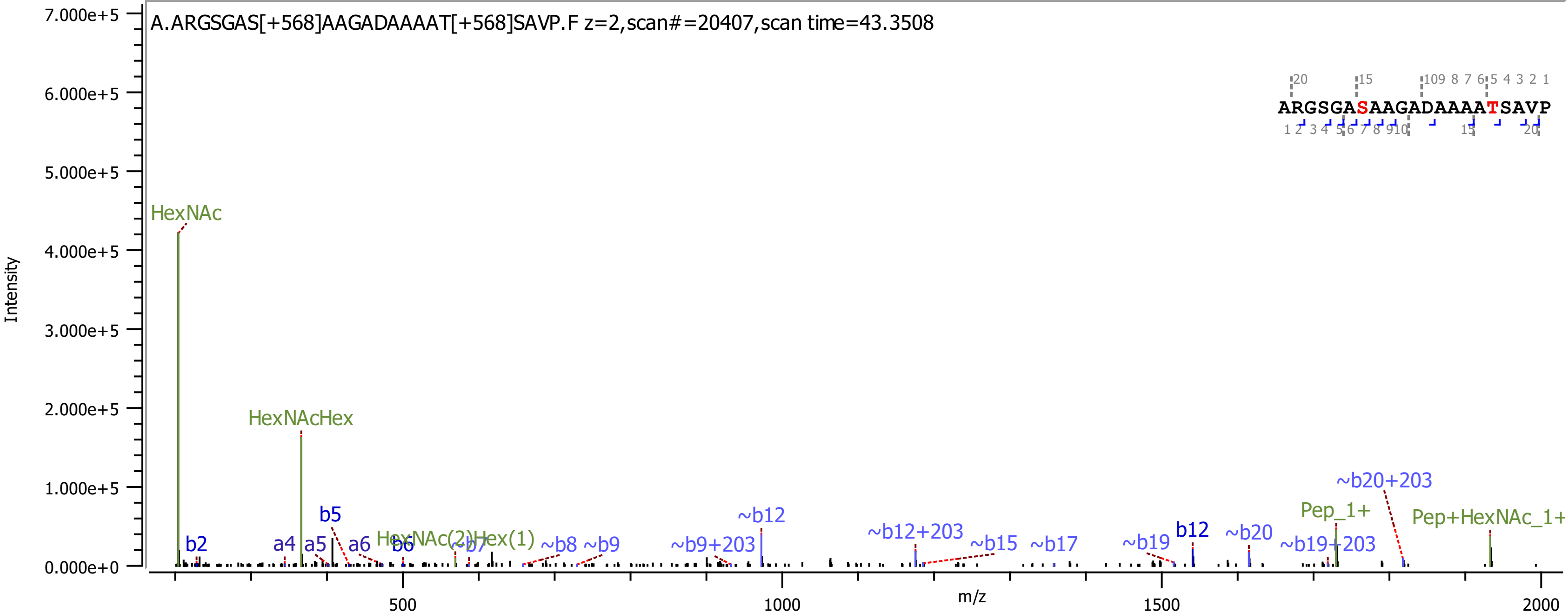
A.ARGSGAS[+568]AAGADAAAAT[+568]SAVP.F z=2,scan#=20407,scan time=43.3508
ARGSGASAAGADAAAATSAVP
Intensity
m/z
HexNAc
HexNAcHex
HexNAc(2)Hex(1)
b2
a4
a5
b5
a6
b6
~b7
~b8
~b9
~b9+203
~b12
~b12+203
~b15
~b17
~b19
b12
~b20
~b19+203
Pep_1+
~b20+203
Pep+HexNAc_1+
0.000e+0
1.000e+5
2.000e+5
3.000e+5
4.000e+5
5.000e+5
6.000e+5
7.000e+5
500
1000
1500
2000

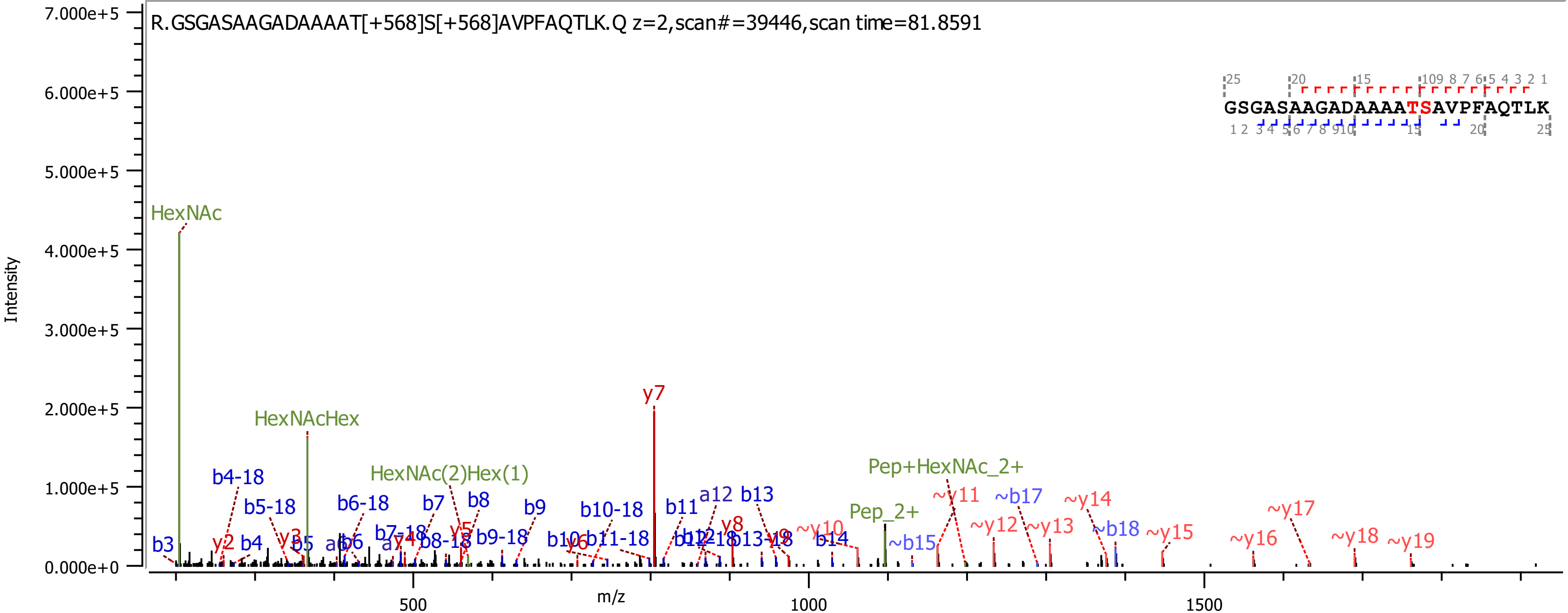
R.GSGASAAGADAAAAT[+568]S[+568]AVPFAQTLK.Q z=2,scan#=39446,scan time=81.8591
GSGASAAGADAAAATSAVPFAQTLK
Intensity
m/z
HexNAc
HexNAcHex
HexNAc(2)Hex(1)
Pep+HexNAc_2+
Pep_2+
y7
y8
~y11
~b17
~y14
~y17
~y12
~y13
~b18
~y15
~y16
~y18
~y19
~b15
~y10
b14
a12
b13
b11
b10-18
b9
b8
b7
b6-18
b5-18
b4-18
b3
y2
b4
y3
b5
b6
b7-18
y4
b8-18
y5
b9-18
b10
y6
b11-18
b12-18
b13-18
7.000e+5
6.000e+5
5.000e+5
4.000e+5
3.000e+5
2.000e+5
1.000e+5
0.000e+0
500
1000
1500

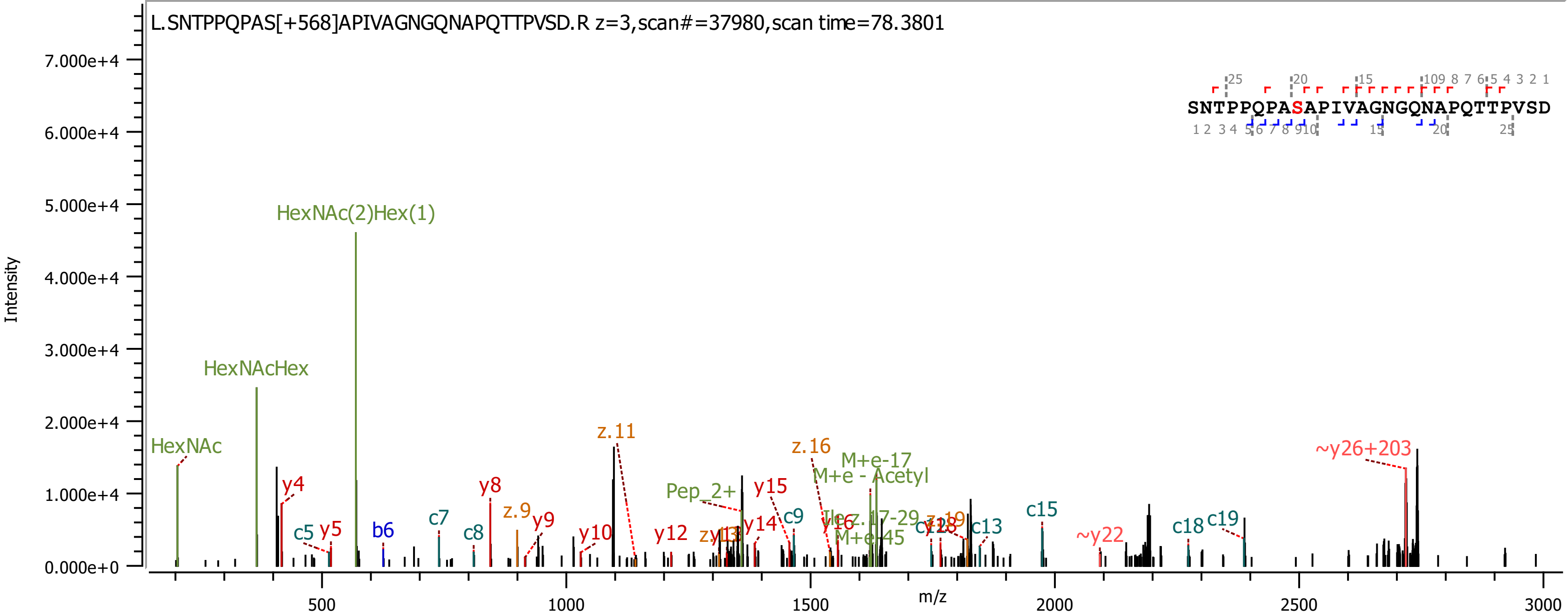

L.SNTPPQPAS[+568]APIVAGNGQNAPQTTPVSD.R z=3,scan#=37980,scan time=78.3801
SNTPPQPASAPIVAGNGQNAPQTTPVSD
Intensity
m/z
HexNAc(2)Hex(1)
HexNAcHex
HexNAc
y4
c5
y5
b6
c7
c8
y8
z.9
y9
y10
z.11
y12
y14
Pep_2+
y15
c9
z.16
M+e-17
M+e - Acetyl
M+e-45
c13
c15
~y22
c18
c19
~y26+203

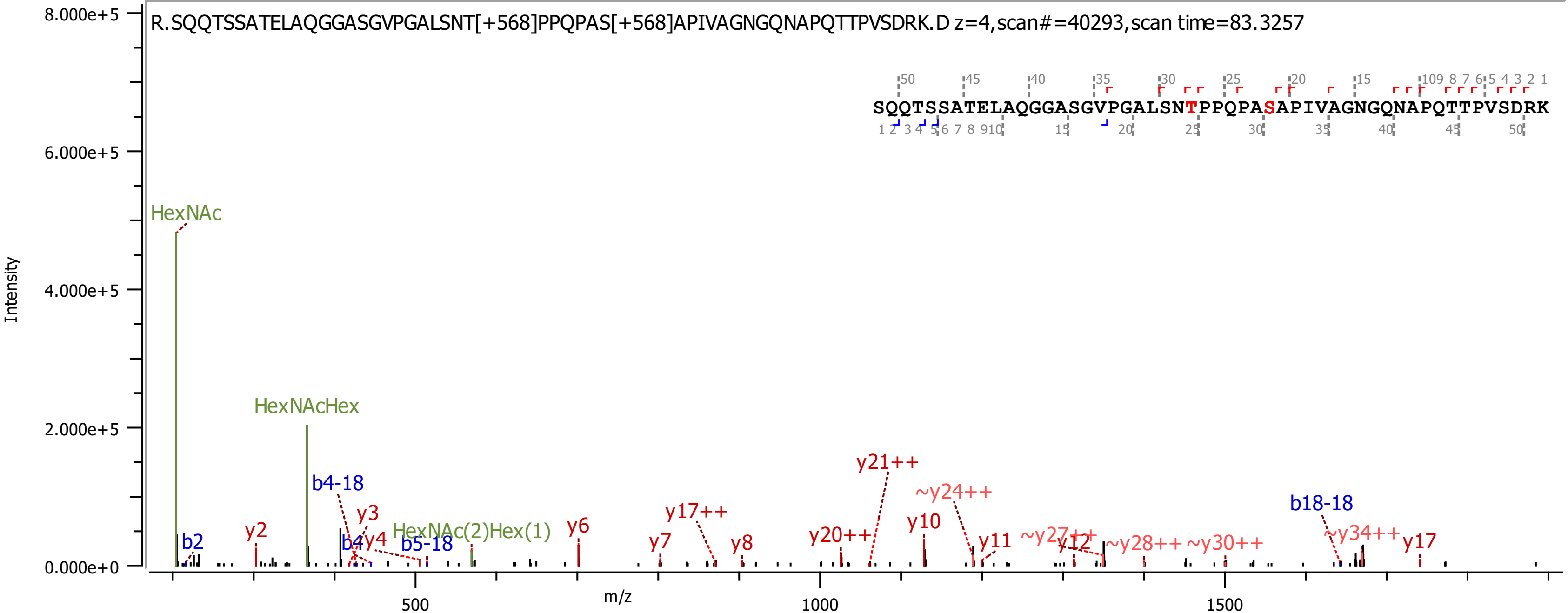
R.SQQTSSATELAQGGASGVPGALSNT[+568]PPQPAS[+568]APIVAGNGQNAPQTTPVSDRK.D z=4,scan#=40293,scan time=83.3257
SQQTSSATELAQGGASGVPGALSNTPPQPASAPIVAGNGQNAPQTTPVSDRK
Intensity
m/z
HexNAc
HexNAcHex
HexNAc(2)Hex(1)
b2
y2
b4-18
y3
b4
y4
b5-18
y6
y7
y17++
y8
y20++
y21++
y10
~y24++
y11
~y27++
y12
~y28++
~y30++
b18-18
~y34++
y17
8.000e+5
6.000e+5
4.000e+5
2.000e+5
0.000e+0
500
1000
1500

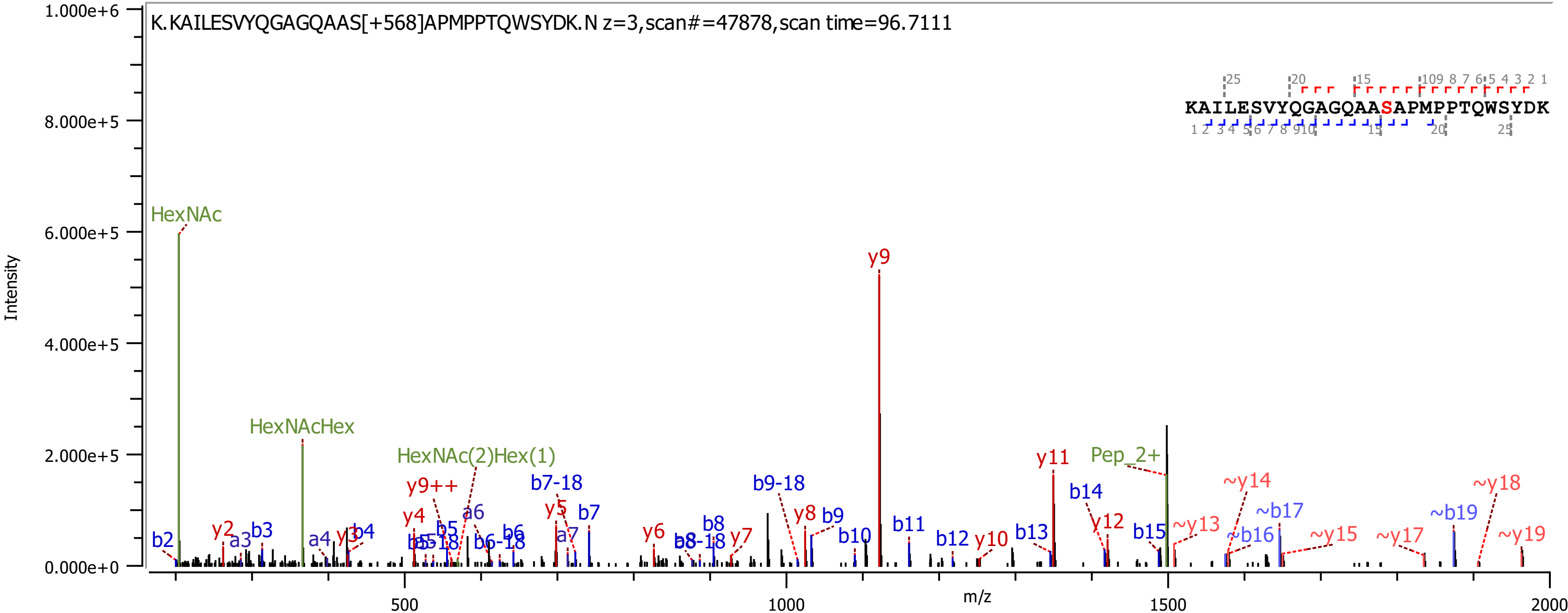
K.KAILESVYQGAGQAAS[+568]APMPPTQWSYDK.N z=3,scan#=47878,scan time=96.7111
KAILESVYQGAGQAASAPMPPTQWSYDK
Intensity
m/z
HexNAc
HexNAcHex
HexNAc(2)Hex(1)
Pep_2+
y9
y11
b14
y12
b15
~y14
~b17
~y18
~b19
~y19
~y17
~y15
~b16
~y13
b13
y10
b12
b11
b10
b9
y8
b9-18
b8
y7
b8-18
y6
b7
b7-18
y5
a7
b6
b6-18
a6
y9++
b5
b5-18
y4
a4
y3
b4
b3
a3
y2
b2
0.000e+0
2.000e+5
4.000e+5
6.000e+5
8.000e+5
1.000e+6
500
1000
1500
2000

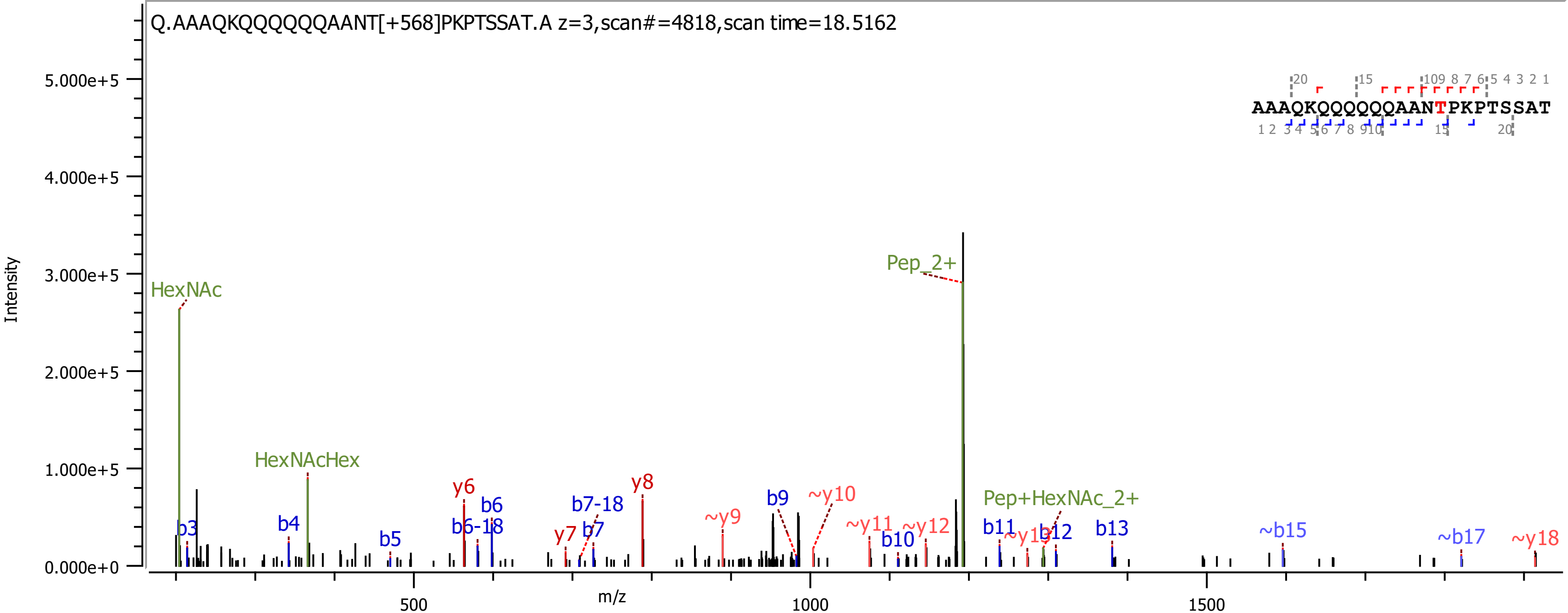

Q.AAAQKQQQQQQAANT[+568]PKPTSSAT.A z=3,scan#=4818,scan time=18.5162
AAAQKQQQQQQAANTPKPTSSAT
Intensity
m/z
5.000e+5
4.000e+5
3.000e+5
2.000e+5
1.000e+5
0.000e+0
500
1000
1500
HexNAc
HexNAcHex
Pep_2+
Pep+HexNAc_2+
b3
b4
b5
b6
b6-18
b7
b7-18
b9
b10
b11
b12
b13
~b15
~b17
y6
y7
y8
~y9
~y10
~y11
~y12
~y18

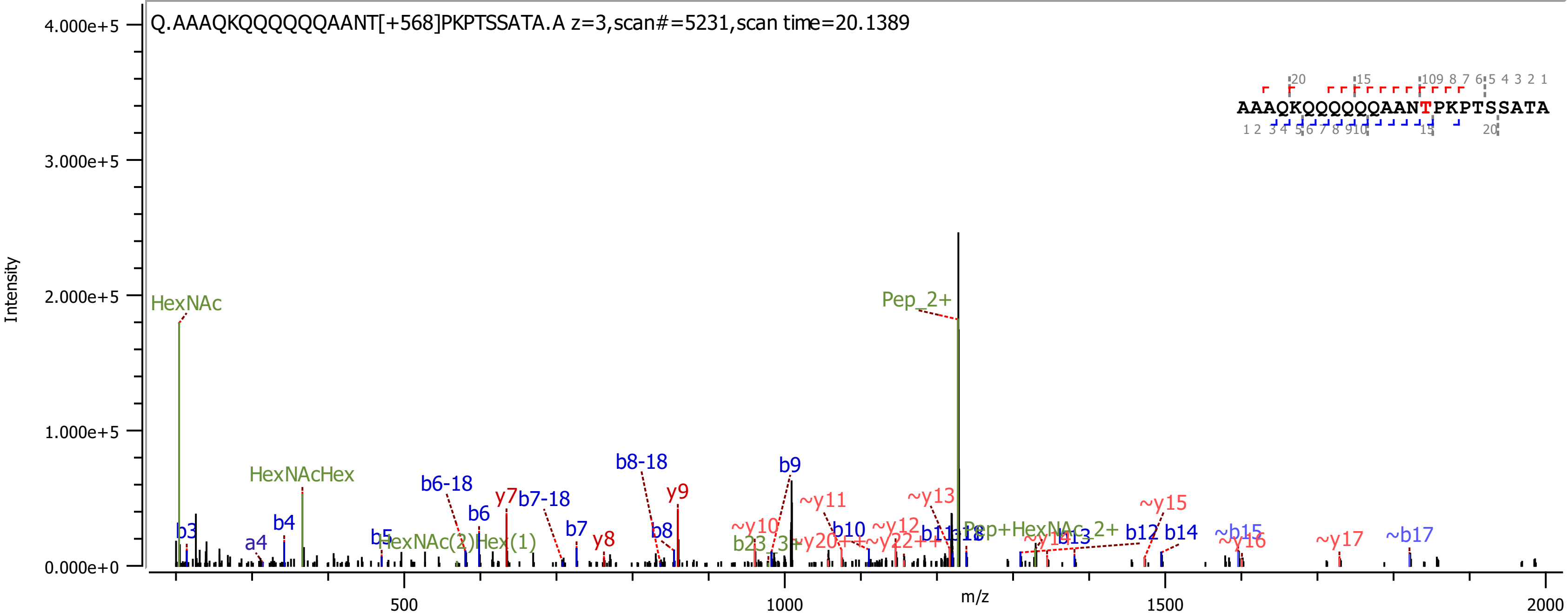

Q.AAAQKQQQQQQAANT[+568]PKPTSSATA.A z=3,scan#=5231,scan time=20.1389
AAAQKQQQQQQAANTPKPTSSATA
Intensity
m/z
4.000e+5
3.000e+5
2.000e+5
1.000e+5
0.000e+0
500
1000
1500
2000
HexNAc
HexNAcHex
HexNAc(2)Hex(1)
Pep_2+
b3
a4
b4
b5
b6-18
b6
y7
b7-18
b7
y8
b8-18
b8
y9
b9
~y10
~y11
b10
~y12
~y13
~y15
b12
b14
~b15
~y16
~y17
~b17

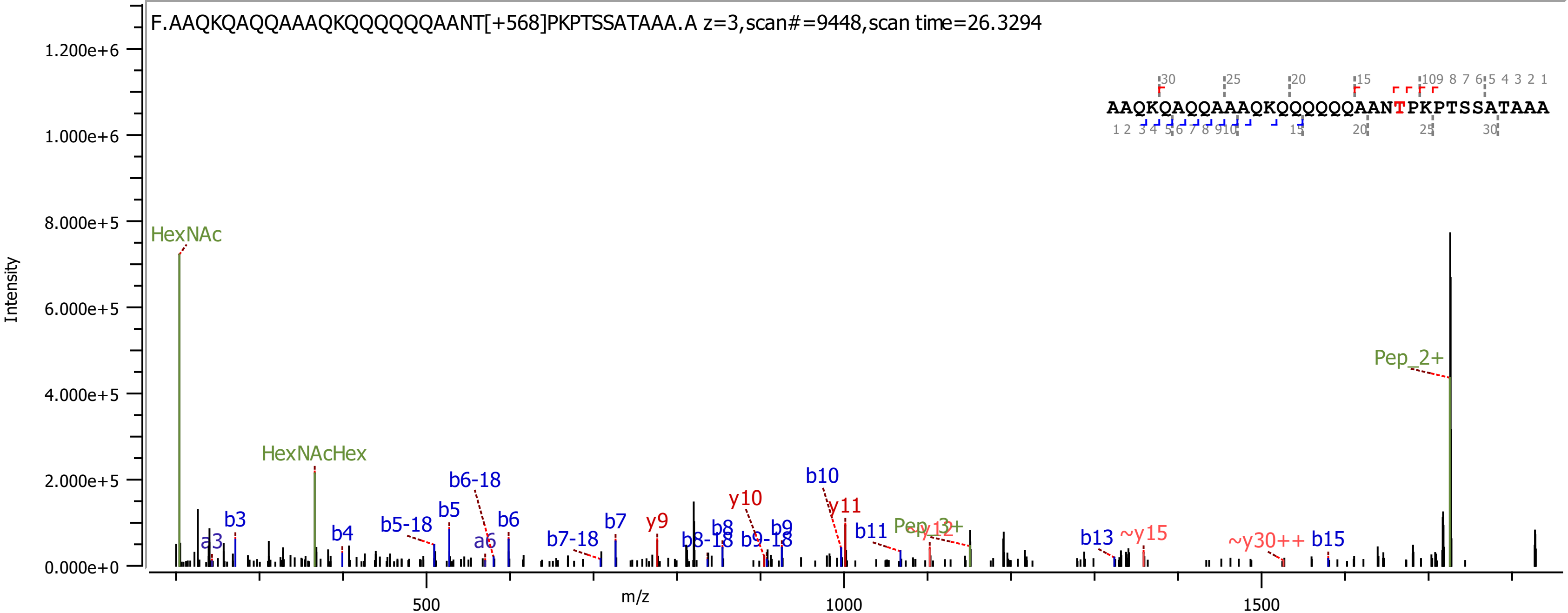

F.AAQKQAQQAAAQKQQQQQQAANT[+568]PKPTSSATAAA.A z=3,scan#=9448,scan time=26.3294
AAQKQAQQAAAQKQQQQQQAANTPKPTSSATAAA
Intensity
m/z
HexNAc
HexNAcHex
Pep_2+
Pep_3+
b3
a3
b4
b5-18
b5
b6-18
a6
b6
b7-18
b7
y9
b8-18
b8
y10
b9-18
b9
b10
y11
b11
~y12
b13
~y15
~y30++
b15

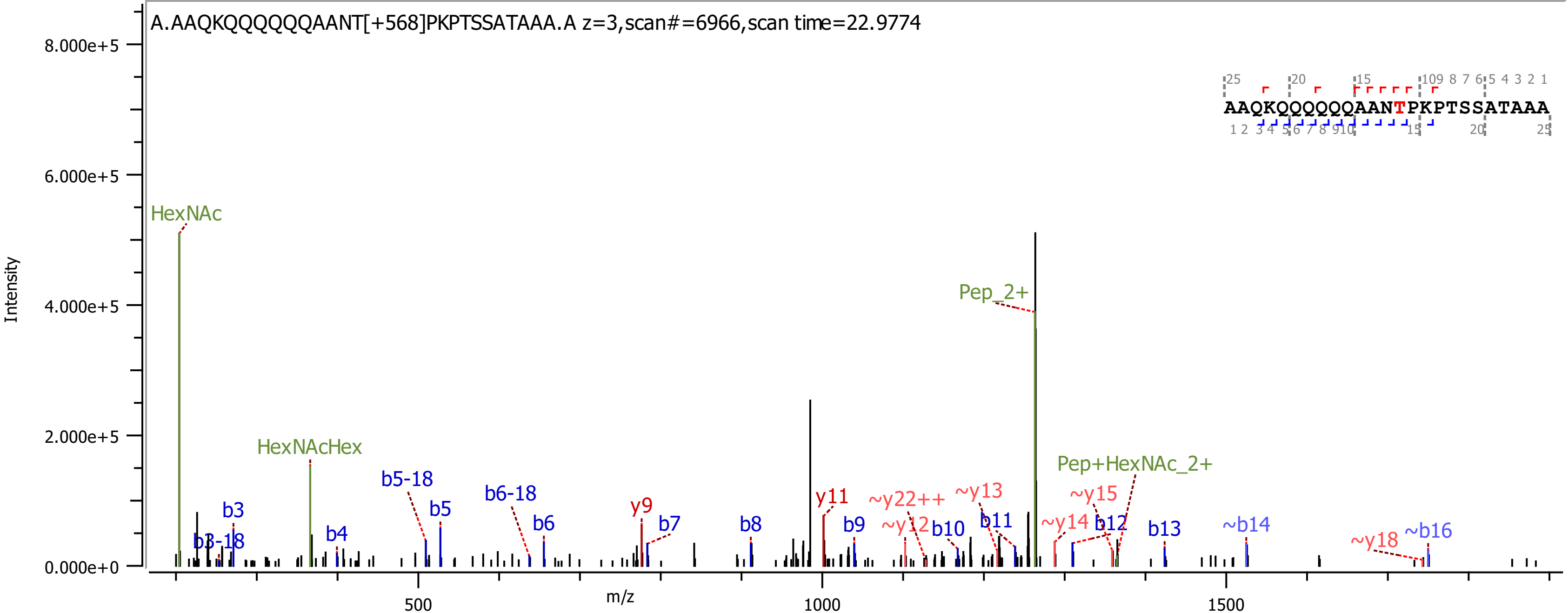

A.AAQKQQQQQQAANT[+568]PKPTSSATAAA.A z=3,scan#=6966,scan time=22.9774
AAQKQQQQQQAANTPKPTSSATAAA
Intensity
m/z
8.000e+5
6.000e+5
4.000e+5
2.000e+5
0.000e+0
500
1000
1500
HexNAc
HexNAcHex
Pep_2+
Pep+HexNAc_2+
b3
b3-18
b4
b5-18
b5
b6-18
b6
y9
b7
b8
y11
b9
~y22++
~y12
b10
~y13
b11
~y14
~y15
b12
b13
~b14
~y18
~b16

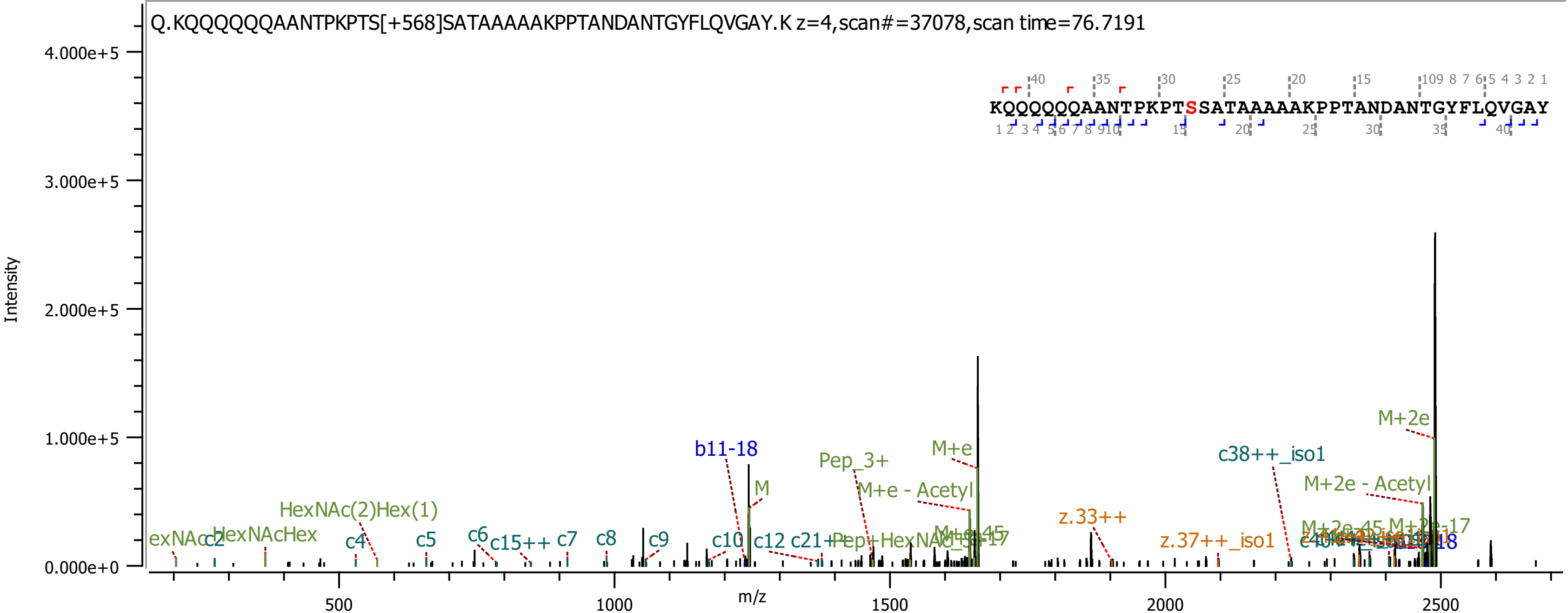

Q.KQQQQQQAANTPKPTS[+568]SATAAAAAKPPTANDANTGYFLQVGAY.K z=4,scan#=37078,scan time=76.7191
KQQQQQQAANTPKPTSSATAAAAAKPPTANDANTGYFLQVGAY
Intensity
4.000e+5
3.000e+5
2.000e+5
1.000e+5
0.000e+0
m/z
500
1000
1500
2000
2500
b11-18
M
HexNAc(2)Hex(1)
HexNAcHex
c2
c4
c5
c6
c15++
c7
c8
c9
c10
c12
Pep_3+
M+e
M+e - Acetyl
z.33++
z.37++_iso1
c38++_iso1
M+2e
M+2e - Acetyl
M+2e-17

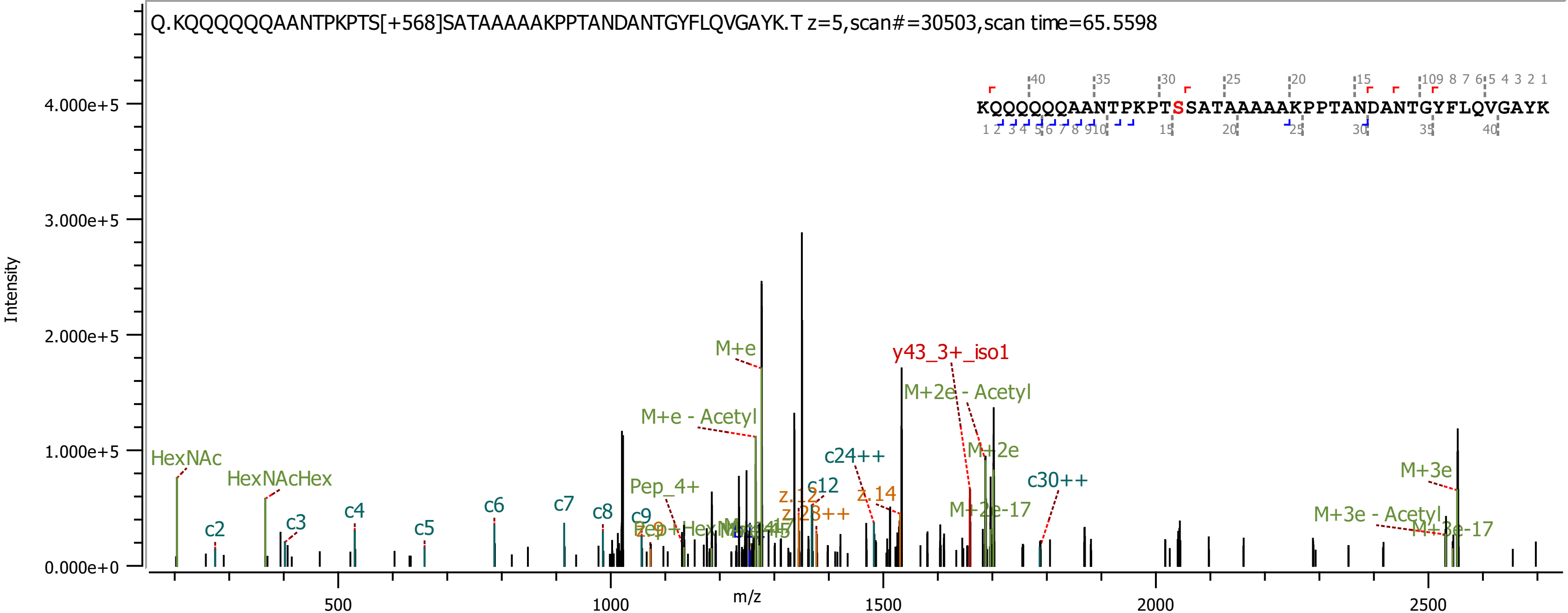
Q.KQQQQQQAANTPKPTS[+568]SATAAAAAKPPTANDANTGYFLQVGAYK.T z=5,scan#=30503,scan time=65.5598
KQQQQQQAANTPKPTSSATAAAAAKPPTANDANTGYFLQVGAYK
Intensity
m/z
HexNAc
HexNAcHex
M+e
M+e - Acetyl
Pep_4+
y43_3+_iso1
M+2e - Acetyl
M+2e
M+2e-17
c24++
c30++
M+3e
M+3e - Acetyl
M+3e-17
c2
c3
c4
c5
c6
c7
c8
c9
c12
z.14
z.28++

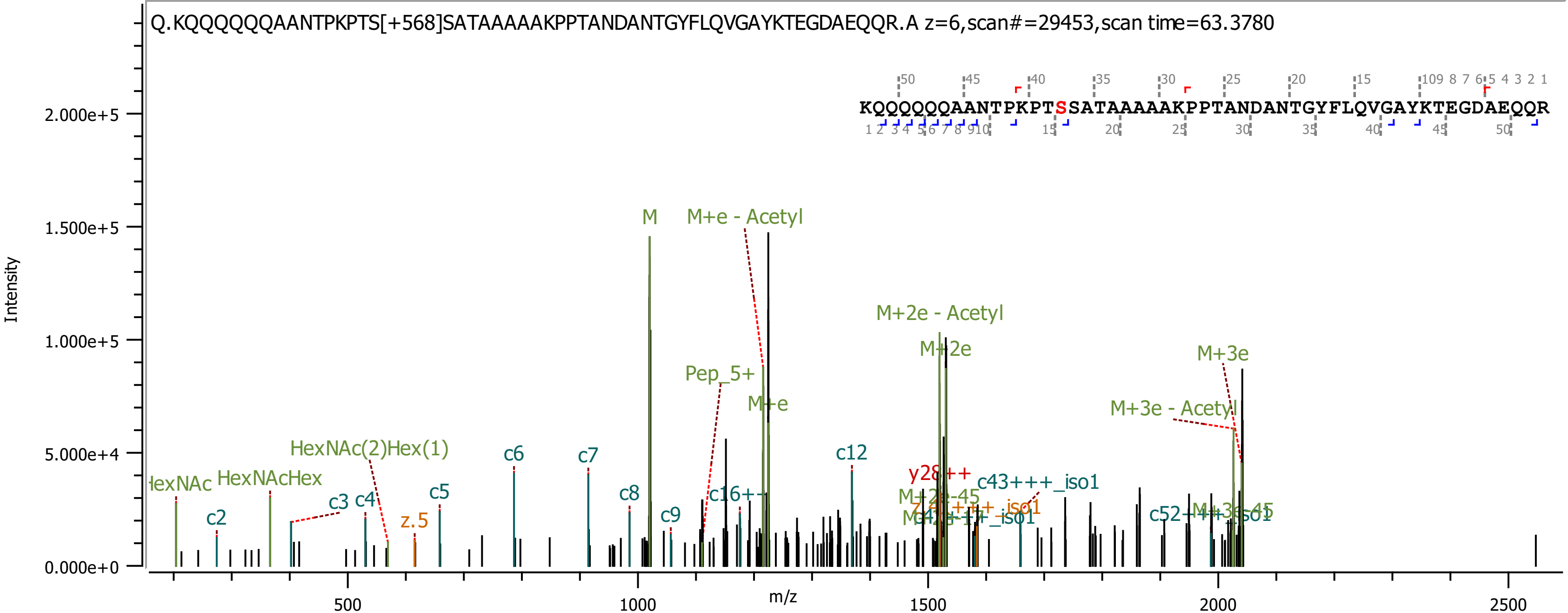

Q.KQQQQQQAANTPKPTS[+568]SATAAAAAKPPTANDANTGYFLQVGAYKTEGDAEQQR.A z=6,scan#=29453,scan time=63.3780
KQQQQQQAANTPKPTSSATAAAAAKPPTANDANTGYFLQVGAYKTEGDAEQQR
Intensity
m/z
M
M+e - Acetyl
M+2e - Acetyl
M+2e
M+3e
M+3e - Acetyl
Pep_5+
M+e
HexNAc(2)Hex(1)
HexNAcHex
c2
c3
c4
z.5
c5
c6
c7
c8
c9
c12
c16++
y28++
c43+++_iso1
c52+++_iso1
2.000e+5
1.500e+5
1.000e+5
5.000e+4
0.000e+0
500
1000
1500
2000
2500

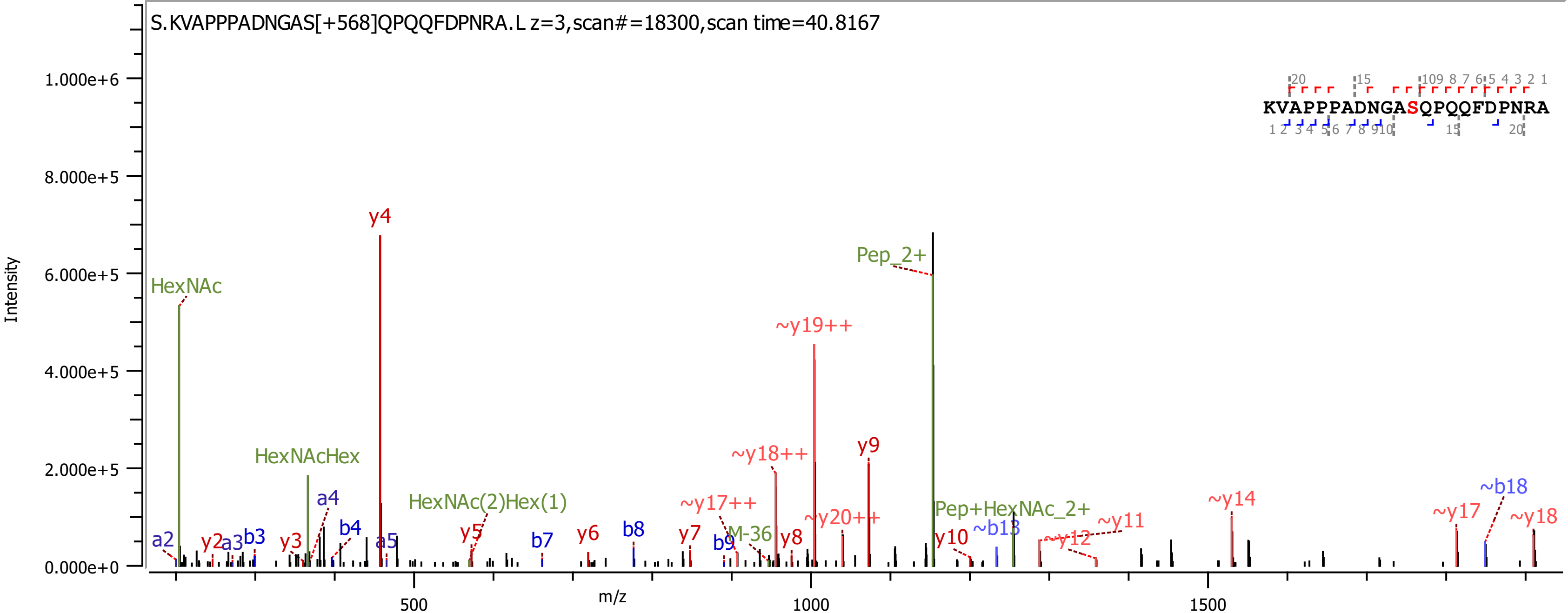

S.KVAPPPADNGAS[+568]QPQQFDPNRA.L z=3,scan#=18300,scan time=40.8167
KVAPPPADNGASQPQQFDPNRA
Intensity
m/z
1.000e+6
8.000e+5
6.000e+5
4.000e+5
2.000e+5
0.000e+0
500
1000
1500
HexNAc
HexNAcHex
HexNAc(2)Hex(1)
Pep_2+
Pep+HexNAc_2+
M-36
y4
~y19++
~y18++
y9
~y17++
~y20++
~y14
~y17
~b18
~y18
~y11
~y12
~b13
y10
y8
y7
y6
y5
y3
y2
a2
a3
b3
a4
b4
a5
b7
b8
b9

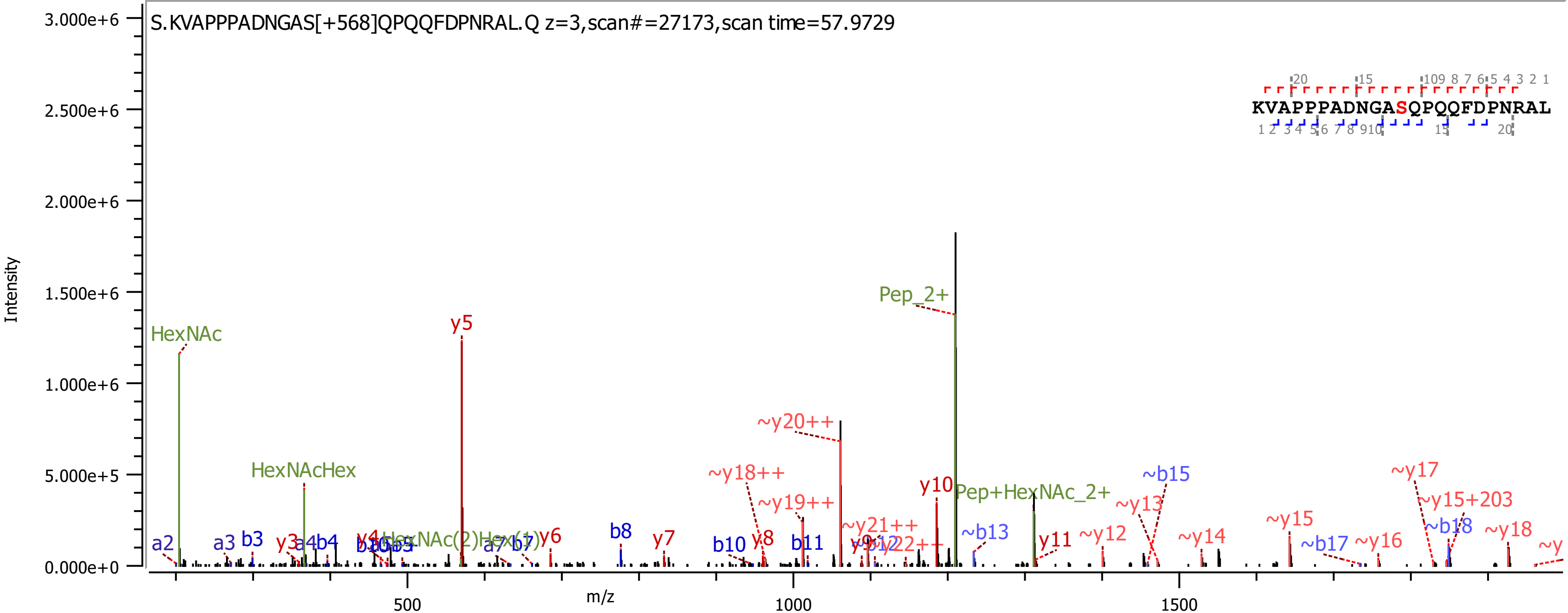

S.KVAPPPADNGAS[+568]QPQQFDPNRAL.Q z=3,scan#=27173,scan time=57.9729
KVAPPPADNGASQPQQFDPNRAL
Intensity
m/z
HexNAc
HexNAcHex
y5
Pep_2+
~y20++
~y18++
~y19++
~y21++
~y22++
y10
Pep+HexNAc_2+
~b15
~y13
~y17
~y15+203
~b18
~y18
~y15
~b17
~y16
~y14
~y12
y11
~b13
b8
y7
b10
y8
b11
y6
b7
a7
HexNAc(2)Hex(1)
y4
b5
a2
a3
b3
y3
a4
b4

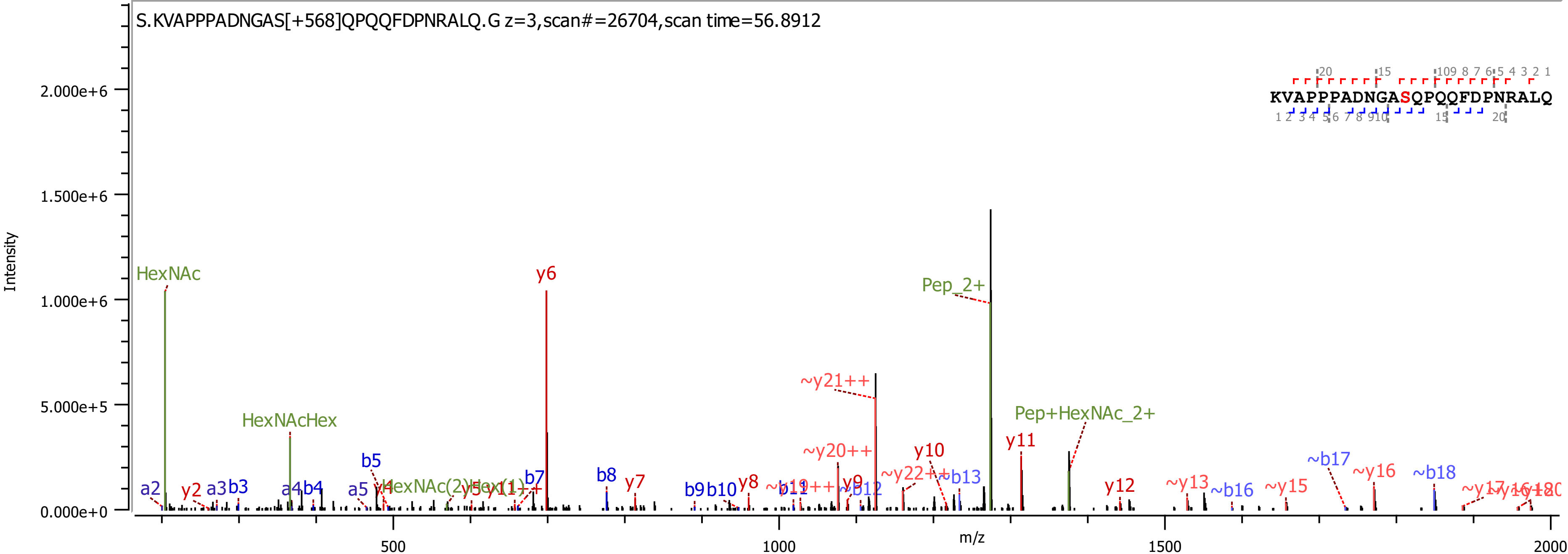
S.KVAPPPADNGAS[+568]QPQQFDPNRALQ.G z=3,scan#=26704,scan time=56.8912
KVAPPPADNGASQPQQFDPNRALQ
Intensity
m/z
0.000e+0
5.000e+5
1.000e+6
1.500e+6
2.000e+6
500
1000
1500
2000
HexNAc
HexNAcHex
y6
Pep_2+
~y21++
Pep+HexNAc_2+
~y20++
y10
y11
~y22++
~b13
~b17
~y16
~b18
y12
~y13
~b16
~y15
b5
b7
b8
y7
y8
y9
b9
b10
a2
y2
a3
b3
a4
b4
a5
y4
y5
b11
~b12

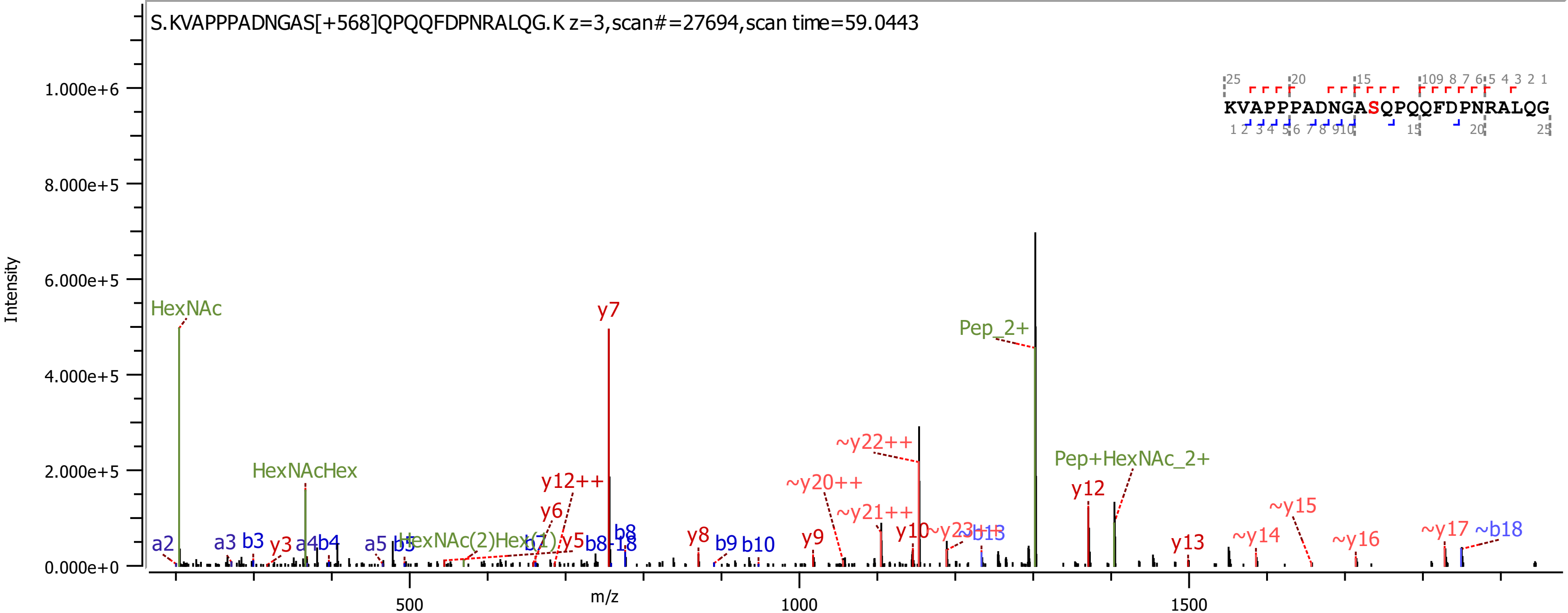

S.KVAPPPADNGAS[+568]QPQQFDPNRALQG.K z=3,scan#=27694,scan time=59.0443
Intensity
m/z
HexNAc
HexNAcHex
y7
Pep_2+
~y22++
Pep+HexNAc_2+
y12++
~y20++
y12
y6
~y21++
~y15
HexNAc(2)Hex(1)
y10
~y23+++
~b13
y13
~y14
~y16
~y17
~b18
a2
a3
b3
y3
a4
b4
a5
b5
b7
y5
b8-18
b8
y8
b9
b10
y9
KVAPPPADNGASQPQQFDPNRALQG

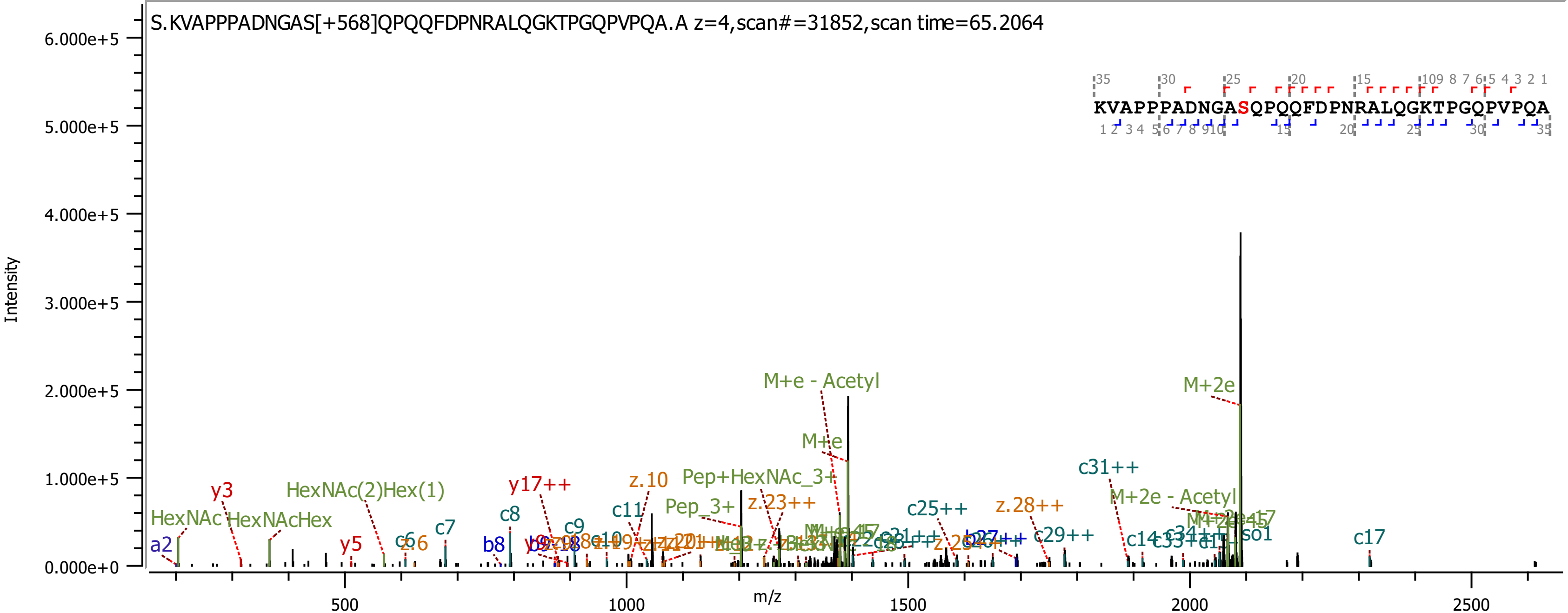

S.KVAPPPADNGAS[+568]QPQQFDPNRALQGKTPGQPVPQA.A z=4,scan#=31852,scan time=65.2064
KVAPPPADNGASQPQQFDPNRALQGKTPGQPVPQA
Intensity
6.000e+5
5.000e+5
4.000e+5
3.000e+5
2.000e+5
1.000e+5
0.000e+0
m/z
500
1000
1500
2000
2500
M+e - Acetyl
M+2e
M+e
Pep+HexNAc_3+
Pep_3+
c31++
M+2e - Acetyl
y3
HexNAc(2)Hex(1)
y17++
z.10
z.23++
c25++
z.28++
HexNAc
HexNAcHex
c8
c11
c9
c7
c6
z.6
y5
a2
b8
c10
c29++
c17
c14
z.25

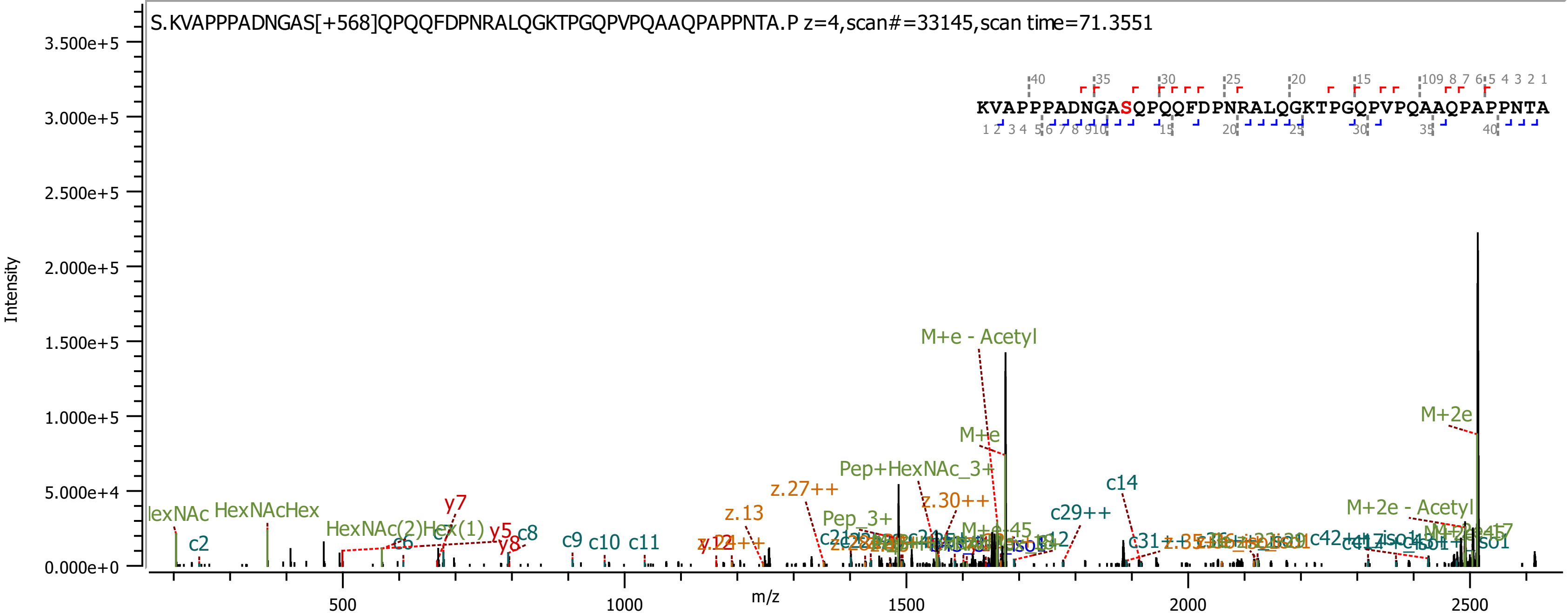
S.KVAPPPADNGAS[+568]QPQQFDPNRALQGKTPGQPVPQAAQPAPPNTA.P z=4,scan#=33145,scan time=71.3551
KVAPPPADNGASQPQQFDPNRALQGKTPGQPVPQAAQPAPPNTA
Intensity
3.500e+5
3.000e+5
2.500e+5
2.000e+5
1.500e+5
1.000e+5
5.000e+4
0.000e+0
m/z
500
1000
1500
2000
2500
M+e - Acetyl
M+e
M+2e
M+2e - Acetyl
Pep+HexNAc_3+
Pep_3+
c14
c29++
z.27++
z.30++
z.13
HexNAcHex
HexNAc(2)Hex(1)
y7
y5
y8
c2
c6
c8
c9
c10
c11

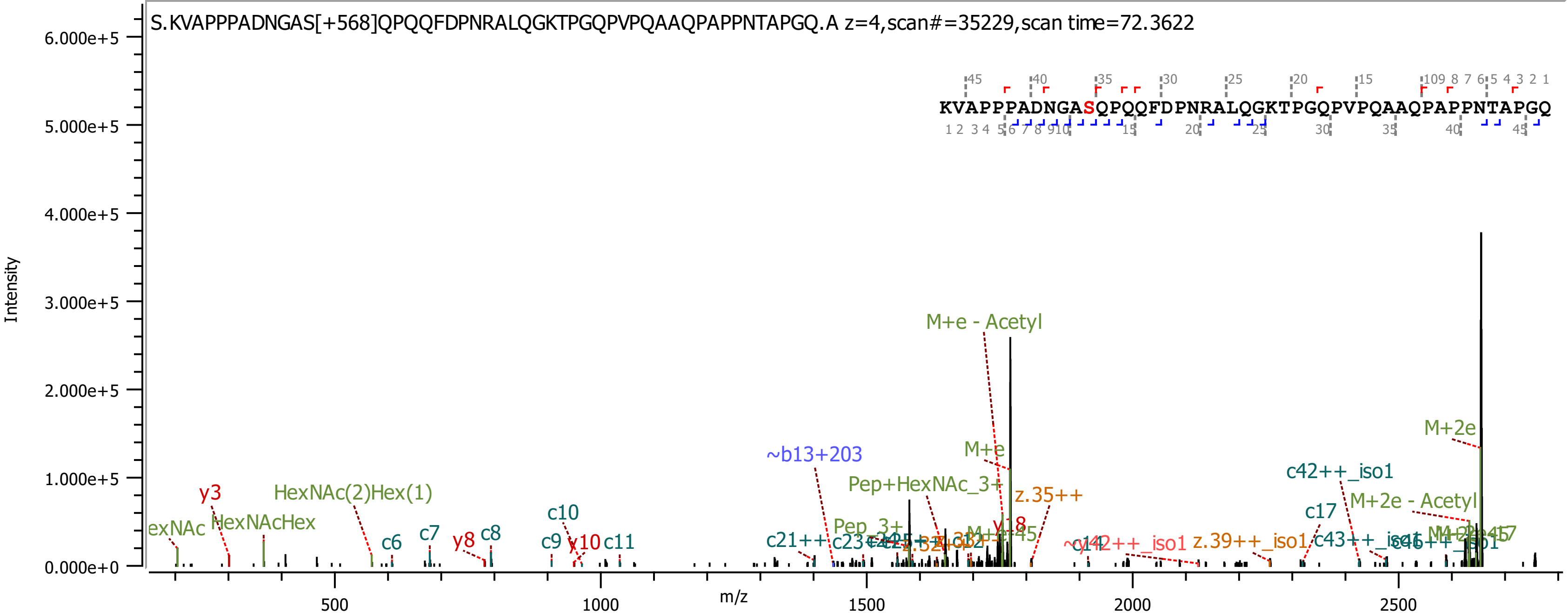
S.KVAPPPADNGAS[+568]QPQQFDPNRALQGKTPGQPVPQAAQPAPPNTAPGQ.A z=4,scan#=35229,scan time=72.3622
KVAPPPADNGASQPQQFDPNRALQGKTPGQPVPQAAQPAPPNTAPGQ
Intensity
m/z
M+e - Acetyl
M+2e
M+e
~b13+203
c42++_iso1
Pep+HexNAc_3+
z.35++
M+2e - Acetyl
c17
HexNAc(2)Hex(1)
y3
c10
HexNAcHex
c7
c8
y8
c9
y10
c11
c6
c21++
Pep_3+
z.39++_iso1
c43++_iso1

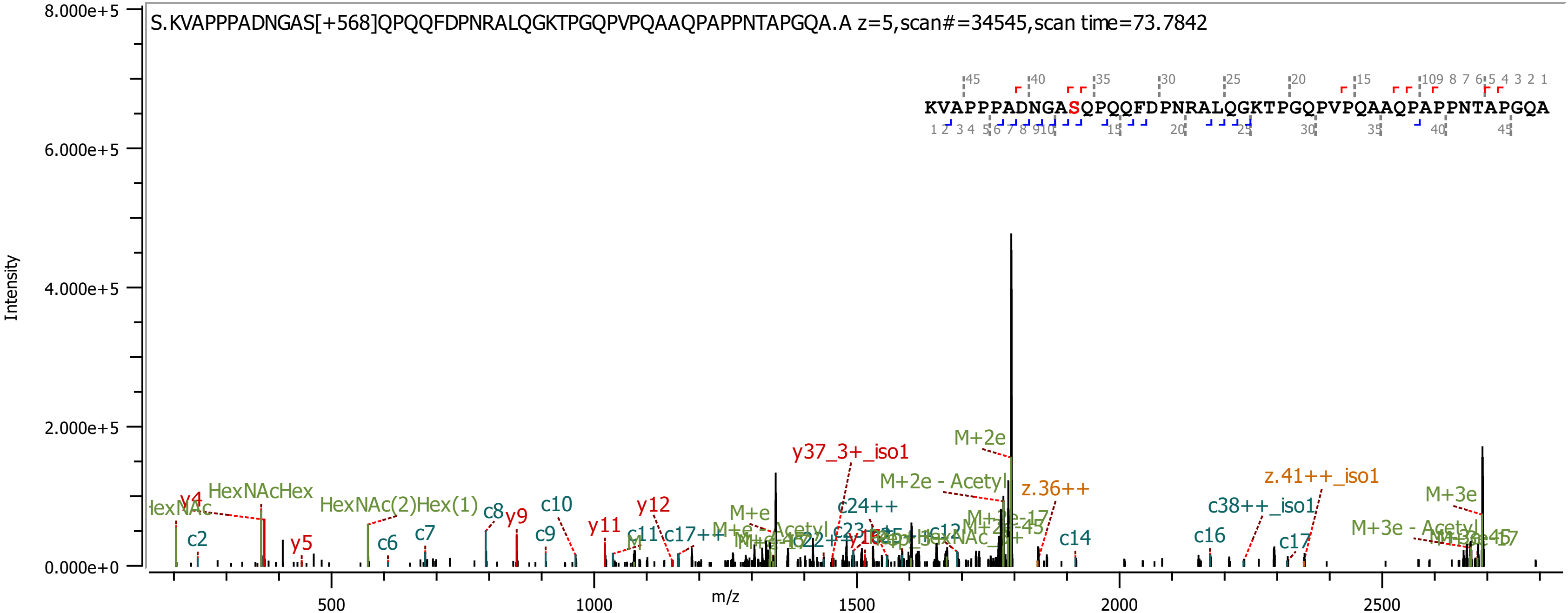
S.KVAPPPADNGAS[+568]QPQQFDPNRALQGKTPGQPVPQAAQPAPPNTAPGQA.A z=5,scan#=34545,scan time=73.7842
KVAPPPADNGASQPQQFDPNRALQGKTPGQPVPQAAQPAPPNTAPGQA
Intensity
m/z
8.000e+5
6.000e+5
4.000e+5
2.000e+5
0.000e+0
500
1000
1500
2000
2500
y37_3+_iso1
M+2e
M+2e - Acetyl
z.36++
z.41++_iso1
M+3e
HexNAcHex
HexNAc(2)Hex(1)
c24++
c38++_iso1
c10
y12
c8
y9
y11
c9
c7
c6
y5
c2
y4
c14
c16
c17
M+3e - Acetyl
c17++
c11
c23
c12
M+e

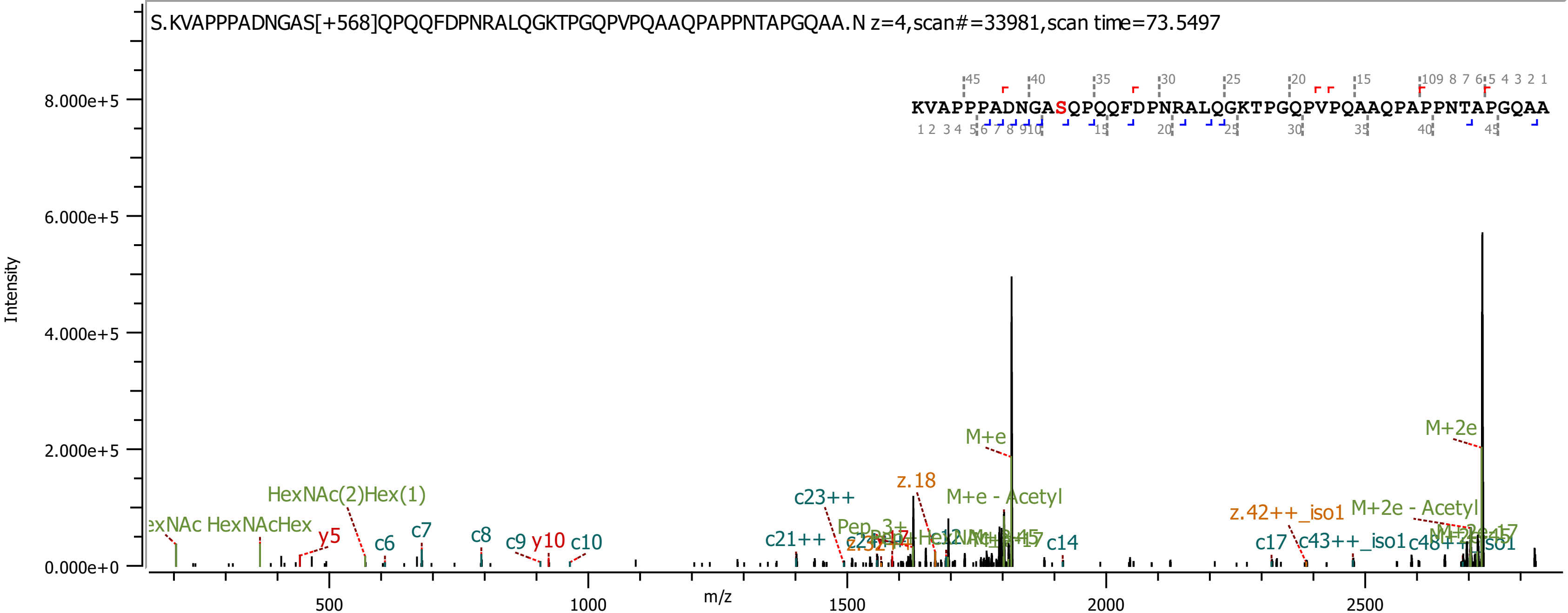

S.KVAPPPADNGAS[+568]QPQQFDPNRALQGKTPGQPVPQAAQPAPPNTAPGQAA.N z=4,scan#=33981,scan time=73.5497
KVAPPPADNGASQPQQFDPNRALQGKTPGQPVPQAAQPAPPNTAPGQAA
Intensity
8.000e+5
6.000e+5
4.000e+5
2.000e+5
0.000e+0
m/z
500
1000
1500
2000
2500
HexNAc(2)Hex(1)
HexNAcHex
y5
c6
c7
c8
c9
y10
c10
c21++
c23++
z.18
M+e
M+e - Acetyl
c14
c17
c43++_iso1
z.42++_iso1
M+2e - Acetyl
M+2e

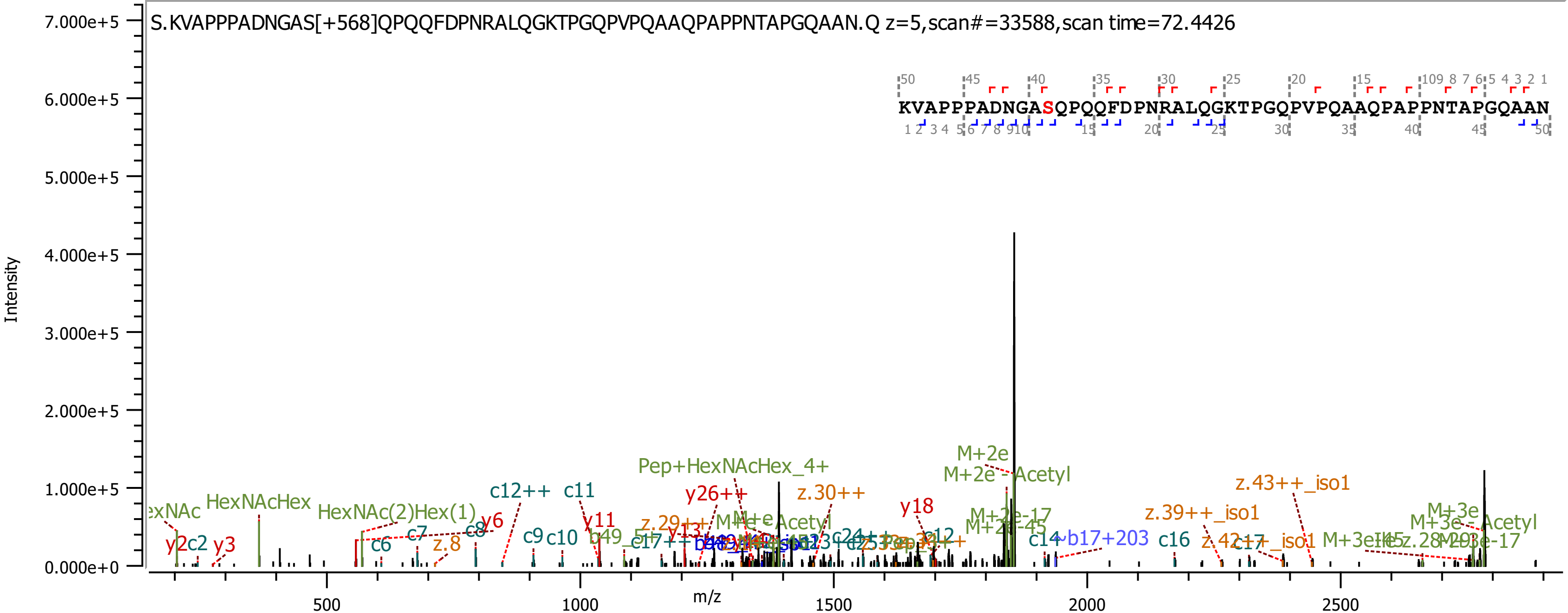
S.KVAPPPADNGAS[+568]QPQQFDPNRALQGKTPGQPVPQAAQPAPPNTAPGQAAN.Q z=5,scan#=33588,scan time=72.4426
KVAPPPADNGASQPQQFDPNRALQGKTPGQPVPQAAQPAPPNTAPGQAAN
Intensity
m/z
7.000e+5
6.000e+5
5.000e+5
4.000e+5
3.000e+5
2.000e+5
1.000e+5
0.000e+0
500
1000
1500
2000
2500
HexNAcHex
HexNAc(2)Hex(1)
Pep+HexNAcHex_4+
M+2e
M+2e-Acetyl
z.43++_iso1
z.39++_iso1
M+3e
M+3e-Acetyl
c12++
c11
y26++
z.30++
y18
y6
y11
y2
c2
y3
c6
c7
z.8
c8
c9
c10
c14
b17+203
c16
c17

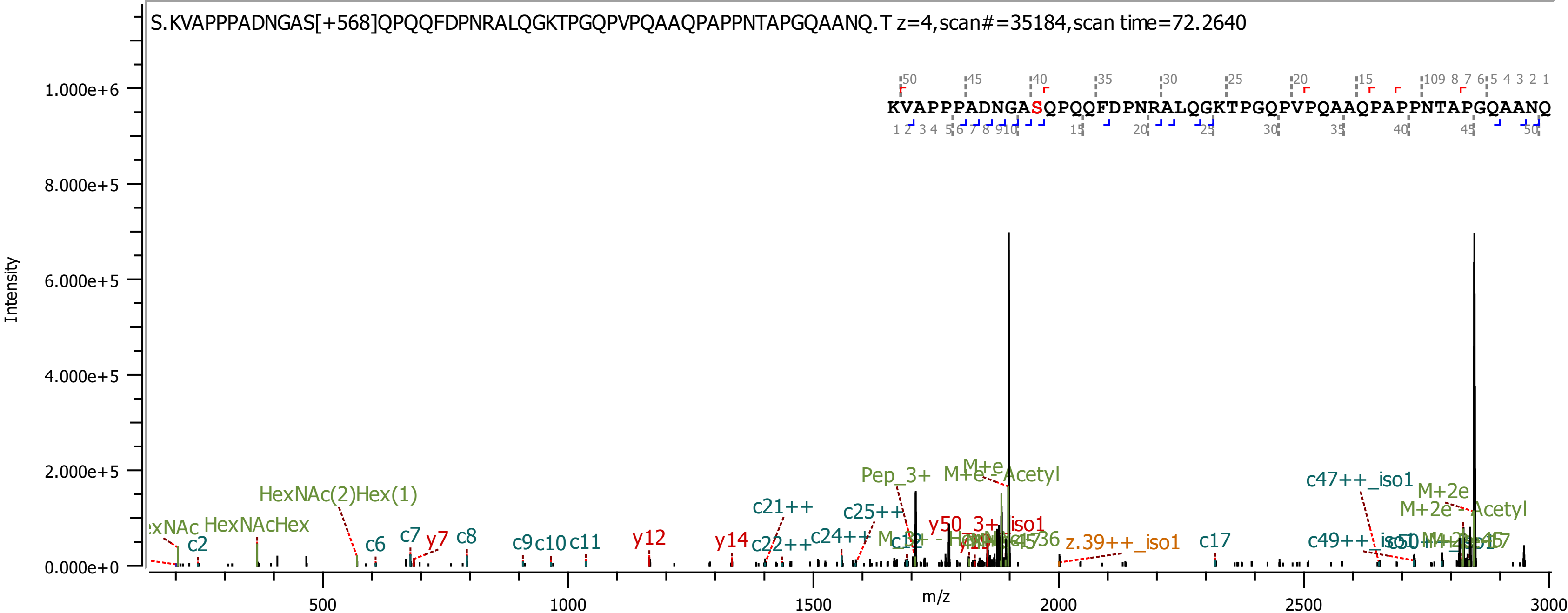
S.KVAPPPADNGAS[+568]QPQQFDPNRALQGKTPGQPVPQAAQPAPPNTAPGQAANQ.T z=4,scan#=35184,scan time=72.2640
KVAPPPADNGASQPQQFDPNRALQGKTPGQPVPQAAQPAPPNTAPGQAANQ
Intensity
1.000e+6
8.000e+5
6.000e+5
4.000e+5
2.000e+5
0.000e+0
m/z
500
1000
1500
2000
2500
3000
HexNAcHex
HexNAc(2)Hex(1)
c2
c6
c7
y7
c8
c9
c10
c11
y12
y14
c21++
c22++
c24++
c25++
Pep_3+
M+e
M+e - Acetyl
y50_3+_iso1
z.39++_iso1
c17
c47++_iso1
c49++_iso1
M+2e
M+2e - Acetyl

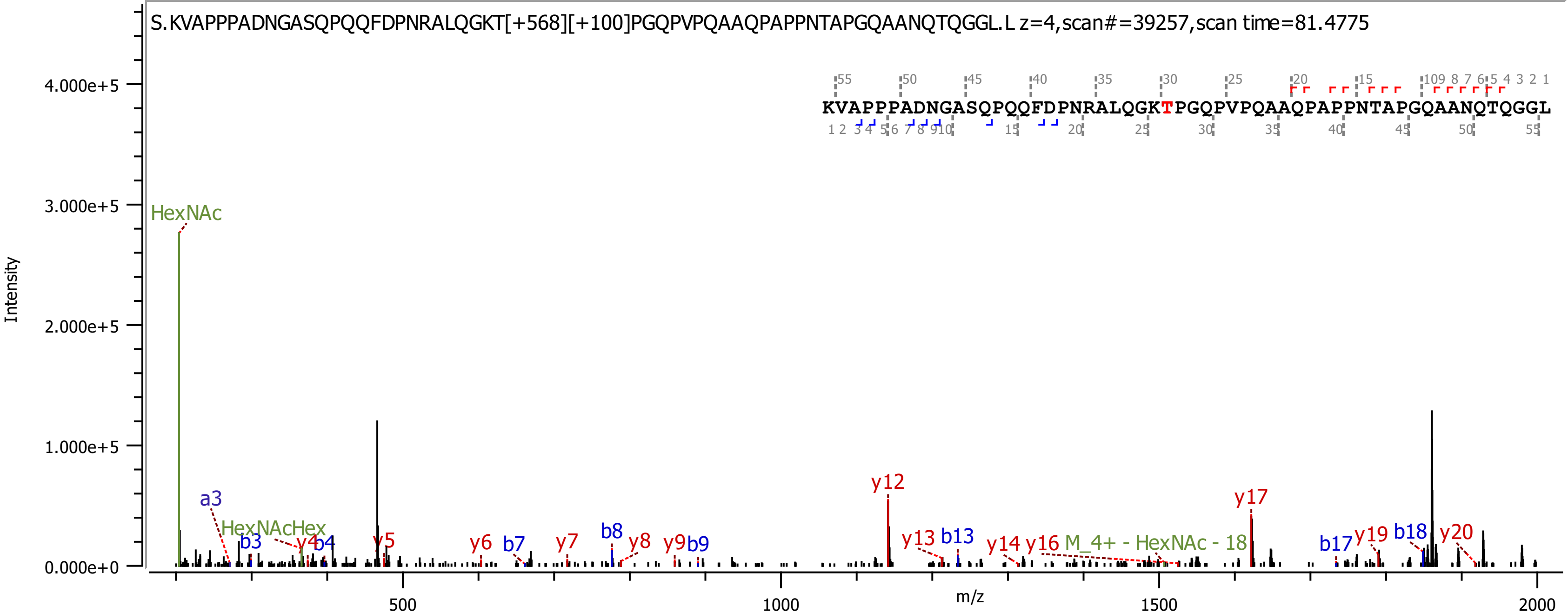

S.KVAPPPADNGASQPQQFDPNRALQGKT[+568][+100]PGQPVPQAAQPAPPNTAPGQAANQTQGGL.L z=4,scan#=39257,scan time=81.4775
KVAPPPADNGASQPQQFDPNRALQGKTPGQPVPQAAQPAPPNTAPGQAANQTQGGL
Intensity
m/z
HexNAc
HexNAcHex
M_4+ - HexNAc - 18
a3
b3
b4
b7
b8
b9
b13
b17
b18
y4
y5
y6
y7
y8
y9
y12
y13
y14
y16
y17
y19
y20

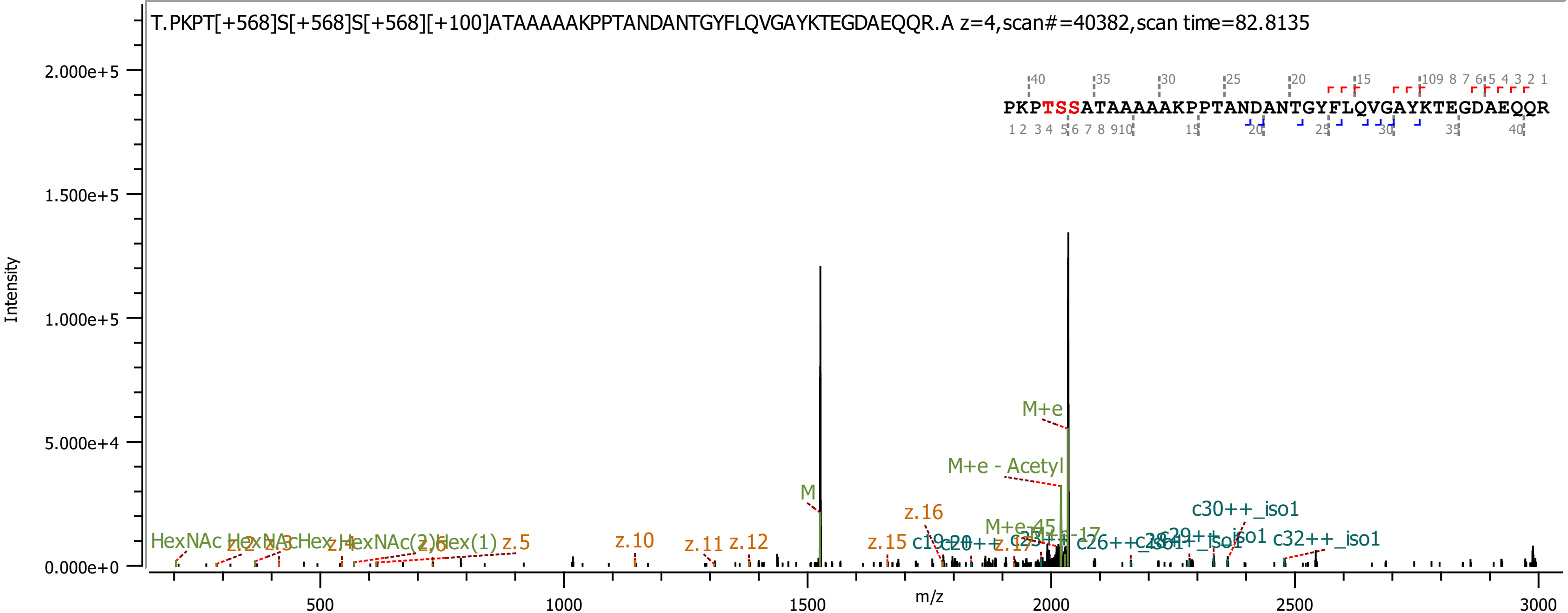
T.PKPT[+568]S[+568]S[+568][+100]ATAAAAAKPPTANDANTGYFLQVGAYKTEGDAEQQR.A z=4,scan#=40382,scan time=82.8135
PKPTSSATAAAAAKPPTANDANTGYFLQVGAYKTEGDAEQQR
Intensity
m/z
M+e
M+e - Acetyl
M
z.16
z.15
z.10
z.11
z.12
z.5
c30++_iso1
c32++_iso1
HexNAc
0.000e+0
5.000e+4
1.000e+5
1.500e+5
2.000e+5
500
1000
1500
2000
2500
3000

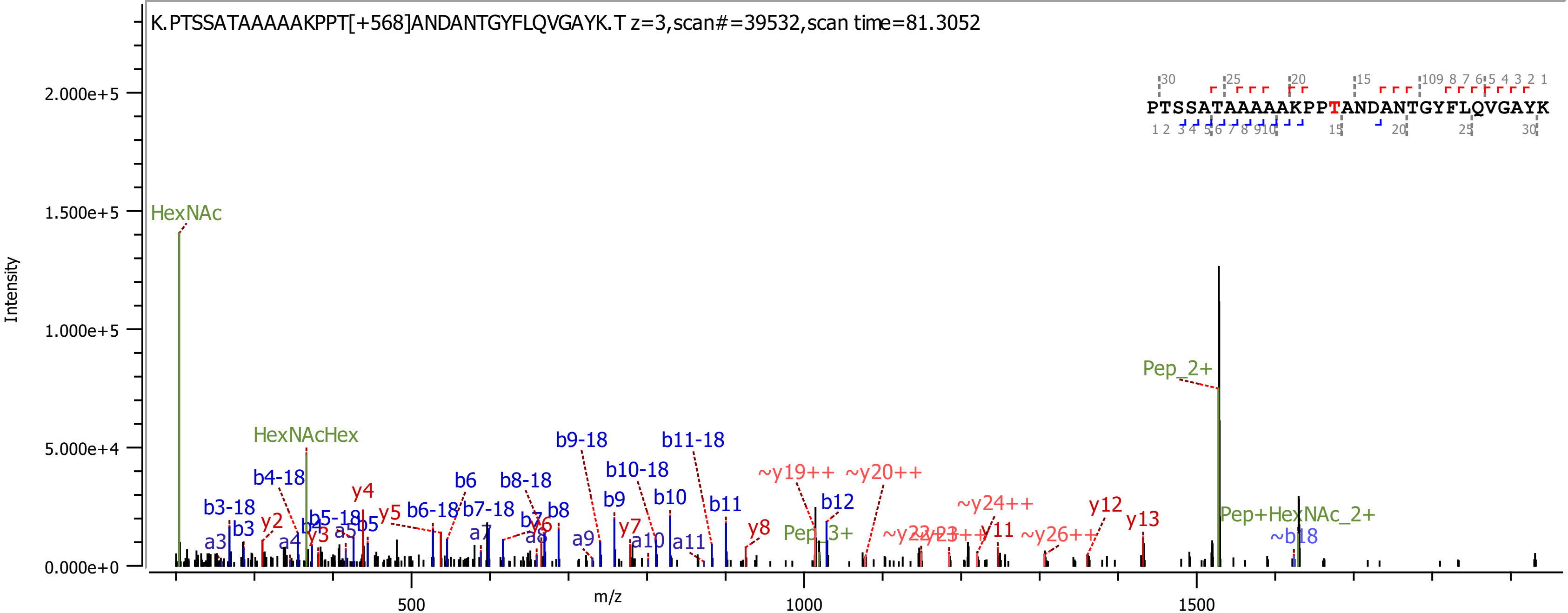

K.PTSSATAAAAAKPPT[+568]ANDANTGYFLQVGAYK.T z=3,scan#=39532,scan time=81.3052
PTSSATAAAAAKPPTANDANTGYFLQVGAYK
Intensity
m/z
HexNAc
HexNAcHex
Pep_2+
Pep+HexNAc_2+
Pep_3+

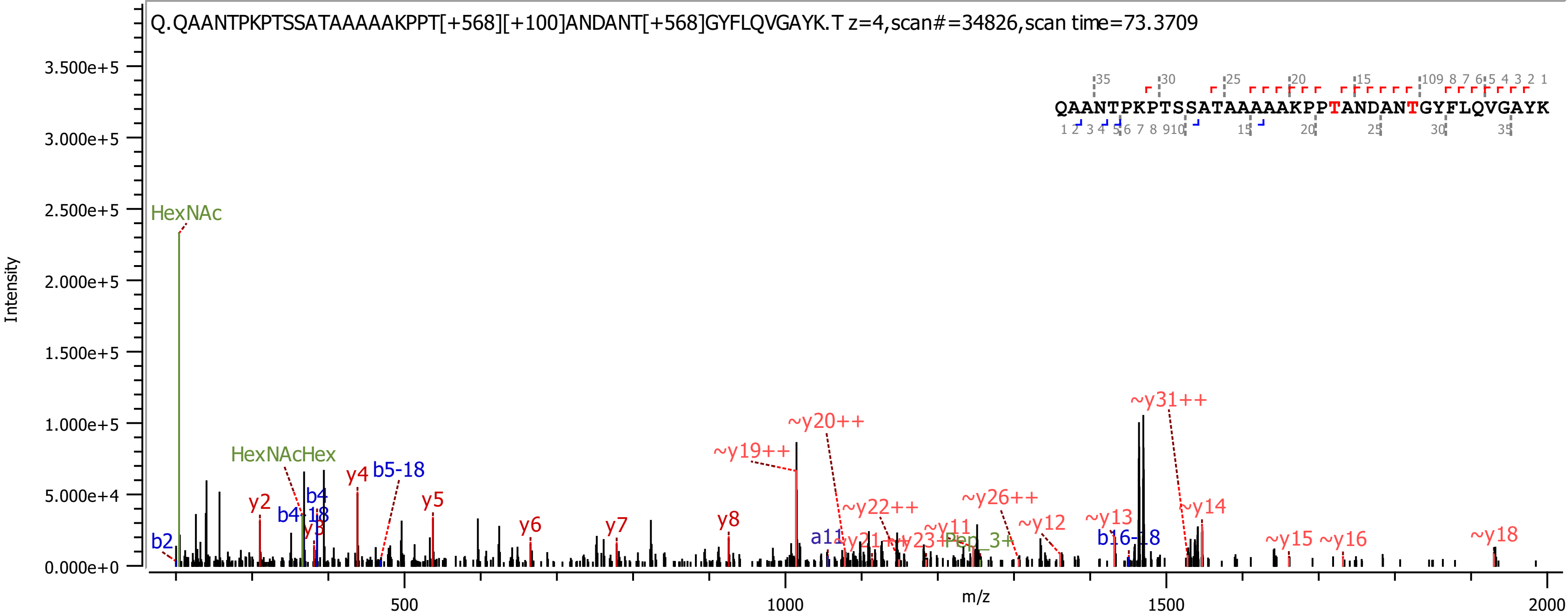

Q.QAANTPKPTSSATAAAAAKPPT[+568][+100]ANDANT[+568]GYFLQVGAYK.T z=4,scan#=34826,scan time=73.3709
QAANTPKPTSSATAAAAAKPPTANDANTGYFLQVGAYK
Intensity
m/z
HexNAc
HexNAcHex
b2
y2
b4-18
b4
y3
y4
b5-18
y5
y6
y7
y8
~y19++
~y20++
a11
~y21++
~y22++
~y23++
~y11
Pep_3+
~y26++
~y12
~y13
b16-18
~y31++
~y14
~y15
~y16
~y18

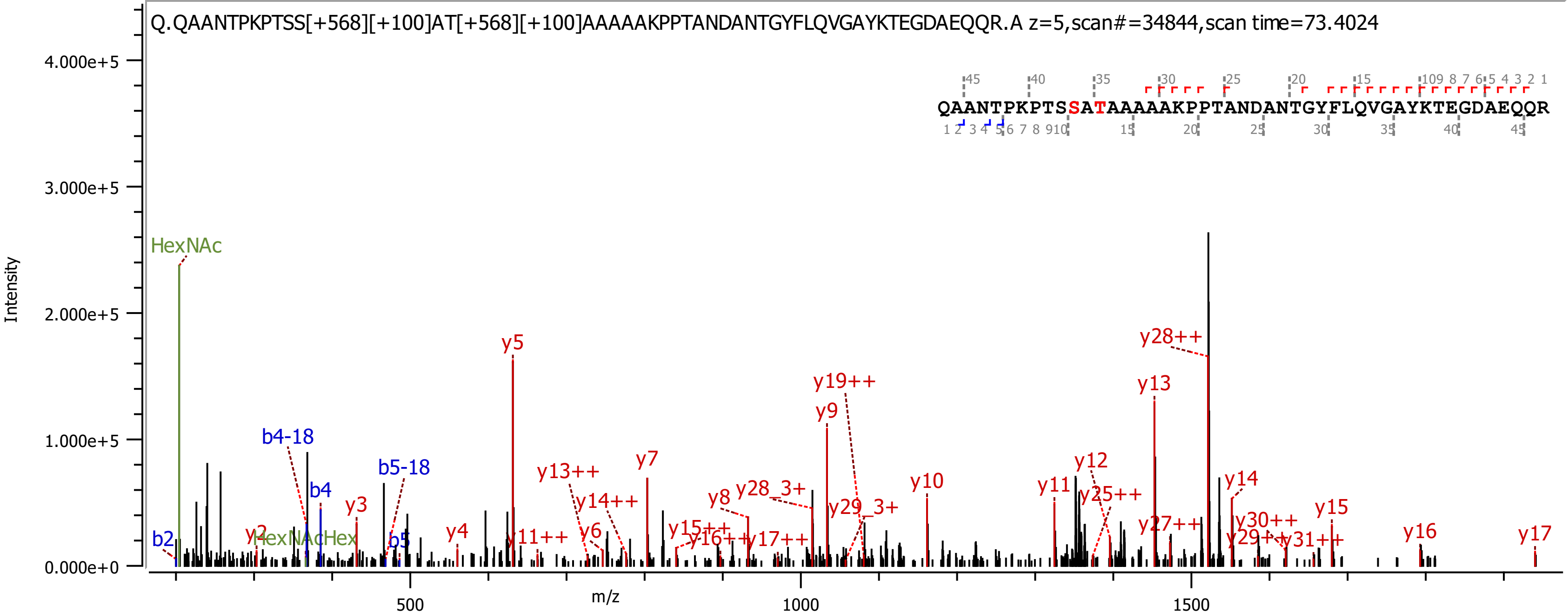

Q.QAANTPKPTSS[+568][+100]AT[+568][+100]AAAAAKPPTANDANTGYFLQVGAYKTEGDAEQQR.A z=5,scan#=34844,scan time=73.4024
QAANTPKPTSSATAAAAAKPPTANDANTGYFLQVGAYKTEGDAEQQR
Intensity
m/z
HexNAc
HexNAcHex
b2
b4-18
b4
b5-18
b5
y2
y3
y4
y5
y6
y7
y8
y9
y10
y11
y12
y13
y14
y15
y16
y17
y11++
y13++
y14++
y15++
y16++
y17++
y19++
y25++
y27++
y28++
y29++
y30++
y31++
y28_3+
y29_3+
4.000e+5
3.000e+5
2.000e+5
1.000e+5
0.000e+0
500
1000
1500

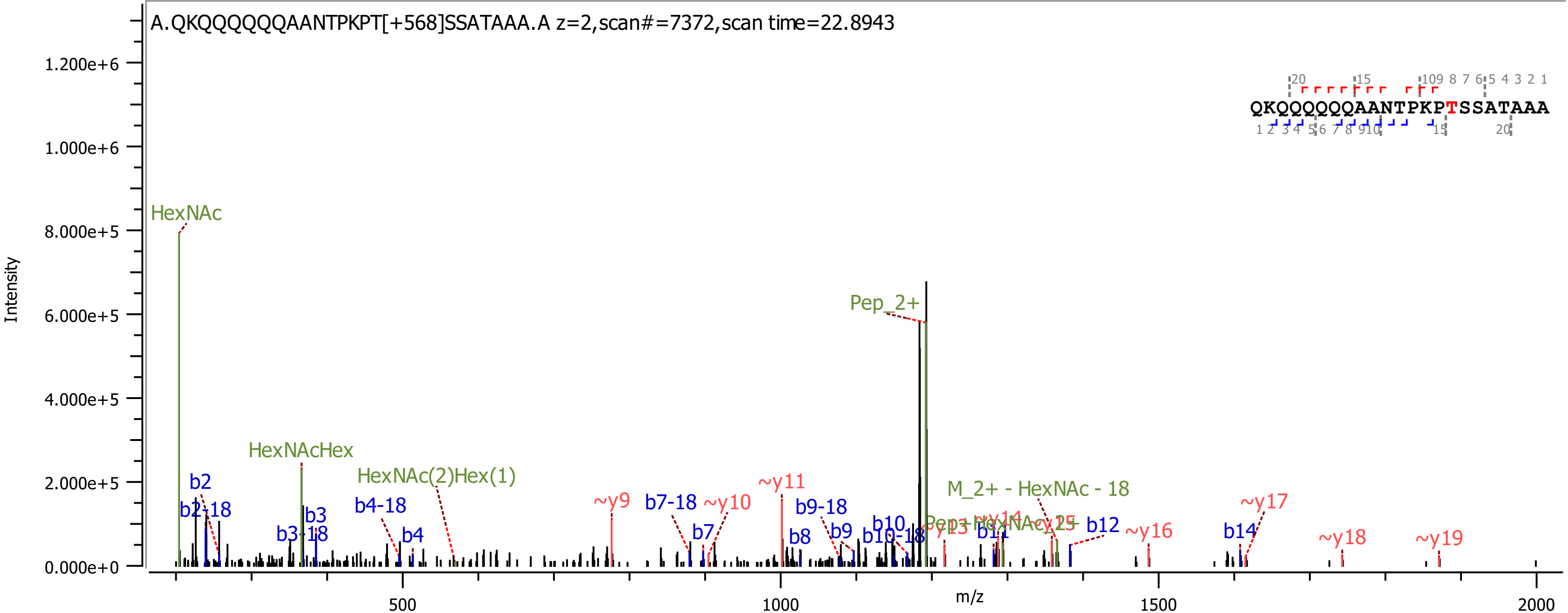
A.QKQQQQQQAANTPKPT[+568]SSATAAA.A z=2,scan#=7372,scan time=22.8943
QKQQQQQQAANTPKPTSSATAAA
Intensity
m/z
HexNAc
HexNAcHex
HexNAc(2)Hex(1)
Pep_2+
M_2+ - HexNAc - 18
b2
b2-18
b3
b3-18
b4-18
b4
~y9
b7-18
~y10
~y11
b7
b8
b9-18
b9
b10
b10-18
b11
b12
b14
~y16
~y17
~y18
~y19

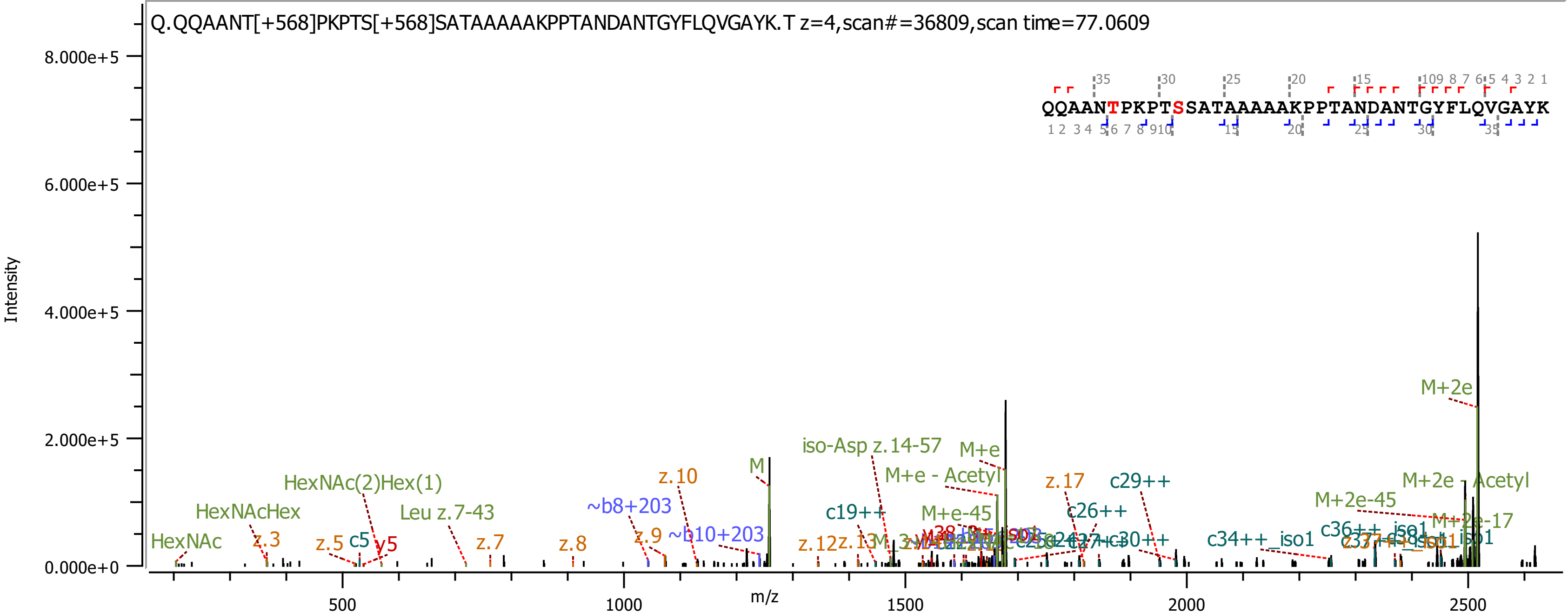
Q.QQAANT[+568]PKPTS[+568]SATAAAAAKPPTANDANTGYFLQVGAYK.T z=4,scan#=36809,scan time=77.0609
QQAANTPKPTSSATAAAAAKPPTANDANTGYFLQVGAYK
Intensity
8.000e+5
6.000e+5
4.000e+5
2.000e+5
0.000e+0
m/z
500
1000
1500
2000
2500
HexNAc
HexNAcHex
HexNAc(2)Hex(1)
z.3
z.5
c5
y5
Leu z.7-43
z.7
z.8
~b8+203
z.9
~b10+203
z.10
M
iso-Asp z.14-57
M+e
M+e - Acetyl
c19++
M+e-45
z.12
z.13
z.17
c29++
c26++
c30++
c34++_iso1
c36++_iso1
M+2e-45
M+2e
M+2e - Acetyl
M+2e-17

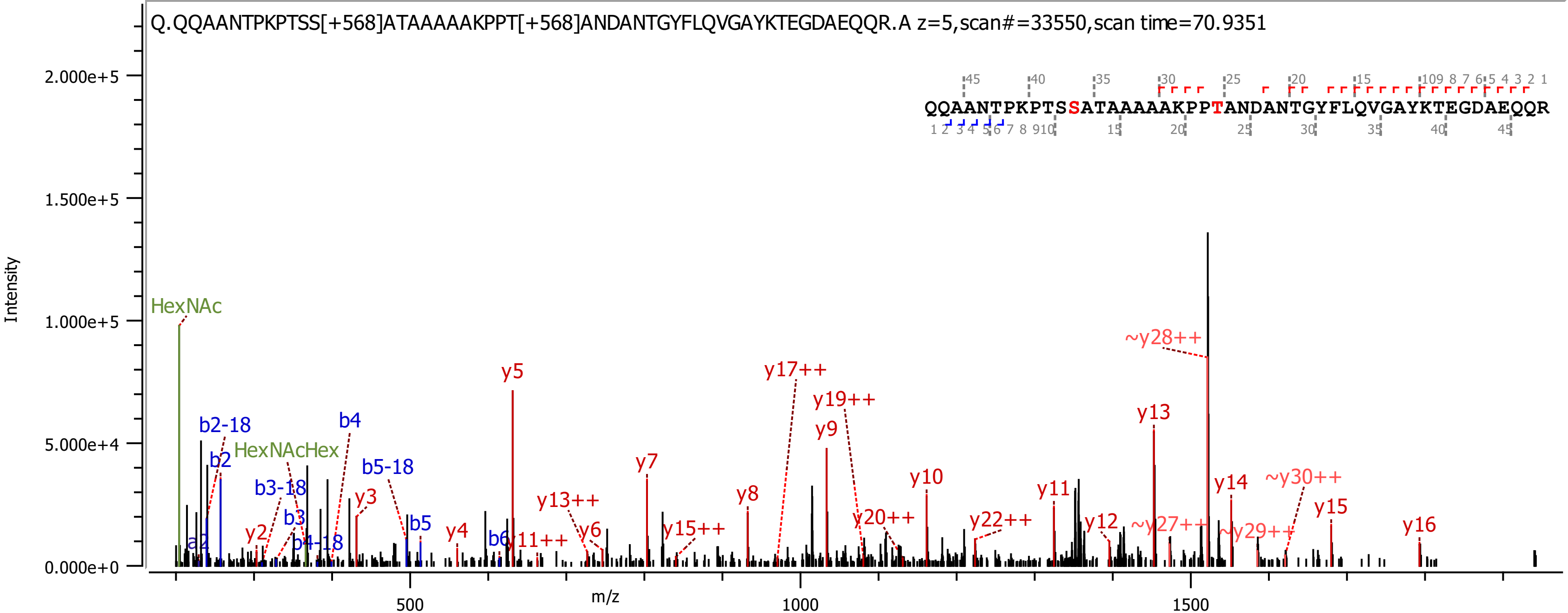

Q.QQAANTPKPTSS[+568]ATAAAAAKPPT[+568]ANDANTGYFLQVGAYKTEGDAEQQR.A z=5,scan#=33550,scan time=70.9351
QQAANTPKPTSSATAAAAAKPPTANDANTGYFLQVGAYKTEGDAEQQR
Intensity
m/z
2.000e+5
1.500e+5
1.000e+5
5.000e+4
0.000e+0
500
1000
1500
HexNAc
HexNAcHex
b2-18
b2
b3-18
b3
b4-18
b4
b5-18
b5
b6
a2
y2
y3
y4
y5
y6
y7
y8
y9
y10
y11
y12
y13
y14
y15
y16
y11++
y13++
y15++
y17++
y19++
y20++
y22++
~y27++
~y28++
~y29++
~y30++

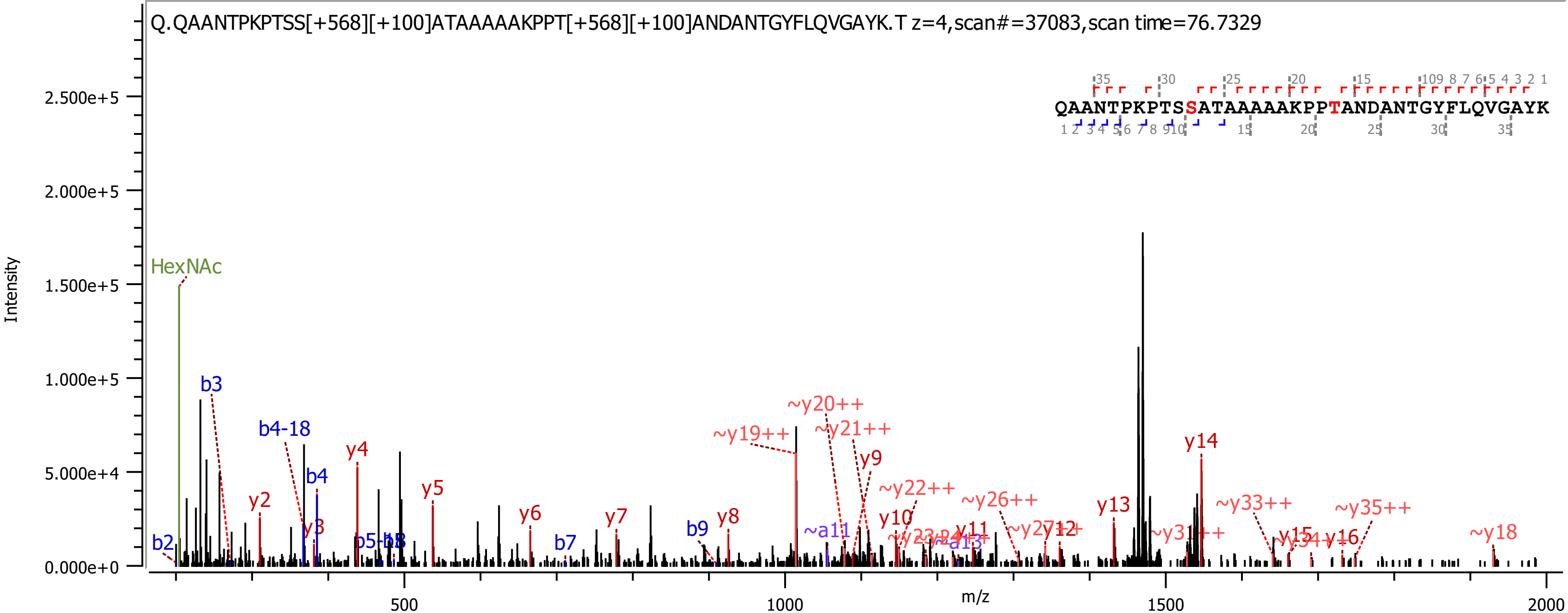
Q.QAANTPKPTSS[+568][+100]ATAAAAAKPPT[+568][+100]ANDANTGYFLQVGAYK.T z=4,scan#=37083,scan time=76.7329
QAANTPKPTSSATAAAAAKPPTANDANTGYFLQVGAYK
Intensity
m/z
HexNAc
b2
b3
b4-18
b4
b7
b9
y2
y3
y4
y5
y6
y7
y8
y9
y10
y11
y12
y13
y14
y15
y16
~y18
~y19++
~y20++
~y21++
~y22++
~y26++
~y33++
~y35++
~a11

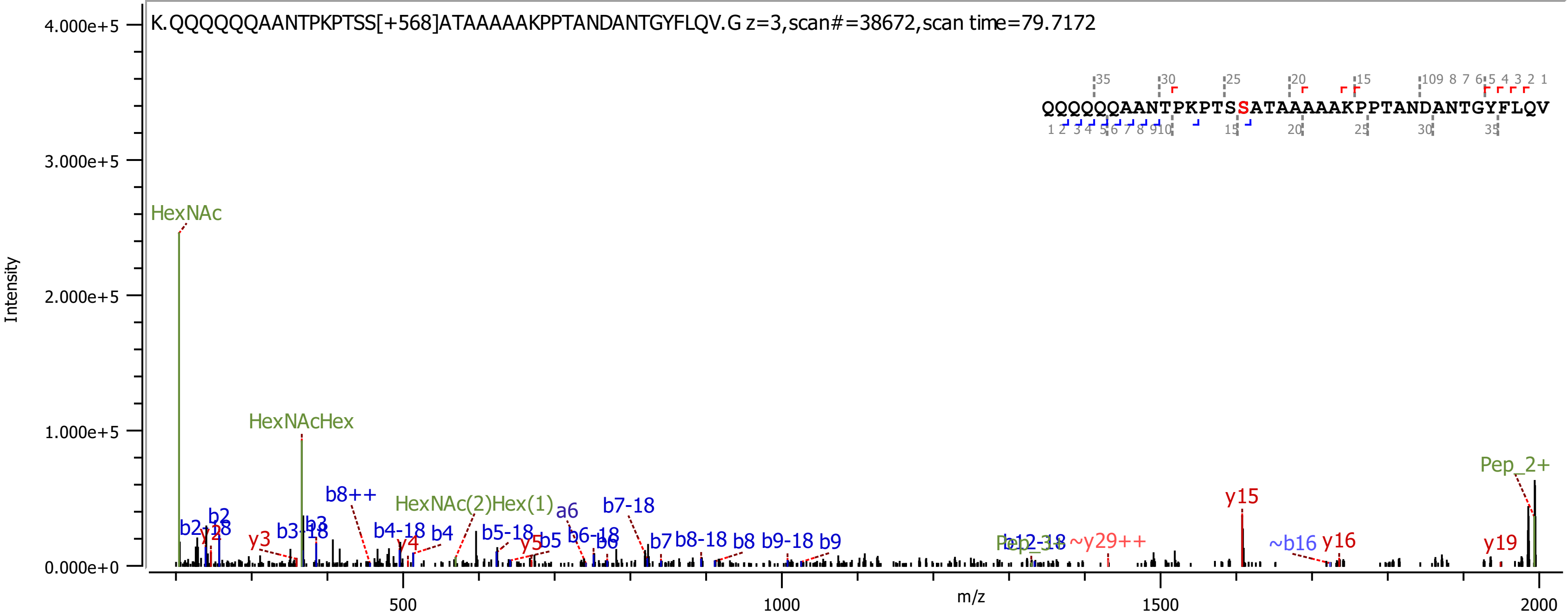

K.QQQQQQAANTPKPTSS[+568]ATAAAAAKPPTANDANTGYFLQV.G z=3,scan#=38672,scan time=79.7172
QQQQQQAANTPKPTSSATAAAAAKPPTANDANTGYFLQV
Intensity
4.000e+5
3.000e+5
2.000e+5
1.000e+5
0.000e+0
m/z
500
1000
1500
2000
HexNAc
HexNAcHex
HexNAc(2)Hex(1)
b8++
a6
b7-18
y3
b3-18
b3
b4-18
b4
y4
b5-18
b5
y5
b6-18
b6
b7
b8-18
b8
b9-18
b9
b12-18
~y29++
y15
~b16
y16
y19
Pep_2+

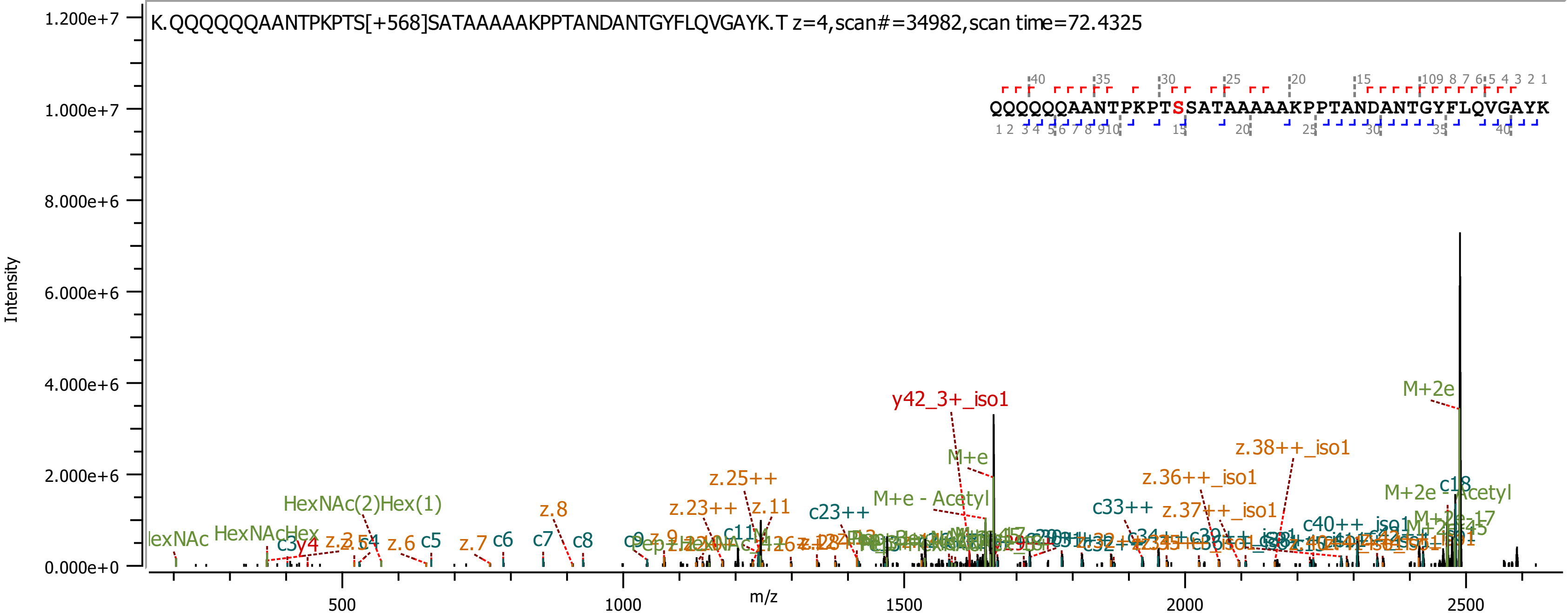
K.QQQQQQAANTPKPTS[+568]SATAAAAAKPPTANDANTGYFLQVGAYK.T z=4,scan#=34982,scan time=72.4325
QQQQQQAANTPKPTSSATAAAAAKPPTANDANTGYFLQVGAYK
Intensity
m/z
y42_3+_iso1
M+2e
M+e
z.38++_iso1
z.25++
z.36++_iso1
M+e - Acetyl
HexNAc(2)Hex(1)
z.8
z.23++
z.11
c23++
c33++
z.37++_iso1
M+2e - Acetyl
c18
c40++_iso1
HexNAcHex
c5
c6
c7
c8
z.6
z.7

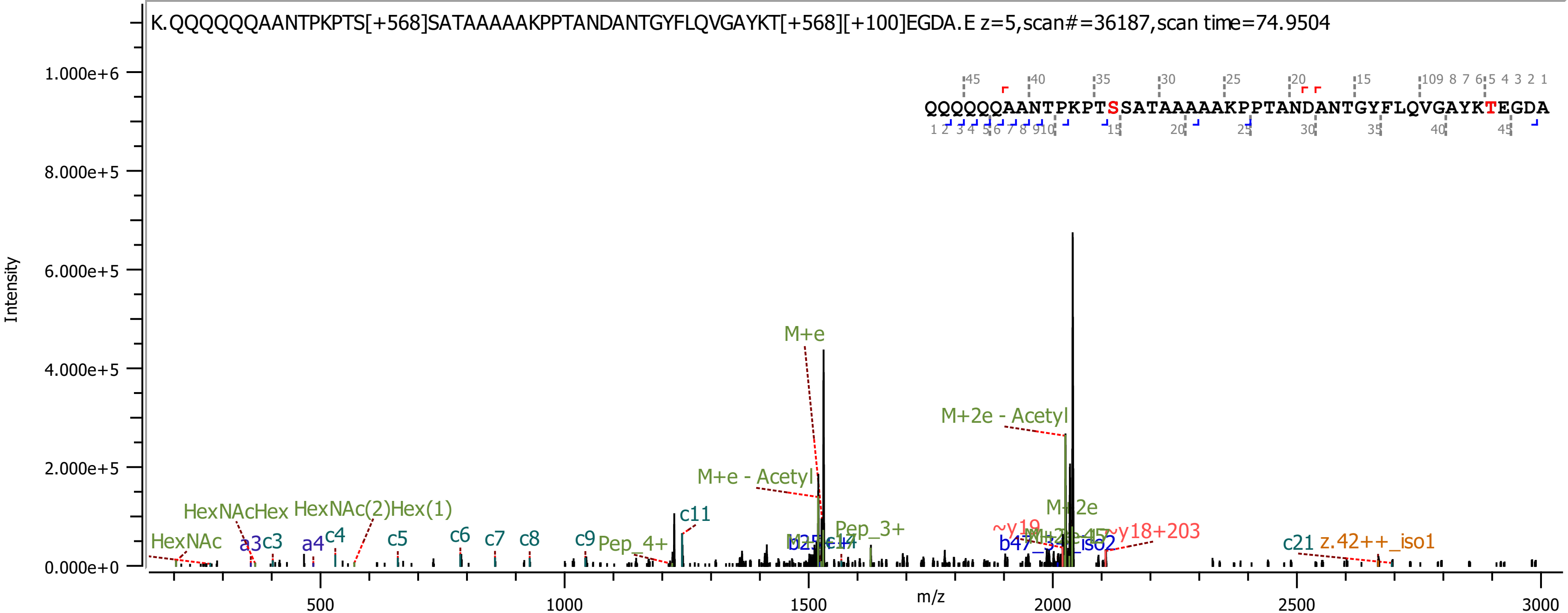
K.QQQQQQAANTPKPTS[+568]SATAAAAAKPPTANDANTGYFLQVGAYKT[+568][+100]EGDA.E z=5,scan#=36187,scan time=74.9504
QQQQQQAANTPKPTSSATAAAAAKPPTANDANTGYFLQVGAYKTEGDA
Intensity
m/z
M+e
M+2e - Acetyl
M+e - Acetyl
M+2e
Pep_3+
Pep_4+
HexNAc
HexNAcHex
HexNAc(2)Hex(1)
a3
c3
a4
c4
c5
c6
c7
c8
c9
c11
c21
~y19
~y18+203
z.42++_iso1

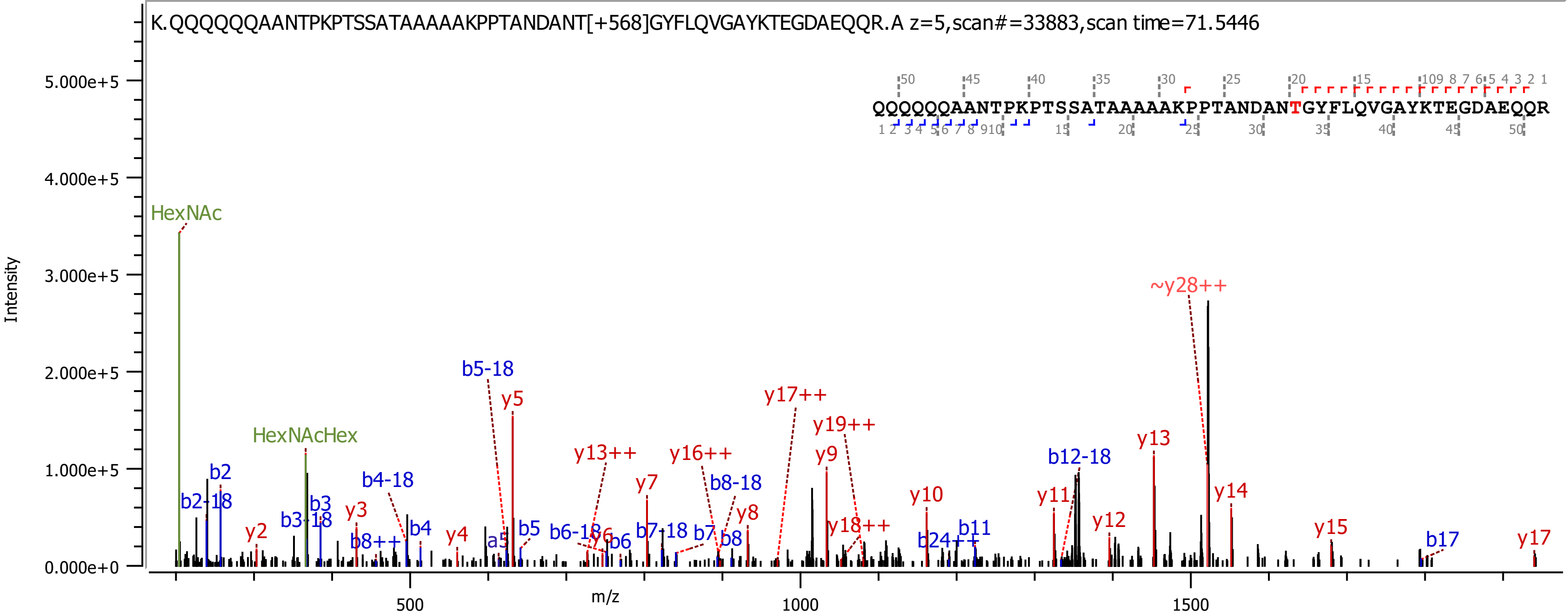

K.QQQQQQAANTPKPTSSATAAAAAKPPTANDANT[+568]GYFLQVGAYKTEGDAEQQR.A z=5,scan#=33883,scan time=71.5446
QQQQQQAANTPKPTSSATAAAAAKPPTANDANTGYFLQVGAYKTEGDAEQQR
Intensity
m/z
HexNAc
HexNAcHex
b2
b2-18
y2
b3
b3-18
y3
b4-18
b8++
b4
y4
b5-18
y5
a5
b5
b6-18
y6
b6
y13++
y7
b7-18
b7
y16++
b8-18
b8
y8
y17++
y9
y19++
y18++
y10
b24++
b11
y11
b12-18
y12
y13
~y28++
y14
y15
b17
y17

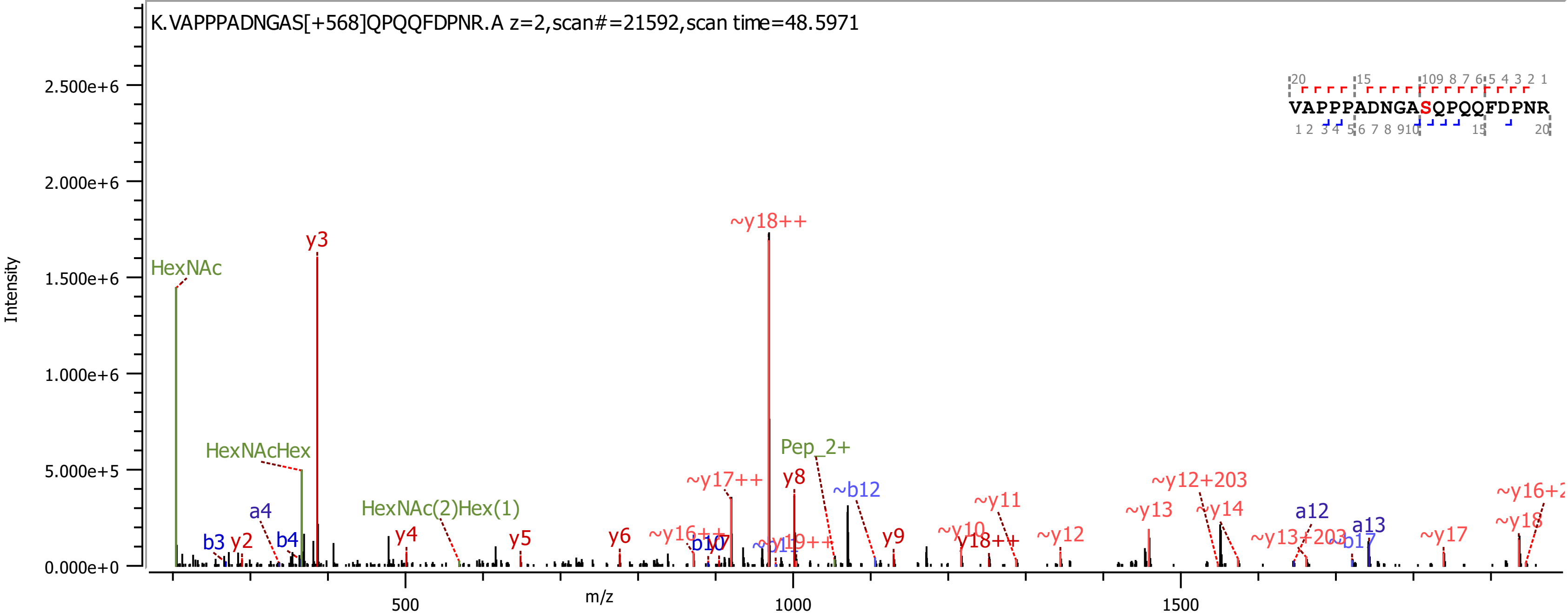
K.VAPPPADNGAS[+568]QPQQFDPNR.A z=2,scan#=21592,scan time=48.5971
VAPPPADNGASQPQQFDPNR
Intensity
m/z
2.500e+6
2.000e+6
1.500e+6
1.000e+6
5.000e+5
0.000e+0
500
1000
1500
HexNAc
y3
~y18++
HexNAcHex
Pep_2+
~y17++
y8
~b12
a4
HexNAc(2)Hex(1)
~y11
~y12+203
~y16+2
~y13
~y14
a12
a13
~y18
b3
y2
b4
y4
y5
y6
~y16++
b10
~y19++
y9
~y10
y18++
~y12
~y13+203
~b17
~y17

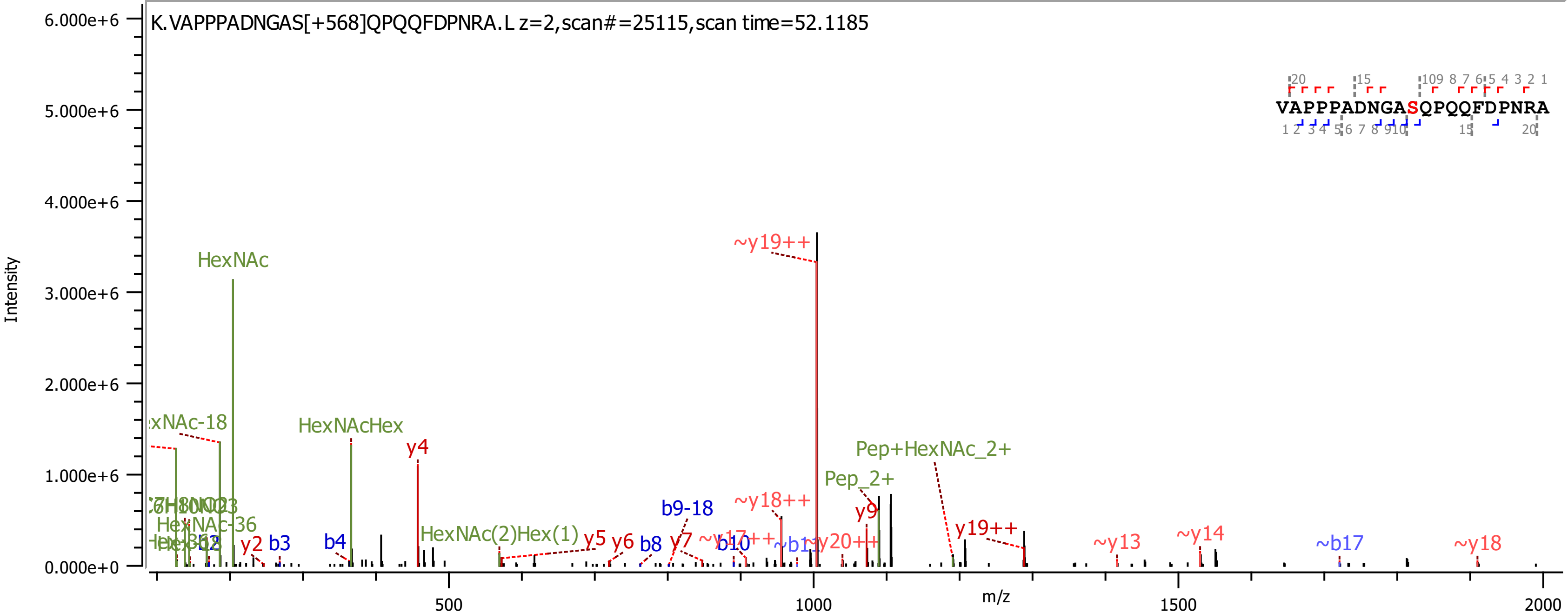
K.VAPPPADNGAS[+568]QPQQFDPNRA.L z=2,scan#=25115,scan time=52.1185
VAPPPADNGASQPQQFDPNRA
Intensity
m/z
6.000e+6
5.000e+6
4.000e+6
3.000e+6
2.000e+6
1.000e+6
0.000e+0
500
1000
1500
2000
HexNAc
HexNAcHex
HexNAc(2)Hex(1)
Pep+HexNAc_2+
Pep_2+
~y19++
~y18++
y19++
y4
y2
b3
b4
y5
y6
b8
y7
b9-18
b10
y9
~y13
~y14
~b17
~y18
HexNAc-36

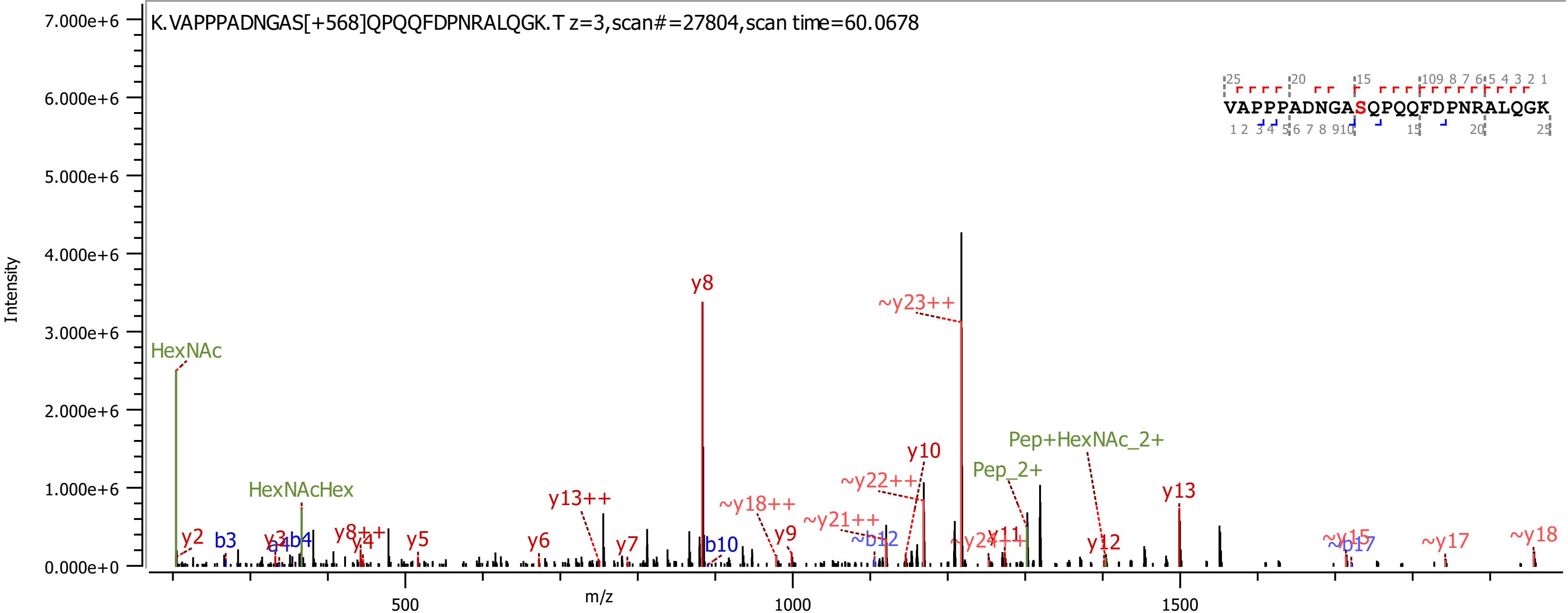

K.VAPPPADNGAS[+568]QPQQFDPNRALQGK.T z=3,scan#=27804,scan time=60.0678
VAPPPADNGASQPQQFDPNRALQGK
Intensity
m/z
HexNAc
HexNAcHex
y8
~y23++
y10
~y22++
Pep+HexNAc_2+
Pep_2+
y13
y13++
~y18++
~y21++
y2
b3
y3
a4
b4
y8++
y4
y5
y6
y7
b10
y9
~b12
~y24++
y11
y12
~y15
~b17
~y17
~y18

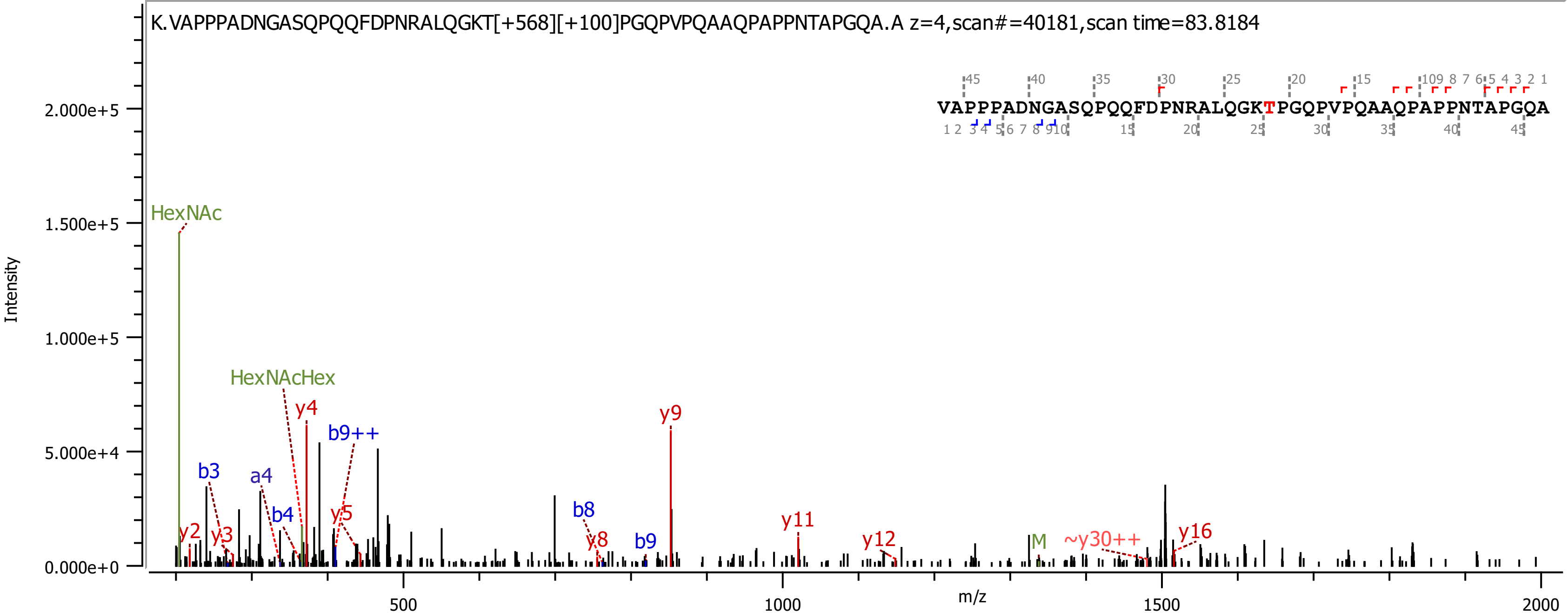

K.VAPPPADNGASQPQQFDPNRALQGKT[+568][+100]PGQPVPQAAQPAPPNTAPGQA.A z=4,scan#=40181,scan time=83.8184
VAPPPADNGASQPQQFDPNRALQGKTPGQPVPQAAQPAPPNTAPGQA
Intensity
m/z
HexNAc
HexNAcHex
y2
y3
b3
a4
b4
y4
b9++
y5
b8
y8
b9
y9
y11
y12
M
~y30++
y16

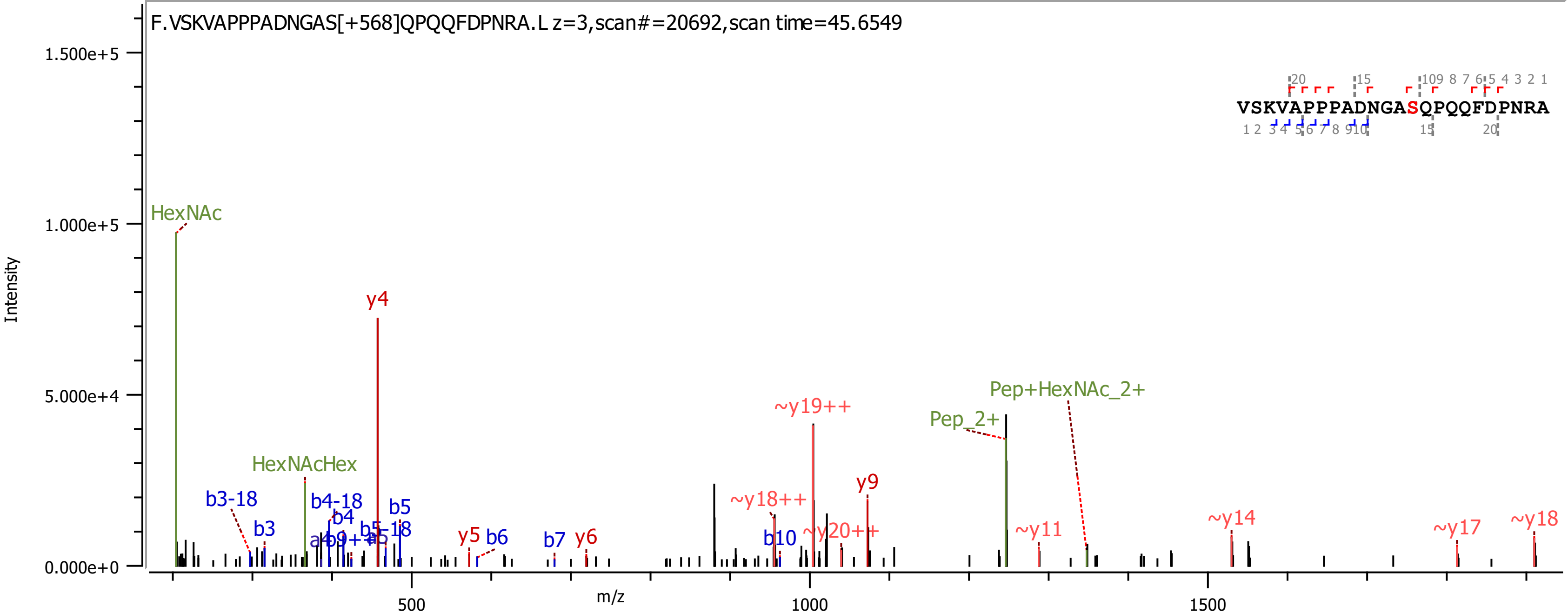

F.VSKVAPPPADNGAS[+568]QPQQFDPNRA.L z=3,scan#=20692,scan time=45.6549
VSKVAPPPADNGASQPQQFDPNRA
Intensity
m/z
1.500e+5
1.000e+5
5.000e+4
0.000e+0
500
1000
1500
HexNAc
HexNAcHex
y4
b3-18
b3
b4-18
b4
b5
b5-18
y5
b6
b7
y6
~y19++
~y18++
b10
~y20++
y9
Pep_2+
Pep+HexNAc_2+
~y11
~y14
~y17
~y18

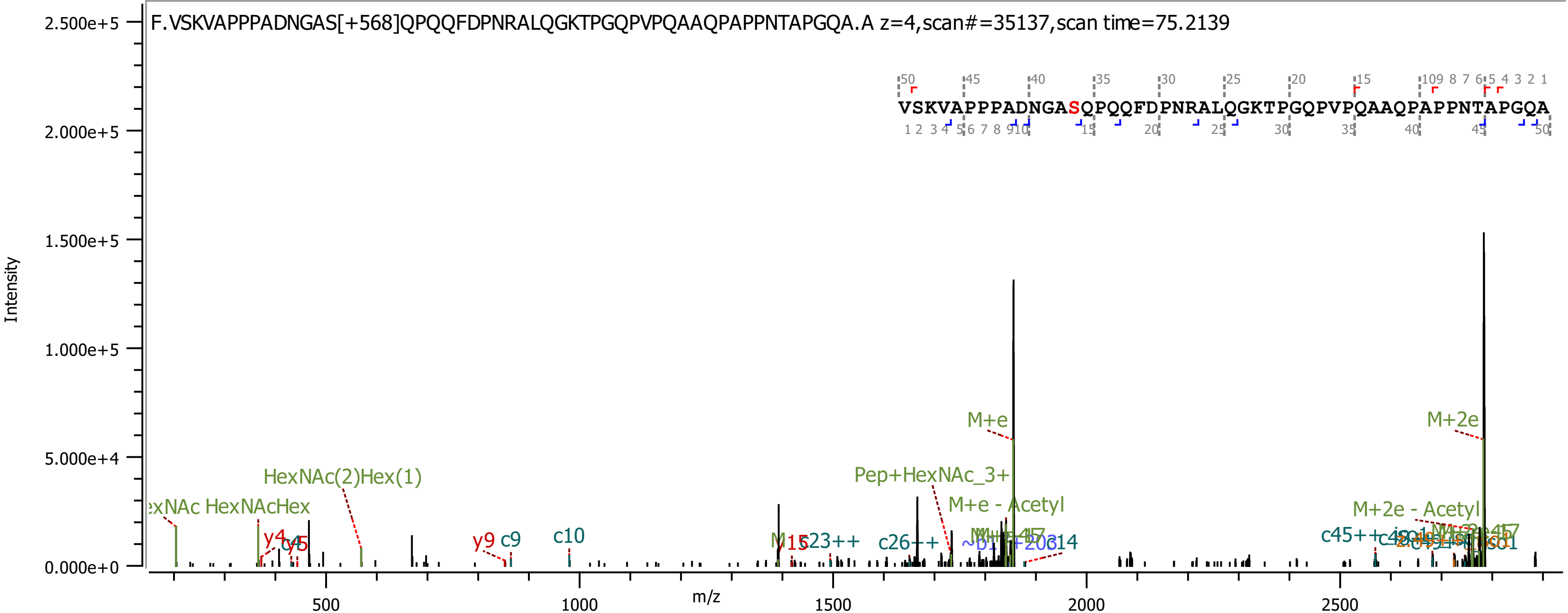
F.VSKVAPPPADNGAS[+568]QPQQFDPNRALQGKTPGQPVPQAAQPAPPNTAPGQA.A z=4,scan#=35137,scan time=75.2139
VSKVAPPPADNGASQPQQFDPNRALQGKTPGQPVPQAAQPAPPNTAPGQA
Intensity
2.500e+5
2.000e+5
1.500e+5
1.000e+5
5.000e+4
0.000e+0
m/z
500
1000
1500
2000
2500
HexNAc(2)Hex(1)
HexNAcHex
y4
y5
y9
c9
c10
c23++
Pep+HexNAc_3+
M+e - Acetyl
M+e
c26++
M+2e
M+2e - Acetyl
c45++

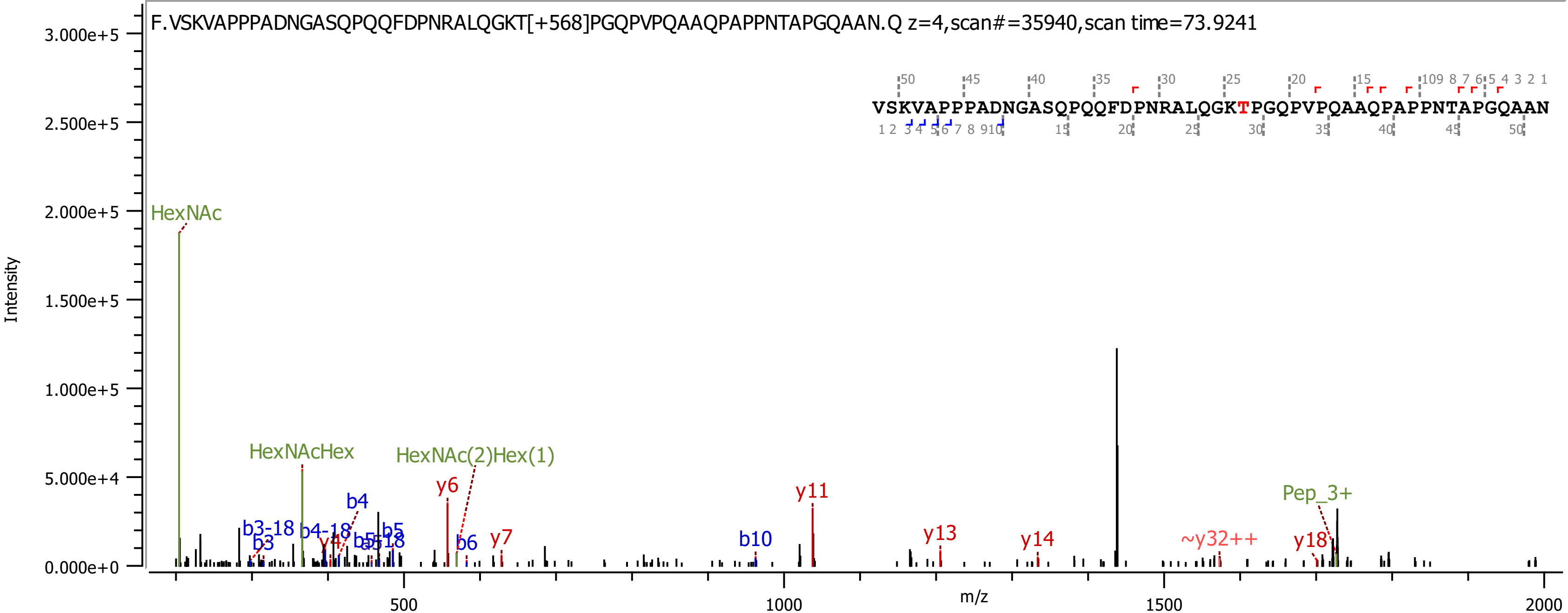
F.VSKVAPPPADNGASQPQQFDPNRALQGKT[+568]PGQPVPQAAQPAPPNTAPGQAAN.Q z=4,scan#=35940,scan time=73.9241
VSKVAPPPADNGASQPQQFDPNRALQGKTPGQPVPQAAQPAPPNTAPGQAAN
Intensity
m/z
HexNAc
HexNAcHex
HexNAc(2)Hex(1)
b3-18
b3
b4-18
b4
y4
b5
a5
y6
b6
y7
b10
y11
y13
y14
~y32++
y18
Pep_3+

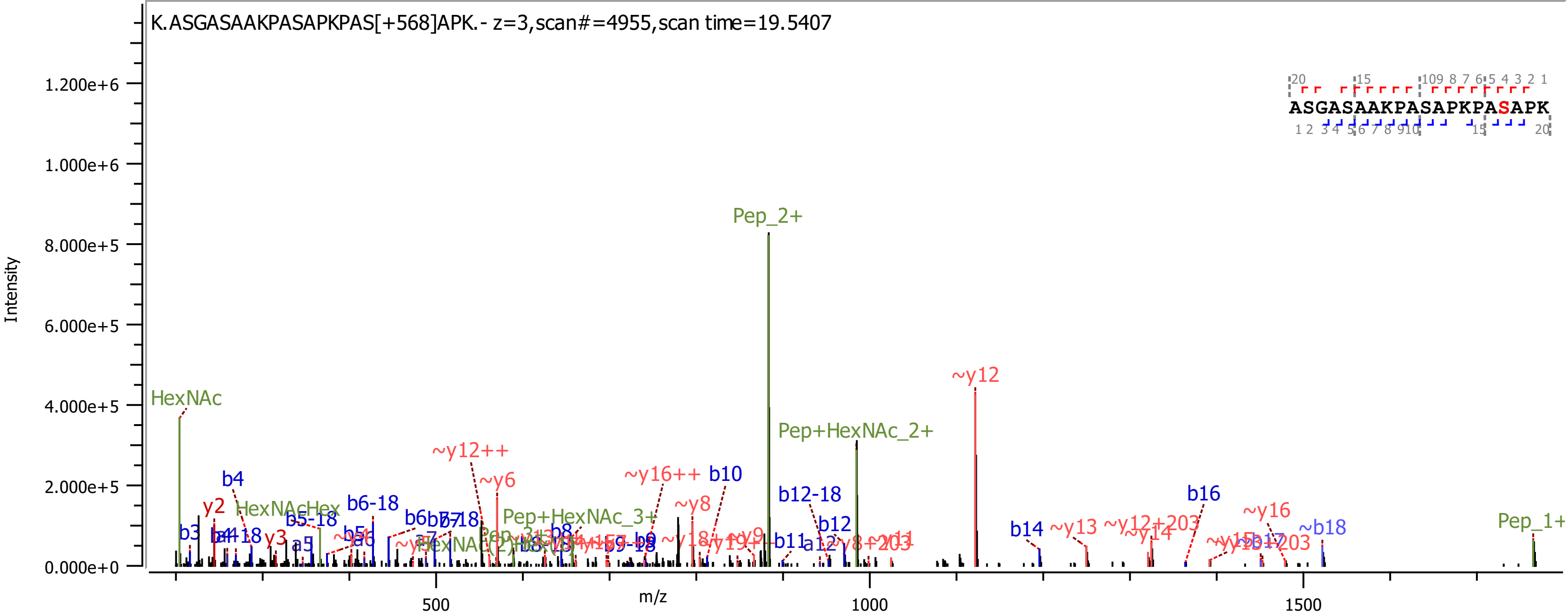
K.ASGASAAKPASAPKPAS[+568]APK.- z=3,scan#=4955,scan time=19.5407
ASGASAAKPASAPKPASAPK
Intensity
m/z
1.200e+6
1.000e+6
8.000e+5
6.000e+5
4.000e+5
2.000e+5
0.000e+0
500
1000
1500
Pep_2+
~y12
HexNAc
Pep+HexNAc_2+
~y12++
~y6
~y16++
b10
b4
y2
b6-18
b5-18
b12-18
b16
~y16
~y8
b12
b14
~y13
~y12+203
~b18
Pep_1+
Pep+HexNAc_3+
b3
y3
a5
b8
b11
a12
~y14

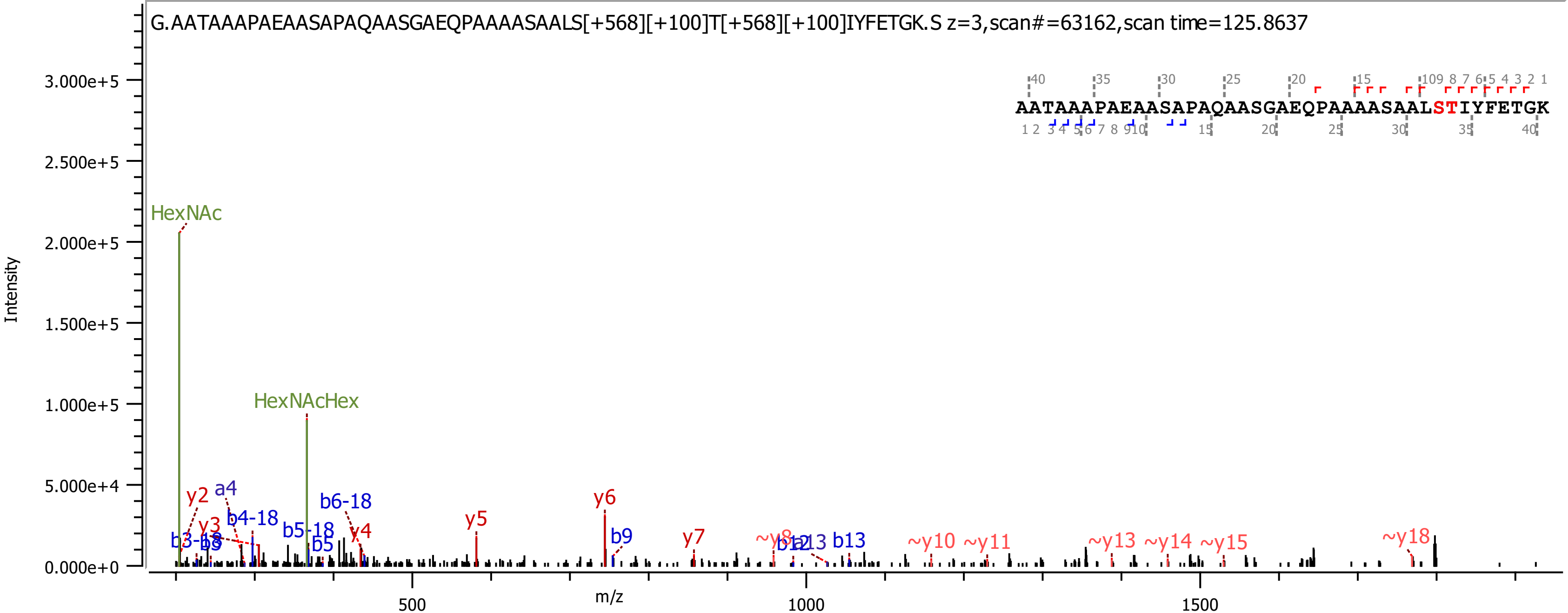

G.AATAAAPAEAASAPAQAASGAEQPAAAASAALS[+568][+100]T[+568][+100]IYFETGK.S z=3,scan#=63162,scan time=125.8637
AATAAAPAEAASAPAQAASGAEQPAAAASAALSTIYFETGK
Intensity
m/z
3.000e+5
2.500e+5
2.000e+5
1.500e+5
1.000e+5
5.000e+4
0.000e+0
500
1000
1500
HexNAc
HexNAcHex
y2
a4
y3
b4-18
b3-18
b3
b5-18
b5
b6-18
y4
y5
y6
b9
y7
~y8
b12
b13
~y10
~y11
~y13
~y14
~y15
~y18

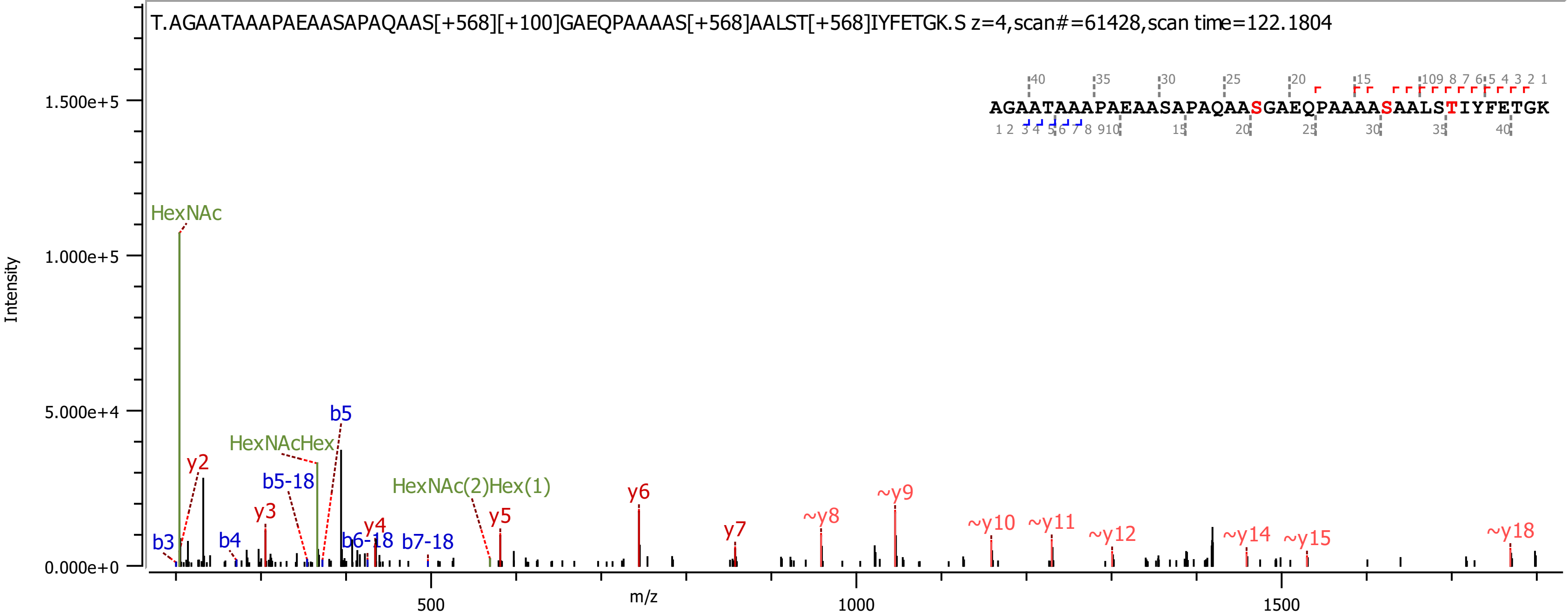

T.AGAATAAAPAEAASAPAQAAS[+568][+100]GAEQPAAAAS[+568]AALST[+568]IYFETGK.S z=4,scan#=61428,scan time=122.1804
AGAATAAAPAEAASAPAQAASGAEQPAAAASAALSTIYFETGK
Intensity
m/z
HexNAc
HexNAcHex
HexNAc(2)Hex(1)
b3
b4
b5
b5-18
b6-18
b7-18
y2
y3
y4
y5
y6
y7
~y8
~y9
~y10
~y11
~y12
~y14
~y15
~y18

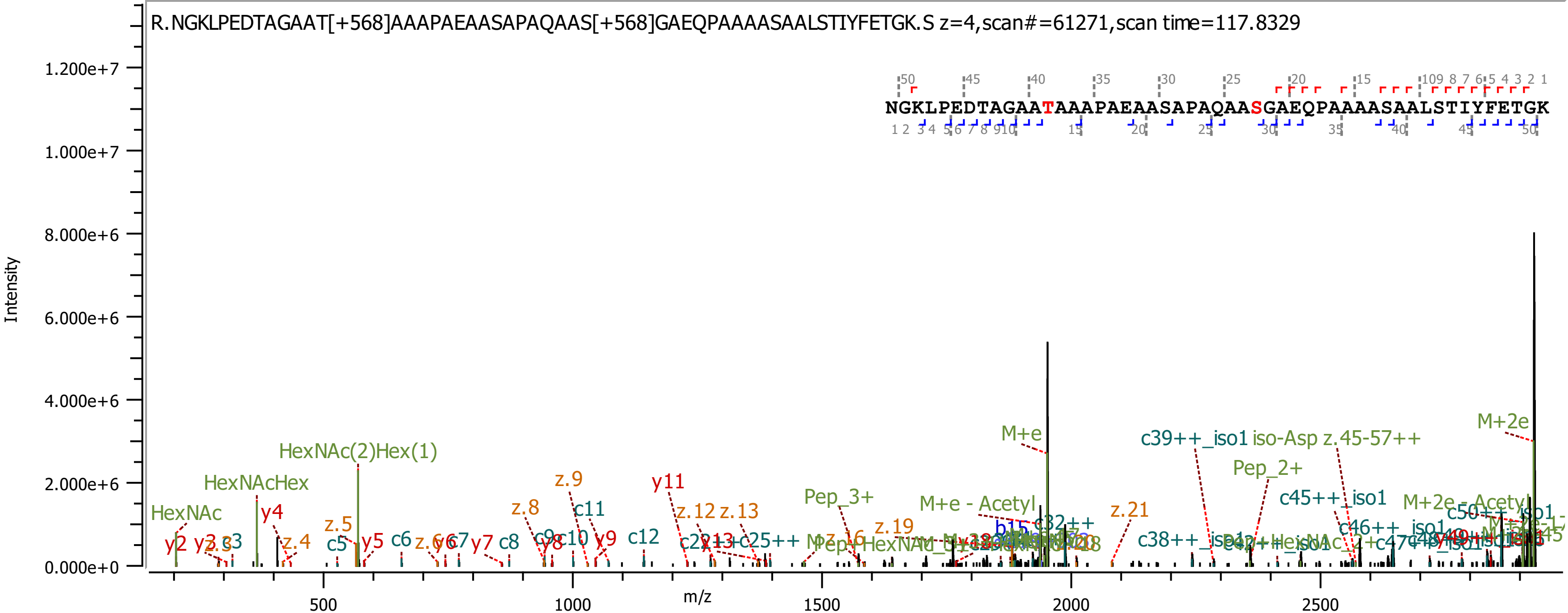
R.NGKLPEDTAGAAT[+568]AAAPAEAASAPAQAAS[+568]GAEQPAAAASAALSTIYFETGK.S z=4,scan#=61271,scan time=117.8329
NGKLPEDTAGAATAAAPAEAASAPAQAASGAEQPAAAASAALSTIYFETGK
Intensity
m/z
1.200e+7
1.000e+7
8.000e+6
6.000e+6
4.000e+6
2.000e+6
0.000e+0
500
1000
1500
2000
2500
HexNAc
HexNAcHex
HexNAc(2)Hex(1)
M+e
M+2e
M+e - Acetyl
M+2e - Acetyl
Pep_3+
Pep_2+
c39++_iso1 iso-Asp z.45-57++
c45++_iso1
c46++_iso1
c38++_iso1
y2
y4
y5
y7
y9
y11
z.4
z.5
z.8
z.9
z.12 z.13
z.19
z.21
c5
c6
c8
c11
c12

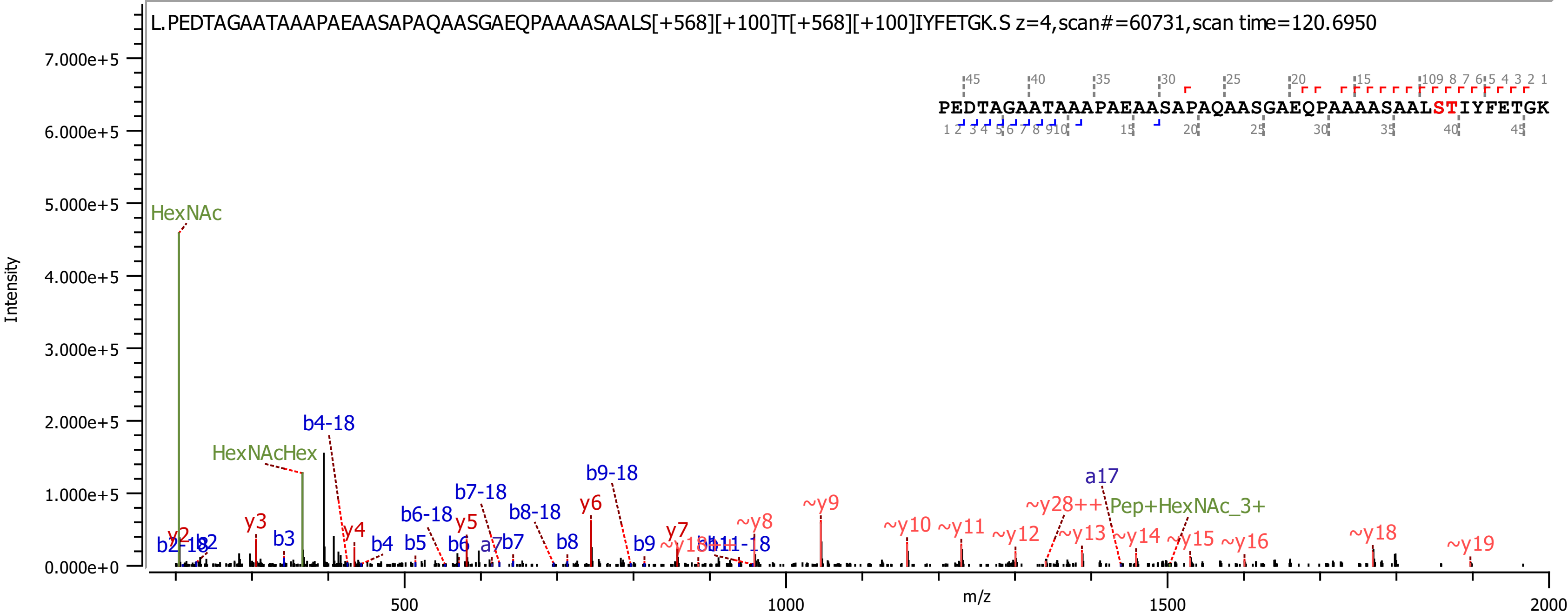
L.PEDTAGAATAAAPAEAASAPAQAASGAEQPAAAASAALS[+568][+100]T[+568][+100]IYFETGK.S z=4,scan#=60731,scan time=120.6950
PEDTAGAATAAAPAEAASAPAQAASGAEQPAAAASAALSTIYFETGK
Intensity
m/z
HexNAc
HexNAcHex
b4-18
b9-18
b7-18
b6-18
b8-18
a17
~y28++
Pep+HexNAc_3+
y6
~y9
~y8
~y10
~y11
~y12
~y13
~y14
~y15
~y16
~y18
~y19
y2
b2-18
b2
y3
b3
y4
b4
b5
y5
b6
a7
b7
b8
b9
y7
b11-18

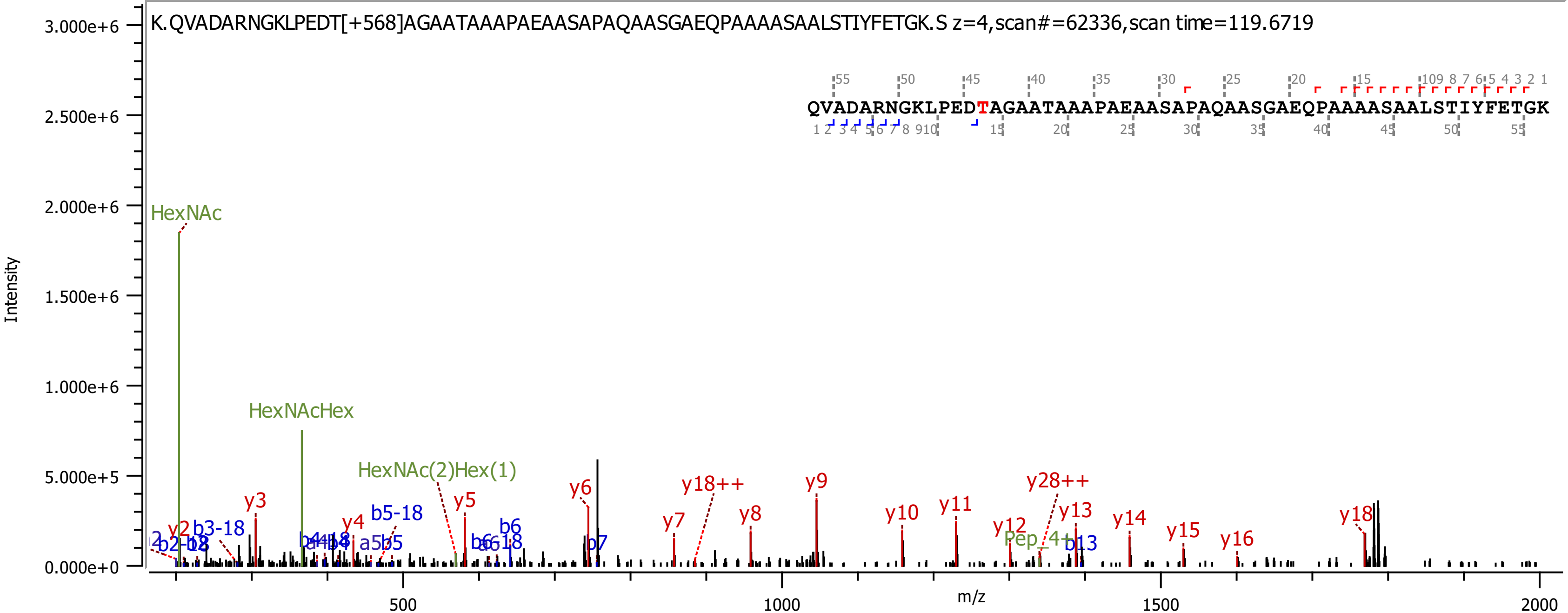

K.QVADARNGKLPEDT[+568]AGAATAAAPAEAASAPAQAASGAEQPAAAASAALSTIYFETGK.S z=4,scan#=62336,scan time=119.6719
QVADARNGKLPEDTAGAATAAAPAEAASAPAQAASGAEQPAAAASAALSTIYFETGK
Intensity
m/z
3.000e+6
2.500e+6
2.000e+6
1.500e+6
1.000e+6
5.000e+5
0.000e+0
500
1000
1500
2000
HexNAc
HexNAcHex
HexNAc(2)Hex(1)
y2
y3
y4
y5
y6
y7
y8
y9
y10
y11
y12
y13
y14
y15
y16
y18
y18++
y28++
b3-18
b5-18
b6
b6-18
b7
b13
a5
Pep_4+

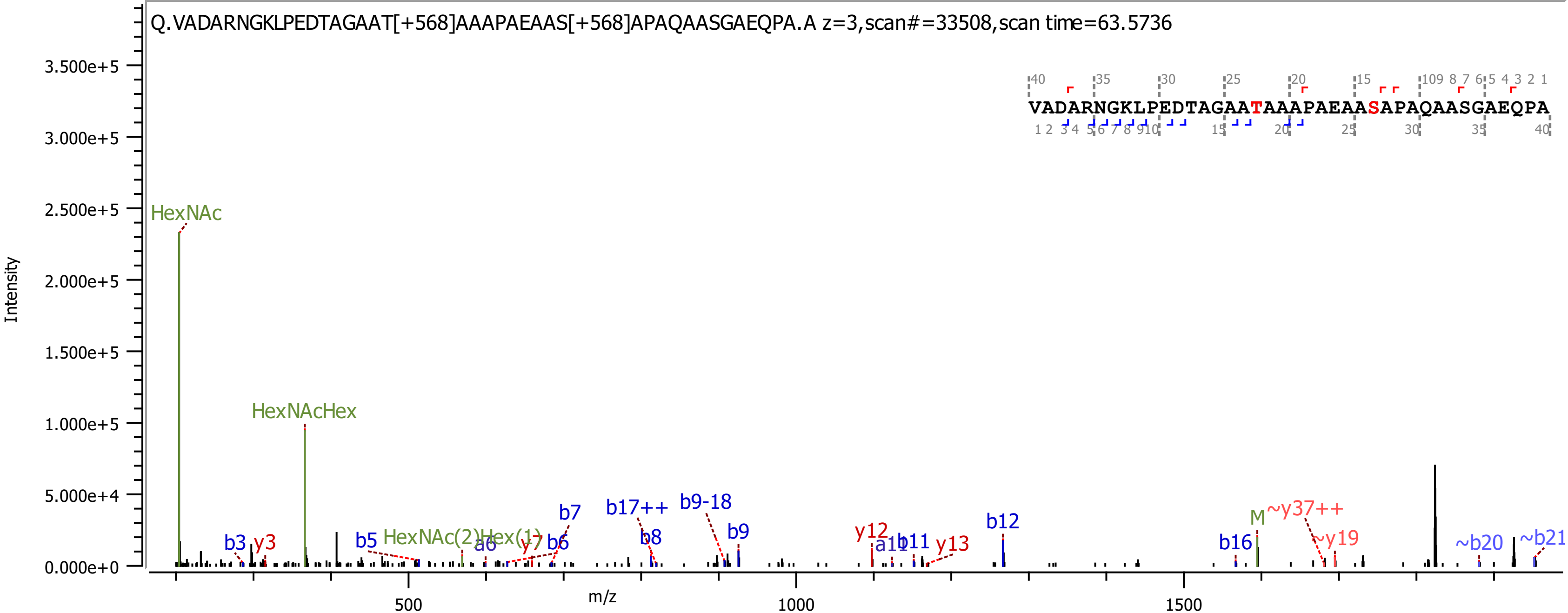

Q.VADARNGKLPEDTAGAAT[+568]AAAPAEAAS[+568]APAQAASGAEQPA.A z=3,scan#=33508,scan time=63.5736
VADARNGKLPEDTAGAATAAAPAEAASAPAQAASGAEQPA
Intensity
m/z
HexNAc
HexNAcHex
HexNAc(2)Hex(1)
b3
y3
b5
a6
y7
b6
b7
b17++
b8
b9-18
b9
y12
a11
b11
y13
b12
b16
M
~y37++
~y19
~b20
~b21

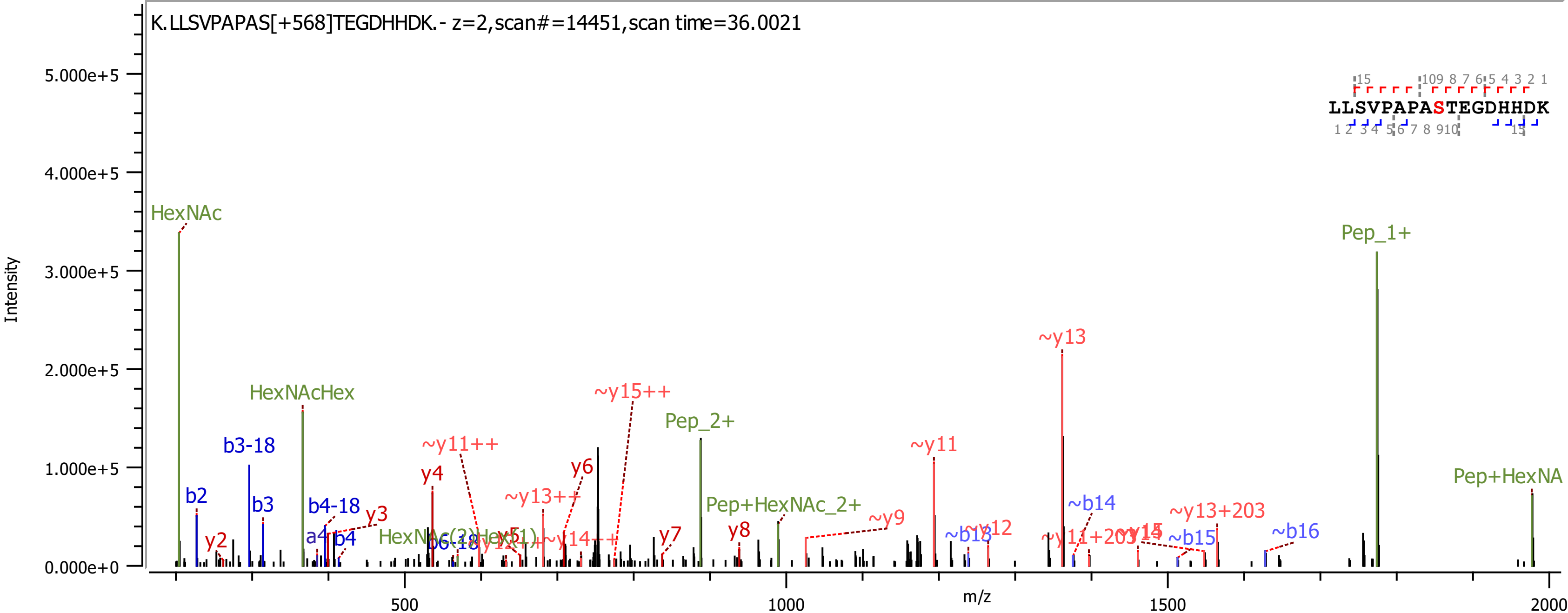
K.LLSVPAPAS[+568]TEGDHHDK.- z=2,scan#=14451,scan time=36.0021
LLSVPAPASTEGDHHDK
Intensity
5.000e+5
4.000e+5
3.000e+5
2.000e+5
1.000e+5
0.000e+0
m/z
500
1000
1500
2000
HexNAc
HexNAcHex
Pep_1+
Pep_2+
Pep+HexNAc_2+
Pep+HexNA
b2
b3-18
b3
b4-18
a4
b4
y2
y3
y4
y6
y7
y8
~y11++
~y13++
~y15++
~y14++
~y11
~y13
~y9
~y12
~b13
~b14
~b15
~b16
~y11+203
~y13+203
HexNAc(2)Hex(1)

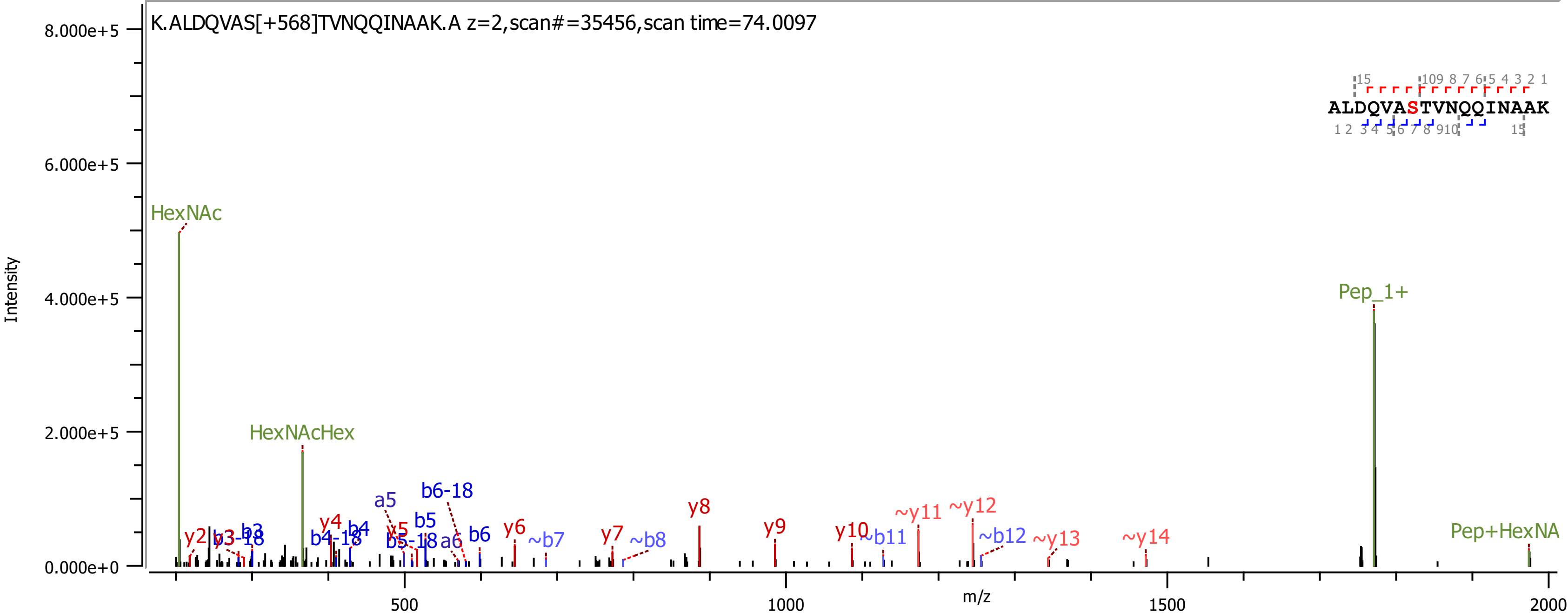

K.ALDQVAS[+568]TVNQQINAAK.A z=2,scan#=35456,scan time=74.0097
ALDQVASTVNQQINAAK
Intensity
8.000e+5
6.000e+5
4.000e+5
2.000e+5
0.000e+0
HexNAc
HexNAcHex
Pep_1+
Pep+HexNA
y2
b3-18
b3
y3
b4-18
y4
b4
a5
y5
b5-18
b5
b6-18
a6
b6
y6
~b7
y7
~b8
y8
y9
y10
~b11
~y11
~y12
~b12
~y13
~y14
500
1000
1500
2000
m/z

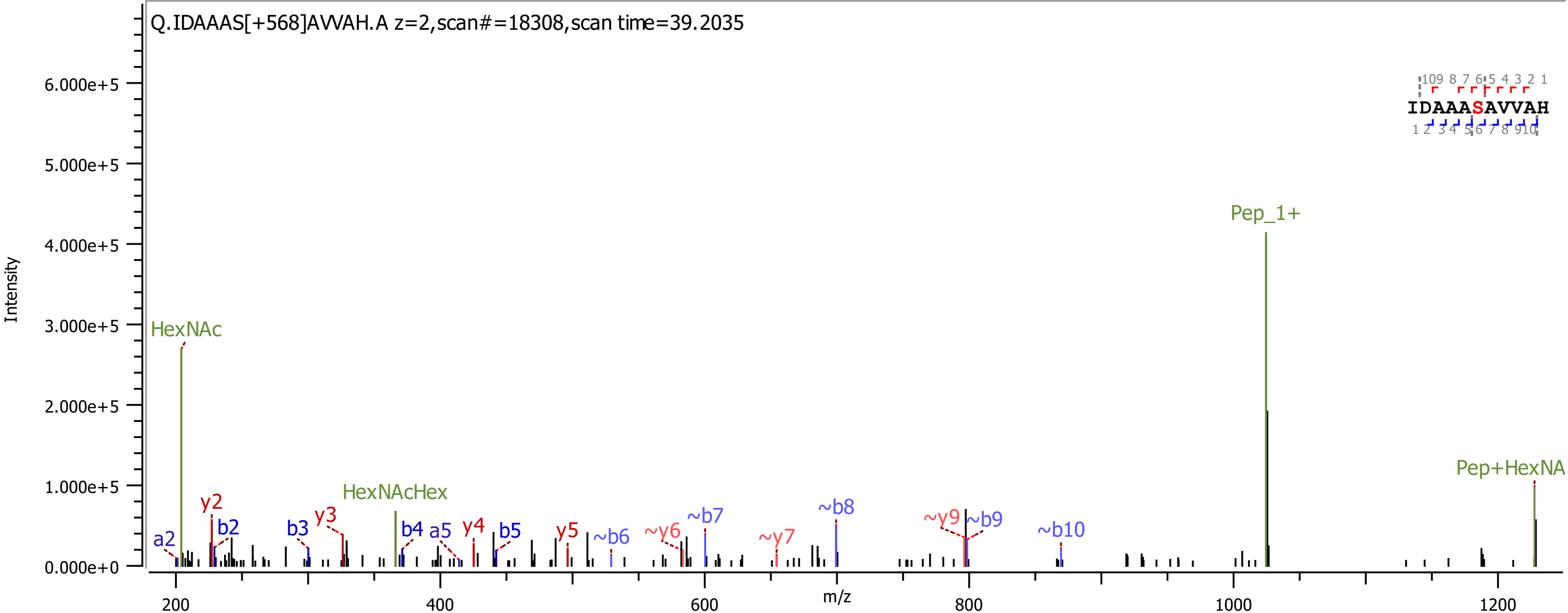
Q.IDAAAS[+568]AVVAH.A z=2,scan#=18308,scan time=39.2035
Intensity
m/z
IDAAASAVVAH
HexNAc
HexNAcHex
Pep_1+
Pep+HexNA
a2
y2
b2
b3
y3
b4
a5
y4
b5
y5
~b6
~y6
~b7
~y7
~b8
~y9
~b9
~b10

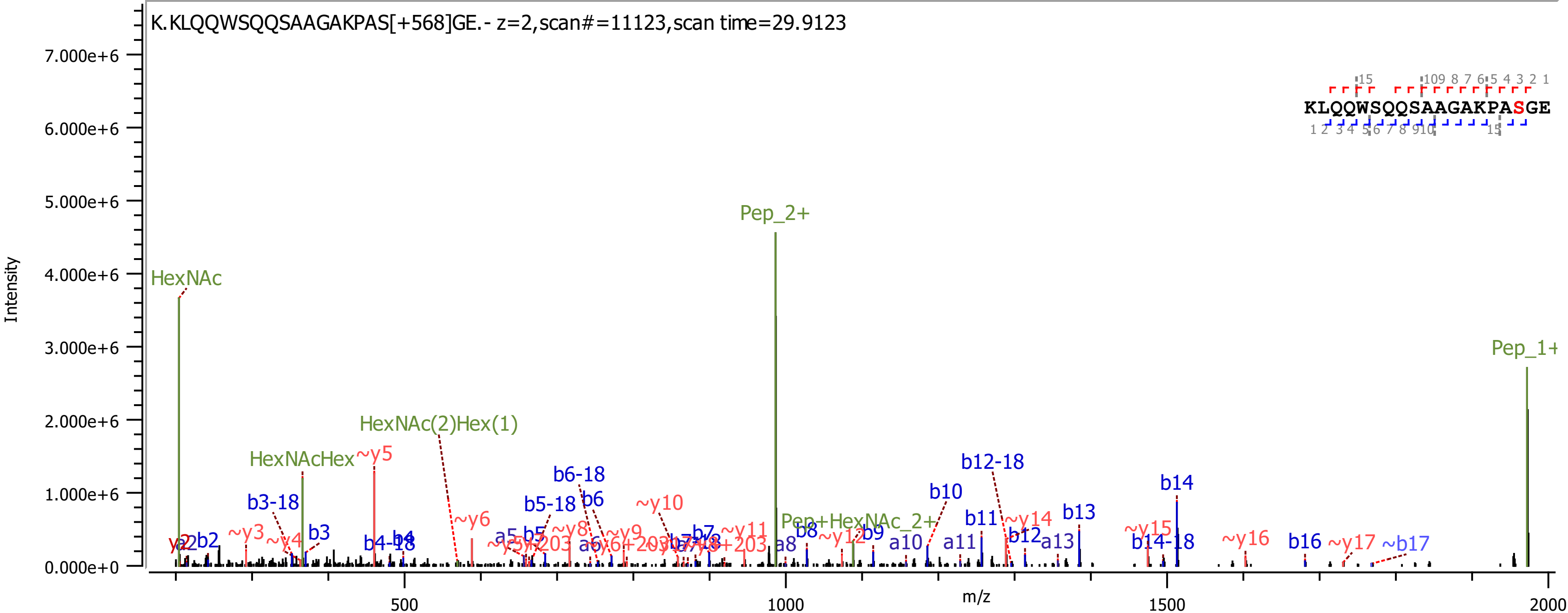

K.KLQQWSQQSAAGAKPAS[+568]GE.- z=2,scan#=11123,scan time=29.9123
KLQQWSQQSAAGAKPASGE
Intensity
m/z
7.000e+6
6.000e+6
5.000e+6
4.000e+6
3.000e+6
2.000e+6
1.000e+6
0.000e+0
500
1000
1500
2000
HexNAc
Pep_2+
Pep_1+
HexNAc(2)Hex(1)
HexNAcHex
~y5
b3-18
b2
~y3
~y4
b3
b4-18
b4
b6-18
b5-18
b6
~y6
a5
b5
~y8
~y9
~y10
b7
~y11
Pep+HexNAc_2+
b8
a8
~y12
b9
a10
a11
b10
b11
b12-18
~y14
b12
a13
b13
b14
~y15
b14-18
~y16
b16
~y17
~b17

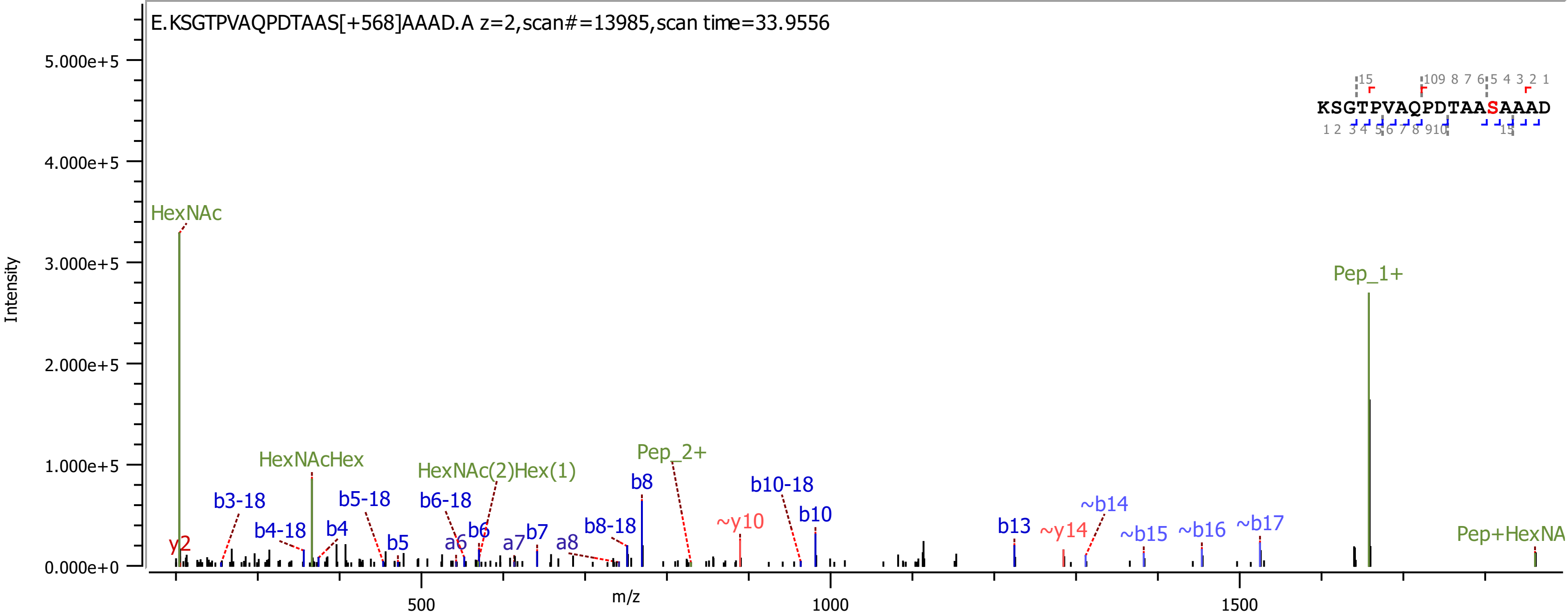

E.KSGTPVAQPDTAAS[+568]AAAD.A z=2,scan#=13985,scan time=33.9556
Intensity
m/z
HexNAc
Pep_1+
HexNAcHex
HexNAc(2)Hex(1)
Pep_2+
Pep+HexNA
y2
b3-18
b4-18
b4
b5-18
b5
b6-18
a6
b6
a7
b7
a8
b8-18
b8
~y10
b10-18
b10
b13
~y14
~b14
~b15
~b16
~b17
KSGTPVAQPDTAASAAAD

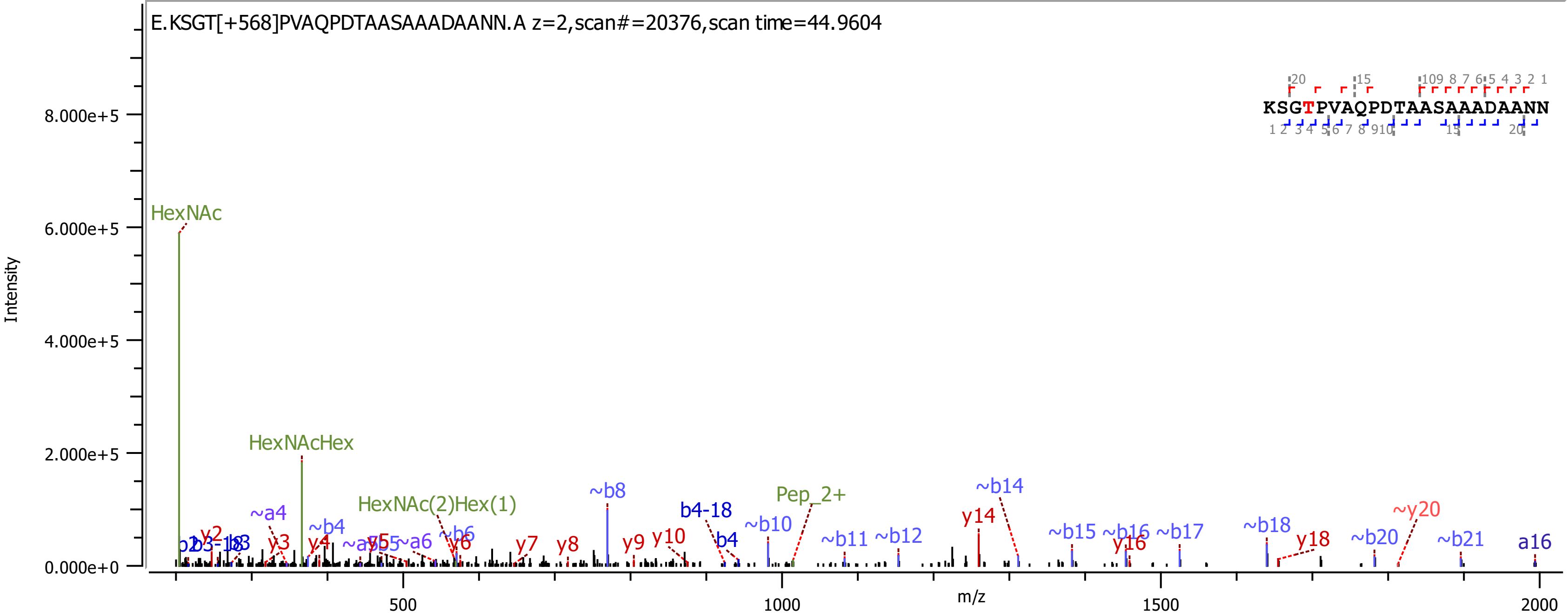
E.KSGT[+568]PVAQPDTAASAAADAANN.A z=2,scan#=20376,scan time=44.9604
KSGTPVAQPDTAASAAADAANN
Intensity
8.000e+5
6.000e+5
4.000e+5
2.000e+5
0.000e+0
HexNAc
HexNAcHex
HexNAc(2)Hex(1)
Pep_2+
~a4
~b4
~a6
~b6
y2
y3
y4
y5
y6
y7
y8
y9
y10
~b8
b4-18
b4
~b10
~b11
~b12
y14
~b14
~b15
~b16
y16
~b17
~b18
y18
~b20
~y20
~b21
a16
m/z
500
1000
1500
2000

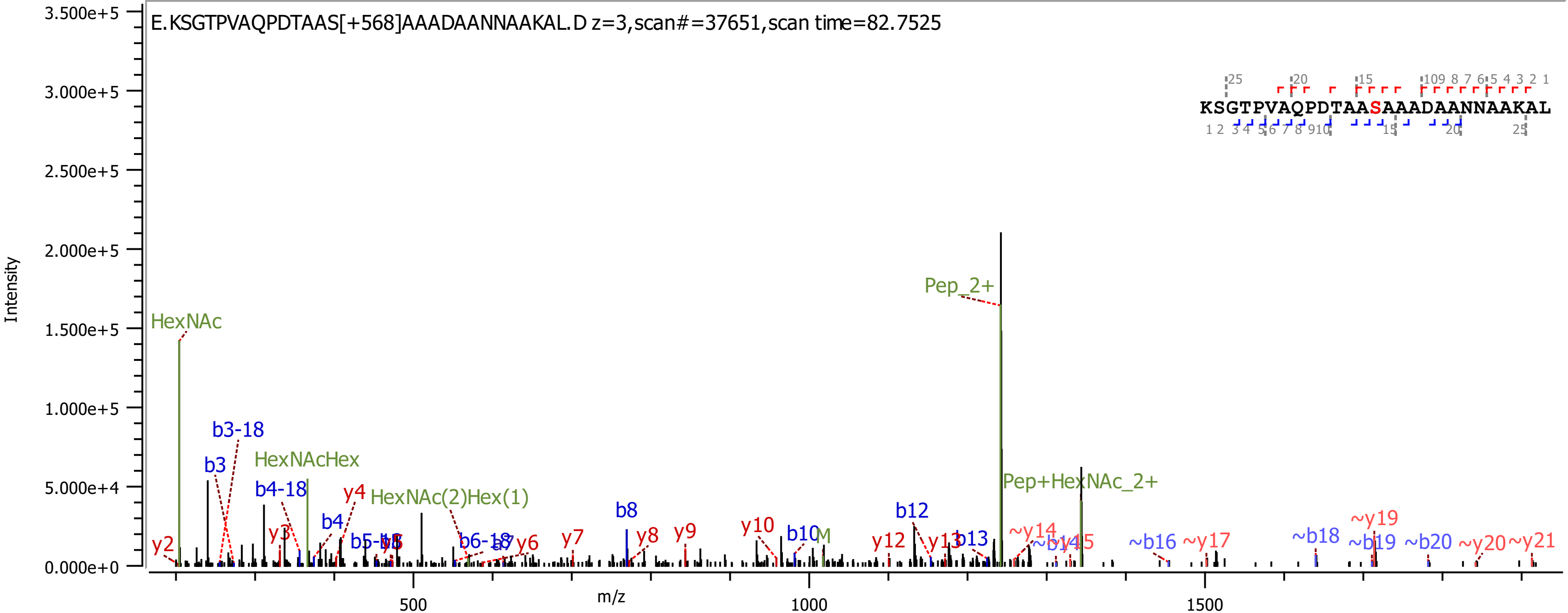

E.KSGTPVAQPDTAAS[+568]AAADAANNAAKAL.D z=3,scan#=37651,scan time=82.7525
KSGTPVAQPDTAASAAADAANNAAKAL
Intensity
m/z
3.500e+5
3.000e+5
2.500e+5
2.000e+5
1.500e+5
1.000e+5
5.000e+4
0.000e+0
500
1000
1500
HexNAc
b3-18
b3
HexNAcHex
b4-18
y4
HexNAc(2)Hex(1)
y2
y3
b4
b5-18
b6-18
a7
y6
y7
b8
y8
y9
y10
b10
M
y12
b12
y13
b13
Pep_2+
Pep+HexNAc_2+
~y14
~y15
~b16
~y17
~b18
~y19
~b19
~b20
~y20
~y21

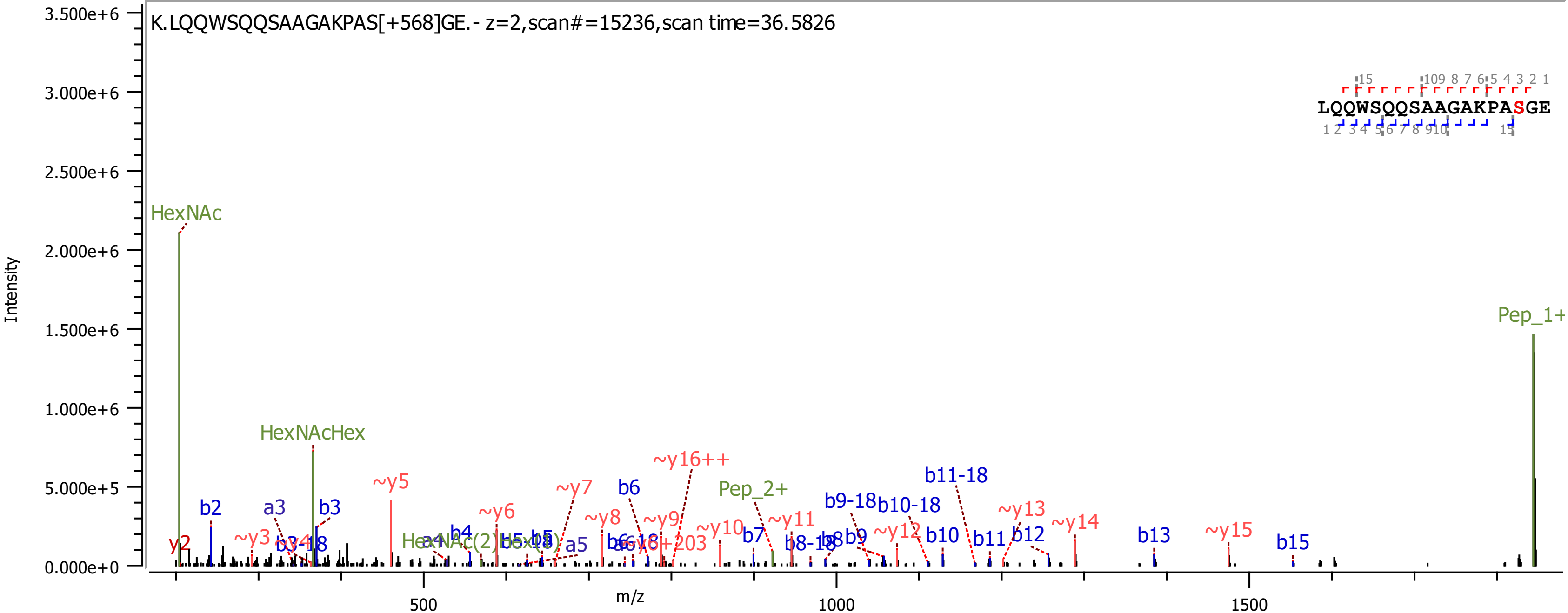
K.LQQWSQQSAAGAKPAS[+568]GE.- z=2,scan#=15236,scan time=36.5826
LQQWSQQSAAGAKPASGE
Intensity
m/z
3.500e+6
3.000e+6
2.500e+6
2.000e+6
1.500e+6
1.000e+6
5.000e+5
0.000e+0
500
1000
1500
HexNAc
HexNAcHex
Pep_1+
Pep_2+
~y16++
~y5
~y7
~y6
~y8
~y9
~y10
~y11
~y12
~y13
~y14
~y15
~y3
y2
b2
a3
b3
b4
a5
b6
b7
b8-18
b9
b9-18
b10-18
b10
b11-18
b11
b12
b13
b15

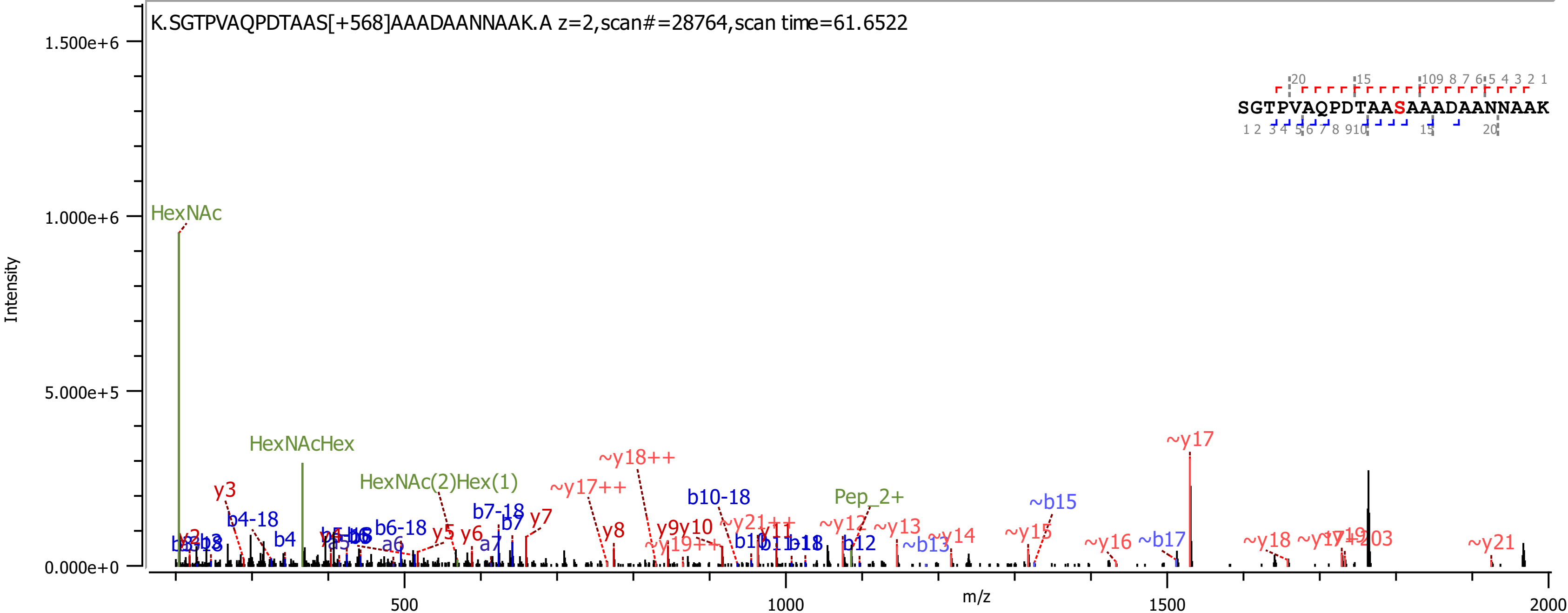

K.SGTPVAQPDTAAS[+568]AAADAANNAAK.A z=2,scan#=28764,scan time=61.6522
SGTPVAQPDTAASAAADAANNAAK
Intensity
m/z
HexNAc
HexNAcHex
HexNAc(2)Hex(1)
Pep_2+
~y17
~y18++
~y17++
b10-18
~b15
y3
b4-18
b7-18
y7
b7
b6-18
y5
y6
a7
a6
b4
y8
y9y10
~y19++
~y21++
y11
b12
~y12
~y13
~y14
~y15
~y16
~b17
~b13
~y18
~y21

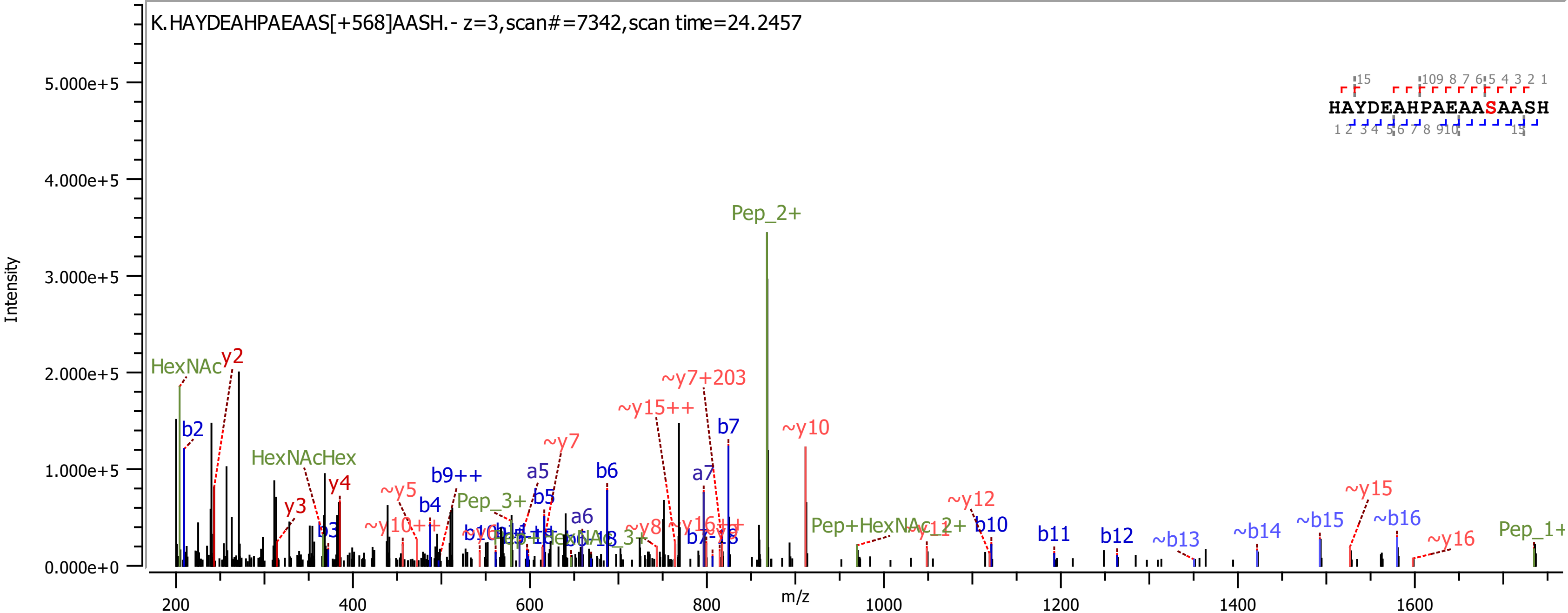
K.HAYDEAHPAEAAS[+568]AASH.- z=3,scan#=7342,scan time=24.2457
HAYDEAHPAEAASAASH
Intensity
m/z
Pep_2+
HexNAc
y2
b2
HexNAcHex
y3
y4
b3
~y5
b9++
b4
Pep_3+
a5
b5
~y7
b6
a6
~y15++
~y7+203
b7
a7
~y10
~y12
b10
b11
b12
~b13
~b14
~b15
~y15
~b16
~y16
Pep_1+
Pep+HexNAc_2+
~y8
~y11
~y10++
b7-18
0.000e+0
1.000e+5
2.000e+5
3.000e+5
4.000e+5
5.000e+5
200
400
600
800
1000
1200
1400
1600

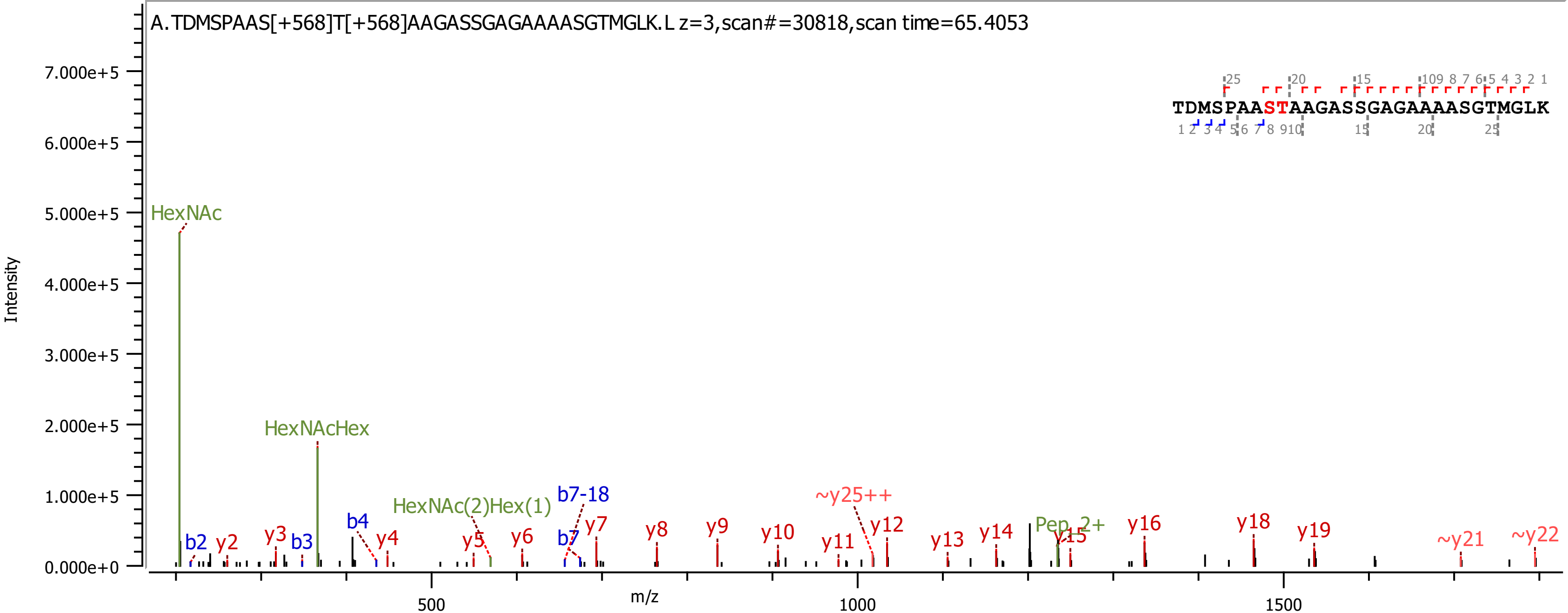

A.TDMSPAAS[+568]T[+568]AAGASSGAGAAAASGTMGLK.L z=3,scan#=30818,scan time=65.4053
TDMSPAASTAAGASSGAGAAAASGTMGLK
Intensity
m/z
HexNAc
HexNAcHex
HexNAc(2)Hex(1)
b2
y2
y3
b3
b4
y4
y5
y6
b7-18
b7
y7
y8
y9
y10
~y25++
y11
y12
y13
y14
Pep_2+
y15
y16
y18
y19
~y21
~y22

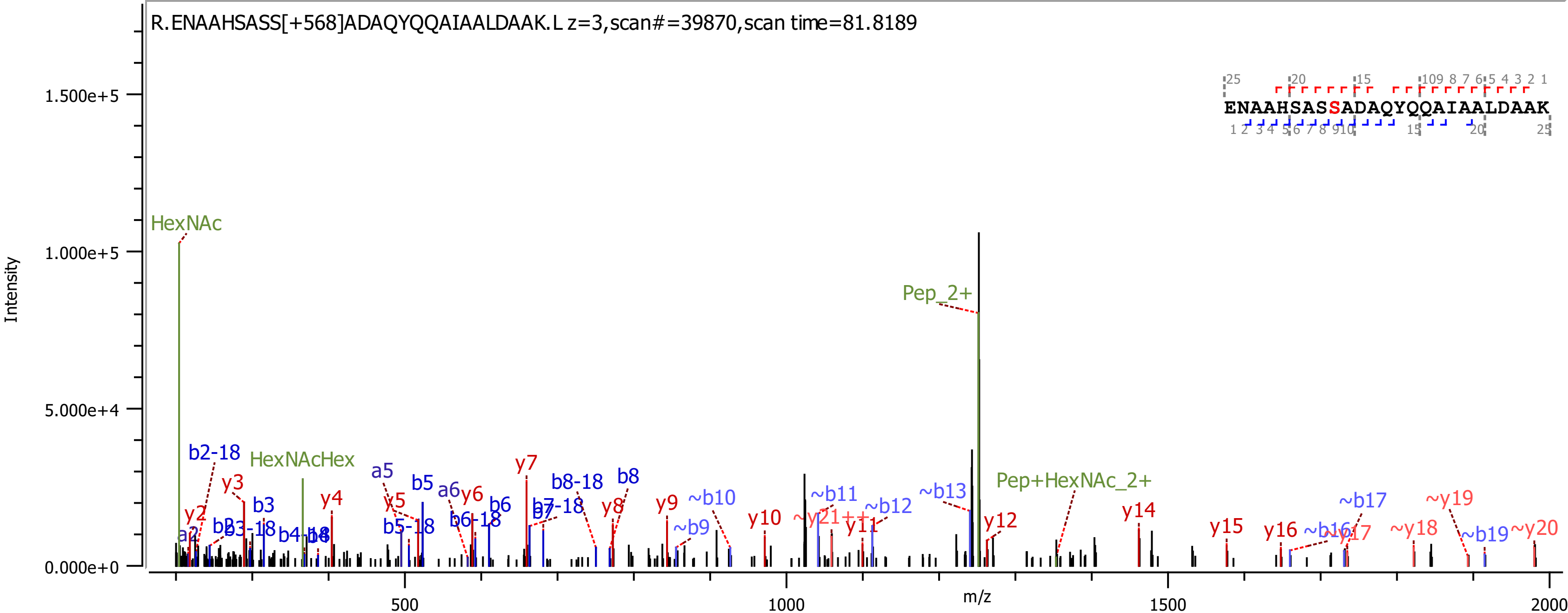
R.ENAAHSASS[+568]ADAQYQQAIAALDAAK.L z=3,scan#=39870,scan time=81.8189
ENAAHSASSADAQYQQAIAALDAAK
Intensity
1.500e+5
1.000e+5
5.000e+4
0.000e+0
m/z
500
1000
1500
2000
HexNAc
HexNAcHex
Pep_2+
Pep+HexNAc_2+
b2-18
y2
a2
y3
b2
b3-18
b3
b4
b4-18
y4
a5
y5
b5-18
b5
a6
y6
b6-18
b6
y7
b7
b7-18
b8-18
b8
y8
y9
~b9
~b10
y10
~y21++
~b11
y11
~b12
~b13
y12
y14
y15
y16
~b16
~y17
~b17
~y18
~y19
~b19
~y20

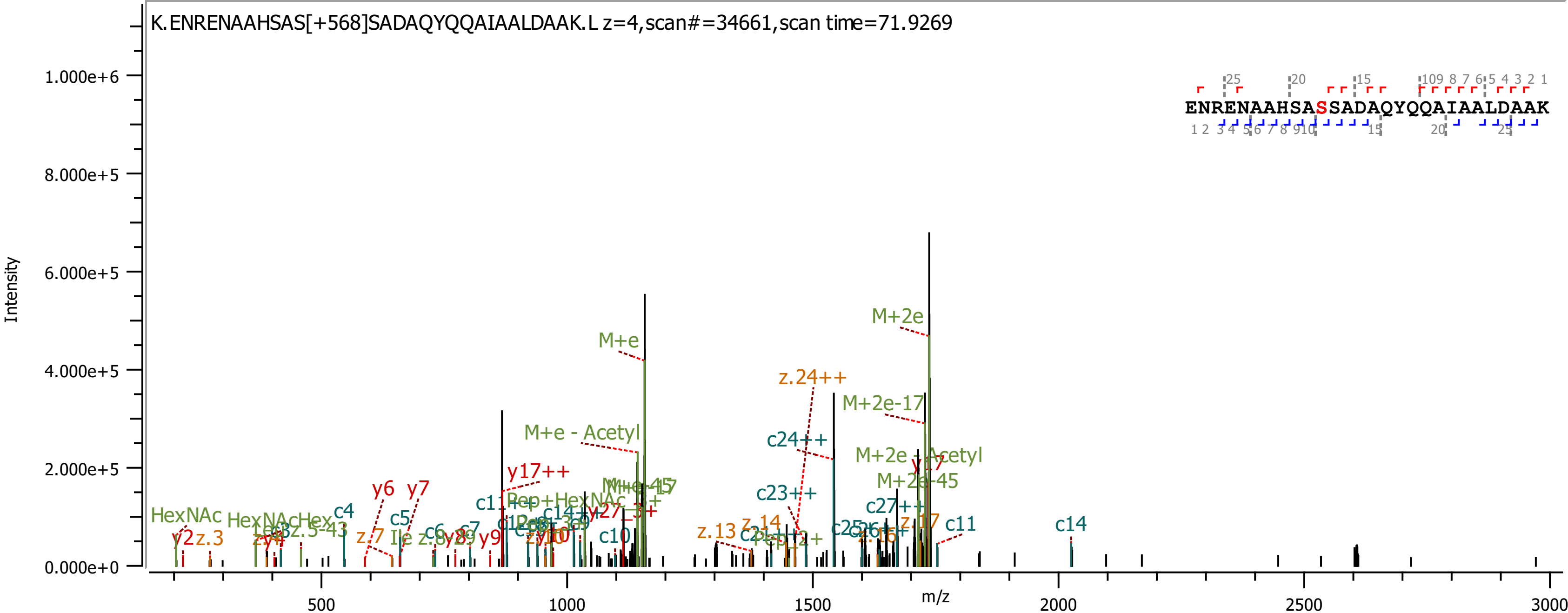
K.ENRENAAHSAS[+568]SADAQYQQAIAALDAAK.L z=4,scan#=34661,scan time=71.9269
ENRENAAHSASSADAQYQQAIAALDAAK
Intensity
m/z
1.000e+6
8.000e+5
6.000e+5
4.000e+5
2.000e+5
0.000e+0
500
1000
1500
2000
2500
3000
M+2e
M+e
z.24++
M+2e-17
M+e - Acetyl
c24++
M+2e - Acetyl
y17++
M+2e-45
c23++
c27++
y6
y7
c4
c5
HexNAc
z.3
y2
z.7
c6
c7
y9
c10
z.13
z.14
z.17
c11
c14

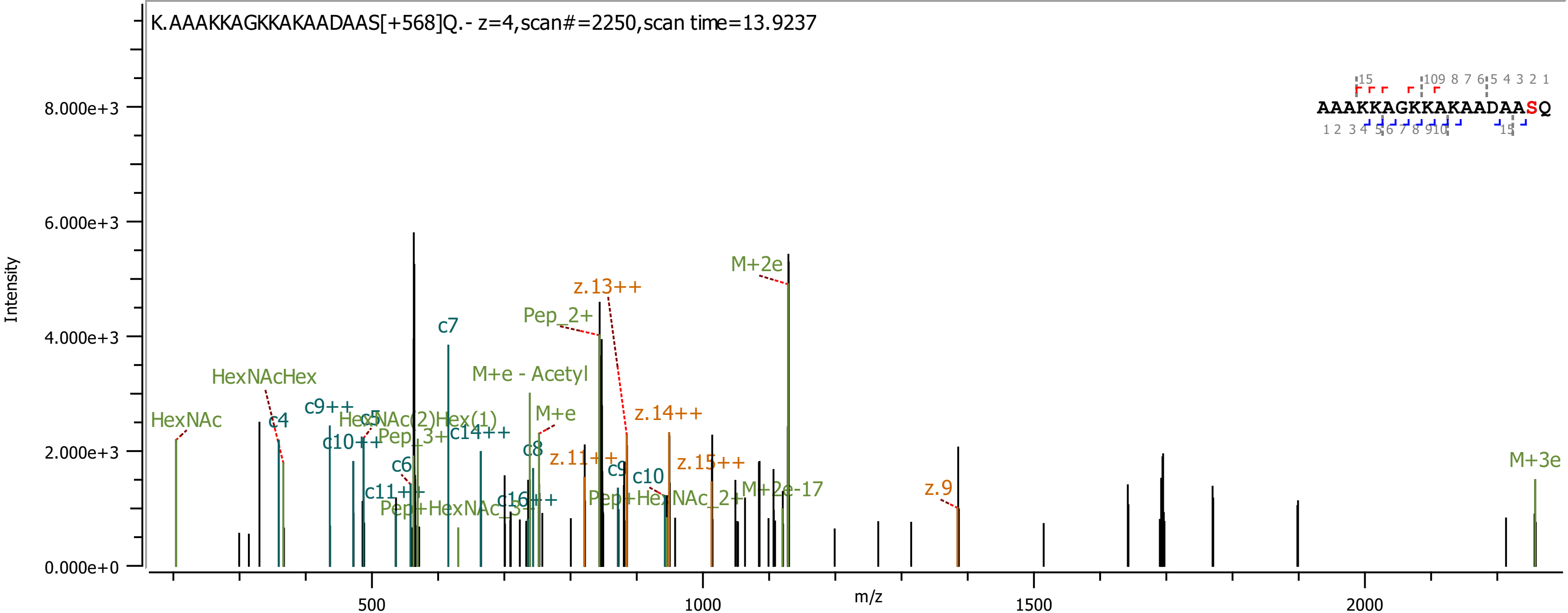

K.AAAKKAGKKAKAADAAS[+568]Q.- z=4,scan#=2250,scan time=13.9237
AAAKKAGKKAKAADAASQ
Intensity
m/z
8.000e+3
6.000e+3
4.000e+3
2.000e+3
0.000e+0
500
1000
1500
2000
HexNAc
HexNAcHex
c4
c9++
c5
HexNAc(2)Hex(1)
c10++
Pep_3+
c14++
c6
c11++
Pep+HexNAc_3+
c16++
c7
M+e - Acetyl
M+e
c8
Pep_2+
z.13++
z.11++
c9
z.14++
c10
Pep+HexNAc_2+
z.15++
M+2e-17
M+2e
z.9
M+3e

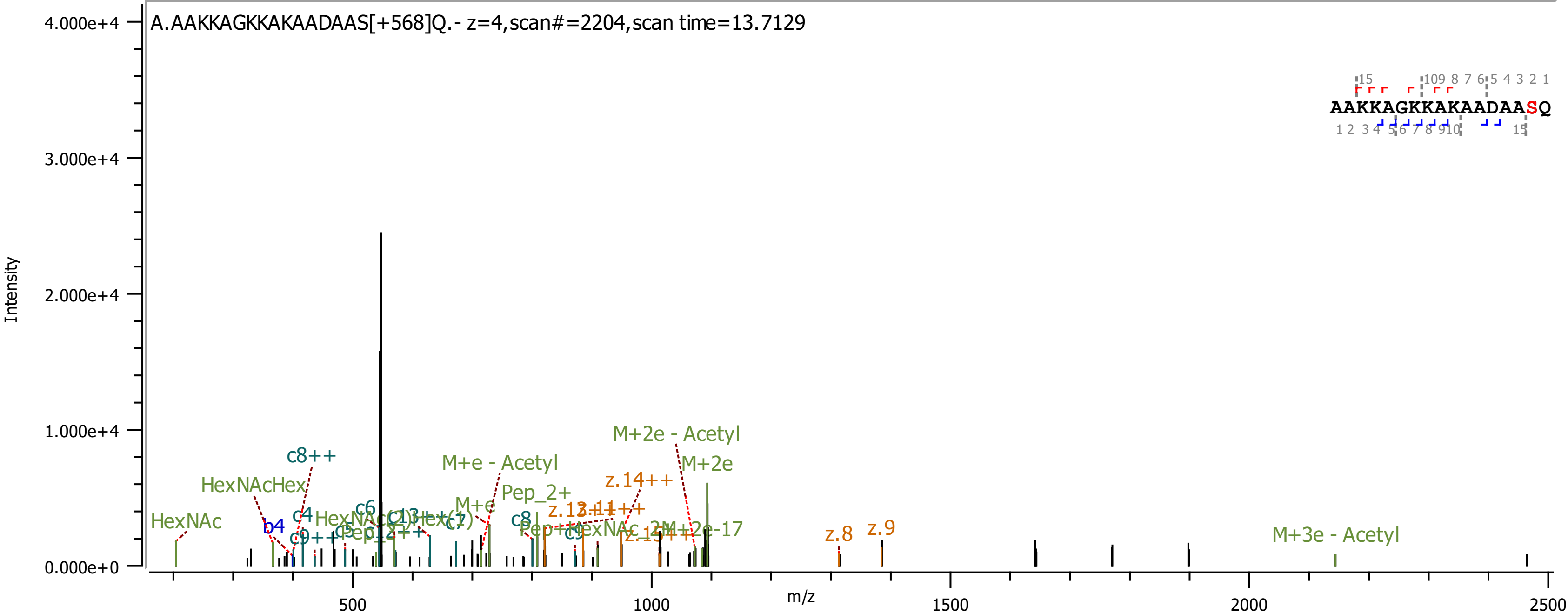

A.AAKKAGKKAKAADAAS[+568]Q.- z=4,scan#=2204,scan time=13.7129
Intensity
m/z
4.000e+4
3.000e+4
2.000e+4
1.000e+4
0.000e+0
500
1000
1500
2000
2500
AAKKAGKKAKAADAASQ
HexNAc
HexNAcHex
b4
c4
c8++
c9++
c6
c7
c8
c9
M+e - Acetyl
M+e
Pep_2+
M+2e - Acetyl
M+2e
M+2e-17
z.14++
z.8
z.9
M+3e - Acetyl

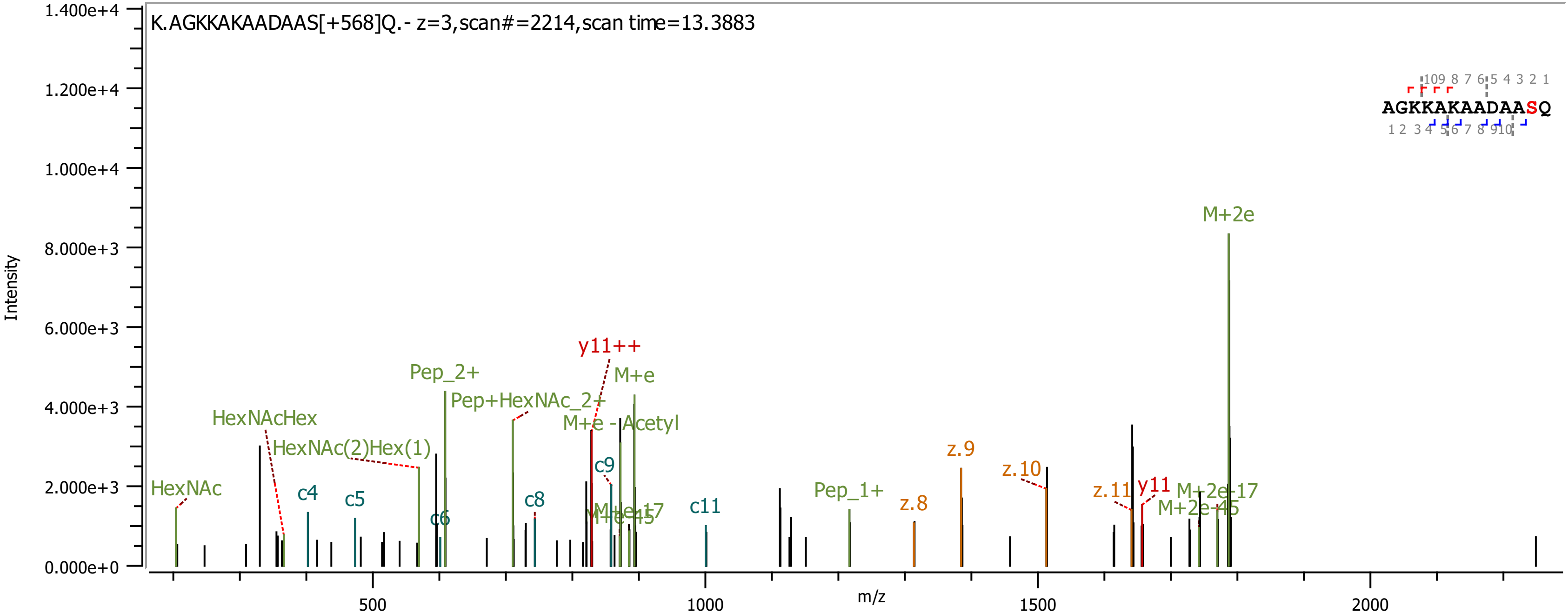

K.AGKKAKAADAAS[+568]Q.- z=3,scan#=2214,scan time=13.3883
Intensity
m/z
AGKKAKAADAASQ
HexNAc
HexNAcHex
HexNAc(2)Hex(1)
c4
c5
c6
Pep_2+
Pep+HexNAc_2+
c8
y11++
M+e
M+e - Acetyl
c9
M+e-17
M+e-45
c11
Pep_1+
z.8
z.9
z.10
z.11
y11
M+2e-17
M+2e-45
M+2e

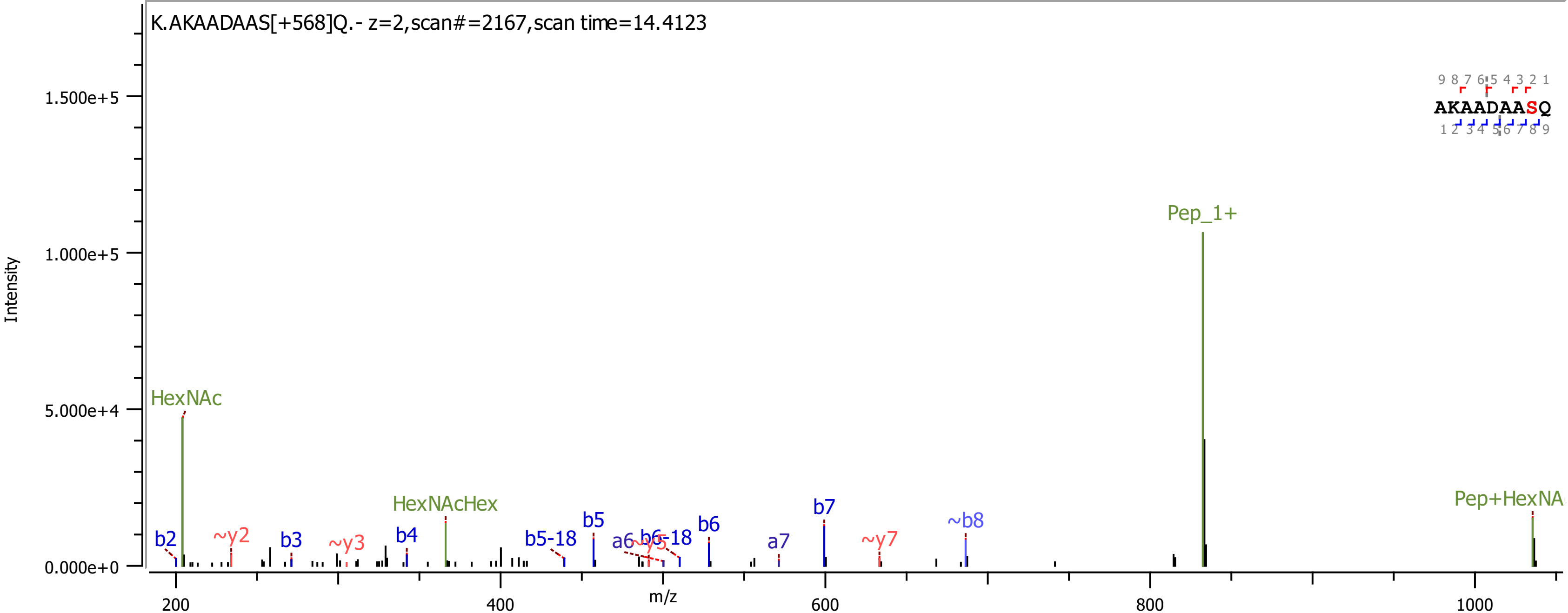
K.AKAADAAS[+568]Q.- z=2,scan#=2167,scan time=14.4123
9 8 7 6 5 4 3 2 1
AKAADAASQ
1 2 3 4 5 6 7 8 9
Intensity
1.500e+5
1.000e+5
5.000e+4
0.000e+0
Pep_1+
HexNAc
HexNAcHex
Pep+HexNA
b2
~y2
b3
~y3
b4
b5-18
b5
a6
~y5
b6-18
b6
a7
b7
~y7
~b8
200
400
600
800
1000
m/z

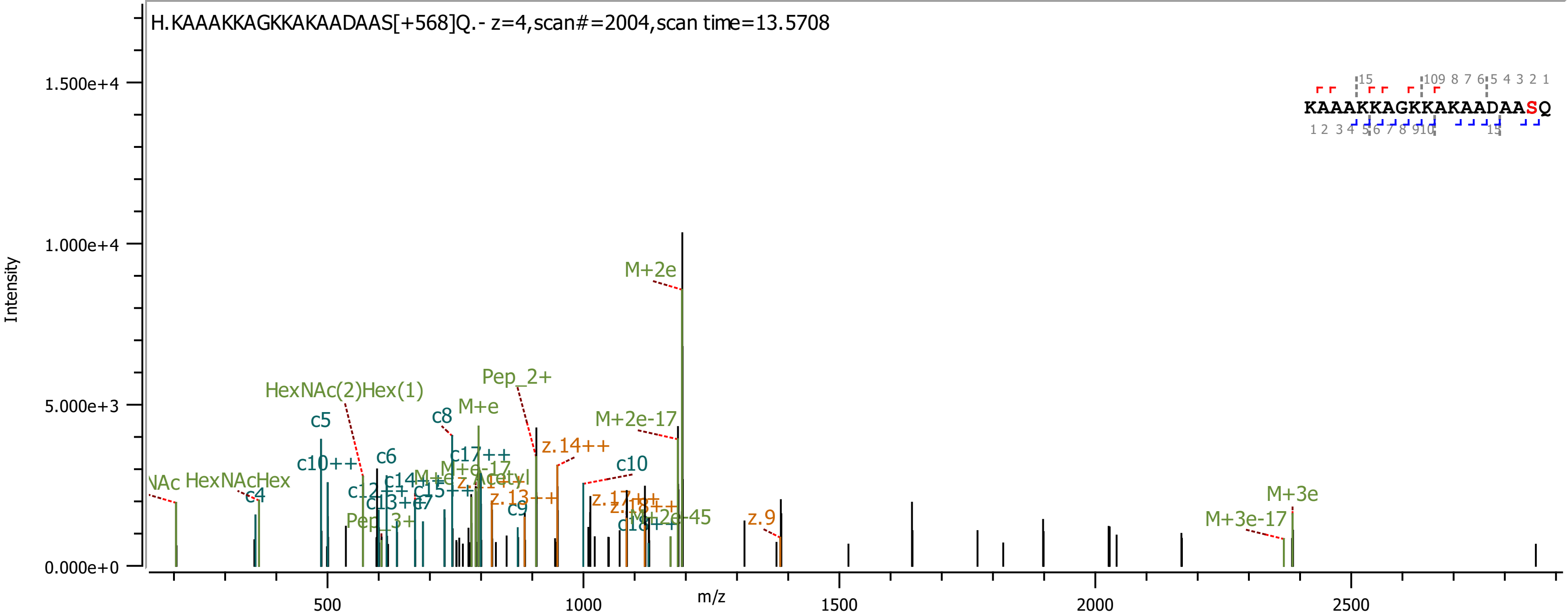
H.KAAAKKAGKKAKAADAAS[+568]Q.- z=4,scan#=2004,scan time=13.5708
KAAAKKAGKKAKAADAASQ
Intensity
1.500e+4
1.000e+4
5.000e+3
0.000e+0
m/z
500
1000
1500
2000
2500
M+2e
Pep_2+
HexNAc(2)Hex(1)
c5
c8
M+e
M+2e-17
z.14++
c6
c10++
c17++
c10
HexNAcHex
c4
z.9
M+3e
M+3e-17
Pep_3+
c9
M+2e-45

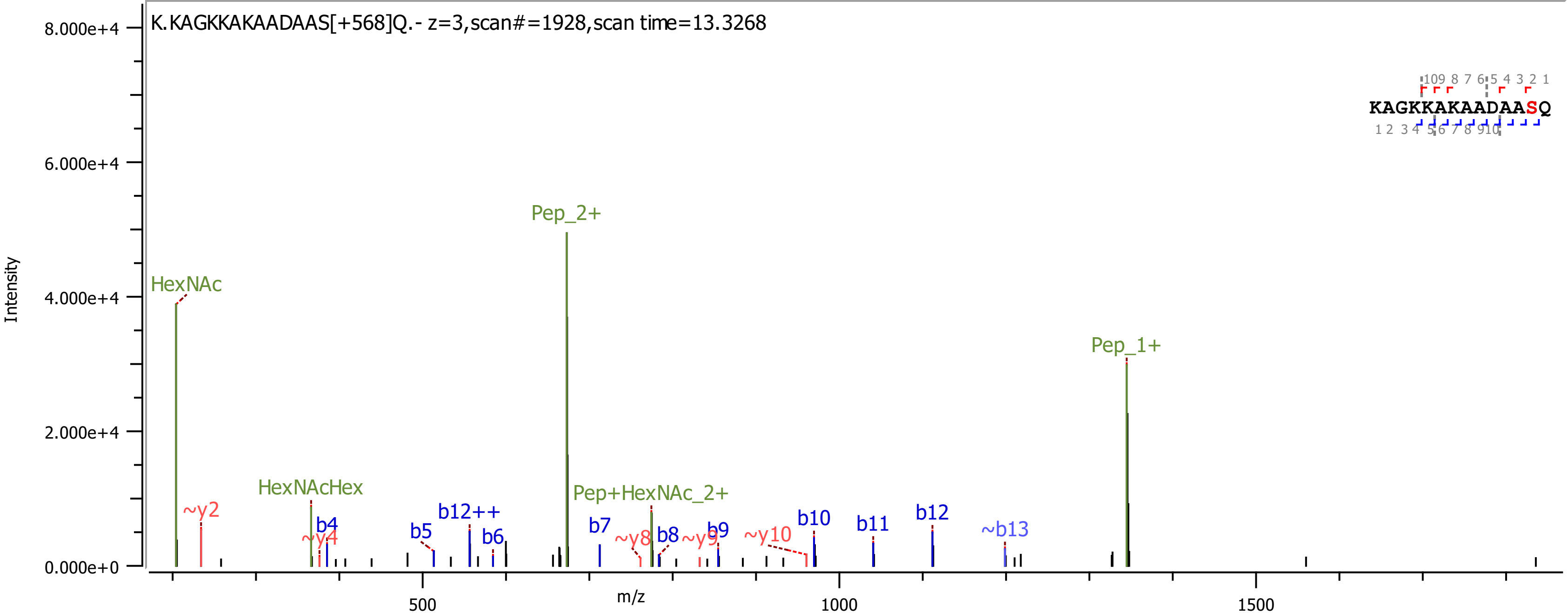
K.KAGKKAKAADAAS[+568]Q.- z=3,scan#=1928,scan time=13.3268
KAGKKAKAADAASQ
Intensity
m/z
HexNAc
~y2
HexNAcHex
~y4
b4
b5
b12++
b6
Pep_2+
Pep+HexNAc_2+
b7
~y8
b8
~y9
b9
~y10
b10
b11
b12
~b13
Pep_1+

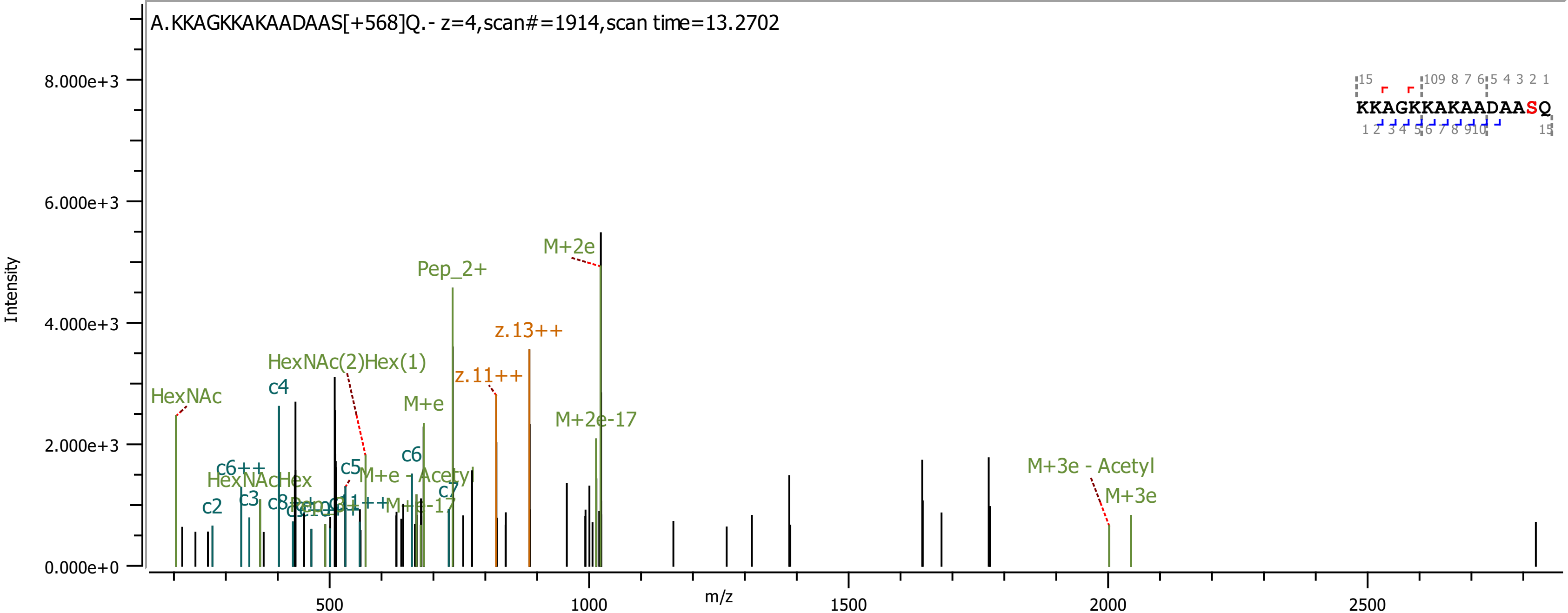

A.KKAGKKAKAADAAS[+568]Q.- z=4,scan#=1914,scan time=13.2702
Intensity
m/z
8.000e+3
6.000e+3
4.000e+3
2.000e+3
0.000e+0
500
1000
1500
2000
2500
KKAGKKAKAADAASQ
HexNAc
HexNAc(2)Hex(1)
c4
Pep_2+
M+2e
z.13++
z.11++
M+e
M+2e-17
c6
c6++
c5
HexNAcHex
M+e - Acetyl
c7
c2
c3
c8
M+e-17
M+3e - Acetyl
M+3e

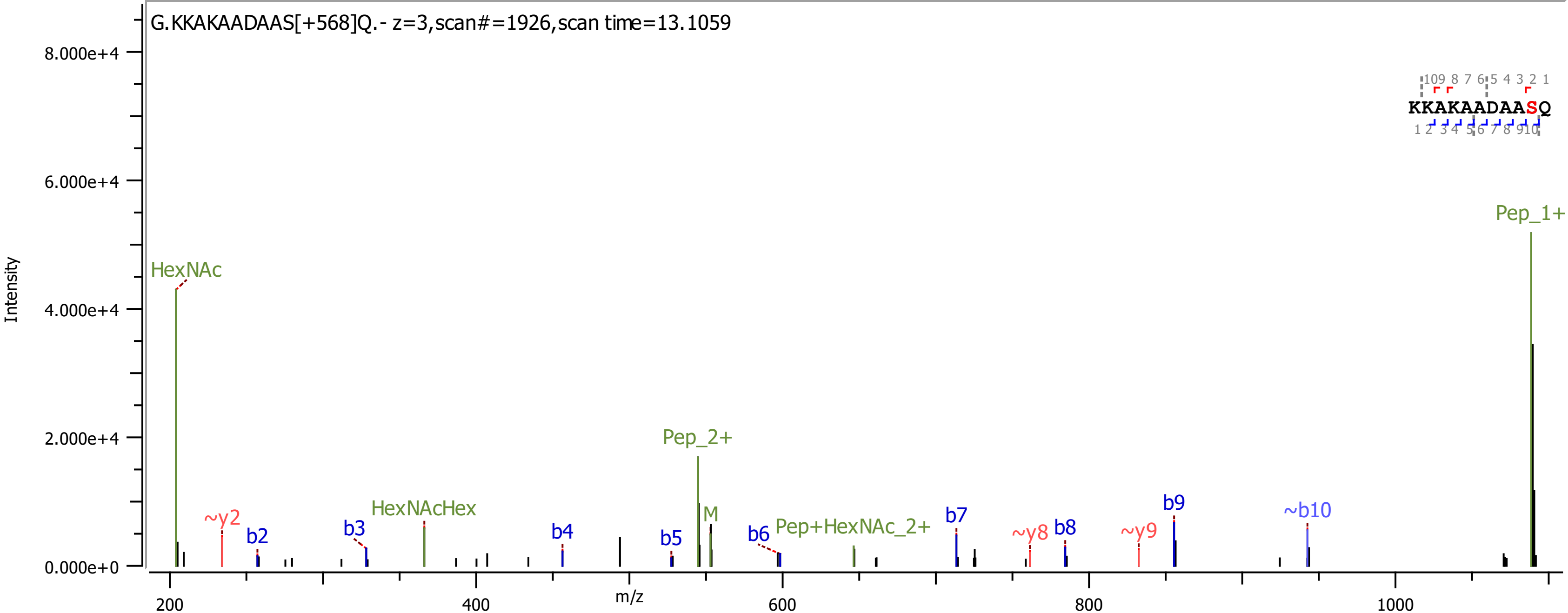

G.KKAKAADAAS[+568]Q.- z=3,scan#=1926,scan time=13.1059
Intensity
m/z
HexNAc
~y2
b2
b3
HexNAcHex
b4
b5
Pep_2+
M
b6
Pep+HexNAc_2+
b7
~y8
b8
~y9
b9
~b10
Pep_1+
KKAKAADAASQ

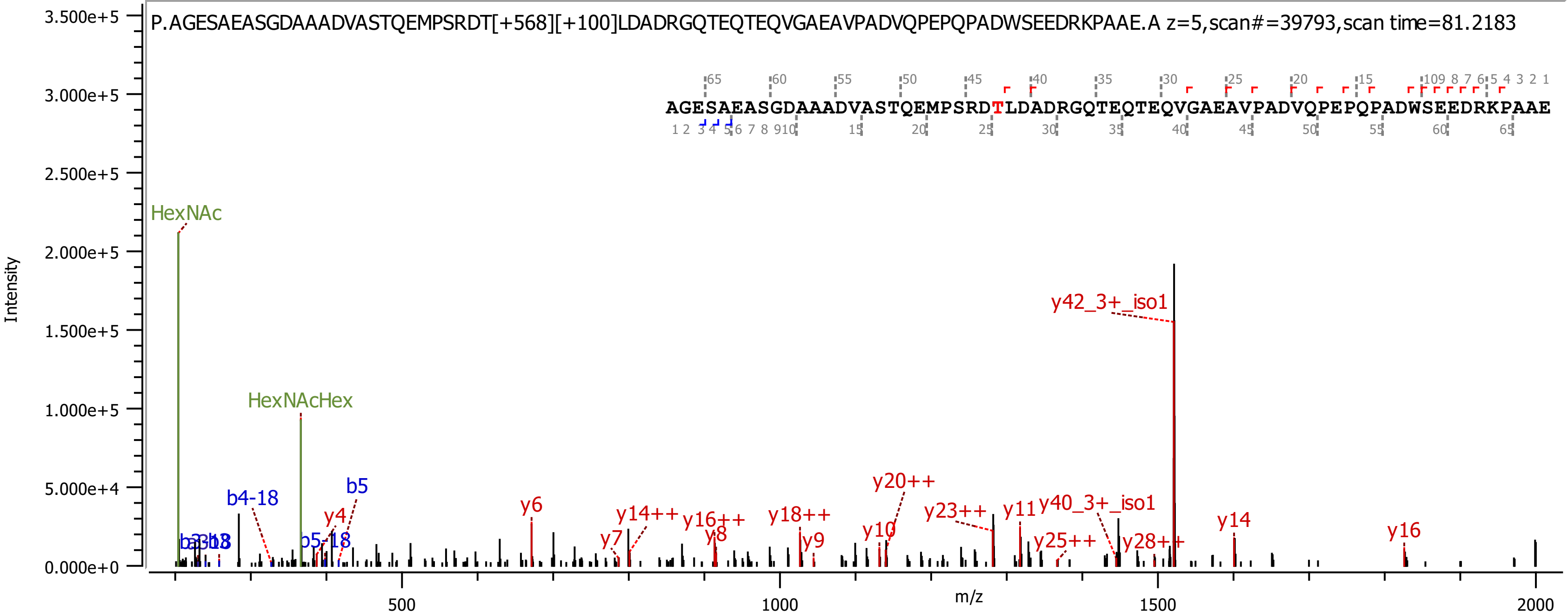
P.AGESAEASGDAAADVASTQEMPSRDT[+568][+100]LDADRGQTEQTEQVGAEAVPADVQPEPQPADWSEEDRKPAAE.A z=5,scan#=39793,scan time=81.2183
AGESAEASGDAAADVASTQEMPSRDTLDADRGQTEQTEQVGAEAVPADVQPEPQPADWSEEDRKPAAE
Intensity
m/z
HexNAc
HexNAcHex
b4-18
b5
b5-18
y4
y6
y7
y14++
y16++
y8
y18++
y9
y10
y20++
y23++
y11
y40_3+_iso1
y42_3+_iso1
y25++
y28++
y14
y16

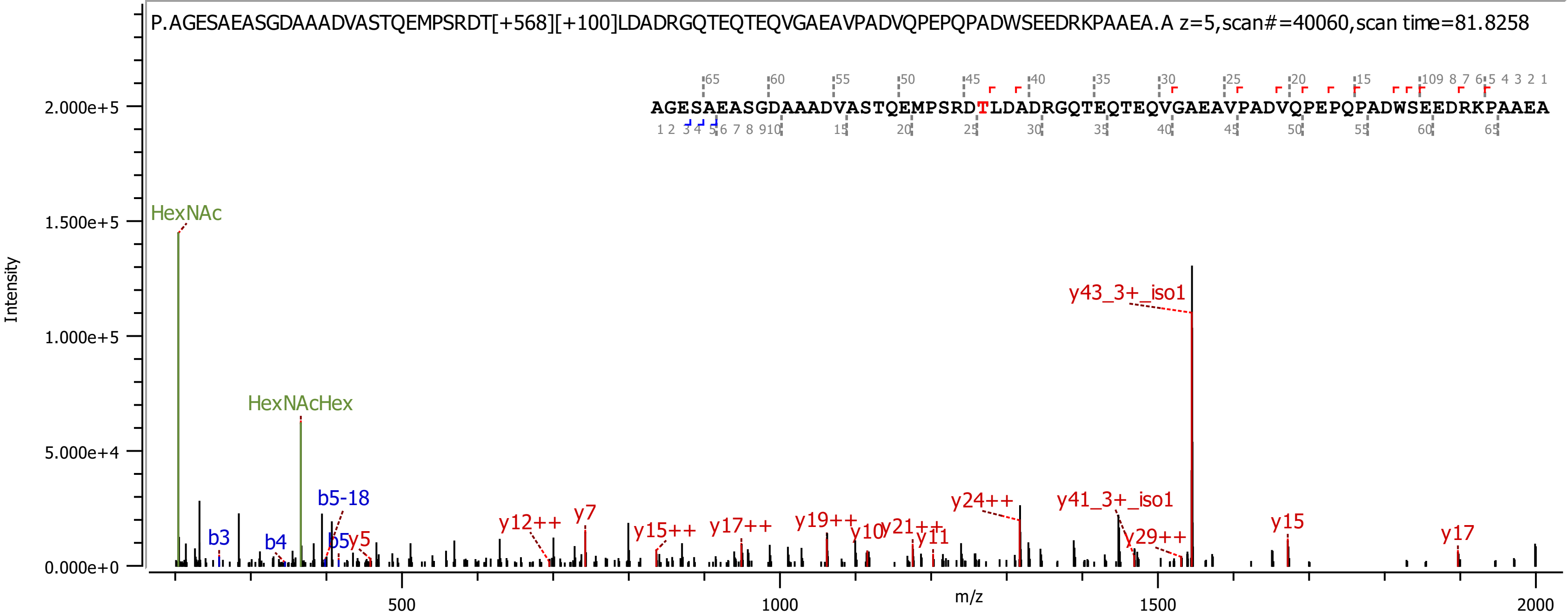

P.AGESAEASGDAAADVASTQEMPSRDT[+568][+100]LDADRGQTEQTEQVGAEAVPADVQPEPQPADWSEEDRKPAAEA.A z=5,scan#=40060,scan time=81.8258
AGESAEASGDAAADVASTQEMPSRDTLDADRGQTEQTEQVGAEAVPADVQPEPQPADWSEEDRKPAAEA
Intensity
m/z
HexNAc
HexNAcHex
b3
b4
b5-18
b5
y5
y12++
y7
y15++
y17++
y19++
y10
y21++
y11
y24++
y43_3+_iso1
y41_3+_iso1
y29++
y15
y17

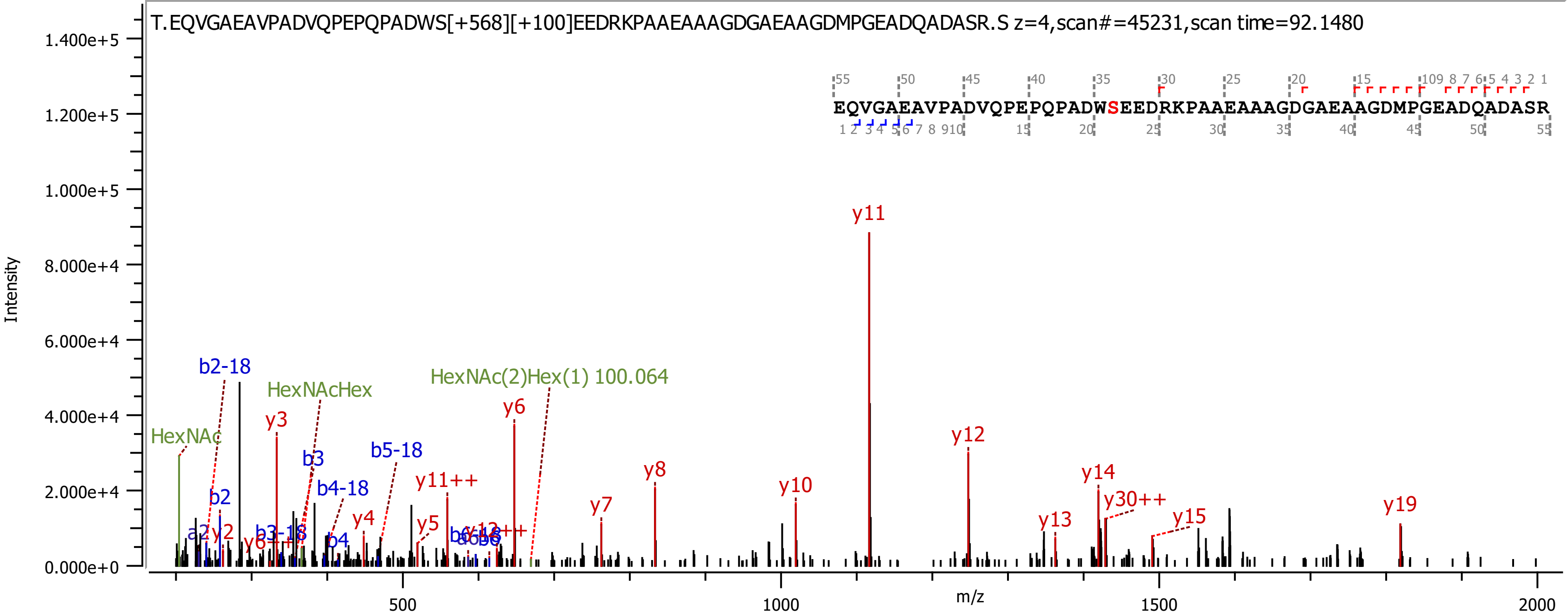

T.EQVGAEAVPADVQPEPQPADWS[+568][+100]EEDRKPAAEAAAGDGAEAAGDMPGEADQADASR.S z=4,scan#=45231,scan time=92.1480
EQVGAEAVPADVQPEPQPADWSEEDRKPAAEAAAGDGAEAAGDMPGEADQADASR
Intensity
m/z
HexNAc
HexNAcHex
HexNAc(2)Hex(1) 100.064
b2-18
b2
y2
y3
b3
b3-18
b4-18
b4
b5-18
y4
y11++
y5
y6
y7
y8
y10
y11
y12
y13
y14
y30++
y15
y19

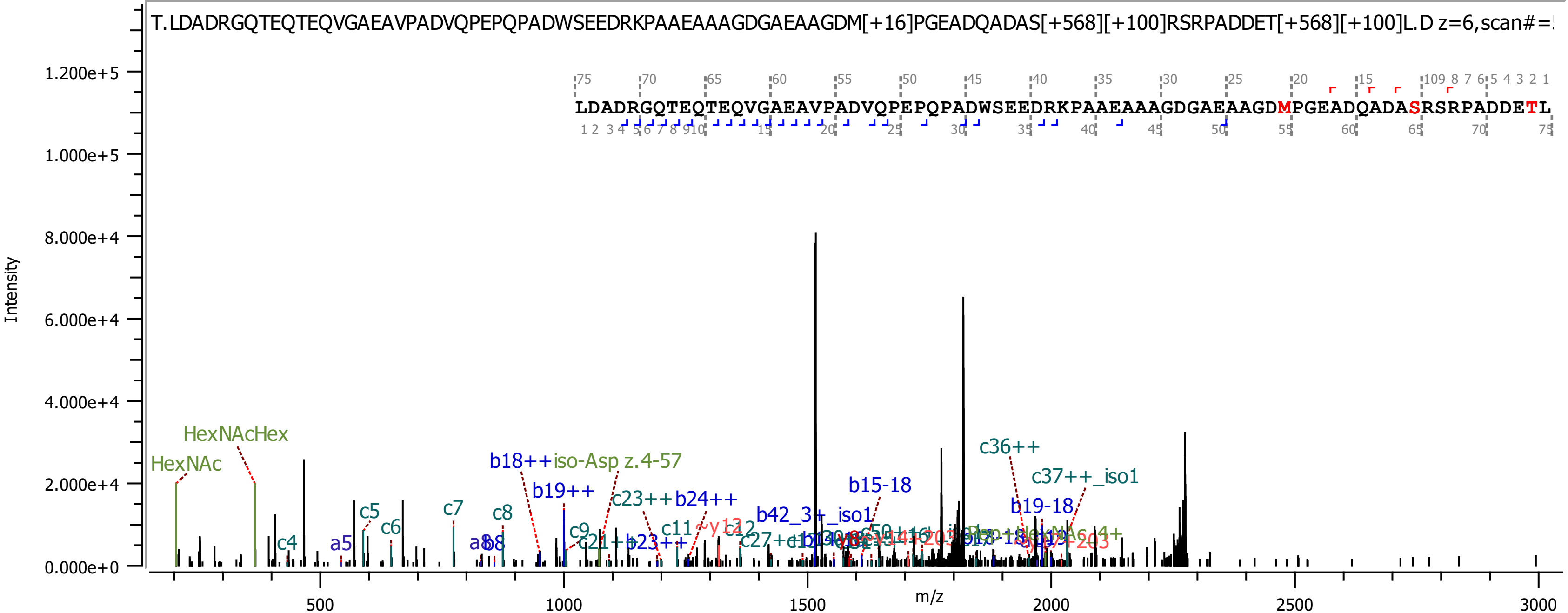

T.LDADRGQTEQTEQVGAEAVPADVQPEPQPADWSEEDRKPAAEAAAGDGAEAAGDM[+16]PGEADQADAS[+568][+100]RSRPADDET[+568][+100]L.D z=6,scan#=
LDADRGQTEQTEQVGAEAVPADVQPEPQPADWSEEDRKPAAEAAAGDGAEAAGDMPGEADQADASRSRPADDETL
Intensity
m/z
1.200e+5
1.000e+5
8.000e+4
6.000e+4
4.000e+4
2.000e+4
0.000e+0
500
1000
1500
2000
2500
3000
HexNAc
HexNAcHex
c4
a5
c5
c6
c7
a8
b8
c8
b18++
iso-Asp z.4-57
b19++
c9
c21++
b23++
c23++
b24++
c11
c12
c27++
b42_3+_iso1
b15-18
c36++
c37++_iso1
b19-18

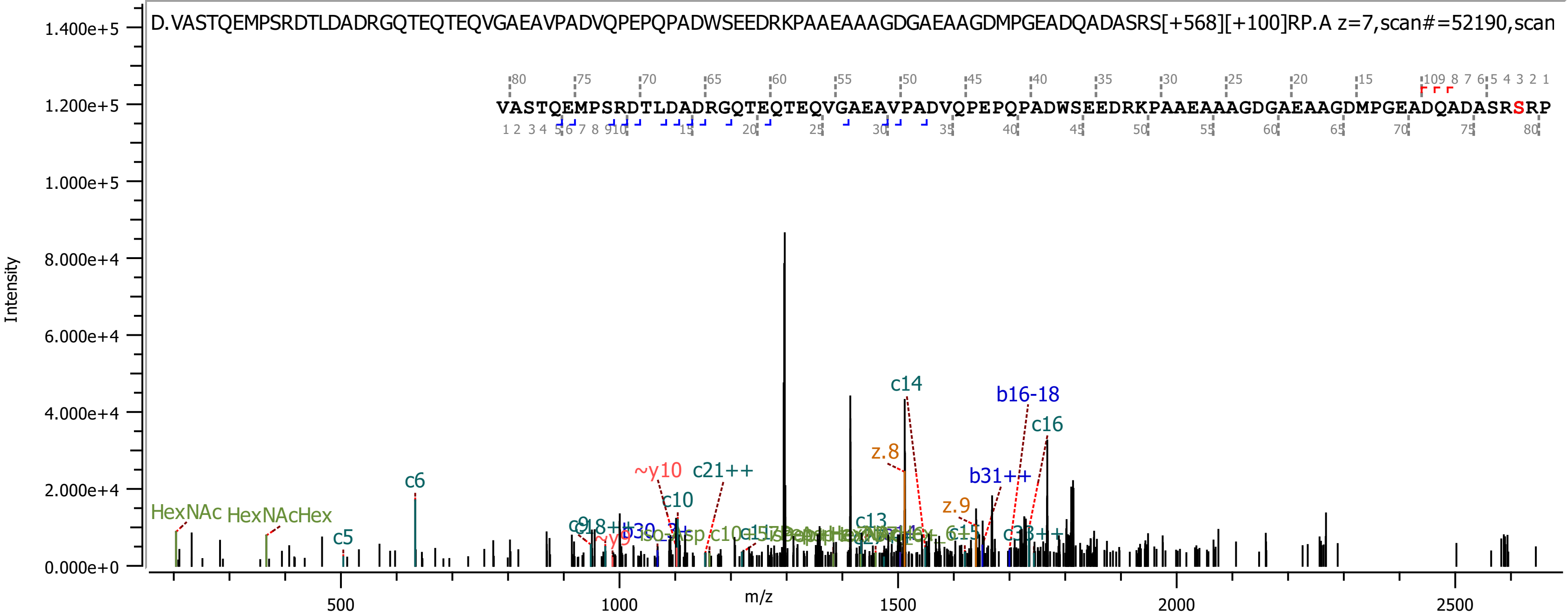

D.VASTQEMPSRDTLDADRGQTEQTEQVGAEAVPADVQPEPQPADWSEEDRKPAAEAAAGDGAEAAGDMPGEADQADASRS[+568][+100]RP.A z=7,scan#=52190,scan
VASTQEMPSRDTLDADRGQTEQTEQVGAEAVPADVQPEPQPADWSEEDRKPAAEAAAGDGAEAAGDMPGEADQADASRSRP
Intensity
m/z
1.400e+5
1.200e+5
1.000e+5
8.000e+4
6.000e+4
4.000e+4
2.000e+4
0.000e+0
500
1000
1500
2000
2500
HexNAc
HexNAcHex
c5
c6
~y10
c21++
c10
c14
z.8
b16-18
c16
b31++
z.9
c13

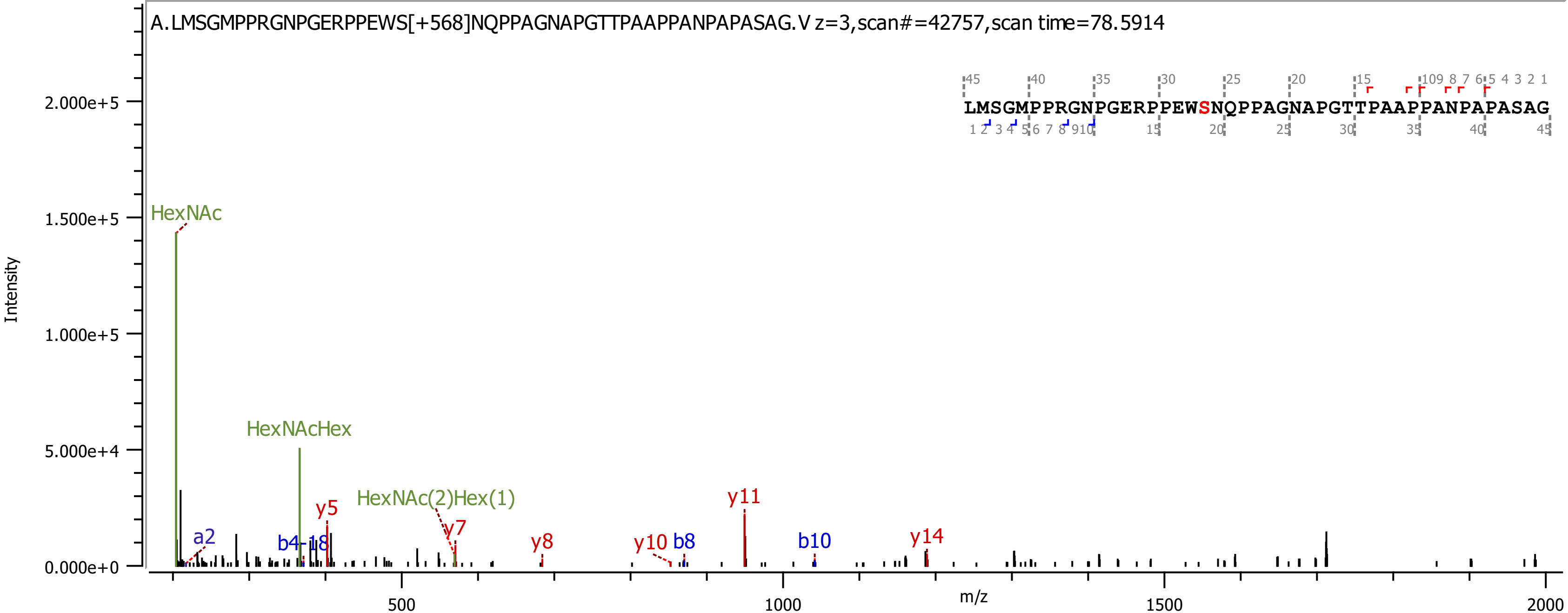

A.LMSGMPPRGNPGERPPEWS[+568]NQPPAGNAPGTTPAAPPANPAPASAG.V z=3,scan#=42757,scan time=78.5914
LMSGMPPRGNPGERPPEWSNQPPAGNAPGTTPAAPPANPAPASAG
Intensity
m/z
2.000e+5
1.500e+5
1.000e+5
5.000e+4
0.000e+0
500
1000
1500
2000
HexNAc
HexNAcHex
HexNAc(2)Hex(1)
a2
b4-18
y5
y7
y8
y10
b8
y11
b10
y14

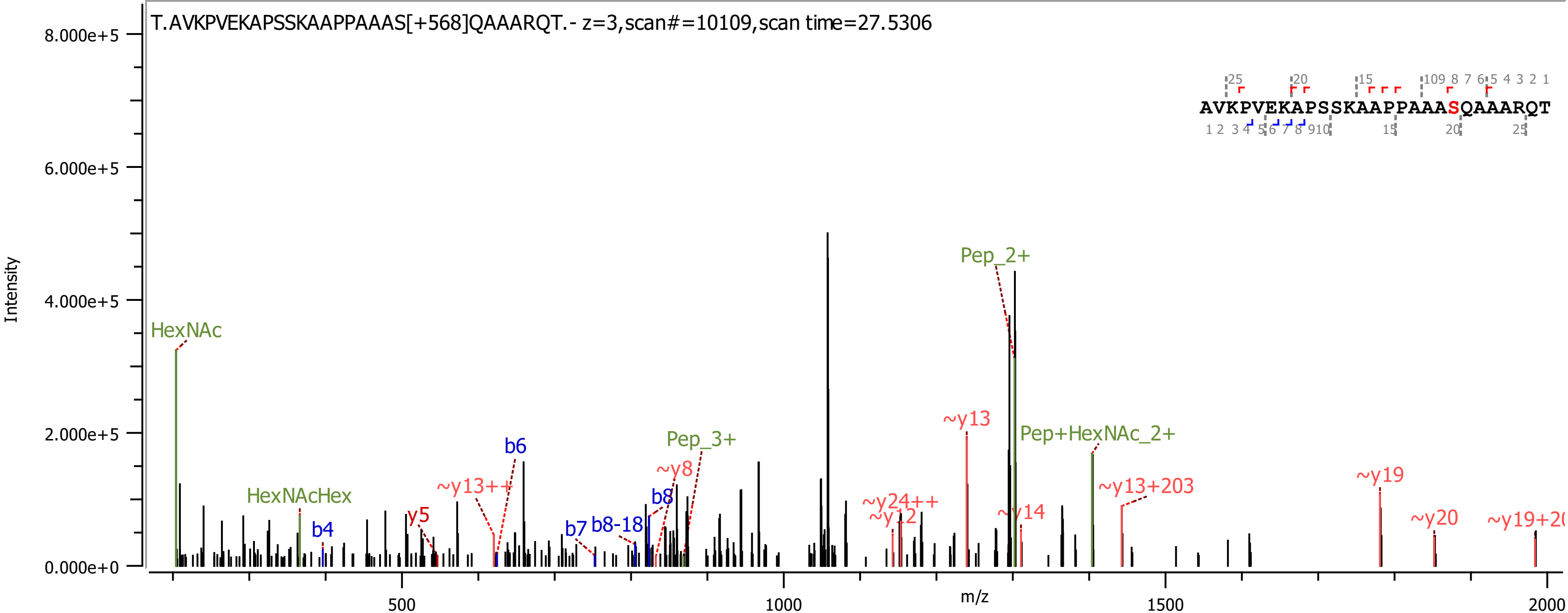
T.AVKPVEKAPSSKAAPPAAAS[+568]QAAARQT.- z=3,scan#=10109,scan time=27.5306
AVKPVEKAPSSKAAPPAAASQAAARQT
Intensity
m/z
HexNAc
HexNAcHex
b4
y5
~y13++
b6
b7
b8-18
b8
~y8
Pep_3+
~y24++
~y12
~y13
~y14
Pep_2+
Pep+HexNAc_2+
~y13+203
~y19
~y20
8.000e+5
6.000e+5
4.000e+5
2.000e+5
0.000e+0
500
1000
1500
2000

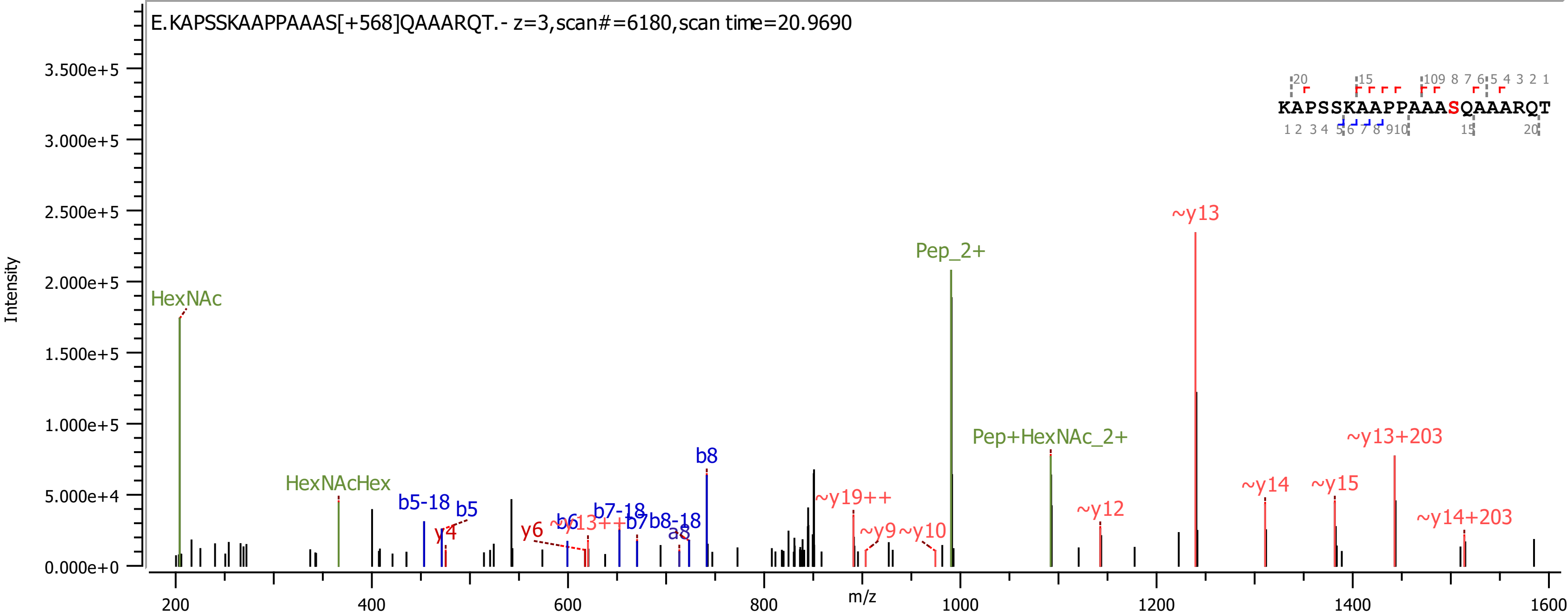

E.KAPSSKAAPPAAAS[+568]QAAARQT.- z=3,scan#=6180,scan time=20.9690
Intensity
m/z
KAPSSKAAPPAAASQAAARQT
HexNAc
HexNAcHex
b5-18
b5
y4
y6
b6
~y13++
b7-18
b7
b8-18
a8
b8
~y19++
~y9
~y10
Pep_2+
Pep+HexNAc_2+
~y12
~y13
~y14
~y15
~y13+203
~y14+203

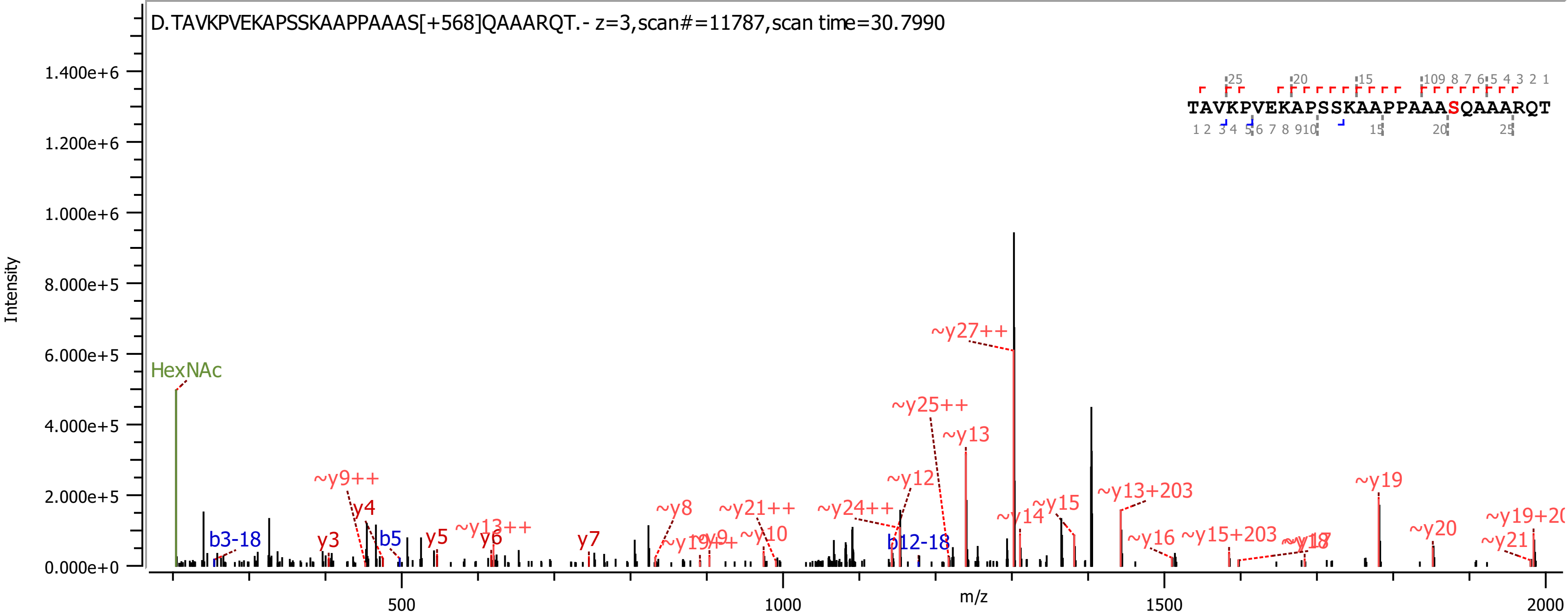
D.TAVKPVEKAPSSKAAPPAAAS[+568]QAAARQT.- z=3,scan#=11787,scan time=30.7990
TAVKPVEKAPSSKAAPPAAASQAAARQT
Intensity
m/z
HexNAc
b3-18
b5
b12-18
y3
y4
y5
y6
y7
~y8
~y9++
~y10
~y12
~y13
~y13++
~y14
~y15
~y16
~y19
~y20
~y21
~y21++
~y24++
~y25++
~y27++
~y13+203
~y15+203

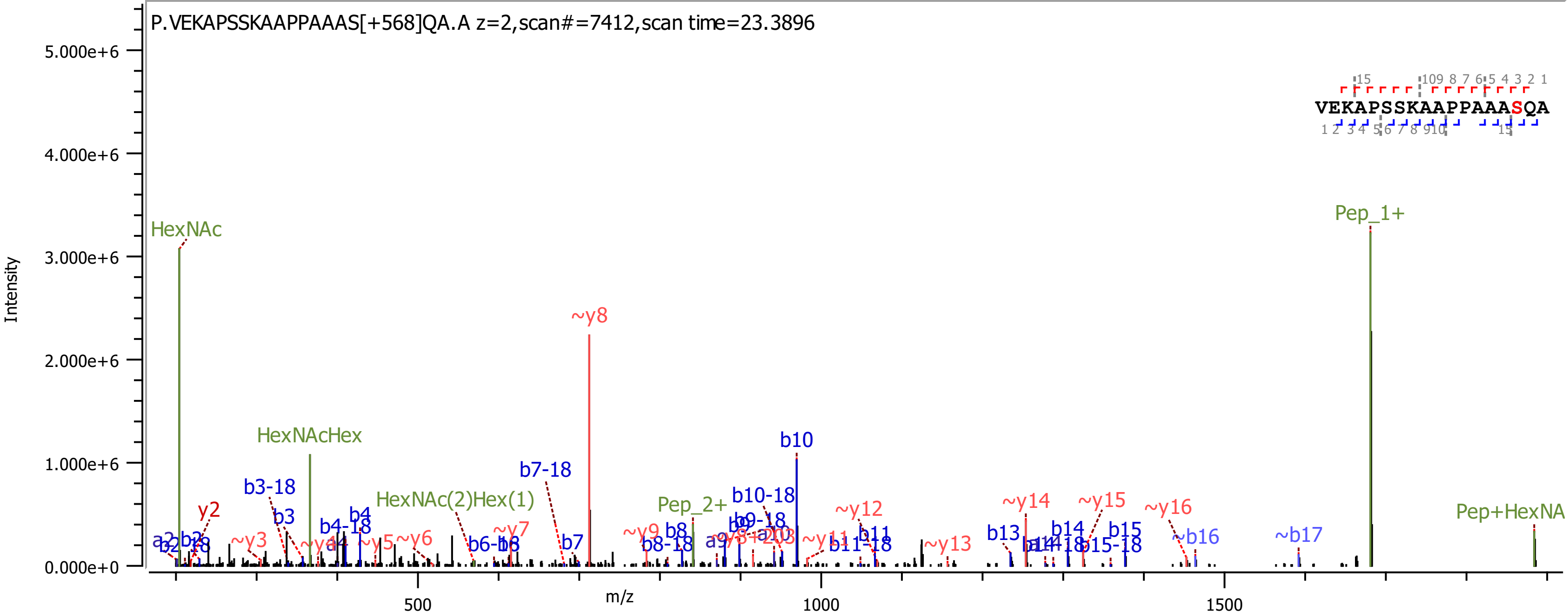

P.VEKAPSSKAAPPAAAS[+568]QA.A z=2,scan#=7412,scan time=23.3896
VEKAPSSKAAPPAAASQA
Intensity
m/z
5.000e+6
4.000e+6
3.000e+6
2.000e+6
1.000e+6
0.000e+0
500
1000
1500
HexNAc
HexNAcHex
HexNAc(2)Hex(1)
Pep_1+
Pep_2+
Pep+HexNA
~y8
b10
b7-18
b10-18
b3-18
b3
y2
~y3
~y7
~y9
~y12
~y13
~y14
~y15
~y16
b4
b4-18
b7
b8
b8-18
b9-18
b11
b11-18
b13
b14
b15
b15-18
~b16
~b17

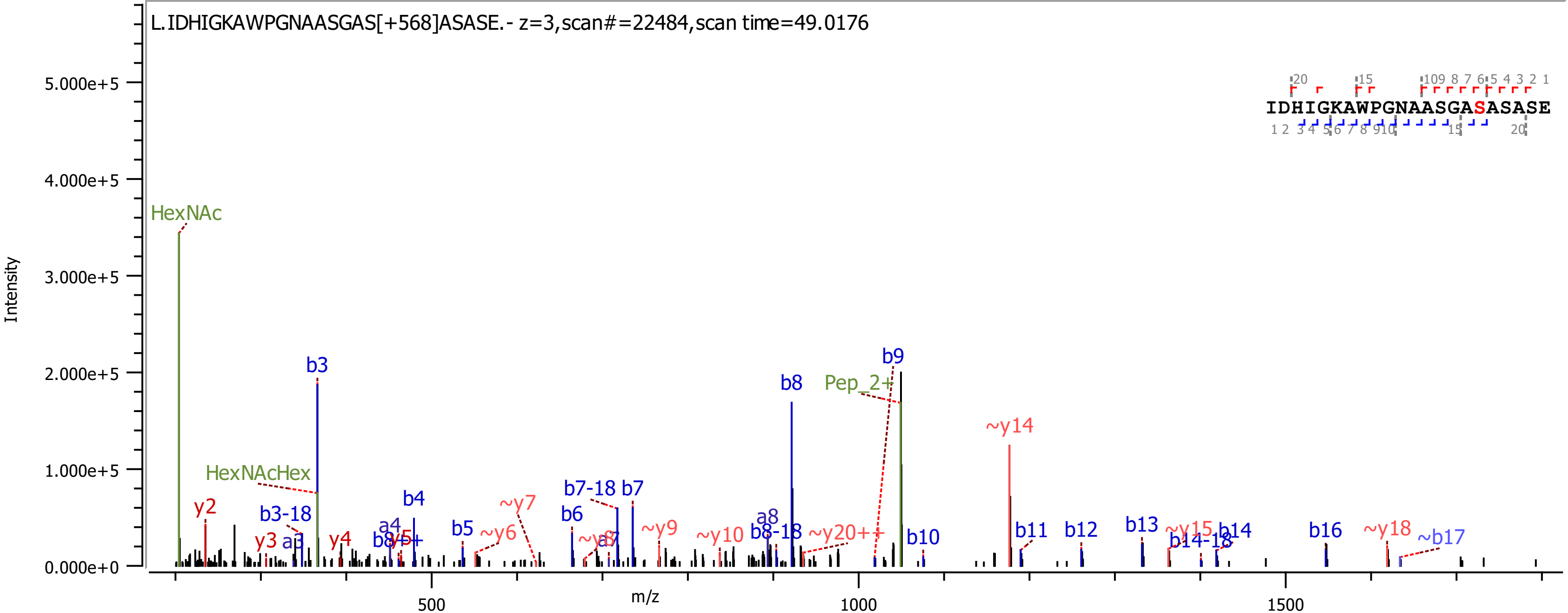

L.IDHIGKAWPGNAASGAS[+568]ASASE.- z=3,scan#=22484,scan time=49.0176
IDHIGKAWPGNAASGASASASE
Intensity
m/z
HexNAc
HexNAcHex
Pep_2+
b3
b3-18
b4
b5
b6
b7-18
b7
b8
b8-18
b9
b10
b11
b12
b13
b14
b14-18
b16
~b17
y2
y3
y4
~y6
~y7
~y9
~y10
~y14
~y15
~y18
~y20++
a3
a4
a8

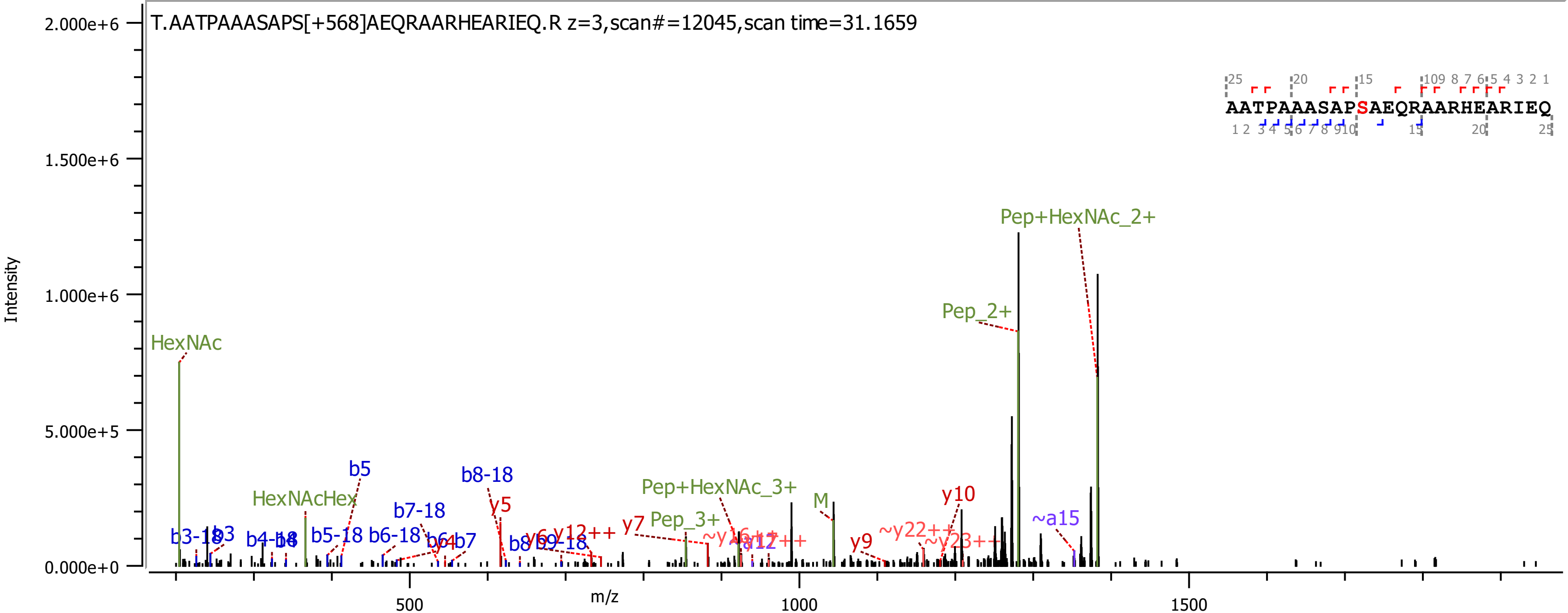
T.AATPAAASAPS[+568]AEQRAARHEARIEQ.R z=3,scan#=12045,scan time=31.1659
Intensity
m/z
AATPAAASAPSAEQRAARHEARIEQ
Pep+HexNAc_2+
Pep_2+
HexNAc
HexNAcHex
Pep+HexNAc_3+
Pep_3+
M
b5
b8-18
b7-18
y5
y10
~a15
y7
y9
~y22++
~y23++
b3-18
b3
b4-18
b5-18
b6-18
b6
b7
b8
b9-18
y6
y12++
~a12

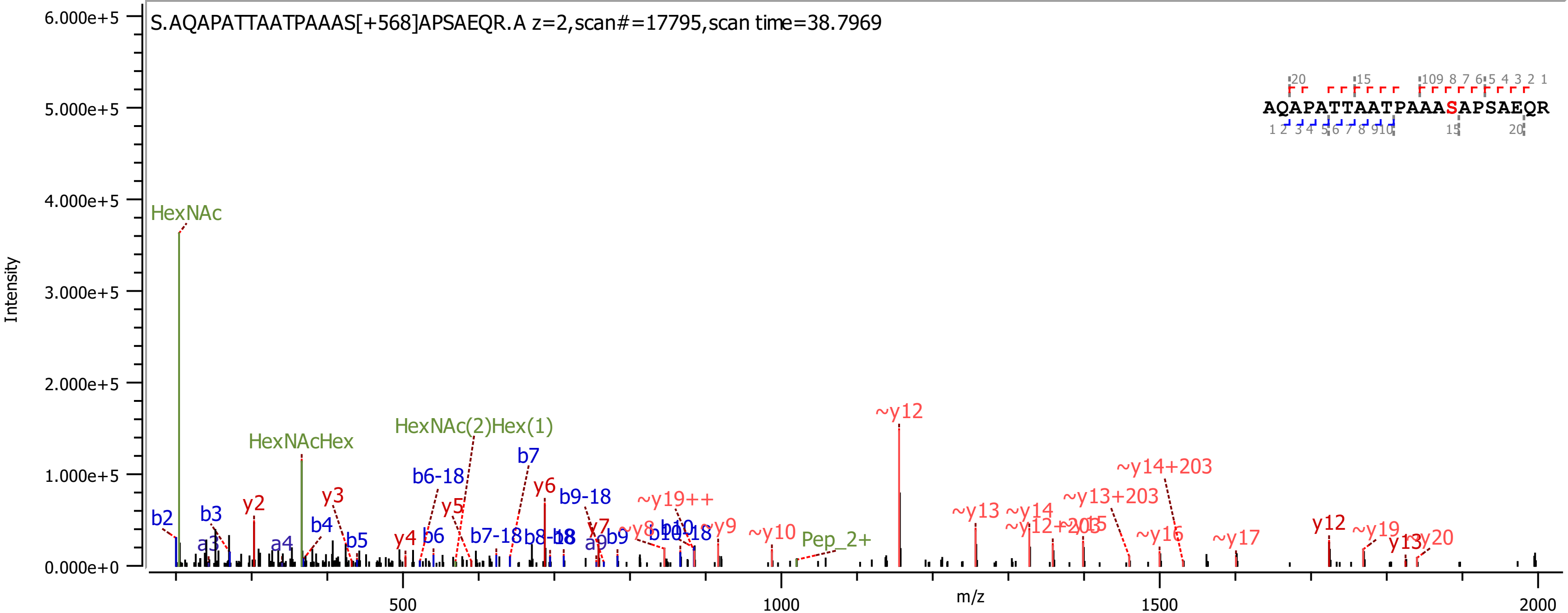

S.AQAPATTAATPAAAS[+568]APSAEQR.A z=2,scan#=17795,scan time=38.7969
AQAPATTAATPAAASAPSAEQR
Intensity
m/z
HexNAc
HexNAcHex
HexNAc(2)Hex(1)
Pep_2+
~y12
~y14+203
~y13+203
~y13
~y14
~y12+203
~y15
~y16
~y17
~y19
~y20
~y19++
~y10
~y9
~y8
y2
y3
y4
y5
y6
y7
y12
y13
b2
b3
b4
b5
b6
b7
b9
b10
b6-18
b7-18
b9-18
b10-18
a3
a4
a9

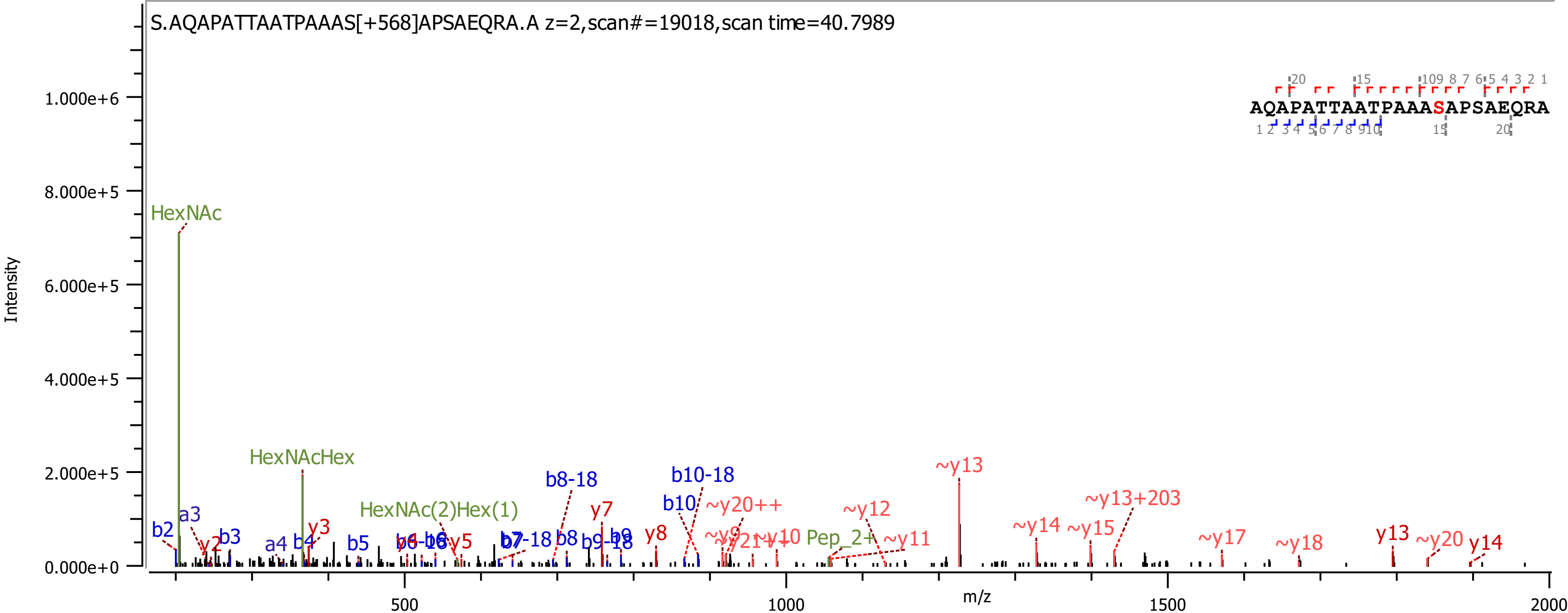

S.AQAPATTAATPAAAS[+568]APSAEQRA.A z=2,scan#=19018,scan time=40.7989
AQAPATTAATPAAASAPSAEQRA
Intensity
m/z
HexNAc
HexNAcHex
HexNAc(2)Hex(1)
Pep_2+
b2
a3
y2
b3
a4
b4
y3
b5
y5
b7-18
b8
b8-18
y7
b9-18
b9
y8
b10
b10-18
~y20++
~y9
~y10
~y12
~y11
~y13
~y14
~y15
~y13+203
~y17
~y18
y13
~y20
y14
0.000e+0
2.000e+5
4.000e+5
6.000e+5
8.000e+5
1.000e+6
500
1000
1500
2000

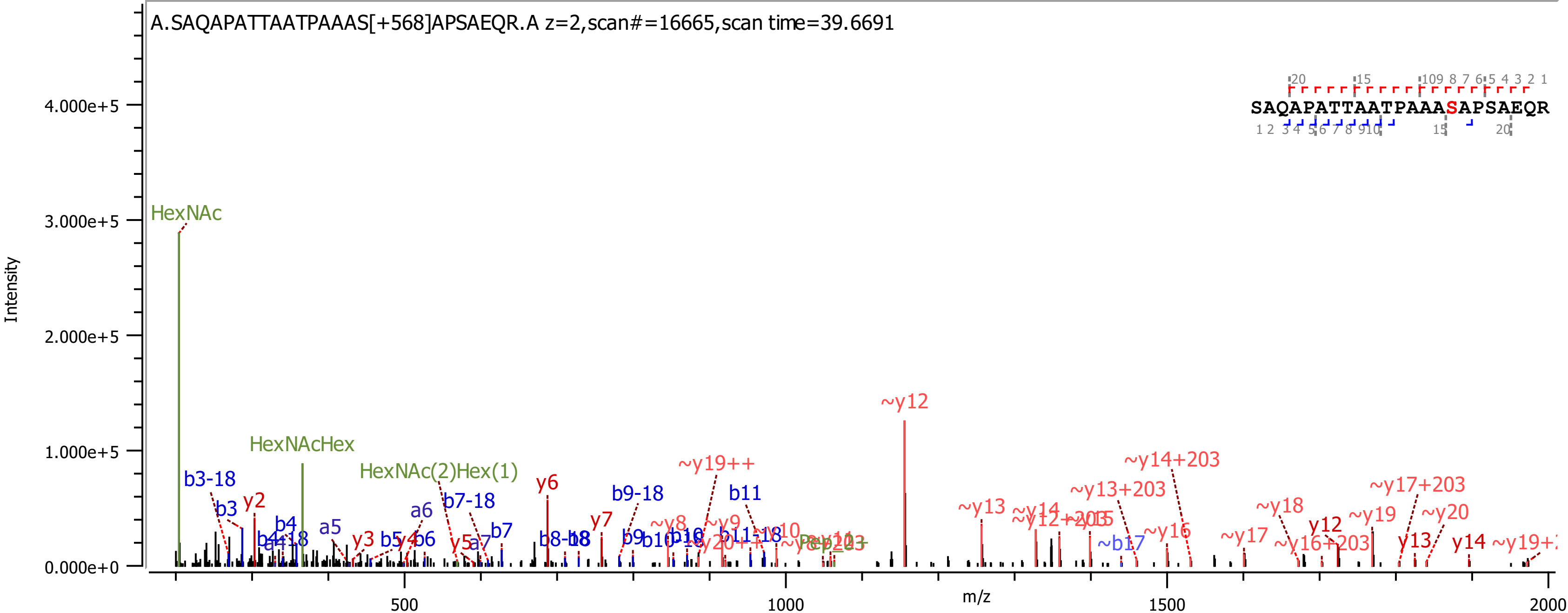

A.SAQAPATTAATPAAAS[+568]APSAEQR.A z=2,scan#=16665,scan time=39.6691
SAQAPATTAATPAAASAPSAEQR
Intensity
4.000e+5
3.000e+5
2.000e+5
1.000e+5
0.000e+0
m/z
500
1000
1500
2000
HexNAc
HexNAcHex
HexNAc(2)Hex(1)
b3-18
b3
y2
b4
a5
y3
b5
y4
b6
a6
b7-18
y5
a7
b7
y6
y7
b9-18
b9
b11
~y19++
~y8
~y9
~y12
~y13
~y14
~y12+203
~y13+203
~y14+203
~y16
~b17
~y17
~y18
~y16+203
y12
~y19
~y17+203
y13
~y20
y14

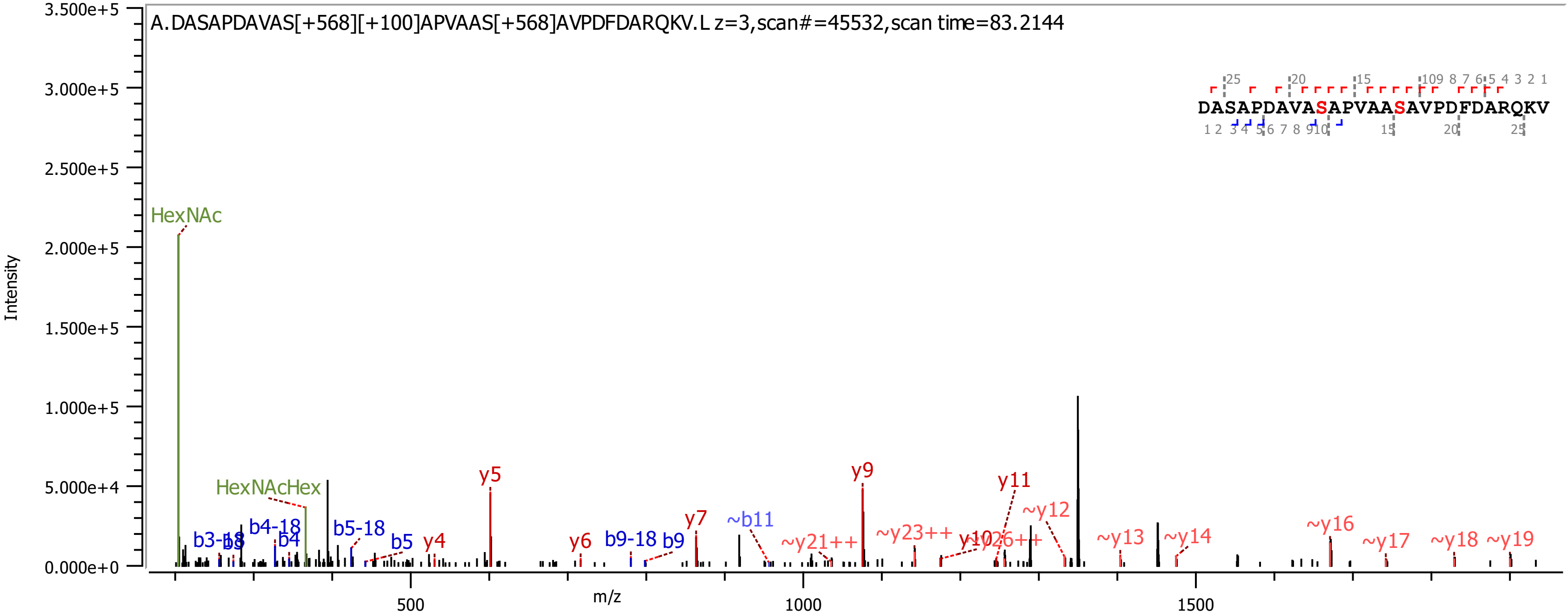

A.DASAPDAVAS[+568][+100]APVAAS[+568]AVPDFDARQKV.L z=3,scan#=45532,scan time=83.2144
DASAPDAVASAPVAASAVPDFDARQKV
Intensity
m/z
HexNAc
HexNAcHex
b3-18
b3
b4-18
b4
b5-18
b5
b9-18
b9
~b11
y4
y5
y6
y7
y9
y10
y11
~y12
~y13
~y14
~y16
~y17
~y18
~y19
~y21++
~y23++
~y26++

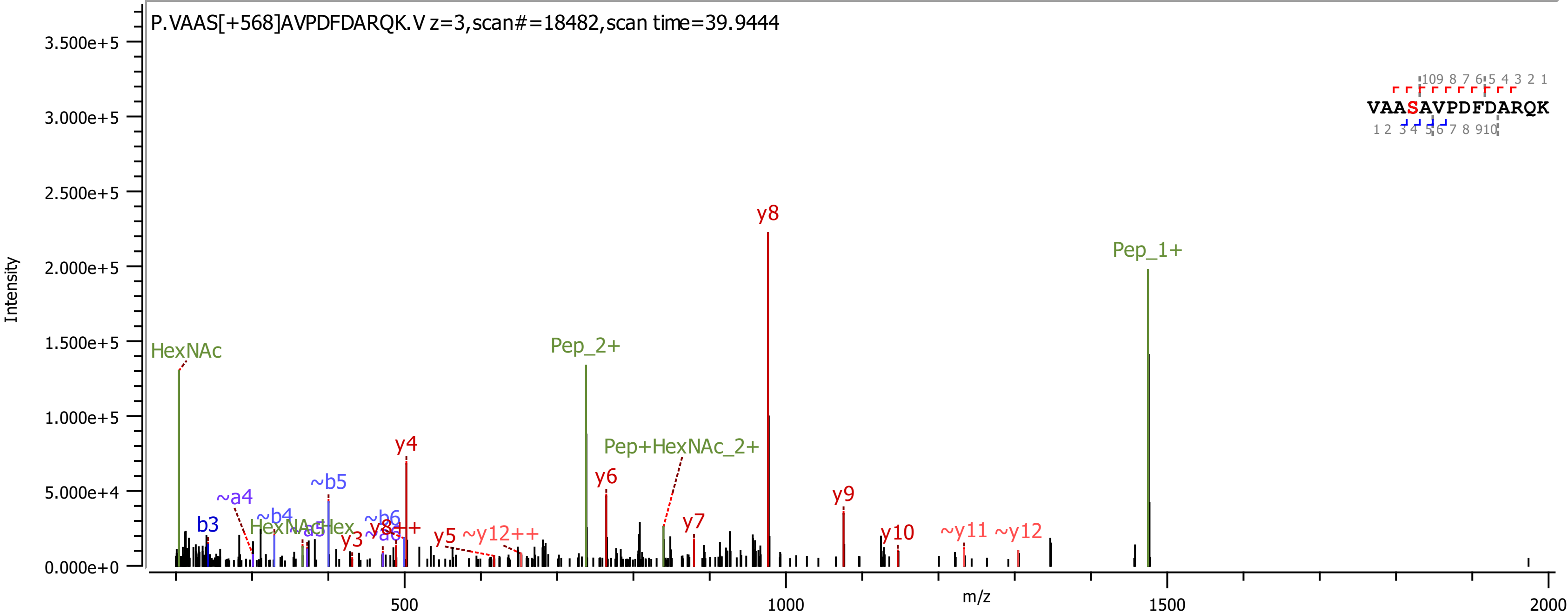

P.VAAS[+568]AVPDFDARQK.V z=3,scan#=18482,scan time=39.9444
Intensity
m/z
3.500e+5
3.000e+5
2.500e+5
2.000e+5
1.500e+5
1.000e+5
5.000e+4
0.000e+0
500
1000
1500
2000
VAASAVPDFDARQK
HexNAc
Pep_2+
Pep_1+
Pep+HexNAc_2+
y8
y4
y6
y9
y7
y10
~y11
~y12
~y12++
y5
y3
~b5
~a4
~b4
b3
~b6
~a6

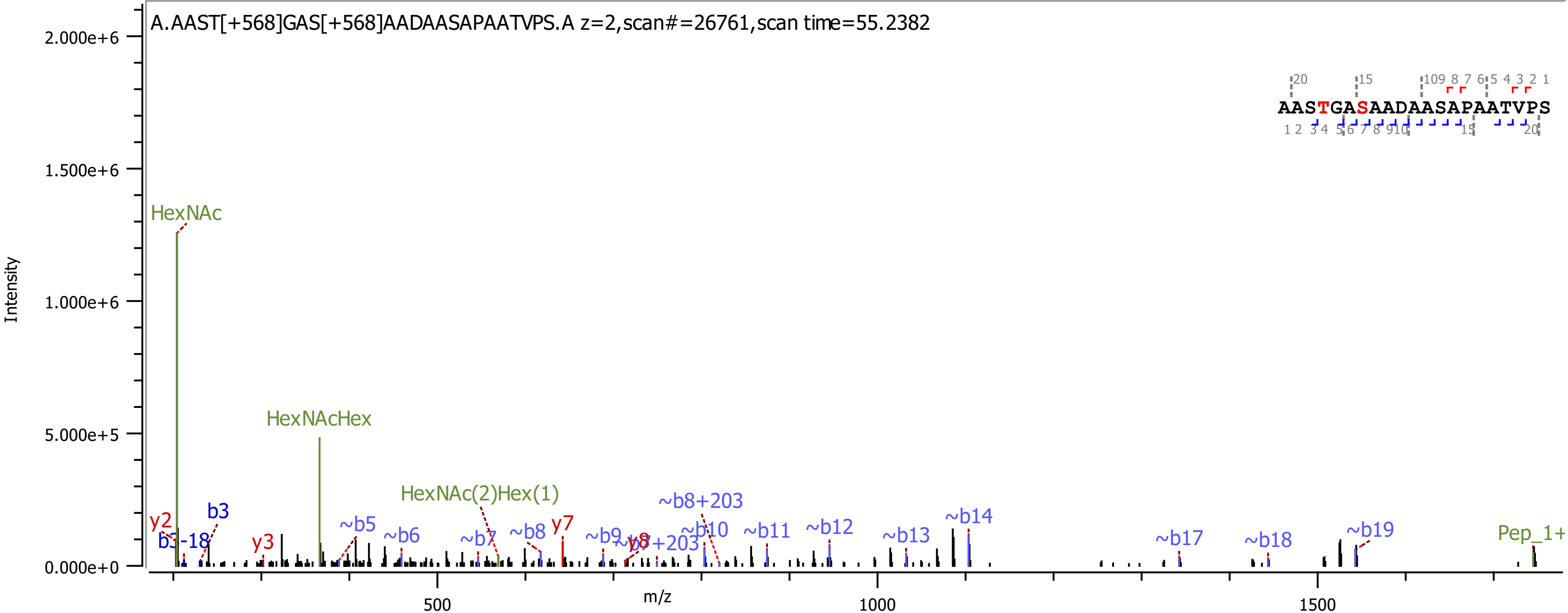
A.AAST[+568]GAS[+568]AADAASAPAATVPS.A z=2,scan#=26761,scan time=55.2382
AASTGASAADAASAPAATVPS
Intensity
m/z
HexNAc
HexNAcHex
HexNAc(2)Hex(1)
y2
b3
b3-18
y3
~b5
~b6
~b7
~b8
y7
~b9
y8
~b8+203
~b7+203
~b10
~b11
~b12
~b13
~b14
~b17
~b18
~b19
Pep_1+

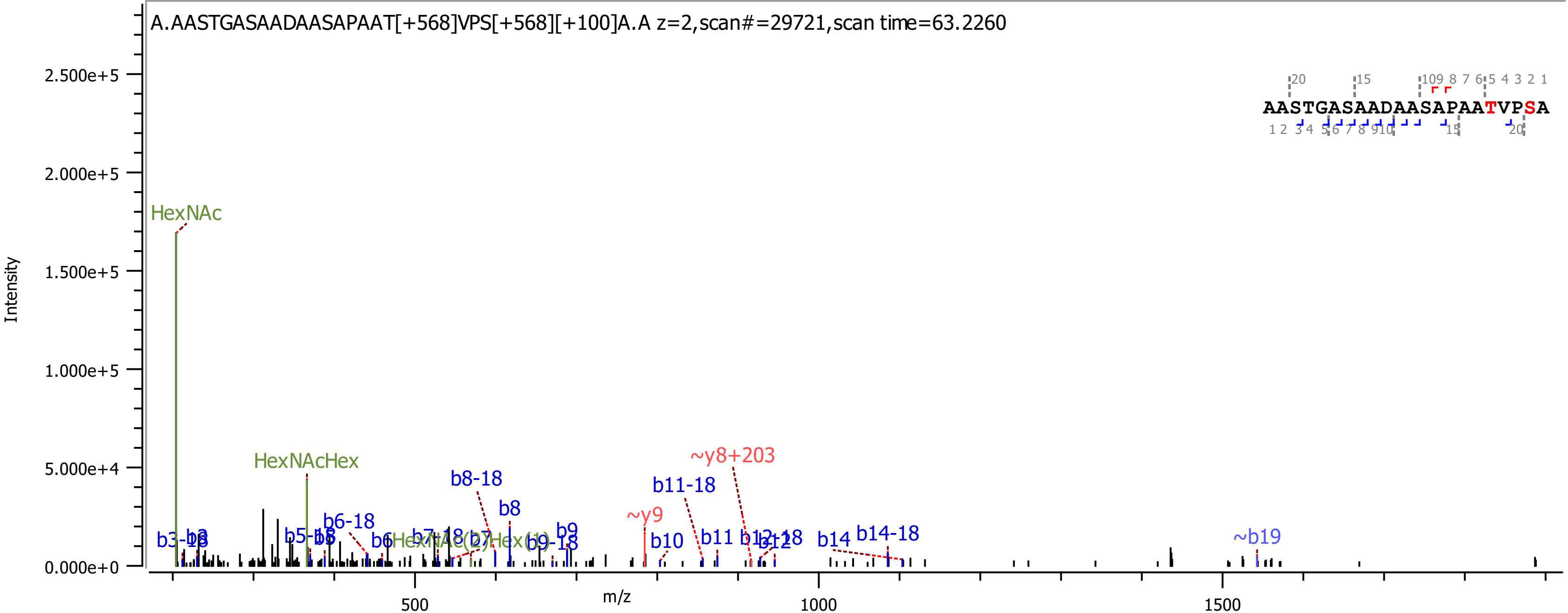

A.AASTGASAADAASAPAAT[+568]VPS[+568][+100]A.A z=2,scan#=29721,scan time=63.2260
Intensity
m/z
AASTGASAADAASAPAATVPSA
HexNAc
HexNAcHex
b8-18
b8
b6-18
b5-18
b11-18
~y8+203
~y9
b10
b11
b14
b14-18
~b19
b9
b9-18
b6
b3-18
0.000e+0
5.000e+4
1.000e+5
1.500e+5
2.000e+5
2.500e+5
500
1000
1500

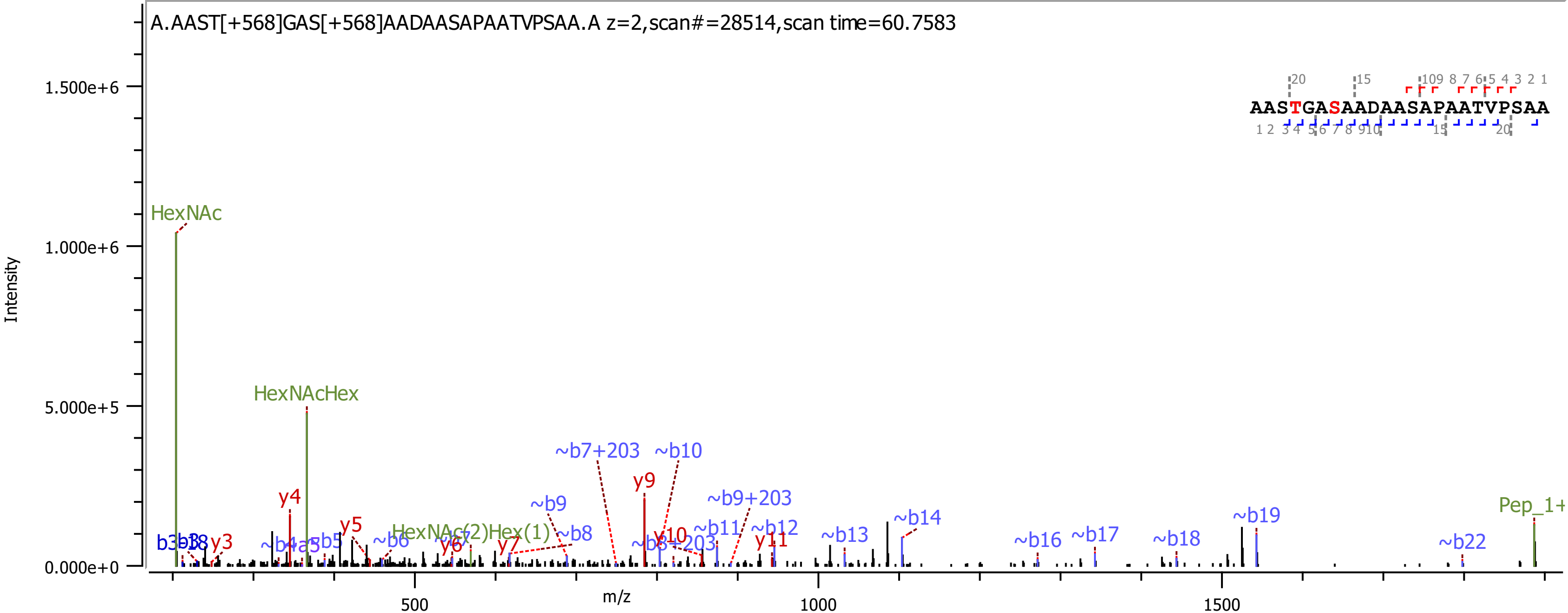
A.AAST[+568]GAS[+568]AADAASAPAATVPSAA.A z=2,scan#=28514,scan time=60.7583
AASTGASAADAASAPAATVPSAA
HexNAc
HexNAcHex
HexNAc(2)Hex(1)
Pep_1+
y3
y4
y5
y6
y7
y9
y10
y11
~b5
~b6
~b8
~b9
~b7+203
~b10
~b9+203
~b11
~b12
~b13
~b14
~b16
~b17
~b18
~b19
~b22
Intensity
m/z
1.500e+6
1.000e+6
5.000e+5
0.000e+0
500
1000
1500

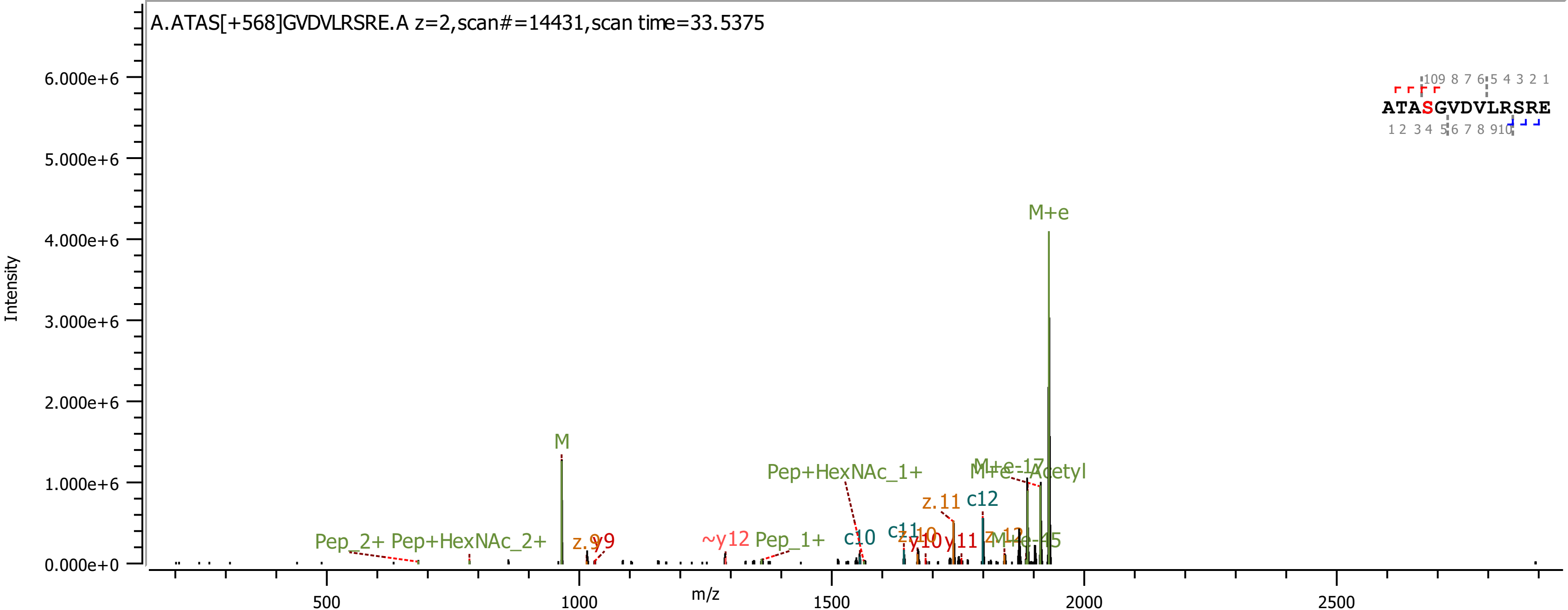
A.ATAS[+568]GVDVLRSRE.A z=2,scan#=14431,scan time=33.5375
ATASGVDVLRSRE
Intensity
m/z
6.000e+6
5.000e+6
4.000e+6
3.000e+6
2.000e+6
1.000e+6
0.000e+0
500
1000
1500
2000
2500
M+e
M
Pep+HexNAc_1+
M+e-Acetyl
z.11
c12
Pep_2+
Pep+HexNAc_2+
~y12
Pep_1+
c10
y11

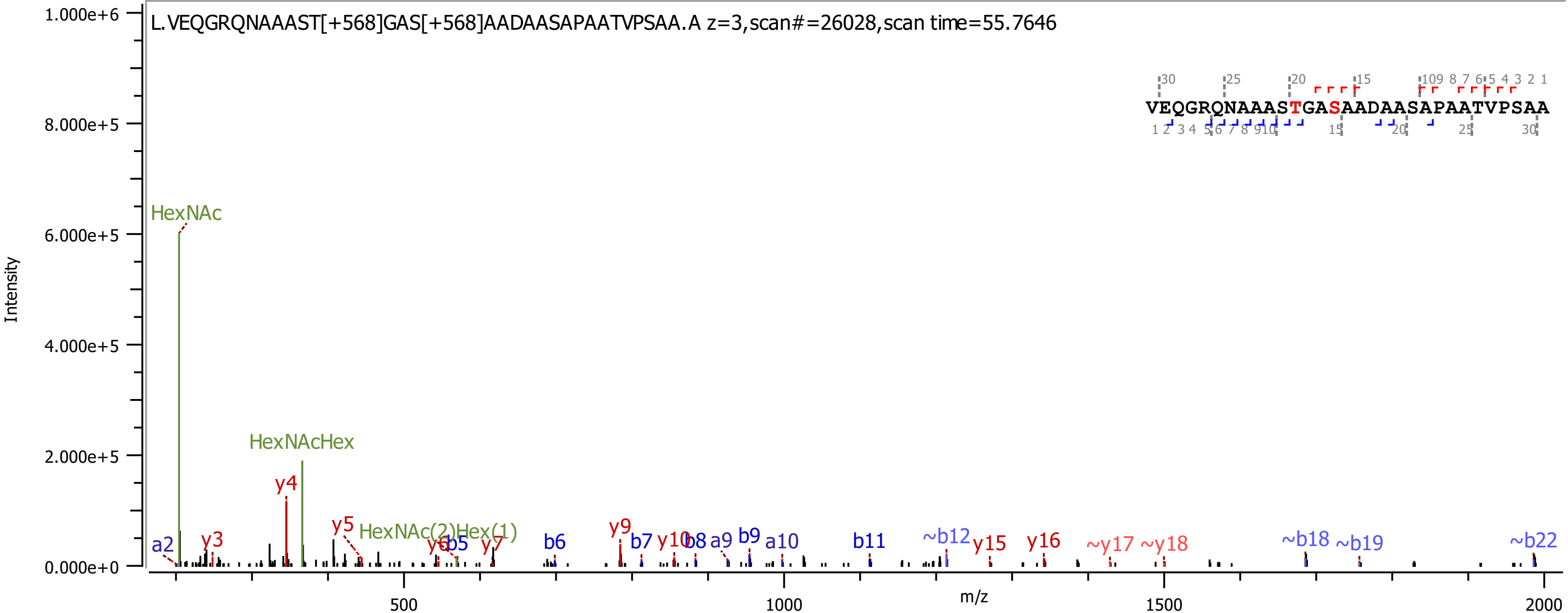

L.VEQGRQNAAAST[+568]GAS[+568]AADAASAPAATVPSAA.A z=3,scan#=26028,scan time=55.7646
VEQGRQNAAASTGASAADAASAPAATVPSAA
Intensity
m/z
1.000e+6
8.000e+5
6.000e+5
4.000e+5
2.000e+5
0.000e+0
500
1000
1500
2000
HexNAc
HexNAcHex
HexNAc(2)Hex(1)
a2
y3
y4
y5
y6
b5
y7
b6
y9
b7
y10
b8
a9
b9
a10
b11
~b12
y15
y16
~y17
~y18
~b18
~b19
~b22

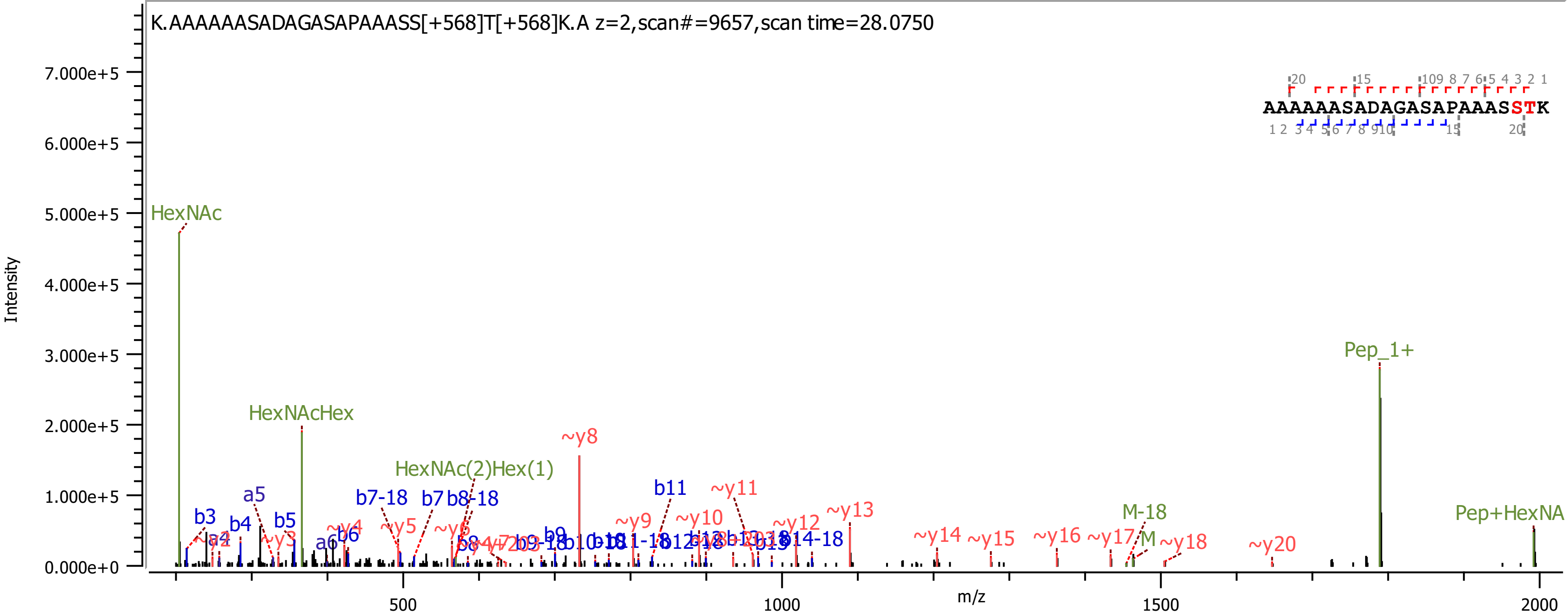

K.AAAAAASADAGASAPAAASS[+568]T[+568]K.A z=2,scan#=9657,scan time=28.0750
AAAAAASADAGASAPAAASSTK
Intensity
m/z
0.000e+0
1.000e+5
2.000e+5
3.000e+5
4.000e+5
5.000e+5
6.000e+5
7.000e+5
500
1000
1500
2000
HexNAc
HexNAcHex
HexNAc(2)Hex(1)
Pep_1+
Pep+HexNA
M-18
M
~y8
~y9
~y10
~y11
~y12
~y13
~y14
~y15
~y16
~y17
~y18
~y20
b3
b4
b5
b6
b7-18
b7
b8-18
b9
b11
a5
a6

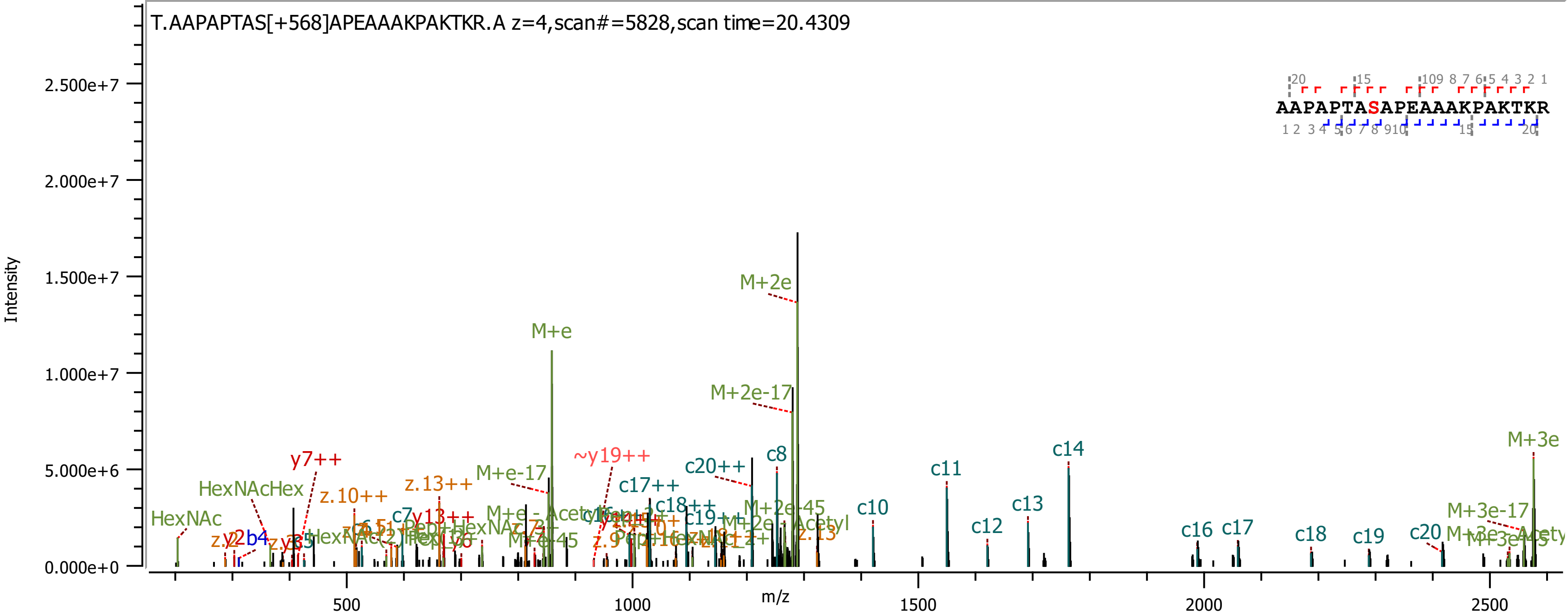
T.AAPAPTAS[+568]APEAAAKPAKTKR.A z=4,scan#=5828,scan time=20.4309
AAPAPTASAPEAAAKPAKTKR
Intensity
m/z
2.500e+7
2.000e+7
1.500e+7
1.000e+7
5.000e+6
0.000e+0
500
1000
1500
2000
2500
M+2e
M+e
M+2e-17
M+3e
c8
c10
c11
c12
c13
c14
c16
c17
c18
c19
c20
c20++
c17++
c18++
~y19++
y7++
y13++
z.10++
z.13++
M+e-17
HexNAcHex
HexNAc
M+2e-45
M+3e-17
b4
c7

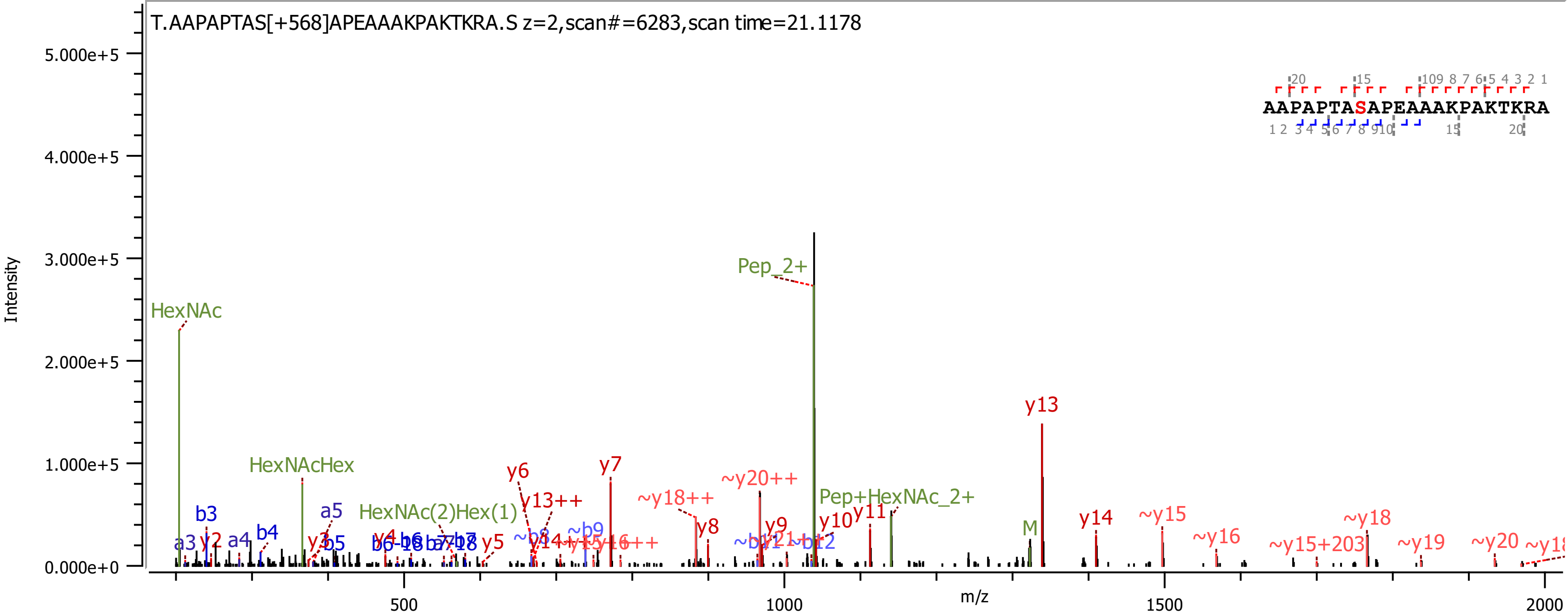

T.AAPAPTAS[+568]APEAAAKPAKTKRA.S z=2,scan#=6283,scan time=21.1178
AAPAPTASAPEAAAKPAKTKRA
Intensity
m/z
5.000e+5
4.000e+5
3.000e+5
2.000e+5
1.000e+5
0.000e+0
500
1000
1500
2000
HexNAc
HexNAcHex
HexNAc(2)Hex(1)
Pep_2+
Pep+HexNAc_2+
M
y13
y7
y6
y13++
~y18++
~y20++
y8
y9
y10
y11
y14
~y15
~y16
~y18
~y19
~y20
~y15+203
b3
a4
b4
a5
b5
y2
y3
y4
y5
~b9

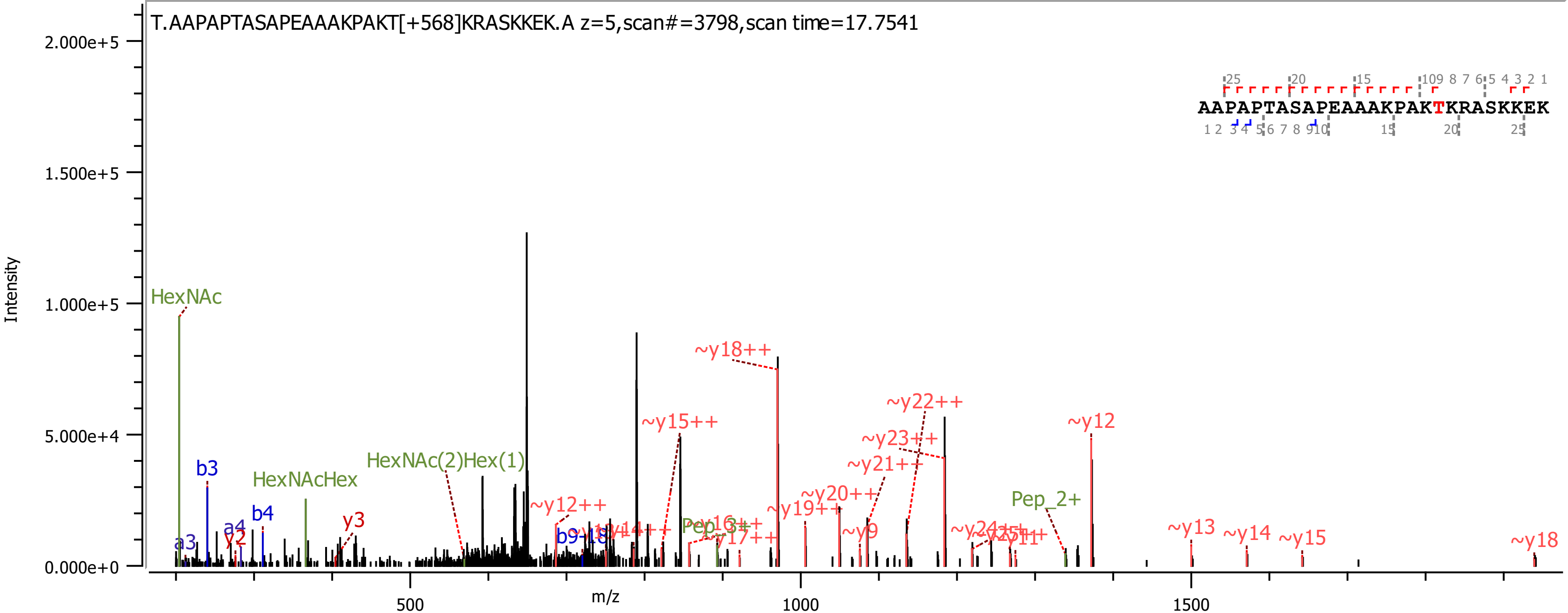
T.AAPAPTASAPEAAAKPAKT[+568]KRASKKEK.A z=5,scan#=3798,scan time=17.7541
AAPAPTASAPEAAAKPAKTKRASKKEK
Intensity
m/z
2.000e+5
1.500e+5
1.000e+5
5.000e+4
0.000e+0
500
1000
1500
HexNAc
HexNAcHex
HexNAc(2)Hex(1)
Pep_2+
b3
b4
a3
a4
y2
y3
~y18++
~y15++
~y22++
~y23++
~y21++
~y20++
~y19++
~y12++
~y17++
~y9
~y12
~y13
~y14
~y15
~y18

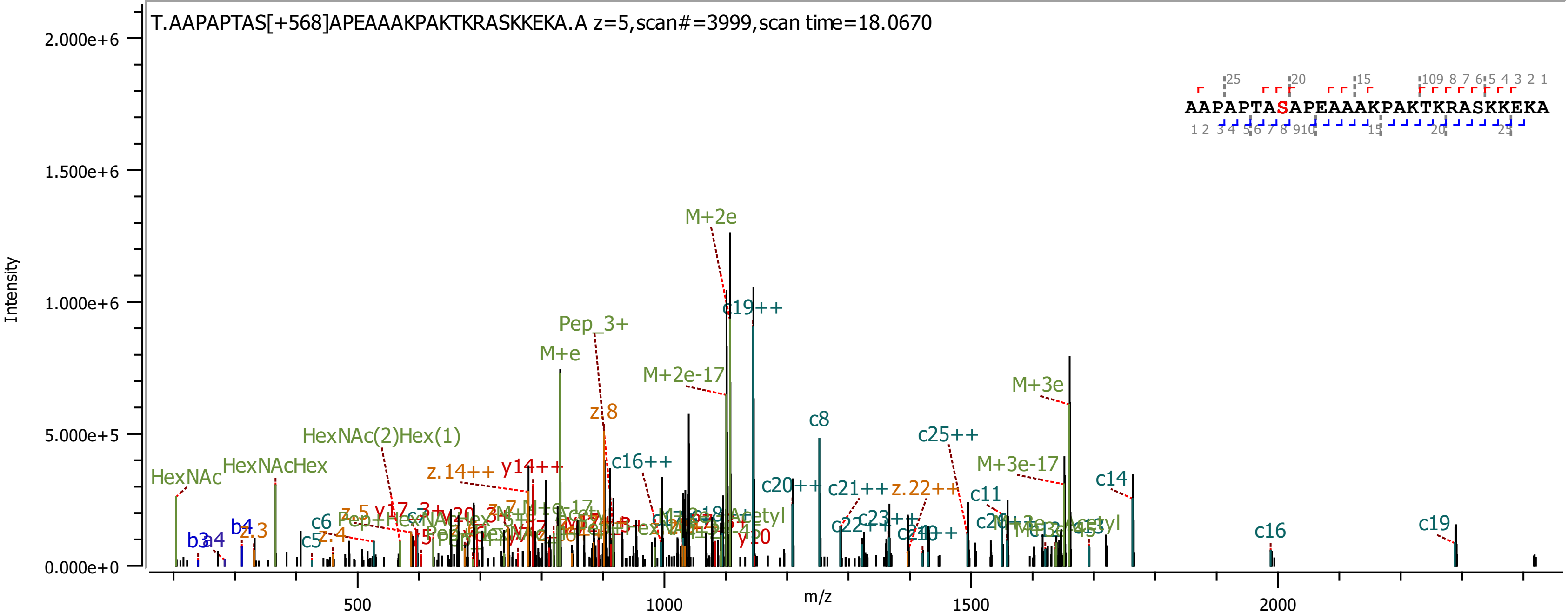

T.AAPAPTAS[+568]APEAAAKPAKTKRASKKEKA.A z=5,scan#=3999,scan time=18.0670
Intensity
m/z
2.000e+6
1.500e+6
1.000e+6
5.000e+5
0.000e+0
500
1000
1500
2000
AAPAPTASAPEAAAKPAKTKRASKKEKA
M+2e
c19++
Pep_3+
M+e
M+2e-17
z.8
c8
c25++
M+3e
M+3e-17
HexNAc(2)Hex(1)
HexNAcHex
HexNAc
z.14++
y14++
c16++
c20++
c21++
z.22++
c11
c14
c16
c19
b3
a4
b4
z.3
c5
z.4
c6
y20

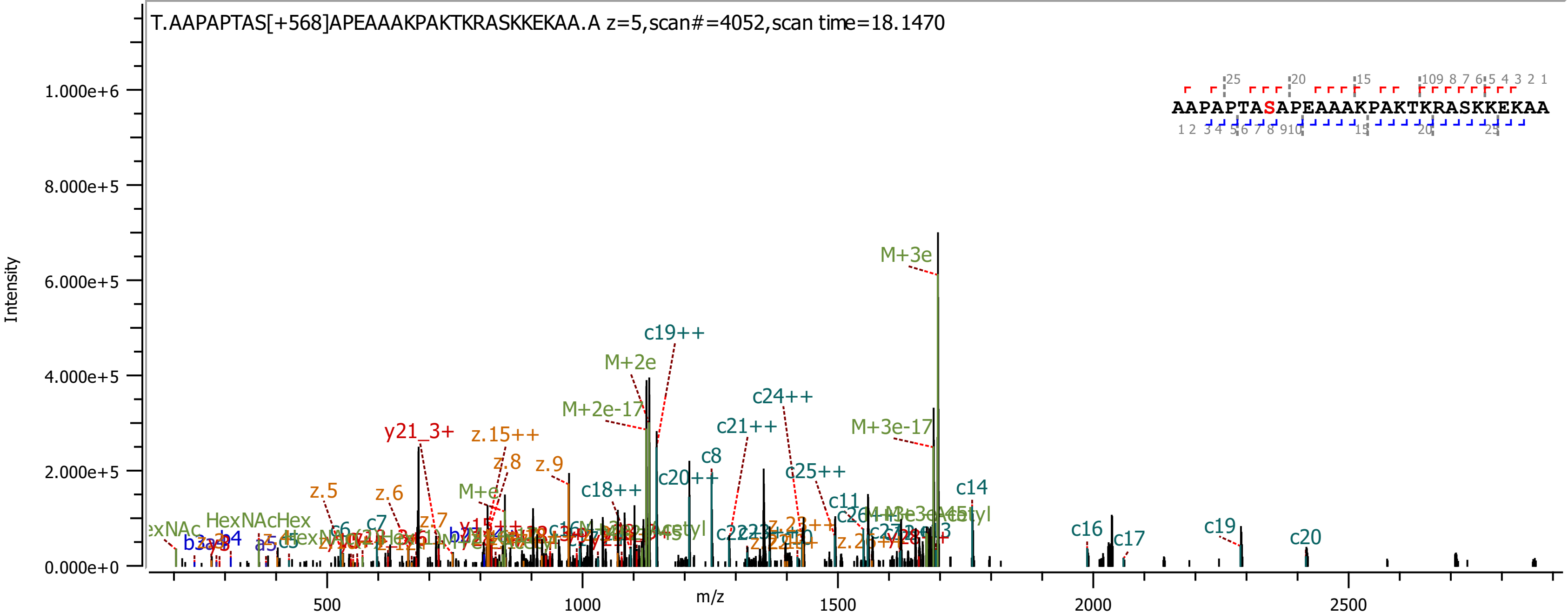
T.AAPAPTAS[+568]APEAAAKPAKTKRASKKEKAA.A z=5,scan#=4052,scan time=18.1470
AAPAPTASAPEAAAKPAKTKRASKKEKAA
Intensity
m/z
M+3e
c19++
M+2e
M+2e-17
c24++
c21++
M+3e-17
y21_3+
z.15++
z.8
z.9
c8
c20++
c25++
c18++
z.5
z.6
M+e
c14
c11
z.7
c16
c17
c19
c20
HexNAcHex

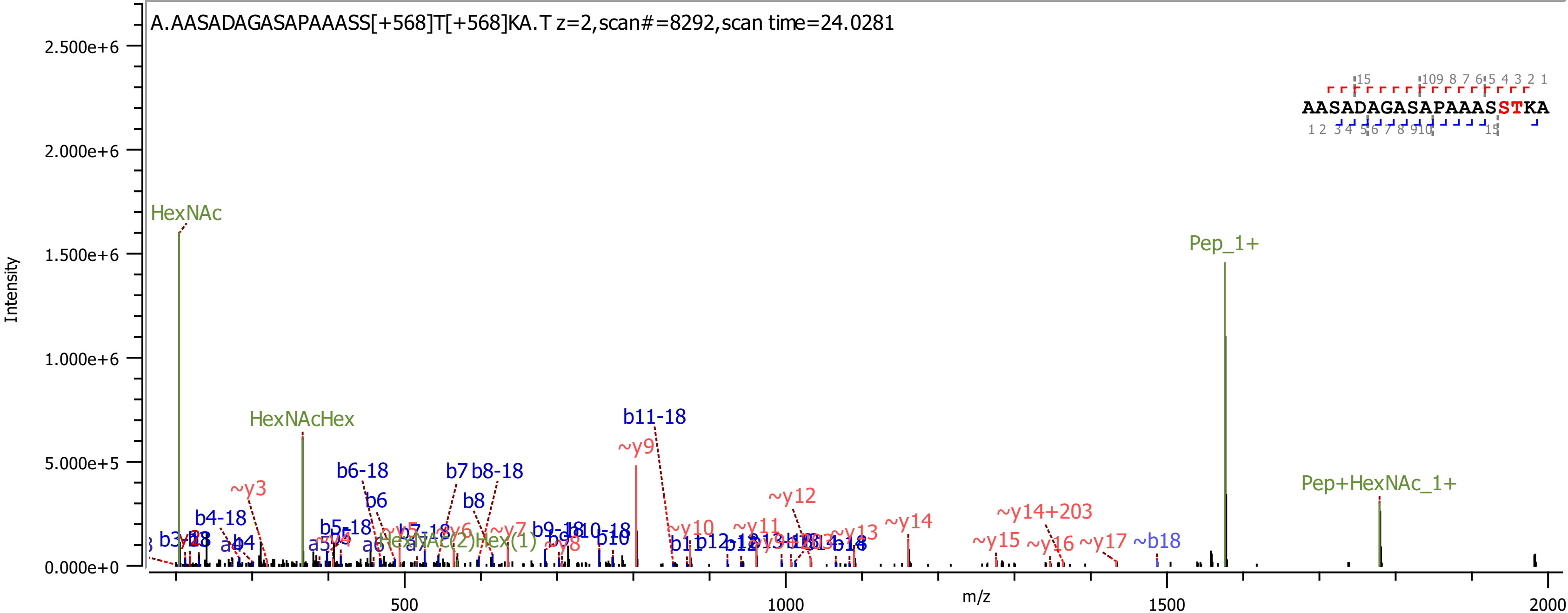

A.AASADAGASAPAAASS[+568]T[+568]KA.T z=2,scan#=8292,scan time=24.0281
AASADAGASAPAAASSTKA
Intensity
m/z
2.500e+6
2.000e+6
1.500e+6
1.000e+6
5.000e+5
0.000e+0
500
1000
1500
2000
HexNAc
HexNAcHex
Pep_1+
Pep+HexNAc_1+
b11-18
~y9
b6-18
b6
b7
b8-18
b8
~y3
b4-18
b4
b5-18
b9-18
b10-18
b10
~y10
~y11
~y12
~y13
~y14
~y14+203
~y15
~y16
~y17
~b18
b11
b12
b18
~y7
~y8
HexNAc(2)Hex(1)

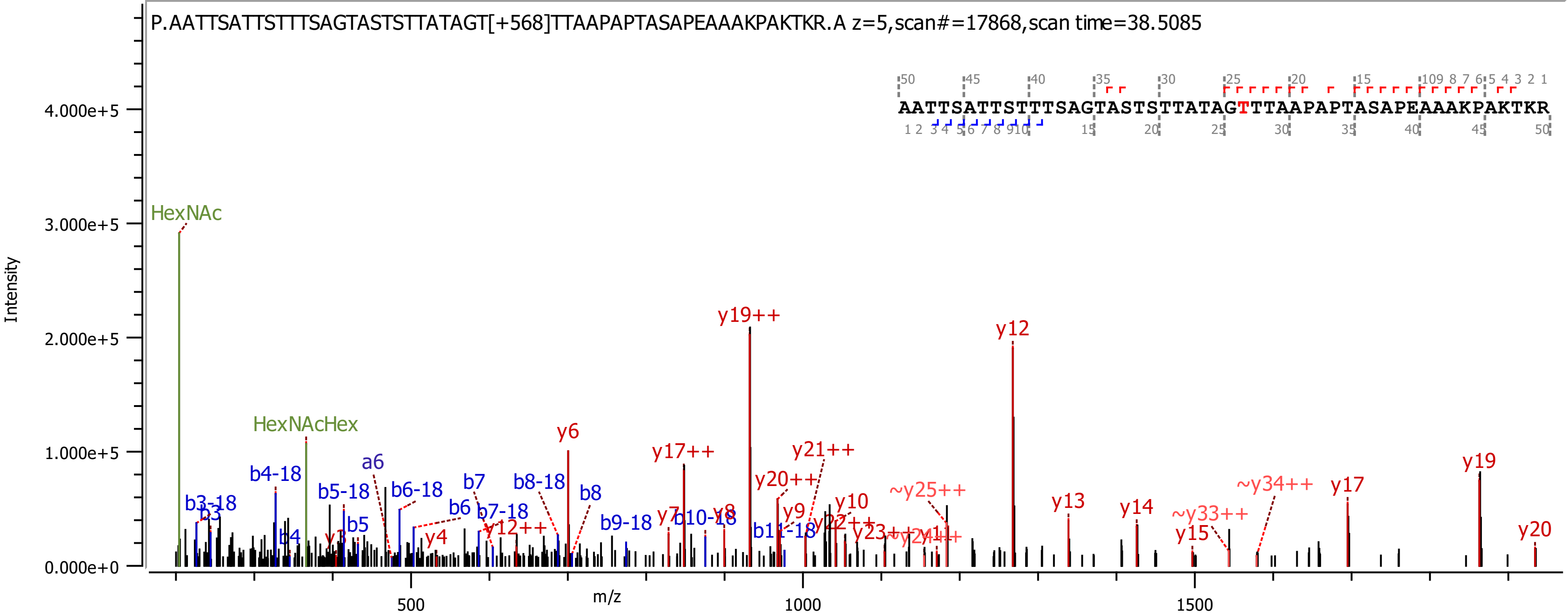
P.AATTSATTSTTTSAGTASTSTTATAGT[+568]TTAAPAPTASAPEAAAKPAKTKR.A z=5,scan#=17868,scan time=38.5085
AATTSATTSTTTSAGTASTSTTATAGTTTAAPAPTASAPEAAAKPAKTKR
Intensity
m/z
HexNAc
HexNAcHex
y19++
y12
y6
y17++
y21++
y20++
y19
y17
y13
y14
y15
y20
y10
y9
y8
y7
y4
y3
~y25++
~y34++
~y33++
b4-18
b5-18
b6-18
b8-18
b9-18
b10-18
b3-18
b3
b4
b5
b6
b7
b8
a6
b7-18

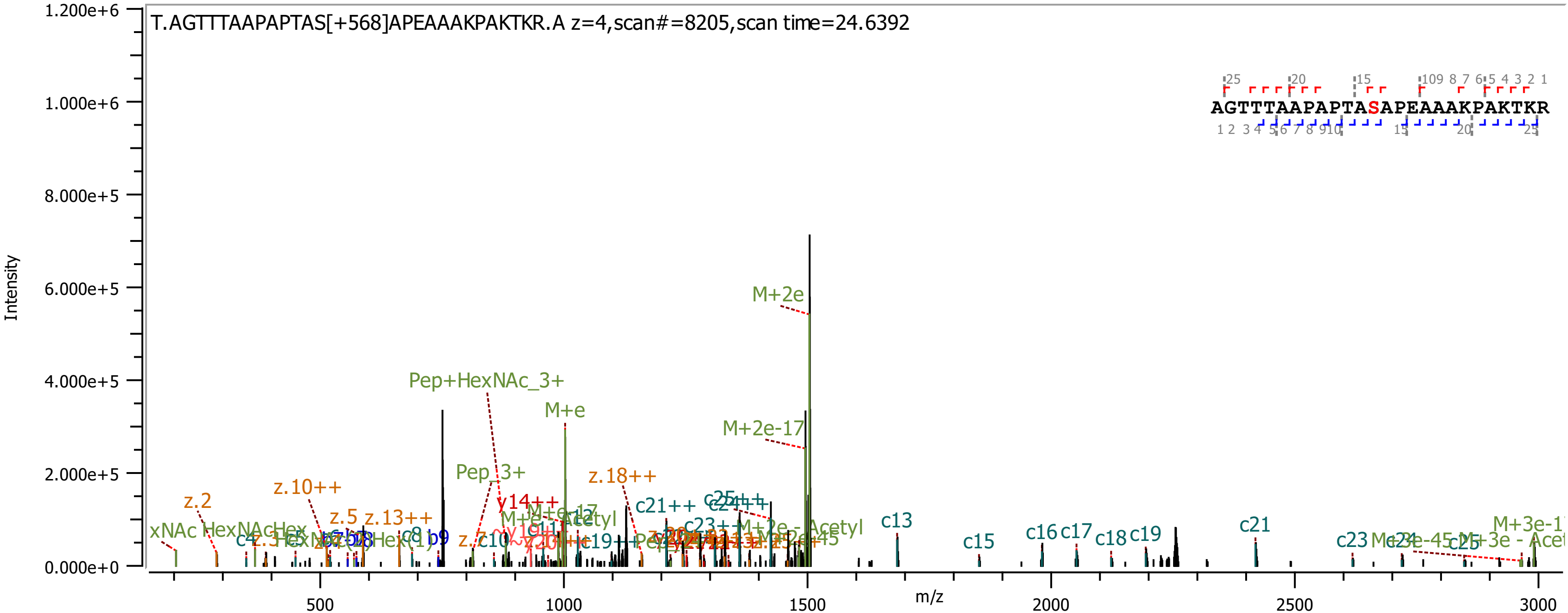
T.AGTTTAAPAPTAS[+568]APEAAAKPAKTKR.A z=4,scan#=8205,scan time=24.6392
AGTTTAAPAPTASAPEAAAKPAKTKR
Intensity
m/z
1.200e+6
1.000e+6
8.000e+5
6.000e+5
4.000e+5
2.000e+5
0.000e+0
500
1000
1500
2000
2500
3000
M+2e
Pep+HexNAc_3+
M+e
M+2e-17
Pep_3+
z.18++
z.10++
z.2
y14++
c21++
z.5
z.13++
c13
c15
c16
c17
c18
c19
c21
c23
c25

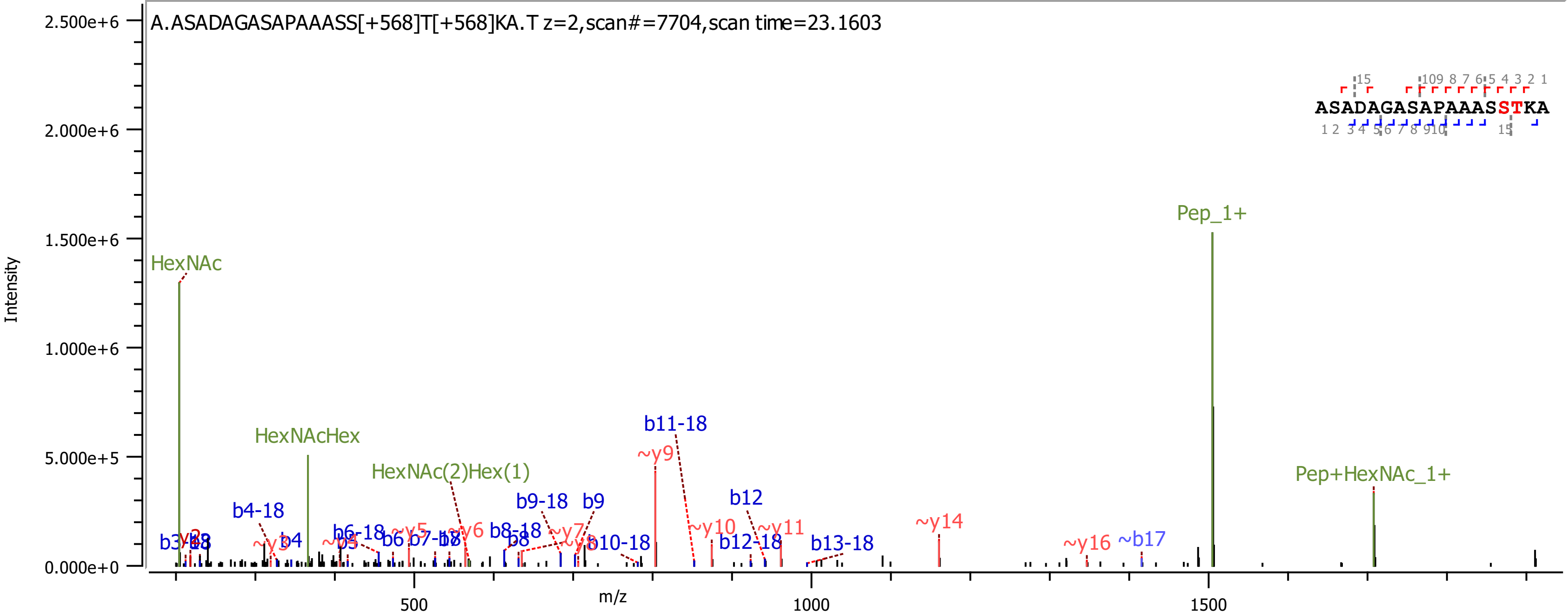
A.ASADAGASAPAAASS[+568]T[+568]KA.T z=2,scan#=7704,scan time=23.1603
ASADAGASAPAAASSTKA
Intensity
m/z
HexNAc
HexNAcHex
HexNAc(2)Hex(1)
Pep_1+
Pep+HexNAc_1+
b11-18
~y9
b12
b9-18
b9
b4-18
b4
b6-18
b6
b7-18
b8-18
b8
b10-18
b12-18
b13-18
~y10
~y11
~y14
~y16
~b17
~y5
~y6
~y7
~y8
0.000e+0
5.000e+5
1.000e+6
1.500e+6
2.000e+6
2.500e+6
500
1000
1500

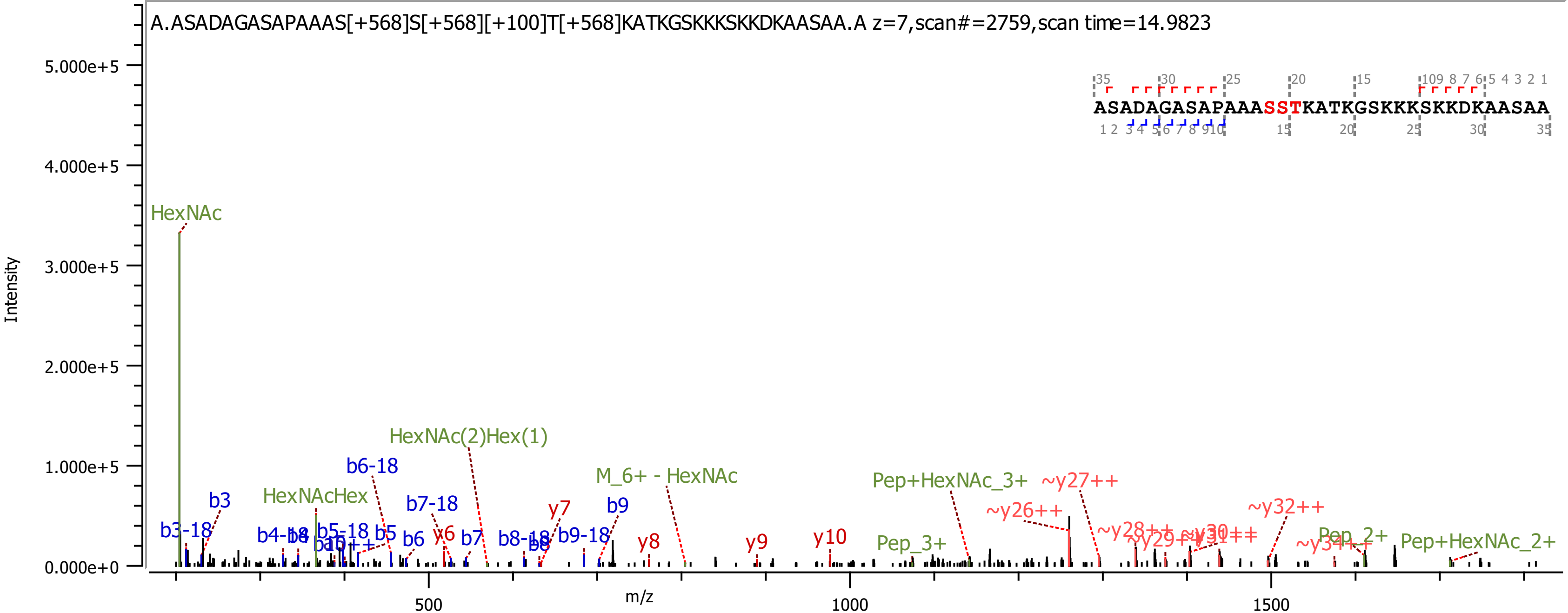
A.ASADAGASAPAAAS[+568]S[+568][+100]T[+568]KATKGSKKKSKKDKAASAA.A z=7,scan#=2759,scan time=14.9823
ASADAGASAPAAASSTKATKGSKKKSKKDKAASAA
Intensity
m/z
HexNAc
HexNAcHex
HexNAc(2)Hex(1)
M_6+ - HexNAc
Pep+HexNAc_3+
Pep_3+
Pep_2+
Pep+HexNAc_2+
b3-18
b3
b4-18
b5-18
b5
b6
b6-18
b7-18
b7
b8-18
b8
b9-18
b9
y6
y7
y8
y9
y10
~y26++
~y27++
~y28++
~y29++
~y30++
~y31++
~y32++
~y34++
0.000e+0
1.000e+5
2.000e+5
3.000e+5
4.000e+5
5.000e+5
500
1000
1500

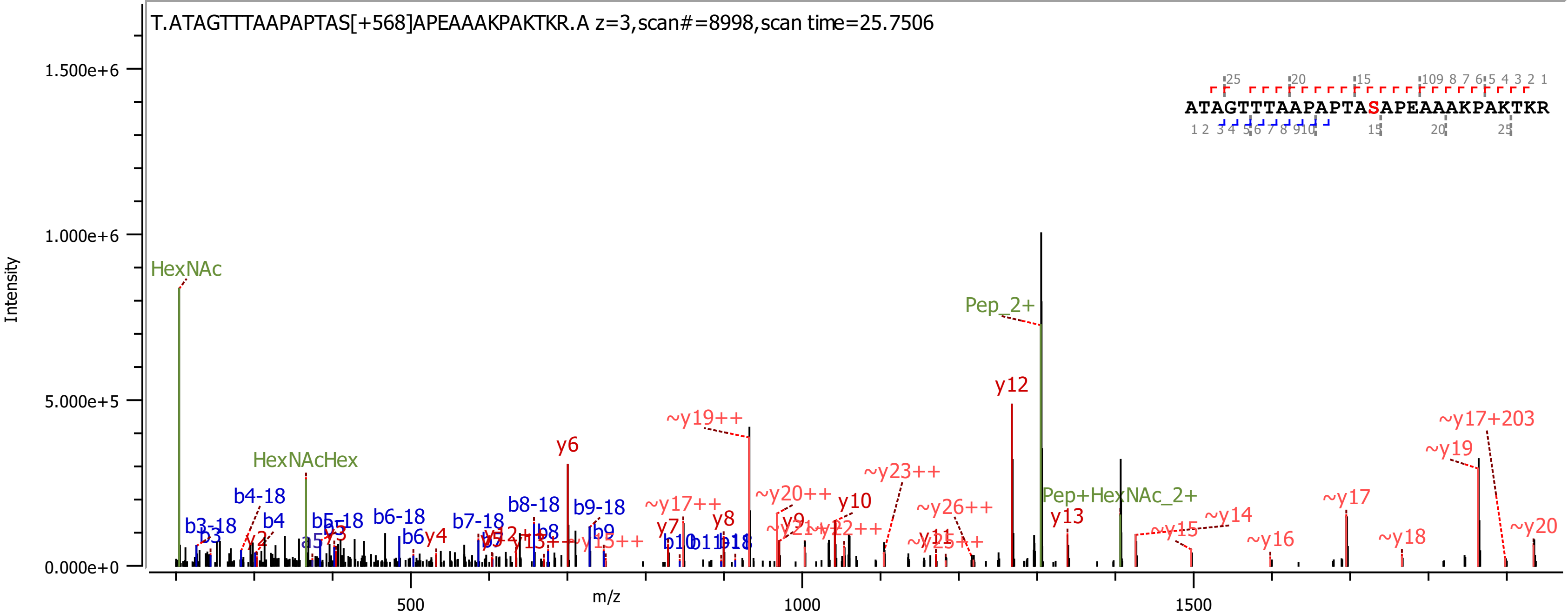

T.ATAGTTTAAPAPTAS[+568]APEAAAKPAKTKR.A z=3,scan#=8998,scan time=25.7506
ATAGTTTAAPAPTASAPEAAAKPAKTKR
Intensity
m/z
HexNAc
HexNAcHex
Pep_2+
Pep+HexNAc_2+
y6
y12
y13
~y19++
~y17+203
~y19
~y20
~y17
~y18
~y16
~y14
~y15
~y23++
~y20++
~y26++
~y17++
y10
y9
y8
y7
b4-18
b3-18
b4
b5-18
b6-18
b7-18
b8-18
b9-18
b9
b8
b6
y4
y3
y2
b3
b10

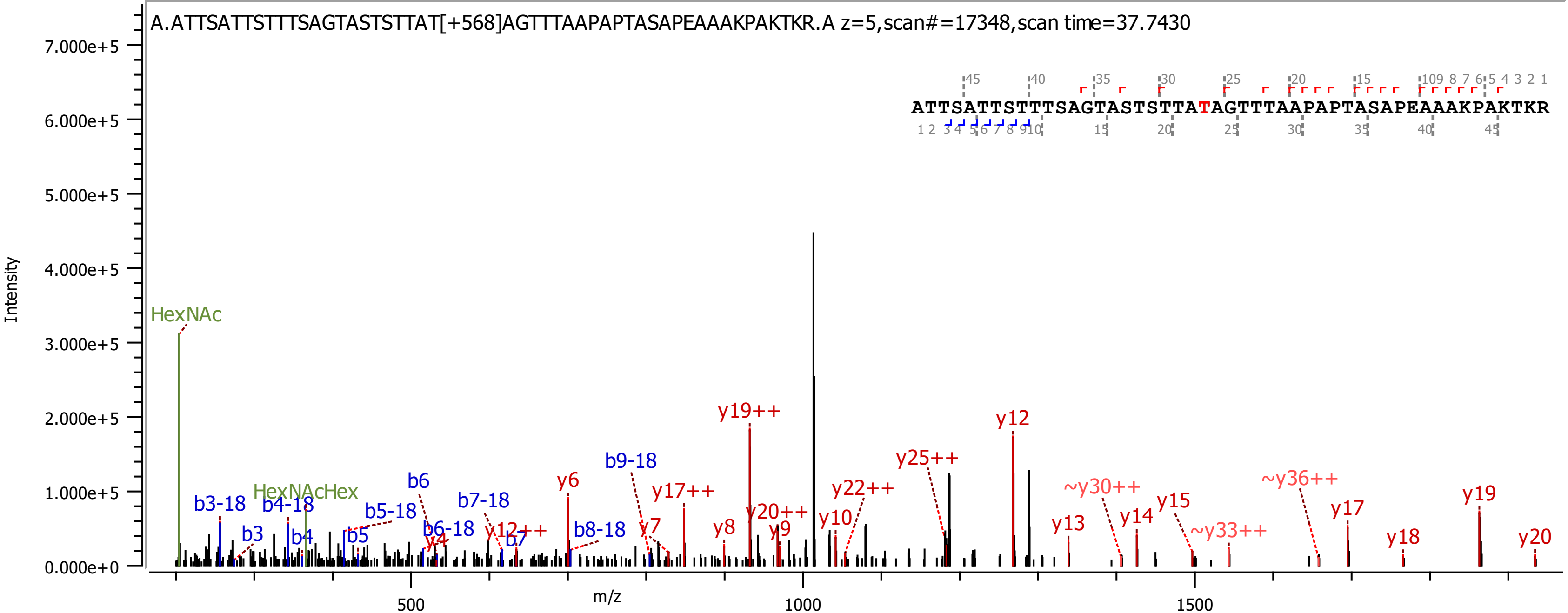
A.ATTSATTSTTTSAGTASTSTTAT[+568]AGTTTAAPAPTASAPEAAAKPAKTKR.A z=5,scan#=17348,scan time=37.7430
ATTSATTSTTTSAGTASTSTTATAGTTTAAPAPTASAPEAAAKPAKTKR
Intensity
m/z
HexNAc
HexNAcHex
b3-18
b3
b4-18
b4
b5-18
b5
b6
b6-18
b7-18
b7
b8-18
b9-18
y4
y6
y7
y8
y9
y10
y12++
y17++
y19++
y20++
y22++
y25++
y12
y13
y14
y15
~y30++
~y33++
~y36++
y17
y18
y19
y20

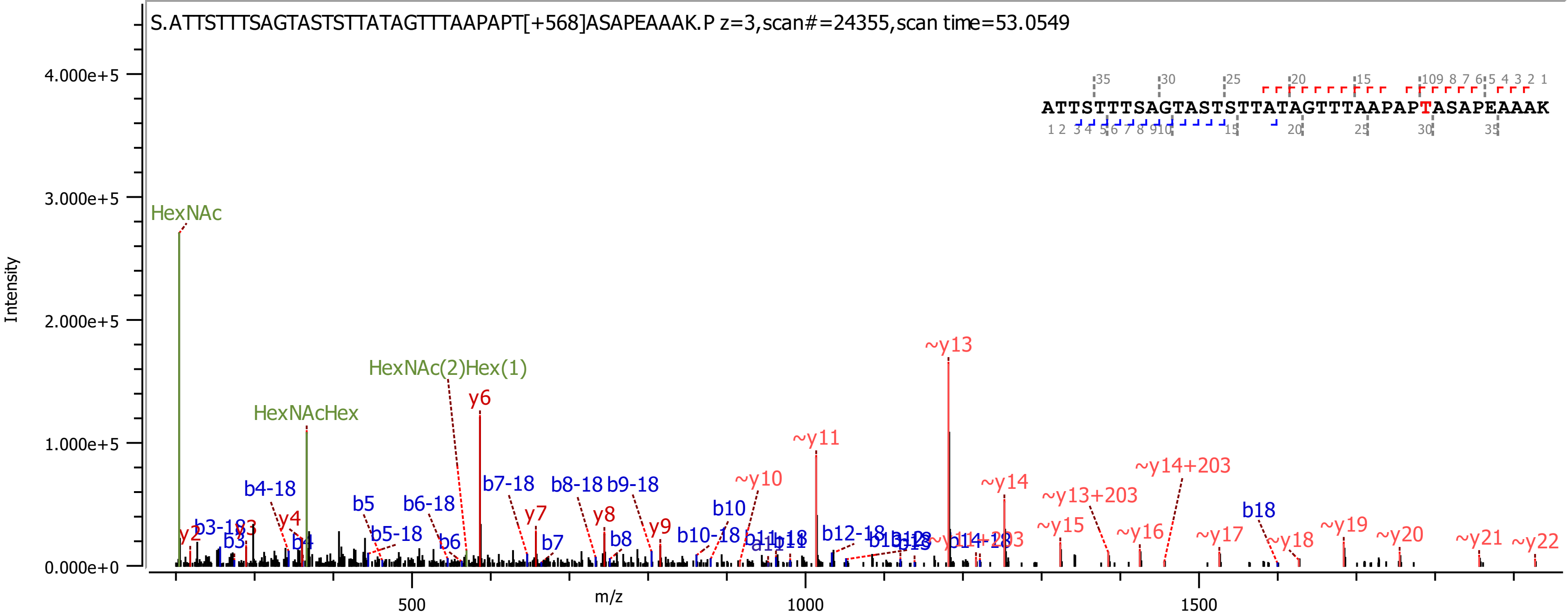
S.ATTSTTTSAGTASTSTTATAGTTTAAPAPT[+568]ASAPEAAAK.P z=3,scan#=24355,scan time=53.0549
ATTSTTTSAGTASTSTTATAGTTTAAPAPTASAPEAAAK
Intensity
m/z
4.000e+5
3.000e+5
2.000e+5
1.000e+5
0.000e+0
500
1000
1500
HexNAc
HexNAc(2)Hex(1)
HexNAcHex
y6
~y13
~y11
~y10
~y14
~y14+203
~y13+203
~y15
~y16
~y17
~y18
~y19
~y20
~y21
~y22
b18
b4-18
b5
b6-18
b7-18
b8-18
b9-18
b10
b10-18
b12-18
b5-18
b6
b7
b8
y7
y8
y9
y4
b4
b3
y2

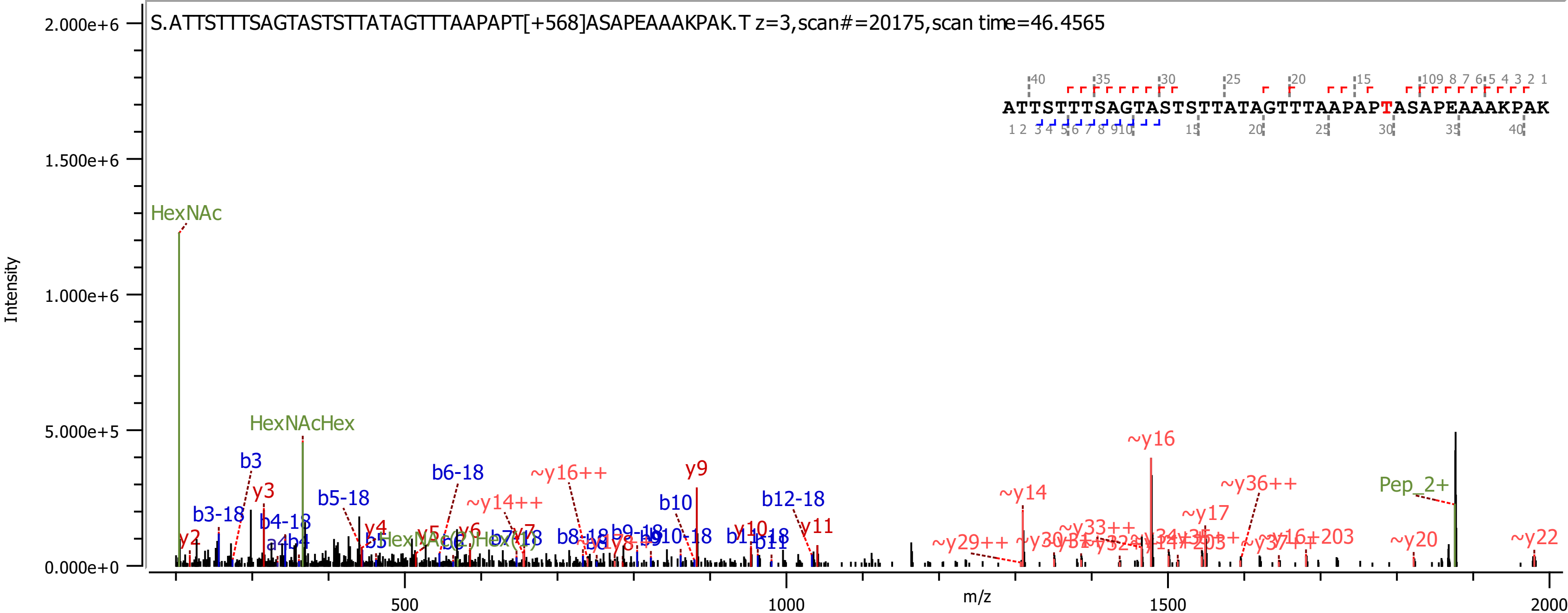
S.ATTSTTTSAGTASTSTTATAGTTTAAPAPT[+568]ASAPEAAAKPAK.T z=3,scan#=20175,scan time=46.4565
ATTSTTTSAGTASTSTTATAGTTTAAPAPTASAPEAAAKPAK
Intensity
m/z
2.000e+6
1.500e+6
1.000e+6
5.000e+5
0.000e+0
500
1000
1500
2000
HexNAc
HexNAcHex
Pep_2+
b3
b3-18
y3
b4-18
y2
y4
b5-18
b6-18
~y16++
~y14++
y9
b10
b12-18
y10
y11
b11
~y16
~y14
~y36++
~y17
~y33++
~y29++
~y16+203
~y20
~y22

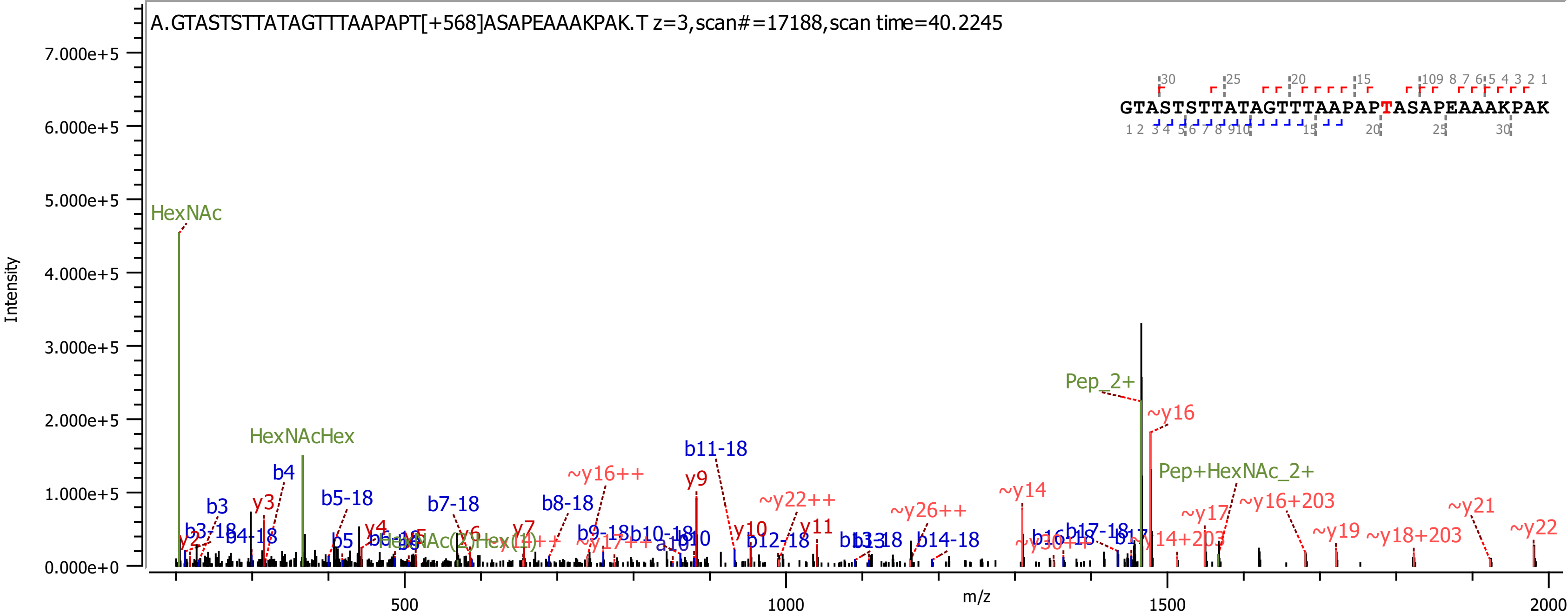

A.GTASTSTTATAGTTTAAPAPT[+568]ASAPEAAAKPAK.T z=3,scan#=17188,scan time=40.2245
GTASTSTTATAGTTTAAPAPTASAPEAAAKPAK
Intensity
m/z
HexNAc
HexNAcHex
Pep_2+
~y16
Pep+HexNAc_2+
b11-18
y9
~y16++
~y22++
~y26++
~y14
~y17
~y16+203
~y19
~y18+203
~y21
~y22
b3
b4
y3
b5-18
b7-18
b8-18
b12-18
b14-18
y10
y11

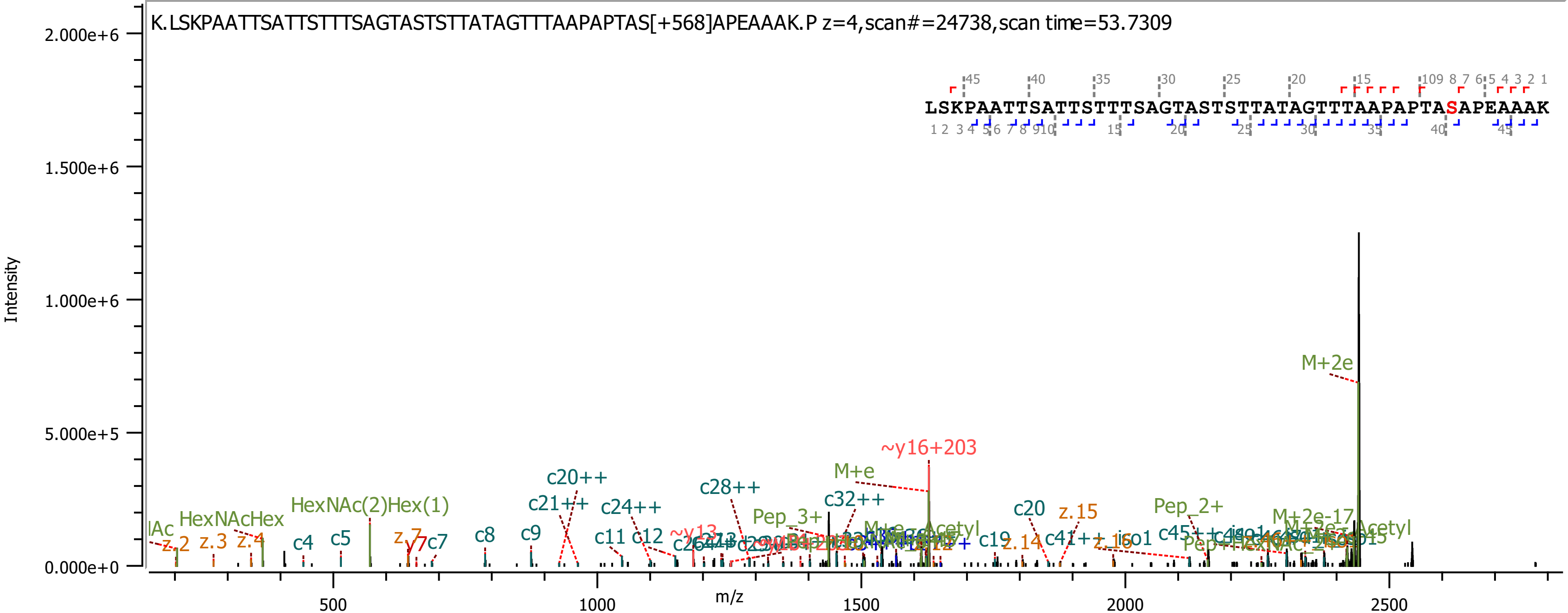
K.LSKPAATTSATTSTTTSAGTASTSTTATAGTTTAAPAPTAS[+568]APEAAAK.P z=4,scan#=24738,scan time=53.7309
LSKPAATTSATTSTTTSAGTASTSTTATAGTTTAAPAPTASAPEAAAK
Intensity
m/z
2.000e+6
1.500e+6
1.000e+6
5.000e+5
0.000e+0
500
1000
1500
2000
2500
M+2e
~y16+203
M+e
c28++
c20++
c21++
c24++
c32++
Pep_3+
HexNAc(2)Hex(1)
HexNAcHex
z.2
z.3
z.4
c4
c5
z.7
y7
c7
c8
c9
c11
c12
c20
z.15
Pep_2+
M+2e-17
c19
z.14
c41++
z.16
iso1
c45++

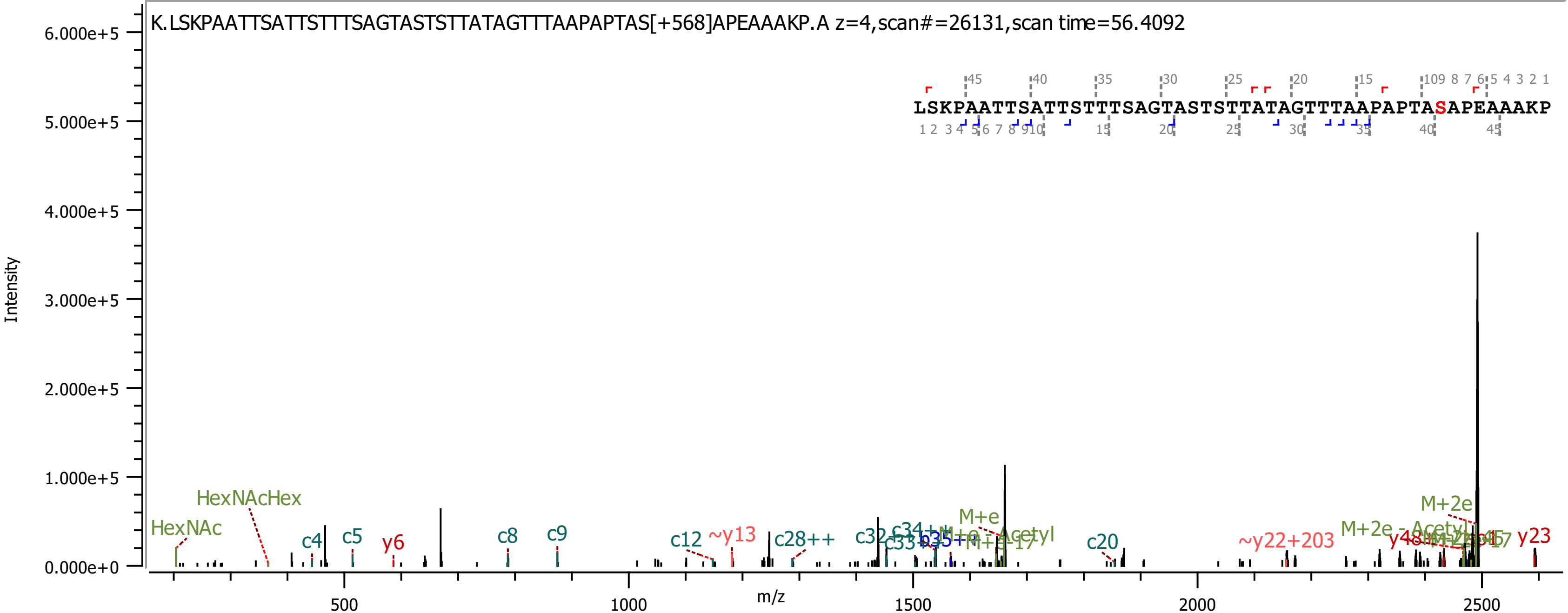
K.LSKPAATTSATTSTTTSAGTASTSTTATAGTTTAAPAPTAS[+568]APEAAAKP.A z=4,scan#=26131,scan time=56.4092
LSKPAATTSATTSTTTSAGTASTSTTATAGTTTAAPAPTASAPEAAAKP
Intensity
m/z
6.000e+5
5.000e+5
4.000e+5
3.000e+5
2.000e+5
1.000e+5
0.000e+0
500
1000
1500
2000
2500
HexNAc
HexNAcHex
c4
c5
y6
c8
c9
c12
~y13
c28++
c20
~y22+203
y23
M+e
M+2e
M+2e - Acetyl

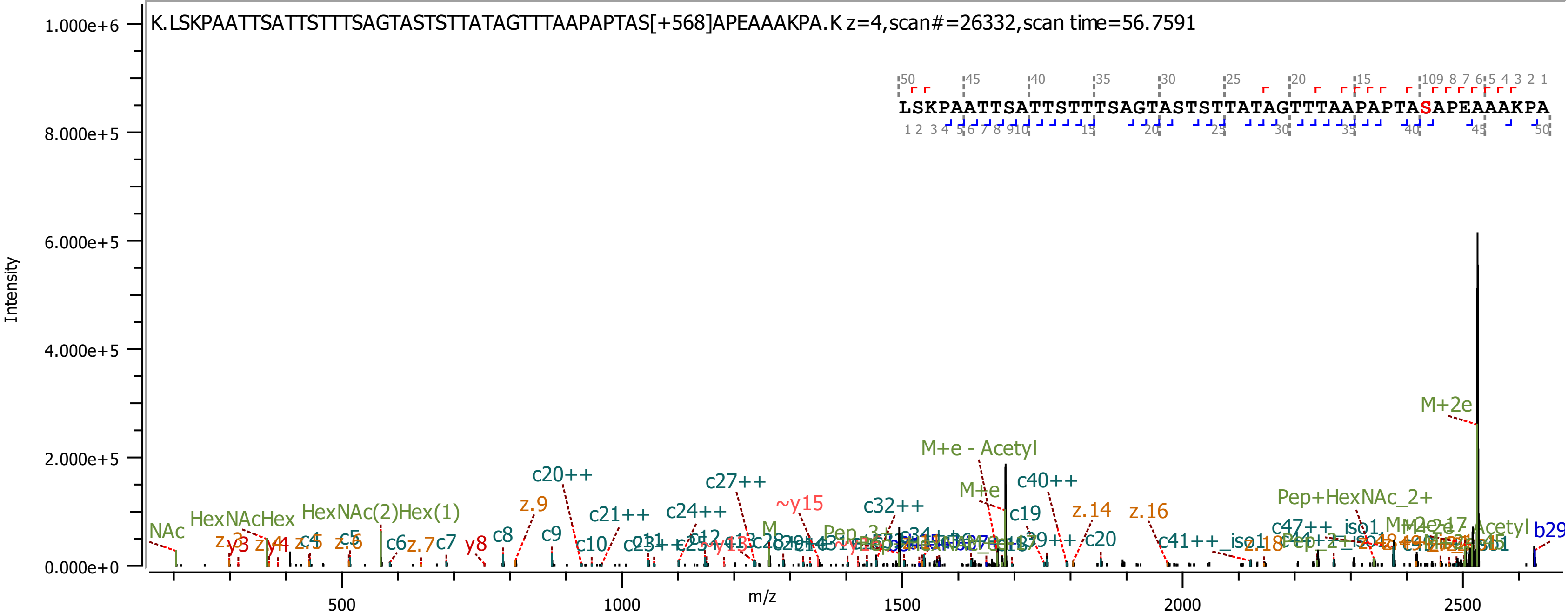
K.LSKPAATTSATTSTTTSAGTASTSTTATAGTTTAAPAPTAS[+568]APEAAAKPA.K z=4,scan#=26332,scan time=56.7591
LSKPAATTSATTSTTTSAGTASTSTTATAGTTTAAPAPTASAPEAAAKPA
Intensity
m/z
1.000e+6
8.000e+5
6.000e+5
4.000e+5
2.000e+5
0.000e+0
500
1000
1500
2000
2500
M+2e
M+e - Acetyl
M+e
c20++
c27++
c40++
c32++
c24++
c21++
~y15
z.9
z.14
z.16
c19
c20
c8
c9
c10
c7
c6
y8
z.7
HexNAcHex
HexNAc(2)Hex(1)
NAc
Pep+HexNAc_2+
c47++_iso1
c41++_iso1
z.18
b29

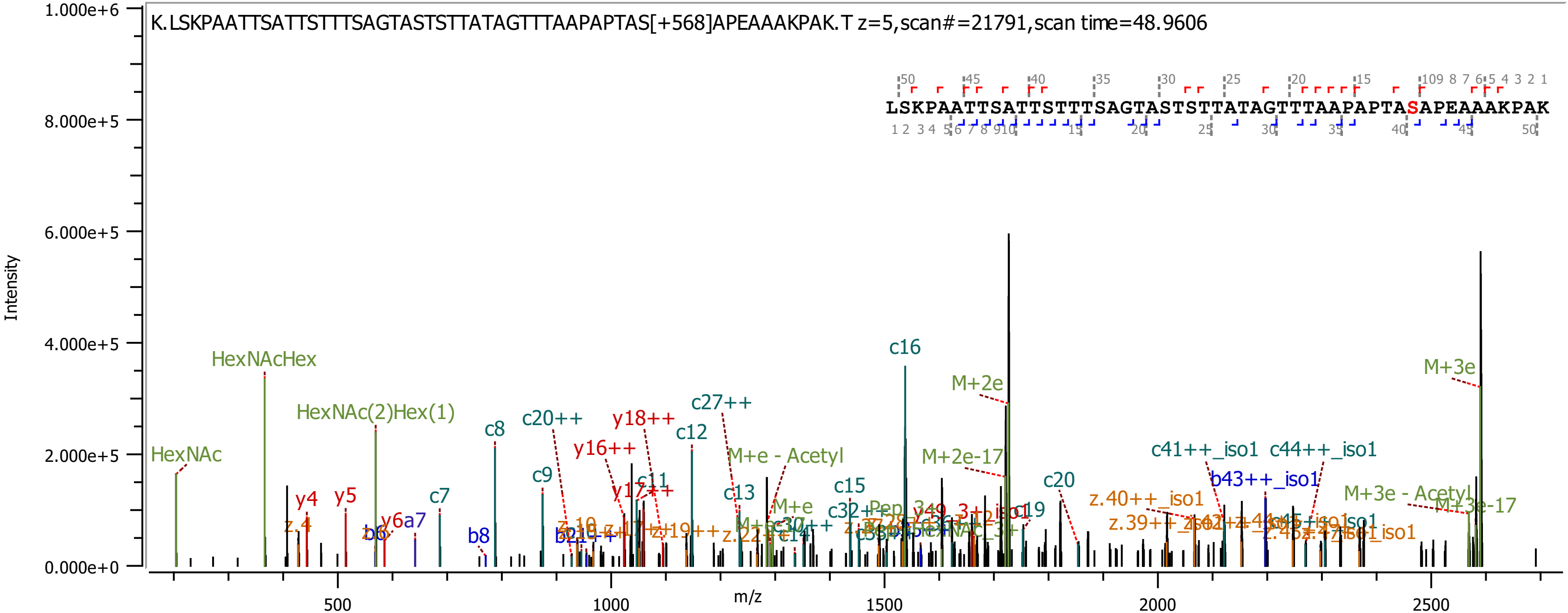
K.LSKPAATTSATTSTTTSAGTASTSTTATAGTTTAAPAPTAS[+568]APEAAAKPAK.T z=5,scan#=21791,scan time=48.9606
LSKPAATTSATTSTTTSAGTASTSTTATAGTTTAAPAPTASAPEAAAKPAK
Intensity
1.000e+6
8.000e+5
6.000e+5
4.000e+5
2.000e+5
0.000e+0
m/z
500
1000
1500
2000
2500
HexNAc
HexNAcHex
HexNAc(2)Hex(1)
y4
y5
z.4
b6
y6
a7
c7
b8
c8
c9
c20++
y16++
y18++
y17++
c11
c12
c27++
c13
M+e - Acetyl
M+e
c14
c15
c16
M+2e
M+2e-17
c19
c20
z.40++_iso1
z.39++_iso1
c41++_iso1
c44++_iso1
b43++_iso1
M+3e
M+3e - Acetyl
M+3e-17

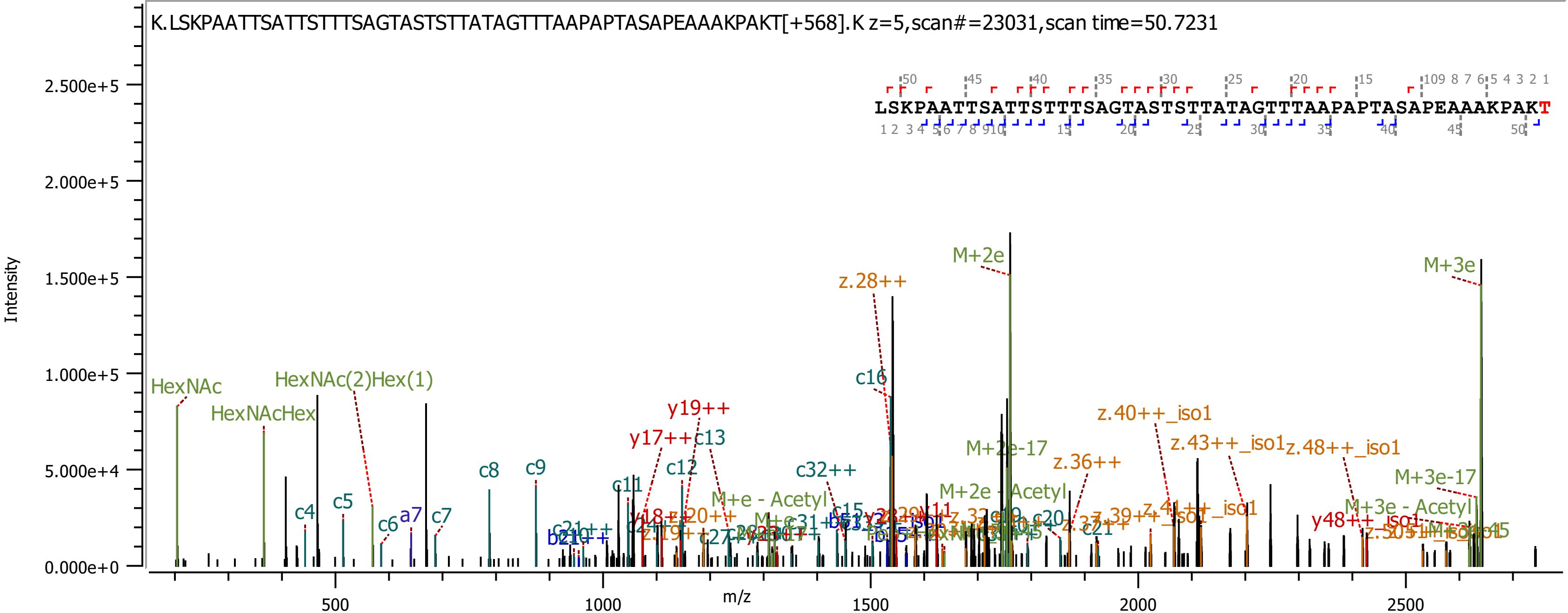
K.LSKPAATTSATTSTTTSAGTASTSTTATAGTTTAAPAPTASAPEAAAKPAKT[+568].K z=5,scan#=23031,scan time=50.7231
LSKPAATTSATTSTTTSAGTASTSTTATAGTTTAAPAPTASAPEAAAKPAKT
Intensity
m/z
2.500e+5
2.000e+5
1.500e+5
1.000e+5
5.000e+4
0.000e+0
500
1000
1500
2000
2500
HexNAc
HexNAcHex
HexNAc(2)Hex(1)
M+2e
M+3e
z.28++
c16
y19++
y17++
c13
c12
c11
c8
c9
c4
c5
c6
a7
c7
c32++
M+e - Acetyl
M+2e-17
M+2e - Acetyl
z.36++
z.40++_iso1
z.43++_iso1
z.48++_iso1
M+3e-17
M+3e - Acetyl
y48++
c20
c21

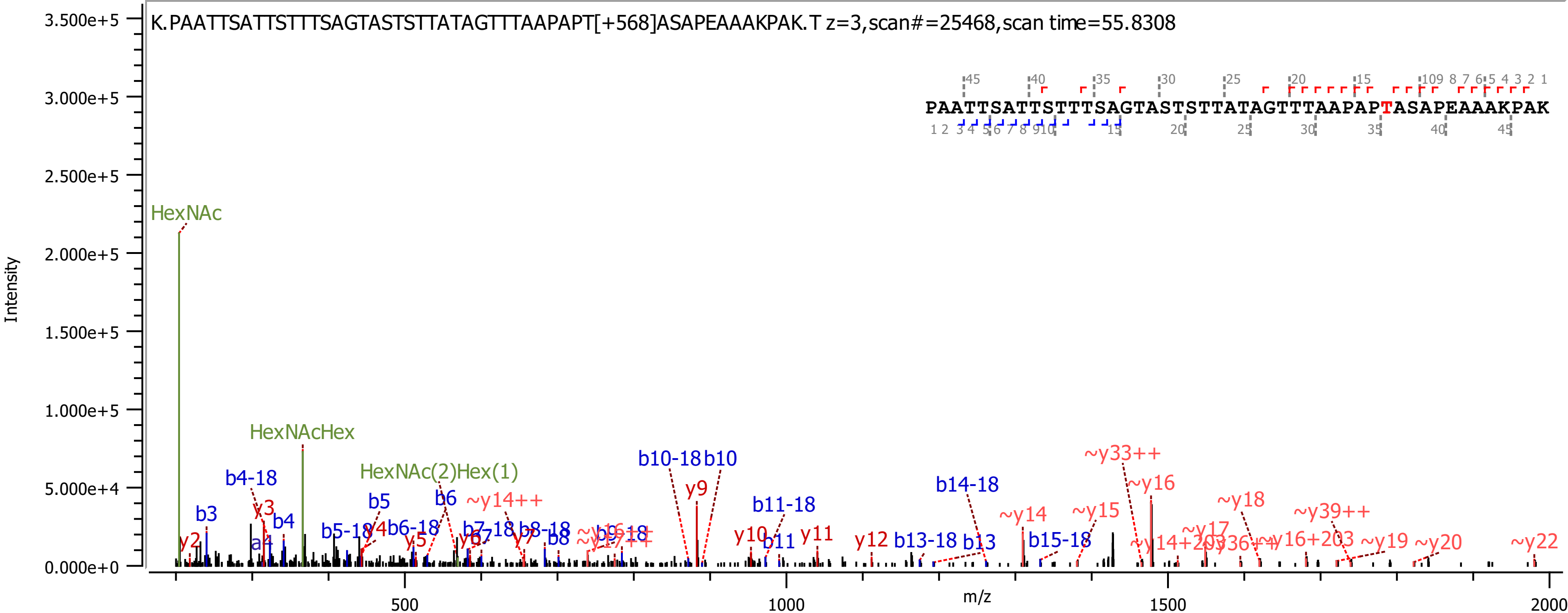
K.PAATTSATTSTTTSAGTASTSTTATAGTTTAAPAPT[+568]ASAPEAAAKPAK.T z=3,scan#=25468,scan time=55.8308
PAATTSATTSTTTSAGTASTSTTATAGTTTAAPAPTASAPEAAAKPAK
Intensity
m/z
3.500e+5
3.000e+5
2.500e+5
2.000e+5
1.500e+5
1.000e+5
5.000e+4
0.000e+0
500
1000
1500
2000
HexNAc
HexNAcHex
HexNAc(2)Hex(1)
b3
b4-18
y3
b4
a4
y2
b5-18
b5
y4
b6-18
b6
y5
~y14++
b7-18
b8-18
b8
b10-18
b10
y9
b11-18
b11
y10
y11
y12
b13-18
b13
b14-18
~y14
b15-18
~y15
~y33++
~y16
~y18
~y17
~y39++
~y16+203
~y19
~y20
~y22

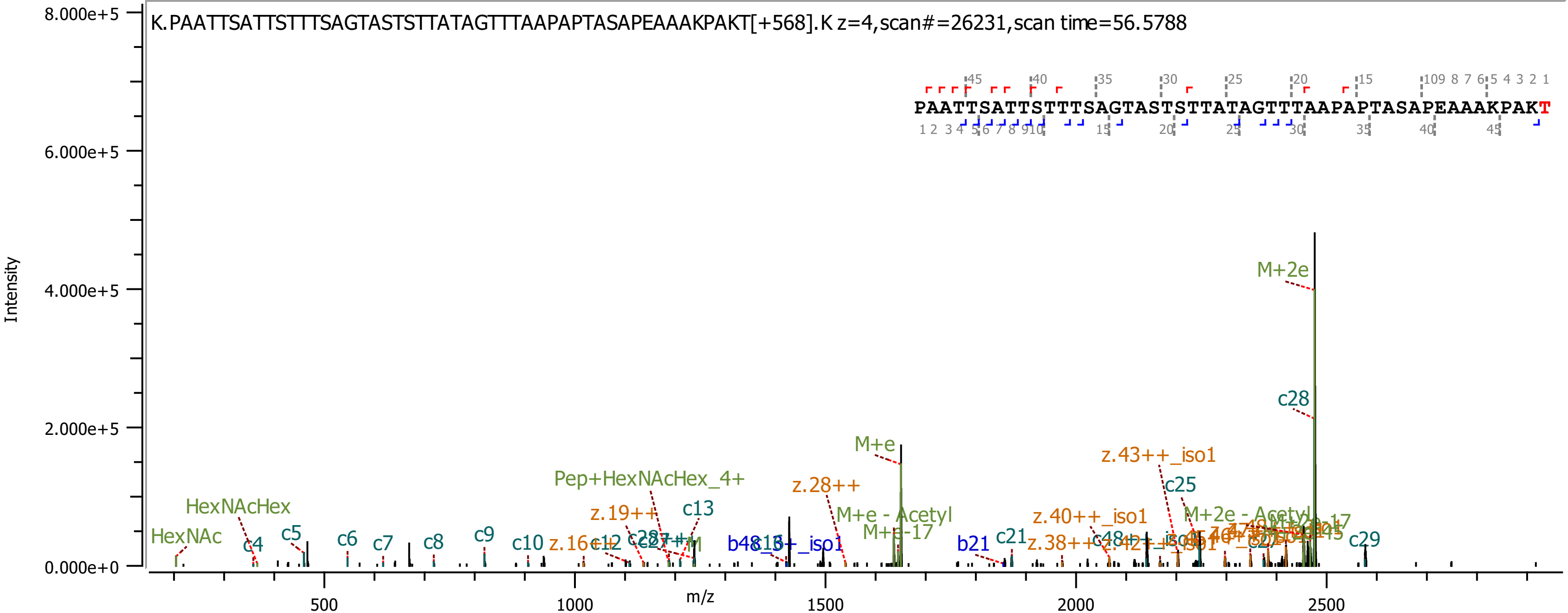
K.PAATTSATTSTTTSAGTASTSTTATAGTTTAAPAPTASAPEAAAKPAKT[+568].K z=4,scan#=26231,scan time=56.5788
PAATTSATTSTTTSAGTASTSTTATAGTTTAAPAPTASAPEAAAKPAKT
Intensity
m/z
8.000e+5
6.000e+5
4.000e+5
2.000e+5
0.000e+0
500
1000
1500
2000
2500
M+2e
c28
M+e
z.43++_iso1
c25
z.28++
M+e - Acetyl
M+e-17
M+2e - Acetyl
Pep+HexNAcHex_4+
HexNAcHex
HexNAc
c4
c5
c6
c7
c8
c9
c10
z.16++
c12
c13
z.19++
z.40++_iso1
z.38++
b21
c21
c29

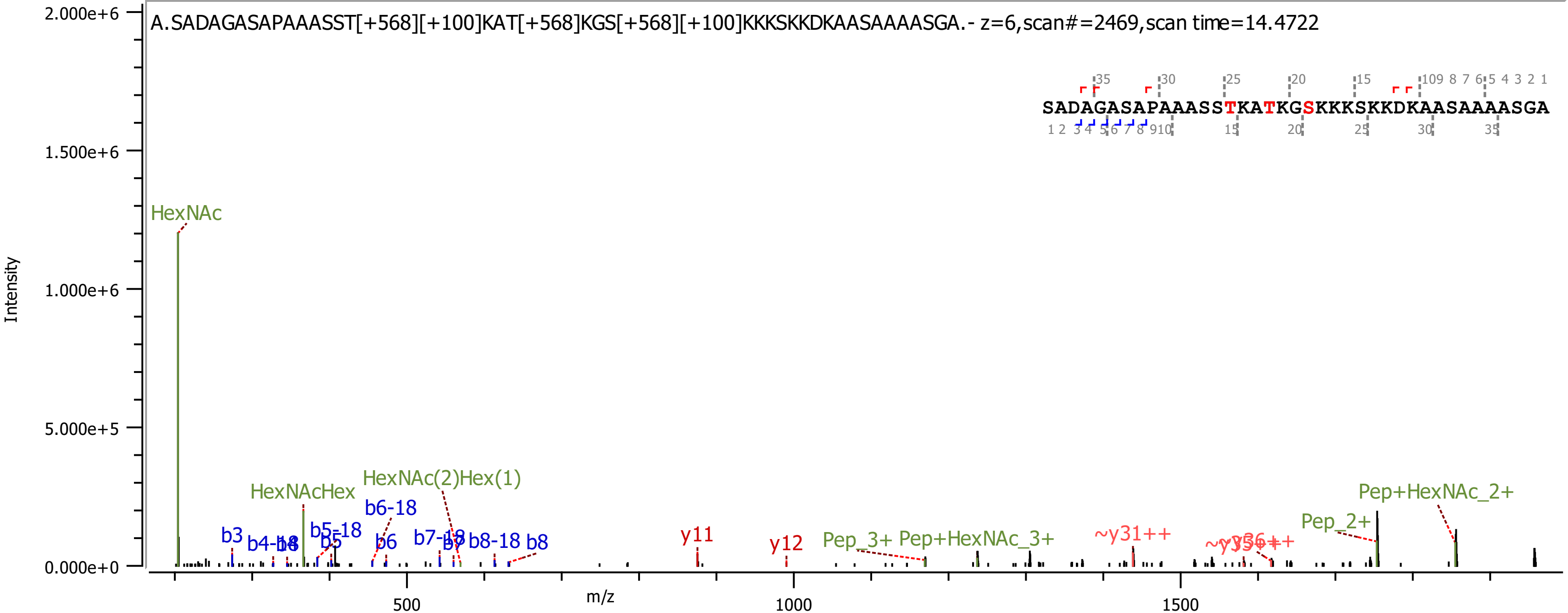

A.SADAGASAPAAASST[+568][+100]KAT[+568]KGS[+568][+100]KKKSKKDKAASAAAASGA.- z=6,scan#=2469,scan time=14.4722
SADAGASAPAAASSTKATKGSKKKSKKDKAASAAAASGA
Intensity
m/z
2.000e+6
1.500e+6
1.000e+6
5.000e+5
0.000e+0
500
1000
1500
HexNAc
HexNAcHex
HexNAc(2)Hex(1)
b3
b5-18
b5
b6-18
b6
b7-18
b7
b8-18
b8
y11
y12
Pep_3+
Pep+HexNAc_3+
~y31++
Pep_2+
Pep+HexNAc_2+

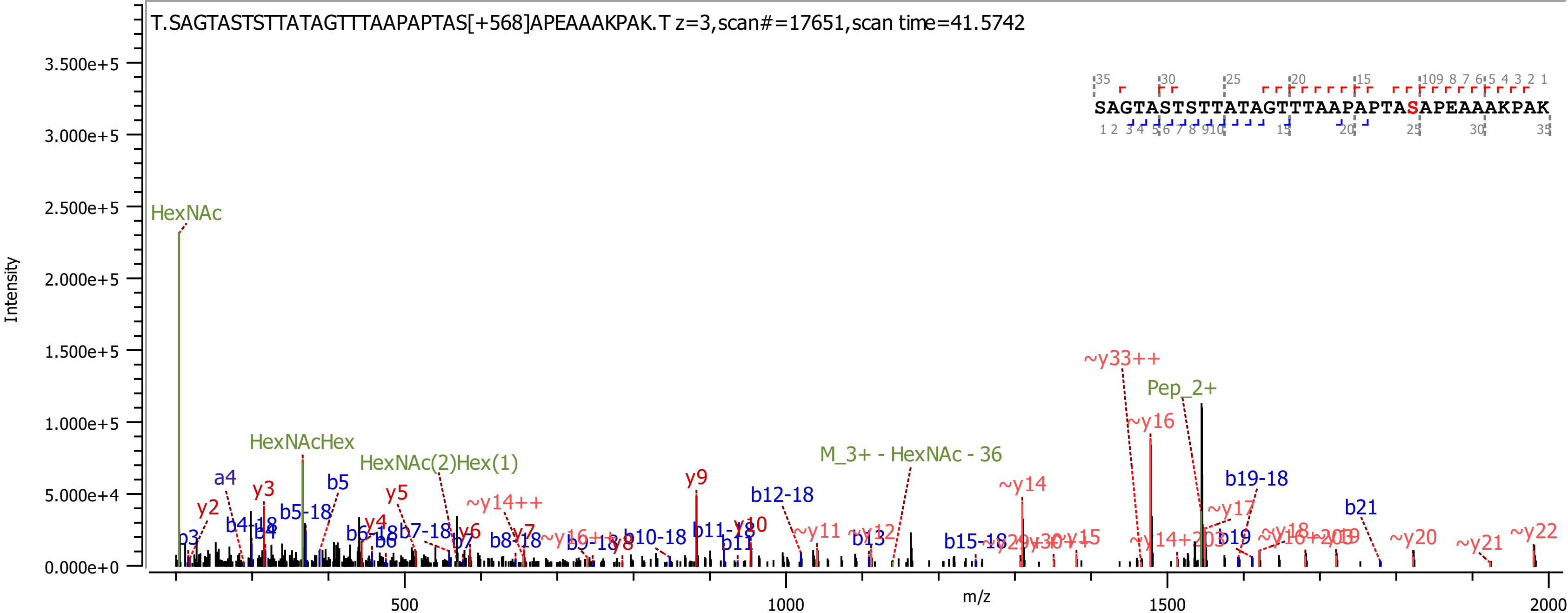
T.SAGTASTSTTATAGTTTAAPAPTAS[+568]APEAAAKPAK.T z=3,scan#=17651,scan time=41.5742
SAGTASTSTTATAGTTTAAPAPTASAPEAAAKPAK
Intensity
m/z
HexNAc
HexNAcHex
HexNAc(2)Hex(1)
M_3+ - HexNAc - 36
Pep_2+
~y33++
~y16
~y14
b19-18
b21
~y17
~y22
~y20
~y21
y9
y10
b12-18
b11-18
b11
b13
b15-18
a4
y2
y3
y4
y5
y6
y7
y8
b3
b4-18
b4
b5
b5-18
b6-18
b6
b7-18
b7
b8-18
b9-18
b10-18
~y14++
~y11
~y12
~y15
b19

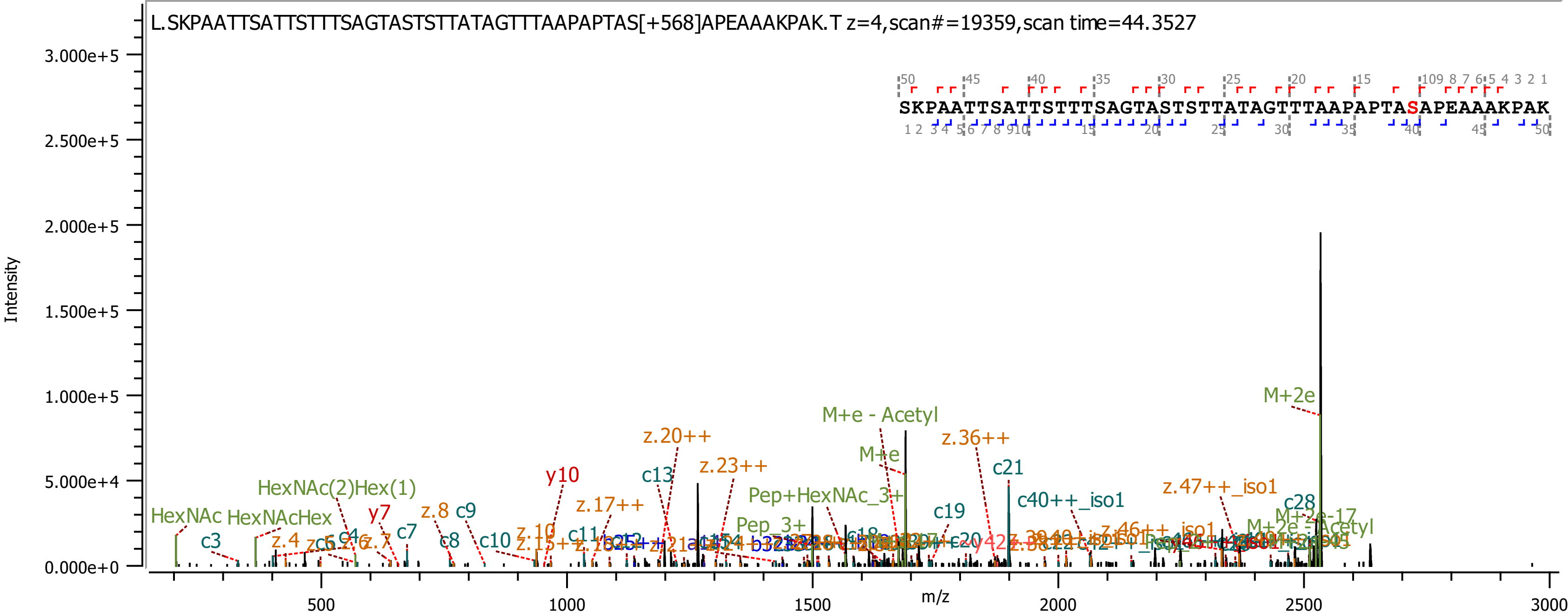

L.SKPAATTSATTSTTTSAGTASTSTTATAGTTTAAPAPTAS[+568]APEAAAKPAK.T z=4,scan#=19359,scan time=44.3527
SKPAATTSATTSTTTSAGTASTSTTATAGTTTAAPAPTASAPEAAAKPAK
Intensity
m/z
M+2e
M+e - Acetyl
M+e
z.36++
z.20++
z.23++
c21
c13
y10
HexNAc(2)Hex(1)
c40++_iso1
z.47++_iso1
c28
M+2e-17
M+2e - Acetyl
Pep+HexNAc_3+
Pep_3+
c19
z.17++
c9
z.8
y7
HexNAc
HexNAcHex
c3
c7
c8
c10
c11

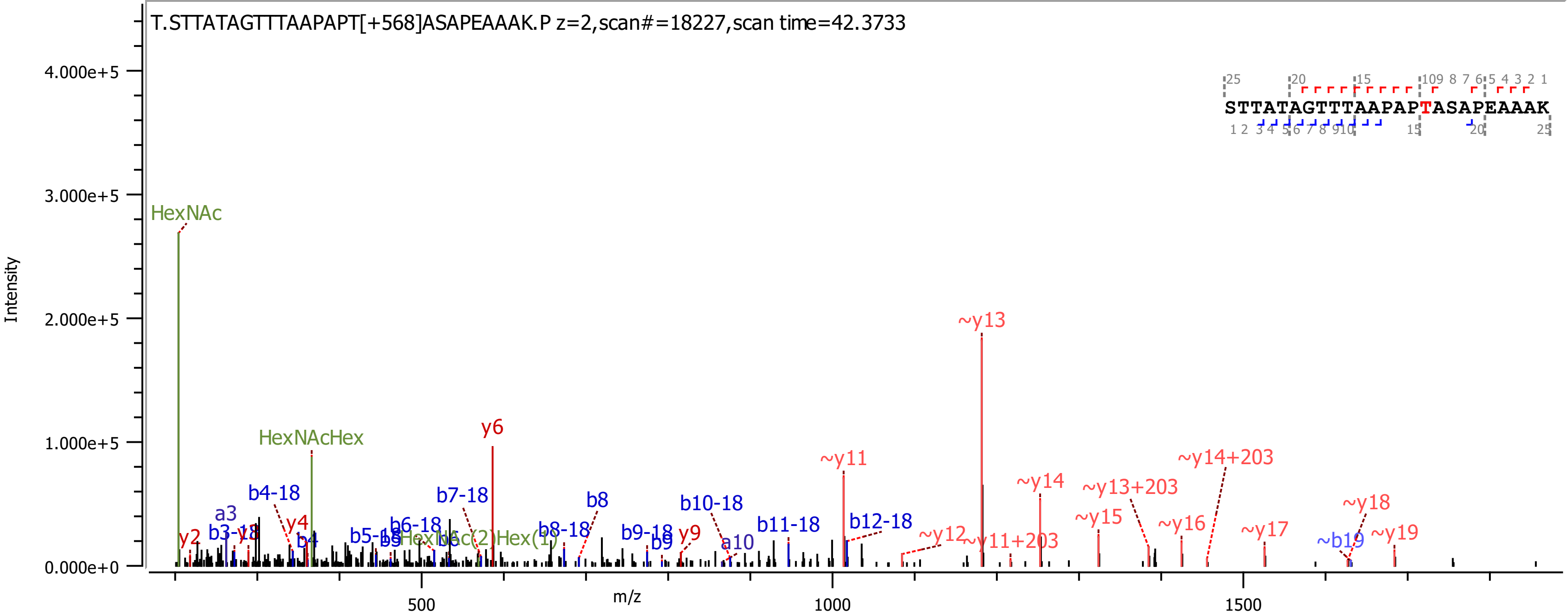

T.STTATAGTTTAAPAPT[+568]ASAPEAAAK.P z=2,scan#=18227,scan time=42.3733
STTATAGTTTAAPAPTASAPEAAAK
Intensity
m/z
4.000e+5
3.000e+5
2.000e+5
1.000e+5
0.000e+0
500
1000
1500
HexNAc
HexNAcHex
y6
~y13
~y11
~y14
~y14+203
~y13+203
~y15
~y16
~y17
~y18
~y19
~b19
~y12
~y11+203
b12-18
b11-18
b10-18
a10
y9
b9-18
b9
b8
b8-18
b7-18
b6-18
b5-18
b4-18
y4
b4
a3
b3-18
y2

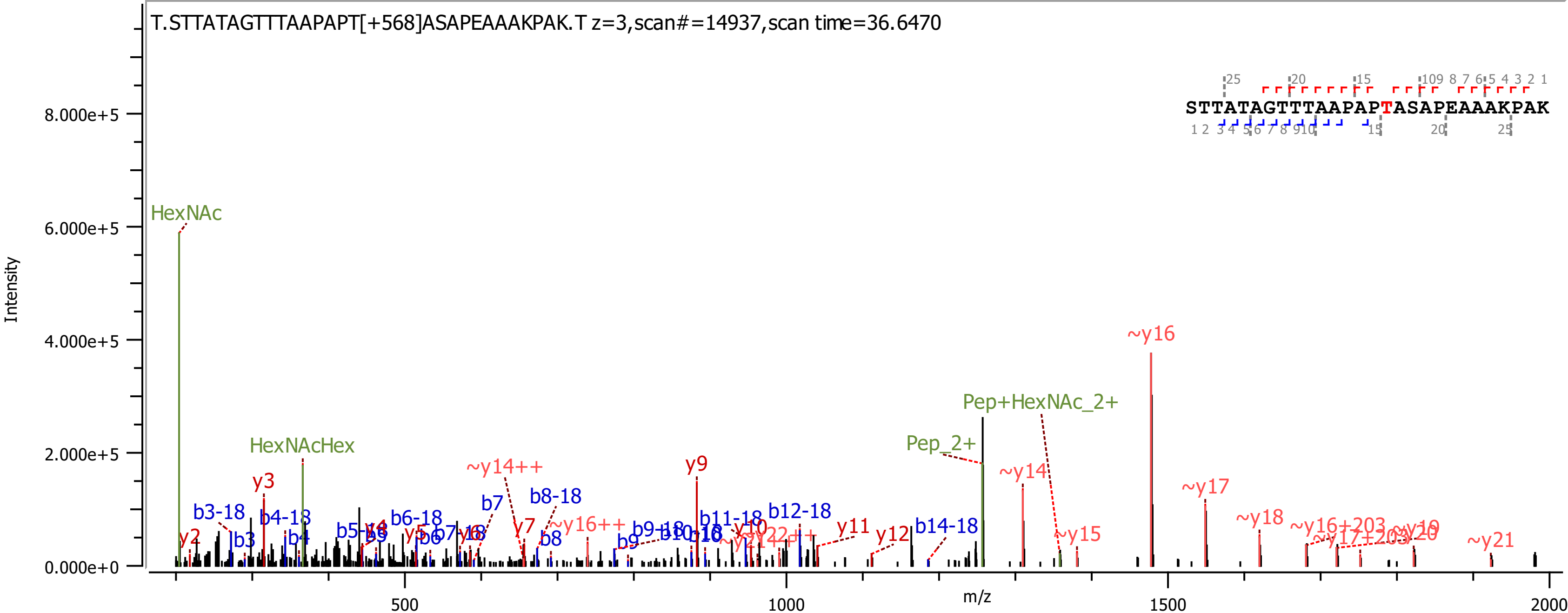

T.STTATAGTTTAAPAPT[+568]ASAPEAAAKPAK.T z=3,scan#=14937,scan time=36.6470
STTATAGTTTAAPAPTASAPEAAAKPAK
Intensity
m/z
HexNAc
HexNAcHex
Pep_2+
Pep+HexNAc_2+
~y16
~y14
~y17
~y18
~y15
~y21
~y14++
~y16++
y2
y3
y7
y9
y11
y12
b3-18
b4-18
b6-18
b7
b8-18
b8
b9
b11-18
b12-18
b14-18

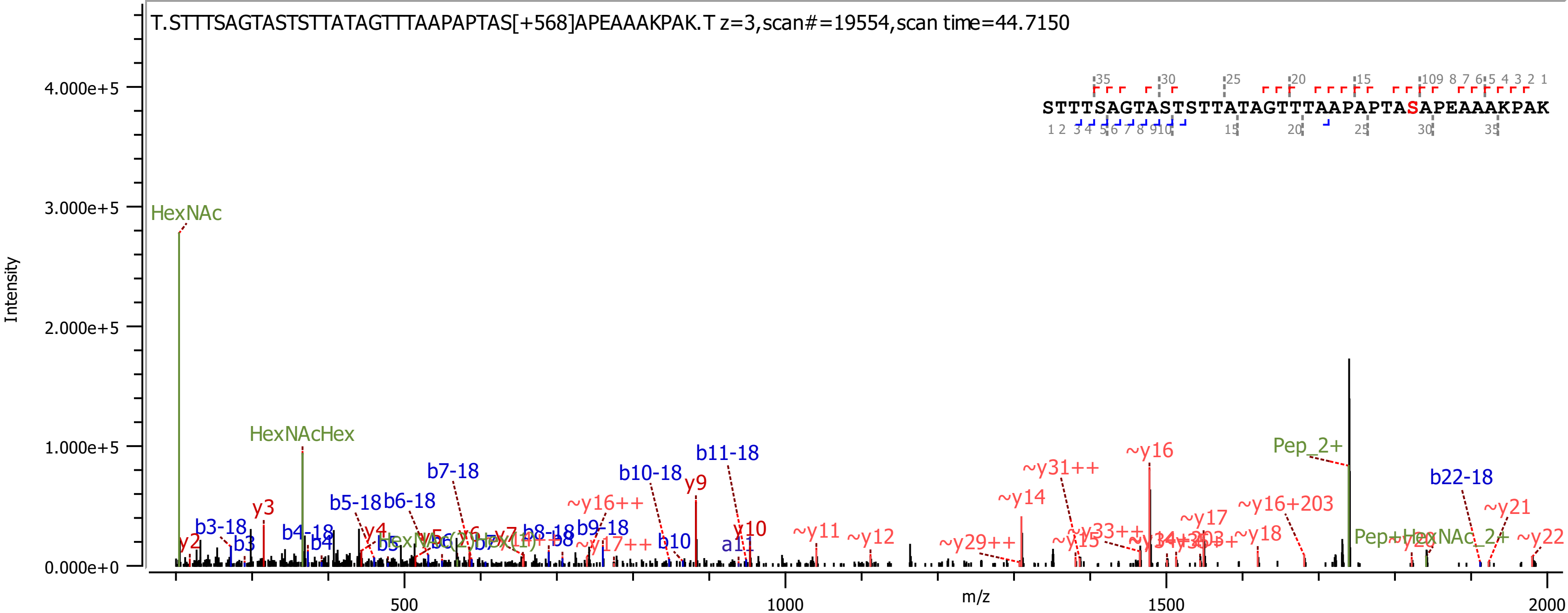
T.STTTSAGTASTSTTATAGTTTAAPAPTAS[+568]APEAAAKPAK.T z=3,scan#=19554,scan time=44.7150
STTTSAGTASTSTTATAGTTTAAPAPTASAPEAAAKPAK
Intensity
m/z
HexNAc
HexNAcHex
Pep_2+
Pep+HexNAc_2+
~y16
~y31++
~y14
~y16++
~y16+203
~y17
~y18
~y21
~y22
~y11
~y12
~y29++
~y15
~y33++
~y17++
b22-18
b11-18
b10-18
b7-18
b5-18
b6-18
b3-18
b4-18
b9-18
b8-18
b3
b4
b5
b8
b10
a11
y2
y3
y4
y5
y7
y9
y10
4.000e+5
3.000e+5
2.000e+5
1.000e+5
0.000e+0
500
1000
1500
2000

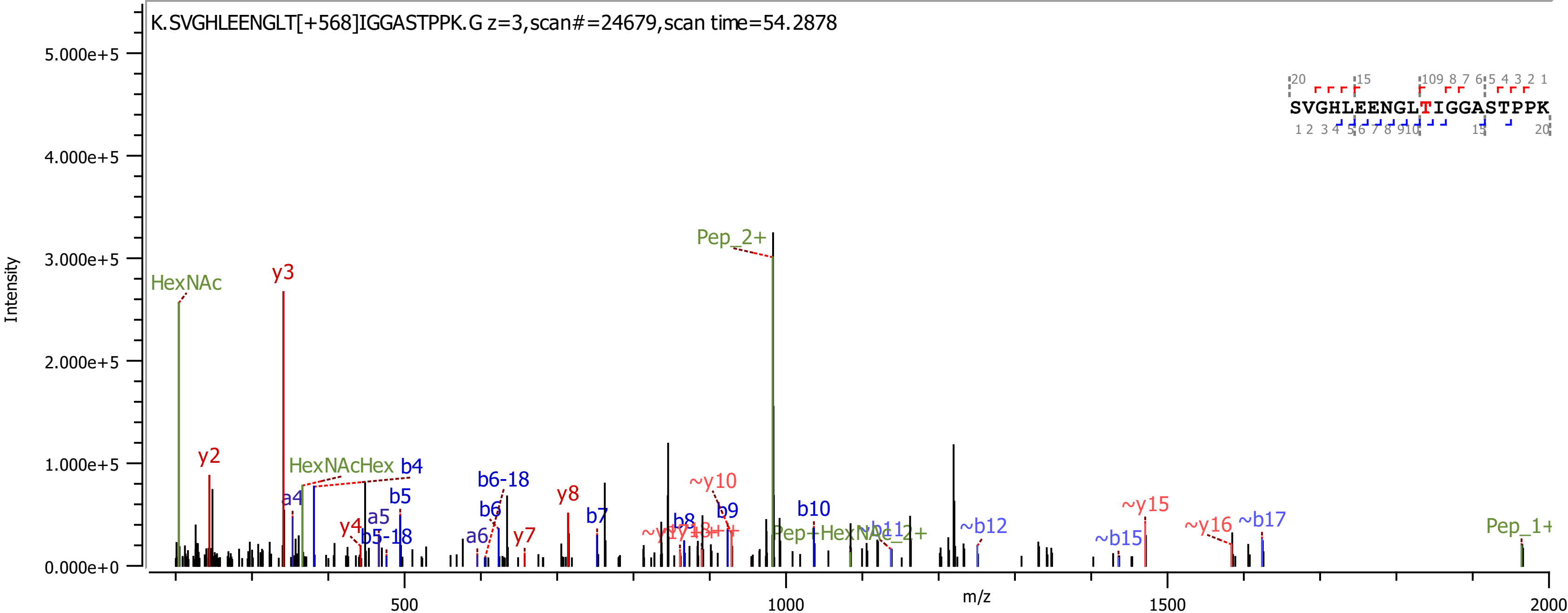

K.SVGHLEENGLT[+568]IGGASTPPK.G z=3,scan#=24679,scan time=54.2878
SVGHLEENGLTIGGASTPPK
Intensity
m/z
HexNAc
y3
y2
HexNAcHex
b4
a4
b5
a5
y4
b5-18
b6-18
b6
a6
y7
y8
b7
Pep_2+
~y10
b8
b9
b10
Pep+HexNAc_2+
~b11
~b12
~y15
~b15
~y16
~b17
Pep_1+

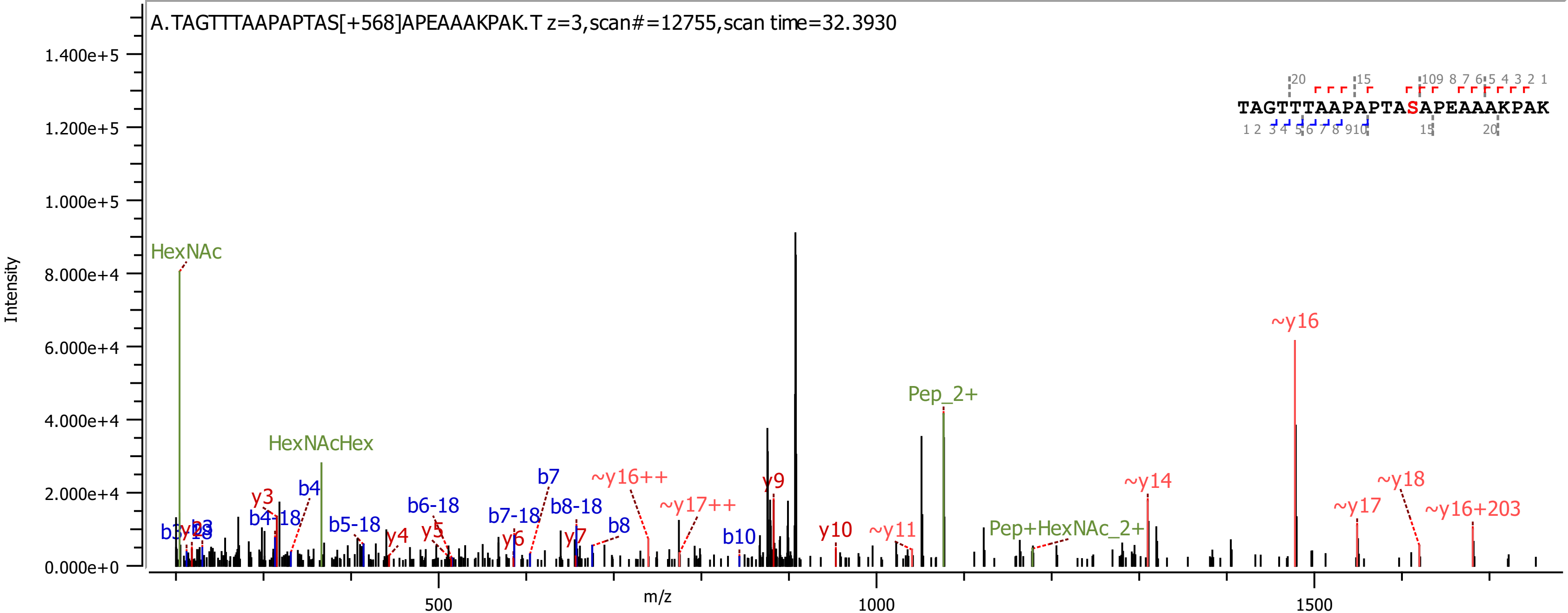

A.TAGTTTAAPAPTAS[+568]APEAAAKPAK.T z=3,scan#=12755,scan time=32.3930
TAGTTTAAPAPTASAPEAAAKPAK
Intensity
m/z
HexNAc
HexNAcHex
Pep_2+
Pep+HexNAc_2+
~y16
~y16++
~y17++
~y14
~y17
~y18
~y16+203
~y11
y3
y4
y5
y6
y7
y9
y10
b4
b4-18
b5-18
b6-18
b7
b7-18
b8-18
b8
b10

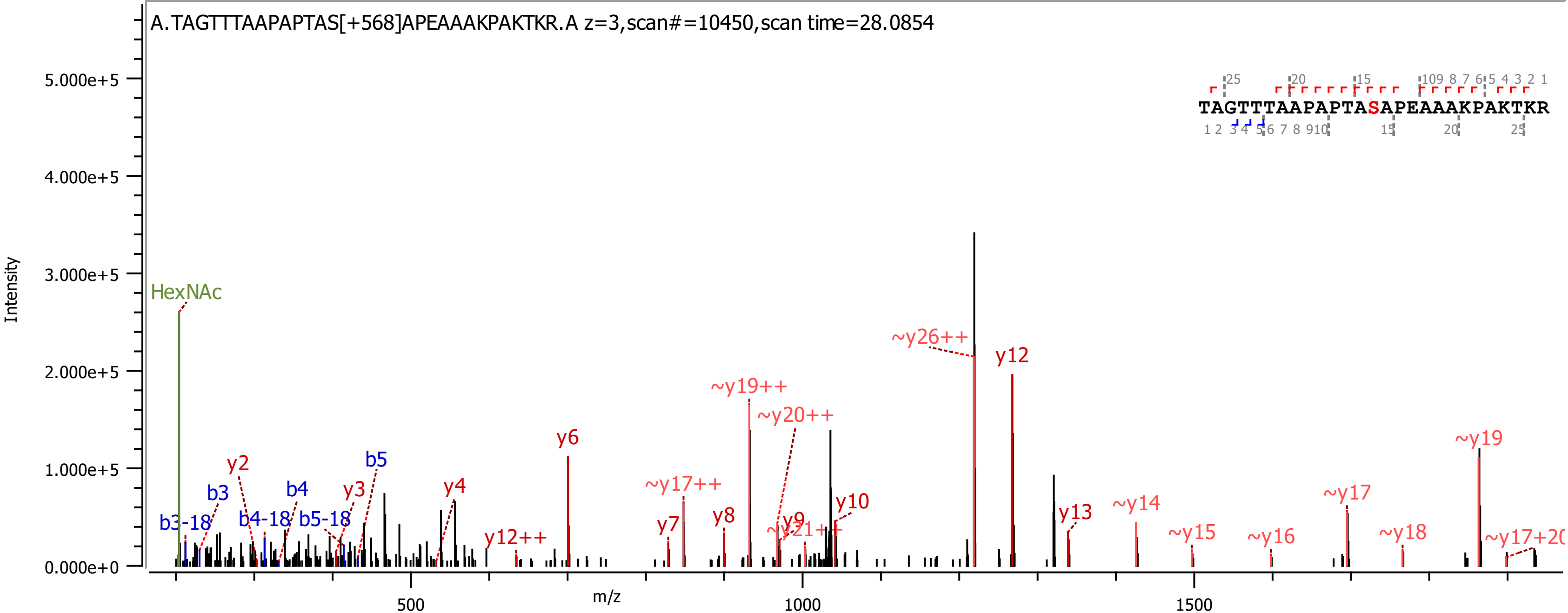
A.TAGTTTAAPAPTAS[+568]APEAAAKPAKTKR.A z=3,scan#=10450,scan time=28.0854
TAGTTTAAPAPTASAPEAAAKPAKTKR
Intensity
m/z
HexNAc
b3-18
b3
y2
b4-18
b4
b5-18
y3
b5
y4
y6
y12++
~y17++
y7
y8
~y19++
~y20++
y9
~y21++
y10
~y26++
y12
y13
~y14
~y15
~y16
~y17
~y18
~y19
~y17+2
0.000e+0
1.000e+5
2.000e+5
3.000e+5
4.000e+5
5.000e+5
500
1000
1500

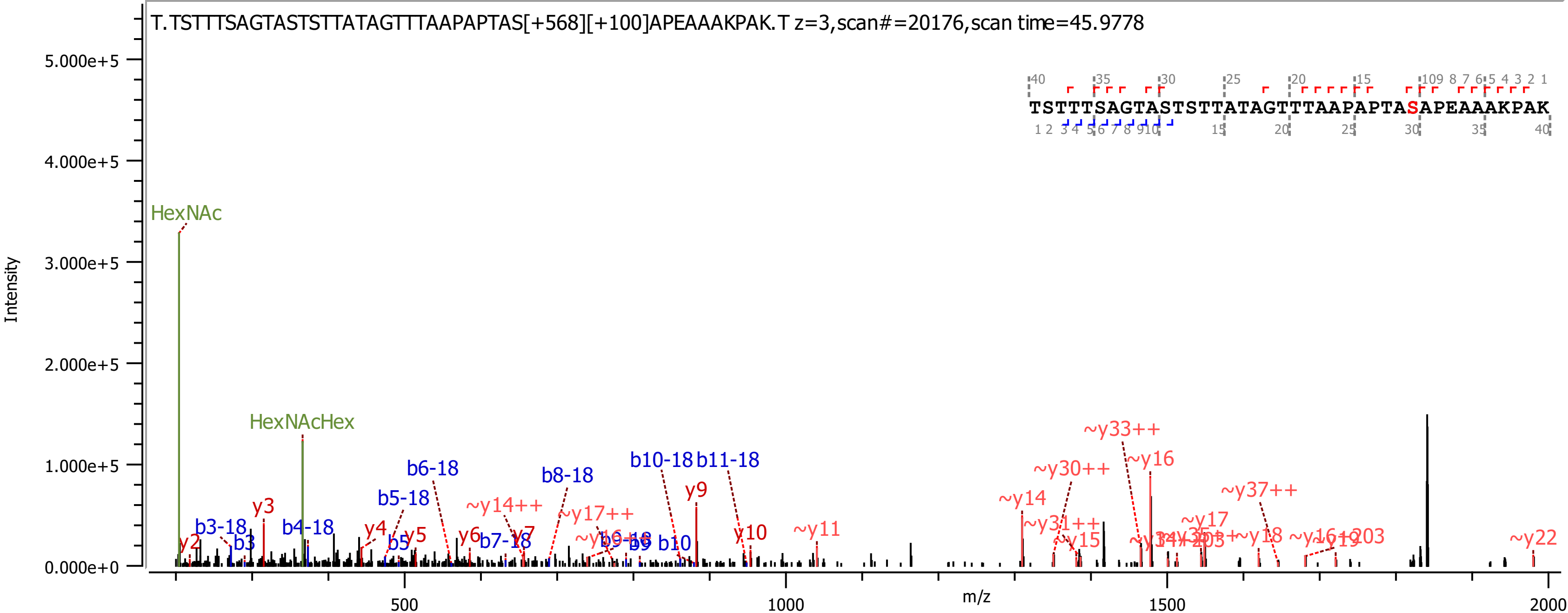
T.TSTTTSAGTASTSTTATAGTTTAAPAPTAS[+568][+100]APEAAAKPAK.T z=3,scan#=20176,scan time=45.9778
TSTTTSAGTASTSTTATAGTTTAAPAPTASAPEAAAKPAK
Intensity
m/z
5.000e+5
4.000e+5
3.000e+5
2.000e+5
1.000e+5
0.000e+0
500
1000
1500
2000
HexNAc
HexNAcHex
y2
b3-18
b3
y3
b4-18
y4
b5-18
b5
y5
b6-18
y6
b7-18
~y14++
y7
b8-18
~y17++
b9-18
b9
b10-18
b10
b11-18
y9
y10
~y11
~y14
~y30++
~y31++
~y15
~y33++
~y16
~y17
~y37++
~y18
~y22

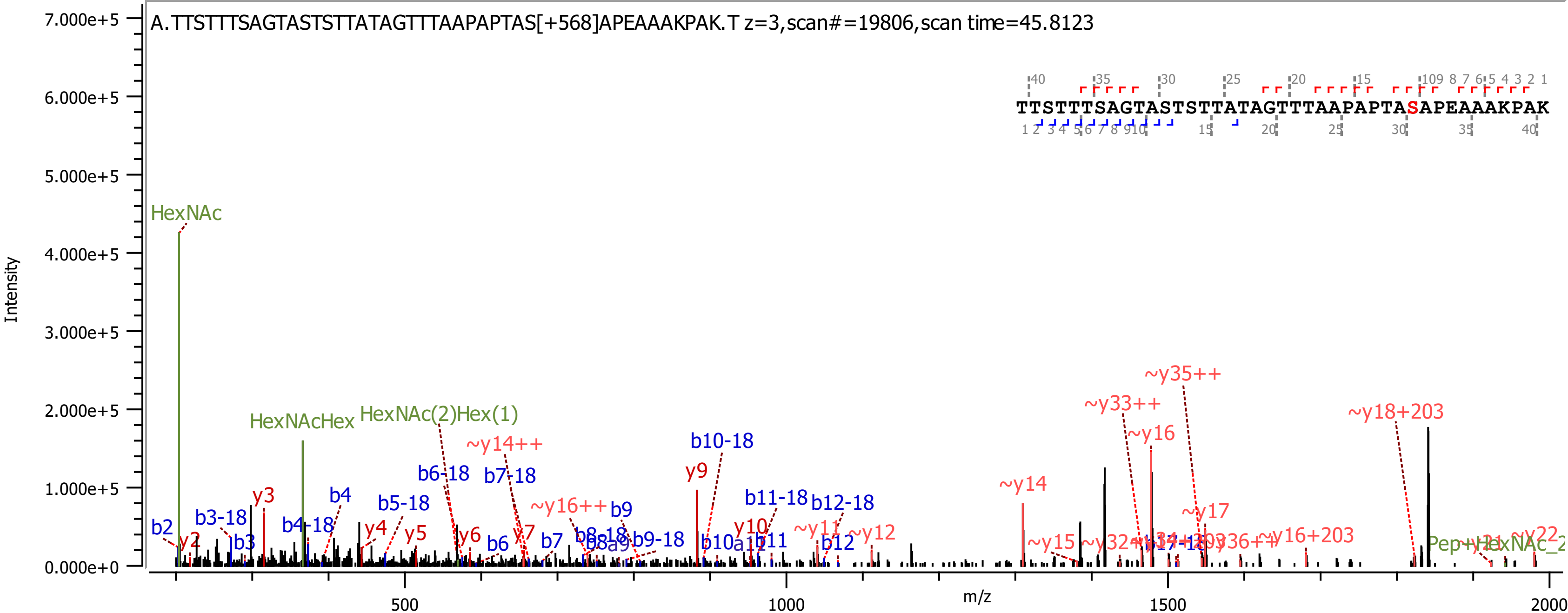
A.TTSTTTSAGTASTSTTATAGTTTAAPAPTAS[+568]APEAAAKPAK.T z=3,scan#=19806,scan time=45.8123
TTSTTTSAGTASTSTTATAGTTTAAPAPTASAPEAAAKPAK
Intensity
m/z
HexNAc
HexNAcHex
HexNAc(2)Hex(1)
~y35++
~y33++
~y16
~y18+203
~y14++
b10-18
y9
~y14
b6-18
b7-18
b4
y3
b5-18
~y16++
b9
b11-18
b12-18
~y17
b3-18
b4-18
y4
y5
y6
y7
y10
~y11
~y12
~y16+203
b2
y2
b3
b6
b7
b9-18
b11
b12
~y15
Pep+HexNAc_2

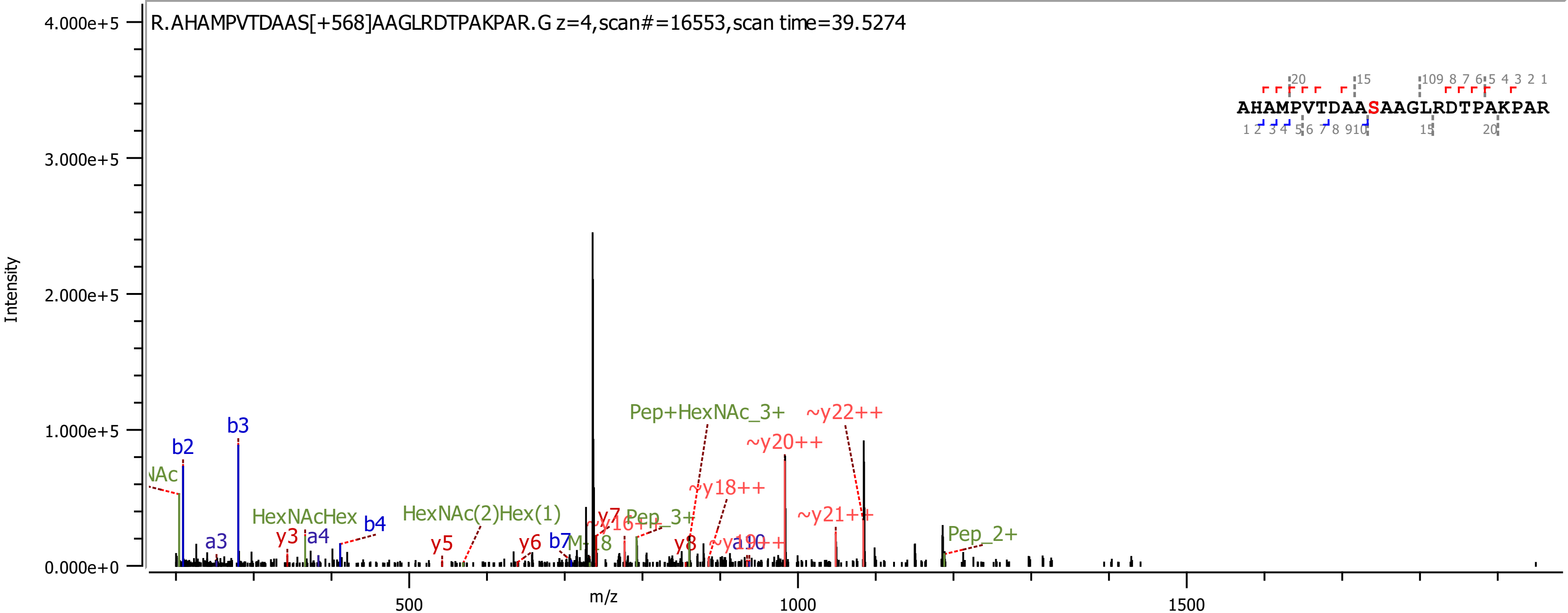

R.AHAMPVTDAAS[+568]AAGLRDTPAKPAR.G z=4,scan#=16553,scan time=39.5274
AHAMPVTDAASAAGLRDTPAKPAR
Intensity
m/z
4.000e+5
3.000e+5
2.000e+5
1.000e+5
0.000e+0
500
1000
1500
b2
b3
a3
a4
b4
b7
y3
y5
y6
y7
y8
HexNAcHex
HexNAc(2)Hex(1)
Pep+HexNAc_3+
Pep_3+
Pep_2+
~y16++
~y18++
~y19++
~y20++
~y21++
~y22++

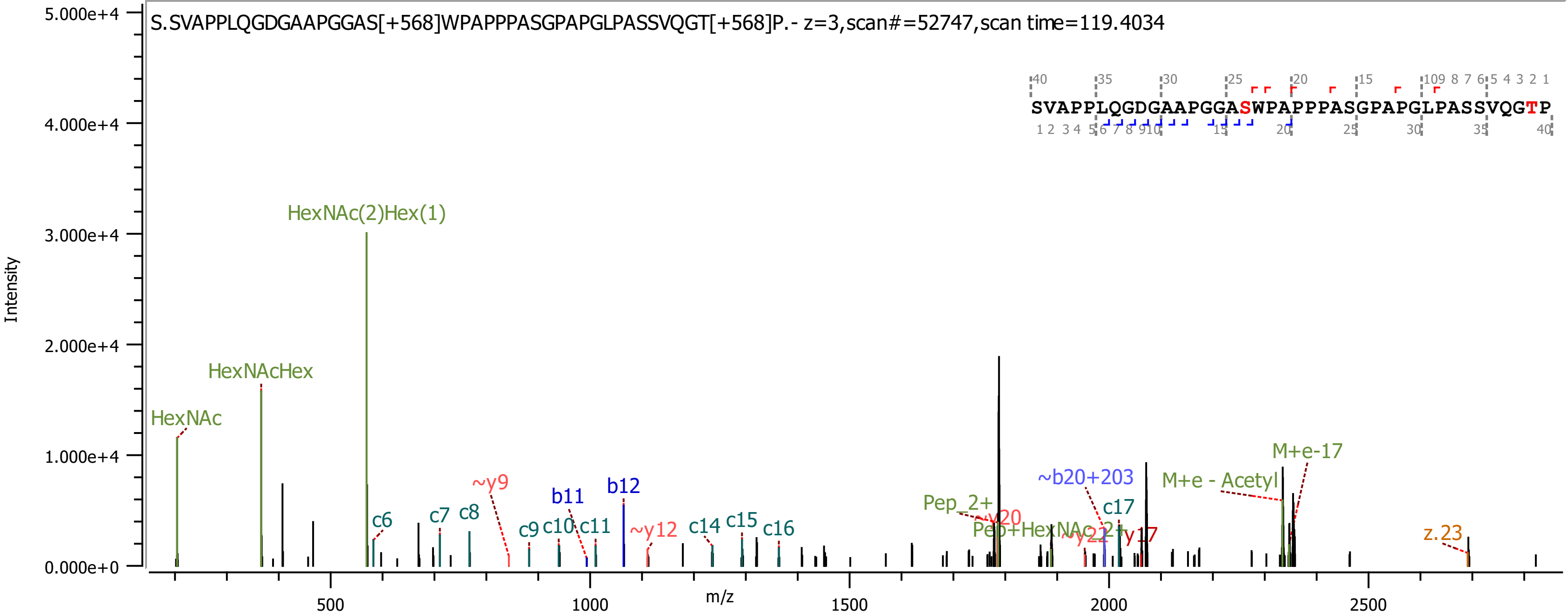

S.SVAPPLQGDGAAPGGAS[+568]WPAPPPASGPAPGLPASSVQGT[+568]P.- z=3,scan#=52747,scan time=119.4034
SVAPPLQGDGAAPGGASWPAPPPASGPAPGLPASSVQGTP
Intensity
m/z
HexNAc
HexNAcHex
HexNAc(2)Hex(1)
c6
c7
c8
~y9
c9
c10
c11
b11
b12
~y12
c14
c15
c16
Pep_2+
~y20
Pep+HexNAc_2+
~y22
~b20+203
c17
y17
M+e - Acetyl
M+e-17
z.23

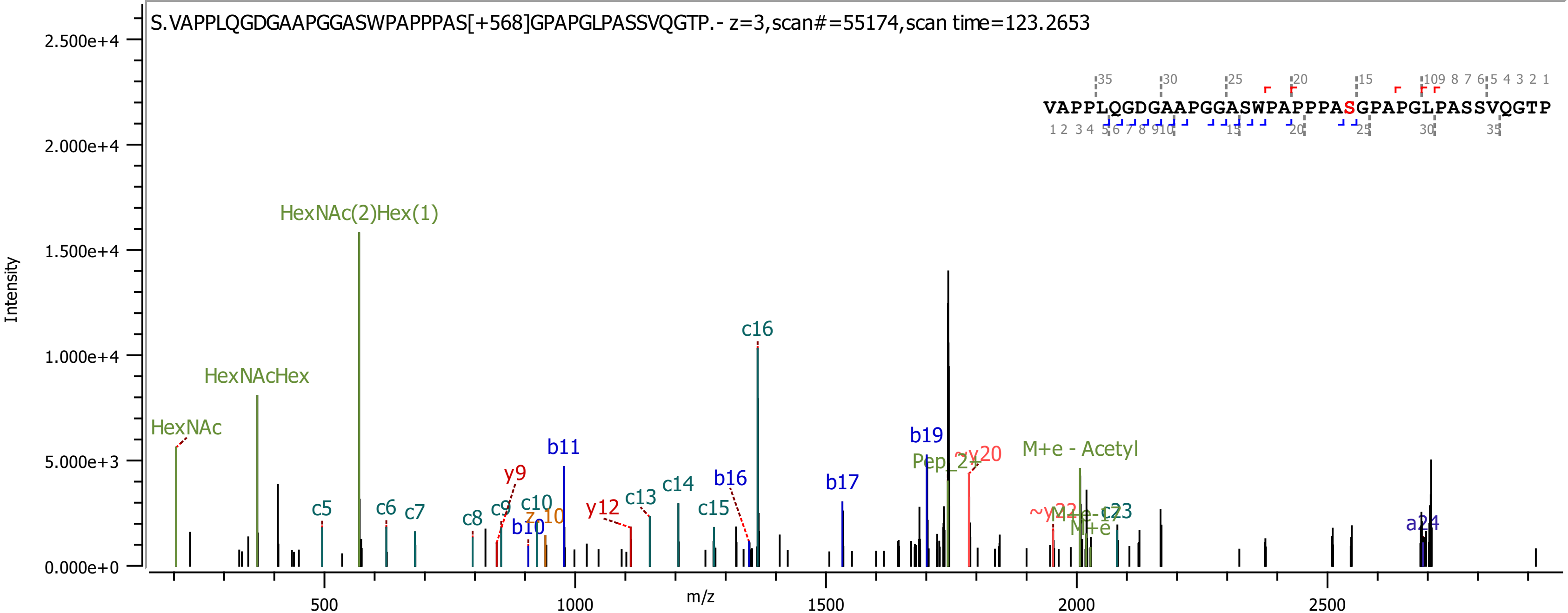

S.VAPPLQGDGAAPGGASWPAPPPAS[+568]GPAPGLPASSVQGTP.- z=3,scan#=55174,scan time=123.2653
VAPPLQGDGAAPGGASWPAPPPASGPAPGLPASSVQGTP
Intensity
m/z
HexNAc(2)Hex(1)
HexNAcHex
HexNAc
c5
c6
c7
c8
c9
c10
b10
z10
b11
y9
y12
c13
c14
c15
c16
b16
b17
b19
~y20
Pep_2+
M+e - Acetyl
~y22
c23
a24

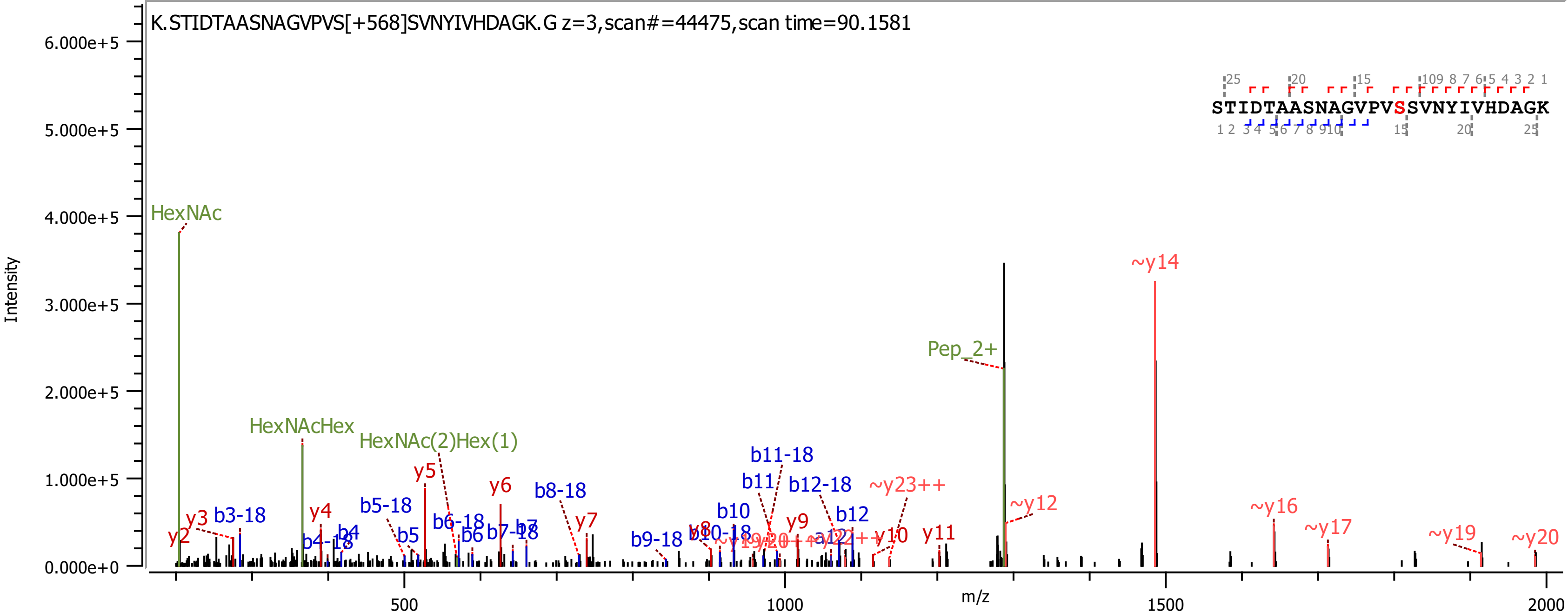

K.STIDTAASNAGVPVS[+568]SVNYIVHDAGK.G z=3,scan#=44475,scan time=90.1581
STIDTAASNAGVPVSSVNYIVHDAGK
Intensity
m/z
HexNAc
HexNAcHex
HexNAc(2)Hex(1)
Pep_2+
~y14
~y12
~y16
~y17
~y19
~y20
~y23++
y2
y3
y4
y5
y6
y7
y9
y10
y11
b3-18
b4-18
b4
b5-18
b5
b6-18
b6
b7-18
b8-18
b9-18
b10
b11
b11-18
b12-18
b12
6.000e+5
5.000e+5
4.000e+5
3.000e+5
2.000e+5
1.000e+5
0.000e+0
500
1000
1500
2000

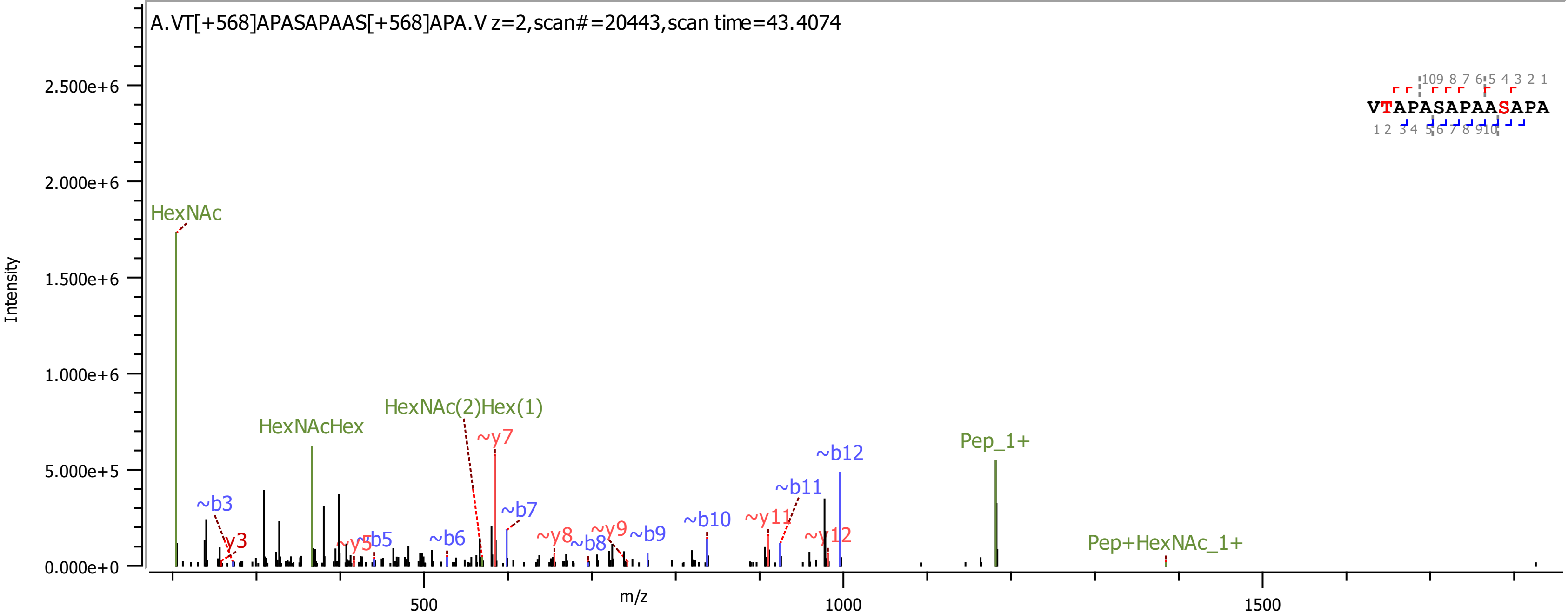
A.VT[+568]APASAPAAS[+568]APA.V z=2,scan#=20443,scan time=43.4074
Intensity
m/z
HexNAc
HexNAcHex
HexNAc(2)Hex(1)
~y7
~b12
~b11
~b3
y3
~y5
~b5
~b6
~b7
~y8
~b8
~y9
~b9
~b10
~y11
~y12
Pep_1+
Pep+HexNAc_1+
VTAPASAPAASAPA

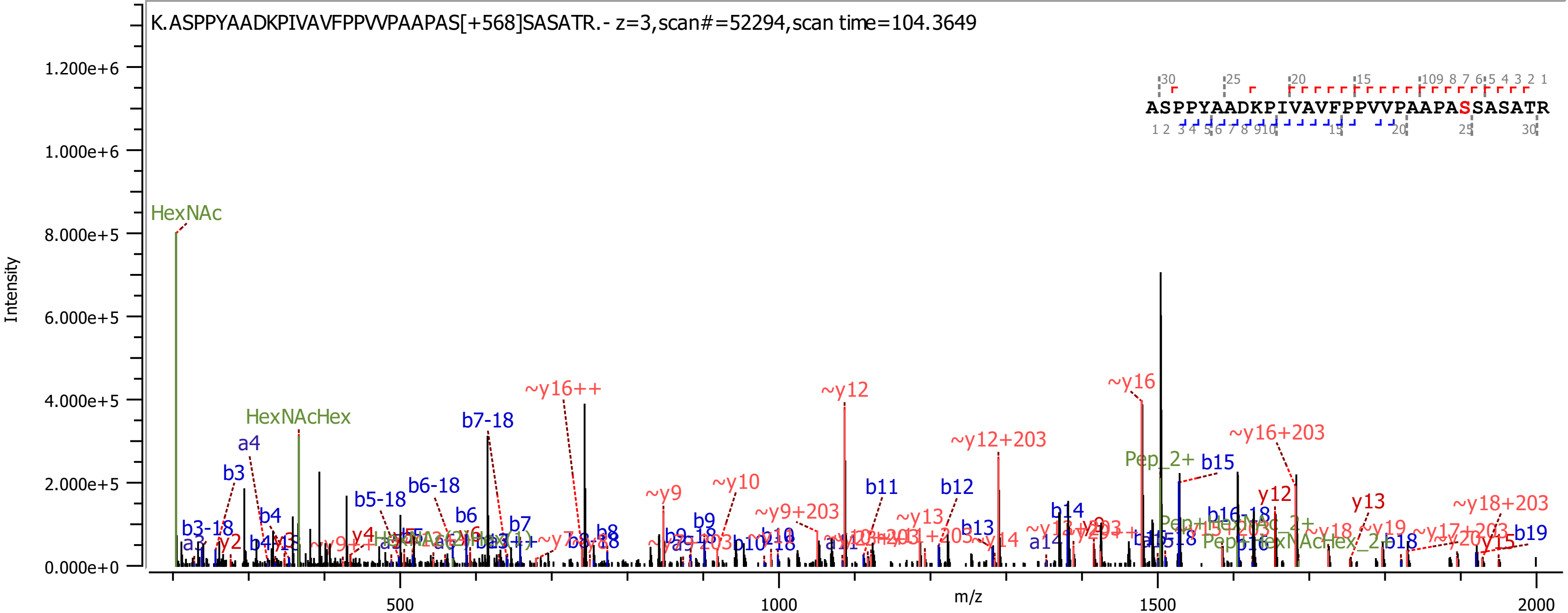
K.ASPPYAADKPIVAVFPPVVPAAPAS[+568]SASATR.- z=3,scan#=52294,scan time=104.3649
ASPPYAADKPIVAVFPPVVPAAPASSASATR
Intensity
m/z
0.000e+0
2.000e+5
4.000e+5
6.000e+5
8.000e+5
1.000e+6
1.200e+6
500
1000
1500
2000
HexNAc
HexNAcHex
a3
a4
b3
b3-18
b4
y2
y4
b5-18
b6-18
b6
b7-18
b7
~y16++
b8
~y9
~y10
b9
b9-18
~y9+203
b10
b10-18
b11
~y12
~y12+203
b12
~y13
b13
~y14
a14
b14
~y16
Pep_2+
b15
~y16+203
b16-18
y12
y13
~y18
~y19
b18
~y18+203
b19
y15

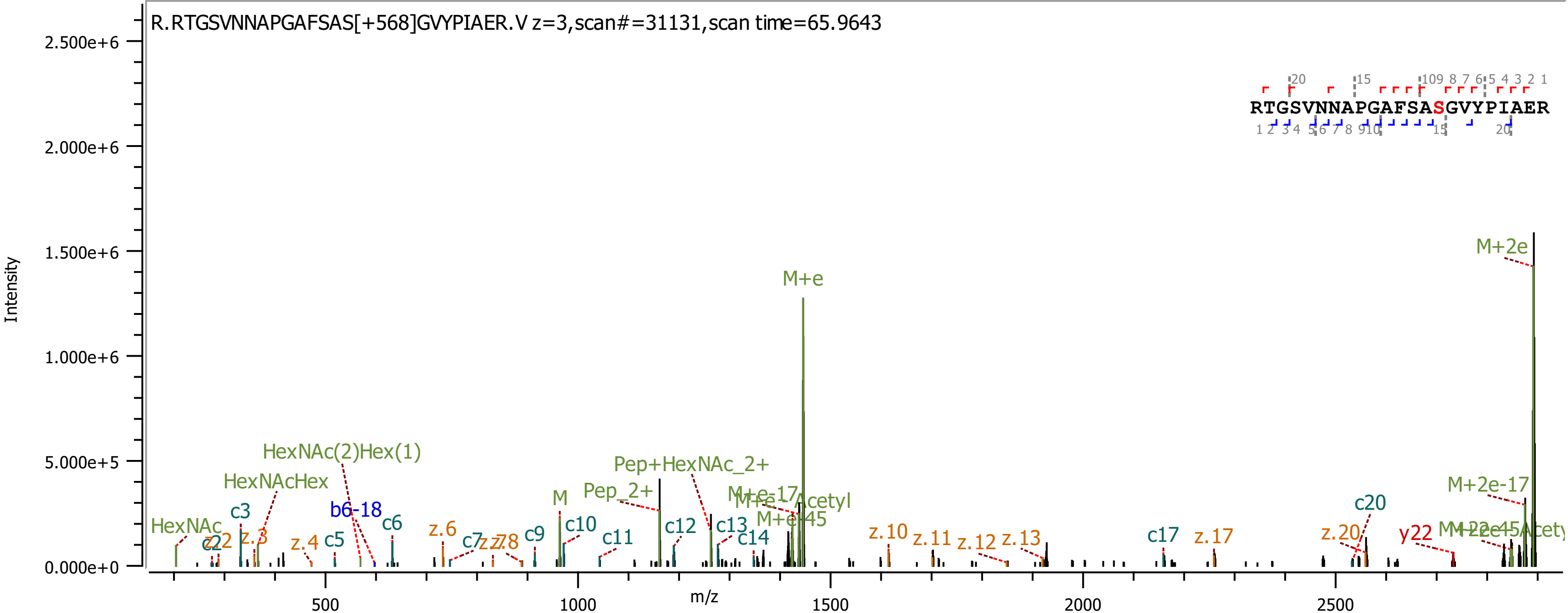

R.RTGSVNNAPGAFSAS[+568]GVYPIAER.V z=3,scan#=31131,scan time=65.9643
RTGSVNNAPGAFSASGVYPIAER
Intensity
m/z
M+2e
M+e
HexNAc(2)Hex(1)
HexNAcHex
HexNAc
Pep+HexNAc_2+
Pep_2+
M+2e-17
c20
y22
c17
z.17
z.20
z.10
z.11
z.12
z.13
c3
c5
c6
c9
c10
c11
c12
c13
c14
b6-18
z.3
z.4
z.6

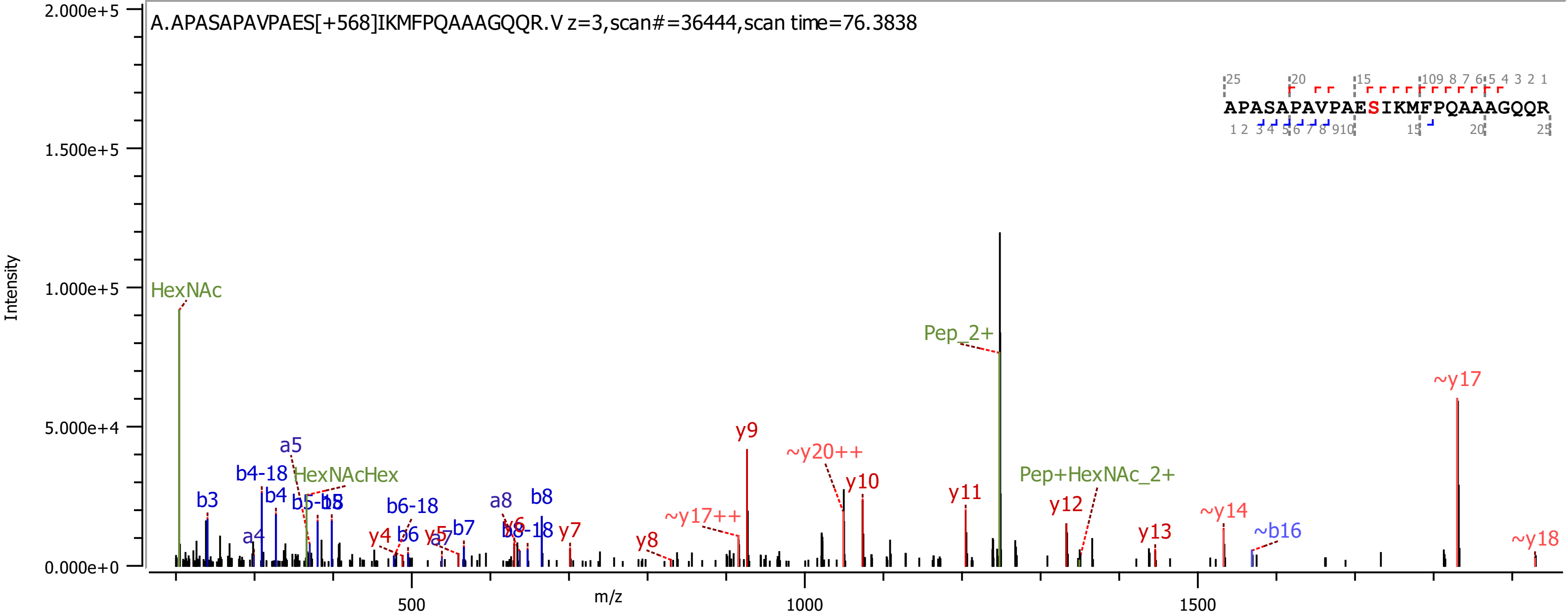

A.APASAPAVPAES[+568]IKMFPQAAAGQQR.V z=3,scan#=36444,scan time=76.3838
APASAPAVPAESIKMFPQAAAGQQR
Intensity
m/z
2.000e+5
1.500e+5
1.000e+5
5.000e+4
0.000e+0
500
1000
1500
HexNAc
HexNAcHex
Pep_2+
Pep+HexNAc_2+
b3
b4-18
b4
a4
a5
b5-18
b6-18
b6
b7
a7
a8
b8
b8-18
y4
y5
y6
y7
y8
~y17++
y9
~y20++
y10
y11
y12
y13
~y14
~b16
~y17
~y18

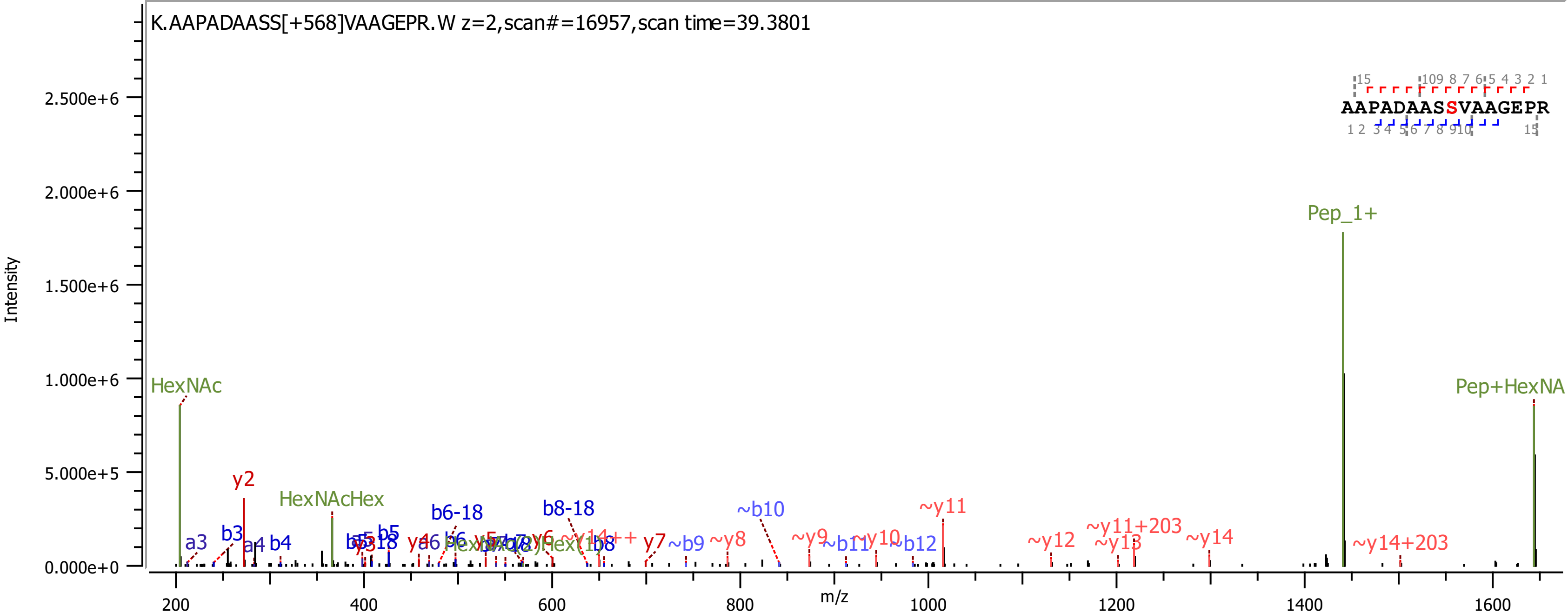
K.AAPADAASS[+568]VAAGEPR.W z=2,scan#=16957,scan time=39.3801
AAPADAASSVAAGEPR
Intensity
m/z
Pep_1+
Pep+HexNA
HexNAc
HexNAcHex
y2
a3
b3
a4
b4
b5
b6-18
b8-18
b6
b8
y7
~b9
~y8
~b10
~y9
~b11
~y10
~b12
~y11
~y12
~y11+203
~y13
~y14
~y14+203
0.000e+0
5.000e+5
1.000e+6
1.500e+6
2.000e+6
2.500e+6
200
400
600
800
1000
1200
1400
1600

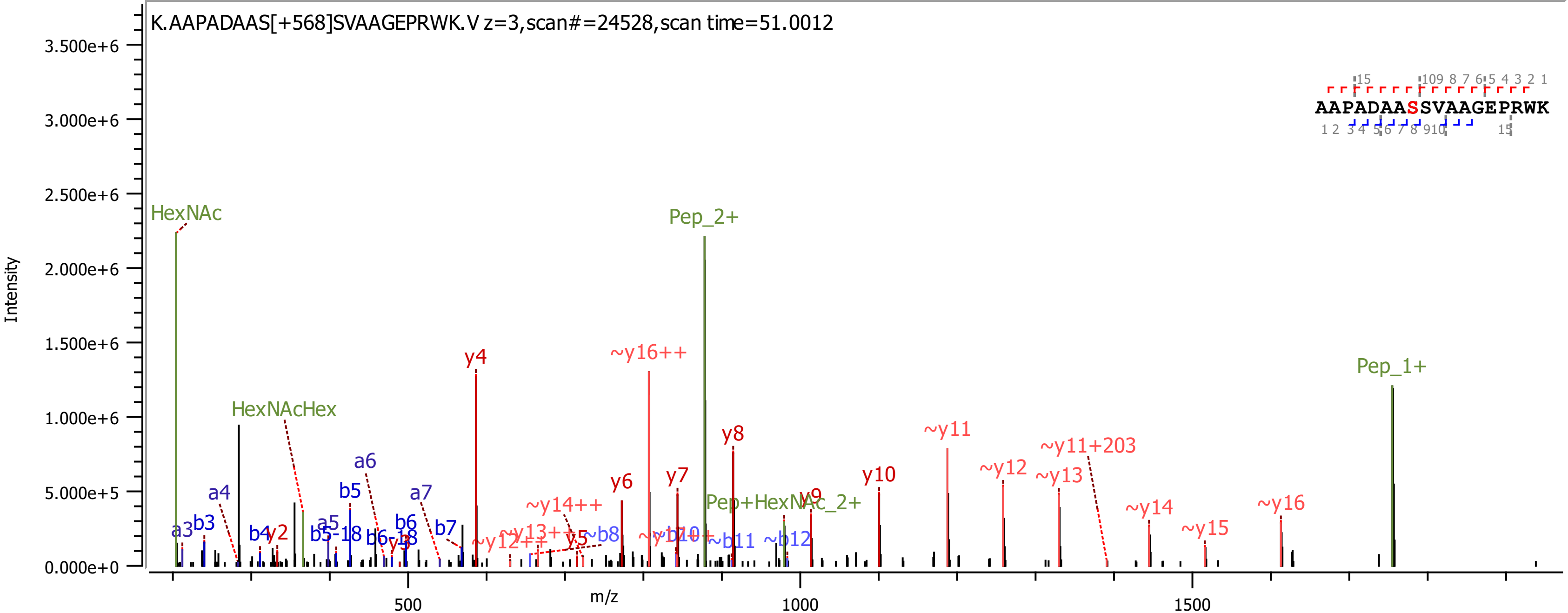

K.AAPADAAS[+568]SVAAGEPRWK.V z=3,scan#=24528,scan time=51.0012
AAPADAASSVAAGEPRWK
Intensity
m/z
HexNAc
Pep_2+
Pep_1+
HexNAcHex
Pep+HexNAc_2+
y4
~y16++
y8
~y11
~y11+203
~y12
~y13
y10
y6
y7
y9
~y14
~y15
~y16
~y14++
~y13++
~y12++
y5
y3
y2
a3
b3
a4
b4
a5
b5
b5-18
a6
b6
b6-18
a7
b7
~b8
~b10
~b11
~b12

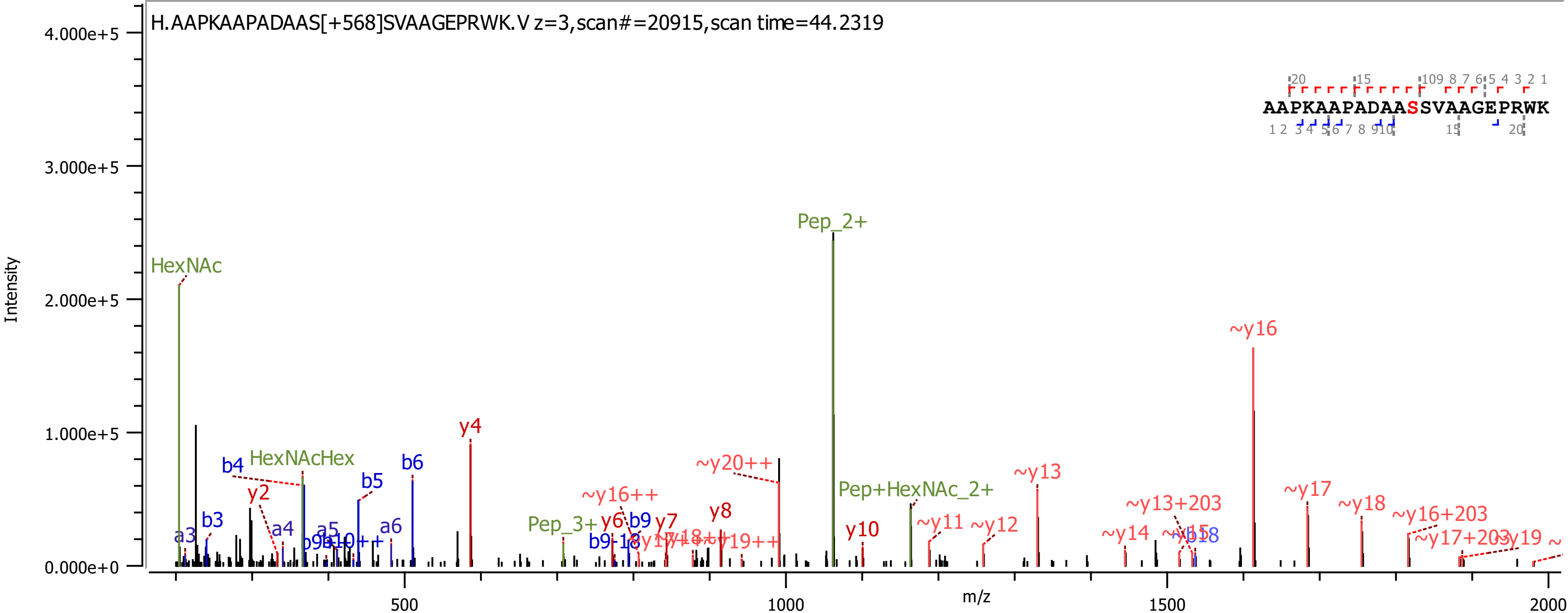
H.AAPKAAPADAAS[+568]SVAAGEPRWK.V z=3,scan#=20915,scan time=44.2319
AAPKAAPADAASSVAAGEPRWK
Intensity
m/z
4.000e+5
3.000e+5
2.000e+5
1.000e+5
0.000e+0
500
1000
1500
2000
HexNAc
HexNAcHex
Pep_2+
Pep_3+
Pep+HexNAc_2+
b3
b4
b5
b6
b9
a3
a4
a5
a6
y2
y4
y6
y7
y8
y10
~y11
~y12
~y13
~y14
~y16
~y17
~y18
~y19
~y16++
~y20++
~y13+203
~y16+203
~y17+203

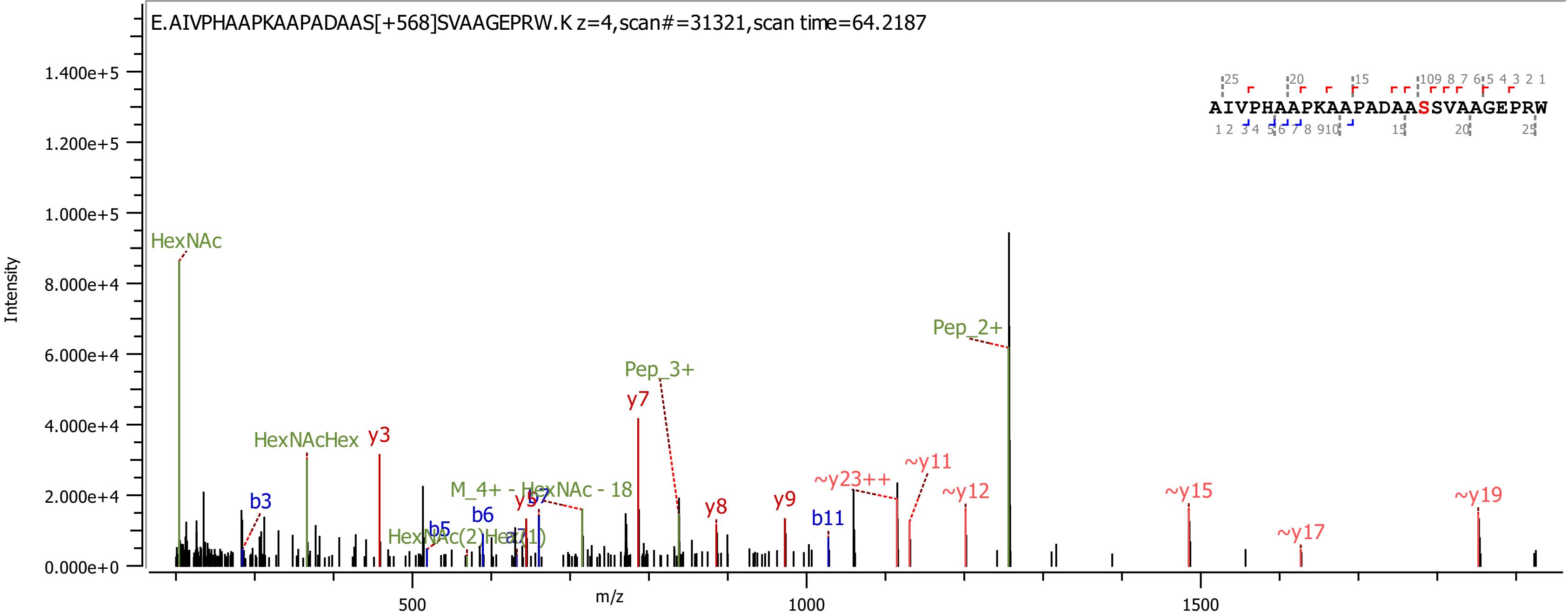
E.AIVPHAAPKAAPADAAS[+568]SVAAGEPRW.K z=4,scan#=31321,scan time=64.2187
AIVPHAAPKAAPADAASSVAAGEPRW
Intensity
m/z
HexNAc
HexNAcHex
y3
b3
b5
b6
y5
b7
M_4+ - HexNAc - 18
HexNAc(2)Hex(1)
y7
Pep_3+
y8
y9
b11
~y23++
~y11
~y12
Pep_2+
~y15
~y17
~y19

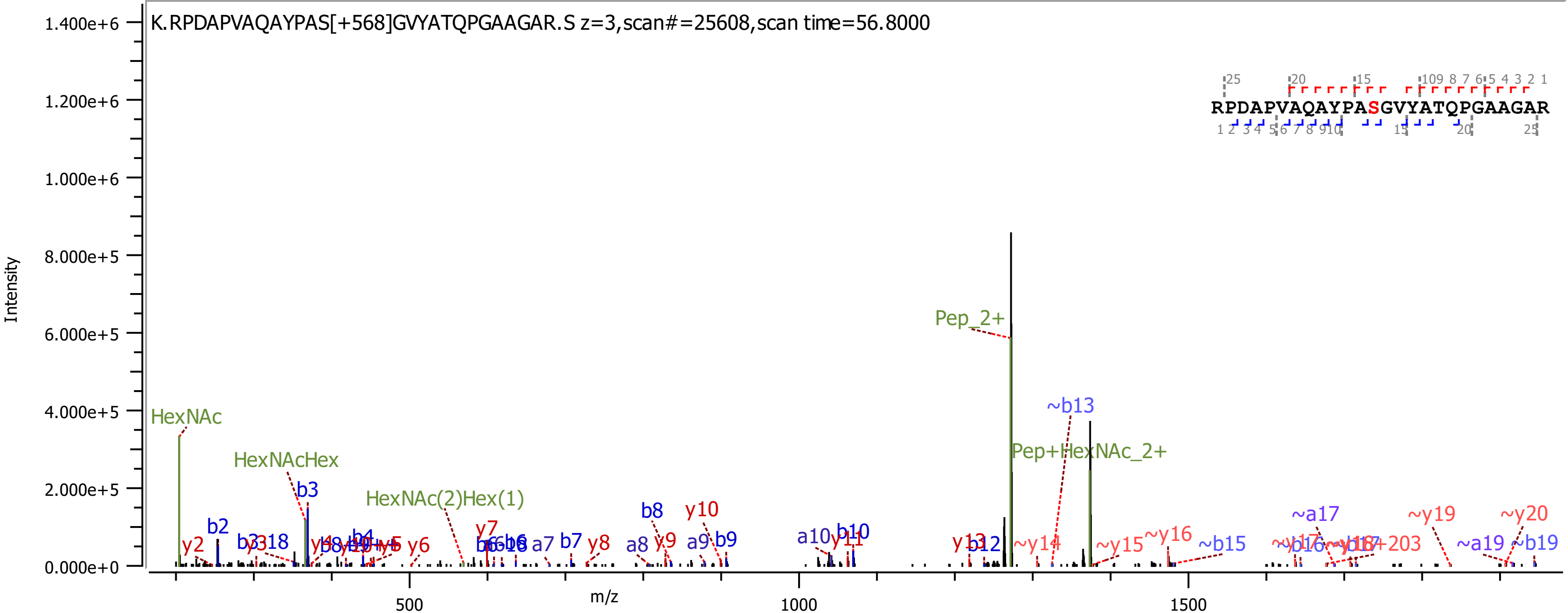
K.RPDAPVAQAYPAS[+568]GVYATQPGAAGAR.S z=3,scan#=25608,scan time=56.8000
RPDAPVAQAYPASGVYATQPGAAGAR
Intensity
m/z
1.400e+6
1.200e+6
1.000e+6
8.000e+5
6.000e+5
4.000e+5
2.000e+5
0.000e+0
500
1000
1500
HexNAc
HexNAcHex
HexNAc(2)Hex(1)
Pep_2+
Pep+HexNAc_2+
~b13
b2
b3
b4
b7
b8
b9
b10
b12
a7
a8
a9
a10
y2
y6
y7
y8
y9
y10
y11
y13
~y14
~y15
~y16
~b15
~a17
~y19
~y20
~a19
~b19

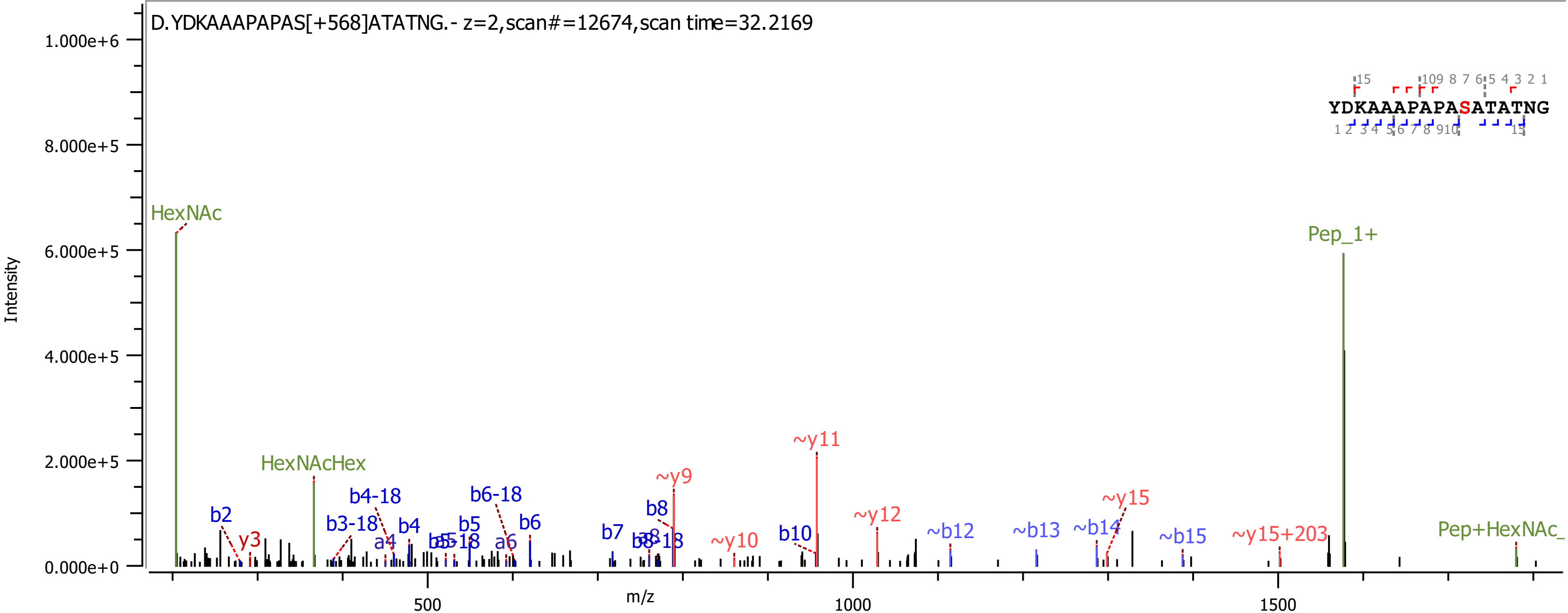

D.YDKAAAPAPAS[+568]ATATNG.- z=2,scan#=12674,scan time=32.2169
YDKAAAPAPASATATNG
Intensity
m/z
HexNAc
HexNAcHex
Pep_1+
Pep+HexNAc_
b2
y3
b3-18
b4-18
a4
b4
b5
b6-18
a6
b6
b7
b8
b8-18
~y9
~y10
b10
~y11
~y12
~b12
~b13
~b14
~y15
~b15
~y15+203

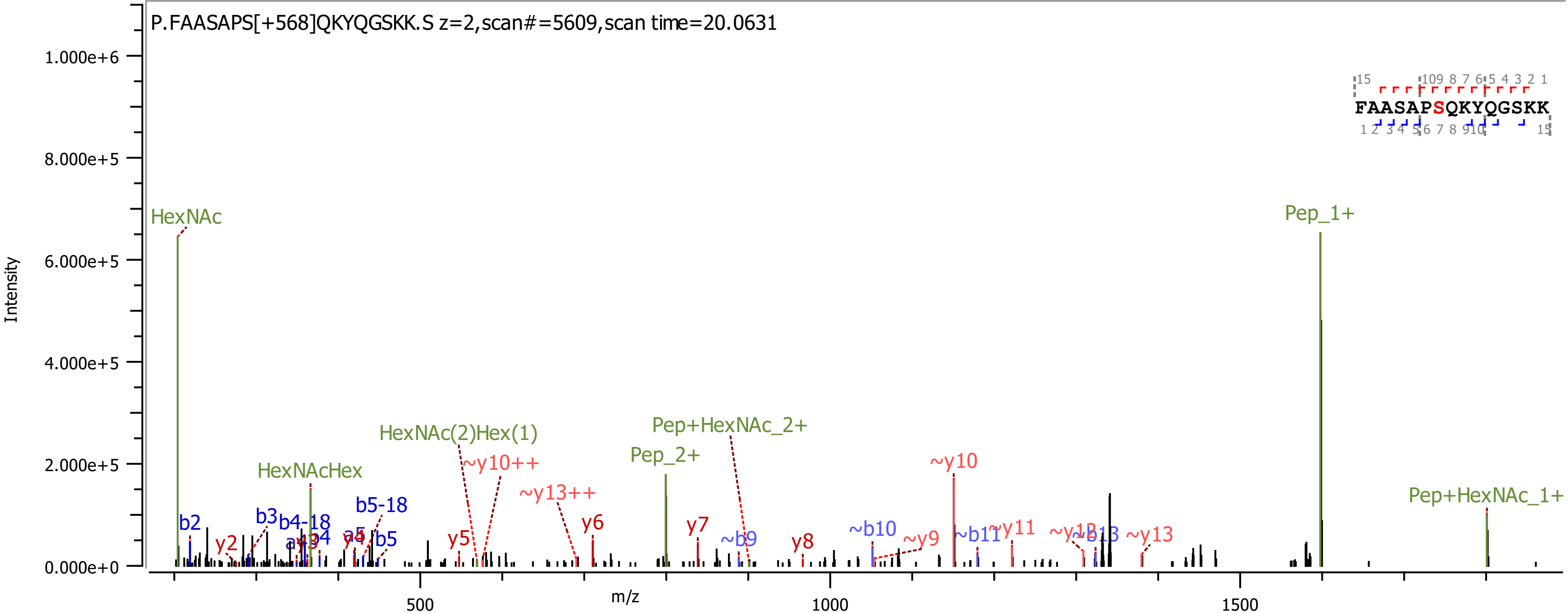
P.FAASAPS[+568]QKYQGSKK.S z=2,scan#=5609,scan time=20.0631
FAASAPSQKYQGSKK
Intensity
m/z
HexNAc
Pep_1+
HexNAcHex
HexNAc(2)Hex(1)
Pep_2+
Pep+HexNAc_2+
Pep+HexNAc_1+
~y10++
~y13++
~y10
~y9
~y11
~y13
~b9
~b10
~b11
b2
b3
b4-18
b5-18
b5
y2
y5
y6
y7
y8

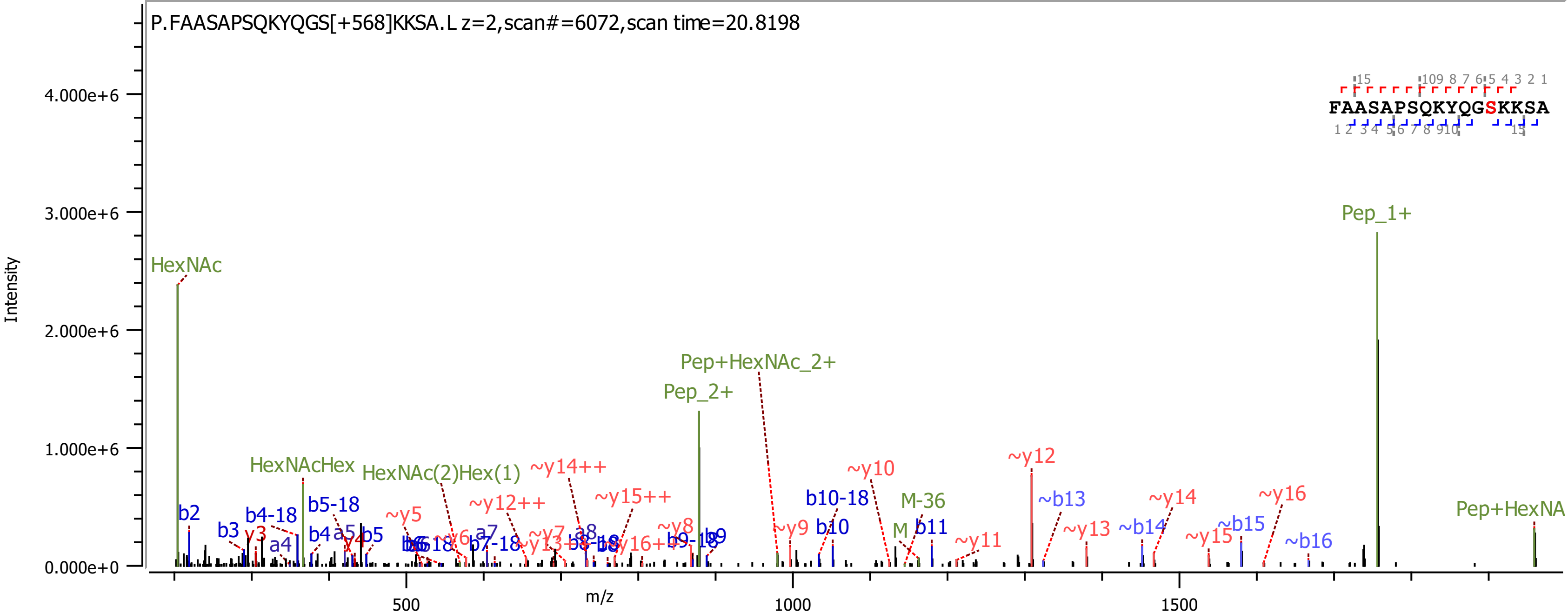

P.FAASAPSQKYQGS[+568]KKSA.L z=2,scan#=6072,scan time=20.8198
Intensity
m/z
FAASAPSQKYQGSKKSA
HexNAc
HexNAcHex
HexNAc(2)Hex(1)
Pep_2+
Pep+HexNAc_2+
Pep_1+
M-36
~y12
~y10
~y14++
~y12++
~y15++
~y5
~y8
~y9
~y11
~y13
~y14
~y15
~y16
~b13
~b14
~b15
~b16
b2
b3
b4-18
b5-18
b10-18
b10
b11
b4
b5
a4
a5
a7
a8
y3
y4
b7-18
b9-18
b9

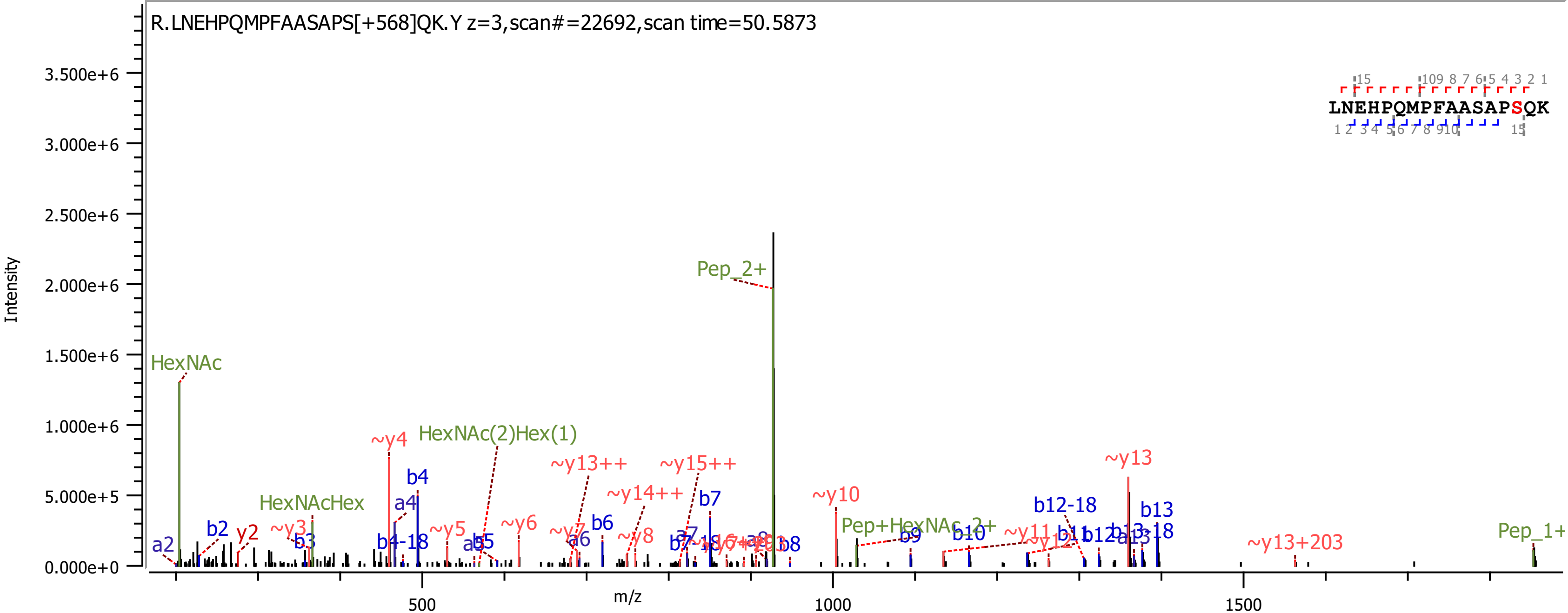

R.LNEHPQMPFAASAPS[+568]QK.Y z=3,scan#=22692,scan time=50.5873
LNEHPQMPFAASAPSQK
Intensity
m/z
3.500e+6
3.000e+6
2.500e+6
2.000e+6
1.500e+6
1.000e+6
5.000e+5
0.000e+0
500
1000
1500
HexNAc
HexNAcHex
HexNAc(2)Hex(1)
Pep_2+
Pep+HexNAc_2+
Pep_1+
a2
b2
y2
~y3
b3
~y4
a4
b4
b4-18
~y5
~y6
~y13++
~y14++
~y15++
b6
a6
~y8
b7
~y10
b9
b10
b12-18
~y13
b13
~y13+203

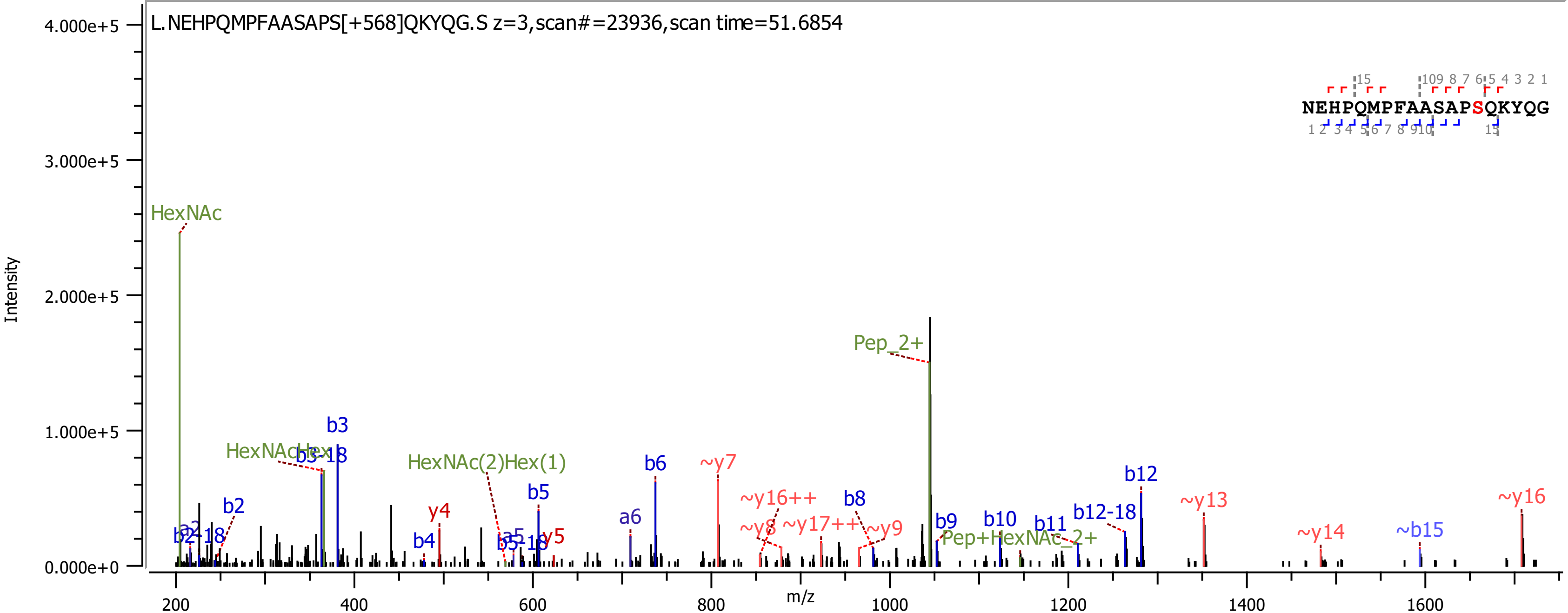
L.NEHPQMPFAASAPS[+568]QKYQG.S z=3,scan#=23936,scan time=51.6854
NEHPQMPFAASAPSQKYQG
Intensity
m/z
HexNAc
HexNAcHex
b3-18
b3
HexNAc(2)Hex(1)
b6
~y7
b12
~y16++
b8
~y13
~y16
Pep_2+
b2
a2
b2-18
b4
y4
b5
a5
b5-18
y5
a6
~y8
~y17++
~y9
b9
b10
b11
b12-18
Pep+HexNAc_2+
~y14
~b15

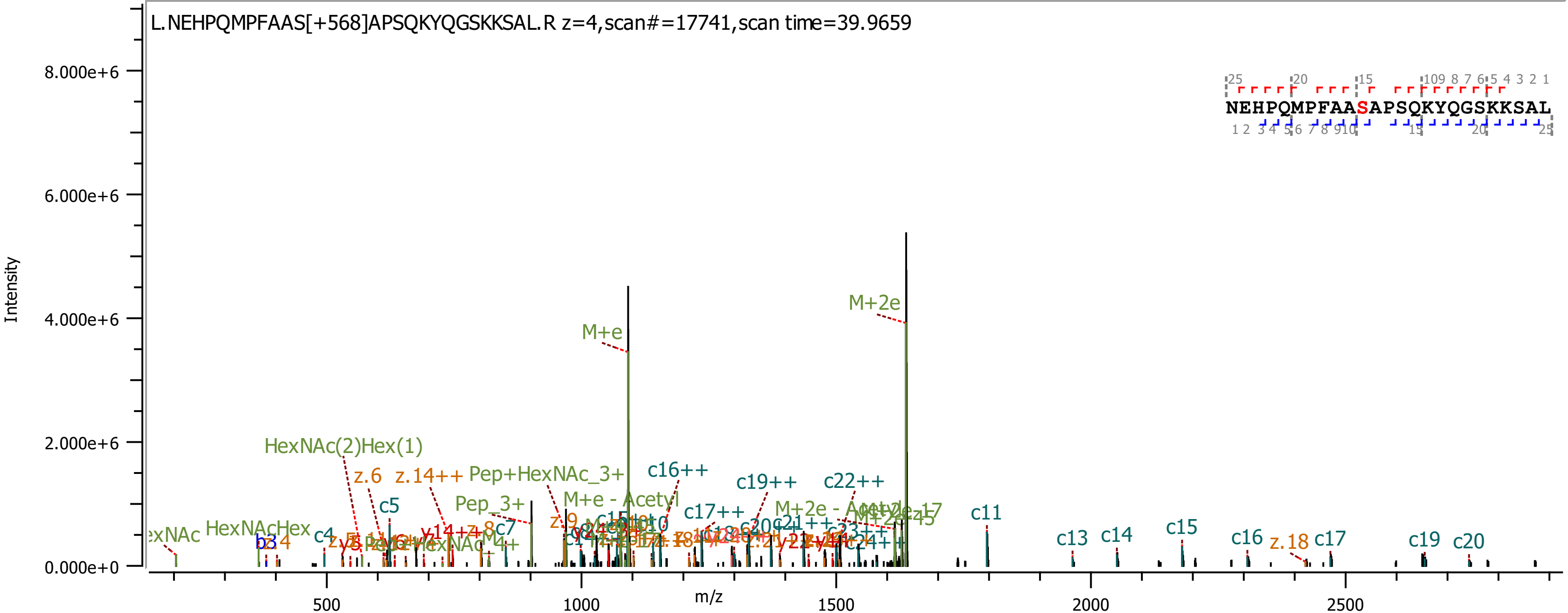

L.NEHPQMPFAAS[+568]APSQKYQGSKKSAL.R z=4,scan#=17741,scan time=39.9659
NEHPQMPFAASAPSQKYQGSKKSAL
Intensity
m/z
8.000e+6
6.000e+6
4.000e+6
2.000e+6
0.000e+0
500
1000
1500
2000
2500
M+e
M+2e
HexNAc(2)Hex(1)
z.6
z.14++
Pep+HexNAc_3+
c16++
c19++
c22++
c5
Pep_3+
M+e - Acetyl
c17++
c11
c13
c14
c15
c16
z.18
c17
c19
c20
HexNAcHex
c4
c7

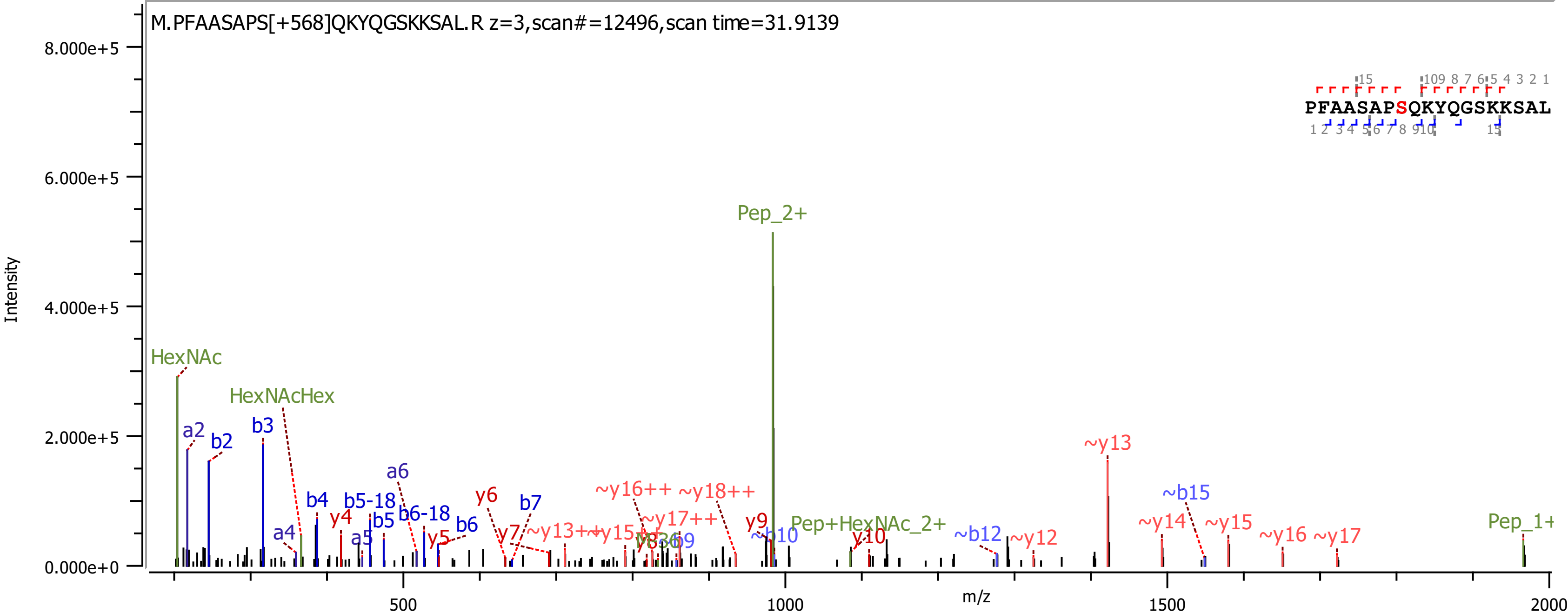
M.PFAASAPS[+568]QKYQGSKKSAL.R z=3,scan#=12496,scan time=31.9139
PFAASAPSQKYQGSKKSAL
Intensity
m/z
Pep_2+
HexNAc
HexNAcHex
a2
b2
b3
a4
b4
a5
b5
b5-18
a6
b6-18
b6
y4
y5
y6
b7
y7
~y13++
~y16++
~y18++
~y17++
y9
~b10
y10
Pep+HexNAc_2+
~b12
~y12
~y13
~y14
~b15
~y15
~y16
~y17
Pep_1+
0.000e+0
2.000e+5
4.000e+5
6.000e+5
8.000e+5
500
1000
1500
2000

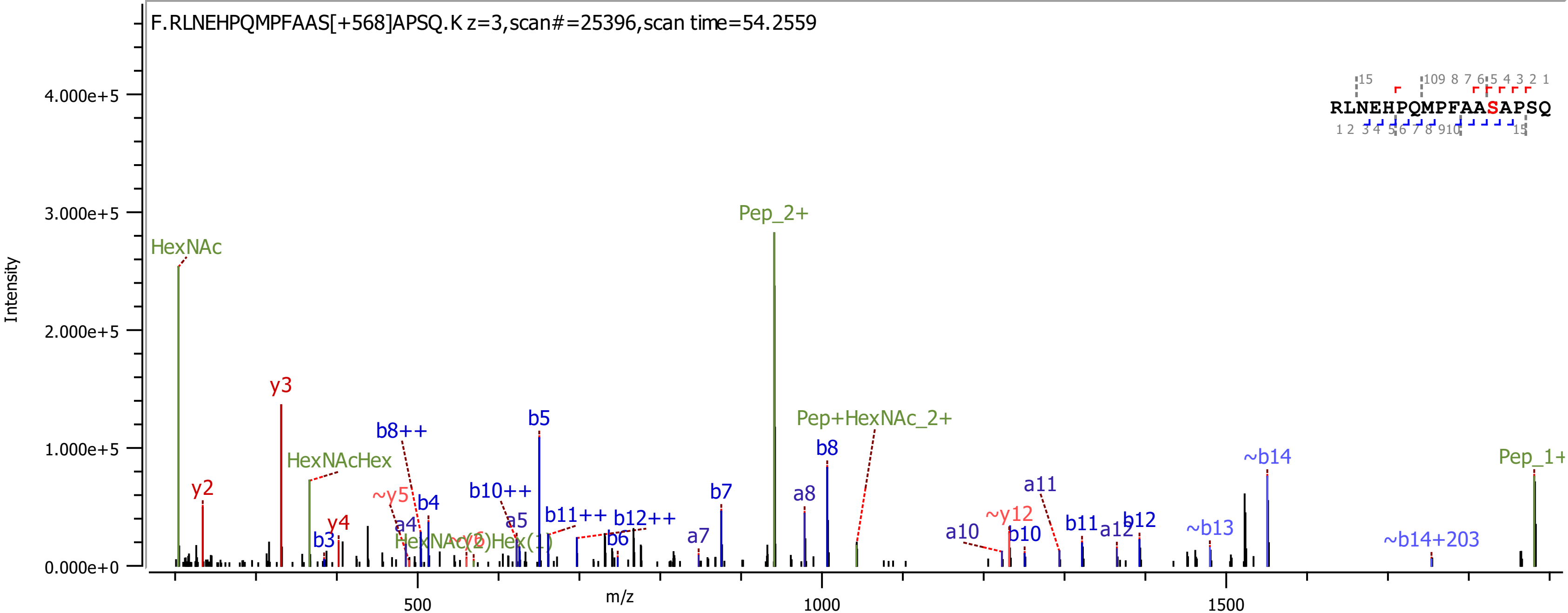
F.RLNEHPQMPFAAS[+568]APSQ.K z=3,scan#=25396,scan time=54.2559
RLNEHPQMPFAASAPSQ
Intensity
m/z
4.000e+5
3.000e+5
2.000e+5
1.000e+5
0.000e+0
500
1000
1500
HexNAc
y2
y3
HexNAcHex
y4
b3
~y5
a4
b8++
b4
HexNAc(2)Hex(1)
b10++
a5
b5
b11++
b12++
b6
a7
b7
Pep_2+
a8
b8
Pep+HexNAc_2+
a10
~y12
b10
a11
b11
a12
b12
~b13
~b14
~b14+203
Pep_1+

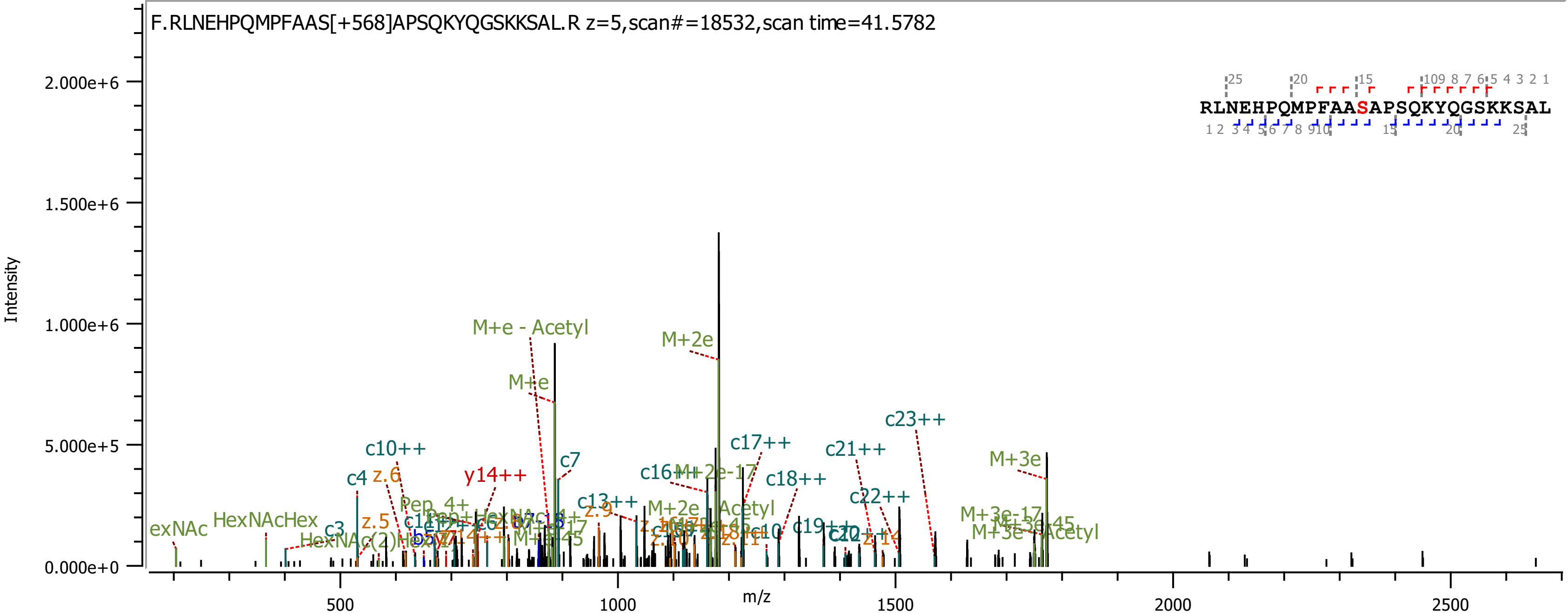
F.RLNEHPQMPFAAS[+568]APSQKYQGSKKSAL.R z=5,scan#=18532,scan time=41.5782
RLNEHPQMPFAASAPSQKYQGSKKSAL
Intensity
m/z
2.000e+6
1.500e+6
1.000e+6
5.000e+5
0.000e+0
500
1000
1500
2000
2500
M+e - Acetyl
M+2e
M+e
c7
c10++
c4
z.6
y14++
c17++
c16++
c18++
c21++
c23++
c22++
c13++
z.9
M+3e
HexNAcHex
c3
z.5
c19++
M+3e-17
M+3e-Acetyl

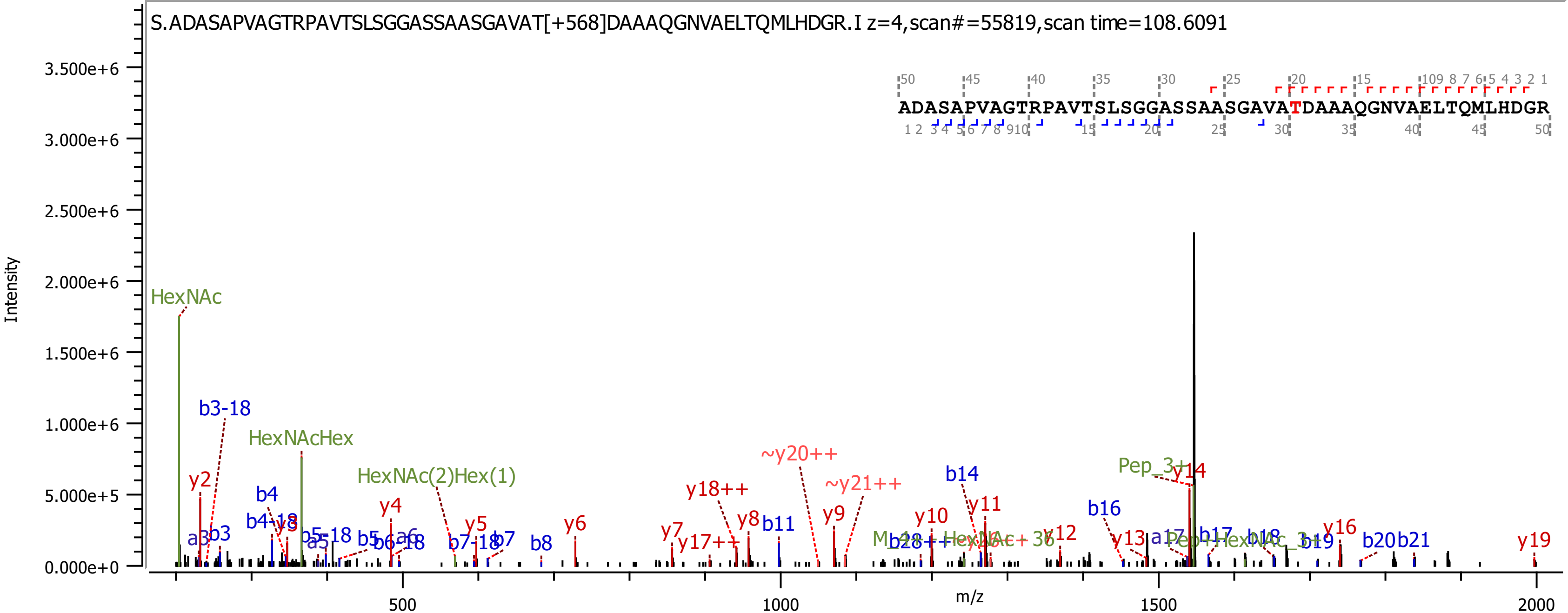
S.ADASAPVAGTRPAVTSLSGGASSAASGAVAT[+568]DAAAQGNVAELTQMLHDGR.I z=4,scan#=55819,scan time=108.6091
ADASAPVAGTRPAVTSLSGGASSAASGAVATDAAAQGNVAELTQMLHDGR
Intensity
m/z
0.000e+0
5.000e+5
1.000e+6
1.500e+6
2.000e+6
2.500e+6
3.000e+6
3.500e+6
500
1000
1500
2000
HexNAc
HexNAcHex
HexNAc(2)Hex(1)
b3-18
y2
a3
b3
b4
b4-18
y3
b5-18
a5
b5
b6-18
a6
y4
y5
b7-18
b7
b8
y6
y7
y17++
y18++
y8
b11
~y20++
~y21++
y9
y10
b14
y11
y12
b16
y13
a17
Pep_3+
y14
b17
b18
b19
y16
b20
b21
y19

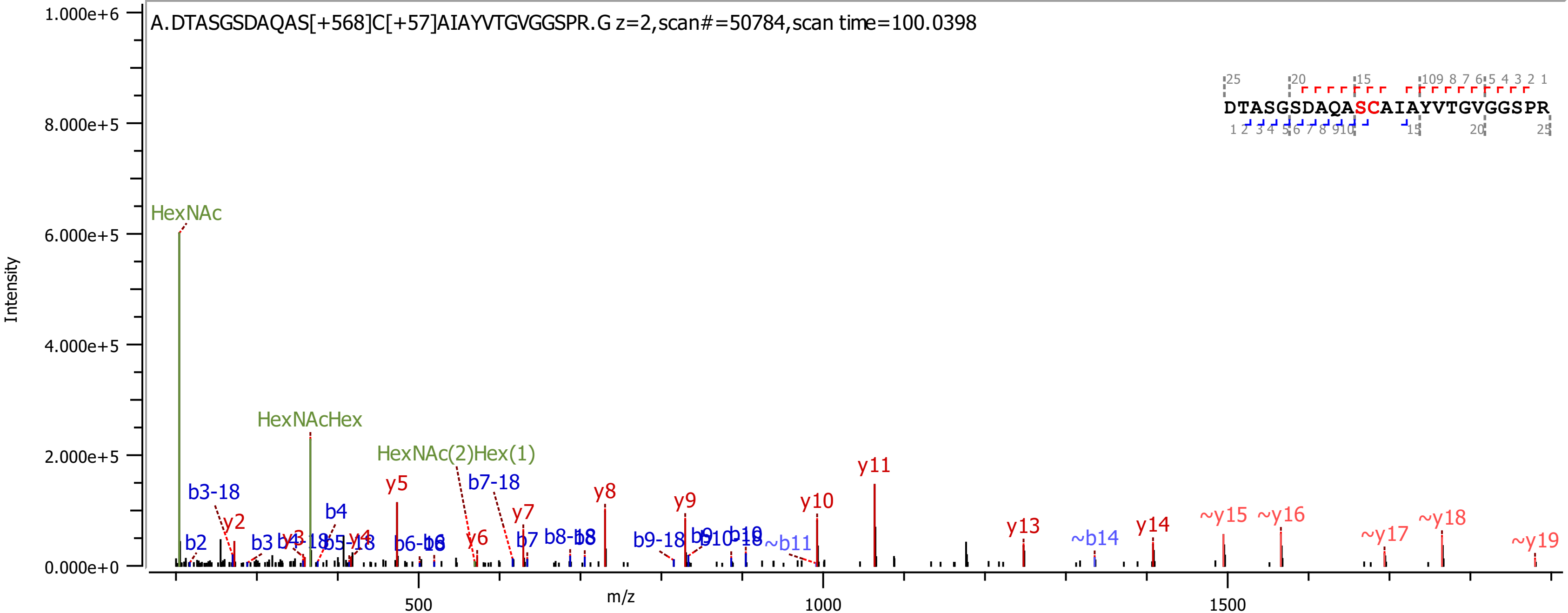
A.DTASGSDAQAS[+568]C[+57]AIAYVTGVGGSPR.G z=2,scan#=50784,scan time=100.0398
DTASGSDAQASCAIAYVTGVGGSPR
Intensity
m/z
HexNAc
HexNAcHex
HexNAc(2)Hex(1)
y2
y3
y4
y5
y6
y7
y8
y9
y10
y11
y13
y14
~y15
~y16
~y17
~y18
~y19
b2
b3
b3-18
b4
b4-18
b5-18
b6-18
b7
b7-18
b8-18
b9-18
b9
b10-18
~b11
~b14
0.000e+0
2.000e+5
4.000e+5
6.000e+5
8.000e+5
1.000e+6
500
1000
1500

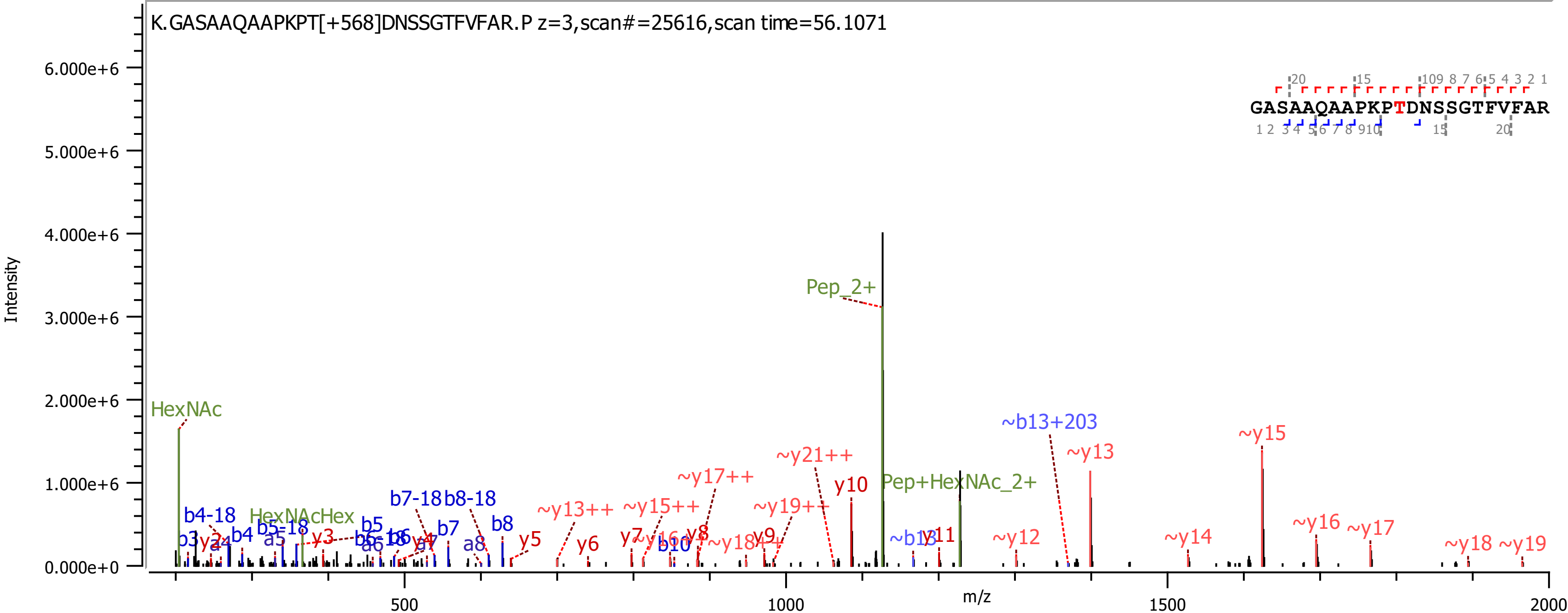

K.GASAAQAAPKPT[+568]DNSSGTFVFAR.P z=3,scan#=25616,scan time=56.1071
GASAAQAAPKPTDNSSGTFVFAR
Intensity
m/z
6.000e+6
5.000e+6
4.000e+6
3.000e+6
2.000e+6
1.000e+6
0.000e+0
500
1000
1500
2000
HexNAc
HexNAcHex
Pep_2+
Pep+HexNAc_2+
~b13+203
~y15
~y13
~y21++
~y17++
y10
~y19++
~y15++
~y13++
~y16
~y17
~y14
~y12
~y18
~y19
y11
y9
y8
y7
y6
y5
y4
y3
b8
b7
b7-18
b8-18
b5
b4
b4-18
b5-18
b3
a5
a6
a8
b10

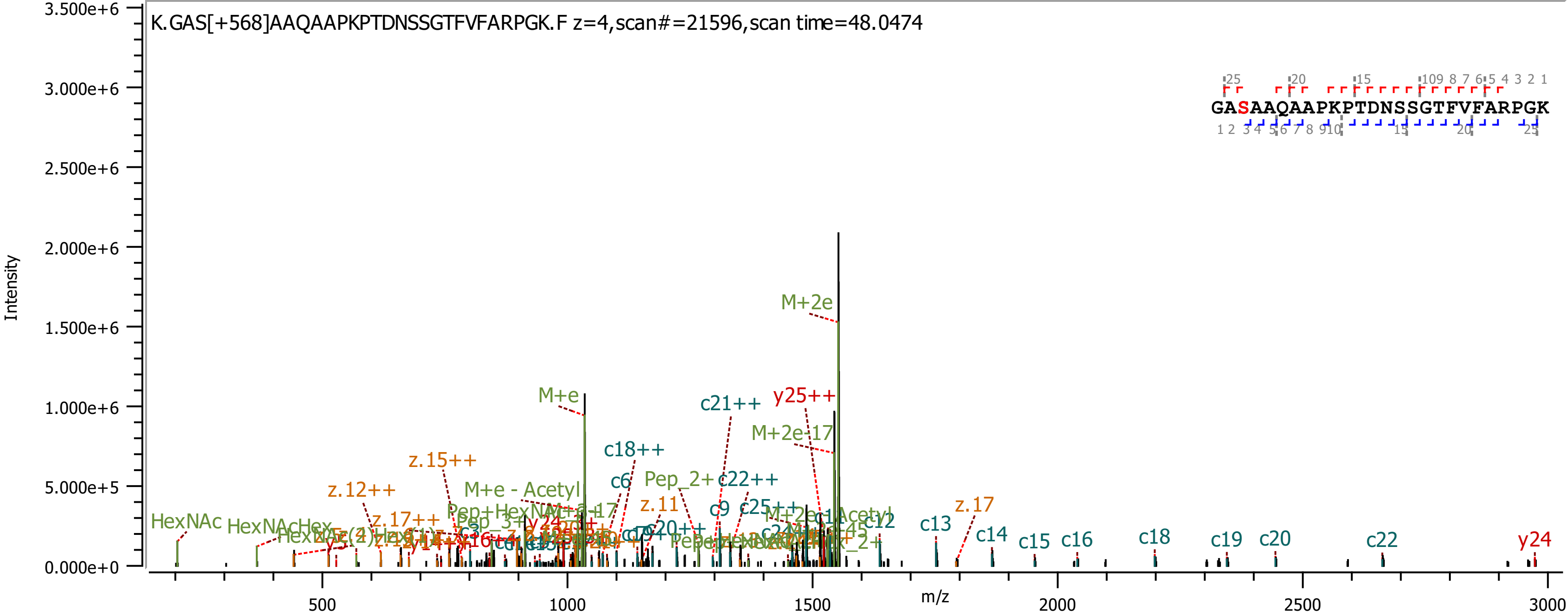

K.GAS[+568]AAQAAPKPTDNSSGTFVFARPGK.F z=4,scan#=21596,scan time=48.0474
GASAAQAAPKPTDNSSGTFVFARPGK
Intensity
m/z
3.500e+6
3.000e+6
2.500e+6
2.000e+6
1.500e+6
1.000e+6
5.000e+5
0.000e+0
500
1000
1500
2000
2500
3000
M+2e
M+e
c21++
y25++
M+2e-17
c18++
z.15++
Pep_2+
c22++
c6
z.12++
M+e - Acetyl
z.11
c9
c25++
HexNAc
HexNAcHex
z.17++
c13
z.17
c12
c14
c15
c16
c18
c19
c20
c22
y24

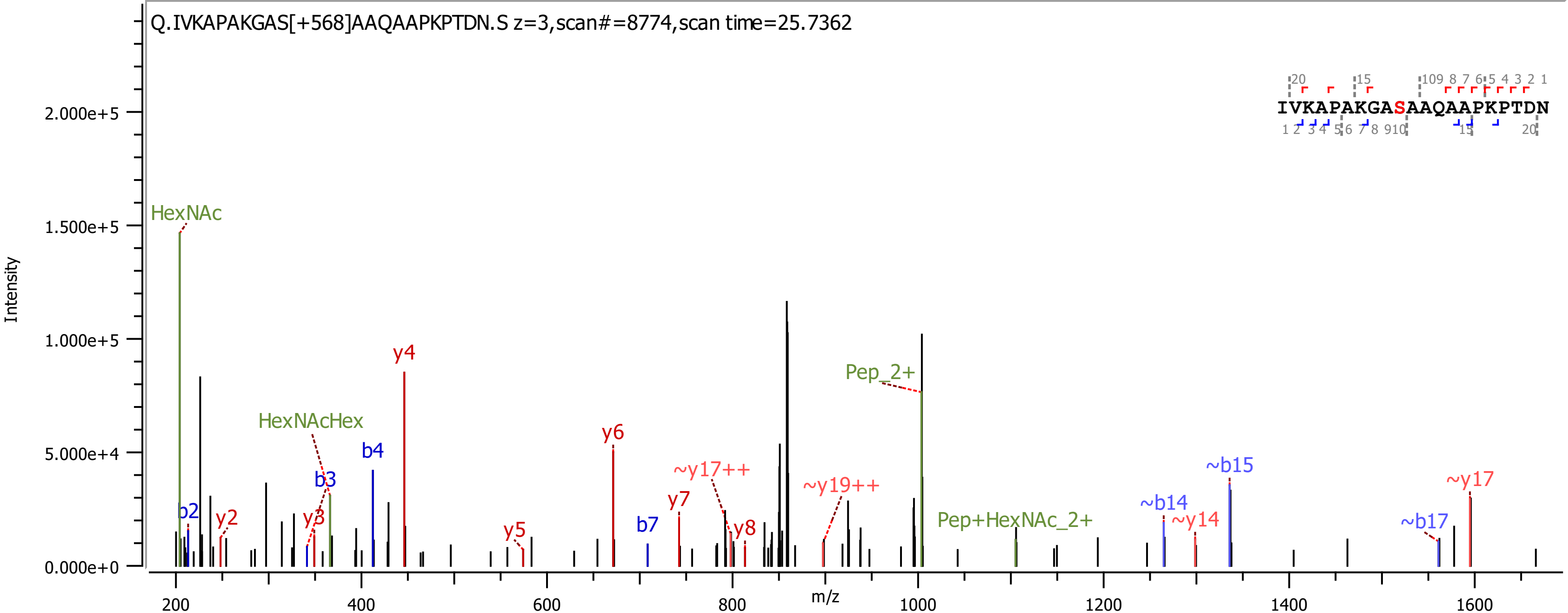

Q.IVKAPAKGAS[+568]AAQAAPKPTDN.S z=3,scan#=8774,scan time=25.7362
IVKAPAKGASAAQAAPKPTDN
Intensity
m/z
HexNAc
HexNAcHex
b2
y2
y3
b3
b4
y4
y5
y6
b7
y7
~y17++
y8
~y19++
Pep_2+
Pep+HexNAc_2+
~b14
~y14
~b15
~b17
~y17

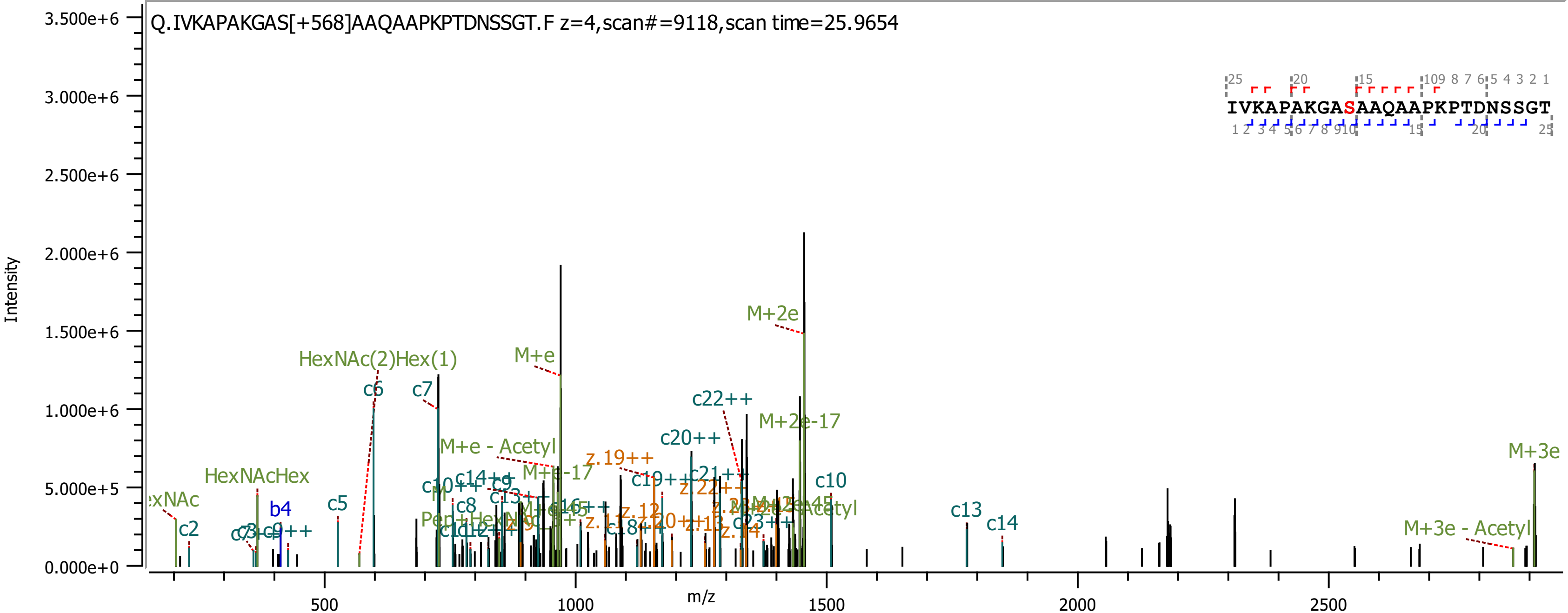
Q.IVKAPAKGAS[+568]AAQAAPKPTDNSSGT.F z=4,scan#=9118,scan time=25.9654
IVKAPAKGASAAQAAPKPTDNSSGT
Intensity
m/z
M+2e
M+e
HexNAc(2)Hex(1)
c6
c7
c22++
M+2e-17
c20++
M+e - Acetyl
z.19++
M+3e
M+3e - Acetyl
HexNAcHex
b4
c5
c2
c10
c13
c14
0.000e+0
5.000e+5
1.000e+6
1.500e+6
2.000e+6
2.500e+6
3.000e+6
3.500e+6
500
1000
1500
2000
2500

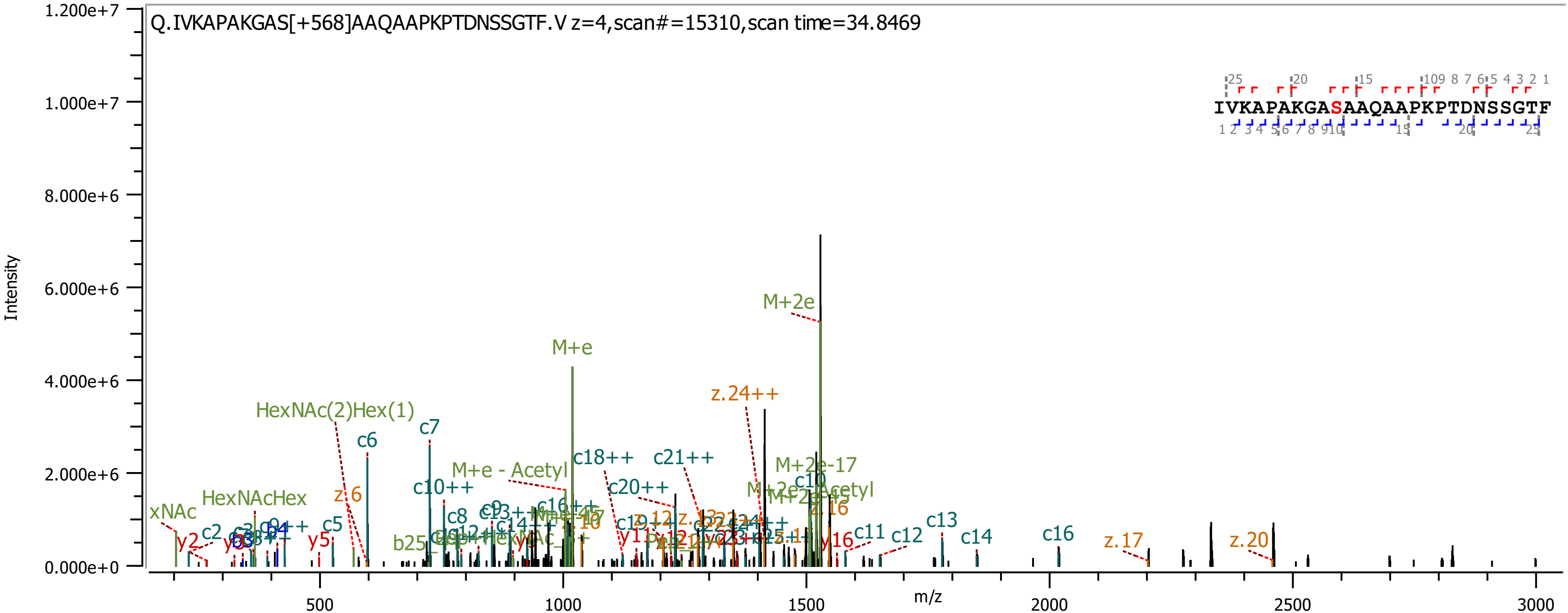
Q.IVKAPAKGAS[+568]AAQAAPKPTDNSSGTF.V z=4,scan#=15310,scan time=34.8469
IVKAPAKGASAAQAAPKPTDNSSGTF
Intensity
m/z
1.200e+7
1.000e+7
8.000e+6
6.000e+6
4.000e+6
2.000e+6
0.000e+0
500
1000
1500
2000
2500
3000
M+2e
M+e
z.24++
HexNAc(2)Hex(1)
c7
c6
M+e - Acetyl
c18++
c21++
M+2e-17
c10++
c20++
HexNAcHex
z.6
c5
y2
c2
y5
y16
c11
c12
c13
c14
c16
z.17
z.20
z.16

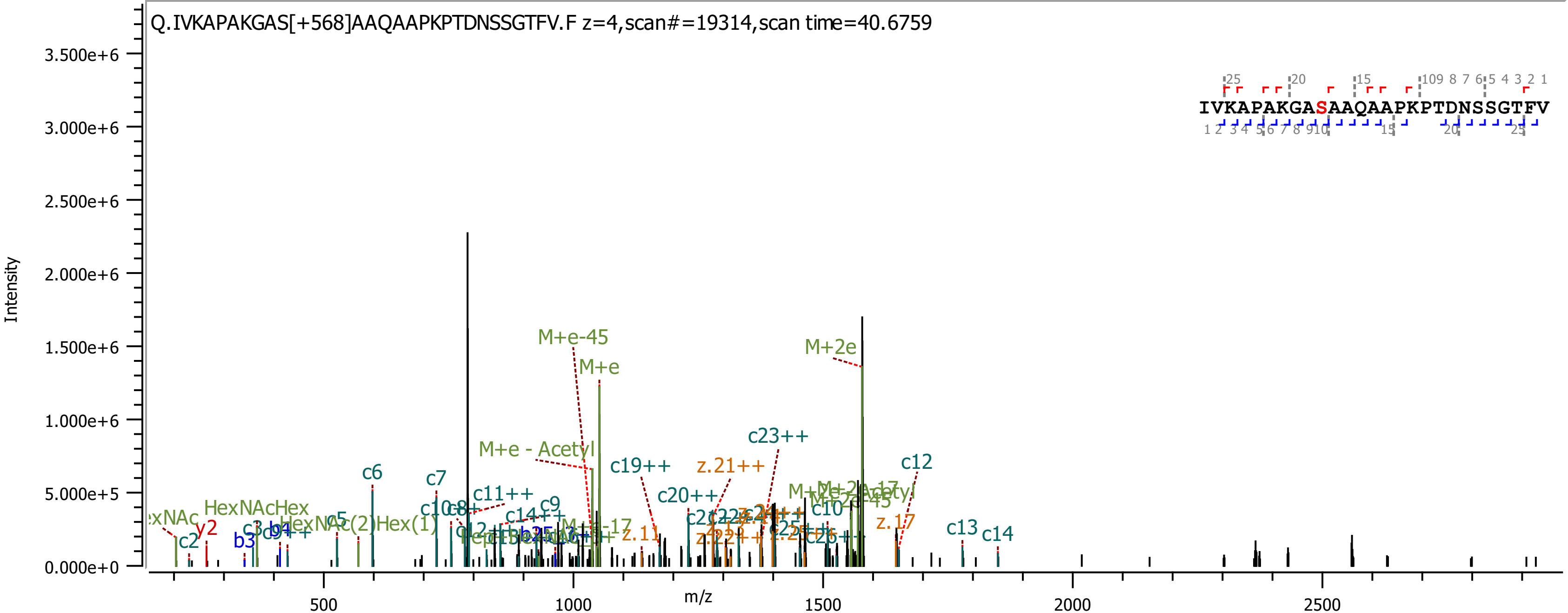
Q.IVKAPAKGAS[+568]AAQAAPKPTDNSSGTFV.F z=4,scan#=19314,scan time=40.6759
IVKAPAKGASAAQAAPKPTDNSSGTFV
Intensity
3.500e+6
3.000e+6
2.500e+6
2.000e+6
1.500e+6
1.000e+6
5.000e+5
0.000e+0
m/z
500
1000
1500
2000
2500
M+e-45
M+e
M+2e
M+e - Acetyl
c23++
c19++
z.21++
c12
c6
c7
c11++
c9
c14++
c20++
c13
c14
HexNAcHex
y2
c2
b3
c5
z.11
z.17

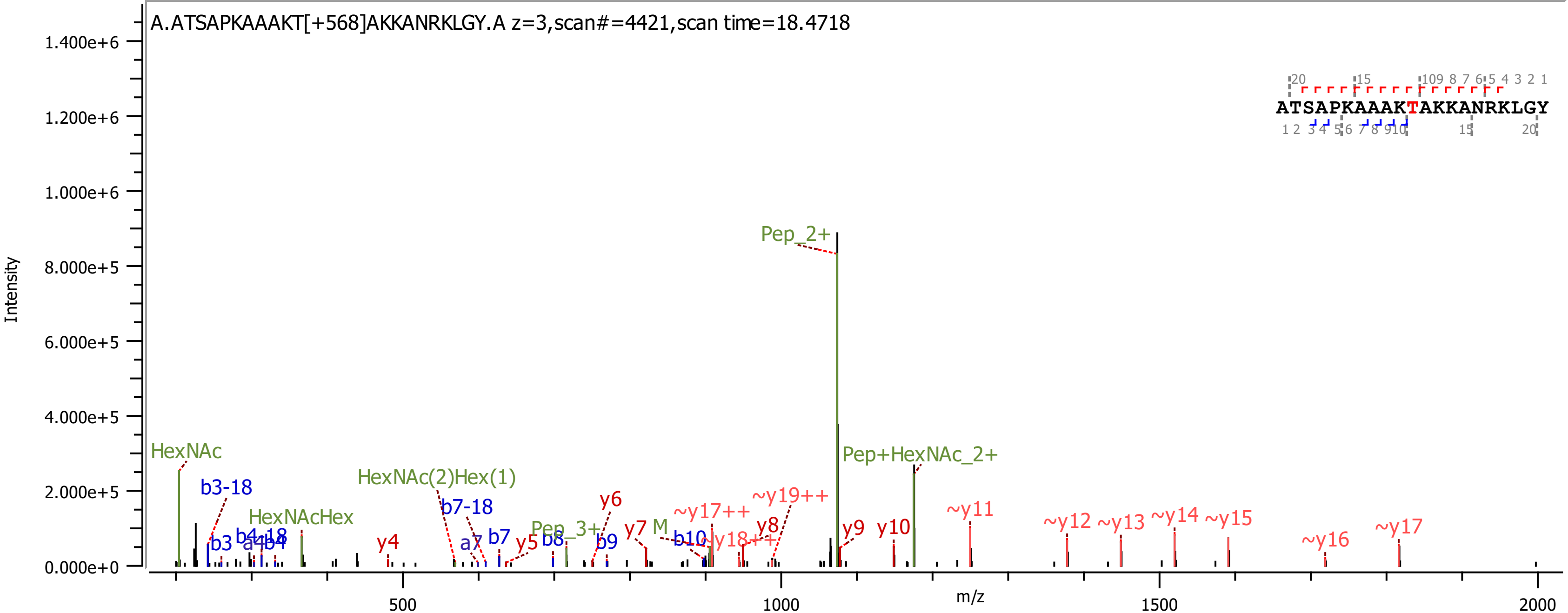
A.ATSAPKAAAKT[+568]AKKANRKLGY.A z=3,scan#=4421,scan time=18.4718
ATSAPKAAAKTAKKANRKLGY
Intensity
m/z
Pep_2+
Pep+HexNAc_2+
HexNAc
HexNAc(2)Hex(1)
HexNAcHex
Pep_3+
b3-18
b3
b4-18
a4
b4
b7-18
a7
b7
b8
b9
b10
y4
y5
y6
y7
M
y8
y9
y10
~y17++
~y18++
~y19++
~y11
~y12
~y13
~y14
~y15
~y16
~y17

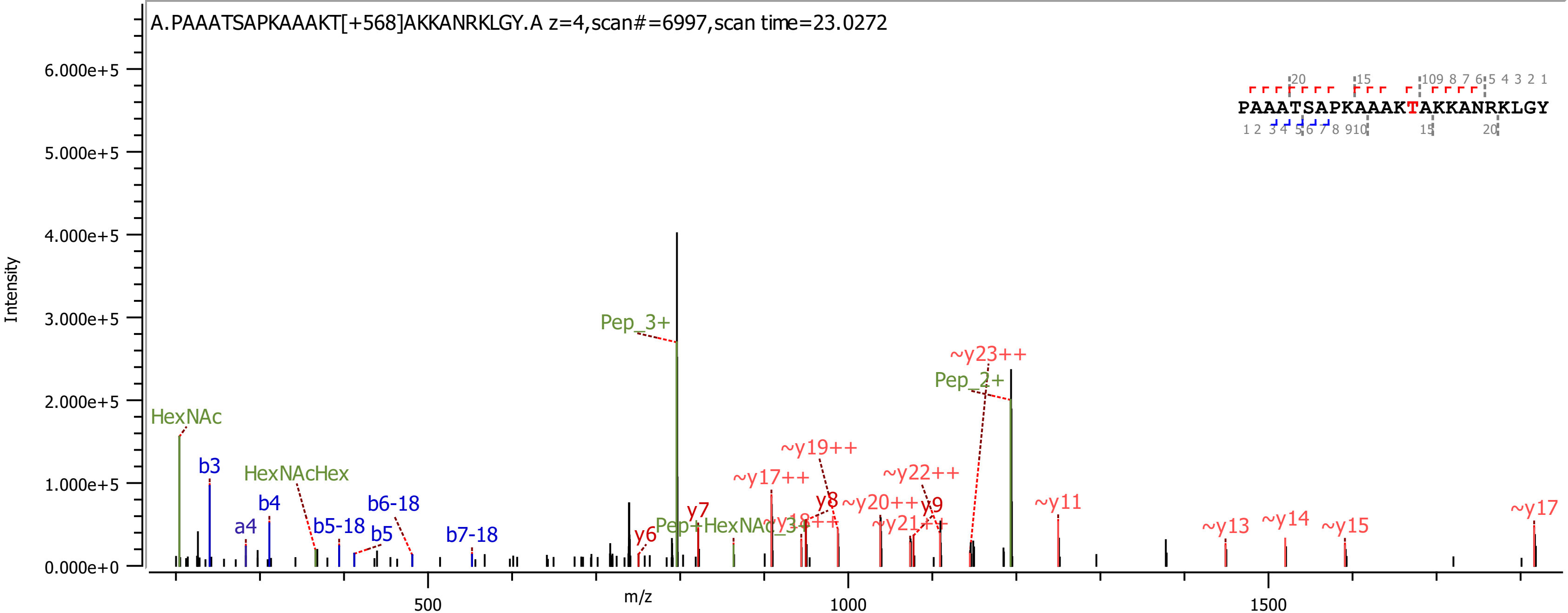

A.PAAATSAPKAAAKT[+568]AKKANRKLGY.A z=4,scan#=6997,scan time=23.0272
PAAATSAPKAAAKTAKKANRKLGY
Intensity
m/z
6.000e+5
5.000e+5
4.000e+5
3.000e+5
2.000e+5
1.000e+5
0.000e+0
500
1000
1500
HexNAc
b3
a4
b4
HexNAcHex
b5-18
b5
b6-18
b7-18
Pep_3+
y6
y7
Pep+HexNAc_3+
~y17++
~y18++
~y19++
y8
~y20++
~y21++
~y22++
y9
~y23++
Pep_2+
~y11
~y13
~y14
~y15
~y17

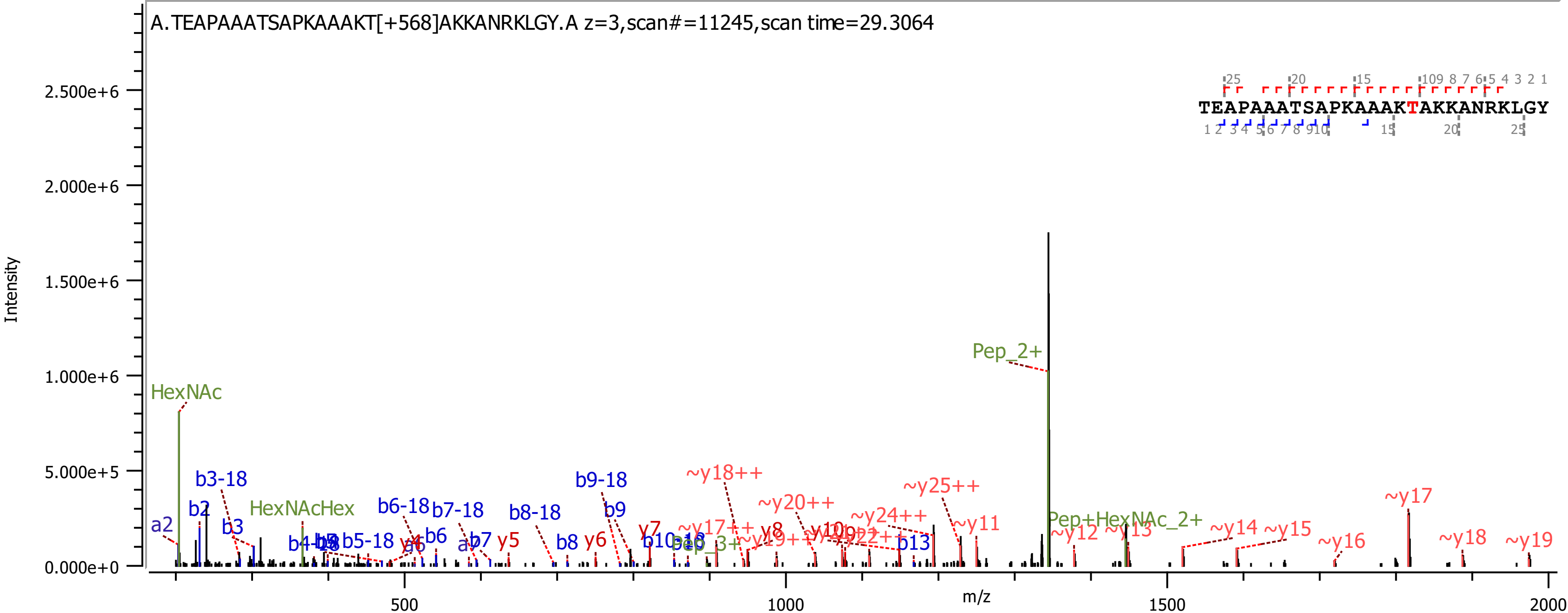

A.TEAPAAATSAPKAAAKT[+568]AKKANRKLGY.A z=3,scan#=11245,scan time=29.3064
TEAPAAATSAPKAAAKTAKKANRKLGY
Intensity
m/z
2.500e+6
2.000e+6
1.500e+6
1.000e+6
5.000e+5
0.000e+0
500
1000
1500
2000
HexNAc
HexNAcHex
Pep_2+
Pep+HexNAc_2+
Pep_3+
a2
b2
b3
b3-18
b6-18
b7-18
b8-18
b9-18
b5-18
b6
b7
b8
b9
b13
y5
y6
y7
y8
~y18++
~y20++
~y24++
~y25++
~y17++
~y11
~y12
~y13
~y14
~y15
~y16
~y17
~y18
~y19

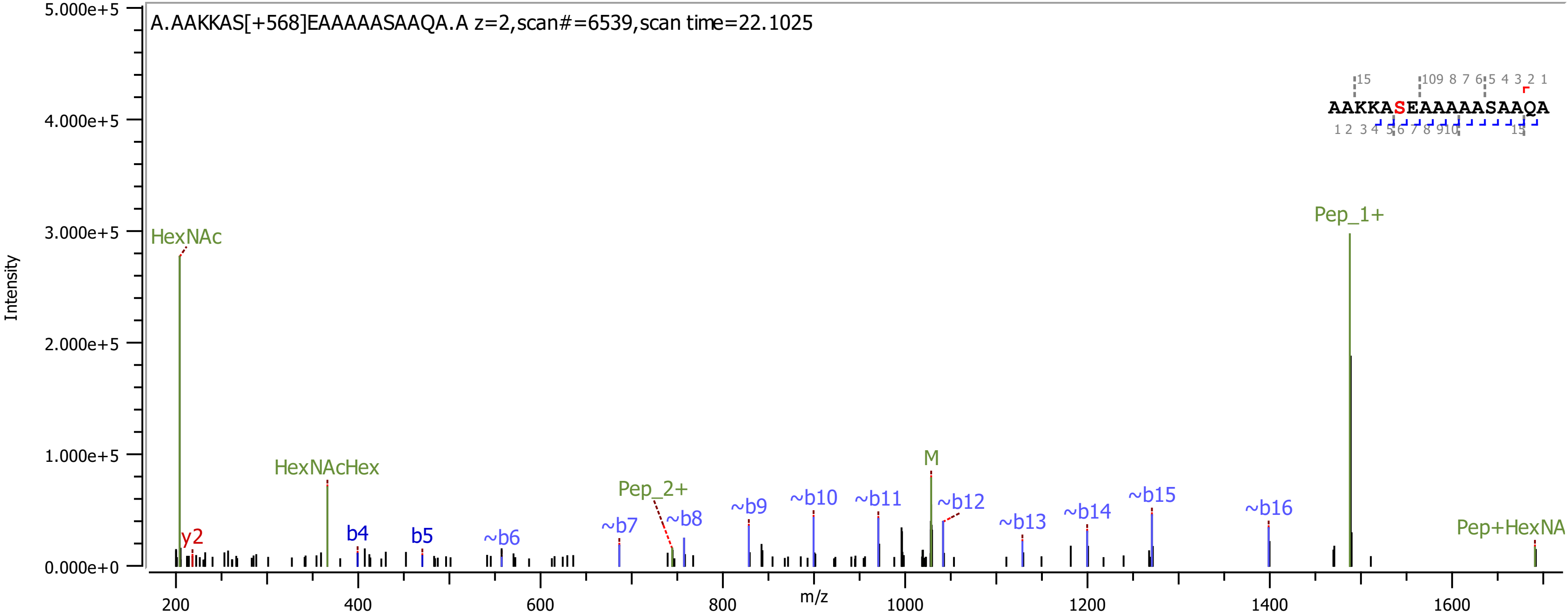

A.AAKKAS[+568]EAAAAASAAQA.A z=2,scan#=6539,scan time=22.1025
AAKKASEAAAAASAAQA
Intensity
m/z
HexNAc
HexNAcHex
y2
b4
b5
~b6
~b7
Pep_2+
~b8
~b9
~b10
~b11
M
~b12
~b13
~b14
~b15
~b16
Pep_1+
Pep+HexNA

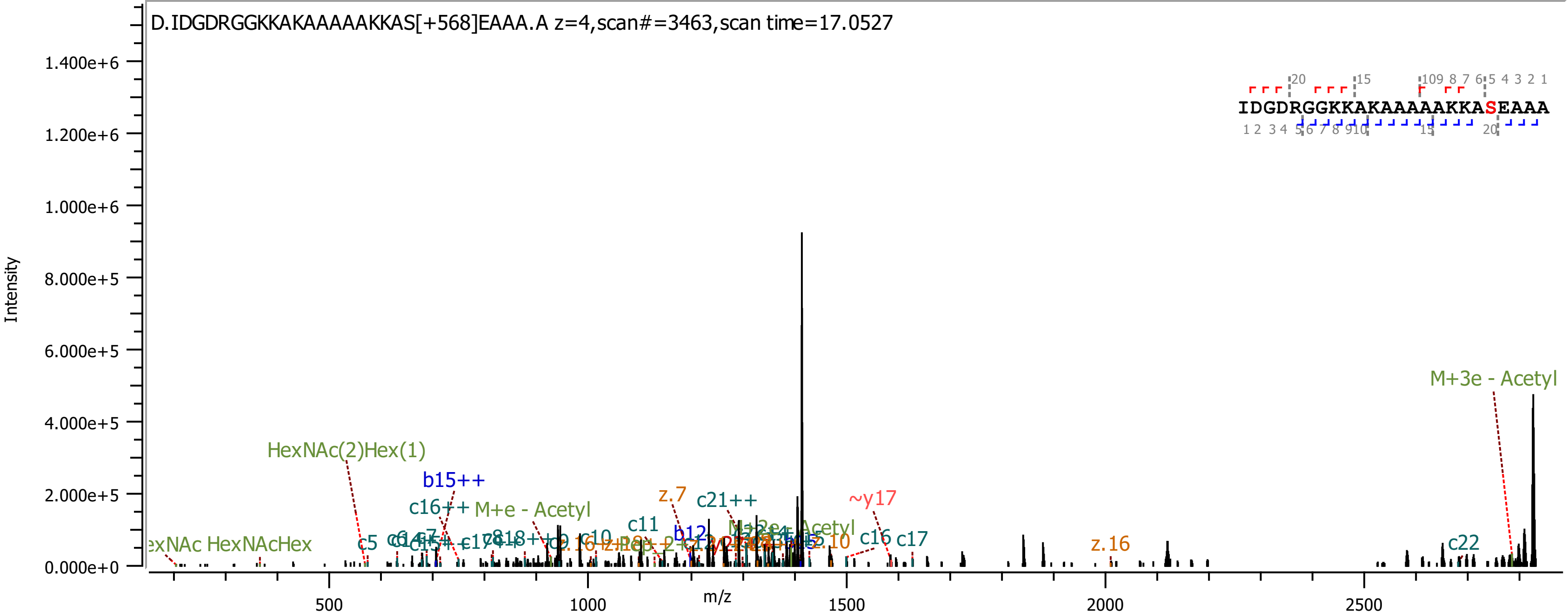

D.IDGDRGGKKAKAAAAAKKAS[+568]EAAA.A z=4,scan#=3463,scan time=17.0527
IDGDRGGKKAKAAAAAKKASEAAA
Intensity
m/z
M+3e - Acetyl
HexNAc(2)Hex(1)
b15++
c16++
M+e - Acetyl
z.7
c21++
~y17
c11
c16
c17
z.16
c22
c5
HexNAcHex

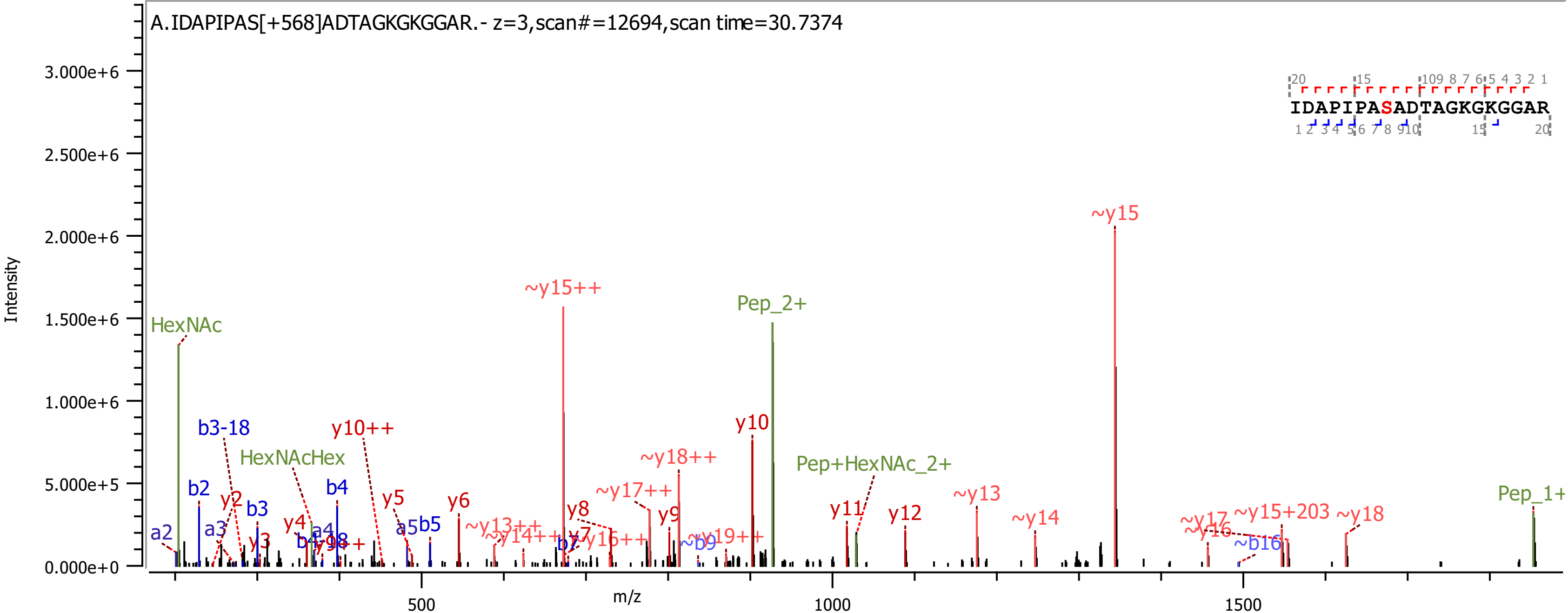
A.IDAPIPAS[+568]ADTAGKGKGGAR.- z=3,scan#=12694,scan time=30.7374
Intensity
m/z
3.000e+6
2.500e+6
2.000e+6
1.500e+6
1.000e+6
5.000e+5
0.000e+0
500
1000
1500
IDAPIPASADTAGKGKGGAR
HexNAc
HexNAcHex
b3-18
y10++
~y15++
Pep_2+
~y15
y10
~y18++
Pep+HexNAc_2+
~y17++
~y13
y11
y12
~y14
~y17
~y15+203
~y18
~y16
~b16
Pep_1+
a2
b2
a3
y2
b3
y3
y4
a4
b4
y5
a5
b5
y6
y8
y9
y7
~b9
~y19++
~y16++
~y14++
~y13++

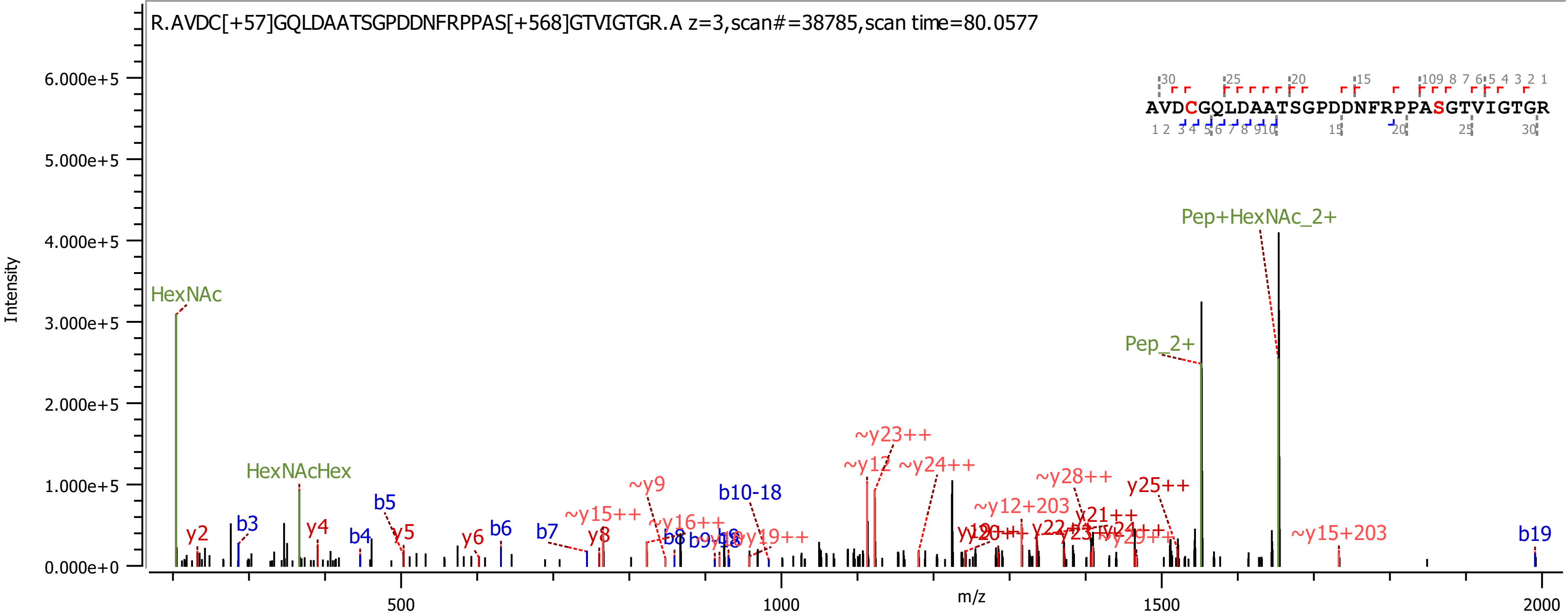
R.AVDC[+57]GQLDAATSGPDDNFRPPAS[+568]GTVIGTGR.A z=3,scan#=38785,scan time=80.0577
AVDCGQLDAATSGPDDNFRPPASGTVIGTGR
Intensity
m/z
HexNAc
HexNAcHex
Pep+HexNAc_2+
Pep_2+
y2
b3
y4
b4
b5
y5
y6
b6
b7
y8
~y9
~y15++
~y16++
b8
b10-18
~y19++
~y23++
~y12
~y24++
~y12+203
~y28++
y25++
y21++
~y15+203
b19
0.000e+0
1.000e+5
2.000e+5
3.000e+5
4.000e+5
5.000e+5
6.000e+5
500
1000
1500
2000

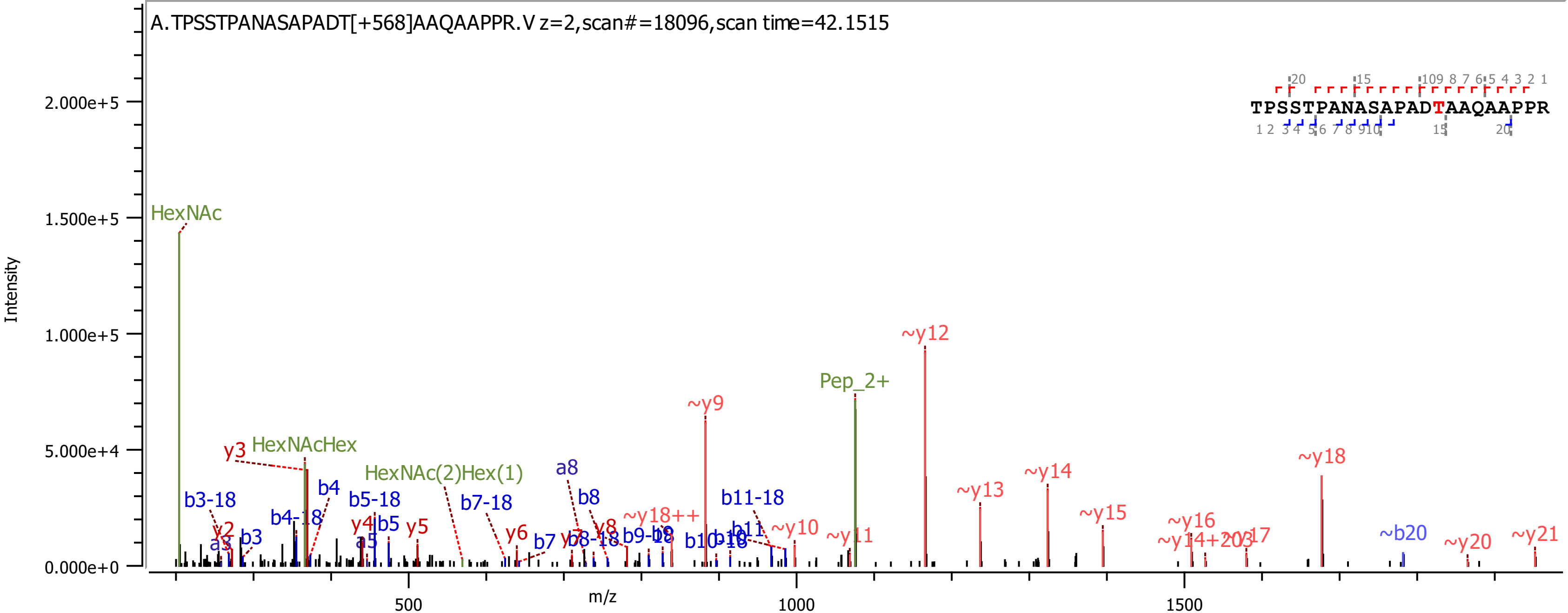
A.TPSSTPANASAPADT[+568]AAQAAPPR.V z=2,scan#=18096,scan time=42.1515
TPSSTPANASAPADTAAQAAPPR
Intensity
m/z
HexNAc
HexNAcHex
HexNAc(2)Hex(1)
Pep_2+
~y9
~y12
~y13
~y14
~y15
~y16
~y18
~y20
~y21
~b20
~y10
~y11
~y18++
b11-18
b11
b10-18
b9-18
b8
b8-18
a8
b7
b7-18
b5-18
b5
b4
b4-18
b3-18
b3
a3
a5
y2
y3
y4
y5
y6
y7
y8
~y14+203
~y17
0.000e+0
5.000e+4
1.000e+5
1.500e+5
2.000e+5
500
1000
1500

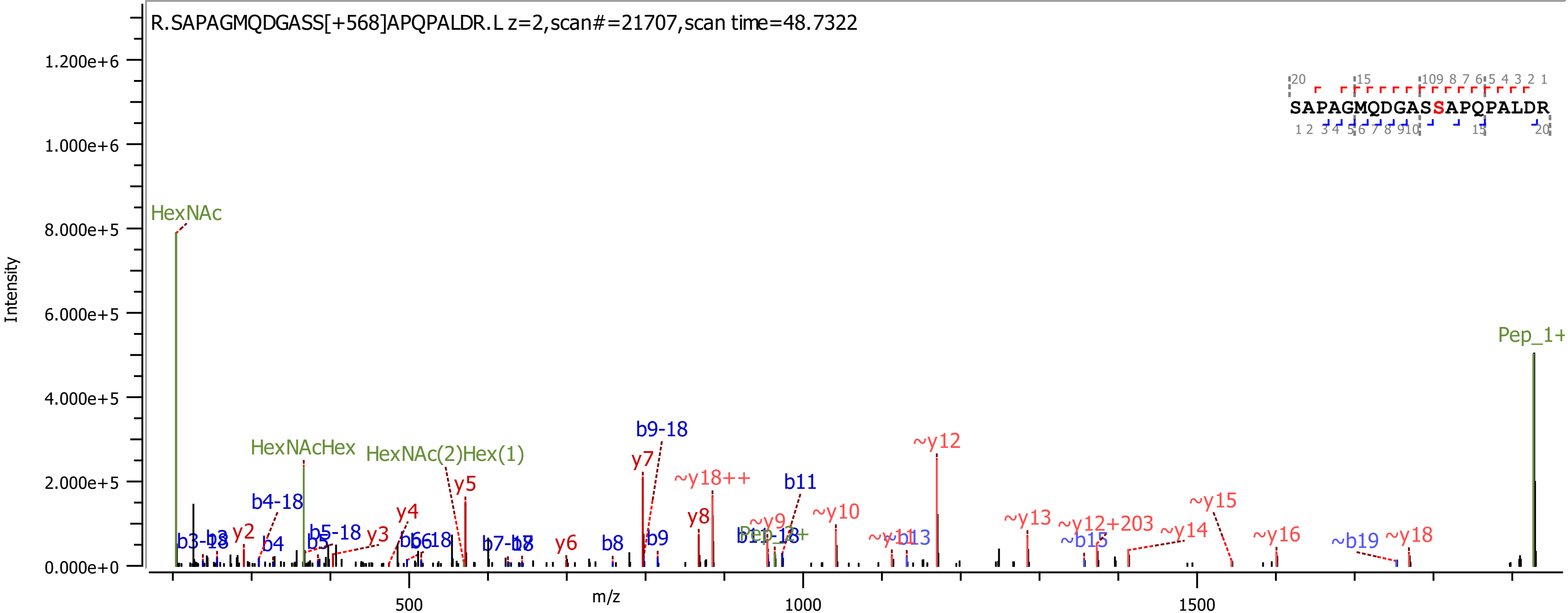

R.SAPAGMQDGASS[+568]APQPALDR.L z=2,scan#=21707,scan time=48.7322
SAPAGMQDGASSAPQPALDR
Intensity
m/z
HexNAc
HexNAcHex
HexNAc(2)Hex(1)
Pep_1+
b9-18
~y12
y7
~y18++
b11
y5
b4-18
y4
~y10
y8
~y15
~y13
~y12+203
~y14
~y16
~y18
~b19
~b15
~y9
y2
y3
b4
b5-18
b5
y6
b8
b9
b3-18
b3
b7-18
b7
b6-18
b6
~y11
~b13

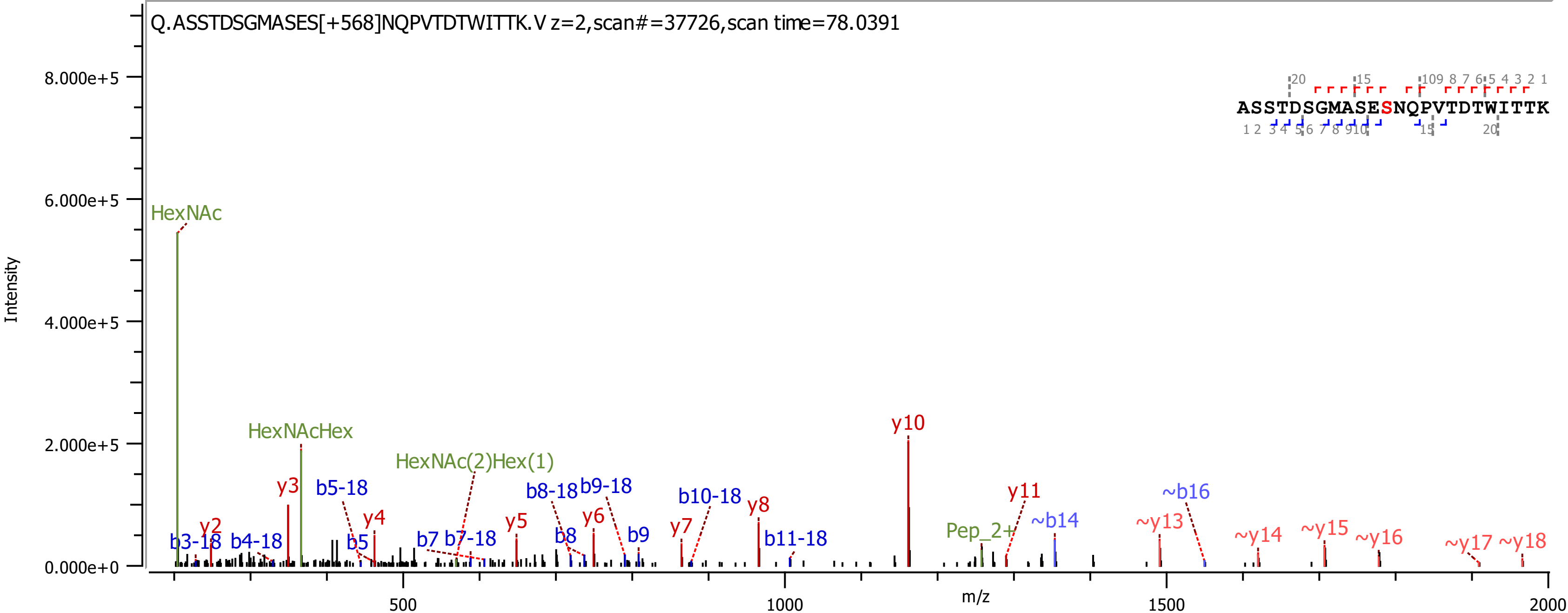

Q.ASSTDSGMASES[+568]NQPVTDTWITTK.V z=2,scan#=37726,scan time=78.0391
Intensity
m/z
ASSTDSGMASESNQPVTDTWITTK
HexNAc
HexNAcHex
HexNAc(2)Hex(1)
Pep_2+
y2
y3
y4
y5
y6
y7
y8
y10
y11
~y13
~y14
~y15
~y16
~y17
~y18
b3-18
b4-18
b5-18
b5
b7
b7-18
b8-18
b9-18
b8
b9
b10-18
b11-18
~b14
~b16
8.000e+5
6.000e+5
4.000e+5
2.000e+5
0.000e+0
500
1000
1500
2000

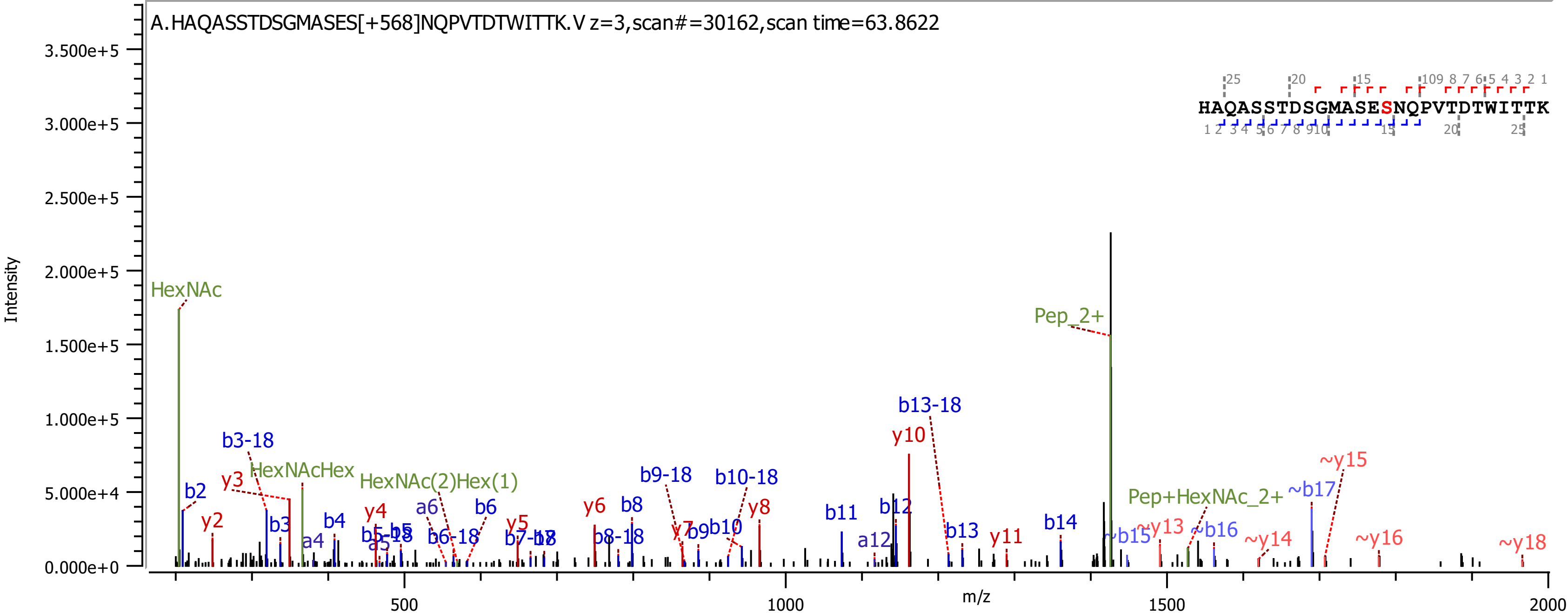
A.HAQASSTDSGMASES[+568]NQPVTDTWITTK.V z=3,scan#=30162,scan time=63.8622
HAQASSTDSGMASESNQPVTDTWITTK
Intensity
m/z
HexNAc
HexNAcHex
HexNAc(2)Hex(1)
Pep_2+
Pep+HexNAc_2+
b2
b3-18
y2
y3
b3
a4
b4
y4
a6
b6
b5-18
a5
b5
b6-18
y5
b7-18
b7
y6
b8
b8-18
b9-18
y7
b9
b10
b10-18
y8
b11
a12
b12
b13-18
y10
b13
y11
b14
~b15
~y13
~b16
~y14
~b17
~y15
~y16
~y18
0.000e+0
5.000e+4
1.000e+5
1.500e+5
2.000e+5
2.500e+5
3.000e+5
3.500e+5
500
1000
1500
2000

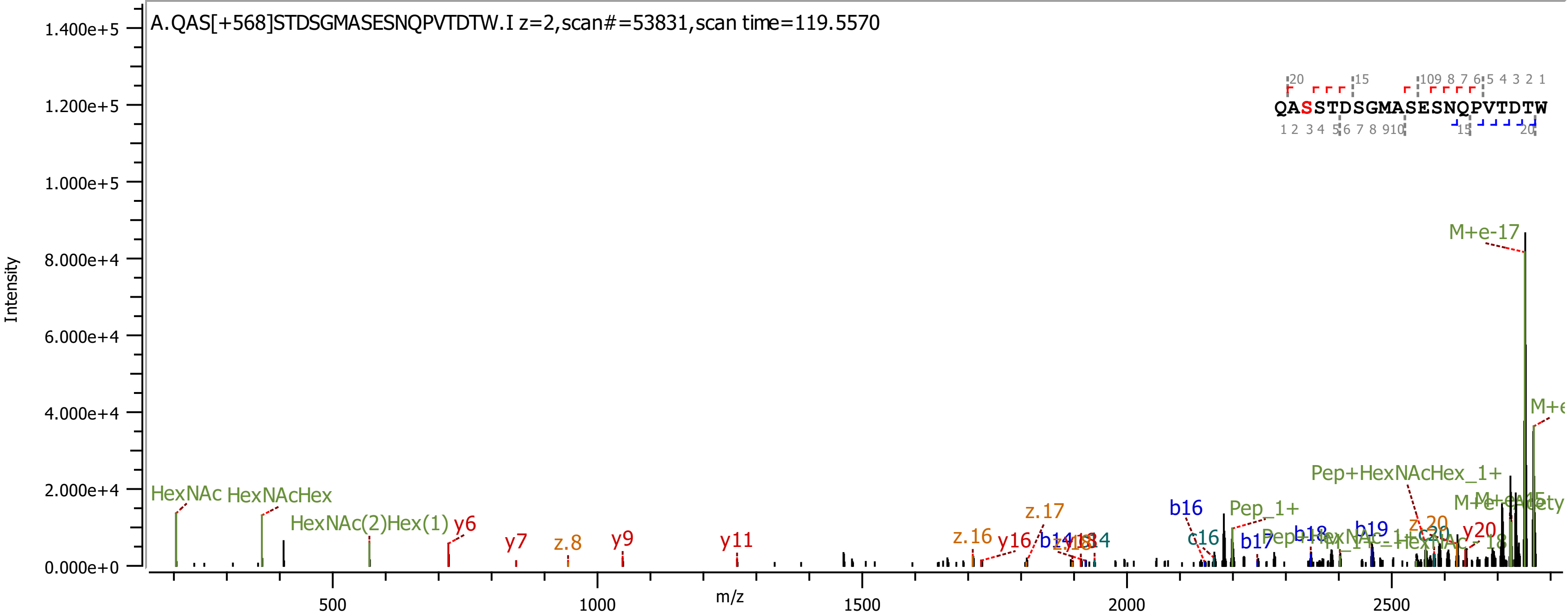

A.QAS[+568]STDSGMASESNQPVTDTW.I z=2,scan#=53831,scan time=119.5570
QASSTDSGMASESNQPVTDTW
Intensity
m/z
HexNAc
HexNAcHex
HexNAc(2)Hex(1)
y6
y7
z.8
y9
y11
z.16
y16
z.17
b16
c16
Pep_1+
b17
b18
b19
Pep+HexNAcHex_1+
z.20
y20
M+e-17

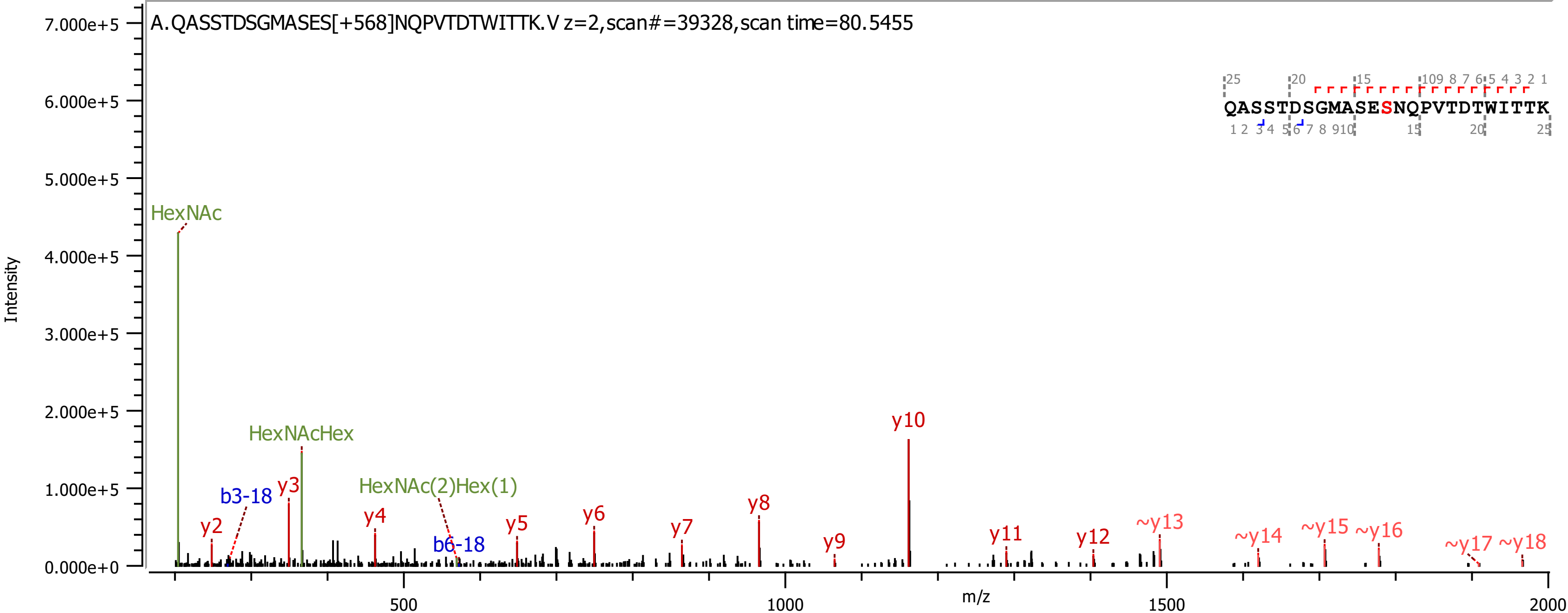

A.QASSTDSGMASES[+568]NQPVTDTWITTK.V z=2,scan#=39328,scan time=80.5455
QASSTDSGMASESNQPVTDTWITTK
HexNAc
HexNAcHex
HexNAc(2)Hex(1)
b3-18
b6-18
y2
y3
y4
y5
y6
y7
y8
y9
y10
y11
y12
~y13
~y14
~y15
~y16
~y17
~y18
Intensity
m/z
0.000e+0
1.000e+5
2.000e+5
3.000e+5
4.000e+5
5.000e+5
6.000e+5
7.000e+5
500
1000
1500
2000

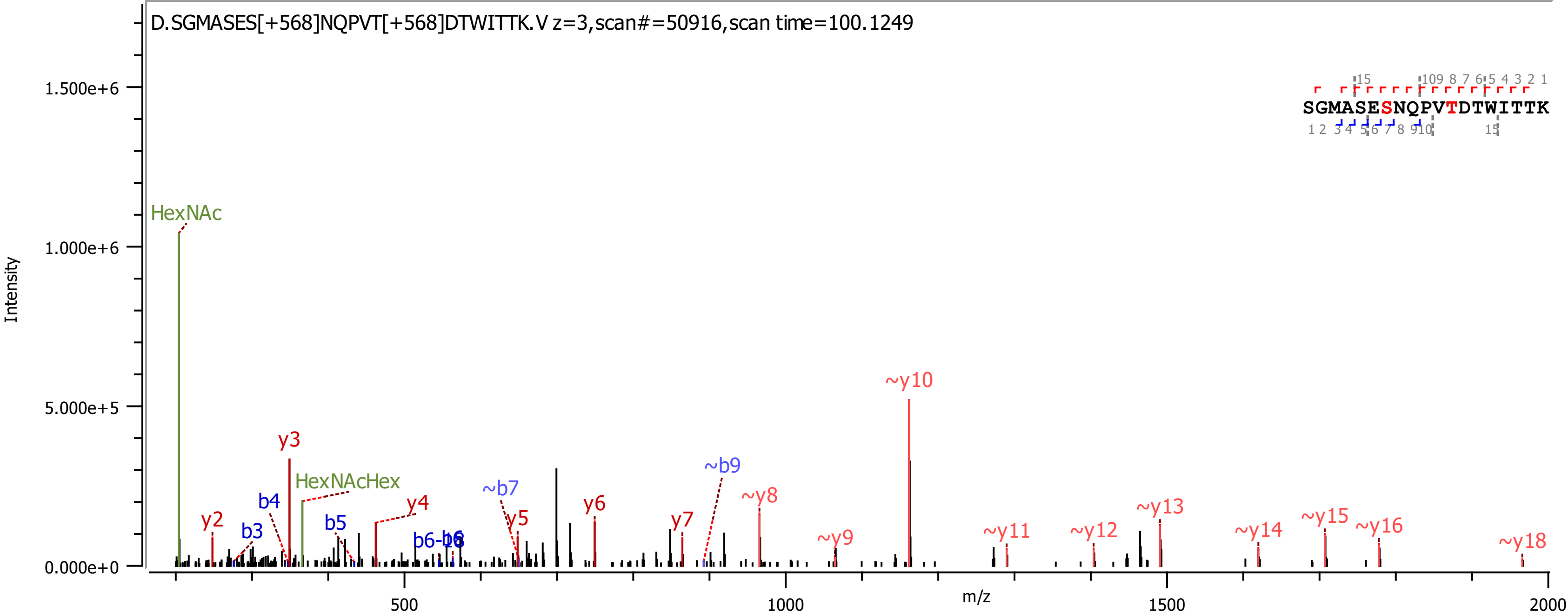
D.SGMASES[+568]NQPVT[+568]DTWITTK.V z=3,scan#=50916,scan time=100.1249
SGMASESNQPVTDTWITTK
Intensity
1.500e+6
1.000e+6
5.000e+5
0.000e+0
m/z
500
1000
1500
2000
HexNAc
HexNAcHex
y2
y3
y4
y5
y6
y7
b3
b4
b5
~b7
~b9
~y8
~y9
~y10
~y11
~y12
~y13
~y14
~y15
~y16
~y18

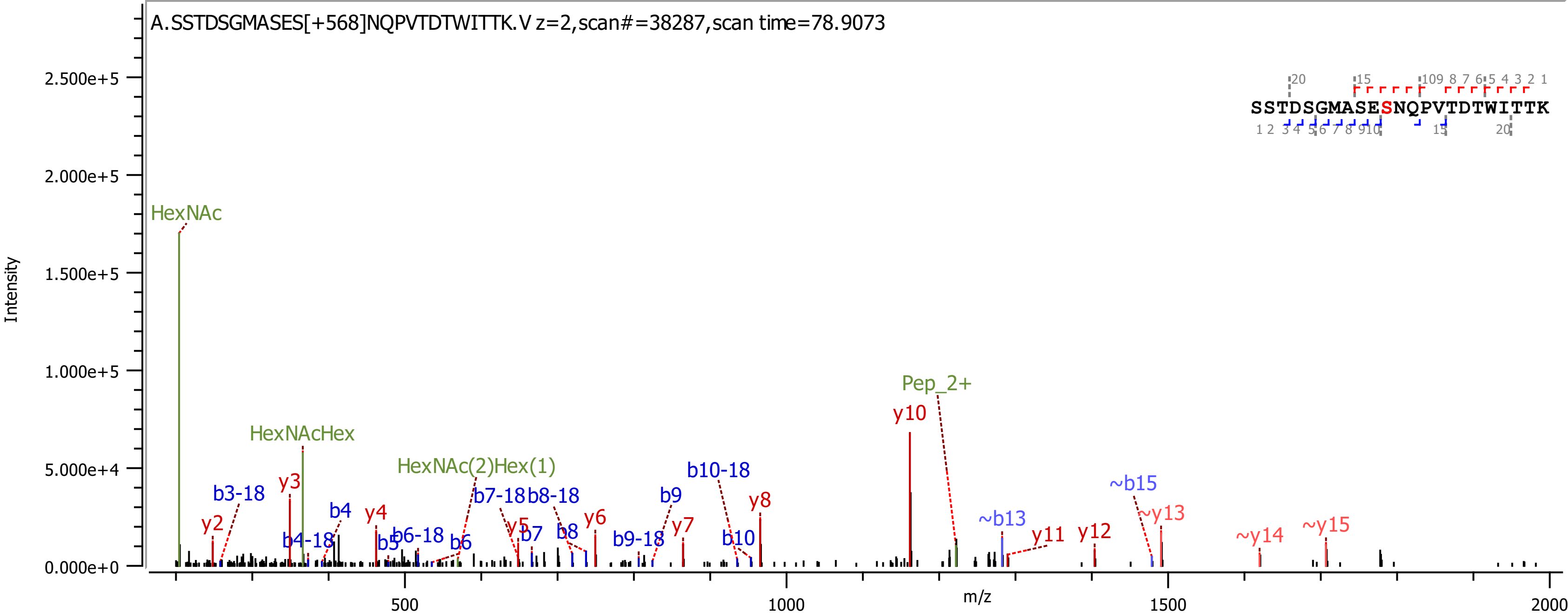
A.SSTDSGMASES[+568]NQPVTDTWITTK.V z=2,scan#=38287,scan time=78.9073
SSTDSGMASESNQPVTDTWITTK
Intensity
m/z
2.500e+5
2.000e+5
1.500e+5
1.000e+5
5.000e+4
0.000e+0
500
1000
1500
2000
HexNAc
HexNAcHex
HexNAc(2)Hex(1)
Pep_2+
y2
y3
y4
y5
y6
y7
y8
y10
y11
y12
~y13
~y14
~y15
b3-18
b4-18
b4
b5
b6-18
b6
b7-18
b7
b8-18
b8
b9-18
b9
b10-18
b10
~b13
~b15

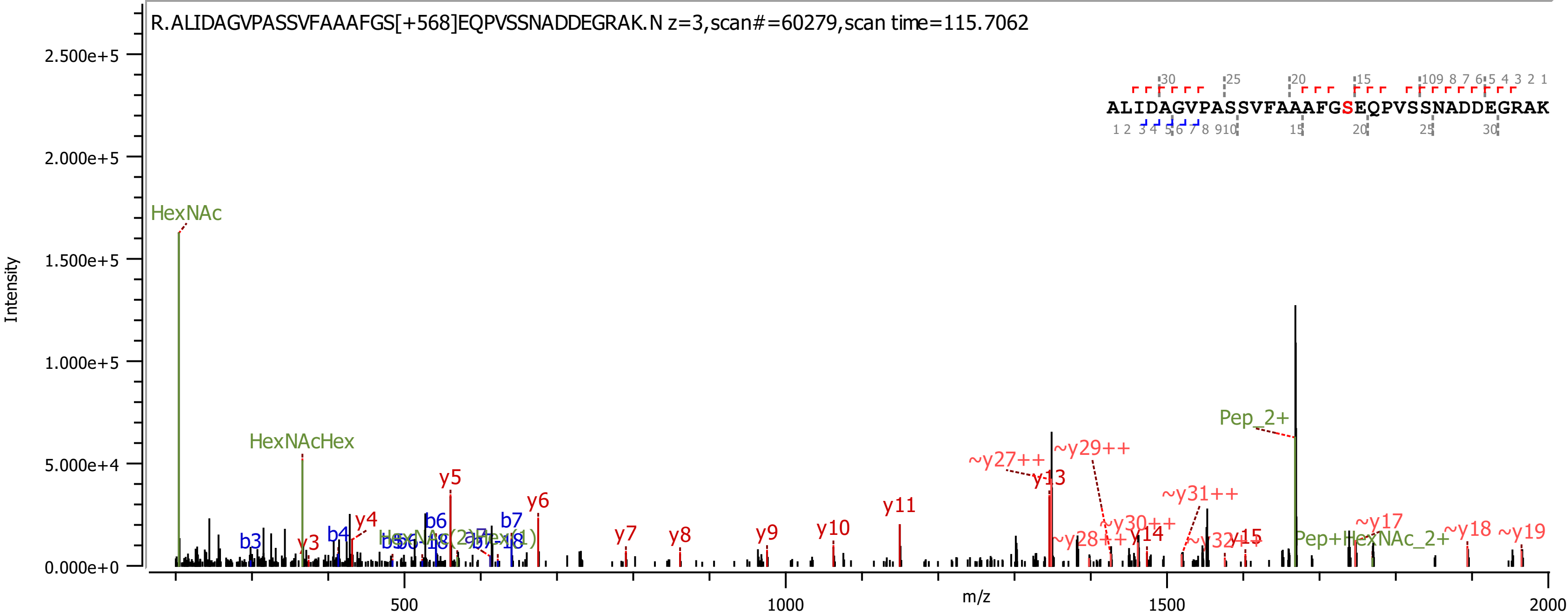
R.ALIDAGVPASSVFAAAFGS[+568]EQPVSSNADDEGRAK.N z=3,scan#=60279,scan time=115.7062
ALIDAGVPASSVFAAAFGSEQPVSSNADDEGRAK
Intensity
m/z
HexNAc
HexNAcHex
Pep_2+
Pep+HexNAc_2+
y3
y4
y5
y6
y7
y8
y9
y10
y11
y13
y14
y15
~y17
~y18
~y19
~y27++
~y28++
~y29++
~y30++
~y31++
~y32++
b3
b4
b6
b7

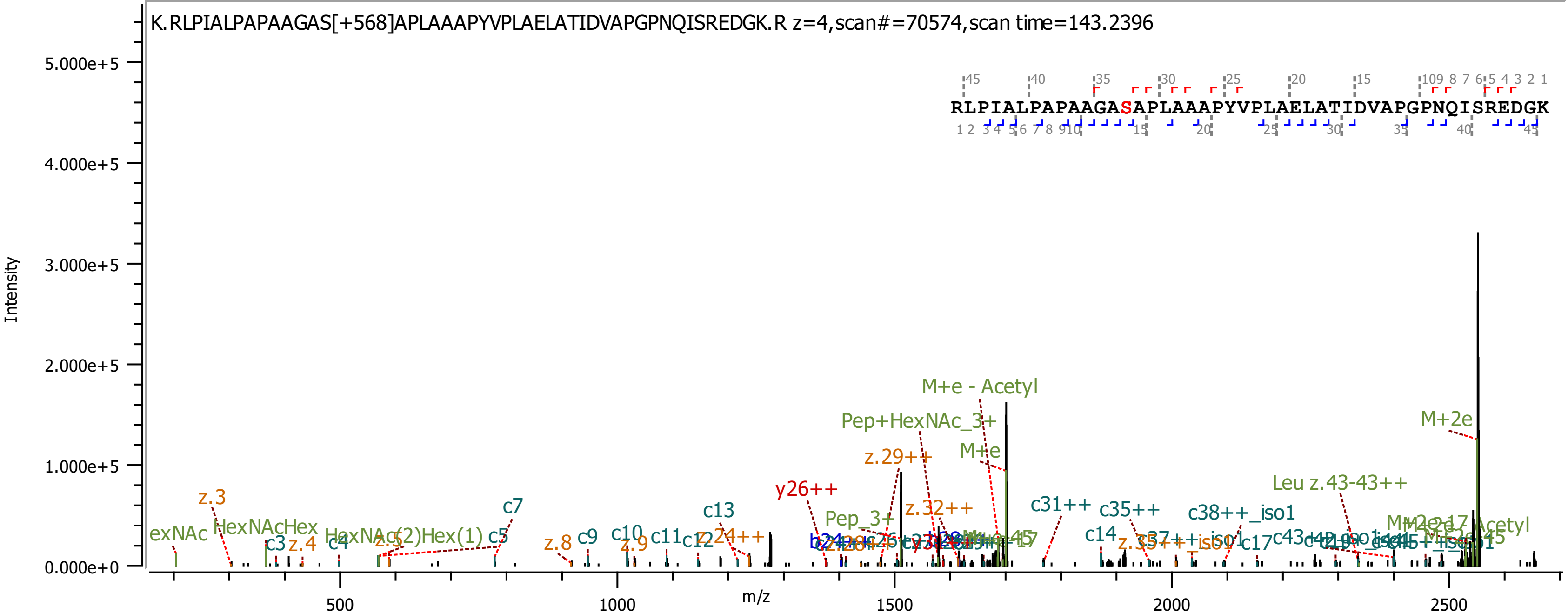

K.RLPIALPAPAAGAS[+568]APLAAAPYVPLAELATIDVAPGPNQISREDGK.R z=4,scan#=70574,scan time=143.2396
RLPIALPAPAAGASAPLAAAPYVPLAELATIDVAPGPNQISREDGK
Intensity
m/z
5.000e+5
4.000e+5
3.000e+5
2.000e+5
1.000e+5
0.000e+0
500
1000
1500
2000
2500
M+e - Acetyl
Pep+HexNAc_3+
M+e
z.29++
M+2e
Leu z.43-43++
y26++
z.32++
c31++
c35++
c38++_iso1
z.3
c7
c13
Pep_3+
HexNAcHex
HexNAc(2)Hex(1)
c3
z.4
c4
c5
z.8
c9
c10
z.9
c11
c12
z.24++
c14
z.35++
c17

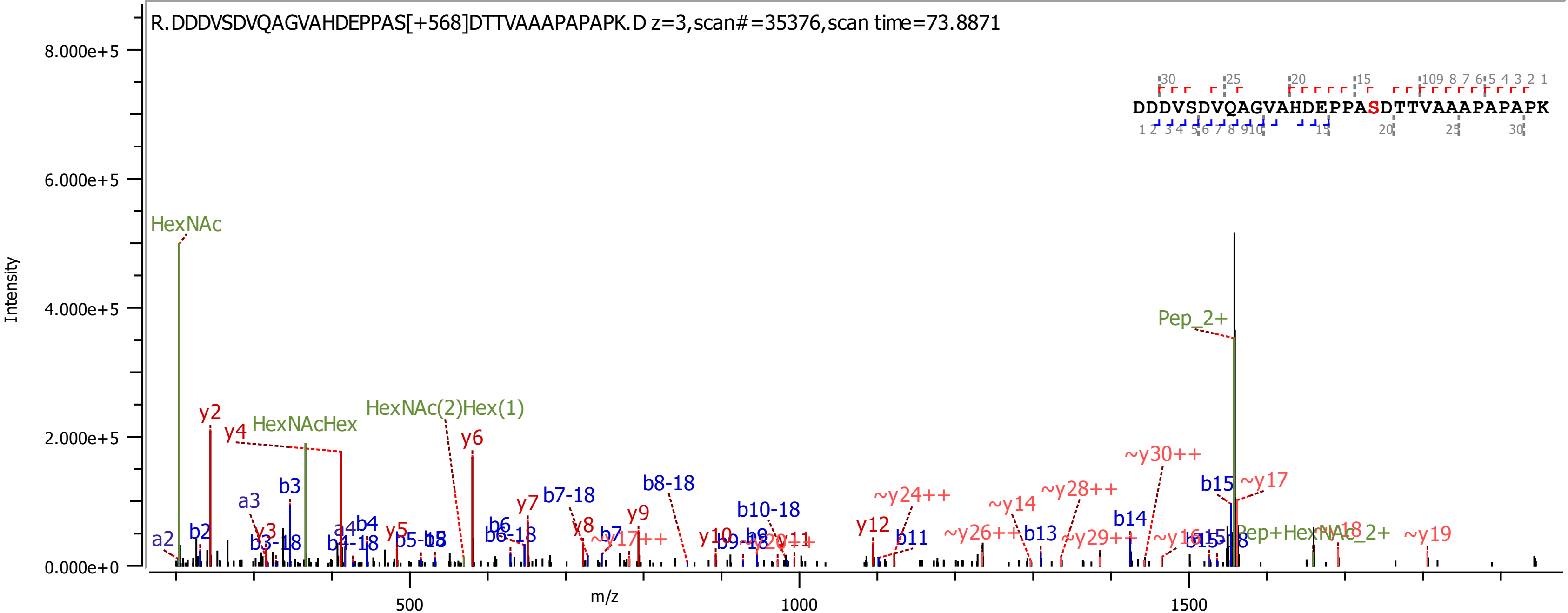

R.DDDVSDVQAGVAHDEPPAS[+568]DTTVAAAPAPAPK.D z=3,scan#=35376,scan time=73.8871
DDDVSDVQAGVAHDEPPASDTTVAAAPAPAPK
Intensity
m/z
8.000e+5
6.000e+5
4.000e+5
2.000e+5
0.000e+0
500
1000
1500
HexNAc
HexNAcHex
HexNAc(2)Hex(1)
Pep_2+
Pep+HexNAc_2+
y2
y4
y6
y7
y8
y9
y12
b3
a3
b7-18
b8-18
b10-18
b14
b15
b11
b13
~y17
~y19
~y30++
~y24++
~y28++
~y14
~y26++
~y29++

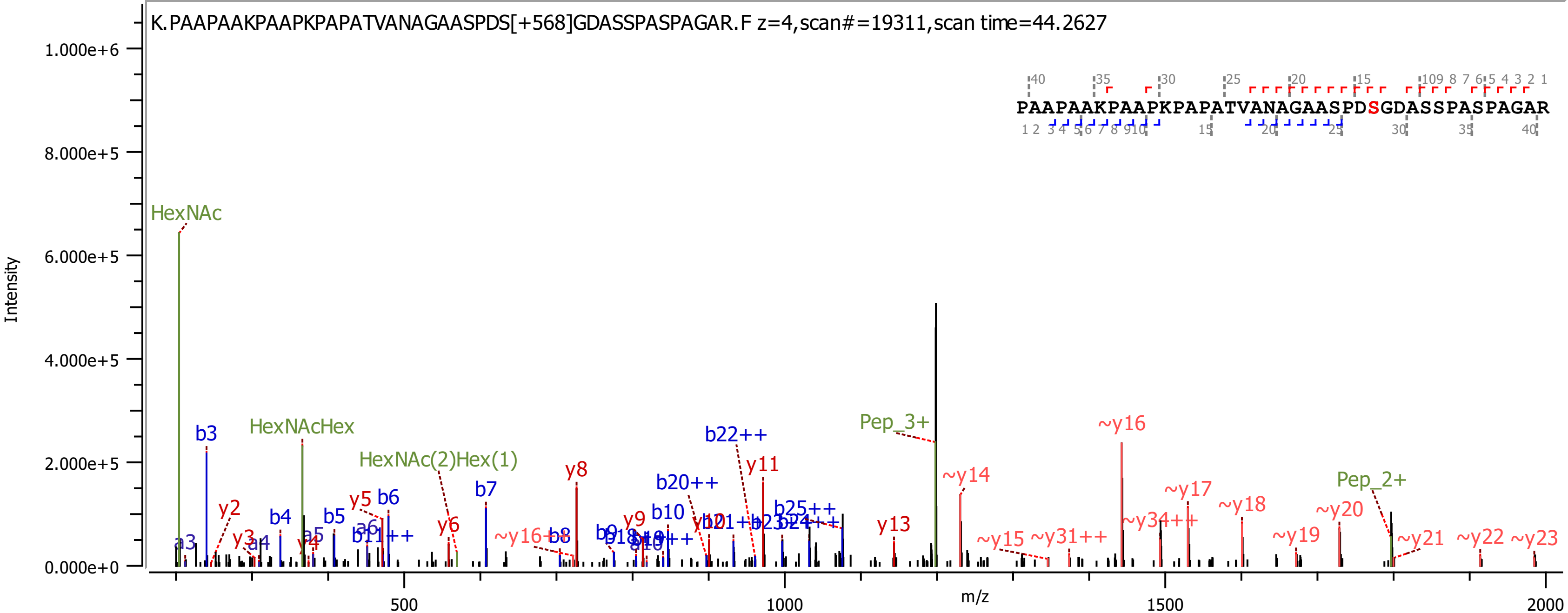
K.PAAPAAKPAAPKPAPATVANAGAASPDS[+568]GDASSPASPAGAR.F z=4,scan#=19311,scan time=44.2627
PAAPAAKPAAPKPAPATVANAGAASPDSGDASSPASPAGAR
HexNAc
HexNAcHex
HexNAc(2)Hex(1)
Pep_3+
Pep_2+
b3
b4
b5
b6
b7
b8
b10
b20++
b22++
b25++
y2
y3
y4
y5
y6
y8
y9
y11
y13
~y14
~y15
~y16
~y17
~y18
~y19
~y20
~y21
~y22
~y23
~y31++
~y34++
a3
a4
a5
a6
Intensity
m/z
0.000e+0
2.000e+5
4.000e+5
6.000e+5
8.000e+5
1.000e+6
500
1000
1500
2000

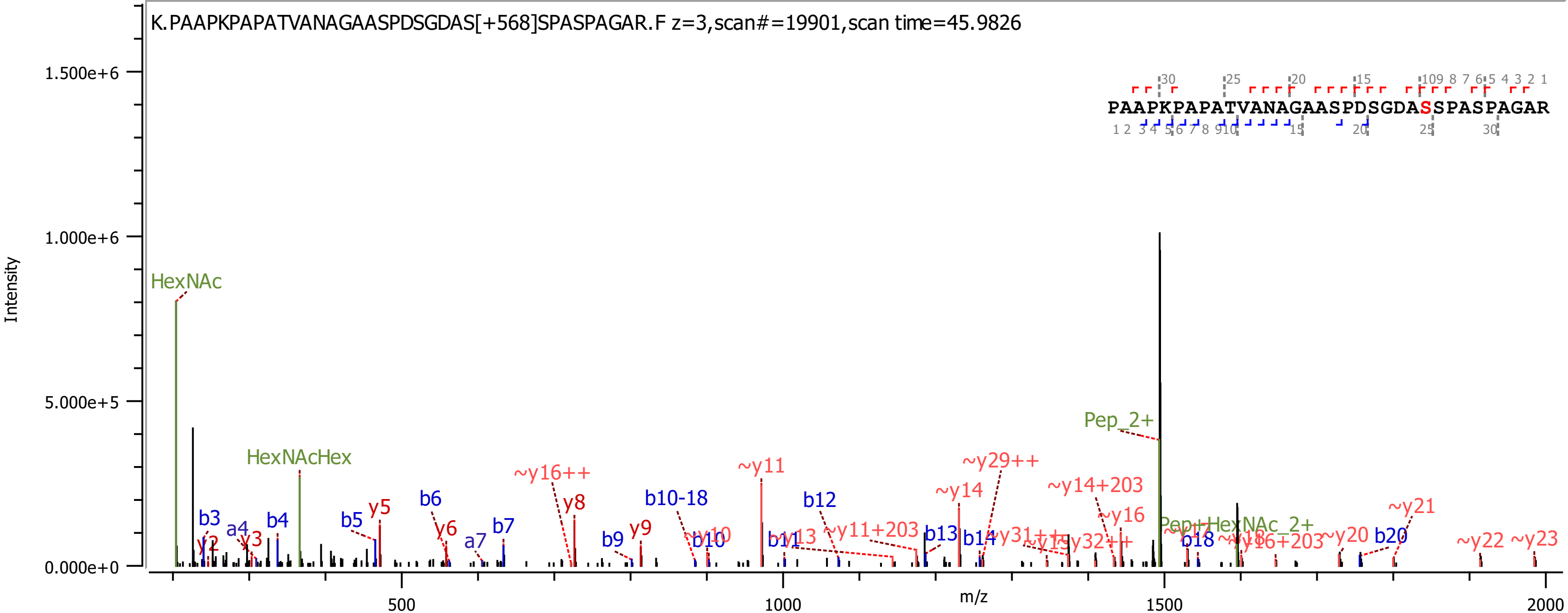
K.PAAPKPAPATVANAGAASPDSGDAS[+568]SPASPAGAR.F z=3,scan#=19901,scan time=45.9826
PAAPKPAPATVANAGAASPDSGDASSPASPAGAR
Intensity
m/z
1.500e+6
1.000e+6
5.000e+5
0.000e+0
500
1000
1500
2000
HexNAc
HexNAcHex
Pep_2+
Pep+HexNAc_2+
b3
a4
y2
y3
b4
b5
y5
b6
y6
a7
b7
~y16++
y8
b9
y9
b10-18
~y10
b10
~y11
b11
~y13
b12
~y11+203
b13
~y14
b14
~y29++
~y31++
~y15
y32++
~y14+203
~y16
~y17
b18
~y18
~y16+203
~y20
b20
~y21
~y22
~y23

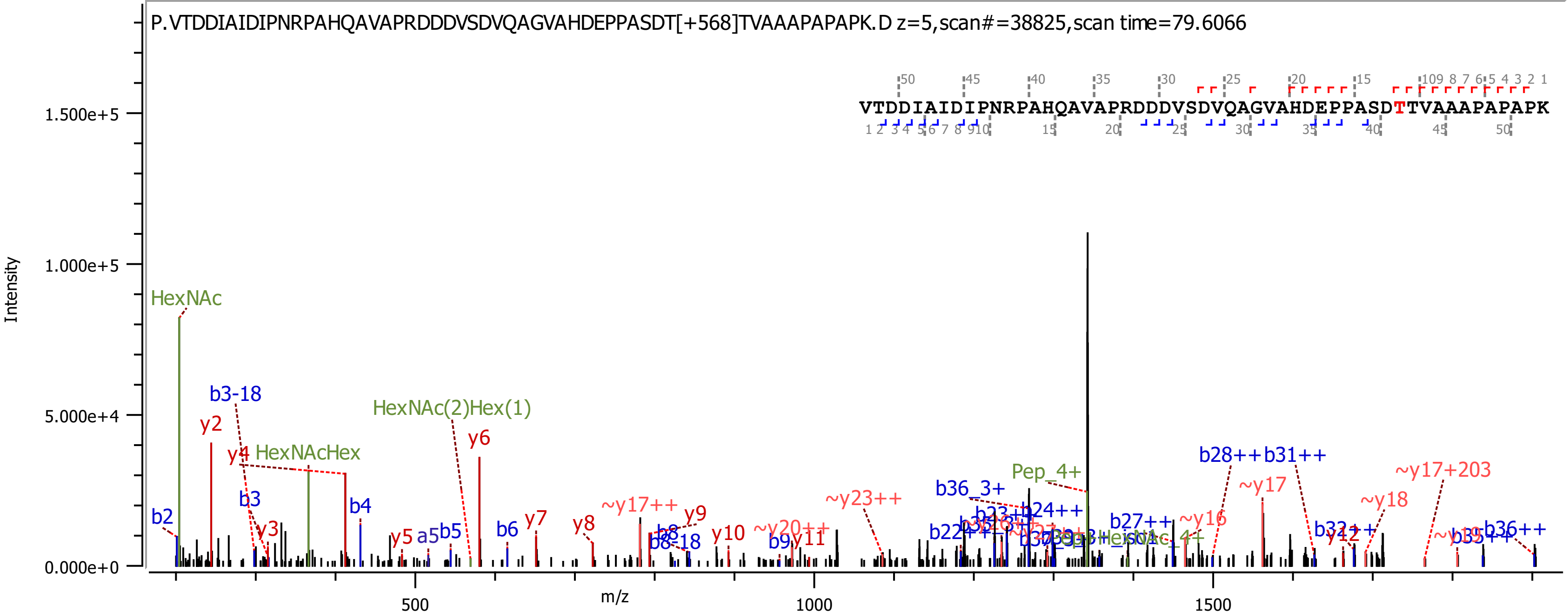
P.VTDDIAIDIPNRPAHQAVAPRDDDVSDVQAGVAHDEPPASDT[+568]TVAAAPAPAPK.D z=5,scan#=38825,scan time=79.6066
VTDDIAIDIPNRPAHQAVAPRDDDVSDVQAGVAHDEPPASDTTVAAAPAPAPK
Intensity
m/z
HexNAc
HexNAc(2)Hex(1)
HexNAcHex
Pep_4+
b28++b31++
~y17+203
~y17
~y18
b36_3+
~y23++
~y17++
b3-18
y2
y4
y6
b3
b4
y3
y5
a5
b5
b6
y7
y8
y9
y10
b9
y11
b2
b27++
~y16
b32++
y12
b36++

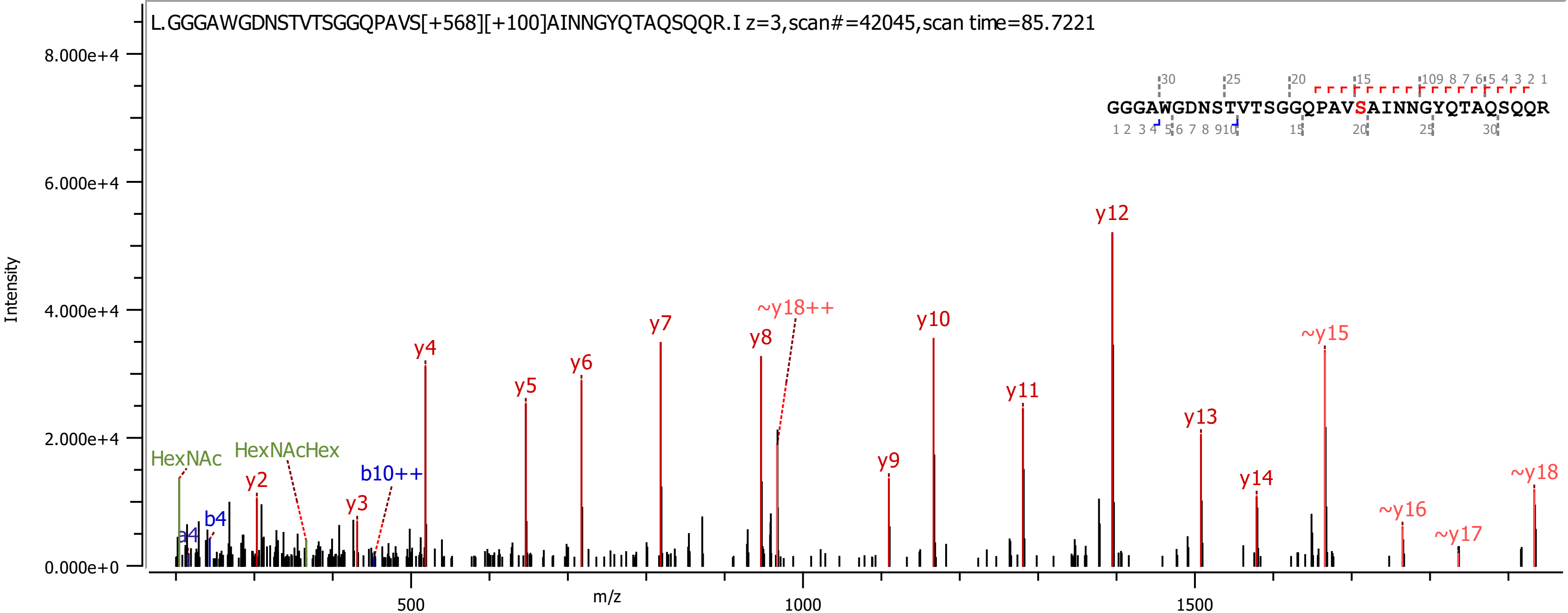

L.GGGAWGDNSTVTSGGQPAVS[+568][+100]AINNGYQTAQSQQR.I z=3,scan#=42045,scan time=85.7221
GGGAWGDNSTVTSGGQPAVSAINNGYQTAQSQQR
Intensity
m/z
HexNAc
HexNAcHex
a4
b4
b10++
y2
y3
y4
y5
y6
y7
y8
~y18++
y9
y10
y11
y12
y13
y14
~y15
~y16
~y17
~y18

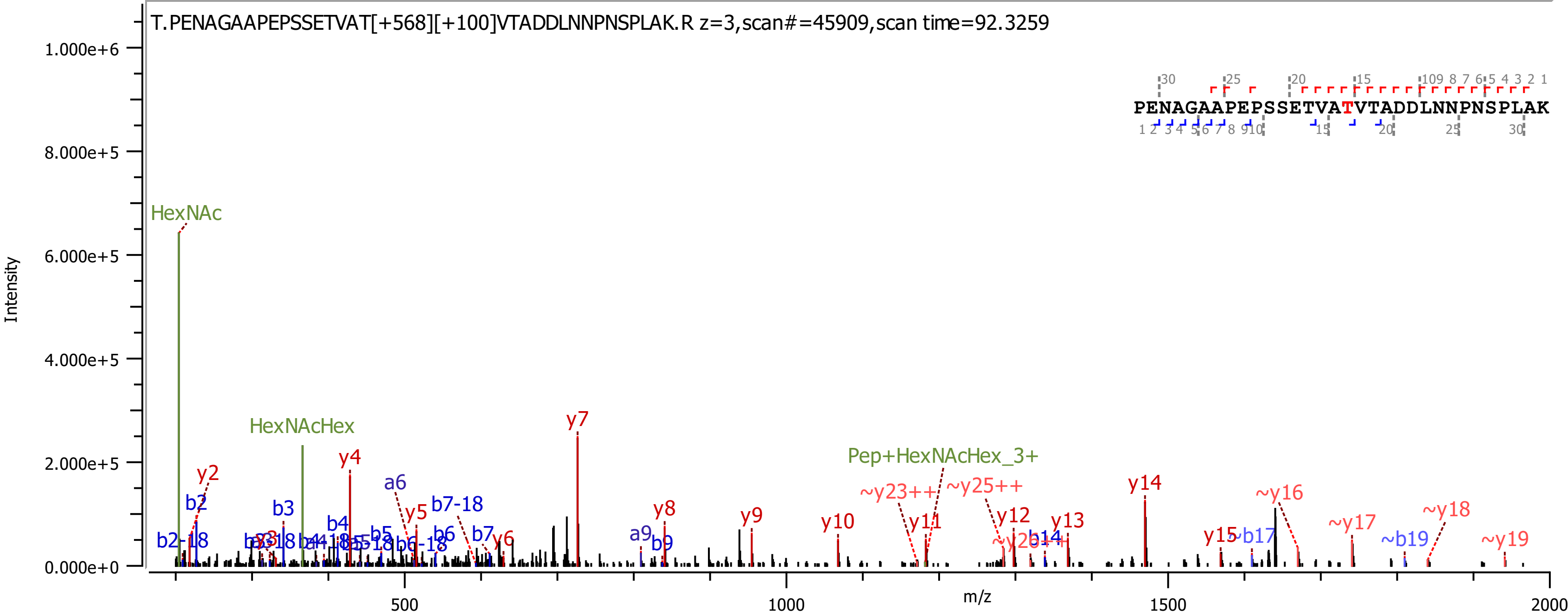

T.PENAGAAPEPSSETVAT[+568][+100]VTADDLNNPNSPLAK.R z=3,scan#=45909,scan time=92.3259
PENAGAAPEPSSETVATVTADDLNNPNSPLAK
Intensity
m/z
1.000e+6
8.000e+5
6.000e+5
4.000e+5
2.000e+5
0.000e+0
500
1000
1500
2000
HexNAc
HexNAcHex
Pep+HexNAcHex_3+
y2
y4
y5
y6
y7
y8
y9
y10
y11
y12
y13
y14
y15
~y16
~y17
~y18
~y19
~y23++
~y25++
b2
b2-18
b3
b4
b5
b6
b6-18
b7
b7-18
b9
~b17
~b19
a6
a9

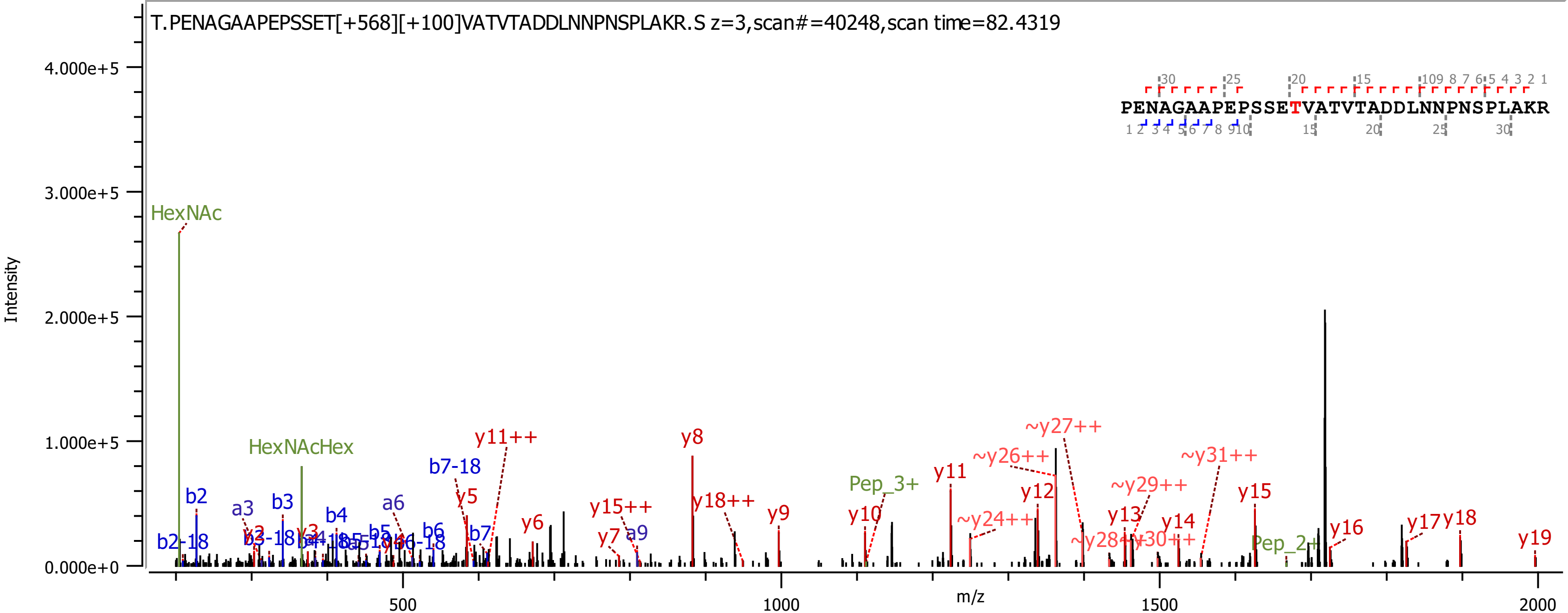

T.PENAGAAPEPSSET[+568][+100]VATVTADDLNNPNSPLAKR.S z=3,scan#=40248,scan time=82.4319
PENAGAAPEPSSETVATVTADDLNNPNSPLAKR
Intensity
4.000e+5
3.000e+5
2.000e+5
1.000e+5
0.000e+0
m/z
500
1000
1500
2000
HexNAc
HexNAcHex
b2
b2-18
a3
b3
b4
a6
b5
b6
b7
b7-18
y5
y11++
y6
y7
a9
y15++
y8
y18++
y9
y10
Pep_3+
y11
~y26++
~y27++
~y24++
y12
y13
~y29++
~y31++
y14
y15
Pep_2+
y16
y17
y18
y19

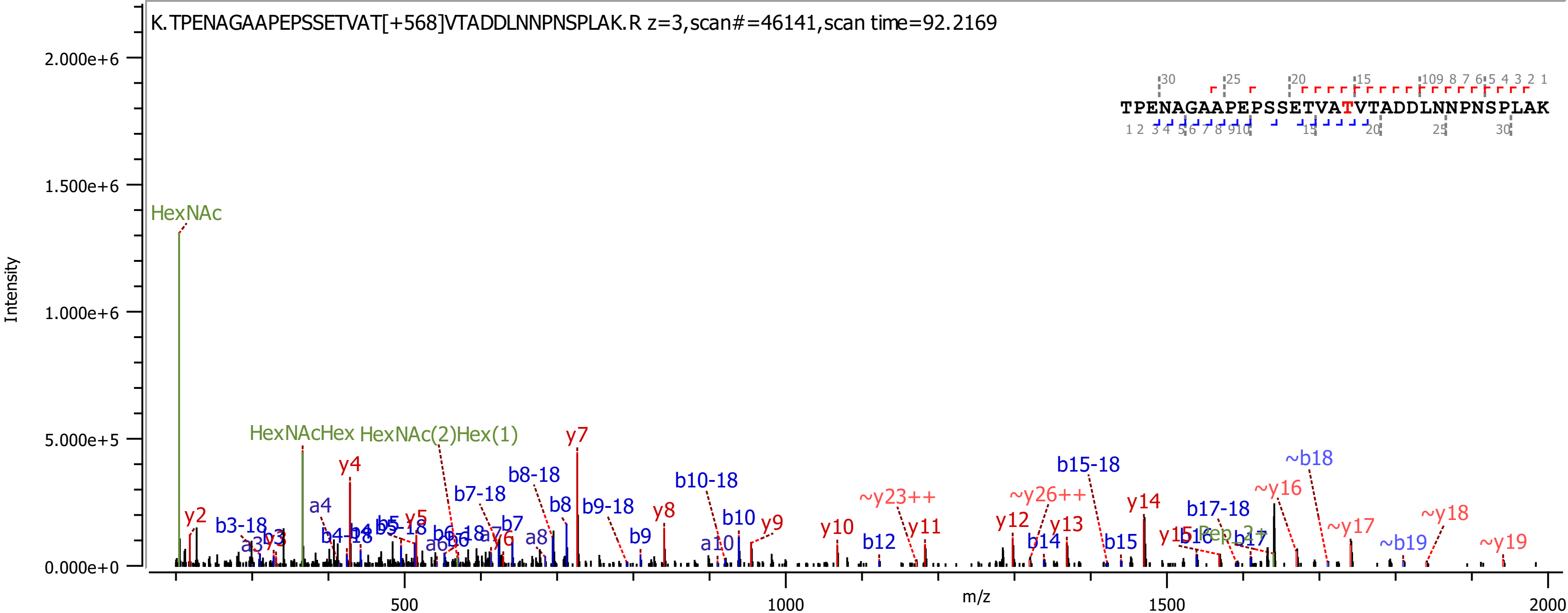
K.TPENAGAAPEPSSETVAT[+568]VTADDLNNPNSPLAK.R z=3,scan#=46141,scan time=92.2169
TPENAGAAPEPSSETVATVTADDLNNPNSPLAK
Intensity
m/z
HexNAc
HexNAcHex
HexNAc(2)Hex(1)
y2
y4
y5
y7
y8
y9
y10
y11
y12
y13
y14
b3-18
b7-18
b8-18
b9-18
b10-18
b15-18
b17-18
b7
b8
b9
b10
b12
b14
b15
a4
a8
a10
~y23++
~y26++
~y16
~y17
~y18
~y19
~b18
~b19
2.000e+6
1.500e+6
1.000e+6
5.000e+5
0.000e+0
500
1000
1500
2000

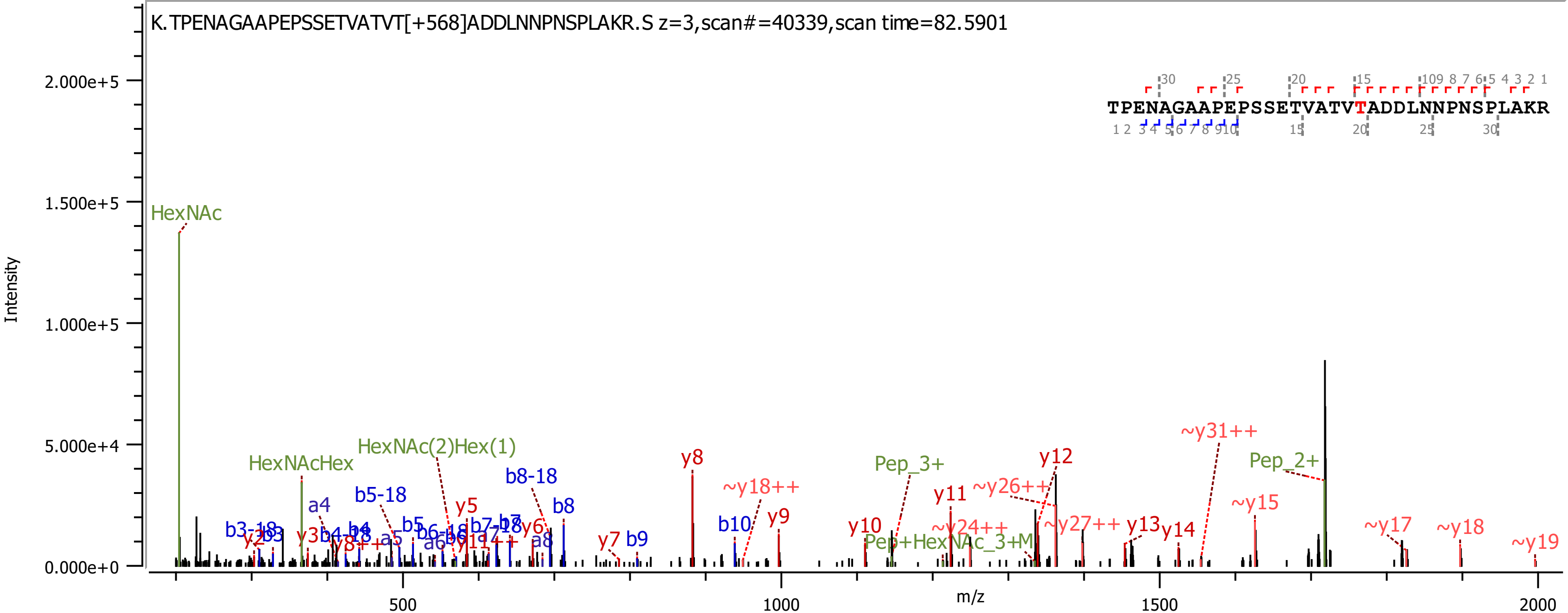

K.TPENAGAAPEPSSETVATVT[+568]ADDLNNPNSPLAKR.S z=3,scan#=40339,scan time=82.5901
TPENAGAAPEPSSETVATVTADDLNNPNSPLAKR
Intensity
m/z
0.000e+0
5.000e+4
1.000e+5
1.500e+5
2.000e+5
500
1000
1500
2000
HexNAc
HexNAcHex
HexNAc(2)Hex(1)
Pep_3+
Pep_2+
Pep+HexNAc_3+M
a4
b5-18
b8-18
b8
y5
y6
a8
y7
b9
y8
~y18++
b10
y9
y10
y11
y12
~y26++
~y24++
~y27++
y13
y14
~y31++
~y15
~y17
~y18
~y19

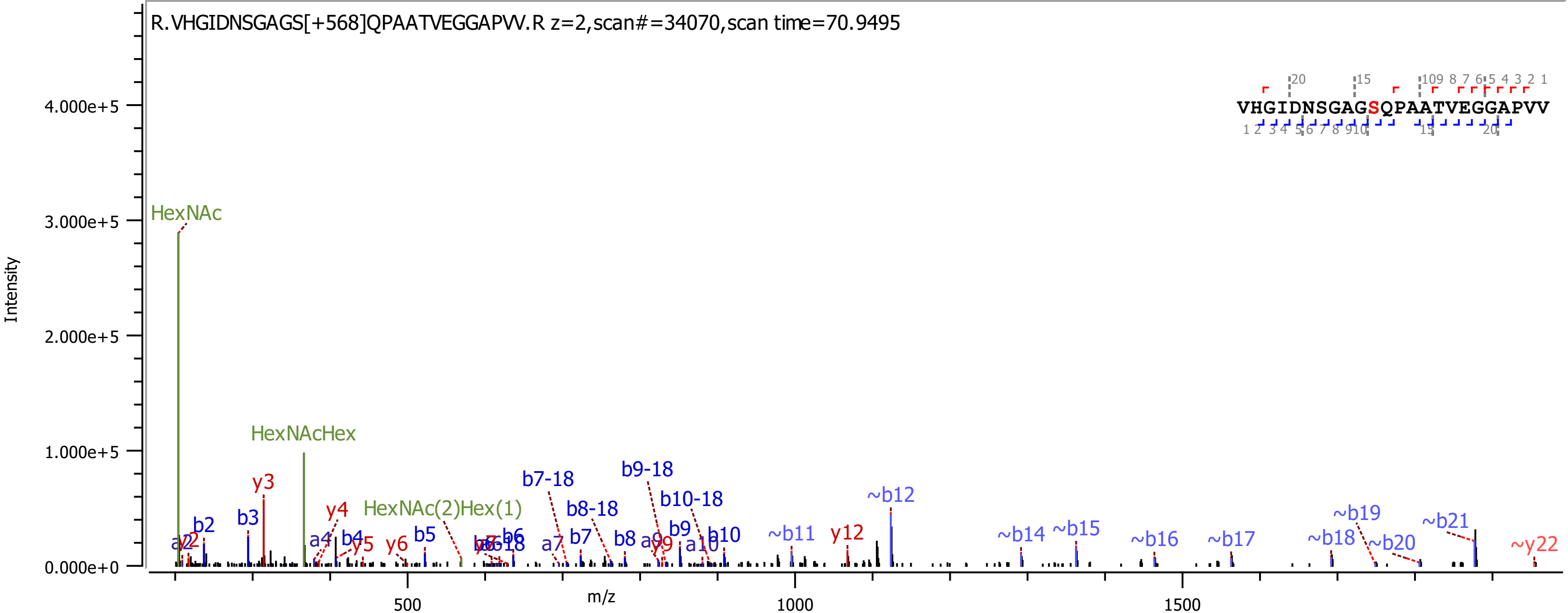
R.VHGIDNSGAGS[+568]QPAATVEGGAPVV.R z=2,scan#=34070,scan time=70.9495
VHGIDNSGAGSQPAATVEGGAPVV
Intensity
m/z
4.000e+5
3.000e+5
2.000e+5
1.000e+5
0.000e+0
500
1000
1500
HexNAc
HexNAcHex
HexNAc(2)Hex(1)
y3
y4
y5
y6
y9
y12
~y22
b2
b3
b4
b5
b6
b7
b8
b9
b10
a4
a7
a9
a10
b7-18
b8-18
b9-18
b10-18
~b11
~b12
~b14
~b15
~b16
~b17
~b18
~b19
~b20
~b21

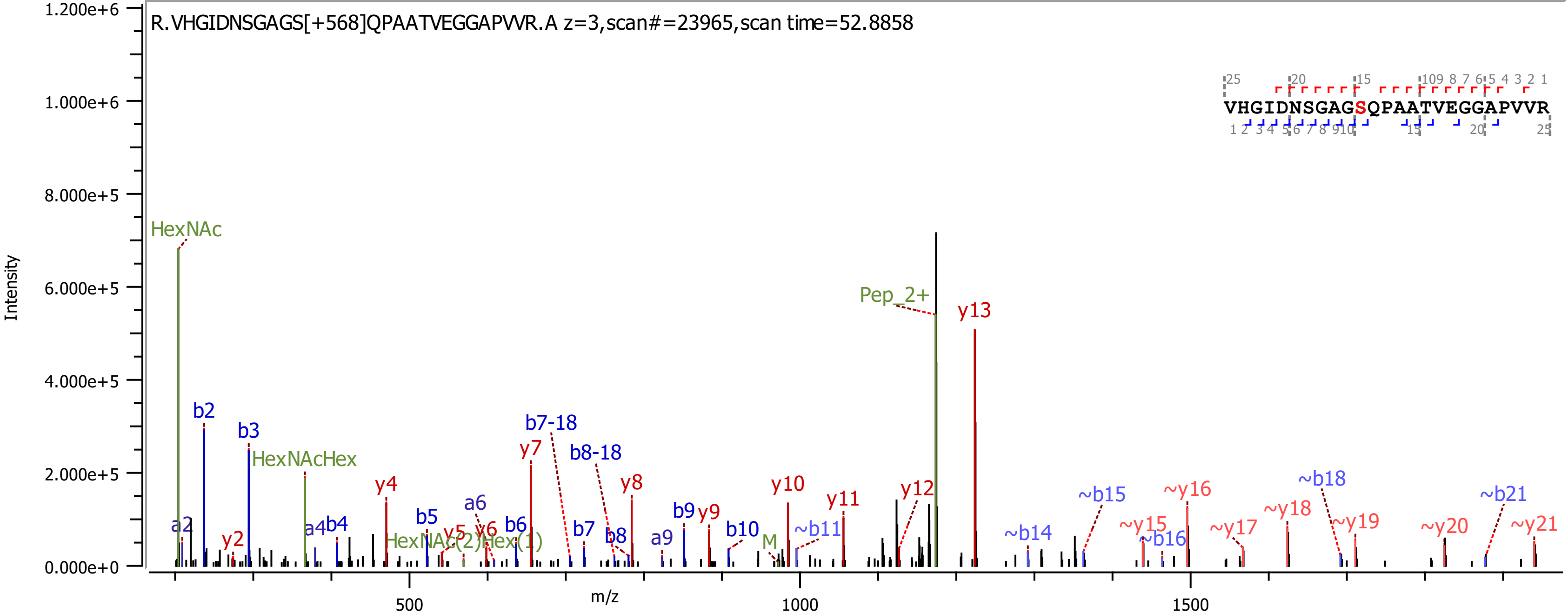
R.VHGIDNSGAGS[+568]QPAATVEGGAPVVR.A z=3,scan#=23965,scan time=52.8858
VHGIDNSGAGSQPAATVEGGAPVVR
Intensity
m/z
0.000e+0
2.000e+5
4.000e+5
6.000e+5
8.000e+5
1.000e+6
1.200e+6
500
1000
1500
HexNAc
HexNAcHex
HexNAc(2)Hex(1)
Pep_2+
M
a2
b2
b3
y2
a4
b4
y4
b5
y5
a6
y6
b6
y7
b7-18
b8-18
b7
b8
y8
a9
b9
y9
b10
y10
~b11
y11
y12
y13
~b14
~b15
~y15
~b16
~y16
~y17
~y18
~b18
~y19
~y20
~b21
~y21

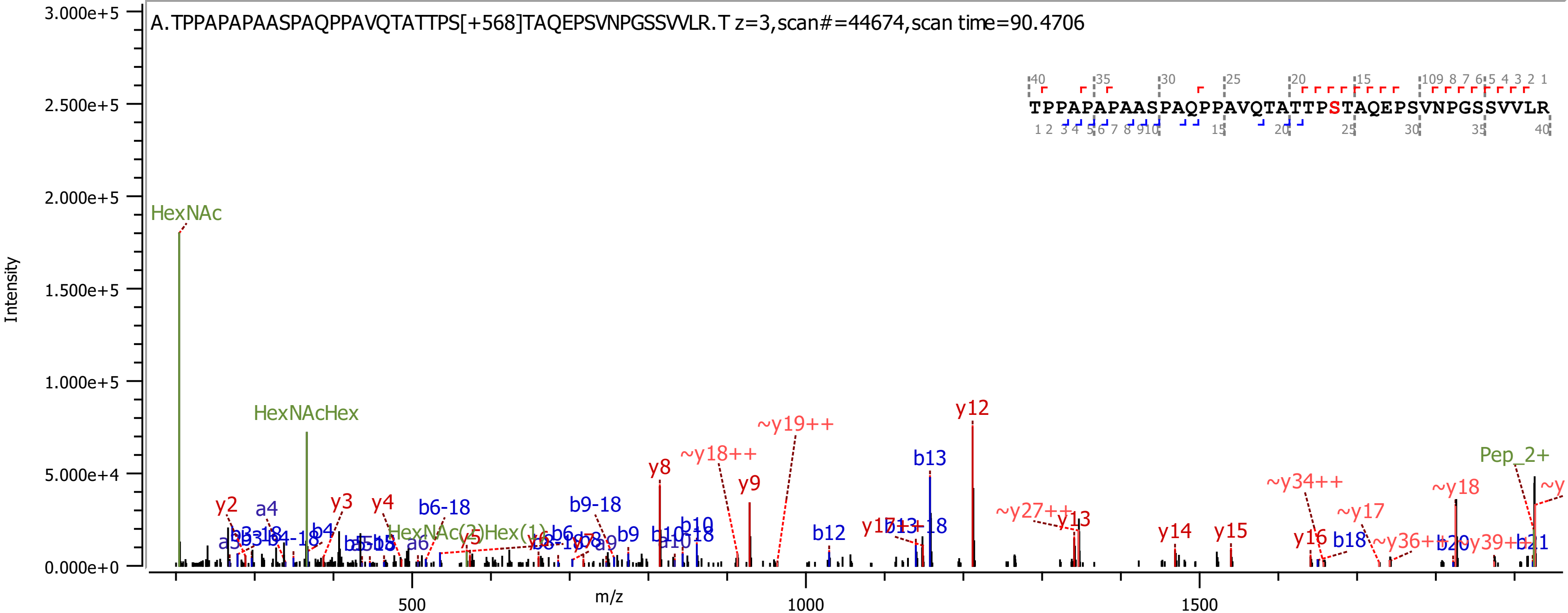

A.TPPAPAPAASPAQPPAVQTATTPS[+568]TAQEPSVNPGSSVVLR.T z=3,scan#=44674,scan time=90.4706
TPPAPAPAASPAQPPAVQTATTPSTAQEPSVNPGSSVVLR
Intensity
m/z
3.000e+5
2.500e+5
2.000e+5
1.500e+5
1.000e+5
5.000e+4
0.000e+0
500
1000
1500
HexNAc
HexNAcHex
HexNAc(2)Hex(1)
Pep_2+
y2
y3
y4
y5
y8
y9
y12
y13
y14
y15
y16
a4
b4
a6
b6-18
b6
b9-18
b9
b10
b10-18
b12
b13
b18
b20
b21
~y18++
~y19++
~y27++
~y34++
~y17
~y18
~y36++

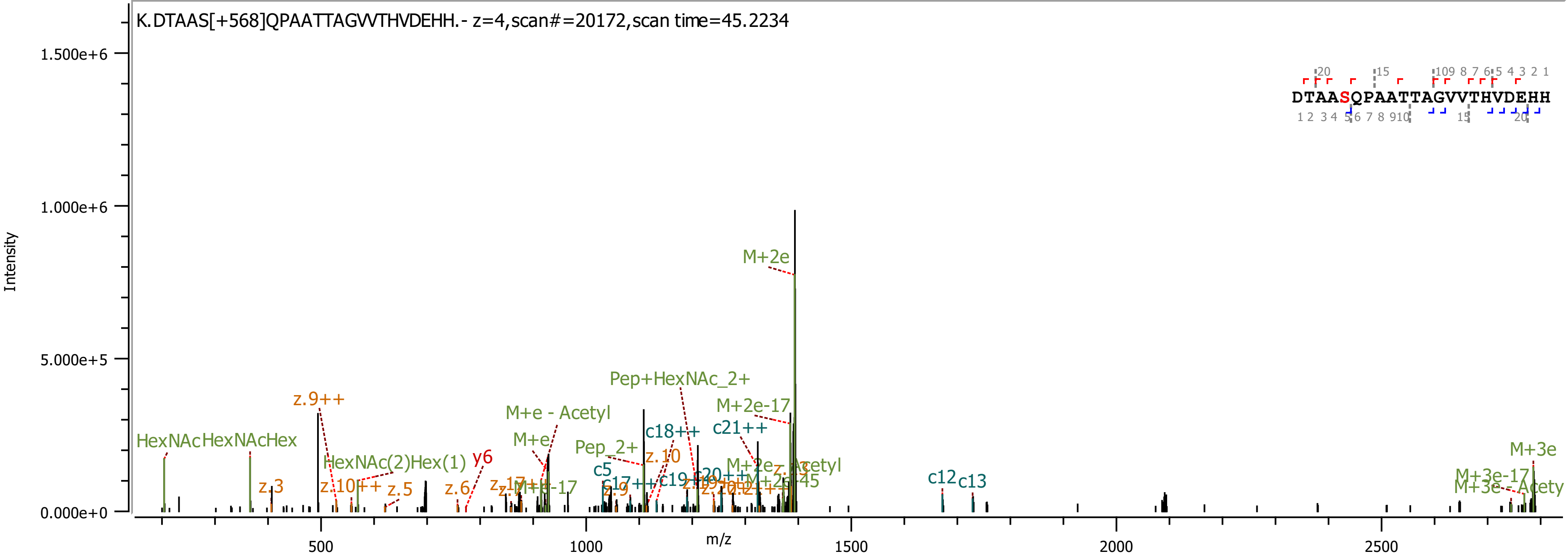

K.DTAAS[+568]QPAATTAGVVTHVDEHH.- z=4,scan#=20172,scan time=45.2234
Intensity
m/z
DTAASQPAATTAGVVTHVDEHH
M+2e
Pep+HexNAc_2+
M+2e-17
z.9++
M+e - Acetyl
c18++
c21++
HexNAc
HexNAcHex
M+e
Pep_2+
M+3e
HexNAc(2)Hex(1)
y6
z.10
c5
c12
c13
M+3e-17
M+3e - Acety
z.3
z.10++
z.5
z.6

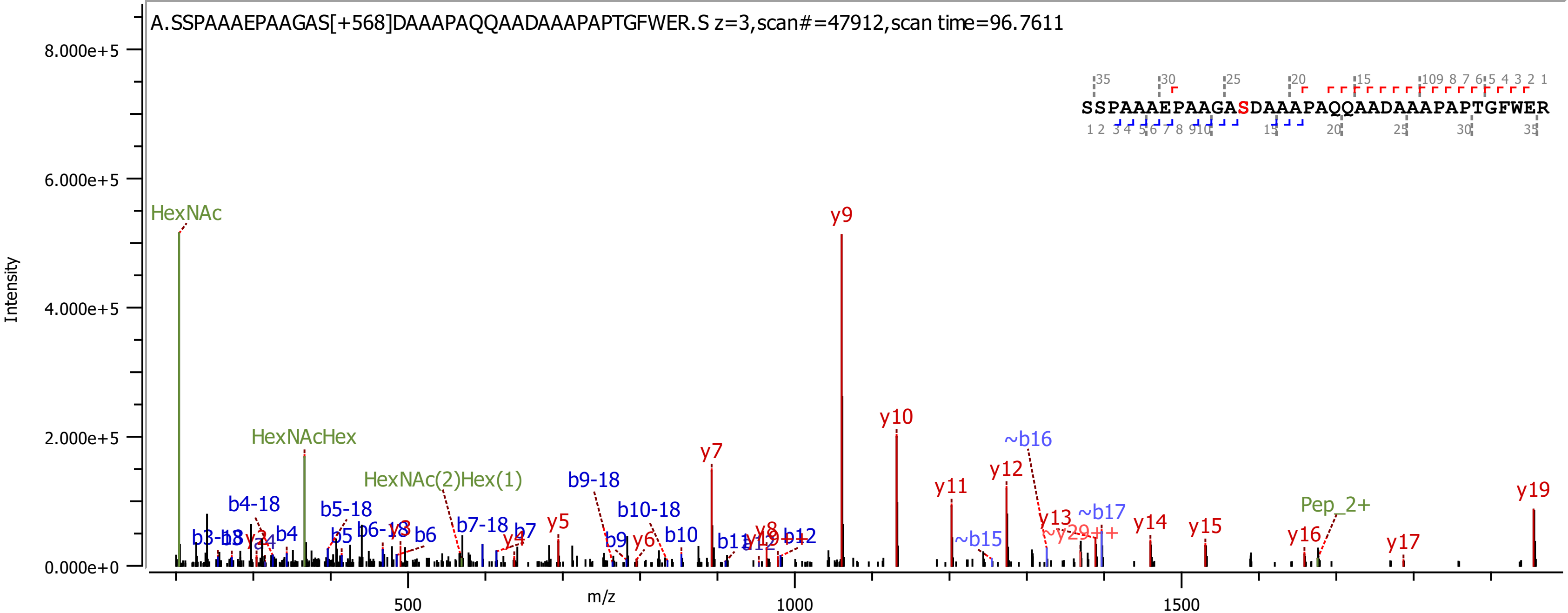

A.SSPAAAEPAAGAS[+568]DAAAPAQQAADAAAPAPTGFWER.S z=3,scan#=47912,scan time=96.7611
SSPAAAEPAAGASDAAAPAQQAADAAAPAPTGFWER
Intensity
m/z
8.000e+5
6.000e+5
4.000e+5
2.000e+5
0.000e+0
500
1000
1500
HexNAc
HexNAcHex
HexNAc(2)Hex(1)
Pep_2+
y9
y10
y7
y11
y12
y13
y14
y15
y16
y17
y19
y5
y6
y8
~b16
~b17
~b15
b9-18
b10-18
b4-18
b5-18
b7-18
b6-18
b4
b5
b6
b7
b9
b10
b12

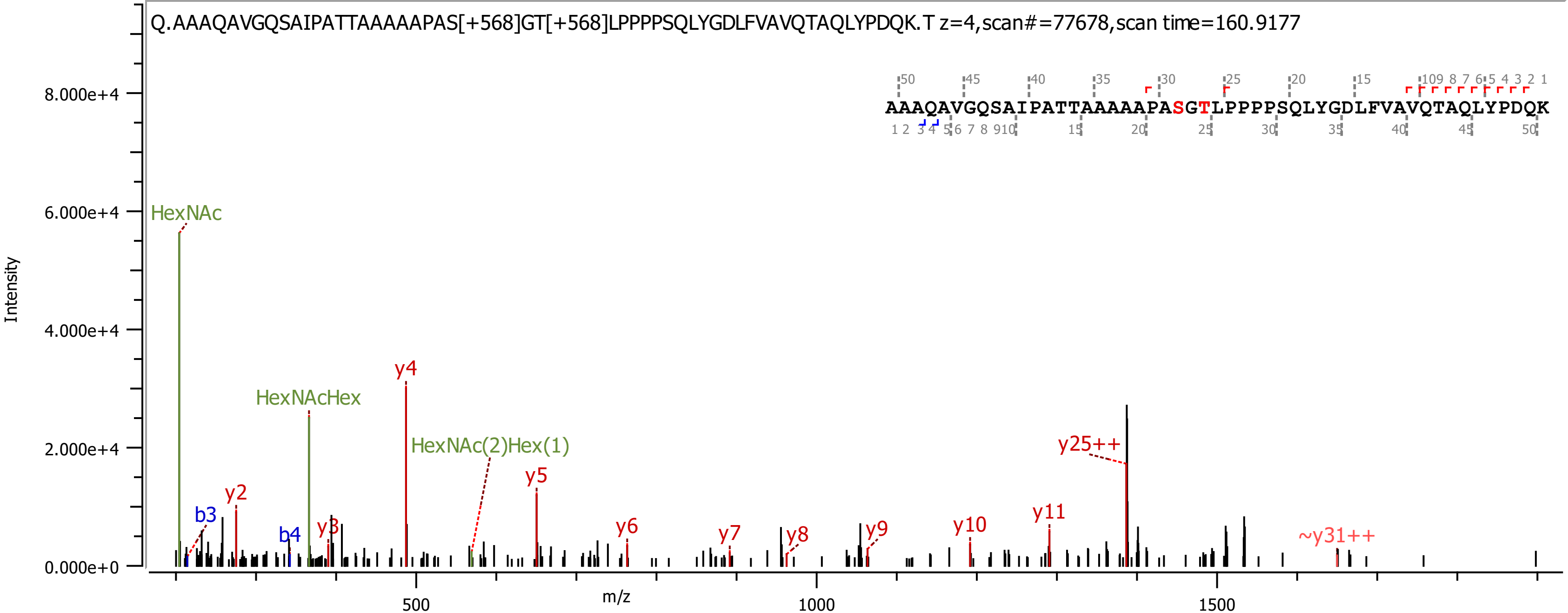

Q.AAAQAVGQSAIPATTAAAAAPAS[+568]GT[+568]LPPPPSQLYGDLFVAVQTAQLYPDQK.T z=4,scan#=77678,scan time=160.9177
AAAQAVGQSAIPATTAAAAAPASGTLPPPPSQLYGDLFVAVQTAQLYPDQK
Intensity
m/z
HexNAc
HexNAcHex
HexNAc(2)Hex(1)
b3
b4
y2
y3
y4
y5
y6
y7
y8
y9
y10
y11
y25++
~y31++

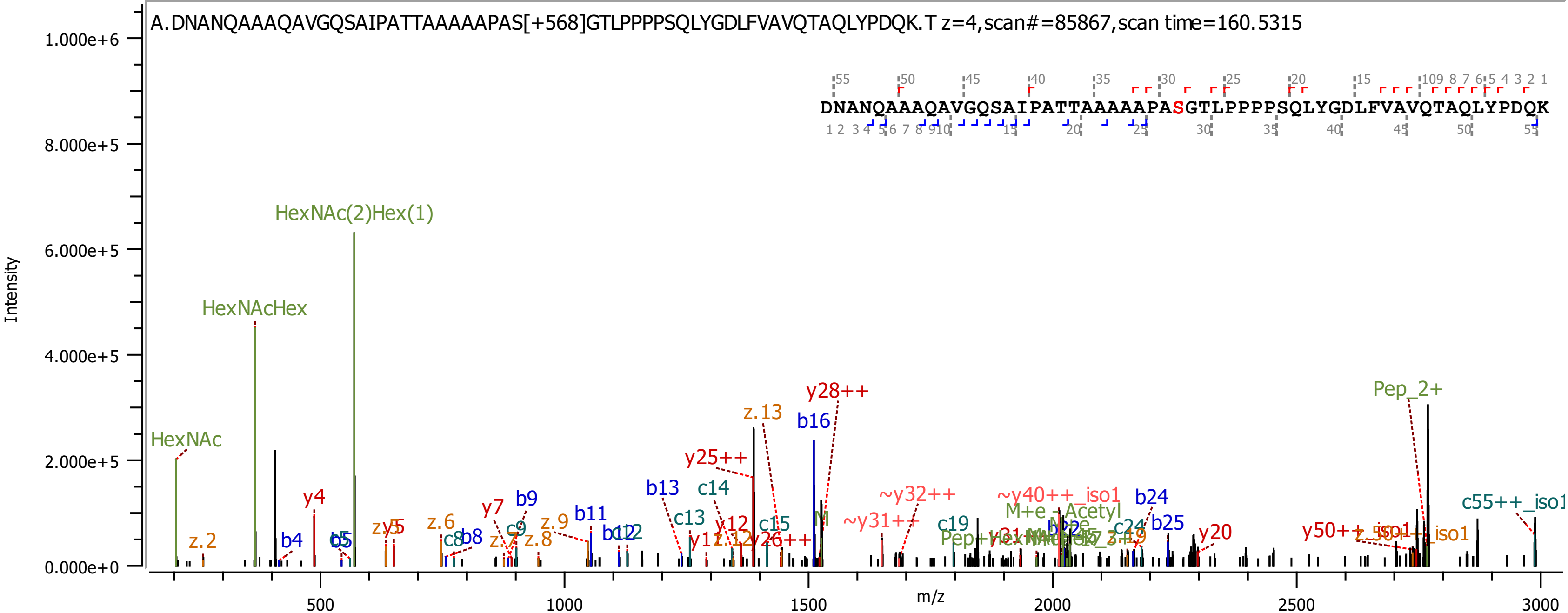

A.DNANQAAAQAVGQSAIPATTAAAAAPAS[+568]GTLPPPPSQLYGDLFVAVQTAQLYPDQK.T z=4,scan#=85867,scan time=160.5315
DNANQAAAQAVGQSAIPATTAAAAAPASGTLPPPPSQLYGDLFVAVQTAQLYPDQK
Intensity
m/z
1.000e+6
8.000e+5
6.000e+5
4.000e+5
2.000e+5
0.000e+0
500
1000
1500
2000
2500
3000
HexNAc(2)Hex(1)
HexNAcHex
HexNAc
Pep_2+
z.2
b4
b5
y4
y5
z.6
c8
b8
y7
b9
c9
z.7
z.8
z.9
b11
b12
b13
c13
c14
y12
z.12
y26++
c15
y25++
z.13
b16
y28++
~y31++
~y32++
c19
~y40++_iso1
M+e -Acetyl
b24
b25
c24
y20
y50++
c55++_iso1

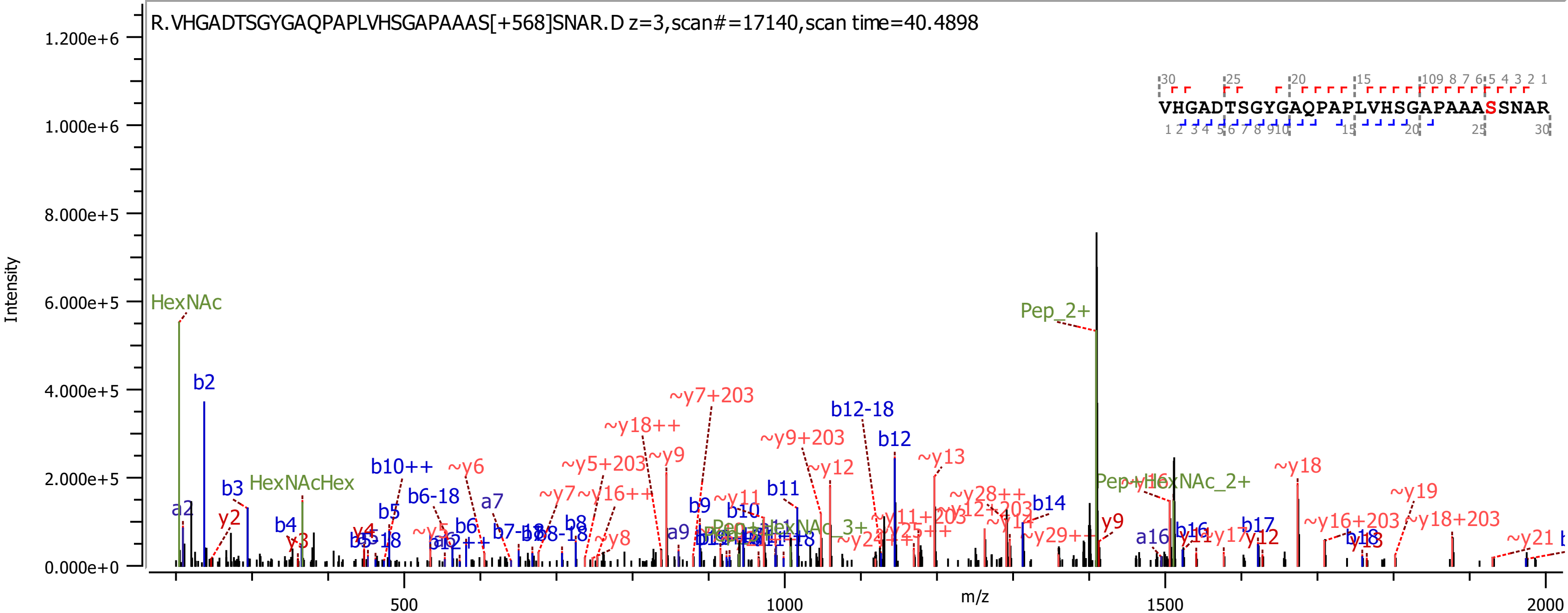
R.VHGADTSGYGAQPAPLVHSGAPAAAS[+568]SNAR.D z=3,scan#=17140,scan time=40.4898
VHGADTSGYGAQPAPLVHSGAPAAASSNAR
Intensity
m/z
1.200e+6
1.000e+6
8.000e+5
6.000e+5
4.000e+5
2.000e+5
0.000e+0
500
1000
1500
2000
HexNAc
b2
HexNAcHex
Pep_2+
Pep+HexNAc_2+
Pep+HexNAc_3+
b3
a2
y2
b4
y3
b5
b10++
b6-18
~y6
a7
b6
b8
~y7
~y16++
~y5+203
~y8
~y18++
~y9
~y7+203
a9
b9
~y11
b10
b11
~y9+203
~y12
b12-18
b12
~y13
~y28++
~y12+203
~y11+203
~y14
~y25++
~y24++
~y29++
b14
y9
a16
b16
y11
~y17
b17
y12
~y18
~y16+203
b18
y18
~y19
~y18+203
~y21

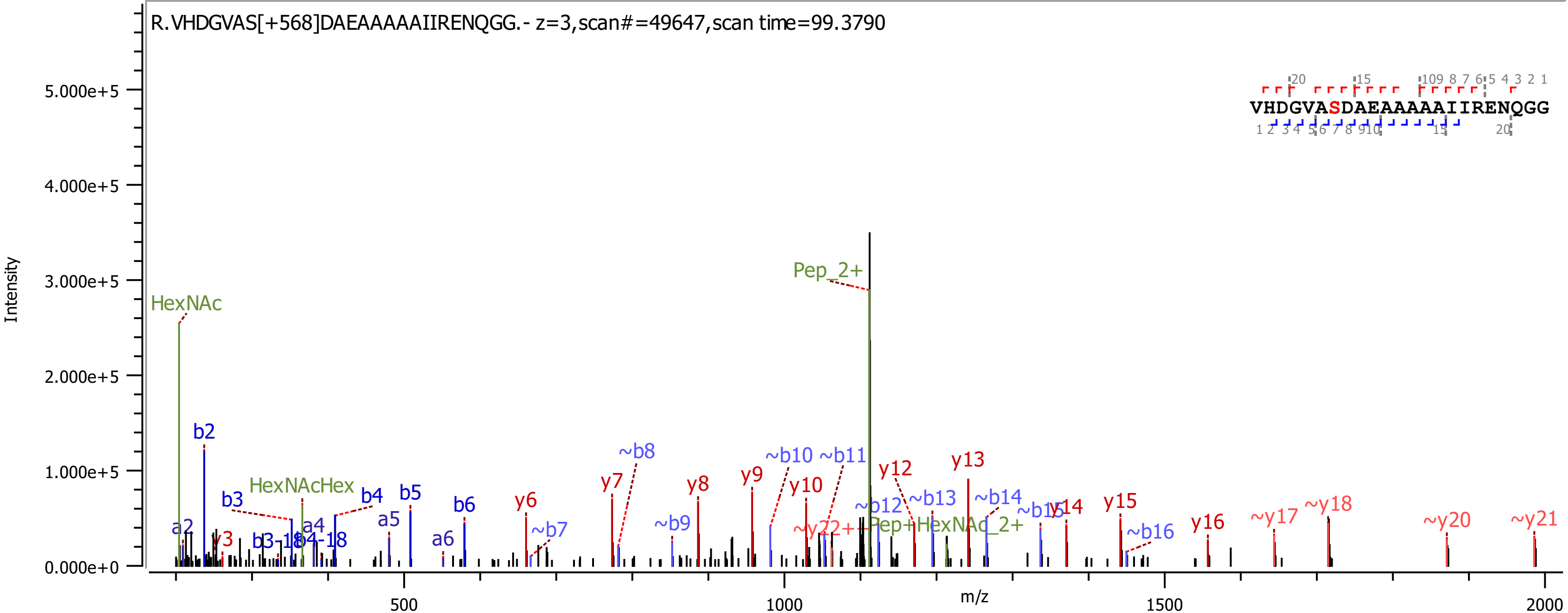
R.VHDGVAS[+568]DAEAAAAAIIRENQGG.- z=3,scan#=49647,scan time=99.3790
VHDGVASDAEAAAAAIIRENQGG
Intensity
m/z
HexNAc
Pep_2+
HexNAcHex
Pep+HexNAc_2+

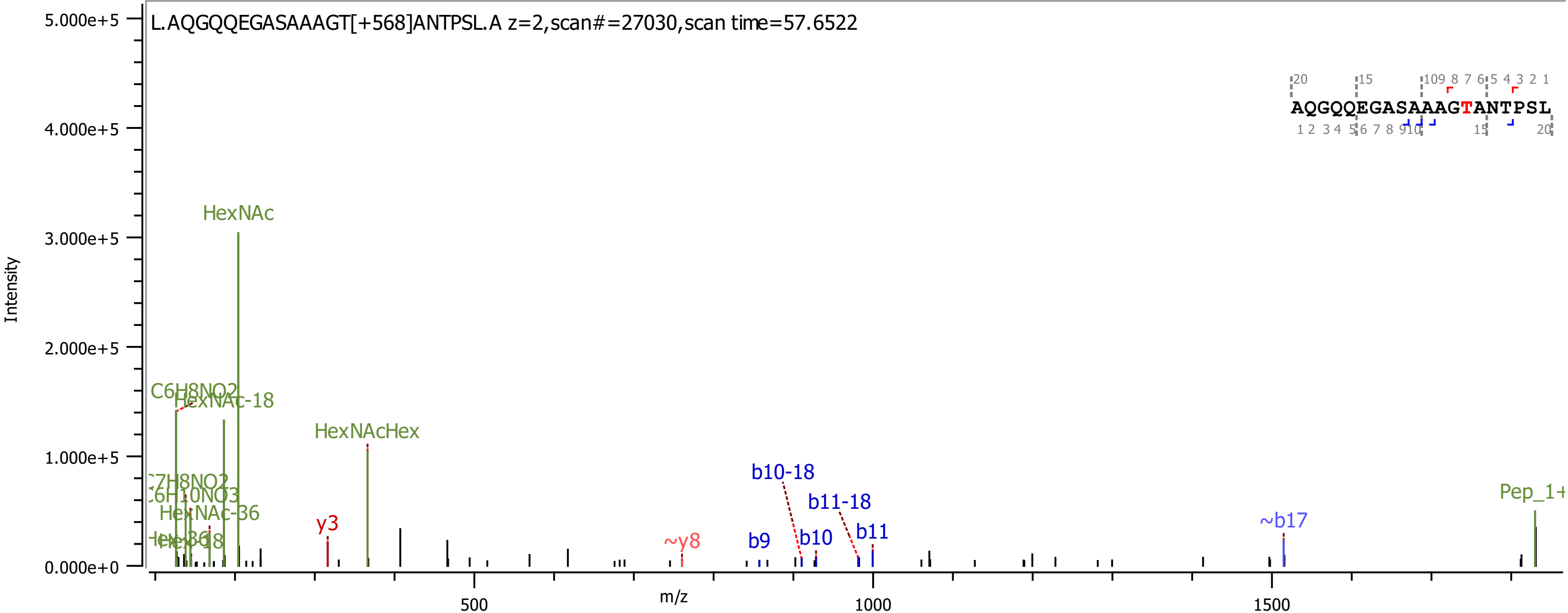

L.AQGQQEGASAAAGT[+568]ANTPSL.A z=2,scan#=27030,scan time=57.6522
AQGQQEGASAAAGTANTPSL
Intensity
m/z
HexNAc
HexNAc-18
C6H8NO2
HexNAcHex
C7H8NO2
C6H10NO3
HexNAc-36
y3
~y8
b9
b10-18
b10
b11-18
b11
~b17
Pep_1+

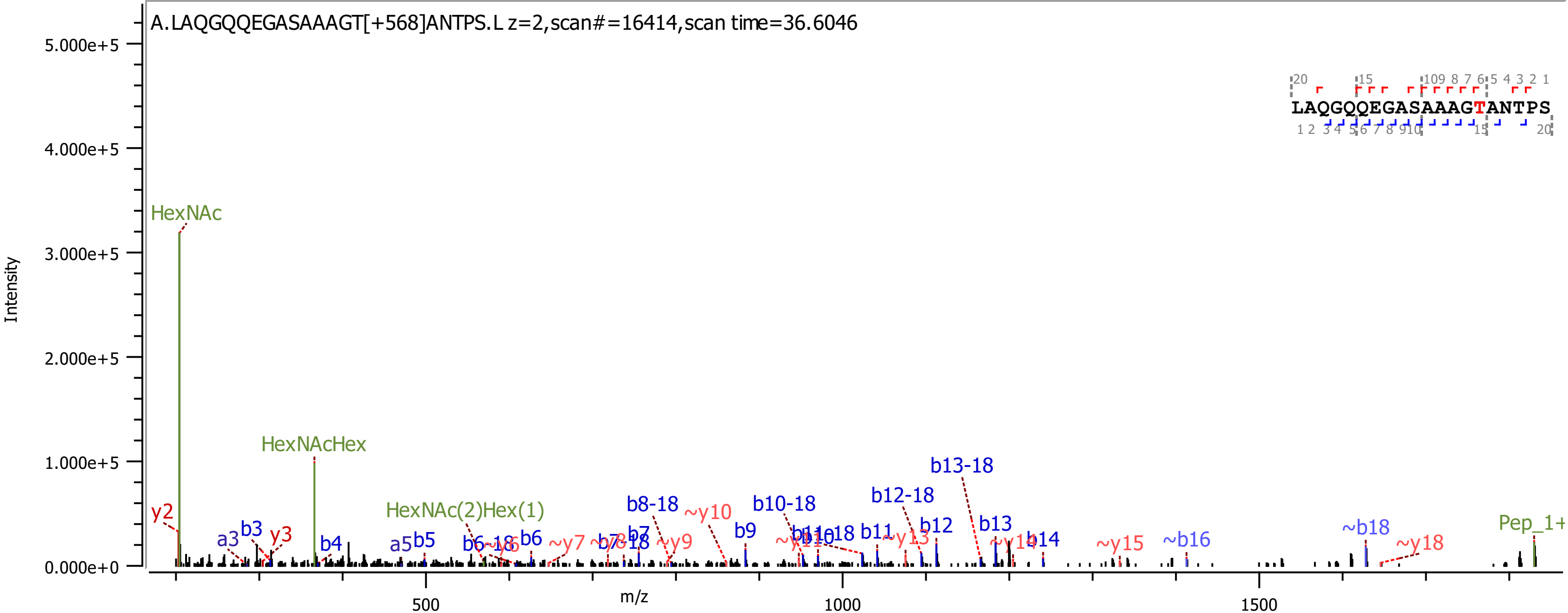

A.LAQGQQEGASAAAGT[+568]ANTPS.L z=2,scan#=16414,scan time=36.6046
Intensity
m/z
HexNAc
HexNAcHex
HexNAc(2)Hex(1)
Pep_1+
LAQGQQEGASAAAGTANTPS
y2
a3
b3
y3
b4
a5
b5
b6
b8-18
b9
b10-18
b11
b12-18
b12
b13-18
b13
b14
~y10
~y15
~b16
~b18
~y18

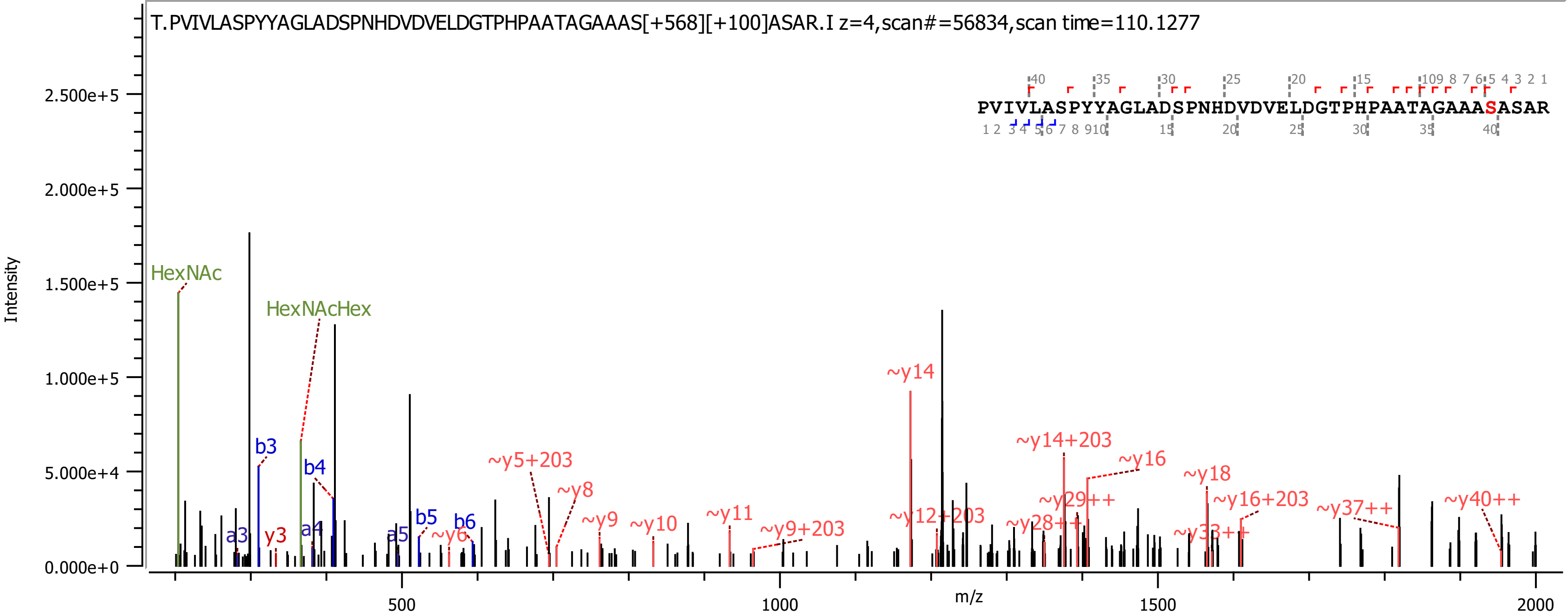

T.PVIVLASPYYAGLADSPNHDVDVELDGTPHPAATAGAAAS[+568][+100]ASAR.I z=4,scan#=56834,scan time=110.1277
PVIVLASPYYAGLADSPNHDVDVELDGTPHPAATAGAAASASAR
Intensity
m/z
HexNAc
HexNAcHex
b3
b4
a3
y3
a4
a5
b5
b6
~y6
~y5+203
~y8
~y9
~y10
~y11
~y9+203
~y14
~y12+203
~y14+203
~y16
~y29++
~y28++
~y18
~y16+203
~y33++
~y37++
~y40++

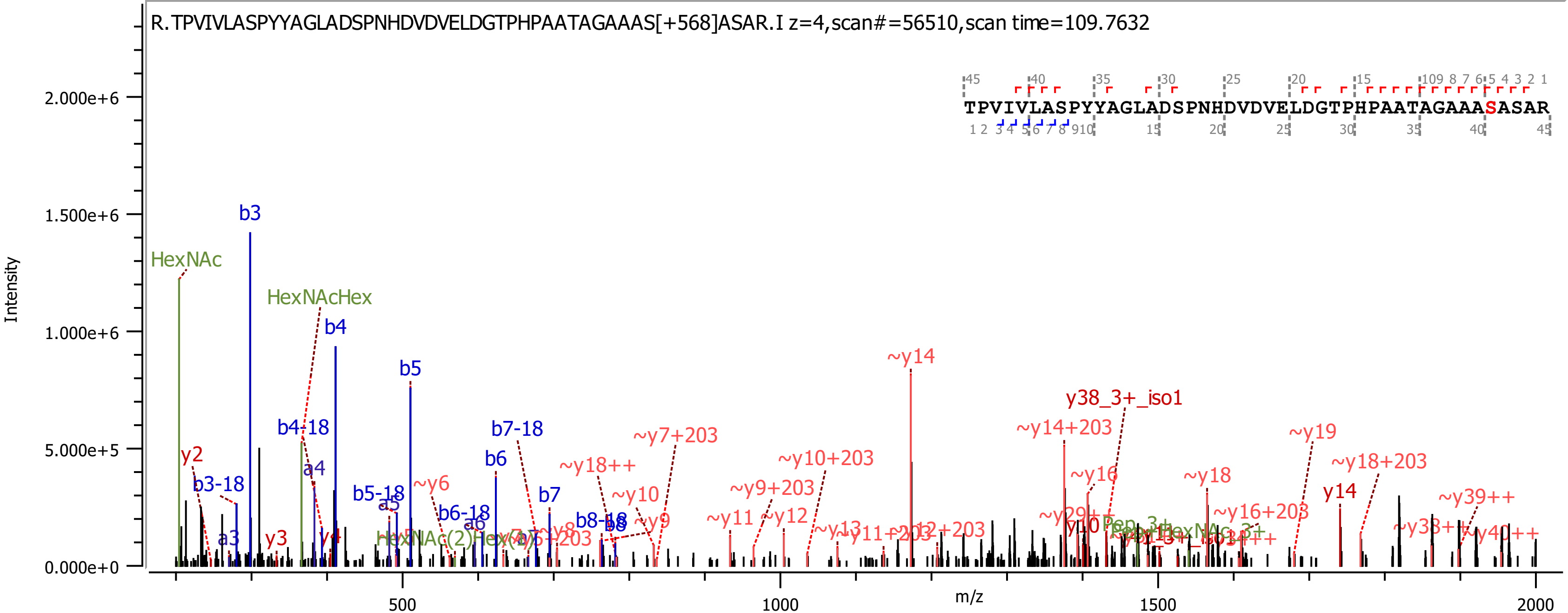
R.TPVIVLASPYYAGLADSPNHDVDVELDGTPHPAATAGAAAS[+568]ASAR.I z=4,scan#=56510,scan time=109.7632
TPVIVLASPYYAGLADSPNHDVDVELDGTPHPAATAGAAASASAR
Intensity
m/z
2.000e+6
1.500e+6
1.000e+6
5.000e+5
0.000e+0
500
1000
1500
2000
HexNAc
HexNAcHex
b3
b4
b5
b4-18
b3-18
b5-18
b6
b7
b7-18
b6-18
b8-18
y2
y3
y4
a4
a5
a6
~y6
~y7+203
~y8
~y9
~y10
~y11
~y12
~y13
~y14
~y16
~y18
~y19
~y18++
~y9+203
~y10+203
~y14+203
~y16+203
~y18+203
~y39++
y38_3+_iso1
y14
y10

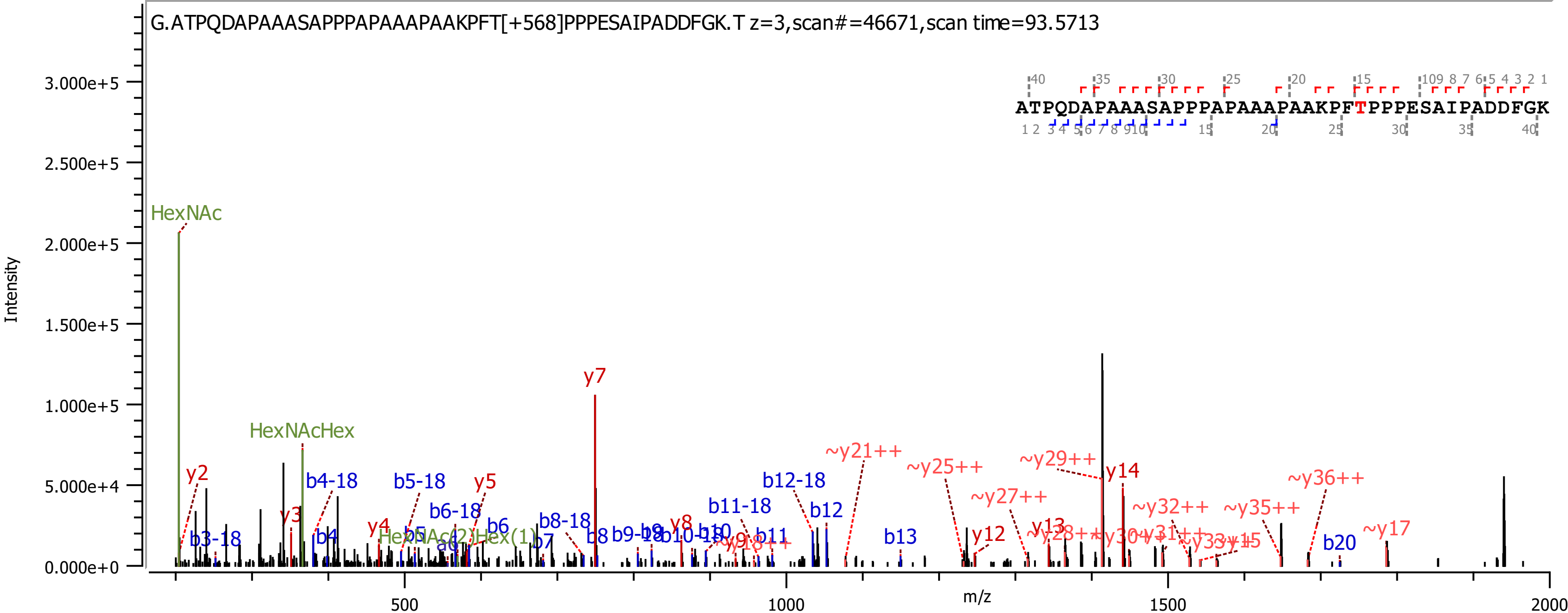
G.ATPQDAPAAASAPPPAPAAAPAAKPFT[+568]PPPESAIPADDFGK.T z=3,scan#=46671,scan time=93.5713
ATPQDAPAAASAPPPAPAAAPAAKPFTPPPESAIPADDFGK
Intensity
3.000e+5
2.500e+5
2.000e+5
1.500e+5
1.000e+5
5.000e+4
0.000e+0
m/z
500
1000
1500
2000
HexNAc
HexNAcHex
HexNAc(2)Hex(1)
y2
y3
y4
y5
y7
y8
y12
y13
y14
b3-18
b4
b4-18
b5
b5-18
b6
b6-18
b7
b8
b8-18
b9-18
b10-18
b10
b11
b11-18
b12
b12-18
b13
b20
~y21++
~y25++
~y27++
~y29++
~y32++
~y35++
~y36++
~y17

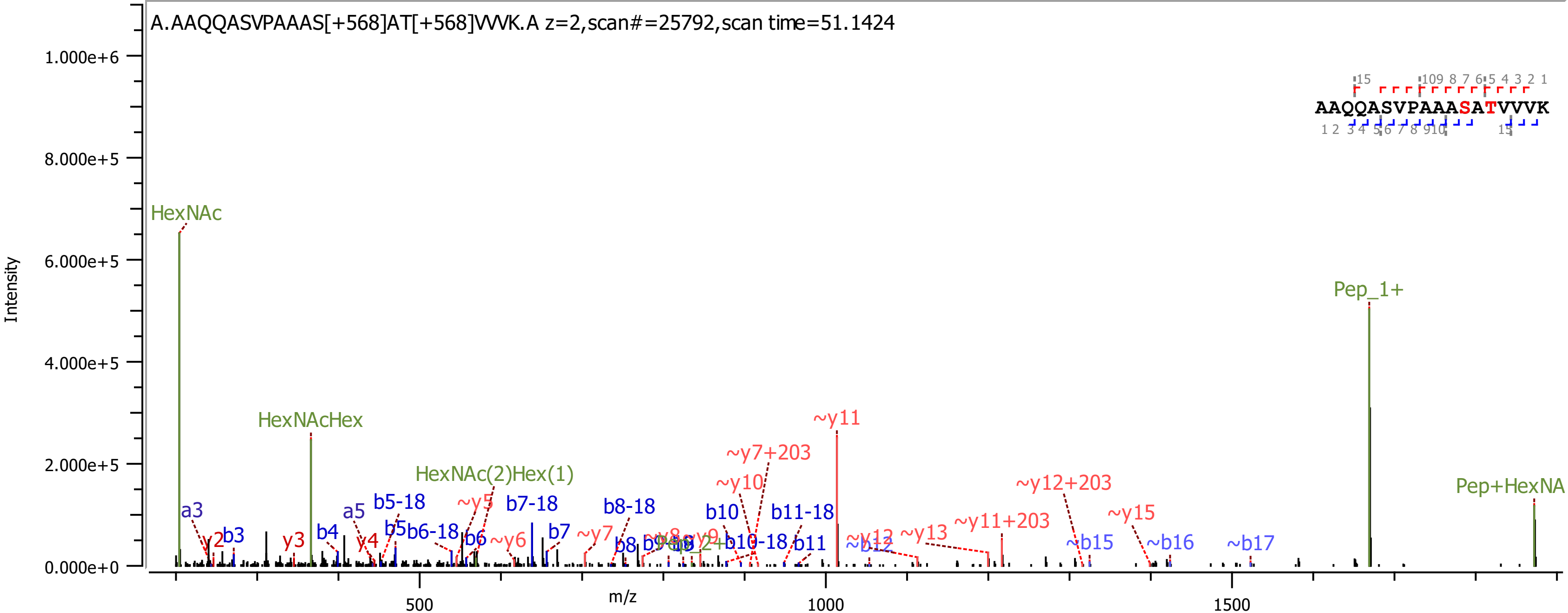

A.AAQQASVPAAAS[+568]AT[+568]VVVK.A z=2,scan#=25792,scan time=51.1424
AAQQASVPAAASATVVVK
Intensity
m/z
HexNAc
HexNAcHex
HexNAc(2)Hex(1)
Pep_1+
Pep+HexNA
~y11
~y7+203
~y10
~y12+203
~y11+203
~y15
~y13
~y12
~b15
~b16
~b17
~b12
b10
b11-18
b11
b10-18
b8-18
b7-18
b7
b6
b6-18
b5-18
b5
a5
b4
y4
y3
b3
y2
a3
~y5
~y6
~y7

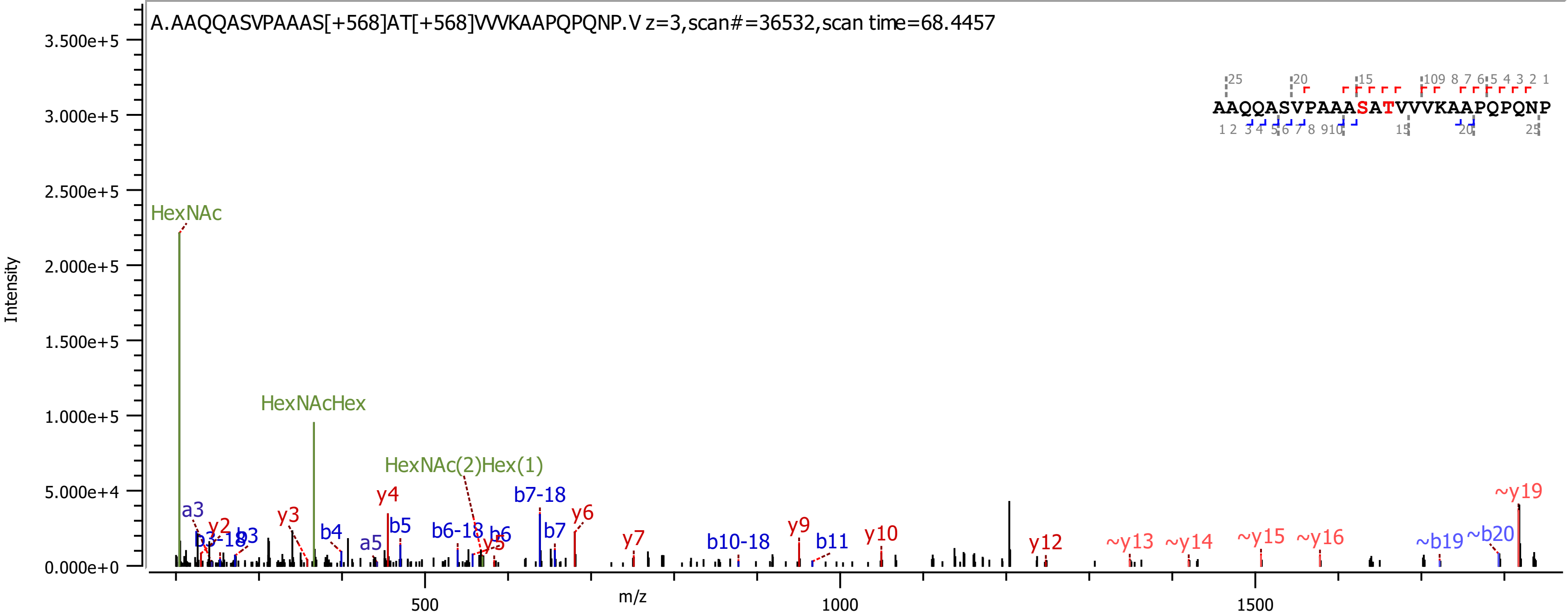

A.AAQQASVPAAAS[+568]AT[+568]VVVKAAPQPQNP.V z=3,scan#=36532,scan time=68.4457
AAQQASVPAAASATVVVKAAPQPQNP
Intensity
m/z
HexNAc
HexNAcHex
HexNAc(2)Hex(1)
a3
y2
b3-18
b3
y3
b4
a5
y4
b5
b6-18
b6
y5
b7-18
b7
y6
y7
b10-18
y9
b11
y10
y12
~y13
~y14
~y15
~y16
~b19
~b20
~y19

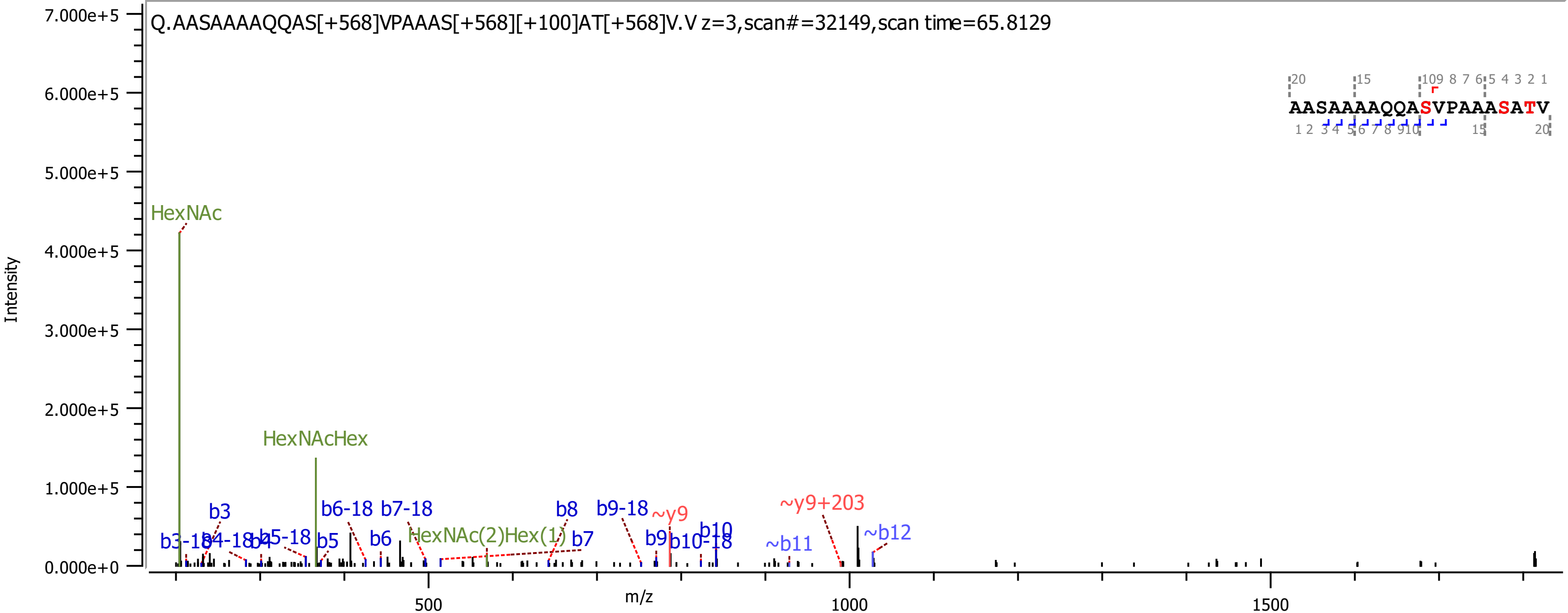
Q.AASAAAAQQAS[+568]VPAAAS[+568][+100]AT[+568]V.V z=3,scan#=32149,scan time=65.8129
Intensity
m/z
HexNAc
HexNAcHex
HexNAc(2)Hex(1)
b3
b3-18
b4-18
b4
b5-18
b5
b6-18
b7-18
b6
b7
b8
b9-18
b9
b10
b10-18
~y9
~b11
~y9+203
~b12
AASAAAAQQASVPAAASATV

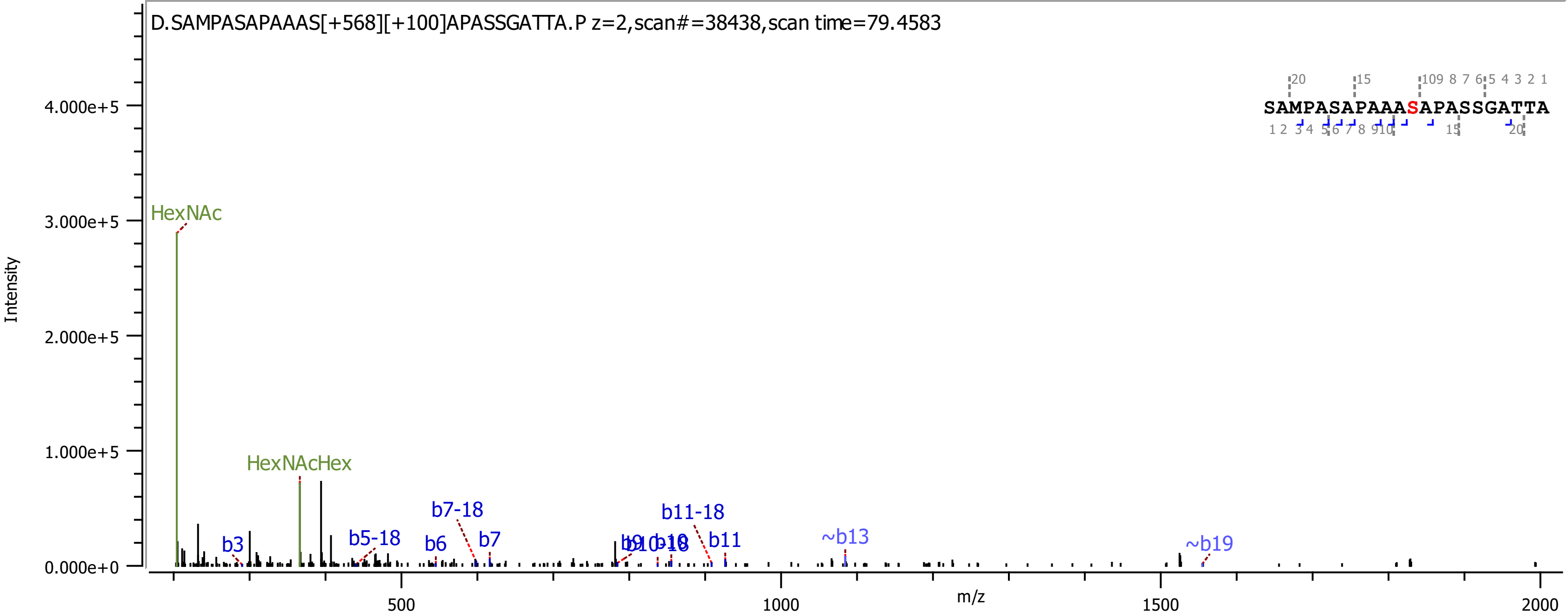

D.SAMPASAPAAAS[+568][+100]APASSGATTA.P z=2,scan#=38438,scan time=79.4583
SAMPASAPAAASAPASSGATTA
Intensity
m/z
HexNAc
HexNAcHex
b3
b5-18
b6
b7-18
b7
b9
b10-18
b11-18
b11
~b13
~b19

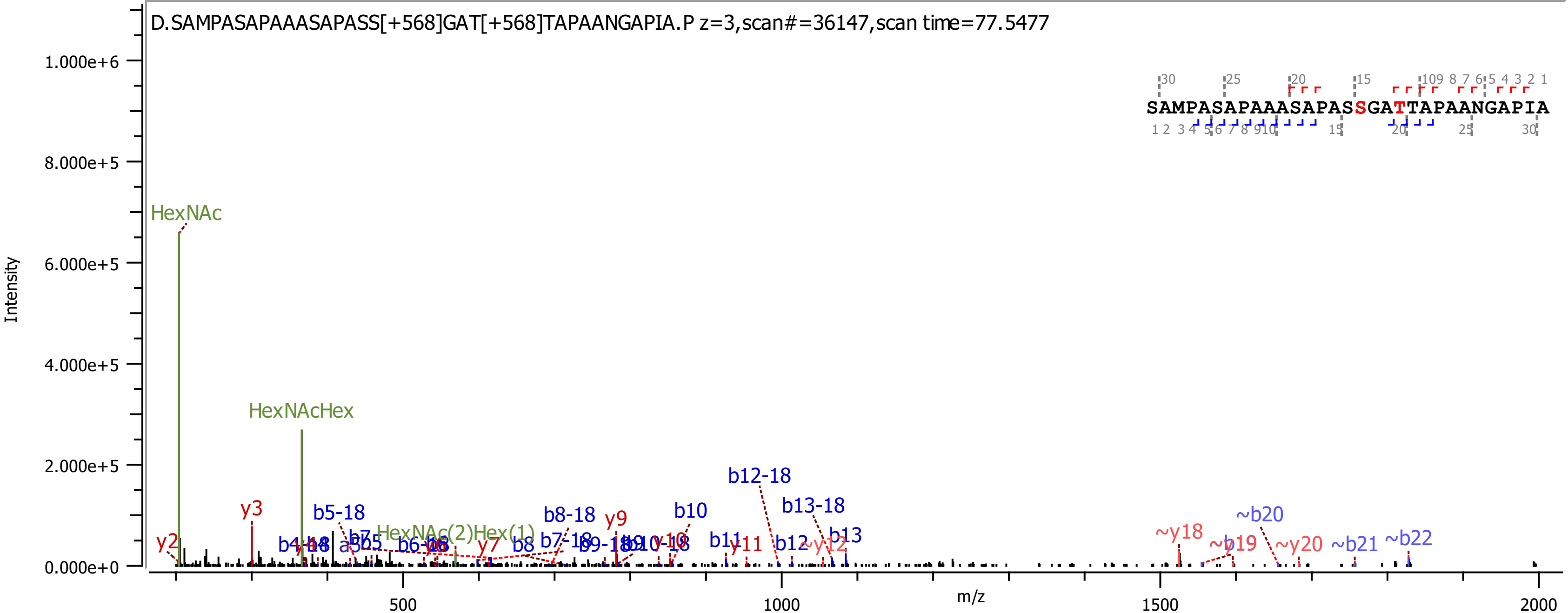
D.SAMPASAPAAASAPASS[+568]GAT[+568]TAPAANGAPIA.P z=3,scan#=36147,scan time=77.5477
SAMPASAPAAASAPASSGATTAPAANGAPIA
Intensity
m/z
HexNAc
HexNAcHex
HexNAc(2)Hex(1)
y2
y3
b5-18
b8-18
y9
b10
b12-18
b13-18
b13
y7
b8
b7-18
b11
y11
b12
~y12
~y18
~b20
~y19
~y20
~b21
~b22

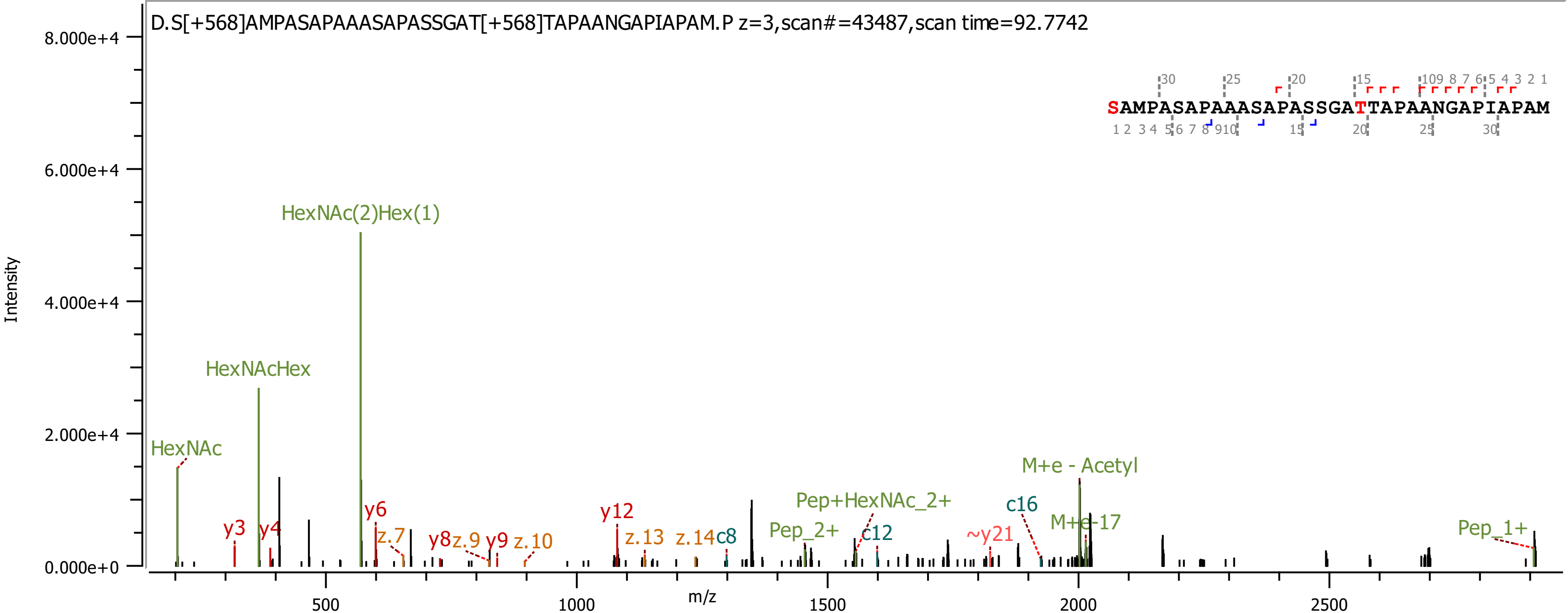

D.S[+568]AMPASAPAAASAPASSGAT[+568]TAPAANGAPIAPAM.P z=3,scan#=43487,scan time=92.7742
SAMPASAPAAASAPASSGATTAPAANGAPIAPAM
Intensity
8.000e+4
6.000e+4
4.000e+4
2.000e+4
0.000e+0
HexNAc(2)Hex(1)
HexNAcHex
HexNAc
y3
y4
y6
z.7
y8
z.9
y9
z.10
y12
z.13
z.14
c8
Pep_2+
Pep+HexNAc_2+
c12
c16
~y21
M+e - Acetyl
M+e-17
Pep_1+
500
1000
1500
2000
2500
m/z

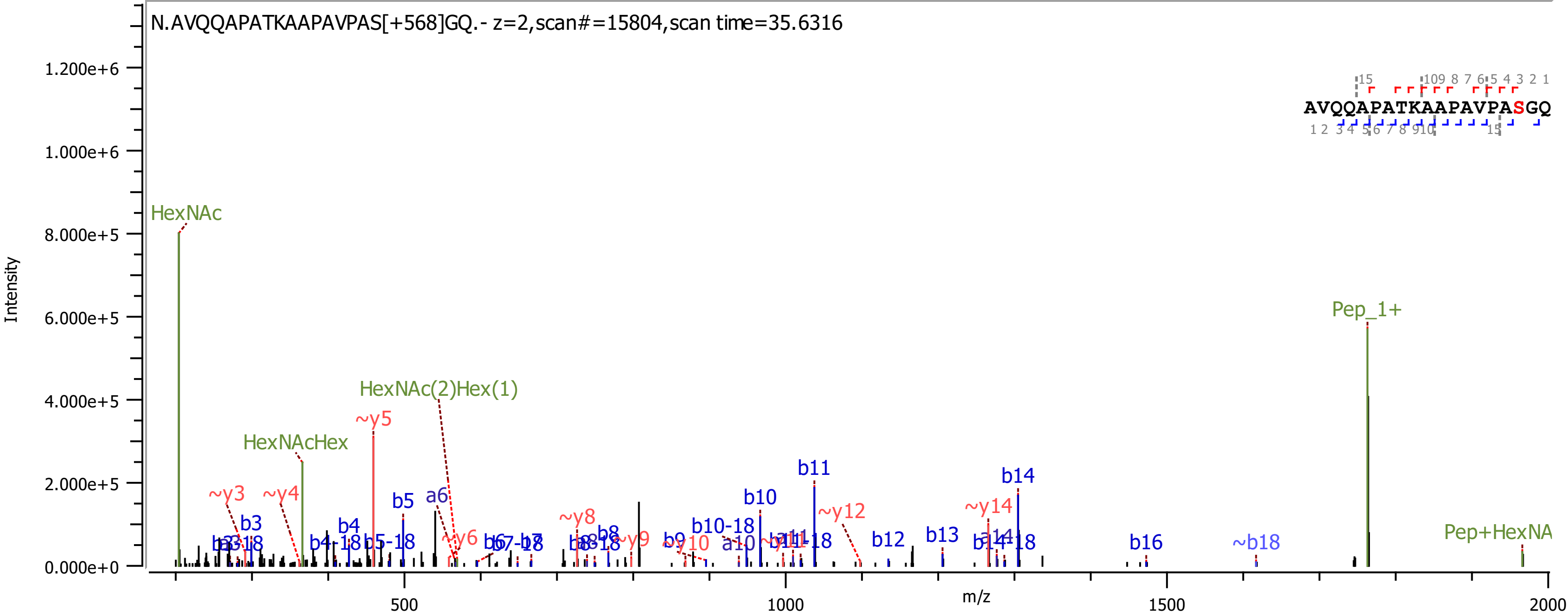

N.AVQQAPATKAAPAVPAS[+568]GQ.- z=2,scan#=15804,scan time=35.6316
AVQQAPATKAAPAVPASGQ
Intensity
m/z
1.200e+6
1.000e+6
8.000e+5
6.000e+5
4.000e+5
2.000e+5
0.000e+0
500
1000
1500
2000
HexNAc
HexNAcHex
HexNAc(2)Hex(1)
Pep_1+
Pep+HexNA
~y3
~y4
~y5
~y6
~y8
~y9
~y10
~y12
~y14
b3
b4
b4-18
b5
b5-18
a6
b8
b9
b10
b10-18
a10
b11
b12
b13
b14
b14-18
b16
~b18

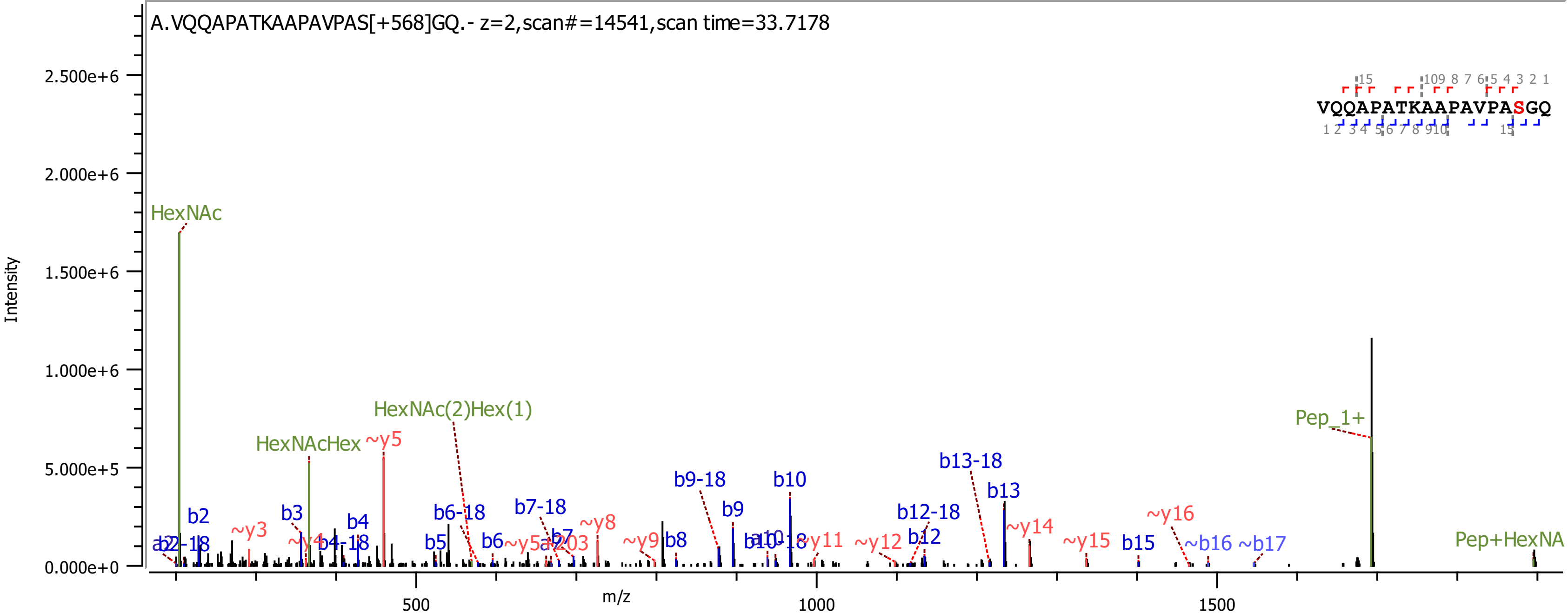

A.VQQAPATKAAPAVPAS[+568]GQ.- z=2,scan#=14541,scan time=33.7178
VQQAPATKAAPAVPASGQ
Intensity
m/z
HexNAc
HexNAcHex
HexNAc(2)Hex(1)
Pep_1+
Pep+HexNA
b2
b3
b4
b5
b6
b7
b8
b9
b10
b12
b13
b15
b4-18
b6-18
b7-18
b9-18
b10-18
b12-18
b13-18
~y3
~y4
~y5
~y8
~y9
~y11
~y12
~y14
~y15
~y16
~b16
~b17

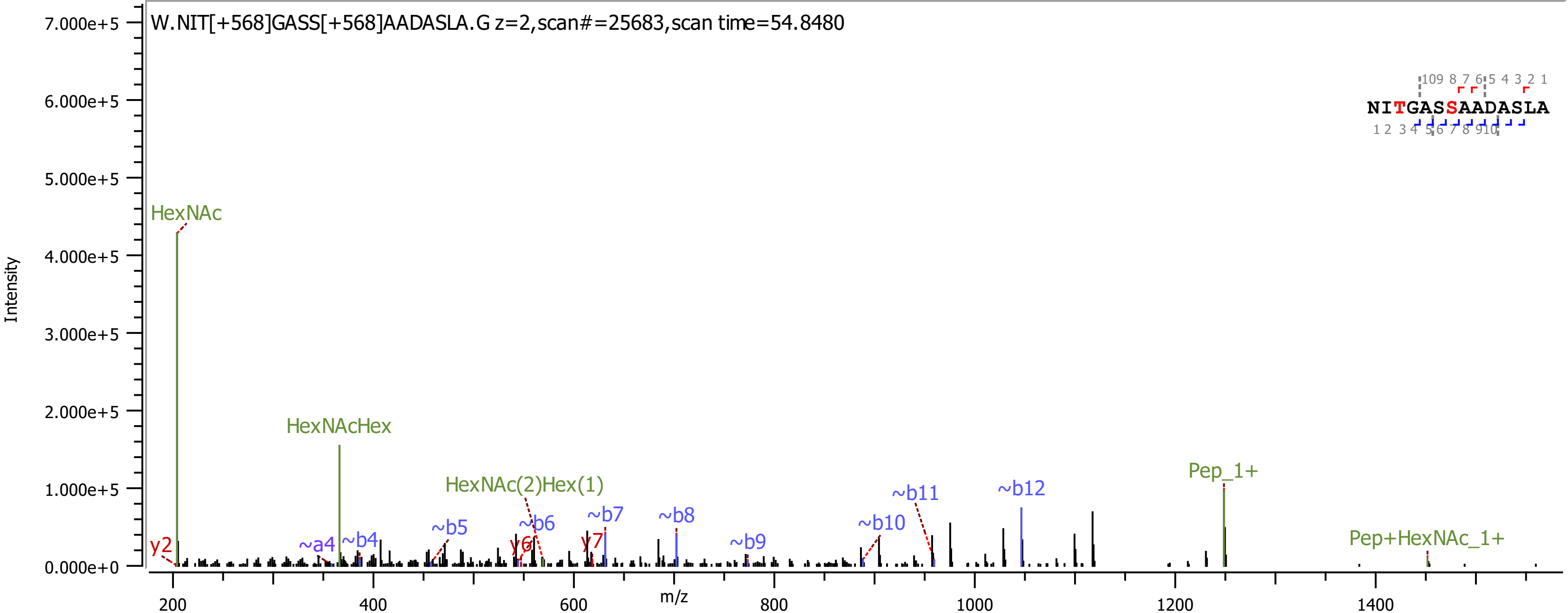

W.NIT[+568]GASS[+568]AADASLA.G z=2,scan#=25683,scan time=54.8480
Intensity
m/z
HexNAc
HexNAcHex
HexNAc(2)Hex(1)
Pep_1+
Pep+HexNAc_1+
y2
~a4
~b4
~b5
~b6
y6
~b7
y7
~b8
~b9
~b10
~b11
~b12
NITGASSAADASLA

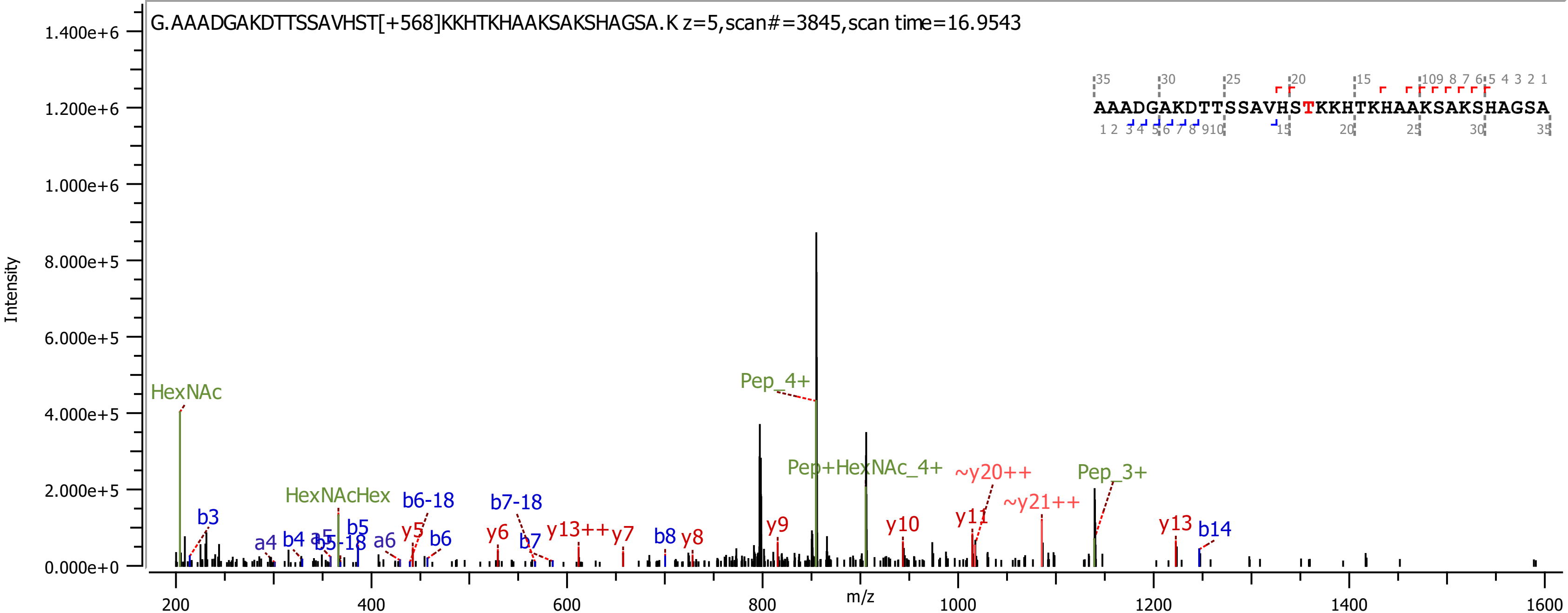

G.AAADGAKDTTSSAVHST[+568]KKHTKHAAKSAKSHAGSA.K z=5,scan#=3845,scan time=16.9543
AAADGAKDTTSSAVHSTKKHTKHAAKSAKSHAGSA
Intensity
m/z
HexNAc
HexNAcHex
Pep_4+
Pep+HexNAc_4+
Pep_3+
b3
a4
b4
a5
b5-18
b5
a6
b6-18
b6
y5
y6
b7-18
b7
y13++
y7
b8
y8
y9
y10
y11
~y20++
~y21++
y13
b14

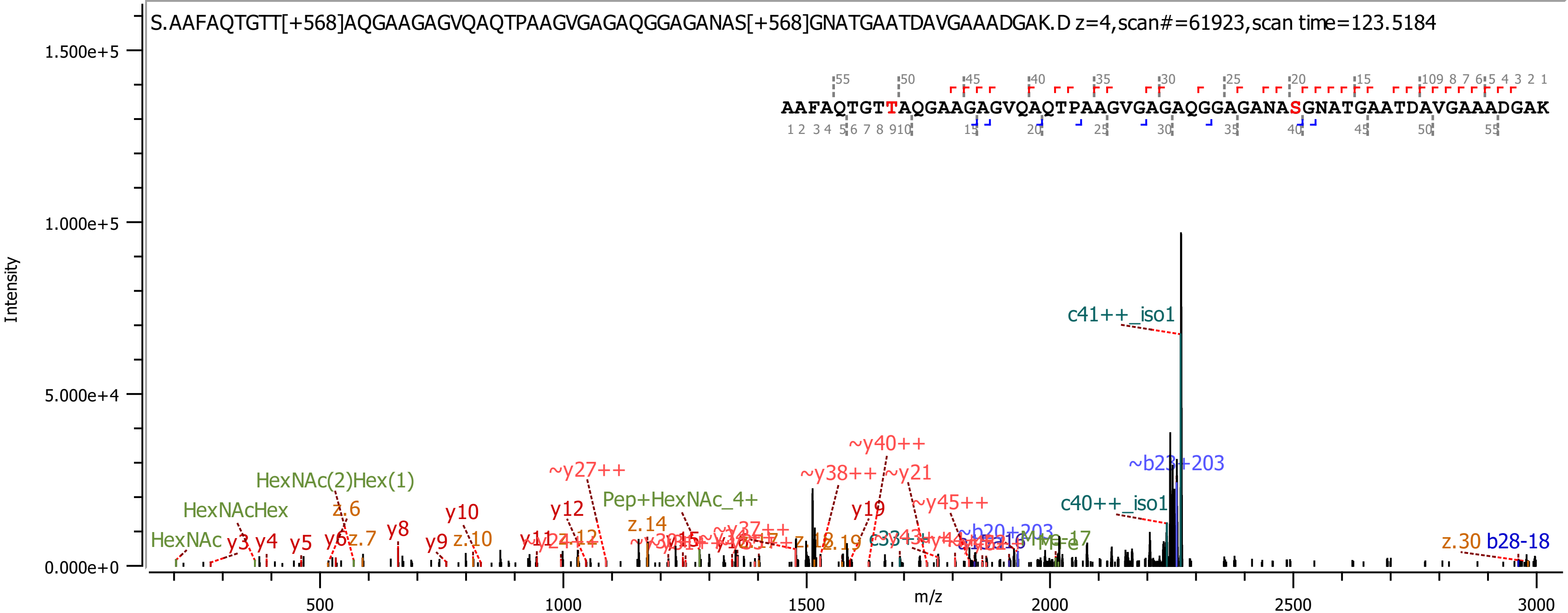
S.AAFAQTGTT[+568]AQGAAGAGVQAQTPAAGVGAGAQGGAGANAS[+568]GNATGAATDAVGAAADGAK.D z=4,scan#=61923,scan time=123.5184
AAFAQTGTTAQGAAGAGVQAQTPAAGVGAGAQGGAGANASGNATGAATDAVGAAADGAK
Intensity
m/z
c41++_iso1
c40++_iso1
~b23+203
~y40++
~y38++
~y21
~y27++
~y45++
y19
y12
y10
y8
y3
y4
y5
y9
z.6
z.7
z.10
z.14
z.30
b28-18
HexNAc
HexNAcHex
HexNAc(2)Hex(1)
Pep+HexNAc_4+

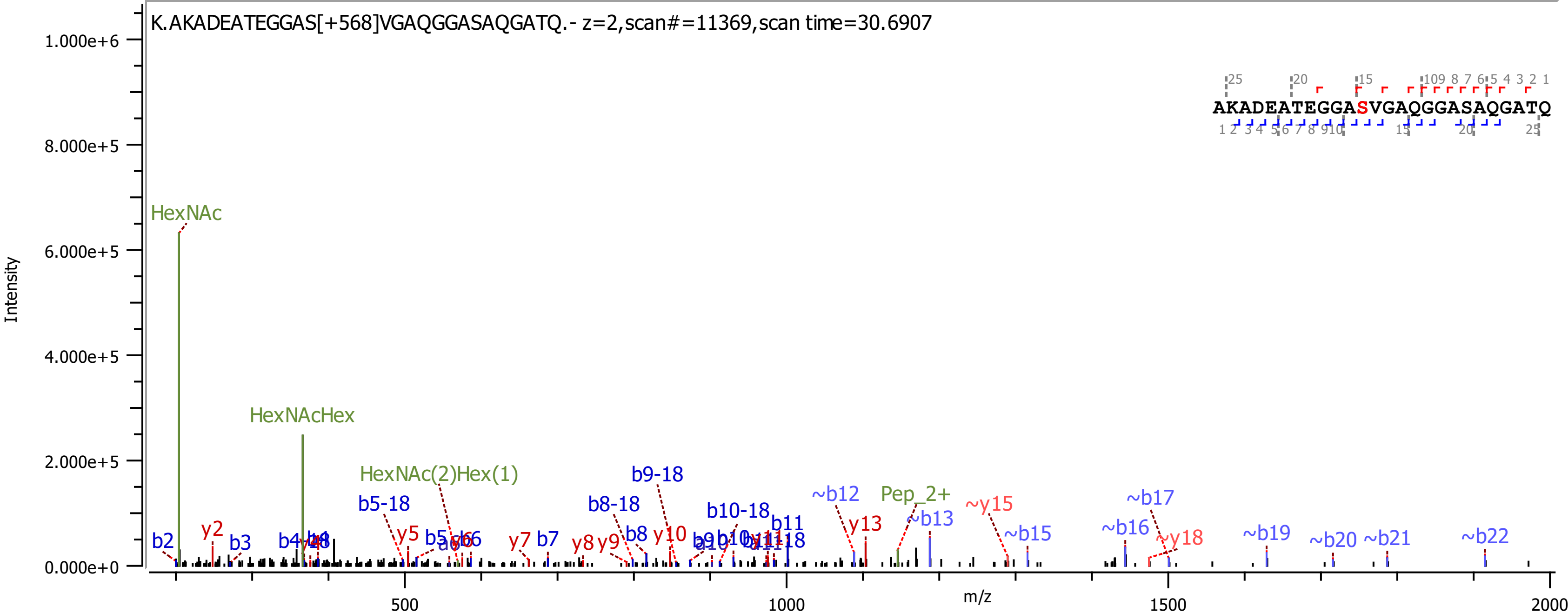

K.AKADEATEGGAS[+568]VGAQGGASAQGATQ.- z=2,scan#=11369,scan time=30.6907
AKADEATEGGASVGAQGGASAQGATQ
Intensity
m/z
HexNAc
HexNAcHex
HexNAc(2)Hex(1)
Pep_2+
b2
y2
b3
b5-18
y5
b7
y7
y8
y9
b8
b8-18
b9-18
y10
b10-18
b11
~b12
y13
~b13
~y15
~b15
~b16
~b17
~y18
~b19
~b20
~b21
~b22

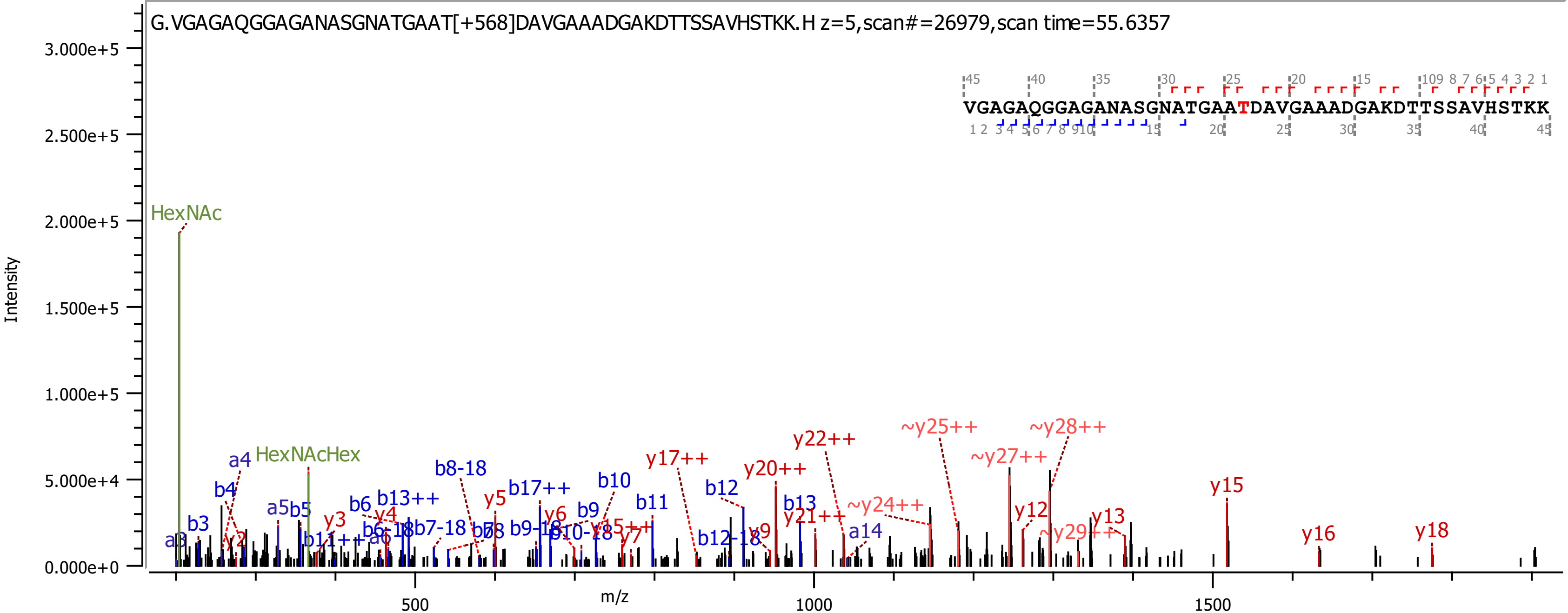
G.VGAGAQGGAGANASGNATGAAT[+568]DAVGAAADGAKDTTSSAVHSTKK.H z=5,scan#=26979,scan time=55.6357
VGAGAQGGAGANASGNATGAATDAVGAAADGAKDTTSSAVHSTKK
Intensity
m/z
HexNAc
HexNAcHex
a3
b3
b4
a4
y2
a5
b5
y3
b6
b13++
y4
b6-18
b7-18
a6
b11++
b8-18
y5
b17++
b9-18
y6
b10-18
b9
b10
y15++
y7
b11
b12
b12-18
y9
b13
y17++
y20++
y21++
y22++
a14
~y24++
~y25++
~y27++
~y28++
y12
~y29++
y13
y15
y16
y18
500
1000
1500

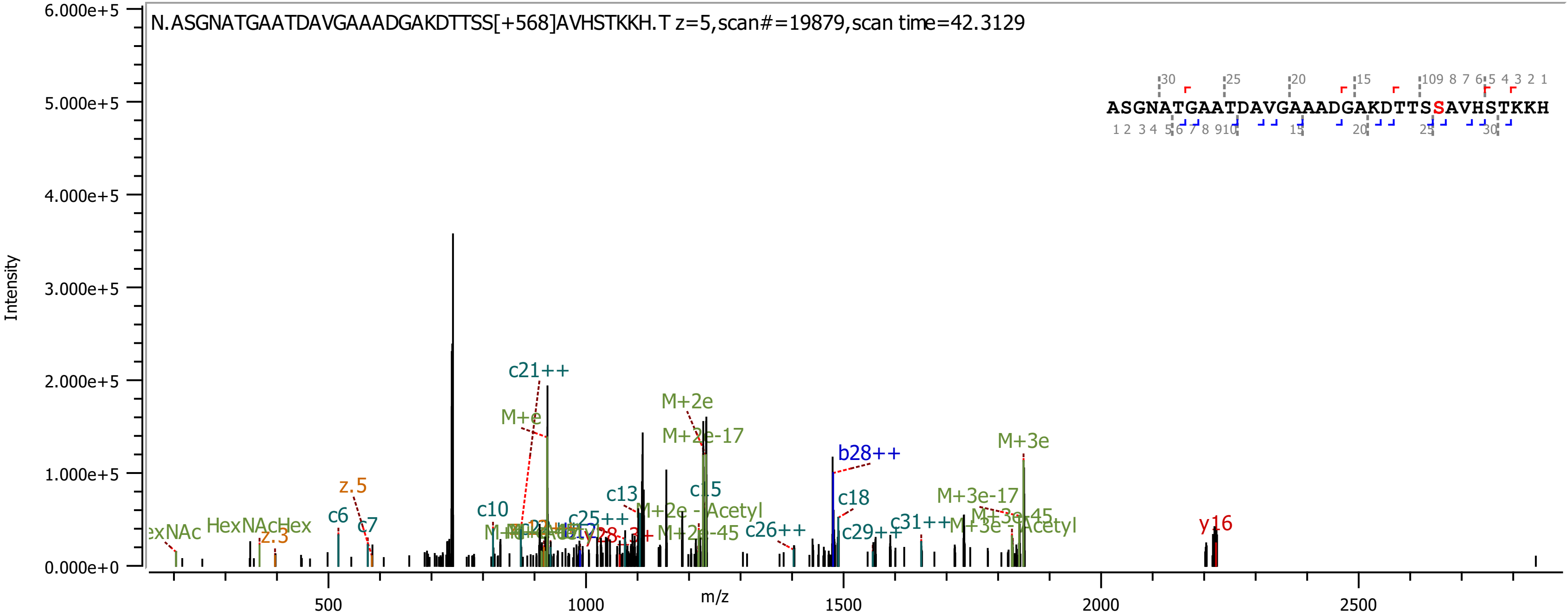
N.ASGNATGAATDAVGAAADGAKDTTSS[+568]AVHSTKKH.T z=5,scan#=19879,scan time=42.3129
ASGNATGAATDAVGAAADGAKDTTSSAVHSTKKH
Intensity
m/z
6.000e+5
5.000e+5
4.000e+5
3.000e+5
2.000e+5
1.000e+5
0.000e+0
500
1000
1500
2000
2500
c21++
M+e
M+2e
M+2e-17
b28++
M+3e
c15
c13
c18
M+3e-17
c10
z.5
c6
c7
z.3
HexNAcHex
c25++
M+2e - Acetyl
M+2e-45
c26++
c29++
c31++
M+3e - Acetyl
y16

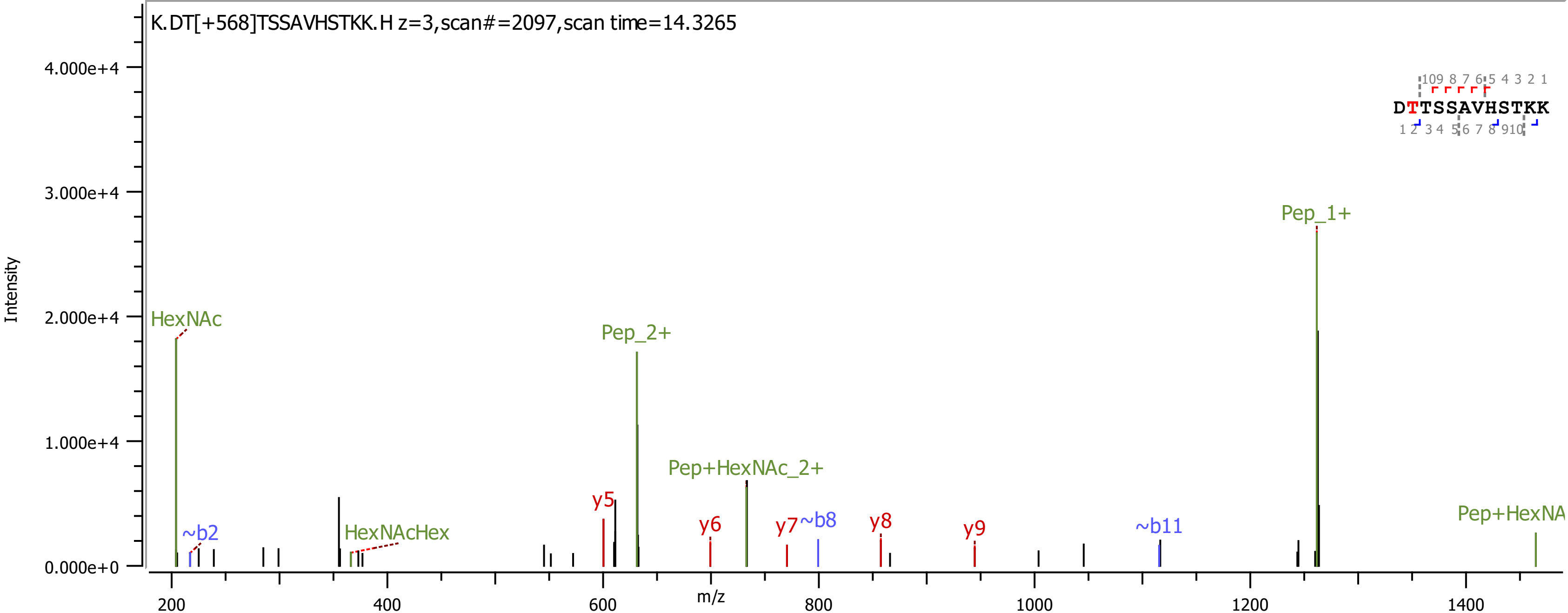

K.DT[+568]TSSAVHSTKK.H z=3,scan#=2097,scan time=14.3265
DTTSSAVHSTKK
Intensity
m/z
HexNAc
~b2
HexNAcHex
y5
Pep_2+
Pep+HexNAc_2+
y6
y7
~b8
y8
y9
~b11
Pep_1+
Pep+HexNA

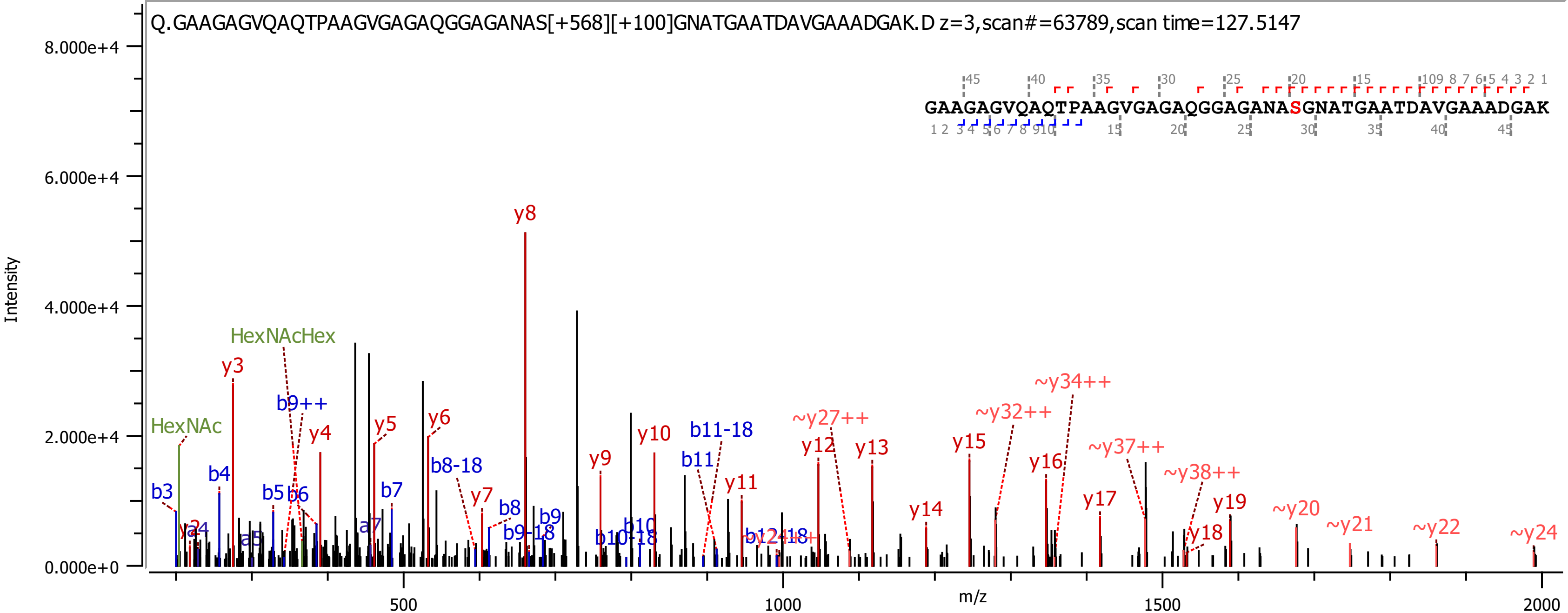
Q.GAAGAGVQAQTPAAGVGAGAQGGAGANAS[+568][+100]GNATGAATDAVGAAADGAK.D z=3,scan#=63789,scan time=127.5147
GAAGAGVQAQTPAAGVGAGAQGGAGANASGNATGAATDAVGAAADGAK
Intensity
m/z
8.000e+4
6.000e+4
4.000e+4
2.000e+4
0.000e+0
500
1000
1500
2000
HexNAcHex
HexNAc
y3
b9++
y4
y5
y6
y8
b8-18
y7
b8
b9
b9-18
b3
b4
b5
b6
b7
a7
a5
y9
y10
b11-18
b11
b10
b10-18
y11
y12
~y27++
y13
~y32++
~y34++
y15
y14
y16
~y37++
y17
~y38++
y19
y18
~y20
~y21
~y22
~y24

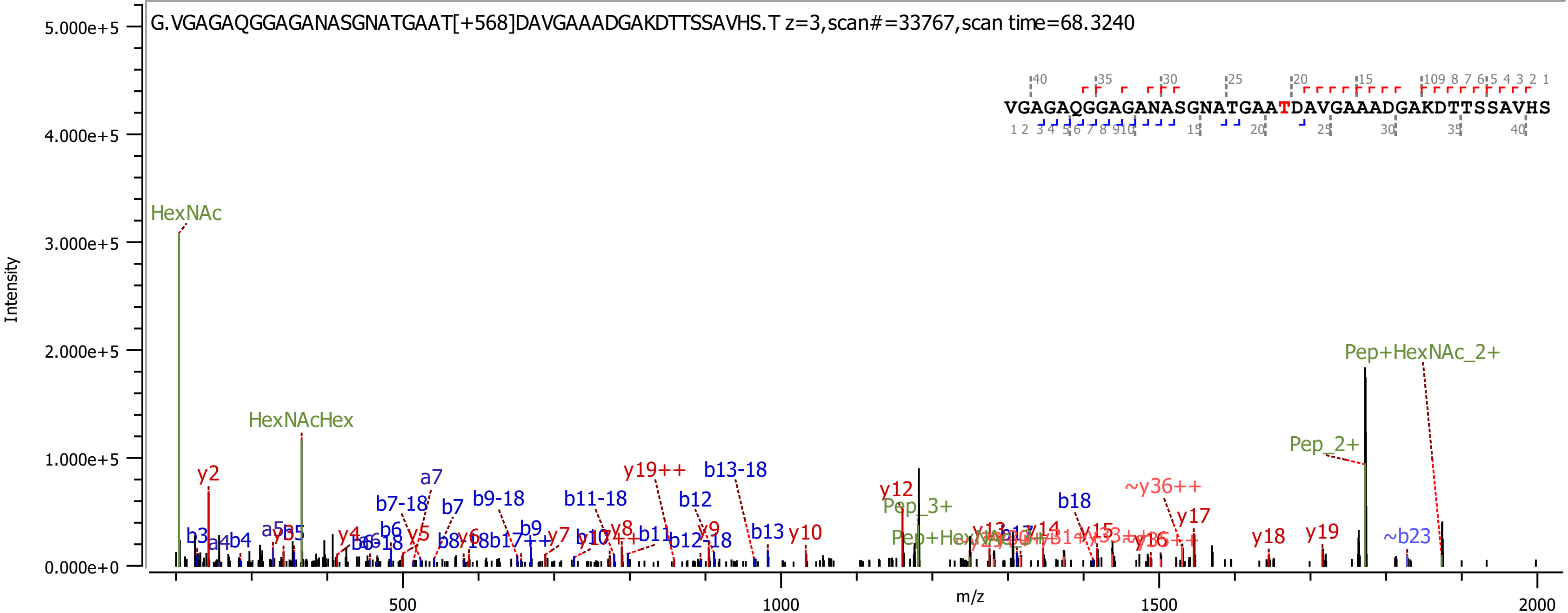
G.VGAGAQGGAGANASGNATGAAT[+568]DAVGAAADGAKDTTSSAVHS.T z=3,scan#=33767,scan time=68.3240
VGAGAQGGAGANASGNATGAATDAVGAAADGAKDTTSSAVHS
Intensity
m/z
5.000e+5
4.000e+5
3.000e+5
2.000e+5
1.000e+5
0.000e+0
500
1000
1500
2000
HexNAc
HexNAcHex
Pep+HexNAc_2+
Pep_2+
Pep_3+
y2
b3
a4
b4
a5
b5
y4
b6
b6-18
y5
a7
b7-18
b7
b9-18
b9
y7
b11-18
y19++
b13-18
b12
y9
b13
y10
y12
b18
~y36++
y17
y18
y19
~b23

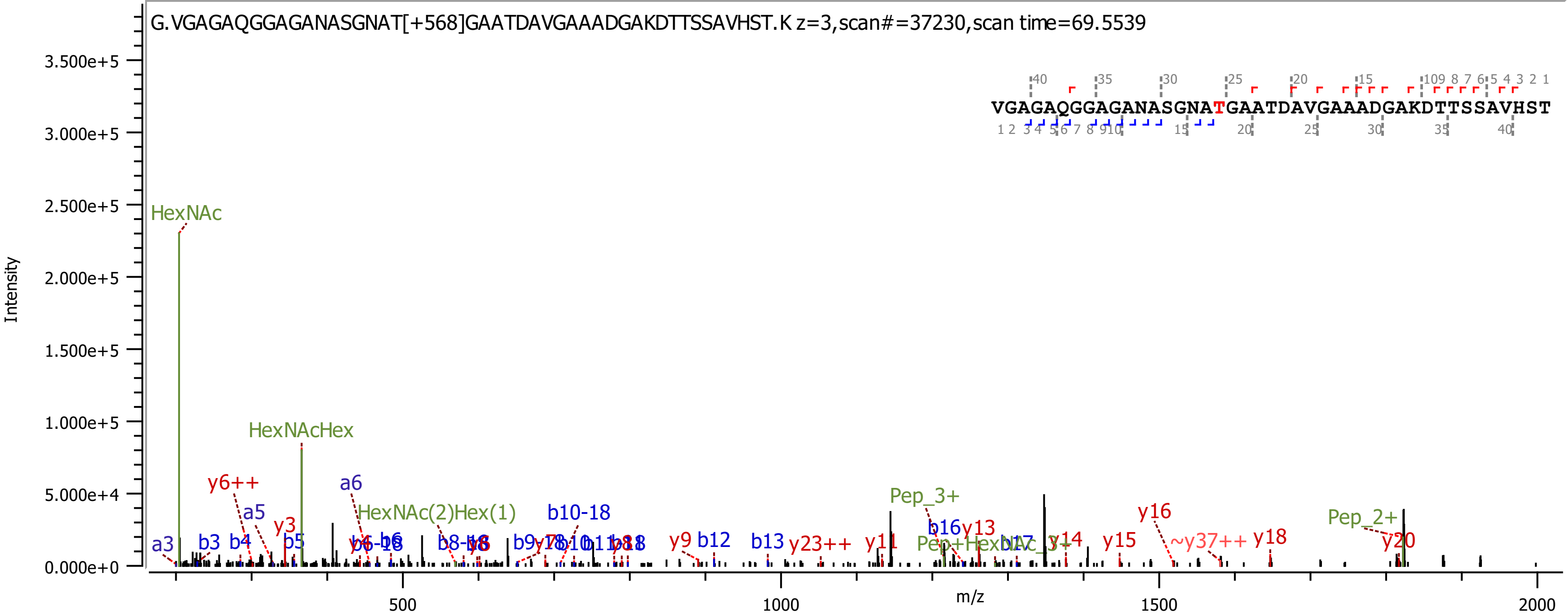
G.VGAGAQGGAGANASGNAT[+568]GAATDAVGAAADGAKDTTSSAVHST.K z=3,scan#=37230,scan time=69.5539
VGAGAQGGAGANASGNATGAATDAVGAAADGAKDTTSSAVHST
Intensity
m/z
HexNAc
HexNAcHex
HexNAc(2)Hex(1)
Pep_3+
Pep_2+
y6++
a5
a6
y3
a3
b3
b4
b5
b10-18
b12
b13
b16
y13
y9
y11
y14
y15
y16
y18
y20
y23++
~y37++

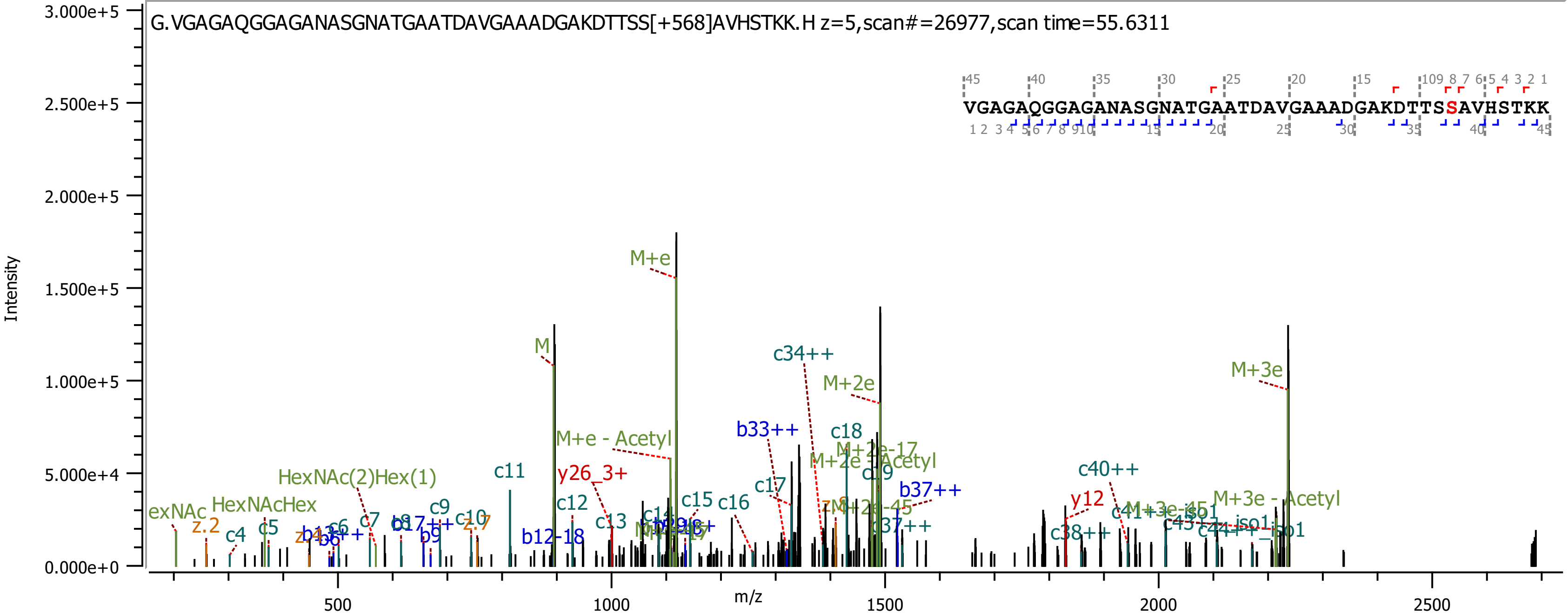
G.VGAGAQGGAGANASGNATGAATDAVGAAADGAKDTTSS[+568]AVHSTKK.H z=5,scan#=26977,scan time=55.6311
VGAGAQGGAGANASGNATGAATDAVGAAADGAKDTTSSAVHSTKK
Intensity
m/z
3.000e+5
2.500e+5
2.000e+5
1.500e+5
1.000e+5
5.000e+4
0.000e+0
500
1000
1500
2000
2500
M+e
M
M+e - Acetyl
M+2e
M+3e
M+3e - Acetyl
c34++
b33++
c18
c11
y26_3+
c12
c13
c15
c16
c17
b37++
c40++
y12
HexNAc(2)HexNAc(1)
HexNAcHex
z.2
c4
c5
c7
c9
b12-18
c38++

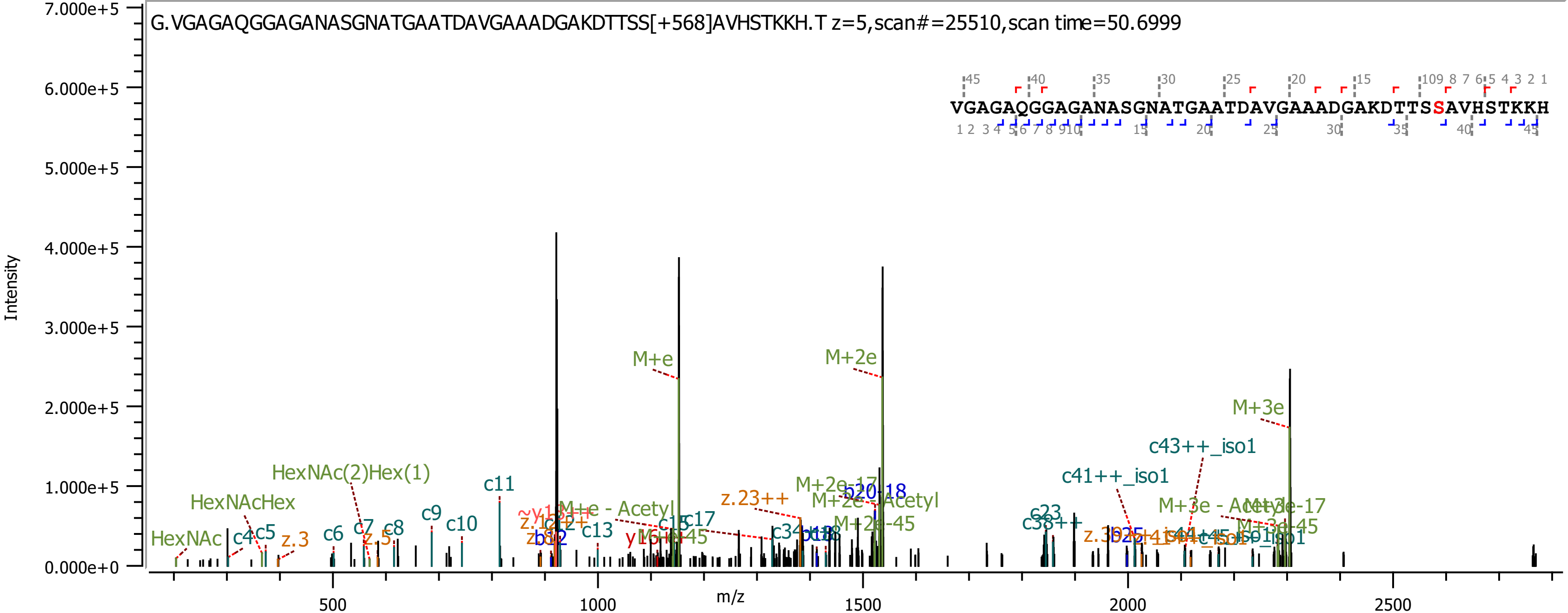
G.VGAGAQGGAGANASGNATGAATDAVGAAADGAKDTTSS[+568]AVHSTKKH.T z=5,scan#=25510,scan time=50.6999
VGAGAQGGAGANASGNATGAATDAVGAAADGAKDTTSSAVHSTKKH
Intensity
m/z
7.000e+5
6.000e+5
5.000e+5
4.000e+5
3.000e+5
2.000e+5
1.000e+5
0.000e+0
500
1000
1500
2000
2500
M+e
M+2e
M+3e
c43++_iso1
c41++_iso1
HexNAc(2)Hex(1)
HexNAcHex
HexNAc
c4
c5
z.3
c6
c7
z.5
c8
c9
c10
c11
c13
z.23++
M+2e-17
M+2e-45
c23
c38++
M+3e-17
M+3e-45
c17

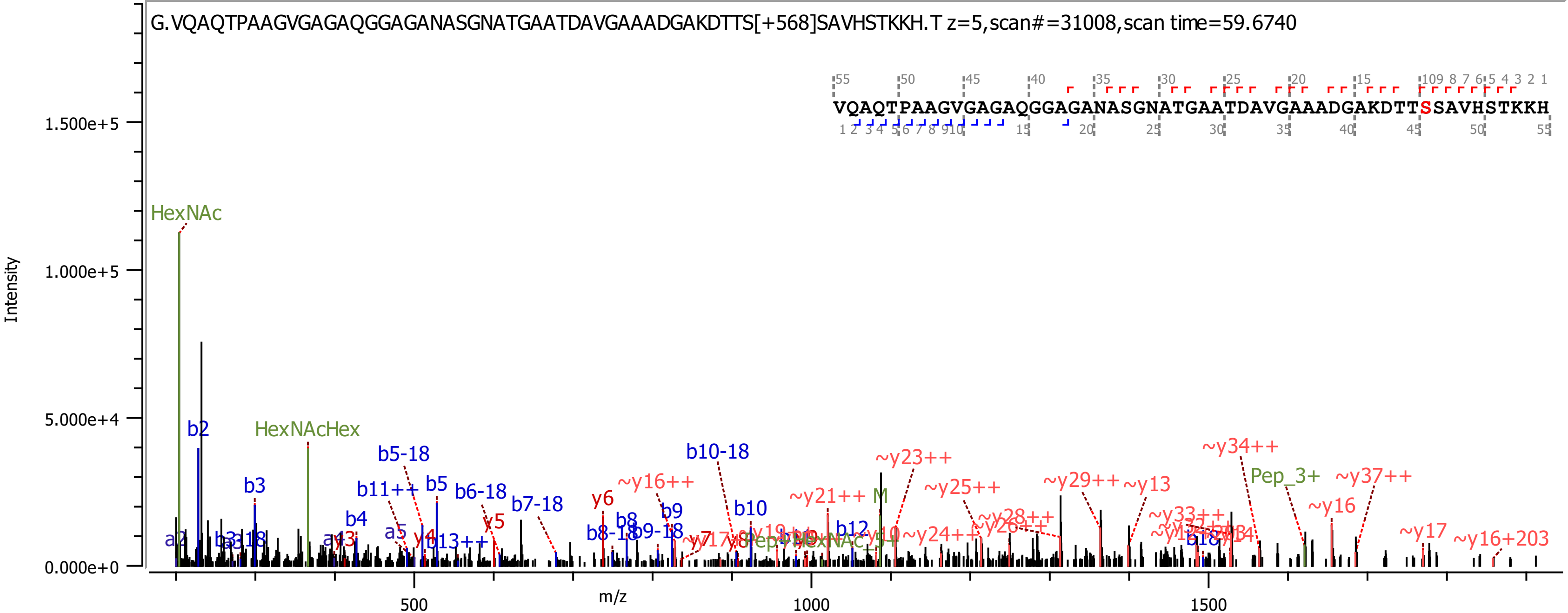
G.VQAQTPAAGVGAGAQGGAGANASGNATGAATDAVGAAADGAKDTTS[+568]SAVHSTKKH.T z=5,scan#=31008,scan time=59.6740
VQAQTPAAGVGAGAQGGAGANASGNATGAATDAVGAAADGAKDTTSSAVHSTKKH
Intensity
1.500e+5
1.000e+5
5.000e+4
0.000e+0
m/z
500
1000
1500
HexNAc
HexNAcHex
b2
b3
b4
b5
b5-18
b6-18
b7-18
b8
b9
b10
b10-18
b11++
b12
b13++
y4
y5
y6
~y16++
~y21++
~y23++
~y24++
~y25++
~y29++
~y13
~y33++
~y34++
Pep_3+
~y37++
~y16
~y17
~y16+203

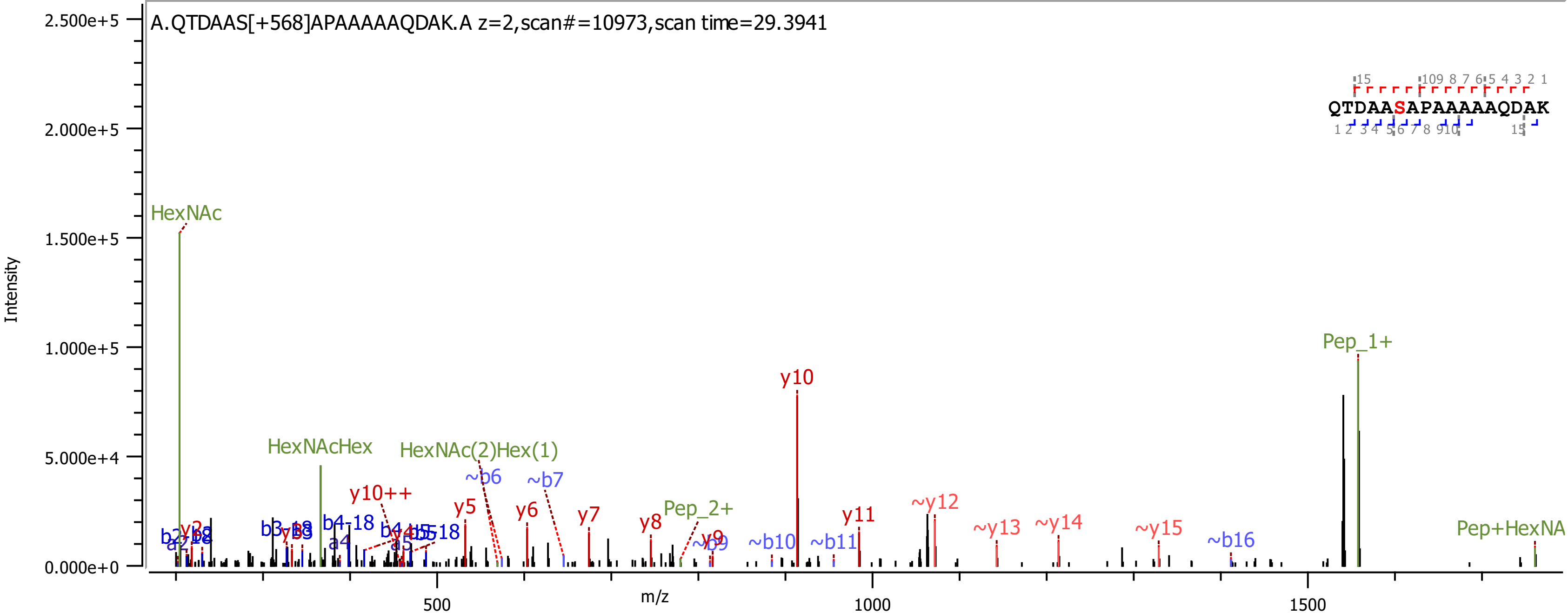
A.QTDAAS[+568]APAAAAAQDAK.A z=2,scan#=10973,scan time=29.3941
QTDAASAPAAAAAQDAK
Intensity
m/z
2.500e+5
2.000e+5
1.500e+5
1.000e+5
5.000e+4
0.000e+0
500
1000
1500
HexNAc
HexNAcHex
HexNAc(2)Hex(1)
y10++
~b6
~b7
y5
y6
y7
y8
Pep_2+
y10
y11
~y12
~y13
~y14
~y15
~b10
~b11
~b16
Pep_1+
b4-18
Pep+HexNA
