# Supplementary material for: Burkholderia PglL enzymes are Serine preferring oligosaccharyltransferases which target conserved proteins across the Burkholderia genus
Source: Commun Biol. 2021 Sep 7;4:1045. doi: 10.1038/s42003-021-02588-y (PMC8423747; doi:10.1038/s42003-021-02588-y)
Supplement: Supplementary file 6 — Supplementary Data 3 [file 42003_2021_2588_MOESM6_ESM.zip › Supplementary_Data_3C_H111_Best_localisation_glycopeptides.pdf]

Burkholderia cenocepacia H111 Best Localised Unique glycopeptides

J2315 numbers	Protein Name	Peptide < ProteinMetrics Confidential >	Glycans NHFAGNa	Localised site (Yes / No / Partial) ?	Observed m/z	z	Observed (M+H)	Calc. mass (M+H)	Mass error (ppm)	Score	Delta	Delta Mod	Comment	Scan Time	Enzyme	Replicate	Site	Page
BCAL0080	>tr|A0A1V2WZJ2|A0A1V2WZJ2_9BURK Class I cytochrome c OS=Burkholderia cenocepacia OX=95486 GN=A8E72_32710 PE=4 SV=1	Y.MANNDGANFPEPAAPAANAAQPAS[+568.212]GAPASGADASNAQA.A	HexNAc(2)Hex(1)	Yes	1345.2598	3	4033.7649	4033.7476	4.3	661.09	592.27	106.51	Nsco_20191108_BC_ZIC_HILIC_H111_thermolysin_B3.42890.42890.3	84.731	Thermolysin	B3	S176	16
BCAL0163	>tr|A0A144S4M8|A0A144S4M8_9BURK BON domain protein OS=Burkholderia cenocepacia OX=95486 GN=A8E72_32405 PE=4 SV=1	S.VQSTPLQPPAPISNSS[+568.212]SVHPGNPKAKAQ.-	HexNAc(2)Hex(1)	Yes	852.1834	4	3405.7117	3405.702	2.8	783.02	702.53	22.83	Nsco_20191108_BC_ZIC_HILIC_H111_pepsin_B2.21640.21640.4	45.203	Pepsin	B2	S262	17
BCAL0163	>tr|A0A144S4M8|A0A144S4M8_9BURK BON domain protein OS=Burkholderia cenocepacia OX=95486 GN=A8E72_32405 PE=4 SV=1	A.SVQSTPLQPPAPISNS[+568.212]SSVHPGNPKAKAQ.-	HexNAc(2)Hex(1)	Partial	873.9426	4	3492.7486	3492.734	4.2	310.05	310.05	1.6	Nsco_20191108_BC_ZIC_HILIC_H111_pepsin_B3.22644.22644.4	46.765	Pepsin	B3	NA	18
BCAL0163	>tr|A0A144S4M8|A0A144S4M8_9BURK BON domain protein OS=Burkholderia cenocepacia OX=95486 GN=A8E72_32405 PE=4 SV=1	A.LQDASPASGASGAQAAAAPADNATVGAVPDASVQSTPLQPPAPIS[+568.212]NSSSVHPGNPKAKAQ.-	HexNAc(2)Hex(1)	Partial	1549.7634	4	6196.0319	6196.0076	3.9	391.83	391.83	102.18	Nsco_20191108_BC_ZIC_HILIC_H111_thermolysin_B1.44265.44265.4	83.556	Thermolysin	B1	S224	19
BCAL0163	>tr|A0A144S4M8|A0A144S4M8_9BURK BON domain protein OS=Burkholderia cenocepacia OX=95486 GN=A8E72_32405 PE=4 SV=1	Y.VKPQDAQALQDASPAS[+568.212]GASGAQA.A	HexNAc(2)Hex(1)	Yes	1368.6424	2	2736.2775	2736.2694	3	280.25	83.45	62.99	Nsco_20191108_BC_ZIC_HILIC_H111_thermolysin_B1.26079.26079.2	51.977	Thermolysin	B1	S224	20
BCAL0163	>tr|A0A144S4M8|A0A144S4M8_9BURK BON domain protein OS=Burkholderia cenocepacia OX=95486 GN=A8E72_32405 PE=4 SV=1	Y.VKPQDAQALQDASPAS[+568.212]GASGAQ.A	HexNAc(2)Hex(1)	Yes	1333.1246	2	2665.242	2665.2323	3.6	191.34	46.96	34.32	Nsco_20191108_BC_ZIC_HILIC_H111_thermolysin_B3.23057.23057.2	49.251	Thermolysin	B3	S224	21
BCAL0163	>tr|A0A144S4M8|A0A144S4M8_9BURK BON domain protein OS=Burkholderia cenocepacia OX=95486 GN=A8E72_32405 PE=4 SV=1	Y.VKPQDAQALQDASPASGAS[+568.212]GAQAAAAPADNATVGAVPDASVQSTPLQPPAPISNSSSVHPGNPK.A	HexNAc(2)Hex(1)	Yes	1659.8156	4	6636.2404	6635.2143	3.4	901.28	550.13	85.05	Nsco_20191108_BC_ZIC_HILIC_H111_typsin_B2.47144.47144.4	91.282	Trypsin	B2	S224	22
BCAL0163	>tr|A0A144S4M8|A0A144S4M8_9BURK BON domain protein OS=Burkholderia cenocepacia OX=95486 GN=A8E72_32405 PE=4 SV=1	K.PQDAQALQDASPASGASGAQAAAAPADNATVGAVPDASVQST[+568.212]PLQPPAPISNSSSVHPGNPK.A	HexNAc(2)Hex(1)	Yes	1603.0234	4	6409.0719	6408.051	2.7	807.19	807.19	121.77	Nsco_20191108_BC_ZIC_HILIC_H111_typsin_B2.50883.50883.4	97.728	Trypsin	B2	S224	23
BCAL0163	>tr|A0A144S4M8|A0A144S4M8_9BURK BON domain protein OS=Burkholderia cenocepacia OX=95486 GN=A8E72_32405 PE=4 SV=1	K.VFQYVKPQDAQALQDASPASGASGAQAAAAPADNATVGAVPDASVQST[+568.212]PLQPPAPISNSSSVHPGNPK.A	HexNAc(2)Hex(1)	Yes	1436.1076	5	7176.5088	7172.4731	3.1	1109.8	690.41	100.78	Nsco_20191108_BC_ZIC_HILIC_H111_typsin_B2.52856.52856.5	**101.38**	Trypsin	B2	S224	24
BCAL0163	>tr|A0A144S4M8|A0A144S4M8_9BURK BON domain protein OS=Burkholderia cenocepacia OX=95486 GN=A8E72_32405 PE=4 SV=1	V.KVFQYVKPQDAQALQDASPASGAS[+568.212]GAQAAAAPADNATVGAVPDASVQSTPLQPPAPISNSSSVHPGNPK.A	HexNAc(2)Hex(1)	Yes	1460.9255	5	7300.5982	7300.568	4.1	1304.2	1304.2	54.21	Nsco_20191108_BC_ZIC_HILIC_H111_typsin_B1.47879.47879.5	**93.267**	Trypsin	B1	S224	25

<table>
<tr><td>BCAL0193</td><td>>tr|A0A071MDE2|A0A071MDE2_9BURK Uncharacterized protein OS=Burkholderia cenocepacia OX=95486 GN=A8E72_00390 PE=4 SV=1</td><td>Q.LKHHGSKKGQAKAAAAS[+568.212]AAGTNDAGTQN.-</td><td>HexNAc(2)Hex(1)</td><td>Yes</td><td>652.5254</td><td>5</td><td>3258.5977</td><td>3258.5833</td><td>4.4</td><td>882.97</td><td>882.97</td><td>102.81</td><td>Nsco_20191108_BC_ZIC_HILIC_H111_thermolysin_B1.2860.2860.5</td><td>16.086</td><td>Thermolysin</td><td>B1</td><td>S66</td><td>26</td></tr>
<tr><td>BCAL0193</td><td>>tr|A0A071MDE2|A0A071MDE2_9BURK Uncharacterized protein OS=Burkholderia cenocepacia OX=95486 GN=A8E72_00390 PE=4 SV=1</td><td>Q.ASAPAADT[+568.212]SAAAPAPAKK.D</td><td>HexNAc(2)Hex(1)</td><td>Yes</td><td>1082.5309</td><td>2</td><td>2164.0546</td><td>2164.0503</td><td>2</td><td>391.31</td><td>227.38</td><td>152.76</td><td>Nsco_20191108_BC_ZIC_HILIC_H111_typsin_B1.6901.6901.2</td><td>22.631</td><td>Trypsin</td><td>B1</td><td>S25</td><td>27</td></tr>
<tr><td>BCAL0272</td><td>>tr|A0A142PJB0|A0A142PJB0_9BURK Lipoprotein OS=Burkholderia cenocepacia OX=95486 GN=A8E72_20920 PE=4 SV=1</td><td>K.PIQPQNTPPSDVKPTDENASSDESPDTSGSPLTLSPELSSTSTMPAPAS[+568.212]GPAATK.-</td><td>HexNAc(2)Hex(1)</td><td>Yes</td><td>1515.9615</td><td>4</td><td>6060.8244</td><td>6058.798</td><td>3.2</td><td>944.57</td><td>888.07</td><td>96.92</td><td>Nsco_20191108_BC_ZIC_HILIC_H111_typsin_B2.51007.51007.4</td><td>97.936</td><td>Trypsin</td><td>B2</td><td>S85</td><td>28</td></tr>
<tr><td>BCAL0303</td><td>>tr|A0A144S0T9|A0A144S0T9_9BURK Outer membrane lipid asymmetry maintenance protein MlaD OS=Burkholderia cenocepacia OX=95486 GN=yrbD PE=4 SV=1</td><td>S.KAADAGGAKPAAGAS[+568.212]AAPAAPAPVAV.P</td><td>HexNAc(2)Hex(1)</td><td>Yes</td><td>909.4655</td><td>3</td><td>2726.3819</td><td>2726.3731</td><td>3.2</td><td>741.46</td><td>731.06</td><td>731.06</td><td>Nsco_20191108_BC_ZIC_HILIC_H111_pepsin_B2.23564.23564.3</td><td>48.96</td><td>Pepsin</td><td>B2</td><td>S164</td><td>29</td></tr>
<tr><td>BCAL0303</td><td>>tr|A0A144S0T9|A0A144S0T9_9BURK Outer membrane lipid asymmetry maintenance protein MlaD OS=Burkholderia cenocepacia OX=95486 GN=yrbD PE=4 SV=1</td><td>Y.SKAADAGGAKPAAGAS[+568.212]AAPAAPAPVAV.P</td><td>HexNAc(2)Hex(1)</td><td>Yes</td><td>938.4753</td><td>3</td><td>2813.4114</td><td>2813.4051</td><td>2.2</td><td>386.22</td><td>312.9</td><td>280.27</td><td>Nsco_20191108_BC_ZIC_HILIC_H111_pepsin_B2.23609.23609.3</td><td>49.036</td><td>Pepsin</td><td>B2</td><td>S164</td><td>30</td></tr>
<tr><td>BCAL0303</td><td>>tr|A0A144S0T9|A0A144S0T9_9BURK Outer membrane lipid asymmetry maintenance protein MlaD OS=Burkholderia cenocepacia OX=95486 GN=yrbD PE=4 SV=1</td><td>L.YSKAADAGGAKPAAGAS[+568.212]AAPAAPAPVAV.P</td><td>HexNAc(2)Hex(1)</td><td>Yes</td><td>744.8754</td><td>4</td><td>2976.48</td><td>2976.4684</td><td>3.9</td><td>843.54</td><td>806.59</td><td>509.24</td><td>Nsco_20191108_BC_ZIC_HILIC_H111_pepsin_B3.25168.25168.4</td><td>51.945</td><td>Pepsin</td><td>B3</td><td>S164</td><td>31</td></tr>
<tr><td>BCAL0303</td><td>>tr|A0A144S0T9|A0A144S0T9_9BURK Outer membrane lipid asymmetry maintenance protein MlaD OS=Burkholderia cenocepacia OX=95486 GN=yrbD PE=4 SV=1</td><td>K.PAAGASAAPAAPAPVAVPAS[+568.212][+100.064]AVS[+568.212]GSAGQ.-</td><td>HexNAc(2)Hex(1)100.064,HexNAc(2)Hex(1)</td><td>Yes (1 of 2)</td><td>1180.2254</td><td>3</td><td>3538.6616</td><td>3537.6705</td><td>-3.4</td><td>247.45</td><td>206.29</td><td>1.76</td><td>Nsco_20191108_BC_ZIC_HILIC_H111_typsin_B2.42457.42457.3</td><td>82.978</td><td>Trypsin</td><td>B2</td><td>S178</td><td>32</td></tr>
<tr><td>BCAL0304</td><td>>tr|A0A2N9CYS8|A0A2N9CYS8_9BURK Surface lipoprotein OS=Burkholderia cenocepacia OX=95486 GN=F01_50090 PE=4 SV=1</td><td>A.VAAPASGTAESPNPAS[+568.212]ET[+568.212]NV.P</td><td>HexNAc(2)Hex(1),HexNAc(2)Hex(1)</td><td>Yes (1 of 2)</td><td>1002.7773</td><td>3</td><td>3006.3172</td><td>3006.3056</td><td>3.9</td><td>162.23</td><td>111.11</td><td>25.82</td><td>Nsco_20191108_BC_ZIC_HILIC_H111_pepsin_B3.27461.27461.3</td><td>56.738</td><td>Pepsin</td><td>B3</td><td>S287</td><td>33</td></tr>
<tr><td>BCAL0304</td><td>>tr|A0A2N9CYS8|A0A2N9CYS8_9BURK Surface lipoprotein OS=Burkholderia cenocepacia OX=95486 GN=F01_50090 PE=4 SV=1</td><td>A.VAAPASGTAESPNPAS[+568.212]ET[+568.212]NVPAM.Q</td><td>HexNAc(2)Hex(1),HexNAc(2)Hex(1)</td><td>Yes (1 of 2)</td><td>1102.4867</td><td>3</td><td>3305.4456</td><td>3305.436</td><td>2.9</td><td>194.56</td><td>117.32</td><td>9.93</td><td>Nsco_20191108_BC_ZIC_HILIC_H111_pepsin_B2.36550.36550.3</td><td>76.601</td><td>Pepsin</td><td>B2</td><td>S297</td><td>34</td></tr>
<tr><td>BCAL0304</td><td>>tr|A0A2N9CYS8|A0A2N9CYS8_9BURK Surface lipoprotein OS=Burkholderia cenocepacia OX=95486 GN=F01_50090 PE=4 SV=1</td><td>A.VAAPASGT[+568.212]AES[+568.212]PNPASETNVPAMQ.V</td><td>HexNAc(2)Hex(1),HexNAc(2)Hex(1)</td><td>Yes (1 of 2)</td><td>1717.2566</td><td>2</td><td>3433.506</td><td>3433.4946</td><td>3.3</td><td>328.67</td><td>298.7</td><td>8.49</td><td>Nsco_20191108_BC_ZIC_HILIC_H111_thermolysin_B3.36251.36251.2</td><td>72.229</td><td>Thermolysin</td><td>B3</td><td>S287</td><td>35</td></tr>
<tr><td>BCAL0340</td><td>>tr|A0A1V2W5P4|A0A1V2W5P4_9BURK Uncharacterized protein OS=Burkholderia cenocepacia OX=95486 GN=A8E72_11900 PE=4 SV=1</td><td>K.AAAAPAAEAAASAAPT[+568.212]PPAAQK.G</td><td>HexNAc(2)Hex(1)</td><td>Yes</td><td>1236.6064</td><td>2</td><td>2472.2055</td><td>2472.1988</td><td>2.7</td><td>548.17</td><td>380.52</td><td>57.59</td><td>Nsco_20191108_BC_ZIC_HILIC_H111_typsin_B1.21380.21380.2</td><td>47.155</td><td>Trypsin</td><td>B1</td><td>S187</td><td>36</td></tr>
<tr><td>BCAL0349 (type</td><td>>tr|A0A2N9D2R1|A0A2N9D2R1_9BURK Outer membrane protein OS=Burkholderia cenocepacia OX=95486 GN=F01_70026 PE=3 SV=1</td><td>T.TALPQANPNAGGAS[+568.212]GTVVHGTAGTLTPPPANAAPGQ.V</td><td>HexNAc(2)Hex(1)</td><td>Yes</td><td>958.7218</td><td>4</td><td>3831.8655</td><td>3831.8519</td><td>3.6</td><td>376.21</td><td>355.68</td><td>52.55</td><td>Nsco_20191108_BC_ZIC_HILIC_H111_pepsin_B1.36442.36442.4</td><td>77.931</td><td>Pepsin</td><td>B1</td><td>S67</td><td>37</td></tr>
<tr><td>BCAL0349 (type</td><td>>tr|A0A2N9D2R1|A0A2N9D2R1_9BURK Outer membrane protein OS=Burkholderia cenocepacia OX=95486 GN=F01_70026 PE=3 SV=1</td><td>L.PQANPNAGGASGTVVHGT[+568.212]AGTLTPPPANAAPGQ.V</td><td>HexNAc(2)Hex(1)</td><td>Yes</td><td>1182.9037</td><td>3</td><td>3546.6967</td><td>3546.6831</td><td>3.8</td><td>487.19</td><td>462.86</td><td>57.94</td><td>Nsco_20191108_BC_ZIC_HILIC_H111_pepsin_B2.30908.30908.3</td><td>63.716</td><td>Pepsin</td><td>B2</td><td>S67</td><td>38</td></tr>
</table>

BCAL0349 (type	>tr|A0A2N9D2R1|A0A2N9D2R1_9BURK Outer membrane protein OS=Burkholderia cenocepacia OX=95486 GN=F01_70026 PE=3 SV=1	A.TTALPQANPNAGGASGT[+568.212]VVHGTAGTLTPPPANAAPGQ.V	HexNAc(2)Hex(1)	Yes	1311.6434	3	3932.9155	3932.8996	4.1	295.56	200.94	20.99	Nsco_20191108_BC_ZIC_HILIC_H111_pepsin_B2.37365.37365.3	78.601	Pepsin	B2	S67	39
BCAL0349 (type	>tr|A0A2N9D2R1|A0A2N9D2R1_9BURK Outer membrane protein OS=Burkholderia cenocepacia OX=95486 GN=F01_70026 PE=3 SV=1	T.ALPQANPNAGGAS[+568.212]GTVVHGTAGTLTPPPANAAPGQVVVGGKVPDEATKA.A	HexNAc(2)Hex(1)	Yes	1296.4121	4	5182.6266	5181.6189	0.8	199.81	199.81	91.51	**Nsco_20191108_BC_ZIC_HILIC_H**	**78.307**	Thermolysin	B1	S67	40
BCAL0349 (type	>tr|A0A2N9D2R1|A0A2N9D2R1_9BURK Outer membrane protein OS=Burkholderia cenocepacia OX=95486 GN=F01_70026 PE=3 SV=1	T.ALPQANPNAGGAS[+568.212][+100.064]GTVVHGTAGTLTPPPANAAPGQVVVGGKVPDEATKAAVLQK.L	HexNAc(2)Hex(1) 100.064	Yes	1145.1952	5	5721.9468	5720.9621	-3.2	803.05	467.38	82.36	Nsco_20191108_BC_ZIC_HILIC_H111_thermolysin_B1.44302.44302.5	**83.627**	Thermolysin	B1	S67	41
BCAL0349 (type	>tr|A0A2N9D2R1|A0A2N9D2R1_9BURK Outer membrane protein OS=Burkholderia cenocepacia OX=95486 GN=F01_70026 PE=3 SV=1	T.TALPQANPNAGGAS[+568.212]GTVVHGTAGTLTPPPANAAPGQVVVGGKVPDEATKA.A	HexNAc(2)Hex(1)	Yes	1296.4056	4	5182.6007	5182.6026	-0.4	747.56	107.6	99.01	Nsco_20191108_BC_ZIC_HILIC_H111_thermolysin_B2.40555.40555.4	80.2	Thermolysin	B2	S67	42
BCAL0349 (type	>tr|A0A2N9D2R1|A0A2N9D2R1_9BURK Outer membrane protein OS=Burkholderia cenocepacia OX=95486 GN=F01_70026 PE=3 SV=1	T.VATTALPQANPNAGGAS[+568.212]GTVVHGTAGTLTPPPANAAPGQVVVGGKVPDEATKA.A	HexNAc(2)Hex(1)	Yes	1364.2004	4	5453.7799	5453.7558	4.4	1043.8	1043.8	116.92	Nsco_20191108_BC_ZIC_HILIC_H111_thermolysin_B3.42314.42314.4	83.58	Thermolysin	B3	S67	43
BCAL0349 (type	>tr|A0A2N9D2R1|A0A2N9D2R1_9BURK Outer membrane protein OS=Burkholderia cenocepacia OX=95486 GN=F01_70026 PE=3 SV=1	T.VATTALPQANPNAGGAS[+568.212]GTVVHGTAGTLTPPPAN.A	HexNAc(2)Hex(1)	Yes	1227.2776	3	3679.8184	3678.7981	4.6	367.42	345.16	47.16	Nsco_20191108_BC_ZIC_HILIC_H111_thermolysin_B2.39792.39792.3	78.721	Thermolysin	B2	S67	44
BCAL0349 (type	>tr|A0A2N9D2R1|A0A2N9D2R1_9BURK Outer membrane protein OS=Burkholderia cenocepacia OX=95486 GN=F01_70026 PE=3 SV=1	T.ALPQANPNAGGASGTVVHGTAGT[+568.212]LTPPPANAAPGQVVVGGK.V	HexNAc(2)Hex(1)	Yes	1424.0583	3	4270.1603	4270.1474	3	208.24	167.3	47.75	Nsco_20191108_BC_ZIC_HILIC_H111_typsin_B1.38426.38426.3	**77.247**	Trypsin	B1	S67	45
BCAL0349 (type	>tr|A0A2N9D2R1|A0A2N9D2R1_9BURK Outer membrane protein OS=Burkholderia cenocepacia OX=95486 GN=F01_70026 PE=3 SV=1	T.ALPQANPNAGGAS[+568.212]GTVVHGTAGTLTPPPANAAPGQVVVGGKVPDEATK.A	HexNAc(2)Hex(1)	Yes	1253.3899	4	5010.5378	5010.5178	4	974.71	974.71	93.18	Nsco_20191108_BC_ZIC_HILIC_H111_typsin_B2.39145.39145.4	77.158	Trypsin	B2	S67	46
BCAL0426	>tr|A0A1V6KWZ1|A0A1V6KWZ1_9BURK Membrane protein insertase YidC OS=Burkholderia cenocepacia OX=95486 GN=yidC PE=3 SV=1	R.DHGRPSMFFPSATHTAPAAAGGAS[+568.212]GTGATTTAGDVPAAAAGAAPSTTAPAAQAQLVK.F	HexNAc(2)Hex(1)	Yes	1446.9513	4	5784.7834	5784.757	4.6	1083.6	1083.6	48.54	Nsco_20191108_BC_ZIC_HILIC_H111_typsin_B1.42478.42478.4	84.003	Trypsin	B1	S49	47
BCAL0479	>tr|A0A2N9CSF4|A0A2N9CSF4_9BURK Peptidoglycan D,D-transpeptidase MrdA OS=Burkholderia cenocepacia OX=95486 GN=mrdA PE=3 SV=1	R.RPAS[+568.212]DAQPVVATPR.D	HexNAc(2)Hex(1)	Yes	1017.0083	2	2033.0094	2033.0033	3	632.91	349.99	39.47	Nsco_20191108_BC_ZIC_HILIC_H111_typsin_B1.9404.9404.2	27.408	Trypsin	B1	S681	48
BCAL0525	>tr|A0A119PFA4|A0A119PFA4_9BURK Flagellar M-ring protein OS=Burkholderia cenocepacia OX=95486 GN=fliF PE=3 SV=1	L.SNTPPQPAS[+568.212]APIVAGNGQNAPQTTPVSD.R	HexNAc(2)Hex(1)	Yes	1095.5181	3	3284.5397	3284.5288	3.3	328.95	301.17	24.3	Nsco_20191108_BC_ZIC_HILIC_H111_pepsin_B1.36638.36638.3	78.434	Pepsin	B1	S358	49
BCAL0525	>tr|A0A119PFA4|A0A119PFA4_9BURK Flagellar M-ring protein OS=Burkholderia cenocepacia OX=95486 GN=fliF PE=3 SV=1	R.SQQTSSATELAQGGASGVPGALS[568.212]NTPPQPAS[+568.212][+100.064]APIVAGNGQNAPQTTPVSDRK.D	HexNAc(2)Hex(1),HexNAc(2)Hex(1) 100.064	Partial	1560.2444	4	6237.9557	6234.9659	-3.2	161.4	161.4	41.1	Nsco_20191108_BC_ZIC_HILIC_H111_typsin_B3.41556.41556.4	86.147	Trypsin	B3	NA	50
BCAL0544	>tr|A0A2N9CS86|A0A2N9CS86_9BURK Dipeptide transport system substrate-binding protein OS=Burkholderia cenocepacia OX=95486 GN=dppA PE=4 SV=1	S.VYQGAGQAASAPMPPT[+568.212]QWSYDKN.L	HexNAc(2)Hex(1)	Partial	1518.1818	2	3035.3563	3035.3463	3.3	336.38	270.08	45.08	Nsco_20191108_BC_ZIC_HILIC_H111_thermolysin_B3.39157.39157.2	77.717	Thermolysin	B3	NA	51

BCAL0544	>tr|A0A2N9CS86|A0A2N9CS86_9BURK Dipeptide transport system substrate-binding protein OS=Burkholderia cenocepacia OX=95486 GN=dppA PE=4 SV=1	K.KAILESVYQGAGQAAS[+568.212]APMPPTQWSYDK.N	HexNAc(2)Hex(1)	Yes	1188.2358	3	3562.6928	3562.6782	4.1	568.83	554.97	117.74	Nsco_20191108_BC_ZIC_HILIC_H111_typsin_B2.50148.50148.3	96.466	Trypsin	B2	S343	52
BCAL0678	>tr|A0A142PL18|A0A142PL18_9BURK Cell division protein OS=Burkholderia cenocepacia OX=95486 GN=A8E72_03155 PE=4 SV=1	S.KVAPPPADNGAS[+568.212]QPQQFDPNRAL.Q	HexNAc(2)Hex(1)	Yes	996.151	3	2986.4384	2986.4276	3.6	652.56	631.5	631.5	Nsco_20191108_BC_ZIC_HILIC_H111_pepsin_B1.27669.27669.3	58.535	Pepsin	B1	S39	53
BCAL0678	>tr|A0A142PL18|A0A142PL18_9BURK Cell division protein OS=Burkholderia cenocepacia OX=95486 GN=A8E72_03155 PE=4 SV=1	S.KVAPPPADNGAS[+568.212]QPQQFDPNRALQGKTPGQPVPQAAQPAPPNTAPGQA.A	HexNAc(2)Hex(1)	Yes	1344.6699	4	5375.6579	5375.6415	3.1	753.23	753.23	297.94	Nsco_20191108_BC_ZIC_HILIC_H1	73.703	Pepsin	B1	S39	54
BCAL0678	>tr|A0A142PL18|A0A142PL18_9BURK Cell division protein OS=Burkholderia cenocepacia OX=95486 GN=A8E72_03155 PE=4 SV=1	S.KVAPPPADNGASQPQQFDPNRALQGKT[+568.212][+100.064]PGQPVPQAAQPAPPNTAPGQAANQ.T	HexNAc(2)Hex(1) 100.064	Yes	1422.9559	4	5688.8019	5688.7801	3.8	593.48	593.48	309.84	Nsco_20191108_BC_ZIC_HILIC_H111_pepsin_B3.34118.34118.4	72.856	Pepsin	B3	S39	55
BCAL0678	>tr|A0A142PL18|A0A142PL18_9BURK Cell division protein OS=Burkholderia cenocepacia OX=95486 GN=A8E72_03155 PE=4 SV=1	S.KVAPPPADNGAS[+568.212]QPQQFDPNRALQGKTPGQPVPQA.A	HexNAc(2)Hex(1)	Yes	1044.523	4	4175.0701	4175.0527	4.2	776.89	776.89	543.75	Nsco_20191108_BC_ZIC_HILIC_H111_pepsin_B2.31728.31728.4	65.589	Pepsin	B2	S39	56
BCAL0678	>tr|A0A142PL18|A0A142PL18_9BURK Cell division protein OS=Burkholderia cenocepacia OX=95486 GN=A8E72_03155 PE=4 SV=1	S.KVAPPPADNGAS[+568.212]QPQQFDPNRALQGKTPGQPVPQAAQPAPPNTAPGQAAN.Q	HexNAc(2)Hex(1)	Yes	1113.3546	5	5562.744	5560.7215	2.8	701.01	701.01	371.37	Nsco_20191108_BC_ZIC_HILIC_H111_pepsin_B3.34070.34070.5	72.729	Pepsin	B3	S39	57
BCAL0678	>tr|A0A142PL18|A0A142PL18_9BURK Cell division protein OS=Burkholderia cenocepacia OX=95486 GN=A8E72_03155 PE=4 SV=1	S.KVAPPPADNGAS[+568.212]QPQQFDPNRALQGKTPGQPVPQAAQPAPPNTAPGQ.A	HexNAc(2)Hex(1)	Yes	1326.9117	4	5304.6252	5304.6044	3.9	691.37	691.37	332.32	Nsco_20191108_BC_ZIC_HILIC_H111_pepsin_B1.34429.34429.4	73.067	Pepsin	B1	S39	58
BCAL0678	>tr|A0A142PL18|A0A142PL18_9BURK Cell division protein OS=Burkholderia cenocepacia OX=95486 GN=A8E72_03155 PE=4 SV=1	S.KVAPPPADNGAS[+568.212]QPQQFDPNRALQGKTPGQPVPQAAQPAPPNTA.P	HexNAc(2)Hex(1)	Yes	1256.3777	4	5022.4889	5022.4716	3.5	602.85	602.85	265.96	Nsco_20191108_BC_ZIC_HILIC_H111_pepsin_B3.33636.33636.4	71.592	Pepsin	B3	S39	59
BCAL0678	>tr|A0A142PL18|A0A142PL18_9BURK Cell division protein OS=Burkholderia cenocepacia OX=95486 GN=A8E72_03155 PE=4 SV=1	S.KVAPPPADNGAS[+568.212]QPQQFDPNRALQGKTPGQPVPQAAQPAPPNTAPGQAA.N	HexNAc(2)Hex(1)	Yes	1362.4308	4	5446.7013	5446.6786	4.2	780.89	780.89	370.32	Nsco_20191108_BC_ZIC_HILIC_H111_pepsin_B3.34360.34360.4	73.518	Pepsin	B3	S39	60
BCAL0678	>tr|A0A142PL18|A0A142PL18_9BURK Cell division protein OS=Burkholderia cenocepacia OX=95486 GN=A8E72_03155 PE=4 SV=1	A.QKQQQQQQAANTPKPTSSAT[+568.212]AAA.A	HexNAc(2)Hex(1)	Partial	984.4789	3	2951.4222	2951.4076	4.9	274.7	274.7	13.8	Nsco_20191108_BC_ZIC_HILIC_H111_pepsin_B3.7849.7849.3	22.125	Pepsin	B3	NA	61
BCAL0678	>tr|A0A142PL18|A0A142PL18_9BURK Cell division protein OS=Burkholderia cenocepacia OX=95486 GN=A8E72_03155 PE=4 SV=1	K.QQQQQQAANTPKPTS[+568.212]SATAAAAAKPPTANDANTGYFLQVGAYK.T	HexNAc(2)Hex(1)	Yes	1244.1075	4	4973.4084	4973.3923	3.2	1214.1	1143.8	28.53	Nsco_20191108_BC_ZIC_HILIC_H111_typsin_B1.35749.35749.4	72.554	Trypsin	B1	S180	62
BCAL0678	>tr|A0A142PL18|A0A142PL18_9BURK Cell division protein OS=Burkholderia cenocepacia OX=95486 GN=A8E72_03155 PE=4 SV=1	Q.KQQQQQQAANTPKPTS[+568.212]SATAAAAAKPPTANDANTGYFLQVGAYKTEGDAEQQR.A	HexNAc(2)Hex(1)	Yes	1037.5025	6	6219.9785	6215.9879	-3.7	1196.8	1084.2	19.24	Nsco_20191108_BC_ZIC_HILIC_H111_typsin_B2.32751.32751.6	65.913	Trypsin	B2	S180	63

BCAL0678	>tr|A0A142PL18|A0A142PL18_9BURK Cell division protein OS=Burkholderia cenocepacia OX=95486 GN=A8E72_03155 PE=4 SV=1	Q.KQQQQQQAANTPKPTS[+568.212]SATAAAAAKPPTANDANTGYFLQVGAYK.T	HexNAc(2)Hex(1)	Yes	1021.5052	5	5103.4971	5101.4873	0.6	1343.4	1328.3	42.28	Nsco_20191108_BC_ZIC_HILIC_H111_typsin_B3.30947.30947.5	65.433	Trypsin	B3	S180	64
BCAL0749	>tr|A0A2N9D2V0|A0A2N9D2V0_9BURK Cytochrome c oxidase subunit 2 OS=Burkholderia cenocepacia OX=95486 GN=ctaC_1 PE=3 SV=1	A.GAATAAAPAEAASAPAQAAS[+568.212]GAEQPAAAAS[+568.212][+100.064]AALSTIYFETGK.S	HexNAc(2)Hex(1),HexNAc(2)Hex(1) 100.064	Partial	1676.4614	3	5027.3696	5026.3437	4.5	318.21	294.02	30.22	Nsco_20191108_BC_ZIC_HILIC_H111_typsin_B1.67136.67136.3	124.42	Trypsin	B1	NA	65
BCAL0749	>tr|A0A2N9D2V0|A0A2N9D2V0_9BURK Cytochrome c oxidase subunit 2 OS=Burkholderia cenocepacia OX=95486 GN=ctaC_1 PE=3 SV=1	G.KLPEDTAGAAT[+568.212][+100.064]AAAPAEAASAPAQAAS[+568.212]GAEQPAAAASAALSTIYFETGK.S	HexNAc(2)Hex(1) 100.064,HexNAc(2)Hex(1)	Yes (1 of 2)	1447.1952	4	5785.7589	5780.7299	2.1	233.5	217.8	21.97	Nsco_20191108_BC_ZIC_HILIC_H111_typsin_B2.63795.63795.4	123.3	Trypsin	B2	S410	66
BCAL0749	>tr|A0A2N9D2V0|A0A2N9D2V0_9BURK Cytochrome c oxidase subunit 2 OS=Burkholderia cenocepacia OX=95486 GN=ctaC_1 PE=3 SV=1	L.PEDTAGAATAAAPAEAASAPAQAASGAEQPAAAASAALS[+568.212][+100.064]T[+568.212][+100.064]IYFETGK.S	HexNAc(2)Hex(1) 100.064,HexNAc(2)Hex(1) 100.064	Yes (1 of 2)	1411.1638	4	5641.6335	5639.6148	2.1	379.32	178.79	11.1	Nsco_20191108_BC_ZIC_HILIC_H111_typsin_B3.58958.58958.4	120.69	Trypsin	B3	S410	67
BCAL0786	>tr|A0A1V2XQK6|A0A1V2XQK6_9BURK Membrane protein OS=Burkholderia cenocepacia OX=95486 GN=A8E72_26595 PE=4 SV=1	K.LLSVPAPAST[+568.212]EGDHHDK.-	HexNAc(2)Hex(1)	Yes	586.2802	4	2342.0991	2342.0882	4.7	445.92	357.63	52.5	Nsco_20191108_BC_ZIC_HILIC_H111_typsin_B2.15426.15426.4	**35.4**	Trypsin	B2	S80	68
BCAL1081	>tr|A0A1V2XQC3|A0A1V2XQC3_9BURK Multidrug efflux system membrane fusion protein OS=Burkholderia cenocepacia OX=95486 GN=mdtA PE=3 SV=1	K.RYQTLLSQDSIAS[+568.212]QTVDTQASLVK.Q	HexNAc(2)Hex(1)	Yes	1074.5406	3	3221.6074	3220.5955	2.7	420.73	382.31	53.27	Nsco_20191108_BC_ZIC_HILIC_H111_typsin_B3.39616.39616.3	82.427	Trypsin	B3	S183	69
BCAL1086	>tr|A0A107L3U1|A0A107L3U1_9BURK Putative lipoprotein OS=Burkholderia cenocepacia OX=95486 GN=A8E72_07105 PE=4 SV=1	K.LQQWSQQSAAGAKPAS[+568.212]GE.-	HexNAc(2)Hex(1)	Yes	1206.5597	2	2412.1122	2412.1049	3	260.84	151.11	64.75	Nsco_20191108_BC_ZIC_HILIC_H111_typsin_B1.15214.15214.2	36.547	Trypsin	B1	S140	70
BCAL1086	>tr|A0A107L3U1|A0A107L3U1_9BURK Putative lipoprotein OS=Burkholderia cenocepacia OX=95486 GN=A8E72_07105 PE=4 SV=1	K.KLQQWSQQS[+568.212]AAGAKPASGE.-	HexNAc(2)Hex(1)	Yes	1270.6075	2	2540.2077	2540.1998	3.1	311.96	187.6	187.6	Nsco_20191108_BC_ZIC_HILIC_H111_typsin_B3.11478.11478.2	29.282	Trypsin	B3	S140	71
BCAL1086	>tr|A0A107L3U1|A0A107L3U1_9BURK Putative lipoprotein OS=Burkholderia cenocepacia OX=95486 GN=A8E72_07105 PE=4 SV=1	K.ALDQVAST[+568.212]VNQQINAAK.A	HexNAc(2)Hex(1)	Yes	1170.0812	2	2339.1552	2339.146	3.9	468.08	292.44	38.51	Nsco_20191108_BC_ZIC_HILIC_H111_typsin_B3.35313.35313.2	**73.921**	Trypsin	B3	S63	72
BCAL1093	>tr|A0A2N9CGE7|A0A2N9CGE7_9BURK Signal peptide transmembrane protein OS=Burkholderia cenocepacia OX=95486 GN=DFS07_119133 PE=4 SV=1	K.HAYDEAHPAEAAS[+568.212]AASH.-	HexNAc(2)Hex(1)	Yes	768.6602	3	2303.9659	2302.9582	1.9	353.8	272.78	95.02	Nsco_20191108_BC_ZIC_HILIC_H111_typsin_B2.7957.7957.3	23.545	Trypsin	B2	S49	73
BCAL1389	>tr|A0A2N9CG52|A0A2N9CG52_9BURK Cyclic di-GMP-binding protein OS=Burkholderia cenocepacia OX=95486 GN=bcsB PE=3 SV=1	P.AVPAAAS[+568.212]T[+568.212][+100.064]AAAGHAAAADVAPAAGPVAAPAAASAPAAGLPATTVHVPFASLGAFDPLR.L	HexNAc(2)Hex(1),HexNAc(2)Hex(1) 100.064	Yes (1 of 2)	1598.0516	4	6389.1847	6389.1838	0.1	770.16	495.83	33.82	Nsco_20191108_BC_ZIC_HILIC_H111_typsin_B2.65504.65504.4	127.09	Trypsin	B2	S62	74
BCAL1389	>tr|A0A2N9CG52|A0A2N9CG52_9BURK Cyclic di-GMP-binding protein OS=Burkholderia cenocepacia OX=95486 GN=bcsB PE=3 SV=1	A.APMPAVPAAAST[+568.212]AAAGHAAAADVAPAAGPVAAPAAAS[+568.212]APAAGLPATTVHVPFASLGAFDPLR.L	HexNAc(2)Hex(1),HexNAc(2)Hex(1)	Yes (1 of 2)	1672.0869	4	6685.3257	6685.3029	3.4	1064.7	1061.8	159.46	Nsco_20191108_BC_ZIC_HILIC_H111_typsin_B2.67298.67298.4	131.09	Trypsin	B2	S62	75

BCAL1496	>tr|A0A0M1IFG9|A0A0M1IFG9_9BURK Signal peptide protein OS=Burkholderia cenocepacia OX=95486 GN=A8F55_16750 PE=4 SV=1	K.KAGKKAKAADAAS[+568.212]Q.-	HexNAc(2)Hex(1)	Yes	638.3309	3	1912.9781	1912.971	3.8	431.43	251.88	251.88	Nsco_20191108_BC_ZIC_HILIC_H111_pepsin_B1.2102.2102.3	**13.295**	Pepsin	B1	S90	76
BCAL1496	>tr|A0A0M1IFG9|A0A0M1IFG9_9BURK Signal peptide protein OS=Burkholderia cenocepacia OX=95486 GN=A8F55_16750 PE=4 SV=1	H.KAAAKKAGKKAKAADAAS[+568.212]Q.-	HexNAc(2)Hex(1)	Yes	596.3257	4	2382.281	2382.2722	3.7	638.78	549.42	549.42	Nsco_20191108_BC_ZIC_HILIC_H111_pepsin_B2.2867.2867.4	14.063	Pepsin	B2	S90	77
BCAL1496	>tr|A0A0M1IFG9|A0A0M1IFG9_9BURK Signal peptide protein OS=Burkholderia cenocepacia OX=95486 GN=A8F55_16750 PE=4 SV=1	G.KKAKAADAAS[+568.212]Q.-	HexNAc(2)Hex(1)	Yes	552.946	3	1656.8236	1656.8174	3.7	308.48	196.93	196.93	Nsco_20191108_BC_ZIC_HILIC_H111_pepsin_B3.2523.2523.3	13.147	Pepsin	B3	S90	78
BCAL1496	>tr|A0A0M1IFG9|A0A0M1IFG9_9BURK Signal peptide protein OS=Burkholderia cenocepacia OX=95486 GN=A8F55_16750 PE=4 SV=1	A.KKAGKKAKAADAAS[+568.212]Q.-	HexNAc(2)Hex(1)	Yes	511.0242	4	2041.0751	2041.0659	4.5	308.02	306.43	306.43	Nsco_20191108_BC_ZIC_HILIC_H111_pepsin_B3.2614.2614.4	13.491	Pepsin	B3	S90	79
BCAL1496	>tr|A0A0M1IFG9|A0A0M1IFG9_9BURK Signal peptide protein OS=Burkholderia cenocepacia OX=95486 GN=A8F55_16750 PE=4 SV=1	A.AAKKAGKKAKAADAAS[+568.212]Q.-	HexNAc(2)Hex(1)	Yes	546.5425	4	2183.1482	2183.1401	3.7	645.31	568.86	568.86	Nsco_20191108_BC_ZIC_HILIC_H111_thermolysin_B1.2030.2030.4	13.248	Thermolysin	B1	S90	80
BCAL1496	>tr|A0A0M1IFG9|A0A0M1IFG9_9BURK Signal peptide protein OS=Burkholderia cenocepacia OX=95486 GN=A8F55_16750 PE=4 SV=1	K.AAAKKAGKKAKAADAAS[+568.212]Q.-	HexNAc(2)Hex(1)	Yes	564.3017	4	2254.1848	2254.1773	3.3	621.5	587.33	587.33	Nsco_20191108_BC_ZIC_HILIC_H111_thermolysin_B2.2380.2380.4	13.86	Thermolysin	B2	S90	81
BCAL1496	>tr|A0A0M1IFG9|A0A0M1IFG9_9BURK Signal peptide protein OS=Burkholderia cenocepacia OX=95486 GN=A8F55_16750 PE=4 SV=1	K.AKAADAAS[+568.212]Q.-	HexNAc(2)Hex(1)	Yes	700.819	2	1400.6308	1400.6275	2.4	314.51	138.87	138.87	Nsco_20191108_BC_ZIC_HILIC_H111_typsin_B1.2165.2165.2	14.283	Trypsin	B1	S90	82
BCAL1674	>tr|A0A2N9CH15|A0A2N9CH15_9BURK Multidrug efflux system OS=Burkholderia cenocepacia OX=95486 GN=acrA PE=3 SV=1	Q.FAPDTAVKPVEKAPPS[+568.212]KAAPPAAASQA.A	HexNAc(2)Hex(1)	Yes	1062.88	3	3186.6255	3185.61	3.8	693.94	656.34	21.6	Nsco_20191108_BC_ZIC_HILIC_H111_thermolysin_B2.20980.20980.3	45.3	Thermolysin	B2	S404	83
BCAL1746	>tr|A0A1V2Y428|A0A1V2Y428_9BURK Uncharacterized protein OS=Burkholderia cenocepacia OX=95486 GN=A8F55_03700 PE=4 SV=1	L.IDHIGKAWPGNAAS[+568.212]GASASE.-	HexNAc(2)Hex(1)	Yes	836.3885	3	2507.1508	2507.142	3.5	313.93	234.39	59.12	Nsco_20191108_BC_ZIC_HILIC_H111_pepsin_B3.22612.22612.3	46.702	Pepsin	B3	S140	84
BCAL1917	>tr|A0A2N9CHU5|A0A2N9CHU5_9BURK Uncharacterized protein OS=Burkholderia cenocepacia OX=95486 GN=F01_210059 PE=4 SV=1	T.AAPAPTAS[+568.212]APEAAAKPAKTKRASKKEK.A	HexNAc(2)Hex(1)	Yes	649.7541	5	3244.7412	3244.7271	4.4	796.81	784.32	121.44	Nsco_20191108_BC_ZIC_HILIC_H111_thermolysin_B1.3500.3500.5	17.346	Thermolysin	B1	S151	85
BCAL1917	>tr|A0A2N9CHU5|A0A2N9CHU5_9BURK Uncharacterized protein OS=Burkholderia cenocepacia OX=95486 GN=F01_210059 PE=4 SV=1	T.AAPAPTAS[+568.212]APEAAAKPAKTKRASKKEKA.A	HexNAc(2)Hex(1)	Yes	663.9616	5	3315.7788	3315.7642	4.4	923.36	897.24	145.29	Nsco_20191108_BC_ZIC_HILIC_H111_thermolysin_B1.3693.3693.5	17.662	Thermolysin	B1	S151	86
BCAL1917	>tr|A0A2N9CHU5|A0A2N9CHU5_9BURK Uncharacterized protein OS=Burkholderia cenocepacia OX=95486 GN=F01_210059 PE=4 SV=1	T.AAPAPTAS[+568.212]APEAAAKPAKTKRASKKEKAAAA.A	HexNAc(2)Hex(1)	Yes	882.9772	4	3528.887	3528.8755	3.3	580.06	580.06	133.19	Nsco_20191108_BC_ZIC_HILIC_H111_thermolysin_B1.4372.4372.4	18.616	Thermolysin	B1	S151	87
BCAL1917	>tr|A0A2N9CHU5|A0A2N9CHU5_9BURK Uncharacterized protein OS=Burkholderia cenocepacia OX=95486 GN=F01_210059 PE=4 SV=1	T.AAPAPTAS[+568.212]APEAAAKPAKTKRAS.K	HexNAc(2)Hex(1)	Yes	683.6077	4	2731.4092	2731.3996	3.5	695.41	663.67	96.64	Nsco_20191108_BC_ZIC_HILIC_H111_thermolysin_B2.5786.5786.4	20.941	Thermolysin	B2	S151	88

BCAL1917	>tr|A0A2N9CHU5|A0A2N9CHU5_9BURK Uncharacterized protein OS=Burkholderia cenocepacia OX=95486 GN=F01_210059 PE=4 SV=1	T.ATAGTTTAAPAPTAS[+568.212]APEAAAKPAKTKR.A	HexNAc(2)Hex(1)	Yes	1059.8811	3	3177.6288	3176.6169	2.7	165.83	165.83	32.16	Nsco_20191108_BC_ZIC_HILIC_H111_thermolysin_B2.9223.9223.3	25.928	Thermolysin	B2	S151	89
BCAL1917	>tr|A0A2N9CHU5|A0A2N9CHU5_9BURK Uncharacterized protein OS=Burkholderia cenocepacia OX=95486 GN=F01_210059 PE=4 SV=1	T.AAPAPTAS[+568.212]APEAAAKPAKTKRASKKEKAAA.A	HexNAc(2)Hex(1)	Yes	692.3761	5	3457.8512	3457.8384	3.7	749.03	741.46	142.57	Nsco_20191108_BC_ZIC_HILIC_H111_thermolysin_B1.4098.4098.5	18.251	Thermolysin	B1	S151	90
BCAL1917	>tr|A0A2N9CHU5|A0A2N9CHU5_9BURK Uncharacterized protein OS=Burkholderia cenocepacia OX=95486 GN=F01_210059 PE=4 SV=1	T.AAPAPTAS[+568.212]APEAAAKPAKTKRASKKEKAA.A	HexNAc(2)Hex(1)	Yes	847.4583	4	3386.8115	3386.8013	3	800.88	800.88	134.79	Nsco_20191108_BC_ZIC_HILIC_H111_thermolysin_B2.3946.3946.4	17.862	Thermolysin	B2	S151	91
BCAL1917	>tr|A0A2N9CHU5|A0A2N9CHU5_9BURK Uncharacterized protein OS=Burkholderia cenocepacia OX=95486 GN=F01_210059 PE=4 SV=1	T.AAPAPTAS[+568.212]APEAAAKPAKTKR.A	HexNAc(2)Hex(1)	Yes	644.0901	4	2573.3386	2573.3305	3.2	915.51	699.84	132.94	Nsco_20191108_BC_ZIC_HILIC_H111_thermolysin_B2.5666.5666.4	20.778	Thermolysin	B2	S151	92
BCAL1917	>tr|A0A2N9CHU5|A0A2N9CHU5_9BURK Uncharacterized protein OS=Burkholderia cenocepacia OX=95486 GN=F01_210059 PE=4 SV=1	T.STTATAGTTTAAPAPT[+568.212]ASAPEAAAKPAK.T	HexNAc(2)Hex(1)	Yes	1027.5093	3	3080.5134	3080.5005	4.2	353.44	288.43	42.43	Nsco_20191108_BC_ZIC_HILIC_H111_typsin_B1.15164.15164.3	36.476	Trypsin	B1	S151	93
BCAL1917	>tr|A0A2N9CHU5|A0A2N9CHU5_9BURK Uncharacterized protein OS=Burkholderia cenocepacia OX=95486 GN=F01_210059 PE=4 SV=1	A.TAGTTTAAPAPTAS[+568.212]APEAAAKPAK.T	HexNAc(2)Hex(1)	Yes	907.4542	3	2720.348	2720.336	4.4	344.84	260.24	49.3	Nsco_20191108_BC_ZIC_HILIC_H111_typsin_B2.13385.13385.3	32.05	Trypsin	B2	S151	94
BCAL1917	>tr|A0A2N9CHU5|A0A2N9CHU5_9BURK Uncharacterized protein OS=Burkholderia cenocepacia OX=95486 GN=F01_210059 PE=4 SV=1	T.SAGTASTSTTATAGTTTAAPAPTAS+568.212]APEAAAKPAK.T	HexNAc(2)Hex(1)	Yes	1219.262	3	3655.7715	3655.7556	4.3	335.99	302.12	43.51	Nsco_20191108_BC_ZIC_HILIC_H111_typsin_B2.19049.19049.3	41.427	Trypsin	B2	S151	95
BCAL1917	>tr|A0A2N9CHU5|A0A2N9CHU5_9BURK Uncharacterized protein OS=Burkholderia cenocepacia OX=95486 GN=F01_210059 PE=4 SV=1	S.KPAATTSATTSTTTSAGTASTSTTAGTTTAAPAPTAS[+568.212]APEAAAKPAK.T	HexNAc(2)Hex(1)	Yes	1244.8641	4	4976.4347	4975.4125	3.8	454.51	454.51	25.96	Nsco_20191108_BC_ZIC_HILIC_H111_typsin_B2.20241.20241.4	43.54	Trypsin	B2	S151	96
BCAL1917	>tr|A0A2N9CHU5|A0A2N9CHU5_9BURK Uncharacterized protein OS=Burkholderia cenocepacia OX=95486 GN=F01_210059 PE=4 SV=1	T.TSATTSTTTSAGTASTS[+568.212][+100.064]TTATAGTTTAAPAPT[+568.212][+100.064]ASAPEAAAKPAK.T	HexNAc(2)Hex(1) 100.064,HexNAc(2)Hex(1) 100.064	Yes (1 of 2)	1319.631	4	5275.5021	5275.4824	3.7	225.08	225.08	24.52	Nsco_20191108_BC_ZIC_HILIC_H111_typsin_B2.43074.43074.4	84.038	Trypsin	B2	S151	97
BCAL1917	>tr|A0A2N9CHU5|A0A2N9CHU5_9BURK Uncharacterized protein OS=Burkholderia cenocepacia OX=95486 GN=F01_210059 PE=4 SV=1	A.GTASTSTTATAGTTTAAPAPT[+568.212]ASAPEAAAKPAK.T	HexNAc(2)Hex(1)	Yes	1166.5723	3	3497.7024	3497.6865	4.6	317.9	257.84	44.9	Nsco_20191108_BC_ZIC_HILIC_H111_typsin_B3.17816.17816.3	40.101	Trypsin	B3	S151	98
BCAL1917	>tr|A0A2N9CHU5|A0A2N9CHU5_9BURK Uncharacterized protein OS=Burkholderia cenocepacia OX=95486 GN=F01_210059 PE=4 SV=1	K.LSKPAATTSATTSTTTSAGTASTSTTAGTTTAAPAPTAS[+568.212]APEAAAK.P	HexNAc(2)Hex(1)	Partial	1220.5935	4	4879.3524	4879.3437	1.8	758.27	736.58	79.08	Nsco_20191108_BC_ZIC_HILIC_H111_typsin_B2.25663.25663.4	53.316	Trypsin	B2	NA	99
BCAL1917	>tr|A0A2N9CHU5|A0A2N9CHU5_9BURK Uncharacterized protein OS=Burkholderia cenocepacia OX=95486 GN=F01_210059 PE=4 SV=1	K.PAATTSATTSTTTSAGTASTSTTATGTTTAAPAPT[+568.212]ASAPEAAAKPAK.T	HexNAc(2)Hex(1)	Partial	1616.4455	3	4847.3219	4847.3175	0.9	324.17	249.96	9.1	Nsco_20191108_BC_ZIC_HILIC_H111_typsin_B2.26810.26810.3	55.497	Trypsin	B2	NA	100

BCAL1917	>tr|A0A2N9CHU5|A0A2N9CHU5_9BURK Uncharacterized protein OS=Burkholderia cenocepacia OX=95486 GN=F01_210059 PE=4 SV=1	A.STSTTATAGTTTAAPAPT[+568.212]ASAPEAAAKPAK.T	HexNAc(2)Hex(1)	Yes	1090.2005	3	3268.5869	3268.5802	2	292.48	253.17	40.26	Nsco_20191108_BC_ZIC_HILIC_H111_typsin_B3.16579.16579.3	37.887	Trypsin	B3	S151	101
BCAL1917	>tr|A0A2N9CHU5|A0A2N9CHU5_9BURK Uncharacterized protein OS=Burkholderia cenocepacia OX=95486 GN=F01_210059 PE=4 SV=1	T.STTTSAGTASTSTTATAGTTTAAPAT[+568.212]ASAPEAAAKPAK.T	HexNAc(2)Hex(1)	Yes	1349.3188	3	4045.9418	4045.9307	2.7	382.44	294.2	50.7	Nsco_20191108_BC_ZIC_HILIC_H111_typsin_B3.20202.20202.3	44.553	Trypsin	B3	S151	102
BCAL1917	>tr|A0A2N9CHU5|A0A2N9CHU5_9BURK Uncharacterized protein OS=Burkholderia cenocepacia OX=95486 GN=F01_210059 PE=4 SV=1	A.TTSTTTSAGTASTSTTATAGTTTAAAPT[+568.212]ASAPEAAAKPAK.T	HexNAc(2)Hex(1)	Yes	1417.6876	3	4251.0483	4248.026	2.9	231.98	192.01	32.92	Nsco_20191108_BC_ZIC_HILIC_H111_typsin_B3.20807.20807.3	45.733	Trypsin	B3	S151	103
BCAL1917	>tr|A0A2N9CHU5|A0A2N9CHU5_9BURK Uncharacterized protein OS=Burkholderia cenocepacia OX=95486 GN=F01_210059 PE=4 SV=1	S.ATTSTTTSAGTASTSTTATAGTTTAAPT[+568.212]ASAPEAAAKPAK.T	HexNAc(2)Hex(1)	Yes	1440.3658	3	4319.0828	4319.0631	4.5	151.41	115.69	115.69	Nsco_20191108_BC_ZIC_HILIC_H111_typsin_B3.21063.21063.3	46.286	Trypsin	B3	S151	104
BCAL1917	>tr|A0A2N9CHU5|A0A2N9CHU5_9BURK Uncharacterized protein OS=Burkholderia cenocepacia OX=95486 GN=F01_210059 PE=4 SV=1	K.SVGHLEENGLT[+568.212]IGGASTPPK.G	HexNAc(2)Hex(1)	Yes	844.7475	3	2532.2279	2532.2199	3.2	233.62	164.31	11.1	Nsco_20191108_BC_ZIC_HILIC_H111_typsin_B3.25307.25307.3	54.409	Trypsin	B3	S104	105
BCAL1917	>tr|A0A2N9CHU5|A0A2N9CHU5_9BURK Uncharacterized protein OS=Burkholderia cenocepacia OX=95486 GN=F01_210059 PE=4 SV=1	K.LSKPAATTSATTSTTTSAGTASTSTTTAGTTTAAPAPTAS[+568.212]APEAAAKPAK.T	HexNAc(2)Hex(1)	Partial	1319.6501	4	5275.5788	5275.5926	-2.6	591.05	591.05	205.17	Nsco_20191108_BC_ZIC_HILIC_H111_typsin_B3.75172.75172.4	160.88	Trypsin	B3	NA	106
BCAL2107	>tr|A0A2N9CIG6|A0A2N9CIG6_9BURK Fused putative transporter subunits of ABC superfamily: ATP-binding components OS=Burkholderia cenocepacia OX=95486 GN=yheS PE=4 SV=1	A.DS[+568.212]AAAK.A	HexNAc(2)Hex(1)	Yes	565.7523	2	1130.4974	1130.4947	2.4	241.82	23.12	23.12	Nsco_20191108_BC_ZIC_HILIC_H111_typsin_B1.1961.1961.2	13.168	Trypsin	B1	NA	107
BCAL2161	>tr|A0A2N9CIP1|A0A2N9CIP1_9BURK Putative exported protein OS=Burkholderia cenocepacia OX=95486 GN=F01_230051 PE=4 SV=1	S.VAPPLQGDGAAPGGAS[+568.212]WPAPPPASGPAPGLPASSVQGT[+568.212]P.-	HexNAc(2)Hex(1),HexNAc(2)Hex(1)	Yes (1 of 2)	1541.0685	3	4621.1911	4621.1727	4	465.76	361.02	34.77	Nsco_20191108_BC_ZIC_HILIC_H111_thermolysin_B1.62166.62166.3	117.73	Thermolysin	B1	S391	108
BCAL2276	>tr|A0A2N9CJ70|A0A2N9CJ70_9BURK Putative membrane protein OS=Burkholderia cenocepacia OX=95486 GN=DFS07_11429 PE=4 SV=1	K.STIDTAASNAGVPVS[+568.212]SVNYIVHDAGK.G	HexNAc(2)Hex(1)	Yes	1048.1735	3	3142.5059	3141.4958	2.2	182.01	146.66	18.54	Nsco_20191108_BC_ZIC_HILIC_H111_typsin_B1.45598.45598.3	89.504	Trypsin	B1	S353	109
BCAL2398	>tr|A0A2N9CKD9|A0A2N9CKD9_9BURK Periplasmic protein TonB, links inner and outer membranes OS=Burkholderia cenocepacia OX=95486 GN=F01_260105 PE=4 SV=1	K.ASPPYAADKPIVAVFPPVVPAAPAS[+568.212]SASATR.-	HexNAc(2)Hex(1)	Yes	1191.9487	3	3573.8314	3573.8211	2.9	382.56	364.3	20.38	Nsco_20191108_BC_ZIC_HILIC_H111_typsin_B2.54241.54241.3	104.04	Trypsin	B2	S315	110
BCAL2449	>tr|A0A144SYR9|A0A144SYR9_9BURK Diguanylate cyclase (GGDEF) domain protein OS=Burkholderia cenocepacia OX=95486 GN=gmr_4 PE=4 SV=1	R.RTGSVNNAPGAFS[+568.212]ASGVYPIAER.V	HexNAc(2)Hex(1)	Yes	963.8006	3	2889.3871	2889.3749	4.3	382.29	269.92	29.81	Nsco_20191108_BC_ZIC_HILIC_H111_typsin_B2.32822.32822.3	66.025	Trypsin	B2	S254	111
BCAL2466	>tr|A0A144SXW2|A0A144SXW2_9BURK Ecotin OS=Burkholderia cenocepacia OX=95486 GN=eco PE=4 SV=1	A.APASAPAVPAES[+568.212]IKMFPQ.A	HexNAc(2)Hex(1)	Yes	1190.5817	2	2380.1561	2380.1476	3.6	360.93	166	166	Nsco_20191108_BC_ZIC_HILIC_H111_thermolysin_B1.47928.47928.2	89.914	Thermolysin	B1	S21	112

<table>
<tr><td>BCAL2466</td><td>>tr|A0A144SXW2|A0A144SXW2_9BURK Ecotin OS=Burkholderia cenocepacia OX=95486 GN=eco PE=4 SV=1</td><td>A.APASAPAVPAES[+568.212]IKMFPQA.A</td><td>HexNAc(2)Hex(1)</td><td>Yes</td><td>1226.5984</td><td>2</td><td>2452.1896</td><td>2451.1847</td><td>0.6</td><td>272.18</td><td>109.18</td><td>109.18</td><td>Nsco_20191108_BC_ZIC_HILIC_H111_thermolysin_B1.49472.49472.2</td><td>92.599</td><td>Thermolysin</td><td>B1</td><td>S21</td><td>113</td></tr>
<tr><td>BCAL2466</td><td>>tr|A0A144SXW2|A0A144SXW2_9BURK Ecotin OS=Burkholderia cenocepacia OX=95486 GN=eco PE=4 SV=1</td><td>A.APASAPAVPAES[+568.212]IKMFPQAAAGQQR.V</td><td>HexNAc(2)Hex(1)</td><td>Yes</td><td>1021.8416</td><td>3</td><td>3063.5102</td><td>3062.4987</td><td>2.7</td><td>298.81</td><td>255.28</td><td>113.68</td><td>Nsco_20191108_BC_ZIC_HILIC_H111_typsin_B2.38632.38632.3</td><td>76.266</td><td>Trypsin</td><td>B2</td><td>S21</td><td>114</td></tr>
<tr><td>BCAL2607</td><td>>tr|A0A1V2WZX0|A0A1V2WZX0_9BURK DNA repair ATPase OS=Burkholderia cenocepacia OX=95486 GN=F01_260195 PE=4 SV=1</td><td>M.KERWEQHRAAAKGAS[+568.212]APAQ.-</td><td>HexNAc(2)Hex(1)</td><td>Yes</td><td>665.8308</td><td>4</td><td>2660.3013</td><td>2660.2911</td><td>3.8</td><td>659.69</td><td>477.63</td><td>477.63</td><td>Nsco_20191108_BC_ZIC_HILIC_H111_pepsin_B2.4597.4597.4</td><td>17.427</td><td>Pepsin</td><td>B2</td><td>S180</td><td>115</td></tr>
<tr><td>BCAL2640</td><td>>tr|A0A144SWF6|A0A144SWF6_9BURK Putative exported protein OS=Burkholderia cenocepacia OX=95486 GN=A8E72_18590 PE=4 SV=1</td><td>R.GAADRYAPPPAAVPVAAT[+568.212]SGAQGGAAAAAAPAGTKPANAPR.E</td><td>HexNAc(2)Hex(1)</td><td>Partial</td><td>1052.5328</td><td>4</td><td>4207.1092</td><td>4207.0902</td><td>4.5</td><td>473.05</td><td>473.05</td><td>26.56</td><td>Nsco_20191108_BC_ZIC_HILIC_H111_typsin_B1.29090.29090.4</td><td>60.942</td><td>Trypsin</td><td>B1</td><td>NA</td><td>116</td></tr>
<tr><td>BCAL2640</td><td>>tr|A0A144SWF6|A0A144SWF6_9BURK Putative exported protein OS=Burkholderia cenocepacia OX=95486 GN=A8E72_18590 PE=4 SV=1</td><td>R.YAPPPAAVPVAATSGAQGGAAAAAAPAGT[+568.212]KPANAPREPAVRR.V</td><td>HexNAc(2)Hex(1)</td><td>Yes</td><td>890.0622</td><td>5</td><td>4446.282</td><td>4445.2695</td><td>2</td><td>378.48</td><td>378.48</td><td>33.62</td><td>Nsco_20191108_BC_ZIC_HILIC_H111_typsin_B3.25543.25543.5</td><td>54.935</td><td>Trypsin</td><td>B3</td><td>S165</td><td>117</td></tr>
<tr><td>BCAL2640</td><td>>tr|A0A144SWF6|A0A144SWF6_9BURK Putative exported protein OS=Burkholderia cenocepacia OX=95486 GN=A8E72_18590 PE=4 SV=1</td><td>R.GAADRYAPPPAAVPVAAT[+568.212]SGAQGGAAAAAAPAGTKPANAPREPAVR.R</td><td>HexNAc(2)Hex(1)</td><td>Yes</td><td>952.6888</td><td>5</td><td>4759.4149</td><td>4759.3922</td><td>4.8</td><td>918.5</td><td>887.07</td><td>40.6</td><td>Nsco_20191108_BC_ZIC_HILIC_H111_typsin_B3.28495.28495.5</td><td>60.698</td><td>Trypsin</td><td>B3</td><td>S165</td><td>118</td></tr>
<tr><td>BCAL2640</td><td>>tr|A0A144SWF6|A0A144SWF6_9BURK Putative exported protein OS=Burkholderia cenocepacia OX=95486 GN=A8E72_18590 PE=4 SV=1</td><td>R.YAPPPAAVPVAATSGAQGGAAAAAAPAGT[+568.212]KPANAPREPAVR.R</td><td>HexNAc(2)Hex(1)</td><td>Yes</td><td>1073.0513</td><td>4</td><td>4289.1832</td><td>4289.1684</td><td>3.5</td><td>611.4</td><td>611.4</td><td>13.02</td><td>Nsco_20191108_BC_ZIC_HILIC_H111_typsin_B2.30519.30519.4</td><td>62.009</td><td>Trypsin</td><td>B2</td><td>S165</td><td>119</td></tr>
<tr><td>BCAL2640</td><td>>tr|A0A144SWF6|A0A144SWF6_9BURK Putative exported protein OS=Burkholderia cenocepacia OX=95486 GN=A8E72_18590 PE=4 SV=1</td><td>R.GAADRYAPPPAAVPVAAT[+568.212]SGAQGGAAAAAAPAGTK.P</td><td>HexNAc(2)Hex(1)</td><td>Partial</td><td>1200.9278</td><td>3</td><td>3600.7688</td><td>3600.7664</td><td>0.7</td><td>394.34</td><td>293.58</td><td>4.05</td><td>Nsco_20191108_BC_ZIC_HILIC_H111_typsin_B2.32987.32987.5</td><td>66.318</td><td>Trypsin</td><td>B2</td><td>NA</td><td>120</td></tr>
<tr><td>BCAL2820</td><td>>tr|A0A1V2WC66|A0A1V2WC66_9BURK Multidrug transporter OS=Burkholderia cenocepacia OX=95486 GN=oprM PE=3 SV=1</td><td>D.YDKAAAPAPASAT[+568.212]ATNG.-</td><td>HexNAc(2)Hex(1)</td><td>Yes</td><td>1072.9942</td><td>2</td><td>2144.9811</td><td>2144.9717</td><td>4.4</td><td>309.82</td><td>162.79</td><td>97.49</td><td>Nsco_20191108_BC_ZIC_HILIC_H111_pepsin_B1.12911.12911.2</td><td>31.741</td><td>Pepsin</td><td>B1</td><td>S501</td><td>121</td></tr>
<tr><td>BCAL2820</td><td>>tr|A0A1V2WC66|A0A1V2WC66_9BURK Multidrug transporter OS=Burkholderia cenocepacia OX=95486 GN=oprM PE=3 SV=1</td><td>A.LGGGWIQRAGETPRAPDAPVDYDKAAAPAPAS[+568.212]ATATNG.-</td><td>HexNAc(2)Hex(1)</td><td>Yes</td><td>1073.021</td><td>4</td><td>4289.0623</td><td>4289.048</td><td>3.3</td><td>572.87</td><td>533.13</td><td>60.25</td><td>Nsco_20191108_BC_ZIC_HILIC_H111_thermolysin_B1.40111.40111.4</td><td>76.557</td><td>Thermolysin</td><td>B1</td><td>S501</td><td>122</td></tr>
<tr><td>BCAL2820</td><td>>tr|A0A1V2WC66|A0A1V2WC66_9BURK Multidrug transporter OS=Burkholderia cenocepacia OX=95486 GN=oprM PE=3 SV=1</td><td>P.VDYDKAAAPAPAS[+568.212]ATATNG.-</td><td>HexNAc(2)Hex(1)</td><td>Yes</td><td>1180.5431</td><td>2</td><td>2360.0789</td><td>2359.0671</td><td>3.6</td><td>376.57</td><td>201.95</td><td>75.8</td><td>Nsco_20191108_BC_ZIC_HILIC_H111_thermolysin_B2.20923.20923.2</td><td>45.214</td><td>Thermolysin</td><td>B2</td><td>S501</td><td>123</td></tr>
<tr><td>BCAL2820</td><td>>tr|A0A1V2WC66|A0A1V2WC66_9BURK Multidrug transporter OS=Burkholderia cenocepacia OX=95486 GN=oprM PE=3 SV=1</td><td>W.IQRAGETPRAPDAPVDYDKAAAPAPAS[+568.212]ATATNG.-</td><td>HexNAc(2)Hex(1)</td><td>Yes</td><td>955.4648</td><td>4</td><td>3818.8372</td><td>3818.8203</td><td>4.4</td><td>569.12</td><td>554.4</td><td>74.73</td><td>Nsco_20191108_BC_ZIC_HILIC_H111_thermolysin_B3.25289.25289.4</td><td>53.253</td><td>Thermolysin</td><td>B3</td><td>S501</td><td>124</td></tr>
<tr><td>BCAL2820</td><td>>tr|A0A1V2WC66|A0A1V2WC66_9BURK Multidrug transporter OS=Burkholderia cenocepacia OX=95486 GN=oprM PE=3 SV=1</td><td>K.AAAPAPASAT[+568.212]ATNG.-</td><td>HexNAc(2)Hex(1)</td><td>Yes</td><td>869.9</td><td>2</td><td>1738.7927</td><td>1738.7865</td><td>3.5</td><td>211.91</td><td>115.51</td><td>82.44</td><td>Nsco_20191108_BC_ZIC_HILIC_H111_typsin_B1.11030.11030.2</td><td>29.902</td><td>Trypsin</td><td>B1</td><td>S501</td><td>125</td></tr>
<tr><td>BCAL2820</td><td>>tr|A0A1V2WC66|A0A1V2WC66_9BURK Multidrug transporter OS=Burkholderia cenocepacia OX=95486 GN=oprM PE=3 SV=1</td><td>K.RPDAPVAQAYPAS[+568.212]GVYATQPGAAGAR.S</td><td>HexNAc(2)Hex(1)</td><td>Yes</td><td>1037.5055</td><td>3</td><td>3110.502</td><td>3110.4913</td><td>3.5</td><td>605.08</td><td>488.25</td><td>177.02</td><td>Nsco_20191108_BC_ZIC_HILIC_H111_typsin_B1.26449.26449.3</td><td>56.523</td><td>Trypsin</td><td>B1</td><td>S39</td><td>126</td></tr>
</table>

BCAL2974	>tr|A0A144U3Q1|A0A144U3Q1_9BURK Uncharacterized protein OS=Burkholderia cenocepacia OX=95486 GN=A8E72_12460 PE=4 SV=1	M.PFAASAPS[+568.212]QKYQGSKKSAL.R	HexNAc(2)Hex(1)	Yes	845.4247	3	2534.2597	2534.2508	3.5	281.45	203.96	50.36	Nsco_20191108_BC_ZIC_HILIC_H111_pepsin_B1.12677.12677.3	31.412	Pepsin	B1	S91	127
BCAL2974	>tr|A0A144U3Q1|A0A144U3Q1_9BURK Uncharacterized protein OS=Burkholderia cenocepacia OX=95486 GN=A8E72_12460 PE=4 SV=1	F.RLNEHPQMPFAAS[+568.212]APSQ.K	HexNAc(2)Hex(1)	Yes	817.0482	3	2449.1302	2449.1188	4.7	170.39	100.54	34.72	Nsco_20191108_BC_ZIC_HILIC_H111_pepsin_B1.26118.26118.3	55.491	Pepsin	B1	S91	128
BCAL2974	>tr|A0A144U3Q1|A0A144U3Q1_9BURK Uncharacterized protein OS=Burkholderia cenocepacia OX=95486 GN=A8E72_12460 PE=4 SV=1	L.NEHPQMPFAASAPS[+568.212]QKYQG.S	HexNAc(2)Hex(1)	Yes	886.0625	3	2656.1728	2656.1719	0.4	286.11	251.7	13.48	Nsco_20191108_BC_ZIC_HILIC_H111_pepsin_B2.24696.24696.3	51.18	Pepsin	B2	S91	129
BCAL2974	>tr|A0A144U3Q1|A0A144U3Q1_9BURK Uncharacterized protein OS=Burkholderia cenocepacia OX=95486 GN=A8E72_12460 PE=4 SV=1	F.RLNEHPQMPFAAS[+568.212]APSQKYQGSKKSAL.R	HexNAc(2)Hex(1)	Yes	708.9552	5	3540.7467	3539.7323	3.1	1074.9	1068.2	124.4	Nsco_20191108_BC_ZIC_HILIC_H111_pepsin_B1.18696.18696.5	41.048	Pepsin	B1	S91	130
BCAL2974	>tr|A0A144U3Q1|A0A144U3Q1_9BURK Uncharacterized protein OS=Burkholderia cenocepacia OX=95486 GN=A8E72_12460 PE=4 SV=1	L.NEHPQMPFAAS[+568.212]APSQKYQGSKKSAL.R	HexNAc(2)Hex(1)	Yes	818.3957	4	3270.5608	3270.5471	4.2	1164.9	1137.3	171.47	Nsco_20191108_BC_ZIC_HILIC_H111_pepsin_B3.19176.19176.4	40.001	Pepsin	B3	S91	131
BCAL2974	>tr|A0A144U3Q1|A0A144U3Q1_9BURK Uncharacterized protein OS=Burkholderia cenocepacia OX=95486 GN=A8E72_12460 PE=4 SV=1	P.FAASAPSQKYQGS[+568.212]KKSA.L	HexNAc(2)Hex(1)	Yes	581.7866	4	2324.1247	2324.114	4.6	261.25	199.88	86.69	Nsco_20191108_BC_ZIC_HILIC_H111_thermolysin_B1.1703.1703.4	11.115	Thermolysin	B1	S91	132
BCAL2974	>tr|A0A144U3Q1|A0A144U3Q1_9BURK Uncharacterized protein OS=Burkholderia cenocepacia OX=95486 GN=A8E72_12460 PE=4 SV=1	R.LNEHPQMPFAASAPS[+568.212]QK.Y	HexNAc(2)Hex(1)	Yes	1211.0634	2	2421.1194	2421.1126	2.8	435.34	227.13	75.7	Nsco_20191108_BC_ZIC_HILIC_H111_typsin_B3.23325.23325.2	50.601	Trypsin	B3	S91	133
BCAL2983A	>tr|A0A2N9CL86|A0A2N9CL86_9BURK Putative lipoprotein OS=Burkholderia cenocepacia OX=95486 GN=F01_310136 PE=4 SV=1	S.ADASAPVAGTRPAVTSLSGGASSAASGAVAT[+568.212]DAAAQGNVAELTQMLHDGR.I	HexNAc(2)Hex(1)	Yes	1301.8806	4	5204.5006	5204.4772	4.5	369.95	369.95	5.1	Nsco_20191108_BC_ZIC_HILIC_H111_typsin_B3.53184.53184.5	108.58	Trypsin	B3	S55	134
BCAL3033	>tr|A0A2N9CM16|A0A2N9CM16_9BURK Outer-membrane lipoprotein carrier protein OS=Burkholderia cenocepacia OX=95486 GN=lolA PE=3 SV=1	Q.IVKAPAKGAS[+568.212]AAQAAPKPTDN.S	HexNAc(2)Hex(1)	Yes	644.5867	4	2575.3249	2574.3145	2.8	729.64	648.17	408.82	Nsco_20191108_BC_ZIC_HILIC_H111_pepsin_B3.10005.10005.4	25.473	Pepsin	B3	S68	135
BCAL3033	>tr|A0A2N9CM16|A0A2N9CM16_9BURK Outer-membrane lipoprotein carrier protein OS=Burkholderia cenocepacia OX=95486 GN=lolA PE=3 SV=1	Q.IVKAPAKGAS[+568.212]AAQAAPKPTDNSSGTF.V	HexNAc(2)Hex(1)	Yes	764.1379	4	3053.5298	3053.5161	4.5	1082.9	1032.6	506.95	Nsco_20191108_BC_ZIC_HILIC_H111_pepsin_B3.16451.16451.4	35.47	Pepsin	B3	S68	136
BCAL3033	>tr|A0A2N9CM16|A0A2N9CM16_9BURK Outer-membrane lipoprotein carrier protein OS=Burkholderia cenocepacia OX=95486 GN=lolA PE=3 SV=1	Q.IVKAPAKGAS[+568.212]AAQAAPKPTDNSSGTFV.F	HexNAc(2)Hex(1)	Yes	788.9051	4	3152.5986	3152.5845	4.5	805.9	805.9	378.72	Nsco_20191108_BC_ZIC_HILIC_H111_pepsin_B3.19759.19759.4	41.093	Pepsin	B3	S68	137
BCAL3033	>tr|A0A2N9CM16|A0A2N9CM16_9BURK Outer-membrane lipoprotein carrier protein OS=Burkholderia cenocepacia OX=95486 GN=lolA PE=3 SV=1	Q.IVKAPAKGAS[+568.212]AAQAAPKPTDNSSGT.F	HexNAc(2)Hex(1)	Yes	727.371	4	2906.4621	2906.4477	5	727.77	543.13	403.44	Nsco_20191108_BC_ZIC_HILIC_H111_thermolysin_B1.9738.9738.4	26.669	Thermolysin	B1	S68	138

BCAL3033	>tr|A0A2N9CM16|A0A2N9CM16_9BURK Outer-membrane lipoprotein carrier protein OS=Burkholderia cenocepacia OX=95486 GN=lolA PE=3 SV=1	Q.IVKAPAKGAS[+568.212]AAQAAPKPTDNSSGTFVF.A	HexNAc(2)Hex(1)	Yes	825.6715	4	3299.6643	3299.6529	3.4	451.66	424.44	313.42	Nsco_20191108_BC_ZIC_HILIC_H111_thermolysin_B3.26971.26971.4	55.985	Thermolysin	B3	S68	139
BCAL3033	>tr|A0A2N9CM16|A0A2N9CM16_9BURK Outer-membrane lipoprotein carrier protein OS=Burkholderia cenocepacia OX=95486 GN=lolA PE=3 SV=1	K.GAS[+568.212]AAQAAPKPTDNSSGTFVFARPGK.F	HexNAc(2)Hex(1)	Yes	776.3766	4	3102.4846	3101.4909	-3.1	376.92	362.3	362.3	Nsco_20191108_BC_ZIC_HILIC_H111_typsin_B3.24977.24977.4	53.711	Trypsin	B3	S68	140
BCAL3033	>tr|A0A2N9CM16|A0A2N9CM16_9BURK Outer-membrane lipoprotein carrier protein OS=Burkholderia cenocepacia OX=95486 GN=lolA PE=3 SV=1	K.GASAAQAAPKPT[+568.212]DNSSGTFVFAR.P	HexNAc(2)Hex(1)	Yes	940.448	3	2819.3293	2819.3218	2.7	537.54	363.25	188.19	Nsco_20191108_BC_ZIC_HILIC_H111_typsin_B3.26102.26102.3	56.04	Trypsin	B3	S68	141
BCAL3166	>tr|A0A1V2WZ65|A0A1V2WZ65_9BURK BON domain-containing protein OS=Burkholderia cenocepacia OX=95486 GN=A8E72_33585 PE=4 SV=1	A.TEAPAAATSAPKAAAKT[+568.212]AKKANRKLGY.A	HexNAc(2)Hex(1)	Yes	1085.9139	3	3255.7273	3254.7114	3.8	197.65	97.64	15.49	Nsco_20191108_BC_ZIC_HILIC_H111_pepsin_B3.12081.12081.3	28.676	Pepsin	B3	S40	142
BCAL3377	>tr|A0A142PIH6|A0A142PIH6_9BURK Outer membrane protein assembly factor BamE OS=Burkholderia cenocepacia OX=95486 GN=bamE PE=3 SV=1	D.IDGDRGGKKAKAAAAAKKAS[+568.212]EAAA.A	HexNAc(2)Hex(1)	Yes	565.6979	5	2824.4605	2824.4534	2.5	649.03	597.74	597.74	Nsco_20191108_BC_ZIC_HILIC_H111_pepsin_B2.4588.4588.5	17.418	Pepsin	B2	S145	143
BCAL3377	>tr|A0A142PIH6|A0A142PIH6_9BURK Outer membrane protein assembly factor BamE OS=Burkholderia cenocepacia OX=95486 GN=bamE PE=3 SV=1	A.AAAASPATVPAS[+568.212]GAAVDQDANAQAARAANRATNQ.V	HexNAc(2)Hex(1)	Yes	1249.5988	3	3746.7819	3746.77	3.2	149.53	95.59	48.93	Nsco_20191108_BC_ZIC_HILIC_H111_thermolysin_B1.28240.28240.3	55.852	Thermolysin	B1	S168	144
BCAL3377	>tr|A0A142PIH6|A0A142PIH6_9BURK Outer membrane protein assembly factor BamE OS=Burkholderia cenocepacia OX=95486 GN=bamE PE=3 SV=1	A.AASPATVPAS[+568.212]GAAVDQDANAQAARAANRATNQ.V	HexNAc(2)Hex(1)	Yes	1202.2402	3	3604.706	3604.6957	2.8	151.2	73.1	18.05	Nsco_20191108_BC_ZIC_HILIC_H111_thermolysin_B3.25905.25905.3	54.325	Thermolysin	B3	S168	145
BCAL3377	>tr|A0A142PIH6|A0A142PIH6_9BURK Outer membrane protein assembly factor BamE OS=Burkholderia cenocepacia OX=95486 GN=bamE PE=3 SV=1	A.AAASPATVPAS[+568.212]GAAVDQDANAQAARAANR.A	HexNAc(2)Hex(1)	Yes	1088.1919	3	3262.5612	3261.5466	3.5	183.94	140.81	140.81	Nsco_20191108_BC_ZIC_HILIC_H111_thermolysin_B2.22542.22542.3	48.016	Thermolysin	B2	S168	146
BCAL3377	>tr|A0A142PIH6|A0A142PIH6_9BURK Outer membrane protein assembly factor BamE OS=Burkholderia cenocepacia OX=95486 GN=bamE PE=3 SV=1	A.AAASPATVPAS[+568.212]GAAVDQDANAQAARAANRATNQ.V	HexNAc(2)Hex(1)	Yes	1225.9203	3	3675.7465	3675.7329	3.7	169.36	108.65	25.51	Nsco_20191108_BC_ZIC_HILIC_H111_thermolysin_B3.26466.26466.3	55.222	Thermolysin	B3	S168	147
BCAL3377	>tr|A0A142PIH6|A0A142PIH6_9BURK Outer membrane protein assembly factor BamE OS=Burkholderia cenocepacia OX=95486 GN=bamE PE=3 SV=1	K.KASEAAAAAS[+568.212]AAQAAAAASPATVPASGAAVDQDANAQAAR.A	HexNAc(2)Hex(1)	Yes	1172.5524	4	4687.1878	4687.1712	3.5	203.54	203.54	203.54	Nsco_20191108_BC_ZIC_HILIC_H111_typsin_B1.36344.36344.4	73.494	Trypsin	B1	S152	148
BCAL3377	>tr|A0A142PIH6|A0A142PIH6_9BURK Outer membrane protein assembly factor BamE OS=Burkholderia cenocepacia OX=95486 GN=bamE PE=3 SV=1	K.ASEAAAAASAAQAAAAASPAT[+568.212]VPAS[+568.212]GAAVDQDANAQAAR.A	HexNAc(2)Hex(1),HexNAc(2)Hex(1)	Yes (1 of 2)	1520.3688	3	4559.0919	4559.0762	3.4	519.3	454.69	61	Nsco_20191108_BC_ZIC_HILIC_H111_typsin_B2.43375.43375.3	84.583	Trypsin	B2	S145	149
BCAL3377	>tr|A0A142PIH6|A0A142PIH6_9BURK Outer membrane protein assembly factor BamE OS=Burkholderia cenocepacia OX=95486 GN=bamE PE=3 SV=1	A.SEAAAAASAAQAAAAASPAT[+568.212][+100.064]VPAS[+568.212]GAAVDQDANAQAAR.A	HexNAc(2)Hex(1) 100.064,HexNAc(2)Hex(1)	Yes	1340.6367	3	4019.8956	4019.8915	1	260.99	221.31	5.54	Nsco_20191108_BC_ZIC_HILIC_H111_typsin_B3.45346.45346.3	93.292	Trypsin	B3	S145	150

BCAL3377	>tr|A0A142PIH6|A0A142PIH6_9BURK Outer membrane protein assembly factor BamE OS=Burkholderia cenocepacia OX=95486 GN=bamE PE=3 SV=1	A.KKASEAAAAAS[+568.212]AAQAAAAASPAT[+568.212]VPASGAAVDQDANAQAAR.A	HexNAc(2)Hex(1),HexNAc(2)Hex(1)	Yes (1 of 2)	1204.5761	4	4815.2825	4815.2662	3.4	813.2	757.03	150.58	Nsco_20191108_BC_ZIC_HILIC_H111_typsin_B3.30336.30336.4	64.258	Trypsin	B3	S145	151
BCAL3377	>tr|A0A142PIH6|A0A142PIH6_9BURK Outer membrane protein assembly factor BamE OS=Burkholderia cenocepacia OX=95486 GN=bamE PE=3 SV=1	A.SAAQAAAAAS[+568.212]PAT[+568.212]VPAS[+568.212][+100.064]GAAVDQDANAQAAR.A	HexNAc(2)Hex(1),HexNAc(2)Hex(1),HexNAc(2)Hex(1) 100.064	Partial	1529.6973	3	4587.0774	4585.0545	3.5	201.77	105.38	0.38	Nsco_20191108_BC_ZIC_HILIC_H111_typsin_B3.42021.42021.3	87.039	Trypsin	B3	NA	152
BCAL3469	>tr|A0A142PJ18|A0A142PJ18_9BURK Cell division protein FtsL OS=Burkholderia cenocepacia OX=95486 GN=ftsL PE=3 SV=1	A.IDAPIPAS[+568.212]ADTAGKGKGGAR.-	HexNAc(2)Hex(1)	Yes	606.057	4	2421.206	2421.1991	2.9	540.75	477.57	169.63	Nsco_20191108_BC_ZIC_HILIC_H111_thermolysin_B2.12795.12795.4	31.856	Thermolysin	B2	S101	153
BCAM0505	>tr|A0A1V2WB29|A0A1V2WB29_9BURK BON domain protein OS=Burkholderia cenocepacia OX=95486 GN=osmY_2 PE=4 SV=1	A.QAS[+568.212]STDSGMASESNQPVTDTWITTKVKGELATTDGVKSTIISVKT.V	HexNAc(2)Hex(1)	Partial	1311.1262	4	5241.4831	5240.4897	-1.9	319.81	319.81	1.4	Nsco_20191108_BC_ZIC_HILIC_H111_thermolysin_B1.55291.55291.4	103.16	Thermolysin	B1	NA	154
BCAM0505	>tr|A0A1V2WB29|A0A1V2WB29_9BURK BON domain protein OS=Burkholderia cenocepacia OX=95486 GN=osmY_2 PE=4 SV=1	A.QASSTDSGMAS[+568.212]ESNQPVTDTWITTK.V	HexNAc(2)Hex(1)	Partial	1606.2012	2	3211.3952	3210.4002	-2.6	512.3	410.81	35.46	Nsco_20191108_BC_ZIC_HILIC_H111_typsin_B2.41051.41051.2	80.551	Trypsin	B2	NA	155
BCAM0505	>tr|A0A1V2WB29|A0A1V2WB29_9BURK BON domain protein OS=Burkholderia cenocepacia OX=95486 GN=osmY_2 PE=4 SV=1	Q.ASSTDSGMASES[+568.212]NQPVTDTWITTK.V	HexNAc(2)Hex(1)	Yes	1541.6788	2	3082.3502	3082.3416	2.8	589.8	443.36	24.29	Nsco_20191108_BC_ZIC_HILIC_H111_typsin_B2.39568.39568.2	77.91	Trypsin	B2	S32	156
BCAM0505	>tr|A0A1V2WB29|A0A1V2WB29_9BURK BON domain protein OS=Burkholderia cenocepacia OX=95486 GN=osmY_2 PE=4 SV=1	A.HAQASSTDSGMASES[+568.212]NQPVTDTWITTK.V	HexNAc(2)Hex(1)	Yes	1140.1757	3	3418.5125	3418.4962	4.8	520.24	516.19	35.19	Nsco_20191108_BC_ZIC_HILIC_H111_typsin_B3.29876.29876.3	63.391	Trypsin	B3	S32	157
BCAM0690	>tr|A0A1V2Y2A4|A0A1V2Y2A4_9BURK Flagellar motor protein OS=Burkholderia cenocepacia OX=95486 GN=A8F55_06330 PE=4 SV=1	R.ALIDAGVPASSVFAAAFGS[+568.212]EQPVSSNADDEGRAK.N	HexNAc(2)Hex(1)	Yes	1301.6209	3	3902.8482	3902.8302	4.6	167.6	138.68	22.81	Nsco_20191108_BC_ZIC_HILIC_H111_typsin_B2.60235.60235.3	115.65	Trypsin	B2	S169	158
BCAM0712	>tr|A0A2N9CPU2|A0A2N9CPU2_9BURK Cobalt-zinc-cadmium resistance protein CzcB OS=Burkholderia cenocepacia OX=95486 GN=czcB PE=3 SV=1	G.GGGASAPT[+568.212]AAEVAQPAAGAGAR.G	HexNAc(2)Hex(1)	Yes	1204.0726	2	2407.138	2406.1267	3.3	387.01	160.83	160.83	Nsco_20191108_BC_ZIC_HILIC_H111_typsin_B2.19918.19918.2	42.9	Trypsin	B2	S34	159
BCAM0936	>tr|A0A1V2XJK6|A0A1V2XJK6_9BURK Mechanosensitive ion channel protein OS=Burkholderia cenocepacia OX=95486 GN=A8F55_37840 PE=4 SV=1	A.AAPAPAAASAAS[+568.212]DAAPALT[+568.212]PQQAR.Q	HexNAc(2)Hex(1),HexNAc(2)Hex(1)	Yes	1656.7818	2	3312.5563	3311.5384	4.4	206.61	87.05	87.05	Nsco_20191108_BC_ZIC_HILIC_H111_typsin_B2.28233.28233.2	57.819	Trypsin	B2	S28	160
BCAM0988	>tr|A0A2N9CRH5|A0A2N9CRH5_9BURK Putative exported protein OS=Burkholderia cenocepacia OX=95486 GN=F01_420505 PE=4 SV=1	Q.PAAPTAGAS[+568.212]GPHVWSGA.I	HexNAc(2)Hex(1)	Yes	1051.4849	2	2101.9625	2101.956	3.1	385.97	222.78	76.31	Nsco_20191108_BC_ZIC_HILIC_H111_pepsin_B2.25602.25602.2	52.855	Pepsin	B2	S216	161
BCAM0996	>tr|A0A2N9CRL1|A0A2N9CRL1_9BURK Sporulation related OS=Burkholderia cenocepacia OX=95486 GN=F01_420513 PE=4 SV=1	A.VAPRDDDVSDVQAGVAHDEPPAS[+568.212]DTT.V	HexNAc(2)Hex(1)	Yes	1078.4798	3	3233.4248	3232.4135	2.5	215.74	117.92	27.68	Nsco_20191108_BC_ZIC_HILIC_H111_thermolysin_B3.26931.26931.3	55.918	Thermolysin	B3	S104	162
BCAM0996	>tr|A0A2N9CRL1|A0A2N9CRL1_9BURK Sporulation related OS=Burkholderia cenocepacia OX=95486 GN=F01_420513 PE=4 SV=1	K.DAAKPAAKPDTTTTASVTPPKPAPKPAAPAAKPAAPKPAPATVANAGPAS[+568.212]PDSGDASSPASPAGAR.F	HexNAc(2)Hex(1)	Partial	1106.2348	6	6632.3723	6628.3541	0.7	431.46	429.91	31.05	Nsco_20191108_BC_ZIC_HILIC_H111_typsin_B1.20631.20631.6	45.778	Trypsin	B1	NA	163

BCAM0996	>tr|A0A2N9CRL1|A0A2N9CRL1_9BURK Sporulation related OS=Burkholderia cenocepacia OX=95486 GN=F01_420513 PE=4 SV=1	K.PAAPAAKPAAPKPAPAT[+568.212]VANAGPASPDSGDASSPASPAGAR.F	HexNAc(2)Hex(1)	Yes	1048.2676	4	4190.0487	4186.0422	-1.7	428.13	63.16	40.31	Nsco_20191108_BC_ZIC_HILIC_H111_typsin_B1.19521.19521.4	43.77	Trypsin	B1	S178	164
BCAM0996	>tr|A0A2N9CRL1|A0A2N9CRL1_9BURK Sporulation related OS=Burkholderia cenocepacia OX=95486 GN=F01_420513 PE=4 SV=1	R.DDDVSDVQAGVAHDEPPAS[+568.212]DTTVAAAPAPAPK.D	HexNAc(2)Hex(1)	Yes	1228.2275	3	3682.6679	3682.6614	1.8	528.54	497.7	75.72	Nsco_20191108_BC_ZIC_HILIC_H111_typsin_B2.37296.37296.3	73.805	Trypsin	B2	S104	165
BCAM0996	>tr|A0A2N9CRL1|A0A2N9CRL1_9BURK Sporulation related OS=Burkholderia cenocepacia OX=95486 GN=F01_420513 PE=4 SV=1	P.AAPKPAPAT[+568.212][+100.064]VANAGPASPDSGDASSPASPAGAR.F	HexNAc(2)Hex(1) 100.064	Yes	1195.2382	3	3583.7001	3582.7045	-2.2	221.21	171.96	42.4	Nsco_20191108_BC_ZIC_HILIC_H111_typsin_B3.20792.20792.3	45.704	Trypsin	B3	T159	166
BCAM0996	>tr|A0A2N9CRL1|A0A2N9CRL1_9BURK Sporulation related OS=Burkholderia cenocepacia OX=95486 GN=F01_420513 PE=4 SV=1	P.AAPAAKPAAPKPAPAT[+568.212][+100.064]VANAGPASPDSGDASSPASPAGAR.F	HexNAc(2)Hex(1) 100.064	Partial	1048.7652	4	4192.0389	4189.0535	-5.9	255.61	226.89	37.83	Nsco_20191108_BC_ZIC_HILIC_H111_typsin_B3.19863.19863.4	43.892	Trypsin	B3	NA	167
BCAM1550	>tr|A0A142PC85|A0A142PC85_9BURK Peptidoglycan-associated protein OS=Burkholderia cenocepacia OX=95486 GN=pal PE=3 SV=1	K.TPENAGAAPEPSSETVATVT[+568.212]ADDLNNPNSPLAKR.S	HexNAc(2)Hex(1)	Yes	1335.3021	3	4003.8918	4002.8786	2.5	193.07	163.28	74.77	Nsco_20191108_BC_ZIC_HILIC_H111_typsin_B1.41312.41312.3	82.212	Trypsin	B1	S37	168
BCAM1550	>tr|A0A142PC85|A0A142PC85_9BURK Peptidoglycan-associated protein OS=Burkholderia cenocepacia OX=95486 GN=pal PE=3 SV=1	K.TPENAGAAPEPSSETVAT[+568.212]VTADDLNNPNSPLAK.R	HexNAc(2)Hex(1)	Yes	1282.9354	3	3846.7915	3846.7775	3.7	737.69	370.44	26.1	Nsco_20191108_BC_ZIC_HILIC_H111_typsin_B1.47218.47218.3	92.175	Trypsin	B1	S37	169
BCAM1669	>tr|A0A1V2X7H1|A0A1V2X7H1_9BURK Purine nucleoside phosphorylase OS=Burkholderia cenocepacia OX=95486 GN=A8E72_24650 PE=4 SV=1	Q.ARVHGIDNS[+568.212]GAGSQPAAT.V	HexNAc(2)Hex(1)	Yes	1139.0308	2	2277.0543	2277.0477	2.9	244.81	57.03	57.03	Nsco_20191108_BC_ZIC_HILIC_H111_pepsin_B1.8943.8943.2	25.52	Pepsin	B1	S73	170
BCAM1669	>tr|A0A1V2X7H1|A0A1V2X7H1_9BURK Purine nucleoside phosphorylase OS=Burkholderia cenocepacia OX=95486 GN=A8E72_24650 PE=4 SV=1	A.RVHGIDNSGAGS[+568.212]QPAA.T	HexNAc(2)Hex(1)	Yes	1052.9897	2	2104.9721	2104.9629	4.3	181.24	18.99	18.99	Nsco_20191108_BC_ZIC_HILIC_H111_pepsin_B3.8744.8744.2	23.545	Pepsin	B3	S73	171
BCAM1669	>tr|A0A1V2X7H1|A0A1V2X7H1_9BURK Purine nucleoside phosphorylase OS=Burkholderia cenocepacia OX=95486 GN=A8E72_24650 PE=4 SV=1	R.VHGIDNSGAGSQPAAT[+568.212]VEGGAPVVRAQNPRDSVYF.G	HexNAc(2)Hex(1)	Yes	1024.2471	4	4093.9665	4091.9429	4.1	402.09	402.09	43.31	Nsco_20191108_BC_ZIC_HILIC_H111_thermolysin_B1.39133.39133.4	74.9	Thermolysin	B1	S73	172
BCAM1669	>tr|A0A1V2X7H1|A0A1V2X7H1_9BURK Purine nucleoside phosphorylase OS=Burkholderia cenocepacia OX=95486 GN=A8E72_24650 PE=4 SV=1	R.VHGIDNSGAGSQPAAT[+568.212]VEGGAPVVRAQNPRDSVY.F	HexNAc(2)Hex(1)	Yes	987.2284	4	3945.8917	3944.8744	3.5	888.69	843.46	140.13	Nsco_20191108_BC_ZIC_HILIC_H111_thermolysin_B1.31465.31465.4	61.34	Thermolysin	B1	S73	173
BCAM1669	>tr|A0A1V2X7H1|A0A1V2X7H1_9BURK Purine nucleoside phosphorylase OS=Burkholderia cenocepacia OX=95486 GN=A8E72_24650 PE=4 SV=1	R.VHGIDNSGAGS[+568.212]QPAATVEGGAP.V	HexNAc(2)Hex(1)	Partial	1280.5865	2	2560.1657	2560.1533	4.8	240.26	124.47	124.47	Nsco_20191108_BC_ZIC_HILIC_H111_thermolysin_B3.24497.24497.2	51.861	Thermolysin	B3	NA	174
BCAM1669	>tr|A0A1V2X7H1|A0A1V2X7H1_9BURK Purine nucleoside phosphorylase OS=Burkholderia cenocepacia OX=95486 GN=A8E72_24650 PE=4 SV=1	R.VHGIDNSGAGSQPAAT[+568.212]VEGGAPVVR.A	HexNAc(2)Hex(1)	Yes	729.3552	4	2914.3992	2914.3912	2.7	958.01	894.26	149.49	Nsco_20191108_BC_ZIC_HILIC_H111_typsin_B2.25408.25408.4	52.904	Trypsin	B2	S73	175

BCAM1737	>tr|A0A2N9CVE8|A0A2N9CVE8_9BURK Putative exported glycoprotein OS=Burkholderia cenocepacia OX=95486 GN=F01_460192 PE=4 SV=1	E.RAISWSQAGSAAPADT[+568.212]PASAAPSAS[+568.212]ATPATRA.A	HexNAc(2)Hex(1)	Yes (1 of 2)	1378.3116	3	4132.9202	4132.9052	3.6	279.49	249.53	11.89	Nsco_20191108_BC_ZIC_HILIC_H111_pepsin_B1.27720.27720.3	58.638	Pepsin	B1	S1820	176
BCAM1737	>tr|A0A2N9CVE8|A0A2N9CVE8_9BURK Putative exported glycoprotein OS=Burkholderia cenocepacia OX=95486 GN=F01_460192 PE=4 SV=1	A.ISWSQAGSAAPADT[+568.212]PASAAPS[+568.212]ASATPATR.A	HexNAc(2)Hex(1),HexNAc(2)Hex(1)	Yes (1 of 2)	1279.2545	3	3835.749	3834.7299	4.1	229.96	143.42	11.45	Nsco_20191108_BC_ZIC_HILIC_H111_thermolysin_B2.34278.34278.3	68.064	Thermolysin	B2	S1820	177
BCAM1737	>tr|A0A2N9CVE8|A0A2N9CVE8_9BURK Putative exported glycoprotein OS=Burkholderia cenocepacia OX=95486 GN=F01_460192 PE=4 SV=1	R.AISWSQAGSAAPADTPASAAPSAS[+568.212]ATPATR.A	HexNAc(2)Hex(1)	Yes	1113.193	3	3337.5645	3337.5554	2.7	421.21	401.86	26.82	Nsco_20191108_BC_ZIC_HILIC_H111_typsin_B1.36326.36326.3	73.457	Trypsin	B1	S1820	178
BCAM2054 (typ	>tr|A0A1V2XAF9|A0A1V2XAF9_9BURK Peptide-binding protein OS=Burkholderia cenocepacia OX=95486 GN=bcscD PE=4 SV=1	R.APEQAPAPPVAPLAS[+568.212]GAAAGVAQPVPGPTLLPREPAAGVSTK.E	HexNAc(2)Hex(1)	Yes	1493.1215	3	4477.35	4477.3348	3.4	407.81	400.18	212.66	Nsco_20191108_BC_ZIC_HILIC_H111_typsin_B1.55635.55635.3	105.77	Trypsin	B1	S416	179
BCAM2055	>tr|A0A1V2VXC7|A0A1V2VXC7_9BURK EscC/YscC/HrcC family type III secretion system outer membrane ring protein OS=Burkholderia cenocepacia OX=95486 GN=A8E72_28425 PE=3 SV=1	D.VTVPLTPKPLPGTKFGPPALPPPKDTAAS[+568.212]QPAA.T	HexNAc(2)Hex(1)	Partial	958.2687	4	3830.053	3830.0361	4.4	296.76	296.76	44.15	Nsco_20191108_BC_ZIC_HILIC_H111_pepsin_B1.40237.40237.4	88.189	Pepsin	B1	NA	180
BCAM2063	>tr|A0A2N9CW94|A0A2N9CW94_9BURK Porin OS=Burkholderia cenocepacia OX=95486 GN=F01_460501 PE=3 SV=1	A.SSPAAAEPAAGASDAAAPAQQAADAAAPAPT[+568.212]GFWERSN.L	HexNAc(2)Hex(1)	Yes	1374.963	3	4122.8745	4119.8538	2.6	470.77	441.26	113.52	Nsco_20191108_BC_ZIC_HILIC_H111_thermolysin_B3.49036.49036.3	96.753	Thermolysin	B3	S46	181
BCAM2064	>tr|A0A2N9CWA7|A0A2N9CWA7_9BURK Periplasmic trehalase OS=Burkholderia cenocepacia OX=95486 GN=treA PE=3 SV=1	A.DNANQAAAQAAGQSAIPATTAAAAAPASGT[+568.212]LPPPSQLYGDLFVAVQTAQLYPDQK.T	HexNAc(2)Hex(1)	Yes	1494.9893	4	5976.9352	5976.9149	3.4	469.92	469.92	2.7	Nsco_20191108_BC_ZIC_HILIC_H111_typsin_B2.79283.79283.4	160.56	Trypsin	B2	S80	182
BCAM2289	>tr|A0A142PAR8|A0A142PAR8_9BURK Purine nucleoside phosphorylase OS=Burkholderia cenocepacia OX=95486 GN=A8E72_27535 PE=4 SV=1	D.TSGYGAQPAPLVHSGAPAAAS[+568.212]SNARD.S	HexNAc(2)Hex(1)	Yes	1007.8045	3	3021.3989	3021.392	2.3	359.5	236.46	20.44	Nsco_20191108_BC_ZIC_HILIC_H111_pepsin_B1.19533.19533.3	42.532	Pepsin	B1	S63	183
BCAM2289	>tr|A0A142PAR8|A0A142PAR8_9BURK Purine nucleoside phosphorylase OS=Burkholderia cenocepacia OX=95486 GN=A8E72_27535 PE=4 SV=1	R.VHGADTSGYGAQPAPLVHSGAPAAAS[+568.212]SNAR.D	HexNAc(2)Hex(1)	Yes	847.1534	4	3385.5918	3385.5779	4.1	1294.2	1230.2	71.23	Nsco_20191108_BC_ZIC_HILIC_H111_typsin_B3.17844.17844.4	40.144	Trypsin	B3	S63	184
BCAM2443	>tr|A0A2N9CXV2|A0A2N9CXV2_9BURK Cytochrome c family protein OS=Burkholderia cenocepacia OX=95486 GN=F01_480355 PE=4 SV=1	G.ATPQDAPAAAS[+568.212]APPPAPAAAAAPAAKPFTPPPES.A	HexNAc(2)Hex(1)	Yes	1224.9368	3	3672.7959	3672.7803	4.3	356.59	69.23	69.23	Nsco_20191108_BC_ZIC_HILIC_H111_pepsin_B2.38607.38607.3	81.685	Pepsin	B2	S38	185
BCAM2681	>tr|A0A1V6L0G1|A0A1V6L0G1_9BURK Putative lipoprotein OS=Burkholderia cenocepacia OX=95486 GN=F01_490209 PE=4 SV=1	R.GASVAVHAGSAPSEAVGGGT[+568.212]PAEQVAALDPK.A	HexNAc(2)Hex(1)	Yes	1124.2145	3	3370.6289	3369.618	2.2	344.3	294.43	32.01	Nsco_20191108_BC_ZIC_HILIC_H111_typsin_B1.35984.35984.3	72.914	Trypsin	B1	S232	186
BCAM2828	>tr|A0A2N9CZJ3|A0A2N9CZJ3_9BURK Hopanoid biosynthesis associated RND transporter like protein HpnN OS=Burkholderia cenocepacia OX=95486 GN=hpnN PE=4 SV=1	K.RAAIAAAAS[+568.212]ELLPALTQPAAPPATDAQR.V	HexNAc(2)Hex(1)	Yes	1104.2439	3	3310.7172	3310.7013	4.8	435.85	424.66	122.25	Nsco_20191108_BC_ZIC_HILIC_H111_typsin_B3.48545.48545.3	99.338	Trypsin	B3	S558	187
BCAS0453	>tr|A0A142PSQ3|A0A142PSQ3_9BURK Uncharacterized protein OS=Burkholderia cenocepacia OX=95486 GN=A8E72_07995 PE=4 SV=1	R.DSLGNGVALDWPAS[+568.212]GVGGVADERQK.L	HexNAc(2)Hex(1)	Yes	1023.1548	3	3067.4498	3066.4386	2.6	229.31	189.06	74.03	Nsco_20191108_BC_ZIC_HILIC_H111_typsin_B1.47978.47978.3	93.418	Trypsin	B1	S98	188

I35_6143	>tr|A0A2N9CX34|A0A2N9CX34_9BURK Uncharacterized protein OS=Burkholderia cenocepacia OX=95486 GN=F01_480128 PE=4 SV=1	A.PAERSPFDTPDTRPLRVTS[+568.212]DAL.V	HexNAc(2)Hex(1)	Partial	753.1206	4	3009.4607	3009.4535	2.4	684.21	615.43	14.88	Nsco_20191108_BC_ZIC_HILIC_H111_pepsin_B1.34820.34820.4	73.999	Pepsin	B1	NA	189
I35_6143	>tr|A0A2N9CX34|A0A2N9CX34_9BURK Uncharacterized protein OS=Burkholderia cenocepacia OX=95486 GN=F01_480128 PE=4 SV=1	F.AAPAERSPFDTPDTRPLRVT[+568.212]SDAL.V	HexNAc(2)Hex(1)	Partial	788.6396	4	3151.5366	3151.5277	2.8	221.14	221.14	8.73	Nsco_20191108_BC_ZIC_HILIC_H111_pepsin_B1.35214.35214.4	74.891	Pepsin	B1	NA	190

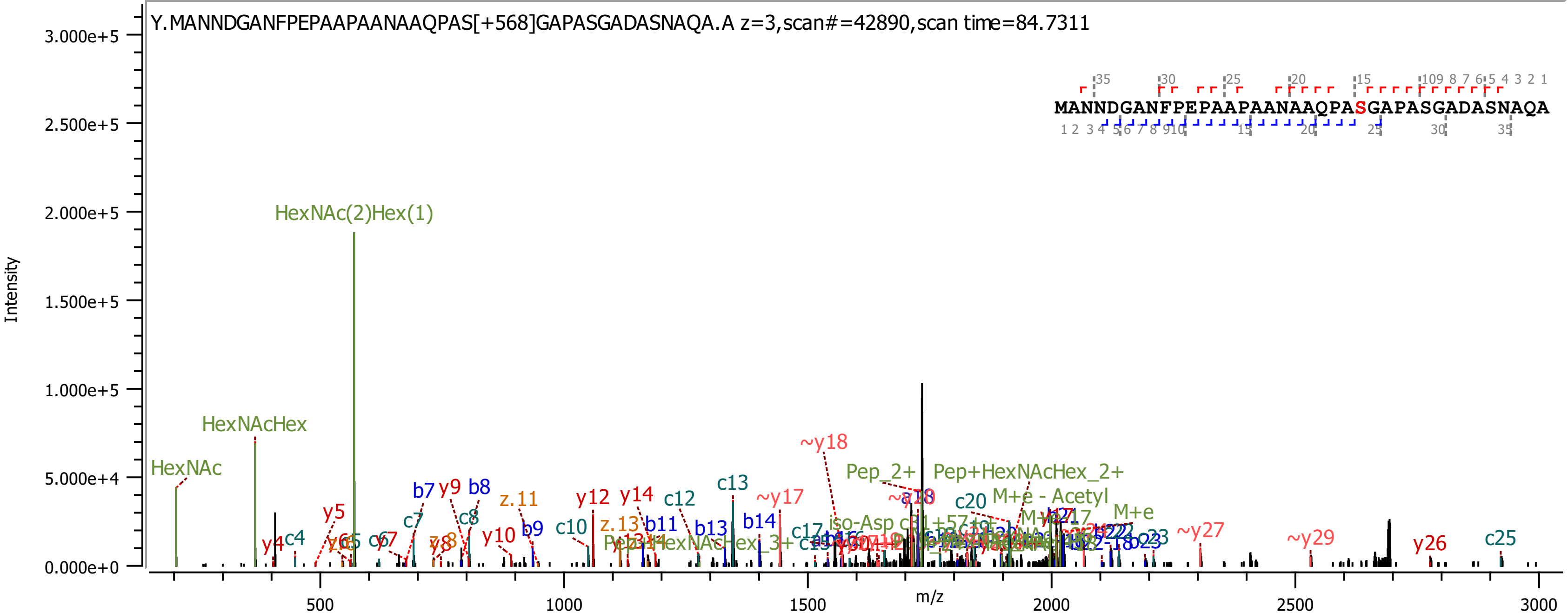

Y.MANNDGANFPEPAAPAANAAQPAS[+568]GAPASGADASNAQA.A z=3,scan#=42890,scan time=84.7311
MANNDGANFPEPAAPAANAAQPASGAPASGADASNAQA
Intensity
m/z
3.000e+5
2.500e+5
2.000e+5
1.500e+5
1.000e+5
5.000e+4
0.000e+0
500
1000
1500
2000
2500
3000
HexNAc(2)Hex(1)
HexNAcHex
HexNAc
Pep_2+
Pep+HexNAcHex_2+
M+e - Acetyl
M+e
~y18
~y17
~y27
~y29
y26
c25
c20
c13
c12
c10
b14
b13
b11
b9
b8
b7
y9
y12
y14
y10
y5
y4
c4
c7
c8
z.11
z.13

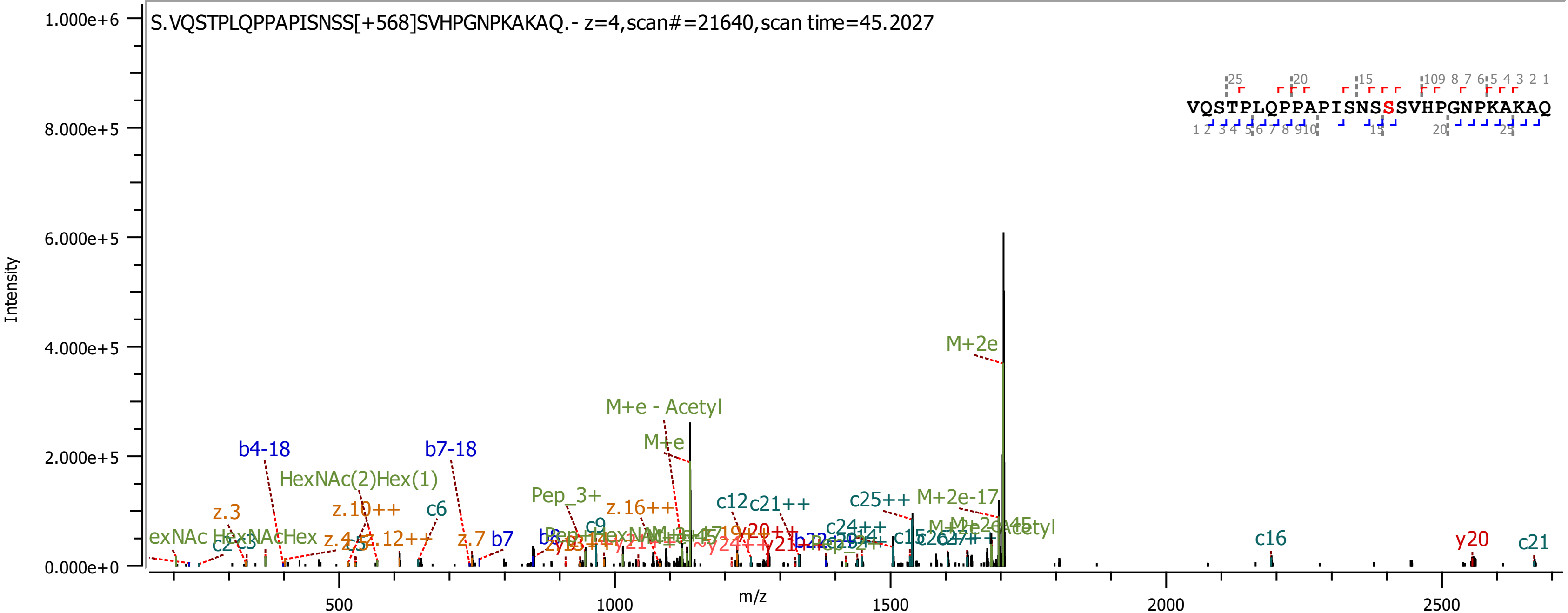
S.VQSTPLQPPAPISNSS[+568]SVHPGNPKAKAQ.- z=4,scan#=21640,scan time=45.2027
VQSTPLQPPAPISNSSSVHPGNPKAKAQ
Intensity
m/z
1.000e+6
8.000e+5
6.000e+5
4.000e+5
2.000e+5
0.000e+0
500
1000
1500
2000
2500
M+2e
M+e - Acetyl
M+e
b4-18
b7-18
HexNAc(2)Hex(1)
Pep_3+
z.3
z.10++
z.12++
z.16++
z.7
c6
b7
c9
c12
c21++
c25++
M+2e-17
c24++
c16
y20
c21

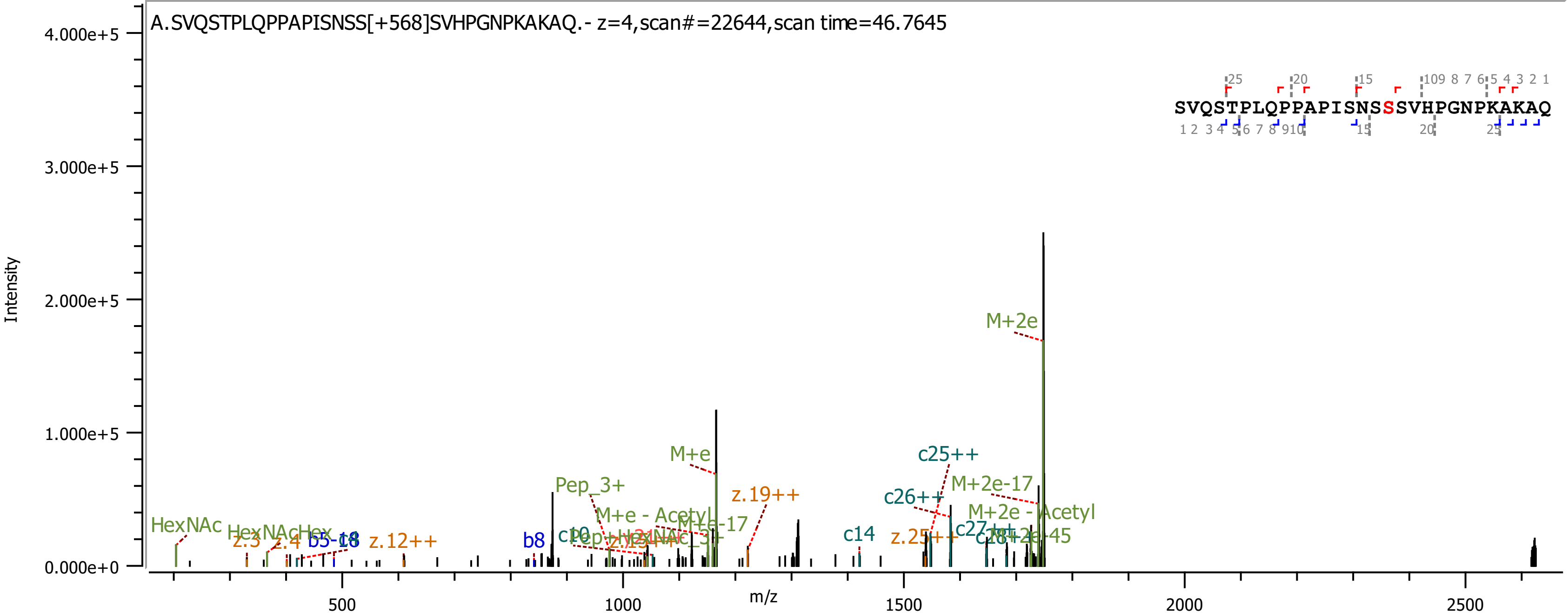
A.SVQSTPLQPPAPISNSS[+568]SVHPGNPKAKAQ.- z=4,scan#=22644,scan time=46.7645
SVQSTPLQPPAPISNSSSVHPGNPKAKAQ
Intensity
m/z
4.000e+5
3.000e+5
2.000e+5
1.000e+5
0.000e+0
500
1000
1500
2000
2500
M+2e
M+e
c25++
M+2e-17
c26++
M+2e - Acetyl
z.19++
Pep_3+
M+e - Acetyl
M+e-17
c14
z.25++
HexNAc
HexNAcHex
z.3
z.4
z.12++
b8

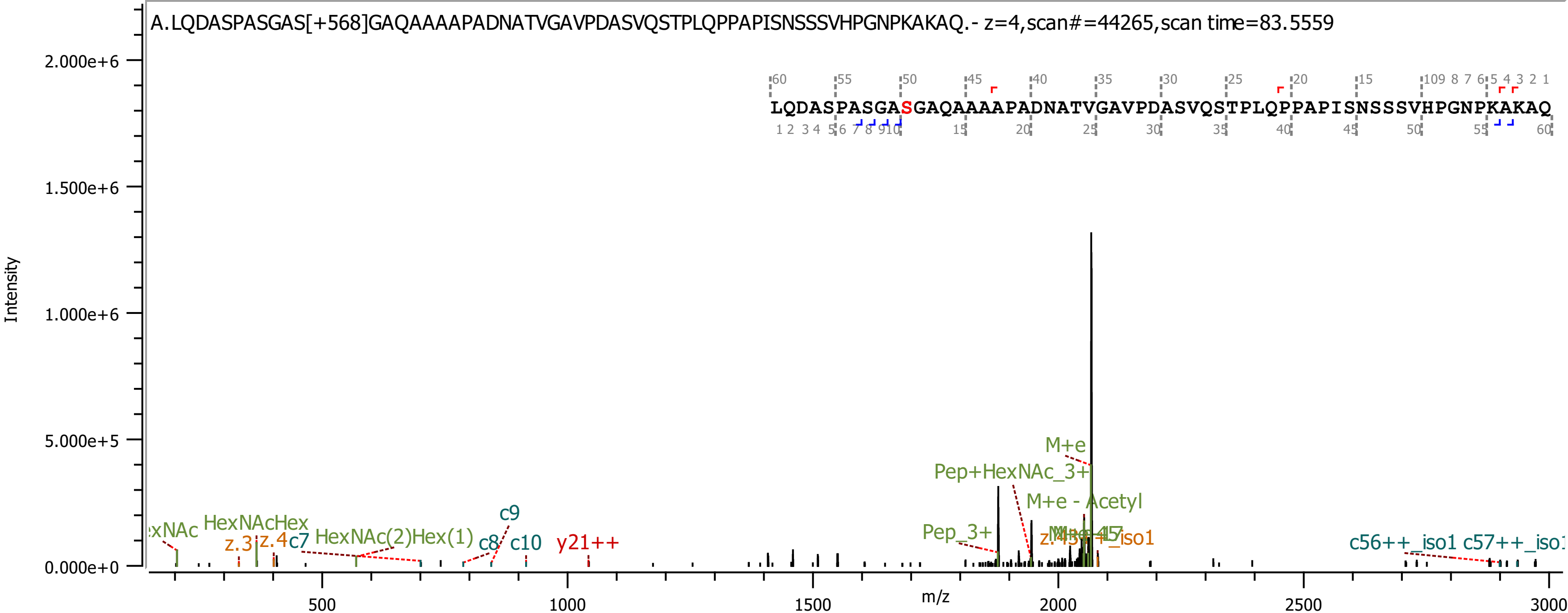
A.LQDASPASGAS[+568]GAQAAAAPADNATVGAVPDASVQSTPLQPPAPISNSSSVHPGNPKAKAQ.- z=4,scan#=44265,scan time=83.5559
LQDASPASGASGAQAAAAPADNATVGAVPDASVQSTPLQPPAPISNSSSVHPGNPKAKAQ
Intensity
m/z
M+e
Pep+HexNAc_3+
M+e - Acetyl
Pep_3+
HexNAcHex
HexNAc(2)Hex(1)
z.3
z.4
c7
c8
c9
c10
y21++
c56++_iso1

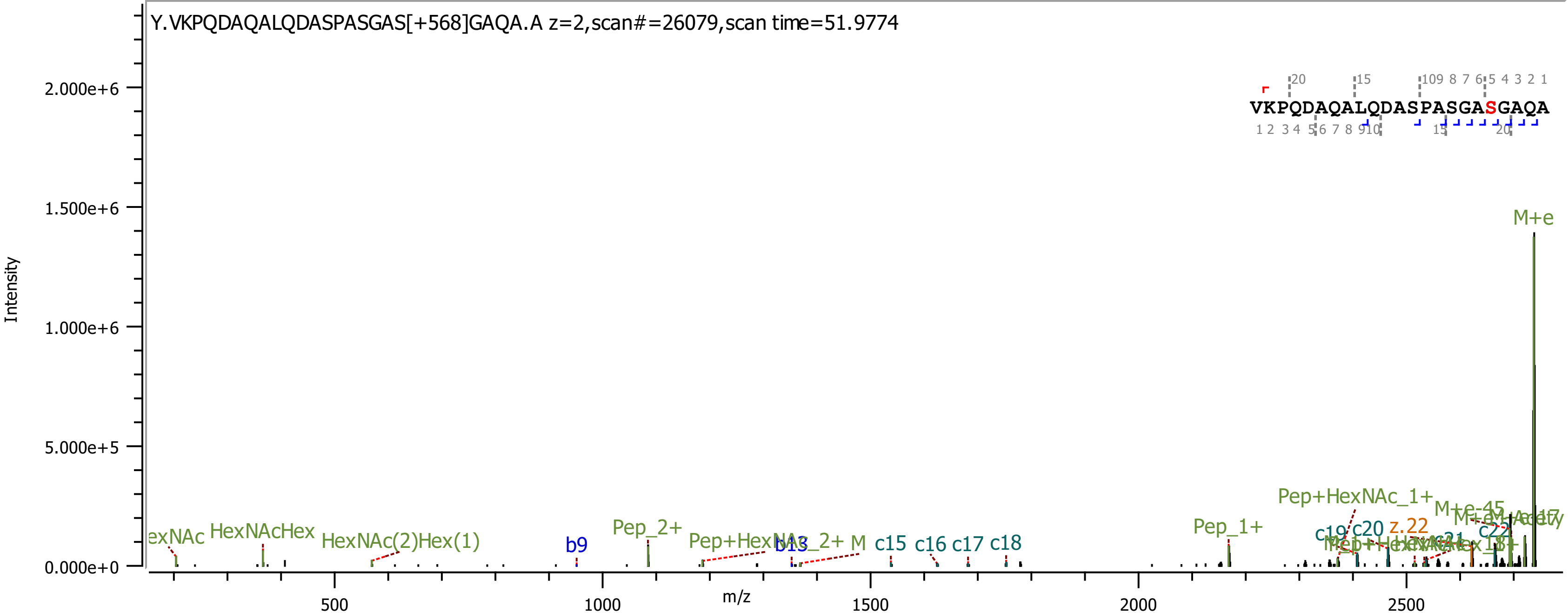
Y.VKPQDAQALQDASPASGAS[+568]GAQA.A z=2,scan#=26079,scan time=51.9774
Intensity
m/z
VKPQDAQALQDASPASGASGAQA
M+e
Pep+HexNAc_1+
Pep_1+
Pep_2+
HexNAcHex
HexNAc(2)Hex(1)
b9
b13
c15
c16
c17
c18
c19
c20
z.22
M+e-45
c22
2.000e+6
1.500e+6
1.000e+6
5.000e+5
0.000e+0
500
1000
1500
2000
2500

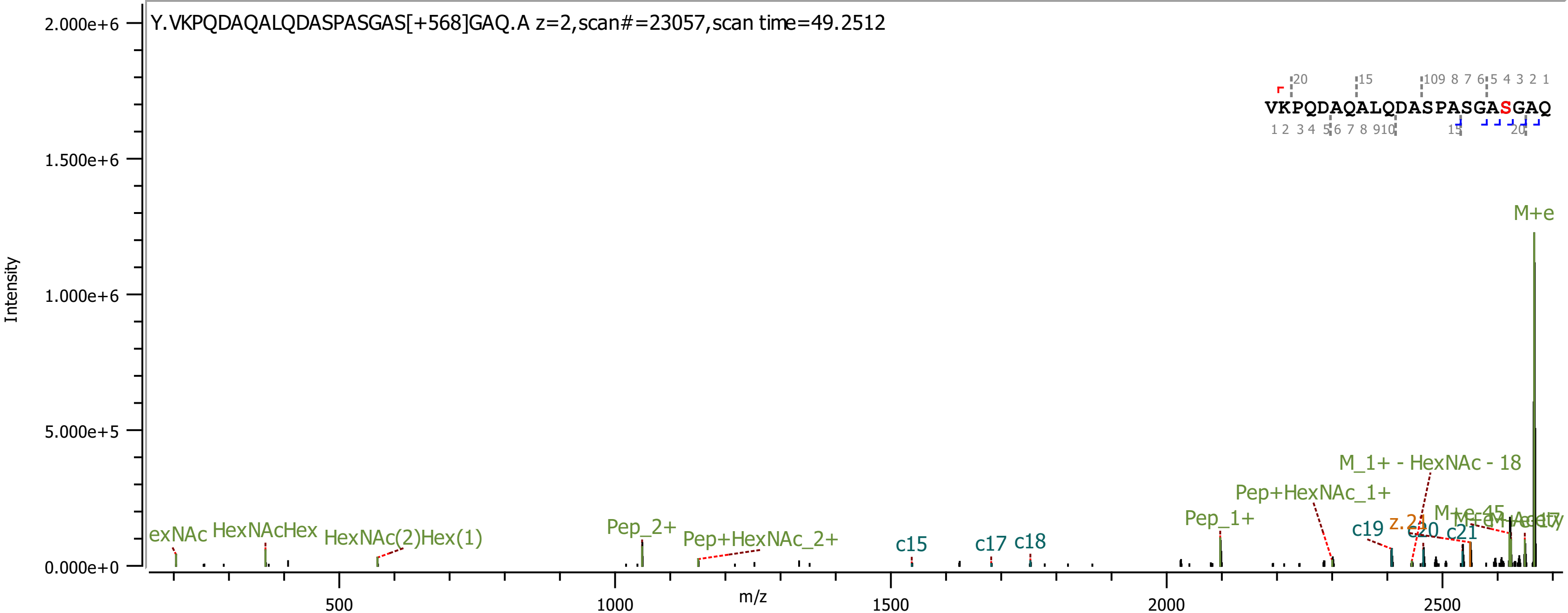

Y.VKPQDAQALQDASPASGAS[+568]GAQ.A z=2,scan#=23057,scan time=49.2512
Intensity
m/z
2.000e+6
1.500e+6
1.000e+6
5.000e+5
0.000e+0
500
1000
1500
2000
2500
VKPQDAQALQDASPASGASGAQ
M+e
M_1+ - HexNAc - 18
Pep+HexNAc_1+
Pep_1+
Pep_2+
Pep+HexNAc_2+
HexNAcHex
HexNAc(2)Hex(1)
c15
c17
c18
c19
c21

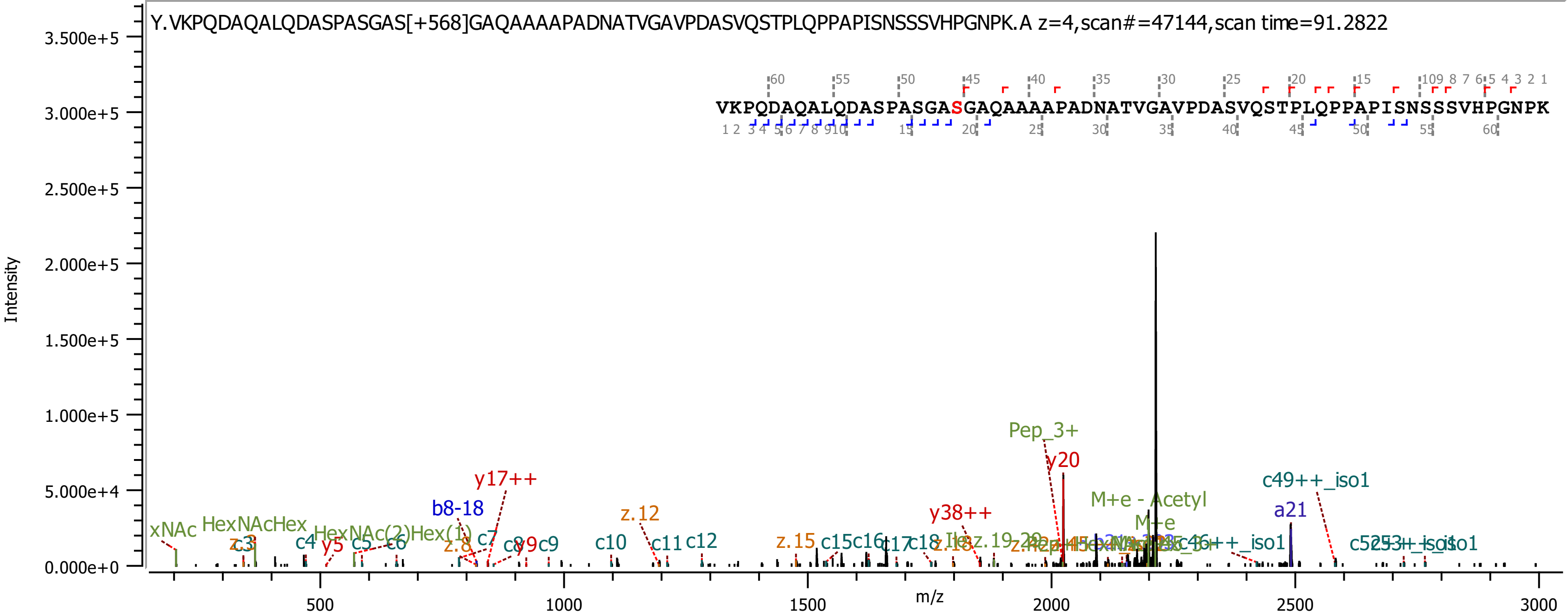
Y.VKPQDAQALQDASPASGAS[+568]GAQAAAAPADNATVGAVPDASVQSTPLQPPAPISNSSSVHPGNPK.A z=4,scan#=47144,scan time=91.2822
VKPQDAQALQDASPASGASGAQAAAAPADNATVGAVPDASVQSTPLQPPAPISNSSSVHPGNPK
Intensity
m/z
Pep_3+
y20
c49++_iso1
M+e - Acetyl
M+e
a21
y38++
y17++
b8-18
z.12
HexNAcHex
HexNAc(2)Hex(1)
c10
c11
c12
z.15
c15
c16
c17
c18
z.18
c4
c5
c6
c7
c9
y5
y9
z.8

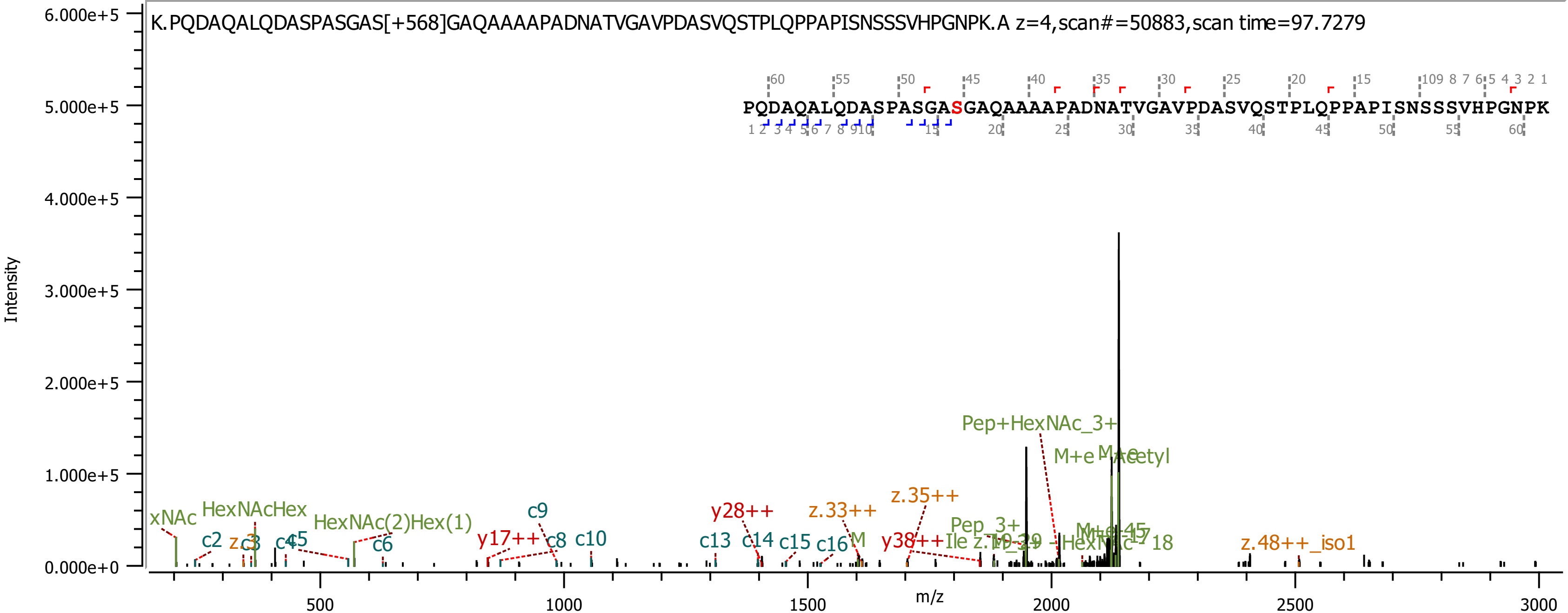
K.PQDAQALQDASPASGAS[+568]GAQAAAAPADNATVGAVPDASVQSTPLQPPAPISNSSSVHPGNPK.A z=4,scan#=50883,scan time=97.7279
PQDAQALQDASPASGASGAQAAAAPADNATVGAVPDASVQSTPLQPPAPISNSSSVHPGNPK
Intensity
m/z
Pep+HexNAc_3+
M+e
M-Acetyl
HexNAcHex
HexNAc(2)Hex(1)
xNAc
c2
c3
c4
c5
c6
c8
c9
c10
c13
c14
c15
c16
y17++
y28++
y38++
z.33++
z.35++
z.48++_iso1
Pep_3+
-HexNAc
18

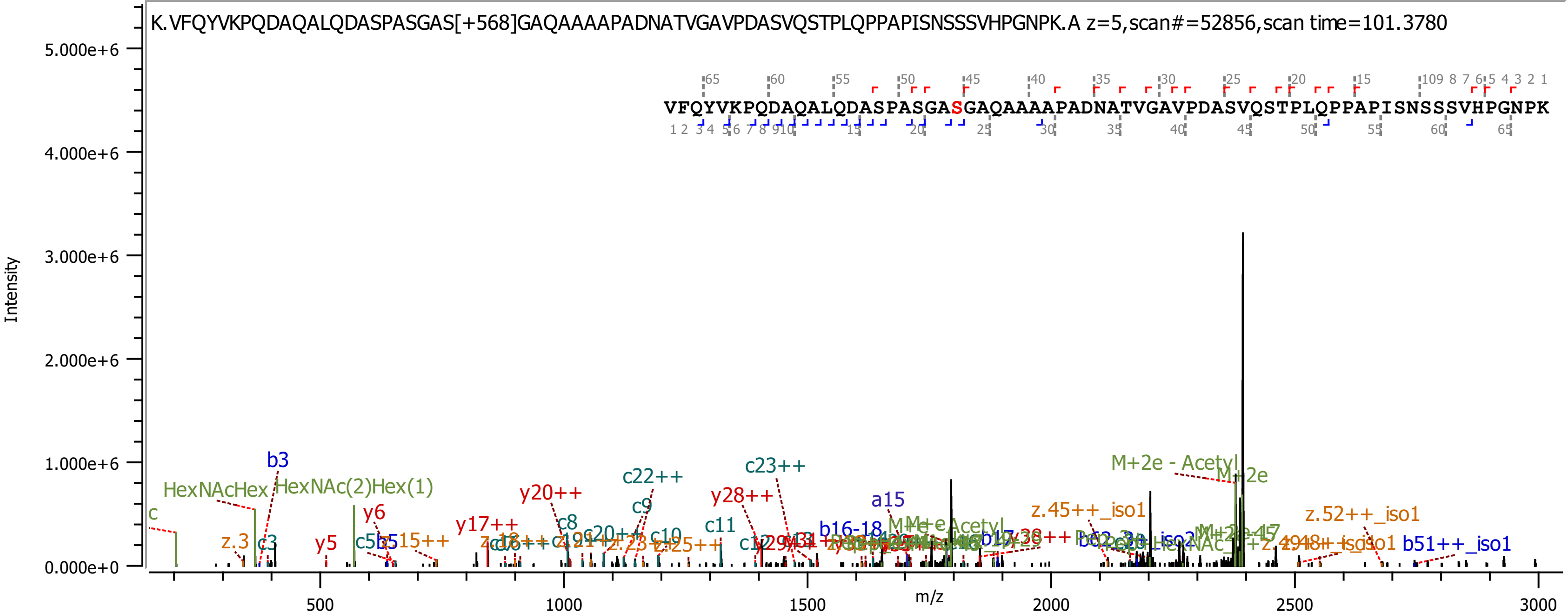
K.VFQYVKPQDAQALQDASPASGAS[+568]GAQAAAAPADNATVGAVPDASVQSTPLQPPAPISNSSSVHPGNPK.A z=5,scan#=52856,scan time=101.3780
VFQYVKPQDAQALQDASPASGASGAQAAAAPADNATVGAVPDASVQSTPLQPPAPISNSSSVHPGNPK
Intensity
5.000e+6
4.000e+6
3.000e+6
2.000e+6
1.000e+6
0.000e+0
m/z
500
1000
1500
2000
2500
3000
HexNAcHex
HexNAc(2)Hex(1)
b3
c3
y5
y6
c5
b5
y20++
y17++
c8
c22++
c9
c10
c11
y28++
c23++
a15
b16-18
M+2e - Acetyl
M+2e
z.45++_iso1
z.52++_iso1
b51++_iso1

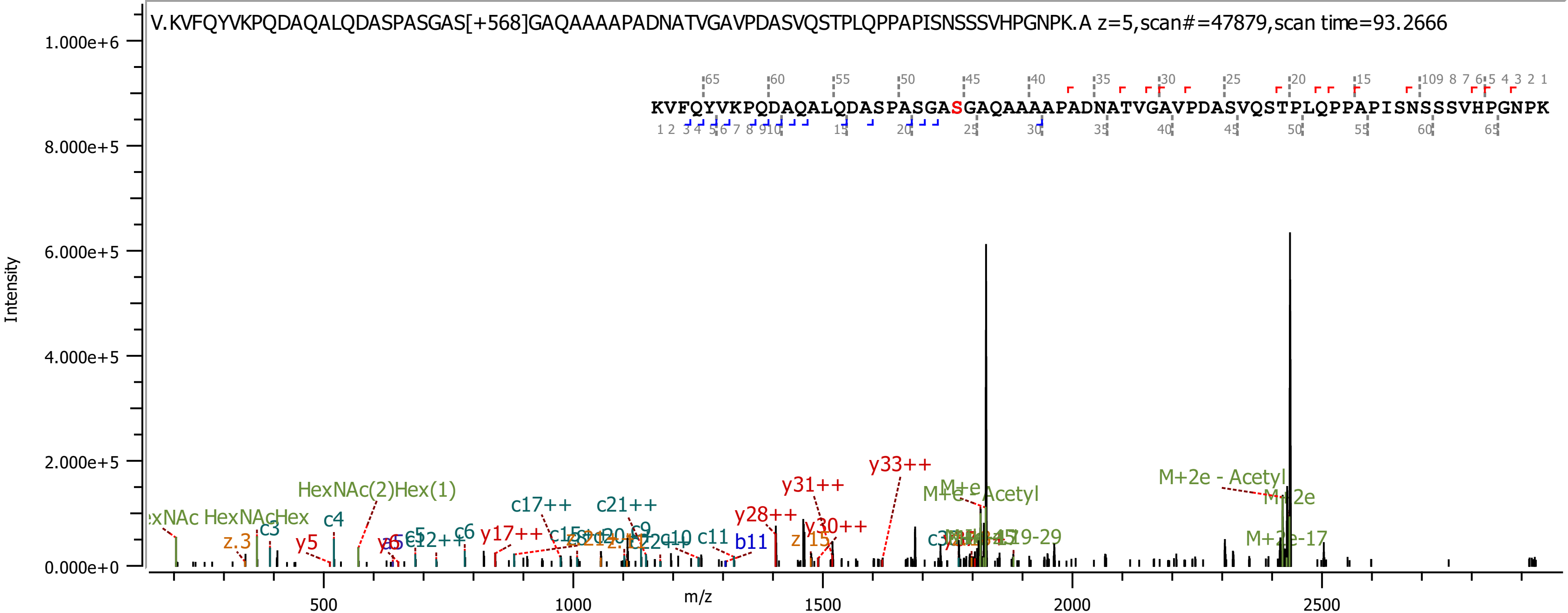

V.KVFQYVKPQDAQALQDASPASGAS[+568]GAQAAAAPADNATVGAVPDASVQSTPLQPPAPISNSSSVHPGNPK.A z=5,scan#=47879,scan time=93.2666
KVFQYVKPQDAQALQDASPASGASGAQAAAAPADNATVGAVPDASVQSTPLQPPAPISNSSSVHPGNPK
Intensity
m/z
HexNAc(2)Hex(1)
HexNAcHex
c3
c4
z.3
y5
c5
c6
c12++
y17++
c17++
c21++
c9
c10
c11
b11
y28++
y31++
y30++
y33++
z.15
M+e
M+e - Acetyl
M+2e - Acetyl
M+2e
M+2e-17

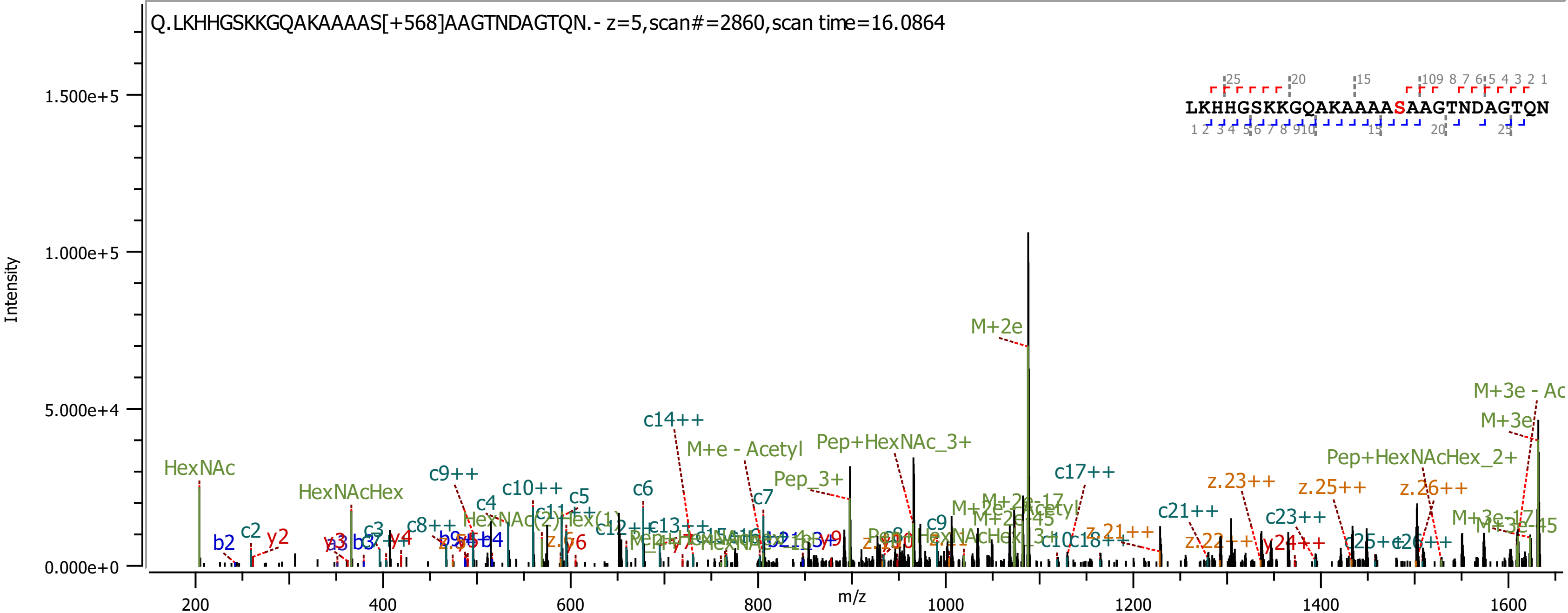
Q.LKHHGSKKGQAKAAAAS[+568]AAGTNDAGTQN.- z=5,scan#=2860,scan time=16.0864
LKHHGSKKGQAKAAAASAAGTNDAGTQN
Intensity
1.500e+5
1.000e+5
5.000e+4
0.000e+0
m/z
200
400
600
800
1000
1200
1400
1600
M+2e
M+3e - Ac
M+3e
HexNAc
HexNAcHex
c14++
M+e - Acetyl
Pep+HexNAc_3+
Pep_3+
Pep+HexNAcHex_2+
c17++
z.23++
z.25++
z.26++
c9++
c10++
c5
c6
c4
c7
c9
c21++
c23++
b2
c2
y2
z.21++
z.22++
y24++
c25++
c26++
M+3e-17
M+3e-45

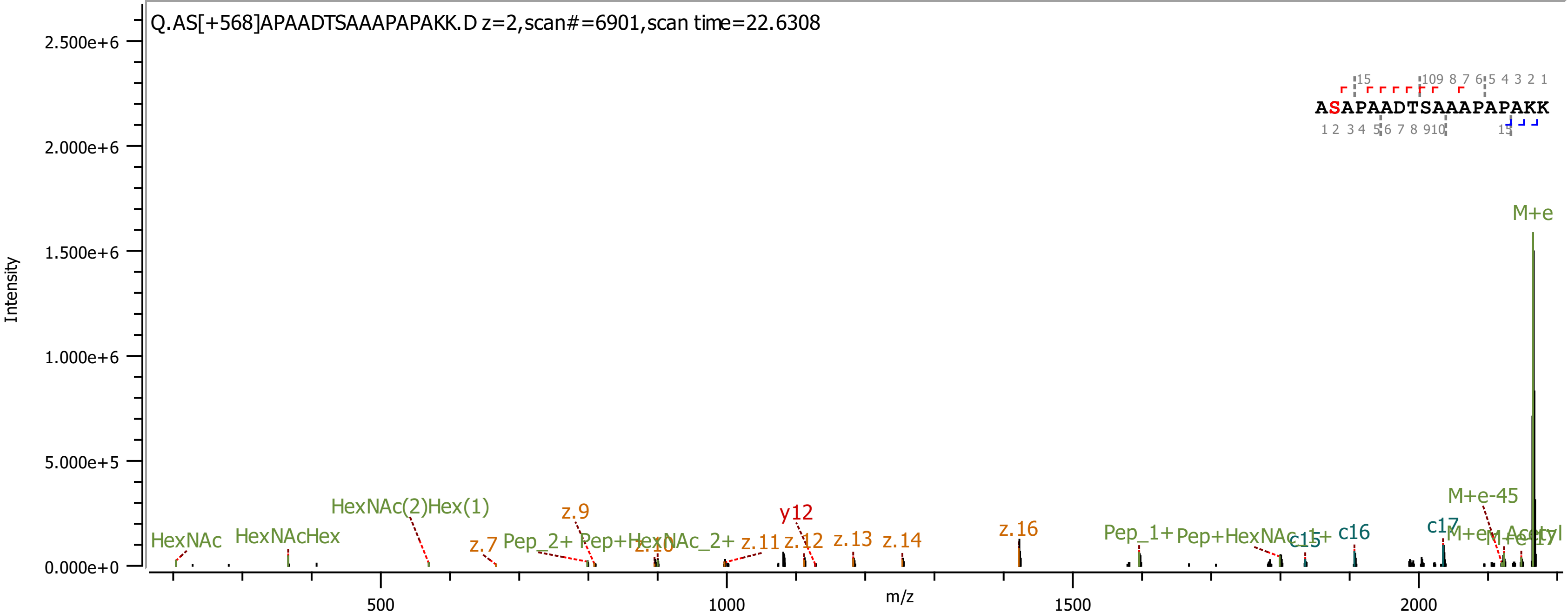
Q.AS[+568]APAADTSAAAPAPAKK.D z=2,scan#=6901,scan time=22.6308
ASAPAADTSAAAPAPAKK
Intensity
m/z
2.500e+6
2.000e+6
1.500e+6
1.000e+6
5.000e+5
0.000e+0
500
1000
1500
2000
M+e
M+e-45
HexNAc
HexNAcHex
HexNAc(2)Hex(1)
z.7
z.9
Pep_2+
Pep+HexNAc_2+
z.10
z.11
y12
z.12
z.13
z.14
z.16
Pep_1+
Pep+HexNAc_1+
c15
c16
c17
M+e-Acetyl

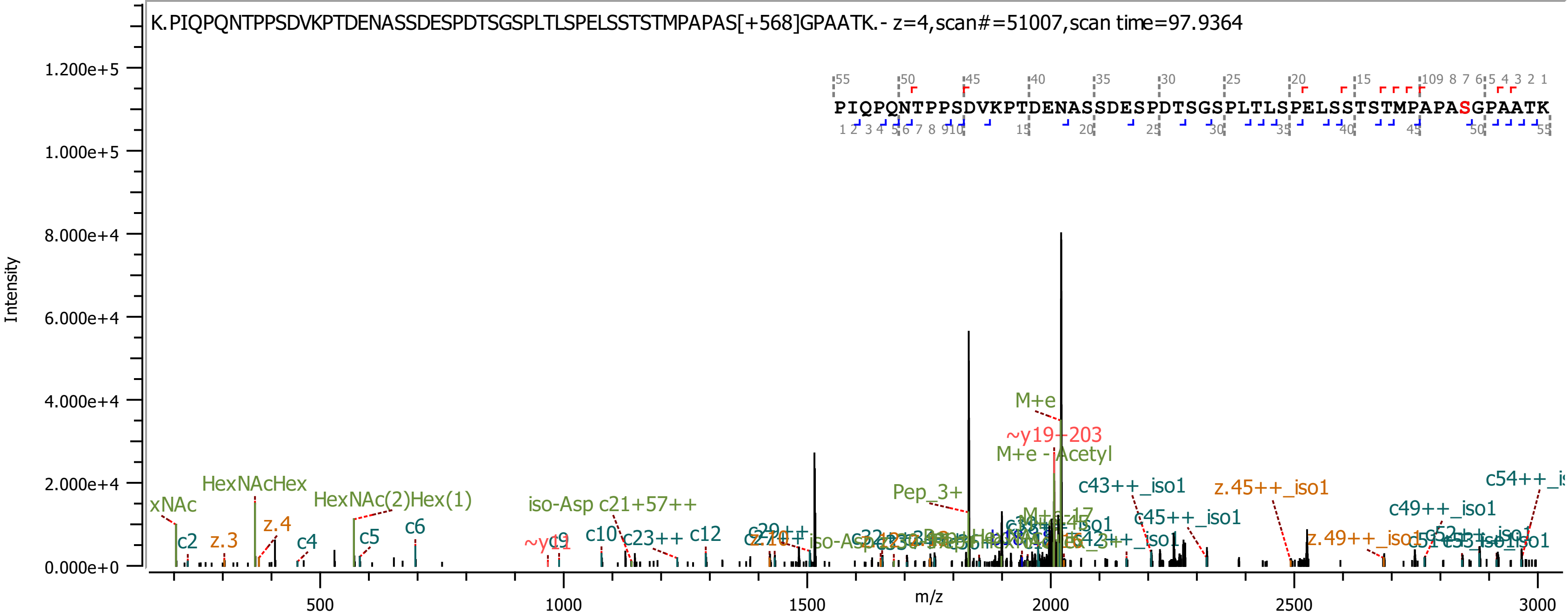
K.PIQPQNTPPSDVKPTDENASSDESPDTSGSPLTLSPELSSTSTMPAPAS[+568]GPAATK.- z=4,scan#=51007,scan time=97.9364
PIQPQNTPPSDVKPTDENASSDESPDTSGSPLTLSPELSSTSTMPAPASGPAATK
Intensity
1.200e+5
1.000e+5
8.000e+4
6.000e+4
4.000e+4
2.000e+4
0.000e+0
m/z
500
1000
1500
2000
2500
3000
xNAc
HexNAcHex
HexNAc(2)Hex(1)
iso-Asp c21+57++
Pep_3+
M+e
~y19-203
M+e - Acetyl
c43++_iso1
z.45++_iso1
c45++_iso1
c49++_iso1
c54++_i
c2
z.3
z.4
c4
c5
c6
c10
c23++
c12
c20++
z.49++_iso1
c51
c53_iso1

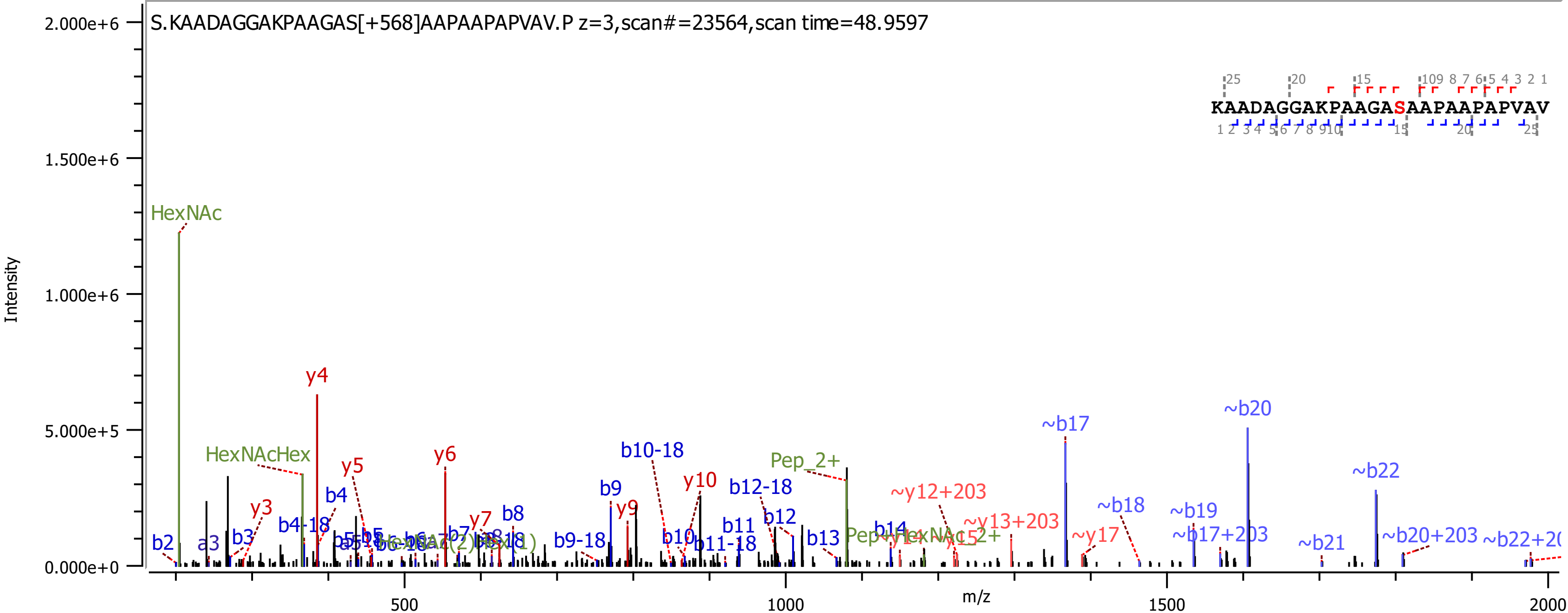

S.KAADAGGAKPAAGAS[+568]AAPAAPAPVAV.P z=3,scan#=23564,scan time=48.9597
KAADAGGAKPAAGASAAPAAPAPVAV
Intensity
m/z
2.000e+6
1.500e+6
1.000e+6
5.000e+5
0.000e+0
500
1000
1500
2000
HexNAc
HexNAcHex
Pep_2+
y4
y5
y6
y3
y7
y9
y10
b2
a3
b3
b4
b4-18
b8
b9
b9-18
b10
b10-18
b11
b11-18
b12
b12-18
b13
b14
~y12+203
~y13+203
~y17
~b17
~b18
~b19
~b17+203
~b20
~b20+203
~b21
~b22

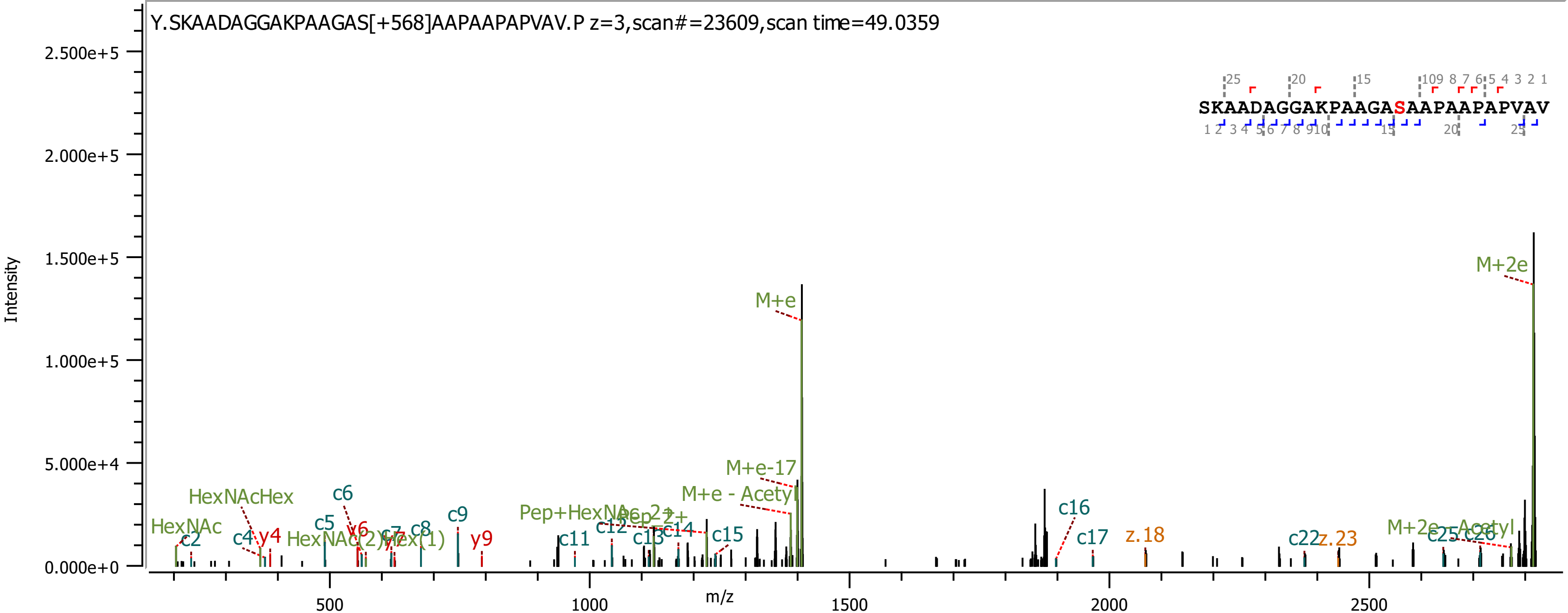

Y.SKAADAGGAKPAAGAS[+568]AAPAAPAPVAV.P z=3,scan#=23609,scan time=49.0359
SKAADAGGAKPAAGASAAPAAPAPVAV
Intensity
m/z
M+e
M+2e
M+e-17
M+e - Acetyl
M+2e - Acetyl
HexNAcHex
HexNAc
HexNAc(2)Hex(1)
Pep+HexNAc 2+
Pep 2+
c2
c4
c5
c6
c7
c8
c9
c11
c12
c13
c14
c15
c16
c17
c22
c25
c26
y4
y6
y7
y9
z.18
z.23

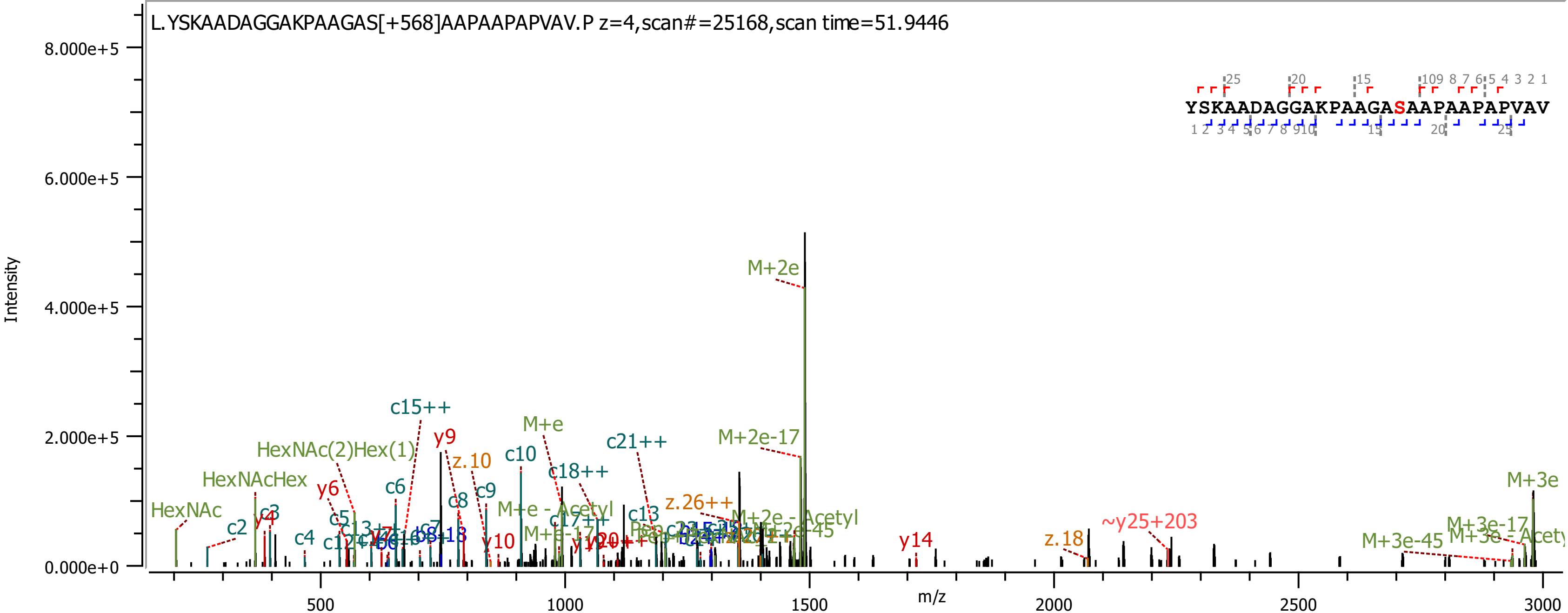
L.YSKAADAGGAKPAAGAS[+568]AAPAAPAPVAV.P z=4,scan#=25168,scan time=51.9446
Intensity
m/z
8.000e+5
6.000e+5
4.000e+5
2.000e+5
0.000e+0
500
1000
1500
2000
2500
3000
YSKAADAGGAKPAAGASAAPAAPAPVAV
M+2e
M+2e-17
c15++
y9
z.10
c10
M+e
c21++
HexNAc(2)Hex(1)
HexNAcHex
y6
c6
c8
c9
c18++
M+e - Acetyl
c13
z.26++
M+2e - Acetyl
HexNAc
c2
y4
c3
c4
c5
c17++
M+e-17
y10
y14
z.18
~y25+203
M+3e
M+3e-17
M+3e-45

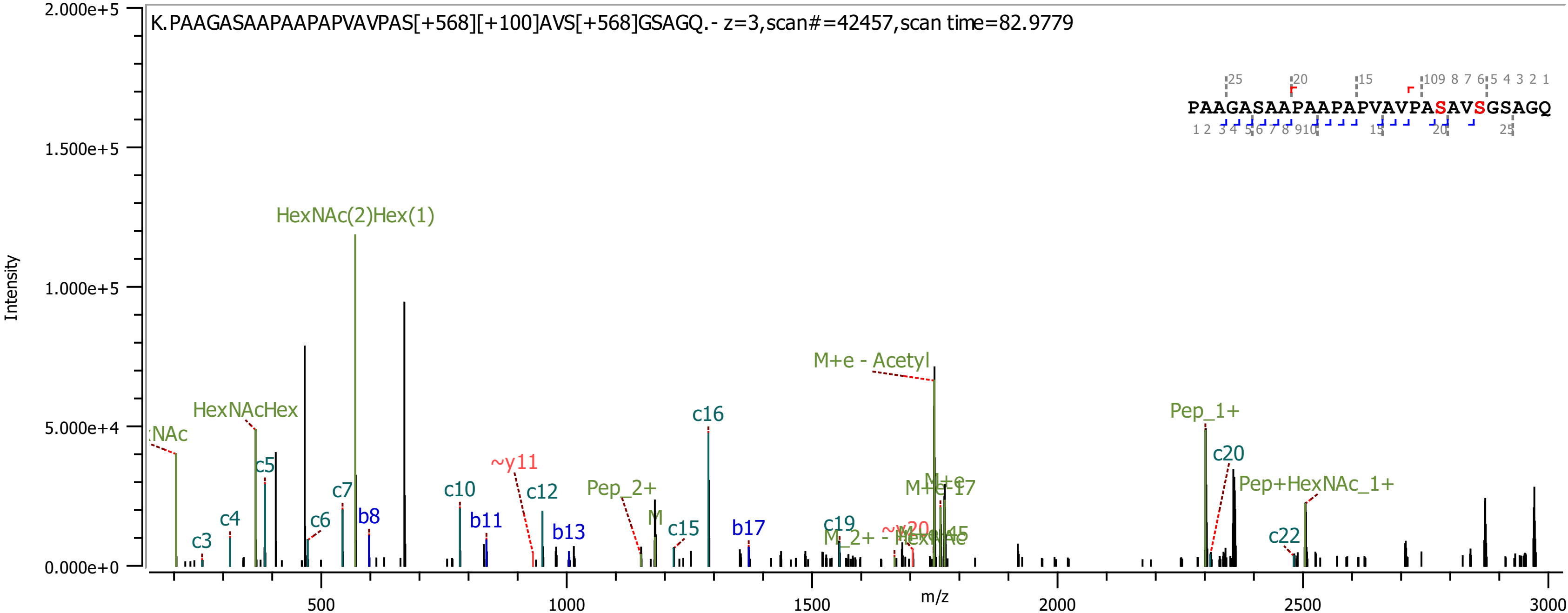

K.PAAGASAAPAAPAPVAVPAS[+568][+100]AVS[+568]GSAGQ.- z=3,scan#=42457,scan time=82.9779
PAAGASAAPAAPAPVAVPASAVSGSAGQ
Intensity
m/z
HexNAc(2)Hex(1)
HexNAcHex
M+e - Acetyl
Pep_1+
c20
Pep+HexNAc_1+
c16
~y11
c12
c10
b11
c7
c6
b8
c5
c4
c3
b13
Pep_2+
M
c15
b17
c19
M_2+ - HexNAc
c22

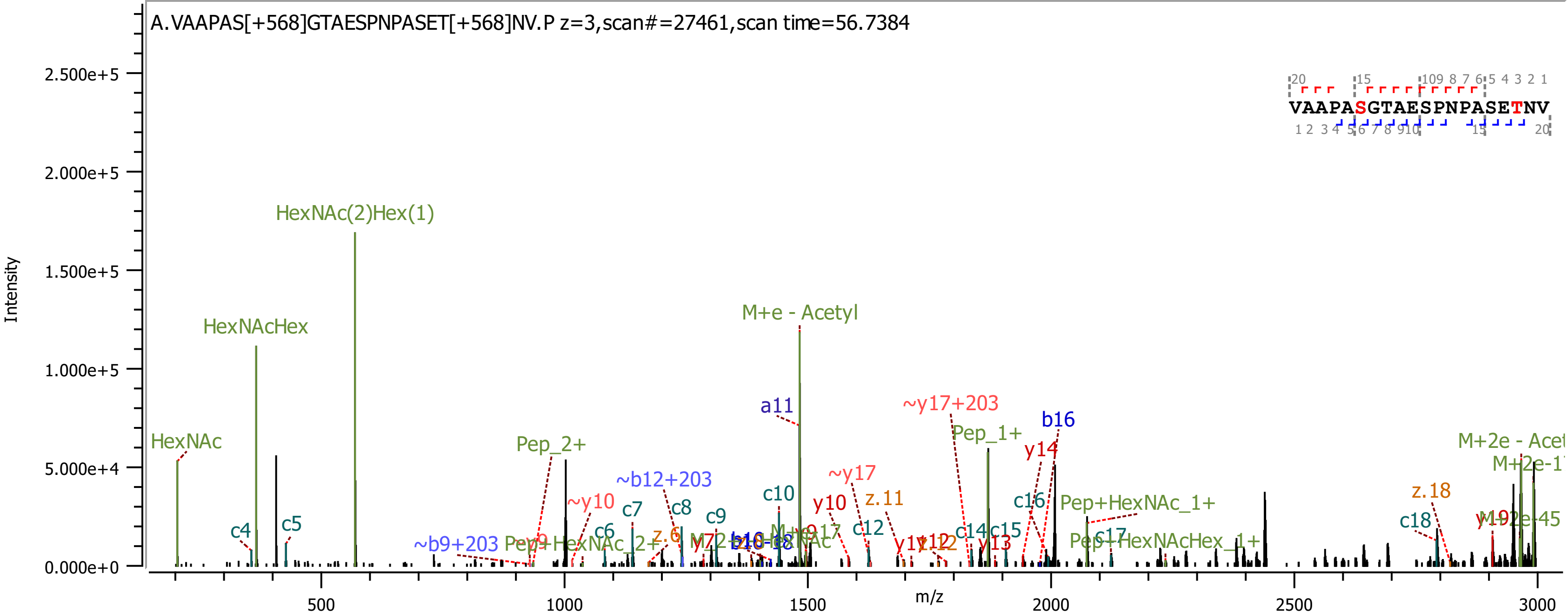
A.VAAPAS[+568]GTAESPNPASET[+568]NV.P z=3,scan#=27461,scan time=56.7384
Intensity
m/z
HexNAc
HexNAcHex
HexNAc(2)Hex(1)
M+e - Acetyl
Pep_2+
Pep_1+
Pep+HexNAc_1+
Pep+HexNAcHex_1+
a11
b16
~y17+203
~b12+203
~b9+203
y14
~y17
~y10
y10
z.11
z.18
c4
c5
c6
c7
c8
c9
c10
c12
c14
c15
c16
c17
c18
y13
VAAPASGTAESPNPASETNV

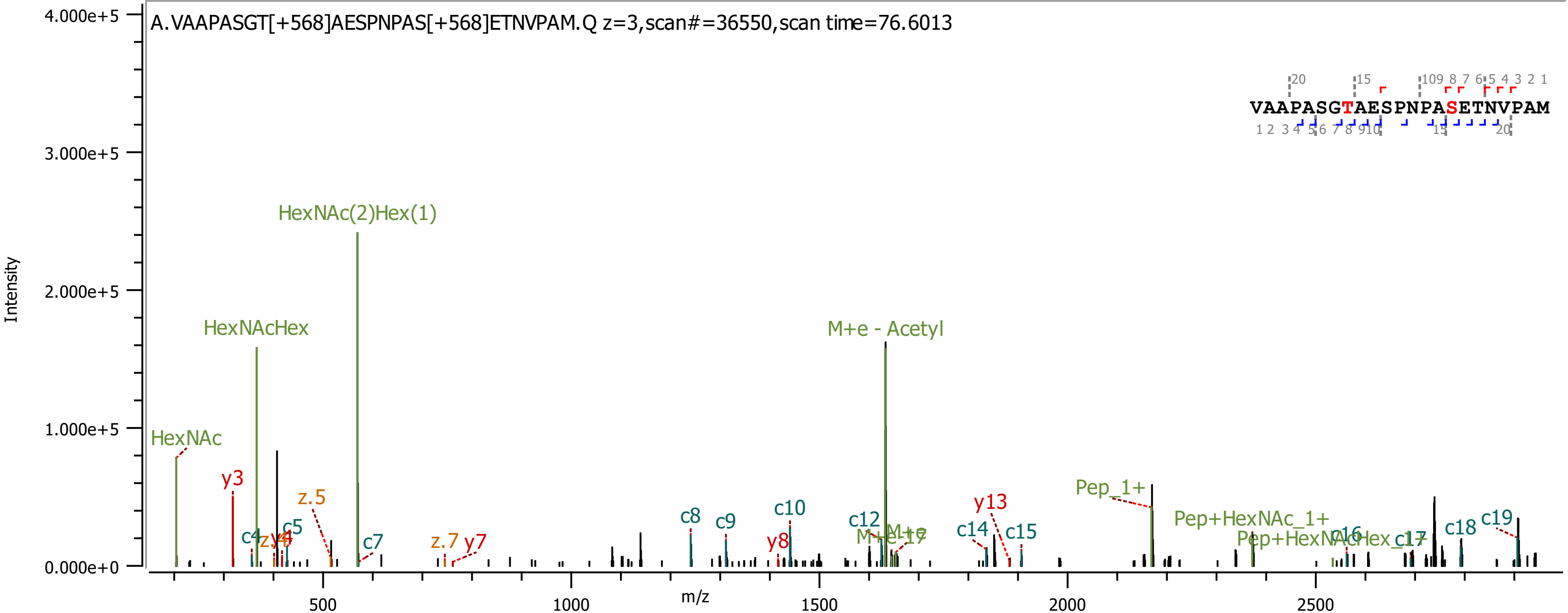

A.VAAPASGT[+568]AESPNPAS[+568]ETNVPAM.Q z=3,scan#=36550,scan time=76.6013
Intensity
m/z
4.000e+5
3.000e+5
2.000e+5
1.000e+5
0.000e+0
500
1000
1500
2000
2500
VAAPASGTAESPNPASETNVPAM
HexNAc
HexNAcHex
HexNAc(2)Hex(1)
M+e - Acetyl
Pep_1+
Pep+HexNAc_1+
Pep+HexNAcHex_1+
y3
y7
y8
y13
z.5
z.7
c4
c5
c7
c8
c9
c10
c12
c14
c15
c16
c17
c18
c19

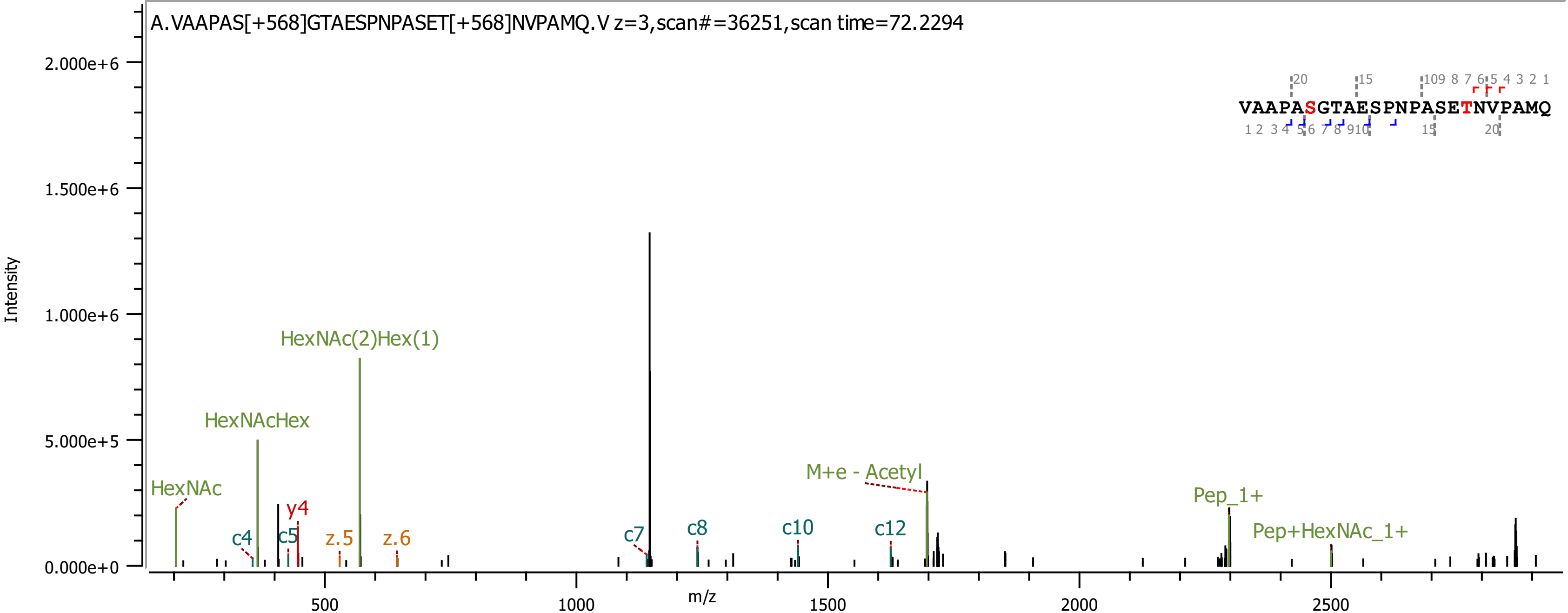
A.VAAPAS[+568]GTAESPNPASET[+568]NVPAMQ.V z=3,scan#=36251,scan time=72.2294
VAAPASGTAESPNPASETNVPAMQ
Intensity
m/z
HexNAc
HexNAcHex
HexNAc(2)Hex(1)
c4
c5
y4
z.5
z.6
c7
c8
c10
c12
M+e - Acetyl
Pep_1+
Pep+HexNAc_1+

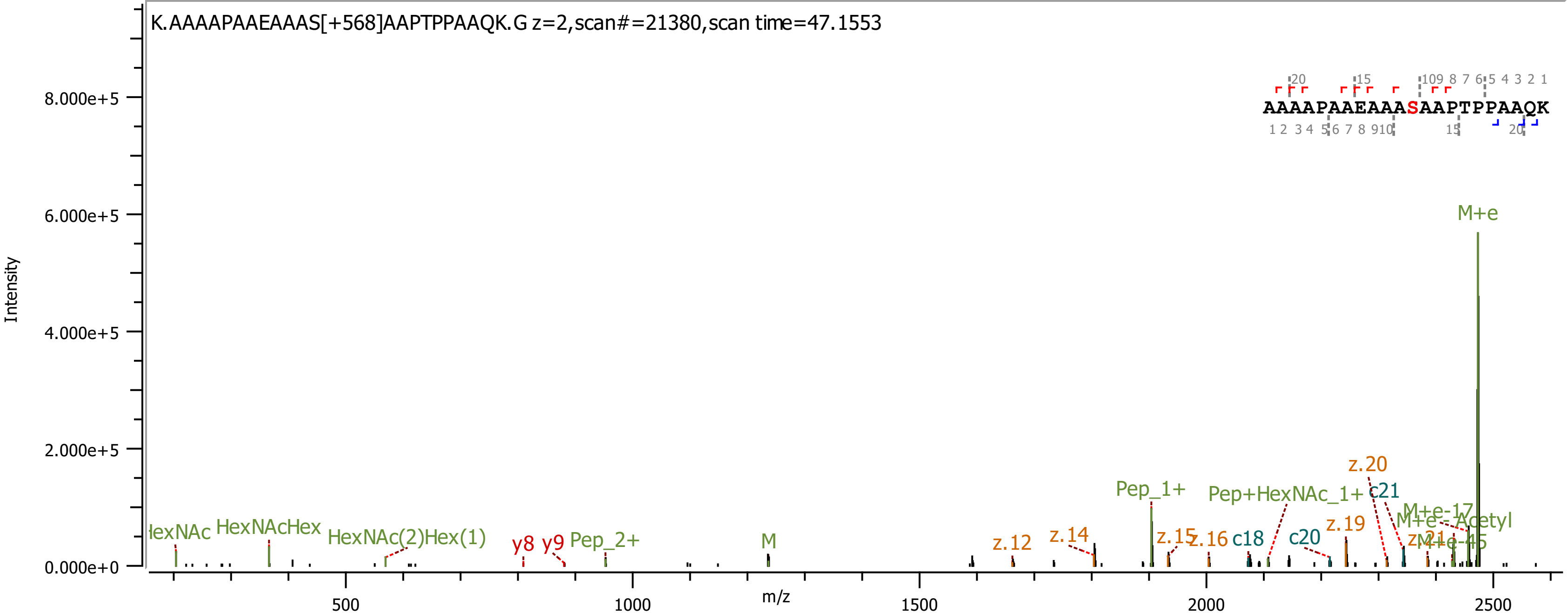
K.AAAAPAAEAAAS[+568]AAPTPPAAQK.G z=2,scan#=21380,scan time=47.1553
AAAAPAAEAAASAAPTPPAAQK
Intensity
m/z
M+e
Pep_1+
Pep+HexNAc_1+
HexNAcHex
HexNAc(2)Hex(1)
y8
y9
Pep_2+
M
z.12
z.14
z.15
z.16
c18
c20
z.19
z.20
c21
M+e-17
M+e-Acetyl
M+e-45
z.21

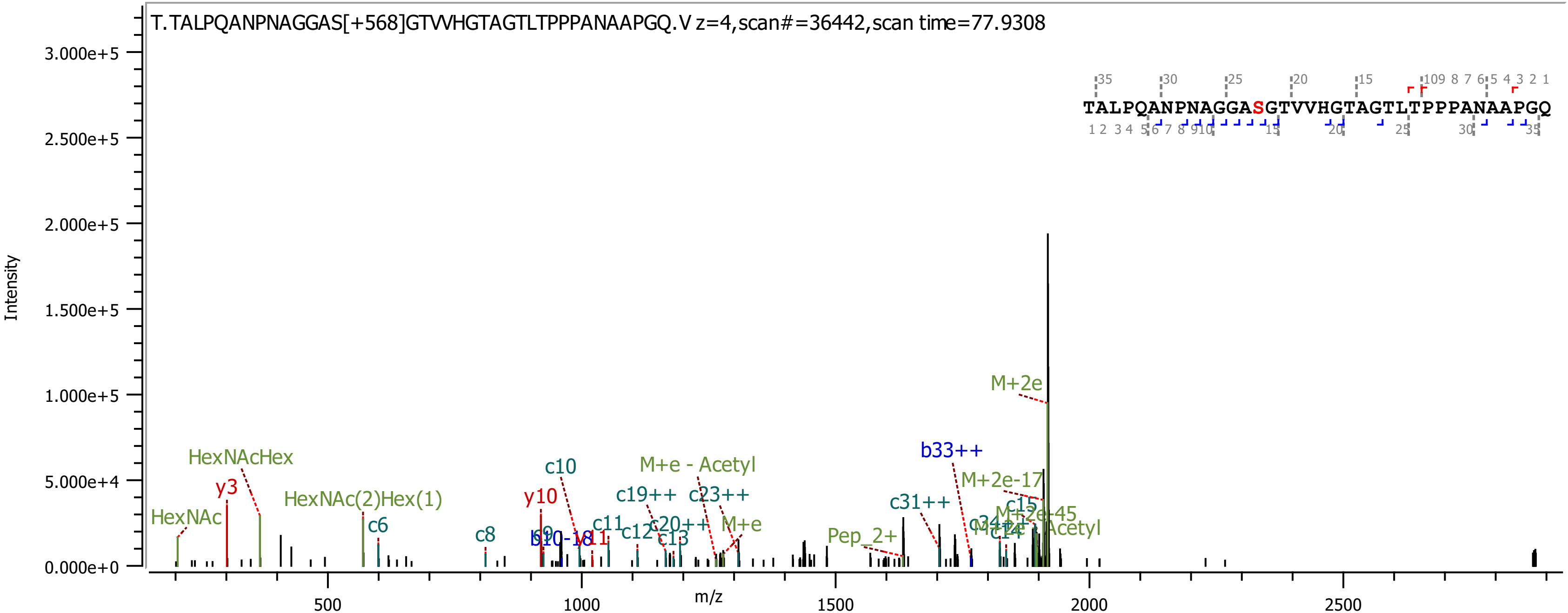
T.TALPQANPNAGGAS[+568]GTVVHGTAGTLTPPPANAAPGQ.V z=4,scan#=36442,scan time=77.9308
TALPQANPNAGGASGTVVHGTAGTLTPPPANAAPGQ
Intensity
m/z
HexNAc
HexNAcHex
y3
HexNAc(2)Hex(1)
c6
c8
c10
y10
c11
c12
c13
c19++
c20++
c23++
M+e - Acetyl
M+e
Pep_2+
c31++
b33++
M+2e-17
M+2e-45
c15
c14
M+2e
Acetyl

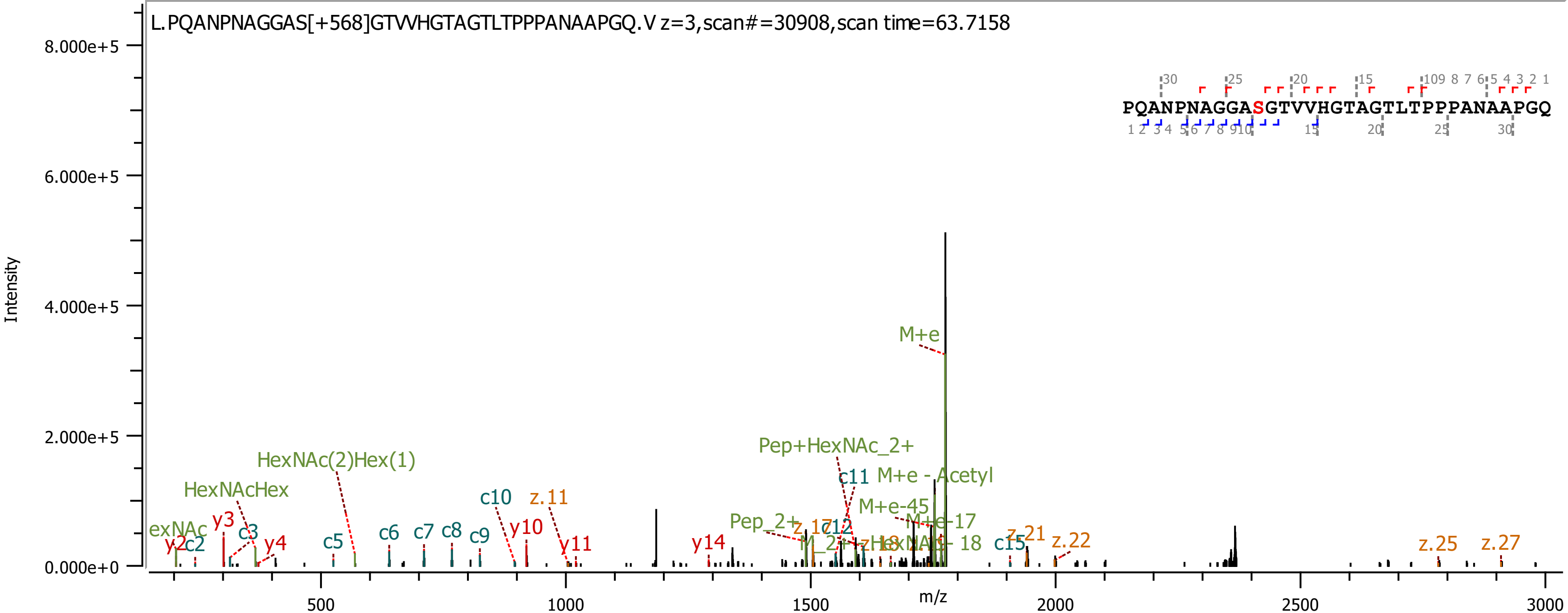

L.PQANPNAGGAS[+568]GTVVHGTAGTLTPPPANAAPGQ.V z=3,scan#=30908,scan time=63.7158
PQANPNAGGASGTVVHGTAGTLTPPPANAAPGQ
Intensity
m/z
M+e
Pep+HexNAc_2+
M+e - Acetyl
M+e-45
M+e-17
HexNAc(2)Hex(1)
HexNAcHex
exNAc
c2
c3
c5
c6
c7
c8
c9
c10
c11
c12
c15
y2
y3
y4
y10
y11
y14
z.11
z.17
z.21
z.22
z.25
z.27
Pep_2+

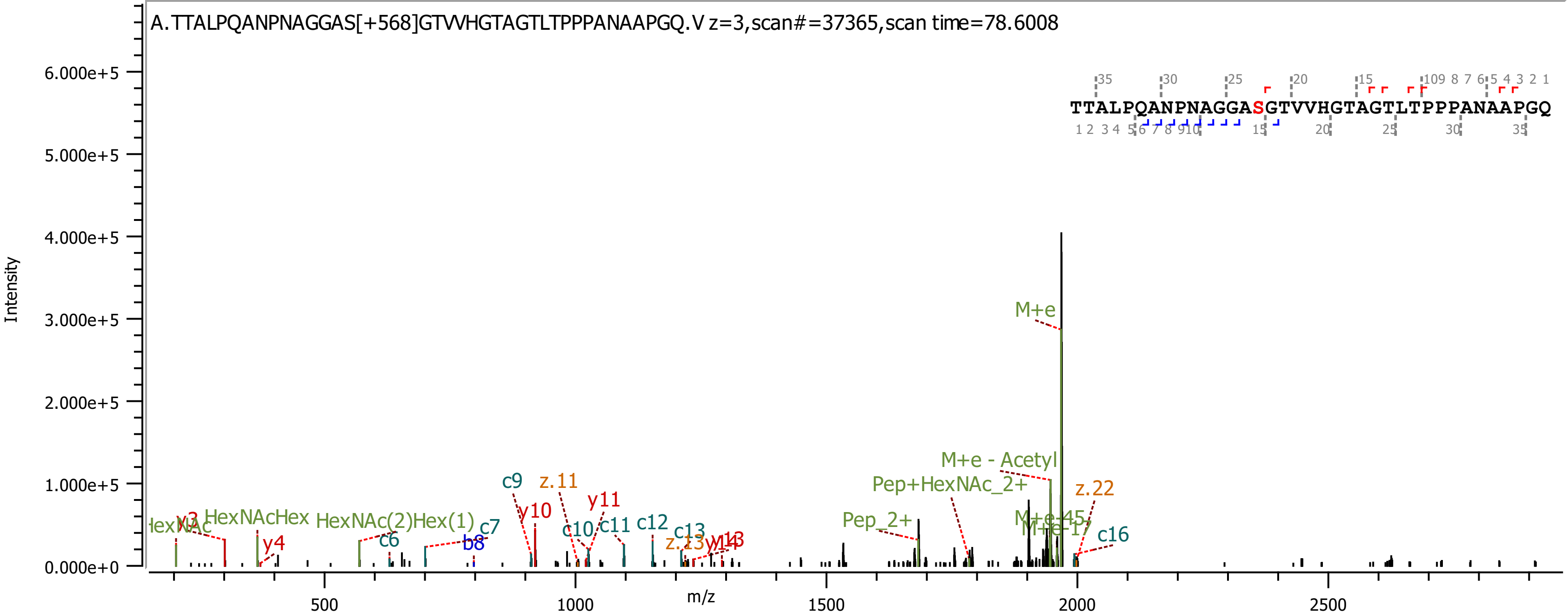

A.TTALPQANPNAGGAS[+568]GTVVHGTAGTLTPPPANAAPGQ.V z=3,scan#=37365,scan time=78.6008
TTALPQANPNAGGASGTVVHGTAGTLTPPPANAAPGQ
Intensity
m/z
M+e
M+e - Acetyl
Pep+HexNAc_2+
Pep_2+
z.22
c16
HexNAcHex
HexNAc(2)Hex(1)
y4
c6
c7
b8
c9
z.11
y10
y11
c10
c11
c12
c13

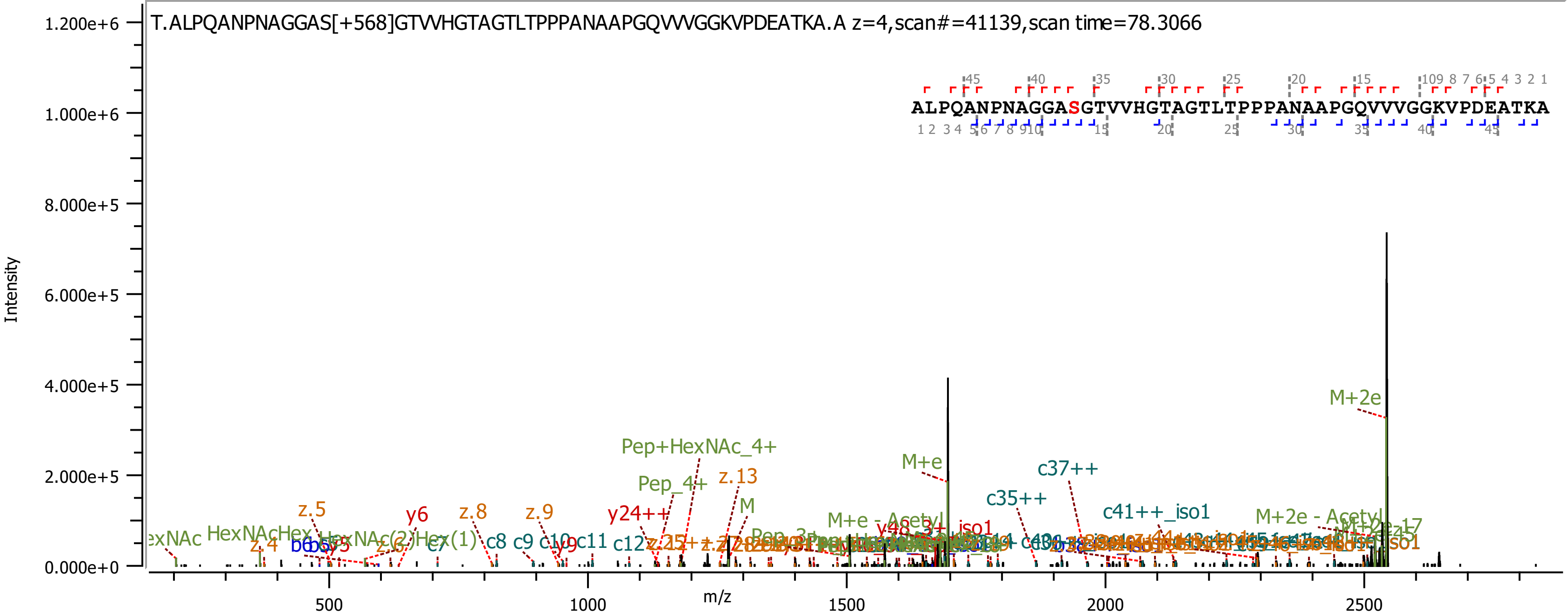

T.ALPQANPNAGGAS[+568]GTVVHGTAGTLTPPPANAAPGQVVVGGKVPDEATKA.A z=4,scan#=41139,scan time=78.3066
ALPQANPNAGGASGTVVHGTAGTLTPPPANAAPGQVVVGGKVPDEATKA
Intensity
m/z
1.200e+6
1.000e+6
8.000e+5
6.000e+5
4.000e+5
2.000e+5
0.000e+0
500
1000
1500
2000
2500
M+2e
Pep+HexNAc_4+
M+e
c37++
z.13
Pep_4+
c35++
z.5
z.8
z.9
y6
y24++
M
c41++_iso1
M+2e - Acetyl
M+e - Acetyl
HexNAcHex
HexNAc(2)Hex(1)
c8
c9
c11
c12
z.4
c7

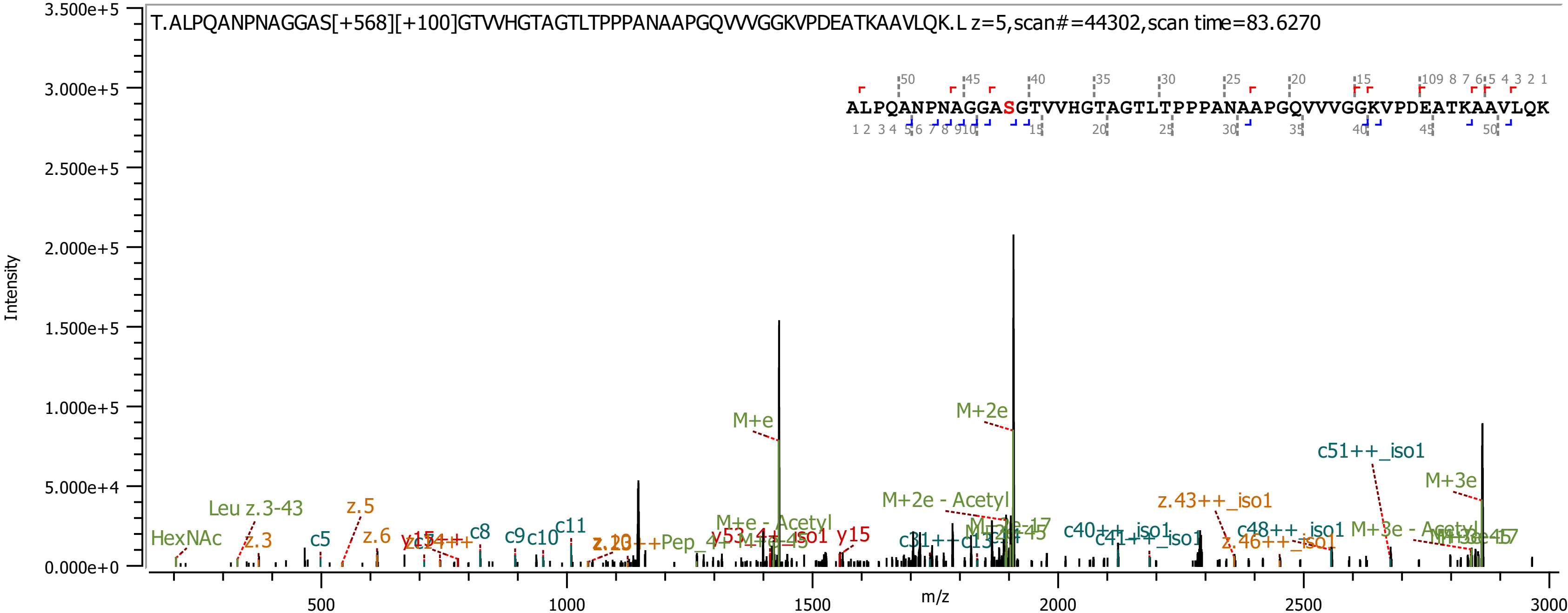
T.ALPQANPNAGGAS[+568][+100]GTVVHGTAGTLTPPPANAAPGQVVVGGKVPDEATKAAVLQK.L z=5,scan#=44302,scan time=83.6270
ALPQANPNAGGASGTVVHGTAGTLTPPPANAAPGQVVVGGKVPDEATKAAVLQK
Intensity
m/z
3.500e+5
3.000e+5
2.500e+5
2.000e+5
1.500e+5
1.000e+5
5.000e+4
0.000e+0
500
1000
1500
2000
2500
3000
M+e
M+2e
M+3e
c51++_iso1
M+2e - Acetyl
M+e - Acetyl
M+3e - Acetyl
z.43++_iso1
z.46++_iso1
c40++_iso1
c41++_iso1
c48++_iso1
HexNAc
Leu z.3-43
z.3
z.5
z.6
c5
c8
c9
c10
c11
y15

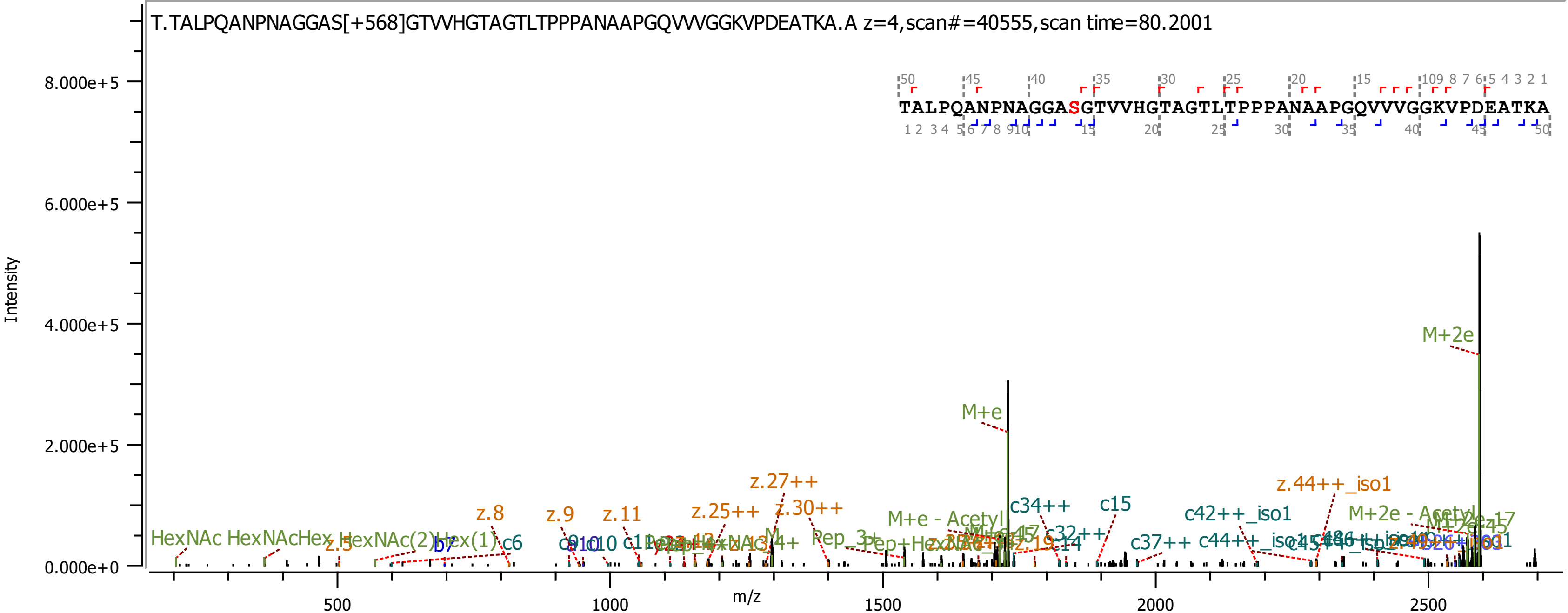
T.TALPQANPNAGGAS[+568]GTVVHGTAGTLTPPPANAAPGQVVVGGKVPDEATKA.A z=4,scan#=40555,scan time=80.2001
TALPQANPNAGGASGTVVHGTAGTLTPPPANAAPGQVVVGGKVPDEATKA
Intensity
m/z
0.000e+0
2.000e+5
4.000e+5
6.000e+5
8.000e+5
500
1000
1500
2000
2500
M+2e
M+e
z.44++_iso1
z.27++
z.30++
z.25++
z.8
z.9
z.11
z.5
c34++
c15
c42++_iso1
c37++
c44++_iso1
c32++
c6
b7
HexNAc
HexNAcHex
HexNAc(2)Hex(1)
M+e - Acetyl
M+2e - Acetyl
Pep_3+

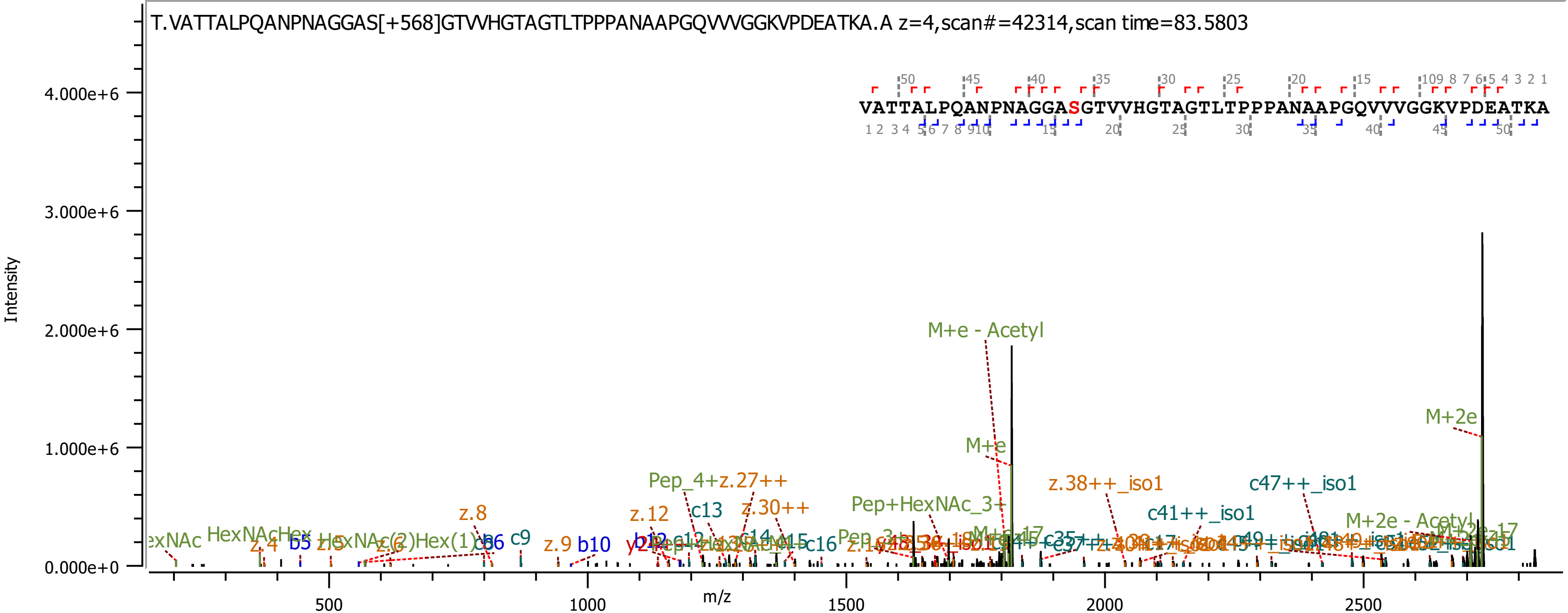
T.VATTALPQANPNAGGAS[+568]GTVVHGTAGTLTPPPANAAPGQVVVGGKVPDEATKA.A z=4,scan#=42314,scan time=83.5803
VATTALPQANPNAGGASGTVVHGTAGTLTPPPANAAPGQVVVGGKVPDEATKA
Intensity
4.000e+6
3.000e+6
2.000e+6
1.000e+6
0.000e+0
m/z
500
1000
1500
2000
2500
M+e - Acetyl
M+2e
M+e
Pep_4+z.27++
z.38++_iso1
c47++_iso1
Pep+HexNAc_3+
z.30++
c41++_iso1
M+2e - Acetyl
z.8
z.12
c13
c9
z.9
b10
HexNAcHex
b5
c16

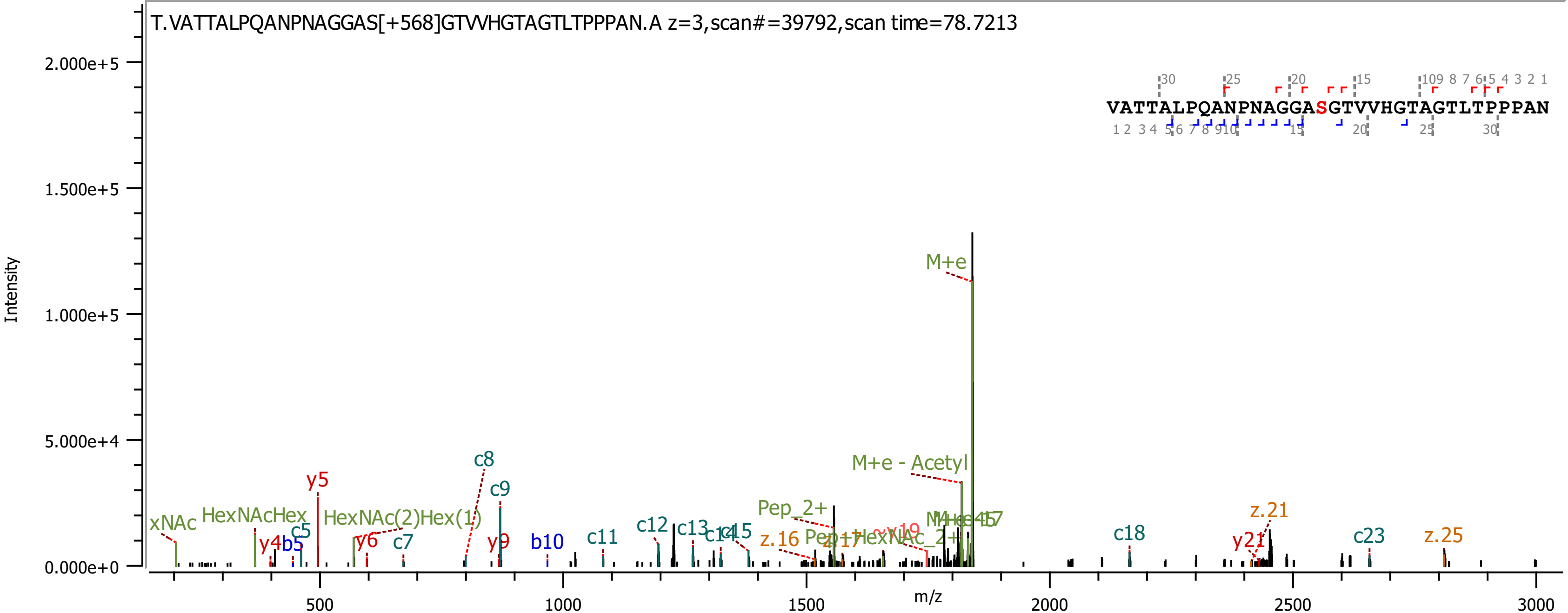

T.VATTALPQANPNAGGAS[+568]GTVVHGTAGTLTPPPAN.A z=3,scan#=39792,scan time=78.7213
VATTALPQANPNAGGASGTVVHGTAGTLTPPPAN
Intensity
m/z
2.000e+5
1.500e+5
1.000e+5
5.000e+4
0.000e+0
500
1000
1500
2000
2500
3000
M+e
M+e - Acetyl
Pep_2+
y5
c8
c9
HexNAcHex
HexNAc(2)Hex(1)
y4
b5
c5
y6
c7
y9
b10
c11
c12
c13
z.16
c18
z.21
y21
c23
z.25

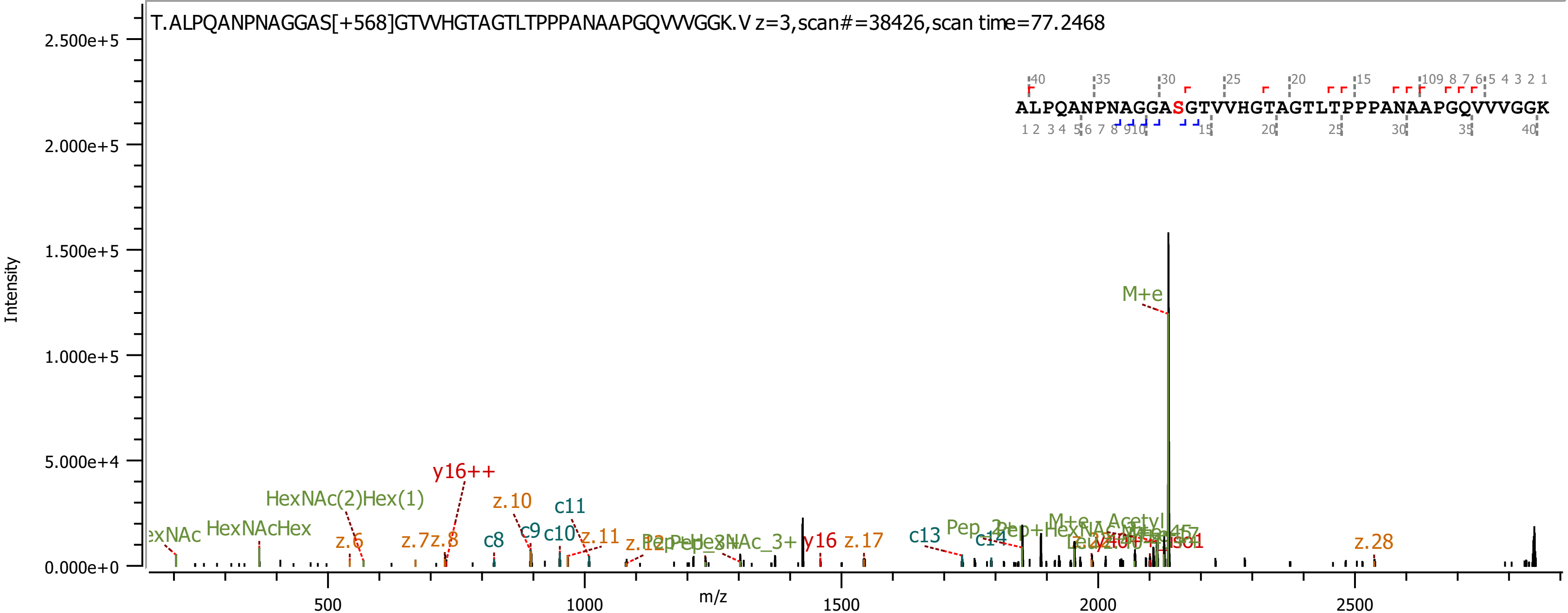

T.ALPQANPNAGGAS[+568]GTVVHGTAGTLTPPPANAAPGQVVVGGK.V z=3,scan#=38426,scan time=77.2468
ALPQANPNAGGASGTVVHGTAGTLTPPPANAAPGQVVVGGK
Intensity
m/z
M+e
y16++
HexNAc(2)Hex(1)
HexNAcHex
z.10
c11
c9
c10
z.11
c8
z.6
z.7
z.8
y16
z.17
c13
c14
z.28
M+e - Acetyl

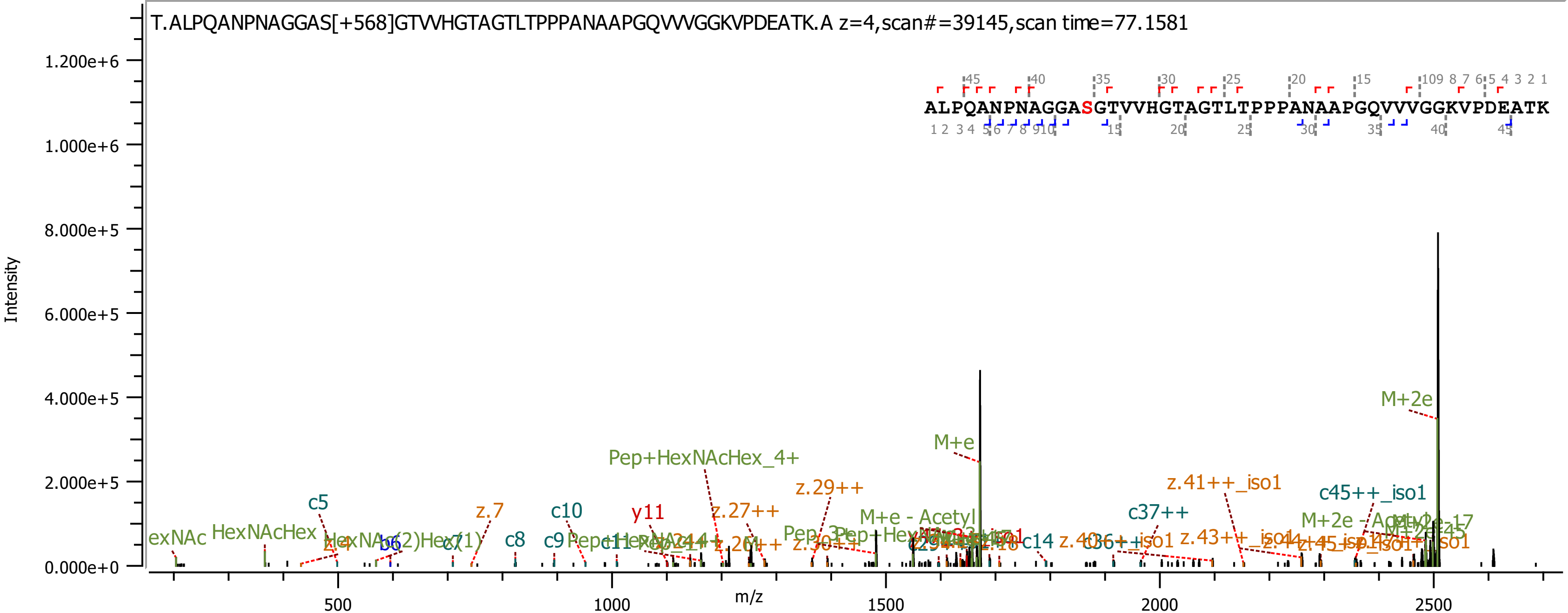

T.ALPQANPNAGGAS[+568]GTVVHGTAGTLTPPPANAAPGQVVVGGKVPDEATK.A z=4,scan#=39145,scan time=77.1581
ALPQANPNAGGASGTVVHGTAGTLTPPPANAAPGQVVVGGKVPDEATK
Intensity
m/z
M+2e
M+e
Pep+HexNAcHex_4+
z.29++
z.41++_iso1
c45++_iso1
c37++
M+e - Acetyl
M+2e - Acetyl
c5
z.7
c10
y11
z.27++
HexNAcHex
c8
c9
c14

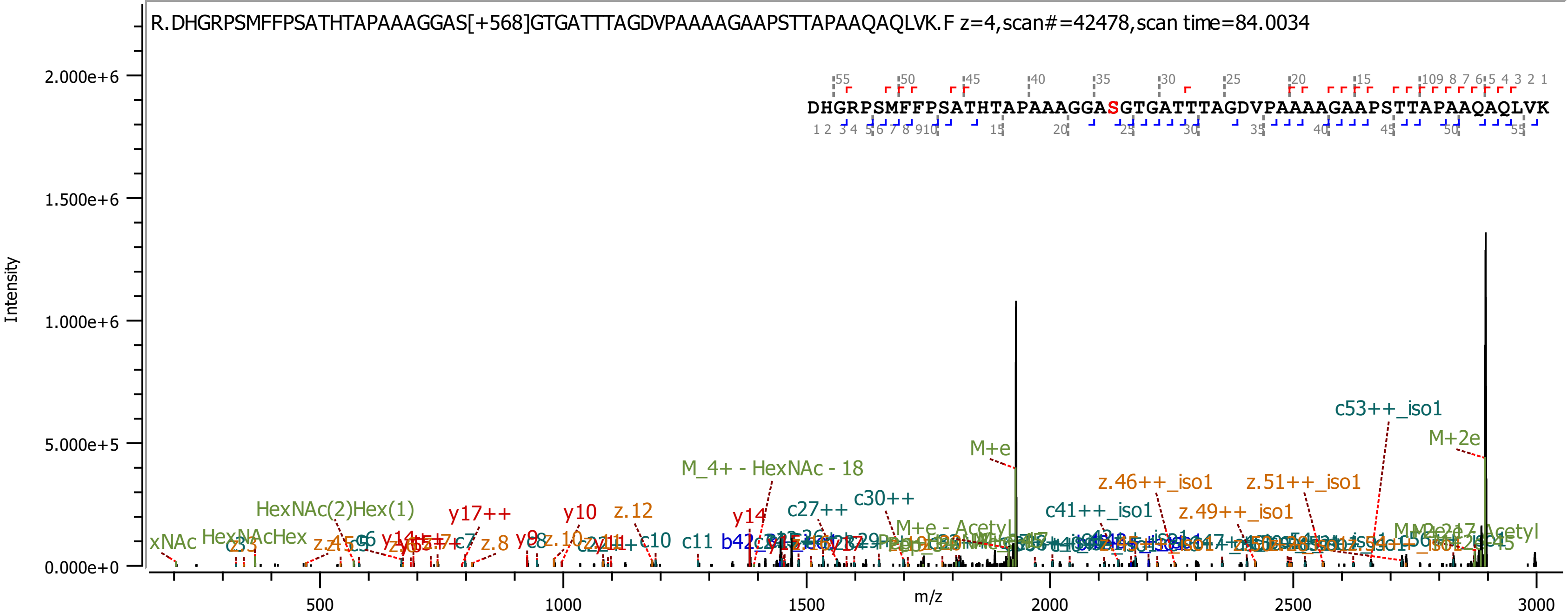
R.DHGRPSMFFPSATHTAPAAAGGAS[+568]GTGATTTAGDVPAAAAGAAPSTTAPAAQAQLVK.F z=4,scan#=42478,scan time=84.0034
DHGRPSMFFPSATHTAPAAAGGASGTGATTTAGDVPAAAAGAAPSTTAPAAQAQLVK
Intensity
m/z
2.000e+6
1.500e+6
1.000e+6
5.000e+5
0.000e+0
500
1000
1500
2000
2500
3000
c53++_iso1
M+2e
M+e
M_4+ - HexNAc - 18
z.46++_iso1
z.51++_iso1
c30++
c27++
c41++_iso1
z.49++_iso1
HexNAc(2)Hex(1)
y17++
y10
z.12
y14
HexNAcHex
xNAc
c6
c7
z.8
y9
c8
c10
c11
M+e - Acetyl

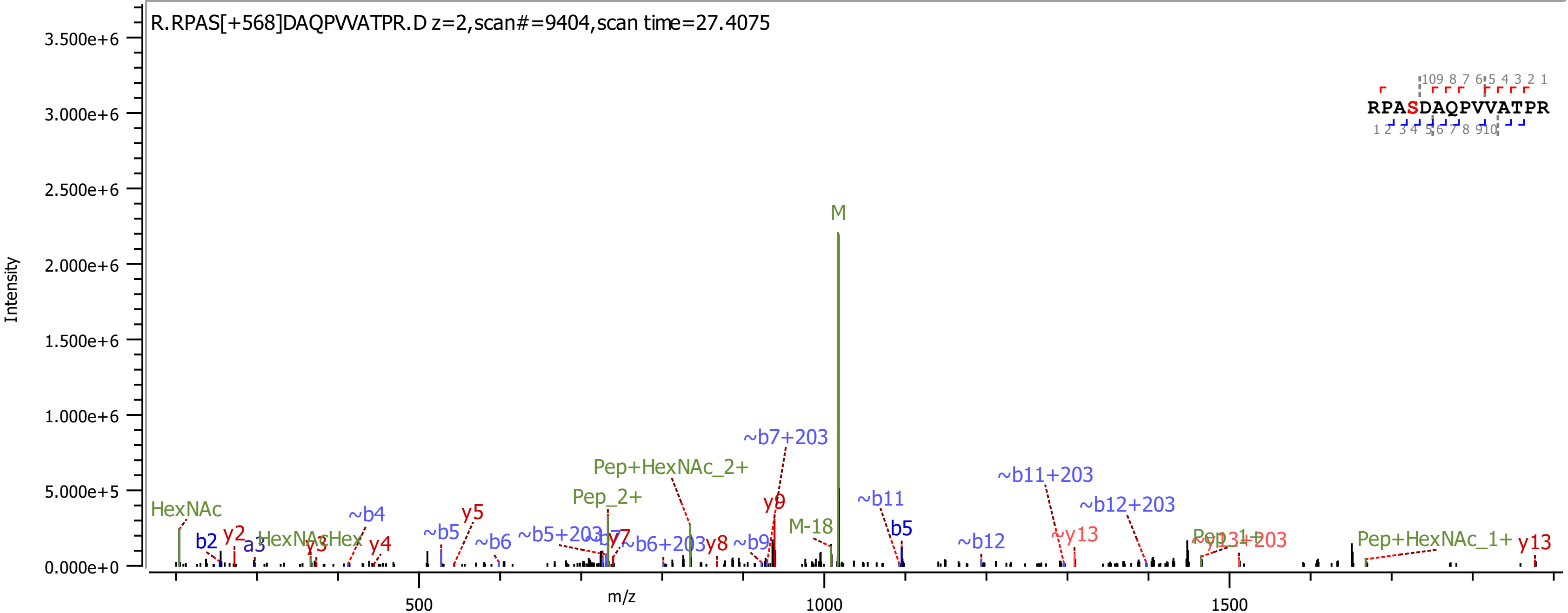
R.RPAS[+568]DAQPVVATPR.D z=2,scan#=9404,scan time=27.4075
Intensity
m/z
RPASDAQPVVATPR
M
~b7+203
Pep+HexNAc_2+
Pep_2+
HexNAc
~b11+203
~b12+203
~b11
y9
M-18
b5
~y13
y13
~b12
Pep+HexNAc_1+
~b4
y5
~b5
~b6
~b5+203
y7
~b6+203
y8
~b9
b2
y2
a3
y3
y4
HexNAcHex

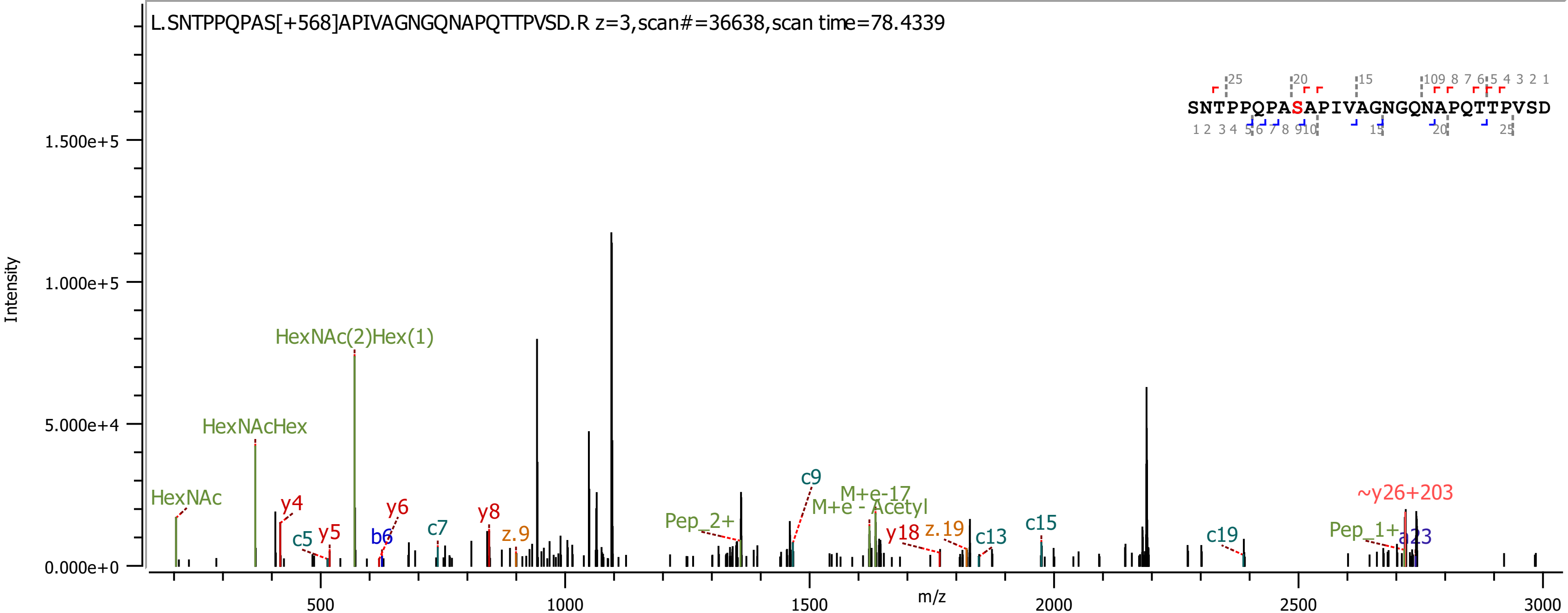

L.SNTPPQPAS[+568]APIVAGNGQNAPQTTPVSD.R z=3,scan#=36638,scan time=78.4339
SNTPPQPASAPIVAGNGQNAPQTTPVSD
Intensity
1.500e+5
1.000e+5
5.000e+4
0.000e+0
HexNAc(2)Hex(1)
HexNAcHex
HexNAc
y4
y5
c5
b6
y6
c7
y8
z.9
Pep_2+
c9
M+e-17
M+e - Acetyl
y18
z.19
c13
c15
c19
~y26+203
Pep_1+
a23
500
1000
1500
2000
2500
3000
m/z

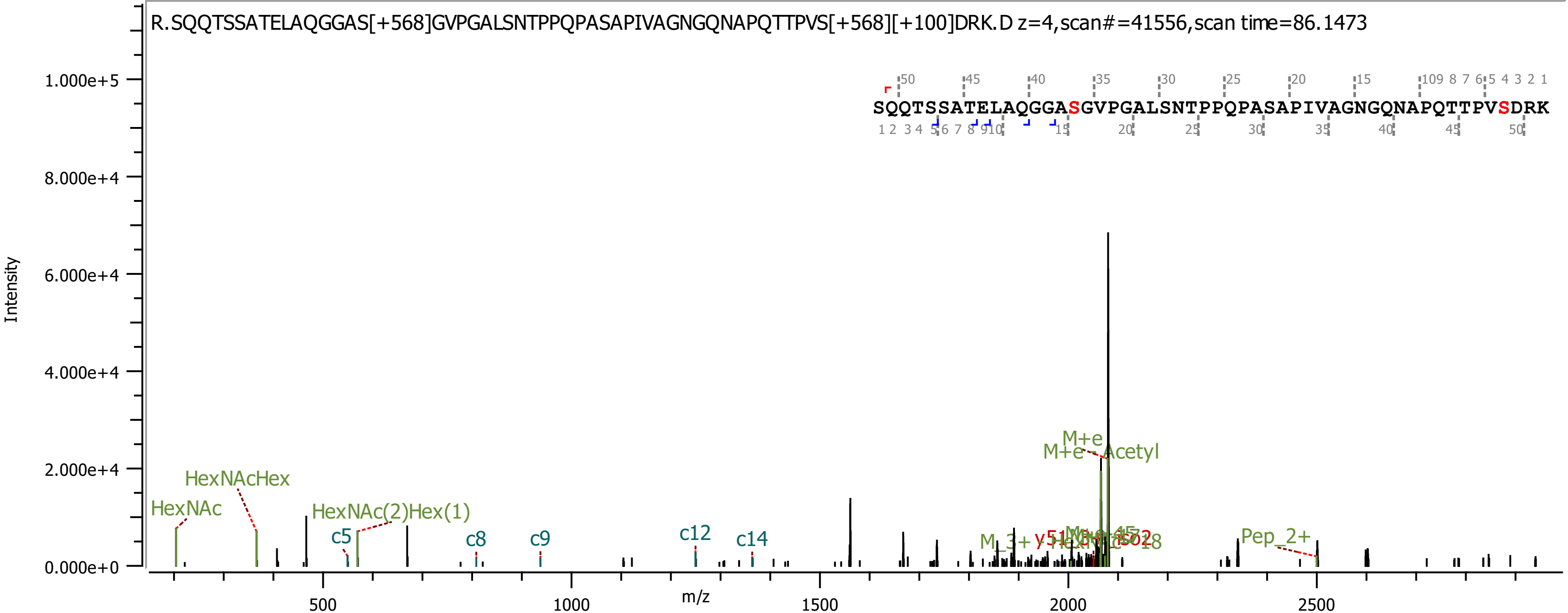
R.SQQTSSATELAQGGAS[+568]GVPGALSNTPPQPASAPIVAGNGQNAPQTTPVS[+568][+100]DRK.D z=4,scan#=41556,scan time=86.1473
SQQTSSATELAQGGASGVPGALSNTPPQPASAPIVAGNGQNAPQTTPVSDRK
Intensity
1.000e+5
8.000e+4
6.000e+4
4.000e+4
2.000e+4
0.000e+0
m/z
500
1000
1500
2000
2500
HexNAc
HexNAcHex
HexNAc(2)Hex(1)
c5
c8
c9
c12
c14
M+e
M+e - Acetyl
Pep_2+

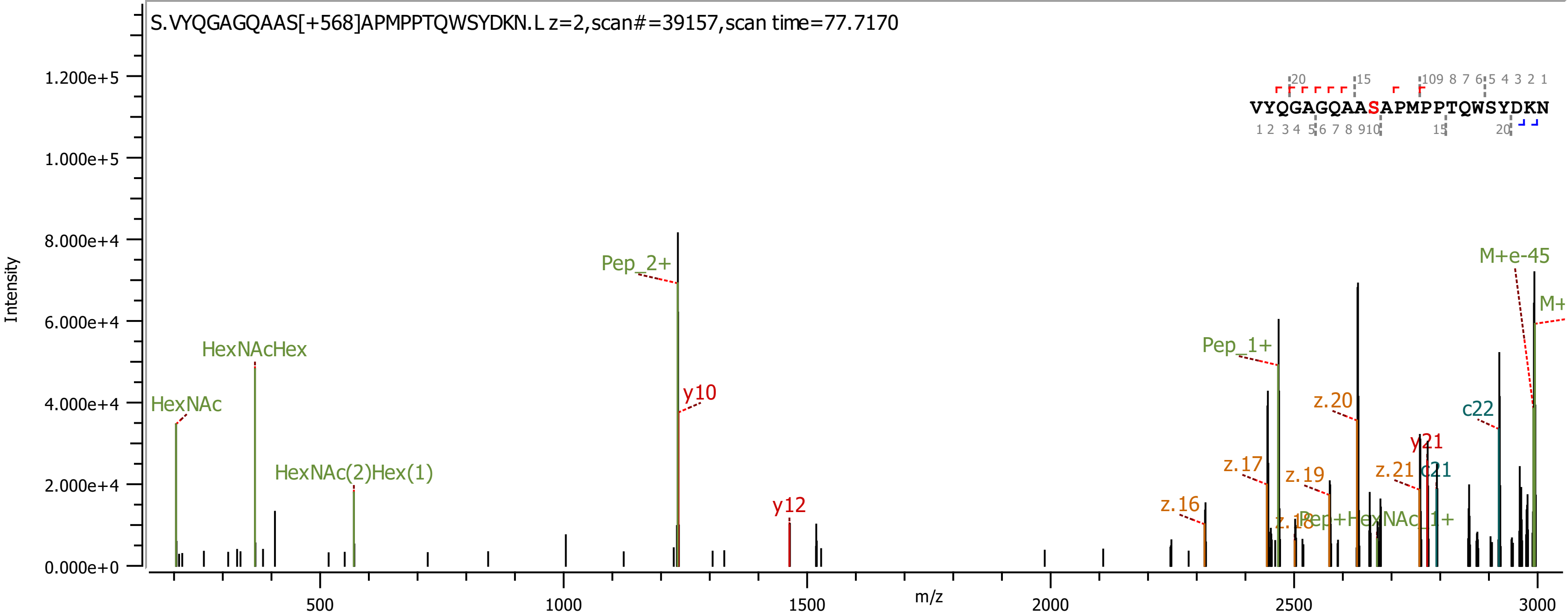
S.VYQGAGQAAS[+568]APMPPTQWSYDKN.L z=2,scan#=39157,scan time=77.7170
VYQGAGQAASAPMPPTQWSYDKN
Intensity
m/z
HexNAc
HexNAcHex
HexNAc(2)Hex(1)
Pep_2+
y10
y12
z.16
z.17
Pep_1+
z.18
z.19
Pep+HexNAc_1+
z.20
z.21
y21
c21
c22
M+e-45
M+

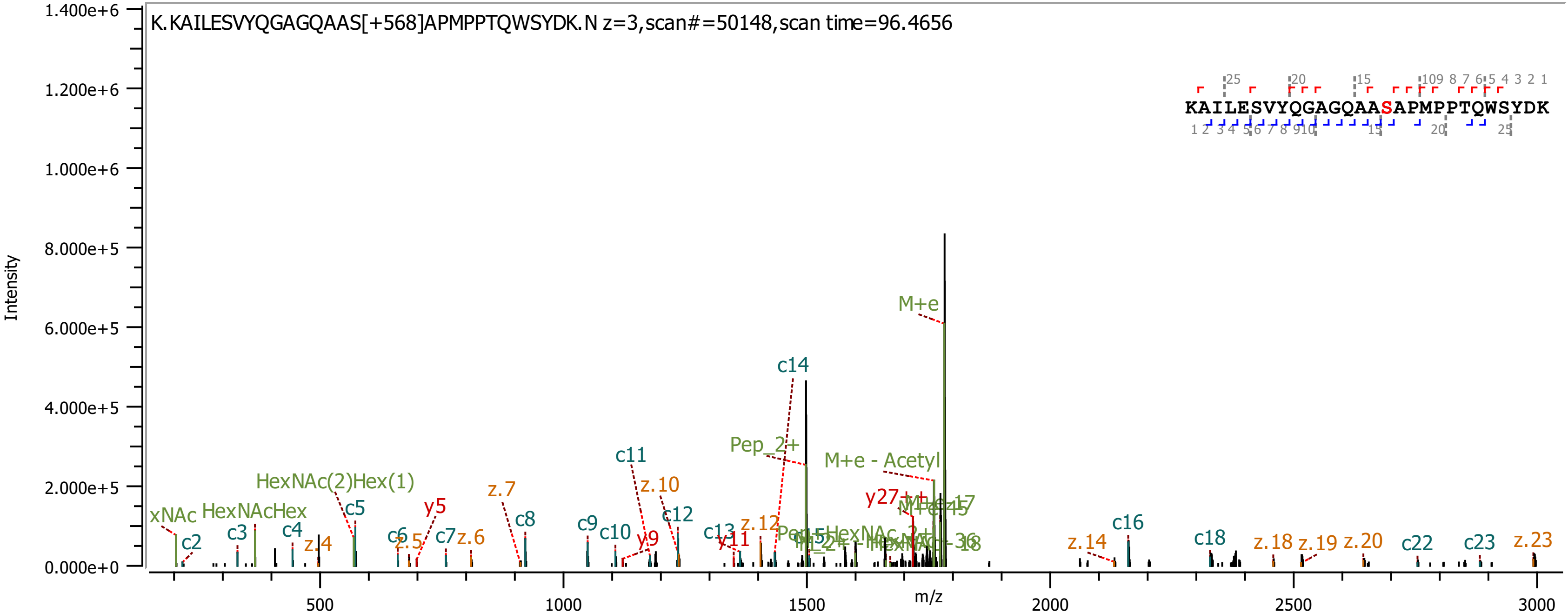
K.KAILESVYQGAGQAAS[+568]APMPPTQWSYDK.N z=3,scan#=50148,scan time=96.4656
KAILESVYQGAGQAASAPMPPTQWSYDK
Intensity
m/z
1.400e+6
1.200e+6
1.000e+6
8.000e+5
6.000e+5
4.000e+5
2.000e+5
0.000e+0
500
1000
1500
2000
2500
3000
M+e
c14
Pep_2+
M+e - Acetyl
c11
z.10
c12
HexNAc(2)Hex(1)
HexNAcHex
xNAc
c2
c3
c4
z.4
c5
y5
c6
z.5
c7
z.6
z.7
c8
c9
c10
y9
c13
y11
z.12
y27++
c16
c18
z.14
z.18
z.19
z.20
c22
c23
z.23

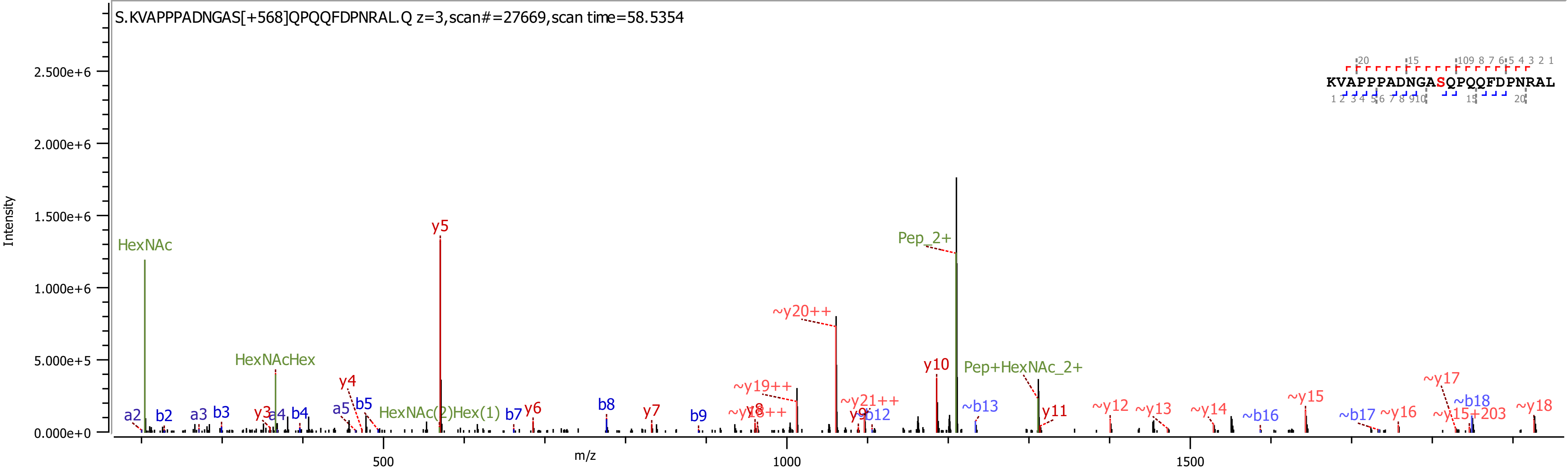
S.KVAPPPADNGAS[+568]QPQQFDPNRAL.Q z=3,scan#=27669,scan time=58.5354
KVAPPPADNGASQPQQFDPNRAL
Intensity
2.500e+6
2.000e+6
1.500e+6
1.000e+6
5.000e+5
0.000e+0
m/z
500
1000
1500
HexNAc
HexNAcHex
HexNAc(2)Hex(1)
y5
Pep_2+
~y20++
y10
Pep+HexNAc_2+
~y19++
~y21++
y4
y6
b8
y7
b9
a2
b2
a3
b3
y3
a4
b4
a5
b5
b7
y9
b12
~b13
y11
~y12
~y13
~y14
~b16
~y15
~b17
~y16
~y17
~b18
~y15+203
~y18

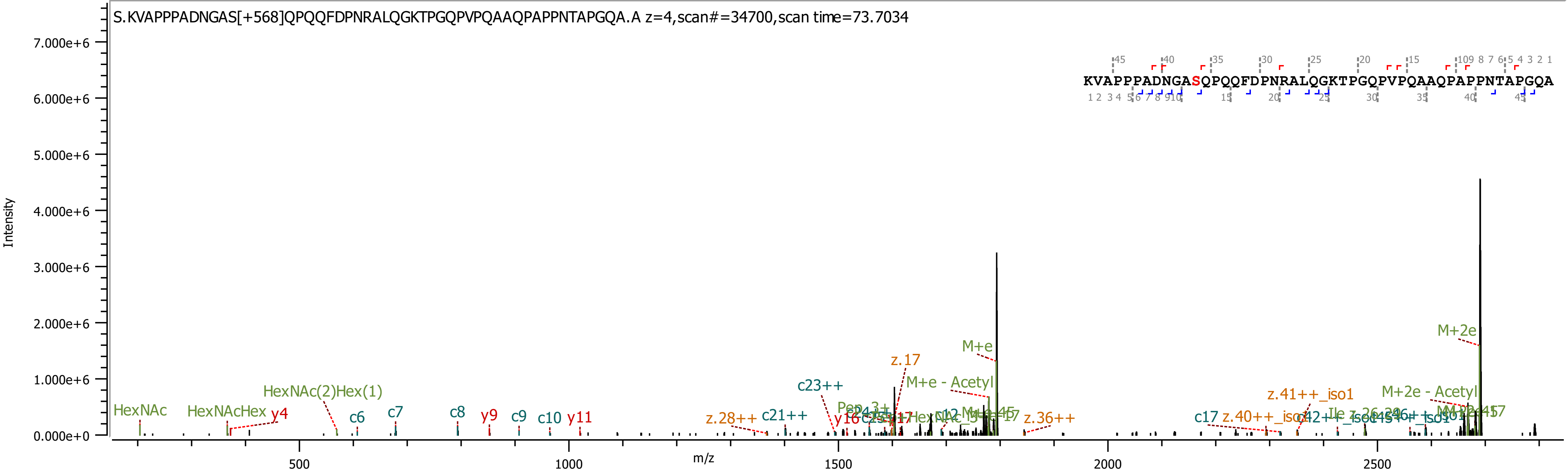
S.KVAPPPADNGAS[+568]QPQQFDPNRALQGKTPGQPVPQAAQPAPPNTAPGQA.A z=4,scan#=34700,scan time=73.7034
KVAPPPADNGASQPQQFDPNRALQGKTPGQPVPQAAQPAPPNTAPGQA
Intensity
m/z
0.000e+0
1.000e+6
2.000e+6
3.000e+6
4.000e+6
5.000e+6
6.000e+6
7.000e+6
500
1000
1500
2000
2500
HexNAc
HexNAcHex
y4
HexNAc(2)Hex(1)
c6
c7
c8
y9
c9
c10
y11
z.28++
c21++
c23++
y16
z.17
M+e - Acetyl
M+e
z.36++
c17
z.40++_iso1
z.41++_iso1
M+2e - Acetyl
M+2e

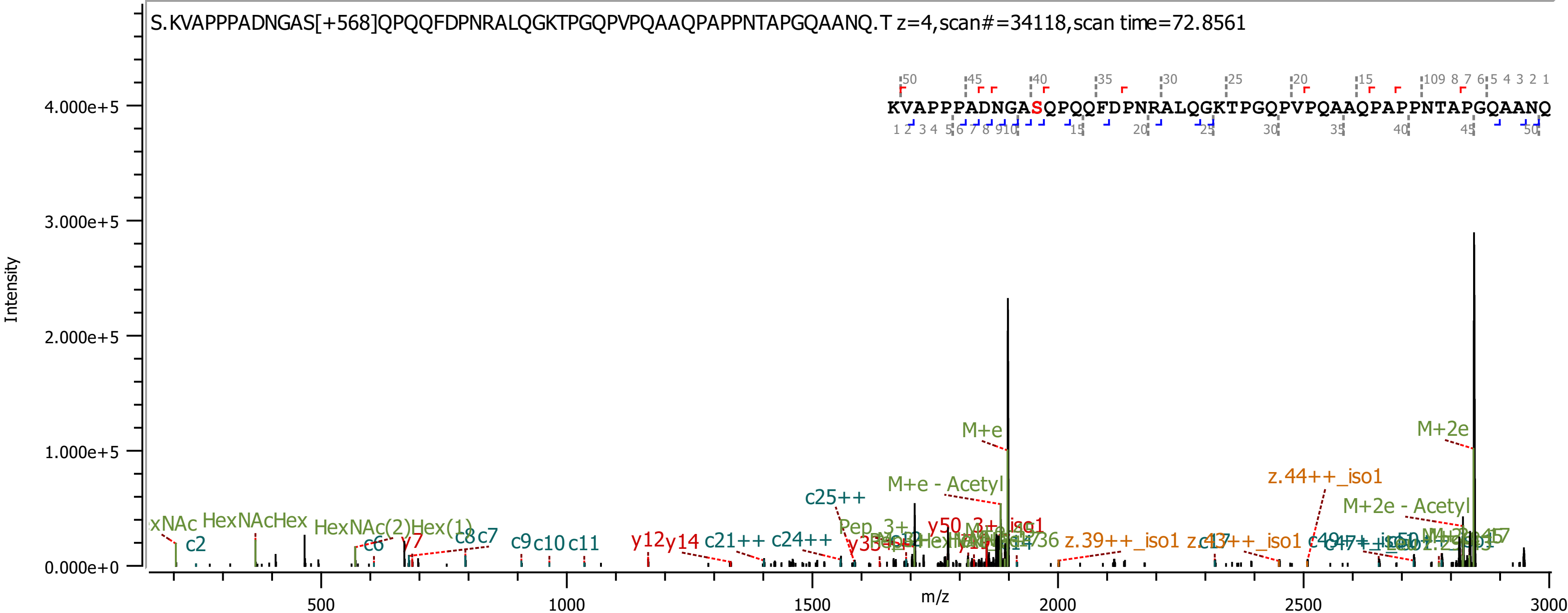
S.KVAPPPADNGAS[+568]QPQQFDPNRALQGKTPGQPVPQAAQPAPPNTAPGQAANQ.T z=4,scan#=34118,scan time=72.8561
KVAPPPADNGASQPQQFDPNRALQGKTPGQPVPQAAQPAPPNTAPGQAANQ
Intensity
m/z
4.000e+5
3.000e+5
2.000e+5
1.000e+5
0.000e+0
500
1000
1500
2000
2500
3000
M+e
M+2e
M+e - Acetyl
M+2e - Acetyl
z.44++_iso1
c25++
HexNAcHex
HexNAc(2)Hex(1)
c2
c6
y7
c8
c7
c9
c10
c11
y12
y14
c21++
c24++
z.39++_iso1

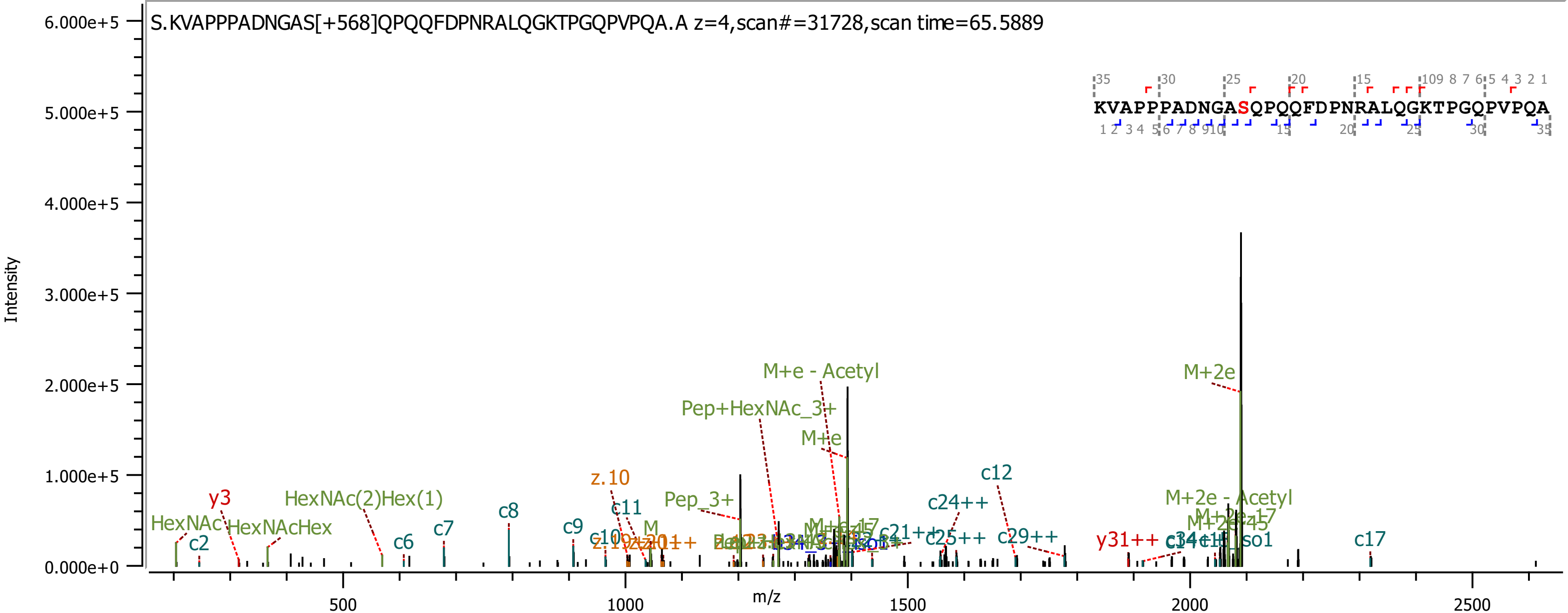

S.KVAPPPADNGAS[+568]QPQQFDPNRALQGKTPGQPVPQA.A z=4,scan#=31728,scan time=65.5889
KVAPPPADNGASQPQQFDPNRALQGKTPGQPVPQA
Intensity
m/z
6.000e+5
5.000e+5
4.000e+5
3.000e+5
2.000e+5
1.000e+5
0.000e+0
500
1000
1500
2000
2500
M+e - Acetyl
Pep+HexNAc_3+
M+e
M+2e
c12
z.10
y3
HexNAc(2)Hex(1)
c8
c11
Pep_3+
c24++
M+2e - Acetyl
HexNAc
HexNAcHex
c2
c6
c7
c9
c10
c25++
c29++
y31++
c17

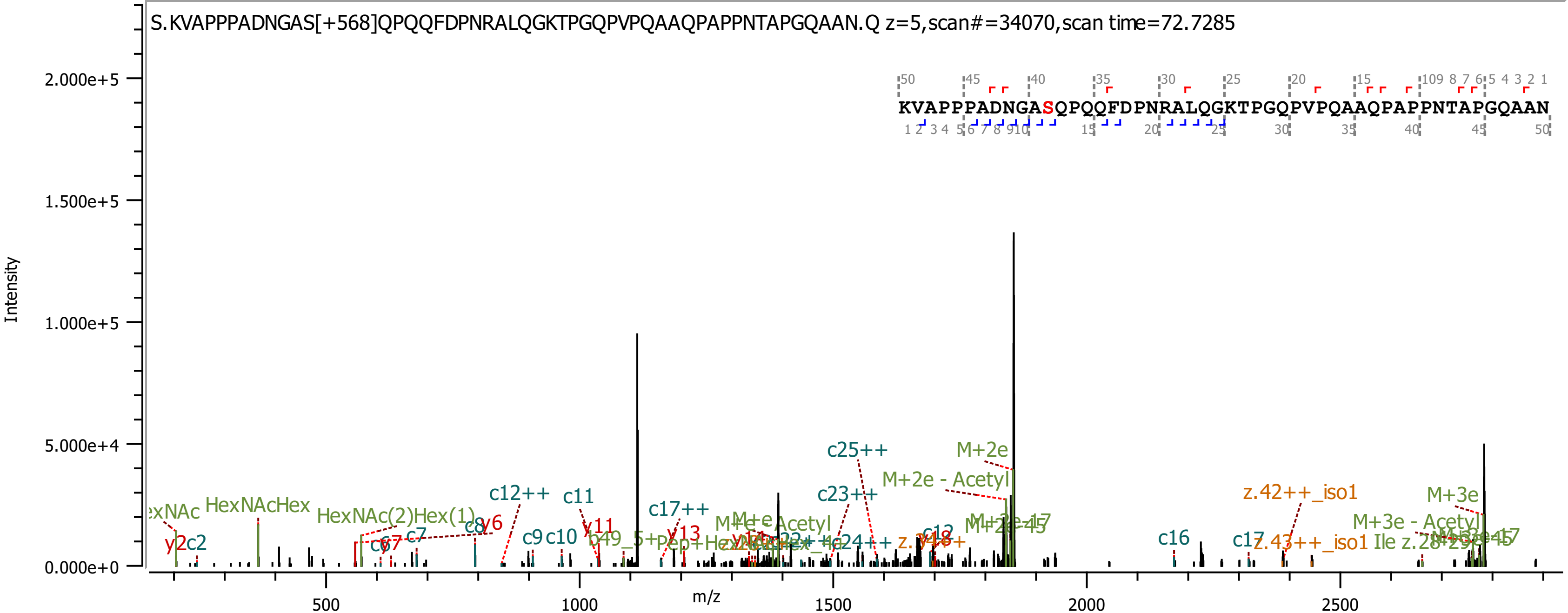

S.KVAPPPADNGAS[+568]QPQQFDPNRALQGKTPGQPVPQAAQPAPPNTAPGQAAN.Q z=5,scan#=34070,scan time=72.7285
KVAPPPADNGASQPQQFDPNRALQGKTPGQPVPQAAQPAPPNTAPGQAAN
Intensity
2.000e+5
1.500e+5
1.000e+5
5.000e+4
0.000e+0
m/z
500
1000
1500
2000
2500
HexNAcHex
HexNAc(2)Hex(1)
y2
c2
c7
c8
y6
c12++
c11
c9
c10
y11
c17++
y13
c25++
c23++
c24++
M+2e
M+2e - Acetyl
c13
c16
c17
z.42++_iso1
z.43++_iso1
M+3e
M+3e - Acetyl

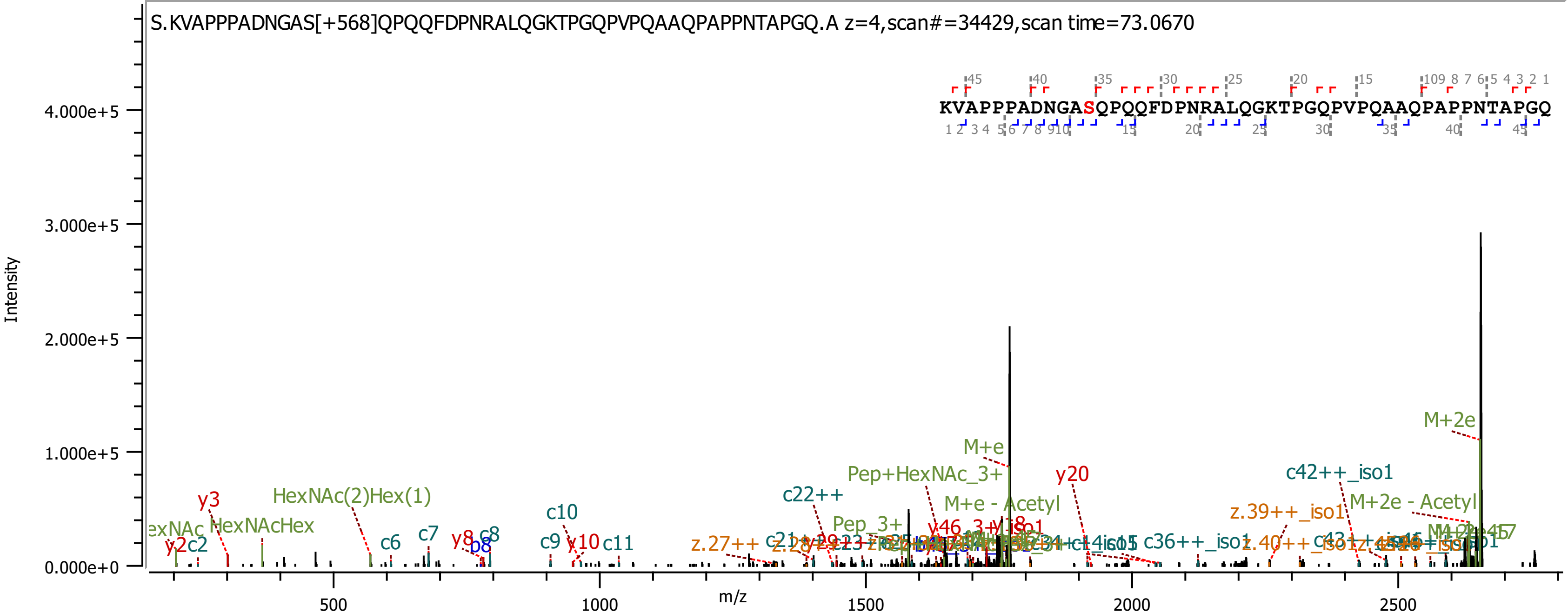
S.KVAPPPADNGAS[+568]QPQQFDPNRALQGKTPGQPVPQAAQPAPPNTAPGQ.A z=4,scan#=34429,scan time=73.0670
KVAPPPADNGASQPQQFDPNRALQGKTPGQPVPQAAQPAPPNTAPGQ
Intensity
4.000e+5
3.000e+5
2.000e+5
1.000e+5
0.000e+0
m/z
500
1000
1500
2000
2500
M+2e
M+e
Pep+HexNAc_3+
y20
c42++_iso1
M+e - Acetyl
M+2e - Acetyl
z.39++_iso1
c22++
Pep_3+
HexNAc(2)Hex(1)
HexNAcHex
y3
y2
c2
c6
c7
y8
b8
c8
c9
c10
y10
c11
z.27++
c36++_iso1
z.40++_iso1

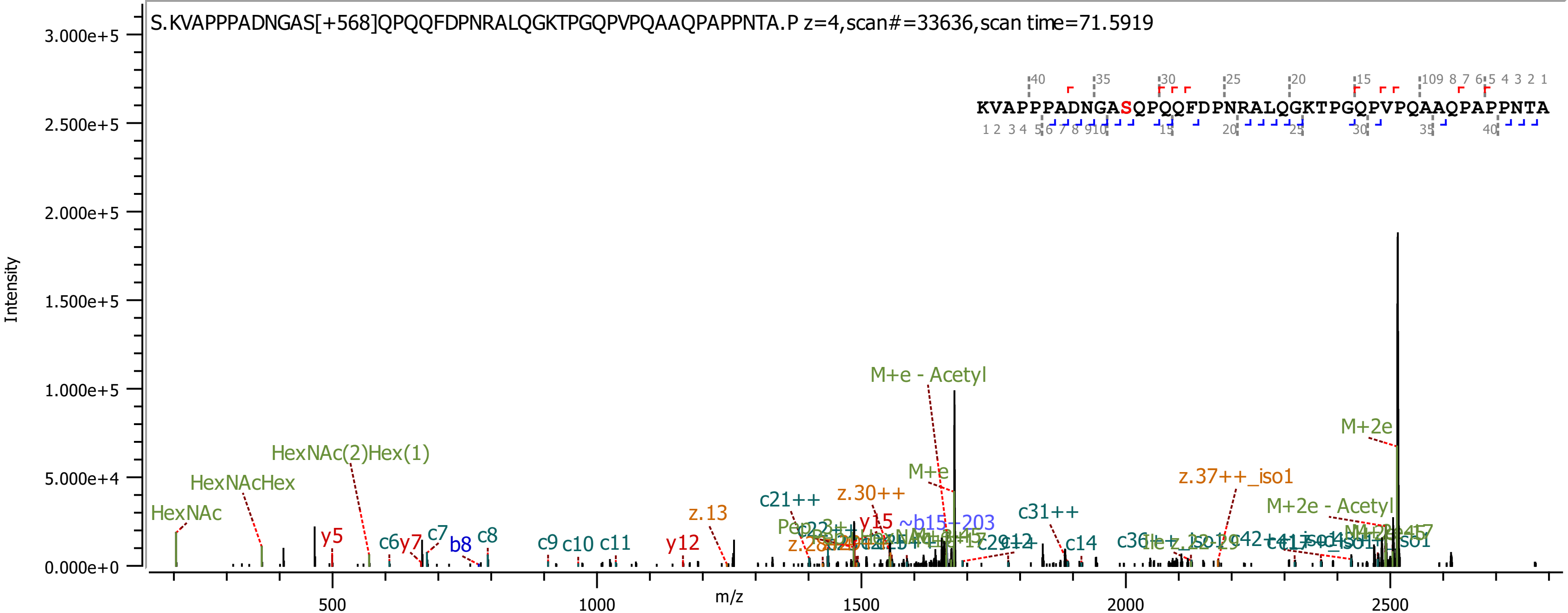
S.KVAPPPADNGAS[+568]QPQQFDPNRALQGKTPGQPVPQAAQPAPPNTA.P z=4,scan#=33636,scan time=71.5919
KVAPPPADNGASQPQQFDPNRALQGKTPGQPVPQAAQPAPPNTA
Intensity
m/z
HexNAc
HexNAcHex
HexNAc(2)Hex(1)
y5
c6
y7
c7
b8
c8
c9
c10
c11
y12
z.13
c21++
z.30++
y15
~b15--203
M+e - Acetyl
M+e
c31++
c14
z.37++_iso1
M+2e - Acetyl
M+2e

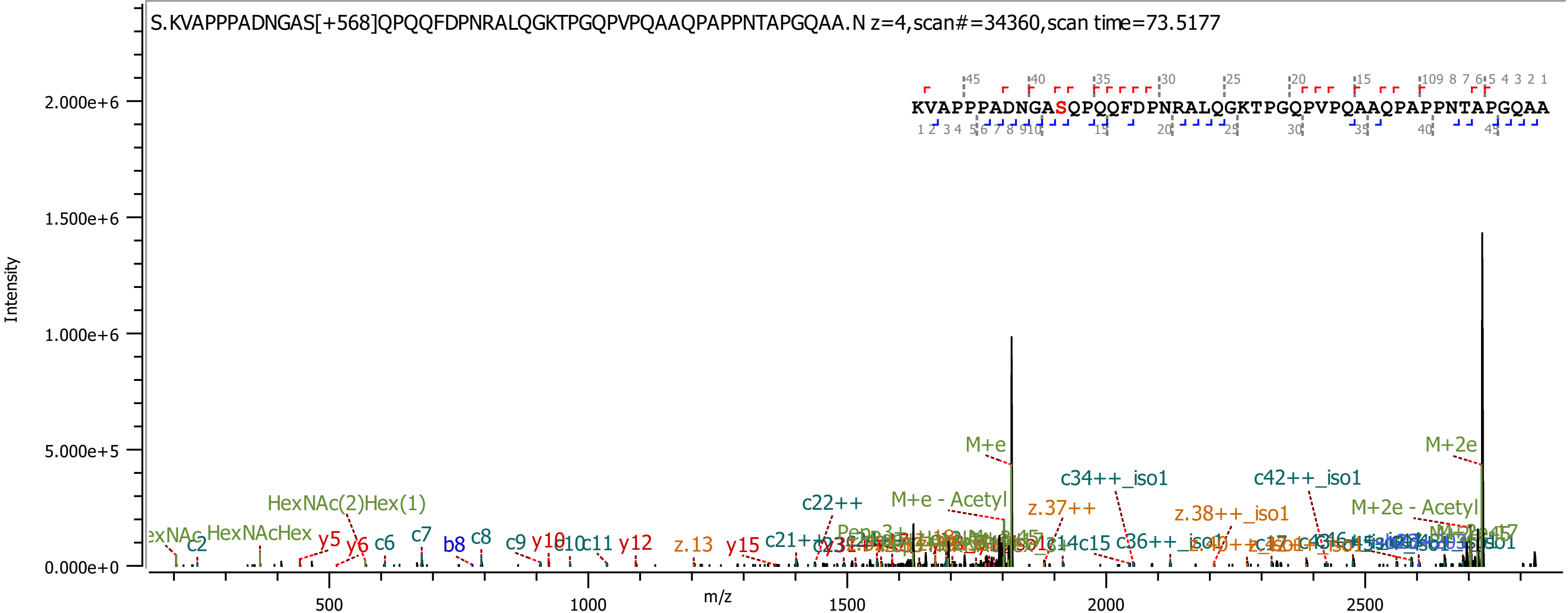
S.KVAPPPADNGAS[+568]QPQQFDPNRALQGKTPGQPVPQAAQPAPPNTAPGQAA.N z=4,scan#=34360,scan time=73.5177
KVAPPPADNGASQPQQFDPNRALQGKTPGQPVPQAAQPAPPNTAPGQAA
Intensity
m/z
M+e
M+2e
M+e - Acetyl
M+2e - Acetyl
c34++_iso1
c42++_iso1
HexNAc(2)Hex(1)
HexNAcHex
c22++
z.37++
z.38++_iso1
y5
y6
c2
c6
c7
b8
c8
c9
y10
c11
y12
z.13
y15
c15
c36++_iso1

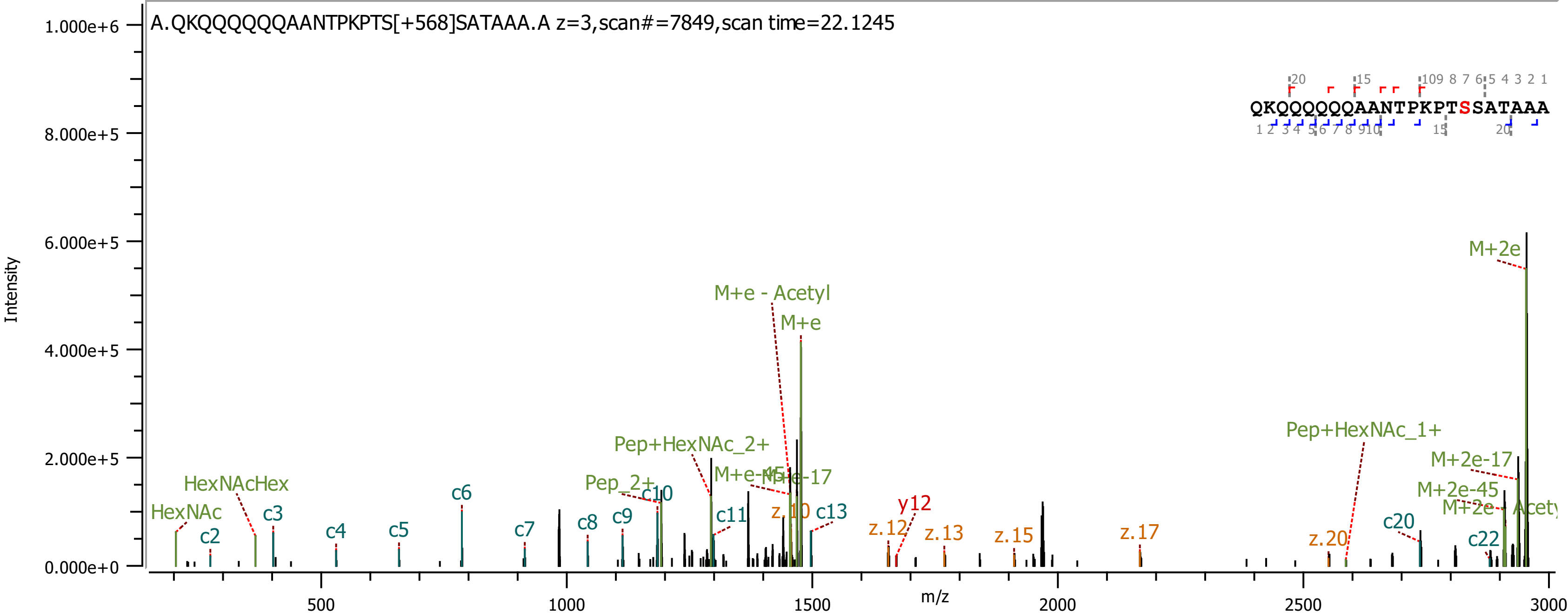

A.QKQQQQQQAANTPKPTS[+568]SATAAA.A z=3,scan#=7849,scan time=22.1245
QKQQQQQQAANTPKPTSSATAAA
Intensity
m/z
1.000e+6
8.000e+5
6.000e+5
4.000e+5
2.000e+5
0.000e+0
500
1000
1500
2000
2500
3000
HexNAc
HexNAcHex
c2
c3
c4
c5
c6
c7
c8
c9
c10
c11
c13
c20
c22
Pep_2+
Pep+HexNAc_2+
M+e - Acetyl
M+e
M+e-45
M+e-17
z.10
z.12
z.13
z.15
z.17
z.20
y12
Pep+HexNAc_1+
M+2e
M+2e-17
M+2e-45

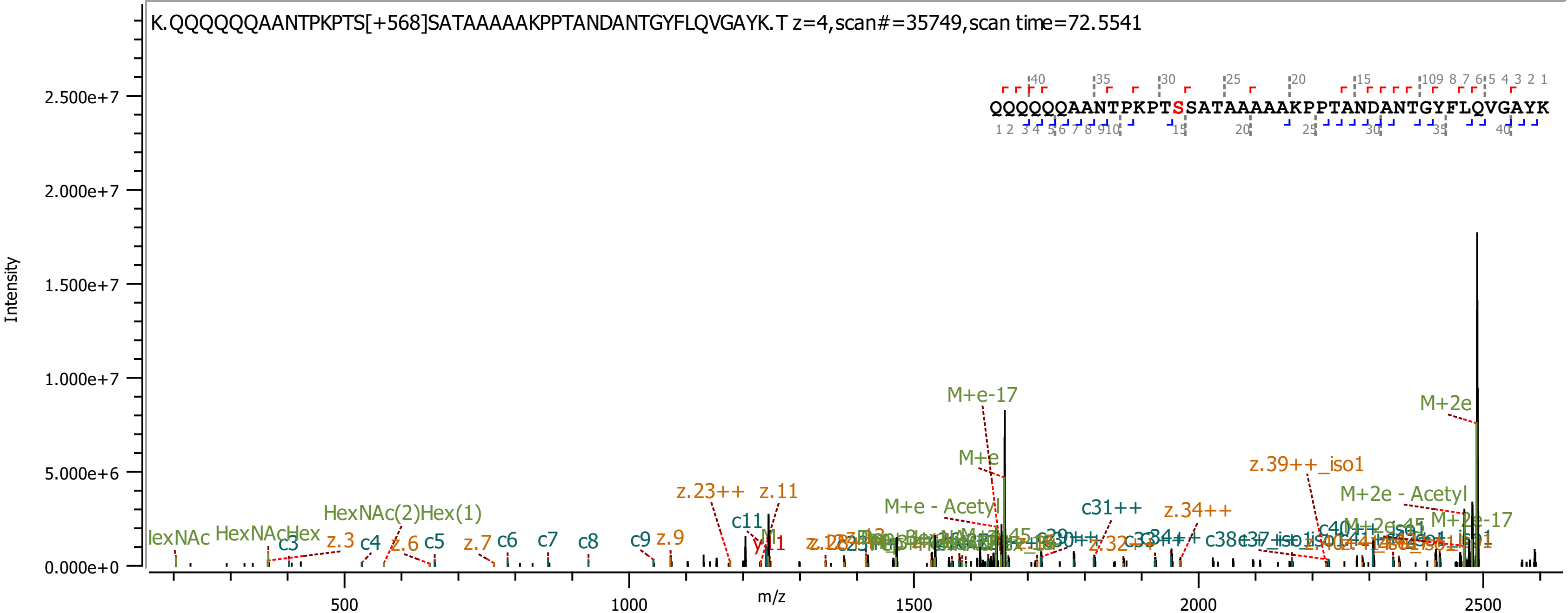
K.QQQQQQAANTPKPTS[+568]SATAAAAAKPPTANDANTGYFLQVGAYK.T z=4,scan#=35749,scan time=72.5541
QQQQQQAANTPKPTSSATAAAAAKPPTANDANTGYFLQVGAYK
Intensity
m/z
M+e-17
M+2e
M+e
z.39++_iso1
M+2e - Acetyl
M+e - Acetyl
c31++
z.34++
M+2e-17
z.23++
z.11
HexNAc(2)Hex(1)
c11
HexNAcHex
z.3
c3
c4
z.6
c5
z.7
c6
c7
c8
c9
z.9
z.32++
c38

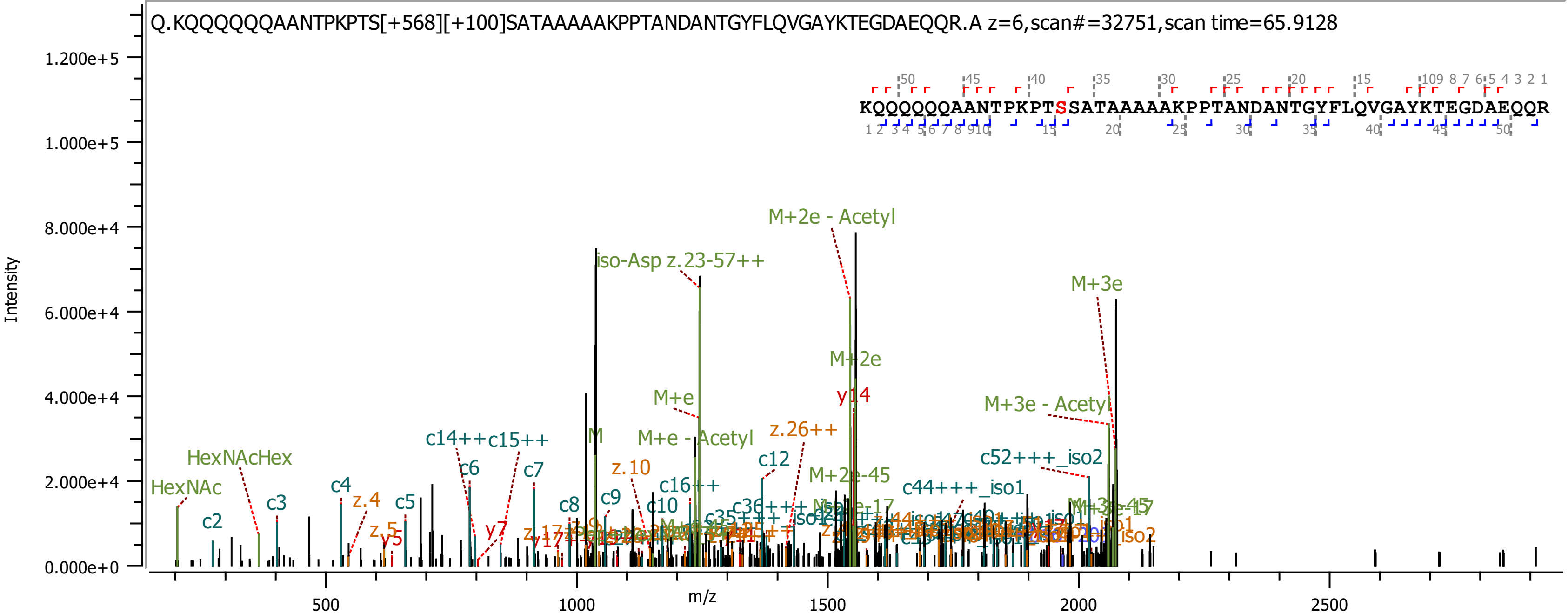

Q.KQQQQQQAANTPKPTS[+568][+100]SATAAAAAKPPTANDANTGYFLQVGAYKTEGDAEQQR.A z=6,scan#=32751,scan time=65.9128
KQQQQQQAANTPKPTSSATAAAAAKPPTANDANTGYFLQVGAYKTEGDAEQQR
Intensity
m/z
M+2e - Acetyl
iso-Asp z.23-57++
M+3e
M+2e
y14
M+e
M+3e - Acetyl
M+e - Acetyl
z.26++
M
c14++
c15++
HexNAcHex
c6
c7
z.10
c12
M+2e-45
c52+++_iso2
HexNAc
c4
c16++
c44+++_iso1
c3
z.4
c5
c8
c9
c10
c2
y7
y5
z.5

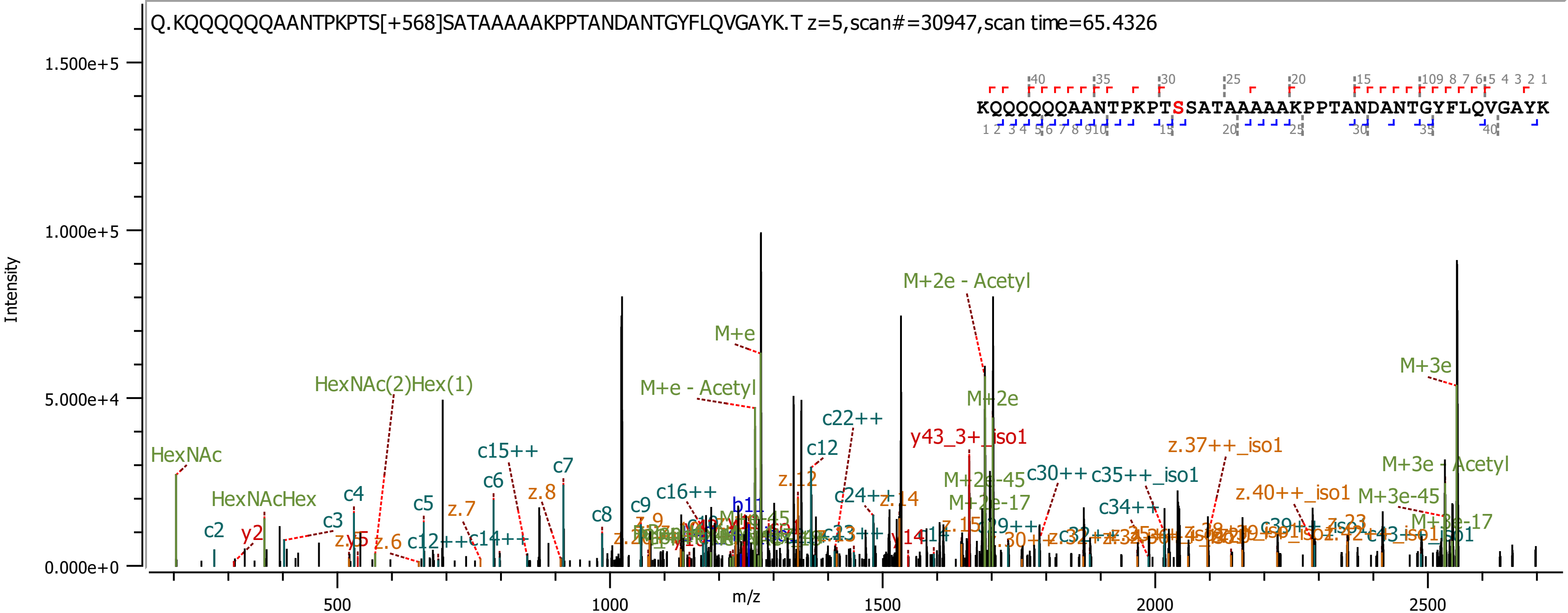

Q.KQQQQQQAANTPKPTS[+568]SATAAAAAKPPTANDANTGYFLQVGAYK.T z=5,scan#=30947,scan time=65.4326
KQQQQQQAANTPKPTSSATAAAAAKPPTANDANTGYFLQVGAYK
Intensity
m/z
1.500e+5
1.000e+5
5.000e+4
0.000e+0
500
1000
1500
2000
2500
HexNAc
HexNAcHex
HexNAc(2)Hex(1)
M+e
M+e - Acetyl
M+2e - Acetyl
M+2e
M+3e
M+3e - Acetyl
M+3e-45
M+3e-17
M+2e-45
M+2e-17
y43_3+_iso1
z.37++_iso1
z.40++_iso1
c22++
c12
c15++
c6
c7
c4
c5
c3
c2
y2
c8
c9
c16++
c24++
c30++
c35++_iso1
c34++
z.8
z.7
z.6
z.12
z.14
y14
c12++
c14++
b11

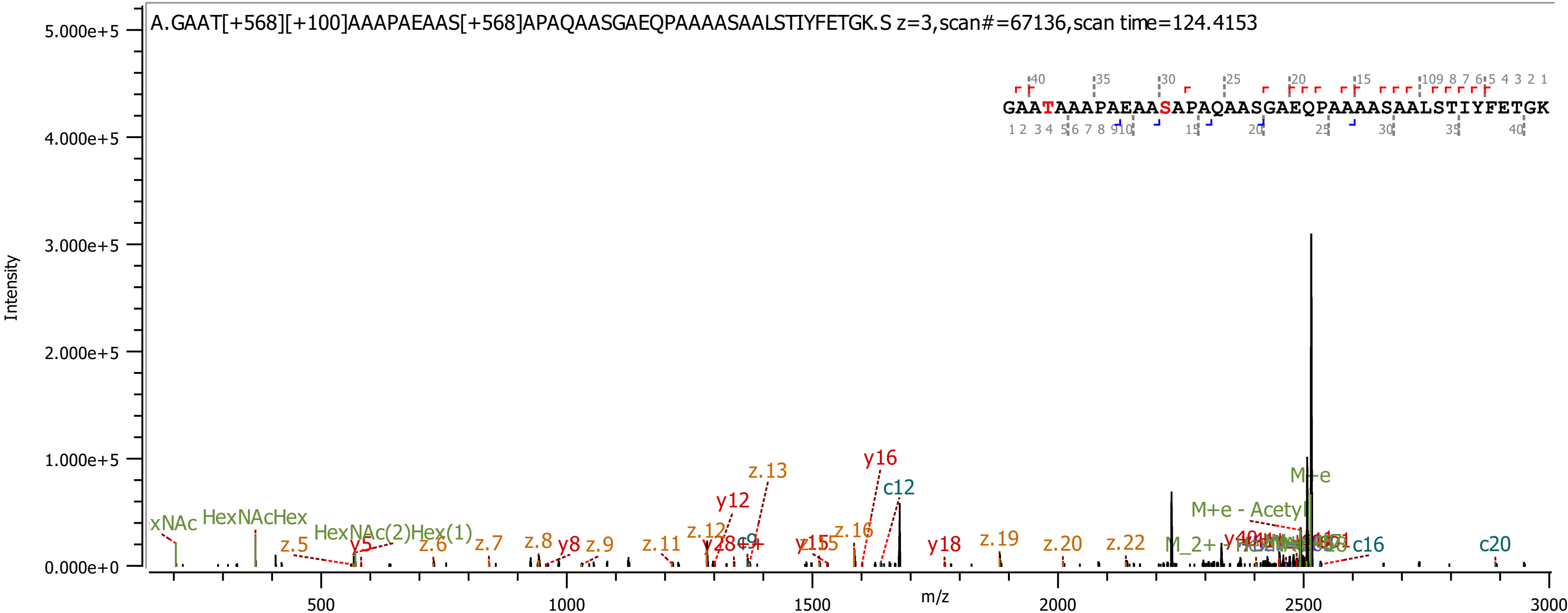
A.GAAT[+568][+100]AAAPAEAAS[+568]APAQAASGAEQPAAAASAALSTIYFETGK.S z=3,scan#=67136,scan time=124.4153
GAATAAAPAEAASAPAQAASGAEQPAAAASAALSTIYFETGK
Intensity
m/z
5.000e+5
4.000e+5
3.000e+5
2.000e+5
1.000e+5
0.000e+0
500
1000
1500
2000
2500
3000
HexNAcHex
HexNAc(2)Hex(1)
z.5
y5
z.6
z.7
z.8
y8
z.9
z.11
z.12
y12
z.13
y16
c12
z.16
y18
z.19
z.20
z.22
M+e
M+e - Acetyl
c16
c20

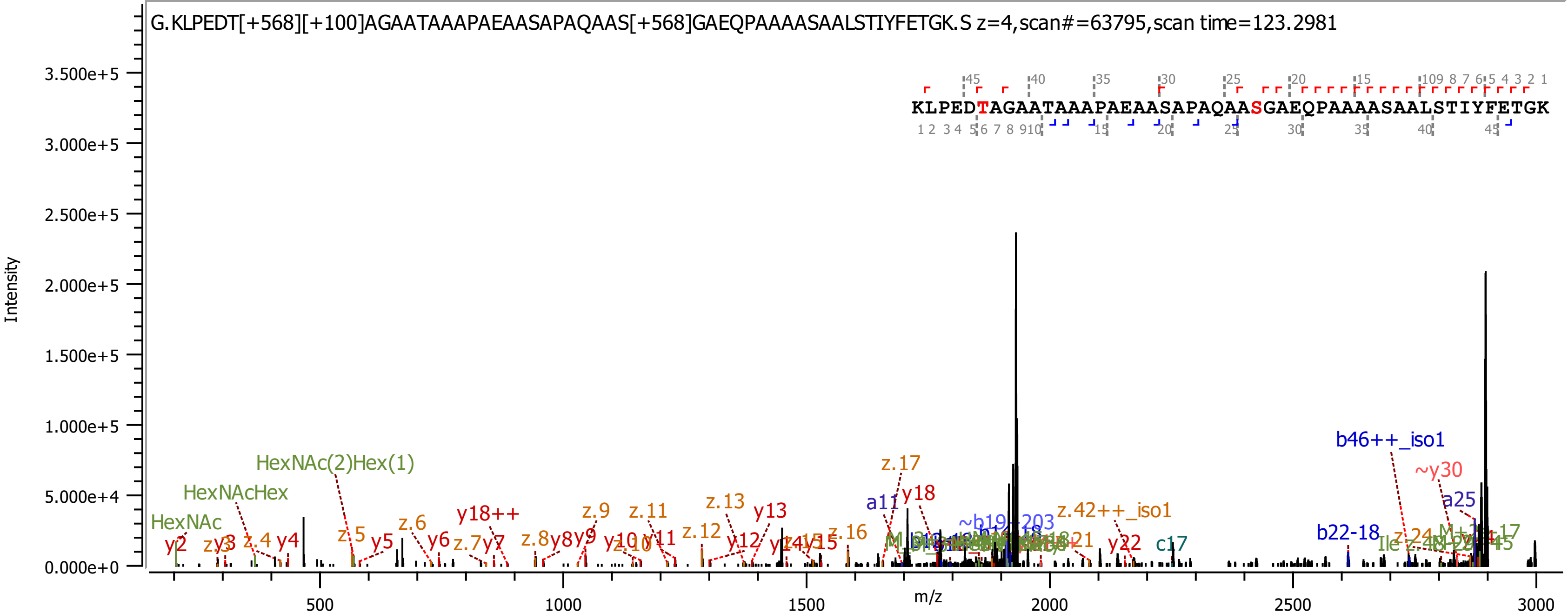
G.KLPEDT[+568][+100]AGAATAAAPAEAASAPAQAAS[+568]GAEQPAAAASAALSTIYFETGK.S z=4,scan#=63795,scan time=123.2981
KLPEDTAGAATAAAPAEAASAPAQAASGAEQPAAAASAALSTIYFETGK
Intensity
m/z
3.500e+5
3.000e+5
2.500e+5
2.000e+5
1.500e+5
1.000e+5
5.000e+4
0.000e+0
500
1000
1500
2000
2500
3000
HexNAc(2)Hex(1)
HexNAcHex
HexNAc
y2
z.3
y3
z.4
y4
z.5
y5
z.6
y6
z.7
y7
y18++
z.8
y8
y9
z.9
y10
z.10
z.11
y11
z.12
z.13
y12
y13
y15
z.16
z.17
a11
y18
~b19
z.42++_iso1
y22
c17
b22-18
b46++_iso1
~y30
a25

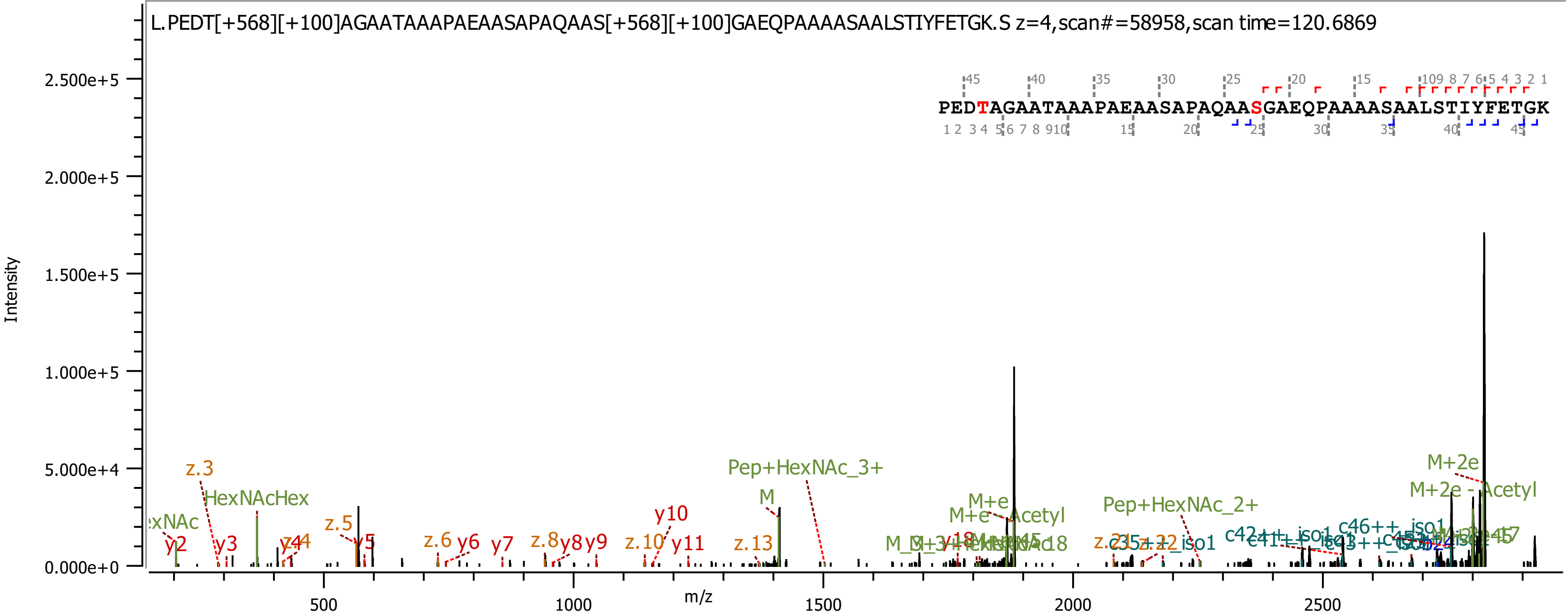
L.PEDT[+568][+100]AGAATAAAPAEAASAPAQAAS[+568][+100]GAEQPAAAASAALSTIYFETGK.S z=4,scan#=58958,scan time=120.6869
PEDTAGAATAAAPAEAASAPAQAASGAEQPAAAASAALSTIYFETGK
Intensity
m/z
Pep+HexNAc_3+
Pep+HexNAc_2+
HexNAcHex
M+2e
M+2e - Acetyl
M+e
M+e - Acetyl
y10
y11
z.13
z.3
z.5
z.6
z.8
z.10
y2
y3
y5
y6
y7
y9

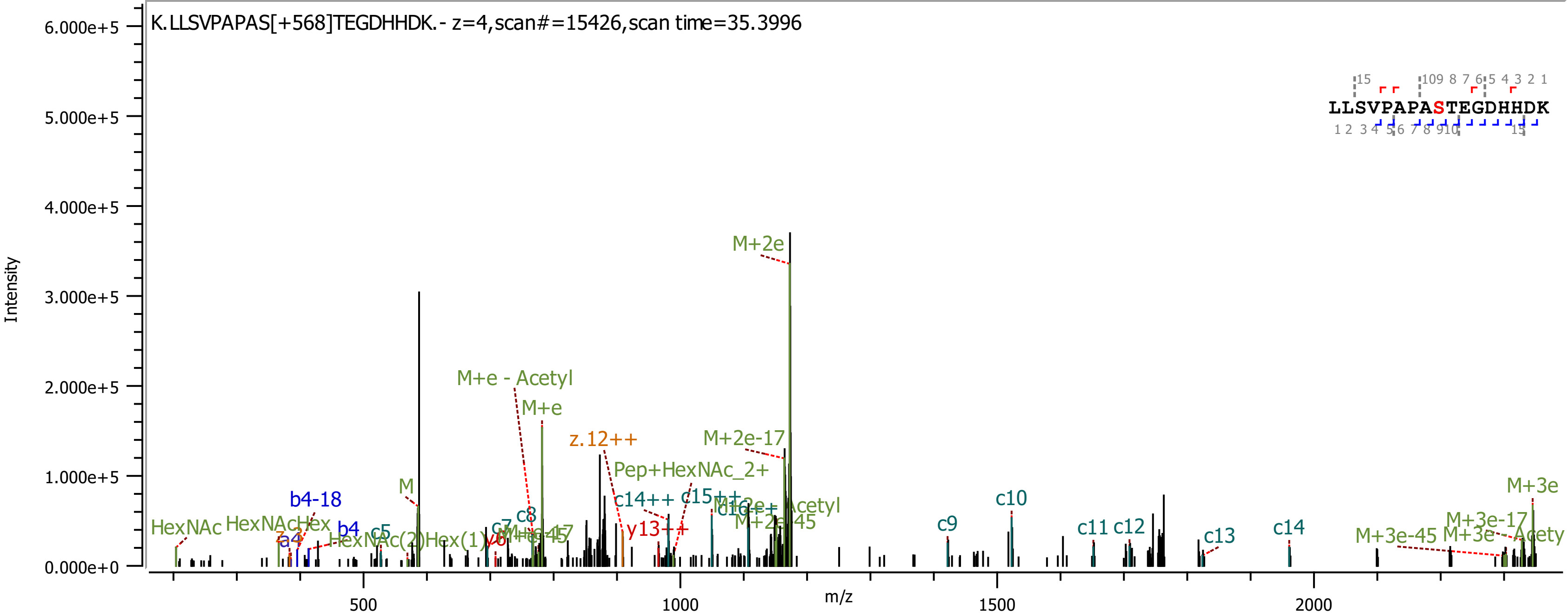

K.LLSVPAPAS[+568]TEGDHHDK.- z=4,scan#=15426,scan time=35.3996
LLSVPAPASTEGDHHDK
Intensity
m/z
M+2e
M+e - Acetyl
M+e
z.12++
M+2e-17
Pep+HexNAc_2+
c14++
c15++
y13++
M
b4-18
HexNAc
HexNAcHex
b4
c5
a4
HexNAc(2)Hex(1)
y6
c7
c8
c9
c10
c11
c12
c13
c14
M+3e
M+3e-17
M+3e-45
M+2e-45

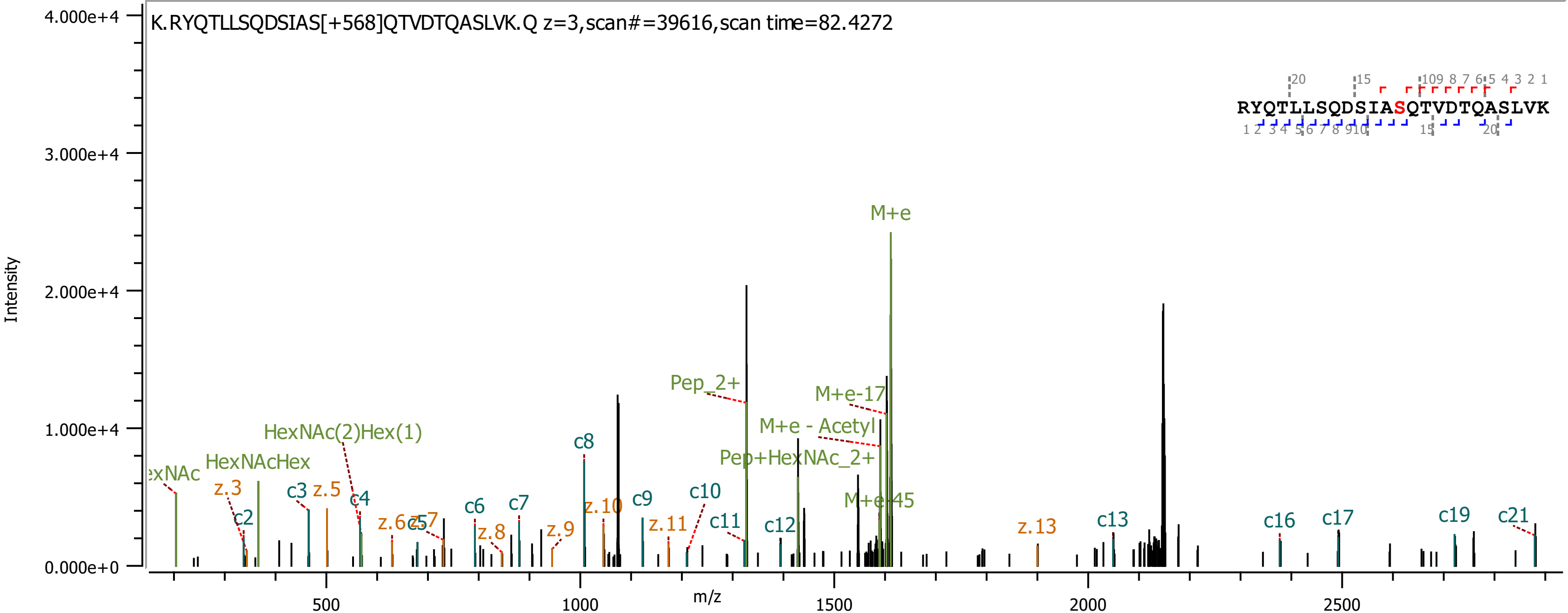
K.RYQTLLSQDSIAS[+568]QTVDTQASLVK.Q z=3,scan#=39616,scan time=82.4272
RYQTLLSQDSIASQTVDTQASLVK
Intensity
m/z
M+e
Pep_2+
M+e-17
M+e - Acetyl
Pep+HexNAc_2+
M+e-45
HexNAc(2)Hex(1)
HexNAcHex
c2
c3
c4
c5
c6
c7
c8
c9
c10
c11
c12
c13
c16
c17
c19
c21
z.3
z.5
z.6
z.7
z.8
z.9
z.10
z.11
z.13

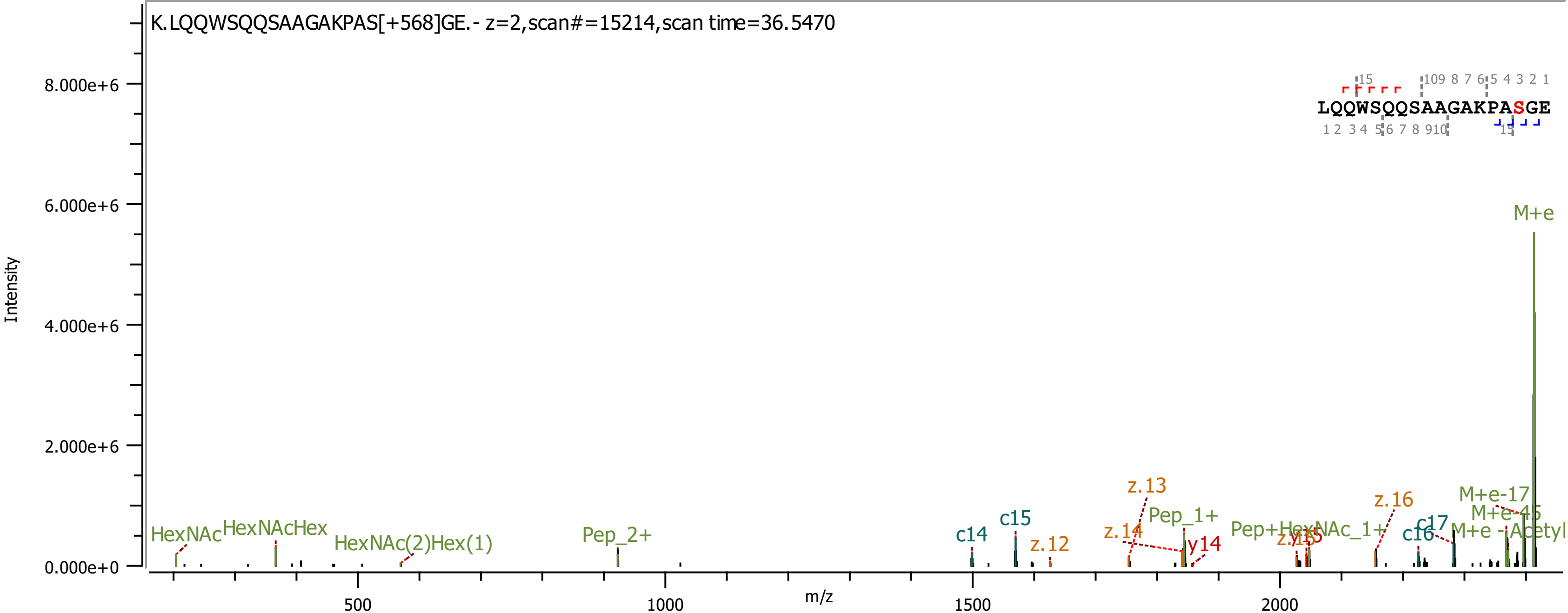

K.LQQWSQQSAAGAKPAS[+568]GE.- z=2,scan#=15214,scan time=36.5470
LQQWSQQSAAGAKPASGE
Intensity
m/z
8.000e+6
6.000e+6
4.000e+6
2.000e+6
0.000e+0
500
1000
1500
2000
HexNAc
HexNAcHex
HexNAc(2)Hex(1)
Pep_2+
c14
c15
z.12
z.14
z.13
Pep_1+
y14
Pep+HexNAc_1+
z.16
c16
c17
M+e-17
M+e-45
M+e

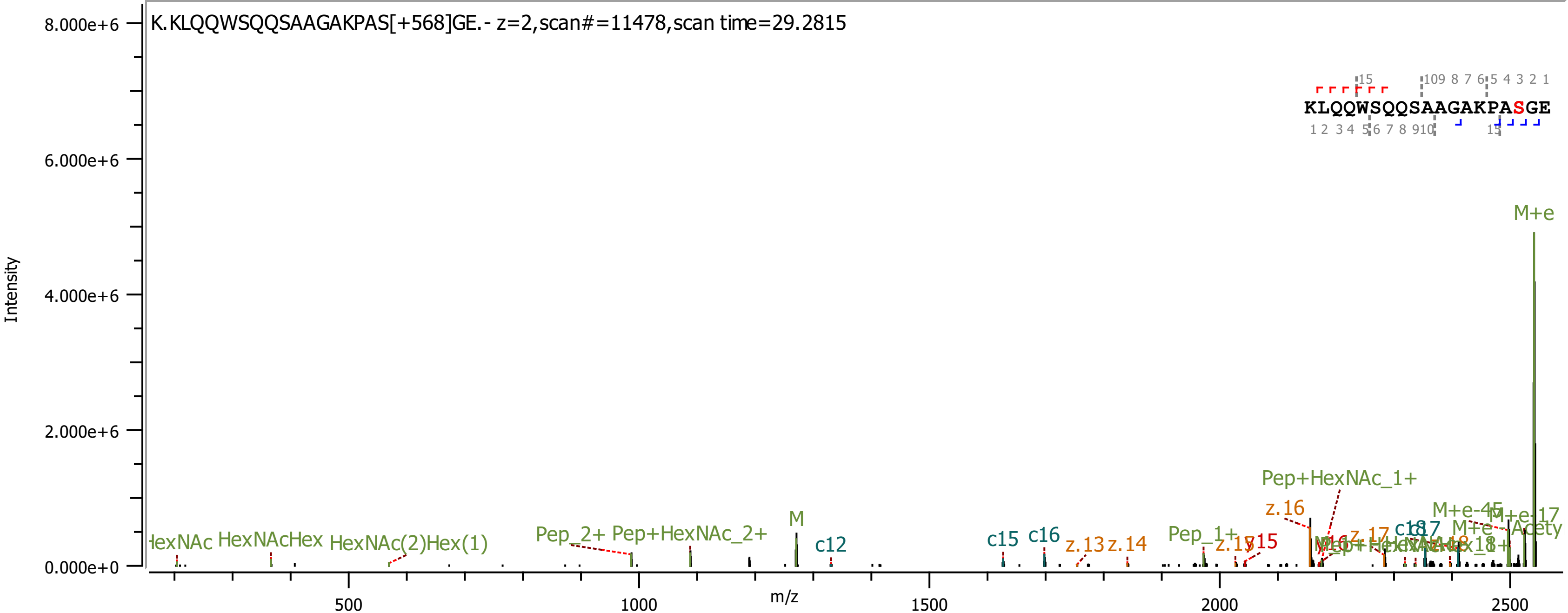
K.KLQQWSQQSAAGAKPAS[+568]GE.- z=2,scan#=11478,scan time=29.2815
KLQQWSQQSAAGAKPASGE
Intensity
m/z
8.000e+6
6.000e+6
4.000e+6
2.000e+6
0.000e+0
500
1000
1500
2000
2500
M+e
Pep+HexNAc_1+
z.16
M
c12
c15
c16
z.13
z.14
Pep_1+
Pep_2+
Pep+HexNAc_2+
HexNAcHex
HexNAc(2)Hex(1)
M+e-45
M+e-17

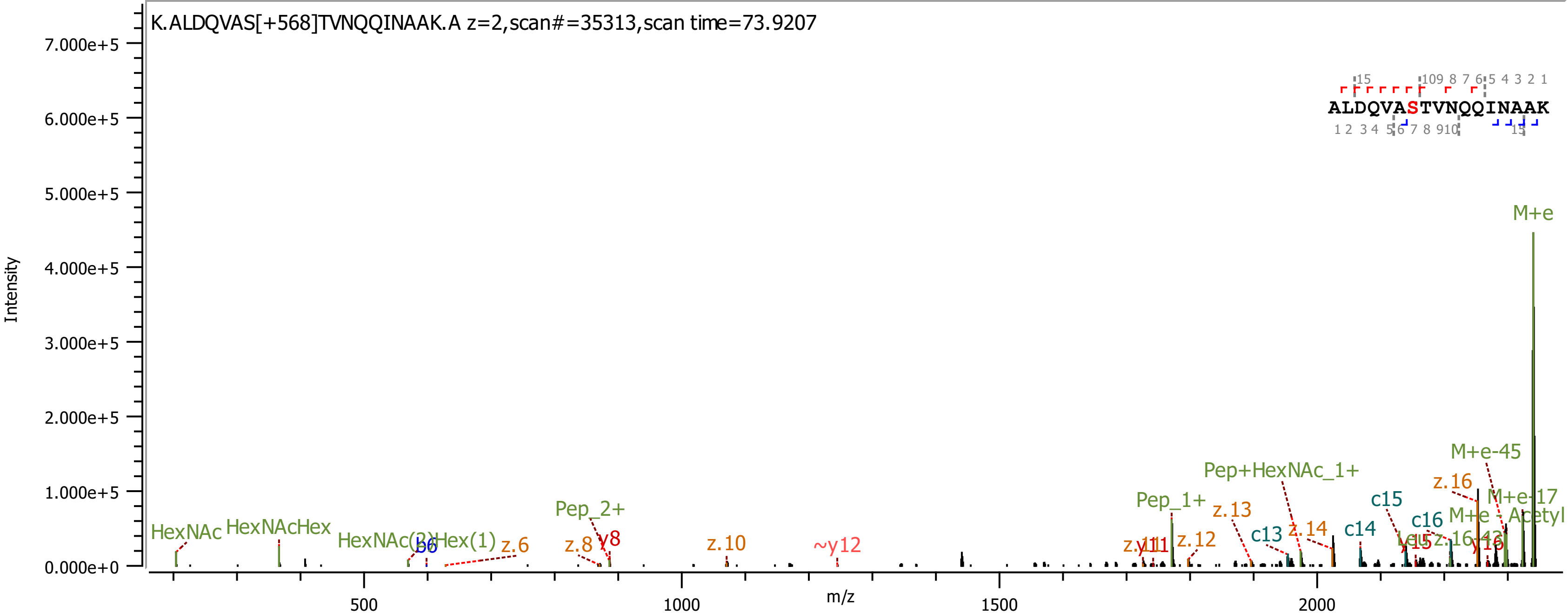

K.ALDQVAS[+568]TVNQQINAAK.A z=2,scan#=35313,scan time=73.9207
Intensity
m/z
ALDQVASTVNQQINAAK
M+e
M+e-45
M+e-17
M+e - Acetyl
Pep+HexNAc_1+
Pep_1+
Pep_2+
HexNAc
HexNAcHex
HexNAc(2)Hex(1)
z.6
z.8
y8
z.10
~y12
z.11
y11
z.12
z.13
z.14
z.16
c13
c14
c15
c16
y15
y16

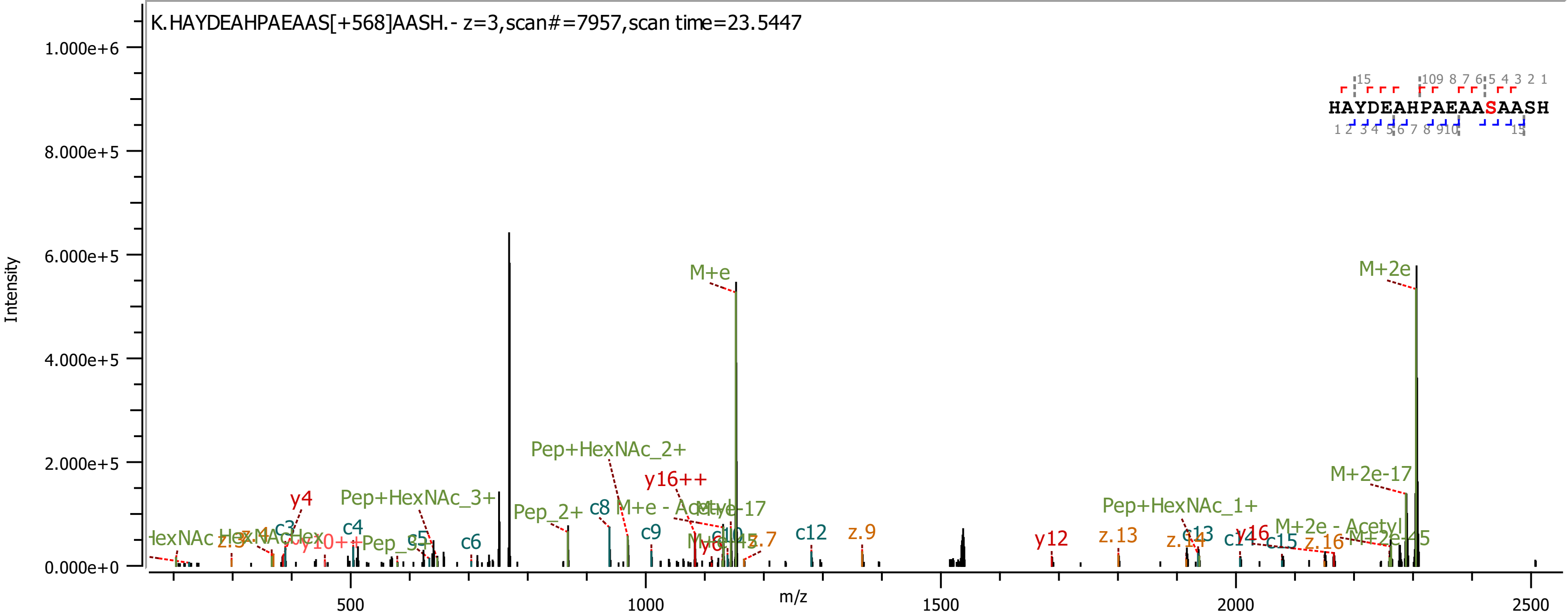
K.HAYDEAHPAEAAS[+568]AASH.- z=3,scan#=7957,scan time=23.5447
HAYDEAHPAEAASAASH
Intensity
m/z
1.000e+6
8.000e+5
6.000e+5
4.000e+5
2.000e+5
0.000e+0
500
1000
1500
2000
2500
M+e
M+2e
M+2e-17
Pep+HexNAc_2+
Pep+HexNAc_3+
Pep+HexNAc_1+
Pep_2+
y16++
y4
y12
y16
c3
c4
c5
c6
c8
c9
c10
c12
c13
c14
c15
z.9
z.13
z.14
z.16
z.7
z.3
M+2e - Acetyl
M+2e-45

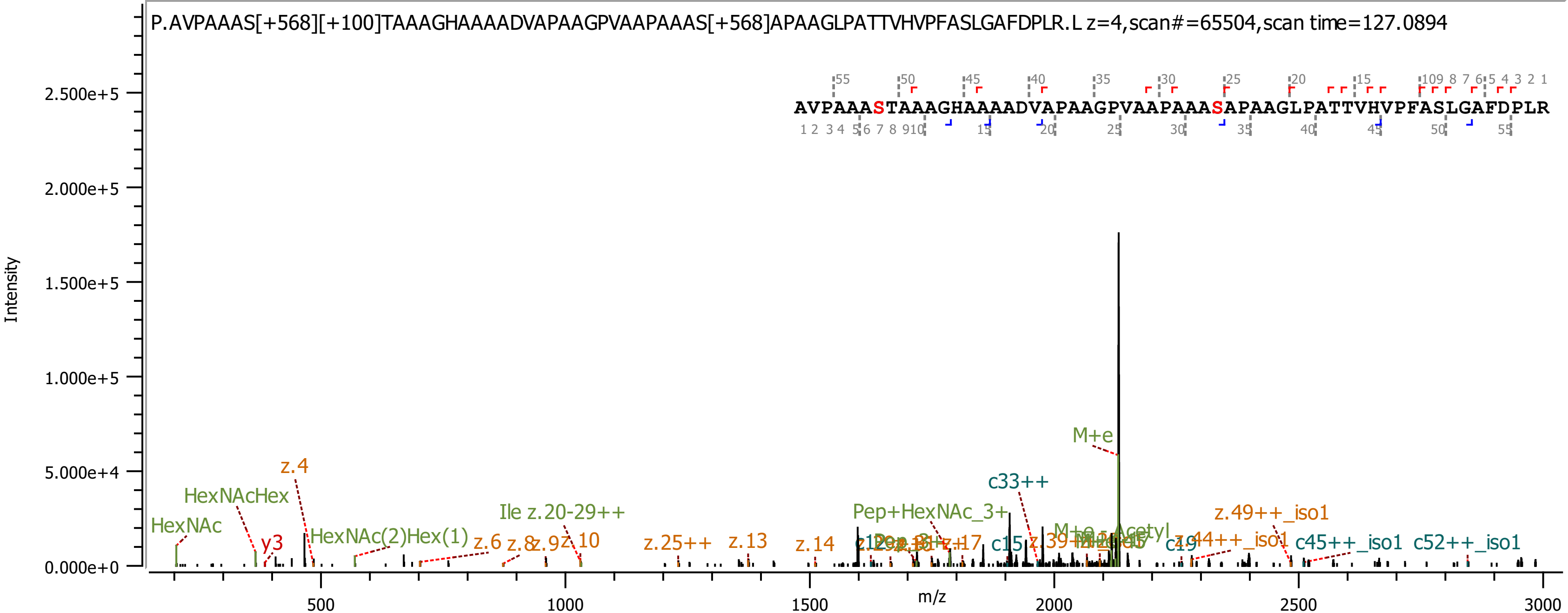
P.AVPAAAS[+568][+100]TAAAGHAAAADVAPAAGPVAAPAAAS[+568]APAAGLPATTVHVPFASLGAFDPLR.L z=4,scan#=65504,scan time=127.0894
AVPAAASTAAAGHAAAADVAPAAGPVAAPAAASAPAAGLPATTVHVPFASLGAFDPLR
Intensity
m/z
2.500e+5
2.000e+5
1.500e+5
1.000e+5
5.000e+4
0.000e+0
500
1000
1500
2000
2500
3000
M+e
c33++
HexNAcHex
HexNAc
z.4
y3
HexNAc(2)Hex(1)
Ile z.20-29++
z.6
z.8
z.9
z.10
z.25++
z.13
z.14
Pep+HexNAc_3+
z.17
c15
c19
z.49++_iso1
c45++_iso1
c52++_iso1

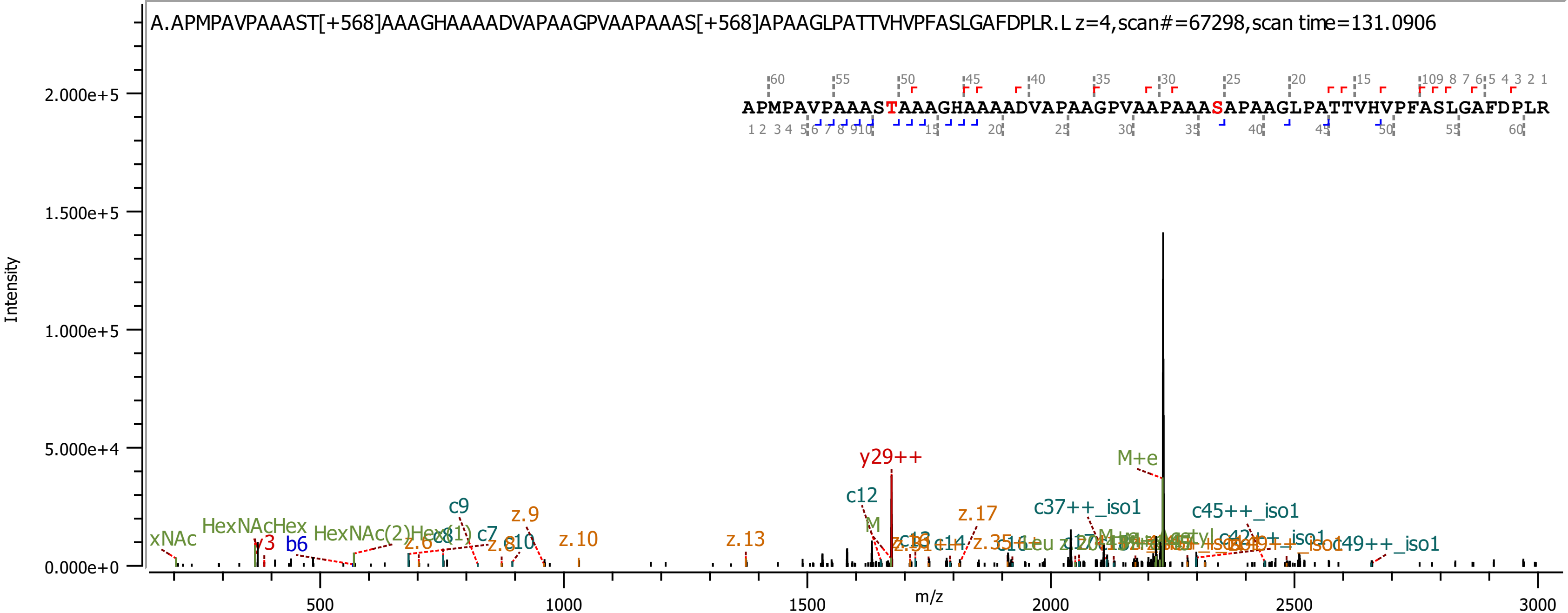

A.APMPAVPAAAST[+568]AAAGHAAAADVAPAAGPVAAPAAAS[+568]APAAGLPATTVHVPFASLGAFDPLR.L z=4,scan#=67298,scan time=131.0906
APMPAVPAAASTAAAGHAAAADVAPAAGPVAAPAAASAPAAGLPATTVHVPFASLGAFDPLR
Intensity
m/z
y29++
M+e
c12
c9
z.9
z.17
c37++_iso1
c45++_iso1
xNAc
HexNAcHex
HexNAc(2)Hex(1)
y3
b6
c7
z.10
z.13
c49++_iso1

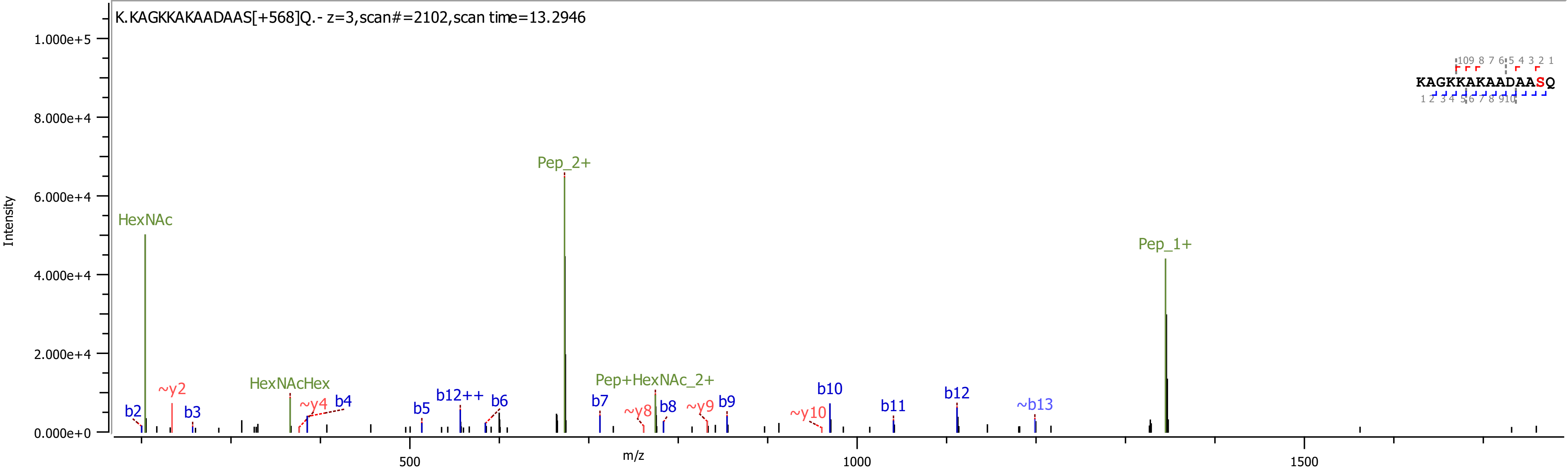
K.KAGKKAKAADAAS[+568]Q.- z=3,scan#=2102,scan time=13.2946
KAGKKAKAADAASQ
Intensity
1.000e+5
8.000e+4
6.000e+4
4.000e+4
2.000e+4
0.000e+0
HexNAc
b2
~y2
b3
HexNAcHex
~y4
b4
b5
b12++
b6
Pep_2+
b7
~y8
Pep+HexNAc_2+
b8
~y9
b9
~y10
b10
b11
b12
~b13
Pep_1+
500
1000
1500
m/z

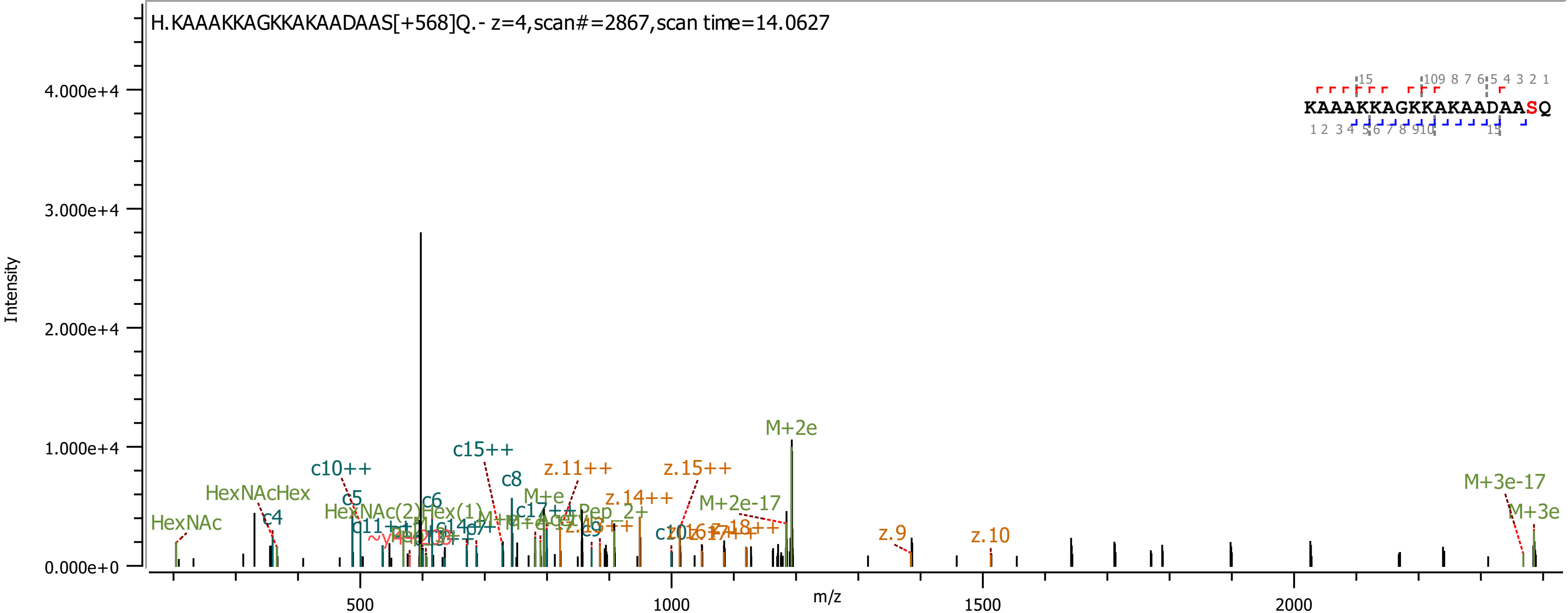
H.KAAAKKAGKKAKAADAAS[+568]Q.- z=4,scan#=2867,scan time=14.0627
Intensity
m/z
4.000e+4
3.000e+4
2.000e+4
1.000e+4
0.000e+0
500
1000
1500
2000
KAAAKKAGKKAKAADAASQ
HexNAc
HexNAcHex
c4
c10++
c5
HexNAc(2)Hex(1)
c6
c15++
c8
M+e
z.11++
z.15++
z.14++
M+2e-17
M+2e
z.9
z.10
M+3e-17
M+3e

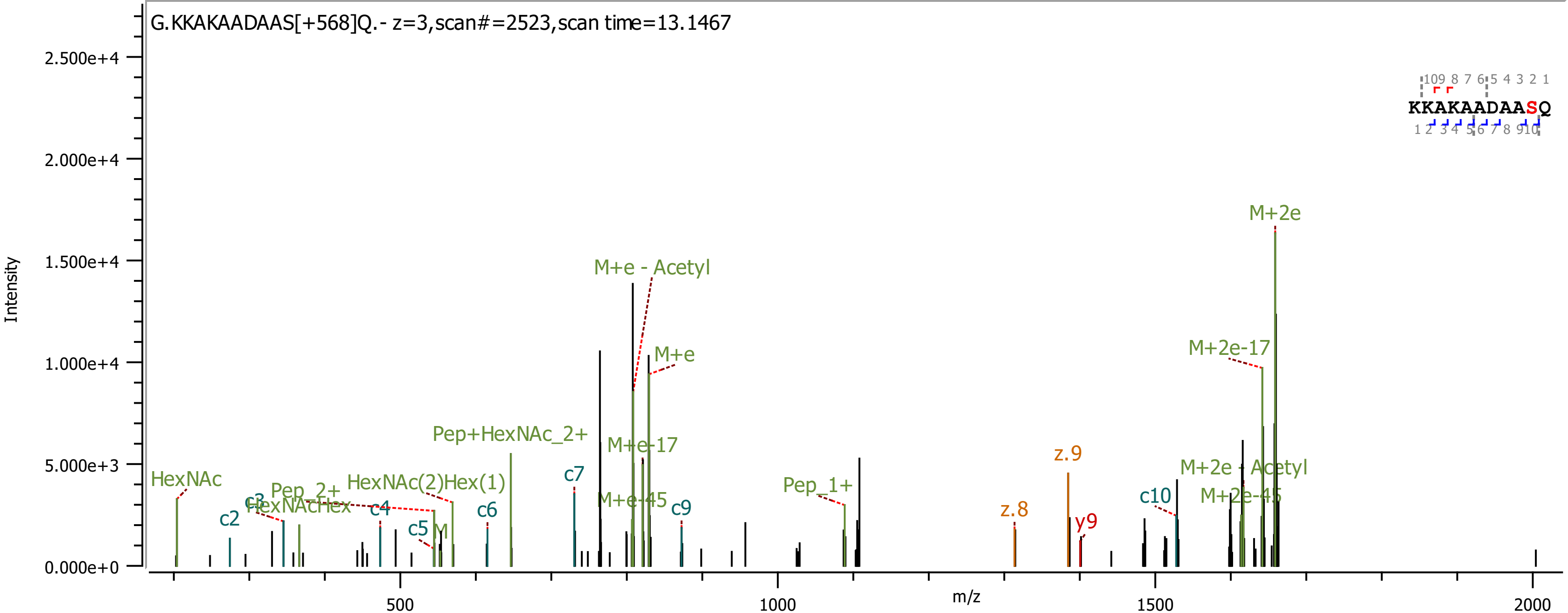
G.KKAKAADAAS[+568]Q.- z=3,scan#=2523,scan time=13.1467
Intensity
m/z
KKAKAADAASQ
HexNAc
c2
c3
HexNAcHex
Pep_2+
c4
HexNAc(2)Hex(1)
c5
M
c6
Pep+HexNAc_2+
c7
M+e - Acetyl
M+e
M+e-17
M+e-45
c9
Pep_1+
z.8
z.9
y9
c10
M+2e-17
M+2e - Acetyl
M+2e-45
M+2e

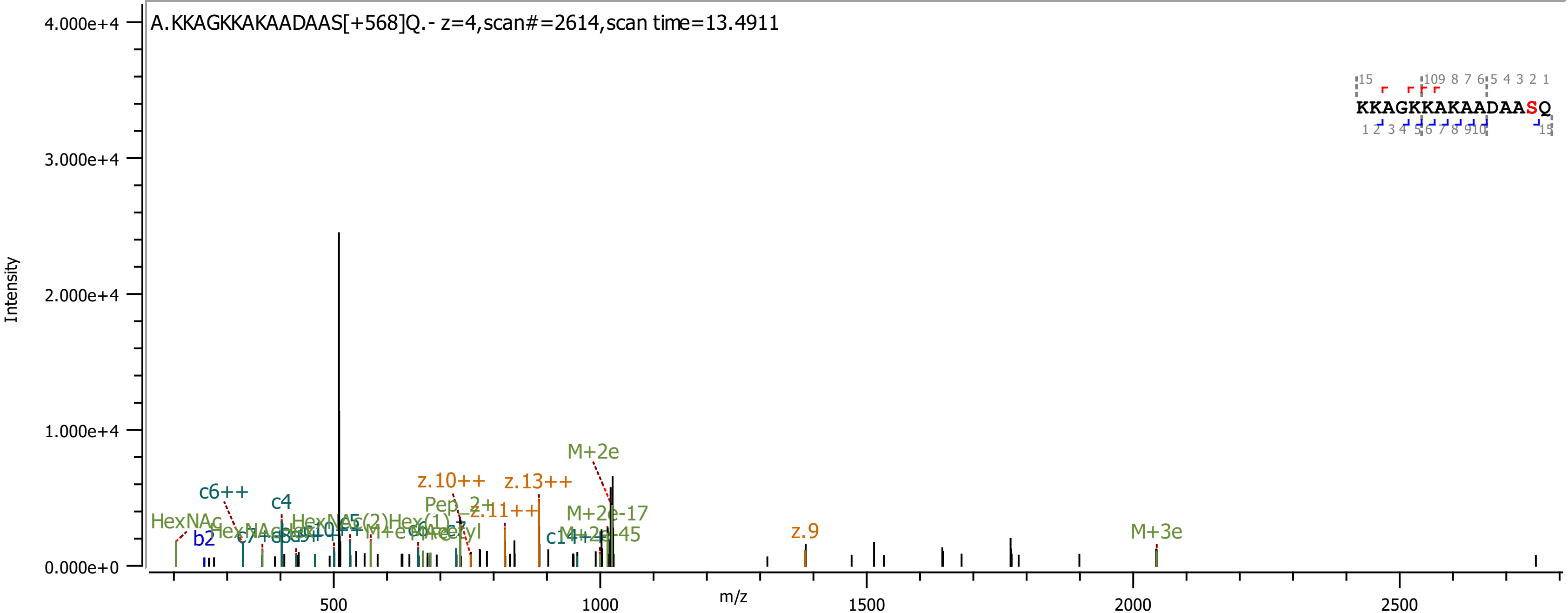

A.KKAGKKAKAADAAS[+568]Q.- z=4,scan#=2614,scan time=13.4911
Intensity
4.000e+4
3.000e+4
2.000e+4
1.000e+4
0.000e+0
m/z
500
1000
1500
2000
2500
KKAGKKAKAADAASQ
HexNAc
b2
c6++
c4
z.10++
z.13++
z.11++
Pep_2+
M+2e
M+2e-17
M+2e-45
z.9
M+3e

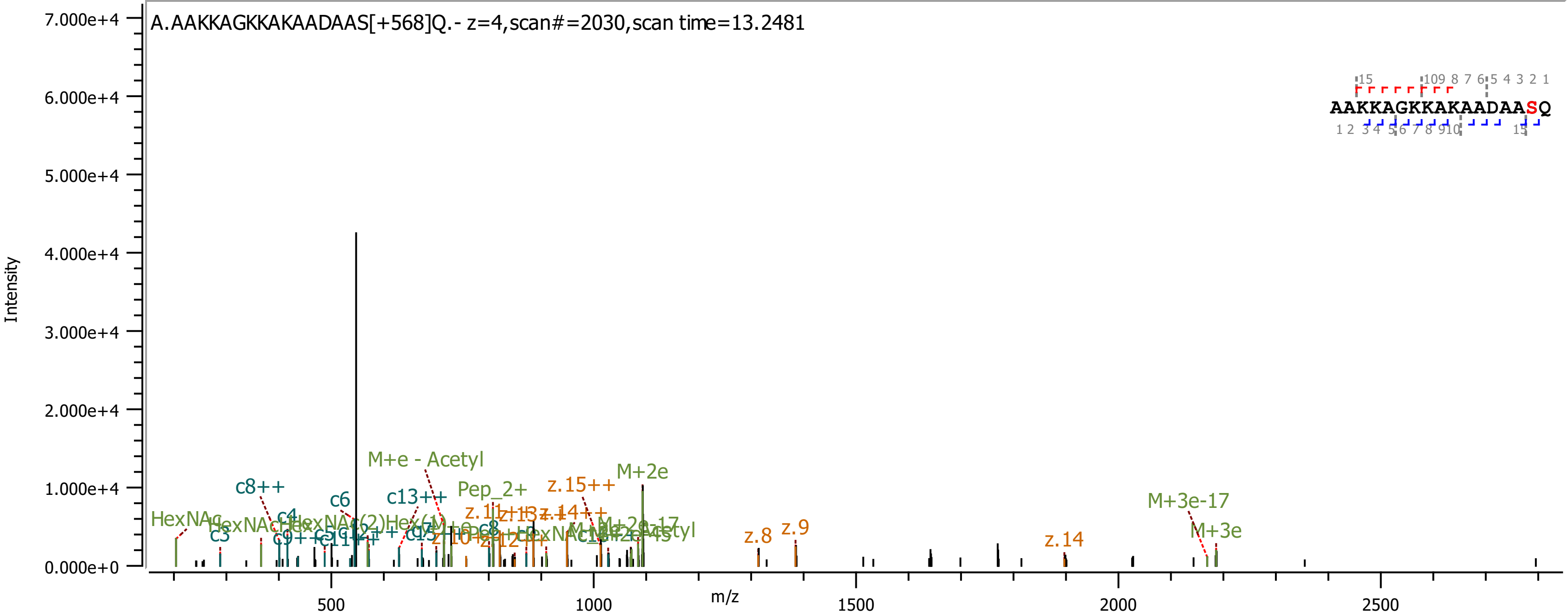
A.AAKKAGKKAKAADAAS[+568]Q.- z=4,scan#=2030,scan time=13.2481
AAKKAGKKAKAADAASQ
Intensity
7.000e+4
6.000e+4
5.000e+4
4.000e+4
3.000e+4
2.000e+4
1.000e+4
0.000e+0
m/z
500
1000
1500
2000
2500
HexNAc
c3
c8++
c4
c6
M+e - Acetyl
c13++
Pep_2+
z.15++
M+2e
z.8
z.9
z.14
M+3e-17
M+3e

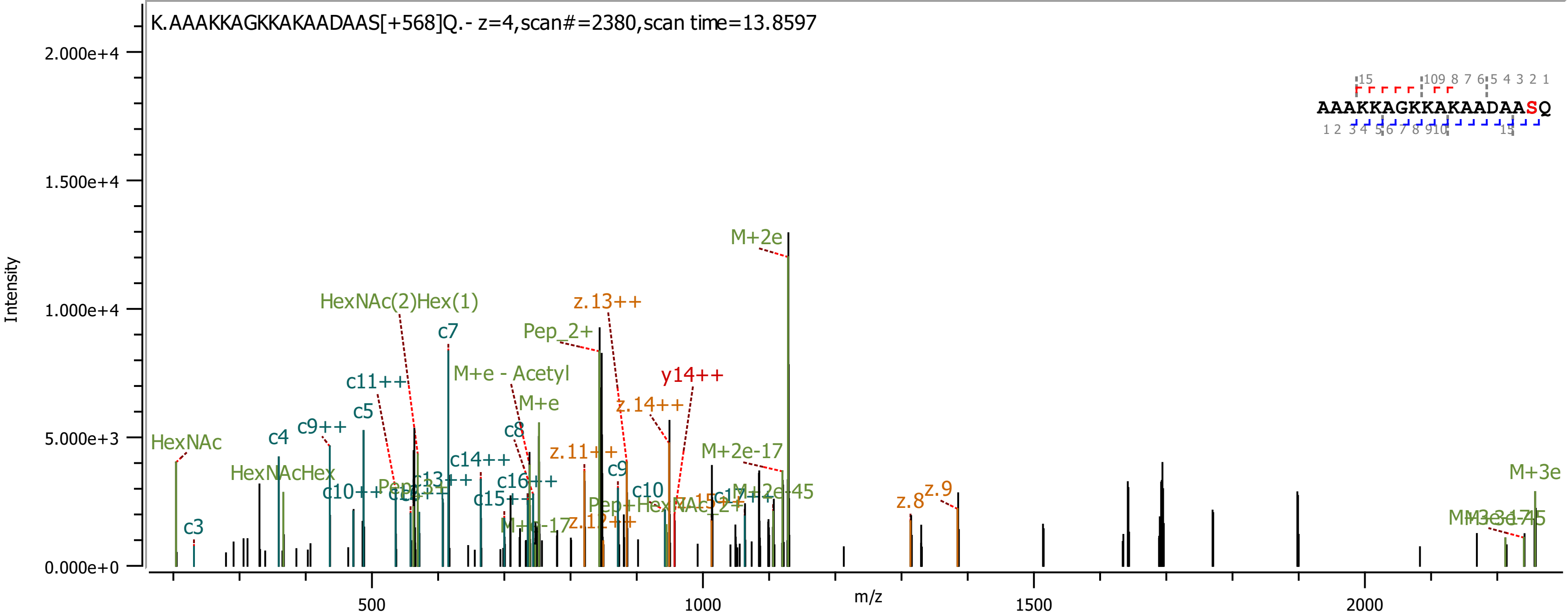

K.AAAKKAGKKAKAADAAS[+568]Q.- z=4,scan#=2380,scan time=13.8597
AAAKKAGKKAKAADAASQ
Intensity
2.000e+4
1.500e+4
1.000e+4
5.000e+3
0.000e+0
m/z
500
1000
1500
2000
HexNAc
HexNAcHex
HexNAc(2)Hex(1)
c3
c4
c5
c7
c8
c9
c10
c9++
c10++
c11++
c14++
c15++
c16++
M+e - Acetyl
M+e
Pep_2+
z.13++
z.14++
z.11++
z.12++
y14++
M+2e
M+2e-17
z.8
z.9
M+3e

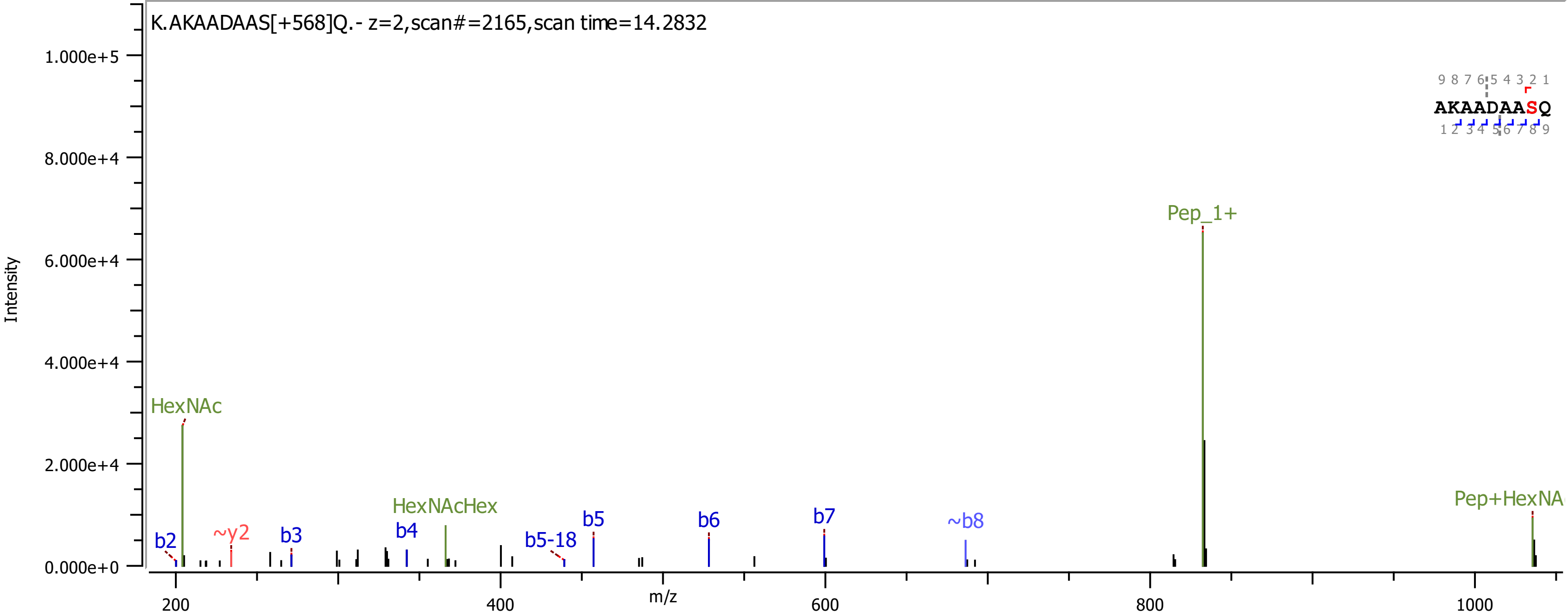
K.AKAADAAS[+568]Q.- z=2,scan#=2165,scan time=14.2832
Intensity
1.000e+5
8.000e+4
6.000e+4
4.000e+4
2.000e+4
0.000e+0
m/z
200
400
600
800
1000
HexNAc
b2
~y2
b3
b4
HexNAcHex
b5-18
b5
b6
b7
~b8
Pep_1+
Pep+HexNA
9 8 7 6 5 4 3 2 1
AKAADAASQ
1 2 3 4 5 6 7 8 9

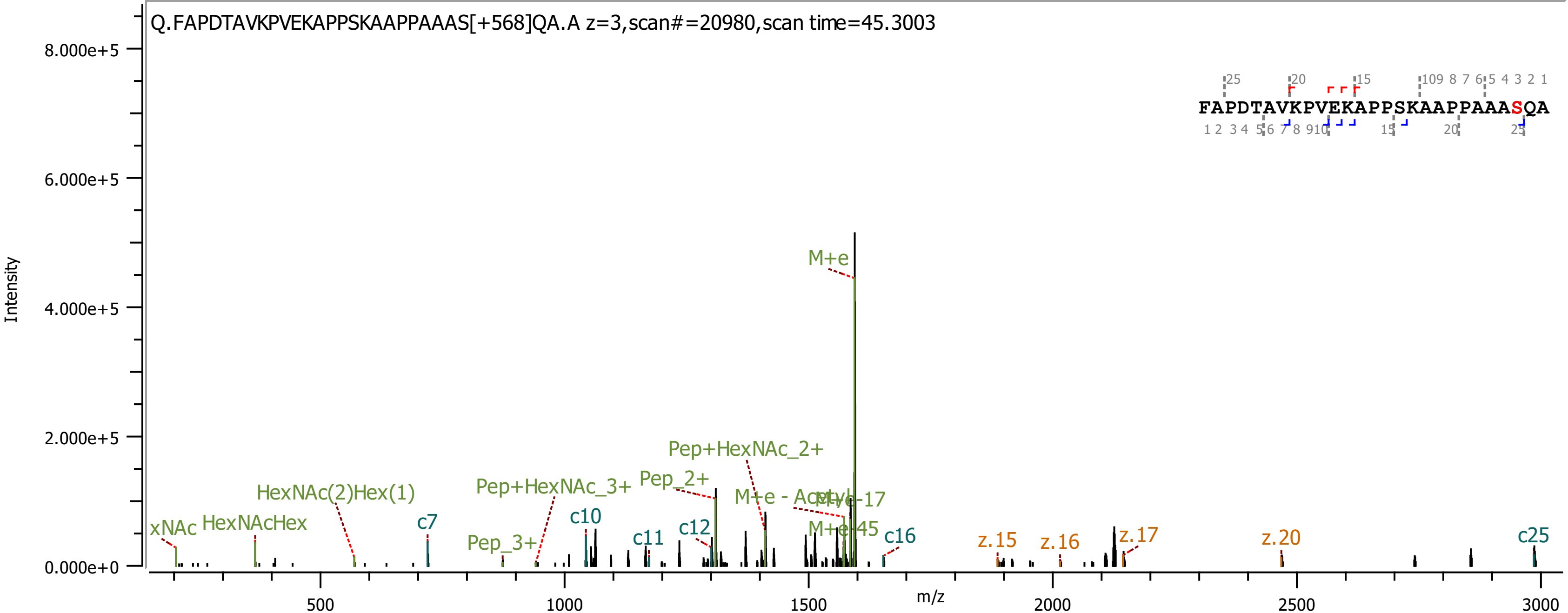

Q.FAPDTAVKPVEKAPPSKAAPPAAAS[+568]QA.A z=3,scan#=20980,scan time=45.3003
Intensity
m/z
8.000e+5
6.000e+5
4.000e+5
2.000e+5
0.000e+0
500
1000
1500
2000
2500
3000
FAPDTAVKPVEKAPPSKAAPPAAASQA
M+e
Pep+HexNAc_2+
Pep_2+
HexNAc(2)Hex(1)
Pep+HexNAc_3+
xNAc
HexNAcHex
c7
Pep_3+
c10
c11
c12
M+e-45
c16
z.15
z.16
z.17
z.20
c25

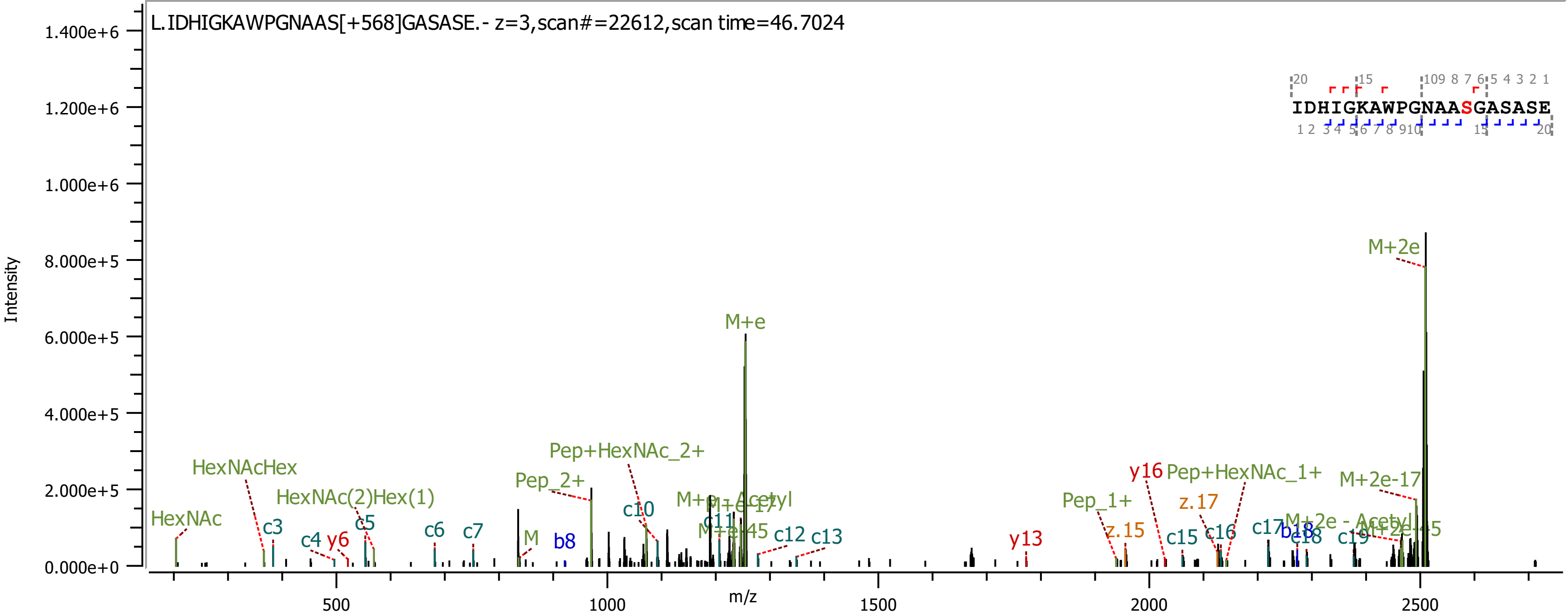
L.IDHIGKAWPGNAAS[+568]GASASE.- z=3,scan#=22612,scan time=46.7024
IDHIGKAWPGNAAS GASASE
Intensity
m/z
M+2e
M+e
Pep+HexNAc_2+
Pep_2+
HexNAcHex
HexNAc(2)Hex(1)
HexNAc
y16
Pep+HexNAc_1+
M+2e-17
Pep_1+
z.17
z.15
y13
y6
b8
M
c3
c4
c5
c6
c7
c10
c11
c12
c13
c15
c16
c17
c19
b18
c18
M+e - Acetyl
M+e-17
M+e-45
M+2e - Acetyl

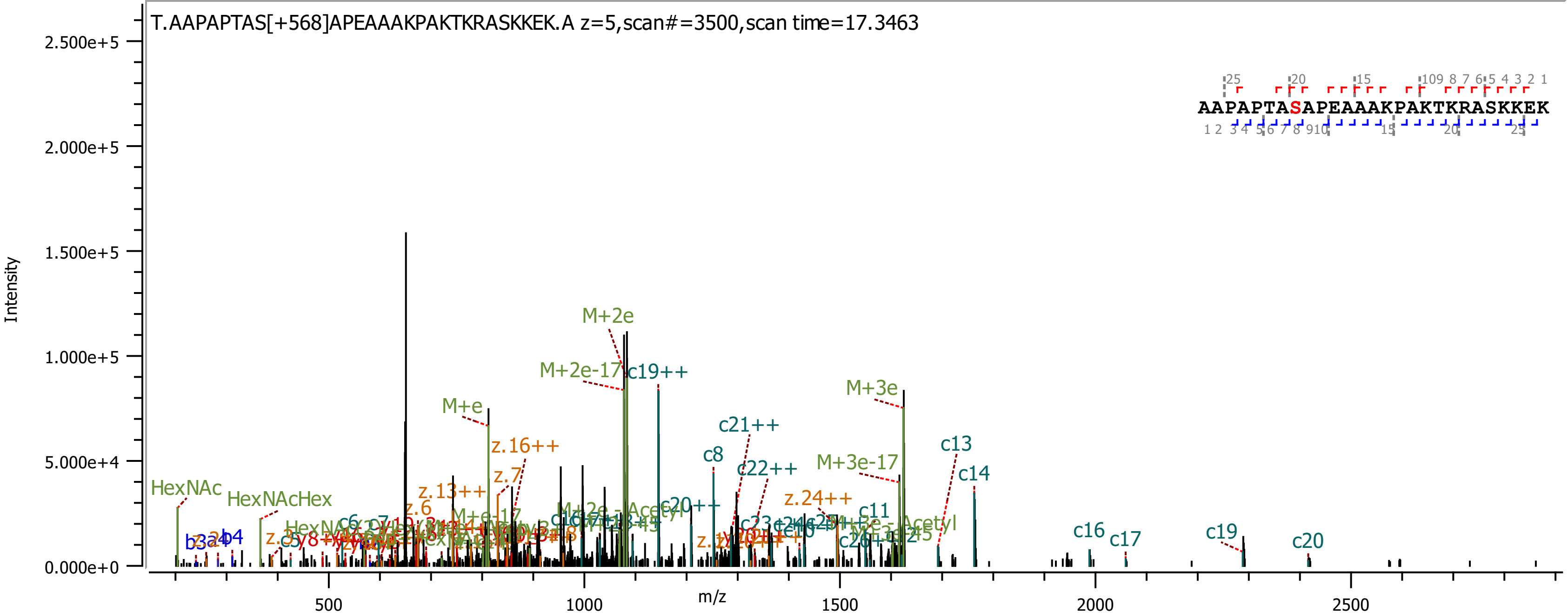
T.AAPAPTAS[+568]APEAAAKPAKTKRASKKEK.A z=5,scan#=3500,scan time=17.3463
AAPAPTASAPEAAAKPAKTKRASKKEK
Intensity
m/z
2.500e+5
2.000e+5
1.500e+5
1.000e+5
5.000e+4
0.000e+0
500
1000
1500
2000
2500
M+2e
M+2e-17
c19++
M+e
z.16++
M+3e
c21++
c8
c22++
M+3e-17
c13
c14
z.7
z.13++
z.6
HexNAc
HexNAcHex
c20++
z.24++
c11
c16
c17
c19
c20
b3
b4

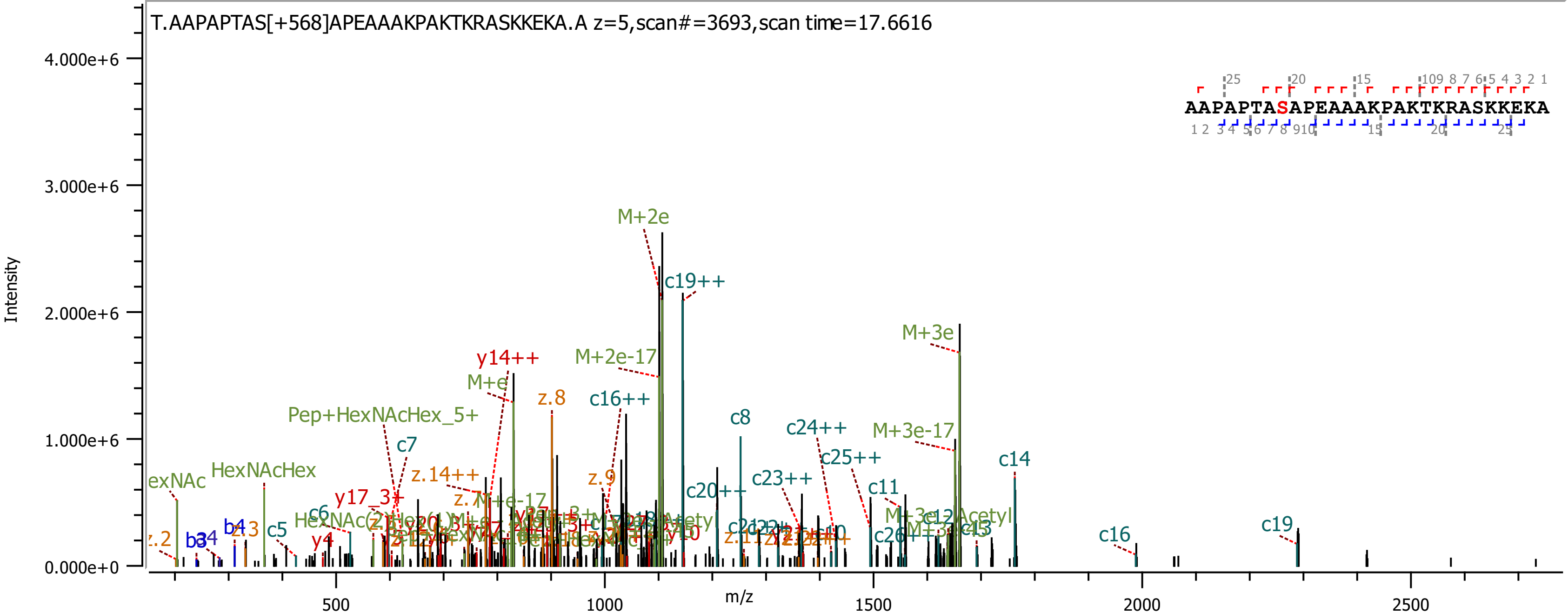
T.AAPAPTAS[+568]APEAAAKPAKTKRASKKEKA.A z=5,scan#=3693,scan time=17.6616
Intensity
m/z
4.000e+6
3.000e+6
2.000e+6
1.000e+6
0.000e+0
500
1000
1500
2000
2500
AAPAPTASAPEAAAKPAKTKRASKKEKA
M+2e
c19++
M+3e
y14++
M+2e-17
M+e
z.8
c16++
Pep+HexNAcHex_5+
c7
c8
c24++
M+3e-17
c25++
c14
HexNAcHex
z.14++
z.9
c20++
c23++
c11
y17_3+
c6
c5
b4
z.3
b3
y4
c16
c19

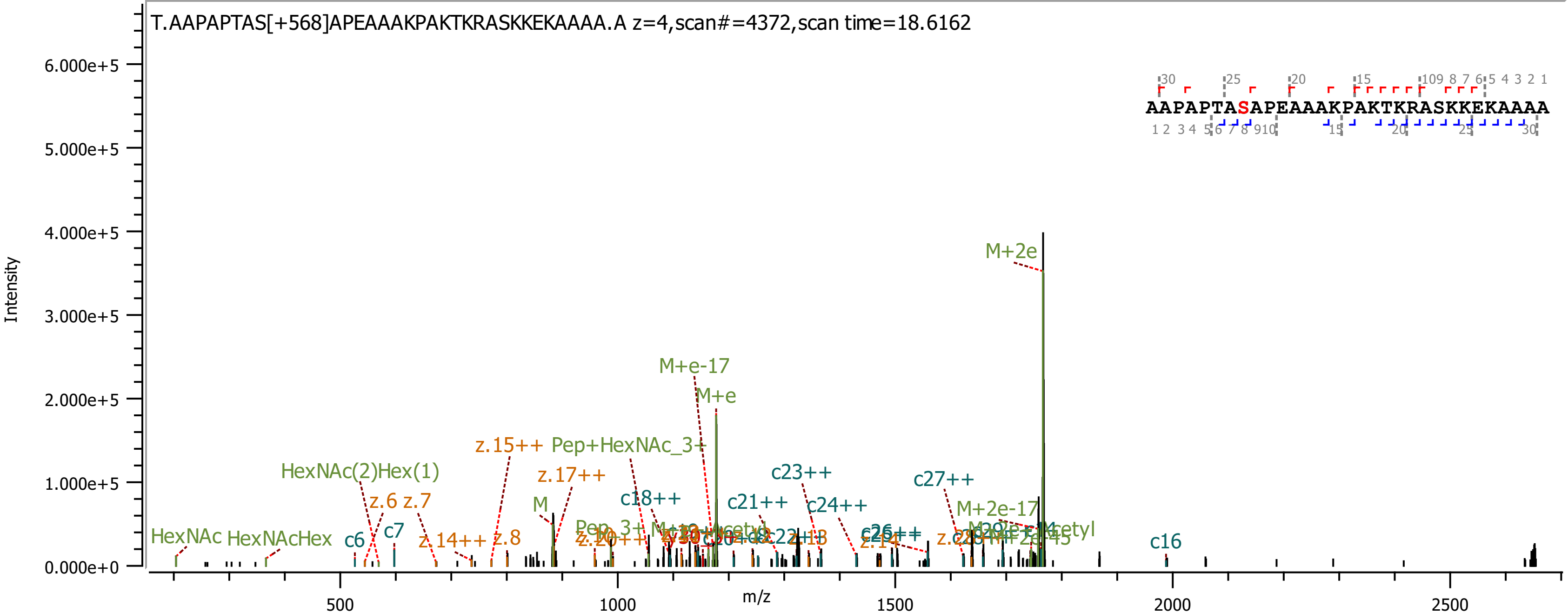
T.AAPAPTAS[+568]APEAAAKPAKTKRASKKEKAAAA.A z=4,scan#=4372,scan time=18.6162
AAPAPTASAPEAAAKPAKTKRASKKEKAAAA
Intensity
6.000e+5
5.000e+5
4.000e+5
3.000e+5
2.000e+5
1.000e+5
0.000e+0
m/z
500
1000
1500
2000
2500
M+2e
M+e-17
M+e
z.15++
Pep+HexNAc_3+
HexNAc(2)Hex(1)
z.17++
c23++
c27++
z.6
z.7
M
c18++
c21++
c24++
M+2e-17
HexNAc
HexNAcHex
c6
c7
z.14++
z.8
c16

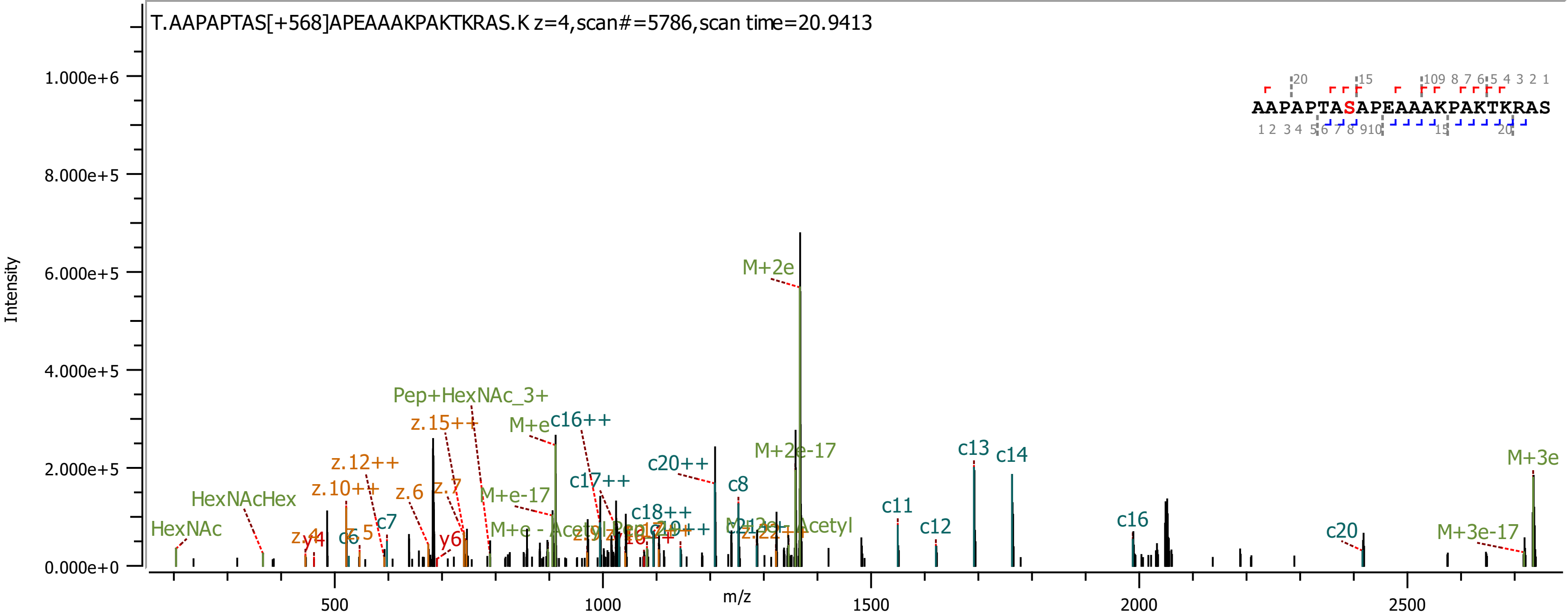

T.AAPAPTAS[+568]APEAAAKPAKTKRAS.K z=4,scan#=5786,scan time=20.9413
AAPAPTASAPEAAAKPAKTKRAS
Intensity
m/z
M+2e
M+3e
M+2e-17
M+3e-17
c13
c14
c11
c12
c16
c20
c8
c7
c6
c16++
c17++
c18++
c20++
z.15++
z.12++
z.10++
z.6
z.7
z.5
z.4
y4
y6
M+e
M+e-17
Pep+HexNAc_3+
HexNAc
HexNAcHex

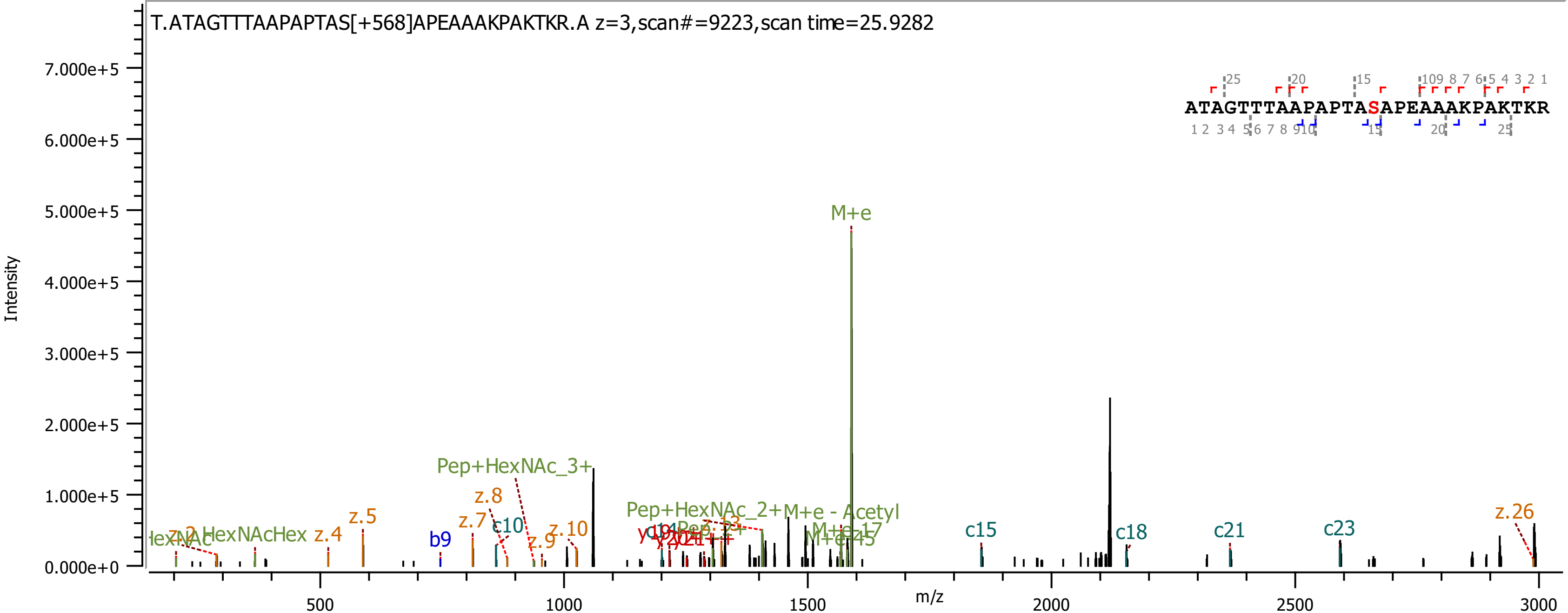

T.ATAGTTTAAPAPTAS[+568]APEAAAKPAKTKR.A z=3,scan#=9223,scan time=25.9282
ATAGTTTAAPAPTASAPEAAAKPAKTKR
Intensity
m/z
M+e
Pep+HexNAc_3+
Pep+HexNAc_2+
M+e - Acetyl
z.8
z.7
z.5
z.4
z.9
z.10
z.26
c10
c15
c18
c21
c23
b9

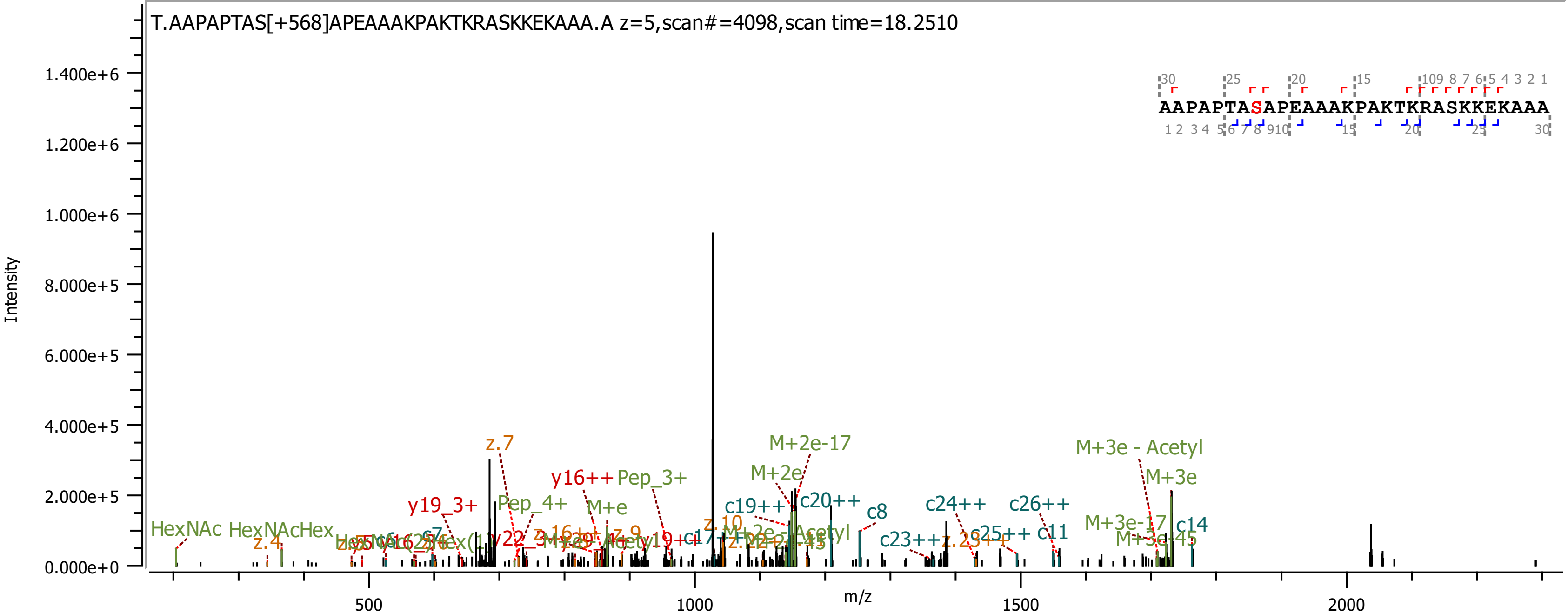
T.AAPAPTAS[+568]APEAAAKPAKTKRASKKEKAAA.A z=5,scan#=4098,scan time=18.2510
AAPAPTASAPEAAAKPAKTKRASKKEKAAA
Intensity
m/z
1.400e+6
1.200e+6
1.000e+6
8.000e+5
6.000e+5
4.000e+5
2.000e+5
0.000e+0
500
1000
1500
2000
HexNAc
HexNAcHex
z.4
z.7
y19_3+
Pep_4+
y16++
Pep_3+
M+e
z.9
z.10
M+2e
M+2e-17
c19++
c20++
M+2e - Acetyl
c8
c23++
c24++
c26++
c25++
c11
M+3e - Acetyl
M+3e
M+3e-17
c14
M+3e-45

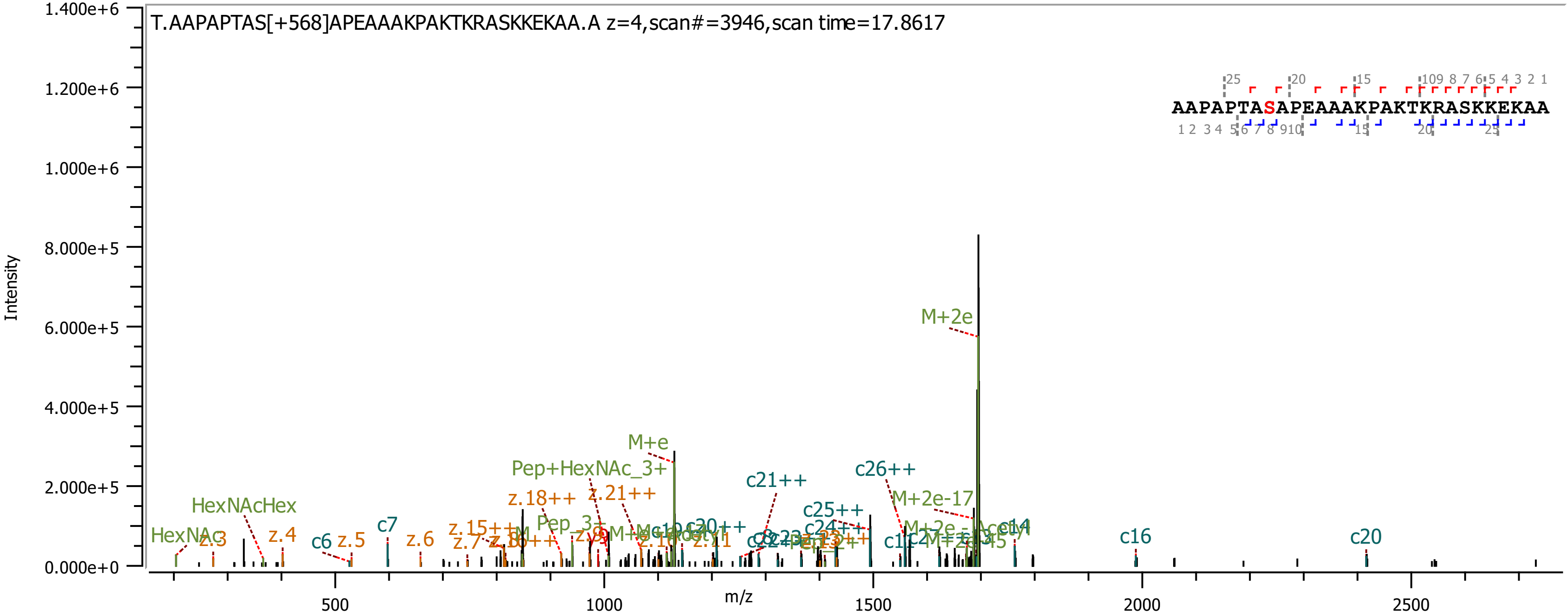
T.AAPAPTAS[+568]APEAAAKPAKTKRASKKEKAA.A z=4,scan#=3946,scan time=17.8617
AAPAPTASAPEAAAKPAKTKRASKKEKAA
Intensity
m/z
1.400e+6
1.200e+6
1.000e+6
8.000e+5
6.000e+5
4.000e+5
2.000e+5
0.000e+0
500
1000
1500
2000
2500
M+2e
M+e
Pep+HexNAc_3+
c21++
c26++
c25++
M+2e-17
c24++
z.18++
z.21++
HexNAcHex
HexNAc
z.3
z.4
c6
z.5
c7
z.6
z.7
z.15++
c16
c20
c14

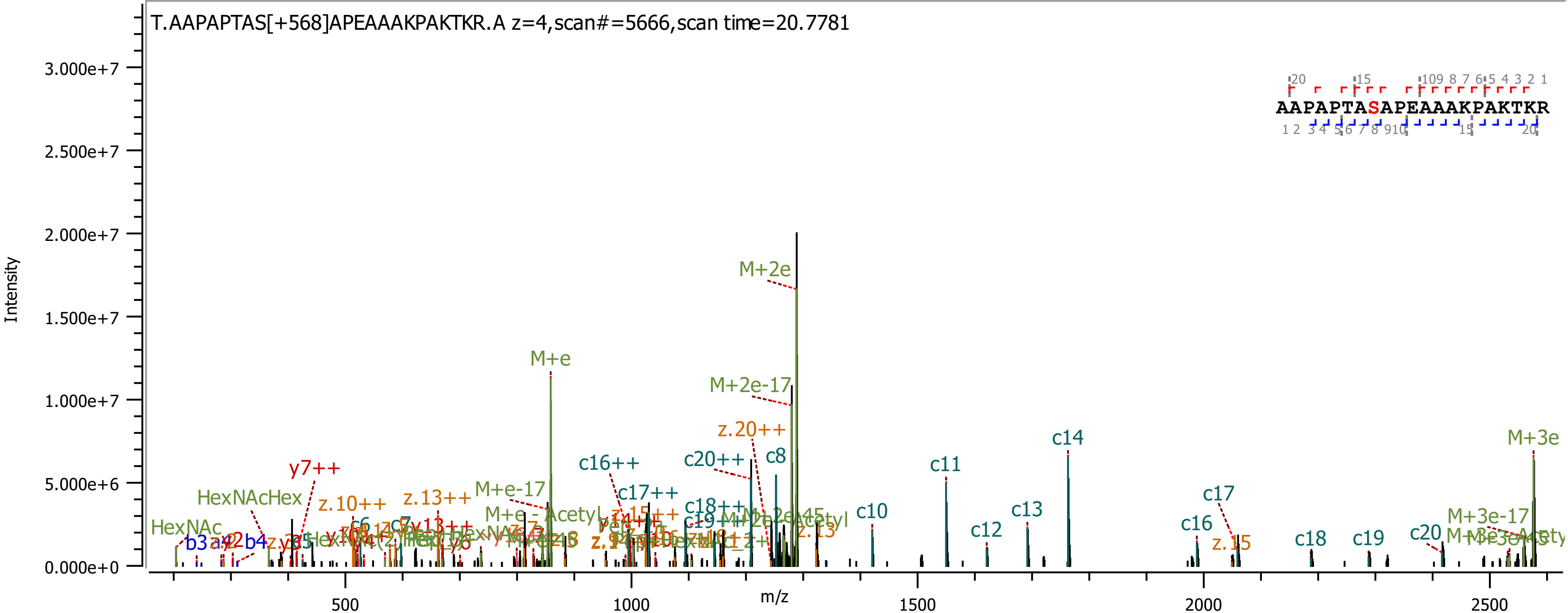
T.AAPAPTAS[+568]APEAAAKPAKTKR.A z=4,scan#=5666,scan time=20.7781
AAPAPTASAPEAAAKPAKTKR
Intensity
m/z
3.000e+7
2.500e+7
2.000e+7
1.500e+7
1.000e+7
5.000e+6
0.000e+0
500
1000
1500
2000
2500
M+2e
M+e
M+2e-17
z.20++
c8
c20++
c16++
c17++
c18++
c14
c11
c10
c12
c13
c16
c17
z.15
c18
c19
c20
M+3e
M+3e-17
y7++
z.10++
z.13++
M+e-17
HexNAcHex
HexNAc
b3
b4
c6
c7
z.13

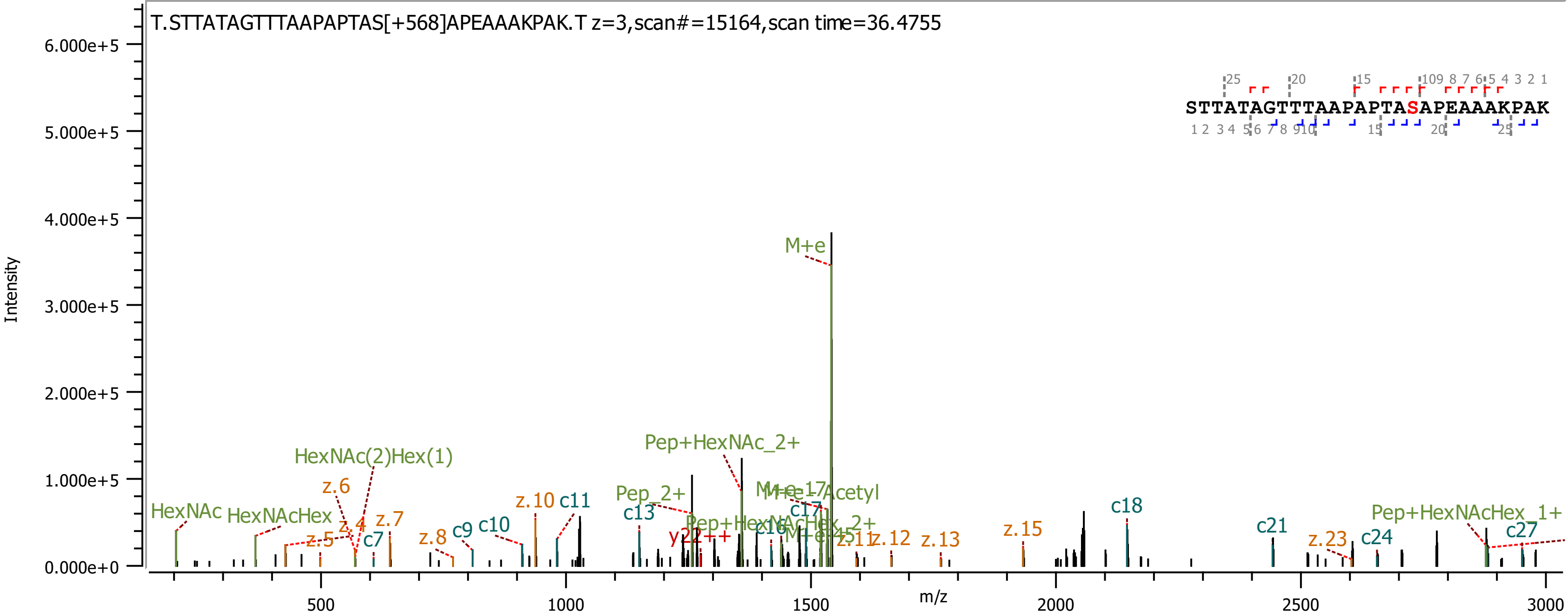

T.STTATAGTTTAAPAPTAS[+568]APEAAAKPAK.T z=3,scan#=15164,scan time=36.4755
STTATAGTTTAAPAPTASAPEAAAKPAK
Intensity
m/z
M+e
Pep+HexNAc_2+
HexNAc(2)Hex(1)
HexNAc
HexNAcHex
Pep_2+
M+e-Acetyl
Pep+HexNAcHex_2+
Pep+HexNAcHex_1+
y22++
z.4
z.5
z.6
z.7
z.8
z.10
z.11
z.12
z.13
z.15
z.23
c7
c9
c10
c11
c13
c16
c17
c18
c21
c24
c27

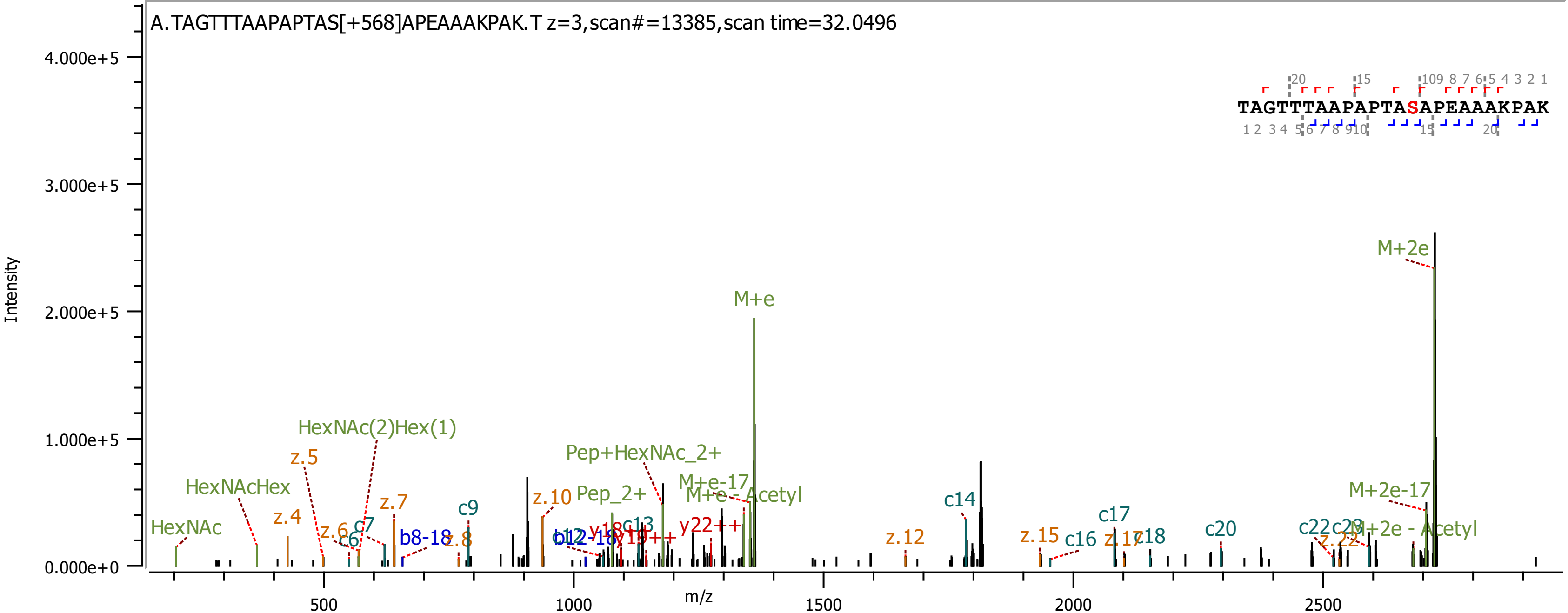
A.TAGTTTAAPAPTAS[+568]APEAAAKPAK.T z=3,scan#=13385,scan time=32.0496
TAGTTTAAPAPTASAPEAAAKPAK
Intensity
4.000e+5
3.000e+5
2.000e+5
1.000e+5
0.000e+0
m/z
500
1000
1500
2000
2500
M+2e
M+e
HexNAc(2)Hex(1)
Pep+HexNAc_2+
HexNAcHex
HexNAc
z.5
z.4
z.7
c9
z.10
Pep_2+
M+e-17
M+e - Acetyl
c14
M+2e-17
c13
y22++
y19++
b8-18
z.8
c7
c6
z.6
z.12
z.15
c16
c17
z.17
c18
c20
c22
M+2e - Acetyl

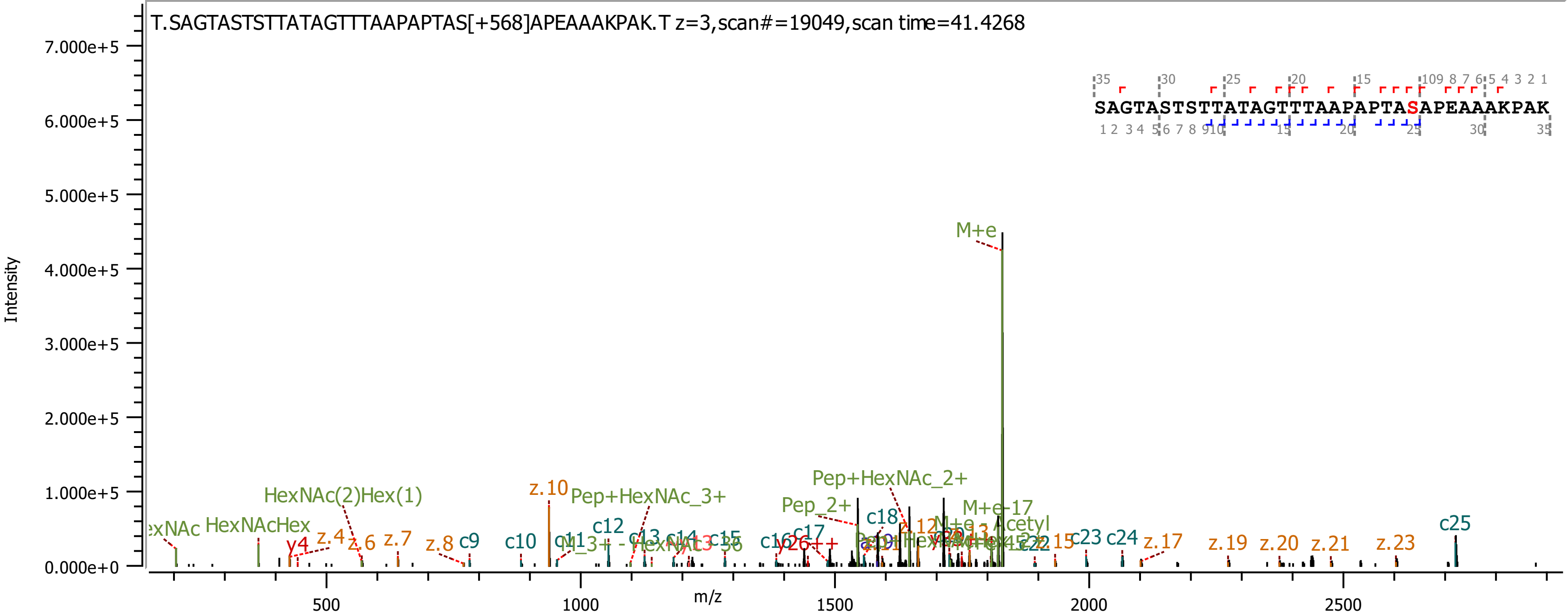

T.SAGTASTSTTATAGTTTAAPAPTAS[+568]APEAAAKPAK.T z=3,scan#=19049,scan time=41.4268
SAGTASTSTTATAGTTTAAPAPTASAPEAAAKPAK
Intensity
m/z
M+e
Pep+HexNAc_2+
Pep_2+
Pep+HexNAc_3+
HexNAc(2)Hex(1)
HexNAcHex
M+e-17
c9
c10
c12
c17
c18
c23
c24
c25
z.4
z.6
z.7
z.8
z.10
z.17
z.19
z.20
z.21
z.23
y4

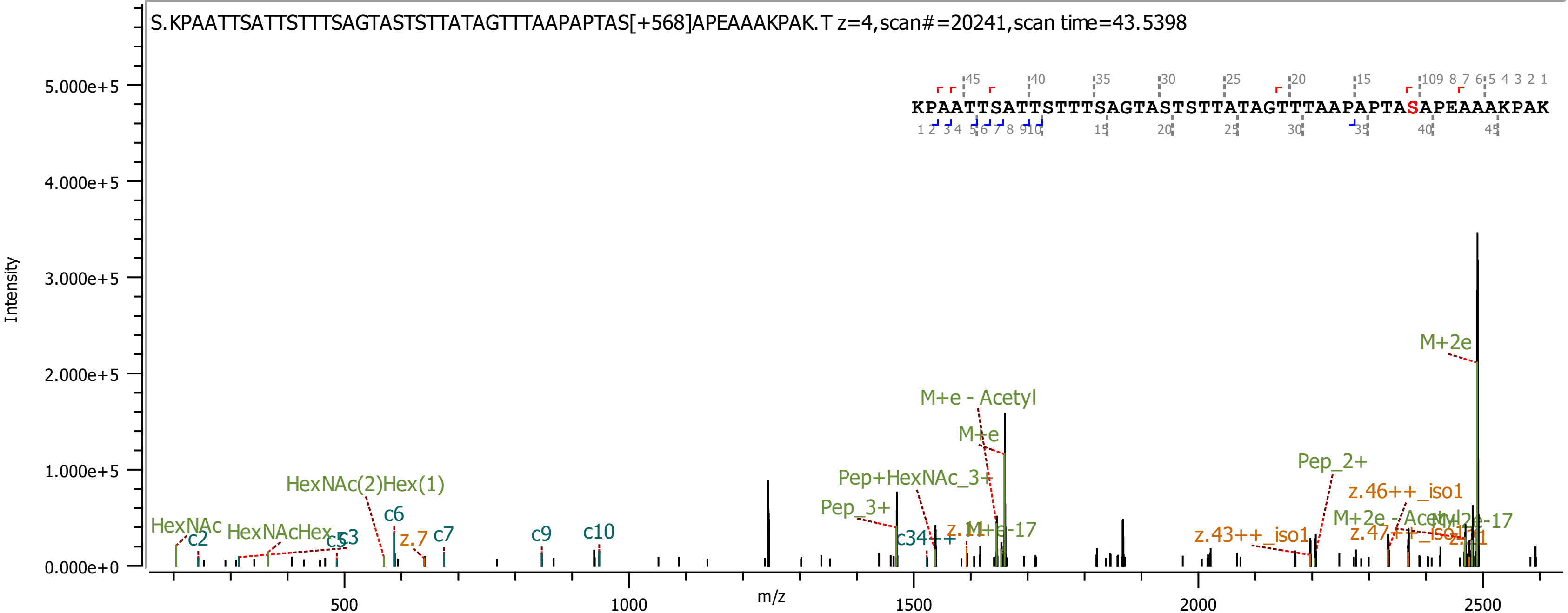

S.KPAATTSATTSTTTSAGTASTSTTATAGTTTAAPAPTAS[+568]APEAAAKPAK.T z=4,scan#=20241,scan time=43.5398
KPAATTSATTSTTTSAGTASTSTTATAGTTTAAPAPTASAPEAAAKPAK
Intensity
m/z
M+2e
M+e - Acetyl
M+e
Pep_2+
Pep+HexNAc_3+
Pep_3+
z.46++_iso1
z.43++_iso1
M+2e - Acetyl
M+2e-17
HexNAc(2)Hex(1)
HexNAc
HexNAcHex
c2
c3
c5
c6
c7
c9
c10
z.7
c34++

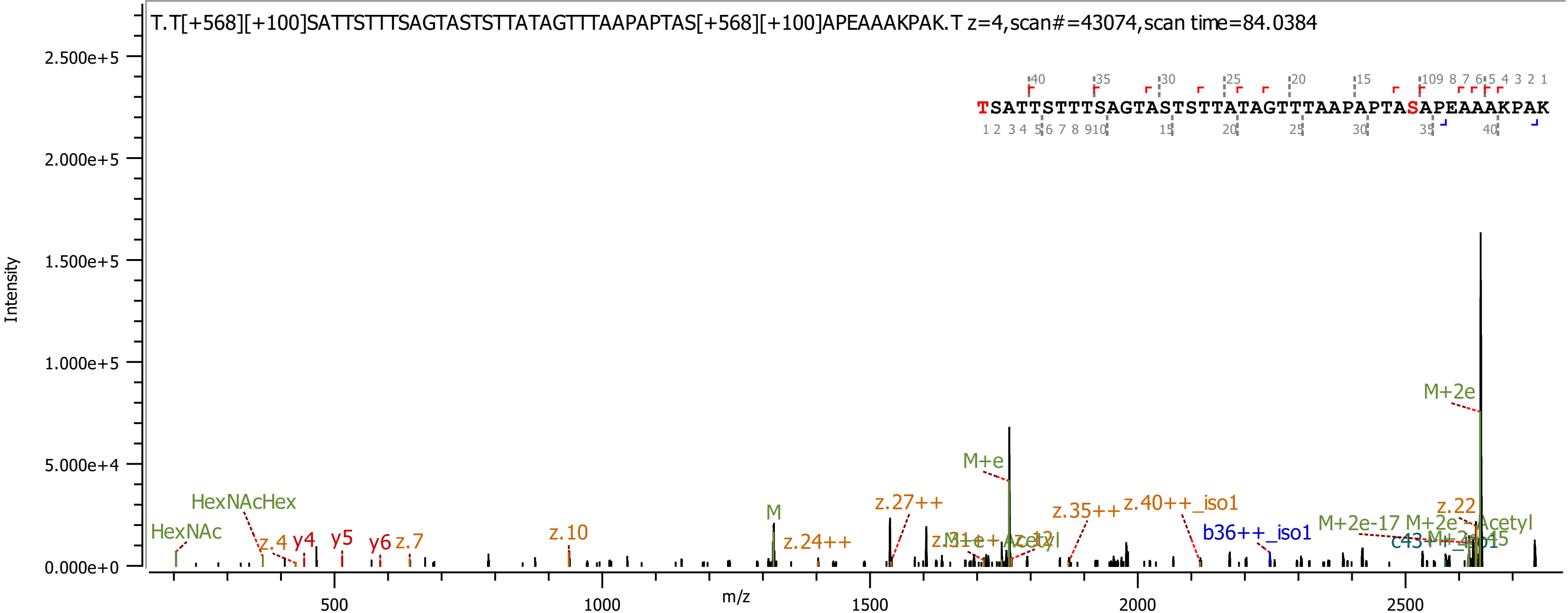
T.T[+568][+100]SATTSTTTSAGTASTSTTATAGTTTAAPAPTAS[+568][+100]APEAAAKPAK.T z=4,scan#=43074,scan time=84.0384
TSATTSTTTSAGTASTSTTATAGTTTAAPAPTASAPEAAAKPAK
Intensity
2.500e+5
2.000e+5
1.500e+5
1.000e+5
5.000e+4
0.000e+0
m/z
500
1000
1500
2000
2500
HexNAc
HexNAcHex
z.4
y4
y5
y6
z.7
z.10
M
z.24++
z.27++
M+e
z.35++
z.40++_iso1
b36++_iso1
M+2e-17
z.22
M+2e

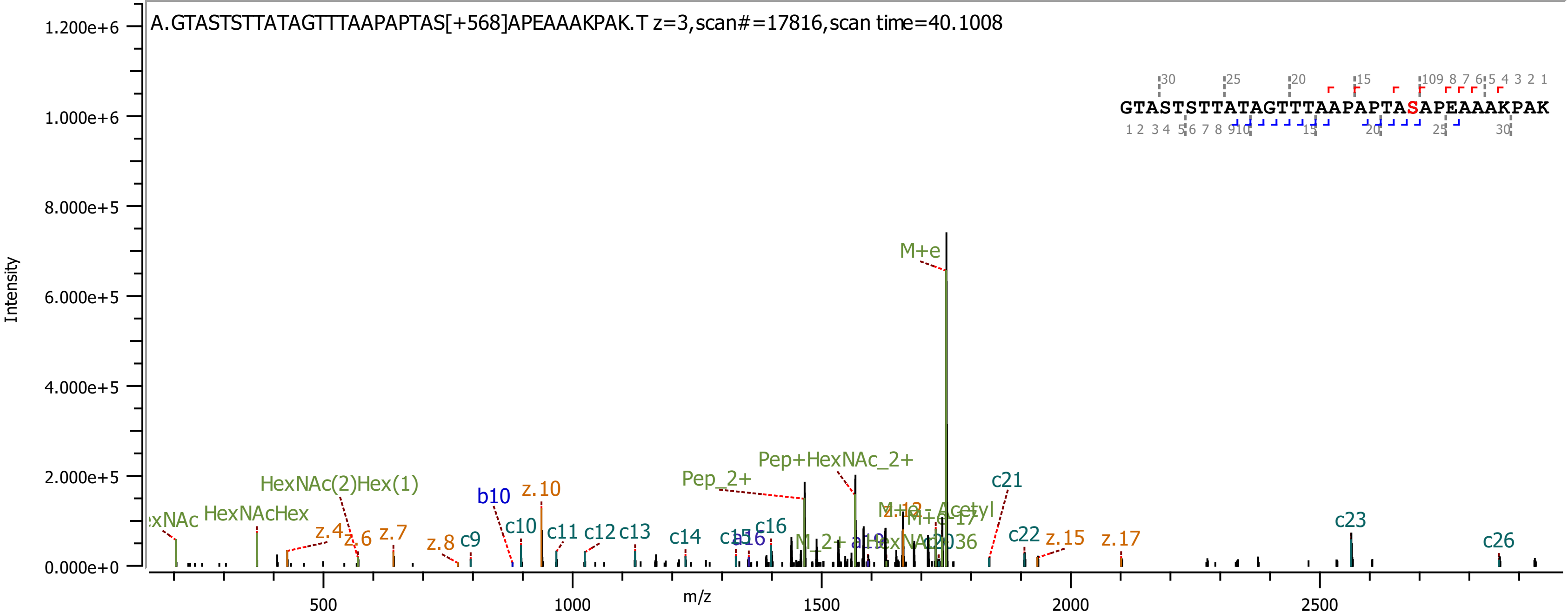
A.GTASTSTTATAGTTTAAPAPTAS[+568]APEAAAKPAK.T z=3,scan#=17816,scan time=40.1008
GTASTSTTATAGTTTAAPAPTASAPEAAAKPAK
Intensity
m/z
1.200e+6
1.000e+6
8.000e+5
6.000e+5
4.000e+5
2.000e+5
0.000e+0
500
1000
1500
2000
2500
M+e
Pep+HexNAc_2+
Pep_2+
HexNAc(2)Hex(1)
HexNAcHex
b10
z.10
c10
c11
c12
c13
c14
c16
c21
c22
c23
c26
c9
z.4
z.6
z.7
z.8
z.15
z.17

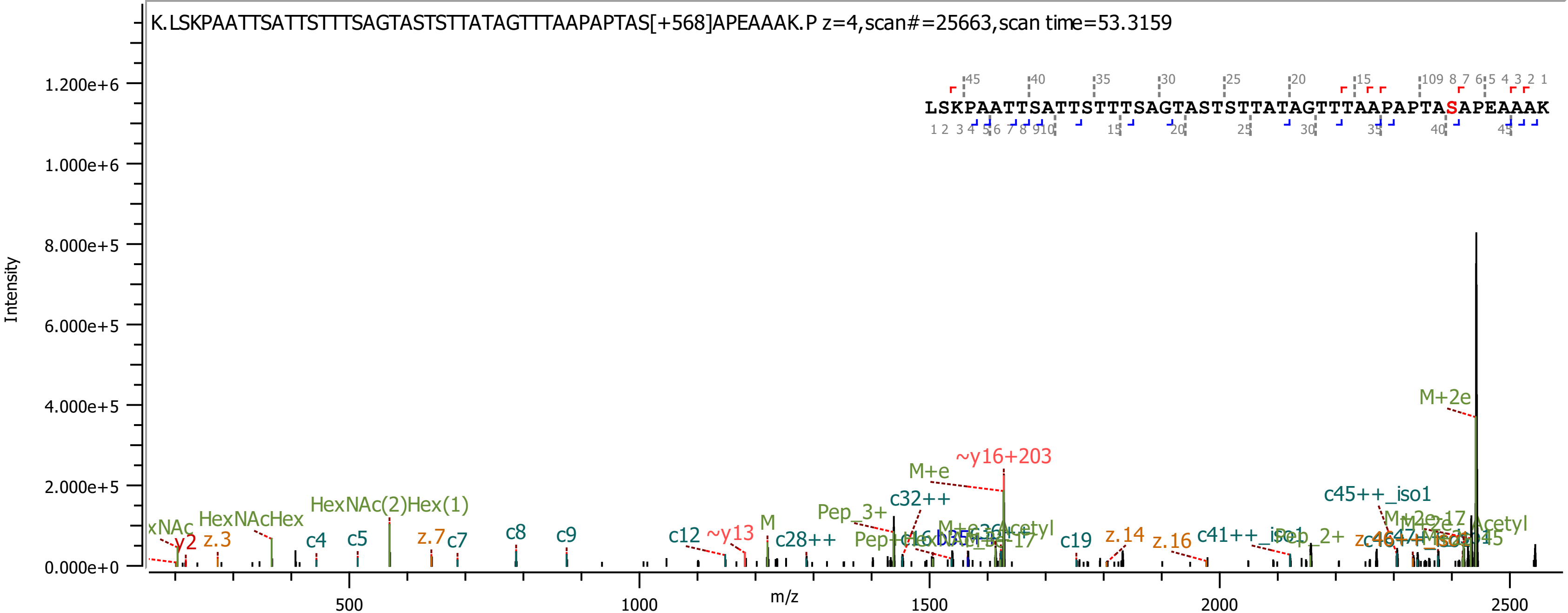

K.LSKPAATTSATTSTTTSAGTASTSTTATAGTTTAAPAPTAS[+568]APEAAAK.P z=4,scan#=25663,scan time=53.3159
LSKPAATTSATTSTTTSAGTASTSTTATAGTTTAAPAPTASAPEAAAK
Intensity
m/z
1.200e+6
1.000e+6
8.000e+5
6.000e+5
4.000e+5
2.000e+5
0.000e+0
500
1000
1500
2000
2500
M+2e
~y16+203
M+e
c32++
Pep_3+
HexNAc(2)Hex(1)
HexNAcHex
y2
z.3
c4
c5
z.7
c7
c8
c9
c12
~y13
M
c28++
c19
z.14
z.16
c41++_iso1
c45++_iso1

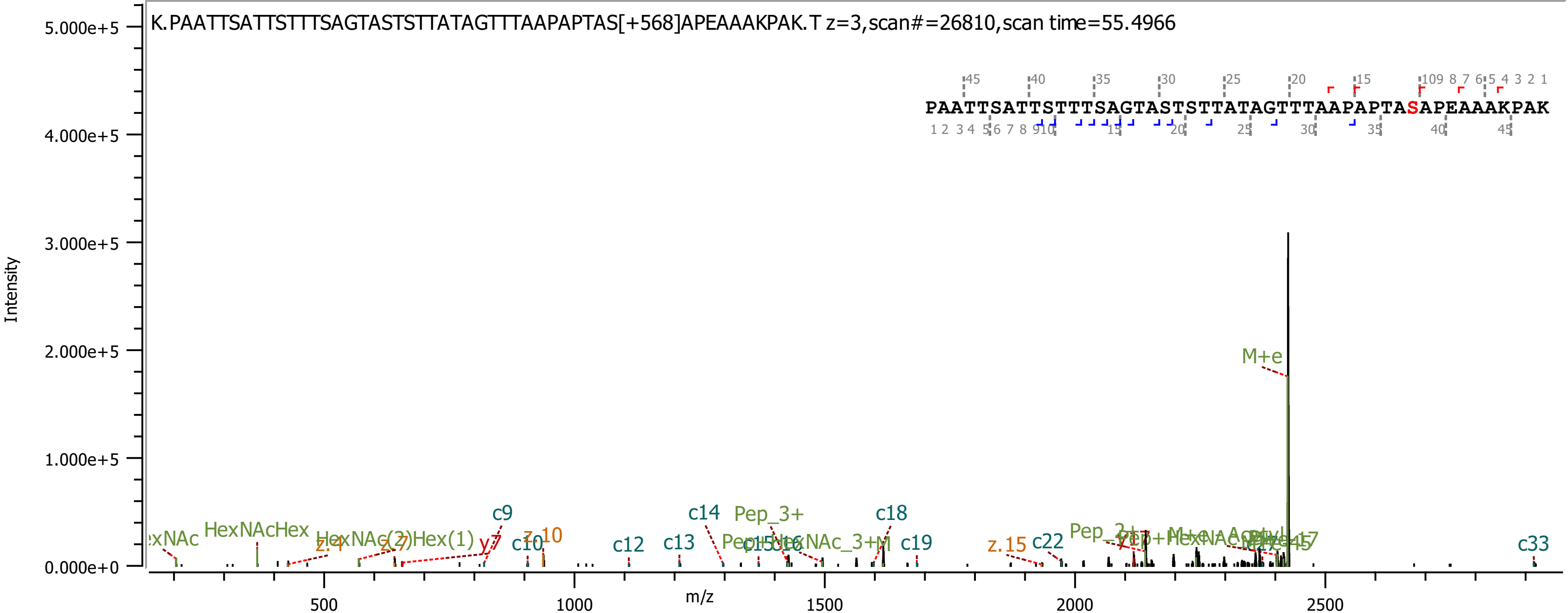
K.PAATTSATTSTTTSAGTASTSTTATAGTTTAAPAPTAS[+568]APEAAAKPAK.T z=3,scan#=26810,scan time=55.4966
PAATTSATTSTTTSAGTASTSTTATAGTTTAAPAPTASAPEAAAKPAK
Intensity
m/z
5.000e+5
4.000e+5
3.000e+5
2.000e+5
1.000e+5
0.000e+0
500
1000
1500
2000
2500
M+e
HexNAcHex
c9
c10
z.10
c12
c13
c14
Pep_3+
c18
c19
z.15
c22
c33
y7

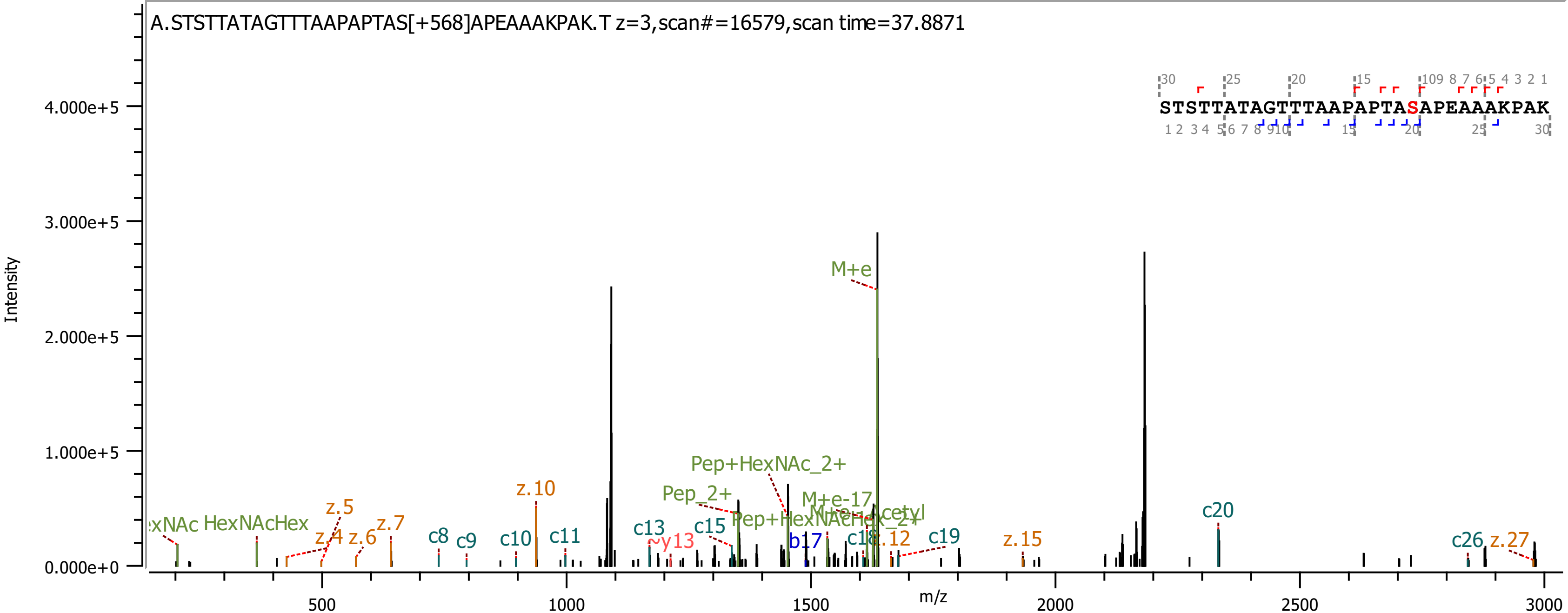

A.STSTTATAGTTTAAPAPTAS[+568]APEAAAKPAK.T z=3,scan#=16579,scan time=37.8871
STSTTATAGTTTAAPAPTASAPEAAAKPAK
Intensity
m/z
M+e
Pep+HexNAc_2+
Pep_2+
M+e-17
Pep+HexNAcHex_2+
HexNAcHex
z.10
z.5
z.4
z.6
z.7
z.12
z.15
z.27
c8
c9
c10
c11
c13
c15
c19
c20
c26
y13
b17

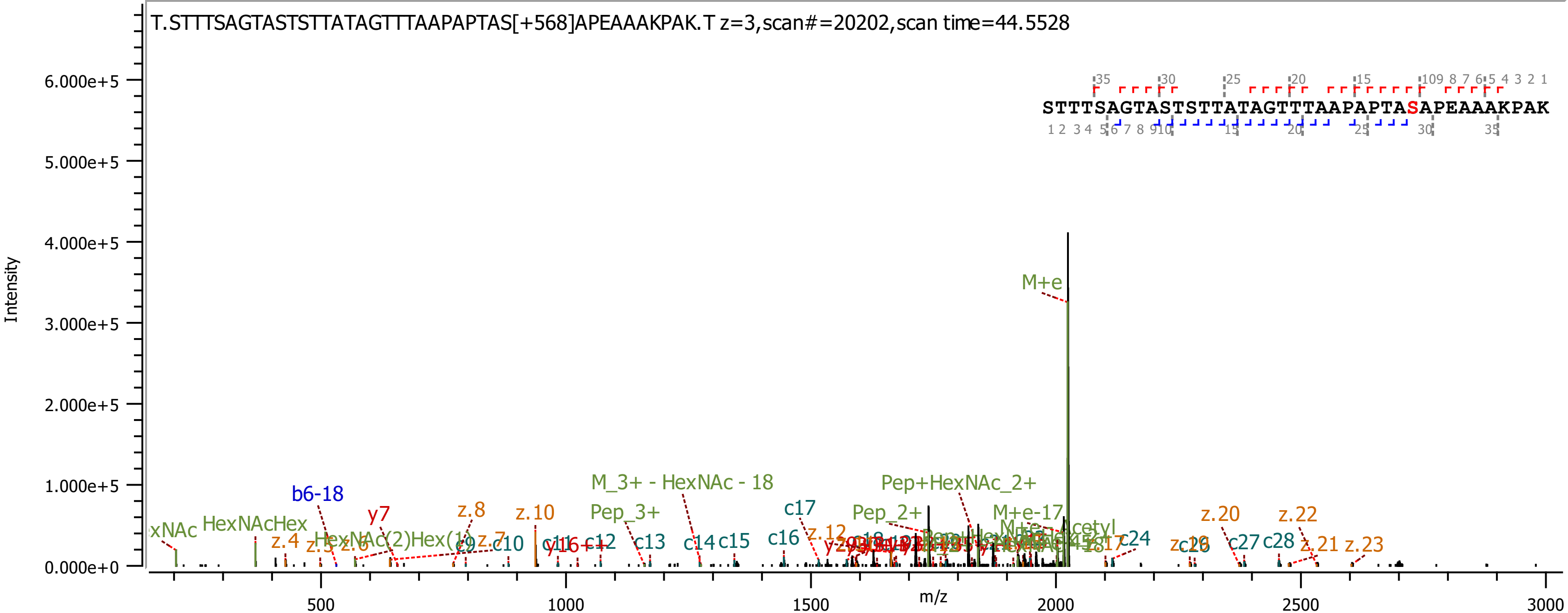

T.STTTSAGTASTSTTATAGTTTAAPAPTAS[+568]APEAAAKPAK.T z=3,scan#=20202,scan time=44.5528
STTTSAGTASTSTTATAGTTTAAPAPTASAPEAAAKPAK
Intensity
m/z
M+e
M+e-17
Pep+HexNAc_2+
M_3+ - HexNAc - 18
Pep_3+
Pep_2+
HexNAcHex
HexNAc(2)Hex(1)
b6-18
y7
y16++
z.4
z.5
z.6
z.7
z.8
z.10
z.12
z.20
z.21
z.22
z.23
c9
c10
c12
c13
c14
c15
c16
c17
c24
c27
c28

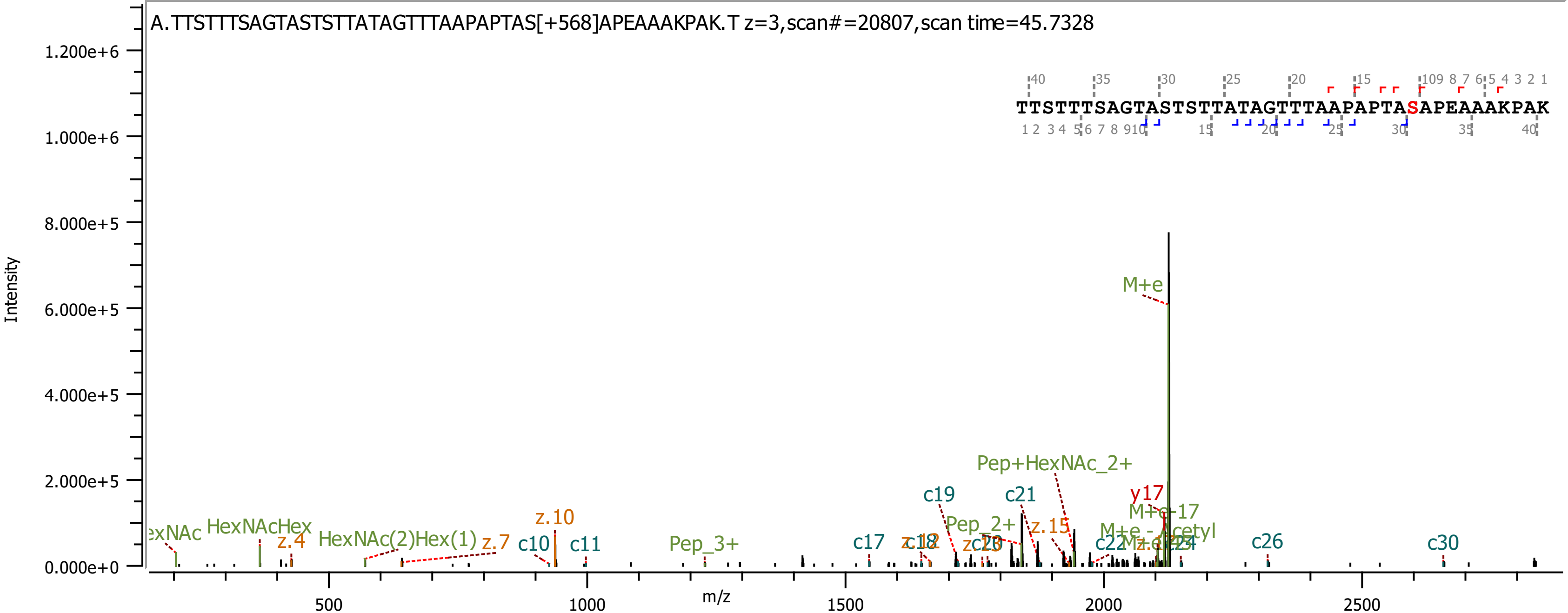
A.TTSTTTSAGTASTSTTATAGTTTAAPAPTAS[+568]APEAAAKPAK.T z=3,scan#=20807,scan time=45.7328
TTSTTTSAGTASTSTTATAGTTTAAPAPTASAPEAAAKPAK
Intensity
m/z
1.200e+6
1.000e+6
8.000e+5
6.000e+5
4.000e+5
2.000e+5
0.000e+0
500
1000
1500
2000
2500
M+e
Pep+HexNAc_2+
y17
M+e-17
c19
c21
Pep_2+
z.15
M+e - Acetyl
HexNAcHex
HexNAc(2)Hex(1)
z.4
z.7
z.10
c10
c11
Pep_3+
c17
c22
c26
c30

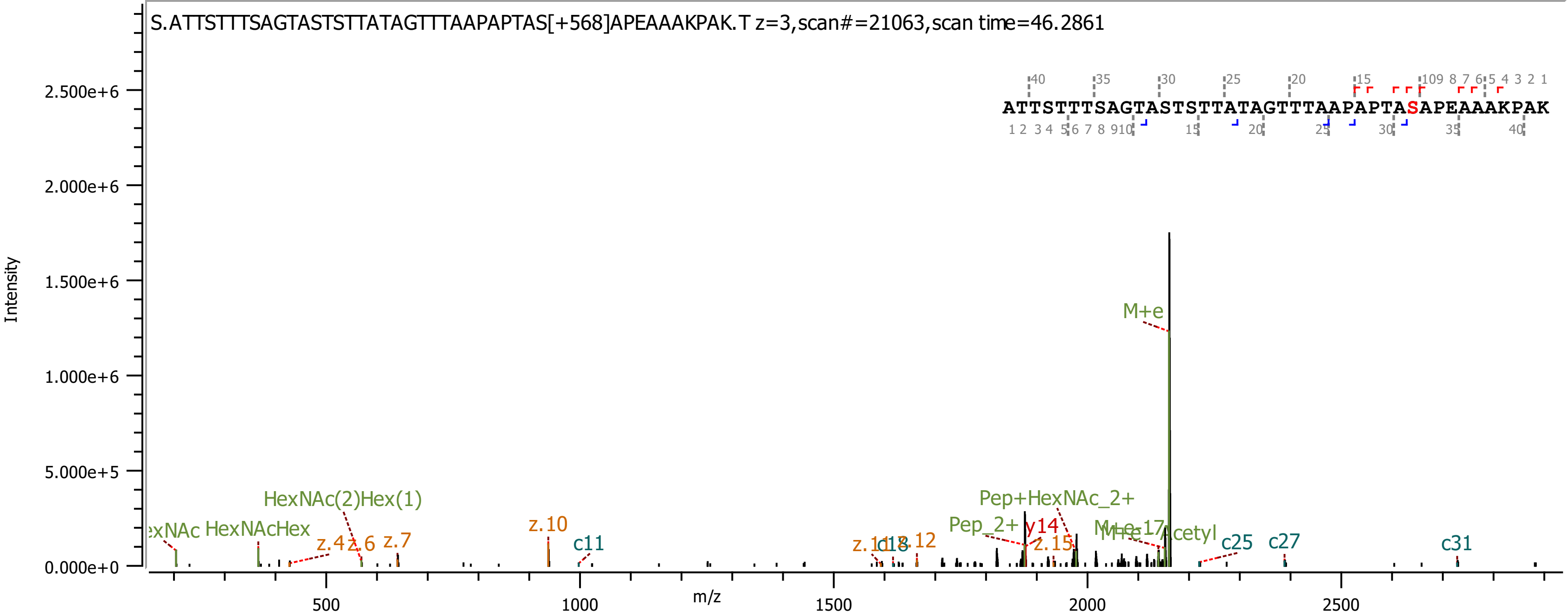

S.ATTSTTTSAGTASTSTTATAGTTTAAPAPTAS[+568]APEAAAKPAK.T z=3,scan#=21063,scan time=46.2861
ATTSTTTSAGTASTSTTATAGTTTAAPAPTASAPEAAAKPAK
Intensity
m/z
2.500e+6
2.000e+6
1.500e+6
1.000e+6
5.000e+5
0.000e+0
500
1000
1500
2000
2500
M+e
HexNAc(2)Hex(1)
HexNAcHex
Pep+HexNAc_2+
Pep_2+
y14
z.15
z.10
c11
z.4
z.6
z.7
z.12
c18
c25
c27
c31

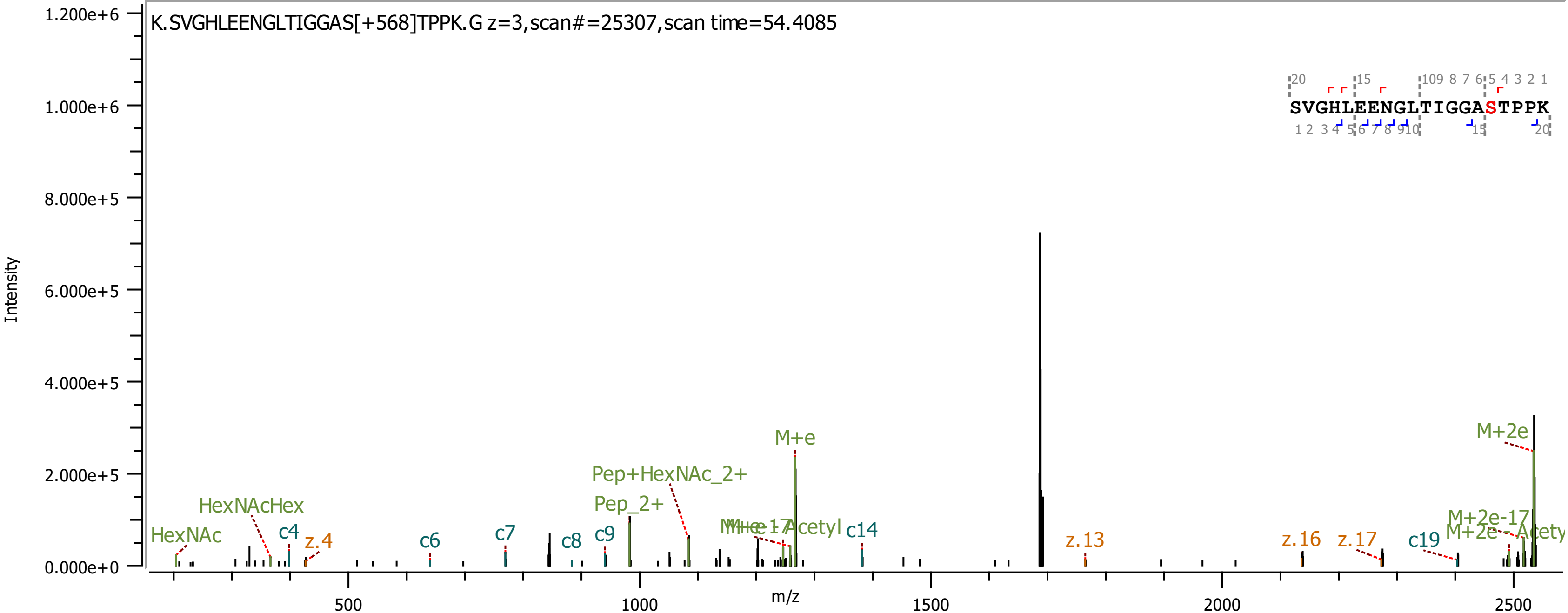
K.SVGHLEENGLTIGGAS[+568]TPPK.G z=3,scan#=25307,scan time=54.4085
SVGHLEENGLTIGGASTPPK
Intensity
m/z
HexNAc
HexNAcHex
c4
z.4
c6
c7
c8
c9
Pep_2+
Pep+HexNAc_2+
M+e
c14
z.13
z.16
z.17
c19
M+2e
M+2e-17
1.200e+6
1.000e+6
8.000e+5
6.000e+5
4.000e+5
2.000e+5
0.000e+0
500
1000
1500
2000
2500

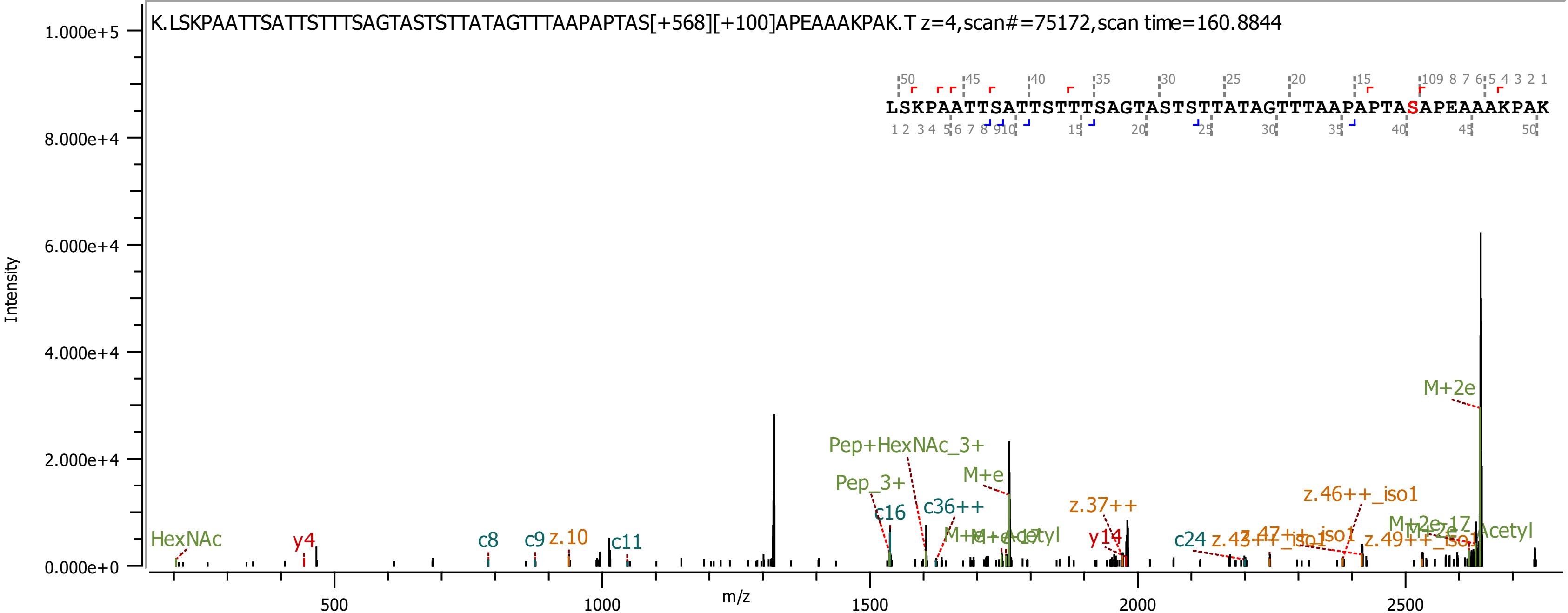
K.LSKPAATTSATTSTTTSAGTASTSTTATAGTTTAAPAPTAS[+568][+100]APEAAAKPAK.T z=4,scan#=75172,scan time=160.8844
LSKPAATTSATTSTTTSAGTASTSTTATAGTTTAAPAPTASAPEAAAKPAK
Intensity
1.000e+5
8.000e+4
6.000e+4
4.000e+4
2.000e+4
0.000e+0
m/z
500
1000
1500
2000
2500
HexNAc
y4
c8
c9
z.10
c11
Pep+HexNAc_3+
Pep_3+
c16
c36++
M+e
z.37++
y14
c24
z.46++_iso1
M+2e

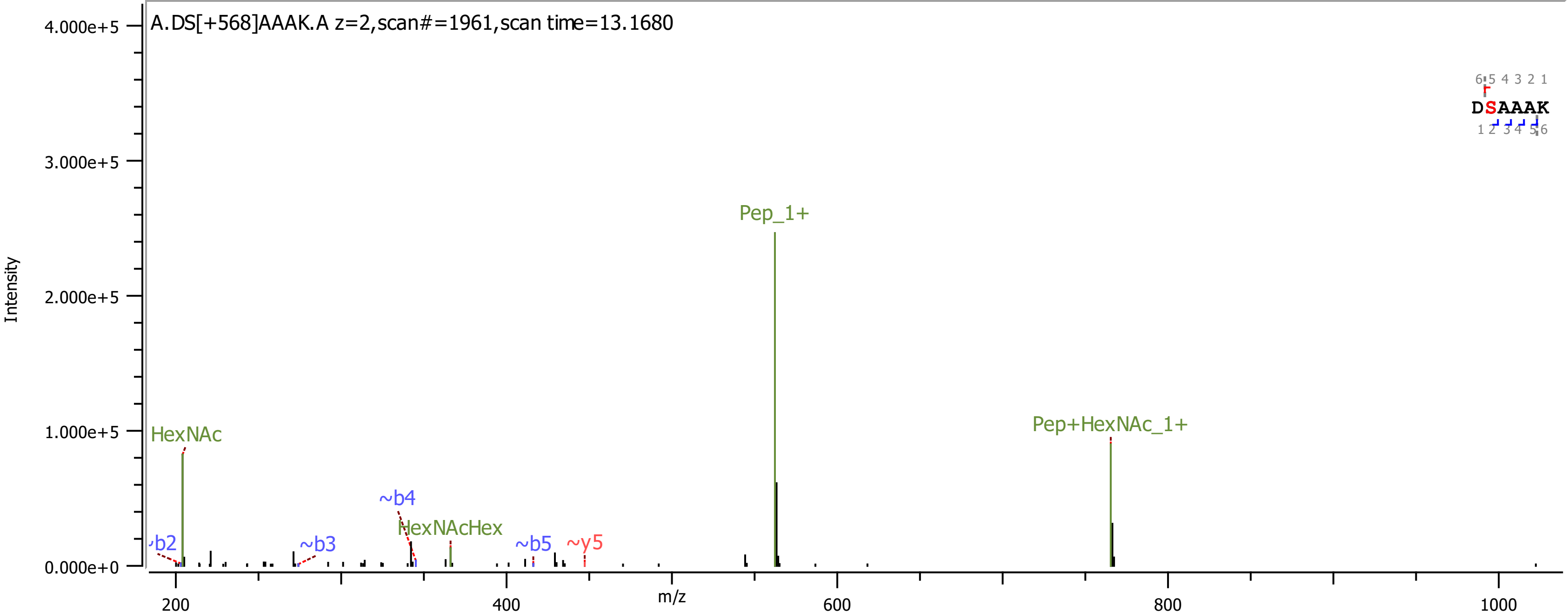

A.DS[+568]AAAK.A z=2,scan#=1961,scan time=13.1680
Intensity
4.000e+5
3.000e+5
2.000e+5
1.000e+5
0.000e+0
6 5 4 3 2 1
DSAAAK
1 2 3 4 5 6
Pep_1+
Pep+HexNAc_1+
HexNAc
~b4
HexNAcHex
~b2
~b3
~b5
~y5
200
400
600
800
1000
m/z

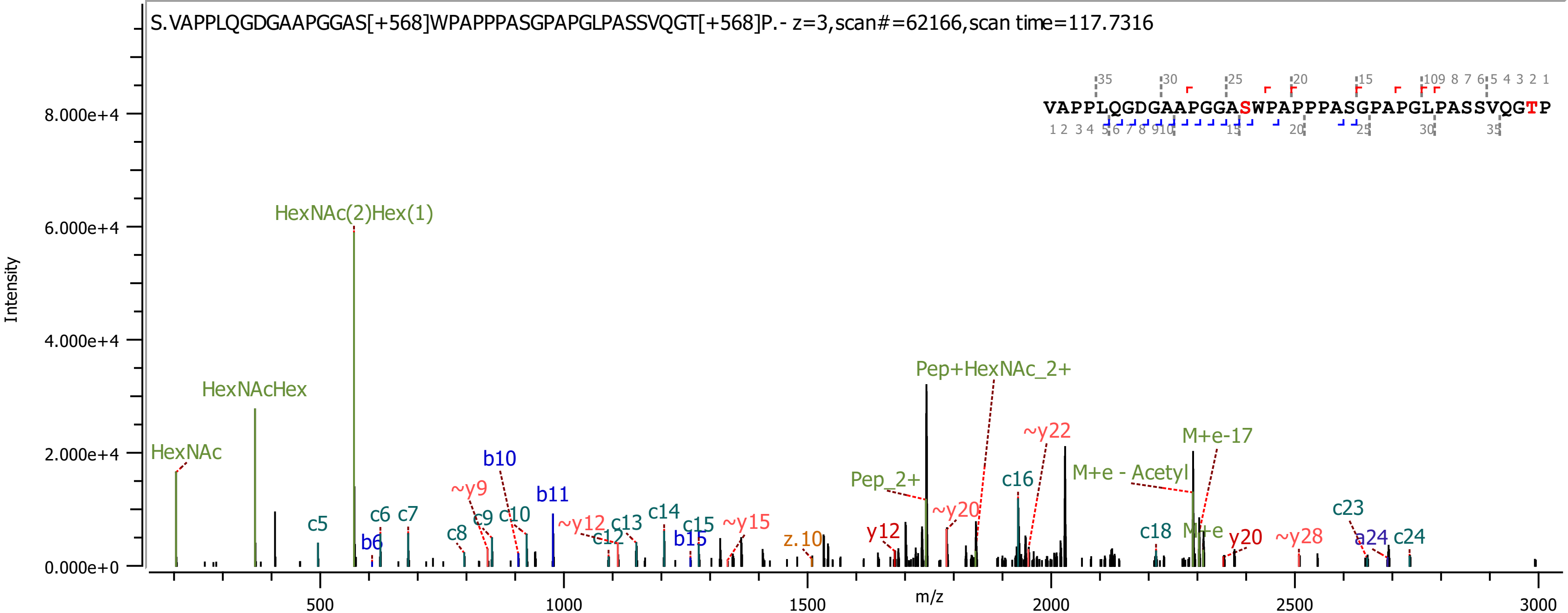

S.VAPPLQGDGAAPGGAS[+568]WPAPPPASGPAPGLPASSVQGT[+568]P.- z=3,scan#=62166,scan time=117.7316
VAPPLQGDGAAPGGASWPAPPPASGPAPGLPASSVQGTP
Intensity
m/z
8.000e+4
6.000e+4
4.000e+4
2.000e+4
0.000e+0
500
1000
1500
2000
2500
3000
HexNAc(2)Hex(1)
HexNAcHex
HexNAc
Pep+HexNAc_2+
Pep_2+
~y22
M+e-17
M+e - Acetyl
c16
b10
b11
~y9
c5
c6
c7
c8
c9
c10
b6
~y12
c12
c13
c14
c15
b15
~y15
z.10
y12
~y20
c18
M+e
y20
~y28
c23
a24
c24

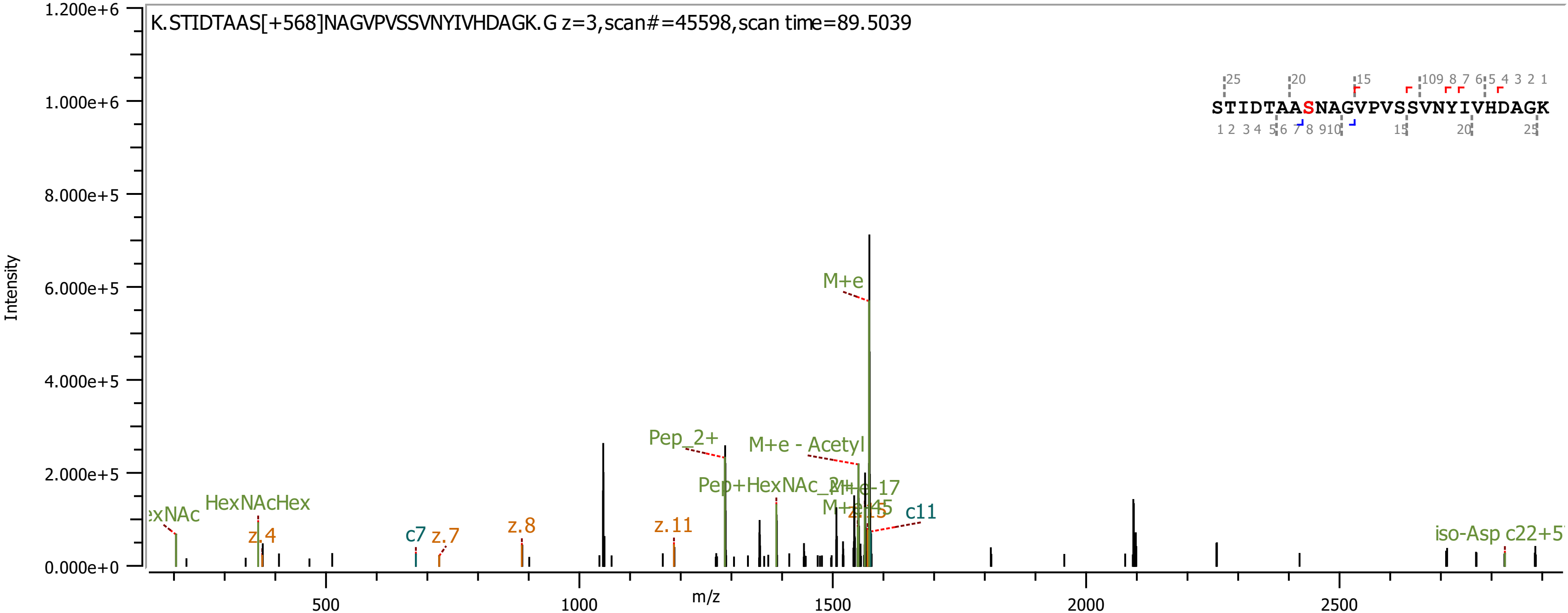
K.STIDTAAS[+568]NAGVPVSSVNYIVHDAGK.G z=3,scan#=45598,scan time=89.5039
STIDTAASNAGVPVSSVNYIVHDAGK
Intensity
m/z
1.200e+6
1.000e+6
8.000e+5
6.000e+5
4.000e+5
2.000e+5
0.000e+0
500
1000
1500
2000
2500
M+e
Pep_2+
M+e - Acetyl
Pep+HexNAc_2+
M+e-17
M+e-45
c11
z.15
HexNAcHex
z.4
c7
z.7
z.8
z.11
iso-Asp c22+57

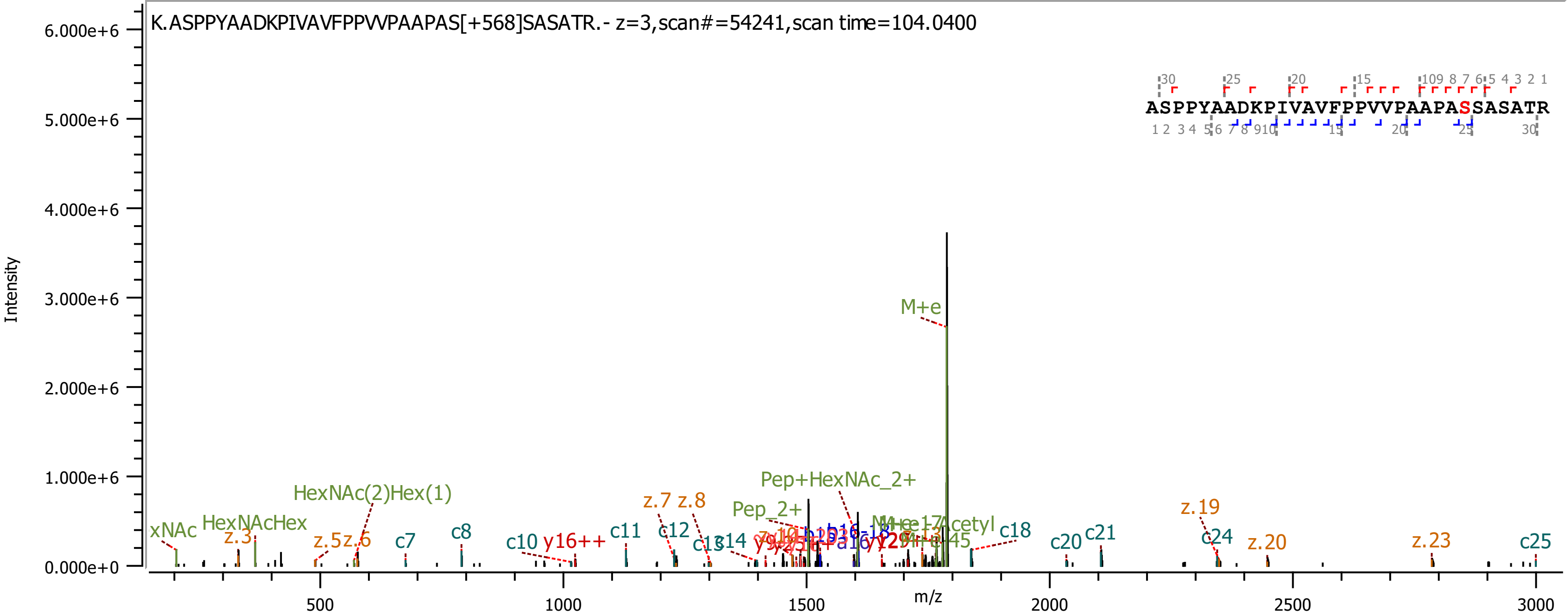

K.ASPPYAADKPIVAVFPPVVPAAPAS[+568]SASATR.- z=3,scan#=54241,scan time=104.0400
ASPPYAADKPIVAVFPPVVPAAPASSASATR
Intensity
m/z
6.000e+6
5.000e+6
4.000e+6
3.000e+6
2.000e+6
1.000e+6
0.000e+0
500
1000
1500
2000
2500
3000
M+e
Pep+HexNAc_2+
Pep_2+
HexNAc(2)Hex(1)
HexNAcHex
xNAc
z.3
z.5
z.6
c7
c8
c10
y16++
c11
c12
z.7
z.8
c13
c14
c18
c20
c21
z.19
c24
z.20
z.23
c25

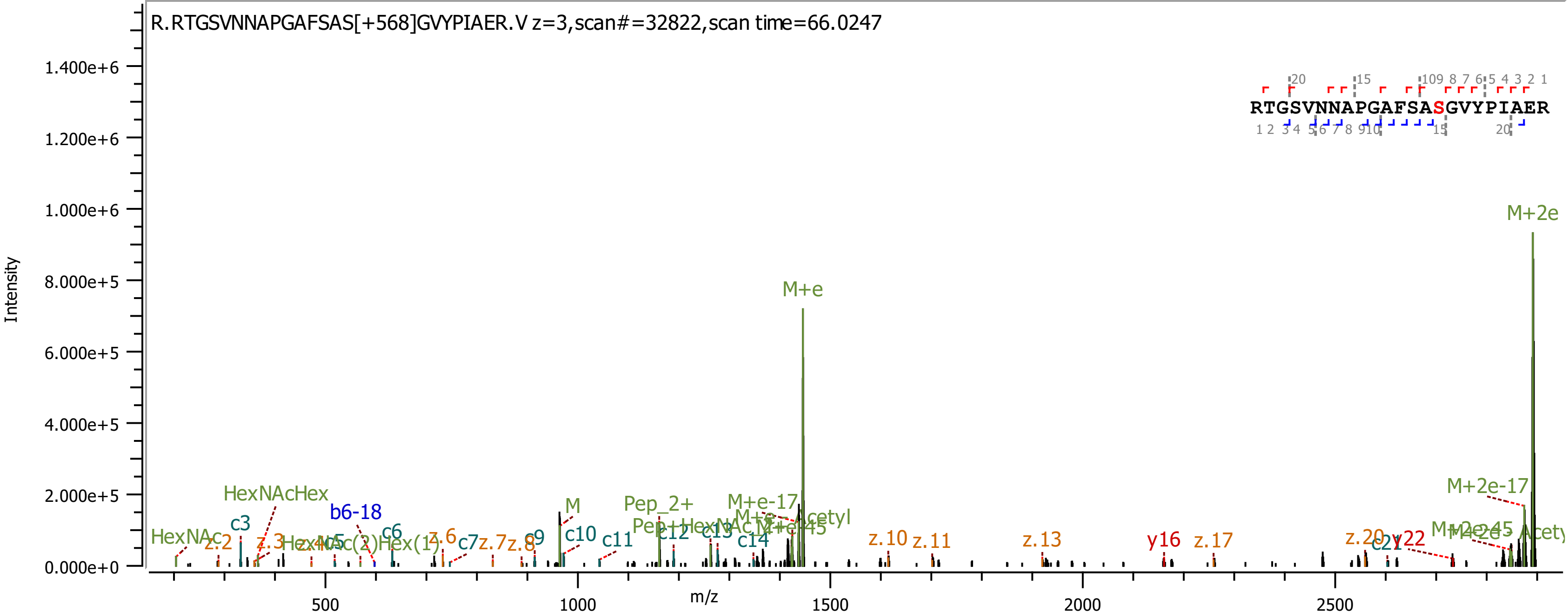
R.RTGSVNNAPGAFSAS[+568]GVYPIAER.V z=3,scan#=32822,scan time=66.0247
RTGSVNNAPGAFSASGVYPIAER
Intensity
m/z
M+2e
M+e
M+2e-17
M+e-17
HexNAcHex
HexNAc
b6-18
c3
c6
c7
c9
c10
c11
c12
c13
c14
c21
z.2
z.3
z.6
z.7
z.8
z.10
z.11
z.13
z.17
z.20
y16
y22
M
Pep_2+
Acetyl

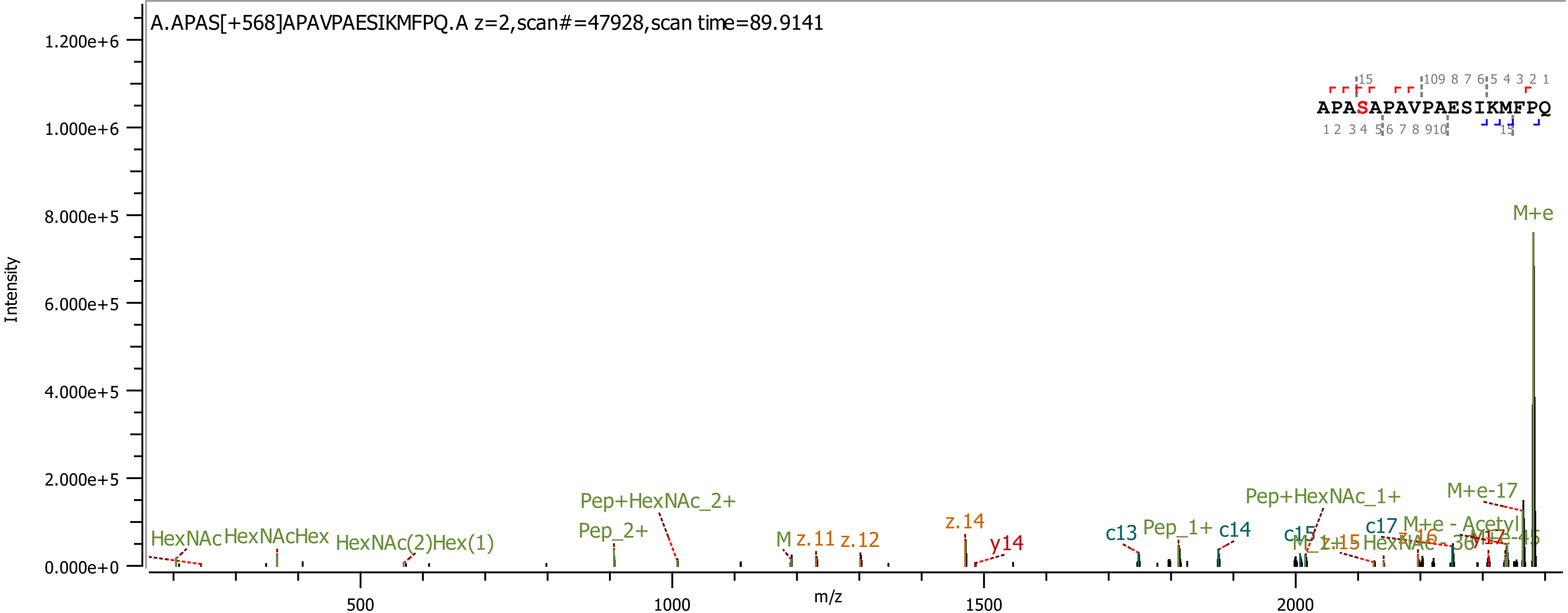

A.APAS[+568]APAVPAESIKMFPQ.A z=2,scan#=47928,scan time=89.9141
Intensity
m/z
APASAPAVPAESIKMFPQ
M+e
M+e-17
Pep+HexNAc_2+
Pep_2+
Pep+HexNAc_1+
Pep_1+
HexNAc
HexNAcHex
HexNAc(2)Hex(1)
z.11
z.12
z.14
y14
c13
c14
c15
c17
z.15
z.16

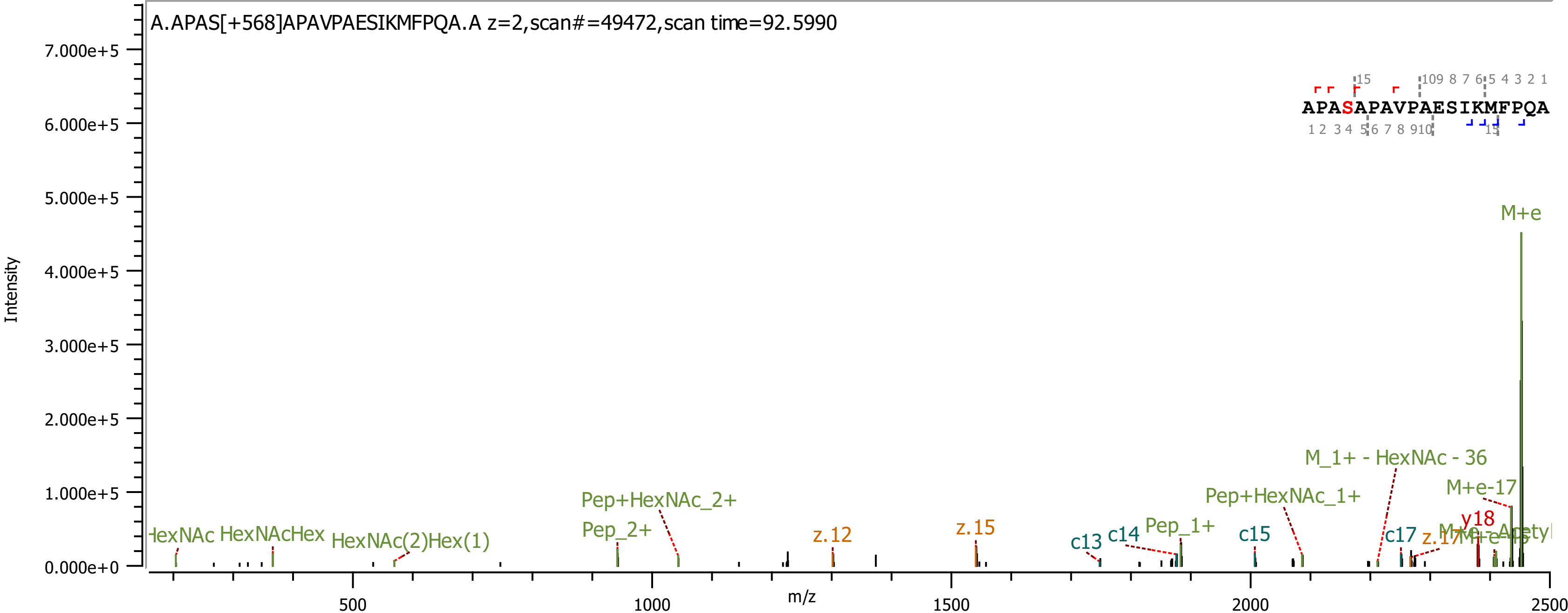
A.APAS[+568]APAVPAESIKMFPQA.A z=2,scan#=49472,scan time=92.5990
APASAPAVPAESIKMFPQA
Intensity
m/z
M+e
M_1+ - HexNAc - 36
M+e-17
Pep+HexNAc_1+
Pep+HexNAc_2+
Pep_2+
Pep_1+
HexNAc
HexNAcHex
HexNAc(2)Hex(1)
z.12
z.15
z.17
c13
c14
c15
c17
y18
7.000e+5
6.000e+5
5.000e+5
4.000e+5
3.000e+5
2.000e+5
1.000e+5
0.000e+0
500
1000
1500
2000
2500

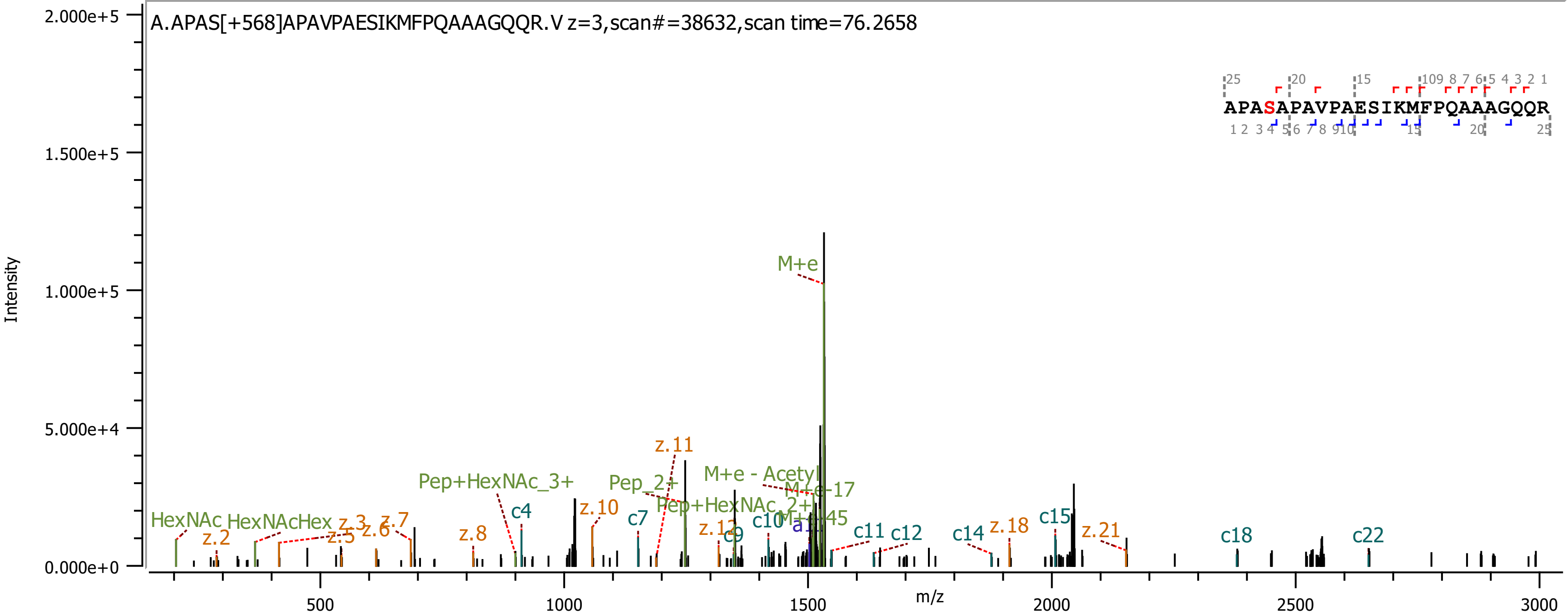

A.APAS[+568]APAVPAESIKMFPQAAAGQQR.V z=3,scan#=38632,scan time=76.2658
APASAPAVPAESIKMFPQAAAGQQR
Intensity
m/z
2.000e+5
1.500e+5
1.000e+5
5.000e+4
0.000e+0
500
1000
1500
2000
2500
3000
M+e
HexNAc
HexNAcHex
z.2
z.3
z.5
z.6
z.7
z.8
Pep+HexNAc_3+
c4
z.10
c7
Pep_2+
z.11
M+e - Acetyl
M+e-17
Pep+HexNAc_2+
z.12
c9
c10
M+e-45
c11
c12
c14
z.18
c15
z.21
c18
c22

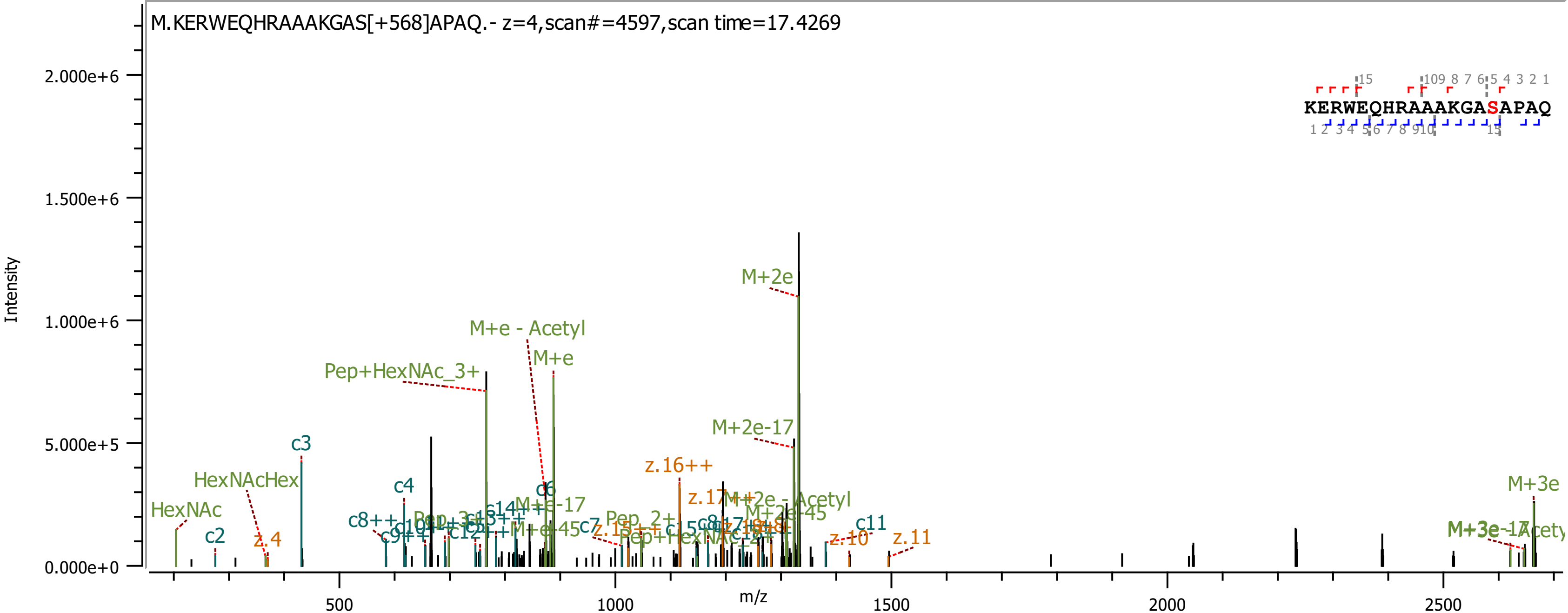
M.KERWEQHRAAAKGAS[+568]APAQ.- z=4,scan#=4597,scan time=17.4269
KERWEQHRAAAKGASAPAQ
Intensity
m/z
M+2e
M+e - Acetyl
M+e
Pep+HexNAc_3+
M+2e-17
c3
HexNAcHex
HexNAc
c2
z.4
c4
z.16++
M+3e
c11
z.10
z.11
M+2e - Acetyl
M+2e-45
M+e-17
M+e-45
c7
c6
c8++
c9++
Pep_2+
Pep+HexNAc_2+
z.17

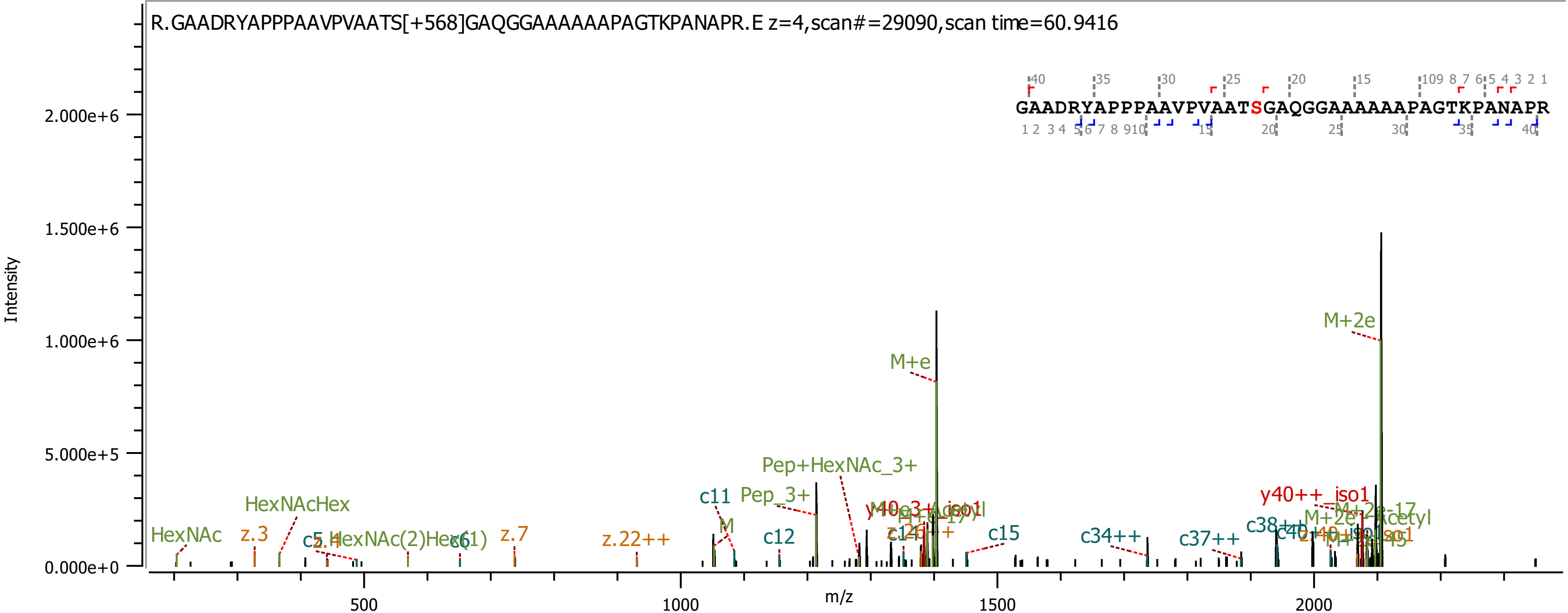

R.GAADRYAPPPAAVPVAATS[+568]GAQGGAAAAAAPAGTKPANAPR.E z=4,scan#=29090,scan time=60.9416
GAADRYAPPPAAVPVAATSGAQGGAAAAAAPAGTKPANAPR
Intensity
m/z
0.000e+0
5.000e+5
1.000e+6
1.500e+6
2.000e+6
500
1000
1500
2000
M+2e
M+e
Pep+HexNAc_3+
Pep_3+
c11
c12
c15
c34++
c37++
c38++
y40++_iso1
HexNAcHex
HexNAc
z.3
z.4
z.7
z.22++
c5
c6
HexNAc(2)Hex(1)
M

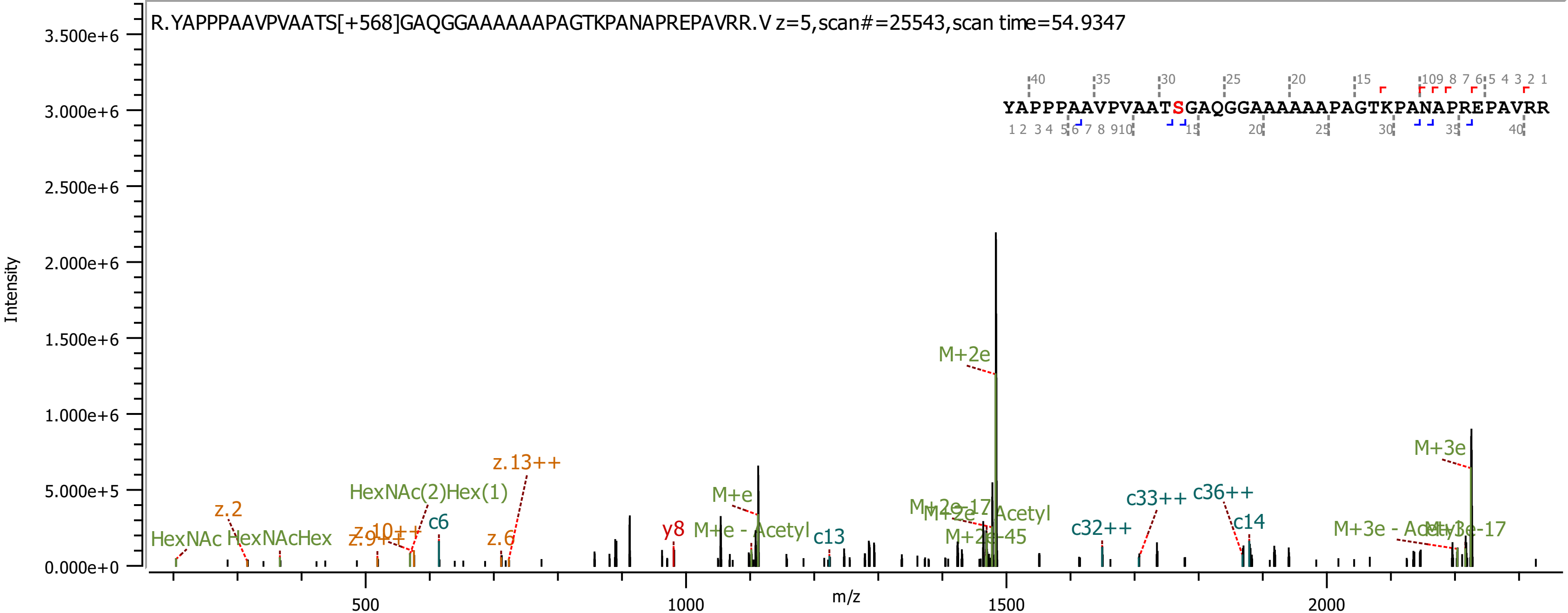

R.YAPPPAAVPVAATS[+568]GAQGGAAAAAAPAGTKPANAPREPAVRR.V z=5,scan#=25543,scan time=54.9347
YAPPPAAVPVAATSGAQGGAAAAAAPAGTKPANAPREPAVRR
Intensity
m/z
M+2e
M+3e
M+e
M+e - Acetyl
M+2e-17
M+2e-Acetyl
M+2e-45
M+3e - Acetyl
M+3e-17
HexNAc
HexNAcHex
HexNAc(2)Hex(1)
z.2
z.6
z.9++
z.10++
z.13++
c6
c13
c14
c32++
c33++
c36++
y8

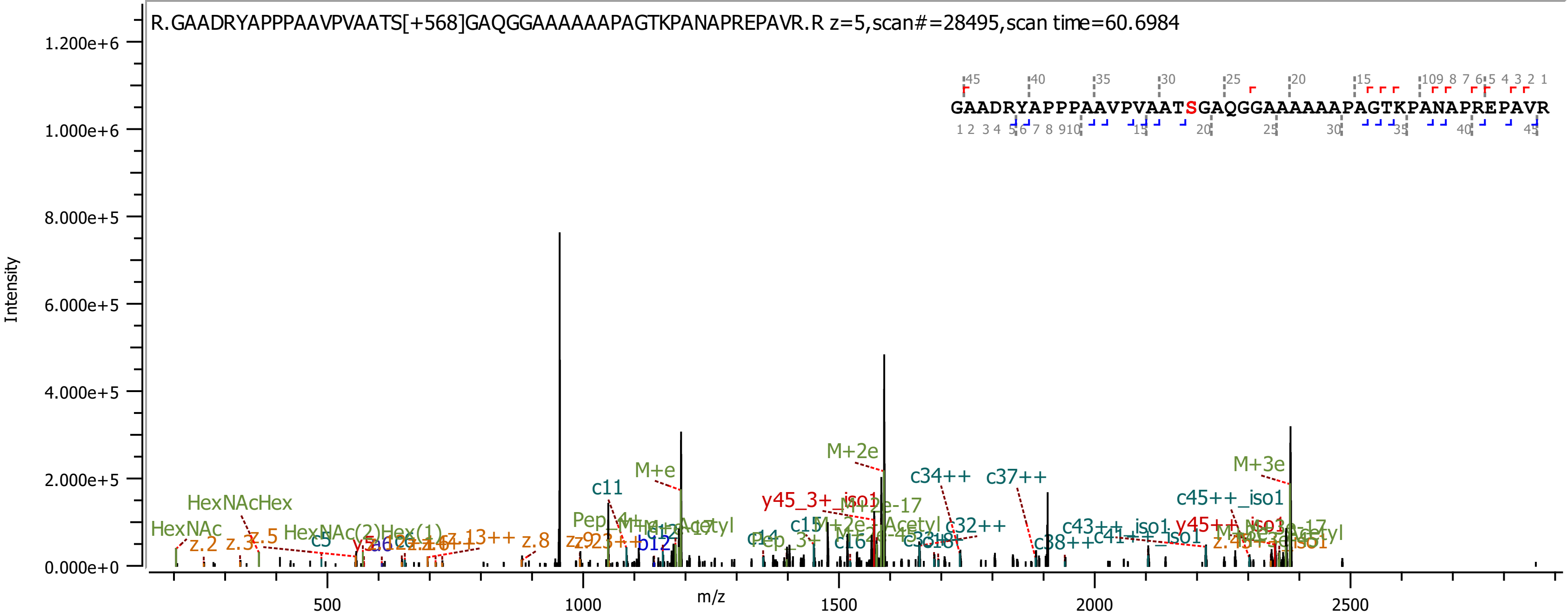

R.GAADRYAPPPAAVPVAATS[+568]GAQGGAAAAAAPAGTKPANAPREPAVR.R z=5,scan#=28495,scan time=60.6984
GAADRYAPPPAAVPVAATSGAQGGAAAAAAPAGTKPANAPREPAVR
Intensity
m/z
1.200e+6
1.000e+6
8.000e+5
6.000e+5
4.000e+5
2.000e+5
0.000e+0
500
1000
1500
2000
2500
M+2e
M+3e
M+e
c34++
c37++
c11
c45++_iso1
HexNAcHex
HexNAc
c43++_iso1
c38++
c32++
c15
z.2
z.3
z.5
z.8
c5
b12

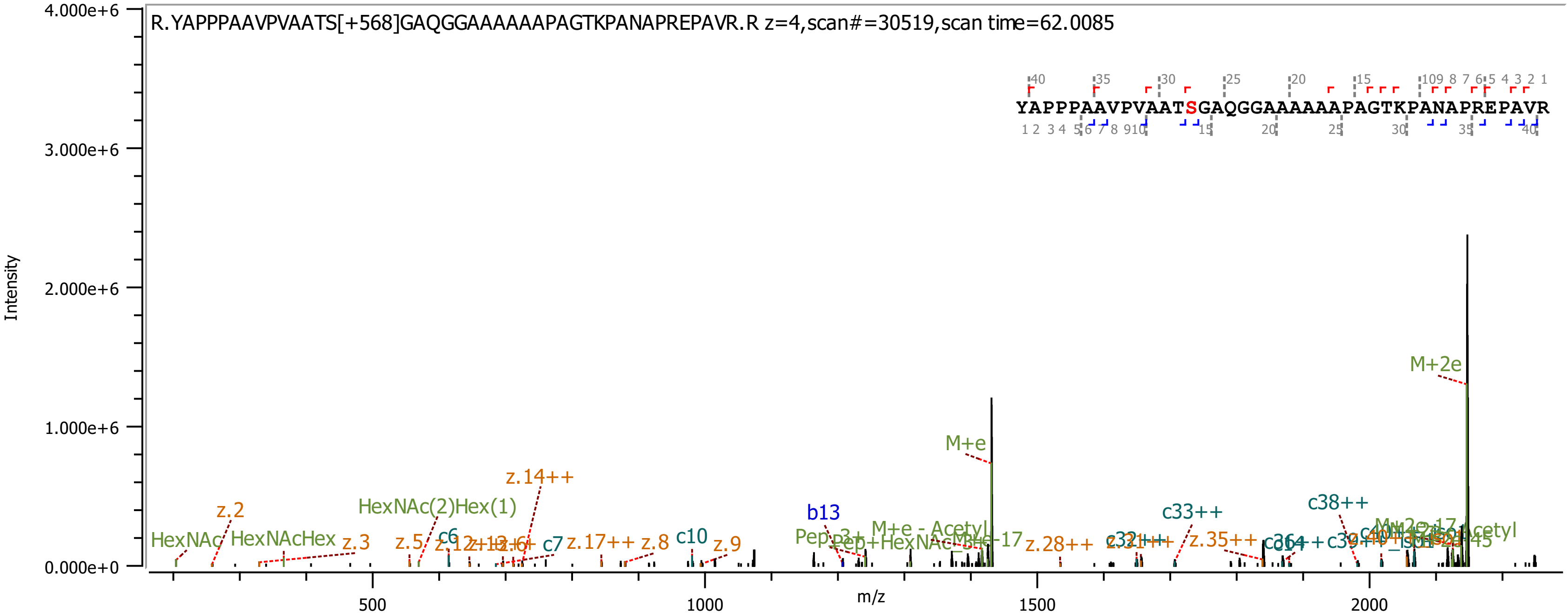

R.YAPPPAAVPVAATS[+568]GAQGGAAAAAAPAGTKPANAPREPAVR.R z=4,scan#=30519,scan time=62.0085
YAPPPAAVPVAATSGAQGGAAAAAAPAGTKPANAPREPAVR
Intensity
m/z
4.000e+6
3.000e+6
2.000e+6
1.000e+6
0.000e+0
500
1000
1500
2000
M+2e
M+e
z.14++
z.2
HexNAc(2)Hex(1)
HexNAc
HexNAcHex
z.3
z.5
c6
c7
z.17++
z.8
c10
z.9
b13
M+e - Acetyl
z.28++
c33++
c38++
z.35++

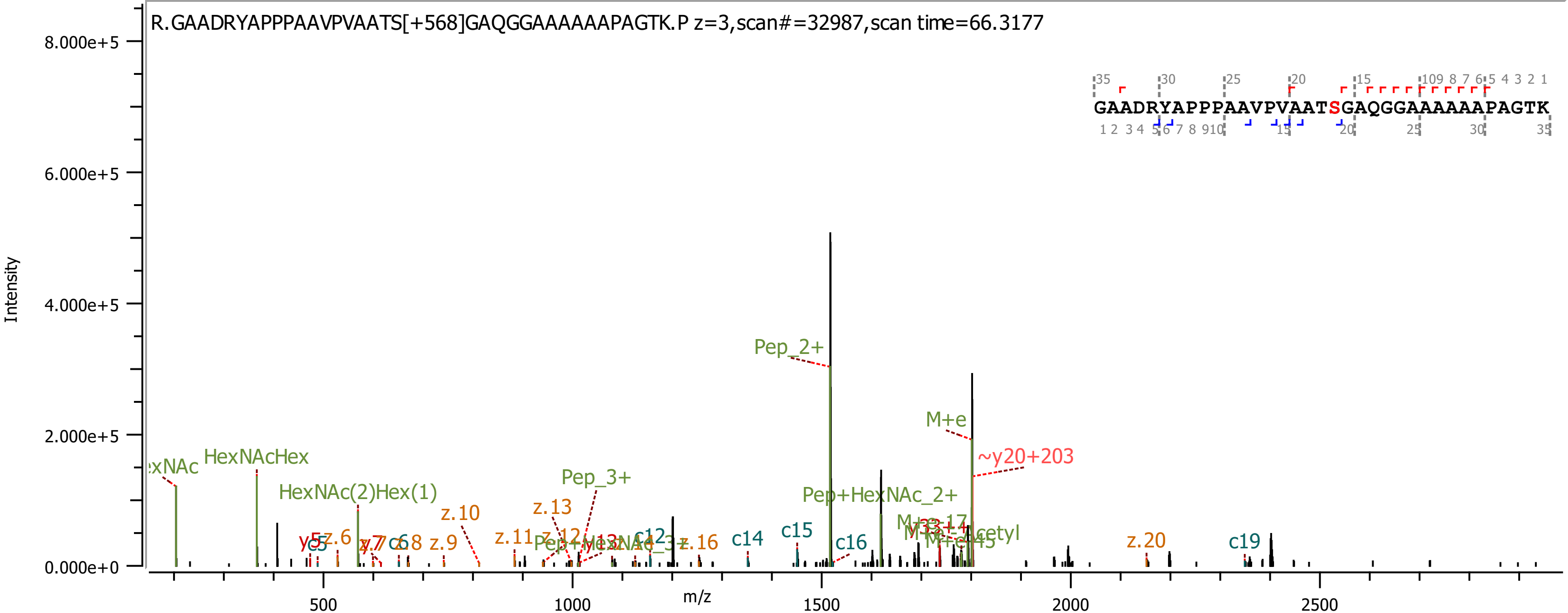
R.GAADRYAPPPAAVPVAATS[+568]GAQGGAAAAAAPAGTK.P z=3,scan#=32987,scan time=66.3177
GAADRYAPPPAAVPVAATSGAQGGAAAAAAPAGTK
Intensity
m/z
8.000e+5
6.000e+5
4.000e+5
2.000e+5
0.000e+0
500
1000
1500
2000
2500
HexNAcHex
HexNAc(2)Hex(1)
Pep_2+
Pep_3+
Pep+HexNAc_2+
M+e
~y20+203
z.10
z.13
z.9
z.11
z.16
z.20
c12
c14
c15
c16
c19

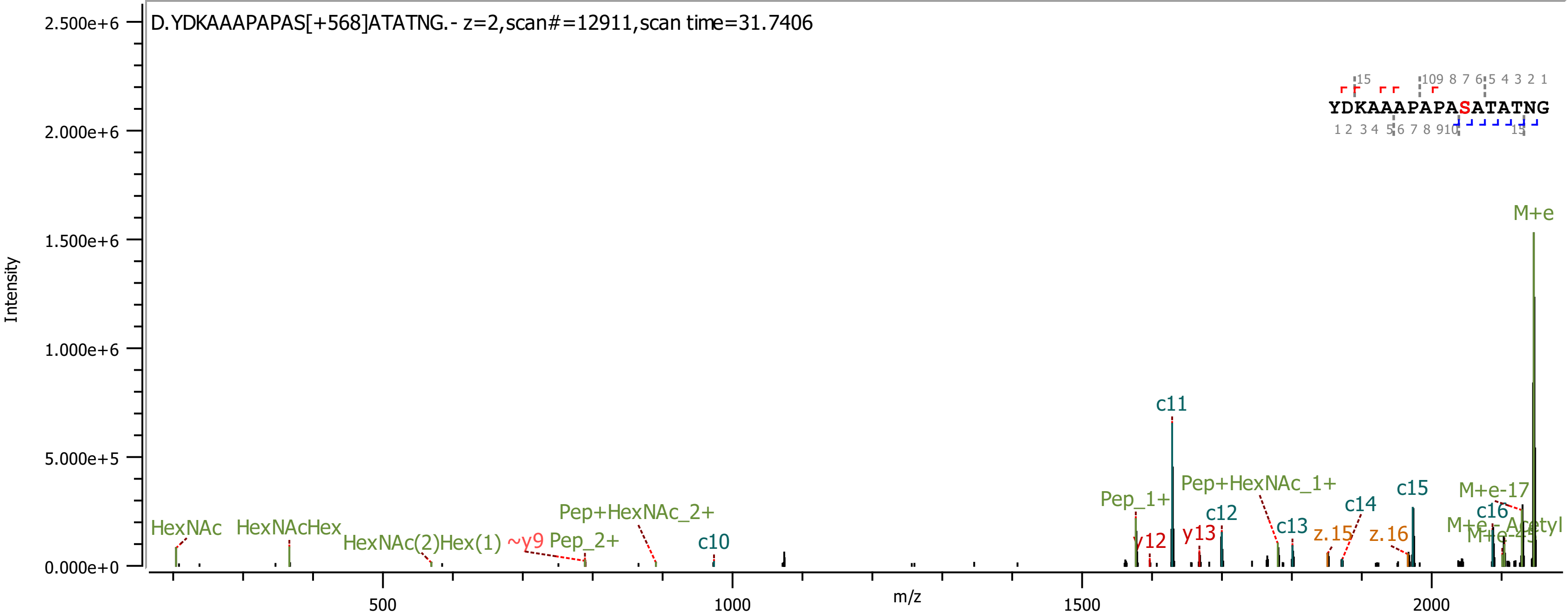
D.YDKAAAPAPAS[+568]ATATNG.- z=2,scan#=12911,scan time=31.7406
Intensity
m/z
YDKAAAPAPASATATNG
M+e
c11
Pep_1+
Pep+HexNAc_1+
c12
c13
c14
c15
c16
M+e-17
y12
y13
z.15
z.16
c10
Pep+HexNAc_2+
Pep_2+
~y9
HexNAc(2)Hex(1)
HexNAcHex
HexNAc

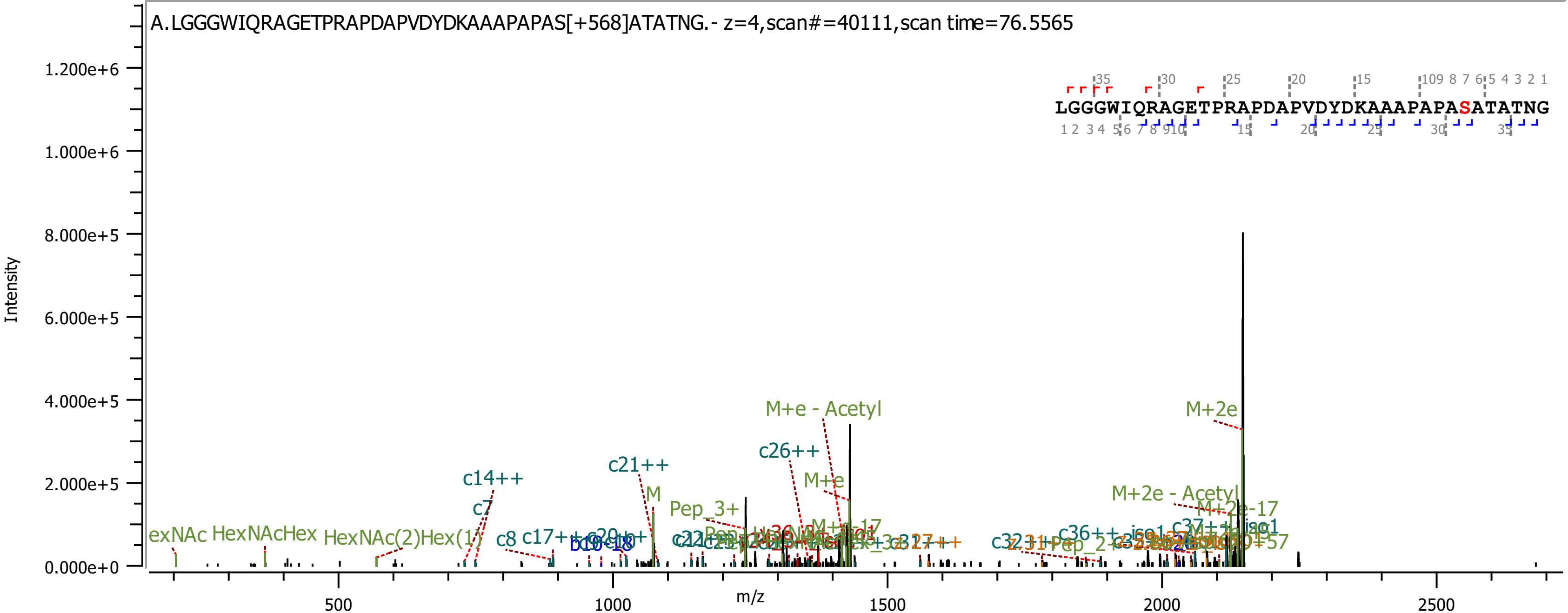
A.LGGGWIQRAGETPRAPDAPVDYDKAAAPAPAS[+568]ATATNG.- z=4,scan#=40111,scan time=76.5565
LGGGWIQRAGETPRAPDAPVDYDKAAAPAPASATATNG
Intensity
m/z
M+e - Acetyl
M+2e
c26++
c21++
c14++
M+e
M
Pep_3+
M+2e - Acetyl
c7
c8
HexNAcHex
HexNAc(2)Hex(1)
0.000e+0
2.000e+5
4.000e+5
6.000e+5
8.000e+5
1.000e+6
1.200e+6
500
1000
1500
2000
2500

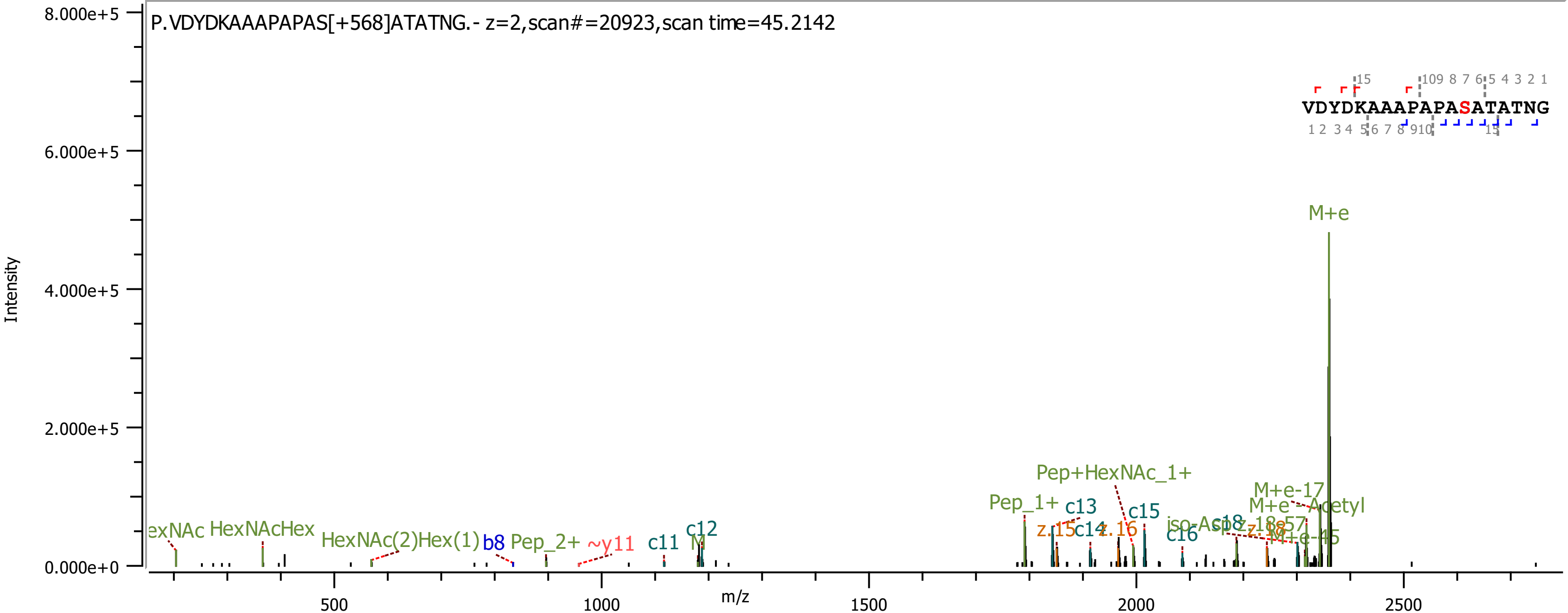
P.VDYDKAAAPAPAS[+568]ATATNG.- z=2,scan#=20923,scan time=45.2142
VDYDKAAAPAPASATATNG
Intensity
m/z
M+e
Pep+HexNAc_1+
M+e-17
M+e-Acetyl
Pep_1+
c13
c15
z.15
c14
z.16
iso-Asp
c18
z.18
c16
M+e-45
HexNAcHex
HexNAc(2)Hex(1)
b8
Pep_2+
~y11
c11
c12
8.000e+5
6.000e+5
4.000e+5
2.000e+5
0.000e+0
500
1000
1500
2000
2500

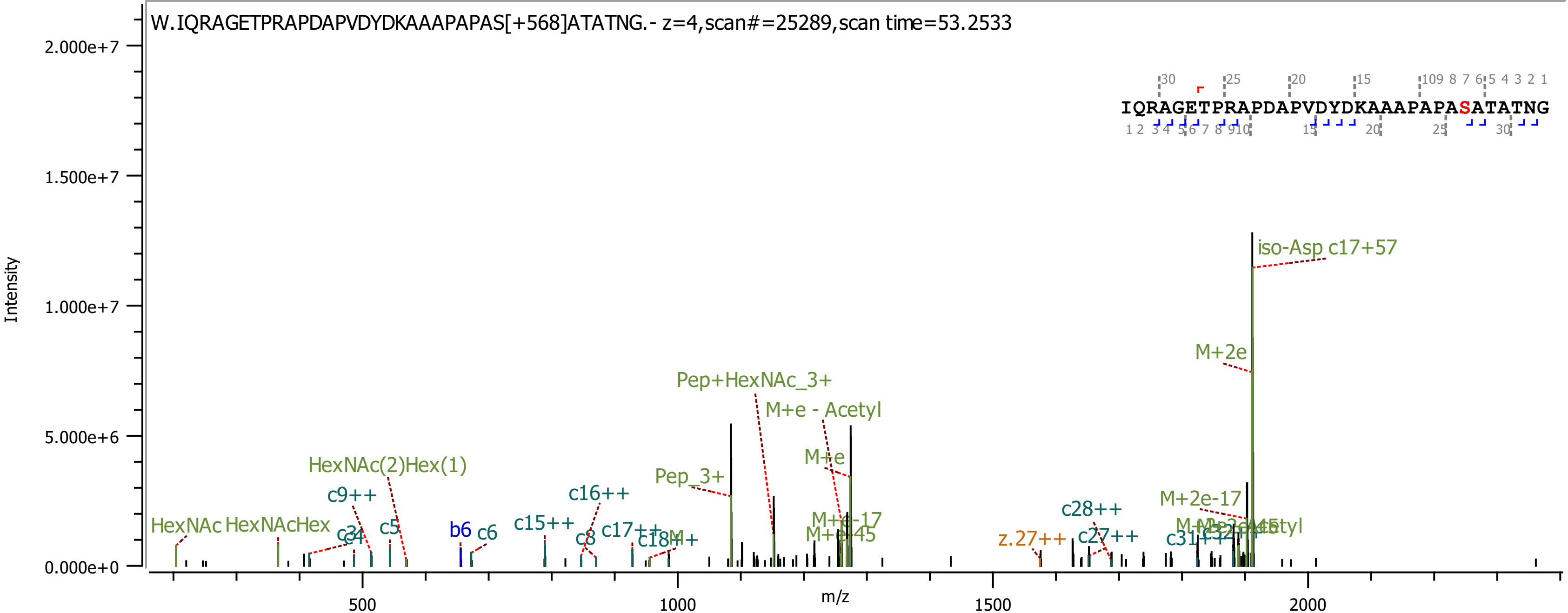

W.IQRAGETPRAPDAPVDYDKAAAPAPAS[+568]ATATNG.- z=4,scan#=25289,scan time=53.2533
IQRAGETPRAPDAPVDYDKAAAPAPASATATNG
iso-Asp c17+57
M+2e
Pep+HexNAc_3+
M+e - Acetyl
M+e
Pep_3+
HexNAc(2)Hex(1)
c9++
c16++
c15++
c17++
c18
M+
HexNAc
HexNAcHex
c3
c4
c5
b6
c6
c8
M+e-17
M+e-45
c28++
M+2e-17
z.27++
c27++
c31
Intensity
m/z
2.000e+7
1.500e+7
1.000e+7
5.000e+6
0.000e+0
500
1000
1500
2000

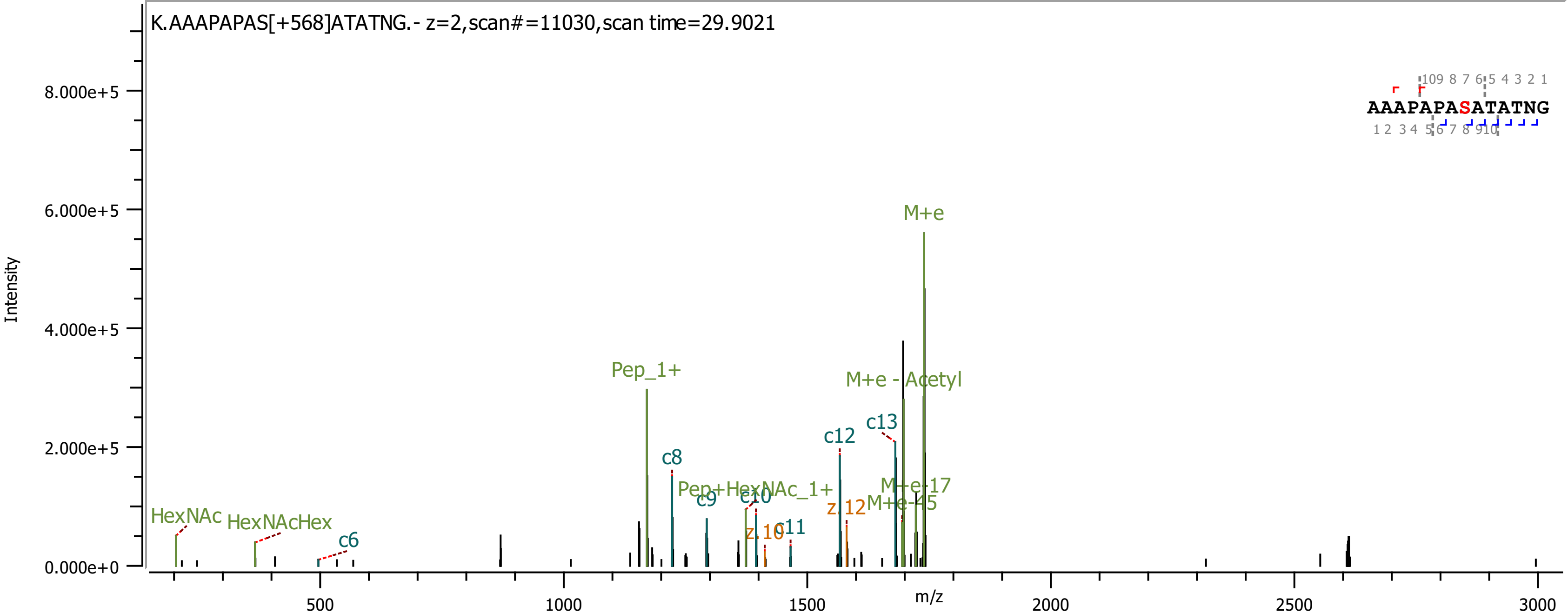
K.AAAPAPAS[+568]ATATNG.- z=2,scan#=11030,scan time=29.9021
Intensity
m/z
8.000e+5
6.000e+5
4.000e+5
2.000e+5
0.000e+0
500
1000
1500
2000
2500
3000
AAAPAPASATATNG
M+e
Pep_1+
M+e - Acetyl
c13
c12
c8
Pep+HexNAc_1+
M+e-17
M+e-45
c9
c10
z 12
z 10
c11
HexNAc
HexNAcHex
c6

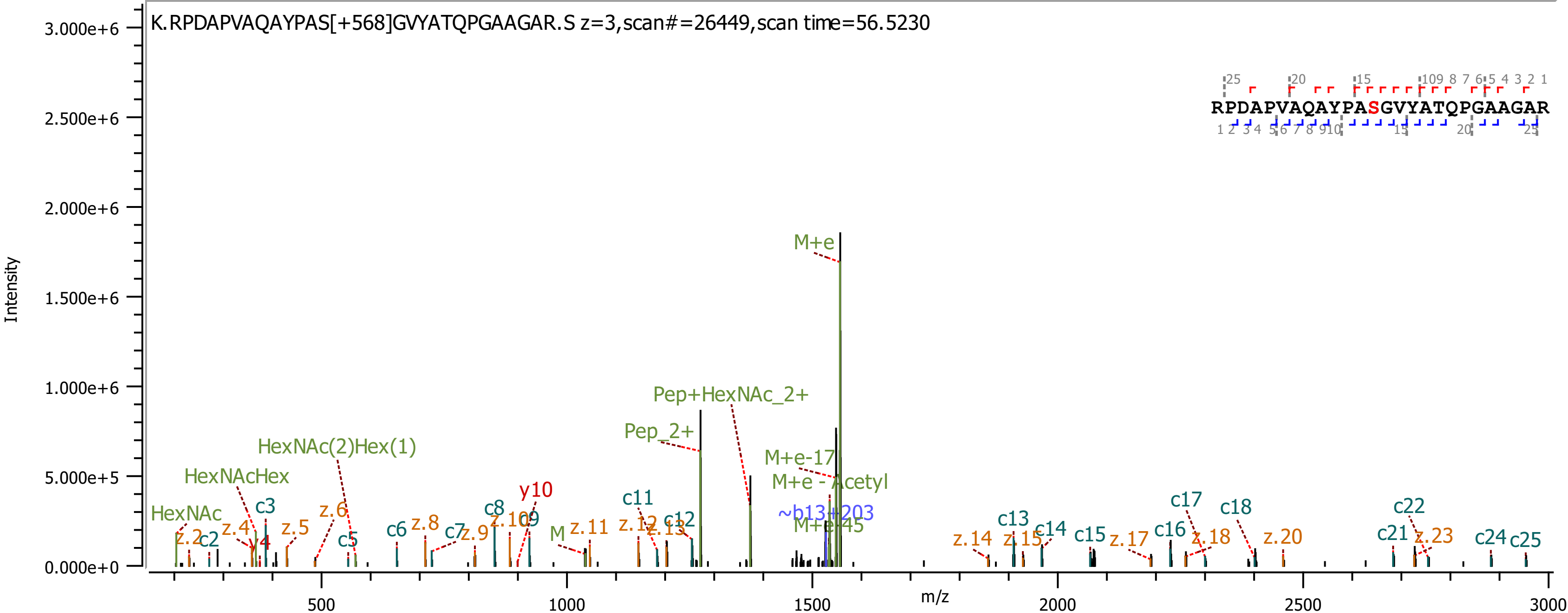

K.RPDAPVAQAYPAS[+568]GVYATQPGAAGAR.S z=3,scan#=26449,scan time=56.5230
RPDAPVAQAYPASGVYATQPGAAGAR
Intensity
m/z
M+e
Pep+HexNAc_2+
Pep_2+
HexNAc(2)Hex(1)
HexNAcHex
HexNAc
M+e-17
M+e - Acetyl
~b13+203
M+e-45
y10
y4
M
c2
c3
c5
c6
c7
c8
c9
c11
c12
c13
c14
c15
c16
c17
c18
c21
c22
c24
c25
z.2
z.4
z.5
z.6
z.8
z.9
z.10
z.11
z.12
z.13
z.14
z.15
z.17
z.18
z.20
z.23

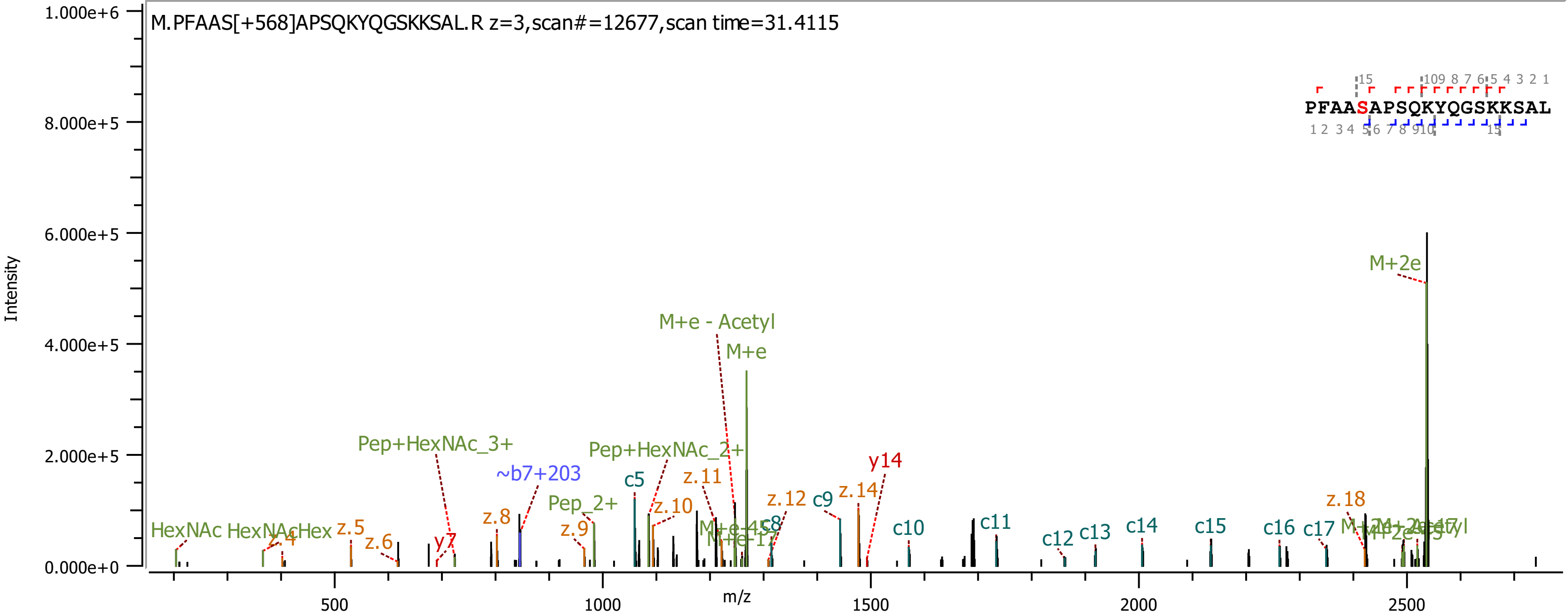

M.PFAAS[+568]APSQKYQGSKKSAL.R z=3,scan#=12677,scan time=31.4115
PFAASAPSQKYQGSKKSAL
Intensity
m/z
M+2e
M+e - Acetyl
M+e
Pep+HexNAc_3+
Pep+HexNAc_2+
~b7+203
y14
y7
c5
c9
c10
c11
c12
c13
c14
c15
c16
c17
z.5
z.6
z.8
z.9
z.10
z.11
z.12
z.14
z.18
Pep_2+
HexNAc
HexNAcHex

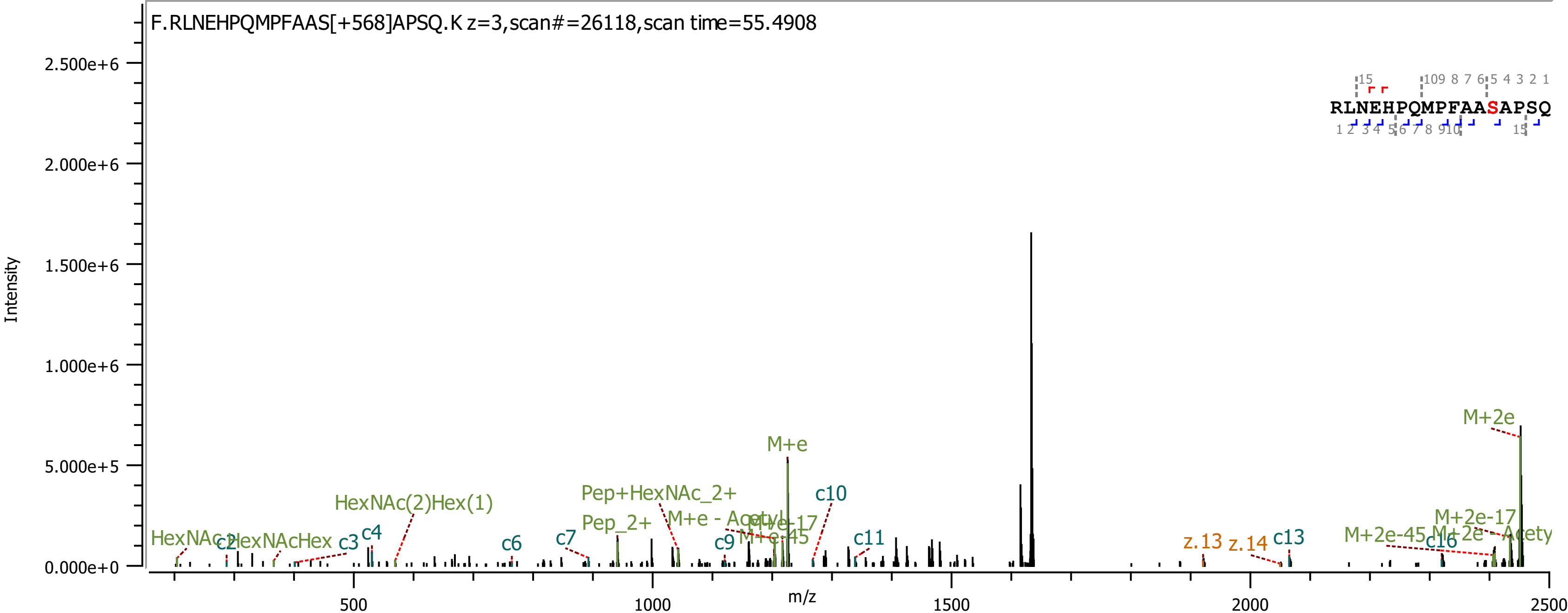
F.RLNEHPQMPFAAS[+568]APSQ.K z=3,scan#=26118,scan time=55.4908
RLNEHPQMPFAASAPSQ
Intensity
m/z
M+2e
M+e
HexNAc(2)Hex(1)
Pep+HexNAc_2+
Pep_2+
M+e - Acetyl
c10
HexNAc
c2
HexNAcHex
c3
c4
c6
c7
c9
c11
z.13
z.14
c13
M+2e-17
M+2e-45
c16
0.000e+0
5.000e+5
1.000e+6
1.500e+6
2.000e+6
2.500e+6
500
1000
1500
2000
2500

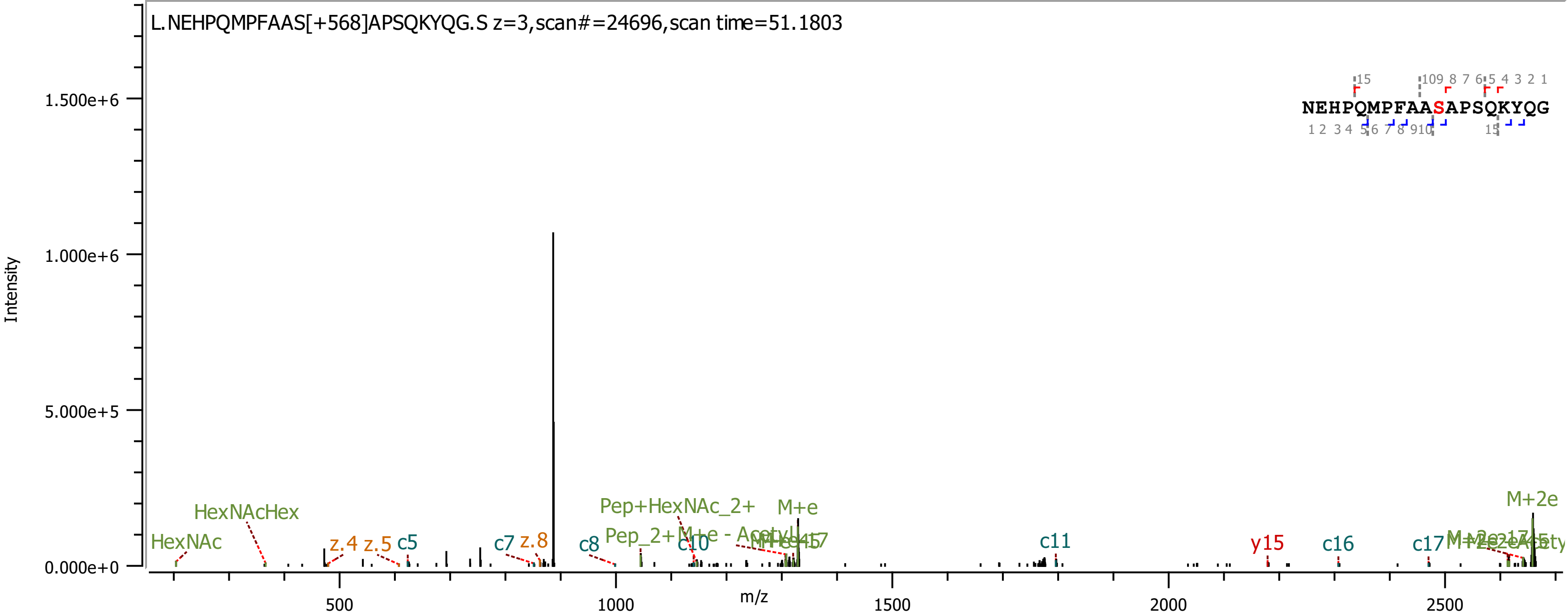

L.NEHPQMPFAAS[+568]APSQKYQG.S z=3,scan#=24696,scan time=51.1803
NEHPQMPFAASAPSQKYQG
Intensity
m/z
0.000e+0
5.000e+5
1.000e+6
1.500e+6
500
1000
1500
2000
2500
HexNAc
HexNAcHex
z.4
z.5
c5
c7
z.8
c8
Pep+HexNAc_2+
Pep_2+
M+e
c10
c11
y15
c16
c17
M+2e

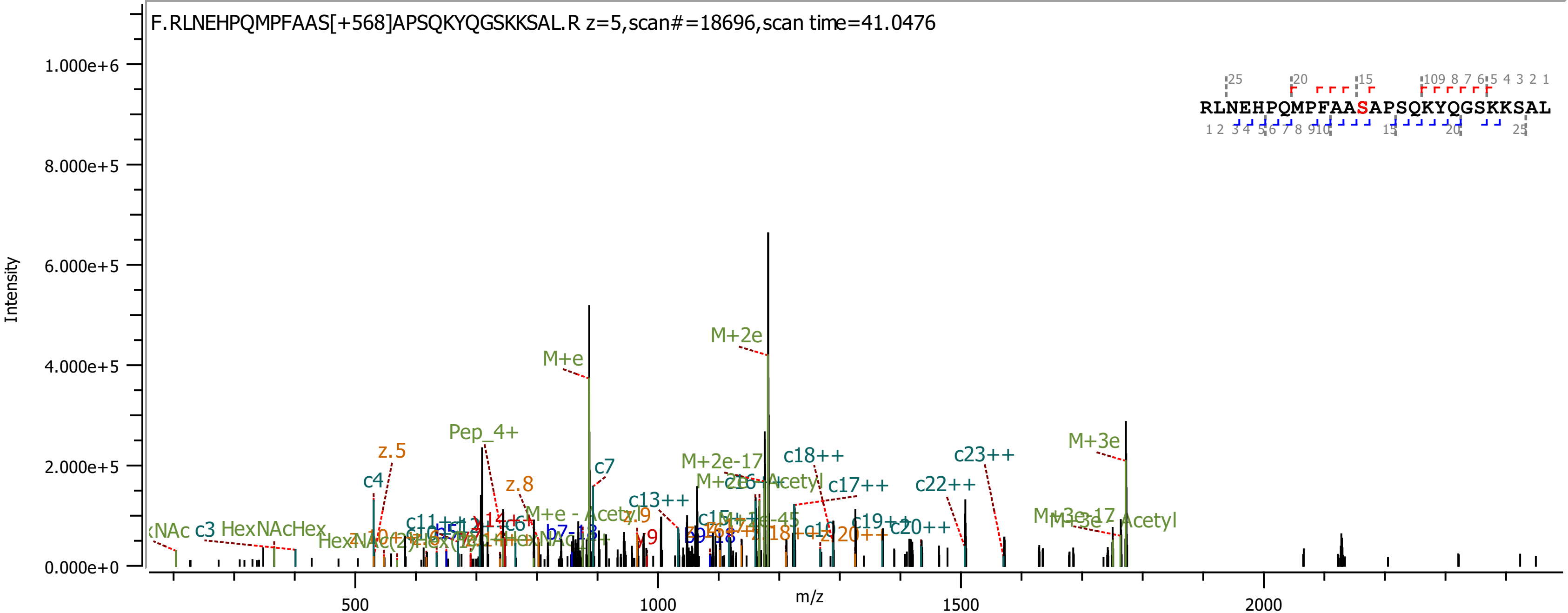
F.RLNEHPQMPFAAS[+568]APSQKYQGSKKSAL.R z=5,scan#=18696,scan time=41.0476
RLNEHPQMPFAASAPSQKYQGSKKSAL
Intensity
m/z
M+e
M+2e
M+3e
Pep_4+
c4
c7
z.5
z.8
c13++
c17++
c18++
c22++
c23++
c20++
M+2e-17
c3
HexNAcHex
M+e - Acetyl
z.9

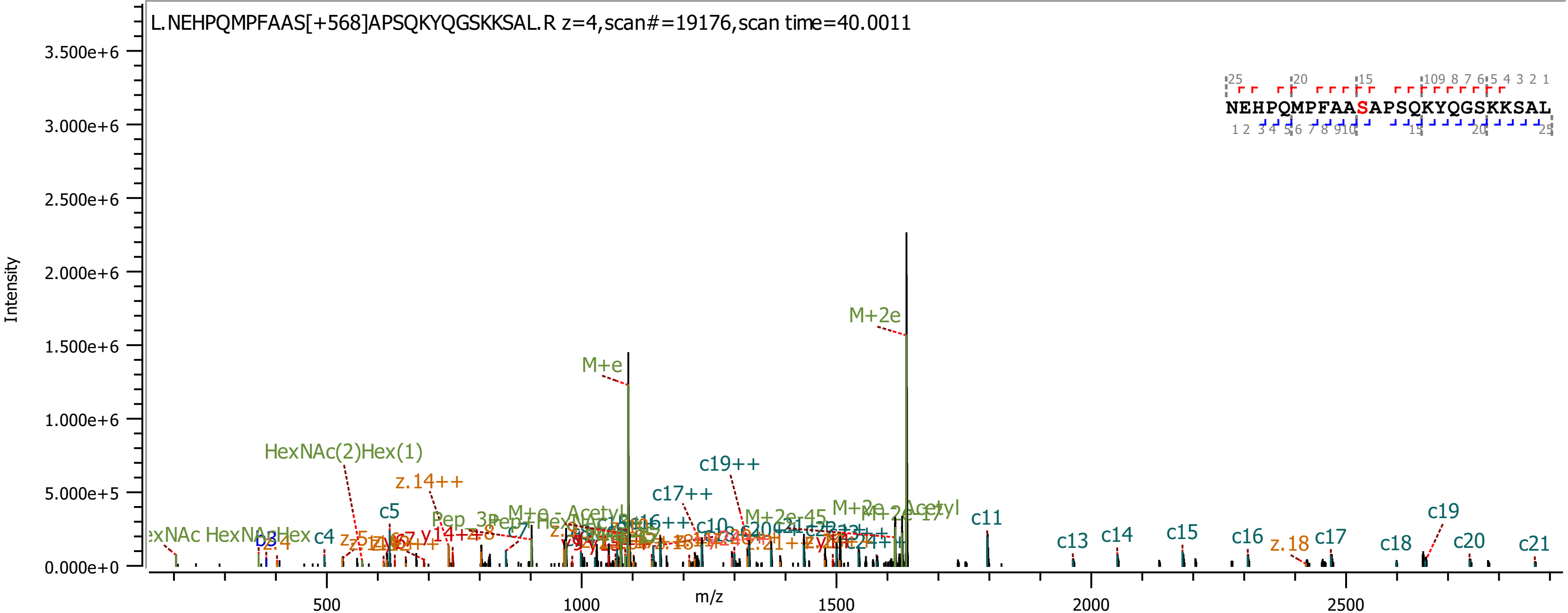

L.NEHPQMPFAAS[+568]APSQKYQGSKKSAL.R z=4,scan#=19176,scan time=40.0011
Intensity
m/z
NEHPQMPFAASAPSQKYQGSKKSAL
M+2e
M+e
HexNAc(2)Hex(1)
z.14++
c19++
c17++
c5
c4
c11
c13
c14
c15
c16
z.18
c17
c18
c19
c20
c21

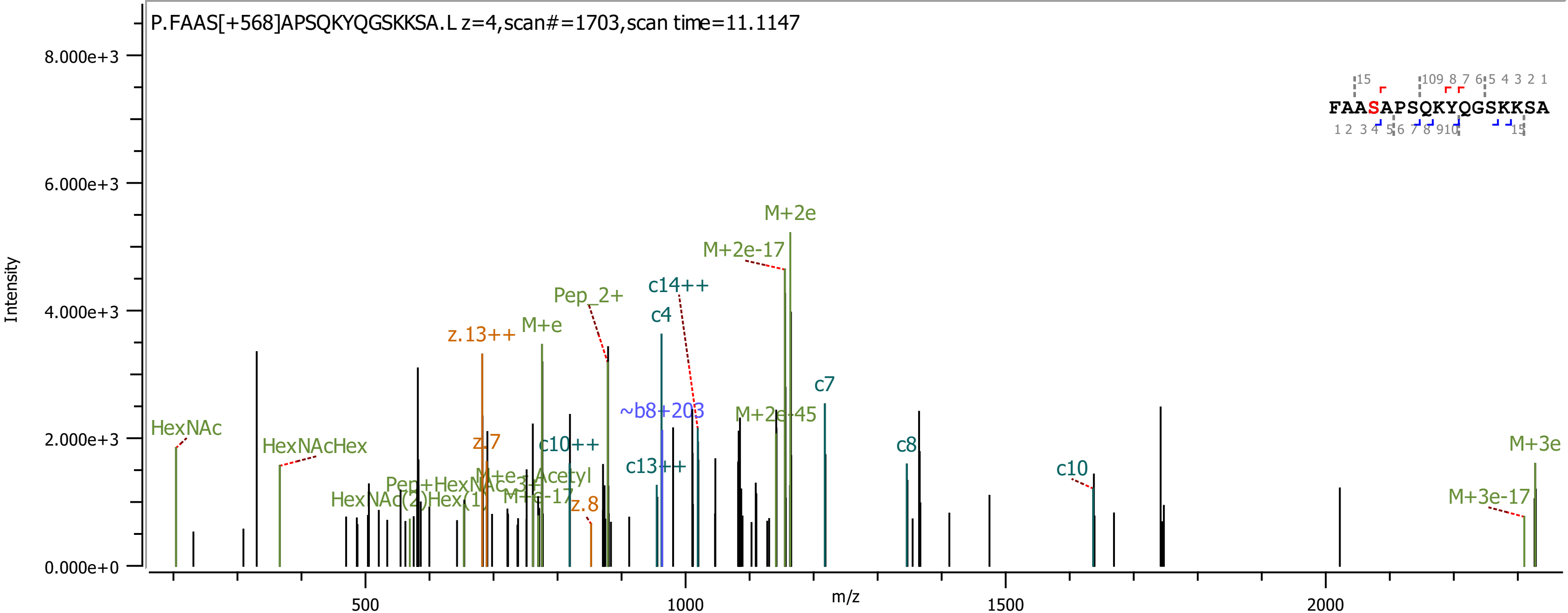
P.FAAS[+568]APSQKYQGSKKSA.L z=4,scan#=1703,scan time=11.1147
Intensity
m/z
FAASAPSQKYQGSKKSA
M+2e
M+2e-17
c14++
Pep_2+
c4
M+e
z.13++
c7
~b8+203
M+2e-45
HexNAc
HexNAcHex
z.7
c10++
c8
M+3e
c13++
c10
M+e-Acetyl
Pep+HexNAc3+
M+e-17
z.8
HexNAc(2)Hex(1)
M+3e-17
8.000e+3
6.000e+3
4.000e+3
2.000e+3
0.000e+0
500
1000
1500
2000

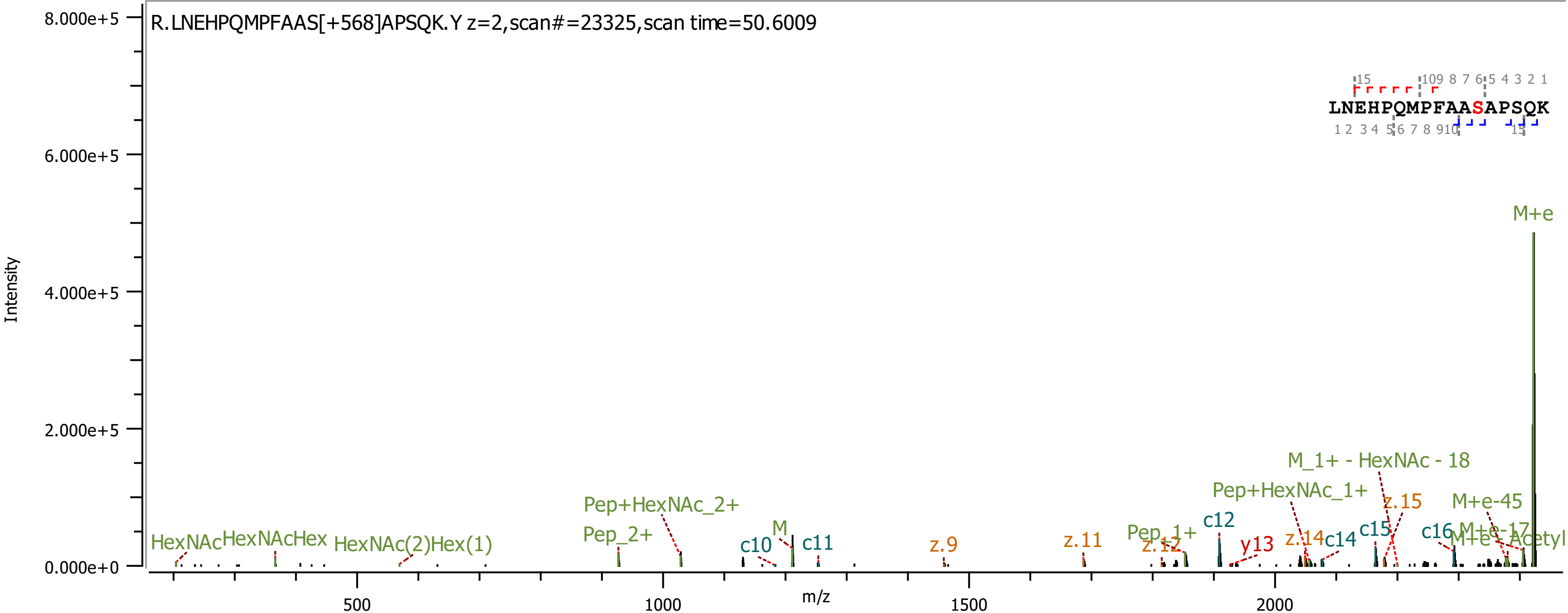
R.LNEHPQMPFAAS[+568]APSQK.Y z=2,scan#=23325,scan time=50.6009
LNEHPQMPFAASAPSQK
Intensity
m/z
8.000e+5
6.000e+5
4.000e+5
2.000e+5
0.000e+0
500
1000
1500
2000
HexNAc
HexNAcHex
HexNAc(2)Hex(1)
Pep+HexNAc_2+
Pep_2+
M
c10
c11
z.9
z.11
Pep_1+
z.12
c12
y13
Pep+HexNAc_1+
M_1+ - HexNAc - 18
z.14
c14
c15
z.15
c16
M+e-45
M+e-17
M+e-Acetyl
M+e

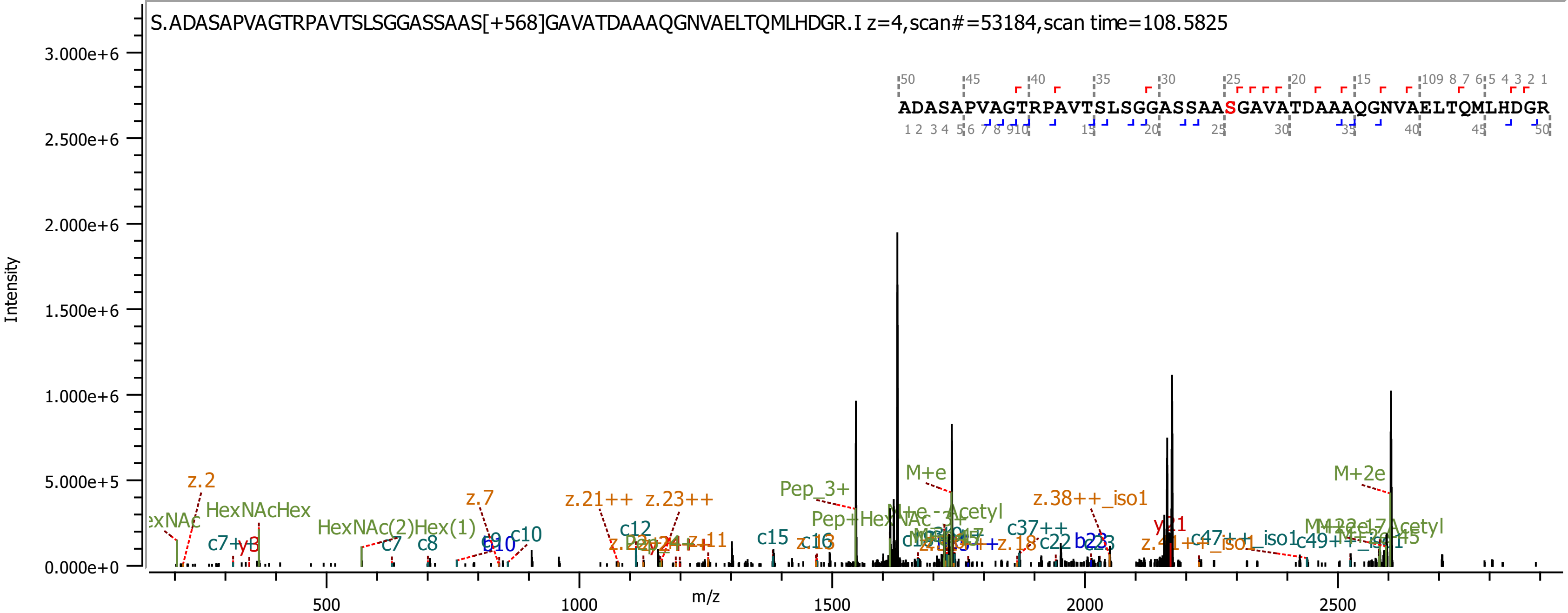
S.ADASAPVAGTRPAVTSLSGGASSAAS[+568]GAVATDAAAQGNVAELTQMLHDGR.I z=4,scan#=53184,scan time=108.5825
ADASAPVAGTRPAVTSLSGGASSAASGAVATDAAAQGNVAELTQMLHDGR
Intensity
3.000e+6
2.500e+6
2.000e+6
1.500e+6
1.000e+6
5.000e+5
0.000e+0
m/z
500
1000
1500
2000
2500
z.2
HexNAcHex
HexNAc(2)Hex(1)
c7+
y3
c7
c8
c10
z.7
z.21++
z.23++
c12
z.11
c15
c16
Pep_3+
M+e
M+e - Acetyl
c37++
z.38++_iso1
y21
c22
b23
c47++_iso1
c49++_iso1
M+2e

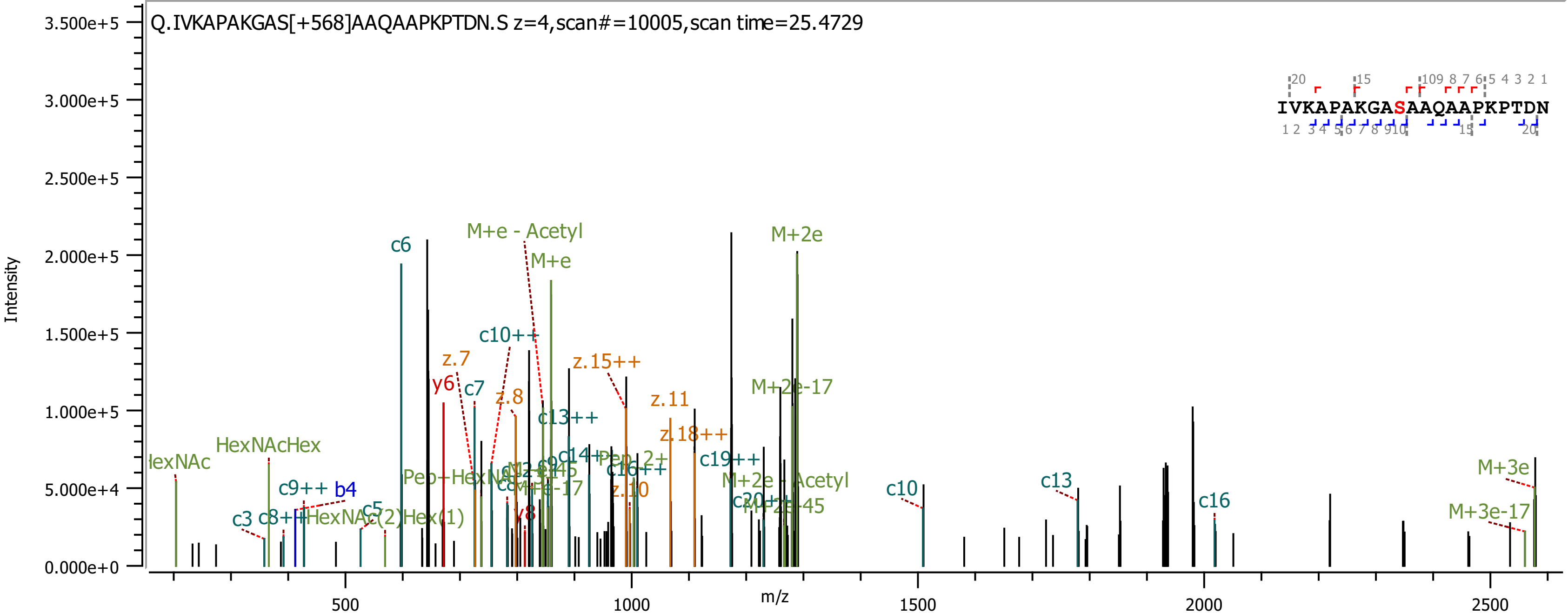
Q.IVKAPAKGAS[+568]AAQAAPKPTDN.S z=4,scan#=10005,scan time=25.4729
IVKAPAKGASAAQAAPKPTDN
Intensity
m/z
c6
M+e - Acetyl
M+e
M+2e
c10++
z.7
y6
c7
z.8
z.15++
z.11
M+2e-17
c13++
z.18++
HexNAcHex
c14++
c19++
c16++
c9++
b4
M+2e - Acetyl
z.10
c10
c13
c16
M+3e
M+3e-17
c3
c5
y8
HexNAc(2)Hex(1)
M+2e-45
M+e-17

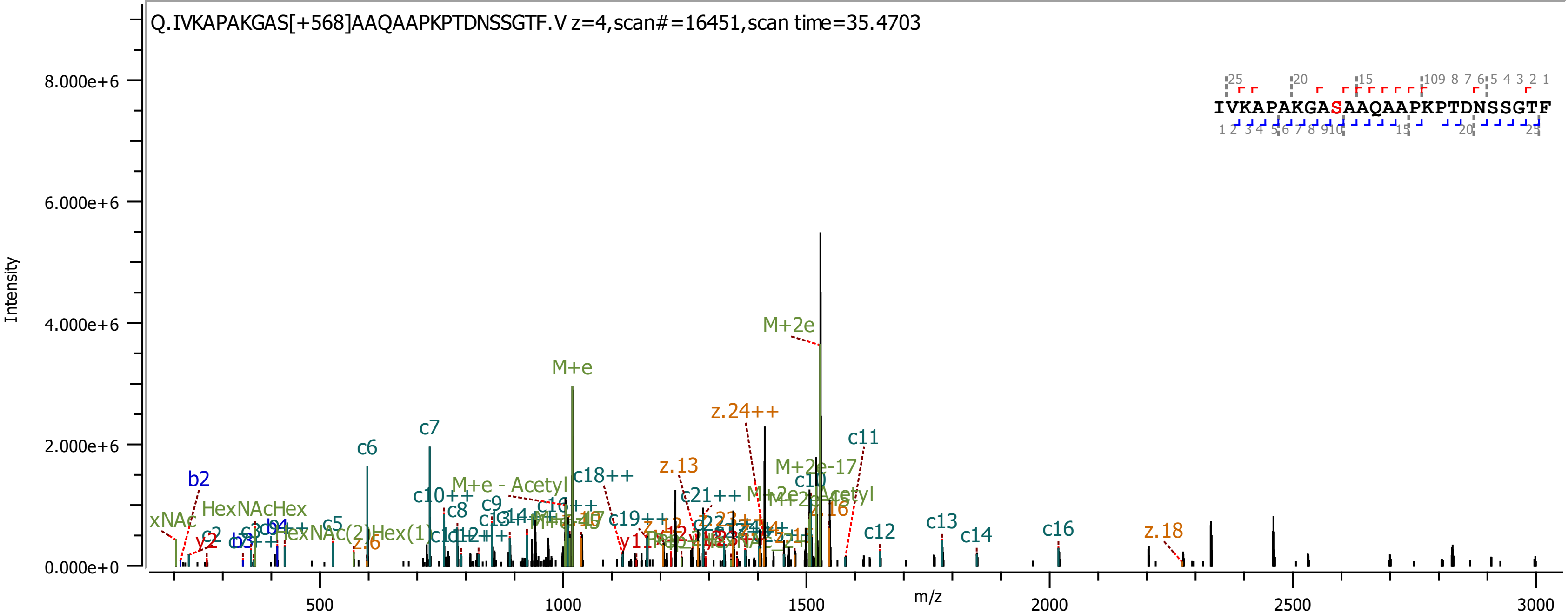
Q.IVKAPAKGAS[+568]AAQAAPKPTDNSSGTF.V z=4,scan#=16451,scan time=35.4703
IVKAPAKGASAAQAAPKPTDNSSGTF
Intensity
m/z
M+2e
M+e
z.24++
c11
c7
c6
b2
c10++
c8
c9
c18++
z.13
c21++
M+2e-17
c12
c13
c14
c16
z.18
HexNAcHex
xNAc
c5
z.6
M+e - Acetyl

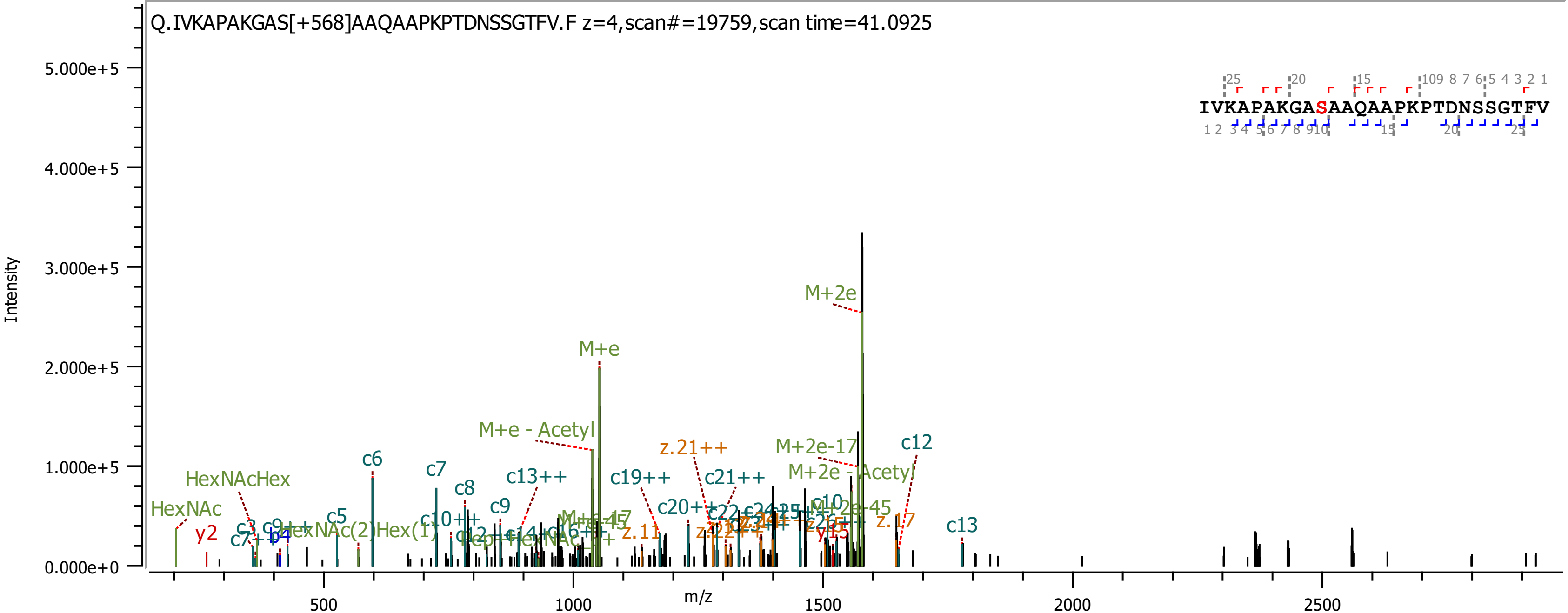
Q.IVKAPAKGAS[+568]AAQAAPKPTDNSSGTFV.F z=4,scan#=19759,scan time=41.0925
IVKAPAKGASAAQAAPKPTDNSSGTFV
Intensity
m/z
M+2e
M+e
M+e - Acetyl
z.21++
M+2e-17
c12
M+2e - Acetyl
HexNAcHex
HexNAc
c6
c7
c8
c9
c13++
c19++
c21++
c20++
c5
y2
c13
z.17
z.11
M+2e-45

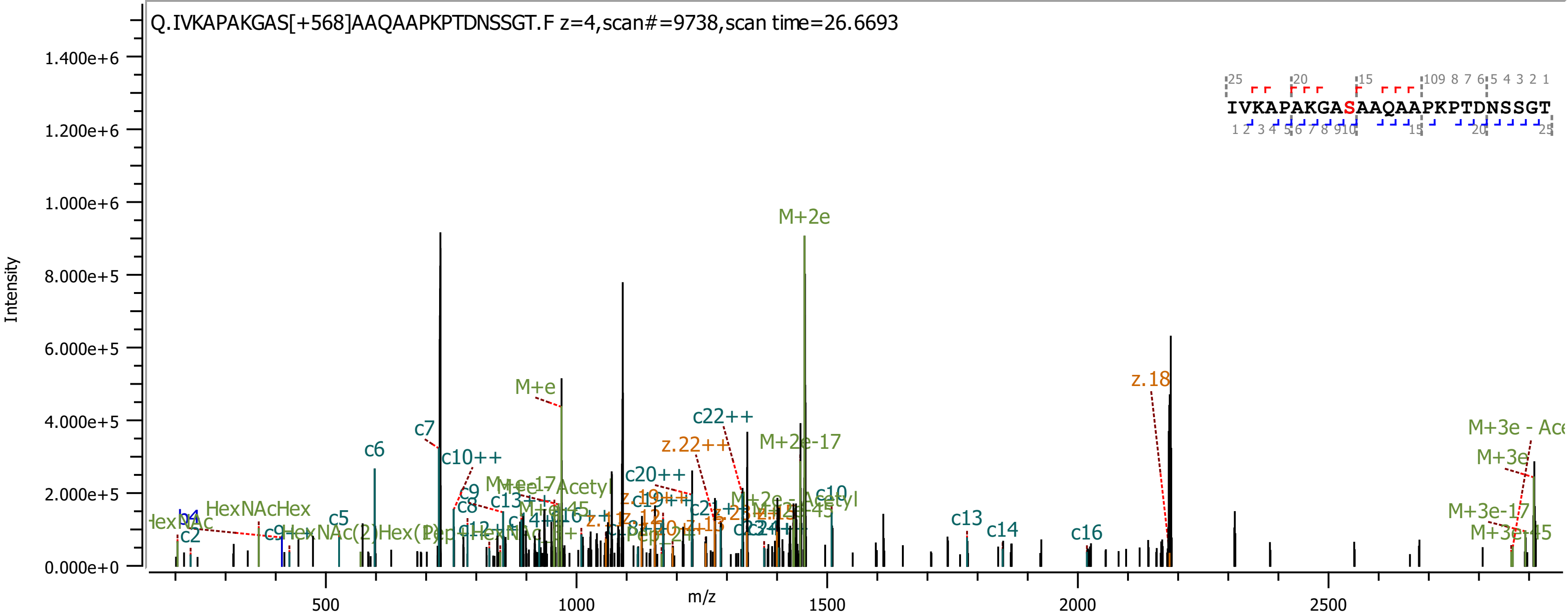
Q.IVKAPAKGAS[+568]AAQAAPKPTDNSSGT.F z=4,scan#=9738,scan time=26.6693
Intensity
m/z
IVKAPAKGASAAQAAPKPTDNSSGT
M+2e
M+e
z.18
c22++
z.22++
M+2e-17
c7
c6
c10++
c20++
c5
c2
HexNAcHex
c13
c14
c16
M+3e
M+3e-17
M+3e-45
0.000e+0
2.000e+5
4.000e+5
6.000e+5
8.000e+5
1.000e+6
1.200e+6
1.400e+6
500
1000
1500
2000
2500

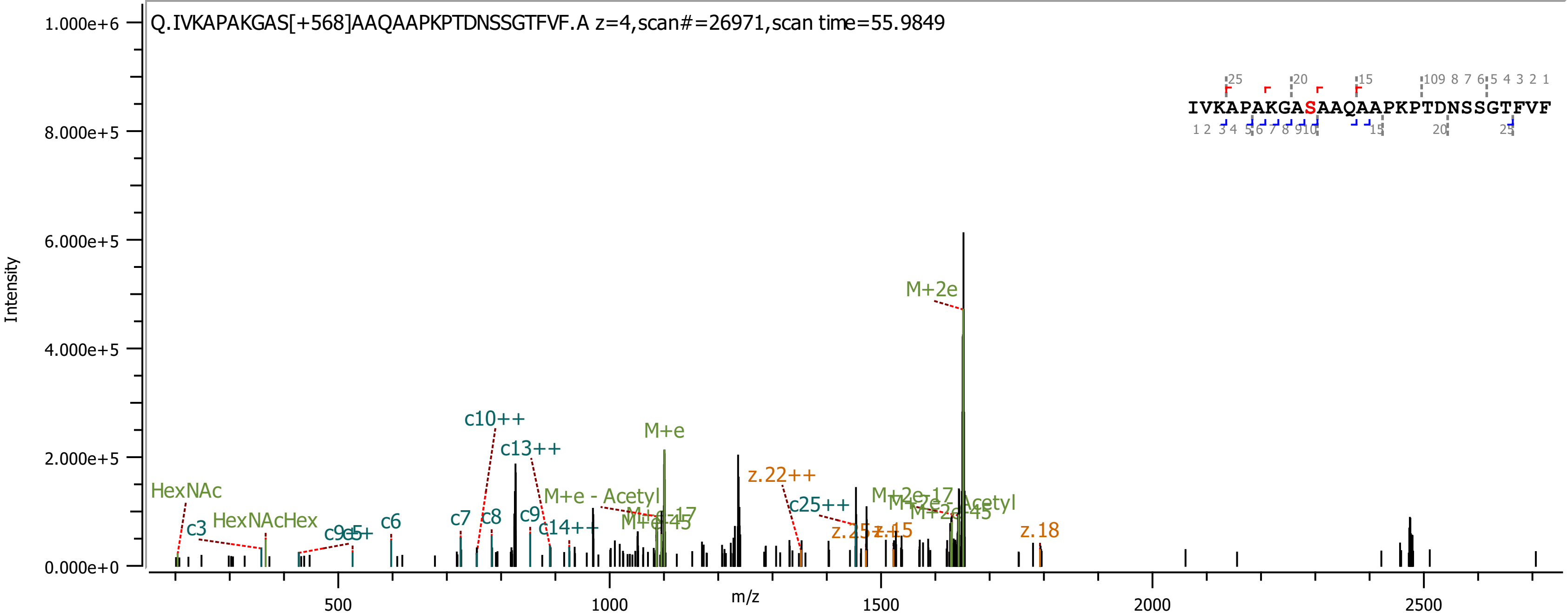
Q.IVKAPAKGAS[+568]AAQAAPKPTDNSSGTFVF.A z=4,scan#=26971,scan time=55.9849
IVKAPAKGASAAQAAPKPTDNSSGTFVF
Intensity
m/z
M+2e
M+e
c10++
c13++
z.22++
c25++
HexNAc
HexNAcHex
c3
c6
c7
c8
c9
c14++
M+e - Acetyl
z.18
z.15
1.000e+6
8.000e+5
6.000e+5
4.000e+5
2.000e+5
0.000e+0
500
1000
1500
2000
2500

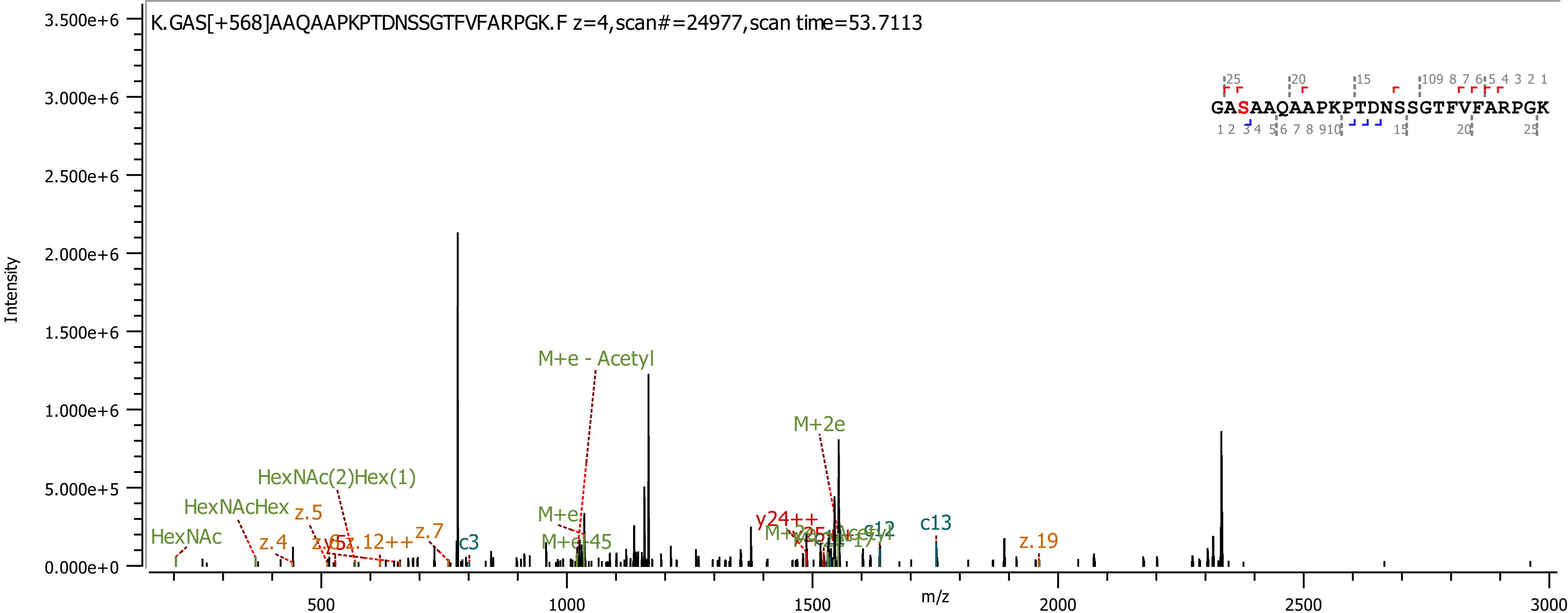

K.GAS[+568]AAQAAPKPTDNSSGTFVFARPGK.F z=4,scan#=24977,scan time=53.7113
GASAAQAAPKPTDNSSGTFVFARPGK
Intensity
m/z
M+e - Acetyl
M+2e
HexNAc(2)Hex(1)
HexNAcHex
HexNAc
z.5
z.4
z.7
z.12++
c3
M+e
M+e-45
y24++
c12
c13
z.19

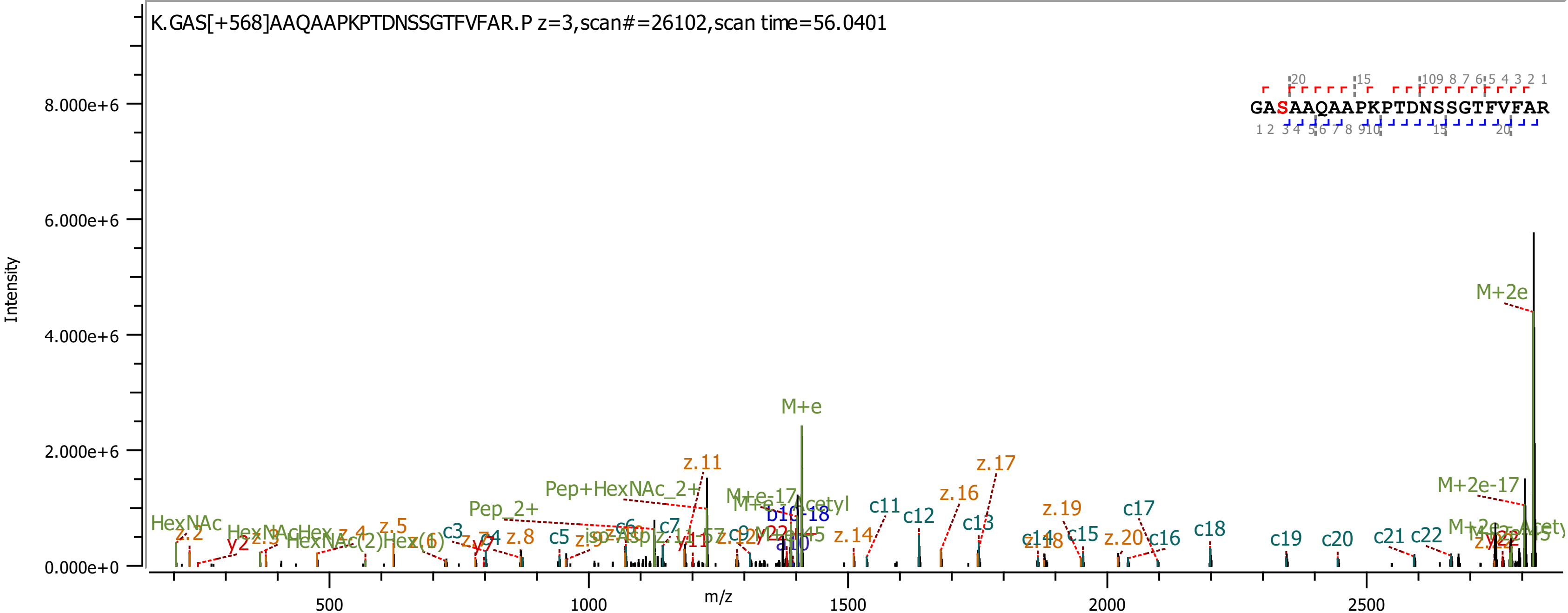
K.GAS[+568]AAQAAPKPTDNSSGTFVFAR.P z=3,scan#=26102,scan time=56.0401
GASAAQAAPKPTDNSSGTFVFAR
Intensity
m/z
8.000e+6
6.000e+6
4.000e+6
2.000e+6
0.000e+0
500
1000
1500
2000
2500
M+2e
M+e
M+2e-17
z.11
z.17
z.16
z.19
Pep+HexNAc_2+
Pep_2+
HexNAc
c11
c12
c13
c14
c15
c16
c17
c18
c19
c20
c21
c22
c3
c5
c7
z.2
z.5
z.8
z.14
z.20
y2
a10

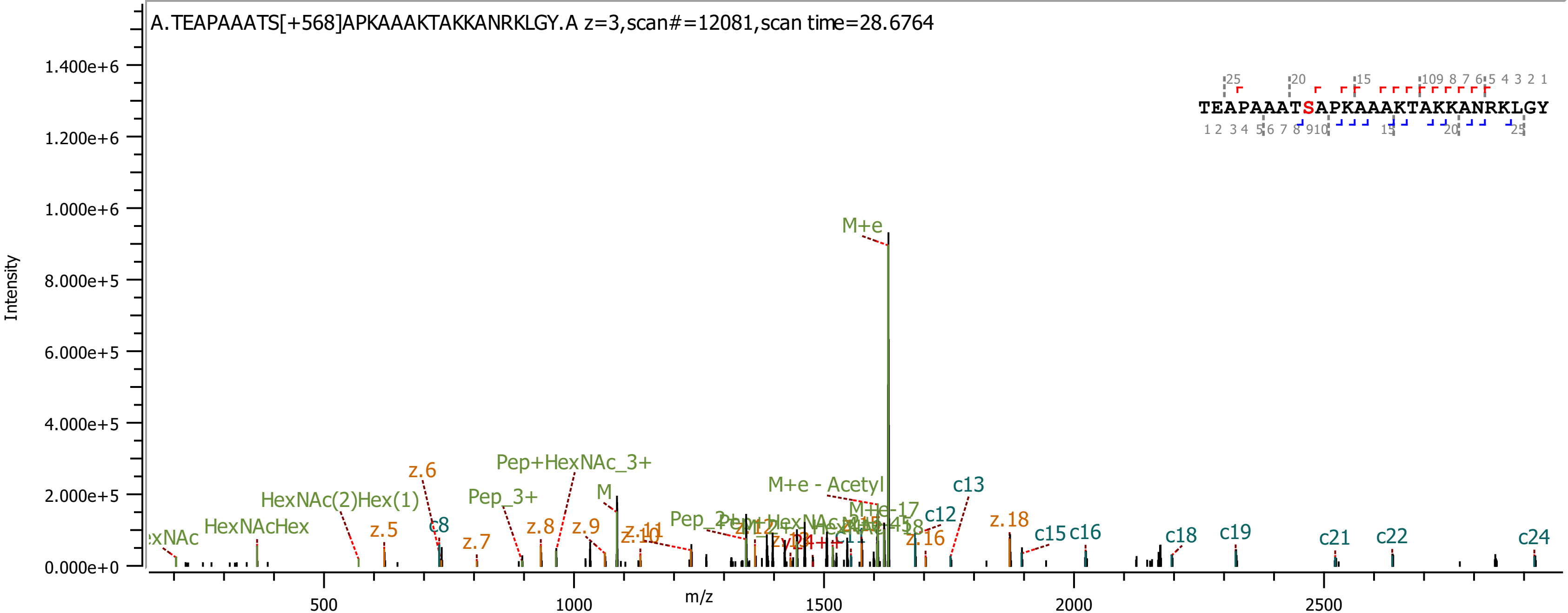
A.TEAPAAATS[+568]APKAAAKTAKKANRKLGY.A z=3,scan#=12081,scan time=28.6764
TEAPAAATSAPKAAAKTAKKANRKLGY
Intensity
m/z
M+e
M+e - Acetyl
M
Pep_3+
Pep+HexNAc_3+
HexNAc(2)Hex(1)
HexNAcHex
z.5
z.6
z.7
z.8
z.9
z.18
z.16
c8
c12
c13
c15
c16
c18
c19
c21
c22
c24
0.000e+0
2.000e+5
4.000e+5
6.000e+5
8.000e+5
1.000e+6
1.200e+6
1.400e+6
500
1000
1500
2000
2500

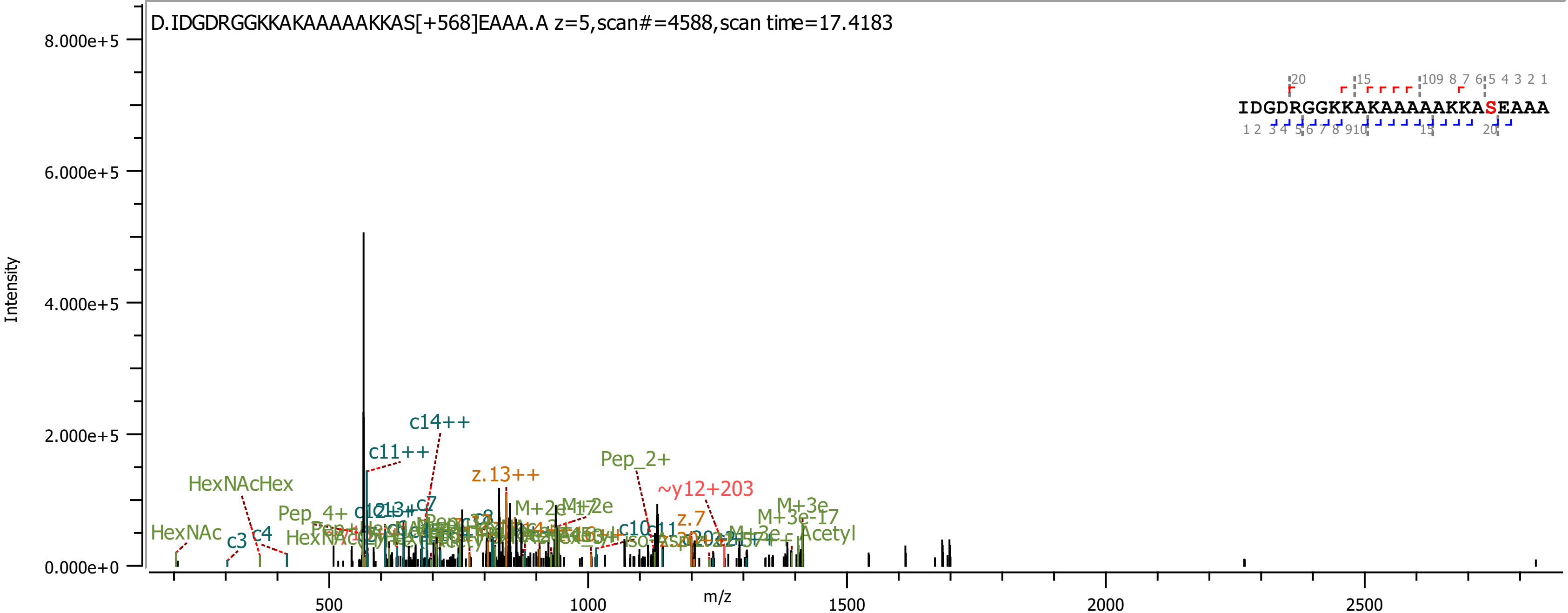
D.IDGDRGGKKAKAAAAAKKAS[+568]EAAA.A z=5,scan#=4588,scan time=17.4183
IDGDRGGKKAKAAAAAKKASEAAA
Intensity
8.000e+5
6.000e+5
4.000e+5
2.000e+5
0.000e+0
m/z
500
1000
1500
2000
2500
c14++
c11++
z.13++
Pep_2+
~y12+203
HexNAcHex
HexNAc
c3
c4
Pep_4+
c7
M+2e
M+3e
M+3e-17
z.7
c10
c11
Acetyl

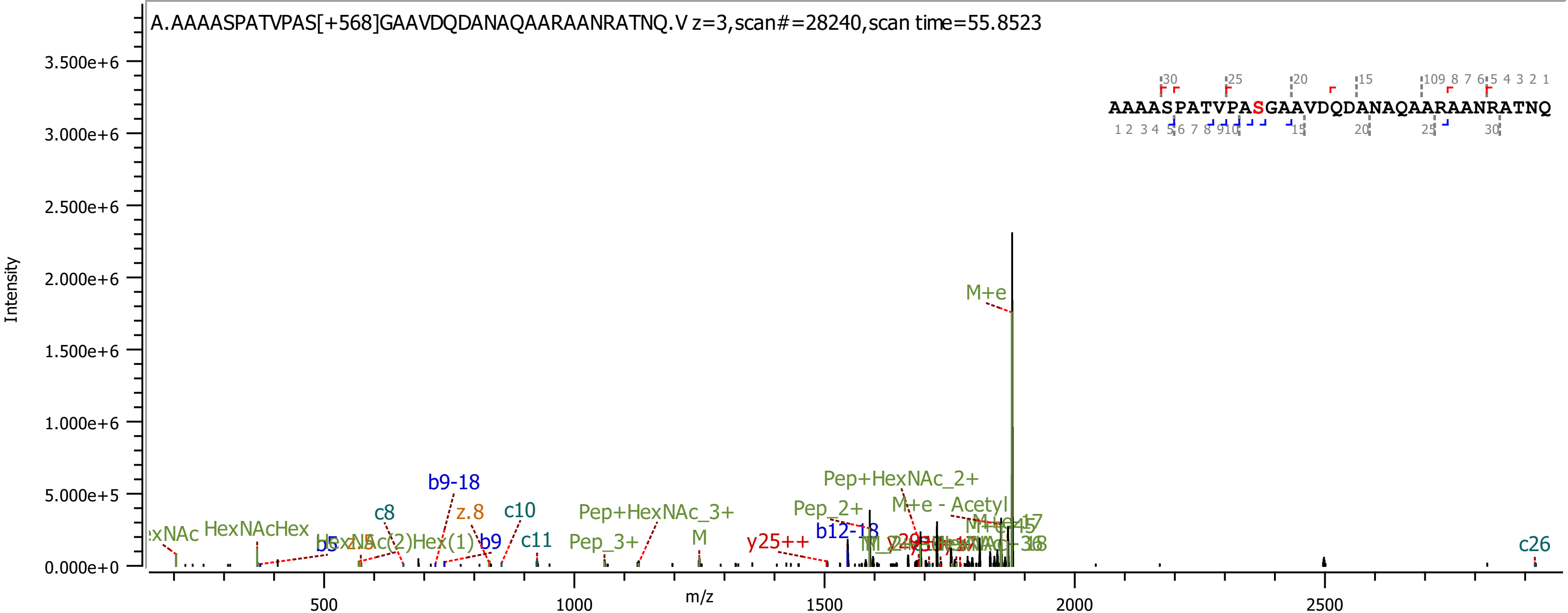
A.AAAASPATVPAS[+568]GAAVDQDANAQAARAANRATNQ.V z=3,scan#=28240,scan time=55.8523
AAAASPATVPASGAAVDQDANAQAARAANRATNQ
Intensity
m/z
M+e
Pep+HexNAc_2+
M+e - Acetyl
Pep_2+
b12-18
y25++
Pep+HexNAc_3+
Pep_3+
M
c10
c11
b9-18
z.8
b9
c8
HexNAcHex
b5
c26

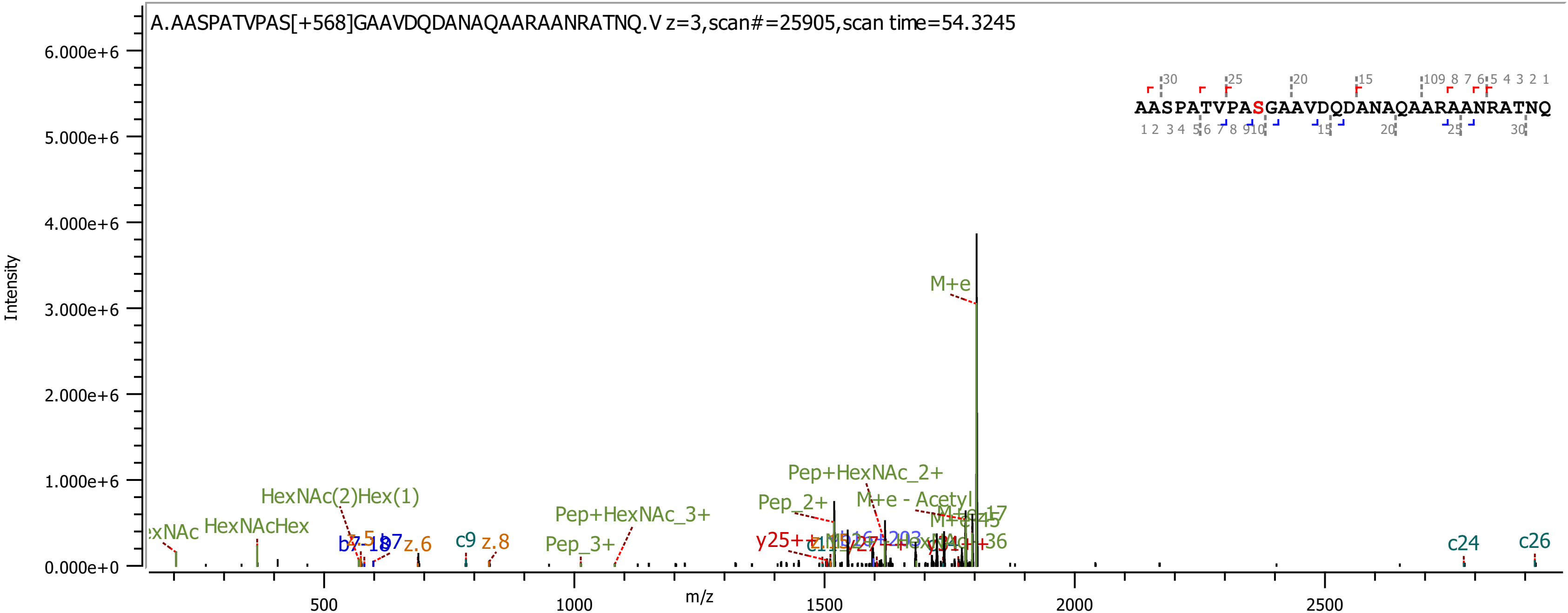
A.AASPATVPAS[+568]GAAVDQDANAQAARAANRATNQ.V z=3,scan#=25905,scan time=54.3245
AASPATVPASGAAVDQDANAQAARAANRATNQ
Intensity
m/z
M+e
Pep+HexNAc_2+
M+e - Acetyl
Pep_2+
HexNAc(2)Hex(1)
Pep+HexNAc_3+
HexNAcHex
Pep_3+
c9
z.8
z.6
c24
c26
y25++

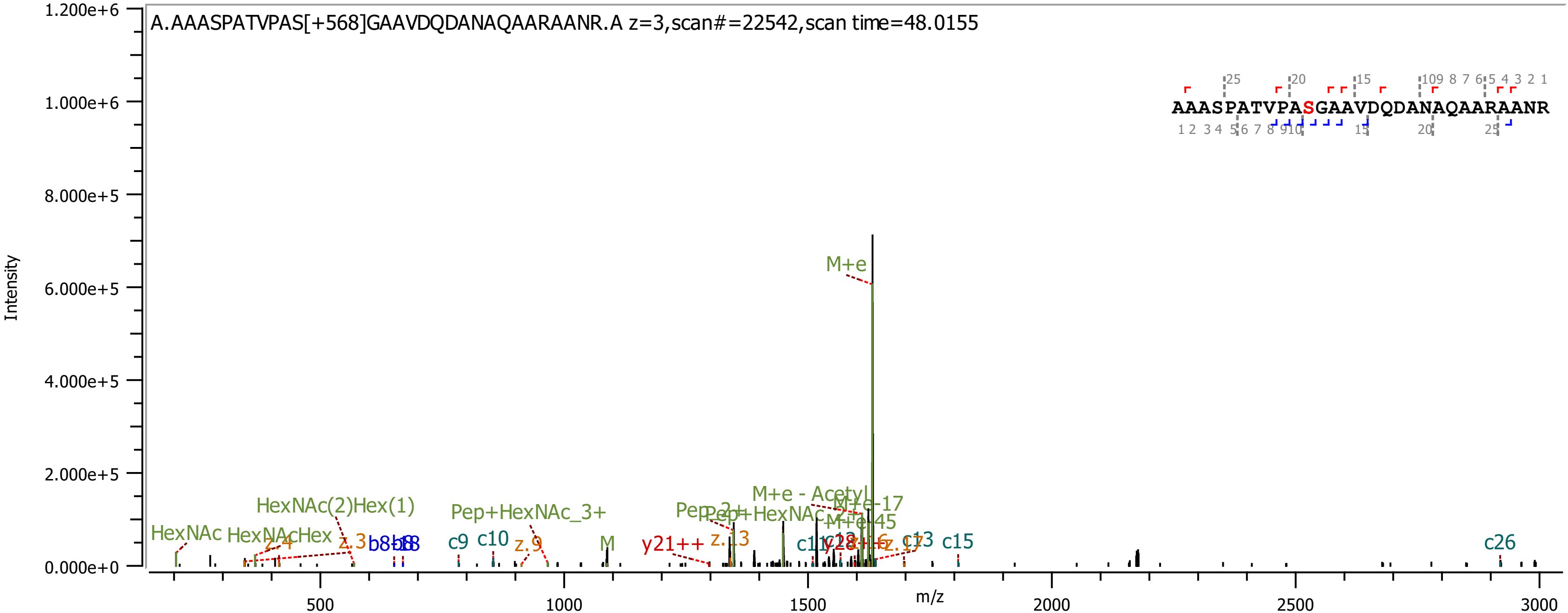
A.AAASPATVPAS[+568]GAAVDQDANAQAARAANR.A z=3,scan#=22542,scan time=48.0155
AAASPATVPASGAAVDQDANAQAARAANR
Intensity
m/z
1.200e+6
1.000e+6
8.000e+5
6.000e+5
4.000e+5
2.000e+5
0.000e+0
500
1000
1500
2000
2500
3000
M+e
HexNAc
HexNAcHex
HexNAc(2)Hex(1)
Pep+HexNAc_3+
M
y21++
M+e - Acetyl
M+e-17
M+e-45
c9
c10
c13
c15
c26
z.3
z.4
z.9
z.13
z.17

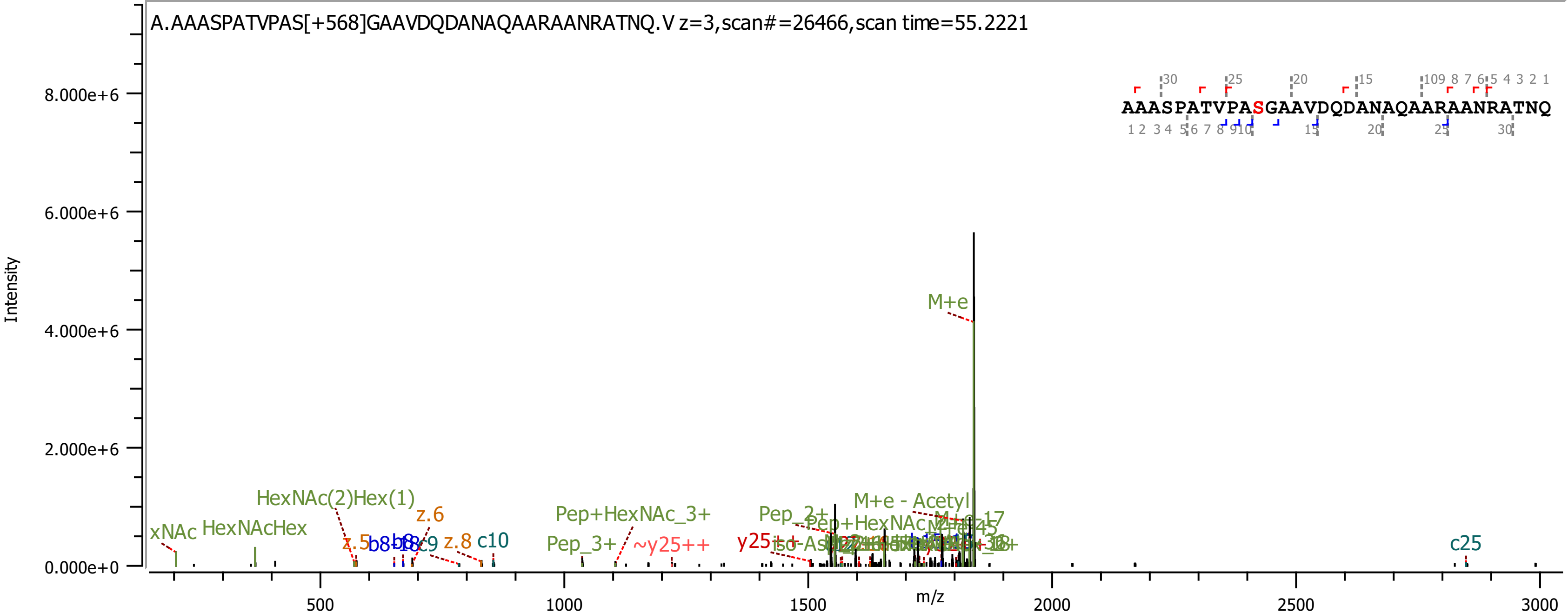

A.AAASPATVPAS[+568]GAAVDQDANAQAARAANRATNQ.V z=3,scan#=26466,scan time=55.2221
AAASPATVPASGAAVDQDANAQAARAANRATNQ
Intensity
8.000e+6
6.000e+6
4.000e+6
2.000e+6
0.000e+0
m/z
500
1000
1500
2000
2500
3000
M+e
M+e - Acetyl
HexNAc(2)Hex(1)
HexNAcHex
z.6
z.5
z.8
c9
c10
c25
Pep+HexNAc_3+
Pep_3+
~y25++
y25++
Pep_2+

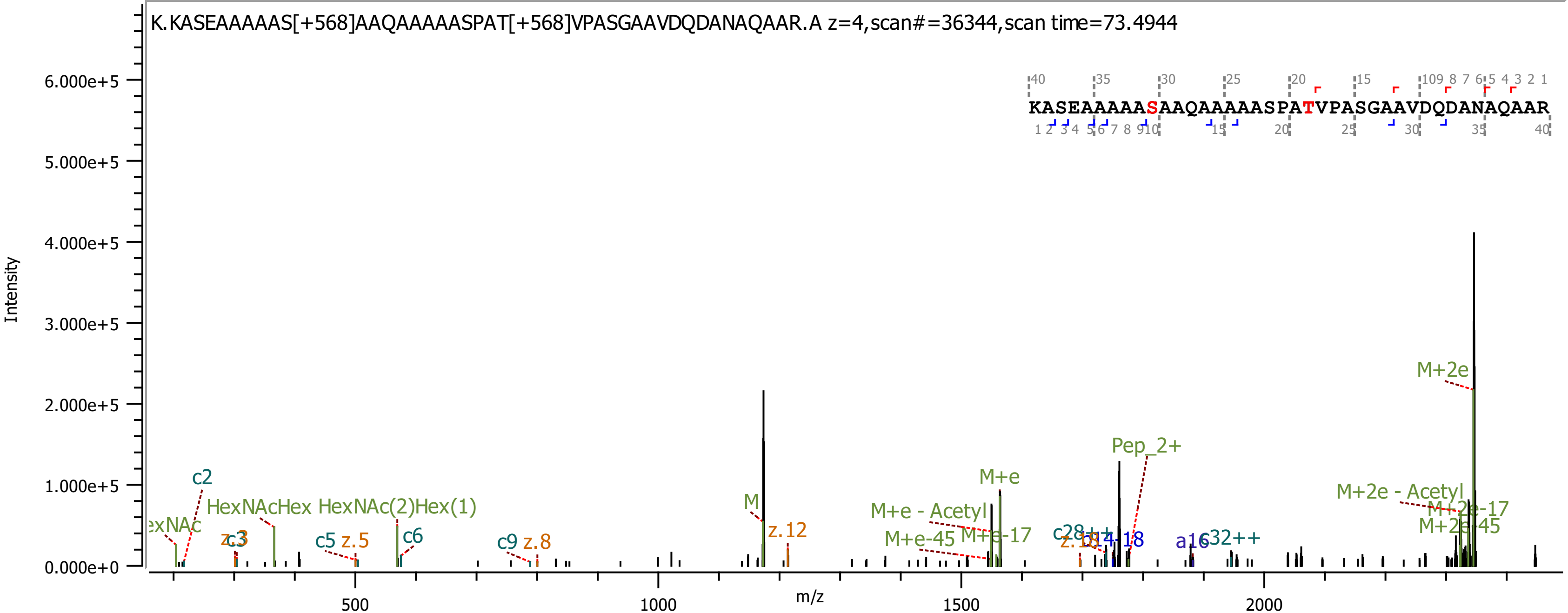
K.KASEAAAAAS[+568]AAQAAAAASPAT[+568]VPASGAAVDQDANAQAAR.A z=4,scan#=36344,scan time=73.4944
KASEAAAAASAAQAAAAASPATVPASGAAVDQDANAQAAR
Intensity
m/z
0.000e+0
1.000e+5
2.000e+5
3.000e+5
4.000e+5
5.000e+5
6.000e+5
500
1000
1500
2000
c2
HexNAcHex
HexNAc(2)Hex(1)
z.3
c5
z.5
c6
c9
z.8
M
z.12
M+e - Acetyl
M+e-45
M+e
M+e-17
Pep_2+
a16
c32++
M+2e
M+2e - Acetyl
M+2e-17
M+2e-45

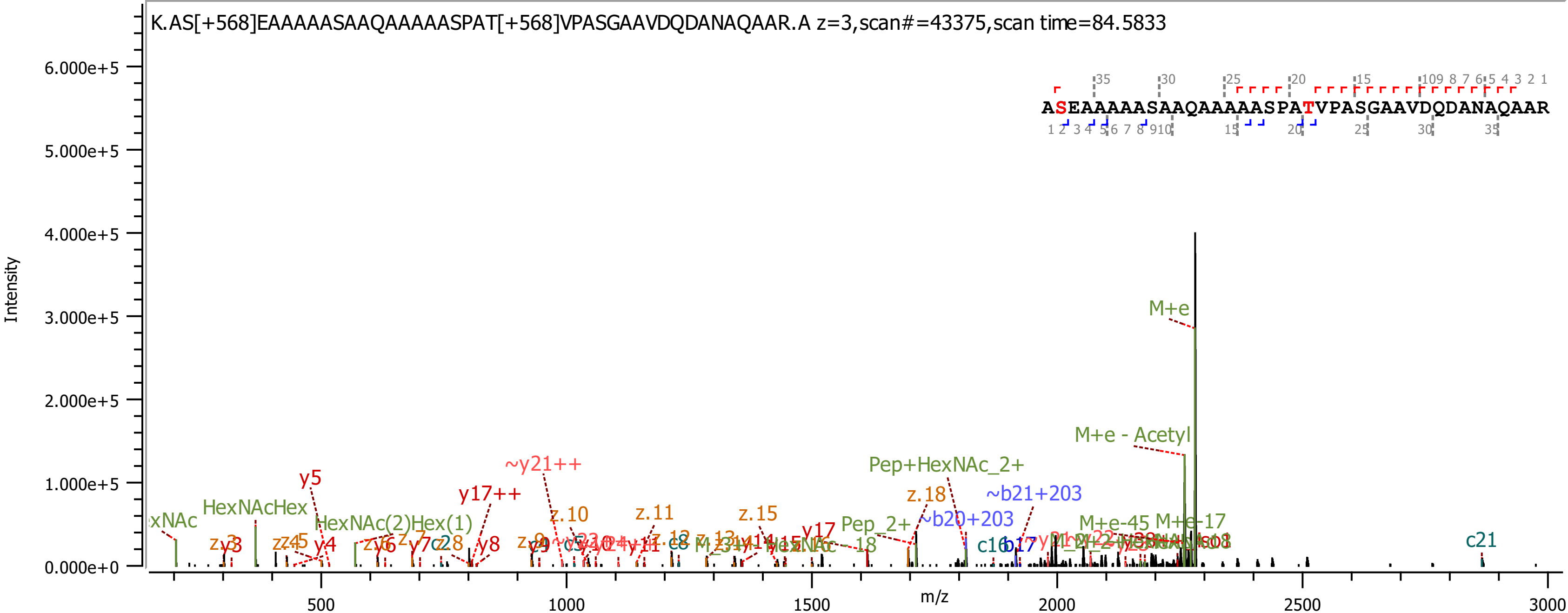
K.AS[+568]EAAAAASAAQAAAAASPAT[+568]VPASGAAVDQDANAQAAR.A z=3,scan#=43375,scan time=84.5833
ASEAAAAASAAQAAAAASPATVPASGAAVDQDANAQAAR
Intensity
m/z
6.000e+5
5.000e+5
4.000e+5
3.000e+5
2.000e+5
1.000e+5
0.000e+0
500
1000
1500
2000
2500
3000
M+e
M+e - Acetyl
Pep+HexNAc_2+
~b21+203
~b20+203
M+e-45
M+e-17
Pep_2+
z.18
z.15
z.11
z.10
y17
y17++
~y21++
y5
y4
y8
HexNAcHex
HexNAc(2)Hex(1)
c16
b17
c21
c8

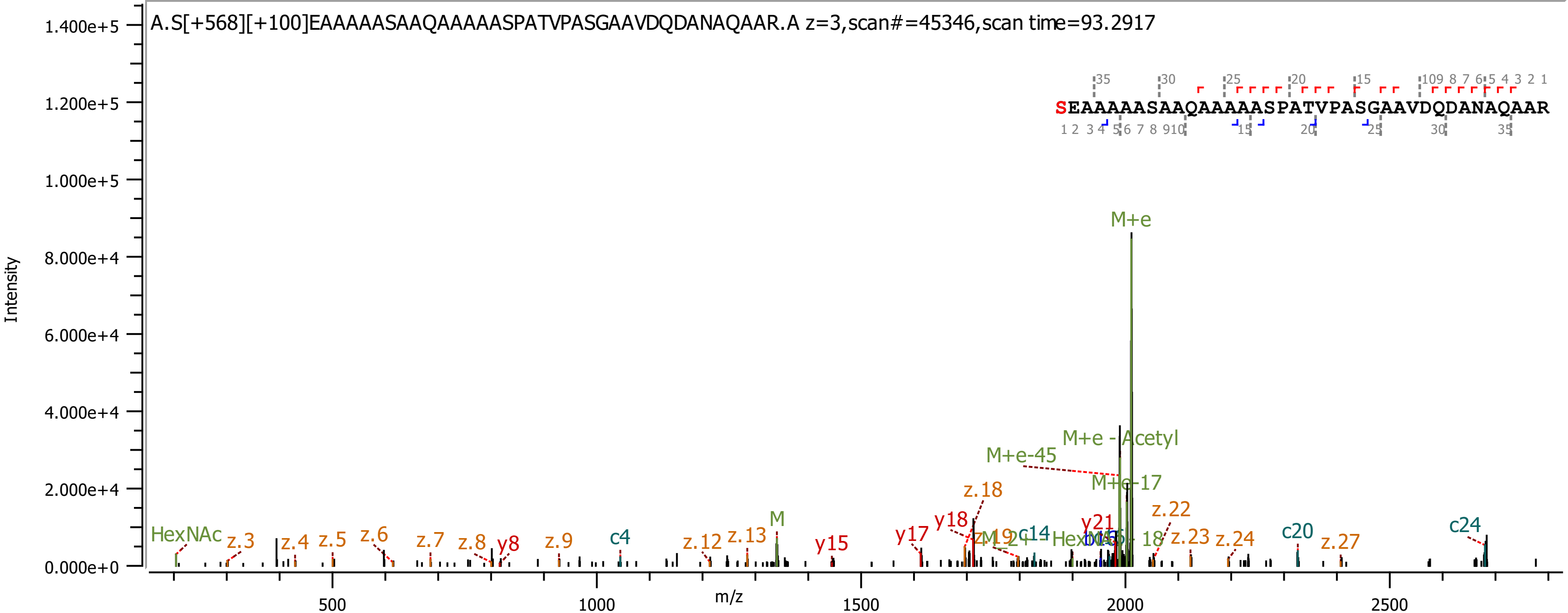
A.S[+568][+100]EAAAAASAAQAAAAASPATVPASGAAVDQDANAQAAR.A z=3,scan#=45346,scan time=93.2917
SEAAAAASAAQAAAAASPATVPASGAAVDQDANAQAAR
Intensity
m/z
M+e
M+e - Acetyl
M+e-45
M+e-17
M
HexNAc
z.3
z.4
z.5
z.6
z.7
z.8
y8
z.9
c4
z.12
z.13
y15
y17
y18
z.18
c14
y21
z.22
z.23
z.24
c20
z.27
c24

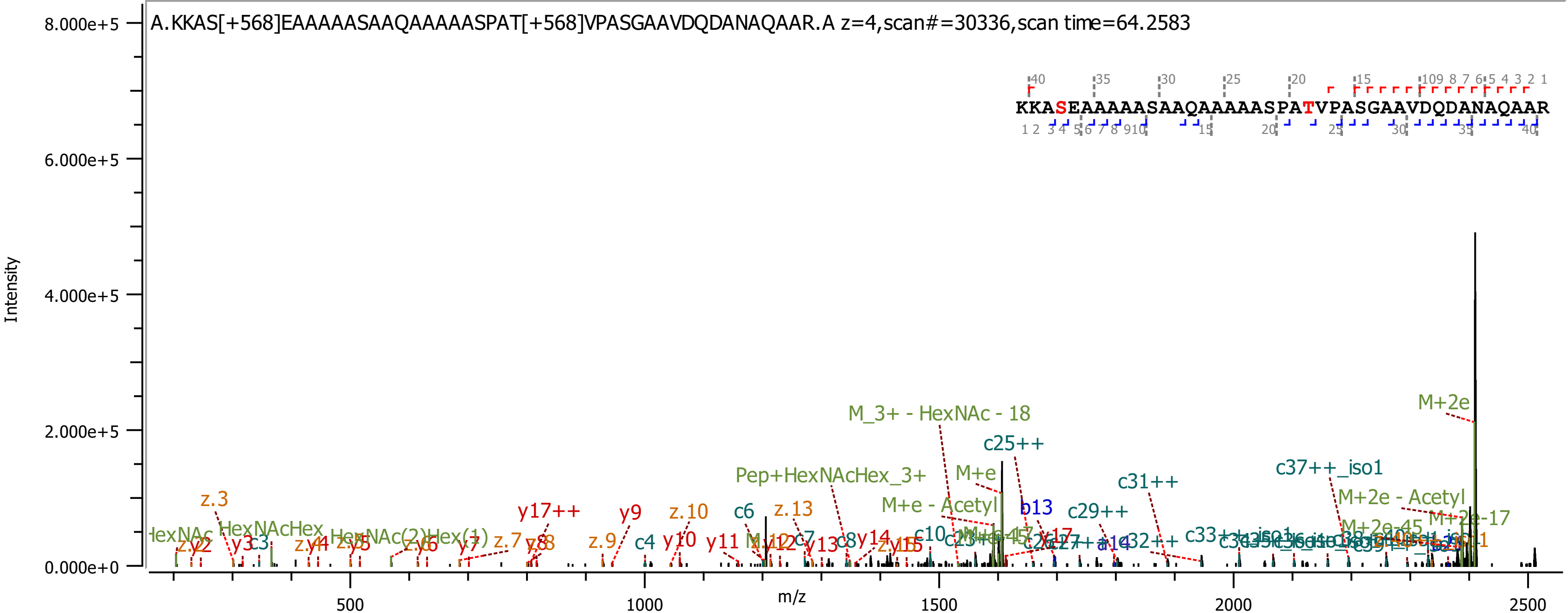

A.KKAS[+568]EAAAAASAAQAAAAASPAT[+568]VPASGAAVDQDANAQAAR.A z=4,scan#=30336,scan time=64.2583
KKASEAAAAASAAQAAAAASPATVPASGAAVDQDANAQAAR
Intensity
m/z
8.000e+5
6.000e+5
4.000e+5
2.000e+5
0.000e+0
500
1000
1500
2000
2500
M+2e
M_3+ - HexNAc - 18
c25++
c37++_iso1
Pep+HexNAcHex_3+
M+e
c31++
M+2e - Acetyl
M+e - Acetyl
b13
c29++
M+2e-17
z.3
y17++
y9
z.10
c6
z.13
HexNAcHex
HexNAc(2)Hex(1)

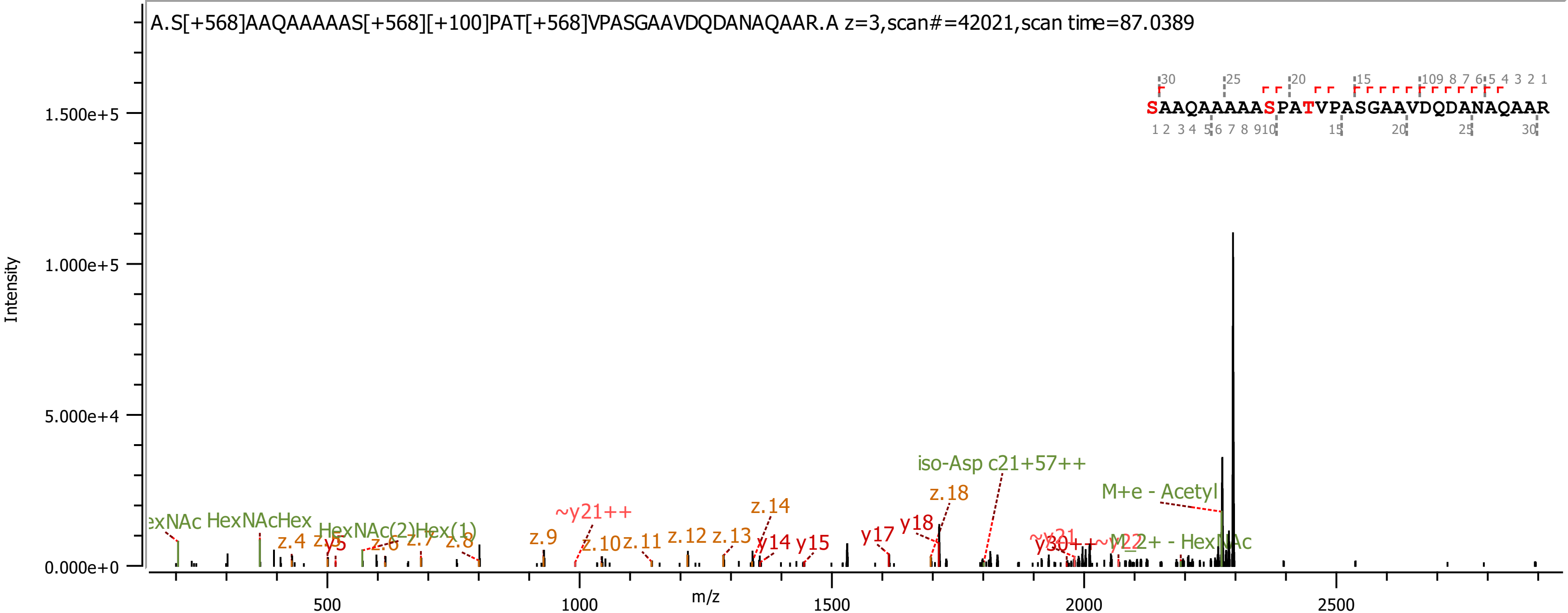

A.S[+568]AAQAAAAAS[+568][+100]PAT[+568]VPASGAAVDQDANAQAAR.A z=3,scan#=42021,scan time=87.0389
SAAQAAAAASPATVPASGAAVDQDANAQAAR
Intensity
1.500e+5
1.000e+5
5.000e+4
0.000e+0
m/z
500
1000
1500
2000
2500
HexNAcHex
HexNAc(2)Hex(1)
z.4
z.6
z.7
z.8
z.9
z.10
z.11
z.12
z.13
z.14
z.18
y5
y14
y15
y17
y18
~y21++
iso-Asp c21+57++
M+e - Acetyl

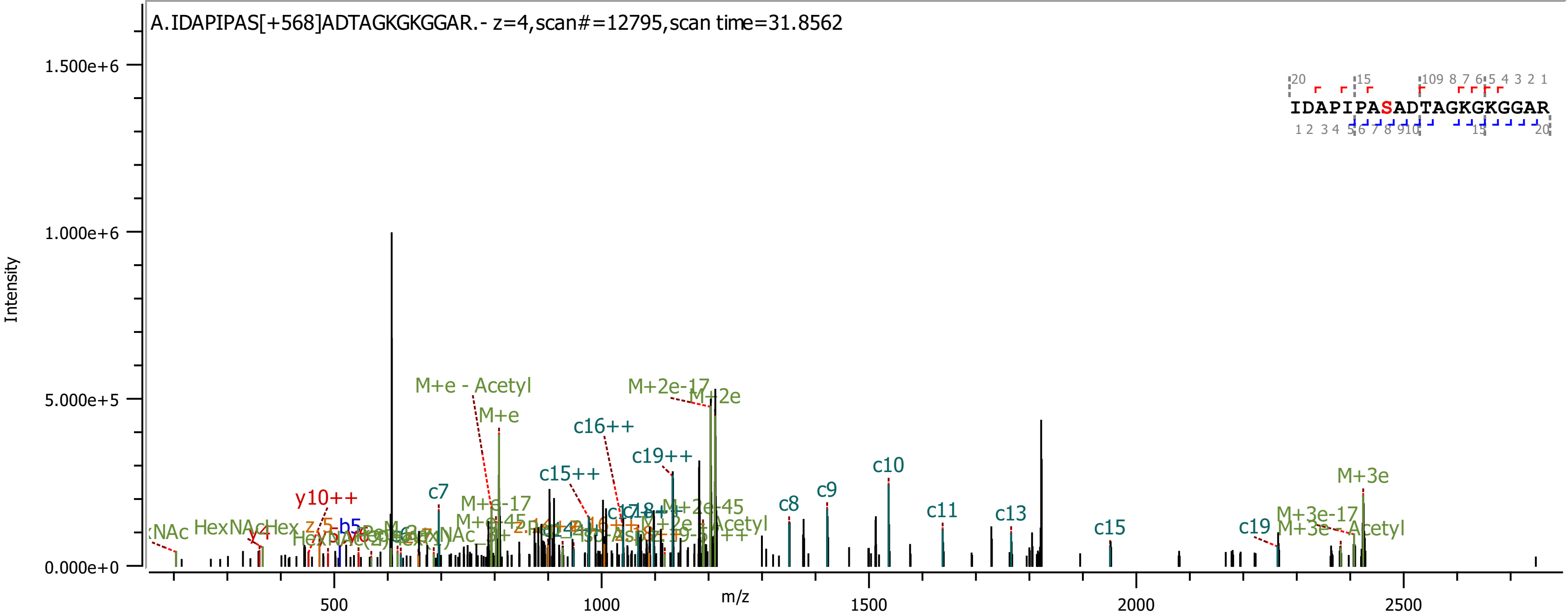

A.IDAPIPAS[+568]ADTAGKGKGGAR.- z=4,scan#=12795,scan time=31.8562
Intensity
m/z
1.500e+6
1.000e+6
5.000e+5
0.000e+0
500
1000
1500
2000
2500
IDAPIPASADTAGKGKGGAR
M+e - Acetyl
M+e
M+2e-17
M+2e
c16++
c19++
c15++
c7
c8
c9
c10
c11
c13
c15
c19
y10++
M+e-17
M+e-45
M+2e-45
M+2e - Acetyl
M+3e
M+3e-17
M+3e - Acetyl
HexNAcHex
b5
y4

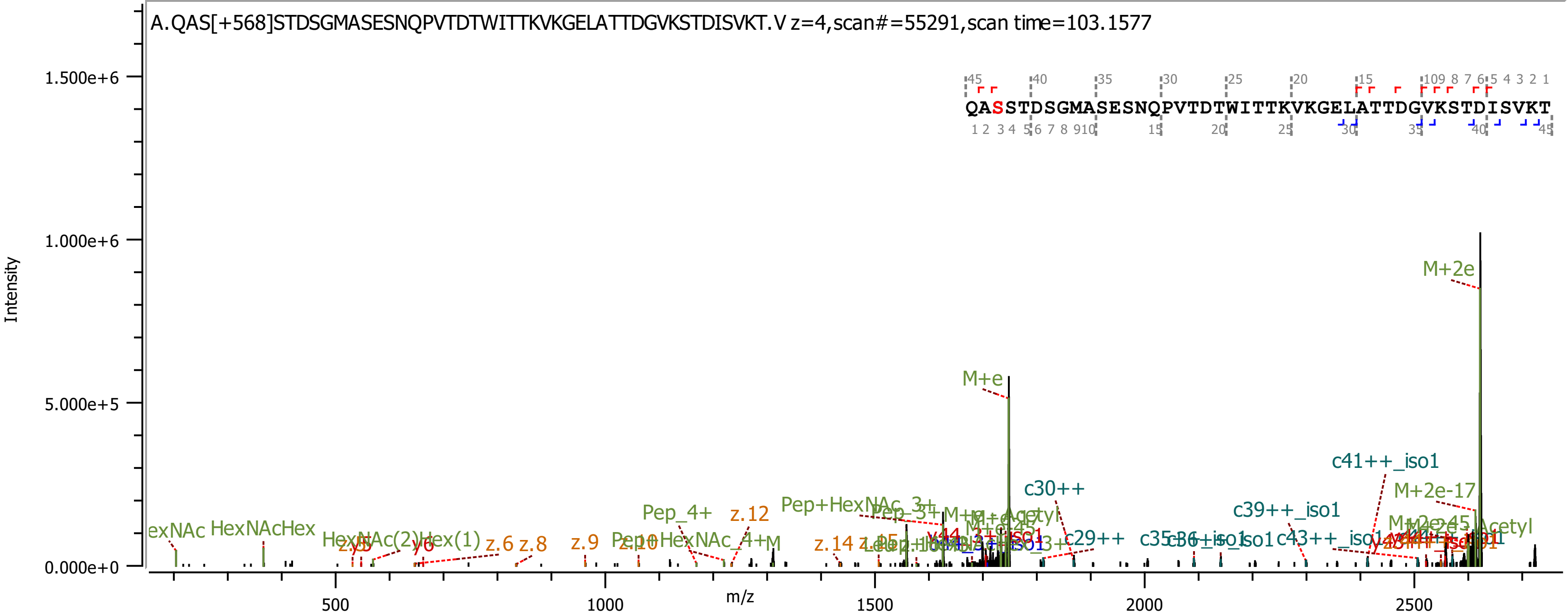

A.QAS[+568]STDSGMASESNQPVTDTWITTKVKGELATTDGVKSTDISVKT.V z=4,scan#=55291,scan time=103.1577
QASSTDSGMASESNQPVTDTWITTKVKGELATTDGVKSTDISVKT
Intensity
1.500e+6
1.000e+6
5.000e+5
0.000e+0
m/z
500
1000
1500
2000
2500
M+2e
M+e
c41++_iso1
M+2e-17
c30++
c39++_iso1
Pep+HexNAc_3+
Pep_4+
z.12
HexNAcHex
HexNAc(2)Hex(1)
y6
z.6
z.8
z.9
z.10
z.14
c29++
c43++_iso1

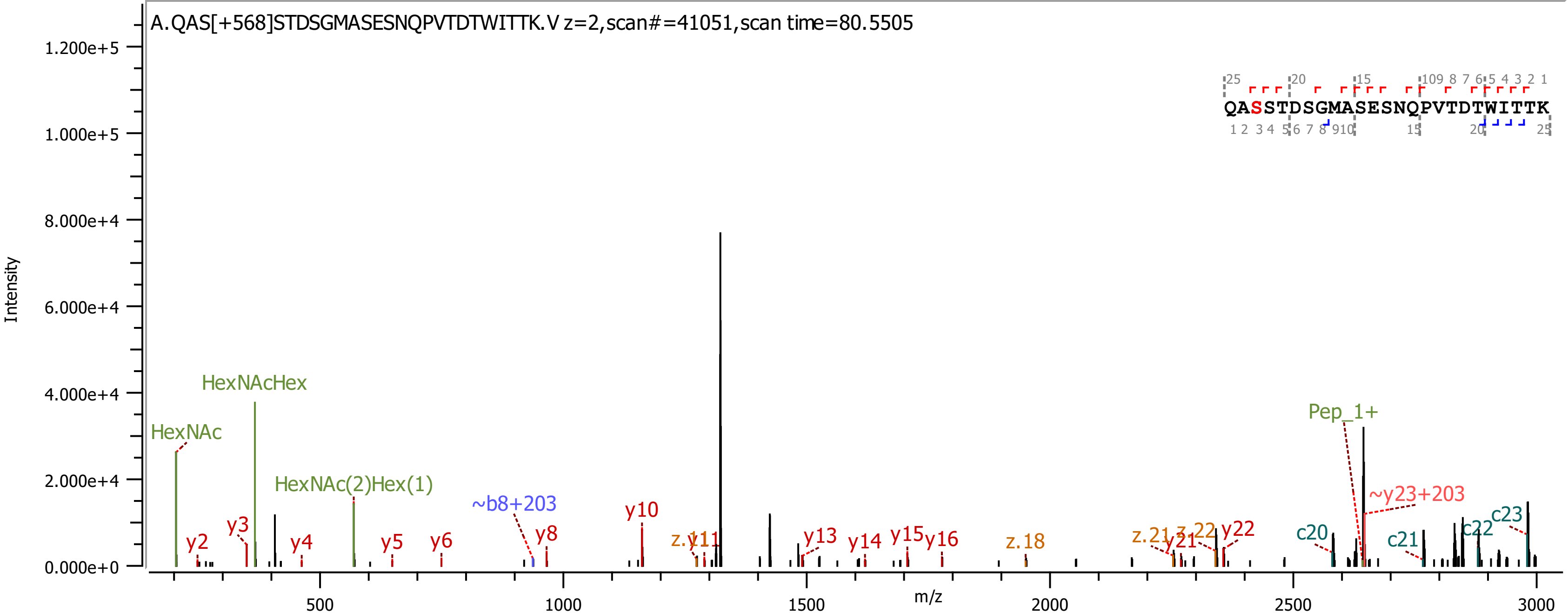
A.QAS[+568]STDSGMASESNQPVTDTWITTK.V z=2,scan#=41051,scan time=80.5505
QASSTDSGMASESNQPVTDTWITTK
Intensity
m/z
HexNAc
HexNAcHex
HexNAc(2)Hex(1)
~b8+203
y2
y3
y4
y5
y6
y8
y10
z.y11
y13
y14
y15
y16
z.18
z.21
y21
z.22
y22
Pep_1+
~y23+203
c20
c21
c22
c23

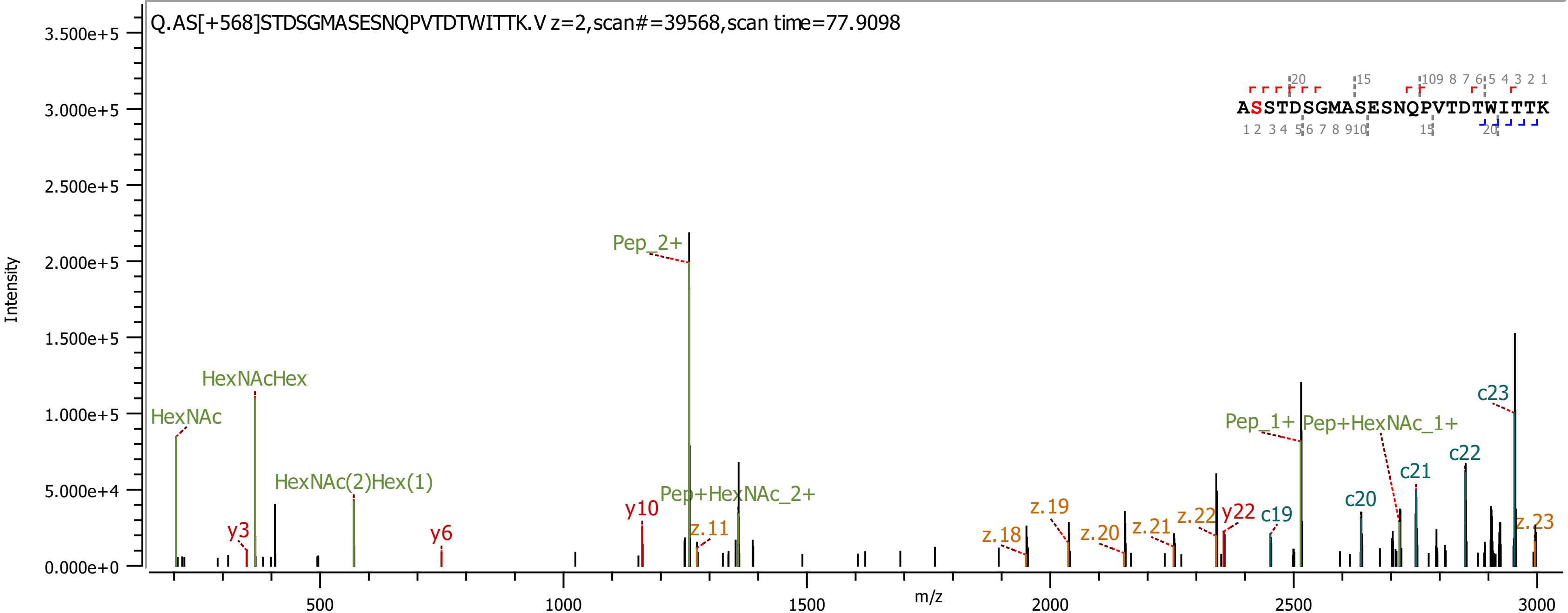
Q.AS[+568]STDSGMASESNQPVTDTWITTK.V z=2,scan#=39568,scan time=77.9098
ASSTDSGMASESNQPVTDTWITTK
Intensity
m/z
HexNAc
HexNAcHex
HexNAc(2)Hex(1)
y3
y6
y10
Pep_2+
Pep+HexNAc_2+
z.11
z.18
z.19
z.20
z.21
z.22
y22
c19
Pep_1+
Pep+HexNAc_1+
c20
c21
c22
c23
z.23

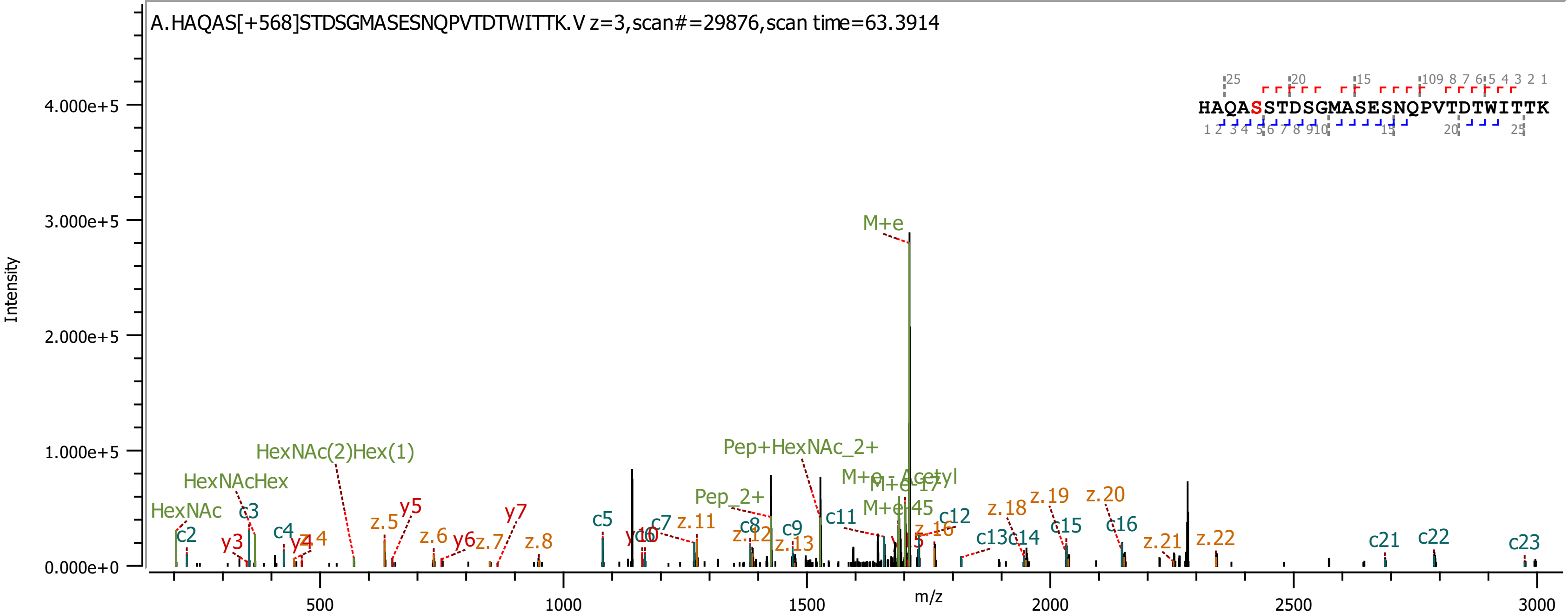
A.HAQAS[+568]STDSGMASESNQPVTDTWITTK.V z=3,scan#=29876,scan time=63.3914
HAQASSTDSGMASESNQPVTDTWITTK
Intensity
m/z
M+e
Pep+HexNAc_2+
HexNAc(2)Hex(1)
HexNAcHex
HexNAc
Pep_2+
M+e-17
M+e-45
c2
c3
c4
c5
c7
c8
c9
c11
c12
c13
c14
c15
c16
c21
c22
c23
y3
y4
y5
y6
y7
y10
z.4
z.5
z.6
z.7
z.8
z.11
z.12
z.13
z.16
z.18
z.19
z.20
z.21
z.22

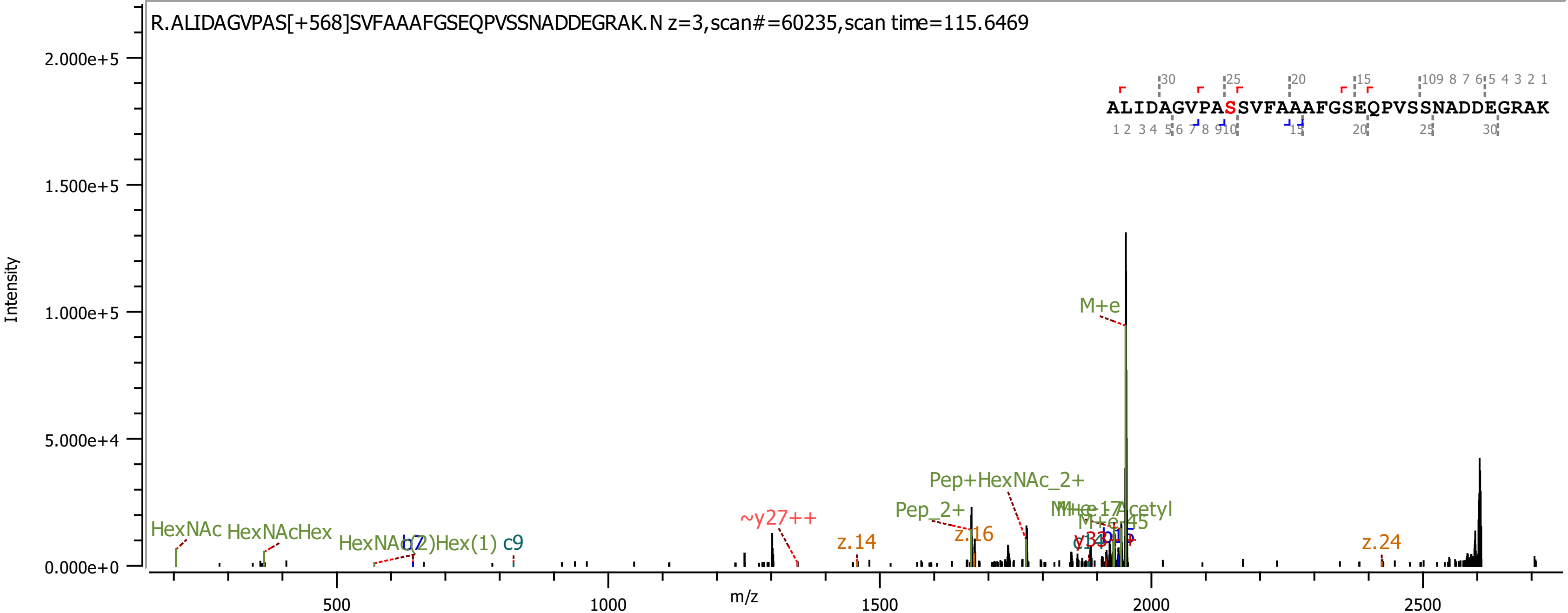
R.ALIDAGVPAS[+568]SVFAAAFGSEQPVSSNADDEGRAK.N z=3,scan#=60235,scan time=115.6469
ALIDAGVPASSVFAAAFGSEQPVSSNADDEGRAK
Intensity
m/z
2.000e+5
1.500e+5
1.000e+5
5.000e+4
0.000e+0
500
1000
1500
2000
2500
M+e
Pep+HexNAc_2+
Pep_2+
z.16
~y27++
z.14
z.24
HexNAc
HexNAcHex
c9

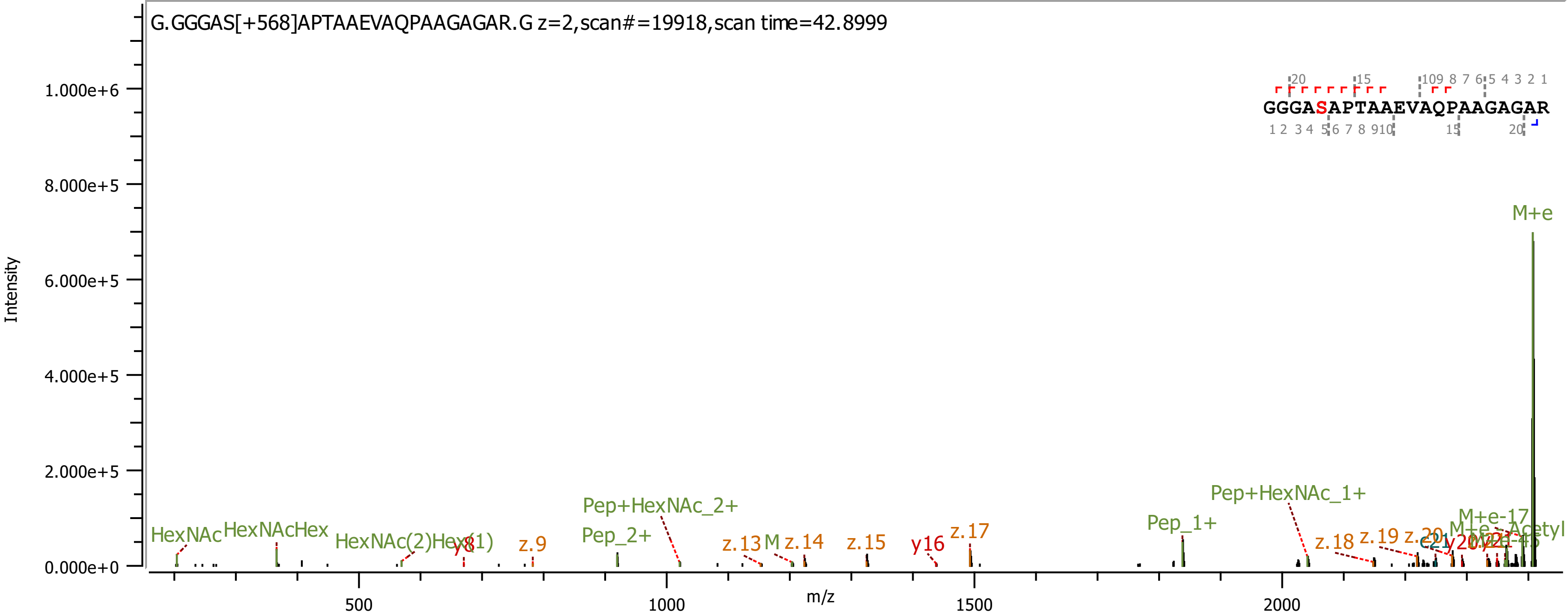

G.GGGAS[+568]APTAAEVAQPAAGAGAR.G z=2,scan#=19918,scan time=42.8999
GGGASAPTAAEVAQPAAGAGAR
Intensity
1.000e+6
8.000e+5
6.000e+5
4.000e+5
2.000e+5
0.000e+0
m/z
500
1000
1500
2000
M+e
HexNAc
HexNAcHex
HexNAc(2)Hex(1)
y8
z.9
Pep+HexNAc_2+
Pep_2+
z.13
z.14
z.15
y16
z.17
Pep_1+
Pep+HexNAc_1+
z.18
z.19
M+e-17

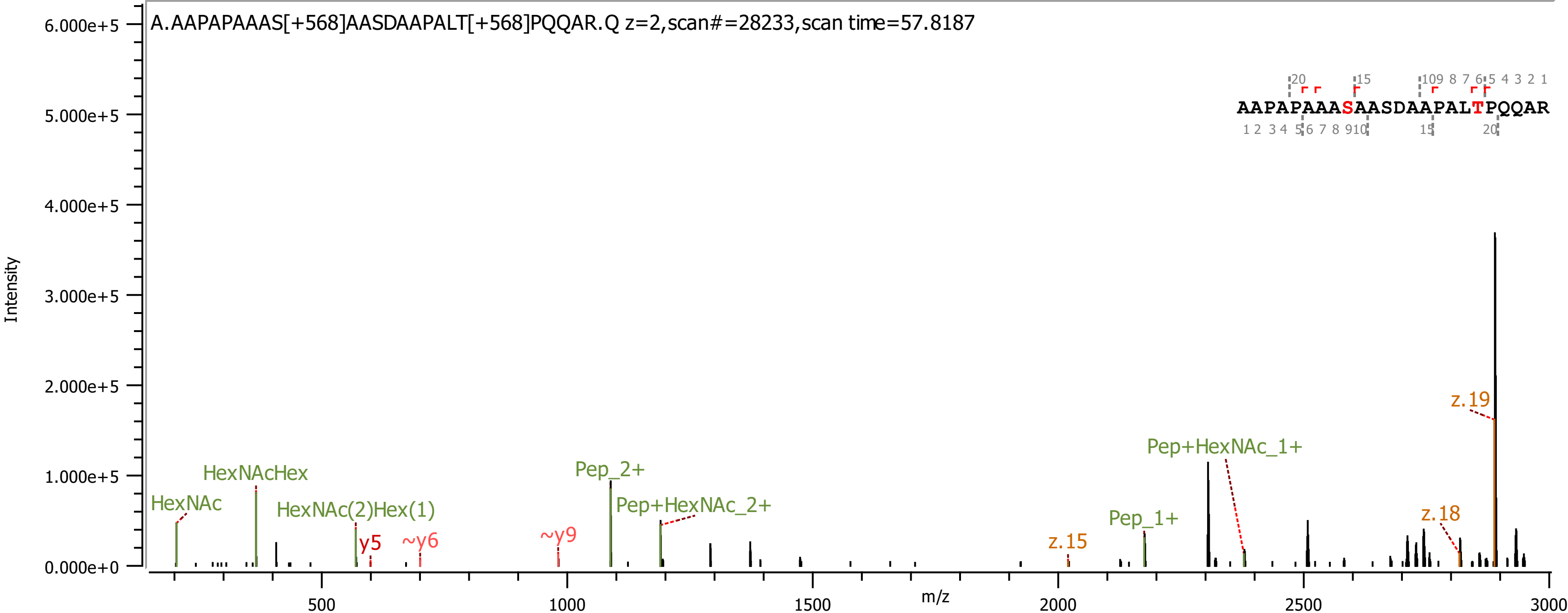

A.AAPAPAAAS[+568]AASDAAPALT[+568]PQQAR.Q z=2,scan#=28233,scan time=57.8187
AAPAPAAASAASDAAPALTPQQAR
Intensity
m/z
HexNAc
HexNAcHex
HexNAc(2)Hex(1)
y5
~y6
~y9
Pep_2+
Pep+HexNAc_2+
z.15
Pep_1+
Pep+HexNAc_1+
z.18
z.19

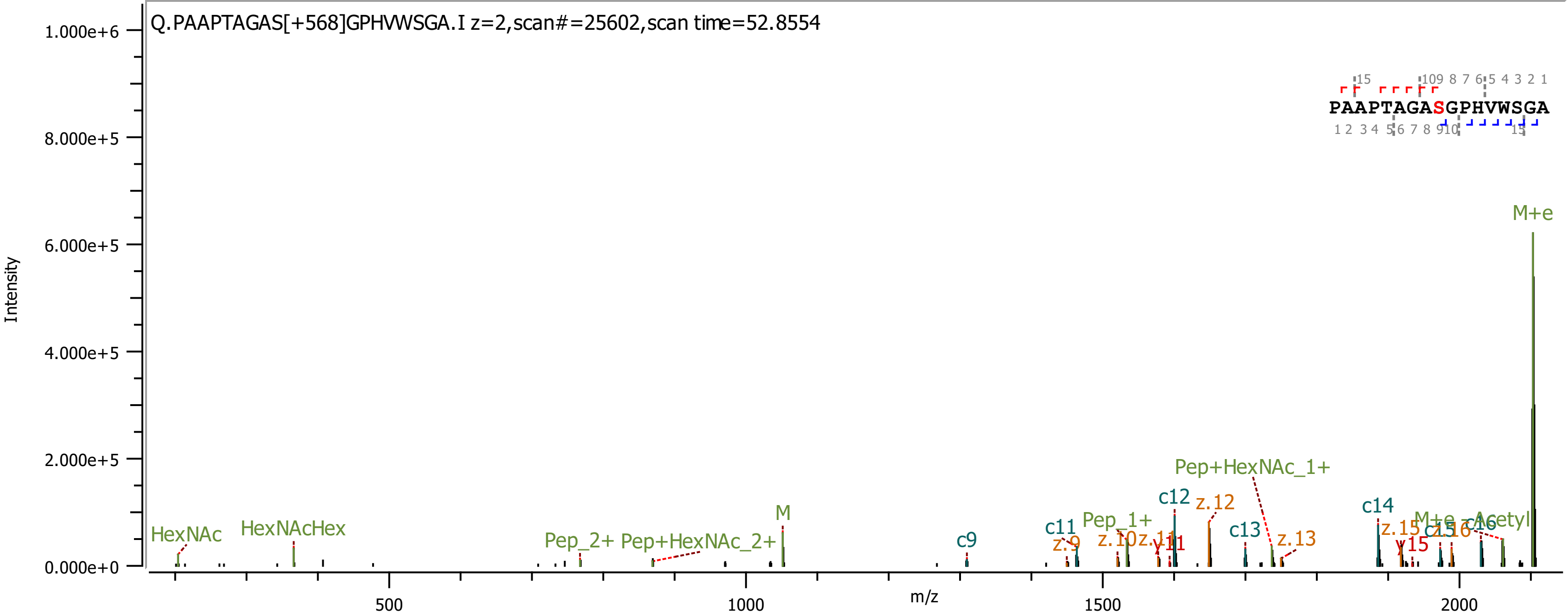

Q.PAAPTAGAS[+568]GPHVWSGA.I z=2,scan#=25602,scan time=52.8554
Intensity
m/z
PAAPTAGASGPHVWSGA
M+e
Pep+HexNAc_1+
c12
z.12
c14
M+e - Acetyl
HexNAc
HexNAcHex
Pep_2+
Pep+HexNAc_2+
M
c9
c11
Pep_1+
c13
z.13
z.15
y15
y11

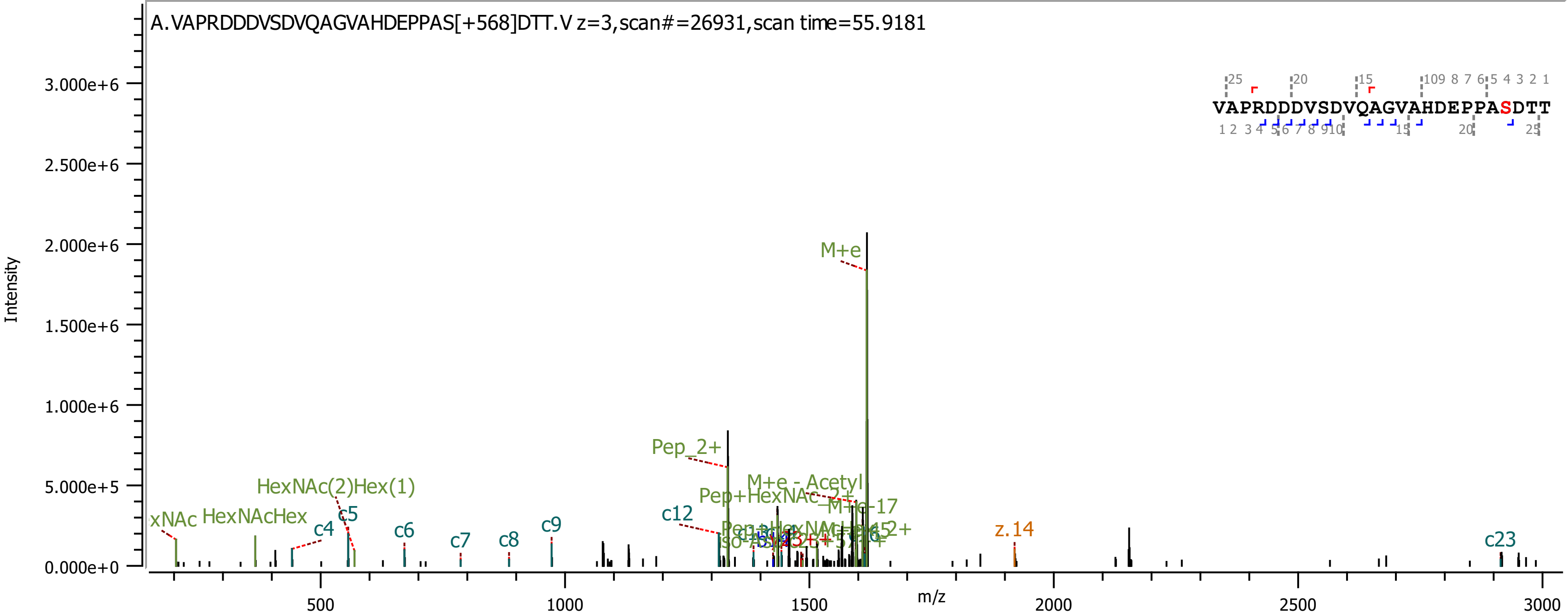
A.VAPRDDDVSDVQAGVAHDEPPAS[+568]DTT.V z=3,scan#=26931,scan time=55.9181
VAPRDDDVSDVQAGVAHDEPPASDTT
Intensity
m/z
M+e
Pep_2+
M+e - Acetyl
Pep+HexNAc_2+
M+e-17
HexNAc(2)Hex(1)
HexNAcHex
xNAc
c4
c5
c6
c7
c8
c9
c12
z.14
c23
0.000e+0
5.000e+5
1.000e+6
1.500e+6
2.000e+6
2.500e+6
3.000e+6
500
1000
1500
2000
2500
3000

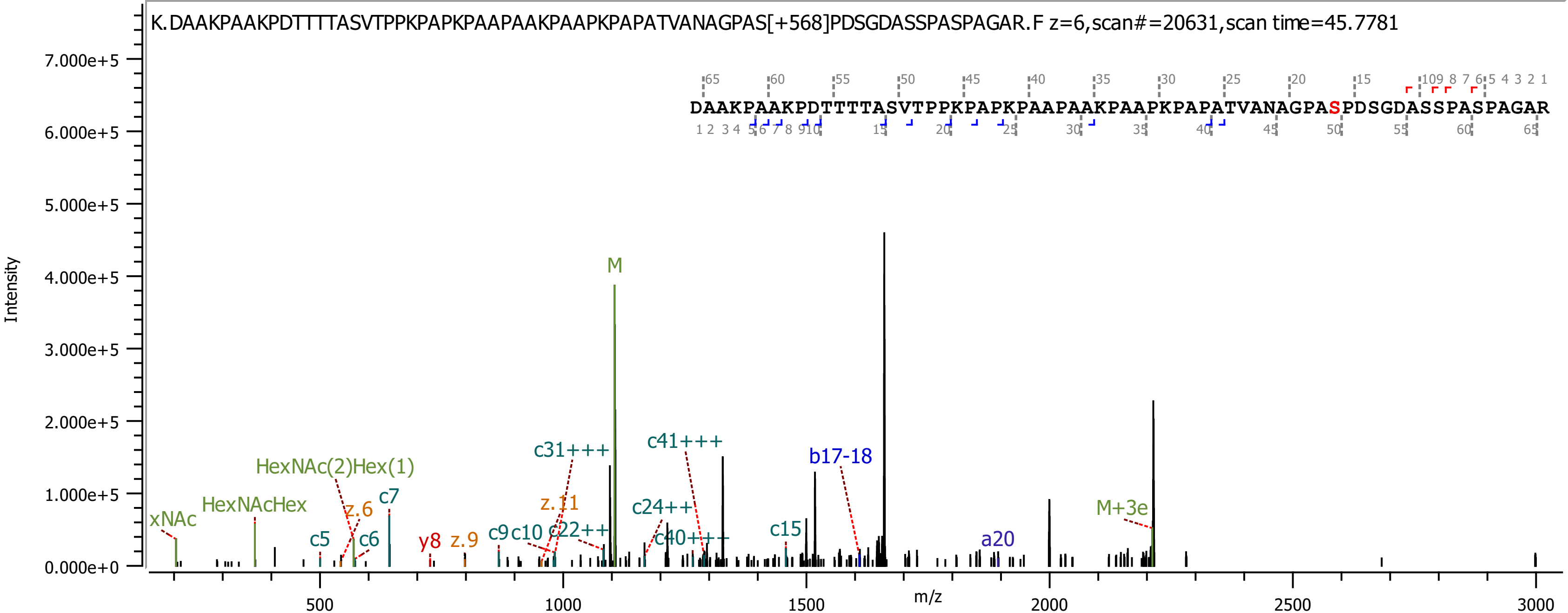
K.DAAKPAAKPDTTTTASVTPPKPAPKPAAPAAKPAAPKPAPATVANAGPAS[+568]PDSGDASSPASPAGAR.F z=6,scan#=20631,scan time=45.7781
DAAKPAAKPDTTTTASVTPPKPAPKPAAPAAKPAAPKPAPATVANAGPASPDSGDASSPASPAGAR
Intensity
m/z
7.000e+5
6.000e+5
5.000e+5
4.000e+5
3.000e+5
2.000e+5
1.000e+5
0.000e+0
500
1000
1500
2000
2500
3000
M
c31+++
c41+++
b17-18
HexNAc(2)Hex(1)
HexNAcHex
xNAc
z.6
c7
c5
c6
y8
z.9
c9
c10
z.11
c22++
c24++
c40+++
c15
a20
M+3e

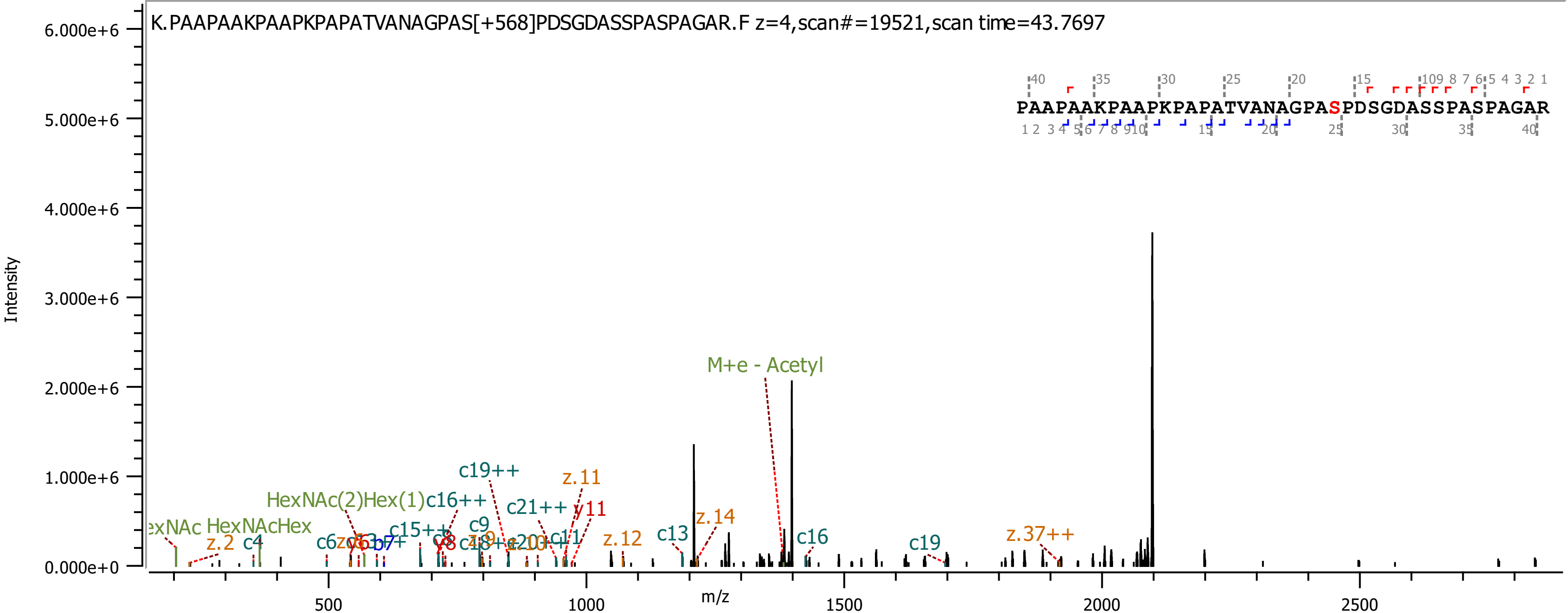
K.PAAPAAKPAAPKPAPATVANAGPAS[+568]PDSGDASSPASPAGAR.F z=4,scan#=19521,scan time=43.7697
PAAPAAKPAAPKPAPATVANAGPASPDSGDASSPASPAGAR
Intensity
m/z
M+e - Acetyl
HexNAcHex
HexNAc(2)Hex(1)c16++
c19++
z.11
c21++
y11
z.14
z.12
c13
c16
c19
z.37++
z.2
c4
c9
c15++

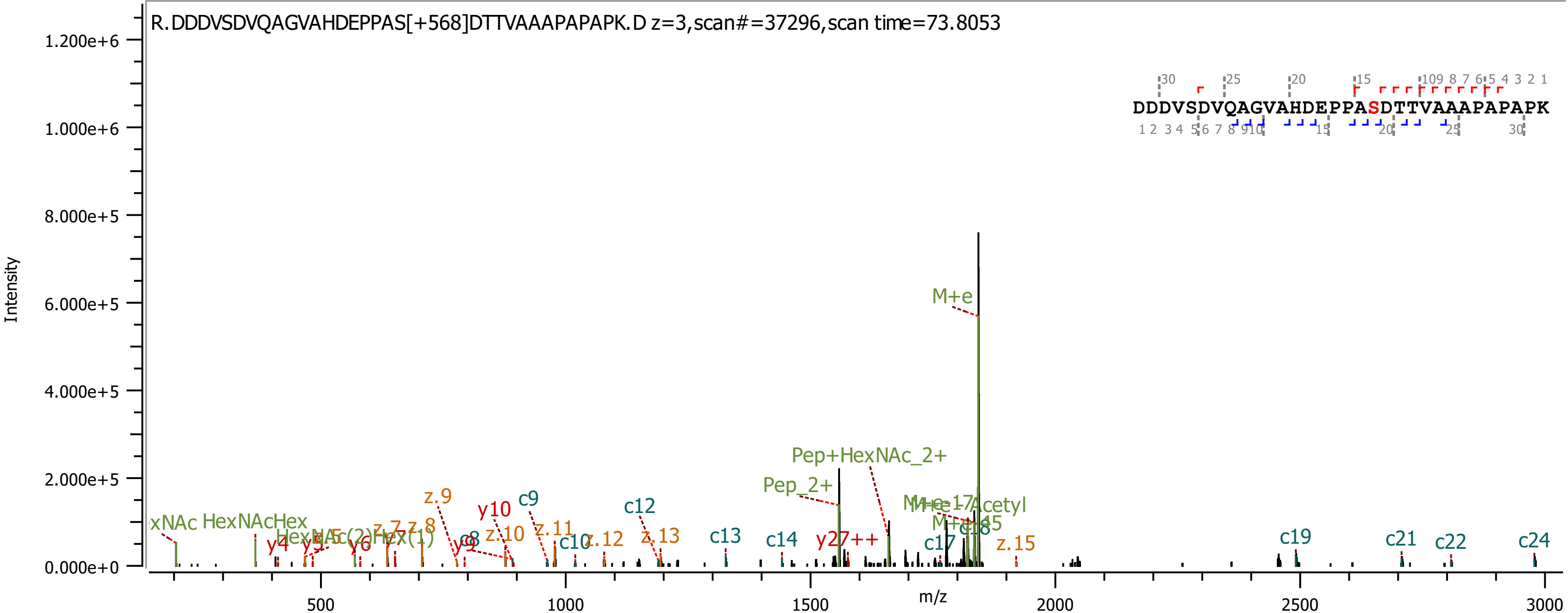
R.DDDVSDVQAGVAHDEPPAS[+568]DTTVAAAPAPAPK.D z=3,scan#=37296,scan time=73.8053
DDDVSDVQAGVAHDEPPASDTTVAAAPAPAPK
Intensity
m/z
1.200e+6
1.000e+6
8.000e+5
6.000e+5
4.000e+5
2.000e+5
0.000e+0
500
1000
1500
2000
2500
3000
M+e
Pep+HexNAc_2+
Pep_2+
HexNAcHex
z.9
y10
c9
c12
z.10
z.11
z.12
z.13
c13
c14
y27++
c17
c19
c21
c22
c24
z.15
c10
y4
y5
y6
y9
c8
M+e-Acetyl

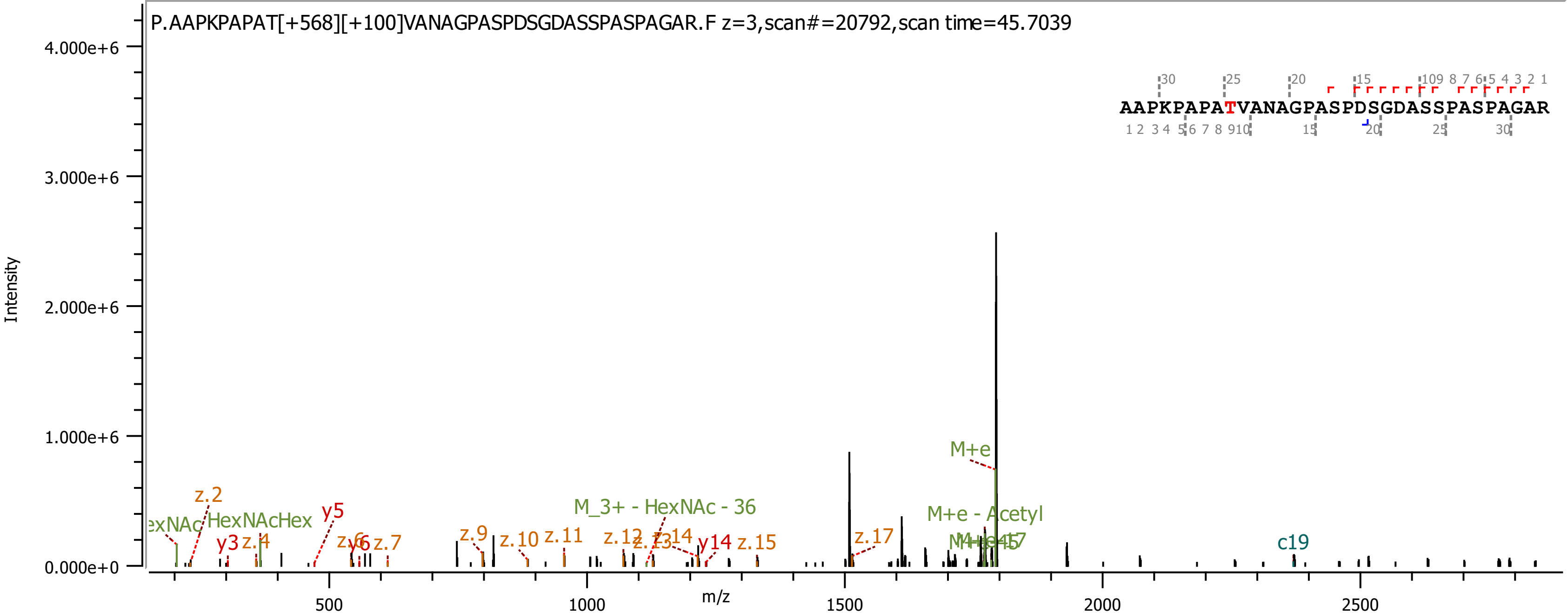

P.AAPKPAPAT[+568][+100]VANAGPASPDSGDASSPASPAGAR.F z=3,scan#=20792,scan time=45.7039
AAPKPAPATVANAGPASPDSGDASSPASPAGAR
Intensity
m/z
4.000e+6
3.000e+6
2.000e+6
1.000e+6
0.000e+0
500
1000
1500
2000
2500
M+e
M_3+ - HexNAc - 36
M+e - Acetyl
HexNAcHex
z.2
y5
y3
z.4
z.6
y6
z.7
z.9
z.10
z.11
z.12
z.13
z.14
y14
z.15
z.17
c19

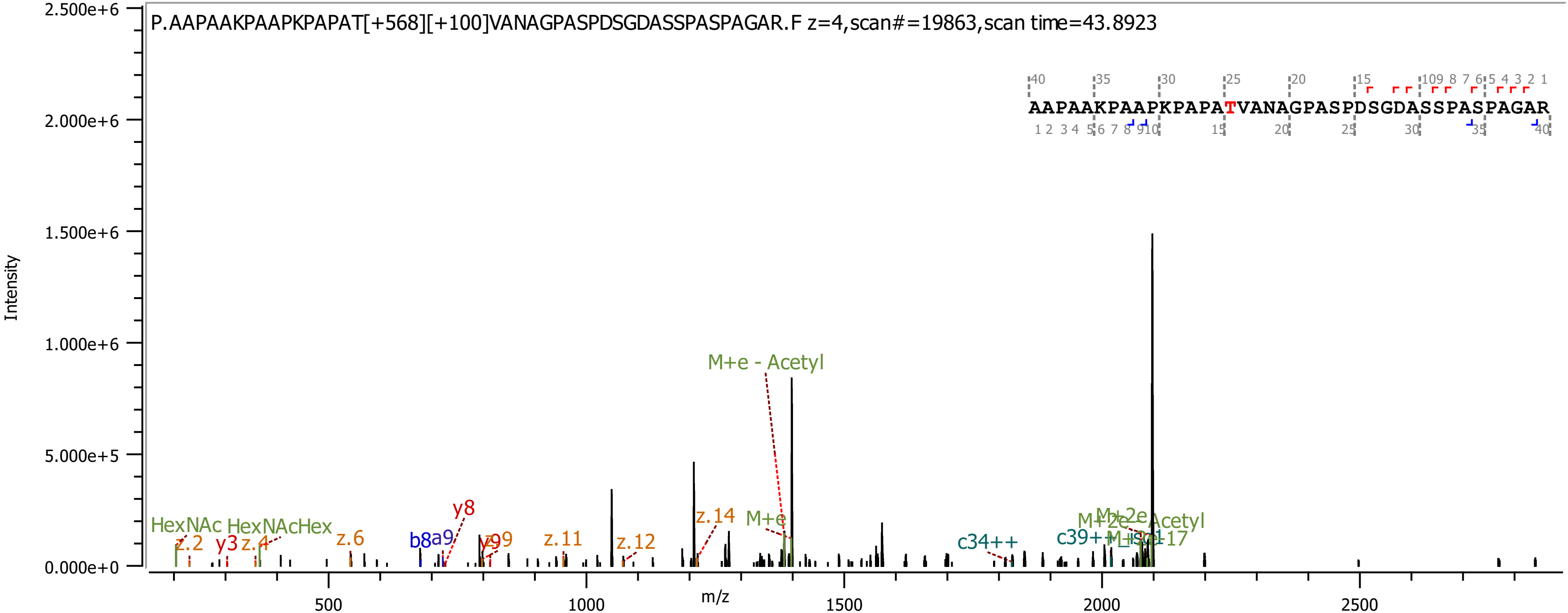

P.AAPAAKPAAPKPAPAT[+568][+100]VANAGPASPDSGDASSPASPAGAR.F z=4,scan#=19863,scan time=43.8923
AAPAAKPAAPKPAPATVANAGPASPDSGDASSPASPAGAR
Intensity
m/z
M+e - Acetyl
M+e
HexNAc
HexNAcHex
z.2
y3
z.4
z.6
b8
a9
y8
y9
z.11
z.12
z.14
c34++
c39++
M+2e
M+2e - Acetyl

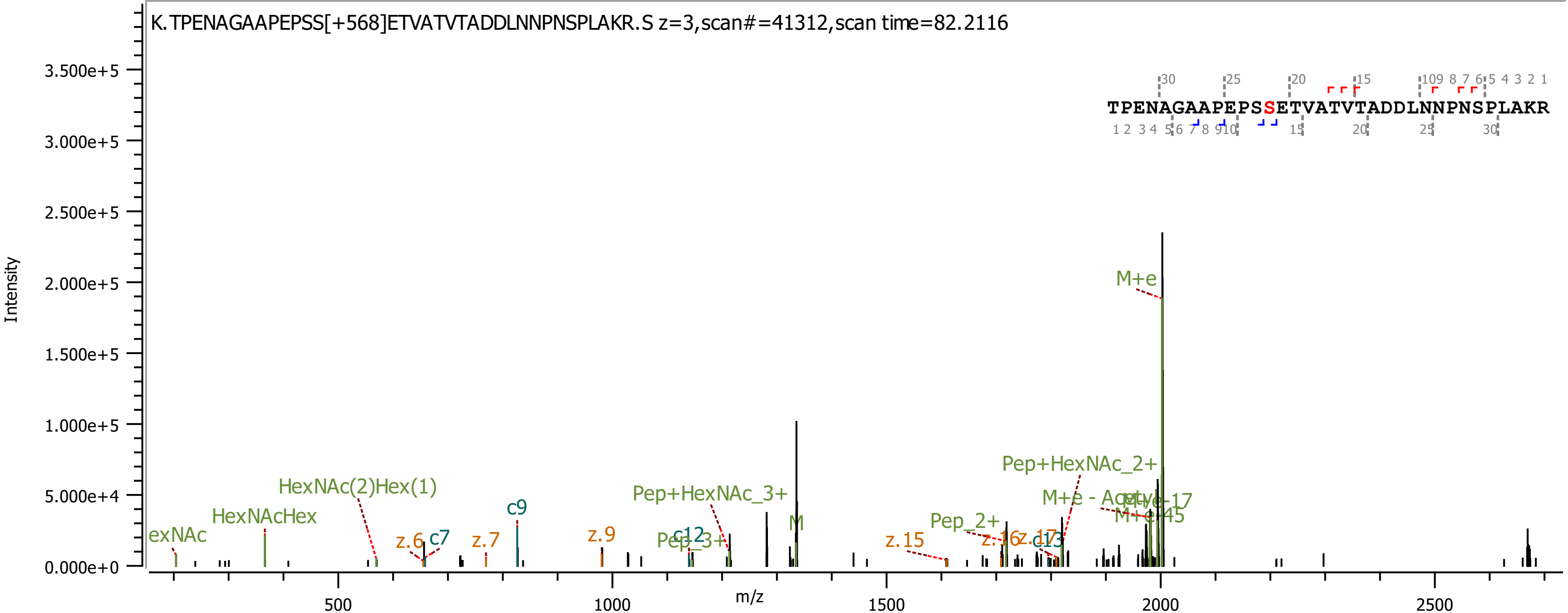
K.TPENAGAAPEPSS[+568]ETVATVTADDLNNPNSPLAKR.S z=3,scan#=41312,scan time=82.2116
TPENAGAAPEPSSETVATVTADDLNNPNSPLAKR
Intensity
m/z
M+e
Pep+HexNAc_2+
M+e - Acetyl
M+e-17
M+e-45
Pep_2+
z.15
z.16
z.17
c13
Pep+HexNAc_3+
Pep_3+
c12
M
z.9
c9
z.7
z.6
c7
HexNAc(2)Hex(1)
HexNAcHex
exNAc

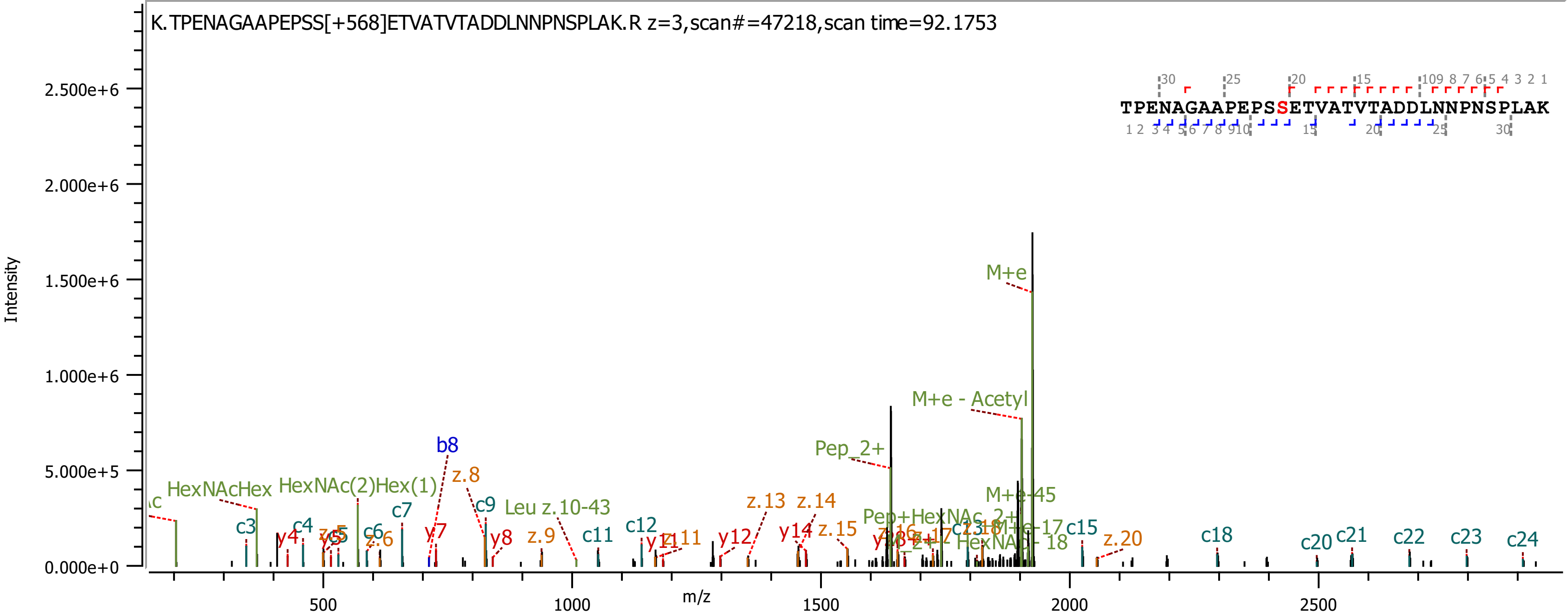

K.TPENAGAAPEPSS[+568]ETVATVTADDLNNPNSPLAK.R z=3,scan#=47218,scan time=92.1753
TPENAGAAPEPSSETVATVTADDLNNPNSPLAK
Intensity
m/z
2.500e+6
2.000e+6
1.500e+6
1.000e+6
5.000e+5
0.000e+0
500
1000
1500
2000
2500
M+e
M+e - Acetyl
Pep_2+
M+e-45
Pep+HexNAc_2+
M+e-17
HexNAc - 18
b8
z.8
HexNAcHex
HexNAc(2)Hex(1)
Leu z.10-43
c3
c4
c6
c7
c9
c11
c12
c15
c18
c20
c21
c22
c23
c24
y4
y5
y7
y8
y12
y14
z.6
z.9
z.11
z.13
z.14
z.15
z.20

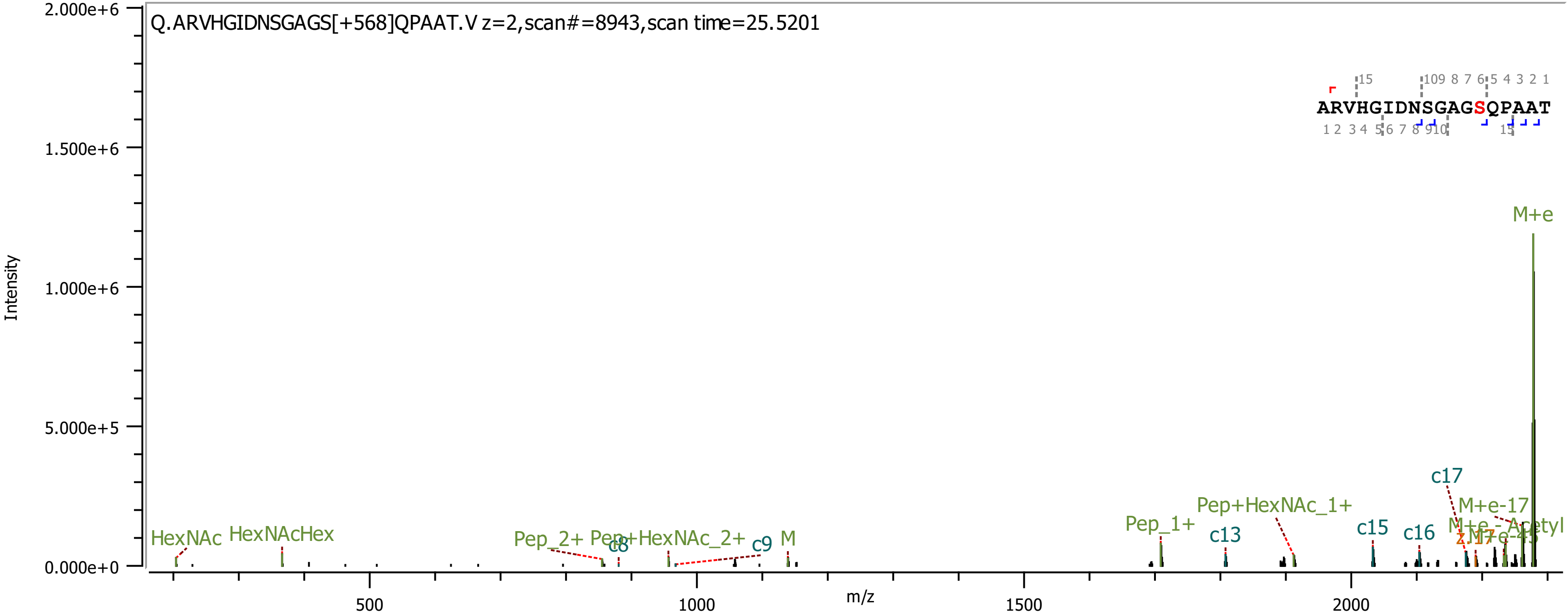

Q.ARVHGIDNSGAGS[+568]QPAAT.V z=2,scan#=8943,scan time=25.5201
ARVHGIDNSGAGSQPAAT
Intensity
2.000e+6
1.500e+6
1.000e+6
5.000e+5
0.000e+0
m/z
500
1000
1500
2000
HexNAc
HexNAcHex
Pep_2+
Pep+HexNAc_2+
c8
c9
M
Pep_1+
c13
Pep+HexNAc_1+
c15
c16
c17
M+e-17
M+e

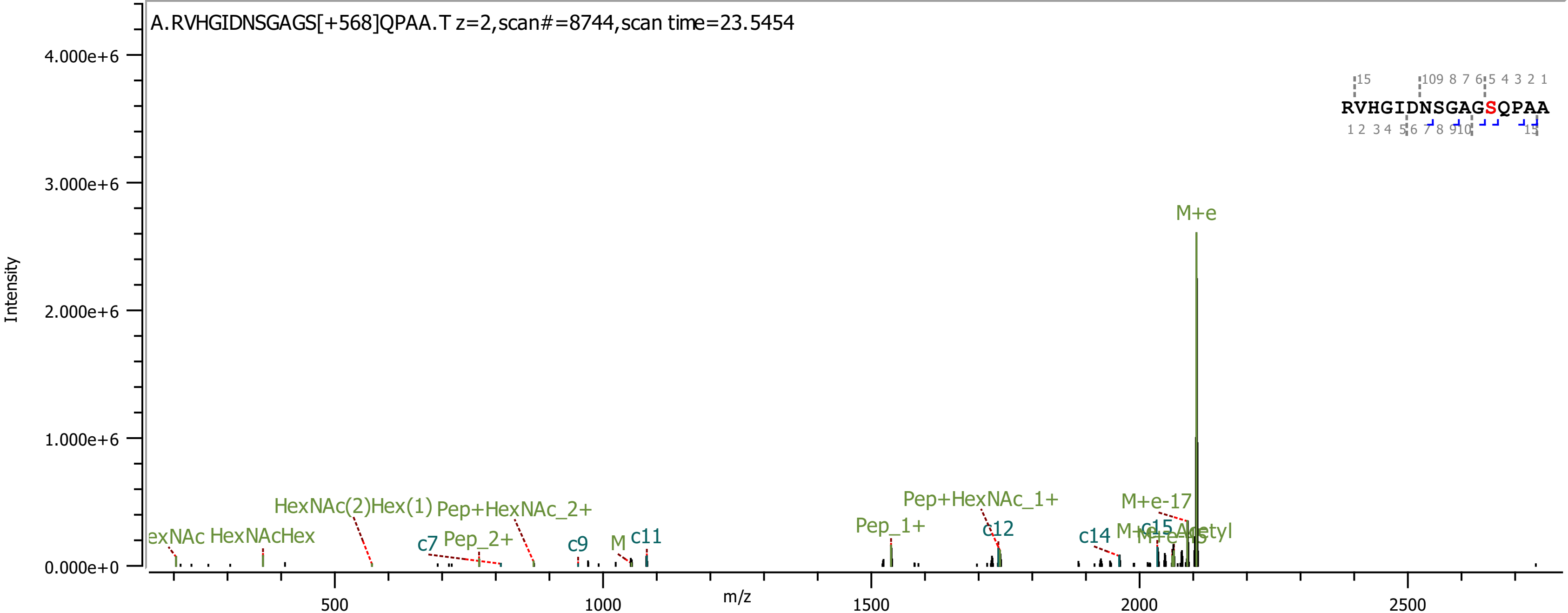

A.RVHGIDNSGAGS[+568]QPAA.T z=2,scan#=8744,scan time=23.5454
Intensity
m/z
RVHGIDNSGAGSQPAA
M+e
M+e-17
Pep+HexNAc_1+
Pep_1+
c12
c14
c11
c9
c7
Pep_2+
Pep+HexNAc_2+
HexNAc(2)Hex(1)
HexNAcHex

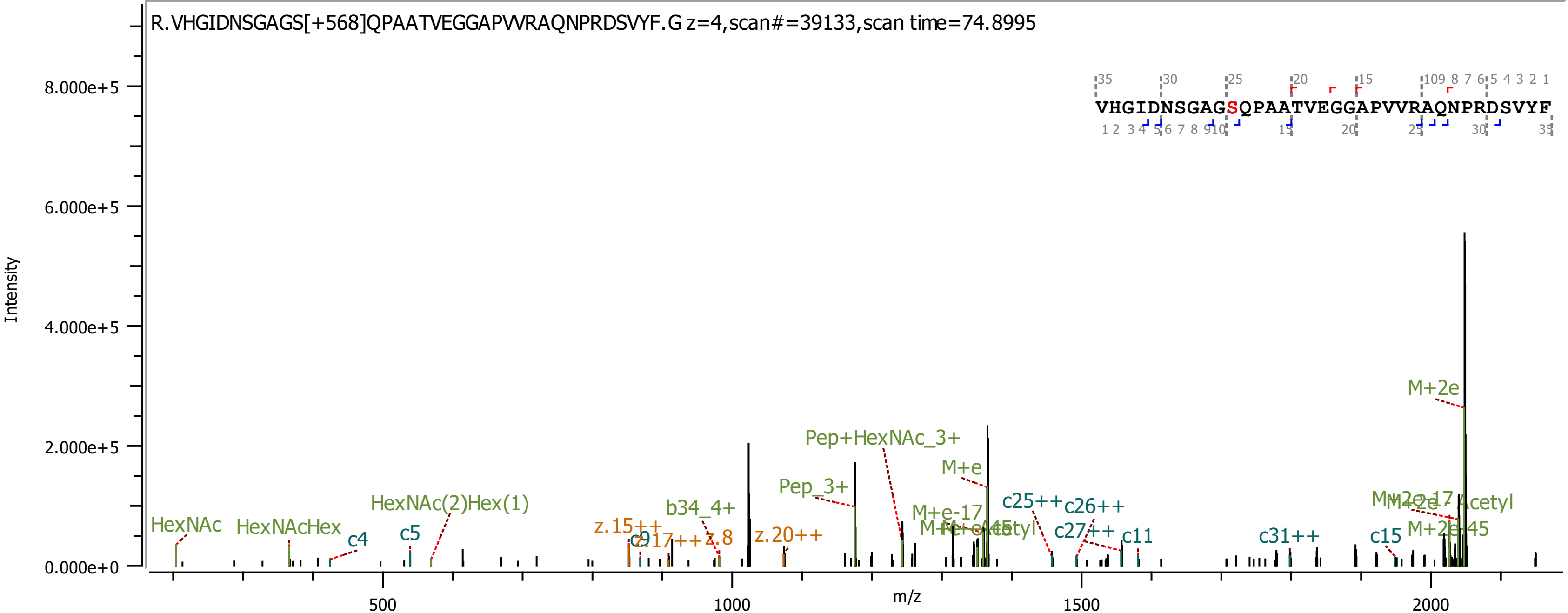
R.VHGIDNSGAGS[+568]QPAATVEGGAPVVRAQNPRDSVYF.G z=4,scan#=39133,scan time=74.8995
VHGIDNSGAGSQPAATVEGGAPVVRAQNPRDSVYF
Intensity
m/z
HexNAc
HexNAcHex
HexNAc(2)Hex(1)
c4
c5
z.15++
b34_4+
Pep_3+
Pep+HexNAc_3+
M+e
M+e-17
z.20++
c25++
c26++
c27++
c11
c31++
c15
M+2e
M+2e-45
8.000e+5
6.000e+5
4.000e+5
2.000e+5
0.000e+0
500
1000
1500
2000

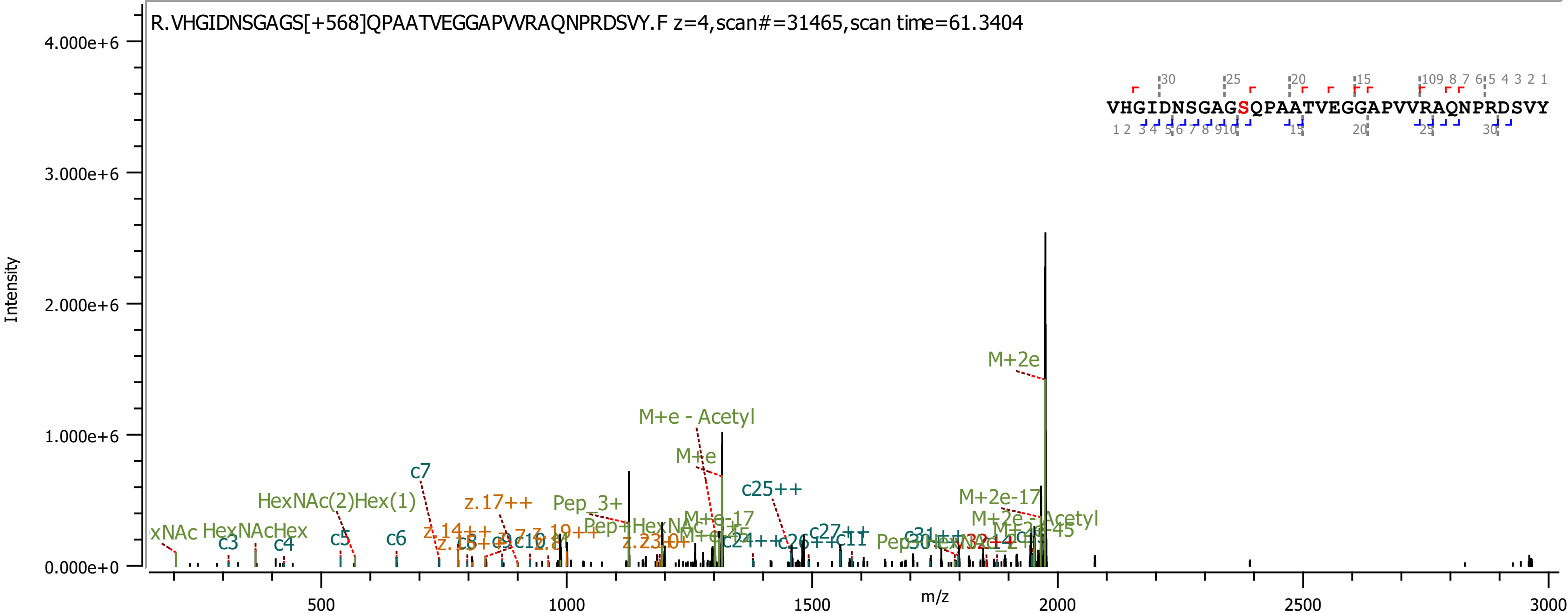
R.VHGIDNSGAGS[+568]QPAATVEGGAPVVRAQNPRDSVY.F z=4,scan#=31465,scan time=61.3404
Intensity
m/z
VHGIDNSGAGSQPAATVEGGAPVVRAQNPRDSVY
M+2e
M+e - Acetyl
M+e
c7
c25++
HexNAc(2)Hex(1)
z.17++
Pep_3+
M+2e-17
M+2e-Acetyl
HexNAcHex
c3
c4
c5
c6

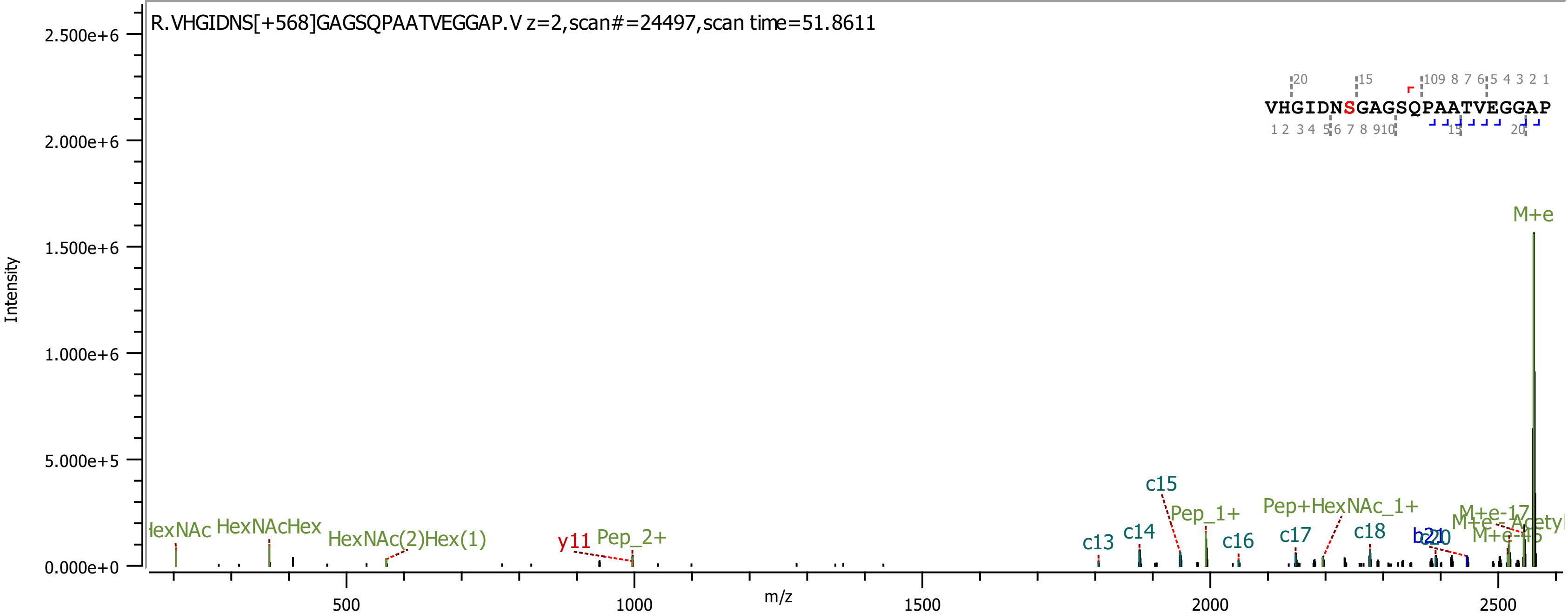
R.VHGIDNS[+568]GAGSQPAATVEGGAP.V z=2,scan#=24497,scan time=51.8611
VHGIDNSGAGSQPAATVEGGAP
Intensity
m/z
M+e
c15
Pep_1+
Pep+HexNAc_1+
HexNAcHex
HexNAc(2)Hex(1)
y11
Pep_2+
c13
c14
c16
c17
c18
M+e-17
2.500e+6
2.000e+6
1.500e+6
1.000e+6
5.000e+5
0.000e+0
500
1000
1500
2000
2500

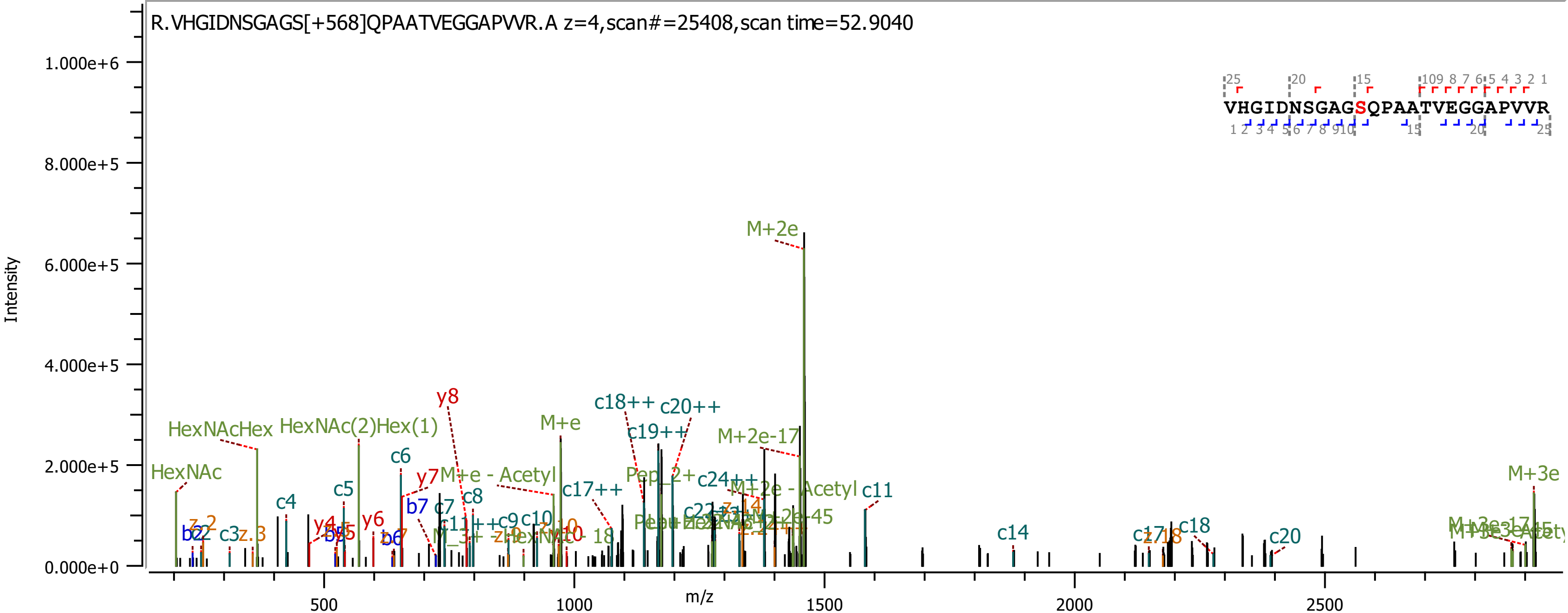

R.VHGIDNSGAGS[+568]QPAATVEGGAPVVR.A z=4,scan#=25408,scan time=52.9040
VHGIDNSGAGSQPAATVEGGAPVVR
Intensity
m/z
M+2e
M+2e-17
M+e
M+3e
HexNAc
HexNAcHex
HexNAc(2)Hex(1)
y8
y7
y6
c4
c5
c6
c7
c8
c11
c14
c18
c20
c18++
c20++
c19++
c17++
b7
M+e - Acetyl
M+2e - Acetyl

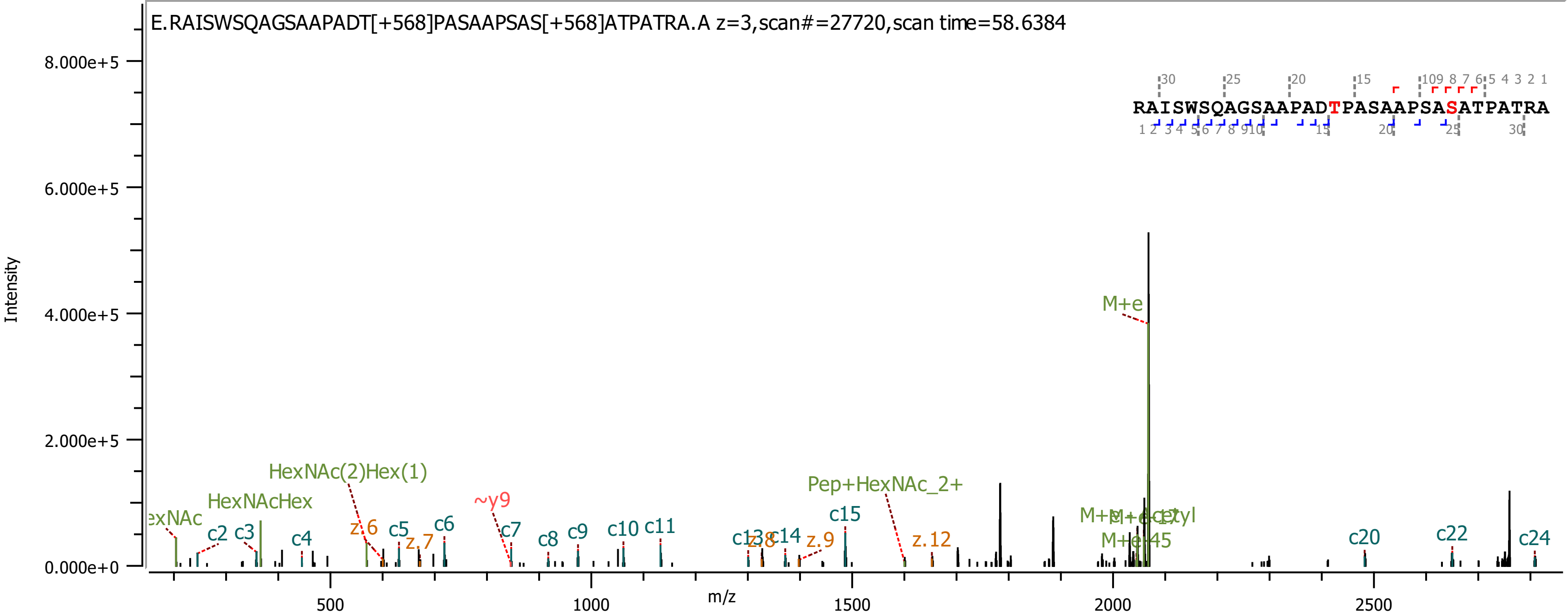

E.RAISWSQAGSAAPADT[+568]PASAAPSAS[+568]ATPATRA.A z=3,scan#=27720,scan time=58.6384
RAISWSQAGSAAPADTPASAAPSASATPATRA
Intensity
m/z
8.000e+5
6.000e+5
4.000e+5
2.000e+5
0.000e+0
500
1000
1500
2000
2500
M+e
HexNAc(2)Hex(1)
HexNAcHex
Pep+HexNAc_2+
~y9
c2
c3
c4
z.6
c5
z.7
c6
c7
c8
c9
c10
c11
c14
z.9
c15
z.12
M+e-45
c20
c22
c24

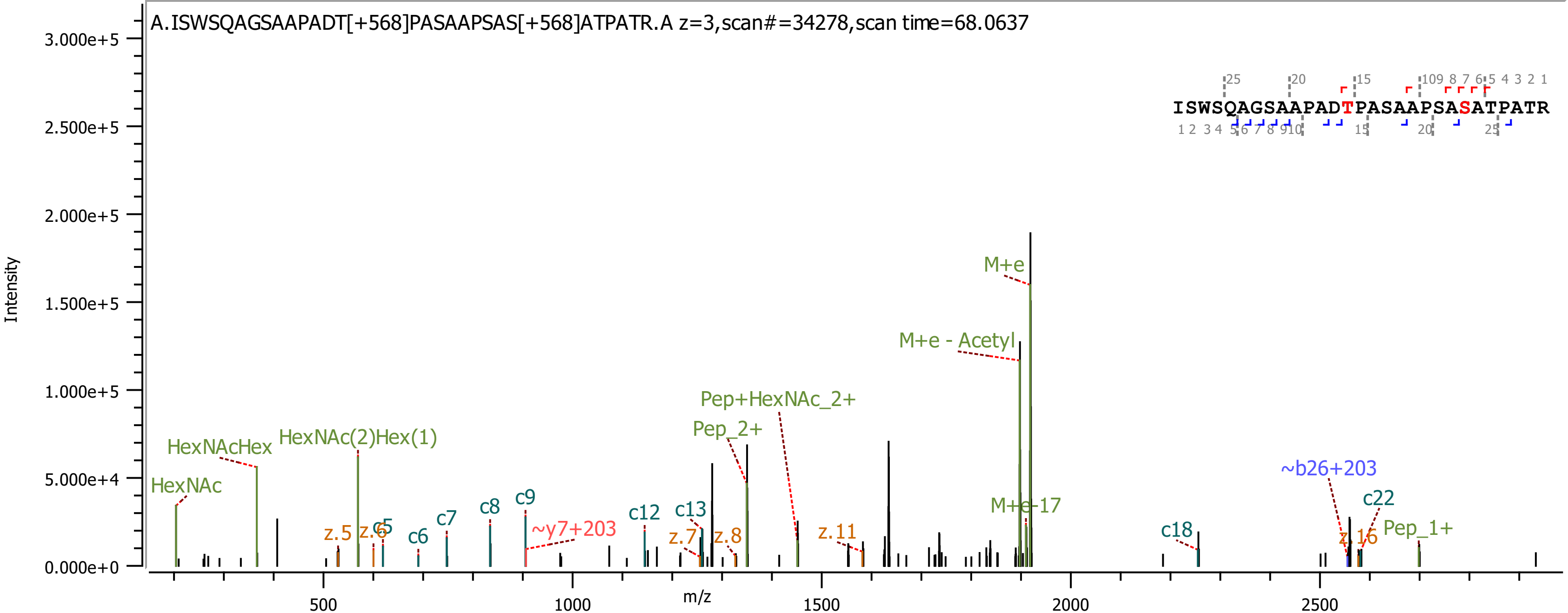
A.ISWSQAGSAAPADT[+568]PASAAPSAS[+568]ATPATR.A z=3,scan#=34278,scan time=68.0637
ISWSQAGSAAPADTPASAAPSASATPATR
Intensity
m/z
HexNAc
HexNAcHex
HexNAc(2)Hex(1)
z.5
z.6
c5
c6
c7
c8
c9
~y7+203
c12
c13
z.7
z.8
Pep_2+
Pep+HexNAc_2+
z.11
M+e - Acetyl
M+e
M+e-17
c18
~b26+203
z.16
c22
Pep_1+

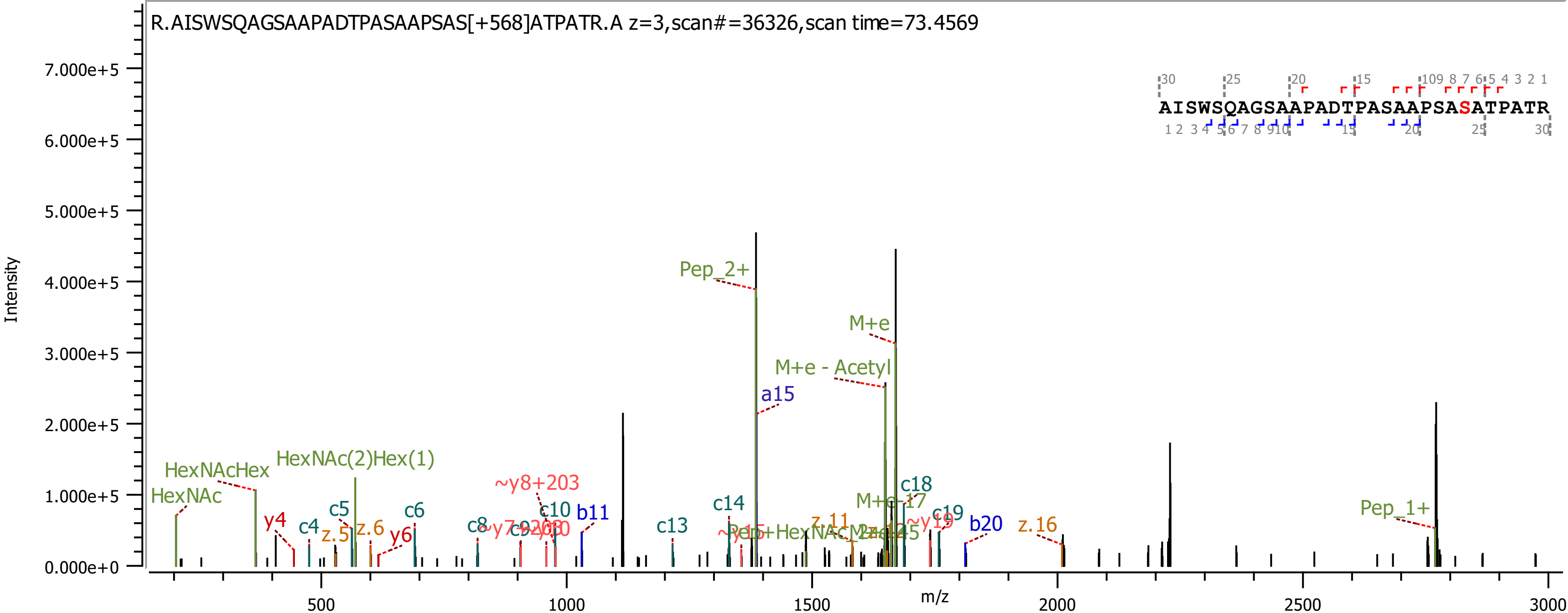

R.AISWSQAGSAAPADTPASAAPSAS[+568]ATPATR.A z=3,scan#=36326,scan time=73.4569
AISWSQAGSAAPADTPASAAPSASATPATR
Intensity
m/z
Pep_2+
M+e
M+e - Acetyl
a15
HexNAcHex
HexNAc(2)Hex(1)
HexNAc
y4
c4
c5
z.5
z.6
y6
c6
~y8+203
c8
c10
b11
c13
c14
c18
c19
z.11
~y19
b20
z.16
Pep_1+

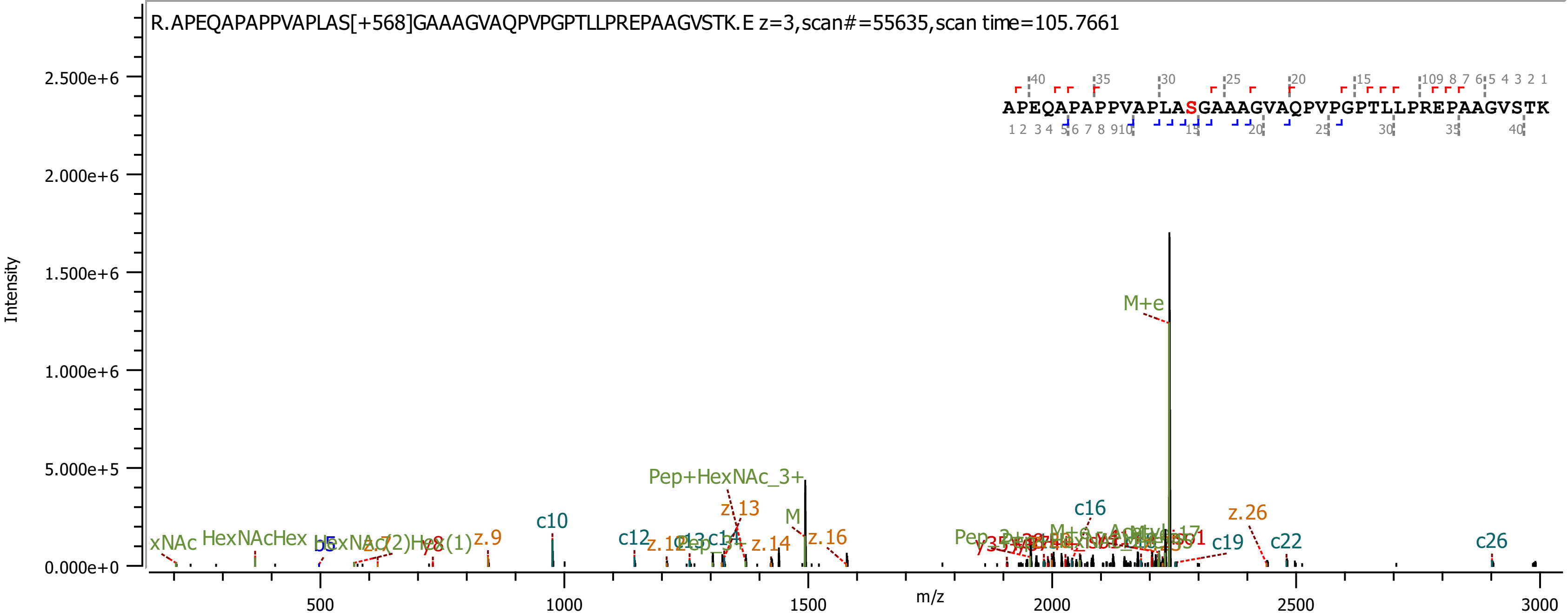
R.APEQAPAPPVAPLAS[+568]GAAAGVAQPVPGPTLLPREPAAGVSTK.E z=3,scan#=55635,scan time=105.7661
APEQAPAPPVAPLASGAAAGVAQPVPGPTLLPREPAAGVSTK
Intensity
m/z
2.500e+6
2.000e+6
1.500e+6
1.000e+6
5.000e+5
0.000e+0
500
1000
1500
2000
2500
3000
M+e
Pep+HexNAc_3+
z.13
M
z.16
c16
z.26
c10
c12
z.14
c19
c22
c26
HexNAcHex
z.9
b5

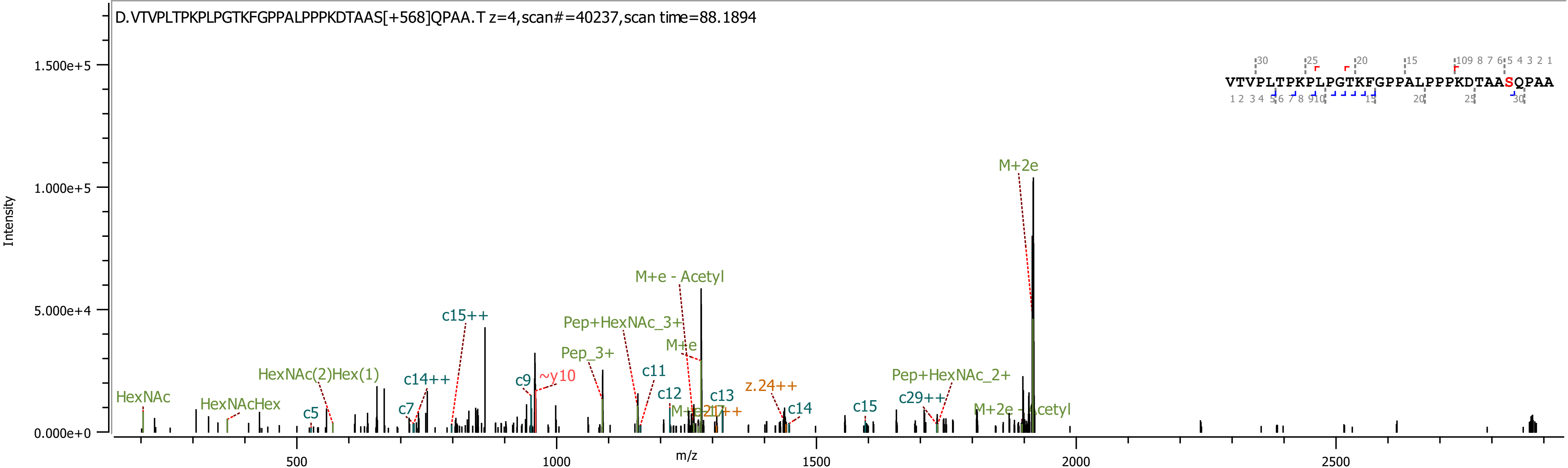

D.VTVPLTPKPLPGTKFGPPALPPPKDTAAS[+568]QPAA.T z=4,scan#=40237,scan time=88.1894
VTVPLTPKPLPGTKFGPPALPPPKDTAASQPAA
Intensity
m/z
M+2e
M+e - Acetyl
Pep+HexNAc_3+
M+e
Pep_3+
c15++
c14++
c11
c9
~y10
HexNAc(2)Hex(1)
Pep+HexNAc_2+
z.24++
c12
c13
c29++
c15
c14
HexNAc
HexNAcHex
c5
c7
M+2e - Acetyl

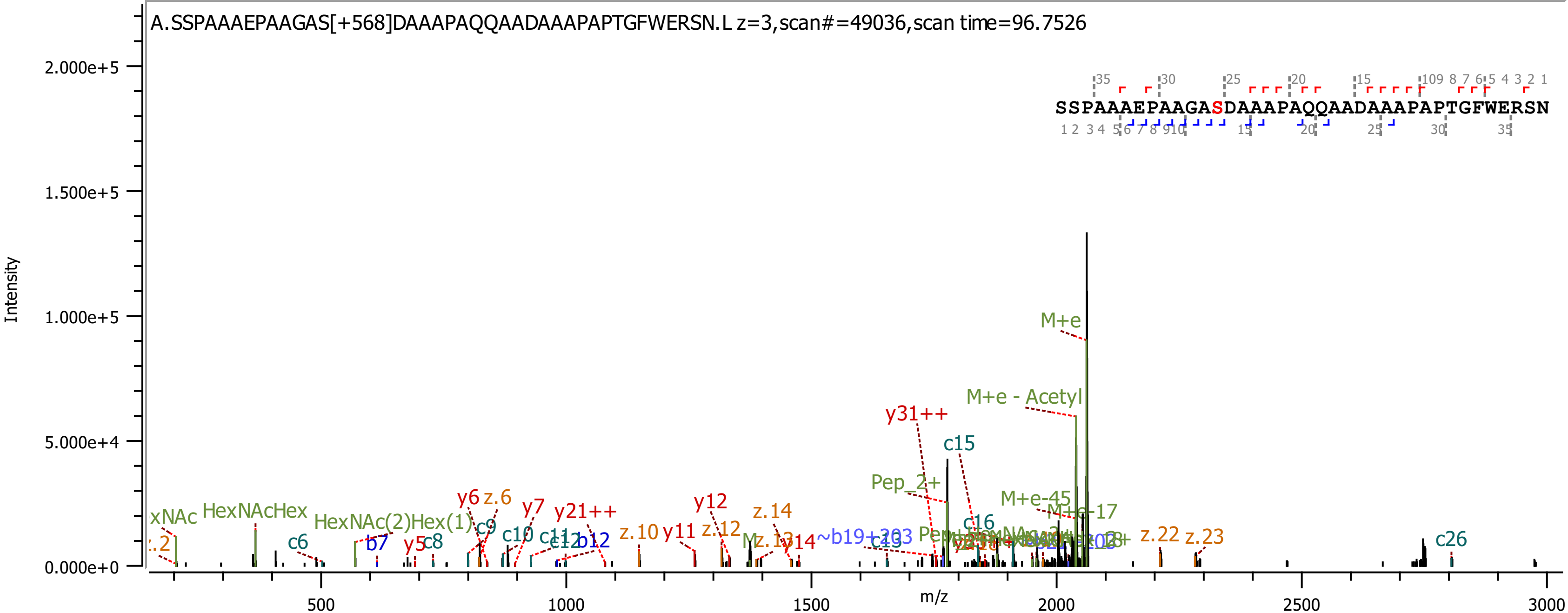

A.SSPAAAEPAAGAS[+568]DAAAPAQQAADAAAPAPTGFWERSN.L z=3,scan#=49036,scan time=96.7526
SSPAAAEPAAGASDAAAPAQQAADAAAPAPTGFWERSN
Intensity
m/z
2.000e+5
1.500e+5
1.000e+5
5.000e+4
0.000e+0
500
1000
1500
2000
2500
3000
M+e
M+e - Acetyl
y31++
c15
Pep_2+
M+e-45
M+e-17
c16
HexNAcHex
HexNAc(2)Hex(1)
c6
b7
y5
c8
y6
z.6
c9
y7
c10
y21++
c11
c12
b12
z.10
y11
y12
z.12
z.14
y14
c13
z.22
z.23
c26

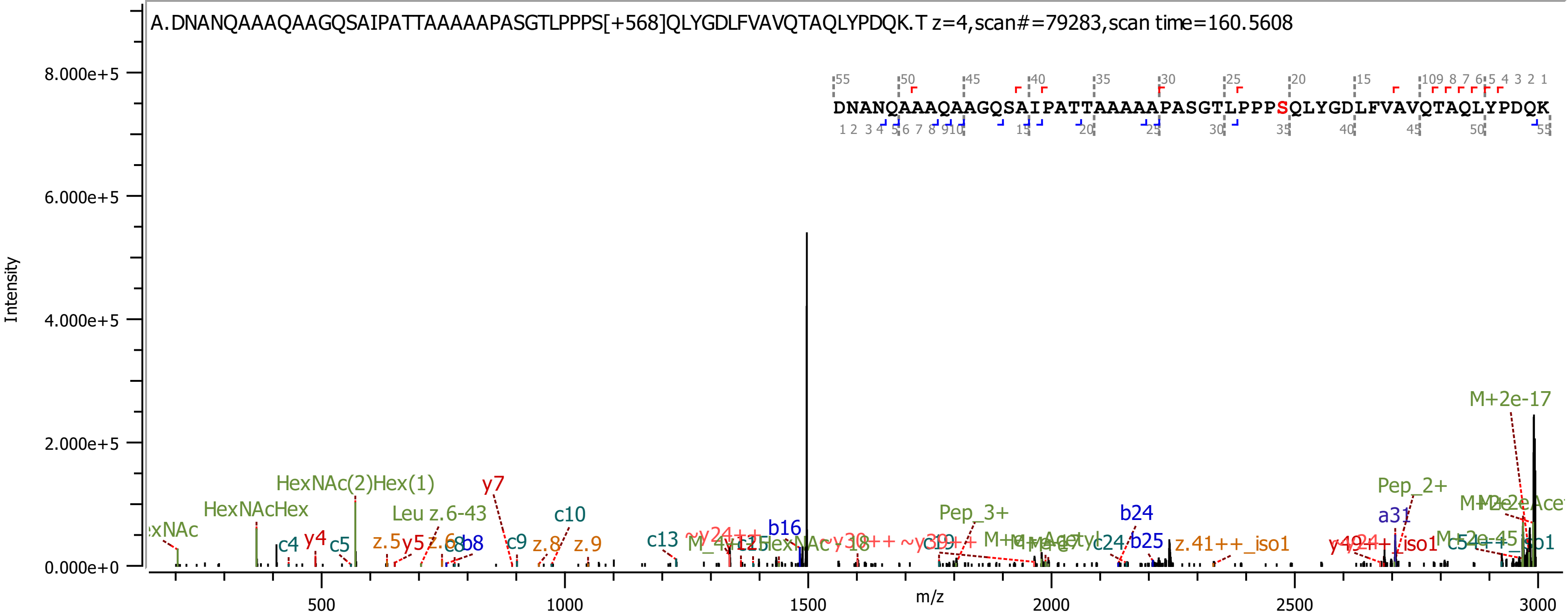

A.DNANQAAAQAAGQSAIPATTAAAAAPASGTLPPPS[+568]QLYGDLFVAVQTAQLYPDQK.T z=4,scan#=79283,scan time=160.5608
DNANQAAAQAAGQSAIPATTAAAAAPASGTLPPPSQLYGDLFVAVQTAQLYPDQK
Intensity
m/z
8.000e+5
6.000e+5
4.000e+5
2.000e+5
0.000e+0
500
1000
1500
2000
2500
3000
M+2e-17
HexNAc(2)Hex(1)
HexNAcHex
y7
Leu z.6-43
c10
Pep_3+
b24
Pep_2+
a31
b16
c4
y4
c5
z.5
y5
b8
c9
z.8
z.9
c13
b25
z.41++_iso1

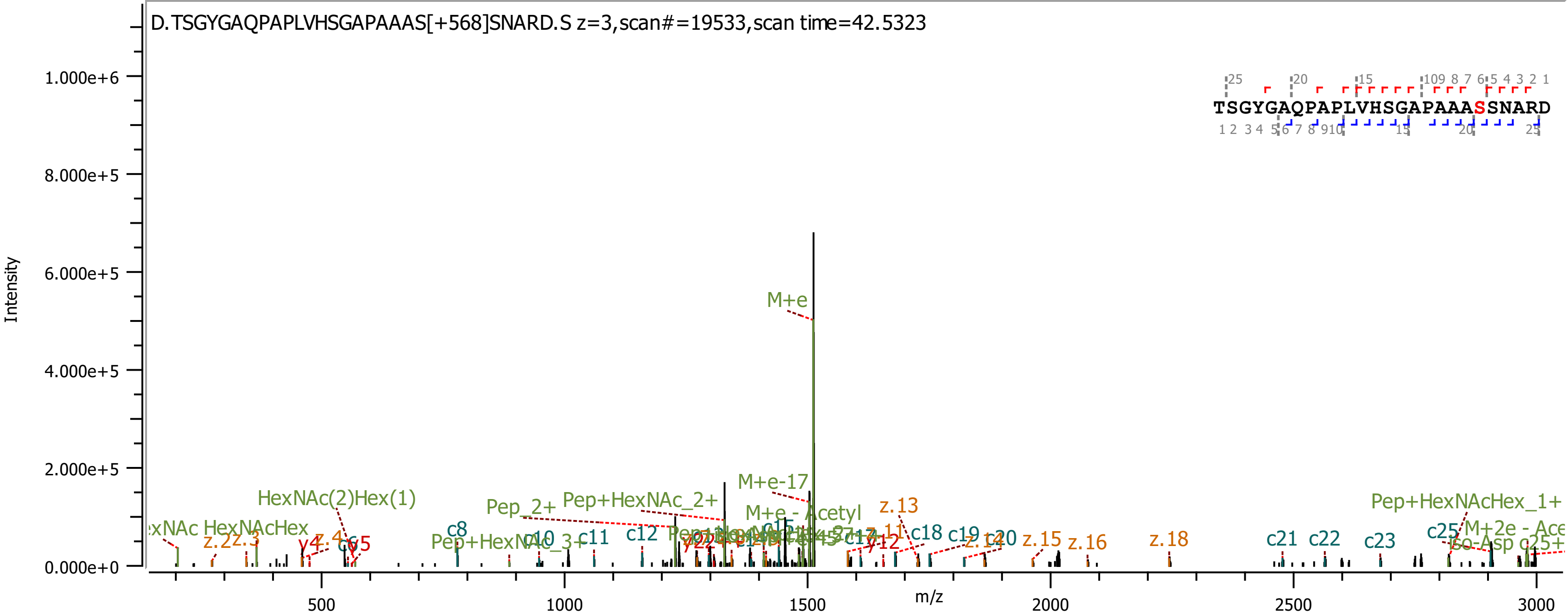
D.TSGYGAQPAPLVHSGAPAAAS[+568]SNARD.S z=3,scan#=19533,scan time=42.5323
TSGYGAQPAPLVHSGAPAAASSNARD
Intensity
m/z
M+e
M+e-17
M+e - Acetyl
HexNAc(2)Hex(1)
Pep_2+
Pep+HexNAc_2+
Pep+HexNAc_3+
Pep+HexNAcHex_1+
M+2e - Ace
iso-Asp
z.2
z.3
z.13
z.15
z.16
z.18
y4
c5
c8
c10
c11
c12
c18
c19
c20
c21
c22
c23
c25
y12
1.000e+6
8.000e+5
6.000e+5
4.000e+5
2.000e+5
0.000e+0
500
1000
1500
2000
2500
3000

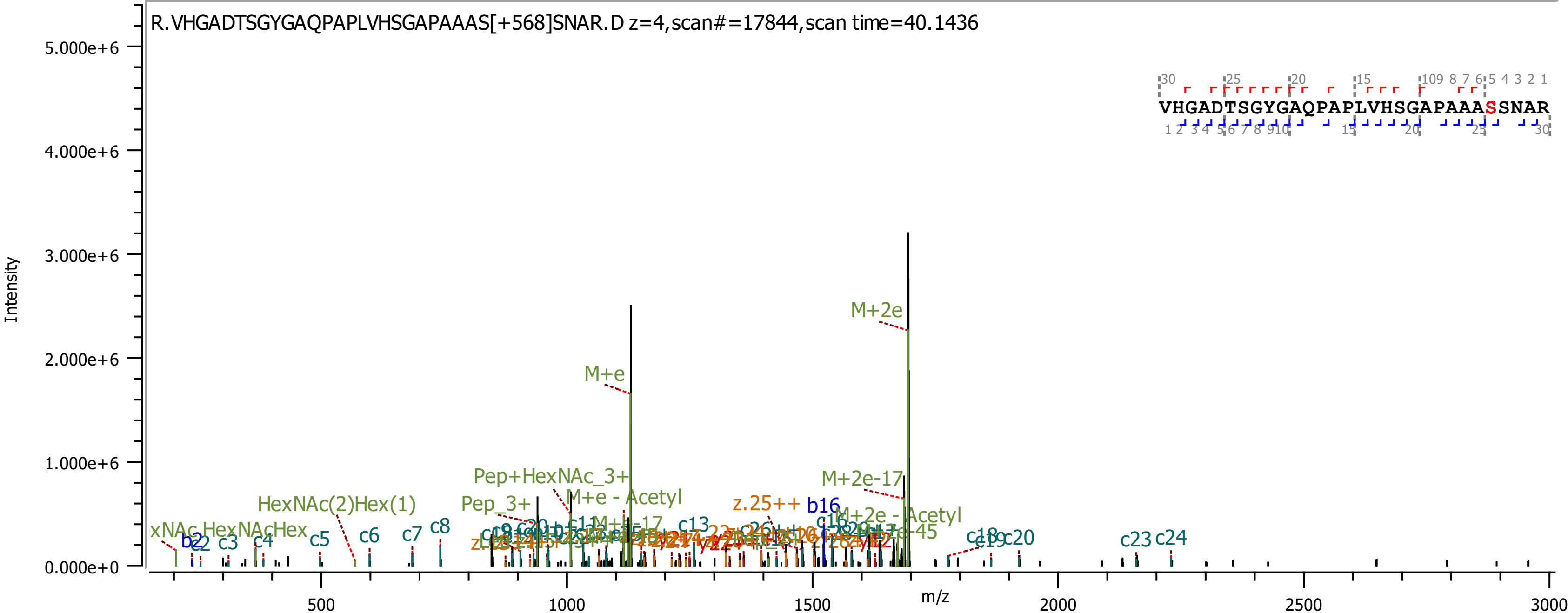
R.VHGADTSGYGAQPAPLVHSGAPAAAS[+568]SNAR.D z=4,scan#=17844,scan time=40.1436
VHGADTSGYGAQPAPLVHSGAPAAASSNAR
Intensity
m/z
5.000e+6
4.000e+6
3.000e+6
2.000e+6
1.000e+6
0.000e+0
500
1000
1500
2000
2500
3000
M+2e
M+e
M+2e-17
Pep+HexNAc_3+
M+e - Acetyl
HexNAc(2)Hex(1)
Pep_3+
z.25++
b16
M+2e - Acetyl
HexNAc HexNAcHex
b2
c2
c3
c4
c5
c6
c7
c8
c13
c18
c19
c20
c23
c24

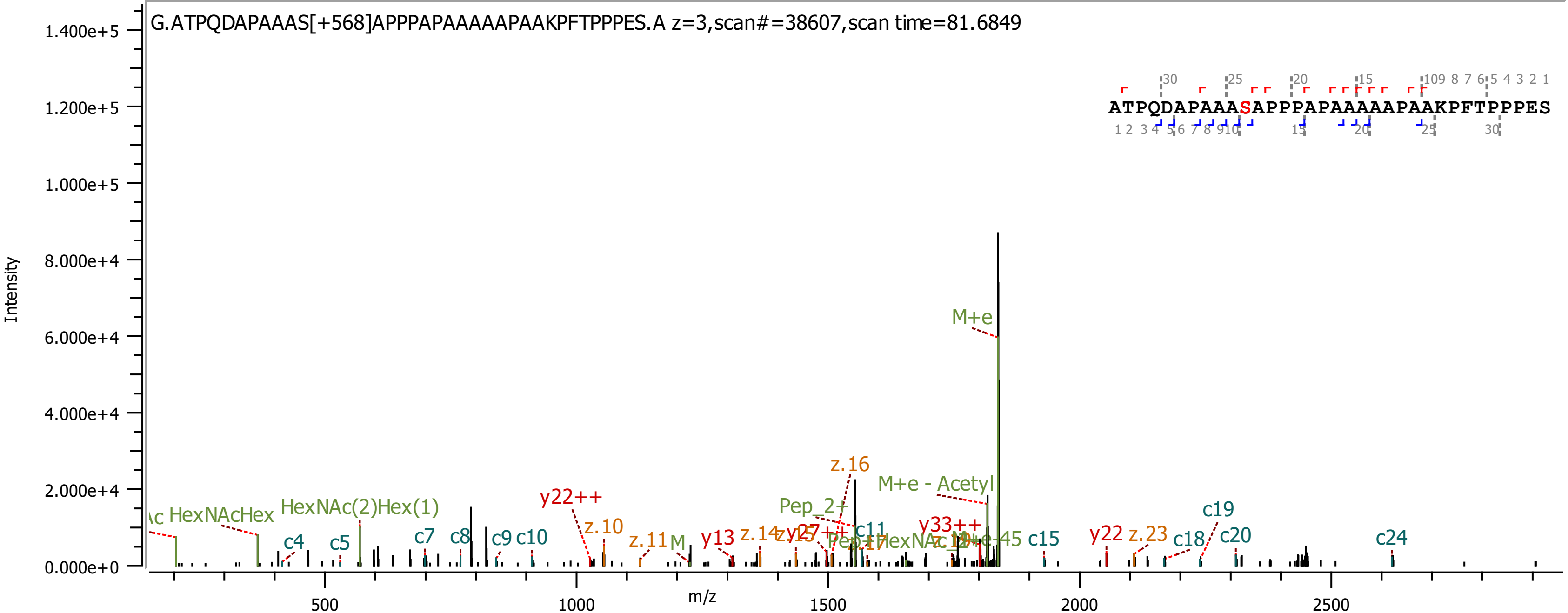
G.ATPQDAPAAAS[+568]APPPAPAAAAAPAAKPFTPPPES.A z=3,scan#=38607,scan time=81.6849
ATPQDAPAAASAPPPAPAAAAAPAAKPFTPPPES
Intensity
m/z
M+e
M+e - Acetyl
z.16
Pep_2+
y22++
z.10
HexNAcHex
HexNAc(2)Hex(1)
c4
c5
c7
c8
c9
c10
z.11
y13
z.14
y33++
c15
y22
z.23
c18
c19
c20
c24

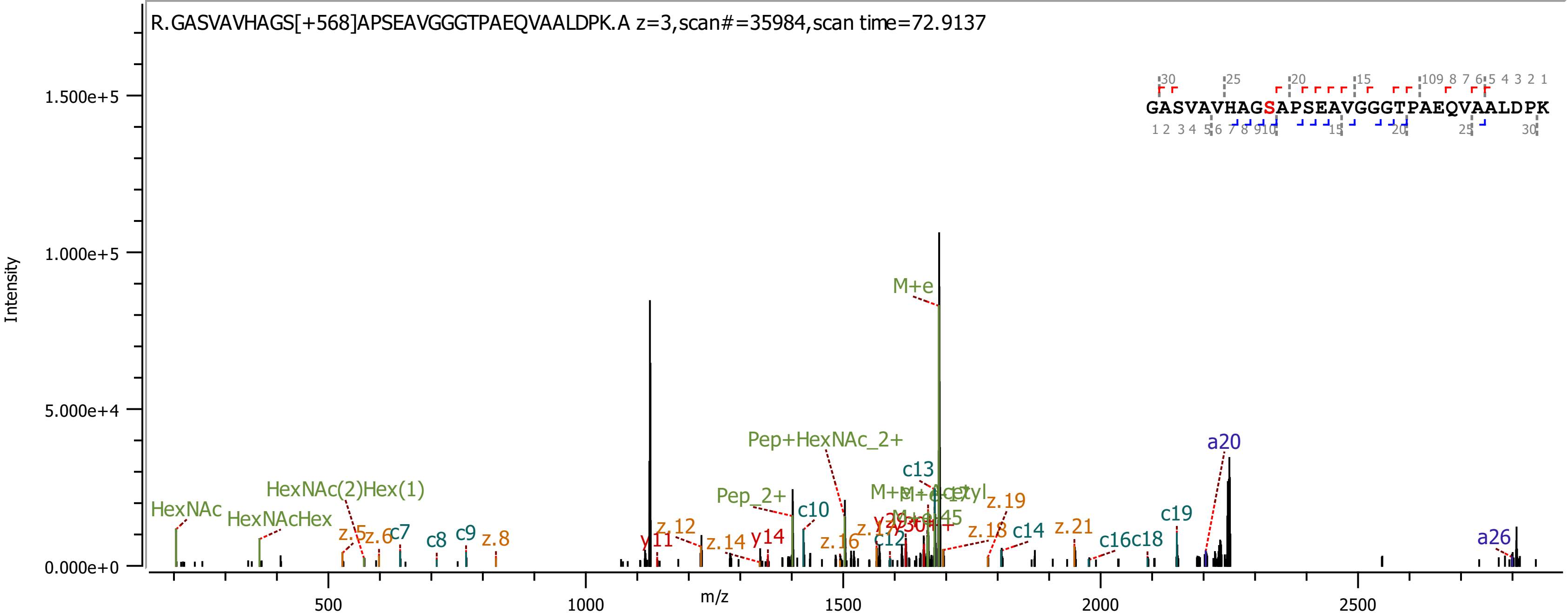
R.GASVAVHAGS[+568]APSEAVGGGTPAEQVAALDPK.A z=3,scan#=35984,scan time=72.9137
GASVAVHAGSAPSEAVGGGTPAEQVAALDPK
Intensity
m/z
HexNAc
HexNAcHex
HexNAc(2)Hex(1)
Pep_2+
Pep+HexNAc_2+
M+e
a20
a26
c7
c8
c9
c10
c13
c14
c19
z.8
z.12
z.14
z.19
z.21
y11
y14

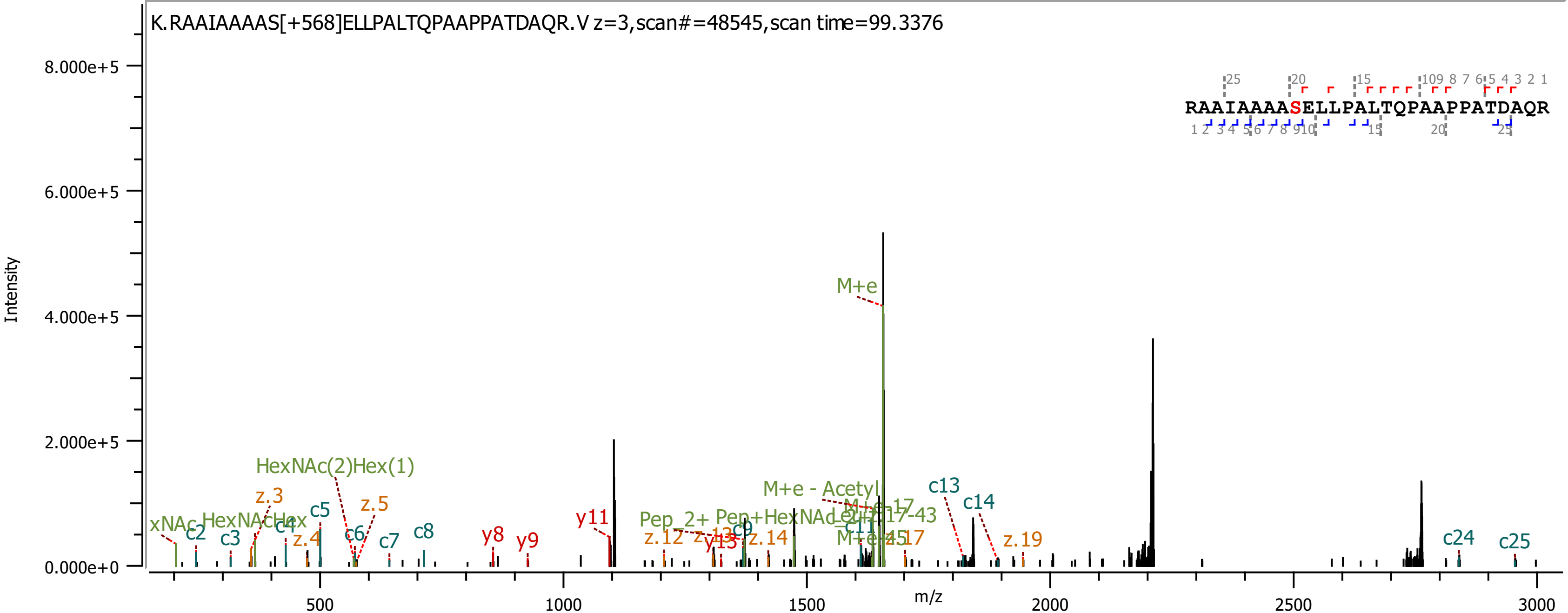

K.RAAIAAAAS[+568]ELLPALTQPAAPPATDAQR.V z=3,scan#=48545,scan time=99.3376
RAAIAAAASELLPALTQPAAPPATDAQR
Intensity
m/z
8.000e+5
6.000e+5
4.000e+5
2.000e+5
0.000e+0
500
1000
1500
2000
2500
3000
M+e
HexNAc(2)Hex(1)
z.3
z.5
c5
xNAc
HexNAcHex
c2
c3
c4
z.4
c6
c7
c8
y8
y9
y11
Pep_2+
z.12
y13
c9
z.14
M+e - Acetyl
c11
M+e-45
c13
c14
z.19
c24
c25

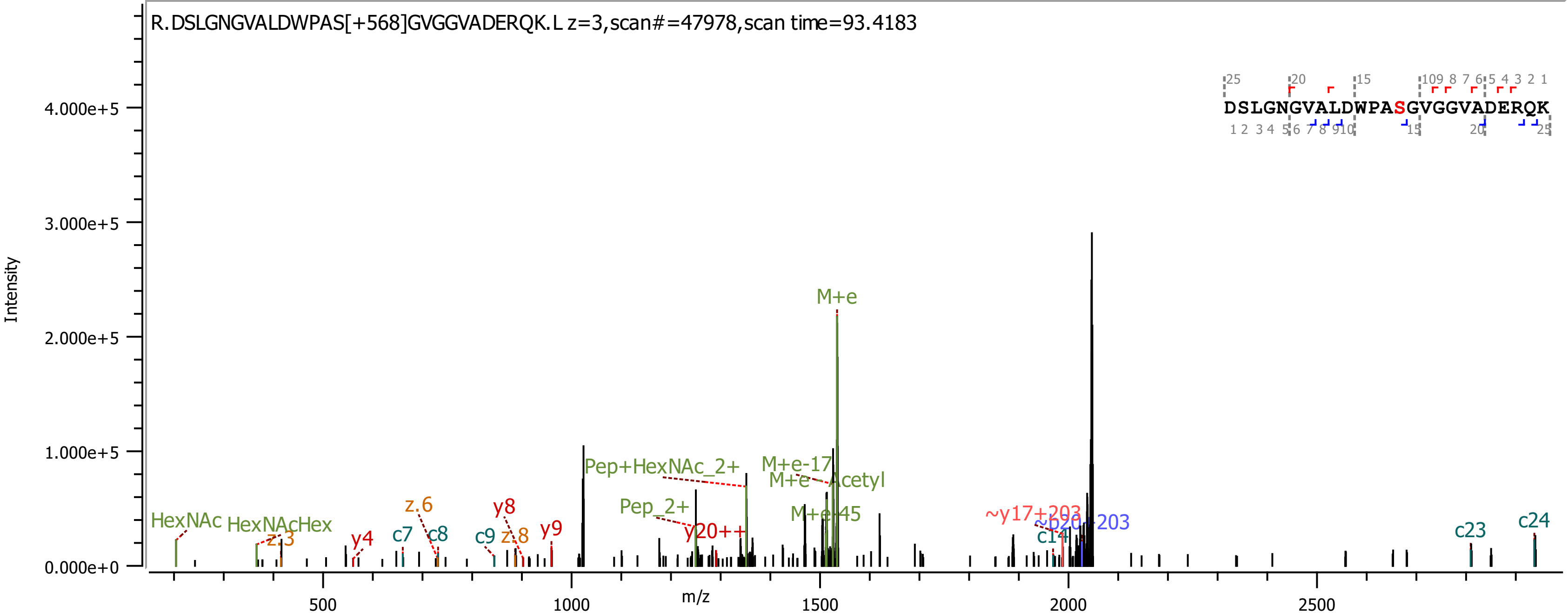
R.DSLGNGVALDWPAS[+568]GVGGVADERQK.L z=3,scan#=47978,scan time=93.4183
DSLGNGVALDWPASGVGGVADERQK
Intensity
m/z
4.000e+5
3.000e+5
2.000e+5
1.000e+5
0.000e+0
500
1000
1500
2000
2500
M+e
Pep+HexNAc_2+
M+e-17
M+e-Acetyl
Pep_2+
M+e-45
HexNAc
HexNAcHex
z.3
y4
c7
z.6
c8
y8
c9
z.8
y9
y20++
~y17+203
~b20+203
c14
c23
c24

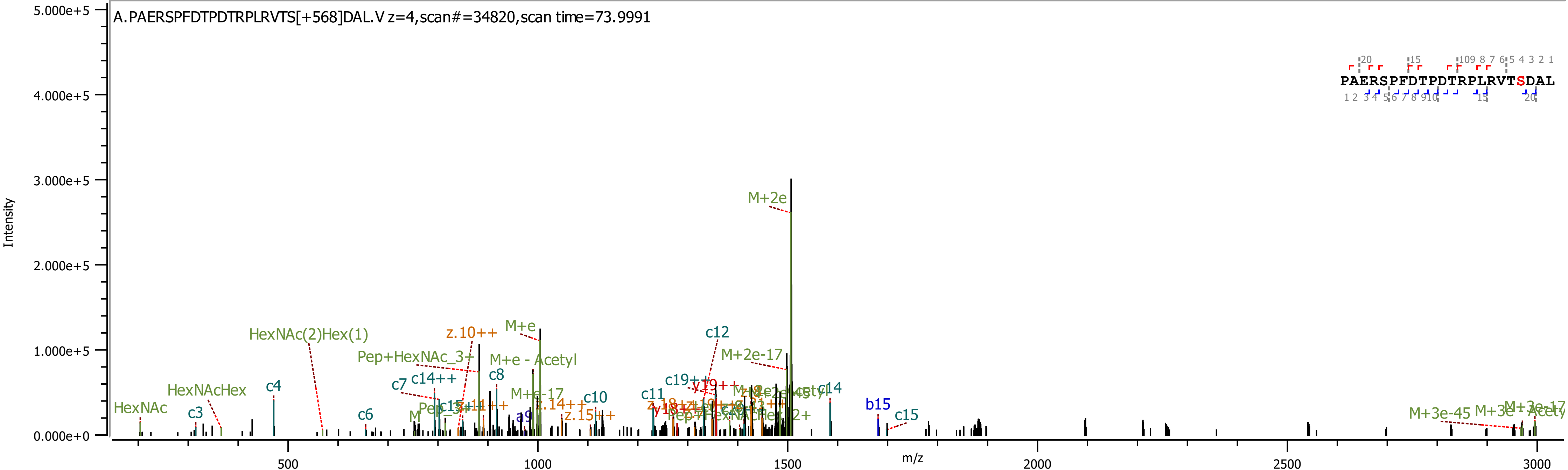
A.PAERSPFDTPDTRPLRVTS[+568]DAL.V z=4,scan#=34820,scan time=73.9991
Intensity
m/z
5.000e+5
4.000e+5
3.000e+5
2.000e+5
1.000e+5
0.000e+0
500
1000
1500
2000
2500
3000
PAERSPFDTPDTRPLRVTSDAL
M+2e
M+e
M+e - Acetyl
M+2e-17
HexNAc
HexNAcHex
HexNAc(2)Hex(1)
Pep+HexNAc_3+
z.10++
c3
c4
c6
c7
c8
c10
c11
c12
c14
c14++
c15
b15
a9
z.14++
z.15++
M+3e-45

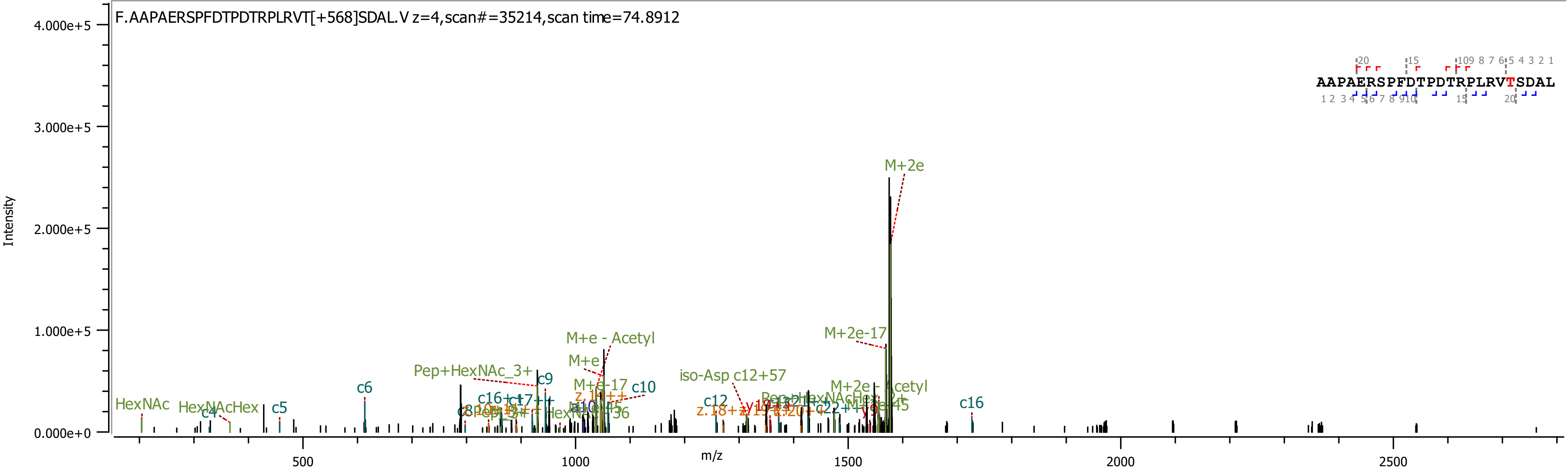
F.AAPAERSPFDTPDTRPLRVT[+568]SDAL.V z=4,scan#=35214,scan time=74.8912
AAPAERSPFDTPDTRPLRVTSDAL
Intensity
m/z
4.000e+5
3.000e+5
2.000e+5
1.000e+5
0.000e+0
500
1000
1500
2000
2500
M+2e
M+2e-17
M+e - Acetyl
M+e
Pep+HexNAc_3+
iso-Asp c12+57
HexNAc
HexNAcHex
c5
c6
c9
c10
c12
c16
