# Supplementary material for: Burkholderia PglL enzymes are Serine preferring oligosaccharyltransferases which target conserved proteins across the Burkholderia genus
Source: Commun Biol. 2021 Sep 7;4:1045. doi: 10.1038/s42003-021-02588-y (PMC8423747; doi:10.1038/s42003-021-02588-y)
Supplement: Supplementary file 16 — Reporting Summary [file 42003_2021_2588_MOESM16_ESM.pdf]

nature research

Corresponding author(s): COMMSBIO-21-1013, Corresponding author Nichollas Scott

Last updated by author(s): Jul 22, 2021

Reporting Summary

Nature Research wishes to improve the reproducibility of the work that we publish. This form provides structure for consistency and transparency in reporting. For further information on Nature Research policies, see our Editorial Policies and the Editorial Policy Checklist.

Statistics

For all statistical analyses, confirm that the following items are present in the figure legend, table legend, main text, or Methods section.

n/a	Confirmed	
☐	☒	The exact sample size (*n*) for each experimental group/condition, given as a discrete number and unit of measurement
☐	☒	A statement on whether measurements were taken from distinct samples or whether the same sample was measured repeatedly
☐	☒	The statistical test(s) used AND whether they are one- or two-sided<br>*Only common tests should be described solely by name; describe more complex techniques in the Methods section.*
☒	☐	A description of all covariates tested
☐	☒	A description of any assumptions or corrections, such as tests of normality and adjustment for multiple comparisons
☒	☐	A full description of the statistical parameters including central tendency (e.g. means) or other basic estimates (e.g. regression coefficient) AND variation (e.g. standard deviation) or associated estimates of uncertainty (e.g. confidence intervals)
☐	☒	For null hypothesis testing, the test statistic (e.g. *F*, *t*, *r*) with confidence intervals, effect sizes, degrees of freedom and *P* value noted<br>*Give P values as exact values whenever suitable.*
☒	☐	For Bayesian analysis, information on the choice of priors and Markov chain Monte Carlo settings
☒	☐	For hierarchical and complex designs, identification of the appropriate level for tests and full reporting of outcomes
☒	☐	Estimates of effect sizes (e.g. Cohen's *d*, Pearson's *r*), indicating how they were calculated

Our web collection on statistics for biologists contains articles on many of the points above.

Software and code

Policy information about availability of computer code

Data collection	MS data was collected using Thermo scientific instruments using Xcalibur (version 4.3.73.11)
Data analysis	Statistical test undertaken in<br>Prism (version 7.0e)<br>Perseus (v1.5.0.9)<br><br>MS database searching undertaken with<br>MaxQuant (v1.5.5.1 or 1.6.3.4.)<br>MetaMorpheus (non-public release version MM0523)<br>Byonic (v3.5.3)<br><br>annotation of MS/MS spectra undertaken with the<br>Interactive Peptide Spectral Annotator (http://www.interactivepeptidespectralannotator.com/PeptideAnnotator.html)

For manuscripts utilizing custom algorithms or software that are central to the research but not yet described in published literature, software must be made available to editors and reviewers. We strongly encourage code deposition in a community repository (e.g. GitHub). See the Nature Research guidelines for submitting code & software for further information.

Data

Policy information about availability of data

All manuscripts must include a data availability statement. This statement should provide the following information, where applicable:

- Accession codes, unique identifiers, or web links for publicly available datasets
- A list of figures that have associated raw data
- A description of any restrictions on data availability

Mass spectrometry data (Raw data files, Byonic/Maxquant/O-pair search outputs, R Scripts and output tables) have been deposited into the PRIDE ProteomeXchange Consortium repository and are now publicly accessible.

-The glycoproteomic datasets are available with the identifier: PXD024090

-the H111 proteome analysis is available with the identifier: PXD023755

-the DsbA1Nm-his6 (B. cenocepacia K56-2) associated analysis is available with the identifier: PXD023955

-the DsbA1Nm-his6 (B. humptydooensis MSMB43 and B. ubonensis MSMB22) associated analysis is available with the identifier: PXD024056

Field-specific reporting

Please select the one below that is the best fit for your research. If you are not sure, read the appropriate sections before making your selection.

[x] Life sciences [ ] Behavioural & social sciences [ ] Ecological, evolutionary & environmental sciences

For a reference copy of the document with all sections, see nature.com/documents/nr-reporting-summary-flat.pdf

Life sciences study design

All studies must disclose on these points even when the disclosure is negative.

Sample size	no-sample size calculations were undertaken for these studies with wet lab experiments undertaken three independent times to enable statistical analysis. The sample size used for genomic analysis was govern by the availability of closed genomes for Burkholderia cenocepacia and available Burkholderia species.
Data exclusions	No data exclusion criteria were used for our analysis
Replication	Three biological replicates were generate to enable statistical analysis. Biological samples were regenerated for different experimental comparisons (i.e the samples used to compare DsbA1Nm-his6 variants within Burkholderia cenocepacia overexpressing pglL were unique to those used to compare DsbA1Nm-his6 variants within WT and pglL Burkholderia cenocepacia).
Randomization	For the collection of MS data samples were randomized to limit any batch effects.
Blinding	Blinding was not undertaken for these studies

Reporting for specific materials, systems and methods

We require information from authors about some types of materials, experimental systems and methods used in many studies. Here, indicate whether each material, system or method listed is relevant to your study. If you are not sure if a list item applies to your research, read the appropriate section before selecting a response.

Materials & experimental systems

n/a	Involved in the study	
[ ]	[x]	Antibodies
[x]	[ ]	Eukaryotic cell lines
[x]	[ ]	Palaeontology and archaeology
[x]	[ ]	Animals and other organisms
[x]	[ ]	Human research participants
[x]	[ ]	Clinical data
[x]	[ ]	Dual use research of concern

Methods

n/a	Involved in the study	
[x]	[ ]	ChIP-seq
[x]	[ ]	Flow cytometry
[x]	[ ]	MRI-based neuroimaging

Antibodies

Antibodies used	Primary antibodies<br>mouse monoclonal anti-His (1:2,000; AD1.1.10, Biorad)<br>mouse anti-RNA pol (1:5,000; 4RA2, Neoclone)<br><br>Secondary antibodies<br>anti-mouse IgG horseradish peroxidase (HRP)-conjugated antibodies (1:3,000; catalog number NEF822001EA, Perkin-Elmer)
Validation	These antibodies have been previously used with the lab and published within Oppy et al 2019. All raw western images are now provided as Supplementary Figure 19
